# Foreign peptide triggers boost in pneumococcal metabolism and growth

**DOI:** 10.1186/s12866-018-1167-y

**Published:** 2018-03-27

**Authors:** Fauzy Nasher, Sunniva Förster, Efe C. Yildirim, Denis Grandgirard, Stephen L. Leib, Manfred Heller, Lucy J. Hathaway

**Affiliations:** 1 Institute for Infectious Diseases, Faculty of Medicine, University of Bern, Friedbühlstrasse 51, CH-3001 Bern, Switzerland; 2 Graduate School for Cellular and Biomedical Sciences, University of Bern, Bern, Switzerland; 3 Institute of Social and Preventive Medicine, University of Bern, Bern, Switzerland; 4 Proteomics and Mass Spectrometry Core Facility, Department for BioMedical Research, University of Bern, CH-3010 Bern, Switzerland

**Keywords:** *Streptococcus pneumoniae*, Peptide, Nonencapsulated, *aliB-like* ORF 2, Transcriptome, Proteome

## Abstract

**Background:**

Nonencapsulated *Streptococcus pneumoniae* bacteria are successful colonizers of the human nasopharynx and often possess genes *aliB-like* ORF 1 and 2 in place of capsule genes. AliB-like ORF 2 binds peptide FPPQSV, found in *Prevotella* species, resulting in enhanced colonization. How this response is mediated is so far unknown.

**Results:**

Here we show that the peptide increases expression of genes involved in release of host carbohydrates, carbohydrate uptake and carbohydrate metabolism. In particular, the peptide increased expression of 1,5-anhydro-D-fructose reductase, a metabolic enzyme of an alternative starch and glycogen degrading pathway found in many organisms, in both transcriptomic and proteomic data. The peptide enhanced pneumococcal growth giving a competitive advantage to a strain with *aliB-like* ORF 2, over its mutant lacking the gene.

Possession of *aliB-like* ORF 2 did not affect release of inflammatory cytokine CXCL8 from epithelial cells in culture and the nonencapsulated wild type strain was not able to establish disease or inflammation in an infant rat model of meningitis.

**Conclusions:**

We propose that AliB-like ORF 2 confers an advantage in colonization by enhancing carbohydrate metabolism resulting in a boost in growth. This may explain the widespread presence of *aliB-like* ORF 2 in the nonencapsulated pneumococcal population in the human nasopharynx.

**Electronic supplementary material:**

The online version of this article (10.1186/s12866-018-1167-y) contains supplementary material, which is available to authorized users.

## Background

*Streptococcus pneumoniae* causes several human diseases including bacterial meningitis, pneumonia, sepsis and otitis media but more often inhabits the human nasopharynx without causing disease. It shares this niche with many other organisms and we have previously proposed that pneumococcus senses its bacterial neighbours by recognizing peptide fragments derived from them [1]. Of pneumococcal strains that lack genes to synthesize a polysaccharide capsule, the majority possess in their stead two genes named *aliB-like* ORF 1 and ORF 2 which we predict encode substrate-binding proteins of an ATP-binding cassette (ABC) transporter [2, 3]. Nonencapsulated pneumococci which lack capsule genes but have *aliB-like* ORF 1 and ORF 2 are commonly of multilocus sequence types 344 or 448 and close relatives, which make up a separate phylogenetic group that clusters away from the majority of encapsulated pneumococci [3–5]. As well as being efficient colonizers of the nasophynyx, these nonencapsulated strains, particularly ST448, are associated with conjunctivitis and have been proposed to be adapted to these niches by distinct virulence factors and metabolic features which differ from those of encapsulated pneumococci [6]. The *aliB-like* ORF 1 and ORF 2 genes are also found in strains of the commensal streptococci *S. mitis, S. oralis* and *S. pseudopneumoniae* [7–9]. By expressing AliB-like ORF 1 and ORF 2 proteins, we have previously shown that they bind to peptides SETTFGRDFN (found in the 50S ribsosomal subunit protein L4 of Enterobacteriaceae) and FPPQSV (found in proteins of *Prevotella* species) respectively. Binding of the ORF 1 ligand was associated with upregulation of competence for genetic transformation whereas binding of ORF 2 ligand aided the pneumococcal colonization of the nasopharynx [1]. Understanding the communication that occurs between bacterial species in their natural environment may provide novel targets with which to interfere.

Here, we investigated how binding of AliB-like ORF 2 to its ligand causes phenotypic changes to the pneumococcus. We determined the changes caused by the peptide ligand to the pneumococcal transcriptome and proteome and the effect on growth in vitro. We also determined the effect of pneumococci with and without AliB-like ORF 2 on host cells in vitro and in an infant rat model of meningitis.

## Methods

### Ethics statement

All animal studies were approved by the Animal Care and Experimentation Committee of the Canton of Bern, Switzerland (licence BE76/14) and followed the Swiss national guidelines for the performance of animal experiments.

### Bacterial culture and strains

Bacteria were stored and cultured as described previously [1] i.e., storage using Protect bacterial preservers (Technical Service Consultants, Heywood, U.K.) at − 80 °C. To grow the pneumococci they were first streaked onto Columbia sheep blood agar (CSBA) plates and then incubated at 37 °C, 5% CO_2_ overnight. Cultures of 3 to 10 colonies were next prepared in 5 ml brain heart infusion (BHI) broth (Becton Dickinson and Company, le Pont de Claix, France) containing 5% fetal calf serum (FCS) (Biochrom KG, Berlin, Germany) for overnight culture.

The Swiss nonencapsulated nasopharyngeal pneumococcal isolate 110.58 of multilocus sequence type (MLST) ST344 [2], and the construction of its mutants in which one or both of its *aliB-like* ORFs have been inactivated to give mutants ΔORF 2 and ΔORF 1 + 2 has been previously described [1, 2].

### Preparation of bacteria and measurement of growth

Following overnight growth on CSBA plates at 37 °C, 5% CO_2,_ the bacteria were subcultured in BHI + FCS medium, grown to OD_600nm_ 0.5 and subsequently centrifuged at 3000 g for 5 min before resuspension in chemically defined CDM medium which contains 5.5 mM glucose [10]. Growth was monitored as described previously [11] in sterile flat-bottomed 96-well microtitre plates (Nunclon Surface, Nunc, Denmark) based on the method of Brewster [12] as follows: 200 μl bacteria culture was incubated per well at 37 °C and OD_450nm_ was measured at 30 min intervals by a VERSAmax microplate reader (Molecular Devices) over 20 h with 5 s of automatic shaking preceding each reading. Condensation was prevented by pre-treating the plate lids with 3 ml 0.05% Triton X-100 in 20% ethanol [11].

### Growth competition assay

Wild type strain 110.58 and mutant ΔORF 2 were streaked onto CSBA plates and incubated at 37 °C in a 5% CO_2_-enriched atmosphere overnight then subcultured in BHI + FCS medium to OD_600nm_ 0.5, centrifuged at 3000 g for 5 min and resuspended in chemically defined CDM medium. 250 μl of each bacterial culture was transferred to 4.5 ml CDM pre-warmed to 37 °C and AliB-like ORF 2 ligand FPPQSV (synthesized by PolyPeptide Group, Strasbourg, France) was added to a final concentration of 0.07 mg/ml (104 μM) and the culture incubated to an OD_600nm_ 0.3. Serial dilutions in PBS were plated onto CSBA plates with and without 3 μg/ml chloramphenicol to differentiate between wild type (chloramphenicol susceptible) and mutant ΔORF 2 (resistant) strains. After overnight incubation, the number of colonies was counted and the colony forming units (CFU) for each strain calculated. (CFU for mutant was calculated from the number of colonies on the chloramphenicol plates, CFU for wild type was calculated from the number of colonies on the CSBA plates minus the number on the chloramphenicol plates).

### Gene expression analysis by RNA-Seq and real-time RT-PCR

RNA-Seq was used to identify differentially expressed genes between wild type and ΔORF 2 mutant as well as wild type compared to wild type exposed to the peptide ligand and the ΔORF 2 mutant compared to ORF 2 treated with the ligand.

110.58 and ΔORF 2 were plated out on CSBA plates and incubated at 37 °C, 5% CO_2_ overnight, colonies picked and cultured overnight in 5 ml BHI + FCS as described above until OD_600nm_ = 0.4 then centrifuged at 2000 g for 5 min and the pellet resuspended in CDM. The centrifugation was repeated and the pellet resuspended in 5 ml CDM. This 5 ml bacterial suspension was added to 10 ml CDM. For each strain, at exactly OD_600nm_ = 0.2 the culture was split into two tubes, each containing 5 ml. To one tube for each strain the peptide FPPQSV was added to give a final concentration of 0.07 mg/ml, mixed and all tubes incubated at 37 °C for exactly 15 min. Transcription was stopped by adding RNAprotect (Qiagen) and RNA extracted as described previously [13]. RNA was isolated from three separate experiments performed on different days for RNA-Seq and from a further three separate experiments performed on different days for real-time RT-PCR.

For RNA-Seq, ribosomal RNA was depleted using Ribominus (Invitrogen) and then the RNA purified using RNA Clean & Concentration™-5 kit (Zymo Research) according to the manufacturers’ instructions, eluting in 6 μl. Libraries were prepared using TruSeq® Stranded mRNA (Illumina). 13 μl Fragment, Prime, Finish Mix was added to 5 μl of the mRNA. Fragmentation was performed for 40 s at 94 °C. Raw reads were obtained from an Illumina 3000 Hi-seq paired end sequencing platform. Reads were mapped to *Streptococcus pneumoniae* strain NT_110_58 assembly gca 000817005 ASM81700v1). Preprocessing and adapter triming was done using the Trimommatic tool (0.35) [14]. Alignments were performed with Bowtie (bowtie2–2.2.8) [15]. Cufflinks (cufflinks-2.2.1) and Cuffdiff were used to count reads and calculate differential gene expression [16]. Quality control and coverage were examined using FastQC (0.11.5) and Qualimap 2.2.1 [17]. The sequencing and coverage was on average 3557 times the genome size (ranging between 543 and 5220). The mean length of sequenced fragments was 141 bp. Mapping to the reference genome was above 99.09% for all samples.

Expression of genes of interest identified by RNA-Seq was quantified by real-time RT-PCR as described previously [13] and normalized against 16S using the following primers and probes: 16S forward primer 5’-GACGATACATAGCCGACCTGAGA-3′; reverse primer 5’-GTAGGAGTCTGGGCCGTGTCT-3′; probe 6-carboxyfluorescein (6-FAM)-CCAGTGTGGCCGATC-minor groove binder (MGB); *afr* forward primer 5′- TCTTCATCACCGAAATGTTCACCTT-3′; reverse primer 5′- ATGCCTGAAACTGTAACCATGACA-3′; probe, (6FAM)- ATGGGCCACATTTCCA-(MGB); *adh*E forward primer 5’-GAAGGAAGTTTCATCCATTGCATGT-3′; reverse primer 5’-ACGTTAGTGCCATTAACCTCTTGAA-3′; probe (6-FAM)-CCGTCTTCCGACTTTT-(MGB); *nanA_*3 forward primer 5’-CACCACTTCACCAGCAGATGTATAA-3′; reverse primer 5’-GAGACTAAAGTTCCAATAACGACTGGTT-3′; probe (6-FAM)-CACGCACCATTTTCTT--(MGB); *nanB* forward primer 5’-GTTAACCCAACTTTAGCAATGGCAAT-3′; reverse primer 5’-GGAGCAATCATGTCGAGACTACT-3′; probe (6-FAM)-TTCCCACCAATTTTG-(MGB); *yesO_2* forward primer 5’-ACTTGGTTAGGAAGAGCTGTACTGA-3′; reverse primer 5’-GAATGACTTCTATACTAAATGGACTACAGGTT-3′; probe (6-FAM)-ATCTGGCACATTTCC-(MGB); *ycjO* forward primer 5’-GAAGTGGCGTACTCTGTGAAGA-3′; reverse primer 5’-TGGACCGTTTTCTCATTAGTTGGT-3′; probe (6-FAM)-AAGCCAATACAAACCC-(MGB).

### Proteomic analysis

Sample processing, LC-MS/MS and data interpretation was essentially done as described previously [18] with the following minor changes. LC-MS/MS analysis was carried out on an Ultimate3000 nanoLC coupled to an Orbitrap Fusion Lumos instrument (ThermoFisher Scientific) acquiring full MS scans in the m/z range 400–1400 in the orbitrap at resolution 120′000 with AGC set to 4e5 and maximal ion injection time of 50 ms. Peptide precursors with charge 2–8 were fragmented once in the iontrap then excluded for 30s. The iontrap setting were data-dependent MS2 cycle time of 3 s, isolation width of 1.6 m/z, fragmentation HCD mode with 30% normalized collision energy, AGC of 1e4 with maximal ion injection time of 35 ms. The LC-MS/MS data was processed with MaxQuant (version 1.5.4.1) using default settings for peak detection, strict trypsin cleavage rule allowing for up to three missed cleavages, variable oxidation on methionine and acetylation of protein N-termini with strict carbamidomethylation of cysteines. Match between runs was activated with a retention time window of 0.7 min. The fragment spectra were interpreted with the *Streptococcus pneumoniae* ensemble database (version gca_000817005_ASM81700v1). For data analysis, peptide feature intensities reported in the evidence file were median normalized and missing values were imputed from the low end of the LOG2 transformed intensity distribution of each LC-MS/MS run using Perseus (version 1.5.5.3) as suggested by Lazar et al. [19]. The three most intense peptide feature intensities were summed for the individual protein group intensity.

### Detroit cell culture and CXCL8 (IL-8) cytokine assay

CXCL8 levels were measured as described previously [20]. The human pharyngeal epithelial cell line Detroit 562 (ATCC CCL-138) was cultured in complete medium at 37 °C at 5% CO_2._ This was composed of Minimum Essential Media (MEM) with 10% heat-inactivated fetal calf serum (FCS), 2 mM of L-glutamine, 1% sodium bicarbonate, 1× MEM non-essential amino acid solution, 1 mM sodium pyruvate (all from Gibco, Life Technologies, Switzerland), 100 μg/ml streptomycin and 100 U/ml penicillin. 0.05% Trypsin-EDTA (Gibco, Switzerland) was used to harvest the cells when they reached 70–90% confluence. The production of CXCL8 in response to exposure to *S. pneumoniae* was determined as described previously [20] as follows.

3 × 10^5^ Detroit cells in 1 ml MEM without antibiotics, was added per well of a 24-well plate (TPP tissue culture plates, Sigma-Aldrich,) which was then incubated overnight at 37 °C, 5% CO_2._ Integrity of the monolayer on the following day was checked by microscopy. The medium was replaced in each well by 0.5 ml MEM without FCS or antibiotics. A suspension of bacteria (strain 110.58 or one of its mutants ΔORF 1 + 2 or ΔORF 2) of approximately 6 × 10^6^ CFU/ml was made to give an estimated MOI of 10. Serial dilutions of the suspension were made and plated out to enable accurate quantification of CFU/ml, and therefore the MOI. 0.5 ml MEM containing the bacteria (3 × 10^6^ CFU) was added per well. For those wells which received ORF2 ligand peptide FPPQSV, it was added to give a concentration of 0.07 mg/ml. The plate was centrifuged at 120 g for 3 min at 25 °C and then incubated at 37 °C, 5% CO_2_. After an incubation of a total of 24 h at 37 °C, 5% CO_2,_ the supernatant was collected in 1.5 ml tubes, spun at 20000 g for 3 min at room temperature and the supernatant collected and stored at − 80 °C for later analysis. CXCL8 concentrations were measured by ELISA (R&D systems ELISA kits, Abingdon, United Kingdom). Experiments were performed in triplicate on three different days.

### Infant rat model of pneumococcal meningitis

A well-established infant rat model of bacterial meningitis [21] adapted to strains not previously passaged in animals [22] was used as follows. As in our previous publication [22] a litter of 12 nursing Wistar rats with their dam was obtained from Charles River (Sulzfeld, Germany) and given 5 days to acclimatize. At 11 days of age rats weighing 24.1 ± 2 g were infected intracisternally with 10 μl 0.85% NaCl containing either 1.25 × 10^7^ colony forming units (CFU)/ml live *S. pneumoniae* strain 110.58 or 5 × 10^6^ colony forming units (CFU)/ml live *S. pneumoniae* mutant ΔORF 1 + 2. Samples of cerebrospinal fluid (CSF) were taken at 21 h post infection (hpi) for quantitative analysis of bacterial cultures to determine whether there was any bacterial meningitis. At 21 and 29 hpi the antibiotic ceftriaxone (Rocephine®, Roche Pharma, Basel, Switzerland; 2 × 100 mg/kg/d i.p.) was administered. Disease severity was assessed and CSF sampled as described previously for determination of cytokine concentrations at 21 and 45 hpi [22]. Animals were sacrificed with an overdose of pentobarbital (150 mg/kg) at 45 hpi and perfused with 4% paraformaldehyde (PFA) in PBS and then the brains were removed and fixed in PFA for histological analysis. The concentration of the cytokines IL-6, IL-1β, TNFα, IL-10, IFN-γ in the CSF samples was determined using the microsphere-based multiplex assays (MILLIPLEX® MAP Kit, Rat Cytokine/Chemokine Magnetic Bead Panel, Millipore Corporation, Billerica, MA, USA) as described previously [23]. Cortical damage and apoptosis in the hippocampus was assessed as previously described in animals sacrificed at 45 hpi [21, 23].

### Statistical analysis

Statistical analysis of RNA-Seq data was done as previously described [16]. FDR was taken into account with Benjamini-Hochberg correction for multiple testing. Differentially expressed genes were considered significant when the *p*-value of three independent biological experiments was below 0.5.

For proteomics data analysis *t-*tests were performed within Perseus, including a permutation-based false discovery rate estimation (cutoff at 1% and using SO function set at 0.5) to correct for multiple testing.

For other experiments student *t-*tests were performed to obtain *p*-values using the software GraphPad Prism (Version 7, GraphPad Software, Inc.).

## Results

### AliB-like ORF 2 peptide ligand FPPQSV affects gene expression

Transcriptome analysis by RNA-Seq was performed for wild type nonencapsulated pneumococcal strain 110.58, which possesses *aliB-like* ORF 1 and ORF 2 in place of capsule genes, and its mutant lacking *aliB-like* ORF 2 expression (ΔORF 2) in the presence and absence of AliB-like ORF 2 peptide ligand FPPQSV. There was a modest difference in gene expression between the wild type strain 110.58 and its mutant ΔORF 2 without treatment but exposure to the ORF 2 ligand FPPQSV caused significant changes in gene expression in strain 110.58 and in mutant ΔORF 2 (Additional file 1: Figure S1 and Additional files 2: Table S1, Additional file 3: Table S2, Additional file 4: Table S3, Additional file 5: Table S4). The top twenty most upregulated genes in the wild type following exposure to the ORF 2 ligand peptide are shown in Table 1. This table was produced following exclusion of those genes for which the wild type treated with the peptide gave expression values of less than 20 as even such small values could give statistically significant results if the control group had an even smaller value. Genes for which the change in expression was not significant were also excluded. Of these 20 most upregulated genes, at least 12 are associated with carbohydrate metabolism or uptake (indicated in Table 1 in bold).

**Table 1:** Summary of top 20 genes with expression upregulated in wild type strain 110.58 by exposure to ORF 2 ligand peptide FPPQSV

Gene number	Gene name	Function	Ratio^a^	q
SpnNT_02141		tRNA-Phe	infinite^b^	0.03
SpnNT_02309		tRNA-Asn	infinite^b^	0.03
SpnNT_01742	***afr***	**1,5-anhydro-D-fructose reductase**	20.2	0.001
SpnNT_01743	***nanB***	**Sialidase**	11.9	0.001
SpnNT_01746	***yesO_2***	**Putative ABC transporter for carbohydrate**	10.1	0.001
SpnNT_01745	***ycjO***	**Putative ABC transporter for carbohydrate**	7.5	0.001
SpnNT_01744	***ycjP***	**Putative ABC transporter for carbohydrate**	7.5	0.001
SpnNT_00088	***sorB_1***	**Sorbose-specific phosphotransferase enzyme IIB component**	6.6	0.04
SpnNT_01738	***yesO_1***	**Putative ABC transporter for carbohydrate**	6.3	0.001
SpnNT_01747	*tabA*	Toxin-antitoxin biofilm protein TabA	6.2	0.001
SpnNT_01751	***nanA_3***	**Sialidase**	5.3	0.001
SpnNT_01537		CsbD-like protein, general stress response protein	4.6	0.001
SpnNT_01944		tRNA-Leu	4.5	0.005
SpnNT_00467	*grpE*	HSP-70 cofactor, heat shock protein	4.4	0.001
SpnNT_01021	*pyrP*	Uracil transporter	4.2	0.001
SpnNT_00091	***manX_1***	**EIIAB-Man, carbohydrate transport**	4.1	0.001
SpnNT_00092	***agaS***	**Putative tagatose-6-phosphate ketose/aldose isomerase**	4.1	0.001
SpnNT_00089	***agaC_1***	**PTS system N-acetylgalactosamine-specific EIIC component 1**	4.1	0.001
SpnNT_02080	***adhE***	**Aldehyde-alcohol dehydrogenase**	4.1	0.001
SpnNT_01032	*pyrR*	Pyrimidine operon attenuation protein/uracil phosphoribosyltransferase	3.8	0.001

Genes names in bold indicate an association with carbohydrate metabolism or uptake. For the complete dataset for the wild type and ΔORF 2 mutant see Additional files 2: Table S1, Additional file 3: Table S2, Additional file 4: Table S3, Additional file 5: Table S4

^a^Ratio represents the fold increase in the expression of genes in the presence of peptide FPPQSV compared to in its absence

^b^Expression in the absence of peptide was 0 so fold increase in the presence of peptide could not be calculated

Kegg pathways with genes that were significantly overrepresented were “ribosomal proteins”, “alanine, aspartate and glutamate” and “purine metabolism”. Branched chain amino acid transporters (*liv* operon) were also differentially regulated (Additional file 1: Figure S2).

**Fig. 1:**
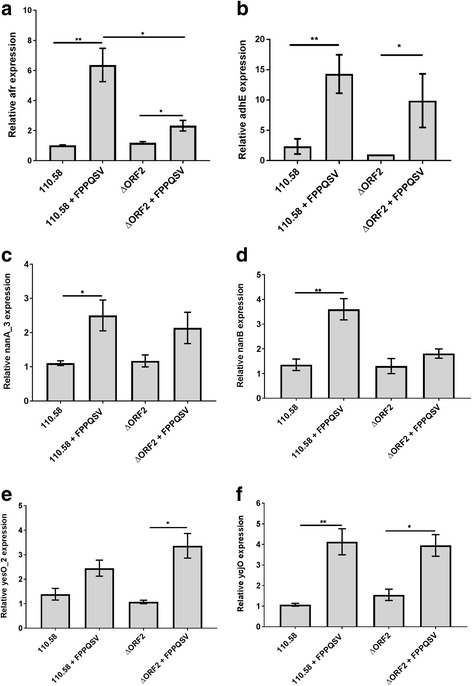
Relative gene expression. **a** *afr,* **b** *adhE,* **c** *nan_A3,* **d** *nanB,* **e** *yesO_2* and **f** *ycjO* expression was determined by real-time RT-PCR and is displayed relative to the value of the lowest expression, after normalization using 16S RNA gene expression. The values are the means of four (*afr*) or three (all other genes) separate experiments. Error bars indicate standard error., *p ≤ 0.05, **p ≤ 0.01

To verify the RNA-Seq results, we performed real-time RT-PCR on several genes as follows. The first was *afr* which encodes 1,5-anhydro-D-fructose reductase. We also determined expression of *adhE* which encodes a bifunctional acetaldehyde-alcohol dehydrogenase, primarily a fermentative enzyme. AdhE has been proposed to act as a pneumococcal virulence factor by increasing expression of pneumolysin and therefore haemolytic activity [24]. We also tested by real-time RT-PCR the expression of genes *nanA_3* and *nanB* which encode sialidases as well as *yesO_2,* encoding a substrate binding protein, and *ycjO,* encoding a permease, of an ABC transporter for carbohydrate (Fig. 1). *nanA_3, nanB, yesO_2* and *ycjO* are located in close proximity to each other in the genome. According to RNA-Seq results in Table 1 *afr* had an increase in expression of 20.2-fold and *adhE* of 4.1-fold in the wild type strain when it was exposed to the peptide. Real-time RT-PCR indicated a significant 6-fold increase in the expression of *afr* and 14-fold expression in *adhE* when exposed to the peptide (Fig. 1a and b), in line with the RNA-Seq result. Expression of both genes was also increased in the ΔORF 2 mutant by exposure to the peptide but to a lesser extent (Fig. 1a and b). The two sialidases (*nanA_3* and *nanB*) also showed upregulation in response to the ORF 2 peptide in the RT-PCR experiments (Fig. 1c and d) which was significant in the wild type strain but not the ΔORF 2 mutant. For the carbohydrate ABC transporter genes, both the substrate binding protein (*yesO_2*) and permease (*ycjO*) were upregulated in both the wild type and the mutant in response to the peptide (Fig. 1e and f). This upregulation was statistically significant for the mutant only for *yesO_2* and for both the wild type and mutant for *ycjO* in contrast to the RNA-Seq results where upregulation was significant for both *yesO_2* and *ycjO* for the wild type but not the mutant (Additional file 2: Table S1).

### Proteomic data indicate peptide activates metabolism

To determine whether changes seen in gene transcription are translated into changes at the protein level, proteomic analysis was performed by LC-MS/MS on wild type strain 110.58 and mutant ΔORF 2 in the presence and absence of AliB-like ORF 2 peptide ligand FPPQSV. Table 2 shows all proteins significantly upregulated in 110.58 following exposure to peptide FPPQSV (see Additional file 6: Table S5, Additional file 7: Table S6, Additional file 8: Table S7, Additional file 9: Table S8 for all data). The proteomic and RNA-Seq datasets were comparable (Additional file 1: Figure S3) with the top of the list of proteins being Afr (1,5- anhydro-D-fructose reductase), supporting the RNA expression data that the peptide may boost carbohydrate metabolism. In addition, the uracil transporter PyrP was also found to be upregulated by 3.8 fold in both the RNA-Seq data (Table 1) and the proteomics data (Table 2). NanA_3 and NanB, which were upregulated by ORF 2 peptide in the RNA-Seq and real-time RT-PCR data were not found in the proteomics data.

**Table 2:** Proteins upregulated in wildtype strain 110.58 by exposure to ORF 2 ligand peptide FPPQSV

Protein number	Protein name	Function	Ratio^a^	q
AJD72675	Afr	1,5-anhydro-D-fructose reductase	infinite^b^	0
AJD71621	DivlB	Cell division protein DivIB	7.1	0.004
AJD71961	PyrP	Uracil transporter	3.8	0.01
AJD71716		ASCH domain protein, Predicted RNA-binding protein YhfF, could regulate translation	3.8	0.01
AJD73085		Hypothetical protein, N-ethylammeline chlorohydrolase, hydrolase activity, acting on carbon-nitrogen (but not peptide) bonds	3.8	0.01
AJD71419	CitS	Sensor protein	2.6	0.01
AJD71122	RibE	Riboflavin synthase	2.3	0.01
AJD71434	trmB	tRNA (guanine-N(7)-)-methyltransferase	2.3	0.01
AJD71098		Hypothetical protein, transcriptional activator, Rgg/GadR/MutR family	1.7	0.02
AJD71572		Hypothetical protein	1.4	0.05
AJD71120	RibH	6,7-dimethyl-8-ribityllumazine synthase	1.4	0.04

For the complete dataset for the wild type and ΔORF 2 mutant see Additional file 6: Table S5, Additional file 7: Table S6, Additional file 8: Table S7, Additional file 9: Table S8

^a^Ratio represents the fold increase in the protein in the presence of peptide FPPQSV compared to in its absence

^b^The value in the absence of peptide was 0 so fold increase in the presence of peptide could not be calculated

Since RNA-Seq and proteomic data indicated a boost in metabolism, we next looked at the effect of ORF 2 peptide on pneumococcal growth.

### ORF 2 peptide boosts growth

**Fig. 2:**
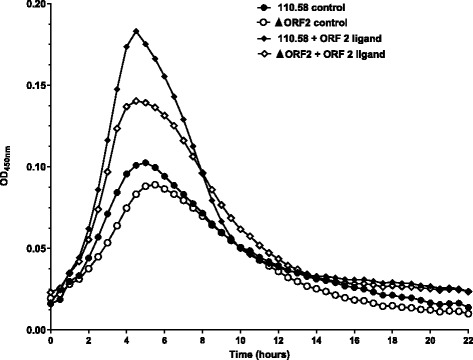
Growth of wild type strain 110.58 and mutant ΔORF 2 in CDM with and without 0.062 mg/ml ORF2 ligand peptide FPPQSV. Curves show the mean values for three independent experiments

In CDM, wild type strain 110.58 and mutant ΔORF 2 had a similar pattern of growth. Addition of ORF 2 peptide ligand FPPQSV reduced the lag phase of growth and increased the maximum OD for both, but to a greater degree for 110.58 (Fig. 2). For clarity, no error bars are shown in Fig. 2. Growth curves were performed with 4 different doses of ORF 2 peptide, plus no peptide controls, in three independent experiments on three different days. The mean values for all doses, with error bars showing standard error, are shown in Additional file 1: Figure S4A. To confirm that the effect is specific to ORF 2 peptide, growth curves with and without a control peptide of sequence LRRASLG are shown in Additional file 1: Figure S4B and indicate no difference in growth between wildtype and mutant ΔORF 2.

**Fig. 3:**
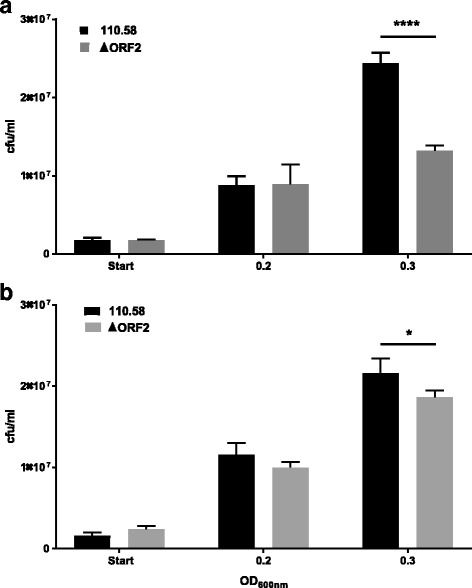
Competition assay in the presence or absence of ORF 2 peptide. A mixture of wild type strain 110.58 and its mutant ΔORF2 was incubated in (**a**) the presence of 0.07 mg/ml of ORF2 ligand peptide FPPQSV or (**b**) the absence of peptide. At the start and at OD_600nm_ = 0.2 and 0.3 CFU/ml were determined to show that the wild type has a competitive advantage and that this is greater in the presence of the peptide. ****p < 0.0001, *p = 0.015. Results are the mean of three independent experiments, error bars show the standard error

To determine whether AliB-like ORF 2 gives a competitive advantage to bacteria which possess it when ORF 2 peptide is present, we next cultured the wildtype strain 110.58 and its ΔORF 2 mutant together. Figure 3 shows that when equal numbers of the two strains were mixed together the wild type 110.58, which has the ORF 2 binding protein, has a competitive advantage and that this advantage is significantly greater in the presence of the peptide.

### No difference in inflammation or meningitis in response to 110.58 or mutant ΔORF 2

**Fig. 4:**
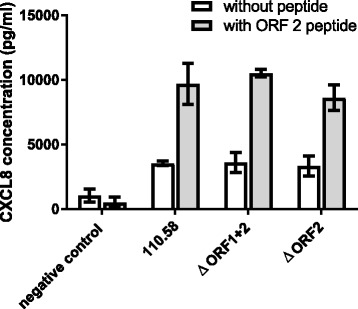
Effect of wild type pneumococcal strain 110.58 and its mutants ΔORF 1 + 2 and ΔORF 2 on CXCL8 induction in human nasopharyngeal Detroit 562 epithelial cells. ORF2 ligand peptide FPPQSV was either not added (open bars) or added to give a final a concentration of 0.07 mg/ml (shaded bars). No significant differences were observed between the strains under the same conditions. The bars indicate the mean of three independent experiments and error bars indicate standard error. Negative control refers to Detroit cells in the absence of bacteria

Because ORF 2 peptide enhanced growth and AdhE has been linked to inflammation, we tested whether the wild type strain and mutants ΔORF 1 + 2 and ΔORF 2 differed in the inflammatory responses they induced in vitro and in vivo. Figure 4 shows that Detroit epithelial cells did not differ in their CXCL8 response to the strains in the presence of 0.07 mg/ml ORF 2 ligand peptide FPPQSV. For all strains the peptide increased CXCL8 response to the bacteria but we interpret this as being due to increased bacterial growth due to the presence of the peptide. Peptide in the absence of bacteria had no effect on the release of CXCL8 from the Detroit cells as shown in the negative control in Fig. 4.

We also tested whether 110.58 and ΔORF 1 + 2 differed in their ability to cause disease and inflammation in an infant rat model of meningitis. 110.58 was not able to establish a detectable infection in the CSF at 21 h post infection (hpi) in any rat. For the rats which received the mutant ΔORF 1 + 2, four had no detectable infection in the CSF at 21 hpi, while two had very low levels: 9.5 and 6 × 10^4^ CFU/ml. No disease, cortical damage, apoptosis in the hippocampus or detection of any of the cytokines IL-6, IL-1β, TNFα, IL-10, IFN-γ in the CSF at 21 or 45 hpi was detected in any rat (data not shown).

## Discussion

Nonencapsulated pneumococci of ST344 which have the genes *aliB-like* ORF 1 and 2 in place of capsule genes, such as Swiss strain 110.58, are highly successful colonizers of the human nasopharynx, an environment where the concentration of free sugar is considered to be limited [3]. AliB-like ORF 2 appears to aid the early stages of nasopharyngeal colonization, at least in a mouse model [1]. AliB-like ORF 2 can bind peptide FPPQSV, a sequence found in *Prevotella* species, likely neighbours in the nasopharyngeal niche, indicating a potential route of interspecies communication [1]. For *E. coli,* secreted proteins include ribosomal associated proteins [25] and we speculate that *Prevotella* too can release proteins usually considered to be intracellular. Here, we aimed to answer the question of how binding of AliB-like ORF 2 to its ligand may lead to improved colonization. We propose that peptide FPPQSV acts as a specific signal and that following the specific binding, pneumococci respond by upregulating sialidase to produce more free carbohydrate, increasing carbohydrate uptake and increasing carbohydrate metabolism. A permease for the update of AliB-like ORF 2 peptide has not yet been identified but due to the sequence homology of AliB-like ORF 2 to substrate binding protein AliB, after which it is named, we speculate that the Ami-AliA/AliB transporter may be responsible for uptake. This oligopeptide permease has also been proposed to be the route of entry into pneumococcus for Phr peptide of the TprA/PhrA quorum sensing system [26]. TprA/PhrA in strain D39 has also been shown to be activated by PhrA2 which is found in the pneumococcal lineage PMEN1 but not in D39 indicating interstrain gene regulation [27]. Other peptides involved in quorum sensing in pneumococcus include virulence peptide 1 (VP1) which promotes biofilm development on chinchilla middle ear epithelial cells [28] and a small hydrophobic peptide (SHP) which induces capsule expression [29]. However, the crucial difference between these quorum-sensing studies and the work we present here is that we describe the effect of non-streptococcal peptides on pneumococcus i.e. interspecies communication.

In both the transcriptome and proteome data, multiple genes related to carbohydrate metabolism were upregulated by the presence of the ORF 2 peptide ligand. There was a good correlation between these two datasets with *afr*, encoding 1,5-anhydro-D-fructose reductase, increasing in expression significantly in both when ORF 2 peptide was present. 1,5-anhydro-D-fructose reductase is a metabolic enzyme which acts on 1,5-anhydro-D-fructose, the central intermediate of the anhydrofructose pathway, an alternative starch- and glycogen degrading pathway in bacteria, fungi, plants and mammals [30]. In *E. coli* the anhydrofructose pathway is proposed to be involved in the stress response related to carbon starvation or reactive oxygen species [31]. Trappetti et al. have also described a link between an interspecies signaling molecule and carbohydrate metabolism in pneumococcus: They found that autoinducer 2 (AI-2), is recognized by FruA (a fructose-specific phosphoenolpyruvate-phosphotransferase system) to enable pneumococcus to use galactose, the predominant sugar source in the nasopharynx, as a carbon source [32].

Neuraminidase-encoding genes *nanA* and *nanB* both had increased expression in response to ORF 2 peptide. Neuraminidases are thought to cleave sialic acid from cell surface glycans and mucin and both NanA and NanB have been shown to be essential for successful colonization in a mouse model [33]. Exposure of galactose on epithelial cells by NanA promotes biofilm formation during colonization [34]. In addition, deglycosylation of host glycoconjugates by neuraminidases releasing monosaccharides provides *S. pneumoniae* with a source of carbohydrate which can be used to sustain growth [35].

Upregulation of *yesO* and *ycj* genes was also noted in the presence of ORF 2 peptide. These are thought to encode components of an ABC transporter for carbohydrate uptake and are found immediately downstream of *nanB*. Other genes upregulated by ORF 2 peptide believed to be involved in carbohydrate transport and metabolism were *sorB_1, manX_1, agaS, agaC_1* and *adhE*. *tabA*, proposed to encode a component of a toxin-antitoxin involved in biofilm formation, was also upregulated which is compatible with stepping up colonization. The uracil transporter encoded by *pyrP* was also of note as this was upregulated in both the RNA and protein data. Cell division protein DivIB was upregulated at the protein level as were RibE and RibH, involved in riboflavin synthesis. Other genes were differentially regulated as well and the strength of gene expression is not necessary related to functional importance. Therefore, it is possible that other pathways that are not discussed here are also involved. In particular, genes belonging to amino acid metabolism and transport were upregulated.

These findings prompted us to look at the effect of ORF 2 peptide on growth. Previously, we had failed to detect a direct effect of the peptide on growth [1] but here we analyzed growth over time in a defined medium devoid of peptides until we added our ORF 2 peptide. This confirmed that the ORF 2 peptide promotes growth. This effect was also seen in the mutant lacking the ORF 2 peptide receptor, although to a lesser extent. This suggests that AliB-like ORF 2 is not the only receptor for the peptide. However, in a competition assay in the presence of the peptide, the wild type strain significantly outcompeted the mutant lacking *aliB-like* ORF 2 indicating that in nature with multiple strains present, having AliB-like ORF 2 would give an advantage in growth and therefore colonization.

Nonencapsulated strains with *aliB-like* ORF 1 and 2 are associated with nasopharyngeal colonization [3] and conjunctivitis [36] but not virulent disease. Our results using an in vivo model of meningitis supported this as neither the wild type nor mutant strain caused disease when administered intracisternally. This may be due to these nonencapsulated pneumococci being removed by the immune system before any growth advantage of the wild type strain becomes apparent. We also looked at the effect of 110.58 and its mutants on human respiratory cells in vitro and found no difference in their ability to stimulate the epithelial cells to release CXCL8, a marker of an inflammatory response.

## Conclusion

A peptide which matches proteins found in *Prevotella* species acts on nonencapsulated pneumococci, which have a specific receptor for it, to trigger a boost in carbohydrate metabolism and thus growth. This would seem to be the mechanism by which possession of AliB-like ORF 2 aids colonization. Pneumococci, including virulent encapsulated strains, possess other ABC transporters of oligopeptides which warrant investigation as potential sensors of signals from members of the microbiota which could trigger responses in pneumococci during colonization and disease.

## Additional files

Additional file 1: **Figure S1.** Venn diagram of RNA-Seq analysis results showing number of genes with different levels of gene expression in untreated wildtype strain 110.58 vs mutant ΔORF 2 (purple), wild type with and without ORF 2 ligand peptide FPPQSV (blue) and mutant ΔORF 2 with and without peptide FPPQSV (green). The corresponding gene lists, including their expression values, can be found in Additional files 2: **Table S1.** Additional file 3: **Table S2.** Additional file 4: **Table S3.** Additional file 5: **Table S4. Figure S2.** Network of differentially regulated gene products. Nodes represent genes with significantly different expression in wild type and wild type treated with the peptide ligand (inferred from TIGR4 from STRING database). Edges represent evidence for protein-protein interactions. Proteins belonging to the KEGG pathway “purine metabolism” (FDR 3 × 10^− 5^) are coloured in blue, KEGG pathway “ribosomal proteins” (FDR 4 × 10^− 34^) in green, KEGG pathway “alanine, aspartate and glutamate” related genes in lilac, branched-chain amino acid transporter proteins [37] (FDR 4 × 10^− 34^) in yellow and pathogenesis related genes [38] in red. Other genes are shown in white. **Figure S3.** Correlation between RNA-Seq and proteome expression data. The Pearson correlation between RNA-Seq and proteomics data across all samples was 0.68. **Figure S4.** Growth of wildtype strain 110.58 and mutant ΔORF 2 in CDM with and without ORF2 ligand peptide FPPQSV at the concentrations indicated. Curves show the mean values for three independent experiments, error bars indicated SEM. (PDF 658 kb)

Additional file 2: **Table S1.** Complete RNA-Seq data. (PDF 7151 kb)

Additional file 3: **Table S2.** RNA-Seq data for wild type with and without ORF 2 peptide. Table shows only significant changes in expression. A significant change in expression was observed for 210 genes of which 159 were upregulated by the ORF 2 peptide and 51 were downregulated by the peptide. (PDF 209 kb)

Additional file 4: **Table S3.** RNA-Seq data for ΔORF 2 mutant with and without ORF 2 peptide. Table shows only significant changes in expression. A significant change in expression was observed for 249 genes of which 177 were upregulated by the ORF 2 peptide and 72 were downregulated by the peptide. (PDF 231 kb)

Additional file 5: **Table S4.** RNA-Seq data for wild type and ΔORF 2 mutant without ORF 2 peptide. Table shows only significant differences in expression. A significant difference in expression was observed for 20 genes of which 6 were more highly expressed in the wildtype and 14 were more highly expressed in the mutant. (PDF 108 kb)

Additional file 6: **Table S5.** Complete proteomic data. (PDF 3560 kb)

Additional file 7: **Table S6.** Proteomic data for wild type with and without ORF 2 peptide. Table shows only significant changes in expression. A significant change in expression was observed for 12 proteins of which 11 were upregulated by the ORF 2 peptide (also shown in Table 2 in the main text) and 1 was downregulated by the peptide. (PDF 34 kb)

Additional file 8: **Table S7.** Proteomic data for ΔORF 2 with and without ORF 2 peptide. Table shows only significant changes in expression. A significant change in expression was observed for 22 proteins of which 20 were upregulated by the ORF 2 peptide and 2 were downregulated by the peptide. (PDF 52 kb)

Additional file 9: **Table S8.** Proteomic data for wild type and ΔORF 2 mutant without ORF 2 peptide. Table shows only significant changes in expression. A significant difference in expression was observed for 19 proteins of which 17 were more highly expressed in the wildtype and 2 were more highly expressed in the mutant. (PDF 46 kb)

## Abbreviations

*adhE*: aldehyde-alcohol dehydrogenase
*afr*: 1,5-anhydro-D-fructose reductase
BHI: Brain heart infusion broth
CFU: Colony forming units
CSBA: Columbia sheep blood agar
CSF: Cerebrospinal fluid
FCS: Fetal calf serum
FDR: False discovery rate
hpi: hours post infection
LC-MS/MS: Liquid chromatography-tandem mass spectrometry
MEM: Minimum Essential Medium
PFA: Paraformaldehyde
