# Supplementary material for: Foreign peptide triggers boost in pneumococcal metabolism and growth
Source: BMC Microbiol. 2018 Mar 27;18:23. doi: 10.1186/s12866-018-1167-y (PMC5870813; doi:10.1186/s12866-018-1167-y)
Supplement: Supplementary file 1 — Figure S1. Venn diagram of RNA-Seq analysis results showing number of genes with different levels of gene expression in untreated wildtype strain 110.58 vs mutant ΔORF 2 (purple), wild type with and without ORF 2 ligand peptide FPPQSV (blue) and mutant ΔORF 2 with and without peptide FPPQSV (green). The corresponding gene lists, including their expression values, can be found in Additional files 2: Table S1. Additional file 3: Table S2. Additional file 4: Table S3. Additional file 5: Table S4. Figure S2. Network of differentially regulated gene products. Nodes represent genes with significantly different expression in wild type and wild type treated with the peptide ligand (inferred from TIGR4 from STRING database). Edges represent evidence for protein-protein interactions. Proteins belonging to the KEGG pathway “purine metabolism” (FDR 3 × 10− 5) are coloured in blue, KEGG pathway “ribosomal proteins” (FDR 4 × 10− 34) in green, KEGG pathway “alanine, aspartate and glutamate” related genes in lilac, branched-chain amino acid transporter proteins [37] (FDR 4 × 10− 34) in yellow and pathogenesis related genes [38] in red. Other genes are shown in white. Figure S3. Correlation between RNA-Seq and proteome expression data. The Pearson correlation between RNA-Seq and proteomics data across all samples was 0.68. Figure S4. Growth of wildtype strain 110.58 and mutant ΔORF 2 in CDM with and without ORF2 ligand peptide FPPQSV at the concentrations indicated. Curves show the mean values for three independent experiments, error bars indicated SEM. (PDF 658 kb) [file 12866_2018_1167_MOESM1_ESM.pdf]

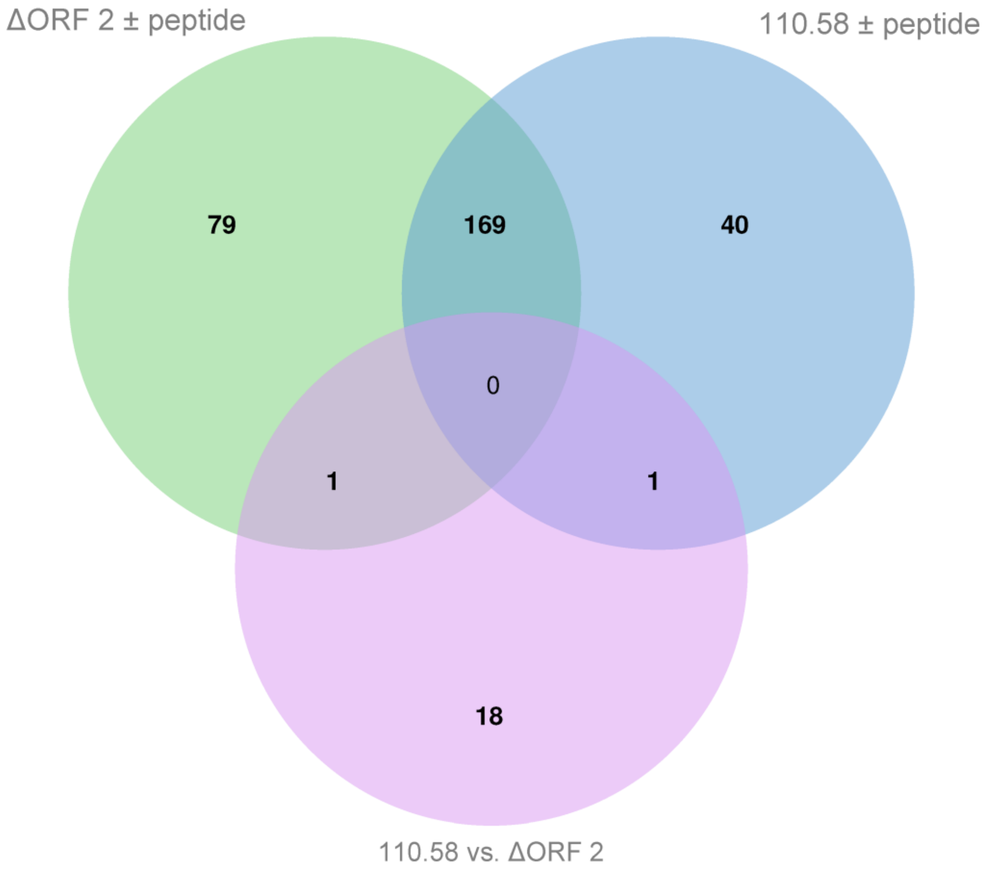

Supplementary Figure S1 – **Venn diagram of RNA-Seq analysis results** showing number of genes with different levels of gene expression in untreated wildtype strain 110.58 vs mutant ΔORF 2 (purple), wildtype with and without ORF 2 ligand peptide FPPQSV (blue) and mutant ΔORF 2 with and without peptide FPPQSV (green). The corresponding gene lists, including their expression values, can be found in Supplementary Table S1.

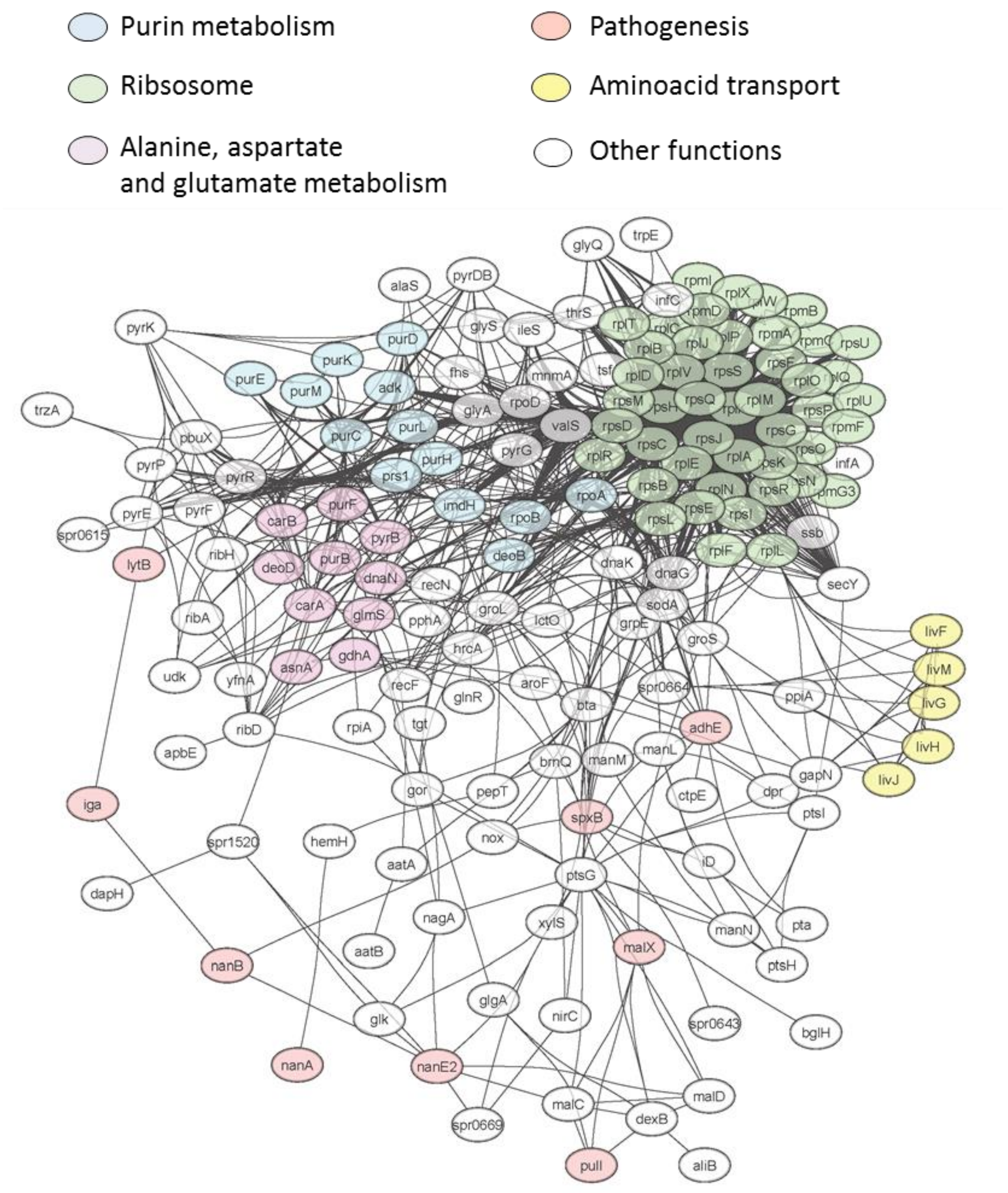

Supplementary Figure S2 – Network of differentially regulated gene products. Nodes represent genes with significantly different expression in wild type and wild type treated with the peptide ligand (inferred from TIGR4 from STRING database). Edges represent evidence for protein-protein interactions. Proteins belonging to the KEGG pathway "purine metabolism" (FDR 3x10^{-5}) are coloured in blue, KEGG pathway "ribosomal proteins" (FDR 4x10^{-34}) in green, KEGG pathway "alanine, aspartate and glutamate" related genes in lilac, branched-chain amino acid transporter proteins [1] (FDR 4x10^{-34}) in yellow and pathogenesis related genes [2] in red. Other genes are shown in white.

1. Basavanna S, Khandavilli S, Yuste J, Cohen J, Hosie A, Webb A, Thomas G, Brown J. 2009 Screening of *Streptococcus pneumoniae* ABC transporter mutants demonstrates that LivJHMGF, a branched-chain amino acid ABC transporter, is necessary for disease pathogenesis. *Infection and Immunity* **77**, 3412-3423.
2. Kadioglu A, Weiser J, Paton J, Andrew P. 2008 The role of *Streptococcus pneumoniae* virulence factors in host respiratory colonization and disease. *Nature Reviews Microbiology* **6**, 288-301.

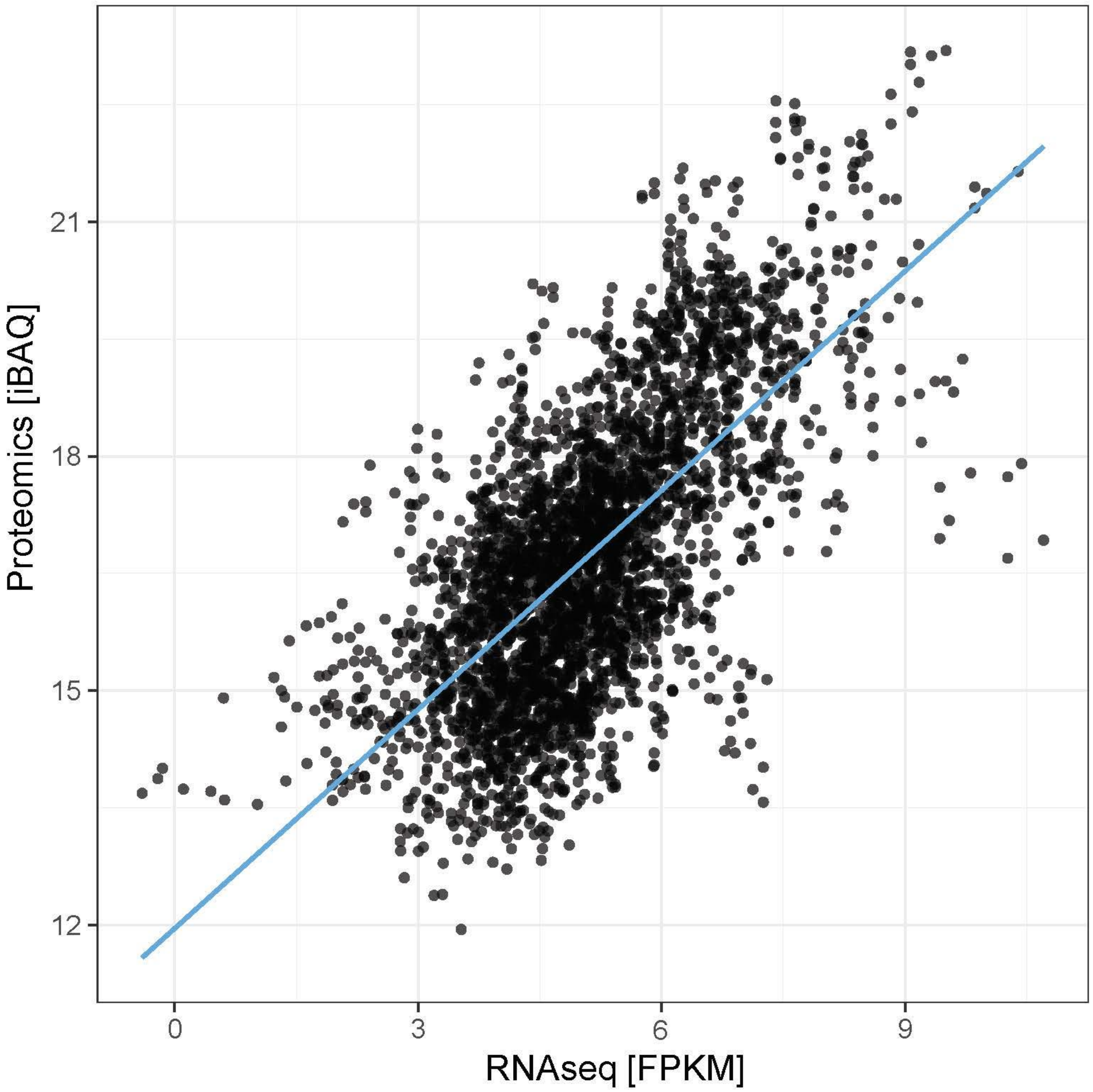

Supplementary Figure S3 – Correlation between RNA-Seq and proteome expression data. The Pearson correlation between RNA-Seq and proteomics data across all samples was 0.68.

A)

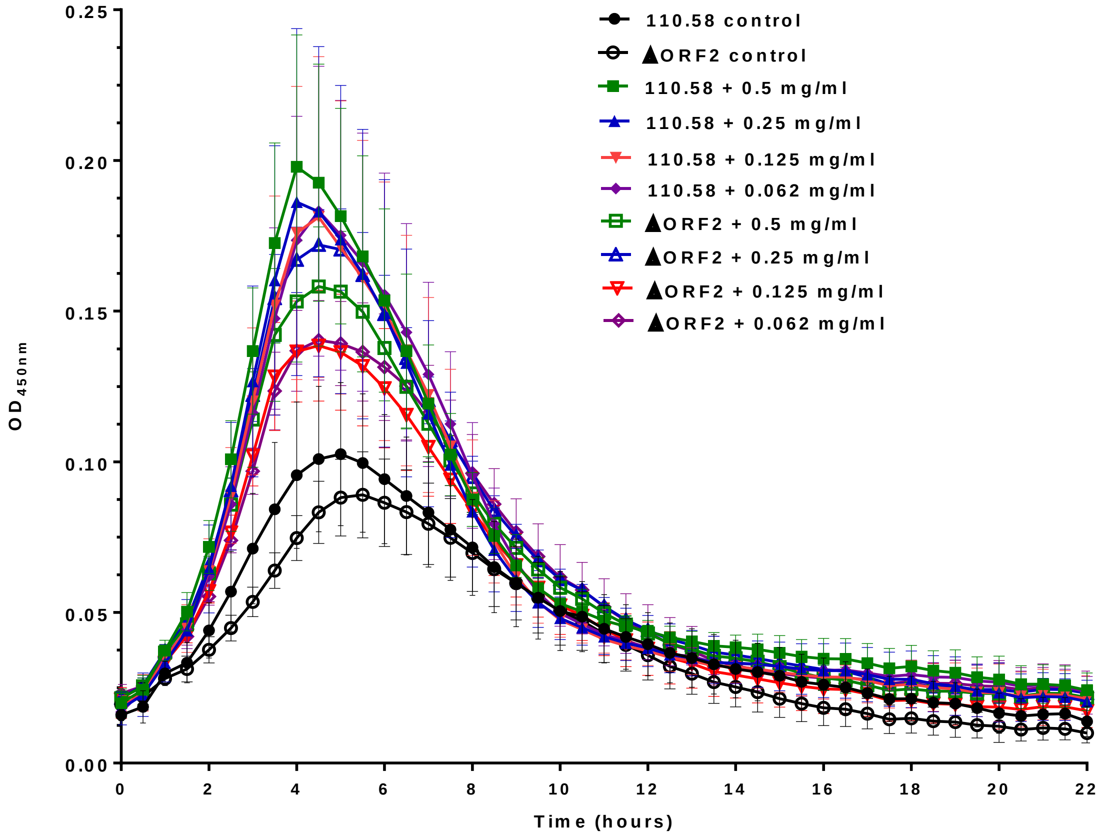

B)

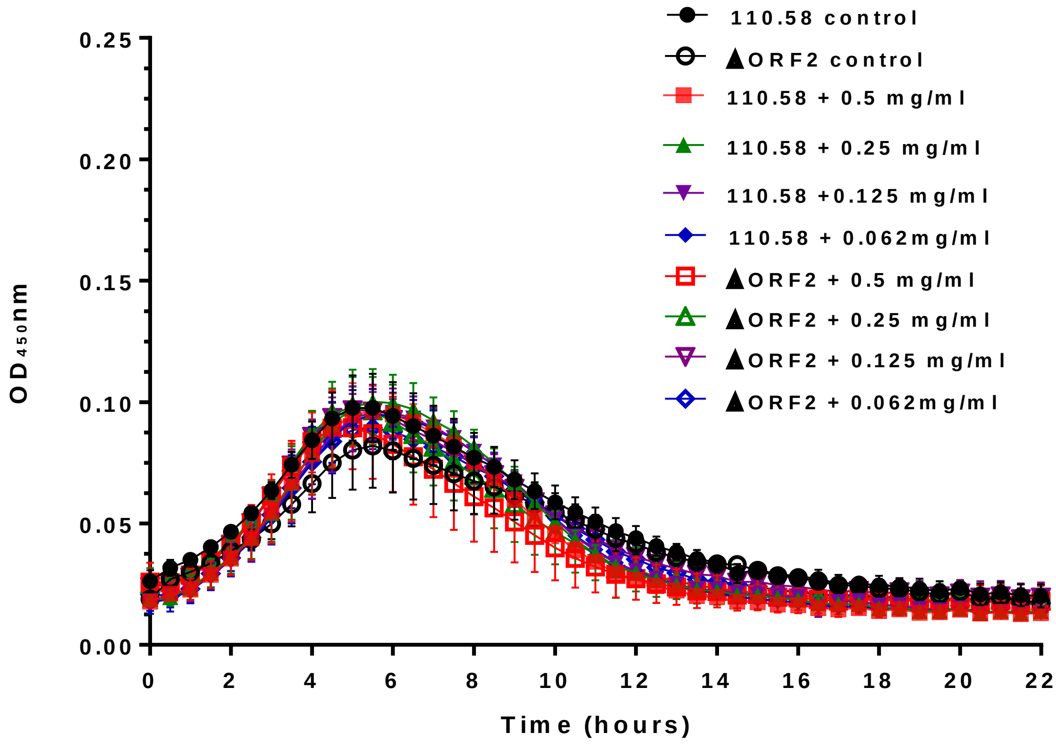

Supplementary Figure S4 – Growth of wildtype strain 110.58 and mutant ΔORF 2 in CDM with and without (A) ORF2 ligand peptide FPPQSV and (B) control peptide LRRASLG at the concentrations indicated. Curves show the mean values for three independent experiments, error bars indicated SEM.
