# Supplementary material for: Foreign peptide triggers boost in pneumococcal metabolism and growth
Source: BMC Microbiol. 2018 Mar 27;18:23. doi: 10.1186/s12866-018-1167-y (PMC5870813; doi:10.1186/s12866-018-1167-y)
Supplement: Supplementary file 2 — Table S1. Complete RNA-Seq data. (PDF 7151 kb) [file 12866_2018_1167_MOESM2_ESM.pdf]

gene_id	gene	locus	sample_1	sample_2	status	value_1	value_2	log2_fold_chan	test_stat	p_value	q_value	signif
gene:SpnNT_00001	dnaA	Chromosome:185-1547	110.58	ΔORF2	OK	35.2254	44.6381	0.341657	0.725154	0.20755	0.693134	no
gene:SpnNT_00001	dnaA	Chromosome:185-1547	110.58	110.58+peptide	OK	35.2254	24.5357	-0.521734	-1.05956	0.06455	0.368753	no
gene:SpnNT_00001	dnaA	Chromosome:185-1547	ΔORF2	110.58+peptide	OK	44.6381	24.5357	-0.863391	-1.76972	0.00255	0.0352222	yes
gene:SpnNT_00001	dnaA	Chromosome:185-1547	110.58	ΔORF2+peptide	OK	35.2254	28.7801	-0.291548	-0.603874	0.28555	0.78895	no
gene:SpnNT_00001	dnaA	Chromosome:185-1547	ΔORF2	ΔORF2+peptide	OK	44.6381	28.7801	-0.633205	-1.32422	0.0226	0.183627	no
gene:SpnNT_00001	dnaA	Chromosome:185-1547	110.58+peptide	ΔORF2+peptide	OK	24.5357	28.7801	0.230186	0.461179	0.41835	0.903487	no
gene:SpnNT_00002	dnaN	Chromosome:1705-2842	110.58	ΔORF2	OK	64.7453	79.4161	0.294655	0.647636	0.2569	0.756517	no
gene:SpnNT_00002	dnaN	Chromosome:1705-2842	110.58	110.58+peptide	OK	64.7453	41.0659	-0.656833	-1.39583	0.0169	0.146377	no
gene:SpnNT_00002	dnaN	Chromosome:1705-2842	ΔORF2	110.58+peptide	OK	79.4161	41.0659	-0.951489	-2.02388	7.00E-04	0.012687	yes
gene:SpnNT_00002	dnaN	Chromosome:1705-2842	110.58	ΔORF2+peptide	OK	64.7453	39.3211	-0.719473	-1.51156	0.0107	0.105409	no
gene:SpnNT_00002	dnaN	Chromosome:1705-2842	ΔORF2	ΔORF2+peptide	OK	79.4161	39.3211	-1.01413	-2.13255	0.00065	0.0118958	yes
gene:SpnNT_00002	dnaN	Chromosome:1705-2842	110.58+peptide	ΔORF2+peptide	OK	41.0659	39.3211	-0.0626401	-0.127708	0.8265	0.994748	no
gene:SpnNT_00003	NA	Chromosome:2906-3101	110.58	ΔORF2	OK	360.691	375.206	0.0569176	0.0962693	0.86415	0.994748	no
gene:SpnNT_00003	NA	Chromosome:2906-3101	110.58	110.58+peptide	OK	360.691	403.186	0.160681	0.265601	0.63215	0.980887	no
gene:SpnNT_00003	NA	Chromosome:2906-3101	ΔORF2	110.58+peptide	OK	375.206	403.186	0.103763	0.171569	0.76125	0.994748	no
gene:SpnNT_00003	NA	Chromosome:2906-3101	110.58	ΔORF2+peptide	OK	360.691	375.678	0.0587331	0.0975154	0.86285	0.994748	no
gene:SpnNT_00003	NA	Chromosome:2906-3101	ΔORF2	ΔORF2+peptide	OK	375.206	375.678	0.00181543	0.00301509	0.99615	0.99927	no
gene:SpnNT_00003	NA	Chromosome:2906-3101	110.58+peptide	ΔORF2+peptide	OK	403.186	375.678	-0.101948	-0.165605	0.7715	0.994748	no
gene:SpnNT_00004	NA	Chromosome:3531-3678	110.58	ΔORF2	OK	8.84643	2.51989	-1.81173	-0.926297	0.23375	0.72774	no
gene:SpnNT_00004	NA	Chromosome:3531-3678	110.58	110.58+peptide	OK	8.84643	11.0839	0.325304	0.182229	0.72765	0.994748	no
gene:SpnNT_00004	NA	Chromosome:3531-3678	ΔORF2	110.58+peptide	OK	2.51989	11.0839	2.13704	2.58894	0.26145	0.761688	no
gene:SpnNT_00004	NA	Chromosome:3531-3678	110.58	ΔORF2+peptide	OK	8.84643	13.8116	0.642709	0.357391	0.52685	0.956161	no
gene:SpnNT_00004	NA	Chromosome:3531-3678	ΔORF2	ΔORF2+peptide	OK	2.51989	13.8116	2.45444	2.87534	0.2119	0.697569	no
gene:SpnNT_00004	NA	Chromosome:3531-3678	110.58+peptide	ΔORF2+peptide	OK	11.0839	13.8116	0.317405	1.05868	0.81795	0.994748	no
gene:SpnNT_00005	NA	Chromosome:3731-4124	110.58	ΔORF2	OK	17.256	11.8869	-0.537735	-0.766996	0.1832	0.653781	no
gene:SpnNT_00005	NA	Chromosome:3731-4124	110.58	110.58+peptide	OK	17.256	13.6776	-0.335287	-0.486478	0.4007	0.893669	no
gene:SpnNT_00005	NA	Chromosome:3731-4124	ΔORF2	110.58+peptide	OK	11.8869	13.6776	0.202448	0.28299	0.61865	0.980887	no
gene:SpnNT_00005	NA	Chromosome:3731-4124	110.58	ΔORF2+peptide	OK	17.256	9.431	-0.871618	-1.2064	0.04435	0.289245	no
gene:SpnNT_00005	NA	Chromosome:3731-4124	ΔORF2	ΔORF2+peptide	OK	11.8869	9.431	-0.333884	-0.446663	0.4337	0.913361	no
gene:SpnNT_00005	NA	Chromosome:3731-4124	110.58+peptide	ΔORF2+peptide	OK	13.6776	9.431	-0.536331	-0.728338	0.21205	0.697713	no
gene:SpnNT_00006	NA	Chromosome:4280-4964	110.58	ΔORF2	OK	13.5698	9.63378	-0.494223	-0.786661	0.1722	0.632235	no
gene:SpnNT_00006	NA	Chromosome:4280-4964	110.58	110.58+peptide	OK	13.5698	13.1656	-0.043623	-0.0712308	0.90345	0.994748	no
gene:SpnNT_00006	NA	Chromosome:4280-4964	ΔORF2	110.58+peptide	OK	9.63378	13.1656	0.4506	0.724729	0.19915	0.681762	no
gene:SpnNT_00006	NA	Chromosome:4280-4964	110.58	ΔORF2+peptide	OK	13.5698	9.71954	-0.481437	-0.761086	0.184	0.655388	no
gene:SpnNT_00006	NA	Chromosome:4280-4964	ΔORF2	ΔORF2+peptide	OK	9.63378	9.71954	0.0127862	0.0199284	0.97185	0.99536	no
gene:SpnNT_00006	NA	Chromosome:4280-4964	110.58+peptide	ΔORF2+peptide	OK	13.1656	9.71954	-0.437814	-0.699264	0.2163	0.703941	no
gene:SpnNT_00007	NA	Chromosome:5102-6198	110.58	ΔORF2	OK	14.4771	8.90932	-0.70038	-0.479142	0.39175	0.886453	no
gene:SpnNT_00007	NA	Chromosome:5102-6198	110.58	110.58+peptide	OK	14.4771	12.2289	-0.243475	-0.168854	0.767	0.994748	no
gene:SpnNT_00007	NA	Chromosome:5102-6198	ΔORF2	110.58+peptide	OK	8.90932	12.2289	0.456905	0.340818	0.5745	0.969538	no
gene:SpnNT_00007	NA	Chromosome:5102-6198	110.58	ΔORF2+peptide	OK	14.4771	7.18841	-1.01002	-0.753237	0.32415	0.829465	no
gene:SpnNT_00007	NA	Chromosome:5102-6198	ΔORF2	ΔORF2+peptide	OK	8.90932	7.18841	-0.309644	-0.251475	0.75425	0.994748	no
gene:SpnNT_00007	NA	Chromosome:5102-6198	110.58+peptide	ΔORF2+peptide	OK	12.2289	7.18841	-0.766549	-0.634702	0.4444	0.917673	no
gene:SpnNT_00008	NA	Chromosome:5102-6198	110.58	ΔORF2	OK	11.8657	9.2738	-0.355568	-0.335904	0.5787	0.969538	no
gene:SpnNT_00008	NA	Chromosome:5102-6198	110.58	110.58+peptide	OK	11.8657	9.76107	-0.28169	-0.268907	0.65475	0.982966	no

gene:SpnNT_00008	NA	Chromosome:5102-6198	ΔORF2	110.58+peptide	OK	9.2738	9.76107	0.0738781	0.0689039	0.9076	0.994748	no
gene:SpnNT_00008	NA	Chromosome:5102-6198	110.58	ΔORF2+peptide	OK	11.8657	8.1334	-0.54487	-0.477216	0.42615	0.908545	no
gene:SpnNT_00008	NA	Chromosome:5102-6198	ΔORF2	ΔORF2+peptide	OK	9.2738	8.1334	-0.189301	-0.16257	0.78585	0.994748	no
gene:SpnNT_00008	NA	Chromosome:5102-6198	110.58+peptide	ΔORF2+peptide	OK	9.76107	8.1334	-0.26318	-0.227973	0.69365	0.98828	no
gene:SpnNT_00009	NA	Chromosome:5102-6198	110.58	ΔORF2	OK	8.23405	7.49213	-0.136228	-0.0865762	0.884	0.994748	no
gene:SpnNT_00009	NA	Chromosome:5102-6198	110.58	110.58+peptide	OK	8.23405	9.25045	0.16792	0.101887	0.8652	0.994748	no
gene:SpnNT_00009	NA	Chromosome:5102-6198	ΔORF2	110.58+peptide	OK	7.49213	9.25045	0.304148	0.208714	0.7193	0.993716	no
gene:SpnNT_00009	NA	Chromosome:5102-6198	110.58	ΔORF2+peptide	OK	8.23405	8.35672	0.0213342	0.0135164	0.97635	0.99536	no
gene:SpnNT_00009	NA	Chromosome:5102-6198	ΔORF2	ΔORF2+peptide	OK	7.49213	8.35672	0.157562	0.114348	0.8488	0.994748	no
gene:SpnNT_00009	NA	Chromosome:5102-6198	110.58+peptide	ΔORF2+peptide	OK	9.25045	8.35672	-0.146586	-0.100228	0.862	0.994748	no
gene:SpnNT_00010	NA	Chromosome:5102-6198	110.58	ΔORF2	OK	6.92085	1.39181	-2.31399	-1.02169	0.43565	0.914071	no
gene:SpnNT_00010	NA	Chromosome:5102-6198	110.58	110.58+peptide	OK	6.92085	2.10017	-1.72044	-0.776219	0.5035	0.945197	no
gene:SpnNT_00010	NA	Chromosome:5102-6198	ΔORF2	110.58+peptide	OK	1.39181	2.10017	0.593545	0.297308	0.7361	0.994748	no
gene:SpnNT_00010	NA	Chromosome:5102-6198	110.58	ΔORF2+peptide	OK	6.92085	1.18246	-2.54915	-1.30547	0.4081	0.897087	no
gene:SpnNT_00010	NA	Chromosome:5102-6198	ΔORF2	ΔORF2+peptide	NOTEST	1.39181	1.18246	-0.235167	0	1	1	no
gene:SpnNT_00010	NA	Chromosome:5102-6198	110.58+peptide	ΔORF2+peptide	OK	2.10017	1.18246	-0.828712	-0.507263	0.7202	0.994053	no
gene:SpnNT_00011	NA	Chromosome:6199-6400	110.58	ΔORF2	OK	8.49077	8.54615	0.00937824	0.00815243	0.93355	0.994855	no
gene:SpnNT_00011	NA	Chromosome:6199-6400	110.58	110.58+peptide	OK	8.49077	10.4042	0.293194	0.301145	0.70585	0.990422	no
gene:SpnNT_00011	NA	Chromosome:6199-6400	ΔORF2	110.58+peptide	OK	8.54615	10.4042	0.283816	0.200855	0.73155	0.994748	no
gene:SpnNT_00011	NA	Chromosome:6199-6400	110.58	ΔORF2+peptide	OK	8.49077	0.98534	-3.1072	-3.46409	0.1623	0.615876	no
gene:SpnNT_00011	NA	Chromosome:6199-6400	ΔORF2	ΔORF2+peptide	OK	8.54615	0.98534	-3.11658	-2.28929	0.16365	0.618317	no
gene:SpnNT_00011	NA	Chromosome:6199-6400	110.58+peptide	ΔORF2+peptide	OK	10.4042	0.98534	-3.4004	-2.79708	0.1609	0.613759	no
gene:SpnNT_00012	NA	Chromosome:6411-6912	110.58	ΔORF2	OK	8.43384	6.2137	-0.440739	-0.5971	0.2946	0.799202	no
gene:SpnNT_00012	NA	Chromosome:6411-6912	110.58	110.58+peptide	OK	8.43384	9.30467	0.141765	0.202113	0.72055	0.994327	no
gene:SpnNT_00012	NA	Chromosome:6411-6912	ΔORF2	110.58+peptide	OK	6.2137	9.30467	0.582504	0.800605	0.1711	0.629959	no
gene:SpnNT_00012	NA	Chromosome:6411-6912	110.58	ΔORF2+peptide	OK	8.43384	8.06918	-0.0637678	-0.0851694	0.88	0.994748	no
gene:SpnNT_00012	NA	Chromosome:6411-6912	ΔORF2	ΔORF2+peptide	OK	6.2137	8.06918	0.376971	0.487494	0.40785	0.897051	no
gene:SpnNT_00012	NA	Chromosome:6411-6912	110.58+peptide	ΔORF2+peptide	OK	9.30467	8.06918	-0.205532	-0.27838	0.63255	0.980887	no
gene:SpnNT_00013	NA	Chromosome:6931-7048	110.58	ΔORF2	NOTEST	0	3.06448	Inf	0	1	1	no
gene:SpnNT_00013	NA	Chromosome:6931-7048	110.58	110.58+peptide	NOTEST	0	8.33932	Inf	0	1	1	no
gene:SpnNT_00013	NA	Chromosome:6931-7048	ΔORF2	110.58+peptide	NOTEST	3.06448	8.33932	1.44429	0	1	1	no
gene:SpnNT_00013	NA	Chromosome:6931-7048	110.58	ΔORF2+peptide	NOTEST	0	0	0	0	1	1	no
gene:SpnNT_00013	NA	Chromosome:6931-7048	ΔORF2	ΔORF2+peptide	NOTEST	3.06448	0	#NAME?	0	1	1	no
gene:SpnNT_00013	NA	Chromosome:6931-7048	110.58+peptide	ΔORF2+peptide	NOTEST	8.33932	0	#NAME?	0	1	1	no
gene:SpnNT_00014	NA	Chromosome:7345-7925	110.58	ΔORF2	OK	5.61633	0	#NAME?	NA	0.2369	0.731436	no
gene:SpnNT_00014	NA	Chromosome:7345-7925	110.58	110.58+peptide	OK	5.61633	1.6651	-1.75402	-1.91609	0.5311	0.957488	no
gene:SpnNT_00014	NA	Chromosome:7345-7925	ΔORF2	110.58+peptide	NOTEST	0	1.6651	Inf	0	1	1	no
gene:SpnNT_00014	NA	Chromosome:7345-7925	110.58	ΔORF2+peptide	OK	5.61633	0	#NAME?	NA	0.2341	0.72774	no
gene:SpnNT_00014	NA	Chromosome:7345-7925	ΔORF2	ΔORF2+peptide	NOTEST	0	0	0	0	1	1	no
gene:SpnNT_00014	NA	Chromosome:7345-7925	110.58+peptide	ΔORF2+peptide	NOTEST	1.6651	0	#NAME?	0	1	1	no
gene:SpnNT_00015	NA	Chromosome:7345-7925	110.58	ΔORF2	OK	11.3117	6.99298	-0.693836	-0.93494	0.11675	0.517915	no
gene:SpnNT_00015	NA	Chromosome:7345-7925	110.58	110.58+peptide	OK	11.3117	13.1483	0.217055	0.317372	0.5822	0.970851	no
gene:SpnNT_00015	NA	Chromosome:7345-7925	ΔORF2	110.58+peptide	OK	6.99298	13.1483	0.910891	1.25174	0.0348	0.246835	no
gene:SpnNT_00015	NA	Chromosome:7345-7925	110.58	ΔORF2+peptide	OK	11.3117	6.42088	-0.816972	-1.05728	0.07175	0.394099	no
gene:SpnNT_00015	NA	Chromosome:7345-7925	ΔORF2	ΔORF2+peptide	OK	6.99298	6.42088	-0.123136	-0.151698	0.79145	0.994748	no

gene:SpnNT_00015	NA	Chromosome:7345-7925	110.58+peptide	ΔORF2+peptide	OK	13.1483	6.42088	-1.03403	-1.36258	0.0232	0.186653	no
gene:SpnNT_00016	NA	Chromosome:8079-8250	110.58	ΔORF2	OK	90.8951	150.745	0.729836	0.944872	0.0971	0.47086	no
gene:SpnNT_00016	NA	Chromosome:8079-8250	110.58	110.58+peptide	OK	90.8951	74.2762	-0.291302	-0.364846	0.5327	0.958424	no
gene:SpnNT_00016	NA	Chromosome:8079-8250	ΔORF2	110.58+peptide	OK	150.745	74.2762	-1.02114	-1.27333	0.0293	0.2187	no
gene:SpnNT_00016	NA	Chromosome:8079-8250	110.58	ΔORF2+peptide	OK	90.8951	76.6638	-0.245658	-0.284275	0.6027	0.976761	no
gene:SpnNT_00016	NA	Chromosome:8079-8250	ΔORF2	ΔORF2+peptide	OK	150.745	76.6638	-0.975495	-1.12461	0.04185	0.278901	no
gene:SpnNT_00016	NA	Chromosome:8079-8250	110.58+peptide	ΔORF2+peptide	OK	74.2762	76.6638	0.0456439	0.0512482	0.92805	0.994748	no
gene:SpnNT_00017	NA	Chromosome:8478-8739	110.58	ΔORF2	OK	25.8795	18.9396	-0.450405	-0.568704	0.3371	0.84149	no
gene:SpnNT_00017	NA	Chromosome:8478-8739	110.58	110.58+peptide	OK	25.8795	22.8305	-0.180851	-0.232694	0.6899	0.98828	no
gene:SpnNT_00017	NA	Chromosome:8478-8739	ΔORF2	110.58+peptide	OK	18.9396	22.8305	0.269554	0.32499	0.57635	0.969538	no
gene:SpnNT_00017	NA	Chromosome:8478-8739	110.58	ΔORF2+peptide	OK	25.8795	17.5595	-0.559561	-0.66208	0.2396	0.73493	no
gene:SpnNT_00017	NA	Chromosome:8478-8739	ΔORF2	ΔORF2+peptide	OK	18.9396	17.5595	-0.109156	-0.122179	0.83195	0.994748	no
gene:SpnNT_00017	NA	Chromosome:8478-8739	110.58+peptide	ΔORF2+peptide	OK	22.8305	17.5595	-0.378711	-0.430189	0.43835	0.915991	no
gene:SpnNT_00018	NA	Chromosome:8750-9176	110.58	ΔORF2	OK	6.38603	3.703	-0.786225	-0.906619	0.12705	0.540931	no
gene:SpnNT_00018	NA	Chromosome:8750-9176	110.58	110.58+peptide	OK	6.38603	5.81976	-0.13396	-0.163431	0.7729	0.994748	no
gene:SpnNT_00018	NA	Chromosome:8750-9176	ΔORF2	110.58+peptide	OK	3.703	5.81976	0.652265	0.730353	0.2121	0.697713	no
gene:SpnNT_00018	NA	Chromosome:8750-9176	110.58	ΔORF2+peptide	OK	6.38603	5.04613	-0.339744	-0.410279	0.4786	0.933291	no
gene:SpnNT_00018	NA	Chromosome:8750-9176	ΔORF2	ΔORF2+peptide	OK	3.703	5.04613	0.446481	0.495648	0.4093	0.897533	no
gene:SpnNT_00018	NA	Chromosome:8750-9176	110.58+peptide	ΔORF2+peptide	OK	5.81976	5.04613	-0.205784	-0.240643	0.68035	0.986361	no
gene:SpnNT_00019	NA	Chromosome:9197-9356	110.58	ΔORF2	OK	10.2763	0.972588	-3.40135	-1.97437	0.114	0.512122	no
gene:SpnNT_00019	NA	Chromosome:9197-9356	110.58	110.58+peptide	OK	10.2763	7.44253	-0.465461	-0.216991	0.66785	0.984334	no
gene:SpnNT_00019	NA	Chromosome:9197-9356	ΔORF2	110.58+peptide	OK	0.972588	7.44253	2.93589	1.48287	0.1336	0.556521	no
gene:SpnNT_00019	NA	Chromosome:9197-9356	110.58	ΔORF2+peptide	OK	10.2763	2.20424	-2.22097	-1.55075	0.21755	0.705186	no
gene:SpnNT_00019	NA	Chromosome:9197-9356	ΔORF2	ΔORF2+peptide	NOTEST	0.972588	2.20424	1.18038	0	1	1	no
gene:SpnNT_00019	NA	Chromosome:9197-9356	110.58+peptide	ΔORF2+peptide	OK	7.44253	2.20424	-1.75551	-1.01301	0.3005	0.806342	no
gene:SpnNT_00020	NA	Chromosome:9445-9679	110.58	ΔORF2	OK	5.25916	3.99162	-0.39786	-0.355029	0.6639	0.982966	no
gene:SpnNT_00020	NA	Chromosome:9445-9679	110.58	110.58+peptide	OK	5.25916	6.07024	0.20692	0.159892	0.7945	0.994748	no
gene:SpnNT_00020	NA	Chromosome:9445-9679	ΔORF2	110.58+peptide	OK	3.99162	6.07024	0.60478	0.580933	0.4758	0.930746	no
gene:SpnNT_00020	NA	Chromosome:9445-9679	110.58	ΔORF2+peptide	OK	5.25916	1.99489	-1.39852	-1.1573	0.19885	0.681625	no
gene:SpnNT_00020	NA	Chromosome:9445-9679	ΔORF2	ΔORF2+peptide	OK	3.99162	1.99489	-1.00066	-1.07323	0.3176	0.823463	no
gene:SpnNT_00020	NA	Chromosome:9445-9679	110.58+peptide	ΔORF2+peptide	OK	6.07024	1.99489	-1.60544	-1.41447	0.1226	0.530068	no
gene:SpnNT_00021	NA	Chromosome:9836-10793	110.58	ΔORF2	OK	14.1626	18.5873	0.392232	0.700204	0.22315	0.712784	no
gene:SpnNT_00021	NA	Chromosome:9836-10793	110.58	110.58+peptide	OK	14.1626	17.6095	0.314268	0.559288	0.32355	0.829061	no
gene:SpnNT_00021	NA	Chromosome:9836-10793	ΔORF2	110.58+peptide	OK	18.5873	17.6095	-0.0779646	-0.139419	0.8103	0.994748	no
gene:SpnNT_00021	NA	Chromosome:9836-10793	110.58	ΔORF2+peptide	OK	14.1626	25.6456	0.856626	1.57158	0.0068	0.0755543	no
gene:SpnNT_00021	NA	Chromosome:9836-10793	ΔORF2	ΔORF2+peptide	OK	18.5873	25.6456	0.464394	0.856349	0.13	0.547452	no
gene:SpnNT_00021	NA	Chromosome:9836-10793	110.58+peptide	ΔORF2+peptide	OK	17.6095	25.6456	0.542359	0.996818	0.077	0.410729	no
gene:SpnNT_00022	NA	Chromosome:10941-12398	110.58	ΔORF2	OK	23.2892	32.0723	0.46167	0.819483	0.1558	0.60112	no
gene:SpnNT_00022	NA	Chromosome:10941-12398	110.58	110.58+peptide	OK	23.2892	32.9023	0.49853	0.898239	0.11875	0.522198	no
gene:SpnNT_00022	NA	Chromosome:10941-12398	ΔORF2	110.58+peptide	OK	32.0723	32.9023	0.0368601	0.066473	0.90985	0.994748	no
gene:SpnNT_00022	NA	Chromosome:10941-12398	110.58	ΔORF2+peptide	OK	23.2892	37.3575	0.681737	1.22729	0.0329	0.237205	no
gene:SpnNT_00022	NA	Chromosome:10941-12398	ΔORF2	ΔORF2+peptide	OK	32.0723	37.3575	0.220067	0.396526	0.49435	0.942197	no
gene:SpnNT_00022	NA	Chromosome:10941-12398	110.58+peptide	ΔORF2+peptide	OK	32.9023	37.3575	0.183207	0.335236	0.5623	0.968621	no
gene:SpnNT_00023	NA	Chromosome:10941-12398	110.58	ΔORF2	OK	14.6406	19.6229	0.422565	0.310942	0.58715	0.973049	no
gene:SpnNT_00023	NA	Chromosome:10941-12398	110.58	110.58+peptide	OK	14.6406	21.9506	0.584288	0.440931	0.45275	0.921244	no

gene:SpnNT_00023	NA	Chromosome:10941-12398	ΔORF2	110.58+peptide	OK	19.6229	21.9506	0.161722	0.118836	0.8357	0.994748	no
gene:SpnNT_00023	NA	Chromosome:10941-12398	110.58	ΔORF2+peptide	OK	14.6406	26.7698	0.870634	0.685081	0.24	0.735469	no
gene:SpnNT_00023	NA	Chromosome:10941-12398	ΔORF2	ΔORF2+peptide	OK	19.6229	26.7698	0.448068	0.342534	0.5421	0.961494	no
gene:SpnNT_00023	NA	Chromosome:10941-12398	110.58+peptide	ΔORF2+peptide	OK	21.9506	26.7698	0.286346	0.224959	0.69635	0.989512	no
gene:SpnNT_00024	NA	Chromosome:12493-12646	110.58	ΔORF2	OK	22.3011	12.1919	-0.871189	-0.527793	0.34675	0.850954	no
gene:SpnNT_00024	NA	Chromosome:12493-12646	110.58	110.58+peptide	OK	22.3011	30.1401	0.43457	0.332738	0.55625	0.966729	no
gene:SpnNT_00024	NA	Chromosome:12493-12646	ΔORF2	110.58+peptide	OK	12.1919	30.1401	1.30576	0.80121	0.17065	0.629539	no
gene:SpnNT_00024	NA	Chromosome:12493-12646	110.58	ΔORF2+peptide	OK	22.3011	11.5083	-0.954439	-0.981247	0.3267	0.831577	no
gene:SpnNT_00024	NA	Chromosome:12493-12646	ΔORF2	ΔORF2+peptide	OK	12.1919	11.5083	-0.0832503	-0.0604535	0.88255	0.994748	no
gene:SpnNT_00024	NA	Chromosome:12493-12646	110.58+peptide	ΔORF2+peptide	OK	30.1401	11.5083	-1.38901	-1.48273	0.14365	0.57877	no
gene:SpnNT_00025	NA	Chromosome:12763-13660	110.58	ΔORF2	OK	13.9674	13.5277	-0.0461539	-0.0803383	0.88665	0.994748	no
gene:SpnNT_00025	NA	Chromosome:12763-13660	110.58	110.58+peptide	OK	13.9674	15.6684	0.165791	0.288155	0.6114	0.979429	no
gene:SpnNT_00025	NA	Chromosome:12763-13660	ΔORF2	110.58+peptide	OK	13.5277	15.6684	0.211945	0.366682	0.51265	0.950409	no
gene:SpnNT_00025	NA	Chromosome:12763-13660	110.58	ΔORF2+peptide	OK	13.9674	20.6382	0.563253	1.01014	0.07505	0.404272	no
gene:SpnNT_00025	NA	Chromosome:12763-13660	ΔORF2	ΔORF2+peptide	OK	13.5277	20.6382	0.609407	1.08758	0.052	0.322621	no
gene:SpnNT_00025	NA	Chromosome:12763-13660	110.58+peptide	ΔORF2+peptide	OK	15.6684	20.6382	0.397462	0.708218	0.2119	0.697569	no
gene:SpnNT_00026	ychF	Chromosome:13779-14895	110.58	ΔORF2	OK	169.888	191.815	0.175134	0.399049	0.4873	0.938799	no
gene:SpnNT_00026	ychF	Chromosome:13779-14895	110.58	110.58+peptide	OK	169.888	237.723	0.4847	1.10798	0.05235	0.323874	no
gene:SpnNT_00026	ychF	Chromosome:13779-14895	ΔORF2	110.58+peptide	OK	191.815	237.723	0.309566	0.702588	0.22005	0.70824	no
gene:SpnNT_00026	ychF	Chromosome:13779-14895	110.58	ΔORF2+peptide	OK	169.888	258.819	0.607362	1.38686	0.01425	0.129404	no
gene:SpnNT_00026	ychF	Chromosome:13779-14895	ΔORF2	ΔORF2+peptide	OK	191.815	258.819	0.432228	0.979925	0.08745	0.445293	no
gene:SpnNT_00026	ychF	Chromosome:13779-14895	110.58+peptide	ΔORF2+peptide	OK	237.723	258.819	0.122661	0.278984	0.62945	0.980887	no
gene:SpnNT_00027	pth	Chromosome:14965-15535	110.58	ΔORF2	OK	58.4382	68.5911	0.23111	0.449785	0.4396	0.91631	no
gene:SpnNT_00027	pth	Chromosome:14965-15535	110.58	110.58+peptide	OK	58.4382	74.9508	0.359033	0.707329	0.21785	0.705473	no
gene:SpnNT_00027	pth	Chromosome:14965-15535	ΔORF2	110.58+peptide	OK	68.5911	74.9508	0.127923	0.252817	0.655	0.982966	no
gene:SpnNT_00027	pth	Chromosome:14965-15535	110.58	ΔORF2+peptide	OK	58.4382	91.5141	0.647083	1.27739	0.02955	0.219691	no
gene:SpnNT_00027	pth	Chromosome:14965-15535	ΔORF2	ΔORF2+peptide	OK	68.5911	91.5141	0.415973	0.823772	0.1545	0.59963	no
gene:SpnNT_00027	pth	Chromosome:14965-15535	110.58+peptide	ΔORF2+peptide	OK	74.9508	91.5141	0.288051	0.577699	0.3073	0.81331	no
gene:SpnNT_00028	mfd	Chromosome:15535-19051	110.58	ΔORF2	OK	37.0921	40.8003	0.137468	0.311026	0.58755	0.973341	no
gene:SpnNT_00028	mfd	Chromosome:15535-19051	110.58	110.58+peptide	OK	37.0921	50.43	0.443169	1.00365	0.08355	0.433852	no
gene:SpnNT_00028	mfd	Chromosome:15535-19051	ΔORF2	110.58+peptide	OK	40.8003	50.43	0.305701	0.694215	0.22755	0.71915	no
gene:SpnNT_00028	mfd	Chromosome:15535-19051	110.58	ΔORF2+peptide	OK	37.0921	63.6958	0.780086	1.76924	0.00245	0.0342374	yes
gene:SpnNT_00028	mfd	Chromosome:15535-19051	ΔORF2	ΔORF2+peptide	OK	40.8003	63.6958	0.642618	1.46145	0.0124	0.117822	no
gene:SpnNT_00028	mfd	Chromosome:15535-19051	110.58+peptide	ΔORF2+peptide	OK	50.43	63.6958	0.336917	0.766967	0.18305	0.653778	no
gene:SpnNT_00029	hslR	Chromosome:19108-19736	110.58	ΔORF2	OK	238.228	283.065	0.248792	0.358898	0.54275	0.961518	no
gene:SpnNT_00029	hslR	Chromosome:19108-19736	110.58	110.58+peptide	OK	238.228	447.125	0.908333	1.38924	0.01825	0.155101	no
gene:SpnNT_00029	hslR	Chromosome:19108-19736	ΔORF2	110.58+peptide	OK	283.065	447.125	0.659542	0.989549	0.0969	0.470411	no
gene:SpnNT_00029	hslR	Chromosome:19108-19736	110.58	ΔORF2+peptide	OK	238.228	496.356	1.05903	1.56778	0.00705	0.077805	no
gene:SpnNT_00029	hslR	Chromosome:19108-19736	ΔORF2	ΔORF2+peptide	OK	283.065	496.356	0.810241	1.17807	0.04655	0.29884	no
gene:SpnNT_00029	hslR	Chromosome:19108-19736	110.58+peptide	ΔORF2+peptide	OK	447.125	496.356	0.150699	0.232538	0.6807	0.986541	no
gene:SpnNT_00030	NA	Chromosome:19108-19736	110.58	ΔORF2	OK	93.5955	114.016	0.284721	0.380428	0.49615	0.943191	no
gene:SpnNT_00030	NA	Chromosome:19108-19736	110.58	110.58+peptide	OK	93.5955	147.78	0.658938	0.881958	0.1201	0.524705	no
gene:SpnNT_00030	NA	Chromosome:19108-19736	ΔORF2	110.58+peptide	OK	114.016	147.78	0.374217	0.502228	0.37755	0.875767	no
gene:SpnNT_00030	NA	Chromosome:19108-19736	110.58	ΔORF2+peptide	OK	93.5955	179.859	0.942353	1.24024	0.02935	0.218948	no
gene:SpnNT_00030	NA	Chromosome:19108-19736	ΔORF2	ΔORF2+peptide	OK	114.016	179.859	0.657632	0.867779	0.1197	0.52453	no

gene:SpnNT_00030	NA	Chromosome:19108-19736	110.58+peptide	ΔORF2+peptide	OK	147.78	179.859	0.283415	0.37461	0.49665	0.943637	no
gene:SpnNT_00031	NA	Chromosome:19740-22398	110.58	ΔORF2	OK	70.079	72.4189	0.0473839	0.00578741	0.9602	0.994855	no
gene:SpnNT_00031	NA	Chromosome:19740-22398	110.58	110.58+peptide	OK	70.079	141.134	1.01001	0.128048	0.57175	0.969025	no
gene:SpnNT_00031	NA	Chromosome:19740-22398	ΔORF2	110.58+peptide	OK	72.4189	141.134	0.962625	0.119804	0.55595	0.966729	no
gene:SpnNT_00031	NA	Chromosome:19740-22398	110.58	ΔORF2+peptide	OK	70.079	100.842	0.525035	0.0914624	0.80375	0.994748	no
gene:SpnNT_00031	NA	Chromosome:19740-22398	ΔORF2	ΔORF2+peptide	OK	72.4189	100.842	0.477651	0.0803976	0.7917	0.994748	no
gene:SpnNT_00031	NA	Chromosome:19740-22398	110.58+peptide	ΔORF2+peptide	OK	141.134	100.842	-0.484974	-0.0878443	0.79805	0.994748	no
gene:SpnNT_00032	NA	Chromosome:19740-22398	110.58	ΔORF2	OK	58.3008	69.5062	0.253625	0.383863	0.49985	0.944017	no
gene:SpnNT_00032	NA	Chromosome:19740-22398	110.58	110.58+peptide	OK	58.3008	98.521	0.756914	1.10868	0.0578	0.342262	no
gene:SpnNT_00032	NA	Chromosome:19740-22398	ΔORF2	110.58+peptide	OK	69.5062	98.521	0.50329	0.778267	0.1805	0.648022	no
gene:SpnNT_00032	NA	Chromosome:19740-22398	110.58	ΔORF2+peptide	OK	58.3008	136.267	1.22485	1.87805	0.0024	0.0337543	yes
gene:SpnNT_00032	NA	Chromosome:19740-22398	ΔORF2	ΔORF2+peptide	OK	69.5062	136.267	0.971228	1.58085	0.0066	0.0739583	no
gene:SpnNT_00032	NA	Chromosome:19740-22398	110.58+peptide	ΔORF2+peptide	OK	98.521	136.267	0.467938	0.733478	0.21115	0.697158	no
gene:SpnNT_00033	tilS	Chromosome:19740-22398	110.58	ΔORF2	OK	58.8651	59.75	0.0215262	0.0319589	0.95585	0.994855	no
gene:SpnNT_00033	tilS	Chromosome:19740-22398	110.58	110.58+peptide	OK	58.8651	89.2378	0.600242	0.86467	0.1305	0.548853	no
gene:SpnNT_00033	tilS	Chromosome:19740-22398	ΔORF2	110.58+peptide	OK	59.75	89.2378	0.578716	0.841338	0.1401	0.571462	no
gene:SpnNT_00033	tilS	Chromosome:19740-22398	110.58	ΔORF2+peptide	OK	58.8651	106.75	0.858746	1.28053	0.0272	0.208969	no
gene:SpnNT_00033	tilS	Chromosome:19740-22398	ΔORF2	ΔORF2+peptide	OK	59.75	106.75	0.83722	1.26076	0.0292	0.218078	no
gene:SpnNT_00033	tilS	Chromosome:19740-22398	110.58+peptide	ΔORF2+peptide	OK	89.2378	106.75	0.258504	0.377394	0.5098	0.948082	no
gene:SpnNT_00034	hpt	Chromosome:22401-22944	110.58	ΔORF2	OK	364.165	528.631	0.537669	1.16665	0.0422	0.280805	no
gene:SpnNT_00034	hpt	Chromosome:22401-22944	110.58	110.58+peptide	OK	364.165	480.589	0.400214	0.894965	0.123	0.530748	no
gene:SpnNT_00034	hpt	Chromosome:22401-22944	ΔORF2	110.58+peptide	OK	528.631	480.589	-0.137456	-0.295995	0.5975	0.97629	no
gene:SpnNT_00034	hpt	Chromosome:22401-22944	110.58	ΔORF2+peptide	OK	364.165	575.083	0.65918	1.48866	0.0105	0.103986	no
gene:SpnNT_00034	hpt	Chromosome:22401-22944	ΔORF2	ΔORF2+peptide	OK	528.631	575.083	0.12151	0.264059	0.63925	0.980887	no
gene:SpnNT_00034	hpt	Chromosome:22401-22944	110.58+peptide	ΔORF2+peptide	OK	480.589	575.083	0.258966	0.580043	0.3127	0.819012	no
gene:SpnNT_00035	ftsH	Chromosome:22959-24918	110.58	ΔORF2	OK	356.198	393.473	0.143584	0.319805	0.57685	0.969538	no
gene:SpnNT_00035	ftsH	Chromosome:22959-24918	110.58	110.58+peptide	OK	356.198	447.516	0.32926	0.731058	0.19845	0.680978	no
gene:SpnNT_00035	ftsH	Chromosome:22959-24918	ΔORF2	110.58+peptide	OK	393.473	447.516	0.185676	0.411734	0.47315	0.928984	no
gene:SpnNT_00035	ftsH	Chromosome:22959-24918	110.58	ΔORF2+peptide	OK	356.198	498.237	0.484153	1.07283	0.05965	0.35037	no
gene:SpnNT_00035	ftsH	Chromosome:22959-24918	ΔORF2	ΔORF2+peptide	OK	393.473	498.237	0.340569	0.753709	0.18285	0.65342	no
gene:SpnNT_00035	ftsH	Chromosome:22959-24918	110.58+peptide	ΔORF2+peptide	OK	447.516	498.237	0.154894	0.34173	0.54505	0.961568	no
gene:SpnNT_00036	NA	Chromosome:25039-25519	110.58	ΔORF2	OK	8.59621	9.92028	0.20668	0.282247	0.6274	0.980887	no
gene:SpnNT_00036	NA	Chromosome:25039-25519	110.58	110.58+peptide	OK	8.59621	6.69077	-0.361528	-0.488627	0.3924	0.887312	no
gene:SpnNT_00036	NA	Chromosome:25039-25519	ΔORF2	110.58+peptide	OK	9.92028	6.69077	-0.568207	-0.749235	0.20035	0.683196	no
gene:SpnNT_00036	NA	Chromosome:25039-25519	110.58	ΔORF2+peptide	OK	8.59621	5.11234	-0.749718	-0.962011	0.09685	0.470343	no
gene:SpnNT_00036	NA	Chromosome:25039-25519	ΔORF2	ΔORF2+peptide	OK	9.92028	5.11234	-0.956398	-1.20014	0.04345	0.285647	no
gene:SpnNT_00036	NA	Chromosome:25039-25519	110.58+peptide	ΔORF2+peptide	OK	6.69077	5.11234	-0.38819	-0.482876	0.40885	0.897533	no
gene:SpnNT_00037	SpnNT_00037	Chromosome:25612-25684	110.58	ΔORF2	NOTEST	233.477	0	#NAME?	0	1	1	no
gene:SpnNT_00037	SpnNT_00037	Chromosome:25612-25684	110.58	110.58+peptide	OK	233.477	1531.94	2.71401	232.369	0.1539	0.598363	no
gene:SpnNT_00037	SpnNT_00037	Chromosome:25612-25684	ΔORF2	110.58+peptide	OK	0	1531.94	Inf	NA	0.00425	0.0530119	no
gene:SpnNT_00037	SpnNT_00037	Chromosome:25612-25684	110.58	ΔORF2+peptide	NOTEST	233.477	0	#NAME?	0	1	1	no
gene:SpnNT_00037	SpnNT_00037	Chromosome:25612-25684	ΔORF2	ΔORF2+peptide	NOTEST	0	0	0	0	1	1	no
gene:SpnNT_00037	SpnNT_00037	Chromosome:25612-25684	110.58+peptide	ΔORF2+peptide	OK	1531.94	0	#NAME?	NA	0.0049	0.0593153	no
gene:SpnNT_00038	SpnNT_00038	Chromosome:25940-27477	110.58	ΔORF2	HIDATA	0	0	0	0	1	1	no
gene:SpnNT_00038	SpnNT_00038	Chromosome:25940-27477	110.58	110.58+peptide	HIDATA	0	0	0	0	1	1	no

gene:SpnNT_00038	SpnNT_00038	Chromosome:25940-27477	ΔORF2	110.58+peptide	HIDATA	0	0	0	0	1	1	no
gene:SpnNT_00038	SpnNT_00038	Chromosome:25940-27477	110.58	ΔORF2+peptide	HIDATA	0	0	0	0	1	1	no
gene:SpnNT_00038	SpnNT_00038	Chromosome:25940-27477	ΔORF2	ΔORF2+peptide	HIDATA	0	0	0	0	1	1	no
gene:SpnNT_00038	SpnNT_00038	Chromosome:25940-27477	110.58+peptide	ΔORF2+peptide	HIDATA	0	0	0	0	1	1	no
gene:SpnNT_00039	SpnNT_00039	Chromosome:27531-27606	110.58	ΔORF2	OK	891.992	1772.17	0.990413	0.457972	0.335	0.840029	no
gene:SpnNT_00039	SpnNT_00039	Chromosome:27531-27606	110.58	110.58+peptide	OK	891.992	789.573	-0.17596	-0.0985959	0.85275	0.994748	no
gene:SpnNT_00039	SpnNT_00039	Chromosome:27531-27606	ΔORF2	110.58+peptide	OK	1772.17	789.573	-1.16637	-0.954883	0.32215	0.827899	no
gene:SpnNT_00039	SpnNT_00039	Chromosome:27531-27606	110.58	ΔORF2+peptide	OK	891.992	971.663	0.123425	0.0691593	0.86915	0.994748	no
gene:SpnNT_00039	SpnNT_00039	Chromosome:27531-27606	ΔORF2	ΔORF2+peptide	OK	1772.17	971.663	-0.866988	-0.709789	0.45615	0.921244	no
gene:SpnNT_00039	SpnNT_00039	Chromosome:27531-27606	110.58+peptide	ΔORF2+peptide	OK	789.573	971.663	0.299385	28.068	0.82865	0.994748	no
gene:SpnNT_00040	SpnNT_00040	Chromosome:27730-30629	110.58	ΔORF2	HIDATA	0	0	0	0	1	1	no
gene:SpnNT_00040	SpnNT_00040	Chromosome:27730-30629	110.58	110.58+peptide	HIDATA	0	0	0	0	1	1	no
gene:SpnNT_00040	SpnNT_00040	Chromosome:27730-30629	ΔORF2	110.58+peptide	HIDATA	0	0	0	0	1	1	no
gene:SpnNT_00040	SpnNT_00040	Chromosome:27730-30629	110.58	ΔORF2+peptide	HIDATA	0	0	0	0	1	1	no
gene:SpnNT_00040	SpnNT_00040	Chromosome:27730-30629	ΔORF2	ΔORF2+peptide	HIDATA	0	0	0	0	1	1	no
gene:SpnNT_00040	SpnNT_00040	Chromosome:27730-30629	110.58+peptide	ΔORF2+peptide	HIDATA	0	0	0	0	1	1	no
gene:SpnNT_00041	SpnNT_00041	Chromosome:30710-30822	110.58	ΔORF2	HIDATA	4568770	0	0	0	1	1	no
gene:SpnNT_00041	SpnNT_00041	Chromosome:30710-30822	110.58	110.58+peptide	OK	4568770	5614180	0.297269	0.479744	0.3992	0.892609	no
gene:SpnNT_00041	SpnNT_00041	Chromosome:30710-30822	ΔORF2	110.58+peptide	HIDATA	0	5614180	0	0	1	1	no
gene:SpnNT_00041	SpnNT_00041	Chromosome:30710-30822	110.58	ΔORF2+peptide	OK	4568770	6621810	0.535421	0.91145	0.12735	0.54133	no
gene:SpnNT_00041	SpnNT_00041	Chromosome:30710-30822	ΔORF2	ΔORF2+peptide	HIDATA	0	6621810	0	0	1	1	no
gene:SpnNT_00041	SpnNT_00041	Chromosome:30710-30822	110.58+peptide	ΔORF2+peptide	OK	5614180	6621810	0.238151	0.412355	0.48635	0.938374	no
gene:SpnNT_00042	SpnNT_00042	Chromosome:30828-30902	110.58	ΔORF2	OK	200.052	1292.85	2.69211	1.81818	0.1662	0.621332	no
gene:SpnNT_00042	SpnNT_00042	Chromosome:30828-30902	110.58	110.58+peptide	OK	200.052	776.03	1.95574	148.214	0.43165	0.913083	no
gene:SpnNT_00042	SpnNT_00042	Chromosome:30828-30902	ΔORF2	110.58+peptide	OK	1292.85	776.03	-0.736369	-0.497335	0.572	0.969077	no
gene:SpnNT_00042	SpnNT_00042	Chromosome:30828-30902	110.58	ΔORF2+peptide	OK	200.052	850.172	2.08738	164.764	0.225	0.716396	no
gene:SpnNT_00042	SpnNT_00042	Chromosome:30828-30902	ΔORF2	ΔORF2+peptide	OK	1292.85	850.172	-0.604726	-0.408426	0.59095	0.975034	no
gene:SpnNT_00042	SpnNT_00042	Chromosome:30828-30902	110.58+peptide	ΔORF2+peptide	OK	776.03	850.172	0.131644	17.0915	0.7398	0.994748	no
gene:SpnNT_00043	NA	Chromosome:32388-32625	110.58	ΔORF2	OK	13.149	19.2712	0.551495	0.600286	0.2984	0.803696	no
gene:SpnNT_00043	NA	Chromosome:32388-32625	110.58	110.58+peptide	OK	13.149	7.17496	-0.87391	-0.807097	0.2057	0.690314	no
gene:SpnNT_00043	NA	Chromosome:32388-32625	ΔORF2	110.58+peptide	OK	19.2712	7.17496	-1.4254	-1.33065	0.05325	0.327282	no
gene:SpnNT_00043	NA	Chromosome:32388-32625	110.58	ΔORF2+peptide	OK	13.149	6.84719	-0.94137	-1.20062	0.16505	0.620569	no
gene:SpnNT_00043	NA	Chromosome:32388-32625	ΔORF2	ΔORF2+peptide	OK	19.2712	6.84719	-1.49286	-1.94382	0.03995	0.270497	no
gene:SpnNT_00043	NA	Chromosome:32388-32625	110.58+peptide	ΔORF2+peptide	OK	7.17496	6.84719	-0.0674594	-0.0704	0.93985	0.994855	no
gene:SpnNT_00044	purA	Chromosome:32813-34142	110.58	ΔORF2	OK	337.598	381.962	0.178123	0.403284	0.47595	0.930901	no
gene:SpnNT_00044	purA	Chromosome:32813-34142	110.58	110.58+peptide	OK	337.598	430.763	0.351588	0.788579	0.1702	0.62894	no
gene:SpnNT_00044	purA	Chromosome:32813-34142	ΔORF2	110.58+peptide	OK	381.962	430.763	0.173465	0.38897	0.49725	0.943998	no
gene:SpnNT_00044	purA	Chromosome:32813-34142	110.58	ΔORF2+peptide	OK	337.598	416.617	0.303418	0.686627	0.22505	0.716396	no
gene:SpnNT_00044	purA	Chromosome:32813-34142	ΔORF2	ΔORF2+peptide	OK	381.962	416.617	0.125295	0.283468	0.61635	0.980293	no
gene:SpnNT_00044	purA	Chromosome:32813-34142	110.58+peptide	ΔORF2+peptide	OK	430.763	416.617	-0.0481703	-0.107964	0.849	0.994748	no
gene:SpnNT_00045	tadA	Chromosome:34342-34810	110.58	ΔORF2	OK	57.4026	55.7139	-0.0430786	-0.0796785	0.8869	0.994748	no
gene:SpnNT_00045	tadA	Chromosome:34342-34810	110.58	110.58+peptide	OK	57.4026	48.9609	-0.229487	-0.416513	0.46645	0.927106	no
gene:SpnNT_00045	tadA	Chromosome:34342-34810	ΔORF2	110.58+peptide	OK	55.7139	48.9609	-0.186408	-0.335089	0.55385	0.966053	no
gene:SpnNT_00045	tadA	Chromosome:34342-34810	110.58	ΔORF2+peptide	OK	57.4026	45.1791	-0.345459	-0.628145	0.27375	0.775958	no
gene:SpnNT_00045	tadA	Chromosome:34342-34810	ΔORF2	ΔORF2+peptide	OK	55.7139	45.1791	-0.302381	-0.544537	0.3332	0.839007	no

gene:SpnNT_00045	tadA	Chromosome:34342-34810	110.58+peptide	ΔORF2+peptide	OK	48.9609	45.1791	-0.115973	-0.205134	0.71685	0.992452	no
gene:SpnNT_00046	dut	Chromosome:34996-35957	110.58	ΔORF2	OK	41.2045	43.4528	0.0766457	0.107219	0.8494	0.994748	no
gene:SpnNT_00046	dut	Chromosome:34996-35957	110.58	110.58+peptide	OK	41.2045	41.7183	0.017879	0.0243236	0.9674	0.994855	no
gene:SpnNT_00046	dut	Chromosome:34996-35957	ΔORF2	110.58+peptide	OK	43.4528	41.7183	-0.0587667	-0.0802698	0.89075	0.994748	no
gene:SpnNT_00046	dut	Chromosome:34996-35957	110.58	ΔORF2+peptide	OK	41.2045	45.6253	0.14703	0.193073	0.73255	0.994748	no
gene:SpnNT_00046	dut	Chromosome:34996-35957	ΔORF2	ΔORF2+peptide	OK	43.4528	45.6253	0.0703847	0.0927707	0.8719	0.994748	no
gene:SpnNT_00046	dut	Chromosome:34996-35957	110.58+peptide	ΔORF2+peptide	OK	41.7183	45.6253	0.129151	0.166058	0.77185	0.994748	no
gene:SpnNT_00047	NA	Chromosome:34996-35957	110.58	ΔORF2	OK	24.7678	23.3355	-0.0859381	-0.110384	0.8458	0.994748	no
gene:SpnNT_00047	NA	Chromosome:34996-35957	110.58	110.58+peptide	OK	24.7678	24.6763	-0.00533988	-0.00659663	0.99	0.997703	no
gene:SpnNT_00047	NA	Chromosome:34996-35957	ΔORF2	110.58+peptide	OK	23.3355	24.6763	0.0805983	0.0981278	0.86265	0.994748	no
gene:SpnNT_00047	NA	Chromosome:34996-35957	110.58	ΔORF2+peptide	OK	24.7678	36.3575	0.553789	0.742608	0.20765	0.693134	no
gene:SpnNT_00047	NA	Chromosome:34996-35957	ΔORF2	ΔORF2+peptide	OK	23.3355	36.3575	0.639727	0.843291	0.15255	0.595231	no
gene:SpnNT_00047	NA	Chromosome:34996-35957	110.58+peptide	ΔORF2+peptide	OK	24.6763	36.3575	0.559129	0.707459	0.2345	0.728242	no
gene:SpnNT_00048	NA	Chromosome:36108-37332	110.58	ΔORF2	OK	36.9888	33.3542	-0.149223	-0.307174	0.58715	0.973049	no
gene:SpnNT_00048	NA	Chromosome:36108-37332	110.58	110.58+peptide	OK	36.9888	41.5905	0.169165	0.35079	0.54745	0.962356	no
gene:SpnNT_00048	NA	Chromosome:36108-37332	ΔORF2	110.58+peptide	OK	33.3542	41.5905	0.318388	0.657279	0.25385	0.752773	no
gene:SpnNT_00048	NA	Chromosome:36108-37332	110.58	ΔORF2+peptide	OK	36.9888	44.1385	0.254947	0.532463	0.35365	0.856246	no
gene:SpnNT_00048	NA	Chromosome:36108-37332	ΔORF2	ΔORF2+peptide	OK	33.3542	44.1385	0.40417	0.840296	0.1338	0.556521	no
gene:SpnNT_00048	NA	Chromosome:36108-37332	110.58+peptide	ΔORF2+peptide	OK	41.5905	44.1385	0.0857822	0.179688	0.7569	0.994748	no
gene:SpnNT_00049	mtcA1	Chromosome:37404-37902	110.58	ΔORF2	OK	402.16	439.302	0.127444	0.27928	0.6269	0.980887	no
gene:SpnNT_00049	mtcA1	Chromosome:37404-37902	110.58	110.58+peptide	OK	402.16	181.215	-1.15007	-2.43141	5.00E-05	0.0013612	yes
gene:SpnNT_00049	mtcA1	Chromosome:37404-37902	ΔORF2	110.58+peptide	OK	439.302	181.215	-1.27751	-2.71446	5.00E-05	0.0013612	yes
gene:SpnNT_00049	mtcA1	Chromosome:37404-37902	110.58	ΔORF2+peptide	OK	402.16	145.759	-1.46418	-3.07593	5.00E-05	0.0013612	yes
gene:SpnNT_00049	mtcA1	Chromosome:37404-37902	ΔORF2	ΔORF2+peptide	OK	439.302	145.759	-1.59162	-3.3603	5.00E-05	0.0013612	yes
gene:SpnNT_00049	mtcA1	Chromosome:37404-37902	110.58+peptide	ΔORF2+peptide	OK	181.215	145.759	-0.314112	-0.641384	0.25135	0.749233	no
gene:SpnNT_00050	NA	Chromosome:37926-38742	110.58	ΔORF2	OK	208.649	276.308	0.405201	0.914121	0.10635	0.496269	no
gene:SpnNT_00050	NA	Chromosome:37926-38742	110.58	110.58+peptide	OK	208.649	86.799	-1.26533	-2.72967	5.00E-05	0.0013612	yes
gene:SpnNT_00050	NA	Chromosome:37926-38742	ΔORF2	110.58+peptide	OK	276.308	86.799	-1.67053	-3.66085	5.00E-05	0.0013612	yes
gene:SpnNT_00050	NA	Chromosome:37926-38742	110.58	ΔORF2+peptide	OK	208.649	119.6	-0.802868	-1.76943	0.0019	0.0281715	yes
gene:SpnNT_00050	NA	Chromosome:37926-38742	ΔORF2	ΔORF2+peptide	OK	276.308	119.6	-1.20807	-2.70647	5.00E-05	0.0013612	yes
gene:SpnNT_00050	NA	Chromosome:37926-38742	110.58+peptide	ΔORF2+peptide	OK	86.799	119.6	0.46246	0.991327	0.08565	0.439538	no
gene:SpnNT_00051	prs	Chromosome:38886-39855	110.58	ΔORF2	OK	163.929	175.744	0.100399	0.23076	0.68155	0.986574	no
gene:SpnNT_00051	prs	Chromosome:38886-39855	110.58	110.58+peptide	OK	163.929	265.612	0.696248	1.60218	0.00595	0.068608	no
gene:SpnNT_00051	prs	Chromosome:38886-39855	ΔORF2	110.58+peptide	OK	175.744	265.612	0.595849	1.37299	0.0166	0.144638	no
gene:SpnNT_00051	prs	Chromosome:38886-39855	110.58	ΔORF2+peptide	OK	163.929	295.962	0.852338	1.95902	6.00E-04	0.0111203	yes
gene:SpnNT_00051	prs	Chromosome:38886-39855	ΔORF2	ΔORF2+peptide	OK	175.744	295.962	0.751939	1.73058	0.0025	0.0347143	yes
gene:SpnNT_00051	prs	Chromosome:38886-39855	110.58+peptide	ΔORF2+peptide	OK	265.612	295.962	0.15609	0.359667	0.51975	0.954494	no
gene:SpnNT_00052	NA	Chromosome:39972-40326	110.58	ΔORF2	OK	125.007	139.003	0.153097	0.294799	0.6022	0.976761	no
gene:SpnNT_00052	NA	Chromosome:39972-40326	110.58	110.58+peptide	OK	125.007	138.721	0.150177	0.29038	0.606	0.976937	no
gene:SpnNT_00052	NA	Chromosome:39972-40326	ΔORF2	110.58+peptide	OK	139.003	138.721	-0.00292037	-0.0056685	0.99215	0.99849	no
gene:SpnNT_00052	NA	Chromosome:39972-40326	110.58	ΔORF2+peptide	OK	125.007	137.228	0.134559	0.2563	0.6541	0.982798	no
gene:SpnNT_00052	NA	Chromosome:39972-40326	ΔORF2	ΔORF2+peptide	OK	139.003	137.228	-0.0185378	-0.0354413	0.9504	0.994855	no
gene:SpnNT_00052	NA	Chromosome:39972-40326	110.58+peptide	ΔORF2+peptide	OK	138.721	137.228	-0.0156174	-0.0299805	0.96045	0.994855	no
gene:SpnNT_00053	NA	Chromosome:40649-40871	110.58	ΔORF2	OK	106.404	102.357	-0.0559517	-0.0869508	0.87605	0.994748	no
gene:SpnNT_00053	NA	Chromosome:40649-40871	110.58	110.58+peptide	OK	106.404	121.836	0.195386	0.299138	0.5993	0.97629	no

gene:SpnNT_00053	NA	Chromosome:40649-40871	ΔORF2	110.58+peptide	OK	102.357	121.836	0.251338	0.38614	0.4928	0.941402	no
gene:SpnNT_00053	NA	Chromosome:40649-40871	110.58	ΔORF2+peptide	OK	106.404	113.126	0.0883729	0.134253	0.81165	0.994748	no
gene:SpnNT_00053	NA	Chromosome:40649-40871	ΔORF2	ΔORF2+peptide	OK	102.357	113.126	0.144325	0.220004	0.7002	0.990367	no
gene:SpnNT_00053	NA	Chromosome:40649-40871	110.58+peptide	ΔORF2+peptide	OK	121.836	113.126	-0.107013	-0.1608	0.77625	0.994748	no
gene:SpnNT_00054	polA	Chromosome:41337-44007	110.58	ΔORF2	OK	195.576	194.448	-0.00834607	-0.0188588	0.9729	0.99536	no
gene:SpnNT_00054	polA	Chromosome:41337-44007	110.58	110.58+peptide	OK	195.576	190.799	-0.0356718	-0.08065	0.8884	0.994748	no
gene:SpnNT_00054	polA	Chromosome:41337-44007	ΔORF2	110.58+peptide	OK	194.448	190.799	-0.0273258	-0.0617924	0.9152	0.994748	no
gene:SpnNT_00054	polA	Chromosome:41337-44007	110.58	ΔORF2+peptide	OK	195.576	191.289	-0.0319748	-0.0723194	0.8953	0.994748	no
gene:SpnNT_00054	polA	Chromosome:41337-44007	ΔORF2	ΔORF2+peptide	OK	194.448	191.289	-0.0236287	-0.0534529	0.92285	0.994748	no
gene:SpnNT_00054	polA	Chromosome:41337-44007	110.58+peptide	ΔORF2+peptide	OK	190.799	191.289	0.00369702	0.00836813	0.98905	0.997703	no
gene:SpnNT_00055	NA	Chromosome:44091-44529	110.58	ΔORF2	OK	662.449	704.051	0.0878711	0.190624	0.73225	0.994748	no
gene:SpnNT_00055	NA	Chromosome:44091-44529	110.58	110.58+peptide	OK	662.449	1018.7	0.620842	1.30362	0.0227	0.184211	no
gene:SpnNT_00055	NA	Chromosome:44091-44529	ΔORF2	110.58+peptide	OK	704.051	1018.7	0.532971	1.11797	0.05135	0.31995	no
gene:SpnNT_00055	NA	Chromosome:44091-44529	110.58	ΔORF2+peptide	OK	662.449	846.683	0.354014	0.77028	0.1699	0.628008	no
gene:SpnNT_00055	NA	Chromosome:44091-44529	ΔORF2	ΔORF2+peptide	OK	704.051	846.683	0.266143	0.578455	0.30795	0.813881	no
gene:SpnNT_00055	NA	Chromosome:44091-44529	110.58+peptide	ΔORF2+peptide	OK	1018.7	846.683	-0.266828	-0.561268	0.32305	0.82836	no
gene:SpnNT_00056	NA	Chromosome:44756-45767	110.58	ΔORF2	OK	15.3213	14.4204	-0.0874234	-0.156189	0.784	0.994748	no
gene:SpnNT_00056	NA	Chromosome:44756-45767	110.58	110.58+peptide	OK	15.3213	14.7974	-0.050192	-0.0894289	0.8769	0.994748	no
gene:SpnNT_00056	NA	Chromosome:44756-45767	ΔORF2	110.58+peptide	OK	14.4204	14.7974	0.0372314	0.0661495	0.9095	0.994748	no
gene:SpnNT_00056	NA	Chromosome:44756-45767	110.58	ΔORF2+peptide	OK	15.3213	13.3108	-0.202941	-0.361429	0.5314	0.957488	no
gene:SpnNT_00056	NA	Chromosome:44756-45767	ΔORF2	ΔORF2+peptide	OK	14.4204	13.3108	-0.115518	-0.205153	0.72395	0.994748	no
gene:SpnNT_00056	NA	Chromosome:44756-45767	110.58+peptide	ΔORF2+peptide	OK	14.7974	13.3108	-0.152749	-0.270547	0.6357	0.980887	no
gene:SpnNT_00057	patA	Chromosome:45915-48841	110.58	ΔORF2	OK	125.407	126.953	0.0176761	0.0264561	0.96475	0.994855	no
gene:SpnNT_00057	patA	Chromosome:45915-48841	110.58	110.58+peptide	OK	125.407	98.9584	-0.341722	-0.502679	0.38575	0.881745	no
gene:SpnNT_00057	patA	Chromosome:45915-48841	ΔORF2	110.58+peptide	OK	126.953	98.9584	-0.359398	-0.530548	0.35185	0.854885	no
gene:SpnNT_00057	patA	Chromosome:45915-48841	110.58	ΔORF2+peptide	OK	125.407	114.632	-0.12961	-0.195428	0.7398	0.994748	no
gene:SpnNT_00057	patA	Chromosome:45915-48841	ΔORF2	ΔORF2+peptide	OK	126.953	114.632	-0.147286	-0.222905	0.6967	0.989512	no
gene:SpnNT_00057	patA	Chromosome:45915-48841	110.58+peptide	ΔORF2+peptide	OK	98.9584	114.632	0.212112	0.315383	0.57875	0.969538	no
gene:SpnNT_00058	recO	Chromosome:45915-48841	110.58	ΔORF2	OK	98.546	96.738	-0.0267144	-0.0269942	0.9642	0.994855	no
gene:SpnNT_00058	recO	Chromosome:45915-48841	110.58	110.58+peptide	OK	98.546	83.1357	-0.245329	-0.250105	0.66405	0.982966	no
gene:SpnNT_00058	recO	Chromosome:45915-48841	ΔORF2	110.58+peptide	OK	96.738	83.1357	-0.218614	-0.220196	0.70005	0.990367	no
gene:SpnNT_00058	recO	Chromosome:45915-48841	110.58	ΔORF2+peptide	OK	98.546	86.1543	-0.193874	-0.188043	0.7457	0.994748	no
gene:SpnNT_00058	recO	Chromosome:45915-48841	ΔORF2	ΔORF2+peptide	OK	96.738	86.1543	-0.16716	-0.160368	0.7783	0.994748	no
gene:SpnNT_00058	recO	Chromosome:45915-48841	110.58+peptide	ΔORF2+peptide	OK	83.1357	86.1543	0.0514548	0.0497599	0.9318	0.994855	no
gene:SpnNT_00059	plsX	Chromosome:45915-48841	110.58	ΔORF2	OK	132.208	118.928	-0.15272	-0.204658	0.72485	0.994748	no
gene:SpnNT_00059	plsX	Chromosome:45915-48841	110.58	110.58+peptide	OK	132.208	121.398	-0.123056	-0.172957	0.7656	0.994748	no
gene:SpnNT_00059	plsX	Chromosome:45915-48841	ΔORF2	110.58+peptide	OK	118.928	121.398	0.0296632	0.0406043	0.94365	0.994855	no
gene:SpnNT_00059	plsX	Chromosome:45915-48841	110.58	ΔORF2+peptide	OK	132.208	119.928	-0.140632	-0.194172	0.73775	0.994748	no
gene:SpnNT_00059	plsX	Chromosome:45915-48841	ΔORF2	ΔORF2+peptide	OK	118.928	119.928	0.0120874	0.0162685	0.9783	0.995765	no
gene:SpnNT_00059	plsX	Chromosome:45915-48841	110.58+peptide	ΔORF2+peptide	OK	121.398	119.928	-0.0175758	-0.0248209	0.96675	0.994855	no
gene:SpnNT_00060	acpP	Chromosome:48846-49080	110.58	ΔORF2	OK	89.2783	87.4373	-0.0300609	-0.0471855	0.9345	0.994855	no
gene:SpnNT_00060	acpP	Chromosome:48846-49080	110.58	110.58+peptide	OK	89.2783	85.2518	-0.0665801	-0.104955	0.8587	0.994748	no
gene:SpnNT_00060	acpP	Chromosome:48846-49080	ΔORF2	110.58+peptide	OK	87.4373	85.2518	-0.0365192	-0.0574923	0.9222	0.994748	no
gene:SpnNT_00060	acpP	Chromosome:48846-49080	110.58	ΔORF2+peptide	OK	89.2783	112.298	0.330956	0.530727	0.36385	0.863839	no
gene:SpnNT_00060	acpP	Chromosome:48846-49080	ΔORF2	ΔORF2+peptide	OK	87.4373	112.298	0.361017	0.578147	0.3231	0.82836	no

gene:SpnNT_00060	acpP	Chromosome:48846-49080	110.58+peptide	ΔORF2+peptide	OK	85.2518	112.298	0.397536	0.639464	0.27875	0.781158	no
gene:SpnNT_00061	NA	Chromosome:49122-49416	110.58	ΔORF2	OK	13.9549	9.21006	-0.599491	-0.687642	0.22015	0.708363	no
gene:SpnNT_00061	NA	Chromosome:49122-49416	110.58	110.58+peptide	OK	13.9549	10.5403	-0.40486	-0.464498	0.40075	0.893669	no
gene:SpnNT_00061	NA	Chromosome:49122-49416	ΔORF2	110.58+peptide	OK	9.21006	10.5403	0.194631	0.209515	0.70095	0.990367	no
gene:SpnNT_00061	NA	Chromosome:49122-49416	110.58	ΔORF2+peptide	OK	13.9549	9.57157	-0.543946	-0.624493	0.27375	0.775958	no
gene:SpnNT_00061	NA	Chromosome:49122-49416	ΔORF2	ΔORF2+peptide	OK	9.21006	9.57157	0.0555451	0.0598281	0.90925	0.994748	no
gene:SpnNT_00061	NA	Chromosome:49122-49416	110.58+peptide	ΔORF2+peptide	OK	10.5403	9.57157	-0.139086	-0.149841	0.7902	0.994748	no
gene:SpnNT_00062	NA	Chromosome:49618-49849	110.58	ΔORF2	OK	126.266	124.514	-0.0201567	-0.0314723	0.9554	0.994855	no
gene:SpnNT_00062	NA	Chromosome:49618-49849	110.58	110.58+peptide	OK	126.266	108.99	-0.212266	-0.333264	0.54555	0.961816	no
gene:SpnNT_00062	NA	Chromosome:49618-49849	ΔORF2	110.58+peptide	OK	124.514	108.99	-0.19211	-0.294017	0.59445	0.975539	no
gene:SpnNT_00062	NA	Chromosome:49618-49849	110.58	ΔORF2+peptide	OK	126.266	79.1637	-0.673554	-0.96421	0.07585	0.406744	no
gene:SpnNT_00062	NA	Chromosome:49618-49849	ΔORF2	ΔORF2+peptide	OK	124.514	79.1637	-0.653397	-0.915636	0.0856	0.439454	no
gene:SpnNT_00062	NA	Chromosome:49618-49849	110.58+peptide	ΔORF2+peptide	OK	108.99	79.1637	-0.461287	-0.649302	0.2182	0.706084	no
gene:SpnNT_00063	NA	Chromosome:49851-49977	110.58	ΔORF2	OK	0	12.5222	Inf	NA	0.12225	0.529253	no
gene:SpnNT_00063	NA	Chromosome:49851-49977	110.58	110.58+peptide	NOTEST	0	3.33784	Inf	0	1	1	no
gene:SpnNT_00063	NA	Chromosome:49851-49977	ΔORF2	110.58+peptide	OK	12.5222	3.33784	-1.9075	-4.20286	0.41105	0.898655	no
gene:SpnNT_00063	NA	Chromosome:49851-49977	110.58	ΔORF2+peptide	NOTEST	0	0	0	0	1	1	no
gene:SpnNT_00063	NA	Chromosome:49851-49977	ΔORF2	ΔORF2+peptide	OK	12.5222	0	#NAME?	NA	0.12565	0.536537	no
gene:SpnNT_00063	NA	Chromosome:49851-49977	110.58+peptide	ΔORF2+peptide	NOTEST	3.33784	0	#NAME?	0	1	1	no
gene:SpnNT_00064	NA	Chromosome:50602-51496	110.58	ΔORF2	OK	151.191	244.451	0.693172	1.50359	0.008	0.0859754	no
gene:SpnNT_00064	NA	Chromosome:50602-51496	110.58	110.58+peptide	OK	151.191	183.29	0.277754	0.614723	0.29265	0.79754	no
gene:SpnNT_00064	NA	Chromosome:50602-51496	ΔORF2	110.58+peptide	OK	244.451	183.29	-0.415418	-0.884641	0.12635	0.538475	no
gene:SpnNT_00064	NA	Chromosome:50602-51496	110.58	ΔORF2+peptide	OK	151.191	233.525	0.627201	1.42872	0.0128	0.120144	no
gene:SpnNT_00064	NA	Chromosome:50602-51496	ΔORF2	ΔORF2+peptide	OK	244.451	233.525	-0.0659708	-0.144278	0.8	0.994748	no
gene:SpnNT_00064	NA	Chromosome:50602-51496	110.58+peptide	ΔORF2+peptide	OK	183.29	233.525	0.349447	0.780021	0.1764	0.640664	no
gene:SpnNT_00065	NA	Chromosome:52038-52335	110.58	ΔORF2	OK	23.9212	25.5578	0.0954737	0.131946	0.8152	0.994748	no
gene:SpnNT_00065	NA	Chromosome:52038-52335	110.58	110.58+peptide	OK	23.9212	52.2429	1.12694	1.56517	0.0068	0.0755543	no
gene:SpnNT_00065	NA	Chromosome:52038-52335	ΔORF2	110.58+peptide	OK	25.5578	52.2429	1.03147	1.47175	0.011	0.107477	no
gene:SpnNT_00065	NA	Chromosome:52038-52335	110.58	ΔORF2+peptide	OK	23.9212	44.2141	0.886217	1.27161	0.027	0.207919	no
gene:SpnNT_00065	NA	Chromosome:52038-52335	ΔORF2	ΔORF2+peptide	OK	25.5578	44.2141	0.790743	1.16783	0.04315	0.284816	no
gene:SpnNT_00065	NA	Chromosome:52038-52335	110.58+peptide	ΔORF2+peptide	OK	52.2429	44.2141	-0.240727	-0.357537	0.53365	0.958867	no
gene:SpnNT_00066	NA	Chromosome:52597-55026	110.58	ΔORF2	OK	0.537084	1.51208	1.49332	0.860384	0.16835	0.624742	no
gene:SpnNT_00066	NA	Chromosome:52597-55026	110.58	110.58+peptide	OK	0.537084	1.50279	1.48443	0.874137	0.26115	0.761443	no
gene:SpnNT_00066	NA	Chromosome:52597-55026	ΔORF2	110.58+peptide	OK	1.51208	1.50279	-0.00888808	-0.00611225	0.98415	0.996246	no
gene:SpnNT_00066	NA	Chromosome:52597-55026	110.58	ΔORF2+peptide	OK	0.537084	1.60988	1.58373	0.903778	0.23195	0.724662	no
gene:SpnNT_00066	NA	Chromosome:52597-55026	ΔORF2	ΔORF2+peptide	OK	1.51208	1.60988	0.0904137	0.0595979	0.9265	0.994748	no
gene:SpnNT_00066	NA	Chromosome:52597-55026	110.58+peptide	ΔORF2+peptide	OK	1.50279	1.60988	0.0993017	0.067367	0.9346	0.994855	no
gene:SpnNT_00067	NA	Chromosome:52597-55026	110.58	ΔORF2	OK	1.89743	2.6191	0.465021	0.271669	0.63695	0.980887	no
gene:SpnNT_00067	NA	Chromosome:52597-55026	110.58	110.58+peptide	OK	1.89743	3.25963	0.78066	0.496852	0.36145	0.862354	no
gene:SpnNT_00067	NA	Chromosome:52597-55026	ΔORF2	110.58+peptide	OK	2.6191	3.25963	0.315639	0.182692	0.74635	0.994748	no
gene:SpnNT_00067	NA	Chromosome:52597-55026	110.58	ΔORF2+peptide	OK	1.89743	3.23264	0.768665	0.49766	0.401	0.893669	no
gene:SpnNT_00067	NA	Chromosome:52597-55026	ΔORF2	ΔORF2+peptide	OK	2.6191	3.23264	0.303644	0.178246	0.7713	0.994748	no
gene:SpnNT_00067	NA	Chromosome:52597-55026	110.58+peptide	ΔORF2+peptide	OK	3.25963	3.23264	-0.0119955	-0.00767831	0.97885	0.995765	no
gene:SpnNT_00068	kpsT	Chromosome:52597-55026	110.58	ΔORF2	OK	1.77897	3.52285	0.9857	0.79964	0.18325	0.653781	no
gene:SpnNT_00068	kpsT	Chromosome:52597-55026	110.58	110.58+peptide	OK	1.77897	3.18691	0.841115	0.63852	0.2526	0.750933	no

gene:SpnNT_00068	kpsT	Chromosome:52597-55026	ΔORF2	110.58+peptide	OK	3.52285	3.18691	-0.144585	-0.108268	0.8436	0.994748	no
gene:SpnNT_00068	kpsT	Chromosome:52597-55026	110.58	ΔORF2+peptide	OK	1.77897	4.46196	1.32663	1.11693	0.06545	0.372112	no
gene:SpnNT_00068	kpsT	Chromosome:52597-55026	ΔORF2	ΔORF2+peptide	OK	3.52285	4.46196	0.340933	0.282267	0.629	0.980887	no
gene:SpnNT_00068	kpsT	Chromosome:52597-55026	110.58+peptide	ΔORF2+peptide	OK	3.18691	4.46196	0.485519	0.375186	0.4914	0.940662	no
gene:SpnNT_00069	NA	Chromosome:52597-55026	110.58	ΔORF2	OK	0.18762	1.01354	2.43353	0.768465	0.51485	0.951798	no
gene:SpnNT_00069	NA	Chromosome:52597-55026	110.58	110.58+peptide	NOTEST	0.18762	0.729744	1.95958	0	1	1	no
gene:SpnNT_00069	NA	Chromosome:52597-55026	ΔORF2	110.58+peptide	OK	1.01354	0.729744	-0.473948	-0.184662	0.7152	0.992053	no
gene:SpnNT_00069	NA	Chromosome:52597-55026	110.58	ΔORF2+peptide	OK	0.18762	1.27539	2.76505	0.820652	0.3076	0.813712	no
gene:SpnNT_00069	NA	Chromosome:52597-55026	ΔORF2	ΔORF2+peptide	OK	1.01354	1.27539	0.331525	0.1139	0.76145	0.994748	no
gene:SpnNT_00069	NA	Chromosome:52597-55026	110.58+peptide	ΔORF2+peptide	OK	0.729744	1.27539	0.805472	0.286367	0.64925	0.981391	no
gene:SpnNT_00070	lagD_1	Chromosome:55405-57559	110.58	ΔORF2	OK	6.05482	5.60337	-0.111789	-0.198548	0.7286	0.994748	no
gene:SpnNT_00070	lagD_1	Chromosome:55405-57559	110.58	110.58+peptide	OK	6.05482	3.12785	-0.952912	-1.55332	0.0066	0.0739583	no
gene:SpnNT_00070	lagD_1	Chromosome:55405-57559	ΔORF2	110.58+peptide	OK	5.60337	3.12785	-0.841123	-1.37375	0.0173	0.148957	no
gene:SpnNT_00070	lagD_1	Chromosome:55405-57559	110.58	ΔORF2+peptide	OK	6.05482	2.95043	-1.03716	-1.70074	0.0037	0.0473672	yes
gene:SpnNT_00070	lagD_1	Chromosome:55405-57559	ΔORF2	ΔORF2+peptide	OK	5.60337	2.95043	-0.925372	-1.52041	0.0098	0.0986919	no
gene:SpnNT_00070	lagD_1	Chromosome:55405-57559	110.58+peptide	ΔORF2+peptide	OK	3.12785	2.95043	-0.0842484	-0.128511	0.82035	0.994748	no
gene:SpnNT_00071	lcnD_1	Chromosome:57571-58921	110.58	ΔORF2	OK	6.91294	6.31085	-0.131466	-0.220015	0.69935	0.990367	no
gene:SpnNT_00071	lcnD_1	Chromosome:57571-58921	110.58	110.58+peptide	OK	6.91294	3.72875	-0.890606	-1.40564	0.01365	0.125607	no
gene:SpnNT_00071	lcnD_1	Chromosome:57571-58921	ΔORF2	110.58+peptide	OK	6.31085	3.72875	-0.75914	-1.20563	0.0379	0.261474	no
gene:SpnNT_00071	lcnD_1	Chromosome:57571-58921	110.58	ΔORF2+peptide	OK	6.91294	3.14893	-1.13444	-1.73144	0.00275	0.037317	yes
gene:SpnNT_00071	lcnD_1	Chromosome:57571-58921	ΔORF2	ΔORF2+peptide	OK	6.31085	3.14893	-1.00297	-1.53973	0.0099	0.09947	no
gene:SpnNT_00071	lcnD_1	Chromosome:57571-58921	110.58+peptide	ΔORF2+peptide	OK	3.72875	3.14893	-0.243831	-0.356152	0.5385	0.960342	no
gene:SpnNT_00072	purC	Chromosome:59090-59798	110.58	ΔORF2	OK	10.348	12.6205	0.286412	0.463497	0.41425	0.900412	no
gene:SpnNT_00072	purC	Chromosome:59090-59798	110.58	110.58+peptide	OK	10.348	1.11288	-3.21699	-3.72326	5.00E-05	0.0013612	yes
gene:SpnNT_00072	purC	Chromosome:59090-59798	ΔORF2	110.58+peptide	OK	12.6205	1.11288	-3.5034	-4.08696	5.00E-05	0.0013612	yes
gene:SpnNT_00072	purC	Chromosome:59090-59798	110.58	ΔORF2+peptide	OK	10.348	0.860569	-3.58792	-4.10063	5.00E-05	0.0013612	yes
gene:SpnNT_00072	purC	Chromosome:59090-59798	ΔORF2	ΔORF2+peptide	OK	12.6205	0.860569	-3.87433	-4.46227	5.00E-05	0.0013612	yes
gene:SpnNT_00072	purC	Chromosome:59090-59798	110.58+peptide	ΔORF2+peptide	OK	1.11288	0.860569	-0.370931	-0.350727	0.5769	0.969538	no
gene:SpnNT_00073	purL	Chromosome:59999-63725	110.58	ΔORF2	OK	17.7458	18.2885	0.0434605	0.0917493	0.87475	0.994748	no
gene:SpnNT_00073	purL	Chromosome:59999-63725	110.58	110.58+peptide	OK	17.7458	2.77224	-2.67836	-5.03976	5.00E-05	0.0013612	yes
gene:SpnNT_00073	purL	Chromosome:59999-63725	ΔORF2	110.58+peptide	OK	18.2885	2.77224	-2.72182	-5.07469	5.00E-05	0.0013612	yes
gene:SpnNT_00073	purL	Chromosome:59999-63725	110.58	ΔORF2+peptide	OK	17.7458	1.84753	-3.26381	-6.04798	5.00E-05	0.0013612	yes
gene:SpnNT_00073	purL	Chromosome:59999-63725	ΔORF2	ΔORF2+peptide	OK	18.2885	1.84753	-3.30727	-6.07412	5.00E-05	0.0013612	yes
gene:SpnNT_00073	purL	Chromosome:59999-63725	110.58+peptide	ΔORF2+peptide	OK	2.77224	1.84753	-0.585455	-0.983273	0.085	0.438777	no
gene:SpnNT_00074	purF	Chromosome:63817-65260	110.58	ΔORF2	OK	16.8536	16.4773	-0.0325737	-0.0603454	0.9153	0.994748	no
gene:SpnNT_00074	purF	Chromosome:63817-65260	110.58	110.58+peptide	OK	16.8536	3.35618	-2.32816	-3.86726	5.00E-05	0.0013612	yes
gene:SpnNT_00074	purF	Chromosome:63817-65260	ΔORF2	110.58+peptide	OK	16.4773	3.35618	-2.29559	-3.7666	5.00E-05	0.0013612	yes
gene:SpnNT_00074	purF	Chromosome:63817-65260	110.58	ΔORF2+peptide	OK	16.8536	2.03969	-3.04663	-4.82961	5.00E-05	0.0013612	yes
gene:SpnNT_00074	purF	Chromosome:63817-65260	ΔORF2	ΔORF2+peptide	OK	16.4773	2.03969	-3.01406	-4.72476	5.00E-05	0.0013612	yes
gene:SpnNT_00074	purF	Chromosome:63817-65260	110.58+peptide	ΔORF2+peptide	OK	3.35618	2.03969	-0.718469	-1.03918	0.0727	0.397264	no
gene:SpnNT_00075	purM	Chromosome:65296-66861	110.58	ΔORF2	OK	20.8546	19.9093	-0.0669232	-0.105821	0.854	0.994748	no
gene:SpnNT_00075	purM	Chromosome:65296-66861	110.58	110.58+peptide	OK	20.8546	3.78042	-2.46375	-3.42677	5.00E-05	0.0013612	yes
gene:SpnNT_00075	purM	Chromosome:65296-66861	ΔORF2	110.58+peptide	OK	19.9093	3.78042	-2.39683	-3.31905	5.00E-05	0.0013612	yes
gene:SpnNT_00075	purM	Chromosome:65296-66861	110.58	ΔORF2+peptide	OK	20.8546	2.80511	-2.89424	-3.82529	5.00E-05	0.0013612	yes
gene:SpnNT_00075	purM	Chromosome:65296-66861	ΔORF2	ΔORF2+peptide	OK	19.9093	2.80511	-2.82731	-3.72202	5.00E-05	0.0013612	yes

gene:SpnNT_00075	purM	Chromosome:65296-66861	110.58+peptide	ΔORF2+peptide	OK	3.78042	2.80511	-0.430489	-0.516757	0.3668	0.864634	no
gene:SpnNT_00076	purN	Chromosome:65296-66861	110.58	ΔORF2	OK	19.9024	18.0268	-0.142802	-0.13818	0.80415	0.994748	no
gene:SpnNT_00076	purN	Chromosome:65296-66861	110.58	110.58+peptide	OK	19.9024	4.39661	-2.17848	-1.97865	0.0017	0.025969	yes
gene:SpnNT_00076	purN	Chromosome:65296-66861	ΔORF2	110.58+peptide	OK	18.0268	4.39661	-2.03568	-1.84629	0.00285	0.0382781	yes
gene:SpnNT_00076	purN	Chromosome:65296-66861	110.58	ΔORF2+peptide	OK	19.9024	1.73971	-3.51602	-2.5237	0.0364	0.254335	no
gene:SpnNT_00076	purN	Chromosome:65296-66861	ΔORF2	ΔORF2+peptide	OK	18.0268	1.73971	-3.37322	-2.41902	0.03935	0.268513	no
gene:SpnNT_00076	purN	Chromosome:65296-66861	110.58+peptide	ΔORF2+peptide	OK	4.39661	1.73971	-1.33754	-0.925492	0.21055	0.696806	no
gene:SpnNT_00077	NA	Chromosome:66944-67454	110.58	ΔORF2	OK	2.06465	3.57093	0.790404	0.835865	0.16295	0.61656	no
gene:SpnNT_00077	NA	Chromosome:66944-67454	110.58	110.58+peptide	OK	2.06465	1.32597	-0.638851	-0.591925	0.32015	0.825861	no
gene:SpnNT_00077	NA	Chromosome:66944-67454	ΔORF2	110.58+peptide	OK	3.57093	1.32597	-1.42925	-1.4209	0.0245	0.194606	no
gene:SpnNT_00077	NA	Chromosome:66944-67454	110.58	ΔORF2+peptide	OK	2.06465	0.656258	-1.65356	-1.37364	0.05515	0.334417	no
gene:SpnNT_00077	NA	Chromosome:66944-67454	ΔORF2	ΔORF2+peptide	OK	3.57093	0.656258	-2.44396	-2.14677	0.0135	0.125016	no
gene:SpnNT_00077	NA	Chromosome:66944-67454	110.58+peptide	ΔORF2+peptide	OK	1.32597	0.656258	-1.01471	-0.810678	0.19125	0.671187	no
gene:SpnNT_00078	purH	Chromosome:67478-69026	110.58	ΔORF2	OK	44.911	45.9005	0.0314403	0.0665089	0.90485	0.994748	no
gene:SpnNT_00078	purH	Chromosome:67478-69026	110.58	110.58+peptide	OK	44.911	10.4894	-2.09814	-4.05106	5.00E-05	0.0013612	yes
gene:SpnNT_00078	purH	Chromosome:67478-69026	ΔORF2	110.58+peptide	OK	45.9005	10.4894	-2.12958	-4.1068	5.00E-05	0.0013612	yes
gene:SpnNT_00078	purH	Chromosome:67478-69026	110.58	ΔORF2+peptide	OK	44.911	7.86369	-2.51379	-4.80716	5.00E-05	0.0013612	yes
gene:SpnNT_00078	purH	Chromosome:67478-69026	ΔORF2	ΔORF2+peptide	OK	45.9005	7.86369	-2.54523	-4.86151	5.00E-05	0.0013612	yes
gene:SpnNT_00078	purH	Chromosome:67478-69026	110.58+peptide	ΔORF2+peptide	OK	10.4894	7.86369	-0.415652	-0.736067	0.20355	0.688187	no
gene:SpnNT_00079	purD	Chromosome:69147-70410	110.58	ΔORF2	OK	45.0527	45.9717	0.029132	0.0600383	0.9153	0.994748	no
gene:SpnNT_00079	purD	Chromosome:69147-70410	110.58	110.58+peptide	OK	45.0527	8.84793	-2.3482	-4.40533	5.00E-05	0.0013612	yes
gene:SpnNT_00079	purD	Chromosome:69147-70410	ΔORF2	110.58+peptide	OK	45.9717	8.84793	-2.37734	-4.47172	5.00E-05	0.0013612	yes
gene:SpnNT_00079	purD	Chromosome:69147-70410	110.58	ΔORF2+peptide	OK	45.0527	6.3938	-2.81687	-5.14906	5.00E-05	0.0013612	yes
gene:SpnNT_00079	purD	Chromosome:69147-70410	ΔORF2	ΔORF2+peptide	OK	45.9717	6.3938	-2.846	-5.21531	5.00E-05	0.0013612	yes
gene:SpnNT_00079	purD	Chromosome:69147-70410	110.58+peptide	ΔORF2+peptide	OK	8.84793	6.3938	-0.468666	-0.796208	0.1613	0.614666	no
gene:SpnNT_00080	purE	Chromosome:70812-72379	110.58	ΔORF2	OK	39.2474	36.4253	-0.107655	-0.106725	0.85865	0.994748	no
gene:SpnNT_00080	purE	Chromosome:70812-72379	110.58	110.58+peptide	OK	39.2474	7.45245	-2.39681	-2.23309	5.00E-04	0.0095781	yes
gene:SpnNT_00080	purE	Chromosome:70812-72379	ΔORF2	110.58+peptide	OK	36.4253	7.45245	-2.28916	-2.08249	0.00085	0.0148124	yes
gene:SpnNT_00080	purE	Chromosome:70812-72379	110.58	ΔORF2+peptide	OK	39.2474	5.05896	-2.95568	-2.48936	6.00E-04	0.0111203	yes
gene:SpnNT_00080	purE	Chromosome:70812-72379	ΔORF2	ΔORF2+peptide	OK	36.4253	5.05896	-2.84803	-2.35216	0.00075	0.0133173	yes
gene:SpnNT_00080	purE	Chromosome:70812-72379	110.58+peptide	ΔORF2+peptide	OK	7.45245	5.05896	-0.558872	-0.441749	0.4495	0.920321	no
gene:SpnNT_00081	purK	Chromosome:70812-72379	110.58	ΔORF2	OK	34.6546	34.9025	0.0102831	0.0178337	0.97345	0.99536	no
gene:SpnNT_00081	purK	Chromosome:70812-72379	110.58	110.58+peptide	OK	34.6546	6.52987	-2.40792	-3.77105	5.00E-05	0.0013612	yes
gene:SpnNT_00081	purK	Chromosome:70812-72379	ΔORF2	110.58+peptide	OK	34.9025	6.52987	-2.41821	-3.7545	5.00E-05	0.0013612	yes
gene:SpnNT_00081	purK	Chromosome:70812-72379	110.58	ΔORF2+peptide	OK	34.6546	5.63025	-2.62178	-4.08043	5.00E-05	0.0013612	yes
gene:SpnNT_00081	purK	Chromosome:70812-72379	ΔORF2	ΔORF2+peptide	OK	34.9025	5.63025	-2.63206	-4.06154	5.00E-05	0.0013612	yes
gene:SpnNT_00081	purK	Chromosome:70812-72379	110.58+peptide	ΔORF2+peptide	OK	6.52987	5.63025	-0.213853	-0.303896	0.596	0.976247	no
gene:SpnNT_00082	NA	Chromosome:72388-72616	110.58	ΔORF2	OK	111.81	93.0818	-0.264484	-0.419624	0.471	0.928416	no
gene:SpnNT_00082	NA	Chromosome:72388-72616	110.58	110.58+peptide	OK	111.81	76.9236	-0.539557	-0.82557	0.15735	0.605143	no
gene:SpnNT_00082	NA	Chromosome:72388-72616	ΔORF2	110.58+peptide	OK	93.0818	76.9236	-0.275073	-0.417584	0.4679	0.92732	no
gene:SpnNT_00082	NA	Chromosome:72388-72616	110.58	ΔORF2+peptide	OK	111.81	66.3734	-0.752377	-1.02233	0.05665	0.338806	no
gene:SpnNT_00082	NA	Chromosome:72388-72616	ΔORF2	ΔORF2+peptide	OK	93.0818	66.3734	-0.487893	-0.658837	0.20725	0.692699	no
gene:SpnNT_00082	NA	Chromosome:72388-72616	110.58+peptide	ΔORF2+peptide	OK	76.9236	66.3734	-0.21282	-0.279864	0.5947	0.975539	no
gene:SpnNT_00083	purB	Chromosome:72678-73977	110.58	ΔORF2	OK	141.84	129.114	-0.135622	-0.305998	0.5918	0.975468	no
gene:SpnNT_00083	purB	Chromosome:72678-73977	110.58	110.58+peptide	OK	141.84	90.756	-0.644201	-1.45839	0.01215	0.115782	no

gene:SpnNT_00083	purB	Chromosome:72678-73977	ΔORF2	110.58+peptide	OK	129.114	90.756	-0.508579	-1.14418	0.0453	0.29311	no
gene:SpnNT_00083	purB	Chromosome:72678-73977	110.58	ΔORF2+peptide	OK	141.84	72.3035	-0.97213	-2.18515	0.00015	0.00355289	yes
gene:SpnNT_00083	purB	Chromosome:72678-73977	ΔORF2	ΔORF2+peptide	OK	129.114	72.3035	-0.836508	-1.86874	8.00E-04	0.0140342	yes
gene:SpnNT_00083	purB	Chromosome:72678-73977	110.58+peptide	ΔORF2+peptide	OK	90.756	72.3035	-0.327928	-0.735008	0.2003	0.683196	no
gene:SpnNT_00084	strH_1	Chromosome:74031-75288	110.58	ΔORF2	OK	8.03233	6.82737	-0.234488	-0.395215	0.48775	0.938811	no
gene:SpnNT_00084	strH_1	Chromosome:74031-75288	110.58	110.58+peptide	OK	8.03233	10.6707	0.409766	0.709112	0.2151	0.701601	no
gene:SpnNT_00084	strH_1	Chromosome:74031-75288	ΔORF2	110.58+peptide	OK	6.82737	10.6707	0.644254	1.11432	0.0567	0.338806	no
gene:SpnNT_00084	strH_1	Chromosome:74031-75288	110.58	ΔORF2+peptide	OK	8.03233	9.47685	0.23859	0.409613	0.472	0.928525	no
gene:SpnNT_00084	strH_1	Chromosome:74031-75288	ΔORF2	ΔORF2+peptide	OK	6.82737	9.47685	0.473078	0.81177	0.1596	0.610575	no
gene:SpnNT_00084	strH_1	Chromosome:74031-75288	110.58+peptide	ΔORF2+peptide	OK	10.6707	9.47685	-0.171176	-0.301884	0.60335	0.976761	no
gene:SpnNT_00085	strH_2	Chromosome:75342-77967	110.58	ΔORF2	OK	7.83705	5.57095	-0.492388	-0.899333	0.11865	0.522108	no
gene:SpnNT_00085	strH_2	Chromosome:75342-77967	110.58	110.58+peptide	OK	7.83705	9.67878	0.304516	0.573951	0.32025	0.825861	no
gene:SpnNT_00085	strH_2	Chromosome:75342-77967	ΔORF2	110.58+peptide	OK	5.57095	9.67878	0.796904	1.50339	0.0082	0.08748	no
gene:SpnNT_00085	strH_2	Chromosome:75342-77967	110.58	ΔORF2+peptide	OK	7.83705	8.09833	0.0473149	0.088386	0.87665	0.994748	no
gene:SpnNT_00085	strH_2	Chromosome:75342-77967	ΔORF2	ΔORF2+peptide	OK	5.57095	8.09833	0.539703	1.0091	0.07305	0.398405	no
gene:SpnNT_00085	strH_2	Chromosome:75342-77967	110.58+peptide	ΔORF2+peptide	OK	9.67878	8.09833	-0.257201	-0.497028	0.38295	0.88051	no
gene:SpnNT_00086	frlR	Chromosome:78283-79000	110.58	ΔORF2	OK	324.65	341.095	0.0712877	0.160606	0.7819	0.994748	no
gene:SpnNT_00086	frlR	Chromosome:78283-79000	110.58	110.58+peptide	OK	324.65	376.162	0.212469	0.482595	0.3993	0.892609	no
gene:SpnNT_00086	frlR	Chromosome:78283-79000	ΔORF2	110.58+peptide	OK	341.095	376.162	0.141181	0.323997	0.57015	0.968621	no
gene:SpnNT_00086	frlR	Chromosome:78283-79000	110.58	ΔORF2+peptide	OK	324.65	373.437	0.20198	0.458117	0.4187	0.903797	no
gene:SpnNT_00086	frlR	Chromosome:78283-79000	ΔORF2	ΔORF2+peptide	OK	341.095	373.437	0.130693	0.29949	0.5996	0.976536	no
gene:SpnNT_00086	frlR	Chromosome:78283-79000	110.58+peptide	ΔORF2+peptide	OK	376.162	373.437	-0.0104884	-0.0242385	0.96465	0.994855	no
gene:SpnNT_00087	bga	Chromosome:79347-81608	110.58	ΔORF2	OK	8.88371	5.92316	-0.584794	-0.959683	0.0999	0.479559	no
gene:SpnNT_00087	bga	Chromosome:79347-81608	110.58	110.58+peptide	OK	8.88371	28.9271	1.70319	2.38439	5.00E-05	0.0013612	yes
gene:SpnNT_00087	bga	Chromosome:79347-81608	ΔORF2	110.58+peptide	OK	5.92316	28.9271	2.28798	3.12751	5.00E-05	0.0013612	yes
gene:SpnNT_00087	bga	Chromosome:79347-81608	110.58	ΔORF2+peptide	OK	8.88371	8.85951	-0.0039339	-0.00672219	0.99075	0.998179	no
gene:SpnNT_00087	bga	Chromosome:79347-81608	ΔORF2	ΔORF2+peptide	OK	5.92316	8.85951	0.58086	0.958268	0.0971	0.47086	no
gene:SpnNT_00087	bga	Chromosome:79347-81608	110.58+peptide	ΔORF2+peptide	OK	28.9271	8.85951	-1.70712	-2.39907	5.00E-05	0.0013612	yes
gene:SpnNT_00088	sorB_1	Chromosome:79347-81608	110.58	ΔORF2	OK	10.7965	8.00458	-0.431661	-0.29193	0.60775	0.978275	no
gene:SpnNT_00088	sorB_1	Chromosome:79347-81608	110.58	110.58+peptide	OK	10.7965	72.4926	2.74727	2.02404	0.0032	0.0422439	yes
gene:SpnNT_00088	sorB_1	Chromosome:79347-81608	ΔORF2	110.58+peptide	OK	8.00458	72.4926	3.17894	2.37645	0.00055	0.0103545	yes
gene:SpnNT_00088	sorB_1	Chromosome:79347-81608	110.58	ΔORF2+peptide	OK	10.7965	8.77475	-0.29913	-0.195786	0.72335	0.994748	no
gene:SpnNT_00088	sorB_1	Chromosome:79347-81608	ΔORF2	ΔORF2+peptide	OK	8.00458	8.77475	0.132531	0.0877447	0.8735	0.994748	no
gene:SpnNT_00088	sorB_1	Chromosome:79347-81608	110.58+peptide	ΔORF2+peptide	OK	72.4926	8.77475	-3.0464	-2.18872	0.002	0.0294545	yes
gene:SpnNT_00089	agaC_1	Chromosome:81635-83343	110.58	ΔORF2	OK	6.69293	6.17229	-0.116832	-0.140696	0.80845	0.994748	no
gene:SpnNT_00089	agaC_1	Chromosome:81635-83343	110.58	110.58+peptide	OK	6.69293	28.0236	2.06593	2.31094	5.00E-05	0.0013612	yes
gene:SpnNT_00089	agaC_1	Chromosome:81635-83343	ΔORF2	110.58+peptide	OK	6.17229	28.0236	2.18276	2.52911	5.00E-05	0.0013612	yes
gene:SpnNT_00089	agaC_1	Chromosome:81635-83343	110.58	ΔORF2+peptide	OK	6.69293	7.06728	0.0785184	0.0935656	0.872	0.994748	no
gene:SpnNT_00089	agaC_1	Chromosome:81635-83343	ΔORF2	ΔORF2+peptide	OK	6.17229	7.06728	0.19535	0.242322	0.6734	0.984845	no
gene:SpnNT_00089	agaC_1	Chromosome:81635-83343	110.58+peptide	ΔORF2+peptide	OK	28.0236	7.06728	-1.98741	-2.2804	0.00015	0.00355289	yes
gene:SpnNT_00090	manZ_1	Chromosome:81635-83343	110.58	ΔORF2	OK	8.43764	5.95235	-0.503378	-0.579434	0.315	0.821264	no
gene:SpnNT_00090	manZ_1	Chromosome:81635-83343	110.58	110.58+peptide	OK	8.43764	31.8994	1.91862	2.22249	3.00E-04	0.00631878	yes
gene:SpnNT_00090	manZ_1	Chromosome:81635-83343	ΔORF2	110.58+peptide	OK	5.95235	31.8994	2.422	2.6793	5.00E-05	0.0013612	yes
gene:SpnNT_00090	manZ_1	Chromosome:81635-83343	110.58	ΔORF2+peptide	OK	8.43764	8.18235	-0.0443231	-0.0531391	0.92435	0.994748	no
gene:SpnNT_00090	manZ_1	Chromosome:81635-83343	ΔORF2	ΔORF2+peptide	OK	5.95235	8.18235	0.459055	0.523948	0.3642	0.864245	no

gene:SpnNT_00090	manZ_1	Chromosome:81635-83343	110.58+peptide	ΔORF2+peptide	OK	31.8994	8.18235	-1.96294	-2.25438	5.00E-04	0.0095781	yes
gene:SpnNT_00091	manX_1	Chromosome:83349-83742	110.58	ΔORF2	OK	9.18859	5.25385	-0.806467	-0.975397	0.09575	0.468643	no
gene:SpnNT_00091	manX_1	Chromosome:83349-83742	110.58	110.58+peptide	OK	9.18859	38.8093	2.07849	2.61476	5.00E-05	0.0013612	yes
gene:SpnNT_00091	manX_1	Chromosome:83349-83742	ΔORF2	110.58+peptide	OK	5.25385	38.8093	2.88496	3.37917	5.00E-05	0.0013612	yes
gene:SpnNT_00091	manX_1	Chromosome:83349-83742	110.58	ΔORF2+peptide	OK	9.18859	8.83717	-0.056259	-0.0721929	0.89815	0.994748	no
gene:SpnNT_00091	manX_1	Chromosome:83349-83742	ΔORF2	ΔORF2+peptide	OK	5.25385	8.83717	0.750208	0.893931	0.13545	0.559979	no
gene:SpnNT_00091	manX_1	Chromosome:83349-83742	110.58+peptide	ΔORF2+peptide	OK	38.8093	8.83717	-2.13475	-2.64263	5.00E-05	0.0013612	yes
gene:SpnNT_00092	agaS	Chromosome:84039-85206	110.58	ΔORF2	OK	7.26321	5.46269	-0.410995	-0.669092	0.24105	0.736034	no
gene:SpnNT_00092	agaS	Chromosome:84039-85206	110.58	110.58+peptide	OK	7.26321	30.5708	2.07348	3.3307	5.00E-05	0.0013612	yes
gene:SpnNT_00092	agaS	Chromosome:84039-85206	ΔORF2	110.58+peptide	OK	5.46269	30.5708	2.48447	3.91701	5.00E-05	0.0013612	yes
gene:SpnNT_00092	agaS	Chromosome:84039-85206	110.58	ΔORF2+peptide	OK	7.26321	6.53376	-0.152694	-0.252887	0.649	0.981391	no
gene:SpnNT_00092	agaS	Chromosome:84039-85206	ΔORF2	ΔORF2+peptide	OK	5.46269	6.53376	0.258301	0.419386	0.46275	0.9258	no
gene:SpnNT_00092	agaS	Chromosome:84039-85206	110.58+peptide	ΔORF2+peptide	OK	30.5708	6.53376	-2.22617	-3.56668	5.00E-05	0.0013612	yes
gene:SpnNT_00093	lytA_1	Chromosome:85504-86692	110.58	ΔORF2	OK	6.42341	4.82682	-0.412264	-0.657687	0.25565	0.7547	no
gene:SpnNT_00093	lytA_1	Chromosome:85504-86692	110.58	110.58+peptide	OK	6.42341	22.2158	1.79018	2.82361	5.00E-05	0.0013612	yes
gene:SpnNT_00093	lytA_1	Chromosome:85504-86692	ΔORF2	110.58+peptide	OK	4.82682	22.2158	2.20244	3.41735	5.00E-05	0.0013612	yes
gene:SpnNT_00093	lytA_1	Chromosome:85504-86692	110.58	ΔORF2+peptide	OK	6.42341	6.90278	0.10384	0.170084	0.7668	0.994748	no
gene:SpnNT_00093	lytA_1	Chromosome:85504-86692	ΔORF2	ΔORF2+peptide	OK	4.82682	6.90278	0.516104	0.830545	0.14635	0.583178	no
gene:SpnNT_00093	lytA_1	Chromosome:85504-86692	110.58+peptide	ΔORF2+peptide	OK	22.2158	6.90278	-1.68634	-2.68256	5.00E-05	0.0013612	yes
gene:SpnNT_00094	NA	Chromosome:87136-88870	110.58	ΔORF2	OK	28.9028	32.6408	0.175468	0.37208	0.51295	0.950747	no
gene:SpnNT_00094	NA	Chromosome:87136-88870	110.58	110.58+peptide	OK	28.9028	28.3987	-0.0253864	-0.0529965	0.92585	0.994748	no
gene:SpnNT_00094	NA	Chromosome:87136-88870	ΔORF2	110.58+peptide	OK	32.6408	28.3987	-0.200854	-0.422	0.46205	0.925228	no
gene:SpnNT_00094	NA	Chromosome:87136-88870	110.58	ΔORF2+peptide	OK	28.9028	36.8073	0.348781	0.740922	0.19915	0.681762	no
gene:SpnNT_00094	NA	Chromosome:87136-88870	ΔORF2	ΔORF2+peptide	OK	32.6408	36.8073	0.173314	0.370627	0.5207	0.954832	no
gene:SpnNT_00094	NA	Chromosome:87136-88870	110.58+peptide	ΔORF2+peptide	OK	28.3987	36.8073	0.374167	0.787525	0.17365	0.634017	no
gene:SpnNT_00095	NA	Chromosome:89343-89757	110.58	ΔORF2	OK	102.558	97.462	-0.0735263	-0.140929	0.801	0.994748	no
gene:SpnNT_00095	NA	Chromosome:89343-89757	110.58	110.58+peptide	OK	102.558	105.873	0.0459037	0.0884171	0.8754	0.994748	no
gene:SpnNT_00095	NA	Chromosome:89343-89757	ΔORF2	110.58+peptide	OK	97.462	105.873	0.11943	0.228266	0.6857	0.988184	no
gene:SpnNT_00095	NA	Chromosome:89343-89757	110.58	ΔORF2+peptide	OK	102.558	93.9721	-0.126134	-0.23766	0.6715	0.984845	no
gene:SpnNT_00095	NA	Chromosome:89343-89757	ΔORF2	ΔORF2+peptide	OK	97.462	93.9721	-0.0526075	-0.0983909	0.8569	0.994748	no
gene:SpnNT_00095	NA	Chromosome:89343-89757	110.58+peptide	ΔORF2+peptide	OK	105.873	93.9721	-0.172038	-0.323265	0.567	0.968621	no
gene:SpnNT_00096	iga_1	Chromosome:89872-94723	110.58	ΔORF2	OK	244.645	277.19	0.180185	0.389579	0.49285	0.941402	no
gene:SpnNT_00096	iga_1	Chromosome:89872-94723	110.58	110.58+peptide	OK	244.645	254.275	0.0557	0.120192	0.8336	0.994748	no
gene:SpnNT_00096	iga_1	Chromosome:89872-94723	ΔORF2	110.58+peptide	OK	277.19	254.275	-0.124485	-0.267431	0.63755	0.980887	no
gene:SpnNT_00096	iga_1	Chromosome:89872-94723	110.58	ΔORF2+peptide	OK	244.645	321.598	0.39457	0.852861	0.1338	0.556521	no
gene:SpnNT_00096	iga_1	Chromosome:89872-94723	ΔORF2	ΔORF2+peptide	OK	277.19	321.598	0.214385	0.461331	0.421	0.904877	no
gene:SpnNT_00096	iga_1	Chromosome:89872-94723	110.58+peptide	ΔORF2+peptide	OK	254.275	321.598	0.33887	0.727789	0.1941	0.675407	no
gene:SpnNT_00097	catE	Chromosome:95078-95933	110.58	ΔORF2	OK	20.4227	15.9681	-0.354977	-0.549565	0.32275	0.828143	no
gene:SpnNT_00097	catE	Chromosome:95078-95933	110.58	110.58+peptide	OK	20.4227	19.5989	-0.0593978	-0.0901374	0.87625	0.994748	no
gene:SpnNT_00097	catE	Chromosome:95078-95933	ΔORF2	110.58+peptide	OK	15.9681	19.5989	0.295579	0.456994	0.41355	0.899934	no
gene:SpnNT_00097	catE	Chromosome:95078-95933	110.58	ΔORF2+peptide	OK	20.4227	20.8836	0.0322003	0.0498779	0.9289	0.994748	no
gene:SpnNT_00097	catE	Chromosome:95078-95933	ΔORF2	ΔORF2+peptide	OK	15.9681	20.8836	0.387177	0.611514	0.2714	0.773525	no
gene:SpnNT_00097	catE	Chromosome:95078-95933	110.58+peptide	ΔORF2+peptide	OK	19.5989	20.8836	0.0915981	0.141694	0.7931	0.994748	no
gene:SpnNT_00098	maa	Chromosome:96038-96596	110.58	ΔORF2	OK	87.142	95.3875	0.130431	0.261604	0.6331	0.980887	no
gene:SpnNT_00098	maa	Chromosome:96038-96596	110.58	110.58+peptide	OK	87.142	68.9554	-0.337705	-0.653803	0.2369	0.731436	no

gene:SpnNT_00098	maa	Chromosome:96038-96596	ΔORF2	110.58+peptide	OK	95.3875	68.9554	-0.468136	-0.918793	0.0995	0.478431	no
gene:SpnNT_00098	maa	Chromosome:96038-96596	110.58	ΔORF2+peptide	OK	87.142	75.0787	-0.214964	-0.420903	0.4529	0.921244	no
gene:SpnNT_00098	maa	Chromosome:96038-96596	ΔORF2	ΔORF2+peptide	OK	95.3875	75.0787	-0.345396	-0.685813	0.223	0.712784	no
gene:SpnNT_00098	maa	Chromosome:96038-96596	110.58+peptide	ΔORF2+peptide	OK	68.9554	75.0787	0.12274	0.235406	0.66995	0.984845	no
gene:SpnNT_00099	deoD_1	Chromosome:96812-97577	110.58	ΔORF2	OK	136.808	138.184	0.0144366	0.0313582	0.95655	0.994855	no
gene:SpnNT_00099	deoD_1	Chromosome:96812-97577	110.58	110.58+peptide	OK	136.808	56.3856	-1.27876	-2.66644	5.00E-05	0.0013612	yes
gene:SpnNT_00099	deoD_1	Chromosome:96812-97577	ΔORF2	110.58+peptide	OK	138.184	56.3856	-1.29319	-2.66519	5.00E-05	0.0013612	yes
gene:SpnNT_00099	deoD_1	Chromosome:96812-97577	110.58	ΔORF2+peptide	OK	136.808	55.624	-1.29838	-2.73373	5.00E-05	0.0013612	yes
gene:SpnNT_00099	deoD_1	Chromosome:96812-97577	ΔORF2	ΔORF2+peptide	OK	138.184	55.624	-1.31281	-2.73137	5.00E-05	0.0013612	yes
gene:SpnNT_00099	deoD_1	Chromosome:96812-97577	110.58+peptide	ΔORF2+peptide	OK	56.3856	55.624	-0.0196182	-0.0393103	0.94525	0.994855	no
gene:SpnNT_00100	NA	Chromosome:97816-98149	110.58	ΔORF2	OK	109.69	103.892	-0.0783502	-0.144423	0.79745	0.994748	no
gene:SpnNT_00100	NA	Chromosome:97816-98149	110.58	110.58+peptide	OK	109.69	68.5721	-0.677735	-1.20116	0.03795	0.261682	no
gene:SpnNT_00100	NA	Chromosome:97816-98149	ΔORF2	110.58+peptide	OK	103.892	68.5721	-0.599385	-1.04932	0.0659	0.373376	no
gene:SpnNT_00100	NA	Chromosome:97816-98149	110.58	ΔORF2+peptide	OK	109.69	53.2438	-1.04274	-1.79798	0.00295	0.0393393	yes
gene:SpnNT_00100	NA	Chromosome:97816-98149	ΔORF2	ΔORF2+peptide	OK	103.892	53.2438	-0.964391	-1.64363	0.0049	0.0593153	no
gene:SpnNT_00100	NA	Chromosome:97816-98149	110.58+peptide	ΔORF2+peptide	OK	68.5721	53.2438	-0.365006	-0.601438	0.29945	0.805036	no
gene:SpnNT_00101	ktrB	Chromosome:98366-99746	110.58	ΔORF2	OK	118.002	123.546	0.0662368	0.151198	0.78745	0.994748	no
gene:SpnNT_00101	ktrB	Chromosome:98366-99746	110.58	110.58+peptide	OK	118.002	89.1155	-0.405066	-0.906144	0.1177	0.519495	no
gene:SpnNT_00101	ktrB	Chromosome:98366-99746	ΔORF2	110.58+peptide	OK	123.546	89.1155	-0.471302	-1.05972	0.0646	0.368878	no
gene:SpnNT_00101	ktrB	Chromosome:98366-99746	110.58	ΔORF2+peptide	OK	118.002	105.884	-0.156325	-0.354253	0.5394	0.960991	no
gene:SpnNT_00101	ktrB	Chromosome:98366-99746	ΔORF2	ΔORF2+peptide	OK	123.546	105.884	-0.222562	-0.507007	0.3735	0.870682	no
gene:SpnNT_00101	ktrB	Chromosome:98366-99746	110.58+peptide	ΔORF2+peptide	OK	89.1155	105.884	0.24874	0.555352	0.34515	0.849092	no
gene:SpnNT_00102	ktrA	Chromosome:99759-100425	110.58	ΔORF2	OK	298.478	318.731	0.0947157	0.217645	0.69765	0.990209	no
gene:SpnNT_00102	ktrA	Chromosome:99759-100425	110.58	110.58+peptide	OK	298.478	226.225	-0.399866	-0.910622	0.115	0.514676	no
gene:SpnNT_00102	ktrA	Chromosome:99759-100425	ΔORF2	110.58+peptide	OK	318.731	226.225	-0.494582	-1.12596	0.0535	0.327438	no
gene:SpnNT_00102	ktrA	Chromosome:99759-100425	110.58	ΔORF2+peptide	OK	298.478	243.393	-0.29434	-0.668369	0.2409	0.735993	no
gene:SpnNT_00102	ktrA	Chromosome:99759-100425	ΔORF2	ΔORF2+peptide	OK	318.731	243.393	-0.389055	-0.883164	0.12025	0.524749	no
gene:SpnNT_00102	ktrA	Chromosome:99759-100425	110.58+peptide	ΔORF2+peptide	OK	226.225	243.393	0.105527	0.237455	0.67345	0.984845	no
gene:SpnNT_00103	epsH	Chromosome:100742-101273	110.58	ΔORF2	OK	39.8062	33.1792	-0.262714	-0.473512	0.39815	0.891861	no
gene:SpnNT_00103	epsH	Chromosome:100742-101273	110.58	110.58+peptide	OK	39.8062	38.4276	-0.0508504	-0.0919124	0.8734	0.994748	no
gene:SpnNT_00103	epsH	Chromosome:100742-101273	ΔORF2	110.58+peptide	OK	33.1792	38.4276	0.211863	0.377909	0.50825	0.947338	no
gene:SpnNT_00103	epsH	Chromosome:100742-101273	110.58	ΔORF2+peptide	OK	39.8062	40.6962	0.031901	0.0574877	0.92315	0.994748	no
gene:SpnNT_00103	epsH	Chromosome:100742-101273	ΔORF2	ΔORF2+peptide	OK	33.1792	40.6962	0.294615	0.523975	0.36105	0.862183	no
gene:SpnNT_00103	epsH	Chromosome:100742-101273	110.58+peptide	ΔORF2+peptide	OK	38.4276	40.6962	0.0827514	0.147581	0.80165	0.994748	no
gene:SpnNT_00104	NA	Chromosome:101339-101750	110.58	ΔORF2	OK	51.516	39.395	-0.387007	-0.672328	0.24525	0.742198	no
gene:SpnNT_00104	NA	Chromosome:101339-101750	110.58	110.58+peptide	OK	51.516	59.4132	0.205764	0.364784	0.5275	0.956161	no
gene:SpnNT_00104	NA	Chromosome:101339-101750	ΔORF2	110.58+peptide	OK	39.395	59.4132	0.592771	1.02285	0.0724	0.396177	no
gene:SpnNT_00104	NA	Chromosome:101339-101750	110.58	ΔORF2+peptide	OK	51.516	49.0891	-0.0696173	-0.12085	0.83715	0.994748	no
gene:SpnNT_00104	NA	Chromosome:101339-101750	ΔORF2	ΔORF2+peptide	OK	39.395	49.0891	0.31739	0.536849	0.35605	0.857745	no
gene:SpnNT_00104	NA	Chromosome:101339-101750	110.58+peptide	ΔORF2+peptide	OK	59.4132	49.0891	-0.275381	-0.474822	0.4078	0.897051	no
gene:SpnNT_00105	NA	Chromosome:101865-103950	110.58	ΔORF2	OK	4.41908	3.59141	-0.299197	-0.496562	0.38725	0.881745	no
gene:SpnNT_00105	NA	Chromosome:101865-103950	110.58	110.58+peptide	OK	4.41908	3.56869	-0.308353	-0.5153	0.36785	0.865399	no
gene:SpnNT_00105	NA	Chromosome:101865-103950	ΔORF2	110.58+peptide	OK	3.59141	3.56869	-0.00915571	-0.0149328	0.9801	0.995963	no
gene:SpnNT_00105	NA	Chromosome:101865-103950	110.58	ΔORF2+peptide	OK	4.41908	2.95724	-0.579497	-0.951217	0.1045	0.490608	no
gene:SpnNT_00105	NA	Chromosome:101865-103950	ΔORF2	ΔORF2+peptide	OK	3.59141	2.95724	-0.2803	-0.449421	0.4349	0.913666	no

gene:SpnNT_00105	NA	Chromosome:101865-103950	110.58+peptide	ΔORF2+peptide	OK	3.56869	2.95724	-0.271144	-0.437547	0.44355	0.917631	no
gene:SpnNT_00106	saeR	Chromosome:104116-105864	110.58	ΔORF2	OK	52.1429	56.1021	0.105584	0.157204	0.78445	0.994748	no
gene:SpnNT_00106	saeR	Chromosome:104116-105864	110.58	110.58+peptide	OK	52.1429	39.8381	-0.388321	-0.558199	0.33835	0.842721	no
gene:SpnNT_00106	saeR	Chromosome:104116-105864	ΔORF2	110.58+peptide	OK	56.1021	39.8381	-0.493904	-0.718129	0.21195	0.697569	no
gene:SpnNT_00106	saeR	Chromosome:104116-105864	110.58	ΔORF2+peptide	OK	52.1429	49.293	-0.0810883	-0.118093	0.8426	0.994748	no
gene:SpnNT_00106	saeR	Chromosome:104116-105864	ΔORF2	ΔORF2+peptide	OK	56.1021	49.293	-0.186672	-0.275066	0.6367	0.980887	no
gene:SpnNT_00106	saeR	Chromosome:104116-105864	110.58+peptide	ΔORF2+peptide	OK	39.8381	49.293	0.307232	0.437383	0.44975	0.920402	no
gene:SpnNT_00107	saeS	Chromosome:104116-105864	110.58	ΔORF2	OK	33.9603	35.0891	0.0471748	0.0742336	0.8974	0.994748	no
gene:SpnNT_00107	saeS	Chromosome:104116-105864	110.58	110.58+peptide	OK	33.9603	28.1631	-0.270043	-0.424439	0.4546	0.921244	no
gene:SpnNT_00107	saeS	Chromosome:104116-105864	ΔORF2	110.58+peptide	OK	35.0891	28.1631	-0.317218	-0.497278	0.3832	0.880579	no
gene:SpnNT_00107	saeS	Chromosome:104116-105864	110.58	ΔORF2+peptide	OK	33.9603	33.7885	-0.00731641	-0.0115619	0.9829	0.996246	no
gene:SpnNT_00107	saeS	Chromosome:104116-105864	ΔORF2	ΔORF2+peptide	OK	35.0891	33.7885	-0.0544912	-0.0858826	0.882	0.994748	no
gene:SpnNT_00107	saeS	Chromosome:104116-105864	110.58+peptide	ΔORF2+peptide	OK	28.1631	33.7885	0.262726	0.413593	0.4708	0.928415	no
gene:SpnNT_00108	rpsD	Chromosome:106058-106670	110.58	ΔORF2	OK	1549.31	1567.8	0.017115	0.037754	0.948	0.994855	no
gene:SpnNT_00108	rpsD	Chromosome:106058-106670	110.58	110.58+peptide	OK	1549.31	3497.97	1.17489	2.54089	5.00E-05	0.0013612	yes
gene:SpnNT_00108	rpsD	Chromosome:106058-106670	ΔORF2	110.58+peptide	OK	1567.8	3497.97	1.15778	2.52597	5.00E-05	0.0013612	yes
gene:SpnNT_00108	rpsD	Chromosome:106058-106670	110.58	ΔORF2+peptide	OK	1549.31	3427.95	1.14572	2.49303	5.00E-05	0.0013612	yes
gene:SpnNT_00108	rpsD	Chromosome:106058-106670	ΔORF2	ΔORF2+peptide	OK	1567.8	3427.95	1.1286	2.47772	5.00E-05	0.0013612	yes
gene:SpnNT_00108	rpsD	Chromosome:106058-106670	110.58+peptide	ΔORF2+peptide	OK	3497.97	3427.95	-0.029173	-0.0628021	0.91085	0.994748	no
gene:SpnNT_00109	yteP	Chromosome:107871-108801	110.58	ΔORF2	OK	0.94237	0.842444	-0.161713	-0.160121	0.77785	0.994748	no
gene:SpnNT_00109	yteP	Chromosome:107871-108801	110.58	110.58+peptide	OK	0.94237	1.15634	0.295195	0.303486	0.58495	0.971855	no
gene:SpnNT_00109	yteP	Chromosome:107871-108801	ΔORF2	110.58+peptide	OK	0.842444	1.15634	0.456908	0.470096	0.40825	0.897087	no
gene:SpnNT_00109	yteP	Chromosome:107871-108801	110.58	ΔORF2+peptide	OK	0.94237	0.760812	-0.308753	-0.300646	0.6134	0.979616	no
gene:SpnNT_00109	yteP	Chromosome:107871-108801	ΔORF2	ΔORF2+peptide	OK	0.842444	0.760812	-0.147041	-0.143277	0.81365	0.994748	no
gene:SpnNT_00109	yteP	Chromosome:107871-108801	110.58+peptide	ΔORF2+peptide	OK	1.15634	0.760812	-0.603949	-0.61028	0.3136	0.81957	no
gene:SpnNT_00110	araQ_1	Chromosome:108814-109738	110.58	ΔORF2	OK	1.70431	1.60076	-0.090426	-0.104453	0.84725	0.994748	no
gene:SpnNT_00110	araQ_1	Chromosome:108814-109738	110.58	110.58+peptide	OK	1.70431	1.81006	0.086849	0.102081	0.8529	0.994748	no
gene:SpnNT_00110	araQ_1	Chromosome:108814-109738	ΔORF2	110.58+peptide	OK	1.60076	1.81006	0.177275	0.20713	0.7145	0.991924	no
gene:SpnNT_00110	araQ_1	Chromosome:108814-109738	110.58	ΔORF2+peptide	OK	1.70431	1.56014	-0.127511	-0.146806	0.7922	0.994748	no
gene:SpnNT_00110	araQ_1	Chromosome:108814-109738	ΔORF2	ΔORF2+peptide	OK	1.60076	1.56014	-0.0370851	-0.0424536	0.9393	0.994855	no
gene:SpnNT_00110	araQ_1	Chromosome:108814-109738	110.58+peptide	ΔORF2+peptide	OK	1.81006	1.56014	-0.21436	-0.249618	0.66515	0.983559	no
gene:SpnNT_00111	NA	Chromosome:109995-111471	110.58	ΔORF2	OK	1.86466	1.61551	-0.206924	-0.278718	0.62705	0.980887	no
gene:SpnNT_00111	NA	Chromosome:109995-111471	110.58	110.58+peptide	OK	1.86466	1.50493	-0.309216	-0.414465	0.47885	0.933291	no
gene:SpnNT_00111	NA	Chromosome:109995-111471	ΔORF2	110.58+peptide	OK	1.61551	1.50493	-0.102292	-0.135279	0.8188	0.994748	no
gene:SpnNT_00111	NA	Chromosome:109995-111471	110.58	ΔORF2+peptide	OK	1.86466	1.05318	-0.82416	-1.04949	0.0705	0.390464	no
gene:SpnNT_00111	NA	Chromosome:109995-111471	ΔORF2	ΔORF2+peptide	OK	1.61551	1.05318	-0.617235	-0.776505	0.1873	0.662288	no
gene:SpnNT_00111	NA	Chromosome:109995-111471	110.58+peptide	ΔORF2+peptide	OK	1.50493	1.05318	-0.514944	-0.645054	0.27395	0.776073	no
gene:SpnNT_00112	NA	Chromosome:111742-112729	110.58	ΔORF2	OK	412.141	467.321	0.181277	0.339135	0.55185	0.964534	no
gene:SpnNT_00112	NA	Chromosome:111742-112729	110.58	110.58+peptide	OK	412.141	970.812	1.23605	2.35608	5.00E-05	0.0013612	yes
gene:SpnNT_00112	NA	Chromosome:111742-112729	ΔORF2	110.58+peptide	OK	467.321	970.812	1.05478	2.11299	6.00E-04	0.0111203	yes
gene:SpnNT_00112	NA	Chromosome:111742-112729	110.58	ΔORF2+peptide	OK	412.141	976.022	1.24378	2.39752	5.00E-05	0.0013612	yes
gene:SpnNT_00112	NA	Chromosome:111742-112729	ΔORF2	ΔORF2+peptide	OK	467.321	976.022	1.0625	2.155	0.00045	0.00885292	yes
gene:SpnNT_00112	NA	Chromosome:111742-112729	110.58+peptide	ΔORF2+peptide	OK	970.812	976.022	0.00772181	0.0160109	0.97575	0.99536	no
gene:SpnNT_00113	NA	Chromosome:113003-113839	110.58	ΔORF2	OK	189.713	239.965	0.339005	0.696196	0.2187	0.707017	no
gene:SpnNT_00113	NA	Chromosome:113003-113839	110.58	110.58+peptide	OK	189.713	191.475	0.0133319	0.027151	0.9612	0.994855	no

gene:SpnNT_00113	NA	Chromosome:113003-113839	ΔORF2	110.58+peptide	OK	239.965	191.475	-0.325673	-0.667558	0.2365	0.730717	no
gene:SpnNT_00113	NA	Chromosome:113003-113839	110.58	ΔORF2+peptide	OK	189.713	190.718	0.00762326	0.0155935	0.9764	0.99536	no
gene:SpnNT_00113	NA	Chromosome:113003-113839	ΔORF2	ΔORF2+peptide	OK	239.965	190.718	-0.331382	-0.682286	0.2311	0.72461	no
gene:SpnNT_00113	NA	Chromosome:113003-113839	110.58+peptide	ΔORF2+peptide	OK	191.475	190.718	-0.00570859	-0.0116552	0.9831	0.996246	no
gene:SpnNT_00114	NA	Chromosome:113003-113839	110.58	ΔORF2	OK	162.107	198.628	0.293122	0.259354	0.6472	0.981345	no
gene:SpnNT_00114	NA	Chromosome:113003-113839	110.58	110.58+peptide	OK	162.107	159.746	-0.0211706	-0.0184228	0.973	0.99536	no
gene:SpnNT_00114	NA	Chromosome:113003-113839	ΔORF2	110.58+peptide	OK	198.628	159.746	-0.314293	-0.266704	0.6388	0.980887	no
gene:SpnNT_00114	NA	Chromosome:113003-113839	110.58	ΔORF2+peptide	OK	162.107	186.421	0.20162	0.186458	0.7447	0.994748	no
gene:SpnNT_00114	NA	Chromosome:113003-113839	ΔORF2	ΔORF2+peptide	OK	198.628	186.421	-0.0915019	-0.0822572	0.88685	0.994748	no
gene:SpnNT_00114	NA	Chromosome:113003-113839	110.58+peptide	ΔORF2+peptide	OK	159.746	186.421	0.222791	0.196875	0.73995	0.994748	no
gene:SpnNT_00115	NA	Chromosome:114037-115102	110.58	ΔORF2	OK	92.1025	89.0629	-0.0484154	-0.104931	0.85215	0.994748	no
gene:SpnNT_00115	NA	Chromosome:114037-115102	110.58	110.58+peptide	OK	92.1025	127.847	0.473111	1.0473	0.06435	0.368252	no
gene:SpnNT_00115	NA	Chromosome:114037-115102	ΔORF2	110.58+peptide	OK	89.0629	127.847	0.521526	1.14217	0.0479	0.304822	no
gene:SpnNT_00115	NA	Chromosome:114037-115102	110.58	ΔORF2+peptide	OK	92.1025	86.4389	-0.0915601	-0.198892	0.72825	0.994748	no
gene:SpnNT_00115	NA	Chromosome:114037-115102	ΔORF2	ΔORF2+peptide	OK	89.0629	86.4389	-0.0431447	-0.0927586	0.8744	0.994748	no
gene:SpnNT_00115	NA	Chromosome:114037-115102	110.58+peptide	ΔORF2+peptide	OK	127.847	86.4389	-0.564671	-1.23955	0.0301	0.222645	no
gene:SpnNT_00116	NA	Chromosome:115163-116974	110.58	ΔORF2	OK	50.473	47.4127	-0.090239	-0.15726	0.78655	0.994748	no
gene:SpnNT_00116	NA	Chromosome:115163-116974	110.58	110.58+peptide	OK	50.473	71.5465	0.503368	0.893838	0.12415	0.533256	no
gene:SpnNT_00116	NA	Chromosome:115163-116974	ΔORF2	110.58+peptide	OK	47.4127	71.5465	0.593607	1.04164	0.06885	0.38461	no
gene:SpnNT_00116	NA	Chromosome:115163-116974	110.58	ΔORF2+peptide	OK	50.473	51.3241	0.0241226	0.0412287	0.94315	0.994855	no
gene:SpnNT_00116	NA	Chromosome:115163-116974	ΔORF2	ΔORF2+peptide	OK	47.4127	51.3241	0.114362	0.193319	0.73525	0.994748	no
gene:SpnNT_00116	NA	Chromosome:115163-116974	110.58+peptide	ΔORF2+peptide	OK	71.5465	51.3241	-0.479245	-0.824539	0.15525	0.60041	no
gene:SpnNT_00117	NA	Chromosome:115163-116974	110.58	ΔORF2	OK	25.6029	25.2222	-0.0216133	-0.0193609	0.9733	0.99536	no
gene:SpnNT_00117	NA	Chromosome:115163-116974	110.58	110.58+peptide	OK	25.6029	40.1842	0.650318	0.613025	0.30915	0.814396	no
gene:SpnNT_00117	NA	Chromosome:115163-116974	ΔORF2	110.58+peptide	OK	25.2222	40.1842	0.671931	0.610765	0.30845	0.814396	no
gene:SpnNT_00117	NA	Chromosome:115163-116974	110.58	ΔORF2+peptide	OK	25.6029	29.371	0.198082	0.177735	0.76375	0.994748	no
gene:SpnNT_00117	NA	Chromosome:115163-116974	ΔORF2	ΔORF2+peptide	OK	25.2222	29.371	0.219695	0.190714	0.74755	0.994748	no
gene:SpnNT_00117	NA	Chromosome:115163-116974	110.58+peptide	ΔORF2+peptide	OK	40.1842	29.371	-0.452236	-0.411774	0.4978	0.944017	no
gene:SpnNT_00118	NA	Chromosome:115163-116974	110.58	ΔORF2	OK	46.9391	42.4897	-0.143677	-0.0841638	0.88245	0.994748	no
gene:SpnNT_00118	NA	Chromosome:115163-116974	110.58	110.58+peptide	OK	46.9391	67.337	0.52061	0.32578	0.5702	0.968621	no
gene:SpnNT_00118	NA	Chromosome:115163-116974	ΔORF2	110.58+peptide	OK	42.4897	67.337	0.664288	0.386383	0.5066	0.945874	no
gene:SpnNT_00118	NA	Chromosome:115163-116974	110.58	ΔORF2+peptide	OK	46.9391	66.4141	0.5007	0.346194	0.54995	0.964281	no
gene:SpnNT_00118	NA	Chromosome:115163-116974	ΔORF2	ΔORF2+peptide	OK	42.4897	66.4141	0.644377	0.408043	0.47805	0.933077	no
gene:SpnNT_00118	NA	Chromosome:115163-116974	110.58+peptide	ΔORF2+peptide	OK	67.337	66.4141	-0.0199101	-0.0136315	0.98275	0.996246	no
gene:SpnNT_00119	yycB	Chromosome:117112-118279	110.58	ΔORF2	OK	58.7525	39.508	-0.572505	-1.17183	0.04025	0.272248	no
gene:SpnNT_00119	yycB	Chromosome:117112-118279	110.58	110.58+peptide	OK	58.7525	53.4218	-0.137221	-0.280572	0.61705	0.980654	no
gene:SpnNT_00119	yycB	Chromosome:117112-118279	ΔORF2	110.58+peptide	OK	39.508	53.4218	0.435284	0.903137	0.1164	0.517109	no
gene:SpnNT_00119	yycB	Chromosome:117112-118279	110.58	ΔORF2+peptide	OK	58.7525	39.4829	-0.573422	-1.17472	0.03945	0.268637	no
gene:SpnNT_00119	yycB	Chromosome:117112-118279	ΔORF2	ΔORF2+peptide	OK	39.508	39.4829	-0.000916852	-0.00190609	0.9988	0.999459	no
gene:SpnNT_00119	yycB	Chromosome:117112-118279	110.58+peptide	ΔORF2+peptide	OK	53.4218	39.4829	-0.436201	-0.905841	0.11705	0.518813	no
gene:SpnNT_00120	pgaC	Chromosome:118429-119497	110.58	ΔORF2	OK	45.649	54.8585	0.265133	0.555784	0.3287	0.834598	no
gene:SpnNT_00120	pgaC	Chromosome:118429-119497	110.58	110.58+peptide	OK	45.649	49.9198	0.129031	0.268576	0.64005	0.980887	no
gene:SpnNT_00120	pgaC	Chromosome:118429-119497	ΔORF2	110.58+peptide	OK	54.8585	49.9198	-0.136102	-0.285745	0.61245	0.979616	no
gene:SpnNT_00120	pgaC	Chromosome:118429-119497	110.58	ΔORF2+peptide	OK	45.649	69.6957	0.610489	1.28991	0.02645	0.204524	no
gene:SpnNT_00120	pgaC	Chromosome:118429-119497	ΔORF2	ΔORF2+peptide	OK	54.8585	69.6957	0.345356	0.736208	0.2014	0.684478	no

gene:SpnNT_00120	pgaC	Chromosome:118429-119497	110.58+peptide	ΔORF2+peptide	OK	49.9198	69.6957	0.481458	1.01888	0.08045	0.422943	no
gene:SpnNT_00121	pglF	Chromosome:119535-121386	110.58	ΔORF2	OK	120.94	124.926	0.0467823	0.107816	0.8475	0.994748	no
gene:SpnNT_00121	pglF	Chromosome:119535-121386	110.58	110.58+peptide	OK	120.94	137.15	0.181464	0.418015	0.45585	0.921244	no
gene:SpnNT_00121	pglF	Chromosome:119535-121386	ΔORF2	110.58+peptide	OK	124.926	137.15	0.134682	0.310068	0.5747	0.969538	no
gene:SpnNT_00121	pglF	Chromosome:119535-121386	110.58	ΔORF2+peptide	OK	120.94	136.324	0.172744	0.397664	0.48145	0.935095	no
gene:SpnNT_00121	pglF	Chromosome:119535-121386	ΔORF2	ΔORF2+peptide	OK	124.926	136.324	0.125962	0.2898	0.60365	0.976761	no
gene:SpnNT_00121	pglF	Chromosome:119535-121386	110.58+peptide	ΔORF2+peptide	OK	137.15	136.324	-0.00872008	-0.020053	0.97075	0.99536	no
gene:SpnNT_00122	NA	Chromosome:121428-122103	110.58	ΔORF2	NOTEST	1.27461	1.26954	-0.00574513	0	1	1	no
gene:SpnNT_00122	NA	Chromosome:121428-122103	110.58	110.58+peptide	NOTEST	1.27461	0.420512	-1.59984	0	1	1	no
gene:SpnNT_00122	NA	Chromosome:121428-122103	ΔORF2	110.58+peptide	NOTEST	1.26954	0.420512	-1.59409	0	1	1	no
gene:SpnNT_00122	NA	Chromosome:121428-122103	110.58	ΔORF2+peptide	NOTEST	1.27461	0	#NAME?	0	1	1	no
gene:SpnNT_00122	NA	Chromosome:121428-122103	ΔORF2	ΔORF2+peptide	NOTEST	1.26954	0	#NAME?	0	1	1	no
gene:SpnNT_00122	NA	Chromosome:121428-122103	110.58+peptide	ΔORF2+peptide	NOTEST	0.420512	0	#NAME?	0	1	1	no
gene:SpnNT_00123	NA	Chromosome:121428-122103	110.58	ΔORF2	NOTEST	1.08256	1.22048	0.173002	0	1	1	no
gene:SpnNT_00123	NA	Chromosome:121428-122103	110.58	110.58+peptide	NOTEST	1.08256	0.693763	-0.641935	0	1	1	no
gene:SpnNT_00123	NA	Chromosome:121428-122103	ΔORF2	110.58+peptide	NOTEST	1.22048	0.693763	-0.814938	0	1	1	no
gene:SpnNT_00123	NA	Chromosome:121428-122103	110.58	ΔORF2+peptide	OK	1.08256	5.81255	2.42472	0.809676	0.47005	0.927937	no
gene:SpnNT_00123	NA	Chromosome:121428-122103	ΔORF2	ΔORF2+peptide	OK	1.22048	5.81255	2.25172	0.72803	0.5032	0.945186	no
gene:SpnNT_00123	NA	Chromosome:121428-122103	110.58+peptide	ΔORF2+peptide	OK	0.693763	5.81255	3.06666	0.787516	0.4574	0.921722	no
gene:SpnNT_00124	NA	Chromosome:121428-122103	110.58	ΔORF2	OK	321.798	291.156	-0.144363	-0.253322	0.6547	0.982966	no
gene:SpnNT_00124	NA	Chromosome:121428-122103	110.58	110.58+peptide	OK	321.798	453.492	0.494923	0.857783	0.123	0.530748	no
gene:SpnNT_00124	NA	Chromosome:121428-122103	ΔORF2	110.58+peptide	OK	291.156	453.492	0.639286	1.11974	0.04955	0.311844	no
gene:SpnNT_00124	NA	Chromosome:121428-122103	110.58	ΔORF2+peptide	OK	321.798	475.516	0.563339	0.973318	0.08525	0.439454	no
gene:SpnNT_00124	NA	Chromosome:121428-122103	ΔORF2	ΔORF2+peptide	OK	291.156	475.516	0.707702	1.23563	0.0312	0.228336	no
gene:SpnNT_00124	NA	Chromosome:121428-122103	110.58+peptide	ΔORF2+peptide	OK	453.492	475.516	0.0684159	0.117998	0.8313	0.994748	no
gene:SpnNT_00125	NA	Chromosome:122152-122785	110.58	ΔORF2	OK	44.1233	45.5066	0.0445363	0.0848291	0.8817	0.994748	no
gene:SpnNT_00125	NA	Chromosome:122152-122785	110.58	110.58+peptide	OK	44.1233	51.7806	0.230873	0.444651	0.43485	0.913666	no
gene:SpnNT_00125	NA	Chromosome:122152-122785	ΔORF2	110.58+peptide	OK	45.5066	51.7806	0.186336	0.359218	0.52605	0.95607	no
gene:SpnNT_00125	NA	Chromosome:122152-122785	110.58	ΔORF2+peptide	OK	44.1233	49.4797	0.165296	0.316022	0.5637	0.968621	no
gene:SpnNT_00125	NA	Chromosome:122152-122785	ΔORF2	ΔORF2+peptide	OK	45.5066	49.4797	0.12076	0.231092	0.67255	0.984845	no
gene:SpnNT_00125	NA	Chromosome:122152-122785	110.58+peptide	ΔORF2+peptide	OK	51.7806	49.4797	-0.0655764	-0.126903	0.82285	0.994748	no
gene:SpnNT_00126	sdhA	Chromosome:122806-123679	110.58	ΔORF2	OK	41.5665	35.6336	-0.222183	-0.435233	0.4446	0.917735	no
gene:SpnNT_00126	sdhA	Chromosome:122806-123679	110.58	110.58+peptide	OK	41.5665	47.7562	0.200267	0.399852	0.48405	0.936747	no
gene:SpnNT_00126	sdhA	Chromosome:122806-123679	ΔORF2	110.58+peptide	OK	35.6336	47.7562	0.42245	0.829668	0.1425	0.576108	no
gene:SpnNT_00126	sdhA	Chromosome:122806-123679	110.58	ΔORF2+peptide	OK	41.5665	37.8986	-0.133275	-0.260229	0.6403	0.980887	no
gene:SpnNT_00126	sdhA	Chromosome:122806-123679	ΔORF2	ΔORF2+peptide	OK	35.6336	37.8986	0.0889081	0.170881	0.75745	0.994748	no
gene:SpnNT_00126	sdhA	Chromosome:122806-123679	110.58+peptide	ΔORF2+peptide	OK	47.7562	37.8986	-0.333542	-0.652933	0.24565	0.743066	no
gene:SpnNT_00127	sdhB	Chromosome:123687-124359	110.58	ΔORF2	OK	65.5943	64.8849	-0.0156872	-0.0315949	0.95785	0.994855	no
gene:SpnNT_00127	sdhB	Chromosome:123687-124359	110.58	110.58+peptide	OK	65.5943	72.4567	0.143548	0.28901	0.61715	0.980654	no
gene:SpnNT_00127	sdhB	Chromosome:123687-124359	ΔORF2	110.58+peptide	OK	64.8849	72.4567	0.159235	0.320634	0.57995	0.969538	no
gene:SpnNT_00127	sdhB	Chromosome:123687-124359	110.58	ΔORF2+peptide	OK	65.5943	74.7345	0.188203	0.37988	0.5147	0.951747	no
gene:SpnNT_00127	sdhB	Chromosome:123687-124359	ΔORF2	ΔORF2+peptide	OK	64.8849	74.7345	0.203891	0.411596	0.4818	0.935095	no
gene:SpnNT_00127	sdhB	Chromosome:123687-124359	110.58+peptide	ΔORF2+peptide	OK	72.4567	74.7345	0.0446553	0.0901137	0.877	0.994748	no
gene:SpnNT_00128	lytN	Chromosome:124600-125188	110.58	ΔORF2	OK	3539.94	4340.31	0.294075	0.609182	0.2802	0.783628	no
gene:SpnNT_00128	lytN	Chromosome:124600-125188	110.58	110.58+peptide	OK	3539.94	4190.91	0.243541	0.501504	0.37475	0.872472	no

gene:SpnNT_00128	lytN	Chromosome:124600-125188	ΔORF2	110.58+peptide	OK	4340.31	4190.91	-0.0505335	-0.103083	0.85735	0.994748	no
gene:SpnNT_00128	lytN	Chromosome:124600-125188	110.58	ΔORF2+peptide	OK	3539.94	4129.92	0.22239	0.46844	0.4117	0.899339	no
gene:SpnNT_00128	lytN	Chromosome:124600-125188	ΔORF2	ΔORF2+peptide	OK	4340.31	4129.92	-0.0716852	-0.149515	0.7956	0.994748	no
gene:SpnNT_00128	lytN	Chromosome:124600-125188	110.58+peptide	ΔORF2+peptide	OK	4190.91	4129.92	-0.0211517	-0.0438509	0.93995	0.994855	no
gene:SpnNT_00129	NA	Chromosome:125239-126103	110.58	ΔORF2	OK	13.5074	14.691	0.121186	0.211256	0.7089	0.990577	no
gene:SpnNT_00129	NA	Chromosome:125239-126103	110.58	110.58+peptide	OK	13.5074	9.58567	-0.494797	-0.840609	0.13895	0.569071	no
gene:SpnNT_00129	NA	Chromosome:125239-126103	ΔORF2	110.58+peptide	OK	14.691	9.58567	-0.615983	-1.04968	0.0672	0.378942	no
gene:SpnNT_00129	NA	Chromosome:125239-126103	110.58	ΔORF2+peptide	OK	13.5074	11.6927	-0.208145	-0.356442	0.52425	0.954832	no
gene:SpnNT_00129	NA	Chromosome:125239-126103	ΔORF2	ΔORF2+peptide	OK	14.691	11.6927	-0.329332	-0.565714	0.3248	0.830123	no
gene:SpnNT_00129	NA	Chromosome:125239-126103	110.58+peptide	ΔORF2+peptide	OK	9.58567	11.6927	0.286651	0.480226	0.39605	0.890349	no
gene:SpnNT_00130	lagD_2	Chromosome:126568-129553	110.58	ΔORF2	OK	4.81411	4.71165	-0.0310388	-0.048915	0.93645	0.994855	no
gene:SpnNT_00130	lagD_2	Chromosome:126568-129553	110.58	110.58+peptide	OK	4.81411	3.45473	-0.478696	-0.723973	0.2103	0.696806	no
gene:SpnNT_00130	lagD_2	Chromosome:126568-129553	ΔORF2	110.58+peptide	OK	4.71165	3.45473	-0.447657	-0.68944	0.22565	0.716568	no
gene:SpnNT_00130	lagD_2	Chromosome:126568-129553	110.58	ΔORF2+peptide	OK	4.81411	3.61311	-0.414027	-0.638307	0.26775	0.768869	no
gene:SpnNT_00130	lagD_2	Chromosome:126568-129553	ΔORF2	ΔORF2+peptide	OK	4.71165	3.61311	-0.382988	-0.601712	0.28945	0.79393	no
gene:SpnNT_00130	lagD_2	Chromosome:126568-129553	110.58+peptide	ΔORF2+peptide	OK	3.45473	3.61311	0.0646683	0.0975272	0.86375	0.994748	no
gene:SpnNT_00131	NA	Chromosome:126568-129553	110.58	ΔORF2	OK	2.67645	3.59328	0.424985	0.192328	0.8212	0.994748	no
gene:SpnNT_00131	NA	Chromosome:126568-129553	110.58	110.58+peptide	OK	2.67645	3.13427	0.227812	0.106113	0.8861	0.994748	no
gene:SpnNT_00131	NA	Chromosome:126568-129553	ΔORF2	110.58+peptide	OK	3.59328	3.13427	-0.197173	-0.0884323	0.9045	0.994748	no
gene:SpnNT_00131	NA	Chromosome:126568-129553	110.58	ΔORF2+peptide	OK	2.67645	1.43142	-0.902872	-0.407022	0.63695	0.980887	no
gene:SpnNT_00131	NA	Chromosome:126568-129553	ΔORF2	ΔORF2+peptide	OK	3.59328	1.43142	-1.32786	-0.577721	0.6002	0.976665	no
gene:SpnNT_00131	NA	Chromosome:126568-129553	110.58+peptide	ΔORF2+peptide	OK	3.13427	1.43142	-1.13068	-0.505193	0.6311	0.980887	no
gene:SpnNT_00132	NA	Chromosome:126568-129553	110.58	ΔORF2	OK	13.6462	8.36406	-0.706222	-0.385886	0.54755	0.962356	no
gene:SpnNT_00132	NA	Chromosome:126568-129553	110.58	110.58+peptide	OK	13.6462	11.4174	-0.257259	-0.145046	0.789	0.994748	no
gene:SpnNT_00132	NA	Chromosome:126568-129553	ΔORF2	110.58+peptide	OK	8.36406	11.4174	0.448962	0.233853	0.7278	0.994748	no
gene:SpnNT_00132	NA	Chromosome:126568-129553	110.58	ΔORF2+peptide	OK	13.6462	7.21574	-0.919279	-0.430453	0.45405	0.921244	no
gene:SpnNT_00132	NA	Chromosome:126568-129553	ΔORF2	ΔORF2+peptide	OK	8.36406	7.21574	-0.213057	-0.0943355	0.89495	0.994748	no
gene:SpnNT_00132	NA	Chromosome:126568-129553	110.58+peptide	ΔORF2+peptide	OK	11.4174	7.21574	-0.662019	-0.299154	0.6262	0.980887	no
gene:SpnNT_00133	NA	Chromosome:129752-130586	110.58	ΔORF2	OK	6.74168	6.68716	-0.0117128	-0.0177894	0.97215	0.99536	no
gene:SpnNT_00133	NA	Chromosome:129752-130586	110.58	110.58+peptide	OK	6.74168	5.83397	-0.208629	-0.314139	0.5801	0.969538	no
gene:SpnNT_00133	NA	Chromosome:129752-130586	ΔORF2	110.58+peptide	OK	6.68716	5.83397	-0.196916	-0.300799	0.5947	0.975539	no
gene:SpnNT_00133	NA	Chromosome:129752-130586	110.58	ΔORF2+peptide	OK	6.74168	4.15179	-0.699375	-0.994983	0.08405	0.435071	no
gene:SpnNT_00133	NA	Chromosome:129752-130586	ΔORF2	ΔORF2+peptide	OK	6.68716	4.15179	-0.687663	-0.990945	0.08135	0.425798	no
gene:SpnNT_00133	NA	Chromosome:129752-130586	110.58+peptide	ΔORF2+peptide	OK	5.83397	4.15179	-0.490747	-0.701698	0.20985	0.696422	no
gene:SpnNT_00134	NA	Chromosome:130730-131048	110.58	ΔORF2	OK	12.8368	12.1457	-0.0798415	-0.1004	0.86005	0.994748	no
gene:SpnNT_00134	NA	Chromosome:130730-131048	110.58	110.58+peptide	OK	12.8368	8.40886	-0.610306	-0.738977	0.20075	0.683494	no
gene:SpnNT_00134	NA	Chromosome:130730-131048	ΔORF2	110.58+peptide	OK	12.1457	8.40886	-0.530464	-0.63202	0.27245	0.774448	no
gene:SpnNT_00134	NA	Chromosome:130730-131048	110.58	ΔORF2+peptide	OK	12.8368	4.18344	-1.61753	-2.12001	0.01255	0.118903	no
gene:SpnNT_00134	NA	Chromosome:130730-131048	ΔORF2	ΔORF2+peptide	OK	12.1457	4.18344	-1.53768	-1.97771	0.01605	0.141158	no
gene:SpnNT_00134	NA	Chromosome:130730-131048	110.58+peptide	ΔORF2+peptide	OK	8.40886	4.18344	-1.00722	-1.24529	0.093	0.460334	no
gene:SpnNT_00135	NA	Chromosome:131056-131956	110.58	ΔORF2	OK	3.50331	4.13519	0.239236	0.337725	0.55985	0.968621	no
gene:SpnNT_00135	NA	Chromosome:131056-131956	110.58	110.58+peptide	OK	3.50331	2.20577	-0.667436	-0.870501	0.1397	0.570362	no
gene:SpnNT_00135	NA	Chromosome:131056-131956	ΔORF2	110.58+peptide	OK	4.13519	2.20577	-0.906672	-1.20864	0.042	0.279758	no
gene:SpnNT_00135	NA	Chromosome:131056-131956	110.58	ΔORF2+peptide	OK	3.50331	3.23124	-0.116631	-0.159524	0.7835	0.994748	no
gene:SpnNT_00135	NA	Chromosome:131056-131956	ΔORF2	ΔORF2+peptide	OK	4.13519	3.23124	-0.355867	-0.498604	0.38645	0.881745	no

gene:SpnNT_00135	NA	Chromosome:131056-131956	110.58+peptide	ΔORF2+peptide	OK	2.20577	3.23124	0.550805	0.713781	0.22675	0.718873	no
gene:SpnNT_00136	NA	Chromosome:132011-132392	110.58	ΔORF2	OK	41.5465	35.698	-0.218881	-0.362151	0.51845	0.954018	no
gene:SpnNT_00136	NA	Chromosome:132011-132392	110.58	110.58+peptide	OK	41.5465	36.8791	-0.171922	-0.271692	0.6249	0.980887	no
gene:SpnNT_00136	NA	Chromosome:132011-132392	ΔORF2	110.58+peptide	OK	35.698	36.8791	0.0469586	0.0732987	0.8906	0.994748	no
gene:SpnNT_00136	NA	Chromosome:132011-132392	110.58	ΔORF2+peptide	OK	41.5465	30.3008	-0.45537	-0.709882	0.19935	0.68192	no
gene:SpnNT_00136	NA	Chromosome:132011-132392	ΔORF2	ΔORF2+peptide	OK	35.698	30.3008	-0.236489	-0.36426	0.50335	0.945186	no
gene:SpnNT_00136	NA	Chromosome:132011-132392	110.58+peptide	ΔORF2+peptide	OK	36.8791	30.3008	-0.283447	-0.419461	0.43645	0.914104	no
gene:SpnNT_00137	NA	Chromosome:133970-134105	110.58	ΔORF2	OK	24.4409	17.5696	-0.476214	-0.279291	0.64235	0.980887	no
gene:SpnNT_00137	NA	Chromosome:133970-134105	110.58	110.58+peptide	OK	24.4409	0	#NAME?	NA	0.00135	0.021343	yes
gene:SpnNT_00137	NA	Chromosome:133970-134105	ΔORF2	110.58+peptide	OK	17.5696	0	#NAME?	NA	0.00615	0.0705422	no
gene:SpnNT_00137	NA	Chromosome:133970-134105	110.58	ΔORF2+peptide	OK	24.4409	26.5655	0.120254	0.10435	0.89405	0.994748	no
gene:SpnNT_00137	NA	Chromosome:133970-134105	ΔORF2	ΔORF2+peptide	OK	17.5696	26.5655	0.596468	0.46779	0.5297	0.9574	no
gene:SpnNT_00137	NA	Chromosome:133970-134105	110.58+peptide	ΔORF2+peptide	OK	0	26.5655	Inf	NA	0.00155	0.0240131	yes
gene:SpnNT_00138	NA	Chromosome:134936-135062	110.58	ΔORF2	OK	24.0819	6.79958	-1.82443	-1.35213	0.3135	0.819472	no
gene:SpnNT_00138	NA	Chromosome:134936-135062	110.58	110.58+peptide	OK	24.0819	5.87961	-2.03416	-1.54313	0.2341	0.72774	no
gene:SpnNT_00138	NA	Chromosome:134936-135062	ΔORF2	110.58+peptide	OK	6.79958	5.87961	-0.209726	-0.482732	0.7814	0.994748	no
gene:SpnNT_00138	NA	Chromosome:134936-135062	110.58	ΔORF2+peptide	OK	24.0819	0	#NAME?	NA	0.00755	0.082285	no
gene:SpnNT_00138	NA	Chromosome:134936-135062	ΔORF2	ΔORF2+peptide	OK	6.79958	0	#NAME?	NA	0.11215	0.509744	no
gene:SpnNT_00138	NA	Chromosome:134936-135062	110.58+peptide	ΔORF2+peptide	NOTEST	5.87961	0	#NAME?	0	1	1	no
gene:SpnNT_00139	NA	Chromosome:135366-135789	110.58	ΔORF2	OK	10.6634	10.5622	-0.0137518	-0.018983	0.9809	0.99608	no
gene:SpnNT_00139	NA	Chromosome:135366-135789	110.58	110.58+peptide	OK	10.6634	8.79301	-0.278232	-0.368986	0.53945	0.960991	no
gene:SpnNT_00139	NA	Chromosome:135366-135789	ΔORF2	110.58+peptide	OK	10.5622	8.79301	-0.26448	-0.35144	0.55085	0.964281	no
gene:SpnNT_00139	NA	Chromosome:135366-135789	110.58	ΔORF2+peptide	OK	10.6634	12.7371	0.256377	0.356816	0.5275	0.956161	no
gene:SpnNT_00139	NA	Chromosome:135366-135789	ΔORF2	ΔORF2+peptide	OK	10.5622	12.7371	0.270129	0.376772	0.49905	0.944017	no
gene:SpnNT_00139	NA	Chromosome:135366-135789	110.58+peptide	ΔORF2+peptide	OK	8.79301	12.7371	0.534609	0.715799	0.22925	0.721915	no
gene:SpnNT_00140	NA	Chromosome:135817-136426	110.58	ΔORF2	OK	2.10096	2.69855	0.361136	0.407221	0.481	0.935095	no
gene:SpnNT_00140	NA	Chromosome:135817-136426	110.58	110.58+peptide	OK	2.10096	3.07645	0.550218	0.620741	0.2827	0.785349	no
gene:SpnNT_00140	NA	Chromosome:135817-136426	ΔORF2	110.58+peptide	OK	2.69855	3.07645	0.189082	0.220422	0.7065	0.990422	no
gene:SpnNT_00140	NA	Chromosome:135817-136426	110.58	ΔORF2+peptide	OK	2.10096	4.99261	1.24875	1.48081	0.01375	0.12635	no
gene:SpnNT_00140	NA	Chromosome:135817-136426	ΔORF2	ΔORF2+peptide	OK	2.69855	4.99261	0.88761	1.0915	0.0643	0.368126	no
gene:SpnNT_00140	NA	Chromosome:135817-136426	110.58+peptide	ΔORF2+peptide	OK	3.07645	4.99261	0.698528	0.859494	0.1474	0.585229	no
gene:SpnNT_00141	NA	Chromosome:136676-136946	110.58	ΔORF2	OK	19.2544	26.9014	0.482494	0.621543	0.28645	0.789829	no
gene:SpnNT_00141	NA	Chromosome:136676-136946	110.58	110.58+peptide	OK	19.2544	24.451	0.344707	0.448677	0.43635	0.914104	no
gene:SpnNT_00141	NA	Chromosome:136676-136946	ΔORF2	110.58+peptide	OK	26.9014	24.451	-0.137787	-0.18251	0.7567	0.994748	no
gene:SpnNT_00141	NA	Chromosome:136676-136946	110.58	ΔORF2+peptide	OK	19.2544	39.9913	1.0545	1.42879	0.0142	0.129128	no
gene:SpnNT_00141	NA	Chromosome:136676-136946	ΔORF2	ΔORF2+peptide	OK	26.9014	39.9913	0.572007	0.789884	0.17895	0.645815	no
gene:SpnNT_00141	NA	Chromosome:136676-136946	110.58+peptide	ΔORF2+peptide	OK	24.451	39.9913	0.709795	0.991925	0.08955	0.449782	no
gene:SpnNT_00142	NA	Chromosome:137280-139014	110.58	ΔORF2	OK	28.927	31.8545	0.139079	0.293923	0.59255	0.975468	no
gene:SpnNT_00142	NA	Chromosome:137280-139014	110.58	110.58+peptide	OK	28.927	28.5482	-0.0190175	-0.0397382	0.94235	0.994855	no
gene:SpnNT_00142	NA	Chromosome:137280-139014	ΔORF2	110.58+peptide	OK	31.8545	28.5482	-0.158097	-0.331711	0.55655	0.966907	no
gene:SpnNT_00142	NA	Chromosome:137280-139014	110.58	ΔORF2+peptide	OK	28.927	36.5859	0.338872	0.720708	0.20735	0.692856	no
gene:SpnNT_00142	NA	Chromosome:137280-139014	ΔORF2	ΔORF2+peptide	OK	31.8545	36.5859	0.199793	0.426727	0.4557	0.921244	no
gene:SpnNT_00142	NA	Chromosome:137280-139014	110.58+peptide	ΔORF2+peptide	OK	28.5482	36.5859	0.35789	0.755613	0.1888	0.665974	no
gene:SpnNT_00143	lytB_1	Chromosome:139270-141529	110.58	ΔORF2	OK	2083.5	2541.24	0.286528	0.55008	0.33455	0.839863	no
gene:SpnNT_00143	lytB_1	Chromosome:139270-141529	110.58	110.58+peptide	OK	2083.5	1650.19	-0.336377	-0.653071	0.2448	0.7415	no

gene:SpnNT_00143	lytB_1	Chromosome:139270-141529	ΔORF2	110.58+peptide	OK	2541.24	1650.19	-0.622905	-1.21633	0.03355	0.24057	no
gene:SpnNT_00143	lytB_1	Chromosome:139270-141529	110.58	ΔORF2+peptide	OK	2083.5	2242.24	0.105935	0.20329	0.7155	0.992053	no
gene:SpnNT_00143	lytB_1	Chromosome:139270-141529	ΔORF2	ΔORF2+peptide	OK	2541.24	2242.24	-0.180593	-0.348508	0.5439	0.961568	no
gene:SpnNT_00143	lytB_1	Chromosome:139270-141529	110.58+peptide	ΔORF2+peptide	OK	1650.19	2242.24	0.442312	0.863314	0.13075	0.549552	no
gene:SpnNT_00144	NA	Chromosome:141635-141797	110.58	ΔORF2	OK	5.85327	7.07983	0.274473	0.149848	0.7766	0.994748	no
gene:SpnNT_00144	NA	Chromosome:141635-141797	110.58	110.58+peptide	OK	5.85327	6.11833	0.0638951	0.0346142	0.8657	0.994748	no
gene:SpnNT_00144	NA	Chromosome:141635-141797	ΔORF2	110.58+peptide	OK	7.07983	6.11833	-0.210578	-0.379443	0.8503	0.994748	no
gene:SpnNT_00144	NA	Chromosome:141635-141797	110.58	ΔORF2+peptide	OK	5.85327	2.05836	-1.50774	-0.808649	0.3077	0.813712	no
gene:SpnNT_00144	NA	Chromosome:141635-141797	ΔORF2	ΔORF2+peptide	OK	7.07983	2.05836	-1.78222	-2.90261	0.2919	0.796956	no
gene:SpnNT_00144	NA	Chromosome:141635-141797	110.58+peptide	ΔORF2+peptide	OK	6.11833	2.05836	-1.57164	-2.39843	0.41265	0.899511	no
gene:SpnNT_00145	mnmA	Chromosome:141928-143050	110.58	ΔORF2	OK	1157.91	1116.8	-0.0521584	-0.109801	0.85085	0.994748	no
gene:SpnNT_00145	mnmA	Chromosome:141928-143050	110.58	110.58+peptide	OK	1157.91	662.917	-0.804626	-1.74253	0.0021	0.0307204	yes
gene:SpnNT_00145	mnmA	Chromosome:141928-143050	ΔORF2	110.58+peptide	OK	1116.8	662.917	-0.752468	-1.58203	0.00585	0.0677526	no
gene:SpnNT_00145	mnmA	Chromosome:141928-143050	110.58	ΔORF2+peptide	OK	1157.91	575.111	-1.00961	-2.2163	0.00035	0.00717609	yes
gene:SpnNT_00145	mnmA	Chromosome:141928-143050	ΔORF2	ΔORF2+peptide	OK	1116.8	575.111	-0.957456	-2.03887	0.00115	0.0187457	yes
gene:SpnNT_00145	mnmA	Chromosome:141928-143050	110.58+peptide	ΔORF2+peptide	OK	662.917	575.111	-0.204988	-0.449361	0.431	0.912485	no
gene:SpnNT_00146	NA	Chromosome:143189-143639	110.58	ΔORF2	OK	110.316	97.577	-0.177035	-0.342637	0.5462	0.962045	no
gene:SpnNT_00146	NA	Chromosome:143189-143639	110.58	110.58+peptide	OK	110.316	135.931	0.30123	0.598237	0.2847	0.787901	no
gene:SpnNT_00146	NA	Chromosome:143189-143639	ΔORF2	110.58+peptide	OK	97.577	135.931	0.478264	0.919316	0.1094	0.502467	no
gene:SpnNT_00146	NA	Chromosome:143189-143639	110.58	ΔORF2+peptide	OK	110.316	109.799	-0.00678529	-0.0132995	0.9809	0.99608	no
gene:SpnNT_00146	NA	Chromosome:143189-143639	ΔORF2	ΔORF2+peptide	OK	97.577	109.799	0.170249	0.323244	0.578	0.969538	no
gene:SpnNT_00146	NA	Chromosome:143189-143639	110.58+peptide	ΔORF2+peptide	OK	135.931	109.799	-0.308015	-0.599493	0.287	0.790183	no
gene:SpnNT_00147	mnmG	Chromosome:143655-145569	110.58	ΔORF2	OK	179.778	151.828	-0.243782	-0.551827	0.3285	0.834251	no
gene:SpnNT_00147	mnmG	Chromosome:143655-145569	110.58	110.58+peptide	OK	179.778	239.195	0.411972	0.925581	0.10195	0.484355	no
gene:SpnNT_00147	mnmG	Chromosome:143655-145569	ΔORF2	110.58+peptide	OK	151.828	239.195	0.655754	1.46247	0.0094	0.0963647	no
gene:SpnNT_00147	mnmG	Chromosome:143655-145569	110.58	ΔORF2+peptide	OK	179.778	209.679	0.221965	0.504234	0.37765	0.875802	no
gene:SpnNT_00147	mnmG	Chromosome:143655-145569	ΔORF2	ΔORF2+peptide	OK	151.828	209.679	0.465747	1.05009	0.064	0.367209	no
gene:SpnNT_00147	mnmG	Chromosome:143655-145569	110.58+peptide	ΔORF2+peptide	OK	239.195	209.679	-0.190007	-0.425223	0.4474	0.919465	no
gene:SpnNT_00148	NA	Chromosome:145920-147600	110.58	ΔORF2	OK	442.021	425.226	-0.0558869	-0.122252	0.83125	0.994748	no
gene:SpnNT_00148	NA	Chromosome:145920-147600	110.58	110.58+peptide	OK	442.021	344.961	-0.357682	-0.796006	0.16075	0.613543	no
gene:SpnNT_00148	NA	Chromosome:145920-147600	ΔORF2	110.58+peptide	OK	425.226	344.961	-0.301795	-0.666561	0.239	0.734291	no
gene:SpnNT_00148	NA	Chromosome:145920-147600	110.58	ΔORF2+peptide	OK	442.021	321.634	-0.458696	-1.02028	0.07205	0.395086	no
gene:SpnNT_00148	NA	Chromosome:145920-147600	ΔORF2	ΔORF2+peptide	OK	425.226	321.634	-0.402809	-0.889215	0.11595	0.516286	no
gene:SpnNT_00148	NA	Chromosome:145920-147600	110.58+peptide	ΔORF2+peptide	OK	344.961	321.634	-0.101014	-0.226938	0.68195	0.986608	no
gene:SpnNT_00149	NA	Chromosome:147601-147835	110.58	ΔORF2	OK	106.01	103.574	-0.0335376	-0.052735	0.92635	0.994748	no
gene:SpnNT_00149	NA	Chromosome:147601-147835	110.58	110.58+peptide	OK	106.01	82.5193	-0.361395	-0.567737	0.32315	0.82836	no
gene:SpnNT_00149	NA	Chromosome:147601-147835	ΔORF2	110.58+peptide	OK	103.574	82.5193	-0.327857	-0.513609	0.36665	0.864634	no
gene:SpnNT_00149	NA	Chromosome:147601-147835	110.58	ΔORF2+peptide	OK	106.01	92.3575	-0.198897	-0.3205	0.594	0.975539	no
gene:SpnNT_00149	NA	Chromosome:147601-147835	ΔORF2	ΔORF2+peptide	OK	103.574	92.3575	-0.165359	-0.265673	0.66145	0.982966	no
gene:SpnNT_00149	NA	Chromosome:147601-147835	110.58+peptide	ΔORF2+peptide	OK	82.5193	92.3575	0.162498	0.260824	0.6668	0.983929	no
gene:SpnNT_00150	NA	Chromosome:148114-148222	110.58	ΔORF2	NOTEST	5.44264	0	#NAME?	0	1	1	no
gene:SpnNT_00150	NA	Chromosome:148114-148222	110.58	110.58+peptide	NOTEST	5.44264	0	#NAME?	0	1	1	no
gene:SpnNT_00150	NA	Chromosome:148114-148222	ΔORF2	110.58+peptide	NOTEST	0	0	0	0	1	1	no
gene:SpnNT_00150	NA	Chromosome:148114-148222	110.58	ΔORF2+peptide	NOTEST	5.44264	0	#NAME?	0	1	1	no
gene:SpnNT_00150	NA	Chromosome:148114-148222	ΔORF2	ΔORF2+peptide	NOTEST	0	0	0	0	1	1	no

gene:SpnNT_00150	NA	Chromosome:148114-148222	110.58+peptide	ΔORF2+peptide	NOTEST	0	0	0	0	1	1	no
gene:SpnNT_00151	NA	Chromosome:148510-149653	110.58	ΔORF2	OK	6.81203	5.92826	-0.200477	-0.325043	0.57395	0.969538	no
gene:SpnNT_00151	NA	Chromosome:148510-149653	110.58	110.58+peptide	OK	6.81203	5.28121	-0.367216	-0.593606	0.29985	0.805452	no
gene:SpnNT_00151	NA	Chromosome:148510-149653	ΔORF2	110.58+peptide	OK	5.92826	5.28121	-0.166739	-0.265164	0.6457	0.980887	no
gene:SpnNT_00151	NA	Chromosome:148510-149653	110.58	ΔORF2+peptide	OK	6.81203	5.82888	-0.224866	-0.36404	0.5238	0.954832	no
gene:SpnNT_00151	NA	Chromosome:148510-149653	ΔORF2	ΔORF2+peptide	OK	5.92826	5.82888	-0.0243893	-0.0388424	0.94495	0.994855	no
gene:SpnNT_00151	NA	Chromosome:148510-149653	110.58+peptide	ΔORF2+peptide	OK	5.28121	5.82888	0.14235	0.226052	0.6891	0.98828	no
gene:SpnNT_00152	NA	Chromosome:149996-150149	110.58	ΔORF2	OK	53.275	40.6405	-0.390543	-0.362783	0.51955	0.954494	no
gene:SpnNT_00152	NA	Chromosome:149996-150149	110.58	110.58+peptide	OK	53.275	67.1905	0.334797	0.340272	0.5502	0.964281	no
gene:SpnNT_00152	NA	Chromosome:149996-150149	ΔORF2	110.58+peptide	OK	40.6405	67.1905	0.725339	0.681714	0.21285	0.698345	no
gene:SpnNT_00152	NA	Chromosome:149996-150149	110.58	ΔORF2+peptide	OK	53.275	68.6012	0.364774	0.372514	0.5237	0.954832	no
gene:SpnNT_00152	NA	Chromosome:149996-150149	ΔORF2	ΔORF2+peptide	OK	40.6405	68.6012	0.755317	0.712791	0.2069	0.692058	no
gene:SpnNT_00152	NA	Chromosome:149996-150149	110.58+peptide	ΔORF2+peptide	OK	67.1905	68.6012	0.0299773	0.0310505	0.95555	0.994855	no
gene:SpnNT_00153	NA	Chromosome:150150-150336	110.58	ΔORF2	OK	126.922	93.9576	-0.433866	-0.624869	0.29595	0.800323	no
gene:SpnNT_00153	NA	Chromosome:150150-150336	110.58	110.58+peptide	OK	126.922	160.099	0.335019	0.502427	0.38925	0.883652	no
gene:SpnNT_00153	NA	Chromosome:150150-150336	ΔORF2	110.58+peptide	OK	93.9576	160.099	0.768885	1.10426	0.0575	0.341564	no
gene:SpnNT_00153	NA	Chromosome:150150-150336	110.58	ΔORF2+peptide	OK	126.922	139.055	0.131713	0.184415	0.74305	0.994748	no
gene:SpnNT_00153	NA	Chromosome:150150-150336	ΔORF2	ΔORF2+peptide	OK	93.9576	139.055	0.565578	0.762413	0.1739	0.634412	no
gene:SpnNT_00153	NA	Chromosome:150150-150336	110.58+peptide	ΔORF2+peptide	OK	160.099	139.055	-0.203306	-0.283898	0.6047	0.976761	no
gene:SpnNT_00154	NA	Chromosome:150513-152631	110.58	ΔORF2	OK	170.157	154.637	-0.13798	-0.186168	0.7422	0.994748	no
gene:SpnNT_00154	NA	Chromosome:150513-152631	110.58	110.58+peptide	OK	170.157	186.284	0.130636	0.176178	0.7549	0.994748	no
gene:SpnNT_00154	NA	Chromosome:150513-152631	ΔORF2	110.58+peptide	OK	154.637	186.284	0.268616	0.357493	0.5314	0.957488	no
gene:SpnNT_00154	NA	Chromosome:150513-152631	110.58	ΔORF2+peptide	OK	170.157	156.518	-0.120537	-0.163985	0.7777	0.994748	no
gene:SpnNT_00154	NA	Chromosome:150513-152631	ΔORF2	ΔORF2+peptide	OK	154.637	156.518	0.0174431	0.0234128	0.967	0.994855	no
gene:SpnNT_00154	NA	Chromosome:150513-152631	110.58+peptide	ΔORF2+peptide	OK	186.284	156.518	-0.251173	-0.33698	0.56235	0.968621	no
gene:SpnNT_00155	NA	Chromosome:150513-152631	110.58	ΔORF2	OK	143.704	122.192	-0.233952	-0.194653	0.7301	0.994748	no
gene:SpnNT_00155	NA	Chromosome:150513-152631	110.58	110.58+peptide	OK	143.704	155.845	0.117014	0.097796	0.86355	0.994748	no
gene:SpnNT_00155	NA	Chromosome:150513-152631	ΔORF2	110.58+peptide	OK	122.192	155.845	0.350967	0.30143	0.5649	0.968621	no
gene:SpnNT_00155	NA	Chromosome:150513-152631	110.58	ΔORF2+peptide	OK	143.704	144.753	0.010491	0.00853824	0.9891	0.997703	no
gene:SpnNT_00155	NA	Chromosome:150513-152631	ΔORF2	ΔORF2+peptide	OK	122.192	144.753	0.244443	0.204146	0.70835	0.990441	no
gene:SpnNT_00155	NA	Chromosome:150513-152631	110.58+peptide	ΔORF2+peptide	OK	155.845	144.753	-0.106523	-0.0893655	0.87275	0.994748	no
gene:SpnNT_00156	gcp	Chromosome:150513-152631	110.58	ΔORF2	OK	143.582	122.239	-0.232171	-0.373027	0.5102	0.948413	no
gene:SpnNT_00156	gcp	Chromosome:150513-152631	110.58	110.58+peptide	OK	143.582	156.155	0.121106	0.194314	0.7342	0.994748	no
gene:SpnNT_00156	gcp	Chromosome:150513-152631	ΔORF2	110.58+peptide	OK	122.239	156.155	0.353277	0.562017	0.31805	0.824141	no
gene:SpnNT_00156	gcp	Chromosome:150513-152631	110.58	ΔORF2+peptide	OK	143.582	132.633	-0.114436	-0.186219	0.7412	0.994748	no
gene:SpnNT_00156	gcp	Chromosome:150513-152631	ΔORF2	ΔORF2+peptide	OK	122.239	132.633	0.117735	0.189914	0.7331	0.994748	no
gene:SpnNT_00156	gcp	Chromosome:150513-152631	110.58+peptide	ΔORF2+peptide	OK	156.155	132.633	-0.235542	-0.379422	0.50365	0.945208	no
gene:SpnNT_00157	NA	Chromosome:152734-153598	110.58	ΔORF2	OK	204.772	224.461	0.132444	0.303735	0.5845	0.971855	no
gene:SpnNT_00157	NA	Chromosome:152734-153598	110.58	110.58+peptide	OK	204.772	266.315	0.379113	0.874138	0.1246	0.533791	no
gene:SpnNT_00157	NA	Chromosome:152734-153598	ΔORF2	110.58+peptide	OK	224.461	266.315	0.246669	0.567742	0.31445	0.820974	no
gene:SpnNT_00157	NA	Chromosome:152734-153598	110.58	ΔORF2+peptide	OK	204.772	284.497	0.474395	1.09109	0.05485	0.33306	no
gene:SpnNT_00157	NA	Chromosome:152734-153598	ΔORF2	ΔORF2+peptide	OK	224.461	284.497	0.341951	0.785079	0.1635	0.617928	no
gene:SpnNT_00157	NA	Chromosome:152734-153598	110.58+peptide	ΔORF2+peptide	OK	266.315	284.497	0.0952825	0.219947	0.70195	0.990367	no
gene:SpnNT_00158	NA	Chromosome:154095-157689	110.58	ΔORF2	OK	44.5565	66.463	0.576914	1.03956	0.0792	0.419057	no
gene:SpnNT_00158	NA	Chromosome:154095-157689	110.58	110.58+peptide	OK	44.5565	39.6197	-0.169421	-0.278756	0.6316	0.980887	no

gene:SpnNT_00158	NA	Chromosome:154095-157689	ΔORF2	110.58+peptide	OK	66.463	39.6197	-0.746334	-1.26227	0.03415	0.243541	no
gene:SpnNT_00158	NA	Chromosome:154095-157689	110.58	ΔORF2+peptide	OK	44.5565	67.4937	0.599115	1.08391	0.0625	0.3611	no
gene:SpnNT_00158	NA	Chromosome:154095-157689	ΔORF2	ΔORF2+peptide	OK	66.463	67.4937	0.0222008	0.0415337	0.94385	0.994855	no
gene:SpnNT_00158	NA	Chromosome:154095-157689	110.58+peptide	ΔORF2+peptide	OK	39.6197	67.4937	0.768535	1.30443	0.03085	0.226786	no
gene:SpnNT_00159	NA	Chromosome:154095-157689	110.58	ΔORF2	OK	98.5036	85.2709	-0.208123	-0.0582623	0.91455	0.994748	no
gene:SpnNT_00159	NA	Chromosome:154095-157689	110.58	110.58+peptide	OK	98.5036	94.9441	-0.0530987	-0.0173683	0.96885	0.994855	no
gene:SpnNT_00159	NA	Chromosome:154095-157689	ΔORF2	110.58+peptide	OK	85.2709	94.9441	0.155024	0.0439313	0.9399	0.994855	no
gene:SpnNT_00159	NA	Chromosome:154095-157689	110.58	ΔORF2+peptide	OK	98.5036	117.24	0.251215	0.0727398	0.89915	0.994748	no
gene:SpnNT_00159	NA	Chromosome:154095-157689	ΔORF2	ΔORF2+peptide	OK	85.2709	117.24	0.459338	0.11847	0.80045	0.994748	no
gene:SpnNT_00159	NA	Chromosome:154095-157689	110.58+peptide	ΔORF2+peptide	OK	94.9441	117.24	0.304313	0.0892753	0.88445	0.994748	no
gene:SpnNT_00160	NA	Chromosome:154095-157689	110.58	ΔORF2	OK	41.9838	43.1216	0.0385789	0.040929	0.93905	0.994855	no
gene:SpnNT_00160	NA	Chromosome:154095-157689	110.58	110.58+peptide	OK	41.9838	40.7503	-0.0430199	-0.0464093	0.93085	0.994855	no
gene:SpnNT_00160	NA	Chromosome:154095-157689	ΔORF2	110.58+peptide	OK	43.1216	40.7503	-0.0815988	-0.0807149	0.88055	0.994748	no
gene:SpnNT_00160	NA	Chromosome:154095-157689	110.58	ΔORF2+peptide	OK	41.9838	52.8845	0.333014	0.395864	0.486	0.937975	no
gene:SpnNT_00160	NA	Chromosome:154095-157689	ΔORF2	ΔORF2+peptide	OK	43.1216	52.8845	0.294435	0.31559	0.56595	0.968621	no
gene:SpnNT_00160	NA	Chromosome:154095-157689	110.58+peptide	ΔORF2+peptide	OK	40.7503	52.8845	0.376033	0.409985	0.4577	0.921722	no
gene:SpnNT_00161	ygaZ	Chromosome:158132-158762	110.58	ΔORF2	OK	67.6039	62.7188	-0.108208	-0.215394	0.6919	0.98828	no
gene:SpnNT_00161	ygaZ	Chromosome:158132-158762	110.58	110.58+peptide	OK	67.6039	79.6424	0.236432	0.473548	0.4064	0.895965	no
gene:SpnNT_00161	ygaZ	Chromosome:158132-158762	ΔORF2	110.58+peptide	OK	62.7188	79.6424	0.34464	0.682842	0.2202	0.708363	no
gene:SpnNT_00161	ygaZ	Chromosome:158132-158762	110.58	ΔORF2+peptide	OK	67.6039	75.3263	0.156048	0.314929	0.5845	0.971855	no
gene:SpnNT_00161	ygaZ	Chromosome:158132-158762	ΔORF2	ΔORF2+peptide	OK	62.7188	75.3263	0.264256	0.527479	0.3489	0.853357	no
gene:SpnNT_00161	ygaZ	Chromosome:158132-158762	110.58+peptide	ΔORF2+peptide	OK	79.6424	75.3263	-0.0803839	-0.161454	0.77715	0.994748	no
gene:SpnNT_00162	NA	Chromosome:158779-159103	110.58	ΔORF2	OK	48.155	39.2705	-0.294239	-0.477525	0.4052	0.894933	no
gene:SpnNT_00162	NA	Chromosome:158779-159103	110.58	110.58+peptide	OK	48.155	42.2821	-0.187637	-0.303852	0.5935	0.975539	no
gene:SpnNT_00162	NA	Chromosome:158779-159103	ΔORF2	110.58+peptide	OK	39.2705	42.2821	0.106601	0.168435	0.7652	0.994748	no
gene:SpnNT_00162	NA	Chromosome:158779-159103	110.58	ΔORF2+peptide	OK	48.155	51.3298	0.0921112	0.153351	0.7961	0.994748	no
gene:SpnNT_00162	NA	Chromosome:158779-159103	ΔORF2	ΔORF2+peptide	OK	39.2705	51.3298	0.38635	0.626745	0.28825	0.792622	no
gene:SpnNT_00162	NA	Chromosome:158779-159103	110.58+peptide	ΔORF2+peptide	OK	42.2821	51.3298	0.279749	0.45282	0.4435	0.917631	no
gene:SpnNT_00163	tcyA	Chromosome:159206-160037	110.58	ΔORF2	OK	324.123	368.839	0.18645	0.419331	0.46595	0.926452	no
gene:SpnNT_00163	tcyA	Chromosome:159206-160037	110.58	110.58+peptide	OK	324.123	310.933	-0.0599399	-0.136052	0.8125	0.994748	no
gene:SpnNT_00163	tcyA	Chromosome:159206-160037	ΔORF2	110.58+peptide	OK	368.839	310.933	-0.24639	-0.553633	0.3292	0.83499	no
gene:SpnNT_00163	tcyA	Chromosome:159206-160037	110.58	ΔORF2+peptide	OK	324.123	303.787	-0.093483	-0.212235	0.7185	0.993483	no
gene:SpnNT_00163	tcyA	Chromosome:159206-160037	ΔORF2	ΔORF2+peptide	OK	368.839	303.787	-0.279933	-0.629141	0.2737	0.775958	no
gene:SpnNT_00163	tcyA	Chromosome:159206-160037	110.58+peptide	ΔORF2+peptide	OK	310.933	303.787	-0.0335431	-0.0760826	0.89755	0.994748	no
gene:SpnNT_00164	metQ	Chromosome:160190-161045	110.58	ΔORF2	OK	117.506	127.486	0.117606	0.263131	0.64675	0.981116	no
gene:SpnNT_00164	metQ	Chromosome:160190-161045	110.58	110.58+peptide	OK	117.506	98.5415	-0.253931	-0.562395	0.3214	0.827268	no
gene:SpnNT_00164	metQ	Chromosome:160190-161045	ΔORF2	110.58+peptide	OK	127.486	98.5415	-0.371537	-0.824545	0.14985	0.591417	no
gene:SpnNT_00164	metQ	Chromosome:160190-161045	110.58	ΔORF2+peptide	OK	117.506	107.04	-0.134585	-0.297761	0.6017	0.976761	no
gene:SpnNT_00164	metQ	Chromosome:160190-161045	ΔORF2	ΔORF2+peptide	OK	127.486	107.04	-0.252191	-0.559096	0.3291	0.834967	no
gene:SpnNT_00164	metQ	Chromosome:160190-161045	110.58+peptide	ΔORF2+peptide	OK	98.5415	107.04	0.119346	0.261957	0.64475	0.980887	no
gene:SpnNT_00165	dapE	Chromosome:161144-163572	110.58	ΔORF2	OK	43.5854	38.6525	-0.173283	-0.221165	0.70215	0.990367	no
gene:SpnNT_00165	dapE	Chromosome:161144-163572	110.58	110.58+peptide	OK	43.5854	39.5043	-0.141833	-0.181825	0.7468	0.994748	no
gene:SpnNT_00165	dapE	Chromosome:161144-163572	ΔORF2	110.58+peptide	OK	38.6525	39.5043	0.0314508	0.0411617	0.9435	0.994855	no
gene:SpnNT_00165	dapE	Chromosome:161144-163572	110.58	ΔORF2+peptide	OK	43.5854	37.6827	-0.209941	-0.268334	0.64375	0.980887	no
gene:SpnNT_00165	dapE	Chromosome:161144-163572	ΔORF2	ΔORF2+peptide	OK	38.6525	37.6827	-0.0366574	-0.0478267	0.934	0.994855	no

gene:SpnNT_00165	dapE	Chromosome:161144-163572	110.58+peptide	ΔORF2+peptide	OK	39.5043	37.6827	-0.0681082	-0.0892716	0.8796	0.994748	no
gene:SpnNT_00166	metN	Chromosome:161144-163572	110.58	ΔORF2	OK	119.054	104.924	-0.182261	-0.338547	0.5545	0.966389	no
gene:SpnNT_00166	metN	Chromosome:161144-163572	110.58	110.58+peptide	OK	119.054	103.479	-0.202271	-0.373425	0.5087	0.947472	no
gene:SpnNT_00166	metN	Chromosome:161144-163572	ΔORF2	110.58+peptide	OK	104.924	103.479	-0.0200093	-0.0368619	0.94845	0.994855	no
gene:SpnNT_00166	metN	Chromosome:161144-163572	110.58	ΔORF2+peptide	OK	119.054	95.5291	-0.317601	-0.587342	0.3035	0.809137	no
gene:SpnNT_00166	metN	Chromosome:161144-163572	ΔORF2	ΔORF2+peptide	OK	104.924	95.5291	-0.13534	-0.24975	0.66455	0.983188	no
gene:SpnNT_00166	metN	Chromosome:161144-163572	110.58+peptide	ΔORF2+peptide	OK	103.479	95.5291	-0.11533	-0.211546	0.7115	0.991007	no
gene:SpnNT_00167	metP	Chromosome:163573-164266	110.58	ΔORF2	OK	166.91	153.561	-0.120264	-0.268651	0.62835	0.980887	no
gene:SpnNT_00167	metP	Chromosome:163573-164266	110.58	110.58+peptide	OK	166.91	145.241	-0.200627	-0.444681	0.42655	0.908594	no
gene:SpnNT_00167	metP	Chromosome:163573-164266	ΔORF2	110.58+peptide	OK	153.561	145.241	-0.0803627	-0.177309	0.7506	0.994748	no
gene:SpnNT_00167	metP	Chromosome:163573-164266	110.58	ΔORF2+peptide	OK	166.91	129.43	-0.366897	-0.812405	0.14975	0.591417	no
gene:SpnNT_00167	metP	Chromosome:163573-164266	ΔORF2	ΔORF2+peptide	OK	153.561	129.43	-0.246632	-0.543625	0.3353	0.840108	no
gene:SpnNT_00167	metP	Chromosome:163573-164266	110.58+peptide	ΔORF2+peptide	OK	145.241	129.43	-0.16627	-0.363712	0.52305	0.954832	no
gene:SpnNT_00168	NA	Chromosome:164295-164865	110.58	ΔORF2	OK	25.7386	21.6603	-0.248883	-0.421869	0.46605	0.926452	no
gene:SpnNT_00168	NA	Chromosome:164295-164865	110.58	110.58+peptide	OK	25.7386	48.3209	0.908713	1.61728	0.0054	0.0637793	no
gene:SpnNT_00168	NA	Chromosome:164295-164865	ΔORF2	110.58+peptide	OK	21.6603	48.3209	1.1576	2.07089	0.00035	0.00717609	yes
gene:SpnNT_00168	NA	Chromosome:164295-164865	110.58	ΔORF2+peptide	OK	25.7386	50.4205	0.970077	1.71807	0.0029	0.0388304	yes
gene:SpnNT_00168	NA	Chromosome:164295-164865	ΔORF2	ΔORF2+peptide	OK	21.6603	50.4205	1.21896	2.16993	1.00E-04	0.0025332	yes
gene:SpnNT_00168	NA	Chromosome:164295-164865	110.58+peptide	ΔORF2+peptide	OK	48.3209	50.4205	0.0613636	0.115304	0.8461	0.994748	no
gene:SpnNT_00169	NA	Chromosome:164999-165768	110.58	ΔORF2	OK	40.4237	43.438	0.103758	0.194002	0.7382	0.994748	no
gene:SpnNT_00169	NA	Chromosome:164999-165768	110.58	110.58+peptide	OK	40.4237	67.9403	0.749066	1.43688	0.01455	0.13113	no
gene:SpnNT_00169	NA	Chromosome:164999-165768	ΔORF2	110.58+peptide	OK	43.438	67.9403	0.645308	1.24473	0.03185	0.231907	no
gene:SpnNT_00169	NA	Chromosome:164999-165768	110.58	ΔORF2+peptide	OK	40.4237	77.1411	0.932298	1.80863	0.0028	0.0378	yes
gene:SpnNT_00169	NA	Chromosome:164999-165768	ΔORF2	ΔORF2+peptide	OK	43.438	77.1411	0.828541	1.61648	0.0055	0.064496	no
gene:SpnNT_00169	NA	Chromosome:164999-165768	110.58+peptide	ΔORF2+peptide	OK	67.9403	77.1411	0.183232	0.367609	0.5169	0.953305	no
gene:SpnNT_00170	NA	Chromosome:164999-165768	110.58	ΔORF2	OK	3.95162	3.15532	-0.324659	-0.132037	0.8439	0.994748	no
gene:SpnNT_00170	NA	Chromosome:164999-165768	110.58	110.58+peptide	OK	3.95162	7.41858	0.9087	0.193269	0.7103	0.990984	no
gene:SpnNT_00170	NA	Chromosome:164999-165768	ΔORF2	110.58+peptide	OK	3.15532	7.41858	1.23336	0.257075	0.57835	0.969538	no
gene:SpnNT_00170	NA	Chromosome:164999-165768	110.58	ΔORF2+peptide	OK	3.95162	0.601351	-2.71616	-0.756783	0.40525	0.894933	no
gene:SpnNT_00170	NA	Chromosome:164999-165768	ΔORF2	ΔORF2+peptide	OK	3.15532	0.601351	-2.39151	-0.643938	0.4166	0.901786	no
gene:SpnNT_00170	NA	Chromosome:164999-165768	110.58+peptide	ΔORF2+peptide	OK	7.41858	0.601351	-3.62486	-0.663432	0.33935	0.844054	no
gene:SpnNT_00171	NA	Chromosome:165859-167248	110.58	ΔORF2	OK	35.3915	31.908	-0.149484	-0.311151	0.5968	0.97629	no
gene:SpnNT_00171	NA	Chromosome:165859-167248	110.58	110.58+peptide	OK	35.3915	58.433	0.723379	1.54113	0.00855	0.0898983	no
gene:SpnNT_00171	NA	Chromosome:165859-167248	ΔORF2	110.58+peptide	OK	31.908	58.433	0.872863	1.85893	0.00165	0.0253528	yes
gene:SpnNT_00171	NA	Chromosome:165859-167248	110.58	ΔORF2+peptide	OK	35.3915	57.9627	0.711721	1.52175	0.00895	0.0931339	no
gene:SpnNT_00171	NA	Chromosome:165859-167248	ΔORF2	ΔORF2+peptide	OK	31.908	57.9627	0.861205	1.8407	0.00175	0.0265167	yes
gene:SpnNT_00171	NA	Chromosome:165859-167248	110.58+peptide	ΔORF2+peptide	OK	58.433	57.9627	-0.0116578	-0.0255362	0.9664	0.994855	no
gene:SpnNT_00172	NA	Chromosome:167259-168546	110.58	ΔORF2	OK	32.7433	29.5316	-0.148938	-0.30369	0.59405	0.975539	no
gene:SpnNT_00172	NA	Chromosome:167259-168546	110.58	110.58+peptide	OK	32.7433	53.5319	0.7092	1.49744	0.0092	0.0949075	no
gene:SpnNT_00172	NA	Chromosome:167259-168546	ΔORF2	110.58+peptide	OK	29.5316	53.5319	0.858138	1.79478	0.0028	0.0378	yes
gene:SpnNT_00172	NA	Chromosome:167259-168546	110.58	ΔORF2+peptide	OK	32.7433	54.5019	0.735109	1.55065	0.006	0.0690632	no
gene:SpnNT_00172	NA	Chromosome:167259-168546	ΔORF2	ΔORF2+peptide	OK	29.5316	54.5019	0.884046	1.84722	0.0019	0.0281715	yes
gene:SpnNT_00172	NA	Chromosome:167259-168546	110.58+peptide	ΔORF2+peptide	OK	53.5319	54.5019	0.0259086	0.0561605	0.92075	0.994748	no
gene:SpnNT_00173	NA	Chromosome:168606-169140	110.58	ΔORF2	OK	1214.22	1725.9	0.507319	1.09222	0.0575	0.341564	no
gene:SpnNT_00173	NA	Chromosome:168606-169140	110.58	110.58+peptide	OK	1214.22	1190.8	-0.028104	-0.0609408	0.91345	0.994748	no

gene:SpnNT_00173	NA	Chromosome:168606-169140	ΔORF2	110.58+peptide	OK	1725.9	1190.8	-0.535423	-1.13175	0.0449	0.291528	no
gene:SpnNT_00173	NA	Chromosome:168606-169140	110.58	ΔORF2+peptide	OK	1214.22	1115.01	-0.122968	-0.275701	0.62825	0.980887	no
gene:SpnNT_00173	NA	Chromosome:168606-169140	ΔORF2	ΔORF2+peptide	OK	1725.9	1115.01	-0.630288	-1.37516	0.0155	0.137612	no
gene:SpnNT_00173	NA	Chromosome:168606-169140	110.58+peptide	ΔORF2+peptide	OK	1190.8	1115.01	-0.0948643	-0.208503	0.7029	0.990367	no
gene:SpnNT_00174	NA	Chromosome:169217-169688	110.58	ΔORF2	OK	189.46	175.788	-0.108055	-0.229847	0.6931	0.98828	no
gene:SpnNT_00174	NA	Chromosome:169217-169688	110.58	110.58+peptide	OK	189.46	101.032	-0.907079	-1.84816	0.00115	0.0187457	yes
gene:SpnNT_00174	NA	Chromosome:169217-169688	ΔORF2	110.58+peptide	OK	175.788	101.032	-0.799024	-1.62944	0.0041	0.0516317	no
gene:SpnNT_00174	NA	Chromosome:169217-169688	110.58	ΔORF2+peptide	OK	189.46	85.0357	-1.15575	-2.31593	5.00E-05	0.0013612	yes
gene:SpnNT_00174	NA	Chromosome:169217-169688	ΔORF2	ΔORF2+peptide	OK	175.788	85.0357	-1.0477	-2.1012	2.00E-04	0.00450928	yes
gene:SpnNT_00174	NA	Chromosome:169217-169688	110.58+peptide	ΔORF2+peptide	OK	101.032	85.0357	-0.248673	-0.47991	0.38415	0.881416	no
gene:SpnNT_00175	NA	Chromosome:169980-171243	110.58	ΔORF2	OK	73.5206	64.294	-0.193465	-0.395947	0.48165	0.935095	no
gene:SpnNT_00175	NA	Chromosome:169980-171243	110.58	110.58+peptide	OK	73.5206	285.471	1.95712	4.02675	5.00E-05	0.0013612	yes
gene:SpnNT_00175	NA	Chromosome:169980-171243	ΔORF2	110.58+peptide	OK	64.294	285.471	2.15059	4.51552	5.00E-05	0.0013612	yes
gene:SpnNT_00175	NA	Chromosome:169980-171243	110.58	ΔORF2+peptide	OK	73.5206	281.989	1.93942	4.12647	5.00E-05	0.0013612	yes
gene:SpnNT_00175	NA	Chromosome:169980-171243	ΔORF2	ΔORF2+peptide	OK	64.294	281.989	2.13288	4.63781	5.00E-05	0.0013612	yes
gene:SpnNT_00175	NA	Chromosome:169980-171243	110.58+peptide	ΔORF2+peptide	OK	285.471	281.989	-0.0177053	-0.0387303	0.946	0.994855	no
gene:SpnNT_00176	NA	Chromosome:171553-172449	110.58	ΔORF2	OK	8.67166	8.88951	0.0357962	0.0425203	0.9369	0.994855	no
gene:SpnNT_00176	NA	Chromosome:171553-172449	110.58	110.58+peptide	OK	8.67166	8.5143	-0.0264199	-0.0303201	0.9569	0.994855	no
gene:SpnNT_00176	NA	Chromosome:171553-172449	ΔORF2	110.58+peptide	OK	8.88951	8.5143	-0.0622162	-0.0717177	0.90185	0.994748	no
gene:SpnNT_00176	NA	Chromosome:171553-172449	110.58	ΔORF2+peptide	OK	8.67166	5.27543	-0.71702	-0.680107	0.24725	0.743921	no
gene:SpnNT_00176	NA	Chromosome:171553-172449	ΔORF2	ΔORF2+peptide	OK	8.88951	5.27543	-0.752816	-0.716221	0.2254	0.716568	no
gene:SpnNT_00176	NA	Chromosome:171553-172449	110.58+peptide	ΔORF2+peptide	OK	8.5143	5.27543	-0.6906	-0.642497	0.26795	0.768869	no
gene:SpnNT_00177	NA	Chromosome:171553-172449	110.58	ΔORF2	OK	3.56926	3.49478	-0.0304213	-0.0204827	0.9746	0.99536	no
gene:SpnNT_00177	NA	Chromosome:171553-172449	110.58	110.58+peptide	OK	3.56926	4.55696	0.352449	0.253568	0.6745	0.985063	no
gene:SpnNT_00177	NA	Chromosome:171553-172449	ΔORF2	110.58+peptide	OK	3.49478	4.55696	0.38287	0.264286	0.65705	0.982966	no
gene:SpnNT_00177	NA	Chromosome:171553-172449	110.58	ΔORF2+peptide	OK	3.56926	4.82638	0.435317	0.306219	0.5958	0.976245	no
gene:SpnNT_00177	NA	Chromosome:171553-172449	ΔORF2	ΔORF2+peptide	OK	3.49478	4.82638	0.465738	0.314886	0.5801	0.969538	no
gene:SpnNT_00177	NA	Chromosome:171553-172449	110.58+peptide	ΔORF2+peptide	OK	4.55696	4.82638	0.0828682	0.0599027	0.91425	0.994748	no
gene:SpnNT_00178	mutL	Chromosome:172654-174604	110.58	ΔORF2	OK	80.9961	76.7278	-0.0781022	-0.17819	0.7542	0.994748	no
gene:SpnNT_00178	mutL	Chromosome:172654-174604	110.58	110.58+peptide	OK	80.9961	64.1392	-0.336646	-0.770828	0.1703	0.628955	no
gene:SpnNT_00178	mutL	Chromosome:172654-174604	ΔORF2	110.58+peptide	OK	76.7278	64.1392	-0.258543	-0.588837	0.2953	0.799613	no
gene:SpnNT_00178	mutL	Chromosome:172654-174604	110.58	ΔORF2+peptide	OK	80.9961	64.1127	-0.337241	-0.771405	0.16905	0.626454	no
gene:SpnNT_00178	mutL	Chromosome:172654-174604	ΔORF2	ΔORF2+peptide	OK	76.7278	64.1127	-0.259139	-0.589598	0.2943	0.798553	no
gene:SpnNT_00178	mutL	Chromosome:172654-174604	110.58+peptide	ΔORF2+peptide	OK	64.1392	64.1127	-0.000595284	-0.00135926	0.9983	0.999412	no
gene:SpnNT_00179	ribH	Chromosome:174928-175396	110.58	ΔORF2	OK	23.3309	27.242	0.223593	0.365681	0.524	0.954832	no
gene:SpnNT_00179	ribH	Chromosome:174928-175396	110.58	110.58+peptide	OK	23.3309	54.833	1.2328	2.11014	0.00065	0.0118958	yes
gene:SpnNT_00179	ribH	Chromosome:174928-175396	ΔORF2	110.58+peptide	OK	27.242	54.833	1.00921	1.75351	0.00265	0.0362222	yes
gene:SpnNT_00179	ribH	Chromosome:174928-175396	110.58	ΔORF2+peptide	OK	23.3309	66.7966	1.51753	2.60139	0.00015	0.00355289	yes
gene:SpnNT_00179	ribH	Chromosome:174928-175396	ΔORF2	ΔORF2+peptide	OK	27.242	66.7966	1.29394	2.25169	0.00015	0.00355289	yes
gene:SpnNT_00179	ribH	Chromosome:174928-175396	110.58+peptide	ΔORF2+peptide	OK	54.833	66.7966	0.284731	0.521862	0.34925	0.853357	no
gene:SpnNT_00180	ribBA	Chromosome:175396-176602	110.58	ΔORF2	OK	17.7309	20.2701	0.193084	0.363781	0.51785	0.953876	no
gene:SpnNT_00180	ribBA	Chromosome:175396-176602	110.58	110.58+peptide	OK	17.7309	39.3466	1.14997	2.23849	0.00015	0.00355289	yes
gene:SpnNT_00180	ribBA	Chromosome:175396-176602	ΔORF2	110.58+peptide	OK	20.2701	39.3466	0.956888	1.89698	0.00115	0.0187457	yes
gene:SpnNT_00180	ribBA	Chromosome:175396-176602	110.58	ΔORF2+peptide	OK	17.7309	58.4787	1.72164	3.4118	5.00E-05	0.0013612	yes
gene:SpnNT_00180	ribBA	Chromosome:175396-176602	ΔORF2	ΔORF2+peptide	OK	20.2701	58.4787	1.52856	3.0871	5.00E-05	0.0013612	yes

gene:SpnNT_00180	ribBA	Chromosome:175396-176602	110.58+peptide	ΔORF2+peptide	OK	39.3466	58.4787	0.571673	1.19891	0.0359	0.252049	no
gene:SpnNT_00181	ribE	Chromosome:176621-178342	110.58	ΔORF2	OK	17.58	20.3733	0.212751	0.22491	0.6979	0.990209	no
gene:SpnNT_00181	ribE	Chromosome:176621-178342	110.58	110.58+peptide	OK	17.58	41.2274	1.22967	1.35837	0.0232	0.186653	no
gene:SpnNT_00181	ribE	Chromosome:176621-178342	ΔORF2	110.58+peptide	OK	20.3733	41.2274	1.01692	1.1989	0.04405	0.287717	no
gene:SpnNT_00181	ribE	Chromosome:176621-178342	110.58	ΔORF2+peptide	OK	17.58	51.5803	1.55289	1.67848	0.00555	0.0650242	no
gene:SpnNT_00181	ribE	Chromosome:176621-178342	ΔORF2	ΔORF2+peptide	OK	20.3733	51.5803	1.34014	1.54139	0.0089	0.0927608	no
gene:SpnNT_00181	ribE	Chromosome:176621-178342	110.58+peptide	ΔORF2+peptide	OK	41.2274	51.5803	0.323217	0.391785	0.49895	0.944017	no
gene:SpnNT_00182	ribD_1	Chromosome:176621-178342	110.58	ΔORF2	OK	19.3887	18.9401	-0.0337717	-0.0542208	0.92315	0.994748	no
gene:SpnNT_00182	ribD_1	Chromosome:176621-178342	110.58	110.58+peptide	OK	19.3887	34.1429	0.816372	1.33469	0.0218	0.179011	no
gene:SpnNT_00182	ribD_1	Chromosome:176621-178342	ΔORF2	110.58+peptide	OK	18.9401	34.1429	0.850144	1.38793	0.0158	0.139615	no
gene:SpnNT_00182	ribD_1	Chromosome:176621-178342	110.58	ΔORF2+peptide	OK	19.3887	55.5954	1.51975	2.58613	5.00E-05	0.0013612	yes
gene:SpnNT_00182	ribD_1	Chromosome:176621-178342	ΔORF2	ΔORF2+peptide	OK	18.9401	55.5954	1.55353	2.63954	5.00E-05	0.0013612	yes
gene:SpnNT_00182	ribD_1	Chromosome:176621-178342	110.58+peptide	ΔORF2+peptide	OK	34.1429	55.5954	0.703382	1.21968	0.0354	0.249607	no
gene:SpnNT_00183	ruvA	Chromosome:178745-179339	110.58	ΔORF2	OK	73.8485	68.4778	-0.108933	-0.215027	0.69975	0.990367	no
gene:SpnNT_00183	ruvA	Chromosome:178745-179339	110.58	110.58+peptide	OK	73.8485	57.6557	-0.357105	-0.701886	0.2164	0.70408	no
gene:SpnNT_00183	ruvA	Chromosome:178745-179339	ΔORF2	110.58+peptide	OK	68.4778	57.6557	-0.248173	-0.480525	0.401	0.893669	no
gene:SpnNT_00183	ruvA	Chromosome:178745-179339	110.58	ΔORF2+peptide	OK	73.8485	54.3197	-0.443094	-0.863004	0.12575	0.53679	no
gene:SpnNT_00183	ruvA	Chromosome:178745-179339	ΔORF2	ΔORF2+peptide	OK	68.4778	54.3197	-0.334162	-0.641329	0.25285	0.751101	no
gene:SpnNT_00183	ruvA	Chromosome:178745-179339	110.58+peptide	ΔORF2+peptide	OK	57.6557	54.3197	-0.0859888	-0.164362	0.76385	0.994748	no
gene:SpnNT_00184	tag	Chromosome:179348-180586	110.58	ΔORF2	OK	61.0947	57.7017	-0.0824335	-0.117399	0.83915	0.994748	no
gene:SpnNT_00184	tag	Chromosome:179348-180586	110.58	110.58+peptide	OK	61.0947	50.6771	-0.269714	-0.407788	0.4818	0.935095	no
gene:SpnNT_00184	tag	Chromosome:179348-180586	ΔORF2	110.58+peptide	OK	57.7017	50.6771	-0.18728	-0.263158	0.6508	0.981475	no
gene:SpnNT_00184	tag	Chromosome:179348-180586	110.58	ΔORF2+peptide	OK	61.0947	48.0941	-0.345186	-0.475286	0.40825	0.897087	no
gene:SpnNT_00184	tag	Chromosome:179348-180586	ΔORF2	ΔORF2+peptide	OK	57.7017	48.0941	-0.262753	-0.340212	0.5606	0.968621	no
gene:SpnNT_00184	tag	Chromosome:179348-180586	110.58+peptide	ΔORF2+peptide	OK	50.6771	48.0941	-0.0754727	-0.10262	0.85835	0.994748	no
gene:SpnNT_00185	NA	Chromosome:179348-180586	110.58	ΔORF2	OK	33.9644	43.13	0.344669	0.464767	0.424	0.907286	no
gene:SpnNT_00185	NA	Chromosome:179348-180586	110.58	110.58+peptide	OK	33.9644	35.8049	0.0761345	0.105964	0.8535	0.994748	no
gene:SpnNT_00185	NA	Chromosome:179348-180586	ΔORF2	110.58+peptide	OK	43.13	35.8049	-0.268534	-0.37283	0.5195	0.954494	no
gene:SpnNT_00185	NA	Chromosome:179348-180586	110.58	ΔORF2+peptide	OK	33.9644	41.5943	0.292365	0.400261	0.4788	0.933291	no
gene:SpnNT_00185	NA	Chromosome:179348-180586	ΔORF2	ΔORF2+peptide	OK	43.13	41.5943	-0.0523037	-0.0714362	0.9	0.994748	no
gene:SpnNT_00185	NA	Chromosome:179348-180586	110.58+peptide	ΔORF2+peptide	OK	35.8049	41.5943	0.216231	0.305081	0.5889	0.974101	no
gene:SpnNT_00186	mccF	Chromosome:180722-181754	110.58	ΔORF2	OK	65.9014	72.7026	0.141699	0.307881	0.5873	0.973049	no
gene:SpnNT_00186	mccF	Chromosome:180722-181754	110.58	110.58+peptide	OK	65.9014	29.602	-1.15462	-2.38305	5.00E-05	0.0013612	yes
gene:SpnNT_00186	mccF	Chromosome:180722-181754	ΔORF2	110.58+peptide	OK	72.7026	29.602	-1.29631	-2.68497	5.00E-05	0.0013612	yes
gene:SpnNT_00186	mccF	Chromosome:180722-181754	110.58	ΔORF2+peptide	OK	65.9014	29.62	-1.15374	-2.37657	5.00E-05	0.0013612	yes
gene:SpnNT_00186	mccF	Chromosome:180722-181754	ΔORF2	ΔORF2+peptide	OK	72.7026	29.62	-1.29544	-2.67785	5.00E-05	0.0013612	yes
gene:SpnNT_00186	mccF	Chromosome:180722-181754	110.58+peptide	ΔORF2+peptide	OK	29.602	29.62	0.000878025	0.00173212	0.9973	0.999412	no
gene:SpnNT_00187	NA	Chromosome:182008-182692	110.58	ΔORF2	OK	99.0717	94.8134	-0.0633826	-0.134446	0.8108	0.994748	no
gene:SpnNT_00187	NA	Chromosome:182008-182692	110.58	110.58+peptide	OK	99.0717	119.349	0.268641	0.578017	0.31175	0.817666	no
gene:SpnNT_00187	NA	Chromosome:182008-182692	ΔORF2	110.58+peptide	OK	94.8134	119.349	0.332023	0.710576	0.21345	0.698651	no
gene:SpnNT_00187	NA	Chromosome:182008-182692	110.58	ΔORF2+peptide	OK	99.0717	118.196	0.254639	0.546291	0.3466	0.850868	no
gene:SpnNT_00187	NA	Chromosome:182008-182692	ΔORF2	ΔORF2+peptide	OK	94.8134	118.196	0.318022	0.678645	0.24325	0.739024	no
gene:SpnNT_00187	NA	Chromosome:182008-182692	110.58+peptide	ΔORF2+peptide	OK	119.349	118.196	-0.0140013	-0.0303125	0.9576	0.994855	no
gene:SpnNT_00188	corA	Chromosome:182703-183648	110.58	ΔORF2	OK	41.5225	39.4021	-0.0756203	-0.152305	0.7929	0.994748	no
gene:SpnNT_00188	corA	Chromosome:182703-183648	110.58	110.58+peptide	OK	41.5225	51.5651	0.3125	0.641561	0.26775	0.768869	no

gene:SpnNT_00188	corA	Chromosome:182703-183648	ΔORF2	110.58+peptide	OK	39.4021	51.5651	0.38812	0.793234	0.16615	0.621332	no
gene:SpnNT_00188	corA	Chromosome:182703-183648	110.58	ΔORF2+peptide	OK	41.5225	53.0738	0.354106	0.725402	0.2122	0.697867	no
gene:SpnNT_00188	corA	Chromosome:182703-183648	ΔORF2	ΔORF2+peptide	OK	39.4021	53.0738	0.429726	0.876381	0.1288	0.544145	no
gene:SpnNT_00188	corA	Chromosome:182703-183648	110.58+peptide	ΔORF2+peptide	OK	51.5651	53.0738	0.0416056	0.0865324	0.8813	0.994748	no
gene:SpnNT_00189	uvrA	Chromosome:183779-187665	110.58	ΔORF2	OK	58.066	54.5619	-0.0897997	-0.172318	0.76025	0.994748	no
gene:SpnNT_00189	uvrA	Chromosome:183779-187665	110.58	110.58+peptide	OK	58.066	56.7973	-0.0318728	-0.061599	0.91755	0.994748	no
gene:SpnNT_00189	uvrA	Chromosome:183779-187665	ΔORF2	110.58+peptide	OK	54.5619	56.7973	0.0579269	0.110377	0.8475	0.994748	no
gene:SpnNT_00189	uvrA	Chromosome:183779-187665	110.58	ΔORF2+peptide	OK	58.066	56.7856	-0.0321686	-0.0621018	0.91395	0.994748	no
gene:SpnNT_00189	uvrA	Chromosome:183779-187665	ΔORF2	ΔORF2+peptide	OK	54.5619	56.7856	0.0576311	0.109695	0.8471	0.994748	no
gene:SpnNT_00189	uvrA	Chromosome:183779-187665	110.58+peptide	ΔORF2+peptide	OK	56.7973	56.7856	-0.000295837	-0.000567062	0.99885	0.999459	no
gene:SpnNT_00190	NA	Chromosome:183779-187665	110.58	ΔORF2	OK	72.0181	70.1165	-0.038607	-0.0460033	0.9309	0.994855	no
gene:SpnNT_00190	NA	Chromosome:183779-187665	110.58	110.58+peptide	OK	72.0181	73.6478	0.032282	0.0398954	0.94235	0.994855	no
gene:SpnNT_00190	NA	Chromosome:183779-187665	ΔORF2	110.58+peptide	OK	70.1165	73.6478	0.070889	0.084249	0.88365	0.994748	no
gene:SpnNT_00190	NA	Chromosome:183779-187665	110.58	ΔORF2+peptide	OK	72.0181	76.457	0.0862889	0.107965	0.8473	0.994748	no
gene:SpnNT_00190	NA	Chromosome:183779-187665	ΔORF2	ΔORF2+peptide	OK	70.1165	76.457	0.124896	0.150138	0.7918	0.994748	no
gene:SpnNT_00190	NA	Chromosome:183779-187665	110.58+peptide	ΔORF2+peptide	OK	73.6478	76.457	0.0540069	0.067379	0.90815	0.994748	no
gene:SpnNT_00191	NA	Chromosome:187851-189585	110.58	ΔORF2	OK	28.6411	32.1616	0.167252	0.353114	0.5378	0.959663	no
gene:SpnNT_00191	NA	Chromosome:187851-189585	110.58	110.58+peptide	OK	28.6411	29.2065	0.0282016	0.0588088	0.9162	0.994748	no
gene:SpnNT_00191	NA	Chromosome:187851-189585	ΔORF2	110.58+peptide	OK	32.1616	29.2065	-0.139051	-0.293032	0.61085	0.979429	no
gene:SpnNT_00191	NA	Chromosome:187851-189585	110.58	ΔORF2+peptide	OK	28.6411	37.7096	0.396844	0.840945	0.15165	0.593484	no
gene:SpnNT_00191	NA	Chromosome:187851-189585	ΔORF2	ΔORF2+peptide	OK	32.1616	37.7096	0.229591	0.491845	0.39595	0.890349	no
gene:SpnNT_00191	NA	Chromosome:187851-189585	110.58+peptide	ΔORF2+peptide	OK	29.2065	37.7096	0.368642	0.779733	0.1778	0.644322	no
gene:SpnNT_00192	NA	Chromosome:189680-189971	110.58	ΔORF2	OK	39.0015	34.879	-0.161173	-0.236569	0.67525	0.985207	no
gene:SpnNT_00192	NA	Chromosome:189680-189971	110.58	110.58+peptide	OK	39.0015	41.3573	0.0846101	0.128545	0.81675	0.994748	no
gene:SpnNT_00192	NA	Chromosome:189680-189971	ΔORF2	110.58+peptide	OK	34.879	41.3573	0.245783	0.359439	0.5198	0.954494	no
gene:SpnNT_00192	NA	Chromosome:189680-189971	110.58	ΔORF2+peptide	OK	39.0015	32.5338	-0.261593	-0.382098	0.4908	0.940324	no
gene:SpnNT_00192	NA	Chromosome:189680-189971	ΔORF2	ΔORF2+peptide	OK	34.879	32.5338	-0.10042	-0.141586	0.8035	0.994748	no
gene:SpnNT_00192	NA	Chromosome:189680-189971	110.58+peptide	ΔORF2+peptide	OK	41.3573	32.5338	-0.346203	-0.503852	0.3633	0.862964	no
gene:SpnNT_00193	spxA_1	Chromosome:190102-190501	110.58	ΔORF2	OK	1043.87	1211.79	0.215191	0.492396	0.3833	0.880579	no
gene:SpnNT_00193	spxA_1	Chromosome:190102-190501	110.58	110.58+peptide	OK	1043.87	1078.79	0.0474742	0.109199	0.843	0.994748	no
gene:SpnNT_00193	spxA_1	Chromosome:190102-190501	ΔORF2	110.58+peptide	OK	1211.79	1078.79	-0.167717	-0.382788	0.496	0.943191	no
gene:SpnNT_00193	spxA_1	Chromosome:190102-190501	110.58	ΔORF2+peptide	OK	1043.87	1220.02	0.224963	0.521985	0.3513	0.854885	no
gene:SpnNT_00193	spxA_1	Chromosome:190102-190501	ΔORF2	ΔORF2+peptide	OK	1211.79	1220.02	0.00977241	0.0224963	0.968	0.994855	no
gene:SpnNT_00193	spxA_1	Chromosome:190102-190501	110.58+peptide	ΔORF2+peptide	OK	1078.79	1220.02	0.177489	0.41075	0.46115	0.924555	no
gene:SpnNT_00194	NA	Chromosome:190566-191136	110.58	ΔORF2	OK	234.601	266.22	0.182406	0.410401	0.47005	0.927937	no
gene:SpnNT_00194	NA	Chromosome:190566-191136	110.58	110.58+peptide	OK	234.601	230.138	-0.0277103	-0.061765	0.9111	0.994748	no
gene:SpnNT_00194	NA	Chromosome:190566-191136	ΔORF2	110.58+peptide	OK	266.22	230.138	-0.210116	-0.46844	0.4171	0.902489	no
gene:SpnNT_00194	NA	Chromosome:190566-191136	110.58	ΔORF2+peptide	OK	234.601	242.351	0.0468866	0.104695	0.85405	0.994748	no
gene:SpnNT_00194	NA	Chromosome:190566-191136	ΔORF2	ΔORF2+peptide	OK	266.22	242.351	-0.135519	-0.302672	0.5926	0.975468	no
gene:SpnNT_00194	NA	Chromosome:190566-191136	110.58+peptide	ΔORF2+peptide	OK	230.138	242.351	0.0745968	0.165075	0.76655	0.994748	no
gene:SpnNT_00195	NA	Chromosome:191222-191489	110.58	ΔORF2	OK	1504.6	1778.45	0.241243	0.535242	0.33575	0.840108	no
gene:SpnNT_00195	NA	Chromosome:191222-191489	110.58	110.58+peptide	OK	1504.6	1449.95	-0.053373	-0.11098	0.8428	0.994748	no
gene:SpnNT_00195	NA	Chromosome:191222-191489	ΔORF2	110.58+peptide	OK	1778.45	1449.95	-0.294616	-0.615584	0.27135	0.773525	no
gene:SpnNT_00195	NA	Chromosome:191222-191489	110.58	ΔORF2+peptide	OK	1504.6	1134.15	-0.407772	-0.87946	0.11675	0.517915	no
gene:SpnNT_00195	NA	Chromosome:191222-191489	ΔORF2	ΔORF2+peptide	OK	1778.45	1134.15	-0.649015	-1.40708	0.0135	0.125016	no

gene:SpnNT_00195	NA	Chromosome:191222-191489	110.58+peptide	ΔORF2+peptide	OK	1449.95	1134.15	-0.354399	-0.722077	0.2008	0.683494	no
gene:SpnNT_00196	yrrK	Chromosome:191492-191912	110.58	ΔORF2	OK	704.508	739.335	0.0696113	0.156873	0.78315	0.994748	no
gene:SpnNT_00196	yrrK	Chromosome:191492-191912	110.58	110.58+peptide	OK	704.508	575.159	-0.292655	-0.63402	0.25585	0.754781	no
gene:SpnNT_00196	yrrK	Chromosome:191492-191912	ΔORF2	110.58+peptide	OK	739.335	575.159	-0.362266	-0.785524	0.16295	0.61656	no
gene:SpnNT_00196	yrrK	Chromosome:191492-191912	110.58	ΔORF2+peptide	OK	704.508	475.281	-0.567836	-1.25869	0.0295	0.219444	no
gene:SpnNT_00196	yrrK	Chromosome:191492-191912	ΔORF2	ΔORF2+peptide	OK	739.335	475.281	-0.637448	-1.41431	0.0143	0.129589	no
gene:SpnNT_00196	yrrK	Chromosome:191492-191912	110.58+peptide	ΔORF2+peptide	OK	575.159	475.281	-0.275181	-0.587628	0.2922	0.797277	no
gene:SpnNT_00197	NA	Chromosome:191927-192233	110.58	ΔORF2	OK	2078.56	2132.94	0.0372577	0.0826975	0.88755	0.994748	no
gene:SpnNT_00197	NA	Chromosome:191927-192233	110.58	110.58+peptide	OK	2078.56	2127.77	0.0337616	0.0697598	0.9	0.994748	no
gene:SpnNT_00197	NA	Chromosome:191927-192233	ΔORF2	110.58+peptide	OK	2132.94	2127.77	-0.00349609	-0.00728285	0.98945	0.997703	no
gene:SpnNT_00197	NA	Chromosome:191927-192233	110.58	ΔORF2+peptide	OK	2078.56	1801.06	-0.206735	-0.433418	0.4338	0.913361	no
gene:SpnNT_00197	NA	Chromosome:191927-192233	ΔORF2	ΔORF2+peptide	OK	2132.94	1801.06	-0.243993	-0.515836	0.3487	0.853357	no
gene:SpnNT_00197	NA	Chromosome:191927-192233	110.58+peptide	ΔORF2+peptide	OK	2127.77	1801.06	-0.240497	-0.476271	0.3784	0.875802	no
gene:SpnNT_00198	fgs_1	Chromosome:192539-193790	110.58	ΔORF2	OK	103.594	98.7421	-0.0692065	-0.156329	0.7881	0.994748	no
gene:SpnNT_00198	fgs_1	Chromosome:192539-193790	110.58	110.58+peptide	OK	103.594	91.5332	-0.178576	-0.403708	0.4787	0.933291	no
gene:SpnNT_00198	fgs_1	Chromosome:192539-193790	ΔORF2	110.58+peptide	OK	98.7421	91.5332	-0.10937	-0.247368	0.6645	0.983188	no
gene:SpnNT_00198	fgs_1	Chromosome:192539-193790	110.58	ΔORF2+peptide	OK	103.594	95.6935	-0.11445	-0.258715	0.6578	0.982966	no
gene:SpnNT_00198	fgs_1	Chromosome:192539-193790	ΔORF2	ΔORF2+peptide	OK	98.7421	95.6935	-0.0452431	-0.10232	0.8564	0.994748	no
gene:SpnNT_00198	fgs_1	Chromosome:192539-193790	110.58+peptide	ΔORF2+peptide	OK	91.5332	95.6935	0.0641268	0.145144	0.802	0.994748	no
gene:SpnNT_00199	NA	Chromosome:193873-194332	110.58	ΔORF2	OK	216.439	196.367	-0.140403	-0.300172	0.59615	0.97629	no
gene:SpnNT_00199	NA	Chromosome:193873-194332	110.58	110.58+peptide	OK	216.439	226.012	0.0624388	0.132527	0.81715	0.994748	no
gene:SpnNT_00199	NA	Chromosome:193873-194332	ΔORF2	110.58+peptide	OK	196.367	226.012	0.202842	0.425609	0.46005	0.924043	no
gene:SpnNT_00199	NA	Chromosome:193873-194332	110.58	ΔORF2+peptide	OK	216.439	180.076	-0.265355	-0.551159	0.33575	0.840108	no
gene:SpnNT_00199	NA	Chromosome:193873-194332	ΔORF2	ΔORF2+peptide	OK	196.367	180.076	-0.124952	-0.256689	0.6532	0.982158	no
gene:SpnNT_00199	NA	Chromosome:193873-194332	110.58+peptide	ΔORF2+peptide	OK	226.012	180.076	-0.327794	-0.668896	0.23535	0.729189	no
gene:SpnNT_00200	cls	Chromosome:194477-196010	110.58	ΔORF2	OK	34.543	41.5817	0.267554	0.57257	0.32035	0.825861	no
gene:SpnNT_00200	cls	Chromosome:194477-196010	110.58	110.58+peptide	OK	34.543	25.8302	-0.419336	-0.877826	0.12815	0.542797	no
gene:SpnNT_00200	cls	Chromosome:194477-196010	ΔORF2	110.58+peptide	OK	41.5817	25.8302	-0.68689	-1.44421	0.01135	0.109915	no
gene:SpnNT_00200	cls	Chromosome:194477-196010	110.58	ΔORF2+peptide	OK	34.543	26.4778	-0.383611	-0.799794	0.15625	0.602148	no
gene:SpnNT_00200	cls	Chromosome:194477-196010	ΔORF2	ΔORF2+peptide	OK	41.5817	26.4778	-0.651165	-1.36352	0.01845	0.156295	no
gene:SpnNT_00200	cls	Chromosome:194477-196010	110.58+peptide	ΔORF2+peptide	OK	25.8302	26.4778	0.035725	0.0732435	0.89345	0.994748	no
gene:SpnNT_00201	NA	Chromosome:196087-196231	110.58	ΔORF2	OK	23.8818	12.0212	-0.990337	-0.688527	0.378	0.875802	no
gene:SpnNT_00201	NA	Chromosome:196087-196231	110.58	110.58+peptide	OK	23.8818	20.1044	-0.248404	-0.213192	0.78915	0.994748	no
gene:SpnNT_00201	NA	Chromosome:196087-196231	ΔORF2	110.58+peptide	OK	12.0212	20.1044	0.741933	0.405508	0.45045	0.920951	no
gene:SpnNT_00201	NA	Chromosome:196087-196231	110.58	ΔORF2+peptide	OK	23.8818	6.77738	-1.81711	-6.90113	0.19625	0.677648	no
gene:SpnNT_00201	NA	Chromosome:196087-196231	ΔORF2	ΔORF2+peptide	OK	12.0212	6.77738	-0.826778	-0.576142	0.55665	0.966953	no
gene:SpnNT_00201	NA	Chromosome:196087-196231	110.58+peptide	ΔORF2+peptide	OK	20.1044	6.77738	-1.56871	-1.35109	0.28315	0.785683	no
gene:SpnNT_00202	NA	Chromosome:196267-197806	110.58	ΔORF2	OK	93.9098	95.981	0.0314733	0.0722042	0.89915	0.994748	no
gene:SpnNT_00202	NA	Chromosome:196267-197806	110.58	110.58+peptide	OK	93.9098	90.0329	-0.0608241	-0.138329	0.8057	0.994748	no
gene:SpnNT_00202	NA	Chromosome:196267-197806	ΔORF2	110.58+peptide	OK	95.981	90.0329	-0.0922974	-0.209995	0.7143	0.991857	no
gene:SpnNT_00202	NA	Chromosome:196267-197806	110.58	ΔORF2+peptide	OK	93.9098	87.6934	-0.0988082	-0.226485	0.68405	0.98747	no
gene:SpnNT_00202	NA	Chromosome:196267-197806	ΔORF2	ΔORF2+peptide	OK	95.981	87.6934	-0.130282	-0.298753	0.6012	0.976678	no
gene:SpnNT_00202	NA	Chromosome:196267-197806	110.58+peptide	ΔORF2+peptide	OK	90.0329	87.6934	-0.0379841	-0.0863479	0.87695	0.994748	no
gene:SpnNT_00203	nrdD_1	Chromosome:197925-200133	110.58	ΔORF2	OK	122.536	101.335	-0.274084	-0.630348	0.26725	0.768517	no
gene:SpnNT_00203	nrdD_1	Chromosome:197925-200133	110.58	110.58+peptide	OK	122.536	94.3306	-0.377413	-0.867687	0.11995	0.524705	no

gene:SpnNT_00203	nrdD_1	Chromosome:197925-200133	ΔORF2	110.58+peptide	OK	101.335	94.3306	-0.103328	-0.238285	0.67125	0.984845	no
gene:SpnNT_00203	nrdD_1	Chromosome:197925-200133	110.58	ΔORF2+peptide	OK	122.536	80.2343	-0.61092	-1.40244	0.014	0.127841	no
gene:SpnNT_00203	nrdD_1	Chromosome:197925-200133	ΔORF2	ΔORF2+peptide	OK	101.335	80.2343	-0.336836	-0.775609	0.1661	0.621332	no
gene:SpnNT_00203	nrdD_1	Chromosome:197925-200133	110.58+peptide	ΔORF2+peptide	OK	94.3306	80.2343	-0.233507	-0.537495	0.3416	0.84575	no
gene:SpnNT_00204	NA	Chromosome:200328-202032	110.58	ΔORF2	OK	87.1661	77.0079	-0.178761	-0.214657	0.70135	0.990367	no
gene:SpnNT_00204	NA	Chromosome:200328-202032	110.58	110.58+peptide	OK	87.1661	62.8862	-0.471023	-0.550011	0.3338	0.839391	no
gene:SpnNT_00204	NA	Chromosome:200328-202032	ΔORF2	110.58+peptide	OK	77.0079	62.8862	-0.292262	-0.343581	0.54005	0.961277	no
gene:SpnNT_00204	NA	Chromosome:200328-202032	110.58	ΔORF2+peptide	OK	87.1661	58.3108	-0.580003	-0.672292	0.23665	0.731008	no
gene:SpnNT_00204	NA	Chromosome:200328-202032	ΔORF2	ΔORF2+peptide	OK	77.0079	58.3108	-0.401243	-0.468185	0.4024	0.894197	no
gene:SpnNT_00204	NA	Chromosome:200328-202032	110.58+peptide	ΔORF2+peptide	OK	62.8862	58.3108	-0.10898	-0.123844	0.82675	0.994748	no
gene:SpnNT_00205	pflA_1	Chromosome:200328-202032	110.58	ΔORF2	OK	70.48	57.5179	-0.293204	-0.344074	0.5435	0.961568	no
gene:SpnNT_00205	pflA_1	Chromosome:200328-202032	110.58	110.58+peptide	OK	70.48	54.9218	-0.359835	-0.426401	0.44795	0.919648	no
gene:SpnNT_00205	pflA_1	Chromosome:200328-202032	ΔORF2	110.58+peptide	OK	57.5179	54.9218	-0.0666312	-0.0793171	0.88965	0.994748	no
gene:SpnNT_00205	pflA_1	Chromosome:200328-202032	110.58	ΔORF2+peptide	OK	70.48	45.2382	-0.639672	-0.722614	0.20915	0.69532	no
gene:SpnNT_00205	pflA_1	Chromosome:200328-202032	ΔORF2	ΔORF2+peptide	OK	57.5179	45.2382	-0.346468	-0.393012	0.48925	0.939137	no
gene:SpnNT_00205	pflA_1	Chromosome:200328-202032	110.58+peptide	ΔORF2+peptide	OK	54.9218	45.2382	-0.279837	-0.320332	0.5682	0.968621	no
gene:SpnNT_00206	NA	Chromosome:200328-202032	110.58	ΔORF2	OK	98.8936	79.9425	-0.306914	-0.442477	0.4427	0.917631	no
gene:SpnNT_00206	NA	Chromosome:200328-202032	110.58	110.58+peptide	OK	98.8936	69.6492	-0.505769	-0.719182	0.21235	0.698185	no
gene:SpnNT_00206	NA	Chromosome:200328-202032	ΔORF2	110.58+peptide	OK	79.9425	69.6492	-0.198856	-0.278614	0.62965	0.980887	no
gene:SpnNT_00206	NA	Chromosome:200328-202032	110.58	ΔORF2+peptide	OK	98.8936	60.9621	-0.697964	-0.972475	0.095	0.466015	no
gene:SpnNT_00206	NA	Chromosome:200328-202032	ΔORF2	ΔORF2+peptide	OK	79.9425	60.9621	-0.39105	-0.537168	0.34815	0.852918	no
gene:SpnNT_00206	NA	Chromosome:200328-202032	110.58+peptide	ΔORF2+peptide	OK	69.6492	60.9621	-0.192195	-0.260721	0.64595	0.980887	no
gene:SpnNT_00207	rpsJ	Chromosome:202298-202607	110.58	ΔORF2	OK	754.256	661.585	-0.189128	-0.412322	0.47055	0.928415	no
gene:SpnNT_00207	rpsJ	Chromosome:202298-202607	110.58	110.58+peptide	OK	754.256	1362.39	0.853012	1.88489	9.00E-04	0.0154579	yes
gene:SpnNT_00207	rpsJ	Chromosome:202298-202607	ΔORF2	110.58+peptide	OK	661.585	1362.39	1.04214	2.23535	5.00E-05	0.0013612	yes
gene:SpnNT_00207	rpsJ	Chromosome:202298-202607	110.58	ΔORF2+peptide	OK	754.256	1486.78	0.979069	2.24238	5.00E-05	0.0013612	yes
gene:SpnNT_00207	rpsJ	Chromosome:202298-202607	ΔORF2	ΔORF2+peptide	OK	661.585	1486.78	1.1682	2.59162	5.00E-05	0.0013612	yes
gene:SpnNT_00207	rpsJ	Chromosome:202298-202607	110.58+peptide	ΔORF2+peptide	OK	1362.39	1486.78	0.126058	0.283587	0.6199	0.980887	no
gene:SpnNT_00208	rplC	Chromosome:202823-203450	110.58	ΔORF2	OK	576.013	546.627	-0.0755446	-0.172294	0.7628	0.994748	no
gene:SpnNT_00208	rplC	Chromosome:202823-203450	110.58	110.58+peptide	OK	576.013	1087.21	0.916459	2.06158	0.00045	0.00885292	yes
gene:SpnNT_00208	rplC	Chromosome:202823-203450	ΔORF2	110.58+peptide	OK	546.627	1087.21	0.992003	2.21759	0.00025	0.00542231	yes
gene:SpnNT_00208	rplC	Chromosome:202823-203450	110.58	ΔORF2+peptide	OK	576.013	1201.88	1.06112	2.41718	1.00E-04	0.0025332	yes
gene:SpnNT_00208	rplC	Chromosome:202823-203450	ΔORF2	ΔORF2+peptide	OK	546.627	1201.88	1.13667	2.57271	5.00E-05	0.0013612	yes
gene:SpnNT_00208	rplC	Chromosome:202823-203450	110.58+peptide	ΔORF2+peptide	OK	1087.21	1201.88	0.144662	0.323014	0.564	0.968621	no
gene:SpnNT_00209	rplD	Chromosome:203474-204394	110.58	ΔORF2	OK	482.406	449.29	-0.102601	-0.214491	0.7131	0.991272	no
gene:SpnNT_00209	rplD	Chromosome:203474-204394	110.58	110.58+peptide	OK	482.406	834.071	0.789923	1.64312	0.0051	0.061228	no
gene:SpnNT_00209	rplD	Chromosome:203474-204394	ΔORF2	110.58+peptide	OK	449.29	834.071	0.892524	1.80967	0.00165	0.0253528	yes
gene:SpnNT_00209	rplD	Chromosome:203474-204394	110.58	ΔORF2+peptide	OK	482.406	934.73	0.954302	2.03914	3.00E-04	0.00631878	yes
gene:SpnNT_00209	rplD	Chromosome:203474-204394	ΔORF2	ΔORF2+peptide	OK	449.29	934.73	1.0569	2.19834	0.00015	0.00355289	yes
gene:SpnNT_00209	rplD	Chromosome:203474-204394	110.58+peptide	ΔORF2+peptide	OK	834.071	934.73	0.164379	0.340215	0.54965	0.964281	no
gene:SpnNT_00210	rplW	Chromosome:203474-204394	110.58	ΔORF2	OK	303.561	324.588	0.0966216	0.076287	0.8986	0.994748	no
gene:SpnNT_00210	rplW	Chromosome:203474-204394	110.58	110.58+peptide	OK	303.561	595.717	0.97264	0.754549	0.19515	0.676554	no
gene:SpnNT_00210	rplW	Chromosome:203474-204394	ΔORF2	110.58+peptide	OK	324.588	595.717	0.876019	0.722423	0.20865	0.694195	no
gene:SpnNT_00210	rplW	Chromosome:203474-204394	110.58	ΔORF2+peptide	OK	303.561	647.619	1.09316	0.789593	0.1868	0.661768	no
gene:SpnNT_00210	rplW	Chromosome:203474-204394	ΔORF2	ΔORF2+peptide	OK	324.588	647.619	0.996536	0.758631	0.19705	0.678125	no

gene:SpnNT_00210	rplW	Chromosome:203474-204394	110.58+peptide	ΔORF2+peptide	OK	595.717	647.619	0.120517	0.090256	0.87365	0.994748	no
gene:SpnNT_00211	rplB	Chromosome:204411-205245	110.58	ΔORF2	OK	811.342	768.608	-0.0780617	-0.169126	0.76295	0.994748	no
gene:SpnNT_00211	rplB	Chromosome:204411-205245	110.58	110.58+peptide	OK	811.342	1469.84	0.857275	1.84142	0.0017	0.025969	yes
gene:SpnNT_00211	rplB	Chromosome:204411-205245	ΔORF2	110.58+peptide	OK	768.608	1469.84	0.935337	1.93607	0.0012	0.0193683	yes
gene:SpnNT_00211	rplB	Chromosome:204411-205245	110.58	ΔORF2+peptide	OK	811.342	1529.09	0.914293	2.03199	5.00E-04	0.0095781	yes
gene:SpnNT_00211	rplB	Chromosome:204411-205245	ΔORF2	ΔORF2+peptide	OK	768.608	1529.09	0.992355	2.11998	2.00E-04	0.00450928	yes
gene:SpnNT_00211	rplB	Chromosome:204411-205245	110.58+peptide	ΔORF2+peptide	OK	1469.84	1529.09	0.057018	0.120793	0.83505	0.994748	no
gene:SpnNT_00212	rpsS	Chromosome:205348-205630	110.58	ΔORF2	OK	1432.03	1222.48	-0.228257	-0.48733	0.3857	0.881745	no
gene:SpnNT_00212	rpsS	Chromosome:205348-205630	110.58	110.58+peptide	OK	1432.03	2153.78	0.588805	1.27877	0.0237	0.189629	no
gene:SpnNT_00212	rpsS	Chromosome:205348-205630	ΔORF2	110.58+peptide	OK	1222.48	2153.78	0.817062	1.69971	0.0023	0.0329123	yes
gene:SpnNT_00212	rpsS	Chromosome:205348-205630	110.58	ΔORF2+peptide	OK	1432.03	2059.55	0.524268	1.17105	0.0383	0.263403	no
gene:SpnNT_00212	rpsS	Chromosome:205348-205630	ΔORF2	ΔORF2+peptide	OK	1222.48	2059.55	0.752525	1.60623	0.0047	0.0572109	no
gene:SpnNT_00212	rpsS	Chromosome:205348-205630	110.58+peptide	ΔORF2+peptide	OK	2153.78	2059.55	-0.0645371	-0.140125	0.8061	0.994748	no
gene:SpnNT_00213	rplV	Chromosome:205641-205986	110.58	ΔORF2	OK	487.248	451.212	-0.110852	-0.235054	0.67475	0.985099	no
gene:SpnNT_00213	rplV	Chromosome:205641-205986	110.58	110.58+peptide	OK	487.248	732.037	0.58726	1.27125	0.02605	0.202745	no
gene:SpnNT_00213	rplV	Chromosome:205641-205986	ΔORF2	110.58+peptide	OK	451.212	732.037	0.698113	1.4456	0.01095	0.107389	no
gene:SpnNT_00213	rplV	Chromosome:205641-205986	110.58	ΔORF2+peptide	OK	487.248	732.535	0.58824	1.30799	0.0205	0.170686	no
gene:SpnNT_00213	rplV	Chromosome:205641-205986	ΔORF2	ΔORF2+peptide	OK	451.212	732.535	0.699092	1.48351	0.0085	0.08966	no
gene:SpnNT_00213	rplV	Chromosome:205641-205986	110.58+peptide	ΔORF2+peptide	OK	732.037	732.535	0.000979788	0.00212265	0.99705	0.999412	no
gene:SpnNT_00214	rpsC	Chromosome:205998-206652	110.58	ΔORF2	OK	953.89	899.993	-0.0839097	-0.182015	0.7467	0.994748	no
gene:SpnNT_00214	rpsC	Chromosome:205998-206652	110.58	110.58+peptide	OK	953.89	1584.25	0.731902	1.59461	0.0039	0.0495409	yes
gene:SpnNT_00214	rpsC	Chromosome:205998-206652	ΔORF2	110.58+peptide	OK	899.993	1584.25	0.815812	1.72352	0.0024	0.0337543	yes
gene:SpnNT_00214	rpsC	Chromosome:205998-206652	110.58	ΔORF2+peptide	OK	953.89	1581.19	0.729114	1.62611	0.0037	0.0473672	yes
gene:SpnNT_00214	rpsC	Chromosome:205998-206652	ΔORF2	ΔORF2+peptide	OK	899.993	1581.19	0.813023	1.75575	0.00275	0.037317	yes
gene:SpnNT_00214	rpsC	Chromosome:205998-206652	110.58+peptide	ΔORF2+peptide	OK	1584.25	1581.19	-0.00278884	-0.00604886	0.9922	0.99849	no
gene:SpnNT_00215	rplP	Chromosome:206655-207069	110.58	ΔORF2	OK	909.609	804.393	-0.177345	-0.383161	0.4932	0.941402	no
gene:SpnNT_00215	rplP	Chromosome:206655-207069	110.58	110.58+peptide	OK	909.609	1428.25	0.650934	1.43405	0.01275	0.119932	no
gene:SpnNT_00215	rplP	Chromosome:206655-207069	ΔORF2	110.58+peptide	OK	804.393	1428.25	0.828279	1.74426	0.00235	0.0334454	yes
gene:SpnNT_00215	rplP	Chromosome:206655-207069	110.58	ΔORF2+peptide	OK	909.609	1462.76	0.685371	1.5589	0.00755	0.082285	no
gene:SpnNT_00215	rplP	Chromosome:206655-207069	ΔORF2	ΔORF2+peptide	OK	804.393	1462.76	0.862717	1.8704	0.00105	0.0174849	yes
gene:SpnNT_00215	rplP	Chromosome:206655-207069	110.58+peptide	ΔORF2+peptide	OK	1428.25	1462.76	0.0344375	0.0761422	0.89105	0.994748	no
gene:SpnNT_00216	rpmC	Chromosome:207078-207285	110.58	ΔORF2	OK	369.196	359.624	-0.0378954	-0.0681592	0.9068	0.994748	no
gene:SpnNT_00216	rpmC	Chromosome:207078-207285	110.58	110.58+peptide	OK	369.196	524.811	0.507412	0.940472	0.10165	0.48363	no
gene:SpnNT_00216	rpmC	Chromosome:207078-207285	ΔORF2	110.58+peptide	OK	359.624	524.811	0.545307	1.00057	0.0845	0.437055	no
gene:SpnNT_00216	rpmC	Chromosome:207078-207285	110.58	ΔORF2+peptide	OK	369.196	781.305	1.0815	2.03901	5.00E-04	0.0095781	yes
gene:SpnNT_00216	rpmC	Chromosome:207078-207285	ΔORF2	ΔORF2+peptide	OK	359.624	781.305	1.1194	2.08856	4.00E-04	0.00811252	yes
gene:SpnNT_00216	rpmC	Chromosome:207078-207285	110.58+peptide	ΔORF2+peptide	OK	524.811	781.305	0.574088	1.10641	0.05995	0.351346	no
gene:SpnNT_00217	rpsQ	Chromosome:207309-207570	110.58	ΔORF2	OK	441.136	386.838	-0.189492	-0.372911	0.5094	0.948	no
gene:SpnNT_00217	rpsQ	Chromosome:207309-207570	110.58	110.58+peptide	OK	441.136	651.658	0.562893	1.1426	0.0437	0.286572	no
gene:SpnNT_00217	rpsQ	Chromosome:207309-207570	ΔORF2	110.58+peptide	OK	386.838	651.658	0.752385	1.50186	0.00895	0.0931339	no
gene:SpnNT_00217	rpsQ	Chromosome:207309-207570	110.58	ΔORF2+peptide	OK	441.136	746.959	0.759807	1.57085	0.00665	0.0743916	no
gene:SpnNT_00217	rpsQ	Chromosome:207309-207570	ΔORF2	ΔORF2+peptide	OK	386.838	746.959	0.949299	1.9288	0.00075	0.0133173	yes
gene:SpnNT_00217	rpsQ	Chromosome:207309-207570	110.58+peptide	ΔORF2+peptide	OK	651.658	746.959	0.196914	0.413553	0.46765	0.92732	no
gene:SpnNT_00218	rplN	Chromosome:207595-207964	110.58	ΔORF2	OK	767.49	719.627	-0.0929002	-0.203214	0.7187	0.993515	no
gene:SpnNT_00218	rplN	Chromosome:207595-207964	110.58	110.58+peptide	OK	767.49	1277.72	0.735351	1.63267	0.00545	0.0642542	no

gene:SpnNT_00218	rplN	Chromosome:207595-207964	ΔORF2	110.58+peptide	OK	719.627	1277.72	0.828251	1.79277	0.00215	0.0312429	yes
gene:SpnNT_00218	rplN	Chromosome:207595-207964	110.58	ΔORF2+peptide	OK	767.49	1201.68	0.646838	1.45915	0.011	0.107477	no
gene:SpnNT_00218	rplN	Chromosome:207595-207964	ΔORF2	ΔORF2+peptide	OK	719.627	1201.68	0.739738	1.62553	0.0045	0.0552376	no
gene:SpnNT_00218	rplN	Chromosome:207595-207964	110.58+peptide	ΔORF2+peptide	OK	1277.72	1201.68	-0.0885133	-0.197448	0.7303	0.994748	no
gene:SpnNT_00219	rplX	Chromosome:208041-208347	110.58	ΔORF2	OK	509.601	475.815	-0.0989672	-0.207606	0.7067	0.990422	no
gene:SpnNT_00219	rplX	Chromosome:208041-208347	110.58	110.58+peptide	OK	509.601	793.435	0.638743	1.37371	0.01665	0.144978	no
gene:SpnNT_00219	rplX	Chromosome:208041-208347	ΔORF2	110.58+peptide	OK	475.815	793.435	0.73771	1.53754	0.0085	0.08966	no
gene:SpnNT_00219	rplX	Chromosome:208041-208347	110.58	ΔORF2+peptide	OK	509.601	878.016	0.784879	1.75025	0.0023	0.0329123	yes
gene:SpnNT_00219	rplX	Chromosome:208041-208347	ΔORF2	ΔORF2+peptide	OK	475.815	878.016	0.883846	1.9057	0.00095	0.0161266	yes
gene:SpnNT_00219	rplX	Chromosome:208041-208347	110.58+peptide	ΔORF2+peptide	OK	793.435	878.016	0.146136	0.323506	0.57135	0.968888	no
gene:SpnNT_00220	rplE	Chromosome:208370-208913	110.58	ΔORF2	OK	497.127	459.71	-0.112891	-0.257646	0.6523	0.98163	no
gene:SpnNT_00220	rplE	Chromosome:208370-208913	110.58	110.58+peptide	OK	497.127	766.284	0.624266	1.42211	0.01195	0.11471	no
gene:SpnNT_00220	rplE	Chromosome:208370-208913	ΔORF2	110.58+peptide	OK	459.71	766.284	0.737157	1.66792	0.0037	0.0473672	yes
gene:SpnNT_00220	rplE	Chromosome:208370-208913	110.58	ΔORF2+peptide	OK	497.127	908.625	0.870071	2.0001	0.00045	0.00885292	yes
gene:SpnNT_00220	rplE	Chromosome:208370-208913	ΔORF2	ΔORF2+peptide	OK	459.71	908.625	0.982961	2.24404	5.00E-05	0.0013612	yes
gene:SpnNT_00220	rplE	Chromosome:208370-208913	110.58+peptide	ΔORF2+peptide	OK	766.284	908.625	0.245804	0.560122	0.33135	0.837459	no
gene:SpnNT_00221	rpsN2	Chromosome:208930-209200	110.58	ΔORF2	OK	388.077	357.605	-0.117977	-0.236607	0.6719	0.984845	no
gene:SpnNT_00221	rpsN2	Chromosome:208930-209200	110.58	110.58+peptide	OK	388.077	635.023	0.710465	1.46333	0.0114	0.110074	no
gene:SpnNT_00221	rpsN2	Chromosome:208930-209200	ΔORF2	110.58+peptide	OK	357.605	635.023	0.828442	1.68191	0.00325	0.0427748	yes
gene:SpnNT_00221	rpsN2	Chromosome:208930-209200	110.58	ΔORF2+peptide	OK	388.077	858.283	1.14511	2.45079	5.00E-05	0.0013612	yes
gene:SpnNT_00221	rpsN2	Chromosome:208930-209200	ΔORF2	ΔORF2+peptide	OK	357.605	858.283	1.26309	2.6616	5.00E-05	0.0013612	yes
gene:SpnNT_00221	rpsN2	Chromosome:208930-209200	110.58+peptide	ΔORF2+peptide	OK	635.023	858.283	0.434645	0.943311	0.11165	0.508705	no
gene:SpnNT_00222	NA	Chromosome:209259-209349	110.58	ΔORF2	NOTEST	0	0	0	0	1	1	no
gene:SpnNT_00222	NA	Chromosome:209259-209349	110.58	110.58+peptide	NOTEST	0	51.7806	Inf	0	1	1	no
gene:SpnNT_00222	NA	Chromosome:209259-209349	ΔORF2	110.58+peptide	NOTEST	0	51.7806	Inf	0	1	1	no
gene:SpnNT_00222	NA	Chromosome:209259-209349	110.58	ΔORF2+peptide	NOTEST	0	27.3756	Inf	0	1	1	no
gene:SpnNT_00222	NA	Chromosome:209259-209349	ΔORF2	ΔORF2+peptide	NOTEST	0	27.3756	Inf	0	1	1	no
gene:SpnNT_00222	NA	Chromosome:209259-209349	110.58+peptide	ΔORF2+peptide	NOTEST	51.7806	27.3756	-0.919522	0	1	1	no
gene:SpnNT_00223	rpsH	Chromosome:209413-209812	110.58	ΔORF2	OK	1134.01	1059.91	-0.0974933	-0.22135	0.69005	0.98828	no
gene:SpnNT_00223	rpsH	Chromosome:209413-209812	110.58	110.58+peptide	OK	1134.01	1810.15	0.674675	1.49708	0.009	0.0935059	no
gene:SpnNT_00223	rpsH	Chromosome:209413-209812	ΔORF2	110.58+peptide	OK	1059.91	1810.15	0.772168	1.72869	0.0024	0.0337543	yes
gene:SpnNT_00223	rpsH	Chromosome:209413-209812	110.58	ΔORF2+peptide	OK	1134.01	1746.97	0.623418	1.40296	0.015	0.133716	no
gene:SpnNT_00223	rpsH	Chromosome:209413-209812	ΔORF2	ΔORF2+peptide	OK	1059.91	1746.97	0.720911	1.63724	0.0037	0.0473672	yes
gene:SpnNT_00223	rpsH	Chromosome:209413-209812	110.58+peptide	ΔORF2+peptide	OK	1810.15	1746.97	-0.0512573	-0.11377	0.841	0.994748	no
gene:SpnNT_00224	rplF	Chromosome:210003-210540	110.58	ΔORF2	OK	927.819	902.549	-0.0398377	-0.0904014	0.87265	0.994748	no
gene:SpnNT_00224	rplF	Chromosome:210003-210540	110.58	110.58+peptide	OK	927.819	1414.45	0.608325	1.3799	0.0165	0.144246	no
gene:SpnNT_00224	rplF	Chromosome:210003-210540	ΔORF2	110.58+peptide	OK	902.549	1414.45	0.648163	1.47	0.0104	0.103229	no
gene:SpnNT_00224	rplF	Chromosome:210003-210540	110.58	ΔORF2+peptide	OK	927.819	1641.03	0.822686	1.85392	0.0012	0.0193683	yes
gene:SpnNT_00224	rplF	Chromosome:210003-210540	ΔORF2	ΔORF2+peptide	OK	902.549	1641.03	0.862523	1.94335	7.00E-04	0.012687	yes
gene:SpnNT_00224	rplF	Chromosome:210003-210540	110.58+peptide	ΔORF2+peptide	OK	1414.45	1641.03	0.214361	0.48279	0.4031	0.894399	no
gene:SpnNT_00225	rplR	Chromosome:210623-210980	110.58	ΔORF2	OK	627.258	580.284	-0.1123	-0.241629	0.67245	0.984845	no
gene:SpnNT_00225	rplR	Chromosome:210623-210980	110.58	110.58+peptide	OK	627.258	938.338	0.581049	1.27536	0.0255	0.199411	no
gene:SpnNT_00225	rplR	Chromosome:210623-210980	ΔORF2	110.58+peptide	OK	580.284	938.338	0.693349	1.45886	0.00975	0.0985666	no
gene:SpnNT_00225	rplR	Chromosome:210623-210980	110.58	ΔORF2+peptide	OK	627.258	926.915	0.563378	1.27175	0.02815	0.214152	no
gene:SpnNT_00225	rplR	Chromosome:210623-210980	ΔORF2	ΔORF2+peptide	OK	580.284	926.915	0.675678	1.45872	0.01205	0.115248	no

gene:SpnNT_00225	rplR	Chromosome:210623-210980	110.58+peptide	ΔORF2+peptide	OK	938.338	926.915	-0.0176708	-0.0389221	0.94835	0.994855	no
gene:SpnNT_00226	rpsE	Chromosome:210997-211492	110.58	ΔORF2	OK	432.664	408.264	-0.0837434	-0.190379	0.7361	0.994748	no
gene:SpnNT_00226	rpsE	Chromosome:210997-211492	110.58	110.58+peptide	OK	432.664	683.659	0.660031	1.50982	0.0087	0.0910378	no
gene:SpnNT_00226	rpsE	Chromosome:210997-211492	ΔORF2	110.58+peptide	OK	408.264	683.659	0.743775	1.68765	0.00325	0.0427748	yes
gene:SpnNT_00226	rpsE	Chromosome:210997-211492	110.58	ΔORF2+peptide	OK	432.664	765.445	0.823053	1.89933	0.0011	0.0181562	yes
gene:SpnNT_00226	rpsE	Chromosome:210997-211492	ΔORF2	ΔORF2+peptide	OK	408.264	765.445	0.906796	2.0754	3.00E-04	0.00631878	yes
gene:SpnNT_00226	rpsE	Chromosome:210997-211492	110.58+peptide	ΔORF2+peptide	OK	683.659	765.445	0.163021	0.37546	0.5079	0.94682	no
gene:SpnNT_00227	rpmD	Chromosome:211505-211688	110.58	ΔORF2	OK	2079.09	1883.51	-0.142528	-0.301601	0.6061	0.976937	no
gene:SpnNT_00227	rpmD	Chromosome:211505-211688	110.58	110.58+peptide	OK	2079.09	4116.71	0.985541	2.08471	5.00E-04	0.0095781	yes
gene:SpnNT_00227	rpmD	Chromosome:211505-211688	ΔORF2	110.58+peptide	OK	1883.51	4116.71	1.12807	2.36131	5.00E-05	0.0013612	yes
gene:SpnNT_00227	rpmD	Chromosome:211505-211688	110.58	ΔORF2+peptide	OK	2079.09	4250	1.03151	2.21116	3.00E-04	0.00631878	yes
gene:SpnNT_00227	rpmD	Chromosome:211505-211688	ΔORF2	ΔORF2+peptide	OK	1883.51	4250	1.17404	2.48974	5.00E-05	0.0013612	yes
gene:SpnNT_00227	rpmD	Chromosome:211505-211688	110.58+peptide	ΔORF2+peptide	OK	4116.71	4250	0.0459716	0.0974536	0.86545	0.994748	no
gene:SpnNT_00228	rplO	Chromosome:211832-212273	110.58	ΔORF2	OK	1040.06	961.026	-0.11402	-0.257909	0.6428	0.980887	no
gene:SpnNT_00228	rplO	Chromosome:211832-212273	110.58	110.58+peptide	OK	1040.06	1501.54	0.529774	1.19301	0.03555	0.250396	no
gene:SpnNT_00228	rplO	Chromosome:211832-212273	ΔORF2	110.58+peptide	OK	961.026	1501.54	0.643793	1.42816	0.01135	0.109915	no
gene:SpnNT_00228	rplO	Chromosome:211832-212273	110.58	ΔORF2+peptide	OK	1040.06	1592.69	0.6148	1.40844	0.01415	0.128942	no
gene:SpnNT_00228	rplO	Chromosome:211832-212273	ΔORF2	ΔORF2+peptide	OK	961.026	1592.69	0.72882	1.64391	0.0036	0.0465411	yes
gene:SpnNT_00228	rplO	Chromosome:211832-212273	110.58+peptide	ΔORF2+peptide	OK	1501.54	1592.69	0.0850268	0.190937	0.7409	0.994748	no
gene:SpnNT_00229	NA	Chromosome:212285-213596	110.58	ΔORF2	OK	729.486	731.366	0.0037147	0.00818272	0.98795	0.997703	no
gene:SpnNT_00229	NA	Chromosome:212285-213596	110.58	110.58+peptide	OK	729.486	1207.4	0.726952	1.57168	0.00675	0.0753176	no
gene:SpnNT_00229	NA	Chromosome:212285-213596	ΔORF2	110.58+peptide	OK	731.366	1207.4	0.723237	1.56471	0.00725	0.0796771	no
gene:SpnNT_00229	NA	Chromosome:212285-213596	110.58	ΔORF2+peptide	OK	729.486	1476.72	1.01744	2.16318	2.00E-04	0.00450928	yes
gene:SpnNT_00229	NA	Chromosome:212285-213596	ΔORF2	ΔORF2+peptide	OK	731.366	1476.72	1.01373	2.15669	2.00E-04	0.00450928	yes
gene:SpnNT_00229	NA	Chromosome:212285-213596	110.58+peptide	ΔORF2+peptide	OK	1207.4	1476.72	0.290489	0.607321	0.2926	0.79754	no
gene:SpnNT_00230	adk	Chromosome:213746-214385	110.58	ΔORF2	OK	207.721	223.317	0.104443	0.232098	0.68725	0.98828	no
gene:SpnNT_00230	adk	Chromosome:213746-214385	110.58	110.58+peptide	OK	207.721	319.64	0.621802	1.39348	0.01445	0.130498	no
gene:SpnNT_00230	adk	Chromosome:213746-214385	ΔORF2	110.58+peptide	OK	223.317	319.64	0.517359	1.17173	0.03945	0.268637	no
gene:SpnNT_00230	adk	Chromosome:213746-214385	110.58	ΔORF2+peptide	OK	207.721	382.157	0.879516	1.96472	9.00E-04	0.0154579	yes
gene:SpnNT_00230	adk	Chromosome:213746-214385	ΔORF2	ΔORF2+peptide	OK	223.317	382.157	0.775073	1.74968	0.0029	0.0388304	yes
gene:SpnNT_00230	adk	Chromosome:213746-214385	110.58+peptide	ΔORF2+peptide	OK	319.64	382.157	0.257714	0.586848	0.30265	0.808834	no
gene:SpnNT_00231	infA	Chromosome:214501-214720	110.58	ΔORF2	OK	428.94	459.058	0.0979017	0.18431	0.7382	0.994748	no
gene:SpnNT_00231	infA	Chromosome:214501-214720	110.58	110.58+peptide	OK	428.94	727.24	0.761656	1.44995	0.01075	0.105743	no
gene:SpnNT_00231	infA	Chromosome:214501-214720	ΔORF2	110.58+peptide	OK	459.058	727.24	0.663754	1.27668	0.02225	0.18157	no
gene:SpnNT_00231	infA	Chromosome:214501-214720	110.58	ΔORF2+peptide	OK	428.94	876.709	1.03132	2.02102	9.00E-04	0.0154579	yes
gene:SpnNT_00231	infA	Chromosome:214501-214720	ΔORF2	ΔORF2+peptide	OK	459.058	876.709	0.933421	1.84929	0.0012	0.0193683	yes
gene:SpnNT_00231	infA	Chromosome:214501-214720	110.58+peptide	ΔORF2+peptide	OK	727.24	876.709	0.269667	0.5409	0.34785	0.852697	no
gene:SpnNT_00232	rpmJ	Chromosome:214744-214861	110.58	ΔORF2	OK	2562.01	2042.05	-0.327253	-0.586668	0.3505	0.854722	no
gene:SpnNT_00232	rpmJ	Chromosome:214744-214861	110.58	110.58+peptide	OK	2562.01	2755.19	0.104877	0.173125	0.76175	0.994748	no
gene:SpnNT_00232	rpmJ	Chromosome:214744-214861	ΔORF2	110.58+peptide	OK	2042.05	2755.19	0.43213	0.752461	0.2112	0.697158	no
gene:SpnNT_00232	rpmJ	Chromosome:214744-214861	110.58	ΔORF2+peptide	OK	2562.01	3494.48	0.447806	0.719955	0.20235	0.6861	no
gene:SpnNT_00232	rpmJ	Chromosome:214744-214861	ΔORF2	ΔORF2+peptide	OK	2042.05	3494.48	0.775059	1.31064	0.02885	0.216326	no
gene:SpnNT_00232	rpmJ	Chromosome:214744-214861	110.58+peptide	ΔORF2+peptide	OK	2755.19	3494.48	0.342929	0.538514	0.326	0.831443	no
gene:SpnNT_00233	rpsM	Chromosome:214878-215244	110.58	ΔORF2	OK	904.769	860.354	-0.0726194	-0.160521	0.77495	0.994748	no
gene:SpnNT_00233	rpsM	Chromosome:214878-215244	110.58	110.58+peptide	OK	904.769	1473.37	0.703503	1.56192	0.0071	0.0782909	no

gene:SpnNT_00233	rpsM	Chromosome:214878-215244	ΔORF2	110.58+peptide	OK	860.354	1473.37	0.776122	1.70764	0.00325	0.0427748	yes
gene:SpnNT_00233	rpsM	Chromosome:214878-215244	110.58	ΔORF2+peptide	OK	904.769	1614.11	0.835115	1.87851	0.00095	0.0161266	yes
gene:SpnNT_00233	rpsM	Chromosome:214878-215244	ΔORF2	ΔORF2+peptide	OK	860.354	1614.11	0.907734	2.023	0.00035	0.00717609	yes
gene:SpnNT_00233	rpsM	Chromosome:214878-215244	110.58+peptide	ΔORF2+peptide	OK	1473.37	1614.11	0.131612	0.294631	0.6037	0.976761	no
gene:SpnNT_00234	rpsK	Chromosome:215261-215645	110.58	ΔORF2	OK	896.221	790.313	-0.181431	-0.398292	0.48205	0.935166	no
gene:SpnNT_00234	rpsK	Chromosome:215261-215645	110.58	110.58+peptide	OK	896.221	1399.12	0.64259	1.43142	0.01115	0.108378	no
gene:SpnNT_00234	rpsK	Chromosome:215261-215645	ΔORF2	110.58+peptide	OK	790.313	1399.12	0.824021	1.79573	0.00175	0.0265167	yes
gene:SpnNT_00234	rpsK	Chromosome:215261-215645	110.58	ΔORF2+peptide	OK	896.221	1437.84	0.68198	1.5354	0.0076	0.082624	no
gene:SpnNT_00234	rpsK	Chromosome:215261-215645	ΔORF2	ΔORF2+peptide	OK	790.313	1437.84	0.863411	1.9008	0.00085	0.0148124	yes
gene:SpnNT_00234	rpsK	Chromosome:215261-215645	110.58+peptide	ΔORF2+peptide	OK	1399.12	1437.84	0.0393897	0.0879999	0.88155	0.994748	no
gene:SpnNT_00235	rpoA	Chromosome:215687-216623	110.58	ΔORF2	OK	733.176	778.703	0.086914	0.195537	0.72355	0.994748	no
gene:SpnNT_00235	rpoA	Chromosome:215687-216623	110.58	110.58+peptide	OK	733.176	1300.51	0.826841	1.82859	0.00115	0.0187457	yes
gene:SpnNT_00235	rpoA	Chromosome:215687-216623	ΔORF2	110.58+peptide	OK	778.703	1300.51	0.739927	1.63596	0.00305	0.0405492	yes
gene:SpnNT_00235	rpoA	Chromosome:215687-216623	110.58	ΔORF2+peptide	OK	733.176	1727.15	1.23616	2.72247	5.00E-05	0.0013612	yes
gene:SpnNT_00235	rpoA	Chromosome:215687-216623	ΔORF2	ΔORF2+peptide	OK	778.703	1727.15	1.14925	2.53042	5.00E-05	0.0013612	yes
gene:SpnNT_00235	rpoA	Chromosome:215687-216623	110.58+peptide	ΔORF2+peptide	OK	1300.51	1727.15	0.409324	0.88656	0.1198	0.52453	no
gene:SpnNT_00236	rplQ	Chromosome:216634-217021	110.58	ΔORF2	OK	1420.94	1431.66	0.0108397	0.0245701	0.96695	0.994855	no
gene:SpnNT_00236	rplQ	Chromosome:216634-217021	110.58	110.58+peptide	OK	1420.94	2667.74	0.908765	2.03415	0.00045	0.00885292	yes
gene:SpnNT_00236	rplQ	Chromosome:216634-217021	ΔORF2	110.58+peptide	OK	1431.66	2667.74	0.897925	2.01465	0.00045	0.00885292	yes
gene:SpnNT_00236	rplQ	Chromosome:216634-217021	110.58	ΔORF2+peptide	OK	1420.94	2867.4	1.01289	2.29752	1.00E-04	0.0025332	yes
gene:SpnNT_00236	rplQ	Chromosome:216634-217021	ΔORF2	ΔORF2+peptide	OK	1431.66	2867.4	1.00205	2.27847	1.00E-04	0.0025332	yes
gene:SpnNT_00236	rplQ	Chromosome:216634-217021	110.58+peptide	ΔORF2+peptide	OK	2667.74	2867.4	0.104127	0.233788	0.6784	0.985276	no
gene:SpnNT_00237	NA	Chromosome:217285-217552	110.58	ΔORF2	OK	77.8557	101.693	0.385342	0.635808	0.271	0.772864	no
gene:SpnNT_00237	NA	Chromosome:217285-217552	110.58	110.58+peptide	OK	77.8557	42.719	-0.865926	-1.31208	0.0254	0.198984	no
gene:SpnNT_00237	NA	Chromosome:217285-217552	ΔORF2	110.58+peptide	OK	101.693	42.719	-1.25127	-1.89514	0.0017	0.025969	yes
gene:SpnNT_00237	NA	Chromosome:217285-217552	110.58	ΔORF2+peptide	OK	77.8557	37.5363	-1.05252	-1.59916	0.00585	0.0677526	no
gene:SpnNT_00237	NA	Chromosome:217285-217552	ΔORF2	ΔORF2+peptide	OK	101.693	37.5363	-1.43786	-2.18368	0.00035	0.00717609	yes
gene:SpnNT_00237	NA	Chromosome:217285-217552	110.58+peptide	ΔORF2+peptide	OK	42.719	37.5363	-0.186589	-0.263403	0.64915	0.981391	no
gene:SpnNT_00238	NA	Chromosome:217561-218899	110.58	ΔORF2	OK	193.78	200.705	0.0506576	0.114613	0.83915	0.994748	no
gene:SpnNT_00238	NA	Chromosome:217561-218899	110.58	110.58+peptide	OK	193.78	79.3568	-1.288	-2.89832	5.00E-05	0.0013612	yes
gene:SpnNT_00238	NA	Chromosome:217561-218899	ΔORF2	110.58+peptide	OK	200.705	79.3568	-1.33865	-3.00896	5.00E-05	0.0013612	yes
gene:SpnNT_00238	NA	Chromosome:217561-218899	110.58	ΔORF2+peptide	OK	193.78	90.6627	-1.09584	-2.48061	5.00E-05	0.0013612	yes
gene:SpnNT_00238	NA	Chromosome:217561-218899	ΔORF2	ΔORF2+peptide	OK	200.705	90.6627	-1.1465	-2.59236	5.00E-05	0.0013612	yes
gene:SpnNT_00238	NA	Chromosome:217561-218899	110.58+peptide	ΔORF2+peptide	OK	79.3568	90.6627	0.192156	0.432134	0.45205	0.921244	no
gene:SpnNT_00239	gpmA_1	Chromosome:219142-219835	110.58	ΔORF2	OK	330.303	303.004	-0.124453	-0.280519	0.6222	0.980887	no
gene:SpnNT_00239	gpmA_1	Chromosome:219142-219835	110.58	110.58+peptide	OK	330.303	286.114	-0.207199	-0.465908	0.41195	0.899511	no
gene:SpnNT_00239	gpmA_1	Chromosome:219142-219835	ΔORF2	110.58+peptide	OK	303.004	286.114	-0.0827467	-0.184587	0.73955	0.994748	no
gene:SpnNT_00239	gpmA_1	Chromosome:219142-219835	110.58	ΔORF2+peptide	OK	330.303	238.358	-0.470663	-1.0657	0.06575	0.373171	no
gene:SpnNT_00239	gpmA_1	Chromosome:219142-219835	ΔORF2	ΔORF2+peptide	OK	303.004	238.358	-0.34621	-0.777596	0.1761	0.639929	no
gene:SpnNT_00239	gpmA_1	Chromosome:219142-219835	110.58+peptide	ΔORF2+peptide	OK	286.114	238.358	-0.263464	-0.590331	0.2991	0.80426	no
gene:SpnNT_00240	phnV_1	Chromosome:219937-221583	110.58	ΔORF2	OK	1.79259	1.48495	-0.271635	-0.288755	0.61155	0.979429	no
gene:SpnNT_00240	phnV_1	Chromosome:219937-221583	110.58	110.58+peptide	OK	1.79259	1.66571	-0.105909	-0.106525	0.85035	0.994748	no
gene:SpnNT_00240	phnV_1	Chromosome:219937-221583	ΔORF2	110.58+peptide	OK	1.48495	1.66571	0.165726	0.173548	0.7599	0.994748	no
gene:SpnNT_00240	phnV_1	Chromosome:219937-221583	110.58	ΔORF2+peptide	OK	1.79259	1.60637	-0.158248	-0.158695	0.7872	0.994748	no
gene:SpnNT_00240	phnV_1	Chromosome:219937-221583	ΔORF2	ΔORF2+peptide	OK	1.48495	1.60637	0.113387	0.118355	0.8377	0.994748	no

gene:SpnNT_00240	phnV_1	Chromosome:219937-221583	110.58+peptide	ΔORF2+peptide	OK	1.66571	1.60637	-0.0523395	-0.0517901	0.926	0.994748	no
gene:SpnNT_00241	phnU	Chromosome:219937-221583	110.58	ΔORF2	OK	1.61675	0.946312	-0.772708	-0.499237	0.3949	0.889898	no
gene:SpnNT_00241	phnU	Chromosome:219937-221583	110.58	110.58+peptide	OK	1.61675	2.38036	0.558084	0.418562	0.4718	0.928525	no
gene:SpnNT_00241	phnU	Chromosome:219937-221583	ΔORF2	110.58+peptide	OK	0.946312	2.38036	1.33079	0.952049	0.10925	0.502305	no
gene:SpnNT_00241	phnU	Chromosome:219937-221583	110.58	ΔORF2+peptide	OK	1.61675	1.475	-0.13238	-0.0797071	0.88455	0.994748	no
gene:SpnNT_00241	phnU	Chromosome:219937-221583	ΔORF2	ΔORF2+peptide	OK	0.946312	1.475	0.640328	0.373799	0.4988	0.944017	no
gene:SpnNT_00241	phnU	Chromosome:219937-221583	110.58+peptide	ΔORF2+peptide	OK	2.38036	1.475	-0.690464	-0.453641	0.39925	0.892609	no
gene:SpnNT_00242	NA	Chromosome:221710-221977	110.58	ΔORF2	OK	0.897449	1.95671	1.12453	0.755939	0.36275	0.86287	no
gene:SpnNT_00242	NA	Chromosome:221710-221977	110.58	110.58+peptide	OK	0.897449	2.34333	1.38466	1.10422	0.2811	0.784473	no
gene:SpnNT_00242	NA	Chromosome:221710-221977	ΔORF2	110.58+peptide	OK	1.95671	2.34333	0.260128	0.150921	0.7997	0.994748	no
gene:SpnNT_00242	NA	Chromosome:221710-221977	110.58	ΔORF2+peptide	NOTEST	0.897449	0	#NAME?	0	1	1	no
gene:SpnNT_00242	NA	Chromosome:221710-221977	ΔORF2	ΔORF2+peptide	OK	1.95671	0	#NAME?	NA	0.0025	0.0347143	yes
gene:SpnNT_00242	NA	Chromosome:221710-221977	110.58+peptide	ΔORF2+peptide	OK	2.34333	0	#NAME?	NA	7.00E-04	0.012687	yes
gene:SpnNT_00243	cysA_1	Chromosome:222013-222535	110.58	ΔORF2	OK	0.910817	0.901526	-0.0147924	-0.0121457	0.92755	0.994748	no
gene:SpnNT_00243	cysA_1	Chromosome:222013-222535	110.58	110.58+peptide	OK	0.910817	1.54654	0.76381	0.656731	0.31145	0.817206	no
gene:SpnNT_00243	cysA_1	Chromosome:222013-222535	ΔORF2	110.58+peptide	OK	0.901526	1.54654	0.778603	0.625289	0.27995	0.783263	no
gene:SpnNT_00243	cysA_1	Chromosome:222013-222535	110.58	ΔORF2+peptide	NOTEST	0.910817	0.847524	-0.103908	0	1	1	no
gene:SpnNT_00243	cysA_1	Chromosome:222013-222535	ΔORF2	ΔORF2+peptide	OK	0.901526	0.847524	-0.0891151	-0.0699867	0.9192	0.994748	no
gene:SpnNT_00243	cysA_1	Chromosome:222013-222535	110.58+peptide	ΔORF2+peptide	OK	1.54654	0.847524	-0.867718	-0.710694	0.2517	0.749765	no
gene:SpnNT_00244	potD_1	Chromosome:222663-223113	110.58	ΔORF2	NOTEST	0.712866	0.706571	-0.0127972	0	1	1	no
gene:SpnNT_00244	potD_1	Chromosome:222663-223113	110.58	110.58+peptide	OK	0.712866	1.47474	1.04876	0.855987	0.2127	0.698345	no
gene:SpnNT_00244	potD_1	Chromosome:222663-223113	ΔORF2	110.58+peptide	OK	0.706571	1.47474	1.06155	0.853437	0.2083	0.693901	no
gene:SpnNT_00244	potD_1	Chromosome:222663-223113	110.58	ΔORF2+peptide	OK	0.712866	2.23833	1.65072	1.47197	0.0734	0.399141	no
gene:SpnNT_00244	potD_1	Chromosome:222663-223113	ΔORF2	ΔORF2+peptide	OK	0.706571	2.23833	1.66352	1.45694	0.06755	0.380099	no
gene:SpnNT_00244	potD_1	Chromosome:222663-223113	110.58+peptide	ΔORF2+peptide	OK	1.47474	2.23833	0.601964	0.56152	0.3797	0.877496	no
gene:SpnNT_00245	NA	Chromosome:223176-223785	110.58	ΔORF2	OK	0.903428	0.330788	-1.4495	-1.07514	0.1267	0.539616	no
gene:SpnNT_00245	NA	Chromosome:223176-223785	110.58	110.58+peptide	OK	0.903428	0.307784	-1.55349	-1.10775	0.13485	0.558731	no
gene:SpnNT_00245	NA	Chromosome:223176-223785	ΔORF2	110.58+peptide	NOTEST	0.330788	0.307784	-0.10399	0	1	1	no
gene:SpnNT_00245	NA	Chromosome:223176-223785	110.58	ΔORF2+peptide	OK	0.903428	0.403347	-1.16339	-0.859988	0.18855	0.665453	no
gene:SpnNT_00245	NA	Chromosome:223176-223785	ΔORF2	ΔORF2+peptide	NOTEST	0.330788	0.403347	0.286111	0	1	1	no
gene:SpnNT_00245	NA	Chromosome:223176-223785	110.58+peptide	ΔORF2+peptide	NOTEST	0.307784	0.403347	0.390102	0	1	1	no
gene:SpnNT_00246	NA	Chromosome:224001-224199	110.58	ΔORF2	OK	6.64886	7.49436	0.172699	0.11974	0.82895	0.994748	no
gene:SpnNT_00246	NA	Chromosome:224001-224199	110.58	110.58+peptide	OK	6.64886	4.05428	-0.713661	-0.61976	0.4732	0.928984	no
gene:SpnNT_00246	NA	Chromosome:224001-224199	ΔORF2	110.58+peptide	OK	7.49436	4.05428	-0.88636	-0.811102	0.36155	0.862361	no
gene:SpnNT_00246	NA	Chromosome:224001-224199	110.58	ΔORF2+peptide	OK	6.64886	18.8489	1.5033	1.39952	0.08995	0.450333	no
gene:SpnNT_00246	NA	Chromosome:224001-224199	ΔORF2	ΔORF2+peptide	OK	7.49436	18.8489	1.3306	1.3162	0.11595	0.516286	no
gene:SpnNT_00246	NA	Chromosome:224001-224199	110.58+peptide	ΔORF2+peptide	OK	4.05428	18.8489	2.21696	4.28404	0.0563	0.3381	no
gene:SpnNT_00247	hpdA	Chromosome:224273-225050	110.58	ΔORF2	OK	6.80014	8.44939	0.313282	0.485642	0.39015	0.884392	no
gene:SpnNT_00247	hpdA	Chromosome:224273-225050	110.58	110.58+peptide	OK	6.80014	5.34336	-0.347818	-0.512631	0.3752	0.872746	no
gene:SpnNT_00247	hpdA	Chromosome:224273-225050	ΔORF2	110.58+peptide	OK	8.44939	5.34336	-0.6611	-0.988388	0.0888	0.448684	no
gene:SpnNT_00247	hpdA	Chromosome:224273-225050	110.58	ΔORF2+peptide	OK	6.80014	6.08746	-0.159724	-0.240626	0.67415	0.985063	no
gene:SpnNT_00247	hpdA	Chromosome:224273-225050	ΔORF2	ΔORF2+peptide	OK	8.44939	6.08746	-0.473006	-0.72332	0.21115	0.697158	no
gene:SpnNT_00247	hpdA	Chromosome:224273-225050	110.58+peptide	ΔORF2+peptide	OK	5.34336	6.08746	0.188094	0.273825	0.6403	0.980887	no
gene:SpnNT_00248	srlR	Chromosome:225171-225918	110.58	ΔORF2	OK	46.5468	75.0626	0.689414	1.31362	0.0244	0.194047	no
gene:SpnNT_00248	srlR	Chromosome:225171-225918	110.58	110.58+peptide	OK	46.5468	67.6999	0.540473	1.08185	0.06175	0.35885	no

gene:SpnNT_00248	srlR	Chromosome:225171-225918	ΔORF2	110.58+peptide	OK	75.0626	67.6999	-0.148941	-0.289065	0.62445	0.980887	no
gene:SpnNT_00248	srlR	Chromosome:225171-225918	110.58	ΔORF2+peptide	OK	46.5468	88.7676	0.931352	1.89406	0.00135	0.021343	yes
gene:SpnNT_00248	srlR	Chromosome:225171-225918	ΔORF2	ΔORF2+peptide	OK	75.0626	88.7676	0.241938	0.476598	0.40585	0.895655	no
gene:SpnNT_00248	srlR	Chromosome:225171-225918	110.58+peptide	ΔORF2+peptide	OK	67.6999	88.7676	0.390879	0.811803	0.15885	0.608415	no
gene:SpnNT_00249	sorC	Chromosome:225930-226911	110.58	ΔORF2	OK	24.0298	37.9158	0.657978	1.2098	0.0341	0.243541	no
gene:SpnNT_00249	sorC	Chromosome:225930-226911	110.58	110.58+peptide	OK	24.0298	36.8374	0.616347	1.1764	0.04075	0.274498	no
gene:SpnNT_00249	sorC	Chromosome:225930-226911	ΔORF2	110.58+peptide	OK	37.9158	36.8374	-0.0416304	-0.0778393	0.89125	0.994748	no
gene:SpnNT_00249	sorC	Chromosome:225930-226911	110.58	ΔORF2+peptide	OK	24.0298	49.986	1.0567	2.07943	5.00E-04	0.0095781	yes
gene:SpnNT_00249	sorC	Chromosome:225930-226911	ΔORF2	ΔORF2+peptide	OK	37.9158	49.986	0.398725	0.767671	0.1709	0.62976	no
gene:SpnNT_00249	sorC	Chromosome:225930-226911	110.58+peptide	ΔORF2+peptide	OK	36.8374	49.986	0.440356	0.883408	0.12135	0.52727	no
gene:SpnNT_00250	NA	Chromosome:226960-228201	110.58	ΔORF2	OK	6.75042	7.04466	0.0615522	0.0709869	0.90115	0.994748	no
gene:SpnNT_00250	NA	Chromosome:226960-228201	110.58	110.58+peptide	OK	6.75042	6.91455	0.0346593	0.0401766	0.94395	0.994855	no
gene:SpnNT_00250	NA	Chromosome:226960-228201	ΔORF2	110.58+peptide	OK	7.04466	6.91455	-0.0268929	-0.0341348	0.95295	0.994855	no
gene:SpnNT_00250	NA	Chromosome:226960-228201	110.58	ΔORF2+peptide	OK	6.75042	6.21846	-0.118419	-0.132264	0.82105	0.994748	no
gene:SpnNT_00250	NA	Chromosome:226960-228201	ΔORF2	ΔORF2+peptide	OK	7.04466	6.21846	-0.179971	-0.218552	0.7056	0.990422	no
gene:SpnNT_00250	NA	Chromosome:226960-228201	110.58+peptide	ΔORF2+peptide	OK	6.91455	6.21846	-0.153078	-0.186951	0.75665	0.994748	no
gene:SpnNT_00251	NA	Chromosome:226960-228201	110.58	ΔORF2	OK	15.6068	13.7386	-0.183938	-0.18498	0.75225	0.994748	no
gene:SpnNT_00251	NA	Chromosome:226960-228201	110.58	110.58+peptide	OK	15.6068	12.5196	-0.317994	-0.31479	0.5837	0.971584	no
gene:SpnNT_00251	NA	Chromosome:226960-228201	ΔORF2	110.58+peptide	OK	13.7386	12.5196	-0.134056	-0.134585	0.8197	0.994748	no
gene:SpnNT_00251	NA	Chromosome:226960-228201	110.58	ΔORF2+peptide	OK	15.6068	13.5843	-0.200233	-0.197508	0.7354	0.994748	no
gene:SpnNT_00251	NA	Chromosome:226960-228201	ΔORF2	ΔORF2+peptide	OK	13.7386	13.5843	-0.0162948	-0.016299	0.97635	0.99536	no
gene:SpnNT_00251	NA	Chromosome:226960-228201	110.58+peptide	ΔORF2+peptide	OK	12.5196	13.5843	0.117761	0.115967	0.84195	0.994748	no
gene:SpnNT_00252	licA	Chromosome:228547-228868	110.58	ΔORF2	OK	0.493275	1.40746	1.51263	1.05085	0.3293	0.83499	no
gene:SpnNT_00252	licA	Chromosome:228547-228868	110.58	110.58+peptide	NOTEST	0.493275	0.215776	-1.19285	0	1	1	no
gene:SpnNT_00252	licA	Chromosome:228547-228868	ΔORF2	110.58+peptide	OK	1.40746	0.215776	-2.70548	-1.23057	0.18195	0.651623	no
gene:SpnNT_00252	licA	Chromosome:228547-228868	110.58	ΔORF2+peptide	NOTEST	0.493275	0.270923	-0.86451	0	1	1	no
gene:SpnNT_00252	licA	Chromosome:228547-228868	ΔORF2	ΔORF2+peptide	OK	1.40746	0.270923	-2.37714	-1.27299	0.18635	0.661065	no
gene:SpnNT_00252	licA	Chromosome:228547-228868	110.58+peptide	ΔORF2+peptide	NOTEST	0.215776	0.270923	0.328343	0	1	1	no
gene:SpnNT_00253	gmuB	Chromosome:228913-229222	110.58	ΔORF2	NOTEST	0.60393	0.673692	0.157708	0	1	1	no
gene:SpnNT_00253	gmuB	Chromosome:228913-229222	110.58	110.58+peptide	NOTEST	0.60393	0.833356	0.464552	0	1	1	no
gene:SpnNT_00253	gmuB	Chromosome:228913-229222	ΔORF2	110.58+peptide	NOTEST	0.673692	0.833356	0.306844	0	1	1	no
gene:SpnNT_00253	gmuB	Chromosome:228913-229222	110.58	ΔORF2+peptide	NOTEST	0.60393	0.509117	-0.246385	0	1	1	no
gene:SpnNT_00253	gmuB	Chromosome:228913-229222	ΔORF2	ΔORF2+peptide	NOTEST	0.673692	0.509117	-0.404093	0	1	1	no
gene:SpnNT_00253	gmuB	Chromosome:228913-229222	110.58+peptide	ΔORF2+peptide	NOTEST	0.833356	0.509117	-0.710937	0	1	1	no
gene:SpnNT_00254	licC_1	Chromosome:229238-230531	110.58	ΔORF2	OK	0.866983	0.9782	0.174125	0.195214	0.7371	0.994748	no
gene:SpnNT_00254	licC_1	Chromosome:229238-230531	110.58	110.58+peptide	OK	0.866983	0.812637	-0.093392	-0.101569	0.85475	0.994748	no
gene:SpnNT_00254	licC_1	Chromosome:229238-230531	ΔORF2	110.58+peptide	OK	0.9782	0.812637	-0.267517	-0.298399	0.6037	0.976761	no
gene:SpnNT_00254	licC_1	Chromosome:229238-230531	110.58	ΔORF2+peptide	OK	0.866983	0.876975	0.0165325	0.0178	0.96705	0.994855	no
gene:SpnNT_00254	licC_1	Chromosome:229238-230531	ΔORF2	ΔORF2+peptide	OK	0.9782	0.876975	-0.157593	-0.173934	0.76515	0.994748	no
gene:SpnNT_00254	licC_1	Chromosome:229238-230531	110.58+peptide	ΔORF2+peptide	OK	0.812637	0.876975	0.109925	0.117799	0.8311	0.994748	no
gene:SpnNT_00255	bssA	Chromosome:230687-233126	110.58	ΔORF2	OK	2.7754	3.05949	0.140598	0.230606	0.68495	0.988009	no
gene:SpnNT_00255	bssA	Chromosome:230687-233126	110.58	110.58+peptide	OK	2.7754	3.18341	0.197881	0.32568	0.5702	0.968621	no
gene:SpnNT_00255	bssA	Chromosome:230687-233126	ΔORF2	110.58+peptide	OK	3.05949	3.18341	0.0572827	0.0960551	0.8644	0.994748	no
gene:SpnNT_00255	bssA	Chromosome:230687-233126	110.58	ΔORF2+peptide	OK	2.7754	3.55117	0.3556	0.587847	0.3071	0.813241	no
gene:SpnNT_00255	bssA	Chromosome:230687-233126	ΔORF2	ΔORF2+peptide	OK	3.05949	3.55117	0.215001	0.362182	0.52845	0.956161	no

gene:SpnNT_00255	bssA	Chromosome:230687-233126	110.58+peptide	ΔORF2+peptide	OK	3.18341	3.55117	0.157719	0.266654	0.64505	0.980887	no
gene:SpnNT_00256	fsaA	Chromosome:233144-233813	110.58	ΔORF2	OK	2.17766	2.08442	-0.0631369	-0.0708213	0.9067	0.994748	no
gene:SpnNT_00256	fsaA	Chromosome:233144-233813	110.58	110.58+peptide	OK	2.17766	2.04737	-0.0890121	-0.100648	0.86315	0.994748	no
gene:SpnNT_00256	fsaA	Chromosome:233144-233813	ΔORF2	110.58+peptide	OK	2.08442	2.04737	-0.0258752	-0.0288468	0.96965	0.995105	no
gene:SpnNT_00256	fsaA	Chromosome:233144-233813	110.58	ΔORF2+peptide	OK	2.17766	2.0545	-0.0839947	-0.0945192	0.87155	0.994748	no
gene:SpnNT_00256	fsaA	Chromosome:233144-233813	ΔORF2	ΔORF2+peptide	OK	2.08442	2.0545	-0.0208578	-0.0231447	0.9677	0.994855	no
gene:SpnNT_00256	fsaA	Chromosome:233144-233813	110.58+peptide	ΔORF2+peptide	OK	2.04737	2.0545	0.00501739	0.00561127	0.967	0.994855	no
gene:SpnNT_00257	gldA	Chromosome:233830-234919	110.58	ΔORF2	OK	2.65088	3.35999	0.341982	0.477917	0.39825	0.891933	no
gene:SpnNT_00257	gldA	Chromosome:233830-234919	110.58	110.58+peptide	OK	2.65088	3.43229	0.372698	0.522278	0.35625	0.857745	no
gene:SpnNT_00257	gldA	Chromosome:233830-234919	ΔORF2	110.58+peptide	OK	3.35999	3.43229	0.030716	0.0448024	0.9377	0.994855	no
gene:SpnNT_00257	gldA	Chromosome:233830-234919	110.58	ΔORF2+peptide	OK	2.65088	4.09482	0.627326	0.880687	0.119	0.522771	no
gene:SpnNT_00257	gldA	Chromosome:233830-234919	ΔORF2	ΔORF2+peptide	OK	3.35999	4.09482	0.285345	0.417017	0.4645	0.926394	no
gene:SpnNT_00257	gldA	Chromosome:233830-234919	110.58+peptide	ΔORF2+peptide	OK	3.43229	4.09482	0.254629	0.37325	0.5153	0.951959	no
gene:SpnNT_00258	leuS	Chromosome:235373-237875	110.58	ΔORF2	OK	139.511	139.348	-0.00168173	-0.00380475	0.9949	0.999241	no
gene:SpnNT_00258	leuS	Chromosome:235373-237875	110.58	110.58+peptide	OK	139.511	117.469	-0.248097	-0.567099	0.3149	0.821168	no
gene:SpnNT_00258	leuS	Chromosome:235373-237875	ΔORF2	110.58+peptide	OK	139.348	117.469	-0.246415	-0.559099	0.31655	0.822529	no
gene:SpnNT_00258	leuS	Chromosome:235373-237875	110.58	ΔORF2+peptide	OK	139.511	114.43	-0.285906	-0.654621	0.2467	0.74363	no
gene:SpnNT_00258	leuS	Chromosome:235373-237875	ΔORF2	ΔORF2+peptide	OK	139.348	114.43	-0.284224	-0.645954	0.2475	0.743991	no
gene:SpnNT_00258	leuS	Chromosome:235373-237875	110.58+peptide	ΔORF2+peptide	OK	117.469	114.43	-0.0378087	-0.086824	0.877	0.994748	no
gene:SpnNT_00259	ydaF_1	Chromosome:238068-238764	110.58	ΔORF2	OK	51.2528	56.0903	0.13012	0.258179	0.646	0.980887	no
gene:SpnNT_00259	ydaF_1	Chromosome:238068-238764	110.58	110.58+peptide	OK	51.2528	34.6204	-0.56601	-1.08876	0.05665	0.338806	no
gene:SpnNT_00259	ydaF_1	Chromosome:238068-238764	ΔORF2	110.58+peptide	OK	56.0903	34.6204	-0.69613	-1.34334	0.01965	0.164969	no
gene:SpnNT_00259	ydaF_1	Chromosome:238068-238764	110.58	ΔORF2+peptide	OK	51.2528	36.4584	-0.491378	-0.946117	0.09485	0.465627	no
gene:SpnNT_00259	ydaF_1	Chromosome:238068-238764	ΔORF2	ΔORF2+peptide	OK	56.0903	36.4584	-0.621498	-1.20049	0.03705	0.257642	no
gene:SpnNT_00259	ydaF_1	Chromosome:238068-238764	110.58+peptide	ΔORF2+peptide	OK	34.6204	36.4584	0.0746318	0.139977	0.8093	0.994748	no
gene:SpnNT_00260	NA	Chromosome:238775-239192	110.58	ΔORF2	OK	111.955	132.399	0.241967	0.477871	0.40905	0.897533	no
gene:SpnNT_00260	NA	Chromosome:238775-239192	110.58	110.58+peptide	OK	111.955	85.8022	-0.383838	-0.73448	0.20795	0.693596	no
gene:SpnNT_00260	NA	Chromosome:238775-239192	ΔORF2	110.58+peptide	OK	132.399	85.8022	-0.625804	-1.21324	0.03485	0.246923	no
gene:SpnNT_00260	NA	Chromosome:238775-239192	110.58	ΔORF2+peptide	OK	111.955	82.3152	-0.443693	-0.84526	0.14725	0.584811	no
gene:SpnNT_00260	NA	Chromosome:238775-239192	ΔORF2	ΔORF2+peptide	OK	132.399	82.3152	-0.68566	-1.32324	0.0226	0.183627	no
gene:SpnNT_00260	NA	Chromosome:238775-239192	110.58+peptide	ΔORF2+peptide	OK	85.8022	82.3152	-0.0598552	-0.112076	0.8488	0.994748	no
gene:SpnNT_00261	ruvB	Chromosome:241084-242636	110.58	ΔORF2	OK	88.2212	78.2561	-0.172923	-0.342309	0.5584	0.968033	no
gene:SpnNT_00261	ruvB	Chromosome:241084-242636	110.58	110.58+peptide	OK	88.2212	91.0769	0.0459598	0.0928651	0.8717	0.994748	no
gene:SpnNT_00261	ruvB	Chromosome:241084-242636	ΔORF2	110.58+peptide	OK	78.2561	91.0769	0.218883	0.432544	0.4512	0.921244	no
gene:SpnNT_00261	ruvB	Chromosome:241084-242636	110.58	ΔORF2+peptide	OK	88.2212	85.227	-0.0498152	-0.097291	0.86925	0.994748	no
gene:SpnNT_00261	ruvB	Chromosome:241084-242636	ΔORF2	ΔORF2+peptide	OK	78.2561	85.227	0.123108	0.235485	0.6828	0.987186	no
gene:SpnNT_00261	ruvB	Chromosome:241084-242636	110.58+peptide	ΔORF2+peptide	OK	91.0769	85.227	-0.095775	-0.18674	0.74645	0.994748	no
gene:SpnNT_00262	NA	Chromosome:241084-242636	110.58	ΔORF2	OK	45.7432	45.0313	-0.0226291	-0.0209316	0.9723	0.99536	no
gene:SpnNT_00262	NA	Chromosome:241084-242636	110.58	110.58+peptide	OK	45.7432	48.2098	0.0757696	0.0721895	0.9008	0.994748	no
gene:SpnNT_00262	NA	Chromosome:241084-242636	ΔORF2	110.58+peptide	OK	45.0313	48.2098	0.0983987	0.0952264	0.87225	0.994748	no
gene:SpnNT_00262	NA	Chromosome:241084-242636	110.58	ΔORF2+peptide	OK	45.7432	66.2265	0.533851	0.52667	0.36495	0.864498	no
gene:SpnNT_00262	NA	Chromosome:241084-242636	ΔORF2	ΔORF2+peptide	OK	45.0313	66.2265	0.556481	0.558284	0.3349	0.840029	no
gene:SpnNT_00262	NA	Chromosome:241084-242636	110.58+peptide	ΔORF2+peptide	OK	48.2098	66.2265	0.458082	0.475926	0.413	0.899511	no
gene:SpnNT_00263	uppS	Chromosome:242865-243624	110.58	ΔORF2	OK	150.387	142.532	-0.077398	-0.172997	0.75745	0.994748	no
gene:SpnNT_00263	uppS	Chromosome:242865-243624	110.58	110.58+peptide	OK	150.387	133.39	-0.173029	-0.385963	0.4961	0.943191	no

gene:SpnNT_00263	uppS	Chromosome:242865-243624	ΔORF2	110.58+peptide	OK	142.532	133.39	-0.0956309	-0.212068	0.71135	0.991007	no
gene:SpnNT_00263	uppS	Chromosome:242865-243624	110.58	ΔORF2+peptide	OK	150.387	148.155	-0.0215748	-0.0483959	0.92895	0.994748	no
gene:SpnNT_00263	uppS	Chromosome:242865-243624	ΔORF2	ΔORF2+peptide	OK	142.532	148.155	0.0558233	0.12448	0.8217	0.994748	no
gene:SpnNT_00263	uppS	Chromosome:242865-243624	110.58+peptide	ΔORF2+peptide	OK	133.39	148.155	0.151454	0.337045	0.5495	0.964281	no
gene:SpnNT_00264	cdsA	Chromosome:243632-244436	110.58	ΔORF2	OK	119.173	116.543	-0.0321998	-0.0708008	0.9045	0.994748	no
gene:SpnNT_00264	cdsA	Chromosome:243632-244436	110.58	110.58+peptide	OK	119.173	132.202	0.149683	0.331464	0.56545	0.968621	no
gene:SpnNT_00264	cdsA	Chromosome:243632-244436	ΔORF2	110.58+peptide	OK	116.543	132.202	0.181882	0.403565	0.48385	0.936747	no
gene:SpnNT_00264	cdsA	Chromosome:243632-244436	110.58	ΔORF2+peptide	OK	119.173	122.901	0.0444413	0.0982377	0.86455	0.994748	no
gene:SpnNT_00264	cdsA	Chromosome:243632-244436	ΔORF2	ΔORF2+peptide	OK	116.543	122.901	0.0766411	0.169749	0.7662	0.994748	no
gene:SpnNT_00264	cdsA	Chromosome:243632-244436	110.58+peptide	ΔORF2+peptide	OK	132.202	122.901	-0.105241	-0.234778	0.67445	0.985063	no
gene:SpnNT_00265	NA	Chromosome:244457-245717	110.58	ΔORF2	OK	142.498	136.437	-0.0627114	-0.143844	0.79995	0.994748	no
gene:SpnNT_00265	NA	Chromosome:244457-245717	110.58	110.58+peptide	OK	142.498	144.17	0.0168258	0.038629	0.94575	0.994855	no
gene:SpnNT_00265	NA	Chromosome:244457-245717	ΔORF2	110.58+peptide	OK	136.437	144.17	0.0795372	0.18262	0.74335	0.994748	no
gene:SpnNT_00265	NA	Chromosome:244457-245717	110.58	ΔORF2+peptide	OK	142.498	152.129	0.0943564	0.21658	0.70225	0.990367	no
gene:SpnNT_00265	NA	Chromosome:244457-245717	ΔORF2	ΔORF2+peptide	OK	136.437	152.129	0.157068	0.360557	0.52225	0.954832	no
gene:SpnNT_00265	NA	Chromosome:244457-245717	110.58+peptide	ΔORF2+peptide	OK	144.17	152.129	0.0775306	0.178137	0.7554	0.994748	no
gene:SpnNT_00266	proS	Chromosome:245729-247583	110.58	ΔORF2	OK	286.69	258.25	-0.150726	-0.33794	0.55915	0.968621	no
gene:SpnNT_00266	proS	Chromosome:245729-247583	110.58	110.58+peptide	OK	286.69	268.593	-0.0940727	-0.212126	0.71155	0.991007	no
gene:SpnNT_00266	proS	Chromosome:245729-247583	ΔORF2	110.58+peptide	OK	258.25	268.593	0.0566531	0.126587	0.8233	0.994748	no
gene:SpnNT_00266	proS	Chromosome:245729-247583	110.58	ΔORF2+peptide	OK	286.69	253.709	-0.176315	-0.399897	0.4859	0.93792	no
gene:SpnNT_00266	proS	Chromosome:245729-247583	ΔORF2	ΔORF2+peptide	OK	258.25	253.709	-0.0255897	-0.0575057	0.9189	0.994748	no
gene:SpnNT_00266	proS	Chromosome:245729-247583	110.58+peptide	ΔORF2+peptide	OK	268.593	253.709	-0.0822428	-0.18588	0.73585	0.994748	no
gene:SpnNT_00267	gmuD_1	Chromosome:247681-249061	110.58	ΔORF2	OK	30.7714	26.4798	-0.216699	-0.440851	0.44495	0.917756	no
gene:SpnNT_00267	gmuD_1	Chromosome:247681-249061	110.58	110.58+peptide	OK	30.7714	24.2198	-0.345404	-0.695681	0.21505	0.701601	no
gene:SpnNT_00267	gmuD_1	Chromosome:247681-249061	ΔORF2	110.58+peptide	OK	26.4798	24.2198	-0.128705	-0.25656	0.64925	0.981391	no
gene:SpnNT_00267	gmuD_1	Chromosome:247681-249061	110.58	ΔORF2+peptide	OK	30.7714	22.9462	-0.423337	-0.857038	0.13	0.547452	no
gene:SpnNT_00267	gmuD_1	Chromosome:247681-249061	ΔORF2	ΔORF2+peptide	OK	26.4798	22.9462	-0.206637	-0.41399	0.4659	0.926452	no
gene:SpnNT_00267	gmuD_1	Chromosome:247681-249061	110.58+peptide	ΔORF2+peptide	OK	24.2198	22.9462	-0.0779323	-0.154624	0.77985	0.994748	no
gene:SpnNT_00268	glmS	Chromosome:249253-251062	110.58	ΔORF2	OK	250.502	196.938	-0.347081	-0.781857	0.17435	0.634977	no
gene:SpnNT_00268	glmS	Chromosome:249253-251062	110.58	110.58+peptide	OK	250.502	446.921	0.835196	1.85995	0.00205	0.0300896	yes
gene:SpnNT_00268	glmS	Chromosome:249253-251062	ΔORF2	110.58+peptide	OK	196.938	446.921	1.18228	2.6617	5.00E-05	0.0013612	yes
gene:SpnNT_00268	glmS	Chromosome:249253-251062	110.58	ΔORF2+peptide	OK	250.502	384.973	0.619936	1.38021	0.01725	0.148722	no
gene:SpnNT_00268	glmS	Chromosome:249253-251062	ΔORF2	ΔORF2+peptide	OK	196.938	384.973	0.967017	2.17649	0.00025	0.00542231	yes
gene:SpnNT_00268	glmS	Chromosome:249253-251062	110.58+peptide	ΔORF2+peptide	OK	446.921	384.973	-0.21526	-0.478974	0.40515	0.894933	no
gene:SpnNT_00269	mtlA	Chromosome:251506-253276	110.58	ΔORF2	OK	1.16534	0.870689	-0.420523	-0.535557	0.35875	0.859746	no
gene:SpnNT_00269	mtlA	Chromosome:251506-253276	110.58	110.58+peptide	OK	1.16534	1.21206	0.0567039	0.0732214	0.89585	0.994748	no
gene:SpnNT_00269	mtlA	Chromosome:251506-253276	ΔORF2	110.58+peptide	OK	0.870689	1.21206	0.477227	0.597852	0.30375	0.809137	no
gene:SpnNT_00269	mtlA	Chromosome:251506-253276	110.58	ΔORF2+peptide	OK	1.16534	1.17054	0.00641612	0.00809814	0.9803	0.99608	no
gene:SpnNT_00269	mtlA	Chromosome:251506-253276	ΔORF2	ΔORF2+peptide	OK	0.870689	1.17054	0.426939	0.523471	0.3712	0.868447	no
gene:SpnNT_00269	mtlA	Chromosome:251506-253276	110.58+peptide	ΔORF2+peptide	OK	1.21206	1.17054	-0.0502878	-0.062453	0.9158	0.994748	no
gene:SpnNT_00270	mtlR	Chromosome:253299-255255	110.58	ΔORF2	NOTEST	0.632558	0.595322	-0.0875274	0	1	1	no
gene:SpnNT_00270	mtlR	Chromosome:253299-255255	110.58	110.58+peptide	OK	0.632558	0.875539	0.468973	0.533151	0.35125	0.854885	no
gene:SpnNT_00270	mtlR	Chromosome:253299-255255	ΔORF2	110.58+peptide	OK	0.595322	0.875539	0.556501	0.65938	0.25965	0.759841	no
gene:SpnNT_00270	mtlR	Chromosome:253299-255255	110.58	ΔORF2+peptide	NOTEST	0.632558	0.585164	-0.112356	0	1	1	no
gene:SpnNT_00270	mtlR	Chromosome:253299-255255	ΔORF2	ΔORF2+peptide	NOTEST	0.595322	0.585164	-0.0248284	0	1	1	no

gene:SpnNT_00270	mtlR	Chromosome:253299-255255	110.58+peptide	ΔORF2+peptide	OK	0.875539	0.585164	-0.581329	-0.678858	0.2486	0.745119	no
gene:SpnNT_00271	mtlF	Chromosome:255256-255694	110.58	ΔORF2	OK	0.829625	1.34011	0.691822	0.533082	0.3962	0.890381	no
gene:SpnNT_00271	mtlF	Chromosome:255256-255694	110.58	110.58+peptide	OK	0.829625	0.866496	0.0627336	0.0431939	0.9078	0.994748	no
gene:SpnNT_00271	mtlF	Chromosome:255256-255694	ΔORF2	110.58+peptide	OK	1.34011	0.866496	-0.629088	-0.432468	0.4354	0.913985	no
gene:SpnNT_00271	mtlF	Chromosome:255256-255694	110.58	ΔORF2+peptide	NOTEST	0.829625	0.632864	-0.390565	0	1	1	no
gene:SpnNT_00271	mtlF	Chromosome:255256-255694	ΔORF2	ΔORF2+peptide	OK	1.34011	0.632864	-1.08239	-0.766528	0.2314	0.724662	no
gene:SpnNT_00271	mtlF	Chromosome:255256-255694	110.58+peptide	ΔORF2+peptide	OK	0.866496	0.632864	-0.453298	-0.291447	0.63545	0.980887	no
gene:SpnNT_00272	mtlD	Chromosome:255755-256892	110.58	ΔORF2	OK	0.871266	0.739139	-0.237268	-0.246275	0.66335	0.982966	no
gene:SpnNT_00272	mtlD	Chromosome:255755-256892	110.58	110.58+peptide	OK	0.871266	1.12676	0.37099	0.404263	0.4717	0.928525	no
gene:SpnNT_00272	mtlD	Chromosome:255755-256892	ΔORF2	110.58+peptide	OK	0.739139	1.12676	0.608258	0.646969	0.25585	0.754781	no
gene:SpnNT_00272	mtlD	Chromosome:255755-256892	110.58	ΔORF2+peptide	OK	0.871266	0.551224	-0.660476	-0.651477	0.26325	0.763902	no
gene:SpnNT_00272	mtlD	Chromosome:255755-256892	ΔORF2	ΔORF2+peptide	NOTEST	0.739139	0.551224	-0.423208	0	1	1	no
gene:SpnNT_00272	mtlD	Chromosome:255755-256892	110.58+peptide	ΔORF2+peptide	OK	1.12676	0.551224	-1.03147	-1.04006	0.07625	0.408056	no
gene:SpnNT_00273	limB	Chromosome:257143-257656	110.58	ΔORF2	OK	1.94986	1.55691	-0.32469	-0.30134	0.5922	0.975468	no
gene:SpnNT_00273	limB	Chromosome:257143-257656	110.58	110.58+peptide	OK	1.94986	1.98356	0.0247227	0.0235592	0.95725	0.994855	no
gene:SpnNT_00273	limB	Chromosome:257143-257656	ΔORF2	110.58+peptide	OK	1.55691	1.98356	0.349413	0.311984	0.58335	0.971331	no
gene:SpnNT_00273	limB	Chromosome:257143-257656	110.58	ΔORF2+peptide	OK	1.94986	0.823367	-1.24377	-1.0977	0.10565	0.494415	no
gene:SpnNT_00273	limB	Chromosome:257143-257656	ΔORF2	ΔORF2+peptide	OK	1.55691	0.823367	-0.919075	-0.766707	0.2395	0.734795	no
gene:SpnNT_00273	limB	Chromosome:257143-257656	110.58+peptide	ΔORF2+peptide	OK	1.98356	0.823367	-1.26849	-1.08091	0.1026	0.485509	no
gene:SpnNT_00274	NA	Chromosome:257668-258193	110.58	ΔORF2	OK	4.24271	2.09115	-1.02069	-1.12576	0.05635	0.3381	no
gene:SpnNT_00274	NA	Chromosome:257668-258193	110.58	110.58+peptide	OK	4.24271	2.97671	-0.511266	-0.589909	0.2956	0.800007	no
gene:SpnNT_00274	NA	Chromosome:257668-258193	ΔORF2	110.58+peptide	OK	2.09115	2.97671	0.509426	0.533976	0.34385	0.84812	no
gene:SpnNT_00274	NA	Chromosome:257668-258193	110.58	ΔORF2+peptide	OK	4.24271	3.37432	-0.330391	-0.388521	0.49995	0.94407	no
gene:SpnNT_00274	NA	Chromosome:257668-258193	ΔORF2	ΔORF2+peptide	OK	2.09115	3.37432	0.690301	0.734958	0.2056	0.690314	no
gene:SpnNT_00274	NA	Chromosome:257668-258193	110.58+peptide	ΔORF2+peptide	OK	2.97671	3.37432	0.180874	0.200816	0.72365	0.994748	no
gene:SpnNT_00275	pulA	Chromosome:258292-262063	110.58	ΔORF2	OK	6.98016	5.88422	-0.246409	-0.478101	0.39745	0.891359	no
gene:SpnNT_00275	pulA	Chromosome:258292-262063	110.58	110.58+peptide	OK	6.98016	9.05592	0.375601	0.749433	0.19065	0.669443	no
gene:SpnNT_00275	pulA	Chromosome:258292-262063	ΔORF2	110.58+peptide	OK	5.88422	9.05592	0.62201	1.23316	0.03085	0.226786	no
gene:SpnNT_00275	pulA	Chromosome:258292-262063	110.58	ΔORF2+peptide	OK	6.98016	8.69631	0.317144	0.6291	0.2797	0.782731	no
gene:SpnNT_00275	pulA	Chromosome:258292-262063	ΔORF2	ΔORF2+peptide	OK	5.88422	8.69631	0.563552	1.11083	0.05455	0.332006	no
gene:SpnNT_00275	pulA	Chromosome:258292-262063	110.58+peptide	ΔORF2+peptide	OK	9.05592	8.69631	-0.0584571	-0.118603	0.8351	0.994748	no
gene:SpnNT_00276	NA	Chromosome:262349-263696	110.58	ΔORF2	OK	2.77506	2.58339	-0.103256	-0.148165	0.80145	0.994748	no
gene:SpnNT_00276	NA	Chromosome:262349-263696	110.58	110.58+peptide	OK	2.77506	2.67355	-0.0537645	-0.0770366	0.89495	0.994748	no
gene:SpnNT_00276	NA	Chromosome:262349-263696	ΔORF2	110.58+peptide	OK	2.58339	2.67355	0.0494917	0.0714544	0.90195	0.994748	no
gene:SpnNT_00276	NA	Chromosome:262349-263696	110.58	ΔORF2+peptide	OK	2.77506	2.82802	0.0272729	0.0387272	0.9455	0.994855	no
gene:SpnNT_00276	NA	Chromosome:262349-263696	ΔORF2	ΔORF2+peptide	OK	2.58339	2.82802	0.130529	0.186735	0.7461	0.994748	no
gene:SpnNT_00276	NA	Chromosome:262349-263696	110.58+peptide	ΔORF2+peptide	OK	2.67355	2.82802	0.0810374	0.115766	0.8451	0.994748	no
gene:SpnNT_00277	rpsL	Chromosome:264187-264601	110.58	ΔORF2	OK	625.73	593.925	-0.075259	-0.16514	0.7758	0.994748	no
gene:SpnNT_00277	rpsL	Chromosome:264187-264601	110.58	110.58+peptide	OK	625.73	1012.54	0.69436	1.56168	0.00625	0.0710065	no
gene:SpnNT_00277	rpsL	Chromosome:264187-264601	ΔORF2	110.58+peptide	OK	593.925	1012.54	0.769619	1.68173	0.00295	0.0393393	yes
gene:SpnNT_00277	rpsL	Chromosome:264187-264601	110.58	ΔORF2+peptide	OK	625.73	1045.38	0.740413	1.68038	0.0041	0.0516317	no
gene:SpnNT_00277	rpsL	Chromosome:264187-264601	ΔORF2	ΔORF2+peptide	OK	593.925	1045.38	0.815672	1.79763	0.00195	0.0288477	yes
gene:SpnNT_00277	rpsL	Chromosome:264187-264601	110.58+peptide	ΔORF2+peptide	OK	1012.54	1045.38	0.0460529	0.104052	0.85765	0.994748	no
gene:SpnNT_00278	rpsG	Chromosome:264620-265091	110.58	ΔORF2	OK	1680.18	1529.43	-0.135623	-0.293727	0.60075	0.976678	no
gene:SpnNT_00278	rpsG	Chromosome:264620-265091	110.58	110.58+peptide	OK	1680.18	2888.11	0.781509	1.70685	0.00265	0.0362222	yes

gene:SpnNT_00278	rpsG	Chromosome:264620-265091	ΔORF2	110.58+peptide	OK	1529.43	2888.11	0.917132	1.95678	0.00035	0.00717609	yes
gene:SpnNT_00278	rpsG	Chromosome:264620-265091	110.58	ΔORF2+peptide	OK	1680.18	2684.82	0.676207	1.50196	0.00935	0.0961526	no
gene:SpnNT_00278	rpsG	Chromosome:264620-265091	ΔORF2	ΔORF2+peptide	OK	1529.43	2684.82	0.81183	1.76016	0.00205	0.0300896	yes
gene:SpnNT_00278	rpsG	Chromosome:264620-265091	110.58+peptide	ΔORF2+peptide	OK	2888.11	2684.82	-0.105302	-0.23024	0.6878	0.98828	no
gene:SpnNT_00279	fus	Chromosome:265515-267597	110.58	ΔORF2	OK	1018.3	971.018	-0.0685929	-0.142633	0.80065	0.994748	no
gene:SpnNT_00279	fus	Chromosome:265515-267597	110.58	110.58+peptide	OK	1018.3	1428.15	0.487988	1.0043	0.0713	0.391792	no
gene:SpnNT_00279	fus	Chromosome:265515-267597	ΔORF2	110.58+peptide	OK	971.018	1428.15	0.556581	1.13528	0.0439	0.287166	no
gene:SpnNT_00279	fus	Chromosome:265515-267597	110.58	ΔORF2+peptide	OK	1018.3	1334.23	0.38985	0.808117	0.1533	0.596738	no
gene:SpnNT_00279	fus	Chromosome:265515-267597	ΔORF2	ΔORF2+peptide	OK	971.018	1334.23	0.458442	0.941735	0.10155	0.483329	no
gene:SpnNT_00279	fus	Chromosome:265515-267597	110.58+peptide	ΔORF2+peptide	OK	1428.15	1334.23	-0.0981386	-0.199573	0.7221	0.994748	no
gene:SpnNT_00280	polC_1	Chromosome:267700-272092	110.58	ΔORF2	OK	62.0579	59.8821	-0.0514888	-0.117402	0.8358	0.994748	no
gene:SpnNT_00280	polC_1	Chromosome:267700-272092	110.58	110.58+peptide	OK	62.0579	60.6428	-0.0332786	-0.0765201	0.8899	0.994748	no
gene:SpnNT_00280	polC_1	Chromosome:267700-272092	ΔORF2	110.58+peptide	OK	59.8821	60.6428	0.0182102	0.041505	0.94055	0.994855	no
gene:SpnNT_00280	polC_1	Chromosome:267700-272092	110.58	ΔORF2+peptide	OK	62.0579	63.2894	0.028349	0.0652187	0.90635	0.994748	no
gene:SpnNT_00280	polC_1	Chromosome:267700-272092	ΔORF2	ΔORF2+peptide	OK	59.8821	63.2894	0.0798378	0.18206	0.74605	0.994748	no
gene:SpnNT_00280	polC_1	Chromosome:267700-272092	110.58+peptide	ΔORF2+peptide	OK	60.6428	63.2894	0.0616276	0.14172	0.80405	0.994748	no
gene:SpnNT_00281	NA	Chromosome:272193-272728	110.58	ΔORF2	OK	464.318	506.058	0.12419	0.241799	0.67625	0.98524	no
gene:SpnNT_00281	NA	Chromosome:272193-272728	110.58	110.58+peptide	OK	464.318	562.484	0.276699	0.543367	0.35705	0.858254	no
gene:SpnNT_00281	NA	Chromosome:272193-272728	ΔORF2	110.58+peptide	OK	506.058	562.484	0.152509	0.302218	0.6109	0.979429	no
gene:SpnNT_00281	NA	Chromosome:272193-272728	110.58	ΔORF2+peptide	OK	464.318	782.438	0.752863	1.50409	0.01005	0.100592	no
gene:SpnNT_00281	NA	Chromosome:272193-272728	ΔORF2	ΔORF2+peptide	OK	506.058	782.438	0.628673	1.26783	0.0333	0.239432	no
gene:SpnNT_00281	NA	Chromosome:272193-272728	110.58+peptide	ΔORF2+peptide	OK	562.484	782.438	0.476164	0.969135	0.1081	0.499996	no
gene:SpnNT_00282	yafQ	Chromosome:272193-272728	110.58	ΔORF2	OK	78.595	73.5752	-0.0952183	-0.0776532	0.8925	0.994748	no
gene:SpnNT_00282	yafQ	Chromosome:272193-272728	110.58	110.58+peptide	OK	78.595	84.6512	0.107093	0.0831845	0.888	0.994748	no
gene:SpnNT_00282	yafQ	Chromosome:272193-272728	ΔORF2	110.58+peptide	OK	73.5752	84.6512	0.202311	0.155501	0.7846	0.994748	no
gene:SpnNT_00282	yafQ	Chromosome:272193-272728	110.58	ΔORF2+peptide	OK	78.595	112.144	0.512839	0.429884	0.4678	0.92732	no
gene:SpnNT_00282	yafQ	Chromosome:272193-272728	ΔORF2	ΔORF2+peptide	OK	73.5752	112.144	0.608057	0.503505	0.38215	0.879865	no
gene:SpnNT_00282	yafQ	Chromosome:272193-272728	110.58+peptide	ΔORF2+peptide	OK	84.6512	112.144	0.405746	0.319546	0.5796	0.969538	no
gene:SpnNT_00283	NA	Chromosome:272748-272901	110.58	ΔORF2	OK	30.3325	35.3867	0.222342	0.193108	0.7546	0.994748	no
gene:SpnNT_00283	NA	Chromosome:272748-272901	110.58	110.58+peptide	OK	30.3325	20.6193	-0.556872	-0.43081	0.45515	0.921244	no
gene:SpnNT_00283	NA	Chromosome:272748-272901	ΔORF2	110.58+peptide	OK	35.3867	20.6193	-0.779214	-0.605915	0.3082	0.814346	no
gene:SpnNT_00283	NA	Chromosome:272748-272901	110.58	ΔORF2+peptide	OK	30.3325	8.89197	-1.77029	-2.11339	0.13085	0.549796	no
gene:SpnNT_00283	NA	Chromosome:272748-272901	ΔORF2	ΔORF2+peptide	OK	35.3867	8.89197	-1.99263	-2.40823	0.11765	0.519495	no
gene:SpnNT_00283	NA	Chromosome:272748-272901	110.58+peptide	ΔORF2+peptide	OK	20.6193	8.89197	-1.21342	-1.19573	0.2824	0.785208	no
gene:SpnNT_00284	pepS	Chromosome:272922-274164	110.58	ΔORF2	OK	276.371	250.667	-0.140836	-0.31737	0.57215	0.969077	no
gene:SpnNT_00284	pepS	Chromosome:272922-274164	110.58	110.58+peptide	OK	276.371	259.041	-0.0934279	-0.211946	0.7023	0.990367	no
gene:SpnNT_00284	pepS	Chromosome:272922-274164	ΔORF2	110.58+peptide	OK	250.667	259.041	0.0474081	0.1058	0.8507	0.994748	no
gene:SpnNT_00284	pepS	Chromosome:272922-274164	110.58	ΔORF2+peptide	OK	276.371	219.494	-0.332424	-0.762871	0.1697	0.627799	no
gene:SpnNT_00284	pepS	Chromosome:272922-274164	ΔORF2	ΔORF2+peptide	OK	250.667	219.494	-0.191588	-0.432362	0.44485	0.917756	no
gene:SpnNT_00284	pepS	Chromosome:272922-274164	110.58+peptide	ΔORF2+peptide	OK	259.041	219.494	-0.238996	-0.542968	0.32805	0.833593	no
gene:SpnNT_00285	NA	Chromosome:274173-274404	110.58	ΔORF2	OK	761.367	807.39	0.0846724	0.176132	0.76305	0.994748	no
gene:SpnNT_00285	NA	Chromosome:274173-274404	110.58	110.58+peptide	OK	761.367	748.289	-0.0249969	-0.0520036	0.92815	0.994748	no
gene:SpnNT_00285	NA	Chromosome:274173-274404	ΔORF2	110.58+peptide	OK	807.39	748.289	-0.109669	-0.228289	0.69465	0.988704	no
gene:SpnNT_00285	NA	Chromosome:274173-274404	110.58	ΔORF2+peptide	OK	761.367	835.443	0.133948	0.274443	0.6402	0.980887	no
gene:SpnNT_00285	NA	Chromosome:274173-274404	ΔORF2	ΔORF2+peptide	OK	807.39	835.443	0.0492754	0.101016	0.86315	0.994748	no

gene:SpnNT_00285	NA	Chromosome:274173-274404	110.58+peptide	ΔORF2+peptide	OK	748.289	835.443	0.158945	0.325879	0.57675	0.969538	no
gene:SpnNT_00286	rsuA_1	Chromosome:274671-275394	110.58	ΔORF2	OK	119.99	134.063	0.159993	0.350033	0.5427	0.961518	no
gene:SpnNT_00286	rsuA_1	Chromosome:274671-275394	110.58	110.58+peptide	OK	119.99	122.211	0.0264612	0.0575774	0.9177	0.994748	no
gene:SpnNT_00286	rsuA_1	Chromosome:274671-275394	ΔORF2	110.58+peptide	OK	134.063	122.211	-0.133532	-0.291045	0.607	0.977869	no
gene:SpnNT_00286	rsuA_1	Chromosome:274671-275394	110.58	ΔORF2+peptide	OK	119.99	121.522	0.0183046	0.03975	0.94195	0.994855	no
gene:SpnNT_00286	rsuA_1	Chromosome:274671-275394	ΔORF2	ΔORF2+peptide	OK	134.063	121.522	-0.141689	-0.308208	0.589	0.974104	no
gene:SpnNT_00286	rsuA_1	Chromosome:274671-275394	110.58+peptide	ΔORF2+peptide	OK	122.211	121.522	-0.00815668	-0.0176475	0.9727	0.99536	no
gene:SpnNT_00287	pepC	Chromosome:275610-276945	110.58	ΔORF2	OK	319.085	332.343	0.0587313	0.132857	0.81035	0.994748	no
gene:SpnNT_00287	pepC	Chromosome:275610-276945	110.58	110.58+peptide	OK	319.085	201.261	-0.664875	-1.50316	0.0091	0.0941721	no
gene:SpnNT_00287	pepC	Chromosome:275610-276945	ΔORF2	110.58+peptide	OK	332.343	201.261	-0.723606	-1.64806	0.0045	0.0552376	no
gene:SpnNT_00287	pepC	Chromosome:275610-276945	110.58	ΔORF2+peptide	OK	319.085	187.824	-0.76456	-1.73872	0.0022	0.0317584	yes
gene:SpnNT_00287	pepC	Chromosome:275610-276945	ΔORF2	ΔORF2+peptide	OK	332.343	187.824	-0.823291	-1.88632	0.0011	0.0181562	yes
gene:SpnNT_00287	pepC	Chromosome:275610-276945	110.58+peptide	ΔORF2+peptide	OK	201.261	187.824	-0.0996847	-0.228263	0.6908	0.98828	no
gene:SpnNT_00288	manZ_2	Chromosome:277000-277912	110.58	ΔORF2	OK	388.93	427.606	0.136769	0.314624	0.57145	0.968932	no
gene:SpnNT_00288	manZ_2	Chromosome:277000-277912	110.58	110.58+peptide	OK	388.93	918.111	1.23916	2.79625	5.00E-05	0.0013612	yes
gene:SpnNT_00288	manZ_2	Chromosome:277000-277912	ΔORF2	110.58+peptide	OK	427.606	918.111	1.10239	2.48816	5.00E-05	0.0013612	yes
gene:SpnNT_00288	manZ_2	Chromosome:277000-277912	110.58	ΔORF2+peptide	OK	388.93	1009.84	1.37654	3.09744	5.00E-05	0.0013612	yes
gene:SpnNT_00288	manZ_2	Chromosome:277000-277912	ΔORF2	ΔORF2+peptide	OK	427.606	1009.84	1.23977	2.79029	5.00E-05	0.0013612	yes
gene:SpnNT_00288	manZ_2	Chromosome:277000-277912	110.58+peptide	ΔORF2+peptide	OK	918.111	1009.84	0.137387	0.303564	0.5849	0.971855	no
gene:SpnNT_00289	manY	Chromosome:277935-278739	110.58	ΔORF2	OK	248.599	228.845	-0.119444	-0.267217	0.63355	0.980887	no
gene:SpnNT_00289	manY	Chromosome:277935-278739	110.58	110.58+peptide	OK	248.599	556.784	1.1633	2.65157	5.00E-05	0.0013612	yes
gene:SpnNT_00289	manY	Chromosome:277935-278739	ΔORF2	110.58+peptide	OK	228.845	556.784	1.28274	2.85763	5.00E-05	0.0013612	yes
gene:SpnNT_00289	manY	Chromosome:277935-278739	110.58	ΔORF2+peptide	OK	248.599	568.053	1.19221	2.71386	5.00E-05	0.0013612	yes
gene:SpnNT_00289	manY	Chromosome:277935-278739	ΔORF2	ΔORF2+peptide	OK	228.845	568.053	1.31165	2.91833	5.00E-05	0.0013612	yes
gene:SpnNT_00289	manY	Chromosome:277935-278739	110.58+peptide	ΔORF2+peptide	OK	556.784	568.053	0.0289086	0.0655186	0.90805	0.994748	no
gene:SpnNT_00290	manX_2	Chromosome:278766-279756	110.58	ΔORF2	OK	248.993	256.28	0.0416166	0.0954286	0.86175	0.994748	no
gene:SpnNT_00290	manX_2	Chromosome:278766-279756	110.58	110.58+peptide	OK	248.993	583.152	1.22777	2.79259	5.00E-05	0.0013612	yes
gene:SpnNT_00290	manX_2	Chromosome:278766-279756	ΔORF2	110.58+peptide	OK	256.28	583.152	1.18615	2.69151	5.00E-05	0.0013612	yes
gene:SpnNT_00290	manX_2	Chromosome:278766-279756	110.58	ΔORF2+peptide	OK	248.993	678.385	1.446	3.29161	5.00E-05	0.0013612	yes
gene:SpnNT_00290	manX_2	Chromosome:278766-279756	ΔORF2	ΔORF2+peptide	OK	256.28	678.385	1.40438	3.18925	5.00E-05	0.0013612	yes
gene:SpnNT_00290	manX_2	Chromosome:278766-279756	110.58+peptide	ΔORF2+peptide	OK	583.152	678.385	0.218231	0.491661	0.37165	0.868684	no
gene:SpnNT_00291	adhA	Chromosome:279988-281008	110.58	ΔORF2	OK	163.677	137.225	-0.254307	-0.576905	0.30665	0.81251	no
gene:SpnNT_00291	adhA	Chromosome:279988-281008	110.58	110.58+peptide	OK	163.677	230.1	0.491407	1.01691	0.08005	0.421873	no
gene:SpnNT_00291	adhA	Chromosome:279988-281008	ΔORF2	110.58+peptide	OK	137.225	230.1	0.745714	1.53022	0.01065	0.105075	no
gene:SpnNT_00291	adhA	Chromosome:279988-281008	110.58	ΔORF2+peptide	OK	163.677	234.481	0.518622	1.09471	0.0535	0.327438	no
gene:SpnNT_00291	adhA	Chromosome:279988-281008	ΔORF2	ΔORF2+peptide	OK	137.225	234.481	0.772929	1.61727	0.00575	0.066949	no
gene:SpnNT_00291	adhA	Chromosome:279988-281008	110.58+peptide	ΔORF2+peptide	OK	230.1	234.481	0.0272148	0.0526084	0.92725	0.994748	no
gene:SpnNT_00292	yidA_1	Chromosome:281346-282159	110.58	ΔORF2	OK	66.2499	71.469	0.109398	0.230593	0.6816	0.986574	no
gene:SpnNT_00292	yidA_1	Chromosome:281346-282159	110.58	110.58+peptide	OK	66.2499	47.3046	-0.485938	-1.00017	0.08135	0.425798	no
gene:SpnNT_00292	yidA_1	Chromosome:281346-282159	ΔORF2	110.58+peptide	OK	71.469	47.3046	-0.595336	-1.22818	0.03165	0.230728	no
gene:SpnNT_00292	yidA_1	Chromosome:281346-282159	110.58	ΔORF2+peptide	OK	66.2499	51.1411	-0.373435	-0.765743	0.174	0.634412	no
gene:SpnNT_00292	yidA_1	Chromosome:281346-282159	ΔORF2	ΔORF2+peptide	OK	71.469	51.1411	-0.482833	-0.99235	0.081	0.424474	no
gene:SpnNT_00292	yidA_1	Chromosome:281346-282159	110.58+peptide	ΔORF2+peptide	OK	47.3046	51.1411	0.112503	0.226041	0.69395	0.98828	no
gene:SpnNT_00293	pbuO	Chromosome:282348-283767	110.58	ΔORF2	OK	151.235	179.721	0.248962	0.567757	0.3122	0.818356	no
gene:SpnNT_00293	pbuO	Chromosome:282348-283767	110.58	110.58+peptide	OK	151.235	46.415	-1.70413	-3.78017	5.00E-05	0.0013612	yes

gene:SpnNT_00293	pbuO	Chromosome:282348-283767	ΔORF2	110.58+peptide	OK	179.721	46.415	-1.95309	-4.35337	5.00E-05	0.0013612	yes
gene:SpnNT_00293	pbuO	Chromosome:282348-283767	110.58	ΔORF2+peptide	OK	151.235	59.3433	-1.34964	-3.00897	5.00E-05	0.0013612	yes
gene:SpnNT_00293	pbuO	Chromosome:282348-283767	ΔORF2	ΔORF2+peptide	OK	179.721	59.3433	-1.5986	-3.58142	5.00E-05	0.0013612	yes
gene:SpnNT_00293	pbuO	Chromosome:282348-283767	110.58+peptide	ΔORF2+peptide	OK	46.415	59.3433	0.354495	0.773237	0.17295	0.632687	no
gene:SpnNT_00294	NA	Chromosome:283819-284527	110.58	ΔORF2	OK	19.2161	28.9423	0.590861	1.05344	0.07225	0.395686	no
gene:SpnNT_00294	NA	Chromosome:283819-284527	110.58	110.58+peptide	OK	19.2161	5.35942	-1.84217	-2.88877	5.00E-05	0.0013612	yes
gene:SpnNT_00294	NA	Chromosome:283819-284527	ΔORF2	110.58+peptide	OK	28.9423	5.35942	-2.43303	-3.86338	5.00E-05	0.0013612	yes
gene:SpnNT_00294	NA	Chromosome:283819-284527	110.58	ΔORF2+peptide	OK	19.2161	7.61873	-1.33469	-2.13586	5.00E-04	0.0095781	yes
gene:SpnNT_00294	NA	Chromosome:283819-284527	ΔORF2	ΔORF2+peptide	OK	28.9423	7.61873	-1.92556	-3.12185	5.00E-05	0.0013612	yes
gene:SpnNT_00294	NA	Chromosome:283819-284527	110.58+peptide	ΔORF2+peptide	OK	5.35942	7.61873	0.507473	0.738258	0.1956	0.677365	no
gene:SpnNT_00295	sulA	Chromosome:284650-285598	110.58	ΔORF2	OK	133.444	132.523	-0.00999124	-0.0225419	0.96865	0.994855	no
gene:SpnNT_00295	sulA	Chromosome:284650-285598	110.58	110.58+peptide	OK	133.444	85.1434	-0.648264	-1.44491	0.0123	0.117127	no
gene:SpnNT_00295	sulA	Chromosome:284650-285598	ΔORF2	110.58+peptide	OK	132.523	85.1434	-0.638273	-1.4175	0.01385	0.127002	no
gene:SpnNT_00295	sulA	Chromosome:284650-285598	110.58	ΔORF2+peptide	OK	133.444	97.6094	-0.451138	-1.00907	0.08285	0.43107	no
gene:SpnNT_00295	sulA	Chromosome:284650-285598	ΔORF2	ΔORF2+peptide	OK	132.523	97.6094	-0.441147	-0.983135	0.0877	0.446219	no
gene:SpnNT_00295	sulA	Chromosome:284650-285598	110.58+peptide	ΔORF2+peptide	OK	85.1434	97.6094	0.197126	0.434129	0.45195	0.921244	no
gene:SpnNT_00296	fgs_2	Chromosome:285599-287457	110.58	ΔORF2	OK	204.473	192.195	-0.0893386	-0.177099	0.7498	0.994748	no
gene:SpnNT_00296	fgs_2	Chromosome:285599-287457	110.58	110.58+peptide	OK	204.473	154.92	-0.40038	-0.798847	0.16715	0.622932	no
gene:SpnNT_00296	fgs_2	Chromosome:285599-287457	ΔORF2	110.58+peptide	OK	192.195	154.92	-0.311041	-0.617505	0.2771	0.780208	no
gene:SpnNT_00296	fgs_2	Chromosome:285599-287457	110.58	ΔORF2+peptide	OK	204.473	149.412	-0.452611	-0.899601	0.11325	0.510776	no
gene:SpnNT_00296	fgs_2	Chromosome:285599-287457	ΔORF2	ΔORF2+peptide	OK	192.195	149.412	-0.363272	-0.718463	0.2002	0.683054	no
gene:SpnNT_00296	fgs_2	Chromosome:285599-287457	110.58+peptide	ΔORF2+peptide	OK	154.92	149.412	-0.0522314	-0.103969	0.8546	0.994748	no
gene:SpnNT_00297	folE	Chromosome:285599-287457	110.58	ΔORF2	OK	197.391	182.495	-0.113196	-0.122525	0.8278	0.994748	no
gene:SpnNT_00297	folE	Chromosome:285599-287457	110.58	110.58+peptide	OK	197.391	154.089	-0.357287	-0.393038	0.496	0.943191	no
gene:SpnNT_00297	folE	Chromosome:285599-287457	ΔORF2	110.58+peptide	OK	182.495	154.089	-0.244092	-0.267353	0.6389	0.980887	no
gene:SpnNT_00297	folE	Chromosome:285599-287457	110.58	ΔORF2+peptide	OK	197.391	158.901	-0.31293	-0.324853	0.57645	0.969538	no
gene:SpnNT_00297	folE	Chromosome:285599-287457	ΔORF2	ΔORF2+peptide	OK	182.495	158.901	-0.199734	-0.206544	0.7196	0.99385	no
gene:SpnNT_00297	folE	Chromosome:285599-287457	110.58+peptide	ΔORF2+peptide	OK	154.089	158.901	0.0443573	0.0465505	0.9364	0.994855	no
gene:SpnNT_00298	sulD	Chromosome:287499-288312	110.58	ΔORF2	OK	206.78	202.19	-0.0323807	-0.073632	0.89745	0.994748	no
gene:SpnNT_00298	sulD	Chromosome:287499-288312	110.58	110.58+peptide	OK	206.78	144.827	-0.51377	-1.16593	0.0406	0.273628	no
gene:SpnNT_00298	sulD	Chromosome:287499-288312	ΔORF2	110.58+peptide	OK	202.19	144.827	-0.481389	-1.08367	0.0588	0.346308	no
gene:SpnNT_00298	sulD	Chromosome:287499-288312	110.58	ΔORF2+peptide	OK	206.78	164.637	-0.328805	-0.747809	0.1868	0.661768	no
gene:SpnNT_00298	sulD	Chromosome:287499-288312	ΔORF2	ΔORF2+peptide	OK	202.19	164.637	-0.296424	-0.668731	0.2467	0.74363	no
gene:SpnNT_00298	sulD	Chromosome:287499-288312	110.58+peptide	ΔORF2+peptide	OK	144.827	164.637	0.184965	0.416451	0.46655	0.927165	no
gene:SpnNT_00299	NA	Chromosome:288356-288674	110.58	ΔORF2	OK	127.34	176.117	0.467849	0.877175	0.1273	0.541293	no
gene:SpnNT_00299	NA	Chromosome:288356-288674	110.58	110.58+peptide	OK	127.34	77.1604	-0.722757	-1.28696	0.0259	0.201817	no
gene:SpnNT_00299	NA	Chromosome:288356-288674	ΔORF2	110.58+peptide	OK	176.117	77.1604	-1.19061	-2.12812	3.00E-04	0.00631878	yes
gene:SpnNT_00299	NA	Chromosome:288356-288674	110.58	ΔORF2+peptide	OK	127.34	102.782	-0.309102	-0.566664	0.33285	0.838644	no
gene:SpnNT_00299	NA	Chromosome:288356-288674	ΔORF2	ΔORF2+peptide	OK	176.117	102.782	-0.776951	-1.43012	0.0144	0.130225	no
gene:SpnNT_00299	NA	Chromosome:288356-288674	110.58+peptide	ΔORF2+peptide	OK	77.1604	102.782	0.413655	0.724403	0.2176	0.705186	no
gene:SpnNT_00300	rplM	Chromosome:289030-289477	110.58	ΔORF2	OK	500.827	479.3	-0.0633816	-0.1432	0.80335	0.994748	no
gene:SpnNT_00300	rplM	Chromosome:289030-289477	110.58	110.58+peptide	OK	500.827	897.461	0.841539	1.92007	0.00075	0.0133173	yes
gene:SpnNT_00300	rplM	Chromosome:289030-289477	ΔORF2	110.58+peptide	OK	479.3	897.461	0.90492	2.06411	2.00E-04	0.00450928	yes
gene:SpnNT_00300	rplM	Chromosome:289030-289477	110.58	ΔORF2+peptide	OK	500.827	969.344	0.952698	2.17368	0.00015	0.00355289	yes
gene:SpnNT_00300	rplM	Chromosome:289030-289477	ΔORF2	ΔORF2+peptide	OK	479.3	969.344	1.01608	2.31766	1.00E-04	0.0025332	yes

gene:SpnNT_00300	rplM	Chromosome:289030-289477	110.58+peptide	ΔORF2+peptide	OK	897.461	969.344	0.111159	0.256102	0.6463	0.980887	no
gene:SpnNT_00301	rpsI	Chromosome:289496-289889	110.58	ΔORF2	OK	2354.32	2181.5	-0.109988	-0.239756	0.67255	0.984845	no
gene:SpnNT_00301	rpsI	Chromosome:289496-289889	110.58	110.58+peptide	OK	2354.32	5131.41	1.12405	2.44871	5.00E-05	0.0013612	yes
gene:SpnNT_00301	rpsI	Chromosome:289496-289889	ΔORF2	110.58+peptide	OK	2181.5	5131.41	1.23404	2.65388	5.00E-05	0.0013612	yes
gene:SpnNT_00301	rpsI	Chromosome:289496-289889	110.58	ΔORF2+peptide	OK	2354.32	4867.35	1.04783	2.32458	5.00E-05	0.0013612	yes
gene:SpnNT_00301	rpsI	Chromosome:289496-289889	ΔORF2	ΔORF2+peptide	OK	2181.5	4867.35	1.15782	2.53449	5.00E-05	0.0013612	yes
gene:SpnNT_00301	rpsI	Chromosome:289496-289889	110.58+peptide	ΔORF2+peptide	OK	5131.41	4867.35	-0.0762187	-0.16674	0.77215	0.994748	no
gene:SpnNT_00302	NA	Chromosome:290150-290573	110.58	ΔORF2	NOTEST	0.124026	0	#NAME?	0	1	1	no
gene:SpnNT_00302	NA	Chromosome:290150-290573	110.58	110.58+peptide	NOTEST	0.124026	0	#NAME?	0	1	1	no
gene:SpnNT_00302	NA	Chromosome:290150-290573	ΔORF2	110.58+peptide	NOTEST	0	0	0	0	1	1	no
gene:SpnNT_00302	NA	Chromosome:290150-290573	110.58	ΔORF2+peptide	NOTEST	0.124026	0.76474	2.62432	0	1	1	no
gene:SpnNT_00302	NA	Chromosome:290150-290573	ΔORF2	ΔORF2+peptide	NOTEST	0	0.76474	Inf	0	1	1	no
gene:SpnNT_00302	NA	Chromosome:290150-290573	110.58+peptide	ΔORF2+peptide	NOTEST	0	0.76474	Inf	0	1	1	no
gene:SpnNT_00303	NA	Chromosome:290719-291021	110.58	ΔORF2	OK	484.772	555.12	0.195493	0.327623	0.556	0.966729	no
gene:SpnNT_00303	NA	Chromosome:290719-291021	110.58	110.58+peptide	OK	484.772	455.742	-0.0890899	-0.146977	0.79425	0.994748	no
gene:SpnNT_00303	NA	Chromosome:290719-291021	ΔORF2	110.58+peptide	OK	555.12	455.742	-0.284583	-0.467247	0.41005	0.898184	no
gene:SpnNT_00303	NA	Chromosome:290719-291021	110.58	ΔORF2+peptide	OK	484.772	384.369	-0.334816	-0.507112	0.3444	0.848679	no
gene:SpnNT_00303	NA	Chromosome:290719-291021	ΔORF2	ΔORF2+peptide	OK	555.12	384.369	-0.530309	-0.799963	0.13765	0.565157	no
gene:SpnNT_00303	NA	Chromosome:290719-291021	110.58+peptide	ΔORF2+peptide	OK	455.742	384.369	-0.245726	-0.365972	0.4982	0.944017	no
gene:SpnNT_00304	NA	Chromosome:290719-291021	110.58	ΔORF2	OK	63.5625	60.0417	-0.0822119	-0.0226674	0.9351	0.994855	no
gene:SpnNT_00304	NA	Chromosome:290719-291021	110.58	110.58+peptide	OK	63.5625	56.8831	-0.160177	-0.0462337	0.9201	0.994748	no
gene:SpnNT_00304	NA	Chromosome:290719-291021	ΔORF2	110.58+peptide	OK	60.0417	56.8831	-0.0779655	-0.0211809	0.93055	0.994855	no
gene:SpnNT_00304	NA	Chromosome:290719-291021	110.58	ΔORF2+peptide	OK	63.5625	29.1862	-1.12289	-0.460943	0.65055	0.981475	no
gene:SpnNT_00304	NA	Chromosome:290719-291021	ΔORF2	ΔORF2+peptide	OK	60.0417	29.1862	-1.04068	-0.38049	0.6517	0.98163	no
gene:SpnNT_00304	NA	Chromosome:290719-291021	110.58+peptide	ΔORF2+peptide	OK	56.8831	29.1862	-0.962712	-0.382662	0.7212	0.994748	no
gene:SpnNT_00305	yicI	Chromosome:291297-293511	110.58	ΔORF2	OK	54.3362	75.5642	0.475789	1.05585	0.06715	0.378942	no
gene:SpnNT_00305	yicI	Chromosome:291297-293511	110.58	110.58+peptide	OK	54.3362	33.9098	-0.680212	-1.49372	0.01055	0.104245	no
gene:SpnNT_00305	yicI	Chromosome:291297-293511	ΔORF2	110.58+peptide	OK	75.5642	33.9098	-1.156	-2.50866	5.00E-05	0.0013612	yes
gene:SpnNT_00305	yicI	Chromosome:291297-293511	110.58	ΔORF2+peptide	OK	54.3362	36.7024	-0.56604	-1.25994	0.03005	0.222526	no
gene:SpnNT_00305	yicI	Chromosome:291297-293511	ΔORF2	ΔORF2+peptide	OK	75.5642	36.7024	-1.04183	-2.29096	5.00E-05	0.0013612	yes
gene:SpnNT_00305	yicI	Chromosome:291297-293511	110.58+peptide	ΔORF2+peptide	OK	33.9098	36.7024	0.114173	0.248488	0.6698	0.984845	no
gene:SpnNT_00306	bsaA	Chromosome:293732-294209	110.58	ΔORF2	OK	33.0271	41.0464	0.313602	0.552228	0.3327	0.838588	no
gene:SpnNT_00306	bsaA	Chromosome:293732-294209	110.58	110.58+peptide	OK	33.0271	22.7818	-0.535773	-0.912187	0.112	0.509744	no
gene:SpnNT_00306	bsaA	Chromosome:293732-294209	ΔORF2	110.58+peptide	OK	41.0464	22.7818	-0.849375	-1.45832	0.01295	0.120861	no
gene:SpnNT_00306	bsaA	Chromosome:293732-294209	110.58	ΔORF2+peptide	OK	33.0271	21.6885	-0.606723	-1.0165	0.07815	0.415176	no
gene:SpnNT_00306	bsaA	Chromosome:293732-294209	ΔORF2	ΔORF2+peptide	OK	41.0464	21.6885	-0.920325	-1.55451	0.0075	0.0820125	no
gene:SpnNT_00306	bsaA	Chromosome:293732-294209	110.58+peptide	ΔORF2+peptide	OK	22.7818	21.6885	-0.0709504	-0.116173	0.8435	0.994748	no
gene:SpnNT_00307	NA	Chromosome:294431-294578	110.58	ΔORF2	OK	11.5437	14.5436	0.333277	0.240843	0.743	0.994748	no
gene:SpnNT_00307	NA	Chromosome:294431-294578	110.58	110.58+peptide	OK	11.5437	16.2514	0.493462	0.262036	0.63835	0.980887	no
gene:SpnNT_00307	NA	Chromosome:294431-294578	ΔORF2	110.58+peptide	OK	14.5436	16.2514	0.160185	0.120974	0.8849	0.994748	no
gene:SpnNT_00307	NA	Chromosome:294431-294578	110.58	ΔORF2+peptide	OK	11.5437	33.5607	1.53967	1.12967	0.23385	0.72774	no
gene:SpnNT_00307	NA	Chromosome:294431-294578	ΔORF2	ΔORF2+peptide	OK	14.5436	33.5607	1.20639	4.74914	0.3065	0.81251	no
gene:SpnNT_00307	NA	Chromosome:294431-294578	110.58+peptide	ΔORF2+peptide	OK	16.2514	33.5607	1.04621	0.803345	0.32525	0.830337	no
gene:SpnNT_00308	NA	Chromosome:294785-297461	110.58	ΔORF2	OK	5.14652	4.40891	-0.223175	-0.400923	0.47985	0.934351	no
gene:SpnNT_00308	NA	Chromosome:294785-297461	110.58	110.58+peptide	OK	5.14652	8.84849	0.781834	1.42195	0.01255	0.118903	no

gene:SpnNT_00308	NA	Chromosome:294785-297461	ΔORF2	110.58+peptide	OK	4.40891	8.84849	1.00501	1.80782	0.0022	0.0317584	yes
gene:SpnNT_00308	NA	Chromosome:294785-297461	110.58	ΔORF2+peptide	OK	5.14652	6.2819	0.287604	0.520543	0.35465	0.857011	no
gene:SpnNT_00308	NA	Chromosome:294785-297461	ΔORF2	ΔORF2+peptide	OK	4.40891	6.2819	0.51078	0.914443	0.11095	0.507277	no
gene:SpnNT_00308	NA	Chromosome:294785-297461	110.58+peptide	ΔORF2+peptide	OK	8.84849	6.2819	-0.494229	-0.895715	0.1228	0.530583	no
gene:SpnNT_00309	NA	Chromosome:298188-298404	110.58	ΔORF2	NOTEST	0	0	0	0	1	1	no
gene:SpnNT_00309	NA	Chromosome:298188-298404	110.58	110.58+peptide	NOTEST	0	0	0	0	1	1	no
gene:SpnNT_00309	NA	Chromosome:298188-298404	ΔORF2	110.58+peptide	NOTEST	0	0	0	0	1	1	no
gene:SpnNT_00309	NA	Chromosome:298188-298404	110.58	ΔORF2+peptide	NOTEST	0	0	0	0	1	1	no
gene:SpnNT_00309	NA	Chromosome:298188-298404	ΔORF2	ΔORF2+peptide	NOTEST	0	0	0	0	1	1	no
gene:SpnNT_00309	NA	Chromosome:298188-298404	110.58+peptide	ΔORF2+peptide	NOTEST	0	0	0	0	1	1	no
gene:SpnNT_00310	kdgA	Chromosome:298778-299408	110.58	ΔORF2	OK	1.67871	1.74717	0.0576719	0.0588731	0.913	0.994748	no
gene:SpnNT_00310	kdgA	Chromosome:298778-299408	110.58	110.58+peptide	OK	1.67871	3.35823	1.00035	1.11312	0.05585	0.337095	no
gene:SpnNT_00310	kdgA	Chromosome:298778-299408	ΔORF2	110.58+peptide	OK	1.74717	3.35823	0.94268	1.02874	0.0801	0.421873	no
gene:SpnNT_00310	kdgA	Chromosome:298778-299408	110.58	ΔORF2+peptide	OK	1.67871	0.49269	-1.7686	-1.49582	0.0372	0.258274	no
gene:SpnNT_00310	kdgA	Chromosome:298778-299408	ΔORF2	ΔORF2+peptide	OK	1.74717	0.49269	-1.82627	-1.5272	0.03345	0.240247	no
gene:SpnNT_00310	kdgA	Chromosome:298778-299408	110.58+peptide	ΔORF2+peptide	OK	3.35823	0.49269	-2.76895	-2.4493	0.00695	0.0769603	no
gene:SpnNT_00311	kdgK	Chromosome:299417-300419	110.58	ΔORF2	OK	0.850021	0.920294	0.114597	0.1179	0.84255	0.994748	no
gene:SpnNT_00311	kdgK	Chromosome:299417-300419	110.58	110.58+peptide	OK	0.850021	1.70986	1.00831	1.11771	0.0577	0.342262	no
gene:SpnNT_00311	kdgK	Chromosome:299417-300419	ΔORF2	110.58+peptide	OK	0.920294	1.70986	0.89371	1.01437	0.08365	0.434027	no
gene:SpnNT_00311	kdgK	Chromosome:299417-300419	110.58	ΔORF2+peptide	OK	0.850021	1.21908	0.52022	0.54978	0.34535	0.849107	no
gene:SpnNT_00311	kdgK	Chromosome:299417-300419	ΔORF2	ΔORF2+peptide	OK	0.920294	1.21908	0.405624	0.437959	0.4487	0.919688	no
gene:SpnNT_00311	kdgK	Chromosome:299417-300419	110.58+peptide	ΔORF2+peptide	OK	1.70986	1.21908	-0.488086	-0.572497	0.31825	0.824497	no
gene:SpnNT_00312	NA	Chromosome:300449-301091	110.58	ΔORF2	OK	1.67343	1.08141	-0.629903	-0.599875	0.29475	0.799279	no
gene:SpnNT_00312	NA	Chromosome:300449-301091	110.58	110.58+peptide	OK	1.67343	2.01875	0.270653	0.285521	0.61765	0.980887	no
gene:SpnNT_00312	NA	Chromosome:300449-301091	ΔORF2	110.58+peptide	OK	1.08141	2.01875	0.900556	0.891874	0.1273	0.541293	no
gene:SpnNT_00312	NA	Chromosome:300449-301091	110.58	ΔORF2+peptide	OK	1.67343	2.66019	0.668715	0.69855	0.22515	0.716396	no
gene:SpnNT_00312	NA	Chromosome:300449-301091	ΔORF2	ΔORF2+peptide	OK	1.08141	2.66019	1.29862	1.27499	0.0353	0.249036	no
gene:SpnNT_00312	NA	Chromosome:300449-301091	110.58+peptide	ΔORF2+peptide	OK	2.01875	2.66019	0.398062	0.436052	0.4577	0.921722	no
gene:SpnNT_00313	gno	Chromosome:301109-301925	110.58	ΔORF2	OK	1.96361	1.47979	-0.408112	-0.453816	0.42445	0.907534	no
gene:SpnNT_00313	gno	Chromosome:301109-301925	110.58	110.58+peptide	OK	1.96361	2.211	0.17119	0.201707	0.7257	0.994748	no
gene:SpnNT_00313	gno	Chromosome:301109-301925	ΔORF2	110.58+peptide	OK	1.47979	2.211	0.579302	0.657418	0.25715	0.756914	no
gene:SpnNT_00313	gno	Chromosome:301109-301925	110.58	ΔORF2+peptide	OK	1.96361	1.16003	-0.759347	-0.791847	0.16545	0.621005	no
gene:SpnNT_00313	gno	Chromosome:301109-301925	ΔORF2	ΔORF2+peptide	OK	1.47979	1.16003	-0.351234	-0.355568	0.53395	0.958867	no
gene:SpnNT_00313	gno	Chromosome:301109-301925	110.58+peptide	ΔORF2+peptide	OK	2.211	1.16003	-0.930536	-0.987839	0.0941	0.463158	no
gene:SpnNT_00314	manX_3	Chromosome:302195-302630	110.58	ΔORF2	NOTEST	0.4923	0.376678	-0.386204	0	1	1	no
gene:SpnNT_00314	manX_3	Chromosome:302195-302630	110.58	110.58+peptide	OK	0.4923	0.852172	0.791609	0.494596	0.44045	0.916647	no
gene:SpnNT_00314	manX_3	Chromosome:302195-302630	ΔORF2	110.58+peptide	OK	0.376678	0.852172	1.17781	0.625211	0.2963	0.800504	no
gene:SpnNT_00314	manX_3	Chromosome:302195-302630	110.58	ΔORF2+peptide	OK	0.4923	1.24921	1.3434	0.911986	0.17675	0.64158	no
gene:SpnNT_00314	manX_3	Chromosome:302195-302630	ΔORF2	ΔORF2+peptide	OK	0.376678	1.24921	1.72961	0.973415	0.1426	0.576108	no
gene:SpnNT_00314	manX_3	Chromosome:302195-302630	110.58+peptide	ΔORF2+peptide	OK	0.852172	1.24921	0.551796	0.363668	0.5168	0.953254	no
gene:SpnNT_00315	ugl	Chromosome:302641-303832	110.58	ΔORF2	NOTEST	0.744352	0.489838	-0.603679	0	1	1	no
gene:SpnNT_00315	ugl	Chromosome:302641-303832	110.58	110.58+peptide	OK	0.744352	0.971132	0.383683	0.409909	0.4671	0.92732	no
gene:SpnNT_00315	ugl	Chromosome:302641-303832	ΔORF2	110.58+peptide	OK	0.489838	0.971132	0.987363	0.993654	0.0961	0.46955	no
gene:SpnNT_00315	ugl	Chromosome:302641-303832	110.58	ΔORF2+peptide	NOTEST	0.744352	0.213719	-1.80027	0	1	1	no
gene:SpnNT_00315	ugl	Chromosome:302641-303832	ΔORF2	ΔORF2+peptide	NOTEST	0.489838	0.213719	-1.19659	0	1	1	no

gene:SpnNT_00315	ugl	Chromosome:302641-303832	110.58+peptide	ΔORF2+peptide	OK	0.971132	0.213719	-2.18395	-1.73269	0.02715	0.208707	no
gene:SpnNT_00316	sorB_2	Chromosome:303842-304334	110.58	ΔORF2	OK	1.22775	0.836124	-0.554228	-0.399642	0.46155	0.924892	no
gene:SpnNT_00316	sorB_2	Chromosome:303842-304334	110.58	110.58+peptide	OK	1.22775	1.52875	0.316333	0.273133	0.642	0.980887	no
gene:SpnNT_00316	sorB_2	Chromosome:303842-304334	ΔORF2	110.58+peptide	OK	0.836124	1.52875	0.870561	0.647954	0.2464	0.74356	no
gene:SpnNT_00316	sorB_2	Chromosome:303842-304334	110.58	ΔORF2+peptide	OK	1.22775	0.735324	-0.739565	-0.595135	0.33995	0.844213	no
gene:SpnNT_00316	sorB_2	Chromosome:303842-304334	ΔORF2	ΔORF2+peptide	OK	0.836124	0.735324	-0.185337	-0.13079	0.8386	0.994748	no
gene:SpnNT_00316	sorB_2	Chromosome:303842-304334	110.58+peptide	ΔORF2+peptide	OK	1.52875	0.735324	-1.0559	-0.884175	0.1781	0.644697	no
gene:SpnNT_00317	agaC_2	Chromosome:304348-306226	110.58	ΔORF2	NOTEST	0.577853	0.609478	0.0768714	0	1	1	no
gene:SpnNT_00317	agaC_2	Chromosome:304348-306226	110.58	110.58+peptide	NOTEST	0.577853	0.723513	0.324316	0	1	1	no
gene:SpnNT_00317	agaC_2	Chromosome:304348-306226	ΔORF2	110.58+peptide	NOTEST	0.609478	0.723513	0.247444	0	1	1	no
gene:SpnNT_00317	agaC_2	Chromosome:304348-306226	110.58	ΔORF2+peptide	NOTEST	0.577853	0.520199	-0.151639	0	1	1	no
gene:SpnNT_00317	agaC_2	Chromosome:304348-306226	ΔORF2	ΔORF2+peptide	NOTEST	0.609478	0.520199	-0.228511	0	1	1	no
gene:SpnNT_00317	agaC_2	Chromosome:304348-306226	110.58+peptide	ΔORF2+peptide	NOTEST	0.723513	0.520199	-0.475955	0	1	1	no
gene:SpnNT_00318	manZ_3	Chromosome:304348-306226	110.58	ΔORF2	OK	1.07385	1.14907	0.0976807	0.0789767	0.8914	0.994748	no
gene:SpnNT_00318	manZ_3	Chromosome:304348-306226	110.58	110.58+peptide	OK	1.07385	1.32892	0.307468	0.230718	0.679	0.98542	no
gene:SpnNT_00318	manZ_3	Chromosome:304348-306226	ΔORF2	110.58+peptide	OK	1.14907	1.32892	0.209787	0.171138	0.7546	0.994748	no
gene:SpnNT_00318	manZ_3	Chromosome:304348-306226	110.58	ΔORF2+peptide	OK	1.07385	1.49988	0.482057	0.402899	0.48385	0.936747	no
gene:SpnNT_00318	manZ_3	Chromosome:304348-306226	ΔORF2	ΔORF2+peptide	OK	1.14907	1.49988	0.384376	0.357155	0.5351	0.958867	no
gene:SpnNT_00318	manZ_3	Chromosome:304348-306226	110.58+peptide	ΔORF2+peptide	OK	1.32892	1.49988	0.174589	0.14732	0.7852	0.994748	no
gene:SpnNT_00319	NA	Chromosome:304348-306226	110.58	ΔORF2	OK	5.08046	2.50232	-1.02169	-0.540112	0.3786	0.875802	no
gene:SpnNT_00319	NA	Chromosome:304348-306226	110.58	110.58+peptide	OK	5.08046	4.7563	-0.0951184	-0.0516536	0.9345	0.994855	no
gene:SpnNT_00319	NA	Chromosome:304348-306226	ΔORF2	110.58+peptide	OK	2.50232	4.7563	0.926573	0.501673	0.4775	0.93282	no
gene:SpnNT_00319	NA	Chromosome:304348-306226	110.58	ΔORF2+peptide	OK	5.08046	3.06444	-0.729336	-0.458255	0.5665	0.968621	no
gene:SpnNT_00319	NA	Chromosome:304348-306226	ΔORF2	ΔORF2+peptide	OK	2.50232	3.06444	0.292355	0.182961	0.81565	0.994748	no
gene:SpnNT_00319	NA	Chromosome:304348-306226	110.58+peptide	ΔORF2+peptide	OK	4.7563	3.06444	-0.634218	-0.412312	0.65055	0.981475	no
gene:SpnNT_00320	NA	Chromosome:306247-308149	110.58	ΔORF2	NOTEST	0.583773	0.323294	-0.852558	0	1	1	no
gene:SpnNT_00320	NA	Chromosome:306247-308149	110.58	110.58+peptide	NOTEST	0.583773	0.593176	0.0230533	0	1	1	no
gene:SpnNT_00320	NA	Chromosome:306247-308149	ΔORF2	110.58+peptide	NOTEST	0.323294	0.593176	0.875612	0	1	1	no
gene:SpnNT_00320	NA	Chromosome:306247-308149	110.58	ΔORF2+peptide	NOTEST	0.583773	0.406052	-0.523743	0	1	1	no
gene:SpnNT_00320	NA	Chromosome:306247-308149	ΔORF2	ΔORF2+peptide	NOTEST	0.323294	0.406052	0.328815	0	1	1	no
gene:SpnNT_00320	NA	Chromosome:306247-308149	110.58+peptide	ΔORF2+peptide	NOTEST	0.593176	0.406052	-0.546796	0	1	1	no
gene:SpnNT_00321	kdgR	Chromosome:308208-309210	110.58	ΔORF2	OK	114.233	144.469	0.338783	0.744643	0.19345	0.674905	no
gene:SpnNT_00321	kdgR	Chromosome:308208-309210	110.58	110.58+peptide	OK	114.233	115.794	0.0195878	0.0430304	0.93955	0.994855	no
gene:SpnNT_00321	kdgR	Chromosome:308208-309210	ΔORF2	110.58+peptide	OK	144.469	115.794	-0.319196	-0.688793	0.23045	0.723783	no
gene:SpnNT_00321	kdgR	Chromosome:308208-309210	110.58	ΔORF2+peptide	OK	114.233	111.715	-0.0321558	-0.0713218	0.9009	0.994748	no
gene:SpnNT_00321	kdgR	Chromosome:308208-309210	ΔORF2	ΔORF2+peptide	OK	144.469	111.715	-0.370939	-0.807902	0.1617	0.614666	no
gene:SpnNT_00321	kdgR	Chromosome:308208-309210	110.58+peptide	ΔORF2+peptide	OK	115.794	111.715	-0.0517436	-0.112637	0.8442	0.994748	no
gene:SpnNT_00322	NA	Chromosome:309480-310059	110.58	ΔORF2	OK	14.396	13.3562	-0.108162	-0.169204	0.77305	0.994748	no
gene:SpnNT_00322	NA	Chromosome:309480-310059	110.58	110.58+peptide	OK	14.396	15.4875	0.105432	0.164179	0.76915	0.994748	no
gene:SpnNT_00322	NA	Chromosome:309480-310059	ΔORF2	110.58+peptide	OK	13.3562	15.4875	0.213594	0.335693	0.5492	0.964281	no
gene:SpnNT_00322	NA	Chromosome:309480-310059	110.58	ΔORF2+peptide	OK	14.396	9.42481	-0.611134	-0.927031	0.113	0.510701	no
gene:SpnNT_00322	NA	Chromosome:309480-310059	ΔORF2	ΔORF2+peptide	OK	13.3562	9.42481	-0.502973	-0.769668	0.17845	0.645075	no
gene:SpnNT_00322	NA	Chromosome:309480-310059	110.58+peptide	ΔORF2+peptide	OK	15.4875	9.42481	-0.716567	-1.09171	0.05565	0.336206	no
gene:SpnNT_00323	NA	Chromosome:310071-310413	110.58	ΔORF2	OK	25.9627	24.6477	-0.0749882	-0.109566	0.8489	0.994748	no
gene:SpnNT_00323	NA	Chromosome:310071-310413	110.58	110.58+peptide	OK	25.9627	26.904	0.0513788	0.0732644	0.90015	0.994748	no

gene:SpnNT_00323	NA	Chromosome:310071-310413	ΔORF2	110.58+peptide	OK	24.6477	26.904	0.126367	0.182304	0.75435	0.994748	no
gene:SpnNT_00323	NA	Chromosome:310071-310413	110.58	ΔORF2+peptide	OK	25.9627	17.7568	-0.548068	-0.726924	0.19165	0.672051	no
gene:SpnNT_00323	NA	Chromosome:310071-310413	ΔORF2	ΔORF2+peptide	OK	24.6477	17.7568	-0.47308	-0.633803	0.2481	0.74413	no
gene:SpnNT_00323	NA	Chromosome:310071-310413	110.58+peptide	ΔORF2+peptide	OK	26.904	17.7568	-0.599447	-0.786769	0.1549	0.600007	no
gene:SpnNT_00324	NA	Chromosome:310423-310666	110.58	ΔORF2	OK	10.9793	15.4183	0.489857	0.496796	0.38485	0.881745	no
gene:SpnNT_00324	NA	Chromosome:310423-310666	110.58	110.58+peptide	OK	10.9793	13.1135	0.256265	0.254739	0.6447	0.980887	no
gene:SpnNT_00324	NA	Chromosome:310423-310666	ΔORF2	110.58+peptide	OK	15.4183	13.1135	-0.233592	-0.231512	0.67155	0.984845	no
gene:SpnNT_00324	NA	Chromosome:310423-310666	110.58	ΔORF2+peptide	OK	10.9793	11.743	0.0970093	0.0960319	0.8654	0.994748	no
gene:SpnNT_00324	NA	Chromosome:310423-310666	ΔORF2	ΔORF2+peptide	OK	15.4183	11.743	-0.392848	-0.387745	0.4902	0.93986	no
gene:SpnNT_00324	NA	Chromosome:310423-310666	110.58+peptide	ΔORF2+peptide	OK	13.1135	11.743	-0.159256	-0.154229	0.78025	0.994748	no
gene:SpnNT_00325	NA	Chromosome:310676-311219	110.58	ΔORF2	OK	19.9221	19.4728	-0.0329111	-0.0540821	0.9246	0.994748	no
gene:SpnNT_00325	NA	Chromosome:310676-311219	110.58	110.58+peptide	OK	19.9221	18.2781	-0.124256	-0.199878	0.7327	0.994748	no
gene:SpnNT_00325	NA	Chromosome:310676-311219	ΔORF2	110.58+peptide	OK	19.4728	18.2781	-0.0913452	-0.147878	0.8023	0.994748	no
gene:SpnNT_00325	NA	Chromosome:310676-311219	110.58	ΔORF2+peptide	OK	19.9221	12.7998	-0.638246	-0.966314	0.09185	0.456363	no
gene:SpnNT_00325	NA	Chromosome:310676-311219	ΔORF2	ΔORF2+peptide	OK	19.4728	12.7998	-0.605335	-0.921675	0.1022	0.484665	no
gene:SpnNT_00325	NA	Chromosome:310676-311219	110.58+peptide	ΔORF2+peptide	OK	18.2781	12.7998	-0.51399	-0.768349	0.17935	0.645837	no
gene:SpnNT_00326	NA	Chromosome:311234-311429	110.58	ΔORF2	OK	16.6472	16.4281	-0.0191131	-0.0177709	0.9485	0.994855	no
gene:SpnNT_00326	NA	Chromosome:311234-311429	110.58	110.58+peptide	OK	16.6472	15.528	-0.100405	-0.0903129	0.86305	0.994748	no
gene:SpnNT_00326	NA	Chromosome:311234-311429	ΔORF2	110.58+peptide	OK	16.4281	15.528	-0.0812922	-0.0708888	0.9037	0.994748	no
gene:SpnNT_00326	NA	Chromosome:311234-311429	110.58	ΔORF2+peptide	OK	16.6472	4.09945	-2.02177	-2.31218	0.0524	0.323878	no
gene:SpnNT_00326	NA	Chromosome:311234-311429	ΔORF2	ΔORF2+peptide	OK	16.4281	4.09945	-2.00266	-2.18036	0.05285	0.325739	no
gene:SpnNT_00326	NA	Chromosome:311234-311429	110.58+peptide	ΔORF2+peptide	OK	15.528	4.09945	-1.92137	-2.00004	0.07335	0.399046	no
gene:SpnNT_00327	rsmH	Chromosome:311594-312545	110.58	ΔORF2	OK	153.094	128.187	-0.256174	-0.570029	0.3191	0.825233	no
gene:SpnNT_00327	rsmH	Chromosome:311594-312545	110.58	110.58+peptide	OK	153.094	153.487	0.00370058	0.00824086	0.98705	0.997359	no
gene:SpnNT_00327	rsmH	Chromosome:311594-312545	ΔORF2	110.58+peptide	OK	128.187	153.487	0.259874	0.586272	0.31195	0.818027	no
gene:SpnNT_00327	rsmH	Chromosome:311594-312545	110.58	ΔORF2+peptide	OK	153.094	159.343	0.0577137	0.12859	0.8213	0.994748	no
gene:SpnNT_00327	rsmH	Chromosome:311594-312545	ΔORF2	ΔORF2+peptide	OK	128.187	159.343	0.313887	0.708503	0.21895	0.707128	no
gene:SpnNT_00327	rsmH	Chromosome:311594-312545	110.58+peptide	ΔORF2+peptide	OK	153.487	159.343	0.0540131	0.122016	0.83235	0.994748	no
gene:SpnNT_00328	NA	Chromosome:312556-312874	110.58	ΔORF2	OK	66.1252	67.4735	0.0291202	0.049166	0.93185	0.994855	no
gene:SpnNT_00328	NA	Chromosome:312556-312874	110.58	110.58+peptide	OK	66.1252	83.1378	0.330304	0.568172	0.3218	0.827743	no
gene:SpnNT_00328	NA	Chromosome:312556-312874	ΔORF2	110.58+peptide	OK	67.4735	83.1378	0.301184	0.516374	0.3657	0.864634	no
gene:SpnNT_00328	NA	Chromosome:312556-312874	110.58	ΔORF2+peptide	OK	66.1252	98.3617	0.572897	0.990556	0.0914	0.454989	no
gene:SpnNT_00328	NA	Chromosome:312556-312874	ΔORF2	ΔORF2+peptide	OK	67.4735	98.3617	0.543777	0.937076	0.1022	0.484665	no
gene:SpnNT_00328	NA	Chromosome:312556-312874	110.58+peptide	ΔORF2+peptide	OK	83.1378	98.3617	0.242593	0.426258	0.45475	0.921244	no
gene:SpnNT_00329	pbpX_1	Chromosome:312877-315130	110.58	ΔORF2	OK	74.1298	74.714	0.0113259	0.0259843	0.96265	0.994855	no
gene:SpnNT_00329	pbpX_1	Chromosome:312877-315130	110.58	110.58+peptide	OK	74.1298	88.5748	0.256843	0.590247	0.29065	0.795003	no
gene:SpnNT_00329	pbpX_1	Chromosome:312877-315130	ΔORF2	110.58+peptide	OK	74.714	88.5748	0.245517	0.56407	0.3151	0.821264	no
gene:SpnNT_00329	pbpX_1	Chromosome:312877-315130	110.58	ΔORF2+peptide	OK	74.1298	106.453	0.522087	1.1989	0.0319	0.231907	no
gene:SpnNT_00329	pbpX_1	Chromosome:312877-315130	ΔORF2	ΔORF2+peptide	OK	74.714	106.453	0.510761	1.17258	0.03725	0.258485	no
gene:SpnNT_00329	pbpX_1	Chromosome:312877-315130	110.58+peptide	ΔORF2+peptide	OK	88.5748	106.453	0.265244	0.609955	0.28485	0.787901	no
gene:SpnNT_00330	mraY	Chromosome:315131-316365	110.58	ΔORF2	OK	122.634	134.432	0.132522	0.245053	0.66415	0.982966	no
gene:SpnNT_00330	mraY	Chromosome:315131-316365	110.58	110.58+peptide	OK	122.634	172.737	0.494224	0.772086	0.1646	0.619232	no
gene:SpnNT_00330	mraY	Chromosome:315131-316365	ΔORF2	110.58+peptide	OK	134.432	172.737	0.361702	0.538263	0.3293	0.83499	no
gene:SpnNT_00330	mraY	Chromosome:315131-316365	110.58	ΔORF2+peptide	OK	122.634	207.711	0.760225	1.44384	0.015	0.133716	no
gene:SpnNT_00330	mraY	Chromosome:315131-316365	ΔORF2	ΔORF2+peptide	OK	134.432	207.711	0.627702	1.1113	0.0556	0.336206	no

gene:SpnNT_00330	mraY	Chromosome:315131-316365	110.58+peptide	ΔORF2+peptide	OK	172.737	207.711	0.266001	0.402691	0.4682	0.927504	no
gene:SpnNT_00331	NA	Chromosome:315131-316365	110.58	ΔORF2	OK	83.0964	118.184	0.508173	0.362466	0.5681	0.968621	no
gene:SpnNT_00331	NA	Chromosome:315131-316365	110.58	110.58+peptide	OK	83.0964	255.06	1.61798	1.1673	0.07045	0.390391	no
gene:SpnNT_00331	NA	Chromosome:315131-316365	ΔORF2	110.58+peptide	OK	118.184	255.06	1.10981	0.75539	0.27815	0.781125	no
gene:SpnNT_00331	NA	Chromosome:315131-316365	110.58	ΔORF2+peptide	OK	83.0964	196.571	1.24219	0.900498	0.1273	0.541293	no
gene:SpnNT_00331	NA	Chromosome:315131-316365	ΔORF2	ΔORF2+peptide	OK	118.184	196.571	0.734018	0.501746	0.43045	0.912351	no
gene:SpnNT_00331	NA	Chromosome:315131-316365	110.58+peptide	ΔORF2+peptide	OK	255.06	196.571	-0.375788	-0.259577	0.666	0.983707	no
gene:SpnNT_00332	clpC_1	Chromosome:316451-318557	110.58	ΔORF2	OK	215.75	171.958	-0.327303	-0.715295	0.2131	0.698345	no
gene:SpnNT_00332	clpC_1	Chromosome:316451-318557	110.58	110.58+peptide	OK	215.75	557.608	1.36989	2.46591	5.00E-05	0.0013612	yes
gene:SpnNT_00332	clpC_1	Chromosome:316451-318557	ΔORF2	110.58+peptide	OK	171.958	557.608	1.69719	3.07274	5.00E-05	0.0013612	yes
gene:SpnNT_00332	clpC_1	Chromosome:316451-318557	110.58	ΔORF2+peptide	OK	215.75	240.417	0.156177	0.333668	0.56815	0.968621	no
gene:SpnNT_00332	clpC_1	Chromosome:316451-318557	ΔORF2	ΔORF2+peptide	OK	171.958	240.417	0.48348	1.04138	0.07085	0.390464	no
gene:SpnNT_00332	clpC_1	Chromosome:316451-318557	110.58+peptide	ΔORF2+peptide	OK	557.608	240.417	-1.21371	-2.16327	5.00E-04	0.0095781	yes
gene:SpnNT_00333	luxS	Chromosome:318852-319335	110.58	ΔORF2	OK	118.041	126.126	0.095581	0.195723	0.72445	0.994748	no
gene:SpnNT_00333	luxS	Chromosome:318852-319335	110.58	110.58+peptide	OK	118.041	132.982	0.171941	0.353541	0.5266	0.956161	no
gene:SpnNT_00333	luxS	Chromosome:318852-319335	ΔORF2	110.58+peptide	OK	126.126	132.982	0.0763602	0.156821	0.77775	0.994748	no
gene:SpnNT_00333	luxS	Chromosome:318852-319335	110.58	ΔORF2+peptide	OK	118.041	128.392	0.121272	0.246268	0.66435	0.983151	no
gene:SpnNT_00333	luxS	Chromosome:318852-319335	ΔORF2	ΔORF2+peptide	OK	126.126	128.392	0.0256912	0.0521102	0.92455	0.994748	no
gene:SpnNT_00333	luxS	Chromosome:318852-319335	110.58+peptide	ΔORF2+peptide	OK	132.982	128.392	-0.050669	-0.103189	0.8548	0.994748	no
gene:SpnNT_00334	NA	Chromosome:319429-320914	110.58	ΔORF2	OK	80.6735	82.6126	0.0342676	0.0774183	0.89155	0.994748	no
gene:SpnNT_00334	NA	Chromosome:319429-320914	110.58	110.58+peptide	OK	80.6735	75.9728	-0.086611	-0.195473	0.72795	0.994748	no
gene:SpnNT_00334	NA	Chromosome:319429-320914	ΔORF2	110.58+peptide	OK	82.6126	75.9728	-0.120879	-0.273233	0.63265	0.980887	no
gene:SpnNT_00334	NA	Chromosome:319429-320914	110.58	ΔORF2+peptide	OK	80.6735	76.7299	-0.0723061	-0.161435	0.776	0.994748	no
gene:SpnNT_00334	NA	Chromosome:319429-320914	ΔORF2	ΔORF2+peptide	OK	82.6126	76.7299	-0.106574	-0.238303	0.6793	0.985601	no
gene:SpnNT_00334	NA	Chromosome:319429-320914	110.58+peptide	ΔORF2+peptide	OK	75.9728	76.7299	0.0143049	0.031954	0.95525	0.994855	no
gene:SpnNT_00335	dexB	Chromosome:321065-322676	110.58	ΔORF2	OK	39.1583	37.7615	-0.0524049	-0.113625	0.83765	0.994748	no
gene:SpnNT_00335	dexB	Chromosome:321065-322676	110.58	110.58+peptide	OK	39.1583	60.8449	0.635818	1.40341	0.01375	0.12635	no
gene:SpnNT_00335	dexB	Chromosome:321065-322676	ΔORF2	110.58+peptide	OK	37.7615	60.8449	0.688223	1.51376	0.0076	0.082624	no
gene:SpnNT_00335	dexB	Chromosome:321065-322676	110.58	ΔORF2+peptide	OK	39.1583	62.8778	0.683232	1.50659	0.00795	0.0856485	no
gene:SpnNT_00335	dexB	Chromosome:321065-322676	ΔORF2	ΔORF2+peptide	OK	37.7615	62.8778	0.735637	1.61649	0.0039	0.0495409	yes
gene:SpnNT_00335	dexB	Chromosome:321065-322676	110.58+peptide	ΔORF2+peptide	OK	60.8449	62.8778	0.0474137	0.106114	0.84945	0.994748	no
gene:SpnNT_00336	sarA_1	Chromosome:322951-324919	110.58	ΔORF2	OK	82.6308	86.1991	0.0609936	0.138204	0.80885	0.994748	no
gene:SpnNT_00336	sarA_1	Chromosome:322951-324919	110.58	110.58+peptide	OK	82.6308	67.9078	-0.283103	-0.618101	0.28665	0.789883	no
gene:SpnNT_00336	sarA_1	Chromosome:322951-324919	ΔORF2	110.58+peptide	OK	86.1991	67.9078	-0.344097	-0.750006	0.19485	0.676386	no
gene:SpnNT_00336	sarA_1	Chromosome:322951-324919	110.58	ΔORF2+peptide	OK	82.6308	84.4309	0.0310915	0.0703726	0.90285	0.994748	no
gene:SpnNT_00336	sarA_1	Chromosome:322951-324919	ΔORF2	ΔORF2+peptide	OK	86.1991	84.4309	-0.0299021	-0.0675582	0.90585	0.994748	no
gene:SpnNT_00336	sarA_1	Chromosome:322951-324919	110.58+peptide	ΔORF2+peptide	OK	67.9078	84.4309	0.314195	0.68414	0.23375	0.72774	no
gene:SpnNT_00337	sarA_2	Chromosome:325122-327081	110.58	ΔORF2	OK	91.3827	235.318	1.36462	3.11995	5.00E-05	0.0013612	yes
gene:SpnNT_00337	sarA_2	Chromosome:325122-327081	110.58	110.58+peptide	OK	91.3827	84.324	-0.115979	-0.266124	0.629	0.980887	no
gene:SpnNT_00337	sarA_2	Chromosome:325122-327081	ΔORF2	110.58+peptide	OK	235.318	84.324	-1.4806	-3.38568	5.00E-05	0.0013612	yes
gene:SpnNT_00337	sarA_2	Chromosome:325122-327081	110.58	ΔORF2+peptide	OK	91.3827	232.161	1.34513	3.05174	5.00E-05	0.0013612	yes
gene:SpnNT_00337	sarA_2	Chromosome:325122-327081	ΔORF2	ΔORF2+peptide	OK	235.318	232.161	-0.0194885	-0.0440656	0.9376	0.994855	no
gene:SpnNT_00337	sarA_2	Chromosome:325122-327081	110.58+peptide	ΔORF2+peptide	OK	84.324	232.161	1.46111	3.31541	5.00E-05	0.0013612	yes
gene:SpnNT_00338	glf_1	Chromosome:327403-327805	110.58	ΔORF2	OK	78.8182	212.336	1.42975	2.80976	5.00E-05	0.0013612	yes
gene:SpnNT_00338	glf_1	Chromosome:327403-327805	110.58	110.58+peptide	OK	78.8182	52.2835	-0.592173	-1.082	0.06545	0.372112	no

gene:SpnNT_00338	glf_1	Chromosome:327403-327805	ΔORF2	110.58+peptide	OK	212.336	52.2835	-2.02192	-3.89452	5.00E-05	0.0013612	yes
gene:SpnNT_00338	glf_1	Chromosome:327403-327805	110.58	ΔORF2+peptide	OK	78.8182	134.935	0.77566	1.49198	0.01115	0.108378	no
gene:SpnNT_00338	glf_1	Chromosome:327403-327805	ΔORF2	ΔORF2+peptide	OK	212.336	134.935	-0.654089	-1.33436	0.02085	0.172723	no
gene:SpnNT_00338	glf_1	Chromosome:327403-327805	110.58+peptide	ΔORF2+peptide	OK	52.2835	134.935	1.36783	2.58087	2.00E-04	0.00450928	yes
gene:SpnNT_00339	glf_2	Chromosome:327814-328321	110.58	ΔORF2	OK	108.31	337.58	1.64007	3.51808	5.00E-05	0.0013612	yes
gene:SpnNT_00339	glf_2	Chromosome:327814-328321	110.58	110.58+peptide	OK	108.31	87.0434	-0.315354	-0.638463	0.255	0.754219	no
gene:SpnNT_00339	glf_2	Chromosome:327814-328321	ΔORF2	110.58+peptide	OK	337.58	87.0434	-1.95542	-4.13501	5.00E-05	0.0013612	yes
gene:SpnNT_00339	glf_2	Chromosome:327814-328321	110.58	ΔORF2+peptide	OK	108.31	219.579	1.01958	2.16508	0.00015	0.00355289	yes
gene:SpnNT_00339	glf_2	Chromosome:327814-328321	ΔORF2	ΔORF2+peptide	OK	337.58	219.579	-0.620492	-1.38253	0.0165	0.144246	no
gene:SpnNT_00339	glf_2	Chromosome:327814-328321	110.58+peptide	ΔORF2+peptide	OK	87.0434	219.579	1.33493	2.79529	5.00E-05	0.0013612	yes
gene:SpnNT_00340	doc_1	Chromosome:328432-329058	110.58	ΔORF2	OK	122.703	155.065	0.337698	0.582322	0.3064	0.81251	no
gene:SpnNT_00340	doc_1	Chromosome:328432-329058	110.58	110.58+peptide	OK	122.703	197.369	0.685725	1.1525	0.0453	0.29311	no
gene:SpnNT_00340	doc_1	Chromosome:328432-329058	ΔORF2	110.58+peptide	OK	155.065	197.369	0.348027	0.574434	0.3091	0.814396	no
gene:SpnNT_00340	doc_1	Chromosome:328432-329058	110.58	ΔORF2+peptide	OK	122.703	141.749	0.208162	0.354478	0.5351	0.958867	no
gene:SpnNT_00340	doc_1	Chromosome:328432-329058	ΔORF2	ΔORF2+peptide	OK	155.065	141.749	-0.129536	-0.216527	0.69845	0.990209	no
gene:SpnNT_00340	doc_1	Chromosome:328432-329058	110.58+peptide	ΔORF2+peptide	OK	197.369	141.749	-0.477563	-0.779226	0.16595	0.621332	no
gene:SpnNT_00341	NA	Chromosome:328432-329058	110.58	ΔORF2	OK	86.8352	145.516	0.744827	0.746296	0.1969	0.678125	no
gene:SpnNT_00341	NA	Chromosome:328432-329058	110.58	110.58+peptide	OK	86.8352	107.981	0.314427	0.245065	0.6597	0.982966	no
gene:SpnNT_00341	NA	Chromosome:328432-329058	ΔORF2	110.58+peptide	OK	145.516	107.981	-0.4304	-0.344891	0.5088	0.947472	no
gene:SpnNT_00341	NA	Chromosome:328432-329058	110.58	ΔORF2+peptide	OK	86.8352	148.168	0.770885	0.749521	0.1988	0.681625	no
gene:SpnNT_00341	NA	Chromosome:328432-329058	ΔORF2	ΔORF2+peptide	OK	145.516	148.168	0.0260577	0.0264717	0.9636	0.994855	no
gene:SpnNT_00341	NA	Chromosome:328432-329058	110.58+peptide	ΔORF2+peptide	OK	107.981	148.168	0.456458	0.358729	0.5044	0.945292	no
gene:SpnNT_00342	sarA_3	Chromosome:329276-331256	110.58	ΔORF2	OK	908.844	953.869	0.0697579	0.147185	0.8003	0.994748	no
gene:SpnNT_00342	sarA_3	Chromosome:329276-331256	110.58	110.58+peptide	OK	908.844	775.598	-0.228723	-0.475348	0.40095	0.893669	no
gene:SpnNT_00342	sarA_3	Chromosome:329276-331256	ΔORF2	110.58+peptide	OK	953.869	775.598	-0.298481	-0.620295	0.27085	0.772772	no
gene:SpnNT_00342	sarA_3	Chromosome:329276-331256	110.58	ΔORF2+peptide	OK	908.844	940.924	0.0500459	0.105129	0.85285	0.994748	no
gene:SpnNT_00342	sarA_3	Chromosome:329276-331256	ΔORF2	ΔORF2+peptide	OK	953.869	940.924	-0.0197121	-0.0414065	0.9395	0.994855	no
gene:SpnNT_00342	sarA_3	Chromosome:329276-331256	110.58+peptide	ΔORF2+peptide	OK	775.598	940.924	0.278769	0.576859	0.29715	0.801975	no
gene:SpnNT_00343	NA	Chromosome:331306-332658	110.58	ΔORF2	OK	4.76675	6.224	0.384835	0.405574	0.47575	0.930746	no
gene:SpnNT_00343	NA	Chromosome:331306-332658	110.58	110.58+peptide	OK	4.76675	6.05295	0.344631	0.356629	0.53465	0.958867	no
gene:SpnNT_00343	NA	Chromosome:331306-332658	ΔORF2	110.58+peptide	OK	6.224	6.05295	-0.0402036	-0.0479336	0.93695	0.994855	no
gene:SpnNT_00343	NA	Chromosome:331306-332658	110.58	ΔORF2+peptide	OK	4.76675	6.12644	0.362044	0.375571	0.52705	0.956161	no
gene:SpnNT_00343	NA	Chromosome:331306-332658	ΔORF2	ΔORF2+peptide	OK	6.224	6.12644	-0.0227911	-0.0272621	0.96455	0.994855	no
gene:SpnNT_00343	NA	Chromosome:331306-332658	110.58+peptide	ΔORF2+peptide	OK	6.05295	6.12644	0.0174125	0.0203465	0.97425	0.99536	no
gene:SpnNT_00344	NA	Chromosome:331306-332658	110.58	ΔORF2	OK	11.0185	8.08359	-0.446864	-0.458789	0.43245	0.913334	no
gene:SpnNT_00344	NA	Chromosome:331306-332658	110.58	110.58+peptide	OK	11.0185	8.65292	-0.348673	-0.353847	0.54485	0.961568	no
gene:SpnNT_00344	NA	Chromosome:331306-332658	ΔORF2	110.58+peptide	OK	8.08359	8.65292	0.0981906	0.0978472	0.8684	0.994748	no
gene:SpnNT_00344	NA	Chromosome:331306-332658	110.58	ΔORF2+peptide	OK	11.0185	8.16142	-0.43304	-0.414967	0.4558	0.921244	no
gene:SpnNT_00344	NA	Chromosome:331306-332658	ΔORF2	ΔORF2+peptide	OK	8.08359	8.16142	0.0138233	0.0130323	0.9833	0.996246	no
gene:SpnNT_00344	NA	Chromosome:331306-332658	110.58+peptide	ΔORF2+peptide	OK	8.65292	8.16142	-0.0843673	-0.0787638	0.8885	0.994748	no
gene:SpnNT_00345	ponA	Chromosome:333070-335820	110.58	ΔORF2	OK	147.721	136.348	-0.115584	-0.236178	0.68165	0.986574	no
gene:SpnNT_00345	ponA	Chromosome:333070-335820	110.58	110.58+peptide	OK	147.721	173.152	0.229159	0.469594	0.41385	0.900099	no
gene:SpnNT_00345	ponA	Chromosome:333070-335820	ΔORF2	110.58+peptide	OK	136.348	173.152	0.344743	0.702918	0.21935	0.707723	no
gene:SpnNT_00345	ponA	Chromosome:333070-335820	110.58	ΔORF2+peptide	OK	147.721	163.178	0.14357	0.295482	0.6088	0.978849	no
gene:SpnNT_00345	ponA	Chromosome:333070-335820	ΔORF2	ΔORF2+peptide	OK	136.348	163.178	0.259154	0.530678	0.3554	0.85704	no

gene:SpnNT_00345	ponA	Chromosome:333070-335820	110.58+peptide	ΔORF2+peptide	OK	173.152	163.178	-0.0855891	-0.175768	0.7604	0.994748	no
gene:SpnNT_00346	recU	Chromosome:333070-335820	110.58	ΔORF2	OK	151.092	165.733	0.133435	0.12004	0.8425	0.994748	no
gene:SpnNT_00346	recU	Chromosome:333070-335820	110.58	110.58+peptide	OK	151.092	199.711	0.402485	0.36223	0.542	0.961494	no
gene:SpnNT_00346	recU	Chromosome:333070-335820	ΔORF2	110.58+peptide	OK	165.733	199.711	0.269049	0.250996	0.67185	0.984845	no
gene:SpnNT_00346	recU	Chromosome:333070-335820	110.58	ΔORF2+peptide	OK	151.092	173.081	0.196019	0.173457	0.7653	0.994748	no
gene:SpnNT_00346	recU	Chromosome:333070-335820	ΔORF2	ΔORF2+peptide	OK	165.733	173.081	0.062584	0.057335	0.9193	0.994748	no
gene:SpnNT_00346	recU	Chromosome:333070-335820	110.58+peptide	ΔORF2+peptide	OK	199.711	173.081	-0.206465	-0.18923	0.739	0.994748	no
gene:SpnNT_00347	NA	Chromosome:335886-336426	110.58	ΔORF2	OK	22.3081	20.5803	-0.11631	-0.191493	0.74015	0.994748	no
gene:SpnNT_00347	NA	Chromosome:335886-336426	110.58	110.58+peptide	OK	22.3081	15.8726	-0.491032	-0.796101	0.1662	0.621332	no
gene:SpnNT_00347	NA	Chromosome:335886-336426	ΔORF2	110.58+peptide	OK	20.5803	15.8726	-0.374722	-0.613176	0.28475	0.787901	no
gene:SpnNT_00347	NA	Chromosome:335886-336426	110.58	ΔORF2+peptide	OK	22.3081	15.6036	-0.515689	-0.811662	0.1583	0.60755	no
gene:SpnNT_00347	NA	Chromosome:335886-336426	ΔORF2	ΔORF2+peptide	OK	20.5803	15.6036	-0.39938	-0.634099	0.267	0.768422	no
gene:SpnNT_00347	NA	Chromosome:335886-336426	110.58+peptide	ΔORF2+peptide	OK	15.8726	15.6036	-0.0246575	-0.0385925	0.94745	0.994855	no
gene:SpnNT_00348	gpsB	Chromosome:336495-336825	110.58	ΔORF2	OK	423.71	489.115	0.207096	0.452947	0.42455	0.907534	no
gene:SpnNT_00348	gpsB	Chromosome:336495-336825	110.58	110.58+peptide	OK	423.71	342.564	-0.306707	-0.659744	0.2448	0.7415	no
gene:SpnNT_00348	gpsB	Chromosome:336495-336825	ΔORF2	110.58+peptide	OK	489.115	342.564	-0.513802	-1.11437	0.05335	0.327284	no
gene:SpnNT_00348	gpsB	Chromosome:336495-336825	110.58	ΔORF2+peptide	OK	423.71	371.558	-0.189491	-0.408971	0.47445	0.930243	no
gene:SpnNT_00348	gpsB	Chromosome:336495-336825	ΔORF2	ΔORF2+peptide	OK	489.115	371.558	-0.396586	-0.86307	0.1336	0.556521	no
gene:SpnNT_00348	gpsB	Chromosome:336495-336825	110.58+peptide	ΔORF2+peptide	OK	342.564	371.558	0.117216	0.250924	0.66585	0.98367	no
gene:SpnNT_00349	rlmL	Chromosome:337310-338468	110.58	ΔORF2	OK	131.813	133.951	0.0232134	0.0529748	0.9238	0.994748	no
gene:SpnNT_00349	rlmL	Chromosome:337310-338468	110.58	110.58+peptide	OK	131.813	127.333	-0.0498833	-0.113673	0.84215	0.994748	no
gene:SpnNT_00349	rlmL	Chromosome:337310-338468	ΔORF2	110.58+peptide	OK	133.951	127.333	-0.0730967	-0.167454	0.7718	0.994748	no
gene:SpnNT_00349	rlmL	Chromosome:337310-338468	110.58	ΔORF2+peptide	OK	131.813	147.678	0.163963	0.371555	0.51565	0.952472	no
gene:SpnNT_00349	rlmL	Chromosome:337310-338468	ΔORF2	ΔORF2+peptide	OK	133.951	147.678	0.14075	0.320624	0.57065	0.968621	no
gene:SpnNT_00349	rlmL	Chromosome:337310-338468	110.58+peptide	ΔORF2+peptide	OK	127.333	147.678	0.213847	0.486433	0.39435	0.889648	no
gene:SpnNT_00350	NA	Chromosome:338480-339851	110.58	ΔORF2	OK	226.147	218.961	-0.0465883	-0.107002	0.8472	0.994748	no
gene:SpnNT_00350	NA	Chromosome:338480-339851	110.58	110.58+peptide	OK	226.147	211.916	-0.0937697	-0.215557	0.6986	0.990209	no
gene:SpnNT_00350	NA	Chromosome:338480-339851	ΔORF2	110.58+peptide	OK	218.961	211.916	-0.0471814	-0.108321	0.8424	0.994748	no
gene:SpnNT_00350	NA	Chromosome:338480-339851	110.58	ΔORF2+peptide	OK	226.147	227.19	0.00663641	0.0152522	0.979	0.995765	no
gene:SpnNT_00350	NA	Chromosome:338480-339851	ΔORF2	ΔORF2+peptide	OK	218.961	227.19	0.0532247	0.122167	0.8246	0.994748	no
gene:SpnNT_00350	NA	Chromosome:338480-339851	110.58+peptide	ΔORF2+peptide	OK	211.916	227.19	0.100406	0.230667	0.67735	0.98524	no
gene:SpnNT_00351	gnd	Chromosome:339926-341351	110.58	ΔORF2	OK	675.616	674.588	-0.00219728	-0.00480219	0.99385	0.998618	no
gene:SpnNT_00351	gnd	Chromosome:339926-341351	110.58	110.58+peptide	OK	675.616	547.116	-0.304358	-0.673467	0.234	0.72774	no
gene:SpnNT_00351	gnd	Chromosome:339926-341351	ΔORF2	110.58+peptide	OK	674.588	547.116	-0.302161	-0.665875	0.247	0.743921	no
gene:SpnNT_00351	gnd	Chromosome:339926-341351	110.58	ΔORF2+peptide	OK	675.616	561.475	-0.266984	-0.590021	0.29345	0.797897	no
gene:SpnNT_00351	gnd	Chromosome:339926-341351	ΔORF2	ΔORF2+peptide	OK	674.588	561.475	-0.264786	-0.582781	0.30965	0.814895	no
gene:SpnNT_00351	gnd	Chromosome:339926-341351	110.58+peptide	ΔORF2+peptide	OK	547.116	561.475	0.0373747	0.0832994	0.8813	0.994748	no
gene:SpnNT_00352	arlR_1	Chromosome:341362-342049	110.58	ΔORF2	OK	1429.88	1531.69	0.0992308	0.219488	0.6947	0.988704	no
gene:SpnNT_00352	arlR_1	Chromosome:341362-342049	110.58	110.58+peptide	OK	1429.88	1342.75	-0.0907093	-0.197853	0.72335	0.994748	no
gene:SpnNT_00352	arlR_1	Chromosome:341362-342049	ΔORF2	110.58+peptide	OK	1531.69	1342.75	-0.18994	-0.416224	0.46375	0.926089	no
gene:SpnNT_00352	arlR_1	Chromosome:341362-342049	110.58	ΔORF2+peptide	OK	1429.88	1258.2	-0.184536	-0.405371	0.4714	0.928525	no
gene:SpnNT_00352	arlR_1	Chromosome:341362-342049	ΔORF2	ΔORF2+peptide	OK	1531.69	1258.2	-0.283767	-0.626299	0.26785	0.768869	no
gene:SpnNT_00352	arlR_1	Chromosome:341362-342049	110.58+peptide	ΔORF2+peptide	OK	1342.75	1258.2	-0.0938268	-0.204221	0.72255	0.994748	no
gene:SpnNT_00353	NA	Chromosome:342136-343393	110.58	ΔORF2	OK	12.1912	12.8569	0.0766963	0.138848	0.8027	0.994748	no
gene:SpnNT_00353	NA	Chromosome:342136-343393	110.58	110.58+peptide	OK	12.1912	10.0192	-0.283082	-0.502292	0.38315	0.880579	no

gene:SpnNT_00353	NA	Chromosome:342136-343393	ΔORF2	110.58+peptide	OK	12.8569	10.0192	-0.359778	-0.645095	0.2561	0.755178	no
gene:SpnNT_00353	NA	Chromosome:342136-343393	110.58	ΔORF2+peptide	OK	12.1912	12.5841	0.0457626	0.0820227	0.88265	0.994748	no
gene:SpnNT_00353	NA	Chromosome:342136-343393	ΔORF2	ΔORF2+peptide	OK	12.8569	12.5841	-0.0309338	-0.0560395	0.91955	0.994748	no
gene:SpnNT_00353	NA	Chromosome:342136-343393	110.58+peptide	ΔORF2+peptide	OK	10.0192	12.5841	0.328844	0.583875	0.3092	0.814396	no
gene:SpnNT_00354	lytA_2	Chromosome:343571-344534	110.58	ΔORF2	OK	200.878	273.679	0.446166	1.00708	0.07655	0.408994	no
gene:SpnNT_00354	lytA_2	Chromosome:343571-344534	110.58	110.58+peptide	OK	200.878	213.276	0.0864059	0.197517	0.72965	0.994748	no
gene:SpnNT_00354	lytA_2	Chromosome:343571-344534	ΔORF2	110.58+peptide	OK	273.679	213.276	-0.35976	-0.808107	0.16025	0.612347	no
gene:SpnNT_00354	lytA_2	Chromosome:343571-344534	110.58	ΔORF2+peptide	OK	200.878	295.776	0.558188	1.2859	0.02315	0.186594	no
gene:SpnNT_00354	lytA_2	Chromosome:343571-344534	ΔORF2	ΔORF2+peptide	OK	273.679	295.776	0.112022	0.253518	0.65745	0.982966	no
gene:SpnNT_00354	lytA_2	Chromosome:343571-344534	110.58+peptide	ΔORF2+peptide	OK	213.276	295.776	0.471782	1.08135	0.06185	0.359272	no
gene:SpnNT_00355	lytA_3	Chromosome:344552-345551	110.58	ΔORF2	OK	328.823	359.549	0.128877	0.295887	0.59655	0.97629	no
gene:SpnNT_00355	lytA_3	Chromosome:344552-345551	110.58	110.58+peptide	OK	328.823	310.142	-0.0843826	-0.193832	0.7251	0.994748	no
gene:SpnNT_00355	lytA_3	Chromosome:344552-345551	ΔORF2	110.58+peptide	OK	359.549	310.142	-0.21326	-0.490404	0.3804	0.878145	no
gene:SpnNT_00355	lytA_3	Chromosome:344552-345551	110.58	ΔORF2+peptide	OK	328.823	346.951	0.0774174	0.177771	0.74825	0.994748	no
gene:SpnNT_00355	lytA_3	Chromosome:344552-345551	ΔORF2	ΔORF2+peptide	OK	359.549	346.951	-0.0514596	-0.118294	0.8328	0.994748	no
gene:SpnNT_00355	lytA_3	Chromosome:344552-345551	110.58+peptide	ΔORF2+peptide	OK	310.142	346.951	0.1618	0.372131	0.5036	0.945208	no
gene:SpnNT_00356	NA	Chromosome:345668-346901	110.58	ΔORF2	OK	46.5697	43.9504	-0.0835156	-0.177584	0.7571	0.994748	no
gene:SpnNT_00356	NA	Chromosome:345668-346901	110.58	110.58+peptide	OK	46.5697	43.6625	-0.0929951	-0.197381	0.7284	0.994748	no
gene:SpnNT_00356	NA	Chromosome:345668-346901	ΔORF2	110.58+peptide	OK	43.9504	43.6625	-0.00947952	-0.0199914	0.97125	0.99536	no
gene:SpnNT_00356	NA	Chromosome:345668-346901	110.58	ΔORF2+peptide	OK	46.5697	47.0855	0.0158922	0.0338659	0.95245	0.994855	no
gene:SpnNT_00356	NA	Chromosome:345668-346901	ΔORF2	ΔORF2+peptide	OK	43.9504	47.0855	0.0994078	0.210469	0.7065	0.990422	no
gene:SpnNT_00356	NA	Chromosome:345668-346901	110.58+peptide	ΔORF2+peptide	OK	43.6625	47.0855	0.108887	0.230124	0.6835	0.98747	no
gene:SpnNT_00357	NA	Chromosome:346943-347225	110.58	ΔORF2	OK	49.5008	57.0088	0.203735	0.322514	0.57625	0.969538	no
gene:SpnNT_00357	NA	Chromosome:346943-347225	110.58	110.58+peptide	OK	49.5008	47.9918	-0.0446635	-0.0699914	0.90175	0.994748	no
gene:SpnNT_00357	NA	Chromosome:346943-347225	ΔORF2	110.58+peptide	OK	57.0088	47.9918	-0.248398	-0.391444	0.4997	0.944017	no
gene:SpnNT_00357	NA	Chromosome:346943-347225	110.58	ΔORF2+peptide	OK	49.5008	51.8016	0.065547	0.103694	0.85695	0.994748	no
gene:SpnNT_00357	NA	Chromosome:346943-347225	ΔORF2	ΔORF2+peptide	OK	57.0088	51.8016	-0.138188	-0.21986	0.7098	0.990851	no
gene:SpnNT_00357	NA	Chromosome:346943-347225	110.58+peptide	ΔORF2+peptide	OK	47.9918	51.8016	0.110211	0.173566	0.763	0.994748	no
gene:SpnNT_00358	NA	Chromosome:347270-347531	110.58	ΔORF2	OK	9.89305	14.3017	0.531704	0.579577	0.3149	0.821168	no
gene:SpnNT_00358	NA	Chromosome:347270-347531	110.58	110.58+peptide	OK	9.89305	13.5419	0.452942	0.485999	0.3876	0.881929	no
gene:SpnNT_00358	NA	Chromosome:347270-347531	ΔORF2	110.58+peptide	OK	14.3017	13.5419	-0.0787621	-0.0871924	0.87135	0.994748	no
gene:SpnNT_00358	NA	Chromosome:347270-347531	110.58	ΔORF2+peptide	OK	9.89305	10.9724	0.149393	0.152181	0.78775	0.994748	no
gene:SpnNT_00358	NA	Chromosome:347270-347531	ΔORF2	ΔORF2+peptide	OK	14.3017	10.9724	-0.382311	-0.400532	0.4734	0.929238	no
gene:SpnNT_00358	NA	Chromosome:347270-347531	110.58+peptide	ΔORF2+peptide	OK	13.5419	10.9724	-0.303549	-0.313412	0.5666	0.968621	no
gene:SpnNT_00359	NA	Chromosome:348430-348760	110.58	ΔORF2	OK	172.341	177.244	0.0404689	0.0780409	0.89015	0.994748	no
gene:SpnNT_00359	NA	Chromosome:348430-348760	110.58	110.58+peptide	OK	172.341	149.666	-0.203515	-0.388776	0.4946	0.942197	no
gene:SpnNT_00359	NA	Chromosome:348430-348760	ΔORF2	110.58+peptide	OK	177.244	149.666	-0.243983	-0.469244	0.41035	0.898184	no
gene:SpnNT_00359	NA	Chromosome:348430-348760	110.58	ΔORF2+peptide	OK	172.341	160.79	-0.100085	-0.192105	0.73655	0.994748	no
gene:SpnNT_00359	NA	Chromosome:348430-348760	ΔORF2	ΔORF2+peptide	OK	177.244	160.79	-0.140554	-0.271628	0.63815	0.980887	no
gene:SpnNT_00359	NA	Chromosome:348430-348760	110.58+peptide	ΔORF2+peptide	OK	149.666	160.79	0.10343	0.198	0.72415	0.994748	no
gene:SpnNT_00360	NA	Chromosome:348881-352683	110.58	ΔORF2	OK	23.0276	24.8943	0.112451	0.0965225	0.86455	0.994748	no
gene:SpnNT_00360	NA	Chromosome:348881-352683	110.58	110.58+peptide	OK	23.0276	33.1963	0.527659	0.441626	0.449	0.919933	no
gene:SpnNT_00360	NA	Chromosome:348881-352683	ΔORF2	110.58+peptide	OK	24.8943	33.1963	0.415208	0.363471	0.5368	0.959314	no
gene:SpnNT_00360	NA	Chromosome:348881-352683	110.58	ΔORF2+peptide	OK	23.0276	35.3013	0.616355	0.489578	0.3994	0.89264	no
gene:SpnNT_00360	NA	Chromosome:348881-352683	ΔORF2	ΔORF2+peptide	OK	24.8943	35.3013	0.503903	0.416701	0.4762	0.930974	no

gene:SpnNT_00360	NA	Chromosome:348881-352683	110.58+peptide	ΔORF2+peptide	OK	33.1963	35.3013	0.0886956	0.071645	0.90185	0.994748	no
gene:SpnNT_00361	NA	Chromosome:348881-352683	110.58	ΔORF2	OK	34.1768	31.7017	-0.108457	-0.117084	0.83875	0.994748	no
gene:SpnNT_00361	NA	Chromosome:348881-352683	110.58	110.58+peptide	OK	34.1768	47.5058	0.475087	0.499748	0.3922	0.887166	no
gene:SpnNT_00361	NA	Chromosome:348881-352683	ΔORF2	110.58+peptide	OK	31.7017	47.5058	0.583544	0.612752	0.2801	0.783516	no
gene:SpnNT_00361	NA	Chromosome:348881-352683	110.58	ΔORF2+peptide	OK	34.1768	50.7282	0.569771	0.599193	0.30505	0.811188	no
gene:SpnNT_00361	NA	Chromosome:348881-352683	ΔORF2	ΔORF2+peptide	OK	31.7017	50.7282	0.678229	0.711994	0.21295	0.698345	no
gene:SpnNT_00361	NA	Chromosome:348881-352683	110.58+peptide	ΔORF2+peptide	OK	47.5058	50.7282	0.0946843	0.0969874	0.8672	0.994748	no
gene:SpnNT_00362	NA	Chromosome:348881-352683	110.58	ΔORF2	OK	36.2951	35.4006	-0.0360047	-0.0410621	0.9406	0.994855	no
gene:SpnNT_00362	NA	Chromosome:348881-352683	110.58	110.58+peptide	OK	36.2951	46.8266	0.367553	0.40274	0.48345	0.936747	no
gene:SpnNT_00362	NA	Chromosome:348881-352683	ΔORF2	110.58+peptide	OK	35.4006	46.8266	0.403557	0.441872	0.4418	0.917253	no
gene:SpnNT_00362	NA	Chromosome:348881-352683	110.58	ΔORF2+peptide	OK	36.2951	57.3289	0.659485	0.752985	0.19925	0.681762	no
gene:SpnNT_00362	NA	Chromosome:348881-352683	ΔORF2	ΔORF2+peptide	OK	35.4006	57.3289	0.69549	0.793471	0.17205	0.632215	no
gene:SpnNT_00362	NA	Chromosome:348881-352683	110.58+peptide	ΔORF2+peptide	OK	46.8266	57.3289	0.291933	0.319989	0.58155	0.970261	no
gene:SpnNT_00363	fni	Chromosome:348881-352683	110.58	ΔORF2	OK	51.5133	45.5276	-0.178204	-0.240873	0.67065	0.984845	no
gene:SpnNT_00363	fni	Chromosome:348881-352683	110.58	110.58+peptide	OK	51.5133	78.4844	0.60746	0.831693	0.1535	0.597162	no
gene:SpnNT_00363	fni	Chromosome:348881-352683	ΔORF2	110.58+peptide	OK	45.5276	78.4844	0.785664	1.06584	0.06545	0.372112	no
gene:SpnNT_00363	fni	Chromosome:348881-352683	110.58	ΔORF2+peptide	OK	51.5133	92.5112	0.844683	1.19789	0.0383	0.263403	no
gene:SpnNT_00363	fni	Chromosome:348881-352683	ΔORF2	ΔORF2+peptide	OK	45.5276	92.5112	1.02289	1.43639	0.0114	0.110074	no
gene:SpnNT_00363	fni	Chromosome:348881-352683	110.58+peptide	ΔORF2+peptide	OK	78.4844	92.5112	0.237223	0.337774	0.55425	0.966366	no
gene:SpnNT_00364	NA	Chromosome:352760-354451	110.58	ΔORF2	OK	65.1998	71.6217	0.135529	0.206723	0.7166	0.992452	no
gene:SpnNT_00364	NA	Chromosome:352760-354451	110.58	110.58+peptide	OK	65.1998	86.7594	0.412153	0.633921	0.27055	0.772705	no
gene:SpnNT_00364	NA	Chromosome:352760-354451	ΔORF2	110.58+peptide	OK	71.6217	86.7594	0.276625	0.429932	0.4564	0.921244	no
gene:SpnNT_00364	NA	Chromosome:352760-354451	110.58	ΔORF2+peptide	OK	65.1998	104.52	0.68084	1.05404	0.0687	0.383936	no
gene:SpnNT_00364	NA	Chromosome:352760-354451	ΔORF2	ΔORF2+peptide	OK	71.6217	104.52	0.545311	0.853195	0.1391	0.569233	no
gene:SpnNT_00364	NA	Chromosome:352760-354451	110.58+peptide	ΔORF2+peptide	OK	86.7594	104.52	0.268687	0.424091	0.46305	0.925818	no
gene:SpnNT_00365	liaS	Chromosome:352760-354451	110.58	ΔORF2	OK	46.9365	43.9783	-0.0939194	-0.146923	0.79785	0.994748	no
gene:SpnNT_00365	liaS	Chromosome:352760-354451	110.58	110.58+peptide	OK	46.9365	54.0923	0.204712	0.325342	0.57235	0.969081	no
gene:SpnNT_00365	liaS	Chromosome:352760-354451	ΔORF2	110.58+peptide	OK	43.9783	54.0923	0.298632	0.459134	0.4217	0.905358	no
gene:SpnNT_00365	liaS	Chromosome:352760-354451	110.58	ΔORF2+peptide	OK	46.9365	55.8838	0.251721	0.395832	0.48725	0.938799	no
gene:SpnNT_00365	liaS	Chromosome:352760-354451	ΔORF2	ΔORF2+peptide	OK	43.9783	55.8838	0.34564	0.526157	0.3508	0.854885	no
gene:SpnNT_00365	liaS	Chromosome:352760-354451	110.58+peptide	ΔORF2+peptide	OK	54.0923	55.8838	0.0470082	0.0726366	0.89665	0.994748	no
gene:SpnNT_00366	liaR	Chromosome:354464-355097	110.58	ΔORF2	OK	53.1768	46.4684	-0.194547	-0.37183	0.5181	0.953876	no
gene:SpnNT_00366	liaR	Chromosome:354464-355097	110.58	110.58+peptide	OK	53.1768	62.1733	0.225497	0.443972	0.43485	0.913666	no
gene:SpnNT_00366	liaR	Chromosome:354464-355097	ΔORF2	110.58+peptide	OK	46.4684	62.1733	0.420044	0.807788	0.15675	0.603543	no
gene:SpnNT_00366	liaR	Chromosome:354464-355097	110.58	ΔORF2+peptide	OK	53.1768	52.591	-0.015981	-0.0309511	0.95685	0.994855	no
gene:SpnNT_00366	liaR	Chromosome:354464-355097	ΔORF2	ΔORF2+peptide	OK	46.4684	52.591	0.178566	0.338051	0.5515	0.96439	no
gene:SpnNT_00366	liaR	Chromosome:354464-355097	110.58+peptide	ΔORF2+peptide	OK	62.1733	52.591	-0.241478	-0.470659	0.39925	0.892609	no
gene:SpnNT_00367	NA	Chromosome:356016-356930	110.58	ΔORF2	OK	36.3573	41.3677	0.186261	0.0987264	0.86045	0.994748	no
gene:SpnNT_00367	NA	Chromosome:356016-356930	110.58	110.58+peptide	OK	36.3573	43.0533	0.243879	0.126603	0.825	0.994748	no
gene:SpnNT_00367	NA	Chromosome:356016-356930	ΔORF2	110.58+peptide	OK	41.3677	43.0533	0.0576186	0.0314737	0.95805	0.994855	no
gene:SpnNT_00367	NA	Chromosome:356016-356930	110.58	ΔORF2+peptide	OK	36.3573	55.8349	0.618921	0.322139	0.6301	0.980887	no
gene:SpnNT_00367	NA	Chromosome:356016-356930	ΔORF2	ΔORF2+peptide	OK	41.3677	55.8349	0.432661	0.237025	0.72275	0.994748	no
gene:SpnNT_00367	NA	Chromosome:356016-356930	110.58+peptide	ΔORF2+peptide	OK	43.0533	55.8349	0.375042	0.200946	0.76395	0.994748	no
gene:SpnNT_00368	blaSE	Chromosome:356016-356930	110.58	ΔORF2	OK	47.1967	54.8445	0.216659	0.405724	0.47455	0.930243	no
gene:SpnNT_00368	blaSE	Chromosome:356016-356930	110.58	110.58+peptide	OK	47.1967	54.5739	0.209524	0.392028	0.4994	0.944017	no

gene:SpnNT_00368	blaSE	Chromosome:356016-356930	ΔORF2	110.58+peptide	OK	54.8445	54.5739	-0.00713553	-0.0133246	0.9823	0.996246	no
gene:SpnNT_00368	blaSE	Chromosome:356016-356930	110.58	ΔORF2+peptide	OK	47.1967	61.2686	0.376462	0.711685	0.22335	0.71309	no
gene:SpnNT_00368	blaSE	Chromosome:356016-356930	ΔORF2	ΔORF2+peptide	OK	54.8445	61.2686	0.159802	0.301493	0.60125	0.976678	no
gene:SpnNT_00368	blaSE	Chromosome:356016-356930	110.58+peptide	ΔORF2+peptide	OK	54.5739	61.2686	0.166938	0.314683	0.59045	0.974909	no
gene:SpnNT_00369	NA	Chromosome:357222-357621	110.58	ΔORF2	NOTEST	0.139291	0.152987	0.13531	0	1	1	no
gene:SpnNT_00369	NA	Chromosome:357222-357621	110.58	110.58+peptide	NOTEST	0.139291	0	#NAME?	0	1	1	no
gene:SpnNT_00369	NA	Chromosome:357222-357621	ΔORF2	110.58+peptide	NOTEST	0.152987	0	#NAME?	0	1	1	no
gene:SpnNT_00369	NA	Chromosome:357222-357621	110.58	ΔORF2+peptide	NOTEST	0.139291	0.378145	1.44084	0	1	1	no
gene:SpnNT_00369	NA	Chromosome:357222-357621	ΔORF2	ΔORF2+peptide	NOTEST	0.152987	0.378145	1.30553	0	1	1	no
gene:SpnNT_00369	NA	Chromosome:357222-357621	110.58+peptide	ΔORF2+peptide	NOTEST	0	0.378145	Inf	0	1	1	no
gene:SpnNT_00370	tig	Chromosome:357781-359065	110.58	ΔORF2	OK	518.123	638.916	0.302331	0.667523	0.2414	0.736283	no
gene:SpnNT_00370	tig	Chromosome:357781-359065	110.58	110.58+peptide	OK	518.123	750.798	0.535129	1.18278	0.038	0.261751	no
gene:SpnNT_00370	tig	Chromosome:357781-359065	ΔORF2	110.58+peptide	OK	638.916	750.798	0.232798	0.512672	0.3614	0.862354	no
gene:SpnNT_00370	tig	Chromosome:357781-359065	110.58	ΔORF2+peptide	OK	518.123	845.868	0.707137	1.56372	0.0062	0.0706832	no
gene:SpnNT_00370	tig	Chromosome:357781-359065	ΔORF2	ΔORF2+peptide	OK	638.916	845.868	0.404806	0.8919	0.1128	0.510701	no
gene:SpnNT_00370	tig	Chromosome:357781-359065	110.58+peptide	ΔORF2+peptide	OK	750.798	845.868	0.172008	0.379383	0.50285	0.944788	no
gene:SpnNT_00371	recD	Chromosome:359112-361479	110.58	ΔORF2	OK	41.6853	36.1559	-0.205307	-0.451994	0.4161	0.901298	no
gene:SpnNT_00371	recD	Chromosome:359112-361479	110.58	110.58+peptide	OK	41.6853	37.0975	-0.168218	-0.368702	0.51465	0.951747	no
gene:SpnNT_00371	recD	Chromosome:359112-361479	ΔORF2	110.58+peptide	OK	36.1559	37.0975	0.0370891	0.082106	0.88835	0.994748	no
gene:SpnNT_00371	recD	Chromosome:359112-361479	110.58	ΔORF2+peptide	OK	41.6853	30.7914	-0.437009	-0.956827	0.09155	0.45539	no
gene:SpnNT_00371	recD	Chromosome:359112-361479	ΔORF2	ΔORF2+peptide	OK	36.1559	30.7914	-0.231702	-0.512376	0.3682	0.865399	no
gene:SpnNT_00371	recD	Chromosome:359112-361479	110.58+peptide	ΔORF2+peptide	OK	37.0975	30.7914	-0.268791	-0.591741	0.3086	0.814396	no
gene:SpnNT_00372	spsB	Chromosome:361606-362221	110.58	ΔORF2	OK	205.258	181.849	-0.174701	-0.387431	0.4988	0.944017	no
gene:SpnNT_00372	spsB	Chromosome:361606-362221	110.58	110.58+peptide	OK	205.258	222.57	0.116817	0.262169	0.64295	0.980887	no
gene:SpnNT_00372	spsB	Chromosome:361606-362221	ΔORF2	110.58+peptide	OK	181.849	222.57	0.291519	0.644538	0.2615	0.761688	no
gene:SpnNT_00372	spsB	Chromosome:361606-362221	110.58	ΔORF2+peptide	OK	205.258	194.891	-0.0747699	-0.166258	0.77235	0.994748	no
gene:SpnNT_00372	spsB	Chromosome:361606-362221	ΔORF2	ΔORF2+peptide	OK	181.849	194.891	0.0999314	0.218969	0.7041	0.990367	no
gene:SpnNT_00372	spsB	Chromosome:361606-362221	110.58+peptide	ΔORF2+peptide	OK	222.57	194.891	-0.191587	-0.424716	0.4613	0.924573	no
gene:SpnNT_00373	rnhC	Chromosome:362232-363105	110.58	ΔORF2	OK	114.777	124.101	0.11269	0.249505	0.658	0.982966	no
gene:SpnNT_00373	rnhC	Chromosome:362232-363105	110.58	110.58+peptide	OK	114.777	121.57	0.0829647	0.181657	0.74735	0.994748	no
gene:SpnNT_00373	rnhC	Chromosome:362232-363105	ΔORF2	110.58+peptide	OK	124.101	121.57	-0.0297249	-0.0658951	0.9061	0.994748	no
gene:SpnNT_00373	rnhC	Chromosome:362232-363105	110.58	ΔORF2+peptide	OK	114.777	142.956	0.316739	0.701757	0.21665	0.704171	no
gene:SpnNT_00373	rnhC	Chromosome:362232-363105	ΔORF2	ΔORF2+peptide	OK	124.101	142.956	0.20405	0.457853	0.4235	0.9067	no
gene:SpnNT_00373	rnhC	Chromosome:362232-363105	110.58+peptide	ΔORF2+peptide	OK	121.57	142.956	0.233775	0.518587	0.36	0.860974	no
gene:SpnNT_00374	NA	Chromosome:363191-364039	110.58	ΔORF2	OK	598.544	597.314	-0.00296712	-0.00449944	0.9934	0.998522	no
gene:SpnNT_00374	NA	Chromosome:363191-364039	110.58	110.58+peptide	OK	598.544	758.71	0.342092	0.499382	0.3853	0.881745	no
gene:SpnNT_00374	NA	Chromosome:363191-364039	ΔORF2	110.58+peptide	OK	597.314	758.71	0.345059	0.508537	0.3806	0.878145	no
gene:SpnNT_00374	NA	Chromosome:363191-364039	110.58	ΔORF2+peptide	OK	598.544	785.56	0.392264	0.545117	0.34295	0.846855	no
gene:SpnNT_00374	NA	Chromosome:363191-364039	ΔORF2	ΔORF2+peptide	OK	597.314	785.56	0.395231	0.554001	0.3409	0.844955	no
gene:SpnNT_00374	NA	Chromosome:363191-364039	110.58+peptide	ΔORF2+peptide	OK	758.71	785.56	0.050172	0.0680639	0.9021	0.994748	no
gene:SpnNT_00375	NA	Chromosome:363191-364039	110.58	ΔORF2	OK	205.002	205	-1.32E-05	-2.07E-05	0.99955	0.999752	no
gene:SpnNT_00375	NA	Chromosome:363191-364039	110.58	110.58+peptide	OK	205.002	273.414	0.41545	0.648199	0.2599	0.760041	no
gene:SpnNT_00375	NA	Chromosome:363191-364039	ΔORF2	110.58+peptide	OK	205	273.414	0.415463	0.649573	0.2564	0.755871	no
gene:SpnNT_00375	NA	Chromosome:363191-364039	110.58	ΔORF2+peptide	OK	205.002	300.361	0.551058	0.869412	0.1343	0.557156	no
gene:SpnNT_00375	NA	Chromosome:363191-364039	ΔORF2	ΔORF2+peptide	OK	205	300.361	0.551072	0.871288	0.1315	0.551468	no

gene:SpnNT_00375	NA	Chromosome:363191-364039	110.58+peptide	ΔORF2+peptide	OK	273.414	300.361	0.135609	0.213037	0.71175	0.991007	no
gene:SpnNT_00376	mutS2	Chromosome:364142-366479	110.58	ΔORF2	OK	78.6876	76.7689	-0.0356154	-0.081884	0.8808	0.994748	no
gene:SpnNT_00376	mutS2	Chromosome:364142-366479	110.58	110.58+peptide	OK	78.6876	80.914	0.0402521	0.0928913	0.8683	0.994748	no
gene:SpnNT_00376	mutS2	Chromosome:364142-366479	ΔORF2	110.58+peptide	OK	76.7689	80.914	0.0758676	0.174287	0.7538	0.994748	no
gene:SpnNT_00376	mutS2	Chromosome:364142-366479	110.58	ΔORF2+peptide	OK	78.6876	80.4021	0.0310957	0.0717559	0.89635	0.994748	no
gene:SpnNT_00376	mutS2	Chromosome:364142-366479	ΔORF2	ΔORF2+peptide	OK	76.7689	80.4021	0.0667111	0.153242	0.7848	0.994748	no
gene:SpnNT_00376	mutS2	Chromosome:364142-366479	110.58+peptide	ΔORF2+peptide	OK	80.914	80.4021	-0.00915643	-0.021112	0.96915	0.994929	no
gene:SpnNT_00377	alsT	Chromosome:366821-368144	110.58	ΔORF2	OK	26.1388	23.1184	-0.177149	-0.347785	0.5281	0.956161	no
gene:SpnNT_00377	alsT	Chromosome:366821-368144	110.58	110.58+peptide	OK	26.1388	69.5722	1.41232	2.85478	5.00E-05	0.0013612	yes
gene:SpnNT_00377	alsT	Chromosome:366821-368144	ΔORF2	110.58+peptide	OK	23.1184	69.5722	1.58947	3.23981	5.00E-05	0.0013612	yes
gene:SpnNT_00377	alsT	Chromosome:366821-368144	110.58	ΔORF2+peptide	OK	26.1388	80.6922	1.62624	3.34357	5.00E-05	0.0013612	yes
gene:SpnNT_00377	alsT	Chromosome:366821-368144	ΔORF2	ΔORF2+peptide	OK	23.1184	80.6922	1.80339	3.73998	5.00E-05	0.0013612	yes
gene:SpnNT_00377	alsT	Chromosome:366821-368144	110.58+peptide	ΔORF2+peptide	OK	69.5722	80.6922	0.213918	0.458366	0.41455	0.900591	no
gene:SpnNT_00378	NA	Chromosome:368361-368910	110.58	ΔORF2	OK	22.8271	19.137	-0.254388	-0.420883	0.46925	0.927614	no
gene:SpnNT_00378	NA	Chromosome:368361-368910	110.58	110.58+peptide	OK	22.8271	15.2441	-0.582503	-0.955339	0.09925	0.477579	no
gene:SpnNT_00378	NA	Chromosome:368361-368910	ΔORF2	110.58+peptide	OK	19.137	15.2441	-0.328114	-0.52371	0.3616	0.862361	no
gene:SpnNT_00378	NA	Chromosome:368361-368910	110.58	ΔORF2+peptide	OK	22.8271	11.6785	-0.966892	-1.51016	0.00835	0.0885762	no
gene:SpnNT_00378	NA	Chromosome:368361-368910	ΔORF2	ΔORF2+peptide	OK	19.137	11.6785	-0.712504	-1.0857	0.05135	0.31995	no
gene:SpnNT_00378	NA	Chromosome:368361-368910	110.58+peptide	ΔORF2+peptide	OK	15.2441	11.6785	-0.384389	-0.581383	0.299	0.80426	no
gene:SpnNT_00379	NA	Chromosome:369019-369919	110.58	ΔORF2	OK	62.8958	68.7589	0.128583	0.268138	0.64305	0.980887	no
gene:SpnNT_00379	NA	Chromosome:369019-369919	110.58	110.58+peptide	OK	62.8958	59.5248	-0.0794738	-0.165158	0.7712	0.994748	no
gene:SpnNT_00379	NA	Chromosome:369019-369919	ΔORF2	110.58+peptide	OK	68.7589	59.5248	-0.208056	-0.434055	0.4495	0.920321	no
gene:SpnNT_00379	NA	Chromosome:369019-369919	110.58	ΔORF2+peptide	OK	62.8958	43.5629	-0.529865	-1.08515	0.064	0.367209	no
gene:SpnNT_00379	NA	Chromosome:369019-369919	ΔORF2	ΔORF2+peptide	OK	68.7589	43.5629	-0.658447	-1.35359	0.02185	0.179309	no
gene:SpnNT_00379	NA	Chromosome:369019-369919	110.58+peptide	ΔORF2+peptide	OK	59.5248	43.5629	-0.450391	-0.922774	0.1107	0.507195	no
gene:SpnNT_00380	serS	Chromosome:369943-371218	110.58	ΔORF2	OK	80.4944	86.5908	0.105326	0.236334	0.67795	0.98524	no
gene:SpnNT_00380	serS	Chromosome:369943-371218	110.58	110.58+peptide	OK	80.4944	86.4269	0.102591	0.230596	0.6887	0.98828	no
gene:SpnNT_00380	serS	Chromosome:369943-371218	ΔORF2	110.58+peptide	OK	86.5908	86.4269	-0.00273457	-0.00615574	0.9901	0.997703	no
gene:SpnNT_00380	serS	Chromosome:369943-371218	110.58	ΔORF2+peptide	OK	80.4944	94.948	0.238248	0.53661	0.352	0.854885	no
gene:SpnNT_00380	serS	Chromosome:369943-371218	ΔORF2	ΔORF2+peptide	OK	86.5908	94.948	0.132923	0.299832	0.6	0.976542	no
gene:SpnNT_00380	serS	Chromosome:369943-371218	110.58+peptide	ΔORF2+peptide	OK	86.4269	94.948	0.135657	0.306536	0.59755	0.97629	no
gene:SpnNT_00381	NA	Chromosome:371431-371803	110.58	ΔORF2	OK	98.5808	101.113	0.036588	0.0686095	0.9018	0.994748	no
gene:SpnNT_00381	NA	Chromosome:371431-371803	110.58	110.58+peptide	OK	98.5808	79.8524	-0.30397	-0.567474	0.3252	0.830337	no
gene:SpnNT_00381	NA	Chromosome:371431-371803	ΔORF2	110.58+peptide	OK	101.113	79.8524	-0.340558	-0.636107	0.27025	0.772705	no
gene:SpnNT_00381	NA	Chromosome:371431-371803	110.58	ΔORF2+peptide	OK	98.5808	100.829	0.0325284	0.0611583	0.914	0.994748	no
gene:SpnNT_00381	NA	Chromosome:371431-371803	ΔORF2	ΔORF2+peptide	OK	101.113	100.829	-0.00405958	-0.00763661	0.98935	0.997703	no
gene:SpnNT_00381	NA	Chromosome:371431-371803	110.58+peptide	ΔORF2+peptide	OK	79.8524	100.829	0.336498	0.630173	0.27015	0.772648	no
gene:SpnNT_00382	yclM	Chromosome:371805-373170	110.58	ΔORF2	OK	97.3557	110.959	0.188692	0.432474	0.44065	0.916647	no
gene:SpnNT_00382	yclM	Chromosome:371805-373170	110.58	110.58+peptide	OK	97.3557	83.6761	-0.218449	-0.497336	0.3818	0.879865	no
gene:SpnNT_00382	yclM	Chromosome:371805-373170	ΔORF2	110.58+peptide	OK	110.959	83.6761	-0.407141	-0.928833	0.0999	0.479559	no
gene:SpnNT_00382	yclM	Chromosome:371805-373170	110.58	ΔORF2+peptide	OK	97.3557	96.0727	-0.0191385	-0.0435516	0.94095	0.994855	no
gene:SpnNT_00382	yclM	Chromosome:371805-373170	ΔORF2	ΔORF2+peptide	OK	110.959	96.0727	-0.207831	-0.473912	0.4083	0.897087	no
gene:SpnNT_00382	yclM	Chromosome:371805-373170	110.58+peptide	ΔORF2+peptide	OK	83.6761	96.0727	0.199311	0.451483	0.43835	0.915991	no
gene:SpnNT_00383	NA	Chromosome:373456-375190	110.58	ΔORF2	OK	27.7317	31.5133	0.184426	0.387997	0.49385	0.941904	no
gene:SpnNT_00383	NA	Chromosome:373456-375190	110.58	110.58+peptide	OK	27.7317	27.863	0.00681544	0.0141699	0.97965	0.995814	no

gene:SpnNT_00383	NA	Chromosome:373456-375190	ΔORF2	110.58+peptide	OK	31.5133	27.863	-0.17761	-0.372835	0.5069	0.946031	no
gene:SpnNT_00383	NA	Chromosome:373456-375190	110.58	ΔORF2+peptide	OK	27.7317	34.4127	0.311406	0.657285	0.25225	0.750402	no
gene:SpnNT_00383	NA	Chromosome:373456-375190	ΔORF2	ΔORF2+peptide	OK	31.5133	34.4127	0.12698	0.270688	0.63215	0.980887	no
gene:SpnNT_00383	NA	Chromosome:373456-375190	110.58+peptide	ΔORF2+peptide	OK	27.863	34.4127	0.30459	0.641472	0.26265	0.76315	no
gene:SpnNT_00384	NA	Chromosome:375570-398615	110.58	ΔORF2	OK	354.321	182.357	-0.958296	-1.54286	0.00845	0.089348	no
gene:SpnNT_00384	NA	Chromosome:375570-398615	110.58	110.58+peptide	OK	354.321	428.595	0.274557	0.420896	0.46045	0.924377	no
gene:SpnNT_00384	NA	Chromosome:375570-398615	ΔORF2	110.58+peptide	OK	182.357	428.595	1.23285	1.92598	0.001	0.0168447	yes
gene:SpnNT_00384	NA	Chromosome:375570-398615	110.58	ΔORF2+peptide	OK	354.321	219.26	-0.692418	-1.08665	0.06105	0.355727	no
gene:SpnNT_00384	NA	Chromosome:375570-398615	ΔORF2	ΔORF2+peptide	OK	182.357	219.26	0.265878	0.425603	0.4617	0.924951	no
gene:SpnNT_00384	NA	Chromosome:375570-398615	110.58+peptide	ΔORF2+peptide	OK	428.595	219.26	-0.966975	-1.47464	0.00965	0.0978573	no
gene:SpnNT_00385	NA	Chromosome:375570-398615	110.58	ΔORF2	OK	297.578	138.641	-1.10191	-0.817101	0.1663	0.621352	no
gene:SpnNT_00385	NA	Chromosome:375570-398615	110.58	110.58+peptide	OK	297.578	368.14	0.306989	0.225691	0.69095	0.98828	no
gene:SpnNT_00385	NA	Chromosome:375570-398615	ΔORF2	110.58+peptide	OK	138.641	368.14	1.4089	1.05304	0.07075	0.390464	no
gene:SpnNT_00385	NA	Chromosome:375570-398615	110.58	ΔORF2+peptide	OK	297.578	162.47	-0.873094	-0.643719	0.26075	0.761099	no
gene:SpnNT_00385	NA	Chromosome:375570-398615	ΔORF2	ΔORF2+peptide	OK	138.641	162.47	0.228821	0.171531	0.76585	0.994748	no
gene:SpnNT_00385	NA	Chromosome:375570-398615	110.58+peptide	ΔORF2+peptide	OK	368.14	162.47	-1.18008	-0.876885	0.11805	0.52069	no
gene:SpnNT_00386	NA	Chromosome:398807-400089	110.58	ΔORF2	OK	2.17976	2.22367	0.02877	0.01651	0.97465	0.99536	no
gene:SpnNT_00386	NA	Chromosome:398807-400089	110.58	110.58+peptide	OK	2.17976	4.0269	0.885501	0.549576	0.32185	0.827743	no
gene:SpnNT_00386	NA	Chromosome:398807-400089	ΔORF2	110.58+peptide	OK	2.22367	4.0269	0.856731	0.581665	0.30965	0.814895	no
gene:SpnNT_00386	NA	Chromosome:398807-400089	110.58	ΔORF2+peptide	OK	2.17976	6.47918	1.57164	1.01226	0.0862	0.440466	no
gene:SpnNT_00386	NA	Chromosome:398807-400089	ΔORF2	ΔORF2+peptide	OK	2.22367	6.47918	1.54287	1.0954	0.06385	0.367151	no
gene:SpnNT_00386	NA	Chromosome:398807-400089	110.58+peptide	ΔORF2+peptide	OK	4.0269	6.47918	0.686141	0.552299	0.35365	0.856246	no
gene:SpnNT_00387	NA	Chromosome:398807-400089	110.58	ΔORF2	OK	6.24701	5.59095	-0.16007	-0.0872589	0.8884	0.994748	no
gene:SpnNT_00387	NA	Chromosome:398807-400089	110.58	110.58+peptide	OK	6.24701	16.9974	1.44408	0.93815	0.08645	0.4414	no
gene:SpnNT_00387	NA	Chromosome:398807-400089	ΔORF2	110.58+peptide	OK	5.59095	16.9974	1.60415	1.08446	0.15305	0.596118	no
gene:SpnNT_00387	NA	Chromosome:398807-400089	110.58	ΔORF2+peptide	OK	6.24701	25.4564	2.02679	1.32013	0.0283	0.21416	no
gene:SpnNT_00387	NA	Chromosome:398807-400089	ΔORF2	ΔORF2+peptide	OK	5.59095	25.4564	2.18686	1.48255	0.0899	0.450255	no
gene:SpnNT_00387	NA	Chromosome:398807-400089	110.58+peptide	ΔORF2+peptide	OK	16.9974	25.4564	0.582714	0.536414	0.37845	0.875802	no
gene:SpnNT_00388	NA	Chromosome:398807-400089	110.58	ΔORF2	OK	3.59582	3.40871	-0.0770942	-0.0673477	0.91535	0.994748	no
gene:SpnNT_00388	NA	Chromosome:398807-400089	110.58	110.58+peptide	OK	3.59582	5.0881	0.500808	0.441773	0.4989	0.944017	no
gene:SpnNT_00388	NA	Chromosome:398807-400089	ΔORF2	110.58+peptide	OK	3.40871	5.0881	0.577902	0.534953	0.3622	0.862575	no
gene:SpnNT_00388	NA	Chromosome:398807-400089	110.58	ΔORF2+peptide	OK	3.59582	6.74737	0.908006	0.782251	0.23925	0.734543	no
gene:SpnNT_00388	NA	Chromosome:398807-400089	ΔORF2	ΔORF2+peptide	OK	3.40871	6.74737	0.9851	0.888503	0.1342	0.557156	no
gene:SpnNT_00388	NA	Chromosome:398807-400089	110.58+peptide	ΔORF2+peptide	OK	5.0881	6.74737	0.407197	0.371101	0.5318	0.957897	no
gene:SpnNT_00389	NA	Chromosome:400429-400792	110.58	ΔORF2	OK	1124.95	1288.48	0.195806	0.433221	0.4408	0.916668	no
gene:SpnNT_00389	NA	Chromosome:400429-400792	110.58	110.58+peptide	OK	1124.95	1285.19	0.192112	0.421812	0.456	0.921244	no
gene:SpnNT_00389	NA	Chromosome:400429-400792	ΔORF2	110.58+peptide	OK	1288.48	1285.19	-0.00369386	-0.00810678	0.98915	0.997703	no
gene:SpnNT_00389	NA	Chromosome:400429-400792	110.58	ΔORF2+peptide	OK	1124.95	1103.36	-0.0279671	-0.0624139	0.9101	0.994748	no
gene:SpnNT_00389	NA	Chromosome:400429-400792	ΔORF2	ΔORF2+peptide	OK	1288.48	1103.36	-0.223773	-0.499158	0.38065	0.878145	no
gene:SpnNT_00389	NA	Chromosome:400429-400792	110.58+peptide	ΔORF2+peptide	OK	1285.19	1103.36	-0.220079	-0.487119	0.3841	0.881416	no
gene:SpnNT_00390	fabM	Chromosome:401141-401927	110.58	ΔORF2	OK	253.373	266.765	0.0743111	0.167696	0.76765	0.994748	no
gene:SpnNT_00390	fabM	Chromosome:401141-401927	110.58	110.58+peptide	OK	253.373	257.747	0.0246983	0.0555477	0.91975	0.994748	no
gene:SpnNT_00390	fabM	Chromosome:401141-401927	ΔORF2	110.58+peptide	OK	266.765	257.747	-0.0496128	-0.111737	0.845	0.994748	no
gene:SpnNT_00390	fabM	Chromosome:401141-401927	110.58	ΔORF2+peptide	OK	253.373	293.182	0.210539	0.476346	0.40415	0.894762	no
gene:SpnNT_00390	fabM	Chromosome:401141-401927	ΔORF2	ΔORF2+peptide	OK	266.765	293.182	0.136228	0.30865	0.5888	0.974101	no

gene:SpnNT_00390	fabM	Chromosome:401141-401927	110.58+peptide	ΔORF2+peptide	OK	257.747	293.182	0.185841	0.419623	0.45635	0.921244	no
gene:SpnNT_00391	NA	Chromosome:402059-403793	110.58	ΔORF2	OK	29.3119	32.3041	0.14023	0.297084	0.59915	0.97629	no
gene:SpnNT_00391	NA	Chromosome:402059-403793	110.58	110.58+peptide	OK	29.3119	28.1283	-0.0594651	-0.124527	0.82655	0.994748	no
gene:SpnNT_00391	NA	Chromosome:402059-403793	ΔORF2	110.58+peptide	OK	32.3041	28.1283	-0.199695	-0.420568	0.47095	0.928415	no
gene:SpnNT_00391	NA	Chromosome:402059-403793	110.58	ΔORF2+peptide	OK	29.3119	33.8806	0.208969	0.44343	0.43625	0.914104	no
gene:SpnNT_00391	NA	Chromosome:402059-403793	ΔORF2	ΔORF2+peptide	OK	32.3041	33.8806	0.0687391	0.146717	0.79765	0.994748	no
gene:SpnNT_00391	NA	Chromosome:402059-403793	110.58+peptide	ΔORF2+peptide	OK	28.1283	33.8806	0.268434	0.566244	0.3221	0.827899	no
gene:SpnNT_00392	marR	Chromosome:404065-405474	110.58	ΔORF2	OK	680.911	674.167	-0.0143615	-0.0158253	0.9782	0.995765	no
gene:SpnNT_00392	marR	Chromosome:404065-405474	110.58	110.58+peptide	OK	680.911	738.528	0.117187	0.131885	0.819	0.994748	no
gene:SpnNT_00392	marR	Chromosome:404065-405474	ΔORF2	110.58+peptide	OK	674.167	738.528	0.131548	0.1487	0.79375	0.994748	no
gene:SpnNT_00392	marR	Chromosome:404065-405474	110.58	ΔORF2+peptide	OK	680.911	716.379	0.0732556	0.0807084	0.89065	0.994748	no
gene:SpnNT_00392	marR	Chromosome:404065-405474	ΔORF2	ΔORF2+peptide	OK	674.167	716.379	0.0876171	0.0969386	0.8676	0.994748	no
gene:SpnNT_00392	marR	Chromosome:404065-405474	110.58+peptide	ΔORF2+peptide	OK	738.528	716.379	-0.043931	-0.0496501	0.9302	0.994855	no
gene:SpnNT_00393	fabH	Chromosome:404065-405474	110.58	ΔORF2	OK	469.82	469.584	-0.000723068	-0.00130598	0.9982	0.999412	no
gene:SpnNT_00393	fabH	Chromosome:404065-405474	110.58	110.58+peptide	OK	469.82	523.951	0.157324	0.286653	0.62165	0.980887	no
gene:SpnNT_00393	fabH	Chromosome:404065-405474	ΔORF2	110.58+peptide	OK	469.584	523.951	0.158047	0.28559	0.6218	0.980887	no
gene:SpnNT_00393	fabH	Chromosome:404065-405474	110.58	ΔORF2+peptide	OK	469.82	558.701	0.249968	0.466283	0.41635	0.901542	no
gene:SpnNT_00393	fabH	Chromosome:404065-405474	ΔORF2	ΔORF2+peptide	OK	469.584	558.701	0.250691	0.463582	0.42	0.904223	no
gene:SpnNT_00393	fabH	Chromosome:404065-405474	110.58+peptide	ΔORF2+peptide	OK	523.951	558.701	0.0926444	0.1729	0.767	0.994748	no
gene:SpnNT_00394	acpA	Chromosome:405533-405758	110.58	ΔORF2	OK	2169.85	2418.84	0.156719	0.32174	0.5674	0.968621	no
gene:SpnNT_00394	acpA	Chromosome:405533-405758	110.58	110.58+peptide	OK	2169.85	3040.69	0.486804	0.952357	0.08795	0.446625	no
gene:SpnNT_00394	acpA	Chromosome:405533-405758	ΔORF2	110.58+peptide	OK	2418.84	3040.69	0.330084	0.632816	0.26365	0.764218	no
gene:SpnNT_00394	acpA	Chromosome:405533-405758	110.58	ΔORF2+peptide	OK	2169.85	2919.47	0.428112	0.897618	0.11095	0.507277	no
gene:SpnNT_00394	acpA	Chromosome:405533-405758	ΔORF2	ΔORF2+peptide	OK	2418.84	2919.47	0.271393	0.555984	0.32485	0.830123	no
gene:SpnNT_00394	acpA	Chromosome:405533-405758	110.58+peptide	ΔORF2+peptide	OK	3040.69	2919.47	-0.0586912	-0.1146	0.83715	0.994748	no
gene:SpnNT_00395	NA	Chromosome:405876-407764	110.58	ΔORF2	OK	330.726	327.602	-0.0136919	-0.0224108	0.9678	0.994855	no
gene:SpnNT_00395	NA	Chromosome:405876-407764	110.58	110.58+peptide	OK	330.726	290.989	-0.18467	-0.299292	0.602	0.976761	no
gene:SpnNT_00395	NA	Chromosome:405876-407764	ΔORF2	110.58+peptide	OK	327.602	290.989	-0.170978	-0.275668	0.6245	0.980887	no
gene:SpnNT_00395	NA	Chromosome:405876-407764	110.58	ΔORF2+peptide	OK	330.726	307.477	-0.105159	-0.169519	0.7645	0.994748	no
gene:SpnNT_00395	NA	Chromosome:405876-407764	ΔORF2	ΔORF2+peptide	OK	327.602	307.477	-0.0914674	-0.146692	0.7914	0.994748	no
gene:SpnNT_00395	NA	Chromosome:405876-407764	110.58+peptide	ΔORF2+peptide	OK	290.989	307.477	0.0795109	0.126312	0.8219	0.994748	no
gene:SpnNT_00396	fabD	Chromosome:405876-407764	110.58	ΔORF2	OK	306.756	291.845	-0.0718872	-0.108034	0.84215	0.994748	no
gene:SpnNT_00396	fabD	Chromosome:405876-407764	110.58	110.58+peptide	OK	306.756	297.461	-0.0443874	-0.0678365	0.90225	0.994748	no
gene:SpnNT_00396	fabD	Chromosome:405876-407764	ΔORF2	110.58+peptide	OK	291.845	297.461	0.0274998	0.0415828	0.94105	0.994855	no
gene:SpnNT_00396	fabD	Chromosome:405876-407764	110.58	ΔORF2+peptide	OK	306.756	304.961	-0.00846484	-0.0130635	0.9828	0.996246	no
gene:SpnNT_00396	fabD	Chromosome:405876-407764	ΔORF2	ΔORF2+peptide	OK	291.845	304.961	0.0634224	0.0968219	0.8646	0.994748	no
gene:SpnNT_00396	fabD	Chromosome:405876-407764	110.58+peptide	ΔORF2+peptide	OK	297.461	304.961	0.0359226	0.0557994	0.9249	0.994748	no
gene:SpnNT_00397	fabG	Chromosome:407797-408529	110.58	ΔORF2	OK	346.36	349.115	0.011428	0.0261161	0.9628	0.994855	no
gene:SpnNT_00397	fabG	Chromosome:407797-408529	110.58	110.58+peptide	OK	346.36	339.021	-0.0308987	-0.070648	0.90545	0.994748	no
gene:SpnNT_00397	fabG	Chromosome:407797-408529	ΔORF2	110.58+peptide	OK	349.115	339.021	-0.0423267	-0.0960844	0.8704	0.994748	no
gene:SpnNT_00397	fabG	Chromosome:407797-408529	110.58	ΔORF2+peptide	OK	346.36	367.396	0.0850642	0.195744	0.7265	0.994748	no
gene:SpnNT_00397	fabG	Chromosome:407797-408529	ΔORF2	ΔORF2+peptide	OK	349.115	367.396	0.0736362	0.168218	0.7643	0.994748	no
gene:SpnNT_00397	fabG	Chromosome:407797-408529	110.58+peptide	ΔORF2+peptide	OK	339.021	367.396	0.115963	0.265047	0.6355	0.980887	no
gene:SpnNT_00398	fabF	Chromosome:408550-409786	110.58	ΔORF2	OK	418.696	410.694	-0.0278412	-0.0616806	0.91265	0.994748	no
gene:SpnNT_00398	fabF	Chromosome:408550-409786	110.58	110.58+peptide	OK	418.696	392.515	-0.0931546	-0.207564	0.71095	0.991007	no

gene:SpnNT_00398	fabF	Chromosome:408550-409786	ΔORF2	110.58+peptide	OK	410.694	392.515	-0.0653134	-0.144591	0.797	0.994748	no
gene:SpnNT_00398	fabF	Chromosome:408550-409786	110.58	ΔORF2+peptide	OK	418.696	417.341	-0.00467859	-0.0104814	0.98495	0.99657	no
gene:SpnNT_00398	fabF	Chromosome:408550-409786	ΔORF2	ΔORF2+peptide	OK	410.694	417.341	0.0231626	0.0515525	0.92365	0.994748	no
gene:SpnNT_00398	fabF	Chromosome:408550-409786	110.58+peptide	ΔORF2+peptide	OK	392.515	417.341	0.088476	0.198061	0.7218	0.994748	no
gene:SpnNT_00399	accB	Chromosome:409788-410693	110.58	ΔORF2	OK	332.763	326.129	-0.0290509	-0.047342	0.93615	0.994855	no
gene:SpnNT_00399	accB	Chromosome:409788-410693	110.58	110.58+peptide	OK	332.763	301.65	-0.141619	-0.224554	0.7035	0.990367	no
gene:SpnNT_00399	accB	Chromosome:409788-410693	ΔORF2	110.58+peptide	OK	326.129	301.65	-0.112568	-0.174258	0.7685	0.994748	no
gene:SpnNT_00399	accB	Chromosome:409788-410693	110.58	ΔORF2+peptide	OK	332.763	312.385	-0.0911714	-0.151709	0.7984	0.994748	no
gene:SpnNT_00399	accB	Chromosome:409788-410693	ΔORF2	ΔORF2+peptide	OK	326.129	312.385	-0.0621205	-0.100679	0.86195	0.994748	no
gene:SpnNT_00399	accB	Chromosome:409788-410693	110.58+peptide	ΔORF2+peptide	OK	301.65	312.385	0.0504477	0.0795761	0.8897	0.994748	no
gene:SpnNT_00400	fabZ	Chromosome:409788-410693	110.58	ΔORF2	OK	370.428	362.669	-0.0305389	-0.0455118	0.93565	0.994855	no
gene:SpnNT_00400	fabZ	Chromosome:409788-410693	110.58	110.58+peptide	OK	370.428	374.904	0.0173294	0.0263652	0.962	0.994855	no
gene:SpnNT_00400	fabZ	Chromosome:409788-410693	ΔORF2	110.58+peptide	OK	362.669	374.904	0.0478683	0.0715276	0.89955	0.994748	no
gene:SpnNT_00400	fabZ	Chromosome:409788-410693	110.58	ΔORF2+peptide	OK	370.428	394.391	0.0904353	0.142136	0.81015	0.994748	no
gene:SpnNT_00400	fabZ	Chromosome:409788-410693	ΔORF2	ΔORF2+peptide	OK	362.669	394.391	0.120974	0.186519	0.7475	0.994748	no
gene:SpnNT_00400	fabZ	Chromosome:409788-410693	110.58+peptide	ΔORF2+peptide	OK	374.904	394.391	0.0731059	0.11524	0.84345	0.994748	no
gene:SpnNT_00401	accC	Chromosome:410704-412072	110.58	ΔORF2	OK	377.355	362.357	-0.0585119	-0.128623	0.8214	0.994748	no
gene:SpnNT_00401	accC	Chromosome:410704-412072	110.58	110.58+peptide	OK	377.355	366.028	-0.0439667	-0.098171	0.8601	0.994748	no
gene:SpnNT_00401	accC	Chromosome:410704-412072	ΔORF2	110.58+peptide	OK	362.357	366.028	0.0145453	0.0316977	0.9534	0.994855	no
gene:SpnNT_00401	accC	Chromosome:410704-412072	110.58	ΔORF2+peptide	OK	377.355	363.724	-0.0530791	-0.12021	0.83235	0.994748	no
gene:SpnNT_00401	accC	Chromosome:410704-412072	ΔORF2	ΔORF2+peptide	OK	362.357	363.724	0.00543284	0.0120003	0.98375	0.996246	no
gene:SpnNT_00401	accC	Chromosome:410704-412072	110.58+peptide	ΔORF2+peptide	OK	366.028	363.724	-0.00911242	-0.020448	0.9723	0.99536	no
gene:SpnNT_00402	accD	Chromosome:412108-413739	110.58	ΔORF2	OK	381.789	351.953	-0.117394	-0.180927	0.7571	0.994748	no
gene:SpnNT_00402	accD	Chromosome:412108-413739	110.58	110.58+peptide	OK	381.789	375.442	-0.0241853	-0.0376305	0.94885	0.994855	no
gene:SpnNT_00402	accD	Chromosome:412108-413739	ΔORF2	110.58+peptide	OK	351.953	375.442	0.0932089	0.142539	0.8076	0.994748	no
gene:SpnNT_00402	accD	Chromosome:412108-413739	110.58	ΔORF2+peptide	OK	381.789	407.735	0.0948555	0.147926	0.79985	0.994748	no
gene:SpnNT_00402	accD	Chromosome:412108-413739	ΔORF2	ΔORF2+peptide	OK	351.953	407.735	0.21225	0.325299	0.57355	0.969538	no
gene:SpnNT_00402	accD	Chromosome:412108-413739	110.58+peptide	ΔORF2+peptide	OK	375.442	407.735	0.119041	0.184169	0.7535	0.994748	no
gene:SpnNT_00403	accA	Chromosome:412108-413739	110.58	ΔORF2	OK	462.303	454.054	-0.0259732	-0.0407625	0.9405	0.994855	no
gene:SpnNT_00403	accA	Chromosome:412108-413739	110.58	110.58+peptide	OK	462.303	460.249	-0.00642421	-0.00994442	0.98685	0.997339	no
gene:SpnNT_00403	accA	Chromosome:412108-413739	ΔORF2	110.58+peptide	OK	454.054	460.249	0.019549	0.0303736	0.95795	0.994855	no
gene:SpnNT_00403	accA	Chromosome:412108-413739	110.58	ΔORF2+peptide	OK	462.303	505.953	0.130166	0.202319	0.7214	0.994748	no
gene:SpnNT_00403	accA	Chromosome:412108-413739	ΔORF2	ΔORF2+peptide	OK	454.054	505.953	0.156139	0.243601	0.6746	0.985063	no
gene:SpnNT_00403	accA	Chromosome:412108-413739	110.58+peptide	ΔORF2+peptide	OK	460.249	505.953	0.13659	0.210223	0.71675	0.992452	no
gene:SpnNT_00404	NA	Chromosome:413897-414083	110.58	ΔORF2	OK	11.5384	23.0647	0.999242	0.855015	0.1598	0.611162	no
gene:SpnNT_00404	NA	Chromosome:413897-414083	110.58	110.58+peptide	OK	11.5384	14.608	0.340313	0.263791	0.6387	0.980887	no
gene:SpnNT_00404	NA	Chromosome:413897-414083	ΔORF2	110.58+peptide	OK	23.0647	14.608	-0.658929	-0.572733	0.3111	0.816778	no
gene:SpnNT_00404	NA	Chromosome:413897-414083	110.58	ΔORF2+peptide	OK	11.5384	22.2954	0.950304	0.80835	0.1824	0.652167	no
gene:SpnNT_00404	NA	Chromosome:413897-414083	ΔORF2	ΔORF2+peptide	OK	23.0647	22.2954	-0.0489377	-0.0479552	0.94665	0.994855	no
gene:SpnNT_00404	NA	Chromosome:413897-414083	110.58+peptide	ΔORF2+peptide	OK	14.608	22.2954	0.609991	0.526974	0.37675	0.875032	no
gene:SpnNT_00405	NA	Chromosome:414301-414580	110.58	ΔORF2	OK	40.7103	47.4049	0.219642	0.33116	0.56545	0.968621	no
gene:SpnNT_00405	NA	Chromosome:414301-414580	110.58	110.58+peptide	OK	40.7103	43.5974	0.0988482	0.149916	0.79815	0.994748	no
gene:SpnNT_00405	NA	Chromosome:414301-414580	ΔORF2	110.58+peptide	OK	47.4049	43.5974	-0.120794	-0.187743	0.7474	0.994748	no
gene:SpnNT_00405	NA	Chromosome:414301-414580	110.58	ΔORF2+peptide	OK	40.7103	47.3946	0.21933	0.324244	0.57075	0.968621	no
gene:SpnNT_00405	NA	Chromosome:414301-414580	ΔORF2	ΔORF2+peptide	OK	47.4049	47.3946	-0.000312044	-0.000472156	0.98975	0.997703	no

gene:SpnNT_00405	NA	Chromosome:414301-414580	110.58+peptide	ΔORF2+peptide	OK	43.5974	47.3946	0.120482	0.183387	0.7537	0.994748	no
gene:SpnNT_00406	NA	Chromosome:414877-415682	110.58	ΔORF2	OK	555.474	644.21	0.213811	0.42573	0.46215	0.925287	no
gene:SpnNT_00406	NA	Chromosome:414877-415682	110.58	110.58+peptide	OK	555.474	496.624	-0.161566	-0.324546	0.5772	0.969538	no
gene:SpnNT_00406	NA	Chromosome:414877-415682	ΔORF2	110.58+peptide	OK	644.21	496.624	-0.375377	-0.756541	0.19285	0.674461	no
gene:SpnNT_00406	NA	Chromosome:414877-415682	110.58	ΔORF2+peptide	OK	555.474	542.709	-0.0335401	-0.0670798	0.90555	0.994748	no
gene:SpnNT_00406	NA	Chromosome:414877-415682	ΔORF2	ΔORF2+peptide	OK	644.21	542.709	-0.247351	-0.496325	0.3902	0.884392	no
gene:SpnNT_00406	NA	Chromosome:414877-415682	110.58+peptide	ΔORF2+peptide	OK	496.624	542.709	0.128026	0.259199	0.64615	0.980887	no
gene:SpnNT_00407	NA	Chromosome:414877-415682	110.58	ΔORF2	OK	215.533	245.283	0.186539	0.209224	0.7141	0.991753	no
gene:SpnNT_00407	NA	Chromosome:414877-415682	110.58	110.58+peptide	OK	215.533	175.161	-0.299224	-0.32592	0.56675	0.968621	no
gene:SpnNT_00407	NA	Chromosome:414877-415682	ΔORF2	110.58+peptide	OK	245.283	175.161	-0.485763	-0.520123	0.36755	0.864955	no
gene:SpnNT_00407	NA	Chromosome:414877-415682	110.58	ΔORF2+peptide	OK	215.533	195.463	-0.141013	-0.15697	0.78815	0.994748	no
gene:SpnNT_00407	NA	Chromosome:414877-415682	ΔORF2	ΔORF2+peptide	OK	245.283	195.463	-0.327552	-0.358161	0.5435	0.961568	no
gene:SpnNT_00407	NA	Chromosome:414877-415682	110.58+peptide	ΔORF2+peptide	OK	175.161	195.463	0.158211	0.168236	0.7769	0.994748	no
gene:SpnNT_00408	efp	Chromosome:415703-416264	110.58	ΔORF2	OK	477.319	530.335	0.151952	0.346184	0.5358	0.958874	no
gene:SpnNT_00408	efp	Chromosome:415703-416264	110.58	110.58+peptide	OK	477.319	439.253	-0.119901	-0.272254	0.6276	0.980887	no
gene:SpnNT_00408	efp	Chromosome:415703-416264	ΔORF2	110.58+peptide	OK	530.335	439.253	-0.271853	-0.624543	0.26325	0.763902	no
gene:SpnNT_00408	efp	Chromosome:415703-416264	110.58	ΔORF2+peptide	OK	477.319	437.564	-0.125458	-0.285046	0.6086	0.978849	no
gene:SpnNT_00408	efp	Chromosome:415703-416264	ΔORF2	ΔORF2+peptide	OK	530.335	437.564	-0.27741	-0.637708	0.25205	0.750147	no
gene:SpnNT_00408	efp	Chromosome:415703-416264	110.58+peptide	ΔORF2+peptide	OK	439.253	437.564	-0.0055574	-0.012732	0.98075	0.99608	no
gene:SpnNT_00409	gutB	Chromosome:416594-417638	110.58	ΔORF2	OK	15.5773	14.9401	-0.0602571	-0.109685	0.84395	0.994748	no
gene:SpnNT_00409	gutB	Chromosome:416594-417638	110.58	110.58+peptide	OK	15.5773	32.0804	1.04225	1.97382	3.00E-04	0.00631878	yes
gene:SpnNT_00409	gutB	Chromosome:416594-417638	ΔORF2	110.58+peptide	OK	14.9401	32.0804	1.1025	2.08006	4.00E-04	0.00811252	yes
gene:SpnNT_00409	gutB	Chromosome:416594-417638	110.58	ΔORF2+peptide	OK	15.5773	39.6159	1.34663	2.58554	1.00E-04	0.0025332	yes
gene:SpnNT_00409	gutB	Chromosome:416594-417638	ΔORF2	ΔORF2+peptide	OK	14.9401	39.6159	1.40689	2.69077	5.00E-05	0.0013612	yes
gene:SpnNT_00409	gutB	Chromosome:416594-417638	110.58+peptide	ΔORF2+peptide	OK	32.0804	39.6159	0.304387	0.608291	0.2965	0.80085	no
gene:SpnNT_00410	gatB	Chromosome:417761-420972	110.58	ΔORF2	OK	128.21	130.026	0.0202961	0.0381497	0.9464	0.994855	no
gene:SpnNT_00410	gatB	Chromosome:417761-420972	110.58	110.58+peptide	OK	128.21	103.997	-0.301964	-0.566328	0.3248	0.830123	no
gene:SpnNT_00410	gatB	Chromosome:417761-420972	ΔORF2	110.58+peptide	OK	130.026	103.997	-0.32226	-0.600003	0.29425	0.798553	no
gene:SpnNT_00410	gatB	Chromosome:417761-420972	110.58	ΔORF2+peptide	OK	128.21	108.454	-0.241422	-0.452417	0.4326	0.913334	no
gene:SpnNT_00410	gatB	Chromosome:417761-420972	ΔORF2	ΔORF2+peptide	OK	130.026	108.454	-0.261718	-0.486894	0.39515	0.890116	no
gene:SpnNT_00410	gatB	Chromosome:417761-420972	110.58+peptide	ΔORF2+peptide	OK	103.997	108.454	0.060542	0.112385	0.84635	0.994748	no
gene:SpnNT_00411	gatA	Chromosome:417761-420972	110.58	ΔORF2	OK	52.2264	50.6546	-0.0440881	-0.0510683	0.9283	0.994748	no
gene:SpnNT_00411	gatA	Chromosome:417761-420972	110.58	110.58+peptide	OK	52.2264	49.8915	-0.0659856	-0.0807279	0.8887	0.994748	no
gene:SpnNT_00411	gatA	Chromosome:417761-420972	ΔORF2	110.58+peptide	OK	50.6546	49.8915	-0.0218975	-0.0266389	0.9635	0.994855	no
gene:SpnNT_00411	gatA	Chromosome:417761-420972	110.58	ΔORF2+peptide	OK	52.2264	49.1672	-0.0870855	-0.104642	0.85715	0.994748	no
gene:SpnNT_00411	gatA	Chromosome:417761-420972	ΔORF2	ΔORF2+peptide	OK	50.6546	49.1672	-0.0429974	-0.0513852	0.9284	0.994748	no
gene:SpnNT_00411	gatA	Chromosome:417761-420972	110.58+peptide	ΔORF2+peptide	OK	49.8915	49.1672	-0.0210999	-0.0267328	0.9625	0.994855	no
gene:SpnNT_00412	gatC_1	Chromosome:417761-420972	110.58	ΔORF2	OK	72.1354	61.0036	-0.241813	-0.095972	0.8145	0.994748	no
gene:SpnNT_00412	gatC_1	Chromosome:417761-420972	110.58	110.58+peptide	OK	72.1354	61.6752	-0.226015	-0.0959214	0.7526	0.994748	no
gene:SpnNT_00412	gatC_1	Chromosome:417761-420972	ΔORF2	110.58+peptide	OK	61.0036	61.6752	0.0157983	0.00625937	0.8589	0.994748	no
gene:SpnNT_00412	gatC_1	Chromosome:417761-420972	110.58	ΔORF2+peptide	OK	72.1354	57.0429	-0.33866	-0.136044	0.8006	0.994748	no
gene:SpnNT_00412	gatC_1	Chromosome:417761-420972	ΔORF2	ΔORF2+peptide	OK	61.0036	57.0429	-0.0968465	-0.0365649	0.919	0.994748	no
gene:SpnNT_00412	gatC_1	Chromosome:417761-420972	110.58+peptide	ΔORF2+peptide	OK	61.6752	57.0429	-0.112645	-0.0451715	0.8393	0.994748	no
gene:SpnNT_00413	prfC	Chromosome:421184-422729	110.58	ΔORF2	OK	113.58	107.369	-0.0811302	-0.186087	0.7381	0.994748	no
gene:SpnNT_00413	prfC	Chromosome:421184-422729	110.58	110.58+peptide	OK	113.58	152.643	0.42646	0.980713	0.0839	0.434809	no

gene:SpnNT_00413	prfC	Chromosome:421184-422729	ΔORF2	110.58+peptide	OK	107.369	152.643	0.50759	1.16681	0.04115	0.275917	no
gene:SpnNT_00413	prfC	Chromosome:421184-422729	110.58	ΔORF2+peptide	OK	113.58	159.743	0.492046	1.1323	0.046	0.296033	no
gene:SpnNT_00413	prfC	Chromosome:421184-422729	ΔORF2	ΔORF2+peptide	OK	107.369	159.743	0.573176	1.31847	0.0195	0.164025	no
gene:SpnNT_00413	prfC	Chromosome:421184-422729	110.58+peptide	ΔORF2+peptide	OK	152.643	159.743	0.0655856	0.151261	0.7882	0.994748	no
gene:SpnNT_00414	NA	Chromosome:423143-423341	110.58	ΔORF2	OK	6.30074	10.3912	0.721774	0.700671	0.3783	0.875802	no
gene:SpnNT_00414	NA	Chromosome:423143-423341	110.58	110.58+peptide	OK	6.30074	10.8445	0.783367	0.838442	0.33185	0.837894	no
gene:SpnNT_00414	NA	Chromosome:423143-423341	ΔORF2	110.58+peptide	OK	10.3912	10.8445	0.0615929	0.0489177	0.93315	0.994855	no
gene:SpnNT_00414	NA	Chromosome:423143-423341	110.58	ΔORF2+peptide	OK	6.30074	14.3512	1.18757	1.35492	0.1595	0.61037	no
gene:SpnNT_00414	NA	Chromosome:423143-423341	ΔORF2	ΔORF2+peptide	OK	10.3912	14.3512	0.4658	0.3828	0.52235	0.954832	no
gene:SpnNT_00414	NA	Chromosome:423143-423341	110.58+peptide	ΔORF2+peptide	OK	10.8445	14.3512	0.404207	0.355546	0.5791	0.969538	no
gene:SpnNT_00415	rpmB	Chromosome:423743-423932	110.58	ΔORF2	OK	4161.6	6004.64	0.528941	1.07995	0.0547	0.332606	no
gene:SpnNT_00415	rpmB	Chromosome:423743-423932	110.58	110.58+peptide	OK	4161.6	13394.7	1.68645	3.40749	5.00E-05	0.0013612	yes
gene:SpnNT_00415	rpmB	Chromosome:423743-423932	ΔORF2	110.58+peptide	OK	6004.64	13394.7	1.15751	2.26347	0.00015	0.00355289	yes
gene:SpnNT_00415	rpmB	Chromosome:423743-423932	110.58	ΔORF2+peptide	OK	4161.6	11754	1.49794	3.35379	5.00E-05	0.0013612	yes
gene:SpnNT_00415	rpmB	Chromosome:423743-423932	ΔORF2	ΔORF2+peptide	OK	6004.64	11754	0.969004	2.08469	0.00025	0.00542231	yes
gene:SpnNT_00415	rpmB	Chromosome:423743-423932	110.58+peptide	ΔORF2+peptide	OK	13394.7	11754	-0.188506	-0.400877	0.484	0.936747	no
gene:SpnNT_00416	NA	Chromosome:424088-424454	110.58	ΔORF2	OK	297.077	313.339	0.0768841	0.16462	0.775	0.994748	no
gene:SpnNT_00416	NA	Chromosome:424088-424454	110.58	110.58+peptide	OK	297.077	261.463	-0.184232	-0.390629	0.4865	0.938388	no
gene:SpnNT_00416	NA	Chromosome:424088-424454	ΔORF2	110.58+peptide	OK	313.339	261.463	-0.261117	-0.554928	0.32185	0.827743	no
gene:SpnNT_00416	NA	Chromosome:424088-424454	110.58	ΔORF2+peptide	OK	297.077	286.959	-0.0499956	-0.106336	0.8511	0.994748	no
gene:SpnNT_00416	NA	Chromosome:424088-424454	ΔORF2	ΔORF2+peptide	OK	313.339	286.959	-0.12688	-0.27049	0.63155	0.980887	no
gene:SpnNT_00416	NA	Chromosome:424088-424454	110.58+peptide	ΔORF2+peptide	OK	261.463	286.959	0.134237	0.283413	0.613	0.979616	no
gene:SpnNT_00417	NA	Chromosome:424456-426124	110.58	ΔORF2	OK	628.518	643.146	0.0331914	0.072644	0.8949	0.994748	no
gene:SpnNT_00417	NA	Chromosome:424456-426124	110.58	110.58+peptide	OK	628.518	682.435	0.118737	0.257861	0.65115	0.981475	no
gene:SpnNT_00417	NA	Chromosome:424456-426124	ΔORF2	110.58+peptide	OK	643.146	682.435	0.0855454	0.186467	0.73255	0.994748	no
gene:SpnNT_00417	NA	Chromosome:424456-426124	110.58	ΔORF2+peptide	OK	628.518	624.074	-0.0102371	-0.0224041	0.96885	0.994855	no
gene:SpnNT_00417	NA	Chromosome:424456-426124	ΔORF2	ΔORF2+peptide	OK	643.146	624.074	-0.0434285	-0.0954014	0.86175	0.994748	no
gene:SpnNT_00417	NA	Chromosome:424456-426124	110.58+peptide	ΔORF2+peptide	OK	682.435	624.074	-0.128974	-0.281115	0.6156	0.979616	no
gene:SpnNT_00418	ilvB	Chromosome:426323-428493	110.58	ΔORF2	OK	240.223	232.37	-0.0479519	-0.10013	0.86175	0.994748	no
gene:SpnNT_00418	ilvB	Chromosome:426323-428493	110.58	110.58+peptide	OK	240.223	218.018	-0.139927	-0.285976	0.61235	0.979616	no
gene:SpnNT_00418	ilvB	Chromosome:426323-428493	ΔORF2	110.58+peptide	OK	232.37	218.018	-0.0919747	-0.188754	0.7423	0.994748	no
gene:SpnNT_00418	ilvB	Chromosome:426323-428493	110.58	ΔORF2+peptide	OK	240.223	218.149	-0.13906	-0.289349	0.6041	0.976761	no
gene:SpnNT_00418	ilvB	Chromosome:426323-428493	ΔORF2	ΔORF2+peptide	OK	232.37	218.149	-0.0911077	-0.190388	0.7368	0.994748	no
gene:SpnNT_00418	ilvB	Chromosome:426323-428493	110.58+peptide	ΔORF2+peptide	OK	218.018	218.149	0.000867039	0.0017733	0.99785	0.999412	no
gene:SpnNT_00419	ilvH	Chromosome:426323-428493	110.58	ΔORF2	OK	187.004	174.974	-0.0959301	-0.0692095	0.9063	0.994748	no
gene:SpnNT_00419	ilvH	Chromosome:426323-428493	110.58	110.58+peptide	OK	187.004	172.839	-0.113635	-0.0803364	0.89395	0.994748	no
gene:SpnNT_00419	ilvH	Chromosome:426323-428493	ΔORF2	110.58+peptide	OK	174.974	172.839	-0.0177051	-0.0126943	0.9821	0.996246	no
gene:SpnNT_00419	ilvH	Chromosome:426323-428493	110.58	ΔORF2+peptide	OK	187.004	180.568	-0.0505285	-0.0371232	0.95225	0.994855	no
gene:SpnNT_00419	ilvH	Chromosome:426323-428493	ΔORF2	ΔORF2+peptide	OK	174.974	180.568	0.0454016	0.0338678	0.95615	0.994855	no
gene:SpnNT_00419	ilvH	Chromosome:426323-428493	110.58+peptide	ΔORF2+peptide	OK	172.839	180.568	0.0631067	0.0460665	0.9375	0.994855	no
gene:SpnNT_00420	ilvC	Chromosome:428558-429581	110.58	ΔORF2	OK	243.81	246.994	0.0187209	0.042558	0.9403	0.994855	no
gene:SpnNT_00420	ilvC	Chromosome:428558-429581	110.58	110.58+peptide	OK	243.81	240.897	-0.0173364	-0.0390776	0.94475	0.994855	no
gene:SpnNT_00420	ilvC	Chromosome:428558-429581	ΔORF2	110.58+peptide	OK	246.994	240.897	-0.0360573	-0.0812376	0.8864	0.994748	no
gene:SpnNT_00420	ilvC	Chromosome:428558-429581	110.58	ΔORF2+peptide	OK	243.81	255.261	0.0662203	0.145423	0.80095	0.994748	no
gene:SpnNT_00420	ilvC	Chromosome:428558-429581	ΔORF2	ΔORF2+peptide	OK	246.994	255.261	0.0474994	0.104264	0.8512	0.994748	no

gene:SpnNT_00420	ilvC	Chromosome:428558-429581	110.58+peptide	ΔORF2+peptide	OK	240.897	255.261	0.0835567	0.181966	0.75255	0.994748	no
gene:SpnNT_00421	NA	Chromosome:429687-429924	110.58	ΔORF2	OK	110.653	114.402	0.0480684	0.0792532	0.88745	0.994748	no
gene:SpnNT_00421	NA	Chromosome:429687-429924	110.58	110.58+peptide	OK	110.653	118.091	0.0938572	0.154934	0.78235	0.994748	no
gene:SpnNT_00421	NA	Chromosome:429687-429924	ΔORF2	110.58+peptide	OK	114.402	118.091	0.0457889	0.0759477	0.8907	0.994748	no
gene:SpnNT_00421	NA	Chromosome:429687-429924	110.58	ΔORF2+peptide	OK	110.653	144.888	0.3889	0.663761	0.2686	0.770416	no
gene:SpnNT_00421	NA	Chromosome:429687-429924	ΔORF2	ΔORF2+peptide	OK	114.402	144.888	0.340832	0.584699	0.3322	0.837972	no
gene:SpnNT_00421	NA	Chromosome:429687-429924	110.58+peptide	ΔORF2+peptide	OK	118.091	144.888	0.295043	0.506808	0.40335	0.89446	no
gene:SpnNT_00422	ilvA	Chromosome:430298-431549	110.58	ΔORF2	OK	96.0651	96.8317	0.0114677	0.0259071	0.962	0.994855	no
gene:SpnNT_00422	ilvA	Chromosome:430298-431549	110.58	110.58+peptide	OK	96.0651	110.725	0.204902	0.462362	0.41605	0.901298	no
gene:SpnNT_00422	ilvA	Chromosome:430298-431549	ΔORF2	110.58+peptide	OK	96.8317	110.725	0.193435	0.436469	0.4408	0.916668	no
gene:SpnNT_00422	ilvA	Chromosome:430298-431549	110.58	ΔORF2+peptide	OK	96.0651	109.9	0.194106	0.429712	0.45645	0.921244	no
gene:SpnNT_00422	ilvA	Chromosome:430298-431549	ΔORF2	ΔORF2+peptide	OK	96.8317	109.9	0.182639	0.404311	0.483	0.936455	no
gene:SpnNT_00422	ilvA	Chromosome:430298-431549	110.58+peptide	ΔORF2+peptide	OK	110.725	109.9	-0.0107958	-0.0238722	0.96585	0.994855	no
gene:SpnNT_00423	NA	Chromosome:432419-432635	110.58	ΔORF2	OK	769.295	772.169	0.00538031	0.0109768	0.98295	0.996246	no
gene:SpnNT_00423	NA	Chromosome:432419-432635	110.58	110.58+peptide	OK	769.295	851.162	0.145898	0.295301	0.6021	0.976761	no
gene:SpnNT_00423	NA	Chromosome:432419-432635	ΔORF2	110.58+peptide	OK	772.169	851.162	0.140517	0.284025	0.6142	0.979616	no
gene:SpnNT_00423	NA	Chromosome:432419-432635	110.58	ΔORF2+peptide	OK	769.295	714.781	-0.106036	-0.215305	0.708	0.990441	no
gene:SpnNT_00423	NA	Chromosome:432419-432635	ΔORF2	ΔORF2+peptide	OK	772.169	714.781	-0.111417	-0.225921	0.69705	0.989794	no
gene:SpnNT_00423	NA	Chromosome:432419-432635	110.58+peptide	ΔORF2+peptide	OK	851.162	714.781	-0.251934	-0.506852	0.3923	0.887239	no
gene:SpnNT_00424	glnQ_1	Chromosome:432743-435049	110.58	ΔORF2	OK	59.9502	61.437	0.0353425	0.0527793	0.9273	0.994748	no
gene:SpnNT_00424	glnQ_1	Chromosome:432743-435049	110.58	110.58+peptide	OK	59.9502	48.8102	-0.296584	-0.431986	0.45295	0.921244	no
gene:SpnNT_00424	glnQ_1	Chromosome:432743-435049	ΔORF2	110.58+peptide	OK	61.437	48.8102	-0.331926	-0.478478	0.4022	0.894197	no
gene:SpnNT_00424	glnQ_1	Chromosome:432743-435049	110.58	ΔORF2+peptide	OK	59.9502	47.861	-0.324915	-0.475736	0.41385	0.900099	no
gene:SpnNT_00424	glnQ_1	Chromosome:432743-435049	ΔORF2	ΔORF2+peptide	OK	61.437	47.861	-0.360258	-0.521988	0.3638	0.863839	no
gene:SpnNT_00424	glnQ_1	Chromosome:432743-435049	110.58+peptide	ΔORF2+peptide	OK	48.8102	47.861	-0.0283315	-0.0400954	0.9451	0.994855	no
gene:SpnNT_00425	yecS_1	Chromosome:432743-435049	110.58	ΔORF2	OK	27.208	29.0488	0.0944433	0.151677	0.7898	0.994748	no
gene:SpnNT_00425	yecS_1	Chromosome:432743-435049	110.58	110.58+peptide	OK	27.208	24.8744	-0.129369	-0.209452	0.7157	0.992121	no
gene:SpnNT_00425	yecS_1	Chromosome:432743-435049	ΔORF2	110.58+peptide	OK	29.0488	24.8744	-0.223812	-0.364619	0.53015	0.957488	no
gene:SpnNT_00425	yecS_1	Chromosome:432743-435049	110.58	ΔORF2+peptide	OK	27.208	22.6152	-0.266743	-0.426409	0.4618	0.92501	no
gene:SpnNT_00425	yecS_1	Chromosome:432743-435049	ΔORF2	ΔORF2+peptide	OK	29.0488	22.6152	-0.361186	-0.580894	0.3169	0.822753	no
gene:SpnNT_00425	yecS_1	Chromosome:432743-435049	110.58+peptide	ΔORF2+peptide	OK	24.8744	22.6152	-0.137374	-0.222736	0.70145	0.990367	no
gene:SpnNT_00426	NA	Chromosome:435187-437077	110.58	ΔORF2	OK	59.2453	60.5975	0.0325564	0.0735484	0.89935	0.994748	no
gene:SpnNT_00426	NA	Chromosome:435187-437077	110.58	110.58+peptide	OK	59.2453	44.9963	-0.396896	-0.879753	0.1237	0.532019	no
gene:SpnNT_00426	NA	Chromosome:435187-437077	ΔORF2	110.58+peptide	OK	60.5975	44.9963	-0.429453	-0.952419	0.0977	0.472721	no
gene:SpnNT_00426	NA	Chromosome:435187-437077	110.58	ΔORF2+peptide	OK	59.2453	54.336	-0.124793	-0.28132	0.626	0.980887	no
gene:SpnNT_00426	NA	Chromosome:435187-437077	ΔORF2	ΔORF2+peptide	OK	60.5975	54.336	-0.157349	-0.354905	0.5381	0.95989	no
gene:SpnNT_00426	NA	Chromosome:435187-437077	110.58+peptide	ΔORF2+peptide	OK	44.9963	54.336	0.272103	0.602219	0.28855	0.792622	no
gene:SpnNT_00427	NA	Chromosome:437455-438397	110.58	ΔORF2	OK	164.69	178.77	0.118351	0.271738	0.632	0.980887	no
gene:SpnNT_00427	NA	Chromosome:437455-438397	110.58	110.58+peptide	OK	164.69	174.569	0.0840444	0.191528	0.74215	0.994748	no
gene:SpnNT_00427	NA	Chromosome:437455-438397	ΔORF2	110.58+peptide	OK	178.77	174.569	-0.0343063	-0.078261	0.89365	0.994748	no
gene:SpnNT_00427	NA	Chromosome:437455-438397	110.58	ΔORF2+peptide	OK	164.69	180.551	0.132652	0.30247	0.599	0.97629	no
gene:SpnNT_00427	NA	Chromosome:437455-438397	ΔORF2	ΔORF2+peptide	OK	178.77	180.551	0.014301	0.0326426	0.9563	0.994855	no
gene:SpnNT_00427	NA	Chromosome:437455-438397	110.58+peptide	ΔORF2+peptide	OK	174.569	180.551	0.0486074	0.110129	0.84705	0.994748	no
gene:SpnNT_00428	uppP	Chromosome:438651-439497	110.58	ΔORF2	OK	193.656	211.827	0.129391	0.29507	0.60595	0.976937	no
gene:SpnNT_00428	uppP	Chromosome:438651-439497	110.58	110.58+peptide	OK	193.656	166.744	-0.215863	-0.485768	0.3962	0.890381	no

gene:SpnNT_00428	uppP	Chromosome:438651-439497	ΔORF2	110.58+peptide	OK	211.827	166.744	-0.345255	-0.772512	0.17795	0.644332	no
gene:SpnNT_00428	uppP	Chromosome:438651-439497	110.58	ΔORF2+peptide	OK	193.656	195.625	0.0145978	0.0332838	0.95505	0.994855	no
gene:SpnNT_00428	uppP	Chromosome:438651-439497	ΔORF2	ΔORF2+peptide	OK	211.827	195.625	-0.114794	-0.260203	0.6529	0.981932	no
gene:SpnNT_00428	uppP	Chromosome:438651-439497	110.58+peptide	ΔORF2+peptide	OK	166.744	195.625	0.230461	0.515575	0.37085	0.867896	no
gene:SpnNT_00429	dinB	Chromosome:439643-440705	110.58	ΔORF2	OK	17.5333	16.5836	-0.0803373	-0.145378	0.8001	0.994748	no
gene:SpnNT_00429	dinB	Chromosome:439643-440705	110.58	110.58+peptide	OK	17.5333	16.533	-0.0847472	-0.153954	0.78695	0.994748	no
gene:SpnNT_00429	dinB	Chromosome:439643-440705	ΔORF2	110.58+peptide	OK	16.5836	16.533	-0.00440998	-0.00790383	0.99	0.997703	no
gene:SpnNT_00429	dinB	Chromosome:439643-440705	110.58	ΔORF2+peptide	OK	17.5333	24.4996	0.482663	0.905643	0.1089	0.502103	no
gene:SpnNT_00429	dinB	Chromosome:439643-440705	ΔORF2	ΔORF2+peptide	OK	16.5836	24.4996	0.563	1.04129	0.0708	0.390464	no
gene:SpnNT_00429	dinB	Chromosome:439643-440705	110.58+peptide	ΔORF2+peptide	OK	16.533	24.4996	0.56741	1.05371	0.06785	0.381133	no
gene:SpnNT_00430	pflB	Chromosome:441052-443377	110.58	ΔORF2	OK	548.548	661.022	0.269081	0.55963	0.32275	0.828143	no
gene:SpnNT_00430	pflB	Chromosome:441052-443377	110.58	110.58+peptide	OK	548.548	865.545	0.657992	1.33408	0.0211	0.174244	no
gene:SpnNT_00430	pflB	Chromosome:441052-443377	ΔORF2	110.58+peptide	OK	661.022	865.545	0.388911	0.795103	0.1663	0.621352	no
gene:SpnNT_00430	pflB	Chromosome:441052-443377	110.58	ΔORF2+peptide	OK	548.548	797.743	0.540307	1.1063	0.05335	0.327284	no
gene:SpnNT_00430	pflB	Chromosome:441052-443377	ΔORF2	ΔORF2+peptide	OK	661.022	797.743	0.271226	0.560073	0.31525	0.821264	no
gene:SpnNT_00430	pflB	Chromosome:441052-443377	110.58+peptide	ΔORF2+peptide	OK	865.545	797.743	-0.117685	-0.236992	0.6765	0.98524	no
gene:SpnNT_00431	NA	Chromosome:443659-443878	110.58	ΔORF2	OK	22.7991	44.7714	0.973602	1.19972	0.0439	0.287166	no
gene:SpnNT_00431	NA	Chromosome:443659-443878	110.58	110.58+peptide	OK	22.7991	33.4886	0.554698	0.676068	0.27155	0.773617	no
gene:SpnNT_00431	NA	Chromosome:443659-443878	ΔORF2	110.58+peptide	OK	44.7714	33.4886	-0.418904	-0.540729	0.3656	0.864634	no
gene:SpnNT_00431	NA	Chromosome:443659-443878	110.58	ΔORF2+peptide	OK	22.7991	35.875	0.654005	0.773604	0.19585	0.677365	no
gene:SpnNT_00431	NA	Chromosome:443659-443878	ΔORF2	ΔORF2+peptide	OK	44.7714	35.875	-0.319597	-0.398973	0.48865	0.939082	no
gene:SpnNT_00431	NA	Chromosome:443659-443878	110.58+peptide	ΔORF2+peptide	OK	33.4886	35.875	0.0993067	0.122583	0.84155	0.994748	no
gene:SpnNT_00432	NA	Chromosome:443973-444499	110.58	ΔORF2	OK	48.1999	61.2519	0.345725	0.330363	0.56385	0.968621	no
gene:SpnNT_00432	NA	Chromosome:443973-444499	110.58	110.58+peptide	OK	48.1999	47.9424	-0.00772672	-0.00803471	0.9914	0.998323	no
gene:SpnNT_00432	NA	Chromosome:443973-444499	ΔORF2	110.58+peptide	OK	61.2519	47.9424	-0.353451	-0.338559	0.5432	0.961568	no
gene:SpnNT_00432	NA	Chromosome:443973-444499	110.58	ΔORF2+peptide	OK	48.1999	44.0072	-0.131291	-0.123767	0.8328	0.994748	no
gene:SpnNT_00432	NA	Chromosome:443973-444499	ΔORF2	ΔORF2+peptide	OK	61.2519	44.0072	-0.477016	-0.419926	0.47175	0.928525	no
gene:SpnNT_00432	NA	Chromosome:443973-444499	110.58+peptide	ΔORF2+peptide	OK	47.9424	44.0072	-0.123564	-0.116756	0.83515	0.994748	no
gene:SpnNT_00433	NA	Chromosome:443973-444499	110.58	ΔORF2	OK	62.7759	78.5118	0.322699	0.442009	0.4504	0.920951	no
gene:SpnNT_00433	NA	Chromosome:443973-444499	110.58	110.58+peptide	OK	62.7759	53.4878	-0.231001	-0.313927	0.5776	0.969538	no
gene:SpnNT_00433	NA	Chromosome:443973-444499	ΔORF2	110.58+peptide	OK	78.5118	53.4878	-0.5537	-0.735115	0.2113	0.697158	no
gene:SpnNT_00433	NA	Chromosome:443973-444499	110.58	ΔORF2+peptide	OK	62.7759	56.71	-0.146608	-0.196142	0.7305	0.994748	no
gene:SpnNT_00433	NA	Chromosome:443973-444499	ΔORF2	ΔORF2+peptide	OK	78.5118	56.71	-0.469307	-0.613819	0.30185	0.807816	no
gene:SpnNT_00433	NA	Chromosome:443973-444499	110.58+peptide	ΔORF2+peptide	OK	53.4878	56.71	0.0843929	0.109589	0.8479	0.994748	no
gene:SpnNT_00434	NA	Chromosome:446086-446798	110.58	ΔORF2	OK	23.733	20.395	-0.218676	-0.347213	0.54535	0.96171	no
gene:SpnNT_00434	NA	Chromosome:446086-446798	110.58	110.58+peptide	OK	23.733	17.9825	-0.400296	-0.627313	0.2775	0.780208	no
gene:SpnNT_00434	NA	Chromosome:446086-446798	ΔORF2	110.58+peptide	OK	20.395	17.9825	-0.18162	-0.282082	0.62515	0.980887	no
gene:SpnNT_00434	NA	Chromosome:446086-446798	110.58	ΔORF2+peptide	OK	23.733	19.444	-0.287566	-0.458143	0.42555	0.908126	no
gene:SpnNT_00434	NA	Chromosome:446086-446798	ΔORF2	ΔORF2+peptide	OK	20.395	19.444	-0.0688907	-0.108743	0.85065	0.994748	no
gene:SpnNT_00434	NA	Chromosome:446086-446798	110.58+peptide	ΔORF2+peptide	OK	17.9825	19.444	0.112729	0.175652	0.7632	0.994748	no
gene:SpnNT_00435	NA	Chromosome:446086-446798	110.58	ΔORF2	OK	24.0679	25.5538	0.086425	0.0439507	0.94635	0.994855	no
gene:SpnNT_00435	NA	Chromosome:446086-446798	110.58	110.58+peptide	OK	24.0679	20.5617	-0.22715	-0.122163	0.8499	0.994748	no
gene:SpnNT_00435	NA	Chromosome:446086-446798	ΔORF2	110.58+peptide	OK	25.5538	20.5617	-0.313575	-0.175647	0.7592	0.994748	no
gene:SpnNT_00435	NA	Chromosome:446086-446798	110.58	ΔORF2+peptide	OK	24.0679	18.8163	-0.355126	-0.188012	0.7835	0.994748	no
gene:SpnNT_00435	NA	Chromosome:446086-446798	ΔORF2	ΔORF2+peptide	OK	25.5538	18.8163	-0.441551	-0.243158	0.6847	0.987974	no

gene:SpnNT_00435	NA	Chromosome:446086-446798	110.58+peptide	ΔORF2+peptide	OK	20.5617	18.8163	-0.127976	-0.0753049	0.8988	0.994748	no
gene:SpnNT_00436	trkG	Chromosome:447047-448487	110.58	ΔORF2	OK	20.6414	22.5786	0.12942	0.25636	0.65065	0.981475	no
gene:SpnNT_00436	trkG	Chromosome:447047-448487	110.58	110.58+peptide	OK	20.6414	16.9467	-0.284531	-0.552123	0.33035	0.836198	no
gene:SpnNT_00436	trkG	Chromosome:447047-448487	ΔORF2	110.58+peptide	OK	22.5786	16.9467	-0.413951	-0.812173	0.1502	0.591869	no
gene:SpnNT_00436	trkG	Chromosome:447047-448487	110.58	ΔORF2+peptide	OK	20.6414	17.9056	-0.205128	-0.397055	0.47865	0.933291	no
gene:SpnNT_00436	trkG	Chromosome:447047-448487	ΔORF2	ΔORF2+peptide	OK	22.5786	17.9056	-0.334548	-0.654717	0.244	0.740122	no
gene:SpnNT_00436	trkG	Chromosome:447047-448487	110.58+peptide	ΔORF2+peptide	OK	16.9467	17.9056	0.0794032	0.1523	0.7839	0.994748	no
gene:SpnNT_00437	trkA	Chromosome:448490-449840	110.58	ΔORF2	OK	65.98	67.7441	0.0380657	0.0849815	0.8802	0.994748	no
gene:SpnNT_00437	trkA	Chromosome:448490-449840	110.58	110.58+peptide	OK	65.98	49.023	-0.428569	-0.94473	0.0941	0.463158	no
gene:SpnNT_00437	trkA	Chromosome:448490-449840	ΔORF2	110.58+peptide	OK	67.7441	49.023	-0.466635	-1.02927	0.0649	0.369947	no
gene:SpnNT_00437	trkA	Chromosome:448490-449840	110.58	ΔORF2+peptide	OK	65.98	46.484	-0.505294	-1.10244	0.05605	0.337535	no
gene:SpnNT_00437	trkA	Chromosome:448490-449840	ΔORF2	ΔORF2+peptide	OK	67.7441	46.484	-0.54336	-1.1862	0.0375	0.259533	no
gene:SpnNT_00437	trkA	Chromosome:448490-449840	110.58+peptide	ΔORF2+peptide	OK	49.023	46.484	-0.0767253	-0.165479	0.77395	0.994748	no
gene:SpnNT_00438	NA	Chromosome:450028-450877	110.58	ΔORF2	OK	56.7588	58.0224	0.031766	0.0657988	0.9112	0.994748	no
gene:SpnNT_00438	NA	Chromosome:450028-450877	110.58	110.58+peptide	OK	56.7588	49.4169	-0.199842	-0.410865	0.4689	0.927613	no
gene:SpnNT_00438	NA	Chromosome:450028-450877	ΔORF2	110.58+peptide	OK	58.0224	49.4169	-0.231608	-0.477721	0.40335	0.89446	no
gene:SpnNT_00438	NA	Chromosome:450028-450877	110.58	ΔORF2+peptide	OK	56.7588	49.4149	-0.199898	-0.407771	0.4801	0.934519	no
gene:SpnNT_00438	NA	Chromosome:450028-450877	ΔORF2	ΔORF2+peptide	OK	58.0224	49.4149	-0.231664	-0.474082	0.4196	0.904223	no
gene:SpnNT_00438	NA	Chromosome:450028-450877	110.58+peptide	ΔORF2+peptide	OK	49.4169	49.4149	-5.62E-05	-0.000114073	0.99825	0.999412	no
gene:SpnNT_00439	NA	Chromosome:450891-451440	110.58	ΔORF2	OK	49.2256	52.0894	0.0815815	0.153111	0.79445	0.994748	no
gene:SpnNT_00439	NA	Chromosome:450891-451440	110.58	110.58+peptide	OK	49.2256	36.4603	-0.433083	-0.794756	0.16665	0.621775	no
gene:SpnNT_00439	NA	Chromosome:450891-451440	ΔORF2	110.58+peptide	OK	52.0894	36.4603	-0.514664	-0.94297	0.10205	0.484479	no
gene:SpnNT_00439	NA	Chromosome:450891-451440	110.58	ΔORF2+peptide	OK	49.2256	46.0738	-0.0954627	-0.178452	0.75625	0.994748	no
gene:SpnNT_00439	NA	Chromosome:450891-451440	ΔORF2	ΔORF2+peptide	OK	52.0894	46.0738	-0.177044	-0.330411	0.56755	0.968621	no
gene:SpnNT_00439	NA	Chromosome:450891-451440	110.58+peptide	ΔORF2+peptide	OK	36.4603	46.0738	0.33762	0.616248	0.2822	0.785039	no
gene:SpnNT_00440	NA	Chromosome:451446-451704	110.58	ΔORF2	OK	2.99986	0.73674	-2.02567	-1.27927	0.27335	0.775829	no
gene:SpnNT_00440	NA	Chromosome:451446-451704	110.58	110.58+peptide	OK	2.99986	2.00335	-0.582482	-0.378072	0.5766	0.969538	no
gene:SpnNT_00440	NA	Chromosome:451446-451704	ΔORF2	110.58+peptide	OK	0.73674	2.00335	1.44319	0.878102	0.389	0.88343	no
gene:SpnNT_00440	NA	Chromosome:451446-451704	110.58	ΔORF2+peptide	OK	2.99986	0	#NAME?	NA	6.00E-04	0.0111203	yes
gene:SpnNT_00440	NA	Chromosome:451446-451704	ΔORF2	ΔORF2+peptide	NOTEST	0.73674	0	#NAME?	0	1	1	no
gene:SpnNT_00440	NA	Chromosome:451446-451704	110.58+peptide	ΔORF2+peptide	OK	2.00335	0	#NAME?	NA	0.00505	0.0609063	no
gene:SpnNT_00441	ykoD_1	Chromosome:451805-454303	110.58	ΔORF2	OK	45.1068	45.1706	0.00203964	0.00407197	0.99335	0.998522	no
gene:SpnNT_00441	ykoD_1	Chromosome:451805-454303	110.58	110.58+peptide	OK	45.1068	48.2982	0.0986234	0.194328	0.73565	0.994748	no
gene:SpnNT_00441	ykoD_1	Chromosome:451805-454303	ΔORF2	110.58+peptide	OK	45.1706	48.2982	0.0965838	0.190307	0.7418	0.994748	no
gene:SpnNT_00441	ykoD_1	Chromosome:451805-454303	110.58	ΔORF2+peptide	OK	45.1068	57.065	0.339261	0.675049	0.23785	0.732745	no
gene:SpnNT_00441	ykoD_1	Chromosome:451805-454303	ΔORF2	ΔORF2+peptide	OK	45.1706	57.065	0.337222	0.670985	0.2433	0.739024	no
gene:SpnNT_00441	ykoD_1	Chromosome:451805-454303	110.58+peptide	ΔORF2+peptide	OK	48.2982	57.065	0.240638	0.47261	0.41	0.898184	no
gene:SpnNT_00442	ecfT_1	Chromosome:451805-454303	110.58	ΔORF2	OK	28.663	28.8931	0.0115343	0.0119579	0.98525	0.996796	no
gene:SpnNT_00442	ecfT_1	Chromosome:451805-454303	110.58	110.58+peptide	OK	28.663	36.4254	0.345756	0.364583	0.52825	0.956161	no
gene:SpnNT_00442	ecfT_1	Chromosome:451805-454303	ΔORF2	110.58+peptide	OK	28.8931	36.4254	0.334222	0.352113	0.54135	0.961494	no
gene:SpnNT_00442	ecfT_1	Chromosome:451805-454303	110.58	ΔORF2+peptide	OK	28.663	43.3293	0.596151	0.629008	0.27725	0.780208	no
gene:SpnNT_00442	ecfT_1	Chromosome:451805-454303	ΔORF2	ΔORF2+peptide	OK	28.8931	43.3293	0.584617	0.616298	0.286	0.789224	no
gene:SpnNT_00442	ecfT_1	Chromosome:451805-454303	110.58+peptide	ΔORF2+peptide	OK	36.4254	43.3293	0.250395	0.268635	0.6389	0.980887	no
gene:SpnNT_00443	NA	Chromosome:454495-454999	110.58	ΔORF2	OK	27.8953	26.0881	-0.0966294	-0.164511	0.77645	0.994748	no
gene:SpnNT_00443	NA	Chromosome:454495-454999	110.58	110.58+peptide	OK	27.8953	50.3211	0.851144	1.51	0.00935	0.0961526	no

gene:SpnNT_00443	NA	Chromosome:454495-454999	ΔORF2	110.58+peptide	OK	26.0881	50.3211	0.947774	1.68217	0.00365	0.0469562	yes
gene:SpnNT_00443	NA	Chromosome:454495-454999	110.58	ΔORF2+peptide	OK	27.8953	68.56	1.29735	2.32733	2.00E-04	0.00450928	yes
gene:SpnNT_00443	NA	Chromosome:454495-454999	ΔORF2	ΔORF2+peptide	OK	26.0881	68.56	1.39398	2.50181	1.00E-04	0.0025332	yes
gene:SpnNT_00443	NA	Chromosome:454495-454999	110.58+peptide	ΔORF2+peptide	OK	50.3211	68.56	0.446203	0.8385	0.15085	0.593025	no
gene:SpnNT_00444	ribU	Chromosome:455435-456639	110.58	ΔORF2	OK	183.407	199.119	0.118581	0.215093	0.7132	0.991272	no
gene:SpnNT_00444	ribU	Chromosome:455435-456639	110.58	110.58+peptide	OK	183.407	310.691	0.760429	1.40359	0.01425	0.129404	no
gene:SpnNT_00444	ribU	Chromosome:455435-456639	ΔORF2	110.58+peptide	OK	199.119	310.691	0.641847	1.18499	0.04155	0.277606	no
gene:SpnNT_00444	ribU	Chromosome:455435-456639	110.58	ΔORF2+peptide	OK	183.407	347.696	0.922776	1.73398	0.00265	0.0362222	yes
gene:SpnNT_00444	ribU	Chromosome:455435-456639	ΔORF2	ΔORF2+peptide	OK	199.119	347.696	0.804195	1.51152	0.0085	0.08966	no
gene:SpnNT_00444	ribU	Chromosome:455435-456639	110.58+peptide	ΔORF2+peptide	OK	310.691	347.696	0.162348	0.310913	0.58935	0.974354	no
gene:SpnNT_00445	NA	Chromosome:455435-456639	110.58	ΔORF2	OK	80.3774	84.5215	0.0725289	0.0936095	0.8725	0.994748	no
gene:SpnNT_00445	NA	Chromosome:455435-456639	110.58	110.58+peptide	OK	80.3774	100.098	0.316545	0.365356	0.5107	0.948939	no
gene:SpnNT_00445	NA	Chromosome:455435-456639	ΔORF2	110.58+peptide	OK	84.5215	100.098	0.244017	0.276984	0.62785	0.980887	no
gene:SpnNT_00445	NA	Chromosome:455435-456639	110.58	ΔORF2+peptide	OK	80.3774	113.35	0.495922	0.633365	0.26865	0.770416	no
gene:SpnNT_00445	NA	Chromosome:455435-456639	ΔORF2	ΔORF2+peptide	OK	84.5215	113.35	0.423393	0.529843	0.3578	0.858956	no
gene:SpnNT_00445	NA	Chromosome:455435-456639	110.58+peptide	ΔORF2+peptide	OK	100.098	113.35	0.179377	0.201957	0.7136	0.991514	no
gene:SpnNT_00446	NA	Chromosome:456666-457056	110.58	ΔORF2	OK	142.774	141.892	-0.0089377	-0.0177756	0.97365	0.99536	no
gene:SpnNT_00446	NA	Chromosome:456666-457056	110.58	110.58+peptide	OK	142.774	199.419	0.482071	0.958114	0.0927	0.459716	no
gene:SpnNT_00446	NA	Chromosome:456666-457056	ΔORF2	110.58+peptide	OK	141.892	199.419	0.491008	0.968326	0.08305	0.431597	no
gene:SpnNT_00446	NA	Chromosome:456666-457056	110.58	ΔORF2+peptide	OK	142.774	208.705	0.547735	1.09297	0.05695	0.339527	no
gene:SpnNT_00446	NA	Chromosome:456666-457056	ΔORF2	ΔORF2+peptide	OK	141.892	208.705	0.556673	1.10214	0.0523	0.323717	no
gene:SpnNT_00446	NA	Chromosome:456666-457056	110.58+peptide	ΔORF2+peptide	OK	199.419	208.705	0.0656644	0.129921	0.81775	0.994748	no
gene:SpnNT_00447	NA	Chromosome:457060-457510	110.58	ΔORF2	OK	160.462	171.505	0.0960145	0.198927	0.7253	0.994748	no
gene:SpnNT_00447	NA	Chromosome:457060-457510	110.58	110.58+peptide	OK	160.462	227.944	0.506447	1.0666	0.06685	0.378106	no
gene:SpnNT_00447	NA	Chromosome:457060-457510	ΔORF2	110.58+peptide	OK	171.505	227.944	0.410433	0.864931	0.13415	0.557156	no
gene:SpnNT_00447	NA	Chromosome:457060-457510	110.58	ΔORF2+peptide	OK	160.462	260.11	0.696887	1.46414	0.0115	0.110795	no
gene:SpnNT_00447	NA	Chromosome:457060-457510	ΔORF2	ΔORF2+peptide	OK	171.505	260.11	0.600873	1.2632	0.0274	0.209891	no
gene:SpnNT_00447	NA	Chromosome:457060-457510	110.58+peptide	ΔORF2+peptide	OK	227.944	260.11	0.19044	0.407166	0.48045	0.934687	no
gene:SpnNT_00448	rpoE	Chromosome:457637-458225	110.58	ΔORF2	OK	856.648	955.824	0.158043	0.350654	0.53485	0.958867	no
gene:SpnNT_00448	rpoE	Chromosome:457637-458225	110.58	110.58+peptide	OK	856.648	1073.94	0.326141	0.702811	0.2179	0.705473	no
gene:SpnNT_00448	rpoE	Chromosome:457637-458225	ΔORF2	110.58+peptide	OK	955.824	1073.94	0.168098	0.362385	0.52345	0.954832	no
gene:SpnNT_00448	rpoE	Chromosome:457637-458225	110.58	ΔORF2+peptide	OK	856.648	831.564	-0.0428751	-0.0944601	0.86745	0.994748	no
gene:SpnNT_00448	rpoE	Chromosome:457637-458225	ΔORF2	ΔORF2+peptide	OK	955.824	831.564	-0.200918	-0.44284	0.43335	0.913334	no
gene:SpnNT_00448	rpoE	Chromosome:457637-458225	110.58+peptide	ΔORF2+peptide	OK	1073.94	831.564	-0.369016	-0.790249	0.167	0.622903	no
gene:SpnNT_00449	pyrG	Chromosome:458614-460222	110.58	ΔORF2	OK	57.6678	56.612	-0.026656	-0.0591627	0.9177	0.994748	no
gene:SpnNT_00449	pyrG	Chromosome:458614-460222	110.58	110.58+peptide	OK	57.6678	106.3	0.882307	1.96976	0.001	0.0168447	yes
gene:SpnNT_00449	pyrG	Chromosome:458614-460222	ΔORF2	110.58+peptide	OK	56.612	106.3	0.908963	2.00948	0.00075	0.0133173	yes
gene:SpnNT_00449	pyrG	Chromosome:458614-460222	110.58	ΔORF2+peptide	OK	57.6678	116.749	1.01757	2.29403	5.00E-05	0.0013612	yes
gene:SpnNT_00449	pyrG	Chromosome:458614-460222	ΔORF2	ΔORF2+peptide	OK	56.612	116.749	1.04423	2.33071	5.00E-05	0.0013612	yes
gene:SpnNT_00449	pyrG	Chromosome:458614-460222	110.58+peptide	ΔORF2+peptide	OK	106.3	116.749	0.135268	0.303709	0.60115	0.976678	no
gene:SpnNT_00450	NA	Chromosome:460780-462411	110.58	ΔORF2	OK	34.6434	33.1494	-0.0635961	-0.120186	0.8314	0.994748	no
gene:SpnNT_00450	NA	Chromosome:460780-462411	110.58	110.58+peptide	OK	34.6434	42.1603	0.283306	0.537205	0.3465	0.850818	no
gene:SpnNT_00450	NA	Chromosome:460780-462411	ΔORF2	110.58+peptide	OK	33.1494	42.1603	0.346902	0.660829	0.2505	0.747909	no
gene:SpnNT_00450	NA	Chromosome:460780-462411	110.58	ΔORF2+peptide	OK	34.6434	37.4611	0.112815	0.212946	0.70975	0.990851	no
gene:SpnNT_00450	NA	Chromosome:460780-462411	ΔORF2	ΔORF2+peptide	OK	33.1494	37.4611	0.176411	0.334509	0.56145	0.968621	no

gene:SpnNT_00450	NA	Chromosome:460780-462411	110.58+peptide	ΔORF2+peptide	OK	42.1603	37.4611	-0.170491	-0.32438	0.57565	0.969538	no
gene:SpnNT_00451	NA	Chromosome:460780-462411	110.58	ΔORF2	OK	33.808	27.0468	-0.32191	-0.269833	0.6406	0.980887	no
gene:SpnNT_00451	NA	Chromosome:460780-462411	110.58	110.58+peptide	OK	33.808	33.9278	0.00509956	0.00437333	0.99325	0.998522	no
gene:SpnNT_00451	NA	Chromosome:460780-462411	ΔORF2	110.58+peptide	OK	27.0468	33.9278	0.327009	0.26517	0.6506	0.981475	no
gene:SpnNT_00451	NA	Chromosome:460780-462411	110.58	ΔORF2+peptide	OK	33.808	38.4973	0.18739	0.16988	0.7701	0.994748	no
gene:SpnNT_00451	NA	Chromosome:460780-462411	ΔORF2	ΔORF2+peptide	OK	27.0468	38.4973	0.5093	0.433878	0.44985	0.920463	no
gene:SpnNT_00451	NA	Chromosome:460780-462411	110.58+peptide	ΔORF2+peptide	OK	33.9278	38.4973	0.182291	0.159005	0.78855	0.994748	no
gene:SpnNT_00452	nanA_1	Chromosome:462699-466614	110.58	ΔORF2	OK	6.12768	6.13712	0.00222166	0.00430364	0.99325	0.998522	no
gene:SpnNT_00452	nanA_1	Chromosome:462699-466614	110.58	110.58+peptide	OK	6.12768	5.33077	-0.200996	-0.386905	0.49385	0.941904	no
gene:SpnNT_00452	nanA_1	Chromosome:462699-466614	ΔORF2	110.58+peptide	OK	6.13712	5.33077	-0.203218	-0.391302	0.4875	0.938799	no
gene:SpnNT_00452	nanA_1	Chromosome:462699-466614	110.58	ΔORF2+peptide	OK	6.12768	4.58589	-0.418139	-0.795955	0.1659	0.621332	no
gene:SpnNT_00452	nanA_1	Chromosome:462699-466614	ΔORF2	ΔORF2+peptide	OK	6.13712	4.58589	-0.420361	-0.800424	0.1657	0.621332	no
gene:SpnNT_00452	nanA_1	Chromosome:462699-466614	110.58+peptide	ΔORF2+peptide	OK	5.33077	4.58589	-0.217143	-0.410956	0.4686	0.927504	no
gene:SpnNT_00453	strH_3	Chromosome:466627-467575	110.58	ΔORF2	OK	7.36437	5.3582	-0.458815	-0.716634	0.21195	0.697569	no
gene:SpnNT_00453	strH_3	Chromosome:466627-467575	110.58	110.58+peptide	OK	7.36437	4.4248	-0.734952	-1.12797	0.0541	0.330494	no
gene:SpnNT_00453	strH_3	Chromosome:466627-467575	ΔORF2	110.58+peptide	OK	5.3582	4.4248	-0.276137	-0.415862	0.46475	0.926394	no
gene:SpnNT_00453	strH_3	Chromosome:466627-467575	110.58	ΔORF2+peptide	OK	7.36437	5.23191	-0.493225	-0.759143	0.1961	0.677521	no
gene:SpnNT_00453	strH_3	Chromosome:466627-467575	ΔORF2	ΔORF2+peptide	OK	5.3582	5.23191	-0.0344108	-0.0519653	0.92665	0.994748	no
gene:SpnNT_00453	strH_3	Chromosome:466627-467575	110.58+peptide	ΔORF2+peptide	OK	4.4248	5.23191	0.241726	0.359094	0.5424	0.961518	no
gene:SpnNT_00454	pgk	Chromosome:467664-468861	110.58	ΔORF2	OK	876.249	706.575	-0.310499	-0.666945	0.24995	0.747607	no
gene:SpnNT_00454	pgk	Chromosome:467664-468861	110.58	110.58+peptide	OK	876.249	855.476	-0.0346137	-0.0722128	0.89995	0.994748	no
gene:SpnNT_00454	pgk	Chromosome:467664-468861	ΔORF2	110.58+peptide	OK	706.575	855.476	0.275885	0.581745	0.30365	0.809137	no
gene:SpnNT_00454	pgk	Chromosome:467664-468861	110.58	ΔORF2+peptide	OK	876.249	792.004	-0.145834	-0.315544	0.5706	0.968621	no
gene:SpnNT_00454	pgk	Chromosome:467664-468861	ΔORF2	ΔORF2+peptide	OK	706.575	792.004	0.164665	0.36041	0.5203	0.954744	no
gene:SpnNT_00454	pgk	Chromosome:467664-468861	110.58+peptide	ΔORF2+peptide	OK	855.476	792.004	-0.11122	-0.23618	0.6683	0.984776	no
gene:SpnNT_00455	NA	Chromosome:468996-469524	110.58	ΔORF2	OK	113.556	197.609	0.799248	1.6683	0.00405	0.0512479	no
gene:SpnNT_00455	NA	Chromosome:468996-469524	110.58	110.58+peptide	OK	113.556	84.6707	-0.423464	-0.857328	0.13155	0.551501	no
gene:SpnNT_00455	NA	Chromosome:468996-469524	ΔORF2	110.58+peptide	OK	197.609	84.6707	-1.22271	-2.49237	5.00E-05	0.0013612	yes
gene:SpnNT_00455	NA	Chromosome:468996-469524	110.58	ΔORF2+peptide	OK	113.556	115.976	0.0304252	0.0623561	0.91435	0.994748	no
gene:SpnNT_00455	NA	Chromosome:468996-469524	ΔORF2	ΔORF2+peptide	OK	197.609	115.976	-0.768823	-1.58673	0.0064	0.0723972	no
gene:SpnNT_00455	NA	Chromosome:468996-469524	110.58+peptide	ΔORF2+peptide	OK	84.6707	115.976	0.453889	0.909187	0.11955	0.524135	no
gene:SpnNT_00456	glnR	Chromosome:469600-469957	110.58	ΔORF2	OK	320.087	442.64	0.46767	1.00653	0.0793	0.419248	no
gene:SpnNT_00456	glnR	Chromosome:469600-469957	110.58	110.58+peptide	OK	320.087	208.095	-0.621225	-1.27926	0.02765	0.211682	no
gene:SpnNT_00456	glnR	Chromosome:469600-469957	ΔORF2	110.58+peptide	OK	442.64	208.095	-1.08889	-2.26641	2.00E-04	0.00450928	yes
gene:SpnNT_00456	glnR	Chromosome:469600-469957	110.58	ΔORF2+peptide	OK	320.087	225.409	-0.505923	-1.05846	0.06785	0.381133	no
gene:SpnNT_00456	glnR	Chromosome:469600-469957	ΔORF2	ΔORF2+peptide	OK	442.64	225.409	-0.973592	-2.0595	0.00095	0.0161266	yes
gene:SpnNT_00456	glnR	Chromosome:469600-469957	110.58+peptide	ΔORF2+peptide	OK	208.095	225.409	0.115302	0.233705	0.6877	0.98828	no
gene:SpnNT_00457	glnA	Chromosome:469993-471340	110.58	ΔORF2	OK	213.297	245.751	0.204335	0.467206	0.4124	0.899511	no
gene:SpnNT_00457	glnA	Chromosome:469993-471340	110.58	110.58+peptide	OK	213.297	155.558	-0.455407	-1.04543	0.064	0.367209	no
gene:SpnNT_00457	glnA	Chromosome:469993-471340	ΔORF2	110.58+peptide	OK	245.751	155.558	-0.659742	-1.50488	0.00875	0.091488	no
gene:SpnNT_00457	glnA	Chromosome:469993-471340	110.58	ΔORF2+peptide	OK	213.297	171.09	-0.318109	-0.728171	0.2028	0.686746	no
gene:SpnNT_00457	glnA	Chromosome:469993-471340	ΔORF2	ΔORF2+peptide	OK	245.751	171.09	-0.522444	-1.18835	0.0408	0.274553	no
gene:SpnNT_00457	glnA	Chromosome:469993-471340	110.58+peptide	ΔORF2+peptide	OK	155.558	171.09	0.137297	0.313529	0.58025	0.969538	no
gene:SpnNT_00458	NA	Chromosome:471519-471669	110.58	ΔORF2	NOTEST	0	0	0	0	1	1	no
gene:SpnNT_00458	NA	Chromosome:471519-471669	110.58	110.58+peptide	NOTEST	0	0	0	0	1	1	no

gene:SpnNT_00458	NA	Chromosome:471519-471669	ΔORF2	110.58+peptide	NOTEST	0	0	0	0	1	1	no
gene:SpnNT_00458	NA	Chromosome:471519-471669	110.58	ΔORF2+peptide	NOTEST	0	0	0	0	1	1	no
gene:SpnNT_00458	NA	Chromosome:471519-471669	ΔORF2	ΔORF2+peptide	NOTEST	0	0	0	0	1	1	no
gene:SpnNT_00458	NA	Chromosome:471519-471669	110.58+peptide	ΔORF2+peptide	NOTEST	0	0	0	0	1	1	no
gene:SpnNT_00459	hsdS_1	Chromosome:472135-473419	110.58	ΔORF2	OK	10.9811	12.9835	0.241662	0.435786	0.43625	0.914104	no
gene:SpnNT_00459	hsdS_1	Chromosome:472135-473419	110.58	110.58+peptide	OK	10.9811	11.811	0.105116	0.186725	0.74045	0.994748	no
gene:SpnNT_00459	hsdS_1	Chromosome:472135-473419	ΔORF2	110.58+peptide	OK	12.9835	11.811	-0.136547	-0.245544	0.6576	0.982966	no
gene:SpnNT_00459	hsdS_1	Chromosome:472135-473419	110.58	ΔORF2+peptide	OK	10.9811	18.2967	0.736562	1.35688	0.0166	0.144638	no
gene:SpnNT_00459	hsdS_1	Chromosome:472135-473419	ΔORF2	ΔORF2+peptide	OK	12.9835	18.2967	0.494899	0.923777	0.1	0.479559	no
gene:SpnNT_00459	hsdS_1	Chromosome:472135-473419	110.58+peptide	ΔORF2+peptide	OK	11.811	18.2967	0.631446	1.15984	0.0398	0.270039	no
gene:SpnNT_00460	xerC_1	Chromosome:473475-474273	110.58	ΔORF2	OK	26.5726	30.5333	0.200446	0.365235	0.51035	0.948516	no
gene:SpnNT_00460	xerC_1	Chromosome:473475-474273	110.58	110.58+peptide	OK	26.5726	29.1546	0.133785	0.246037	0.66905	0.984845	no
gene:SpnNT_00460	xerC_1	Chromosome:473475-474273	ΔORF2	110.58+peptide	OK	30.5333	29.1546	-0.0666615	-0.121185	0.8337	0.994748	no
gene:SpnNT_00460	xerC_1	Chromosome:473475-474273	110.58	ΔORF2+peptide	OK	26.5726	29.9113	0.170754	0.317681	0.57745	0.969538	no
gene:SpnNT_00460	xerC_1	Chromosome:473475-474273	ΔORF2	ΔORF2+peptide	OK	30.5333	29.9113	-0.0296923	-0.0545918	0.9246	0.994748	no
gene:SpnNT_00460	xerC_1	Chromosome:473475-474273	110.58+peptide	ΔORF2+peptide	OK	29.1546	29.9113	0.0369692	0.0686147	0.9054	0.994748	no
gene:SpnNT_00461	NA	Chromosome:474592-477850	110.58	ΔORF2	OK	9.72181	18.9971	0.966479	0.313593	0.69255	0.98828	no
gene:SpnNT_00461	NA	Chromosome:474592-477850	110.58	110.58+peptide	OK	9.72181	13.9479	0.520755	0.167764	0.81275	0.994748	no
gene:SpnNT_00461	NA	Chromosome:474592-477850	ΔORF2	110.58+peptide	OK	18.9971	13.9479	-0.445724	-0.159892	0.85005	0.994748	no
gene:SpnNT_00461	NA	Chromosome:474592-477850	110.58	ΔORF2+peptide	OK	9.72181	17.9833	0.887365	0.273306	0.68735	0.98828	no
gene:SpnNT_00461	NA	Chromosome:474592-477850	ΔORF2	ΔORF2+peptide	OK	18.9971	17.9833	-0.0791144	-0.0268576	0.9565	0.994855	no
gene:SpnNT_00461	NA	Chromosome:474592-477850	110.58+peptide	ΔORF2+peptide	OK	13.9479	17.9833	0.366609	0.123485	0.867	0.994748	no
gene:SpnNT_00462	hsdS_2	Chromosome:474592-477850	110.58	ΔORF2	OK	15.7931	21.8463	0.468094	0.604875	0.29225	0.797277	no
gene:SpnNT_00462	hsdS_2	Chromosome:474592-477850	110.58	110.58+peptide	OK	15.7931	15.1312	-0.0617644	-0.0742682	0.8976	0.994748	no
gene:SpnNT_00462	hsdS_2	Chromosome:474592-477850	ΔORF2	110.58+peptide	OK	21.8463	15.1312	-0.529858	-0.665875	0.2544	0.75336	no
gene:SpnNT_00462	hsdS_2	Chromosome:474592-477850	110.58	ΔORF2+peptide	OK	15.7931	26.6277	0.753633	0.998356	0.08245	0.42967	no
gene:SpnNT_00462	hsdS_2	Chromosome:474592-477850	ΔORF2	ΔORF2+peptide	OK	21.8463	26.6277	0.285539	0.399287	0.4808	0.935095	no
gene:SpnNT_00462	hsdS_2	Chromosome:474592-477850	110.58+peptide	ΔORF2+peptide	OK	15.1312	26.6277	0.815397	1.04905	0.0743	0.401882	no
gene:SpnNT_00463	NA	Chromosome:474592-477850	110.58	ΔORF2	OK	26.4801	32.04	0.274966	0.440954	0.44375	0.917631	no
gene:SpnNT_00463	NA	Chromosome:474592-477850	110.58	110.58+peptide	OK	26.4801	25.7563	-0.0399822	-0.0624113	0.9135	0.994748	no
gene:SpnNT_00463	NA	Chromosome:474592-477850	ΔORF2	110.58+peptide	OK	32.04	25.7563	-0.314948	-0.497393	0.3896	0.883875	no
gene:SpnNT_00463	NA	Chromosome:474592-477850	110.58	ΔORF2+peptide	OK	26.4801	35.2648	0.413322	0.664247	0.24805	0.74413	no
gene:SpnNT_00463	NA	Chromosome:474592-477850	ΔORF2	ΔORF2+peptide	OK	32.04	35.2648	0.138356	0.225118	0.6966	0.989512	no
gene:SpnNT_00463	NA	Chromosome:474592-477850	110.58+peptide	ΔORF2+peptide	OK	25.7563	35.2648	0.453304	0.717379	0.2125	0.698345	no
gene:SpnNT_00464	hsdR_1	Chromosome:477862-480196	110.58	ΔORF2	OK	47.639	50.9877	0.0980075	0.221696	0.69375	0.98828	no
gene:SpnNT_00464	hsdR_1	Chromosome:477862-480196	110.58	110.58+peptide	OK	47.639	46.7522	-0.0271091	-0.0604489	0.9158	0.994748	no
gene:SpnNT_00464	hsdR_1	Chromosome:477862-480196	ΔORF2	110.58+peptide	OK	50.9877	46.7522	-0.125117	-0.27916	0.62675	0.980887	no
gene:SpnNT_00464	hsdR_1	Chromosome:477862-480196	110.58	ΔORF2+peptide	OK	47.639	48.1268	0.0146963	0.0331691	0.9537	0.994855	no
gene:SpnNT_00464	hsdR_1	Chromosome:477862-480196	ΔORF2	ΔORF2+peptide	OK	50.9877	48.1268	-0.0833113	-0.188149	0.74545	0.994748	no
gene:SpnNT_00464	hsdR_1	Chromosome:477862-480196	110.58+peptide	ΔORF2+peptide	OK	46.7522	48.1268	0.0418053	0.0930734	0.87155	0.994748	no
gene:SpnNT_00465	NA	Chromosome:480803-481313	110.58	ΔORF2	OK	1.77117	0.714493	-1.30971	-1.12419	0.1065	0.496269	no
gene:SpnNT_00465	NA	Chromosome:480803-481313	110.58	110.58+peptide	OK	1.77117	1.25497	-0.497049	-0.436962	0.4564	0.921244	no
gene:SpnNT_00465	NA	Chromosome:480803-481313	ΔORF2	110.58+peptide	OK	0.714493	1.25497	0.812662	0.66464	0.3036	0.809137	no
gene:SpnNT_00465	NA	Chromosome:480803-481313	110.58	ΔORF2+peptide	OK	1.77117	0.987676	-0.842593	-0.756824	0.25095	0.748571	no
gene:SpnNT_00465	NA	Chromosome:480803-481313	ΔORF2	ΔORF2+peptide	NOTEST	0.714493	0.987676	0.467118	0	1	1	no

gene:SpnNT_00465	NA	Chromosome:480803-481313	110.58+peptide	ΔORF2+peptide	OK	1.25497	0.987676	-0.345544	-0.294442	0.6501	0.981475	no
gene:SpnNT_00466	hrcA	Chromosome:481476-482511	110.58	ΔORF2	OK	368.212	367.162	-0.00412294	-0.00931339	0.98665	0.997339	no
gene:SpnNT_00466	hrcA	Chromosome:481476-482511	110.58	110.58+peptide	OK	368.212	944.415	1.35888	3.00978	5.00E-05	0.0013612	yes
gene:SpnNT_00466	hrcA	Chromosome:481476-482511	ΔORF2	110.58+peptide	OK	367.162	944.415	1.36301	3.04895	5.00E-05	0.0013612	yes
gene:SpnNT_00466	hrcA	Chromosome:481476-482511	110.58	ΔORF2+peptide	OK	368.212	997.246	1.43741	3.19663	5.00E-05	0.0013612	yes
gene:SpnNT_00466	hrcA	Chromosome:481476-482511	ΔORF2	ΔORF2+peptide	OK	367.162	997.246	1.44153	3.23795	5.00E-05	0.0013612	yes
gene:SpnNT_00466	hrcA	Chromosome:481476-482511	110.58+peptide	ΔORF2+peptide	OK	944.415	997.246	0.0785283	0.172989	0.75605	0.994748	no
gene:SpnNT_00467	grpE	Chromosome:482537-483062	110.58	ΔORF2	OK	656.282	547.778	-0.260727	-0.594569	0.29355	0.797915	no
gene:SpnNT_00467	grpE	Chromosome:482537-483062	110.58	110.58+peptide	OK	656.282	2919.89	2.15352	3.89856	5.00E-05	0.0013612	yes
gene:SpnNT_00467	grpE	Chromosome:482537-483062	ΔORF2	110.58+peptide	OK	547.778	2919.89	2.41425	4.39626	5.00E-05	0.0013612	yes
gene:SpnNT_00467	grpE	Chromosome:482537-483062	110.58	ΔORF2+peptide	OK	656.282	1178.29	0.844305	1.91577	0.00105	0.0174849	yes
gene:SpnNT_00467	grpE	Chromosome:482537-483062	ΔORF2	ΔORF2+peptide	OK	547.778	1178.29	1.10503	2.53065	5.00E-05	0.0013612	yes
gene:SpnNT_00467	grpE	Chromosome:482537-483062	110.58+peptide	ΔORF2+peptide	OK	2919.89	1178.29	-1.30922	-2.37643	1.00E-04	0.0025332	yes
gene:SpnNT_00468	dnaK	Chromosome:483541-485365	110.58	ΔORF2	OK	509.029	496.75	-0.0352275	-0.076784	0.891	0.994748	no
gene:SpnNT_00468	dnaK	Chromosome:483541-485365	110.58	110.58+peptide	OK	509.029	977.915	0.941962	2.02434	5.00E-04	0.0095781	yes
gene:SpnNT_00468	dnaK	Chromosome:483541-485365	ΔORF2	110.58+peptide	OK	496.75	977.915	0.977189	2.11041	0.00015	0.00355289	yes
gene:SpnNT_00468	dnaK	Chromosome:483541-485365	110.58	ΔORF2+peptide	OK	509.029	1032.5	1.02032	2.18677	0.00035	0.00717609	yes
gene:SpnNT_00468	dnaK	Chromosome:483541-485365	ΔORF2	ΔORF2+peptide	OK	496.75	1032.5	1.05555	2.27337	3.00E-04	0.00631878	yes
gene:SpnNT_00468	dnaK	Chromosome:483541-485365	110.58+peptide	ΔORF2+peptide	OK	977.915	1032.5	0.0783568	0.166447	0.77345	0.994748	no
gene:SpnNT_00469	dnaJ	Chromosome:486123-487260	110.58	ΔORF2	OK	372.767	323.749	-0.203402	-0.449125	0.42275	0.905982	no
gene:SpnNT_00469	dnaJ	Chromosome:486123-487260	110.58	110.58+peptide	OK	372.767	537.042	0.526761	1.17753	0.03945	0.268637	no
gene:SpnNT_00469	dnaJ	Chromosome:486123-487260	ΔORF2	110.58+peptide	OK	323.749	537.042	0.730162	1.62375	0.00455	0.0556951	no
gene:SpnNT_00469	dnaJ	Chromosome:486123-487260	110.58	ΔORF2+peptide	OK	372.767	518.134	0.475051	1.061	0.0603	0.35261	no
gene:SpnNT_00469	dnaJ	Chromosome:486123-487260	ΔORF2	ΔORF2+peptide	OK	323.749	518.134	0.678453	1.50743	0.008	0.0859754	no
gene:SpnNT_00469	dnaJ	Chromosome:486123-487260	110.58+peptide	ΔORF2+peptide	OK	537.042	518.134	-0.0517096	-0.116333	0.82905	0.994748	no
gene:SpnNT_00470	NA	Chromosome:487985-488273	110.58	ΔORF2	OK	338.265	372.653	0.139676	0.276258	0.6237	0.980887	no
gene:SpnNT_00470	NA	Chromosome:487985-488273	110.58	110.58+peptide	OK	338.265	297.06	-0.1874	-0.360036	0.5136	0.95097	no
gene:SpnNT_00470	NA	Chromosome:487985-488273	ΔORF2	110.58+peptide	OK	372.653	297.06	-0.327076	-0.63897	0.25985	0.760041	no
gene:SpnNT_00470	NA	Chromosome:487985-488273	110.58	ΔORF2+peptide	OK	338.265	291.99	-0.212238	-0.409519	0.4598	0.923884	no
gene:SpnNT_00470	NA	Chromosome:487985-488273	ΔORF2	ΔORF2+peptide	OK	372.653	291.99	-0.351915	-0.690568	0.2269	0.71894	no
gene:SpnNT_00470	NA	Chromosome:487985-488273	110.58+peptide	ΔORF2+peptide	OK	297.06	291.99	-0.0248388	-0.047367	0.9326	0.994855	no
gene:SpnNT_00471	NA	Chromosome:488282-488693	110.58	ΔORF2	OK	72.3633	75.4858	0.0609471	0.111916	0.8389	0.994748	no
gene:SpnNT_00471	NA	Chromosome:488282-488693	110.58	110.58+peptide	OK	72.3633	42.5002	-0.767789	-1.37903	0.0172	0.148486	no
gene:SpnNT_00471	NA	Chromosome:488282-488693	ΔORF2	110.58+peptide	OK	75.4858	42.5002	-0.828736	-1.4779	0.0096	0.0977279	no
gene:SpnNT_00471	NA	Chromosome:488282-488693	110.58	ΔORF2+peptide	OK	72.3633	40.7156	-0.829676	-1.44808	0.01055	0.104245	no
gene:SpnNT_00471	NA	Chromosome:488282-488693	ΔORF2	ΔORF2+peptide	OK	75.4858	40.7156	-0.890623	-1.544	0.00625	0.0710065	no
gene:SpnNT_00471	NA	Chromosome:488282-488693	110.58+peptide	ΔORF2+peptide	OK	42.5002	40.7156	-0.0618868	-0.105188	0.8518	0.994748	no
gene:SpnNT_00472	ecsA_1	Chromosome:488760-490538	110.58	ΔORF2	OK	107.351	113.213	0.0767037	0.109644	0.84925	0.994748	no
gene:SpnNT_00472	ecsA_1	Chromosome:488760-490538	110.58	110.58+peptide	OK	107.351	76.9168	-0.480963	-0.640113	0.26185	0.762031	no
gene:SpnNT_00472	ecsA_1	Chromosome:488760-490538	ΔORF2	110.58+peptide	OK	113.213	76.9168	-0.557666	-0.74314	0.19705	0.678125	no
gene:SpnNT_00472	ecsA_1	Chromosome:488760-490538	110.58	ΔORF2+peptide	OK	107.351	72.7018	-0.562271	-0.740297	0.19395	0.675249	no
gene:SpnNT_00472	ecsA_1	Chromosome:488760-490538	ΔORF2	ΔORF2+peptide	OK	113.213	72.7018	-0.638975	-0.842331	0.1449	0.580489	no
gene:SpnNT_00472	ecsA_1	Chromosome:488760-490538	110.58+peptide	ΔORF2+peptide	OK	76.9168	72.7018	-0.0813087	-0.100804	0.85975	0.994748	no
gene:SpnNT_00473	NA	Chromosome:488760-490538	110.58	ΔORF2	OK	98.2383	107.348	0.127942	0.226392	0.69535	0.98895	no
gene:SpnNT_00473	NA	Chromosome:488760-490538	110.58	110.58+peptide	OK	98.2383	81.9898	-0.260841	-0.465599	0.42595	0.908389	no

gene:SpnNT_00473	NA	Chromosome:488760-490538	ΔORF2	110.58+peptide	OK	107.348	81.9898	-0.388783	-0.694198	0.23165	0.724662	no
gene:SpnNT_00473	NA	Chromosome:488760-490538	110.58	ΔORF2+peptide	OK	98.2383	94.7443	-0.0522456	-0.0941754	0.8707	0.994748	no
gene:SpnNT_00473	NA	Chromosome:488760-490538	ΔORF2	ΔORF2+peptide	OK	107.348	94.7443	-0.180188	-0.324904	0.5771	0.969538	no
gene:SpnNT_00473	NA	Chromosome:488760-490538	110.58+peptide	ΔORF2+peptide	OK	81.9898	94.7443	0.208596	0.379552	0.50995	0.948082	no
gene:SpnNT_00474	NA	Chromosome:490598-490928	110.58	ΔORF2	OK	4.59927	2.9603	-0.635664	-0.565616	0.33085	0.836657	no
gene:SpnNT_00474	NA	Chromosome:490598-490928	110.58	110.58+peptide	OK	4.59927	1.7162	-1.42219	-1.14663	0.07	0.388882	no
gene:SpnNT_00474	NA	Chromosome:490598-490928	ΔORF2	110.58+peptide	OK	2.9603	1.7162	-0.786528	-0.592602	0.3219	0.827743	no
gene:SpnNT_00474	NA	Chromosome:490598-490928	110.58	ΔORF2+peptide	OK	4.59927	2.70964	-0.763306	-0.821564	0.25965	0.759841	no
gene:SpnNT_00474	NA	Chromosome:490598-490928	ΔORF2	ΔORF2+peptide	OK	2.9603	2.70964	-0.127643	-0.122463	0.8532	0.994748	no
gene:SpnNT_00474	NA	Chromosome:490598-490928	110.58+peptide	ΔORF2+peptide	OK	1.7162	2.70964	0.658886	0.564623	0.41655	0.901786	no
gene:SpnNT_00475	NA	Chromosome:491182-491521	110.58	ΔORF2	OK	48.9614	66.6838	0.445692	0.756346	0.1897	0.667533	no
gene:SpnNT_00475	NA	Chromosome:491182-491521	110.58	110.58+peptide	OK	48.9614	33.207	-0.560157	-0.916059	0.11525	0.515092	no
gene:SpnNT_00475	NA	Chromosome:491182-491521	ΔORF2	110.58+peptide	OK	66.6838	33.207	-1.00585	-1.65073	0.0056	0.0654347	no
gene:SpnNT_00475	NA	Chromosome:491182-491521	110.58	ΔORF2+peptide	OK	48.9614	45.0415	-0.120389	-0.19102	0.7366	0.994748	no
gene:SpnNT_00475	NA	Chromosome:491182-491521	ΔORF2	ΔORF2+peptide	OK	66.6838	45.0415	-0.566081	-0.901178	0.1159	0.516286	no
gene:SpnNT_00475	NA	Chromosome:491182-491521	110.58+peptide	ΔORF2+peptide	OK	33.207	45.0415	0.439768	0.677562	0.2394	0.734795	no
gene:SpnNT_00476	NA	Chromosome:491525-492264	110.58	ΔORF2	OK	96.9141	114.781	0.244102	0.141485	0.81425	0.994748	no
gene:SpnNT_00476	NA	Chromosome:491525-492264	110.58	110.58+peptide	OK	96.9141	79.571	-0.284464	-0.147315	0.81305	0.994748	no
gene:SpnNT_00476	NA	Chromosome:491525-492264	ΔORF2	110.58+peptide	OK	114.781	79.571	-0.528566	-0.29037	0.64805	0.981391	no
gene:SpnNT_00476	NA	Chromosome:491525-492264	110.58	ΔORF2+peptide	OK	96.9141	80.1507	-0.273992	-0.153873	0.7979	0.994748	no
gene:SpnNT_00476	NA	Chromosome:491525-492264	ΔORF2	ΔORF2+peptide	OK	114.781	80.1507	-0.518095	-0.312111	0.6146	0.979616	no
gene:SpnNT_00476	NA	Chromosome:491525-492264	110.58+peptide	ΔORF2+peptide	OK	79.571	80.1507	0.0104717	0.00559131	0.98985	0.997703	no
gene:SpnNT_00477	agrA_1	Chromosome:491525-492264	110.58	ΔORF2	OK	59.2898	61.6131	0.055453	0.104123	0.8562	0.994748	no
gene:SpnNT_00477	agrA_1	Chromosome:491525-492264	110.58	110.58+peptide	OK	59.2898	33.805	-0.810546	-1.45176	0.01115	0.108378	no
gene:SpnNT_00477	agrA_1	Chromosome:491525-492264	ΔORF2	110.58+peptide	OK	61.6131	33.805	-0.865999	-1.55091	0.00775	0.0837689	no
gene:SpnNT_00477	agrA_1	Chromosome:491525-492264	110.58	ΔORF2+peptide	OK	59.2898	42.6798	-0.47423	-0.86894	0.12815	0.542797	no
gene:SpnNT_00477	agrA_1	Chromosome:491525-492264	ΔORF2	ΔORF2+peptide	OK	61.6131	42.6798	-0.529683	-0.970436	0.0964	0.470071	no
gene:SpnNT_00477	agrA_1	Chromosome:491525-492264	110.58+peptide	ΔORF2+peptide	OK	33.805	42.6798	0.336317	0.589025	0.3063	0.81251	no
gene:SpnNT_00478	citS	Chromosome:492277-493618	110.58	ΔORF2	OK	66.0265	75.408	0.191671	0.425744	0.45465	0.921244	no
gene:SpnNT_00478	citS	Chromosome:492277-493618	110.58	110.58+peptide	OK	66.0265	47.6376	-0.470944	-1.02381	0.07875	0.41735	no
gene:SpnNT_00478	citS	Chromosome:492277-493618	ΔORF2	110.58+peptide	OK	75.408	47.6376	-0.662616	-1.45304	0.01185	0.113916	no
gene:SpnNT_00478	citS	Chromosome:492277-493618	110.58	ΔORF2+peptide	OK	66.0265	55.4408	-0.252099	-0.552301	0.33385	0.839391	no
gene:SpnNT_00478	citS	Chromosome:492277-493618	ΔORF2	ΔORF2+peptide	OK	75.408	55.4408	-0.44377	-0.980814	0.08345	0.433504	no
gene:SpnNT_00478	citS	Chromosome:492277-493618	110.58+peptide	ΔORF2+peptide	OK	47.6376	55.4408	0.218846	0.473497	0.39815	0.891861	no
gene:SpnNT_00479	NA	Chromosome:493659-493815	110.58	ΔORF2	OK	21.8913	15.7301	-0.476827	-0.416507	0.56515	0.968621	no
gene:SpnNT_00479	NA	Chromosome:493659-493815	110.58	110.58+peptide	OK	21.8913	5.00395	-2.12922	-4.82236	0.2643	0.764751	no
gene:SpnNT_00479	NA	Chromosome:493659-493815	ΔORF2	110.58+peptide	OK	15.7301	5.00395	-1.65239	-1.38879	0.2975	0.802589	no
gene:SpnNT_00479	NA	Chromosome:493659-493815	110.58	ΔORF2+peptide	OK	21.8913	22.4712	0.0377183	0.12651	0.92815	0.994748	no
gene:SpnNT_00479	NA	Chromosome:493659-493815	ΔORF2	ΔORF2+peptide	OK	15.7301	22.4712	0.514545	0.449631	0.54745	0.962356	no
gene:SpnNT_00479	NA	Chromosome:493659-493815	110.58+peptide	ΔORF2+peptide	OK	5.00395	22.4712	2.16694	4.92077	0.26325	0.763902	no
gene:SpnNT_00480	lcnD_2	Chromosome:493871-495233	110.58	ΔORF2	OK	3.1297	3.21897	0.0405764	0.0603384	0.9199	0.994748	no
gene:SpnNT_00480	lcnD_2	Chromosome:493871-495233	110.58	110.58+peptide	OK	3.1297	2.13275	-0.553311	-0.787357	0.1727	0.632681	no
gene:SpnNT_00480	lcnD_2	Chromosome:493871-495233	ΔORF2	110.58+peptide	OK	3.21897	2.13275	-0.593887	-0.849312	0.1456	0.581072	no
gene:SpnNT_00480	lcnD_2	Chromosome:493871-495233	110.58	ΔORF2+peptide	OK	3.1297	1.28575	-1.28341	-1.70306	0.0044	0.0544687	no
gene:SpnNT_00480	lcnD_2	Chromosome:493871-495233	ΔORF2	ΔORF2+peptide	OK	3.21897	1.28575	-1.32399	-1.76452	0.00365	0.0469562	yes

gene:SpnNT_00480	lcnD_2	Chromosome:493871-495233	110.58+peptide	ΔORF2+peptide	OK	2.13275	1.28575	-0.7301	-0.938938	0.09825	0.474682	no
gene:SpnNT_00481	lagD_3	Chromosome:495243-497397	110.58	ΔORF2	OK	2.65172	3.03403	0.194305	0.306269	0.59185	0.975468	no
gene:SpnNT_00481	lagD_3	Chromosome:495243-497397	110.58	110.58+peptide	OK	2.65172	2.24303	-0.241482	-0.37122	0.5162	0.95301	no
gene:SpnNT_00481	lagD_3	Chromosome:495243-497397	ΔORF2	110.58+peptide	OK	3.03403	2.24303	-0.435787	-0.694422	0.22515	0.716396	no
gene:SpnNT_00481	lagD_3	Chromosome:495243-497397	110.58	ΔORF2+peptide	OK	2.65172	2.1379	-0.310732	-0.475234	0.40605	0.895795	no
gene:SpnNT_00481	lagD_3	Chromosome:495243-497397	ΔORF2	ΔORF2+peptide	OK	3.03403	2.1379	-0.505037	-0.800356	0.1606	0.613149	no
gene:SpnNT_00481	lagD_3	Chromosome:495243-497397	110.58+peptide	ΔORF2+peptide	OK	2.24303	2.1379	-0.0692498	-0.107001	0.84855	0.994748	no
gene:SpnNT_00482	lafA	Chromosome:497677-497932	110.58	ΔORF2	OK	51.2909	38.4026	-0.417497	-0.602167	0.28855	0.792622	no
gene:SpnNT_00482	lafA	Chromosome:497677-497932	110.58	110.58+peptide	OK	51.2909	38.5593	-0.411623	-0.586834	0.3101	0.815784	no
gene:SpnNT_00482	lafA	Chromosome:497677-497932	ΔORF2	110.58+peptide	OK	38.4026	38.5593	0.00587322	0.00814122	0.98475	0.996444	no
gene:SpnNT_00482	lafA	Chromosome:497677-497932	110.58	ΔORF2+peptide	OK	51.2909	36.8589	-0.476687	-0.674107	0.2434	0.73908	no
gene:SpnNT_00482	lafA	Chromosome:497677-497932	ΔORF2	ΔORF2+peptide	OK	38.4026	36.8589	-0.0591906	-0.0814211	0.8899	0.994748	no
gene:SpnNT_00482	lafA	Chromosome:497677-497932	110.58+peptide	ΔORF2+peptide	OK	38.5593	36.8589	-0.0650638	-0.0885577	0.8838	0.994748	no
gene:SpnNT_00483	NA	Chromosome:497947-498142	110.58	ΔORF2	OK	7.53068	8.98364	0.25452	0.245622	0.7672	0.994748	no
gene:SpnNT_00483	NA	Chromosome:497947-498142	110.58	110.58+peptide	OK	7.53068	9.87482	0.390975	0.296307	0.62725	0.980887	no
gene:SpnNT_00483	NA	Chromosome:497947-498142	ΔORF2	110.58+peptide	OK	8.98364	9.87482	0.136455	0.143851	0.8781	0.994748	no
gene:SpnNT_00483	NA	Chromosome:497947-498142	110.58	ΔORF2+peptide	OK	7.53068	5.72847	-0.394629	-0.371085	0.6772	0.98524	no
gene:SpnNT_00483	NA	Chromosome:497947-498142	ΔORF2	ΔORF2+peptide	OK	8.98364	5.72847	-0.649149	-1.20614	0.4839	0.936747	no
gene:SpnNT_00483	NA	Chromosome:497947-498142	110.58+peptide	ΔORF2+peptide	OK	9.87482	5.72847	-0.785604	-0.803075	0.3722	0.869505	no
gene:SpnNT_00484	NA	Chromosome:499055-499621	110.58	ΔORF2	OK	2.56248	8.72621	1.76781	1.67599	0.5243	0.954832	no
gene:SpnNT_00484	NA	Chromosome:499055-499621	110.58	110.58+peptide	NOTEST	2.56248	1.76154	-0.540702	0	1	1	no
gene:SpnNT_00484	NA	Chromosome:499055-499621	ΔORF2	110.58+peptide	OK	8.72621	1.76154	-2.30852	-2.02711	0.4585	0.922502	no
gene:SpnNT_00484	NA	Chromosome:499055-499621	110.58	ΔORF2+peptide	OK	2.56248	5.6367	1.13731	1.2272	0.6088	0.978849	no
gene:SpnNT_00484	NA	Chromosome:499055-499621	ΔORF2	ΔORF2+peptide	OK	8.72621	5.6367	-0.630505	-0.61662	0.7472	0.994748	no
gene:SpnNT_00484	NA	Chromosome:499055-499621	110.58+peptide	ΔORF2+peptide	OK	1.76154	5.6367	1.67801	1.6429	0.5295	0.957393	no
gene:SpnNT_00485	NA	Chromosome:499055-499621	110.58	ΔORF2	OK	11.9433	14.4943	0.279281	0.385448	0.50595	0.945292	no
gene:SpnNT_00485	NA	Chromosome:499055-499621	110.58	110.58+peptide	OK	11.9433	10.8394	-0.139924	-0.190185	0.74135	0.994748	no
gene:SpnNT_00485	NA	Chromosome:499055-499621	ΔORF2	110.58+peptide	OK	14.4943	10.8394	-0.419205	-0.565527	0.3352	0.840108	no
gene:SpnNT_00485	NA	Chromosome:499055-499621	110.58	ΔORF2+peptide	OK	11.9433	7.23808	-0.722524	-0.859355	0.11925	0.523344	no
gene:SpnNT_00485	NA	Chromosome:499055-499621	ΔORF2	ΔORF2+peptide	OK	14.4943	7.23808	-1.00181	-1.18469	0.0366	0.25546	no
gene:SpnNT_00485	NA	Chromosome:499055-499621	110.58+peptide	ΔORF2+peptide	OK	10.8394	7.23808	-0.5826	-0.681235	0.21655	0.704171	no
gene:SpnNT_00486	NA	Chromosome:499781-500808	110.58	ΔORF2	OK	0.852008	0.625999	-0.444708	-0.130319	0.817	0.994748	no
gene:SpnNT_00486	NA	Chromosome:499781-500808	110.58	110.58+peptide	OK	0.852008	0.925698	0.119674	0.0347648	0.932	0.994855	no
gene:SpnNT_00486	NA	Chromosome:499781-500808	ΔORF2	110.58+peptide	OK	0.625999	0.925698	0.564382	0.146545	0.7689	0.994748	no
gene:SpnNT_00486	NA	Chromosome:499781-500808	110.58	ΔORF2+peptide	OK	0.852008	0	#NAME?	NA	0.142	0.575633	no
gene:SpnNT_00486	NA	Chromosome:499781-500808	ΔORF2	ΔORF2+peptide	NOTEST	0.625999	0	#NAME?	0	1	1	no
gene:SpnNT_00486	NA	Chromosome:499781-500808	110.58+peptide	ΔORF2+peptide	OK	0.925698	0	#NAME?	NA	0.1294	0.545626	no
gene:SpnNT_00487	NA	Chromosome:499781-500808	110.58	ΔORF2	OK	3.78653	4.86828	0.362536	0.440791	0.4461	0.918643	no
gene:SpnNT_00487	NA	Chromosome:499781-500808	110.58	110.58+peptide	OK	3.78653	4.89973	0.371826	0.443152	0.4491	0.919933	no
gene:SpnNT_00487	NA	Chromosome:499781-500808	ΔORF2	110.58+peptide	OK	4.86828	4.89973	0.00928971	0.0111696	0.98465	0.99642	no
gene:SpnNT_00487	NA	Chromosome:499781-500808	110.58	ΔORF2+peptide	OK	3.78653	3.90448	0.0442555	0.0534092	0.93145	0.994855	no
gene:SpnNT_00487	NA	Chromosome:499781-500808	ΔORF2	ΔORF2+peptide	OK	4.86828	3.90448	-0.318281	-0.387594	0.50215	0.944784	no
gene:SpnNT_00487	NA	Chromosome:499781-500808	110.58+peptide	ΔORF2+peptide	OK	4.89973	3.90448	-0.32757	-0.391	0.5024	0.944788	no
gene:SpnNT_00488	NA	Chromosome:501053-501287	110.58	ΔORF2	OK	6.13433	0.844522	-2.8607	-1.89551	0.2278	0.719706	no
gene:SpnNT_00488	NA	Chromosome:501053-501287	110.58	110.58+peptide	OK	6.13433	1.64738	-1.89673	-0.929966	0.0399	0.270438	no

gene:SpnNT_00488	NA	Chromosome:501053-501287	ΔORF2	110.58+peptide	OK	0.844522	1.64738	0.963966	0.437592	0.49815	0.944017	no
gene:SpnNT_00488	NA	Chromosome:501053-501287	110.58	ΔORF2+peptide	OK	6.13433	1.68352	-1.86543	-1.50014	0.12065	0.526145	no
gene:SpnNT_00488	NA	Chromosome:501053-501287	ΔORF2	ΔORF2+peptide	NOTEST	0.844522	1.68352	0.995274	0	1	1	no
gene:SpnNT_00488	NA	Chromosome:501053-501287	110.58+peptide	ΔORF2+peptide	OK	1.64738	1.68352	0.0313086	0.0154222	0.86915	0.994748	no
gene:SpnNT_00489	NA	Chromosome:501318-502008	110.58	ΔORF2	OK	5.32858	4.97214	-0.0998866	-0.139799	0.8004	0.994748	no
gene:SpnNT_00489	NA	Chromosome:501318-502008	110.58	110.58+peptide	OK	5.32858	4.30127	-0.308988	-0.429337	0.44395	0.917631	no
gene:SpnNT_00489	NA	Chromosome:501318-502008	ΔORF2	110.58+peptide	OK	4.97214	4.30127	-0.209102	-0.287714	0.6043	0.976761	no
gene:SpnNT_00489	NA	Chromosome:501318-502008	110.58	ΔORF2+peptide	OK	5.32858	3.67561	-0.535768	-0.723067	0.2094	0.695632	no
gene:SpnNT_00489	NA	Chromosome:501318-502008	ΔORF2	ΔORF2+peptide	OK	4.97214	3.67561	-0.435882	-0.582847	0.3061	0.812428	no
gene:SpnNT_00489	NA	Chromosome:501318-502008	110.58+peptide	ΔORF2+peptide	OK	4.30127	3.67561	-0.22678	-0.301248	0.60255	0.976761	no
gene:SpnNT_00490	NA	Chromosome:502050-502299	110.58	ΔORF2	OK	1.52523	3.61079	1.24329	1.06247	0.27555	0.777752	no
gene:SpnNT_00490	NA	Chromosome:502050-502299	110.58	110.58+peptide	OK	1.52523	3.19537	1.06696	0.950366	0.33295	0.838735	no
gene:SpnNT_00490	NA	Chromosome:502050-502299	ΔORF2	110.58+peptide	OK	3.61079	3.19537	-0.176329	-0.125864	0.84375	0.994748	no
gene:SpnNT_00490	NA	Chromosome:502050-502299	110.58	ΔORF2+peptide	OK	1.52523	2.45257	0.685265	0.780575	0.5711	0.96884	no
gene:SpnNT_00490	NA	Chromosome:502050-502299	ΔORF2	ΔORF2+peptide	OK	3.61079	2.45257	-0.558021	-0.459788	0.56305	0.968621	no
gene:SpnNT_00490	NA	Chromosome:502050-502299	110.58+peptide	ΔORF2+peptide	OK	3.19537	2.45257	-0.381692	-0.326814	0.6915	0.98828	no
gene:SpnNT_00491	NA	Chromosome:502328-502940	110.58	ΔORF2	OK	6.15458	7.01369	0.188513	0.260686	0.65235	0.98163	no
gene:SpnNT_00491	NA	Chromosome:502328-502940	110.58	110.58+peptide	OK	6.15458	4.57969	-0.426412	-0.554781	0.3423	0.846365	no
gene:SpnNT_00491	NA	Chromosome:502328-502940	ΔORF2	110.58+peptide	OK	7.01369	4.57969	-0.614925	-0.830733	0.14945	0.590866	no
gene:SpnNT_00491	NA	Chromosome:502328-502940	110.58	ΔORF2+peptide	OK	6.15458	5.54681	-0.150002	-0.195013	0.7423	0.994748	no
gene:SpnNT_00491	NA	Chromosome:502328-502940	ΔORF2	ΔORF2+peptide	OK	7.01369	5.54681	-0.338516	-0.456946	0.4315	0.913083	no
gene:SpnNT_00491	NA	Chromosome:502328-502940	110.58+peptide	ΔORF2+peptide	OK	4.57969	5.54681	0.27641	0.351994	0.5495	0.964281	no
gene:SpnNT_00492	NA	Chromosome:503101-504528	110.58	ΔORF2	OK	170.522	181.43	0.0894575	0.142174	0.80025	0.994748	no
gene:SpnNT_00492	NA	Chromosome:503101-504528	110.58	110.58+peptide	OK	170.522	153.6	-0.150783	-0.238748	0.67435	0.985063	no
gene:SpnNT_00492	NA	Chromosome:503101-504528	ΔORF2	110.58+peptide	OK	181.43	153.6	-0.24024	-0.369399	0.5197	0.954494	no
gene:SpnNT_00492	NA	Chromosome:503101-504528	110.58	ΔORF2+peptide	OK	170.522	183.459	0.105499	0.16771	0.7699	0.994748	no
gene:SpnNT_00492	NA	Chromosome:503101-504528	ΔORF2	ΔORF2+peptide	OK	181.43	183.459	0.0160415	0.0247582	0.96505	0.994855	no
gene:SpnNT_00492	NA	Chromosome:503101-504528	110.58+peptide	ΔORF2+peptide	OK	153.6	183.459	0.256282	0.394155	0.49495	0.942632	no
gene:SpnNT_00493	trmB	Chromosome:503101-504528	110.58	ΔORF2	OK	219.728	238.497	0.118255	0.181351	0.75345	0.994748	no
gene:SpnNT_00493	trmB	Chromosome:503101-504528	110.58	110.58+peptide	OK	219.728	249.386	0.182664	0.296193	0.60395	0.976761	no
gene:SpnNT_00493	trmB	Chromosome:503101-504528	ΔORF2	110.58+peptide	OK	238.497	249.386	0.0644089	0.10104	0.8612	0.994748	no
gene:SpnNT_00493	trmB	Chromosome:503101-504528	110.58	ΔORF2+peptide	OK	219.728	281.491	0.357372	0.579051	0.30905	0.814396	no
gene:SpnNT_00493	trmB	Chromosome:503101-504528	ΔORF2	ΔORF2+peptide	OK	238.497	281.491	0.239117	0.374847	0.5178	0.953876	no
gene:SpnNT_00493	trmB	Chromosome:503101-504528	110.58+peptide	ΔORF2+peptide	OK	249.386	281.491	0.174708	0.290356	0.6167	0.980533	no
gene:SpnNT_00494	rimP	Chromosome:504653-505133	110.58	ΔORF2	OK	157.145	169.166	0.106343	0.22139	0.6927	0.98828	no
gene:SpnNT_00494	rimP	Chromosome:504653-505133	110.58	110.58+peptide	OK	157.145	113.35	-0.471308	-0.972007	0.091	0.453591	no
gene:SpnNT_00494	rimP	Chromosome:504653-505133	ΔORF2	110.58+peptide	OK	169.166	113.35	-0.577651	-1.1759	0.04175	0.278659	no
gene:SpnNT_00494	rimP	Chromosome:504653-505133	110.58	ΔORF2+peptide	OK	157.145	124.599	-0.334806	-0.692253	0.22635	0.717936	no
gene:SpnNT_00494	rimP	Chromosome:504653-505133	ΔORF2	ΔORF2+peptide	OK	169.166	124.599	-0.441149	-0.90026	0.11395	0.512073	no
gene:SpnNT_00494	rimP	Chromosome:504653-505133	110.58+peptide	ΔORF2+peptide	OK	113.35	124.599	0.136502	0.276056	0.62845	0.980887	no
gene:SpnNT_00495	nusA	Chromosome:505188-506313	110.58	ΔORF2	OK	166.1	171.138	0.0431075	0.0954979	0.86795	0.994748	no
gene:SpnNT_00495	nusA	Chromosome:505188-506313	110.58	110.58+peptide	OK	166.1	144.764	-0.198346	-0.448117	0.43205	0.913334	no
gene:SpnNT_00495	nusA	Chromosome:505188-506313	ΔORF2	110.58+peptide	OK	171.138	144.764	-0.241454	-0.531488	0.3492	0.853357	no
gene:SpnNT_00495	nusA	Chromosome:505188-506313	110.58	ΔORF2+peptide	OK	166.1	159.645	-0.0571818	-0.129863	0.81895	0.994748	no
gene:SpnNT_00495	nusA	Chromosome:505188-506313	ΔORF2	ΔORF2+peptide	OK	171.138	159.645	-0.100289	-0.22185	0.6958	0.989304	no

gene:SpnNT_00495	nusA	Chromosome:505188-506313	110.58+peptide	ΔORF2+peptide	OK	144.764	159.645	0.141164	0.318443	0.57705	0.969538	no
gene:SpnNT_00496	NA	Chromosome:506334-506920	110.58	ΔORF2	OK	144.405	184.533	0.353757	0.55516	0.3263	0.831527	no
gene:SpnNT_00496	NA	Chromosome:506334-506920	110.58	110.58+peptide	OK	144.405	130.629	-0.144641	-0.224232	0.6917	0.98828	no
gene:SpnNT_00496	NA	Chromosome:506334-506920	ΔORF2	110.58+peptide	OK	184.533	130.629	-0.498398	-0.767859	0.1723	0.632248	no
gene:SpnNT_00496	NA	Chromosome:506334-506920	110.58	ΔORF2+peptide	OK	144.405	148.355	0.0389287	0.059818	0.91375	0.994748	no
gene:SpnNT_00496	NA	Chromosome:506334-506920	ΔORF2	ΔORF2+peptide	OK	184.533	148.355	-0.314828	-0.480821	0.39555	0.890245	no
gene:SpnNT_00496	NA	Chromosome:506334-506920	110.58+peptide	ΔORF2+peptide	OK	130.629	148.355	0.18357	0.277128	0.63165	0.980887	no
gene:SpnNT_00497	rplGA	Chromosome:506334-506920	110.58	ΔORF2	OK	71.5716	83.7429	0.226579	0.244463	0.66955	0.984845	no
gene:SpnNT_00497	rplGA	Chromosome:506334-506920	110.58	110.58+peptide	OK	71.5716	62.5445	-0.194506	-0.206944	0.71725	0.992452	no
gene:SpnNT_00497	rplGA	Chromosome:506334-506920	ΔORF2	110.58+peptide	OK	83.7429	62.5445	-0.421085	-0.449545	0.4218	0.905409	no
gene:SpnNT_00497	rplGA	Chromosome:506334-506920	110.58	ΔORF2+peptide	OK	71.5716	75.5658	0.0783466	0.0829417	0.8902	0.994748	no
gene:SpnNT_00497	rplGA	Chromosome:506334-506920	ΔORF2	ΔORF2+peptide	OK	83.7429	75.5658	-0.148233	-0.157458	0.7827	0.994748	no
gene:SpnNT_00497	rplGA	Chromosome:506334-506920	110.58+peptide	ΔORF2+peptide	OK	62.5445	75.5658	0.272852	0.28593	0.6167	0.980533	no
gene:SpnNT_00498	infB	Chromosome:506936-509729	110.58	ΔORF2	OK	279.672	287.725	0.0409592	0.087898	0.876	0.994748	no
gene:SpnNT_00498	infB	Chromosome:506936-509729	110.58	110.58+peptide	OK	279.672	246.144	-0.184229	-0.405581	0.4729	0.928812	no
gene:SpnNT_00498	infB	Chromosome:506936-509729	ΔORF2	110.58+peptide	OK	287.725	246.144	-0.225188	-0.483193	0.38685	0.881745	no
gene:SpnNT_00498	infB	Chromosome:506936-509729	110.58	ΔORF2+peptide	OK	279.672	250.767	-0.157388	-0.348138	0.5413	0.961494	no
gene:SpnNT_00498	infB	Chromosome:506936-509729	ΔORF2	ΔORF2+peptide	OK	287.725	250.767	-0.198347	-0.427521	0.4443	0.917673	no
gene:SpnNT_00498	infB	Chromosome:506936-509729	110.58+peptide	ΔORF2+peptide	OK	246.144	250.767	0.0268411	0.059364	0.91915	0.994748	no
gene:SpnNT_00499	rbfA	Chromosome:509979-510330	110.58	ΔORF2	OK	162.029	200.596	0.308043	0.613977	0.27715	0.780208	no
gene:SpnNT_00499	rbfA	Chromosome:509979-510330	110.58	110.58+peptide	OK	162.029	186.423	0.202335	0.399	0.47195	0.928525	no
gene:SpnNT_00499	rbfA	Chromosome:509979-510330	ΔORF2	110.58+peptide	OK	200.596	186.423	-0.105708	-0.211358	0.71045	0.990984	no
gene:SpnNT_00499	rbfA	Chromosome:509979-510330	110.58	ΔORF2+peptide	OK	162.029	178.751	0.141702	0.272554	0.61535	0.979616	no
gene:SpnNT_00499	rbfA	Chromosome:509979-510330	ΔORF2	ΔORF2+peptide	OK	200.596	178.751	-0.166341	-0.324181	0.5633	0.968621	no
gene:SpnNT_00499	rbfA	Chromosome:509979-510330	110.58+peptide	ΔORF2+peptide	OK	186.423	178.751	-0.0606327	-0.116966	0.82865	0.994748	no
gene:SpnNT_00500	NA	Chromosome:510608-511392	110.58	ΔORF2	OK	1.42623	1.0766	-0.405731	-0.295029	0.60845	0.978801	no
gene:SpnNT_00500	NA	Chromosome:510608-511392	110.58	110.58+peptide	OK	1.42623	1.52863	0.100031	0.0720353	0.8941	0.994748	no
gene:SpnNT_00500	NA	Chromosome:510608-511392	ΔORF2	110.58+peptide	OK	1.0766	1.52863	0.505762	0.371189	0.5256	0.955517	no
gene:SpnNT_00500	NA	Chromosome:510608-511392	110.58	ΔORF2+peptide	OK	1.42623	1.97969	0.473061	0.361743	0.55465	0.966389	no
gene:SpnNT_00500	NA	Chromosome:510608-511392	ΔORF2	ΔORF2+peptide	OK	1.0766	1.97969	0.878792	0.686565	0.2822	0.785039	no
gene:SpnNT_00500	NA	Chromosome:510608-511392	110.58+peptide	ΔORF2+peptide	OK	1.52863	1.97969	0.37303	0.288191	0.63895	0.980887	no
gene:SpnNT_00501	NA	Chromosome:510608-511392	110.58	ΔORF2	OK	1.82718	1.38884	-0.395738	-0.227749	0.80345	0.994748	no
gene:SpnNT_00501	NA	Chromosome:510608-511392	110.58	110.58+peptide	OK	1.82718	3.78152	1.04935	0.779663	0.3674	0.864913	no
gene:SpnNT_00501	NA	Chromosome:510608-511392	ΔORF2	110.58+peptide	OK	1.38884	3.78152	1.44509	0.785863	0.24045	0.735646	no
gene:SpnNT_00501	NA	Chromosome:510608-511392	110.58	ΔORF2+peptide	OK	1.82718	5.64192	1.62657	1.23211	0.1832	0.653781	no
gene:SpnNT_00501	NA	Chromosome:510608-511392	ΔORF2	ΔORF2+peptide	OK	1.38884	5.64192	2.02231	1.1111	0.1106	0.507091	no
gene:SpnNT_00501	NA	Chromosome:510608-511392	110.58+peptide	ΔORF2+peptide	OK	3.78152	5.64192	0.57722	0.397856	0.5074	0.946425	no
gene:SpnNT_00502	NA	Chromosome:511393-511576	110.58	ΔORF2	NOTEST	1.52666	1.16852	-0.385701	0	1	1	no
gene:SpnNT_00502	NA	Chromosome:511393-511576	110.58	110.58+peptide	NOTEST	1.52666	1.10511	-0.466194	0	1	1	no
gene:SpnNT_00502	NA	Chromosome:511393-511576	ΔORF2	110.58+peptide	NOTEST	1.16852	1.10511	-0.0804925	0	1	1	no
gene:SpnNT_00502	NA	Chromosome:511393-511576	110.58	ΔORF2+peptide	NOTEST	1.52666	1.33902	-0.189199	0	1	1	no
gene:SpnNT_00502	NA	Chromosome:511393-511576	ΔORF2	ΔORF2+peptide	NOTEST	1.16852	1.33902	0.196502	0	1	1	no
gene:SpnNT_00502	NA	Chromosome:511393-511576	110.58+peptide	ΔORF2+peptide	NOTEST	1.10511	1.33902	0.276995	0	1	1	no
gene:SpnNT_00503	NA	Chromosome:511990-513558	110.58	ΔORF2	OK	46.3867	35.4629	-0.387403	-0.173778	0.85675	0.994748	no
gene:SpnNT_00503	NA	Chromosome:511990-513558	110.58	110.58+peptide	OK	46.3867	36.4743	-0.346832	-0.159998	0.8698	0.994748	no

gene:SpnNT_00503	NA	Chromosome:511990-513558	ΔORF2	110.58+peptide	OK	35.4629	36.4743	0.0405706	0.0177044	0.97065	0.99536	no
gene:SpnNT_00503	NA	Chromosome:511990-513558	110.58	ΔORF2+peptide	OK	46.3867	32.0085	-0.535259	-0.229158	0.8321	0.994748	no
gene:SpnNT_00503	NA	Chromosome:511990-513558	ΔORF2	ΔORF2+peptide	OK	35.4629	32.0085	-0.147856	-0.0603218	0.92935	0.994774	no
gene:SpnNT_00503	NA	Chromosome:511990-513558	110.58+peptide	ΔORF2+peptide	OK	36.4743	32.0085	-0.188427	-0.0786666	0.91735	0.994748	no
gene:SpnNT_00504	NA	Chromosome:511990-513558	110.58	ΔORF2	OK	37.0876	39.3487	0.0853808	0.174624	0.7614	0.994748	no
gene:SpnNT_00504	NA	Chromosome:511990-513558	110.58	110.58+peptide	OK	37.0876	37.4544	0.0141991	0.029034	0.9584	0.994855	no
gene:SpnNT_00504	NA	Chromosome:511990-513558	ΔORF2	110.58+peptide	OK	39.3487	37.4544	-0.0711817	-0.146151	0.79415	0.994748	no
gene:SpnNT_00504	NA	Chromosome:511990-513558	110.58	ΔORF2+peptide	OK	37.0876	40.7001	0.134097	0.277364	0.62965	0.980887	no
gene:SpnNT_00504	NA	Chromosome:511990-513558	ΔORF2	ΔORF2+peptide	OK	39.3487	40.7001	0.0487167	0.10119	0.8588	0.994748	no
gene:SpnNT_00504	NA	Chromosome:511990-513558	110.58+peptide	ΔORF2+peptide	OK	37.4544	40.7001	0.119898	0.248983	0.66325	0.982966	no
gene:SpnNT_00505	NA	Chromosome:513567-513822	110.58	ΔORF2	OK	65.9634	74.3992	0.173621	0.271131	0.63335	0.980887	no
gene:SpnNT_00505	NA	Chromosome:513567-513822	110.58	110.58+peptide	OK	65.9634	55.565	-0.24749	-0.382692	0.49405	0.942012	no
gene:SpnNT_00505	NA	Chromosome:513567-513822	ΔORF2	110.58+peptide	OK	74.3992	55.565	-0.42111	-0.65852	0.2478	0.74404	no
gene:SpnNT_00505	NA	Chromosome:513567-513822	110.58	ΔORF2+peptide	OK	65.9634	62.7865	-0.0712121	-0.108509	0.8511	0.994748	no
gene:SpnNT_00505	NA	Chromosome:513567-513822	ΔORF2	ΔORF2+peptide	OK	74.3992	62.7865	-0.244833	-0.377155	0.51825	0.953876	no
gene:SpnNT_00505	NA	Chromosome:513567-513822	110.58+peptide	ΔORF2+peptide	OK	55.565	62.7865	0.176278	0.268953	0.64485	0.980887	no
gene:SpnNT_00506	NA	Chromosome:513917-514313	110.58	ΔORF2	OK	72.442	81.947	0.177864	0.330012	0.5656	0.968621	no
gene:SpnNT_00506	NA	Chromosome:513917-514313	110.58	110.58+peptide	OK	72.442	126.609	0.805479	1.54543	0.0081	0.0866951	no
gene:SpnNT_00506	NA	Chromosome:513917-514313	ΔORF2	110.58+peptide	OK	81.947	126.609	0.627615	1.21115	0.03575	0.251265	no
gene:SpnNT_00506	NA	Chromosome:513917-514313	110.58	ΔORF2+peptide	OK	72.442	132.6	0.872186	1.65839	0.00565	0.0658431	no
gene:SpnNT_00506	NA	Chromosome:513917-514313	ΔORF2	ΔORF2+peptide	OK	81.947	132.6	0.694321	1.32771	0.023	0.186185	no
gene:SpnNT_00506	NA	Chromosome:513917-514313	110.58+peptide	ΔORF2+peptide	OK	126.609	132.6	0.0667069	0.132192	0.81625	0.994748	no
gene:SpnNT_00507	NA	Chromosome:514725-518944	110.58	ΔORF2	OK	46.6586	50.2889	0.108097	0.0500452	0.75455	0.994748	no
gene:SpnNT_00507	NA	Chromosome:514725-518944	110.58	110.58+peptide	OK	46.6586	83.6104	0.84154	0.384523	0.62195	0.980887	no
gene:SpnNT_00507	NA	Chromosome:514725-518944	ΔORF2	110.58+peptide	OK	50.2889	83.6104	0.733442	0.343258	0.74685	0.994748	no
gene:SpnNT_00507	NA	Chromosome:514725-518944	110.58	ΔORF2+peptide	OK	46.6586	115.11	1.30279	0.617669	0.4586	0.922502	no
gene:SpnNT_00507	NA	Chromosome:514725-518944	ΔORF2	ΔORF2+peptide	OK	50.2889	115.11	1.19469	0.58125	0.5342	0.958867	no
gene:SpnNT_00507	NA	Chromosome:514725-518944	110.58+peptide	ΔORF2+peptide	OK	83.6104	115.11	0.461252	0.221187	0.7865	0.994748	no
gene:SpnNT_00508	ydaF_2	Chromosome:514725-518944	110.58	ΔORF2	OK	41.5885	43.6624	0.0702045	0.0357407	0.96265	0.994855	no
gene:SpnNT_00508	ydaF_2	Chromosome:514725-518944	110.58	110.58+peptide	OK	41.5885	84.3591	1.02036	0.54733	0.4107	0.898351	no
gene:SpnNT_00508	ydaF_2	Chromosome:514725-518944	ΔORF2	110.58+peptide	OK	43.6624	84.3591	0.950154	0.501294	0.4477	0.919505	no
gene:SpnNT_00508	ydaF_2	Chromosome:514725-518944	110.58	ΔORF2+peptide	OK	41.5885	98.2128	1.23973	0.664786	0.3231	0.82836	no
gene:SpnNT_00508	ydaF_2	Chromosome:514725-518944	ΔORF2	ΔORF2+peptide	OK	43.6624	98.2128	1.16952	0.616837	0.3555	0.85704	no
gene:SpnNT_00508	ydaF_2	Chromosome:514725-518944	110.58+peptide	ΔORF2+peptide	OK	84.3591	98.2128	0.219367	0.122404	0.82335	0.994748	no
gene:SpnNT_00509	NA	Chromosome:514725-518944	110.58	ΔORF2	OK	21.1643	21.7434	0.0389487	0.0150584	0.9277	0.994748	no
gene:SpnNT_00509	NA	Chromosome:514725-518944	110.58	110.58+peptide	OK	21.1643	35.5027	0.746298	0.279773	0.7877	0.994748	no
gene:SpnNT_00509	NA	Chromosome:514725-518944	ΔORF2	110.58+peptide	OK	21.7434	35.5027	0.70735	0.269361	0.79405	0.994748	no
gene:SpnNT_00509	NA	Chromosome:514725-518944	110.58	ΔORF2+peptide	OK	21.1643	49.5939	1.22853	0.484435	0.69955	0.990367	no
gene:SpnNT_00509	NA	Chromosome:514725-518944	ΔORF2	ΔORF2+peptide	OK	21.7434	49.5939	1.18958	0.477295	0.7098	0.990851	no
gene:SpnNT_00509	NA	Chromosome:514725-518944	110.58+peptide	ΔORF2+peptide	OK	35.5027	49.5939	0.482232	0.187179	0.82635	0.994748	no
gene:SpnNT_00510	valS	Chromosome:514725-518944	110.58	ΔORF2	OK	105.54	102.152	-0.0470665	-0.0986938	0.86245	0.994748	no
gene:SpnNT_00510	valS	Chromosome:514725-518944	110.58	110.58+peptide	OK	105.54	175.054	0.730019	1.5276	0.00785	0.0847103	no
gene:SpnNT_00510	valS	Chromosome:514725-518944	ΔORF2	110.58+peptide	OK	102.152	175.054	0.777085	1.60498	0.00535	0.0633027	no
gene:SpnNT_00510	valS	Chromosome:514725-518944	110.58	ΔORF2+peptide	OK	105.54	190.079	0.848818	1.76842	0.0022	0.0317584	yes
gene:SpnNT_00510	valS	Chromosome:514725-518944	ΔORF2	ΔORF2+peptide	OK	102.152	190.079	0.895884	1.84246	0.0017	0.025969	yes

gene:SpnNT_00510	valS	Chromosome:514725-518944	110.58+peptide	ΔORF2+peptide	OK	175.054	190.079	0.118799	0.243832	0.6667	0.983929	no
gene:SpnNT_00511	NA	Chromosome:519038-520872	110.58	ΔORF2	OK	90.918	155.431	0.77364	1.4485	0.01265	0.119248	no
gene:SpnNT_00511	NA	Chromosome:519038-520872	110.58	110.58+peptide	OK	90.918	104.891	0.206249	0.388963	0.4994	0.944017	no
gene:SpnNT_00511	NA	Chromosome:519038-520872	ΔORF2	110.58+peptide	OK	155.431	104.891	-0.567391	-1.04547	0.0678	0.381133	no
gene:SpnNT_00511	NA	Chromosome:519038-520872	110.58	ΔORF2+peptide	OK	90.918	142.36	0.646906	1.25441	0.0319	0.231907	no
gene:SpnNT_00511	NA	Chromosome:519038-520872	ΔORF2	ΔORF2+peptide	OK	155.431	142.36	-0.126734	-0.239799	0.6746	0.985063	no
gene:SpnNT_00511	NA	Chromosome:519038-520872	110.58+peptide	ΔORF2+peptide	OK	104.891	142.36	0.440656	0.839951	0.14155	0.574874	no
gene:SpnNT_00512	NA	Chromosome:519038-520872	110.58	ΔORF2	OK	54.8153	88.7151	0.694603	0.763048	0.1798	0.64657	no
gene:SpnNT_00512	NA	Chromosome:519038-520872	110.58	110.58+peptide	OK	54.8153	66.3647	0.27584	0.316843	0.5786	0.969538	no
gene:SpnNT_00512	NA	Chromosome:519038-520872	ΔORF2	110.58+peptide	OK	88.7151	66.3647	-0.418763	-0.461504	0.41365	0.900003	no
gene:SpnNT_00512	NA	Chromosome:519038-520872	110.58	ΔORF2+peptide	OK	54.8153	89.4164	0.705961	0.826386	0.1405	0.572204	no
gene:SpnNT_00512	NA	Chromosome:519038-520872	ΔORF2	ΔORF2+peptide	OK	88.7151	89.4164	0.0113584	0.0127373	0.9821	0.996246	no
gene:SpnNT_00512	NA	Chromosome:519038-520872	110.58+peptide	ΔORF2+peptide	OK	66.3647	89.4164	0.430122	0.50533	0.369	0.866165	no
gene:SpnNT_00513	NA	Chromosome:521079-522813	110.58	ΔORF2	OK	27.8885	32.6588	0.227799	0.47836	0.39945	0.89264	no
gene:SpnNT_00513	NA	Chromosome:521079-522813	110.58	110.58+peptide	OK	27.8885	28.4868	0.0306233	0.0638134	0.9114	0.994748	no
gene:SpnNT_00513	NA	Chromosome:521079-522813	ΔORF2	110.58+peptide	OK	32.6588	28.4868	-0.197176	-0.415628	0.46945	0.927729	no
gene:SpnNT_00513	NA	Chromosome:521079-522813	110.58	ΔORF2+peptide	OK	27.8885	33.9788	0.284966	0.601469	0.2981	0.803352	no
gene:SpnNT_00513	NA	Chromosome:521079-522813	ΔORF2	ΔORF2+peptide	OK	32.6588	33.9788	0.0571662	0.12209	0.83135	0.994748	no
gene:SpnNT_00513	NA	Chromosome:521079-522813	110.58+peptide	ΔORF2+peptide	OK	28.4868	33.9788	0.254342	0.538895	0.35645	0.857875	no
gene:SpnNT_00514	NA	Chromosome:523018-523774	110.58	ΔORF2	OK	18.7307	28.5718	0.609187	1.0847	0.05705	0.339649	no
gene:SpnNT_00514	NA	Chromosome:523018-523774	110.58	110.58+peptide	OK	18.7307	17.8008	-0.0734585	-0.128094	0.8252	0.994748	no
gene:SpnNT_00514	NA	Chromosome:523018-523774	ΔORF2	110.58+peptide	OK	28.5718	17.8008	-0.682645	-1.20365	0.03805	0.261958	no
gene:SpnNT_00514	NA	Chromosome:523018-523774	110.58	ΔORF2+peptide	OK	18.7307	26.6792	0.510314	0.918157	0.10335	0.488002	no
gene:SpnNT_00514	NA	Chromosome:523018-523774	ΔORF2	ΔORF2+peptide	OK	28.5718	26.6792	-0.0988728	-0.180007	0.7545	0.994748	no
gene:SpnNT_00514	NA	Chromosome:523018-523774	110.58+peptide	ΔORF2+peptide	OK	17.8008	26.6792	0.583773	1.03988	0.0708	0.390464	no
gene:SpnNT_00515	NA	Chromosome:524052-524856	110.58	ΔORF2	OK	34.1456	41.0294	0.26496	0.516888	0.35535	0.85704	no
gene:SpnNT_00515	NA	Chromosome:524052-524856	110.58	110.58+peptide	OK	34.1456	25.8273	-0.402801	-0.762292	0.18505	0.658055	no
gene:SpnNT_00515	NA	Chromosome:524052-524856	ΔORF2	110.58+peptide	OK	41.0294	25.8273	-0.667761	-1.27917	0.02535	0.198711	no
gene:SpnNT_00515	NA	Chromosome:524052-524856	110.58	ΔORF2+peptide	OK	34.1456	34.8035	0.0275362	0.0526777	0.9291	0.994748	no
gene:SpnNT_00515	NA	Chromosome:524052-524856	ΔORF2	ΔORF2+peptide	OK	41.0294	34.8035	-0.237424	-0.459874	0.42025	0.904235	no
gene:SpnNT_00515	NA	Chromosome:524052-524856	110.58+peptide	ΔORF2+peptide	OK	25.8273	34.8035	0.430337	0.808947	0.1641	0.618436	no
gene:SpnNT_00516	NA	Chromosome:524971-525313	110.58	ΔORF2	OK	4.86763	3.37938	-0.526461	-0.505902	0.38045	0.878145	no
gene:SpnNT_00516	NA	Chromosome:524971-525313	110.58	110.58+peptide	OK	4.86763	1.64931	-1.56136	-1.31184	0.0421	0.280282	no
gene:SpnNT_00516	NA	Chromosome:524971-525313	ΔORF2	110.58+peptide	OK	3.37938	1.64931	-1.0349	-0.831963	0.15165	0.593484	no
gene:SpnNT_00516	NA	Chromosome:524971-525313	110.58	ΔORF2+peptide	OK	4.86763	1.74333	-1.48137	-1.47674	0.0535	0.327438	no
gene:SpnNT_00516	NA	Chromosome:524971-525313	ΔORF2	ΔORF2+peptide	OK	3.37938	1.74333	-0.954914	-0.895525	0.20045	0.683196	no
gene:SpnNT_00516	NA	Chromosome:524971-525313	110.58+peptide	ΔORF2+peptide	OK	1.64931	1.74333	0.0799836	0.0659536	0.91795	0.994748	no
gene:SpnNT_00517	NA	Chromosome:525322-525972	110.58	ΔORF2	OK	3.11284	4.7449	0.60815	0.677156	0.24065	0.735706	no
gene:SpnNT_00517	NA	Chromosome:525322-525972	110.58	110.58+peptide	OK	3.11284	3.20322	0.041292	0.0440952	0.9474	0.994855	no
gene:SpnNT_00517	NA	Chromosome:525322-525972	ΔORF2	110.58+peptide	OK	4.7449	3.20322	-0.566858	-0.633623	0.2854	0.788923	no
gene:SpnNT_00517	NA	Chromosome:525322-525972	110.58	ΔORF2+peptide	OK	3.11284	4.90295	0.655422	0.74076	0.20375	0.688187	no
gene:SpnNT_00517	NA	Chromosome:525322-525972	ΔORF2	ΔORF2+peptide	OK	4.7449	4.90295	0.0472724	0.0562476	0.92095	0.994748	no
gene:SpnNT_00517	NA	Chromosome:525322-525972	110.58+peptide	ΔORF2+peptide	OK	3.20322	4.90295	0.61413	0.696862	0.24175	0.736324	no
gene:SpnNT_00518	NA	Chromosome:525322-525972	110.58	ΔORF2	OK	5.03557	7.46073	0.567162	0.729831	0.7989	0.994748	no
gene:SpnNT_00518	NA	Chromosome:525322-525972	110.58	110.58+peptide	OK	5.03557	4.82385	-0.06197	-0.0662353	0.86055	0.994748	no

gene:SpnNT_00518	NA	Chromosome:525322-525972	ΔORF2	110.58+peptide	OK	7.46073	4.82385	-0.629132	-0.704829	0.74285	0.994748	no
gene:SpnNT_00518	NA	Chromosome:525322-525972	110.58	ΔORF2+peptide	OK	5.03557	1.80421	-1.48079	-1.85051	0.5865	0.973049	no
gene:SpnNT_00518	NA	Chromosome:525322-525972	ΔORF2	ΔORF2+peptide	OK	7.46073	1.80421	-2.04795	-2.73252	0.50675	0.945979	no
gene:SpnNT_00518	NA	Chromosome:525322-525972	110.58+peptide	ΔORF2+peptide	OK	4.82385	1.80421	-1.41882	-1.5544	0.52855	0.956161	no
gene:SpnNT_00519	NA	Chromosome:526107-526377	110.58	ΔORF2	NOTEST	0	0	0	0	1	1	no
gene:SpnNT_00519	NA	Chromosome:526107-526377	110.58	110.58+peptide	NOTEST	0	0.315604	Inf	0	1	1	no
gene:SpnNT_00519	NA	Chromosome:526107-526377	ΔORF2	110.58+peptide	NOTEST	0	0.315604	Inf	0	1	1	no
gene:SpnNT_00519	NA	Chromosome:526107-526377	110.58	ΔORF2+peptide	NOTEST	0	0	0	0	1	1	no
gene:SpnNT_00519	NA	Chromosome:526107-526377	ΔORF2	ΔORF2+peptide	NOTEST	0	0	0	0	1	1	no
gene:SpnNT_00519	NA	Chromosome:526107-526377	110.58+peptide	ΔORF2+peptide	NOTEST	0.315604	0	#NAME?	0	1	1	no
gene:SpnNT_00520	NA	Chromosome:526566-529246	110.58	ΔORF2	OK	18.3499	24.6397	0.425211	0.530526	0.3649	0.864498	no
gene:SpnNT_00520	NA	Chromosome:526566-529246	110.58	110.58+peptide	OK	18.3499	15.0908	-0.282101	-0.345333	0.54705	0.962122	no
gene:SpnNT_00520	NA	Chromosome:526566-529246	ΔORF2	110.58+peptide	OK	24.6397	15.0908	-0.707312	-0.888384	0.12815	0.542797	no
gene:SpnNT_00520	NA	Chromosome:526566-529246	110.58	ΔORF2+peptide	OK	18.3499	16.853	-0.122772	-0.147672	0.79985	0.994748	no
gene:SpnNT_00520	NA	Chromosome:526566-529246	ΔORF2	ΔORF2+peptide	OK	24.6397	16.853	-0.547983	-0.675662	0.2555	0.7547	no
gene:SpnNT_00520	NA	Chromosome:526566-529246	110.58+peptide	ΔORF2+peptide	OK	15.0908	16.853	0.15933	0.192832	0.739	0.994748	no
gene:SpnNT_00521	NA	Chromosome:526566-529246	110.58	ΔORF2	OK	8.05653	9.11234	0.177663	0.257611	0.6537	0.982684	no
gene:SpnNT_00521	NA	Chromosome:526566-529246	110.58	110.58+peptide	OK	8.05653	5.4256	-0.570375	-0.81399	0.1592	0.609578	no
gene:SpnNT_00521	NA	Chromosome:526566-529246	ΔORF2	110.58+peptide	OK	9.11234	5.4256	-0.748039	-1.03619	0.07035	0.390331	no
gene:SpnNT_00521	NA	Chromosome:526566-529246	110.58	ΔORF2+peptide	OK	8.05653	5.59177	-0.526853	-0.728416	0.2019	0.685467	no
gene:SpnNT_00521	NA	Chromosome:526566-529246	ΔORF2	ΔORF2+peptide	OK	9.11234	5.59177	-0.704516	-0.947136	0.09285	0.460112	no
gene:SpnNT_00521	NA	Chromosome:526566-529246	110.58+peptide	ΔORF2+peptide	OK	5.4256	5.59177	0.0435223	0.0577141	0.921	0.994748	no
gene:SpnNT_00522	licT_1	Chromosome:529544-530384	110.58	ΔORF2	OK	1.37887	4.49977	1.70636	2.08442	0.0014	0.0220273	yes
gene:SpnNT_00522	licT_1	Chromosome:529544-530384	110.58	110.58+peptide	OK	1.37887	1.41821	0.040582	0.0447225	0.94595	0.994855	no
gene:SpnNT_00522	licT_1	Chromosome:529544-530384	ΔORF2	110.58+peptide	OK	4.49977	1.41821	-1.66578	-2.04125	0.0012	0.0193683	yes
gene:SpnNT_00522	licT_1	Chromosome:529544-530384	110.58	ΔORF2+peptide	OK	1.37887	2.39706	0.797778	0.932671	0.1242	0.533296	no
gene:SpnNT_00522	licT_1	Chromosome:529544-530384	ΔORF2	ΔORF2+peptide	OK	4.49977	2.39706	-0.908582	-1.19903	0.05175	0.321526	no
gene:SpnNT_00522	licT_1	Chromosome:529544-530384	110.58+peptide	ΔORF2+peptide	OK	1.41821	2.39706	0.757196	0.887779	0.14525	0.580558	no
gene:SpnNT_00523	bglF_1	Chromosome:530401-532240	110.58	ΔORF2	OK	6.6289	8.70104	0.392419	0.698907	0.2271	0.71894	no
gene:SpnNT_00523	bglF_1	Chromosome:530401-532240	110.58	110.58+peptide	OK	6.6289	3.87625	-0.774106	-1.32024	0.02025	0.168819	no
gene:SpnNT_00523	bglF_1	Chromosome:530401-532240	ΔORF2	110.58+peptide	OK	8.70104	3.87625	-1.16653	-1.99825	8.00E-04	0.0140342	yes
gene:SpnNT_00523	bglF_1	Chromosome:530401-532240	110.58	ΔORF2+peptide	OK	6.6289	4.50383	-0.557617	-0.955842	0.0966	0.470343	no
gene:SpnNT_00523	bglF_1	Chromosome:530401-532240	ΔORF2	ΔORF2+peptide	OK	8.70104	4.50383	-0.950036	-1.63574	0.00605	0.0694559	no
gene:SpnNT_00523	bglF_1	Chromosome:530401-532240	110.58+peptide	ΔORF2+peptide	OK	3.87625	4.50383	0.216489	0.357911	0.52105	0.954832	no
gene:SpnNT_00524	bglH_1	Chromosome:532252-533668	110.58	ΔORF2	OK	7.32241	11.172	0.609498	1.0676	0.0679	0.381251	no
gene:SpnNT_00524	bglH_1	Chromosome:532252-533668	110.58	110.58+peptide	OK	7.32241	3.93004	-0.897775	-1.46946	0.01205	0.115248	no
gene:SpnNT_00524	bglH_1	Chromosome:532252-533668	ΔORF2	110.58+peptide	OK	11.172	3.93004	-1.50727	-2.52236	5.00E-05	0.0013612	yes
gene:SpnNT_00524	bglH_1	Chromosome:532252-533668	110.58	ΔORF2+peptide	OK	7.32241	5.09793	-0.522407	-0.86142	0.1369	0.563312	no
gene:SpnNT_00524	bglH_1	Chromosome:532252-533668	ΔORF2	ΔORF2+peptide	OK	11.172	5.09793	-1.1319	-1.90892	0.00125	0.0201258	yes
gene:SpnNT_00524	bglH_1	Chromosome:532252-533668	110.58+peptide	ΔORF2+peptide	OK	3.93004	5.09793	0.375368	0.594302	0.29655	0.80085	no
gene:SpnNT_00525	pheS	Chromosome:534259-535815	110.58	ΔORF2	OK	36.2262	36.9332	0.0278833	0.0480777	0.93175	0.994855	no
gene:SpnNT_00525	pheS	Chromosome:534259-535815	110.58	110.58+peptide	OK	36.2262	43.7676	0.272829	0.494198	0.3957	0.890245	no
gene:SpnNT_00525	pheS	Chromosome:534259-535815	ΔORF2	110.58+peptide	OK	36.9332	43.7676	0.244946	0.416549	0.4723	0.928525	no
gene:SpnNT_00525	pheS	Chromosome:534259-535815	110.58	ΔORF2+peptide	OK	36.2262	51.3633	0.503703	0.930127	0.1074	0.498339	no
gene:SpnNT_00525	pheS	Chromosome:534259-535815	ΔORF2	ΔORF2+peptide	OK	36.9332	51.3633	0.47582	0.822976	0.1536	0.597374	no

gene:SpnNT_00525	pheS	Chromosome:534259-535815	110.58+peptide	ΔORF2+peptide	OK	43.7676	51.3633	0.230874	0.419634	0.46775	0.92732	no
gene:SpnNT_00526	paiA	Chromosome:534259-535815	110.58	ΔORF2	OK	24.5993	34.1763	0.47438	0.395683	0.4875	0.938799	no
gene:SpnNT_00526	paiA	Chromosome:534259-535815	110.58	110.58+peptide	OK	24.5993	40.99	0.736657	0.691842	0.2351	0.729138	no
gene:SpnNT_00526	paiA	Chromosome:534259-535815	ΔORF2	110.58+peptide	OK	34.1763	40.99	0.262277	0.234745	0.68305	0.98733	no
gene:SpnNT_00526	paiA	Chromosome:534259-535815	110.58	ΔORF2+peptide	OK	24.5993	52.6922	1.09897	1.02526	0.08555	0.439454	no
gene:SpnNT_00526	paiA	Chromosome:534259-535815	ΔORF2	ΔORF2+peptide	OK	34.1763	52.6922	0.624595	0.555654	0.3401	0.844266	no
gene:SpnNT_00526	paiA	Chromosome:534259-535815	110.58+peptide	ΔORF2+peptide	OK	40.99	52.6922	0.362318	0.3698	0.5365	0.959314	no
gene:SpnNT_00527	pheT_1	Chromosome:535891-538297	110.58	ΔORF2	OK	53.1064	52.1223	-0.0269851	-0.0601892	0.91535	0.994748	no
gene:SpnNT_00527	pheT_1	Chromosome:535891-538297	110.58	110.58+peptide	OK	53.1064	63.5768	0.259614	0.585641	0.3051	0.811188	no
gene:SpnNT_00527	pheT_1	Chromosome:535891-538297	ΔORF2	110.58+peptide	OK	52.1223	63.5768	0.286599	0.63908	0.26355	0.764218	no
gene:SpnNT_00527	pheT_1	Chromosome:535891-538297	110.58	ΔORF2+peptide	OK	53.1064	68.0443	0.357589	0.810548	0.15745	0.60535	no
gene:SpnNT_00527	pheT_1	Chromosome:535891-538297	ΔORF2	ΔORF2+peptide	OK	52.1223	68.0443	0.384575	0.861596	0.1344	0.557395	no
gene:SpnNT_00527	pheT_1	Chromosome:535891-538297	110.58+peptide	ΔORF2+peptide	OK	63.5768	68.0443	0.0979758	0.222022	0.70395	0.990367	no
gene:SpnNT_00528	NA	Chromosome:538364-539132	110.58	ΔORF2	OK	28.9242	29.7896	0.0425317	0.0773978	0.89295	0.994748	no
gene:SpnNT_00528	NA	Chromosome:538364-539132	110.58	110.58+peptide	OK	28.9242	30.0099	0.0531586	0.0980428	0.8678	0.994748	no
gene:SpnNT_00528	NA	Chromosome:538364-539132	ΔORF2	110.58+peptide	OK	29.7896	30.0099	0.010627	0.0196163	0.97445	0.99536	no
gene:SpnNT_00528	NA	Chromosome:538364-539132	110.58	ΔORF2+peptide	OK	28.9242	32.0863	0.149677	0.275985	0.642	0.980887	no
gene:SpnNT_00528	NA	Chromosome:538364-539132	ΔORF2	ΔORF2+peptide	OK	29.7896	32.0863	0.107145	0.197728	0.7425	0.994748	no
gene:SpnNT_00528	NA	Chromosome:538364-539132	110.58+peptide	ΔORF2+peptide	OK	30.0099	32.0863	0.0965181	0.180592	0.76375	0.994748	no
gene:SpnNT_00529	NA	Chromosome:539157-539367	110.58	ΔORF2	OK	14.2465	7.31423	-0.961831	-0.801578	0.1731	0.63306	no
gene:SpnNT_00529	NA	Chromosome:539157-539367	110.58	110.58+peptide	OK	14.2465	8.90733	-0.677543	-0.578388	0.3087	0.814396	no
gene:SpnNT_00529	NA	Chromosome:539157-539367	ΔORF2	110.58+peptide	OK	7.31423	8.90733	0.284288	0.219855	0.70255	0.990367	no
gene:SpnNT_00529	NA	Chromosome:539157-539367	110.58	ΔORF2+peptide	OK	14.2465	5.35614	-1.41134	-1.59831	0.09105	0.453591	no
gene:SpnNT_00529	NA	Chromosome:539157-539367	ΔORF2	ΔORF2+peptide	OK	7.31423	5.35614	-0.449512	-0.432643	0.5945	0.975539	no
gene:SpnNT_00529	NA	Chromosome:539157-539367	110.58+peptide	ΔORF2+peptide	OK	8.90733	5.35614	-0.7338	-0.729454	0.3801	0.878145	no
gene:SpnNT_00530	NA	Chromosome:539521-540016	110.58	ΔORF2	NOTEST	0.161912	0.046582	-1.79737	0	1	1	no
gene:SpnNT_00530	NA	Chromosome:539521-540016	110.58	110.58+peptide	NOTEST	0.161912	0	#NAME?	0	1	1	no
gene:SpnNT_00530	NA	Chromosome:539521-540016	ΔORF2	110.58+peptide	NOTEST	0.046582	0	#NAME?	0	1	1	no
gene:SpnNT_00530	NA	Chromosome:539521-540016	110.58	ΔORF2+peptide	NOTEST	0.161912	0.0546231	-1.56763	0	1	1	no
gene:SpnNT_00530	NA	Chromosome:539521-540016	ΔORF2	ΔORF2+peptide	NOTEST	0.046582	0.0546231	0.22974	0	1	1	no
gene:SpnNT_00530	NA	Chromosome:539521-540016	110.58+peptide	ΔORF2+peptide	NOTEST	0	0.0546231	Inf	0	1	1	no
gene:SpnNT_00531	metE	Chromosome:540727-542977	110.58	ΔORF2	OK	3.29511	3.26002	-0.0154454	-0.0256382	0.9636	0.994855	no
gene:SpnNT_00531	metE	Chromosome:540727-542977	110.58	110.58+peptide	OK	3.29511	3.22398	-0.0314846	-0.052304	0.9239	0.994748	no
gene:SpnNT_00531	metE	Chromosome:540727-542977	ΔORF2	110.58+peptide	OK	3.26002	3.22398	-0.0160393	-0.0265443	0.96085	0.994855	no
gene:SpnNT_00531	metE	Chromosome:540727-542977	110.58	ΔORF2+peptide	OK	3.29511	2.45987	-0.421743	-0.681448	0.2298	0.722779	no
gene:SpnNT_00531	metE	Chromosome:540727-542977	ΔORF2	ΔORF2+peptide	OK	3.26002	2.45987	-0.406298	-0.654137	0.25085	0.748571	no
gene:SpnNT_00531	metE	Chromosome:540727-542977	110.58+peptide	ΔORF2+peptide	OK	3.22398	2.45987	-0.390259	-0.628786	0.2673	0.768517	no
gene:SpnNT_00532	metF	Chromosome:543040-543907	110.58	ΔORF2	OK	6.3934	5.84852	-0.128511	-0.197135	0.72815	0.994748	no
gene:SpnNT_00532	metF	Chromosome:543040-543907	110.58	110.58+peptide	OK	6.3934	6.04288	-0.0813454	-0.125747	0.82775	0.994748	no
gene:SpnNT_00532	metF	Chromosome:543040-543907	ΔORF2	110.58+peptide	OK	5.84852	6.04288	0.0471657	0.072055	0.9005	0.994748	no
gene:SpnNT_00532	metF	Chromosome:543040-543907	110.58	ΔORF2+peptide	OK	6.3934	5.66034	-0.175695	-0.263183	0.6478	0.981391	no
gene:SpnNT_00532	metF	Chromosome:543040-543907	ΔORF2	ΔORF2+peptide	OK	5.84852	5.66034	-0.0471835	-0.0698989	0.90095	0.994748	no
gene:SpnNT_00532	metF	Chromosome:543040-543907	110.58+peptide	ΔORF2+peptide	OK	6.04288	5.66034	-0.0943492	-0.140778	0.8103	0.994748	no
gene:SpnNT_00533	pnp_1	Chromosome:544591-546805	110.58	ΔORF2	OK	59.266	60.7136	0.0348142	0.0790505	0.8933	0.994748	no
gene:SpnNT_00533	pnp_1	Chromosome:544591-546805	110.58	110.58+peptide	OK	59.266	61.6952	0.057954	0.131695	0.81795	0.994748	no

gene:SpnNT_00533	pnp_1	Chromosome:544591-546805	ΔORF2	110.58+peptide	OK	60.7136	61.6952	0.0231399	0.0522009	0.92895	0.994748	no
gene:SpnNT_00533	pnp_1	Chromosome:544591-546805	110.58	ΔORF2+peptide	OK	59.266	69.8018	0.236059	0.540879	0.3395	0.844054	no
gene:SpnNT_00533	pnp_1	Chromosome:544591-546805	ΔORF2	ΔORF2+peptide	OK	60.7136	69.8018	0.201245	0.457702	0.42	0.904223	no
gene:SpnNT_00533	pnp_1	Chromosome:544591-546805	110.58+peptide	ΔORF2+peptide	OK	61.6952	69.8018	0.178105	0.40539	0.47815	0.933077	no
gene:SpnNT_00534	cysE	Chromosome:546820-547438	110.58	ΔORF2	OK	125.597	125.712	0.00131932	0.00277499	0.9971	0.999412	no
gene:SpnNT_00534	cysE	Chromosome:546820-547438	110.58	110.58+peptide	OK	125.597	125.797	0.00229752	0.00491173	0.9935	0.998522	no
gene:SpnNT_00534	cysE	Chromosome:546820-547438	ΔORF2	110.58+peptide	OK	125.712	125.797	0.000978198	0.00206171	0.99675	0.999412	no
gene:SpnNT_00534	cysE	Chromosome:546820-547438	110.58	ΔORF2+peptide	OK	125.597	136.646	0.121635	0.259907	0.65735	0.982966	no
gene:SpnNT_00534	cysE	Chromosome:546820-547438	ΔORF2	ΔORF2+peptide	OK	125.712	136.646	0.120316	0.253462	0.66045	0.982966	no
gene:SpnNT_00534	cysE	Chromosome:546820-547438	110.58+peptide	ΔORF2+peptide	OK	125.797	136.646	0.119338	0.255537	0.65925	0.982966	no
gene:SpnNT_00535	NA	Chromosome:547449-548334	110.58	ΔORF2	OK	93.038	94.0363	0.0153975	0.0338916	0.94975	0.994855	no
gene:SpnNT_00535	NA	Chromosome:547449-548334	110.58	110.58+peptide	OK	93.038	91.5333	-0.0235247	-0.0517698	0.926	0.994748	no
gene:SpnNT_00535	NA	Chromosome:547449-548334	ΔORF2	110.58+peptide	OK	94.0363	91.5333	-0.0389222	-0.0855742	0.8806	0.994748	no
gene:SpnNT_00535	NA	Chromosome:547449-548334	110.58	ΔORF2+peptide	OK	93.038	129.21	0.473829	1.05568	0.066	0.373782	no
gene:SpnNT_00535	NA	Chromosome:547449-548334	ΔORF2	ΔORF2+peptide	OK	94.0363	129.21	0.458432	1.02039	0.07485	0.404024	no
gene:SpnNT_00535	NA	Chromosome:547449-548334	110.58+peptide	ΔORF2+peptide	OK	91.5333	129.21	0.497354	1.10679	0.0558	0.336957	no
gene:SpnNT_00536	cysS	Chromosome:548415-550138	110.58	ΔORF2	OK	132.649	128.029	-0.0511402	-0.108274	0.8496	0.994748	no
gene:SpnNT_00536	cysS	Chromosome:548415-550138	110.58	110.58+peptide	OK	132.649	141.453	0.0927092	0.196693	0.7272	0.994748	no
gene:SpnNT_00536	cysS	Chromosome:548415-550138	ΔORF2	110.58+peptide	OK	128.029	141.453	0.143849	0.304433	0.59045	0.974909	no
gene:SpnNT_00536	cysS	Chromosome:548415-550138	110.58	ΔORF2+peptide	OK	132.649	144.08	0.119263	0.252294	0.66125	0.982966	no
gene:SpnNT_00536	cysS	Chromosome:548415-550138	ΔORF2	ΔORF2+peptide	OK	128.029	144.08	0.170403	0.359587	0.52735	0.956161	no
gene:SpnNT_00536	cysS	Chromosome:548415-550138	110.58+peptide	ΔORF2+peptide	OK	141.453	144.08	0.0265535	0.0561496	0.921	0.994748	no
gene:SpnNT_00537	mrnC	Chromosome:548415-550138	110.58	ΔORF2	OK	144.006	125.614	-0.197137	-0.151535	0.7934	0.994748	no
gene:SpnNT_00537	mrnC	Chromosome:548415-550138	110.58	110.58+peptide	OK	144.006	147.284	0.032471	0.0251789	0.96305	0.994855	no
gene:SpnNT_00537	mrnC	Chromosome:548415-550138	ΔORF2	110.58+peptide	OK	125.614	147.284	0.229608	0.177379	0.75935	0.994748	no
gene:SpnNT_00537	mrnC	Chromosome:548415-550138	110.58	ΔORF2+peptide	OK	144.006	140.772	-0.0327738	-0.0239437	0.9658	0.994855	no
gene:SpnNT_00537	mrnC	Chromosome:548415-550138	ΔORF2	ΔORF2+peptide	OK	125.614	140.772	0.164364	0.119681	0.83595	0.994748	no
gene:SpnNT_00537	mrnC	Chromosome:548415-550138	110.58+peptide	ΔORF2+peptide	OK	147.284	140.772	-0.0652448	-0.0478818	0.9348	0.994855	no
gene:SpnNT_00538	lrp	Chromosome:550141-551026	110.58	ΔORF2	OK	141.2	147.496	0.0629301	0.1424	0.8082	0.994748	no
gene:SpnNT_00538	lrp	Chromosome:550141-551026	110.58	110.58+peptide	OK	141.2	163.64	0.212785	0.482355	0.40365	0.894511	no
gene:SpnNT_00538	lrp	Chromosome:550141-551026	ΔORF2	110.58+peptide	OK	147.496	163.64	0.149855	0.339237	0.55135	0.964385	no
gene:SpnNT_00538	lrp	Chromosome:550141-551026	110.58	ΔORF2+peptide	OK	141.2	186.838	0.404046	0.921323	0.1044	0.490549	no
gene:SpnNT_00538	lrp	Chromosome:550141-551026	ΔORF2	ΔORF2+peptide	OK	147.496	186.838	0.341116	0.776753	0.1723	0.632248	no
gene:SpnNT_00538	lrp	Chromosome:550141-551026	110.58+peptide	ΔORF2+peptide	OK	163.64	186.838	0.191261	0.436306	0.44025	0.916647	no
gene:SpnNT_00539	NA	Chromosome:551684-552659	110.58	ΔORF2	OK	66.0651	71.4087	0.112212	0.240088	0.67865	0.985348	no
gene:SpnNT_00539	NA	Chromosome:551684-552659	110.58	110.58+peptide	OK	66.0651	79.4261	0.265726	0.562895	0.3246	0.830123	no
gene:SpnNT_00539	NA	Chromosome:551684-552659	ΔORF2	110.58+peptide	OK	71.4087	79.4261	0.153514	0.32581	0.5678	0.968621	no
gene:SpnNT_00539	NA	Chromosome:551684-552659	110.58	ΔORF2+peptide	OK	66.0651	72.8861	0.141755	0.300829	0.5992	0.97629	no
gene:SpnNT_00539	NA	Chromosome:551684-552659	ΔORF2	ΔORF2+peptide	OK	71.4087	72.8861	0.0295433	0.0628152	0.91055	0.994748	no
gene:SpnNT_00539	NA	Chromosome:551684-552659	110.58+peptide	ΔORF2+peptide	OK	79.4261	72.8861	-0.123971	-0.261	0.64165	0.980887	no
gene:SpnNT_00540	NA	Chromosome:552813-553023	110.58	ΔORF2	OK	323.533	290.144	-0.157146	-0.272513	0.6207	0.980887	no
gene:SpnNT_00540	NA	Chromosome:552813-553023	110.58	110.58+peptide	OK	323.533	385.75	0.253752	0.421398	0.44315	0.917631	no
gene:SpnNT_00540	NA	Chromosome:552813-553023	ΔORF2	110.58+peptide	OK	290.144	385.75	0.410898	0.684453	0.21415	0.69972	no
gene:SpnNT_00540	NA	Chromosome:552813-553023	110.58	ΔORF2+peptide	OK	323.533	334.325	0.0473379	0.0784799	0.88655	0.994748	no
gene:SpnNT_00540	NA	Chromosome:552813-553023	ΔORF2	ΔORF2+peptide	OK	290.144	334.325	0.204484	0.34004	0.5397	0.961134	no

gene:SpnNT_00540	NA	Chromosome:552813-553023	110.58+peptide	ΔORF2+peptide	OK	385.75	334.325	-0.206414	-0.32981	0.5469	0.962122	no
gene:SpnNT_00541	NA	Chromosome:553467-553758	110.58	ΔORF2	OK	0.944554	1.17022	0.309069	0.164678	0.8223	0.994748	no
gene:SpnNT_00541	NA	Chromosome:553467-553758	110.58	110.58+peptide	OK	0.944554	0.260548	-1.85808	-0.716836	0.29225	0.797277	no
gene:SpnNT_00541	NA	Chromosome:553467-553758	ΔORF2	110.58+peptide	NOTEST	1.17022	0.260548	-2.16715	0	1	1	no
gene:SpnNT_00541	NA	Chromosome:553467-553758	110.58	ΔORF2+peptide	OK	0.944554	0	#NAME?	NA	0.01755	0.150419	no
gene:SpnNT_00541	NA	Chromosome:553467-553758	ΔORF2	ΔORF2+peptide	NOTEST	1.17022	0	#NAME?	0	1	1	no
gene:SpnNT_00541	NA	Chromosome:553467-553758	110.58+peptide	ΔORF2+peptide	NOTEST	0.260548	0	#NAME?	0	1	1	no
gene:SpnNT_00542	NA	Chromosome:554139-554829	110.58	ΔORF2	OK	133.465	143.861	0.108212	0.237611	0.6772	0.98524	no
gene:SpnNT_00542	NA	Chromosome:554139-554829	110.58	110.58+peptide	OK	133.465	106.373	-0.32733	-0.710342	0.21615	0.703627	no
gene:SpnNT_00542	NA	Chromosome:554139-554829	ΔORF2	110.58+peptide	OK	143.861	106.373	-0.435542	-0.946885	0.1003	0.480167	no
gene:SpnNT_00542	NA	Chromosome:554139-554829	110.58	ΔORF2+peptide	OK	133.465	118.035	-0.177249	-0.38525	0.50325	0.945186	no
gene:SpnNT_00542	NA	Chromosome:554139-554829	ΔORF2	ΔORF2+peptide	OK	143.861	118.035	-0.285461	-0.621575	0.28685	0.790183	no
gene:SpnNT_00542	NA	Chromosome:554139-554829	110.58+peptide	ΔORF2+peptide	OK	106.373	118.035	0.150081	0.323035	0.5805	0.969538	no
gene:SpnNT_00543	NA	Chromosome:555058-562378	110.58	ΔORF2	OK	19.1381	77.7203	2.02184	4.49705	5.00E-05	0.0013612	yes
gene:SpnNT_00543	NA	Chromosome:555058-562378	110.58	110.58+peptide	OK	19.1381	27.9034	0.543998	1.2468	0.02625	0.203818	no
gene:SpnNT_00543	NA	Chromosome:555058-562378	ΔORF2	110.58+peptide	OK	77.7203	27.9034	-1.47785	-3.29436	5.00E-05	0.0013612	yes
gene:SpnNT_00543	NA	Chromosome:555058-562378	110.58	ΔORF2+peptide	OK	19.1381	109.537	2.5169	5.51433	5.00E-05	0.0013612	yes
gene:SpnNT_00543	NA	Chromosome:555058-562378	ΔORF2	ΔORF2+peptide	OK	77.7203	109.537	0.495051	1.05738	0.062	0.359666	no
gene:SpnNT_00543	NA	Chromosome:555058-562378	110.58+peptide	ΔORF2+peptide	OK	27.9034	109.537	1.9729	4.33177	5.00E-05	0.0013612	yes
gene:SpnNT_00544	NA	Chromosome:562528-563806	110.58	ΔORF2	OK	60.6865	73.6257	0.278835	0.613667	0.2901	0.794659	no
gene:SpnNT_00544	NA	Chromosome:562528-563806	110.58	110.58+peptide	OK	60.6865	48.446	-0.324998	-0.704576	0.2197	0.707982	no
gene:SpnNT_00544	NA	Chromosome:562528-563806	ΔORF2	110.58+peptide	OK	73.6257	48.446	-0.603833	-1.31315	0.0236	0.189059	no
gene:SpnNT_00544	NA	Chromosome:562528-563806	110.58	ΔORF2+peptide	OK	60.6865	60.6923	0.00013833	0.000304233	0.9991	0.999631	no
gene:SpnNT_00544	NA	Chromosome:562528-563806	ΔORF2	ΔORF2+peptide	OK	73.6257	60.6923	-0.278697	-0.614912	0.2848	0.787901	no
gene:SpnNT_00544	NA	Chromosome:562528-563806	110.58+peptide	ΔORF2+peptide	OK	48.446	60.6923	0.325136	0.706604	0.21935	0.707723	no
gene:SpnNT_00545	NA	Chromosome:563932-564580	110.58	ΔORF2	OK	131.882	154.259	0.2261	0.486567	0.399	0.892547	no
gene:SpnNT_00545	NA	Chromosome:563932-564580	110.58	110.58+peptide	OK	131.882	112.441	-0.230077	-0.493006	0.39375	0.888833	no
gene:SpnNT_00545	NA	Chromosome:563932-564580	ΔORF2	110.58+peptide	OK	154.259	112.441	-0.456177	-0.979964	0.0926	0.459394	no
gene:SpnNT_00545	NA	Chromosome:563932-564580	110.58	ΔORF2+peptide	OK	131.882	108.41	-0.28275	-0.603611	0.29275	0.797646	no
gene:SpnNT_00545	NA	Chromosome:563932-564580	ΔORF2	ΔORF2+peptide	OK	154.259	108.41	-0.50885	-1.08902	0.06055	0.353599	no
gene:SpnNT_00545	NA	Chromosome:563932-564580	110.58+peptide	ΔORF2+peptide	OK	112.441	108.41	-0.0526733	-0.112251	0.8376	0.994748	no
gene:SpnNT_00546	NA	Chromosome:564631-566011	110.58	ΔORF2	OK	64.3248	63.2418	-0.024497	-0.0533163	0.9291	0.994748	no
gene:SpnNT_00546	NA	Chromosome:564631-566011	110.58	110.58+peptide	OK	64.3248	49.8285	-0.368403	-0.804702	0.16415	0.618436	no
gene:SpnNT_00546	NA	Chromosome:564631-566011	ΔORF2	110.58+peptide	OK	63.2418	49.8285	-0.343906	-0.743384	0.197	0.678125	no
gene:SpnNT_00546	NA	Chromosome:564631-566011	110.58	ΔORF2+peptide	OK	64.3248	53.5308	-0.265007	-0.581016	0.31455	0.821071	no
gene:SpnNT_00546	NA	Chromosome:564631-566011	ΔORF2	ΔORF2+peptide	OK	63.2418	53.5308	-0.240509	-0.521786	0.365	0.864498	no
gene:SpnNT_00546	NA	Chromosome:564631-566011	110.58+peptide	ΔORF2+peptide	OK	49.8285	53.5308	0.103396	0.225123	0.6923	0.98828	no
gene:SpnNT_00547	regX3	Chromosome:566289-568271	110.58	ΔORF2	OK	31.5848	24.7684	-0.350728	-0.403194	0.4845	0.936747	no
gene:SpnNT_00547	regX3	Chromosome:566289-568271	110.58	110.58+peptide	OK	31.5848	20.1292	-0.649935	-0.718324	0.21645	0.70408	no
gene:SpnNT_00547	regX3	Chromosome:566289-568271	ΔORF2	110.58+peptide	OK	24.7684	20.1292	-0.299208	-0.312023	0.59745	0.97629	no
gene:SpnNT_00547	regX3	Chromosome:566289-568271	110.58	ΔORF2+peptide	OK	31.5848	20.2328	-0.642533	-0.725729	0.20635	0.691266	no
gene:SpnNT_00547	regX3	Chromosome:566289-568271	ΔORF2	ΔORF2+peptide	OK	24.7684	20.2328	-0.291805	-0.310228	0.5925	0.975468	no
gene:SpnNT_00547	regX3	Chromosome:566289-568271	110.58+peptide	ΔORF2+peptide	OK	20.1292	20.2328	0.00740232	0.00760773	0.99	0.997703	no
gene:SpnNT_00548	hssS	Chromosome:566289-568271	110.58	ΔORF2	OK	21.9568	24.0889	0.133699	0.221512	0.6987	0.990209	no
gene:SpnNT_00548	hssS	Chromosome:566289-568271	110.58	110.58+peptide	OK	21.9568	19.9854	-0.135718	-0.223813	0.69285	0.98828	no

gene:SpnNT_00548	hssS	Chromosome:566289-568271	ΔORF2	110.58+peptide	OK	24.0889	19.9854	-0.269417	-0.465584	0.4124	0.899511	no
gene:SpnNT_00548	hssS	Chromosome:566289-568271	110.58	ΔORF2+peptide	OK	21.9568	19.4049	-0.178248	-0.292	0.61275	0.979616	no
gene:SpnNT_00548	hssS	Chromosome:566289-568271	ΔORF2	ΔORF2+peptide	OK	24.0889	19.4049	-0.311947	-0.535158	0.35395	0.856293	no
gene:SpnNT_00548	hssS	Chromosome:566289-568271	110.58+peptide	ΔORF2+peptide	OK	19.9854	19.4049	-0.0425299	-0.0725987	0.8986	0.994748	no
gene:SpnNT_00549	fba	Chromosome:568411-569293	110.58	ΔORF2	OK	743.501	682.283	-0.123965	-0.267902	0.63715	0.980887	no
gene:SpnNT_00549	fba	Chromosome:568411-569293	110.58	110.58+peptide	OK	743.501	807.929	0.119893	0.253053	0.6582	0.982966	no
gene:SpnNT_00549	fba	Chromosome:568411-569293	ΔORF2	110.58+peptide	OK	682.283	807.929	0.243858	0.528561	0.3553	0.85704	no
gene:SpnNT_00549	fba	Chromosome:568411-569293	110.58	ΔORF2+peptide	OK	743.501	937.997	0.335248	0.721813	0.2077	0.693134	no
gene:SpnNT_00549	fba	Chromosome:568411-569293	ΔORF2	ΔORF2+peptide	OK	682.283	937.997	0.459213	1.01647	0.0778	0.414155	no
gene:SpnNT_00549	fba	Chromosome:568411-569293	110.58+peptide	ΔORF2+peptide	OK	807.929	937.997	0.215355	0.465032	0.40585	0.895655	no
gene:SpnNT_00550	nagAa	Chromosome:569612-570803	110.58	ΔORF2	OK	61.5482	62.1473	0.0139758	0.0302949	0.9549	0.994855	no
gene:SpnNT_00550	nagAa	Chromosome:569612-570803	110.58	110.58+peptide	OK	61.5482	36.1067	-0.769451	-1.63315	0.005	0.06047	no
gene:SpnNT_00550	nagAa	Chromosome:569612-570803	ΔORF2	110.58+peptide	OK	62.1473	36.1067	-0.783427	-1.64978	0.0045	0.0552376	no
gene:SpnNT_00550	nagAa	Chromosome:569612-570803	110.58	ΔORF2+peptide	OK	61.5482	40.0388	-0.620318	-1.3232	0.02205	0.180612	no
gene:SpnNT_00550	nagAa	Chromosome:569612-570803	ΔORF2	ΔORF2+peptide	OK	62.1473	40.0388	-0.634293	-1.3423	0.0226	0.183627	no
gene:SpnNT_00550	nagAa	Chromosome:569612-570803	110.58+peptide	ΔORF2+peptide	OK	36.1067	40.0388	0.149133	0.309319	0.5927	0.975468	no
gene:SpnNT_00551	glnP_1	Chromosome:571096-571756	110.58	ΔORF2	OK	41.3639	33.3963	-0.308686	-0.567351	0.32255	0.828116	no
gene:SpnNT_00551	glnP_1	Chromosome:571096-571756	110.58	110.58+peptide	OK	41.3639	37.6566	-0.13547	-0.249729	0.66325	0.982966	no
gene:SpnNT_00551	glnP_1	Chromosome:571096-571756	ΔORF2	110.58+peptide	OK	33.3963	37.6566	0.173216	0.317711	0.57025	0.968621	no
gene:SpnNT_00551	glnP_1	Chromosome:571096-571756	110.58	ΔORF2+peptide	OK	41.3639	22.9	-0.853024	-1.50943	0.0109	0.106978	no
gene:SpnNT_00551	glnP_1	Chromosome:571096-571756	ΔORF2	ΔORF2+peptide	OK	33.3963	22.9	-0.544339	-0.958756	0.09695	0.47048	no
gene:SpnNT_00551	glnP_1	Chromosome:571096-571756	110.58+peptide	ΔORF2+peptide	OK	37.6566	22.9	-0.717555	-1.2673	0.028	0.213242	no
gene:SpnNT_00552	glnM	Chromosome:571765-572443	110.58	ΔORF2	OK	45.2781	35.8204	-0.338031	-0.634766	0.2657	0.766773	no
gene:SpnNT_00552	glnM	Chromosome:571765-572443	110.58	110.58+peptide	OK	45.2781	41.0362	-0.141915	-0.268111	0.6394	0.980887	no
gene:SpnNT_00552	glnM	Chromosome:571765-572443	ΔORF2	110.58+peptide	OK	35.8204	41.0362	0.196116	0.366716	0.52515	0.955327	no
gene:SpnNT_00552	glnM	Chromosome:571765-572443	110.58	ΔORF2+peptide	OK	45.2781	34.409	-0.396028	-0.74611	0.193	0.674611	no
gene:SpnNT_00552	glnM	Chromosome:571765-572443	ΔORF2	ΔORF2+peptide	OK	35.8204	34.409	-0.0579976	-0.108154	0.8496	0.994748	no
gene:SpnNT_00552	glnM	Chromosome:571765-572443	110.58+peptide	ΔORF2+peptide	OK	41.0362	34.409	-0.254113	-0.476708	0.4123	0.899511	no
gene:SpnNT_00553	glnQ_2	Chromosome:572455-573808	110.58	ΔORF2	OK	46.2762	38.9833	-0.247414	-0.524484	0.35675	0.858005	no
gene:SpnNT_00553	glnQ_2	Chromosome:572455-573808	110.58	110.58+peptide	OK	46.2762	40.9354	-0.176921	-0.376356	0.5139	0.951114	no
gene:SpnNT_00553	glnQ_2	Chromosome:572455-573808	ΔORF2	110.58+peptide	OK	38.9833	40.9354	0.0704922	0.149264	0.79485	0.994748	no
gene:SpnNT_00553	glnQ_2	Chromosome:572455-573808	110.58	ΔORF2+peptide	OK	46.2762	34.3979	-0.427953	-0.901597	0.1207	0.526188	no
gene:SpnNT_00553	glnQ_2	Chromosome:572455-573808	ΔORF2	ΔORF2+peptide	OK	38.9833	34.3979	-0.180539	-0.378636	0.50865	0.947472	no
gene:SpnNT_00553	glnQ_2	Chromosome:572455-573808	110.58+peptide	ΔORF2+peptide	OK	40.9354	34.3979	-0.251032	-0.52827	0.357	0.858254	no
gene:SpnNT_00554	recJ	Chromosome:574083-576306	110.58	ΔORF2	OK	62.2154	64.8787	0.0604742	0.138363	0.80085	0.994748	no
gene:SpnNT_00554	recJ	Chromosome:574083-576306	110.58	110.58+peptide	OK	62.2154	60.7101	-0.0353355	-0.0808443	0.88615	0.994748	no
gene:SpnNT_00554	recJ	Chromosome:574083-576306	ΔORF2	110.58+peptide	OK	64.8787	60.7101	-0.0958097	-0.219259	0.6882	0.98828	no
gene:SpnNT_00554	recJ	Chromosome:574083-576306	110.58	ΔORF2+peptide	OK	62.2154	70.4099	0.178507	0.408334	0.4675	0.92732	no
gene:SpnNT_00554	recJ	Chromosome:574083-576306	ΔORF2	ΔORF2+peptide	OK	64.8787	70.4099	0.118033	0.270067	0.6292	0.980887	no
gene:SpnNT_00554	recJ	Chromosome:574083-576306	110.58+peptide	ΔORF2+peptide	OK	60.7101	70.4099	0.213842	0.489274	0.38335	0.880579	no
gene:SpnNT_00555	rnjB	Chromosome:576480-578142	110.58	ΔORF2	OK	390.514	408.289	0.0642179	0.143009	0.79985	0.994748	no
gene:SpnNT_00555	rnjB	Chromosome:576480-578142	110.58	110.58+peptide	OK	390.514	333.174	-0.229098	-0.510813	0.35935	0.86063	no
gene:SpnNT_00555	rnjB	Chromosome:576480-578142	ΔORF2	110.58+peptide	OK	408.289	333.174	-0.293316	-0.659052	0.23545	0.729189	no
gene:SpnNT_00555	rnjB	Chromosome:576480-578142	110.58	ΔORF2+peptide	OK	390.514	333.755	-0.226586	-0.505678	0.3646	0.864498	no
gene:SpnNT_00555	rnjB	Chromosome:576480-578142	ΔORF2	ΔORF2+peptide	OK	408.289	333.755	-0.290804	-0.654019	0.2401	0.735604	no

gene:SpnNT_00555	rnjB	Chromosome:576480-578142	110.58+peptide	ΔORF2+peptide	OK	333.174	333.755	0.00251177	0.00565606	0.9917	0.998369	no
gene:SpnNT_00556	axe1-6A_1	Chromosome:578210-578990	110.58	ΔORF2	OK	110.7	115.396	0.0599317	0.131648	0.8183	0.994748	no
gene:SpnNT_00556	axe1-6A_1	Chromosome:578210-578990	110.58	110.58+peptide	OK	110.7	74.2833	-0.575551	-1.24007	0.0309	0.226886	no
gene:SpnNT_00556	axe1-6A_1	Chromosome:578210-578990	ΔORF2	110.58+peptide	OK	115.396	74.2833	-0.635483	-1.37	0.01455	0.13113	no
gene:SpnNT_00556	axe1-6A_1	Chromosome:578210-578990	110.58	ΔORF2+peptide	OK	110.7	76.9011	-0.525583	-1.12372	0.0527	0.325273	no
gene:SpnNT_00556	axe1-6A_1	Chromosome:578210-578990	ΔORF2	ΔORF2+peptide	OK	115.396	76.9011	-0.585515	-1.25259	0.02825	0.214152	no
gene:SpnNT_00556	axe1-6A_1	Chromosome:578210-578990	110.58+peptide	ΔORF2+peptide	OK	74.2833	76.9011	0.0499676	0.104951	0.8587	0.994748	no
gene:SpnNT_00557	femX	Chromosome:579101-580322	110.58	ΔORF2	OK	95.725	118.082	0.302821	0.681781	0.22385	0.713991	no
gene:SpnNT_00557	femX	Chromosome:579101-580322	110.58	110.58+peptide	OK	95.725	62.4481	-0.616238	-1.34825	0.0169	0.146377	no
gene:SpnNT_00557	femX	Chromosome:579101-580322	ΔORF2	110.58+peptide	OK	118.082	62.4481	-0.919059	-2.01722	0.00045	0.00885292	yes
gene:SpnNT_00557	femX	Chromosome:579101-580322	110.58	ΔORF2+peptide	OK	95.725	93.7237	-0.0304818	-0.068398	0.9012	0.994748	no
gene:SpnNT_00557	femX	Chromosome:579101-580322	ΔORF2	ΔORF2+peptide	OK	118.082	93.7237	-0.333302	-0.75042	0.1878	0.663696	no
gene:SpnNT_00557	femX	Chromosome:579101-580322	110.58+peptide	ΔORF2+peptide	OK	62.4481	93.7237	0.585756	1.28157	0.02515	0.197971	no
gene:SpnNT_00558	femA	Chromosome:580326-581559	110.58	ΔORF2	OK	127.85	157.002	0.296324	0.673262	0.2416	0.736324	no
gene:SpnNT_00558	femA	Chromosome:580326-581559	110.58	110.58+peptide	OK	127.85	89.6129	-0.512678	-1.14704	0.0492	0.310984	no
gene:SpnNT_00558	femA	Chromosome:580326-581559	ΔORF2	110.58+peptide	OK	157.002	89.6129	-0.809002	-1.81342	0.0021	0.0307204	yes
gene:SpnNT_00558	femA	Chromosome:580326-581559	110.58	ΔORF2+peptide	OK	127.85	121.456	-0.0740252	-0.167742	0.76685	0.994748	no
gene:SpnNT_00558	femA	Chromosome:580326-581559	ΔORF2	ΔORF2+peptide	OK	157.002	121.456	-0.370349	-0.840837	0.14025	0.571896	no
gene:SpnNT_00558	femA	Chromosome:580326-581559	110.58+peptide	ΔORF2+peptide	OK	89.6129	121.456	0.438653	0.980722	0.0875	0.445374	no
gene:SpnNT_00559	NA	Chromosome:581637-581820	110.58	ΔORF2	OK	170.795	187.2	0.132315	0.205848	0.7199	0.993917	no
gene:SpnNT_00559	NA	Chromosome:581637-581820	110.58	110.58+peptide	OK	170.795	166.132	-0.0399387	-0.0610366	0.91605	0.994748	no
gene:SpnNT_00559	NA	Chromosome:581637-581820	ΔORF2	110.58+peptide	OK	187.2	166.132	-0.172254	-0.266516	0.6331	0.980887	no
gene:SpnNT_00559	NA	Chromosome:581637-581820	110.58	ΔORF2+peptide	OK	170.795	148.189	-0.204828	-0.26924	0.59075	0.974909	no
gene:SpnNT_00559	NA	Chromosome:581637-581820	ΔORF2	ΔORF2+peptide	OK	187.2	148.189	-0.337143	-0.447214	0.38245	0.880246	no
gene:SpnNT_00559	NA	Chromosome:581637-581820	110.58+peptide	ΔORF2+peptide	OK	166.132	148.189	-0.164889	-0.215893	0.6708	0.984845	no
gene:SpnNT_00560	NA	Chromosome:581922-582327	110.58	ΔORF2	OK	235.883	231.964	-0.0241713	-0.0506176	0.93395	0.994855	no
gene:SpnNT_00560	NA	Chromosome:581922-582327	110.58	110.58+peptide	OK	235.883	203.345	-0.214139	-0.448271	0.42755	0.909248	no
gene:SpnNT_00560	NA	Chromosome:581922-582327	ΔORF2	110.58+peptide	OK	231.964	203.345	-0.189967	-0.393667	0.49075	0.940324	no
gene:SpnNT_00560	NA	Chromosome:581922-582327	110.58	ΔORF2+peptide	OK	235.883	212.547	-0.15029	-0.315001	0.58045	0.969538	no
gene:SpnNT_00560	NA	Chromosome:581922-582327	ΔORF2	ΔORF2+peptide	OK	231.964	212.547	-0.126119	-0.261671	0.6499	0.981475	no
gene:SpnNT_00560	NA	Chromosome:581922-582327	110.58+peptide	ΔORF2+peptide	OK	203.345	212.547	0.0638488	0.132426	0.81915	0.994748	no
gene:SpnNT_00561	uvrC	Chromosome:582365-585036	110.58	ΔORF2	OK	28.0663	26.6627	-0.0740151	-0.130995	0.82195	0.994748	no
gene:SpnNT_00561	uvrC	Chromosome:582365-585036	110.58	110.58+peptide	OK	28.0663	23.6267	-0.248418	-0.43794	0.44365	0.917631	no
gene:SpnNT_00561	uvrC	Chromosome:582365-585036	ΔORF2	110.58+peptide	OK	26.6627	23.6267	-0.174403	-0.304194	0.5965	0.97629	no
gene:SpnNT_00561	uvrC	Chromosome:582365-585036	110.58	ΔORF2+peptide	OK	28.0663	25.1515	-0.158193	-0.281543	0.6226	0.980887	no
gene:SpnNT_00561	uvrC	Chromosome:582365-585036	ΔORF2	ΔORF2+peptide	OK	26.6627	25.1515	-0.0841778	-0.148195	0.7947	0.994748	no
gene:SpnNT_00561	uvrC	Chromosome:582365-585036	110.58+peptide	ΔORF2+peptide	OK	23.6267	25.1515	0.0902256	0.158227	0.78485	0.994748	no
gene:SpnNT_00562	NA	Chromosome:582365-585036	110.58	ΔORF2	OK	38.8725	40.0313	0.0423782	0.0557712	0.92055	0.994748	no
gene:SpnNT_00562	NA	Chromosome:582365-585036	110.58	110.58+peptide	OK	38.8725	33.1297	-0.230625	-0.295372	0.6051	0.976847	no
gene:SpnNT_00562	NA	Chromosome:582365-585036	ΔORF2	110.58+peptide	OK	40.0313	33.1297	-0.273003	-0.352261	0.5379	0.959663	no
gene:SpnNT_00562	NA	Chromosome:582365-585036	110.58	ΔORF2+peptide	OK	38.8725	34.3309	-0.179243	-0.231404	0.6818	0.986574	no
gene:SpnNT_00562	NA	Chromosome:582365-585036	ΔORF2	ΔORF2+peptide	OK	40.0313	34.3309	-0.221621	-0.288289	0.6119	0.979429	no
gene:SpnNT_00562	NA	Chromosome:582365-585036	110.58+peptide	ΔORF2+peptide	OK	33.1297	34.3309	0.0513823	0.0650861	0.90815	0.994748	no
gene:SpnNT_00563	tcyJ	Chromosome:585221-586022	110.58	ΔORF2	OK	23.7637	22.5442	-0.0760008	-0.139429	0.8075	0.994748	no
gene:SpnNT_00563	tcyJ	Chromosome:585221-586022	110.58	110.58+peptide	OK	23.7637	25.7791	0.117443	0.217672	0.70425	0.990367	no

gene:SpnNT_00563	tcyJ	Chromosome:585221-586022	ΔORF2	110.58+peptide	OK	22.5442	25.7791	0.193444	0.357506	0.53075	0.957488	no
gene:SpnNT_00563	tcyJ	Chromosome:585221-586022	110.58	ΔORF2+peptide	OK	23.7637	31.115	0.388851	0.726016	0.20215	0.685961	no
gene:SpnNT_00563	tcyJ	Chromosome:585221-586022	ΔORF2	ΔORF2+peptide	OK	22.5442	31.115	0.464852	0.865389	0.1261	0.537759	no
gene:SpnNT_00563	tcyJ	Chromosome:585221-586022	110.58+peptide	ΔORF2+peptide	OK	25.7791	31.115	0.271409	0.510615	0.374	0.871383	no
gene:SpnNT_00564	NA	Chromosome:586192-586798	110.58	ΔORF2	OK	90.5489	100.103	0.144711	0.300336	0.6091	0.978849	no
gene:SpnNT_00564	NA	Chromosome:586192-586798	110.58	110.58+peptide	OK	90.5489	108.173	0.256572	0.537823	0.3461	0.850154	no
gene:SpnNT_00564	NA	Chromosome:586192-586798	ΔORF2	110.58+peptide	OK	100.103	108.173	0.111861	0.234606	0.6786	0.985348	no
gene:SpnNT_00564	NA	Chromosome:586192-586798	110.58	ΔORF2+peptide	OK	90.5489	126.66	0.484189	1.01937	0.07635	0.408258	no
gene:SpnNT_00564	NA	Chromosome:586192-586798	ΔORF2	ΔORF2+peptide	OK	100.103	126.66	0.339478	0.715093	0.22025	0.708363	no
gene:SpnNT_00564	NA	Chromosome:586192-586798	110.58+peptide	ΔORF2+peptide	OK	108.173	126.66	0.227618	0.484408	0.39655	0.890558	no
gene:SpnNT_00565	pepV	Chromosome:586810-588211	110.58	ΔORF2	OK	214.906	215.847	0.00630254	0.0144078	0.9808	0.99608	no
gene:SpnNT_00565	pepV	Chromosome:586810-588211	110.58	110.58+peptide	OK	214.906	225.808	0.07139	0.163217	0.7757	0.994748	no
gene:SpnNT_00565	pepV	Chromosome:586810-588211	ΔORF2	110.58+peptide	OK	215.847	225.808	0.0650874	0.148471	0.79955	0.994748	no
gene:SpnNT_00565	pepV	Chromosome:586810-588211	110.58	ΔORF2+peptide	OK	214.906	246.501	0.197889	0.451951	0.42805	0.909574	no
gene:SpnNT_00565	pepV	Chromosome:586810-588211	ΔORF2	ΔORF2+peptide	OK	215.847	246.501	0.191586	0.436568	0.45265	0.921244	no
gene:SpnNT_00565	pepV	Chromosome:586810-588211	110.58+peptide	ΔORF2+peptide	OK	225.808	246.501	0.126499	0.288284	0.62115	0.980887	no
gene:SpnNT_00566	NA	Chromosome:588264-588843	110.58	ΔORF2	OK	34.3498	34.5722	0.00931193	0.0169558	0.9768	0.995385	no
gene:SpnNT_00566	NA	Chromosome:588264-588843	110.58	110.58+peptide	OK	34.3498	44.7498	0.381578	0.707467	0.21915	0.7076	no
gene:SpnNT_00566	NA	Chromosome:588264-588843	ΔORF2	110.58+peptide	OK	34.5722	44.7498	0.372266	0.689762	0.23015	0.723533	no
gene:SpnNT_00566	NA	Chromosome:588264-588843	110.58	ΔORF2+peptide	OK	34.3498	49.587	0.529659	0.979416	0.08945	0.449718	no
gene:SpnNT_00566	NA	Chromosome:588264-588843	ΔORF2	ΔORF2+peptide	OK	34.5722	49.587	0.520347	0.961586	0.09325	0.461224	no
gene:SpnNT_00566	NA	Chromosome:588264-588843	110.58+peptide	ΔORF2+peptide	OK	44.7498	49.587	0.148081	0.27879	0.63635	0.980887	no
gene:SpnNT_00567	NA	Chromosome:589002-589155	110.58	ΔORF2	OK	96.0614	63.7697	-0.591087	-0.661557	0.25195	0.75002	no
gene:SpnNT_00567	NA	Chromosome:589002-589155	110.58	110.58+peptide	OK	96.0614	97.6049	0.0229959	0.0259458	0.9721	0.99536	no
gene:SpnNT_00567	NA	Chromosome:589002-589155	ΔORF2	110.58+peptide	OK	63.7697	97.6049	0.614082	0.670834	0.2465	0.74356	no
gene:SpnNT_00567	NA	Chromosome:589002-589155	110.58	ΔORF2+peptide	OK	96.0614	34.5249	-1.47632	-2.32722	0.028	0.213242	no
gene:SpnNT_00567	NA	Chromosome:589002-589155	ΔORF2	ΔORF2+peptide	OK	63.7697	34.5249	-0.885232	-1.31258	0.16955	0.627598	no
gene:SpnNT_00567	NA	Chromosome:589002-589155	110.58+peptide	ΔORF2+peptide	OK	97.6049	34.5249	-1.49931	-2.25497	0.02875	0.215822	no
gene:SpnNT_00568	brnQ	Chromosome:589460-590786	110.58	ΔORF2	OK	38.9221	37.6157	-0.0492551	-0.0946924	0.86715	0.994748	no
gene:SpnNT_00568	brnQ	Chromosome:589460-590786	110.58	110.58+peptide	OK	38.9221	70.3582	0.854131	1.73731	0.00265	0.0362222	yes
gene:SpnNT_00568	brnQ	Chromosome:589460-590786	ΔORF2	110.58+peptide	OK	37.6157	70.3582	0.903386	1.87965	0.0017	0.025969	yes
gene:SpnNT_00568	brnQ	Chromosome:589460-590786	110.58	ΔORF2+peptide	OK	38.9221	85.4873	1.13512	2.29682	0.00015	0.00355289	yes
gene:SpnNT_00568	brnQ	Chromosome:589460-590786	ΔORF2	ΔORF2+peptide	OK	37.6157	85.4873	1.18438	2.45086	5.00E-05	0.0013612	yes
gene:SpnNT_00568	brnQ	Chromosome:589460-590786	110.58+peptide	ΔORF2+peptide	OK	70.3582	85.4873	0.280992	0.621101	0.28035	0.783714	no
gene:SpnNT_00569	pepA_1	Chromosome:590883-591921	110.58	ΔORF2	OK	144.108	147.043	0.0290934	0.0655847	0.90415	0.994748	no
gene:SpnNT_00569	pepA_1	Chromosome:590883-591921	110.58	110.58+peptide	OK	144.108	141.799	-0.023302	-0.0526642	0.9201	0.994748	no
gene:SpnNT_00569	pepA_1	Chromosome:590883-591921	ΔORF2	110.58+peptide	OK	147.043	141.799	-0.0523955	-0.117884	0.83545	0.994748	no
gene:SpnNT_00569	pepA_1	Chromosome:590883-591921	110.58	ΔORF2+peptide	OK	144.108	155.561	0.110336	0.251683	0.65765	0.982966	no
gene:SpnNT_00569	pepA_1	Chromosome:590883-591921	ΔORF2	ΔORF2+peptide	OK	147.043	155.561	0.0812425	0.184468	0.7462	0.994748	no
gene:SpnNT_00569	pepA_1	Chromosome:590883-591921	110.58+peptide	ΔORF2+peptide	OK	141.799	155.561	0.133638	0.304228	0.60005	0.976542	no
gene:SpnNT_00570	NA	Chromosome:591922-592393	110.58	ΔORF2	OK	80.2324	85.9304	0.0989848	0.192377	0.7378	0.994748	no
gene:SpnNT_00570	NA	Chromosome:591922-592393	110.58	110.58+peptide	OK	80.2324	78.8083	-0.025837	-0.0502359	0.92965	0.99482	no
gene:SpnNT_00570	NA	Chromosome:591922-592393	ΔORF2	110.58+peptide	OK	85.9304	78.8083	-0.124822	-0.243241	0.66995	0.984845	no
gene:SpnNT_00570	NA	Chromosome:591922-592393	110.58	ΔORF2+peptide	OK	80.2324	103.121	0.362082	0.712177	0.2096	0.695944	no
gene:SpnNT_00570	NA	Chromosome:591922-592393	ΔORF2	ΔORF2+peptide	OK	85.9304	103.121	0.263097	0.518674	0.362	0.862575	no

gene:SpnNT_00570	NA	Chromosome:591922-592393	110.58+peptide	ΔORF2+peptide	OK	78.8083	103.121	0.387919	0.765088	0.18075	0.648565	no
gene:SpnNT_00571	vanYB	Chromosome:592395-593112	110.58	ΔORF2	OK	106.561	105.904	-0.00892706	-0.0191646	0.97185	0.99536	no
gene:SpnNT_00571	vanYB	Chromosome:592395-593112	110.58	110.58+peptide	OK	106.561	101.71	-0.0672272	-0.145048	0.7972	0.994748	no
gene:SpnNT_00571	vanYB	Chromosome:592395-593112	ΔORF2	110.58+peptide	OK	105.904	101.71	-0.0583001	-0.124403	0.8252	0.994748	no
gene:SpnNT_00571	vanYB	Chromosome:592395-593112	110.58	ΔORF2+peptide	OK	106.561	119.218	0.161919	0.352959	0.5297	0.9574	no
gene:SpnNT_00571	vanYB	Chromosome:592395-593112	ΔORF2	ΔORF2+peptide	OK	105.904	119.218	0.170846	0.368239	0.5171	0.953322	no
gene:SpnNT_00571	vanYB	Chromosome:592395-593112	110.58+peptide	ΔORF2+peptide	OK	101.71	119.218	0.229146	0.496397	0.38745	0.881894	no
gene:SpnNT_00572	rplK	Chromosome:593430-593856	110.58	ΔORF2	OK	575.984	643.026	0.158848	0.362986	0.5217	0.954832	no
gene:SpnNT_00572	rplK	Chromosome:593430-593856	110.58	110.58+peptide	OK	575.984	1080.78	0.907976	2.08136	0.00035	0.00717609	yes
gene:SpnNT_00572	rplK	Chromosome:593430-593856	ΔORF2	110.58+peptide	OK	643.026	1080.78	0.749128	1.7216	0.0025	0.0347143	yes
gene:SpnNT_00572	rplK	Chromosome:593430-593856	110.58	ΔORF2+peptide	OK	575.984	1423.74	1.30558	2.98765	5.00E-05	0.0013612	yes
gene:SpnNT_00572	rplK	Chromosome:593430-593856	ΔORF2	ΔORF2+peptide	OK	643.026	1423.74	1.14673	2.63079	5.00E-05	0.0013612	yes
gene:SpnNT_00572	rplK	Chromosome:593430-593856	110.58+peptide	ΔORF2+peptide	OK	1080.78	1423.74	0.397606	0.915066	0.1035	0.488069	no
gene:SpnNT_00573	rplA	Chromosome:594064-594754	110.58	ΔORF2	OK	1017.05	1091.84	0.102364	0.228308	0.688	0.98828	no
gene:SpnNT_00573	rplA	Chromosome:594064-594754	110.58	110.58+peptide	OK	1017.05	2052.78	1.01319	2.22403	5.00E-05	0.0013612	yes
gene:SpnNT_00573	rplA	Chromosome:594064-594754	ΔORF2	110.58+peptide	OK	1091.84	2052.78	0.910824	2.01427	3.00E-04	0.00631878	yes
gene:SpnNT_00573	rplA	Chromosome:594064-594754	110.58	ΔORF2+peptide	OK	1017.05	2390.52	1.23293	2.71088	5.00E-05	0.0013612	yes
gene:SpnNT_00573	rplA	Chromosome:594064-594754	ΔORF2	ΔORF2+peptide	OK	1091.84	2390.52	1.13057	2.50444	5.00E-05	0.0013612	yes
gene:SpnNT_00573	rplA	Chromosome:594064-594754	110.58+peptide	ΔORF2+peptide	OK	2052.78	2390.52	0.219743	0.479183	0.407	0.896303	no
gene:SpnNT_00574	NA	Chromosome:595629-596061	110.58	ΔORF2	OK	1.73181	4.23392	1.28971	1.14635	0.05015	0.314264	no
gene:SpnNT_00574	NA	Chromosome:595629-596061	110.58	110.58+peptide	OK	1.73181	2.77724	0.681372	0.598566	0.2752	0.777354	no
gene:SpnNT_00574	NA	Chromosome:595629-596061	ΔORF2	110.58+peptide	OK	4.23392	2.77724	-0.608342	-0.58657	0.29065	0.795003	no
gene:SpnNT_00574	NA	Chromosome:595629-596061	110.58	ΔORF2+peptide	OK	1.73181	3.45871	0.997956	0.949641	0.1525	0.595213	no
gene:SpnNT_00574	NA	Chromosome:595629-596061	ΔORF2	ΔORF2+peptide	OK	4.23392	3.45871	-0.291759	-0.310287	0.6362	0.980887	no
gene:SpnNT_00574	NA	Chromosome:595629-596061	110.58+peptide	ΔORF2+peptide	OK	2.77724	3.45871	0.316584	0.331108	0.60125	0.976678	no
gene:SpnNT_00575	NA	Chromosome:596163-596520	110.58	ΔORF2	NOTEST	0.512927	0.2672	-0.940835	0	1	1	no
gene:SpnNT_00575	NA	Chromosome:596163-596520	110.58	110.58+peptide	NOTEST	0.512927	0.282518	-0.860414	0	1	1	no
gene:SpnNT_00575	NA	Chromosome:596163-596520	ΔORF2	110.58+peptide	NOTEST	0.2672	0.282518	0.080421	0	1	1	no
gene:SpnNT_00575	NA	Chromosome:596163-596520	110.58	ΔORF2+peptide	NOTEST	0.512927	0	#NAME?	0	1	1	no
gene:SpnNT_00575	NA	Chromosome:596163-596520	ΔORF2	ΔORF2+peptide	NOTEST	0.2672	0	#NAME?	0	1	1	no
gene:SpnNT_00575	NA	Chromosome:596163-596520	110.58+peptide	ΔORF2+peptide	NOTEST	0.282518	0	#NAME?	0	1	1	no
gene:SpnNT_00576	NA	Chromosome:596942-598676	110.58	ΔORF2	OK	28.6345	33.9551	0.245876	0.519471	0.3671	0.864634	no
gene:SpnNT_00576	NA	Chromosome:596942-598676	110.58	110.58+peptide	OK	28.6345	28.341	-0.0148619	-0.0310318	0.9558	0.994855	no
gene:SpnNT_00576	NA	Chromosome:596942-598676	ΔORF2	110.58+peptide	OK	33.9551	28.341	-0.260738	-0.550154	0.33325	0.839007	no
gene:SpnNT_00576	NA	Chromosome:596942-598676	110.58	ΔORF2+peptide	OK	28.6345	35.7231	0.319104	0.675869	0.24875	0.745378	no
gene:SpnNT_00576	NA	Chromosome:596942-598676	ΔORF2	ΔORF2+peptide	OK	33.9551	35.7231	0.0732284	0.15678	0.7819	0.994748	no
gene:SpnNT_00576	NA	Chromosome:596942-598676	110.58+peptide	ΔORF2+peptide	OK	28.341	35.7231	0.333966	0.706422	0.22295	0.712784	no
gene:SpnNT_00577	NA	Chromosome:599092-599551	110.58	ΔORF2	OK	181.338	202.297	0.15779	0.331744	0.5638	0.968621	no
gene:SpnNT_00577	NA	Chromosome:599092-599551	110.58	110.58+peptide	OK	181.338	144.358	-0.329031	-0.686059	0.23465	0.728242	no
gene:SpnNT_00577	NA	Chromosome:599092-599551	ΔORF2	110.58+peptide	OK	202.297	144.358	-0.486821	-1.0147	0.07545	0.405594	no
gene:SpnNT_00577	NA	Chromosome:599092-599551	110.58	ΔORF2+peptide	OK	181.338	167.914	-0.110962	-0.232085	0.68405	0.98747	no
gene:SpnNT_00577	NA	Chromosome:599092-599551	ΔORF2	ΔORF2+peptide	OK	202.297	167.914	-0.268752	-0.561908	0.3223	0.828116	no
gene:SpnNT_00577	NA	Chromosome:599092-599551	110.58+peptide	ΔORF2+peptide	OK	144.358	167.914	0.218069	0.452218	0.4303	0.91218	no
gene:SpnNT_00578	NA	Chromosome:599563-600575	110.58	ΔORF2	OK	156.282	166.702	0.0931219	0.165917	0.77	0.994748	no
gene:SpnNT_00578	NA	Chromosome:599563-600575	110.58	110.58+peptide	OK	156.282	122.195	-0.354964	-0.61716	0.29295	0.797831	no

gene:SpnNT_00578	NA	Chromosome:599563-600575	ΔORF2	110.58+peptide	OK	166.702	122.195	-0.448086	-0.784818	0.18055	0.648024	no
gene:SpnNT_00578	NA	Chromosome:599563-600575	110.58	ΔORF2+peptide	OK	156.282	171.06	0.130349	0.235511	0.68565	0.988184	no
gene:SpnNT_00578	NA	Chromosome:599563-600575	ΔORF2	ΔORF2+peptide	OK	166.702	171.06	0.0372275	0.0677981	0.9035	0.994748	no
gene:SpnNT_00578	NA	Chromosome:599563-600575	110.58+peptide	ΔORF2+peptide	OK	122.195	171.06	0.485314	0.861561	0.14505	0.580489	no
gene:SpnNT_00579	NA	Chromosome:599563-600575	110.58	ΔORF2	OK	165.573	182.426	0.139851	0.180324	0.75315	0.994748	no
gene:SpnNT_00579	NA	Chromosome:599563-600575	110.58	110.58+peptide	OK	165.573	135.936	-0.284542	-0.343976	0.54375	0.961568	no
gene:SpnNT_00579	NA	Chromosome:599563-600575	ΔORF2	110.58+peptide	OK	182.426	135.936	-0.424393	-0.527412	0.34945	0.853357	no
gene:SpnNT_00579	NA	Chromosome:599563-600575	110.58	ΔORF2+peptide	OK	165.573	158.382	-0.0640575	-0.075946	0.89045	0.994748	no
gene:SpnNT_00579	NA	Chromosome:599563-600575	ΔORF2	ΔORF2+peptide	OK	182.426	158.382	-0.203908	-0.248256	0.65635	0.982966	no
gene:SpnNT_00579	NA	Chromosome:599563-600575	110.58+peptide	ΔORF2+peptide	OK	135.936	158.382	0.220484	0.253341	0.6571	0.982966	no
gene:SpnNT_00580	ply_1	Chromosome:600585-602001	110.58	ΔORF2	OK	117.259	120.565	0.0401102	0.0882394	0.87495	0.994748	no
gene:SpnNT_00580	ply_1	Chromosome:600585-602001	110.58	110.58+peptide	OK	117.259	74.5409	-0.653593	-1.44731	0.01315	0.122206	no
gene:SpnNT_00580	ply_1	Chromosome:600585-602001	ΔORF2	110.58+peptide	OK	120.565	74.5409	-0.693703	-1.52434	0.0072	0.079194	no
gene:SpnNT_00580	ply_1	Chromosome:600585-602001	110.58	ΔORF2+peptide	OK	117.259	84.6894	-0.469444	-1.04468	0.0659	0.373376	no
gene:SpnNT_00580	ply_1	Chromosome:600585-602001	ΔORF2	ΔORF2+peptide	OK	120.565	84.6894	-0.509554	-1.12515	0.0448	0.291023	no
gene:SpnNT_00580	ply_1	Chromosome:600585-602001	110.58+peptide	ΔORF2+peptide	OK	74.5409	84.6894	0.184149	0.409317	0.4694	0.927729	no
gene:SpnNT_00581	NA	Chromosome:602010-602757	110.58	ΔORF2	OK	219.95	202.866	-0.116645	-0.261941	0.64365	0.980887	no
gene:SpnNT_00581	NA	Chromosome:602010-602757	110.58	110.58+peptide	OK	219.95	235.233	0.0969191	0.218767	0.7031	0.990367	no
gene:SpnNT_00581	NA	Chromosome:602010-602757	ΔORF2	110.58+peptide	OK	202.866	235.233	0.213564	0.479336	0.39855	0.8923	no
gene:SpnNT_00581	NA	Chromosome:602010-602757	110.58	ΔORF2+peptide	OK	219.95	252.434	0.198733	0.451533	0.4191	0.903984	no
gene:SpnNT_00581	NA	Chromosome:602010-602757	ΔORF2	ΔORF2+peptide	OK	202.866	252.434	0.315378	0.712456	0.1983	0.680978	no
gene:SpnNT_00581	NA	Chromosome:602010-602757	110.58+peptide	ΔORF2+peptide	OK	235.233	252.434	0.101814	0.231205	0.6815	0.986574	no
gene:SpnNT_00582	NA	Chromosome:602772-604451	110.58	ΔORF2	OK	761.143	678.357	-0.166123	-0.0842698	0.88365	0.994748	no
gene:SpnNT_00582	NA	Chromosome:602772-604451	110.58	110.58+peptide	OK	761.143	906.209	0.251676	0.124719	0.83105	0.994748	no
gene:SpnNT_00582	NA	Chromosome:602772-604451	ΔORF2	110.58+peptide	OK	678.357	906.209	0.417799	0.201616	0.789	0.994748	no
gene:SpnNT_00582	NA	Chromosome:602772-604451	110.58	ΔORF2+peptide	OK	761.143	771.902	0.02025	0.00981618	0.98195	0.996246	no
gene:SpnNT_00582	NA	Chromosome:602772-604451	ΔORF2	ΔORF2+peptide	OK	678.357	771.902	0.186373	0.0880748	0.9049	0.994748	no
gene:SpnNT_00582	NA	Chromosome:602772-604451	110.58+peptide	ΔORF2+peptide	OK	906.209	771.902	-0.231426	-0.107163	0.8776	0.994748	no
gene:SpnNT_00583	NA	Chromosome:602772-604451	110.58	ΔORF2	OK	389.571	409.502	0.0719834	0.156456	0.78625	0.994748	no
gene:SpnNT_00583	NA	Chromosome:602772-604451	110.58	110.58+peptide	OK	389.571	424.867	0.125123	0.270137	0.635	0.980887	no
gene:SpnNT_00583	NA	Chromosome:602772-604451	ΔORF2	110.58+peptide	OK	409.502	424.867	0.0531396	0.114939	0.84175	0.994748	no
gene:SpnNT_00583	NA	Chromosome:602772-604451	110.58	ΔORF2+peptide	OK	389.571	563.993	0.533789	1.15739	0.04395	0.28735	no
gene:SpnNT_00583	NA	Chromosome:602772-604451	ΔORF2	ΔORF2+peptide	OK	409.502	563.993	0.461806	1.00318	0.077	0.410729	no
gene:SpnNT_00583	NA	Chromosome:602772-604451	110.58+peptide	ΔORF2+peptide	OK	424.867	563.993	0.408666	0.881818	0.11975	0.52453	no
gene:SpnNT_00584	NA	Chromosome:604586-605584	110.58	ΔORF2	OK	21.893	14.1966	-0.624926	-0.309771	0.6555	0.982966	no
gene:SpnNT_00584	NA	Chromosome:604586-605584	110.58	110.58+peptide	OK	21.893	22.0465	0.0100782	0.00511777	0.96575	0.994855	no
gene:SpnNT_00584	NA	Chromosome:604586-605584	ΔORF2	110.58+peptide	OK	14.1966	22.0465	0.635004	0.311078	0.6943	0.988457	no
gene:SpnNT_00584	NA	Chromosome:604586-605584	110.58	ΔORF2+peptide	OK	21.893	23.9932	0.132153	0.0582747	0.9137	0.994748	no
gene:SpnNT_00584	NA	Chromosome:604586-605584	ΔORF2	ΔORF2+peptide	OK	14.1966	23.9932	0.757078	0.324844	0.61555	0.979616	no
gene:SpnNT_00584	NA	Chromosome:604586-605584	110.58+peptide	ΔORF2+peptide	OK	22.0465	23.9932	0.122075	0.0533296	0.932	0.994855	no
gene:SpnNT_00585	NA	Chromosome:604586-605584	110.58	ΔORF2	OK	3.21172	1.0753	-1.57861	-1.54527	0.01855	0.156838	no
gene:SpnNT_00585	NA	Chromosome:604586-605584	110.58	110.58+peptide	OK	3.21172	2.30908	-0.476027	-0.557268	0.332	0.83795	no
gene:SpnNT_00585	NA	Chromosome:604586-605584	ΔORF2	110.58+peptide	OK	1.0753	2.30908	1.10258	1.02692	0.08015	0.421873	no
gene:SpnNT_00585	NA	Chromosome:604586-605584	110.58	ΔORF2+peptide	OK	3.21172	1.12998	-1.50705	-1.42882	0.1173	0.518952	no
gene:SpnNT_00585	NA	Chromosome:604586-605584	ΔORF2	ΔORF2+peptide	OK	1.0753	1.12998	0.0715605	0.0577478	0.93495	0.994855	no

gene:SpnNT_00585	NA	Chromosome:604586-605584	110.58+peptide	ΔORF2+peptide	OK	2.30908	1.12998	-1.03102	-0.932809	0.22375	0.713991	no
gene:SpnNT_00586	hpaIIM_1	Chromosome:605743-607511	110.58	ΔORF2	OK	2.61236	1.01396	-1.36535	-1.60109	0.0081	0.0866951	no
gene:SpnNT_00586	hpaIIM_1	Chromosome:605743-607511	110.58	110.58+peptide	OK	2.61236	2.93587	0.168434	0.209984	0.7116	0.991007	no
gene:SpnNT_00586	hpaIIM_1	Chromosome:605743-607511	ΔORF2	110.58+peptide	OK	1.01396	2.93587	1.53378	1.79067	0.0036	0.0465411	yes
gene:SpnNT_00586	hpaIIM_1	Chromosome:605743-607511	110.58	ΔORF2+peptide	OK	2.61236	1.47367	-0.825939	-1.01304	0.08535	0.439454	no
gene:SpnNT_00586	hpaIIM_1	Chromosome:605743-607511	ΔORF2	ΔORF2+peptide	OK	1.01396	1.47367	0.539407	0.620799	0.2904	0.794875	no
gene:SpnNT_00586	hpaIIM_1	Chromosome:605743-607511	110.58+peptide	ΔORF2+peptide	OK	2.93587	1.47367	-0.994372	-1.21375	0.0423	0.281043	no
gene:SpnNT_00587	NA	Chromosome:605743-607511	110.58	ΔORF2	OK	3.91164	1.35291	-1.53171	-0.826973	0.2503	0.747823	no
gene:SpnNT_00587	NA	Chromosome:605743-607511	110.58	110.58+peptide	OK	3.91164	3.57907	-0.128191	-0.0800081	0.893	0.994748	no
gene:SpnNT_00587	NA	Chromosome:605743-607511	ΔORF2	110.58+peptide	OK	1.35291	3.57907	1.40352	0.739667	0.2859	0.789224	no
gene:SpnNT_00587	NA	Chromosome:605743-607511	110.58	ΔORF2+peptide	OK	3.91164	0.75906	-2.36549	-1.07717	0.10885	0.502049	no
gene:SpnNT_00587	NA	Chromosome:605743-607511	ΔORF2	ΔORF2+peptide	OK	1.35291	0.75906	-0.833776	-0.344552	0.6048	0.976761	no
gene:SpnNT_00587	NA	Chromosome:605743-607511	110.58+peptide	ΔORF2+peptide	OK	3.57907	0.75906	-2.2373	-1.00131	0.12405	0.533175	no
gene:SpnNT_00588	NA	Chromosome:607520-607904	110.58	ΔORF2	OK	2.41864	0.827107	-1.54805	-1.2286	0.10355	0.488069	no
gene:SpnNT_00588	NA	Chromosome:607520-607904	110.58	110.58+peptide	OK	2.41864	1.37486	-0.814914	-0.636869	0.26345	0.764218	no
gene:SpnNT_00588	NA	Chromosome:607520-607904	ΔORF2	110.58+peptide	OK	0.827107	1.37486	0.733138	0.514231	0.4428	0.917631	no
gene:SpnNT_00588	NA	Chromosome:607520-607904	110.58	ΔORF2+peptide	OK	2.41864	0.31811	-2.9266	-2.11489	0.11675	0.517915	no
gene:SpnNT_00588	NA	Chromosome:607520-607904	ΔORF2	ΔORF2+peptide	NOTEST	0.827107	0.31811	-1.37855	0	1	1	no
gene:SpnNT_00588	NA	Chromosome:607520-607904	110.58+peptide	ΔORF2+peptide	OK	1.37486	0.31811	-2.11168	-1.37462	0.1486	0.588569	no
gene:SpnNT_00589	NA	Chromosome:607913-608147	110.58	ΔORF2	OK	2.82204	0	#NAME?	NA	0.0024	0.0337543	yes
gene:SpnNT_00589	NA	Chromosome:607913-608147	110.58	110.58+peptide	OK	2.82204	1.91273	-0.561101	-0.384077	0.625	0.980887	no
gene:SpnNT_00589	NA	Chromosome:607913-608147	ΔORF2	110.58+peptide	OK	0	1.91273	Inf	NA	0.0129	0.120861	no
gene:SpnNT_00589	NA	Chromosome:607913-608147	110.58	ΔORF2+peptide	OK	2.82204	0	#NAME?	NA	0.0024	0.0337543	yes
gene:SpnNT_00589	NA	Chromosome:607913-608147	ΔORF2	ΔORF2+peptide	NOTEST	0	0	0	0	1	1	no
gene:SpnNT_00589	NA	Chromosome:607913-608147	110.58+peptide	ΔORF2+peptide	OK	1.91273	0	#NAME?	NA	0.01115	0.108378	no
gene:SpnNT_00590	NA	Chromosome:608149-608731	110.58	ΔORF2	OK	2.09063	0.790462	-1.40317	-1.18198	0.0476	0.303945	no
gene:SpnNT_00590	NA	Chromosome:608149-608731	110.58	110.58+peptide	OK	2.09063	2.92934	0.486637	0.520777	0.3575	0.858392	no
gene:SpnNT_00590	NA	Chromosome:608149-608731	ΔORF2	110.58+peptide	OK	0.790462	2.92934	1.88981	1.64833	0.0105	0.103986	no
gene:SpnNT_00590	NA	Chromosome:608149-608731	110.58	ΔORF2+peptide	OK	2.09063	1.44048	-0.537388	-0.531695	0.41985	0.904223	no
gene:SpnNT_00590	NA	Chromosome:608149-608731	ΔORF2	ΔORF2+peptide	OK	0.790462	1.44048	0.865782	0.71584	0.2586	0.759455	no
gene:SpnNT_00590	NA	Chromosome:608149-608731	110.58+peptide	ΔORF2+peptide	OK	2.92934	1.44048	-1.02402	-1.06375	0.1169	0.518405	no
gene:SpnNT_00591	NA	Chromosome:608806-611115	110.58	ΔORF2	OK	5.03219	1.31409	-1.93712	-0.952384	0.29265	0.79754	no
gene:SpnNT_00591	NA	Chromosome:608806-611115	110.58	110.58+peptide	OK	5.03219	4.31115	-0.223112	-0.117208	0.8433	0.994748	no
gene:SpnNT_00591	NA	Chromosome:608806-611115	ΔORF2	110.58+peptide	OK	1.31409	4.31115	1.714	0.797528	0.3446	0.848904	no
gene:SpnNT_00591	NA	Chromosome:608806-611115	110.58	ΔORF2+peptide	OK	5.03219	3.10849	-0.694971	-0.418426	0.5089	0.947472	no
gene:SpnNT_00591	NA	Chromosome:608806-611115	ΔORF2	ΔORF2+peptide	OK	1.31409	3.10849	1.24215	0.641102	0.42295	0.906114	no
gene:SpnNT_00591	NA	Chromosome:608806-611115	110.58+peptide	ΔORF2+peptide	OK	4.31115	3.10849	-0.471859	-0.262125	0.65865	0.982966	no
gene:SpnNT_00592	NA	Chromosome:608806-611115	110.58	ΔORF2	OK	6.74045	2.06949	-1.70357	-2.48878	5.00E-05	0.0013612	yes
gene:SpnNT_00592	NA	Chromosome:608806-611115	110.58	110.58+peptide	OK	6.74045	5.86295	-0.201217	-0.320998	0.57315	0.969538	no
gene:SpnNT_00592	NA	Chromosome:608806-611115	ΔORF2	110.58+peptide	OK	2.06949	5.86295	1.50236	2.20816	0.00025	0.00542231	yes
gene:SpnNT_00592	NA	Chromosome:608806-611115	110.58	ΔORF2+peptide	OK	6.74045	2.30421	-1.54857	-2.25843	3.00E-04	0.00631878	yes
gene:SpnNT_00592	NA	Chromosome:608806-611115	ΔORF2	ΔORF2+peptide	OK	2.06949	2.30421	0.155001	0.210906	0.7138	0.991687	no
gene:SpnNT_00592	NA	Chromosome:608806-611115	110.58+peptide	ΔORF2+peptide	OK	5.86295	2.30421	-1.34735	-1.97688	0.00095	0.0161266	yes
gene:SpnNT_00593	NA	Chromosome:611132-611375	110.58	ΔORF2	OK	8.02047	0.99604	-3.00941	-2.42886	0.2459	0.743308	no
gene:SpnNT_00593	NA	Chromosome:611132-611375	110.58	110.58+peptide	OK	8.02047	7.19675	-0.156342	-0.134138	0.8214	0.994748	no

gene:SpnNT_00593	NA	Chromosome:611132-611375	ΔORF2	110.58+peptide	OK	0.99604	7.19675	2.85307	2.17416	0.25015	0.747694	no
gene:SpnNT_00593	NA	Chromosome:611132-611375	110.58	ΔORF2+peptide	OK	8.02047	8.6662	0.111712	0.101137	0.8573	0.994748	no
gene:SpnNT_00593	NA	Chromosome:611132-611375	ΔORF2	ΔORF2+peptide	OK	0.99604	8.6662	3.12112	2.48018	0.25	0.747607	no
gene:SpnNT_00593	NA	Chromosome:611132-611375	110.58+peptide	ΔORF2+peptide	OK	7.19675	8.6662	0.268054	0.225989	0.68705	0.98828	no
gene:SpnNT_00594	NA	Chromosome:611394-612246	110.58	ΔORF2	OK	4.10737	1.55498	-1.40132	-1.72431	0.00415	0.0519126	no
gene:SpnNT_00594	NA	Chromosome:611394-612246	110.58	110.58+peptide	OK	4.10737	4.76931	0.215564	0.294104	0.612	0.979429	no
gene:SpnNT_00594	NA	Chromosome:611394-612246	ΔORF2	110.58+peptide	OK	1.55498	4.76931	1.61689	2.0395	9.00E-04	0.0154579	yes
gene:SpnNT_00594	NA	Chromosome:611394-612246	110.58	ΔORF2+peptide	OK	4.10737	2.06494	-0.992118	-1.20556	0.0453	0.29311	no
gene:SpnNT_00594	NA	Chromosome:611394-612246	ΔORF2	ΔORF2+peptide	OK	1.55498	2.06494	0.409203	0.466773	0.4203	0.904235	no
gene:SpnNT_00594	NA	Chromosome:611394-612246	110.58+peptide	ΔORF2+peptide	OK	4.76931	2.06494	-1.20768	-1.50339	0.014	0.127841	no
gene:SpnNT_00595	NA	Chromosome:612435-617772	110.58	ΔORF2	OK	2.14214	1.43455	-0.578451	-0.170198	0.8209	0.994748	no
gene:SpnNT_00595	NA	Chromosome:612435-617772	110.58	110.58+peptide	OK	2.14214	2.8279	0.400682	0.119187	0.8615	0.994748	no
gene:SpnNT_00595	NA	Chromosome:612435-617772	ΔORF2	110.58+peptide	OK	1.43455	2.8279	0.979133	0.270916	0.6997	0.990367	no
gene:SpnNT_00595	NA	Chromosome:612435-617772	110.58	ΔORF2+peptide	OK	2.14214	1.82765	-0.22906	-0.075025	0.91285	0.994748	no
gene:SpnNT_00595	NA	Chromosome:612435-617772	ΔORF2	ΔORF2+peptide	OK	1.43455	1.82765	0.349391	0.104955	0.87145	0.994748	no
gene:SpnNT_00595	NA	Chromosome:612435-617772	110.58+peptide	ΔORF2+peptide	OK	2.8279	1.82765	-0.629742	-0.191336	0.77395	0.994748	no
gene:SpnNT_00596	NA	Chromosome:612435-617772	110.58	ΔORF2	OK	4.3925	1.53562	-1.51622	-1.67894	0.0062	0.0706832	no
gene:SpnNT_00596	NA	Chromosome:612435-617772	110.58	110.58+peptide	OK	4.3925	4.21692	-0.0588518	-0.0711241	0.8993	0.994748	no
gene:SpnNT_00596	NA	Chromosome:612435-617772	ΔORF2	110.58+peptide	OK	1.53562	4.21692	1.45737	1.62191	0.0081	0.0866951	no
gene:SpnNT_00596	NA	Chromosome:612435-617772	110.58	ΔORF2+peptide	OK	4.3925	1.41877	-1.6304	-1.67084	0.00815	0.0871592	no
gene:SpnNT_00596	NA	Chromosome:612435-617772	ΔORF2	ΔORF2+peptide	OK	1.53562	1.41877	-0.11418	-0.11013	0.84895	0.994748	no
gene:SpnNT_00596	NA	Chromosome:612435-617772	110.58+peptide	ΔORF2+peptide	OK	4.21692	1.41877	-1.57155	-1.61747	0.0109	0.106978	no
gene:SpnNT_00597	NA	Chromosome:612435-617772	110.58	ΔORF2	OK	5.10598	1.83198	-1.47879	-1.87643	0.00185	0.0276489	yes
gene:SpnNT_00597	NA	Chromosome:612435-617772	110.58	110.58+peptide	OK	5.10598	4.8014	-0.0887317	-0.123233	0.83255	0.994748	no
gene:SpnNT_00597	NA	Chromosome:612435-617772	ΔORF2	110.58+peptide	OK	1.83198	4.8014	1.39005	1.7685	0.003	0.0399655	yes
gene:SpnNT_00597	NA	Chromosome:612435-617772	110.58	ΔORF2+peptide	OK	5.10598	1.98401	-1.36377	-1.78671	0.0033	0.0432593	yes
gene:SpnNT_00597	NA	Chromosome:612435-617772	ΔORF2	ΔORF2+peptide	OK	1.83198	1.98401	0.115019	0.139279	0.8087	0.994748	no
gene:SpnNT_00597	NA	Chromosome:612435-617772	110.58+peptide	ΔORF2+peptide	OK	4.8014	1.98401	-1.27504	-1.67517	0.00595	0.068608	no
gene:SpnNT_00598	ltrA_1	Chromosome:618480-620403	110.58	ΔORF2	OK	2.60793	1.52711	-0.772103	-1.10674	0.06085	0.354877	no
gene:SpnNT_00598	ltrA_1	Chromosome:618480-620403	110.58	110.58+peptide	OK	2.60793	2.38229	-0.130554	-0.198442	0.7309	0.994748	no
gene:SpnNT_00598	ltrA_1	Chromosome:618480-620403	ΔORF2	110.58+peptide	OK	1.52711	2.38229	0.641549	0.930409	0.1114	0.508272	no
gene:SpnNT_00598	ltrA_1	Chromosome:618480-620403	110.58	ΔORF2+peptide	OK	2.60793	1.85119	-0.494455	-0.734576	0.20825	0.693901	no
gene:SpnNT_00598	ltrA_1	Chromosome:618480-620403	ΔORF2	ΔORF2+peptide	OK	1.52711	1.85119	0.277649	0.394347	0.501	0.944652	no
gene:SpnNT_00598	ltrA_1	Chromosome:618480-620403	110.58+peptide	ΔORF2+peptide	OK	2.38229	1.85119	-0.363901	-0.547453	0.33595	0.840163	no
gene:SpnNT_00599	NA	Chromosome:620752-622185	110.58	ΔORF2	OK	9.67834	10.955	0.178762	0.256054	0.6582	0.982966	no
gene:SpnNT_00599	NA	Chromosome:620752-622185	110.58	110.58+peptide	OK	9.67834	8.00188	-0.27442	-0.382501	0.50435	0.945292	no
gene:SpnNT_00599	NA	Chromosome:620752-622185	ΔORF2	110.58+peptide	OK	10.955	8.00188	-0.453181	-0.626933	0.27835	0.781125	no
gene:SpnNT_00599	NA	Chromosome:620752-622185	110.58	ΔORF2+peptide	OK	9.67834	7.75534	-0.31957	-0.445033	0.44065	0.916647	no
gene:SpnNT_00599	NA	Chromosome:620752-622185	ΔORF2	ΔORF2+peptide	OK	10.955	7.75534	-0.498332	-0.688782	0.2319	0.724662	no
gene:SpnNT_00599	NA	Chromosome:620752-622185	110.58+peptide	ΔORF2+peptide	OK	8.00188	7.75534	-0.0451502	-0.0608385	0.91855	0.994748	no
gene:SpnNT_00600	NA	Chromosome:620752-622185	110.58	ΔORF2	OK	5.17173	6.69225	0.371844	0.308692	0.60075	0.976678	no
gene:SpnNT_00600	NA	Chromosome:620752-622185	110.58	110.58+peptide	OK	5.17173	5.03864	-0.0376113	-0.0318857	0.95645	0.994855	no
gene:SpnNT_00600	NA	Chromosome:620752-622185	ΔORF2	110.58+peptide	OK	6.69225	5.03864	-0.409455	-0.339199	0.5697	0.968621	no
gene:SpnNT_00600	NA	Chromosome:620752-622185	110.58	ΔORF2+peptide	OK	5.17173	5.94246	0.200413	0.167474	0.76705	0.994748	no
gene:SpnNT_00600	NA	Chromosome:620752-622185	ΔORF2	ΔORF2+peptide	OK	6.69225	5.94246	-0.171431	-0.140074	0.8168	0.994748	no

gene:SpnNT_00600	NA	Chromosome:620752-622185	110.58+peptide	ΔORF2+peptide	OK	5.03864	5.94246	0.238025	0.198479	0.7344	0.994748	no
gene:SpnNT_00601	NA	Chromosome:622378-627283	110.58	ΔORF2	OK	4.05112	1.48794	-1.445	-2.46408	5.00E-05	0.0013612	yes
gene:SpnNT_00601	NA	Chromosome:622378-627283	110.58	110.58+peptide	OK	4.05112	4.03198	-0.00683178	-0.0124375	0.98305	0.996246	no
gene:SpnNT_00601	NA	Chromosome:622378-627283	ΔORF2	110.58+peptide	OK	1.48794	4.03198	1.43817	2.46091	5.00E-05	0.0013612	yes
gene:SpnNT_00601	NA	Chromosome:622378-627283	110.58	ΔORF2+peptide	OK	4.05112	1.7	-1.25279	-2.1731	2.00E-04	0.00450928	yes
gene:SpnNT_00601	NA	Chromosome:622378-627283	ΔORF2	ΔORF2+peptide	OK	1.48794	1.7	0.192217	0.315082	0.5787	0.969538	no
gene:SpnNT_00601	NA	Chromosome:622378-627283	110.58+peptide	ΔORF2+peptide	OK	4.03198	1.7	-1.24596	-2.16898	2.00E-04	0.00450928	yes
gene:SpnNT_00602	NA	Chromosome:627283-628010	110.58	ΔORF2	OK	3.74269	2.91973	-0.358238	-0.279983	0.80385	0.994748	no
gene:SpnNT_00602	NA	Chromosome:627283-628010	110.58	110.58+peptide	OK	3.74269	4.70473	0.330036	0.257555	0.82105	0.994748	no
gene:SpnNT_00602	NA	Chromosome:627283-628010	ΔORF2	110.58+peptide	OK	2.91973	4.70473	0.688274	0.587482	0.75535	0.994748	no
gene:SpnNT_00602	NA	Chromosome:627283-628010	110.58	ΔORF2+peptide	OK	3.74269	0.571077	-2.71232	-1.52582	0.4771	0.932178	no
gene:SpnNT_00602	NA	Chromosome:627283-628010	ΔORF2	ΔORF2+peptide	OK	2.91973	0.571077	-2.35408	-1.38465	0.4389	0.916056	no
gene:SpnNT_00602	NA	Chromosome:627283-628010	110.58+peptide	ΔORF2+peptide	OK	4.70473	0.571077	-3.04236	-1.78796	0.41585	0.901203	no
gene:SpnNT_00603	NA	Chromosome:627283-628010	110.58	ΔORF2	OK	6.87973	2.60496	-1.40109	-1.60542	0.00915	0.0945404	no
gene:SpnNT_00603	NA	Chromosome:627283-628010	110.58	110.58+peptide	OK	6.87973	4.98422	-0.464984	-0.58261	0.31105	0.816778	no
gene:SpnNT_00603	NA	Chromosome:627283-628010	ΔORF2	110.58+peptide	OK	2.60496	4.98422	0.936109	1.04595	0.0737	0.39979	no
gene:SpnNT_00603	NA	Chromosome:627283-628010	110.58	ΔORF2+peptide	OK	6.87973	1.61836	-2.08782	-2.3968	0.1235	0.531508	no
gene:SpnNT_00603	NA	Chromosome:627283-628010	ΔORF2	ΔORF2+peptide	OK	2.60496	1.61836	-0.686729	-0.714868	0.4386	0.916013	no
gene:SpnNT_00603	NA	Chromosome:627283-628010	110.58+peptide	ΔORF2+peptide	OK	4.98422	1.61836	-1.62284	-1.8165	0.16665	0.621775	no
gene:SpnNT_00604	NA	Chromosome:628061-634865	110.58	ΔORF2	OK	1.96122	1.04065	-0.914265	-1.54345	0.00775	0.0837689	no
gene:SpnNT_00604	NA	Chromosome:628061-634865	110.58	110.58+peptide	OK	1.96122	1.99697	0.0260585	0.0457609	0.9359	0.994855	no
gene:SpnNT_00604	NA	Chromosome:628061-634865	ΔORF2	110.58+peptide	OK	1.04065	1.99697	0.940323	1.62232	0.0059	0.0681512	no
gene:SpnNT_00604	NA	Chromosome:628061-634865	110.58	ΔORF2+peptide	OK	1.96122	1.03121	-0.927417	-1.56139	0.0069	0.0765358	no
gene:SpnNT_00604	NA	Chromosome:628061-634865	ΔORF2	ΔORF2+peptide	OK	1.04065	1.03121	-0.0131521	-0.021785	0.96725	0.994855	no
gene:SpnNT_00604	NA	Chromosome:628061-634865	110.58+peptide	ΔORF2+peptide	OK	1.99697	1.03121	-0.953475	-1.64034	0.00525	0.0625141	no
gene:SpnNT_00605	NA	Chromosome:634934-635234	110.58	ΔORF2	OK	9.71218	8.99844	-0.11012	-0.124413	0.828	0.994748	no
gene:SpnNT_00605	NA	Chromosome:634934-635234	110.58	110.58+peptide	OK	9.71218	6.89488	-0.49427	-0.546538	0.3356	0.840108	no
gene:SpnNT_00605	NA	Chromosome:634934-635234	ΔORF2	110.58+peptide	OK	8.99844	6.89488	-0.38415	-0.415803	0.47375	0.929647	no
gene:SpnNT_00605	NA	Chromosome:634934-635234	110.58	ΔORF2+peptide	OK	9.71218	1.82454	-2.41226	-2.67842	0.0198	0.165804	no
gene:SpnNT_00605	NA	Chromosome:634934-635234	ΔORF2	ΔORF2+peptide	OK	8.99844	1.82454	-2.30214	-2.50174	0.02545	0.199138	no
gene:SpnNT_00605	NA	Chromosome:634934-635234	110.58+peptide	ΔORF2+peptide	OK	6.89488	1.82454	-1.91799	-2.04315	0.0409	0.274944	no
gene:SpnNT_00606	NA	Chromosome:635248-635539	110.58	ΔORF2	OK	7.46788	4.25016	-0.813182	-0.773486	0.1882	0.664751	no
gene:SpnNT_00606	NA	Chromosome:635248-635539	110.58	110.58+peptide	OK	7.46788	4.63159	-0.68919	-0.661816	0.2479	0.74404	no
gene:SpnNT_00606	NA	Chromosome:635248-635539	ΔORF2	110.58+peptide	OK	4.25016	4.63159	0.123992	0.109104	0.8446	0.994748	no
gene:SpnNT_00606	NA	Chromosome:635248-635539	110.58	ΔORF2+peptide	OK	7.46788	3.48757	-1.09848	-0.991779	0.11915	0.52308	no
gene:SpnNT_00606	NA	Chromosome:635248-635539	ΔORF2	ΔORF2+peptide	OK	4.25016	3.48757	-0.285295	-0.238257	0.69295	0.98828	no
gene:SpnNT_00606	NA	Chromosome:635248-635539	110.58+peptide	ΔORF2+peptide	OK	4.63159	3.48757	-0.409287	-0.344317	0.5668	0.968621	no
gene:SpnNT_00607	pnp_2	Chromosome:635984-637424	110.58	ΔORF2	OK	17.2136	15.7637	-0.126946	-0.241384	0.676	0.98524	no
gene:SpnNT_00607	pnp_2	Chromosome:635984-637424	110.58	110.58+peptide	OK	17.2136	17.7512	0.0443631	0.0858723	0.8759	0.994748	no
gene:SpnNT_00607	pnp_2	Chromosome:635984-637424	ΔORF2	110.58+peptide	OK	15.7637	17.7512	0.171309	0.327201	0.57075	0.968621	no
gene:SpnNT_00607	pnp_2	Chromosome:635984-637424	110.58	ΔORF2+peptide	OK	17.2136	19.4731	0.177934	0.346326	0.5324	0.958279	no
gene:SpnNT_00607	pnp_2	Chromosome:635984-637424	ΔORF2	ΔORF2+peptide	OK	15.7637	19.4731	0.30488	0.585457	0.2981	0.803352	no
gene:SpnNT_00607	pnp_2	Chromosome:635984-637424	110.58+peptide	ΔORF2+peptide	OK	17.7512	19.4731	0.133571	0.261202	0.64015	0.980887	no
gene:SpnNT_00608	NA	Chromosome:637695-638589	110.58	ΔORF2	OK	1.56085	0.9849	-0.664284	-0.68418	0.2199	0.70824	no
gene:SpnNT_00608	NA	Chromosome:637695-638589	110.58	110.58+peptide	OK	1.56085	0.811706	-0.943303	-0.960415	0.10595	0.495113	no

gene:SpnNT_00608	NA	Chromosome:637695-638589	ΔORF2	110.58+peptide	OK	0.9849	0.811706	-0.279019	-0.268058	0.63595	0.980887	no
gene:SpnNT_00608	NA	Chromosome:637695-638589	110.58	ΔORF2+peptide	OK	1.56085	0.669639	-1.22088	-1.21222	0.04645	0.298344	no
gene:SpnNT_00608	NA	Chromosome:637695-638589	ΔORF2	ΔORF2+peptide	OK	0.9849	0.669639	-0.556594	-0.522881	0.36195	0.862575	no
gene:SpnNT_00608	NA	Chromosome:637695-638589	110.58+peptide	ΔORF2+peptide	OK	0.811706	0.669639	-0.277574	-0.258267	0.6588	0.982966	no
gene:SpnNT_00609	NA	Chromosome:638892-661572	110.58	ΔORF2	OK	24.3995	20.0381	-0.284104	-0.607419	0.2902	0.794659	no
gene:SpnNT_00609	NA	Chromosome:638892-661572	110.58	110.58+peptide	OK	24.3995	26.2189	0.103754	0.225153	0.6912	0.98828	no
gene:SpnNT_00609	NA	Chromosome:638892-661572	ΔORF2	110.58+peptide	OK	20.0381	26.2189	0.387858	0.809717	0.15995	0.611379	no
gene:SpnNT_00609	NA	Chromosome:638892-661572	110.58	ΔORF2+peptide	OK	24.3995	19.4523	-0.326916	-0.733209	0.1997	0.682576	no
gene:SpnNT_00609	NA	Chromosome:638892-661572	ΔORF2	ΔORF2+peptide	OK	20.0381	19.4523	-0.0428116	-0.0921381	0.87205	0.994748	no
gene:SpnNT_00609	NA	Chromosome:638892-661572	110.58+peptide	ΔORF2+peptide	OK	26.2189	19.4523	-0.43067	-0.940963	0.10125	0.482777	no
gene:SpnNT_00610	NA	Chromosome:661708-662965	110.58	ΔORF2	OK	1.62784	1.31398	-0.309015	-0.387316	0.48815	0.938811	no
gene:SpnNT_00610	NA	Chromosome:661708-662965	110.58	110.58+peptide	OK	1.62784	1.89806	0.221564	0.289376	0.61465	0.979616	no
gene:SpnNT_00610	NA	Chromosome:661708-662965	ΔORF2	110.58+peptide	OK	1.31398	1.89806	0.530579	0.676539	0.24615	0.743359	no
gene:SpnNT_00610	NA	Chromosome:661708-662965	110.58	ΔORF2+peptide	OK	1.62784	2.35611	0.533448	0.712495	0.20315	0.687222	no
gene:SpnNT_00610	NA	Chromosome:661708-662965	ΔORF2	ΔORF2+peptide	OK	1.31398	2.35611	0.842463	1.09737	0.0588	0.346308	no
gene:SpnNT_00610	NA	Chromosome:661708-662965	110.58+peptide	ΔORF2+peptide	OK	1.89806	2.35611	0.311884	0.424786	0.4586	0.922502	no
gene:SpnNT_00611	NA	Chromosome:663206-663845	110.58	ΔORF2	OK	3.74544	2.88543	-0.376348	-0.452675	0.43315	0.913334	no
gene:SpnNT_00611	NA	Chromosome:663206-663845	110.58	110.58+peptide	OK	3.74544	4.77448	0.350207	0.443741	0.43885	0.916056	no
gene:SpnNT_00611	NA	Chromosome:663206-663845	ΔORF2	110.58+peptide	OK	2.88543	4.77448	0.726554	0.916281	0.12085	0.526535	no
gene:SpnNT_00611	NA	Chromosome:663206-663845	110.58	ΔORF2+peptide	OK	3.74544	1.78058	-1.07279	-1.18137	0.0515	0.32058	no
gene:SpnNT_00611	NA	Chromosome:663206-663845	ΔORF2	ΔORF2+peptide	OK	2.88543	1.78058	-0.696441	-0.764203	0.1927	0.674291	no
gene:SpnNT_00611	NA	Chromosome:663206-663845	110.58+peptide	ΔORF2+peptide	OK	4.77448	1.78058	-1.423	-1.62995	0.011	0.107477	no
gene:SpnNT_00612	NA	Chromosome:663883-664972	110.58	ΔORF2	OK	1.59978	0.806236	-0.988604	-1.09944	0.06215	0.360348	no
gene:SpnNT_00612	NA	Chromosome:663883-664972	110.58	110.58+peptide	OK	1.59978	1.92087	0.263884	0.326287	0.56095	0.968621	no
gene:SpnNT_00612	NA	Chromosome:663883-664972	ΔORF2	110.58+peptide	OK	0.806236	1.92087	1.25249	1.43769	0.01915	0.161391	no
gene:SpnNT_00612	NA	Chromosome:663883-664972	110.58	ΔORF2+peptide	OK	1.59978	1.67183	0.0635512	0.0756412	0.89625	0.994748	no
gene:SpnNT_00612	NA	Chromosome:663883-664972	ΔORF2	ΔORF2+peptide	OK	0.806236	1.67183	1.05215	1.16851	0.05115	0.31931	no
gene:SpnNT_00612	NA	Chromosome:663883-664972	110.58+peptide	ΔORF2+peptide	OK	1.92087	1.67183	-0.200333	-0.247287	0.67535	0.985207	no
gene:SpnNT_00613	NA	Chromosome:665025-665651	110.58	ΔORF2	OK	5.90951	2.84171	-1.05628	-0.515951	0.4652	0.926405	no
gene:SpnNT_00613	NA	Chromosome:665025-665651	110.58	110.58+peptide	OK	5.90951	9.03296	0.612161	0.343524	0.57585	0.969538	no
gene:SpnNT_00613	NA	Chromosome:665025-665651	ΔORF2	110.58+peptide	OK	2.84171	9.03296	1.66844	0.855175	0.28325	0.785794	no
gene:SpnNT_00613	NA	Chromosome:665025-665651	110.58	ΔORF2+peptide	OK	5.90951	0.311372	-4.24633	-1.66882	0.25475	0.753908	no
gene:SpnNT_00613	NA	Chromosome:665025-665651	ΔORF2	ΔORF2+peptide	OK	2.84171	0.311372	-3.19005	-1.19675	0.37795	0.875802	no
gene:SpnNT_00613	NA	Chromosome:665025-665651	110.58+peptide	ΔORF2+peptide	OK	9.03296	0.311372	-4.85849	-1.96881	0.25295	0.751101	no
gene:SpnNT_00614	NA	Chromosome:665025-665651	110.58	ΔORF2	OK	6.43676	2.47404	-1.37947	-1.32228	0.0316	0.230492	no
gene:SpnNT_00614	NA	Chromosome:665025-665651	110.58	110.58+peptide	OK	6.43676	6.159	-0.0636385	-0.067559	0.9073	0.994748	no
gene:SpnNT_00614	NA	Chromosome:665025-665651	ΔORF2	110.58+peptide	OK	2.47404	6.159	1.31583	1.21647	0.0414	0.277027	no
gene:SpnNT_00614	NA	Chromosome:665025-665651	110.58	ΔORF2+peptide	OK	6.43676	3.30323	-0.962455	-1.00122	0.09725	0.471239	no
gene:SpnNT_00614	NA	Chromosome:665025-665651	ΔORF2	ΔORF2+peptide	OK	2.47404	3.30323	0.41701	0.379604	0.50285	0.944788	no
gene:SpnNT_00614	NA	Chromosome:665025-665651	110.58+peptide	ΔORF2+peptide	OK	6.159	3.30323	-0.898817	-0.89625	0.12525	0.535526	no
gene:SpnNT_00615	NA	Chromosome:665666-665984	110.58	ΔORF2	OK	2.39573	1.38308	-0.792579	-0.571792	0.391	0.885288	no
gene:SpnNT_00615	NA	Chromosome:665666-665984	110.58	110.58+peptide	OK	2.39573	1.37989	-0.795906	-0.592122	0.38925	0.883652	no
gene:SpnNT_00615	NA	Chromosome:665666-665984	ΔORF2	110.58+peptide	OK	1.38308	1.37989	-0.00332764	-0.00194901	0.89735	0.994748	no
gene:SpnNT_00615	NA	Chromosome:665666-665984	110.58	ΔORF2+peptide	OK	2.39573	0.969856	-1.30462	-1.39636	0.2627	0.76315	no
gene:SpnNT_00615	NA	Chromosome:665666-665984	ΔORF2	ΔORF2+peptide	OK	1.38308	0.969856	-0.512043	-0.363783	0.6591	0.982966	no

gene:SpnNT_00615	NA	Chromosome:665666-665984	110.58+peptide	ΔORF2+peptide	OK	1.37989	0.969856	-0.508716	-0.372347	0.65815	0.982966	no
gene:SpnNT_00616	pezA_1	Chromosome:666052-667311	110.58	ΔORF2	OK	56.9518	50.8003	-0.164905	-0.241434	0.67685	0.98524	no
gene:SpnNT_00616	pezA_1	Chromosome:666052-667311	110.58	110.58+peptide	OK	56.9518	57.7115	0.0191175	0.0280909	0.96225	0.994855	no
gene:SpnNT_00616	pezA_1	Chromosome:666052-667311	ΔORF2	110.58+peptide	OK	50.8003	57.7115	0.184023	0.268505	0.645	0.980887	no
gene:SpnNT_00616	pezA_1	Chromosome:666052-667311	110.58	ΔORF2+peptide	OK	56.9518	40.3431	-0.497422	-0.714704	0.21395	0.699241	no
gene:SpnNT_00616	pezA_1	Chromosome:666052-667311	ΔORF2	ΔORF2+peptide	OK	50.8003	40.3431	-0.332517	-0.474563	0.4131	0.899511	no
gene:SpnNT_00616	pezA_1	Chromosome:666052-667311	110.58+peptide	ΔORF2+peptide	OK	57.7115	40.3431	-0.51654	-0.739735	0.19905	0.681762	no
gene:SpnNT_00617	pezT_1	Chromosome:666052-667311	110.58	ΔORF2	OK	16.5829	18.624	0.16747	0.196587	0.7336	0.994748	no
gene:SpnNT_00617	pezT_1	Chromosome:666052-667311	110.58	110.58+peptide	OK	16.5829	16.5104	-0.00632097	-0.00696357	0.9911	0.998249	no
gene:SpnNT_00617	pezT_1	Chromosome:666052-667311	ΔORF2	110.58+peptide	OK	18.624	16.5104	-0.173791	-0.201151	0.71725	0.992452	no
gene:SpnNT_00617	pezT_1	Chromosome:666052-667311	110.58	ΔORF2+peptide	OK	16.5829	15.9694	-0.0543816	-0.0652416	0.9097	0.994748	no
gene:SpnNT_00617	pezT_1	Chromosome:666052-667311	ΔORF2	ΔORF2+peptide	OK	18.624	15.9694	-0.221852	-0.282366	0.61875	0.980887	no
gene:SpnNT_00617	pezT_1	Chromosome:666052-667311	110.58+peptide	ΔORF2+peptide	OK	16.5104	15.9694	-0.0480606	-0.0568159	0.9182	0.994748	no
gene:SpnNT_00618	NA	Chromosome:667507-669999	110.58	ΔORF2	OK	5.95513	10.1697	0.77207	0.36057	0.5582	0.968033	no
gene:SpnNT_00618	NA	Chromosome:667507-669999	110.58	110.58+peptide	OK	5.95513	6.6068	0.14982	0.0788229	0.8832	0.994748	no
gene:SpnNT_00618	NA	Chromosome:667507-669999	ΔORF2	110.58+peptide	OK	10.1697	6.6068	-0.622249	-0.286622	0.61255	0.979616	no
gene:SpnNT_00618	NA	Chromosome:667507-669999	110.58	ΔORF2+peptide	OK	5.95513	11.3477	0.930193	0.517067	0.3626	0.86287	no
gene:SpnNT_00618	NA	Chromosome:667507-669999	ΔORF2	ΔORF2+peptide	OK	10.1697	11.3477	0.158123	0.0759304	0.897	0.994748	no
gene:SpnNT_00618	NA	Chromosome:667507-669999	110.58+peptide	ΔORF2+peptide	OK	6.6068	11.3477	0.780372	0.425442	0.4155	0.901067	no
gene:SpnNT_00619	NA	Chromosome:667507-669999	110.58	ΔORF2	NOTEST	0	0	0	0	1	1	no
gene:SpnNT_00619	NA	Chromosome:667507-669999	110.58	110.58+peptide	NOTEST	0	0	0	0	1	1	no
gene:SpnNT_00619	NA	Chromosome:667507-669999	ΔORF2	110.58+peptide	NOTEST	0	0	0	0	1	1	no
gene:SpnNT_00619	NA	Chromosome:667507-669999	110.58	ΔORF2+peptide	NOTEST	0	0	0	0	1	1	no
gene:SpnNT_00619	NA	Chromosome:667507-669999	ΔORF2	ΔORF2+peptide	NOTEST	0	0	0	0	1	1	no
gene:SpnNT_00619	NA	Chromosome:667507-669999	110.58+peptide	ΔORF2+peptide	NOTEST	0	0	0	0	1	1	no
gene:SpnNT_00620	NA	Chromosome:667507-669999	110.58	ΔORF2	OK	18.4672	29.5524	0.678312	1.26599	0.0283	0.21416	no
gene:SpnNT_00620	NA	Chromosome:667507-669999	110.58	110.58+peptide	OK	18.4672	21.2997	0.20587	0.390037	0.50385	0.945292	no
gene:SpnNT_00620	NA	Chromosome:667507-669999	ΔORF2	110.58+peptide	OK	29.5524	21.2997	-0.472443	-0.869256	0.1303	0.548187	no
gene:SpnNT_00620	NA	Chromosome:667507-669999	110.58	ΔORF2+peptide	OK	18.4672	25.7761	0.481068	0.938358	0.1069	0.497426	no
gene:SpnNT_00620	NA	Chromosome:667507-669999	ΔORF2	ΔORF2+peptide	OK	29.5524	25.7761	-0.197244	-0.373004	0.50985	0.948082	no
gene:SpnNT_00620	NA	Chromosome:667507-669999	110.58+peptide	ΔORF2+peptide	OK	21.2997	25.7761	0.275198	0.528496	0.36125	0.862347	no
gene:SpnNT_00621	NA	Chromosome:670332-670695	110.58	ΔORF2	OK	1.38833	0.100477	-3.78841	-0.937123	0.10535	0.493363	no
gene:SpnNT_00621	NA	Chromosome:670332-670695	110.58	110.58+peptide	OK	1.38833	0.529549	-1.39051	-0.819022	0.2212	0.710374	no
gene:SpnNT_00621	NA	Chromosome:670332-670695	ΔORF2	110.58+peptide	NOTEST	0.100477	0.529549	2.39789	0	1	1	no
gene:SpnNT_00621	NA	Chromosome:670332-670695	110.58	ΔORF2+peptide	OK	1.38833	0.203763	-2.76839	-1.25691	0.16835	0.624742	no
gene:SpnNT_00621	NA	Chromosome:670332-670695	ΔORF2	ΔORF2+peptide	NOTEST	0.100477	0.203763	1.02002	0	1	1	no
gene:SpnNT_00621	NA	Chromosome:670332-670695	110.58+peptide	ΔORF2+peptide	NOTEST	0.529549	0.203763	-1.37787	0	1	1	no
gene:SpnNT_00622	NA	Chromosome:670697-672936	110.58	ΔORF2	OK	1.7913	0.879267	-1.02663	-0.358074	0.65285	0.981932	no
gene:SpnNT_00622	NA	Chromosome:670697-672936	110.58	110.58+peptide	OK	1.7913	1.16799	-0.616975	-0.221507	0.78555	0.994748	no
gene:SpnNT_00622	NA	Chromosome:670697-672936	ΔORF2	110.58+peptide	OK	0.879267	1.16799	0.409655	0.137454	0.85115	0.994748	no
gene:SpnNT_00622	NA	Chromosome:670697-672936	110.58	ΔORF2+peptide	OK	1.7913	0.40013	-2.16246	-0.849645	0.52265	0.954832	no
gene:SpnNT_00622	NA	Chromosome:670697-672936	ΔORF2	ΔORF2+peptide	OK	0.879267	0.40013	-1.13583	-0.411963	0.6093	0.978849	no
gene:SpnNT_00622	NA	Chromosome:670697-672936	110.58+peptide	ΔORF2+peptide	OK	1.16799	0.40013	-1.54549	-0.578395	0.5811	0.96988	no
gene:SpnNT_00623	NA	Chromosome:670697-672936	110.58	ΔORF2	OK	1.58961	1.38799	-0.195676	-0.253822	0.65865	0.982966	no
gene:SpnNT_00623	NA	Chromosome:670697-672936	110.58	110.58+peptide	OK	1.58961	1.8933	0.252235	0.343353	0.54535	0.96171	no

gene:SpnNT_00623	NA	Chromosome:670697-672936	ΔORF2	110.58+peptide	OK	1.38799	1.8933	0.447911	0.615608	0.29035	0.794875	no
gene:SpnNT_00623	NA	Chromosome:670697-672936	110.58	ΔORF2+peptide	OK	1.58961	1.87378	0.23728	0.315519	0.5855	0.9724	no
gene:SpnNT_00623	NA	Chromosome:670697-672936	ΔORF2	ΔORF2+peptide	OK	1.38799	1.87378	0.432956	0.581022	0.32265	0.828143	no
gene:SpnNT_00623	NA	Chromosome:670697-672936	110.58+peptide	ΔORF2+peptide	OK	1.8933	1.87378	-0.014955	-0.0211363	0.9672	0.994855	no
gene:SpnNT_00624	NA	Chromosome:673342-673935	110.58	ΔORF2	OK	12.0916	19.2292	0.669295	0.539743	0.3408	0.844885	no
gene:SpnNT_00624	NA	Chromosome:673342-673935	110.58	110.58+peptide	OK	12.0916	19.889	0.717969	0.619113	0.3251	0.830337	no
gene:SpnNT_00624	NA	Chromosome:673342-673935	ΔORF2	110.58+peptide	OK	19.2292	19.889	0.0486739	0.041777	0.9466	0.994855	no
gene:SpnNT_00624	NA	Chromosome:673342-673935	110.58	ΔORF2+peptide	OK	12.0916	27.0288	1.1605	1.0127	0.09405	0.463158	no
gene:SpnNT_00624	NA	Chromosome:673342-673935	ΔORF2	ΔORF2+peptide	OK	19.2292	27.0288	0.491201	0.426603	0.4529	0.921244	no
gene:SpnNT_00624	NA	Chromosome:673342-673935	110.58+peptide	ΔORF2+peptide	OK	19.889	27.0288	0.442527	0.41575	0.5076	0.946529	no
gene:SpnNT_00625	patB_1	Chromosome:673342-673935	110.58	ΔORF2	OK	10.7803	11.3235	0.0709246	0.0769483	0.8965	0.994748	no
gene:SpnNT_00625	patB_1	Chromosome:673342-673935	110.58	110.58+peptide	OK	10.7803	10.9906	0.0278641	0.0299558	0.95505	0.994855	no
gene:SpnNT_00625	patB_1	Chromosome:673342-673935	ΔORF2	110.58+peptide	OK	11.3235	10.9906	-0.0430605	-0.0437396	0.9418	0.994855	no
gene:SpnNT_00625	patB_1	Chromosome:673342-673935	110.58	ΔORF2+peptide	OK	10.7803	12.2828	0.188241	0.202508	0.71885	0.993549	no
gene:SpnNT_00625	patB_1	Chromosome:673342-673935	ΔORF2	ΔORF2+peptide	OK	11.3235	12.2828	0.117316	0.119238	0.8397	0.994748	no
gene:SpnNT_00625	patB_1	Chromosome:673342-673935	110.58+peptide	ΔORF2+peptide	OK	10.9906	12.2828	0.160377	0.161701	0.7722	0.994748	no
gene:SpnNT_00626	NA	Chromosome:674054-674324	110.58	ΔORF2	OK	82.9979	65.8247	-0.334446	-0.506048	0.36705	0.864634	no
gene:SpnNT_00626	NA	Chromosome:674054-674324	110.58	110.58+peptide	OK	82.9979	91.426	0.139531	0.204418	0.70485	0.990367	no
gene:SpnNT_00626	NA	Chromosome:674054-674324	ΔORF2	110.58+peptide	OK	65.8247	91.426	0.473977	0.697006	0.21105	0.697158	no
gene:SpnNT_00626	NA	Chromosome:674054-674324	110.58	ΔORF2+peptide	OK	82.9979	62.5531	-0.407993	-0.564225	0.2963	0.800504	no
gene:SpnNT_00626	NA	Chromosome:674054-674324	ΔORF2	ΔORF2+peptide	OK	65.8247	62.5531	-0.0735465	-0.10205	0.8529	0.994748	no
gene:SpnNT_00626	NA	Chromosome:674054-674324	110.58+peptide	ΔORF2+peptide	OK	91.426	62.5531	-0.547524	-0.739277	0.16665	0.621775	no
gene:SpnNT_00627	NA	Chromosome:674374-674941	110.58	ΔORF2	OK	43.983	38.1072	-0.206883	-0.381732	0.50055	0.944482	no
gene:SpnNT_00627	NA	Chromosome:674374-674941	110.58	110.58+peptide	OK	43.983	48.4573	0.139767	0.257894	0.6499	0.981475	no
gene:SpnNT_00627	NA	Chromosome:674374-674941	ΔORF2	110.58+peptide	OK	38.1072	48.4573	0.34665	0.6358	0.25795	0.757909	no
gene:SpnNT_00627	NA	Chromosome:674374-674941	110.58	ΔORF2+peptide	OK	43.983	33.9163	-0.374968	-0.679173	0.23545	0.729189	no
gene:SpnNT_00627	NA	Chromosome:674374-674941	ΔORF2	ΔORF2+peptide	OK	38.1072	33.9163	-0.168085	-0.302693	0.59385	0.975539	no
gene:SpnNT_00627	NA	Chromosome:674374-674941	110.58+peptide	ΔORF2+peptide	OK	48.4573	33.9163	-0.514735	-0.926958	0.1049	0.491958	no
gene:SpnNT_00628	NA	Chromosome:675385-675727	110.58	ΔORF2	OK	81.2	56.5083	-0.523015	-0.900027	0.11315	0.510751	no
gene:SpnNT_00628	NA	Chromosome:675385-675727	110.58	110.58+peptide	OK	81.2	96.6795	0.25173	0.422526	0.4409	0.91669	no
gene:SpnNT_00628	NA	Chromosome:675385-675727	ΔORF2	110.58+peptide	OK	56.5083	96.6795	0.774746	1.28553	0.0246	0.195164	no
gene:SpnNT_00628	NA	Chromosome:675385-675727	110.58	ΔORF2+peptide	OK	81.2	60.3409	-0.428343	-0.714281	0.2028	0.686746	no
gene:SpnNT_00628	NA	Chromosome:675385-675727	ΔORF2	ΔORF2+peptide	OK	56.5083	60.3409	0.094672	0.156088	0.7864	0.994748	no
gene:SpnNT_00628	NA	Chromosome:675385-675727	110.58+peptide	ΔORF2+peptide	OK	96.6795	60.3409	-0.680073	-1.09585	0.05125	0.31963	no
gene:SpnNT_00629	NA	Chromosome:675950-676136	110.58	ΔORF2	OK	39.2559	28.549	-0.459471	-0.496506	0.38265	0.880283	no
gene:SpnNT_00629	NA	Chromosome:675950-676136	110.58	110.58+peptide	OK	39.2559	36.6133	-0.10054	-0.111522	0.8427	0.994748	no
gene:SpnNT_00629	NA	Chromosome:675950-676136	ΔORF2	110.58+peptide	OK	28.549	36.6133	0.358931	0.382004	0.5051	0.945292	no
gene:SpnNT_00629	NA	Chromosome:675950-676136	110.58	ΔORF2+peptide	OK	39.2559	34.7912	-0.174186	-0.199176	0.75165	0.994748	no
gene:SpnNT_00629	NA	Chromosome:675950-676136	ΔORF2	ΔORF2+peptide	OK	28.549	34.7912	0.285284	0.312219	0.62875	0.980887	no
gene:SpnNT_00629	NA	Chromosome:675950-676136	110.58+peptide	ΔORF2+peptide	OK	36.6133	34.7912	-0.0736464	-0.0827917	0.89815	0.994748	no
gene:SpnNT_00630	immR_1	Chromosome:676628-676826	110.58	ΔORF2	OK	28727.1	30316.5	0.0776884	0.153084	0.7801	0.994748	no
gene:SpnNT_00630	immR_1	Chromosome:676628-676826	110.58	110.58+peptide	OK	28727.1	44691.9	0.6376	1.32187	0.0221	0.180796	no
gene:SpnNT_00630	immR_1	Chromosome:676628-676826	ΔORF2	110.58+peptide	OK	30316.5	44691.9	0.559912	1.08176	0.06245	0.3611	no
gene:SpnNT_00630	immR_1	Chromosome:676628-676826	110.58	ΔORF2+peptide	OK	28727.1	34034.7	0.244591	0.538863	0.34725	0.851704	no
gene:SpnNT_00630	immR_1	Chromosome:676628-676826	ΔORF2	ΔORF2+peptide	OK	30316.5	34034.7	0.166903	0.33979	0.555	0.966389	no

gene:SpnNT_00630	immR_1	Chromosome:676628-676826	110.58+peptide	ΔORF2+peptide	OK	44691.9	34034.7	-0.393009	-0.844864	0.1499	0.591417	no
gene:SpnNT_00631	NA	Chromosome:677096-677237	110.58	ΔORF2	OK	912.659	488.386	-0.902054	-1.49658	0.01545	0.137261	no
gene:SpnNT_00631	NA	Chromosome:677096-677237	110.58	110.58+peptide	OK	912.659	921.318	0.0136224	0.022233	0.96855	0.994855	no
gene:SpnNT_00631	NA	Chromosome:677096-677237	ΔORF2	110.58+peptide	OK	488.386	921.318	0.915676	1.4682	0.013	0.121155	no
gene:SpnNT_00631	NA	Chromosome:677096-677237	110.58	ΔORF2+peptide	OK	912.659	346.947	-1.39536	-2.07201	0.00115	0.0187457	yes
gene:SpnNT_00631	NA	Chromosome:677096-677237	ΔORF2	ΔORF2+peptide	OK	488.386	346.947	-0.493305	-0.721817	0.2301	0.723533	no
gene:SpnNT_00631	NA	Chromosome:677096-677237	110.58+peptide	ΔORF2+peptide	OK	921.318	346.947	-1.40898	-2.03543	0.0011	0.0181562	yes
gene:SpnNT_00632	NA	Chromosome:677336-681953	110.58	ΔORF2	OK	1358.71	777.506	-0.805313	-0.901146	0.1136	0.5112	no
gene:SpnNT_00632	NA	Chromosome:677336-681953	110.58	110.58+peptide	OK	1358.71	1270.14	-0.0972508	-0.111076	0.8455	0.994748	no
gene:SpnNT_00632	NA	Chromosome:677336-681953	ΔORF2	110.58+peptide	OK	777.506	1270.14	0.708062	0.806807	0.16795	0.623964	no
gene:SpnNT_00632	NA	Chromosome:677336-681953	110.58	ΔORF2+peptide	OK	1358.71	815.361	-0.736728	-0.826489	0.14505	0.580489	no
gene:SpnNT_00632	NA	Chromosome:677336-681953	ΔORF2	ΔORF2+peptide	OK	777.506	815.361	0.0685848	0.0767654	0.89815	0.994748	no
gene:SpnNT_00632	NA	Chromosome:677336-681953	110.58+peptide	ΔORF2+peptide	OK	1270.14	815.361	-0.639478	-0.730572	0.20855	0.694195	no
gene:SpnNT_00633	NA	Chromosome:677336-681953	110.58	ΔORF2	OK	1048.34	623.233	-0.750265	-0.742286	0.18665	0.661768	no
gene:SpnNT_00633	NA	Chromosome:677336-681953	110.58	110.58+peptide	OK	1048.34	905.393	-0.211493	-0.214267	0.7027	0.990367	no
gene:SpnNT_00633	NA	Chromosome:677336-681953	ΔORF2	110.58+peptide	OK	623.233	905.393	0.538772	0.537848	0.3442	0.848346	no
gene:SpnNT_00633	NA	Chromosome:677336-681953	110.58	ΔORF2+peptide	OK	1048.34	650.716	-0.68801	-0.689578	0.2223	0.711816	no
gene:SpnNT_00633	NA	Chromosome:677336-681953	ΔORF2	ΔORF2+peptide	OK	623.233	650.716	0.0622546	0.0615022	0.91595	0.994748	no
gene:SpnNT_00633	NA	Chromosome:677336-681953	110.58+peptide	ΔORF2+peptide	OK	905.393	650.716	-0.476517	-0.482024	0.4016	0.894197	no
gene:SpnNT_00634	NA	Chromosome:677336-681953	110.58	ΔORF2	OK	1412.39	793.172	-0.832431	-0.899673	0.1185	0.521623	no
gene:SpnNT_00634	NA	Chromosome:677336-681953	110.58	110.58+peptide	OK	1412.39	1242.23	-0.185204	-0.20016	0.7229	0.994748	no
gene:SpnNT_00634	NA	Chromosome:677336-681953	ΔORF2	110.58+peptide	OK	793.172	1242.23	0.647227	0.726127	0.2018	0.685467	no
gene:SpnNT_00634	NA	Chromosome:677336-681953	110.58	ΔORF2+peptide	OK	1412.39	878.141	-0.685613	-0.744341	0.1961	0.677521	no
gene:SpnNT_00634	NA	Chromosome:677336-681953	ΔORF2	ΔORF2+peptide	OK	793.172	878.141	0.146817	0.165521	0.7652	0.994748	no
gene:SpnNT_00634	NA	Chromosome:677336-681953	110.58+peptide	ΔORF2+peptide	OK	1242.23	878.141	-0.50041	-0.564143	0.3262	0.831527	no
gene:SpnNT_00635	NA	Chromosome:682068-683400	110.58	ΔORF2	OK	775.627	423.834	-0.871862	-1.88679	0.00105	0.0174849	yes
gene:SpnNT_00635	NA	Chromosome:682068-683400	110.58	110.58+peptide	OK	775.627	673.51	-0.203663	-0.445255	0.4307	0.912439	no
gene:SpnNT_00635	NA	Chromosome:682068-683400	ΔORF2	110.58+peptide	OK	423.834	673.51	0.668199	1.43896	0.0121	0.11539	no
gene:SpnNT_00635	NA	Chromosome:682068-683400	110.58	ΔORF2+peptide	OK	775.627	368.927	-1.07203	-2.3731	1.00E-04	0.0025332	yes
gene:SpnNT_00635	NA	Chromosome:682068-683400	ΔORF2	ΔORF2+peptide	OK	423.834	368.927	-0.200164	-0.436292	0.4433	0.917631	no
gene:SpnNT_00635	NA	Chromosome:682068-683400	110.58+peptide	ΔORF2+peptide	OK	673.51	368.927	-0.868363	-1.91241	0.00105	0.0174849	yes
gene:SpnNT_00636	NA	Chromosome:683497-684143	110.58	ΔORF2	OK	335.668	207.769	-0.692056	-1.21318	0.036	0.252346	no
gene:SpnNT_00636	NA	Chromosome:683497-684143	110.58	110.58+peptide	OK	335.668	239.692	-0.485857	-0.878097	0.1306	0.549097	no
gene:SpnNT_00636	NA	Chromosome:683497-684143	ΔORF2	110.58+peptide	OK	207.769	239.692	0.206198	0.351688	0.53765	0.959663	no
gene:SpnNT_00636	NA	Chromosome:683497-684143	110.58	ΔORF2+peptide	OK	335.668	148.986	-1.17186	-1.98661	0.00115	0.0187457	yes
gene:SpnNT_00636	NA	Chromosome:683497-684143	ΔORF2	ΔORF2+peptide	OK	207.769	148.986	-0.479802	-0.772701	0.17925	0.645832	no
gene:SpnNT_00636	NA	Chromosome:683497-684143	110.58+peptide	ΔORF2+peptide	OK	239.692	148.986	-0.686001	-1.13345	0.0526	0.324961	no
gene:SpnNT_00637	NA	Chromosome:683497-684143	110.58	ΔORF2	OK	268.499	197.608	-0.442272	-0.545564	0.33445	0.839773	no
gene:SpnNT_00637	NA	Chromosome:683497-684143	110.58	110.58+peptide	OK	268.499	191.423	-0.488151	-0.589024	0.30055	0.806342	no
gene:SpnNT_00637	NA	Chromosome:683497-684143	ΔORF2	110.58+peptide	OK	197.608	191.423	-0.0458786	-0.054023	0.92475	0.994748	no
gene:SpnNT_00637	NA	Chromosome:683497-684143	110.58	ΔORF2+peptide	OK	268.499	158.911	-0.756695	-0.94438	0.1224	0.529378	no
gene:SpnNT_00637	NA	Chromosome:683497-684143	ΔORF2	ΔORF2+peptide	OK	197.608	158.911	-0.314423	-0.382303	0.52745	0.956161	no
gene:SpnNT_00637	NA	Chromosome:683497-684143	110.58+peptide	ΔORF2+peptide	OK	191.423	158.911	-0.268544	-0.319595	0.59925	0.97629	no
gene:SpnNT_00638	NA	Chromosome:684708-685088	110.58	ΔORF2	OK	1492.22	1549.7	0.0545294	0.109684	0.84475	0.994748	no
gene:SpnNT_00638	NA	Chromosome:684708-685088	110.58	110.58+peptide	OK	1492.22	1421.49	-0.070061	-0.126906	0.81885	0.994748	no

gene:SpnNT_00638	NA	Chromosome:684708-685088	ΔORF2	110.58+peptide	OK	1549.7	1421.49	-0.12459	-0.224983	0.68495	0.988009	no
gene:SpnNT_00638	NA	Chromosome:684708-685088	110.58	ΔORF2+peptide	OK	1492.22	1281.17	-0.219996	-0.410818	0.4521	0.921244	no
gene:SpnNT_00638	NA	Chromosome:684708-685088	ΔORF2	ΔORF2+peptide	OK	1549.7	1281.17	-0.274525	-0.510969	0.35415	0.856303	no
gene:SpnNT_00638	NA	Chromosome:684708-685088	110.58+peptide	ΔORF2+peptide	OK	1421.49	1281.17	-0.149935	-0.254796	0.6352	0.980887	no
gene:SpnNT_00639	NA	Chromosome:684708-685088	110.58	ΔORF2	OK	483.366	640.337	0.405715	0.23853	0.6738	0.985063	no
gene:SpnNT_00639	NA	Chromosome:684708-685088	110.58	110.58+peptide	OK	483.366	490.325	0.0206222	0.0114799	0.98445	0.996345	no
gene:SpnNT_00639	NA	Chromosome:684708-685088	ΔORF2	110.58+peptide	OK	640.337	490.325	-0.385093	-0.220701	0.68025	0.986361	no
gene:SpnNT_00639	NA	Chromosome:684708-685088	110.58	ΔORF2+peptide	OK	483.366	526.096	0.122211	0.0645366	0.9094	0.994748	no
gene:SpnNT_00639	NA	Chromosome:684708-685088	ΔORF2	ΔORF2+peptide	OK	640.337	526.096	-0.283504	-0.153671	0.78285	0.994748	no
gene:SpnNT_00639	NA	Chromosome:684708-685088	110.58+peptide	ΔORF2+peptide	OK	490.325	526.096	0.101589	0.0525481	0.92185	0.994748	no
gene:SpnNT_00640	prtP	Chromosome:685901-692378	110.58	ΔORF2	OK	15.5768	12.1355	-0.360166	-0.798371	0.17095	0.62976	no
gene:SpnNT_00640	prtP	Chromosome:685901-692378	110.58	110.58+peptide	OK	15.5768	12.2012	-0.352377	-0.777059	0.17695	0.641951	no
gene:SpnNT_00640	prtP	Chromosome:685901-692378	ΔORF2	110.58+peptide	OK	12.1355	12.2012	0.00778892	0.0172701	0.97565	0.99536	no
gene:SpnNT_00640	prtP	Chromosome:685901-692378	110.58	ΔORF2+peptide	OK	15.5768	13.6303	-0.192577	-0.42742	0.45485	0.921244	no
gene:SpnNT_00640	prtP	Chromosome:685901-692378	ΔORF2	ΔORF2+peptide	OK	12.1355	13.6303	0.167589	0.374024	0.50655	0.945874	no
gene:SpnNT_00640	prtP	Chromosome:685901-692378	110.58+peptide	ΔORF2+peptide	OK	12.2012	13.6303	0.1598	0.354767	0.53385	0.958867	no
gene:SpnNT_00641	NA	Chromosome:692861-693170	110.58	ΔORF2	OK	3.85449	5.6611	0.554544	0.598625	0.4044	0.894933	no
gene:SpnNT_00641	NA	Chromosome:692861-693170	110.58	110.58+peptide	OK	3.85449	6.14015	0.671736	0.768248	0.3061	0.812428	no
gene:SpnNT_00641	NA	Chromosome:692861-693170	ΔORF2	110.58+peptide	OK	5.6611	6.14015	0.117191	0.116565	0.8421	0.994748	no
gene:SpnNT_00641	NA	Chromosome:692861-693170	110.58	ΔORF2+peptide	OK	3.85449	3.7571	-0.0369202	-0.0370553	0.94155	0.994855	no
gene:SpnNT_00641	NA	Chromosome:692861-693170	ΔORF2	ΔORF2+peptide	OK	5.6611	3.7571	-0.591464	-0.531371	0.3424	0.846453	no
gene:SpnNT_00641	NA	Chromosome:692861-693170	110.58+peptide	ΔORF2+peptide	OK	6.14015	3.7571	-0.708656	-0.662164	0.2604	0.760848	no
gene:SpnNT_00642	NA	Chromosome:693400-693745	110.58	ΔORF2	OK	102.445	139.981	0.450375	0.847812	0.14325	0.578022	no
gene:SpnNT_00642	NA	Chromosome:693400-693745	110.58	110.58+peptide	OK	102.445	98.5673	-0.055675	-0.103906	0.8621	0.994748	no
gene:SpnNT_00642	NA	Chromosome:693400-693745	ΔORF2	110.58+peptide	OK	139.981	98.5673	-0.50605	-0.949623	0.10955	0.502979	no
gene:SpnNT_00642	NA	Chromosome:693400-693745	110.58	ΔORF2+peptide	OK	102.445	122.661	0.259819	0.490036	0.4016	0.894197	no
gene:SpnNT_00642	NA	Chromosome:693400-693745	ΔORF2	ΔORF2+peptide	OK	139.981	122.661	-0.190556	-0.361416	0.54125	0.961494	no
gene:SpnNT_00642	NA	Chromosome:693400-693745	110.58+peptide	ΔORF2+peptide	OK	98.5673	122.661	0.315494	0.593164	0.32315	0.82836	no
gene:SpnNT_00643	NA	Chromosome:693851-694361	110.58	ΔORF2	OK	95.6917	122.847	0.3604	0.727952	0.21315	0.698345	no
gene:SpnNT_00643	NA	Chromosome:693851-694361	110.58	110.58+peptide	OK	95.6917	88.0792	-0.119592	-0.239061	0.66945	0.984845	no
gene:SpnNT_00643	NA	Chromosome:693851-694361	ΔORF2	110.58+peptide	OK	122.847	88.0792	-0.479992	-0.961447	0.08905	0.448911	no
gene:SpnNT_00643	NA	Chromosome:693851-694361	110.58	ΔORF2+peptide	OK	95.6917	111.157	0.21614	0.439321	0.44555	0.918272	no
gene:SpnNT_00643	NA	Chromosome:693851-694361	ΔORF2	ΔORF2+peptide	OK	122.847	111.157	-0.14426	-0.293839	0.60235	0.976761	no
gene:SpnNT_00643	NA	Chromosome:693851-694361	110.58+peptide	ΔORF2+peptide	OK	88.0792	111.157	0.335732	0.676655	0.22755	0.71915	no
gene:SpnNT_00644	NA	Chromosome:694361-694781	110.58	ΔORF2	OK	80.0002	67.0495	-0.254779	-0.463376	0.4219	0.905409	no
gene:SpnNT_00644	NA	Chromosome:694361-694781	110.58	110.58+peptide	OK	80.0002	75.0674	-0.0918184	-0.168361	0.76075	0.994748	no
gene:SpnNT_00644	NA	Chromosome:694361-694781	ΔORF2	110.58+peptide	OK	67.0495	75.0674	0.16296	0.292795	0.60245	0.976761	no
gene:SpnNT_00644	NA	Chromosome:694361-694781	110.58	ΔORF2+peptide	OK	80.0002	68.1334	-0.231643	-0.427871	0.4543	0.921244	no
gene:SpnNT_00644	NA	Chromosome:694361-694781	ΔORF2	ΔORF2+peptide	OK	67.0495	68.1334	0.0231357	0.0418619	0.9439	0.994855	no
gene:SpnNT_00644	NA	Chromosome:694361-694781	110.58+peptide	ΔORF2+peptide	OK	75.0674	68.1334	-0.139824	-0.25505	0.6503	0.981475	no
gene:SpnNT_00645	NA	Chromosome:694783-695588	110.58	ΔORF2	OK	72.0348	61.6572	-0.224424	-0.427294	0.45525	0.921244	no
gene:SpnNT_00645	NA	Chromosome:694783-695588	110.58	110.58+peptide	OK	72.0348	70.3619	-0.0338989	-0.0641306	0.91075	0.994748	no
gene:SpnNT_00645	NA	Chromosome:694783-695588	ΔORF2	110.58+peptide	OK	61.6572	70.3619	0.190525	0.358371	0.53275	0.958424	no
gene:SpnNT_00645	NA	Chromosome:694783-695588	110.58	ΔORF2+peptide	OK	72.0348	61.4923	-0.228288	-0.432159	0.44995	0.920524	no
gene:SpnNT_00645	NA	Chromosome:694783-695588	ΔORF2	ΔORF2+peptide	OK	61.6572	61.4923	-0.00386441	-0.00727349	0.9895	0.997703	no

gene:SpnNT_00645	NA	Chromosome:694783-695588	110.58+peptide	ΔORF2+peptide	OK	70.3619	61.4923	-0.194389	-0.363593	0.5236	0.954832	no
gene:SpnNT_00646	NA	Chromosome:694783-695588	110.58	ΔORF2	OK	24.637	23.1338	-0.0908232	-0.0422602	0.9457	0.994855	no
gene:SpnNT_00646	NA	Chromosome:694783-695588	110.58	110.58+peptide	OK	24.637	22.7405	-0.115563	-0.0557927	0.93475	0.994855	no
gene:SpnNT_00646	NA	Chromosome:694783-695588	ΔORF2	110.58+peptide	OK	23.1338	22.7405	-0.0247398	-0.0110752	0.9533	0.994855	no
gene:SpnNT_00646	NA	Chromosome:694783-695588	110.58	ΔORF2+peptide	OK	24.637	19.3517	-0.348367	-0.159127	0.8071	0.994748	no
gene:SpnNT_00646	NA	Chromosome:694783-695588	ΔORF2	ΔORF2+peptide	OK	23.1338	19.3517	-0.257544	-0.109893	0.87165	0.994748	no
gene:SpnNT_00646	NA	Chromosome:694783-695588	110.58+peptide	ΔORF2+peptide	OK	22.7405	19.3517	-0.232805	-0.102449	0.87985	0.994748	no
gene:SpnNT_00647	nhaK	Chromosome:695599-697654	110.58	ΔORF2	OK	30.5488	29.0171	-0.0742109	-0.160695	0.7729	0.994748	no
gene:SpnNT_00647	nhaK	Chromosome:695599-697654	110.58	110.58+peptide	OK	30.5488	28.2687	-0.111911	-0.241151	0.6655	0.98367	no
gene:SpnNT_00647	nhaK	Chromosome:695599-697654	ΔORF2	110.58+peptide	OK	29.0171	28.2687	-0.0377001	-0.081076	0.88275	0.994748	no
gene:SpnNT_00647	nhaK	Chromosome:695599-697654	110.58	ΔORF2+peptide	OK	30.5488	31.5407	0.046102	0.100139	0.85715	0.994748	no
gene:SpnNT_00647	nhaK	Chromosome:695599-697654	ΔORF2	ΔORF2+peptide	OK	29.0171	31.5407	0.120313	0.260807	0.6446	0.980887	no
gene:SpnNT_00647	nhaK	Chromosome:695599-697654	110.58+peptide	ΔORF2+peptide	OK	28.2687	31.5407	0.158013	0.340859	0.5554	0.966573	no
gene:SpnNT_00648	NA	Chromosome:697820-698681	110.58	ΔORF2	OK	31.6409	35.2522	0.155922	0.302405	0.59485	0.975541	no
gene:SpnNT_00648	NA	Chromosome:697820-698681	110.58	110.58+peptide	OK	31.6409	34.3036	0.116568	0.226101	0.6922	0.98828	no
gene:SpnNT_00648	NA	Chromosome:697820-698681	ΔORF2	110.58+peptide	OK	35.2522	34.3036	-0.0393534	-0.0769356	0.89825	0.994748	no
gene:SpnNT_00648	NA	Chromosome:697820-698681	110.58	ΔORF2+peptide	OK	31.6409	34.7369	0.134679	0.260656	0.64685	0.981154	no
gene:SpnNT_00648	NA	Chromosome:697820-698681	ΔORF2	ΔORF2+peptide	OK	35.2522	34.7369	-0.0212428	-0.0414371	0.9453	0.994855	no
gene:SpnNT_00648	NA	Chromosome:697820-698681	110.58+peptide	ΔORF2+peptide	OK	34.3036	34.7369	0.0181105	0.0353304	0.95405	0.994855	no
gene:SpnNT_00649	dsbD_1	Chromosome:698891-699602	110.58	ΔORF2	OK	9.66851	9.3259	-0.0520504	-0.0821301	0.88855	0.994748	no
gene:SpnNT_00649	dsbD_1	Chromosome:698891-699602	110.58	110.58+peptide	OK	9.66851	7.28441	-0.408482	-0.627613	0.26935	0.771007	no
gene:SpnNT_00649	dsbD_1	Chromosome:698891-699602	ΔORF2	110.58+peptide	OK	9.3259	7.28441	-0.356432	-0.546877	0.33785	0.842186	no
gene:SpnNT_00649	dsbD_1	Chromosome:698891-699602	110.58	ΔORF2+peptide	OK	9.66851	9.99081	0.0473077	0.0736341	0.89735	0.994748	no
gene:SpnNT_00649	dsbD_1	Chromosome:698891-699602	ΔORF2	ΔORF2+peptide	OK	9.3259	9.99081	0.0993581	0.154429	0.78925	0.994748	no
gene:SpnNT_00649	dsbD_1	Chromosome:698891-699602	110.58+peptide	ΔORF2+peptide	OK	7.28441	9.99081	0.45579	0.690345	0.2292	0.721915	no
gene:SpnNT_00650	msrAB_1	Chromosome:699621-700188	110.58	ΔORF2	OK	19.3299	17.4804	-0.145094	-0.240519	0.67405	0.985063	no
gene:SpnNT_00650	msrAB_1	Chromosome:699621-700188	110.58	110.58+peptide	OK	19.3299	16.5972	-0.219897	-0.363621	0.5203	0.954744	no
gene:SpnNT_00650	msrAB_1	Chromosome:699621-700188	ΔORF2	110.58+peptide	OK	17.4804	16.5972	-0.0748022	-0.122068	0.82585	0.994748	no
gene:SpnNT_00650	msrAB_1	Chromosome:699621-700188	110.58	ΔORF2+peptide	OK	19.3299	15.4094	-0.327025	-0.533317	0.35475	0.857011	no
gene:SpnNT_00650	msrAB_1	Chromosome:699621-700188	ΔORF2	ΔORF2+peptide	OK	17.4804	15.4094	-0.18193	-0.292903	0.6068	0.977825	no
gene:SpnNT_00650	msrAB_1	Chromosome:699621-700188	110.58+peptide	ΔORF2+peptide	OK	16.5972	15.4094	-0.107128	-0.172073	0.76045	0.994748	no
gene:SpnNT_00651	msrAB_2	Chromosome:700198-701311	110.58	ΔORF2	OK	12.9961	11.929	-0.123603	-0.219596	0.70445	0.990367	no
gene:SpnNT_00651	msrAB_2	Chromosome:700198-701311	110.58	110.58+peptide	OK	12.9961	10.8005	-0.266977	-0.473149	0.4103	0.898184	no
gene:SpnNT_00651	msrAB_2	Chromosome:700198-701311	ΔORF2	110.58+peptide	OK	11.929	10.8005	-0.143374	-0.252047	0.65195	0.98163	no
gene:SpnNT_00651	msrAB_2	Chromosome:700198-701311	110.58	ΔORF2+peptide	OK	12.9961	12.916	-0.00892363	-0.0159317	0.97765	0.995765	no
gene:SpnNT_00651	msrAB_2	Chromosome:700198-701311	ΔORF2	ΔORF2+peptide	OK	11.929	12.916	0.114679	0.203068	0.71975	0.99385	no
gene:SpnNT_00651	msrAB_2	Chromosome:700198-701311	110.58+peptide	ΔORF2+peptide	OK	10.8005	12.916	0.258053	0.45583	0.4277	0.909313	no
gene:SpnNT_00652	NA	Chromosome:701759-704185	110.58	ΔORF2	OK	38.663	41.0228	0.0854727	0.0926244	0.87025	0.994748	no
gene:SpnNT_00652	NA	Chromosome:701759-704185	110.58	110.58+peptide	OK	38.663	32.4107	-0.254483	-0.266506	0.63915	0.980887	no
gene:SpnNT_00652	NA	Chromosome:701759-704185	ΔORF2	110.58+peptide	OK	41.0228	32.4107	-0.339956	-0.373008	0.51375	0.95097	no
gene:SpnNT_00652	NA	Chromosome:701759-704185	110.58	ΔORF2+peptide	OK	38.663	41.6485	0.10731	0.11401	0.8464	0.994748	no
gene:SpnNT_00652	NA	Chromosome:701759-704185	ΔORF2	ΔORF2+peptide	OK	41.0228	41.6485	0.0218377	0.024343	0.96805	0.994855	no
gene:SpnNT_00652	NA	Chromosome:701759-704185	110.58+peptide	ΔORF2+peptide	OK	32.4107	41.6485	0.361793	0.388998	0.4993	0.944017	no
gene:SpnNT_00653	yehU	Chromosome:701759-704185	110.58	ΔORF2	OK	41.4528	42.2112	0.0261552	0.0504693	0.927	0.994748	no
gene:SpnNT_00653	yehU	Chromosome:701759-704185	110.58	110.58+peptide	OK	41.4528	38.0351	-0.124138	-0.2395	0.67795	0.98524	no

gene:SpnNT_00653	yehU	Chromosome:701759-704185	ΔORF2	110.58+peptide	OK	42.2112	38.0351	-0.150293	-0.289962	0.6157	0.979616	no
gene:SpnNT_00653	yehU	Chromosome:701759-704185	110.58	ΔORF2+peptide	OK	41.4528	39.2158	-0.0800334	-0.15339	0.78805	0.994748	no
gene:SpnNT_00653	yehU	Chromosome:701759-704185	ΔORF2	ΔORF2+peptide	OK	42.2112	39.2158	-0.106189	-0.203519	0.72315	0.994748	no
gene:SpnNT_00653	yehU	Chromosome:701759-704185	110.58+peptide	ΔORF2+peptide	OK	38.0351	39.2158	0.0441045	0.0845168	0.8846	0.994748	no
gene:SpnNT_00654	NA	Chromosome:704300-704864	110.58	ΔORF2	OK	246.039	257.784	0.0672718	0.152168	0.79515	0.994748	no
gene:SpnNT_00654	NA	Chromosome:704300-704864	110.58	110.58+peptide	OK	246.039	288.763	0.230999	0.520815	0.37145	0.868488	no
gene:SpnNT_00654	NA	Chromosome:704300-704864	ΔORF2	110.58+peptide	OK	257.784	288.763	0.163727	0.369209	0.5179	0.953876	no
gene:SpnNT_00654	NA	Chromosome:704300-704864	110.58	ΔORF2+peptide	OK	246.039	296.138	0.267384	0.604713	0.295	0.799296	no
gene:SpnNT_00654	NA	Chromosome:704300-704864	ΔORF2	ΔORF2+peptide	OK	257.784	296.138	0.200112	0.452653	0.42495	0.907986	no
gene:SpnNT_00654	NA	Chromosome:704300-704864	110.58+peptide	ΔORF2+peptide	OK	288.763	296.138	0.0363845	0.0820335	0.88425	0.994748	no
gene:SpnNT_00655	iga_2	Chromosome:704885-710822	110.58	ΔORF2	OK	180.125	242.842	0.431018	0.92232	0.10805	0.499941	no
gene:SpnNT_00655	iga_2	Chromosome:704885-710822	110.58	110.58+peptide	OK	180.125	160.414	-0.167199	-0.357487	0.5309	0.957488	no
gene:SpnNT_00655	iga_2	Chromosome:704885-710822	ΔORF2	110.58+peptide	OK	242.842	160.414	-0.598218	-1.27306	0.03055	0.225212	no
gene:SpnNT_00655	iga_2	Chromosome:704885-710822	110.58	ΔORF2+peptide	OK	180.125	251.663	0.482492	1.03776	0.06375	0.366737	no
gene:SpnNT_00655	iga_2	Chromosome:704885-710822	ΔORF2	ΔORF2+peptide	OK	242.842	251.663	0.0514741	0.110188	0.8455	0.994748	no
gene:SpnNT_00655	iga_2	Chromosome:704885-710822	110.58+peptide	ΔORF2+peptide	OK	160.414	251.663	0.649692	1.3896	0.0158	0.139615	no
gene:SpnNT_00656	NA	Chromosome:710869-712126	110.58	ΔORF2	OK	10.9656	13.3352	0.282253	0.510982	0.36935	0.866165	no
gene:SpnNT_00656	NA	Chromosome:710869-712126	110.58	110.58+peptide	OK	10.9656	9.27441	-0.241658	-0.428024	0.466	0.926452	no
gene:SpnNT_00656	NA	Chromosome:710869-712126	ΔORF2	110.58+peptide	OK	13.3352	9.27441	-0.52391	-0.941201	0.10175	0.48393	no
gene:SpnNT_00656	NA	Chromosome:710869-712126	110.58	ΔORF2+peptide	OK	10.9656	11.1777	0.0276425	0.0490892	0.93225	0.994855	no
gene:SpnNT_00656	NA	Chromosome:710869-712126	ΔORF2	ΔORF2+peptide	OK	13.3352	11.1777	-0.25461	-0.458644	0.4224	0.905849	no
gene:SpnNT_00656	NA	Chromosome:710869-712126	110.58+peptide	ΔORF2+peptide	OK	9.27441	11.1777	0.2693	0.47471	0.40625	0.895895	no
gene:SpnNT_00657	pabB	Chromosome:712312-714034	110.58	ΔORF2	OK	78.9748	84.5341	0.0981411	0.22318	0.6951	0.98895	no
gene:SpnNT_00657	pabB	Chromosome:712312-714034	110.58	110.58+peptide	OK	78.9748	63.6796	-0.31056	-0.70567	0.2176	0.705186	no
gene:SpnNT_00657	pabB	Chromosome:712312-714034	ΔORF2	110.58+peptide	OK	84.5341	63.6796	-0.408701	-0.92415	0.11355	0.51115	no
gene:SpnNT_00657	pabB	Chromosome:712312-714034	110.58	ΔORF2+peptide	OK	78.9748	69.7168	-0.179886	-0.408107	0.4669	0.92732	no
gene:SpnNT_00657	pabB	Chromosome:712312-714034	ΔORF2	ΔORF2+peptide	OK	84.5341	69.7168	-0.278027	-0.627698	0.2819	0.784843	no
gene:SpnNT_00657	pabB	Chromosome:712312-714034	110.58+peptide	ΔORF2+peptide	OK	63.6796	69.7168	0.130674	0.294788	0.6045	0.976761	no
gene:SpnNT_00658	NA	Chromosome:714034-714652	110.58	ΔORF2	OK	28.3451	34.2157	0.271561	0.49178	0.3867	0.881745	no
gene:SpnNT_00658	NA	Chromosome:714034-714652	110.58	110.58+peptide	OK	28.3451	25.6613	-0.143503	-0.253798	0.6637	0.982966	no
gene:SpnNT_00658	NA	Chromosome:714034-714652	ΔORF2	110.58+peptide	OK	34.2157	25.6613	-0.415064	-0.745708	0.2092	0.69532	no
gene:SpnNT_00658	NA	Chromosome:714034-714652	110.58	ΔORF2+peptide	OK	28.3451	38.7296	0.450338	0.819343	0.1516	0.593484	no
gene:SpnNT_00658	NA	Chromosome:714034-714652	ΔORF2	ΔORF2+peptide	OK	34.2157	38.7296	0.178776	0.330726	0.56685	0.968621	no
gene:SpnNT_00658	NA	Chromosome:714034-714652	110.58+peptide	ΔORF2+peptide	OK	25.6613	38.7296	0.59384	1.0718	0.0691	0.385679	no
gene:SpnNT_00659	lytB_2	Chromosome:714701-715700	110.58	ΔORF2	OK	59.1944	58.0194	-0.0289257	-0.0614888	0.91115	0.994748	no
gene:SpnNT_00659	lytB_2	Chromosome:714701-715700	110.58	110.58+peptide	OK	59.1944	48.1761	-0.297143	-0.624659	0.2737	0.775958	no
gene:SpnNT_00659	lytB_2	Chromosome:714701-715700	ΔORF2	110.58+peptide	OK	58.0194	48.1761	-0.268217	-0.564542	0.33005	0.836084	no
gene:SpnNT_00659	lytB_2	Chromosome:714701-715700	110.58	ΔORF2+peptide	OK	59.1944	53.8733	-0.13589	-0.287092	0.60375	0.976761	no
gene:SpnNT_00659	lytB_2	Chromosome:714701-715700	ΔORF2	ΔORF2+peptide	OK	58.0194	53.8733	-0.106964	-0.226261	0.691	0.98828	no
gene:SpnNT_00659	lytB_2	Chromosome:714701-715700	110.58+peptide	ΔORF2+peptide	OK	48.1761	53.8733	0.161253	0.337358	0.5475	0.962356	no
gene:SpnNT_00660	glcK	Chromosome:715826-716786	110.58	ΔORF2	OK	124.527	125.809	0.0147728	0.0328009	0.9545	0.994855	no
gene:SpnNT_00660	glcK	Chromosome:715826-716786	110.58	110.58+peptide	OK	124.527	117.163	-0.0879432	-0.197432	0.7293	0.994748	no
gene:SpnNT_00660	glcK	Chromosome:715826-716786	ΔORF2	110.58+peptide	OK	125.809	117.163	-0.102716	-0.228199	0.6863	0.98828	no
gene:SpnNT_00660	glcK	Chromosome:715826-716786	110.58	ΔORF2+peptide	OK	124.527	105.794	-0.235207	-0.526963	0.3539	0.856293	no
gene:SpnNT_00660	glcK	Chromosome:715826-716786	ΔORF2	ΔORF2+peptide	OK	125.809	105.794	-0.24998	-0.55426	0.33195	0.83795	no

gene:SpnNT_00660	glcK	Chromosome:715826-716786	110.58+peptide	ΔORF2+peptide	OK	117.163	105.794	-0.147264	-0.330128	0.56555	0.968621	no
gene:SpnNT_00661	thyA	Chromosome:716882-717722	110.58	ΔORF2	OK	76.1597	81.377	0.0955928	0.204531	0.7237	0.994748	no
gene:SpnNT_00661	thyA	Chromosome:716882-717722	110.58	110.58+peptide	OK	76.1597	64.7438	-0.234286	-0.494658	0.3866	0.881745	no
gene:SpnNT_00661	thyA	Chromosome:716882-717722	ΔORF2	110.58+peptide	OK	81.377	64.7438	-0.329879	-0.696626	0.2304	0.723783	no
gene:SpnNT_00661	thyA	Chromosome:716882-717722	110.58	ΔORF2+peptide	OK	76.1597	70.2243	-0.117058	-0.248851	0.66325	0.982966	no
gene:SpnNT_00661	thyA	Chromosome:716882-717722	ΔORF2	ΔORF2+peptide	OK	81.377	70.2243	-0.212651	-0.452161	0.43145	0.913083	no
gene:SpnNT_00661	thyA	Chromosome:716882-717722	110.58+peptide	ΔORF2+peptide	OK	64.7438	70.2243	0.117228	0.24601	0.6742	0.985063	no
gene:SpnNT_00662	NA	Chromosome:717769-717940	110.58	ΔORF2	OK	7663.3	8223.76	0.101833	0.21307	0.71115	0.991007	no
gene:SpnNT_00662	NA	Chromosome:717769-717940	110.58	110.58+peptide	OK	7663.3	5513.84	-0.474907	-0.922292	0.1035	0.488069	no
gene:SpnNT_00662	NA	Chromosome:717769-717940	ΔORF2	110.58+peptide	OK	8223.76	5513.84	-0.57674	-1.13884	0.04745	0.303282	no
gene:SpnNT_00662	NA	Chromosome:717769-717940	110.58	ΔORF2+peptide	OK	7663.3	3977.4	-0.946139	-1.84718	0.00165	0.0253528	yes
gene:SpnNT_00662	NA	Chromosome:717769-717940	ΔORF2	ΔORF2+peptide	OK	8223.76	3977.4	-1.04797	-2.08069	0.00055	0.0103545	yes
gene:SpnNT_00662	NA	Chromosome:717769-717940	110.58+peptide	ΔORF2+peptide	OK	5513.84	3977.4	-0.471232	-0.874448	0.11355	0.51115	no
gene:SpnNT_00663	miaA	Chromosome:718060-720792	110.58	ΔORF2	OK	77.0456	79.7257	0.0493323	0.0684583	0.9061	0.994748	no
gene:SpnNT_00663	miaA	Chromosome:718060-720792	110.58	110.58+peptide	OK	77.0456	88.6734	0.202789	0.265812	0.63905	0.980887	no
gene:SpnNT_00663	miaA	Chromosome:718060-720792	ΔORF2	110.58+peptide	OK	79.7257	88.6734	0.153457	0.204341	0.7187	0.993515	no
gene:SpnNT_00663	miaA	Chromosome:718060-720792	110.58	ΔORF2+peptide	OK	77.0456	85.2103	0.145316	0.195969	0.7363	0.994748	no
gene:SpnNT_00663	miaA	Chromosome:718060-720792	ΔORF2	ΔORF2+peptide	OK	79.7257	85.2103	0.0959839	0.131619	0.8212	0.994748	no
gene:SpnNT_00663	miaA	Chromosome:718060-720792	110.58+peptide	ΔORF2+peptide	OK	88.6734	85.2103	-0.0574729	-0.0745369	0.8995	0.994748	no
gene:SpnNT_00664	hflX	Chromosome:718060-720792	110.58	ΔORF2	OK	60.4805	55.7027	-0.118722	-0.176305	0.7583	0.994748	no
gene:SpnNT_00664	hflX	Chromosome:718060-720792	110.58	110.58+peptide	OK	60.4805	65.9188	0.124221	0.182447	0.75095	0.994748	no
gene:SpnNT_00664	hflX	Chromosome:718060-720792	ΔORF2	110.58+peptide	OK	55.7027	65.9188	0.242943	0.342763	0.55535	0.966573	no
gene:SpnNT_00664	hflX	Chromosome:718060-720792	110.58	ΔORF2+peptide	OK	60.4805	70.9482	0.230298	0.362593	0.5356	0.958867	no
gene:SpnNT_00664	hflX	Chromosome:718060-720792	ΔORF2	ΔORF2+peptide	OK	55.7027	70.9482	0.349019	0.524857	0.3664	0.864634	no
gene:SpnNT_00664	hflX	Chromosome:718060-720792	110.58+peptide	ΔORF2+peptide	OK	65.9188	70.9482	0.106076	0.157724	0.78455	0.994748	no
gene:SpnNT_00665	NA	Chromosome:718060-720792	110.58	ΔORF2	OK	43.3127	43.0056	-0.0102672	-0.00802217	0.98965	0.997703	no
gene:SpnNT_00665	NA	Chromosome:718060-720792	110.58	110.58+peptide	OK	43.3127	44.811	0.0490628	0.0364423	0.94915	0.994855	no
gene:SpnNT_00665	NA	Chromosome:718060-720792	ΔORF2	110.58+peptide	OK	43.0056	44.811	0.05933	0.0470593	0.93465	0.994855	no
gene:SpnNT_00665	NA	Chromosome:718060-720792	110.58	ΔORF2+peptide	OK	43.3127	50.1888	0.212576	0.166405	0.7727	0.994748	no
gene:SpnNT_00665	NA	Chromosome:718060-720792	ΔORF2	ΔORF2+peptide	OK	43.0056	50.1888	0.222843	0.187745	0.7485	0.994748	no
gene:SpnNT_00665	NA	Chromosome:718060-720792	110.58+peptide	ΔORF2+peptide	OK	44.811	50.1888	0.163513	0.129945	0.82425	0.994748	no
gene:SpnNT_00666	rnz	Chromosome:720806-721736	110.58	ΔORF2	OK	48.875	47.0384	-0.0552598	-0.112796	0.8455	0.994748	no
gene:SpnNT_00666	rnz	Chromosome:720806-721736	110.58	110.58+peptide	OK	48.875	51.0592	0.0630718	0.13057	0.82165	0.994748	no
gene:SpnNT_00666	rnz	Chromosome:720806-721736	ΔORF2	110.58+peptide	OK	47.0384	51.0592	0.118332	0.241911	0.67565	0.985207	no
gene:SpnNT_00666	rnz	Chromosome:720806-721736	110.58	ΔORF2+peptide	OK	48.875	55.8544	0.192574	0.399285	0.4906	0.940215	no
gene:SpnNT_00666	rnz	Chromosome:720806-721736	ΔORF2	ΔORF2+peptide	OK	47.0384	55.8544	0.247834	0.507431	0.3856	0.881745	no
gene:SpnNT_00666	rnz	Chromosome:720806-721736	110.58+peptide	ΔORF2+peptide	OK	51.0592	55.8544	0.129502	0.268941	0.6432	0.980887	no
gene:SpnNT_00667	NA	Chromosome:721756-722512	110.58	ΔORF2	OK	76.9908	80.8679	0.0708805	0.149783	0.79395	0.994748	no
gene:SpnNT_00667	NA	Chromosome:721756-722512	110.58	110.58+peptide	OK	76.9908	90.5356	0.2338	0.497802	0.3802	0.878145	no
gene:SpnNT_00667	NA	Chromosome:721756-722512	ΔORF2	110.58+peptide	OK	80.8679	90.5356	0.162919	0.347807	0.537	0.959321	no
gene:SpnNT_00667	NA	Chromosome:721756-722512	110.58	ΔORF2+peptide	OK	76.9908	107.672	0.483883	1.03221	0.07355	0.399141	no
gene:SpnNT_00667	NA	Chromosome:721756-722512	ΔORF2	ΔORF2+peptide	OK	80.8679	107.672	0.413002	0.88336	0.12595	0.537469	no
gene:SpnNT_00667	NA	Chromosome:721756-722512	110.58+peptide	ΔORF2+peptide	OK	90.5356	107.672	0.250083	0.539049	0.3549	0.857011	no
gene:SpnNT_00668	hcaR	Chromosome:722565-723534	110.58	ΔORF2	OK	49.852	51.5373	0.0479679	0.0996929	0.8661	0.994748	no
gene:SpnNT_00668	hcaR	Chromosome:722565-723534	110.58	110.58+peptide	OK	49.852	46.9286	-0.0871838	-0.180366	0.75805	0.994748	no

gene:SpnNT_00668	hcaR	Chromosome:722565-723534	ΔORF2	110.58+peptide	OK	51.5373	46.9286	-0.135152	-0.280525	0.63145	0.980887	no
gene:SpnNT_00668	hcaR	Chromosome:722565-723534	110.58	ΔORF2+peptide	OK	49.852	51.1529	0.0371647	0.0771158	0.8965	0.994748	no
gene:SpnNT_00668	hcaR	Chromosome:722565-723534	ΔORF2	ΔORF2+peptide	OK	51.5373	51.1529	-0.0108032	-0.0224909	0.968	0.994855	no
gene:SpnNT_00668	hcaR	Chromosome:722565-723534	110.58+peptide	ΔORF2+peptide	OK	46.9286	51.1529	0.124348	0.257686	0.65025	0.981475	no
gene:SpnNT_00669	NA	Chromosome:723537-724187	110.58	ΔORF2	OK	635.711	721.155	0.181937	0.297033	0.60415	0.976761	no
gene:SpnNT_00669	NA	Chromosome:723537-724187	110.58	110.58+peptide	OK	635.711	536.355	-0.245182	-0.385898	0.5022	0.944784	no
gene:SpnNT_00669	NA	Chromosome:723537-724187	ΔORF2	110.58+peptide	OK	721.155	536.355	-0.42712	-0.675749	0.24605	0.743359	no
gene:SpnNT_00669	NA	Chromosome:723537-724187	110.58	ΔORF2+peptide	OK	635.711	571.21	-0.15435	-0.229905	0.6886	0.98828	no
gene:SpnNT_00669	NA	Chromosome:723537-724187	ΔORF2	ΔORF2+peptide	OK	721.155	571.21	-0.336288	-0.503231	0.3863	0.881745	no
gene:SpnNT_00669	NA	Chromosome:723537-724187	110.58+peptide	ΔORF2+peptide	OK	536.355	571.21	0.0908322	0.131784	0.8191	0.994748	no
gene:SpnNT_00670	moeZ_1	Chromosome:723537-724187	110.58	ΔORF2	OK	217.857	252.15	0.2109	0.301261	0.5974	0.97629	no
gene:SpnNT_00670	moeZ_1	Chromosome:723537-724187	110.58	110.58+peptide	OK	217.857	205.172	-0.0865527	-0.122824	0.82615	0.994748	no
gene:SpnNT_00670	moeZ_1	Chromosome:723537-724187	ΔORF2	110.58+peptide	OK	252.15	205.172	-0.297453	-0.418004	0.4599	0.923884	no
gene:SpnNT_00670	moeZ_1	Chromosome:723537-724187	110.58	ΔORF2+peptide	OK	217.857	222.88	0.0328827	0.0450172	0.9358	0.994855	no
gene:SpnNT_00670	moeZ_1	Chromosome:723537-724187	ΔORF2	ΔORF2+peptide	OK	252.15	222.88	-0.178018	-0.241506	0.6734	0.984845	no
gene:SpnNT_00670	moeZ_1	Chromosome:723537-724187	110.58+peptide	ΔORF2+peptide	OK	205.172	222.88	0.119435	0.161069	0.7803	0.994748	no
gene:SpnNT_00671	NA	Chromosome:724275-724503	110.58	ΔORF2	OK	224.088	392.442	0.808413	1.41956	0.0126	0.119205	no
gene:SpnNT_00671	NA	Chromosome:724275-724503	110.58	110.58+peptide	OK	224.088	295.092	0.397101	0.727864	0.21725	0.70491	no
gene:SpnNT_00671	NA	Chromosome:724275-724503	ΔORF2	110.58+peptide	OK	392.442	295.092	-0.411312	-0.735979	0.19835	0.680978	no
gene:SpnNT_00671	NA	Chromosome:724275-724503	110.58	ΔORF2+peptide	OK	224.088	388.648	0.794398	1.46339	0.012	0.115105	no
gene:SpnNT_00671	NA	Chromosome:724275-724503	ΔORF2	ΔORF2+peptide	OK	392.442	388.648	-0.0140149	-0.0251973	0.96405	0.994855	no
gene:SpnNT_00671	NA	Chromosome:724275-724503	110.58+peptide	ΔORF2+peptide	OK	295.092	388.648	0.397297	0.747223	0.20775	0.693134	no
gene:SpnNT_00672	rsuA_2	Chromosome:724542-725268	110.58	ΔORF2	OK	52.8134	55.9568	0.0834106	0.167391	0.77195	0.994748	no
gene:SpnNT_00672	rsuA_2	Chromosome:724542-725268	110.58	110.58+peptide	OK	52.8134	38.0303	-0.473753	-0.929804	0.10405	0.489722	no
gene:SpnNT_00672	rsuA_2	Chromosome:724542-725268	ΔORF2	110.58+peptide	OK	55.9568	38.0303	-0.557164	-1.09341	0.05855	0.345456	no
gene:SpnNT_00672	rsuA_2	Chromosome:724542-725268	110.58	ΔORF2+peptide	OK	52.8134	41.1915	-0.358556	-0.706253	0.2198	0.708131	no
gene:SpnNT_00672	rsuA_2	Chromosome:724542-725268	ΔORF2	ΔORF2+peptide	OK	55.9568	41.1915	-0.441967	-0.870468	0.13165	0.551568	no
gene:SpnNT_00672	rsuA_2	Chromosome:724542-725268	110.58+peptide	ΔORF2+peptide	OK	38.0303	41.1915	0.115197	0.222066	0.69775	0.990209	no
gene:SpnNT_00673	typA	Chromosome:725429-727271	110.58	ΔORF2	OK	193.992	202.62	0.0627849	0.142849	0.8066	0.994748	no
gene:SpnNT_00673	typA	Chromosome:725429-727271	110.58	110.58+peptide	OK	193.992	221.172	0.189171	0.429639	0.4595	0.923646	no
gene:SpnNT_00673	typA	Chromosome:725429-727271	ΔORF2	110.58+peptide	OK	202.62	221.172	0.126386	0.286724	0.6144	0.979616	no
gene:SpnNT_00673	typA	Chromosome:725429-727271	110.58	ΔORF2+peptide	OK	193.992	229.894	0.244972	0.555837	0.33725	0.84149	no
gene:SpnNT_00673	typA	Chromosome:725429-727271	ΔORF2	ΔORF2+peptide	OK	202.62	229.894	0.182187	0.412919	0.4676	0.92732	no
gene:SpnNT_00673	typA	Chromosome:725429-727271	110.58+peptide	ΔORF2+peptide	OK	221.172	229.894	0.0558005	0.126246	0.82495	0.994748	no
gene:SpnNT_00674	NA	Chromosome:727290-727545	110.58	ΔORF2	OK	484.874	643.956	0.409352	0.848262	0.13865	0.568197	no
gene:SpnNT_00674	NA	Chromosome:727290-727545	110.58	110.58+peptide	OK	484.874	625.257	0.36684	0.755451	0.1814	0.650541	no
gene:SpnNT_00674	NA	Chromosome:727290-727545	ΔORF2	110.58+peptide	OK	643.956	625.257	-0.0425126	-0.0884406	0.88035	0.994748	no
gene:SpnNT_00674	NA	Chromosome:727290-727545	110.58	ΔORF2+peptide	OK	484.874	764.355	0.656633	1.3937	0.0166	0.144638	no
gene:SpnNT_00674	NA	Chromosome:727290-727545	ΔORF2	ΔORF2+peptide	OK	643.956	764.355	0.24728	0.530541	0.36275	0.86287	no
gene:SpnNT_00674	NA	Chromosome:727290-727545	110.58+peptide	ΔORF2+peptide	OK	625.257	764.355	0.289793	0.61762	0.28665	0.789883	no
gene:SpnNT_00675	NA	Chromosome:727863-728208	110.58	ΔORF2	OK	29.4116	26.0683	-0.174093	-0.266045	0.64285	0.980887	no
gene:SpnNT_00675	NA	Chromosome:727863-728208	110.58	110.58+peptide	OK	29.4116	101.479	1.78672	2.71493	5.00E-05	0.0013612	yes
gene:SpnNT_00675	NA	Chromosome:727863-728208	ΔORF2	110.58+peptide	OK	26.0683	101.479	1.96082	2.96094	5.00E-05	0.0013612	yes
gene:SpnNT_00675	NA	Chromosome:727863-728208	110.58	ΔORF2+peptide	OK	29.4116	70.8279	1.26793	2.0484	9.00E-04	0.0154579	yes
gene:SpnNT_00675	NA	Chromosome:727863-728208	ΔORF2	ΔORF2+peptide	OK	26.0683	70.8279	1.44202	2.3133	0.00025	0.00542231	yes

gene:SpnNT_00675	NA	Chromosome:727863-728208	110.58+peptide	ΔORF2+peptide	OK	101.479	70.8279	-0.518793	-0.827046	0.14935	0.590648	no
gene:SpnNT_00676	NA	Chromosome:728290-730312	110.58	ΔORF2	OK	7.17119	5.66032	-0.34133	-0.604079	0.2846	0.787874	no
gene:SpnNT_00676	NA	Chromosome:728290-730312	110.58	110.58+peptide	OK	7.17119	14.287	0.994417	1.7811	0.00175	0.0265167	yes
gene:SpnNT_00676	NA	Chromosome:728290-730312	ΔORF2	110.58+peptide	OK	5.66032	14.287	1.33575	2.37825	5.00E-05	0.0013612	yes
gene:SpnNT_00676	NA	Chromosome:728290-730312	110.58	ΔORF2+peptide	OK	7.17119	9.41858	0.393297	0.713507	0.2071	0.69255	no
gene:SpnNT_00676	NA	Chromosome:728290-730312	ΔORF2	ΔORF2+peptide	OK	5.66032	9.41858	0.734627	1.32462	0.02065	0.171608	no
gene:SpnNT_00676	NA	Chromosome:728290-730312	110.58+peptide	ΔORF2+peptide	OK	14.287	9.41858	-0.601121	-1.09746	0.0505	0.315703	no
gene:SpnNT_00677	yxdL	Chromosome:730313-730955	110.58	ΔORF2	OK	12.9833	12.0012	-0.113472	-0.179028	0.75265	0.994748	no
gene:SpnNT_00677	yxdL	Chromosome:730313-730955	110.58	110.58+peptide	OK	12.9833	25.7381	0.987249	1.60535	0.0056	0.0654347	no
gene:SpnNT_00677	yxdL	Chromosome:730313-730955	ΔORF2	110.58+peptide	OK	12.0012	25.7381	1.10072	1.77906	0.0019	0.0281715	yes
gene:SpnNT_00677	yxdL	Chromosome:730313-730955	110.58	ΔORF2+peptide	OK	12.9833	15.4665	0.25249	0.404418	0.4867	0.938498	no
gene:SpnNT_00677	yxdL	Chromosome:730313-730955	ΔORF2	ΔORF2+peptide	OK	12.0012	15.4665	0.365963	0.582733	0.30825	0.814346	no
gene:SpnNT_00677	yxdL	Chromosome:730313-730955	110.58+peptide	ΔORF2+peptide	OK	25.7381	15.4665	-0.734758	-1.20654	0.03945	0.268637	no
gene:SpnNT_00678	murD	Chromosome:731058-732411	110.58	ΔORF2	OK	94.1429	102.123	0.117383	0.267114	0.63185	0.980887	no
gene:SpnNT_00678	murD	Chromosome:731058-732411	110.58	110.58+peptide	OK	94.1429	86.8627	-0.116115	-0.262681	0.64395	0.980887	no
gene:SpnNT_00678	murD	Chromosome:731058-732411	ΔORF2	110.58+peptide	OK	102.123	86.8627	-0.233498	-0.530938	0.35375	0.856246	no
gene:SpnNT_00678	murD	Chromosome:731058-732411	110.58	ΔORF2+peptide	OK	94.1429	84.0867	-0.162975	-0.36882	0.5215	0.954832	no
gene:SpnNT_00678	murD	Chromosome:731058-732411	ΔORF2	ΔORF2+peptide	OK	102.123	84.0867	-0.280358	-0.637717	0.2621	0.762589	no
gene:SpnNT_00678	murD	Chromosome:731058-732411	110.58+peptide	ΔORF2+peptide	OK	86.8627	84.0867	-0.0468602	-0.105966	0.84695	0.994748	no
gene:SpnNT_00679	murG	Chromosome:732414-733473	110.58	ΔORF2	OK	36.3918	38.518	0.081921	0.166113	0.76405	0.994748	no
gene:SpnNT_00679	murG	Chromosome:732414-733473	110.58	110.58+peptide	OK	36.3918	38.0372	0.0637994	0.130263	0.81355	0.994748	no
gene:SpnNT_00679	murG	Chromosome:732414-733473	ΔORF2	110.58+peptide	OK	38.518	38.0372	-0.0181216	-0.0368246	0.94895	0.994855	no
gene:SpnNT_00679	murG	Chromosome:732414-733473	110.58	ΔORF2+peptide	OK	36.3918	42.2962	0.216916	0.444698	0.4263	0.908545	no
gene:SpnNT_00679	murG	Chromosome:732414-733473	ΔORF2	ΔORF2+peptide	OK	38.518	42.2962	0.134995	0.275429	0.63015	0.980887	no
gene:SpnNT_00679	murG	Chromosome:732414-733473	110.58+peptide	ΔORF2+peptide	OK	38.0372	42.2962	0.153117	0.314592	0.5796	0.969538	no
gene:SpnNT_00680	divIB	Chromosome:733482-734682	110.58	ΔORF2	OK	48.0921	51.1418	0.0887041	0.189819	0.7418	0.994748	no
gene:SpnNT_00680	divIB	Chromosome:733482-734682	110.58	110.58+peptide	OK	48.0921	45.7373	-0.0724276	-0.15422	0.79005	0.994748	no
gene:SpnNT_00680	divIB	Chromosome:733482-734682	ΔORF2	110.58+peptide	OK	51.1418	45.7373	-0.161132	-0.344057	0.55165	0.964524	no
gene:SpnNT_00680	divIB	Chromosome:733482-734682	110.58	ΔORF2+peptide	OK	48.0921	55.8886	0.216754	0.462501	0.4258	0.908216	no
gene:SpnNT_00680	divIB	Chromosome:733482-734682	ΔORF2	ΔORF2+peptide	OK	51.1418	55.8886	0.12805	0.273995	0.6418	0.980887	no
gene:SpnNT_00680	divIB	Chromosome:733482-734682	110.58+peptide	ΔORF2+peptide	OK	45.7373	55.8886	0.289181	0.61571	0.2891	0.793273	no
gene:SpnNT_00681	NA	Chromosome:734812-734938	110.58	ΔORF2	OK	54.9897	50.9454	-0.110209	-0.0779513	0.89985	0.994748	no
gene:SpnNT_00681	NA	Chromosome:734812-734938	110.58	110.58+peptide	OK	54.9897	110.903	1.01207	0.865647	0.1369	0.563312	no
gene:SpnNT_00681	NA	Chromosome:734812-734938	ΔORF2	110.58+peptide	OK	50.9454	110.903	1.12228	0.848308	0.1255	0.536246	no
gene:SpnNT_00681	NA	Chromosome:734812-734938	110.58	ΔORF2+peptide	OK	54.9897	173.439	1.6572	1.47637	0.0233	0.187228	no
gene:SpnNT_00681	NA	Chromosome:734812-734938	ΔORF2	ΔORF2+peptide	OK	50.9454	173.439	1.7674	1.37873	0.0231	0.18642	no
gene:SpnNT_00681	NA	Chromosome:734812-734938	110.58+peptide	ΔORF2+peptide	OK	110.903	173.439	0.645128	0.64151	0.27735	0.780208	no
gene:SpnNT_00682	NA	Chromosome:734958-735081	110.58	ΔORF2	OK	17.4903	7.48464	-1.22455	-0.602636	0.3756	0.873405	no
gene:SpnNT_00682	NA	Chromosome:734958-735081	110.58	110.58+peptide	OK	17.4903	39.2339	1.16555	0.501561	0.2111	0.697158	no
gene:SpnNT_00682	NA	Chromosome:734958-735081	ΔORF2	110.58+peptide	OK	7.48464	39.2339	2.39009	1.94524	0.25045	0.747909	no
gene:SpnNT_00682	NA	Chromosome:734958-735081	110.58	ΔORF2+peptide	OK	17.4903	29.5922	0.758665	0.378123	0.3298	0.835739	no
gene:SpnNT_00682	NA	Chromosome:734958-735081	ΔORF2	ΔORF2+peptide	OK	7.48464	29.5922	1.98321	5.39602	0.31585	0.821686	no
gene:SpnNT_00682	NA	Chromosome:734958-735081	110.58+peptide	ΔORF2+peptide	OK	39.2339	29.5922	-0.406881	-0.343104	0.70615	0.990422	no
gene:SpnNT_00683	NA	Chromosome:735263-737628	110.58	ΔORF2	OK	13.0363	18.112	0.474412	0.44721	0.4392	0.916225	no
gene:SpnNT_00683	NA	Chromosome:735263-737628	110.58	110.58+peptide	OK	13.0363	26.7557	1.03731	0.898798	0.10235	0.485026	no

gene:SpnNT_00683	NA	Chromosome:735263-737628	ΔORF2	110.58+peptide	OK	18.112	26.7557	0.562895	0.500543	0.36635	0.864634	no
gene:SpnNT_00683	NA	Chromosome:735263-737628	110.58	ΔORF2+peptide	OK	13.0363	42.3687	1.70046	1.60192	0.00645	0.0727121	no
gene:SpnNT_00683	NA	Chromosome:735263-737628	ΔORF2	ΔORF2+peptide	OK	18.112	42.3687	1.22605	1.19112	0.0434	0.285604	no
gene:SpnNT_00683	NA	Chromosome:735263-737628	110.58+peptide	ΔORF2+peptide	OK	26.7557	42.3687	0.663153	0.589357	0.27535	0.777354	no
gene:SpnNT_00684	thiF	Chromosome:735263-737628	110.58	ΔORF2	OK	26.4662	39.1386	0.564442	1.0117	0.07965	0.42059	no
gene:SpnNT_00684	thiF	Chromosome:735263-737628	110.58	110.58+peptide	OK	26.4662	58.4142	1.14217	1.96431	0.0013	0.020854	yes
gene:SpnNT_00684	thiF	Chromosome:735263-737628	ΔORF2	110.58+peptide	OK	39.1386	58.4142	0.577725	1.01596	0.08895	0.448911	no
gene:SpnNT_00684	thiF	Chromosome:735263-737628	110.58	ΔORF2+peptide	OK	26.4662	96.6196	1.86816	3.45858	5.00E-05	0.0013612	yes
gene:SpnNT_00684	thiF	Chromosome:735263-737628	ΔORF2	ΔORF2+peptide	OK	39.1386	96.6196	1.30372	2.47695	5.00E-05	0.0013612	yes
gene:SpnNT_00684	thiF	Chromosome:735263-737628	110.58+peptide	ΔORF2+peptide	OK	58.4142	96.6196	0.725997	1.31703	0.02725	0.209108	no
gene:SpnNT_00685	NA	Chromosome:737629-737791	110.58	ΔORF2	NOTEST	5.61221	1.82356	-1.62181	0	1	1	no
gene:SpnNT_00685	NA	Chromosome:737629-737791	110.58	110.58+peptide	NOTEST	5.61221	0	#NAME?	0	1	1	no
gene:SpnNT_00685	NA	Chromosome:737629-737791	ΔORF2	110.58+peptide	NOTEST	1.82356	0	#NAME?	0	1	1	no
gene:SpnNT_00685	NA	Chromosome:737629-737791	110.58	ΔORF2+peptide	NOTEST	5.61221	0	#NAME?	0	1	1	no
gene:SpnNT_00685	NA	Chromosome:737629-737791	ΔORF2	ΔORF2+peptide	NOTEST	1.82356	0	#NAME?	0	1	1	no
gene:SpnNT_00685	NA	Chromosome:737629-737791	110.58+peptide	ΔORF2+peptide	NOTEST	0	0	0	0	1	1	no
gene:SpnNT_00686	NA	Chromosome:737837-739377	110.58	ΔORF2	OK	47.7768	55.0296	0.2039	0.351814	0.54875	0.964037	no
gene:SpnNT_00686	NA	Chromosome:737837-739377	110.58	110.58+peptide	OK	47.7768	83.8688	0.811825	1.37409	0.0175	0.150186	no
gene:SpnNT_00686	NA	Chromosome:737837-739377	ΔORF2	110.58+peptide	OK	55.0296	83.8688	0.607926	1.02965	0.0707	0.390464	no
gene:SpnNT_00686	NA	Chromosome:737837-739377	110.58	ΔORF2+peptide	OK	47.7768	105.49	1.14272	1.98958	5.00E-04	0.0095781	yes
gene:SpnNT_00686	NA	Chromosome:737837-739377	ΔORF2	ΔORF2+peptide	OK	55.0296	105.49	0.938825	1.63572	0.0038	0.0485054	yes
gene:SpnNT_00686	NA	Chromosome:737837-739377	110.58+peptide	ΔORF2+peptide	OK	83.8688	105.49	0.330899	0.565349	0.31615	0.821977	no
gene:SpnNT_00687	NA	Chromosome:737837-739377	110.58	ΔORF2	OK	27.7038	34.5851	0.320068	0.36244	0.5304	0.957488	no
gene:SpnNT_00687	NA	Chromosome:737837-739377	110.58	110.58+peptide	OK	27.7038	49.7046	0.843297	0.935764	0.1266	0.539365	no
gene:SpnNT_00687	NA	Chromosome:737837-739377	ΔORF2	110.58+peptide	OK	34.5851	49.7046	0.523229	0.593809	0.3315	0.837459	no
gene:SpnNT_00687	NA	Chromosome:737837-739377	110.58	ΔORF2+peptide	OK	27.7038	84.5548	1.6098	1.96194	9.00E-04	0.0154579	yes
gene:SpnNT_00687	NA	Chromosome:737837-739377	ΔORF2	ΔORF2+peptide	OK	34.5851	84.5548	1.28973	1.6153	0.00715	0.07871	no
gene:SpnNT_00687	NA	Chromosome:737837-739377	110.58+peptide	ΔORF2+peptide	OK	49.7046	84.5548	0.766505	0.936574	0.13115	0.55088	no
gene:SpnNT_00688	pyrF	Chromosome:740110-740812	110.58	ΔORF2	OK	21.8499	18.9494	-0.205475	-0.361432	0.52985	0.957488	no
gene:SpnNT_00688	pyrF	Chromosome:740110-740812	110.58	110.58+peptide	OK	21.8499	48.9777	1.1645	2.14871	0.00045	0.00885292	yes
gene:SpnNT_00688	pyrF	Chromosome:740110-740812	ΔORF2	110.58+peptide	OK	18.9494	48.9777	1.36997	2.49679	5.00E-05	0.0013612	yes
gene:SpnNT_00688	pyrF	Chromosome:740110-740812	110.58	ΔORF2+peptide	OK	21.8499	45.2581	1.05055	1.949	0.00095	0.0161266	yes
gene:SpnNT_00688	pyrF	Chromosome:740110-740812	ΔORF2	ΔORF2+peptide	OK	18.9494	45.2581	1.25603	2.30127	2.00E-04	0.00450928	yes
gene:SpnNT_00688	pyrF	Chromosome:740110-740812	110.58+peptide	ΔORF2+peptide	OK	48.9777	45.2581	-0.113948	-0.21994	0.69095	0.98828	no
gene:SpnNT_00689	pyrE	Chromosome:740845-741478	110.58	ΔORF2	OK	29.4782	28.2391	-0.0619585	-0.110233	0.84815	0.994748	no
gene:SpnNT_00689	pyrE	Chromosome:740845-741478	110.58	110.58+peptide	OK	29.4782	74.5554	1.33866	2.48934	5.00E-05	0.0013612	yes
gene:SpnNT_00689	pyrE	Chromosome:740845-741478	ΔORF2	110.58+peptide	OK	28.2391	74.5554	1.40062	2.60844	5.00E-05	0.0013612	yes
gene:SpnNT_00689	pyrE	Chromosome:740845-741478	110.58	ΔORF2+peptide	OK	29.4782	75.1248	1.34964	2.55064	5.00E-05	0.0013612	yes
gene:SpnNT_00689	pyrE	Chromosome:740845-741478	ΔORF2	ΔORF2+peptide	OK	28.2391	75.1248	1.4116	2.67184	5.00E-05	0.0013612	yes
gene:SpnNT_00689	pyrE	Chromosome:740845-741478	110.58+peptide	ΔORF2+peptide	OK	74.5554	75.1248	0.0109748	0.0218454	0.96955	0.995105	no
gene:SpnNT_00690	NA	Chromosome:741710-742061	110.58	ΔORF2	OK	26.3529	24.3133	-0.116218	-0.174481	0.76585	0.994748	no
gene:SpnNT_00690	NA	Chromosome:741710-742061	110.58	110.58+peptide	OK	26.3529	102.51	1.95974	2.98838	5.00E-05	0.0013612	yes
gene:SpnNT_00690	NA	Chromosome:741710-742061	ΔORF2	110.58+peptide	OK	24.3133	102.51	2.07595	3.13242	5.00E-05	0.0013612	yes
gene:SpnNT_00690	NA	Chromosome:741710-742061	110.58	ΔORF2+peptide	OK	26.3529	66.0763	1.32617	2.13569	0.00065	0.0118958	yes
gene:SpnNT_00690	NA	Chromosome:741710-742061	ΔORF2	ΔORF2+peptide	OK	24.3133	66.0763	1.44239	2.29575	0.00015	0.00355289	yes

gene:SpnNT_00690	NA	Chromosome:741710-742061	110.58+peptide	ΔORF2+peptide	OK	102.51	66.0763	-0.633564	-1.02624	0.06935	0.386745	no
gene:SpnNT_00691	NA	Chromosome:742156-743673	110.58	ΔORF2	OK	6.03932	6.80263	0.171708	0.209484	0.7117	0.991007	no
gene:SpnNT_00691	NA	Chromosome:742156-743673	110.58	110.58+peptide	OK	6.03932	15.168	1.32858	1.72662	0.00445	0.054829	no
gene:SpnNT_00691	NA	Chromosome:742156-743673	ΔORF2	110.58+peptide	OK	6.80263	15.168	1.15687	1.464	0.01395	0.127651	no
gene:SpnNT_00691	NA	Chromosome:742156-743673	110.58	ΔORF2+peptide	OK	6.03932	16.5107	1.45095	1.74171	0.0038	0.0485054	yes
gene:SpnNT_00691	NA	Chromosome:742156-743673	ΔORF2	ΔORF2+peptide	OK	6.80263	16.5107	1.27924	1.501	0.0102	0.101628	no
gene:SpnNT_00691	NA	Chromosome:742156-743673	110.58+peptide	ΔORF2+peptide	OK	15.168	16.5107	0.12237	0.152184	0.78605	0.994748	no
gene:SpnNT_00692	NA	Chromosome:742156-743673	110.58	ΔORF2	OK	2.99115	4.2493	0.506524	0.451738	0.443	0.917631	no
gene:SpnNT_00692	NA	Chromosome:742156-743673	110.58	110.58+peptide	OK	2.99115	10.552	1.81875	1.75666	0.0053	0.0629381	no
gene:SpnNT_00692	NA	Chromosome:742156-743673	ΔORF2	110.58+peptide	OK	4.2493	10.552	1.31222	1.31311	0.032	0.232505	no
gene:SpnNT_00692	NA	Chromosome:742156-743673	110.58	ΔORF2+peptide	OK	2.99115	11.9142	1.99391	1.79428	0.00525	0.0625141	no
gene:SpnNT_00692	NA	Chromosome:742156-743673	ΔORF2	ΔORF2+peptide	OK	4.2493	11.9142	1.48739	1.38005	0.02845	0.214799	no
gene:SpnNT_00692	NA	Chromosome:742156-743673	110.58+peptide	ΔORF2+peptide	OK	10.552	11.9142	0.175168	0.177283	0.76255	0.994748	no
gene:SpnNT_00693	NA	Chromosome:743676-745025	110.58	ΔORF2	OK	5.90983	10.3934	0.814486	0.83314	0.1515	0.593484	no
gene:SpnNT_00693	NA	Chromosome:743676-745025	110.58	110.58+peptide	OK	5.90983	15.6028	1.40062	1.29377	0.024	0.191679	no
gene:SpnNT_00693	NA	Chromosome:743676-745025	ΔORF2	110.58+peptide	OK	10.3934	15.6028	0.586129	0.571633	0.29615	0.800428	no
gene:SpnNT_00693	NA	Chromosome:743676-745025	110.58	ΔORF2+peptide	OK	5.90983	21.0296	1.83123	1.74909	0.0032	0.0422439	yes
gene:SpnNT_00693	NA	Chromosome:743676-745025	ΔORF2	ΔORF2+peptide	OK	10.3934	21.0296	1.01675	1.02944	0.0723	0.395795	no
gene:SpnNT_00693	NA	Chromosome:743676-745025	110.58+peptide	ΔORF2+peptide	OK	15.6028	21.0296	0.430616	0.394452	0.4724	0.928525	no
gene:SpnNT_00694	yxlF_1	Chromosome:743676-745025	110.58	ΔORF2	OK	15.3044	18.3758	0.263861	0.371703	0.52295	0.954832	no
gene:SpnNT_00694	yxlF_1	Chromosome:743676-745025	110.58	110.58+peptide	OK	15.3044	37.8852	1.30769	1.91586	0.00145	0.0226781	yes
gene:SpnNT_00694	yxlF_1	Chromosome:743676-745025	ΔORF2	110.58+peptide	OK	18.3758	37.8852	1.04383	1.47161	0.01265	0.119248	no
gene:SpnNT_00694	yxlF_1	Chromosome:743676-745025	110.58	ΔORF2+peptide	OK	15.3044	32.1144	1.06928	1.39416	0.02525	0.198283	no
gene:SpnNT_00694	yxlF_1	Chromosome:743676-745025	ΔORF2	ΔORF2+peptide	OK	18.3758	32.1144	0.805417	1.01839	0.09035	0.451647	no
gene:SpnNT_00694	yxlF_1	Chromosome:743676-745025	110.58+peptide	ΔORF2+peptide	OK	37.8852	32.1144	-0.238412	-0.31106	0.5987	0.97629	no
gene:SpnNT_00695	glnH_1	Chromosome:745111-745561	110.58	ΔORF2	OK	3.95339	5.0056	0.340453	0.394725	0.4869	0.938745	no
gene:SpnNT_00695	glnH_1	Chromosome:745111-745561	110.58	110.58+peptide	OK	3.95339	9.53899	1.27075	1.55243	0.0103	0.102469	no
gene:SpnNT_00695	glnH_1	Chromosome:745111-745561	ΔORF2	110.58+peptide	OK	5.0056	9.53899	0.930294	1.18074	0.04225	0.280996	no
gene:SpnNT_00695	glnH_1	Chromosome:745111-745561	110.58	ΔORF2+peptide	OK	3.95339	10.5061	1.41006	1.74296	0.00485	0.0588186	no
gene:SpnNT_00695	glnH_1	Chromosome:745111-745561	ΔORF2	ΔORF2+peptide	OK	5.0056	10.5061	1.06961	1.37488	0.0196	0.164655	no
gene:SpnNT_00695	glnH_1	Chromosome:745111-745561	110.58+peptide	ΔORF2+peptide	OK	9.53899	10.5061	0.139314	0.191122	0.7427	0.994748	no
gene:SpnNT_00696	lysS	Chromosome:745676-747167	110.58	ΔORF2	OK	187.473	197.183	0.0728562	0.166797	0.7679	0.994748	no
gene:SpnNT_00696	lysS	Chromosome:745676-747167	110.58	110.58+peptide	OK	187.473	230.117	0.295685	0.67697	0.23535	0.729189	no
gene:SpnNT_00696	lysS	Chromosome:745676-747167	ΔORF2	110.58+peptide	OK	197.183	230.117	0.222829	0.506643	0.377	0.875109	no
gene:SpnNT_00696	lysS	Chromosome:745676-747167	110.58	ΔORF2+peptide	OK	187.473	242.28	0.369996	0.844361	0.13485	0.558731	no
gene:SpnNT_00696	lysS	Chromosome:745676-747167	ΔORF2	ΔORF2+peptide	OK	197.183	242.28	0.29714	0.673445	0.2411	0.736034	no
gene:SpnNT_00696	lysS	Chromosome:745676-747167	110.58+peptide	ΔORF2+peptide	OK	230.117	242.28	0.0743107	0.168427	0.76545	0.994748	no
gene:SpnNT_00697	NA	Chromosome:747790-748927	110.58	ΔORF2	OK	469.544	456.786	-0.0397418	-0.0897932	0.8731	0.994748	no
gene:SpnNT_00697	NA	Chromosome:747790-748927	110.58	110.58+peptide	OK	469.544	916.696	0.965184	2.12487	0.00035	0.00717609	yes
gene:SpnNT_00697	NA	Chromosome:747790-748927	ΔORF2	110.58+peptide	OK	456.786	916.696	1.00493	2.22679	2.00E-04	0.00450928	yes
gene:SpnNT_00697	NA	Chromosome:747790-748927	110.58	ΔORF2+peptide	OK	469.544	897.205	0.934178	2.07586	0.00025	0.00542231	yes
gene:SpnNT_00697	NA	Chromosome:747790-748927	ΔORF2	ΔORF2+peptide	OK	456.786	897.205	0.97392	2.17855	0.00015	0.00355289	yes
gene:SpnNT_00697	NA	Chromosome:747790-748927	110.58+peptide	ΔORF2+peptide	OK	916.696	897.205	-0.0310062	-0.0676143	0.90235	0.994748	no
gene:SpnNT_00698	tenA_1	Chromosome:749483-750152	110.58	ΔORF2	OK	34.1531	39.1719	0.197804	0.37015	0.52375	0.954832	no
gene:SpnNT_00698	tenA_1	Chromosome:749483-750152	110.58	110.58+peptide	OK	34.1531	41.1612	0.269269	0.508167	0.36435	0.864245	no

gene:SpnNT_00698	tenA_1	Chromosome:749483-750152	ΔORF2	110.58+peptide	OK	39.1719	41.1612	0.0714649	0.135695	0.80815	0.994748	no
gene:SpnNT_00698	tenA_1	Chromosome:749483-750152	110.58	ΔORF2+peptide	OK	34.1531	51.0997	0.581299	1.10727	0.05795	0.342686	no
gene:SpnNT_00698	tenA_1	Chromosome:749483-750152	ΔORF2	ΔORF2+peptide	OK	39.1719	51.0997	0.383495	0.735048	0.21375	0.698761	no
gene:SpnNT_00698	tenA_1	Chromosome:749483-750152	110.58+peptide	ΔORF2+peptide	OK	41.1612	51.0997	0.312031	0.603411	0.29365	0.797915	no
gene:SpnNT_00699	thiM_1	Chromosome:750378-751161	110.58	ΔORF2	OK	53.3413	65.8928	0.304866	0.626503	0.2689	0.770416	no
gene:SpnNT_00699	thiM_1	Chromosome:750378-751161	110.58	110.58+peptide	OK	53.3413	61.2469	0.199385	0.408774	0.4714	0.928525	no
gene:SpnNT_00699	thiM_1	Chromosome:750378-751161	ΔORF2	110.58+peptide	OK	65.8928	61.2469	-0.105482	-0.218469	0.703	0.990367	no
gene:SpnNT_00699	thiM_1	Chromosome:750378-751161	110.58	ΔORF2+peptide	OK	53.3413	74.1071	0.474358	0.975643	0.09045	0.451974	no
gene:SpnNT_00699	thiM_1	Chromosome:750378-751161	ΔORF2	ΔORF2+peptide	OK	65.8928	74.1071	0.169492	0.352195	0.53585	0.958874	no
gene:SpnNT_00699	thiM_1	Chromosome:750378-751161	110.58+peptide	ΔORF2+peptide	OK	61.2469	74.1071	0.274974	0.570007	0.3185	0.824882	no
gene:SpnNT_00700	thiE_1	Chromosome:751162-751792	110.58	ΔORF2	OK	40.0707	52.8215	0.398575	0.755718	0.18835	0.664926	no
gene:SpnNT_00700	thiE_1	Chromosome:751162-751792	110.58	110.58+peptide	OK	40.0707	48.9477	0.288693	0.547929	0.34485	0.848991	no
gene:SpnNT_00700	thiE_1	Chromosome:751162-751792	ΔORF2	110.58+peptide	OK	52.8215	48.9477	-0.109882	-0.212077	0.70785	0.990441	no
gene:SpnNT_00700	thiE_1	Chromosome:751162-751792	110.58	ΔORF2+peptide	OK	40.0707	70.2594	0.810141	1.56529	0.0081	0.0866951	no
gene:SpnNT_00700	thiE_1	Chromosome:751162-751792	ΔORF2	ΔORF2+peptide	OK	52.8215	70.2594	0.411566	0.809136	0.1569	0.603766	no
gene:SpnNT_00700	thiE_1	Chromosome:751162-751792	110.58+peptide	ΔORF2+peptide	OK	48.9477	70.2594	0.521448	1.02628	0.0755	0.405697	no
gene:SpnNT_00701	ykoE	Chromosome:752365-752926	110.58	ΔORF2	OK	18.9894	14.3517	-0.403977	-0.646496	0.26715	0.768422	no
gene:SpnNT_00701	ykoE	Chromosome:752365-752926	110.58	110.58+peptide	OK	18.9894	16.0672	-0.241074	-0.388136	0.4991	0.944017	no
gene:SpnNT_00701	ykoE	Chromosome:752365-752926	ΔORF2	110.58+peptide	OK	14.3517	16.0672	0.162903	0.258879	0.65615	0.982966	no
gene:SpnNT_00701	ykoE	Chromosome:752365-752926	110.58	ΔORF2+peptide	OK	18.9894	12.3238	-0.623745	-0.988629	0.0909	0.453591	no
gene:SpnNT_00701	ykoE	Chromosome:752365-752926	ΔORF2	ΔORF2+peptide	OK	14.3517	12.3238	-0.219768	-0.343951	0.5519	0.964534	no
gene:SpnNT_00701	ykoE	Chromosome:752365-752926	110.58+peptide	ΔORF2+peptide	OK	16.0672	12.3238	-0.382671	-0.602375	0.29895	0.80426	no
gene:SpnNT_00702	ykoD_2	Chromosome:752926-754312	110.58	ΔORF2	OK	30.4065	27.5641	-0.141587	-0.286078	0.6081	0.978519	no
gene:SpnNT_00702	ykoD_2	Chromosome:752926-754312	110.58	110.58+peptide	OK	30.4065	30.6554	0.0117626	0.0239076	0.96635	0.994855	no
gene:SpnNT_00702	ykoD_2	Chromosome:752926-754312	ΔORF2	110.58+peptide	OK	27.5641	30.6554	0.153349	0.314644	0.5759	0.969538	no
gene:SpnNT_00702	ykoD_2	Chromosome:752926-754312	110.58	ΔORF2+peptide	OK	30.4065	25.9357	-0.229439	-0.461256	0.4129	0.899511	no
gene:SpnNT_00702	ykoD_2	Chromosome:752926-754312	ΔORF2	ΔORF2+peptide	OK	27.5641	25.9357	-0.0878528	-0.178257	0.7492	0.994748	no
gene:SpnNT_00702	ykoD_2	Chromosome:752926-754312	110.58+peptide	ΔORF2+peptide	OK	30.6554	25.9357	-0.241202	-0.492337	0.38365	0.88073	no
gene:SpnNT_00703	NA	Chromosome:754313-754964	110.58	ΔORF2	OK	30.2534	25.846	-0.227158	-0.406987	0.4867	0.938498	no
gene:SpnNT_00703	NA	Chromosome:754313-754964	110.58	110.58+peptide	OK	30.2534	30.767	0.0242868	0.0442431	0.9404	0.994855	no
gene:SpnNT_00703	NA	Chromosome:754313-754964	ΔORF2	110.58+peptide	OK	25.846	30.767	0.251444	0.454396	0.42535	0.908126	no
gene:SpnNT_00703	NA	Chromosome:754313-754964	110.58	ΔORF2+peptide	OK	30.2534	22.82	-0.406798	-0.721188	0.209	0.695007	no
gene:SpnNT_00703	NA	Chromosome:754313-754964	ΔORF2	ΔORF2+peptide	OK	25.846	22.82	-0.179641	-0.316064	0.57695	0.969538	no
gene:SpnNT_00703	NA	Chromosome:754313-754964	110.58+peptide	ΔORF2+peptide	OK	30.767	22.82	-0.431085	-0.770713	0.1733	0.633615	no
gene:SpnNT_00704	tenA_2	Chromosome:754974-755667	110.58	ΔORF2	OK	27.1999	27.9516	0.0393318	0.0704799	0.9014	0.994748	no
gene:SpnNT_00704	tenA_2	Chromosome:754974-755667	110.58	110.58+peptide	OK	27.1999	31.3054	0.20281	0.367968	0.51595	0.952758	no
gene:SpnNT_00704	tenA_2	Chromosome:754974-755667	ΔORF2	110.58+peptide	OK	27.9516	31.3054	0.163478	0.298053	0.6031	0.976761	no
gene:SpnNT_00704	tenA_2	Chromosome:754974-755667	110.58	ΔORF2+peptide	OK	27.1999	29.2678	0.105714	0.188086	0.74165	0.994748	no
gene:SpnNT_00704	tenA_2	Chromosome:754974-755667	ΔORF2	ΔORF2+peptide	OK	27.9516	29.2678	0.0663826	0.118661	0.8354	0.994748	no
gene:SpnNT_00704	tenA_2	Chromosome:754974-755667	110.58+peptide	ΔORF2+peptide	OK	31.3054	29.2678	-0.0970953	-0.175722	0.75905	0.994748	no
gene:SpnNT_00705	NA	Chromosome:755671-757624	110.58	ΔORF2	OK	33.8177	27.6636	-0.28979	-0.255774	0.65585	0.982966	no
gene:SpnNT_00705	NA	Chromosome:755671-757624	110.58	110.58+peptide	OK	33.8177	34.6187	0.0337728	0.0293768	0.9576	0.994855	no
gene:SpnNT_00705	NA	Chromosome:755671-757624	ΔORF2	110.58+peptide	OK	27.6636	34.6187	0.323563	0.28824	0.6119	0.979429	no
gene:SpnNT_00705	NA	Chromosome:755671-757624	110.58	ΔORF2+peptide	OK	33.8177	24.2354	-0.480666	-0.400076	0.47945	0.933711	no
gene:SpnNT_00705	NA	Chromosome:755671-757624	ΔORF2	ΔORF2+peptide	OK	27.6636	24.2354	-0.190876	-0.162374	0.76785	0.994748	no

gene:SpnNT_00705	NA	Chromosome:755671-757624	110.58+peptide	ΔORF2+peptide	OK	34.6187	24.2354	-0.514439	-0.431724	0.4447	0.917756	no
gene:SpnNT_00706	thiM_2	Chromosome:755671-757624	110.58	ΔORF2	OK	37.7214	32.8768	-0.198315	-0.2636	0.64535	0.980887	no
gene:SpnNT_00706	thiM_2	Chromosome:755671-757624	110.58	110.58+peptide	OK	37.7214	39.6022	0.0701971	0.0927041	0.87355	0.994748	no
gene:SpnNT_00706	thiM_2	Chromosome:755671-757624	ΔORF2	110.58+peptide	OK	32.8768	39.6022	0.268512	0.35488	0.5379	0.959663	no
gene:SpnNT_00706	thiM_2	Chromosome:755671-757624	110.58	ΔORF2+peptide	OK	37.7214	34.3629	-0.13453	-0.177948	0.7536	0.994748	no
gene:SpnNT_00706	thiM_2	Chromosome:755671-757624	ΔORF2	ΔORF2+peptide	OK	32.8768	34.3629	0.0637849	0.0844364	0.88125	0.994748	no
gene:SpnNT_00706	thiM_2	Chromosome:755671-757624	110.58+peptide	ΔORF2+peptide	OK	39.6022	34.3629	-0.204728	-0.269278	0.64155	0.980887	no
gene:SpnNT_00707	thiE_2	Chromosome:755671-757624	110.58	ΔORF2	OK	58.8055	48.1364	-0.288822	-0.388168	0.50125	0.944758	no
gene:SpnNT_00707	thiE_2	Chromosome:755671-757624	110.58	110.58+peptide	OK	58.8055	72.084	0.293728	0.415288	0.47005	0.927937	no
gene:SpnNT_00707	thiE_2	Chromosome:755671-757624	ΔORF2	110.58+peptide	OK	48.1364	72.084	0.58255	0.82879	0.153	0.5961	no
gene:SpnNT_00707	thiE_2	Chromosome:755671-757624	110.58	ΔORF2+peptide	OK	58.8055	58.5794	-0.00555758	-0.0077327	0.9899	0.997703	no
gene:SpnNT_00707	thiE_2	Chromosome:755671-757624	ΔORF2	ΔORF2+peptide	OK	48.1364	58.5794	0.283265	0.396514	0.49465	0.942197	no
gene:SpnNT_00707	thiE_2	Chromosome:755671-757624	110.58+peptide	ΔORF2+peptide	OK	72.084	58.5794	-0.299285	-0.442733	0.45075	0.921012	no
gene:SpnNT_00708	thiD	Chromosome:757832-758624	110.58	ΔORF2	OK	67.4517	75.3499	0.159751	0.335947	0.5617	0.968621	no
gene:SpnNT_00708	thiD	Chromosome:757832-758624	110.58	110.58+peptide	OK	67.4517	80.4471	0.254186	0.533731	0.35085	0.854885	no
gene:SpnNT_00708	thiD	Chromosome:757832-758624	ΔORF2	110.58+peptide	OK	75.3499	80.4471	0.0944358	0.199784	0.7289	0.994748	no
gene:SpnNT_00708	thiD	Chromosome:757832-758624	110.58	ΔORF2+peptide	OK	67.4517	74.8093	0.149364	0.312042	0.5927	0.975468	no
gene:SpnNT_00708	thiD	Chromosome:757832-758624	ΔORF2	ΔORF2+peptide	OK	75.3499	74.8093	-0.0103867	-0.0218608	0.97075	0.99536	no
gene:SpnNT_00708	thiD	Chromosome:757832-758624	110.58+peptide	ΔORF2+peptide	OK	80.4471	74.8093	-0.104822	-0.220285	0.69945	0.990367	no
gene:SpnNT_00709	copY	Chromosome:758989-759385	110.58	ΔORF2	OK	18.1047	13.9612	-0.374941	-0.443086	0.41995	0.904223	no
gene:SpnNT_00709	copY	Chromosome:758989-759385	110.58	110.58+peptide	OK	18.1047	19.7119	0.122701	0.136715	0.80305	0.994748	no
gene:SpnNT_00709	copY	Chromosome:758989-759385	ΔORF2	110.58+peptide	OK	13.9612	19.7119	0.497641	0.584375	0.2817	0.784843	no
gene:SpnNT_00709	copY	Chromosome:758989-759385	110.58	ΔORF2+peptide	OK	18.1047	11.0875	-0.707431	-0.765645	0.16285	0.61656	no
gene:SpnNT_00709	copY	Chromosome:758989-759385	ΔORF2	ΔORF2+peptide	OK	13.9612	11.0875	-0.332491	-0.378071	0.48765	0.938811	no
gene:SpnNT_00709	copY	Chromosome:758989-759385	110.58+peptide	ΔORF2+peptide	OK	19.7119	11.0875	-0.830132	-0.893679	0.10245	0.485149	no
gene:SpnNT_00710	NA	Chromosome:759395-759767	110.58	ΔORF2	OK	27.5622	16.6173	-0.730006	-0.878666	0.111	0.507329	no
gene:SpnNT_00710	NA	Chromosome:759395-759767	110.58	110.58+peptide	OK	27.5622	25.6919	-0.101375	-0.116465	0.8302	0.994748	no
gene:SpnNT_00710	NA	Chromosome:759395-759767	ΔORF2	110.58+peptide	OK	16.6173	25.6919	0.628631	0.720199	0.1911	0.670844	no
gene:SpnNT_00710	NA	Chromosome:759395-759767	110.58	ΔORF2+peptide	OK	27.5622	15.9617	-0.788073	-0.885011	0.1065	0.496269	no
gene:SpnNT_00710	NA	Chromosome:759395-759767	ΔORF2	ΔORF2+peptide	OK	16.6173	15.9617	-0.0580674	-0.0650368	0.9045	0.994748	no
gene:SpnNT_00710	NA	Chromosome:759395-759767	110.58+peptide	ΔORF2+peptide	OK	25.6919	15.9617	-0.686698	-0.738529	0.17665	0.641395	no
gene:SpnNT_00711	pacS	Chromosome:759776-762020	110.58	ΔORF2	OK	30.9194	21.4915	-0.524746	-0.847838	0.1442	0.579207	no
gene:SpnNT_00711	pacS	Chromosome:759776-762020	110.58	110.58+peptide	OK	30.9194	28.7971	-0.102585	-0.160717	0.7761	0.994748	no
gene:SpnNT_00711	pacS	Chromosome:759776-762020	ΔORF2	110.58+peptide	OK	21.4915	28.7971	0.422161	0.676895	0.2318	0.724662	no
gene:SpnNT_00711	pacS	Chromosome:759776-762020	110.58	ΔORF2+peptide	OK	30.9194	24.0176	-0.364418	-0.585502	0.30385	0.809208	no
gene:SpnNT_00711	pacS	Chromosome:759776-762020	ΔORF2	ΔORF2+peptide	OK	21.4915	24.0176	0.160328	0.263958	0.64095	0.980887	no
gene:SpnNT_00711	pacS	Chromosome:759776-762020	110.58+peptide	ΔORF2+peptide	OK	28.7971	24.0176	-0.261833	-0.417512	0.455	0.921244	no
gene:SpnNT_00712	pox5	Chromosome:762226-764002	110.58	ΔORF2	OK	501.471	581.371	0.213292	0.466857	0.4092	0.897533	no
gene:SpnNT_00712	pox5	Chromosome:762226-764002	110.58	110.58+peptide	OK	501.471	1377.08	1.45738	3.07853	5.00E-05	0.0013612	yes
gene:SpnNT_00712	pox5	Chromosome:762226-764002	ΔORF2	110.58+peptide	OK	581.371	1377.08	1.24409	2.60877	5.00E-05	0.0013612	yes
gene:SpnNT_00712	pox5	Chromosome:762226-764002	110.58	ΔORF2+peptide	OK	501.471	1643.76	1.71276	3.60946	5.00E-05	0.0013612	yes
gene:SpnNT_00712	pox5	Chromosome:762226-764002	ΔORF2	ΔORF2+peptide	OK	581.371	1643.76	1.49947	3.13698	5.00E-05	0.0013612	yes
gene:SpnNT_00712	pox5	Chromosome:762226-764002	110.58+peptide	ΔORF2+peptide	OK	1377.08	1643.76	0.255386	0.517159	0.3545	0.856991	no
gene:SpnNT_00713	NA	Chromosome:764112-764460	110.58	ΔORF2	OK	463.174	702.588	0.601126	1.25917	0.0253	0.198438	no
gene:SpnNT_00713	NA	Chromosome:764112-764460	110.58	110.58+peptide	OK	463.174	1456.29	1.65267	3.46962	5.00E-05	0.0013612	yes

gene:SpnNT_00713	NA	Chromosome:764112-764460	ΔORF2	110.58+peptide	OK	702.588	1456.29	1.05154	2.19892	0.00015	0.00355289	yes
gene:SpnNT_00713	NA	Chromosome:764112-764460	110.58	ΔORF2+peptide	OK	463.174	1720.02	1.8928	4.05622	5.00E-05	0.0013612	yes
gene:SpnNT_00713	NA	Chromosome:764112-764460	ΔORF2	ΔORF2+peptide	OK	702.588	1720.02	1.29167	2.75667	5.00E-05	0.0013612	yes
gene:SpnNT_00713	NA	Chromosome:764112-764460	110.58+peptide	ΔORF2+peptide	OK	1456.29	1720.02	0.24013	0.51368	0.3508	0.854885	no
gene:SpnNT_00714	NA	Chromosome:764576-765086	110.58	ΔORF2	OK	12.2382	11.78	-0.0550476	-0.0830726	0.8872	0.994748	no
gene:SpnNT_00714	NA	Chromosome:764576-765086	110.58	110.58+peptide	OK	12.2382	15.8275	0.371047	0.571286	0.32795	0.833593	no
gene:SpnNT_00714	NA	Chromosome:764576-765086	ΔORF2	110.58+peptide	OK	11.78	15.8275	0.426095	0.656567	0.2477	0.74404	no
gene:SpnNT_00714	NA	Chromosome:764576-765086	110.58	ΔORF2+peptide	OK	12.2382	19.2437	0.652998	1.0112	0.08785	0.446463	no
gene:SpnNT_00714	NA	Chromosome:764576-765086	ΔORF2	ΔORF2+peptide	OK	11.78	19.2437	0.708046	1.09734	0.0564	0.338245	no
gene:SpnNT_00714	NA	Chromosome:764576-765086	110.58+peptide	ΔORF2+peptide	OK	15.8275	19.2437	0.281951	0.446317	0.44045	0.916647	no
gene:SpnNT_00715	NA	Chromosome:765117-765384	110.58	ΔORF2	OK	3.0093	4.40816	0.550749	0.406218	0.4574	0.921722	no
gene:SpnNT_00715	NA	Chromosome:765117-765384	110.58	110.58+peptide	OK	3.0093	3.35314	0.156087	0.108356	0.83775	0.994748	no
gene:SpnNT_00715	NA	Chromosome:765117-765384	ΔORF2	110.58+peptide	OK	4.40816	3.35314	-0.394662	-0.30632	0.59285	0.975539	no
gene:SpnNT_00715	NA	Chromosome:765117-765384	110.58	ΔORF2+peptide	OK	3.0093	2.2418	-0.424772	-0.341697	0.6128	0.979616	no
gene:SpnNT_00715	NA	Chromosome:765117-765384	ΔORF2	ΔORF2+peptide	OK	4.40816	2.2418	-0.975521	-0.917585	0.2808	0.784388	no
gene:SpnNT_00715	NA	Chromosome:765117-765384	110.58+peptide	ΔORF2+peptide	OK	3.35314	2.2418	-0.580859	-0.49678	0.5348	0.958867	no
gene:SpnNT_00716	NA	Chromosome:765748-766046	110.58	ΔORF2	NOTEST	0	0	0	0	1	1	no
gene:SpnNT_00716	NA	Chromosome:765748-766046	110.58	110.58+peptide	NOTEST	0	0	0	0	1	1	no
gene:SpnNT_00716	NA	Chromosome:765748-766046	ΔORF2	110.58+peptide	NOTEST	0	0	0	0	1	1	no
gene:SpnNT_00716	NA	Chromosome:765748-766046	110.58	ΔORF2+peptide	NOTEST	0	0	0	0	1	1	no
gene:SpnNT_00716	NA	Chromosome:765748-766046	ΔORF2	ΔORF2+peptide	NOTEST	0	0	0	0	1	1	no
gene:SpnNT_00716	NA	Chromosome:765748-766046	110.58+peptide	ΔORF2+peptide	NOTEST	0	0	0	0	1	1	no
gene:SpnNT_00717	bglH_2	Chromosome:765748-766046	110.58	ΔORF2	OK	14.0623	13.4999	-0.0588873	-0.0557725	0.9328	0.994855	no
gene:SpnNT_00717	bglH_2	Chromosome:765748-766046	110.58	110.58+peptide	OK	14.0623	20.2847	0.528557	0.517081	0.3491	0.853357	no
gene:SpnNT_00717	bglH_2	Chromosome:765748-766046	ΔORF2	110.58+peptide	OK	13.4999	20.2847	0.587445	0.575491	0.29345	0.797897	no
gene:SpnNT_00717	bglH_2	Chromosome:765748-766046	110.58	ΔORF2+peptide	OK	14.0623	42.7866	1.60532	1.72684	0.0055	0.064496	no
gene:SpnNT_00717	bglH_2	Chromosome:765748-766046	ΔORF2	ΔORF2+peptide	OK	13.4999	42.7866	1.66421	1.79321	0.00435	0.0540026	no
gene:SpnNT_00717	bglH_2	Chromosome:765748-766046	110.58+peptide	ΔORF2+peptide	OK	20.2847	42.7866	1.07677	1.21041	0.0373	0.258558	no
gene:SpnNT_00718	gmuF	Chromosome:766166-767111	110.58	ΔORF2	OK	84.1906	83.2706	-0.0158509	-0.0345871	0.95445	0.994855	no
gene:SpnNT_00718	gmuF	Chromosome:766166-767111	110.58	110.58+peptide	OK	84.1906	63.3774	-0.409691	-0.88014	0.119	0.522771	no
gene:SpnNT_00718	gmuF	Chromosome:766166-767111	ΔORF2	110.58+peptide	OK	83.2706	63.3774	-0.39384	-0.850882	0.13695	0.563341	no
gene:SpnNT_00718	gmuF	Chromosome:766166-767111	110.58	ΔORF2+peptide	OK	84.1906	66.7827	-0.334183	-0.718535	0.2043	0.689513	no
gene:SpnNT_00718	gmuF	Chromosome:766166-767111	ΔORF2	ΔORF2+peptide	OK	83.2706	66.7827	-0.318332	-0.688339	0.22565	0.716568	no
gene:SpnNT_00718	gmuF	Chromosome:766166-767111	110.58+peptide	ΔORF2+peptide	OK	63.3774	66.7827	0.0755079	0.160793	0.77405	0.994748	no
gene:SpnNT_00719	NA	Chromosome:767151-768602	110.58	ΔORF2	OK	29.524	27.3265	-0.111589	-0.218546	0.6983	0.990209	no
gene:SpnNT_00719	NA	Chromosome:767151-768602	110.58	110.58+peptide	OK	29.524	28.8344	-0.0340981	-0.0660991	0.90405	0.994748	no
gene:SpnNT_00719	NA	Chromosome:767151-768602	ΔORF2	110.58+peptide	OK	27.3265	28.8344	0.0774909	0.150027	0.78555	0.994748	no
gene:SpnNT_00719	NA	Chromosome:767151-768602	110.58	ΔORF2+peptide	OK	29.524	22.6959	-0.379458	-0.734219	0.19655	0.677648	no
gene:SpnNT_00719	NA	Chromosome:767151-768602	ΔORF2	ΔORF2+peptide	OK	27.3265	22.6959	-0.267869	-0.517653	0.3588	0.859746	no
gene:SpnNT_00719	NA	Chromosome:767151-768602	110.58+peptide	ΔORF2+peptide	OK	28.8344	22.6959	-0.34536	-0.660767	0.24955	0.747113	no
gene:SpnNT_00720	NA	Chromosome:767151-768602	110.58	ΔORF2	OK	44.3003	36.9902	-0.260172	-0.127165	0.84155	0.994748	no
gene:SpnNT_00720	NA	Chromosome:767151-768602	110.58	110.58+peptide	OK	44.3003	33.9348	-0.384551	-0.185709	0.76975	0.994748	no
gene:SpnNT_00720	NA	Chromosome:767151-768602	ΔORF2	110.58+peptide	OK	36.9902	33.9348	-0.124379	-0.0566857	0.93045	0.994855	no
gene:SpnNT_00720	NA	Chromosome:767151-768602	110.58	ΔORF2+peptide	OK	44.3003	41.5555	-0.092275	-0.0482151	0.93985	0.994855	no
gene:SpnNT_00720	NA	Chromosome:767151-768602	ΔORF2	ΔORF2+peptide	OK	36.9902	41.5555	0.167897	0.0820298	0.9186	0.994748	no

gene:SpnNT_00720	NA	Chromosome:767151-768602	110.58+peptide	ΔORF2+peptide	OK	33.9348	41.5555	0.292276	0.141091	0.86365	0.994748	no
gene:SpnNT_00721	mta	Chromosome:768706-769456	110.58	ΔORF2	OK	36.038	36.2738	0.00940969	0.0180585	0.972	0.99536	no
gene:SpnNT_00721	mta	Chromosome:768706-769456	110.58	110.58+peptide	OK	36.038	28.1623	-0.355755	-0.671495	0.23805	0.732745	no
gene:SpnNT_00721	mta	Chromosome:768706-769456	ΔORF2	110.58+peptide	OK	36.2738	28.1623	-0.365164	-0.689089	0.2274	0.71915	no
gene:SpnNT_00721	mta	Chromosome:768706-769456	110.58	ΔORF2+peptide	OK	36.038	31.921	-0.175013	-0.33192	0.5582	0.968033	no
gene:SpnNT_00721	mta	Chromosome:768706-769456	ΔORF2	ΔORF2+peptide	OK	36.2738	31.921	-0.184422	-0.349681	0.5425	0.961518	no
gene:SpnNT_00721	mta	Chromosome:768706-769456	110.58+peptide	ΔORF2+peptide	OK	28.1623	31.921	0.180742	0.337188	0.5548	0.966389	no
gene:SpnNT_00722	NA	Chromosome:769595-770886	110.58	ΔORF2	OK	3477.66	7162.75	1.0424	1.85037	0.00185	0.0276489	yes
gene:SpnNT_00722	NA	Chromosome:769595-770886	110.58	110.58+peptide	OK	3477.66	4565.04	0.392511	0.788512	0.17405	0.634412	no
gene:SpnNT_00722	NA	Chromosome:769595-770886	ΔORF2	110.58+peptide	OK	7162.75	4565.04	-0.649886	-1.11767	0.04995	0.31361	no
gene:SpnNT_00722	NA	Chromosome:769595-770886	110.58	ΔORF2+peptide	OK	3477.66	6029.04	0.793812	1.55663	0.007	0.0774486	no
gene:SpnNT_00722	NA	Chromosome:769595-770886	ΔORF2	ΔORF2+peptide	OK	7162.75	6029.04	-0.248585	-0.419966	0.4506	0.920951	no
gene:SpnNT_00722	NA	Chromosome:769595-770886	110.58+peptide	ΔORF2+peptide	OK	4565.04	6029.04	0.401301	0.757308	0.1849	0.657701	no
gene:SpnNT_00723	NA	Chromosome:769595-770886	110.58	ΔORF2	OK	15.7243	15.7076	-0.00153319	-0.00011832	0.998	0.999412	no
gene:SpnNT_00723	NA	Chromosome:769595-770886	110.58	110.58+peptide	OK	15.7243	18.171	0.20864	0.0222997	0.8809	0.994748	no
gene:SpnNT_00723	NA	Chromosome:769595-770886	ΔORF2	110.58+peptide	OK	15.7076	18.171	0.210174	0.0158474	0.87755	0.994748	no
gene:SpnNT_00723	NA	Chromosome:769595-770886	110.58	ΔORF2+peptide	OK	15.7243	18.3762	0.224842	0.021416	0.86895	0.994748	no
gene:SpnNT_00723	NA	Chromosome:769595-770886	ΔORF2	ΔORF2+peptide	OK	15.7076	18.3762	0.226375	0.0160644	0.8638	0.994748	no
gene:SpnNT_00723	NA	Chromosome:769595-770886	110.58+peptide	ΔORF2+peptide	OK	18.171	18.3762	0.0162013	0.00149016	0.99	0.997703	no
gene:SpnNT_00724	mutX_1	Chromosome:770994-771450	110.58	ΔORF2	OK	57.2789	65.1949	0.186754	0.347489	0.54685	0.962122	no
gene:SpnNT_00724	mutX_1	Chromosome:770994-771450	110.58	110.58+peptide	OK	57.2789	58.2544	0.0243634	0.0452219	0.9369	0.994855	no
gene:SpnNT_00724	mutX_1	Chromosome:770994-771450	ΔORF2	110.58+peptide	OK	65.1949	58.2544	-0.16239	-0.303763	0.59715	0.97629	no
gene:SpnNT_00724	mutX_1	Chromosome:770994-771450	110.58	ΔORF2+peptide	OK	57.2789	53.1162	-0.108852	-0.199047	0.7302	0.994748	no
gene:SpnNT_00724	mutX_1	Chromosome:770994-771450	ΔORF2	ΔORF2+peptide	OK	65.1949	53.1162	-0.295606	-0.544622	0.34515	0.849092	no
gene:SpnNT_00724	mutX_1	Chromosome:770994-771450	110.58+peptide	ΔORF2+peptide	OK	58.2544	53.1162	-0.133216	-0.244848	0.67015	0.984845	no
gene:SpnNT_00725	NA	Chromosome:771469-772645	110.58	ΔORF2	OK	40.5205	44.1785	0.124691	0.261135	0.6441	0.980887	no
gene:SpnNT_00725	NA	Chromosome:771469-772645	110.58	110.58+peptide	OK	40.5205	35.8485	-0.17674	-0.361132	0.5248	0.955254	no
gene:SpnNT_00725	NA	Chromosome:771469-772645	ΔORF2	110.58+peptide	OK	44.1785	35.8485	-0.301432	-0.618544	0.2666	0.768019	no
gene:SpnNT_00725	NA	Chromosome:771469-772645	110.58	ΔORF2+peptide	OK	40.5205	45.9333	0.180889	0.379667	0.5045	0.945292	no
gene:SpnNT_00725	NA	Chromosome:771469-772645	ΔORF2	ΔORF2+peptide	OK	44.1785	45.9333	0.0561973	0.118484	0.8313	0.994748	no
gene:SpnNT_00725	NA	Chromosome:771469-772645	110.58+peptide	ΔORF2+peptide	OK	35.8485	45.9333	0.357629	0.735426	0.19485	0.676386	no
gene:SpnNT_00726	NA	Chromosome:772703-773549	110.58	ΔORF2	OK	160.473	168.318	0.0688646	0.155599	0.78555	0.994748	no
gene:SpnNT_00726	NA	Chromosome:772703-773549	110.58	110.58+peptide	OK	160.473	145.815	-0.138186	-0.310069	0.5896	0.974359	no
gene:SpnNT_00726	NA	Chromosome:772703-773549	ΔORF2	110.58+peptide	OK	168.318	145.815	-0.20705	-0.46327	0.4203	0.904235	no
gene:SpnNT_00726	NA	Chromosome:772703-773549	110.58	ΔORF2+peptide	OK	160.473	158.729	-0.0157577	-0.0355635	0.95435	0.994855	no
gene:SpnNT_00726	NA	Chromosome:772703-773549	ΔORF2	ΔORF2+peptide	OK	168.318	158.729	-0.0846223	-0.190435	0.74615	0.994748	no
gene:SpnNT_00726	NA	Chromosome:772703-773549	110.58+peptide	ΔORF2+peptide	OK	145.815	158.729	0.122428	0.273622	0.6372	0.980887	no
gene:SpnNT_00727	mtrR	Chromosome:773673-774231	110.58	ΔORF2	OK	16.6586	20.0865	0.269958	0.441188	0.4451	0.917756	no
gene:SpnNT_00727	mtrR	Chromosome:773673-774231	110.58	110.58+peptide	OK	16.6586	17.5207	0.0727895	0.115604	0.84205	0.994748	no
gene:SpnNT_00727	mtrR	Chromosome:773673-774231	ΔORF2	110.58+peptide	OK	20.0865	17.5207	-0.197168	-0.320395	0.5765	0.969538	no
gene:SpnNT_00727	mtrR	Chromosome:773673-774231	110.58	ΔORF2+peptide	OK	16.6586	19.0437	0.193047	0.309377	0.5954	0.975751	no
gene:SpnNT_00727	mtrR	Chromosome:773673-774231	ΔORF2	ΔORF2+peptide	OK	20.0865	19.0437	-0.0769113	-0.126168	0.8272	0.994748	no
gene:SpnNT_00727	mtrR	Chromosome:773673-774231	110.58+peptide	ΔORF2+peptide	OK	17.5207	19.0437	0.120257	0.19167	0.74405	0.994748	no
gene:SpnNT_00728	ribD_2	Chromosome:774249-774717	110.58	ΔORF2	OK	69.2561	69.992	0.0152478	0.0281451	0.959	0.994855	no
gene:SpnNT_00728	ribD_2	Chromosome:774249-774717	110.58	110.58+peptide	OK	69.2561	56.5473	-0.292485	-0.541926	0.3419	0.846014	no

gene:SpnNT_00728	ribD_2	Chromosome:774249-774717	ΔORF2	110.58+peptide	OK	69.992	56.5473	-0.307732	-0.571067	0.31795	0.824045	no
gene:SpnNT_00728	ribD_2	Chromosome:774249-774717	110.58	ΔORF2+peptide	OK	69.2561	56.2996	-0.298816	-0.549996	0.3248	0.830123	no
gene:SpnNT_00728	ribD_2	Chromosome:774249-774717	ΔORF2	ΔORF2+peptide	OK	69.992	56.2996	-0.314064	-0.57895	0.30295	0.809137	no
gene:SpnNT_00728	ribD_2	Chromosome:774249-774717	110.58+peptide	ΔORF2+peptide	OK	56.5473	56.2996	-0.00633152	-0.0117156	0.9821	0.996246	no
gene:SpnNT_00729	upp	Chromosome:774805-775435	110.58	ΔORF2	OK	319.244	350.099	0.133104	0.301692	0.58295	0.971238	no
gene:SpnNT_00729	upp	Chromosome:774805-775435	110.58	110.58+peptide	OK	319.244	325.067	0.0260784	0.0591556	0.91485	0.994748	no
gene:SpnNT_00729	upp	Chromosome:774805-775435	ΔORF2	110.58+peptide	OK	350.099	325.067	-0.107026	-0.245671	0.6584	0.982966	no
gene:SpnNT_00729	upp	Chromosome:774805-775435	110.58	ΔORF2+peptide	OK	319.244	352.088	0.141278	0.320454	0.5678	0.968621	no
gene:SpnNT_00729	upp	Chromosome:774805-775435	ΔORF2	ΔORF2+peptide	OK	350.099	352.088	0.00817335	0.0187604	0.97335	0.99536	no
gene:SpnNT_00729	upp	Chromosome:774805-775435	110.58+peptide	ΔORF2+peptide	OK	325.067	352.088	0.115199	0.264633	0.63805	0.980887	no
gene:SpnNT_00730	clpP	Chromosome:775608-776199	110.58	ΔORF2	OK	673.673	751.911	0.158514	0.360233	0.52535	0.955327	no
gene:SpnNT_00730	clpP	Chromosome:775608-776199	110.58	110.58+peptide	OK	673.673	667.905	-0.012405	-0.0280829	0.9601	0.994855	no
gene:SpnNT_00730	clpP	Chromosome:775608-776199	ΔORF2	110.58+peptide	OK	751.911	667.905	-0.170919	-0.38793	0.49775	0.944017	no
gene:SpnNT_00730	clpP	Chromosome:775608-776199	110.58	ΔORF2+peptide	OK	673.673	648.853	-0.0541565	-0.122946	0.8289	0.994748	no
gene:SpnNT_00730	clpP	Chromosome:775608-776199	ΔORF2	ΔORF2+peptide	OK	751.911	648.853	-0.21267	-0.484054	0.39795	0.891861	no
gene:SpnNT_00730	clpP	Chromosome:775608-776199	110.58+peptide	ΔORF2+peptide	OK	667.905	648.853	-0.0417515	-0.0946637	0.86775	0.994748	no
gene:SpnNT_00731	NA	Chromosome:776277-776526	110.58	ΔORF2	OK	261.464	381.344	0.54448	1.02301	0.0785	0.416361	no
gene:SpnNT_00731	NA	Chromosome:776277-776526	110.58	110.58+peptide	OK	261.464	213.159	-0.294686	-0.535442	0.35185	0.854885	no
gene:SpnNT_00731	NA	Chromosome:776277-776526	ΔORF2	110.58+peptide	OK	381.344	213.159	-0.839166	-1.52886	0.0094	0.0963647	no
gene:SpnNT_00731	NA	Chromosome:776277-776526	110.58	ΔORF2+peptide	OK	261.464	228.799	-0.192533	-0.35183	0.545	0.961568	no
gene:SpnNT_00731	NA	Chromosome:776277-776526	ΔORF2	ΔORF2+peptide	OK	381.344	228.799	-0.737013	-1.35046	0.0189	0.159592	no
gene:SpnNT_00731	NA	Chromosome:776277-776526	110.58+peptide	ΔORF2+peptide	OK	213.159	228.799	0.102153	0.181303	0.74725	0.994748	no
gene:SpnNT_00732	braC	Chromosome:776627-777788	110.58	ΔORF2	OK	152.651	145.867	-0.0655826	-0.149263	0.7912	0.994748	no
gene:SpnNT_00732	braC	Chromosome:776627-777788	110.58	110.58+peptide	OK	152.651	394.455	1.36962	3.03792	5.00E-05	0.0013612	yes
gene:SpnNT_00732	braC	Chromosome:776627-777788	ΔORF2	110.58+peptide	OK	145.867	394.455	1.43521	3.16614	5.00E-05	0.0013612	yes
gene:SpnNT_00732	braC	Chromosome:776627-777788	110.58	ΔORF2+peptide	OK	152.651	400.962	1.39323	3.17432	5.00E-05	0.0013612	yes
gene:SpnNT_00732	braC	Chromosome:776627-777788	ΔORF2	ΔORF2+peptide	OK	145.867	400.962	1.45881	3.30474	5.00E-05	0.0013612	yes
gene:SpnNT_00732	braC	Chromosome:776627-777788	110.58+peptide	ΔORF2+peptide	OK	394.455	400.962	0.0236042	0.0521245	0.92495	0.994748	no
gene:SpnNT_00733	livH_1	Chromosome:778055-778925	110.58	ΔORF2	OK	47.0072	40.6202	-0.210684	-0.42251	0.4572	0.921705	no
gene:SpnNT_00733	livH_1	Chromosome:778055-778925	110.58	110.58+peptide	OK	47.0072	155.177	1.72296	3.56881	5.00E-05	0.0013612	yes
gene:SpnNT_00733	livH_1	Chromosome:778055-778925	ΔORF2	110.58+peptide	OK	40.6202	155.177	1.93364	3.96196	5.00E-05	0.0013612	yes
gene:SpnNT_00733	livH_1	Chromosome:778055-778925	110.58	ΔORF2+peptide	OK	47.0072	190.524	2.01902	4.29853	5.00E-05	0.0013612	yes
gene:SpnNT_00733	livH_1	Chromosome:778055-778925	ΔORF2	ΔORF2+peptide	OK	40.6202	190.524	2.2297	4.69299	5.00E-05	0.0013612	yes
gene:SpnNT_00733	livH_1	Chromosome:778055-778925	110.58+peptide	ΔORF2+peptide	OK	155.177	190.524	0.296059	0.645803	0.25945	0.759841	no
gene:SpnNT_00734	livH_2	Chromosome:778928-781359	110.58	ΔORF2	OK	50.1429	44.2395	-0.180708	-0.231836	0.68795	0.98828	no
gene:SpnNT_00734	livH_2	Chromosome:778928-781359	110.58	110.58+peptide	OK	50.1429	171.942	1.77781	2.25877	0.00015	0.00355289	yes
gene:SpnNT_00734	livH_2	Chromosome:778928-781359	ΔORF2	110.58+peptide	OK	44.2395	171.942	1.95851	2.53219	5.00E-05	0.0013612	yes
gene:SpnNT_00734	livH_2	Chromosome:778928-781359	110.58	ΔORF2+peptide	OK	50.1429	191.035	1.92972	2.43911	5.00E-05	0.0013612	yes
gene:SpnNT_00734	livH_2	Chromosome:778928-781359	ΔORF2	ΔORF2+peptide	OK	44.2395	191.035	2.11043	2.71399	5.00E-05	0.0013612	yes
gene:SpnNT_00734	livH_2	Chromosome:778928-781359	110.58+peptide	ΔORF2+peptide	OK	171.942	191.035	0.151913	0.193464	0.7444	0.994748	no
gene:SpnNT_00735	lptB	Chromosome:778928-781359	110.58	ΔORF2	OK	63.984	54.0367	-0.243771	-0.303066	0.5983	0.97629	no
gene:SpnNT_00735	lptB	Chromosome:778928-781359	110.58	110.58+peptide	OK	63.984	193.34	1.59536	1.85586	0.00245	0.0342374	yes
gene:SpnNT_00735	lptB	Chromosome:778928-781359	ΔORF2	110.58+peptide	OK	54.0367	193.34	1.83913	2.15104	0.00045	0.00885292	yes
gene:SpnNT_00735	lptB	Chromosome:778928-781359	110.58	ΔORF2+peptide	OK	63.984	242.483	1.9221	2.38471	5.00E-05	0.0013612	yes
gene:SpnNT_00735	lptB	Chromosome:778928-781359	ΔORF2	ΔORF2+peptide	OK	54.0367	242.483	2.16587	2.70374	5.00E-05	0.0013612	yes

gene:SpnNT_00735	lptB	Chromosome:778928-781359	110.58+peptide	ΔORF2+peptide	OK	193.34	242.483	0.326745	0.381462	0.51125	0.949557	no
gene:SpnNT_00736	livF	Chromosome:778928-781359	110.58	ΔORF2	OK	80.994	70.9691	-0.190625	-0.25419	0.6607	0.982966	no
gene:SpnNT_00736	livF	Chromosome:778928-781359	110.58	110.58+peptide	OK	80.994	263.852	1.70384	2.22266	5.00E-05	0.0013612	yes
gene:SpnNT_00736	livF	Chromosome:778928-781359	ΔORF2	110.58+peptide	OK	70.9691	263.852	1.89446	2.48035	1.00E-04	0.0025332	yes
gene:SpnNT_00736	livF	Chromosome:778928-781359	110.58	ΔORF2+peptide	OK	80.994	305.866	1.91701	2.48439	5.00E-05	0.0013612	yes
gene:SpnNT_00736	livF	Chromosome:778928-781359	ΔORF2	ΔORF2+peptide	OK	70.9691	305.866	2.10764	2.74127	5.00E-05	0.0013612	yes
gene:SpnNT_00736	livF	Chromosome:778928-781359	110.58+peptide	ΔORF2+peptide	OK	263.852	305.866	0.213173	0.271523	0.63145	0.980887	no
gene:SpnNT_00737	NA	Chromosome:781666-782323	110.58	ΔORF2	OK	49.8849	51.514	0.0463605	0.0907232	0.87465	0.994748	no
gene:SpnNT_00737	NA	Chromosome:781666-782323	110.58	110.58+peptide	OK	49.8849	107.722	1.11064	2.26881	3.00E-04	0.00631878	yes
gene:SpnNT_00737	NA	Chromosome:781666-782323	ΔORF2	110.58+peptide	OK	51.514	107.722	1.06428	2.17607	3.00E-04	0.00631878	yes
gene:SpnNT_00737	NA	Chromosome:781666-782323	110.58	ΔORF2+peptide	OK	49.8849	120.381	1.27093	2.59358	5.00E-05	0.0013612	yes
gene:SpnNT_00737	NA	Chromosome:781666-782323	ΔORF2	ΔORF2+peptide	OK	51.514	120.381	1.22457	2.50122	5.00E-05	0.0013612	yes
gene:SpnNT_00737	NA	Chromosome:781666-782323	110.58+peptide	ΔORF2+peptide	OK	107.722	120.381	0.160286	0.343139	0.55235	0.964719	no
gene:SpnNT_00738	prfB	Chromosome:782548-783526	110.58	ΔORF2	OK	124.724	121.126	-0.0422307	-0.0948653	0.8669	0.994748	no
gene:SpnNT_00738	prfB	Chromosome:782548-783526	110.58	110.58+peptide	OK	124.724	169.007	0.438346	0.995824	0.0772	0.411629	no
gene:SpnNT_00738	prfB	Chromosome:782548-783526	ΔORF2	110.58+peptide	OK	121.126	169.007	0.480576	1.08726	0.0552	0.334566	no
gene:SpnNT_00738	prfB	Chromosome:782548-783526	110.58	ΔORF2+peptide	OK	124.724	173.829	0.478934	1.09069	0.05605	0.337535	no
gene:SpnNT_00738	prfB	Chromosome:782548-783526	ΔORF2	ΔORF2+peptide	OK	121.126	173.829	0.521165	1.18195	0.0388	0.265727	no
gene:SpnNT_00738	prfB	Chromosome:782548-783526	110.58+peptide	ΔORF2+peptide	OK	169.007	173.829	0.0405886	0.0931128	0.8683	0.994748	no
gene:SpnNT_00739	ftsE	Chromosome:783543-785155	110.58	ΔORF2	OK	127.716	133.181	0.0604526	0.0689388	0.8999	0.994748	no
gene:SpnNT_00739	ftsE	Chromosome:783543-785155	110.58	110.58+peptide	OK	127.716	143.064	0.163717	0.189533	0.7446	0.994748	no
gene:SpnNT_00739	ftsE	Chromosome:783543-785155	ΔORF2	110.58+peptide	OK	133.181	143.064	0.103265	0.109866	0.8472	0.994748	no
gene:SpnNT_00739	ftsE	Chromosome:783543-785155	110.58	ΔORF2+peptide	OK	127.716	198.997	0.639804	0.766173	0.18395	0.655387	no
gene:SpnNT_00739	ftsE	Chromosome:783543-785155	ΔORF2	ΔORF2+peptide	OK	133.181	198.997	0.579351	0.634151	0.264	0.764702	no
gene:SpnNT_00739	ftsE	Chromosome:783543-785155	110.58+peptide	ΔORF2+peptide	OK	143.064	198.997	0.476086	0.528391	0.36145	0.862354	no
gene:SpnNT_00740	ftsX	Chromosome:783543-785155	110.58	ΔORF2	OK	183.264	221.408	0.272783	0.510656	0.3843	0.881452	no
gene:SpnNT_00740	ftsX	Chromosome:783543-785155	110.58	110.58+peptide	OK	183.264	264.829	0.531136	1.00826	0.0805	0.423036	no
gene:SpnNT_00740	ftsX	Chromosome:783543-785155	ΔORF2	110.58+peptide	OK	221.408	264.829	0.258353	0.483895	0.40255	0.894197	no
gene:SpnNT_00740	ftsX	Chromosome:783543-785155	110.58	ΔORF2+peptide	OK	183.264	356.976	0.961905	1.8453	0.0019	0.0281715	yes
gene:SpnNT_00740	ftsX	Chromosome:783543-785155	ΔORF2	ΔORF2+peptide	OK	221.408	356.976	0.689122	1.304	0.0258	0.201277	no
gene:SpnNT_00740	ftsX	Chromosome:783543-785155	110.58+peptide	ΔORF2+peptide	OK	264.829	356.976	0.430769	0.82683	0.14765	0.585867	no
gene:SpnNT_00741	ptsG_1	Chromosome:785440-787621	110.58	ΔORF2	OK	126.397	119.809	-0.0772227	-0.176792	0.7538	0.994748	no
gene:SpnNT_00741	ptsG_1	Chromosome:785440-787621	110.58	110.58+peptide	OK	126.397	230.823	0.868826	1.98367	6.00E-04	0.0111203	yes
gene:SpnNT_00741	ptsG_1	Chromosome:785440-787621	ΔORF2	110.58+peptide	OK	119.809	230.823	0.946049	2.14841	0.00035	0.00717609	yes
gene:SpnNT_00741	ptsG_1	Chromosome:785440-787621	110.58	ΔORF2+peptide	OK	126.397	218.797	0.791634	1.79269	0.0012	0.0193683	yes
gene:SpnNT_00741	ptsG_1	Chromosome:785440-787621	ΔORF2	ΔORF2+peptide	OK	119.809	218.797	0.868856	1.9572	6.00E-04	0.0111203	yes
gene:SpnNT_00741	ptsG_1	Chromosome:785440-787621	110.58+peptide	ΔORF2+peptide	OK	230.823	218.797	-0.0771925	-0.173428	0.7565	0.994748	no
gene:SpnNT_00742	mapP	Chromosome:787672-788488	110.58	ΔORF2	OK	17.2089	15.4265	-0.157742	-0.276773	0.6198	0.980887	no
gene:SpnNT_00742	mapP	Chromosome:787672-788488	110.58	110.58+peptide	OK	17.2089	47.3627	1.46059	2.74554	5.00E-05	0.0013612	yes
gene:SpnNT_00742	mapP	Chromosome:787672-788488	ΔORF2	110.58+peptide	OK	15.4265	47.3627	1.61834	3.00519	5.00E-05	0.0013612	yes
gene:SpnNT_00742	mapP	Chromosome:787672-788488	110.58	ΔORF2+peptide	OK	17.2089	56.3811	1.71205	3.23524	5.00E-05	0.0013612	yes
gene:SpnNT_00742	mapP	Chromosome:787672-788488	ΔORF2	ΔORF2+peptide	OK	15.4265	56.3811	1.8698	3.49007	5.00E-05	0.0013612	yes
gene:SpnNT_00742	mapP	Chromosome:787672-788488	110.58+peptide	ΔORF2+peptide	OK	47.3627	56.3811	0.251459	0.507801	0.375	0.87259	no
gene:SpnNT_00743	cshB	Chromosome:788627-789971	110.58	ΔORF2	OK	120.584	138.326	0.198032	0.448773	0.43525	0.913816	no
gene:SpnNT_00743	cshB	Chromosome:788627-789971	110.58	110.58+peptide	OK	120.584	133.334	0.14501	0.33048	0.55995	0.968621	no

gene:SpnNT_00743	cshB	Chromosome:788627-789971	ΔORF2	110.58+peptide	OK	138.326	133.334	-0.0530214	-0.120923	0.8291	0.994748	no
gene:SpnNT_00743	cshB	Chromosome:788627-789971	110.58	ΔORF2+peptide	OK	120.584	149.246	0.307653	0.703245	0.21555	0.702371	no
gene:SpnNT_00743	cshB	Chromosome:788627-789971	ΔORF2	ΔORF2+peptide	OK	138.326	149.246	0.109622	0.250757	0.65655	0.982966	no
gene:SpnNT_00743	cshB	Chromosome:788627-789971	110.58+peptide	ΔORF2+peptide	OK	133.334	149.246	0.162643	0.374192	0.49915	0.944017	no
gene:SpnNT_00744	metK	Chromosome:790185-791376	110.58	ΔORF2	OK	124.99	127.576	0.0295499	0.0668036	0.9078	0.994748	no
gene:SpnNT_00744	metK	Chromosome:790185-791376	110.58	110.58+peptide	OK	124.99	100.982	-0.307711	-0.695267	0.2238	0.713991	no
gene:SpnNT_00744	metK	Chromosome:790185-791376	ΔORF2	110.58+peptide	OK	127.576	100.982	-0.337261	-0.763811	0.18965	0.667533	no
gene:SpnNT_00744	metK	Chromosome:790185-791376	110.58	ΔORF2+peptide	OK	124.99	103.296	-0.275031	-0.621486	0.2806	0.784246	no
gene:SpnNT_00744	metK	Chromosome:790185-791376	ΔORF2	ΔORF2+peptide	OK	127.576	103.296	-0.304581	-0.689865	0.23765	0.732717	no
gene:SpnNT_00744	metK	Chromosome:790185-791376	110.58+peptide	ΔORF2+peptide	OK	100.982	103.296	0.03268	0.0739787	0.8956	0.994748	no
gene:SpnNT_00745	pyrDA	Chromosome:791928-792864	110.58	ΔORF2	OK	198.675	218.3	0.135903	0.311215	0.57555	0.969538	no
gene:SpnNT_00745	pyrDA	Chromosome:791928-792864	110.58	110.58+peptide	OK	198.675	134.758	-0.560038	-1.26965	0.0258	0.201277	no
gene:SpnNT_00745	pyrDA	Chromosome:791928-792864	ΔORF2	110.58+peptide	OK	218.3	134.758	-0.695941	-1.57744	0.0058	0.0674115	no
gene:SpnNT_00745	pyrDA	Chromosome:791928-792864	110.58	ΔORF2+peptide	OK	198.675	158.187	-0.328785	-0.74867	0.19465	0.676386	no
gene:SpnNT_00745	pyrDA	Chromosome:791928-792864	ΔORF2	ΔORF2+peptide	OK	218.3	158.187	-0.464687	-1.05792	0.0667	0.37742	no
gene:SpnNT_00745	pyrDA	Chromosome:791928-792864	110.58+peptide	ΔORF2+peptide	OK	134.758	158.187	0.231253	0.521272	0.36815	0.865399	no
gene:SpnNT_00746	NA	Chromosome:792897-793935	110.58	ΔORF2	OK	264.131	302.561	0.195976	0.451578	0.4148	0.900591	no
gene:SpnNT_00746	NA	Chromosome:792897-793935	110.58	110.58+peptide	OK	264.131	204.879	-0.36648	-0.844391	0.1288	0.544145	no
gene:SpnNT_00746	NA	Chromosome:792897-793935	ΔORF2	110.58+peptide	OK	302.561	204.879	-0.562456	-1.29399	0.0215	0.177102	no
gene:SpnNT_00746	NA	Chromosome:792897-793935	110.58	ΔORF2+peptide	OK	264.131	217.164	-0.282465	-0.651913	0.24725	0.743921	no
gene:SpnNT_00746	NA	Chromosome:792897-793935	ΔORF2	ΔORF2+peptide	OK	302.561	217.164	-0.478441	-1.10255	0.0524	0.323878	no
gene:SpnNT_00746	NA	Chromosome:792897-793935	110.58+peptide	ΔORF2+peptide	OK	204.879	217.164	0.0840147	0.193592	0.73155	0.994748	no
gene:SpnNT_00747	sodA	Chromosome:794107-794713	110.58	ΔORF2	OK	782.562	980.597	0.325457	0.736107	0.195	0.676386	no
gene:SpnNT_00747	sodA	Chromosome:794107-794713	110.58	110.58+peptide	OK	782.562	1477.46	0.916841	2.04824	0.00045	0.00885292	yes
gene:SpnNT_00747	sodA	Chromosome:794107-794713	ΔORF2	110.58+peptide	OK	980.597	1477.46	0.591385	1.32662	0.02125	0.175262	no
gene:SpnNT_00747	sodA	Chromosome:794107-794713	110.58	ΔORF2+peptide	OK	782.562	1745.16	1.15709	2.57695	5.00E-05	0.0013612	yes
gene:SpnNT_00747	sodA	Chromosome:794107-794713	ΔORF2	ΔORF2+peptide	OK	980.597	1745.16	0.831629	1.85973	0.0015	0.0233488	yes
gene:SpnNT_00747	sodA	Chromosome:794107-794713	110.58+peptide	ΔORF2+peptide	OK	1477.46	1745.16	0.240245	0.5308	0.3533	0.856141	no
gene:SpnNT_00748	NA	Chromosome:794868-795399	110.58	ΔORF2	OK	82.1165	112.675	0.456423	0.92642	0.10045	0.480359	no
gene:SpnNT_00748	NA	Chromosome:794868-795399	110.58	110.58+peptide	OK	82.1165	57.7949	-0.506731	-0.986916	0.08555	0.439454	no
gene:SpnNT_00748	NA	Chromosome:794868-795399	ΔORF2	110.58+peptide	OK	112.675	57.7949	-0.963154	-1.90427	0.00135	0.021343	yes
gene:SpnNT_00748	NA	Chromosome:794868-795399	110.58	ΔORF2+peptide	OK	82.1165	64.1417	-0.356411	-0.695845	0.2112	0.697158	no
gene:SpnNT_00748	NA	Chromosome:794868-795399	ΔORF2	ΔORF2+peptide	OK	112.675	64.1417	-0.812833	-1.61112	0.00585	0.0677526	no
gene:SpnNT_00748	NA	Chromosome:794868-795399	110.58+peptide	ΔORF2+peptide	OK	57.7949	64.1417	0.15032	0.286421	0.6138	0.979616	no
gene:SpnNT_00749	rlmN	Chromosome:795419-796987	110.58	ΔORF2	OK	84.1634	82.5952	-0.0271352	-0.0545051	0.92655	0.994748	no
gene:SpnNT_00749	rlmN	Chromosome:795419-796987	110.58	110.58+peptide	OK	84.1634	58.8453	-0.516266	-1.02832	0.073	0.398405	no
gene:SpnNT_00749	rlmN	Chromosome:795419-796987	ΔORF2	110.58+peptide	OK	82.5952	58.8453	-0.489131	-0.95477	0.09555	0.468188	no
gene:SpnNT_00749	rlmN	Chromosome:795419-796987	110.58	ΔORF2+peptide	OK	84.1634	61.1389	-0.461103	-0.908658	0.11565	0.516049	no
gene:SpnNT_00749	rlmN	Chromosome:795419-796987	ΔORF2	ΔORF2+peptide	OK	82.5952	61.1389	-0.433968	-0.838424	0.14755	0.585648	no
gene:SpnNT_00749	rlmN	Chromosome:795419-796987	110.58+peptide	ΔORF2+peptide	OK	58.8453	61.1389	0.0551626	0.105747	0.8522	0.994748	no
gene:SpnNT_00750	NA	Chromosome:795419-796987	110.58	ΔORF2	OK	40.9545	44.6008	0.123046	0.0845016	0.88495	0.994748	no
gene:SpnNT_00750	NA	Chromosome:795419-796987	110.58	110.58+peptide	OK	40.9545	35.6795	-0.19893	-0.146178	0.8152	0.994748	no
gene:SpnNT_00750	NA	Chromosome:795419-796987	ΔORF2	110.58+peptide	OK	44.6008	35.6795	-0.321976	-0.228859	0.6907	0.98828	no
gene:SpnNT_00750	NA	Chromosome:795419-796987	110.58	ΔORF2+peptide	OK	40.9545	56.3494	0.460375	0.366822	0.54115	0.961494	no
gene:SpnNT_00750	NA	Chromosome:795419-796987	ΔORF2	ΔORF2+peptide	OK	44.6008	56.3494	0.337329	0.258535	0.64295	0.980887	no

gene:SpnNT_00750	NA	Chromosome:795419-796987	110.58+peptide	ΔORF2+peptide	OK	35.6795	56.3494	0.659305	0.550555	0.36715	0.864634	no
gene:SpnNT_00751	NA	Chromosome:796988-798530	110.58	ΔORF2	OK	84.6174	79.1071	-0.0971473	-0.215155	0.6973	0.990042	no
gene:SpnNT_00751	NA	Chromosome:796988-798530	110.58	110.58+peptide	OK	84.6174	67.3948	-0.328317	-0.73091	0.2048	0.689595	no
gene:SpnNT_00751	NA	Chromosome:796988-798530	ΔORF2	110.58+peptide	OK	79.1071	67.3948	-0.23117	-0.507384	0.37335	0.870487	no
gene:SpnNT_00751	NA	Chromosome:796988-798530	110.58	ΔORF2+peptide	OK	84.6174	71.6747	-0.23949	-0.53969	0.34285	0.846768	no
gene:SpnNT_00751	NA	Chromosome:796988-798530	ΔORF2	ΔORF2+peptide	OK	79.1071	71.6747	-0.142343	-0.316139	0.57915	0.969538	no
gene:SpnNT_00751	NA	Chromosome:796988-798530	110.58+peptide	ΔORF2+peptide	OK	67.3948	71.6747	0.0888274	0.198314	0.7276	0.994748	no
gene:SpnNT_00752	NA	Chromosome:798585-799389	110.58	ΔORF2	OK	160.584	184.939	0.203719	0.458649	0.41695	0.902395	no
gene:SpnNT_00752	NA	Chromosome:798585-799389	110.58	110.58+peptide	OK	160.584	97.8695	-0.714395	-1.58022	0.0063	0.0714508	no
gene:SpnNT_00752	NA	Chromosome:798585-799389	ΔORF2	110.58+peptide	OK	184.939	97.8695	-0.918114	-2.04167	4.00E-04	0.00811252	yes
gene:SpnNT_00752	NA	Chromosome:798585-799389	110.58	ΔORF2+peptide	OK	160.584	99.1721	-0.695319	-1.53609	0.00735	0.0805737	no
gene:SpnNT_00752	NA	Chromosome:798585-799389	ΔORF2	ΔORF2+peptide	OK	184.939	99.1721	-0.899039	-1.99671	0.00075	0.0133173	yes
gene:SpnNT_00752	NA	Chromosome:798585-799389	110.58+peptide	ΔORF2+peptide	OK	97.8695	99.1721	0.0190752	0.0416428	0.9408	0.994855	no
gene:SpnNT_00753	NA	Chromosome:799471-799693	110.58	ΔORF2	OK	3.0562	0.642334	-2.25034	-1.01718	0.2127	0.698345	no
gene:SpnNT_00753	NA	Chromosome:799471-799693	110.58	110.58+peptide	OK	3.0562	0.961373	-1.66857	-0.926355	0.31675	0.822722	no
gene:SpnNT_00753	NA	Chromosome:799471-799693	ΔORF2	110.58+peptide	NOTEST	0.642334	0.961373	0.581773	0	1	1	no
gene:SpnNT_00753	NA	Chromosome:799471-799693	110.58	ΔORF2+peptide	OK	3.0562	7.08123	1.21226	0.859839	0.2428	0.738169	no
gene:SpnNT_00753	NA	Chromosome:799471-799693	ΔORF2	ΔORF2+peptide	OK	0.642334	7.08123	3.4626	1.95478	0.1647	0.619431	no
gene:SpnNT_00753	NA	Chromosome:799471-799693	110.58+peptide	ΔORF2+peptide	OK	0.961373	7.08123	2.88083	2.36191	0.23075	0.724032	no
gene:SpnNT_00754	rpsP	Chromosome:800550-800823	110.58	ΔORF2	OK	1814.4	1606.29	-0.175765	-0.373063	0.4915	0.940662	no
gene:SpnNT_00754	rpsP	Chromosome:800550-800823	110.58	110.58+peptide	OK	1814.4	2809.35	0.630739	1.32872	0.01955	0.16434	no
gene:SpnNT_00754	rpsP	Chromosome:800550-800823	ΔORF2	110.58+peptide	OK	1606.29	2809.35	0.806504	1.77031	0.002	0.0294545	yes
gene:SpnNT_00754	rpsP	Chromosome:800550-800823	110.58	ΔORF2+peptide	OK	1814.4	2723.71	0.586076	1.21288	0.0314	0.229544	no
gene:SpnNT_00754	rpsP	Chromosome:800550-800823	ΔORF2	ΔORF2+peptide	OK	1606.29	2723.71	0.761841	1.64035	0.00335	0.0437836	yes
gene:SpnNT_00754	rpsP	Chromosome:800550-800823	110.58+peptide	ΔORF2+peptide	OK	2809.35	2723.71	-0.0446627	-0.0954243	0.86255	0.994748	no
gene:SpnNT_00755	NA	Chromosome:800842-801082	110.58	ΔORF2	OK	1504.52	1750.59	0.218532	0.474748	0.3997	0.892895	no
gene:SpnNT_00755	NA	Chromosome:800842-801082	110.58	110.58+peptide	OK	1504.52	3434.57	1.19082	2.6103	5.00E-05	0.0013612	yes
gene:SpnNT_00755	NA	Chromosome:800842-801082	ΔORF2	110.58+peptide	OK	1750.59	3434.57	0.972289	2.18875	2.00E-04	0.00450928	yes
gene:SpnNT_00755	NA	Chromosome:800842-801082	110.58	ΔORF2+peptide	OK	1504.52	3761.82	1.32212	2.94753	5.00E-05	0.0013612	yes
gene:SpnNT_00755	NA	Chromosome:800842-801082	ΔORF2	ΔORF2+peptide	OK	1750.59	3761.82	1.10359	2.52906	5.00E-05	0.0013612	yes
gene:SpnNT_00755	NA	Chromosome:800842-801082	110.58+peptide	ΔORF2+peptide	OK	3434.57	3761.82	0.131302	0.303924	0.5922	0.975468	no
gene:SpnNT_00756	NA	Chromosome:801658-802432	110.58	ΔORF2	OK	106.459	168.728	0.664408	1.4186	0.0131	0.121914	no
gene:SpnNT_00756	NA	Chromosome:801658-802432	110.58	110.58+peptide	OK	106.459	115.954	0.123262	0.267839	0.64565	0.980887	no
gene:SpnNT_00756	NA	Chromosome:801658-802432	ΔORF2	110.58+peptide	OK	168.728	115.954	-0.541146	-1.14449	0.0464	0.298169	no
gene:SpnNT_00756	NA	Chromosome:801658-802432	110.58	ΔORF2+peptide	OK	106.459	143.502	0.430779	0.951918	0.09635	0.470071	no
gene:SpnNT_00756	NA	Chromosome:801658-802432	ΔORF2	ΔORF2+peptide	OK	168.728	143.502	-0.233629	-0.502035	0.37685	0.875032	no
gene:SpnNT_00756	NA	Chromosome:801658-802432	110.58+peptide	ΔORF2+peptide	OK	115.954	143.502	0.307517	0.67266	0.2374	0.73229	no
gene:SpnNT_00757	rimM	Chromosome:802477-803705	110.58	ΔORF2	OK	134.502	138.67	0.0440227	0.0712555	0.89985	0.994748	no
gene:SpnNT_00757	rimM	Chromosome:802477-803705	110.58	110.58+peptide	OK	134.502	136.87	0.025173	0.040753	0.9397	0.994855	no
gene:SpnNT_00757	rimM	Chromosome:802477-803705	ΔORF2	110.58+peptide	OK	138.67	136.87	-0.0188497	-0.0303731	0.95755	0.994855	no
gene:SpnNT_00757	rimM	Chromosome:802477-803705	110.58	ΔORF2+peptide	OK	134.502	177.626	0.401215	0.640489	0.26055	0.760924	no
gene:SpnNT_00757	rimM	Chromosome:802477-803705	ΔORF2	ΔORF2+peptide	OK	138.67	177.626	0.357193	0.567613	0.3211	0.826982	no
gene:SpnNT_00757	rimM	Chromosome:802477-803705	110.58+peptide	ΔORF2+peptide	OK	136.87	177.626	0.376042	0.597677	0.2949	0.799296	no
gene:SpnNT_00758	trmD	Chromosome:802477-803705	110.58	ΔORF2	OK	73.3645	76.9037	0.0679695	0.104453	0.8552	0.994748	no
gene:SpnNT_00758	trmD	Chromosome:802477-803705	110.58	110.58+peptide	OK	73.3645	80.7095	0.137655	0.214005	0.7051	0.990422	no

gene:SpnNT_00758	trmD	Chromosome:802477-803705	ΔORF2	110.58+peptide	OK	76.9037	80.7095	0.069686	0.108512	0.8471	0.994748	no
gene:SpnNT_00758	trmD	Chromosome:802477-803705	110.58	ΔORF2+peptide	OK	73.3645	98.2416	0.421251	0.655372	0.25445	0.75336	no
gene:SpnNT_00758	trmD	Chromosome:802477-803705	ΔORF2	ΔORF2+peptide	OK	76.9037	98.2416	0.353282	0.550515	0.33495	0.840029	no
gene:SpnNT_00758	trmD	Chromosome:802477-803705	110.58+peptide	ΔORF2+peptide	OK	80.7095	98.2416	0.283596	0.447212	0.4345	0.913666	no
gene:SpnNT_00759	nrdD_2	Chromosome:803716-804055	110.58	ΔORF2	OK	444.105	447.086	0.00965337	0.0193627	0.974	0.99536	no
gene:SpnNT_00759	nrdD_2	Chromosome:803716-804055	110.58	110.58+peptide	OK	444.105	694.834	0.645769	1.25743	0.02855	0.215059	no
gene:SpnNT_00759	nrdD_2	Chromosome:803716-804055	ΔORF2	110.58+peptide	OK	447.086	694.834	0.636116	1.25548	0.02965	0.220185	no
gene:SpnNT_00759	nrdD_2	Chromosome:803716-804055	110.58	ΔORF2+peptide	OK	444.105	518.266	0.222793	0.451683	0.42165	0.905358	no
gene:SpnNT_00759	nrdD_2	Chromosome:803716-804055	ΔORF2	ΔORF2+peptide	OK	447.086	518.266	0.213139	0.438496	0.442	0.917378	no
gene:SpnNT_00759	nrdD_2	Chromosome:803716-804055	110.58+peptide	ΔORF2+peptide	OK	694.834	518.266	-0.422976	-0.843505	0.1281	0.542797	no
gene:SpnNT_00760	NA	Chromosome:804084-804405	110.58	ΔORF2	OK	258.611	325.606	0.332344	0.678979	0.23385	0.72774	no
gene:SpnNT_00760	NA	Chromosome:804084-804405	110.58	110.58+peptide	OK	258.611	217.677	-0.248597	-0.499983	0.37655	0.875032	no
gene:SpnNT_00760	NA	Chromosome:804084-804405	ΔORF2	110.58+peptide	OK	325.606	217.677	-0.580941	-1.16321	0.04695	0.300526	no
gene:SpnNT_00760	NA	Chromosome:804084-804405	110.58	ΔORF2+peptide	OK	258.611	241.962	-0.0960023	-0.195713	0.7241	0.994748	no
gene:SpnNT_00760	NA	Chromosome:804084-804405	ΔORF2	ΔORF2+peptide	OK	325.606	241.962	-0.428346	-0.869253	0.1351	0.559237	no
gene:SpnNT_00760	NA	Chromosome:804084-804405	110.58+peptide	ΔORF2+peptide	OK	217.677	241.962	0.152594	0.304909	0.5924	0.975468	no
gene:SpnNT_00761	NA	Chromosome:804502-804718	110.58	ΔORF2	OK	66.1732	92.8709	0.48898	0.716925	0.20005	0.683054	no
gene:SpnNT_00761	NA	Chromosome:804502-804718	110.58	110.58+peptide	OK	66.1732	69.0734	0.0618834	0.0876078	0.87335	0.994748	no
gene:SpnNT_00761	NA	Chromosome:804502-804718	ΔORF2	110.58+peptide	OK	92.8709	69.0734	-0.427096	-0.625852	0.26245	0.762762	no
gene:SpnNT_00761	NA	Chromosome:804502-804718	110.58	ΔORF2+peptide	OK	66.1732	74.6682	0.174247	0.248961	0.6624	0.982966	no
gene:SpnNT_00761	NA	Chromosome:804502-804718	ΔORF2	ΔORF2+peptide	OK	92.8709	74.6682	-0.314733	-0.465772	0.4172	0.902489	no
gene:SpnNT_00761	NA	Chromosome:804502-804718	110.58+peptide	ΔORF2+peptide	OK	69.0734	74.6682	0.112364	0.16046	0.7752	0.994748	no
gene:SpnNT_00762	bioY2	Chromosome:804923-805460	110.58	ΔORF2	OK	32.11	30.8452	-0.0579789	-0.0997178	0.8614	0.994748	no
gene:SpnNT_00762	bioY2	Chromosome:804923-805460	110.58	110.58+peptide	OK	32.11	27.1719	-0.240909	-0.411476	0.4858	0.937865	no
gene:SpnNT_00762	bioY2	Chromosome:804923-805460	ΔORF2	110.58+peptide	OK	30.8452	27.1719	-0.18293	-0.318953	0.5803	0.969538	no
gene:SpnNT_00762	bioY2	Chromosome:804923-805460	110.58	ΔORF2+peptide	OK	32.11	27.4929	-0.223965	-0.381725	0.51345	0.95097	no
gene:SpnNT_00762	bioY2	Chromosome:804923-805460	ΔORF2	ΔORF2+peptide	OK	30.8452	27.4929	-0.165986	-0.288771	0.619	0.980887	no
gene:SpnNT_00762	bioY2	Chromosome:804923-805460	110.58+peptide	ΔORF2+peptide	OK	27.1719	27.4929	0.0169446	0.0292706	0.9594	0.994855	no
gene:SpnNT_00763	gor	Chromosome:805656-807003	110.58	ΔORF2	OK	393.977	449.731	0.190951	0.422064	0.4571	0.921645	no
gene:SpnNT_00763	gor	Chromosome:805656-807003	110.58	110.58+peptide	OK	393.977	242.877	-0.697888	-1.54934	0.00645	0.0727121	no
gene:SpnNT_00763	gor	Chromosome:805656-807003	ΔORF2	110.58+peptide	OK	449.731	242.877	-0.88884	-1.98898	6.00E-04	0.0111203	yes
gene:SpnNT_00763	gor	Chromosome:805656-807003	110.58	ΔORF2+peptide	OK	393.977	257.34	-0.614438	-1.36926	0.01715	0.148152	no
gene:SpnNT_00763	gor	Chromosome:805656-807003	ΔORF2	ΔORF2+peptide	OK	449.731	257.34	-0.80539	-1.80919	0.00165	0.0253528	yes
gene:SpnNT_00763	gor	Chromosome:805656-807003	110.58+peptide	ΔORF2+peptide	OK	242.877	257.34	0.0834498	0.18831	0.73915	0.994748	no
gene:SpnNT_00764	yknX	Chromosome:807288-809173	110.58	ΔORF2	OK	75.1099	80.6529	0.102723	0.19551	0.72815	0.994748	no
gene:SpnNT_00764	yknX	Chromosome:807288-809173	110.58	110.58+peptide	OK	75.1099	59.3489	-0.339782	-0.638937	0.26445	0.7648	no
gene:SpnNT_00764	yknX	Chromosome:807288-809173	ΔORF2	110.58+peptide	OK	80.6529	59.3489	-0.442506	-0.837435	0.14695	0.584504	no
gene:SpnNT_00764	yknX	Chromosome:807288-809173	110.58	ΔORF2+peptide	OK	75.1099	66.5708	-0.174114	-0.327711	0.56725	0.968621	no
gene:SpnNT_00764	yknX	Chromosome:807288-809173	ΔORF2	ΔORF2+peptide	OK	80.6529	66.5708	-0.276838	-0.524399	0.36315	0.862958	no
gene:SpnNT_00764	yknX	Chromosome:807288-809173	110.58+peptide	ΔORF2+peptide	OK	59.3489	66.5708	0.165668	0.310087	0.59395	0.975539	no
gene:SpnNT_00765	macB_1	Chromosome:807288-809173	110.58	ΔORF2	OK	65.2162	65.7774	0.0123622	0.0145523	0.97925	0.995765	no
gene:SpnNT_00765	macB_1	Chromosome:807288-809173	110.58	110.58+peptide	OK	65.2162	49.2326	-0.405617	-0.476364	0.40355	0.89449	no
gene:SpnNT_00765	macB_1	Chromosome:807288-809173	ΔORF2	110.58+peptide	OK	65.7774	49.2326	-0.41798	-0.482398	0.4008	0.893669	no
gene:SpnNT_00765	macB_1	Chromosome:807288-809173	110.58	ΔORF2+peptide	OK	65.2162	60.393	-0.110848	-0.132751	0.8153	0.994748	no
gene:SpnNT_00765	macB_1	Chromosome:807288-809173	ΔORF2	ΔORF2+peptide	OK	65.7774	60.393	-0.12321	-0.144906	0.80035	0.994748	no

gene:SpnNT_00765	macB_1	Chromosome:807288-809173	110.58+peptide	ΔORF2+peptide	OK	49.2326	60.393	0.294769	0.345868	0.547	0.962122	no
gene:SpnNT_00766	yknZ	Chromosome:809174-810434	110.58	ΔORF2	OK	145.525	173.485	0.253538	0.575527	0.3174	0.823107	no
gene:SpnNT_00766	yknZ	Chromosome:809174-810434	110.58	110.58+peptide	OK	145.525	137.022	-0.0868657	-0.19714	0.73385	0.994748	no
gene:SpnNT_00766	yknZ	Chromosome:809174-810434	ΔORF2	110.58+peptide	OK	173.485	137.022	-0.340404	-0.773357	0.1799	0.646578	no
gene:SpnNT_00766	yknZ	Chromosome:809174-810434	110.58	ΔORF2+peptide	OK	145.525	150.444	0.047959	0.109506	0.847	0.994748	no
gene:SpnNT_00766	yknZ	Chromosome:809174-810434	ΔORF2	ΔORF2+peptide	OK	173.485	150.444	-0.205579	-0.469905	0.40925	0.897533	no
gene:SpnNT_00766	yknZ	Chromosome:809174-810434	110.58+peptide	ΔORF2+peptide	OK	137.022	150.444	0.134825	0.308108	0.58945	0.974359	no
gene:SpnNT_00767	metG	Chromosome:810557-812555	110.58	ΔORF2	OK	113.038	117.26	0.0529088	0.120845	0.82955	0.994748	no
gene:SpnNT_00767	metG	Chromosome:810557-812555	110.58	110.58+peptide	OK	113.038	120.09	0.0873065	0.199128	0.72125	0.994748	no
gene:SpnNT_00767	metG	Chromosome:810557-812555	ΔORF2	110.58+peptide	OK	117.26	120.09	0.0343976	0.0781312	0.8856	0.994748	no
gene:SpnNT_00767	metG	Chromosome:810557-812555	110.58	ΔORF2+peptide	OK	113.038	136.673	0.273927	0.628801	0.26405	0.764702	no
gene:SpnNT_00767	metG	Chromosome:810557-812555	ΔORF2	ΔORF2+peptide	OK	117.26	136.673	0.221018	0.505236	0.36565	0.864634	no
gene:SpnNT_00767	metG	Chromosome:810557-812555	110.58+peptide	ΔORF2+peptide	OK	120.09	136.673	0.186621	0.426001	0.4491	0.919933	no
gene:SpnNT_00768	NA	Chromosome:813201-813522	110.58	ΔORF2	OK	59.8188	60.0511	0.00559371	0.00944115	0.98685	0.997339	no
gene:SpnNT_00768	NA	Chromosome:813201-813522	110.58	110.58+peptide	OK	59.8188	72.8613	0.284556	0.485071	0.40495	0.894933	no
gene:SpnNT_00768	NA	Chromosome:813201-813522	ΔORF2	110.58+peptide	OK	60.0511	72.8613	0.278962	0.472233	0.4085	0.897087	no
gene:SpnNT_00768	NA	Chromosome:813201-813522	110.58	ΔORF2+peptide	OK	59.8188	64.7111	0.113416	0.184724	0.74215	0.994748	no
gene:SpnNT_00768	NA	Chromosome:813201-813522	ΔORF2	ΔORF2+peptide	OK	60.0511	64.7111	0.107822	0.174499	0.7536	0.994748	no
gene:SpnNT_00768	NA	Chromosome:813201-813522	110.58+peptide	ΔORF2+peptide	OK	72.8613	64.7111	-0.17114	-0.279512	0.62225	0.980887	no
gene:SpnNT_00769	NA	Chromosome:813525-814341	110.58	ΔORF2	OK	64.3461	74.6754	0.214781	0.446452	0.44175	0.917253	no
gene:SpnNT_00769	NA	Chromosome:813525-814341	110.58	110.58+peptide	OK	64.3461	89.2625	0.472202	0.985578	0.0888	0.448684	no
gene:SpnNT_00769	NA	Chromosome:813525-814341	ΔORF2	110.58+peptide	OK	74.6754	89.2625	0.257421	0.53573	0.352	0.854885	no
gene:SpnNT_00769	NA	Chromosome:813525-814341	110.58	ΔORF2+peptide	OK	64.3461	76.6855	0.253101	0.530983	0.35585	0.85769	no
gene:SpnNT_00769	NA	Chromosome:813525-814341	ΔORF2	ΔORF2+peptide	OK	74.6754	76.6855	0.03832	0.0801564	0.88925	0.994748	no
gene:SpnNT_00769	NA	Chromosome:813525-814341	110.58+peptide	ΔORF2+peptide	OK	89.2625	76.6855	-0.219101	-0.460219	0.41995	0.904223	no
gene:SpnNT_00770	ydhF	Chromosome:814415-815342	110.58	ΔORF2	OK	46.6206	74.1835	0.67013	1.31039	0.025	0.197145	no
gene:SpnNT_00770	ydhF	Chromosome:814415-815342	110.58	110.58+peptide	OK	46.6206	43.2526	-0.108181	-0.210572	0.70725	0.990441	no
gene:SpnNT_00770	ydhF	Chromosome:814415-815342	ΔORF2	110.58+peptide	OK	74.1835	43.2526	-0.778311	-1.47834	0.01205	0.115248	no
gene:SpnNT_00770	ydhF	Chromosome:814415-815342	110.58	ΔORF2+peptide	OK	46.6206	44.156	-0.0783591	-0.15821	0.77325	0.994748	no
gene:SpnNT_00770	ydhF	Chromosome:814415-815342	ΔORF2	ΔORF2+peptide	OK	74.1835	44.156	-0.748489	-1.47204	0.0119	0.114313	no
gene:SpnNT_00770	ydhF	Chromosome:814415-815342	110.58+peptide	ΔORF2+peptide	OK	43.2526	44.156	0.0298224	0.0583791	0.9197	0.994748	no
gene:SpnNT_00771	NA	Chromosome:815439-816367	110.58	ΔORF2	OK	122.93	122.722	-0.00244577	-0.0023247	0.9963	0.99927	no
gene:SpnNT_00771	NA	Chromosome:815439-816367	110.58	110.58+peptide	OK	122.93	117.457	-0.0657121	-0.0586476	0.9161	0.994748	no
gene:SpnNT_00771	NA	Chromosome:815439-816367	ΔORF2	110.58+peptide	OK	122.722	117.457	-0.0632664	-0.0570442	0.919	0.994748	no
gene:SpnNT_00771	NA	Chromosome:815439-816367	110.58	ΔORF2+peptide	OK	122.93	110.563	-0.152972	-0.144951	0.8061	0.994748	no
gene:SpnNT_00771	NA	Chromosome:815439-816367	ΔORF2	ΔORF2+peptide	OK	122.722	110.563	-0.150526	-0.144287	0.80765	0.994748	no
gene:SpnNT_00771	NA	Chromosome:815439-816367	110.58+peptide	ΔORF2+peptide	OK	117.457	110.563	-0.08726	-0.0784594	0.88965	0.994748	no
gene:SpnNT_00772	cpnA	Chromosome:815439-816367	110.58	ΔORF2	OK	33.4711	38.2992	0.194398	0.317417	0.5823	0.970895	no
gene:SpnNT_00772	cpnA	Chromosome:815439-816367	110.58	110.58+peptide	OK	33.4711	30.4093	-0.138406	-0.21441	0.71875	0.993515	no
gene:SpnNT_00772	cpnA	Chromosome:815439-816367	ΔORF2	110.58+peptide	OK	38.2992	30.4093	-0.332804	-0.532253	0.36705	0.864634	no
gene:SpnNT_00772	cpnA	Chromosome:815439-816367	110.58	ΔORF2+peptide	OK	33.4711	36.537	0.126439	0.207593	0.7209	0.994601	no
gene:SpnNT_00772	cpnA	Chromosome:815439-816367	ΔORF2	ΔORF2+peptide	OK	38.2992	36.537	-0.0679589	-0.11566	0.84345	0.994748	no
gene:SpnNT_00772	cpnA	Chromosome:815439-816367	110.58+peptide	ΔORF2+peptide	OK	30.4093	36.537	0.264845	0.42581	0.4658	0.926452	no
gene:SpnNT_00773	NA	Chromosome:816377-818140	110.58	ΔORF2	OK	42.566	49.7034	0.223643	0.240334	0.6822	0.986608	no
gene:SpnNT_00773	NA	Chromosome:816377-818140	110.58	110.58+peptide	OK	42.566	40.6751	-0.0655583	-0.0691278	0.90445	0.994748	no

gene:SpnNT_00773	NA	Chromosome:816377-818140	ΔORF2	110.58+peptide	OK	49.7034	40.6751	-0.289201	-0.306007	0.60055	0.976678	no
gene:SpnNT_00773	NA	Chromosome:816377-818140	110.58	ΔORF2+peptide	OK	42.566	51.1062	0.263796	0.284569	0.62565	0.980887	no
gene:SpnNT_00773	NA	Chromosome:816377-818140	ΔORF2	ΔORF2+peptide	OK	49.7034	51.1062	0.0401527	0.0434722	0.94025	0.994855	no
gene:SpnNT_00773	NA	Chromosome:816377-818140	110.58+peptide	ΔORF2+peptide	OK	40.6751	51.1062	0.329354	0.349787	0.55085	0.964281	no
gene:SpnNT_00774	ppsA	Chromosome:816377-818140	110.58	ΔORF2	OK	37.4813	42.2728	0.173556	0.264064	0.6524	0.98163	no
gene:SpnNT_00774	ppsA	Chromosome:816377-818140	110.58	110.58+peptide	OK	37.4813	35.374	-0.0834822	-0.125088	0.8322	0.994748	no
gene:SpnNT_00774	ppsA	Chromosome:816377-818140	ΔORF2	110.58+peptide	OK	42.2728	35.374	-0.257038	-0.385193	0.506	0.945292	no
gene:SpnNT_00774	ppsA	Chromosome:816377-818140	110.58	ΔORF2+peptide	OK	37.4813	42.6761	0.187257	0.285541	0.62635	0.980887	no
gene:SpnNT_00774	ppsA	Chromosome:816377-818140	ΔORF2	ΔORF2+peptide	OK	42.2728	42.6761	0.013701	0.0208949	0.9729	0.99536	no
gene:SpnNT_00774	ppsA	Chromosome:816377-818140	110.58+peptide	ΔORF2+peptide	OK	35.374	42.6761	0.270739	0.406596	0.48965	0.939492	no
gene:SpnNT_00775	NA	Chromosome:816377-818140	110.58	ΔORF2	OK	50.4087	51.4727	0.0301345	0.0303807	0.95715	0.994855	no
gene:SpnNT_00775	NA	Chromosome:816377-818140	110.58	110.58+peptide	OK	50.4087	58.9955	0.226933	0.237086	0.67425	0.985063	no
gene:SpnNT_00775	NA	Chromosome:816377-818140	ΔORF2	110.58+peptide	OK	51.4727	58.9955	0.196798	0.203636	0.72405	0.994748	no
gene:SpnNT_00775	NA	Chromosome:816377-818140	110.58	ΔORF2+peptide	OK	50.4087	62.1671	0.302479	0.302332	0.60465	0.976761	no
gene:SpnNT_00775	NA	Chromosome:816377-818140	ΔORF2	ΔORF2+peptide	OK	51.4727	62.1671	0.272345	0.269826	0.646	0.980887	no
gene:SpnNT_00775	NA	Chromosome:816377-818140	110.58+peptide	ΔORF2+peptide	OK	58.9955	62.1671	0.0755467	0.077465	0.89525	0.994748	no
gene:SpnNT_00776	pepN	Chromosome:818262-820809	110.58	ΔORF2	OK	151.661	156.256	0.0430571	0.0977654	0.86075	0.994748	no
gene:SpnNT_00776	pepN	Chromosome:818262-820809	110.58	110.58+peptide	OK	151.661	173.436	0.193555	0.438198	0.43975	0.916373	no
gene:SpnNT_00776	pepN	Chromosome:818262-820809	ΔORF2	110.58+peptide	OK	156.256	173.436	0.150498	0.339597	0.5415	0.961494	no
gene:SpnNT_00776	pepN	Chromosome:818262-820809	110.58	ΔORF2+peptide	OK	151.661	180.374	0.250143	0.567641	0.31865	0.824882	no
gene:SpnNT_00776	pepN	Chromosome:818262-820809	ΔORF2	ΔORF2+peptide	OK	156.256	180.374	0.207086	0.468377	0.40255	0.894197	no
gene:SpnNT_00776	pepN	Chromosome:818262-820809	110.58+peptide	ΔORF2+peptide	OK	173.436	180.374	0.0565875	0.127615	0.8177	0.994748	no
gene:SpnNT_00777	arlR_2	Chromosome:820917-822916	110.58	ΔORF2	OK	160.033	199.277	0.316405	0.446138	0.4412	0.916774	no
gene:SpnNT_00777	arlR_2	Chromosome:820917-822916	110.58	110.58+peptide	OK	160.033	157.725	-0.0209625	-0.0289049	0.9622	0.994855	no
gene:SpnNT_00777	arlR_2	Chromosome:820917-822916	ΔORF2	110.58+peptide	OK	199.277	157.725	-0.337367	-0.477416	0.4156	0.901067	no
gene:SpnNT_00777	arlR_2	Chromosome:820917-822916	110.58	ΔORF2+peptide	OK	160.033	207.168	0.372427	0.518447	0.3704	0.867152	no
gene:SpnNT_00777	arlR_2	Chromosome:820917-822916	ΔORF2	ΔORF2+peptide	OK	199.277	207.168	0.056022	0.0800773	0.88895	0.994748	no
gene:SpnNT_00777	arlR_2	Chromosome:820917-822916	110.58+peptide	ΔORF2+peptide	OK	157.725	207.168	0.393389	0.549558	0.3435	0.847735	no
gene:SpnNT_00778	arlS	Chromosome:820917-822916	110.58	ΔORF2	OK	108.149	122.438	0.179029	0.320697	0.5822	0.970851	no
gene:SpnNT_00778	arlS	Chromosome:820917-822916	110.58	110.58+peptide	OK	108.149	114.928	0.087705	0.159108	0.78275	0.994748	no
gene:SpnNT_00778	arlS	Chromosome:820917-822916	ΔORF2	110.58+peptide	OK	122.438	114.928	-0.0913245	-0.164474	0.77535	0.994748	no
gene:SpnNT_00778	arlS	Chromosome:820917-822916	110.58	ΔORF2+peptide	OK	108.149	134.848	0.318308	0.571412	0.3271	0.832145	no
gene:SpnNT_00778	arlS	Chromosome:820917-822916	ΔORF2	ΔORF2+peptide	OK	122.438	134.848	0.139278	0.248253	0.66125	0.982966	no
gene:SpnNT_00778	arlS	Chromosome:820917-822916	110.58+peptide	ΔORF2+peptide	OK	114.928	134.848	0.230603	0.416215	0.46515	0.926405	no
gene:SpnNT_00779	NA	Chromosome:822949-823237	110.58	ΔORF2	OK	682.098	1087.09	0.672418	1.42447	0.013	0.121155	no
gene:SpnNT_00779	NA	Chromosome:822949-823237	110.58	110.58+peptide	OK	682.098	913.496	0.421419	0.922717	0.11095	0.507277	no
gene:SpnNT_00779	NA	Chromosome:822949-823237	ΔORF2	110.58+peptide	OK	1087.09	913.496	-0.251	-0.526555	0.3575	0.858392	no
gene:SpnNT_00779	NA	Chromosome:822949-823237	110.58	ΔORF2+peptide	OK	682.098	1082.07	0.665736	1.46915	0.00955	0.0975974	no
gene:SpnNT_00779	NA	Chromosome:822949-823237	ΔORF2	ΔORF2+peptide	OK	1087.09	1082.07	-0.00668225	-0.0141195	0.9791	0.995765	no
gene:SpnNT_00779	NA	Chromosome:822949-823237	110.58+peptide	ΔORF2+peptide	OK	913.496	1082.07	0.244317	0.533478	0.35195	0.854885	no
gene:SpnNT_00780	yhbU_1	Chromosome:823376-824306	110.58	ΔORF2	OK	162.614	171.236	0.0745374	0.169842	0.76125	0.994748	no
gene:SpnNT_00780	yhbU_1	Chromosome:823376-824306	110.58	110.58+peptide	OK	162.614	154.639	-0.072543	-0.163999	0.7754	0.994748	no
gene:SpnNT_00780	yhbU_1	Chromosome:823376-824306	ΔORF2	110.58+peptide	OK	171.236	154.639	-0.14708	-0.33437	0.5582	0.968033	no
gene:SpnNT_00780	yhbU_1	Chromosome:823376-824306	110.58	ΔORF2+peptide	OK	162.614	154.634	-0.0725912	-0.164457	0.77555	0.994748	no
gene:SpnNT_00780	yhbU_1	Chromosome:823376-824306	ΔORF2	ΔORF2+peptide	OK	171.236	154.634	-0.147129	-0.3352	0.55855	0.968033	no

gene:SpnNT_00780	yhbU_1	Chromosome:823376-824306	110.58+peptide	ΔORF2+peptide	OK	154.639	154.634	-4.82E-05	-0.000108842	0.9996	0.999752	no
gene:SpnNT_00781	dinG	Chromosome:824505-826956	110.58	ΔORF2	OK	59.9262	65.4802	0.12787	0.293834	0.59585	0.976245	no
gene:SpnNT_00781	dinG	Chromosome:824505-826956	110.58	110.58+peptide	OK	59.9262	52.3636	-0.194623	-0.44415	0.43435	0.913666	no
gene:SpnNT_00781	dinG	Chromosome:824505-826956	ΔORF2	110.58+peptide	OK	65.4802	52.3636	-0.322493	-0.734668	0.1955	0.677231	no
gene:SpnNT_00781	dinG	Chromosome:824505-826956	110.58	ΔORF2+peptide	OK	59.9262	63.4434	0.0822825	0.188019	0.739	0.994748	no
gene:SpnNT_00781	dinG	Chromosome:824505-826956	ΔORF2	ΔORF2+peptide	OK	65.4802	63.4434	-0.0455875	-0.103986	0.85375	0.994748	no
gene:SpnNT_00781	dinG	Chromosome:824505-826956	110.58+peptide	ΔORF2+peptide	OK	52.3636	63.4434	0.276906	0.627344	0.2786	0.781125	no
gene:SpnNT_00782	rodA	Chromosome:826992-828216	110.58	ΔORF2	OK	37.9278	40.944	0.110396	0.230512	0.6815	0.986574	no
gene:SpnNT_00782	rodA	Chromosome:826992-828216	110.58	110.58+peptide	OK	37.9278	33.8534	-0.163954	-0.330339	0.56675	0.968621	no
gene:SpnNT_00782	rodA	Chromosome:826992-828216	ΔORF2	110.58+peptide	OK	40.944	33.8534	-0.274351	-0.555923	0.33395	0.839481	no
gene:SpnNT_00782	rodA	Chromosome:826992-828216	110.58	ΔORF2+peptide	OK	37.9278	45.2979	0.256186	0.53764	0.34725	0.851704	no
gene:SpnNT_00782	rodA	Chromosome:826992-828216	ΔORF2	ΔORF2+peptide	OK	40.944	45.2979	0.145789	0.307854	0.5824	0.970938	no
gene:SpnNT_00782	rodA	Chromosome:826992-828216	110.58+peptide	ΔORF2+peptide	OK	33.8534	45.2979	0.42014	0.855407	0.1436	0.57877	no
gene:SpnNT_00783	yajL	Chromosome:828226-828781	110.58	ΔORF2	OK	196.673	190.967	-0.0424784	-0.0927918	0.8703	0.994748	no
gene:SpnNT_00783	yajL	Chromosome:828226-828781	110.58	110.58+peptide	OK	196.673	185.356	-0.0854991	-0.187585	0.74205	0.994748	no
gene:SpnNT_00783	yajL	Chromosome:828226-828781	ΔORF2	110.58+peptide	OK	190.967	185.356	-0.0430207	-0.0943989	0.86845	0.994748	no
gene:SpnNT_00783	yajL	Chromosome:828226-828781	110.58	ΔORF2+peptide	OK	196.673	168.474	-0.223272	-0.485586	0.38945	0.883687	no
gene:SpnNT_00783	yajL	Chromosome:828226-828781	ΔORF2	ΔORF2+peptide	OK	190.967	168.474	-0.180793	-0.393249	0.47875	0.933291	no
gene:SpnNT_00783	yajL	Chromosome:828226-828781	110.58+peptide	ΔORF2+peptide	OK	185.356	168.474	-0.137773	-0.300972	0.5937	0.975539	no
gene:SpnNT_00784	gph	Chromosome:828856-829429	110.58	ΔORF2	OK	72.4309	89.8066	0.310215	0.617297	0.28315	0.785683	no
gene:SpnNT_00784	gph	Chromosome:828856-829429	110.58	110.58+peptide	OK	72.4309	67.8259	-0.0947688	-0.182226	0.75385	0.994748	no
gene:SpnNT_00784	gph	Chromosome:828856-829429	ΔORF2	110.58+peptide	OK	89.8066	67.8259	-0.404984	-0.787083	0.17125	0.630335	no
gene:SpnNT_00784	gph	Chromosome:828856-829429	110.58	ΔORF2+peptide	OK	72.4309	100.98	0.479393	0.961009	0.1006	0.480726	no
gene:SpnNT_00784	gph	Chromosome:828856-829429	ΔORF2	ΔORF2+peptide	OK	89.8066	100.98	0.169178	0.343103	0.55565	0.966729	no
gene:SpnNT_00784	gph	Chromosome:828856-829429	110.58+peptide	ΔORF2+peptide	OK	67.8259	100.98	0.574162	1.12376	0.05875	0.346308	no
gene:SpnNT_00785	gyrB	Chromosome:829443-831390	110.58	ΔORF2	OK	103.633	103.147	-0.00678508	-0.0155803	0.9783	0.995765	no
gene:SpnNT_00785	gyrB	Chromosome:829443-831390	110.58	110.58+peptide	OK	103.633	110.236	0.0891108	0.204783	0.7153	0.992053	no
gene:SpnNT_00785	gyrB	Chromosome:829443-831390	ΔORF2	110.58+peptide	OK	103.147	110.236	0.0958959	0.22085	0.69395	0.98828	no
gene:SpnNT_00785	gyrB	Chromosome:829443-831390	110.58	ΔORF2+peptide	OK	103.633	119.792	0.209052	0.480741	0.3871	0.881745	no
gene:SpnNT_00785	gyrB	Chromosome:829443-831390	ΔORF2	ΔORF2+peptide	OK	103.147	119.792	0.215837	0.497414	0.37375	0.870955	no
gene:SpnNT_00785	gyrB	Chromosome:829443-831390	110.58+peptide	ΔORF2+peptide	OK	110.236	119.792	0.119942	0.276635	0.61815	0.980887	no
gene:SpnNT_00786	ezrA	Chromosome:831471-833199	110.58	ΔORF2	OK	141.351	172.108	0.28403	0.648394	0.25595	0.754906	no
gene:SpnNT_00786	ezrA	Chromosome:831471-833199	110.58	110.58+peptide	OK	141.351	188.271	0.413528	0.944506	0.09605	0.46955	no
gene:SpnNT_00786	ezrA	Chromosome:831471-833199	ΔORF2	110.58+peptide	OK	172.108	188.271	0.129498	0.29251	0.6022	0.976761	no
gene:SpnNT_00786	ezrA	Chromosome:831471-833199	110.58	ΔORF2+peptide	OK	141.351	245.712	0.797687	1.82749	0.0018	0.0270557	yes
gene:SpnNT_00786	ezrA	Chromosome:831471-833199	ΔORF2	ΔORF2+peptide	OK	172.108	245.712	0.513657	1.16371	0.04045	0.272897	no
gene:SpnNT_00786	ezrA	Chromosome:831471-833199	110.58+peptide	ΔORF2+peptide	OK	188.271	245.712	0.384159	0.870775	0.1214	0.527312	no
gene:SpnNT_00787	NA	Chromosome:833717-834224	110.58	ΔORF2	OK	33.0623	37.7938	0.192962	0.340803	0.54575	0.961816	no
gene:SpnNT_00787	NA	Chromosome:833717-834224	110.58	110.58+peptide	OK	33.0623	30.9084	-0.097188	-0.170297	0.76525	0.994748	no
gene:SpnNT_00787	NA	Chromosome:833717-834224	ΔORF2	110.58+peptide	OK	37.7938	30.9084	-0.29015	-0.513584	0.36975	0.866249	no
gene:SpnNT_00787	NA	Chromosome:833717-834224	110.58	ΔORF2+peptide	OK	33.0623	34.3723	0.0560608	0.0978996	0.8616	0.994748	no
gene:SpnNT_00787	NA	Chromosome:833717-834224	ΔORF2	ΔORF2+peptide	OK	37.7938	34.3723	-0.136901	-0.241487	0.6734	0.984845	no
gene:SpnNT_00787	NA	Chromosome:833717-834224	110.58+peptide	ΔORF2+peptide	OK	30.9084	34.3723	0.153249	0.268197	0.63545	0.980887	no
gene:SpnNT_00788	NA	Chromosome:834255-834651	110.58	ΔORF2	OK	16.2152	16.0818	-0.0119111	-0.0175362	0.97985	0.995863	no
gene:SpnNT_00788	NA	Chromosome:834255-834651	110.58	110.58+peptide	OK	16.2152	13.7607	-0.236789	-0.337415	0.5657	0.968621	no

gene:SpnNT_00788	NA	Chromosome:834255-834651	ΔORF2	110.58+peptide	OK	16.0818	13.7607	-0.224878	-0.319355	0.5799	0.969538	no
gene:SpnNT_00788	NA	Chromosome:834255-834651	110.58	ΔORF2+peptide	OK	16.2152	19.3537	0.255262	0.380134	0.51245	0.950409	no
gene:SpnNT_00788	NA	Chromosome:834255-834651	ΔORF2	ΔORF2+peptide	OK	16.0818	19.3537	0.267173	0.396397	0.489	0.939082	no
gene:SpnNT_00788	NA	Chromosome:834255-834651	110.58+peptide	ΔORF2+peptide	OK	13.7607	19.3537	0.492052	0.706242	0.22565	0.716568	no
gene:SpnNT_00789	cshA_1	Chromosome:834828-836885	110.58	ΔORF2	OK	43.4448	49.0649	0.175507	0.300391	0.60265	0.976761	no
gene:SpnNT_00789	cshA_1	Chromosome:834828-836885	110.58	110.58+peptide	OK	43.4448	47.5486	0.130218	0.22498	0.6919	0.98828	no
gene:SpnNT_00789	cshA_1	Chromosome:834828-836885	ΔORF2	110.58+peptide	OK	49.0649	47.5486	-0.0452886	-0.0785602	0.8886	0.994748	no
gene:SpnNT_00789	cshA_1	Chromosome:834828-836885	110.58	ΔORF2+peptide	OK	43.4448	52.5982	0.275827	0.47109	0.4103	0.898184	no
gene:SpnNT_00789	cshA_1	Chromosome:834828-836885	ΔORF2	ΔORF2+peptide	OK	49.0649	52.5982	0.10032	0.172011	0.76095	0.994748	no
gene:SpnNT_00789	cshA_1	Chromosome:834828-836885	110.58+peptide	ΔORF2+peptide	OK	47.5486	52.5982	0.145609	0.252029	0.66055	0.982966	no
gene:SpnNT_00790	ycjS	Chromosome:834828-836885	110.58	ΔORF2	OK	31.8005	37.4871	0.237345	0.330855	0.5508	0.964281	no
gene:SpnNT_00790	ycjS	Chromosome:834828-836885	110.58	110.58+peptide	OK	31.8005	31.8798	0.00359491	0.00480114	0.99315	0.998522	no
gene:SpnNT_00790	ycjS	Chromosome:834828-836885	ΔORF2	110.58+peptide	OK	37.4871	31.8798	-0.23375	-0.315244	0.58185	0.970638	no
gene:SpnNT_00790	ycjS	Chromosome:834828-836885	110.58	ΔORF2+peptide	OK	31.8005	31.8066	0.000278457	0.000366056	0.9983	0.999412	no
gene:SpnNT_00790	ycjS	Chromosome:834828-836885	ΔORF2	ΔORF2+peptide	OK	37.4871	31.8066	-0.237067	-0.314605	0.58155	0.970261	no
gene:SpnNT_00790	ycjS	Chromosome:834828-836885	110.58+peptide	ΔORF2+peptide	OK	31.8798	31.8066	-0.00331645	-0.00423296	0.9939	0.998618	no
gene:SpnNT_00791	xynD	Chromosome:837678-839121	110.58	ΔORF2	OK	342.687	409.987	0.258686	0.572636	0.3129	0.819111	no
gene:SpnNT_00791	xynD	Chromosome:837678-839121	110.58	110.58+peptide	OK	342.687	399.604	0.221679	0.48921	0.38665	0.881745	no
gene:SpnNT_00791	xynD	Chromosome:837678-839121	ΔORF2	110.58+peptide	OK	409.987	399.604	-0.0370072	-0.0803045	0.88865	0.994748	no
gene:SpnNT_00791	xynD	Chromosome:837678-839121	110.58	ΔORF2+peptide	OK	342.687	404.013	0.237509	0.534939	0.33705	0.84149	no
gene:SpnNT_00791	xynD	Chromosome:837678-839121	ΔORF2	ΔORF2+peptide	OK	409.987	404.013	-0.0211773	-0.0468684	0.93355	0.994855	no
gene:SpnNT_00791	xynD	Chromosome:837678-839121	110.58+peptide	ΔORF2+peptide	OK	399.604	404.013	0.0158298	0.0349261	0.94955	0.994855	no
gene:SpnNT_00792	yvgN	Chromosome:839234-840077	110.58	ΔORF2	OK	129.183	147.341	0.189741	0.422899	0.46285	0.9258	no
gene:SpnNT_00792	yvgN	Chromosome:839234-840077	110.58	110.58+peptide	OK	129.183	94.1664	-0.45613	-0.999567	0.0858	0.439964	no
gene:SpnNT_00792	yvgN	Chromosome:839234-840077	ΔORF2	110.58+peptide	OK	147.341	94.1664	-0.645871	-1.4355	0.01315	0.122206	no
gene:SpnNT_00792	yvgN	Chromosome:839234-840077	110.58	ΔORF2+peptide	OK	129.183	114.82	-0.170044	-0.376014	0.51635	0.95301	no
gene:SpnNT_00792	yvgN	Chromosome:839234-840077	ΔORF2	ΔORF2+peptide	OK	147.341	114.82	-0.359785	-0.807109	0.1615	0.614666	no
gene:SpnNT_00792	yvgN	Chromosome:839234-840077	110.58+peptide	ΔORF2+peptide	OK	94.1664	114.82	0.286086	0.630872	0.26765	0.768869	no
gene:SpnNT_00793	NA	Chromosome:840349-841111	110.58	ΔORF2	OK	28.1171	33.2494	0.241879	0.453089	0.43675	0.914104	no
gene:SpnNT_00793	NA	Chromosome:840349-841111	110.58	110.58+peptide	OK	28.1171	81.0592	1.52753	2.93295	5.00E-05	0.0013612	yes
gene:SpnNT_00793	NA	Chromosome:840349-841111	ΔORF2	110.58+peptide	OK	33.2494	81.0592	1.28565	2.48942	5.00E-05	0.0013612	yes
gene:SpnNT_00793	NA	Chromosome:840349-841111	110.58	ΔORF2+peptide	OK	28.1171	121.831	2.11536	4.16045	5.00E-05	0.0013612	yes
gene:SpnNT_00793	NA	Chromosome:840349-841111	ΔORF2	ΔORF2+peptide	OK	33.2494	121.831	1.87348	3.71747	5.00E-05	0.0013612	yes
gene:SpnNT_00793	NA	Chromosome:840349-841111	110.58+peptide	ΔORF2+peptide	OK	81.0592	121.831	0.587832	1.19931	0.0411	0.275864	no
gene:SpnNT_00794	glyQ	Chromosome:841303-842221	110.58	ΔORF2	OK	56.7058	56.911	0.00521127	0.0108112	0.98385	0.996246	no
gene:SpnNT_00794	glyQ	Chromosome:841303-842221	110.58	110.58+peptide	OK	56.7058	136.885	1.27139	2.69917	5.00E-05	0.0013612	yes
gene:SpnNT_00794	glyQ	Chromosome:841303-842221	ΔORF2	110.58+peptide	OK	56.911	136.885	1.26618	2.68678	5.00E-05	0.0013612	yes
gene:SpnNT_00794	glyQ	Chromosome:841303-842221	110.58	ΔORF2+peptide	OK	56.7058	167.381	1.56157	3.3707	5.00E-05	0.0013612	yes
gene:SpnNT_00794	glyQ	Chromosome:841303-842221	ΔORF2	ΔORF2+peptide	OK	56.911	167.381	1.55635	3.35773	5.00E-05	0.0013612	yes
gene:SpnNT_00794	glyQ	Chromosome:841303-842221	110.58+peptide	ΔORF2+peptide	OK	136.885	167.381	0.290171	0.641873	0.2667	0.768138	no
gene:SpnNT_00795	glyS	Chromosome:842480-844517	110.58	ΔORF2	OK	71.6761	76.9567	0.102554	0.232715	0.68935	0.98828	no
gene:SpnNT_00795	glyS	Chromosome:842480-844517	110.58	110.58+peptide	OK	71.6761	179.763	1.32653	2.97157	5.00E-05	0.0013612	yes
gene:SpnNT_00795	glyS	Chromosome:842480-844517	ΔORF2	110.58+peptide	OK	76.9567	179.763	1.22398	2.74843	5.00E-05	0.0013612	yes
gene:SpnNT_00795	glyS	Chromosome:842480-844517	110.58	ΔORF2+peptide	OK	71.6761	233.648	1.70477	3.83377	5.00E-05	0.0013612	yes
gene:SpnNT_00795	glyS	Chromosome:842480-844517	ΔORF2	ΔORF2+peptide	OK	76.9567	233.648	1.60222	3.61186	5.00E-05	0.0013612	yes

gene:SpnNT_00795	glyS	Chromosome:842480-844517	110.58+peptide	ΔORF2+peptide	OK	179.763	233.648	0.378239	0.841866	0.14425	0.579207	no
gene:SpnNT_00796	NA	Chromosome:844558-844816	110.58	ΔORF2	OK	493.496	422.93	-0.222619	-0.431285	0.44395	0.917631	no
gene:SpnNT_00796	NA	Chromosome:844558-844816	110.58	110.58+peptide	OK	493.496	1763.59	1.83741	3.61296	5.00E-05	0.0013612	yes
gene:SpnNT_00796	NA	Chromosome:844558-844816	ΔORF2	110.58+peptide	OK	422.93	1763.59	2.06003	4.11332	5.00E-05	0.0013612	yes
gene:SpnNT_00796	NA	Chromosome:844558-844816	110.58	ΔORF2+peptide	OK	493.496	1644.1	1.73619	3.45417	5.00E-05	0.0013612	yes
gene:SpnNT_00796	NA	Chromosome:844558-844816	ΔORF2	ΔORF2+peptide	OK	422.93	1644.1	1.95881	3.95877	5.00E-05	0.0013612	yes
gene:SpnNT_00796	NA	Chromosome:844558-844816	110.58+peptide	ΔORF2+peptide	OK	1763.59	1644.1	-0.101216	-0.207899	0.70445	0.990367	no
gene:SpnNT_00797	azr_1	Chromosome:844932-846177	110.58	ΔORF2	OK	56.5963	45.919	-0.301618	-0.641894	0.26085	0.761099	no
gene:SpnNT_00797	azr_1	Chromosome:844932-846177	110.58	110.58+peptide	OK	56.5963	101.89	0.848228	1.75868	0.00245	0.0342374	yes
gene:SpnNT_00797	azr_1	Chromosome:844932-846177	ΔORF2	110.58+peptide	OK	45.919	101.89	1.14985	2.34768	5.00E-05	0.0013612	yes
gene:SpnNT_00797	azr_1	Chromosome:844932-846177	110.58	ΔORF2+peptide	OK	56.5963	84.6997	0.581649	1.25523	0.02655	0.205055	no
gene:SpnNT_00797	azr_1	Chromosome:844932-846177	ΔORF2	ΔORF2+peptide	OK	45.919	84.6997	0.883267	1.8747	0.0013	0.020854	yes
gene:SpnNT_00797	azr_1	Chromosome:844932-846177	110.58+peptide	ΔORF2+peptide	OK	101.89	84.6997	-0.266579	-0.551308	0.337	0.84149	no
gene:SpnNT_00798	azr_2	Chromosome:846194-846800	110.58	ΔORF2	OK	14.1691	13.362	-0.0846145	-0.134165	0.8134	0.994748	no
gene:SpnNT_00798	azr_2	Chromosome:846194-846800	110.58	110.58+peptide	OK	14.1691	30.4331	1.10289	1.74522	0.00345	0.0449116	yes
gene:SpnNT_00798	azr_2	Chromosome:846194-846800	ΔORF2	110.58+peptide	OK	13.362	30.4331	1.18751	1.87983	0.00135	0.021343	yes
gene:SpnNT_00798	azr_2	Chromosome:846194-846800	110.58	ΔORF2+peptide	OK	14.1691	29.9439	1.07951	1.75217	0.00315	0.0417097	yes
gene:SpnNT_00798	azr_2	Chromosome:846194-846800	ΔORF2	ΔORF2+peptide	OK	13.362	29.9439	1.16413	1.89026	9.00E-04	0.0154579	yes
gene:SpnNT_00798	azr_2	Chromosome:846194-846800	110.58+peptide	ΔORF2+peptide	OK	30.4331	29.9439	-0.0233812	-0.0378851	0.9469	0.994855	no
gene:SpnNT_00799	apbE	Chromosome:846859-847783	110.58	ΔORF2	OK	16.6393	14.8627	-0.162894	-0.290125	0.61475	0.979616	no
gene:SpnNT_00799	apbE	Chromosome:846859-847783	110.58	110.58+peptide	OK	16.6393	30.9862	0.897035	1.61153	0.0062	0.0706832	no
gene:SpnNT_00799	apbE	Chromosome:846859-847783	ΔORF2	110.58+peptide	OK	14.8627	30.9862	1.05993	1.89281	0.00165	0.0253528	yes
gene:SpnNT_00799	apbE	Chromosome:846859-847783	110.58	ΔORF2+peptide	OK	16.6393	30.1688	0.858466	1.52128	0.0092	0.0949075	no
gene:SpnNT_00799	apbE	Chromosome:846859-847783	ΔORF2	ΔORF2+peptide	OK	14.8627	30.1688	1.02136	1.79944	0.00265	0.0362222	yes
gene:SpnNT_00799	apbE	Chromosome:846859-847783	110.58+peptide	ΔORF2+peptide	OK	30.9862	30.1688	-0.0385686	-0.0685264	0.90445	0.994748	no
gene:SpnNT_00800	NA	Chromosome:848077-849457	110.58	ΔORF2	OK	298.017	328.938	0.142419	0.323837	0.56905	0.968621	no
gene:SpnNT_00800	NA	Chromosome:848077-849457	110.58	110.58+peptide	OK	298.017	543.081	0.865771	1.92993	0.00105	0.0174849	yes
gene:SpnNT_00800	NA	Chromosome:848077-849457	ΔORF2	110.58+peptide	OK	328.938	543.081	0.723351	1.60961	0.0055	0.064496	no
gene:SpnNT_00800	NA	Chromosome:848077-849457	110.58	ΔORF2+peptide	OK	298.017	650.852	1.12693	2.52757	5.00E-05	0.0013612	yes
gene:SpnNT_00800	NA	Chromosome:848077-849457	ΔORF2	ΔORF2+peptide	OK	328.938	650.852	0.984514	2.2042	1.00E-04	0.0025332	yes
gene:SpnNT_00800	NA	Chromosome:848077-849457	110.58+peptide	ΔORF2+peptide	OK	543.081	650.852	0.261163	0.573562	0.32035	0.825861	no
gene:SpnNT_00801	pdxS	Chromosome:849608-850484	110.58	ΔORF2	OK	24.3449	23.561	-0.0472162	-0.0879017	0.87575	0.994748	no
gene:SpnNT_00801	pdxS	Chromosome:849608-850484	110.58	110.58+peptide	OK	24.3449	25.6432	0.0749578	0.139604	0.8089	0.994748	no
gene:SpnNT_00801	pdxS	Chromosome:849608-850484	ΔORF2	110.58+peptide	OK	23.561	25.6432	0.122174	0.227663	0.69385	0.98828	no
gene:SpnNT_00801	pdxS	Chromosome:849608-850484	110.58	ΔORF2+peptide	OK	24.3449	27.7639	0.18959	0.354138	0.53525	0.958867	no
gene:SpnNT_00801	pdxS	Chromosome:849608-850484	ΔORF2	ΔORF2+peptide	OK	23.561	27.7639	0.236807	0.442571	0.43855	0.916013	no
gene:SpnNT_00801	pdxS	Chromosome:849608-850484	110.58+peptide	ΔORF2+peptide	OK	25.6432	27.7639	0.114632	0.214325	0.7094	0.990851	no
gene:SpnNT_00802	pdxT	Chromosome:850484-851066	110.58	ΔORF2	OK	38.8952	38.1118	-0.0293574	-0.0541143	0.92535	0.994748	no
gene:SpnNT_00802	pdxT	Chromosome:850484-851066	110.58	110.58+peptide	OK	38.8952	45.7939	0.235563	0.439723	0.4473	0.919465	no
gene:SpnNT_00802	pdxT	Chromosome:850484-851066	ΔORF2	110.58+peptide	OK	38.1118	45.7939	0.264921	0.492397	0.38995	0.88421	no
gene:SpnNT_00802	pdxT	Chromosome:850484-851066	110.58	ΔORF2+peptide	OK	38.8952	50.065	0.364209	0.679996	0.2418	0.736324	no
gene:SpnNT_00802	pdxT	Chromosome:850484-851066	ΔORF2	ΔORF2+peptide	OK	38.1118	50.065	0.393566	0.731645	0.2054	0.69003	no
gene:SpnNT_00802	pdxT	Chromosome:850484-851066	110.58+peptide	ΔORF2+peptide	OK	45.7939	50.065	0.128645	0.242242	0.68645	0.98828	no
gene:SpnNT_00803	NA	Chromosome:851086-852177	110.58	ΔORF2	OK	63.0809	70.4172	0.158726	0.305062	0.59375	0.975539	no
gene:SpnNT_00803	NA	Chromosome:851086-852177	110.58	110.58+peptide	OK	63.0809	45.2194	-0.48026	-0.895667	0.12525	0.535526	no

gene:SpnNT_00803	NA	Chromosome:851086-852177	ΔORF2	110.58+peptide	OK	70.4172	45.2194	-0.638986	-1.20354	0.03965	0.269579	no
gene:SpnNT_00803	NA	Chromosome:851086-852177	110.58	ΔORF2+peptide	OK	63.0809	40.6051	-0.635543	-1.16409	0.0437	0.286572	no
gene:SpnNT_00803	NA	Chromosome:851086-852177	ΔORF2	ΔORF2+peptide	OK	70.4172	40.6051	-0.794269	-1.46877	0.0114	0.110074	no
gene:SpnNT_00803	NA	Chromosome:851086-852177	110.58+peptide	ΔORF2+peptide	OK	45.2194	40.6051	-0.155282	-0.279243	0.628	0.980887	no
gene:SpnNT_00804	NA	Chromosome:851086-852177	110.58	ΔORF2	OK	16.357	16.4142	0.00503666	0.00293988	0.9958	0.99927	no
gene:SpnNT_00804	NA	Chromosome:851086-852177	110.58	110.58+peptide	OK	16.357	12.0309	-0.443159	-0.271696	0.64435	0.980887	no
gene:SpnNT_00804	NA	Chromosome:851086-852177	ΔORF2	110.58+peptide	OK	16.4142	12.0309	-0.448196	-0.261792	0.6544	0.982966	no
gene:SpnNT_00804	NA	Chromosome:851086-852177	110.58	ΔORF2+peptide	OK	16.357	13.5644	-0.270077	-0.171467	0.7678	0.994748	no
gene:SpnNT_00804	NA	Chromosome:851086-852177	ΔORF2	ΔORF2+peptide	OK	16.4142	13.5644	-0.275114	-0.165854	0.7696	0.994748	no
gene:SpnNT_00804	NA	Chromosome:851086-852177	110.58+peptide	ΔORF2+peptide	OK	12.0309	13.5644	0.173082	0.109977	0.8468	0.994748	no
gene:SpnNT_00805	NA	Chromosome:852311-853686	110.58	ΔORF2	OK	126.19	127.892	0.0193319	0.0273355	0.9622	0.994855	no
gene:SpnNT_00805	NA	Chromosome:852311-853686	110.58	110.58+peptide	OK	126.19	71.7076	-0.815399	-1.18559	0.0397	0.269639	no
gene:SpnNT_00805	NA	Chromosome:852311-853686	ΔORF2	110.58+peptide	OK	127.892	71.7076	-0.834731	-1.16519	0.0447	0.290805	no
gene:SpnNT_00805	NA	Chromosome:852311-853686	110.58	ΔORF2+peptide	OK	126.19	78.1082	-0.692052	-0.946342	0.10615	0.495694	no
gene:SpnNT_00805	NA	Chromosome:852311-853686	ΔORF2	ΔORF2+peptide	OK	127.892	78.1082	-0.711384	-0.938154	0.1133	0.510776	no
gene:SpnNT_00805	NA	Chromosome:852311-853686	110.58+peptide	ΔORF2+peptide	OK	71.7076	78.1082	0.123346	0.166645	0.76925	0.994748	no
gene:SpnNT_00806	ogt	Chromosome:852311-853686	110.58	ΔORF2	OK	74.2472	77.81	0.0676181	0.081368	0.88545	0.994748	no
gene:SpnNT_00806	ogt	Chromosome:852311-853686	110.58	110.58+peptide	OK	74.2472	46.6862	-0.669341	-0.826716	0.1509	0.593025	no
gene:SpnNT_00806	ogt	Chromosome:852311-853686	ΔORF2	110.58+peptide	OK	77.81	46.6862	-0.736959	-0.884968	0.1213	0.52727	no
gene:SpnNT_00806	ogt	Chromosome:852311-853686	110.58	ΔORF2+peptide	OK	74.2472	51.1842	-0.536637	-0.63667	0.2624	0.762762	no
gene:SpnNT_00806	ogt	Chromosome:852311-853686	ΔORF2	ΔORF2+peptide	OK	77.81	51.1842	-0.604256	-0.698475	0.21825	0.706084	no
gene:SpnNT_00806	ogt	Chromosome:852311-853686	110.58+peptide	ΔORF2+peptide	OK	46.6862	51.1842	0.132704	0.157122	0.78455	0.994748	no
gene:SpnNT_00807	mgsR	Chromosome:852311-853686	110.58	ΔORF2	OK	98.326	104.33	0.0855118	0.0789614	0.88755	0.994748	no
gene:SpnNT_00807	mgsR	Chromosome:852311-853686	110.58	110.58+peptide	OK	98.326	55.2261	-0.832223	-0.744448	0.19585	0.677365	no
gene:SpnNT_00807	mgsR	Chromosome:852311-853686	ΔORF2	110.58+peptide	OK	104.33	55.2261	-0.917734	-0.805285	0.1616	0.614666	no
gene:SpnNT_00807	mgsR	Chromosome:852311-853686	110.58	ΔORF2+peptide	OK	98.326	62.516	-0.653347	-0.605743	0.2902	0.794659	no
gene:SpnNT_00807	mgsR	Chromosome:852311-853686	ΔORF2	ΔORF2+peptide	OK	104.33	62.516	-0.738859	-0.67102	0.2411	0.736034	no
gene:SpnNT_00807	mgsR	Chromosome:852311-853686	110.58+peptide	ΔORF2+peptide	OK	55.2261	62.516	0.178875	0.157532	0.78645	0.994748	no
gene:SpnNT_00808	yxeN	Chromosome:853843-855387	110.58	ΔORF2	OK	110.929	114.085	0.0404782	0.0584771	0.92165	0.994748	no
gene:SpnNT_00808	yxeN	Chromosome:853843-855387	110.58	110.58+peptide	OK	110.929	60.5689	-0.872985	-1.26798	0.03025	0.223503	no
gene:SpnNT_00808	yxeN	Chromosome:853843-855387	ΔORF2	110.58+peptide	OK	114.085	60.5689	-0.913463	-1.28846	0.0277	0.211941	no
gene:SpnNT_00808	yxeN	Chromosome:853843-855387	110.58	ΔORF2+peptide	OK	110.929	62.6513	-0.824218	-1.21152	0.03625	0.253827	no
gene:SpnNT_00808	yxeN	Chromosome:853843-855387	ΔORF2	ΔORF2+peptide	OK	114.085	62.6513	-0.864696	-1.23347	0.0329	0.237205	no
gene:SpnNT_00808	yxeN	Chromosome:853843-855387	110.58+peptide	ΔORF2+peptide	OK	60.5689	62.6513	0.0487671	0.0699321	0.89975	0.994748	no
gene:SpnNT_00809	tcyC	Chromosome:853843-855387	110.58	ΔORF2	OK	135.742	161.053	0.246671	0.403439	0.48315	0.936607	no
gene:SpnNT_00809	tcyC	Chromosome:853843-855387	110.58	110.58+peptide	OK	135.742	79.9485	-0.763725	-1.23465	0.0333	0.239432	no
gene:SpnNT_00809	tcyC	Chromosome:853843-855387	ΔORF2	110.58+peptide	OK	161.053	79.9485	-1.0104	-1.63214	0.0051	0.061228	no
gene:SpnNT_00809	tcyC	Chromosome:853843-855387	110.58	ΔORF2+peptide	OK	135.742	96.3848	-0.49399	-0.822515	0.15505	0.600007	no
gene:SpnNT_00809	tcyC	Chromosome:853843-855387	ΔORF2	ΔORF2+peptide	OK	161.053	96.3848	-0.740661	-1.23222	0.0325	0.235096	no
gene:SpnNT_00809	tcyC	Chromosome:853843-855387	110.58+peptide	ΔORF2+peptide	OK	79.9485	96.3848	0.269735	0.443381	0.4442	0.917631	no
gene:SpnNT_00810	NA	Chromosome:855464-855689	110.58	ΔORF2	OK	47.901	62.279	0.378689	0.492786	0.3914	0.885814	no
gene:SpnNT_00810	NA	Chromosome:855464-855689	110.58	110.58+peptide	OK	47.901	44.9132	-0.0929193	-0.119438	0.83195	0.994748	no
gene:SpnNT_00810	NA	Chromosome:855464-855689	ΔORF2	110.58+peptide	OK	62.279	44.9132	-0.471608	-0.615408	0.2885	0.792622	no
gene:SpnNT_00810	NA	Chromosome:855464-855689	110.58	ΔORF2+peptide	OK	47.901	34.3781	-0.478569	-0.61718	0.2929	0.797831	no
gene:SpnNT_00810	NA	Chromosome:855464-855689	ΔORF2	ΔORF2+peptide	OK	62.279	34.3781	-0.857258	-1.12245	0.0619	0.359403	no

gene:SpnNT_00810	NA	Chromosome:855464-855689	110.58+peptide	ΔORF2+peptide	OK	44.9132	34.3781	-0.38565	-0.498707	0.4024	0.894197	no
gene:SpnNT_00811	trxB	Chromosome:855754-856666	110.58	ΔORF2	OK	244.408	251.649	0.0421222	0.0951632	0.86895	0.994748	no
gene:SpnNT_00811	trxB	Chromosome:855754-856666	110.58	110.58+peptide	OK	244.408	180.158	-0.44003	-0.976806	0.08605	0.440385	no
gene:SpnNT_00811	trxB	Chromosome:855754-856666	ΔORF2	110.58+peptide	OK	251.649	180.158	-0.482152	-1.08469	0.05735	0.340982	no
gene:SpnNT_00811	trxB	Chromosome:855754-856666	110.58	ΔORF2+peptide	OK	244.408	190.801	-0.357223	-0.792555	0.16165	0.614666	no
gene:SpnNT_00811	trxB	Chromosome:855754-856666	ΔORF2	ΔORF2+peptide	OK	251.649	190.801	-0.399345	-0.897901	0.1154	0.515587	no
gene:SpnNT_00811	trxB	Chromosome:855754-856666	110.58+peptide	ΔORF2+peptide	OK	180.158	190.801	0.0828074	0.182974	0.75265	0.994748	no
gene:SpnNT_00812	NA	Chromosome:856704-857433	110.58	ΔORF2	OK	348.162	389.31	0.161162	0.370729	0.5057	0.945292	no
gene:SpnNT_00812	NA	Chromosome:856704-857433	110.58	110.58+peptide	OK	348.162	313.887	-0.149512	-0.344068	0.53875	0.960657	no
gene:SpnNT_00812	NA	Chromosome:856704-857433	ΔORF2	110.58+peptide	OK	389.31	313.887	-0.310674	-0.715311	0.20185	0.685467	no
gene:SpnNT_00812	NA	Chromosome:856704-857433	110.58	ΔORF2+peptide	OK	348.162	310.777	-0.163875	-0.376884	0.4998	0.944017	no
gene:SpnNT_00812	NA	Chromosome:856704-857433	ΔORF2	ΔORF2+peptide	OK	389.31	310.777	-0.325038	-0.747909	0.18235	0.652166	no
gene:SpnNT_00812	NA	Chromosome:856704-857433	110.58+peptide	ΔORF2+peptide	OK	313.887	310.777	-0.0143631	-0.0330627	0.95505	0.994855	no
gene:SpnNT_00813	def_1	Chromosome:857478-858090	110.58	ΔORF2	OK	378.488	410.804	0.118201	0.269716	0.63665	0.980887	no
gene:SpnNT_00813	def_1	Chromosome:857478-858090	110.58	110.58+peptide	OK	378.488	373.89	-0.0176341	-0.0392423	0.9463	0.994855	no
gene:SpnNT_00813	def_1	Chromosome:857478-858090	ΔORF2	110.58+peptide	OK	410.804	373.89	-0.135835	-0.304145	0.58725	0.973049	no
gene:SpnNT_00813	def_1	Chromosome:857478-858090	110.58	ΔORF2+peptide	OK	378.488	307.842	-0.298058	-0.669741	0.24265	0.738169	no
gene:SpnNT_00813	def_1	Chromosome:857478-858090	ΔORF2	ΔORF2+peptide	OK	410.804	307.842	-0.416259	-0.941216	0.0985	0.475715	no
gene:SpnNT_00813	def_1	Chromosome:857478-858090	110.58+peptide	ΔORF2+peptide	OK	373.89	307.842	-0.280424	-0.618655	0.2709	0.772772	no
gene:SpnNT_00814	NA	Chromosome:858347-858470	110.58	ΔORF2	OK	25.0916	14.9164	-0.750313	-0.535209	0.534	0.958867	no
gene:SpnNT_00814	NA	Chromosome:858347-858470	110.58	110.58+peptide	OK	25.0916	23.4322	-0.0987121	-0.0702634	0.87075	0.994748	no
gene:SpnNT_00814	NA	Chromosome:858347-858470	ΔORF2	110.58+peptide	OK	14.9164	23.4322	0.6516	3.16175	0.61575	0.979616	no
gene:SpnNT_00814	NA	Chromosome:858347-858470	110.58	ΔORF2+peptide	OK	25.0916	20.5436	-0.288519	-0.204979	0.8215	0.994748	no
gene:SpnNT_00814	NA	Chromosome:858347-858470	ΔORF2	ΔORF2+peptide	OK	14.9164	20.5436	0.461794	2.06588	0.7285	0.994748	no
gene:SpnNT_00814	NA	Chromosome:858347-858470	110.58+peptide	ΔORF2+peptide	OK	23.4322	20.5436	-0.189807	-0.785892	0.8575	0.994748	no
gene:SpnNT_00815	capA_1	Chromosome:858612-859116	110.58	ΔORF2	OK	9.8211	11.3179	0.204647	0.299081	0.60815	0.978519	no
gene:SpnNT_00815	capA_1	Chromosome:858612-859116	110.58	110.58+peptide	OK	9.8211	15.1594	0.626259	0.945515	0.0997	0.479042	no
gene:SpnNT_00815	capA_1	Chromosome:858612-859116	ΔORF2	110.58+peptide	OK	11.3179	15.1594	0.421612	0.642037	0.26555	0.766773	no
gene:SpnNT_00815	capA_1	Chromosome:858612-859116	110.58	ΔORF2+peptide	OK	9.8211	22.8433	1.21782	1.83272	0.0029	0.0388304	yes
gene:SpnNT_00815	capA_1	Chromosome:858612-859116	ΔORF2	ΔORF2+peptide	OK	11.3179	22.8433	1.01317	1.53782	0.0096	0.0977279	no
gene:SpnNT_00815	capA_1	Chromosome:858612-859116	110.58+peptide	ΔORF2+peptide	OK	15.1594	22.8433	0.591556	0.930038	0.1127	0.510701	no
gene:SpnNT_00816	capA_2	Chromosome:859163-859670	110.58	ΔORF2	OK	17.5276	14.816	-0.242468	-0.380867	0.50535	0.945292	no
gene:SpnNT_00816	capA_2	Chromosome:859163-859670	110.58	110.58+peptide	OK	17.5276	22.9544	0.389147	0.627642	0.28095	0.784388	no
gene:SpnNT_00816	capA_2	Chromosome:859163-859670	ΔORF2	110.58+peptide	OK	14.816	22.9544	0.631614	1.01564	0.0845	0.437055	no
gene:SpnNT_00816	capA_2	Chromosome:859163-859670	110.58	ΔORF2+peptide	OK	17.5276	26.649	0.604456	0.961514	0.0937	0.462752	no
gene:SpnNT_00816	capA_2	Chromosome:859163-859670	ΔORF2	ΔORF2+peptide	OK	14.816	26.649	0.846923	1.34327	0.02175	0.178712	no
gene:SpnNT_00816	capA_2	Chromosome:859163-859670	110.58+peptide	ΔORF2+peptide	OK	22.9544	26.649	0.215309	0.350824	0.5413	0.961494	no
gene:SpnNT_00817	ybiV	Chromosome:859691-860519	110.58	ΔORF2	OK	25.0862	20.5119	-0.290428	-0.533142	0.34795	0.852783	no
gene:SpnNT_00817	ybiV	Chromosome:859691-860519	110.58	110.58+peptide	OK	25.0862	32.1927	0.359843	0.674283	0.24575	0.743197	no
gene:SpnNT_00817	ybiV	Chromosome:859691-860519	ΔORF2	110.58+peptide	OK	20.5119	32.1927	0.650271	1.20293	0.0371	0.257853	no
gene:SpnNT_00817	ybiV	Chromosome:859691-860519	110.58	ΔORF2+peptide	OK	25.0862	35.5278	0.502057	0.944797	0.1013	0.48284	no
gene:SpnNT_00817	ybiV	Chromosome:859691-860519	ΔORF2	ΔORF2+peptide	OK	20.5119	35.5278	0.792486	1.47212	0.00865	0.0908042	no
gene:SpnNT_00817	ybiV	Chromosome:859691-860519	110.58+peptide	ΔORF2+peptide	OK	32.1927	35.5278	0.142215	0.2698	0.63405	0.980887	no
gene:SpnNT_00818	NA	Chromosome:860520-861156	110.58	ΔORF2	OK	13.3061	13.0634	-0.0265592	-0.0432496	0.93755	0.994855	no
gene:SpnNT_00818	NA	Chromosome:860520-861156	110.58	110.58+peptide	OK	13.3061	21.4403	0.688243	1.13811	0.04585	0.295648	no

gene:SpnNT_00818	NA	Chromosome:860520-861156	ΔORF2	110.58+peptide	OK	13.0634	21.4403	0.714802	1.18115	0.0385	0.263948	no
gene:SpnNT_00818	NA	Chromosome:860520-861156	110.58	ΔORF2+peptide	OK	13.3061	23.0052	0.789876	1.31	0.01905	0.160652	no
gene:SpnNT_00818	NA	Chromosome:860520-861156	ΔORF2	ΔORF2+peptide	OK	13.0634	23.0052	0.816435	1.35303	0.01495	0.133716	no
gene:SpnNT_00818	NA	Chromosome:860520-861156	110.58+peptide	ΔORF2+peptide	OK	21.4403	23.0052	0.101634	0.171136	0.7592	0.994748	no
gene:SpnNT_00819	NA	Chromosome:861239-862897	110.58	ΔORF2	OK	76.4631	114.378	0.580978	0.918359	0.11255	0.510502	no
gene:SpnNT_00819	NA	Chromosome:861239-862897	110.58	110.58+peptide	OK	76.4631	94.161	0.300366	0.481377	0.4026	0.894197	no
gene:SpnNT_00819	NA	Chromosome:861239-862897	ΔORF2	110.58+peptide	OK	114.378	94.161	-0.280612	-0.447831	0.4531	0.921244	no
gene:SpnNT_00819	NA	Chromosome:861239-862897	110.58	ΔORF2+peptide	OK	76.4631	148.452	0.957158	1.56162	0.00695	0.0769603	no
gene:SpnNT_00819	NA	Chromosome:861239-862897	ΔORF2	ΔORF2+peptide	OK	114.378	148.452	0.376181	0.611076	0.3018	0.807816	no
gene:SpnNT_00819	NA	Chromosome:861239-862897	110.58+peptide	ΔORF2+peptide	OK	94.161	148.452	0.656793	1.08255	0.06585	0.373376	no
gene:SpnNT_00820	pbpX_2	Chromosome:861239-862897	110.58	ΔORF2	OK	53.9436	60.846	0.173712	0.241547	0.66035	0.982966	no
gene:SpnNT_00820	pbpX_2	Chromosome:861239-862897	110.58	110.58+peptide	OK	53.9436	57.8645	0.101226	0.152013	0.791	0.994748	no
gene:SpnNT_00820	pbpX_2	Chromosome:861239-862897	ΔORF2	110.58+peptide	OK	60.846	57.8645	-0.0724853	-0.0972614	0.8607	0.994748	no
gene:SpnNT_00820	pbpX_2	Chromosome:861239-862897	110.58	ΔORF2+peptide	OK	53.9436	73.2125	0.440638	0.656128	0.25555	0.7547	no
gene:SpnNT_00820	pbpX_2	Chromosome:861239-862897	ΔORF2	ΔORF2+peptide	OK	60.846	73.2125	0.266927	0.355744	0.5284	0.956161	no
gene:SpnNT_00820	pbpX_2	Chromosome:861239-862897	110.58+peptide	ΔORF2+peptide	OK	57.8645	73.2125	0.339412	0.485253	0.3997	0.892895	no
gene:SpnNT_00821	yicL_1	Chromosome:862905-863802	110.58	ΔORF2	OK	79.6017	82.9836	0.0600263	0.130514	0.81855	0.994748	no
gene:SpnNT_00821	yicL_1	Chromosome:862905-863802	110.58	110.58+peptide	OK	79.6017	92.0029	0.208879	0.455089	0.41785	0.90311	no
gene:SpnNT_00821	yicL_1	Chromosome:862905-863802	ΔORF2	110.58+peptide	OK	82.9836	92.0029	0.148852	0.325143	0.56785	0.968621	no
gene:SpnNT_00821	yicL_1	Chromosome:862905-863802	110.58	ΔORF2+peptide	OK	79.6017	107.373	0.431765	0.944936	0.09925	0.477579	no
gene:SpnNT_00821	yicL_1	Chromosome:862905-863802	ΔORF2	ΔORF2+peptide	OK	82.9836	107.373	0.371738	0.815679	0.15635	0.602356	no
gene:SpnNT_00821	yicL_1	Chromosome:862905-863802	110.58+peptide	ΔORF2+peptide	OK	92.0029	107.373	0.222886	0.490081	0.3904	0.884466	no
gene:SpnNT_00822	yvoA	Chromosome:863844-864543	110.58	ΔORF2	OK	28.7906	37.3269	0.374616	0.701499	0.2132	0.698345	no
gene:SpnNT_00822	yvoA	Chromosome:863844-864543	110.58	110.58+peptide	OK	28.7906	24.4201	-0.237529	-0.429919	0.45535	0.921244	no
gene:SpnNT_00822	yvoA	Chromosome:863844-864543	ΔORF2	110.58+peptide	OK	37.3269	24.4201	-0.612145	-1.12344	0.05155	0.320739	no
gene:SpnNT_00822	yvoA	Chromosome:863844-864543	110.58	ΔORF2+peptide	OK	28.7906	30.4872	0.0826031	0.151317	0.79255	0.994748	no
gene:SpnNT_00822	yvoA	Chromosome:863844-864543	ΔORF2	ΔORF2+peptide	OK	37.3269	30.4872	-0.292013	-0.542584	0.34115	0.844955	no
gene:SpnNT_00822	yvoA	Chromosome:863844-864543	110.58+peptide	ΔORF2+peptide	OK	24.4201	30.4872	0.320132	0.575234	0.32405	0.829371	no
gene:SpnNT_00823	guaA	Chromosome:864684-866247	110.58	ΔORF2	OK	208.978	220.336	0.0763569	0.172735	0.7569	0.994748	no
gene:SpnNT_00823	guaA	Chromosome:864684-866247	110.58	110.58+peptide	OK	208.978	132.091	-0.661814	-1.50599	0.00735	0.0805737	no
gene:SpnNT_00823	guaA	Chromosome:864684-866247	ΔORF2	110.58+peptide	OK	220.336	132.091	-0.73817	-1.68296	0.0033	0.0432593	yes
gene:SpnNT_00823	guaA	Chromosome:864684-866247	110.58	ΔORF2+peptide	OK	208.978	132.457	-0.657824	-1.49071	0.0087	0.0910378	no
gene:SpnNT_00823	guaA	Chromosome:864684-866247	ΔORF2	ΔORF2+peptide	OK	220.336	132.457	-0.734181	-1.66691	0.0043	0.0535338	no
gene:SpnNT_00823	guaA	Chromosome:864684-866247	110.58+peptide	ΔORF2+peptide	OK	132.091	132.457	0.00398935	0.00911138	0.98865	0.997703	no
gene:SpnNT_00824	NA	Chromosome:866432-866825	110.58	ΔORF2	OK	15.6551	27.0386	0.78839	1.11342	0.0542	0.330796	no
gene:SpnNT_00824	NA	Chromosome:866432-866825	110.58	110.58+peptide	OK	15.6551	13.3838	-0.22614	-0.296956	0.59215	0.975468	no
gene:SpnNT_00824	NA	Chromosome:866432-866825	ΔORF2	110.58+peptide	OK	27.0386	13.3838	-1.01453	-1.32672	0.02295	0.185895	no
gene:SpnNT_00824	NA	Chromosome:866432-866825	110.58	ΔORF2+peptide	OK	15.6551	15.9942	0.0309216	0.0431212	0.93705	0.994855	no
gene:SpnNT_00824	NA	Chromosome:866432-866825	ΔORF2	ΔORF2+peptide	OK	27.0386	15.9942	-0.757468	-1.05139	0.0709	0.390523	no
gene:SpnNT_00824	NA	Chromosome:866432-866825	110.58+peptide	ΔORF2+peptide	OK	13.3838	15.9942	0.257062	0.332535	0.55765	0.968033	no
gene:SpnNT_00825	NA	Chromosome:866845-868210	110.58	ΔORF2	OK	3.72251	8.99838	1.27339	1.93535	0.00095	0.0161266	yes
gene:SpnNT_00825	NA	Chromosome:866845-868210	110.58	110.58+peptide	OK	3.72251	3.39633	-0.132299	-0.190378	0.743	0.994748	no
gene:SpnNT_00825	NA	Chromosome:866845-868210	ΔORF2	110.58+peptide	OK	8.99838	3.39633	-1.40569	-2.04274	8.00E-04	0.0140342	yes
gene:SpnNT_00825	NA	Chromosome:866845-868210	110.58	ΔORF2+peptide	OK	3.72251	7.39944	0.991139	1.57329	0.00705	0.077805	no
gene:SpnNT_00825	NA	Chromosome:866845-868210	ΔORF2	ΔORF2+peptide	OK	8.99838	7.39944	-0.282249	-0.453427	0.43215	0.913334	no

gene:SpnNT_00825	NA	Chromosome:866845-868210	110.58+peptide	ΔORF2+peptide	OK	3.39633	7.39944	1.12344	1.69849	0.0045	0.0552376	no
gene:SpnNT_00826	NA	Chromosome:868324-868540	110.58	ΔORF2	OK	64.4622	80.7076	0.324251	0.457735	0.41275	0.899511	no
gene:SpnNT_00826	NA	Chromosome:868324-868540	110.58	110.58+peptide	OK	64.4622	54.5703	-0.240338	-0.325629	0.5617	0.968621	no
gene:SpnNT_00826	NA	Chromosome:868324-868540	ΔORF2	110.58+peptide	OK	80.7076	54.5703	-0.564588	-0.762757	0.18535	0.658231	no
gene:SpnNT_00826	NA	Chromosome:868324-868540	110.58	ΔORF2+peptide	OK	64.4622	35.7467	-0.850644	-1.01997	0.063	0.363378	no
gene:SpnNT_00826	NA	Chromosome:868324-868540	ΔORF2	ΔORF2+peptide	OK	80.7076	35.7467	-1.17489	-1.40559	0.01345	0.124817	no
gene:SpnNT_00826	NA	Chromosome:868324-868540	110.58+peptide	ΔORF2+peptide	OK	54.5703	35.7467	-0.610306	-0.708687	0.1976	0.679305	no
gene:SpnNT_00827	NA	Chromosome:868544-869231	110.58	ΔORF2	OK	7.82022	20.0891	1.36113	1.9602	0.00055	0.0103545	yes
gene:SpnNT_00827	NA	Chromosome:868544-869231	110.58	110.58+peptide	OK	7.82022	9.57739	0.292424	0.421983	0.4522	0.921244	no
gene:SpnNT_00827	NA	Chromosome:868544-869231	ΔORF2	110.58+peptide	OK	20.0891	9.57739	-1.06871	-1.56214	0.00855	0.0898983	no
gene:SpnNT_00827	NA	Chromosome:868544-869231	110.58	ΔORF2+peptide	OK	7.82022	12.574	0.685163	1.01183	0.07355	0.399141	no
gene:SpnNT_00827	NA	Chromosome:868544-869231	ΔORF2	ΔORF2+peptide	OK	20.0891	12.574	-0.675971	-1.01178	0.07925	0.419153	no
gene:SpnNT_00827	NA	Chromosome:868544-869231	110.58+peptide	ΔORF2+peptide	OK	9.57739	12.574	0.392739	0.589135	0.30235	0.80833	no
gene:SpnNT_00828	NA	Chromosome:869415-871259	110.58	ΔORF2	NOTEST	0.115974	0.695337	2.58391	0	1	1	no
gene:SpnNT_00828	NA	Chromosome:869415-871259	110.58	110.58+peptide	NOTEST	0.115974	0.182401	0.653304	0	1	1	no
gene:SpnNT_00828	NA	Chromosome:869415-871259	ΔORF2	110.58+peptide	NOTEST	0.695337	0.182401	-1.9306	0	1	1	no
gene:SpnNT_00828	NA	Chromosome:869415-871259	110.58	ΔORF2+peptide	NOTEST	0.115974	0.137592	0.24659	0	1	1	no
gene:SpnNT_00828	NA	Chromosome:869415-871259	ΔORF2	ΔORF2+peptide	NOTEST	0.695337	0.137592	-2.33732	0	1	1	no
gene:SpnNT_00828	NA	Chromosome:869415-871259	110.58+peptide	ΔORF2+peptide	NOTEST	0.182401	0.137592	-0.406714	0	1	1	no
gene:SpnNT_00829	NA	Chromosome:869415-871259	110.58	ΔORF2	NOTEST	0.416027	0.742311	0.835347	0	1	1	no
gene:SpnNT_00829	NA	Chromosome:869415-871259	110.58	110.58+peptide	NOTEST	0.416027	0.587591	0.498136	0	1	1	no
gene:SpnNT_00829	NA	Chromosome:869415-871259	ΔORF2	110.58+peptide	NOTEST	0.742311	0.587591	-0.337211	0	1	1	no
gene:SpnNT_00829	NA	Chromosome:869415-871259	110.58	ΔORF2+peptide	NOTEST	0.416027	0.221192	-0.911379	0	1	1	no
gene:SpnNT_00829	NA	Chromosome:869415-871259	ΔORF2	ΔORF2+peptide	NOTEST	0.742311	0.221192	-1.74673	0	1	1	no
gene:SpnNT_00829	NA	Chromosome:869415-871259	110.58+peptide	ΔORF2+peptide	NOTEST	0.587591	0.221192	-1.40951	0	1	1	no
gene:SpnNT_00830	NA	Chromosome:871362-872175	110.58	ΔORF2	NOTEST	0.357969	0.286877	-0.319405	0	1	1	no
gene:SpnNT_00830	NA	Chromosome:871362-872175	110.58	110.58+peptide	NOTEST	0.357969	0.436861	0.287339	0	1	1	no
gene:SpnNT_00830	NA	Chromosome:871362-872175	ΔORF2	110.58+peptide	NOTEST	0.286877	0.436861	0.606745	0	1	1	no
gene:SpnNT_00830	NA	Chromosome:871362-872175	110.58	ΔORF2+peptide	NOTEST	0.357969	0.241812	-0.565948	0	1	1	no
gene:SpnNT_00830	NA	Chromosome:871362-872175	ΔORF2	ΔORF2+peptide	NOTEST	0.286877	0.241812	-0.246543	0	1	1	no
gene:SpnNT_00830	NA	Chromosome:871362-872175	110.58+peptide	ΔORF2+peptide	NOTEST	0.436861	0.241812	-0.853287	0	1	1	no
gene:SpnNT_00831	NA	Chromosome:872182-872377	110.58	ΔORF2	NOTEST	0	0	0	0	1	1	no
gene:SpnNT_00831	NA	Chromosome:872182-872377	110.58	110.58+peptide	NOTEST	0	0.871073	Inf	0	1	1	no
gene:SpnNT_00831	NA	Chromosome:872182-872377	ΔORF2	110.58+peptide	NOTEST	0	0.871073	Inf	0	1	1	no
gene:SpnNT_00831	NA	Chromosome:872182-872377	110.58	ΔORF2+peptide	NOTEST	0	0	0	0	1	1	no
gene:SpnNT_00831	NA	Chromosome:872182-872377	ΔORF2	ΔORF2+peptide	NOTEST	0	0	0	0	1	1	no
gene:SpnNT_00831	NA	Chromosome:872182-872377	110.58+peptide	ΔORF2+peptide	NOTEST	0.871073	0	#NAME?	0	1	1	no
gene:SpnNT_00832	xerC_2	Chromosome:872380-873607	110.58	ΔORF2	OK	25.3713	34.7786	0.455002	0.898248	0.1163	0.517109	no
gene:SpnNT_00832	xerC_2	Chromosome:872380-873607	110.58	110.58+peptide	OK	25.3713	30.7109	0.27555	0.547885	0.32595	0.831443	no
gene:SpnNT_00832	xerC_2	Chromosome:872380-873607	ΔORF2	110.58+peptide	OK	34.7786	30.7109	-0.179452	-0.354595	0.5366	0.959314	no
gene:SpnNT_00832	xerC_2	Chromosome:872380-873607	110.58	ΔORF2+peptide	OK	25.3713	32.7882	0.369978	0.74578	0.18235	0.652166	no
gene:SpnNT_00832	xerC_2	Chromosome:872380-873607	ΔORF2	ΔORF2+peptide	OK	34.7786	32.7882	-0.0850239	-0.170293	0.76585	0.994748	no
gene:SpnNT_00832	xerC_2	Chromosome:872380-873607	110.58+peptide	ΔORF2+peptide	OK	30.7109	32.7882	0.0944282	0.190526	0.7326	0.994748	no
gene:SpnNT_00833	dpnA_1	Chromosome:873787-875163	110.58	ΔORF2	OK	25.0897	51.8324	1.04676	1.92661	0.00095	0.0161266	yes
gene:SpnNT_00833	dpnA_1	Chromosome:873787-875163	110.58	110.58+peptide	OK	25.0897	30.0803	0.261724	0.491911	0.39275	0.887798	no

gene:SpnNT_00833	dpnA_1	Chromosome:873787-875163	ΔORF2	110.58+peptide	OK	51.8324	30.0803	-0.785037	-1.42932	0.0142	0.129128	no
gene:SpnNT_00833	dpnA_1	Chromosome:873787-875163	110.58	ΔORF2+peptide	OK	25.0897	52.0491	1.05278	2.08006	0.00025	0.00542231	yes
gene:SpnNT_00833	dpnA_1	Chromosome:873787-875163	ΔORF2	ΔORF2+peptide	OK	51.8324	52.0491	0.00601898	0.0114831	0.98285	0.996246	no
gene:SpnNT_00833	dpnA_1	Chromosome:873787-875163	110.58+peptide	ΔORF2+peptide	OK	30.0803	52.0491	0.791055	1.54358	0.0082	0.08748	no
gene:SpnNT_00834	NA	Chromosome:873787-875163	110.58	ΔORF2	OK	35.9985	52.2134	0.536485	0.191725	0.80765	0.994748	no
gene:SpnNT_00834	NA	Chromosome:873787-875163	110.58	110.58+peptide	OK	35.9985	46.1015	0.356877	0.137497	0.84075	0.994748	no
gene:SpnNT_00834	NA	Chromosome:873787-875163	ΔORF2	110.58+peptide	OK	52.2134	46.1015	-0.179608	-0.0642474	0.891	0.994748	no
gene:SpnNT_00834	NA	Chromosome:873787-875163	110.58	ΔORF2+peptide	OK	35.9985	29.3005	-0.297011	-0.144044	0.8865	0.994748	no
gene:SpnNT_00834	NA	Chromosome:873787-875163	ΔORF2	ΔORF2+peptide	OK	52.2134	29.3005	-0.833496	-0.361023	0.7749	0.994748	no
gene:SpnNT_00834	NA	Chromosome:873787-875163	110.58+peptide	ΔORF2+peptide	OK	46.1015	29.3005	-0.653887	-0.317672	0.80005	0.994748	no
gene:SpnNT_00835	NA	Chromosome:875346-875529	110.58	ΔORF2	NOTEST	1.69199	1.21054	-0.483079	0	1	1	no
gene:SpnNT_00835	NA	Chromosome:875346-875529	110.58	110.58+peptide	NOTEST	1.69199	2.25037	0.411435	0	1	1	no
gene:SpnNT_00835	NA	Chromosome:875346-875529	ΔORF2	110.58+peptide	NOTEST	1.21054	2.25037	0.894514	0	1	1	no
gene:SpnNT_00835	NA	Chromosome:875346-875529	110.58	ΔORF2+peptide	NOTEST	1.69199	1.33902	-0.337542	0	1	1	no
gene:SpnNT_00835	NA	Chromosome:875346-875529	ΔORF2	ΔORF2+peptide	NOTEST	1.21054	1.33902	0.145536	0	1	1	no
gene:SpnNT_00835	NA	Chromosome:875346-875529	110.58+peptide	ΔORF2+peptide	NOTEST	2.25037	1.33902	-0.748978	0	1	1	no
gene:SpnNT_00836	yhbU_2	Chromosome:876152-877439	110.58	ΔORF2	OK	84.947	87.0466	0.0352263	0.0736406	0.8962	0.994748	no
gene:SpnNT_00836	yhbU_2	Chromosome:876152-877439	110.58	110.58+peptide	OK	84.947	52.9634	-0.681567	-1.44534	0.01275	0.119932	no
gene:SpnNT_00836	yhbU_2	Chromosome:876152-877439	ΔORF2	110.58+peptide	OK	87.0466	52.9634	-0.716794	-1.48163	0.0106	0.10466	no
gene:SpnNT_00836	yhbU_2	Chromosome:876152-877439	110.58	ΔORF2+peptide	OK	84.947	48.9384	-0.795595	-1.70917	0.0041	0.0516317	no
gene:SpnNT_00836	yhbU_2	Chromosome:876152-877439	ΔORF2	ΔORF2+peptide	OK	87.0466	48.9384	-0.830821	-1.7386	0.00415	0.0519126	no
gene:SpnNT_00836	yhbU_2	Chromosome:876152-877439	110.58+peptide	ΔORF2+peptide	OK	52.9634	48.9384	-0.114028	-0.242062	0.6751	0.985207	no
gene:SpnNT_00837	NA	Chromosome:878027-878753	110.58	ΔORF2	OK	0.802222	1.18917	0.567877	0.523728	0.3668	0.864634	no
gene:SpnNT_00837	NA	Chromosome:878027-878753	110.58	110.58+peptide	NOTEST	0.802222	0.685652	-0.226524	0	1	1	no
gene:SpnNT_00837	NA	Chromosome:878027-878753	ΔORF2	110.58+peptide	OK	1.18917	0.685652	-0.794401	-0.717972	0.22255	0.712269	no
gene:SpnNT_00837	NA	Chromosome:878027-878753	110.58	ΔORF2+peptide	NOTEST	0.802222	0.377125	-1.08896	0	1	1	no
gene:SpnNT_00837	NA	Chromosome:878027-878753	ΔORF2	ΔORF2+peptide	OK	1.18917	0.377125	-1.65683	-1.30591	0.05205	0.322779	no
gene:SpnNT_00837	NA	Chromosome:878027-878753	110.58+peptide	ΔORF2+peptide	NOTEST	0.685652	0.377125	-0.862434	0	1	1	no
gene:SpnNT_00838	yxlF_2	Chromosome:879135-879981	110.58	ΔORF2	OK	13.6807	23.5372	0.782801	1.33809	0.0227	0.184211	no
gene:SpnNT_00838	yxlF_2	Chromosome:879135-879981	110.58	110.58+peptide	OK	13.6807	15.4916	0.179341	0.305285	0.5976	0.97629	no
gene:SpnNT_00838	yxlF_2	Chromosome:879135-879981	ΔORF2	110.58+peptide	OK	23.5372	15.4916	-0.603459	-1.01301	0.08675	0.442587	no
gene:SpnNT_00838	yxlF_2	Chromosome:879135-879981	110.58	ΔORF2+peptide	OK	13.6807	16.0723	0.232433	0.396704	0.48095	0.935095	no
gene:SpnNT_00838	yxlF_2	Chromosome:879135-879981	ΔORF2	ΔORF2+peptide	OK	23.5372	16.0723	-0.550368	-0.926258	0.1071	0.498004	no
gene:SpnNT_00838	yxlF_2	Chromosome:879135-879981	110.58+peptide	ΔORF2+peptide	OK	15.4916	16.0723	0.0530919	0.0889926	0.8761	0.994748	no
gene:SpnNT_00839	NA	Chromosome:879996-880500	110.58	ΔORF2	OK	17.3756	24.9063	0.519448	0.836069	0.15125	0.593484	no
gene:SpnNT_00839	NA	Chromosome:879996-880500	110.58	110.58+peptide	OK	17.3756	17.0848	-0.0243473	-0.0386782	0.94695	0.994855	no
gene:SpnNT_00839	NA	Chromosome:879996-880500	ΔORF2	110.58+peptide	OK	24.9063	17.0848	-0.543795	-0.87828	0.1255	0.536246	no
gene:SpnNT_00839	NA	Chromosome:879996-880500	110.58	ΔORF2+peptide	OK	17.3756	18.0653	0.0561533	0.0886403	0.8767	0.994748	no
gene:SpnNT_00839	NA	Chromosome:879996-880500	ΔORF2	ΔORF2+peptide	OK	24.9063	18.0653	-0.463295	-0.743367	0.19635	0.677648	no
gene:SpnNT_00839	NA	Chromosome:879996-880500	110.58+peptide	ΔORF2+peptide	OK	17.0848	18.0653	0.0805007	0.127496	0.82435	0.994748	no
gene:SpnNT_00840	NA	Chromosome:881103-881791	110.58	ΔORF2	OK	22.3301	27.3524	0.292675	0.182972	0.75095	0.994748	no
gene:SpnNT_00840	NA	Chromosome:881103-881791	110.58	110.58+peptide	OK	22.3301	29.6771	0.41036	0.23151	0.68835	0.98828	no
gene:SpnNT_00840	NA	Chromosome:881103-881791	ΔORF2	110.58+peptide	OK	27.3524	29.6771	0.117685	0.0723177	0.8962	0.994748	no
gene:SpnNT_00840	NA	Chromosome:881103-881791	110.58	ΔORF2+peptide	OK	22.3301	21.9519	-0.0246427	-0.013757	0.97905	0.995765	no
gene:SpnNT_00840	NA	Chromosome:881103-881791	ΔORF2	ΔORF2+peptide	OK	27.3524	21.9519	-0.317318	-0.192578	0.7342	0.994748	no

gene:SpnNT_00840	NA	Chromosome:881103-881791	110.58+peptide	ΔORF2+peptide	OK	29.6771	21.9519	-0.435003	-0.239521	0.6816	0.986574	no
gene:SpnNT_00841	NA	Chromosome:881103-881791	110.58	ΔORF2	OK	12.163	15.7468	0.372555	0.513472	0.37255	0.869703	no
gene:SpnNT_00841	NA	Chromosome:881103-881791	110.58	110.58+peptide	OK	12.163	13.4739	0.147672	0.194404	0.74335	0.994748	no
gene:SpnNT_00841	NA	Chromosome:881103-881791	ΔORF2	110.58+peptide	OK	15.7468	13.4739	-0.224883	-0.307604	0.59335	0.975539	no
gene:SpnNT_00841	NA	Chromosome:881103-881791	110.58	ΔORF2+peptide	OK	12.163	18.538	0.607989	0.841826	0.14625	0.582956	no
gene:SpnNT_00841	NA	Chromosome:881103-881791	ΔORF2	ΔORF2+peptide	OK	15.7468	18.538	0.235434	0.340148	0.545	0.961568	no
gene:SpnNT_00841	NA	Chromosome:881103-881791	110.58+peptide	ΔORF2+peptide	OK	13.4739	18.538	0.460317	0.632504	0.28085	0.784388	no
gene:SpnNT_00842	pncB2	Chromosome:881906-884188	110.58	ΔORF2	OK	83.6061	69.3066	-0.270616	-0.500492	0.3768	0.875032	no
gene:SpnNT_00842	pncB2	Chromosome:881906-884188	110.58	110.58+peptide	OK	83.6061	76.4436	-0.129213	-0.241215	0.6722	0.984845	no
gene:SpnNT_00842	pncB2	Chromosome:881906-884188	ΔORF2	110.58+peptide	OK	69.3066	76.4436	0.141403	0.261533	0.647	0.981268	no
gene:SpnNT_00842	pncB2	Chromosome:881906-884188	110.58	ΔORF2+peptide	OK	83.6061	69.7069	-0.262308	-0.490539	0.3893	0.883652	no
gene:SpnNT_00842	pncB2	Chromosome:881906-884188	ΔORF2	ΔORF2+peptide	OK	69.3066	69.7069	0.00830843	0.0153935	0.9781	0.995765	no
gene:SpnNT_00842	pncB2	Chromosome:881906-884188	110.58+peptide	ΔORF2+peptide	OK	76.4436	69.7069	-0.133095	-0.248913	0.66625	0.983855	no
gene:SpnNT_00843	nadE	Chromosome:881906-884188	110.58	ΔORF2	OK	84.0185	66.8705	-0.329338	-0.425417	0.45625	0.921244	no
gene:SpnNT_00843	nadE	Chromosome:881906-884188	110.58	110.58+peptide	OK	84.0185	71.8326	-0.226068	-0.287406	0.61785	0.980887	no
gene:SpnNT_00843	nadE	Chromosome:881906-884188	ΔORF2	110.58+peptide	OK	66.8705	71.8326	0.10327	0.129489	0.817	0.994748	no
gene:SpnNT_00843	nadE	Chromosome:881906-884188	110.58	ΔORF2+peptide	OK	84.0185	68.9618	-0.284909	-0.367323	0.52275	0.954832	no
gene:SpnNT_00843	nadE	Chromosome:881906-884188	ΔORF2	ΔORF2+peptide	OK	66.8705	68.9618	0.0444289	0.0564733	0.91945	0.994748	no
gene:SpnNT_00843	nadE	Chromosome:881906-884188	110.58+peptide	ΔORF2+peptide	OK	71.8326	68.9618	-0.058841	-0.0736472	0.8976	0.994748	no
gene:SpnNT_00844	ydaF_3	Chromosome:884436-884988	110.58	ΔORF2	OK	79.1708	69.8531	-0.180644	-0.357224	0.5335	0.958867	no
gene:SpnNT_00844	ydaF_3	Chromosome:884436-884988	110.58	110.58+peptide	OK	79.1708	69.8149	-0.181434	-0.358553	0.52745	0.956161	no
gene:SpnNT_00844	ydaF_3	Chromosome:884436-884988	ΔORF2	110.58+peptide	OK	69.8531	69.8149	-0.00079002	-0.00154824	0.99745	0.999412	no
gene:SpnNT_00844	ydaF_3	Chromosome:884436-884988	110.58	ΔORF2+peptide	OK	79.1708	76.047	-0.0580776	-0.115158	0.8331	0.994748	no
gene:SpnNT_00844	ydaF_3	Chromosome:884436-884988	ΔORF2	ΔORF2+peptide	OK	69.8531	76.047	0.122567	0.24099	0.66845	0.984845	no
gene:SpnNT_00844	ydaF_3	Chromosome:884436-884988	110.58+peptide	ΔORF2+peptide	OK	69.8149	76.047	0.123357	0.242388	0.66635	0.983892	no
gene:SpnNT_00845	NA	Chromosome:885604-885706	110.58	ΔORF2	OK	68.558	12.9518	-2.40418	-15.5626	0.1958	0.677365	no
gene:SpnNT_00845	NA	Chromosome:885604-885706	110.58	110.58+peptide	OK	68.558	69.5031	0.0197526	0.18493	0.88195	0.994748	no
gene:SpnNT_00845	NA	Chromosome:885604-885706	ΔORF2	110.58+peptide	OK	12.9518	69.5031	2.42393	16.5331	0.20015	0.683054	no
gene:SpnNT_00845	NA	Chromosome:885604-885706	110.58	ΔORF2+peptide	OK	68.558	23.5219	-1.54332	-14.0636	0.3929	0.887984	no
gene:SpnNT_00845	NA	Chromosome:885604-885706	ΔORF2	ΔORF2+peptide	NOTEST	12.9518	23.5219	0.860859	0	1	1	no
gene:SpnNT_00845	NA	Chromosome:885604-885706	110.58+peptide	ΔORF2+peptide	OK	69.5031	23.5219	-1.56307	-15.8938	0.4114	0.898833	no
gene:SpnNT_00846	lytA_4	Chromosome:886440-886830	110.58	ΔORF2	OK	86.5135	67.655	-0.354728	-0.642496	0.25745	0.757287	no
gene:SpnNT_00846	lytA_4	Chromosome:886440-886830	110.58	110.58+peptide	OK	86.5135	64.737	-0.418334	-0.76335	0.1828	0.653419	no
gene:SpnNT_00846	lytA_4	Chromosome:886440-886830	ΔORF2	110.58+peptide	OK	67.655	64.737	-0.0636057	-0.113631	0.8431	0.994748	no
gene:SpnNT_00846	lytA_4	Chromosome:886440-886830	110.58	ΔORF2+peptide	OK	86.5135	50.5656	-0.774768	-1.36402	0.01775	0.151736	no
gene:SpnNT_00846	lytA_4	Chromosome:886440-886830	ΔORF2	ΔORF2+peptide	OK	67.655	50.5656	-0.42004	-0.725041	0.20265	0.686593	no
gene:SpnNT_00846	lytA_4	Chromosome:886440-886830	110.58+peptide	ΔORF2+peptide	OK	64.737	50.5656	-0.356434	-0.619412	0.2856	0.78895	no
gene:SpnNT_00847	NA	Chromosome:886965-888293	110.58	ΔORF2	OK	2.64806	2.56758	-0.0445268	-0.0319397	0.96275	0.994855	no
gene:SpnNT_00847	NA	Chromosome:886965-888293	110.58	110.58+peptide	OK	2.64806	4.5476	0.780167	0.561725	0.35375	0.856246	no
gene:SpnNT_00847	NA	Chromosome:886965-888293	ΔORF2	110.58+peptide	OK	2.56758	4.5476	0.824693	0.604557	0.32395	0.829371	no
gene:SpnNT_00847	NA	Chromosome:886965-888293	110.58	ΔORF2+peptide	OK	2.64806	4.88899	0.884597	0.519044	0.38135	0.879103	no
gene:SpnNT_00847	NA	Chromosome:886965-888293	ΔORF2	ΔORF2+peptide	OK	2.56758	4.88899	0.929124	0.551679	0.36045	0.861221	no
gene:SpnNT_00847	NA	Chromosome:886965-888293	110.58+peptide	ΔORF2+peptide	OK	4.5476	4.88899	0.10443	0.0621661	0.91205	0.994748	no
gene:SpnNT_00848	NA	Chromosome:886965-888293	110.58	ΔORF2	NOTEST	0	0	0	0	1	1	no
gene:SpnNT_00848	NA	Chromosome:886965-888293	110.58	110.58+peptide	NOTEST	0	0	0	0	1	1	no

gene:SpnNT_00848	NA	Chromosome:886965-888293	ΔORF2	110.58+peptide	NOTEST	0	0	0	0	1	1	no
gene:SpnNT_00848	NA	Chromosome:886965-888293	110.58	ΔORF2+peptide	NOTEST	0	9.69348	Inf	0	1	1	no
gene:SpnNT_00848	NA	Chromosome:886965-888293	ΔORF2	ΔORF2+peptide	NOTEST	0	9.69348	Inf	0	1	1	no
gene:SpnNT_00848	NA	Chromosome:886965-888293	110.58+peptide	ΔORF2+peptide	NOTEST	0	9.69348	Inf	0	1	1	no
gene:SpnNT_00849	NA	Chromosome:886965-888293	110.58	ΔORF2	OK	7.79731	6.01863	-0.373542	-0.225377	0.675	0.985207	no
gene:SpnNT_00849	NA	Chromosome:886965-888293	110.58	110.58+peptide	OK	7.79731	18.7227	1.26374	0.950113	0.0988	0.476287	no
gene:SpnNT_00849	NA	Chromosome:886965-888293	ΔORF2	110.58+peptide	OK	6.01863	18.7227	1.63728	1.08841	0.0695	0.387088	no
gene:SpnNT_00849	NA	Chromosome:886965-888293	110.58	ΔORF2+peptide	OK	7.79731	27.042	1.79415	1.37699	0.02775	0.211953	no
gene:SpnNT_00849	NA	Chromosome:886965-888293	ΔORF2	ΔORF2+peptide	OK	6.01863	27.042	2.16769	1.46432	0.02865	0.215689	no
gene:SpnNT_00849	NA	Chromosome:886965-888293	110.58+peptide	ΔORF2+peptide	OK	18.7227	27.042	0.530411	0.481483	0.44005	0.916416	no
gene:SpnNT_00850	NA	Chromosome:886965-888293	110.58	ΔORF2	OK	2.50849	3.97231	0.663158	0.537439	0.38455	0.881717	no
gene:SpnNT_00850	NA	Chromosome:886965-888293	110.58	110.58+peptide	OK	2.50849	5.16095	1.04082	0.829061	0.19275	0.674291	no
gene:SpnNT_00850	NA	Chromosome:886965-888293	ΔORF2	110.58+peptide	OK	3.97231	5.16095	0.377658	0.354097	0.5507	0.964281	no
gene:SpnNT_00850	NA	Chromosome:886965-888293	110.58	ΔORF2+peptide	OK	2.50849	5.80983	1.21167	0.957105	0.14	0.571231	no
gene:SpnNT_00850	NA	Chromosome:886965-888293	ΔORF2	ΔORF2+peptide	OK	3.97231	5.80983	0.548517	0.50838	0.40025	0.893627	no
gene:SpnNT_00850	NA	Chromosome:886965-888293	110.58+peptide	ΔORF2+peptide	OK	5.16095	5.80983	0.17086	0.154839	0.7921	0.994748	no
gene:SpnNT_00851	queA	Chromosome:888983-890012	110.58	ΔORF2	OK	22.3607	25.0389	0.163208	0.309694	0.58485	0.971855	no
gene:SpnNT_00851	queA	Chromosome:888983-890012	110.58	110.58+peptide	OK	22.3607	15.9629	-0.486244	-0.894274	0.1202	0.524705	no
gene:SpnNT_00851	queA	Chromosome:888983-890012	ΔORF2	110.58+peptide	OK	25.0389	15.9629	-0.649452	-1.20101	0.0355	0.250178	no
gene:SpnNT_00851	queA	Chromosome:888983-890012	110.58	ΔORF2+peptide	OK	22.3607	15.2771	-0.549593	-1.00834	0.0807	0.423409	no
gene:SpnNT_00851	queA	Chromosome:888983-890012	ΔORF2	ΔORF2+peptide	OK	25.0389	15.2771	-0.712801	-1.31493	0.02075	0.172221	no
gene:SpnNT_00851	queA	Chromosome:888983-890012	110.58+peptide	ΔORF2+peptide	OK	15.9629	15.2771	-0.0633495	-0.113456	0.83995	0.994748	no
gene:SpnNT_00852	nagB	Chromosome:890162-890870	110.58	ΔORF2	OK	25.3463	28.9053	0.189561	0.344063	0.55365	0.965961	no
gene:SpnNT_00852	nagB	Chromosome:890162-890870	110.58	110.58+peptide	OK	25.3463	19.9662	-0.344212	-0.608437	0.28915	0.793273	no
gene:SpnNT_00852	nagB	Chromosome:890162-890870	ΔORF2	110.58+peptide	OK	28.9053	19.9662	-0.533773	-0.959346	0.0965	0.470343	no
gene:SpnNT_00852	nagB	Chromosome:890162-890870	110.58	ΔORF2+peptide	OK	25.3463	19.7869	-0.357232	-0.62922	0.2727	0.774707	no
gene:SpnNT_00852	nagB	Chromosome:890162-890870	ΔORF2	ΔORF2+peptide	OK	28.9053	19.7869	-0.546793	-0.979156	0.08905	0.448911	no
gene:SpnNT_00852	nagB	Chromosome:890162-890870	110.58+peptide	ΔORF2+peptide	OK	19.9662	19.7869	-0.0130201	-0.0227218	0.96595	0.994855	no
gene:SpnNT_00853	rpsU	Chromosome:891009-891186	110.58	ΔORF2	OK	5245.19	5858.35	0.159497	0.314791	0.5711	0.96884	no
gene:SpnNT_00853	rpsU	Chromosome:891009-891186	110.58	110.58+peptide	OK	5245.19	16486.3	1.6522	3.16128	5.00E-05	0.0013612	yes
gene:SpnNT_00853	rpsU	Chromosome:891009-891186	ΔORF2	110.58+peptide	OK	5858.35	16486.3	1.49271	2.85055	5.00E-05	0.0013612	yes
gene:SpnNT_00853	rpsU	Chromosome:891009-891186	110.58	ΔORF2+peptide	OK	5245.19	14743.1	1.49097	3.06715	5.00E-05	0.0013612	yes
gene:SpnNT_00853	rpsU	Chromosome:891009-891186	ΔORF2	ΔORF2+peptide	OK	5858.35	14743.1	1.33148	2.73288	5.00E-05	0.0013612	yes
gene:SpnNT_00853	rpsU	Chromosome:891009-891186	110.58+peptide	ΔORF2+peptide	OK	16486.3	14743.1	-0.161229	-0.320037	0.5582	0.968033	no
gene:SpnNT_00854	hprK	Chromosome:891315-893032	110.58	ΔORF2	OK	186.678	198.093	0.0856264	0.151251	0.79305	0.994748	no
gene:SpnNT_00854	hprK	Chromosome:891315-893032	110.58	110.58+peptide	OK	186.678	142.434	-0.390255	-0.680131	0.24045	0.735646	no
gene:SpnNT_00854	hprK	Chromosome:891315-893032	ΔORF2	110.58+peptide	OK	198.093	142.434	-0.475881	-0.833832	0.1486	0.588569	no
gene:SpnNT_00854	hprK	Chromosome:891315-893032	110.58	ΔORF2+peptide	OK	186.678	162.332	-0.201603	-0.351372	0.5419	0.961494	no
gene:SpnNT_00854	hprK	Chromosome:891315-893032	ΔORF2	ΔORF2+peptide	OK	198.093	162.332	-0.28723	-0.503309	0.37965	0.877496	no
gene:SpnNT_00854	hprK	Chromosome:891315-893032	110.58+peptide	ΔORF2+peptide	OK	142.434	162.332	0.188651	0.32622	0.5728	0.969538	no
gene:SpnNT_00855	lgt	Chromosome:891315-893032	110.58	ΔORF2	OK	155.298	162.235	0.0630433	0.0891266	0.87785	0.994748	no
gene:SpnNT_00855	lgt	Chromosome:891315-893032	110.58	110.58+peptide	OK	155.298	110.063	-0.496718	-0.689943	0.2404	0.735646	no
gene:SpnNT_00855	lgt	Chromosome:891315-893032	ΔORF2	110.58+peptide	OK	162.235	110.063	-0.559762	-0.788292	0.1778	0.644322	no
gene:SpnNT_00855	lgt	Chromosome:891315-893032	110.58	ΔORF2+peptide	OK	155.298	160.911	0.0512157	0.0746918	0.8985	0.994748	no
gene:SpnNT_00855	lgt	Chromosome:891315-893032	ΔORF2	ΔORF2+peptide	OK	162.235	160.911	-0.0118276	-0.0175133	0.97655	0.99536	no

gene:SpnNT_00855	lgt	Chromosome:891315-893032	110.58+peptide	ΔORF2+peptide	OK	110.063	160.911	0.547934	0.795803	0.1744	0.634982	no
gene:SpnNT_00856	NA	Chromosome:893032-893416	110.58	ΔORF2	OK	180.72	166.126	-0.12148	-0.241054	0.6703	0.984845	no
gene:SpnNT_00856	NA	Chromosome:893032-893416	110.58	110.58+peptide	OK	180.72	155.027	-0.221234	-0.432341	0.445	0.917756	no
gene:SpnNT_00856	NA	Chromosome:893032-893416	ΔORF2	110.58+peptide	OK	166.126	155.027	-0.0997543	-0.195261	0.7264	0.994748	no
gene:SpnNT_00856	NA	Chromosome:893032-893416	110.58	ΔORF2+peptide	OK	180.72	133.106	-0.441181	-0.856172	0.1251	0.535526	no
gene:SpnNT_00856	NA	Chromosome:893032-893416	ΔORF2	ΔORF2+peptide	OK	166.126	133.106	-0.319701	-0.621426	0.26315	0.763902	no
gene:SpnNT_00856	NA	Chromosome:893032-893416	110.58+peptide	ΔORF2+peptide	OK	155.027	133.106	-0.219947	-0.4213	0.454	0.921244	no
gene:SpnNT_00857	NA	Chromosome:893431-893821	110.58	ΔORF2	OK	492.241	600.277	0.286263	0.623068	0.2819	0.784843	no
gene:SpnNT_00857	NA	Chromosome:893431-893821	110.58	110.58+peptide	OK	492.241	390.472	-0.334147	-0.731647	0.20135	0.684478	no
gene:SpnNT_00857	NA	Chromosome:893431-893821	ΔORF2	110.58+peptide	OK	600.277	390.472	-0.62041	-1.35402	0.0207	0.171915	no
gene:SpnNT_00857	NA	Chromosome:893431-893821	110.58	ΔORF2+peptide	OK	492.241	487.566	-0.0137677	-0.0303239	0.9572	0.994855	no
gene:SpnNT_00857	NA	Chromosome:893431-893821	ΔORF2	ΔORF2+peptide	OK	600.277	487.566	-0.300031	-0.658647	0.24755	0.743991	no
gene:SpnNT_00857	NA	Chromosome:893431-893821	110.58+peptide	ΔORF2+peptide	OK	390.472	487.566	0.320379	0.707604	0.2155	0.702371	no
gene:SpnNT_00858	hemN	Chromosome:893904-895035	110.58	ΔORF2	OK	90.186	99.7108	0.144846	0.325868	0.57005	0.968621	no
gene:SpnNT_00858	hemN	Chromosome:893904-895035	110.58	110.58+peptide	OK	90.186	75.6509	-0.253547	-0.564104	0.3258	0.831256	no
gene:SpnNT_00858	hemN	Chromosome:893904-895035	ΔORF2	110.58+peptide	OK	99.7108	75.6509	-0.398393	-0.887993	0.12195	0.528994	no
gene:SpnNT_00858	hemN	Chromosome:893904-895035	110.58	ΔORF2+peptide	OK	90.186	93.9471	0.0589445	0.132308	0.8192	0.994748	no
gene:SpnNT_00858	hemN	Chromosome:893904-895035	ΔORF2	ΔORF2+peptide	OK	99.7108	93.9471	-0.0859018	-0.193178	0.738	0.994748	no
gene:SpnNT_00858	hemN	Chromosome:893904-895035	110.58+peptide	ΔORF2+peptide	OK	75.6509	93.9471	0.312491	0.694965	0.2218	0.711082	no
gene:SpnNT_00859	NA	Chromosome:895039-895777	110.58	ΔORF2	OK	48.7387	54.5925	0.163635	0.327667	0.56775	0.968621	no
gene:SpnNT_00859	NA	Chromosome:895039-895777	110.58	110.58+peptide	OK	48.7387	42.886	-0.184562	-0.364218	0.5232	0.954832	no
gene:SpnNT_00859	NA	Chromosome:895039-895777	ΔORF2	110.58+peptide	OK	54.5925	42.886	-0.348196	-0.691721	0.23245	0.725549	no
gene:SpnNT_00859	NA	Chromosome:895039-895777	110.58	ΔORF2+peptide	OK	48.7387	45.0239	-0.114378	-0.2254	0.69505	0.98895	no
gene:SpnNT_00859	NA	Chromosome:895039-895777	ΔORF2	ΔORF2+peptide	OK	54.5925	45.0239	-0.278012	-0.551512	0.34055	0.844744	no
gene:SpnNT_00859	NA	Chromosome:895039-895777	110.58+peptide	ΔORF2+peptide	OK	42.886	45.0239	0.0701839	0.137248	0.8122	0.994748	no
gene:SpnNT_00860	yutF	Chromosome:895787-897168	110.58	ΔORF2	OK	106.701	102.29	-0.0609012	-0.113455	0.84475	0.994748	no
gene:SpnNT_00860	yutF	Chromosome:895787-897168	110.58	110.58+peptide	OK	106.701	81.0784	-0.396182	-0.725008	0.20865	0.694195	no
gene:SpnNT_00860	yutF	Chromosome:895787-897168	ΔORF2	110.58+peptide	OK	102.29	81.0784	-0.33528	-0.606313	0.291	0.795269	no
gene:SpnNT_00860	yutF	Chromosome:895787-897168	110.58	ΔORF2+peptide	OK	106.701	80.7592	-0.401874	-0.734552	0.20315	0.687222	no
gene:SpnNT_00860	yutF	Chromosome:895787-897168	ΔORF2	ΔORF2+peptide	OK	102.29	80.7592	-0.340973	-0.615893	0.28385	0.786627	no
gene:SpnNT_00860	yutF	Chromosome:895787-897168	110.58+peptide	ΔORF2+peptide	OK	81.0784	80.7592	-0.00569247	-0.010111	0.9867	0.997339	no
gene:SpnNT_00861	NA	Chromosome:895787-897168	110.58	ΔORF2	OK	48.0509	59.8093	0.315808	0.336126	0.55045	0.964281	no
gene:SpnNT_00861	NA	Chromosome:895787-897168	110.58	110.58+peptide	OK	48.0509	43.6167	-0.139683	-0.146976	0.80205	0.994748	no
gene:SpnNT_00861	NA	Chromosome:895787-897168	ΔORF2	110.58+peptide	OK	59.8093	43.6167	-0.455491	-0.495168	0.39465	0.889826	no
gene:SpnNT_00861	NA	Chromosome:895787-897168	110.58	ΔORF2+peptide	OK	48.0509	47.2947	-0.022884	-0.0248391	0.96295	0.994855	no
gene:SpnNT_00861	NA	Chromosome:895787-897168	ΔORF2	ΔORF2+peptide	OK	59.8093	47.2947	-0.338692	-0.380645	0.5048	0.945292	no
gene:SpnNT_00861	NA	Chromosome:895787-897168	110.58+peptide	ΔORF2+peptide	OK	43.6167	47.2947	0.116799	0.129602	0.82205	0.994748	no
gene:SpnNT_00862	NA	Chromosome:897439-899173	110.58	ΔORF2	OK	27.4244	33.6464	0.294993	0.621918	0.2773	0.780208	no
gene:SpnNT_00862	NA	Chromosome:897439-899173	110.58	110.58+peptide	OK	27.4244	28.7785	0.0695309	0.145209	0.8016	0.994748	no
gene:SpnNT_00862	NA	Chromosome:897439-899173	ΔORF2	110.58+peptide	OK	33.6464	28.7785	-0.225462	-0.476346	0.40855	0.897087	no
gene:SpnNT_00862	NA	Chromosome:897439-899173	110.58	ΔORF2+peptide	OK	27.4244	35.8648	0.387107	0.817563	0.15845	0.607592	no
gene:SpnNT_00862	NA	Chromosome:897439-899173	ΔORF2	ΔORF2+peptide	OK	33.6464	35.8648	0.0921142	0.196865	0.73135	0.994748	no
gene:SpnNT_00862	NA	Chromosome:897439-899173	110.58+peptide	ΔORF2+peptide	OK	28.7785	35.8648	0.317576	0.672154	0.2462	0.743359	no
gene:SpnNT_00863	spxA_2	Chromosome:899430-900873	110.58	ΔORF2	OK	331.56	571.826	0.786309	1.30003	0.0294	0.218948	no
gene:SpnNT_00863	spxA_2	Chromosome:899430-900873	110.58	110.58+peptide	OK	331.56	378.521	0.191103	0.294285	0.6147	0.979616	no

gene:SpnNT_00863	spxA_2	Chromosome:899430-900873	ΔORF2	110.58+peptide	OK	571.826	378.521	-0.595205	-0.924267	0.1331	0.556045	no
gene:SpnNT_00863	spxA_2	Chromosome:899430-900873	110.58	ΔORF2+peptide	OK	331.56	304.686	-0.121948	-0.181402	0.75135	0.994748	no
gene:SpnNT_00863	spxA_2	Chromosome:899430-900873	ΔORF2	ΔORF2+peptide	OK	571.826	304.686	-0.908256	-1.36164	0.024	0.191679	no
gene:SpnNT_00863	spxA_2	Chromosome:899430-900873	110.58+peptide	ΔORF2+peptide	OK	378.521	304.686	-0.313051	-0.442368	0.4607	0.924377	no
gene:SpnNT_00864	NA	Chromosome:899430-900873	110.58	ΔORF2	OK	70.7015	73.5213	0.0564209	0.0273923	0.9606	0.994855	no
gene:SpnNT_00864	NA	Chromosome:899430-900873	110.58	110.58+peptide	OK	70.7015	50.4232	-0.487653	-0.186296	0.72115	0.994748	no
gene:SpnNT_00864	NA	Chromosome:899430-900873	ΔORF2	110.58+peptide	OK	73.5213	50.4232	-0.544074	-0.196085	0.807	0.994748	no
gene:SpnNT_00864	NA	Chromosome:899430-900873	110.58	ΔORF2+peptide	OK	70.7015	52.1551	-0.438932	-0.21077	0.74	0.994748	no
gene:SpnNT_00864	NA	Chromosome:899430-900873	ΔORF2	ΔORF2+peptide	OK	73.5213	52.1551	-0.495353	-0.217565	0.81435	0.994748	no
gene:SpnNT_00864	NA	Chromosome:899430-900873	110.58+peptide	ΔORF2+peptide	OK	50.4232	52.1551	0.0487206	0.0174523	0.9715	0.99536	no
gene:SpnNT_00865	suhB	Chromosome:899430-900873	110.58	ΔORF2	OK	65.4511	68.1083	0.0574134	0.0529754	0.91795	0.994748	no
gene:SpnNT_00865	suhB	Chromosome:899430-900873	110.58	110.58+peptide	OK	65.4511	60.1823	-0.121079	-0.115507	0.82625	0.994748	no
gene:SpnNT_00865	suhB	Chromosome:899430-900873	ΔORF2	110.58+peptide	OK	68.1083	60.1823	-0.178492	-0.14417	0.77835	0.994748	no
gene:SpnNT_00865	suhB	Chromosome:899430-900873	110.58	ΔORF2+peptide	OK	65.4511	80.2724	0.294486	0.337559	0.55775	0.968033	no
gene:SpnNT_00865	suhB	Chromosome:899430-900873	ΔORF2	ΔORF2+peptide	OK	68.1083	80.2724	0.237073	0.21686	0.6784	0.985276	no
gene:SpnNT_00865	suhB	Chromosome:899430-900873	110.58+peptide	ΔORF2+peptide	OK	60.1823	80.2724	0.415565	0.392789	0.4688	0.927562	no
gene:SpnNT_00866	rsmF	Chromosome:900873-902178	110.58	ΔORF2	OK	32.1514	34.2269	0.0902458	0.186543	0.7413	0.994748	no
gene:SpnNT_00866	rsmF	Chromosome:900873-902178	110.58	110.58+peptide	OK	32.1514	36.549	0.18495	0.382683	0.50455	0.945292	no
gene:SpnNT_00866	rsmF	Chromosome:900873-902178	ΔORF2	110.58+peptide	OK	34.2269	36.549	0.0947043	0.197238	0.7319	0.994748	no
gene:SpnNT_00866	rsmF	Chromosome:900873-902178	110.58	ΔORF2+peptide	OK	32.1514	40.9757	0.349886	0.729134	0.19465	0.676386	no
gene:SpnNT_00866	rsmF	Chromosome:900873-902178	ΔORF2	ΔORF2+peptide	OK	34.2269	40.9757	0.25964	0.544667	0.33905	0.843895	no
gene:SpnNT_00866	rsmF	Chromosome:900873-902178	110.58+peptide	ΔORF2+peptide	OK	36.549	40.9757	0.164936	0.346353	0.54985	0.964281	no
gene:SpnNT_00867	pstS1	Chromosome:902751-905421	110.58	ΔORF2	OK	166.104	170.059	0.0339533	0.04786	0.9336	0.994855	no
gene:SpnNT_00867	pstS1	Chromosome:902751-905421	110.58	110.58+peptide	OK	166.104	155.979	-0.090733	-0.127048	0.82285	0.994748	no
gene:SpnNT_00867	pstS1	Chromosome:902751-905421	ΔORF2	110.58+peptide	OK	170.059	155.979	-0.124686	-0.168918	0.76415	0.994748	no
gene:SpnNT_00867	pstS1	Chromosome:902751-905421	110.58	ΔORF2+peptide	OK	166.104	176.117	0.0844475	0.117461	0.83695	0.994748	no
gene:SpnNT_00867	pstS1	Chromosome:902751-905421	ΔORF2	ΔORF2+peptide	OK	170.059	176.117	0.0504942	0.0679806	0.90355	0.994748	no
gene:SpnNT_00867	pstS1	Chromosome:902751-905421	110.58+peptide	ΔORF2+peptide	OK	155.979	176.117	0.17518	0.234419	0.68455	0.987866	no
gene:SpnNT_00868	pstC_1	Chromosome:902751-905421	110.58	ΔORF2	OK	122.492	125.555	0.0356323	0.0444912	0.9412	0.994855	no
gene:SpnNT_00868	pstC_1	Chromosome:902751-905421	110.58	110.58+peptide	OK	122.492	112.228	-0.12626	-0.153837	0.7916	0.994748	no
gene:SpnNT_00868	pstC_1	Chromosome:902751-905421	ΔORF2	110.58+peptide	OK	125.555	112.228	-0.161892	-0.197237	0.7347	0.994748	no
gene:SpnNT_00868	pstC_1	Chromosome:902751-905421	110.58	ΔORF2+peptide	OK	122.492	129.624	0.0816443	0.101602	0.86155	0.994748	no
gene:SpnNT_00868	pstC_1	Chromosome:902751-905421	ΔORF2	ΔORF2+peptide	OK	125.555	129.624	0.0460121	0.0572552	0.9194	0.994748	no
gene:SpnNT_00868	pstC_1	Chromosome:902751-905421	110.58+peptide	ΔORF2+peptide	OK	112.228	129.624	0.207905	0.252489	0.65945	0.982966	no
gene:SpnNT_00869	pstA_1	Chromosome:902751-905421	110.58	ΔORF2	OK	114.019	124.515	0.127043	0.150661	0.7966	0.994748	no
gene:SpnNT_00869	pstA_1	Chromosome:902751-905421	110.58	110.58+peptide	OK	114.019	111.696	-0.0296936	-0.0353002	0.9509	0.994855	no
gene:SpnNT_00869	pstA_1	Chromosome:902751-905421	ΔORF2	110.58+peptide	OK	124.515	111.696	-0.156736	-0.187403	0.74735	0.994748	no
gene:SpnNT_00869	pstA_1	Chromosome:902751-905421	110.58	ΔORF2+peptide	OK	114.019	136.721	0.26196	0.319427	0.5806	0.969538	no
gene:SpnNT_00869	pstA_1	Chromosome:902751-905421	ΔORF2	ΔORF2+peptide	OK	124.515	136.721	0.134917	0.165512	0.7762	0.994748	no
gene:SpnNT_00869	pstA_1	Chromosome:902751-905421	110.58+peptide	ΔORF2+peptide	OK	111.696	136.721	0.291653	0.358729	0.5369	0.959314	no
gene:SpnNT_00870	pstB3_1	Chromosome:905431-906235	110.58	ΔORF2	OK	123.411	128.983	0.0637156	0.137318	0.81035	0.994748	no
gene:SpnNT_00870	pstB3_1	Chromosome:905431-906235	110.58	110.58+peptide	OK	123.411	121.968	-0.0169656	-0.0370817	0.94635	0.994855	no
gene:SpnNT_00870	pstB3_1	Chromosome:905431-906235	ΔORF2	110.58+peptide	OK	128.983	121.968	-0.0806811	-0.170553	0.7644	0.994748	no
gene:SpnNT_00870	pstB3_1	Chromosome:905431-906235	110.58	ΔORF2+peptide	OK	123.411	121.259	-0.0253781	-0.0563082	0.92015	0.994748	no
gene:SpnNT_00870	pstB3_1	Chromosome:905431-906235	ΔORF2	ΔORF2+peptide	OK	128.983	121.259	-0.0890937	-0.190997	0.73605	0.994748	no

gene:SpnNT_00870	pstB3_1	Chromosome:905431-906235	110.58+peptide	ΔORF2+peptide	OK	121.968	121.259	-0.00841254	-0.0182874	0.975	0.99536	no
gene:SpnNT_00871	pstB3_2	Chromosome:906247-907006	110.58	ΔORF2	OK	137.629	144.147	0.0667588	0.14728	0.79805	0.994748	no
gene:SpnNT_00871	pstB3_2	Chromosome:906247-907006	110.58	110.58+peptide	OK	137.629	130.675	-0.0747965	-0.165974	0.7772	0.994748	no
gene:SpnNT_00871	pstB3_2	Chromosome:906247-907006	ΔORF2	110.58+peptide	OK	144.147	130.675	-0.141555	-0.310115	0.5858	0.972529	no
gene:SpnNT_00871	pstB3_2	Chromosome:906247-907006	110.58	ΔORF2+peptide	OK	137.629	152.395	0.147036	0.329348	0.55855	0.968033	no
gene:SpnNT_00871	pstB3_2	Chromosome:906247-907006	ΔORF2	ΔORF2+peptide	OK	144.147	152.395	0.0802776	0.177484	0.75065	0.994748	no
gene:SpnNT_00871	pstB3_2	Chromosome:906247-907006	110.58+peptide	ΔORF2+peptide	OK	130.675	152.395	0.221833	0.493316	0.38715	0.881745	no
gene:SpnNT_00872	phoU_2	Chromosome:907017-907671	110.58	ΔORF2	OK	376.284	410.884	0.12691	0.291758	0.60425	0.976761	no
gene:SpnNT_00872	phoU_2	Chromosome:907017-907671	110.58	110.58+peptide	OK	376.284	374.146	-0.00821847	-0.0189573	0.97345	0.99536	no
gene:SpnNT_00872	phoU_2	Chromosome:907017-907671	ΔORF2	110.58+peptide	OK	410.884	374.146	-0.135128	-0.312055	0.5782	0.969538	no
gene:SpnNT_00872	phoU_2	Chromosome:907017-907671	110.58	ΔORF2+peptide	OK	376.284	395.071	0.0702932	0.161817	0.7736	0.994748	no
gene:SpnNT_00872	phoU_2	Chromosome:907017-907671	ΔORF2	ΔORF2+peptide	OK	410.884	395.071	-0.0566165	-0.130483	0.81765	0.994748	no
gene:SpnNT_00872	phoU_2	Chromosome:907017-907671	110.58+peptide	ΔORF2+peptide	OK	374.146	395.071	0.0785117	0.181555	0.75	0.994748	no
gene:SpnNT_00873	glnH_2	Chromosome:907827-908643	110.58	ΔORF2	OK	431.854	504.752	0.22503	0.50656	0.38035	0.878145	no
gene:SpnNT_00873	glnH_2	Chromosome:907827-908643	110.58	110.58+peptide	OK	431.854	163.028	-1.40542	-3.13879	5.00E-05	0.0013612	yes
gene:SpnNT_00873	glnH_2	Chromosome:907827-908643	ΔORF2	110.58+peptide	OK	504.752	163.028	-1.63045	-3.68932	5.00E-05	0.0013612	yes
gene:SpnNT_00873	glnH_2	Chromosome:907827-908643	110.58	ΔORF2+peptide	OK	431.854	144.532	-1.57916	-3.5114	5.00E-05	0.0013612	yes
gene:SpnNT_00873	glnH_2	Chromosome:907827-908643	ΔORF2	ΔORF2+peptide	OK	504.752	144.532	-1.80419	-4.06414	5.00E-05	0.0013612	yes
gene:SpnNT_00873	glnH_2	Chromosome:907827-908643	110.58+peptide	ΔORF2+peptide	OK	163.028	144.532	-0.173736	-0.388274	0.4914	0.940662	no
gene:SpnNT_00874	yjiR	Chromosome:908662-909934	110.58	ΔORF2	OK	25.6523	25.7024	0.00281317	0.00559962	0.99175	0.998369	no
gene:SpnNT_00874	yjiR	Chromosome:908662-909934	110.58	110.58+peptide	OK	25.6523	17.2774	-0.570199	-1.10385	0.05455	0.332006	no
gene:SpnNT_00874	yjiR	Chromosome:908662-909934	ΔORF2	110.58+peptide	OK	25.7024	17.2774	-0.573012	-1.10618	0.05125	0.31963	no
gene:SpnNT_00874	yjiR	Chromosome:908662-909934	110.58	ΔORF2+peptide	OK	25.6523	18.5775	-0.465531	-0.908306	0.10925	0.502305	no
gene:SpnNT_00874	yjiR	Chromosome:908662-909934	ΔORF2	ΔORF2+peptide	OK	25.7024	18.5775	-0.468344	-0.911189	0.10395	0.489427	no
gene:SpnNT_00874	yjiR	Chromosome:908662-909934	110.58+peptide	ΔORF2+peptide	OK	17.2774	18.5775	0.104668	0.198291	0.7234	0.994748	no
gene:SpnNT_00875	aldC	Chromosome:910135-910807	110.58	ΔORF2	OK	63.1788	66.874	0.0820046	0.164049	0.77445	0.994748	no
gene:SpnNT_00875	aldC	Chromosome:910135-910807	110.58	110.58+peptide	OK	63.1788	49.4277	-0.354121	-0.701308	0.22075	0.709102	no
gene:SpnNT_00875	aldC	Chromosome:910135-910807	ΔORF2	110.58+peptide	OK	66.874	49.4277	-0.436125	-0.860775	0.13345	0.556521	no
gene:SpnNT_00875	aldC	Chromosome:910135-910807	110.58	ΔORF2+peptide	OK	63.1788	47.4207	-0.413922	-0.816291	0.1522	0.594749	no
gene:SpnNT_00875	aldC	Chromosome:910135-910807	ΔORF2	ΔORF2+peptide	OK	66.874	47.4207	-0.495927	-0.974714	0.0881	0.446868	no
gene:SpnNT_00875	aldC	Chromosome:910135-910807	110.58+peptide	ΔORF2+peptide	OK	49.4277	47.4207	-0.0598012	-0.116397	0.8373	0.994748	no
gene:SpnNT_00876	NA	Chromosome:910816-911569	110.58	ΔORF2	OK	70.2641	82.6029	0.233406	0.486453	0.4084	0.897087	no
gene:SpnNT_00876	NA	Chromosome:910816-911569	110.58	110.58+peptide	OK	70.2641	71.3929	0.0229932	0.0478962	0.93375	0.994855	no
gene:SpnNT_00876	NA	Chromosome:910816-911569	ΔORF2	110.58+peptide	OK	82.6029	71.3929	-0.210413	-0.438737	0.4461	0.918643	no
gene:SpnNT_00876	NA	Chromosome:910816-911569	110.58	ΔORF2+peptide	OK	70.2641	87.0666	0.309333	0.645548	0.27745	0.780208	no
gene:SpnNT_00876	NA	Chromosome:910816-911569	ΔORF2	ΔORF2+peptide	OK	82.6029	87.0666	0.0759269	0.15861	0.78605	0.994748	no
gene:SpnNT_00876	NA	Chromosome:910816-911569	110.58+peptide	ΔORF2+peptide	OK	71.3929	87.0666	0.28634	0.597844	0.30335	0.809137	no
gene:SpnNT_00877	murB	Chromosome:911680-912586	110.58	ΔORF2	OK	49.2879	49.7559	0.013634	0.0280836	0.96125	0.994855	no
gene:SpnNT_00877	murB	Chromosome:911680-912586	110.58	110.58+peptide	OK	49.2879	69.4437	0.494608	1.03801	0.0695	0.387088	no
gene:SpnNT_00877	murB	Chromosome:911680-912586	ΔORF2	110.58+peptide	OK	49.7559	69.4437	0.480974	1.00983	0.0801	0.421873	no
gene:SpnNT_00877	murB	Chromosome:911680-912586	110.58	ΔORF2+peptide	OK	49.2879	71.9714	0.546188	1.14646	0.0457	0.294971	no
gene:SpnNT_00877	murB	Chromosome:911680-912586	ΔORF2	ΔORF2+peptide	OK	49.7559	71.9714	0.532554	1.11832	0.05305	0.326511	no
gene:SpnNT_00877	murB	Chromosome:911680-912586	110.58+peptide	ΔORF2+peptide	OK	69.4437	71.9714	0.0515801	0.110439	0.84655	0.994748	no
gene:SpnNT_00878	potA	Chromosome:912745-916527	110.58	ΔORF2	OK	67.0285	61.4223	-0.126012	-0.193554	0.73135	0.994748	no
gene:SpnNT_00878	potA	Chromosome:912745-916527	110.58	110.58+peptide	OK	67.0285	104.509	0.640775	0.969017	0.09475	0.46531	no

gene:SpnNT_00878	potA	Chromosome:912745-916527	ΔORF2	110.58+peptide	OK	61.4223	104.509	0.766787	1.14657	0.0518	0.321685	no
gene:SpnNT_00878	potA	Chromosome:912745-916527	110.58	ΔORF2+peptide	OK	67.0285	113.988	0.766035	1.1726	0.04255	0.282276	no
gene:SpnNT_00878	potA	Chromosome:912745-916527	ΔORF2	ΔORF2+peptide	OK	61.4223	113.988	0.892047	1.34981	0.02215	0.180866	no
gene:SpnNT_00878	potA	Chromosome:912745-916527	110.58+peptide	ΔORF2+peptide	OK	104.509	113.988	0.12526	0.186694	0.74285	0.994748	no
gene:SpnNT_00879	potB	Chromosome:912745-916527	110.58	ΔORF2	OK	29.5569	27.895	-0.0834872	-0.0666343	0.90835	0.994748	no
gene:SpnNT_00879	potB	Chromosome:912745-916527	110.58	110.58+peptide	OK	29.5569	53.6963	0.861329	0.725896	0.22745	0.71915	no
gene:SpnNT_00879	potB	Chromosome:912745-916527	ΔORF2	110.58+peptide	OK	27.895	53.6963	0.944816	0.760548	0.1994	0.68192	no
gene:SpnNT_00879	potB	Chromosome:912745-916527	110.58	ΔORF2+peptide	OK	29.5569	59.6694	1.0135	0.808951	0.17785	0.644326	no
gene:SpnNT_00879	potB	Chromosome:912745-916527	ΔORF2	ΔORF2+peptide	OK	27.895	59.6694	1.09698	0.840125	0.15675	0.603543	no
gene:SpnNT_00879	potB	Chromosome:912745-916527	110.58+peptide	ΔORF2+peptide	OK	53.6963	59.6694	0.152167	0.122496	0.837	0.994748	no
gene:SpnNT_00880	ydcV	Chromosome:912745-916527	110.58	ΔORF2	OK	27.1206	27.4027	0.014928	0.0112026	0.98405	0.996246	no
gene:SpnNT_00880	ydcV	Chromosome:912745-916527	110.58	110.58+peptide	OK	27.1206	48.8207	0.848105	0.638589	0.27435	0.77651	no
gene:SpnNT_00880	ydcV	Chromosome:912745-916527	ΔORF2	110.58+peptide	OK	27.4027	48.8207	0.833177	0.597795	0.31125	0.816845	no
gene:SpnNT_00880	ydcV	Chromosome:912745-916527	110.58	ΔORF2+peptide	OK	27.1206	56.067	1.04777	0.82454	0.1677	0.623936	no
gene:SpnNT_00880	ydcV	Chromosome:912745-916527	ΔORF2	ΔORF2+peptide	OK	27.4027	56.067	1.03284	0.771235	0.19635	0.677648	no
gene:SpnNT_00880	ydcV	Chromosome:912745-916527	110.58+peptide	ΔORF2+peptide	OK	48.8207	56.067	0.19966	0.149584	0.7978	0.994748	no
gene:SpnNT_00881	potD_2	Chromosome:912745-916527	110.58	ΔORF2	OK	38.085	39.109	0.0382776	0.044675	0.93815	0.994855	no
gene:SpnNT_00881	potD_2	Chromosome:912745-916527	110.58	110.58+peptide	OK	38.085	67.9429	0.835102	0.975829	0.0905	0.452052	no
gene:SpnNT_00881	potD_2	Chromosome:912745-916527	ΔORF2	110.58+peptide	OK	39.109	67.9429	0.796825	0.940983	0.1025	0.485211	no
gene:SpnNT_00881	potD_2	Chromosome:912745-916527	110.58	ΔORF2+peptide	OK	38.085	80.6132	1.08179	1.24496	0.0333	0.239432	no
gene:SpnNT_00881	potD_2	Chromosome:912745-916527	ΔORF2	ΔORF2+peptide	OK	39.109	80.6132	1.04352	1.21327	0.0384	0.263814	no
gene:SpnNT_00881	potD_2	Chromosome:912745-916527	110.58+peptide	ΔORF2+peptide	OK	67.9429	80.6132	0.246692	0.28716	0.61435	0.979616	no
gene:SpnNT_00882	NA	Chromosome:917301-917787	110.58	ΔORF2	OK	24.0687	26.0851	0.11607	0.195272	0.7342	0.994748	no
gene:SpnNT_00882	NA	Chromosome:917301-917787	110.58	110.58+peptide	OK	24.0687	61.3143	1.34907	2.37662	1.00E-04	0.0025332	yes
gene:SpnNT_00882	NA	Chromosome:917301-917787	ΔORF2	110.58+peptide	OK	26.0851	61.3143	1.233	2.178	0.00025	0.00542231	yes
gene:SpnNT_00882	NA	Chromosome:917301-917787	110.58	ΔORF2+peptide	OK	24.0687	88.3408	1.87592	3.37223	5.00E-05	0.0013612	yes
gene:SpnNT_00882	NA	Chromosome:917301-917787	ΔORF2	ΔORF2+peptide	OK	26.0851	88.3408	1.75985	3.17247	5.00E-05	0.0013612	yes
gene:SpnNT_00882	NA	Chromosome:917301-917787	110.58+peptide	ΔORF2+peptide	OK	61.3143	88.3408	0.526856	1.00172	0.0837	0.434115	no
gene:SpnNT_00883	alaS	Chromosome:917808-920427	110.58	ΔORF2	OK	69.1295	68.9088	-0.00461378	-0.0105186	0.98425	0.996246	no
gene:SpnNT_00883	alaS	Chromosome:917808-920427	110.58	110.58+peptide	OK	69.1295	150.043	1.118	2.52603	5.00E-05	0.0013612	yes
gene:SpnNT_00883	alaS	Chromosome:917808-920427	ΔORF2	110.58+peptide	OK	68.9088	150.043	1.12262	2.51868	5.00E-05	0.0013612	yes
gene:SpnNT_00883	alaS	Chromosome:917808-920427	110.58	ΔORF2+peptide	OK	69.1295	172.886	1.32245	3.0196	5.00E-05	0.0013612	yes
gene:SpnNT_00883	alaS	Chromosome:917808-920427	ΔORF2	ΔORF2+peptide	OK	68.9088	172.886	1.32706	3.00846	5.00E-05	0.0013612	yes
gene:SpnNT_00883	alaS	Chromosome:917808-920427	110.58+peptide	ΔORF2+peptide	OK	150.043	172.886	0.204442	0.459368	0.42545	0.908126	no
gene:SpnNT_00884	amyS	Chromosome:920516-921971	110.58	ΔORF2	OK	12.747	12.542	-0.0233922	-0.0436162	0.941	0.994855	no
gene:SpnNT_00884	amyS	Chromosome:920516-921971	110.58	110.58+peptide	OK	12.747	9.31204	-0.452991	-0.828834	0.1581	0.607315	no
gene:SpnNT_00884	amyS	Chromosome:920516-921971	ΔORF2	110.58+peptide	OK	12.542	9.31204	-0.429599	-0.783527	0.17845	0.645075	no
gene:SpnNT_00884	amyS	Chromosome:920516-921971	110.58	ΔORF2+peptide	OK	12.747	9.39114	-0.440788	-0.806281	0.16735	0.623323	no
gene:SpnNT_00884	amyS	Chromosome:920516-921971	ΔORF2	ΔORF2+peptide	OK	12.542	9.39114	-0.417395	-0.761059	0.1874	0.662463	no
gene:SpnNT_00884	amyS	Chromosome:920516-921971	110.58+peptide	ΔORF2+peptide	OK	9.31204	9.39114	0.0122034	0.0218527	0.9697	0.995105	no
gene:SpnNT_00885	ecsA_2	Chromosome:922149-924514	110.58	ΔORF2	OK	20.062	22.2183	0.147283	0.16708	0.7729	0.994748	no
gene:SpnNT_00885	ecsA_2	Chromosome:922149-924514	110.58	110.58+peptide	OK	20.062	17.2638	-0.216716	-0.250133	0.66915	0.984845	no
gene:SpnNT_00885	ecsA_2	Chromosome:922149-924514	ΔORF2	110.58+peptide	OK	22.2183	17.2638	-0.363999	-0.416864	0.4727	0.928558	no
gene:SpnNT_00885	ecsA_2	Chromosome:922149-924514	110.58	ΔORF2+peptide	OK	20.062	14.5604	-0.462414	-0.488581	0.40065	0.893669	no
gene:SpnNT_00885	ecsA_2	Chromosome:922149-924514	ΔORF2	ΔORF2+peptide	OK	22.2183	14.5604	-0.609697	-0.639998	0.2769	0.780052	no

gene:SpnNT_00885	ecsA_2	Chromosome:922149-924514	110.58+peptide	ΔORF2+peptide	OK	17.2638	14.5604	-0.245698	-0.261745	0.6492	0.981391	no
gene:SpnNT_00886	NA	Chromosome:922149-924514	110.58	ΔORF2	OK	16.4964	18.947	0.199815	0.340623	0.55965	0.968621	no
gene:SpnNT_00886	NA	Chromosome:922149-924514	110.58	110.58+peptide	OK	16.4964	13.0614	-0.336844	-0.557368	0.33925	0.844054	no
gene:SpnNT_00886	NA	Chromosome:922149-924514	ΔORF2	110.58+peptide	OK	18.947	13.0614	-0.536659	-0.901255	0.1217	0.528441	no
gene:SpnNT_00886	NA	Chromosome:922149-924514	110.58	ΔORF2+peptide	OK	16.4964	12.8798	-0.357041	-0.588557	0.31255	0.818913	no
gene:SpnNT_00886	NA	Chromosome:922149-924514	ΔORF2	ΔORF2+peptide	OK	18.947	12.8798	-0.556856	-0.931536	0.1085	0.501139	no
gene:SpnNT_00886	NA	Chromosome:922149-924514	110.58+peptide	ΔORF2+peptide	OK	13.0614	12.8798	-0.0201971	-0.0328305	0.95605	0.994855	no
gene:SpnNT_00887	rlmI	Chromosome:925200-927882	110.58	ΔORF2	OK	147.305	167.739	0.187412	0.313781	0.58435	0.971855	no
gene:SpnNT_00887	rlmI	Chromosome:925200-927882	110.58	110.58+peptide	OK	147.305	167.538	0.185685	0.300256	0.61095	0.979429	no
gene:SpnNT_00887	rlmI	Chromosome:925200-927882	ΔORF2	110.58+peptide	OK	167.739	167.538	-0.00172734	-0.00277025	0.99605	0.99927	no
gene:SpnNT_00887	rlmI	Chromosome:925200-927882	110.58	ΔORF2+peptide	OK	147.305	218.253	0.567198	0.978656	0.08865	0.448445	no
gene:SpnNT_00887	rlmI	Chromosome:925200-927882	ΔORF2	ΔORF2+peptide	OK	167.739	218.253	0.379786	0.649185	0.2584	0.759061	no
gene:SpnNT_00887	rlmI	Chromosome:925200-927882	110.58+peptide	ΔORF2+peptide	OK	167.538	218.253	0.381513	0.628939	0.2803	0.783714	no
gene:SpnNT_00888	aroD	Chromosome:925200-927882	110.58	ΔORF2	OK	138.267	124.38	-0.152712	-0.160342	0.78105	0.994748	no
gene:SpnNT_00888	aroD	Chromosome:925200-927882	110.58	110.58+peptide	OK	138.267	144.063	0.0592415	0.0627799	0.91635	0.994748	no
gene:SpnNT_00888	aroD	Chromosome:925200-927882	ΔORF2	110.58+peptide	OK	124.38	144.063	0.211954	0.212	0.71	0.990919	no
gene:SpnNT_00888	aroD	Chromosome:925200-927882	110.58	ΔORF2+peptide	OK	138.267	167.814	0.279405	0.300143	0.6069	0.977866	no
gene:SpnNT_00888	aroD	Chromosome:925200-927882	ΔORF2	ΔORF2+peptide	OK	124.38	167.814	0.432118	0.437466	0.456	0.921244	no
gene:SpnNT_00888	aroD	Chromosome:925200-927882	110.58+peptide	ΔORF2+peptide	OK	144.063	167.814	0.220164	0.224815	0.6967	0.989512	no
gene:SpnNT_00889	aroE	Chromosome:925200-927882	110.58	ΔORF2	OK	81.6835	80.986	-0.0123708	-0.0123446	0.9845	0.996345	no
gene:SpnNT_00889	aroE	Chromosome:925200-927882	110.58	110.58+peptide	OK	81.6835	106.169	0.378243	0.368609	0.52115	0.954832	no
gene:SpnNT_00889	aroE	Chromosome:925200-927882	ΔORF2	110.58+peptide	OK	80.986	106.169	0.390614	0.377595	0.50445	0.945292	no
gene:SpnNT_00889	aroE	Chromosome:925200-927882	110.58	ΔORF2+peptide	OK	81.6835	103.473	0.341134	0.333408	0.57705	0.969538	no
gene:SpnNT_00889	aroE	Chromosome:925200-927882	ΔORF2	ΔORF2+peptide	OK	80.986	103.473	0.353505	0.342696	0.5616	0.968621	no
gene:SpnNT_00889	aroE	Chromosome:925200-927882	110.58+peptide	ΔORF2+peptide	OK	106.169	103.473	-0.0371094	-0.0351788	0.952	0.994855	no
gene:SpnNT_00890	aroB	Chromosome:927900-928968	110.58	ΔORF2	OK	109.719	107.775	-0.025795	-0.0582582	0.92	0.994748	no
gene:SpnNT_00890	aroB	Chromosome:927900-928968	110.58	110.58+peptide	OK	109.719	109.124	-0.00785285	-0.0177515	0.9767	0.99536	no
gene:SpnNT_00890	aroB	Chromosome:927900-928968	ΔORF2	110.58+peptide	OK	107.775	109.124	0.0179422	0.0406056	0.9455	0.994855	no
gene:SpnNT_00890	aroB	Chromosome:927900-928968	110.58	ΔORF2+peptide	OK	109.719	121.768	0.150317	0.340179	0.5534	0.96591	no
gene:SpnNT_00890	aroB	Chromosome:927900-928968	ΔORF2	ΔORF2+peptide	OK	107.775	121.768	0.176112	0.399017	0.48555	0.937658	no
gene:SpnNT_00890	aroB	Chromosome:927900-928968	110.58+peptide	ΔORF2+peptide	OK	109.124	121.768	0.15817	0.358688	0.532	0.958126	no
gene:SpnNT_00891	aroC	Chromosome:928977-930144	110.58	ΔORF2	OK	155.484	155.869	0.00357287	0.00817203	0.9895	0.997703	no
gene:SpnNT_00891	aroC	Chromosome:928977-930144	110.58	110.58+peptide	OK	155.484	158.43	0.0270837	0.0620121	0.91145	0.994748	no
gene:SpnNT_00891	aroC	Chromosome:928977-930144	ΔORF2	110.58+peptide	OK	155.869	158.43	0.0235108	0.0538867	0.9232	0.994748	no
gene:SpnNT_00891	aroC	Chromosome:928977-930144	110.58	ΔORF2+peptide	OK	155.484	170.641	0.134199	0.307716	0.58215	0.970851	no
gene:SpnNT_00891	aroC	Chromosome:928977-930144	ΔORF2	ΔORF2+peptide	OK	155.869	170.641	0.130626	0.299831	0.59745	0.97629	no
gene:SpnNT_00891	aroC	Chromosome:928977-930144	110.58+peptide	ΔORF2+peptide	OK	158.43	170.641	0.107116	0.246126	0.66535	0.98367	no
gene:SpnNT_00892	tyrC	Chromosome:930153-931257	110.58	ΔORF2	OK	141.604	137.14	-0.046216	-0.105264	0.8529	0.994748	no
gene:SpnNT_00892	tyrC	Chromosome:930153-931257	110.58	110.58+peptide	OK	141.604	140.291	-0.0134387	-0.030749	0.9587	0.994855	no
gene:SpnNT_00892	tyrC	Chromosome:930153-931257	ΔORF2	110.58+peptide	OK	137.14	140.291	0.0327773	0.0746297	0.89515	0.994748	no
gene:SpnNT_00892	tyrC	Chromosome:930153-931257	110.58	ΔORF2+peptide	OK	141.604	144.595	0.0301476	0.0690739	0.9047	0.994748	no
gene:SpnNT_00892	tyrC	Chromosome:930153-931257	ΔORF2	ΔORF2+peptide	OK	137.14	144.595	0.0763636	0.174103	0.7633	0.994748	no
gene:SpnNT_00892	tyrC	Chromosome:930153-931257	110.58+peptide	ΔORF2+peptide	OK	140.291	144.595	0.0435863	0.09983	0.86695	0.994748	no
gene:SpnNT_00893	NA	Chromosome:931267-931606	110.58	ΔORF2	OK	436.825	432.043	-0.0158802	-0.0332093	0.95285	0.994855	no
gene:SpnNT_00893	NA	Chromosome:931267-931606	110.58	110.58+peptide	OK	436.825	516.288	0.241123	0.484876	0.3885	0.883172	no

gene:SpnNT_00893	NA	Chromosome:931267-931606	ΔORF2	110.58+peptide	OK	432.043	516.288	0.257004	0.52406	0.3463	0.850486	no
gene:SpnNT_00893	NA	Chromosome:931267-931606	110.58	ΔORF2+peptide	OK	436.825	451.468	0.0475704	0.0964265	0.86025	0.994748	no
gene:SpnNT_00893	NA	Chromosome:931267-931606	ΔORF2	ΔORF2+peptide	OK	432.043	451.468	0.0634506	0.13045	0.8139	0.994748	no
gene:SpnNT_00893	NA	Chromosome:931267-931606	110.58+peptide	ΔORF2+peptide	OK	516.288	451.468	-0.193553	-0.38313	0.48725	0.938799	no
gene:SpnNT_00894	aroA	Chromosome:931697-935566	110.58	ΔORF2	OK	152.198	153.913	0.0161664	0.0219823	0.97085	0.99536	no
gene:SpnNT_00894	aroA	Chromosome:931697-935566	110.58	110.58+peptide	OK	152.198	83.456	-0.866859	-1.17977	0.04325	0.285189	no
gene:SpnNT_00894	aroA	Chromosome:931697-935566	ΔORF2	110.58+peptide	OK	153.913	83.456	-0.883025	-1.18853	0.0425	0.282086	no
gene:SpnNT_00894	aroA	Chromosome:931697-935566	110.58	ΔORF2+peptide	OK	152.198	90.0876	-0.756547	-1.02479	0.0746	0.403172	no
gene:SpnNT_00894	aroA	Chromosome:931697-935566	ΔORF2	ΔORF2+peptide	OK	153.913	90.0876	-0.772713	-1.03526	0.07505	0.404272	no
gene:SpnNT_00894	aroA	Chromosome:931697-935566	110.58+peptide	ΔORF2+peptide	OK	83.456	90.0876	0.110312	0.147922	0.80095	0.994748	no
gene:SpnNT_00895	aroL	Chromosome:931697-935566	110.58	ΔORF2	OK	149.923	159.651	0.0906995	0.0594085	0.91705	0.994748	no
gene:SpnNT_00895	aroL	Chromosome:931697-935566	110.58	110.58+peptide	OK	149.923	88.9764	-0.75273	-0.513167	0.3513	0.854885	no
gene:SpnNT_00895	aroL	Chromosome:931697-935566	ΔORF2	110.58+peptide	OK	159.651	88.9764	-0.843429	-0.587062	0.28895	0.793193	no
gene:SpnNT_00895	aroL	Chromosome:931697-935566	110.58	ΔORF2+peptide	OK	149.923	98.301	-0.608947	-0.414307	0.4464	0.918705	no
gene:SpnNT_00895	aroL	Chromosome:931697-935566	ΔORF2	ΔORF2+peptide	OK	159.651	98.301	-0.699647	-0.48596	0.37415	0.871501	no
gene:SpnNT_00895	aroL	Chromosome:931697-935566	110.58+peptide	ΔORF2+peptide	OK	88.9764	98.301	0.143782	0.104488	0.8431	0.994748	no
gene:SpnNT_00896	pheA	Chromosome:931697-935566	110.58	ΔORF2	OK	104.555	111.718	0.0955969	0.0858155	0.8825	0.994748	no
gene:SpnNT_00896	pheA	Chromosome:931697-935566	110.58	110.58+peptide	OK	104.555	60.19	-0.796672	-0.717244	0.21945	0.707834	no
gene:SpnNT_00896	pheA	Chromosome:931697-935566	ΔORF2	110.58+peptide	OK	111.718	60.19	-0.892269	-0.799166	0.1719	0.632018	no
gene:SpnNT_00896	pheA	Chromosome:931697-935566	110.58	ΔORF2+peptide	OK	104.555	67.1189	-0.639477	-0.568206	0.33315	0.839007	no
gene:SpnNT_00896	pheA	Chromosome:931697-935566	ΔORF2	ΔORF2+peptide	OK	111.718	67.1189	-0.735074	-0.649866	0.27045	0.772705	no
gene:SpnNT_00896	pheA	Chromosome:931697-935566	110.58+peptide	ΔORF2+peptide	OK	60.19	67.1189	0.157196	0.139367	0.8082	0.994748	no
gene:SpnNT_00897	msrR	Chromosome:931697-935566	110.58	ΔORF2	OK	144.365	149.315	0.0486362	0.0642654	0.91075	0.994748	no
gene:SpnNT_00897	msrR	Chromosome:931697-935566	110.58	110.58+peptide	OK	144.365	81.4531	-0.825678	-1.10685	0.0599	0.35121	no
gene:SpnNT_00897	msrR	Chromosome:931697-935566	ΔORF2	110.58+peptide	OK	149.315	81.4531	-0.874314	-1.15761	0.04835	0.306942	no
gene:SpnNT_00897	msrR	Chromosome:931697-935566	110.58	ΔORF2+peptide	OK	144.365	86.1043	-0.745563	-0.991494	0.08835	0.44779	no
gene:SpnNT_00897	msrR	Chromosome:931697-935566	ΔORF2	ΔORF2+peptide	OK	149.315	86.1043	-0.794199	-1.04337	0.07225	0.395686	no
gene:SpnNT_00897	msrR	Chromosome:931697-935566	110.58+peptide	ΔORF2+peptide	OK	81.4531	86.1043	0.0801152	0.10676	0.85255	0.994748	no
gene:SpnNT_00898	NA	Chromosome:935853-937783	110.58	ΔORF2	OK	95.0653	105.429	0.149279	0.250957	0.6574	0.982966	no
gene:SpnNT_00898	NA	Chromosome:935853-937783	110.58	110.58+peptide	OK	95.0653	70.1031	-0.439441	-0.689078	0.22785	0.719706	no
gene:SpnNT_00898	NA	Chromosome:935853-937783	ΔORF2	110.58+peptide	OK	105.429	70.1031	-0.58872	-0.938034	0.09755	0.472169	no
gene:SpnNT_00898	NA	Chromosome:935853-937783	110.58	ΔORF2+peptide	OK	95.0653	77.1532	-0.301193	-0.455444	0.42755	0.909248	no
gene:SpnNT_00898	NA	Chromosome:935853-937783	ΔORF2	ΔORF2+peptide	OK	105.429	77.1532	-0.450472	-0.691362	0.21865	0.707017	no
gene:SpnNT_00898	NA	Chromosome:935853-937783	110.58+peptide	ΔORF2+peptide	OK	70.1031	77.1532	0.138248	0.200085	0.72475	0.994748	no
gene:SpnNT_00899	pglJ	Chromosome:935853-937783	110.58	ΔORF2	OK	52.5276	58.3724	0.15221	0.224893	0.6913	0.98828	no
gene:SpnNT_00899	pglJ	Chromosome:935853-937783	110.58	110.58+peptide	OK	52.5276	47.9654	-0.131082	-0.192531	0.7379	0.994748	no
gene:SpnNT_00899	pglJ	Chromosome:935853-937783	ΔORF2	110.58+peptide	OK	58.3724	47.9654	-0.283291	-0.427582	0.45925	0.923527	no
gene:SpnNT_00899	pglJ	Chromosome:935853-937783	110.58	ΔORF2+peptide	OK	52.5276	65.5176	0.318806	0.48755	0.3937	0.888833	no
gene:SpnNT_00899	pglJ	Chromosome:935853-937783	ΔORF2	ΔORF2+peptide	OK	58.3724	65.5176	0.166596	0.262427	0.64235	0.980887	no
gene:SpnNT_00899	pglJ	Chromosome:935853-937783	110.58+peptide	ΔORF2+peptide	OK	47.9654	65.5176	0.449887	0.703922	0.227	0.71894	no
gene:SpnNT_00900	epsJ	Chromosome:937784-938771	110.58	ΔORF2	OK	42.1008	48.6594	0.208873	0.43168	0.4462	0.918643	no
gene:SpnNT_00900	epsJ	Chromosome:937784-938771	110.58	110.58+peptide	OK	42.1008	46.1046	0.131064	0.269921	0.6247	0.980887	no
gene:SpnNT_00900	epsJ	Chromosome:937784-938771	ΔORF2	110.58+peptide	OK	48.6594	46.1046	-0.077809	-0.161345	0.77855	0.994748	no
gene:SpnNT_00900	epsJ	Chromosome:937784-938771	110.58	ΔORF2+peptide	OK	42.1008	55.2895	0.39316	0.817806	0.1449	0.580489	no
gene:SpnNT_00900	epsJ	Chromosome:937784-938771	ΔORF2	ΔORF2+peptide	OK	48.6594	55.2895	0.184287	0.386019	0.5046	0.945292	no

gene:SpnNT_00900	epsJ	Chromosome:937784-938771	110.58+peptide	ΔORF2+peptide	OK	46.1046	55.2895	0.262096	0.547023	0.3362	0.840308	no
gene:SpnNT_00901	NA	Chromosome:938786-941283	110.58	ΔORF2	OK	42.4746	50.5489	0.25108	0.275568	0.6144	0.979616	no
gene:SpnNT_00901	NA	Chromosome:938786-941283	110.58	110.58+peptide	OK	42.4746	44.5519	0.0688869	0.0779578	0.88915	0.994748	no
gene:SpnNT_00901	NA	Chromosome:938786-941283	ΔORF2	110.58+peptide	OK	50.5489	44.5519	-0.182193	-0.184997	0.7413	0.994748	no
gene:SpnNT_00901	NA	Chromosome:938786-941283	110.58	ΔORF2+peptide	OK	42.4746	59.5282	0.486975	0.620693	0.2845	0.787874	no
gene:SpnNT_00901	NA	Chromosome:938786-941283	ΔORF2	ΔORF2+peptide	OK	50.5489	59.5282	0.235895	0.262976	0.6358	0.980887	no
gene:SpnNT_00901	NA	Chromosome:938786-941283	110.58+peptide	ΔORF2+peptide	OK	44.5519	59.5282	0.418088	0.481071	0.4003	0.893627	no
gene:SpnNT_00902	NA	Chromosome:938786-941283	110.58	ΔORF2	OK	134.954	207.776	0.622561	1.15061	0.049	0.310168	no
gene:SpnNT_00902	NA	Chromosome:938786-941283	110.58	110.58+peptide	OK	134.954	159.991	0.24552	0.451603	0.43995	0.916416	no
gene:SpnNT_00902	NA	Chromosome:938786-941283	ΔORF2	110.58+peptide	OK	207.776	159.991	-0.37704	-0.676238	0.2522	0.750402	no
gene:SpnNT_00902	NA	Chromosome:938786-941283	110.58	ΔORF2+peptide	OK	134.954	176.289	0.385472	0.716159	0.21305	0.698345	no
gene:SpnNT_00902	NA	Chromosome:938786-941283	ΔORF2	ΔORF2+peptide	OK	207.776	176.289	-0.237089	-0.429294	0.4579	0.921942	no
gene:SpnNT_00902	NA	Chromosome:938786-941283	110.58+peptide	ΔORF2+peptide	OK	159.991	176.289	0.139952	0.252247	0.6666	0.983929	no
gene:SpnNT_00903	mecA	Chromosome:941482-942220	110.58	ΔORF2	OK	286.646	335.56	0.227298	0.516302	0.3694	0.866165	no
gene:SpnNT_00903	mecA	Chromosome:941482-942220	110.58	110.58+peptide	OK	286.646	236.101	-0.279867	-0.634111	0.2666	0.768019	no
gene:SpnNT_00903	mecA	Chromosome:941482-942220	ΔORF2	110.58+peptide	OK	335.56	236.101	-0.507165	-1.15171	0.04785	0.304652	no
gene:SpnNT_00903	mecA	Chromosome:941482-942220	110.58	ΔORF2+peptide	OK	286.646	237.159	-0.273414	-0.617874	0.28385	0.786627	no
gene:SpnNT_00903	mecA	Chromosome:941482-942220	ΔORF2	ΔORF2+peptide	OK	335.56	237.159	-0.500712	-1.13407	0.05215	0.323094	no
gene:SpnNT_00903	mecA	Chromosome:941482-942220	110.58+peptide	ΔORF2+peptide	OK	236.101	237.159	0.00645291	0.0145787	0.97945	0.995765	no
gene:SpnNT_00904	hom	Chromosome:942370-943657	110.58	ΔORF2	OK	263.228	257.433	-0.0321156	-0.0733235	0.8938	0.994748	no
gene:SpnNT_00904	hom	Chromosome:942370-943657	110.58	110.58+peptide	OK	263.228	257.691	-0.0306744	-0.06993	0.90025	0.994748	no
gene:SpnNT_00904	hom	Chromosome:942370-943657	ΔORF2	110.58+peptide	OK	257.433	257.691	0.00144117	0.00330833	0.99605	0.99927	no
gene:SpnNT_00904	hom	Chromosome:942370-943657	110.58	ΔORF2+peptide	OK	263.228	269.308	0.0329449	0.0749076	0.8943	0.994748	no
gene:SpnNT_00904	hom	Chromosome:942370-943657	ΔORF2	ΔORF2+peptide	OK	257.433	269.308	0.0650605	0.148952	0.7956	0.994748	no
gene:SpnNT_00904	hom	Chromosome:942370-943657	110.58+peptide	ΔORF2+peptide	OK	257.691	269.308	0.0636194	0.145437	0.80035	0.994748	no
gene:SpnNT_00905	thrB	Chromosome:943658-944528	110.58	ΔORF2	OK	220.163	215.317	-0.0321047	-0.0725575	0.8999	0.994748	no
gene:SpnNT_00905	thrB	Chromosome:943658-944528	110.58	110.58+peptide	OK	220.163	226.61	0.0416424	0.0945573	0.86935	0.994748	no
gene:SpnNT_00905	thrB	Chromosome:943658-944528	ΔORF2	110.58+peptide	OK	215.317	226.61	0.0737471	0.167359	0.7689	0.994748	no
gene:SpnNT_00905	thrB	Chromosome:943658-944528	110.58	ΔORF2+peptide	OK	220.163	232.9	0.0811412	0.183996	0.75215	0.994748	no
gene:SpnNT_00905	thrB	Chromosome:943658-944528	ΔORF2	ΔORF2+peptide	OK	215.317	232.9	0.113246	0.256646	0.6512	0.981475	no
gene:SpnNT_00905	thrB	Chromosome:943658-944528	110.58+peptide	ΔORF2+peptide	OK	226.61	232.9	0.0394988	0.0899402	0.87275	0.994748	no
gene:SpnNT_00906	msrAB1	Chromosome:944822-945761	110.58	ΔORF2	OK	302.741	299.633	-0.0148879	-0.0340636	0.9506	0.994855	no
gene:SpnNT_00906	msrAB1	Chromosome:944822-945761	110.58	110.58+peptide	OK	302.741	313.87	0.0520829	0.119196	0.83225	0.994748	no
gene:SpnNT_00906	msrAB1	Chromosome:944822-945761	ΔORF2	110.58+peptide	OK	299.633	313.87	0.0669708	0.154459	0.7854	0.994748	no
gene:SpnNT_00906	msrAB1	Chromosome:944822-945761	110.58	ΔORF2+peptide	OK	302.741	362.876	0.261391	0.596433	0.2899	0.7945	no
gene:SpnNT_00906	msrAB1	Chromosome:944822-945761	ΔORF2	ΔORF2+peptide	OK	299.633	362.876	0.276279	0.635269	0.25965	0.759841	no
gene:SpnNT_00906	msrAB1	Chromosome:944822-945761	110.58+peptide	ΔORF2+peptide	OK	313.87	362.876	0.209308	0.481403	0.3872	0.881745	no
gene:SpnNT_00907	bmrA	Chromosome:945820-947545	110.58	ΔORF2	OK	84.3872	92.8231	0.13746	0.314625	0.5777	0.969538	no
gene:SpnNT_00907	bmrA	Chromosome:945820-947545	110.58	110.58+peptide	OK	84.3872	77.1696	-0.128991	-0.295179	0.6039	0.976761	no
gene:SpnNT_00907	bmrA	Chromosome:945820-947545	ΔORF2	110.58+peptide	OK	92.8231	77.1696	-0.266451	-0.608812	0.28175	0.784843	no
gene:SpnNT_00907	bmrA	Chromosome:945820-947545	110.58	ΔORF2+peptide	OK	84.3872	94.3471	0.160954	0.369049	0.51365	0.95097	no
gene:SpnNT_00907	bmrA	Chromosome:945820-947545	ΔORF2	ΔORF2+peptide	OK	92.8231	94.3471	0.023494	0.0537868	0.92185	0.994748	no
gene:SpnNT_00907	bmrA	Chromosome:945820-947545	110.58+peptide	ΔORF2+peptide	OK	77.1696	94.3471	0.289945	0.663657	0.23405	0.72774	no
gene:SpnNT_00908	NA	Chromosome:947546-949295	110.58	ΔORF2	OK	106.901	112.55	0.0742896	0.171268	0.76045	0.994748	no
gene:SpnNT_00908	NA	Chromosome:947546-949295	110.58	110.58+peptide	OK	106.901	102.43	-0.0616323	-0.141956	0.8031	0.994748	no

gene:SpnNT_00908	NA	Chromosome:947546-949295	ΔORF2	110.58+peptide	OK	112.55	102.43	-0.135922	-0.31291	0.5834	0.971331	no
gene:SpnNT_00908	NA	Chromosome:947546-949295	110.58	ΔORF2+peptide	OK	106.901	125.434	0.230658	0.531326	0.34505	0.849092	no
gene:SpnNT_00908	NA	Chromosome:947546-949295	ΔORF2	ΔORF2+peptide	OK	112.55	125.434	0.156368	0.360019	0.522	0.954832	no
gene:SpnNT_00908	NA	Chromosome:947546-949295	110.58+peptide	ΔORF2+peptide	OK	102.43	125.434	0.29229	0.672341	0.2332	0.727198	no
gene:SpnNT_00909	mtaD	Chromosome:949389-950856	110.58	ΔORF2	OK	158.001	173.123	0.131858	0.30396	0.58615	0.972864	no
gene:SpnNT_00909	mtaD	Chromosome:949389-950856	110.58	110.58+peptide	OK	158.001	77.8727	-1.02075	-2.33226	1.00E-04	0.0025332	yes
gene:SpnNT_00909	mtaD	Chromosome:949389-950856	ΔORF2	110.58+peptide	OK	173.123	77.8727	-1.1526	-2.63045	5.00E-05	0.0013612	yes
gene:SpnNT_00909	mtaD	Chromosome:949389-950856	110.58	ΔORF2+peptide	OK	158.001	92.3677	-0.774476	-1.76639	0.002	0.0294545	yes
gene:SpnNT_00909	mtaD	Chromosome:949389-950856	ΔORF2	ΔORF2+peptide	OK	173.123	92.3677	-0.906334	-2.06472	0.00075	0.0133173	yes
gene:SpnNT_00909	mtaD	Chromosome:949389-950856	110.58+peptide	ΔORF2+peptide	OK	77.8727	92.3677	0.24627	0.556186	0.3358	0.840108	no
gene:SpnNT_00910	rplJ	Chromosome:951105-951606	110.58	ΔORF2	OK	330.379	364.225	0.140709	0.317353	0.5766	0.969538	no
gene:SpnNT_00910	rplJ	Chromosome:951105-951606	110.58	110.58+peptide	OK	330.379	961.294	1.54086	3.4987	5.00E-05	0.0013612	yes
gene:SpnNT_00910	rplJ	Chromosome:951105-951606	ΔORF2	110.58+peptide	OK	364.225	961.294	1.40015	3.1705	5.00E-05	0.0013612	yes
gene:SpnNT_00910	rplJ	Chromosome:951105-951606	110.58	ΔORF2+peptide	OK	330.379	1346.82	2.02737	4.57376	5.00E-05	0.0013612	yes
gene:SpnNT_00910	rplJ	Chromosome:951105-951606	ΔORF2	ΔORF2+peptide	OK	364.225	1346.82	1.88666	4.24481	5.00E-05	0.0013612	yes
gene:SpnNT_00910	rplJ	Chromosome:951105-951606	110.58+peptide	ΔORF2+peptide	OK	961.294	1346.82	0.486511	1.10196	0.0559	0.337095	no
gene:SpnNT_00911	rplL	Chromosome:951681-952186	110.58	ΔORF2	OK	1743.95	1755.08	0.00917643	0.0201185	0.9733	0.99536	no
gene:SpnNT_00911	rplL	Chromosome:951681-952186	110.58	110.58+peptide	OK	1743.95	4822.11	1.46731	3.09817	5.00E-05	0.0013612	yes
gene:SpnNT_00911	rplL	Chromosome:951681-952186	ΔORF2	110.58+peptide	OK	1755.08	4822.11	1.45813	3.11335	5.00E-05	0.0013612	yes
gene:SpnNT_00911	rplL	Chromosome:951681-952186	110.58	ΔORF2+peptide	OK	1743.95	4738.12	1.44196	3.06873	5.00E-05	0.0013612	yes
gene:SpnNT_00911	rplL	Chromosome:951681-952186	ΔORF2	ΔORF2+peptide	OK	1755.08	4738.12	1.43278	3.08398	5.00E-05	0.0013612	yes
gene:SpnNT_00911	rplL	Chromosome:951681-952186	110.58+peptide	ΔORF2+peptide	OK	4822.11	4738.12	-0.0253499	-0.0526187	0.9245	0.994748	no
gene:SpnNT_00912	NA	Chromosome:952251-953201	110.58	ΔORF2	NOTEST	0	2.15961	Inf	0	1	1	no
gene:SpnNT_00912	NA	Chromosome:952251-953201	110.58	110.58+peptide	NOTEST	0	0.936615	Inf	0	1	1	no
gene:SpnNT_00912	NA	Chromosome:952251-953201	ΔORF2	110.58+peptide	NOTEST	2.15961	0.936615	-1.20524	0	1	1	no
gene:SpnNT_00912	NA	Chromosome:952251-953201	110.58	ΔORF2+peptide	NOTEST	0	0	0	0	1	1	no
gene:SpnNT_00912	NA	Chromosome:952251-953201	ΔORF2	ΔORF2+peptide	NOTEST	2.15961	0	#NAME?	0	1	1	no
gene:SpnNT_00912	NA	Chromosome:952251-953201	110.58+peptide	ΔORF2+peptide	NOTEST	0.936615	0	#NAME?	0	1	1	no
gene:SpnNT_00913	NA	Chromosome:952251-953201	110.58	ΔORF2	NOTEST	0.046206	0.0907031	0.973079	0	1	1	no
gene:SpnNT_00913	NA	Chromosome:952251-953201	110.58	110.58+peptide	NOTEST	0.046206	0.0967202	1.06574	0	1	1	no
gene:SpnNT_00913	NA	Chromosome:952251-953201	ΔORF2	110.58+peptide	NOTEST	0.090703	0.0967202	0.092665	0	1	1	no
gene:SpnNT_00913	NA	Chromosome:952251-953201	110.58	ΔORF2+peptide	NOTEST	0.046206	0.106437	1.20385	0	1	1	no
gene:SpnNT_00913	NA	Chromosome:952251-953201	ΔORF2	ΔORF2+peptide	NOTEST	0.090703	0.106437	0.230774	0	1	1	no
gene:SpnNT_00913	NA	Chromosome:952251-953201	110.58+peptide	ΔORF2+peptide	NOTEST	0.09672	0.106437	0.13811	0	1	1	no
gene:SpnNT_00914	NA	Chromosome:953201-955464	110.58	ΔORF2	NOTEST	0	0	0	0	1	1	no
gene:SpnNT_00914	NA	Chromosome:953201-955464	110.58	110.58+peptide	NOTEST	0	0	0	0	1	1	no
gene:SpnNT_00914	NA	Chromosome:953201-955464	ΔORF2	110.58+peptide	NOTEST	0	0	0	0	1	1	no
gene:SpnNT_00914	NA	Chromosome:953201-955464	110.58	ΔORF2+peptide	NOTEST	0	0	0	0	1	1	no
gene:SpnNT_00914	NA	Chromosome:953201-955464	ΔORF2	ΔORF2+peptide	NOTEST	0	0	0	0	1	1	no
gene:SpnNT_00914	NA	Chromosome:953201-955464	110.58+peptide	ΔORF2+peptide	NOTEST	0	0	0	0	1	1	no
gene:SpnNT_00915	hpaIIM_2	Chromosome:953201-955464	110.58	ΔORF2	NOTEST	0.318546	0.280147	-0.185317	0	1	1	no
gene:SpnNT_00915	hpaIIM_2	Chromosome:953201-955464	110.58	110.58+peptide	NOTEST	0.318546	0.320053	0.00680954	0	1	1	no
gene:SpnNT_00915	hpaIIM_2	Chromosome:953201-955464	ΔORF2	110.58+peptide	NOTEST	0.280147	0.320053	0.192127	0	1	1	no
gene:SpnNT_00915	hpaIIM_2	Chromosome:953201-955464	110.58	ΔORF2+peptide	NOTEST	0.318546	0.114065	-1.48164	0	1	1	no
gene:SpnNT_00915	hpaIIM_2	Chromosome:953201-955464	ΔORF2	ΔORF2+peptide	NOTEST	0.280147	0.114065	-1.29632	0	1	1	no

gene:SpnNT_00915	hpaIIM_2	Chromosome:953201-955464	110.58+peptide	ΔORF2+peptide	NOTEST	0.320053	0.114065	-1.48845	0	1	1	no
gene:SpnNT_00916	NA	Chromosome:953201-955464	110.58	ΔORF2	NOTEST	0.303402	0	#NAME?	0	1	1	no
gene:SpnNT_00916	NA	Chromosome:953201-955464	110.58	110.58+peptide	NOTEST	0.303402	0.324537	0.0971544	0	1	1	no
gene:SpnNT_00916	NA	Chromosome:953201-955464	ΔORF2	110.58+peptide	NOTEST	0	0.324537	Inf	0	1	1	no
gene:SpnNT_00916	NA	Chromosome:953201-955464	110.58	ΔORF2+peptide	NOTEST	0.303402	0	#NAME?	0	1	1	no
gene:SpnNT_00916	NA	Chromosome:953201-955464	ΔORF2	ΔORF2+peptide	NOTEST	0	0	0	0	1	1	no
gene:SpnNT_00916	NA	Chromosome:953201-955464	110.58+peptide	ΔORF2+peptide	NOTEST	0.324537	0	#NAME?	0	1	1	no
gene:SpnNT_00917	spxA_3	Chromosome:953201-955464	110.58	ΔORF2	NOTEST	0.534812	0.0908132	-2.55806	0	1	1	no
gene:SpnNT_00917	spxA_3	Chromosome:953201-955464	110.58	110.58+peptide	NOTEST	0.534812	0.303091	-0.81928	0	1	1	no
gene:SpnNT_00917	spxA_3	Chromosome:953201-955464	ΔORF2	110.58+peptide	NOTEST	0.090813	0.303091	1.73878	0	1	1	no
gene:SpnNT_00917	spxA_3	Chromosome:953201-955464	110.58	ΔORF2+peptide	NOTEST	0.534812	0	#NAME?	0	1	1	no
gene:SpnNT_00917	spxA_3	Chromosome:953201-955464	ΔORF2	ΔORF2+peptide	NOTEST	0.090813	0	#NAME?	0	1	1	no
gene:SpnNT_00917	spxA_3	Chromosome:953201-955464	110.58+peptide	ΔORF2+peptide	NOTEST	0.303091	0	#NAME?	0	1	1	no
gene:SpnNT_00918	NA	Chromosome:955476-955710	110.58	ΔORF2	NOTEST	0	0.261303	Inf	0	1	1	no
gene:SpnNT_00918	NA	Chromosome:955476-955710	110.58	110.58+peptide	NOTEST	0	0	0	0	1	1	no
gene:SpnNT_00918	NA	Chromosome:955476-955710	ΔORF2	110.58+peptide	NOTEST	0.261303	0	#NAME?	0	1	1	no
gene:SpnNT_00918	NA	Chromosome:955476-955710	110.58	ΔORF2+peptide	NOTEST	0	0	0	0	1	1	no
gene:SpnNT_00918	NA	Chromosome:955476-955710	ΔORF2	ΔORF2+peptide	NOTEST	0.261303	0	#NAME?	0	1	1	no
gene:SpnNT_00918	NA	Chromosome:955476-955710	110.58+peptide	ΔORF2+peptide	NOTEST	0	0	0	0	1	1	no
gene:SpnNT_00919	NA	Chromosome:955712-956300	110.58	ΔORF2	NOTEST	0.104797	0	#NAME?	0	1	1	no
gene:SpnNT_00919	NA	Chromosome:955712-956300	110.58	110.58+peptide	NOTEST	0.104797	0.157651	0.589138	0	1	1	no
gene:SpnNT_00919	NA	Chromosome:955712-956300	ΔORF2	110.58+peptide	NOTEST	0	0.157651	Inf	0	1	1	no
gene:SpnNT_00919	NA	Chromosome:955712-956300	110.58	ΔORF2+peptide	NOTEST	0.104797	0.151781	0.534393	0	1	1	no
gene:SpnNT_00919	NA	Chromosome:955712-956300	ΔORF2	ΔORF2+peptide	NOTEST	0	0.151781	Inf	0	1	1	no
gene:SpnNT_00919	NA	Chromosome:955712-956300	110.58+peptide	ΔORF2+peptide	NOTEST	0.157651	0.151781	-0.0547448	0	1	1	no
gene:SpnNT_00920	NA	Chromosome:956676-956991	110.58	ΔORF2	NOTEST	0	0	0	0	1	1	no
gene:SpnNT_00920	NA	Chromosome:956676-956991	110.58	110.58+peptide	NOTEST	0	0	0	0	1	1	no
gene:SpnNT_00920	NA	Chromosome:956676-956991	ΔORF2	110.58+peptide	NOTEST	0	0	0	0	1	1	no
gene:SpnNT_00920	NA	Chromosome:956676-956991	110.58	ΔORF2+peptide	NOTEST	0	0	0	0	1	1	no
gene:SpnNT_00920	NA	Chromosome:956676-956991	ΔORF2	ΔORF2+peptide	NOTEST	0	0	0	0	1	1	no
gene:SpnNT_00920	NA	Chromosome:956676-956991	110.58+peptide	ΔORF2+peptide	NOTEST	0	0	0	0	1	1	no
gene:SpnNT_00921	NA	Chromosome:957009-957393	110.58	ΔORF2	NOTEST	0.107653	0.228158	1.08365	0	1	1	no
gene:SpnNT_00921	NA	Chromosome:957009-957393	110.58	110.58+peptide	NOTEST	0.107653	0.141853	0.398011	0	1	1	no
gene:SpnNT_00921	NA	Chromosome:957009-957393	ΔORF2	110.58+peptide	NOTEST	0.228158	0.141853	-0.685637	0	1	1	no
gene:SpnNT_00921	NA	Chromosome:957009-957393	110.58	ΔORF2+peptide	NOTEST	0.107653	0	#NAME?	0	1	1	no
gene:SpnNT_00921	NA	Chromosome:957009-957393	ΔORF2	ΔORF2+peptide	NOTEST	0.228158	0	#NAME?	0	1	1	no
gene:SpnNT_00921	NA	Chromosome:957009-957393	110.58+peptide	ΔORF2+peptide	NOTEST	0.141853	0	#NAME?	0	1	1	no
gene:SpnNT_00922	ftsK_1	Chromosome:957421-958804	110.58	ΔORF2	NOTEST	0.093517	0.0245957	-1.92683	0	1	1	no
gene:SpnNT_00922	ftsK_1	Chromosome:957421-958804	110.58	110.58+peptide	NOTEST	0.093517	0.0900564	-0.0544048	0	1	1	no
gene:SpnNT_00922	ftsK_1	Chromosome:957421-958804	ΔORF2	110.58+peptide	NOTEST	0.024596	0.0900564	1.87242	0	1	1	no
gene:SpnNT_00922	ftsK_1	Chromosome:957421-958804	110.58	ΔORF2+peptide	NOTEST	0.093517	0.100426	0.102827	0	1	1	no
gene:SpnNT_00922	ftsK_1	Chromosome:957421-958804	ΔORF2	ΔORF2+peptide	NOTEST	0.024596	0.100426	2.02965	0	1	1	no
gene:SpnNT_00922	ftsK_1	Chromosome:957421-958804	110.58+peptide	ΔORF2+peptide	NOTEST	0.090056	0.100426	0.157232	0	1	1	no
gene:SpnNT_00923	NA	Chromosome:958980-960429	110.58	ΔORF2	OK	12.7883	12.2431	-0.0628582	-0.113748	0.83695	0.994748	no
gene:SpnNT_00923	NA	Chromosome:958980-960429	110.58	110.58+peptide	OK	12.7883	8.35896	-0.613428	-1.06217	0.0622	0.360348	no

gene:SpnNT_00923	NA	Chromosome:958980-960429	ΔORF2	110.58+peptide	OK	12.2431	8.35896	-0.55057	-0.963075	0.08575	0.439879	no
gene:SpnNT_00923	NA	Chromosome:958980-960429	110.58	ΔORF2+peptide	OK	12.7883	6.51965	-0.971957	-1.63783	0.00385	0.0490483	yes
gene:SpnNT_00923	NA	Chromosome:958980-960429	ΔORF2	ΔORF2+peptide	OK	12.2431	6.51965	-0.909099	-1.54673	0.00605	0.0694559	no
gene:SpnNT_00923	NA	Chromosome:958980-960429	110.58+peptide	ΔORF2+peptide	OK	8.35896	6.51965	-0.358529	-0.586559	0.2876	0.791337	no
gene:SpnNT_00924	erm_1	Chromosome:960657-961443	110.58	ΔORF2	OK	23.4885	28.4806	0.278025	0.510092	0.3618	0.862562	no
gene:SpnNT_00924	erm_1	Chromosome:960657-961443	110.58	110.58+peptide	OK	23.4885	25.105	0.0960191	0.176043	0.75475	0.994748	no
gene:SpnNT_00924	erm_1	Chromosome:960657-961443	ΔORF2	110.58+peptide	OK	28.4806	25.105	-0.182006	-0.333708	0.55025	0.964281	no
gene:SpnNT_00924	erm_1	Chromosome:960657-961443	110.58	ΔORF2+peptide	OK	23.4885	25.5878	0.123501	0.225408	0.6845	0.987866	no
gene:SpnNT_00924	erm_1	Chromosome:960657-961443	ΔORF2	ΔORF2+peptide	OK	28.4806	25.5878	-0.154524	-0.282042	0.6091	0.978849	no
gene:SpnNT_00924	erm_1	Chromosome:960657-961443	110.58+peptide	ΔORF2+peptide	OK	25.105	25.5878	0.0274818	0.050126	0.92825	0.994748	no
gene:SpnNT_00925	NA	Chromosome:961780-962323	110.58	ΔORF2	OK	128.365	130.241	0.0209301	0.0442763	0.93905	0.994855	no
gene:SpnNT_00925	NA	Chromosome:961780-962323	110.58	110.58+peptide	OK	128.365	108.654	-0.240512	-0.504335	0.37865	0.875802	no
gene:SpnNT_00925	NA	Chromosome:961780-962323	ΔORF2	110.58+peptide	OK	130.241	108.654	-0.261442	-0.547934	0.33495	0.840029	no
gene:SpnNT_00925	NA	Chromosome:961780-962323	110.58	ΔORF2+peptide	OK	128.365	136.245	0.0859558	0.182241	0.75115	0.994748	no
gene:SpnNT_00925	NA	Chromosome:961780-962323	ΔORF2	ΔORF2+peptide	OK	130.241	136.245	0.0650258	0.137791	0.81	0.994748	no
gene:SpnNT_00925	NA	Chromosome:961780-962323	110.58+peptide	ΔORF2+peptide	OK	108.654	136.245	0.326468	0.685716	0.23635	0.73058	no
gene:SpnNT_00926	aphA	Chromosome:962415-963210	110.58	ΔORF2	OK	234.594	236.346	0.010733	0.0243608	0.96685	0.994855	no
gene:SpnNT_00926	aphA	Chromosome:962415-963210	110.58	110.58+peptide	OK	234.594	195.646	-0.261921	-0.594169	0.3014	0.807631	no
gene:SpnNT_00926	aphA	Chromosome:962415-963210	ΔORF2	110.58+peptide	OK	236.346	195.646	-0.272654	-0.618174	0.28395	0.786738	no
gene:SpnNT_00926	aphA	Chromosome:962415-963210	110.58	ΔORF2+peptide	OK	234.594	235.329	0.00451131	0.0102885	0.9856	0.99692	no
gene:SpnNT_00926	aphA	Chromosome:962415-963210	ΔORF2	ΔORF2+peptide	OK	236.346	235.329	-0.00622173	-0.0141813	0.9785	0.995765	no
gene:SpnNT_00926	aphA	Chromosome:962415-963210	110.58+peptide	ΔORF2+peptide	OK	195.646	235.329	0.266433	0.606961	0.28585	0.789224	no
gene:SpnNT_00927	NA	Chromosome:963693-964059	110.58	ΔORF2	OK	188.862	181.189	-0.0598374	-0.120871	0.8367	0.994748	no
gene:SpnNT_00927	NA	Chromosome:963693-964059	110.58	110.58+peptide	OK	188.862	162.651	-0.215545	-0.426466	0.4604	0.924377	no
gene:SpnNT_00927	NA	Chromosome:963693-964059	ΔORF2	110.58+peptide	OK	181.189	162.651	-0.155708	-0.307831	0.59425	0.975539	no
gene:SpnNT_00927	NA	Chromosome:963693-964059	110.58	ΔORF2+peptide	OK	188.862	143.041	-0.4009	-0.765661	0.17505	0.636996	no
gene:SpnNT_00927	NA	Chromosome:963693-964059	ΔORF2	ΔORF2+peptide	OK	181.189	143.041	-0.341063	-0.6509	0.2495	0.747113	no
gene:SpnNT_00927	NA	Chromosome:963693-964059	110.58+peptide	ΔORF2+peptide	OK	162.651	143.041	-0.185355	-0.347241	0.53295	0.958521	no
gene:SpnNT_00928	NA	Chromosome:964302-964770	110.58	ΔORF2	OK	5.04105	5.72538	0.183647	0.226868	0.6954	0.98895	no
gene:SpnNT_00928	NA	Chromosome:964302-964770	110.58	110.58+peptide	OK	5.04105	4.64747	-0.117277	-0.142203	0.80865	0.994748	no
gene:SpnNT_00928	NA	Chromosome:964302-964770	ΔORF2	110.58+peptide	OK	5.72538	4.64747	-0.300924	-0.371647	0.5137	0.95097	no
gene:SpnNT_00928	NA	Chromosome:964302-964770	110.58	ΔORF2+peptide	OK	5.04105	3.68257	-0.453012	-0.565789	0.3972	0.89095	no
gene:SpnNT_00928	NA	Chromosome:964302-964770	ΔORF2	ΔORF2+peptide	OK	5.72538	3.68257	-0.636659	-0.81082	0.2196	0.707834	no
gene:SpnNT_00928	NA	Chromosome:964302-964770	110.58+peptide	ΔORF2+peptide	OK	4.64747	3.68257	-0.335735	-0.419201	0.5263	0.956161	no
gene:SpnNT_00929	NA	Chromosome:964785-965654	110.58	ΔORF2	OK	6.3602	6.96612	0.131283	0.0976675	0.86135	0.994748	no
gene:SpnNT_00929	NA	Chromosome:964785-965654	110.58	110.58+peptide	OK	6.3602	7.39669	0.217808	0.168839	0.7709	0.994748	no
gene:SpnNT_00929	NA	Chromosome:964785-965654	ΔORF2	110.58+peptide	OK	6.96612	7.39669	0.0865247	0.0692312	0.90735	0.994748	no
gene:SpnNT_00929	NA	Chromosome:964785-965654	110.58	ΔORF2+peptide	OK	6.3602	8.71509	0.454442	0.351568	0.54195	0.961494	no
gene:SpnNT_00929	NA	Chromosome:964785-965654	ΔORF2	ΔORF2+peptide	OK	6.96612	8.71509	0.323159	0.258019	0.66225	0.982966	no
gene:SpnNT_00929	NA	Chromosome:964785-965654	110.58+peptide	ΔORF2+peptide	OK	7.39669	8.71509	0.236635	0.198161	0.7411	0.994748	no
gene:SpnNT_00930	NA	Chromosome:964785-965654	110.58	ΔORF2	OK	11.3649	9.05793	-0.32733	-0.438818	0.43455	0.913666	no
gene:SpnNT_00930	NA	Chromosome:964785-965654	110.58	110.58+peptide	OK	11.3649	7.51693	-0.596368	-0.787493	0.1678	0.623936	no
gene:SpnNT_00930	NA	Chromosome:964785-965654	ΔORF2	110.58+peptide	OK	9.05793	7.51693	-0.269038	-0.342787	0.55405	0.966274	no
gene:SpnNT_00930	NA	Chromosome:964785-965654	110.58	ΔORF2+peptide	OK	11.3649	7.57607	-0.585062	-0.748234	0.1925	0.673955	no
gene:SpnNT_00930	NA	Chromosome:964785-965654	ΔORF2	ΔORF2+peptide	OK	9.05793	7.57607	-0.257732	-0.318723	0.58675	0.973049	no

gene:SpnNT_00930	NA	Chromosome:964785-965654	110.58+peptide	ΔORF2+peptide	OK	7.51693	7.57607	0.0113061	0.0138025	0.98705	0.997359	no
gene:SpnNT_00931	NA	Chromosome:965834-966098	110.58	ΔORF2	OK	181.308	188.487	0.056024	0.0994355	0.86465	0.994748	no
gene:SpnNT_00931	NA	Chromosome:965834-966098	110.58	110.58+peptide	OK	181.308	125.321	-0.532818	-0.890887	0.11105	0.507381	no
gene:SpnNT_00931	NA	Chromosome:965834-966098	ΔORF2	110.58+peptide	OK	188.487	125.321	-0.588842	-0.987688	0.0807	0.423409	no
gene:SpnNT_00931	NA	Chromosome:965834-966098	110.58	ΔORF2+peptide	OK	181.308	128.362	-0.498225	-0.782622	0.14255	0.576108	no
gene:SpnNT_00931	NA	Chromosome:965834-966098	ΔORF2	ΔORF2+peptide	OK	188.487	128.362	-0.554249	-0.873066	0.10655	0.496326	no
gene:SpnNT_00931	NA	Chromosome:965834-966098	110.58+peptide	ΔORF2+peptide	OK	125.321	128.362	0.0345927	0.0519582	0.924	0.994748	no
gene:SpnNT_00932	erm_2	Chromosome:966326-967064	110.58	ΔORF2	OK	23.5686	27.1606	0.204652	0.368448	0.51195	0.950278	no
gene:SpnNT_00932	erm_2	Chromosome:966326-967064	110.58	110.58+peptide	OK	23.5686	27.7209	0.234111	0.427185	0.44675	0.919137	no
gene:SpnNT_00932	erm_2	Chromosome:966326-967064	ΔORF2	110.58+peptide	OK	27.1606	27.7209	0.0294589	0.0533537	0.9267	0.994748	no
gene:SpnNT_00932	erm_2	Chromosome:966326-967064	110.58	ΔORF2+peptide	OK	23.5686	33.1572	0.492459	0.905539	0.1119	0.509667	no
gene:SpnNT_00932	erm_2	Chromosome:966326-967064	ΔORF2	ΔORF2+peptide	OK	27.1606	33.1572	0.287807	0.525221	0.3603	0.861019	no
gene:SpnNT_00932	erm_2	Chromosome:966326-967064	110.58+peptide	ΔORF2+peptide	OK	27.7209	33.1572	0.258348	0.478017	0.39705	0.89095	no
gene:SpnNT_00933	NA	Chromosome:967068-967200	110.58	ΔORF2	OK	40.5515	40.6288	0.00274646	0.00200855	0.9212	0.994748	no
gene:SpnNT_00933	NA	Chromosome:967068-967200	110.58	110.58+peptide	OK	40.5515	63.6511	0.650429	0.496683	0.3911	0.885288	no
gene:SpnNT_00933	NA	Chromosome:967068-967200	ΔORF2	110.58+peptide	OK	40.6288	63.6511	0.647682	0.504217	0.38715	0.881745	no
gene:SpnNT_00933	NA	Chromosome:967068-967200	110.58	ΔORF2+peptide	OK	40.5515	157.589	1.95834	1.74563	0.0175	0.150186	no
gene:SpnNT_00933	NA	Chromosome:967068-967200	ΔORF2	ΔORF2+peptide	OK	40.6288	157.589	1.95559	1.78994	0.017	0.147146	no
gene:SpnNT_00933	NA	Chromosome:967068-967200	110.58+peptide	ΔORF2+peptide	OK	63.6511	157.589	1.30791	1.28324	0.0571	0.339649	no
gene:SpnNT_00934	NA	Chromosome:967320-968568	110.58	ΔORF2	OK	8.95678	11.1523	0.316289	0.558265	0.3305	0.836255	no
gene:SpnNT_00934	NA	Chromosome:967320-968568	110.58	110.58+peptide	OK	8.95678	13.8008	0.623702	1.10602	0.0571	0.339649	no
gene:SpnNT_00934	NA	Chromosome:967320-968568	ΔORF2	110.58+peptide	OK	11.1523	13.8008	0.307414	0.550931	0.3442	0.848346	no
gene:SpnNT_00934	NA	Chromosome:967320-968568	110.58	ΔORF2+peptide	OK	8.95678	20.9064	1.22289	2.24359	0.00015	0.00355289	yes
gene:SpnNT_00934	NA	Chromosome:967320-968568	ΔORF2	ΔORF2+peptide	OK	11.1523	20.9064	0.906602	1.68224	0.00365	0.0469562	yes
gene:SpnNT_00934	NA	Chromosome:967320-968568	110.58+peptide	ΔORF2+peptide	OK	13.8008	20.9064	0.599189	1.11758	0.0576	0.341849	no
gene:SpnNT_00935	NA	Chromosome:968747-968969	110.58	ΔORF2	NOTEST	0.404114	0	#NAME?	0	1	1	no
gene:SpnNT_00935	NA	Chromosome:968747-968969	110.58	110.58+peptide	NOTEST	0.404114	0.5587	0.467313	0	1	1	no
gene:SpnNT_00935	NA	Chromosome:968747-968969	ΔORF2	110.58+peptide	NOTEST	0	0.5587	Inf	0	1	1	no
gene:SpnNT_00935	NA	Chromosome:968747-968969	110.58	ΔORF2+peptide	NOTEST	0.404114	1.08927	1.43053	0	1	1	no
gene:SpnNT_00935	NA	Chromosome:968747-968969	ΔORF2	ΔORF2+peptide	NOTEST	0	1.08927	Inf	0	1	1	no
gene:SpnNT_00935	NA	Chromosome:968747-968969	110.58+peptide	ΔORF2+peptide	NOTEST	0.5587	1.08927	0.963215	0	1	1	no
gene:SpnNT_00936	NA	Chromosome:969085-972495	110.58	ΔORF2	NOTEST	0.182779	0.503569	1.46209	0	1	1	no
gene:SpnNT_00936	NA	Chromosome:969085-972495	110.58	110.58+peptide	NOTEST	0.182779	0.391243	1.09797	0	1	1	no
gene:SpnNT_00936	NA	Chromosome:969085-972495	ΔORF2	110.58+peptide	NOTEST	0.503569	0.391243	-0.364123	0	1	1	no
gene:SpnNT_00936	NA	Chromosome:969085-972495	110.58	ΔORF2+peptide	NOTEST	0.182779	0.25193	0.462927	0	1	1	no
gene:SpnNT_00936	NA	Chromosome:969085-972495	ΔORF2	ΔORF2+peptide	NOTEST	0.503569	0.25193	-0.999164	0	1	1	no
gene:SpnNT_00936	NA	Chromosome:969085-972495	110.58+peptide	ΔORF2+peptide	NOTEST	0.391243	0.25193	-0.635041	0	1	1	no
gene:SpnNT_00937	NA	Chromosome:969085-972495	110.58	ΔORF2	NOTEST	0.200083	0.142627	-0.488348	0	1	1	no
gene:SpnNT_00937	NA	Chromosome:969085-972495	110.58	110.58+peptide	NOTEST	0.200083	0.0924391	-1.11402	0	1	1	no
gene:SpnNT_00937	NA	Chromosome:969085-972495	ΔORF2	110.58+peptide	NOTEST	0.142627	0.0924391	-0.625674	0	1	1	no
gene:SpnNT_00937	NA	Chromosome:969085-972495	110.58	ΔORF2+peptide	NOTEST	0.200083	0.156984	-0.349977	0	1	1	no
gene:SpnNT_00937	NA	Chromosome:969085-972495	ΔORF2	ΔORF2+peptide	NOTEST	0.142627	0.156984	0.138371	0	1	1	no
gene:SpnNT_00937	NA	Chromosome:969085-972495	110.58+peptide	ΔORF2+peptide	NOTEST	0.092439	0.156984	0.764045	0	1	1	no
gene:SpnNT_00938	NA	Chromosome:969085-972495	110.58	ΔORF2	NOTEST	0.206591	0.162455	-0.346735	0	1	1	no
gene:SpnNT_00938	NA	Chromosome:969085-972495	110.58	110.58+peptide	NOTEST	0.206591	0.143432	-0.526413	0	1	1	no

gene:SpnNT_00938	NA	Chromosome:969085-972495	ΔORF2	110.58+peptide	NOTEST	0.162455	0.143432	-0.179678	0	1	1	no
gene:SpnNT_00938	NA	Chromosome:969085-972495	110.58	ΔORF2+peptide	NOTEST	0.206591	0.174618	-0.242575	0	1	1	no
gene:SpnNT_00938	NA	Chromosome:969085-972495	ΔORF2	ΔORF2+peptide	NOTEST	0.162455	0.174618	0.10416	0	1	1	no
gene:SpnNT_00938	NA	Chromosome:969085-972495	110.58+peptide	ΔORF2+peptide	NOTEST	0.143432	0.174618	0.283838	0	1	1	no
gene:SpnNT_00939	NA	Chromosome:972563-975673	110.58	ΔORF2	NOTEST	0.045537	0.148718	1.70747	0	1	1	no
gene:SpnNT_00939	NA	Chromosome:972563-975673	110.58	110.58+peptide	NOTEST	0.045537	0.0771752	0.761096	0	1	1	no
gene:SpnNT_00939	NA	Chromosome:972563-975673	ΔORF2	110.58+peptide	NOTEST	0.148718	0.0771752	-0.946374	0	1	1	no
gene:SpnNT_00939	NA	Chromosome:972563-975673	110.58	ΔORF2+peptide	NOTEST	0.045537	0.202578	2.15336	0	1	1	no
gene:SpnNT_00939	NA	Chromosome:972563-975673	ΔORF2	ΔORF2+peptide	NOTEST	0.148718	0.202578	0.445893	0	1	1	no
gene:SpnNT_00939	NA	Chromosome:972563-975673	110.58+peptide	ΔORF2+peptide	NOTEST	0.077175	0.202578	1.39227	0	1	1	no
gene:SpnNT_00940	iap	Chromosome:972563-975673	110.58	ΔORF2	NOTEST	0.095132	0.0364873	-1.38254	0	1	1	no
gene:SpnNT_00940	iap	Chromosome:972563-975673	110.58	110.58+peptide	NOTEST	0.095132	0	#NAME?	0	1	1	no
gene:SpnNT_00940	iap	Chromosome:972563-975673	ΔORF2	110.58+peptide	NOTEST	0.036487	0	#NAME?	0	1	1	no
gene:SpnNT_00940	iap	Chromosome:972563-975673	110.58	ΔORF2+peptide	NOTEST	0.095132	0	#NAME?	0	1	1	no
gene:SpnNT_00940	iap	Chromosome:972563-975673	ΔORF2	ΔORF2+peptide	NOTEST	0.036487	0	#NAME?	0	1	1	no
gene:SpnNT_00940	iap	Chromosome:972563-975673	110.58+peptide	ΔORF2+peptide	NOTEST	0	0	0	0	1	1	no
gene:SpnNT_00941	NA	Chromosome:975687-976605	110.58	ΔORF2	NOTEST	0.095705	0.162681	0.765387	0	1	1	no
gene:SpnNT_00941	NA	Chromosome:975687-976605	110.58	110.58+peptide	NOTEST	0.095705	0.0405803	-1.23781	0	1	1	no
gene:SpnNT_00941	NA	Chromosome:975687-976605	ΔORF2	110.58+peptide	NOTEST	0.162681	0.0405803	-2.0032	0	1	1	no
gene:SpnNT_00941	NA	Chromosome:975687-976605	110.58	ΔORF2+peptide	NOTEST	0.095705	0.0817189	-0.227919	0	1	1	no
gene:SpnNT_00941	NA	Chromosome:975687-976605	ΔORF2	ΔORF2+peptide	NOTEST	0.162681	0.0817189	-0.993306	0	1	1	no
gene:SpnNT_00941	NA	Chromosome:975687-976605	110.58+peptide	ΔORF2+peptide	NOTEST	0.04058	0.0817189	1.00989	0	1	1	no
gene:SpnNT_00942	tetM	Chromosome:976980-978900	110.58	ΔORF2	OK	6.95905	8.32522	0.258597	0.462563	0.42695	0.908839	no
gene:SpnNT_00942	tetM	Chromosome:976980-978900	110.58	110.58+peptide	OK	6.95905	13.7842	0.986055	1.84028	0.00215	0.0312429	yes
gene:SpnNT_00942	tetM	Chromosome:976980-978900	ΔORF2	110.58+peptide	OK	8.32522	13.7842	0.727458	1.35988	0.0204	0.169961	no
gene:SpnNT_00942	tetM	Chromosome:976980-978900	110.58	ΔORF2+peptide	OK	6.95905	17.3085	1.31452	2.4827	1.00E-04	0.0025332	yes
gene:SpnNT_00942	tetM	Chromosome:976980-978900	ΔORF2	ΔORF2+peptide	OK	8.32522	17.3085	1.05592	1.99765	0.00055	0.0103545	yes
gene:SpnNT_00942	tetM	Chromosome:976980-978900	110.58+peptide	ΔORF2+peptide	OK	13.7842	17.3085	0.328463	0.651782	0.25655	0.755996	no
gene:SpnNT_00943	NA	Chromosome:979245-979599	110.58	ΔORF2	NOTEST	0.444767	0.551035	0.309095	0	1	1	no
gene:SpnNT_00943	NA	Chromosome:979245-979599	110.58	110.58+peptide	NOTEST	0.444767	0.504417	0.181567	0	1	1	no
gene:SpnNT_00943	NA	Chromosome:979245-979599	ΔORF2	110.58+peptide	NOTEST	0.551035	0.504417	-0.127527	0	1	1	no
gene:SpnNT_00943	NA	Chromosome:979245-979599	110.58	ΔORF2+peptide	NOTEST	0.444767	0	#NAME?	0	1	1	no
gene:SpnNT_00943	NA	Chromosome:979245-979599	ΔORF2	ΔORF2+peptide	NOTEST	0.551035	0	#NAME?	0	1	1	no
gene:SpnNT_00943	NA	Chromosome:979245-979599	110.58+peptide	ΔORF2+peptide	NOTEST	0.504417	0	#NAME?	0	1	1	no
gene:SpnNT_00944	NA	Chromosome:980103-980753	110.58	ΔORF2	NOTEST	0	0	0	0	1	1	no
gene:SpnNT_00944	NA	Chromosome:980103-980753	110.58	110.58+peptide	NOTEST	0	0.236723	Inf	0	1	1	no
gene:SpnNT_00944	NA	Chromosome:980103-980753	ΔORF2	110.58+peptide	NOTEST	0	0.236723	Inf	0	1	1	no
gene:SpnNT_00944	NA	Chromosome:980103-980753	110.58	ΔORF2+peptide	NOTEST	0	0	0	0	1	1	no
gene:SpnNT_00944	NA	Chromosome:980103-980753	ΔORF2	ΔORF2+peptide	NOTEST	0	0	0	0	1	1	no
gene:SpnNT_00944	NA	Chromosome:980103-980753	110.58+peptide	ΔORF2+peptide	NOTEST	0.236723	0	#NAME?	0	1	1	no
gene:SpnNT_00945	NA	Chromosome:980103-980753	110.58	ΔORF2	NOTEST	0	0	0	0	1	1	no
gene:SpnNT_00945	NA	Chromosome:980103-980753	110.58	110.58+peptide	NOTEST	0	0	0	0	1	1	no
gene:SpnNT_00945	NA	Chromosome:980103-980753	ΔORF2	110.58+peptide	NOTEST	0	0	0	0	1	1	no
gene:SpnNT_00945	NA	Chromosome:980103-980753	110.58	ΔORF2+peptide	NOTEST	0	0	0	0	1	1	no
gene:SpnNT_00945	NA	Chromosome:980103-980753	ΔORF2	ΔORF2+peptide	NOTEST	0	0	0	0	1	1	no

gene:SpnNT_00945	NA	Chromosome:980103-980753	110.58+peptide	ΔORF2+peptide	NOTEST	0	0	0	0	1	1	no
gene:SpnNT_00946	NA	Chromosome:981213-981417	110.58	ΔORF2	NOTEST	0	0	0	0	1	1	no
gene:SpnNT_00946	NA	Chromosome:981213-981417	110.58	110.58+peptide	NOTEST	0	0	0	0	1	1	no
gene:SpnNT_00946	NA	Chromosome:981213-981417	ΔORF2	110.58+peptide	NOTEST	0	0	0	0	1	1	no
gene:SpnNT_00946	NA	Chromosome:981213-981417	110.58	ΔORF2+peptide	NOTEST	0	0	0	0	1	1	no
gene:SpnNT_00946	NA	Chromosome:981213-981417	ΔORF2	ΔORF2+peptide	NOTEST	0	0	0	0	1	1	no
gene:SpnNT_00946	NA	Chromosome:981213-981417	110.58+peptide	ΔORF2+peptide	NOTEST	0	0	0	0	1	1	no
gene:SpnNT_00947	NA	Chromosome:981498-982716	110.58	ΔORF2	OK	9.80662	12.6598	0.36843	0.651045	0.2513	0.749233	no
gene:SpnNT_00947	NA	Chromosome:981498-982716	110.58	110.58+peptide	OK	9.80662	8.5737	-0.193837	-0.331665	0.56495	0.968621	no
gene:SpnNT_00947	NA	Chromosome:981498-982716	ΔORF2	110.58+peptide	OK	12.6598	8.5737	-0.562267	-0.987502	0.08205	0.428266	no
gene:SpnNT_00947	NA	Chromosome:981498-982716	110.58	ΔORF2+peptide	OK	9.80662	9.7368	-0.0103075	-0.0178962	0.9772	0.995534	no
gene:SpnNT_00947	NA	Chromosome:981498-982716	ΔORF2	ΔORF2+peptide	OK	12.6598	9.7368	-0.378738	-0.675498	0.23455	0.728242	no
gene:SpnNT_00947	NA	Chromosome:981498-982716	110.58+peptide	ΔORF2+peptide	OK	8.5737	9.7368	0.183529	0.31677	0.58325	0.971331	no
gene:SpnNT_00948	NA	Chromosome:982985-984412	110.58	ΔORF2	OK	13.9362	21.5601	0.629525	0.583095	0.3061	0.812428	no
gene:SpnNT_00948	NA	Chromosome:982985-984412	110.58	110.58+peptide	OK	13.9362	14.188	0.0258311	0.0226952	0.96865	0.994855	no
gene:SpnNT_00948	NA	Chromosome:982985-984412	ΔORF2	110.58+peptide	OK	21.5601	14.188	-0.603694	-0.564258	0.335	0.840029	no
gene:SpnNT_00948	NA	Chromosome:982985-984412	110.58	ΔORF2+peptide	OK	13.9362	15.4439	0.148197	0.131962	0.8201	0.994748	no
gene:SpnNT_00948	NA	Chromosome:982985-984412	ΔORF2	ΔORF2+peptide	OK	21.5601	15.4439	-0.481328	-0.456774	0.43285	0.913334	no
gene:SpnNT_00948	NA	Chromosome:982985-984412	110.58+peptide	ΔORF2+peptide	OK	14.188	15.4439	0.122366	0.109877	0.8525	0.994748	no
gene:SpnNT_00949	NA	Chromosome:982985-984412	110.58	ΔORF2	OK	28.0047	38.6292	0.464024	0.789435	0.1722	0.632235	no
gene:SpnNT_00949	NA	Chromosome:982985-984412	110.58	110.58+peptide	OK	28.0047	27.138	-0.0453537	-0.0761155	0.88885	0.994748	no
gene:SpnNT_00949	NA	Chromosome:982985-984412	ΔORF2	110.58+peptide	OK	38.6292	27.138	-0.509377	-0.850818	0.14115	0.573605	no
gene:SpnNT_00949	NA	Chromosome:982985-984412	110.58	ΔORF2+peptide	OK	28.0047	35.1215	0.326687	0.568723	0.32185	0.827743	no
gene:SpnNT_00949	NA	Chromosome:982985-984412	ΔORF2	ΔORF2+peptide	OK	38.6292	35.1215	-0.137336	-0.237867	0.68185	0.986574	no
gene:SpnNT_00949	NA	Chromosome:982985-984412	110.58+peptide	ΔORF2+peptide	OK	27.138	35.1215	0.372041	0.635348	0.26695	0.768422	no
gene:SpnNT_00950	NA	Chromosome:984694-987349	110.58	ΔORF2	NOTEST	0.760859	0.134146	-2.50382	0	1	1	no
gene:SpnNT_00950	NA	Chromosome:984694-987349	110.58	110.58+peptide	NOTEST	0.760859	0.251743	-1.59568	0	1	1	no
gene:SpnNT_00950	NA	Chromosome:984694-987349	ΔORF2	110.58+peptide	NOTEST	0.134146	0.251743	0.908141	0	1	1	no
gene:SpnNT_00950	NA	Chromosome:984694-987349	110.58	ΔORF2+peptide	NOTEST	0.760859	0.159829	-2.2511	0	1	1	no
gene:SpnNT_00950	NA	Chromosome:984694-987349	ΔORF2	ΔORF2+peptide	NOTEST	0.134146	0.159829	0.252722	0	1	1	no
gene:SpnNT_00950	NA	Chromosome:984694-987349	110.58+peptide	ΔORF2+peptide	NOTEST	0.251743	0.159829	-0.655419	0	1	1	no
gene:SpnNT_00951	NA	Chromosome:984694-987349	110.58	ΔORF2	OK	1.59079	1.19106	-0.417492	-0.225677	0.70405	0.990367	no
gene:SpnNT_00951	NA	Chromosome:984694-987349	110.58	110.58+peptide	OK	1.59079	0.766425	-1.05353	-0.580318	0.32125	0.827206	no
gene:SpnNT_00951	NA	Chromosome:984694-987349	ΔORF2	110.58+peptide	OK	1.19106	0.766425	-0.636033	-0.318388	0.57535	0.969538	no
gene:SpnNT_00951	NA	Chromosome:984694-987349	110.58	ΔORF2+peptide	OK	1.59079	1.46652	-0.117347	-0.0768461	0.9236	0.994748	no
gene:SpnNT_00951	NA	Chromosome:984694-987349	ΔORF2	ΔORF2+peptide	OK	1.19106	1.46652	0.300145	0.172521	0.82595	0.994748	no
gene:SpnNT_00951	NA	Chromosome:984694-987349	110.58+peptide	ΔORF2+peptide	OK	0.766425	1.46652	0.936178	0.549724	0.48885	0.939082	no
gene:SpnNT_00952	NA	Chromosome:984694-987349	110.58	ΔORF2	NOTEST	0.705068	0.69907	-0.012326	0	1	1	no
gene:SpnNT_00952	NA	Chromosome:984694-987349	110.58	110.58+peptide	NOTEST	0.705068	0.47248	-0.577508	0	1	1	no
gene:SpnNT_00952	NA	Chromosome:984694-987349	ΔORF2	110.58+peptide	NOTEST	0.69907	0.47248	-0.565182	0	1	1	no
gene:SpnNT_00952	NA	Chromosome:984694-987349	110.58	ΔORF2+peptide	NOTEST	0.705068	0.306986	-1.19959	0	1	1	no
gene:SpnNT_00952	NA	Chromosome:984694-987349	ΔORF2	ΔORF2+peptide	NOTEST	0.69907	0.306986	-1.18726	0	1	1	no
gene:SpnNT_00952	NA	Chromosome:984694-987349	110.58+peptide	ΔORF2+peptide	NOTEST	0.47248	0.306986	-0.622081	0	1	1	no
gene:SpnNT_00953	NA	Chromosome:987369-987612	110.58	ΔORF2	OK	1.90944	0.23473	-3.02408	-0.987887	0.1131	0.510701	no
gene:SpnNT_00953	NA	Chromosome:987369-987612	110.58	110.58+peptide	OK	1.90944	0.742569	-1.36255	-0.721414	0.4078	0.897051	no

gene:SpnNT_00953	NA	Chromosome:987369-987612	ΔORF2	110.58+peptide	NOTEST	0.23473	0.742569	1.66152	0	1	1	no
gene:SpnNT_00953	NA	Chromosome:987369-987612	110.58	ΔORF2+peptide	OK	1.90944	1.22938	-0.635221	-0.358872	0.59335	0.975539	no
gene:SpnNT_00953	NA	Chromosome:987369-987612	ΔORF2	ΔORF2+peptide	NOTEST	0.23473	1.22938	2.38886	0	1	1	no
gene:SpnNT_00953	NA	Chromosome:987369-987612	110.58+peptide	ΔORF2+peptide	NOTEST	0.742569	1.22938	0.727334	0	1	1	no
gene:SpnNT_00954	NA	Chromosome:987628-988483	110.58	ΔORF2	OK	1.05742	0.405103	-1.38419	-1.14616	0.0568	0.339094	no
gene:SpnNT_00954	NA	Chromosome:987628-988483	110.58	110.58+peptide	OK	1.05742	0.705304	-0.584227	-0.554258	0.35975	0.860959	no
gene:SpnNT_00954	NA	Chromosome:987628-988483	ΔORF2	110.58+peptide	NOTEST	0.405103	0.705304	0.799958	0	1	1	no
gene:SpnNT_00954	NA	Chromosome:987628-988483	110.58	ΔORF2+peptide	OK	1.05742	0.802134	-0.39863	-0.378378	0.5104	0.948516	no
gene:SpnNT_00954	NA	Chromosome:987628-988483	ΔORF2	ΔORF2+peptide	NOTEST	0.405103	0.802134	0.985556	0	1	1	no
gene:SpnNT_00954	NA	Chromosome:987628-988483	110.58+peptide	ΔORF2+peptide	NOTEST	0.705304	0.802134	0.185598	0	1	1	no
gene:SpnNT_00955	NA	Chromosome:988536-989629	110.58	ΔORF2	NOTEST	0.453045	0	#NAME?	0	1	1	no
gene:SpnNT_00955	NA	Chromosome:988536-989629	110.58	110.58+peptide	NOTEST	0.453045	0.324468	-0.48158	0	1	1	no
gene:SpnNT_00955	NA	Chromosome:988536-989629	ΔORF2	110.58+peptide	NOTEST	0	0.324468	Inf	0	1	1	no
gene:SpnNT_00955	NA	Chromosome:988536-989629	110.58	ΔORF2+peptide	NOTEST	0.453045	0.103792	-2.12595	0	1	1	no
gene:SpnNT_00955	NA	Chromosome:988536-989629	ΔORF2	ΔORF2+peptide	NOTEST	0	0.103792	Inf	0	1	1	no
gene:SpnNT_00955	NA	Chromosome:988536-989629	110.58+peptide	ΔORF2+peptide	NOTEST	0.324468	0.103792	-1.64437	0	1	1	no
gene:SpnNT_00956	NA	Chromosome:988536-989629	110.58	ΔORF2	NOTEST	0.045963	0.117881	1.3588	0	1	1	no
gene:SpnNT_00956	NA	Chromosome:988536-989629	110.58	110.58+peptide	NOTEST	0.045963	0.0271794	-0.757941	0	1	1	no
gene:SpnNT_00956	NA	Chromosome:988536-989629	ΔORF2	110.58+peptide	NOTEST	0.117881	0.0271794	-2.11674	0	1	1	no
gene:SpnNT_00956	NA	Chromosome:988536-989629	110.58	ΔORF2+peptide	NOTEST	0.045963	0	#NAME?	0	1	1	no
gene:SpnNT_00956	NA	Chromosome:988536-989629	ΔORF2	ΔORF2+peptide	NOTEST	0.117881	0	#NAME?	0	1	1	no
gene:SpnNT_00956	NA	Chromosome:988536-989629	110.58+peptide	ΔORF2+peptide	NOTEST	0.027179	0	#NAME?	0	1	1	no
gene:SpnNT_00957	NA	Chromosome:989635-991204	110.58	ΔORF2	NOTEST	0.113715	0.0520072	-1.12864	0	1	1	no
gene:SpnNT_00957	NA	Chromosome:989635-991204	110.58	110.58+peptide	NOTEST	0.113715	0.0399576	-1.50888	0	1	1	no
gene:SpnNT_00957	NA	Chromosome:989635-991204	ΔORF2	110.58+peptide	NOTEST	0.052007	0.0399576	-0.380243	0	1	1	no
gene:SpnNT_00957	NA	Chromosome:989635-991204	110.58	ΔORF2+peptide	NOTEST	0.113715	0.0109661	-3.3743	0	1	1	no
gene:SpnNT_00957	NA	Chromosome:989635-991204	ΔORF2	ΔORF2+peptide	NOTEST	0.052007	0.0109661	-2.24566	0	1	1	no
gene:SpnNT_00957	NA	Chromosome:989635-991204	110.58+peptide	ΔORF2+peptide	NOTEST	0.039958	0.0109661	-1.86542	0	1	1	no
gene:SpnNT_00958	NA	Chromosome:991215-993921	110.58	ΔORF2	NOTEST	0.206872	0.184282	-0.166825	0	1	1	no
gene:SpnNT_00958	NA	Chromosome:991215-993921	110.58	110.58+peptide	NOTEST	0.206872	0.238127	0.202995	0	1	1	no
gene:SpnNT_00958	NA	Chromosome:991215-993921	ΔORF2	110.58+peptide	NOTEST	0.184282	0.238127	0.36982	0	1	1	no
gene:SpnNT_00958	NA	Chromosome:991215-993921	110.58	ΔORF2+peptide	NOTEST	0.206872	0.265402	0.359442	0	1	1	no
gene:SpnNT_00958	NA	Chromosome:991215-993921	ΔORF2	ΔORF2+peptide	NOTEST	0.184282	0.265402	0.526267	0	1	1	no
gene:SpnNT_00958	NA	Chromosome:991215-993921	110.58+peptide	ΔORF2+peptide	NOTEST	0.238127	0.265402	0.156448	0	1	1	no
gene:SpnNT_00959	ltrA_2	Chromosome:994629-996552	110.58	ΔORF2	OK	2.55323	1.38317	-0.884342	-1.27054	0.03225	0.233804	no
gene:SpnNT_00959	ltrA_2	Chromosome:994629-996552	110.58	110.58+peptide	OK	2.55323	2.68584	0.0730506	0.11268	0.8435	0.994748	no
gene:SpnNT_00959	ltrA_2	Chromosome:994629-996552	ΔORF2	110.58+peptide	OK	1.38317	2.68584	0.957393	1.40217	0.01765	0.151177	no
gene:SpnNT_00959	ltrA_2	Chromosome:994629-996552	110.58	ΔORF2+peptide	OK	2.55323	1.66666	-0.615358	-0.906578	0.1202	0.524705	no
gene:SpnNT_00959	ltrA_2	Chromosome:994629-996552	ΔORF2	ΔORF2+peptide	OK	1.38317	1.66666	0.268984	0.3779	0.5087	0.947472	no
gene:SpnNT_00959	ltrA_2	Chromosome:994629-996552	110.58+peptide	ΔORF2+peptide	OK	2.68584	1.66666	-0.688409	-1.03492	0.0738	0.400167	no
gene:SpnNT_00960	NA	Chromosome:996999-997757	110.58	ΔORF2	OK	11.513	8.62075	-0.417379	-0.503053	0.3879	0.882306	no
gene:SpnNT_00960	NA	Chromosome:996999-997757	110.58	110.58+peptide	OK	11.513	8.66217	-0.410464	-0.507283	0.38855	0.883172	no
gene:SpnNT_00960	NA	Chromosome:996999-997757	ΔORF2	110.58+peptide	OK	8.62075	8.66217	0.00691552	0.00805286	0.98405	0.996246	no
gene:SpnNT_00960	NA	Chromosome:996999-997757	110.58	ΔORF2+peptide	OK	11.513	8.30783	-0.470722	-0.555091	0.33285	0.838644	no
gene:SpnNT_00960	NA	Chromosome:996999-997757	ΔORF2	ΔORF2+peptide	OK	8.62075	8.30783	-0.0533426	-0.0595686	0.9181	0.994748	no

gene:SpnNT_00960	NA	Chromosome:996999-997757	110.58+peptide	ΔORF2+peptide	OK	8.66217	8.30783	-0.0602581	-0.0687506	0.90415	0.994748	no
gene:SpnNT_00961	NA	Chromosome:996999-997757	110.58	ΔORF2	OK	3.84332	6.14033	0.675964	0.414876	0.4565	0.921244	no
gene:SpnNT_00961	NA	Chromosome:996999-997757	110.58	110.58+peptide	OK	3.84332	6.01638	0.646544	0.409606	0.4705	0.928415	no
gene:SpnNT_00961	NA	Chromosome:996999-997757	ΔORF2	110.58+peptide	OK	6.14033	6.01638	-0.02942	-0.0213139	0.97815	0.995765	no
gene:SpnNT_00961	NA	Chromosome:996999-997757	110.58	ΔORF2+peptide	OK	3.84332	5.75518	0.582509	0.359266	0.56865	0.968621	no
gene:SpnNT_00961	NA	Chromosome:996999-997757	ΔORF2	ΔORF2+peptide	OK	6.14033	5.75518	-0.0934552	-0.0653892	0.9136	0.994748	no
gene:SpnNT_00961	NA	Chromosome:996999-997757	110.58+peptide	ΔORF2+peptide	OK	6.01638	5.75518	-0.0640352	-0.0467086	0.9413	0.994855	no
gene:SpnNT_00962	NA	Chromosome:997772-998240	110.58	ΔORF2	OK	4.77977	5.32912	0.156955	0.19071	0.73825	0.994748	no
gene:SpnNT_00962	NA	Chromosome:997772-998240	110.58	110.58+peptide	OK	4.77977	3.71505	-0.363559	-0.425435	0.4539	0.921244	no
gene:SpnNT_00962	NA	Chromosome:997772-998240	ΔORF2	110.58+peptide	OK	5.32912	3.71505	-0.520513	-0.60551	0.29325	0.797897	no
gene:SpnNT_00962	NA	Chromosome:997772-998240	110.58	ΔORF2+peptide	OK	4.77977	4.6309	-0.0456511	-0.0545062	0.9254	0.994748	no
gene:SpnNT_00962	NA	Chromosome:997772-998240	ΔORF2	ΔORF2+peptide	OK	5.32912	4.6309	-0.202606	-0.240421	0.67875	0.985348	no
gene:SpnNT_00962	NA	Chromosome:997772-998240	110.58+peptide	ΔORF2+peptide	OK	3.71505	4.6309	0.317908	0.363924	0.52865	0.956161	no
gene:SpnNT_00963	hlyB_1	Chromosome:998245-999559	110.58	ΔORF2	OK	3.13634	3.70486	0.240334	0.355234	0.5372	0.959458	no
gene:SpnNT_00963	hlyB_1	Chromosome:998245-999559	110.58	110.58+peptide	OK	3.13634	2.23082	-0.491512	-0.686526	0.2347	0.728242	no
gene:SpnNT_00963	hlyB_1	Chromosome:998245-999559	ΔORF2	110.58+peptide	OK	3.70486	2.23082	-0.731846	-1.03547	0.08235	0.429319	no
gene:SpnNT_00963	hlyB_1	Chromosome:998245-999559	110.58	ΔORF2+peptide	OK	3.13634	4.97901	0.666775	1.01521	0.07475	0.40365	no
gene:SpnNT_00963	hlyB_1	Chromosome:998245-999559	ΔORF2	ΔORF2+peptide	OK	3.70486	4.97901	0.42644	0.659327	0.2546	0.753634	no
gene:SpnNT_00963	hlyB_1	Chromosome:998245-999559	110.58+peptide	ΔORF2+peptide	OK	2.23082	4.97901	1.15829	1.68386	0.0053	0.0629381	no
gene:SpnNT_00964	NA	Chromosome:999694-1000543	110.58	ΔORF2	OK	22.3003	20.866	-0.0959124	-0.174168	0.76025	0.994748	no
gene:SpnNT_00964	NA	Chromosome:999694-1000543	110.58	110.58+peptide	OK	22.3003	20.2814	-0.13691	-0.249112	0.66755	0.984113	no
gene:SpnNT_00964	NA	Chromosome:999694-1000543	ΔORF2	110.58+peptide	OK	20.866	20.2814	-0.0409975	-0.0732803	0.89875	0.994748	no
gene:SpnNT_00964	NA	Chromosome:999694-1000543	110.58	ΔORF2+peptide	OK	22.3003	20.0107	-0.156291	-0.28468	0.62055	0.980887	no
gene:SpnNT_00964	NA	Chromosome:999694-1000543	ΔORF2	ΔORF2+peptide	OK	20.866	20.0107	-0.0603785	-0.108034	0.85195	0.994748	no
gene:SpnNT_00964	NA	Chromosome:999694-1000543	110.58+peptide	ΔORF2+peptide	OK	20.2814	20.0107	-0.019381	-0.0347451	0.954	0.994855	no
gene:SpnNT_00965	NA	Chromosome:1000557-1000965	110.58	ΔORF2	OK	12.1245	12.6202	0.0578093	0.0804246	0.88705	0.994748	no
gene:SpnNT_00965	NA	Chromosome:1000557-1000965	110.58	110.58+peptide	OK	12.1245	12.6362	0.0596374	0.0844157	0.88145	0.994748	no
gene:SpnNT_00965	NA	Chromosome:1000557-1000965	ΔORF2	110.58+peptide	OK	12.6202	12.6362	0.00182807	0.00254565	0.98545	0.996845	no
gene:SpnNT_00965	NA	Chromosome:1000557-1000965	110.58	ΔORF2+peptide	OK	12.1245	16.1892	0.417103	0.602989	0.29975	0.805348	no
gene:SpnNT_00965	NA	Chromosome:1000557-1000965	ΔORF2	ΔORF2+peptide	OK	12.6202	16.1892	0.359294	0.510643	0.3866	0.881745	no
gene:SpnNT_00965	NA	Chromosome:1000557-1000965	110.58+peptide	ΔORF2+peptide	OK	12.6362	16.1892	0.357466	0.517308	0.37725	0.875535	no
gene:SpnNT_00966	NA	Chromosome:1001507-1002149	110.58	ΔORF2	NOTEST	0.293018	0.195321	-0.585141	0	1	1	no
gene:SpnNT_00966	NA	Chromosome:1001507-1002149	110.58	110.58+peptide	NOTEST	0.293018	0.759588	1.37423	0	1	1	no
gene:SpnNT_00966	NA	Chromosome:1001507-1002149	ΔORF2	110.58+peptide	NOTEST	0.195321	0.759588	1.95937	0	1	1	no
gene:SpnNT_00966	NA	Chromosome:1001507-1002149	110.58	ΔORF2+peptide	NOTEST	0.293018	0.561939	0.939426	0	1	1	no
gene:SpnNT_00966	NA	Chromosome:1001507-1002149	ΔORF2	ΔORF2+peptide	NOTEST	0.195321	0.561939	1.52457	0	1	1	no
gene:SpnNT_00966	NA	Chromosome:1001507-1002149	110.58+peptide	ΔORF2+peptide	NOTEST	0.759588	0.561939	-0.434804	0	1	1	no
gene:SpnNT_00967	NA	Chromosome:1002158-1002656	110.58	ΔORF2	NOTEST	0.298048	0.287207	-0.0534533	0	1	1	no
gene:SpnNT_00967	NA	Chromosome:1002158-1002656	110.58	110.58+peptide	NOTEST	0.298048	0.285298	-0.063073	0	1	1	no
gene:SpnNT_00967	NA	Chromosome:1002158-1002656	ΔORF2	110.58+peptide	NOTEST	0.287207	0.285298	-0.00961964	0	1	1	no
gene:SpnNT_00967	NA	Chromosome:1002158-1002656	110.58	ΔORF2+peptide	NOTEST	0.298048	0	#NAME?	0	1	1	no
gene:SpnNT_00967	NA	Chromosome:1002158-1002656	ΔORF2	ΔORF2+peptide	NOTEST	0.287207	0	#NAME?	0	1	1	no
gene:SpnNT_00967	NA	Chromosome:1002158-1002656	110.58+peptide	ΔORF2+peptide	NOTEST	0.285298	0	#NAME?	0	1	1	no
gene:SpnNT_00968	NA	Chromosome:1002791-1003244	110.58	ΔORF2	NOTEST	0.323257	0.328682	0.0240124	0	1	1	no
gene:SpnNT_00968	NA	Chromosome:1002791-1003244	110.58	110.58+peptide	NOTEST	0.323257	0.2381	-0.441116	0	1	1	no

gene:SpnNT_00968	NA	Chromosome:1002791-1003244	ΔORF2	110.58+peptide	NOTEST	0.328682	0.2381	-0.465128	0	1	1	no
gene:SpnNT_00968	NA	Chromosome:1002791-1003244	110.58	ΔORF2+peptide	NOTEST	0.323257	0.128868	-1.32679	0	1	1	no
gene:SpnNT_00968	NA	Chromosome:1002791-1003244	ΔORF2	ΔORF2+peptide	NOTEST	0.328682	0.128868	-1.3508	0	1	1	no
gene:SpnNT_00968	NA	Chromosome:1002791-1003244	110.58+peptide	ΔORF2+peptide	NOTEST	0.2381	0.128868	-0.885675	0	1	1	no
gene:SpnNT_00969	NA	Chromosome:1003294-1003908	110.58	ΔORF2	OK	2.90742	2.37972	-0.28895	-0.119747	0.833	0.994748	no
gene:SpnNT_00969	NA	Chromosome:1003294-1003908	110.58	110.58+peptide	OK	2.90742	3.47396	0.256842	0.131928	0.86545	0.994748	no
gene:SpnNT_00969	NA	Chromosome:1003294-1003908	ΔORF2	110.58+peptide	OK	2.37972	3.47396	0.545792	0.311501	0.7042	0.990367	no
gene:SpnNT_00969	NA	Chromosome:1003294-1003908	110.58	ΔORF2+peptide	OK	2.90742	5.99271	1.04347	0.478983	0.41025	0.898184	no
gene:SpnNT_00969	NA	Chromosome:1003294-1003908	ΔORF2	ΔORF2+peptide	OK	2.37972	5.99271	1.33242	0.664078	0.29565	0.800007	no
gene:SpnNT_00969	NA	Chromosome:1003294-1003908	110.58+peptide	ΔORF2+peptide	OK	3.47396	5.99271	0.786626	0.55717	0.5629	0.968621	no
gene:SpnNT_00970	NA	Chromosome:1003294-1003908	110.58	ΔORF2	OK	3.63628	2.31538	-0.651215	-0.572135	0.32485	0.830123	no
gene:SpnNT_00970	NA	Chromosome:1003294-1003908	110.58	110.58+peptide	OK	3.63628	3.20674	-0.181355	-0.163023	0.78195	0.994748	no
gene:SpnNT_00970	NA	Chromosome:1003294-1003908	ΔORF2	110.58+peptide	OK	2.31538	3.20674	0.469861	0.402773	0.48985	0.939739	no
gene:SpnNT_00970	NA	Chromosome:1003294-1003908	110.58	ΔORF2+peptide	OK	3.63628	1.21916	-1.57658	-1.25695	0.1508	0.593025	no
gene:SpnNT_00970	NA	Chromosome:1003294-1003908	ΔORF2	ΔORF2+peptide	OK	2.31538	1.21916	-0.92536	-0.710438	0.33845	0.842721	no
gene:SpnNT_00970	NA	Chromosome:1003294-1003908	110.58+peptide	ΔORF2+peptide	OK	3.20674	1.21916	-1.39522	-1.08996	0.18005	0.64676	no
gene:SpnNT_00971	NA	Chromosome:1003945-1004218	110.58	ΔORF2	NOTEST	0.227893	0.194382	-0.229464	0	1	1	no
gene:SpnNT_00971	NA	Chromosome:1003945-1004218	110.58	110.58+peptide	NOTEST	0.227893	0.830653	1.86589	0	1	1	no
gene:SpnNT_00971	NA	Chromosome:1003945-1004218	ΔORF2	110.58+peptide	NOTEST	0.194382	0.830653	2.09535	0	1	1	no
gene:SpnNT_00971	NA	Chromosome:1003945-1004218	110.58	ΔORF2+peptide	NOTEST	0.227893	0	#NAME?	0	1	1	no
gene:SpnNT_00971	NA	Chromosome:1003945-1004218	ΔORF2	ΔORF2+peptide	NOTEST	0.194382	0	#NAME?	0	1	1	no
gene:SpnNT_00971	NA	Chromosome:1003945-1004218	110.58+peptide	ΔORF2+peptide	NOTEST	0.830653	0	#NAME?	0	1	1	no
gene:SpnNT_00972	pezA_2	Chromosome:1004287-1005543	110.58	ΔORF2	OK	18.9562	28.7517	0.600976	0.828993	0.1551	0.600007	no
gene:SpnNT_00972	pezA_2	Chromosome:1004287-1005543	110.58	110.58+peptide	OK	18.9562	17.5539	-0.110878	-0.144182	0.8	0.994748	no
gene:SpnNT_00972	pezA_2	Chromosome:1004287-1005543	ΔORF2	110.58+peptide	OK	28.7517	17.5539	-0.711855	-0.933029	0.10735	0.498339	no
gene:SpnNT_00972	pezA_2	Chromosome:1004287-1005543	110.58	ΔORF2+peptide	OK	18.9562	27.009	0.510769	0.663336	0.2543	0.75336	no
gene:SpnNT_00972	pezA_2	Chromosome:1004287-1005543	ΔORF2	ΔORF2+peptide	OK	28.7517	27.009	-0.0902075	-0.118082	0.83745	0.994748	no
gene:SpnNT_00972	pezA_2	Chromosome:1004287-1005543	110.58+peptide	ΔORF2+peptide	OK	17.5539	27.009	0.621647	0.771389	0.186	0.660182	no
gene:SpnNT_00973	pezT_2	Chromosome:1004287-1005543	110.58	ΔORF2	OK	3.83955	5.19094	0.435059	0.365892	0.5136	0.95097	no
gene:SpnNT_00973	pezT_2	Chromosome:1004287-1005543	110.58	110.58+peptide	OK	3.83955	5.99827	0.64361	0.607773	0.2947	0.799279	no
gene:SpnNT_00973	pezT_2	Chromosome:1004287-1005543	ΔORF2	110.58+peptide	OK	5.19094	5.99827	0.208552	0.189353	0.72735	0.994748	no
gene:SpnNT_00973	pezT_2	Chromosome:1004287-1005543	110.58	ΔORF2+peptide	OK	3.83955	5.45478	0.506584	0.391049	0.49965	0.944017	no
gene:SpnNT_00973	pezT_2	Chromosome:1004287-1005543	ΔORF2	ΔORF2+peptide	OK	5.19094	5.45478	0.0715256	0.0537643	0.92515	0.994748	no
gene:SpnNT_00973	pezT_2	Chromosome:1004287-1005543	110.58+peptide	ΔORF2+peptide	OK	5.99827	5.45478	-0.137026	-0.112732	0.83735	0.994748	no
gene:SpnNT_00974	NA	Chromosome:1005544-1005871	110.58	ΔORF2	OK	16.0824	19.8046	0.300353	0.413801	0.46355	0.926089	no
gene:SpnNT_00974	NA	Chromosome:1005544-1005871	110.58	110.58+peptide	OK	16.0824	15.5626	-0.047403	-0.0615913	0.91145	0.994748	no
gene:SpnNT_00974	NA	Chromosome:1005544-1005871	ΔORF2	110.58+peptide	OK	19.8046	15.5626	-0.347756	-0.46161	0.41455	0.900591	no
gene:SpnNT_00974	NA	Chromosome:1005544-1005871	110.58	ΔORF2+peptide	OK	16.0824	11.8532	-0.440211	-0.556678	0.33565	0.840108	no
gene:SpnNT_00974	NA	Chromosome:1005544-1005871	ΔORF2	ΔORF2+peptide	OK	19.8046	11.8532	-0.740564	-0.955636	0.0968	0.470343	no
gene:SpnNT_00974	NA	Chromosome:1005544-1005871	110.58+peptide	ΔORF2+peptide	OK	15.5626	11.8532	-0.392808	-0.481317	0.4055	0.895334	no
gene:SpnNT_00975	NA	Chromosome:1006185-1006941	110.58	ΔORF2	OK	6.20898	11.2948	0.863237	1.30067	0.02875	0.215822	no
gene:SpnNT_00975	NA	Chromosome:1006185-1006941	110.58	110.58+peptide	OK	6.20898	8.10843	0.385067	0.578874	0.32025	0.825861	no
gene:SpnNT_00975	NA	Chromosome:1006185-1006941	ΔORF2	110.58+peptide	OK	11.2948	8.10843	-0.47817	-0.729196	0.2104	0.696806	no
gene:SpnNT_00975	NA	Chromosome:1006185-1006941	110.58	ΔORF2+peptide	OK	6.20898	10.8258	0.802049	1.23214	0.0364	0.254335	no
gene:SpnNT_00975	NA	Chromosome:1006185-1006941	ΔORF2	ΔORF2+peptide	OK	11.2948	10.8258	-0.0611872	-0.0954139	0.8698	0.994748	no

gene:SpnNT_00975	NA	Chromosome:1006185-1006941	110.58+peptide	ΔORF2+peptide	OK	8.10843	10.8258	0.416983	0.64865	0.255	0.754219	no
gene:SpnNT_00976	NA	Chromosome:1006942-1010283	110.58	ΔORF2	OK	6.67632	13.7556	1.04289	0.727061	0.2115	0.697312	no
gene:SpnNT_00976	NA	Chromosome:1006942-1010283	110.58	110.58+peptide	OK	6.67632	7.09266	0.0872732	0.0578243	0.9231	0.994748	no
gene:SpnNT_00976	NA	Chromosome:1006942-1010283	ΔORF2	110.58+peptide	OK	13.7556	7.09266	-0.955618	-0.739018	0.2113	0.697158	no
gene:SpnNT_00976	NA	Chromosome:1006942-1010283	110.58	ΔORF2+peptide	OK	6.67632	10.1458	0.603753	0.406624	0.48265	0.935914	no
gene:SpnNT_00976	NA	Chromosome:1006942-1010283	ΔORF2	ΔORF2+peptide	OK	13.7556	10.1458	-0.439138	-0.347302	0.55415	0.96632	no
gene:SpnNT_00976	NA	Chromosome:1006942-1010283	110.58+peptide	ΔORF2+peptide	OK	7.09266	10.1458	0.51648	0.382922	0.51525	0.951959	no
gene:SpnNT_00977	NA	Chromosome:1006942-1010283	110.58	ΔORF2	OK	7.62164	11.7994	0.630539	1.10746	0.05775	0.342262	no
gene:SpnNT_00977	NA	Chromosome:1006942-1010283	110.58	110.58+peptide	OK	7.62164	7.425	-0.0377112	-0.065142	0.9107	0.994748	no
gene:SpnNT_00977	NA	Chromosome:1006942-1010283	ΔORF2	110.58+peptide	OK	11.7994	7.425	-0.668251	-1.15392	0.0486	0.308082	no
gene:SpnNT_00977	NA	Chromosome:1006942-1010283	110.58	ΔORF2+peptide	OK	7.62164	10.6396	0.481264	0.859224	0.1346	0.558048	no
gene:SpnNT_00977	NA	Chromosome:1006942-1010283	ΔORF2	ΔORF2+peptide	OK	11.7994	10.6396	-0.149275	-0.266407	0.64475	0.980887	no
gene:SpnNT_00977	NA	Chromosome:1006942-1010283	110.58+peptide	ΔORF2+peptide	OK	7.425	10.6396	0.518975	0.910434	0.11565	0.516049	no
gene:SpnNT_00978	NA	Chromosome:1010671-1011031	110.58	ΔORF2	OK	4.1039	9.08983	1.14726	1.24452	0.03985	0.270239	no
gene:SpnNT_00978	NA	Chromosome:1010671-1011031	110.58	110.58+peptide	OK	4.1039	4.8703	0.247013	0.254388	0.6622	0.982966	no
gene:SpnNT_00978	NA	Chromosome:1010671-1011031	ΔORF2	110.58+peptide	OK	9.08983	4.8703	-0.900244	-1.02464	0.09	0.450412	no
gene:SpnNT_00978	NA	Chromosome:1010671-1011031	110.58	ΔORF2+peptide	OK	4.1039	3.47541	-0.239812	-0.263338	0.7034	0.990367	no
gene:SpnNT_00978	NA	Chromosome:1010671-1011031	ΔORF2	ΔORF2+peptide	OK	9.08983	3.47541	-1.38707	-1.70948	0.02825	0.214152	no
gene:SpnNT_00978	NA	Chromosome:1010671-1011031	110.58+peptide	ΔORF2+peptide	OK	4.8703	3.47541	-0.486825	-0.561605	0.4185	0.903662	no
gene:SpnNT_00979	NA	Chromosome:1011040-1011406	110.58	ΔORF2	OK	5.90529	7.71832	0.386278	0.453912	0.4366	0.914104	no
gene:SpnNT_00979	NA	Chromosome:1011040-1011406	110.58	110.58+peptide	OK	5.90529	6.14228	0.0567643	0.0641218	0.9082	0.994748	no
gene:SpnNT_00979	NA	Chromosome:1011040-1011406	ΔORF2	110.58+peptide	OK	7.71832	6.14228	-0.329514	-0.379874	0.52255	0.954832	no
gene:SpnNT_00979	NA	Chromosome:1011040-1011406	110.58	ΔORF2+peptide	OK	5.90529	4.42516	-0.416279	-0.428412	0.4534	0.921244	no
gene:SpnNT_00979	NA	Chromosome:1011040-1011406	ΔORF2	ΔORF2+peptide	OK	7.71832	4.42516	-0.802557	-0.839966	0.15835	0.607564	no
gene:SpnNT_00979	NA	Chromosome:1011040-1011406	110.58+peptide	ΔORF2+peptide	OK	6.14228	4.42516	-0.473043	-0.479711	0.4122	0.899511	no
gene:SpnNT_00980	NA	Chromosome:1011717-1013259	110.58	ΔORF2	OK	4.15211	5.30273	0.352889	0.581113	0.30305	0.809137	no
gene:SpnNT_00980	NA	Chromosome:1011717-1013259	110.58	110.58+peptide	OK	4.15211	3.00807	-0.465007	-0.727425	0.2036	0.688187	no
gene:SpnNT_00980	NA	Chromosome:1011717-1013259	ΔORF2	110.58+peptide	OK	5.30273	3.00807	-0.817896	-1.2969	0.02775	0.211953	no
gene:SpnNT_00980	NA	Chromosome:1011717-1013259	110.58	ΔORF2+peptide	OK	4.15211	4.90975	0.241805	0.396303	0.48465	0.936747	no
gene:SpnNT_00980	NA	Chromosome:1011717-1013259	ΔORF2	ΔORF2+peptide	OK	5.30273	4.90975	-0.111085	-0.184791	0.7485	0.994748	no
gene:SpnNT_00980	NA	Chromosome:1011717-1013259	110.58+peptide	ΔORF2+peptide	OK	3.00807	4.90975	0.706812	1.11584	0.05565	0.336206	no
gene:SpnNT_00981	NA	Chromosome:1013303-1014179	110.58	ΔORF2	OK	26.1149	43.1584	0.724768	1.35715	0.01775	0.151736	no
gene:SpnNT_00981	NA	Chromosome:1013303-1014179	110.58	110.58+peptide	OK	26.1149	24.657	-0.0828742	-0.15274	0.79565	0.994748	no
gene:SpnNT_00981	NA	Chromosome:1013303-1014179	ΔORF2	110.58+peptide	OK	43.1584	24.657	-0.807643	-1.48959	0.00955	0.0975974	no
gene:SpnNT_00981	NA	Chromosome:1013303-1014179	110.58	ΔORF2+peptide	OK	26.1149	23.6423	-0.1435	-0.267619	0.64625	0.980887	no
gene:SpnNT_00981	NA	Chromosome:1013303-1014179	ΔORF2	ΔORF2+peptide	OK	43.1584	23.6423	-0.868268	-1.62046	0.0052	0.0621443	no
gene:SpnNT_00981	NA	Chromosome:1013303-1014179	110.58+peptide	ΔORF2+peptide	OK	24.657	23.6423	-0.0606259	-0.111376	0.84755	0.994748	no
gene:SpnNT_00982	NA	Chromosome:1014380-1016059	110.58	ΔORF2	OK	0.922873	1.53384	0.732941	0.375358	0.6694	0.984845	no
gene:SpnNT_00982	NA	Chromosome:1014380-1016059	110.58	110.58+peptide	OK	0.922873	0.891802	-0.0494082	-0.0237089	0.9522	0.994855	no
gene:SpnNT_00982	NA	Chromosome:1014380-1016059	ΔORF2	110.58+peptide	OK	1.53384	0.891802	-0.782349	-0.379809	0.6426	0.980887	no
gene:SpnNT_00982	NA	Chromosome:1014380-1016059	110.58	ΔORF2+peptide	OK	0.922873	1.44446	0.646324	0.331391	0.61795	0.980887	no
gene:SpnNT_00982	NA	Chromosome:1014380-1016059	ΔORF2	ΔORF2+peptide	OK	1.53384	1.44446	-0.086617	-0.0450061	0.9442	0.994855	no
gene:SpnNT_00982	NA	Chromosome:1014380-1016059	110.58+peptide	ΔORF2+peptide	OK	0.891802	1.44446	0.695732	0.338118	0.59705	0.97629	no
gene:SpnNT_00983	lagD_4	Chromosome:1014380-1016059	110.58	ΔORF2	OK	1.54652	2.21214	0.516415	0.574487	0.30875	0.814396	no
gene:SpnNT_00983	lagD_4	Chromosome:1014380-1016059	110.58	110.58+peptide	OK	1.54652	2.05383	0.409288	0.466328	0.4142	0.900412	no

gene:SpnNT_00983	lagD_4	Chromosome:1014380-1016059	ΔORF2	110.58+peptide	OK	2.21214	2.05383	-0.107127	-0.127954	0.8209	0.994748	no
gene:SpnNT_00983	lagD_4	Chromosome:1014380-1016059	110.58	ΔORF2+peptide	OK	1.54652	1.92292	0.314269	0.350697	0.5472	0.962257	no
gene:SpnNT_00983	lagD_4	Chromosome:1014380-1016059	ΔORF2	ΔORF2+peptide	OK	2.21214	1.92292	-0.202146	-0.236	0.67815	0.98524	no
gene:SpnNT_00983	lagD_4	Chromosome:1014380-1016059	110.58+peptide	ΔORF2+peptide	OK	2.05383	1.92292	-0.0950185	-0.113898	0.84525	0.994748	no
gene:SpnNT_00984	hlyB_2	Chromosome:1016121-1016763	110.58	ΔORF2	OK	2.24198	2.21159	-0.0196872	-0.0223233	0.9661	0.994855	no
gene:SpnNT_00984	hlyB_2	Chromosome:1016121-1016763	110.58	110.58+peptide	OK	2.24198	1.83652	-0.287802	-0.319008	0.57775	0.969538	no
gene:SpnNT_00984	hlyB_2	Chromosome:1016121-1016763	ΔORF2	110.58+peptide	OK	2.21159	1.83652	-0.268115	-0.294996	0.6072	0.977869	no
gene:SpnNT_00984	hlyB_2	Chromosome:1016121-1016763	110.58	ΔORF2+peptide	OK	2.24198	2.14393	-0.0645134	-0.0727657	0.8953	0.994748	no
gene:SpnNT_00984	hlyB_2	Chromosome:1016121-1016763	ΔORF2	ΔORF2+peptide	OK	2.21159	2.14393	-0.0448262	-0.0501744	0.92795	0.994748	no
gene:SpnNT_00984	hlyB_2	Chromosome:1016121-1016763	110.58+peptide	ΔORF2+peptide	OK	1.83652	2.14393	0.223289	0.244455	0.66405	0.982966	no
gene:SpnNT_00985	NA	Chromosome:1016775-1016946	110.58	ΔORF2	NOTEST	0.981934	1.51485	0.625478	0	1	1	no
gene:SpnNT_00985	NA	Chromosome:1016775-1016946	110.58	110.58+peptide	NOTEST	0.981934	1.51124	0.622036	0	1	1	no
gene:SpnNT_00985	NA	Chromosome:1016775-1016946	ΔORF2	110.58+peptide	NOTEST	1.51485	1.51124	-0.00344214	0	1	1	no
gene:SpnNT_00985	NA	Chromosome:1016775-1016946	110.58	ΔORF2+peptide	NOTEST	0.981934	0	#NAME?	0	1	1	no
gene:SpnNT_00985	NA	Chromosome:1016775-1016946	ΔORF2	ΔORF2+peptide	NOTEST	1.51485	0	#NAME?	0	1	1	no
gene:SpnNT_00985	NA	Chromosome:1016775-1016946	110.58+peptide	ΔORF2+peptide	NOTEST	1.51124	0	#NAME?	0	1	1	no
gene:SpnNT_00986	yxlF_3	Chromosome:1016979-1018438	110.58	ΔORF2	OK	1.34192	3.7199	1.47097	1.46949	0.01815	0.154753	no
gene:SpnNT_00986	yxlF_3	Chromosome:1016979-1018438	110.58	110.58+peptide	OK	1.34192	2.02003	0.590081	0.570614	0.3173	0.823011	no
gene:SpnNT_00986	yxlF_3	Chromosome:1016979-1018438	ΔORF2	110.58+peptide	OK	3.7199	2.02003	-0.880885	-0.994464	0.08585	0.440048	no
gene:SpnNT_00986	yxlF_3	Chromosome:1016979-1018438	110.58	ΔORF2+peptide	OK	1.34192	2.32469	0.792741	0.647754	0.2729	0.775081	no
gene:SpnNT_00986	yxlF_3	Chromosome:1016979-1018438	ΔORF2	ΔORF2+peptide	OK	3.7199	2.32469	-0.678225	-0.615808	0.27465	0.777024	no
gene:SpnNT_00986	yxlF_3	Chromosome:1016979-1018438	110.58+peptide	ΔORF2+peptide	OK	2.02003	2.32469	0.20266	0.179102	0.7424	0.994748	no
gene:SpnNT_00987	NA	Chromosome:1016979-1018438	110.58	ΔORF2	OK	0.380828	1.06927	1.48941	0.803346	0.2468	0.74363	no
gene:SpnNT_00987	NA	Chromosome:1016979-1018438	110.58	110.58+peptide	NOTEST	0.380828	0.522883	0.457348	0	1	1	no
gene:SpnNT_00987	NA	Chromosome:1016979-1018438	ΔORF2	110.58+peptide	OK	1.06927	0.522883	-1.03206	-0.561447	0.3559	0.85769	no
gene:SpnNT_00987	NA	Chromosome:1016979-1018438	110.58	ΔORF2+peptide	OK	0.380828	1.50041	1.97814	1.01926	0.1729	0.632681	no
gene:SpnNT_00987	NA	Chromosome:1016979-1018438	ΔORF2	ΔORF2+peptide	OK	1.06927	1.50041	0.488734	0.285819	0.64155	0.980887	no
gene:SpnNT_00987	NA	Chromosome:1016979-1018438	110.58+peptide	ΔORF2+peptide	OK	0.522883	1.50041	1.52079	0.789746	0.22705	0.71894	no
gene:SpnNT_00988	NA	Chromosome:1018450-1019161	110.58	ΔORF2	OK	0.29502	0.864755	1.55148	0.967713	0.10695	0.497482	no
gene:SpnNT_00988	NA	Chromosome:1018450-1019161	110.58	110.58+peptide	NOTEST	0.29502	0.400473	0.440893	0	1	1	no
gene:SpnNT_00988	NA	Chromosome:1018450-1019161	ΔORF2	110.58+peptide	OK	0.864755	0.400473	-1.11059	-0.768414	0.1839	0.655387	no
gene:SpnNT_00988	NA	Chromosome:1018450-1019161	110.58	ΔORF2+peptide	NOTEST	0.29502	0.327842	0.15219	0	1	1	no
gene:SpnNT_00988	NA	Chromosome:1018450-1019161	ΔORF2	ΔORF2+peptide	OK	0.864755	0.327842	-1.39929	-0.999161	0.13235	0.55397	no
gene:SpnNT_00988	NA	Chromosome:1018450-1019161	110.58+peptide	ΔORF2+peptide	NOTEST	0.400473	0.327842	-0.288704	0	1	1	no
gene:SpnNT_00989	immR_2	Chromosome:1019703-1019925	110.58	ΔORF2	OK	92.6937	29.9242	-1.63116	-1.95923	0.00285	0.0382781	yes
gene:SpnNT_00989	immR_2	Chromosome:1019703-1019925	110.58	110.58+peptide	OK	92.6937	130.021	0.488197	0.662474	0.23185	0.724662	no
gene:SpnNT_00989	immR_2	Chromosome:1019703-1019925	ΔORF2	110.58+peptide	OK	29.9242	130.021	2.11935	2.4055	0.00035	0.00717609	yes
gene:SpnNT_00989	immR_2	Chromosome:1019703-1019925	110.58	ΔORF2+peptide	OK	92.6937	25.8633	-1.84156	-2.14385	8.00E-04	0.0140342	yes
gene:SpnNT_00989	immR_2	Chromosome:1019703-1019925	ΔORF2	ΔORF2+peptide	OK	29.9242	25.8633	-0.210406	-0.21352	0.70565	0.990422	no
gene:SpnNT_00989	immR_2	Chromosome:1019703-1019925	110.58+peptide	ΔORF2+peptide	OK	130.021	25.8633	-2.32976	-2.57126	0.00015	0.00355289	yes
gene:SpnNT_00990	NA	Chromosome:1020471-1021657	110.58	ΔORF2	OK	9.5058	13.7668	0.534316	0.313445	0.60205	0.976761	no
gene:SpnNT_00990	NA	Chromosome:1020471-1021657	110.58	110.58+peptide	OK	9.5058	5.02989	-0.918283	-0.465884	0.5458	0.961816	no
gene:SpnNT_00990	NA	Chromosome:1020471-1021657	ΔORF2	110.58+peptide	OK	13.7668	5.02989	-1.4526	-0.78968	0.34055	0.844744	no
gene:SpnNT_00990	NA	Chromosome:1020471-1021657	110.58	ΔORF2+peptide	OK	9.5058	13.9056	0.548782	0.331599	0.5634	0.968621	no
gene:SpnNT_00990	NA	Chromosome:1020471-1021657	ΔORF2	ΔORF2+peptide	OK	13.7668	13.9056	0.0144658	0.00967077	0.98775	0.997703	no

gene:SpnNT_00990	NA	Chromosome:1020471-1021657	110.58+peptide	ΔORF2+peptide	OK	5.02989	13.9056	1.46706	0.817982	0.31085	0.816775	no
gene:SpnNT_00991	xerD_1	Chromosome:1020471-1021657	110.58	ΔORF2	OK	28.3622	34.7612	0.293507	0.519515	0.3668	0.864634	no
gene:SpnNT_00991	xerD_1	Chromosome:1020471-1021657	110.58	110.58+peptide	OK	28.3622	21.9831	-0.367572	-0.637166	0.2666	0.768019	no
gene:SpnNT_00991	xerD_1	Chromosome:1020471-1021657	ΔORF2	110.58+peptide	OK	34.7612	21.9831	-0.661079	-1.14977	0.045	0.292033	no
gene:SpnNT_00991	xerD_1	Chromosome:1020471-1021657	110.58	ΔORF2+peptide	OK	28.3622	23.0578	-0.298715	-0.498214	0.3847	0.881745	no
gene:SpnNT_00991	xerD_1	Chromosome:1020471-1021657	ΔORF2	ΔORF2+peptide	OK	34.7612	23.0578	-0.592222	-0.990794	0.08675	0.442587	no
gene:SpnNT_00991	xerD_1	Chromosome:1020471-1021657	110.58+peptide	ΔORF2+peptide	OK	21.9831	23.0578	0.0688572	0.113065	0.83915	0.994748	no
gene:SpnNT_00992	gdhA	Chromosome:1022113-1023460	110.58	ΔORF2	OK	480.103	433.525	-0.147226	-0.331599	0.54875	0.964037	no
gene:SpnNT_00992	gdhA	Chromosome:1022113-1023460	110.58	110.58+peptide	OK	480.103	1679.11	1.80629	3.7587	5.00E-05	0.0013612	yes
gene:SpnNT_00992	gdhA	Chromosome:1022113-1023460	ΔORF2	110.58+peptide	OK	433.525	1679.11	1.95351	4.06608	5.00E-05	0.0013612	yes
gene:SpnNT_00992	gdhA	Chromosome:1022113-1023460	110.58	ΔORF2+peptide	OK	480.103	1844.27	1.94164	4.07594	5.00E-05	0.0013612	yes
gene:SpnNT_00992	gdhA	Chromosome:1022113-1023460	ΔORF2	ΔORF2+peptide	OK	433.525	1844.27	2.08886	4.38611	5.00E-05	0.0013612	yes
gene:SpnNT_00992	gdhA	Chromosome:1022113-1023460	110.58+peptide	ΔORF2+peptide	OK	1679.11	1844.27	0.135354	0.265134	0.6396	0.980887	no
gene:SpnNT_00993	NA	Chromosome:1024094-1024439	110.58	ΔORF2	NOTEST	0	0	0	0	1	1	no
gene:SpnNT_00993	NA	Chromosome:1024094-1024439	110.58	110.58+peptide	NOTEST	0	0	0	0	1	1	no
gene:SpnNT_00993	NA	Chromosome:1024094-1024439	ΔORF2	110.58+peptide	NOTEST	0	0	0	0	1	1	no
gene:SpnNT_00993	NA	Chromosome:1024094-1024439	110.58	ΔORF2+peptide	NOTEST	0	0	0	0	1	1	no
gene:SpnNT_00993	NA	Chromosome:1024094-1024439	ΔORF2	ΔORF2+peptide	NOTEST	0	0	0	0	1	1	no
gene:SpnNT_00993	NA	Chromosome:1024094-1024439	110.58+peptide	ΔORF2+peptide	NOTEST	0	0	0	0	1	1	no
gene:SpnNT_00994	NA	Chromosome:1024591-1025918	110.58	ΔORF2	OK	2.18242	1.76087	-0.309634	-0.189655	0.7369	0.994748	no
gene:SpnNT_00994	NA	Chromosome:1024591-1025918	110.58	110.58+peptide	OK	2.18242	2.58667	0.245172	0.147244	0.79055	0.994748	no
gene:SpnNT_00994	NA	Chromosome:1024591-1025918	ΔORF2	110.58+peptide	OK	1.76087	2.58667	0.554805	0.342604	0.5376	0.959663	no
gene:SpnNT_00994	NA	Chromosome:1024591-1025918	110.58	ΔORF2+peptide	OK	2.18242	2.55213	0.225775	0.122895	0.8242	0.994748	no
gene:SpnNT_00994	NA	Chromosome:1024591-1025918	ΔORF2	ΔORF2+peptide	OK	1.76087	2.55213	0.535408	0.298141	0.59755	0.97629	no
gene:SpnNT_00994	NA	Chromosome:1024591-1025918	110.58+peptide	ΔORF2+peptide	OK	2.58667	2.55213	-0.0193968	-0.0106262	0.9781	0.995765	no
gene:SpnNT_00995	NA	Chromosome:1024591-1025918	110.58	ΔORF2	OK	8.00391	5.30461	-0.593457	-0.347131	0.5323	0.958279	no
gene:SpnNT_00995	NA	Chromosome:1024591-1025918	110.58	110.58+peptide	OK	8.00391	11.0749	0.468516	0.338582	0.55495	0.966389	no
gene:SpnNT_00995	NA	Chromosome:1024591-1025918	ΔORF2	110.58+peptide	OK	5.30461	11.0749	1.06197	0.629549	0.2809	0.784388	no
gene:SpnNT_00995	NA	Chromosome:1024591-1025918	110.58	ΔORF2+peptide	OK	8.00391	14.2049	0.827608	0.45627	0.38775	0.882118	no
gene:SpnNT_00995	NA	Chromosome:1024591-1025918	ΔORF2	ΔORF2+peptide	OK	5.30461	14.2049	1.42107	0.691695	0.24175	0.736324	no
gene:SpnNT_00995	NA	Chromosome:1024591-1025918	110.58+peptide	ΔORF2+peptide	OK	11.0749	14.2049	0.359092	0.200336	0.69905	0.990367	no
gene:SpnNT_00996	NA	Chromosome:1024591-1025918	110.58	ΔORF2	OK	5.77588	5.523	-0.0645881	-0.0700307	0.908	0.994748	no
gene:SpnNT_00996	NA	Chromosome:1024591-1025918	110.58	110.58+peptide	OK	5.77588	10.057	0.800083	0.900628	0.1343	0.557156	no
gene:SpnNT_00996	NA	Chromosome:1024591-1025918	ΔORF2	110.58+peptide	OK	5.523	10.057	0.864671	1.01383	0.09175	0.456039	no
gene:SpnNT_00996	NA	Chromosome:1024591-1025918	110.58	ΔORF2+peptide	OK	5.77588	15.9621	1.46654	1.67579	0.008	0.0859754	no
gene:SpnNT_00996	NA	Chromosome:1024591-1025918	ΔORF2	ΔORF2+peptide	OK	5.523	15.9621	1.53113	1.82476	0.00345	0.0449116	yes
gene:SpnNT_00996	NA	Chromosome:1024591-1025918	110.58+peptide	ΔORF2+peptide	OK	10.057	15.9621	0.666457	0.831353	0.1762	0.640115	no
gene:SpnNT_00997	lytA_5	Chromosome:1026024-1027236	110.58	ΔORF2	OK	870.318	903.139	0.0534059	0.116255	0.83475	0.994748	no
gene:SpnNT_00997	lytA_5	Chromosome:1026024-1027236	110.58	110.58+peptide	OK	870.318	929.394	0.094747	0.2073	0.71255	0.991034	no
gene:SpnNT_00997	lytA_5	Chromosome:1026024-1027236	ΔORF2	110.58+peptide	OK	903.139	929.394	0.041341	0.0905193	0.87585	0.994748	no
gene:SpnNT_00997	lytA_5	Chromosome:1026024-1027236	110.58	ΔORF2+peptide	OK	870.318	929.243	0.0945139	0.206576	0.71305	0.991272	no
gene:SpnNT_00997	lytA_5	Chromosome:1026024-1027236	ΔORF2	ΔORF2+peptide	OK	903.139	929.243	0.0411079	0.0899154	0.87325	0.994748	no
gene:SpnNT_00997	lytA_5	Chromosome:1026024-1027236	110.58+peptide	ΔORF2+peptide	OK	929.394	929.243	-0.000233137	-0.000512569	0.99925	0.999631	no
gene:SpnNT_00998	NA	Chromosome:1027582-1028850	110.58	ΔORF2	OK	1.57214	2.13759	0.44326	0.0224389	0.78285	0.994748	no
gene:SpnNT_00998	NA	Chromosome:1027582-1028850	110.58	110.58+peptide	OK	1.57214	0.897309	-0.80905	-0.0249103	0.6144	0.979616	no

gene:SpnNT_00998	NA	Chromosome:1027582-1028850	ΔORF2	110.58+peptide	OK	2.13759	0.897309	-1.25231	-0.0388345	0.39765	0.891655	no
gene:SpnNT_00998	NA	Chromosome:1027582-1028850	110.58	ΔORF2+peptide	OK	1.57214	2.33532	0.570892	0.0325704	0.77625	0.994748	no
gene:SpnNT_00998	NA	Chromosome:1027582-1028850	ΔORF2	ΔORF2+peptide	OK	2.13759	2.33532	0.127631	0.00746562	0.9422	0.994855	no
gene:SpnNT_00998	NA	Chromosome:1027582-1028850	110.58+peptide	ΔORF2+peptide	OK	0.897309	2.33532	1.37994	0.0446096	0.4461	0.918643	no
gene:SpnNT_00999	NA	Chromosome:1027582-1028850	110.58	ΔORF2	OK	7.20907	7.06842	-0.0284267	-0.00254911	0.98275	0.996246	no
gene:SpnNT_00999	NA	Chromosome:1027582-1028850	110.58	110.58+peptide	OK	7.20907	7.26889	0.0119208	0.000929411	0.9932	0.998522	no
gene:SpnNT_00999	NA	Chromosome:1027582-1028850	ΔORF2	110.58+peptide	OK	7.06842	7.26889	0.0403475	0.00301591	0.97575	0.99536	no
gene:SpnNT_00999	NA	Chromosome:1027582-1028850	110.58	ΔORF2+peptide	OK	7.20907	6.27148	-0.201007	-0.0178951	0.8886	0.994748	no
gene:SpnNT_00999	NA	Chromosome:1027582-1028850	ΔORF2	ΔORF2+peptide	OK	7.06842	6.27148	-0.17258	-0.0145528	0.9018	0.994748	no
gene:SpnNT_00999	NA	Chromosome:1027582-1028850	110.58+peptide	ΔORF2+peptide	OK	7.26889	6.27148	-0.212928	-0.0158361	0.8782	0.994748	no
gene:SpnNT_01000	rpmE2	Chromosome:1027582-1028850	110.58	ΔORF2	OK	19136.5	22858.7	0.256419	0.514022	0.36305	0.862958	no
gene:SpnNT_01000	rpmE2	Chromosome:1027582-1028850	110.58	110.58+peptide	OK	19136.5	32703.6	0.773121	1.42384	0.01135	0.109915	no
gene:SpnNT_01000	rpmE2	Chromosome:1027582-1028850	ΔORF2	110.58+peptide	OK	22858.7	32703.6	0.516702	0.954661	0.08625	0.44055	no
gene:SpnNT_01000	rpmE2	Chromosome:1027582-1028850	110.58	ΔORF2+peptide	OK	19136.5	22158.5	0.211531	0.406917	0.46515	0.926405	no
gene:SpnNT_01000	rpmE2	Chromosome:1027582-1028850	ΔORF2	ΔORF2+peptide	OK	22858.7	22158.5	-0.0448877	-0.0866527	0.878	0.994748	no
gene:SpnNT_01000	rpmE2	Chromosome:1027582-1028850	110.58+peptide	ΔORF2+peptide	OK	32703.6	22158.5	-0.56159	-1.00168	0.06805	0.38193	no
gene:SpnNT_01001	nrnA_1	Chromosome:1028945-1029881	110.58	ΔORF2	OK	57.0778	66.3538	0.21725	0.459376	0.41555	0.901067	no
gene:SpnNT_01001	nrnA_1	Chromosome:1028945-1029881	110.58	110.58+peptide	OK	57.0778	54.3434	-0.0708262	-0.148052	0.79275	0.994748	no
gene:SpnNT_01001	nrnA_1	Chromosome:1028945-1029881	ΔORF2	110.58+peptide	OK	66.3538	54.3434	-0.288076	-0.605779	0.28845	0.792622	no
gene:SpnNT_01001	nrnA_1	Chromosome:1028945-1029881	110.58	ΔORF2+peptide	OK	57.0778	57.6901	0.0153948	0.0323568	0.9533	0.994855	no
gene:SpnNT_01001	nrnA_1	Chromosome:1028945-1029881	ΔORF2	ΔORF2+peptide	OK	66.3538	57.6901	-0.201855	-0.426821	0.45655	0.921244	no
gene:SpnNT_01001	nrnA_1	Chromosome:1028945-1029881	110.58+peptide	ΔORF2+peptide	OK	54.3434	57.6901	0.086221	0.180232	0.75745	0.994748	no
gene:SpnNT_01002	NA	Chromosome:1029984-1030428	110.58	ΔORF2	OK	1509.33	1766.71	0.227153	0.502088	0.36875	0.866071	no
gene:SpnNT_01002	NA	Chromosome:1029984-1030428	110.58	110.58+peptide	OK	1509.33	2041.87	0.435975	0.951889	0.09615	0.46955	no
gene:SpnNT_01002	NA	Chromosome:1029984-1030428	ΔORF2	110.58+peptide	OK	1766.71	2041.87	0.208823	0.458718	0.42245	0.905849	no
gene:SpnNT_01002	NA	Chromosome:1029984-1030428	110.58	ΔORF2+peptide	OK	1509.33	1782.39	0.239898	0.535777	0.3447	0.848904	no
gene:SpnNT_01002	NA	Chromosome:1029984-1030428	ΔORF2	ΔORF2+peptide	OK	1766.71	1782.39	0.0127455	0.0286471	0.95885	0.994855	no
gene:SpnNT_01002	NA	Chromosome:1029984-1030428	110.58+peptide	ΔORF2+peptide	OK	2041.87	1782.39	-0.196077	-0.435145	0.451	0.921244	no
gene:SpnNT_01003	NA	Chromosome:1030543-1031504	110.58	ΔORF2	OK	335.728	393.793	0.230142	0.347513	0.5429	0.961568	no
gene:SpnNT_01003	NA	Chromosome:1030543-1031504	110.58	110.58+peptide	OK	335.728	143.321	-1.22804	-1.66153	0.00565	0.0658431	no
gene:SpnNT_01003	NA	Chromosome:1030543-1031504	ΔORF2	110.58+peptide	OK	393.793	143.321	-1.45818	-1.95805	0.00175	0.0265167	yes
gene:SpnNT_01003	NA	Chromosome:1030543-1031504	110.58	ΔORF2+peptide	OK	335.728	150.261	-1.15982	-1.45029	0.0199	0.166429	no
gene:SpnNT_01003	NA	Chromosome:1030543-1031504	ΔORF2	ΔORF2+peptide	OK	393.793	150.261	-1.38996	-1.72687	0.00655	0.0736496	no
gene:SpnNT_01003	NA	Chromosome:1030543-1031504	110.58+peptide	ΔORF2+peptide	OK	143.321	150.261	0.0682211	0.0784842	0.89325	0.994748	no
gene:SpnNT_01004	NA	Chromosome:1030543-1031504	110.58	ΔORF2	OK	111.548	122.966	0.140598	0.17856	0.7595	0.994748	no
gene:SpnNT_01004	NA	Chromosome:1030543-1031504	110.58	110.58+peptide	OK	111.548	57.5528	-0.954702	-1.1832	0.053	0.326357	no
gene:SpnNT_01004	NA	Chromosome:1030543-1031504	ΔORF2	110.58+peptide	OK	122.966	57.5528	-1.0953	-1.33738	0.0279	0.212851	no
gene:SpnNT_01004	NA	Chromosome:1030543-1031504	110.58	ΔORF2+peptide	OK	111.548	73.0703	-0.610304	-0.762188	0.19605	0.677521	no
gene:SpnNT_01004	NA	Chromosome:1030543-1031504	ΔORF2	ΔORF2+peptide	OK	122.966	73.0703	-0.750902	-0.923708	0.1187	0.522153	no
gene:SpnNT_01004	NA	Chromosome:1030543-1031504	110.58+peptide	ΔORF2+peptide	OK	57.5528	73.0703	0.344398	0.414036	0.49255	0.941402	no
gene:SpnNT_01005	NA	Chromosome:1030543-1031504	110.58	ΔORF2	OK	53.2747	60.2591	0.177729	0.138729	0.8148	0.994748	no
gene:SpnNT_01005	NA	Chromosome:1030543-1031504	110.58	110.58+peptide	OK	53.2747	27.192	-0.970268	-0.733265	0.21765	0.705186	no
gene:SpnNT_01005	NA	Chromosome:1030543-1031504	ΔORF2	110.58+peptide	OK	60.2591	27.192	-1.148	-0.882586	0.1448	0.580489	no
gene:SpnNT_01005	NA	Chromosome:1030543-1031504	110.58	ΔORF2+peptide	OK	53.2747	38.8414	-0.455855	-0.353988	0.5577	0.968033	no
gene:SpnNT_01005	NA	Chromosome:1030543-1031504	ΔORF2	ΔORF2+peptide	OK	60.2591	38.8414	-0.633584	-0.500998	0.41235	0.899511	no

gene:SpnNT_01005	NA	Chromosome:1030543-1031504	110.58+peptide	ΔORF2+peptide	OK	27.192	38.8414	0.514412	0.393503	0.5151	0.951959	no
gene:SpnNT_01006	rplS	Chromosome:1031622-1031970	110.58	ΔORF2	OK	2100.75	2478.65	0.238653	0.542459	0.34665	0.850868	no
gene:SpnNT_01006	rplS	Chromosome:1031622-1031970	110.58	110.58+peptide	OK	2100.75	4044.99	0.945235	2.13139	0.00045	0.00885292	yes
gene:SpnNT_01006	rplS	Chromosome:1031622-1031970	ΔORF2	110.58+peptide	OK	2478.65	4044.99	0.706582	1.59207	0.0064	0.0723972	no
gene:SpnNT_01006	rplS	Chromosome:1031622-1031970	110.58	ΔORF2+peptide	OK	2100.75	3578.24	0.768349	1.75889	0.00275	0.037317	yes
gene:SpnNT_01006	rplS	Chromosome:1031622-1031970	ΔORF2	ΔORF2+peptide	OK	2478.65	3578.24	0.529696	1.21164	0.0351	0.248025	no
gene:SpnNT_01006	rplS	Chromosome:1031622-1031970	110.58+peptide	ΔORF2+peptide	OK	4044.99	3578.24	-0.176887	-0.401347	0.4846	0.936747	no
gene:SpnNT_01007	SpnNT_01007	Chromosome:1032011-1032083	110.58	ΔORF2	OK	700.432	0	#NAME?	NA	0.0494	0.311514	no
gene:SpnNT_01007	SpnNT_01007	Chromosome:1032011-1032083	110.58	110.58+peptide	OK	700.432	3127.83	2.15884	1.8128	0.14085	0.572741	no
gene:SpnNT_01007	SpnNT_01007	Chromosome:1032011-1032083	ΔORF2	110.58+peptide	OK	0	3127.83	Inf	NA	0.00015	0.00355289	yes
gene:SpnNT_01007	SpnNT_01007	Chromosome:1032011-1032083	110.58	ΔORF2+peptide	OK	700.432	0	#NAME?	NA	0.0494	0.311514	no
gene:SpnNT_01007	SpnNT_01007	Chromosome:1032011-1032083	ΔORF2	ΔORF2+peptide	NOTEST	0	0	0	0	1	1	no
gene:SpnNT_01007	SpnNT_01007	Chromosome:1032011-1032083	110.58+peptide	ΔORF2+peptide	OK	3127.83	0	#NAME?	NA	0.00015	0.00355289	yes
gene:SpnNT_01008	NA	Chromosome:1032249-1032417	110.58	ΔORF2	OK	6.3864	0	#NAME?	NA	0.0136	0.125322	no
gene:SpnNT_01008	NA	Chromosome:1032249-1032417	110.58	110.58+peptide	OK	6.3864	1.6302	-1.96996	-2.76499	0.2933	0.797897	no
gene:SpnNT_01008	NA	Chromosome:1032249-1032417	ΔORF2	110.58+peptide	NOTEST	0	1.6302	Inf	0	1	1	no
gene:SpnNT_01008	NA	Chromosome:1032249-1032417	110.58	ΔORF2+peptide	OK	6.3864	0	#NAME?	NA	0.0136	0.125322	no
gene:SpnNT_01008	NA	Chromosome:1032249-1032417	ΔORF2	ΔORF2+peptide	NOTEST	0	0	0	0	1	1	no
gene:SpnNT_01008	NA	Chromosome:1032249-1032417	110.58+peptide	ΔORF2+peptide	NOTEST	1.6302	0	#NAME?	0	1	1	no
gene:SpnNT_01009	NA	Chromosome:1032648-1033503	110.58	ΔORF2	OK	1.57974	2.55234	0.692135	0.750962	0.18785	0.663696	no
gene:SpnNT_01009	NA	Chromosome:1032648-1033503	110.58	110.58+peptide	OK	1.57974	1.1223	-0.493229	-0.502448	0.38205	0.879865	no
gene:SpnNT_01009	NA	Chromosome:1032648-1033503	ΔORF2	110.58+peptide	OK	2.55234	1.1223	-1.18536	-1.29729	0.02825	0.214152	no
gene:SpnNT_01009	NA	Chromosome:1032648-1033503	110.58	ΔORF2+peptide	OK	1.57974	2.15176	0.445832	0.488385	0.3958	0.890245	no
gene:SpnNT_01009	NA	Chromosome:1032648-1033503	ΔORF2	ΔORF2+peptide	OK	2.55234	2.15176	-0.246303	-0.29343	0.61075	0.979429	no
gene:SpnNT_01009	NA	Chromosome:1032648-1033503	110.58+peptide	ΔORF2+peptide	OK	1.1223	2.15176	0.939061	1.0378	0.07625	0.408056	no
gene:SpnNT_01010	lytA_6	Chromosome:1033596-1034817	110.58	ΔORF2	OK	98.4461	88.8657	-0.147707	-0.277418	0.6134	0.979616	no
gene:SpnNT_01010	lytA_6	Chromosome:1033596-1034817	110.58	110.58+peptide	OK	98.4461	52.3515	-0.911104	-1.73787	0.0025	0.0347143	yes
gene:SpnNT_01010	lytA_6	Chromosome:1033596-1034817	ΔORF2	110.58+peptide	OK	88.8657	52.3515	-0.763397	-1.468	0.0091	0.0941721	no
gene:SpnNT_01010	lytA_6	Chromosome:1033596-1034817	110.58	ΔORF2+peptide	OK	98.4461	58.3957	-0.753471	-1.42279	0.01495	0.133716	no
gene:SpnNT_01010	lytA_6	Chromosome:1033596-1034817	ΔORF2	ΔORF2+peptide	OK	88.8657	58.3957	-0.605764	-1.15301	0.0439	0.287166	no
gene:SpnNT_01010	lytA_6	Chromosome:1033596-1034817	110.58+peptide	ΔORF2+peptide	OK	52.3515	58.3957	0.157633	0.304842	0.58825	0.973845	no
gene:SpnNT_01011	lytB_3	Chromosome:1034829-1035948	110.58	ΔORF2	OK	277.248	240.783	-0.203443	-0.392039	0.4881	0.938811	no
gene:SpnNT_01011	lytB_3	Chromosome:1034829-1035948	110.58	110.58+peptide	OK	277.248	159.793	-0.794968	-1.60124	0.0066	0.0739583	no
gene:SpnNT_01011	lytB_3	Chromosome:1034829-1035948	ΔORF2	110.58+peptide	OK	240.783	159.793	-0.591525	-1.19304	0.03845	0.263881	no
gene:SpnNT_01011	lytB_3	Chromosome:1034829-1035948	110.58	ΔORF2+peptide	OK	277.248	124.891	-1.1505	-2.25569	2.00E-04	0.00450928	yes
gene:SpnNT_01011	lytB_3	Chromosome:1034829-1035948	ΔORF2	ΔORF2+peptide	OK	240.783	124.891	-0.947059	-1.85914	0.00155	0.0240131	yes
gene:SpnNT_01011	lytB_3	Chromosome:1034829-1035948	110.58+peptide	ΔORF2+peptide	OK	159.793	124.891	-0.355533	-0.730795	0.2096	0.695944	no
gene:SpnNT_01012	NA	Chromosome:1036433-1036724	110.58	ΔORF2	OK	42.9051	64.7148	0.592947	0.887066	0.12725	0.541293	no
gene:SpnNT_01012	NA	Chromosome:1036433-1036724	110.58	110.58+peptide	OK	42.9051	56.4955	0.396987	0.584427	0.30375	0.809137	no
gene:SpnNT_01012	NA	Chromosome:1036433-1036724	ΔORF2	110.58+peptide	OK	64.7148	56.4955	-0.19596	-0.288587	0.61205	0.979429	no
gene:SpnNT_01012	NA	Chromosome:1036433-1036724	110.58	ΔORF2+peptide	OK	42.9051	76.777	0.839525	1.29335	0.02435	0.193766	no
gene:SpnNT_01012	NA	Chromosome:1036433-1036724	ΔORF2	ΔORF2+peptide	OK	64.7148	76.777	0.246578	0.380022	0.50495	0.945292	no
gene:SpnNT_01012	NA	Chromosome:1036433-1036724	110.58+peptide	ΔORF2+peptide	OK	56.4955	76.777	0.442538	0.670498	0.22795	0.719706	no
gene:SpnNT_01013	NA	Chromosome:1036733-1038085	110.58	ΔORF2	OK	5.90038	6.63263	0.168772	0.196944	0.7316	0.994748	no
gene:SpnNT_01013	NA	Chromosome:1036733-1038085	110.58	110.58+peptide	OK	5.90038	6.09522	0.0468703	0.0534553	0.9257	0.994748	no

gene:SpnNT_01013	NA	Chromosome:1036733-1038085	ΔORF2	110.58+peptide	OK	6.63263	6.09522	-0.121901	-0.148336	0.80045	0.994748	no
gene:SpnNT_01013	NA	Chromosome:1036733-1038085	110.58	ΔORF2+peptide	OK	5.90038	6.56047	0.152989	0.173544	0.76435	0.994748	no
gene:SpnNT_01013	NA	Chromosome:1036733-1038085	ΔORF2	ΔORF2+peptide	OK	6.63263	6.56047	-0.0157821	-0.019087	0.9753	0.99536	no
gene:SpnNT_01013	NA	Chromosome:1036733-1038085	110.58+peptide	ΔORF2+peptide	OK	6.09522	6.56047	0.106119	0.125226	0.83265	0.994748	no
gene:SpnNT_01014	NA	Chromosome:1036733-1038085	110.58	ΔORF2	OK	12.2726	7.92228	-0.631458	-0.677595	0.2392	0.734543	no
gene:SpnNT_01014	NA	Chromosome:1036733-1038085	110.58	110.58+peptide	OK	12.2726	8.48532	-0.532405	-0.574091	0.31105	0.816778	no
gene:SpnNT_01014	NA	Chromosome:1036733-1038085	ΔORF2	110.58+peptide	OK	7.92228	8.48532	0.0990533	0.0972771	0.86855	0.994748	no
gene:SpnNT_01014	NA	Chromosome:1036733-1038085	110.58	ΔORF2+peptide	OK	12.2726	8.05906	-0.606763	-0.626976	0.26785	0.768869	no
gene:SpnNT_01014	NA	Chromosome:1036733-1038085	ΔORF2	ΔORF2+peptide	OK	7.92228	8.05906	0.024695	0.023404	0.96755	0.994855	no
gene:SpnNT_01014	NA	Chromosome:1036733-1038085	110.58+peptide	ΔORF2+peptide	OK	8.48532	8.05906	-0.0743583	-0.0707388	0.90265	0.994748	no
gene:SpnNT_01015	NA	Chromosome:1038318-1039545	110.58	ΔORF2	NOTEST	0.325281	0.20845	-0.641983	0	1	1	no
gene:SpnNT_01015	NA	Chromosome:1038318-1039545	110.58	110.58+peptide	NOTEST	0.325281	0.305055	-0.0926176	0	1	1	no
gene:SpnNT_01015	NA	Chromosome:1038318-1039545	ΔORF2	110.58+peptide	NOTEST	0.20845	0.305055	0.549366	0	1	1	no
gene:SpnNT_01015	NA	Chromosome:1038318-1039545	110.58	ΔORF2+peptide	NOTEST	0.325281	0.117729	-1.46622	0	1	1	no
gene:SpnNT_01015	NA	Chromosome:1038318-1039545	ΔORF2	ΔORF2+peptide	NOTEST	0.20845	0.117729	-0.824233	0	1	1	no
gene:SpnNT_01015	NA	Chromosome:1038318-1039545	110.58+peptide	ΔORF2+peptide	NOTEST	0.305055	0.117729	-1.3736	0	1	1	no
gene:SpnNT_01016	NA	Chromosome:1039845-1040652	110.58	ΔORF2	OK	50.4707	57.8938	0.197964	0.400959	0.4769	0.932178	no
gene:SpnNT_01016	NA	Chromosome:1039845-1040652	110.58	110.58+peptide	OK	50.4707	42.9572	-0.232545	-0.456863	0.4182	0.903487	no
gene:SpnNT_01016	NA	Chromosome:1039845-1040652	ΔORF2	110.58+peptide	OK	57.8938	42.9572	-0.430508	-0.851289	0.12195	0.528994	no
gene:SpnNT_01016	NA	Chromosome:1039845-1040652	110.58	ΔORF2+peptide	OK	50.4707	33.5742	-0.588094	-1.141	0.0498	0.313117	no
gene:SpnNT_01016	NA	Chromosome:1039845-1040652	ΔORF2	ΔORF2+peptide	OK	57.8938	33.5742	-0.786058	-1.53476	0.0081	0.0866951	no
gene:SpnNT_01016	NA	Chromosome:1039845-1040652	110.58+peptide	ΔORF2+peptide	OK	42.9572	33.5742	-0.355549	-0.674778	0.2356	0.729482	no
gene:SpnNT_01017	NA	Chromosome:1040664-1041969	110.58	ΔORF2	OK	14.6023	15.8755	0.120602	0.222989	0.6966	0.989512	no
gene:SpnNT_01017	NA	Chromosome:1040664-1041969	110.58	110.58+peptide	OK	14.6023	13.0537	-0.161743	-0.298064	0.6029	0.976761	no
gene:SpnNT_01017	NA	Chromosome:1040664-1041969	ΔORF2	110.58+peptide	OK	15.8755	13.0537	-0.282345	-0.524557	0.3631	0.862958	no
gene:SpnNT_01017	NA	Chromosome:1040664-1041969	110.58	ΔORF2+peptide	OK	14.6023	13.3294	-0.131591	-0.242109	0.66865	0.984845	no
gene:SpnNT_01017	NA	Chromosome:1040664-1041969	ΔORF2	ΔORF2+peptide	OK	15.8755	13.3294	-0.252193	-0.467772	0.41525	0.900944	no
gene:SpnNT_01017	NA	Chromosome:1040664-1041969	110.58+peptide	ΔORF2+peptide	OK	13.0537	13.3294	0.030152	0.0557395	0.9183	0.994748	no
gene:SpnNT_01018	NA	Chromosome:1042040-1042418	110.58	ΔORF2	OK	71.9424	92.038	0.355386	0.648782	0.24485	0.741501	no
gene:SpnNT_01018	NA	Chromosome:1042040-1042418	110.58	110.58+peptide	OK	71.9424	58.6484	-0.294753	-0.519905	0.36425	0.864245	no
gene:SpnNT_01018	NA	Chromosome:1042040-1042418	ΔORF2	110.58+peptide	OK	92.038	58.6484	-0.650139	-1.15483	0.0468	0.299712	no
gene:SpnNT_01018	NA	Chromosome:1042040-1042418	110.58	ΔORF2+peptide	OK	71.9424	65.3552	-0.138541	-0.249339	0.6623	0.982966	no
gene:SpnNT_01018	NA	Chromosome:1042040-1042418	ΔORF2	ΔORF2+peptide	OK	92.038	65.3552	-0.493928	-0.895464	0.11445	0.513089	no
gene:SpnNT_01018	NA	Chromosome:1042040-1042418	110.58+peptide	ΔORF2+peptide	OK	58.6484	65.3552	0.156211	0.273756	0.63185	0.980887	no
gene:SpnNT_01019	NA	Chromosome:1042508-1042841	110.58	ΔORF2	OK	97.744	159.729	0.708546	1.29237	0.02325	0.18694	no
gene:SpnNT_01019	NA	Chromosome:1042508-1042841	110.58	110.58+peptide	OK	97.744	113.071	0.210148	0.384298	0.5018	0.944784	no
gene:SpnNT_01019	NA	Chromosome:1042508-1042841	ΔORF2	110.58+peptide	OK	159.729	113.071	-0.498398	-0.913534	0.11735	0.518998	no
gene:SpnNT_01019	NA	Chromosome:1042508-1042841	110.58	ΔORF2+peptide	OK	97.744	130.786	0.420129	0.758661	0.18375	0.655031	no
gene:SpnNT_01019	NA	Chromosome:1042508-1042841	ΔORF2	ΔORF2+peptide	OK	159.729	130.786	-0.288416	-0.521993	0.3622	0.862575	no
gene:SpnNT_01019	NA	Chromosome:1042508-1042841	110.58+peptide	ΔORF2+peptide	OK	113.071	130.786	0.209982	0.381007	0.5115	0.949753	no
gene:SpnNT_01020	ffh	Chromosome:1042852-1044424	110.58	ΔORF2	OK	140.056	149.391	0.0930919	0.212483	0.70015	0.990367	no
gene:SpnNT_01020	ffh	Chromosome:1042852-1044424	110.58	110.58+peptide	OK	140.056	121.074	-0.210116	-0.477987	0.3954	0.890245	no
gene:SpnNT_01020	ffh	Chromosome:1042852-1044424	ΔORF2	110.58+peptide	OK	149.391	121.074	-0.303208	-0.697465	0.21895	0.707128	no
gene:SpnNT_01020	ffh	Chromosome:1042852-1044424	110.58	ΔORF2+peptide	OK	140.056	136.191	-0.0403783	-0.0919206	0.8655	0.994748	no
gene:SpnNT_01020	ffh	Chromosome:1042852-1044424	ΔORF2	ΔORF2+peptide	OK	149.391	136.191	-0.13347	-0.307242	0.58505	0.971898	no

gene:SpnNT_01020	ffh	Chromosome:1042852-1044424	110.58+peptide	ΔORF2+peptide	OK	121.074	136.191	0.169738	0.3894	0.48965	0.939492	no
gene:SpnNT_01021	pyrP	Chromosome:1044618-1045902	110.58	ΔORF2	OK	48.1634	48.2595	0.00287703	0.00615882	0.99125	0.998249	no
gene:SpnNT_01021	pyrP	Chromosome:1044618-1045902	110.58	110.58+peptide	OK	48.1634	205.416	2.09254	4.59883	5.00E-05	0.0013612	yes
gene:SpnNT_01021	pyrP	Chromosome:1044618-1045902	ΔORF2	110.58+peptide	OK	48.2595	205.416	2.08966	4.58933	5.00E-05	0.0013612	yes
gene:SpnNT_01021	pyrP	Chromosome:1044618-1045902	110.58	ΔORF2+peptide	OK	48.1634	220.529	2.19496	4.84486	5.00E-05	0.0013612	yes
gene:SpnNT_01021	pyrP	Chromosome:1044618-1045902	ΔORF2	ΔORF2+peptide	OK	48.2595	220.529	2.19208	4.83514	5.00E-05	0.0013612	yes
gene:SpnNT_01021	pyrP	Chromosome:1044618-1045902	110.58+peptide	ΔORF2+peptide	OK	205.416	220.529	0.102423	0.232324	0.68425	0.98765	no
gene:SpnNT_01022	rsmG	Chromosome:1046302-1047016	110.58	ΔORF2	OK	42.0999	51.0461	0.277985	0.545546	0.3305	0.836255	no
gene:SpnNT_01022	rsmG	Chromosome:1046302-1047016	110.58	110.58+peptide	OK	42.0999	41.0042	-0.0380434	-0.0738122	0.89385	0.994748	no
gene:SpnNT_01022	rsmG	Chromosome:1046302-1047016	ΔORF2	110.58+peptide	OK	51.0461	41.0042	-0.316029	-0.619523	0.2706	0.772705	no
gene:SpnNT_01022	rsmG	Chromosome:1046302-1047016	110.58	ΔORF2+peptide	OK	42.0999	44.4775	0.0792596	0.15407	0.78735	0.994748	no
gene:SpnNT_01022	rsmG	Chromosome:1046302-1047016	ΔORF2	ΔORF2+peptide	OK	51.0461	44.4775	-0.198726	-0.390319	0.492	0.941111	no
gene:SpnNT_01022	rsmG	Chromosome:1046302-1047016	110.58+peptide	ΔORF2+peptide	OK	41.0042	44.4775	0.117303	0.227775	0.6903	0.98828	no
gene:SpnNT_01023	NA	Chromosome:1047109-1047670	110.58	ΔORF2	OK	212.97	240.771	0.177017	0.395336	0.49035	0.940011	no
gene:SpnNT_01023	NA	Chromosome:1047109-1047670	110.58	110.58+peptide	OK	212.97	150.439	-0.501466	-1.09986	0.0498	0.313117	no
gene:SpnNT_01023	NA	Chromosome:1047109-1047670	ΔORF2	110.58+peptide	OK	240.771	150.439	-0.678484	-1.49937	0.0084	0.088891	no
gene:SpnNT_01023	NA	Chromosome:1047109-1047670	110.58	ΔORF2+peptide	OK	212.97	155.463	-0.454075	-0.988005	0.08285	0.43107	no
gene:SpnNT_01023	NA	Chromosome:1047109-1047670	ΔORF2	ΔORF2+peptide	OK	240.771	155.463	-0.631093	-1.3834	0.0181	0.154427	no
gene:SpnNT_01023	NA	Chromosome:1047109-1047670	110.58+peptide	ΔORF2+peptide	OK	150.439	155.463	0.047391	0.102088	0.85365	0.994748	no
gene:SpnNT_01024	htpX	Chromosome:1047671-1048571	110.58	ΔORF2	OK	208.416	226.435	0.119634	0.274129	0.6254	0.980887	no
gene:SpnNT_01024	htpX	Chromosome:1047671-1048571	110.58	110.58+peptide	OK	208.416	149.324	-0.481023	-1.0947	0.05755	0.341706	no
gene:SpnNT_01024	htpX	Chromosome:1047671-1048571	ΔORF2	110.58+peptide	OK	226.435	149.324	-0.600657	-1.36681	0.01845	0.156295	no
gene:SpnNT_01024	htpX	Chromosome:1047671-1048571	110.58	ΔORF2+peptide	OK	208.416	159.775	-0.383426	-0.874436	0.1256	0.536498	no
gene:SpnNT_01024	htpX	Chromosome:1047671-1048571	ΔORF2	ΔORF2+peptide	OK	226.435	159.775	-0.50306	-1.14715	0.04505	0.292068	no
gene:SpnNT_01024	htpX	Chromosome:1047671-1048571	110.58+peptide	ΔORF2+peptide	OK	149.324	159.775	0.0975973	0.221052	0.70115	0.990367	no
gene:SpnNT_01025	yheS_1	Chromosome:1048614-1049817	110.58	ΔORF2	OK	32.6155	37.1925	0.189458	0.390851	0.4944	0.942197	no
gene:SpnNT_01025	yheS_1	Chromosome:1048614-1049817	110.58	110.58+peptide	OK	32.6155	41.4814	0.34691	0.717331	0.20835	0.693901	no
gene:SpnNT_01025	yheS_1	Chromosome:1048614-1049817	ΔORF2	110.58+peptide	OK	37.1925	41.4814	0.157452	0.328468	0.57385	0.969538	no
gene:SpnNT_01025	yheS_1	Chromosome:1048614-1049817	110.58	ΔORF2+peptide	OK	32.6155	40.8402	0.324434	0.669001	0.2382	0.732863	no
gene:SpnNT_01025	yheS_1	Chromosome:1048614-1049817	ΔORF2	ΔORF2+peptide	OK	37.1925	40.8402	0.134977	0.280788	0.63015	0.980887	no
gene:SpnNT_01025	yheS_1	Chromosome:1048614-1049817	110.58+peptide	ΔORF2+peptide	OK	41.4814	40.8402	-0.0224756	-0.0468653	0.9365	0.994855	no
gene:SpnNT_01026	NA	Chromosome:1049864-1050173	110.58	ΔORF2	OK	71.4172	81.9568	0.198591	0.336418	0.56415	0.968621	no
gene:SpnNT_01026	NA	Chromosome:1049864-1050173	110.58	110.58+peptide	OK	71.4172	109.925	0.622175	1.087	0.05985	0.351074	no
gene:SpnNT_01026	NA	Chromosome:1049864-1050173	ΔORF2	110.58+peptide	OK	81.9568	109.925	0.423584	0.743474	0.1992	0.681762	no
gene:SpnNT_01026	NA	Chromosome:1049864-1050173	110.58	ΔORF2+peptide	OK	71.4172	105.878	0.568064	0.992905	0.08595	0.440217	no
gene:SpnNT_01026	NA	Chromosome:1049864-1050173	ΔORF2	ΔORF2+peptide	OK	81.9568	105.878	0.369473	0.648793	0.26095	0.761099	no
gene:SpnNT_01026	NA	Chromosome:1049864-1050173	110.58+peptide	ΔORF2+peptide	OK	109.925	105.878	-0.054111	-0.0982279	0.86695	0.994748	no
gene:SpnNT_01027	NA	Chromosome:1050767-1052501	110.58	ΔORF2	OK	22.243	24.2508	0.124677	0.254456	0.6556	0.982966	no
gene:SpnNT_01027	NA	Chromosome:1050767-1052501	110.58	110.58+peptide	OK	22.243	22.3533	0.00713291	0.014451	0.9807	0.99608	no
gene:SpnNT_01027	NA	Chromosome:1050767-1052501	ΔORF2	110.58+peptide	OK	24.2508	22.3533	-0.117544	-0.240183	0.67275	0.984845	no
gene:SpnNT_01027	NA	Chromosome:1050767-1052501	110.58	ΔORF2+peptide	OK	22.243	27.8516	0.324409	0.66616	0.24745	0.743991	no
gene:SpnNT_01027	NA	Chromosome:1050767-1052501	ΔORF2	ΔORF2+peptide	OK	24.2508	27.8516	0.199732	0.413757	0.46745	0.92732	no
gene:SpnNT_01027	NA	Chromosome:1050767-1052501	110.58+peptide	ΔORF2+peptide	OK	22.3533	27.8516	0.317276	0.652295	0.26445	0.7648	no
gene:SpnNT_01028	NA	Chromosome:1052652-1052970	110.58	ΔORF2	OK	10.9188	7.0476	-0.631616	-0.726947	0.20485	0.689595	no
gene:SpnNT_01028	NA	Chromosome:1052652-1052970	110.58	110.58+peptide	OK	10.9188	7.30944	-0.578986	-0.66433	0.2412	0.736034	no

gene:SpnNT_01028	NA	Chromosome:1052652-1052970	ΔORF2	110.58+peptide	OK	7.0476	7.30944	0.0526295	0.0567412	0.9107	0.994748	no
gene:SpnNT_01028	NA	Chromosome:1052652-1052970	110.58	ΔORF2+peptide	OK	10.9188	8.67439	-0.331985	-0.394141	0.5047	0.945292	no
gene:SpnNT_01028	NA	Chromosome:1052652-1052970	ΔORF2	ΔORF2+peptide	OK	7.0476	8.67439	0.29963	0.332878	0.57125	0.968844	no
gene:SpnNT_01028	NA	Chromosome:1052652-1052970	110.58+peptide	ΔORF2+peptide	OK	7.30944	8.67439	0.247001	0.273624	0.63385	0.980887	no
gene:SpnNT_01029	NA	Chromosome:1053139-1054311	110.58	ΔORF2	OK	89.4191	115.208	0.365583	0.576135	0.3196	0.825861	no
gene:SpnNT_01029	NA	Chromosome:1053139-1054311	110.58	110.58+peptide	OK	89.4191	99.0067	0.146943	0.236221	0.6892	0.98828	no
gene:SpnNT_01029	NA	Chromosome:1053139-1054311	ΔORF2	110.58+peptide	OK	115.208	99.0067	-0.21864	-0.347472	0.5467	0.962122	no
gene:SpnNT_01029	NA	Chromosome:1053139-1054311	110.58	ΔORF2+peptide	OK	89.4191	111.601	0.319693	0.514847	0.36965	0.866169	no
gene:SpnNT_01029	NA	Chromosome:1053139-1054311	ΔORF2	ΔORF2+peptide	OK	115.208	111.601	-0.0458904	-0.0730583	0.9003	0.994748	no
gene:SpnNT_01029	NA	Chromosome:1053139-1054311	110.58+peptide	ΔORF2+peptide	OK	99.0067	111.601	0.17275	0.280658	0.6178	0.980887	no
gene:SpnNT_01030	pdg	Chromosome:1053139-1054311	110.58	ΔORF2	OK	59.0354	60.0435	0.0244268	0.0325783	0.9545	0.994855	no
gene:SpnNT_01030	pdg	Chromosome:1053139-1054311	110.58	110.58+peptide	OK	59.0354	54.575	-0.113341	-0.15523	0.7814	0.994748	no
gene:SpnNT_01030	pdg	Chromosome:1053139-1054311	ΔORF2	110.58+peptide	OK	60.0435	54.575	-0.137768	-0.174909	0.75465	0.994748	no
gene:SpnNT_01030	pdg	Chromosome:1053139-1054311	110.58	ΔORF2+peptide	OK	59.0354	60.6141	0.0380713	0.0529653	0.9265	0.994748	no
gene:SpnNT_01030	pdg	Chromosome:1053139-1054311	ΔORF2	ΔORF2+peptide	OK	60.0435	60.6141	0.0136445	0.0175573	0.97605	0.99536	no
gene:SpnNT_01030	pdg	Chromosome:1053139-1054311	110.58+peptide	ΔORF2+peptide	OK	54.575	60.6141	0.151413	0.199697	0.73255	0.994748	no
gene:SpnNT_01031	NA	Chromosome:1054459-1056193	110.58	ΔORF2	OK	28.0626	31.9165	0.185656	0.390963	0.49625	0.943191	no
gene:SpnNT_01031	NA	Chromosome:1054459-1056193	110.58	110.58+peptide	OK	28.0626	28.118	0.00284899	0.00590858	0.9912	0.998249	no
gene:SpnNT_01031	NA	Chromosome:1054459-1056193	ΔORF2	110.58+peptide	OK	31.9165	28.118	-0.182807	-0.382852	0.4989	0.944017	no
gene:SpnNT_01031	NA	Chromosome:1054459-1056193	110.58	ΔORF2+peptide	OK	28.0626	36.7553	0.389305	0.826907	0.1528	0.595675	no
gene:SpnNT_01031	NA	Chromosome:1054459-1056193	ΔORF2	ΔORF2+peptide	OK	31.9165	36.7553	0.203649	0.437023	0.4442	0.917631	no
gene:SpnNT_01031	NA	Chromosome:1054459-1056193	110.58+peptide	ΔORF2+peptide	OK	28.118	36.7553	0.386456	0.816276	0.1492	0.590233	no
gene:SpnNT_01032	pyrR	Chromosome:1056577-1057099	110.58	ΔORF2	OK	43.4849	43.039	-0.0148694	-0.0268388	0.9627	0.994855	no
gene:SpnNT_01032	pyrR	Chromosome:1056577-1057099	110.58	110.58+peptide	OK	43.4849	169.244	1.96052	3.83957	5.00E-05	0.0013612	yes
gene:SpnNT_01032	pyrR	Chromosome:1056577-1057099	ΔORF2	110.58+peptide	OK	43.039	169.244	1.97539	3.81619	5.00E-05	0.0013612	yes
gene:SpnNT_01032	pyrR	Chromosome:1056577-1057099	110.58	ΔORF2+peptide	OK	43.4849	155.552	1.83881	3.60109	5.00E-05	0.0013612	yes
gene:SpnNT_01032	pyrR	Chromosome:1056577-1057099	ΔORF2	ΔORF2+peptide	OK	43.039	155.552	1.85368	3.58094	5.00E-05	0.0013612	yes
gene:SpnNT_01032	pyrR	Chromosome:1056577-1057099	110.58+peptide	ΔORF2+peptide	OK	169.244	155.552	-0.121709	-0.258466	0.6511	0.981475	no
gene:SpnNT_01033	pyrB	Chromosome:1057117-1058041	110.58	ΔORF2	OK	54.8878	49.8619	-0.138547	-0.287345	0.61915	0.980887	no
gene:SpnNT_01033	pyrB	Chromosome:1057117-1058041	110.58	110.58+peptide	OK	54.8878	205.724	1.90615	4.10718	5.00E-05	0.0013612	yes
gene:SpnNT_01033	pyrB	Chromosome:1057117-1058041	ΔORF2	110.58+peptide	OK	49.8619	205.724	2.0447	4.39231	5.00E-05	0.0013612	yes
gene:SpnNT_01033	pyrB	Chromosome:1057117-1058041	110.58	ΔORF2+peptide	OK	54.8878	234.304	2.09383	4.56695	5.00E-05	0.0013612	yes
gene:SpnNT_01033	pyrB	Chromosome:1057117-1058041	ΔORF2	ΔORF2+peptide	OK	49.8619	234.304	2.23237	4.85398	5.00E-05	0.0013612	yes
gene:SpnNT_01033	pyrB	Chromosome:1057117-1058041	110.58+peptide	ΔORF2+peptide	OK	205.724	234.304	0.187674	0.425631	0.4484	0.919688	no
gene:SpnNT_01034	carA	Chromosome:1058090-1059170	110.58	ΔORF2	OK	51.7615	47.9782	-0.109501	-0.230157	0.68555	0.988184	no
gene:SpnNT_01034	carA	Chromosome:1058090-1059170	110.58	110.58+peptide	OK	51.7615	183.712	1.82749	3.9601	5.00E-05	0.0013612	yes
gene:SpnNT_01034	carA	Chromosome:1058090-1059170	ΔORF2	110.58+peptide	OK	47.9782	183.712	1.937	4.1457	5.00E-05	0.0013612	yes
gene:SpnNT_01034	carA	Chromosome:1058090-1059170	110.58	ΔORF2+peptide	OK	51.7615	186.535	1.8495	4.0806	5.00E-05	0.0013612	yes
gene:SpnNT_01034	carA	Chromosome:1058090-1059170	ΔORF2	ΔORF2+peptide	OK	47.9782	186.535	1.959	4.26707	5.00E-05	0.0013612	yes
gene:SpnNT_01034	carA	Chromosome:1058090-1059170	110.58+peptide	ΔORF2+peptide	OK	183.712	186.535	0.0220014	0.0495228	0.9309	0.994855	no
gene:SpnNT_01035	carB	Chromosome:1059482-1062659	110.58	ΔORF2	OK	51.943	48.4182	-0.10138	-0.231104	0.6832	0.987438	no
gene:SpnNT_01035	carB	Chromosome:1059482-1062659	110.58	110.58+peptide	OK	51.943	175.052	1.75278	3.9137	5.00E-05	0.0013612	yes
gene:SpnNT_01035	carB	Chromosome:1059482-1062659	ΔORF2	110.58+peptide	OK	48.4182	175.052	1.85416	4.11287	5.00E-05	0.0013612	yes
gene:SpnNT_01035	carB	Chromosome:1059482-1062659	110.58	ΔORF2+peptide	OK	51.943	191.72	1.884	4.25629	5.00E-05	0.0013612	yes
gene:SpnNT_01035	carB	Chromosome:1059482-1062659	ΔORF2	ΔORF2+peptide	OK	48.4182	191.72	1.98538	4.45518	5.00E-05	0.0013612	yes

gene:SpnNT_01035	carB	Chromosome:1059482-1062659	110.58+peptide	ΔORF2+peptide	OK	175.052	191.72	0.131214	0.288587	0.6157	0.979616	no
gene:SpnNT_01036	NA	Chromosome:1062933-1063743	110.58	ΔORF2	OK	39.7799	46.7167	0.231898	0.46063	0.41485	0.900591	no
gene:SpnNT_01036	NA	Chromosome:1062933-1063743	110.58	110.58+peptide	OK	39.7799	30.8022	-0.369005	-0.711736	0.20685	0.692058	no
gene:SpnNT_01036	NA	Chromosome:1062933-1063743	ΔORF2	110.58+peptide	OK	46.7167	30.8022	-0.600904	-1.16555	0.0457	0.294971	no
gene:SpnNT_01036	NA	Chromosome:1062933-1063743	110.58	ΔORF2+peptide	OK	39.7799	38.9372	-0.0308918	-0.0604294	0.9147	0.994748	no
gene:SpnNT_01036	NA	Chromosome:1062933-1063743	ΔORF2	ΔORF2+peptide	OK	46.7167	38.9372	-0.26279	-0.517038	0.36665	0.864634	no
gene:SpnNT_01036	NA	Chromosome:1062933-1063743	110.58+peptide	ΔORF2+peptide	OK	30.8022	38.9372	0.338114	0.64631	0.26635	0.767804	no
gene:SpnNT_01037	NA	Chromosome:1063744-1064548	110.58	ΔORF2	OK	11.8926	14.6548	0.301302	0.504296	0.38705	0.881745	no
gene:SpnNT_01037	NA	Chromosome:1063744-1064548	110.58	110.58+peptide	OK	11.8926	11.5643	-0.0403854	-0.0649108	0.9103	0.994748	no
gene:SpnNT_01037	NA	Chromosome:1063744-1064548	ΔORF2	110.58+peptide	OK	14.6548	11.5643	-0.341687	-0.561686	0.3334	0.839028	no
gene:SpnNT_01037	NA	Chromosome:1063744-1064548	110.58	ΔORF2+peptide	OK	11.8926	20.2044	0.764605	1.30595	0.02565	0.200464	no
gene:SpnNT_01037	NA	Chromosome:1063744-1064548	ΔORF2	ΔORF2+peptide	OK	14.6548	20.2044	0.463302	0.811748	0.1614	0.614666	no
gene:SpnNT_01037	NA	Chromosome:1063744-1064548	110.58+peptide	ΔORF2+peptide	OK	11.5643	20.2044	0.80499	1.34941	0.0221	0.180796	no
gene:SpnNT_01038	NA	Chromosome:1064557-1066045	110.58	ΔORF2	OK	19.5798	23.8582	0.285119	0.567869	0.32675	0.831577	no
gene:SpnNT_01038	NA	Chromosome:1064557-1066045	110.58	110.58+peptide	OK	19.5798	19.0065	-0.042878	-0.0839129	0.88025	0.994748	no
gene:SpnNT_01038	NA	Chromosome:1064557-1066045	ΔORF2	110.58+peptide	OK	23.8582	19.0065	-0.327997	-0.650885	0.2594	0.759841	no
gene:SpnNT_01038	NA	Chromosome:1064557-1066045	110.58	ΔORF2+peptide	OK	19.5798	20.59	0.0725771	0.143004	0.79925	0.994748	no
gene:SpnNT_01038	NA	Chromosome:1064557-1066045	ΔORF2	ΔORF2+peptide	OK	23.8582	20.59	-0.212542	-0.424734	0.4557	0.921244	no
gene:SpnNT_01038	NA	Chromosome:1064557-1066045	110.58+peptide	ΔORF2+peptide	OK	19.0065	20.59	0.115455	0.226677	0.6924	0.98828	no
gene:SpnNT_01039	ispD2	Chromosome:1066380-1067088	110.58	ΔORF2	OK	97.1053	103.696	0.0947384	0.202941	0.72585	0.994748	no
gene:SpnNT_01039	ispD2	Chromosome:1066380-1067088	110.58	110.58+peptide	OK	97.1053	65.9381	-0.558438	-1.17031	0.04145	0.277079	no
gene:SpnNT_01039	ispD2	Chromosome:1066380-1067088	ΔORF2	110.58+peptide	OK	103.696	65.9381	-0.653176	-1.36981	0.01905	0.160652	no
gene:SpnNT_01039	ispD2	Chromosome:1066380-1067088	110.58	ΔORF2+peptide	OK	97.1053	75.7739	-0.357849	-0.753675	0.1955	0.677231	no
gene:SpnNT_01039	ispD2	Chromosome:1066380-1067088	ΔORF2	ΔORF2+peptide	OK	103.696	75.7739	-0.452587	-0.953882	0.1027	0.485807	no
gene:SpnNT_01039	ispD2	Chromosome:1066380-1067088	110.58+peptide	ΔORF2+peptide	OK	65.9381	75.7739	0.200589	0.413883	0.46315	0.925853	no
gene:SpnNT_01040	yjjN	Chromosome:1067092-1068115	110.58	ΔORF2	OK	116.158	122.093	0.0718927	0.162468	0.7761	0.994748	no
gene:SpnNT_01040	yjjN	Chromosome:1067092-1068115	110.58	110.58+peptide	OK	116.158	84.4141	-0.460529	-1.02996	0.07355	0.399141	no
gene:SpnNT_01040	yjjN	Chromosome:1067092-1068115	ΔORF2	110.58+peptide	OK	122.093	84.4141	-0.532421	-1.19024	0.0402	0.27205	no
gene:SpnNT_01040	yjjN	Chromosome:1067092-1068115	110.58	ΔORF2+peptide	OK	116.158	94.7001	-0.294645	-0.661416	0.2471	0.743921	no
gene:SpnNT_01040	yjjN	Chromosome:1067092-1068115	ΔORF2	ΔORF2+peptide	OK	122.093	94.7001	-0.366538	-0.822449	0.15065	0.593025	no
gene:SpnNT_01040	yjjN	Chromosome:1067092-1068115	110.58+peptide	ΔORF2+peptide	OK	84.4141	94.7001	0.165883	0.368414	0.5201	0.954744	no
gene:SpnNT_01041	NA	Chromosome:1068131-1069869	110.58	ΔORF2	OK	72.6133	84.7124	0.222339	0.337714	0.5633	0.968621	no
gene:SpnNT_01041	NA	Chromosome:1068131-1069869	110.58	110.58+peptide	OK	72.6133	54.8467	-0.404829	-0.594075	0.309	0.814396	no
gene:SpnNT_01041	NA	Chromosome:1068131-1069869	ΔORF2	110.58+peptide	OK	84.7124	54.8467	-0.627167	-0.924326	0.11335	0.510776	no
gene:SpnNT_01041	NA	Chromosome:1068131-1069869	110.58	ΔORF2+peptide	OK	72.6133	76.3153	0.0717386	0.108287	0.85135	0.994748	no
gene:SpnNT_01041	NA	Chromosome:1068131-1069869	ΔORF2	ΔORF2+peptide	OK	84.7124	76.3153	-0.1506	-0.228366	0.69365	0.98828	no
gene:SpnNT_01041	NA	Chromosome:1068131-1069869	110.58+peptide	ΔORF2+peptide	OK	54.8467	76.3153	0.476567	0.698255	0.22935	0.722056	no
gene:SpnNT_01042	NA	Chromosome:1068131-1069869	110.58	ΔORF2	OK	89.4498	93.8875	0.0698545	0.114236	0.8387	0.994748	no
gene:SpnNT_01042	NA	Chromosome:1068131-1069869	110.58	110.58+peptide	OK	89.4498	72.2846	-0.30739	-0.505544	0.37835	0.875802	no
gene:SpnNT_01042	NA	Chromosome:1068131-1069869	ΔORF2	110.58+peptide	OK	93.8875	72.2846	-0.377244	-0.605106	0.2949	0.799296	no
gene:SpnNT_01042	NA	Chromosome:1068131-1069869	110.58	ΔORF2+peptide	OK	89.4498	89.5056	0.000900222	0.00151001	0.99755	0.999412	no
gene:SpnNT_01042	NA	Chromosome:1068131-1069869	ΔORF2	ΔORF2+peptide	OK	93.8875	89.5056	-0.0689542	-0.112695	0.8429	0.994748	no
gene:SpnNT_01042	NA	Chromosome:1068131-1069869	110.58+peptide	ΔORF2+peptide	OK	72.2846	89.5056	0.30829	0.506714	0.37315	0.870292	no
gene:SpnNT_01043	gtaB_1	Chromosome:1069880-1070570	110.58	ΔORF2	OK	209.669	232.118	0.146747	0.331161	0.56705	0.968621	no
gene:SpnNT_01043	gtaB_1	Chromosome:1069880-1070570	110.58	110.58+peptide	OK	209.669	233.565	0.155711	0.352178	0.54045	0.961468	no

gene:SpnNT_01043	gtaB_1	Chromosome:1069880-1070570	ΔORF2	110.58+peptide	OK	232.118	233.565	0.008964	0.0202233	0.972	0.99536	no
gene:SpnNT_01043	gtaB_1	Chromosome:1069880-1070570	110.58	ΔORF2+peptide	OK	209.669	231.814	0.144854	0.326915	0.56895	0.968621	no
gene:SpnNT_01043	gtaB_1	Chromosome:1069880-1070570	ΔORF2	ΔORF2+peptide	OK	232.118	231.814	-0.00189256	-0.00426053	0.9938	0.998618	no
gene:SpnNT_01043	gtaB_1	Chromosome:1069880-1070570	110.58+peptide	ΔORF2+peptide	OK	233.565	231.814	-0.0108566	-0.0244948	0.96435	0.994855	no
gene:SpnNT_01044	NA	Chromosome:1070666-1071515	110.58	ΔORF2	OK	2.18129	2.43966	0.161502	0.202421	0.71925	0.993716	no
gene:SpnNT_01044	NA	Chromosome:1070666-1071515	110.58	110.58+peptide	OK	2.18129	1.81879	-0.262203	-0.314202	0.5806	0.969538	no
gene:SpnNT_01044	NA	Chromosome:1070666-1071515	ΔORF2	110.58+peptide	OK	2.43966	1.81879	-0.423705	-0.515199	0.36935	0.866165	no
gene:SpnNT_01044	NA	Chromosome:1070666-1071515	110.58	ΔORF2+peptide	OK	2.18129	1.6839	-0.373373	-0.443874	0.4336	0.913361	no
gene:SpnNT_01044	NA	Chromosome:1070666-1071515	ΔORF2	ΔORF2+peptide	OK	2.43966	1.6839	-0.534875	-0.64507	0.2659	0.767012	no
gene:SpnNT_01044	NA	Chromosome:1070666-1071515	110.58+peptide	ΔORF2+peptide	OK	1.81879	1.6839	-0.11117	-0.128594	0.8237	0.994748	no
gene:SpnNT_01045	NA	Chromosome:1072398-1073442	110.58	ΔORF2	OK	46.8885	56.2964	0.263811	0.548605	0.3411	0.844955	no
gene:SpnNT_01045	NA	Chromosome:1072398-1073442	110.58	110.58+peptide	OK	46.8885	83.9414	0.840149	1.77715	0.0022	0.0317584	yes
gene:SpnNT_01045	NA	Chromosome:1072398-1073442	ΔORF2	110.58+peptide	OK	56.2964	83.9414	0.576338	1.22384	0.03495	0.247365	no
gene:SpnNT_01045	NA	Chromosome:1072398-1073442	110.58	ΔORF2+peptide	OK	46.8885	129.722	1.46812	3.17089	5.00E-05	0.0013612	yes
gene:SpnNT_01045	NA	Chromosome:1072398-1073442	ΔORF2	ΔORF2+peptide	OK	56.2964	129.722	1.20431	2.61162	5.00E-05	0.0013612	yes
gene:SpnNT_01045	NA	Chromosome:1072398-1073442	110.58+peptide	ΔORF2+peptide	OK	83.9414	129.722	0.62797	1.3873	0.01555	0.137963	no
gene:SpnNT_01046	topA	Chromosome:1073608-1075696	110.58	ΔORF2	OK	154.85	164.051	0.0832737	0.190246	0.73295	0.994748	no
gene:SpnNT_01046	topA	Chromosome:1073608-1075696	110.58	110.58+peptide	OK	154.85	214.999	0.473456	1.08174	0.05625	0.3381	no
gene:SpnNT_01046	topA	Chromosome:1073608-1075696	ΔORF2	110.58+peptide	OK	164.051	214.999	0.390182	0.886423	0.12425	0.533336	no
gene:SpnNT_01046	topA	Chromosome:1073608-1075696	110.58	ΔORF2+peptide	OK	154.85	260.154	0.748494	1.70593	0.00265	0.0362222	yes
gene:SpnNT_01046	topA	Chromosome:1073608-1075696	ΔORF2	ΔORF2+peptide	OK	164.051	260.154	0.66522	1.50757	0.00775	0.0837689	no
gene:SpnNT_01046	topA	Chromosome:1073608-1075696	110.58+peptide	ΔORF2+peptide	OK	214.999	260.154	0.275038	0.623363	0.27235	0.774383	no
gene:SpnNT_01047	ybaN	Chromosome:1075916-1076276	110.58	ΔORF2	OK	151.863	159.698	0.0725726	0.142759	0.80275	0.994748	no
gene:SpnNT_01047	ybaN	Chromosome:1075916-1076276	110.58	110.58+peptide	OK	151.863	112.544	-0.432276	-0.841535	0.14075	0.572689	no
gene:SpnNT_01047	ybaN	Chromosome:1075916-1076276	ΔORF2	110.58+peptide	OK	159.698	112.544	-0.504848	-0.979832	0.09665	0.470343	no
gene:SpnNT_01047	ybaN	Chromosome:1075916-1076276	110.58	ΔORF2+peptide	OK	151.863	114.296	-0.409999	-0.791555	0.16375	0.618339	no
gene:SpnNT_01047	ybaN	Chromosome:1075916-1076276	ΔORF2	ΔORF2+peptide	OK	159.698	114.296	-0.482571	-0.928882	0.1109	0.507277	no
gene:SpnNT_01047	ybaN	Chromosome:1075916-1076276	110.58+peptide	ΔORF2+peptide	OK	112.544	114.296	0.022277	0.0424546	0.94165	0.994855	no
gene:SpnNT_01048	NA	Chromosome:1076368-1077001	110.58	ΔORF2	OK	163.761	184.226	0.169884	0.37604	0.51255	0.950409	no
gene:SpnNT_01048	NA	Chromosome:1076368-1077001	110.58	110.58+peptide	OK	163.761	126.924	-0.367624	-0.798523	0.16445	0.619023	no
gene:SpnNT_01048	NA	Chromosome:1076368-1077001	ΔORF2	110.58+peptide	OK	184.226	126.924	-0.537508	-1.1738	0.03785	0.261474	no
gene:SpnNT_01048	NA	Chromosome:1076368-1077001	110.58	ΔORF2+peptide	OK	163.761	141.018	-0.215713	-0.471447	0.4199	0.904223	no
gene:SpnNT_01048	NA	Chromosome:1076368-1077001	ΔORF2	ΔORF2+peptide	OK	184.226	141.018	-0.385597	-0.847314	0.1404	0.571974	no
gene:SpnNT_01048	NA	Chromosome:1076368-1077001	110.58+peptide	ΔORF2+peptide	OK	126.924	141.018	0.151911	0.327657	0.56685	0.968621	no
gene:SpnNT_01049	NA	Chromosome:1077044-1077701	110.58	ΔORF2	OK	57.9902	61.5268	0.0854061	0.168013	0.7637	0.994748	no
gene:SpnNT_01049	NA	Chromosome:1077044-1077701	110.58	110.58+peptide	OK	57.9902	34.74	-0.739209	-1.41896	0.01295	0.120861	no
gene:SpnNT_01049	NA	Chromosome:1077044-1077701	ΔORF2	110.58+peptide	OK	61.5268	34.74	-0.824615	-1.57237	0.00755	0.082285	no
gene:SpnNT_01049	NA	Chromosome:1077044-1077701	110.58	ΔORF2+peptide	OK	57.9902	33.4368	-0.794373	-1.51135	0.0082	0.08748	no
gene:SpnNT_01049	NA	Chromosome:1077044-1077701	ΔORF2	ΔORF2+peptide	OK	61.5268	33.4368	-0.879779	-1.6629	0.00445	0.054829	no
gene:SpnNT_01049	NA	Chromosome:1077044-1077701	110.58+peptide	ΔORF2+peptide	OK	34.74	33.4368	-0.0551645	-0.10193	0.86155	0.994748	no
gene:SpnNT_01050	leuA	Chromosome:1077719-1078814	110.58	ΔORF2	OK	23.5088	22.3303	-0.0741976	-0.141154	0.80335	0.994748	no
gene:SpnNT_01050	leuA	Chromosome:1077719-1078814	110.58	110.58+peptide	OK	23.5088	14.9651	-0.651602	-1.21276	0.03595	0.25213	no
gene:SpnNT_01050	leuA	Chromosome:1077719-1078814	ΔORF2	110.58+peptide	OK	22.3303	14.9651	-0.577404	-1.063	0.0697	0.387873	no
gene:SpnNT_01050	leuA	Chromosome:1077719-1078814	110.58	ΔORF2+peptide	OK	23.5088	18.2816	-0.362807	-0.686918	0.2275	0.71915	no
gene:SpnNT_01050	leuA	Chromosome:1077719-1078814	ΔORF2	ΔORF2+peptide	OK	22.3303	18.2816	-0.28861	-0.540304	0.3453	0.849107	no

gene:SpnNT_01050	leuA	Chromosome:1077719-1078814	110.58+peptide	ΔORF2+peptide	OK	14.9651	18.2816	0.288794	0.529298	0.35525	0.85704	no
gene:SpnNT_01051	leuB	Chromosome:1078825-1080129	110.58	ΔORF2	OK	28.9033	26.2245	-0.140315	-0.264394	0.6433	0.980887	no
gene:SpnNT_01051	leuB	Chromosome:1078825-1080129	110.58	110.58+peptide	OK	28.9033	18.7316	-0.625757	-1.15317	0.0432	0.285003	no
gene:SpnNT_01051	leuB	Chromosome:1078825-1080129	ΔORF2	110.58+peptide	OK	26.2245	18.7316	-0.485442	-0.880942	0.12345	0.531467	no
gene:SpnNT_01051	leuB	Chromosome:1078825-1080129	110.58	ΔORF2+peptide	OK	28.9033	18.2616	-0.662422	-1.24342	0.02915	0.217952	no
gene:SpnNT_01051	leuB	Chromosome:1078825-1080129	ΔORF2	ΔORF2+peptide	OK	26.2245	18.2616	-0.522106	-0.964541	0.09545	0.468047	no
gene:SpnNT_01051	leuB	Chromosome:1078825-1080129	110.58+peptide	ΔORF2+peptide	OK	18.7316	18.2616	-0.0366646	-0.0663004	0.90925	0.994748	no
gene:SpnNT_01052	NA	Chromosome:1078825-1080129	110.58	ΔORF2	OK	11.8464	13.6177	0.201038	0.0809692	0.80695	0.994748	no
gene:SpnNT_01052	NA	Chromosome:1078825-1080129	110.58	110.58+peptide	OK	11.8464	7.48302	-0.662752	-0.247937	0.7811	0.994748	no
gene:SpnNT_01052	NA	Chromosome:1078825-1080129	ΔORF2	110.58+peptide	OK	13.6177	7.48302	-0.86379	-0.330336	0.7299	0.994748	no
gene:SpnNT_01052	NA	Chromosome:1078825-1080129	110.58	ΔORF2+peptide	OK	11.8464	5.94314	-0.995147	-0.462671	0.74545	0.994748	no
gene:SpnNT_01052	NA	Chromosome:1078825-1080129	ΔORF2	ΔORF2+peptide	OK	13.6177	5.94314	-1.19618	-0.575608	0.6899	0.98828	no
gene:SpnNT_01052	NA	Chromosome:1078825-1080129	110.58+peptide	ΔORF2+peptide	OK	7.48302	5.94314	-0.332395	-0.144395	0.85015	0.994748	no
gene:SpnNT_01053	leuD	Chromosome:1080132-1080492	110.58	ΔORF2	OK	16.2878	19.1282	0.231904	0.326239	0.56555	0.968621	no
gene:SpnNT_01053	leuD	Chromosome:1080132-1080492	110.58	110.58+peptide	OK	16.2878	12.1286	-0.425387	-0.583816	0.31325	0.819308	no
gene:SpnNT_01053	leuD	Chromosome:1080132-1080492	ΔORF2	110.58+peptide	OK	19.1282	12.1286	-0.657291	-0.889334	0.11795	0.520424	no
gene:SpnNT_01053	leuD	Chromosome:1080132-1080492	110.58	ΔORF2+peptide	OK	16.2878	13.9778	-0.220659	-0.30794	0.5982	0.97629	no
gene:SpnNT_01053	leuD	Chromosome:1080132-1080492	ΔORF2	ΔORF2+peptide	OK	19.1282	13.9778	-0.452564	-0.622346	0.28795	0.791968	no
gene:SpnNT_01053	leuD	Chromosome:1080132-1080492	110.58+peptide	ΔORF2+peptide	OK	12.1286	13.9778	0.204728	0.274954	0.64195	0.980887	no
gene:SpnNT_01054	NA	Chromosome:1080710-1080854	110.58	ΔORF2	OK	25.7659	25.168	-0.0338729	-0.0250167	0.91745	0.994748	no
gene:SpnNT_01054	NA	Chromosome:1080710-1080854	110.58	110.58+peptide	OK	25.7659	10.3038	-1.32229	-1.32547	0.2218	0.711082	no
gene:SpnNT_01054	NA	Chromosome:1080710-1080854	ΔORF2	110.58+peptide	OK	25.168	10.3038	-1.28841	-1.27963	0.2602	0.760602	no
gene:SpnNT_01054	NA	Chromosome:1080710-1080854	110.58	ΔORF2+peptide	OK	25.7659	3.18702	-3.01518	-2.95372	0.16305	0.61676	no
gene:SpnNT_01054	NA	Chromosome:1080710-1080854	ΔORF2	ΔORF2+peptide	OK	25.168	3.18702	-2.98131	-2.89487	0.16535	0.620808	no
gene:SpnNT_01054	NA	Chromosome:1080710-1080854	110.58+peptide	ΔORF2+peptide	OK	10.3038	3.18702	-1.6929	-3.58917	0.319	0.825137	no
gene:SpnNT_01055	NA	Chromosome:1081107-1081224	110.58	ΔORF2	NOTEST	0	0	0	0	1	1	no
gene:SpnNT_01055	NA	Chromosome:1081107-1081224	110.58	110.58+peptide	NOTEST	0	0	0	0	1	1	no
gene:SpnNT_01055	NA	Chromosome:1081107-1081224	ΔORF2	110.58+peptide	NOTEST	0	0	0	0	1	1	no
gene:SpnNT_01055	NA	Chromosome:1081107-1081224	110.58	ΔORF2+peptide	NOTEST	0	3.76543	Inf	0	1	1	no
gene:SpnNT_01055	NA	Chromosome:1081107-1081224	ΔORF2	ΔORF2+peptide	NOTEST	0	3.76543	Inf	0	1	1	no
gene:SpnNT_01055	NA	Chromosome:1081107-1081224	110.58+peptide	ΔORF2+peptide	NOTEST	0	3.76543	Inf	0	1	1	no
gene:SpnNT_01056	mcrB	Chromosome:1081545-1084780	110.58	ΔORF2	OK	17.7517	20.9115	0.236339	0.381685	0.5077	0.946581	no
gene:SpnNT_01056	mcrB	Chromosome:1081545-1084780	110.58	110.58+peptide	OK	17.7517	6.37854	-1.47666	-2.15732	0.00035	0.00717609	yes
gene:SpnNT_01056	mcrB	Chromosome:1081545-1084780	ΔORF2	110.58+peptide	OK	20.9115	6.37854	-1.713	-2.52194	5.00E-05	0.0013612	yes
gene:SpnNT_01056	mcrB	Chromosome:1081545-1084780	110.58	ΔORF2+peptide	OK	17.7517	9.28165	-0.935507	-1.36824	0.021	0.173747	no
gene:SpnNT_01056	mcrB	Chromosome:1081545-1084780	ΔORF2	ΔORF2+peptide	OK	20.9115	9.28165	-1.17185	-1.72718	0.00445	0.054829	no
gene:SpnNT_01056	mcrB	Chromosome:1081545-1084780	110.58+peptide	ΔORF2+peptide	OK	6.37854	9.28165	0.541155	0.732732	0.2067	0.691918	no
gene:SpnNT_01057	NA	Chromosome:1081545-1084780	110.58	ΔORF2	OK	20.7244	25.6892	0.309832	0.442891	0.4388	0.916056	no
gene:SpnNT_01057	NA	Chromosome:1081545-1084780	110.58	110.58+peptide	OK	20.7244	9.06261	-1.19333	-1.61571	0.0062	0.0706832	no
gene:SpnNT_01057	NA	Chromosome:1081545-1084780	ΔORF2	110.58+peptide	OK	25.6892	9.06261	-1.50316	-2.07926	5.00E-04	0.0095781	yes
gene:SpnNT_01057	NA	Chromosome:1081545-1084780	110.58	ΔORF2+peptide	OK	20.7244	12.0026	-0.787984	-1.02899	0.0732	0.398891	no
gene:SpnNT_01057	NA	Chromosome:1081545-1084780	ΔORF2	ΔORF2+peptide	OK	25.6892	12.0026	-1.09782	-1.46238	0.01265	0.119248	no
gene:SpnNT_01057	NA	Chromosome:1081545-1084780	110.58+peptide	ΔORF2+peptide	OK	9.06261	12.0026	0.405345	0.51493	0.36625	0.864634	no
gene:SpnNT_01058	guaC	Chromosome:1084953-1085940	110.58	ΔORF2	OK	267.689	282.296	0.0766529	0.172619	0.75145	0.994748	no
gene:SpnNT_01058	guaC	Chromosome:1084953-1085940	110.58	110.58+peptide	OK	267.689	217.738	-0.297965	-0.662728	0.24055	0.735706	no

gene:SpnNT_01058	guaC	Chromosome:1084953-1085940	ΔORF2	110.58+peptide	OK	282.296	217.738	-0.374618	-0.847059	0.1276	0.541728	no
gene:SpnNT_01058	guaC	Chromosome:1084953-1085940	110.58	ΔORF2+peptide	OK	267.689	202.511	-0.402555	-0.907059	0.10835	0.500854	no
gene:SpnNT_01058	guaC	Chromosome:1084953-1085940	ΔORF2	ΔORF2+peptide	OK	282.296	202.511	-0.479207	-1.0982	0.04945	0.311514	no
gene:SpnNT_01058	guaC	Chromosome:1084953-1085940	110.58+peptide	ΔORF2+peptide	OK	217.738	202.511	-0.104589	-0.236627	0.67005	0.984845	no
gene:SpnNT_01059	SpnNT_01059	Chromosome:1086002-1086076	110.58	ΔORF2	OK	1166.92	1394.39	0.256922	0.174762	0.8459	0.994748	no
gene:SpnNT_01059	SpnNT_01059	Chromosome:1086002-1086076	110.58	110.58+peptide	OK	1166.92	1069.34	-0.125993	-0.0555943	0.86815	0.994748	no
gene:SpnNT_01059	SpnNT_01059	Chromosome:1086002-1086076	ΔORF2	110.58+peptide	OK	1394.39	1069.34	-0.382915	-0.222009	0.75845	0.994748	no
gene:SpnNT_01059	SpnNT_01059	Chromosome:1086002-1086076	110.58	ΔORF2+peptide	OK	1166.92	633.559	-0.881157	-0.599376	0.5626	0.968621	no
gene:SpnNT_01059	SpnNT_01059	Chromosome:1086002-1086076	ΔORF2	ΔORF2+peptide	OK	1394.39	633.559	-1.13808	-188.811	0.43215	0.913334	no
gene:SpnNT_01059	SpnNT_01059	Chromosome:1086002-1086076	110.58+peptide	ΔORF2+peptide	OK	1069.34	633.559	-0.755165	-0.437835	0.5973	0.97629	no
gene:SpnNT_01060	NA	Chromosome:1086471-1088205	110.58	ΔORF2	OK	29.3237	33.4956	0.191901	0.406492	0.471	0.928415	no
gene:SpnNT_01060	NA	Chromosome:1086471-1088205	110.58	110.58+peptide	OK	29.3237	28.5785	-0.0371396	-0.0779754	0.89115	0.994748	no
gene:SpnNT_01060	NA	Chromosome:1086471-1088205	ΔORF2	110.58+peptide	OK	33.4956	28.5785	-0.22904	-0.483838	0.40145	0.894197	no
gene:SpnNT_01060	NA	Chromosome:1086471-1088205	110.58	ΔORF2+peptide	OK	29.3237	36.6669	0.322407	0.684551	0.23155	0.724662	no
gene:SpnNT_01060	NA	Chromosome:1086471-1088205	ΔORF2	ΔORF2+peptide	OK	33.4956	36.6669	0.130506	0.278844	0.63105	0.980887	no
gene:SpnNT_01060	NA	Chromosome:1086471-1088205	110.58+peptide	ΔORF2+peptide	OK	28.5785	36.6669	0.359546	0.761314	0.19315	0.674611	no
gene:SpnNT_01061	rnc	Chromosome:1088432-1094270	110.58	ΔORF2	OK	41.8485	56.1076	0.423019	0.328018	0.5751	0.969538	no
gene:SpnNT_01061	rnc	Chromosome:1088432-1094270	110.58	110.58+peptide	OK	41.8485	46.4644	0.150949	0.105406	0.852	0.994748	no
gene:SpnNT_01061	rnc	Chromosome:1088432-1094270	ΔORF2	110.58+peptide	OK	56.1076	46.4644	-0.27207	-0.191978	0.7365	0.994748	no
gene:SpnNT_01061	rnc	Chromosome:1088432-1094270	110.58	ΔORF2+peptide	OK	41.8485	61.5864	0.557435	0.388457	0.5045	0.945292	no
gene:SpnNT_01061	rnc	Chromosome:1088432-1094270	ΔORF2	ΔORF2+peptide	OK	56.1076	61.5864	0.134416	0.0946494	0.8716	0.994748	no
gene:SpnNT_01061	rnc	Chromosome:1088432-1094270	110.58+peptide	ΔORF2+peptide	OK	46.4644	61.5864	0.406486	0.262139	0.652	0.98163	no
gene:SpnNT_01062	smc	Chromosome:1088432-1094270	110.58	ΔORF2	OK	30.2025	32.6533	0.112559	0.20277	0.7255	0.994748	no
gene:SpnNT_01062	smc	Chromosome:1088432-1094270	110.58	110.58+peptide	OK	30.2025	41.7082	0.465663	0.832673	0.15035	0.592282	no
gene:SpnNT_01062	smc	Chromosome:1088432-1094270	ΔORF2	110.58+peptide	OK	32.6533	41.7082	0.353104	0.630641	0.27735	0.780208	no
gene:SpnNT_01062	smc	Chromosome:1088432-1094270	110.58	ΔORF2+peptide	OK	30.2025	55.3564	0.874081	1.58838	0.0059	0.0681512	no
gene:SpnNT_01062	smc	Chromosome:1088432-1094270	ΔORF2	ΔORF2+peptide	OK	32.6533	55.3564	0.761521	1.38212	0.0182	0.155078	no
gene:SpnNT_01062	smc	Chromosome:1088432-1094270	110.58+peptide	ΔORF2+peptide	OK	41.7082	55.3564	0.408417	0.735696	0.2024	0.6861	no
gene:SpnNT_01063	ybjI	Chromosome:1088432-1094270	110.58	ΔORF2	OK	32.0965	34.2977	0.0956976	0.0689711	0.9036	0.994748	no
gene:SpnNT_01063	ybjI	Chromosome:1088432-1094270	110.58	110.58+peptide	OK	32.0965	52.1619	0.700583	0.539211	0.35215	0.855054	no
gene:SpnNT_01063	ybjI	Chromosome:1088432-1094270	ΔORF2	110.58+peptide	OK	34.2977	52.1619	0.604885	0.474575	0.41595	0.901271	no
gene:SpnNT_01063	ybjI	Chromosome:1088432-1094270	110.58	ΔORF2+peptide	OK	32.0965	70.3994	1.13315	0.879622	0.1175	0.519311	no
gene:SpnNT_01063	ybjI	Chromosome:1088432-1094270	ΔORF2	ΔORF2+peptide	OK	34.2977	70.3994	1.03745	0.821212	0.14235	0.576108	no
gene:SpnNT_01063	ybjI	Chromosome:1088432-1094270	110.58+peptide	ΔORF2+peptide	OK	52.1619	70.3994	0.432565	0.37107	0.50145	0.944784	no
gene:SpnNT_01064	yidA_2	Chromosome:1088432-1094270	110.58	ΔORF2	OK	30.2741	32.453	0.100266	0.0714481	0.9053	0.994748	no
gene:SpnNT_01064	yidA_2	Chromosome:1088432-1094270	110.58	110.58+peptide	OK	30.2741	47.9751	0.6642	0.494224	0.3944	0.889648	no
gene:SpnNT_01064	yidA_2	Chromosome:1088432-1094270	ΔORF2	110.58+peptide	OK	32.453	47.9751	0.563934	0.415674	0.47605	0.930958	no
gene:SpnNT_01064	yidA_2	Chromosome:1088432-1094270	110.58	ΔORF2+peptide	OK	30.2741	47.3155	0.644229	0.435401	0.45315	0.921244	no
gene:SpnNT_01064	yidA_2	Chromosome:1088432-1094270	ΔORF2	ΔORF2+peptide	OK	32.453	47.3155	0.543963	0.364779	0.53215	0.958264	no
gene:SpnNT_01064	yidA_2	Chromosome:1088432-1094270	110.58+peptide	ΔORF2+peptide	OK	47.9751	47.3155	-0.0199717	-0.0139134	0.98095	0.99608	no
gene:SpnNT_01065	ftsY	Chromosome:1094273-1095563	110.58	ΔORF2	OK	33.526	32.3792	-0.0502093	-0.103659	0.85565	0.994748	no
gene:SpnNT_01065	ftsY	Chromosome:1094273-1095563	110.58	110.58+peptide	OK	33.526	48.5565	0.534386	1.1247	0.05475	0.332606	no
gene:SpnNT_01065	ftsY	Chromosome:1094273-1095563	ΔORF2	110.58+peptide	OK	32.3792	48.5565	0.584595	1.22824	0.0353	0.249036	no
gene:SpnNT_01065	ftsY	Chromosome:1094273-1095563	110.58	ΔORF2+peptide	OK	33.526	54.9248	0.712179	1.51147	0.0094	0.0963647	no
gene:SpnNT_01065	ftsY	Chromosome:1094273-1095563	ΔORF2	ΔORF2+peptide	OK	32.3792	54.9248	0.762389	1.61518	0.00505	0.0609063	no

gene:SpnNT_01065	ftsY	Chromosome:1094273-1095563	110.58+peptide	ΔORF2+peptide	OK	48.5565	54.9248	0.177794	0.384391	0.50445	0.945292	no
gene:SpnNT_01066	zwf	Chromosome:1095608-1097096	110.58	ΔORF2	OK	289.25	314.652	0.121442	0.276161	0.62675	0.980887	no
gene:SpnNT_01066	zwf	Chromosome:1095608-1097096	110.58	110.58+peptide	OK	289.25	240.088	-0.268752	-0.610858	0.28965	0.794313	no
gene:SpnNT_01066	zwf	Chromosome:1095608-1097096	ΔORF2	110.58+peptide	OK	314.652	240.088	-0.390194	-0.88828	0.12455	0.533791	no
gene:SpnNT_01066	zwf	Chromosome:1095608-1097096	110.58	ΔORF2+peptide	OK	289.25	232.505	-0.315056	-0.715847	0.2114	0.697158	no
gene:SpnNT_01066	zwf	Chromosome:1095608-1097096	ΔORF2	ΔORF2+peptide	OK	314.652	232.505	-0.436498	-0.993332	0.082	0.428175	no
gene:SpnNT_01066	zwf	Chromosome:1095608-1097096	110.58+peptide	ΔORF2+peptide	OK	240.088	232.505	-0.046304	-0.105324	0.85705	0.994748	no
gene:SpnNT_01067	artM_1	Chromosome:1097244-1100150	110.58	ΔORF2	OK	558.371	600.985	0.106107	0.150721	0.7917	0.994748	no
gene:SpnNT_01067	artM_1	Chromosome:1097244-1100150	110.58	110.58+peptide	OK	558.371	604.165	0.113719	0.159941	0.77905	0.994748	no
gene:SpnNT_01067	artM_1	Chromosome:1097244-1100150	ΔORF2	110.58+peptide	OK	600.985	604.165	0.00761211	0.010818	0.9868	0.997339	no
gene:SpnNT_01067	artM_1	Chromosome:1097244-1100150	110.58	ΔORF2+peptide	OK	558.371	507.701	-0.137244	-0.187308	0.74495	0.994748	no
gene:SpnNT_01067	artM_1	Chromosome:1097244-1100150	ΔORF2	ΔORF2+peptide	OK	600.985	507.701	-0.243351	-0.335386	0.561	0.968621	no
gene:SpnNT_01067	artM_1	Chromosome:1097244-1100150	110.58+peptide	ΔORF2+peptide	OK	604.165	507.701	-0.250963	-0.342665	0.55055	0.964281	no
gene:SpnNT_01068	glnP_2	Chromosome:1097244-1100150	110.58	ΔORF2	OK	188.596	216.217	0.197183	0.315662	0.5785	0.969538	no
gene:SpnNT_01068	glnP_2	Chromosome:1097244-1100150	110.58	110.58+peptide	OK	188.596	172.563	-0.128175	-0.189785	0.73845	0.994748	no
gene:SpnNT_01068	glnP_2	Chromosome:1097244-1100150	ΔORF2	110.58+peptide	OK	216.217	172.563	-0.325357	-0.493058	0.388	0.882381	no
gene:SpnNT_01068	glnP_2	Chromosome:1097244-1100150	110.58	ΔORF2+peptide	OK	188.596	194.344	0.0433158	0.0691916	0.90495	0.994748	no
gene:SpnNT_01068	glnP_2	Chromosome:1097244-1100150	ΔORF2	ΔORF2+peptide	OK	216.217	194.344	-0.153867	-0.252538	0.66145	0.982966	no
gene:SpnNT_01068	glnP_2	Chromosome:1097244-1100150	110.58+peptide	ΔORF2+peptide	OK	172.563	194.344	0.17149	0.259375	0.65105	0.981475	no
gene:SpnNT_01069	NA	Chromosome:1100322-1101276	110.58	ΔORF2	OK	10.2552	12.1735	0.247394	0.422455	0.4687	0.927504	no
gene:SpnNT_01069	NA	Chromosome:1100322-1101276	110.58	110.58+peptide	OK	10.2552	9.57153	-0.099533	-0.166264	0.779	0.994748	no
gene:SpnNT_01069	NA	Chromosome:1100322-1101276	ΔORF2	110.58+peptide	OK	12.1735	9.57153	-0.346927	-0.581879	0.31885	0.825137	no
gene:SpnNT_01069	NA	Chromosome:1100322-1101276	110.58	ΔORF2+peptide	OK	10.2552	13.5736	0.40445	0.687918	0.23995	0.735469	no
gene:SpnNT_01069	NA	Chromosome:1100322-1101276	ΔORF2	ΔORF2+peptide	OK	12.1735	13.5736	0.157056	0.268259	0.6466	0.981002	no
gene:SpnNT_01069	NA	Chromosome:1100322-1101276	110.58+peptide	ΔORF2+peptide	OK	9.57153	13.5736	0.503983	0.842077	0.14915	0.590213	no
gene:SpnNT_01070	uvrB	Chromosome:1101352-1103341	110.58	ΔORF2	OK	35.2085	37.6294	0.0959347	0.210268	0.71245	0.991034	no
gene:SpnNT_01070	uvrB	Chromosome:1101352-1103341	110.58	110.58+peptide	OK	35.2085	28.3268	-0.313758	-0.68122	0.2375	0.732427	no
gene:SpnNT_01070	uvrB	Chromosome:1101352-1103341	ΔORF2	110.58+peptide	OK	37.6294	28.3268	-0.409692	-0.885167	0.12325	0.531129	no
gene:SpnNT_01070	uvrB	Chromosome:1101352-1103341	110.58	ΔORF2+peptide	OK	35.2085	31.635	-0.154404	-0.337852	0.54965	0.964281	no
gene:SpnNT_01070	uvrB	Chromosome:1101352-1103341	ΔORF2	ΔORF2+peptide	OK	37.6294	31.635	-0.250339	-0.545051	0.3342	0.839627	no
gene:SpnNT_01070	uvrB	Chromosome:1101352-1103341	110.58+peptide	ΔORF2+peptide	OK	28.3268	31.635	0.159353	0.34373	0.5419	0.961494	no
gene:SpnNT_01071	NA	Chromosome:1103386-1104389	110.58	ΔORF2	OK	24.769	31.5847	0.350689	0.481718	0.4023	0.894197	no
gene:SpnNT_01071	NA	Chromosome:1103386-1104389	110.58	110.58+peptide	OK	24.769	21.2456	-0.221372	-0.298537	0.60765	0.978234	no
gene:SpnNT_01071	NA	Chromosome:1103386-1104389	ΔORF2	110.58+peptide	OK	31.5847	21.2456	-0.572061	-0.77399	0.17975	0.64657	no
gene:SpnNT_01071	NA	Chromosome:1103386-1104389	110.58	ΔORF2+peptide	OK	24.769	31.1433	0.330386	0.452908	0.4343	0.913666	no
gene:SpnNT_01071	NA	Chromosome:1103386-1104389	ΔORF2	ΔORF2+peptide	OK	31.5847	31.1433	-0.020303	-0.0279264	0.96	0.994855	no
gene:SpnNT_01071	NA	Chromosome:1103386-1104389	110.58+peptide	ΔORF2+peptide	OK	21.2456	31.1433	0.551758	0.74505	0.20475	0.689595	no
gene:SpnNT_01072	yjaB	Chromosome:1103386-1104389	110.58	ΔORF2	OK	20.5418	23.36	0.185478	0.221528	0.69835	0.990209	no
gene:SpnNT_01072	yjaB	Chromosome:1103386-1104389	110.58	110.58+peptide	OK	20.5418	16.424	-0.322751	-0.374323	0.5099	0.948082	no
gene:SpnNT_01072	yjaB	Chromosome:1103386-1104389	ΔORF2	110.58+peptide	OK	23.36	16.424	-0.508229	-0.581832	0.32025	0.825861	no
gene:SpnNT_01072	yjaB	Chromosome:1103386-1104389	110.58	ΔORF2+peptide	OK	20.5418	26.9796	0.393308	0.486885	0.3948	0.889826	no
gene:SpnNT_01072	yjaB	Chromosome:1103386-1104389	ΔORF2	ΔORF2+peptide	OK	23.36	26.9796	0.20783	0.253506	0.6677	0.984224	no
gene:SpnNT_01072	yjaB	Chromosome:1103386-1104389	110.58+peptide	ΔORF2+peptide	OK	16.424	26.9796	0.71606	0.847104	0.1451	0.580488	no
gene:SpnNT_01073	mutX_2	Chromosome:1104441-1105421	110.58	ΔORF2	OK	51.8316	56.6535	0.128331	0.138515	0.8086	0.994748	no
gene:SpnNT_01073	mutX_2	Chromosome:1104441-1105421	110.58	110.58+peptide	OK	51.8316	52.5473	0.0197848	0.020679	0.9713	0.99536	no

gene:SpnNT_01073	mutX_2	Chromosome:1104441-1105421	ΔORF2	110.58+peptide	OK	56.6535	52.5473	-0.108547	-0.118275	0.83635	0.994748	no
gene:SpnNT_01073	mutX_2	Chromosome:1104441-1105421	110.58	ΔORF2+peptide	OK	51.8316	51.6967	-0.0037594	-0.00399969	0.99455	0.999116	no
gene:SpnNT_01073	mutX_2	Chromosome:1104441-1105421	ΔORF2	ΔORF2+peptide	OK	56.6535	51.6967	-0.132091	-0.146737	0.80175	0.994748	no
gene:SpnNT_01073	mutX_2	Chromosome:1104441-1105421	110.58+peptide	ΔORF2+peptide	OK	52.5473	51.6967	-0.0235442	-0.0252805	0.9624	0.994855	no
gene:SpnNT_01074	niaR	Chromosome:1104441-1105421	110.58	ΔORF2	OK	123.851	123.331	-0.00607596	-0.0111593	0.98385	0.996246	no
gene:SpnNT_01074	niaR	Chromosome:1104441-1105421	110.58	110.58+peptide	OK	123.851	127.85	0.0458386	0.0841129	0.8814	0.994748	no
gene:SpnNT_01074	niaR	Chromosome:1104441-1105421	ΔORF2	110.58+peptide	OK	123.331	127.85	0.0519146	0.09472	0.86705	0.994748	no
gene:SpnNT_01074	niaR	Chromosome:1104441-1105421	110.58	ΔORF2+peptide	OK	123.851	107.757	-0.200825	-0.363906	0.5245	0.954973	no
gene:SpnNT_01074	niaR	Chromosome:1104441-1105421	ΔORF2	ΔORF2+peptide	OK	123.331	107.757	-0.194749	-0.350937	0.54175	0.961494	no
gene:SpnNT_01074	niaR	Chromosome:1104441-1105421	110.58+peptide	ΔORF2+peptide	OK	127.85	107.757	-0.246664	-0.4441	0.4417	0.917253	no
gene:SpnNT_01075	niaX	Chromosome:1105432-1105960	110.58	ΔORF2	OK	37.2153	46.3404	0.316375	0.576215	0.3155	0.821264	no
gene:SpnNT_01075	niaX	Chromosome:1105432-1105960	110.58	110.58+peptide	OK	37.2153	37.4272	0.0081921	0.0145973	0.9788	0.995765	no
gene:SpnNT_01075	niaX	Chromosome:1105432-1105960	ΔORF2	110.58+peptide	OK	46.3404	37.4272	-0.308183	-0.556574	0.3394	0.844054	no
gene:SpnNT_01075	niaX	Chromosome:1105432-1105960	110.58	ΔORF2+peptide	OK	37.2153	40.3602	0.117038	0.210899	0.71335	0.991272	no
gene:SpnNT_01075	niaX	Chromosome:1105432-1105960	ΔORF2	ΔORF2+peptide	OK	46.3404	40.3602	-0.199337	-0.364171	0.52845	0.956161	no
gene:SpnNT_01075	niaX	Chromosome:1105432-1105960	110.58+peptide	ΔORF2+peptide	OK	37.4272	40.3602	0.108846	0.194522	0.7384	0.994748	no
gene:SpnNT_01076	panT	Chromosome:1106241-1107340	110.58	ΔORF2	OK	186.746	267.875	0.520484	1.07704	0.0655	0.372235	no
gene:SpnNT_01076	panT	Chromosome:1106241-1107340	110.58	110.58+peptide	OK	186.746	126.378	-0.563332	-1.14983	0.0487	0.308567	no
gene:SpnNT_01076	panT	Chromosome:1106241-1107340	ΔORF2	110.58+peptide	OK	267.875	126.378	-1.08382	-2.20508	0.00015	0.00355289	yes
gene:SpnNT_01076	panT	Chromosome:1106241-1107340	110.58	ΔORF2+peptide	OK	186.746	118.403	-0.657372	-1.32577	0.0242	0.192924	no
gene:SpnNT_01076	panT	Chromosome:1106241-1107340	ΔORF2	ΔORF2+peptide	OK	267.875	118.403	-1.17786	-2.36801	1.00E-04	0.0025332	yes
gene:SpnNT_01076	panT	Chromosome:1106241-1107340	110.58+peptide	ΔORF2+peptide	OK	126.378	118.403	-0.0940396	-0.186627	0.73835	0.994748	no
gene:SpnNT_01077	coaBC_1	Chromosome:1106241-1107340	110.58	ΔORF2	OK	30.7677	34.5172	0.165897	0.109717	0.8405	0.994748	no
gene:SpnNT_01077	coaBC_1	Chromosome:1106241-1107340	110.58	110.58+peptide	OK	30.7677	19.1893	-0.681118	-0.478688	0.41115	0.898655	no
gene:SpnNT_01077	coaBC_1	Chromosome:1106241-1107340	ΔORF2	110.58+peptide	OK	34.5172	19.1893	-0.847016	-0.533059	0.3426	0.846557	no
gene:SpnNT_01077	coaBC_1	Chromosome:1106241-1107340	110.58	ΔORF2+peptide	OK	30.7677	23.5219	-0.387416	-0.30447	0.5991	0.97629	no
gene:SpnNT_01077	coaBC_1	Chromosome:1106241-1107340	ΔORF2	ΔORF2+peptide	OK	34.5172	23.5219	-0.553313	-0.380079	0.48455	0.936747	no
gene:SpnNT_01077	coaBC_1	Chromosome:1106241-1107340	110.58+peptide	ΔORF2+peptide	OK	19.1893	23.5219	0.293703	0.215492	0.706	0.990422	no
gene:SpnNT_01078	coaBC_2	Chromosome:1107351-1108041	110.58	ΔORF2	OK	125.392	142.176	0.181231	0.397354	0.4884	0.939082	no
gene:SpnNT_01078	coaBC_2	Chromosome:1107351-1108041	110.58	110.58+peptide	OK	125.392	82.6888	-0.600679	-1.28228	0.02655	0.205055	no
gene:SpnNT_01078	coaBC_2	Chromosome:1107351-1108041	ΔORF2	110.58+peptide	OK	142.176	82.6888	-0.78191	-1.67879	0.00455	0.0556951	no
gene:SpnNT_01078	coaBC_2	Chromosome:1107351-1108041	110.58	ΔORF2+peptide	OK	125.392	90.0568	-0.477536	-1.02401	0.06965	0.387759	no
gene:SpnNT_01078	coaBC_2	Chromosome:1107351-1108041	ΔORF2	ΔORF2+peptide	OK	142.176	90.0568	-0.658767	-1.42086	0.0147	0.132209	no
gene:SpnNT_01078	coaBC_2	Chromosome:1107351-1108041	110.58+peptide	ΔORF2+peptide	OK	82.6888	90.0568	0.123143	0.258816	0.64845	0.981391	no
gene:SpnNT_01079	fhs1	Chromosome:1108271-1109942	110.58	ΔORF2	OK	167.968	176.686	0.073004	0.163929	0.7744	0.994748	no
gene:SpnNT_01079	fhs1	Chromosome:1108271-1109942	110.58	110.58+peptide	OK	167.968	45.4634	-1.88541	-4.1678	5.00E-05	0.0013612	yes
gene:SpnNT_01079	fhs1	Chromosome:1108271-1109942	ΔORF2	110.58+peptide	OK	176.686	45.4634	-1.95841	-4.37604	5.00E-05	0.0013612	yes
gene:SpnNT_01079	fhs1	Chromosome:1108271-1109942	110.58	ΔORF2+peptide	OK	167.968	44.3548	-1.92102	-4.22963	5.00E-05	0.0013612	yes
gene:SpnNT_01079	fhs1	Chromosome:1108271-1109942	ΔORF2	ΔORF2+peptide	OK	176.686	44.3548	-1.99403	-4.4375	5.00E-05	0.0013612	yes
gene:SpnNT_01079	fhs1	Chromosome:1108271-1109942	110.58+peptide	ΔORF2+peptide	OK	45.4634	44.3548	-0.0356141	-0.0780443	0.89	0.994748	no
gene:SpnNT_01080	yfhQ	Chromosome:1110269-1111445	110.58	ΔORF2	OK	69.6274	81.0087	0.218422	0.483455	0.403	0.894399	no
gene:SpnNT_01080	yfhQ	Chromosome:1110269-1111445	110.58	110.58+peptide	OK	69.6274	54.243	-0.360218	-0.783014	0.17825	0.644885	no
gene:SpnNT_01080	yfhQ	Chromosome:1110269-1111445	ΔORF2	110.58+peptide	OK	81.0087	54.243	-0.57864	-1.26754	0.02825	0.214152	no
gene:SpnNT_01080	yfhQ	Chromosome:1110269-1111445	110.58	ΔORF2+peptide	OK	69.6274	74.3337	0.0943622	0.205528	0.72635	0.994748	no
gene:SpnNT_01080	yfhQ	Chromosome:1110269-1111445	ΔORF2	ΔORF2+peptide	OK	81.0087	74.3337	-0.12406	-0.272313	0.63105	0.980887	no

gene:SpnNT_01080	yfhQ	Chromosome:1110269-1111445	110.58+peptide	ΔORF2+peptide	OK	54.243	74.3337	0.45458	0.980211	0.09285	0.460112	no
gene:SpnNT_01081	yycF	Chromosome:1111500-1113547	110.58	ΔORF2	OK	189.331	217.295	0.198748	0.272869	0.6339	0.980887	no
gene:SpnNT_01081	yycF	Chromosome:1111500-1113547	110.58	110.58+peptide	OK	189.331	179.706	-0.075269	-0.102939	0.85635	0.994748	no
gene:SpnNT_01081	yycF	Chromosome:1111500-1113547	ΔORF2	110.58+peptide	OK	217.295	179.706	-0.274017	-0.371176	0.5213	0.954832	no
gene:SpnNT_01081	yycF	Chromosome:1111500-1113547	110.58	ΔORF2+peptide	OK	189.331	184.706	-0.0356773	-0.0473513	0.9287	0.994748	no
gene:SpnNT_01081	yycF	Chromosome:1111500-1113547	ΔORF2	ΔORF2+peptide	OK	217.295	184.706	-0.234425	-0.308335	0.58985	0.974567	no
gene:SpnNT_01081	yycF	Chromosome:1111500-1113547	110.58+peptide	ΔORF2+peptide	OK	179.706	184.706	0.0395917	0.0518886	0.92605	0.994748	no
gene:SpnNT_01082	yycG	Chromosome:1111500-1113547	110.58	ΔORF2	OK	142.991	160.219	0.164118	0.29728	0.6117	0.979429	no
gene:SpnNT_01082	yycG	Chromosome:1111500-1113547	110.58	110.58+peptide	OK	142.991	144.965	0.01978	0.0356802	0.9523	0.994855	no
gene:SpnNT_01082	yycG	Chromosome:1111500-1113547	ΔORF2	110.58+peptide	OK	160.219	144.965	-0.144338	-0.262187	0.64595	0.980887	no
gene:SpnNT_01082	yycG	Chromosome:1111500-1113547	110.58	ΔORF2+peptide	OK	142.991	169.377	0.244305	0.451394	0.433	0.913334	no
gene:SpnNT_01082	yycG	Chromosome:1111500-1113547	ΔORF2	ΔORF2+peptide	OK	160.219	169.377	0.0801871	0.149248	0.7992	0.994748	no
gene:SpnNT_01082	yycG	Chromosome:1111500-1113547	110.58+peptide	ΔORF2+peptide	OK	144.965	169.377	0.224525	0.416063	0.4684	0.927504	no
gene:SpnNT_01083	yycJ	Chromosome:1113548-1114358	110.58	ΔORF2	OK	163.649	175.976	0.104771	0.235494	0.67905	0.98542	no
gene:SpnNT_01083	yycJ	Chromosome:1113548-1114358	110.58	110.58+peptide	OK	163.649	186.142	0.185796	0.415844	0.45705	0.921645	no
gene:SpnNT_01083	yycJ	Chromosome:1113548-1114358	ΔORF2	110.58+peptide	OK	175.976	186.142	0.0810249	0.182041	0.7425	0.994748	no
gene:SpnNT_01083	yycJ	Chromosome:1113548-1114358	110.58	ΔORF2+peptide	OK	163.649	201.293	0.29869	0.668708	0.24075	0.735706	no
gene:SpnNT_01083	yycJ	Chromosome:1113548-1114358	ΔORF2	ΔORF2+peptide	OK	175.976	201.293	0.19392	0.435807	0.4442	0.917631	no
gene:SpnNT_01083	yycJ	Chromosome:1113548-1114358	110.58+peptide	ΔORF2+peptide	OK	186.142	201.293	0.112895	0.252641	0.6569	0.982966	no
gene:SpnNT_01084	NA	Chromosome:1114448-1114947	110.58	ΔORF2	OK	43.7963	65.7043	0.585178	0.604314	0.30055	0.806342	no
gene:SpnNT_01084	NA	Chromosome:1114448-1114947	110.58	110.58+peptide	OK	43.7963	39.3732	-0.153596	-0.163276	0.7807	0.994748	no
gene:SpnNT_01084	NA	Chromosome:1114448-1114947	ΔORF2	110.58+peptide	OK	65.7043	39.3732	-0.738774	-0.750873	0.20115	0.683983	no
gene:SpnNT_01084	NA	Chromosome:1114448-1114947	110.58	ΔORF2+peptide	OK	43.7963	44.5216	0.0236983	0.0217363	0.97015	0.99536	no
gene:SpnNT_01084	NA	Chromosome:1114448-1114947	ΔORF2	ΔORF2+peptide	OK	65.7043	44.5216	-0.56148	-0.497887	0.3958	0.890245	no
gene:SpnNT_01084	NA	Chromosome:1114448-1114947	110.58+peptide	ΔORF2+peptide	OK	39.3732	44.5216	0.177294	0.160578	0.776	0.994748	no
gene:SpnNT_01085	NA	Chromosome:1114448-1114947	110.58	ΔORF2	OK	39.752	57.8336	0.540882	0.590462	0.3066	0.81251	no
gene:SpnNT_01085	NA	Chromosome:1114448-1114947	110.58	110.58+peptide	OK	39.752	36.8278	-0.110232	-0.128312	0.82695	0.994748	no
gene:SpnNT_01085	NA	Chromosome:1114448-1114947	ΔORF2	110.58+peptide	OK	57.8336	36.8278	-0.651114	-0.724917	0.2105	0.696806	no
gene:SpnNT_01085	NA	Chromosome:1114448-1114947	110.58	ΔORF2+peptide	OK	39.752	42.5525	0.0982174	0.101138	0.8631	0.994748	no
gene:SpnNT_01085	NA	Chromosome:1114448-1114947	ΔORF2	ΔORF2+peptide	OK	57.8336	42.5525	-0.442665	-0.440079	0.45055	0.920951	no
gene:SpnNT_01085	NA	Chromosome:1114448-1114947	110.58+peptide	ΔORF2+peptide	OK	36.8278	42.5525	0.208449	0.218429	0.70595	0.990422	no
gene:SpnNT_01086	kcsA	Chromosome:1114993-1115770	110.58	ΔORF2	OK	30.0657	41.2754	0.457164	0.859105	0.14285	0.576585	no
gene:SpnNT_01086	kcsA	Chromosome:1114993-1115770	110.58	110.58+peptide	OK	30.0657	26.2495	-0.195826	-0.360291	0.53685	0.959314	no
gene:SpnNT_01086	kcsA	Chromosome:1114993-1115770	ΔORF2	110.58+peptide	OK	41.2754	26.2495	-0.65299	-1.20814	0.04035	0.272783	no
gene:SpnNT_01086	kcsA	Chromosome:1114993-1115770	110.58	ΔORF2+peptide	OK	30.0657	31.9669	0.0884592	0.165623	0.7747	0.994748	no
gene:SpnNT_01086	kcsA	Chromosome:1114993-1115770	ΔORF2	ΔORF2+peptide	OK	41.2754	31.9669	-0.368705	-0.694341	0.23215	0.724785	no
gene:SpnNT_01086	kcsA	Chromosome:1114993-1115770	110.58+peptide	ΔORF2+peptide	OK	26.2495	31.9669	0.284285	0.524105	0.36555	0.864634	no
gene:SpnNT_01087	ldh	Chromosome:1116025-1117012	110.58	ΔORF2	OK	1152.37	1183.05	0.037898	0.0821176	0.88295	0.994748	no
gene:SpnNT_01087	ldh	Chromosome:1116025-1117012	110.58	110.58+peptide	OK	1152.37	892.484	-0.368711	-0.810548	0.15995	0.611379	no
gene:SpnNT_01087	ldh	Chromosome:1116025-1117012	ΔORF2	110.58+peptide	OK	1183.05	892.484	-0.406609	-0.892276	0.1184	0.521357	no
gene:SpnNT_01087	ldh	Chromosome:1116025-1117012	110.58	ΔORF2+peptide	OK	1152.37	880.429	-0.388332	-0.8519	0.1388	0.568634	no
gene:SpnNT_01087	ldh	Chromosome:1116025-1117012	ΔORF2	ΔORF2+peptide	OK	1183.05	880.429	-0.42623	-0.933388	0.1019	0.484293	no
gene:SpnNT_01087	ldh	Chromosome:1116025-1117012	110.58+peptide	ΔORF2+peptide	OK	892.484	880.429	-0.0196202	-0.0436046	0.94045	0.994855	no
gene:SpnNT_01088	gyrA	Chromosome:1117205-1120417	110.58	ΔORF2	OK	179.971	185.374	0.042671	0.0797642	0.88905	0.994748	no
gene:SpnNT_01088	gyrA	Chromosome:1117205-1120417	110.58	110.58+peptide	OK	179.971	173.895	-0.0495501	-0.0945326	0.8694	0.994748	no

gene:SpnNT_01088	gyrA	Chromosome:1117205-1120417	ΔORF2	110.58+peptide	OK	185.374	173.895	-0.0922212	-0.167054	0.76655	0.994748	no
gene:SpnNT_01088	gyrA	Chromosome:1117205-1120417	110.58	ΔORF2+peptide	OK	179.971	188.52	0.0669481	0.130985	0.8212	0.994748	no
gene:SpnNT_01088	gyrA	Chromosome:1117205-1120417	ΔORF2	ΔORF2+peptide	OK	185.374	188.52	0.0242771	0.0449848	0.93605	0.994855	no
gene:SpnNT_01088	gyrA	Chromosome:1117205-1120417	110.58+peptide	ΔORF2+peptide	OK	173.895	188.52	0.116498	0.220239	0.70345	0.990367	no
gene:SpnNT_01089	NA	Chromosome:1117205-1120417	110.58	ΔORF2	OK	219.046	277.428	0.340875	0.333261	0.5655	0.968621	no
gene:SpnNT_01089	NA	Chromosome:1117205-1120417	110.58	110.58+peptide	OK	219.046	246.191	0.168545	0.171473	0.7674	0.994748	no
gene:SpnNT_01089	NA	Chromosome:1117205-1120417	ΔORF2	110.58+peptide	OK	277.428	246.191	-0.17233	-0.163626	0.7751	0.994748	no
gene:SpnNT_01089	NA	Chromosome:1117205-1120417	110.58	ΔORF2+peptide	OK	219.046	284.422	0.376796	0.409738	0.4782	0.933077	no
gene:SpnNT_01089	NA	Chromosome:1117205-1120417	ΔORF2	ΔORF2+peptide	OK	277.428	284.422	0.0359208	0.0361245	0.9492	0.994855	no
gene:SpnNT_01089	NA	Chromosome:1117205-1120417	110.58+peptide	ΔORF2+peptide	OK	246.191	284.422	0.208251	0.218466	0.70505	0.990422	no
gene:SpnNT_01090	NA	Chromosome:1120660-1121457	110.58	ΔORF2	OK	13.1051	11.911	-0.137837	-0.069227	0.9044	0.994748	no
gene:SpnNT_01090	NA	Chromosome:1120660-1121457	110.58	110.58+peptide	OK	13.1051	21.7903	0.733555	0.373714	0.59375	0.975539	no
gene:SpnNT_01090	NA	Chromosome:1120660-1121457	ΔORF2	110.58+peptide	OK	11.911	21.7903	0.871392	0.457479	0.5019	0.944784	no
gene:SpnNT_01090	NA	Chromosome:1120660-1121457	110.58	ΔORF2+peptide	OK	13.1051	21.5593	0.718177	0.445009	0.6637	0.982966	no
gene:SpnNT_01090	NA	Chromosome:1120660-1121457	ΔORF2	ΔORF2+peptide	OK	11.911	21.5593	0.856014	0.554898	0.5857	0.972529	no
gene:SpnNT_01090	NA	Chromosome:1120660-1121457	110.58+peptide	ΔORF2+peptide	OK	21.7903	21.5593	-0.0153778	-0.0102106	0.98195	0.996246	no
gene:SpnNT_01091	NA	Chromosome:1120660-1121457	110.58	ΔORF2	OK	17.4324	16.185	-0.107114	-0.168852	0.7647	0.994748	no
gene:SpnNT_01091	NA	Chromosome:1120660-1121457	110.58	110.58+peptide	OK	17.4324	30.1861	0.792117	1.26143	0.0322	0.233699	no
gene:SpnNT_01091	NA	Chromosome:1120660-1121457	ΔORF2	110.58+peptide	OK	16.185	30.1861	0.899232	1.42274	0.0154	0.137003	no
gene:SpnNT_01091	NA	Chromosome:1120660-1121457	110.58	ΔORF2+peptide	OK	17.4324	36.0856	1.04965	1.65671	0.0035	0.0454723	yes
gene:SpnNT_01091	NA	Chromosome:1120660-1121457	ΔORF2	ΔORF2+peptide	OK	16.185	36.0856	1.15677	1.81416	0.00175	0.0265167	yes
gene:SpnNT_01091	NA	Chromosome:1120660-1121457	110.58+peptide	ΔORF2+peptide	OK	30.1861	36.0856	0.257538	0.407982	0.477	0.932178	no
gene:SpnNT_01092	NA	Chromosome:1121604-1121727	110.58	ΔORF2	NOTEST	0	7.45818	Inf	0	1	1	no
gene:SpnNT_01092	NA	Chromosome:1121604-1121727	110.58	110.58+peptide	NOTEST	0	6.22855	Inf	0	1	1	no
gene:SpnNT_01092	NA	Chromosome:1121604-1121727	ΔORF2	110.58+peptide	NOTEST	7.45818	6.22855	-0.259925	0	1	1	no
gene:SpnNT_01092	NA	Chromosome:1121604-1121727	110.58	ΔORF2+peptide	NOTEST	0	0	0	0	1	1	no
gene:SpnNT_01092	NA	Chromosome:1121604-1121727	ΔORF2	ΔORF2+peptide	NOTEST	7.45818	0	#NAME?	0	1	1	no
gene:SpnNT_01092	NA	Chromosome:1121604-1121727	110.58+peptide	ΔORF2+peptide	NOTEST	6.22855	0	#NAME?	0	1	1	no
gene:SpnNT_01093	mdeA	Chromosome:1121748-1122864	110.58	ΔORF2	OK	8.4087	7.65463	-0.135551	-0.226549	0.68725	0.98828	no
gene:SpnNT_01093	mdeA	Chromosome:1121748-1122864	110.58	110.58+peptide	OK	8.4087	4.19166	-1.00436	-1.58566	0.00565	0.0658431	no
gene:SpnNT_01093	mdeA	Chromosome:1121748-1122864	ΔORF2	110.58+peptide	OK	7.65463	4.19166	-0.86881	-1.35652	0.01715	0.148152	no
gene:SpnNT_01093	mdeA	Chromosome:1121748-1122864	110.58	ΔORF2+peptide	OK	8.4087	4.16995	-1.01185	-1.61723	0.0052	0.0621443	no
gene:SpnNT_01093	mdeA	Chromosome:1121748-1122864	ΔORF2	ΔORF2+peptide	OK	7.65463	4.16995	-0.876303	-1.38474	0.01485	0.133284	no
gene:SpnNT_01093	mdeA	Chromosome:1121748-1122864	110.58+peptide	ΔORF2+peptide	OK	4.19166	4.16995	-0.00749287	-0.0112491	0.98935	0.997703	no
gene:SpnNT_01094	NA	Chromosome:1122959-1124234	110.58	ΔORF2	OK	33.797	40.9297	0.276254	0.57509	0.30665	0.81251	no
gene:SpnNT_01094	NA	Chromosome:1122959-1124234	110.58	110.58+peptide	OK	33.797	35.1662	0.0572954	0.118084	0.8347	0.994748	no
gene:SpnNT_01094	NA	Chromosome:1122959-1124234	ΔORF2	110.58+peptide	OK	40.9297	35.1662	-0.218959	-0.456919	0.42555	0.908126	no
gene:SpnNT_01094	NA	Chromosome:1122959-1124234	110.58	ΔORF2+peptide	OK	33.797	37.772	0.160426	0.331799	0.56455	0.968621	no
gene:SpnNT_01094	NA	Chromosome:1122959-1124234	ΔORF2	ΔORF2+peptide	OK	40.9297	37.772	-0.115829	-0.242584	0.6722	0.984845	no
gene:SpnNT_01094	NA	Chromosome:1122959-1124234	110.58+peptide	ΔORF2+peptide	OK	35.1662	37.772	0.10313	0.213808	0.7034	0.990367	no
gene:SpnNT_01095	truB	Chromosome:1124243-1125122	110.58	ΔORF2	OK	47.849	43.6116	-0.133778	-0.269845	0.6418	0.980887	no
gene:SpnNT_01095	truB	Chromosome:1124243-1125122	110.58	110.58+peptide	OK	47.849	36.9897	-0.371365	-0.743652	0.19375	0.674905	no
gene:SpnNT_01095	truB	Chromosome:1124243-1125122	ΔORF2	110.58+peptide	OK	43.6116	36.9897	-0.237587	-0.474275	0.40195	0.894197	no
gene:SpnNT_01095	truB	Chromosome:1124243-1125122	110.58	ΔORF2+peptide	OK	47.849	42.1203	-0.183972	-0.37039	0.5167	0.953204	no
gene:SpnNT_01095	truB	Chromosome:1124243-1125122	ΔORF2	ΔORF2+peptide	OK	43.6116	42.1203	-0.0501942	-0.100736	0.85755	0.994748	no

gene:SpnNT_01095	truB	Chromosome:1124243-1125122	110.58+peptide	ΔORF2+peptide	OK	36.9897	42.1203	0.187393	0.373383	0.5073	0.946373	no
gene:SpnNT_01096	NA	Chromosome:1125270-1125665	110.58	ΔORF2	OK	0	30.5349	Inf	NA	0.2057	0.690314	no
gene:SpnNT_01096	NA	Chromosome:1125270-1125665	110.58	110.58+peptide	NOTEST	0	27.026	Inf	0	1	1	no
gene:SpnNT_01096	NA	Chromosome:1125270-1125665	ΔORF2	110.58+peptide	OK	30.5349	27.026	-0.176111	-0.378268	0.8153	0.994748	no
gene:SpnNT_01096	NA	Chromosome:1125270-1125665	110.58	ΔORF2+peptide	NOTEST	0	0	0	0	1	1	no
gene:SpnNT_01096	NA	Chromosome:1125270-1125665	ΔORF2	ΔORF2+peptide	OK	30.5349	0	#NAME?	NA	0.20625	0.691266	no
gene:SpnNT_01096	NA	Chromosome:1125270-1125665	110.58+peptide	ΔORF2+peptide	NOTEST	27.026	0	#NAME?	0	1	1	no
gene:SpnNT_01097	NA	Chromosome:1125270-1125665	110.58	ΔORF2	OK	316.196	344.9	0.125359	0.255661	0.64885	0.981391	no
gene:SpnNT_01097	NA	Chromosome:1125270-1125665	110.58	110.58+peptide	OK	316.196	352.37	0.156272	0.32401	0.56545	0.968621	no
gene:SpnNT_01097	NA	Chromosome:1125270-1125665	ΔORF2	110.58+peptide	OK	344.9	352.37	0.0309129	0.064198	0.91045	0.994748	no
gene:SpnNT_01097	NA	Chromosome:1125270-1125665	110.58	ΔORF2+peptide	OK	316.196	385.843	0.287194	0.586238	0.31375	0.819635	no
gene:SpnNT_01097	NA	Chromosome:1125270-1125665	ΔORF2	ΔORF2+peptide	OK	344.9	385.843	0.161835	0.330867	0.566	0.968621	no
gene:SpnNT_01097	NA	Chromosome:1125270-1125665	110.58+peptide	ΔORF2+peptide	OK	352.37	385.843	0.130922	0.272144	0.6377	0.980887	no
gene:SpnNT_01098	udk	Chromosome:1125772-1126411	110.58	ΔORF2	OK	177.633	168.173	-0.078952	-0.173041	0.764	0.994748	no
gene:SpnNT_01098	udk	Chromosome:1125772-1126411	110.58	110.58+peptide	OK	177.633	331.094	0.898342	1.99623	0.00065	0.0118958	yes
gene:SpnNT_01098	udk	Chromosome:1125772-1126411	ΔORF2	110.58+peptide	OK	168.173	331.094	0.977294	2.17178	2.00E-04	0.00450928	yes
gene:SpnNT_01098	udk	Chromosome:1125772-1126411	110.58	ΔORF2+peptide	OK	177.633	296.938	0.741264	1.65045	0.00415	0.0519126	no
gene:SpnNT_01098	udk	Chromosome:1125772-1126411	ΔORF2	ΔORF2+peptide	OK	168.173	296.938	0.820216	1.82633	0.00135	0.021343	yes
gene:SpnNT_01098	udk	Chromosome:1125772-1126411	110.58+peptide	ΔORF2+peptide	OK	331.094	296.938	-0.157079	-0.35477	0.5308	0.957488	no
gene:SpnNT_01099	xseA	Chromosome:1126538-1130173	110.58	ΔORF2	OK	81.4468	87.7384	0.107351	0.166643	0.7679	0.994748	no
gene:SpnNT_01099	xseA	Chromosome:1126538-1130173	110.58	110.58+peptide	OK	81.4468	92.2243	0.179288	0.267481	0.64065	0.980887	no
gene:SpnNT_01099	xseA	Chromosome:1126538-1130173	ΔORF2	110.58+peptide	OK	87.7384	92.2243	0.0719375	0.109546	0.8488	0.994748	no
gene:SpnNT_01099	xseA	Chromosome:1126538-1130173	110.58	ΔORF2+peptide	OK	81.4468	100.654	0.30548	0.453884	0.42995	0.91188	no
gene:SpnNT_01099	xseA	Chromosome:1126538-1130173	ΔORF2	ΔORF2+peptide	OK	87.7384	100.654	0.198129	0.300425	0.5995	0.976494	no
gene:SpnNT_01099	xseA	Chromosome:1126538-1130173	110.58+peptide	ΔORF2+peptide	OK	92.2243	100.654	0.126191	0.184221	0.75605	0.994748	no
gene:SpnNT_01100	xseB	Chromosome:1126538-1130173	110.58	ΔORF2	OK	233.326	171.798	-0.441639	-0.198921	0.78445	0.994748	no
gene:SpnNT_01100	xseB	Chromosome:1126538-1130173	110.58	110.58+peptide	OK	233.326	259.46	0.153165	0.0719825	0.9264	0.994748	no
gene:SpnNT_01100	xseB	Chromosome:1126538-1130173	ΔORF2	110.58+peptide	OK	171.798	259.46	0.594804	0.258279	0.7707	0.994748	no
gene:SpnNT_01100	xseB	Chromosome:1126538-1130173	110.58	ΔORF2+peptide	OK	233.326	196.66	-0.246644	-0.0964406	0.87465	0.994748	no
gene:SpnNT_01100	xseB	Chromosome:1126538-1130173	ΔORF2	ΔORF2+peptide	OK	171.798	196.66	0.194996	0.0720889	0.91255	0.994748	no
gene:SpnNT_01100	xseB	Chromosome:1126538-1130173	110.58+peptide	ΔORF2+peptide	OK	259.46	196.66	-0.399809	-0.152039	0.8379	0.994748	no
gene:SpnNT_01101	NA	Chromosome:1126538-1130173	110.58	ΔORF2	OK	75.9533	70.2574	-0.112461	-0.125303	0.82735	0.994748	no
gene:SpnNT_01101	NA	Chromosome:1126538-1130173	110.58	110.58+peptide	OK	75.9533	97.8381	0.365285	0.415059	0.46925	0.927613	no
gene:SpnNT_01101	NA	Chromosome:1126538-1130173	ΔORF2	110.58+peptide	OK	70.2574	97.8381	0.477746	0.537711	0.34955	0.853357	no
gene:SpnNT_01101	NA	Chromosome:1126538-1130173	110.58	ΔORF2+peptide	OK	75.9533	107.251	0.497806	0.56988	0.3224	0.828116	no
gene:SpnNT_01101	NA	Chromosome:1126538-1130173	ΔORF2	ΔORF2+peptide	OK	70.2574	107.251	0.610267	0.691919	0.2318	0.724662	no
gene:SpnNT_01101	NA	Chromosome:1126538-1130173	110.58+peptide	ΔORF2+peptide	OK	97.8381	107.251	0.132522	0.153338	0.7919	0.994748	no
gene:SpnNT_01102	tlyA	Chromosome:1126538-1130173	110.58	ΔORF2	OK	63.2397	59.6377	-0.0846061	-0.0810851	0.8888	0.994748	no
gene:SpnNT_01102	tlyA	Chromosome:1126538-1130173	110.58	110.58+peptide	OK	63.2397	94.8608	0.584982	0.590497	0.30345	0.809137	no
gene:SpnNT_01102	tlyA	Chromosome:1126538-1130173	ΔORF2	110.58+peptide	OK	59.6377	94.8608	0.669588	0.687721	0.232	0.724662	no
gene:SpnNT_01102	tlyA	Chromosome:1126538-1130173	110.58	ΔORF2+peptide	OK	63.2397	97.4549	0.623905	0.620493	0.2818	0.784843	no
gene:SpnNT_01102	tlyA	Chromosome:1126538-1130173	ΔORF2	ΔORF2+peptide	OK	59.6377	97.4549	0.708511	0.71659	0.2131	0.698345	no
gene:SpnNT_01102	tlyA	Chromosome:1126538-1130173	110.58+peptide	ΔORF2+peptide	OK	94.8608	97.4549	0.0389236	0.0417244	0.94455	0.994855	no
gene:SpnNT_01103	argR_1	Chromosome:1126538-1130173	110.58	ΔORF2	OK	56.9548	63.232	0.150837	0.0804449	0.89235	0.994748	no
gene:SpnNT_01103	argR_1	Chromosome:1126538-1130173	110.58	110.58+peptide	OK	56.9548	82.9322	0.542115	0.293804	0.61	0.979374	no

gene:SpnNT_01103	argR_1	Chromosome:1126538-1130173	ΔORF2	110.58+peptide	OK	63.232	82.9322	0.391278	0.20617	0.7275	0.994748	no
gene:SpnNT_01103	argR_1	Chromosome:1126538-1130173	110.58	ΔORF2+peptide	OK	56.9548	106.319	0.900514	0.471186	0.4141	0.900384	no
gene:SpnNT_01103	argR_1	Chromosome:1126538-1130173	ΔORF2	ΔORF2+peptide	OK	63.232	106.319	0.749677	0.382083	0.5182	0.953876	no
gene:SpnNT_01103	argR_1	Chromosome:1126538-1130173	110.58+peptide	ΔORF2+peptide	OK	82.9322	106.319	0.358399	0.185359	0.7604	0.994748	no
gene:SpnNT_01104	recN	Chromosome:1130179-1131847	110.58	ΔORF2	OK	53.2623	54.4027	0.0305626	0.0682601	0.906	0.994748	no
gene:SpnNT_01104	recN	Chromosome:1130179-1131847	110.58	110.58+peptide	OK	53.2623	84.1328	0.659552	1.49144	0.00775	0.0837689	no
gene:SpnNT_01104	recN	Chromosome:1130179-1131847	ΔORF2	110.58+peptide	OK	54.4027	84.1328	0.628989	1.42356	0.01365	0.125607	no
gene:SpnNT_01104	recN	Chromosome:1130179-1131847	110.58	ΔORF2+peptide	OK	53.2623	99.7871	0.905738	2.04885	0.00015	0.00355289	yes
gene:SpnNT_01104	recN	Chromosome:1130179-1131847	ΔORF2	ΔORF2+peptide	OK	54.4027	99.7871	0.875175	1.98143	5.00E-04	0.0095781	yes
gene:SpnNT_01104	recN	Chromosome:1130179-1131847	110.58+peptide	ΔORF2+peptide	OK	84.1328	99.7871	0.246186	0.564517	0.3194	0.825683	no
gene:SpnNT_01105	pphA	Chromosome:1131848-1132577	110.58	ΔORF2	OK	55.4973	52.498	-0.0801536	-0.160497	0.7736	0.994748	no
gene:SpnNT_01105	pphA	Chromosome:1131848-1132577	110.58	110.58+peptide	OK	55.4973	89.3297	0.686724	1.42164	0.01445	0.130498	no
gene:SpnNT_01105	pphA	Chromosome:1131848-1132577	ΔORF2	110.58+peptide	OK	52.498	89.3297	0.766878	1.58244	0.00705	0.077805	no
gene:SpnNT_01105	pphA	Chromosome:1131848-1132577	110.58	ΔORF2+peptide	OK	55.4973	98.716	0.830867	1.72109	0.0028	0.0378	yes
gene:SpnNT_01105	pphA	Chromosome:1131848-1132577	ΔORF2	ΔORF2+peptide	OK	52.498	98.716	0.911021	1.88101	0.00115	0.0187457	yes
gene:SpnNT_01105	pphA	Chromosome:1131848-1132577	110.58+peptide	ΔORF2+peptide	OK	89.3297	98.716	0.144143	0.308367	0.5883	0.973845	no
gene:SpnNT_01106	lepA	Chromosome:1132655-1134479	110.58	ΔORF2	OK	150.951	154.946	0.0376843	0.0859406	0.8762	0.994748	no
gene:SpnNT_01106	lepA	Chromosome:1132655-1134479	110.58	110.58+peptide	OK	150.951	135.976	-0.150723	-0.346781	0.5312	0.957488	no
gene:SpnNT_01106	lepA	Chromosome:1132655-1134479	ΔORF2	110.58+peptide	OK	154.946	135.976	-0.188408	-0.431328	0.4471	0.919424	no
gene:SpnNT_01106	lepA	Chromosome:1132655-1134479	110.58	ΔORF2+peptide	OK	150.951	145.552	-0.0525434	-0.120881	0.8276	0.994748	no
gene:SpnNT_01106	lepA	Chromosome:1132655-1134479	ΔORF2	ΔORF2+peptide	OK	154.946	145.552	-0.0902277	-0.206546	0.72275	0.994748	no
gene:SpnNT_01106	lepA	Chromosome:1132655-1134479	110.58+peptide	ΔORF2+peptide	OK	135.976	145.552	0.0981801	0.22676	0.6858	0.988184	no
gene:SpnNT_01107	NA	Chromosome:1134891-1135368	110.58	ΔORF2	OK	7.74417	7.14429	-0.116319	-0.149668	0.7977	0.994748	no
gene:SpnNT_01107	NA	Chromosome:1134891-1135368	110.58	110.58+peptide	OK	7.74417	22.5453	1.54165	2.0868	2.00E-04	0.00450928	yes
gene:SpnNT_01107	NA	Chromosome:1134891-1135368	ΔORF2	110.58+peptide	OK	7.14429	22.5453	1.65797	2.14583	9.00E-04	0.0154579	yes
gene:SpnNT_01107	NA	Chromosome:1134891-1135368	110.58	ΔORF2+peptide	OK	7.74417	11.1435	0.525017	0.730866	0.201	0.68382	no
gene:SpnNT_01107	NA	Chromosome:1134891-1135368	ΔORF2	ΔORF2+peptide	OK	7.14429	11.1435	0.641336	0.851537	0.1469	0.584482	no
gene:SpnNT_01107	NA	Chromosome:1134891-1135368	110.58+peptide	ΔORF2+peptide	OK	22.5453	11.1435	-1.01663	-1.42497	0.01235	0.117432	no
gene:SpnNT_01108	NA	Chromosome:1135406-1135712	110.58	ΔORF2	OK	5.94109	4.8267	-0.299691	-0.297225	0.6117	0.979429	no
gene:SpnNT_01108	NA	Chromosome:1135406-1135712	110.58	110.58+peptide	OK	5.94109	7.87057	0.40574	0.431963	0.45235	0.921244	no
gene:SpnNT_01108	NA	Chromosome:1135406-1135712	ΔORF2	110.58+peptide	OK	4.8267	7.87057	0.705431	0.719972	0.21355	0.698761	no
gene:SpnNT_01108	NA	Chromosome:1135406-1135712	110.58	ΔORF2+peptide	OK	5.94109	9.15532	0.623882	0.66931	0.2661	0.767252	no
gene:SpnNT_01108	NA	Chromosome:1135406-1135712	ΔORF2	ΔORF2+peptide	OK	4.8267	9.15532	0.923572	0.949265	0.1199	0.524705	no
gene:SpnNT_01108	NA	Chromosome:1135406-1135712	110.58+peptide	ΔORF2+peptide	OK	7.87057	9.15532	0.218142	0.24205	0.679	0.98542	no
gene:SpnNT_01109	gatC_2	Chromosome:1135759-1136158	110.58	ΔORF2	OK	6.21124	6.28219	0.0163864	0.0195269	0.9739	0.99536	no
gene:SpnNT_01109	gatC_2	Chromosome:1135759-1136158	110.58	110.58+peptide	OK	6.21124	20.9831	1.75628	2.24944	0.00025	0.00542231	yes
gene:SpnNT_01109	gatC_2	Chromosome:1135759-1136158	ΔORF2	110.58+peptide	OK	6.28219	20.9831	1.73989	2.20603	0.00045	0.00885292	yes
gene:SpnNT_01109	gatC_2	Chromosome:1135759-1136158	110.58	ΔORF2+peptide	OK	6.21124	7.63715	0.298154	0.363475	0.5267	0.956161	no
gene:SpnNT_01109	gatC_2	Chromosome:1135759-1136158	ΔORF2	ΔORF2+peptide	OK	6.28219	7.63715	0.281768	0.340363	0.55625	0.966729	no
gene:SpnNT_01109	gatC_2	Chromosome:1135759-1136158	110.58+peptide	ΔORF2+peptide	OK	20.9831	7.63715	-1.45812	-1.89717	0.00115	0.0187457	yes
gene:SpnNT_01110	gatC_3	Chromosome:1136161-1137235	110.58	ΔORF2	OK	7.56271	6.5441	-0.208708	-0.342722	0.54355	0.961568	no
gene:SpnNT_01110	gatC_3	Chromosome:1136161-1137235	110.58	110.58+peptide	OK	7.56271	14.5326	0.942321	1.60679	0.00625	0.0710065	no
gene:SpnNT_01110	gatC_3	Chromosome:1136161-1137235	ΔORF2	110.58+peptide	OK	6.5441	14.5326	1.15103	1.94308	8.00E-04	0.0140342	yes
gene:SpnNT_01110	gatC_3	Chromosome:1136161-1137235	110.58	ΔORF2+peptide	OK	7.56271	8.72405	0.206096	0.343995	0.54485	0.961568	no
gene:SpnNT_01110	gatC_3	Chromosome:1136161-1137235	ΔORF2	ΔORF2+peptide	OK	6.5441	8.72405	0.414805	0.685725	0.22555	0.716568	no

gene:SpnNT_01110	gatC_3	Chromosome:1136161-1137235	110.58+peptide	ΔORF2+peptide	OK	14.5326	8.72405	-0.736225	-1.26445	0.02595	0.202087	no
gene:SpnNT_01111	NA	Chromosome:1137485-1138382	110.58	ΔORF2	OK	2.01743	2.42184	0.263586	0.330961	0.56465	0.968621	no
gene:SpnNT_01111	NA	Chromosome:1137485-1138382	110.58	110.58+peptide	OK	2.01743	3.51627	0.801528	1.03867	0.0739	0.400378	no
gene:SpnNT_01111	NA	Chromosome:1137485-1138382	ΔORF2	110.58+peptide	OK	2.42184	3.51627	0.537942	0.707651	0.2257	0.716568	no
gene:SpnNT_01111	NA	Chromosome:1137485-1138382	110.58	ΔORF2+peptide	OK	2.01743	3.49834	0.794153	1.03956	0.0708	0.390464	no
gene:SpnNT_01111	NA	Chromosome:1137485-1138382	ΔORF2	ΔORF2+peptide	OK	2.42184	3.49834	0.530567	0.705254	0.21805	0.705785	no
gene:SpnNT_01111	NA	Chromosome:1137485-1138382	110.58+peptide	ΔORF2+peptide	OK	3.51627	3.49834	-0.00737483	-0.0101572	0.9832	0.996246	no
gene:SpnNT_01112	lacA	Chromosome:1138539-1138965	110.58	ΔORF2	OK	47.0702	43.3851	-0.117616	-0.201907	0.7236	0.994748	no
gene:SpnNT_01112	lacA	Chromosome:1138539-1138965	110.58	110.58+peptide	OK	47.0702	58.8789	0.322937	0.567555	0.3152	0.821264	no
gene:SpnNT_01112	lacA	Chromosome:1138539-1138965	ΔORF2	110.58+peptide	OK	43.3851	58.8789	0.440553	0.762088	0.1818	0.651442	no
gene:SpnNT_01112	lacA	Chromosome:1138539-1138965	110.58	ΔORF2+peptide	OK	47.0702	38.1718	-0.302306	-0.518859	0.3572	0.858392	no
gene:SpnNT_01112	lacA	Chromosome:1138539-1138965	ΔORF2	ΔORF2+peptide	OK	43.3851	38.1718	-0.18469	-0.312232	0.58725	0.973049	no
gene:SpnNT_01112	lacA	Chromosome:1138539-1138965	110.58+peptide	ΔORF2+peptide	OK	58.8789	38.1718	-0.625243	-1.08136	0.06235	0.360898	no
gene:SpnNT_01113	lacB	Chromosome:1138995-1139511	110.58	ΔORF2	OK	44.328	47.8658	0.110775	0.203909	0.71755	0.992553	no
gene:SpnNT_01113	lacB	Chromosome:1138995-1139511	110.58	110.58+peptide	OK	44.328	54.0596	0.286331	0.526849	0.35095	0.854885	no
gene:SpnNT_01113	lacB	Chromosome:1138995-1139511	ΔORF2	110.58+peptide	OK	47.8658	54.0596	0.175556	0.325636	0.56215	0.968621	no
gene:SpnNT_01113	lacB	Chromosome:1138995-1139511	110.58	ΔORF2+peptide	OK	44.328	45.8648	0.0491687	0.0898786	0.86945	0.994748	no
gene:SpnNT_01113	lacB	Chromosome:1138995-1139511	ΔORF2	ΔORF2+peptide	OK	47.8658	45.8648	-0.0616066	-0.113513	0.83515	0.994748	no
gene:SpnNT_01113	lacB	Chromosome:1138995-1139511	110.58+peptide	ΔORF2+peptide	OK	54.0596	45.8648	-0.237162	-0.436808	0.44005	0.916416	no
gene:SpnNT_01114	lacC_1	Chromosome:1139521-1140451	110.58	ΔORF2	OK	28.6193	29.6107	0.0491296	0.0950728	0.8714	0.994748	no
gene:SpnNT_01114	lacC_1	Chromosome:1139521-1140451	110.58	110.58+peptide	OK	28.6193	33.8729	0.243141	0.474144	0.40085	0.893669	no
gene:SpnNT_01114	lacC_1	Chromosome:1139521-1140451	ΔORF2	110.58+peptide	OK	29.6107	33.8729	0.194011	0.379212	0.4994	0.944017	no
gene:SpnNT_01114	lacC_1	Chromosome:1139521-1140451	110.58	ΔORF2+peptide	OK	28.6193	35.5062	0.311084	0.605247	0.2899	0.7945	no
gene:SpnNT_01114	lacC_1	Chromosome:1139521-1140451	ΔORF2	ΔORF2+peptide	OK	29.6107	35.5062	0.261954	0.510832	0.37295	0.870018	no
gene:SpnNT_01114	lacC_1	Chromosome:1139521-1140451	110.58+peptide	ΔORF2+peptide	OK	33.8729	35.5062	0.067943	0.133533	0.81125	0.994748	no
gene:SpnNT_01115	lacD	Chromosome:1140452-1141433	110.58	ΔORF2	OK	44.5514	39.4351	-0.175991	-0.357976	0.5279	0.956161	no
gene:SpnNT_01115	lacD	Chromosome:1140452-1141433	110.58	110.58+peptide	OK	44.5514	45.8151	0.0403531	0.0828278	0.88315	0.994748	no
gene:SpnNT_01115	lacD	Chromosome:1140452-1141433	ΔORF2	110.58+peptide	OK	39.4351	45.8151	0.216344	0.440305	0.43805	0.915885	no
gene:SpnNT_01115	lacD	Chromosome:1140452-1141433	110.58	ΔORF2+peptide	OK	44.5514	39.755	-0.164334	-0.333771	0.56115	0.968621	no
gene:SpnNT_01115	lacD	Chromosome:1140452-1141433	ΔORF2	ΔORF2+peptide	OK	39.4351	39.755	0.011657	0.02348	0.96835	0.994855	no
gene:SpnNT_01115	lacD	Chromosome:1140452-1141433	110.58+peptide	ΔORF2+peptide	OK	45.8151	39.755	-0.204687	-0.415964	0.463	0.925818	no
gene:SpnNT_01116	NA	Chromosome:1141691-1142309	110.58	ΔORF2	OK	191.292	196.892	0.0416284	0.0932554	0.87225	0.994748	no
gene:SpnNT_01116	NA	Chromosome:1141691-1142309	110.58	110.58+peptide	OK	191.292	365.159	0.932749	2.06644	0.00045	0.00885292	yes
gene:SpnNT_01116	NA	Chromosome:1141691-1142309	ΔORF2	110.58+peptide	OK	196.892	365.159	0.891121	1.976	5.00E-04	0.0095781	yes
gene:SpnNT_01116	NA	Chromosome:1141691-1142309	110.58	ΔORF2+peptide	OK	191.292	325.11	0.765155	1.71672	0.0028	0.0378	yes
gene:SpnNT_01116	NA	Chromosome:1141691-1142309	ΔORF2	ΔORF2+peptide	OK	196.892	325.11	0.723527	1.62483	0.0052	0.0621443	no
gene:SpnNT_01116	NA	Chromosome:1141691-1142309	110.58+peptide	ΔORF2+peptide	OK	365.159	325.11	-0.167594	-0.372188	0.50585	0.945292	no
gene:SpnNT_01117	licT_2	Chromosome:1142706-1143543	110.58	ΔORF2	OK	2.11802	3.66793	0.792247	1.00805	0.0849	0.438605	no
gene:SpnNT_01117	licT_2	Chromosome:1142706-1143543	110.58	110.58+peptide	OK	2.11802	4.52637	1.09564	1.45988	0.01455	0.13113	no
gene:SpnNT_01117	licT_2	Chromosome:1142706-1143543	ΔORF2	110.58+peptide	OK	3.66793	4.52637	0.30339	0.41604	0.47445	0.930243	no
gene:SpnNT_01117	licT_2	Chromosome:1142706-1143543	110.58	ΔORF2+peptide	OK	2.11802	3.51743	0.731803	0.908222	0.1182	0.520826	no
gene:SpnNT_01117	licT_2	Chromosome:1142706-1143543	ΔORF2	ΔORF2+peptide	OK	3.66793	3.51743	-0.0604442	-0.0769024	0.89495	0.994748	no
gene:SpnNT_01117	licT_2	Chromosome:1142706-1143543	110.58+peptide	ΔORF2+peptide	OK	4.52637	3.51743	-0.363834	-0.484746	0.4001	0.893627	no
gene:SpnNT_01118	lacF_1	Chromosome:1143578-1145587	110.58	ΔORF2	OK	3.79786	4.12833	0.12037	0.0532884	0.90755	0.994748	no
gene:SpnNT_01118	lacF_1	Chromosome:1143578-1145587	110.58	110.58+peptide	OK	3.79786	8.25786	1.12058	0.519271	0.4958	0.943191	no

gene:SpnNT_01118	lacF_1	Chromosome:1143578-1145587	ΔORF2	110.58+peptide	OK	4.12833	8.25786	1.00021	0.532983	0.3504	0.854722	no
gene:SpnNT_01118	lacF_1	Chromosome:1143578-1145587	110.58	ΔORF2+peptide	OK	3.79786	8.65745	1.18876	0.546536	0.5356	0.958867	no
gene:SpnNT_01118	lacF_1	Chromosome:1143578-1145587	ΔORF2	ΔORF2+peptide	OK	4.12833	8.65745	1.06838	0.56342	0.389	0.88343	no
gene:SpnNT_01118	lacF_1	Chromosome:1143578-1145587	110.58+peptide	ΔORF2+peptide	OK	8.25786	8.65745	0.0681737	0.0384097	0.9518	0.994855	no
gene:SpnNT_01119	lacE	Chromosome:1143578-1145587	110.58	ΔORF2	OK	2.83876	2.74908	-0.0463161	-0.0682372	0.90395	0.994748	no
gene:SpnNT_01119	lacE	Chromosome:1143578-1145587	110.58	110.58+peptide	OK	2.83876	4.83516	0.768301	1.17545	0.04355	0.285874	no
gene:SpnNT_01119	lacE	Chromosome:1143578-1145587	ΔORF2	110.58+peptide	OK	2.74908	4.83516	0.814617	1.24683	0.0297	0.220307	no
gene:SpnNT_01119	lacE	Chromosome:1143578-1145587	110.58	ΔORF2+peptide	OK	2.83876	4.18335	0.559395	0.841922	0.1455	0.581026	no
gene:SpnNT_01119	lacE	Chromosome:1143578-1145587	ΔORF2	ΔORF2+peptide	OK	2.74908	4.18335	0.605712	0.911997	0.10925	0.502305	no
gene:SpnNT_01119	lacE	Chromosome:1143578-1145587	110.58+peptide	ΔORF2+peptide	OK	4.83516	4.18335	-0.208905	-0.327204	0.5664	0.968621	no
gene:SpnNT_01120	lacG	Chromosome:1145801-1147208	110.58	ΔORF2	OK	3.13615	2.88609	-0.119876	-0.179591	0.7524	0.994748	no
gene:SpnNT_01120	lacG	Chromosome:1145801-1147208	110.58	110.58+peptide	OK	3.13615	3.44867	0.137047	0.210716	0.7083	0.990441	no
gene:SpnNT_01120	lacG	Chromosome:1145801-1147208	ΔORF2	110.58+peptide	OK	2.88609	3.44867	0.256923	0.388429	0.50345	0.945197	no
gene:SpnNT_01120	lacG	Chromosome:1145801-1147208	110.58	ΔORF2+peptide	OK	3.13615	3.6693	0.226512	0.347864	0.54375	0.961568	no
gene:SpnNT_01120	lacG	Chromosome:1145801-1147208	ΔORF2	ΔORF2+peptide	OK	2.88609	3.6693	0.346388	0.523092	0.36335	0.862964	no
gene:SpnNT_01120	lacG	Chromosome:1145801-1147208	110.58+peptide	ΔORF2+peptide	OK	3.44867	3.6693	0.0894652	0.138718	0.81	0.994748	no
gene:SpnNT_01121	NA	Chromosome:1147250-1147508	110.58	ΔORF2	OK	4.6135	2.94165	-0.649236	-0.449322	0.4186	0.90373	no
gene:SpnNT_01121	NA	Chromosome:1147250-1147508	110.58	110.58+peptide	OK	4.6135	3.11097	-0.568496	-0.4156	0.47525	0.930746	no
gene:SpnNT_01121	NA	Chromosome:1147250-1147508	ΔORF2	110.58+peptide	OK	2.94165	3.11097	0.08074	0.0527128	0.9087	0.994748	no
gene:SpnNT_01121	NA	Chromosome:1147250-1147508	110.58	ΔORF2+peptide	OK	4.6135	1.18355	-1.96274	-1.70671	0.14255	0.576108	no
gene:SpnNT_01121	NA	Chromosome:1147250-1147508	ΔORF2	ΔORF2+peptide	OK	2.94165	1.18355	-1.31351	-0.979714	0.28845	0.792622	no
gene:SpnNT_01121	NA	Chromosome:1147250-1147508	110.58+peptide	ΔORF2+peptide	OK	3.11097	1.18355	-1.39425	-1.10892	0.25085	0.748571	no
gene:SpnNT_01122	lacR_1	Chromosome:1147647-1148409	110.58	ΔORF2	OK	55.2631	78.0448	0.497986	0.995119	0.0887	0.448525	no
gene:SpnNT_01122	lacR_1	Chromosome:1147647-1148409	110.58	110.58+peptide	OK	55.2631	62.8519	0.18564	0.370944	0.5197	0.954494	no
gene:SpnNT_01122	lacR_1	Chromosome:1147647-1148409	ΔORF2	110.58+peptide	OK	78.0448	62.8519	-0.312346	-0.617102	0.29195	0.796956	no
gene:SpnNT_01122	lacR_1	Chromosome:1147647-1148409	110.58	ΔORF2+peptide	OK	55.2631	69.2526	0.325552	0.660731	0.24635	0.74356	no
gene:SpnNT_01122	lacR_1	Chromosome:1147647-1148409	ΔORF2	ΔORF2+peptide	OK	78.0448	69.2526	-0.172434	-0.345905	0.54975	0.964281	no
gene:SpnNT_01122	lacR_1	Chromosome:1147647-1148409	110.58+peptide	ΔORF2+peptide	OK	62.8519	69.2526	0.139912	0.280652	0.6232	0.980887	no
gene:SpnNT_01123	nrdF	Chromosome:1148590-1149553	110.58	ΔORF2	OK	67.2224	67.4592	0.00507161	0.0108322	0.98335	0.996246	no
gene:SpnNT_01123	nrdF	Chromosome:1148590-1149553	110.58	110.58+peptide	OK	67.2224	68.9911	0.0374673	0.0800148	0.89145	0.994748	no
gene:SpnNT_01123	nrdF	Chromosome:1148590-1149553	ΔORF2	110.58+peptide	OK	67.4592	68.9911	0.0323956	0.0693562	0.9057	0.994748	no
gene:SpnNT_01123	nrdF	Chromosome:1148590-1149553	110.58	ΔORF2+peptide	OK	67.2224	68.4689	0.0265052	0.0566176	0.9216	0.994748	no
gene:SpnNT_01123	nrdF	Chromosome:1148590-1149553	ΔORF2	ΔORF2+peptide	OK	67.4592	68.4689	0.0214335	0.0458982	0.9343	0.994855	no
gene:SpnNT_01123	nrdF	Chromosome:1148590-1149553	110.58+peptide	ΔORF2+peptide	OK	68.9911	68.4689	-0.0109621	-0.0234716	0.96635	0.994855	no
gene:SpnNT_01124	nrdE2	Chromosome:1149740-1151900	110.58	ΔORF2	OK	146.852	131.653	-0.157627	-0.35793	0.53535	0.958867	no
gene:SpnNT_01124	nrdE2	Chromosome:1149740-1151900	110.58	110.58+peptide	OK	146.852	107.094	-0.455494	-1.04158	0.0719	0.394758	no
gene:SpnNT_01124	nrdE2	Chromosome:1149740-1151900	ΔORF2	110.58+peptide	OK	131.653	107.094	-0.297866	-0.678715	0.23175	0.724662	no
gene:SpnNT_01124	nrdE2	Chromosome:1149740-1151900	110.58	ΔORF2+peptide	OK	146.852	103.211	-0.508775	-1.16461	0.0434	0.285604	no
gene:SpnNT_01124	nrdE2	Chromosome:1149740-1151900	ΔORF2	ΔORF2+peptide	OK	131.653	103.211	-0.351148	-0.800934	0.15875	0.608209	no
gene:SpnNT_01124	nrdE2	Chromosome:1149740-1151900	110.58+peptide	ΔORF2+peptide	OK	107.094	103.211	-0.0532814	-0.122392	0.8309	0.994748	no
gene:SpnNT_01125	nrdH	Chromosome:1151980-1152199	110.58	ΔORF2	OK	282.933	225.73	-0.325862	-0.569037	0.30675	0.81251	no
gene:SpnNT_01125	nrdH	Chromosome:1151980-1152199	110.58	110.58+peptide	OK	282.933	245.909	-0.202333	-0.346237	0.5375	0.959663	no
gene:SpnNT_01125	nrdH	Chromosome:1151980-1152199	ΔORF2	110.58+peptide	OK	225.73	245.909	0.123529	0.210899	0.7097	0.990851	no
gene:SpnNT_01125	nrdH	Chromosome:1151980-1152199	110.58	ΔORF2+peptide	OK	282.933	248.409	-0.187744	-0.322853	0.5636	0.968621	no
gene:SpnNT_01125	nrdH	Chromosome:1151980-1152199	ΔORF2	ΔORF2+peptide	OK	225.73	248.409	0.138118	0.236963	0.67125	0.984845	no

gene:SpnNT_01125	nrdH	Chromosome:1151980-1152199	110.58+peptide	ΔORF2+peptide	OK	245.909	248.409	0.0145894	0.0245452	0.9637	0.994855	no
gene:SpnNT_01126	ptsH	Chromosome:1152548-1152812	110.58	ΔORF2	OK	2470.72	2243.68	-0.139062	-0.304505	0.58375	0.971584	no
gene:SpnNT_01126	ptsH	Chromosome:1152548-1152812	110.58	110.58+peptide	OK	2470.72	4742.86	0.940824	1.98734	0.00055	0.0103545	yes
gene:SpnNT_01126	ptsH	Chromosome:1152548-1152812	ΔORF2	110.58+peptide	OK	2243.68	4742.86	1.07989	2.30982	1.00E-04	0.0025332	yes
gene:SpnNT_01126	ptsH	Chromosome:1152548-1152812	110.58	ΔORF2+peptide	OK	2470.72	4094.72	0.728831	1.55797	0.00585	0.0677526	no
gene:SpnNT_01126	ptsH	Chromosome:1152548-1152812	ΔORF2	ΔORF2+peptide	OK	2243.68	4094.72	0.867894	1.87918	0.00105	0.0174849	yes
gene:SpnNT_01126	ptsH	Chromosome:1152548-1152812	110.58+peptide	ΔORF2+peptide	OK	4742.86	4094.72	-0.211993	-0.443134	0.4296	0.911728	no
gene:SpnNT_01127	ptsI	Chromosome:1152826-1154551	110.58	ΔORF2	OK	890.777	882.941	-0.0127478	-0.0270663	0.9623	0.994855	no
gene:SpnNT_01127	ptsI	Chromosome:1152826-1154551	110.58	110.58+peptide	OK	890.777	1439.79	0.692723	1.45077	0.0098	0.0986919	no
gene:SpnNT_01127	ptsI	Chromosome:1152826-1154551	ΔORF2	110.58+peptide	OK	882.941	1439.79	0.705471	1.46001	0.01035	0.102811	no
gene:SpnNT_01127	ptsI	Chromosome:1152826-1154551	110.58	ΔORF2+peptide	OK	890.777	1614.13	0.857618	1.79766	0.00235	0.0334454	yes
gene:SpnNT_01127	ptsI	Chromosome:1152826-1154551	ΔORF2	ΔORF2+peptide	OK	882.941	1614.13	0.870366	1.80279	0.0022	0.0317584	yes
gene:SpnNT_01127	ptsI	Chromosome:1152826-1154551	110.58+peptide	ΔORF2+peptide	OK	1439.79	1614.13	0.164895	0.337116	0.55225	0.964719	no
gene:SpnNT_01128	NA	Chromosome:1154686-1155181	110.58	ΔORF2	OK	26.2214	28.1982	0.104857	0.177494	0.7607	0.994748	no
gene:SpnNT_01128	NA	Chromosome:1154686-1155181	110.58	110.58+peptide	OK	26.2214	23.8312	-0.137889	-0.23004	0.6906	0.98828	no
gene:SpnNT_01128	NA	Chromosome:1154686-1155181	ΔORF2	110.58+peptide	OK	28.1982	23.8312	-0.242746	-0.407466	0.48875	0.939082	no
gene:SpnNT_01128	NA	Chromosome:1154686-1155181	110.58	ΔORF2+peptide	OK	26.2214	31.1433	0.248179	0.420456	0.46415	0.926394	no
gene:SpnNT_01128	NA	Chromosome:1154686-1155181	ΔORF2	ΔORF2+peptide	OK	28.1982	31.1433	0.143322	0.244354	0.67535	0.985207	no
gene:SpnNT_01128	NA	Chromosome:1154686-1155181	110.58+peptide	ΔORF2+peptide	OK	23.8312	31.1433	0.386068	0.648585	0.26585	0.767012	no
gene:SpnNT_01129	NA	Chromosome:1155353-1157681	110.58	ΔORF2	OK	14.3701	15.1135	0.0727652	0.0773577	0.8913	0.994748	no
gene:SpnNT_01129	NA	Chromosome:1155353-1157681	110.58	110.58+peptide	OK	14.3701	9.87574	-0.541109	-0.559273	0.33015	0.836176	no
gene:SpnNT_01129	NA	Chromosome:1155353-1157681	ΔORF2	110.58+peptide	OK	15.1135	9.87574	-0.613874	-0.646681	0.2526	0.750933	no
gene:SpnNT_01129	NA	Chromosome:1155353-1157681	110.58	ΔORF2+peptide	OK	14.3701	9.45292	-0.604238	-0.639438	0.2725	0.774448	no
gene:SpnNT_01129	NA	Chromosome:1155353-1157681	ΔORF2	ΔORF2+peptide	OK	15.1135	9.45292	-0.677003	-0.730905	0.2024	0.6861	no
gene:SpnNT_01129	NA	Chromosome:1155353-1157681	110.58+peptide	ΔORF2+peptide	OK	9.87574	9.45292	-0.0631293	-0.0662047	0.9079	0.994748	no
gene:SpnNT_01130	NA	Chromosome:1155353-1157681	110.58	ΔORF2	OK	26.1296	21.3423	-0.291969	-0.377555	0.51305	0.950747	no
gene:SpnNT_01130	NA	Chromosome:1155353-1157681	110.58	110.58+peptide	OK	26.1296	17.2048	-0.602876	-0.796324	0.17095	0.62976	no
gene:SpnNT_01130	NA	Chromosome:1155353-1157681	ΔORF2	110.58+peptide	OK	21.3423	17.2048	-0.310907	-0.391572	0.50275	0.944788	no
gene:SpnNT_01130	NA	Chromosome:1155353-1157681	110.58	ΔORF2+peptide	OK	26.1296	18.4151	-0.504798	-0.674327	0.2435	0.73908	no
gene:SpnNT_01130	NA	Chromosome:1155353-1157681	ΔORF2	ΔORF2+peptide	OK	21.3423	18.4151	-0.21283	-0.270804	0.6373	0.980887	no
gene:SpnNT_01130	NA	Chromosome:1155353-1157681	110.58+peptide	ΔORF2+peptide	OK	17.2048	18.4151	0.0980774	0.127383	0.82165	0.994748	no
gene:SpnNT_01131	NA	Chromosome:1155353-1157681	110.58	ΔORF2	OK	61.005	63.0113	0.0466829	0.0494737	0.9315	0.994855	no
gene:SpnNT_01131	NA	Chromosome:1155353-1157681	110.58	110.58+peptide	OK	61.005	35.5649	-0.778471	-0.787713	0.1829	0.653421	no
gene:SpnNT_01131	NA	Chromosome:1155353-1157681	ΔORF2	110.58+peptide	OK	63.0113	35.5649	-0.825154	-0.876344	0.1382	0.566706	no
gene:SpnNT_01131	NA	Chromosome:1155353-1157681	110.58	ΔORF2+peptide	OK	61.005	29.3439	-1.05586	-0.984529	0.1084	0.500854	no
gene:SpnNT_01131	NA	Chromosome:1155353-1157681	ΔORF2	ΔORF2+peptide	OK	63.0113	29.3439	-1.10255	-1.07085	0.08275	0.430891	no
gene:SpnNT_01131	NA	Chromosome:1155353-1157681	110.58+peptide	ΔORF2+peptide	OK	35.5649	29.3439	-0.277394	-0.259079	0.6656	0.98367	no
gene:SpnNT_01132	NA	Chromosome:1157789-1158329	110.58	ΔORF2	OK	30.0852	34.1483	0.182761	0.32525	0.57045	0.968621	no
gene:SpnNT_01132	NA	Chromosome:1157789-1158329	110.58	110.58+peptide	OK	30.0852	24.5028	-0.29611	-0.515096	0.3654	0.864634	no
gene:SpnNT_01132	NA	Chromosome:1157789-1158329	ΔORF2	110.58+peptide	OK	34.1483	24.5028	-0.478871	-0.837786	0.15195	0.594303	no
gene:SpnNT_01132	NA	Chromosome:1157789-1158329	110.58	ΔORF2+peptide	OK	30.0852	31.7416	0.0773184	0.135596	0.8129	0.994748	no
gene:SpnNT_01132	NA	Chromosome:1157789-1158329	ΔORF2	ΔORF2+peptide	OK	34.1483	31.7416	-0.105442	-0.185994	0.7475	0.994748	no
gene:SpnNT_01132	NA	Chromosome:1157789-1158329	110.58+peptide	ΔORF2+peptide	OK	24.5028	31.7416	0.373428	0.644113	0.2699	0.772101	no
gene:SpnNT_01133	dhaQ	Chromosome:1158532-1160049	110.58	ΔORF2	OK	27.8963	30.1368	0.111449	0.196908	0.7272	0.994748	no
gene:SpnNT_01133	dhaQ	Chromosome:1158532-1160049	110.58	110.58+peptide	OK	27.8963	25.1108	-0.151767	-0.266195	0.6382	0.980887	no

gene:SpnNT_01133	dhaQ	Chromosome:1158532-1160049	ΔORF2	110.58+peptide	OK	30.1368	25.1108	-0.263216	-0.459828	0.42065	0.904691	no
gene:SpnNT_01133	dhaQ	Chromosome:1158532-1160049	110.58	ΔORF2+peptide	OK	27.8963	26.5602	-0.0708125	-0.124916	0.8282	0.994748	no
gene:SpnNT_01133	dhaQ	Chromosome:1158532-1160049	ΔORF2	ΔORF2+peptide	OK	30.1368	26.5602	-0.182261	-0.320215	0.57585	0.969538	no
gene:SpnNT_01133	dhaQ	Chromosome:1158532-1160049	110.58+peptide	ΔORF2+peptide	OK	25.1108	26.5602	0.0809543	0.141207	0.8068	0.994748	no
gene:SpnNT_01134	dhaS	Chromosome:1158532-1160049	110.58	ΔORF2	OK	14.8068	20.791	0.489693	0.409775	0.4855	0.937658	no
gene:SpnNT_01134	dhaS	Chromosome:1158532-1160049	110.58	110.58+peptide	OK	14.8068	11.6014	-0.351969	-0.275675	0.64575	0.980887	no
gene:SpnNT_01134	dhaS	Chromosome:1158532-1160049	ΔORF2	110.58+peptide	OK	20.791	11.6014	-0.841662	-0.700459	0.2442	0.740215	no
gene:SpnNT_01134	dhaS	Chromosome:1158532-1160049	110.58	ΔORF2+peptide	OK	14.8068	16.4705	0.153618	0.127207	0.83025	0.994748	no
gene:SpnNT_01134	dhaS	Chromosome:1158532-1160049	ΔORF2	ΔORF2+peptide	OK	20.791	16.4705	-0.336076	-0.297979	0.61315	0.979616	no
gene:SpnNT_01134	dhaS	Chromosome:1158532-1160049	110.58+peptide	ΔORF2+peptide	OK	11.6014	16.4705	0.505586	0.416427	0.4826	0.935914	no
gene:SpnNT_01135	dhaK	Chromosome:1160202-1161192	110.58	ΔORF2	OK	27.1535	28.178	0.0534324	0.103312	0.857	0.994748	no
gene:SpnNT_01135	dhaK	Chromosome:1160202-1161192	110.58	110.58+peptide	OK	27.1535	34.061	0.326981	0.634851	0.2642	0.764751	no
gene:SpnNT_01135	dhaK	Chromosome:1160202-1161192	ΔORF2	110.58+peptide	OK	28.178	34.061	0.273548	0.53426	0.35175	0.854885	no
gene:SpnNT_01135	dhaK	Chromosome:1160202-1161192	110.58	ΔORF2+peptide	OK	27.1535	30.4077	0.163299	0.31609	0.58485	0.971855	no
gene:SpnNT_01135	dhaK	Chromosome:1160202-1161192	ΔORF2	ΔORF2+peptide	OK	28.178	30.4077	0.109867	0.213918	0.71235	0.991034	no
gene:SpnNT_01135	dhaK	Chromosome:1160202-1161192	110.58+peptide	ΔORF2+peptide	OK	34.061	30.4077	-0.163682	-0.320045	0.58455	0.971855	no
gene:SpnNT_01136	dhaL	Chromosome:1161213-1162163	110.58	ΔORF2	OK	38.2789	41.7839	0.126398	0.158149	0.78235	0.994748	no
gene:SpnNT_01136	dhaL	Chromosome:1161213-1162163	110.58	110.58+peptide	OK	38.2789	50.5319	0.400645	0.495491	0.3845	0.881717	no
gene:SpnNT_01136	dhaL	Chromosome:1161213-1162163	ΔORF2	110.58+peptide	OK	41.7839	50.5319	0.274247	0.338013	0.55705	0.967478	no
gene:SpnNT_01136	dhaL	Chromosome:1161213-1162163	110.58	ΔORF2+peptide	OK	38.2789	44.0165	0.201497	0.253339	0.6619	0.982966	no
gene:SpnNT_01136	dhaL	Chromosome:1161213-1162163	ΔORF2	ΔORF2+peptide	OK	41.7839	44.0165	0.0750984	0.0940873	0.87445	0.994748	no
gene:SpnNT_01136	dhaL	Chromosome:1161213-1162163	110.58+peptide	ΔORF2+peptide	OK	50.5319	44.0165	-0.199148	-0.246612	0.6651	0.983559	no
gene:SpnNT_01137	dhaM	Chromosome:1161213-1162163	110.58	ΔORF2	OK	118.905	139.275	0.228117	0.352721	0.5417	0.961494	no
gene:SpnNT_01137	dhaM	Chromosome:1161213-1162163	110.58	110.58+peptide	OK	118.905	179.613	0.595079	0.941516	0.1084	0.500854	no
gene:SpnNT_01137	dhaM	Chromosome:1161213-1162163	ΔORF2	110.58+peptide	OK	139.275	179.613	0.366962	0.58915	0.30885	0.814396	no
gene:SpnNT_01137	dhaM	Chromosome:1161213-1162163	110.58	ΔORF2+peptide	OK	118.905	132.698	0.158337	0.239397	0.67745	0.98524	no
gene:SpnNT_01137	dhaM	Chromosome:1161213-1162163	ΔORF2	ΔORF2+peptide	OK	139.275	132.698	-0.0697804	-0.106921	0.8497	0.994748	no
gene:SpnNT_01137	dhaM	Chromosome:1161213-1162163	110.58+peptide	ΔORF2+peptide	OK	179.613	132.698	-0.436742	-0.684463	0.23865	0.733731	no
gene:SpnNT_01138	NA	Chromosome:1162650-1163846	110.58	ΔORF2	OK	24.357	37.198	0.610888	1.15085	0.0482	0.306138	no
gene:SpnNT_01138	NA	Chromosome:1162650-1163846	110.58	110.58+peptide	OK	24.357	13.9611	-0.80292	-1.41298	0.01735	0.149192	no
gene:SpnNT_01138	NA	Chromosome:1162650-1163846	ΔORF2	110.58+peptide	OK	37.198	13.9611	-1.41381	-2.53178	5.00E-05	0.0013612	yes
gene:SpnNT_01138	NA	Chromosome:1162650-1163846	110.58	ΔORF2+peptide	OK	24.357	19.6856	-0.307198	-0.558383	0.33675	0.84149	no
gene:SpnNT_01138	NA	Chromosome:1162650-1163846	ΔORF2	ΔORF2+peptide	OK	37.198	19.6856	-0.918086	-1.70015	0.0031	0.0411306	yes
gene:SpnNT_01138	NA	Chromosome:1162650-1163846	110.58+peptide	ΔORF2+peptide	OK	13.9611	19.6856	0.495721	0.859374	0.1427	0.576334	no
gene:SpnNT_01139	NA	Chromosome:1162650-1163846	110.58	ΔORF2	OK	34.4252	39.633	0.203241	0.0868034	0.91225	0.994748	no
gene:SpnNT_01139	NA	Chromosome:1162650-1163846	110.58	110.58+peptide	OK	34.4252	19.5736	-0.814551	-0.334295	0.64375	0.980887	no
gene:SpnNT_01139	NA	Chromosome:1162650-1163846	ΔORF2	110.58+peptide	OK	39.633	19.5736	-1.01779	-0.397656	0.5354	0.958867	no
gene:SpnNT_01139	NA	Chromosome:1162650-1163846	110.58	ΔORF2+peptide	OK	34.4252	12.7125	-1.43721	-0.802619	0.62885	0.980887	no
gene:SpnNT_01139	NA	Chromosome:1162650-1163846	ΔORF2	ΔORF2+peptide	OK	39.633	12.7125	-1.64045	-0.839303	0.5357	0.958867	no
gene:SpnNT_01139	NA	Chromosome:1162650-1163846	110.58+peptide	ΔORF2+peptide	OK	19.5736	12.7125	-0.622662	-0.301141	0.72735	0.994748	no
gene:SpnNT_01140	yfnB	Chromosome:1163884-1164598	110.58	ΔORF2	OK	23.654	24.8158	0.0691798	0.123528	0.83035	0.994748	no
gene:SpnNT_01140	yfnB	Chromosome:1163884-1164598	110.58	110.58+peptide	OK	23.654	13.3575	-0.824426	-1.41128	0.0183	0.155426	no
gene:SpnNT_01140	yfnB	Chromosome:1163884-1164598	ΔORF2	110.58+peptide	OK	24.8158	13.3575	-0.893606	-1.52752	0.0112	0.108783	no
gene:SpnNT_01140	yfnB	Chromosome:1163884-1164598	110.58	ΔORF2+peptide	OK	23.654	18.4431	-0.358996	-0.617694	0.2873	0.790678	no
gene:SpnNT_01140	yfnB	Chromosome:1163884-1164598	ΔORF2	ΔORF2+peptide	OK	24.8158	18.4431	-0.428176	-0.735663	0.2118	0.697569	no

gene:SpnNT_01140	yfnB	Chromosome:1163884-1164598	110.58+peptide	ΔORF2+peptide	OK	13.3575	18.4431	0.46543	0.768939	0.1894	0.667017	no
gene:SpnNT_01141	NA	Chromosome:1164611-1165061	110.58	ΔORF2	OK	13.9501	21.5222	0.625551	0.944167	0.0982	0.474615	no
gene:SpnNT_01141	NA	Chromosome:1164611-1165061	110.58	110.58+peptide	OK	13.9501	11.8243	-0.238523	-0.347239	0.54525	0.96171	no
gene:SpnNT_01141	NA	Chromosome:1164611-1165061	ΔORF2	110.58+peptide	OK	21.5222	11.8243	-0.864073	-1.27943	0.03095	0.226886	no
gene:SpnNT_01141	NA	Chromosome:1164611-1165061	110.58	ΔORF2+peptide	OK	13.9501	20.7108	0.570106	0.865638	0.1384	0.567349	no
gene:SpnNT_01141	NA	Chromosome:1164611-1165061	ΔORF2	ΔORF2+peptide	OK	21.5222	20.7108	-0.0554443	-0.0857558	0.8811	0.994748	no
gene:SpnNT_01141	NA	Chromosome:1164611-1165061	110.58+peptide	ΔORF2+peptide	OK	11.8243	20.7108	0.808629	1.20423	0.04475	0.290842	no
gene:SpnNT_01142	ung	Chromosome:1165106-1165760	110.58	ΔORF2	OK	69.342	80.6424	0.217809	0.445912	0.4288	0.910619	no
gene:SpnNT_01142	ung	Chromosome:1165106-1165760	110.58	110.58+peptide	OK	69.342	44.4266	-0.642305	-1.26353	0.0301	0.222645	no
gene:SpnNT_01142	ung	Chromosome:1165106-1165760	ΔORF2	110.58+peptide	OK	80.6424	44.4266	-0.860113	-1.69773	0.0041	0.0516317	no
gene:SpnNT_01142	ung	Chromosome:1165106-1165760	110.58	ΔORF2+peptide	OK	69.342	49.7959	-0.477702	-0.940179	0.10625	0.495985	no
gene:SpnNT_01142	ung	Chromosome:1165106-1165760	ΔORF2	ΔORF2+peptide	OK	80.6424	49.7959	-0.695511	-1.3735	0.01995	0.166741	no
gene:SpnNT_01142	ung	Chromosome:1165106-1165760	110.58+peptide	ΔORF2+peptide	OK	44.4266	49.7959	0.164603	0.313178	0.5938	0.975539	no
gene:SpnNT_01143	mutX_3	Chromosome:1165769-1166234	110.58	ΔORF2	OK	74.4454	70.1857	-0.0850051	-0.157327	0.77735	0.994748	no
gene:SpnNT_01143	mutX_3	Chromosome:1165769-1166234	110.58	110.58+peptide	OK	74.4454	46.9039	-0.666474	-1.21596	0.03485	0.246923	no
gene:SpnNT_01143	mutX_3	Chromosome:1165769-1166234	ΔORF2	110.58+peptide	OK	70.1857	46.9039	-0.581469	-1.04824	0.0647	0.368967	no
gene:SpnNT_01143	mutX_3	Chromosome:1165769-1166234	110.58	ΔORF2+peptide	OK	74.4454	58.8896	-0.338169	-0.624694	0.2628	0.763272	no
gene:SpnNT_01143	mutX_3	Chromosome:1165769-1166234	ΔORF2	ΔORF2+peptide	OK	70.1857	58.8896	-0.253164	-0.46196	0.4039	0.894511	no
gene:SpnNT_01143	mutX_3	Chromosome:1165769-1166234	110.58+peptide	ΔORF2+peptide	OK	46.9039	58.8896	0.328304	0.590783	0.2942	0.798553	no
gene:SpnNT_01144	pyrC	Chromosome:1166246-1167515	110.58	ΔORF2	OK	90.6468	92.2728	0.0256504	0.0574991	0.92105	0.994748	no
gene:SpnNT_01144	pyrC	Chromosome:1166246-1167515	110.58	110.58+peptide	OK	90.6468	57.604	-0.654088	-1.45015	0.011	0.107477	no
gene:SpnNT_01144	pyrC	Chromosome:1166246-1167515	ΔORF2	110.58+peptide	OK	92.2728	57.604	-0.679738	-1.4992	0.00825	0.0878705	no
gene:SpnNT_01144	pyrC	Chromosome:1166246-1167515	110.58	ΔORF2+peptide	OK	90.6468	64.0081	-0.502002	-1.11824	0.05175	0.321526	no
gene:SpnNT_01144	pyrC	Chromosome:1166246-1167515	ΔORF2	ΔORF2+peptide	OK	92.2728	64.0081	-0.527652	-1.16923	0.0389	0.265996	no
gene:SpnNT_01144	pyrC	Chromosome:1166246-1167515	110.58+peptide	ΔORF2+peptide	OK	57.604	64.0081	0.152086	0.333393	0.5646	0.968621	no
gene:SpnNT_01145	NA	Chromosome:1167528-1167789	110.58	ΔORF2	OK	10.694	11.728	0.133152	0.143733	0.8063	0.994748	no
gene:SpnNT_01145	NA	Chromosome:1167528-1167789	110.58	110.58+peptide	OK	10.694	7.433	-0.52479	-0.51585	0.3612	0.862347	no
gene:SpnNT_01145	NA	Chromosome:1167528-1167789	ΔORF2	110.58+peptide	OK	11.728	7.433	-0.657942	-0.642915	0.26185	0.762031	no
gene:SpnNT_01145	NA	Chromosome:1167528-1167789	110.58	ΔORF2+peptide	OK	10.694	13.3962	0.325022	0.361584	0.5515	0.96439	no
gene:SpnNT_01145	NA	Chromosome:1167528-1167789	ΔORF2	ΔORF2+peptide	OK	11.728	13.3962	0.19187	0.211843	0.74095	0.994748	no
gene:SpnNT_01145	NA	Chromosome:1167528-1167789	110.58+peptide	ΔORF2+peptide	OK	7.433	13.3962	0.849812	0.851047	0.156	0.601537	no
gene:SpnNT_01146	norM_1	Chromosome:1167799-1169161	110.58	ΔORF2	OK	68.4965	76.224	0.154215	0.342187	0.55285	0.965295	no
gene:SpnNT_01146	norM_1	Chromosome:1167799-1169161	110.58	110.58+peptide	OK	68.4965	50.5412	-0.438571	-0.959997	0.0941	0.463158	no
gene:SpnNT_01146	norM_1	Chromosome:1167799-1169161	ΔORF2	110.58+peptide	OK	76.224	50.5412	-0.592786	-1.28894	0.02515	0.197971	no
gene:SpnNT_01146	norM_1	Chromosome:1167799-1169161	110.58	ΔORF2+peptide	OK	68.4965	56.7526	-0.271343	-0.59773	0.287	0.790183	no
gene:SpnNT_01146	norM_1	Chromosome:1167799-1169161	ΔORF2	ΔORF2+peptide	OK	76.224	56.7526	-0.425558	-0.931137	0.1006	0.480726	no
gene:SpnNT_01146	norM_1	Chromosome:1167799-1169161	110.58+peptide	ΔORF2+peptide	OK	50.5412	56.7526	0.167227	0.361091	0.52175	0.954832	no
gene:SpnNT_01147	acoA	Chromosome:1169314-1170283	110.58	ΔORF2	OK	56.7049	61.9912	0.12859	0.268499	0.64235	0.980887	no
gene:SpnNT_01147	acoA	Chromosome:1169314-1170283	110.58	110.58+peptide	OK	56.7049	70.1932	0.307859	0.630995	0.27505	0.777354	no
gene:SpnNT_01147	acoA	Chromosome:1169314-1170283	ΔORF2	110.58+peptide	OK	61.9912	70.1932	0.179268	0.366783	0.52425	0.954832	no
gene:SpnNT_01147	acoA	Chromosome:1169314-1170283	110.58	ΔORF2+peptide	OK	56.7049	71.0848	0.326069	0.682336	0.23715	0.732035	no
gene:SpnNT_01147	acoA	Chromosome:1169314-1170283	ΔORF2	ΔORF2+peptide	OK	61.9912	71.0848	0.197479	0.412484	0.47175	0.928525	no
gene:SpnNT_01147	acoA	Chromosome:1169314-1170283	110.58+peptide	ΔORF2+peptide	OK	70.1932	71.0848	0.0182102	0.0373368	0.9486	0.994855	no
gene:SpnNT_01148	bfmBAB_1	Chromosome:1170298-1171266	110.58	ΔORF2	OK	43.1228	45.381	0.0736358	0.0942486	0.8661	0.994748	no
gene:SpnNT_01148	bfmBAB_1	Chromosome:1170298-1171266	110.58	110.58+peptide	OK	43.1228	60.9858	0.500022	0.643957	0.266	0.767132	no

gene:SpnNT_01148	bfmBAB_1	Chromosome:1170298-1171266	ΔORF2	110.58+peptide	OK	45.381	60.9858	0.426386	0.531433	0.36005	0.860974	no
gene:SpnNT_01148	bfmBAB_1	Chromosome:1170298-1171266	110.58	ΔORF2+peptide	OK	43.1228	54.309	0.332739	0.435762	0.44865	0.919688	no
gene:SpnNT_01148	bfmBAB_1	Chromosome:1170298-1171266	ΔORF2	ΔORF2+peptide	OK	45.381	54.309	0.259103	0.32804	0.5738	0.969538	no
gene:SpnNT_01148	bfmBAB_1	Chromosome:1170298-1171266	110.58+peptide	ΔORF2+peptide	OK	60.9858	54.309	-0.167283	-0.213074	0.7155	0.992053	no
gene:SpnNT_01149	bfmBAB_2	Chromosome:1170298-1171266	110.58	ΔORF2	OK	37.491	38.9887	0.05651	0.0782535	0.8902	0.994748	no
gene:SpnNT_01149	bfmBAB_2	Chromosome:1170298-1171266	110.58	110.58+peptide	OK	37.491	49.8902	0.412212	0.571543	0.3203	0.825861	no
gene:SpnNT_01149	bfmBAB_2	Chromosome:1170298-1171266	ΔORF2	110.58+peptide	OK	38.9887	49.8902	0.355702	0.480895	0.39645	0.890558	no
gene:SpnNT_01149	bfmBAB_2	Chromosome:1170298-1171266	110.58	ΔORF2+peptide	OK	37.491	52.8396	0.495074	0.715299	0.21665	0.704171	no
gene:SpnNT_01149	bfmBAB_2	Chromosome:1170298-1171266	ΔORF2	ΔORF2+peptide	OK	38.9887	52.8396	0.438564	0.616552	0.28095	0.784388	no
gene:SpnNT_01149	bfmBAB_2	Chromosome:1170298-1171266	110.58+peptide	ΔORF2+peptide	OK	49.8902	52.8396	0.0828616	0.116642	0.8351	0.994748	no
gene:SpnNT_01150	pdhC	Chromosome:1171482-1171626	110.58	ΔORF2	NOTEST	2.93223	2.74702	-0.0941299	0	1	1	no
gene:SpnNT_01150	pdhC	Chromosome:1171482-1171626	110.58	110.58+peptide	NOTEST	2.93223	3.20021	0.126169	0	1	1	no
gene:SpnNT_01150	pdhC	Chromosome:1171482-1171626	ΔORF2	110.58+peptide	NOTEST	2.74702	3.20021	0.220298	0	1	1	no
gene:SpnNT_01150	pdhC	Chromosome:1171482-1171626	110.58	ΔORF2+peptide	NOTEST	2.93223	1.59351	-0.879789	0	1	1	no
gene:SpnNT_01150	pdhC	Chromosome:1171482-1171626	ΔORF2	ΔORF2+peptide	NOTEST	2.74702	1.59351	-0.785659	0	1	1	no
gene:SpnNT_01150	pdhC	Chromosome:1171482-1171626	110.58+peptide	ΔORF2+peptide	NOTEST	3.20021	1.59351	-1.00596	0	1	1	no
gene:SpnNT_01151	NA	Chromosome:1171638-1173342	110.58	ΔORF2	OK	3.27065	3.45903	0.0807892	0.126048	0.82545	0.994748	no
gene:SpnNT_01151	NA	Chromosome:1171638-1173342	110.58	110.58+peptide	OK	3.27065	3.46563	0.0835419	0.128104	0.8253	0.994748	no
gene:SpnNT_01151	NA	Chromosome:1171638-1173342	ΔORF2	110.58+peptide	OK	3.45903	3.46563	0.00275261	0.00429341	0.9922	0.99849	no
gene:SpnNT_01151	NA	Chromosome:1171638-1173342	110.58	ΔORF2+peptide	OK	3.27065	2.78029	-0.23434	-0.360994	0.53515	0.958867	no
gene:SpnNT_01151	NA	Chromosome:1171638-1173342	ΔORF2	ΔORF2+peptide	OK	3.45903	2.78029	-0.31513	-0.493867	0.39475	0.889826	no
gene:SpnNT_01151	NA	Chromosome:1171638-1173342	110.58+peptide	ΔORF2+peptide	OK	3.46563	2.78029	-0.317882	-0.489549	0.3969	0.89095	no
gene:SpnNT_01152	lplJ	Chromosome:1173412-1173766	110.58	ΔORF2	OK	4.37405	5.05466	0.208642	0.211855	0.715	0.992053	no
gene:SpnNT_01152	lplJ	Chromosome:1173412-1173766	110.58	110.58+peptide	OK	4.37405	4.97301	0.185147	0.192411	0.73965	0.994748	no
gene:SpnNT_01152	lplJ	Chromosome:1173412-1173766	ΔORF2	110.58+peptide	OK	5.05466	4.97301	-0.0234955	-0.0242987	0.96315	0.994855	no
gene:SpnNT_01152	lplJ	Chromosome:1173412-1173766	110.58	ΔORF2+peptide	OK	4.37405	3.05117	-0.519606	-0.493064	0.408	0.897087	no
gene:SpnNT_01152	lplJ	Chromosome:1173412-1173766	ΔORF2	ΔORF2+peptide	OK	5.05466	3.05117	-0.728249	-0.688245	0.24185	0.736324	no
gene:SpnNT_01152	lplJ	Chromosome:1173412-1173766	110.58+peptide	ΔORF2+peptide	OK	4.97301	3.05117	-0.704753	-0.679522	0.24735	0.743921	no
gene:SpnNT_01153	ltrA_3	Chromosome:1173930-1174989	110.58	ΔORF2	OK	1.8599	2.24146	0.269212	0.346025	0.54575	0.961816	no
gene:SpnNT_01153	ltrA_3	Chromosome:1173930-1174989	110.58	110.58+peptide	OK	1.8599	1.51635	-0.294621	-0.359095	0.52335	0.954832	no
gene:SpnNT_01153	ltrA_3	Chromosome:1173930-1174989	ΔORF2	110.58+peptide	OK	2.24146	1.51635	-0.563834	-0.700859	0.22945	0.722182	no
gene:SpnNT_01153	ltrA_3	Chromosome:1173930-1174989	110.58	ΔORF2+peptide	OK	1.8599	1.91438	0.0416525	0.0517603	0.92805	0.994748	no
gene:SpnNT_01153	ltrA_3	Chromosome:1173930-1174989	ΔORF2	ΔORF2+peptide	OK	2.24146	1.91438	-0.22756	-0.288622	0.61885	0.980887	no
gene:SpnNT_01153	ltrA_3	Chromosome:1173930-1174989	110.58+peptide	ΔORF2+peptide	OK	1.51635	1.91438	0.336274	0.404981	0.4794	0.933711	no
gene:SpnNT_01154	xerS	Chromosome:1175689-1176760	110.58	ΔORF2	OK	82.902	84.7371	0.0315866	0.0686165	0.90675	0.994748	no
gene:SpnNT_01154	xerS	Chromosome:1175689-1176760	110.58	110.58+peptide	OK	82.902	79.2557	-0.0648935	-0.135059	0.81295	0.994748	no
gene:SpnNT_01154	xerS	Chromosome:1175689-1176760	ΔORF2	110.58+peptide	OK	84.7371	79.2557	-0.0964801	-0.202973	0.7229	0.994748	no
gene:SpnNT_01154	xerS	Chromosome:1175689-1176760	110.58	ΔORF2+peptide	OK	82.902	68.4911	-0.275491	-0.584992	0.29805	0.803352	no
gene:SpnNT_01154	xerS	Chromosome:1175689-1176760	ΔORF2	ΔORF2+peptide	OK	84.7371	68.4911	-0.307077	-0.659421	0.2483	0.744389	no
gene:SpnNT_01154	xerS	Chromosome:1175689-1176760	110.58+peptide	ΔORF2+peptide	OK	79.2557	68.4911	-0.210597	-0.433682	0.44205	0.917378	no
gene:SpnNT_01155	clcA	Chromosome:1177343-1178894	110.58	ΔORF2	OK	34.1941	38.2029	0.159935	0.340265	0.547	0.962122	no
gene:SpnNT_01155	clcA	Chromosome:1177343-1178894	110.58	110.58+peptide	OK	34.1941	40.7257	0.252193	0.531784	0.35625	0.857745	no
gene:SpnNT_01155	clcA	Chromosome:1177343-1178894	ΔORF2	110.58+peptide	OK	38.2029	40.7257	0.0922587	0.195162	0.7308	0.994748	no
gene:SpnNT_01155	clcA	Chromosome:1177343-1178894	110.58	ΔORF2+peptide	OK	34.1941	49.1093	0.522247	1.11622	0.05475	0.332606	no
gene:SpnNT_01155	clcA	Chromosome:1177343-1178894	ΔORF2	ΔORF2+peptide	OK	38.2029	49.1093	0.362313	0.776926	0.1789	0.645815	no

gene:SpnNT_01155	clcA	Chromosome:1177343-1178894	110.58+peptide	ΔORF2+peptide	OK	40.7257	49.1093	0.270054	0.573869	0.32525	0.830337	no
gene:SpnNT_01156	rnhB	Chromosome:1178909-1180527	110.58	ΔORF2	OK	22.9828	24.411	0.0869799	0.0990203	0.86265	0.994748	no
gene:SpnNT_01156	rnhB	Chromosome:1178909-1180527	110.58	110.58+peptide	OK	22.9828	25.3005	0.138615	0.159081	0.77655	0.994748	no
gene:SpnNT_01156	rnhB	Chromosome:1178909-1180527	ΔORF2	110.58+peptide	OK	24.411	25.3005	0.0516352	0.0562826	0.92125	0.994748	no
gene:SpnNT_01156	rnhB	Chromosome:1178909-1180527	110.58	ΔORF2+peptide	OK	22.9828	34.5668	0.588833	0.703741	0.2184	0.706395	no
gene:SpnNT_01156	rnhB	Chromosome:1178909-1180527	ΔORF2	ΔORF2+peptide	OK	24.411	34.5668	0.501853	0.56732	0.33095	0.836749	no
gene:SpnNT_01156	rnhB	Chromosome:1178909-1180527	110.58+peptide	ΔORF2+peptide	OK	25.3005	34.5668	0.450217	0.513012	0.3749	0.87255	no
gene:SpnNT_01157	rbgA	Chromosome:1178909-1180527	110.58	ΔORF2	OK	41.7031	50.9929	0.290144	0.500062	0.3867	0.881745	no
gene:SpnNT_01157	rbgA	Chromosome:1178909-1180527	110.58	110.58+peptide	OK	41.7031	48.7036	0.223876	0.385095	0.5048	0.945292	no
gene:SpnNT_01157	rbgA	Chromosome:1178909-1180527	ΔORF2	110.58+peptide	OK	50.9929	48.7036	-0.0662686	-0.114143	0.8379	0.994748	no
gene:SpnNT_01157	rbgA	Chromosome:1178909-1180527	110.58	ΔORF2+peptide	OK	41.7031	58.1207	0.478898	0.823847	0.15225	0.594768	no
gene:SpnNT_01157	rbgA	Chromosome:1178909-1180527	ΔORF2	ΔORF2+peptide	OK	50.9929	58.1207	0.188753	0.325147	0.5623	0.968621	no
gene:SpnNT_01157	rbgA	Chromosome:1178909-1180527	110.58+peptide	ΔORF2+peptide	OK	48.7036	58.1207	0.255022	0.438444	0.44975	0.920402	no
gene:SpnNT_01158	iga_3	Chromosome:1180786-1182703	110.58	ΔORF2	OK	19.895	22.4138	0.171987	0.355412	0.53145	0.957488	no
gene:SpnNT_01158	iga_3	Chromosome:1180786-1182703	110.58	110.58+peptide	OK	19.895	31.797	0.676485	1.41937	0.0136	0.125322	no
gene:SpnNT_01158	iga_3	Chromosome:1180786-1182703	ΔORF2	110.58+peptide	OK	22.4138	31.797	0.504498	1.06701	0.0703	0.390219	no
gene:SpnNT_01158	iga_3	Chromosome:1180786-1182703	110.58	ΔORF2+peptide	OK	19.895	39.5477	0.991191	2.10404	2.00E-04	0.00450928	yes
gene:SpnNT_01158	iga_3	Chromosome:1180786-1182703	ΔORF2	ΔORF2+peptide	OK	22.4138	39.5477	0.819204	1.75325	0.0032	0.0422439	yes
gene:SpnNT_01158	iga_3	Chromosome:1180786-1182703	110.58+peptide	ΔORF2+peptide	OK	31.797	39.5477	0.314706	0.684608	0.23785	0.732745	no
gene:SpnNT_01159	iga_4	Chromosome:1182877-1185841	110.58	ΔORF2	OK	61.753	66.6046	0.109112	0.24925	0.66225	0.982966	no
gene:SpnNT_01159	iga_4	Chromosome:1182877-1185841	110.58	110.58+peptide	OK	61.753	87.8214	0.508063	1.15191	0.0443	0.289062	no
gene:SpnNT_01159	iga_4	Chromosome:1182877-1185841	ΔORF2	110.58+peptide	OK	66.6046	87.8214	0.398951	0.911612	0.1051	0.492368	no
gene:SpnNT_01159	iga_4	Chromosome:1182877-1185841	110.58	ΔORF2+peptide	OK	61.753	94.6757	0.616484	1.40653	0.0158	0.139615	no
gene:SpnNT_01159	iga_4	Chromosome:1182877-1185841	ΔORF2	ΔORF2+peptide	OK	66.6046	94.6757	0.507372	1.16677	0.0384	0.263814	no
gene:SpnNT_01159	iga_4	Chromosome:1182877-1185841	110.58+peptide	ΔORF2+peptide	OK	87.8214	94.6757	0.108421	0.247438	0.66165	0.982966	no
gene:SpnNT_01160	iga_5	Chromosome:1185949-1191166	110.58	ΔORF2	OK	41.0323	50.05	0.286608	0.655791	0.24645	0.74356	no
gene:SpnNT_01160	iga_5	Chromosome:1185949-1191166	110.58	110.58+peptide	OK	41.0323	58.8805	0.52103	1.18411	0.034	0.243	no
gene:SpnNT_01160	iga_5	Chromosome:1185949-1191166	ΔORF2	110.58+peptide	OK	50.05	58.8805	0.234422	0.529786	0.34905	0.853357	no
gene:SpnNT_01160	iga_5	Chromosome:1185949-1191166	110.58	ΔORF2+peptide	OK	41.0323	83.7541	1.0294	2.3561	1.00E-04	0.0025332	yes
gene:SpnNT_01160	iga_5	Chromosome:1185949-1191166	ΔORF2	ΔORF2+peptide	OK	50.05	83.7541	0.74279	1.6905	0.00365	0.0469562	yes
gene:SpnNT_01160	iga_5	Chromosome:1185949-1191166	110.58+peptide	ΔORF2+peptide	OK	58.8805	83.7541	0.508368	1.14924	0.0429	0.28388	no
gene:SpnNT_01161	NA	Chromosome:1191404-1192583	110.58	ΔORF2	OK	13.6858	15.3053	0.161352	0.293895	0.6071	0.977869	no
gene:SpnNT_01161	NA	Chromosome:1191404-1192583	110.58	110.58+peptide	OK	13.6858	20.6736	0.595105	1.09564	0.06055	0.353599	no
gene:SpnNT_01161	NA	Chromosome:1191404-1192583	ΔORF2	110.58+peptide	OK	15.3053	20.6736	0.433753	0.809823	0.1614	0.614666	no
gene:SpnNT_01161	NA	Chromosome:1191404-1192583	110.58	ΔORF2+peptide	OK	13.6858	30.7774	1.16919	2.21283	2.00E-04	0.00450928	yes
gene:SpnNT_01161	NA	Chromosome:1191404-1192583	ΔORF2	ΔORF2+peptide	OK	15.3053	30.7774	1.00784	1.93587	0.00095	0.0161266	yes
gene:SpnNT_01161	NA	Chromosome:1191404-1192583	110.58+peptide	ΔORF2+peptide	OK	20.6736	30.7774	0.574084	1.11595	0.05025	0.31474	no
gene:SpnNT_01162	addA	Chromosome:1192600-1199523	110.58	ΔORF2	OK	16.9336	16.1669	-0.0668419	-0.108609	0.8496	0.994748	no
gene:SpnNT_01162	addA	Chromosome:1192600-1199523	110.58	110.58+peptide	OK	16.9336	24.4485	0.529861	0.86413	0.1338	0.556521	no
gene:SpnNT_01162	addA	Chromosome:1192600-1199523	ΔORF2	110.58+peptide	OK	16.1669	24.4485	0.596703	0.981445	0.08975	0.450098	no
gene:SpnNT_01162	addA	Chromosome:1192600-1199523	110.58	ΔORF2+peptide	OK	16.9336	27.3555	0.691947	1.13763	0.0467	0.299217	no
gene:SpnNT_01162	addA	Chromosome:1192600-1199523	ΔORF2	ΔORF2+peptide	OK	16.1669	27.3555	0.758789	1.25834	0.02855	0.215059	no
gene:SpnNT_01162	addA	Chromosome:1192600-1199523	110.58+peptide	ΔORF2+peptide	OK	24.4485	27.3555	0.162085	0.26983	0.63545	0.980887	no
gene:SpnNT_01163	rexB	Chromosome:1192600-1199523	110.58	ΔORF2	OK	16.111	16.5438	0.0382425	0.0586154	0.9218	0.994748	no
gene:SpnNT_01163	rexB	Chromosome:1192600-1199523	110.58	110.58+peptide	OK	16.111	21.4108	0.410292	0.602172	0.30675	0.81251	no

gene:SpnNT_01163	rexB	Chromosome:1192600-1199523	ΔORF2	110.58+peptide	OK	16.5438	21.4108	0.37205	0.556916	0.34225	0.846365	no
gene:SpnNT_01163	rexB	Chromosome:1192600-1199523	110.58	ΔORF2+peptide	OK	16.111	25.7246	0.675101	1.01125	0.0804	0.422849	no
gene:SpnNT_01163	rexB	Chromosome:1192600-1199523	ΔORF2	ΔORF2+peptide	OK	16.5438	25.7246	0.636858	0.973774	0.091	0.453591	no
gene:SpnNT_01163	rexB	Chromosome:1192600-1199523	110.58+peptide	ΔORF2+peptide	OK	21.4108	25.7246	0.264809	0.387789	0.50535	0.945292	no
gene:SpnNT_01164	NA	Chromosome:1200033-1200402	110.58	ΔORF2	OK	86.0441	74.6005	-0.20589	-0.366741	0.52395	0.954832	no
gene:SpnNT_01164	NA	Chromosome:1200033-1200402	110.58	110.58+peptide	OK	86.0441	71.504	-0.267052	-0.453864	0.4245	0.907534	no
gene:SpnNT_01164	NA	Chromosome:1200033-1200402	ΔORF2	110.58+peptide	OK	74.6005	71.504	-0.0611622	-0.103955	0.85785	0.994748	no
gene:SpnNT_01164	NA	Chromosome:1200033-1200402	110.58	ΔORF2+peptide	OK	86.0441	66.6603	-0.368249	-0.614853	0.272	0.774059	no
gene:SpnNT_01164	NA	Chromosome:1200033-1200402	ΔORF2	ΔORF2+peptide	OK	74.6005	66.6603	-0.162359	-0.271105	0.62615	0.980887	no
gene:SpnNT_01164	NA	Chromosome:1200033-1200402	110.58+peptide	ΔORF2+peptide	OK	71.504	66.6603	-0.101197	-0.162109	0.7682	0.994748	no
gene:SpnNT_01165	eno	Chromosome:1201491-1202796	110.58	ΔORF2	OK	1762.95	1577.83	-0.160052	-0.327661	0.56065	0.968621	no
gene:SpnNT_01165	eno	Chromosome:1201491-1202796	110.58	110.58+peptide	OK	1762.95	1877.28	0.0906474	0.183756	0.7532	0.994748	no
gene:SpnNT_01165	eno	Chromosome:1201491-1202796	ΔORF2	110.58+peptide	OK	1577.83	1877.28	0.250699	0.522825	0.36005	0.860974	no
gene:SpnNT_01165	eno	Chromosome:1201491-1202796	110.58	ΔORF2+peptide	OK	1762.95	1584.33	-0.154126	-0.316564	0.5773	0.969538	no
gene:SpnNT_01165	eno	Chromosome:1201491-1202796	ΔORF2	ΔORF2+peptide	OK	1577.83	1584.33	0.00592591	0.0125313	0.9833	0.996246	no
gene:SpnNT_01165	eno	Chromosome:1201491-1202796	110.58+peptide	ΔORF2+peptide	OK	1877.28	1584.33	-0.244773	-0.512204	0.37275	0.869823	no
gene:SpnNT_01166	NA	Chromosome:1202959-1204518	110.58	ΔORF2	OK	45.144	66.7253	0.563698	0.848286	0.15245	0.595195	no
gene:SpnNT_01166	NA	Chromosome:1202959-1204518	110.58	110.58+peptide	OK	45.144	77.9236	0.787525	1.20284	0.0427	0.282842	no
gene:SpnNT_01166	NA	Chromosome:1202959-1204518	ΔORF2	110.58+peptide	OK	66.7253	77.9236	0.223826	0.35229	0.56085	0.968621	no
gene:SpnNT_01166	NA	Chromosome:1202959-1204518	110.58	ΔORF2+peptide	OK	45.144	122.024	1.43456	2.29487	1.00E-04	0.0025332	yes
gene:SpnNT_01166	NA	Chromosome:1202959-1204518	ΔORF2	ΔORF2+peptide	OK	66.7253	122.024	0.870863	1.43993	0.01585	0.139962	no
gene:SpnNT_01166	NA	Chromosome:1202959-1204518	110.58+peptide	ΔORF2+peptide	OK	77.9236	122.024	0.647036	1.08926	0.06455	0.368753	no
gene:SpnNT_01167	glxK	Chromosome:1202959-1204518	110.58	ΔORF2	OK	6.16609	6.50758	0.0777651	0.0734461	0.8929	0.994748	no
gene:SpnNT_01167	glxK	Chromosome:1202959-1204518	110.58	110.58+peptide	OK	6.16609	7.31707	0.246908	0.226135	0.69525	0.98895	no
gene:SpnNT_01167	glxK	Chromosome:1202959-1204518	ΔORF2	110.58+peptide	OK	6.50758	7.31707	0.169143	0.142648	0.79525	0.994748	no
gene:SpnNT_01167	glxK	Chromosome:1202959-1204518	110.58	ΔORF2+peptide	OK	6.16609	8.41128	0.447968	0.423083	0.45575	0.921244	no
gene:SpnNT_01167	glxK	Chromosome:1202959-1204518	ΔORF2	ΔORF2+peptide	OK	6.50758	8.41128	0.370203	0.320416	0.55605	0.966729	no
gene:SpnNT_01167	glxK	Chromosome:1202959-1204518	110.58+peptide	ΔORF2+peptide	OK	7.31707	8.41128	0.20106	0.169563	0.76465	0.994748	no
gene:SpnNT_01168	NA	Chromosome:1204774-1205242	110.58	ΔORF2	OK	6.75575	5.13018	-0.397108	-0.497367	0.38615	0.881745	no
gene:SpnNT_01168	NA	Chromosome:1204774-1205242	110.58	110.58+peptide	OK	6.75575	5.82268	-0.214432	-0.276083	0.6371	0.980887	no
gene:SpnNT_01168	NA	Chromosome:1204774-1205242	ΔORF2	110.58+peptide	OK	5.13018	5.82268	0.182675	0.22735	0.6935	0.98828	no
gene:SpnNT_01168	NA	Chromosome:1204774-1205242	110.58	ΔORF2+peptide	OK	6.75575	7.18052	0.0879729	0.113704	0.84465	0.994748	no
gene:SpnNT_01168	NA	Chromosome:1204774-1205242	ΔORF2	ΔORF2+peptide	OK	5.13018	7.18052	0.485081	0.605892	0.2907	0.795003	no
gene:SpnNT_01168	NA	Chromosome:1204774-1205242	110.58+peptide	ΔORF2+peptide	OK	5.82268	7.18052	0.302405	0.388226	0.4984	0.944017	no
gene:SpnNT_01169	NA	Chromosome:1205257-1206126	110.58	ΔORF2	OK	7.16932	7.13722	-0.00647441	-0.00409747	0.98425	0.996246	no
gene:SpnNT_01169	NA	Chromosome:1205257-1206126	110.58	110.58+peptide	OK	7.16932	6.6192	-0.115179	-0.0744994	0.8956	0.994748	no
gene:SpnNT_01169	NA	Chromosome:1205257-1206126	ΔORF2	110.58+peptide	OK	7.13722	6.6192	-0.108705	-0.0732776	0.8956	0.994748	no
gene:SpnNT_01169	NA	Chromosome:1205257-1206126	110.58	ΔORF2+peptide	OK	7.16932	8.18308	0.190807	0.126531	0.8257	0.994748	no
gene:SpnNT_01169	NA	Chromosome:1205257-1206126	ΔORF2	ΔORF2+peptide	OK	7.13722	8.18308	0.197281	0.136645	0.8099	0.994748	no
gene:SpnNT_01169	NA	Chromosome:1205257-1206126	110.58+peptide	ΔORF2+peptide	OK	6.6192	8.18308	0.305987	0.217566	0.71125	0.991007	no
gene:SpnNT_01170	NA	Chromosome:1205257-1206126	110.58	ΔORF2	OK	12.6027	11.5433	-0.126673	-0.170305	0.76845	0.994748	no
gene:SpnNT_01170	NA	Chromosome:1205257-1206126	110.58	110.58+peptide	OK	12.6027	10.0982	-0.319637	-0.43207	0.46285	0.9258	no
gene:SpnNT_01170	NA	Chromosome:1205257-1206126	ΔORF2	110.58+peptide	OK	11.5433	10.0982	-0.192965	-0.255277	0.66065	0.982966	no
gene:SpnNT_01170	NA	Chromosome:1205257-1206126	110.58	ΔORF2+peptide	OK	12.6027	11.1746	-0.173508	-0.234851	0.68745	0.98828	no
gene:SpnNT_01170	NA	Chromosome:1205257-1206126	ΔORF2	ΔORF2+peptide	OK	11.5433	11.1746	-0.046835	-0.0620378	0.9136	0.994748	no

gene:SpnNT_01170	NA	Chromosome:1205257-1206126	110.58+peptide	ΔORF2+peptide	OK	10.0982	11.1746	0.14613	0.194584	0.73575	0.994748	no
gene:SpnNT_01171	serB	Chromosome:1206242-1206614	110.58	ΔORF2	OK	34.927	29.4111	-0.247978	-0.389412	0.4928	0.941402	no
gene:SpnNT_01171	serB	Chromosome:1206242-1206614	110.58	110.58+peptide	OK	34.927	16.3383	-1.09608	-1.64761	0.00645	0.0727121	no
gene:SpnNT_01171	serB	Chromosome:1206242-1206614	ΔORF2	110.58+peptide	OK	29.4111	16.3383	-0.848102	-1.22499	0.0353	0.249036	no
gene:SpnNT_01171	serB	Chromosome:1206242-1206614	110.58	ΔORF2+peptide	OK	34.927	12.6588	-1.4642	-2.09514	0.00095	0.0161266	yes
gene:SpnNT_01171	serB	Chromosome:1206242-1206614	ΔORF2	ΔORF2+peptide	OK	29.4111	12.6588	-1.21622	-1.67829	0.0046	0.0562022	no
gene:SpnNT_01171	serB	Chromosome:1206242-1206614	110.58+peptide	ΔORF2+peptide	OK	16.3383	12.6588	-0.368119	-0.490953	0.40305	0.894399	no
gene:SpnNT_01172	glgA	Chromosome:1206672-1210242	110.58	ΔORF2	OK	230.259	224.084	-0.039223	-0.0528598	0.9272	0.994748	no
gene:SpnNT_01172	glgA	Chromosome:1206672-1210242	110.58	110.58+peptide	OK	230.259	84.6431	-1.4438	-2.02289	8.00E-04	0.0140342	yes
gene:SpnNT_01172	glgA	Chromosome:1206672-1210242	ΔORF2	110.58+peptide	OK	224.084	84.6431	-1.40457	-1.98592	0.0014	0.0220273	yes
gene:SpnNT_01172	glgA	Chromosome:1206672-1210242	110.58	ΔORF2+peptide	OK	230.259	71.5362	-1.68652	-2.28499	0.00015	0.00355289	yes
gene:SpnNT_01172	glgA	Chromosome:1206672-1210242	ΔORF2	ΔORF2+peptide	OK	224.084	71.5362	-1.64729	-2.2509	0.00035	0.00717609	yes
gene:SpnNT_01172	glgA	Chromosome:1206672-1210242	110.58+peptide	ΔORF2+peptide	OK	84.6431	71.5362	-0.24272	-0.345196	0.5547	0.966389	no
gene:SpnNT_01173	glgD	Chromosome:1206672-1210242	110.58	ΔORF2	OK	253.77	238.973	-0.0866708	-0.0841234	0.8834	0.994748	no
gene:SpnNT_01173	glgD	Chromosome:1206672-1210242	110.58	110.58+peptide	OK	253.77	83.6753	-1.60065	-1.583	0.00785	0.0847103	no
gene:SpnNT_01173	glgD	Chromosome:1206672-1210242	ΔORF2	110.58+peptide	OK	238.973	83.6753	-1.51397	-1.51699	0.00985	0.0990434	no
gene:SpnNT_01173	glgD	Chromosome:1206672-1210242	110.58	ΔORF2+peptide	OK	253.77	85.0124	-1.57777	-1.57847	0.0083	0.0883314	no
gene:SpnNT_01173	glgD	Chromosome:1206672-1210242	ΔORF2	ΔORF2+peptide	OK	238.973	85.0124	-1.4911	-1.51186	0.0097	0.0982125	no
gene:SpnNT_01173	glgD	Chromosome:1206672-1210242	110.58+peptide	ΔORF2+peptide	OK	83.6753	85.0124	0.0228716	0.0236701	0.9651	0.994855	no
gene:SpnNT_01174	gapN	Chromosome:1206672-1210242	110.58	ΔORF2	OK	227.797	223.619	-0.0267017	-0.0347357	0.9515	0.994855	no
gene:SpnNT_01174	gapN	Chromosome:1206672-1210242	110.58	110.58+peptide	OK	227.797	71.7947	-1.6658	-2.18888	3.00E-04	0.00631878	yes
gene:SpnNT_01174	gapN	Chromosome:1206672-1210242	ΔORF2	110.58+peptide	OK	223.619	71.7947	-1.6391	-2.17825	4.00E-04	0.00811252	yes
gene:SpnNT_01174	gapN	Chromosome:1206672-1210242	110.58	ΔORF2+peptide	OK	227.797	65.4001	-1.80038	-2.3209	0.00015	0.00355289	yes
gene:SpnNT_01174	gapN	Chromosome:1206672-1210242	ΔORF2	ΔORF2+peptide	OK	223.619	65.4001	-1.77368	-2.31145	1.00E-04	0.0025332	yes
gene:SpnNT_01174	gapN	Chromosome:1206672-1210242	110.58+peptide	ΔORF2+peptide	OK	71.7947	65.4001	-0.134586	-0.177169	0.75185	0.994748	no
gene:SpnNT_01175	amyX	Chromosome:1210785-1213065	110.58	ΔORF2	OK	41.881	44.5446	0.0889549	0.19982	0.73445	0.994748	no
gene:SpnNT_01175	amyX	Chromosome:1210785-1213065	110.58	110.58+peptide	OK	41.881	63.2872	0.595618	1.35143	0.0184	0.156073	no
gene:SpnNT_01175	amyX	Chromosome:1210785-1213065	ΔORF2	110.58+peptide	OK	44.5446	63.2872	0.506663	1.15158	0.0438	0.286941	no
gene:SpnNT_01175	amyX	Chromosome:1210785-1213065	110.58	ΔORF2+peptide	OK	41.881	75.5187	0.850538	1.92986	0.00075	0.0133173	yes
gene:SpnNT_01175	amyX	Chromosome:1210785-1213065	ΔORF2	ΔORF2+peptide	OK	44.5446	75.5187	0.761583	1.73101	0.002	0.0294545	yes
gene:SpnNT_01175	amyX	Chromosome:1210785-1213065	110.58+peptide	ΔORF2+peptide	OK	63.2872	75.5187	0.25492	0.585395	0.3042	0.809811	no
gene:SpnNT_01176	ligA	Chromosome:1213176-1215135	110.58	ΔORF2	OK	58.2607	58.3159	0.00136611	0.00306825	0.996	0.99927	no
gene:SpnNT_01176	ligA	Chromosome:1213176-1215135	110.58	110.58+peptide	OK	58.2607	68.1401	0.22598	0.51118	0.3597	0.860959	no
gene:SpnNT_01176	ligA	Chromosome:1213176-1215135	ΔORF2	110.58+peptide	OK	58.3159	68.1401	0.224614	0.5106	0.3692	0.866165	no
gene:SpnNT_01176	ligA	Chromosome:1213176-1215135	110.58	ΔORF2+peptide	OK	58.2607	73.9546	0.344116	0.77857	0.1627	0.61656	no
gene:SpnNT_01176	ligA	Chromosome:1213176-1215135	ΔORF2	ΔORF2+peptide	OK	58.3159	73.9546	0.34275	0.779313	0.1698	0.627815	no
gene:SpnNT_01176	ligA	Chromosome:1213176-1215135	110.58+peptide	ΔORF2+peptide	OK	68.1401	73.9546	0.118137	0.270579	0.62885	0.980887	no
gene:SpnNT_01177	NA	Chromosome:1215227-1216406	110.58	ΔORF2	OK	9.40654	11.4069	0.278175	0.486384	0.40175	0.894197	no
gene:SpnNT_01177	NA	Chromosome:1215227-1216406	110.58	110.58+peptide	OK	9.40654	12.4123	0.400038	0.69082	0.2359	0.730203	no
gene:SpnNT_01177	NA	Chromosome:1215227-1216406	ΔORF2	110.58+peptide	OK	11.4069	12.4123	0.121863	0.214536	0.7129	0.991272	no
gene:SpnNT_01177	NA	Chromosome:1215227-1216406	110.58	ΔORF2+peptide	OK	9.40654	16.6402	0.822941	1.46132	0.01055	0.104245	no
gene:SpnNT_01177	NA	Chromosome:1215227-1216406	ΔORF2	ΔORF2+peptide	OK	11.4069	16.6402	0.544765	0.987278	0.082	0.428175	no
gene:SpnNT_01177	NA	Chromosome:1215227-1216406	110.58+peptide	ΔORF2+peptide	OK	12.4123	16.6402	0.422902	0.756269	0.18825	0.664751	no
gene:SpnNT_01178	NA	Chromosome:1216485-1217349	110.58	ΔORF2	OK	60.5104	78.3105	0.372022	0.775486	0.1869	0.661936	no
gene:SpnNT_01178	NA	Chromosome:1216485-1217349	110.58	110.58+peptide	OK	60.5104	67.3248	0.153954	0.322417	0.5806	0.969538	no

gene:SpnNT_01178	NA	Chromosome:1216485-1217349	ΔORF2	110.58+peptide	OK	78.3105	67.3248	-0.218067	-0.456012	0.434	0.91353	no
gene:SpnNT_01178	NA	Chromosome:1216485-1217349	110.58	ΔORF2+peptide	OK	60.5104	90.5271	0.581166	1.23184	0.0348	0.246835	no
gene:SpnNT_01178	NA	Chromosome:1216485-1217349	ΔORF2	ΔORF2+peptide	OK	78.3105	90.5271	0.209145	0.442636	0.44755	0.919505	no
gene:SpnNT_01178	NA	Chromosome:1216485-1217349	110.58+peptide	ΔORF2+peptide	OK	67.3248	90.5271	0.427212	0.908503	0.1212	0.52727	no
gene:SpnNT_01179	yheS_2	Chromosome:1217351-1219253	110.58	ΔORF2	OK	57.1374	64.5765	0.176574	0.39803	0.49255	0.941402	no
gene:SpnNT_01179	yheS_2	Chromosome:1217351-1219253	110.58	110.58+peptide	OK	57.1374	49.0226	-0.220988	-0.493221	0.3932	0.888509	no
gene:SpnNT_01179	yheS_2	Chromosome:1217351-1219253	ΔORF2	110.58+peptide	OK	64.5765	49.0226	-0.397563	-0.888151	0.12335	0.531211	no
gene:SpnNT_01179	yheS_2	Chromosome:1217351-1219253	110.58	ΔORF2+peptide	OK	57.1374	55.5732	-0.0400455	-0.0893009	0.88245	0.994748	no
gene:SpnNT_01179	yheS_2	Chromosome:1217351-1219253	ΔORF2	ΔORF2+peptide	OK	64.5765	55.5732	-0.21662	-0.483514	0.40145	0.894197	no
gene:SpnNT_01179	yheS_2	Chromosome:1217351-1219253	110.58+peptide	ΔORF2+peptide	OK	49.0226	55.5732	0.180943	0.399962	0.49295	0.941402	no
gene:SpnNT_01180	hup	Chromosome:1219678-1219954	110.58	ΔORF2	OK	8665.72	11205.3	0.370794	0.712208	0.20465	0.689595	no
gene:SpnNT_01180	hup	Chromosome:1219678-1219954	110.58	110.58+peptide	OK	8665.72	13438	0.632923	1.17701	0.03445	0.245281	no
gene:SpnNT_01180	hup	Chromosome:1219678-1219954	ΔORF2	110.58+peptide	OK	11205.3	13438	0.262129	0.482554	0.3766	0.875032	no
gene:SpnNT_01180	hup	Chromosome:1219678-1219954	110.58	ΔORF2+peptide	OK	8665.72	11231.9	0.374218	0.757227	0.1729	0.632681	no
gene:SpnNT_01180	hup	Chromosome:1219678-1219954	ΔORF2	ΔORF2+peptide	OK	11205.3	11231.9	0.00342372	0.00684542	0.99035	0.997879	no
gene:SpnNT_01180	hup	Chromosome:1219678-1219954	110.58+peptide	ΔORF2+peptide	OK	13438	11231.9	-0.258706	-0.499494	0.35725	0.858392	no
gene:SpnNT_01181	NA	Chromosome:1220060-1220900	110.58	ΔORF2	OK	151.258	180.393	0.254138	0.575237	0.31595	0.821783	no
gene:SpnNT_01181	NA	Chromosome:1220060-1220900	110.58	110.58+peptide	OK	151.258	184.013	0.282803	0.639301	0.267	0.768422	no
gene:SpnNT_01181	NA	Chromosome:1220060-1220900	ΔORF2	110.58+peptide	OK	180.393	184.013	0.0286656	0.0649925	0.91145	0.994748	no
gene:SpnNT_01181	NA	Chromosome:1220060-1220900	110.58	ΔORF2+peptide	OK	151.258	185.56	0.294879	0.670141	0.23835	0.733153	no
gene:SpnNT_01181	NA	Chromosome:1220060-1220900	ΔORF2	ΔORF2+peptide	OK	180.393	185.56	0.0407419	0.0928663	0.8708	0.994748	no
gene:SpnNT_01181	NA	Chromosome:1220060-1220900	110.58+peptide	ΔORF2+peptide	OK	184.013	185.56	0.0120763	0.0274908	0.9593	0.994855	no
gene:SpnNT_01182	NA	Chromosome:1221040-1221889	110.58	ΔORF2	OK	59.7022	85.3461	0.51554	1.06343	0.06845	0.383192	no
gene:SpnNT_01182	NA	Chromosome:1221040-1221889	110.58	110.58+peptide	OK	59.7022	48.1205	-0.311134	-0.635128	0.26905	0.770678	no
gene:SpnNT_01182	NA	Chromosome:1221040-1221889	ΔORF2	110.58+peptide	OK	85.3461	48.1205	-0.826674	-1.66885	0.00425	0.0530119	no
gene:SpnNT_01182	NA	Chromosome:1221040-1221889	110.58	ΔORF2+peptide	OK	59.7022	53.2087	-0.166122	-0.341048	0.56045	0.968621	no
gene:SpnNT_01182	NA	Chromosome:1221040-1221889	ΔORF2	ΔORF2+peptide	OK	85.3461	53.2087	-0.681663	-1.38379	0.0166	0.144638	no
gene:SpnNT_01182	NA	Chromosome:1221040-1221889	110.58+peptide	ΔORF2+peptide	OK	48.1205	53.2087	0.145011	0.291414	0.6082	0.978519	no
gene:SpnNT_01183	ribF	Chromosome:1221981-1222899	110.58	ΔORF2	OK	104.623	125.091	0.257783	0.553667	0.3361	0.840218	no
gene:SpnNT_01183	ribF	Chromosome:1221981-1222899	110.58	110.58+peptide	OK	104.623	98.5262	-0.0866139	-0.1851	0.739	0.994748	no
gene:SpnNT_01183	ribF	Chromosome:1221981-1222899	ΔORF2	110.58+peptide	OK	125.091	98.5262	-0.344396	-0.738821	0.2002	0.683054	no
gene:SpnNT_01183	ribF	Chromosome:1221981-1222899	110.58	ΔORF2+peptide	OK	104.623	102.274	-0.03276	-0.0710907	0.9047	0.994748	no
gene:SpnNT_01183	ribF	Chromosome:1221981-1222899	ΔORF2	ΔORF2+peptide	OK	125.091	102.274	-0.290543	-0.632983	0.27415	0.776305	no
gene:SpnNT_01183	ribF	Chromosome:1221981-1222899	110.58+peptide	ΔORF2+peptide	OK	98.5262	102.274	0.0538539	0.116724	0.84475	0.994748	no
gene:SpnNT_01184	rpmA	Chromosome:1223852-1224146	110.58	ΔORF2	OK	4293.04	3604.68	-0.252128	-0.525258	0.3453	0.849107	no
gene:SpnNT_01184	rpmA	Chromosome:1223852-1224146	110.58	110.58+peptide	OK	4293.04	9441.48	1.13701	2.16613	1.00E-04	0.0025332	yes
gene:SpnNT_01184	rpmA	Chromosome:1223852-1224146	ΔORF2	110.58+peptide	OK	3604.68	9441.48	1.38914	2.74017	5.00E-05	0.0013612	yes
gene:SpnNT_01184	rpmA	Chromosome:1223852-1224146	110.58	ΔORF2+peptide	OK	4293.04	7564.47	0.817239	1.5519	0.0048	0.0582661	no
gene:SpnNT_01184	rpmA	Chromosome:1223852-1224146	ΔORF2	ΔORF2+peptide	OK	3604.68	7564.47	1.06937	2.1021	0.00025	0.00542231	yes
gene:SpnNT_01184	rpmA	Chromosome:1223852-1224146	110.58+peptide	ΔORF2+peptide	OK	9441.48	7564.47	-0.319773	-0.580059	0.27525	0.777354	no
gene:SpnNT_01185	NA	Chromosome:1224162-1224507	110.58	ΔORF2	OK	489.216	552.513	0.175538	0.387797	0.4943	0.942197	no
gene:SpnNT_01185	NA	Chromosome:1224162-1224507	110.58	110.58+peptide	OK	489.216	1127.88	1.20507	2.70217	5.00E-05	0.0013612	yes
gene:SpnNT_01185	NA	Chromosome:1224162-1224507	ΔORF2	110.58+peptide	OK	552.513	1127.88	1.02953	2.33098	5.00E-05	0.0013612	yes
gene:SpnNT_01185	NA	Chromosome:1224162-1224507	110.58	ΔORF2+peptide	OK	489.216	1275.25	1.38224	3.07489	5.00E-05	0.0013612	yes
gene:SpnNT_01185	NA	Chromosome:1224162-1224507	ΔORF2	ΔORF2+peptide	OK	552.513	1275.25	1.2067	2.71005	5.00E-05	0.0013612	yes

gene:SpnNT_01185	NA	Chromosome:1224162-1224507	110.58+peptide	ΔORF2+peptide	OK	1127.88	1275.25	0.17717	0.404066	0.4754	0.930746	no
gene:SpnNT_01186	rplU	Chromosome:1224522-1224837	110.58	ΔORF2	OK	497.099	564.915	0.184501	0.401654	0.4758	0.930747	no
gene:SpnNT_01186	rplU	Chromosome:1224522-1224837	110.58	110.58+peptide	OK	497.099	1133.64	1.18935	2.62461	5.00E-05	0.0013612	yes
gene:SpnNT_01186	rplU	Chromosome:1224522-1224837	ΔORF2	110.58+peptide	OK	564.915	1133.64	1.00485	2.23266	5.00E-05	0.0013612	yes
gene:SpnNT_01186	rplU	Chromosome:1224522-1224837	110.58	ΔORF2+peptide	OK	497.099	1222.76	1.29854	2.87761	5.00E-05	0.0013612	yes
gene:SpnNT_01186	rplU	Chromosome:1224522-1224837	ΔORF2	ΔORF2+peptide	OK	564.915	1222.76	1.11404	2.48581	5.00E-05	0.0013612	yes
gene:SpnNT_01186	rplU	Chromosome:1224522-1224837	110.58+peptide	ΔORF2+peptide	OK	1133.64	1222.76	0.109182	0.247129	0.66085	0.982966	no
gene:SpnNT_01187	NA	Chromosome:1225018-1225582	110.58	ΔORF2	OK	27.0558	29.3973	0.119746	0.210447	0.7117	0.991007	no
gene:SpnNT_01187	NA	Chromosome:1225018-1225582	110.58	110.58+peptide	OK	27.0558	25.4829	-0.0864057	-0.151464	0.7865	0.994748	no
gene:SpnNT_01187	NA	Chromosome:1225018-1225582	ΔORF2	110.58+peptide	OK	29.3973	25.4829	-0.206152	-0.362136	0.52195	0.954832	no
gene:SpnNT_01187	NA	Chromosome:1225018-1225582	110.58	ΔORF2+peptide	OK	27.0558	33.1499	0.293069	0.515789	0.36665	0.864634	no
gene:SpnNT_01187	NA	Chromosome:1225018-1225582	ΔORF2	ΔORF2+peptide	OK	29.3973	33.1499	0.173323	0.305691	0.59125	0.975366	no
gene:SpnNT_01187	NA	Chromosome:1225018-1225582	110.58+peptide	ΔORF2+peptide	OK	25.4829	33.1499	0.379474	0.667556	0.23805	0.732745	no
gene:SpnNT_01188	NA	Chromosome:1226052-1226352	110.58	ΔORF2	OK	85.0088	91.8771	0.112092	0.192012	0.73395	0.994748	no
gene:SpnNT_01188	NA	Chromosome:1226052-1226352	110.58	110.58+peptide	OK	85.0088	94.3663	0.150658	0.258586	0.64575	0.980887	no
gene:SpnNT_01188	NA	Chromosome:1226052-1226352	ΔORF2	110.58+peptide	OK	91.8771	94.3663	0.0385663	0.0670268	0.9032	0.994748	no
gene:SpnNT_01188	NA	Chromosome:1226052-1226352	110.58	ΔORF2+peptide	OK	85.0088	81.9485	-0.0528956	-0.0898336	0.875	0.994748	no
gene:SpnNT_01188	NA	Chromosome:1226052-1226352	ΔORF2	ΔORF2+peptide	OK	91.8771	81.9485	-0.164988	-0.283651	0.6197	0.980887	no
gene:SpnNT_01188	NA	Chromosome:1226052-1226352	110.58+peptide	ΔORF2+peptide	OK	94.3663	81.9485	-0.203554	-0.350652	0.54245	0.961518	no
gene:SpnNT_01189	NA	Chromosome:1226536-1227812	110.58	ΔORF2	OK	1.76315	2.07993	0.238382	0.155729	0.7813	0.994748	no
gene:SpnNT_01189	NA	Chromosome:1226536-1227812	110.58	110.58+peptide	OK	1.76315	2.86963	0.70271	0.461818	0.4285	0.910129	no
gene:SpnNT_01189	NA	Chromosome:1226536-1227812	ΔORF2	110.58+peptide	OK	2.07993	2.86963	0.464328	0.319136	0.57965	0.969538	no
gene:SpnNT_01189	NA	Chromosome:1226536-1227812	110.58	ΔORF2+peptide	OK	1.76315	3.68286	1.06267	0.738597	0.1864	0.661065	no
gene:SpnNT_01189	NA	Chromosome:1226536-1227812	ΔORF2	ΔORF2+peptide	OK	2.07993	3.68286	0.824288	0.602516	0.2718	0.774059	no
gene:SpnNT_01189	NA	Chromosome:1226536-1227812	110.58+peptide	ΔORF2+peptide	OK	2.86963	3.68286	0.35996	0.265097	0.6359	0.980887	no
gene:SpnNT_01190	NA	Chromosome:1226536-1227812	110.58	ΔORF2	OK	4.9016	8.30169	0.760152	0.553697	0.3548	0.857011	no
gene:SpnNT_01190	NA	Chromosome:1226536-1227812	110.58	110.58+peptide	OK	4.9016	13.0562	1.41342	1.10428	0.07605	0.407484	no
gene:SpnNT_01190	NA	Chromosome:1226536-1227812	ΔORF2	110.58+peptide	OK	8.30169	13.0562	0.653264	0.537496	0.35365	0.856246	no
gene:SpnNT_01190	NA	Chromosome:1226536-1227812	110.58	ΔORF2+peptide	OK	4.9016	12.1482	1.30941	0.942718	0.1123	0.510073	no
gene:SpnNT_01190	NA	Chromosome:1226536-1227812	ΔORF2	ΔORF2+peptide	OK	8.30169	12.1482	0.549262	0.413066	0.4647	0.926394	no
gene:SpnNT_01190	NA	Chromosome:1226536-1227812	110.58+peptide	ΔORF2+peptide	OK	13.0562	12.1482	-0.104001	-0.0843104	0.8794	0.994748	no
gene:SpnNT_01191	NA	Chromosome:1226536-1227812	110.58	ΔORF2	OK	1.62015	2.15922	0.414383	0.270119	0.67245	0.984845	no
gene:SpnNT_01191	NA	Chromosome:1226536-1227812	110.58	110.58+peptide	OK	1.62015	4.11933	1.34628	0.980984	0.1126	0.510552	no
gene:SpnNT_01191	NA	Chromosome:1226536-1227812	ΔORF2	110.58+peptide	OK	2.15922	4.11933	0.931901	0.665087	0.27805	0.781125	no
gene:SpnNT_01191	NA	Chromosome:1226536-1227812	110.58	ΔORF2+peptide	OK	1.62015	2.96102	0.869972	0.61482	0.4266	0.908594	no
gene:SpnNT_01191	NA	Chromosome:1226536-1227812	ΔORF2	ΔORF2+peptide	OK	2.15922	2.96102	0.455588	0.315736	0.68175	0.986574	no
gene:SpnNT_01191	NA	Chromosome:1226536-1227812	110.58+peptide	ΔORF2+peptide	OK	4.11933	2.96102	-0.476312	-0.375141	0.6058	0.976937	no
gene:SpnNT_01192	pta	Chromosome:1228170-1229145	110.58	ΔORF2	OK	54.4825	57.4289	0.0759849	0.160221	0.78185	0.994748	no
gene:SpnNT_01192	pta	Chromosome:1228170-1229145	110.58	110.58+peptide	OK	54.4825	102.776	0.915638	1.98181	8.00E-04	0.0140342	yes
gene:SpnNT_01192	pta	Chromosome:1228170-1229145	ΔORF2	110.58+peptide	OK	57.4289	102.776	0.839653	1.82483	0.00175	0.0265167	yes
gene:SpnNT_01192	pta	Chromosome:1228170-1229145	110.58	ΔORF2+peptide	OK	54.4825	109.157	1.00254	2.17543	2.00E-04	0.00450928	yes
gene:SpnNT_01192	pta	Chromosome:1228170-1229145	ΔORF2	ΔORF2+peptide	OK	57.4289	109.157	0.926552	2.01888	0.00055	0.0103545	yes
gene:SpnNT_01192	pta	Chromosome:1228170-1229145	110.58+peptide	ΔORF2+peptide	OK	102.776	109.157	0.0868984	0.19471	0.72735	0.994748	no
gene:SpnNT_01193	rluD_1	Chromosome:1229188-1231555	110.58	ΔORF2	OK	25.9734	27.3392	0.0739355	0.0795492	0.8884	0.994748	no
gene:SpnNT_01193	rluD_1	Chromosome:1229188-1231555	110.58	110.58+peptide	OK	25.9734	55.5834	1.09762	1.16926	0.04555	0.294292	no

gene:SpnNT_01193	rluD_1	Chromosome:1229188-1231555	ΔORF2	110.58+peptide	OK	27.3392	55.5834	1.02368	1.10971	0.0567	0.338806	no
gene:SpnNT_01193	rluD_1	Chromosome:1229188-1231555	110.58	ΔORF2+peptide	OK	25.9734	67.2385	1.37225	1.54312	0.00835	0.0885762	no
gene:SpnNT_01193	rluD_1	Chromosome:1229188-1231555	ΔORF2	ΔORF2+peptide	OK	27.3392	67.2385	1.29831	1.48873	0.0121	0.11539	no
gene:SpnNT_01193	rluD_1	Chromosome:1229188-1231555	110.58+peptide	ΔORF2+peptide	OK	55.5834	67.2385	0.274633	0.311375	0.5853	0.972191	no
gene:SpnNT_01194	ppnK	Chromosome:1229188-1231555	110.58	ΔORF2	OK	31.6031	30.5768	-0.047629	-0.0529576	0.92505	0.994748	no
gene:SpnNT_01194	ppnK	Chromosome:1229188-1231555	110.58	110.58+peptide	OK	31.6031	55.3268	0.807912	0.909052	0.11205	0.509744	no
gene:SpnNT_01194	ppnK	Chromosome:1229188-1231555	ΔORF2	110.58+peptide	OK	30.5768	55.3268	0.855541	0.911093	0.1157	0.516049	no
gene:SpnNT_01194	ppnK	Chromosome:1229188-1231555	110.58	ΔORF2+peptide	OK	31.6031	66.1306	1.06525	1.20804	0.0368	0.256447	no
gene:SpnNT_01194	ppnK	Chromosome:1229188-1231555	ΔORF2	ΔORF2+peptide	OK	30.5768	66.1306	1.11288	1.19349	0.04045	0.272897	no
gene:SpnNT_01194	ppnK	Chromosome:1229188-1231555	110.58+peptide	ΔORF2+peptide	OK	55.3268	66.1306	0.25734	0.27905	0.62965	0.980887	no
gene:SpnNT_01195	yjbM	Chromosome:1229188-1231555	110.58	ΔORF2	OK	66.1341	75.1657	0.18468	0.268073	0.6417	0.980887	no
gene:SpnNT_01195	yjbM	Chromosome:1229188-1231555	110.58	110.58+peptide	OK	66.1341	134.248	1.02143	1.41669	0.01545	0.137261	no
gene:SpnNT_01195	yjbM	Chromosome:1229188-1231555	ΔORF2	110.58+peptide	OK	75.1657	134.248	0.83675	1.18086	0.04345	0.285647	no
gene:SpnNT_01195	yjbM	Chromosome:1229188-1231555	110.58	ΔORF2+peptide	OK	66.1341	137.522	1.0562	1.45988	0.0136	0.125322	no
gene:SpnNT_01195	yjbM	Chromosome:1229188-1231555	ΔORF2	ΔORF2+peptide	OK	75.1657	137.522	0.871518	1.22555	0.03715	0.258064	no
gene:SpnNT_01195	yjbM	Chromosome:1229188-1231555	110.58+peptide	ΔORF2+peptide	OK	134.248	137.522	0.034768	0.0468417	0.9329	0.994855	no
gene:SpnNT_01196	NA	Chromosome:1231682-1232252	110.58	ΔORF2	OK	18.1796	18.8438	0.0517688	0.0866466	0.88165	0.994748	no
gene:SpnNT_01196	NA	Chromosome:1231682-1232252	110.58	110.58+peptide	OK	18.1796	13.7235	-0.405673	-0.660006	0.25095	0.748571	no
gene:SpnNT_01196	NA	Chromosome:1231682-1232252	ΔORF2	110.58+peptide	OK	18.8438	13.7235	-0.457442	-0.743791	0.1938	0.674905	no
gene:SpnNT_01196	NA	Chromosome:1231682-1232252	110.58	ΔORF2+peptide	OK	18.1796	17.0035	-0.0964839	-0.160068	0.78345	0.994748	no
gene:SpnNT_01196	NA	Chromosome:1231682-1232252	ΔORF2	ΔORF2+peptide	OK	18.8438	17.0035	-0.148253	-0.245802	0.6646	0.983188	no
gene:SpnNT_01196	NA	Chromosome:1231682-1232252	110.58+peptide	ΔORF2+peptide	OK	13.7235	17.0035	0.309189	0.498562	0.3787	0.875802	no
gene:SpnNT_01197	prs2	Chromosome:1232364-1233324	110.58	ΔORF2	OK	91.5871	93.7735	0.0340373	0.0745825	0.89895	0.994748	no
gene:SpnNT_01197	prs2	Chromosome:1232364-1233324	110.58	110.58+peptide	OK	91.5871	77.2797	-0.245053	-0.533577	0.35255	0.855356	no
gene:SpnNT_01197	prs2	Chromosome:1232364-1233324	ΔORF2	110.58+peptide	OK	93.7735	77.2797	-0.279091	-0.603437	0.28145	0.784843	no
gene:SpnNT_01197	prs2	Chromosome:1232364-1233324	110.58	ΔORF2+peptide	OK	91.5871	84.138	-0.122386	-0.266725	0.6481	0.981391	no
gene:SpnNT_01197	prs2	Chromosome:1232364-1233324	ΔORF2	ΔORF2+peptide	OK	93.7735	84.138	-0.156423	-0.338515	0.5496	0.964281	no
gene:SpnNT_01197	prs2	Chromosome:1232364-1233324	110.58+peptide	ΔORF2+peptide	OK	77.2797	84.138	0.122667	0.263832	0.6465	0.980964	no
gene:SpnNT_01198	iscS_1	Chromosome:1233333-1234449	110.58	ΔORF2	OK	80.1412	88.0225	0.135328	0.299891	0.5991	0.97629	no
gene:SpnNT_01198	iscS_1	Chromosome:1233333-1234449	110.58	110.58+peptide	OK	80.1412	78.4583	-0.0306176	-0.0679881	0.90605	0.994748	no
gene:SpnNT_01198	iscS_1	Chromosome:1233333-1234449	ΔORF2	110.58+peptide	OK	88.0225	78.4583	-0.165946	-0.366373	0.52675	0.956161	no
gene:SpnNT_01198	iscS_1	Chromosome:1233333-1234449	110.58	ΔORF2+peptide	OK	80.1412	81.2572	0.0199514	0.0444661	0.9383	0.994855	no
gene:SpnNT_01198	iscS_1	Chromosome:1233333-1234449	ΔORF2	ΔORF2+peptide	OK	88.0225	81.2572	-0.115377	-0.255653	0.6557	0.982966	no
gene:SpnNT_01198	iscS_1	Chromosome:1233333-1234449	110.58+peptide	ΔORF2+peptide	OK	78.4583	81.2572	0.050569	0.11228	0.8424	0.994748	no
gene:SpnNT_01199	NA	Chromosome:1234453-1234801	110.58	ΔORF2	OK	198.102	215.007	0.118139	0.233175	0.66955	0.984845	no
gene:SpnNT_01199	NA	Chromosome:1234453-1234801	110.58	110.58+peptide	OK	198.102	194.329	-0.0277377	-0.053953	0.92205	0.994748	no
gene:SpnNT_01199	NA	Chromosome:1234453-1234801	ΔORF2	110.58+peptide	OK	215.007	194.329	-0.145877	-0.284792	0.60995	0.979374	no
gene:SpnNT_01199	NA	Chromosome:1234453-1234801	110.58	ΔORF2+peptide	OK	198.102	194.309	-0.0278934	-0.0526357	0.9248	0.994748	no
gene:SpnNT_01199	NA	Chromosome:1234453-1234801	ΔORF2	ΔORF2+peptide	OK	215.007	194.309	-0.146033	-0.276523	0.6157	0.979616	no
gene:SpnNT_01199	NA	Chromosome:1234453-1234801	110.58+peptide	ΔORF2+peptide	OK	194.329	194.309	-0.000155749	-0.00029098	0.99825	0.999412	no
gene:SpnNT_01200	NA	Chromosome:1234808-1235015	110.58	ΔORF2	OK	738.387	755.574	0.0331952	0.0588566	0.91625	0.994748	no
gene:SpnNT_01200	NA	Chromosome:1234808-1235015	110.58	110.58+peptide	OK	738.387	949.015	0.362053	0.652064	0.24125	0.736034	no
gene:SpnNT_01200	NA	Chromosome:1234808-1235015	ΔORF2	110.58+peptide	OK	755.574	949.015	0.328858	0.597175	0.2856	0.78895	no
gene:SpnNT_01200	NA	Chromosome:1234808-1235015	110.58	ΔORF2+peptide	OK	738.387	737.741	-0.00126291	-0.00230017	0.99715	0.999412	no
gene:SpnNT_01200	NA	Chromosome:1234808-1235015	ΔORF2	ΔORF2+peptide	OK	755.574	737.741	-0.0344581	-0.0632901	0.9141	0.994748	no

gene:SpnNT_01200	NA	Chromosome:1234808-1235015	110.58+peptide	ΔORF2+peptide	OK	949.015	737.741	-0.363316	-0.67863	0.2348	0.72838	no
gene:SpnNT_01201	rex	Chromosome:1235097-1235739	110.58	ΔORF2	OK	96.2271	94.3264	-0.0287821	-0.0599243	0.91765	0.994748	no
gene:SpnNT_01201	rex	Chromosome:1235097-1235739	110.58	110.58+peptide	OK	96.2271	110.503	0.199576	0.400945	0.4865	0.938388	no
gene:SpnNT_01201	rex	Chromosome:1235097-1235739	ΔORF2	110.58+peptide	OK	94.3264	110.503	0.228358	0.45906	0.4276	0.909248	no
gene:SpnNT_01201	rex	Chromosome:1235097-1235739	110.58	ΔORF2+peptide	OK	96.2271	112.715	0.228162	0.475972	0.41125	0.898655	no
gene:SpnNT_01201	rex	Chromosome:1235097-1235739	ΔORF2	ΔORF2+peptide	OK	94.3264	112.715	0.256944	0.536382	0.355	0.85704	no
gene:SpnNT_01201	rex	Chromosome:1235097-1235739	110.58+peptide	ΔORF2+peptide	OK	110.503	112.715	0.0285857	0.0575706	0.92075	0.994748	no
gene:SpnNT_01202	NA	Chromosome:1235756-1237123	110.58	ΔORF2	OK	105.077	93.5612	-0.167459	-0.297485	0.6049	0.976803	no
gene:SpnNT_01202	NA	Chromosome:1235756-1237123	110.58	110.58+peptide	OK	105.077	108.106	0.0410059	0.0689452	0.9053	0.994748	no
gene:SpnNT_01202	NA	Chromosome:1235756-1237123	ΔORF2	110.58+peptide	OK	93.5612	108.106	0.208465	0.346681	0.56245	0.968621	no
gene:SpnNT_01202	NA	Chromosome:1235756-1237123	110.58	ΔORF2+peptide	OK	105.077	112.054	0.0927516	0.168595	0.76515	0.994748	no
gene:SpnNT_01202	NA	Chromosome:1235756-1237123	ΔORF2	ΔORF2+peptide	OK	93.5612	112.054	0.26021	0.466976	0.4179	0.90311	no
gene:SpnNT_01202	NA	Chromosome:1235756-1237123	110.58+peptide	ΔORF2+peptide	OK	108.106	112.054	0.0517457	0.0877972	0.882	0.994748	no
gene:SpnNT_01203	NA	Chromosome:1235756-1237123	110.58	ΔORF2	OK	49.3463	43.1736	-0.192794	-0.223468	0.70095	0.990367	no
gene:SpnNT_01203	NA	Chromosome:1235756-1237123	110.58	110.58+peptide	OK	49.3463	58.2684	0.23977	0.266295	0.6303	0.980887	no
gene:SpnNT_01203	NA	Chromosome:1235756-1237123	ΔORF2	110.58+peptide	OK	43.1736	58.2684	0.432564	0.467896	0.3972	0.89095	no
gene:SpnNT_01203	NA	Chromosome:1235756-1237123	110.58	ΔORF2+peptide	OK	49.3463	48.7054	-0.0188613	-0.0224051	0.9667	0.994855	no
gene:SpnNT_01203	NA	Chromosome:1235756-1237123	ΔORF2	ΔORF2+peptide	OK	43.1736	48.7054	0.173933	0.200486	0.72895	0.994748	no
gene:SpnNT_01203	NA	Chromosome:1235756-1237123	110.58+peptide	ΔORF2+peptide	OK	58.2684	48.7054	-0.258631	-0.285777	0.60305	0.976761	no
gene:SpnNT_01204	pcrA	Chromosome:1237165-1239457	110.58	ΔORF2	OK	61.2315	61.6505	0.00983816	0.0225138	0.9661	0.994855	no
gene:SpnNT_01204	pcrA	Chromosome:1237165-1239457	110.58	110.58+peptide	OK	61.2315	65.8488	0.104884	0.238883	0.67695	0.98524	no
gene:SpnNT_01204	pcrA	Chromosome:1237165-1239457	ΔORF2	110.58+peptide	OK	61.6505	65.8488	0.0950459	0.216481	0.7034	0.990367	no
gene:SpnNT_01204	pcrA	Chromosome:1237165-1239457	110.58	ΔORF2+peptide	OK	61.2315	73.384	0.261191	0.595086	0.2969	0.80163	no
gene:SpnNT_01204	pcrA	Chromosome:1237165-1239457	ΔORF2	ΔORF2+peptide	OK	61.6505	73.384	0.251353	0.572686	0.3134	0.819373	no
gene:SpnNT_01204	pcrA	Chromosome:1237165-1239457	110.58+peptide	ΔORF2+peptide	OK	65.8488	73.384	0.156307	0.354461	0.53685	0.959314	no
gene:SpnNT_01205	NA	Chromosome:1239648-1239912	110.58	ΔORF2	OK	8.02332	10.0955	0.331439	0.333937	0.55465	0.966389	no
gene:SpnNT_01205	NA	Chromosome:1239648-1239912	110.58	110.58+peptide	OK	8.02332	8.26735	0.0432248	0.0424728	0.954	0.994855	no
gene:SpnNT_01205	NA	Chromosome:1239648-1239912	ΔORF2	110.58+peptide	OK	10.0955	8.26735	-0.288215	-0.286612	0.60315	0.976761	no
gene:SpnNT_01205	NA	Chromosome:1239648-1239912	110.58	ΔORF2+peptide	OK	8.02332	5.01913	-0.676762	-0.806391	0.32515	0.830337	no
gene:SpnNT_01205	NA	Chromosome:1239648-1239912	ΔORF2	ΔORF2+peptide	OK	10.0955	5.01913	-1.0082	-1.22278	0.1337	0.556521	no
gene:SpnNT_01205	NA	Chromosome:1239648-1239912	110.58+peptide	ΔORF2+peptide	OK	8.26735	5.01913	-0.719987	-0.842418	0.29265	0.79754	no
gene:SpnNT_01206	NA	Chromosome:1240192-1240735	110.58	ΔORF2	OK	30.8575	33.7606	0.129716	0.230127	0.6803	0.986361	no
gene:SpnNT_01206	NA	Chromosome:1240192-1240735	110.58	110.58+peptide	OK	30.8575	27.8205	-0.149474	-0.260708	0.6419	0.980887	no
gene:SpnNT_01206	NA	Chromosome:1240192-1240735	ΔORF2	110.58+peptide	OK	33.7606	27.8205	-0.27919	-0.488993	0.4003	0.893627	no
gene:SpnNT_01206	NA	Chromosome:1240192-1240735	110.58	ΔORF2+peptide	OK	30.8575	24.0548	-0.359297	-0.622399	0.27815	0.781125	no
gene:SpnNT_01206	NA	Chromosome:1240192-1240735	ΔORF2	ΔORF2+peptide	OK	33.7606	24.0548	-0.489013	-0.8506	0.1406	0.572434	no
gene:SpnNT_01206	NA	Chromosome:1240192-1240735	110.58+peptide	ΔORF2+peptide	OK	27.8205	24.0548	-0.209823	-0.35905	0.54255	0.961518	no
gene:SpnNT_01207	map	Chromosome:1240833-1241694	110.58	ΔORF2	OK	326.888	313.346	-0.0610401	-0.139583	0.80595	0.994748	no
gene:SpnNT_01207	map	Chromosome:1240833-1241694	110.58	110.58+peptide	OK	326.888	482.909	0.562957	1.25888	0.0292	0.218078	no
gene:SpnNT_01207	map	Chromosome:1240833-1241694	ΔORF2	110.58+peptide	OK	313.346	482.909	0.623997	1.40604	0.0136	0.125322	no
gene:SpnNT_01207	map	Chromosome:1240833-1241694	110.58	ΔORF2+peptide	OK	326.888	415.964	0.347663	0.787985	0.1686	0.62514	no
gene:SpnNT_01207	map	Chromosome:1240833-1241694	ΔORF2	ΔORF2+peptide	OK	313.346	415.964	0.408703	0.933612	0.10315	0.487584	no
gene:SpnNT_01207	map	Chromosome:1240833-1241694	110.58+peptide	ΔORF2+peptide	OK	482.909	415.964	-0.215294	-0.480952	0.4038	0.894511	no
gene:SpnNT_01208	NA	Chromosome:1241709-1243531	110.58	ΔORF2	OK	120.651	121.549	0.0107081	0.0223711	0.96765	0.994855	no
gene:SpnNT_01208	NA	Chromosome:1241709-1243531	110.58	110.58+peptide	OK	120.651	150.51	0.319027	0.672772	0.236	0.730203	no

gene:SpnNT_01208	NA	Chromosome:1241709-1243531	ΔORF2	110.58+peptide	OK	121.549	150.51	0.308319	0.649057	0.2574	0.757287	no
gene:SpnNT_01208	NA	Chromosome:1241709-1243531	110.58	ΔORF2+peptide	OK	120.651	166.842	0.467649	0.972385	0.0898	0.450098	no
gene:SpnNT_01208	NA	Chromosome:1241709-1243531	ΔORF2	ΔORF2+peptide	OK	121.549	166.842	0.456941	0.94851	0.09955	0.478496	no
gene:SpnNT_01208	NA	Chromosome:1241709-1243531	110.58+peptide	ΔORF2+peptide	OK	150.51	166.842	0.148622	0.31137	0.58785	0.973469	no
gene:SpnNT_01209	ydaF_4	Chromosome:1241709-1243531	110.58	ΔORF2	OK	75.5337	81.0949	0.102489	0.0943765	0.87005	0.994748	no
gene:SpnNT_01209	ydaF_4	Chromosome:1241709-1243531	110.58	110.58+peptide	OK	75.5337	77.8766	0.0440684	0.039759	0.94755	0.994855	no
gene:SpnNT_01209	ydaF_4	Chromosome:1241709-1243531	ΔORF2	110.58+peptide	OK	81.0949	77.8766	-0.0584208	-0.0514019	0.93	0.99482	no
gene:SpnNT_01209	ydaF_4	Chromosome:1241709-1243531	110.58	ΔORF2+peptide	OK	75.5337	110.462	0.548362	0.487954	0.39425	0.889648	no
gene:SpnNT_01209	ydaF_4	Chromosome:1241709-1243531	ΔORF2	ΔORF2+peptide	OK	81.0949	110.462	0.445873	0.387182	0.49395	0.941958	no
gene:SpnNT_01209	ydaF_4	Chromosome:1241709-1243531	110.58+peptide	ΔORF2+peptide	OK	77.8766	110.462	0.504294	0.430007	0.4606	0.924377	no
gene:SpnNT_01210	murAB	Chromosome:1243546-1244806	110.58	ΔORF2	OK	294.018	300.605	0.0319648	0.07269	0.9002	0.994748	no
gene:SpnNT_01210	murAB	Chromosome:1243546-1244806	110.58	110.58+peptide	OK	294.018	319.121	0.118197	0.268972	0.6344	0.980887	no
gene:SpnNT_01210	murAB	Chromosome:1243546-1244806	ΔORF2	110.58+peptide	OK	300.605	319.121	0.086232	0.196316	0.73245	0.994748	no
gene:SpnNT_01210	murAB	Chromosome:1243546-1244806	110.58	ΔORF2+peptide	OK	294.018	345.294	0.231917	0.528068	0.35875	0.859746	no
gene:SpnNT_01210	murAB	Chromosome:1243546-1244806	ΔORF2	ΔORF2+peptide	OK	300.605	345.294	0.199953	0.455481	0.4338	0.913361	no
gene:SpnNT_01210	murAB	Chromosome:1243546-1244806	110.58+peptide	ΔORF2+peptide	OK	319.121	345.294	0.113721	0.259228	0.65485	0.982966	no
gene:SpnNT_01211	NA	Chromosome:1246190-1247186	110.58	ΔORF2	OK	3.81763	3.63076	-0.072404	-0.105338	0.857	0.994748	no
gene:SpnNT_01211	NA	Chromosome:1246190-1247186	110.58	110.58+peptide	OK	3.81763	6.33173	0.729923	1.11179	0.05605	0.337535	no
gene:SpnNT_01211	NA	Chromosome:1246190-1247186	ΔORF2	110.58+peptide	OK	3.63076	6.33173	0.802327	1.21609	0.0397	0.269639	no
gene:SpnNT_01211	NA	Chromosome:1246190-1247186	110.58	ΔORF2+peptide	OK	3.81763	6.61948	0.794042	1.20797	0.0385	0.263948	no
gene:SpnNT_01211	NA	Chromosome:1246190-1247186	ΔORF2	ΔORF2+peptide	OK	3.63076	6.61948	0.866446	1.31168	0.025	0.197145	no
gene:SpnNT_01211	NA	Chromosome:1246190-1247186	110.58+peptide	ΔORF2+peptide	OK	6.33173	6.61948	0.0641187	0.10203	0.86325	0.994748	no
gene:SpnNT_01212	NA	Chromosome:1247236-1247395	110.58	ΔORF2	OK	103.195	103.139	-0.000775531	-0.0009859	0.97645	0.99536	no
gene:SpnNT_01212	NA	Chromosome:1247236-1247395	110.58	110.58+peptide	OK	103.195	104.809	0.0223838	0.0275888	0.9711	0.99536	no
gene:SpnNT_01212	NA	Chromosome:1247236-1247395	ΔORF2	110.58+peptide	OK	103.139	104.809	0.0231593	0.0284676	0.96935	0.995057	no
gene:SpnNT_01212	NA	Chromosome:1247236-1247395	110.58	ΔORF2+peptide	OK	103.195	199.168	0.948616	1.24007	0.03235	0.23427	no
gene:SpnNT_01212	NA	Chromosome:1247236-1247395	ΔORF2	ΔORF2+peptide	OK	103.139	199.168	0.949392	1.23731	0.0357	0.251183	no
gene:SpnNT_01212	NA	Chromosome:1247236-1247395	110.58+peptide	ΔORF2+peptide	OK	104.809	199.168	0.926232	1.16857	0.04555	0.294292	no
gene:SpnNT_01213	obg	Chromosome:1247404-1248715	110.58	ΔORF2	OK	229.14	223.805	-0.0339881	-0.0775524	0.88995	0.994748	no
gene:SpnNT_01213	obg	Chromosome:1247404-1248715	110.58	110.58+peptide	OK	229.14	249.637	0.123602	0.282619	0.6093	0.978849	no
gene:SpnNT_01213	obg	Chromosome:1247404-1248715	ΔORF2	110.58+peptide	OK	223.805	249.637	0.15759	0.361858	0.51235	0.950409	no
gene:SpnNT_01213	obg	Chromosome:1247404-1248715	110.58	ΔORF2+peptide	OK	229.14	255.202	0.155409	0.355272	0.5279	0.956161	no
gene:SpnNT_01213	obg	Chromosome:1247404-1248715	ΔORF2	ΔORF2+peptide	OK	223.805	255.202	0.189397	0.434802	0.43465	0.913666	no
gene:SpnNT_01213	obg	Chromosome:1247404-1248715	110.58+peptide	ΔORF2+peptide	OK	249.637	255.202	0.0318067	0.0731739	0.89455	0.994748	no
gene:SpnNT_01214	NA	Chromosome:1248778-1248913	110.58	ΔORF2	OK	142.72	246.923	0.790869	0.944317	0.1092	0.502305	no
gene:SpnNT_01214	NA	Chromosome:1248778-1248913	110.58	110.58+peptide	OK	142.72	208.807	0.548978	0.594286	0.2988	0.80426	no
gene:SpnNT_01214	NA	Chromosome:1248778-1248913	ΔORF2	110.58+peptide	OK	246.923	208.807	-0.241891	-0.291748	0.62065	0.980887	no
gene:SpnNT_01214	NA	Chromosome:1248778-1248913	110.58	ΔORF2+peptide	OK	142.72	65.5189	-1.12321	-1.67629	0.08265	0.430541	no
gene:SpnNT_01214	NA	Chromosome:1248778-1248913	ΔORF2	ΔORF2+peptide	OK	246.923	65.5189	-1.91408	-3.59767	0.0079	0.0851798	no
gene:SpnNT_01214	NA	Chromosome:1248778-1248913	110.58+peptide	ΔORF2+peptide	OK	208.807	65.5189	-1.67219	-2.53542	0.0164	0.143851	no
gene:SpnNT_01215	NA	Chromosome:1248971-1249163	110.58	ΔORF2	OK	60.181	80.9998	0.42861	0.581412	0.31865	0.824882	no
gene:SpnNT_01215	NA	Chromosome:1248971-1249163	110.58	110.58+peptide	OK	60.181	78.8542	0.389878	0.529325	0.3648	0.864498	no
gene:SpnNT_01215	NA	Chromosome:1248971-1249163	ΔORF2	110.58+peptide	OK	80.9998	78.8542	-0.0387315	-0.0541	0.9226	0.994748	no
gene:SpnNT_01215	NA	Chromosome:1248971-1249163	110.58	ΔORF2+peptide	OK	60.181	77.6992	0.36859	0.461937	0.4176	0.902759	no
gene:SpnNT_01215	NA	Chromosome:1248971-1249163	ΔORF2	ΔORF2+peptide	OK	80.9998	77.6992	-0.0600198	-0.0770554	0.89075	0.994748	no

gene:SpnNT_01215	NA	Chromosome:1248971-1249163	110.58+peptide	ΔORF2+peptide	OK	78.8542	77.6992	-0.0212882	-0.0273516	0.9569	0.994855	no
gene:SpnNT_01216	mgtA	Chromosome:1249176-1250502	110.58	ΔORF2	OK	88.1309	88.3092	0.00291572	0.00659826	0.99095	0.998249	no
gene:SpnNT_01216	mgtA	Chromosome:1249176-1250502	110.58	110.58+peptide	OK	88.1309	99.519	0.175324	0.397109	0.49005	0.93986	no
gene:SpnNT_01216	mgtA	Chromosome:1249176-1250502	ΔORF2	110.58+peptide	OK	88.3092	99.519	0.172408	0.390326	0.498	0.944017	no
gene:SpnNT_01216	mgtA	Chromosome:1249176-1250502	110.58	ΔORF2+peptide	OK	88.1309	121.588	0.464279	1.05313	0.0671	0.378942	no
gene:SpnNT_01216	mgtA	Chromosome:1249176-1250502	ΔORF2	ΔORF2+peptide	OK	88.3092	121.588	0.461363	1.04604	0.06895	0.385005	no
gene:SpnNT_01216	mgtA	Chromosome:1249176-1250502	110.58+peptide	ΔORF2+peptide	OK	99.519	121.588	0.288955	0.655722	0.2544	0.75336	no
gene:SpnNT_01217	mshA	Chromosome:1250517-1251561	110.58	ΔORF2	OK	106.15	110.692	0.0604422	0.135945	0.8146	0.994748	no
gene:SpnNT_01217	mshA	Chromosome:1250517-1251561	110.58	110.58+peptide	OK	106.15	106.301	0.00204723	0.00456365	0.99365	0.998597	no
gene:SpnNT_01217	mshA	Chromosome:1250517-1251561	ΔORF2	110.58+peptide	OK	110.692	106.301	-0.058395	-0.129997	0.81775	0.994748	no
gene:SpnNT_01217	mshA	Chromosome:1250517-1251561	110.58	ΔORF2+peptide	OK	106.15	141.8	0.417746	0.942469	0.09865	0.475914	no
gene:SpnNT_01217	mshA	Chromosome:1250517-1251561	ΔORF2	ΔORF2+peptide	OK	110.692	141.8	0.357304	0.80499	0.15865	0.608182	no
gene:SpnNT_01217	mshA	Chromosome:1250517-1251561	110.58+peptide	ΔORF2+peptide	OK	106.301	141.8	0.415699	0.928199	0.10795	0.499941	no
gene:SpnNT_01218	NA	Chromosome:1251675-1252005	110.58	ΔORF2	OK	376.057	369.793	-0.0242358	-0.0509338	0.92785	0.994748	no
gene:SpnNT_01218	NA	Chromosome:1251675-1252005	110.58	110.58+peptide	OK	376.057	721.575	0.940196	1.97871	0.00065	0.0118958	yes
gene:SpnNT_01218	NA	Chromosome:1251675-1252005	ΔORF2	110.58+peptide	OK	369.793	721.575	0.964432	2.0399	0.00025	0.00542231	yes
gene:SpnNT_01218	NA	Chromosome:1251675-1252005	110.58	ΔORF2+peptide	OK	376.057	658.834	0.808961	1.72887	0.00235	0.0334454	yes
gene:SpnNT_01218	NA	Chromosome:1251675-1252005	ΔORF2	ΔORF2+peptide	OK	369.793	658.834	0.833197	1.78988	0.0011	0.0181562	yes
gene:SpnNT_01218	NA	Chromosome:1251675-1252005	110.58+peptide	ΔORF2+peptide	OK	721.575	658.834	-0.131235	-0.282337	0.61805	0.980887	no
gene:SpnNT_01219	sigA	Chromosome:1252018-1253128	110.58	ΔORF2	OK	119.887	114.519	-0.0660778	-0.148902	0.7966	0.994748	no
gene:SpnNT_01219	sigA	Chromosome:1252018-1253128	110.58	110.58+peptide	OK	119.887	192.236	0.681206	1.54828	0.00865	0.0908042	no
gene:SpnNT_01219	sigA	Chromosome:1252018-1253128	ΔORF2	110.58+peptide	OK	114.519	192.236	0.747284	1.70098	0.00335	0.0437836	yes
gene:SpnNT_01219	sigA	Chromosome:1252018-1253128	110.58	ΔORF2+peptide	OK	119.887	205.836	0.779823	1.77918	0.0024	0.0337543	yes
gene:SpnNT_01219	sigA	Chromosome:1252018-1253128	ΔORF2	ΔORF2+peptide	OK	114.519	205.836	0.845901	1.93281	6.00E-04	0.0111203	yes
gene:SpnNT_01219	sigA	Chromosome:1252018-1253128	110.58+peptide	ΔORF2+peptide	OK	192.236	205.836	0.0986171	0.227328	0.6837	0.98747	no
gene:SpnNT_01220	dnaG	Chromosome:1253130-1254891	110.58	ΔORF2	OK	82.7642	80.6101	-0.038046	-0.0872671	0.87415	0.994748	no
gene:SpnNT_01220	dnaG	Chromosome:1253130-1254891	110.58	110.58+peptide	OK	82.7642	141.719	0.775955	1.78736	0.0018	0.0270557	yes
gene:SpnNT_01220	dnaG	Chromosome:1253130-1254891	ΔORF2	110.58+peptide	OK	80.6101	141.719	0.814001	1.87299	0.00135	0.021343	yes
gene:SpnNT_01220	dnaG	Chromosome:1253130-1254891	110.58	ΔORF2+peptide	OK	82.7642	147.745	0.836035	1.92407	0.00075	0.0133173	yes
gene:SpnNT_01220	dnaG	Chromosome:1253130-1254891	ΔORF2	ΔORF2+peptide	OK	80.6101	147.745	0.874081	2.00947	5.00E-04	0.0095781	yes
gene:SpnNT_01220	dnaG	Chromosome:1253130-1254891	110.58+peptide	ΔORF2+peptide	OK	141.719	147.745	0.0600798	0.138708	0.80575	0.994748	no
gene:SpnNT_01221	NA	Chromosome:1255167-1256901	110.58	ΔORF2	OK	21.9821	25.5214	0.215384	0.443325	0.4307	0.912439	no
gene:SpnNT_01221	NA	Chromosome:1255167-1256901	110.58	110.58+peptide	OK	21.9821	22.8481	0.055749	0.113491	0.8424	0.994748	no
gene:SpnNT_01221	NA	Chromosome:1255167-1256901	ΔORF2	110.58+peptide	OK	25.5214	22.8481	-0.159635	-0.327216	0.5657	0.968621	no
gene:SpnNT_01221	NA	Chromosome:1255167-1256901	110.58	ΔORF2+peptide	OK	21.9821	29.3076	0.414948	0.861755	0.1246	0.533791	no
gene:SpnNT_01221	NA	Chromosome:1255167-1256901	ΔORF2	ΔORF2+peptide	OK	25.5214	29.3076	0.199564	0.417424	0.46065	0.924377	no
gene:SpnNT_01221	NA	Chromosome:1255167-1256901	110.58+peptide	ΔORF2+peptide	OK	22.8481	29.3076	0.359199	0.742832	0.19435	0.675924	no
gene:SpnNT_01222	proV	Chromosome:1257049-1258671	110.58	ΔORF2	OK	41.9925	44.3876	0.080022	0.113436	0.84465	0.994748	no
gene:SpnNT_01222	proV	Chromosome:1257049-1258671	110.58	110.58+peptide	OK	41.9925	34.1442	-0.298492	-0.409946	0.47575	0.930746	no
gene:SpnNT_01222	proV	Chromosome:1257049-1258671	ΔORF2	110.58+peptide	OK	44.3876	34.1442	-0.378514	-0.514245	0.36965	0.866169	no
gene:SpnNT_01222	proV	Chromosome:1257049-1258671	110.58	ΔORF2+peptide	OK	41.9925	36.2742	-0.211189	-0.282689	0.6209	0.980887	no
gene:SpnNT_01222	proV	Chromosome:1257049-1258671	ΔORF2	ΔORF2+peptide	OK	44.3876	36.2742	-0.291211	-0.385809	0.4962	0.943191	no
gene:SpnNT_01222	proV	Chromosome:1257049-1258671	110.58+peptide	ΔORF2+peptide	OK	34.1442	36.2742	0.0873028	0.112496	0.8395	0.994748	no
gene:SpnNT_01223	NA	Chromosome:1257049-1258671	110.58	ΔORF2	OK	39.9194	39.8337	-0.00310082	-0.00468953	0.9932	0.998522	no
gene:SpnNT_01223	NA	Chromosome:1257049-1258671	110.58	110.58+peptide	OK	39.9194	36.9145	-0.112903	-0.177287	0.75095	0.994748	no

gene:SpnNT_01223	NA	Chromosome:1257049-1258671	ΔORF2	110.58+peptide	OK	39.8337	36.9145	-0.109803	-0.166807	0.77255	0.994748	no
gene:SpnNT_01223	NA	Chromosome:1257049-1258671	110.58	ΔORF2+peptide	OK	39.9194	41.4119	0.0529551	0.0813991	0.88505	0.994748	no
gene:SpnNT_01223	NA	Chromosome:1257049-1258671	ΔORF2	ΔORF2+peptide	OK	39.8337	41.4119	0.0560559	0.0834733	0.886	0.994748	no
gene:SpnNT_01223	NA	Chromosome:1257049-1258671	110.58+peptide	ΔORF2+peptide	OK	36.9145	41.4119	0.165859	0.256132	0.65795	0.982966	no
gene:SpnNT_01224	NA	Chromosome:1258739-1259744	110.58	ΔORF2	OK	42.2473	47.7928	0.177934	0.364992	0.5195	0.954494	no
gene:SpnNT_01224	NA	Chromosome:1258739-1259744	110.58	110.58+peptide	OK	42.2473	32.1259	-0.39512	-0.79388	0.1551	0.600007	no
gene:SpnNT_01224	NA	Chromosome:1258739-1259744	ΔORF2	110.58+peptide	OK	47.7928	32.1259	-0.573054	-1.15716	0.0408	0.274553	no
gene:SpnNT_01224	NA	Chromosome:1258739-1259744	110.58	ΔORF2+peptide	OK	42.2473	42.0448	-0.00693261	-0.0141496	0.97975	0.995839	no
gene:SpnNT_01224	NA	Chromosome:1258739-1259744	ΔORF2	ΔORF2+peptide	OK	47.7928	42.0448	-0.184867	-0.379268	0.5009	0.944652	no
gene:SpnNT_01224	NA	Chromosome:1258739-1259744	110.58+peptide	ΔORF2+peptide	OK	32.1259	42.0448	0.388187	0.780059	0.1642	0.618436	no
gene:SpnNT_01225	NA	Chromosome:1260101-1260617	110.58	ΔORF2	OK	44.4471	43.0746	-0.0452514	-0.0827578	0.8786	0.994748	no
gene:SpnNT_01225	NA	Chromosome:1260101-1260617	110.58	110.58+peptide	OK	44.4471	38.468	-0.208428	-0.378749	0.51335	0.95097	no
gene:SpnNT_01225	NA	Chromosome:1260101-1260617	ΔORF2	110.58+peptide	OK	43.0746	38.468	-0.163177	-0.294843	0.6048	0.976761	no
gene:SpnNT_01225	NA	Chromosome:1260101-1260617	110.58	ΔORF2+peptide	OK	44.4471	38.9655	-0.189891	-0.342684	0.54575	0.961816	no
gene:SpnNT_01225	NA	Chromosome:1260101-1260617	ΔORF2	ΔORF2+peptide	OK	43.0746	38.9655	-0.14464	-0.259566	0.64745	0.981391	no
gene:SpnNT_01225	NA	Chromosome:1260101-1260617	110.58+peptide	ΔORF2+peptide	OK	38.468	38.9655	0.0185372	0.0330617	0.9541	0.994855	no
gene:SpnNT_01226	NA	Chromosome:1260665-1261355	110.58	ΔORF2	OK	10.8505	7.68621	-0.497418	-0.763564	0.18355	0.654673	no
gene:SpnNT_01226	NA	Chromosome:1260665-1261355	110.58	110.58+peptide	OK	10.8505	11.8249	0.124062	0.197017	0.72915	0.994748	no
gene:SpnNT_01226	NA	Chromosome:1260665-1261355	ΔORF2	110.58+peptide	OK	7.68621	11.8249	0.62148	0.954772	0.0968	0.470343	no
gene:SpnNT_01226	NA	Chromosome:1260665-1261355	110.58	ΔORF2+peptide	OK	10.8505	8.75243	-0.310006	-0.483036	0.397	0.89095	no
gene:SpnNT_01226	NA	Chromosome:1260665-1261355	ΔORF2	ΔORF2+peptide	OK	7.68621	8.75243	0.187412	0.282835	0.61865	0.980887	no
gene:SpnNT_01226	NA	Chromosome:1260665-1261355	110.58+peptide	ΔORF2+peptide	OK	11.8249	8.75243	-0.434068	-0.676904	0.23255	0.725689	no
gene:SpnNT_01227	ppc	Chromosome:1261897-1264594	110.58	ΔORF2	OK	258.297	289.579	0.164928	0.367558	0.52545	0.955377	no
gene:SpnNT_01227	ppc	Chromosome:1261897-1264594	110.58	110.58+peptide	OK	258.297	168.815	-0.613584	-1.3786	0.01655	0.144587	no
gene:SpnNT_01227	ppc	Chromosome:1261897-1264594	ΔORF2	110.58+peptide	OK	289.579	168.815	-0.778512	-1.7489	0.00245	0.0342374	yes
gene:SpnNT_01227	ppc	Chromosome:1261897-1264594	110.58	ΔORF2+peptide	OK	258.297	186.765	-0.467809	-1.04998	0.0672	0.378942	no
gene:SpnNT_01227	ppc	Chromosome:1261897-1264594	ΔORF2	ΔORF2+peptide	OK	289.579	186.765	-0.632737	-1.41994	0.0149	0.13355	no
gene:SpnNT_01227	ppc	Chromosome:1261897-1264594	110.58+peptide	ΔORF2+peptide	OK	168.815	186.765	0.145775	0.329846	0.55645	0.966861	no
gene:SpnNT_01228	ftsW	Chromosome:1264613-1265843	110.58	ΔORF2	OK	128.085	149.534	0.223369	0.509088	0.37195	0.869075	no
gene:SpnNT_01228	ftsW	Chromosome:1264613-1265843	110.58	110.58+peptide	OK	128.085	86.162	-0.571981	-1.26414	0.0289	0.216577	no
gene:SpnNT_01228	ftsW	Chromosome:1264613-1265843	ΔORF2	110.58+peptide	OK	149.534	86.162	-0.79535	-1.76839	0.00225	0.0324089	yes
gene:SpnNT_01228	ftsW	Chromosome:1264613-1265843	110.58	ΔORF2+peptide	OK	128.085	108.591	-0.238195	-0.536855	0.3492	0.853357	no
gene:SpnNT_01228	ftsW	Chromosome:1264613-1265843	ΔORF2	ΔORF2+peptide	OK	149.534	108.591	-0.461564	-1.0468	0.0653	0.372065	no
gene:SpnNT_01228	ftsW	Chromosome:1264613-1265843	110.58+peptide	ΔORF2+peptide	OK	86.162	108.591	0.333786	0.7343	0.19995	0.682911	no
gene:SpnNT_01229	NA	Chromosome:1266629-1267526	110.58	ΔORF2	OK	5.52554	8.90113	0.687873	1.04449	0.07345	0.399141	no
gene:SpnNT_01229	NA	Chromosome:1266629-1267526	110.58	110.58+peptide	OK	5.52554	6.43326	0.219435	0.330625	0.5687	0.968621	no
gene:SpnNT_01229	NA	Chromosome:1266629-1267526	ΔORF2	110.58+peptide	OK	8.90113	6.43326	-0.468438	-0.719374	0.2143	0.699862	no
gene:SpnNT_01229	NA	Chromosome:1266629-1267526	110.58	ΔORF2+peptide	OK	5.52554	7.9866	0.531466	0.802026	0.1679	0.623964	no
gene:SpnNT_01229	NA	Chromosome:1266629-1267526	ΔORF2	ΔORF2+peptide	OK	8.90113	7.9866	-0.156407	-0.240585	0.67665	0.98524	no
gene:SpnNT_01229	NA	Chromosome:1266629-1267526	110.58+peptide	ΔORF2+peptide	OK	6.43326	7.9866	0.312031	0.476162	0.4093	0.897533	no
gene:SpnNT_01230	NA	Chromosome:1267564-1272579	110.58	ΔORF2	OK	5.24786	7.23607	0.463477	0.579069	0.32685	0.831671	no
gene:SpnNT_01230	NA	Chromosome:1267564-1272579	110.58	110.58+peptide	OK	5.24786	6.26018	0.254475	0.3227	0.57985	0.969538	no
gene:SpnNT_01230	NA	Chromosome:1267564-1272579	ΔORF2	110.58+peptide	OK	7.23607	6.26018	-0.209002	-0.261523	0.66045	0.982966	no
gene:SpnNT_01230	NA	Chromosome:1267564-1272579	110.58	ΔORF2+peptide	OK	5.24786	8.69808	0.728969	1.00075	0.08545	0.439454	no
gene:SpnNT_01230	NA	Chromosome:1267564-1272579	ΔORF2	ΔORF2+peptide	OK	7.23607	8.69808	0.265492	0.358833	0.54025	0.961372	no

gene:SpnNT_01230	NA	Chromosome:1267564-1272579	110.58+peptide	ΔORF2+peptide	OK	6.26018	8.69808	0.474494	0.652594	0.26415	0.764751	no
gene:SpnNT_01231	NA	Chromosome:1267564-1272579	110.58	ΔORF2	OK	6.64527	6.93263	0.0610761	0.0347242	0.9507	0.994855	no
gene:SpnNT_01231	NA	Chromosome:1267564-1272579	110.58	110.58+peptide	OK	6.64527	6.19902	-0.100288	-0.0611798	0.9105	0.994748	no
gene:SpnNT_01231	NA	Chromosome:1267564-1272579	ΔORF2	110.58+peptide	OK	6.93263	6.19902	-0.161364	-0.0856561	0.879	0.994748	no
gene:SpnNT_01231	NA	Chromosome:1267564-1272579	110.58	ΔORF2+peptide	OK	6.64527	9.67354	0.541716	0.348822	0.55115	0.964385	no
gene:SpnNT_01231	NA	Chromosome:1267564-1272579	ΔORF2	ΔORF2+peptide	OK	6.93263	9.67354	0.48064	0.265647	0.64505	0.980887	no
gene:SpnNT_01231	NA	Chromosome:1267564-1272579	110.58+peptide	ΔORF2+peptide	OK	6.19902	9.67354	0.642004	0.379162	0.509	0.947524	no
gene:SpnNT_01232	pezT_3	Chromosome:1267564-1272579	110.58	ΔORF2	OK	18.0912	20.9029	0.208409	0.200905	0.70725	0.990441	no
gene:SpnNT_01232	pezT_3	Chromosome:1267564-1272579	110.58	110.58+peptide	OK	18.0912	18.1849	0.00744688	0.00763366	0.9895	0.997703	no
gene:SpnNT_01232	pezT_3	Chromosome:1267564-1272579	ΔORF2	110.58+peptide	OK	20.9029	18.1849	-0.200962	-0.181402	0.7466	0.994748	no
gene:SpnNT_01232	pezT_3	Chromosome:1267564-1272579	110.58	ΔORF2+peptide	OK	18.0912	17.0971	-0.0815359	-0.0785898	0.8867	0.994748	no
gene:SpnNT_01232	pezT_3	Chromosome:1267564-1272579	ΔORF2	ΔORF2+peptide	OK	20.9029	17.0971	-0.289945	-0.249361	0.6551	0.982966	no
gene:SpnNT_01232	pezT_3	Chromosome:1267564-1272579	110.58+peptide	ΔORF2+peptide	OK	18.1849	17.0971	-0.0889828	-0.0803128	0.88545	0.994748	no
gene:SpnNT_01233	pezA_3	Chromosome:1267564-1272579	110.58	ΔORF2	OK	24.4574	35.5884	0.541136	0.467284	0.42035	0.904235	no
gene:SpnNT_01233	pezA_3	Chromosome:1267564-1272579	110.58	110.58+peptide	OK	24.4574	24.844	0.022624	0.0189441	0.97375	0.99536	no
gene:SpnNT_01233	pezA_3	Chromosome:1267564-1272579	ΔORF2	110.58+peptide	OK	35.5884	24.844	-0.518512	-0.437109	0.46195	0.925169	no
gene:SpnNT_01233	pezA_3	Chromosome:1267564-1272579	110.58	ΔORF2+peptide	OK	24.4574	32.5091	0.410572	0.348661	0.54455	0.961568	no
gene:SpnNT_01233	pezA_3	Chromosome:1267564-1272579	ΔORF2	ΔORF2+peptide	OK	35.5884	32.5091	-0.130564	-0.111647	0.8421	0.994748	no
gene:SpnNT_01233	pezA_3	Chromosome:1267564-1272579	110.58+peptide	ΔORF2+peptide	OK	24.844	32.5091	0.387948	0.321869	0.5781	0.969538	no
gene:SpnNT_01234	NA	Chromosome:1273023-1273479	110.58	ΔORF2	OK	10.9106	15.3112	0.488853	0.718094	0.2104	0.696806	no
gene:SpnNT_01234	NA	Chromosome:1273023-1273479	110.58	110.58+peptide	OK	10.9106	7.31219	-0.577358	-0.783037	0.1819	0.651622	no
gene:SpnNT_01234	NA	Chromosome:1273023-1273479	ΔORF2	110.58+peptide	OK	15.3112	7.31219	-1.06621	-1.46347	0.0146	0.1314	no
gene:SpnNT_01234	NA	Chromosome:1273023-1273479	110.58	ΔORF2+peptide	OK	10.9106	9.5143	-0.197565	-0.267553	0.6344	0.980887	no
gene:SpnNT_01234	NA	Chromosome:1273023-1273479	ΔORF2	ΔORF2+peptide	OK	15.3112	9.5143	-0.686417	-0.940755	0.09795	0.473756	no
gene:SpnNT_01234	NA	Chromosome:1273023-1273479	110.58+peptide	ΔORF2+peptide	OK	7.31219	9.5143	0.379794	0.485243	0.40455	0.894933	no
gene:SpnNT_01235	bbmA	Chromosome:1273804-1275493	110.58	ΔORF2	OK	11.767	13.0447	0.148711	0.281155	0.62535	0.980887	no
gene:SpnNT_01235	bbmA	Chromosome:1273804-1275493	110.58	110.58+peptide	OK	11.767	9.82361	-0.260422	-0.483904	0.4049	0.894933	no
gene:SpnNT_01235	bbmA	Chromosome:1273804-1275493	ΔORF2	110.58+peptide	OK	13.0447	9.82361	-0.409133	-0.765879	0.17365	0.634017	no
gene:SpnNT_01235	bbmA	Chromosome:1273804-1275493	110.58	ΔORF2+peptide	OK	11.767	11.6306	-0.0168206	-0.0314338	0.95945	0.994855	no
gene:SpnNT_01235	bbmA	Chromosome:1273804-1275493	ΔORF2	ΔORF2+peptide	OK	13.0447	11.6306	-0.165532	-0.311666	0.57835	0.969538	no
gene:SpnNT_01235	bbmA	Chromosome:1273804-1275493	110.58+peptide	ΔORF2+peptide	OK	9.82361	11.6306	0.243601	0.450846	0.4271	0.908839	no
gene:SpnNT_01236	NA	Chromosome:1275655-1276489	110.58	ΔORF2	OK	101.487	116.366	0.197379	0.434463	0.4502	0.920749	no
gene:SpnNT_01236	NA	Chromosome:1275655-1276489	110.58	110.58+peptide	OK	101.487	107.432	0.0821414	0.180453	0.7577	0.994748	no
gene:SpnNT_01236	NA	Chromosome:1275655-1276489	ΔORF2	110.58+peptide	OK	116.366	107.432	-0.115238	-0.253323	0.65915	0.982966	no
gene:SpnNT_01236	NA	Chromosome:1275655-1276489	110.58	ΔORF2+peptide	OK	101.487	127.838	0.333031	0.736503	0.19975	0.682576	no
gene:SpnNT_01236	NA	Chromosome:1275655-1276489	ΔORF2	ΔORF2+peptide	OK	116.366	127.838	0.135652	0.300192	0.59915	0.97629	no
gene:SpnNT_01236	NA	Chromosome:1275655-1276489	110.58+peptide	ΔORF2+peptide	OK	107.432	127.838	0.25089	0.554111	0.3322	0.837972	no
gene:SpnNT_01237	NA	Chromosome:1276650-1277505	110.58	ΔORF2	OK	56.1205	54.5056	-0.0421248	-0.0870442	0.8779	0.994748	no
gene:SpnNT_01237	NA	Chromosome:1276650-1277505	110.58	110.58+peptide	OK	56.1205	61.3157	0.127728	0.264403	0.6413	0.980887	no
gene:SpnNT_01237	NA	Chromosome:1276650-1277505	ΔORF2	110.58+peptide	OK	54.5056	61.3157	0.169853	0.350053	0.5396	0.961128	no
gene:SpnNT_01237	NA	Chromosome:1276650-1277505	110.58	ΔORF2+peptide	OK	56.1205	60.2889	0.103365	0.214503	0.70955	0.990851	no
gene:SpnNT_01237	NA	Chromosome:1276650-1277505	ΔORF2	ΔORF2+peptide	OK	54.5056	60.2889	0.14549	0.300583	0.5991	0.97629	no
gene:SpnNT_01237	NA	Chromosome:1276650-1277505	110.58+peptide	ΔORF2+peptide	OK	61.3157	60.2889	-0.0243635	-0.0504254	0.9297	0.99482	no
gene:SpnNT_01238	NA	Chromosome:1277562-1278321	110.58	ΔORF2	OK	41.7753	43.488	0.0579644	0.113734	0.8372	0.994748	no
gene:SpnNT_01238	NA	Chromosome:1277562-1278321	110.58	110.58+peptide	OK	41.7753	45.3778	0.119334	0.234436	0.6777	0.98524	no

gene:SpnNT_01238	NA	Chromosome:1277562-1278321	ΔORF2	110.58+peptide	OK	43.488	45.3778	0.0613695	0.120856	0.8263	0.994748	no
gene:SpnNT_01238	NA	Chromosome:1277562-1278321	110.58	ΔORF2+peptide	OK	41.7753	42.3299	0.0190258	0.0370742	0.947	0.994855	no
gene:SpnNT_01238	NA	Chromosome:1277562-1278321	ΔORF2	ΔORF2+peptide	OK	43.488	42.3299	-0.0389386	-0.0760586	0.88945	0.994748	no
gene:SpnNT_01238	NA	Chromosome:1277562-1278321	110.58+peptide	ΔORF2+peptide	OK	45.3778	42.3299	-0.100308	-0.196169	0.728	0.994748	no
gene:SpnNT_01239	NA	Chromosome:1278621-1279188	110.58	ΔORF2	OK	29.4128	32.7369	0.154471	0.274026	0.62755	0.980887	no
gene:SpnNT_01239	NA	Chromosome:1278621-1279188	110.58	110.58+peptide	OK	29.4128	27.7487	-0.0840287	-0.148521	0.7942	0.994748	no
gene:SpnNT_01239	NA	Chromosome:1278621-1279188	ΔORF2	110.58+peptide	OK	32.7369	27.7487	-0.238499	-0.424838	0.45795	0.921942	no
gene:SpnNT_01239	NA	Chromosome:1278621-1279188	110.58	ΔORF2+peptide	OK	29.4128	45.4994	0.629401	1.14076	0.0476	0.303945	no
gene:SpnNT_01239	NA	Chromosome:1278621-1279188	ΔORF2	ΔORF2+peptide	OK	32.7369	45.4994	0.47493	0.867852	0.13385	0.556521	no
gene:SpnNT_01239	NA	Chromosome:1278621-1279188	110.58+peptide	ΔORF2+peptide	OK	27.7487	45.4994	0.713429	1.29863	0.0245	0.194606	no
gene:SpnNT_01240	rlmCD_1	Chromosome:1279455-1280814	110.58	ΔORF2	OK	56.3167	51.9489	-0.116469	-0.254788	0.6503	0.981475	no
gene:SpnNT_01240	rlmCD_1	Chromosome:1279455-1280814	110.58	110.58+peptide	OK	56.3167	51.8497	-0.119226	-0.259671	0.64905	0.981391	no
gene:SpnNT_01240	rlmCD_1	Chromosome:1279455-1280814	ΔORF2	110.58+peptide	OK	51.9489	51.8497	-0.00275747	-0.0059995	0.99165	0.998369	no
gene:SpnNT_01240	rlmCD_1	Chromosome:1279455-1280814	110.58	ΔORF2+peptide	OK	56.3167	56.0514	-0.00681211	-0.0149683	0.97955	0.99579	no
gene:SpnNT_01240	rlmCD_1	Chromosome:1279455-1280814	ΔORF2	ΔORF2+peptide	OK	51.9489	56.0514	0.109657	0.240698	0.68235	0.986644	no
gene:SpnNT_01240	rlmCD_1	Chromosome:1279455-1280814	110.58+peptide	ΔORF2+peptide	OK	51.8497	56.0514	0.112414	0.245655	0.6671	0.983929	no
gene:SpnNT_01241	NA	Chromosome:1280887-1281862	110.58	ΔORF2	OK	595.199	627.934	0.0772401	0.17462	0.75675	0.994748	no
gene:SpnNT_01241	NA	Chromosome:1280887-1281862	110.58	110.58+peptide	OK	595.199	480.972	-0.30742	-0.698071	0.2227	0.712575	no
gene:SpnNT_01241	NA	Chromosome:1280887-1281862	ΔORF2	110.58+peptide	OK	627.934	480.972	-0.384661	-0.873314	0.12825	0.54287	no
gene:SpnNT_01241	NA	Chromosome:1280887-1281862	110.58	ΔORF2+peptide	OK	595.199	483.14	-0.300931	-0.682793	0.23625	0.73046	no
gene:SpnNT_01241	NA	Chromosome:1280887-1281862	ΔORF2	ΔORF2+peptide	OK	627.934	483.14	-0.378171	-0.8579	0.1375	0.564718	no
gene:SpnNT_01241	NA	Chromosome:1280887-1281862	110.58+peptide	ΔORF2+peptide	OK	480.972	483.14	0.00648904	0.0147862	0.981	0.99608	no
gene:SpnNT_01242	NA	Chromosome:1281935-1283524	110.58	ΔORF2	OK	103.54	101.367	-0.0306123	-0.03028	0.9543	0.994855	no
gene:SpnNT_01242	NA	Chromosome:1281935-1283524	110.58	110.58+peptide	OK	103.54	79.2183	-0.38629	-0.42119	0.4683	0.927504	no
gene:SpnNT_01242	NA	Chromosome:1281935-1283524	ΔORF2	110.58+peptide	OK	101.367	79.2183	-0.355677	-0.350322	0.53995	0.961277	no
gene:SpnNT_01242	NA	Chromosome:1281935-1283524	110.58	ΔORF2+peptide	OK	103.54	79.6346	-0.378727	-0.420329	0.469	0.927613	no
gene:SpnNT_01242	NA	Chromosome:1281935-1283524	ΔORF2	ΔORF2+peptide	OK	101.367	79.6346	-0.348115	-0.347852	0.5404	0.961468	no
gene:SpnNT_01242	NA	Chromosome:1281935-1283524	110.58+peptide	ΔORF2+peptide	OK	79.2183	79.6346	0.00756283	0.00834877	0.98965	0.997703	no
gene:SpnNT_01243	NA	Chromosome:1281935-1283524	110.58	ΔORF2	OK	174.968	200.496	0.196484	0.393808	0.4953	0.942888	no
gene:SpnNT_01243	NA	Chromosome:1281935-1283524	110.58	110.58+peptide	OK	174.968	95.7894	-0.869151	-1.6329	0.0055	0.064496	no
gene:SpnNT_01243	NA	Chromosome:1281935-1283524	ΔORF2	110.58+peptide	OK	200.496	95.7894	-1.06564	-2.01425	0.00045	0.00885292	yes
gene:SpnNT_01243	NA	Chromosome:1281935-1283524	110.58	ΔORF2+peptide	OK	174.968	118.011	-0.568167	-1.12206	0.0523	0.323717	no
gene:SpnNT_01243	NA	Chromosome:1281935-1283524	ΔORF2	ΔORF2+peptide	OK	200.496	118.011	-0.764652	-1.52028	0.00905	0.0938768	no
gene:SpnNT_01243	NA	Chromosome:1281935-1283524	110.58+peptide	ΔORF2+peptide	OK	95.7894	118.011	0.300983	0.561477	0.3323	0.837993	no
gene:SpnNT_01244	glyA	Chromosome:1283531-1284788	110.58	ΔORF2	OK	334.12	333.679	-0.00190747	-0.00433914	0.99315	0.998522	no
gene:SpnNT_01244	glyA	Chromosome:1283531-1284788	110.58	110.58+peptide	OK	334.12	204.14	-0.710806	-1.6306	0.00445	0.054829	no
gene:SpnNT_01244	glyA	Chromosome:1283531-1284788	ΔORF2	110.58+peptide	OK	333.679	204.14	-0.708899	-1.62128	0.00415	0.0519126	no
gene:SpnNT_01244	glyA	Chromosome:1283531-1284788	110.58	ΔORF2+peptide	OK	334.12	201.656	-0.72847	-1.67225	0.00415	0.0519126	no
gene:SpnNT_01244	glyA	Chromosome:1283531-1284788	ΔORF2	ΔORF2+peptide	OK	333.679	201.656	-0.726563	-1.66279	0.00375	0.0479605	yes
gene:SpnNT_01244	glyA	Chromosome:1283531-1284788	110.58+peptide	ΔORF2+peptide	OK	204.14	201.656	-0.0176642	-0.0407711	0.9431	0.994855	no
gene:SpnNT_01245	NA	Chromosome:1284848-1285280	110.58	ΔORF2	OK	257.905	245.299	-0.0722945	-0.157354	0.7856	0.994748	no
gene:SpnNT_01245	NA	Chromosome:1284848-1285280	110.58	110.58+peptide	OK	257.905	159.275	-0.695319	-1.48732	0.0101	0.100938	no
gene:SpnNT_01245	NA	Chromosome:1284848-1285280	ΔORF2	110.58+peptide	OK	245.299	159.275	-0.623024	-1.32002	0.0231	0.18642	no
gene:SpnNT_01245	NA	Chromosome:1284848-1285280	110.58	ΔORF2+peptide	OK	257.905	186.895	-0.464609	-1.00003	0.088	0.446706	no
gene:SpnNT_01245	NA	Chromosome:1284848-1285280	ΔORF2	ΔORF2+peptide	OK	245.299	186.895	-0.392315	-0.83631	0.1533	0.596738	no

gene:SpnNT_01245	NA	Chromosome:1284848-1285280	110.58+peptide	ΔORF2+peptide	OK	159.275	186.895	0.230709	0.483668	0.4078	0.897051	no
gene:SpnNT_01246	rimN	Chromosome:1285281-1288373	110.58	ΔORF2	OK	387.103	391.176	0.0151008	0.0171192	0.9757	0.99536	no
gene:SpnNT_01246	rimN	Chromosome:1285281-1288373	110.58	110.58+peptide	OK	387.103	230.891	-0.745504	-0.820731	0.15845	0.607592	no
gene:SpnNT_01246	rimN	Chromosome:1285281-1288373	ΔORF2	110.58+peptide	OK	391.176	230.891	-0.760605	-0.841898	0.1521	0.594536	no
gene:SpnNT_01246	rimN	Chromosome:1285281-1288373	110.58	ΔORF2+peptide	OK	387.103	273.306	-0.502203	-0.561802	0.3344	0.839773	no
gene:SpnNT_01246	rimN	Chromosome:1285281-1288373	ΔORF2	ΔORF2+peptide	OK	391.176	273.306	-0.517304	-0.581937	0.32255	0.828116	no
gene:SpnNT_01246	rimN	Chromosome:1285281-1288373	110.58+peptide	ΔORF2+peptide	OK	230.891	273.306	0.243301	0.265909	0.64745	0.981391	no
gene:SpnNT_01247	prmC	Chromosome:1285281-1288373	110.58	ΔORF2	OK	403.427	415.909	0.0439589	0.0643471	0.90925	0.994748	no
gene:SpnNT_01247	prmC	Chromosome:1285281-1288373	110.58	110.58+peptide	OK	403.427	241.39	-0.740942	-1.05474	0.07075	0.390464	no
gene:SpnNT_01247	prmC	Chromosome:1285281-1288373	ΔORF2	110.58+peptide	OK	415.909	241.39	-0.784901	-1.1374	0.04815	0.306116	no
gene:SpnNT_01247	prmC	Chromosome:1285281-1288373	110.58	ΔORF2+peptide	OK	403.427	234.628	-0.781934	-1.08799	0.06255	0.3611	no
gene:SpnNT_01247	prmC	Chromosome:1285281-1288373	ΔORF2	ΔORF2+peptide	OK	415.909	234.628	-0.825893	-1.16887	0.04265	0.282796	no
gene:SpnNT_01247	prmC	Chromosome:1285281-1288373	110.58+peptide	ΔORF2+peptide	OK	241.39	234.628	-0.0409919	-0.056518	0.9186	0.994748	no
gene:SpnNT_01248	prfA	Chromosome:1285281-1288373	110.58	ΔORF2	OK	119.226	115.76	-0.0425547	-0.0381138	0.94905	0.994855	no
gene:SpnNT_01248	prfA	Chromosome:1285281-1288373	110.58	110.58+peptide	OK	119.226	121.238	0.0241425	0.0248736	0.9657	0.994855	no
gene:SpnNT_01248	prfA	Chromosome:1285281-1288373	ΔORF2	110.58+peptide	OK	115.76	121.238	0.0666972	0.0663497	0.9059	0.994748	no
gene:SpnNT_01248	prfA	Chromosome:1285281-1288373	110.58	ΔORF2+peptide	OK	119.226	124.699	0.0647491	0.0652549	0.91135	0.994748	no
gene:SpnNT_01248	prfA	Chromosome:1285281-1288373	ΔORF2	ΔORF2+peptide	OK	115.76	124.699	0.107304	0.104569	0.8553	0.994748	no
gene:SpnNT_01248	prfA	Chromosome:1285281-1288373	110.58+peptide	ΔORF2+peptide	OK	121.238	124.699	0.0406066	0.046937	0.93605	0.994855	no
gene:SpnNT_01249	tdk	Chromosome:1285281-1288373	110.58	ΔORF2	OK	81.6733	75.5849	-0.111767	-0.0584163	0.92105	0.994748	no
gene:SpnNT_01249	tdk	Chromosome:1285281-1288373	110.58	110.58+peptide	OK	81.6733	81.6221	-0.000904575	-0.000501431	0.99945	0.999752	no
gene:SpnNT_01249	tdk	Chromosome:1285281-1288373	ΔORF2	110.58+peptide	OK	75.5849	81.6221	0.110863	0.0601952	0.924	0.994748	no
gene:SpnNT_01249	tdk	Chromosome:1285281-1288373	110.58	ΔORF2+peptide	OK	81.6733	87.5668	0.100519	0.0563863	0.9134	0.994748	no
gene:SpnNT_01249	tdk	Chromosome:1285281-1288373	ΔORF2	ΔORF2+peptide	OK	75.5849	87.5668	0.212286	0.116586	0.8575	0.994748	no
gene:SpnNT_01249	tdk	Chromosome:1285281-1288373	110.58+peptide	ΔORF2+peptide	OK	81.6221	87.5668	0.101423	0.0594635	0.91835	0.994748	no
gene:SpnNT_01250	xylH	Chromosome:1288495-1288678	110.58	ΔORF2	OK	1888.02	1582.12	-0.255014	-0.503392	0.36335	0.862964	no
gene:SpnNT_01250	xylH	Chromosome:1288495-1288678	110.58	110.58+peptide	OK	1888.02	1569.4	-0.26666	-0.497292	0.3681	0.865399	no
gene:SpnNT_01250	xylH	Chromosome:1288495-1288678	ΔORF2	110.58+peptide	OK	1582.12	1569.4	-0.0116459	-0.0224327	0.96725	0.994855	no
gene:SpnNT_01250	xylH	Chromosome:1288495-1288678	110.58	ΔORF2+peptide	OK	1888.02	1142.06	-0.725238	-1.28799	0.02105	0.17405	no
gene:SpnNT_01250	xylH	Chromosome:1288495-1288678	ΔORF2	ΔORF2+peptide	OK	1582.12	1142.06	-0.470224	-0.859891	0.1152	0.515092	no
gene:SpnNT_01250	xylH	Chromosome:1288495-1288678	110.58+peptide	ΔORF2+peptide	OK	1569.4	1142.06	-0.458578	-0.798359	0.13975	0.570389	no
gene:SpnNT_01251	mnmE	Chromosome:1288814-1290188	110.58	ΔORF2	OK	65.1275	69.5494	0.0947701	0.211584	0.70825	0.990441	no
gene:SpnNT_01251	mnmE	Chromosome:1288814-1290188	110.58	110.58+peptide	OK	65.1275	59.9601	-0.119266	-0.264509	0.6413	0.980887	no
gene:SpnNT_01251	mnmE	Chromosome:1288814-1290188	ΔORF2	110.58+peptide	OK	69.5494	59.9601	-0.214036	-0.476296	0.4126	0.899511	no
gene:SpnNT_01251	mnmE	Chromosome:1288814-1290188	110.58	ΔORF2+peptide	OK	65.1275	56.9624	-0.193258	-0.424939	0.44865	0.919688	no
gene:SpnNT_01251	mnmE	Chromosome:1288814-1290188	ΔORF2	ΔORF2+peptide	OK	69.5494	56.9624	-0.288028	-0.635425	0.2643	0.764751	no
gene:SpnNT_01251	mnmE	Chromosome:1288814-1290188	110.58+peptide	ΔORF2+peptide	OK	59.9601	56.9624	-0.0739925	-0.162181	0.77595	0.994748	no
gene:SpnNT_01252	dapA	Chromosome:1290234-1291170	110.58	ΔORF2	OK	175.528	158.9	-0.143583	-0.323358	0.5642	0.968621	no
gene:SpnNT_01252	dapA	Chromosome:1290234-1291170	110.58	110.58+peptide	OK	175.528	152.381	-0.204021	-0.455982	0.425	0.907986	no
gene:SpnNT_01252	dapA	Chromosome:1290234-1291170	ΔORF2	110.58+peptide	OK	158.9	152.381	-0.0604383	-0.134221	0.812	0.994748	no
gene:SpnNT_01252	dapA	Chromosome:1290234-1291170	110.58	ΔORF2+peptide	OK	175.528	169.122	-0.0536392	-0.120647	0.83625	0.994748	no
gene:SpnNT_01252	dapA	Chromosome:1290234-1291170	ΔORF2	ΔORF2+peptide	OK	158.9	169.122	0.0899437	0.201005	0.72325	0.994748	no
gene:SpnNT_01252	dapA	Chromosome:1290234-1291170	110.58+peptide	ΔORF2+peptide	OK	152.381	169.122	0.150382	0.33356	0.55845	0.968033	no
gene:SpnNT_01253	asd	Chromosome:1291225-1292302	110.58	ΔORF2	OK	227.365	211.473	-0.10454	-0.238517	0.67315	0.984845	no
gene:SpnNT_01253	asd	Chromosome:1291225-1292302	110.58	110.58+peptide	OK	227.365	273.711	0.267644	0.603446	0.29975	0.805348	no

gene:SpnNT_01253	asd	Chromosome:1291225-1292302	ΔORF2	110.58+peptide	OK	211.473	273.711	0.372184	0.842587	0.1404	0.571974	no
gene:SpnNT_01253	asd	Chromosome:1291225-1292302	110.58	ΔORF2+peptide	OK	227.365	302.282	0.410882	0.935914	0.1065	0.496269	no
gene:SpnNT_01253	asd	Chromosome:1291225-1292302	ΔORF2	ΔORF2+peptide	OK	211.473	302.282	0.515423	1.17895	0.0411	0.275864	no
gene:SpnNT_01253	asd	Chromosome:1291225-1292302	110.58+peptide	ΔORF2+peptide	OK	273.711	302.282	0.143239	0.323752	0.5706	0.968621	no
gene:SpnNT_01254	SpnNT_01254	Chromosome:1292886-1292959	110.58	ΔORF2	NOTEST	0	178.177	Inf	0	1	1	no
gene:SpnNT_01254	SpnNT_01254	Chromosome:1292886-1292959	110.58	110.58+peptide	NOTEST	0	0	0	0	1	1	no
gene:SpnNT_01254	SpnNT_01254	Chromosome:1292886-1292959	ΔORF2	110.58+peptide	NOTEST	178.177	0	#NAME?	0	1	1	no
gene:SpnNT_01254	SpnNT_01254	Chromosome:1292886-1292959	110.58	ΔORF2+peptide	NOTEST	0	0	0	0	1	1	no
gene:SpnNT_01254	SpnNT_01254	Chromosome:1292886-1292959	ΔORF2	ΔORF2+peptide	NOTEST	178.177	0	#NAME?	0	1	1	no
gene:SpnNT_01254	SpnNT_01254	Chromosome:1292886-1292959	110.58+peptide	ΔORF2+peptide	NOTEST	0	0	0	0	1	1	no
gene:SpnNT_01255	queT	Chromosome:1293058-1293568	110.58	ΔORF2	OK	76.3541	125.322	0.714861	1.39958	0.01605	0.141158	no
gene:SpnNT_01255	queT	Chromosome:1293058-1293568	110.58	110.58+peptide	OK	76.3541	261.646	1.77684	3.71331	5.00E-05	0.0013612	yes
gene:SpnNT_01255	queT	Chromosome:1293058-1293568	ΔORF2	110.58+peptide	OK	125.322	261.646	1.06198	2.2165	0.00015	0.00355289	yes
gene:SpnNT_01255	queT	Chromosome:1293058-1293568	110.58	ΔORF2+peptide	OK	76.3541	307.193	2.00837	4.17945	5.00E-05	0.0013612	yes
gene:SpnNT_01255	queT	Chromosome:1293058-1293568	ΔORF2	ΔORF2+peptide	OK	125.322	307.193	1.29351	2.68837	5.00E-05	0.0013612	yes
gene:SpnNT_01255	queT	Chromosome:1293058-1293568	110.58+peptide	ΔORF2+peptide	OK	261.646	307.193	0.231529	0.518251	0.36215	0.862575	no
gene:SpnNT_01256	NA	Chromosome:1293690-1294122	110.58	ΔORF2	OK	3.12696	5.01644	0.681903	0.728135	0.2086	0.694195	no
gene:SpnNT_01256	NA	Chromosome:1293690-1294122	110.58	110.58+peptide	OK	3.12696	3.87717	0.310242	0.32578	0.5668	0.968621	no
gene:SpnNT_01256	NA	Chromosome:1293690-1294122	ΔORF2	110.58+peptide	OK	5.01644	3.87717	-0.37166	-0.423253	0.46755	0.92732	no
gene:SpnNT_01256	NA	Chromosome:1293690-1294122	110.58	ΔORF2+peptide	OK	3.12696	3.11832	-0.00399337	-0.00397232	0.95865	0.994855	no
gene:SpnNT_01256	NA	Chromosome:1293690-1294122	ΔORF2	ΔORF2+peptide	OK	5.01644	3.11832	-0.685896	-0.733336	0.20575	0.690314	no
gene:SpnNT_01256	NA	Chromosome:1293690-1294122	110.58+peptide	ΔORF2+peptide	OK	3.87717	3.11832	-0.314236	-0.330381	0.5619	0.968621	no
gene:SpnNT_01257	mscL	Chromosome:1294203-1294581	110.58	ΔORF2	OK	86.9325	146.029	0.748291	1.40052	0.01685	0.146331	no
gene:SpnNT_01257	mscL	Chromosome:1294203-1294581	110.58	110.58+peptide	OK	86.9325	137.199	0.658298	1.25457	0.03415	0.243541	no
gene:SpnNT_01257	mscL	Chromosome:1294203-1294581	ΔORF2	110.58+peptide	OK	146.029	137.199	-0.0899927	-0.175401	0.7634	0.994748	no
gene:SpnNT_01257	mscL	Chromosome:1294203-1294581	110.58	ΔORF2+peptide	OK	86.9325	212.553	1.28986	2.51029	5.00E-05	0.0013612	yes
gene:SpnNT_01257	mscL	Chromosome:1294203-1294581	ΔORF2	ΔORF2+peptide	OK	146.029	212.553	0.541566	1.07898	0.07005	0.388995	no
gene:SpnNT_01257	mscL	Chromosome:1294203-1294581	110.58+peptide	ΔORF2+peptide	OK	137.199	212.553	0.631559	1.28439	0.0316	0.230492	no
gene:SpnNT_01258	hemH	Chromosome:1294622-1295717	110.58	ΔORF2	OK	8.01384	7.9104	-0.0187424	-0.0309101	0.95835	0.994855	no
gene:SpnNT_01258	hemH	Chromosome:1294622-1295717	110.58	110.58+peptide	OK	8.01384	27.9902	1.80436	3.03475	5.00E-05	0.0013612	yes
gene:SpnNT_01258	hemH	Chromosome:1294622-1295717	ΔORF2	110.58+peptide	OK	7.9104	27.9902	1.8231	3.01268	5.00E-05	0.0013612	yes
gene:SpnNT_01258	hemH	Chromosome:1294622-1295717	110.58	ΔORF2+peptide	OK	8.01384	32.18	2.0056	3.40655	5.00E-05	0.0013612	yes
gene:SpnNT_01258	hemH	Chromosome:1294622-1295717	ΔORF2	ΔORF2+peptide	OK	7.9104	32.18	2.02434	3.37712	5.00E-05	0.0013612	yes
gene:SpnNT_01258	hemH	Chromosome:1294622-1295717	110.58+peptide	ΔORF2+peptide	OK	27.9902	32.18	0.201245	0.342543	0.5581	0.968033	no
gene:SpnNT_01259	pepT	Chromosome:1295871-1297095	110.58	ΔORF2	OK	110.037	123.321	0.164438	0.374846	0.5065	0.945874	no
gene:SpnNT_01259	pepT	Chromosome:1295871-1297095	110.58	110.58+peptide	OK	110.037	70.059	-0.651341	-1.46153	0.01	0.100168	no
gene:SpnNT_01259	pepT	Chromosome:1295871-1297095	ΔORF2	110.58+peptide	OK	123.321	70.059	-0.81578	-1.84286	0.001	0.0168447	yes
gene:SpnNT_01259	pepT	Chromosome:1295871-1297095	110.58	ΔORF2+peptide	OK	110.037	64.443	-0.77189	-1.72304	0.00315	0.0417097	yes
gene:SpnNT_01259	pepT	Chromosome:1295871-1297095	ΔORF2	ΔORF2+peptide	OK	123.321	64.443	-0.936328	-2.10406	6.00E-04	0.0111203	yes
gene:SpnNT_01259	pepT	Chromosome:1295871-1297095	110.58+peptide	ΔORF2+peptide	OK	70.059	64.443	-0.120549	-0.266767	0.63755	0.980887	no
gene:SpnNT_01260	NA	Chromosome:1297130-1297760	110.58	ΔORF2	OK	3.23898	3.52799	0.123307	0.153135	0.79215	0.994748	no
gene:SpnNT_01260	NA	Chromosome:1297130-1297760	110.58	110.58+peptide	OK	3.23898	1.77059	-0.87131	-0.98932	0.09855	0.475781	no
gene:SpnNT_01260	NA	Chromosome:1297130-1297760	ΔORF2	110.58+peptide	OK	3.52799	1.77059	-0.994618	-1.14119	0.057	0.339649	no
gene:SpnNT_01260	NA	Chromosome:1297130-1297760	110.58	ΔORF2+peptide	OK	3.23898	3.58947	0.148232	0.183877	0.7499	0.994748	no
gene:SpnNT_01260	NA	Chromosome:1297130-1297760	ΔORF2	ΔORF2+peptide	OK	3.52799	3.58947	0.0249244	0.0313069	0.96025	0.994855	no

gene:SpnNT_01260	NA	Chromosome:1297130-1297760	110.58+peptide	ΔORF2+peptide	OK	1.77059	3.58947	1.01954	1.16864	0.0499	0.313446	no
gene:SpnNT_01261	NA	Chromosome:1298007-1298265	110.58	ΔORF2	NOTEST	0.394284	0.73674	0.901918	0	1	1	no
gene:SpnNT_01261	NA	Chromosome:1298007-1298265	110.58	110.58+peptide	OK	0.394284	2.85557	2.85647	1.51496	0.15485	0.600007	no
gene:SpnNT_01261	NA	Chromosome:1298007-1298265	ΔORF2	110.58+peptide	OK	0.73674	2.85557	1.95455	1.19368	0.2934	0.797897	no
gene:SpnNT_01261	NA	Chromosome:1298007-1298265	110.58	ΔORF2+peptide	NOTEST	0.394284	0.47342	0.263884	0	1	1	no
gene:SpnNT_01261	NA	Chromosome:1298007-1298265	ΔORF2	ΔORF2+peptide	NOTEST	0.73674	0.47342	-0.638034	0	1	1	no
gene:SpnNT_01261	NA	Chromosome:1298007-1298265	110.58+peptide	ΔORF2+peptide	OK	2.85557	0.47342	-2.59259	-1.52862	0.1707	0.629546	no
gene:SpnNT_01262	NA	Chromosome:1298562-1300296	110.58	ΔORF2	OK	27.0638	31.8947	0.236956	0.498333	0.3781	0.875802	no
gene:SpnNT_01262	NA	Chromosome:1298562-1300296	110.58	110.58+peptide	OK	27.0638	27.5197	0.0240999	0.0501266	0.9321	0.994855	no
gene:SpnNT_01262	NA	Chromosome:1298562-1300296	ΔORF2	110.58+peptide	OK	31.8947	27.5197	-0.212857	-0.447063	0.4356	0.914071	no
gene:SpnNT_01262	NA	Chromosome:1298562-1300296	110.58	ΔORF2+peptide	OK	27.0638	37.6325	0.475615	1.00599	0.07855	0.416458	no
gene:SpnNT_01262	NA	Chromosome:1298562-1300296	ΔORF2	ΔORF2+peptide	OK	31.8947	37.6325	0.238659	0.509904	0.37745	0.875689	no
gene:SpnNT_01262	NA	Chromosome:1298562-1300296	110.58+peptide	ΔORF2+peptide	OK	27.5197	37.6325	0.451516	0.953746	0.09455	0.46485	no
gene:SpnNT_01263	NA	Chromosome:1301276-1301366	110.58	ΔORF2	NOTEST	0	0	0	0	1	1	no
gene:SpnNT_01263	NA	Chromosome:1301276-1301366	110.58	110.58+peptide	NOTEST	0	0	0	0	1	1	no
gene:SpnNT_01263	NA	Chromosome:1301276-1301366	ΔORF2	110.58+peptide	NOTEST	0	0	0	0	1	1	no
gene:SpnNT_01263	NA	Chromosome:1301276-1301366	110.58	ΔORF2+peptide	NOTEST	0	0	0	0	1	1	no
gene:SpnNT_01263	NA	Chromosome:1301276-1301366	ΔORF2	ΔORF2+peptide	NOTEST	0	0	0	0	1	1	no
gene:SpnNT_01263	NA	Chromosome:1301276-1301366	110.58+peptide	ΔORF2+peptide	NOTEST	0	0	0	0	1	1	no
gene:SpnNT_01264	NA	Chromosome:1301591-1303973	110.58	ΔORF2	OK	1.01585	0.944045	-0.105753	-0.142434	0.8027	0.994748	no
gene:SpnNT_01264	NA	Chromosome:1301591-1303973	110.58	110.58+peptide	OK	1.01585	0.902934	-0.169988	-0.228143	0.6882	0.98828	no
gene:SpnNT_01264	NA	Chromosome:1301591-1303973	ΔORF2	110.58+peptide	OK	0.944045	0.902934	-0.0642352	-0.0854312	0.88055	0.994748	no
gene:SpnNT_01264	NA	Chromosome:1301591-1303973	110.58	ΔORF2+peptide	OK	1.01585	0.771096	-0.397699	-0.526063	0.36435	0.864245	no
gene:SpnNT_01264	NA	Chromosome:1301591-1303973	ΔORF2	ΔORF2+peptide	OK	0.944045	0.771096	-0.291946	-0.382784	0.50455	0.945292	no
gene:SpnNT_01264	NA	Chromosome:1301591-1303973	110.58+peptide	ΔORF2+peptide	OK	0.902934	0.771096	-0.227711	-0.297566	0.60415	0.976761	no
gene:SpnNT_01265	NA	Chromosome:1304170-1306651	110.58	ΔORF2	OK	7.63406	6.52863	-0.225671	-0.420011	0.46785	0.92732	no
gene:SpnNT_01265	NA	Chromosome:1304170-1306651	110.58	110.58+peptide	OK	7.63406	6.63502	-0.202351	-0.376434	0.512	0.950278	no
gene:SpnNT_01265	NA	Chromosome:1304170-1306651	ΔORF2	110.58+peptide	OK	6.52863	6.63502	0.0233201	0.0430807	0.94	0.994855	no
gene:SpnNT_01265	NA	Chromosome:1304170-1306651	110.58	ΔORF2+peptide	OK	7.63406	6.12003	-0.318913	-0.592033	0.31075	0.816676	no
gene:SpnNT_01265	NA	Chromosome:1304170-1306651	ΔORF2	ΔORF2+peptide	OK	6.52863	6.12003	-0.093242	-0.171896	0.76035	0.994748	no
gene:SpnNT_01265	NA	Chromosome:1304170-1306651	110.58+peptide	ΔORF2+peptide	OK	6.63502	6.12003	-0.116562	-0.21479	0.70025	0.990367	no
gene:SpnNT_01266	adcA_1	Chromosome:1306658-1307576	110.58	ΔORF2	OK	3.42078	3.91467	0.194565	0.273278	0.6332	0.980887	no
gene:SpnNT_01266	adcA_1	Chromosome:1306658-1307576	110.58	110.58+peptide	OK	3.42078	3.96673	0.213624	0.296454	0.6076	0.978234	no
gene:SpnNT_01266	adcA_1	Chromosome:1306658-1307576	ΔORF2	110.58+peptide	OK	3.91467	3.96673	0.0190582	0.0271376	0.9589	0.994855	no
gene:SpnNT_01266	adcA_1	Chromosome:1306658-1307576	110.58	ΔORF2+peptide	OK	3.42078	3.86768	0.17714	0.24244	0.6734	0.984845	no
gene:SpnNT_01266	adcA_1	Chromosome:1306658-1307576	ΔORF2	ΔORF2+peptide	OK	3.91467	3.86768	-0.0174253	-0.0244532	0.96055	0.994855	no
gene:SpnNT_01266	adcA_1	Chromosome:1306658-1307576	110.58+peptide	ΔORF2+peptide	OK	3.96673	3.86768	-0.0364835	-0.0505858	0.9287	0.994748	no
gene:SpnNT_01267	yhdG	Chromosome:1307718-1309110	110.58	ΔORF2	OK	151.693	162.867	0.102544	0.230355	0.6771	0.98524	no
gene:SpnNT_01267	yhdG	Chromosome:1307718-1309110	110.58	110.58+peptide	OK	151.693	76.7204	-0.983471	-2.18349	5.00E-05	0.0013612	yes
gene:SpnNT_01267	yhdG	Chromosome:1307718-1309110	ΔORF2	110.58+peptide	OK	162.867	76.7204	-1.08601	-2.40617	5.00E-05	0.0013612	yes
gene:SpnNT_01267	yhdG	Chromosome:1307718-1309110	110.58	ΔORF2+peptide	OK	151.693	87.2489	-0.797944	-1.78009	0.0023	0.0329123	yes
gene:SpnNT_01267	yhdG	Chromosome:1307718-1309110	ΔORF2	ΔORF2+peptide	OK	162.867	87.2489	-0.900488	-2.00464	0.00055	0.0103545	yes
gene:SpnNT_01267	yhdG	Chromosome:1307718-1309110	110.58+peptide	ΔORF2+peptide	OK	76.7204	87.2489	0.185527	0.40828	0.47455	0.930243	no
gene:SpnNT_01268	resA	Chromosome:1309231-1309798	110.58	ΔORF2	OK	336.329	521.245	0.632088	1.33819	0.01825	0.155101	no
gene:SpnNT_01268	resA	Chromosome:1309231-1309798	110.58	110.58+peptide	OK	336.329	280.979	-0.259413	-0.554336	0.32255	0.828116	no

gene:SpnNT_01268	resA	Chromosome:1309231-1309798	ΔORF2	110.58+peptide	OK	521.245	280.979	-0.891501	-1.82293	0.00155	0.0240131	yes
gene:SpnNT_01268	resA	Chromosome:1309231-1309798	110.58	ΔORF2+peptide	OK	336.329	289.972	-0.213961	-0.470127	0.40475	0.894933	no
gene:SpnNT_01268	resA	Chromosome:1309231-1309798	ΔORF2	ΔORF2+peptide	OK	521.245	289.972	-0.846049	-1.77458	0.0016	0.0247585	yes
gene:SpnNT_01268	resA	Chromosome:1309231-1309798	110.58+peptide	ΔORF2+peptide	OK	280.979	289.972	0.0454522	0.09621	0.866	0.994748	no
gene:SpnNT_01269	dsbD_2	Chromosome:1309808-1310540	110.58	ΔORF2	OK	77.4814	124.052	0.679027	1.41262	0.0149	0.13355	no
gene:SpnNT_01269	dsbD_2	Chromosome:1309808-1310540	110.58	110.58+peptide	OK	77.4814	68.7106	-0.173317	-0.357989	0.5408	0.961494	no
gene:SpnNT_01269	dsbD_2	Chromosome:1309808-1310540	ΔORF2	110.58+peptide	OK	124.052	68.7106	-0.852344	-1.75112	0.00285	0.0382781	yes
gene:SpnNT_01269	dsbD_2	Chromosome:1309808-1310540	110.58	ΔORF2+peptide	OK	77.4814	99.4521	0.360151	0.763646	0.1868	0.661768	no
gene:SpnNT_01269	dsbD_2	Chromosome:1309808-1310540	ΔORF2	ΔORF2+peptide	OK	124.052	99.4521	-0.318876	-0.672321	0.238	0.732745	no
gene:SpnNT_01269	dsbD_2	Chromosome:1309808-1310540	110.58+peptide	ΔORF2+peptide	OK	68.7106	99.4521	0.533468	1.11653	0.0583	0.344135	no
gene:SpnNT_01270	NA	Chromosome:1310746-1311163	110.58	ΔORF2	OK	8.81158	7.37539	-0.25668	-0.332133	0.5586	0.968033	no
gene:SpnNT_01270	NA	Chromosome:1310746-1311163	110.58	110.58+peptide	OK	8.81158	10.9209	0.309623	0.415915	0.47265	0.928558	no
gene:SpnNT_01270	NA	Chromosome:1310746-1311163	ΔORF2	110.58+peptide	OK	7.37539	10.9209	0.566303	0.740674	0.1982	0.680978	no
gene:SpnNT_01270	NA	Chromosome:1310746-1311163	110.58	ΔORF2+peptide	OK	8.81158	9.51877	0.111374	0.148286	0.79755	0.994748	no
gene:SpnNT_01270	NA	Chromosome:1310746-1311163	ΔORF2	ΔORF2+peptide	OK	7.37539	9.51877	0.368055	0.477346	0.4063	0.895895	no
gene:SpnNT_01270	NA	Chromosome:1310746-1311163	110.58+peptide	ΔORF2+peptide	OK	10.9209	9.51877	-0.198248	-0.266969	0.6396	0.980887	no
gene:SpnNT_01271	NA	Chromosome:1311449-1312074	110.58	ΔORF2	OK	21.9335	15.592	-0.492329	-0.775683	0.1725	0.632629	no
gene:SpnNT_01271	NA	Chromosome:1311449-1312074	110.58	110.58+peptide	OK	21.9335	21.7798	-0.0101489	-0.0156987	0.98075	0.99608	no
gene:SpnNT_01271	NA	Chromosome:1311449-1312074	ΔORF2	110.58+peptide	OK	15.592	21.7798	0.48218	0.737646	0.19585	0.677365	no
gene:SpnNT_01271	NA	Chromosome:1311449-1312074	110.58	ΔORF2+peptide	OK	21.9335	16.4203	-0.417657	-0.638499	0.2603	0.760725	no
gene:SpnNT_01271	NA	Chromosome:1311449-1312074	ΔORF2	ΔORF2+peptide	OK	15.592	16.4203	0.0746716	0.112927	0.84025	0.994748	no
gene:SpnNT_01271	NA	Chromosome:1311449-1312074	110.58+peptide	ΔORF2+peptide	OK	21.7798	16.4203	-0.407509	-0.605919	0.27865	0.781125	no
gene:SpnNT_01272	NA	Chromosome:1311449-1312074	110.58	ΔORF2	NOTEST	1.61653	0	#NAME?	0	1	1	no
gene:SpnNT_01272	NA	Chromosome:1311449-1312074	110.58	110.58+peptide	OK	1.61653	7.9066	2.29015	0.590751	0.43695	0.914167	no
gene:SpnNT_01272	NA	Chromosome:1311449-1312074	ΔORF2	110.58+peptide	OK	0	7.9066	Inf	NA	0.1922	0.673264	no
gene:SpnNT_01272	NA	Chromosome:1311449-1312074	110.58	ΔORF2+peptide	NOTEST	1.61653	1.59277	-0.0213686	0	1	1	no
gene:SpnNT_01272	NA	Chromosome:1311449-1312074	ΔORF2	ΔORF2+peptide	NOTEST	0	1.59277	Inf	0	1	1	no
gene:SpnNT_01272	NA	Chromosome:1311449-1312074	110.58+peptide	ΔORF2+peptide	OK	7.9066	1.59277	-2.31152	-0.673736	0.44845	0.919688	no
gene:SpnNT_01273	NA	Chromosome:1312352-1313076	110.58	ΔORF2	OK	19.1265	18.1199	-0.0779962	-0.101692	0.86315	0.994748	no
gene:SpnNT_01273	NA	Chromosome:1312352-1313076	110.58	110.58+peptide	OK	19.1265	15.299	-0.322134	-0.412779	0.4717	0.928525	no
gene:SpnNT_01273	NA	Chromosome:1312352-1313076	ΔORF2	110.58+peptide	OK	18.1199	15.299	-0.244138	-0.309286	0.58785	0.973469	no
gene:SpnNT_01273	NA	Chromosome:1312352-1313076	110.58	ΔORF2+peptide	OK	19.1265	15.5482	-0.298823	-0.349635	0.5313	0.957488	no
gene:SpnNT_01273	NA	Chromosome:1312352-1313076	ΔORF2	ΔORF2+peptide	OK	18.1199	15.5482	-0.220827	-0.255925	0.64395	0.980887	no
gene:SpnNT_01273	NA	Chromosome:1312352-1313076	110.58+peptide	ΔORF2+peptide	OK	15.299	15.5482	0.0233112	0.0266472	0.9615	0.994855	no
gene:SpnNT_01274	NA	Chromosome:1312352-1313076	110.58	ΔORF2	OK	25.9758	23.1222	-0.167891	-0.163375	0.77735	0.994748	no
gene:SpnNT_01274	NA	Chromosome:1312352-1313076	110.58	110.58+peptide	OK	25.9758	20.4849	-0.342607	-0.329954	0.57235	0.969081	no
gene:SpnNT_01274	NA	Chromosome:1312352-1313076	ΔORF2	110.58+peptide	OK	23.1222	20.4849	-0.174716	-0.16382	0.7841	0.994748	no
gene:SpnNT_01274	NA	Chromosome:1312352-1313076	110.58	ΔORF2+peptide	OK	25.9758	27.8241	0.0991649	0.0950696	0.8736	0.994748	no
gene:SpnNT_01274	NA	Chromosome:1312352-1313076	ΔORF2	ΔORF2+peptide	OK	23.1222	27.8241	0.267056	0.249324	0.6746	0.985063	no
gene:SpnNT_01274	NA	Chromosome:1312352-1313076	110.58+peptide	ΔORF2+peptide	OK	20.4849	27.8241	0.441772	0.408522	0.4977	0.944017	no
gene:SpnNT_01275	ugpQ	Chromosome:1313437-1315201	110.58	ΔORF2	OK	40.6739	46.2832	0.186387	0.405946	0.4809	0.935095	no
gene:SpnNT_01275	ugpQ	Chromosome:1313437-1315201	110.58	110.58+peptide	OK	40.6739	55.6578	0.452483	0.997055	0.08465	0.437659	no
gene:SpnNT_01275	ugpQ	Chromosome:1313437-1315201	ΔORF2	110.58+peptide	OK	46.2832	55.6578	0.266096	0.579966	0.3161	0.821977	no
gene:SpnNT_01275	ugpQ	Chromosome:1313437-1315201	110.58	ΔORF2+peptide	OK	40.6739	68.5841	0.753772	1.6798	0.0051	0.061228	no
gene:SpnNT_01275	ugpQ	Chromosome:1313437-1315201	ΔORF2	ΔORF2+peptide	OK	46.2832	68.5841	0.567385	1.25036	0.03245	0.234864	no

gene:SpnNT_01275	ugpQ	Chromosome:1313437-1315201	110.58+peptide	ΔORF2+peptide	OK	55.6578	68.5841	0.301289	0.671938	0.2441	0.740215	no
gene:SpnNT_01276	polC_2	Chromosome:1315209-1315800	110.58	ΔORF2	OK	37.2604	40.4502	0.118502	0.219478	0.70445	0.990367	no
gene:SpnNT_01276	polC_2	Chromosome:1315209-1315800	110.58	110.58+peptide	OK	37.2604	44.3873	0.252504	0.471778	0.40965	0.8977	no
gene:SpnNT_01276	polC_2	Chromosome:1315209-1315800	ΔORF2	110.58+peptide	OK	40.4502	44.3873	0.134002	0.251042	0.6603	0.982966	no
gene:SpnNT_01276	polC_2	Chromosome:1315209-1315800	110.58	ΔORF2+peptide	OK	37.2604	52.5099	0.494946	0.932077	0.10455	0.490667	no
gene:SpnNT_01276	polC_2	Chromosome:1315209-1315800	ΔORF2	ΔORF2+peptide	OK	40.4502	52.5099	0.376445	0.71085	0.2103	0.696806	no
gene:SpnNT_01276	polC_2	Chromosome:1315209-1315800	110.58+peptide	ΔORF2+peptide	OK	44.3873	52.5099	0.242443	0.462	0.4191	0.903984	no
gene:SpnNT_01277	NA	Chromosome:1315897-1316389	110.58	ΔORF2	OK	1379.24	1646.6	0.255609	0.526868	0.35115	0.854885	no
gene:SpnNT_01277	NA	Chromosome:1315897-1316389	110.58	110.58+peptide	OK	1379.24	1774.09	0.3632	0.754508	0.17895	0.645815	no
gene:SpnNT_01277	NA	Chromosome:1315897-1316389	ΔORF2	110.58+peptide	OK	1646.6	1774.09	0.107591	0.216345	0.7016	0.990367	no
gene:SpnNT_01277	NA	Chromosome:1315897-1316389	110.58	ΔORF2+peptide	OK	1379.24	1649.47	0.25812	0.554742	0.3134	0.819373	no
gene:SpnNT_01277	NA	Chromosome:1315897-1316389	ΔORF2	ΔORF2+peptide	OK	1646.6	1649.47	0.00251119	0.00521243	0.99235	0.998514	no
gene:SpnNT_01277	NA	Chromosome:1315897-1316389	110.58+peptide	ΔORF2+peptide	OK	1774.09	1649.47	-0.10508	-0.219848	0.69105	0.98828	no
gene:SpnNT_01278	mtnN	Chromosome:1316465-1317158	110.58	ΔORF2	OK	208.103	231.539	0.153959	0.343627	0.54985	0.964281	no
gene:SpnNT_01278	mtnN	Chromosome:1316465-1317158	110.58	110.58+peptide	OK	208.103	285.75	0.457457	1.02103	0.08015	0.421873	no
gene:SpnNT_01278	mtnN	Chromosome:1316465-1317158	ΔORF2	110.58+peptide	OK	231.539	285.75	0.303498	0.687656	0.23165	0.724662	no
gene:SpnNT_01278	mtnN	Chromosome:1316465-1317158	110.58	ΔORF2+peptide	OK	208.103	237.706	0.191883	0.430229	0.4546	0.921244	no
gene:SpnNT_01278	mtnN	Chromosome:1316465-1317158	ΔORF2	ΔORF2+peptide	OK	231.539	237.706	0.0379242	0.0863314	0.87665	0.994748	no
gene:SpnNT_01278	mtnN	Chromosome:1316465-1317158	110.58+peptide	ΔORF2+peptide	OK	285.75	237.706	-0.265574	-0.604564	0.2911	0.795269	no
gene:SpnNT_01279	NA	Chromosome:1317174-1317489	110.58	ΔORF2	OK	164.779	172.521	0.066235	0.127017	0.82375	0.994748	no
gene:SpnNT_01279	NA	Chromosome:1317174-1317489	110.58	110.58+peptide	OK	164.779	154.459	-0.0933099	-0.178947	0.7509	0.994748	no
gene:SpnNT_01279	NA	Chromosome:1317174-1317489	ΔORF2	110.58+peptide	OK	172.521	154.459	-0.159545	-0.307138	0.5908	0.974909	no
gene:SpnNT_01279	NA	Chromosome:1317174-1317489	110.58	ΔORF2+peptide	OK	164.779	172.143	0.0630707	0.119304	0.8379	0.994748	no
gene:SpnNT_01279	NA	Chromosome:1317174-1317489	ΔORF2	ΔORF2+peptide	OK	172.521	172.143	-0.0031643	-0.00600779	0.99215	0.99849	no
gene:SpnNT_01279	NA	Chromosome:1317174-1317489	110.58+peptide	ΔORF2+peptide	OK	154.459	172.143	0.156381	0.296919	0.6058	0.976937	no
gene:SpnNT_01280	nudF	Chromosome:1317500-1318046	110.58	ΔORF2	OK	128.94	135.937	0.0762349	0.161963	0.77545	0.994748	no
gene:SpnNT_01280	nudF	Chromosome:1317500-1318046	110.58	110.58+peptide	OK	128.94	136.495	0.0821466	0.174744	0.7623	0.994748	no
gene:SpnNT_01280	nudF	Chromosome:1317500-1318046	ΔORF2	110.58+peptide	OK	135.937	136.495	0.00591167	0.0126018	0.98315	0.996246	no
gene:SpnNT_01280	nudF	Chromosome:1317500-1318046	110.58	ΔORF2+peptide	OK	128.94	153.189	0.24861	0.52048	0.3641	0.864245	no
gene:SpnNT_01280	nudF	Chromosome:1317500-1318046	ΔORF2	ΔORF2+peptide	OK	135.937	153.189	0.172375	0.36161	0.53	0.957488	no
gene:SpnNT_01280	nudF	Chromosome:1317500-1318046	110.58+peptide	ΔORF2+peptide	OK	136.495	153.189	0.166463	0.349641	0.5421	0.961494	no
gene:SpnNT_01281	glmU	Chromosome:1318055-1319435	110.58	ΔORF2	OK	183.857	192.167	0.0637768	0.146968	0.7889	0.994748	no
gene:SpnNT_01281	glmU	Chromosome:1318055-1319435	110.58	110.58+peptide	OK	183.857	198.127	0.107837	0.248593	0.65415	0.982798	no
gene:SpnNT_01281	glmU	Chromosome:1318055-1319435	ΔORF2	110.58+peptide	OK	192.167	198.127	0.0440602	0.10164	0.85115	0.994748	no
gene:SpnNT_01281	glmU	Chromosome:1318055-1319435	110.58	ΔORF2+peptide	OK	183.857	217.003	0.239131	0.550505	0.3237	0.829122	no
gene:SpnNT_01281	glmU	Chromosome:1318055-1319435	ΔORF2	ΔORF2+peptide	OK	192.167	217.003	0.175355	0.403959	0.4722	0.928525	no
gene:SpnNT_01281	glmU	Chromosome:1318055-1319435	110.58+peptide	ΔORF2+peptide	OK	198.127	217.003	0.131294	0.302571	0.5873	0.973049	no
gene:SpnNT_01282	NA	Chromosome:1319578-1320379	110.58	ΔORF2	OK	75.5138	94.1775	0.318643	0.679645	0.2398	0.7352	no
gene:SpnNT_01282	NA	Chromosome:1319578-1320379	110.58	110.58+peptide	OK	75.5138	74.4135	-0.0211751	-0.0448709	0.9392	0.994855	no
gene:SpnNT_01282	NA	Chromosome:1319578-1320379	ΔORF2	110.58+peptide	OK	94.1775	74.4135	-0.339818	-0.725015	0.20875	0.694352	no
gene:SpnNT_01282	NA	Chromosome:1319578-1320379	110.58	ΔORF2+peptide	OK	75.5138	83.5767	0.146362	0.311249	0.58685	0.973049	no
gene:SpnNT_01282	NA	Chromosome:1319578-1320379	ΔORF2	ΔORF2+peptide	OK	94.1775	83.5767	-0.172281	-0.368893	0.5189	0.954444	no
gene:SpnNT_01282	NA	Chromosome:1319578-1320379	110.58+peptide	ΔORF2+peptide	OK	74.4135	83.5767	0.167537	0.356379	0.52715	0.956161	no
gene:SpnNT_01283	NA	Chromosome:1320473-1322418	110.58	ΔORF2	OK	22.6247	28.1763	0.316582	0.429228	0.45565	0.921244	no
gene:SpnNT_01283	NA	Chromosome:1320473-1322418	110.58	110.58+peptide	OK	22.6247	22.4237	-0.0128729	-0.0173617	0.97375	0.99536	no

gene:SpnNT_01283	NA	Chromosome:1320473-1322418	ΔORF2	110.58+peptide	OK	28.1763	22.4237	-0.329455	-0.443769	0.4472	0.919465	no
gene:SpnNT_01283	NA	Chromosome:1320473-1322418	110.58	ΔORF2+peptide	OK	22.6247	26.3285	0.218729	0.306016	0.59535	0.975751	no
gene:SpnNT_01283	NA	Chromosome:1320473-1322418	ΔORF2	ΔORF2+peptide	OK	28.1763	26.3285	-0.0978527	-0.136714	0.8148	0.994748	no
gene:SpnNT_01283	NA	Chromosome:1320473-1322418	110.58+peptide	ΔORF2+peptide	OK	22.4237	26.3285	0.231602	0.321778	0.5913	0.975366	no
gene:SpnNT_01284	ybaK	Chromosome:1320473-1322418	110.58	ΔORF2	OK	33.6278	36.0018	0.0984128	0.100689	0.8593	0.994748	no
gene:SpnNT_01284	ybaK	Chromosome:1320473-1322418	110.58	110.58+peptide	OK	33.6278	31.9702	-0.0729269	-0.0728469	0.9013	0.994748	no
gene:SpnNT_01284	ybaK	Chromosome:1320473-1322418	ΔORF2	110.58+peptide	OK	36.0018	31.9702	-0.17134	-0.172673	0.75845	0.994748	no
gene:SpnNT_01284	ybaK	Chromosome:1320473-1322418	110.58	ΔORF2+peptide	OK	33.6278	28.2301	-0.252422	-0.23769	0.6787	0.985348	no
gene:SpnNT_01284	ybaK	Chromosome:1320473-1322418	ΔORF2	ΔORF2+peptide	OK	36.0018	28.2301	-0.350835	-0.332965	0.55195	0.964534	no
gene:SpnNT_01284	ybaK	Chromosome:1320473-1322418	110.58+peptide	ΔORF2+peptide	OK	31.9702	28.2301	-0.179495	-0.166865	0.77225	0.994748	no
gene:SpnNT_01285	pspB	Chromosome:1320473-1322418	110.58	ΔORF2	OK	17.1597	20.1766	0.233666	0.22092	0.702	0.990367	no
gene:SpnNT_01285	pspB	Chromosome:1320473-1322418	110.58	110.58+peptide	OK	17.1597	16.3417	-0.0704584	-0.0628138	0.9172	0.994748	no
gene:SpnNT_01285	pspB	Chromosome:1320473-1322418	ΔORF2	110.58+peptide	OK	20.1766	16.3417	-0.304124	-0.270217	0.6463	0.980887	no
gene:SpnNT_01285	pspB	Chromosome:1320473-1322418	110.58	ΔORF2+peptide	OK	17.1597	19.3742	0.175116	0.170764	0.76535	0.994748	no
gene:SpnNT_01285	pspB	Chromosome:1320473-1322418	ΔORF2	ΔORF2+peptide	OK	20.1766	19.3742	-0.0585501	-0.056866	0.9218	0.994748	no
gene:SpnNT_01285	pspB	Chromosome:1320473-1322418	110.58+peptide	ΔORF2+peptide	OK	16.3417	19.3742	0.245574	0.224214	0.69855	0.990209	no
gene:SpnNT_01286	NA	Chromosome:1323044-1323520	110.58	ΔORF2	NOTEST	0	0	0	0	1	1	no
gene:SpnNT_01286	NA	Chromosome:1323044-1323520	110.58	110.58+peptide	NOTEST	0	0	0	0	1	1	no
gene:SpnNT_01286	NA	Chromosome:1323044-1323520	ΔORF2	110.58+peptide	NOTEST	0	0	0	0	1	1	no
gene:SpnNT_01286	NA	Chromosome:1323044-1323520	110.58	ΔORF2+peptide	NOTEST	0	0	0	0	1	1	no
gene:SpnNT_01286	NA	Chromosome:1323044-1323520	ΔORF2	ΔORF2+peptide	NOTEST	0	0	0	0	1	1	no
gene:SpnNT_01286	NA	Chromosome:1323044-1323520	110.58+peptide	ΔORF2+peptide	NOTEST	0	0	0	0	1	1	no
gene:SpnNT_01287	NA	Chromosome:1323044-1323520	110.58	ΔORF2	NOTEST	0	0	0	0	1	1	no
gene:SpnNT_01287	NA	Chromosome:1323044-1323520	110.58	110.58+peptide	NOTEST	0	0	0	0	1	1	no
gene:SpnNT_01287	NA	Chromosome:1323044-1323520	ΔORF2	110.58+peptide	NOTEST	0	0	0	0	1	1	no
gene:SpnNT_01287	NA	Chromosome:1323044-1323520	110.58	ΔORF2+peptide	NOTEST	0	0	0	0	1	1	no
gene:SpnNT_01287	NA	Chromosome:1323044-1323520	ΔORF2	ΔORF2+peptide	NOTEST	0	0	0	0	1	1	no
gene:SpnNT_01287	NA	Chromosome:1323044-1323520	110.58+peptide	ΔORF2+peptide	NOTEST	0	0	0	0	1	1	no
gene:SpnNT_01288	prsA	Chromosome:1323636-1324578	110.58	ΔORF2	OK	155.878	174.54	0.163141	0.374226	0.5068	0.945979	no
gene:SpnNT_01288	prsA	Chromosome:1323636-1324578	110.58	110.58+peptide	OK	155.878	167.474	0.103519	0.236181	0.6752	0.985207	no
gene:SpnNT_01288	prsA	Chromosome:1323636-1324578	ΔORF2	110.58+peptide	OK	174.54	167.474	-0.0596222	-0.13608	0.8081	0.994748	no
gene:SpnNT_01288	prsA	Chromosome:1323636-1324578	110.58	ΔORF2+peptide	OK	155.878	211.674	0.441431	1.01292	0.07045	0.390391	no
gene:SpnNT_01288	prsA	Chromosome:1323636-1324578	ΔORF2	ΔORF2+peptide	OK	174.54	211.674	0.27829	0.638813	0.2538	0.752773	no
gene:SpnNT_01288	prsA	Chromosome:1323636-1324578	110.58+peptide	ΔORF2+peptide	OK	167.474	211.674	0.337912	0.771494	0.1727	0.632681	no
gene:SpnNT_01289	NA	Chromosome:1324644-1325358	110.58	ΔORF2	OK	15.3587	16.3367	0.089063	0.150623	0.79185	0.994748	no
gene:SpnNT_01289	NA	Chromosome:1324644-1325358	110.58	110.58+peptide	OK	15.3587	44.1889	1.52463	2.75464	5.00E-05	0.0013612	yes
gene:SpnNT_01289	NA	Chromosome:1324644-1325358	ΔORF2	110.58+peptide	OK	16.3367	44.1889	1.43557	2.59667	5.00E-05	0.0013612	yes
gene:SpnNT_01289	NA	Chromosome:1324644-1325358	110.58	ΔORF2+peptide	OK	15.3587	57.3418	1.90053	3.47001	5.00E-05	0.0013612	yes
gene:SpnNT_01289	NA	Chromosome:1324644-1325358	ΔORF2	ΔORF2+peptide	OK	16.3367	57.3418	1.81147	3.31124	5.00E-05	0.0013612	yes
gene:SpnNT_01289	NA	Chromosome:1324644-1325358	110.58+peptide	ΔORF2+peptide	OK	44.1889	57.3418	0.375904	0.742969	0.19335	0.674905	no
gene:SpnNT_01290	pepF1_1	Chromosome:1325359-1327162	110.58	ΔORF2	OK	28.1247	28.5891	0.0236239	0.0500474	0.9296	0.99482	no
gene:SpnNT_01290	pepF1_1	Chromosome:1325359-1327162	110.58	110.58+peptide	OK	28.1247	73.9095	1.39392	3.05388	5.00E-05	0.0013612	yes
gene:SpnNT_01290	pepF1_1	Chromosome:1325359-1327162	ΔORF2	110.58+peptide	OK	28.5891	73.9095	1.3703	2.99729	5.00E-05	0.0013612	yes
gene:SpnNT_01290	pepF1_1	Chromosome:1325359-1327162	110.58	ΔORF2+peptide	OK	28.1247	86.9644	1.62858	3.56582	5.00E-05	0.0013612	yes
gene:SpnNT_01290	pepF1_1	Chromosome:1325359-1327162	ΔORF2	ΔORF2+peptide	OK	28.5891	86.9644	1.60496	3.50845	5.00E-05	0.0013612	yes

gene:SpnNT_01290	pepF1_1	Chromosome:1325359-1327162	110.58+peptide	ΔORF2+peptide	OK	73.9095	86.9644	0.234665	0.531693	0.35885	0.859746	no
gene:SpnNT_01291	NA	Chromosome:1327180-1328134	110.58	ΔORF2	OK	1.54733	1.39097	-0.153692	-0.178591	0.75325	0.994748	no
gene:SpnNT_01291	NA	Chromosome:1327180-1328134	110.58	110.58+peptide	OK	1.54733	0.682672	-1.18052	-1.22704	0.04335	0.285562	no
gene:SpnNT_01291	NA	Chromosome:1327180-1328134	ΔORF2	110.58+peptide	OK	1.39097	0.682672	-1.02683	-1.06199	0.0706	0.390464	no
gene:SpnNT_01291	NA	Chromosome:1327180-1328134	110.58	ΔORF2+peptide	OK	1.54733	1.12752	-0.456632	-0.515987	0.37845	0.875802	no
gene:SpnNT_01291	NA	Chromosome:1327180-1328134	ΔORF2	ΔORF2+peptide	OK	1.39097	1.12752	-0.30294	-0.340309	0.5529	0.965295	no
gene:SpnNT_01291	NA	Chromosome:1327180-1328134	110.58+peptide	ΔORF2+peptide	OK	0.682672	1.12752	0.723887	0.732191	0.21675	0.704171	no
gene:SpnNT_01292	tehB	Chromosome:1328206-1329067	110.58	ΔORF2	OK	198.404	194.293	-0.0302078	-0.0687641	0.90185	0.994748	no
gene:SpnNT_01292	tehB	Chromosome:1328206-1329067	110.58	110.58+peptide	OK	198.404	133.984	-0.56638	-1.27801	0.0282	0.214152	no
gene:SpnNT_01292	tehB	Chromosome:1328206-1329067	ΔORF2	110.58+peptide	OK	194.293	133.984	-0.536172	-1.21599	0.0338	0.241834	no
gene:SpnNT_01292	tehB	Chromosome:1328206-1329067	110.58	ΔORF2+peptide	OK	198.404	141.64	-0.486218	-1.09848	0.0611	0.355861	no
gene:SpnNT_01292	tehB	Chromosome:1328206-1329067	ΔORF2	ΔORF2+peptide	OK	194.293	141.64	-0.456011	-1.03548	0.07215	0.395469	no
gene:SpnNT_01292	tehB	Chromosome:1328206-1329067	110.58+peptide	ΔORF2+peptide	OK	133.984	141.64	0.0801618	0.180442	0.7563	0.994748	no
gene:SpnNT_01293	smpB	Chromosome:1329082-1331867	110.58	ΔORF2	OK	150.776	142.699	-0.0794277	-0.0583471	0.91605	0.994748	no
gene:SpnNT_01293	smpB	Chromosome:1329082-1331867	110.58	110.58+peptide	OK	150.776	101.83	-0.566246	-0.418117	0.46565	0.926452	no
gene:SpnNT_01293	smpB	Chromosome:1329082-1331867	ΔORF2	110.58+peptide	OK	142.699	101.83	-0.486818	-0.374109	0.5016	0.944784	no
gene:SpnNT_01293	smpB	Chromosome:1329082-1331867	110.58	ΔORF2+peptide	OK	150.776	114.109	-0.401993	-0.303698	0.5978	0.97629	no
gene:SpnNT_01293	smpB	Chromosome:1329082-1331867	ΔORF2	ΔORF2+peptide	OK	142.699	114.109	-0.322566	-0.254113	0.65135	0.981476	no
gene:SpnNT_01293	smpB	Chromosome:1329082-1331867	110.58+peptide	ΔORF2+peptide	OK	101.83	114.109	0.164252	0.130168	0.82275	0.994748	no
gene:SpnNT_01294	rnr	Chromosome:1329082-1331867	110.58	ΔORF2	OK	133.937	132.34	-0.0173021	-0.0369574	0.94715	0.994855	no
gene:SpnNT_01294	rnr	Chromosome:1329082-1331867	110.58	110.58+peptide	OK	133.937	91.0437	-0.556923	-1.19567	0.03575	0.251265	no
gene:SpnNT_01294	rnr	Chromosome:1329082-1331867	ΔORF2	110.58+peptide	OK	132.34	91.0437	-0.539621	-1.15701	0.0428	0.283361	no
gene:SpnNT_01294	rnr	Chromosome:1329082-1331867	110.58	ΔORF2+peptide	OK	133.937	85.0965	-0.654381	-1.40043	0.0136	0.125322	no
gene:SpnNT_01294	rnr	Chromosome:1329082-1331867	ΔORF2	ΔORF2+peptide	OK	132.34	85.0965	-0.637079	-1.36162	0.0171	0.147914	no
gene:SpnNT_01294	rnr	Chromosome:1329082-1331867	110.58+peptide	ΔORF2+peptide	OK	91.0437	85.0965	-0.0974585	-0.209363	0.715	0.992053	no
gene:SpnNT_01295	NA	Chromosome:1331968-1332202	110.58	ΔORF2	OK	4956.51	7108.19	0.520157	1.01996	0.0698	0.3881	no
gene:SpnNT_01295	NA	Chromosome:1331968-1332202	110.58	110.58+peptide	OK	4956.51	10543.4	1.08894	2.23333	5.00E-05	0.0013612	yes
gene:SpnNT_01295	NA	Chromosome:1331968-1332202	ΔORF2	110.58+peptide	OK	7108.19	10543.4	0.568783	1.09698	0.0543	0.331253	no
gene:SpnNT_01295	NA	Chromosome:1331968-1332202	110.58	ΔORF2+peptide	OK	4956.51	10668.1	1.10591	2.43267	5.00E-05	0.0013612	yes
gene:SpnNT_01295	NA	Chromosome:1331968-1332202	ΔORF2	ΔORF2+peptide	OK	7108.19	10668.1	0.585752	1.20125	0.04185	0.278901	no
gene:SpnNT_01295	NA	Chromosome:1331968-1332202	110.58+peptide	ΔORF2+peptide	OK	10543.4	10668.1	0.0169686	0.0365585	0.9471	0.994855	no
gene:SpnNT_01296	NA	Chromosome:1332404-1334138	110.58	ΔORF2	OK	21.9602	25.3971	0.209769	0.430859	0.4406	0.916647	no
gene:SpnNT_01296	NA	Chromosome:1332404-1334138	110.58	110.58+peptide	OK	21.9602	22.6704	0.0459161	0.0933694	0.8705	0.994748	no
gene:SpnNT_01296	NA	Chromosome:1332404-1334138	ΔORF2	110.58+peptide	OK	25.3971	22.6704	-0.163853	-0.335382	0.5629	0.968621	no
gene:SpnNT_01296	NA	Chromosome:1332404-1334138	110.58	ΔORF2+peptide	OK	21.9602	30.0627	0.453079	0.939734	0.0925	0.459071	no
gene:SpnNT_01296	NA	Chromosome:1332404-1334138	ΔORF2	ΔORF2+peptide	OK	25.3971	30.0627	0.24331	0.508104	0.3686	0.865873	no
gene:SpnNT_01296	NA	Chromosome:1332404-1334138	110.58+peptide	ΔORF2+peptide	OK	22.6704	30.0627	0.407163	0.841514	0.1395	0.569723	no
gene:SpnNT_01297	tetA	Chromosome:1334398-1337014	110.58	ΔORF2	OK	33.672	36.3144	0.108994	0.141276	0.80805	0.994748	no
gene:SpnNT_01297	tetA	Chromosome:1334398-1337014	110.58	110.58+peptide	OK	33.672	29.8046	-0.176011	-0.219017	0.716	0.992361	no
gene:SpnNT_01297	tetA	Chromosome:1334398-1337014	ΔORF2	110.58+peptide	OK	36.3144	29.8046	-0.285005	-0.365618	0.53365	0.958867	no
gene:SpnNT_01297	tetA	Chromosome:1334398-1337014	110.58	ΔORF2+peptide	OK	33.672	34.8633	0.0501598	0.0657829	0.91005	0.994748	no
gene:SpnNT_01297	tetA	Chromosome:1334398-1337014	ΔORF2	ΔORF2+peptide	OK	36.3144	34.8633	-0.0588343	-0.0798259	0.8896	0.994748	no
gene:SpnNT_01297	tetA	Chromosome:1334398-1337014	110.58+peptide	ΔORF2+peptide	OK	29.8046	34.8633	0.226171	0.293494	0.61485	0.979616	no
gene:SpnNT_01298	coaE	Chromosome:1334398-1337014	110.58	ΔORF2	OK	62.8299	62.9963	0.00381507	0.0041469	0.9951	0.999258	no
gene:SpnNT_01298	coaE	Chromosome:1334398-1337014	110.58	110.58+peptide	OK	62.8299	54.2806	-0.211014	-0.237374	0.6766	0.98524	no

gene:SpnNT_01298	coaE	Chromosome:1334398-1337014	ΔORF2	110.58+peptide	OK	62.9963	54.2806	-0.214829	-0.233391	0.68225	0.986608	no
gene:SpnNT_01298	coaE	Chromosome:1334398-1337014	110.58	ΔORF2+peptide	OK	62.8299	56.9556	-0.141614	-0.156544	0.789	0.994748	no
gene:SpnNT_01298	coaE	Chromosome:1334398-1337014	ΔORF2	ΔORF2+peptide	OK	62.9963	56.9556	-0.145429	-0.155437	0.78805	0.994748	no
gene:SpnNT_01298	coaE	Chromosome:1334398-1337014	110.58+peptide	ΔORF2+peptide	OK	54.2806	56.9556	0.0694002	0.0766754	0.8943	0.994748	no
gene:SpnNT_01299	mutM	Chromosome:1334398-1337014	110.58	ΔORF2	OK	71.4445	67.3125	-0.0859475	-0.124305	0.8267	0.994748	no
gene:SpnNT_01299	mutM	Chromosome:1334398-1337014	110.58	110.58+peptide	OK	71.4445	58.1075	-0.298098	-0.42224	0.4613	0.924573	no
gene:SpnNT_01299	mutM	Chromosome:1334398-1337014	ΔORF2	110.58+peptide	OK	67.3125	58.1075	-0.21215	-0.296302	0.6053	0.976847	no
gene:SpnNT_01299	mutM	Chromosome:1334398-1337014	110.58	ΔORF2+peptide	OK	71.4445	58.6979	-0.283513	-0.395216	0.4894	0.939287	no
gene:SpnNT_01299	mutM	Chromosome:1334398-1337014	ΔORF2	ΔORF2+peptide	OK	67.3125	58.6979	-0.197565	-0.271677	0.63615	0.980887	no
gene:SpnNT_01299	mutM	Chromosome:1334398-1337014	110.58+peptide	ΔORF2+peptide	OK	58.1075	58.6979	0.014585	0.019681	0.97255	0.99536	no
gene:SpnNT_01300	era	Chromosome:1337062-1337962	110.58	ΔORF2	OK	184.88	176.051	-0.0705944	-0.159693	0.77345	0.994748	no
gene:SpnNT_01300	era	Chromosome:1337062-1337962	110.58	110.58+peptide	OK	184.88	181.993	-0.0227015	-0.0514872	0.9261	0.994748	no
gene:SpnNT_01300	era	Chromosome:1337062-1337962	ΔORF2	110.58+peptide	OK	176.051	181.993	0.0478929	0.109548	0.8442	0.994748	no
gene:SpnNT_01300	era	Chromosome:1337062-1337962	110.58	ΔORF2+peptide	OK	184.88	175.854	-0.0722133	-0.163593	0.77285	0.994748	no
gene:SpnNT_01300	era	Chromosome:1337062-1337962	ΔORF2	ΔORF2+peptide	OK	176.051	175.854	-0.00161892	-0.00369874	0.99525	0.999258	no
gene:SpnNT_01300	era	Chromosome:1337062-1337962	110.58+peptide	ΔORF2+peptide	OK	181.993	175.854	-0.0495118	-0.11342	0.8444	0.994748	no
gene:SpnNT_01301	dgkA	Chromosome:1337978-1338852	110.58	ΔORF2	OK	40.8478	40.1122	-0.0262156	-0.0153752	0.97725	0.995534	no
gene:SpnNT_01301	dgkA	Chromosome:1337978-1338852	110.58	110.58+peptide	OK	40.8478	39.9927	-0.0305224	-0.0187196	0.9728	0.99536	no
gene:SpnNT_01301	dgkA	Chromosome:1337978-1338852	ΔORF2	110.58+peptide	OK	40.1122	39.9927	-0.00430687	-0.00248455	0.99775	0.999412	no
gene:SpnNT_01301	dgkA	Chromosome:1337978-1338852	110.58	ΔORF2+peptide	OK	40.8478	46.1791	0.176983	0.111248	0.83995	0.994748	no
gene:SpnNT_01301	dgkA	Chromosome:1337978-1338852	ΔORF2	ΔORF2+peptide	OK	40.1122	46.1791	0.203198	0.119793	0.82625	0.994748	no
gene:SpnNT_01301	dgkA	Chromosome:1337978-1338852	110.58+peptide	ΔORF2+peptide	OK	39.9927	46.1791	0.207505	0.127988	0.813	0.994748	no
gene:SpnNT_01302	ybeY	Chromosome:1337978-1338852	110.58	ΔORF2	OK	235.244	272.963	0.214549	0.44584	0.4332	0.913334	no
gene:SpnNT_01302	ybeY	Chromosome:1337978-1338852	110.58	110.58+peptide	OK	235.244	211.427	-0.153999	-0.309364	0.58645	0.973049	no
gene:SpnNT_01302	ybeY	Chromosome:1337978-1338852	ΔORF2	110.58+peptide	OK	272.963	211.427	-0.368549	-0.74535	0.1938	0.674905	no
gene:SpnNT_01302	ybeY	Chromosome:1337978-1338852	110.58	ΔORF2+peptide	OK	235.244	210.711	-0.158891	-0.320807	0.56865	0.968621	no
gene:SpnNT_01302	ybeY	Chromosome:1337978-1338852	ΔORF2	ΔORF2+peptide	OK	272.963	210.711	-0.37344	-0.759121	0.18225	0.652164	no
gene:SpnNT_01302	ybeY	Chromosome:1337978-1338852	110.58+peptide	ΔORF2+peptide	OK	211.427	210.711	-0.00489175	-0.00962645	0.98685	0.997339	no
gene:SpnNT_01303	NA	Chromosome:1338956-1340612	110.58	ΔORF2	OK	39.9184	42.0212	0.074064	0.162125	0.7762	0.994748	no
gene:SpnNT_01303	NA	Chromosome:1338956-1340612	110.58	110.58+peptide	OK	39.9184	38.7592	-0.0425167	-0.0920049	0.8749	0.994748	no
gene:SpnNT_01303	NA	Chromosome:1338956-1340612	ΔORF2	110.58+peptide	OK	42.0212	38.7592	-0.116581	-0.252985	0.65585	0.982966	no
gene:SpnNT_01303	NA	Chromosome:1338956-1340612	110.58	ΔORF2+peptide	OK	39.9184	39.8817	-0.00132774	-0.00289078	0.99535	0.999258	no
gene:SpnNT_01303	NA	Chromosome:1338956-1340612	ΔORF2	ΔORF2+peptide	OK	42.0212	39.8817	-0.0753917	-0.16461	0.76755	0.994748	no
gene:SpnNT_01303	NA	Chromosome:1338956-1340612	110.58+peptide	ΔORF2+peptide	OK	38.7592	39.8817	0.0411889	0.0889092	0.8823	0.994748	no
gene:SpnNT_01304	lytB_4	Chromosome:1340672-1342649	110.58	ΔORF2	OK	232.249	240.901	0.0527626	0.119053	0.8335	0.994748	no
gene:SpnNT_01304	lytB_4	Chromosome:1340672-1342649	110.58	110.58+peptide	OK	232.249	255.394	0.137049	0.309458	0.58255	0.970941	no
gene:SpnNT_01304	lytB_4	Chromosome:1340672-1342649	ΔORF2	110.58+peptide	OK	240.901	255.394	0.0842863	0.190591	0.73535	0.994748	no
gene:SpnNT_01304	lytB_4	Chromosome:1340672-1342649	110.58	ΔORF2+peptide	OK	232.249	293.165	0.33604	0.751784	0.1901	0.668227	no
gene:SpnNT_01304	lytB_4	Chromosome:1340672-1342649	ΔORF2	ΔORF2+peptide	OK	240.901	293.165	0.283277	0.634631	0.26565	0.766773	no
gene:SpnNT_01304	lytB_4	Chromosome:1340672-1342649	110.58+peptide	ΔORF2+peptide	OK	255.394	293.165	0.198991	0.446119	0.4345	0.913666	no
gene:SpnNT_01305	pyrDB	Chromosome:1342747-1343686	110.58	ΔORF2	OK	34.6086	25.7697	-0.425452	-0.822207	0.14615	0.582735	no
gene:SpnNT_01305	pyrDB	Chromosome:1342747-1343686	110.58	110.58+peptide	OK	34.6086	52.8252	0.610098	1.21253	0.0373	0.258558	no
gene:SpnNT_01305	pyrDB	Chromosome:1342747-1343686	ΔORF2	110.58+peptide	OK	25.7697	52.8252	1.03555	2.03095	0.00045	0.00885292	yes
gene:SpnNT_01305	pyrDB	Chromosome:1342747-1343686	110.58	ΔORF2+peptide	OK	34.6086	53.396	0.625603	1.25903	0.0294	0.218948	no
gene:SpnNT_01305	pyrDB	Chromosome:1342747-1343686	ΔORF2	ΔORF2+peptide	OK	25.7697	53.396	1.05106	2.08666	3.00E-04	0.00631878	yes

gene:SpnNT_01305	pyrDB	Chromosome:1342747-1343686	110.58+peptide	ΔORF2+peptide	OK	52.8252	53.396	0.0155053	0.0317076	0.95625	0.994855	no
gene:SpnNT_01306	pyrK	Chromosome:1343696-1344497	110.58	ΔORF2	OK	32.5501	26.5699	-0.29287	-0.547721	0.3303	0.836198	no
gene:SpnNT_01306	pyrK	Chromosome:1343696-1344497	110.58	110.58+peptide	OK	32.5501	56.7002	0.800692	1.55912	0.00675	0.0753176	no
gene:SpnNT_01306	pyrK	Chromosome:1343696-1344497	ΔORF2	110.58+peptide	OK	26.5699	56.7002	1.09356	2.08884	0.00025	0.00542231	yes
gene:SpnNT_01306	pyrK	Chromosome:1343696-1344497	110.58	ΔORF2+peptide	OK	32.5501	54.8662	0.753257	1.47618	0.0118	0.113519	no
gene:SpnNT_01306	pyrK	Chromosome:1343696-1344497	ΔORF2	ΔORF2+peptide	OK	26.5699	54.8662	1.04613	2.01059	0.00055	0.0103545	yes
gene:SpnNT_01306	pyrK	Chromosome:1343696-1344497	110.58+peptide	ΔORF2+peptide	OK	56.7002	54.8662	-0.0474358	-0.0951487	0.86815	0.994748	no
gene:SpnNT_01307	gloA	Chromosome:1344745-1345126	110.58	ΔORF2	OK	686.521	668.282	-0.0388469	-0.0834313	0.8824	0.994748	no
gene:SpnNT_01307	gloA	Chromosome:1344745-1345126	110.58	110.58+peptide	OK	686.521	1352.23	0.977968	2.05785	0.00025	0.00542231	yes
gene:SpnNT_01307	gloA	Chromosome:1344745-1345126	ΔORF2	110.58+peptide	OK	668.282	1352.23	1.01681	2.17762	2.00E-04	0.00450928	yes
gene:SpnNT_01307	gloA	Chromosome:1344745-1345126	110.58	ΔORF2+peptide	OK	686.521	1045.02	0.606158	1.30581	0.0231	0.18642	no
gene:SpnNT_01307	gloA	Chromosome:1344745-1345126	ΔORF2	ΔORF2+peptide	OK	668.282	1045.02	0.645005	1.41541	0.0128	0.120144	no
gene:SpnNT_01307	gloA	Chromosome:1344745-1345126	110.58+peptide	ΔORF2+peptide	OK	1352.23	1045.02	-0.37181	-0.798687	0.15395	0.59838	no
gene:SpnNT_01308	rplT	Chromosome:1345184-1345544	110.58	ΔORF2	OK	2149.68	1837.9	-0.226068	-0.494985	0.38345	0.880579	no
gene:SpnNT_01308	rplT	Chromosome:1345184-1345544	110.58	110.58+peptide	OK	2149.68	4917.87	1.19391	2.5585	5.00E-05	0.0013612	yes
gene:SpnNT_01308	rplT	Chromosome:1345184-1345544	ΔORF2	110.58+peptide	OK	1837.9	4917.87	1.41998	3.11316	5.00E-05	0.0013612	yes
gene:SpnNT_01308	rplT	Chromosome:1345184-1345544	110.58	ΔORF2+peptide	OK	2149.68	4965.77	1.20789	2.59856	5.00E-05	0.0013612	yes
gene:SpnNT_01308	rplT	Chromosome:1345184-1345544	ΔORF2	ΔORF2+peptide	OK	1837.9	4965.77	1.43396	3.15665	5.00E-05	0.0013612	yes
gene:SpnNT_01308	rplT	Chromosome:1345184-1345544	110.58+peptide	ΔORF2+peptide	OK	4917.87	4965.77	0.0139831	0.0301198	0.95755	0.994855	no
gene:SpnNT_01309	rpmI	Chromosome:1345595-1345796	110.58	ΔORF2	OK	259.59	243.651	-0.0914195	-0.15197	0.78515	0.994748	no
gene:SpnNT_01309	rpmI	Chromosome:1345595-1345796	110.58	110.58+peptide	OK	259.59	546.813	1.07481	1.86226	0.0011	0.0181562	yes
gene:SpnNT_01309	rpmI	Chromosome:1345595-1345796	ΔORF2	110.58+peptide	OK	243.651	546.813	1.16623	2.05038	0.00035	0.00717609	yes
gene:SpnNT_01309	rpmI	Chromosome:1345595-1345796	110.58	ΔORF2+peptide	OK	259.59	704.198	1.43975	2.55815	5.00E-05	0.0013612	yes
gene:SpnNT_01309	rpmI	Chromosome:1345595-1345796	ΔORF2	ΔORF2+peptide	OK	243.651	704.198	1.53117	2.76272	5.00E-05	0.0013612	yes
gene:SpnNT_01309	rpmI	Chromosome:1345595-1345796	110.58+peptide	ΔORF2+peptide	OK	546.813	704.198	0.364932	0.691641	0.2297	0.722638	no
gene:SpnNT_01310	infC	Chromosome:1345828-1346416	110.58	ΔORF2	OK	185.44	192.905	0.0569426	0.126723	0.8226	0.994748	no
gene:SpnNT_01310	infC	Chromosome:1345828-1346416	110.58	110.58+peptide	OK	185.44	499.628	1.4299	3.22458	5.00E-05	0.0013612	yes
gene:SpnNT_01310	infC	Chromosome:1345828-1346416	ΔORF2	110.58+peptide	OK	192.905	499.628	1.37296	3.10408	5.00E-05	0.0013612	yes
gene:SpnNT_01310	infC	Chromosome:1345828-1346416	110.58	ΔORF2+peptide	OK	185.44	618.089	1.73686	3.915	5.00E-05	0.0013612	yes
gene:SpnNT_01310	infC	Chromosome:1345828-1346416	ΔORF2	ΔORF2+peptide	OK	192.905	618.089	1.67992	3.7963	5.00E-05	0.0013612	yes
gene:SpnNT_01310	infC	Chromosome:1345828-1346416	110.58+peptide	ΔORF2+peptide	OK	499.628	618.089	0.306961	0.703209	0.2248	0.716151	no
gene:SpnNT_01311	NA	Chromosome:1346883-1347708	110.58	ΔORF2	OK	6.72413	6.34886	-0.0828509	-0.1244	0.8271	0.994748	no
gene:SpnNT_01311	NA	Chromosome:1346883-1347708	110.58	110.58+peptide	OK	6.72413	5.21826	-0.36578	-0.546082	0.34515	0.849092	no
gene:SpnNT_01311	NA	Chromosome:1346883-1347708	ΔORF2	110.58+peptide	OK	6.34886	5.21826	-0.282929	-0.419754	0.4629	0.9258	no
gene:SpnNT_01311	NA	Chromosome:1346883-1347708	110.58	ΔORF2+peptide	OK	6.72413	6.36443	-0.0793172	-0.120583	0.8298	0.994748	no
gene:SpnNT_01311	NA	Chromosome:1346883-1347708	ΔORF2	ΔORF2+peptide	OK	6.34886	6.36443	0.0035337	0.00533736	0.98815	0.997703	no
gene:SpnNT_01311	NA	Chromosome:1346883-1347708	110.58+peptide	ΔORF2+peptide	OK	5.21826	6.36443	0.286463	0.430182	0.45155	0.921244	no
gene:SpnNT_01312	NA	Chromosome:1347725-1348205	110.58	ΔORF2	OK	4.61967	4.05475	-0.188175	-0.22099	0.7023	0.990367	no
gene:SpnNT_01312	NA	Chromosome:1347725-1348205	110.58	110.58+peptide	OK	4.61967	4.49799	-0.0385084	-0.0456978	0.9278	0.994748	no
gene:SpnNT_01312	NA	Chromosome:1347725-1348205	ΔORF2	110.58+peptide	OK	4.05475	4.49799	0.149667	0.177013	0.7644	0.994748	no
gene:SpnNT_01312	NA	Chromosome:1347725-1348205	110.58	ΔORF2+peptide	OK	4.61967	2.05597	-1.16797	-1.34778	0.05145	0.320421	no
gene:SpnNT_01312	NA	Chromosome:1347725-1348205	ΔORF2	ΔORF2+peptide	OK	4.05475	2.05597	-0.979796	-1.12705	0.1001	0.479559	no
gene:SpnNT_01312	NA	Chromosome:1347725-1348205	110.58+peptide	ΔORF2+peptide	OK	4.49799	2.05597	-1.12946	-1.31227	0.05695	0.339527	no
gene:SpnNT_01313	NA	Chromosome:1348583-1349360	110.58	ΔORF2	OK	77.7652	102.999	0.405427	0.836001	0.1338	0.556521	no
gene:SpnNT_01313	NA	Chromosome:1348583-1349360	110.58	110.58+peptide	OK	77.7652	88.5986	0.188159	0.386817	0.4931	0.941402	no

gene:SpnNT_01313	NA	Chromosome:1348583-1349360	ΔORF2	110.58+peptide	OK	102.999	88.5986	-0.217268	-0.437064	0.44445	0.917673	no
gene:SpnNT_01313	NA	Chromosome:1348583-1349360	110.58	ΔORF2+peptide	OK	77.7652	85.0506	0.129196	0.267942	0.632	0.980887	no
gene:SpnNT_01313	NA	Chromosome:1348583-1349360	ΔORF2	ΔORF2+peptide	OK	102.999	85.0506	-0.276231	-0.560362	0.32425	0.829559	no
gene:SpnNT_01313	NA	Chromosome:1348583-1349360	110.58+peptide	ΔORF2+peptide	OK	88.5986	85.0506	-0.0589629	-0.119263	0.83085	0.994748	no
gene:SpnNT_01314	NA	Chromosome:1349424-1349646	110.58	ΔORF2	OK	185.295	267.133	0.527737	0.852418	0.13925	0.569233	no
gene:SpnNT_01314	NA	Chromosome:1349424-1349646	110.58	110.58+peptide	OK	185.295	283.697	0.614531	1.02908	0.07395	0.400484	no
gene:SpnNT_01314	NA	Chromosome:1349424-1349646	ΔORF2	110.58+peptide	OK	267.133	283.697	0.0867932	0.142548	0.79755	0.994748	no
gene:SpnNT_01314	NA	Chromosome:1349424-1349646	110.58	ΔORF2+peptide	OK	185.295	336.408	0.860393	1.52452	0.01285	0.120527	no
gene:SpnNT_01314	NA	Chromosome:1349424-1349646	ΔORF2	ΔORF2+peptide	OK	267.133	336.408	0.332655	0.576784	0.32945	0.835209	no
gene:SpnNT_01314	NA	Chromosome:1349424-1349646	110.58+peptide	ΔORF2+peptide	OK	283.697	336.408	0.245862	0.444502	0.45315	0.921244	no
gene:SpnNT_01315	NA	Chromosome:1349820-1351554	110.58	ΔORF2	OK	21.5807	24.0811	0.158164	0.324356	0.5688	0.968621	no
gene:SpnNT_01315	NA	Chromosome:1349820-1351554	110.58	110.58+peptide	OK	21.5807	22.6944	0.0725996	0.147831	0.79405	0.994748	no
gene:SpnNT_01315	NA	Chromosome:1349820-1351554	ΔORF2	110.58+peptide	OK	24.0811	22.6944	-0.0855648	-0.174676	0.7627	0.994748	no
gene:SpnNT_01315	NA	Chromosome:1349820-1351554	110.58	ΔORF2+peptide	OK	21.5807	29.4072	0.446428	0.928446	0.0986	0.475847	no
gene:SpnNT_01315	NA	Chromosome:1349820-1351554	ΔORF2	ΔORF2+peptide	OK	24.0811	29.4072	0.288264	0.601101	0.28245	0.785208	no
gene:SpnNT_01315	NA	Chromosome:1349820-1351554	110.58+peptide	ΔORF2+peptide	OK	22.6944	29.4072	0.373829	0.773831	0.17565	0.639002	no
gene:SpnNT_01316	soj	Chromosome:1351604-1352453	110.58	ΔORF2	OK	231.415	299.526	0.372197	0.828972	0.1449	0.580489	no
gene:SpnNT_01316	soj	Chromosome:1351604-1352453	110.58	110.58+peptide	OK	231.415	308.494	0.41476	0.915568	0.1007	0.480853	no
gene:SpnNT_01316	soj	Chromosome:1351604-1352453	ΔORF2	110.58+peptide	OK	299.526	308.494	0.0425635	0.0914266	0.87095	0.994748	no
gene:SpnNT_01316	soj	Chromosome:1351604-1352453	110.58	ΔORF2+peptide	OK	231.415	252.519	0.125906	0.286653	0.61375	0.979616	no
gene:SpnNT_01316	soj	Chromosome:1351604-1352453	ΔORF2	ΔORF2+peptide	OK	299.526	252.519	-0.246291	-0.544712	0.3437	0.84791	no
gene:SpnNT_01316	soj	Chromosome:1351604-1352453	110.58+peptide	ΔORF2+peptide	OK	308.494	252.519	-0.288854	-0.633252	0.2648	0.765354	no
gene:SpnNT_01317	NA	Chromosome:1352528-1354564	110.58	ΔORF2	NOTEST	0.310972	0.413526	0.411192	0	1	1	no
gene:SpnNT_01317	NA	Chromosome:1352528-1354564	110.58	110.58+peptide	NOTEST	0.310972	0.409231	0.39613	0	1	1	no
gene:SpnNT_01317	NA	Chromosome:1352528-1354564	ΔORF2	110.58+peptide	NOTEST	0.413526	0.409231	-0.0150624	0	1	1	no
gene:SpnNT_01317	NA	Chromosome:1352528-1354564	110.58	ΔORF2+peptide	NOTEST	0.310972	0.337521	0.118194	0	1	1	no
gene:SpnNT_01317	NA	Chromosome:1352528-1354564	ΔORF2	ΔORF2+peptide	NOTEST	0.413526	0.337521	-0.292998	0	1	1	no
gene:SpnNT_01317	NA	Chromosome:1352528-1354564	110.58+peptide	ΔORF2+peptide	NOTEST	0.409231	0.337521	-0.277936	0	1	1	no
gene:SpnNT_01318	NA	Chromosome:1352528-1354564	110.58	ΔORF2	NOTEST	0.280293	0.194103	-0.53011	0	1	1	no
gene:SpnNT_01318	NA	Chromosome:1352528-1354564	110.58	110.58+peptide	NOTEST	0.280293	0.20449	-0.454902	0	1	1	no
gene:SpnNT_01318	NA	Chromosome:1352528-1354564	ΔORF2	110.58+peptide	NOTEST	0.194103	0.20449	0.0752078	0	1	1	no
gene:SpnNT_01318	NA	Chromosome:1352528-1354564	110.58	ΔORF2+peptide	NOTEST	0.280293	0.294729	0.0724579	0	1	1	no
gene:SpnNT_01318	NA	Chromosome:1352528-1354564	ΔORF2	ΔORF2+peptide	NOTEST	0.194103	0.294729	0.602567	0	1	1	no
gene:SpnNT_01318	NA	Chromosome:1352528-1354564	110.58+peptide	ΔORF2+peptide	NOTEST	0.20449	0.294729	0.52736	0	1	1	no
gene:SpnNT_01319	NA	Chromosome:1354785-1355094	110.58	ΔORF2	NOTEST	1.03315	0.249443	-2.05027	0	1	1	no
gene:SpnNT_01319	NA	Chromosome:1354785-1355094	110.58	110.58+peptide	NOTEST	1.03315	0.676801	-0.610249	0	1	1	no
gene:SpnNT_01319	NA	Chromosome:1354785-1355094	ΔORF2	110.58+peptide	NOTEST	0.249443	0.676801	1.44002	0	1	1	no
gene:SpnNT_01319	NA	Chromosome:1354785-1355094	110.58	ΔORF2+peptide	NOTEST	1.03315	0.509117	-1.02099	0	1	1	no
gene:SpnNT_01319	NA	Chromosome:1354785-1355094	ΔORF2	ΔORF2+peptide	NOTEST	0.249443	0.509117	1.02928	0	1	1	no
gene:SpnNT_01319	NA	Chromosome:1354785-1355094	110.58+peptide	ΔORF2+peptide	NOTEST	0.676801	0.509117	-0.410736	0	1	1	no
gene:SpnNT_01320	NA	Chromosome:1355142-1356438	110.58	ΔORF2	NOTEST	0.436645	0.445078	0.0275978	0	1	1	no
gene:SpnNT_01320	NA	Chromosome:1355142-1356438	110.58	110.58+peptide	NOTEST	0.436645	0.450545	0.0452092	0	1	1	no
gene:SpnNT_01320	NA	Chromosome:1355142-1356438	ΔORF2	110.58+peptide	NOTEST	0.445078	0.450545	0.0176114	0	1	1	no
gene:SpnNT_01320	NA	Chromosome:1355142-1356438	110.58	ΔORF2+peptide	NOTEST	0.436645	0.439643	0.00987035	0	1	1	no
gene:SpnNT_01320	NA	Chromosome:1355142-1356438	ΔORF2	ΔORF2+peptide	NOTEST	0.445078	0.439643	-0.0177275	0	1	1	no

gene:SpnNT_01320	NA	Chromosome:1355142-1356438	110.58+peptide	ΔORF2+peptide	NOTEST	0.450545	0.439643	-0.0353389	0	1	1	no
gene:SpnNT_01321	NA	Chromosome:1356457-1358524	110.58	ΔORF2	NOTEST	0.514056	0.421544	-0.286242	0	1	1	no
gene:SpnNT_01321	NA	Chromosome:1356457-1358524	110.58	110.58+peptide	NOTEST	0.514056	0.476479	-0.109515	0	1	1	no
gene:SpnNT_01321	NA	Chromosome:1356457-1358524	ΔORF2	110.58+peptide	NOTEST	0.421544	0.476479	0.176727	0	1	1	no
gene:SpnNT_01321	NA	Chromosome:1356457-1358524	110.58	ΔORF2+peptide	NOTEST	0.514056	0.378732	-0.440751	0	1	1	no
gene:SpnNT_01321	NA	Chromosome:1356457-1358524	ΔORF2	ΔORF2+peptide	NOTEST	0.421544	0.378732	-0.154509	0	1	1	no
gene:SpnNT_01321	NA	Chromosome:1356457-1358524	110.58+peptide	ΔORF2+peptide	NOTEST	0.476479	0.378732	-0.331236	0	1	1	no
gene:SpnNT_01322	NA	Chromosome:1358535-1359885	110.58	ΔORF2	NOTEST	0.587481	0.605248	0.0429832	0	1	1	no
gene:SpnNT_01322	NA	Chromosome:1358535-1359885	110.58	110.58+peptide	NOTEST	0.587481	0.710354	0.273996	0	1	1	no
gene:SpnNT_01322	NA	Chromosome:1358535-1359885	ΔORF2	110.58+peptide	NOTEST	0.605248	0.710354	0.231012	0	1	1	no
gene:SpnNT_01322	NA	Chromosome:1358535-1359885	110.58	ΔORF2+peptide	NOTEST	0.587481	0.715336	0.284079	0	1	1	no
gene:SpnNT_01322	NA	Chromosome:1358535-1359885	ΔORF2	ΔORF2+peptide	NOTEST	0.605248	0.715336	0.241095	0	1	1	no
gene:SpnNT_01322	NA	Chromosome:1358535-1359885	110.58+peptide	ΔORF2+peptide	NOTEST	0.710354	0.715336	0.010083	0	1	1	no
gene:SpnNT_01323	NA	Chromosome:1360159-1360633	110.58	ΔORF2	OK	15.7735	32.8558	1.05865	1.59016	0.0062	0.0706832	no
gene:SpnNT_01323	NA	Chromosome:1360159-1360633	110.58	110.58+peptide	OK	15.7735	16.5011	0.065063	0.0991076	0.8636	0.994748	no
gene:SpnNT_01323	NA	Chromosome:1360159-1360633	ΔORF2	110.58+peptide	OK	32.8558	16.5011	-0.993585	-1.47186	0.014	0.127841	no
gene:SpnNT_01323	NA	Chromosome:1360159-1360633	110.58	ΔORF2+peptide	OK	15.7735	25.9391	0.717625	1.12498	0.0414	0.277027	no
gene:SpnNT_01323	NA	Chromosome:1360159-1360633	ΔORF2	ΔORF2+peptide	OK	32.8558	25.9391	-0.341024	-0.519067	0.3625	0.86287	no
gene:SpnNT_01323	NA	Chromosome:1360159-1360633	110.58+peptide	ΔORF2+peptide	OK	16.5011	25.9391	0.652561	1.00765	0.0839	0.434809	no
gene:SpnNT_01324	NA	Chromosome:1360648-1361056	110.58	ΔORF2	OK	25.8906	45.3343	0.808173	1.28828	0.02645	0.204524	no
gene:SpnNT_01324	NA	Chromosome:1360648-1361056	110.58	110.58+peptide	OK	25.8906	28.2794	0.127321	0.198802	0.72915	0.994748	no
gene:SpnNT_01324	NA	Chromosome:1360648-1361056	ΔORF2	110.58+peptide	OK	45.3343	28.2794	-0.680851	-1.06643	0.06345	0.365492	no
gene:SpnNT_01324	NA	Chromosome:1360648-1361056	110.58	ΔORF2+peptide	OK	25.8906	22.6687	-0.191727	-0.292273	0.6045	0.976761	no
gene:SpnNT_01324	NA	Chromosome:1360648-1361056	ΔORF2	ΔORF2+peptide	OK	45.3343	22.6687	-0.9999	-1.52882	0.0084	0.088891	no
gene:SpnNT_01324	NA	Chromosome:1360648-1361056	110.58+peptide	ΔORF2+peptide	OK	28.2794	22.6687	-0.319049	-0.478604	0.39555	0.890245	no
gene:SpnNT_01325	NA	Chromosome:1361396-1363777	110.58	ΔORF2	NOTEST	0.177017	0.0434424	-2.02671	0	1	1	no
gene:SpnNT_01325	NA	Chromosome:1361396-1363777	110.58	110.58+peptide	NOTEST	0.177017	0.0347478	-2.34889	0	1	1	no
gene:SpnNT_01325	NA	Chromosome:1361396-1363777	ΔORF2	110.58+peptide	NOTEST	0.043442	0.0347478	-0.322179	0	1	1	no
gene:SpnNT_01325	NA	Chromosome:1361396-1363777	110.58	ΔORF2+peptide	NOTEST	0.177017	0.0997935	-0.826868	0	1	1	no
gene:SpnNT_01325	NA	Chromosome:1361396-1363777	ΔORF2	ΔORF2+peptide	NOTEST	0.043442	0.0997935	1.19984	0	1	1	no
gene:SpnNT_01325	NA	Chromosome:1361396-1363777	110.58+peptide	ΔORF2+peptide	NOTEST	0.034748	0.0997935	1.52202	0	1	1	no
gene:SpnNT_01326	NA	Chromosome:1361396-1363777	110.58	ΔORF2	OK	0.820503	1.57012	0.936293	0.669783	0.44095	0.91669	no
gene:SpnNT_01326	NA	Chromosome:1361396-1363777	110.58	110.58+peptide	OK	0.820503	2.40002	1.54846	1.16479	0.12765	0.541728	no
gene:SpnNT_01326	NA	Chromosome:1361396-1363777	ΔORF2	110.58+peptide	OK	1.57012	2.40002	0.612169	0.569087	0.4373	0.914608	no
gene:SpnNT_01326	NA	Chromosome:1361396-1363777	110.58	ΔORF2+peptide	OK	0.820503	1.06669	0.378557	0.248626	0.6963	0.989512	no
gene:SpnNT_01326	NA	Chromosome:1361396-1363777	ΔORF2	ΔORF2+peptide	OK	1.57012	1.06669	-0.557736	-0.42674	0.5663	0.968621	no
gene:SpnNT_01326	NA	Chromosome:1361396-1363777	110.58+peptide	ΔORF2+peptide	OK	2.40002	1.06669	-1.16991	-0.948511	0.1131	0.510701	no
gene:SpnNT_01327	NA	Chromosome:1363778-1364910	110.58	ΔORF2	NOTEST	0.457171	0.490508	0.101544	0	1	1	no
gene:SpnNT_01327	NA	Chromosome:1363778-1364910	110.58	110.58+peptide	NOTEST	0.457171	0.629776	0.462105	0	1	1	no
gene:SpnNT_01327	NA	Chromosome:1363778-1364910	ΔORF2	110.58+peptide	NOTEST	0.490508	0.629776	0.360561	0	1	1	no
gene:SpnNT_01327	NA	Chromosome:1363778-1364910	110.58	ΔORF2+peptide	NOTEST	0.457171	0.470145	0.0403735	0	1	1	no
gene:SpnNT_01327	NA	Chromosome:1363778-1364910	ΔORF2	ΔORF2+peptide	NOTEST	0.490508	0.470145	-0.0611706	0	1	1	no
gene:SpnNT_01327	NA	Chromosome:1363778-1364910	110.58+peptide	ΔORF2+peptide	NOTEST	0.629776	0.470145	-0.421731	0	1	1	no
gene:SpnNT_01328	NA	Chromosome:1363778-1364910	110.58	ΔORF2	NOTEST	0.242213	0.243126	0.0054258	0	1	1	no
gene:SpnNT_01328	NA	Chromosome:1363778-1364910	110.58	110.58+peptide	NOTEST	0.242213	0.31655	0.386155	0	1	1	no

gene:SpnNT_01328	NA	Chromosome:1363778-1364910	ΔORF2	110.58+peptide	NOTEST	0.243126	0.31655	0.380729	0	1	1	no
gene:SpnNT_01328	NA	Chromosome:1363778-1364910	110.58	ΔORF2+peptide	NOTEST	0.242213	0	#NAME?	0	1	1	no
gene:SpnNT_01328	NA	Chromosome:1363778-1364910	ΔORF2	ΔORF2+peptide	NOTEST	0.243126	0	#NAME?	0	1	1	no
gene:SpnNT_01328	NA	Chromosome:1363778-1364910	110.58+peptide	ΔORF2+peptide	NOTEST	0.31655	0	#NAME?	0	1	1	no
gene:SpnNT_01329	NA	Chromosome:1364920-1365601	110.58	ΔORF2	OK	0.470759	0.956198	1.02232	0.782664	0.1664	0.621549	no
gene:SpnNT_01329	NA	Chromosome:1364920-1365601	110.58	110.58+peptide	NOTEST	0.470759	0.594996	0.337892	0	1	1	no
gene:SpnNT_01329	NA	Chromosome:1364920-1365601	ΔORF2	110.58+peptide	OK	0.956198	0.594996	-0.684428	-0.532974	0.3524	0.855345	no
gene:SpnNT_01329	NA	Chromosome:1364920-1365601	110.58	ΔORF2+peptide	OK	0.470759	0.952922	1.01737	0.767112	0.1852	0.658231	no
gene:SpnNT_01329	NA	Chromosome:1364920-1365601	ΔORF2	ΔORF2+peptide	OK	0.956198	0.952922	-0.00495108	-0.00422988	0.9471	0.994855	no
gene:SpnNT_01329	NA	Chromosome:1364920-1365601	110.58+peptide	ΔORF2+peptide	OK	0.594996	0.952922	0.679477	0.520858	0.3691	0.866165	no
gene:SpnNT_01330	ltrA_4	Chromosome:1365868-1367791	110.58	ΔORF2	OK	2.82946	1.97842	-0.516179	-0.769944	0.17945	0.645843	no
gene:SpnNT_01330	ltrA_4	Chromosome:1365868-1367791	110.58	110.58+peptide	OK	2.82946	3.20277	0.178798	0.275574	0.6308	0.980887	no
gene:SpnNT_01330	ltrA_4	Chromosome:1365868-1367791	ΔORF2	110.58+peptide	OK	1.97842	3.20277	0.694976	1.06431	0.06915	0.385793	no
gene:SpnNT_01330	ltrA_4	Chromosome:1365868-1367791	110.58	ΔORF2+peptide	OK	2.82946	2.07079	-0.450343	-0.670006	0.24355	0.73908	no
gene:SpnNT_01330	ltrA_4	Chromosome:1365868-1367791	ΔORF2	ΔORF2+peptide	OK	1.97842	2.07079	0.0658356	0.0973663	0.86505	0.994748	no
gene:SpnNT_01330	ltrA_4	Chromosome:1365868-1367791	110.58+peptide	ΔORF2+peptide	OK	3.20277	2.07079	-0.629141	-0.960868	0.1023	0.484964	no
gene:SpnNT_01331	NA	Chromosome:1368499-1371187	110.58	ΔORF2	NOTEST	0.485041	0.563705	0.216832	0	1	1	no
gene:SpnNT_01331	NA	Chromosome:1368499-1371187	110.58	110.58+peptide	NOTEST	0.485041	0.556293	0.197738	0	1	1	no
gene:SpnNT_01331	NA	Chromosome:1368499-1371187	ΔORF2	110.58+peptide	NOTEST	0.563705	0.556293	-0.0190944	0	1	1	no
gene:SpnNT_01331	NA	Chromosome:1368499-1371187	110.58	ΔORF2+peptide	NOTEST	0.485041	0.59574	0.296574	0	1	1	no
gene:SpnNT_01331	NA	Chromosome:1368499-1371187	ΔORF2	ΔORF2+peptide	NOTEST	0.563705	0.59574	0.0797421	0	1	1	no
gene:SpnNT_01331	NA	Chromosome:1368499-1371187	110.58+peptide	ΔORF2+peptide	NOTEST	0.556293	0.59574	0.0988365	0	1	1	no
gene:SpnNT_01332	NA	Chromosome:1371197-1373877	110.58	ΔORF2	NOTEST	0.404301	0.278434	-0.538091	0	1	1	no
gene:SpnNT_01332	NA	Chromosome:1371197-1373877	110.58	110.58+peptide	NOTEST	0.404301	0.358159	-0.17483	0	1	1	no
gene:SpnNT_01332	NA	Chromosome:1371197-1373877	ΔORF2	110.58+peptide	NOTEST	0.278434	0.358159	0.363261	0	1	1	no
gene:SpnNT_01332	NA	Chromosome:1371197-1373877	110.58	ΔORF2+peptide	NOTEST	0.404301	0.515855	0.351537	0	1	1	no
gene:SpnNT_01332	NA	Chromosome:1371197-1373877	ΔORF2	ΔORF2+peptide	NOTEST	0.278434	0.515855	0.889628	0	1	1	no
gene:SpnNT_01332	NA	Chromosome:1371197-1373877	110.58+peptide	ΔORF2+peptide	NOTEST	0.358159	0.515855	0.526367	0	1	1	no
gene:SpnNT_01333	NA	Chromosome:1371197-1373877	110.58	ΔORF2	NOTEST	0.304584	0.07998	-1.92913	0	1	1	no
gene:SpnNT_01333	NA	Chromosome:1371197-1373877	110.58	110.58+peptide	OK	0.304584	0.872246	1.51789	0.482303	0.5056	0.945292	no
gene:SpnNT_01333	NA	Chromosome:1371197-1373877	ΔORF2	110.58+peptide	OK	0.07998	0.872246	3.44702	0.681052	0.3324	0.837993	no
gene:SpnNT_01333	NA	Chromosome:1371197-1373877	110.58	ΔORF2+peptide	NOTEST	0.304584	0.094834	-1.68336	0	1	1	no
gene:SpnNT_01333	NA	Chromosome:1371197-1373877	ΔORF2	ΔORF2+peptide	NOTEST	0.07998	0.094834	0.245765	0	1	1	no
gene:SpnNT_01333	NA	Chromosome:1371197-1373877	110.58+peptide	ΔORF2+peptide	OK	0.872246	0.094834	-3.20126	-0.729004	0.33715	0.84149	no
gene:SpnNT_01334	NA	Chromosome:1373975-1374479	110.58	ΔORF2	NOTEST	0.157099	0.206632	0.395395	0	1	1	no
gene:SpnNT_01334	NA	Chromosome:1373975-1374479	110.58	110.58+peptide	NOTEST	0.157099	0.26902	0.776041	0	1	1	no
gene:SpnNT_01334	NA	Chromosome:1373975-1374479	ΔORF2	110.58+peptide	NOTEST	0.206632	0.26902	0.380647	0	1	1	no
gene:SpnNT_01334	NA	Chromosome:1373975-1374479	110.58	ΔORF2+peptide	NOTEST	0.157099	0	#NAME?	0	1	1	no
gene:SpnNT_01334	NA	Chromosome:1373975-1374479	ΔORF2	ΔORF2+peptide	NOTEST	0.206632	0	#NAME?	0	1	1	no
gene:SpnNT_01334	NA	Chromosome:1373975-1374479	110.58+peptide	ΔORF2+peptide	NOTEST	0.26902	0	#NAME?	0	1	1	no
gene:SpnNT_01335	NA	Chromosome:1374566-1375316	110.58	ΔORF2	NOTEST	0.482135	0.237335	-1.02251	0	1	1	no
gene:SpnNT_01335	NA	Chromosome:1374566-1375316	110.58	110.58+peptide	NOTEST	0.482135	0.339694	-0.505198	0	1	1	no
gene:SpnNT_01335	NA	Chromosome:1374566-1375316	ΔORF2	110.58+peptide	NOTEST	0.237335	0.339694	0.517312	0	1	1	no
gene:SpnNT_01335	NA	Chromosome:1374566-1375316	110.58	ΔORF2+peptide	NOTEST	0.482135	0.134972	-1.83678	0	1	1	no
gene:SpnNT_01335	NA	Chromosome:1374566-1375316	ΔORF2	ΔORF2+peptide	NOTEST	0.237335	0.134972	-0.81427	0	1	1	no

gene:SpnNT_01335	NA	Chromosome:1374566-1375316	110.58+peptide	ΔORF2+peptide	NOTEST	0.339694	0.134972	-1.33158	0	1	1	no
gene:SpnNT_01336	NA	Chromosome:1375325-1375553	110.58	ΔORF2	OK	2.0566	0.87664	-1.2302	-0.866861	0.44675	0.919137	no
gene:SpnNT_01336	NA	Chromosome:1375325-1375553	110.58	110.58+peptide	OK	2.0566	0.529712	-1.95698	-1.33095	0.2924	0.797521	no
gene:SpnNT_01336	NA	Chromosome:1375325-1375553	ΔORF2	110.58+peptide	NOTEST	0.87664	0.529712	-0.726775	0	1	1	no
gene:SpnNT_01336	NA	Chromosome:1375325-1375553	110.58	ΔORF2+peptide	OK	2.0566	0.335657	-2.6152	-1.1921	0.13685	0.563312	no
gene:SpnNT_01336	NA	Chromosome:1375325-1375553	ΔORF2	ΔORF2+peptide	NOTEST	0.87664	0.335657	-1.385	0	1	1	no
gene:SpnNT_01336	NA	Chromosome:1375325-1375553	110.58+peptide	ΔORF2+peptide	NOTEST	0.529712	0.335657	-0.65822	0	1	1	no
gene:SpnNT_01337	NA	Chromosome:1375635-1376055	110.58	ΔORF2	OK	225.268	429.361	0.93055	1.8249	0.00185	0.0276489	yes
gene:SpnNT_01337	NA	Chromosome:1375635-1376055	110.58	110.58+peptide	OK	225.268	257.841	0.194843	0.404677	0.4781	0.933077	no
gene:SpnNT_01337	NA	Chromosome:1375635-1376055	ΔORF2	110.58+peptide	OK	429.361	257.841	-0.735707	-1.45274	0.01295	0.120861	no
gene:SpnNT_01337	NA	Chromosome:1375635-1376055	110.58	ΔORF2+peptide	OK	225.268	287.274	0.350786	0.732426	0.1975	0.679305	no
gene:SpnNT_01337	NA	Chromosome:1375635-1376055	ΔORF2	ΔORF2+peptide	OK	429.361	287.274	-0.579764	-1.1503	0.04765	0.304116	no
gene:SpnNT_01337	NA	Chromosome:1375635-1376055	110.58+peptide	ΔORF2+peptide	OK	257.841	287.274	0.155943	0.328151	0.56385	0.968621	no
gene:SpnNT_01338	spaP_1	Chromosome:1376126-1380422	110.58	ΔORF2	OK	1.81355	1.85294	0.0310042	0.0521122	0.92365	0.994748	no
gene:SpnNT_01338	spaP_1	Chromosome:1376126-1380422	110.58	110.58+peptide	OK	1.81355	1.24959	-0.537362	-0.867173	0.1221	0.528994	no
gene:SpnNT_01338	spaP_1	Chromosome:1376126-1380422	ΔORF2	110.58+peptide	OK	1.85294	1.24959	-0.568366	-0.922866	0.1018	0.483993	no
gene:SpnNT_01338	spaP_1	Chromosome:1376126-1380422	110.58	ΔORF2+peptide	OK	1.81355	1.73441	-0.0643723	-0.105902	0.8531	0.994748	no
gene:SpnNT_01338	spaP_1	Chromosome:1376126-1380422	ΔORF2	ΔORF2+peptide	OK	1.85294	1.73441	-0.0953764	-0.157915	0.7847	0.994748	no
gene:SpnNT_01338	spaP_1	Chromosome:1376126-1380422	110.58+peptide	ΔORF2+peptide	OK	1.24959	1.73441	0.47299	0.752762	0.1849	0.657701	no
gene:SpnNT_01339	spaP_2	Chromosome:1380588-1381095	110.58	ΔORF2	OK	1.09511	0.816714	-0.423172	-0.333	0.56365	0.968621	no
gene:SpnNT_01339	spaP_2	Chromosome:1380588-1381095	110.58	110.58+peptide	OK	1.09511	0.893152	-0.294098	-0.231622	0.68675	0.98828	no
gene:SpnNT_01339	spaP_2	Chromosome:1380588-1381095	ΔORF2	110.58+peptide	OK	0.816714	0.893152	0.129074	0.0961003	0.88785	0.994748	no
gene:SpnNT_01339	spaP_2	Chromosome:1380588-1381095	110.58	ΔORF2+peptide	OK	1.09511	1.85356	0.759226	0.666324	0.25895	0.759841	no
gene:SpnNT_01339	spaP_2	Chromosome:1380588-1381095	ΔORF2	ΔORF2+peptide	OK	0.816714	1.85356	1.1824	0.968651	0.1124	0.510174	no
gene:SpnNT_01339	spaP_2	Chromosome:1380588-1381095	110.58+peptide	ΔORF2+peptide	OK	0.893152	1.85356	1.05332	0.863686	0.14975	0.591417	no
gene:SpnNT_01340	lytB_5	Chromosome:1381240-1383175	110.58	ΔORF2	OK	77.5008	86.4958	0.158419	0.360724	0.5217	0.954832	no
gene:SpnNT_01340	lytB_5	Chromosome:1381240-1383175	110.58	110.58+peptide	OK	77.5008	40.8185	-0.924989	-2.06277	0.00035	0.00717609	yes
gene:SpnNT_01340	lytB_5	Chromosome:1381240-1383175	ΔORF2	110.58+peptide	OK	86.4958	40.8185	-1.08341	-2.43536	5.00E-05	0.0013612	yes
gene:SpnNT_01340	lytB_5	Chromosome:1381240-1383175	110.58	ΔORF2+peptide	OK	77.5008	38.7124	-1.00142	-2.23337	0.00015	0.00355289	yes
gene:SpnNT_01340	lytB_5	Chromosome:1381240-1383175	ΔORF2	ΔORF2+peptide	OK	86.4958	38.7124	-1.15983	-2.60735	5.00E-05	0.0013612	yes
gene:SpnNT_01340	lytB_5	Chromosome:1381240-1383175	110.58+peptide	ΔORF2+peptide	OK	40.8185	38.7124	-0.0764267	-0.168353	0.77115	0.994748	no
gene:SpnNT_01341	NA	Chromosome:1383366-1383681	110.58	ΔORF2	OK	1.24357	0.835435	-0.57389	-0.258146	0.58575	0.972529	no
gene:SpnNT_01341	NA	Chromosome:1383366-1383681	110.58	110.58+peptide	OK	1.24357	0.440829	-1.4962	-0.784415	0.3565	0.857875	no
gene:SpnNT_01341	NA	Chromosome:1383366-1383681	ΔORF2	110.58+peptide	OK	0.835435	0.440829	-0.922308	-0.409648	0.48775	0.938811	no
gene:SpnNT_01341	NA	Chromosome:1383366-1383681	110.58	ΔORF2+peptide	OK	1.24357	1.47925	0.250377	0.164152	0.8139	0.994748	no
gene:SpnNT_01341	NA	Chromosome:1383366-1383681	ΔORF2	ΔORF2+peptide	OK	0.835435	1.47925	0.824267	0.425232	0.3952	0.890116	no
gene:SpnNT_01341	NA	Chromosome:1383366-1383681	110.58+peptide	ΔORF2+peptide	OK	0.440829	1.47925	1.74658	1.1151	0.3121	0.818257	no
gene:SpnNT_01342	NA	Chromosome:1383992-1385087	110.58	ΔORF2	NOTEST	0.118796	0.218397	0.878466	0	1	1	no
gene:SpnNT_01342	NA	Chromosome:1383992-1385087	110.58	110.58+peptide	NOTEST	0.118796	0.165214	0.475847	0	1	1	no
gene:SpnNT_01342	NA	Chromosome:1383992-1385087	ΔORF2	110.58+peptide	NOTEST	0.218397	0.165214	-0.40262	0	1	1	no
gene:SpnNT_01342	NA	Chromosome:1383992-1385087	110.58	ΔORF2+peptide	NOTEST	0.118796	0.0169538	-2.80881	0	1	1	no
gene:SpnNT_01342	NA	Chromosome:1383992-1385087	ΔORF2	ΔORF2+peptide	NOTEST	0.218397	0.0169538	-3.68728	0	1	1	no
gene:SpnNT_01342	NA	Chromosome:1383992-1385087	110.58+peptide	ΔORF2+peptide	NOTEST	0.165214	0.0169538	-3.28466	0	1	1	no
gene:SpnNT_01343	NA	Chromosome:1385091-1385531	110.58	ΔORF2	NOTEST	0	0	0	0	1	1	no
gene:SpnNT_01343	NA	Chromosome:1385091-1385531	110.58	110.58+peptide	NOTEST	0	0	0	0	1	1	no

gene:SpnNT_01343	NA	Chromosome:1385091-1385531	ΔORF2	110.58+peptide	NOTEST	0	0	0	0	1	1	no
gene:SpnNT_01343	NA	Chromosome:1385091-1385531	110.58	ΔORF2+peptide	NOTEST	0	0	0	0	1	1	no
gene:SpnNT_01343	NA	Chromosome:1385091-1385531	ΔORF2	ΔORF2+peptide	NOTEST	0	0	0	0	1	1	no
gene:SpnNT_01343	NA	Chromosome:1385091-1385531	110.58+peptide	ΔORF2+peptide	NOTEST	0	0	0	0	1	1	no
gene:SpnNT_01344	NA	Chromosome:1385091-1385531	110.58	ΔORF2	NOTEST	0.535287	0	#NAME?	0	1	1	no
gene:SpnNT_01344	NA	Chromosome:1385091-1385531	110.58	110.58+peptide	NOTEST	0.535287	0.20498	-1.38483	0	1	1	no
gene:SpnNT_01344	NA	Chromosome:1385091-1385531	ΔORF2	110.58+peptide	NOTEST	0	0.20498	Inf	0	1	1	no
gene:SpnNT_01344	NA	Chromosome:1385091-1385531	110.58	ΔORF2+peptide	NOTEST	0.535287	0.192224	-1.47752	0	1	1	no
gene:SpnNT_01344	NA	Chromosome:1385091-1385531	ΔORF2	ΔORF2+peptide	NOTEST	0	0.192224	Inf	0	1	1	no
gene:SpnNT_01344	NA	Chromosome:1385091-1385531	110.58+peptide	ΔORF2+peptide	NOTEST	0.20498	0.192224	-0.0926928	0	1	1	no
gene:SpnNT_01345	NA	Chromosome:1385534-1385624	110.58	ΔORF2	NOTEST	0	0	0	0	1	1	no
gene:SpnNT_01345	NA	Chromosome:1385534-1385624	110.58	110.58+peptide	NOTEST	0	0	0	0	1	1	no
gene:SpnNT_01345	NA	Chromosome:1385534-1385624	ΔORF2	110.58+peptide	NOTEST	0	0	0	0	1	1	no
gene:SpnNT_01345	NA	Chromosome:1385534-1385624	110.58	ΔORF2+peptide	NOTEST	0	0	0	0	1	1	no
gene:SpnNT_01345	NA	Chromosome:1385534-1385624	ΔORF2	ΔORF2+peptide	NOTEST	0	0	0	0	1	1	no
gene:SpnNT_01345	NA	Chromosome:1385534-1385624	110.58+peptide	ΔORF2+peptide	NOTEST	0	0	0	0	1	1	no
gene:SpnNT_01346	NA	Chromosome:1385797-1385923	110.58	ΔORF2	NOTEST	0	0	0	0	1	1	no
gene:SpnNT_01346	NA	Chromosome:1385797-1385923	110.58	110.58+peptide	NOTEST	0	0	0	0	1	1	no
gene:SpnNT_01346	NA	Chromosome:1385797-1385923	ΔORF2	110.58+peptide	NOTEST	0	0	0	0	1	1	no
gene:SpnNT_01346	NA	Chromosome:1385797-1385923	110.58	ΔORF2+peptide	NOTEST	0	0	0	0	1	1	no
gene:SpnNT_01346	NA	Chromosome:1385797-1385923	ΔORF2	ΔORF2+peptide	NOTEST	0	0	0	0	1	1	no
gene:SpnNT_01346	NA	Chromosome:1385797-1385923	110.58+peptide	ΔORF2+peptide	NOTEST	0	0	0	0	1	1	no
gene:SpnNT_01347	NA	Chromosome:1385926-1386127	110.58	ΔORF2	NOTEST	0	0	0	0	1	1	no
gene:SpnNT_01347	NA	Chromosome:1385926-1386127	110.58	110.58+peptide	NOTEST	0	0	0	0	1	1	no
gene:SpnNT_01347	NA	Chromosome:1385926-1386127	ΔORF2	110.58+peptide	NOTEST	0	0	0	0	1	1	no
gene:SpnNT_01347	NA	Chromosome:1385926-1386127	110.58	ΔORF2+peptide	NOTEST	0	0	0	0	1	1	no
gene:SpnNT_01347	NA	Chromosome:1385926-1386127	ΔORF2	ΔORF2+peptide	NOTEST	0	0	0	0	1	1	no
gene:SpnNT_01347	NA	Chromosome:1385926-1386127	110.58+peptide	ΔORF2+peptide	NOTEST	0	0	0	0	1	1	no
gene:SpnNT_01348	NA	Chromosome:1386345-1388120	110.58	ΔORF2	OK	65.0235	63.0949	-0.0434381	-0.0817012	0.8863	0.994748	no
gene:SpnNT_01348	NA	Chromosome:1386345-1388120	110.58	110.58+peptide	OK	65.0235	77.3755	0.250916	0.448656	0.44395	0.917631	no
gene:SpnNT_01348	NA	Chromosome:1386345-1388120	ΔORF2	110.58+peptide	OK	63.0949	77.3755	0.294354	0.527922	0.36965	0.866169	no
gene:SpnNT_01348	NA	Chromosome:1386345-1388120	110.58	ΔORF2+peptide	OK	65.0235	89.5952	0.46246	0.852121	0.14065	0.572459	no
gene:SpnNT_01348	NA	Chromosome:1386345-1388120	ΔORF2	ΔORF2+peptide	OK	63.0949	89.5952	0.505899	0.935162	0.10505	0.492309	no
gene:SpnNT_01348	NA	Chromosome:1386345-1388120	110.58+peptide	ΔORF2+peptide	OK	77.3755	89.5952	0.211544	0.372362	0.53005	0.957488	no
gene:SpnNT_01349	tagH	Chromosome:1386345-1388120	110.58	ΔORF2	OK	60.5854	57.7218	-0.069854	-0.0734278	0.90025	0.994748	no
gene:SpnNT_01349	tagH	Chromosome:1386345-1388120	110.58	110.58+peptide	OK	60.5854	96.5696	0.6726	0.710505	0.2143	0.699862	no
gene:SpnNT_01349	tagH	Chromosome:1386345-1388120	ΔORF2	110.58+peptide	OK	57.7218	96.5696	0.742454	0.79233	0.16515	0.620589	no
gene:SpnNT_01349	tagH	Chromosome:1386345-1388120	110.58	ΔORF2+peptide	OK	60.5854	82.013	0.436883	0.458772	0.4225	0.905849	no
gene:SpnNT_01349	tagH	Chromosome:1386345-1388120	ΔORF2	ΔORF2+peptide	OK	57.7218	82.013	0.506737	0.537511	0.346	0.850068	no
gene:SpnNT_01349	tagH	Chromosome:1386345-1388120	110.58+peptide	ΔORF2+peptide	OK	96.5696	82.013	-0.235717	-0.251291	0.65665	0.982966	no
gene:SpnNT_01350	NA	Chromosome:1388519-1391394	110.58	ΔORF2	OK	2.02277	2.00055	-0.0159378	-0.0204307	0.973	0.99536	no
gene:SpnNT_01350	NA	Chromosome:1388519-1391394	110.58	110.58+peptide	OK	2.02277	1.51972	-0.412527	-0.524909	0.3668	0.864634	no
gene:SpnNT_01350	NA	Chromosome:1388519-1391394	ΔORF2	110.58+peptide	OK	2.00055	1.51972	-0.396589	-0.510443	0.36795	0.865399	no
gene:SpnNT_01350	NA	Chromosome:1388519-1391394	110.58	ΔORF2+peptide	OK	2.02277	1.73751	-0.21931	-0.285955	0.6228	0.980887	no
gene:SpnNT_01350	NA	Chromosome:1388519-1391394	ΔORF2	ΔORF2+peptide	OK	2.00055	1.73751	-0.203372	-0.268385	0.6366	0.980887	no

gene:SpnNT_01350	NA	Chromosome:1388519-1391394	110.58+peptide	ΔORF2+peptide	OK	1.51972	1.73751	0.193217	0.252986	0.66185	0.982966	no
gene:SpnNT_01351	comEA	Chromosome:1388519-1391394	110.58	ΔORF2	OK	5.09493	4.67587	-0.123828	-0.11486	0.8368	0.994748	no
gene:SpnNT_01351	comEA	Chromosome:1388519-1391394	110.58	110.58+peptide	OK	5.09493	2.94283	-0.791858	-0.697644	0.2285	0.720575	no
gene:SpnNT_01351	comEA	Chromosome:1388519-1391394	ΔORF2	110.58+peptide	OK	4.67587	2.94283	-0.66803	-0.583708	0.31795	0.824045	no
gene:SpnNT_01351	comEA	Chromosome:1388519-1391394	110.58	ΔORF2+peptide	OK	5.09493	2.76514	-0.88171	-0.704846	0.2327	0.725984	no
gene:SpnNT_01351	comEA	Chromosome:1388519-1391394	ΔORF2	ΔORF2+peptide	OK	4.67587	2.76514	-0.757881	-0.601745	0.3094	0.81476	no
gene:SpnNT_01351	comEA	Chromosome:1388519-1391394	110.58+peptide	ΔORF2+peptide	OK	2.94283	2.76514	-0.0898513	-0.068664	0.9058	0.994748	no
gene:SpnNT_01352	ydaF_5	Chromosome:1391460-1392030	110.58	ΔORF2	OK	162.249	182.9	0.172844	0.374454	0.513	0.950747	no
gene:SpnNT_01352	ydaF_5	Chromosome:1391460-1392030	110.58	110.58+peptide	OK	162.249	67.4202	-1.26696	-2.58771	5.00E-05	0.0013612	yes
gene:SpnNT_01352	ydaF_5	Chromosome:1391460-1392030	ΔORF2	110.58+peptide	OK	182.9	67.4202	-1.4398	-2.98037	5.00E-05	0.0013612	yes
gene:SpnNT_01352	ydaF_5	Chromosome:1391460-1392030	110.58	ΔORF2+peptide	OK	162.249	64.8783	-1.3224	-2.69648	5.00E-05	0.0013612	yes
gene:SpnNT_01352	ydaF_5	Chromosome:1391460-1392030	ΔORF2	ΔORF2+peptide	OK	182.9	64.8783	-1.49525	-3.08987	5.00E-05	0.0013612	yes
gene:SpnNT_01352	ydaF_5	Chromosome:1391460-1392030	110.58+peptide	ΔORF2+peptide	OK	67.4202	64.8783	-0.0554441	-0.108563	0.849	0.994748	no
gene:SpnNT_01353	ald_1	Chromosome:1392206-1393318	110.58	ΔORF2	OK	33.2379	34.8809	0.069609	0.122961	0.8327	0.994748	no
gene:SpnNT_01353	ald_1	Chromosome:1392206-1393318	110.58	110.58+peptide	OK	33.2379	25.8613	-0.362035	-0.639917	0.2708	0.772772	no
gene:SpnNT_01353	ald_1	Chromosome:1392206-1393318	ΔORF2	110.58+peptide	OK	34.8809	25.8613	-0.431644	-0.751188	0.19365	0.674905	no
gene:SpnNT_01353	ald_1	Chromosome:1392206-1393318	110.58	ΔORF2+peptide	OK	33.2379	23.6223	-0.492679	-0.872928	0.12765	0.541728	no
gene:SpnNT_01353	ald_1	Chromosome:1392206-1393318	ΔORF2	ΔORF2+peptide	OK	34.8809	23.6223	-0.562288	-0.980824	0.08735	0.444956	no
gene:SpnNT_01353	ald_1	Chromosome:1392206-1393318	110.58+peptide	ΔORF2+peptide	OK	25.8613	23.6223	-0.130644	-0.228026	0.6912	0.98828	no
gene:SpnNT_01354	ald_2	Chromosome:1392206-1393318	110.58	ΔORF2	OK	40.688	30.343	-0.423237	-0.25263	0.6711	0.984845	no
gene:SpnNT_01354	ald_2	Chromosome:1392206-1393318	110.58	110.58+peptide	OK	40.688	25.0389	-0.700435	-0.425187	0.4633	0.925853	no
gene:SpnNT_01354	ald_2	Chromosome:1392206-1393318	ΔORF2	110.58+peptide	OK	30.343	25.0389	-0.277197	-0.155564	0.7956	0.994748	no
gene:SpnNT_01354	ald_2	Chromosome:1392206-1393318	110.58	ΔORF2+peptide	OK	40.688	21.451	-0.923559	-0.484452	0.4272	0.908839	no
gene:SpnNT_01354	ald_2	Chromosome:1392206-1393318	ΔORF2	ΔORF2+peptide	OK	30.343	21.451	-0.500321	-0.247221	0.69175	0.98828	no
gene:SpnNT_01354	ald_2	Chromosome:1392206-1393318	110.58+peptide	ΔORF2+peptide	OK	25.0389	21.451	-0.223124	-0.111524	0.85165	0.994748	no
gene:SpnNT_01355	NA	Chromosome:1393717-1393945	110.58	ΔORF2	OK	166.584	145.869	-0.191572	-0.323802	0.5811	0.96988	no
gene:SpnNT_01355	NA	Chromosome:1393717-1393945	110.58	110.58+peptide	OK	166.584	124.411	-0.42113	-0.693728	0.2364	0.73058	no
gene:SpnNT_01355	NA	Chromosome:1393717-1393945	ΔORF2	110.58+peptide	OK	145.869	124.411	-0.229558	-0.376748	0.51645	0.95301	no
gene:SpnNT_01355	NA	Chromosome:1393717-1393945	110.58	ΔORF2+peptide	OK	166.584	132.646	-0.328666	-0.514057	0.35245	0.855345	no
gene:SpnNT_01355	NA	Chromosome:1393717-1393945	ΔORF2	ΔORF2+peptide	OK	145.869	132.646	-0.137095	-0.213708	0.697	0.989794	no
gene:SpnNT_01355	NA	Chromosome:1393717-1393945	110.58+peptide	ΔORF2+peptide	OK	124.411	132.646	0.0924635	0.141003	0.79965	0.994748	no
gene:SpnNT_01356	NA	Chromosome:1393947-1394448	110.58	ΔORF2	OK	189.78	208.483	0.135605	0.297007	0.6051	0.976847	no
gene:SpnNT_01356	NA	Chromosome:1393947-1394448	110.58	110.58+peptide	OK	189.78	149.515	-0.344033	-0.740242	0.18925	0.666668	no
gene:SpnNT_01356	NA	Chromosome:1393947-1394448	ΔORF2	110.58+peptide	OK	208.483	149.515	-0.479638	-1.03613	0.06835	0.383122	no
gene:SpnNT_01356	NA	Chromosome:1393947-1394448	110.58	ΔORF2+peptide	OK	189.78	175.032	-0.116708	-0.252322	0.65975	0.982966	no
gene:SpnNT_01356	NA	Chromosome:1393947-1394448	ΔORF2	ΔORF2+peptide	OK	208.483	175.032	-0.252313	-0.547694	0.33495	0.840029	no
gene:SpnNT_01356	NA	Chromosome:1393947-1394448	110.58+peptide	ΔORF2+peptide	OK	149.515	175.032	0.227325	0.484915	0.3954	0.890245	no
gene:SpnNT_01357	ybeZ	Chromosome:1394640-1395609	110.58	ΔORF2	OK	213.369	202.065	-0.0785337	-0.177342	0.74955	0.994748	no
gene:SpnNT_01357	ybeZ	Chromosome:1394640-1395609	110.58	110.58+peptide	OK	213.369	166.488	-0.357936	-0.814563	0.153	0.5961	no
gene:SpnNT_01357	ybeZ	Chromosome:1394640-1395609	ΔORF2	110.58+peptide	OK	202.065	166.488	-0.279402	-0.627919	0.2688	0.770416	no
gene:SpnNT_01357	ybeZ	Chromosome:1394640-1395609	110.58	ΔORF2+peptide	OK	213.369	154.081	-0.469663	-1.07446	0.05905	0.347312	no
gene:SpnNT_01357	ybeZ	Chromosome:1394640-1395609	ΔORF2	ΔORF2+peptide	OK	202.065	154.081	-0.391129	-0.883529	0.1123	0.510073	no
gene:SpnNT_01357	ybeZ	Chromosome:1394640-1395609	110.58+peptide	ΔORF2+peptide	OK	166.488	154.081	-0.111727	-0.254346	0.6525	0.981668	no
gene:SpnNT_01358	NA	Chromosome:1395694-1395910	110.58	ΔORF2	OK	116.985	106.323	-0.137868	-0.218048	0.70745	0.990441	no
gene:SpnNT_01358	NA	Chromosome:1395694-1395910	110.58	110.58+peptide	OK	116.985	124.516	0.0900006	0.143617	0.8023	0.994748	no

gene:SpnNT_01358	NA	Chromosome:1395694-1395910	ΔORF2	110.58+peptide	OK	106.323	124.516	0.227869	0.359804	0.5247	0.955204	no
gene:SpnNT_01358	NA	Chromosome:1395694-1395910	110.58	ΔORF2+peptide	OK	116.985	109.216	-0.099145	-0.155024	0.7948	0.994748	no
gene:SpnNT_01358	NA	Chromosome:1395694-1395910	ΔORF2	ΔORF2+peptide	OK	106.323	109.216	0.0387231	0.0599376	0.91905	0.994748	no
gene:SpnNT_01358	NA	Chromosome:1395694-1395910	110.58+peptide	ΔORF2+peptide	OK	124.516	109.216	-0.189146	-0.295279	0.61505	0.979616	no
gene:SpnNT_01359	rnpS1	Chromosome:1395918-1396773	110.58	ΔORF2	OK	87.0196	84.256	-0.0465604	-0.0998521	0.8557	0.994748	no
gene:SpnNT_01359	rnpS1	Chromosome:1395918-1396773	110.58	110.58+peptide	OK	87.0196	85.4794	-0.0257637	-0.0554734	0.9216	0.994748	no
gene:SpnNT_01359	rnpS1	Chromosome:1395918-1396773	ΔORF2	110.58+peptide	OK	84.256	85.4794	0.0207967	0.0444103	0.93785	0.994855	no
gene:SpnNT_01359	rnpS1	Chromosome:1395918-1396773	110.58	ΔORF2+peptide	OK	87.0196	87.7109	0.0114166	0.024758	0.966	0.994855	no
gene:SpnNT_01359	rnpS1	Chromosome:1395918-1396773	ΔORF2	ΔORF2+peptide	OK	84.256	87.7109	0.057977	0.12468	0.8256	0.994748	no
gene:SpnNT_01359	rnpS1	Chromosome:1395918-1396773	110.58+peptide	ΔORF2+peptide	OK	85.4794	87.7109	0.0371803	0.0802786	0.8903	0.994748	no
gene:SpnNT_01360	frr	Chromosome:1396832-1397390	110.58	ΔORF2	OK	720.763	827.748	0.199666	0.444747	0.4316	0.913083	no
gene:SpnNT_01360	frr	Chromosome:1396832-1397390	110.58	110.58+peptide	OK	720.763	679.319	-0.0854351	-0.18852	0.74215	0.994748	no
gene:SpnNT_01360	frr	Chromosome:1396832-1397390	ΔORF2	110.58+peptide	OK	827.748	679.319	-0.285101	-0.632718	0.25755	0.757412	no
gene:SpnNT_01360	frr	Chromosome:1396832-1397390	110.58	ΔORF2+peptide	OK	720.763	685.071	-0.0732712	-0.162639	0.77475	0.994748	no
gene:SpnNT_01360	frr	Chromosome:1396832-1397390	ΔORF2	ΔORF2+peptide	OK	827.748	685.071	-0.272937	-0.609361	0.2715	0.773617	no
gene:SpnNT_01360	frr	Chromosome:1396832-1397390	110.58+peptide	ΔORF2+peptide	OK	679.319	685.071	0.0121639	0.0269016	0.96	0.994855	no
gene:SpnNT_01361	pyrH	Chromosome:1397398-1398136	110.58	ΔORF2	OK	333.127	341.668	0.0365247	0.0824508	0.8815	0.994748	no
gene:SpnNT_01361	pyrH	Chromosome:1397398-1398136	110.58	110.58+peptide	OK	333.127	361.714	0.118778	0.268489	0.63615	0.980887	no
gene:SpnNT_01361	pyrH	Chromosome:1397398-1398136	ΔORF2	110.58+peptide	OK	341.668	361.714	0.082253	0.18637	0.7443	0.994748	no
gene:SpnNT_01361	pyrH	Chromosome:1397398-1398136	110.58	ΔORF2+peptide	OK	333.127	353.78	0.0867813	0.195955	0.7271	0.994748	no
gene:SpnNT_01361	pyrH	Chromosome:1397398-1398136	ΔORF2	ΔORF2+peptide	OK	341.668	353.78	0.0502566	0.113751	0.84095	0.994748	no
gene:SpnNT_01361	pyrH	Chromosome:1397398-1398136	110.58+peptide	ΔORF2+peptide	OK	361.714	353.78	-0.0319963	-0.0725185	0.8967	0.994748	no
gene:SpnNT_01362	trmFO	Chromosome:1398219-1399554	110.58	ΔORF2	OK	58.3252	58.7406	0.0102393	0.0223942	0.96915	0.994929	no
gene:SpnNT_01362	trmFO	Chromosome:1398219-1399554	110.58	110.58+peptide	OK	58.3252	54.0138	-0.110789	-0.243354	0.6733	0.984845	no
gene:SpnNT_01362	trmFO	Chromosome:1398219-1399554	ΔORF2	110.58+peptide	OK	58.7406	54.0138	-0.121029	-0.263539	0.6462	0.980887	no
gene:SpnNT_01362	trmFO	Chromosome:1398219-1399554	110.58	ΔORF2+peptide	OK	58.3252	57.071	-0.0313605	-0.0688113	0.9048	0.994748	no
gene:SpnNT_01362	trmFO	Chromosome:1398219-1399554	ΔORF2	ΔORF2+peptide	OK	58.7406	57.071	-0.0415997	-0.0904883	0.8725	0.994748	no
gene:SpnNT_01362	trmFO	Chromosome:1398219-1399554	110.58+peptide	ΔORF2+peptide	OK	54.0138	57.071	0.0794289	0.173514	0.75945	0.994748	no
gene:SpnNT_01363	NA	Chromosome:1399897-1401783	110.58	ΔORF2	OK	43.7949	55.0535	0.330071	0.645837	0.2613	0.761443	no
gene:SpnNT_01363	NA	Chromosome:1399897-1401783	110.58	110.58+peptide	OK	43.7949	55.8001	0.349503	0.694519	0.22535	0.716568	no
gene:SpnNT_01363	NA	Chromosome:1399897-1401783	ΔORF2	110.58+peptide	OK	55.0535	55.8001	0.0194318	0.0381868	0.9479	0.994855	no
gene:SpnNT_01363	NA	Chromosome:1399897-1401783	110.58	ΔORF2+peptide	OK	43.7949	76.2087	0.799193	1.61156	0.00525	0.0625141	no
gene:SpnNT_01363	NA	Chromosome:1399897-1401783	ΔORF2	ΔORF2+peptide	OK	55.0535	76.2087	0.469121	0.935206	0.10505	0.492309	no
gene:SpnNT_01363	NA	Chromosome:1399897-1401783	110.58+peptide	ΔORF2+peptide	OK	55.8001	76.2087	0.44969	0.910989	0.11115	0.507661	no
gene:SpnNT_01364	NA	Chromosome:1399897-1401783	110.58	ΔORF2	OK	17.482	26.5812	0.604538	0.383382	0.5229	0.954832	no
gene:SpnNT_01364	NA	Chromosome:1399897-1401783	110.58	110.58+peptide	OK	17.482	21.9051	0.325403	0.190061	0.75905	0.994748	no
gene:SpnNT_01364	NA	Chromosome:1399897-1401783	ΔORF2	110.58+peptide	OK	26.5812	21.9051	-0.279135	-0.180657	0.7685	0.994748	no
gene:SpnNT_01364	NA	Chromosome:1399897-1401783	110.58	ΔORF2+peptide	OK	17.482	44.7864	1.35719	0.913469	0.1318	0.55202	no
gene:SpnNT_01364	NA	Chromosome:1399897-1401783	ΔORF2	ΔORF2+peptide	OK	26.5812	44.7864	0.752657	0.583551	0.3377	0.842132	no
gene:SpnNT_01364	NA	Chromosome:1399897-1401783	110.58+peptide	ΔORF2+peptide	OK	21.9051	44.7864	1.03179	0.71059	0.245	0.741784	no
gene:SpnNT_01365	NA	Chromosome:1401800-1402595	110.58	ΔORF2	OK	25.9442	31.4369	0.277044	0.519157	0.368	0.865399	no
gene:SpnNT_01365	NA	Chromosome:1401800-1402595	110.58	110.58+peptide	OK	25.9442	28.1413	0.117277	0.214921	0.70195	0.990367	no
gene:SpnNT_01365	NA	Chromosome:1401800-1402595	ΔORF2	110.58+peptide	OK	31.4369	28.1413	-0.159767	-0.294074	0.60545	0.976937	no
gene:SpnNT_01365	NA	Chromosome:1401800-1402595	110.58	ΔORF2+peptide	OK	25.9442	44.815	0.788568	1.51491	0.00945	0.0967261	no
gene:SpnNT_01365	NA	Chromosome:1401800-1402595	ΔORF2	ΔORF2+peptide	OK	31.4369	44.815	0.511524	0.987437	0.08135	0.425798	no

gene:SpnNT_01365	NA	Chromosome:1401800-1402595	110.58+peptide	ΔORF2+peptide	OK	28.1413	44.815	0.67129	1.26558	0.02525	0.198283	no
gene:SpnNT_01366	haeIIIM	Chromosome:1402619-1405057	110.58	ΔORF2	OK	42.9499	50.6209	0.237079	0.328023	0.5704	0.968621	no
gene:SpnNT_01366	haeIIIM	Chromosome:1402619-1405057	110.58	110.58+peptide	OK	42.9499	49.9154	0.216832	0.303512	0.6	0.976542	no
gene:SpnNT_01366	haeIIIM	Chromosome:1402619-1405057	ΔORF2	110.58+peptide	OK	50.6209	49.9154	-0.0202478	-0.0280923	0.9628	0.994855	no
gene:SpnNT_01366	haeIIIM	Chromosome:1402619-1405057	110.58	ΔORF2+peptide	OK	42.9499	69.7829	0.700221	0.994445	0.08805	0.446787	no
gene:SpnNT_01366	haeIIIM	Chromosome:1402619-1405057	ΔORF2	ΔORF2+peptide	OK	50.6209	69.7829	0.463142	0.651786	0.25795	0.757909	no
gene:SpnNT_01366	haeIIIM	Chromosome:1402619-1405057	110.58+peptide	ΔORF2+peptide	OK	49.9154	69.7829	0.48339	0.688505	0.23735	0.73229	no
gene:SpnNT_01367	NA	Chromosome:1402619-1405057	110.58	ΔORF2	OK	46.6574	52.9024	0.181228	0.318441	0.5782	0.969538	no
gene:SpnNT_01367	NA	Chromosome:1402619-1405057	110.58	110.58+peptide	OK	46.6574	52.9002	0.181168	0.316819	0.57945	0.969538	no
gene:SpnNT_01367	NA	Chromosome:1402619-1405057	ΔORF2	110.58+peptide	OK	52.9024	52.9002	-6.03E-05	-0.000104966	0.9992	0.999631	no
gene:SpnNT_01367	NA	Chromosome:1402619-1405057	110.58	ΔORF2+peptide	OK	46.6574	67.6589	0.536174	0.935823	0.1008	0.481156	no
gene:SpnNT_01367	NA	Chromosome:1402619-1405057	ΔORF2	ΔORF2+peptide	OK	52.9024	67.6589	0.354946	0.616725	0.2837	0.786627	no
gene:SpnNT_01367	NA	Chromosome:1402619-1405057	110.58+peptide	ΔORF2+peptide	OK	52.9002	67.6589	0.355006	0.613957	0.2866	0.789883	no
gene:SpnNT_01368	bspRIM	Chromosome:1405059-1406115	110.58	ΔORF2	OK	58.3493	69.2558	0.247219	0.531553	0.3655	0.864634	no
gene:SpnNT_01368	bspRIM	Chromosome:1405059-1406115	110.58	110.58+peptide	OK	58.3493	66.3119	0.184551	0.397149	0.49985	0.944017	no
gene:SpnNT_01368	bspRIM	Chromosome:1405059-1406115	ΔORF2	110.58+peptide	OK	69.2558	66.3119	-0.0626682	-0.135132	0.8165	0.994748	no
gene:SpnNT_01368	bspRIM	Chromosome:1405059-1406115	110.58	ΔORF2+peptide	OK	58.3493	87.8031	0.589556	1.28145	0.02875	0.215822	no
gene:SpnNT_01368	bspRIM	Chromosome:1405059-1406115	ΔORF2	ΔORF2+peptide	OK	69.2558	87.8031	0.342337	0.745624	0.1997	0.682576	no
gene:SpnNT_01368	bspRIM	Chromosome:1405059-1406115	110.58+peptide	ΔORF2+peptide	OK	66.3119	87.8031	0.405005	0.882896	0.1281	0.542797	no
gene:SpnNT_01369	rsmI	Chromosome:1406332-1407202	110.58	ΔORF2	OK	48.2089	52.773	0.130501	0.267702	0.63115	0.980887	no
gene:SpnNT_01369	rsmI	Chromosome:1406332-1407202	110.58	110.58+peptide	OK	48.2089	53.2947	0.144693	0.297244	0.6012	0.976678	no
gene:SpnNT_01369	rsmI	Chromosome:1406332-1407202	ΔORF2	110.58+peptide	OK	52.773	53.2947	0.0141921	0.0293256	0.9586	0.994855	no
gene:SpnNT_01369	rsmI	Chromosome:1406332-1407202	110.58	ΔORF2+peptide	OK	48.2089	65.0753	0.432812	0.89276	0.1171	0.518813	no
gene:SpnNT_01369	rsmI	Chromosome:1406332-1407202	ΔORF2	ΔORF2+peptide	OK	52.773	65.0753	0.302311	0.627254	0.26975	0.77184	no
gene:SpnNT_01369	rsmI	Chromosome:1406332-1407202	110.58+peptide	ΔORF2+peptide	OK	53.2947	65.0753	0.288119	0.598692	0.3005	0.806342	no
gene:SpnNT_01370	NA	Chromosome:1407204-1407522	110.58	ΔORF2	OK	72.6582	59.9272	-0.277915	-0.455813	0.42305	0.906181	no
gene:SpnNT_01370	NA	Chromosome:1407204-1407522	110.58	110.58+peptide	OK	72.6582	62.6856	-0.212991	-0.348973	0.5377	0.959663	no
gene:SpnNT_01370	NA	Chromosome:1407204-1407522	ΔORF2	110.58+peptide	OK	59.9272	62.6856	0.0649245	0.104222	0.85405	0.994748	no
gene:SpnNT_01370	NA	Chromosome:1407204-1407522	110.58	ΔORF2+peptide	OK	72.6582	71.4539	-0.024112	-0.0401171	0.94335	0.994855	no
gene:SpnNT_01370	NA	Chromosome:1407204-1407522	ΔORF2	ΔORF2+peptide	OK	59.9272	71.4539	0.253803	0.413467	0.46445	0.926394	no
gene:SpnNT_01370	NA	Chromosome:1407204-1407522	110.58+peptide	ΔORF2+peptide	OK	62.6856	71.4539	0.188879	0.307389	0.5811	0.96988	no
gene:SpnNT_01371	dnaX_1	Chromosome:1407561-1409087	110.58	ΔORF2	OK	58.8114	63.0209	0.0997351	0.152679	0.79015	0.994748	no
gene:SpnNT_01371	dnaX_1	Chromosome:1407561-1409087	110.58	110.58+peptide	OK	58.8114	63.9101	0.11995	0.184607	0.7485	0.994748	no
gene:SpnNT_01371	dnaX_1	Chromosome:1407561-1409087	ΔORF2	110.58+peptide	OK	63.0209	63.9101	0.020215	0.0317091	0.95815	0.994855	no
gene:SpnNT_01371	dnaX_1	Chromosome:1407561-1409087	110.58	ΔORF2+peptide	OK	58.8114	78.5764	0.418001	0.642565	0.2624	0.762762	no
gene:SpnNT_01371	dnaX_1	Chromosome:1407561-1409087	ΔORF2	ΔORF2+peptide	OK	63.0209	78.5764	0.318266	0.498622	0.38535	0.881745	no
gene:SpnNT_01371	dnaX_1	Chromosome:1407561-1409087	110.58+peptide	ΔORF2+peptide	OK	63.9101	78.5764	0.298051	0.469569	0.41315	0.899511	no
gene:SpnNT_01372	tmk	Chromosome:1407561-1409087	110.58	ΔORF2	OK	99.0695	104.815	0.0813352	0.129408	0.8256	0.994748	no
gene:SpnNT_01372	tmk	Chromosome:1407561-1409087	110.58	110.58+peptide	OK	99.0695	100.425	0.0196081	0.0309872	0.9596	0.994855	no
gene:SpnNT_01372	tmk	Chromosome:1407561-1409087	ΔORF2	110.58+peptide	OK	104.815	100.425	-0.0617271	-0.0970391	0.8669	0.994748	no
gene:SpnNT_01372	tmk	Chromosome:1407561-1409087	110.58	ΔORF2+peptide	OK	99.0695	120.38	0.281079	0.447763	0.44255	0.917631	no
gene:SpnNT_01372	tmk	Chromosome:1407561-1409087	ΔORF2	ΔORF2+peptide	OK	104.815	120.38	0.199744	0.316506	0.58885	0.974101	no
gene:SpnNT_01372	tmk	Chromosome:1407561-1409087	110.58+peptide	ΔORF2+peptide	OK	100.425	120.38	0.261471	0.411546	0.4815	0.935095	no
gene:SpnNT_01373	proC	Chromosome:1409306-1410104	110.58	ΔORF2	OK	98.6201	97.5407	-0.015877	-0.034585	0.9505	0.994855	no
gene:SpnNT_01373	proC	Chromosome:1409306-1410104	110.58	110.58+peptide	OK	98.6201	78.6007	-0.32734	-0.705153	0.21365	0.698761	no

gene:SpnNT_01373	proC	Chromosome:1409306-1410104	ΔORF2	110.58+peptide	OK	97.5407	78.6007	-0.311463	-0.669211	0.23865	0.733731	no
gene:SpnNT_01373	proC	Chromosome:1409306-1410104	110.58	ΔORF2+peptide	OK	98.6201	84.7304	-0.219003	-0.473275	0.40655	0.896145	no
gene:SpnNT_01373	proC	Chromosome:1409306-1410104	ΔORF2	ΔORF2+peptide	OK	97.5407	84.7304	-0.203126	-0.437818	0.4425	0.917631	no
gene:SpnNT_01373	proC	Chromosome:1409306-1410104	110.58+peptide	ΔORF2+peptide	OK	78.6007	84.7304	0.108337	0.230978	0.6866	0.98828	no
gene:SpnNT_01374	proA	Chromosome:1410107-1411370	110.58	ΔORF2	OK	84.0473	80.7077	-0.0584946	-0.130125	0.8196	0.994748	no
gene:SpnNT_01374	proA	Chromosome:1410107-1411370	110.58	110.58+peptide	OK	84.0473	70.9052	-0.24531	-0.544792	0.3427	0.846557	no
gene:SpnNT_01374	proA	Chromosome:1410107-1411370	ΔORF2	110.58+peptide	OK	80.7077	70.9052	-0.186815	-0.410329	0.4762	0.930974	no
gene:SpnNT_01374	proA	Chromosome:1410107-1411370	110.58	ΔORF2+peptide	OK	84.0473	72.3566	-0.216077	-0.48362	0.39275	0.887798	no
gene:SpnNT_01374	proA	Chromosome:1410107-1411370	ΔORF2	ΔORF2+peptide	OK	80.7077	72.3566	-0.157582	-0.348766	0.53505	0.958867	no
gene:SpnNT_01374	proA	Chromosome:1410107-1411370	110.58+peptide	ΔORF2+peptide	OK	70.9052	72.3566	0.0292332	0.0645927	0.90785	0.994748	no
gene:SpnNT_01375	proB	Chromosome:1411379-1412510	110.58	ΔORF2	OK	87.5509	86.7664	-0.012987	-0.0288104	0.9574	0.994855	no
gene:SpnNT_01375	proB	Chromosome:1411379-1412510	110.58	110.58+peptide	OK	87.5509	65.4071	-0.420675	-0.929899	0.10445	0.490549	no
gene:SpnNT_01375	proB	Chromosome:1411379-1412510	ΔORF2	110.58+peptide	OK	86.7664	65.4071	-0.407688	-0.892443	0.11525	0.515092	no
gene:SpnNT_01375	proB	Chromosome:1411379-1412510	110.58	ΔORF2+peptide	OK	87.5509	76.8046	-0.18893	-0.420114	0.4676	0.92732	no
gene:SpnNT_01375	proB	Chromosome:1411379-1412510	ΔORF2	ΔORF2+peptide	OK	86.7664	76.8046	-0.175943	-0.387393	0.50195	0.944784	no
gene:SpnNT_01375	proB	Chromosome:1411379-1412510	110.58+peptide	ΔORF2+peptide	OK	65.4071	76.8046	0.231746	0.50847	0.3762	0.874645	no
gene:SpnNT_01376	NA	Chromosome:1412690-1413997	110.58	ΔORF2	NOTEST	0.699256	0.439181	-0.671004	0	1	1	no
gene:SpnNT_01376	NA	Chromosome:1412690-1413997	110.58	110.58+peptide	NOTEST	0.699256	0.594492	-0.234165	0	1	1	no
gene:SpnNT_01376	NA	Chromosome:1412690-1413997	ΔORF2	110.58+peptide	NOTEST	0.439181	0.594492	0.43684	0	1	1	no
gene:SpnNT_01376	NA	Chromosome:1412690-1413997	110.58	ΔORF2+peptide	NOTEST	0.699256	0.793163	0.181796	0	1	1	no
gene:SpnNT_01376	NA	Chromosome:1412690-1413997	ΔORF2	ΔORF2+peptide	NOTEST	0.439181	0.793163	0.852801	0	1	1	no
gene:SpnNT_01376	NA	Chromosome:1412690-1413997	110.58+peptide	ΔORF2+peptide	NOTEST	0.594492	0.793163	0.415961	0	1	1	no
gene:SpnNT_01377	NA	Chromosome:1412690-1413997	110.58	ΔORF2	OK	2.3819	1.0236	-1.21847	-0.959692	0.1129	0.510701	no
gene:SpnNT_01377	NA	Chromosome:1412690-1413997	110.58	110.58+peptide	OK	2.3819	1.17983	-1.01353	-0.824335	0.17215	0.632235	no
gene:SpnNT_01377	NA	Chromosome:1412690-1413997	ΔORF2	110.58+peptide	OK	1.0236	1.17983	0.204936	0.14833	0.7989	0.994748	no
gene:SpnNT_01377	NA	Chromosome:1412690-1413997	110.58	ΔORF2+peptide	OK	2.3819	1.07524	-1.14745	-0.868425	0.24275	0.738169	no
gene:SpnNT_01377	NA	Chromosome:1412690-1413997	ΔORF2	ΔORF2+peptide	OK	1.0236	1.07524	0.0710132	0.0485093	0.94395	0.994855	no
gene:SpnNT_01377	NA	Chromosome:1412690-1413997	110.58+peptide	ΔORF2+peptide	OK	1.17983	1.07524	-0.133923	-0.0937021	0.89025	0.994748	no
gene:SpnNT_01378	lytB_6	Chromosome:1414062-1415943	110.58	ΔORF2	OK	86.1199	106.591	0.307673	0.696922	0.21285	0.698345	no
gene:SpnNT_01378	lytB_6	Chromosome:1414062-1415943	110.58	110.58+peptide	OK	86.1199	93.4178	0.11735	0.268122	0.6394	0.980887	no
gene:SpnNT_01378	lytB_6	Chromosome:1414062-1415943	ΔORF2	110.58+peptide	OK	106.591	93.4178	-0.190323	-0.4294	0.44855	0.919688	no
gene:SpnNT_01378	lytB_6	Chromosome:1414062-1415943	110.58	ΔORF2+peptide	OK	86.1199	115.154	0.419145	0.963011	0.08395	0.434896	no
gene:SpnNT_01378	lytB_6	Chromosome:1414062-1415943	ΔORF2	ΔORF2+peptide	OK	106.591	115.154	0.111472	0.252868	0.6489	0.981391	no
gene:SpnNT_01378	lytB_6	Chromosome:1414062-1415943	110.58+peptide	ΔORF2+peptide	OK	93.4178	115.154	0.301796	0.690567	0.2221	0.711509	no
gene:SpnNT_01379	rluD_2	Chromosome:1415945-1418189	110.58	ΔORF2	OK	74.9164	73.8256	-0.021161	-0.0283927	0.9578	0.994855	no
gene:SpnNT_01379	rluD_2	Chromosome:1415945-1418189	110.58	110.58+peptide	OK	74.9164	67.9779	-0.140216	-0.192772	0.7407	0.994748	no
gene:SpnNT_01379	rluD_2	Chromosome:1415945-1418189	ΔORF2	110.58+peptide	OK	73.8256	67.9779	-0.119055	-0.163159	0.77905	0.994748	no
gene:SpnNT_01379	rluD_2	Chromosome:1415945-1418189	110.58	ΔORF2+peptide	OK	74.9164	69.1069	-0.116452	-0.157542	0.7831	0.994748	no
gene:SpnNT_01379	rluD_2	Chromosome:1415945-1418189	ΔORF2	ΔORF2+peptide	OK	73.8256	69.1069	-0.095291	-0.128518	0.82135	0.994748	no
gene:SpnNT_01379	rluD_2	Chromosome:1415945-1418189	110.58+peptide	ΔORF2+peptide	OK	67.9779	69.1069	0.0237638	0.0328486	0.954	0.994855	no
gene:SpnNT_01380	lspA	Chromosome:1415945-1418189	110.58	ΔORF2	OK	99.9868	97.7072	-0.0332728	-0.0313934	0.9567	0.994855	no
gene:SpnNT_01380	lspA	Chromosome:1415945-1418189	110.58	110.58+peptide	OK	99.9868	84.0718	-0.250116	-0.24035	0.68115	0.986574	no
gene:SpnNT_01380	lspA	Chromosome:1415945-1418189	ΔORF2	110.58+peptide	OK	97.7072	84.0718	-0.216843	-0.206099	0.72305	0.994748	no
gene:SpnNT_01380	lspA	Chromosome:1415945-1418189	110.58	ΔORF2+peptide	OK	99.9868	102.373	0.0340265	0.0333659	0.9526	0.994855	no
gene:SpnNT_01380	lspA	Chromosome:1415945-1418189	ΔORF2	ΔORF2+peptide	OK	97.7072	102.373	0.0672993	0.0652422	0.9063	0.994748	no

gene:SpnNT_01380	lspA	Chromosome:1415945-1418189	110.58+peptide	ΔORF2+peptide	OK	84.0718	102.373	0.284143	0.280839	0.62365	0.980887	no
gene:SpnNT_01381	cysB	Chromosome:1415945-1418189	110.58	ΔORF2	OK	89.6482	101.694	0.181894	0.282109	0.62735	0.980887	no
gene:SpnNT_01381	cysB	Chromosome:1415945-1418189	110.58	110.58+peptide	OK	89.6482	67.6736	-0.405681	-0.594599	0.30055	0.806342	no
gene:SpnNT_01381	cysB	Chromosome:1415945-1418189	ΔORF2	110.58+peptide	OK	101.694	67.6736	-0.587574	-0.879943	0.1291	0.544886	no
gene:SpnNT_01381	cysB	Chromosome:1415945-1418189	110.58	ΔORF2+peptide	OK	89.6482	71.5838	-0.324641	-0.47599	0.4085	0.897087	no
gene:SpnNT_01381	cysB	Chromosome:1415945-1418189	ΔORF2	ΔORF2+peptide	OK	101.694	71.5838	-0.506535	-0.758861	0.1926	0.674126	no
gene:SpnNT_01381	cysB	Chromosome:1415945-1418189	110.58+peptide	ΔORF2+peptide	OK	67.6736	71.5838	0.0810395	0.115146	0.8414	0.994748	no
gene:SpnNT_01382	NA	Chromosome:1418430-1419102	110.58	ΔORF2	OK	0.938931	1.42519	0.602067	0.539586	0.3495	0.853357	no
gene:SpnNT_01382	NA	Chromosome:1418430-1419102	110.58	110.58+peptide	OK	0.938931	1.17779	0.326991	0.289357	0.6156	0.979616	no
gene:SpnNT_01382	NA	Chromosome:1418430-1419102	ΔORF2	110.58+peptide	OK	1.42519	1.17779	-0.275076	-0.272627	0.65095	0.981475	no
gene:SpnNT_01382	NA	Chromosome:1418430-1419102	110.58	ΔORF2+peptide	OK	0.938931	0.818852	-0.197417	-0.162903	0.7855	0.994748	no
gene:SpnNT_01382	NA	Chromosome:1418430-1419102	ΔORF2	ΔORF2+peptide	OK	1.42519	0.818852	-0.799484	-0.726915	0.25675	0.756415	no
gene:SpnNT_01382	NA	Chromosome:1418430-1419102	110.58+peptide	ΔORF2+peptide	OK	1.17779	0.818852	-0.524409	-0.470615	0.45285	0.921244	no
gene:SpnNT_01383	NA	Chromosome:1419155-1419377	110.58	ΔORF2	OK	0.867711	1.80092	1.05345	0.48417	0.39815	0.891861	no
gene:SpnNT_01383	NA	Chromosome:1419155-1419377	110.58	110.58+peptide	OK	0.867711	1.9574	1.17366	1.02388	0.48015	0.934519	no
gene:SpnNT_01383	NA	Chromosome:1419155-1419377	ΔORF2	110.58+peptide	OK	1.80092	1.9574	0.120206	0.0553034	0.86795	0.994748	no
gene:SpnNT_01383	NA	Chromosome:1419155-1419377	110.58	ΔORF2+peptide	NOTEST	0.867711	0	#NAME?	0	1	1	no
gene:SpnNT_01383	NA	Chromosome:1419155-1419377	ΔORF2	ΔORF2+peptide	OK	1.80092	0	#NAME?	NA	0.00135	0.021343	yes
gene:SpnNT_01383	NA	Chromosome:1419155-1419377	110.58+peptide	ΔORF2+peptide	OK	1.9574	0	#NAME?	NA	0.02295	0.185895	no
gene:SpnNT_01384	yidA_3	Chromosome:1419648-1420458	110.58	ΔORF2	OK	76.7562	83.9118	0.12859	0.274798	0.63125	0.980887	no
gene:SpnNT_01384	yidA_3	Chromosome:1419648-1420458	110.58	110.58+peptide	OK	76.7562	69.8583	-0.135852	-0.285766	0.61735	0.980734	no
gene:SpnNT_01384	yidA_3	Chromosome:1419648-1420458	ΔORF2	110.58+peptide	OK	83.9118	69.8583	-0.264442	-0.558011	0.33515	0.840108	no
gene:SpnNT_01384	yidA_3	Chromosome:1419648-1420458	110.58	ΔORF2+peptide	OK	76.7562	58.2246	-0.398655	-0.834429	0.15015	0.59185	no
gene:SpnNT_01384	yidA_3	Chromosome:1419648-1420458	ΔORF2	ΔORF2+peptide	OK	83.9118	58.2246	-0.527245	-1.10703	0.05795	0.342686	no
gene:SpnNT_01384	yidA_3	Chromosome:1419648-1420458	110.58+peptide	ΔORF2+peptide	OK	69.8583	58.2246	-0.262803	-0.543436	0.34935	0.853357	no
gene:SpnNT_01385	NA	Chromosome:1420638-1421514	110.58	ΔORF2	OK	33.5503	27.9325	-0.26438	-0.504393	0.3806	0.878145	no
gene:SpnNT_01385	NA	Chromosome:1420638-1421514	110.58	110.58+peptide	OK	33.5503	49.8134	0.57021	1.13302	0.04935	0.311514	no
gene:SpnNT_01385	NA	Chromosome:1420638-1421514	ΔORF2	110.58+peptide	OK	27.9325	49.8134	0.83459	1.61904	0.0051	0.061228	no
gene:SpnNT_01385	NA	Chromosome:1420638-1421514	110.58	ΔORF2+peptide	OK	33.5503	51.8411	0.627771	1.25488	0.02725	0.209108	no
gene:SpnNT_01385	NA	Chromosome:1420638-1421514	ΔORF2	ΔORF2+peptide	OK	27.9325	51.8411	0.892151	1.7406	0.00255	0.0352222	yes
gene:SpnNT_01385	NA	Chromosome:1420638-1421514	110.58+peptide	ΔORF2+peptide	OK	49.8134	51.8411	0.0575611	0.117191	0.842	0.994748	no
gene:SpnNT_01386	aguA	Chromosome:1421523-1425849	110.58	ΔORF2	OK	33.3585	30.8896	-0.110931	-0.130396	0.8209	0.994748	no
gene:SpnNT_01386	aguA	Chromosome:1421523-1425849	110.58	110.58+peptide	OK	33.3585	47.4919	0.509626	0.628263	0.2734	0.775829	no
gene:SpnNT_01386	aguA	Chromosome:1421523-1425849	ΔORF2	110.58+peptide	OK	30.8896	47.4919	0.620557	0.733211	0.21005	0.696723	no
gene:SpnNT_01386	aguA	Chromosome:1421523-1425849	110.58	ΔORF2+peptide	OK	33.3585	44.6669	0.421154	0.506298	0.38215	0.879865	no
gene:SpnNT_01386	aguA	Chromosome:1421523-1425849	ΔORF2	ΔORF2+peptide	OK	30.8896	44.6669	0.532085	0.614289	0.29545	0.799855	no
gene:SpnNT_01386	aguA	Chromosome:1421523-1425849	110.58+peptide	ΔORF2+peptide	OK	47.4919	44.6669	-0.0884725	-0.106933	0.8572	0.994748	no
gene:SpnNT_01387	nspC	Chromosome:1421523-1425849	110.58	ΔORF2	OK	31.0664	29.9505	-0.0527744	-0.0631862	0.91275	0.994748	no
gene:SpnNT_01387	nspC	Chromosome:1421523-1425849	110.58	110.58+peptide	OK	31.0664	41.7952	0.427982	0.519024	0.35535	0.85704	no
gene:SpnNT_01387	nspC	Chromosome:1421523-1425849	ΔORF2	110.58+peptide	OK	29.9505	41.7952	0.480756	0.576248	0.3139	0.819701	no
gene:SpnNT_01387	nspC	Chromosome:1421523-1425849	110.58	ΔORF2+peptide	OK	31.0664	44.7305	0.525904	0.628749	0.27055	0.772705	no
gene:SpnNT_01387	nspC	Chromosome:1421523-1425849	ΔORF2	ΔORF2+peptide	OK	29.9505	44.7305	0.578678	0.684025	0.2378	0.732745	no
gene:SpnNT_01387	nspC	Chromosome:1421523-1425849	110.58+peptide	ΔORF2+peptide	OK	41.7952	44.7305	0.0979221	0.117202	0.8422	0.994748	no
gene:SpnNT_01388	NA	Chromosome:1421523-1425849	110.58	ΔORF2	OK	27.7673	27.4577	-0.0161772	-0.01971	0.9749	0.99536	no
gene:SpnNT_01388	NA	Chromosome:1421523-1425849	110.58	110.58+peptide	OK	27.7673	34.9426	0.331602	0.397697	0.48015	0.934519	no

gene:SpnNT_01388	NA	Chromosome:1421523-1425849	ΔORF2	110.58+peptide	OK	27.4577	34.9426	0.34778	0.41503	0.46525	0.926405	no
gene:SpnNT_01388	NA	Chromosome:1421523-1425849	110.58	ΔORF2+peptide	OK	27.7673	41.891	0.593255	0.73282	0.20055	0.683352	no
gene:SpnNT_01388	NA	Chromosome:1421523-1425849	ΔORF2	ΔORF2+peptide	OK	27.4577	41.891	0.609432	0.748845	0.19485	0.676386	no
gene:SpnNT_01388	NA	Chromosome:1421523-1425849	110.58+peptide	ΔORF2+peptide	OK	34.9426	41.891	0.261653	0.316393	0.5749	0.969538	no
gene:SpnNT_01389	speE	Chromosome:1421523-1425849	110.58	ΔORF2	OK	18.7214	20.3223	0.118372	0.092047	0.8712	0.994748	no
gene:SpnNT_01389	speE	Chromosome:1421523-1425849	110.58	110.58+peptide	OK	18.7214	26.5768	0.505475	0.395071	0.49325	0.941402	no
gene:SpnNT_01389	speE	Chromosome:1421523-1425849	ΔORF2	110.58+peptide	OK	20.3223	26.5768	0.387103	0.29552	0.6044	0.976761	no
gene:SpnNT_01389	speE	Chromosome:1421523-1425849	110.58	ΔORF2+peptide	OK	18.7214	34.134	0.866519	0.727943	0.20295	0.687077	no
gene:SpnNT_01389	speE	Chromosome:1421523-1425849	ΔORF2	ΔORF2+peptide	OK	20.3223	34.134	0.748147	0.611713	0.2774	0.780208	no
gene:SpnNT_01389	speE	Chromosome:1421523-1425849	110.58+peptide	ΔORF2+peptide	OK	26.5768	34.134	0.361045	0.296873	0.60075	0.976678	no
gene:SpnNT_01390	speA	Chromosome:1425849-1427310	110.58	ΔORF2	OK	25.8232	24.3959	-0.082029	-0.166692	0.7603	0.994748	no
gene:SpnNT_01390	speA	Chromosome:1425849-1427310	110.58	110.58+peptide	OK	25.8232	37.6095	0.54243	1.13176	0.0446	0.290442	no
gene:SpnNT_01390	speA	Chromosome:1425849-1427310	ΔORF2	110.58+peptide	OK	24.3959	37.6095	0.624459	1.29297	0.02545	0.199138	no
gene:SpnNT_01390	speA	Chromosome:1425849-1427310	110.58	ΔORF2+peptide	OK	25.8232	37.2926	0.530223	1.10658	0.05085	0.317589	no
gene:SpnNT_01390	speA	Chromosome:1425849-1427310	ΔORF2	ΔORF2+peptide	OK	24.3959	37.2926	0.612252	1.26802	0.0287	0.215822	no
gene:SpnNT_01390	speA	Chromosome:1425849-1427310	110.58+peptide	ΔORF2+peptide	OK	37.6095	37.2926	-0.0122068	-0.0259846	0.9621	0.994855	no
gene:SpnNT_01391	NA	Chromosome:1428017-1428713	110.58	ΔORF2	OK	77.9114	72.8028	-0.0978404	-0.181806	0.7501	0.994748	no
gene:SpnNT_01391	NA	Chromosome:1428017-1428713	110.58	110.58+peptide	OK	77.9114	21.3319	-1.86882	-3.32339	5.00E-05	0.0013612	yes
gene:SpnNT_01391	NA	Chromosome:1428017-1428713	ΔORF2	110.58+peptide	OK	72.8028	21.3319	-1.77098	-3.09172	5.00E-05	0.0013612	yes
gene:SpnNT_01391	NA	Chromosome:1428017-1428713	110.58	ΔORF2+peptide	OK	77.9114	22.7837	-1.77383	-3.20048	5.00E-05	0.0013612	yes
gene:SpnNT_01391	NA	Chromosome:1428017-1428713	ΔORF2	ΔORF2+peptide	OK	72.8028	22.7837	-1.67599	-2.96699	5.00E-05	0.0013612	yes
gene:SpnNT_01391	NA	Chromosome:1428017-1428713	110.58+peptide	ΔORF2+peptide	OK	21.3319	22.7837	0.0949906	0.161563	0.7866	0.994748	no
gene:SpnNT_01392	bceB	Chromosome:1429043-1431032	110.58	ΔORF2	OK	7.2568	7.97224	0.135652	0.246208	0.67135	0.984845	no
gene:SpnNT_01392	bceB	Chromosome:1429043-1431032	110.58	110.58+peptide	OK	7.2568	5.30634	-0.451616	-0.799038	0.1609	0.613759	no
gene:SpnNT_01392	bceB	Chromosome:1429043-1431032	ΔORF2	110.58+peptide	OK	7.97224	5.30634	-0.587268	-1.05003	0.067	0.378628	no
gene:SpnNT_01392	bceB	Chromosome:1429043-1431032	110.58	ΔORF2+peptide	OK	7.2568	4.9646	-0.547656	-0.958444	0.09935	0.477885	no
gene:SpnNT_01392	bceB	Chromosome:1429043-1431032	ΔORF2	ΔORF2+peptide	OK	7.97224	4.9646	-0.683307	-1.20821	0.03895	0.266199	no
gene:SpnNT_01392	bceB	Chromosome:1429043-1431032	110.58+peptide	ΔORF2+peptide	OK	5.30634	4.9646	-0.0960397	-0.165748	0.7709	0.994748	no
gene:SpnNT_01393	bceA_1	Chromosome:1431033-1431792	110.58	ΔORF2	OK	7.29076	6.73617	-0.114141	-0.174068	0.7571	0.994748	no
gene:SpnNT_01393	bceA_1	Chromosome:1431033-1431792	110.58	110.58+peptide	OK	7.29076	6.7884	-0.102997	-0.157667	0.78115	0.994748	no
gene:SpnNT_01393	bceA_1	Chromosome:1431033-1431792	ΔORF2	110.58+peptide	OK	6.73617	6.7884	0.0111438	0.0169011	0.9722	0.99536	no
gene:SpnNT_01393	bceA_1	Chromosome:1431033-1431792	110.58	ΔORF2+peptide	OK	7.29076	6.44128	-0.178723	-0.268288	0.6328	0.980887	no
gene:SpnNT_01393	bceA_1	Chromosome:1431033-1431792	ΔORF2	ΔORF2+peptide	OK	6.73617	6.44128	-0.0645814	-0.0960846	0.8609	0.994748	no
gene:SpnNT_01393	bceA_1	Chromosome:1431033-1431792	110.58+peptide	ΔORF2+peptide	OK	6.7884	6.44128	-0.0757252	-0.113069	0.8386	0.994748	no
gene:SpnNT_01394	NA	Chromosome:1431806-1431917	110.58	ΔORF2	OK	12.4187	15.7025	0.338477	1.50701	0.6783	0.985276	no
gene:SpnNT_01394	NA	Chromosome:1431806-1431917	110.58	110.58+peptide	NOTEST	12.4187	0	#NAME?	0	1	1	no
gene:SpnNT_01394	NA	Chromosome:1431806-1431917	ΔORF2	110.58+peptide	OK	15.7025	0	#NAME?	NA	0.12215	0.528994	no
gene:SpnNT_01394	NA	Chromosome:1431806-1431917	110.58	ΔORF2+peptide	NOTEST	12.4187	24.611	0.986793	0	1	1	no
gene:SpnNT_01394	NA	Chromosome:1431806-1431917	ΔORF2	ΔORF2+peptide	OK	15.7025	24.611	0.648316	3.28329	0.6186	0.980887	no
gene:SpnNT_01394	NA	Chromosome:1431806-1431917	110.58+peptide	ΔORF2+peptide	NOTEST	0	24.611	Inf	0	1	1	no
gene:SpnNT_01395	NA	Chromosome:1432219-1432411	110.58	ΔORF2	OK	20.9084	18.7674	-0.155853	-0.151515	0.7921	0.994748	no
gene:SpnNT_01395	NA	Chromosome:1432219-1432411	110.58	110.58+peptide	OK	20.9084	21.6176	0.0481252	0.0457534	0.9519	0.994855	no
gene:SpnNT_01395	NA	Chromosome:1432219-1432411	ΔORF2	110.58+peptide	OK	18.7674	21.6176	0.203978	0.186511	0.73845	0.994748	no
gene:SpnNT_01395	NA	Chromosome:1432219-1432411	110.58	ΔORF2+peptide	OK	20.9084	11.0088	-0.92543	-1.1956	0.18715	0.661936	no
gene:SpnNT_01395	NA	Chromosome:1432219-1432411	ΔORF2	ΔORF2+peptide	OK	18.7674	11.0088	-0.769578	-0.927247	0.2786	0.781125	no

gene:SpnNT_01395	NA	Chromosome:1432219-1432411	110.58+peptide	ΔORF2+peptide	OK	21.6176	11.0088	-0.973555	-1.13395	0.1791	0.645832	no
gene:SpnNT_01396	NA	Chromosome:1432764-1433037	110.58	ΔORF2	OK	714.807	798.087	0.158992	0.327514	0.56235	0.968621	no
gene:SpnNT_01396	NA	Chromosome:1432764-1433037	110.58	110.58+peptide	OK	714.807	1233.75	0.787424	1.50953	0.0062	0.0706832	no
gene:SpnNT_01396	NA	Chromosome:1432764-1433037	ΔORF2	110.58+peptide	OK	798.087	1233.75	0.628432	1.2138	0.0284	0.214669	no
gene:SpnNT_01396	NA	Chromosome:1432764-1433037	110.58	ΔORF2+peptide	OK	714.807	850.683	0.251066	0.516754	0.35315	0.856141	no
gene:SpnNT_01396	NA	Chromosome:1432764-1433037	ΔORF2	ΔORF2+peptide	OK	798.087	850.683	0.0920742	0.191156	0.726	0.994748	no
gene:SpnNT_01396	NA	Chromosome:1432764-1433037	110.58+peptide	ΔORF2+peptide	OK	1233.75	850.683	-0.536358	-1.03521	0.0579	0.342686	no
gene:SpnNT_01397	NA	Chromosome:1433095-1435661	110.58	ΔORF2	OK	43.9186	44.9394	0.0331477	0.0203493	0.9731	0.99536	no
gene:SpnNT_01397	NA	Chromosome:1433095-1435661	110.58	110.58+peptide	OK	43.9186	47.8016	0.122226	0.0802437	0.88915	0.994748	no
gene:SpnNT_01397	NA	Chromosome:1433095-1435661	ΔORF2	110.58+peptide	OK	44.9394	47.8016	0.089078	0.0534964	0.9224	0.994748	no
gene:SpnNT_01397	NA	Chromosome:1433095-1435661	110.58	ΔORF2+peptide	OK	43.9186	48.6448	0.147453	0.10052	0.86405	0.994748	no
gene:SpnNT_01397	NA	Chromosome:1433095-1435661	ΔORF2	ΔORF2+peptide	OK	44.9394	48.6448	0.114305	0.0708299	0.90075	0.994748	no
gene:SpnNT_01397	NA	Chromosome:1433095-1435661	110.58+peptide	ΔORF2+peptide	OK	47.8016	48.6448	0.0252268	0.01674	0.9763	0.99536	no
gene:SpnNT_01398	rpsA_1	Chromosome:1433095-1435661	110.58	ΔORF2	OK	54.9694	55.0626	0.00244412	0.00531424	0.9927	0.998522	no
gene:SpnNT_01398	rpsA_1	Chromosome:1433095-1435661	110.58	110.58+peptide	OK	54.9694	57.0809	0.0543782	0.118178	0.83535	0.994748	no
gene:SpnNT_01398	rpsA_1	Chromosome:1433095-1435661	ΔORF2	110.58+peptide	OK	55.0626	57.0809	0.0519341	0.112702	0.846	0.994748	no
gene:SpnNT_01398	rpsA_1	Chromosome:1433095-1435661	110.58	ΔORF2+peptide	OK	54.9694	58.7946	0.0970541	0.211489	0.70725	0.990441	no
gene:SpnNT_01398	rpsA_1	Chromosome:1433095-1435661	ΔORF2	ΔORF2+peptide	OK	55.0626	58.7946	0.09461	0.205861	0.7215	0.994748	no
gene:SpnNT_01398	rpsA_1	Chromosome:1433095-1435661	110.58+peptide	ΔORF2+peptide	OK	57.0809	58.7946	0.0426759	0.0928139	0.8729	0.994748	no
gene:SpnNT_01399	NA	Chromosome:1436120-1436252	110.58	ΔORF2	OK	453.016	434.918	-0.0588198	-0.0875414	0.88625	0.994748	no
gene:SpnNT_01399	NA	Chromosome:1436120-1436252	110.58	110.58+peptide	OK	453.016	431.384	-0.0705882	-0.0994173	0.8619	0.994748	no
gene:SpnNT_01399	NA	Chromosome:1436120-1436252	ΔORF2	110.58+peptide	OK	434.918	431.384	-0.0117683	-0.0168028	0.98355	0.996246	no
gene:SpnNT_01399	NA	Chromosome:1436120-1436252	110.58	ΔORF2+peptide	OK	453.016	233.722	-0.95477	-1.17499	0.04145	0.277079	no
gene:SpnNT_01399	NA	Chromosome:1436120-1436252	ΔORF2	ΔORF2+peptide	OK	434.918	233.722	-0.89595	-1.11413	0.05465	0.332461	no
gene:SpnNT_01399	NA	Chromosome:1436120-1436252	110.58+peptide	ΔORF2+peptide	OK	431.384	233.722	-0.884182	-1.05729	0.06315	0.364084	no
gene:SpnNT_01400	NA	Chromosome:1436252-1437970	110.58	ΔORF2	OK	43.8993	54.0191	0.299271	0.354702	0.54185	0.961494	no
gene:SpnNT_01400	NA	Chromosome:1436252-1437970	110.58	110.58+peptide	OK	43.8993	42.6351	-0.0421594	-0.0457606	0.93615	0.994855	no
gene:SpnNT_01400	NA	Chromosome:1436252-1437970	ΔORF2	110.58+peptide	OK	54.0191	42.6351	-0.341431	-0.376691	0.5112	0.949557	no
gene:SpnNT_01400	NA	Chromosome:1436252-1437970	110.58	ΔORF2+peptide	OK	43.8993	45.7793	0.0604968	0.0702198	0.90185	0.994748	no
gene:SpnNT_01400	NA	Chromosome:1436252-1437970	ΔORF2	ΔORF2+peptide	OK	54.0191	45.7793	-0.238774	-0.282381	0.6266	0.980887	no
gene:SpnNT_01400	NA	Chromosome:1436252-1437970	110.58+peptide	ΔORF2+peptide	OK	42.6351	45.7793	0.102656	0.111221	0.85095	0.994748	no
gene:SpnNT_01401	NA	Chromosome:1436252-1437970	110.58	ΔORF2	OK	123.795	130.072	0.0713591	0.133126	0.8147	0.994748	no
gene:SpnNT_01401	NA	Chromosome:1436252-1437970	110.58	110.58+peptide	OK	123.795	127.019	0.0371012	0.0690943	0.90465	0.994748	no
gene:SpnNT_01401	NA	Chromosome:1436252-1437970	ΔORF2	110.58+peptide	OK	130.072	127.019	-0.0342578	-0.0639822	0.91205	0.994748	no
gene:SpnNT_01401	NA	Chromosome:1436252-1437970	110.58	ΔORF2+peptide	OK	123.795	121.088	-0.0318917	-0.0587765	0.9197	0.994748	no
gene:SpnNT_01401	NA	Chromosome:1436252-1437970	ΔORF2	ΔORF2+peptide	OK	130.072	121.088	-0.103251	-0.190827	0.74195	0.994748	no
gene:SpnNT_01401	NA	Chromosome:1436252-1437970	110.58+peptide	ΔORF2+peptide	OK	127.019	121.088	-0.0689929	-0.127293	0.83135	0.994748	no
gene:SpnNT_01402	NA	Chromosome:1438098-1438815	110.58	ΔORF2	OK	67.251	74.185	0.141571	0.289869	0.61165	0.979429	no
gene:SpnNT_01402	NA	Chromosome:1438098-1438815	110.58	110.58+peptide	OK	67.251	38.5399	-0.803202	-1.576	0.0068	0.0755543	no
gene:SpnNT_01402	NA	Chromosome:1438098-1438815	ΔORF2	110.58+peptide	OK	74.185	38.5399	-0.944773	-1.86559	0.0018	0.0270557	yes
gene:SpnNT_01402	NA	Chromosome:1438098-1438815	110.58	ΔORF2+peptide	OK	67.251	47.6755	-0.496309	-0.983828	0.08555	0.439454	no
gene:SpnNT_01402	NA	Chromosome:1438098-1438815	ΔORF2	ΔORF2+peptide	OK	74.185	47.6755	-0.637879	-1.27269	0.02615	0.203162	no
gene:SpnNT_01402	NA	Chromosome:1438098-1438815	110.58+peptide	ΔORF2+peptide	OK	38.5399	47.6755	0.306894	0.587991	0.3088	0.814396	no
gene:SpnNT_01403	NA	Chromosome:1438818-1439130	110.58	ΔORF2	OK	83.6713	90.7894	0.117791	0.203767	0.7176	0.992553	no
gene:SpnNT_01403	NA	Chromosome:1438818-1439130	110.58	110.58+peptide	OK	83.6713	55.1626	-0.601043	-1.01397	0.0712	0.39157	no

gene:SpnNT_01403	NA	Chromosome:1438818-1439130	ΔORF2	110.58+peptide	OK	90.7894	55.1626	-0.718834	-1.19776	0.03415	0.243541	no
gene:SpnNT_01403	NA	Chromosome:1438818-1439130	110.58	ΔORF2+peptide	OK	83.6713	42.4229	-0.97989	-1.56062	0.00625	0.0710065	no
gene:SpnNT_01403	NA	Chromosome:1438818-1439130	ΔORF2	ΔORF2+peptide	OK	90.7894	42.4229	-1.09768	-1.72901	0.0022	0.0317584	yes
gene:SpnNT_01403	NA	Chromosome:1438818-1439130	110.58+peptide	ΔORF2+peptide	OK	55.1626	42.4229	-0.378847	-0.584394	0.2889	0.793193	no
gene:SpnNT_01404	NA	Chromosome:1439272-1439656	110.58	ΔORF2	OK	5.09217	4.55905	-0.159549	-0.172433	0.76825	0.994748	no
gene:SpnNT_01404	NA	Chromosome:1439272-1439656	110.58	110.58+peptide	OK	5.09217	3.81253	-0.417532	-0.432937	0.4394	0.916225	no
gene:SpnNT_01404	NA	Chromosome:1439272-1439656	ΔORF2	110.58+peptide	OK	4.55905	3.81253	-0.257982	-0.256805	0.6427	0.980887	no
gene:SpnNT_01404	NA	Chromosome:1439272-1439656	110.58	ΔORF2+peptide	OK	5.09217	2.31177	-1.13929	-1.1161	0.0687	0.383936	no
gene:SpnNT_01404	NA	Chromosome:1439272-1439656	ΔORF2	ΔORF2+peptide	OK	4.55905	2.31177	-0.979736	-0.925323	0.11935	0.523608	no
gene:SpnNT_01404	NA	Chromosome:1439272-1439656	110.58+peptide	ΔORF2+peptide	OK	3.81253	2.31177	-0.721754	-0.660241	0.2462	0.743359	no
gene:SpnNT_01405	NA	Chromosome:1439762-1440635	110.58	ΔORF2	OK	105.631	115.224	0.125402	0.276434	0.62595	0.980887	no
gene:SpnNT_01405	NA	Chromosome:1439762-1440635	110.58	110.58+peptide	OK	105.631	62.4112	-0.75916	-1.62863	0.0055	0.064496	no
gene:SpnNT_01405	NA	Chromosome:1439762-1440635	ΔORF2	110.58+peptide	OK	115.224	62.4112	-0.884562	-1.89496	0.00075	0.0133173	yes
gene:SpnNT_01405	NA	Chromosome:1439762-1440635	110.58	ΔORF2+peptide	OK	105.631	72.0791	-0.551384	-1.1916	0.0387	0.265181	no
gene:SpnNT_01405	NA	Chromosome:1439762-1440635	ΔORF2	ΔORF2+peptide	OK	115.224	72.0791	-0.676786	-1.4605	0.0114	0.110074	no
gene:SpnNT_01405	NA	Chromosome:1439762-1440635	110.58+peptide	ΔORF2+peptide	OK	62.4112	72.0791	0.207776	0.436844	0.4492	0.919994	no
gene:SpnNT_01406	pyk	Chromosome:1442392-1443898	110.58	ΔORF2	OK	916.462	904.354	-0.0191872	-0.0407231	0.9449	0.994855	no
gene:SpnNT_01406	pyk	Chromosome:1442392-1443898	110.58	110.58+peptide	OK	916.462	951.599	0.0542793	0.116216	0.83875	0.994748	no
gene:SpnNT_01406	pyk	Chromosome:1442392-1443898	ΔORF2	110.58+peptide	OK	904.354	951.599	0.0734665	0.157531	0.7811	0.994748	no
gene:SpnNT_01406	pyk	Chromosome:1442392-1443898	110.58	ΔORF2+peptide	OK	916.462	932.485	0.0250058	0.0535061	0.92545	0.994748	no
gene:SpnNT_01406	pyk	Chromosome:1442392-1443898	ΔORF2	ΔORF2+peptide	OK	904.354	932.485	0.0441929	0.0947021	0.8699	0.994748	no
gene:SpnNT_01406	pyk	Chromosome:1442392-1443898	110.58+peptide	ΔORF2+peptide	OK	951.599	932.485	-0.0292736	-0.0632933	0.9127	0.994748	no
gene:SpnNT_01407	pfkA	Chromosome:1443956-1444964	110.58	ΔORF2	OK	571.168	591.256	0.0498679	0.110617	0.8433	0.994748	no
gene:SpnNT_01407	pfkA	Chromosome:1443956-1444964	110.58	110.58+peptide	OK	571.168	586.191	0.0374549	0.0837163	0.88095	0.994748	no
gene:SpnNT_01407	pfkA	Chromosome:1443956-1444964	ΔORF2	110.58+peptide	OK	591.256	586.191	-0.012413	-0.0274375	0.96195	0.994855	no
gene:SpnNT_01407	pfkA	Chromosome:1443956-1444964	110.58	ΔORF2+peptide	OK	571.168	631.43	0.144708	0.322645	0.5629	0.968621	no
gene:SpnNT_01407	pfkA	Chromosome:1443956-1444964	ΔORF2	ΔORF2+peptide	OK	591.256	631.43	0.0948403	0.209129	0.71045	0.990984	no
gene:SpnNT_01407	pfkA	Chromosome:1443956-1444964	110.58+peptide	ΔORF2+peptide	OK	586.191	631.43	0.107253	0.238284	0.67785	0.98524	no
gene:SpnNT_01408	dnaE	Chromosome:1445046-1448148	110.58	ΔORF2	OK	42.8879	43.5938	0.0235516	0.0539636	0.92275	0.994748	no
gene:SpnNT_01408	dnaE	Chromosome:1445046-1448148	110.58	110.58+peptide	OK	42.8879	48.5144	0.177841	0.404656	0.4818	0.935095	no
gene:SpnNT_01408	dnaE	Chromosome:1445046-1448148	ΔORF2	110.58+peptide	OK	43.5938	48.5144	0.15429	0.351209	0.53565	0.958867	no
gene:SpnNT_01408	dnaE	Chromosome:1445046-1448148	110.58	ΔORF2+peptide	OK	42.8879	49.749	0.214096	0.49038	0.3852	0.881745	no
gene:SpnNT_01408	dnaE	Chromosome:1445046-1448148	ΔORF2	ΔORF2+peptide	OK	43.5938	49.749	0.190544	0.436614	0.4333	0.913334	no
gene:SpnNT_01408	dnaE	Chromosome:1445046-1448148	110.58+peptide	ΔORF2+peptide	OK	48.5144	49.749	0.0362545	0.0824965	0.88795	0.994748	no
gene:SpnNT_01409	pepX	Chromosome:1448493-1450767	110.58	ΔORF2	OK	197.244	213.554	0.114623	0.259388	0.64425	0.980887	no
gene:SpnNT_01409	pepX	Chromosome:1448493-1450767	110.58	110.58+peptide	OK	197.244	159.434	-0.307019	-0.696551	0.22005	0.70824	no
gene:SpnNT_01409	pepX	Chromosome:1448493-1450767	ΔORF2	110.58+peptide	OK	213.554	159.434	-0.421642	-0.953893	0.09315	0.460903	no
gene:SpnNT_01409	pepX	Chromosome:1448493-1450767	110.58	ΔORF2+peptide	OK	197.244	171.836	-0.198943	-0.452309	0.4302	0.91218	no
gene:SpnNT_01409	pepX	Chromosome:1448493-1450767	ΔORF2	ΔORF2+peptide	OK	213.554	171.836	-0.313566	-0.710882	0.21885	0.707128	no
gene:SpnNT_01409	pepX	Chromosome:1448493-1450767	110.58+peptide	ΔORF2+peptide	OK	159.434	171.836	0.108076	0.245647	0.66705	0.983929	no
gene:SpnNT_01410	argR_2	Chromosome:1450783-1451254	110.58	ΔORF2	OK	66.4126	67.1774	0.0165187	0.030926	0.9579	0.994855	no
gene:SpnNT_01410	argR_2	Chromosome:1450783-1451254	110.58	110.58+peptide	OK	66.4126	48.5601	-0.451685	-0.830054	0.1557	0.601087	no
gene:SpnNT_01410	argR_2	Chromosome:1450783-1451254	ΔORF2	110.58+peptide	OK	67.1774	48.5601	-0.468203	-0.854278	0.1419	0.575405	no
gene:SpnNT_01410	argR_2	Chromosome:1450783-1451254	110.58	ΔORF2+peptide	OK	66.4126	44.6527	-0.57271	-1.04923	0.07335	0.399046	no
gene:SpnNT_01410	argR_2	Chromosome:1450783-1451254	ΔORF2	ΔORF2+peptide	OK	67.1774	44.6527	-0.589229	-1.07185	0.0645	0.368753	no

gene:SpnNT_01410	argR_2	Chromosome:1450783-1451254	110.58+peptide	ΔORF2+peptide	OK	48.5601	44.6527	-0.121025	-0.216318	0.7071	0.990441	no
gene:SpnNT_01411	hsdR_2	Chromosome:1451796-1454034	110.58	ΔORF2	OK	30.6984	39.5684	0.366188	0.805436	0.16185	0.614702	no
gene:SpnNT_01411	hsdR_2	Chromosome:1451796-1454034	110.58	110.58+peptide	OK	30.6984	30.582	-0.00547729	-0.0118508	0.9822	0.996246	no
gene:SpnNT_01411	hsdR_2	Chromosome:1451796-1454034	ΔORF2	110.58+peptide	OK	39.5684	30.582	-0.371665	-0.806334	0.1549	0.600007	no
gene:SpnNT_01411	hsdR_2	Chromosome:1451796-1454034	110.58	ΔORF2+peptide	OK	30.6984	40.387	0.39573	0.874749	0.12135	0.52727	no
gene:SpnNT_01411	hsdR_2	Chromosome:1451796-1454034	ΔORF2	ΔORF2+peptide	OK	39.5684	40.387	0.0295424	0.0654882	0.9082	0.994748	no
gene:SpnNT_01411	hsdR_2	Chromosome:1451796-1454034	110.58+peptide	ΔORF2+peptide	OK	30.582	40.387	0.401207	0.874643	0.12235	0.529336	no
gene:SpnNT_01412	NA	Chromosome:1454073-1455147	110.58	ΔORF2	OK	50.5702	58.4578	0.209107	0.446162	0.43685	0.914104	no
gene:SpnNT_01412	NA	Chromosome:1454073-1455147	110.58	110.58+peptide	OK	50.5702	44.9002	-0.171568	-0.361415	0.5306	0.957488	no
gene:SpnNT_01412	NA	Chromosome:1454073-1455147	ΔORF2	110.58+peptide	OK	58.4578	44.9002	-0.380675	-0.806421	0.16735	0.623323	no
gene:SpnNT_01412	NA	Chromosome:1454073-1455147	110.58	ΔORF2+peptide	OK	50.5702	50.5414	-0.000822985	-0.00174034	0.9986	0.999412	no
gene:SpnNT_01412	NA	Chromosome:1454073-1455147	ΔORF2	ΔORF2+peptide	OK	58.4578	50.5414	-0.20993	-0.446449	0.436	0.914104	no
gene:SpnNT_01412	NA	Chromosome:1454073-1455147	110.58+peptide	ΔORF2+peptide	OK	44.9002	50.5414	0.170745	0.358531	0.52825	0.956161	no
gene:SpnNT_01413	NA	Chromosome:1455766-1457500	110.58	ΔORF2	OK	28.8476	33.1331	0.199822	0.423876	0.45455	0.921244	no
gene:SpnNT_01413	NA	Chromosome:1455766-1457500	110.58	110.58+peptide	OK	28.8476	28.4981	-0.0175827	-0.0367191	0.94585	0.994855	no
gene:SpnNT_01413	NA	Chromosome:1455766-1457500	ΔORF2	110.58+peptide	OK	33.1331	28.4981	-0.217404	-0.457328	0.42145	0.905117	no
gene:SpnNT_01413	NA	Chromosome:1455766-1457500	110.58	ΔORF2+peptide	OK	28.8476	34.9075	0.275087	0.584517	0.30425	0.809811	no
gene:SpnNT_01413	NA	Chromosome:1455766-1457500	ΔORF2	ΔORF2+peptide	OK	33.1331	34.9075	0.0752659	0.161135	0.7809	0.994748	no
gene:SpnNT_01413	NA	Chromosome:1455766-1457500	110.58+peptide	ΔORF2+peptide	OK	28.4981	34.9075	0.29267	0.616675	0.28275	0.785349	no
gene:SpnNT_01414	NA	Chromosome:1457621-1458269	110.58	ΔORF2	OK	1.27114	2.35574	0.890058	0.918225	0.1198	0.52453	no
gene:SpnNT_01414	NA	Chromosome:1457621-1458269	110.58	110.58+peptide	OK	1.27114	1.55091	0.286993	0.281077	0.6263	0.980887	no
gene:SpnNT_01414	NA	Chromosome:1457621-1458269	ΔORF2	110.58+peptide	OK	2.35574	1.55091	-0.603065	-0.633684	0.27755	0.780208	no
gene:SpnNT_01414	NA	Chromosome:1457621-1458269	110.58	ΔORF2+peptide	OK	1.27114	2.23643	0.815075	0.839181	0.14995	0.591417	no
gene:SpnNT_01414	NA	Chromosome:1457621-1458269	ΔORF2	ΔORF2+peptide	OK	2.35574	2.23643	-0.0749828	-0.083493	0.88965	0.994748	no
gene:SpnNT_01414	NA	Chromosome:1457621-1458269	110.58+peptide	ΔORF2+peptide	OK	1.55091	2.23643	0.528082	0.553739	0.33605	0.840218	no
gene:SpnNT_01415	xerC_3	Chromosome:1458302-1459257	110.58	ΔORF2	OK	1.08085	0.788949	-0.454165	-0.157172	0.7724	0.994748	no
gene:SpnNT_01415	xerC_3	Chromosome:1458302-1459257	110.58	110.58+peptide	OK	1.08085	0.74894	-0.529248	-0.18547	0.7521	0.994748	no
gene:SpnNT_01415	xerC_3	Chromosome:1458302-1459257	ΔORF2	110.58+peptide	NOTEST	0.788949	0.74894	-0.0750827	0	1	1	no
gene:SpnNT_01415	xerC_3	Chromosome:1458302-1459257	110.58	ΔORF2+peptide	OK	1.08085	0.870176	-0.312789	-0.145943	0.84825	0.994748	no
gene:SpnNT_01415	xerC_3	Chromosome:1458302-1459257	ΔORF2	ΔORF2+peptide	NOTEST	0.788949	0.870176	0.141376	0	1	1	no
gene:SpnNT_01415	xerC_3	Chromosome:1458302-1459257	110.58+peptide	ΔORF2+peptide	NOTEST	0.74894	0.870176	0.216459	0	1	1	no
gene:SpnNT_01416	NA	Chromosome:1458302-1459257	110.58	ΔORF2	OK	2.92962	3.43771	0.230731	0.251342	0.67685	0.98524	no
gene:SpnNT_01416	NA	Chromosome:1458302-1459257	110.58	110.58+peptide	OK	2.92962	3.13025	0.0955618	0.103673	0.8603	0.994748	no
gene:SpnNT_01416	NA	Chromosome:1458302-1459257	ΔORF2	110.58+peptide	OK	3.43771	3.13025	-0.135169	-0.149434	0.8078	0.994748	no
gene:SpnNT_01416	NA	Chromosome:1458302-1459257	110.58	ΔORF2+peptide	OK	2.92962	3.36335	0.199184	0.217086	0.70835	0.990441	no
gene:SpnNT_01416	NA	Chromosome:1458302-1459257	ΔORF2	ΔORF2+peptide	OK	3.43771	3.36335	-0.0315467	-0.0350427	0.9623	0.994855	no
gene:SpnNT_01416	NA	Chromosome:1458302-1459257	110.58+peptide	ΔORF2+peptide	OK	3.13025	3.36335	0.103623	0.114617	0.8457	0.994748	no
gene:SpnNT_01417	doc_2	Chromosome:1459374-1460021	110.58	ΔORF2	OK	65.323	121.67	0.897308	0.894251	0.1137	0.511299	no
gene:SpnNT_01417	doc_2	Chromosome:1459374-1460021	110.58	110.58+peptide	OK	65.323	67.7166	0.0519168	0.049066	0.92925	0.994748	no
gene:SpnNT_01417	doc_2	Chromosome:1459374-1460021	ΔORF2	110.58+peptide	OK	121.67	67.7166	-0.845391	-0.947389	0.10805	0.499941	no
gene:SpnNT_01417	doc_2	Chromosome:1459374-1460021	110.58	ΔORF2+peptide	OK	65.323	74.3508	0.186757	0.184599	0.733	0.994748	no
gene:SpnNT_01417	doc_2	Chromosome:1459374-1460021	ΔORF2	ΔORF2+peptide	OK	121.67	74.3508	-0.710551	-0.849149	0.1466	0.583997	no
gene:SpnNT_01417	doc_2	Chromosome:1459374-1460021	110.58+peptide	ΔORF2+peptide	OK	67.7166	74.3508	0.13484	0.149552	0.7978	0.994748	no
gene:SpnNT_01418	NA	Chromosome:1459374-1460021	110.58	ΔORF2	OK	655.41	862.97	0.396913	0.660619	0.2552	0.754219	no
gene:SpnNT_01418	NA	Chromosome:1459374-1460021	110.58	110.58+peptide	OK	655.41	430.077	-0.607801	-0.934927	0.1057	0.494473	no

gene:SpnNT_01418	NA	Chromosome:1459374-1460021	ΔORF2	110.58+peptide	OK	862.97	430.077	-1.00471	-1.53336	0.00975	0.0985666	no
gene:SpnNT_01418	NA	Chromosome:1459374-1460021	110.58	ΔORF2+peptide	OK	655.41	471.241	-0.475931	-0.761672	0.19025	0.668572	no
gene:SpnNT_01418	NA	Chromosome:1459374-1460021	ΔORF2	ΔORF2+peptide	OK	862.97	471.241	-0.872844	-1.38505	0.01755	0.150419	no
gene:SpnNT_01418	NA	Chromosome:1459374-1460021	110.58+peptide	ΔORF2+peptide	OK	430.077	471.241	0.131869	0.194687	0.7352	0.994748	no
gene:SpnNT_01419	NA	Chromosome:1460309-1460666	110.58	ΔORF2	OK	24.5851	33.529	0.447625	0.693873	0.23415	0.72774	no
gene:SpnNT_01419	NA	Chromosome:1460309-1460666	110.58	110.58+peptide	OK	24.5851	32.1446	0.386793	0.606831	0.29835	0.803696	no
gene:SpnNT_01419	NA	Chromosome:1460309-1460666	ΔORF2	110.58+peptide	OK	33.529	32.1446	-0.0608314	-0.097093	0.8627	0.994748	no
gene:SpnNT_01419	NA	Chromosome:1460309-1460666	110.58	ΔORF2+peptide	OK	24.5851	47.6639	0.955114	1.53267	0.01035	0.102811	no
gene:SpnNT_01419	NA	Chromosome:1460309-1460666	ΔORF2	ΔORF2+peptide	OK	33.529	47.6639	0.50749	0.829166	0.15075	0.593025	no
gene:SpnNT_01419	NA	Chromosome:1460309-1460666	110.58+peptide	ΔORF2+peptide	OK	32.1446	47.6639	0.568321	0.941063	0.11165	0.508705	no
gene:SpnNT_01420	hsdM_1	Chromosome:1460948-1462171	110.58	ΔORF2	OK	33.1161	36.1252	0.125473	0.0767509	0.888	0.994748	no
gene:SpnNT_01420	hsdM_1	Chromosome:1460948-1462171	110.58	110.58+peptide	OK	33.1161	40.4304	0.287908	0.162046	0.756	0.994748	no
gene:SpnNT_01420	hsdM_1	Chromosome:1460948-1462171	ΔORF2	110.58+peptide	OK	36.1252	40.4304	0.162435	0.0883115	0.8733	0.994748	no
gene:SpnNT_01420	hsdM_1	Chromosome:1460948-1462171	110.58	ΔORF2+peptide	OK	33.1161	54.8183	0.727127	0.451046	0.42285	0.906048	no
gene:SpnNT_01420	hsdM_1	Chromosome:1460948-1462171	ΔORF2	ΔORF2+peptide	OK	36.1252	54.8183	0.601653	0.357942	0.5416	0.961494	no
gene:SpnNT_01420	hsdM_1	Chromosome:1460948-1462171	110.58+peptide	ΔORF2+peptide	OK	40.4304	54.8183	0.439218	0.241438	0.6673	0.983929	no
gene:SpnNT_01421	hsdM_2	Chromosome:1460948-1462171	110.58	ΔORF2	OK	31.3005	41.5652	0.40919	0.783071	0.1705	0.629162	no
gene:SpnNT_01421	hsdM_2	Chromosome:1460948-1462171	110.58	110.58+peptide	OK	31.3005	40.0259	0.354747	0.662611	0.2552	0.754219	no
gene:SpnNT_01421	hsdM_2	Chromosome:1460948-1462171	ΔORF2	110.58+peptide	OK	41.5652	40.0259	-0.054443	-0.103632	0.8593	0.994748	no
gene:SpnNT_01421	hsdM_2	Chromosome:1460948-1462171	110.58	ΔORF2+peptide	OK	31.3005	55.9749	0.838593	1.61324	0.00535	0.0633027	no
gene:SpnNT_01421	hsdM_2	Chromosome:1460948-1462171	ΔORF2	ΔORF2+peptide	OK	41.5652	55.9749	0.429403	0.842814	0.1442	0.579207	no
gene:SpnNT_01421	hsdM_2	Chromosome:1460948-1462171	110.58+peptide	ΔORF2+peptide	OK	40.0259	55.9749	0.483846	0.925772	0.11625	0.517109	no
gene:SpnNT_01422	NA	Chromosome:1462678-1463500	110.58	ΔORF2	OK	15.4466	13.1531	-0.231888	-0.399611	0.4887	0.939082	no
gene:SpnNT_01422	NA	Chromosome:1462678-1463500	110.58	110.58+peptide	OK	15.4466	31.4143	1.02413	1.84538	0.0019	0.0281715	yes
gene:SpnNT_01422	NA	Chromosome:1462678-1463500	ΔORF2	110.58+peptide	OK	13.1531	31.4143	1.25602	2.22422	0.00035	0.00717609	yes
gene:SpnNT_01422	NA	Chromosome:1462678-1463500	110.58	ΔORF2+peptide	OK	15.4466	33.2372	1.10551	2.0111	0.00085	0.0148124	yes
gene:SpnNT_01422	NA	Chromosome:1462678-1463500	ΔORF2	ΔORF2+peptide	OK	13.1531	33.2372	1.3374	2.39023	2.00E-04	0.00450928	yes
gene:SpnNT_01422	NA	Chromosome:1462678-1463500	110.58+peptide	ΔORF2+peptide	OK	31.4143	33.2372	0.08138	0.152616	0.7934	0.994748	no
gene:SpnNT_01423	NA	Chromosome:1463557-1463845	110.58	ΔORF2	OK	12.0279	12.1546	0.0151191	0.0177521	0.9714	0.99536	no
gene:SpnNT_01423	NA	Chromosome:1463557-1463845	110.58	110.58+peptide	OK	12.0279	30.6747	1.35066	1.7699	0.00355	0.0460763	yes
gene:SpnNT_01423	NA	Chromosome:1463557-1463845	ΔORF2	110.58+peptide	OK	12.1546	30.6747	1.33554	1.6997	0.0056	0.0654347	no
gene:SpnNT_01423	NA	Chromosome:1463557-1463845	110.58	ΔORF2+peptide	OK	12.0279	26.6104	1.1456	1.46954	0.01275	0.119932	no
gene:SpnNT_01423	NA	Chromosome:1463557-1463845	ΔORF2	ΔORF2+peptide	OK	12.1546	26.6104	1.13048	1.41006	0.01605	0.141158	no
gene:SpnNT_01423	NA	Chromosome:1463557-1463845	110.58+peptide	ΔORF2+peptide	OK	30.6747	26.6104	-0.205059	-0.290066	0.62705	0.980887	no
gene:SpnNT_01424	NA	Chromosome:1463857-1464598	110.58	ΔORF2	OK	23.5461	18.4118	-0.354858	-0.625354	0.27225	0.774266	no
gene:SpnNT_01424	NA	Chromosome:1463857-1464598	110.58	110.58+peptide	OK	23.5461	49.6505	1.07632	2.02965	6.00E-04	0.0111203	yes
gene:SpnNT_01424	NA	Chromosome:1463857-1464598	ΔORF2	110.58+peptide	OK	18.4118	49.6505	1.43118	2.61286	5.00E-05	0.0013612	yes
gene:SpnNT_01424	NA	Chromosome:1463857-1464598	110.58	ΔORF2+peptide	OK	23.5461	46.6984	0.987888	1.85499	0.00105	0.0174849	yes
gene:SpnNT_01424	NA	Chromosome:1463857-1464598	ΔORF2	ΔORF2+peptide	OK	18.4118	46.6984	1.34275	2.44167	1.00E-04	0.0025332	yes
gene:SpnNT_01424	NA	Chromosome:1463857-1464598	110.58+peptide	ΔORF2+peptide	OK	49.6505	46.6984	-0.0884345	-0.17289	0.7624	0.994748	no
gene:SpnNT_01425	axe1-6A_2	Chromosome:1464619-1465444	110.58	ΔORF2	OK	30.3663	24.6223	-0.302502	-0.567099	0.31645	0.822432	no
gene:SpnNT_01425	axe1-6A_2	Chromosome:1464619-1465444	110.58	110.58+peptide	OK	30.3663	61.1156	1.00907	1.96038	0.00125	0.0201258	yes
gene:SpnNT_01425	axe1-6A_2	Chromosome:1464619-1465444	ΔORF2	110.58+peptide	OK	24.6223	61.1156	1.31157	2.5166	5.00E-05	0.0013612	yes
gene:SpnNT_01425	axe1-6A_2	Chromosome:1464619-1465444	110.58	ΔORF2+peptide	OK	30.3663	70.0003	1.20489	2.39368	5.00E-05	0.0013612	yes
gene:SpnNT_01425	axe1-6A_2	Chromosome:1464619-1465444	ΔORF2	ΔORF2+peptide	OK	24.6223	70.0003	1.50739	2.95599	5.00E-05	0.0013612	yes

gene:SpnNT_01425	axe1-6A_2	Chromosome:1464619-1465444	110.58+peptide	ΔORF2+peptide	OK	61.1156	70.0003	0.195819	0.399334	0.48915	0.939082	no
gene:SpnNT_01426	thiI	Chromosome:1465579-1466794	110.58	ΔORF2	OK	69.9155	65.6763	-0.0902397	-0.198913	0.72905	0.994748	no
gene:SpnNT_01426	thiI	Chromosome:1465579-1466794	110.58	110.58+peptide	OK	69.9155	80.0584	0.195442	0.430489	0.4548	0.921244	no
gene:SpnNT_01426	thiI	Chromosome:1465579-1466794	ΔORF2	110.58+peptide	OK	65.6763	80.0584	0.285681	0.628901	0.27675	0.780052	no
gene:SpnNT_01426	thiI	Chromosome:1465579-1466794	110.58	ΔORF2+peptide	OK	69.9155	86.1515	0.301264	0.671079	0.24655	0.74356	no
gene:SpnNT_01426	thiI	Chromosome:1465579-1466794	ΔORF2	ΔORF2+peptide	OK	65.6763	86.1515	0.391504	0.87159	0.13245	0.554212	no
gene:SpnNT_01426	thiI	Chromosome:1465579-1466794	110.58+peptide	ΔORF2+peptide	OK	80.0584	86.1515	0.105823	0.235411	0.68215	0.986608	no
gene:SpnNT_01427	iscS_2	Chromosome:1466802-1468005	110.58	ΔORF2	OK	72.9102	71.551	-0.0271471	-0.0597808	0.91845	0.994748	no
gene:SpnNT_01427	iscS_2	Chromosome:1466802-1468005	110.58	110.58+peptide	OK	72.9102	84.6086	0.214685	0.476824	0.402	0.894197	no
gene:SpnNT_01427	iscS_2	Chromosome:1466802-1468005	ΔORF2	110.58+peptide	OK	71.551	84.6086	0.241832	0.535677	0.3423	0.846365	no
gene:SpnNT_01427	iscS_2	Chromosome:1466802-1468005	110.58	ΔORF2+peptide	OK	72.9102	92.5019	0.343363	0.765807	0.17925	0.645832	no
gene:SpnNT_01427	iscS_2	Chromosome:1466802-1468005	ΔORF2	ΔORF2+peptide	OK	71.551	92.5019	0.37051	0.824116	0.14775	0.586087	no
gene:SpnNT_01427	iscS_2	Chromosome:1466802-1468005	110.58+peptide	ΔORF2+peptide	OK	84.6086	92.5019	0.128678	0.288726	0.61095	0.979429	no
gene:SpnNT_01428	NA	Chromosome:1468082-1468532	110.58	ΔORF2	OK	104.492	126.259	0.272992	0.545221	0.33985	0.844213	no
gene:SpnNT_01428	NA	Chromosome:1468082-1468532	110.58	110.58+peptide	OK	104.492	67.0437	-0.640221	-1.23745	0.03465	0.246304	no
gene:SpnNT_01428	NA	Chromosome:1468082-1468532	ΔORF2	110.58+peptide	OK	126.259	67.0437	-0.913213	-1.77612	0.00285	0.0382781	yes
gene:SpnNT_01428	NA	Chromosome:1468082-1468532	110.58	ΔORF2+peptide	OK	104.492	71.9928	-0.537469	-1.02643	0.07495	0.404272	no
gene:SpnNT_01428	NA	Chromosome:1468082-1468532	ΔORF2	ΔORF2+peptide	OK	126.259	71.9928	-0.810461	-1.55721	0.0069	0.0765358	no
gene:SpnNT_01428	NA	Chromosome:1468082-1468532	110.58+peptide	ΔORF2+peptide	OK	67.0437	71.9928	0.102752	0.191517	0.73915	0.994748	no
gene:SpnNT_01429	ftsK_2	Chromosome:1468575-1470879	110.58	ΔORF2	OK	86.4307	85.3758	-0.0177158	-0.0409887	0.9411	0.994855	no
gene:SpnNT_01429	ftsK_2	Chromosome:1468575-1470879	110.58	110.58+peptide	OK	86.4307	90.4666	0.0658424	0.152137	0.78255	0.994748	no
gene:SpnNT_01429	ftsK_2	Chromosome:1468575-1470879	ΔORF2	110.58+peptide	OK	85.3758	90.4666	0.0835581	0.193224	0.7358	0.994748	no
gene:SpnNT_01429	ftsK_2	Chromosome:1468575-1470879	110.58	ΔORF2+peptide	OK	86.4307	96.4594	0.158379	0.365813	0.51105	0.949455	no
gene:SpnNT_01429	ftsK_2	Chromosome:1468575-1470879	ΔORF2	ΔORF2+peptide	OK	85.3758	96.4594	0.176094	0.407052	0.4717	0.928525	no
gene:SpnNT_01429	ftsK_2	Chromosome:1468575-1470879	110.58+peptide	ΔORF2+peptide	OK	90.4666	96.4594	0.0925362	0.213621	0.7016	0.990367	no
gene:SpnNT_01430	fruA	Chromosome:1470964-1474562	110.58	ΔORF2	OK	26.8776	27.212	0.0178384	0.0317814	0.95605	0.994855	no
gene:SpnNT_01430	fruA	Chromosome:1470964-1474562	110.58	110.58+peptide	OK	26.8776	25.6864	-0.0654045	-0.115727	0.8426	0.994748	no
gene:SpnNT_01430	fruA	Chromosome:1470964-1474562	ΔORF2	110.58+peptide	OK	27.212	25.6864	-0.0832429	-0.148529	0.79815	0.994748	no
gene:SpnNT_01430	fruA	Chromosome:1470964-1474562	110.58	ΔORF2+peptide	OK	26.8776	23.4294	-0.198088	-0.346262	0.55095	0.964328	no
gene:SpnNT_01430	fruA	Chromosome:1470964-1474562	ΔORF2	ΔORF2+peptide	OK	27.212	23.4294	-0.215927	-0.380542	0.50875	0.947472	no
gene:SpnNT_01430	fruA	Chromosome:1470964-1474562	110.58+peptide	ΔORF2+peptide	OK	25.6864	23.4294	-0.132684	-0.232267	0.6876	0.98828	no
gene:SpnNT_01431	lacC_2	Chromosome:1470964-1474562	110.58	ΔORF2	OK	20.6091	20.0707	-0.0381915	-0.036213	0.9487	0.994855	no
gene:SpnNT_01431	lacC_2	Chromosome:1470964-1474562	110.58	110.58+peptide	OK	20.6091	17.8917	-0.203987	-0.193494	0.73735	0.994748	no
gene:SpnNT_01431	lacC_2	Chromosome:1470964-1474562	ΔORF2	110.58+peptide	OK	20.0707	17.8917	-0.165796	-0.15048	0.7991	0.994748	no
gene:SpnNT_01431	lacC_2	Chromosome:1470964-1474562	110.58	ΔORF2+peptide	OK	20.6091	22.1997	0.107262	0.108766	0.85435	0.994748	no
gene:SpnNT_01431	lacC_2	Chromosome:1470964-1474562	ΔORF2	ΔORF2+peptide	OK	20.0707	22.1997	0.145454	0.140285	0.8132	0.994748	no
gene:SpnNT_01431	lacC_2	Chromosome:1470964-1474562	110.58+peptide	ΔORF2+peptide	OK	17.8917	22.1997	0.311249	0.300309	0.6074	0.978071	no
gene:SpnNT_01432	lacR_2	Chromosome:1470964-1474562	110.58	ΔORF2	OK	20.9664	19.299	-0.11955	-0.0974089	0.86705	0.994748	no
gene:SpnNT_01432	lacR_2	Chromosome:1470964-1474562	110.58	110.58+peptide	OK	20.9664	19.3831	-0.113275	-0.0902773	0.8755	0.994748	no
gene:SpnNT_01432	lacR_2	Chromosome:1470964-1474562	ΔORF2	110.58+peptide	OK	19.299	19.3831	0.0062745	0.005108	0.9927	0.998522	no
gene:SpnNT_01432	lacR_2	Chromosome:1470964-1474562	110.58	ΔORF2+peptide	OK	20.9664	20.2451	-0.050502	-0.0407412	0.9469	0.994855	no
gene:SpnNT_01432	lacR_2	Chromosome:1470964-1474562	ΔORF2	ΔORF2+peptide	OK	19.299	20.2451	0.0690478	0.0569293	0.92455	0.994748	no
gene:SpnNT_01432	lacR_2	Chromosome:1470964-1474562	110.58+peptide	ΔORF2+peptide	OK	19.3831	20.2451	0.0627733	0.0505976	0.934	0.994855	no
gene:SpnNT_01433	SpnNT_01433	Chromosome:1474722-1474813	110.58	ΔORF2	OK	1188.99	1444.99	0.281322	0.295692	0.5939	0.975539	no
gene:SpnNT_01433	SpnNT_01433	Chromosome:1474722-1474813	110.58	110.58+peptide	OK	1188.99	1331.63	0.163458	0.161559	0.77145	0.994748	no

gene:SpnNT_01433	SpnNT_01433	Chromosome:1474722-1474813	ΔORF2	110.58+peptide	OK	1444.99	1331.63	-0.117863	-0.123438	0.8259	0.994748	no
gene:SpnNT_01433	SpnNT_01433	Chromosome:1474722-1474813	110.58	ΔORF2+peptide	OK	1188.99	1197.43	0.0102086	0.0106426	0.9543	0.994855	no
gene:SpnNT_01433	SpnNT_01433	Chromosome:1474722-1474813	ΔORF2	ΔORF2+peptide	OK	1444.99	1197.43	-0.271113	-0.301576	0.64045	0.980887	no
gene:SpnNT_01433	SpnNT_01433	Chromosome:1474722-1474813	110.58+peptide	ΔORF2+peptide	OK	1331.63	1197.43	-0.15325	-0.159197	0.8021	0.994748	no
gene:SpnNT_01434	ssrA	Chromosome:1474983-1475328	110.58	ΔORF2	HIDATA	0	0	0	0	1	1	no
gene:SpnNT_01434	ssrA	Chromosome:1474983-1475328	110.58	110.58+peptide	HIDATA	0	0	0	0	1	1	no
gene:SpnNT_01434	ssrA	Chromosome:1474983-1475328	ΔORF2	110.58+peptide	HIDATA	0	0	0	0	1	1	no
gene:SpnNT_01434	ssrA	Chromosome:1474983-1475328	110.58	ΔORF2+peptide	HIDATA	0	0	0	0	1	1	no
gene:SpnNT_01434	ssrA	Chromosome:1474983-1475328	ΔORF2	ΔORF2+peptide	HIDATA	0	0	0	0	1	1	no
gene:SpnNT_01434	ssrA	Chromosome:1474983-1475328	110.58+peptide	ΔORF2+peptide	HIDATA	0	0	0	0	1	1	no
gene:SpnNT_01435	tqsA	Chromosome:1475973-1477083	110.58	ΔORF2	OK	103.063	141.405	0.456301	1.00425	0.0778	0.414155	no
gene:SpnNT_01435	tqsA	Chromosome:1475973-1477083	110.58	110.58+peptide	OK	103.063	105.678	0.0361404	0.0801486	0.8824	0.994748	no
gene:SpnNT_01435	tqsA	Chromosome:1475973-1477083	ΔORF2	110.58+peptide	OK	141.405	105.678	-0.42016	-0.911883	0.10935	0.502466	no
gene:SpnNT_01435	tqsA	Chromosome:1475973-1477083	110.58	ΔORF2+peptide	OK	103.063	115.827	0.168443	0.378918	0.50255	0.944788	no
gene:SpnNT_01435	tqsA	Chromosome:1475973-1477083	ΔORF2	ΔORF2+peptide	OK	141.405	115.827	-0.287858	-0.633327	0.2687	0.770416	no
gene:SpnNT_01435	tqsA	Chromosome:1475973-1477083	110.58+peptide	ΔORF2+peptide	OK	105.678	115.827	0.132302	0.293309	0.60045	0.976678	no
gene:SpnNT_01436	dacA	Chromosome:1477185-1478427	110.58	ΔORF2	OK	117.188	136.222	0.217129	0.494426	0.38505	0.881745	no
gene:SpnNT_01436	dacA	Chromosome:1477185-1478427	110.58	110.58+peptide	OK	117.188	75.3971	-0.636249	-1.42344	0.01295	0.120861	no
gene:SpnNT_01436	dacA	Chromosome:1477185-1478427	ΔORF2	110.58+peptide	OK	136.222	75.3971	-0.853379	-1.90583	0.00135	0.021343	yes
gene:SpnNT_01436	dacA	Chromosome:1477185-1478427	110.58	ΔORF2+peptide	OK	117.188	88.5949	-0.403533	-0.916372	0.10595	0.495113	no
gene:SpnNT_01436	dacA	Chromosome:1477185-1478427	ΔORF2	ΔORF2+peptide	OK	136.222	88.5949	-0.620662	-1.40688	0.01825	0.155101	no
gene:SpnNT_01436	dacA	Chromosome:1477185-1478427	110.58+peptide	ΔORF2+peptide	OK	75.3971	88.5949	0.232716	0.518352	0.3719	0.869075	no
gene:SpnNT_01437	sufB_1	Chromosome:1478657-1480070	110.58	ΔORF2	OK	301.009	341.134	0.180533	0.399803	0.46845	0.927504	no
gene:SpnNT_01437	sufB_1	Chromosome:1478657-1480070	110.58	110.58+peptide	OK	301.009	390.013	0.373717	0.823714	0.14425	0.579207	no
gene:SpnNT_01437	sufB_1	Chromosome:1478657-1480070	ΔORF2	110.58+peptide	OK	341.134	390.013	0.193185	0.431057	0.4517	0.921244	no
gene:SpnNT_01437	sufB_1	Chromosome:1478657-1480070	110.58	ΔORF2+peptide	OK	301.009	466.402	0.631766	1.38503	0.0142	0.129128	no
gene:SpnNT_01437	sufB_1	Chromosome:1478657-1480070	ΔORF2	ΔORF2+peptide	OK	341.134	466.402	0.451233	1.00133	0.07355	0.399141	no
gene:SpnNT_01437	sufB_1	Chromosome:1478657-1480070	110.58+peptide	ΔORF2+peptide	OK	390.013	466.402	0.258049	0.569916	0.31725	0.823011	no
gene:SpnNT_01438	nifU	Chromosome:1480127-1481781	110.58	ΔORF2	OK	181.953	199.325	0.131563	0.0980732	0.8615	0.994748	no
gene:SpnNT_01438	nifU	Chromosome:1480127-1481781	110.58	110.58+peptide	OK	181.953	259.433	0.511797	0.382525	0.51265	0.950409	no
gene:SpnNT_01438	nifU	Chromosome:1480127-1481781	ΔORF2	110.58+peptide	OK	199.325	259.433	0.380233	0.275724	0.6326	0.980887	no
gene:SpnNT_01438	nifU	Chromosome:1480127-1481781	110.58	ΔORF2+peptide	OK	181.953	286.445	0.654697	0.495781	0.3989	0.892547	no
gene:SpnNT_01438	nifU	Chromosome:1480127-1481781	ΔORF2	ΔORF2+peptide	OK	199.325	286.445	0.523133	0.384049	0.50575	0.945292	no
gene:SpnNT_01438	nifU	Chromosome:1480127-1481781	110.58+peptide	ΔORF2+peptide	OK	259.433	286.445	0.1429	0.105177	0.85725	0.994748	no
gene:SpnNT_01439	csd	Chromosome:1480127-1481781	110.58	ΔORF2	OK	264.289	278.31	0.0745776	0.153955	0.78885	0.994748	no
gene:SpnNT_01439	csd	Chromosome:1480127-1481781	110.58	110.58+peptide	OK	264.289	339.99	0.36338	0.745923	0.1891	0.666498	no
gene:SpnNT_01439	csd	Chromosome:1480127-1481781	ΔORF2	110.58+peptide	OK	278.31	339.99	0.288803	0.593712	0.2953	0.799613	no
gene:SpnNT_01439	csd	Chromosome:1480127-1481781	110.58	ΔORF2+peptide	OK	264.289	378.468	0.518056	1.06249	0.0645	0.368753	no
gene:SpnNT_01439	csd	Chromosome:1480127-1481781	ΔORF2	ΔORF2+peptide	OK	278.31	378.468	0.443479	0.910881	0.11335	0.510776	no
gene:SpnNT_01439	csd	Chromosome:1480127-1481781	110.58+peptide	ΔORF2+peptide	OK	339.99	378.468	0.154676	0.315924	0.57395	0.969538	no
gene:SpnNT_01440	sufB_2	Chromosome:1481791-1483054	110.58	ΔORF2	OK	242.082	262.329	0.115884	0.262517	0.64025	0.980887	no
gene:SpnNT_01440	sufB_2	Chromosome:1481791-1483054	110.58	110.58+peptide	OK	242.082	297.724	0.298482	0.675482	0.2403	0.735646	no
gene:SpnNT_01440	sufB_2	Chromosome:1481791-1483054	ΔORF2	110.58+peptide	OK	262.329	297.724	0.182599	0.411178	0.4692	0.927613	no
gene:SpnNT_01440	sufB_2	Chromosome:1481791-1483054	110.58	ΔORF2+peptide	OK	242.082	357.757	0.563487	1.26807	0.03085	0.226786	no
gene:SpnNT_01440	sufB_2	Chromosome:1481791-1483054	ΔORF2	ΔORF2+peptide	OK	262.329	357.757	0.447603	1.00234	0.08035	0.422756	no

gene:SpnNT_01440	sufB_2	Chromosome:1481791-1483054	110.58+peptide	ΔORF2+peptide	OK	297.724	357.757	0.265004	0.59285	0.2976	0.802694	no
gene:SpnNT_01441	NA	Chromosome:1483081-1483852	110.58	ΔORF2	OK	182.165	193.735	0.0888407	0.200565	0.7287	0.994748	no
gene:SpnNT_01441	NA	Chromosome:1483081-1483852	110.58	110.58+peptide	OK	182.165	214.403	0.235083	0.529728	0.35325	0.856141	no
gene:SpnNT_01441	NA	Chromosome:1483081-1483852	ΔORF2	110.58+peptide	OK	193.735	214.403	0.146242	0.330258	0.5605	0.968621	no
gene:SpnNT_01441	NA	Chromosome:1483081-1483852	110.58	ΔORF2+peptide	OK	182.165	265.667	0.544376	1.23273	0.03395	0.242775	no
gene:SpnNT_01441	NA	Chromosome:1483081-1483852	ΔORF2	ΔORF2+peptide	OK	193.735	265.667	0.455535	1.03383	0.07325	0.398998	no
gene:SpnNT_01441	NA	Chromosome:1483081-1483852	110.58+peptide	ΔORF2+peptide	OK	214.403	265.667	0.309293	0.700612	0.2204	0.708672	no
gene:SpnNT_01442	NA	Chromosome:1484001-1484196	110.58	ΔORF2	OK	632.521	890.757	0.493918	0.840579	0.12615	0.537797	no
gene:SpnNT_01442	NA	Chromosome:1484001-1484196	110.58	110.58+peptide	OK	632.521	611.986	-0.0476153	-0.0842374	0.8799	0.994748	no
gene:SpnNT_01442	NA	Chromosome:1484001-1484196	ΔORF2	110.58+peptide	OK	890.757	611.986	-0.541533	-0.928375	0.09335	0.461544	no
gene:SpnNT_01442	NA	Chromosome:1484001-1484196	110.58	ΔORF2+peptide	OK	632.521	745.028	0.23618	0.427831	0.44805	0.919648	no
gene:SpnNT_01442	NA	Chromosome:1484001-1484196	ΔORF2	ΔORF2+peptide	OK	890.757	745.028	-0.257738	-0.451759	0.4175	0.902759	no
gene:SpnNT_01442	NA	Chromosome:1484001-1484196	110.58+peptide	ΔORF2+peptide	OK	611.986	745.028	0.283795	0.518363	0.35895	0.859829	no
gene:SpnNT_01443	dnaX_2	Chromosome:1484223-1486376	110.58	ΔORF2	OK	55.0276	58.9663	0.0997337	0.168951	0.7658	0.994748	no
gene:SpnNT_01443	dnaX_2	Chromosome:1484223-1486376	110.58	110.58+peptide	OK	55.0276	66.5485	0.274251	0.470478	0.41435	0.90048	no
gene:SpnNT_01443	dnaX_2	Chromosome:1484223-1486376	ΔORF2	110.58+peptide	OK	58.9663	66.5485	0.174518	0.304963	0.59985	0.976542	no
gene:SpnNT_01443	dnaX_2	Chromosome:1484223-1486376	110.58	ΔORF2+peptide	OK	55.0276	79.3065	0.527283	0.91715	0.1094	0.502466	no
gene:SpnNT_01443	dnaX_2	Chromosome:1484223-1486376	ΔORF2	ΔORF2+peptide	OK	58.9663	79.3065	0.427549	0.757931	0.189	0.666324	no
gene:SpnNT_01443	dnaX_2	Chromosome:1484223-1486376	110.58+peptide	ΔORF2+peptide	OK	66.5485	79.3065	0.253032	0.454799	0.42565	0.908192	no
gene:SpnNT_01444	msrC	Chromosome:1484223-1486376	110.58	ΔORF2	OK	210.624	220.686	0.0673229	0.0972398	0.8633	0.994748	no
gene:SpnNT_01444	msrC	Chromosome:1484223-1486376	110.58	110.58+peptide	OK	210.624	195.587	-0.106861	-0.147916	0.7944	0.994748	no
gene:SpnNT_01444	msrC	Chromosome:1484223-1486376	ΔORF2	110.58+peptide	OK	220.686	195.587	-0.174184	-0.243014	0.67305	0.984845	no
gene:SpnNT_01444	msrC	Chromosome:1484223-1486376	110.58	ΔORF2+peptide	OK	210.624	193.209	-0.124512	-0.160536	0.7764	0.994748	no
gene:SpnNT_01444	msrC	Chromosome:1484223-1486376	ΔORF2	ΔORF2+peptide	OK	220.686	193.209	-0.191835	-0.249035	0.6622	0.982966	no
gene:SpnNT_01444	msrC	Chromosome:1484223-1486376	110.58+peptide	ΔORF2+peptide	OK	195.587	193.209	-0.0176505	-0.0221328	0.96855	0.994855	no
gene:SpnNT_01445	NA	Chromosome:1486569-1487896	110.58	ΔORF2	OK	2.1467	2.14949	0.00187591	0.00115071	0.974	0.99536	no
gene:SpnNT_01445	NA	Chromosome:1486569-1487896	110.58	110.58+peptide	OK	2.1467	2.60759	0.280595	0.158416	0.80635	0.994748	no
gene:SpnNT_01445	NA	Chromosome:1486569-1487896	ΔORF2	110.58+peptide	OK	2.14949	2.60759	0.278719	0.172118	0.7683	0.994748	no
gene:SpnNT_01445	NA	Chromosome:1486569-1487896	110.58	ΔORF2+peptide	OK	2.1467	3.75887	0.808179	0.473519	0.49265	0.941402	no
gene:SpnNT_01445	NA	Chromosome:1486569-1487896	ΔORF2	ΔORF2+peptide	OK	2.14949	3.75887	0.806303	0.520687	0.40275	0.894379	no
gene:SpnNT_01445	NA	Chromosome:1486569-1487896	110.58+peptide	ΔORF2+peptide	OK	2.60759	3.75887	0.527584	0.311009	0.5965	0.97629	no
gene:SpnNT_01446	NA	Chromosome:1486569-1487896	110.58	ΔORF2	OK	6.19445	5.43485	-0.188737	-0.0949741	0.8711	0.994748	no
gene:SpnNT_01446	NA	Chromosome:1486569-1487896	110.58	110.58+peptide	OK	6.19445	12.7478	1.0412	0.569399	0.34945	0.853357	no
gene:SpnNT_01446	NA	Chromosome:1486569-1487896	ΔORF2	110.58+peptide	OK	5.43485	12.7478	1.22993	0.785972	0.20045	0.683196	no
gene:SpnNT_01446	NA	Chromosome:1486569-1487896	110.58	ΔORF2+peptide	OK	6.19445	8.01099	0.371003	0.201927	0.8063	0.994748	no
gene:SpnNT_01446	NA	Chromosome:1486569-1487896	ΔORF2	ΔORF2+peptide	OK	5.43485	8.01099	0.55974	0.355381	0.6637	0.982966	no
gene:SpnNT_01446	NA	Chromosome:1486569-1487896	110.58+peptide	ΔORF2+peptide	OK	12.7478	8.01099	-0.670195	-0.489393	0.5673	0.968621	no
gene:SpnNT_01447	NA	Chromosome:1486569-1487896	110.58	ΔORF2	OK	4.76846	5.09345	0.0951176	0.102533	0.86165	0.994748	no
gene:SpnNT_01447	NA	Chromosome:1486569-1487896	110.58	110.58+peptide	OK	4.76846	8.35373	0.808895	0.910918	0.1202	0.524705	no
gene:SpnNT_01447	NA	Chromosome:1486569-1487896	ΔORF2	110.58+peptide	OK	5.09345	8.35373	0.713777	0.812412	0.1662	0.621332	no
gene:SpnNT_01447	NA	Chromosome:1486569-1487896	110.58	ΔORF2+peptide	OK	4.76846	12.8995	1.43572	1.53376	0.0098	0.0986919	no
gene:SpnNT_01447	NA	Chromosome:1486569-1487896	ΔORF2	ΔORF2+peptide	OK	5.09345	12.8995	1.34061	1.44593	0.0143	0.129589	no
gene:SpnNT_01447	NA	Chromosome:1486569-1487896	110.58+peptide	ΔORF2+peptide	OK	8.35373	12.8995	0.626829	0.70632	0.2231	0.712784	no
gene:SpnNT_01448	rpsA_2	Chromosome:1487981-1489184	110.58	ΔORF2	OK	2057.37	2217.82	0.108338	0.219193	0.6994	0.990367	no
gene:SpnNT_01448	rpsA_2	Chromosome:1487981-1489184	110.58	110.58+peptide	OK	2057.37	2750.59	0.418938	0.843765	0.1412	0.57363	no

gene:SpnNT_01448	rpsA_2	Chromosome:1487981-1489184	ΔORF2	110.58+peptide	OK	2217.82	2750.59	0.3106	0.636461	0.26705	0.768422	no
gene:SpnNT_01448	rpsA_2	Chromosome:1487981-1489184	110.58	ΔORF2+peptide	OK	2057.37	2956.07	0.522878	1.05111	0.0654	0.372112	no
gene:SpnNT_01448	rpsA_2	Chromosome:1487981-1489184	ΔORF2	ΔORF2+peptide	OK	2217.82	2956.07	0.41454	0.847779	0.13525	0.559505	no
gene:SpnNT_01448	rpsA_2	Chromosome:1487981-1489184	110.58+peptide	ΔORF2+peptide	OK	2750.59	2956.07	0.103941	0.211585	0.6964	0.989512	no
gene:SpnNT_01449	SpnNT_01449	Chromosome:1489345-1489417	110.58	ΔORF2	OK	6763.35	6653.44	-0.0236386	-0.0216296	0.95085	0.994855	no
gene:SpnNT_01449	SpnNT_01449	Chromosome:1489345-1489417	110.58	110.58+peptide	OK	6763.35	6834.6	0.0151174	0.0134587	0.9526	0.994855	no
gene:SpnNT_01449	SpnNT_01449	Chromosome:1489345-1489417	ΔORF2	110.58+peptide	OK	6653.44	6834.6	0.038756	0.0337476	0.9512	0.994855	no
gene:SpnNT_01449	SpnNT_01449	Chromosome:1489345-1489417	110.58	ΔORF2+peptide	OK	6763.35	2406.42	-1.49085	-1.97706	0.108	0.499941	no
gene:SpnNT_01449	SpnNT_01449	Chromosome:1489345-1489417	ΔORF2	ΔORF2+peptide	OK	6653.44	2406.42	-1.46721	-1.85474	0.1142	0.512669	no
gene:SpnNT_01449	SpnNT_01449	Chromosome:1489345-1489417	110.58+peptide	ΔORF2+peptide	OK	6834.6	2406.42	-1.50597	-1.80894	0.1131	0.510701	no
gene:SpnNT_01450	SpnNT_01450	Chromosome:1489426-1489507	110.58	ΔORF2	OK	2558.5	3059.08	0.257801	0.224912	0.703	0.990367	no
gene:SpnNT_01450	SpnNT_01450	Chromosome:1489426-1489507	110.58	110.58+peptide	OK	2558.5	6875.44	1.42616	1.36885	0.02335	0.187285	no
gene:SpnNT_01450	SpnNT_01450	Chromosome:1489426-1489507	ΔORF2	110.58+peptide	OK	3059.08	6875.44	1.16836	1.1306	0.05665	0.338806	no
gene:SpnNT_01450	SpnNT_01450	Chromosome:1489426-1489507	110.58	ΔORF2+peptide	OK	2558.5	6066.28	1.24552	1.18765	0.05705	0.339649	no
gene:SpnNT_01450	SpnNT_01450	Chromosome:1489426-1489507	ΔORF2	ΔORF2+peptide	OK	3059.08	6066.28	0.987716	0.949442	0.1315	0.551468	no
gene:SpnNT_01450	SpnNT_01450	Chromosome:1489426-1489507	110.58+peptide	ΔORF2+peptide	OK	6875.44	6066.28	-0.18064	-0.195486	0.73305	0.994748	no
gene:SpnNT_01451	NA	Chromosome:1489556-1489787	110.58	ΔORF2	OK	1035.23	1425.82	0.461846	0.922839	0.10375	0.488836	no
gene:SpnNT_01451	NA	Chromosome:1489556-1489787	110.58	110.58+peptide	OK	1035.23	1736.84	0.746521	1.51146	0.0093	0.0958638	no
gene:SpnNT_01451	NA	Chromosome:1489556-1489787	ΔORF2	110.58+peptide	OK	1425.82	1736.84	0.284675	0.557772	0.3211	0.826982	no
gene:SpnNT_01451	NA	Chromosome:1489556-1489787	110.58	ΔORF2+peptide	OK	1035.23	1803.51	0.80086	1.60242	0.0044	0.0544687	no
gene:SpnNT_01451	NA	Chromosome:1489556-1489787	ΔORF2	ΔORF2+peptide	OK	1425.82	1803.51	0.339014	0.656921	0.22455	0.715528	no
gene:SpnNT_01451	NA	Chromosome:1489556-1489787	110.58+peptide	ΔORF2+peptide	OK	1736.84	1803.51	0.0543393	0.106608	0.8428	0.994748	no
gene:SpnNT_01452	pcp_1	Chromosome:1489954-1490599	110.58	ΔORF2	OK	621.979	718.466	0.208053	0.470394	0.40695	0.896303	no
gene:SpnNT_01452	pcp_1	Chromosome:1489954-1490599	110.58	110.58+peptide	OK	621.979	536.751	-0.212614	-0.486179	0.3911	0.885288	no
gene:SpnNT_01452	pcp_1	Chromosome:1489954-1490599	ΔORF2	110.58+peptide	OK	718.466	536.751	-0.420666	-0.949089	0.10335	0.488002	no
gene:SpnNT_01452	pcp_1	Chromosome:1489954-1490599	110.58	ΔORF2+peptide	OK	621.979	581.504	-0.0970774	-0.22317	0.6885	0.98828	no
gene:SpnNT_01452	pcp_1	Chromosome:1489954-1490599	ΔORF2	ΔORF2+peptide	OK	718.466	581.504	-0.30513	-0.691999	0.22725	0.71915	no
gene:SpnNT_01452	pcp_1	Chromosome:1489954-1490599	110.58+peptide	ΔORF2+peptide	OK	536.751	581.504	0.115536	0.265024	0.64355	0.980887	no
gene:SpnNT_01453	NA	Chromosome:1490613-1492223	110.58	ΔORF2	OK	301.337	325.787	0.112553	0.191366	0.74325	0.994748	no
gene:SpnNT_01453	NA	Chromosome:1490613-1492223	110.58	110.58+peptide	OK	301.337	266.086	-0.179482	-0.302574	0.6012	0.976678	no
gene:SpnNT_01453	NA	Chromosome:1490613-1492223	ΔORF2	110.58+peptide	OK	325.787	266.086	-0.292035	-0.488663	0.3935	0.888728	no
gene:SpnNT_01453	NA	Chromosome:1490613-1492223	110.58	ΔORF2+peptide	OK	301.337	310.331	0.0424312	0.0708934	0.9023	0.994748	no
gene:SpnNT_01453	NA	Chromosome:1490613-1492223	ΔORF2	ΔORF2+peptide	OK	325.787	310.331	-0.0701214	-0.116303	0.8363	0.994748	no
gene:SpnNT_01453	NA	Chromosome:1490613-1492223	110.58+peptide	ΔORF2+peptide	OK	266.086	310.331	0.221913	0.365093	0.5368	0.959314	no
gene:SpnNT_01454	NA	Chromosome:1490613-1492223	110.58	ΔORF2	OK	342.275	378.367	0.144631	0.209646	0.71545	0.992053	no
gene:SpnNT_01454	NA	Chromosome:1490613-1492223	110.58	110.58+peptide	OK	342.275	311.58	-0.135555	-0.198678	0.73655	0.994748	no
gene:SpnNT_01454	NA	Chromosome:1490613-1492223	ΔORF2	110.58+peptide	OK	378.367	311.58	-0.280186	-0.412208	0.4857	0.93781	no
gene:SpnNT_01454	NA	Chromosome:1490613-1492223	110.58	ΔORF2+peptide	OK	342.275	384.522	0.167908	0.245361	0.67325	0.984845	no
gene:SpnNT_01454	NA	Chromosome:1490613-1492223	ΔORF2	ΔORF2+peptide	OK	378.367	384.522	0.0232774	0.0341425	0.9533	0.994855	no
gene:SpnNT_01454	NA	Chromosome:1490613-1492223	110.58+peptide	ΔORF2+peptide	OK	311.58	384.522	0.303463	0.450183	0.4384	0.915991	no
gene:SpnNT_01455	sarA_4	Chromosome:1492365-1492965	110.58	ΔORF2	OK	319.514	313.772	-0.0261637	-0.0590773	0.9174	0.994748	no
gene:SpnNT_01455	sarA_4	Chromosome:1492365-1492965	110.58	110.58+peptide	OK	319.514	241.609	-0.403204	-0.912748	0.11405	0.512171	no
gene:SpnNT_01455	sarA_4	Chromosome:1492365-1492965	ΔORF2	110.58+peptide	OK	313.772	241.609	-0.37704	-0.853383	0.13585	0.560574	no
gene:SpnNT_01455	sarA_4	Chromosome:1492365-1492965	110.58	ΔORF2+peptide	OK	319.514	282.992	-0.175117	-0.395345	0.49465	0.942197	no
gene:SpnNT_01455	sarA_4	Chromosome:1492365-1492965	ΔORF2	ΔORF2+peptide	OK	313.772	282.992	-0.148954	-0.336224	0.55835	0.968033	no

gene:SpnNT_01455	sarA_4	Chromosome:1492365-1492965	110.58+peptide	ΔORF2+peptide	OK	241.609	282.992	0.228087	0.516156	0.3728	0.869823	no
gene:SpnNT_01456	ilvE	Chromosome:1493047-1494070	110.58	ΔORF2	OK	314.069	313.887	-0.000832858	-0.00189305	0.99735	0.999412	no
gene:SpnNT_01456	ilvE	Chromosome:1493047-1494070	110.58	110.58+peptide	OK	314.069	241.339	-0.380019	-0.85783	0.1392	0.569233	no
gene:SpnNT_01456	ilvE	Chromosome:1493047-1494070	ΔORF2	110.58+peptide	OK	313.887	241.339	-0.379186	-0.855378	0.13725	0.564045	no
gene:SpnNT_01456	ilvE	Chromosome:1493047-1494070	110.58	ΔORF2+peptide	OK	314.069	271.09	-0.212308	-0.484249	0.40185	0.894197	no
gene:SpnNT_01456	ilvE	Chromosome:1493047-1494070	ΔORF2	ΔORF2+peptide	OK	313.887	271.09	-0.211475	-0.48202	0.397	0.89095	no
gene:SpnNT_01456	ilvE	Chromosome:1493047-1494070	110.58+peptide	ΔORF2+peptide	OK	241.339	271.09	0.167711	0.379625	0.5091	0.947576	no
gene:SpnNT_01457	parC	Chromosome:1494204-1496640	110.58	ΔORF2	OK	63.4891	60.4808	-0.0700319	-0.159306	0.7787	0.994748	no
gene:SpnNT_01457	parC	Chromosome:1494204-1496640	110.58	110.58+peptide	OK	63.4891	80.8506	0.34875	0.796833	0.16455	0.619221	no
gene:SpnNT_01457	parC	Chromosome:1494204-1496640	ΔORF2	110.58+peptide	OK	60.4808	80.8506	0.418782	0.948364	0.10025	0.480102	no
gene:SpnNT_01457	parC	Chromosome:1494204-1496640	110.58	ΔORF2+peptide	OK	63.4891	87.7006	0.466078	1.07192	0.05615	0.337827	no
gene:SpnNT_01457	parC	Chromosome:1494204-1496640	ΔORF2	ΔORF2+peptide	OK	60.4808	87.7006	0.53611	1.22191	0.03155	0.230383	no
gene:SpnNT_01457	parC	Chromosome:1494204-1496640	110.58+peptide	ΔORF2+peptide	OK	80.8506	87.7006	0.117328	0.268605	0.63755	0.980887	no
gene:SpnNT_01458	NA	Chromosome:1496849-1496975	110.58	ΔORF2	NOTEST	0	0	0	0	1	1	no
gene:SpnNT_01458	NA	Chromosome:1496849-1496975	110.58	110.58+peptide	NOTEST	0	11.117	Inf	0	1	1	no
gene:SpnNT_01458	NA	Chromosome:1496849-1496975	ΔORF2	110.58+peptide	NOTEST	0	11.117	Inf	0	1	1	no
gene:SpnNT_01458	NA	Chromosome:1496849-1496975	110.58	ΔORF2+peptide	NOTEST	0	0	0	0	1	1	no
gene:SpnNT_01458	NA	Chromosome:1496849-1496975	ΔORF2	ΔORF2+peptide	NOTEST	0	0	0	0	1	1	no
gene:SpnNT_01458	NA	Chromosome:1496849-1496975	110.58+peptide	ΔORF2+peptide	NOTEST	11.117	0	#NAME?	0	1	1	no
gene:SpnNT_01459	NA	Chromosome:1496988-1499039	110.58	ΔORF2	NOTEST	0	0	0	0	1	1	no
gene:SpnNT_01459	NA	Chromosome:1496988-1499039	110.58	110.58+peptide	NOTEST	0	0	0	0	1	1	no
gene:SpnNT_01459	NA	Chromosome:1496988-1499039	ΔORF2	110.58+peptide	NOTEST	0	0	0	0	1	1	no
gene:SpnNT_01459	NA	Chromosome:1496988-1499039	110.58	ΔORF2+peptide	NOTEST	0	0	0	0	1	1	no
gene:SpnNT_01459	NA	Chromosome:1496988-1499039	ΔORF2	ΔORF2+peptide	NOTEST	0	0	0	0	1	1	no
gene:SpnNT_01459	NA	Chromosome:1496988-1499039	110.58+peptide	ΔORF2+peptide	NOTEST	0	0	0	0	1	1	no
gene:SpnNT_01460	parE	Chromosome:1496988-1499039	110.58	ΔORF2	OK	56.7806	54.3356	-0.0635014	-0.140915	0.8088	0.994748	no
gene:SpnNT_01460	parE	Chromosome:1496988-1499039	110.58	110.58+peptide	OK	56.7806	61.0143	0.103749	0.231211	0.6892	0.98828	no
gene:SpnNT_01460	parE	Chromosome:1496988-1499039	ΔORF2	110.58+peptide	OK	54.3356	61.0143	0.16725	0.372564	0.51135	0.949609	no
gene:SpnNT_01460	parE	Chromosome:1496988-1499039	110.58	ΔORF2+peptide	OK	56.7806	66.1293	0.219892	0.494034	0.38295	0.88051	no
gene:SpnNT_01460	parE	Chromosome:1496988-1499039	ΔORF2	ΔORF2+peptide	OK	54.3356	66.1293	0.283393	0.636418	0.26375	0.764339	no
gene:SpnNT_01460	parE	Chromosome:1496988-1499039	110.58+peptide	ΔORF2+peptide	OK	61.0143	66.1293	0.116143	0.261965	0.6434	0.980887	no
gene:SpnNT_01461	plsY	Chromosome:1499174-1499816	110.58	ΔORF2	OK	51.552	54.5849	0.0824723	0.159628	0.7768	0.994748	no
gene:SpnNT_01461	plsY	Chromosome:1499174-1499816	110.58	110.58+peptide	OK	51.552	54.4463	0.0788055	0.153201	0.7838	0.994748	no
gene:SpnNT_01461	plsY	Chromosome:1499174-1499816	ΔORF2	110.58+peptide	OK	54.5849	54.4463	-0.00366685	-0.00718755	0.98835	0.997703	no
gene:SpnNT_01461	plsY	Chromosome:1499174-1499816	110.58	ΔORF2+peptide	OK	51.552	53.0084	0.0401926	0.0778363	0.88875	0.994748	no
gene:SpnNT_01461	plsY	Chromosome:1499174-1499816	ΔORF2	ΔORF2+peptide	OK	54.5849	53.0084	-0.0422797	-0.0825509	0.886	0.994748	no
gene:SpnNT_01461	plsY	Chromosome:1499174-1499816	110.58+peptide	ΔORF2+peptide	OK	54.4463	53.0084	-0.0386128	-0.0757288	0.89245	0.994748	no
gene:SpnNT_01462	NA	Chromosome:1499858-1501210	110.58	ΔORF2	OK	6.7699	5.92923	-0.19129	-0.22492	0.6929	0.98828	no
gene:SpnNT_01462	NA	Chromosome:1499858-1501210	110.58	110.58+peptide	OK	6.7699	6.62559	-0.0310857	-0.0374563	0.9495	0.994855	no
gene:SpnNT_01462	NA	Chromosome:1499858-1501210	ΔORF2	110.58+peptide	OK	5.92923	6.62559	0.160205	0.194047	0.741	0.994748	no
gene:SpnNT_01462	NA	Chromosome:1499858-1501210	110.58	ΔORF2+peptide	OK	6.7699	6.64955	-0.0258775	-0.0316785	0.95735	0.994855	no
gene:SpnNT_01462	NA	Chromosome:1499858-1501210	ΔORF2	ΔORF2+peptide	OK	5.92923	6.64955	0.165413	0.203587	0.7257	0.994748	no
gene:SpnNT_01462	NA	Chromosome:1499858-1501210	110.58+peptide	ΔORF2+peptide	OK	6.62559	6.64955	0.0052082	0.00658482	0.99175	0.998369	no
gene:SpnNT_01463	NA	Chromosome:1499858-1501210	110.58	ΔORF2	OK	9.58804	9.9426	0.0523872	0.0532564	0.92865	0.994748	no
gene:SpnNT_01463	NA	Chromosome:1499858-1501210	110.58	110.58+peptide	OK	9.58804	8.66776	-0.145577	-0.143424	0.8087	0.994748	no

gene:SpnNT_01463	NA	Chromosome:1499858-1501210	ΔORF2	110.58+peptide	OK	9.9426	8.66776	-0.197964	-0.204227	0.7259	0.994748	no
gene:SpnNT_01463	NA	Chromosome:1499858-1501210	110.58	ΔORF2+peptide	OK	9.58804	7.92529	-0.274772	-0.268698	0.65155	0.981589	no
gene:SpnNT_01463	NA	Chromosome:1499858-1501210	ΔORF2	ΔORF2+peptide	OK	9.9426	7.92529	-0.327159	-0.334764	0.5664	0.968621	no
gene:SpnNT_01463	NA	Chromosome:1499858-1501210	110.58+peptide	ΔORF2+peptide	OK	8.66776	7.92529	-0.129196	-0.128067	0.822	0.994748	no
gene:SpnNT_01464	NA	Chromosome:1501293-1502250	110.58	ΔORF2	OK	66.1137	63.8211	-0.0509166	-0.108968	0.84945	0.994748	no
gene:SpnNT_01464	NA	Chromosome:1501293-1502250	110.58	110.58+peptide	OK	66.1137	101.791	0.622596	1.35447	0.01855	0.156838	no
gene:SpnNT_01464	NA	Chromosome:1501293-1502250	ΔORF2	110.58+peptide	OK	63.8211	101.791	0.673513	1.45985	0.01115	0.108378	no
gene:SpnNT_01464	NA	Chromosome:1501293-1502250	110.58	ΔORF2+peptide	OK	66.1137	111.145	0.749426	1.64039	0.00385	0.0490483	yes
gene:SpnNT_01464	NA	Chromosome:1501293-1502250	ΔORF2	ΔORF2+peptide	OK	63.8211	111.145	0.800342	1.74531	0.0028	0.0378	yes
gene:SpnNT_01464	NA	Chromosome:1501293-1502250	110.58+peptide	ΔORF2+peptide	OK	101.791	111.145	0.12683	0.281333	0.62375	0.980887	no
gene:SpnNT_01465	mglC	Chromosome:1502252-1504839	110.58	ΔORF2	OK	67.0253	62.0816	-0.11054	-0.161031	0.77945	0.994748	no
gene:SpnNT_01465	mglC	Chromosome:1502252-1504839	110.58	110.58+peptide	OK	67.0253	98.2096	0.551159	0.809517	0.1642	0.618436	no
gene:SpnNT_01465	mglC	Chromosome:1502252-1504839	ΔORF2	110.58+peptide	OK	62.0816	98.2096	0.661699	0.958313	0.1055	0.493889	no
gene:SpnNT_01465	mglC	Chromosome:1502252-1504839	110.58	ΔORF2+peptide	OK	67.0253	117.782	0.813339	1.19216	0.04045	0.272897	no
gene:SpnNT_01465	mglC	Chromosome:1502252-1504839	ΔORF2	ΔORF2+peptide	OK	62.0816	117.782	0.923879	1.33536	0.02275	0.184503	no
gene:SpnNT_01465	mglC	Chromosome:1502252-1504839	110.58+peptide	ΔORF2+peptide	OK	98.2096	117.782	0.26218	0.382023	0.5164	0.95301	no
gene:SpnNT_01466	mglA	Chromosome:1502252-1504839	110.58	ΔORF2	OK	55.8191	55.2016	-0.0160481	-0.0270901	0.96255	0.994855	no
gene:SpnNT_01466	mglA	Chromosome:1502252-1504839	110.58	110.58+peptide	OK	55.8191	83.045	0.573135	0.965076	0.0955	0.468118	no
gene:SpnNT_01466	mglA	Chromosome:1502252-1504839	ΔORF2	110.58+peptide	OK	55.2016	83.045	0.589183	1.00188	0.08225	0.428968	no
gene:SpnNT_01466	mglA	Chromosome:1502252-1504839	110.58	ΔORF2+peptide	OK	55.8191	102.906	0.882499	1.49071	0.00985	0.0990435	no
gene:SpnNT_01466	mglA	Chromosome:1502252-1504839	ΔORF2	ΔORF2+peptide	OK	55.2016	102.906	0.898547	1.53288	0.00835	0.0885762	no
gene:SpnNT_01466	mglA	Chromosome:1502252-1504839	110.58+peptide	ΔORF2+peptide	OK	83.045	102.906	0.309364	0.526418	0.36715	0.864634	no
gene:SpnNT_01467	tmpC	Chromosome:1504982-1506035	110.58	ΔORF2	OK	470.794	455.187	-0.0486351	-0.110318	0.846	0.994748	no
gene:SpnNT_01467	tmpC	Chromosome:1504982-1506035	110.58	110.58+peptide	OK	470.794	690.21	0.551939	1.24087	0.0323	0.234037	no
gene:SpnNT_01467	tmpC	Chromosome:1504982-1506035	ΔORF2	110.58+peptide	OK	455.187	690.21	0.600575	1.35328	0.02005	0.167364	no
gene:SpnNT_01467	tmpC	Chromosome:1504982-1506035	110.58	ΔORF2+peptide	OK	470.794	808.361	0.779905	1.75202	0.00275	0.037317	yes
gene:SpnNT_01467	tmpC	Chromosome:1504982-1506035	ΔORF2	ΔORF2+peptide	OK	455.187	808.361	0.82854	1.8655	0.0014	0.0220273	yes
gene:SpnNT_01467	tmpC	Chromosome:1504982-1506035	110.58+peptide	ΔORF2+peptide	OK	690.21	808.361	0.227965	0.508796	0.38125	0.879066	no
gene:SpnNT_01468	cdd	Chromosome:1506124-1507163	110.58	ΔORF2	OK	112.26	96.0398	-0.225136	-0.192204	0.73685	0.994748	no
gene:SpnNT_01468	cdd	Chromosome:1506124-1507163	110.58	110.58+peptide	OK	112.26	114.893	0.0334477	0.0295257	0.9592	0.994855	no
gene:SpnNT_01468	cdd	Chromosome:1506124-1507163	ΔORF2	110.58+peptide	OK	96.0398	114.893	0.258584	0.219774	0.7052	0.990422	no
gene:SpnNT_01468	cdd	Chromosome:1506124-1507163	110.58	ΔORF2+peptide	OK	112.26	127.676	0.185653	0.16113	0.78415	0.994748	no
gene:SpnNT_01468	cdd	Chromosome:1506124-1507163	ΔORF2	ΔORF2+peptide	OK	96.0398	127.676	0.410789	0.343687	0.54665	0.962122	no
gene:SpnNT_01468	cdd	Chromosome:1506124-1507163	110.58+peptide	ΔORF2+peptide	OK	114.893	127.676	0.152205	0.131491	0.81585	0.994748	no
gene:SpnNT_01469	deoC1	Chromosome:1506124-1507163	110.58	ΔORF2	OK	254.733	265.964	0.0622466	0.132082	0.8166	0.994748	no
gene:SpnNT_01469	deoC1	Chromosome:1506124-1507163	110.58	110.58+peptide	OK	254.733	249.386	-0.0306066	-0.0644125	0.90805	0.994748	no
gene:SpnNT_01469	deoC1	Chromosome:1506124-1507163	ΔORF2	110.58+peptide	OK	265.964	249.386	-0.0928531	-0.197823	0.7225	0.994748	no
gene:SpnNT_01469	deoC1	Chromosome:1506124-1507163	110.58	ΔORF2+peptide	OK	254.733	263.807	0.0505009	0.105955	0.854	0.994748	no
gene:SpnNT_01469	deoC1	Chromosome:1506124-1507163	ΔORF2	ΔORF2+peptide	OK	265.964	263.807	-0.0117457	-0.0249456	0.96525	0.994855	no
gene:SpnNT_01469	deoC1	Chromosome:1506124-1507163	110.58+peptide	ΔORF2+peptide	OK	249.386	263.807	0.0811074	0.170842	0.75995	0.994748	no
gene:SpnNT_01470	pdp	Chromosome:1507180-1509045	110.58	ΔORF2	OK	68.0454	64.4707	-0.0778543	-0.123132	0.82935	0.994748	no
gene:SpnNT_01470	pdp	Chromosome:1507180-1509045	110.58	110.58+peptide	OK	68.0454	121.582	0.83736	1.48045	0.0115	0.110795	no
gene:SpnNT_01470	pdp	Chromosome:1507180-1509045	ΔORF2	110.58+peptide	OK	64.4707	121.582	0.915215	1.43525	0.01575	0.139549	no
gene:SpnNT_01470	pdp	Chromosome:1507180-1509045	110.58	ΔORF2+peptide	OK	68.0454	128.313	0.915099	1.63408	0.0043	0.0535338	no
gene:SpnNT_01470	pdp	Chromosome:1507180-1509045	ΔORF2	ΔORF2+peptide	OK	64.4707	128.313	0.992953	1.56938	0.0084	0.088891	no

gene:SpnNT_01470	pdp	Chromosome:1507180-1509045	110.58+peptide	ΔORF2+peptide	OK	121.582	128.313	0.0777384	0.137326	0.8126	0.994748	no
gene:SpnNT_01471	rsmC	Chromosome:1507180-1509045	110.58	ΔORF2	OK	114.458	136.847	0.257743	0.331595	0.5661	0.968621	no
gene:SpnNT_01471	rsmC	Chromosome:1507180-1509045	110.58	110.58+peptide	OK	114.458	213.698	0.900755	1.20867	0.03885	0.265931	no
gene:SpnNT_01471	rsmC	Chromosome:1507180-1509045	ΔORF2	110.58+peptide	OK	136.847	213.698	0.643012	0.825253	0.1696	0.627606	no
gene:SpnNT_01471	rsmC	Chromosome:1507180-1509045	110.58	ΔORF2+peptide	OK	114.458	220.192	0.943941	1.27507	0.0303	0.223746	no
gene:SpnNT_01471	rsmC	Chromosome:1507180-1509045	ΔORF2	ΔORF2+peptide	OK	136.847	220.192	0.686198	0.88605	0.13905	0.569233	no
gene:SpnNT_01471	rsmC	Chromosome:1507180-1509045	110.58+peptide	ΔORF2+peptide	OK	213.698	220.192	0.0431859	0.0581798	0.9199	0.994748	no
gene:SpnNT_01472	NA	Chromosome:1509418-1509655	110.58	ΔORF2	NOTEST	0.964584	1.63065	0.757467	0	1	1	no
gene:SpnNT_01472	NA	Chromosome:1509418-1509655	110.58	110.58+peptide	OK	0.964584	3.61208	1.90485	1.16148	0.30155	0.807673	no
gene:SpnNT_01472	NA	Chromosome:1509418-1509655	ΔORF2	110.58+peptide	OK	1.63065	3.61208	1.14738	0.743201	0.32135	0.827268	no
gene:SpnNT_01472	NA	Chromosome:1509418-1509655	110.58	ΔORF2+peptide	OK	0.964584	3.9656	2.03956	1.72479	0.28385	0.786627	no
gene:SpnNT_01472	NA	Chromosome:1509418-1509655	ΔORF2	ΔORF2+peptide	OK	1.63065	3.9656	1.28209	1.22684	0.291	0.795269	no
gene:SpnNT_01472	NA	Chromosome:1509418-1509655	110.58+peptide	ΔORF2+peptide	OK	3.61208	3.9656	0.134711	0.096622	0.9088	0.994748	no
gene:SpnNT_01473	coaA	Chromosome:1509844-1510765	110.58	ΔORF2	OK	16.9819	20.8702	0.297451	0.543232	0.3493	0.853357	no
gene:SpnNT_01473	coaA	Chromosome:1509844-1510765	110.58	110.58+peptide	OK	16.9819	20.7897	0.291871	0.526306	0.36605	0.864634	no
gene:SpnNT_01473	coaA	Chromosome:1509844-1510765	ΔORF2	110.58+peptide	OK	20.8702	20.7897	-0.00558042	-0.0102287	0.98595	0.997197	no
gene:SpnNT_01473	coaA	Chromosome:1509844-1510765	110.58	ΔORF2+peptide	OK	16.9819	26.8545	0.661171	1.21622	0.03845	0.263881	no
gene:SpnNT_01473	coaA	Chromosome:1509844-1510765	ΔORF2	ΔORF2+peptide	OK	20.8702	26.8545	0.363719	0.68056	0.2415	0.736283	no
gene:SpnNT_01473	coaA	Chromosome:1509844-1510765	110.58+peptide	ΔORF2+peptide	OK	20.7897	26.8545	0.3693	0.681846	0.23885	0.734156	no
gene:SpnNT_01474	rpsT	Chromosome:1510832-1511069	110.58	ΔORF2	OK	2626.2	4561.21	0.796438	1.67398	0.0037	0.0473672	yes
gene:SpnNT_01474	rpsT	Chromosome:1510832-1511069	110.58	110.58+peptide	OK	2626.2	6286.19	1.25921	2.79228	5.00E-05	0.0013612	yes
gene:SpnNT_01474	rpsT	Chromosome:1510832-1511069	ΔORF2	110.58+peptide	OK	4561.21	6286.19	0.462769	0.961269	0.0982	0.474615	no
gene:SpnNT_01474	rpsT	Chromosome:1510832-1511069	110.58	ΔORF2+peptide	OK	2626.2	7279.01	1.47076	3.44068	5.00E-05	0.0013612	yes
gene:SpnNT_01474	rpsT	Chromosome:1510832-1511069	ΔORF2	ΔORF2+peptide	OK	4561.21	7279.01	0.674325	1.46759	0.0138	0.126721	no
gene:SpnNT_01474	rpsT	Chromosome:1510832-1511069	110.58+peptide	ΔORF2+peptide	OK	6286.19	7279.01	0.211556	0.487759	0.41835	0.903487	no
gene:SpnNT_01475	NA	Chromosome:1511121-1511646	110.58	ΔORF2	OK	98.075	103.209	0.0736171	0.149886	0.79525	0.994748	no
gene:SpnNT_01475	NA	Chromosome:1511121-1511646	110.58	110.58+peptide	OK	98.075	80.6315	-0.282542	-0.565605	0.3321	0.837972	no
gene:SpnNT_01475	NA	Chromosome:1511121-1511646	ΔORF2	110.58+peptide	OK	103.209	80.6315	-0.356159	-0.716181	0.2128	0.698345	no
gene:SpnNT_01475	NA	Chromosome:1511121-1511646	110.58	ΔORF2+peptide	OK	98.075	120.92	0.302098	0.620202	0.2828	0.785349	no
gene:SpnNT_01475	NA	Chromosome:1511121-1511646	ΔORF2	ΔORF2+peptide	OK	103.209	120.92	0.228481	0.471286	0.40855	0.897087	no
gene:SpnNT_01475	NA	Chromosome:1511121-1511646	110.58+peptide	ΔORF2+peptide	OK	80.6315	120.92	0.58464	1.18517	0.0431	0.284629	no
gene:SpnNT_01476	deoD_2	Chromosome:1511668-1512379	110.58	ΔORF2	OK	80.9349	80.5487	-0.00689924	-0.0144055	0.97865	0.995765	no
gene:SpnNT_01476	deoD_2	Chromosome:1511668-1512379	110.58	110.58+peptide	OK	80.9349	157.402	0.959625	2.05173	0.00045	0.00885292	yes
gene:SpnNT_01476	deoD_2	Chromosome:1511668-1512379	ΔORF2	110.58+peptide	OK	80.5487	157.402	0.966524	2.07492	3.00E-04	0.00631878	yes
gene:SpnNT_01476	deoD_2	Chromosome:1511668-1512379	110.58	ΔORF2+peptide	OK	80.9349	152.982	0.918527	1.97765	0.00065	0.0118958	yes
gene:SpnNT_01476	deoD_2	Chromosome:1511668-1512379	ΔORF2	ΔORF2+peptide	OK	80.5487	152.982	0.925426	2.00075	5.00E-04	0.0095781	yes
gene:SpnNT_01476	deoD_2	Chromosome:1511668-1512379	110.58+peptide	ΔORF2+peptide	OK	157.402	152.982	-0.0410982	-0.0911443	0.87445	0.994748	no
gene:SpnNT_01477	ply_2	Chromosome:1513036-1513549	110.58	ΔORF2	OK	60.7925	59.5698	-0.0293109	-0.055785	0.92265	0.994748	no
gene:SpnNT_01477	ply_2	Chromosome:1513036-1513549	110.58	110.58+peptide	OK	60.7925	105.771	0.798979	1.56606	0.0068	0.0755543	no
gene:SpnNT_01477	ply_2	Chromosome:1513036-1513549	ΔORF2	110.58+peptide	OK	59.5698	105.771	0.82829	1.6215	0.0048	0.0582661	no
gene:SpnNT_01477	ply_2	Chromosome:1513036-1513549	110.58	ΔORF2+peptide	OK	60.7925	111.493	0.87499	1.72704	0.0026	0.0358374	yes
gene:SpnNT_01477	ply_2	Chromosome:1513036-1513549	ΔORF2	ΔORF2+peptide	OK	59.5698	111.493	0.9043	1.78265	0.00215	0.0312429	yes
gene:SpnNT_01477	ply_2	Chromosome:1513036-1513549	110.58+peptide	ΔORF2+peptide	OK	105.771	111.493	0.0760105	0.154658	0.78385	0.994748	no
gene:SpnNT_01478	NA	Chromosome:1513573-1514566	110.58	ΔORF2	OK	49.5393	46.0825	-0.104355	-0.216824	0.7108	0.991007	no
gene:SpnNT_01478	NA	Chromosome:1513573-1514566	110.58	110.58+peptide	OK	49.5393	80.1026	0.693275	1.48443	0.0102	0.101628	no

gene:SpnNT_01478	NA	Chromosome:1513573-1514566	ΔORF2	110.58+peptide	OK	46.0825	80.1026	0.79763	1.70036	0.00395	0.0500308	no
gene:SpnNT_01478	NA	Chromosome:1513573-1514566	110.58	ΔORF2+peptide	OK	49.5393	87.0004	0.812449	1.7384	0.00235	0.0334454	yes
gene:SpnNT_01478	NA	Chromosome:1513573-1514566	ΔORF2	ΔORF2+peptide	OK	46.0825	87.0004	0.916804	1.95307	0.001	0.0168447	yes
gene:SpnNT_01478	NA	Chromosome:1513573-1514566	110.58+peptide	ΔORF2+peptide	OK	80.1026	87.0004	0.119174	0.262043	0.65055	0.981475	no
gene:SpnNT_01479	NA	Chromosome:1514569-1515214	110.58	ΔORF2	OK	67.1197	65.8172	-0.0282732	-0.057004	0.92375	0.994748	no
gene:SpnNT_01479	NA	Chromosome:1514569-1515214	110.58	110.58+peptide	OK	67.1197	110.403	0.717967	1.48965	0.0107	0.105409	no
gene:SpnNT_01479	NA	Chromosome:1514569-1515214	ΔORF2	110.58+peptide	OK	65.8172	110.403	0.74624	1.54492	0.0076	0.082624	no
gene:SpnNT_01479	NA	Chromosome:1514569-1515214	110.58	ΔORF2+peptide	OK	67.1197	122.091	0.863147	1.79299	0.00275	0.037317	yes
gene:SpnNT_01479	NA	Chromosome:1514569-1515214	ΔORF2	ΔORF2+peptide	OK	65.8172	122.091	0.891421	1.84765	0.00155	0.0240131	yes
gene:SpnNT_01479	NA	Chromosome:1514569-1515214	110.58+peptide	ΔORF2+peptide	OK	110.403	122.091	0.14518	0.310188	0.58905	0.974104	no
gene:SpnNT_01480	punA	Chromosome:1515677-1516487	110.58	ΔORF2	OK	55.1816	54.2866	-0.0235919	-0.0481365	0.9311	0.994855	no
gene:SpnNT_01480	punA	Chromosome:1515677-1516487	110.58	110.58+peptide	OK	55.1816	94.708	0.779298	1.63514	0.00365	0.0469562	yes
gene:SpnNT_01480	punA	Chromosome:1515677-1516487	ΔORF2	110.58+peptide	OK	54.2866	94.708	0.80289	1.68293	0.0027	0.0368289	yes
gene:SpnNT_01480	punA	Chromosome:1515677-1516487	110.58	ΔORF2+peptide	OK	55.1816	109.197	0.984669	2.08151	0.00035	0.00717609	yes
gene:SpnNT_01480	punA	Chromosome:1515677-1516487	ΔORF2	ΔORF2+peptide	OK	54.2866	109.197	1.00826	2.12918	2.00E-04	0.00450928	yes
gene:SpnNT_01480	punA	Chromosome:1515677-1516487	110.58+peptide	ΔORF2+peptide	OK	94.708	109.197	0.205371	0.446899	0.4384	0.915991	no
gene:SpnNT_01481	NA	Chromosome:1516502-1517045	110.58	ΔORF2	OK	35.674	39.1681	0.134805	0.24325	0.6695	0.984845	no
gene:SpnNT_01481	NA	Chromosome:1516502-1517045	110.58	110.58+peptide	OK	35.674	61.9816	0.796966	1.47347	0.01305	0.121534	no
gene:SpnNT_01481	NA	Chromosome:1516502-1517045	ΔORF2	110.58+peptide	OK	39.1681	61.9816	0.662161	1.23958	0.0338	0.241834	no
gene:SpnNT_01481	NA	Chromosome:1516502-1517045	110.58	ΔORF2+peptide	OK	35.674	92.5697	1.37567	2.59568	5.00E-05	0.0013612	yes
gene:SpnNT_01481	NA	Chromosome:1516502-1517045	ΔORF2	ΔORF2+peptide	OK	39.1681	92.5697	1.24086	2.37192	5.00E-05	0.0013612	yes
gene:SpnNT_01481	NA	Chromosome:1516502-1517045	110.58+peptide	ΔORF2+peptide	OK	61.9816	92.5697	0.578701	1.13686	0.0528	0.325737	no
gene:SpnNT_01482	deoB	Chromosome:1517046-1518258	110.58	ΔORF2	OK	75.4671	70.2211	-0.103945	-0.230247	0.6872	0.98828	no
gene:SpnNT_01482	deoB	Chromosome:1517046-1518258	110.58	110.58+peptide	OK	75.4671	143.151	0.923618	2.09084	4.00E-04	0.00811252	yes
gene:SpnNT_01482	deoB	Chromosome:1517046-1518258	ΔORF2	110.58+peptide	OK	70.2211	143.151	1.02756	2.30771	1.00E-04	0.0025332	yes
gene:SpnNT_01482	deoB	Chromosome:1517046-1518258	110.58	ΔORF2+peptide	OK	75.4671	156.559	1.05279	2.38492	5.00E-05	0.0013612	yes
gene:SpnNT_01482	deoB	Chromosome:1517046-1518258	ΔORF2	ΔORF2+peptide	OK	70.2211	156.559	1.15673	2.5996	5.00E-05	0.0013612	yes
gene:SpnNT_01482	deoB	Chromosome:1517046-1518258	110.58+peptide	ΔORF2+peptide	OK	143.151	156.559	0.129169	0.296861	0.5944	0.975539	no
gene:SpnNT_01483	rpiA	Chromosome:1518271-1518955	110.58	ΔORF2	OK	79.9838	71.227	-0.167284	-0.345305	0.54635	0.962122	no
gene:SpnNT_01483	rpiA	Chromosome:1518271-1518955	110.58	110.58+peptide	OK	79.9838	161.506	1.01381	2.18373	0.00015	0.00355289	yes
gene:SpnNT_01483	rpiA	Chromosome:1518271-1518955	ΔORF2	110.58+peptide	OK	71.227	161.506	1.18109	2.52199	5.00E-05	0.0013612	yes
gene:SpnNT_01483	rpiA	Chromosome:1518271-1518955	110.58	ΔORF2+peptide	OK	79.9838	172.706	1.11053	2.39591	5.00E-05	0.0013612	yes
gene:SpnNT_01483	rpiA	Chromosome:1518271-1518955	ΔORF2	ΔORF2+peptide	OK	71.227	172.706	1.27782	2.73282	5.00E-05	0.0013612	yes
gene:SpnNT_01483	rpiA	Chromosome:1518271-1518955	110.58+peptide	ΔORF2+peptide	OK	161.506	172.706	0.0967266	0.216574	0.69865	0.990209	no
gene:SpnNT_01484	nnrD	Chromosome:1519236-1520109	110.58	ΔORF2	OK	74.8121	78.7581	0.0741568	0.157749	0.77375	0.994748	no
gene:SpnNT_01484	nnrD	Chromosome:1519236-1520109	110.58	110.58+peptide	OK	74.8121	93.3518	0.319405	0.650483	0.25895	0.759841	no
gene:SpnNT_01484	nnrD	Chromosome:1519236-1520109	ΔORF2	110.58+peptide	OK	78.7581	93.3518	0.245249	0.500855	0.3765	0.875032	no
gene:SpnNT_01484	nnrD	Chromosome:1519236-1520109	110.58	ΔORF2+peptide	OK	74.8121	67.9846	-0.138065	-0.289649	0.6125	0.979616	no
gene:SpnNT_01484	nnrD	Chromosome:1519236-1520109	ΔORF2	ΔORF2+peptide	OK	78.7581	67.9846	-0.212222	-0.446544	0.4348	0.913666	no
gene:SpnNT_01484	nnrD	Chromosome:1519236-1520109	110.58+peptide	ΔORF2+peptide	OK	93.3518	67.9846	-0.457471	-0.922372	0.1132	0.510776	no
gene:SpnNT_01485	folD	Chromosome:1520250-1521171	110.58	ΔORF2	OK	47.9035	42.4855	-0.173162	-0.351915	0.534	0.958867	no
gene:SpnNT_01485	folD	Chromosome:1520250-1521171	110.58	110.58+peptide	OK	47.9035	45.9312	-0.0606578	-0.12413	0.82675	0.994748	no
gene:SpnNT_01485	folD	Chromosome:1520250-1521171	ΔORF2	110.58+peptide	OK	42.4855	45.9312	0.112505	0.22901	0.6937	0.98828	no
gene:SpnNT_01485	folD	Chromosome:1520250-1521171	110.58	ΔORF2+peptide	OK	47.9035	43.4868	-0.139554	-0.28318	0.6141	0.979616	no
gene:SpnNT_01485	folD	Chromosome:1520250-1521171	ΔORF2	ΔORF2+peptide	OK	42.4855	43.4868	0.0336083	0.0678421	0.9063	0.994748	no

gene:SpnNT_01485	folD	Chromosome:1520250-1521171	110.58+peptide	ΔORF2+peptide	OK	45.9312	43.4868	-0.0788964	-0.160352	0.77635	0.994748	no
gene:SpnNT_01486	artM_2	Chromosome:1521261-1522742	110.58	ΔORF2	OK	185.602	177.401	-0.0651933	-0.125399	0.83235	0.994748	no
gene:SpnNT_01486	artM_2	Chromosome:1521261-1522742	110.58	110.58+peptide	OK	185.602	226.341	0.286286	0.527454	0.3685	0.865873	no
gene:SpnNT_01486	artM_2	Chromosome:1521261-1522742	ΔORF2	110.58+peptide	OK	177.401	226.341	0.351479	0.648768	0.25745	0.757287	no
gene:SpnNT_01486	artM_2	Chromosome:1521261-1522742	110.58	ΔORF2+peptide	OK	185.602	191.612	0.0459763	0.0824033	0.88485	0.994748	no
gene:SpnNT_01486	artM_2	Chromosome:1521261-1522742	ΔORF2	ΔORF2+peptide	OK	177.401	191.612	0.11117	0.199599	0.7241	0.994748	no
gene:SpnNT_01486	artM_2	Chromosome:1521261-1522742	110.58+peptide	ΔORF2+peptide	OK	226.341	191.612	-0.240309	-0.415486	0.47235	0.928525	no
gene:SpnNT_01487	artQ	Chromosome:1521261-1522742	110.58	ΔORF2	OK	52.8222	60.8967	0.20522	0.198861	0.7315	0.994748	no
gene:SpnNT_01487	artQ	Chromosome:1521261-1522742	110.58	110.58+peptide	OK	52.8222	63.785	0.272073	0.21704	0.7046	0.990367	no
gene:SpnNT_01487	artQ	Chromosome:1521261-1522742	ΔORF2	110.58+peptide	OK	60.8967	63.785	0.0668532	0.0569113	0.9179	0.994748	no
gene:SpnNT_01487	artQ	Chromosome:1521261-1522742	110.58	ΔORF2+peptide	OK	52.8222	78.5339	0.572171	0.518789	0.37005	0.866642	no
gene:SpnNT_01487	artQ	Chromosome:1521261-1522742	ΔORF2	ΔORF2+peptide	OK	60.8967	78.5339	0.366951	0.362473	0.5211	0.954832	no
gene:SpnNT_01487	artQ	Chromosome:1521261-1522742	110.58+peptide	ΔORF2+peptide	OK	63.785	78.5339	0.300098	0.242511	0.66245	0.982966	no
gene:SpnNT_01488	NA	Chromosome:1522868-1523099	110.58	ΔORF2	OK	3761.66	3745.54	-0.00619578	-0.0130731	0.98105	0.99608	no
gene:SpnNT_01488	NA	Chromosome:1522868-1523099	110.58	110.58+peptide	OK	3761.66	6590.04	0.80892	1.54743	0.00505	0.0609063	no
gene:SpnNT_01488	NA	Chromosome:1522868-1523099	ΔORF2	110.58+peptide	OK	3745.54	6590.04	0.815115	1.58179	0.00535	0.0633027	no
gene:SpnNT_01488	NA	Chromosome:1522868-1523099	110.58	ΔORF2+peptide	OK	3761.66	4747.24	0.335721	0.677789	0.21325	0.698345	no
gene:SpnNT_01488	NA	Chromosome:1522868-1523099	ΔORF2	ΔORF2+peptide	OK	3745.54	4747.24	0.341916	0.701427	0.20665	0.691918	no
gene:SpnNT_01488	NA	Chromosome:1522868-1523099	110.58+peptide	ΔORF2+peptide	OK	6590.04	4747.24	-0.473199	-0.88441	0.1069	0.497426	no
gene:SpnNT_01489	clpE	Chromosome:1523322-1525581	110.58	ΔORF2	OK	167.064	170.928	0.032992	0.0751522	0.8952	0.994748	no
gene:SpnNT_01489	clpE	Chromosome:1523322-1525581	110.58	110.58+peptide	OK	167.064	214.182	0.358437	0.782837	0.17415	0.6346	no
gene:SpnNT_01489	clpE	Chromosome:1523322-1525581	ΔORF2	110.58+peptide	OK	170.928	214.182	0.325445	0.711087	0.217	0.704472	no
gene:SpnNT_01489	clpE	Chromosome:1523322-1525581	110.58	ΔORF2+peptide	OK	167.064	168.713	0.0141667	0.0320624	0.9528	0.994855	no
gene:SpnNT_01489	clpE	Chromosome:1523322-1525581	ΔORF2	ΔORF2+peptide	OK	170.928	168.713	-0.0188253	-0.0426256	0.9379	0.994855	no
gene:SpnNT_01489	clpE	Chromosome:1523322-1525581	110.58+peptide	ΔORF2+peptide	OK	214.182	168.713	-0.34427	-0.747759	0.1821	0.651805	no
gene:SpnNT_01490	NA	Chromosome:1525786-1526125	110.58	ΔORF2	OK	75.3802	80.1559	0.0886213	0.157736	0.7872	0.994748	no
gene:SpnNT_01490	NA	Chromosome:1525786-1526125	110.58	110.58+peptide	OK	75.3802	63.5077	-0.247255	-0.438536	0.44565	0.918272	no
gene:SpnNT_01490	NA	Chromosome:1525786-1526125	ΔORF2	110.58+peptide	OK	80.1559	63.5077	-0.335876	-0.596312	0.30115	0.807126	no
gene:SpnNT_01490	NA	Chromosome:1525786-1526125	110.58	ΔORF2+peptide	OK	75.3802	52.1453	-0.531648	-0.876691	0.13015	0.547908	no
gene:SpnNT_01490	NA	Chromosome:1525786-1526125	ΔORF2	ΔORF2+peptide	OK	80.1559	52.1453	-0.62027	-1.02371	0.0756	0.406068	no
gene:SpnNT_01490	NA	Chromosome:1525786-1526125	110.58+peptide	ΔORF2+peptide	OK	63.5077	52.1453	-0.284394	-0.467948	0.41205	0.899511	no
gene:SpnNT_01491	NA	Chromosome:1526283-1526634	110.58	ΔORF2	OK	287.079	327.801	0.19137	0.40195	0.48545	0.937658	no
gene:SpnNT_01491	NA	Chromosome:1526283-1526634	110.58	110.58+peptide	OK	287.079	230.828	-0.314633	-0.654041	0.24275	0.738169	no
gene:SpnNT_01491	NA	Chromosome:1526283-1526634	ΔORF2	110.58+peptide	OK	327.801	230.828	-0.506003	-1.04629	0.067	0.378628	no
gene:SpnNT_01491	NA	Chromosome:1526283-1526634	110.58	ΔORF2+peptide	OK	287.079	261.674	-0.133681	-0.273824	0.63105	0.980887	no
gene:SpnNT_01491	NA	Chromosome:1526283-1526634	ΔORF2	ΔORF2+peptide	OK	327.801	261.674	-0.325051	-0.662395	0.2476	0.743991	no
gene:SpnNT_01491	NA	Chromosome:1526283-1526634	110.58+peptide	ΔORF2+peptide	OK	230.828	261.674	0.180952	0.365168	0.5199	0.954544	no
gene:SpnNT_01492	NA	Chromosome:1527327-1527627	110.58	ΔORF2	OK	829.267	1145.56	0.466147	1.02895	0.06845	0.383191	no
gene:SpnNT_01492	NA	Chromosome:1527327-1527627	110.58	110.58+peptide	OK	829.267	1024.23	0.304634	0.651596	0.24735	0.743921	no
gene:SpnNT_01492	NA	Chromosome:1527327-1527627	ΔORF2	110.58+peptide	OK	1145.56	1024.23	-0.161514	-0.343413	0.5436	0.961568	no
gene:SpnNT_01492	NA	Chromosome:1527327-1527627	110.58	ΔORF2+peptide	OK	829.267	753.944	-0.137381	-0.305322	0.5896	0.974359	no
gene:SpnNT_01492	NA	Chromosome:1527327-1527627	ΔORF2	ΔORF2+peptide	OK	1145.56	753.944	-0.603528	-1.3327	0.022	0.180315	no
gene:SpnNT_01492	NA	Chromosome:1527327-1527627	110.58+peptide	ΔORF2+peptide	OK	1024.23	753.944	-0.442014	-0.94578	0.10425	0.490311	no
gene:SpnNT_01493	NA	Chromosome:1527887-1528124	110.58	ΔORF2	NOTEST	0	0	0	0	1	1	no
gene:SpnNT_01493	NA	Chromosome:1527887-1528124	110.58	110.58+peptide	NOTEST	0	0	0	0	1	1	no

gene:SpnNT_01493	NA	Chromosome:1527887-1528124	ΔORF2	110.58+peptide	NOTEST	0	0	0	0	1	1	no
gene:SpnNT_01493	NA	Chromosome:1527887-1528124	110.58	ΔORF2+peptide	NOTEST	0	0	0	0	1	1	no
gene:SpnNT_01493	NA	Chromosome:1527887-1528124	ΔORF2	ΔORF2+peptide	NOTEST	0	0	0	0	1	1	no
gene:SpnNT_01493	NA	Chromosome:1527887-1528124	110.58+peptide	ΔORF2+peptide	NOTEST	0	0	0	0	1	1	no
gene:SpnNT_01494	NA	Chromosome:1528374-1528770	110.58	ΔORF2	OK	19.8375	20.1686	0.0238815	0.0366716	0.94455	0.994855	no
gene:SpnNT_01494	NA	Chromosome:1528374-1528770	110.58	110.58+peptide	OK	19.8375	16.4054	-0.274053	-0.41293	0.46455	0.926394	no
gene:SpnNT_01494	NA	Chromosome:1528374-1528770	ΔORF2	110.58+peptide	OK	20.1686	16.4054	-0.297935	-0.448066	0.428	0.909574	no
gene:SpnNT_01494	NA	Chromosome:1528374-1528770	110.58	ΔORF2+peptide	OK	19.8375	16.7384	-0.245065	-0.35671	0.52415	0.954832	no
gene:SpnNT_01494	NA	Chromosome:1528374-1528770	ΔORF2	ΔORF2+peptide	OK	20.1686	16.7384	-0.268947	-0.390782	0.48525	0.937493	no
gene:SpnNT_01494	NA	Chromosome:1528374-1528770	110.58+peptide	ΔORF2+peptide	OK	16.4054	16.7384	0.028988	0.04141	0.9398	0.994855	no
gene:SpnNT_01495	NA	Chromosome:1528801-1529308	110.58	ΔORF2	OK	36.2729	49.8712	0.459314	0.82995	0.1455	0.581026	no
gene:SpnNT_01495	NA	Chromosome:1528801-1529308	110.58	110.58+peptide	OK	36.2729	41.3004	0.187261	0.333721	0.5735	0.969538	no
gene:SpnNT_01495	NA	Chromosome:1528801-1529308	ΔORF2	110.58+peptide	OK	49.8712	41.3004	-0.272053	-0.49291	0.3867	0.881745	no
gene:SpnNT_01495	NA	Chromosome:1528801-1529308	110.58	ΔORF2+peptide	OK	36.2729	44.3984	0.291615	0.518144	0.3707	0.8677	no
gene:SpnNT_01495	NA	Chromosome:1528801-1529308	ΔORF2	ΔORF2+peptide	OK	49.8712	44.3984	-0.167699	-0.302904	0.5979	0.97629	no
gene:SpnNT_01495	NA	Chromosome:1528801-1529308	110.58+peptide	ΔORF2+peptide	OK	41.3004	44.3984	0.104354	0.185902	0.75105	0.994748	no
gene:SpnNT_01496	tuf	Chromosome:1529691-1530888	110.58	ΔORF2	OK	4259.56	4191.72	-0.0231604	-0.0435598	0.93675	0.994855	no
gene:SpnNT_01496	tuf	Chromosome:1529691-1530888	110.58	110.58+peptide	OK	4259.56	5126.83	0.267365	0.510891	0.36695	0.864634	no
gene:SpnNT_01496	tuf	Chromosome:1529691-1530888	ΔORF2	110.58+peptide	OK	4191.72	5126.83	0.290525	0.544459	0.3288	0.83469	no
gene:SpnNT_01496	tuf	Chromosome:1529691-1530888	110.58	ΔORF2+peptide	OK	4259.56	4673.51	0.133804	0.25736	0.654	0.982798	no
gene:SpnNT_01496	tuf	Chromosome:1529691-1530888	ΔORF2	ΔORF2+peptide	OK	4191.72	4673.51	0.156964	0.296021	0.5978	0.97629	no
gene:SpnNT_01496	tuf	Chromosome:1529691-1530888	110.58+peptide	ΔORF2+peptide	OK	5126.83	4673.51	-0.133561	-0.25593	0.65075	0.981475	no
gene:SpnNT_01497	NA	Chromosome:1531214-1532948	110.58	ΔORF2	OK	21.7238	25.3203	0.221018	0.452595	0.4411	0.916711	no
gene:SpnNT_01497	NA	Chromosome:1531214-1532948	110.58	110.58+peptide	OK	21.7238	23.498	0.113261	0.228728	0.6943	0.988457	no
gene:SpnNT_01497	NA	Chromosome:1531214-1532948	ΔORF2	110.58+peptide	OK	25.3203	23.498	-0.107757	-0.220971	0.70235	0.990367	no
gene:SpnNT_01497	NA	Chromosome:1531214-1532948	110.58	ΔORF2+peptide	OK	21.7238	29.5481	0.443786	0.909828	0.1209	0.526535	no
gene:SpnNT_01497	NA	Chromosome:1531214-1532948	ΔORF2	ΔORF2+peptide	OK	25.3203	29.5481	0.222768	0.463977	0.4243	0.907534	no
gene:SpnNT_01497	NA	Chromosome:1531214-1532948	110.58+peptide	ΔORF2+peptide	OK	23.498	29.5481	0.330525	0.678575	0.24605	0.743359	no
gene:SpnNT_01498	gla	Chromosome:1533763-1534633	110.58	ΔORF2	OK	1257.63	1351.22	0.103554	0.225258	0.6927	0.98828	no
gene:SpnNT_01498	gla	Chromosome:1533763-1534633	110.58	110.58+peptide	OK	1257.63	1030.15	-0.287854	-0.628395	0.27535	0.777354	no
gene:SpnNT_01498	gla	Chromosome:1533763-1534633	ΔORF2	110.58+peptide	OK	1351.22	1030.15	-0.391408	-0.853519	0.13935	0.569287	no
gene:SpnNT_01498	gla	Chromosome:1533763-1534633	110.58	ΔORF2+peptide	OK	1257.63	1091.95	-0.203802	-0.447891	0.43095	0.912485	no
gene:SpnNT_01498	gla	Chromosome:1533763-1534633	ΔORF2	ΔORF2+peptide	OK	1351.22	1091.95	-0.307356	-0.674719	0.2407	0.735706	no
gene:SpnNT_01498	gla	Chromosome:1533763-1534633	110.58+peptide	ΔORF2+peptide	OK	1030.15	1091.95	0.0840519	0.185185	0.7438	0.994748	no
gene:SpnNT_01499	NA	Chromosome:1534856-1535887	110.58	ΔORF2	OK	4.64408	3.72247	-0.319135	-0.330191	0.5717	0.969025	no
gene:SpnNT_01499	NA	Chromosome:1534856-1535887	110.58	110.58+peptide	OK	4.64408	4.50589	-0.043582	-0.048154	0.93225	0.994855	no
gene:SpnNT_01499	NA	Chromosome:1534856-1535887	ΔORF2	110.58+peptide	OK	3.72247	4.50589	0.275553	0.292068	0.60585	0.976937	no
gene:SpnNT_01499	NA	Chromosome:1534856-1535887	110.58	ΔORF2+peptide	OK	4.64408	4.35777	-0.0918022	-0.0942014	0.8662	0.994748	no
gene:SpnNT_01499	NA	Chromosome:1534856-1535887	ΔORF2	ΔORF2+peptide	OK	3.72247	4.35777	0.227333	0.225017	0.6923	0.98828	no
gene:SpnNT_01499	NA	Chromosome:1534856-1535887	110.58+peptide	ΔORF2+peptide	OK	4.50589	4.35777	-0.0482202	-0.0506694	0.92965	0.99482	no
gene:SpnNT_01500	inlJ	Chromosome:1534856-1535887	110.58	ΔORF2	OK	3.63898	3.06037	-0.249828	-0.21573	0.7122	0.991034	no
gene:SpnNT_01500	inlJ	Chromosome:1534856-1535887	110.58	110.58+peptide	OK	3.63898	2.15376	-0.756679	-0.568503	0.32645	0.831577	no
gene:SpnNT_01500	inlJ	Chromosome:1534856-1535887	ΔORF2	110.58+peptide	OK	3.06037	2.15376	-0.506851	-0.37922	0.5189	0.954444	no
gene:SpnNT_01500	inlJ	Chromosome:1534856-1535887	110.58	ΔORF2+peptide	OK	3.63898	3.1138	-0.224856	-0.18104	0.7579	0.994748	no
gene:SpnNT_01500	inlJ	Chromosome:1534856-1535887	ΔORF2	ΔORF2+peptide	OK	3.06037	3.1138	0.0249717	0.0200097	0.97525	0.99536	no

gene:SpnNT_01500	inlJ	Chromosome:1534856-1535887	110.58+peptide	ΔORF2+peptide	OK	2.15376	3.1138	0.531823	0.377196	0.5198	0.954494	no
gene:SpnNT_01501	NA	Chromosome:1535974-1536484	110.58	ΔORF2	OK	2.40695	1.88955	-0.349158	-0.337953	0.56545	0.968621	no
gene:SpnNT_01501	NA	Chromosome:1535974-1536484	110.58	110.58+peptide	OK	2.40695	1.77613	-0.43847	-0.424716	0.4683	0.927504	no
gene:SpnNT_01501	NA	Chromosome:1535974-1536484	ΔORF2	110.58+peptide	OK	1.88955	1.77613	-0.0893123	-0.0861912	0.87515	0.994748	no
gene:SpnNT_01501	NA	Chromosome:1535974-1536484	110.58	ΔORF2+peptide	OK	2.40695	1.24659	-0.949212	-0.911975	0.17235	0.632255	no
gene:SpnNT_01501	NA	Chromosome:1535974-1536484	ΔORF2	ΔORF2+peptide	OK	1.88955	1.24659	-0.600054	-0.57442	0.3723	0.869546	no
gene:SpnNT_01501	NA	Chromosome:1535974-1536484	110.58+peptide	ΔORF2+peptide	OK	1.77613	1.24659	-0.510742	-0.48928	0.4569	0.921525	no
gene:SpnNT_01502	NA	Chromosome:1536594-1536849	110.58	ΔORF2	OK	3.46497	2.01289	-0.783575	-0.494847	0.4244	0.907534	no
gene:SpnNT_01502	NA	Chromosome:1536594-1536849	110.58	110.58+peptide	OK	3.46497	2.12162	-0.707676	-0.482345	0.4611	0.924555	no
gene:SpnNT_01502	NA	Chromosome:1536594-1536849	ΔORF2	110.58+peptide	OK	2.01289	2.12162	0.0758991	0.0378421	0.8877	0.994748	no
gene:SpnNT_01502	NA	Chromosome:1536594-1536849	110.58	ΔORF2+peptide	OK	3.46497	4.37375	0.336028	0.468513	0.7073	0.990441	no
gene:SpnNT_01502	NA	Chromosome:1536594-1536849	ΔORF2	ΔORF2+peptide	OK	2.01289	4.37375	1.1196	0.725029	0.27295	0.775081	no
gene:SpnNT_01502	NA	Chromosome:1536594-1536849	110.58+peptide	ΔORF2+peptide	OK	2.12162	4.37375	1.0437	0.732576	0.2819	0.784843	no
gene:SpnNT_01503	NA	Chromosome:1537188-1537722	110.58	ΔORF2	OK	2.17019	2.0584	-0.0763009	-0.0767104	0.8887	0.994748	no
gene:SpnNT_01503	NA	Chromosome:1537188-1537722	110.58	110.58+peptide	OK	2.17019	1.36581	-0.668069	-0.587862	0.2933	0.797897	no
gene:SpnNT_01503	NA	Chromosome:1537188-1537722	ΔORF2	110.58+peptide	OK	2.0584	1.36581	-0.591768	-0.527261	0.3388	0.843433	no
gene:SpnNT_01503	NA	Chromosome:1537188-1537722	110.58	ΔORF2+peptide	OK	2.17019	1.70765	-0.345812	-0.331614	0.5742	0.969538	no
gene:SpnNT_01503	NA	Chromosome:1537188-1537722	ΔORF2	ΔORF2+peptide	OK	2.0584	1.70765	-0.269511	-0.262313	0.65475	0.982966	no
gene:SpnNT_01503	NA	Chromosome:1537188-1537722	110.58+peptide	ΔORF2+peptide	OK	1.36581	1.70765	0.322257	0.27656	0.61435	0.979616	no
gene:SpnNT_01504	NA	Chromosome:1538324-1540058	110.58	ΔORF2	OK	27.81	31.8641	0.196328	0.413546	0.46695	0.92732	no
gene:SpnNT_01504	NA	Chromosome:1538324-1540058	110.58	110.58+peptide	OK	27.81	27.7122	-0.00508144	-0.0105741	0.98415	0.996246	no
gene:SpnNT_01504	NA	Chromosome:1538324-1540058	ΔORF2	110.58+peptide	OK	31.8641	27.7122	-0.20141	-0.423092	0.45645	0.921244	no
gene:SpnNT_01504	NA	Chromosome:1538324-1540058	110.58	ΔORF2+peptide	OK	27.81	36.5962	0.39609	0.838611	0.1456	0.581072	no
gene:SpnNT_01504	NA	Chromosome:1538324-1540058	ΔORF2	ΔORF2+peptide	OK	31.8641	36.5962	0.199762	0.427091	0.4513	0.921244	no
gene:SpnNT_01504	NA	Chromosome:1538324-1540058	110.58+peptide	ΔORF2+peptide	OK	27.7122	36.5962	0.401171	0.847027	0.1375	0.564718	no
gene:SpnNT_01505	pgcA	Chromosome:1540338-1542057	110.58	ΔORF2	OK	156.887	163.942	0.0634584	0.145274	0.791	0.994748	no
gene:SpnNT_01505	pgcA	Chromosome:1540338-1542057	110.58	110.58+peptide	OK	156.887	154.761	-0.019682	-0.0452665	0.9376	0.994855	no
gene:SpnNT_01505	pgcA	Chromosome:1540338-1542057	ΔORF2	110.58+peptide	OK	163.942	154.761	-0.0831404	-0.189786	0.74035	0.994748	no
gene:SpnNT_01505	pgcA	Chromosome:1540338-1542057	110.58	ΔORF2+peptide	OK	156.887	159.028	0.0195549	0.0448177	0.93685	0.994855	no
gene:SpnNT_01505	pgcA	Chromosome:1540338-1542057	ΔORF2	ΔORF2+peptide	OK	163.942	159.028	-0.0439035	-0.0998755	0.8639	0.994748	no
gene:SpnNT_01505	pgcA	Chromosome:1540338-1542057	110.58+peptide	ΔORF2+peptide	OK	154.761	159.028	0.0392369	0.0896684	0.87605	0.994748	no
gene:SpnNT_01506	NA	Chromosome:1542167-1542515	110.58	ΔORF2	OK	975.316	1026.67	0.0740352	0.162898	0.7715	0.994748	no
gene:SpnNT_01506	NA	Chromosome:1542167-1542515	110.58	110.58+peptide	OK	975.316	505.705	-0.947575	-2.01389	0.00055	0.0103545	yes
gene:SpnNT_01506	NA	Chromosome:1542167-1542515	ΔORF2	110.58+peptide	OK	1026.67	505.705	-1.02161	-2.24846	1.00E-04	0.0025332	yes
gene:SpnNT_01506	NA	Chromosome:1542167-1542515	110.58	ΔORF2+peptide	OK	975.316	529.337	-0.881685	-1.84624	0.0023	0.0329123	yes
gene:SpnNT_01506	NA	Chromosome:1542167-1542515	ΔORF2	ΔORF2+peptide	OK	1026.67	529.337	-0.95572	-2.07026	6.00E-04	0.0111203	yes
gene:SpnNT_01506	NA	Chromosome:1542167-1542515	110.58+peptide	ΔORF2+peptide	OK	505.705	529.337	0.0658902	0.138009	0.80775	0.994748	no
gene:SpnNT_01507	fliY	Chromosome:1542792-1543629	110.58	ΔORF2	OK	366.202	451.573	0.302322	0.691216	0.2231	0.712784	no
gene:SpnNT_01507	fliY	Chromosome:1542792-1543629	110.58	110.58+peptide	OK	366.202	118.564	-1.62698	-3.66647	5.00E-05	0.0013612	yes
gene:SpnNT_01507	fliY	Chromosome:1542792-1543629	ΔORF2	110.58+peptide	OK	451.573	118.564	-1.9293	-4.36545	5.00E-05	0.0013612	yes
gene:SpnNT_01507	fliY	Chromosome:1542792-1543629	110.58	ΔORF2+peptide	OK	366.202	131.326	-1.47949	-3.33825	5.00E-05	0.0013612	yes
gene:SpnNT_01507	fliY	Chromosome:1542792-1543629	ΔORF2	ΔORF2+peptide	OK	451.573	131.326	-1.78181	-4.03679	5.00E-05	0.0013612	yes
gene:SpnNT_01507	fliY	Chromosome:1542792-1543629	110.58+peptide	ΔORF2+peptide	OK	118.564	131.326	0.147489	0.329434	0.55805	0.968033	no
gene:SpnNT_01508	glnQ_3	Chromosome:1543641-1544271	110.58	ΔORF2	OK	288.811	304.164	0.0747254	0.165369	0.78015	0.994748	no
gene:SpnNT_01508	glnQ_3	Chromosome:1543641-1544271	110.58	110.58+peptide	OK	288.811	83.7272	-1.78636	-3.8098	5.00E-05	0.0013612	yes

gene:SpnNT_01508	glnQ_3	Chromosome:1543641-1544271	ΔORF2	110.58+peptide	OK	304.164	83.7272	-1.86108	-3.96567	5.00E-05	0.0013612	yes
gene:SpnNT_01508	glnQ_3	Chromosome:1543641-1544271	110.58	ΔORF2+peptide	OK	288.811	86.172	-1.74483	-3.7206	5.00E-05	0.0013612	yes
gene:SpnNT_01508	glnQ_3	Chromosome:1543641-1544271	ΔORF2	ΔORF2+peptide	OK	304.164	86.172	-1.81956	-3.87652	5.00E-05	0.0013612	yes
gene:SpnNT_01508	glnQ_3	Chromosome:1543641-1544271	110.58+peptide	ΔORF2+peptide	OK	83.7272	86.172	0.0415224	0.085476	0.8803	0.994748	no
gene:SpnNT_01509	yecS_2	Chromosome:1544280-1544922	110.58	ΔORF2	OK	211.6	251.826	0.251088	0.552135	0.33405	0.839572	no
gene:SpnNT_01509	yecS_2	Chromosome:1544280-1544922	110.58	110.58+peptide	OK	211.6	51.9972	-2.02483	-4.14391	5.00E-05	0.0013612	yes
gene:SpnNT_01509	yecS_2	Chromosome:1544280-1544922	ΔORF2	110.58+peptide	OK	251.826	51.9972	-2.27592	-4.66703	5.00E-05	0.0013612	yes
gene:SpnNT_01509	yecS_2	Chromosome:1544280-1544922	110.58	ΔORF2+peptide	OK	211.6	57.3928	-1.8824	-3.88173	5.00E-05	0.0013612	yes
gene:SpnNT_01509	yecS_2	Chromosome:1544280-1544922	ΔORF2	ΔORF2+peptide	OK	251.826	57.3928	-2.13348	-4.40838	5.00E-05	0.0013612	yes
gene:SpnNT_01509	yecS_2	Chromosome:1544280-1544922	110.58+peptide	ΔORF2+peptide	OK	51.9972	57.3928	0.142437	0.276085	0.63255	0.980887	no
gene:SpnNT_01510	NA	Chromosome:1545067-1547453	110.58	ΔORF2	OK	103.752	132.348	0.351193	0.628648	0.27045	0.772705	no
gene:SpnNT_01510	NA	Chromosome:1545067-1547453	110.58	110.58+peptide	OK	103.752	104.97	0.0168389	0.0304645	0.95825	0.994855	no
gene:SpnNT_01510	NA	Chromosome:1545067-1547453	ΔORF2	110.58+peptide	OK	132.348	104.97	-0.334354	-0.607174	0.29575	0.800007	no
gene:SpnNT_01510	NA	Chromosome:1545067-1547453	110.58	ΔORF2+peptide	OK	103.752	131.391	0.340725	0.624386	0.2694	0.771007	no
gene:SpnNT_01510	NA	Chromosome:1545067-1547453	ΔORF2	ΔORF2+peptide	OK	132.348	131.391	-0.0104677	-0.0192562	0.97075	0.99536	no
gene:SpnNT_01510	NA	Chromosome:1545067-1547453	110.58+peptide	ΔORF2+peptide	OK	104.97	131.391	0.323886	0.602548	0.2942	0.798553	no
gene:SpnNT_01511	NA	Chromosome:1545067-1547453	110.58	ΔORF2	OK	75.6726	89.313	0.239098	0.347939	0.5352	0.958867	no
gene:SpnNT_01511	NA	Chromosome:1545067-1547453	110.58	110.58+peptide	OK	75.6726	63.2439	-0.258846	-0.36385	0.52305	0.954832	no
gene:SpnNT_01511	NA	Chromosome:1545067-1547453	ΔORF2	110.58+peptide	OK	89.313	63.2439	-0.497943	-0.691077	0.22705	0.71894	no
gene:SpnNT_01511	NA	Chromosome:1545067-1547453	110.58	ΔORF2+peptide	OK	75.6726	69.5422	-0.121882	-0.168631	0.76865	0.994748	no
gene:SpnNT_01511	NA	Chromosome:1545067-1547453	ΔORF2	ΔORF2+peptide	OK	89.313	69.5422	-0.36098	-0.493304	0.3843	0.881452	no
gene:SpnNT_01511	NA	Chromosome:1545067-1547453	110.58+peptide	ΔORF2+peptide	OK	63.2439	69.5422	0.136963	0.181516	0.7564	0.994748	no
gene:SpnNT_01512	pgl	Chromosome:1547596-1548610	110.58	ΔORF2	OK	71.6626	80.0718	0.160073	0.351405	0.5462	0.962045	no
gene:SpnNT_01512	pgl	Chromosome:1547596-1548610	110.58	110.58+peptide	OK	71.6626	53.9828	-0.40872	-0.878822	0.1292	0.545133	no
gene:SpnNT_01512	pgl	Chromosome:1547596-1548610	ΔORF2	110.58+peptide	OK	80.0718	53.9828	-0.568793	-1.22899	0.0329	0.237205	no
gene:SpnNT_01512	pgl	Chromosome:1547596-1548610	110.58	ΔORF2+peptide	OK	71.6626	54.7974	-0.387114	-0.830819	0.144	0.579113	no
gene:SpnNT_01512	pgl	Chromosome:1547596-1548610	ΔORF2	ΔORF2+peptide	OK	80.0718	54.7974	-0.547187	-1.18008	0.03755	0.259742	no
gene:SpnNT_01512	pgl	Chromosome:1547596-1548610	110.58+peptide	ΔORF2+peptide	OK	53.9828	54.7974	0.0216063	0.0456721	0.93685	0.994855	no
gene:SpnNT_01513	atpC	Chromosome:1548655-1549075	110.58	ΔORF2	OK	2473.29	2288.82	-0.111826	-0.235116	0.67285	0.984845	no
gene:SpnNT_01513	atpC	Chromosome:1548655-1549075	110.58	110.58+peptide	OK	2473.29	2361.01	-0.067031	-0.135948	0.8075	0.994748	no
gene:SpnNT_01513	atpC	Chromosome:1548655-1549075	ΔORF2	110.58+peptide	OK	2288.82	2361.01	0.0447954	0.0929209	0.87235	0.994748	no
gene:SpnNT_01513	atpC	Chromosome:1548655-1549075	110.58	ΔORF2+peptide	OK	2473.29	1750.45	-0.498703	-1.02773	0.06755	0.380099	no
gene:SpnNT_01513	atpC	Chromosome:1548655-1549075	ΔORF2	ΔORF2+peptide	OK	2288.82	1750.45	-0.386877	-0.816053	0.15095	0.593044	no
gene:SpnNT_01513	atpC	Chromosome:1548655-1549075	110.58+peptide	ΔORF2+peptide	OK	2361.01	1750.45	-0.431672	-0.878136	0.11955	0.524135	no
gene:SpnNT_01514	atpD	Chromosome:1549085-1550492	110.58	ΔORF2	OK	593.902	610.515	0.0398024	0.0872326	0.87865	0.994748	no
gene:SpnNT_01514	atpD	Chromosome:1549085-1550492	110.58	110.58+peptide	OK	593.902	500.935	-0.245603	-0.544415	0.3418	0.845926	no
gene:SpnNT_01514	atpD	Chromosome:1549085-1550492	ΔORF2	110.58+peptide	OK	610.515	500.935	-0.285405	-0.621135	0.2814	0.784843	no
gene:SpnNT_01514	atpD	Chromosome:1549085-1550492	110.58	ΔORF2+peptide	OK	593.902	508.376	-0.224329	-0.502568	0.3777	0.875802	no
gene:SpnNT_01514	atpD	Chromosome:1549085-1550492	ΔORF2	ΔORF2+peptide	OK	610.515	508.376	-0.264131	-0.580749	0.3097	0.814895	no
gene:SpnNT_01514	atpD	Chromosome:1549085-1550492	110.58+peptide	ΔORF2+peptide	OK	500.935	508.376	0.0212738	0.0473123	0.9328	0.994855	no
gene:SpnNT_01515	atpG	Chromosome:1550577-1551456	110.58	ΔORF2	OK	452.338	479.258	0.0834012	0.189465	0.7413	0.994748	no
gene:SpnNT_01515	atpG	Chromosome:1550577-1551456	110.58	110.58+peptide	OK	452.338	381.993	-0.243855	-0.557414	0.3283	0.834032	no
gene:SpnNT_01515	atpG	Chromosome:1550577-1551456	ΔORF2	110.58+peptide	OK	479.258	381.993	-0.327256	-0.740442	0.1921	0.673094	no
gene:SpnNT_01515	atpG	Chromosome:1550577-1551456	110.58	ΔORF2+peptide	OK	452.338	397.681	-0.185792	-0.427224	0.4456	0.918272	no
gene:SpnNT_01515	atpG	Chromosome:1550577-1551456	ΔORF2	ΔORF2+peptide	OK	479.258	397.681	-0.269193	-0.612629	0.2719	0.774059	no

gene:SpnNT_01515	atpG	Chromosome:1550577-1551456	110.58+peptide	ΔORF2+peptide	OK	381.993	397.681	0.0580629	0.132963	0.81045	0.994748	no
gene:SpnNT_01516	atpA	Chromosome:1551471-1552977	110.58	ΔORF2	OK	457.066	454.544	-0.00798314	-0.017448	0.9749	0.99536	no
gene:SpnNT_01516	atpA	Chromosome:1551471-1552977	110.58	110.58+peptide	OK	457.066	386.925	-0.240347	-0.531942	0.35545	0.85704	no
gene:SpnNT_01516	atpA	Chromosome:1551471-1552977	ΔORF2	110.58+peptide	OK	454.544	386.925	-0.232364	-0.506078	0.36725	0.864715	no
gene:SpnNT_01516	atpA	Chromosome:1551471-1552977	110.58	ΔORF2+peptide	OK	457.066	373.071	-0.292953	-0.654478	0.2511	0.748849	no
gene:SpnNT_01516	atpA	Chromosome:1551471-1552977	ΔORF2	ΔORF2+peptide	OK	454.544	373.071	-0.28497	-0.62631	0.26125	0.761443	no
gene:SpnNT_01516	atpA	Chromosome:1551471-1552977	110.58+peptide	ΔORF2+peptide	OK	386.925	373.071	-0.0526057	-0.117095	0.8352	0.994748	no
gene:SpnNT_01517	atpH	Chromosome:1552991-1554022	110.58	ΔORF2	OK	238.312	315.195	0.403391	0.519922	0.3805	0.878145	no
gene:SpnNT_01517	atpH	Chromosome:1552991-1554022	110.58	110.58+peptide	OK	238.312	232.401	-0.0362378	-0.0480221	0.93535	0.994855	no
gene:SpnNT_01517	atpH	Chromosome:1552991-1554022	ΔORF2	110.58+peptide	OK	315.195	232.401	-0.439629	-0.588455	0.3147	0.821136	no
gene:SpnNT_01517	atpH	Chromosome:1552991-1554022	110.58	ΔORF2+peptide	OK	238.312	293.794	0.301951	0.393766	0.49155	0.940662	no
gene:SpnNT_01517	atpH	Chromosome:1552991-1554022	ΔORF2	ΔORF2+peptide	OK	315.195	293.794	-0.10144	-0.133574	0.8157	0.994748	no
gene:SpnNT_01517	atpH	Chromosome:1552991-1554022	110.58+peptide	ΔORF2+peptide	OK	232.401	293.794	0.338189	0.458438	0.41915	0.903984	no
gene:SpnNT_01518	atpF	Chromosome:1552991-1554022	110.58	ΔORF2	OK	544.192	589.223	0.114697	0.20624	0.71825	0.993347	no
gene:SpnNT_01518	atpF	Chromosome:1552991-1554022	110.58	110.58+peptide	OK	544.192	431.906	-0.333399	-0.611231	0.2823	0.785151	no
gene:SpnNT_01518	atpF	Chromosome:1552991-1554022	ΔORF2	110.58+peptide	OK	589.223	431.906	-0.448095	-0.786063	0.16585	0.621332	no
gene:SpnNT_01518	atpF	Chromosome:1552991-1554022	110.58	ΔORF2+peptide	OK	544.192	532.541	-0.0312239	-0.0565187	0.9185	0.994748	no
gene:SpnNT_01518	atpF	Chromosome:1552991-1554022	ΔORF2	ΔORF2+peptide	OK	589.223	532.541	-0.145921	-0.253005	0.65845	0.982966	no
gene:SpnNT_01518	atpF	Chromosome:1552991-1554022	110.58+peptide	ΔORF2+peptide	OK	431.906	532.541	0.302175	0.533443	0.35855	0.859746	no
gene:SpnNT_01519	atpB	Chromosome:1554035-1554752	110.58	ΔORF2	OK	337.812	400.333	0.244977	0.564969	0.32105	0.826982	no
gene:SpnNT_01519	atpB	Chromosome:1554035-1554752	110.58	110.58+peptide	OK	337.812	270.308	-0.321616	-0.735641	0.1986	0.681315	no
gene:SpnNT_01519	atpB	Chromosome:1554035-1554752	ΔORF2	110.58+peptide	OK	400.333	270.308	-0.566593	-1.29822	0.02525	0.198283	no
gene:SpnNT_01519	atpB	Chromosome:1554035-1554752	110.58	ΔORF2+peptide	OK	337.812	337.45	-0.0015493	-0.003549	0.995	0.999258	no
gene:SpnNT_01519	atpB	Chromosome:1554035-1554752	ΔORF2	ΔORF2+peptide	OK	400.333	337.45	-0.246526	-0.565701	0.32835	0.834032	no
gene:SpnNT_01519	atpB	Chromosome:1554035-1554752	110.58+peptide	ΔORF2+peptide	OK	270.308	337.45	0.320067	0.728498	0.21345	0.698651	no
gene:SpnNT_01520	atpE	Chromosome:1554786-1554987	110.58	ΔORF2	OK	1322.74	1509.61	0.190642	0.402489	0.47545	0.930746	no
gene:SpnNT_01520	atpE	Chromosome:1554786-1554987	110.58	110.58+peptide	OK	1322.74	1288.89	-0.0374065	-0.0757622	0.8908	0.994748	no
gene:SpnNT_01520	atpE	Chromosome:1554786-1554987	ΔORF2	110.58+peptide	OK	1509.61	1288.89	-0.228048	-0.465502	0.4102	0.898184	no
gene:SpnNT_01520	atpE	Chromosome:1554786-1554987	110.58	ΔORF2+peptide	OK	1322.74	1250.02	-0.08158	-0.166367	0.76525	0.994748	no
gene:SpnNT_01520	atpE	Chromosome:1554786-1554987	ΔORF2	ΔORF2+peptide	OK	1509.61	1250.02	-0.272222	-0.559556	0.3211	0.826982	no
gene:SpnNT_01520	atpE	Chromosome:1554786-1554987	110.58+peptide	ΔORF2+peptide	OK	1288.89	1250.02	-0.0441734	-0.0872879	0.87585	0.994748	no
gene:SpnNT_01521	NA	Chromosome:1555061-1556069	110.58	ΔORF2	OK	59.6589	60.2941	0.0152796	0.0325942	0.9523	0.994855	no
gene:SpnNT_01521	NA	Chromosome:1555061-1556069	110.58	110.58+peptide	OK	59.6589	51.8733	-0.201745	-0.424554	0.45765	0.921722	no
gene:SpnNT_01521	NA	Chromosome:1555061-1556069	ΔORF2	110.58+peptide	OK	60.2941	51.8733	-0.217025	-0.457348	0.42915	0.911067	no
gene:SpnNT_01521	NA	Chromosome:1555061-1556069	110.58	ΔORF2+peptide	OK	59.6589	57.8792	-0.0436919	-0.0929503	0.8636	0.994748	no
gene:SpnNT_01521	NA	Chromosome:1555061-1556069	ΔORF2	ΔORF2+peptide	OK	60.2941	57.8792	-0.0589715	-0.125636	0.82355	0.994748	no
gene:SpnNT_01521	NA	Chromosome:1555061-1556069	110.58+peptide	ΔORF2+peptide	OK	51.8733	57.8792	0.158053	0.332195	0.5554	0.966573	no
gene:SpnNT_01522	ywnH	Chromosome:1556525-1556918	110.58	ΔORF2	OK	206.193	234.715	0.186915	0.393482	0.49175	0.94077	no
gene:SpnNT_01522	ywnH	Chromosome:1556525-1556918	110.58	110.58+peptide	OK	206.193	178.546	-0.207697	-0.430493	0.4546	0.921244	no
gene:SpnNT_01522	ywnH	Chromosome:1556525-1556918	ΔORF2	110.58+peptide	OK	234.715	178.546	-0.394612	-0.823892	0.14585	0.581892	no
gene:SpnNT_01522	ywnH	Chromosome:1556525-1556918	110.58	ΔORF2+peptide	OK	206.193	229.653	0.155459	0.325227	0.5718	0.969025	no
gene:SpnNT_01522	ywnH	Chromosome:1556525-1556918	ΔORF2	ΔORF2+peptide	OK	234.715	229.653	-0.0314555	-0.0662964	0.9037	0.994748	no
gene:SpnNT_01522	ywnH	Chromosome:1556525-1556918	110.58+peptide	ΔORF2+peptide	OK	178.546	229.653	0.363156	0.753575	0.18975	0.667533	no
gene:SpnNT_01523	greA	Chromosome:1557276-1557759	110.58	ΔORF2	OK	454.01	489.94	0.109882	0.249249	0.65835	0.982966	no
gene:SpnNT_01523	greA	Chromosome:1557276-1557759	110.58	110.58+peptide	OK	454.01	577.894	0.348081	0.782049	0.17585	0.639375	no

gene:SpnNT_01523	greA	Chromosome:1557276-1557759	ΔORF2	110.58+peptide	OK	489.94	577.894	0.238198	0.538836	0.3398	0.844213	no
gene:SpnNT_01523	greA	Chromosome:1557276-1557759	110.58	ΔORF2+peptide	OK	454.01	549.963	0.276611	0.621911	0.27425	0.77642	no
gene:SpnNT_01523	greA	Chromosome:1557276-1557759	ΔORF2	ΔORF2+peptide	OK	489.94	549.963	0.166728	0.377431	0.497	0.943933	no
gene:SpnNT_01523	greA	Chromosome:1557276-1557759	110.58+peptide	ΔORF2+peptide	OK	577.894	549.963	-0.07147	-0.160257	0.77385	0.994748	no
gene:SpnNT_01524	NA	Chromosome:1557829-1559485	110.58	ΔORF2	OK	393.184	420.693	0.0975644	0.216713	0.70825	0.990441	no
gene:SpnNT_01524	NA	Chromosome:1557829-1559485	110.58	110.58+peptide	OK	393.184	393.016	-0.000615725	-0.00136709	0.99855	0.999412	no
gene:SpnNT_01524	NA	Chromosome:1557829-1559485	ΔORF2	110.58+peptide	OK	420.693	393.016	-0.0981802	-0.218004	0.7046	0.990367	no
gene:SpnNT_01524	NA	Chromosome:1557829-1559485	110.58	ΔORF2+peptide	OK	393.184	428.089	0.122706	0.272392	0.63745	0.980887	no
gene:SpnNT_01524	NA	Chromosome:1557829-1559485	ΔORF2	ΔORF2+peptide	OK	420.693	428.089	0.0251413	0.0558147	0.9238	0.994748	no
gene:SpnNT_01524	NA	Chromosome:1557829-1559485	110.58+peptide	ΔORF2+peptide	OK	393.016	428.089	0.123321	0.273663	0.63495	0.980887	no
gene:SpnNT_01525	NA	Chromosome:1559565-1560521	110.58	ΔORF2	OK	58.537	57.6091	-0.0230511	-0.032721	0.95575	0.994855	no
gene:SpnNT_01525	NA	Chromosome:1559565-1560521	110.58	110.58+peptide	OK	58.537	65.2688	0.157045	0.22072	0.70485	0.990367	no
gene:SpnNT_01525	NA	Chromosome:1559565-1560521	ΔORF2	110.58+peptide	OK	57.6091	65.2688	0.180096	0.254098	0.65725	0.982966	no
gene:SpnNT_01525	NA	Chromosome:1559565-1560521	110.58	ΔORF2+peptide	OK	58.537	60.8347	0.0555452	0.0796208	0.89085	0.994748	no
gene:SpnNT_01525	NA	Chromosome:1559565-1560521	ΔORF2	ΔORF2+peptide	OK	57.6091	60.8347	0.0785963	0.113117	0.841	0.994748	no
gene:SpnNT_01525	NA	Chromosome:1559565-1560521	110.58+peptide	ΔORF2+peptide	OK	65.2688	60.8347	-0.101499	-0.144596	0.7994	0.994748	no
gene:SpnNT_01526	NA	Chromosome:1559565-1560521	110.58	ΔORF2	OK	70.2562	63.7676	-0.139801	-0.199917	0.72535	0.994748	no
gene:SpnNT_01526	NA	Chromosome:1559565-1560521	110.58	110.58+peptide	OK	70.2562	67.8937	-0.0493481	-0.0695308	0.90435	0.994748	no
gene:SpnNT_01526	NA	Chromosome:1559565-1560521	ΔORF2	110.58+peptide	OK	63.7676	67.8937	0.0904526	0.128688	0.82835	0.994748	no
gene:SpnNT_01526	NA	Chromosome:1559565-1560521	110.58	ΔORF2+peptide	OK	70.2562	69.355	-0.0186261	-0.0265341	0.963	0.994855	no
gene:SpnNT_01526	NA	Chromosome:1559565-1560521	ΔORF2	ΔORF2+peptide	OK	63.7676	69.355	0.121175	0.174341	0.76065	0.994748	no
gene:SpnNT_01526	NA	Chromosome:1559565-1560521	110.58+peptide	ΔORF2+peptide	OK	67.8937	69.355	0.030722	0.0435436	0.94235	0.994855	no
gene:SpnNT_01527	murC	Chromosome:1560530-1561865	110.58	ΔORF2	OK	156.503	145.501	-0.105157	-0.241667	0.6708	0.984845	no
gene:SpnNT_01527	murC	Chromosome:1560530-1561865	110.58	110.58+peptide	OK	156.503	172.108	0.137124	0.315399	0.57675	0.969538	no
gene:SpnNT_01527	murC	Chromosome:1560530-1561865	ΔORF2	110.58+peptide	OK	145.501	172.108	0.242281	0.558157	0.3237	0.829122	no
gene:SpnNT_01527	murC	Chromosome:1560530-1561865	110.58	ΔORF2+peptide	OK	156.503	163.099	0.0595587	0.137014	0.81015	0.994748	no
gene:SpnNT_01527	murC	Chromosome:1560530-1561865	ΔORF2	ΔORF2+peptide	OK	145.501	163.099	0.164716	0.37953	0.4979	0.944017	no
gene:SpnNT_01527	murC	Chromosome:1560530-1561865	110.58+peptide	ΔORF2+peptide	OK	172.108	163.099	-0.0775655	-0.178875	0.74955	0.994748	no
gene:SpnNT_01528	NA	Chromosome:1561876-1562494	110.58	ΔORF2	OK	105.119	105.137	0.000258132	0.000547264	0.99865	0.999412	no
gene:SpnNT_01528	NA	Chromosome:1561876-1562494	110.58	110.58+peptide	OK	105.119	115.2	0.132128	0.281791	0.62605	0.980887	no
gene:SpnNT_01528	NA	Chromosome:1561876-1562494	ΔORF2	110.58+peptide	OK	105.137	115.2	0.131869	0.280921	0.62465	0.980887	no
gene:SpnNT_01528	NA	Chromosome:1561876-1562494	110.58	ΔORF2+peptide	OK	105.119	135.634	0.367705	0.788882	0.1629	0.61656	no
gene:SpnNT_01528	NA	Chromosome:1561876-1562494	ΔORF2	ΔORF2+peptide	OK	105.137	135.634	0.367447	0.787424	0.1639	0.618371	no
gene:SpnNT_01528	NA	Chromosome:1561876-1562494	110.58+peptide	ΔORF2+peptide	OK	115.2	135.634	0.235578	0.507904	0.3732	0.870292	no
gene:SpnNT_01529	NA	Chromosome:1562541-1565640	110.58	ΔORF2	OK	66.6133	65.2278	-0.0303233	-0.0692704	0.8986	0.994748	no
gene:SpnNT_01529	NA	Chromosome:1562541-1565640	110.58	110.58+peptide	OK	66.6133	54.4288	-0.29144	-0.655788	0.25645	0.755871	no
gene:SpnNT_01529	NA	Chromosome:1562541-1565640	ΔORF2	110.58+peptide	OK	65.2278	54.4288	-0.261116	-0.589284	0.3037	0.809137	no
gene:SpnNT_01529	NA	Chromosome:1562541-1565640	110.58	ΔORF2+peptide	OK	66.6133	62.5898	-0.0898826	-0.204684	0.7246	0.994748	no
gene:SpnNT_01529	NA	Chromosome:1562541-1565640	ΔORF2	ΔORF2+peptide	OK	65.2278	62.5898	-0.0595593	-0.136039	0.81125	0.994748	no
gene:SpnNT_01529	NA	Chromosome:1562541-1565640	110.58+peptide	ΔORF2+peptide	OK	54.4288	62.5898	0.201557	0.453479	0.43195	0.913334	no
gene:SpnNT_01530	patB_2	Chromosome:1565732-1566899	110.58	ΔORF2	OK	16.6709	16.5778	-0.00808003	-0.0150338	0.9791	0.995765	no
gene:SpnNT_01530	patB_2	Chromosome:1565732-1566899	110.58	110.58+peptide	OK	16.6709	12.3243	-0.435823	-0.793396	0.16585	0.621332	no
gene:SpnNT_01530	patB_2	Chromosome:1565732-1566899	ΔORF2	110.58+peptide	OK	16.5778	12.3243	-0.427743	-0.781041	0.17405	0.634412	no
gene:SpnNT_01530	patB_2	Chromosome:1565732-1566899	110.58	ΔORF2+peptide	OK	16.6709	11.7371	-0.506255	-0.915068	0.10675	0.497081	no
gene:SpnNT_01530	patB_2	Chromosome:1565732-1566899	ΔORF2	ΔORF2+peptide	OK	16.5778	11.7371	-0.498175	-0.903147	0.1159	0.516286	no

gene:SpnNT_01530	patB_2	Chromosome:1565732-1566899	110.58+peptide	ΔORF2+peptide	OK	12.3243	11.7371	-0.0704312	-0.125065	0.82785	0.994748	no
gene:SpnNT_01531	metI	Chromosome:1566906-1568001	110.58	ΔORF2	OK	13.1819	13.673	0.0527709	0.0936061	0.86835	0.994748	no
gene:SpnNT_01531	metI	Chromosome:1566906-1568001	110.58	110.58+peptide	OK	13.1819	8.78388	-0.585623	-1.01933	0.07985	0.421307	no
gene:SpnNT_01531	metI	Chromosome:1566906-1568001	ΔORF2	110.58+peptide	OK	13.673	8.78388	-0.638394	-1.10313	0.0556	0.336206	no
gene:SpnNT_01531	metI	Chromosome:1566906-1568001	110.58	ΔORF2+peptide	OK	13.1819	8.5794	-0.619605	-1.07574	0.0636	0.366195	no
gene:SpnNT_01531	metI	Chromosome:1566906-1568001	ΔORF2	ΔORF2+peptide	OK	13.673	8.5794	-0.672376	-1.15893	0.04505	0.292068	no
gene:SpnNT_01531	metI	Chromosome:1566906-1568001	110.58+peptide	ΔORF2+peptide	OK	8.78388	8.5794	-0.0339814	-0.057534	0.9193	0.994748	no
gene:SpnNT_01532	lmrA	Chromosome:1568158-1569589	110.58	ΔORF2	OK	200.835	215.149	0.0993225	0.219116	0.6938	0.98828	no
gene:SpnNT_01532	lmrA	Chromosome:1568158-1569589	110.58	110.58+peptide	OK	200.835	51.7142	-1.95738	-4.24422	5.00E-05	0.0013612	yes
gene:SpnNT_01532	lmrA	Chromosome:1568158-1569589	ΔORF2	110.58+peptide	OK	215.149	51.7142	-2.0567	-4.47454	5.00E-05	0.0013612	yes
gene:SpnNT_01532	lmrA	Chromosome:1568158-1569589	110.58	ΔORF2+peptide	OK	200.835	59.3975	-1.75754	-3.85228	5.00E-05	0.0013612	yes
gene:SpnNT_01532	lmrA	Chromosome:1568158-1569589	ΔORF2	ΔORF2+peptide	OK	215.149	59.3975	-1.85686	-4.08393	5.00E-05	0.0013612	yes
gene:SpnNT_01532	lmrA	Chromosome:1568158-1569589	110.58+peptide	ΔORF2+peptide	OK	51.7142	59.3975	0.199843	0.432044	0.4476	0.919505	no
gene:SpnNT_01533	NA	Chromosome:1569606-1569840	110.58	ΔORF2	OK	60.0944	81.3698	0.437264	0.659891	0.2526	0.750933	no
gene:SpnNT_01533	NA	Chromosome:1569606-1569840	110.58	110.58+peptide	OK	60.0944	23.528	-1.35285	-1.83649	0.0033	0.0432593	yes
gene:SpnNT_01533	NA	Chromosome:1569606-1569840	ΔORF2	110.58+peptide	OK	81.3698	23.528	-1.79011	-2.43935	5.00E-05	0.0013612	yes
gene:SpnNT_01533	NA	Chromosome:1569606-1569840	110.58	ΔORF2+peptide	OK	60.0944	45.6854	-0.395496	-0.584117	0.34865	0.853357	no
gene:SpnNT_01533	NA	Chromosome:1569606-1569840	ΔORF2	ΔORF2+peptide	OK	81.3698	45.6854	-0.83276	-1.23548	0.05165	0.321209	no
gene:SpnNT_01533	NA	Chromosome:1569606-1569840	110.58+peptide	ΔORF2+peptide	OK	23.528	45.6854	0.957354	1.28173	0.0461	0.296531	no
gene:SpnNT_01534	sarA_5	Chromosome:1570011-1571970	110.58	ΔORF2	OK	1132.49	1228.19	0.117041	0.236411	0.6778	0.98524	no
gene:SpnNT_01534	sarA_5	Chromosome:1570011-1571970	110.58	110.58+peptide	OK	1132.49	437.484	-1.37219	-2.85131	5.00E-05	0.0013612	yes
gene:SpnNT_01534	sarA_5	Chromosome:1570011-1571970	ΔORF2	110.58+peptide	OK	1228.19	437.484	-1.48923	-3.14269	5.00E-05	0.0013612	yes
gene:SpnNT_01534	sarA_5	Chromosome:1570011-1571970	110.58	ΔORF2+peptide	OK	1132.49	478.031	-1.24432	-2.60103	5.00E-05	0.0013612	yes
gene:SpnNT_01534	sarA_5	Chromosome:1570011-1571970	ΔORF2	ΔORF2+peptide	OK	1228.19	478.031	-1.36136	-2.89053	5.00E-05	0.0013612	yes
gene:SpnNT_01534	sarA_5	Chromosome:1570011-1571970	110.58+peptide	ΔORF2+peptide	OK	437.484	478.031	0.127874	0.280168	0.6213	0.980887	no
gene:SpnNT_01535	ytgP	Chromosome:1572171-1573794	110.58	ΔORF2	OK	96.2752	104.253	0.114851	0.262837	0.63825	0.980887	no
gene:SpnNT_01535	ytgP	Chromosome:1572171-1573794	110.58	110.58+peptide	OK	96.2752	93.0853	-0.048611	-0.11075	0.84555	0.994748	no
gene:SpnNT_01535	ytgP	Chromosome:1572171-1573794	ΔORF2	110.58+peptide	OK	104.253	93.0853	-0.163462	-0.37266	0.50585	0.945292	no
gene:SpnNT_01535	ytgP	Chromosome:1572171-1573794	110.58	ΔORF2+peptide	OK	96.2752	103.681	0.106921	0.243338	0.67145	0.984845	no
gene:SpnNT_01535	ytgP	Chromosome:1572171-1573794	ΔORF2	ΔORF2+peptide	OK	104.253	103.681	-0.00792955	-0.0180585	0.97425	0.99536	no
gene:SpnNT_01535	ytgP	Chromosome:1572171-1573794	110.58+peptide	ΔORF2+peptide	OK	93.0853	103.681	0.155532	0.352637	0.54195	0.961494	no
gene:SpnNT_01536	murE	Chromosome:1573899-1575345	110.58	ΔORF2	OK	85.9248	89.4385	0.0578223	0.130218	0.81845	0.994748	no
gene:SpnNT_01536	murE	Chromosome:1573899-1575345	110.58	110.58+peptide	OK	85.9248	86.7484	0.0137621	0.0309701	0.95735	0.994855	no
gene:SpnNT_01536	murE	Chromosome:1573899-1575345	ΔORF2	110.58+peptide	OK	89.4385	86.7484	-0.0440602	-0.0993049	0.8614	0.994748	no
gene:SpnNT_01536	murE	Chromosome:1573899-1575345	110.58	ΔORF2+peptide	OK	85.9248	89.5645	0.0598531	0.134842	0.81365	0.994748	no
gene:SpnNT_01536	murE	Chromosome:1573899-1575345	ΔORF2	ΔORF2+peptide	OK	89.4385	89.5645	0.00203085	0.00458232	0.9934	0.998522	no
gene:SpnNT_01536	murE	Chromosome:1573899-1575345	110.58+peptide	ΔORF2+peptide	OK	86.7484	89.5645	0.046091	0.103921	0.85375	0.994748	no
gene:SpnNT_01537	NA	Chromosome:1575387-1575585	110.58	ΔORF2	OK	2068.24	1912.69	-0.112804	-0.205931	0.7057	0.990422	no
gene:SpnNT_01537	NA	Chromosome:1575387-1575585	110.58	110.58+peptide	OK	2068.24	9578.11	2.21134	3.68634	5.00E-05	0.0013612	yes
gene:SpnNT_01537	NA	Chromosome:1575387-1575585	ΔORF2	110.58+peptide	OK	1912.69	9578.11	2.32414	3.91866	5.00E-05	0.0013612	yes
gene:SpnNT_01537	NA	Chromosome:1575387-1575585	110.58	ΔORF2+peptide	OK	2068.24	7866.66	1.92734	3.31025	5.00E-05	0.0013612	yes
gene:SpnNT_01537	NA	Chromosome:1575387-1575585	ΔORF2	ΔORF2+peptide	OK	1912.69	7866.66	2.04015	3.54653	5.00E-05	0.0013612	yes
gene:SpnNT_01537	NA	Chromosome:1575387-1575585	110.58+peptide	ΔORF2+peptide	OK	9578.11	7866.66	-0.28399	-0.454342	0.38995	0.88421	no
gene:SpnNT_01538	NA	Chromosome:1575685-1576282	110.58	ΔORF2	OK	20.9825	18.8594	-0.153905	-0.256472	0.66175	0.982966	no
gene:SpnNT_01538	NA	Chromosome:1575685-1576282	110.58	110.58+peptide	OK	20.9825	28.994	0.466566	0.814193	0.16155	0.614666	no

gene:SpnNT_01538	NA	Chromosome:1575685-1576282	ΔORF2	110.58+peptide	OK	18.8594	28.994	0.620471	1.06216	0.06755	0.380099	no
gene:SpnNT_01538	NA	Chromosome:1575685-1576282	110.58	ΔORF2+peptide	OK	20.9825	34.6159	0.722246	1.26718	0.0309	0.226886	no
gene:SpnNT_01538	NA	Chromosome:1575685-1576282	ΔORF2	ΔORF2+peptide	OK	18.8594	34.6159	0.876151	1.50763	0.0102	0.101628	no
gene:SpnNT_01538	NA	Chromosome:1575685-1576282	110.58+peptide	ΔORF2+peptide	OK	28.994	34.6159	0.25568	0.462208	0.4192	0.903984	no
gene:SpnNT_01539	NA	Chromosome:1576318-1576831	110.58	ΔORF2	OK	27.8999	24.4076	-0.192928	-0.328233	0.5686	0.968621	no
gene:SpnNT_01539	NA	Chromosome:1576318-1576831	110.58	110.58+peptide	OK	27.8999	34.4628	0.304779	0.531162	0.35485	0.857011	no
gene:SpnNT_01539	NA	Chromosome:1576318-1576831	ΔORF2	110.58+peptide	OK	24.4076	34.4628	0.497707	0.863921	0.1356	0.560423	no
gene:SpnNT_01539	NA	Chromosome:1576318-1576831	110.58	ΔORF2+peptide	OK	27.8999	46.7677	0.745252	1.29885	0.02795	0.213109	no
gene:SpnNT_01539	NA	Chromosome:1576318-1576831	ΔORF2	ΔORF2+peptide	OK	24.4076	46.7677	0.93818	1.62855	0.0059	0.0681512	no
gene:SpnNT_01539	NA	Chromosome:1576318-1576831	110.58+peptide	ΔORF2+peptide	OK	34.4628	46.7677	0.440473	0.784021	0.1737	0.634017	no
gene:SpnNT_01540	ppaC	Chromosome:1576917-1577853	110.58	ΔORF2	OK	455.592	457.916	0.00734045	0.0166782	0.9772	0.995534	no
gene:SpnNT_01540	ppaC	Chromosome:1576917-1577853	110.58	110.58+peptide	OK	455.592	425.399	-0.0989274	-0.225303	0.69375	0.98828	no
gene:SpnNT_01540	ppaC	Chromosome:1576917-1577853	ΔORF2	110.58+peptide	OK	457.916	425.399	-0.106268	-0.242588	0.67565	0.985207	no
gene:SpnNT_01540	ppaC	Chromosome:1576917-1577853	110.58	ΔORF2+peptide	OK	455.592	415.114	-0.134236	-0.306759	0.5869	0.973049	no
gene:SpnNT_01540	ppaC	Chromosome:1576917-1577853	ΔORF2	ΔORF2+peptide	OK	457.916	415.114	-0.141576	-0.324297	0.5694	0.968621	no
gene:SpnNT_01540	ppaC	Chromosome:1576917-1577853	110.58+peptide	ΔORF2+peptide	OK	425.399	415.114	-0.0353085	-0.0810725	0.887	0.994748	no
gene:SpnNT_01541	NA	Chromosome:1577923-1578962	110.58	ΔORF2	OK	156.568	153.396	-0.0295272	-0.0264749	0.9614	0.994855	no
gene:SpnNT_01541	NA	Chromosome:1577923-1578962	110.58	110.58+peptide	OK	156.568	184.029	0.23315	0.224701	0.6991	0.990367	no
gene:SpnNT_01541	NA	Chromosome:1577923-1578962	ΔORF2	110.58+peptide	OK	153.396	184.029	0.262677	0.224396	0.7011	0.990367	no
gene:SpnNT_01541	NA	Chromosome:1577923-1578962	110.58	ΔORF2+peptide	OK	156.568	192.04	0.294621	0.279288	0.62425	0.980887	no
gene:SpnNT_01541	NA	Chromosome:1577923-1578962	ΔORF2	ΔORF2+peptide	OK	153.396	192.04	0.324148	0.273321	0.6377	0.980887	no
gene:SpnNT_01541	NA	Chromosome:1577923-1578962	110.58+peptide	ΔORF2+peptide	OK	184.029	192.04	0.0614706	0.0552192	0.92335	0.994748	no
gene:SpnNT_01542	NA	Chromosome:1577923-1578962	110.58	ΔORF2	OK	91.2425	93.0279	0.0279562	0.051025	0.9292	0.994748	no
gene:SpnNT_01542	NA	Chromosome:1577923-1578962	110.58	110.58+peptide	OK	91.2425	119.719	0.39187	0.745847	0.1924	0.673785	no
gene:SpnNT_01542	NA	Chromosome:1577923-1578962	ΔORF2	110.58+peptide	OK	93.0279	119.719	0.363914	0.667226	0.2415	0.736283	no
gene:SpnNT_01542	NA	Chromosome:1577923-1578962	110.58	ΔORF2+peptide	OK	91.2425	131.2	0.523994	0.998071	0.08495	0.438691	no
gene:SpnNT_01542	NA	Chromosome:1577923-1578962	ΔORF2	ΔORF2+peptide	OK	93.0279	131.2	0.496038	0.910108	0.1137	0.511299	no
gene:SpnNT_01542	NA	Chromosome:1577923-1578962	110.58+peptide	ΔORF2+peptide	OK	119.719	131.2	0.132124	0.252908	0.65755	0.982966	no
gene:SpnNT_01543	yugI	Chromosome:1579016-1579376	110.58	ΔORF2	OK	89.136	111.054	0.317175	0.591816	0.3052	0.811188	no
gene:SpnNT_01543	yugI	Chromosome:1579016-1579376	110.58	110.58+peptide	OK	89.136	122.642	0.460378	0.872121	0.1286	0.54365	no
gene:SpnNT_01543	yugI	Chromosome:1579016-1579376	ΔORF2	110.58+peptide	OK	111.054	122.642	0.143203	0.274047	0.6329	0.980887	no
gene:SpnNT_01543	yugI	Chromosome:1579016-1579376	110.58	ΔORF2+peptide	OK	89.136	170.774	0.93801	1.80269	0.0026	0.0358374	yes
gene:SpnNT_01543	yugI	Chromosome:1579016-1579376	ΔORF2	ΔORF2+peptide	OK	111.054	170.774	0.620835	1.20568	0.04125	0.276306	no
gene:SpnNT_01543	yugI	Chromosome:1579016-1579376	110.58+peptide	ΔORF2+peptide	OK	122.642	170.774	0.477632	0.94293	0.10615	0.495694	no
gene:SpnNT_01544	NA	Chromosome:1579377-1580778	110.58	ΔORF2	OK	140.128	159.612	0.187824	0.433921	0.4441	0.917631	no
gene:SpnNT_01544	NA	Chromosome:1579377-1580778	110.58	110.58+peptide	OK	140.128	170.279	0.281154	0.648874	0.24985	0.747607	no
gene:SpnNT_01544	NA	Chromosome:1579377-1580778	ΔORF2	110.58+peptide	OK	159.612	170.279	0.0933302	0.215312	0.6999	0.990367	no
gene:SpnNT_01544	NA	Chromosome:1579377-1580778	110.58	ΔORF2+peptide	OK	140.128	217.99	0.637515	1.46993	0.01045	0.103647	no
gene:SpnNT_01544	NA	Chromosome:1579377-1580778	ΔORF2	ΔORF2+peptide	OK	159.612	217.99	0.449692	1.03646	0.0686	0.383704	no
gene:SpnNT_01544	NA	Chromosome:1579377-1580778	110.58+peptide	ΔORF2+peptide	OK	170.279	217.99	0.356361	0.820512	0.14405	0.579113	no
gene:SpnNT_01545	NA	Chromosome:1581168-1582234	110.58	ΔORF2	OK	3.3579	1.46817	-1.19354	-0.798981	0.55305	0.965428	no
gene:SpnNT_01545	NA	Chromosome:1581168-1582234	110.58	110.58+peptide	OK	3.3579	3.34218	-0.0067715	-0.00328732	0.81255	0.994748	no
gene:SpnNT_01545	NA	Chromosome:1581168-1582234	ΔORF2	110.58+peptide	OK	1.46817	3.34218	1.18677	0.535739	0.6173	0.980734	no
gene:SpnNT_01545	NA	Chromosome:1581168-1582234	110.58	ΔORF2+peptide	OK	3.3579	9.40349	1.48564	0.951955	0.3667	0.864634	no
gene:SpnNT_01545	NA	Chromosome:1581168-1582234	ΔORF2	ΔORF2+peptide	OK	1.46817	9.40349	2.67917	1.5218	0.2817	0.784843	no

gene:SpnNT_01545	NA	Chromosome:1581168-1582234	110.58+peptide	ΔORF2+peptide	OK	3.34218	9.40349	1.49241	0.660131	0.46515	0.926405	no
gene:SpnNT_01546	NA	Chromosome:1581168-1582234	110.58	ΔORF2	OK	6.79358	5.17229	-0.393368	-0.267138	0.65885	0.982966	no
gene:SpnNT_01546	NA	Chromosome:1581168-1582234	110.58	110.58+peptide	OK	6.79358	11.9869	0.819216	0.5712	0.33155	0.837459	no
gene:SpnNT_01546	NA	Chromosome:1581168-1582234	ΔORF2	110.58+peptide	OK	5.17229	11.9869	1.21258	0.844559	0.1546	0.599841	no
gene:SpnNT_01546	NA	Chromosome:1581168-1582234	110.58	ΔORF2+peptide	OK	6.79358	18.4608	1.44222	1.09929	0.08845	0.447951	no
gene:SpnNT_01546	NA	Chromosome:1581168-1582234	ΔORF2	ΔORF2+peptide	OK	5.17229	18.4608	1.83559	1.39731	0.03425	0.244122	no
gene:SpnNT_01546	NA	Chromosome:1581168-1582234	110.58+peptide	ΔORF2+peptide	OK	11.9869	18.4608	0.623007	0.490344	0.40945	0.8977	no
gene:SpnNT_01547	NA	Chromosome:1581168-1582234	110.58	ΔORF2	OK	3.50952	3.28056	-0.097331	-0.0919088	0.87085	0.994748	no
gene:SpnNT_01547	NA	Chromosome:1581168-1582234	110.58	110.58+peptide	OK	3.50952	7.57507	1.10999	1.10015	0.0622	0.360348	no
gene:SpnNT_01547	NA	Chromosome:1581168-1582234	ΔORF2	110.58+peptide	OK	3.28056	7.57507	1.20732	1.3105	0.02885	0.216326	no
gene:SpnNT_01547	NA	Chromosome:1581168-1582234	110.58	ΔORF2+peptide	OK	3.50952	7.26768	1.05022	0.967968	0.09295	0.46026	no
gene:SpnNT_01547	NA	Chromosome:1581168-1582234	ΔORF2	ΔORF2+peptide	OK	3.28056	7.26768	1.14755	1.14303	0.04945	0.311514	no
gene:SpnNT_01547	NA	Chromosome:1581168-1582234	110.58+peptide	ΔORF2+peptide	OK	7.57507	7.26768	-0.0597654	-0.0628441	0.9126	0.994748	no
gene:SpnNT_01548	rpsR	Chromosome:1582275-1582515	110.58	ΔORF2	OK	2010.07	1780.01	-0.175363	-0.386994	0.49465	0.942197	no
gene:SpnNT_01548	rpsR	Chromosome:1582275-1582515	110.58	110.58+peptide	OK	2010.07	4559.14	1.18151	2.61354	5.00E-05	0.0013612	yes
gene:SpnNT_01548	rpsR	Chromosome:1582275-1582515	ΔORF2	110.58+peptide	OK	1780.01	4559.14	1.35688	2.96702	5.00E-05	0.0013612	yes
gene:SpnNT_01548	rpsR	Chromosome:1582275-1582515	110.58	ΔORF2+peptide	OK	2010.07	4748.41	1.2402	2.73863	5.00E-05	0.0013612	yes
gene:SpnNT_01548	rpsR	Chromosome:1582275-1582515	ΔORF2	ΔORF2+peptide	OK	1780.01	4748.41	1.41556	3.09013	5.00E-05	0.0013612	yes
gene:SpnNT_01548	rpsR	Chromosome:1582275-1582515	110.58+peptide	ΔORF2+peptide	OK	4559.14	4748.41	0.0586839	0.128402	0.82895	0.994748	no
gene:SpnNT_01549	ssb_1	Chromosome:1582546-1583017	110.58	ΔORF2	OK	736.644	726.469	-0.0200663	-0.0448231	0.9395	0.994855	no
gene:SpnNT_01549	ssb_1	Chromosome:1582546-1583017	110.58	110.58+peptide	OK	736.644	1989.34	1.43325	3.11065	5.00E-05	0.0013612	yes
gene:SpnNT_01549	ssb_1	Chromosome:1582546-1583017	ΔORF2	110.58+peptide	OK	726.469	1989.34	1.45332	3.20138	5.00E-05	0.0013612	yes
gene:SpnNT_01549	ssb_1	Chromosome:1582546-1583017	110.58	ΔORF2+peptide	OK	736.644	1929.31	1.38905	3.06456	5.00E-05	0.0013612	yes
gene:SpnNT_01549	ssb_1	Chromosome:1582546-1583017	ΔORF2	ΔORF2+peptide	OK	726.469	1929.31	1.40911	3.15691	5.00E-05	0.0013612	yes
gene:SpnNT_01549	ssb_1	Chromosome:1582546-1583017	110.58+peptide	ΔORF2+peptide	OK	1989.34	1929.31	-0.0442057	-0.0962096	0.86565	0.994748	no
gene:SpnNT_01550	rpsF	Chromosome:1583028-1583319	110.58	ΔORF2	OK	1032.11	1118.24	0.115639	0.253291	0.652	0.98163	no
gene:SpnNT_01550	rpsF	Chromosome:1583028-1583319	110.58	110.58+peptide	OK	1032.11	3008.46	1.54343	3.32732	5.00E-05	0.0013612	yes
gene:SpnNT_01550	rpsF	Chromosome:1583028-1583319	ΔORF2	110.58+peptide	OK	1118.24	3008.46	1.42779	3.13318	5.00E-05	0.0013612	yes
gene:SpnNT_01550	rpsF	Chromosome:1583028-1583319	110.58	ΔORF2+peptide	OK	1032.11	2997.52	1.53818	3.40083	5.00E-05	0.0013612	yes
gene:SpnNT_01550	rpsF	Chromosome:1583028-1583319	ΔORF2	ΔORF2+peptide	OK	1118.24	2997.52	1.42254	3.20452	5.00E-05	0.0013612	yes
gene:SpnNT_01550	rpsF	Chromosome:1583028-1583319	110.58+peptide	ΔORF2+peptide	OK	3008.46	2997.52	-0.0052537	-0.0116377	0.9841	0.996246	no
gene:SpnNT_01551	asnS	Chromosome:1583471-1584815	110.58	ΔORF2	OK	270.186	257.828	-0.0675456	-0.151553	0.7897	0.994748	no
gene:SpnNT_01551	asnS	Chromosome:1583471-1584815	110.58	110.58+peptide	OK	270.186	237.797	-0.184227	-0.41944	0.46535	0.926452	no
gene:SpnNT_01551	asnS	Chromosome:1583471-1584815	ΔORF2	110.58+peptide	OK	257.828	237.797	-0.116682	-0.262146	0.64785	0.981391	no
gene:SpnNT_01551	asnS	Chromosome:1583471-1584815	110.58	ΔORF2+peptide	OK	270.186	245.582	-0.137752	-0.314933	0.5749	0.969538	no
gene:SpnNT_01551	asnS	Chromosome:1583471-1584815	ΔORF2	ΔORF2+peptide	OK	257.828	245.582	-0.0702064	-0.158371	0.7779	0.994748	no
gene:SpnNT_01551	asnS	Chromosome:1583471-1584815	110.58+peptide	ΔORF2+peptide	OK	237.797	245.582	0.0464753	0.106399	0.847	0.994748	no
gene:SpnNT_01552	NA	Chromosome:1584833-1586807	110.58	ΔORF2	OK	85.573	80.1507	-0.0944396	-0.0487583	0.93515	0.994855	no
gene:SpnNT_01552	NA	Chromosome:1584833-1586807	110.58	110.58+peptide	OK	85.573	80.9811	-0.0795697	-0.0423543	0.93305	0.994855	no
gene:SpnNT_01552	NA	Chromosome:1584833-1586807	ΔORF2	110.58+peptide	OK	80.1507	80.9811	0.0148699	0.00770609	0.98965	0.997703	no
gene:SpnNT_01552	NA	Chromosome:1584833-1586807	110.58	ΔORF2+peptide	OK	85.573	92.7505	0.116201	0.0621445	0.9029	0.994748	no
gene:SpnNT_01552	NA	Chromosome:1584833-1586807	ΔORF2	ΔORF2+peptide	OK	80.1507	92.7505	0.21064	0.109649	0.85665	0.994748	no
gene:SpnNT_01552	NA	Chromosome:1584833-1586807	110.58+peptide	ΔORF2+peptide	OK	80.9811	92.7505	0.19577	0.105122	0.8371	0.994748	no
gene:SpnNT_01553	NA	Chromosome:1584833-1586807	110.58	ΔORF2	OK	234.318	234.306	-7.12E-05	-0.000147408	0.9998	0.9998	no
gene:SpnNT_01553	NA	Chromosome:1584833-1586807	110.58	110.58+peptide	OK	234.318	214.393	-0.128207	-0.265452	0.64185	0.980887	no

gene:SpnNT_01553	NA	Chromosome:1584833-1586807	ΔORF2	110.58+peptide	OK	234.306	214.393	-0.128136	-0.263946	0.65225	0.98163	no
gene:SpnNT_01553	NA	Chromosome:1584833-1586807	110.58	ΔORF2+peptide	OK	234.318	222.405	-0.0752787	-0.156499	0.78555	0.994748	no
gene:SpnNT_01553	NA	Chromosome:1584833-1586807	ΔORF2	ΔORF2+peptide	OK	234.306	222.405	-0.0752075	-0.155543	0.79015	0.994748	no
gene:SpnNT_01553	NA	Chromosome:1584833-1586807	110.58+peptide	ΔORF2+peptide	OK	214.393	222.405	0.0529281	0.109407	0.84935	0.994748	no
gene:SpnNT_01554	NA	Chromosome:1584833-1586807	110.58	ΔORF2	OK	121.538	143.183	0.236452	0.172916	0.7552	0.994748	no
gene:SpnNT_01554	NA	Chromosome:1584833-1586807	110.58	110.58+peptide	OK	121.538	138.432	0.187776	0.140285	0.8041	0.994748	no
gene:SpnNT_01554	NA	Chromosome:1584833-1586807	ΔORF2	110.58+peptide	OK	143.183	138.432	-0.0486768	-0.0362661	0.94895	0.994855	no
gene:SpnNT_01554	NA	Chromosome:1584833-1586807	110.58	ΔORF2+peptide	OK	121.538	163.937	0.431733	0.322782	0.57345	0.969538	no
gene:SpnNT_01554	NA	Chromosome:1584833-1586807	ΔORF2	ΔORF2+peptide	OK	143.183	163.937	0.19528	0.145599	0.79955	0.994748	no
gene:SpnNT_01554	NA	Chromosome:1584833-1586807	110.58+peptide	ΔORF2+peptide	OK	138.432	163.937	0.243957	0.185982	0.75395	0.994748	no
gene:SpnNT_01555	NA	Chromosome:1587184-1587733	110.58	ΔORF2	OK	374.679	320.23	-0.226546	-0.439098	0.43465	0.913666	no
gene:SpnNT_01555	NA	Chromosome:1587184-1587733	110.58	110.58+peptide	OK	374.679	337.027	-0.152793	-0.280138	0.6152	0.979616	no
gene:SpnNT_01555	NA	Chromosome:1587184-1587733	ΔORF2	110.58+peptide	OK	320.23	337.027	0.0737532	0.145494	0.7912	0.994748	no
gene:SpnNT_01555	NA	Chromosome:1587184-1587733	110.58	ΔORF2+peptide	OK	374.679	314.454	-0.252807	-0.470342	0.38365	0.88073	no
gene:SpnNT_01555	NA	Chromosome:1587184-1587733	ΔORF2	ΔORF2+peptide	OK	320.23	314.454	-0.0262615	-0.0526936	0.9232	0.994748	no
gene:SpnNT_01555	NA	Chromosome:1587184-1587733	110.58+peptide	ΔORF2+peptide	OK	337.027	314.454	-0.100015	-0.189119	0.7305	0.994748	no
gene:SpnNT_01556	NA	Chromosome:1588099-1588828	110.58	ΔORF2	OK	74.3635	94.9494	0.352565	0.74081	0.1965	0.677648	no
gene:SpnNT_01556	NA	Chromosome:1588099-1588828	110.58	110.58+peptide	OK	74.3635	65.9925	-0.172292	-0.358101	0.53135	0.957488	no
gene:SpnNT_01556	NA	Chromosome:1588099-1588828	ΔORF2	110.58+peptide	OK	94.9494	65.9925	-0.524857	-1.09741	0.05315	0.326973	no
gene:SpnNT_01556	NA	Chromosome:1588099-1588828	110.58	ΔORF2+peptide	OK	74.3635	85.3501	0.198798	0.418177	0.46465	0.926394	no
gene:SpnNT_01556	NA	Chromosome:1588099-1588828	ΔORF2	ΔORF2+peptide	OK	94.9494	85.3501	-0.153767	-0.325435	0.57035	0.968621	no
gene:SpnNT_01556	NA	Chromosome:1588099-1588828	110.58+peptide	ΔORF2+peptide	OK	65.9925	85.3501	0.37109	0.776755	0.17285	0.632681	no
gene:SpnNT_01557	NA	Chromosome:1588906-1589071	110.58	ΔORF2	OK	2084.96	3133.46	0.587737	0.985234	0.063	0.363378	no
gene:SpnNT_01557	NA	Chromosome:1588906-1589071	110.58	110.58+peptide	OK	2084.96	2404.4	0.205659	0.360942	0.5062	0.945531	no
gene:SpnNT_01557	NA	Chromosome:1588906-1589071	ΔORF2	110.58+peptide	OK	3133.46	2404.4	-0.382078	-0.650945	0.22345	0.713235	no
gene:SpnNT_01557	NA	Chromosome:1588906-1589071	110.58	ΔORF2+peptide	OK	2084.96	1377.05	-0.598434	-1.10677	0.05535	0.33532	no
gene:SpnNT_01557	NA	Chromosome:1588906-1589071	ΔORF2	ΔORF2+peptide	OK	3133.46	1377.05	-1.18617	-2.12281	0.00025	0.00542231	yes
gene:SpnNT_01557	NA	Chromosome:1588906-1589071	110.58+peptide	ΔORF2+peptide	OK	2404.4	1377.05	-0.804092	-1.51685	0.01015	0.101361	no
gene:SpnNT_01558	NA	Chromosome:1589136-1590732	110.58	ΔORF2	OK	114.939	111.379	-0.0453956	-0.103761	0.85955	0.994748	no
gene:SpnNT_01558	NA	Chromosome:1589136-1590732	110.58	110.58+peptide	OK	114.939	89.0171	-0.368716	-0.842136	0.1423	0.576108	no
gene:SpnNT_01558	NA	Chromosome:1589136-1590732	ΔORF2	110.58+peptide	OK	111.379	89.0171	-0.32332	-0.734109	0.20485	0.689595	no
gene:SpnNT_01558	NA	Chromosome:1589136-1590732	110.58	ΔORF2+peptide	OK	114.939	97.5726	-0.236322	-0.541914	0.3359	0.840163	no
gene:SpnNT_01558	NA	Chromosome:1589136-1590732	ΔORF2	ΔORF2+peptide	OK	111.379	97.5726	-0.190927	-0.43522	0.44625	0.918643	no
gene:SpnNT_01558	NA	Chromosome:1589136-1590732	110.58+peptide	ΔORF2+peptide	OK	89.0171	97.5726	0.132394	0.301564	0.5945	0.975539	no
gene:SpnNT_01559	def_2	Chromosome:1590743-1591154	110.58	ΔORF2	OK	92.9799	88.1945	-0.0762293	-0.144082	0.8011	0.994748	no
gene:SpnNT_01559	def_2	Chromosome:1590743-1591154	110.58	110.58+peptide	OK	92.9799	81.9173	-0.18275	-0.34629	0.543	0.961568	no
gene:SpnNT_01559	def_2	Chromosome:1590743-1591154	ΔORF2	110.58+peptide	OK	88.1945	81.9173	-0.10652	-0.199556	0.7346	0.994748	no
gene:SpnNT_01559	def_2	Chromosome:1590743-1591154	110.58	ΔORF2+peptide	OK	92.9799	79.378	-0.22818	-0.428696	0.4452	0.917818	no
gene:SpnNT_01559	def_2	Chromosome:1590743-1591154	ΔORF2	ΔORF2+peptide	OK	88.1945	79.378	-0.151951	-0.282297	0.62015	0.980887	no
gene:SpnNT_01559	def_2	Chromosome:1590743-1591154	110.58+peptide	ΔORF2+peptide	OK	81.9173	79.378	-0.0454302	-0.0846071	0.88565	0.994748	no
gene:SpnNT_01560	yghU	Chromosome:1591269-1591956	110.58	ΔORF2	OK	105.84	110.657	0.0642129	0.13798	0.8115	0.994748	no
gene:SpnNT_01560	yghU	Chromosome:1591269-1591956	110.58	110.58+peptide	OK	105.84	124.079	0.229368	0.497745	0.38715	0.881745	no
gene:SpnNT_01560	yghU	Chromosome:1591269-1591956	ΔORF2	110.58+peptide	OK	110.657	124.079	0.165155	0.358656	0.5361	0.959191	no
gene:SpnNT_01560	yghU	Chromosome:1591269-1591956	110.58	ΔORF2+peptide	OK	105.84	163.094	0.62382	1.35502	0.0197	0.165072	no
gene:SpnNT_01560	yghU	Chromosome:1591269-1591956	ΔORF2	ΔORF2+peptide	OK	110.657	163.094	0.559607	1.21642	0.038	0.261751	no

gene:SpnNT_01560	yghU	Chromosome:1591269-1591956	110.58+peptide	ΔORF2+peptide	OK	124.079	163.094	0.394452	0.866115	0.13535	0.559742	no
gene:SpnNT_01561	NA	Chromosome:1592073-1594770	110.58	ΔORF2	OK	106.69	103.015	-0.0505695	-0.114713	0.8363	0.994748	no
gene:SpnNT_01561	NA	Chromosome:1592073-1594770	110.58	110.58+peptide	OK	106.69	116.765	0.130184	0.295509	0.59475	0.975539	no
gene:SpnNT_01561	NA	Chromosome:1592073-1594770	ΔORF2	110.58+peptide	OK	103.015	116.765	0.180754	0.404762	0.47255	0.928558	no
gene:SpnNT_01561	NA	Chromosome:1592073-1594770	110.58	ΔORF2+peptide	OK	106.69	120.941	0.180876	0.41584	0.45515	0.921244	no
gene:SpnNT_01561	NA	Chromosome:1592073-1594770	ΔORF2	ΔORF2+peptide	OK	103.015	120.941	0.231446	0.524741	0.3522	0.855054	no
gene:SpnNT_01561	NA	Chromosome:1592073-1594770	110.58+peptide	ΔORF2+peptide	OK	116.765	120.941	0.0506917	0.115005	0.8376	0.994748	no
gene:SpnNT_01562	fieF	Chromosome:1595073-1596258	110.58	ΔORF2	OK	93.6195	98.4149	0.0720667	0.162352	0.7789	0.994748	no
gene:SpnNT_01562	fieF	Chromosome:1595073-1596258	110.58	110.58+peptide	OK	93.6195	78.557	-0.253069	-0.566771	0.31665	0.822625	no
gene:SpnNT_01562	fieF	Chromosome:1595073-1596258	ΔORF2	110.58+peptide	OK	98.4149	78.557	-0.325136	-0.729938	0.20465	0.689595	no
gene:SpnNT_01562	fieF	Chromosome:1595073-1596258	110.58	ΔORF2+peptide	OK	93.6195	73.5243	-0.348588	-0.774092	0.17505	0.636996	no
gene:SpnNT_01562	fieF	Chromosome:1595073-1596258	ΔORF2	ΔORF2+peptide	OK	98.4149	73.5243	-0.420655	-0.936356	0.1038	0.488896	no
gene:SpnNT_01562	fieF	Chromosome:1595073-1596258	110.58+peptide	ΔORF2+peptide	OK	78.557	73.5243	-0.0955184	-0.211401	0.706	0.990422	no
gene:SpnNT_01563	NA	Chromosome:1596400-1599453	110.58	ΔORF2	OK	160.683	156.788	-0.0354047	-0.0659724	0.9088	0.994748	no
gene:SpnNT_01563	NA	Chromosome:1596400-1599453	110.58	110.58+peptide	OK	160.683	149.159	-0.107367	-0.20216	0.72695	0.994748	no
gene:SpnNT_01563	NA	Chromosome:1596400-1599453	ΔORF2	110.58+peptide	OK	156.788	149.159	-0.0719624	-0.134403	0.81785	0.994748	no
gene:SpnNT_01563	NA	Chromosome:1596400-1599453	110.58	ΔORF2+peptide	OK	160.683	161.561	0.00786304	0.0148277	0.98145	0.996246	no
gene:SpnNT_01563	NA	Chromosome:1596400-1599453	ΔORF2	ΔORF2+peptide	OK	156.788	161.561	0.0432678	0.0809314	0.89115	0.994748	no
gene:SpnNT_01563	NA	Chromosome:1596400-1599453	110.58+peptide	ΔORF2+peptide	OK	149.159	161.561	0.11523	0.21781	0.70115	0.990367	no
gene:SpnNT_01564	cca	Chromosome:1596400-1599453	110.58	ΔORF2	OK	130.749	130.735	-0.000144379	-0.000186727	0.9998	0.9998	no
gene:SpnNT_01564	cca	Chromosome:1596400-1599453	110.58	110.58+peptide	OK	130.749	116.319	-0.168703	-0.214502	0.70845	0.990441	no
gene:SpnNT_01564	cca	Chromosome:1596400-1599453	ΔORF2	110.58+peptide	OK	130.735	116.319	-0.168558	-0.21303	0.7126	0.991034	no
gene:SpnNT_01564	cca	Chromosome:1596400-1599453	110.58	ΔORF2+peptide	OK	130.749	126.039	-0.0529304	-0.0678	0.90435	0.994748	no
gene:SpnNT_01564	cca	Chromosome:1596400-1599453	ΔORF2	ΔORF2+peptide	OK	130.735	126.039	-0.052786	-0.0672026	0.9076	0.994748	no
gene:SpnNT_01564	cca	Chromosome:1596400-1599453	110.58+peptide	ΔORF2+peptide	OK	116.319	126.039	0.115772	0.144978	0.7987	0.994748	no
gene:SpnNT_01565	dapB	Chromosome:1599464-1600232	110.58	ΔORF2	OK	313.14	292.31	-0.0993094	-0.228154	0.68395	0.98747	no
gene:SpnNT_01565	dapB	Chromosome:1599464-1600232	110.58	110.58+peptide	OK	313.14	311.556	-0.00731754	-0.0167796	0.97925	0.995765	no
gene:SpnNT_01565	dapB	Chromosome:1599464-1600232	ΔORF2	110.58+peptide	OK	292.31	311.556	0.0919918	0.211114	0.7069	0.990441	no
gene:SpnNT_01565	dapB	Chromosome:1599464-1600232	110.58	ΔORF2+peptide	OK	313.14	294.546	-0.0883192	-0.202212	0.71915	0.993716	no
gene:SpnNT_01565	dapB	Chromosome:1599464-1600232	ΔORF2	ΔORF2+peptide	OK	292.31	294.546	0.0109902	0.0251831	0.9638	0.994855	no
gene:SpnNT_01565	dapB	Chromosome:1599464-1600232	110.58+peptide	ΔORF2+peptide	OK	311.556	294.546	-0.0810016	-0.185259	0.7381	0.994748	no
gene:SpnNT_01566	NA	Chromosome:1600508-1601357	110.58	ΔORF2	OK	256.618	267.738	0.0612002	0.141135	0.8004	0.994748	no
gene:SpnNT_01566	NA	Chromosome:1600508-1601357	110.58	110.58+peptide	OK	256.618	244.016	-0.0726479	-0.167554	0.7663	0.994748	no
gene:SpnNT_01566	NA	Chromosome:1600508-1601357	ΔORF2	110.58+peptide	OK	267.738	244.016	-0.133848	-0.308369	0.5844	0.971855	no
gene:SpnNT_01566	NA	Chromosome:1600508-1601357	110.58	ΔORF2+peptide	OK	256.618	273.403	0.0914064	0.210465	0.703	0.990367	no
gene:SpnNT_01566	NA	Chromosome:1600508-1601357	ΔORF2	ΔORF2+peptide	OK	267.738	273.403	0.0302062	0.069475	0.90115	0.994748	no
gene:SpnNT_01566	NA	Chromosome:1600508-1601357	110.58+peptide	ΔORF2+peptide	OK	244.016	273.403	0.164054	0.377372	0.5002	0.944135	no
gene:SpnNT_01567	NA	Chromosome:1601358-1601733	110.58	ΔORF2	OK	517.966	583.904	0.172874	0.385732	0.4901	0.93986	no
gene:SpnNT_01567	NA	Chromosome:1601358-1601733	110.58	110.58+peptide	OK	517.966	509.966	-0.022456	-0.0490675	0.931	0.994855	no
gene:SpnNT_01567	NA	Chromosome:1601358-1601733	ΔORF2	110.58+peptide	OK	583.904	509.966	-0.19533	-0.430406	0.44685	0.919157	no
gene:SpnNT_01567	NA	Chromosome:1601358-1601733	110.58	ΔORF2+peptide	OK	517.966	484.163	-0.0973646	-0.21396	0.7072	0.990441	no
gene:SpnNT_01567	NA	Chromosome:1601358-1601733	ΔORF2	ΔORF2+peptide	OK	583.904	484.163	-0.270239	-0.59892	0.29	0.794608	no
gene:SpnNT_01567	NA	Chromosome:1601358-1601733	110.58+peptide	ΔORF2+peptide	OK	509.966	484.163	-0.0749086	-0.162622	0.7711	0.994748	no
gene:SpnNT_01568	glmM	Chromosome:1601806-1603159	110.58	ΔORF2	OK	188.44	191.022	0.019634	0.0448137	0.9385	0.994855	no
gene:SpnNT_01568	glmM	Chromosome:1601806-1603159	110.58	110.58+peptide	OK	188.44	177.042	-0.0900106	-0.206901	0.71105	0.991007	no

gene:SpnNT_01568	glmM	Chromosome:1601806-1603159	ΔORF2	110.58+peptide	OK	191.022	177.042	-0.109645	-0.250989	0.6612	0.982966	no
gene:SpnNT_01568	glmM	Chromosome:1601806-1603159	110.58	ΔORF2+peptide	OK	188.44	181.485	-0.0542532	-0.124497	0.82165	0.994748	no
gene:SpnNT_01568	glmM	Chromosome:1601806-1603159	ΔORF2	ΔORF2+peptide	OK	191.022	181.485	-0.0738871	-0.168852	0.76685	0.994748	no
gene:SpnNT_01568	glmM	Chromosome:1601806-1603159	110.58+peptide	ΔORF2+peptide	OK	177.042	181.485	0.0357574	0.0822959	0.88115	0.994748	no
gene:SpnNT_01569	NA	Chromosome:1603182-1604806	110.58	ΔORF2	OK	123.77	132.083	0.0937902	0.152329	0.7885	0.994748	no
gene:SpnNT_01569	NA	Chromosome:1603182-1604806	110.58	110.58+peptide	OK	123.77	112.81	-0.133768	-0.216194	0.70645	0.990422	no
gene:SpnNT_01569	NA	Chromosome:1603182-1604806	ΔORF2	110.58+peptide	OK	132.083	112.81	-0.227559	-0.36332	0.5329	0.958521	no
gene:SpnNT_01569	NA	Chromosome:1603182-1604806	110.58	ΔORF2+peptide	OK	123.77	120.075	-0.0437277	-0.0712286	0.90205	0.994748	no
gene:SpnNT_01569	NA	Chromosome:1603182-1604806	ΔORF2	ΔORF2+peptide	OK	132.083	120.075	-0.137518	-0.221249	0.70255	0.990367	no
gene:SpnNT_01569	NA	Chromosome:1603182-1604806	110.58+peptide	ΔORF2+peptide	OK	112.81	120.075	0.0900407	0.144166	0.80255	0.994748	no
gene:SpnNT_01570	NA	Chromosome:1603182-1604806	110.58	ΔORF2	OK	84.5777	97.6135	0.206804	0.298387	0.60465	0.976761	no
gene:SpnNT_01570	NA	Chromosome:1603182-1604806	110.58	110.58+peptide	OK	84.5777	81.7468	-0.0491141	-0.0710936	0.89835	0.994748	no
gene:SpnNT_01570	NA	Chromosome:1603182-1604806	ΔORF2	110.58+peptide	OK	97.6135	81.7468	-0.255919	-0.369076	0.52245	0.954832	no
gene:SpnNT_01570	NA	Chromosome:1603182-1604806	110.58	ΔORF2+peptide	OK	84.5777	94.2725	0.156561	0.231085	0.68645	0.98828	no
gene:SpnNT_01570	NA	Chromosome:1603182-1604806	ΔORF2	ΔORF2+peptide	OK	97.6135	94.2725	-0.0502438	-0.073875	0.89605	0.994748	no
gene:SpnNT_01570	NA	Chromosome:1603182-1604806	110.58+peptide	ΔORF2+peptide	OK	81.7468	94.2725	0.205675	0.303428	0.5951	0.975625	no
gene:SpnNT_01571	yumC	Chromosome:1604941-1605910	110.58	ΔORF2	OK	49.2031	52.9336	0.105433	0.218911	0.70605	0.990422	no
gene:SpnNT_01571	yumC	Chromosome:1604941-1605910	110.58	110.58+peptide	OK	49.2031	50.6595	0.0420838	0.0875052	0.8792	0.994748	no
gene:SpnNT_01571	yumC	Chromosome:1604941-1605910	ΔORF2	110.58+peptide	OK	52.9336	50.6595	-0.0633494	-0.131556	0.81945	0.994748	no
gene:SpnNT_01571	yumC	Chromosome:1604941-1605910	110.58	ΔORF2+peptide	OK	49.2031	55.7556	0.180367	0.375052	0.5179	0.953876	no
gene:SpnNT_01571	yumC	Chromosome:1604941-1605910	ΔORF2	ΔORF2+peptide	OK	52.9336	55.7556	0.0749342	0.155619	0.79125	0.994748	no
gene:SpnNT_01571	yumC	Chromosome:1604941-1605910	110.58+peptide	ΔORF2+peptide	OK	50.6595	55.7556	0.138284	0.287595	0.6131	0.979616	no
gene:SpnNT_01572	lytA_7	Chromosome:1606162-1607119	110.58	ΔORF2	OK	37.2078	21.6178	-0.783387	-1.40316	0.0135	0.125016	no
gene:SpnNT_01572	lytA_7	Chromosome:1606162-1607119	110.58	110.58+peptide	OK	37.2078	43.4275	0.223004	0.417183	0.4593	0.923527	no
gene:SpnNT_01572	lytA_7	Chromosome:1606162-1607119	ΔORF2	110.58+peptide	OK	21.6178	43.4275	1.00639	1.81103	0.0018	0.0270557	yes
gene:SpnNT_01572	lytA_7	Chromosome:1606162-1607119	110.58	ΔORF2+peptide	OK	37.2078	23.6418	-0.654262	-1.14056	0.04125	0.276306	no
gene:SpnNT_01572	lytA_7	Chromosome:1606162-1607119	ΔORF2	ΔORF2+peptide	OK	21.6178	23.6418	0.129125	0.217604	0.6909	0.98828	no
gene:SpnNT_01572	lytA_7	Chromosome:1606162-1607119	110.58+peptide	ΔORF2+peptide	OK	43.4275	23.6418	-0.877265	-1.53609	0.00765	0.0829614	no
gene:SpnNT_01573	NA	Chromosome:1607122-1607455	110.58	ΔORF2	OK	32.7212	20.0206	-0.708742	-0.953316	0.09655	0.470343	no
gene:SpnNT_01573	NA	Chromosome:1607122-1607455	110.58	110.58+peptide	OK	32.7212	36.9616	0.175803	0.249436	0.65495	0.982966	no
gene:SpnNT_01573	NA	Chromosome:1607122-1607455	ΔORF2	110.58+peptide	OK	20.0206	36.9616	0.884544	1.19468	0.0418	0.278851	no
gene:SpnNT_01573	NA	Chromosome:1607122-1607455	110.58	ΔORF2+peptide	OK	32.7212	25.97	-0.333379	-0.452496	0.4076	0.897051	no
gene:SpnNT_01573	NA	Chromosome:1607122-1607455	ΔORF2	ΔORF2+peptide	OK	20.0206	25.97	0.375363	0.486928	0.3888	0.88343	no
gene:SpnNT_01573	NA	Chromosome:1607122-1607455	110.58+peptide	ΔORF2+peptide	OK	36.9616	25.97	-0.509181	-0.694011	0.21105	0.697158	no
gene:SpnNT_01574	NA	Chromosome:1607458-1607758	110.58	ΔORF2	OK	13.5776	11.6964	-0.215168	-0.242596	0.66585	0.98367	no
gene:SpnNT_01574	NA	Chromosome:1607458-1607758	110.58	110.58+peptide	OK	13.5776	9.22715	-0.557271	-0.635451	0.26235	0.762762	no
gene:SpnNT_01574	NA	Chromosome:1607458-1607758	ΔORF2	110.58+peptide	OK	11.6964	9.22715	-0.342103	-0.381801	0.5227	0.954832	no
gene:SpnNT_01574	NA	Chromosome:1607458-1607758	110.58	ΔORF2+peptide	OK	13.5776	10.5861	-0.35906	-0.469748	0.505	0.945292	no
gene:SpnNT_01574	NA	Chromosome:1607458-1607758	ΔORF2	ΔORF2+peptide	OK	11.6964	10.5861	-0.143892	-0.183032	0.8009	0.994748	no
gene:SpnNT_01574	NA	Chromosome:1607458-1607758	110.58+peptide	ΔORF2+peptide	OK	9.22715	10.5861	0.198211	0.255793	0.7243	0.994748	no
gene:SpnNT_01575	NA	Chromosome:1607766-1608117	110.58	ΔORF2	OK	38.3094	25.6741	-0.577388	-0.859194	0.1363	0.561724	no
gene:SpnNT_01575	NA	Chromosome:1607766-1608117	110.58	110.58+peptide	OK	38.3094	40.1206	0.0666432	0.10189	0.85985	0.994748	no
gene:SpnNT_01575	NA	Chromosome:1607766-1608117	ΔORF2	110.58+peptide	OK	25.6741	40.1206	0.644031	0.96571	0.0949	0.465699	no
gene:SpnNT_01575	NA	Chromosome:1607766-1608117	110.58	ΔORF2+peptide	OK	38.3094	32.1164	-0.254389	-0.367521	0.49915	0.944017	no
gene:SpnNT_01575	NA	Chromosome:1607766-1608117	ΔORF2	ΔORF2+peptide	OK	25.6741	32.1164	0.322999	0.458602	0.4067	0.896284	no

gene:SpnNT_01575	NA	Chromosome:1607766-1608117	110.58+peptide	ΔORF2+peptide	OK	40.1206	32.1164	-0.321032	-0.46715	0.38565	0.881745	no
gene:SpnNT_01576	NA	Chromosome:1608119-1611614	110.58	ΔORF2	OK	49.966	29.0801	-0.780912	-0.226895	0.75135	0.994748	no
gene:SpnNT_01576	NA	Chromosome:1608119-1611614	110.58	110.58+peptide	OK	49.966	64.5621	0.36974	0.114013	0.8851	0.994748	no
gene:SpnNT_01576	NA	Chromosome:1608119-1611614	ΔORF2	110.58+peptide	OK	29.0801	64.5621	1.15065	0.345032	0.6522	0.98163	no
gene:SpnNT_01576	NA	Chromosome:1608119-1611614	110.58	ΔORF2+peptide	OK	49.966	60.3724	0.272942	0.0849208	0.90525	0.994748	no
gene:SpnNT_01576	NA	Chromosome:1608119-1611614	ΔORF2	ΔORF2+peptide	OK	29.0801	60.3724	1.05385	0.318689	0.6616	0.982966	no
gene:SpnNT_01576	NA	Chromosome:1608119-1611614	110.58+peptide	ΔORF2+peptide	OK	64.5621	60.3724	-0.0967979	-0.0312309	0.9492	0.994855	no
gene:SpnNT_01577	NA	Chromosome:1608119-1611614	110.58	ΔORF2	OK	9.5747	9.2543	-0.0491029	-0.0295708	0.7743	0.994748	no
gene:SpnNT_01577	NA	Chromosome:1608119-1611614	110.58	110.58+peptide	OK	9.5747	20.8598	1.12343	0.102193	0.56345	0.968621	no
gene:SpnNT_01577	NA	Chromosome:1608119-1611614	ΔORF2	110.58+peptide	OK	9.2543	20.8598	1.17253	0.106163	0.55985	0.968621	no
gene:SpnNT_01577	NA	Chromosome:1608119-1611614	110.58	ΔORF2+peptide	NOTEST	9.5747	0	#NAME?	0	1	1	no
gene:SpnNT_01577	NA	Chromosome:1608119-1611614	ΔORF2	ΔORF2+peptide	OK	9.2543	0	#NAME?	NA	0.22435	0.715065	no
gene:SpnNT_01577	NA	Chromosome:1608119-1611614	110.58+peptide	ΔORF2+peptide	OK	20.8598	0	#NAME?	NA	0.17995	0.646578	no
gene:SpnNT_01578	NA	Chromosome:1608119-1611614	110.58	ΔORF2	OK	21.3106	14.2734	-0.57824	-1.11836	0.0519	0.322153	no
gene:SpnNT_01578	NA	Chromosome:1608119-1611614	110.58	110.58+peptide	OK	21.3106	25.2659	0.245619	0.490085	0.39345	0.888728	no
gene:SpnNT_01578	NA	Chromosome:1608119-1611614	ΔORF2	110.58+peptide	OK	14.2734	25.2659	0.823859	1.60995	0.0058	0.0674115	no
gene:SpnNT_01578	NA	Chromosome:1608119-1611614	110.58	ΔORF2+peptide	OK	21.3106	16.1034	-0.404205	-0.785913	0.16945	0.627405	no
gene:SpnNT_01578	NA	Chromosome:1608119-1611614	ΔORF2	ΔORF2+peptide	OK	14.2734	16.1034	0.174035	0.331748	0.5654	0.968621	no
gene:SpnNT_01578	NA	Chromosome:1608119-1611614	110.58+peptide	ΔORF2+peptide	OK	25.2659	16.1034	-0.649824	-1.27674	0.02695	0.207656	no
gene:SpnNT_01579	NA	Chromosome:1611618-1611969	110.58	ΔORF2	OK	10.491	8.19331	-0.356632	-0.422172	0.45965	0.923806	no
gene:SpnNT_01579	NA	Chromosome:1611618-1611969	110.58	110.58+peptide	OK	10.491	15.2705	0.541594	0.677013	0.22915	0.721915	no
gene:SpnNT_01579	NA	Chromosome:1611618-1611969	ΔORF2	110.58+peptide	OK	8.19331	15.2705	0.898226	1.08823	0.06535	0.372112	no
gene:SpnNT_01579	NA	Chromosome:1611618-1611969	110.58	ΔORF2+peptide	OK	10.491	8.50053	-0.303525	-0.355637	0.52955	0.957393	no
gene:SpnNT_01579	NA	Chromosome:1611618-1611969	ΔORF2	ΔORF2+peptide	OK	8.19331	8.50053	0.0531071	0.0605317	0.9156	0.994748	no
gene:SpnNT_01579	NA	Chromosome:1611618-1611969	110.58+peptide	ΔORF2+peptide	OK	15.2705	8.50053	-0.845119	-1.01295	0.07605	0.407484	no
gene:SpnNT_01580	NA	Chromosome:1611977-1613330	110.58	ΔORF2	OK	21.8506	13.2065	-0.726427	-1.33457	0.0217	0.178525	no
gene:SpnNT_01580	NA	Chromosome:1611977-1613330	110.58	110.58+peptide	OK	21.8506	23.8645	0.127196	0.242805	0.67005	0.984845	no
gene:SpnNT_01580	NA	Chromosome:1611977-1613330	ΔORF2	110.58+peptide	OK	13.2065	23.8645	0.853623	1.59015	0.00605	0.0694559	no
gene:SpnNT_01580	NA	Chromosome:1611977-1613330	110.58	ΔORF2+peptide	OK	21.8506	13.2012	-0.727004	-1.29701	0.02085	0.172723	no
gene:SpnNT_01580	NA	Chromosome:1611977-1613330	ΔORF2	ΔORF2+peptide	OK	13.2065	13.2012	-0.000576897	-0.00100741	0.99665	0.999412	no
gene:SpnNT_01580	NA	Chromosome:1611977-1613330	110.58+peptide	ΔORF2+peptide	OK	23.8645	13.2012	-0.8542	-1.54398	0.0064	0.0723972	no
gene:SpnNT_01581	NA	Chromosome:1613378-1614713	110.58	ΔORF2	OK	14.7416	9.27744	-0.668092	-1.12018	0.05665	0.338806	no
gene:SpnNT_01581	NA	Chromosome:1613378-1614713	110.58	110.58+peptide	OK	14.7416	16.7715	0.186124	0.33305	0.56635	0.968621	no
gene:SpnNT_01581	NA	Chromosome:1613378-1614713	ΔORF2	110.58+peptide	OK	9.27744	16.7715	0.854216	1.45599	0.01395	0.127651	no
gene:SpnNT_01581	NA	Chromosome:1613378-1614713	110.58	ΔORF2+peptide	OK	14.7416	11.2136	-0.39464	-0.660493	0.2514	0.749233	no
gene:SpnNT_01581	NA	Chromosome:1613378-1614713	ΔORF2	ΔORF2+peptide	OK	9.27744	11.2136	0.273452	0.438495	0.44835	0.919688	no
gene:SpnNT_01581	NA	Chromosome:1613378-1614713	110.58+peptide	ΔORF2+peptide	OK	16.7715	11.2136	-0.580763	-0.988052	0.0859	0.440133	no
gene:SpnNT_01582	NA	Chromosome:1615020-1615921	110.58	ΔORF2	OK	21.5202	14.3418	-0.585469	-0.693863	0.2606	0.760924	no
gene:SpnNT_01582	NA	Chromosome:1615020-1615921	110.58	110.58+peptide	OK	21.5202	27.0212	0.3284	0.423682	0.461	0.924536	no
gene:SpnNT_01582	NA	Chromosome:1615020-1615921	ΔORF2	110.58+peptide	OK	14.3418	27.0212	0.913868	1.13386	0.0766	0.409095	no
gene:SpnNT_01582	NA	Chromosome:1615020-1615921	110.58	ΔORF2+peptide	OK	21.5202	16.903	-0.348411	-0.420069	0.45615	0.921244	no
gene:SpnNT_01582	NA	Chromosome:1615020-1615921	ΔORF2	ΔORF2+peptide	OK	14.3418	16.903	0.237057	0.276182	0.64535	0.980887	no
gene:SpnNT_01582	NA	Chromosome:1615020-1615921	110.58+peptide	ΔORF2+peptide	OK	27.0212	16.903	-0.676811	-0.855715	0.1191	0.523036	no
gene:SpnNT_01583	NA	Chromosome:1615020-1615921	110.58	ΔORF2	OK	27.5424	21.5813	-0.351873	-0.285624	0.58405	0.971837	no
gene:SpnNT_01583	NA	Chromosome:1615020-1615921	110.58	110.58+peptide	OK	27.5424	28.3224	0.0402855	0.0355945	0.94775	0.994855	no

gene:SpnNT_01583	NA	Chromosome:1615020-1615921	ΔORF2	110.58+peptide	OK	21.5813	28.3224	0.392158	0.319789	0.5559	0.966729	no
gene:SpnNT_01583	NA	Chromosome:1615020-1615921	110.58	ΔORF2+peptide	OK	27.5424	26.9078	-0.0336308	-0.0308117	0.952	0.994855	no
gene:SpnNT_01583	NA	Chromosome:1615020-1615921	ΔORF2	ΔORF2+peptide	OK	21.5813	26.9078	0.318242	0.267607	0.61915	0.980887	no
gene:SpnNT_01583	NA	Chromosome:1615020-1615921	110.58+peptide	ΔORF2+peptide	OK	28.3224	26.9078	-0.0739163	-0.0681178	0.90975	0.994748	no
gene:SpnNT_01584	NA	Chromosome:1615959-1616340	110.58	ΔORF2	OK	49.3783	33.2035	-0.572543	-0.90835	0.1202	0.524705	no
gene:SpnNT_01584	NA	Chromosome:1615959-1616340	110.58	110.58+peptide	OK	49.3783	51.7611	0.06799	0.113332	0.83485	0.994748	no
gene:SpnNT_01584	NA	Chromosome:1615959-1616340	ΔORF2	110.58+peptide	OK	33.2035	51.7611	0.640533	1.00405	0.0798	0.421213	no
gene:SpnNT_01584	NA	Chromosome:1615959-1616340	110.58	ΔORF2+peptide	OK	49.3783	32.4697	-0.604783	-0.978905	0.09355	0.462185	no
gene:SpnNT_01584	NA	Chromosome:1615959-1616340	ΔORF2	ΔORF2+peptide	OK	33.2035	32.4697	-0.0322401	-0.0492361	0.93515	0.994855	no
gene:SpnNT_01584	NA	Chromosome:1615959-1616340	110.58+peptide	ΔORF2+peptide	OK	51.7611	32.4697	-0.672773	-1.07539	0.06065	0.353868	no
gene:SpnNT_01585	NA	Chromosome:1616344-1616758	110.58	ΔORF2	OK	25.5178	14.6104	-0.804506	-1.11302	0.04535	0.293289	no
gene:SpnNT_01585	NA	Chromosome:1616344-1616758	110.58	110.58+peptide	OK	25.5178	29.5611	0.212193	0.305453	0.5759	0.969538	no
gene:SpnNT_01585	NA	Chromosome:1616344-1616758	ΔORF2	110.58+peptide	OK	14.6104	29.5611	1.0167	1.40061	0.01295	0.120861	no
gene:SpnNT_01585	NA	Chromosome:1616344-1616758	110.58	ΔORF2+peptide	OK	25.5178	22.7038	-0.16857	-0.235188	0.6791	0.98542	no
gene:SpnNT_01585	NA	Chromosome:1616344-1616758	ΔORF2	ΔORF2+peptide	OK	14.6104	22.7038	0.635936	0.851275	0.13375	0.556521	no
gene:SpnNT_01585	NA	Chromosome:1616344-1616758	110.58+peptide	ΔORF2+peptide	OK	29.5611	22.7038	-0.380763	-0.528944	0.34045	0.844744	no
gene:SpnNT_01586	NA	Chromosome:1616760-1618246	110.58	ΔORF2	OK	14.7337	14.0746	-0.066031	-0.0398607	0.94455	0.994855	no
gene:SpnNT_01586	NA	Chromosome:1616760-1618246	110.58	110.58+peptide	OK	14.7337	15.6813	0.089922	0.0443833	0.9462	0.994855	no
gene:SpnNT_01586	NA	Chromosome:1616760-1618246	ΔORF2	110.58+peptide	OK	14.0746	15.6813	0.155953	0.0765996	0.92035	0.994748	no
gene:SpnNT_01586	NA	Chromosome:1616760-1618246	110.58	ΔORF2+peptide	OK	14.7337	21.696	0.558308	0.369001	0.5445	0.961568	no
gene:SpnNT_01586	NA	Chromosome:1616760-1618246	ΔORF2	ΔORF2+peptide	OK	14.0746	21.696	0.624339	0.409059	0.6081	0.978519	no
gene:SpnNT_01586	NA	Chromosome:1616760-1618246	110.58+peptide	ΔORF2+peptide	OK	15.6813	21.696	0.468386	0.243826	0.67815	0.98524	no
gene:SpnNT_01587	NA	Chromosome:1616760-1618246	110.58	ΔORF2	OK	27.087	18.9017	-0.51908	-0.571279	0.31035	0.816278	no
gene:SpnNT_01587	NA	Chromosome:1616760-1618246	110.58	110.58+peptide	OK	27.087	39.1624	0.531869	0.629978	0.2645	0.7648	no
gene:SpnNT_01587	NA	Chromosome:1616760-1618246	ΔORF2	110.58+peptide	OK	18.9017	39.1624	1.05095	1.21073	0.0319	0.231907	no
gene:SpnNT_01587	NA	Chromosome:1616760-1618246	110.58	ΔORF2+peptide	OK	27.087	23.6431	-0.196177	-0.219162	0.7045	0.990367	no
gene:SpnNT_01587	NA	Chromosome:1616760-1618246	ΔORF2	ΔORF2+peptide	OK	18.9017	23.6431	0.322903	0.351911	0.5239	0.954832	no
gene:SpnNT_01587	NA	Chromosome:1616760-1618246	110.58+peptide	ΔORF2+peptide	OK	39.1624	23.6431	-0.728047	-0.852629	0.12395	0.53292	no
gene:SpnNT_01588	NA	Chromosome:1616760-1618246	110.58	ΔORF2	OK	31.4431	18.281	-0.782397	-0.571642	0.3476	0.852403	no
gene:SpnNT_01588	NA	Chromosome:1616760-1618246	110.58	110.58+peptide	OK	31.4431	38.7026	0.299686	0.235065	0.70065	0.990367	no
gene:SpnNT_01588	NA	Chromosome:1616760-1618246	ΔORF2	110.58+peptide	OK	18.281	38.7026	1.08208	0.762289	0.2281	0.719848	no
gene:SpnNT_01588	NA	Chromosome:1616760-1618246	110.58	ΔORF2+peptide	OK	31.4431	18.2677	-0.78345	-0.490921	0.43555	0.914071	no
gene:SpnNT_01588	NA	Chromosome:1616760-1618246	ΔORF2	ΔORF2+peptide	OK	18.281	18.2677	-0.00105289	-0.000614428	0.9934	0.998522	no
gene:SpnNT_01588	NA	Chromosome:1616760-1618246	110.58+peptide	ΔORF2+peptide	OK	38.7026	18.2677	-1.08314	-0.660576	0.33065	0.836474	no
gene:SpnNT_01589	NA	Chromosome:1616760-1618246	110.58	ΔORF2	OK	33.9555	19.6118	-0.791924	-0.509153	0.33155	0.837459	no
gene:SpnNT_01589	NA	Chromosome:1616760-1618246	110.58	110.58+peptide	OK	33.9555	35.615	0.0688396	0.0492952	0.92775	0.994748	no
gene:SpnNT_01589	NA	Chromosome:1616760-1618246	ΔORF2	110.58+peptide	OK	19.6118	35.615	0.860763	0.55704	0.2937	0.797915	no
gene:SpnNT_01589	NA	Chromosome:1616760-1618246	110.58	ΔORF2+peptide	OK	33.9555	20.4733	-0.729901	-0.445477	0.4039	0.894511	no
gene:SpnNT_01589	NA	Chromosome:1616760-1618246	ΔORF2	ΔORF2+peptide	OK	19.6118	20.4733	0.0620226	0.0351009	0.94975	0.994855	no
gene:SpnNT_01589	NA	Chromosome:1616760-1618246	110.58+peptide	ΔORF2+peptide	OK	35.615	20.4733	-0.798741	-0.490369	0.35885	0.859746	no
gene:SpnNT_01590	NA	Chromosome:1618247-1618436	110.58	ΔORF2	OK	30.2234	17.7451	-0.76825	-0.734073	0.20745	0.693014	no
gene:SpnNT_01590	NA	Chromosome:1618247-1618436	110.58	110.58+peptide	OK	30.2234	30.2802	0.00270942	0.00257563	0.96735	0.994855	no
gene:SpnNT_01590	NA	Chromosome:1618247-1618436	ΔORF2	110.58+peptide	OK	17.7451	30.2802	0.770959	0.702796	0.21045	0.696806	no
gene:SpnNT_01590	NA	Chromosome:1618247-1618436	110.58	ΔORF2+peptide	OK	30.2234	32.0816	0.0860783	0.0807164	0.88235	0.994748	no
gene:SpnNT_01590	NA	Chromosome:1618247-1618436	ΔORF2	ΔORF2+peptide	OK	17.7451	32.0816	0.854328	0.76905	0.1743	0.634971	no

gene:SpnNT_01590	NA	Chromosome:1618247-1618436	110.58+peptide	ΔORF2+peptide	OK	30.2802	32.0816	0.0833689	0.0747058	0.88615	0.994748	no
gene:SpnNT_01591	NA	Chromosome:1618445-1619471	110.58	ΔORF2	OK	22.3349	16.1859	-0.464562	-0.790443	0.16135	0.614666	no
gene:SpnNT_01591	NA	Chromosome:1618445-1619471	110.58	110.58+peptide	OK	22.3349	25.0292	0.164311	0.290423	0.60895	0.978849	no
gene:SpnNT_01591	NA	Chromosome:1618445-1619471	ΔORF2	110.58+peptide	OK	16.1859	25.0292	0.628873	1.07632	0.0544	0.331554	no
gene:SpnNT_01591	NA	Chromosome:1618445-1619471	110.58	ΔORF2+peptide	OK	22.3349	16.0502	-0.476707	-0.795563	0.1639	0.618371	no
gene:SpnNT_01591	NA	Chromosome:1618445-1619471	ΔORF2	ΔORF2+peptide	OK	16.1859	16.0502	-0.0121448	-0.0196926	0.9726	0.99536	no
gene:SpnNT_01591	NA	Chromosome:1618445-1619471	110.58+peptide	ΔORF2+peptide	OK	25.0292	16.0502	-0.641018	-1.07584	0.06035	0.352745	no
gene:SpnNT_01592	NA	Chromosome:1619493-1620018	110.58	ΔORF2	OK	26.5702	18.2699	-0.540336	-0.781863	0.16275	0.61656	no
gene:SpnNT_01592	NA	Chromosome:1619493-1620018	110.58	110.58+peptide	OK	26.5702	31.1939	0.231459	0.349561	0.52795	0.956161	no
gene:SpnNT_01592	NA	Chromosome:1619493-1620018	ΔORF2	110.58+peptide	OK	18.2699	31.1939	0.771796	1.15254	0.0473	0.302471	no
gene:SpnNT_01592	NA	Chromosome:1619493-1620018	110.58	ΔORF2+peptide	OK	26.5702	22.4418	-0.243616	-0.347711	0.53245	0.958279	no
gene:SpnNT_01592	NA	Chromosome:1619493-1620018	ΔORF2	ΔORF2+peptide	OK	18.2699	22.4418	0.296721	0.41926	0.4646	0.926394	no
gene:SpnNT_01592	NA	Chromosome:1619493-1620018	110.58+peptide	ΔORF2+peptide	OK	31.1939	22.4418	-0.475075	-0.699165	0.2153	0.702079	no
gene:SpnNT_01593	NA	Chromosome:1620331-1621261	110.58	ΔORF2	OK	8.22059	5.8986	-0.478869	-0.693789	0.2215	0.710815	no
gene:SpnNT_01593	NA	Chromosome:1620331-1621261	110.58	110.58+peptide	OK	8.22059	7.84179	-0.0680584	-0.0996349	0.8618	0.994748	no
gene:SpnNT_01593	NA	Chromosome:1620331-1621261	ΔORF2	110.58+peptide	OK	5.8986	7.84179	0.41081	0.592094	0.29335	0.797897	no
gene:SpnNT_01593	NA	Chromosome:1620331-1621261	110.58	ΔORF2+peptide	OK	8.22059	6.28697	-0.386878	-0.551334	0.34005	0.844266	no
gene:SpnNT_01593	NA	Chromosome:1620331-1621261	ΔORF2	ΔORF2+peptide	OK	5.8986	6.28697	0.0919907	0.129168	0.81685	0.994748	no
gene:SpnNT_01593	NA	Chromosome:1620331-1621261	110.58+peptide	ΔORF2+peptide	OK	7.84179	6.28697	-0.31882	-0.452062	0.43675	0.914104	no
gene:SpnNT_01594	NA	Chromosome:1621433-1623261	110.58	ΔORF2	OK	7.57932	7.31099	-0.052002	-0.0247965	0.96695	0.994855	no
gene:SpnNT_01594	NA	Chromosome:1621433-1623261	110.58	110.58+peptide	OK	7.57932	13.1217	0.791809	0.412746	0.5059	0.945292	no
gene:SpnNT_01594	NA	Chromosome:1621433-1623261	ΔORF2	110.58+peptide	OK	7.31099	13.1217	0.843811	0.495709	0.36855	0.865873	no
gene:SpnNT_01594	NA	Chromosome:1621433-1623261	110.58	ΔORF2+peptide	OK	7.57932	5.75734	-0.396666	-0.195963	0.80725	0.994748	no
gene:SpnNT_01594	NA	Chromosome:1621433-1623261	ΔORF2	ΔORF2+peptide	OK	7.31099	5.75734	-0.344664	-0.189311	0.77685	0.994748	no
gene:SpnNT_01594	NA	Chromosome:1621433-1623261	110.58+peptide	ΔORF2+peptide	OK	13.1217	5.75734	-1.18848	-0.737508	0.30715	0.813241	no
gene:SpnNT_01595	NA	Chromosome:1621433-1623261	110.58	ΔORF2	OK	9.51975	6.22177	-0.613599	-0.936404	0.10795	0.499941	no
gene:SpnNT_01595	NA	Chromosome:1621433-1623261	110.58	110.58+peptide	OK	9.51975	9.97812	0.0678436	0.108276	0.8506	0.994748	no
gene:SpnNT_01595	NA	Chromosome:1621433-1623261	ΔORF2	110.58+peptide	OK	6.22177	9.97812	0.681442	1.0425	0.07195	0.394867	no
gene:SpnNT_01595	NA	Chromosome:1621433-1623261	110.58	ΔORF2+peptide	OK	9.51975	7.48475	-0.346971	-0.522563	0.36445	0.864325	no
gene:SpnNT_01595	NA	Chromosome:1621433-1623261	ΔORF2	ΔORF2+peptide	OK	6.22177	7.48475	0.266628	0.386645	0.50275	0.944788	no
gene:SpnNT_01595	NA	Chromosome:1621433-1623261	110.58+peptide	ΔORF2+peptide	OK	9.97812	7.48475	-0.414814	-0.626239	0.2705	0.772705	no
gene:SpnNT_01596	NA	Chromosome:1623272-1624989	110.58	ΔORF2	OK	9.54161	6.76747	-0.495615	-0.727744	0.19295	0.674611	no
gene:SpnNT_01596	NA	Chromosome:1623272-1624989	110.58	110.58+peptide	OK	9.54161	10.8412	0.184216	0.284436	0.6116	0.979429	no
gene:SpnNT_01596	NA	Chromosome:1623272-1624989	ΔORF2	110.58+peptide	OK	6.76747	10.8412	0.679831	1.01511	0.07385	0.400272	no
gene:SpnNT_01596	NA	Chromosome:1623272-1624989	110.58	ΔORF2+peptide	OK	9.54161	7.30755	-0.384844	-0.568674	0.3077	0.813712	no
gene:SpnNT_01596	NA	Chromosome:1623272-1624989	ΔORF2	ΔORF2+peptide	OK	6.76747	7.30755	0.110771	0.158725	0.78395	0.994748	no
gene:SpnNT_01596	NA	Chromosome:1623272-1624989	110.58+peptide	ΔORF2+peptide	OK	10.8412	7.30755	-0.569059	-0.855278	0.12835	0.543118	no
gene:SpnNT_01597	NA	Chromosome:1623272-1624989	110.58	ΔORF2	OK	10.3191	8.39885	-0.297054	-0.218051	0.7079	0.990441	no
gene:SpnNT_01597	NA	Chromosome:1623272-1624989	110.58	110.58+peptide	OK	10.3191	10.4951	0.0243992	0.0176799	0.9765	0.99536	no
gene:SpnNT_01597	NA	Chromosome:1623272-1624989	ΔORF2	110.58+peptide	OK	8.39885	10.4951	0.321453	0.232381	0.69445	0.988563	no
gene:SpnNT_01597	NA	Chromosome:1623272-1624989	110.58	ΔORF2+peptide	OK	10.3191	7.6607	-0.429771	-0.281103	0.64845	0.981391	no
gene:SpnNT_01597	NA	Chromosome:1623272-1624989	ΔORF2	ΔORF2+peptide	OK	8.39885	7.6607	-0.132717	-0.0866407	0.8883	0.994748	no
gene:SpnNT_01597	NA	Chromosome:1623272-1624989	110.58+peptide	ΔORF2+peptide	OK	10.4951	7.6607	-0.45417	-0.293466	0.63605	0.980887	no
gene:SpnNT_01598	NA	Chromosome:1626220-1626682	110.58	ΔORF2	OK	66.9276	44.3202	-0.594637	-1.07374	0.0586	0.345595	no
gene:SpnNT_01598	NA	Chromosome:1626220-1626682	110.58	110.58+peptide	OK	66.9276	68.5289	0.0341112	0.0622831	0.9077	0.994748	no

gene:SpnNT_01598	NA	Chromosome:1626220-1626682	ΔORF2	110.58+peptide	OK	44.3202	68.5289	0.628748	1.12704	0.046	0.296033	no
gene:SpnNT_01598	NA	Chromosome:1626220-1626682	110.58	ΔORF2+peptide	OK	66.9276	40.485	-0.725212	-1.19334	0.03155	0.230383	no
gene:SpnNT_01598	NA	Chromosome:1626220-1626682	ΔORF2	ΔORF2+peptide	OK	44.3202	40.485	-0.130575	-0.211655	0.69745	0.990148	no
gene:SpnNT_01598	NA	Chromosome:1626220-1626682	110.58+peptide	ΔORF2+peptide	OK	68.5289	40.485	-0.759323	-1.24187	0.0235	0.188373	no
gene:SpnNT_01599	NA	Chromosome:1626756-1627071	110.58	ΔORF2	OK	58.2812	41.7327	-0.481853	-0.749006	0.19655	0.677648	no
gene:SpnNT_01599	NA	Chromosome:1626756-1627071	110.58	110.58+peptide	OK	58.2812	54.7713	-0.0896111	-0.14512	0.80435	0.994748	no
gene:SpnNT_01599	NA	Chromosome:1626756-1627071	ΔORF2	110.58+peptide	OK	41.7327	54.7713	0.392242	0.604636	0.3004	0.806342	no
gene:SpnNT_01599	NA	Chromosome:1626756-1627071	110.58	ΔORF2+peptide	OK	58.2812	36.8845	-0.660015	-0.983378	0.083	0.431508	no
gene:SpnNT_01599	NA	Chromosome:1626756-1627071	ΔORF2	ΔORF2+peptide	OK	41.7327	36.8845	-0.178162	-0.254513	0.6575	0.982966	no
gene:SpnNT_01599	NA	Chromosome:1626756-1627071	110.58+peptide	ΔORF2+peptide	OK	54.7713	36.8845	-0.570404	-0.843356	0.13265	0.554518	no
gene:SpnNT_01600	NA	Chromosome:1627263-1628671	110.58	ΔORF2	OK	49.5791	34.6234	-0.517984	-0.462668	0.4253	0.908126	no
gene:SpnNT_01600	NA	Chromosome:1627263-1628671	110.58	110.58+peptide	OK	49.5791	51.2087	0.046657	0.0417415	0.9429	0.994855	no
gene:SpnNT_01600	NA	Chromosome:1627263-1628671	ΔORF2	110.58+peptide	OK	34.6234	51.2087	0.564641	0.513878	0.38105	0.878913	no
gene:SpnNT_01600	NA	Chromosome:1627263-1628671	110.58	ΔORF2+peptide	OK	49.5791	32.8467	-0.593984	-0.5126	0.3871	0.881745	no
gene:SpnNT_01600	NA	Chromosome:1627263-1628671	ΔORF2	ΔORF2+peptide	OK	34.6234	32.8467	-0.0760006	-0.0666395	0.91105	0.994748	no
gene:SpnNT_01600	NA	Chromosome:1627263-1628671	110.58+peptide	ΔORF2+peptide	OK	51.2087	32.8467	-0.640641	-0.562603	0.3437	0.84791	no
gene:SpnNT_01601	NA	Chromosome:1627263-1628671	110.58	ΔORF2	OK	27.0061	4.61983	-2.54737	-2.57872	0.42645	0.908569	no
gene:SpnNT_01601	NA	Chromosome:1627263-1628671	110.58	110.58+peptide	OK	27.0061	12.4971	-1.11169	-1.27323	0.5919	0.975468	no
gene:SpnNT_01601	NA	Chromosome:1627263-1628671	ΔORF2	110.58+peptide	NOTEST	4.61983	12.4971	1.43568	0	1	1	no
gene:SpnNT_01601	NA	Chromosome:1627263-1628671	110.58	ΔORF2+peptide	OK	27.0061	0	#NAME?	NA	0.19645	0.677648	no
gene:SpnNT_01601	NA	Chromosome:1627263-1628671	ΔORF2	ΔORF2+peptide	NOTEST	4.61983	0	#NAME?	0	1	1	no
gene:SpnNT_01601	NA	Chromosome:1627263-1628671	110.58+peptide	ΔORF2+peptide	NOTEST	12.4971	0	#NAME?	0	1	1	no
gene:SpnNT_01602	hhaIM	Chromosome:1627263-1628671	110.58	ΔORF2	OK	57.4523	37.3315	-0.621971	-1.11332	0.05375	0.328508	no
gene:SpnNT_01602	hhaIM	Chromosome:1627263-1628671	110.58	110.58+peptide	OK	57.4523	54.8046	-0.0680671	-0.122972	0.82745	0.994748	no
gene:SpnNT_01602	hhaIM	Chromosome:1627263-1628671	ΔORF2	110.58+peptide	OK	37.3315	54.8046	0.553904	0.977043	0.08835	0.44779	no
gene:SpnNT_01602	hhaIM	Chromosome:1627263-1628671	110.58	ΔORF2+peptide	OK	57.4523	39.2343	-0.55025	-0.981706	0.0862	0.440466	no
gene:SpnNT_01602	hhaIM	Chromosome:1627263-1628671	ΔORF2	ΔORF2+peptide	OK	37.3315	39.2343	0.0717209	0.125006	0.82485	0.994748	no
gene:SpnNT_01602	hhaIM	Chromosome:1627263-1628671	110.58+peptide	ΔORF2+peptide	OK	54.8046	39.2343	-0.482183	-0.847817	0.1337	0.556521	no
gene:SpnNT_01603	NA	Chromosome:1628733-1629532	110.58	ΔORF2	OK	22.1963	12.7616	-0.798508	-0.241981	0.7578	0.994748	no
gene:SpnNT_01603	NA	Chromosome:1628733-1629532	110.58	110.58+peptide	OK	22.1963	21.164	-0.0687106	-0.0222372	0.9166	0.994748	no
gene:SpnNT_01603	NA	Chromosome:1628733-1629532	ΔORF2	110.58+peptide	OK	12.7616	21.164	0.729797	0.21446	0.7734	0.994748	no
gene:SpnNT_01603	NA	Chromosome:1628733-1629532	110.58	ΔORF2+peptide	OK	22.1963	31.1606	0.489404	0.166312	0.817	0.994748	no
gene:SpnNT_01603	NA	Chromosome:1628733-1629532	ΔORF2	ΔORF2+peptide	OK	12.7616	31.1606	1.28791	0.393873	0.5901	0.974817	no
gene:SpnNT_01603	NA	Chromosome:1628733-1629532	110.58+peptide	ΔORF2+peptide	OK	21.164	31.1606	0.558115	0.182521	0.809	0.994748	no
gene:SpnNT_01604	NA	Chromosome:1628733-1629532	110.58	ΔORF2	OK	39.6449	28.339	-0.484348	-0.777496	0.17715	0.642499	no
gene:SpnNT_01604	NA	Chromosome:1628733-1629532	110.58	110.58+peptide	OK	39.6449	42.4776	0.0995646	0.167196	0.76845	0.994748	no
gene:SpnNT_01604	NA	Chromosome:1628733-1629532	ΔORF2	110.58+peptide	OK	28.339	42.4776	0.583913	0.939517	0.10045	0.480359	no
gene:SpnNT_01604	NA	Chromosome:1628733-1629532	110.58	ΔORF2+peptide	OK	39.6449	36.93	-0.102344	-0.164865	0.77445	0.994748	no
gene:SpnNT_01604	NA	Chromosome:1628733-1629532	ΔORF2	ΔORF2+peptide	OK	28.339	36.93	0.382004	0.591556	0.3017	0.807775	no
gene:SpnNT_01604	NA	Chromosome:1628733-1629532	110.58+peptide	ΔORF2+peptide	OK	42.4776	36.93	-0.201908	-0.326021	0.56875	0.968621	no
gene:SpnNT_01605	NA	Chromosome:1628733-1629532	110.58	ΔORF2	OK	76.1548	45.1234	-0.75506	-0.285637	0.65475	0.982966	no
gene:SpnNT_01605	NA	Chromosome:1628733-1629532	110.58	110.58+peptide	OK	76.1548	87.3534	0.197928	0.0856812	0.8985	0.994748	no
gene:SpnNT_01605	NA	Chromosome:1628733-1629532	ΔORF2	110.58+peptide	OK	45.1234	87.3534	0.952988	0.376208	0.5571	0.967478	no
gene:SpnNT_01605	NA	Chromosome:1628733-1629532	110.58	ΔORF2+peptide	OK	76.1548	53.2369	-0.51651	-0.294301	0.74665	0.994748	no
gene:SpnNT_01605	NA	Chromosome:1628733-1629532	ΔORF2	ΔORF2+peptide	OK	45.1234	53.2369	0.23855	0.11695	0.8795	0.994748	no

gene:SpnNT_01605	NA	Chromosome:1628733-1629532	110.58+peptide	ΔORF2+peptide	OK	87.3534	53.2369	-0.714438	-0.451016	0.6464	0.980925	no
gene:SpnNT_01606	NA	Chromosome:1629831-1631169	110.58	ΔORF2	OK	38.1803	24.8931	-0.617087	-1.22919	0.03035	0.223989	no
gene:SpnNT_01606	NA	Chromosome:1629831-1631169	110.58	110.58+peptide	OK	38.1803	35.8232	-0.0919357	-0.189449	0.7392	0.994748	no
gene:SpnNT_01606	NA	Chromosome:1629831-1631169	ΔORF2	110.58+peptide	OK	24.8931	35.8232	0.525152	1.05339	0.0694	0.386859	no
gene:SpnNT_01606	NA	Chromosome:1629831-1631169	110.58	ΔORF2+peptide	OK	38.1803	27.3332	-0.482177	-0.971095	0.08005	0.421873	no
gene:SpnNT_01606	NA	Chromosome:1629831-1631169	ΔORF2	ΔORF2+peptide	OK	24.8931	27.3332	0.13491	0.264792	0.63695	0.980887	no
gene:SpnNT_01606	NA	Chromosome:1629831-1631169	110.58+peptide	ΔORF2+peptide	OK	35.8232	27.3332	-0.390242	-0.791566	0.1575	0.605365	no
gene:SpnNT_01607	NA	Chromosome:1631171-1631993	110.58	ΔORF2	OK	65.6254	44.4517	-0.562016	-1.12443	0.0491	0.310501	no
gene:SpnNT_01607	NA	Chromosome:1631171-1631993	110.58	110.58+peptide	OK	65.6254	64.4952	-0.0250617	-0.0514709	0.92835	0.994748	no
gene:SpnNT_01607	NA	Chromosome:1631171-1631993	ΔORF2	110.58+peptide	OK	44.4517	64.4952	0.536954	1.07303	0.0591	0.347451	no
gene:SpnNT_01607	NA	Chromosome:1631171-1631993	110.58	ΔORF2+peptide	OK	65.6254	45.7708	-0.519826	-1.02985	0.06745	0.380099	no
gene:SpnNT_01607	NA	Chromosome:1631171-1631993	ΔORF2	ΔORF2+peptide	OK	44.4517	45.7708	0.042189	0.0814784	0.8815	0.994748	no
gene:SpnNT_01607	NA	Chromosome:1631171-1631993	110.58+peptide	ΔORF2+peptide	OK	64.4952	45.7708	-0.494765	-0.979071	0.07965	0.42059	no
gene:SpnNT_01608	NA	Chromosome:1632165-1632672	110.58	ΔORF2	OK	78.7083	44.6572	-0.817623	-1.53182	0.0066	0.0739583	no
gene:SpnNT_01608	NA	Chromosome:1632165-1632672	110.58	110.58+peptide	OK	78.7083	84.8288	0.108039	0.207305	0.71195	0.991034	no
gene:SpnNT_01608	NA	Chromosome:1632165-1632672	ΔORF2	110.58+peptide	OK	44.6572	84.8288	0.925662	1.73143	0.0025	0.0347143	yes
gene:SpnNT_01608	NA	Chromosome:1632165-1632672	110.58	ΔORF2+peptide	OK	78.7083	56.5205	-0.477741	-0.880325	0.11585	0.516286	no
gene:SpnNT_01608	NA	Chromosome:1632165-1632672	ΔORF2	ΔORF2+peptide	OK	44.6572	56.5205	0.339882	0.611708	0.27585	0.778264	no
gene:SpnNT_01608	NA	Chromosome:1632165-1632672	110.58+peptide	ΔORF2+peptide	OK	84.8288	56.5205	-0.58578	-1.07772	0.05445	0.331705	no
gene:SpnNT_01609	NA	Chromosome:1632691-1633441	110.58	ΔORF2	OK	50.9602	32.1609	-0.664064	-1.26364	0.0283	0.21416	no
gene:SpnNT_01609	NA	Chromosome:1632691-1633441	110.58	110.58+peptide	OK	50.9602	54.0189	0.0840925	0.16335	0.78295	0.994748	no
gene:SpnNT_01609	NA	Chromosome:1632691-1633441	ΔORF2	110.58+peptide	OK	32.1609	54.0189	0.748156	1.41639	0.01265	0.119248	no
gene:SpnNT_01609	NA	Chromosome:1632691-1633441	110.58	ΔORF2+peptide	OK	50.9602	32.9358	-0.629715	-1.17849	0.0404	0.272897	no
gene:SpnNT_01609	NA	Chromosome:1632691-1633441	ΔORF2	ΔORF2+peptide	OK	32.1609	32.9358	0.0343488	0.0627631	0.91395	0.994748	no
gene:SpnNT_01609	NA	Chromosome:1632691-1633441	110.58+peptide	ΔORF2+peptide	OK	54.0189	32.9358	-0.713808	-1.32927	0.0208	0.172527	no
gene:SpnNT_01610	NA	Chromosome:1633517-1633835	110.58	ΔORF2	OK	35.0457	30.9764	-0.178071	-0.249802	0.6629	0.982966	no
gene:SpnNT_01610	NA	Chromosome:1633517-1633835	110.58	110.58+peptide	OK	35.0457	35.4591	0.0169165	0.0241008	0.9691	0.994929	no
gene:SpnNT_01610	NA	Chromosome:1633517-1633835	ΔORF2	110.58+peptide	OK	30.9764	35.4591	0.194988	0.274038	0.63635	0.980887	no
gene:SpnNT_01610	NA	Chromosome:1633517-1633835	110.58	ΔORF2+peptide	OK	35.0457	22.4208	-0.644399	-0.855902	0.12325	0.531129	no
gene:SpnNT_01610	NA	Chromosome:1633517-1633835	ΔORF2	ΔORF2+peptide	OK	30.9764	22.4208	-0.466327	-0.612078	0.27825	0.781125	no
gene:SpnNT_01610	NA	Chromosome:1633517-1633835	110.58+peptide	ΔORF2+peptide	OK	35.4591	22.4208	-0.661315	-0.879823	0.11305	0.510701	no
gene:SpnNT_01611	NA	Chromosome:1633852-1637294	110.58	ΔORF2	OK	35.7569	26.3865	-0.438423	-0.581095	0.3053	0.811289	no
gene:SpnNT_01611	NA	Chromosome:1633852-1637294	110.58	110.58+peptide	OK	35.7569	33.9236	-0.0759313	-0.100961	0.862	0.994748	no
gene:SpnNT_01611	NA	Chromosome:1633852-1637294	ΔORF2	110.58+peptide	OK	26.3865	33.9236	0.362492	0.473715	0.4096	0.8977	no
gene:SpnNT_01611	NA	Chromosome:1633852-1637294	110.58	ΔORF2+peptide	OK	35.7569	27.3946	-0.384329	-0.50363	0.36625	0.864634	no
gene:SpnNT_01611	NA	Chromosome:1633852-1637294	ΔORF2	ΔORF2+peptide	OK	26.3865	27.3946	0.0540937	0.0697033	0.90165	0.994748	no
gene:SpnNT_01611	NA	Chromosome:1633852-1637294	110.58+peptide	ΔORF2+peptide	OK	33.9236	27.3946	-0.308398	-0.398586	0.4781	0.933077	no
gene:SpnNT_01612	NA	Chromosome:1633852-1637294	110.58	ΔORF2	OK	33.9739	26.7704	-0.343784	-0.232133	0.7017	0.990367	no
gene:SpnNT_01612	NA	Chromosome:1633852-1637294	110.58	110.58+peptide	OK	33.9739	38.2942	0.172702	0.118687	0.839	0.994748	no
gene:SpnNT_01612	NA	Chromosome:1633852-1637294	ΔORF2	110.58+peptide	OK	26.7704	38.2942	0.516486	0.35764	0.5394	0.960991	no
gene:SpnNT_01612	NA	Chromosome:1633852-1637294	110.58	ΔORF2+peptide	OK	33.9739	21.9833	-0.628015	-0.407778	0.4757	0.930746	no
gene:SpnNT_01612	NA	Chromosome:1633852-1637294	ΔORF2	ΔORF2+peptide	OK	26.7704	21.9833	-0.284231	-0.185802	0.74055	0.994748	no
gene:SpnNT_01612	NA	Chromosome:1633852-1637294	110.58+peptide	ΔORF2+peptide	OK	38.2942	21.9833	-0.800717	-0.532141	0.33805	0.842454	no
gene:SpnNT_01613	NA	Chromosome:1633852-1637294	110.58	ΔORF2	OK	50.623	35.4183	-0.515297	-0.276887	0.6325	0.980887	no
gene:SpnNT_01613	NA	Chromosome:1633852-1637294	110.58	110.58+peptide	OK	50.623	45.9543	-0.139593	-0.0741623	0.89875	0.994748	no

gene:SpnNT_01613	NA	Chromosome:1633852-1637294	ΔORF2	110.58+peptide	OK	35.4183	45.9543	0.375705	0.194475	0.73885	0.994748	no
gene:SpnNT_01613	NA	Chromosome:1633852-1637294	110.58	ΔORF2+peptide	OK	50.623	42.5321	-0.251241	-0.14327	0.8064	0.994748	no
gene:SpnNT_01613	NA	Chromosome:1633852-1637294	ΔORF2	ΔORF2+peptide	OK	35.4183	42.5321	0.264056	0.146146	0.8	0.994748	no
gene:SpnNT_01613	NA	Chromosome:1633852-1637294	110.58+peptide	ΔORF2+peptide	OK	45.9543	42.5321	-0.111649	-0.0610555	0.91635	0.994748	no
gene:SpnNT_01614	NA	Chromosome:1633852-1637294	110.58	ΔORF2	OK	43.9361	30.272	-0.537425	-0.418439	0.46495	0.926405	no
gene:SpnNT_01614	NA	Chromosome:1633852-1637294	110.58	110.58+peptide	OK	43.9361	41.9562	-0.0665247	-0.0530093	0.9262	0.994748	no
gene:SpnNT_01614	NA	Chromosome:1633852-1637294	ΔORF2	110.58+peptide	OK	30.272	41.9562	0.4709	0.360004	0.53325	0.958798	no
gene:SpnNT_01614	NA	Chromosome:1633852-1637294	110.58	ΔORF2+peptide	OK	43.9361	28.0499	-0.64741	-0.444937	0.4477	0.919505	no
gene:SpnNT_01614	NA	Chromosome:1633852-1637294	ΔORF2	ΔORF2+peptide	OK	30.272	28.0499	-0.109985	-0.0732709	0.9007	0.994748	no
gene:SpnNT_01614	NA	Chromosome:1633852-1637294	110.58+peptide	ΔORF2+peptide	OK	41.9562	28.0499	-0.580885	-0.393552	0.5153	0.951959	no
gene:SpnNT_01615	NA	Chromosome:1633852-1637294	110.58	ΔORF2	OK	27.919	23.1892	-0.267795	-0.240037	0.68205	0.986608	no
gene:SpnNT_01615	NA	Chromosome:1633852-1637294	110.58	110.58+peptide	OK	27.919	27.5019	-0.0217145	-0.0186355	0.97495	0.99536	no
gene:SpnNT_01615	NA	Chromosome:1633852-1637294	ΔORF2	110.58+peptide	OK	23.1892	27.5019	0.246081	0.215306	0.71935	0.993716	no
gene:SpnNT_01615	NA	Chromosome:1633852-1637294	110.58	ΔORF2+peptide	OK	27.919	20.9424	-0.414816	-0.369712	0.50975	0.948082	no
gene:SpnNT_01615	NA	Chromosome:1633852-1637294	ΔORF2	ΔORF2+peptide	OK	23.1892	20.9424	-0.147021	-0.133797	0.8186	0.994748	no
gene:SpnNT_01615	NA	Chromosome:1633852-1637294	110.58+peptide	ΔORF2+peptide	OK	27.5019	20.9424	-0.393102	-0.342084	0.54225	0.961518	no
gene:SpnNT_01616	NA	Chromosome:1633852-1637294	110.58	ΔORF2	OK	48.7256	25.124	-0.955616	-0.327218	0.72675	0.994748	no
gene:SpnNT_01616	NA	Chromosome:1633852-1637294	110.58	110.58+peptide	OK	48.7256	39.203	-0.313716	-0.112597	0.87895	0.994748	no
gene:SpnNT_01616	NA	Chromosome:1633852-1637294	ΔORF2	110.58+peptide	OK	25.124	39.203	0.6419	0.20915	0.80095	0.994748	no
gene:SpnNT_01616	NA	Chromosome:1633852-1637294	110.58	ΔORF2+peptide	OK	48.7256	36.1103	-0.432269	-0.153405	0.8473	0.994748	no
gene:SpnNT_01616	NA	Chromosome:1633852-1637294	ΔORF2	ΔORF2+peptide	OK	25.124	36.1103	0.523346	0.168939	0.8274	0.994748	no
gene:SpnNT_01616	NA	Chromosome:1633852-1637294	110.58+peptide	ΔORF2+peptide	OK	39.203	36.1103	-0.118554	-0.0398951	0.94145	0.994855	no
gene:SpnNT_01617	NA	Chromosome:1637294-1637636	110.58	ΔORF2	OK	35.4629	27.1243	-0.386724	-0.565901	0.3255	0.830813	no
gene:SpnNT_01617	NA	Chromosome:1637294-1637636	110.58	110.58+peptide	OK	35.4629	44.6343	0.33184	0.482475	0.3822	0.879865	no
gene:SpnNT_01617	NA	Chromosome:1637294-1637636	ΔORF2	110.58+peptide	OK	27.1243	44.6343	0.718564	1.06007	0.05685	0.339239	no
gene:SpnNT_01617	NA	Chromosome:1637294-1637636	110.58	ΔORF2+peptide	OK	35.4629	24.8775	-0.511473	-0.676057	0.21735	0.70491	no
gene:SpnNT_01617	NA	Chromosome:1637294-1637636	ΔORF2	ΔORF2+peptide	OK	27.1243	24.8775	-0.124748	-0.166882	0.76	0.994748	no
gene:SpnNT_01617	NA	Chromosome:1637294-1637636	110.58+peptide	ΔORF2+peptide	OK	44.6343	24.8775	-0.843312	-1.12209	0.0369	0.257007	no
gene:SpnNT_01618	NA	Chromosome:1637647-1638299	110.58	ΔORF2	OK	30.3177	22.3695	-0.438624	-0.237259	0.66945	0.984845	no
gene:SpnNT_01618	NA	Chromosome:1637647-1638299	110.58	110.58+peptide	OK	30.3177	19.9468	-0.603999	-0.296556	0.6628	0.982966	no
gene:SpnNT_01618	NA	Chromosome:1637647-1638299	ΔORF2	110.58+peptide	OK	22.3695	19.9468	-0.165375	-0.0793644	0.87015	0.994748	no
gene:SpnNT_01618	NA	Chromosome:1637647-1638299	110.58	ΔORF2+peptide	OK	30.3177	24.6561	-0.298217	-0.138571	0.80555	0.994748	no
gene:SpnNT_01618	NA	Chromosome:1637647-1638299	ΔORF2	ΔORF2+peptide	OK	22.3695	24.6561	0.140407	0.0639187	0.9093	0.994748	no
gene:SpnNT_01618	NA	Chromosome:1637647-1638299	110.58+peptide	ΔORF2+peptide	OK	19.9468	24.6561	0.305782	0.12973	0.84065	0.994748	no
gene:SpnNT_01619	NA	Chromosome:1637647-1638299	110.58	ΔORF2	OK	32.0232	21.7795	-0.556146	-0.289318	0.64955	0.981475	no
gene:SpnNT_01619	NA	Chromosome:1637647-1638299	110.58	110.58+peptide	OK	32.0232	33.5054	0.0652764	0.0361727	0.9514	0.994855	no
gene:SpnNT_01619	NA	Chromosome:1637647-1638299	ΔORF2	110.58+peptide	OK	21.7795	33.5054	0.621422	0.334053	0.59765	0.97629	no
gene:SpnNT_01619	NA	Chromosome:1637647-1638299	110.58	ΔORF2+peptide	OK	32.0232	37.3549	0.222179	0.159812	0.8639	0.994748	no
gene:SpnNT_01619	NA	Chromosome:1637647-1638299	ΔORF2	ΔORF2+peptide	OK	21.7795	37.3549	0.778325	0.532445	0.60205	0.976761	no
gene:SpnNT_01619	NA	Chromosome:1637647-1638299	110.58+peptide	ΔORF2+peptide	OK	33.5054	37.3549	0.156903	0.120401	0.89755	0.994748	no
gene:SpnNT_01620	NA	Chromosome:1637647-1638299	110.58	ΔORF2	OK	54.041	42.089	-0.36061	-0.460436	0.4203	0.904235	no
gene:SpnNT_01620	NA	Chromosome:1637647-1638299	110.58	110.58+peptide	OK	54.041	52.8017	-0.033469	-0.0434804	0.9356	0.994855	no
gene:SpnNT_01620	NA	Chromosome:1637647-1638299	ΔORF2	110.58+peptide	OK	42.089	52.8017	0.327141	0.406708	0.46595	0.926452	no
gene:SpnNT_01620	NA	Chromosome:1637647-1638299	110.58	ΔORF2+peptide	OK	54.041	37.1482	-0.540762	-0.584919	0.25995	0.760041	no
gene:SpnNT_01620	NA	Chromosome:1637647-1638299	ΔORF2	ΔORF2+peptide	OK	42.089	37.1482	-0.180152	-0.188933	0.71655	0.992452	no

gene:SpnNT_01620	NA	Chromosome:1637647-1638299	110.58+peptide	ΔORF2+peptide	OK	52.8017	37.1482	-0.507293	-0.538237	0.29185	0.796956	no
gene:SpnNT_01621	NA	Chromosome:1638417-1638960	110.58	ΔORF2	OK	12442.9	16649.6	0.420163	0.832987	0.13615	0.561458	no
gene:SpnNT_01621	NA	Chromosome:1638417-1638960	110.58	110.58+peptide	OK	12442.9	18168.7	0.546129	1.06932	0.0534	0.327284	no
gene:SpnNT_01621	NA	Chromosome:1638417-1638960	ΔORF2	110.58+peptide	OK	16649.6	18168.7	0.125966	0.242758	0.66115	0.982966	no
gene:SpnNT_01621	NA	Chromosome:1638417-1638960	110.58	ΔORF2+peptide	OK	12442.9	13967.5	0.166749	0.342415	0.544	0.961568	no
gene:SpnNT_01621	NA	Chromosome:1638417-1638960	ΔORF2	ΔORF2+peptide	OK	16649.6	13967.5	-0.253414	-0.511389	0.36715	0.864634	no
gene:SpnNT_01621	NA	Chromosome:1638417-1638960	110.58+peptide	ΔORF2+peptide	OK	18168.7	13967.5	-0.37938	-0.755778	0.1784	0.645075	no
gene:SpnNT_01622	NA	Chromosome:1638417-1638960	110.58	ΔORF2	OK	3.216	3.70013	0.202307	0.0137777	0.8545	0.994748	no
gene:SpnNT_01622	NA	Chromosome:1638417-1638960	110.58	110.58+peptide	OK	3.216	3.51303	0.127446	0.00888639	0.8575	0.994748	no
gene:SpnNT_01622	NA	Chromosome:1638417-1638960	ΔORF2	110.58+peptide	OK	3.70013	3.51303	-0.0748605	-0.00444316	0.83835	0.994748	no
gene:SpnNT_01622	NA	Chromosome:1638417-1638960	110.58	ΔORF2+peptide	OK	3.216	0	#NAME?	NA	0.1218	0.5287	no
gene:SpnNT_01622	NA	Chromosome:1638417-1638960	ΔORF2	ΔORF2+peptide	OK	3.70013	0	#NAME?	NA	0.12095	0.526578	no
gene:SpnNT_01622	NA	Chromosome:1638417-1638960	110.58+peptide	ΔORF2+peptide	OK	3.51303	0	#NAME?	NA	0.12885	0.544181	no
gene:SpnNT_01623	NA	Chromosome:1638417-1638960	110.58	ΔORF2	OK	74.1168	37.5914	-0.9794	-0.0430717	0.56805	0.968621	no
gene:SpnNT_01623	NA	Chromosome:1638417-1638960	110.58	110.58+peptide	OK	74.1168	73.6692	-0.00873938	-0.000448516	0.9794	0.995765	no
gene:SpnNT_01623	NA	Chromosome:1638417-1638960	ΔORF2	110.58+peptide	OK	37.5914	73.6692	0.97066	0.0390055	0.5737	0.969538	no
gene:SpnNT_01623	NA	Chromosome:1638417-1638960	110.58	ΔORF2+peptide	OK	74.1168	96.0734	0.374336	0.031443	0.8435	0.994748	no
gene:SpnNT_01623	NA	Chromosome:1638417-1638960	ΔORF2	ΔORF2+peptide	OK	37.5914	96.0734	1.35374	0.0693233	0.55375	0.966007	no
gene:SpnNT_01623	NA	Chromosome:1638417-1638960	110.58+peptide	ΔORF2+peptide	OK	73.6692	96.0734	0.383076	0.0245262	0.83805	0.994748	no
gene:SpnNT_01624	NA	Chromosome:1639076-1639384	110.58	ΔORF2	OK	1386.57	1341.53	-0.0476379	-0.0925591	0.8758	0.994748	no
gene:SpnNT_01624	NA	Chromosome:1639076-1639384	110.58	110.58+peptide	OK	1386.57	1512.25	0.125182	0.234962	0.67615	0.98524	no
gene:SpnNT_01624	NA	Chromosome:1639076-1639384	ΔORF2	110.58+peptide	OK	1341.53	1512.25	0.172819	0.32253	0.5628	0.968621	no
gene:SpnNT_01624	NA	Chromosome:1639076-1639384	110.58	ΔORF2+peptide	OK	1386.57	1321.53	-0.0693063	-0.141402	0.8251	0.994748	no
gene:SpnNT_01624	NA	Chromosome:1639076-1639384	ΔORF2	ΔORF2+peptide	OK	1341.53	1321.53	-0.0216684	-0.043912	0.9426	0.994855	no
gene:SpnNT_01624	NA	Chromosome:1639076-1639384	110.58+peptide	ΔORF2+peptide	OK	1512.25	1321.53	-0.194488	-0.379637	0.52715	0.956161	no
gene:SpnNT_01625	NA	Chromosome:1639076-1639384	110.58	ΔORF2	OK	45.0313	49.2412	0.128939	0.0350857	0.9406	0.994855	no
gene:SpnNT_01625	NA	Chromosome:1639076-1639384	110.58	110.58+peptide	OK	45.0313	61.0361	0.438736	0.115382	0.83725	0.994748	no
gene:SpnNT_01625	NA	Chromosome:1639076-1639384	ΔORF2	110.58+peptide	OK	49.2412	61.0361	0.309797	0.0827503	0.87875	0.994748	no
gene:SpnNT_01625	NA	Chromosome:1639076-1639384	110.58	ΔORF2+peptide	OK	45.0313	29.4899	-0.610708	-0.227479	0.7966	0.994748	no
gene:SpnNT_01625	NA	Chromosome:1639076-1639384	ΔORF2	ΔORF2+peptide	OK	49.2412	29.4899	-0.739647	-0.284383	0.7477	0.994748	no
gene:SpnNT_01625	NA	Chromosome:1639076-1639384	110.58+peptide	ΔORF2+peptide	OK	61.0361	29.4899	-1.04944	-0.377756	0.6662	0.983855	no
gene:SpnNT_01626	NA	Chromosome:1639398-1639602	110.58	ΔORF2	OK	17.2743	27.6129	0.676718	0.666029	0.24955	0.747113	no
gene:SpnNT_01626	NA	Chromosome:1639398-1639602	110.58	110.58+peptide	OK	17.2743	28.6768	0.731259	0.767821	0.18985	0.667706	no
gene:SpnNT_01626	NA	Chromosome:1639398-1639602	ΔORF2	110.58+peptide	OK	27.6129	28.6768	0.0545404	0.0564158	0.91615	0.994748	no
gene:SpnNT_01626	NA	Chromosome:1639398-1639602	110.58	ΔORF2+peptide	OK	17.2743	19.8542	0.200818	0.200329	0.7408	0.994748	no
gene:SpnNT_01626	NA	Chromosome:1639398-1639602	ΔORF2	ΔORF2+peptide	OK	27.6129	19.8542	-0.4759	-0.468355	0.42	0.904223	no
gene:SpnNT_01626	NA	Chromosome:1639398-1639602	110.58+peptide	ΔORF2+peptide	OK	28.6768	19.8542	-0.530441	-0.556924	0.3498	0.85365	no
gene:SpnNT_01627	NA	Chromosome:1639768-1640113	110.58	ΔORF2	OK	102.918	141.613	0.46046	0.838329	0.14715	0.584811	no
gene:SpnNT_01627	NA	Chromosome:1639768-1640113	110.58	110.58+peptide	OK	102.918	119.058	0.210173	0.384972	0.501	0.944652	no
gene:SpnNT_01627	NA	Chromosome:1639768-1640113	ΔORF2	110.58+peptide	OK	141.613	119.058	-0.250286	-0.461457	0.42115	0.904877	no
gene:SpnNT_01627	NA	Chromosome:1639768-1640113	110.58	ΔORF2+peptide	OK	102.918	126.284	0.295176	0.546901	0.3495	0.853357	no
gene:SpnNT_01627	NA	Chromosome:1639768-1640113	ΔORF2	ΔORF2+peptide	OK	141.613	126.284	-0.165284	-0.308296	0.595	0.975583	no
gene:SpnNT_01627	NA	Chromosome:1639768-1640113	110.58+peptide	ΔORF2+peptide	OK	119.058	126.284	0.0850024	0.159561	0.78085	0.994748	no
gene:SpnNT_01628	NA	Chromosome:1640172-1640304	110.58	ΔORF2	OK	42.162	29.3391	-0.52312	-0.376242	0.5221	0.954832	no
gene:SpnNT_01628	NA	Chromosome:1640172-1640304	110.58	110.58+peptide	OK	42.162	25.4339	-0.72919	-0.447883	0.41955	0.904223	no

gene:SpnNT_01628	NA	Chromosome:1640172-1640304	ΔORF2	110.58+peptide	OK	29.3391	25.4339	-0.20607	-0.123279	0.84285	0.994748	no
gene:SpnNT_01628	NA	Chromosome:1640172-1640304	110.58	ΔORF2+peptide	OK	42.162	0	#NAME?	NA	5.00E-05	0.0013612	yes
gene:SpnNT_01628	NA	Chromosome:1640172-1640304	ΔORF2	ΔORF2+peptide	OK	29.3391	0	#NAME?	NA	0.001	0.0168447	yes
gene:SpnNT_01628	NA	Chromosome:1640172-1640304	110.58+peptide	ΔORF2+peptide	OK	25.4339	0	#NAME?	NA	0.00295	0.0393393	yes
gene:SpnNT_01629	NA	Chromosome:1640360-1641067	110.58	ΔORF2	OK	40.543	49.5245	0.288691	0.498502	0.38345	0.880579	no
gene:SpnNT_01629	NA	Chromosome:1640360-1641067	110.58	110.58+peptide	OK	40.543	34.5643	-0.230171	-0.397078	0.4909	0.940378	no
gene:SpnNT_01629	NA	Chromosome:1640360-1641067	ΔORF2	110.58+peptide	OK	49.5245	34.5643	-0.518862	-0.890266	0.12515	0.535526	no
gene:SpnNT_01629	NA	Chromosome:1640360-1641067	110.58	ΔORF2+peptide	OK	40.543	31.9004	-0.345878	-0.586491	0.30375	0.809137	no
gene:SpnNT_01629	NA	Chromosome:1640360-1641067	ΔORF2	ΔORF2+peptide	OK	49.5245	31.9004	-0.634569	-1.07038	0.0639	0.367209	no
gene:SpnNT_01629	NA	Chromosome:1640360-1641067	110.58+peptide	ΔORF2+peptide	OK	34.5643	31.9004	-0.115707	-0.194997	0.7326	0.994748	no
gene:SpnNT_01630	NA	Chromosome:1640360-1641067	110.58	ΔORF2	OK	60.2887	56.5487	-0.0923935	-0.0445348	0.91515	0.994748	no
gene:SpnNT_01630	NA	Chromosome:1640360-1641067	110.58	110.58+peptide	OK	60.2887	65.5482	0.120668	0.0583565	0.9218	0.994748	no
gene:SpnNT_01630	NA	Chromosome:1640360-1641067	ΔORF2	110.58+peptide	OK	56.5487	65.5482	0.213061	0.101087	0.88835	0.994748	no
gene:SpnNT_01630	NA	Chromosome:1640360-1641067	110.58	ΔORF2+peptide	OK	60.2887	37.7765	-0.674398	-0.435875	0.78955	0.994748	no
gene:SpnNT_01630	NA	Chromosome:1640360-1641067	ΔORF2	ΔORF2+peptide	OK	56.5487	37.7765	-0.582005	-0.363708	0.82775	0.994748	no
gene:SpnNT_01630	NA	Chromosome:1640360-1641067	110.58+peptide	ΔORF2+peptide	OK	65.5482	37.7765	-0.795066	-0.499639	0.7084	0.990441	no
gene:SpnNT_01631	NA	Chromosome:1641093-1641285	110.58	ΔORF2	OK	26.9666	15.5235	-0.796721	-0.715601	0.1941	0.675407	no
gene:SpnNT_01631	NA	Chromosome:1641093-1641285	110.58	110.58+peptide	OK	26.9666	21.6733	-0.315252	-0.297469	0.57985	0.969538	no
gene:SpnNT_01631	NA	Chromosome:1641093-1641285	ΔORF2	110.58+peptide	OK	15.5235	21.6733	0.481469	0.4253	0.4362	0.914104	no
gene:SpnNT_01631	NA	Chromosome:1641093-1641285	110.58	ΔORF2+peptide	OK	26.9666	11.5799	-1.21956	-1.51838	0.08605	0.440385	no
gene:SpnNT_01631	NA	Chromosome:1641093-1641285	ΔORF2	ΔORF2+peptide	OK	15.5235	11.5799	-0.422836	-0.471693	0.54365	0.961568	no
gene:SpnNT_01631	NA	Chromosome:1641093-1641285	110.58+peptide	ΔORF2+peptide	OK	21.6733	11.5799	-0.904305	-1.09092	0.1917	0.672051	no
gene:SpnNT_01632	NA	Chromosome:1641585-1642375	110.58	ΔORF2	OK	49.2975	45.0536	-0.129873	-0.201161	0.72685	0.994748	no
gene:SpnNT_01632	NA	Chromosome:1641585-1642375	110.58	110.58+peptide	OK	49.2975	40.9365	-0.268128	-0.410144	0.46765	0.92732	no
gene:SpnNT_01632	NA	Chromosome:1641585-1642375	ΔORF2	110.58+peptide	OK	45.0536	40.9365	-0.138255	-0.208285	0.71155	0.991007	no
gene:SpnNT_01632	NA	Chromosome:1641585-1642375	110.58	ΔORF2+peptide	OK	49.2975	25.729	-0.93812	-1.25134	0.02505	0.197421	no
gene:SpnNT_01632	NA	Chromosome:1641585-1642375	ΔORF2	ΔORF2+peptide	OK	45.0536	25.729	-0.808248	-1.06564	0.0534	0.327284	no
gene:SpnNT_01632	NA	Chromosome:1641585-1642375	110.58+peptide	ΔORF2+peptide	OK	40.9365	25.729	-0.669993	-0.87536	0.10355	0.488069	no
gene:SpnNT_01633	NA	Chromosome:1641585-1642375	110.58	ΔORF2	OK	31.2123	20.5489	-0.603051	-0.375396	0.50265	0.944788	no
gene:SpnNT_01633	NA	Chromosome:1641585-1642375	110.58	110.58+peptide	OK	31.2123	18.5658	-0.749466	-0.433586	0.43845	0.915991	no
gene:SpnNT_01633	NA	Chromosome:1641585-1642375	ΔORF2	110.58+peptide	OK	20.5489	18.5658	-0.146415	-0.0761866	0.8919	0.994748	no
gene:SpnNT_01633	NA	Chromosome:1641585-1642375	110.58	ΔORF2+peptide	OK	31.2123	12.0483	-1.37329	-1.16372	0.2334	0.727649	no
gene:SpnNT_01633	NA	Chromosome:1641585-1642375	ΔORF2	ΔORF2+peptide	OK	20.5489	12.0483	-0.770235	-0.531758	0.5315	0.957488	no
gene:SpnNT_01633	NA	Chromosome:1641585-1642375	110.58+peptide	ΔORF2+peptide	OK	18.5658	12.0483	-0.623821	-0.394127	0.6399	0.980887	no
gene:SpnNT_01634	NA	Chromosome:1641585-1642375	110.58	ΔORF2	OK	15.9876	23.3548	0.546765	0.197718	0.8102	0.994748	no
gene:SpnNT_01634	NA	Chromosome:1641585-1642375	110.58	110.58+peptide	OK	15.9876	17.3442	0.117494	0.0413481	0.8605	0.994748	no
gene:SpnNT_01634	NA	Chromosome:1641585-1642375	ΔORF2	110.58+peptide	OK	23.3548	17.3442	-0.429271	-0.159757	0.8101	0.994748	no
gene:SpnNT_01634	NA	Chromosome:1641585-1642375	110.58	ΔORF2+peptide	OK	15.9876	11.3276	-0.497119	-0.229302	0.79845	0.994748	no
gene:SpnNT_01634	NA	Chromosome:1641585-1642375	ΔORF2	ΔORF2+peptide	OK	23.3548	11.3276	-1.04388	-0.532318	0.65735	0.982966	no
gene:SpnNT_01634	NA	Chromosome:1641585-1642375	110.58+peptide	ΔORF2+peptide	OK	17.3442	11.3276	-0.614614	-0.297333	0.75275	0.994748	no
gene:SpnNT_01635	NA	Chromosome:1642468-1643109	110.58	ΔORF2	OK	15.6188	19.8846	0.348368	0.531837	0.357	0.858254	no
gene:SpnNT_01635	NA	Chromosome:1642468-1643109	110.58	110.58+peptide	OK	15.6188	10.1879	-0.61643	-0.878059	0.13565	0.560453	no
gene:SpnNT_01635	NA	Chromosome:1642468-1643109	ΔORF2	110.58+peptide	OK	19.8846	10.1879	-0.964797	-1.40323	0.0193	0.162447	no
gene:SpnNT_01635	NA	Chromosome:1642468-1643109	110.58	ΔORF2+peptide	OK	15.6188	9.86867	-0.662357	-0.94181	0.10135	0.482903	no
gene:SpnNT_01635	NA	Chromosome:1642468-1643109	ΔORF2	ΔORF2+peptide	OK	19.8846	9.86867	-1.01072	-1.46732	0.01135	0.109915	no

gene:SpnNT_01635	NA	Chromosome:1642468-1643109	110.58+peptide	ΔORF2+peptide	OK	10.1879	9.86867	-0.0459275	-0.0625996	0.9133	0.994748	no
gene:SpnNT_01636	NA	Chromosome:1642468-1643109	110.58	ΔORF2	OK	18.2789	22.7487	0.315604	0.122742	0.8833	0.994748	no
gene:SpnNT_01636	NA	Chromosome:1642468-1643109	110.58	110.58+peptide	OK	18.2789	18.6916	0.0322109	0.01156	0.94965	0.994855	no
gene:SpnNT_01636	NA	Chromosome:1642468-1643109	ΔORF2	110.58+peptide	OK	22.7487	18.6916	-0.283394	-0.110135	0.87875	0.994748	no
gene:SpnNT_01636	NA	Chromosome:1642468-1643109	110.58	ΔORF2+peptide	OK	18.2789	7.94942	-1.20126	-0.585621	0.6189	0.980887	no
gene:SpnNT_01636	NA	Chromosome:1642468-1643109	ΔORF2	ΔORF2+peptide	OK	22.7487	7.94942	-1.51686	-0.866485	0.58035	0.969538	no
gene:SpnNT_01636	NA	Chromosome:1642468-1643109	110.58+peptide	ΔORF2+peptide	OK	18.6916	7.94942	-1.23347	-0.600645	0.5303	0.957488	no
gene:SpnNT_01637	NA	Chromosome:1643119-1643500	110.58	ΔORF2	OK	42.0851	38.3561	-0.133855	-0.224671	0.69335	0.98828	no
gene:SpnNT_01637	NA	Chromosome:1643119-1643500	110.58	110.58+peptide	OK	42.0851	39.0012	-0.109791	-0.18474	0.74565	0.994748	no
gene:SpnNT_01637	NA	Chromosome:1643119-1643500	ΔORF2	110.58+peptide	OK	38.3561	39.0012	0.0240646	0.040102	0.9433	0.994855	no
gene:SpnNT_01637	NA	Chromosome:1643119-1643500	110.58	ΔORF2+peptide	OK	42.0851	25.4441	-0.725979	-1.16597	0.0435	0.285832	no
gene:SpnNT_01637	NA	Chromosome:1643119-1643500	ΔORF2	ΔORF2+peptide	OK	38.3561	25.4441	-0.592124	-0.942628	0.09755	0.472169	no
gene:SpnNT_01637	NA	Chromosome:1643119-1643500	110.58+peptide	ΔORF2+peptide	OK	39.0012	25.4441	-0.616189	-0.98314	0.08405	0.435071	no
gene:SpnNT_01638	NA	Chromosome:1643551-1644148	110.58	ΔORF2	OK	924.162	1146.02	0.31041	0.634768	0.25555	0.7547	no
gene:SpnNT_01638	NA	Chromosome:1643551-1644148	110.58	110.58+peptide	OK	924.162	992.421	0.102807	0.205881	0.70885	0.990577	no
gene:SpnNT_01638	NA	Chromosome:1643551-1644148	ΔORF2	110.58+peptide	OK	1146.02	992.421	-0.207603	-0.402312	0.46975	0.927937	no
gene:SpnNT_01638	NA	Chromosome:1643551-1644148	110.58	ΔORF2+peptide	OK	924.162	778.263	-0.247887	-0.522046	0.3497	0.853565	no
gene:SpnNT_01638	NA	Chromosome:1643551-1644148	ΔORF2	ΔORF2+peptide	OK	1146.02	778.263	-0.558297	-1.13397	0.04665	0.299189	no
gene:SpnNT_01638	NA	Chromosome:1643551-1644148	110.58+peptide	ΔORF2+peptide	OK	992.421	778.263	-0.350694	-0.697745	0.22305	0.712784	no
gene:SpnNT_01639	xerD_2	Chromosome:1644297-1645425	110.58	ΔORF2	OK	35.5431	35.8382	0.0119288	0.024169	0.9675	0.994855	no
gene:SpnNT_01639	xerD_2	Chromosome:1644297-1645425	110.58	110.58+peptide	OK	35.5431	23.6003	-0.590762	-1.17063	0.0389	0.265996	no
gene:SpnNT_01639	xerD_2	Chromosome:1644297-1645425	ΔORF2	110.58+peptide	OK	35.8382	23.6003	-0.602691	-1.18101	0.037	0.257431	no
gene:SpnNT_01639	xerD_2	Chromosome:1644297-1645425	110.58	ΔORF2+peptide	OK	35.5431	25.189	-0.496775	-0.992113	0.08225	0.428968	no
gene:SpnNT_01639	xerD_2	Chromosome:1644297-1645425	ΔORF2	ΔORF2+peptide	OK	35.8382	25.189	-0.508704	-1.00448	0.079	0.418337	no
gene:SpnNT_01639	xerD_2	Chromosome:1644297-1645425	110.58+peptide	ΔORF2+peptide	OK	23.6003	25.189	0.0939867	0.181703	0.7472	0.994748	no
gene:SpnNT_01640	whiA	Chromosome:1645512-1648285	110.58	ΔORF2	OK	138.908	141.84	0.0301316	0.0393138	0.94555	0.994855	no
gene:SpnNT_01640	whiA	Chromosome:1645512-1648285	110.58	110.58+peptide	OK	138.908	93.4982	-0.571119	-0.76947	0.17185	0.632011	no
gene:SpnNT_01640	whiA	Chromosome:1645512-1648285	ΔORF2	110.58+peptide	OK	141.84	93.4982	-0.601251	-0.80378	0.15165	0.593484	no
gene:SpnNT_01640	whiA	Chromosome:1645512-1648285	110.58	ΔORF2+peptide	OK	138.908	99.8127	-0.476834	-0.647435	0.254	0.752855	no
gene:SpnNT_01640	whiA	Chromosome:1645512-1648285	ΔORF2	ΔORF2+peptide	OK	141.84	99.8127	-0.506966	-0.682923	0.22915	0.721915	no
gene:SpnNT_01640	whiA	Chromosome:1645512-1648285	110.58+peptide	ΔORF2+peptide	OK	93.4982	99.8127	0.0942851	0.13144	0.81125	0.994748	no
gene:SpnNT_01641	NA	Chromosome:1645512-1648285	110.58	ΔORF2	OK	126.253	123.511	-0.0316803	-0.0415658	0.9428	0.994855	no
gene:SpnNT_01641	NA	Chromosome:1645512-1648285	110.58	110.58+peptide	OK	126.253	84.2099	-0.584254	-0.772115	0.1789	0.645815	no
gene:SpnNT_01641	NA	Chromosome:1645512-1648285	ΔORF2	110.58+peptide	OK	123.511	84.2099	-0.552574	-0.731874	0.2007	0.683494	no
gene:SpnNT_01641	NA	Chromosome:1645512-1648285	110.58	ΔORF2+peptide	OK	126.253	90.7403	-0.476502	-0.633389	0.27865	0.781125	no
gene:SpnNT_01641	NA	Chromosome:1645512-1648285	ΔORF2	ΔORF2+peptide	OK	123.511	90.7403	-0.444822	-0.592609	0.31045	0.816346	no
gene:SpnNT_01641	NA	Chromosome:1645512-1648285	110.58+peptide	ΔORF2+peptide	OK	84.2099	90.7403	0.107752	0.144624	0.8047	0.994748	no
gene:SpnNT_01642	NA	Chromosome:1645512-1648285	110.58	ΔORF2	OK	122.151	120.511	-0.0195069	-0.0236657	0.96775	0.994855	no
gene:SpnNT_01642	NA	Chromosome:1645512-1648285	110.58	110.58+peptide	OK	122.151	73.4517	-0.733802	-0.8725	0.13145	0.551468	no
gene:SpnNT_01642	NA	Chromosome:1645512-1648285	ΔORF2	110.58+peptide	OK	120.511	73.4517	-0.714295	-0.855128	0.1364	0.561959	no
gene:SpnNT_01642	NA	Chromosome:1645512-1648285	110.58	ΔORF2+peptide	OK	122.151	72.4127	-0.754356	-0.897882	0.12235	0.529336	no
gene:SpnNT_01642	NA	Chromosome:1645512-1648285	ΔORF2	ΔORF2+peptide	OK	120.511	72.4127	-0.734849	-0.880673	0.12515	0.535526	no
gene:SpnNT_01642	NA	Chromosome:1645512-1648285	110.58+peptide	ΔORF2+peptide	OK	73.4517	72.4127	-0.0205539	-0.0241532	0.96845	0.994855	no
gene:SpnNT_01643	yabJ	Chromosome:1648336-1648717	110.58	ΔORF2	OK	826.437	1011.14	0.291009	0.640074	0.259	0.759841	no
gene:SpnNT_01643	yabJ	Chromosome:1648336-1648717	110.58	110.58+peptide	OK	826.437	778.204	-0.0867568	-0.189071	0.7377	0.994748	no

gene:SpnNT_01643	yabJ	Chromosome:1648336-1648717	ΔORF2	110.58+peptide	OK	1011.14	778.204	-0.377766	-0.827906	0.13815	0.566678	no
gene:SpnNT_01643	yabJ	Chromosome:1648336-1648717	110.58	ΔORF2+peptide	OK	826.437	720.984	-0.196937	-0.437943	0.44855	0.919688	no
gene:SpnNT_01643	yabJ	Chromosome:1648336-1648717	ΔORF2	ΔORF2+peptide	OK	1011.14	720.984	-0.487947	-1.09144	0.055	0.333816	no
gene:SpnNT_01643	yabJ	Chromosome:1648336-1648717	110.58+peptide	ΔORF2+peptide	OK	778.204	720.984	-0.110181	-0.244116	0.6673	0.983929	no
gene:SpnNT_01644	engB	Chromosome:1648727-1649315	110.58	ΔORF2	OK	140.55	150.954	0.103033	0.221538	0.7008	0.990367	no
gene:SpnNT_01644	engB	Chromosome:1648727-1649315	110.58	110.58+peptide	OK	140.55	113.277	-0.311221	-0.667142	0.23615	0.73046	no
gene:SpnNT_01644	engB	Chromosome:1648727-1649315	ΔORF2	110.58+peptide	OK	150.954	113.277	-0.414253	-0.882068	0.12415	0.533256	no
gene:SpnNT_01644	engB	Chromosome:1648727-1649315	110.58	ΔORF2+peptide	OK	140.55	126.336	-0.153813	-0.329875	0.56005	0.968621	no
gene:SpnNT_01644	engB	Chromosome:1648727-1649315	ΔORF2	ΔORF2+peptide	OK	150.954	126.336	-0.256845	-0.547158	0.341	0.844955	no
gene:SpnNT_01644	engB	Chromosome:1648727-1649315	110.58+peptide	ΔORF2+peptide	OK	113.277	126.336	0.157408	0.334325	0.5536	0.965961	no
gene:SpnNT_01645	clpX	Chromosome:1649323-1650556	110.58	ΔORF2	OK	265.707	275.1	0.0501179	0.113804	0.84435	0.994748	no
gene:SpnNT_01645	clpX	Chromosome:1649323-1650556	110.58	110.58+peptide	OK	265.707	215.823	-0.299992	-0.684193	0.22855	0.720575	no
gene:SpnNT_01645	clpX	Chromosome:1649323-1650556	ΔORF2	110.58+peptide	OK	275.1	215.823	-0.35011	-0.80023	0.1634	0.617728	no
gene:SpnNT_01645	clpX	Chromosome:1649323-1650556	110.58	ΔORF2+peptide	OK	265.707	226.662	-0.229294	-0.523311	0.3541	0.856303	no
gene:SpnNT_01645	clpX	Chromosome:1649323-1650556	ΔORF2	ΔORF2+peptide	OK	275.1	226.662	-0.279412	-0.639079	0.2594	0.759841	no
gene:SpnNT_01645	clpX	Chromosome:1649323-1650556	110.58+peptide	ΔORF2+peptide	OK	215.823	226.662	0.0706979	0.162424	0.77305	0.994748	no
gene:SpnNT_01646	NA	Chromosome:1650587-1651264	110.58	ΔORF2	OK	37.0457	19.304	-0.940405	-0.141927	0.56675	0.968621	no
gene:SpnNT_01646	NA	Chromosome:1650587-1651264	110.58	110.58+peptide	OK	37.0457	27.7257	-0.418079	-0.0762193	0.7854	0.994748	no
gene:SpnNT_01646	NA	Chromosome:1650587-1651264	ΔORF2	110.58+peptide	OK	19.304	27.7257	0.522326	0.0779468	0.7452	0.994748	no
gene:SpnNT_01646	NA	Chromosome:1650587-1651264	110.58	ΔORF2+peptide	OK	37.0457	31.8359	-0.218651	-0.0403306	0.8908	0.994748	no
gene:SpnNT_01646	NA	Chromosome:1650587-1651264	ΔORF2	ΔORF2+peptide	OK	19.304	31.8359	0.721753	0.108551	0.65605	0.982966	no
gene:SpnNT_01646	NA	Chromosome:1650587-1651264	110.58+peptide	ΔORF2+peptide	OK	27.7257	31.8359	0.199427	0.036174	0.89755	0.994748	no
gene:SpnNT_01647	dhfR	Chromosome:1650587-1651264	110.58	ΔORF2	OK	336.01	376.366	0.163631	0.36802	0.5126	0.950409	no
gene:SpnNT_01647	dhfR	Chromosome:1650587-1651264	110.58	110.58+peptide	OK	336.01	254.003	-0.403662	-0.899779	0.11085	0.507277	no
gene:SpnNT_01647	dhfR	Chromosome:1650587-1651264	ΔORF2	110.58+peptide	OK	376.366	254.003	-0.567293	-1.28268	0.024	0.191679	no
gene:SpnNT_01647	dhfR	Chromosome:1650587-1651264	110.58	ΔORF2+peptide	OK	336.01	276.893	-0.27918	-0.62195	0.27225	0.774266	no
gene:SpnNT_01647	dhfR	Chromosome:1650587-1651264	ΔORF2	ΔORF2+peptide	OK	376.366	276.893	-0.442811	-1.00063	0.0781	0.415078	no
gene:SpnNT_01647	dhfR	Chromosome:1650587-1651264	110.58+peptide	ΔORF2+peptide	OK	254.003	276.893	0.124483	0.278766	0.62585	0.980887	no
gene:SpnNT_01648	dps	Chromosome:1651393-1651912	110.58	ΔORF2	OK	1788.5	2550.58	0.512074	1.09551	0.05635	0.3381	no
gene:SpnNT_01648	dps	Chromosome:1651393-1651912	110.58	110.58+peptide	OK	1788.5	893.843	-1.00066	-2.16178	5.00E-04	0.0095781	yes
gene:SpnNT_01648	dps	Chromosome:1651393-1651912	ΔORF2	110.58+peptide	OK	2550.58	893.843	-1.51273	-3.39413	5.00E-05	0.0013612	yes
gene:SpnNT_01648	dps	Chromosome:1651393-1651912	110.58	ΔORF2+peptide	OK	1788.5	769.4	-1.21694	-2.64684	5.00E-05	0.0013612	yes
gene:SpnNT_01648	dps	Chromosome:1651393-1651912	ΔORF2	ΔORF2+peptide	OK	2550.58	769.4	-1.72902	-3.90776	5.00E-05	0.0013612	yes
gene:SpnNT_01648	dps	Chromosome:1651393-1651912	110.58+peptide	ΔORF2+peptide	OK	893.843	769.4	-0.216287	-0.494197	0.3903	0.884392	no
gene:SpnNT_01649	lytB_7	Chromosome:1652407-1653880	110.58	ΔORF2	OK	708.751	759.915	0.100559	0.221388	0.68935	0.98828	no
gene:SpnNT_01649	lytB_7	Chromosome:1652407-1653880	110.58	110.58+peptide	OK	708.751	797.003	0.169306	0.368766	0.5089	0.947472	no
gene:SpnNT_01649	lytB_7	Chromosome:1652407-1653880	ΔORF2	110.58+peptide	OK	759.915	797.003	0.0687471	0.149562	0.78645	0.994748	no
gene:SpnNT_01649	lytB_7	Chromosome:1652407-1653880	110.58	ΔORF2+peptide	OK	708.751	1045.65	0.561053	1.22081	0.0295	0.219444	no
gene:SpnNT_01649	lytB_7	Chromosome:1652407-1653880	ΔORF2	ΔORF2+peptide	OK	759.915	1045.65	0.460494	1.00082	0.0726	0.39694	no
gene:SpnNT_01649	lytB_7	Chromosome:1652407-1653880	110.58+peptide	ΔORF2+peptide	OK	797.003	1045.65	0.391747	0.842563	0.1315	0.551468	no
gene:SpnNT_01650	tpiA	Chromosome:1653949-1654708	110.58	ΔORF2	OK	451.29	463.839	0.0395692	0.0902433	0.8742	0.994748	no
gene:SpnNT_01650	tpiA	Chromosome:1653949-1654708	110.58	110.58+peptide	OK	451.29	531.689	0.236529	0.540527	0.33935	0.844054	no
gene:SpnNT_01650	tpiA	Chromosome:1653949-1654708	ΔORF2	110.58+peptide	OK	463.839	531.689	0.19696	0.44697	0.42975	0.911751	no
gene:SpnNT_01650	tpiA	Chromosome:1653949-1654708	110.58	ΔORF2+peptide	OK	451.29	596.416	0.402264	0.92061	0.1065	0.496269	no
gene:SpnNT_01650	tpiA	Chromosome:1653949-1654708	ΔORF2	ΔORF2+peptide	OK	463.839	596.416	0.362694	0.824261	0.1521	0.594536	no

gene:SpnNT_01650	tpiA	Chromosome:1653949-1654708	110.58+peptide	ΔORF2+peptide	OK	531.689	596.416	0.165735	0.377405	0.50755	0.946529	no
gene:SpnNT_01651	dnaD	Chromosome:1654806-1655484	110.58	ΔORF2	OK	98.3373	87.7408	-0.164491	-0.347951	0.5348	0.958867	no
gene:SpnNT_01651	dnaD	Chromosome:1654806-1655484	110.58	110.58+peptide	OK	98.3373	99.6993	0.0198441	0.0423936	0.9392	0.994855	no
gene:SpnNT_01651	dnaD	Chromosome:1654806-1655484	ΔORF2	110.58+peptide	OK	87.7408	99.6993	0.184335	0.391476	0.496	0.943191	no
gene:SpnNT_01651	dnaD	Chromosome:1654806-1655484	110.58	ΔORF2+peptide	OK	98.3373	100.491	0.0312534	0.0664799	0.90705	0.994748	no
gene:SpnNT_01651	dnaD	Chromosome:1654806-1655484	ΔORF2	ΔORF2+peptide	OK	87.7408	100.491	0.195744	0.413935	0.47515	0.930746	no
gene:SpnNT_01651	dnaD	Chromosome:1654806-1655484	110.58+peptide	ΔORF2+peptide	OK	99.6993	100.491	0.0114093	0.0243665	0.9697	0.995105	no
gene:SpnNT_01652	metA	Chromosome:1655492-1656437	110.58	ΔORF2	OK	69.3279	69.5776	0.00518617	0.0111283	0.98545	0.996845	no
gene:SpnNT_01652	metA	Chromosome:1655492-1656437	110.58	110.58+peptide	OK	69.3279	67.2497	-0.0439096	-0.0932724	0.87195	0.994748	no
gene:SpnNT_01652	metA	Chromosome:1655492-1656437	ΔORF2	110.58+peptide	OK	69.5776	67.2497	-0.0490958	-0.104285	0.85525	0.994748	no
gene:SpnNT_01652	metA	Chromosome:1655492-1656437	110.58	ΔORF2+peptide	OK	69.3279	79.768	0.202373	0.437423	0.4441	0.917631	no
gene:SpnNT_01652	metA	Chromosome:1655492-1656437	ΔORF2	ΔORF2+peptide	OK	69.5776	79.768	0.197187	0.426199	0.4551	0.921244	no
gene:SpnNT_01652	metA	Chromosome:1655492-1656437	110.58+peptide	ΔORF2+peptide	OK	67.2497	79.768	0.246283	0.526887	0.36165	0.862361	no
gene:SpnNT_01653	apt	Chromosome:1656618-1657131	110.58	ΔORF2	OK	506.434	466.867	-0.117361	-0.263396	0.65005	0.981475	no
gene:SpnNT_01653	apt	Chromosome:1656618-1657131	110.58	110.58+peptide	OK	506.434	781.207	0.62533	1.38844	0.01385	0.127002	no
gene:SpnNT_01653	apt	Chromosome:1656618-1657131	ΔORF2	110.58+peptide	OK	466.867	781.207	0.742692	1.65864	0.0044	0.0544687	no
gene:SpnNT_01653	apt	Chromosome:1656618-1657131	110.58	ΔORF2+peptide	OK	506.434	699.792	0.466552	1.04941	0.06425	0.368	no
gene:SpnNT_01653	apt	Chromosome:1656618-1657131	ΔORF2	ΔORF2+peptide	OK	466.867	699.792	0.583913	1.32125	0.0226	0.183627	no
gene:SpnNT_01653	apt	Chromosome:1656618-1657131	110.58+peptide	ΔORF2+peptide	OK	781.207	699.792	-0.158779	-0.355374	0.52705	0.956161	no
gene:SpnNT_01654	rebM	Chromosome:1657217-1657976	110.58	ΔORF2	OK	54.1741	56.5152	0.0610359	0.108332	0.84565	0.994748	no
gene:SpnNT_01654	rebM	Chromosome:1657217-1657976	110.58	110.58+peptide	OK	54.1741	109.599	1.01657	1.95549	0.00075	0.0133173	yes
gene:SpnNT_01654	rebM	Chromosome:1657217-1657976	ΔORF2	110.58+peptide	OK	56.5152	109.599	0.95553	1.82369	0.00215	0.0312429	yes
gene:SpnNT_01654	rebM	Chromosome:1657217-1657976	110.58	ΔORF2+peptide	OK	54.1741	114.184	1.07568	2.10176	4.00E-04	0.00811252	yes
gene:SpnNT_01654	rebM	Chromosome:1657217-1657976	ΔORF2	ΔORF2+peptide	OK	56.5152	114.184	1.01465	1.96649	0.001	0.0168447	yes
gene:SpnNT_01654	rebM	Chromosome:1657217-1657976	110.58+peptide	ΔORF2+peptide	OK	109.599	114.184	0.0591167	0.126316	0.8251	0.994748	no
gene:SpnNT_01655	sugC	Chromosome:1658476-1659607	110.58	ΔORF2	OK	127.481	118.257	-0.108351	-0.244305	0.6723	0.984845	no
gene:SpnNT_01655	sugC	Chromosome:1658476-1659607	110.58	110.58+peptide	OK	127.481	160.386	0.331266	0.739112	0.2034	0.68789	no
gene:SpnNT_01655	sugC	Chromosome:1658476-1659607	ΔORF2	110.58+peptide	OK	118.257	160.386	0.439617	0.979944	0.0907	0.452879	no
gene:SpnNT_01655	sugC	Chromosome:1658476-1659607	110.58	ΔORF2+peptide	OK	127.481	127.75	0.00304555	0.00686612	0.9908	0.998179	no
gene:SpnNT_01655	sugC	Chromosome:1658476-1659607	ΔORF2	ΔORF2+peptide	OK	118.257	127.75	0.111396	0.2509	0.6647	0.983225	no
gene:SpnNT_01655	sugC	Chromosome:1658476-1659607	110.58+peptide	ΔORF2+peptide	OK	160.386	127.75	-0.328221	-0.73154	0.20805	0.693596	no
gene:SpnNT_01656	SpnNT_01656	Chromosome:1659889-1659975	110.58	ΔORF2	OK	407.074	1108.74	1.44556	0.867514	0.14995	0.591417	no
gene:SpnNT_01656	SpnNT_01656	Chromosome:1659889-1659975	110.58	110.58+peptide	OK	407.074	1344.71	1.72393	1.04993	0.09635	0.470071	no
gene:SpnNT_01656	SpnNT_01656	Chromosome:1659889-1659975	ΔORF2	110.58+peptide	OK	1108.74	1344.71	0.278369	0.229697	0.7017	0.990367	no
gene:SpnNT_01656	SpnNT_01656	Chromosome:1659889-1659975	110.58	ΔORF2+peptide	OK	407.074	61.9469	-2.71619	-1.91918	0.1303	0.548187	no
gene:SpnNT_01656	SpnNT_01656	Chromosome:1659889-1659975	ΔORF2	ΔORF2+peptide	OK	1108.74	61.9469	-4.16175	-4.72517	0.09685	0.470343	no
gene:SpnNT_01656	SpnNT_01656	Chromosome:1659889-1659975	110.58+peptide	ΔORF2+peptide	OK	1344.71	61.9469	-4.44012	-5.32572	0.09675	0.470343	no
gene:SpnNT_01657	yecD	Chromosome:1660056-1661420	110.58	ΔORF2	OK	169.543	187.644	0.146344	0.182212	0.75035	0.994748	no
gene:SpnNT_01657	yecD	Chromosome:1660056-1661420	110.58	110.58+peptide	OK	169.543	284.878	0.748695	0.980967	0.0889	0.448911	no
gene:SpnNT_01657	yecD	Chromosome:1660056-1661420	ΔORF2	110.58+peptide	OK	187.644	284.878	0.60235	0.754176	0.19345	0.674905	no
gene:SpnNT_01657	yecD	Chromosome:1660056-1661420	110.58	ΔORF2+peptide	OK	169.543	306.321	0.853392	1.12741	0.0495	0.311679	no
gene:SpnNT_01657	yecD	Chromosome:1660056-1661420	ΔORF2	ΔORF2+peptide	OK	187.644	306.321	0.707048	0.891955	0.12205	0.528994	no
gene:SpnNT_01657	yecD	Chromosome:1660056-1661420	110.58+peptide	ΔORF2+peptide	OK	284.878	306.321	0.104698	0.139187	0.8102	0.994748	no
gene:SpnNT_01658	codY	Chromosome:1660056-1661420	110.58	ΔORF2	OK	229.058	264.219	0.206021	0.391312	0.4917	0.94077	no
gene:SpnNT_01658	codY	Chromosome:1660056-1661420	110.58	110.58+peptide	OK	229.058	314.196	0.455952	0.837213	0.14515	0.580512	no

gene:SpnNT_01658	codY	Chromosome:1660056-1661420	ΔORF2	110.58+peptide	OK	264.219	314.196	0.249931	0.460093	0.42215	0.905583	no
gene:SpnNT_01658	codY	Chromosome:1660056-1661420	110.58	ΔORF2+peptide	OK	229.058	365.252	0.673181	1.24865	0.02875	0.215822	no
gene:SpnNT_01658	codY	Chromosome:1660056-1661420	ΔORF2	ΔORF2+peptide	OK	264.219	365.252	0.467159	0.868776	0.1294	0.545626	no
gene:SpnNT_01658	codY	Chromosome:1660056-1661420	110.58+peptide	ΔORF2+peptide	OK	314.196	365.252	0.217229	0.391069	0.50015	0.944135	no
gene:SpnNT_01659	cshA_2	Chromosome:1661683-1663258	110.58	ΔORF2	OK	262.01	300.957	0.19994	0.452498	0.4254	0.908126	no
gene:SpnNT_01659	cshA_2	Chromosome:1661683-1663258	110.58	110.58+peptide	OK	262.01	214.534	-0.288416	-0.650508	0.24355	0.73908	no
gene:SpnNT_01659	cshA_2	Chromosome:1661683-1663258	ΔORF2	110.58+peptide	OK	300.957	214.534	-0.488356	-1.09898	0.0504	0.315379	no
gene:SpnNT_01659	cshA_2	Chromosome:1661683-1663258	110.58	ΔORF2+peptide	OK	262.01	202.482	-0.371829	-0.841884	0.1359	0.560604	no
gene:SpnNT_01659	cshA_2	Chromosome:1661683-1663258	ΔORF2	ΔORF2+peptide	OK	300.957	202.482	-0.571769	-1.29165	0.02365	0.189344	no
gene:SpnNT_01659	cshA_2	Chromosome:1661683-1663258	110.58+peptide	ΔORF2+peptide	OK	214.534	202.482	-0.0834128	-0.187792	0.73595	0.994748	no
gene:SpnNT_01660	yhjX	Chromosome:1663456-1664683	110.58	ΔORF2	OK	91.0081	118.852	0.385104	0.869761	0.1332	0.556286	no
gene:SpnNT_01660	yhjX	Chromosome:1663456-1664683	110.58	110.58+peptide	OK	91.0081	26.1712	-1.79801	-3.80155	5.00E-05	0.0013612	yes
gene:SpnNT_01660	yhjX	Chromosome:1663456-1664683	ΔORF2	110.58+peptide	OK	118.852	26.1712	-2.18312	-4.61957	5.00E-05	0.0013612	yes
gene:SpnNT_01660	yhjX	Chromosome:1663456-1664683	110.58	ΔORF2+peptide	OK	91.0081	31.3166	-1.53907	-3.27555	5.00E-05	0.0013612	yes
gene:SpnNT_01660	yhjX	Chromosome:1663456-1664683	ΔORF2	ΔORF2+peptide	OK	118.852	31.3166	-1.92417	-4.09856	5.00E-05	0.0013612	yes
gene:SpnNT_01660	yhjX	Chromosome:1663456-1664683	110.58+peptide	ΔORF2+peptide	OK	26.1712	31.3166	0.258946	0.519911	0.3647	0.864498	no
gene:SpnNT_01661	lpd	Chromosome:1664876-1666193	110.58	ΔORF2	OK	422.226	490.083	0.215011	0.477714	0.40695	0.896303	no
gene:SpnNT_01661	lpd	Chromosome:1664876-1666193	110.58	110.58+peptide	OK	422.226	389.843	-0.115123	-0.257131	0.64385	0.980887	no
gene:SpnNT_01661	lpd	Chromosome:1664876-1666193	ΔORF2	110.58+peptide	OK	490.083	389.843	-0.330134	-0.739438	0.1975	0.679305	no
gene:SpnNT_01661	lpd	Chromosome:1664876-1666193	110.58	ΔORF2+peptide	OK	422.226	450.722	0.0942213	0.210392	0.7079	0.990441	no
gene:SpnNT_01661	lpd	Chromosome:1664876-1666193	ΔORF2	ΔORF2+peptide	OK	490.083	450.722	-0.12079	-0.270475	0.6356	0.980887	no
gene:SpnNT_01661	lpd	Chromosome:1664876-1666193	110.58+peptide	ΔORF2+peptide	OK	389.843	450.722	0.209344	0.471281	0.398	0.891861	no
gene:SpnNT_01662	NA	Chromosome:1666291-1668417	110.58	ΔORF2	OK	67.5578	66.8765	-0.0146247	-0.0252856	0.9662	0.994855	no
gene:SpnNT_01662	NA	Chromosome:1666291-1668417	110.58	110.58+peptide	OK	67.5578	65.7258	-0.0396639	-0.0687104	0.9032	0.994748	no
gene:SpnNT_01662	NA	Chromosome:1666291-1668417	ΔORF2	110.58+peptide	OK	66.8765	65.7258	-0.0250392	-0.0427076	0.9412	0.994855	no
gene:SpnNT_01662	NA	Chromosome:1666291-1668417	110.58	ΔORF2+peptide	OK	67.5578	65.026	-0.0551075	-0.0938897	0.866	0.994748	no
gene:SpnNT_01662	NA	Chromosome:1666291-1668417	ΔORF2	ΔORF2+peptide	OK	66.8765	65.026	-0.0404827	-0.0679442	0.90355	0.994748	no
gene:SpnNT_01662	NA	Chromosome:1666291-1668417	110.58+peptide	ΔORF2+peptide	OK	65.7258	65.026	-0.0154436	-0.0259671	0.96435	0.994855	no
gene:SpnNT_01663	NA	Chromosome:1666291-1668417	110.58	ΔORF2	OK	92.0005	97.8404	0.0887889	0.127087	0.82265	0.994748	no
gene:SpnNT_01663	NA	Chromosome:1666291-1668417	110.58	110.58+peptide	OK	92.0005	99.6009	0.114517	0.163496	0.778	0.994748	no
gene:SpnNT_01663	NA	Chromosome:1666291-1668417	ΔORF2	110.58+peptide	OK	97.8404	99.6009	0.0257285	0.0378658	0.9499	0.994855	no
gene:SpnNT_01663	NA	Chromosome:1666291-1668417	110.58	ΔORF2+peptide	OK	92.0005	117.362	0.351252	0.518189	0.36285	0.86287	no
gene:SpnNT_01663	NA	Chromosome:1666291-1668417	ΔORF2	ΔORF2+peptide	OK	97.8404	117.362	0.262463	0.4	0.4846	0.936747	no
gene:SpnNT_01663	NA	Chromosome:1666291-1668417	110.58+peptide	ΔORF2+peptide	OK	99.6009	117.362	0.236734	0.359748	0.52785	0.956161	no
gene:SpnNT_01664	pepQ	Chromosome:1668542-1669625	110.58	ΔORF2	OK	127.462	138.741	0.122332	0.27708	0.617	0.980654	no
gene:SpnNT_01664	pepQ	Chromosome:1668542-1669625	110.58	110.58+peptide	OK	127.462	97.6737	-0.38402	-0.868614	0.12325	0.531129	no
gene:SpnNT_01664	pepQ	Chromosome:1668542-1669625	ΔORF2	110.58+peptide	OK	138.741	97.6737	-0.506352	-1.1352	0.04525	0.29311	no
gene:SpnNT_01664	pepQ	Chromosome:1668542-1669625	110.58	ΔORF2+peptide	OK	127.462	88.8781	-0.520163	-1.16646	0.0416	0.277799	no
gene:SpnNT_01664	pepQ	Chromosome:1668542-1669625	ΔORF2	ΔORF2+peptide	OK	138.741	88.8781	-0.642495	-1.42827	0.0132	0.122583	no
gene:SpnNT_01664	pepQ	Chromosome:1668542-1669625	110.58+peptide	ΔORF2+peptide	OK	97.6737	88.8781	-0.136143	-0.30225	0.5981	0.97629	no
gene:SpnNT_01665	NA	Chromosome:1669948-1670782	110.58	ΔORF2	OK	9.23603	7.9116	-0.223302	-0.354098	0.5376	0.959663	no
gene:SpnNT_01665	NA	Chromosome:1669948-1670782	110.58	110.58+peptide	OK	9.23603	8.5688	-0.108179	-0.172565	0.7629	0.994748	no
gene:SpnNT_01665	NA	Chromosome:1669948-1670782	ΔORF2	110.58+peptide	OK	7.9116	8.5688	0.115123	0.183319	0.7521	0.994748	no
gene:SpnNT_01665	NA	Chromosome:1669948-1670782	110.58	ΔORF2+peptide	OK	9.23603	8.25494	-0.162015	-0.253944	0.64835	0.981391	no
gene:SpnNT_01665	NA	Chromosome:1669948-1670782	ΔORF2	ΔORF2+peptide	OK	7.9116	8.25494	0.0612872	0.095899	0.8645	0.994748	no

gene:SpnNT_01665	NA	Chromosome:1669948-1670782	110.58+peptide	ΔORF2+peptide	OK	8.5688	8.25494	-0.0538358	-0.0847281	0.87985	0.994748	no
gene:SpnNT_01666	NA	Chromosome:1671001-1671214	110.58	ΔORF2	NOTEST	1.73823	1.8658	0.102175	0	1	1	no
gene:SpnNT_01666	NA	Chromosome:1671001-1671214	110.58	110.58+peptide	NOTEST	1.73823	1.29883	-0.420414	0	1	1	no
gene:SpnNT_01666	NA	Chromosome:1671001-1671214	ΔORF2	110.58+peptide	NOTEST	1.8658	1.29883	-0.522589	0	1	1	no
gene:SpnNT_01666	NA	Chromosome:1671001-1671214	110.58	ΔORF2+peptide	NOTEST	1.73823	0.411257	-2.07951	0	1	1	no
gene:SpnNT_01666	NA	Chromosome:1671001-1671214	ΔORF2	ΔORF2+peptide	NOTEST	1.8658	0.411257	-2.18169	0	1	1	no
gene:SpnNT_01666	NA	Chromosome:1671001-1671214	110.58+peptide	ΔORF2+peptide	NOTEST	1.29883	0.411257	-1.6591	0	1	1	no
gene:SpnNT_01667	hmpT	Chromosome:1671440-1673392	110.58	ΔORF2	OK	54.6101	53.2148	-0.0373414	-0.0364311	0.95205	0.994855	no
gene:SpnNT_01667	hmpT	Chromosome:1671440-1673392	110.58	110.58+peptide	OK	54.6101	58.0963	0.0892783	0.0913792	0.8721	0.994748	no
gene:SpnNT_01667	hmpT	Chromosome:1671440-1673392	ΔORF2	110.58+peptide	OK	53.2148	58.0963	0.12662	0.126266	0.82815	0.994748	no
gene:SpnNT_01667	hmpT	Chromosome:1671440-1673392	110.58	ΔORF2+peptide	OK	54.6101	59.377	0.120735	0.125676	0.8272	0.994748	no
gene:SpnNT_01667	hmpT	Chromosome:1671440-1673392	ΔORF2	ΔORF2+peptide	OK	53.2148	59.377	0.158077	0.160174	0.7822	0.994748	no
gene:SpnNT_01667	hmpT	Chromosome:1671440-1673392	110.58+peptide	ΔORF2+peptide	OK	58.0963	59.377	0.0314572	0.033573	0.9566	0.994855	no
gene:SpnNT_01668	pdxK	Chromosome:1671440-1673392	110.58	ΔORF2	OK	46.8443	38.5455	-0.281312	-0.367822	0.52135	0.954832	no
gene:SpnNT_01668	pdxK	Chromosome:1671440-1673392	110.58	110.58+peptide	OK	46.8443	44.6727	-0.0684808	-0.0921813	0.8742	0.994748	no
gene:SpnNT_01668	pdxK	Chromosome:1671440-1673392	ΔORF2	110.58+peptide	OK	38.5455	44.6727	0.212831	0.274032	0.64485	0.980887	no
gene:SpnNT_01668	pdxK	Chromosome:1671440-1673392	110.58	ΔORF2+peptide	OK	46.8443	37.4064	-0.32459	-0.431131	0.4536	0.921244	no
gene:SpnNT_01668	pdxK	Chromosome:1671440-1673392	ΔORF2	ΔORF2+peptide	OK	38.5455	37.4064	-0.0432778	-0.0550453	0.926	0.994748	no
gene:SpnNT_01668	pdxK	Chromosome:1671440-1673392	110.58+peptide	ΔORF2+peptide	OK	44.6727	37.4064	-0.256109	-0.334816	0.5683	0.968621	no
gene:SpnNT_01669	truA	Chromosome:1671440-1673392	110.58	ΔORF2	OK	53.3634	51.0252	-0.0646406	-0.0901902	0.8753	0.994748	no
gene:SpnNT_01669	truA	Chromosome:1671440-1673392	110.58	110.58+peptide	OK	53.3634	45.7145	-0.223198	-0.303084	0.59665	0.97629	no
gene:SpnNT_01669	truA	Chromosome:1671440-1673392	ΔORF2	110.58+peptide	OK	51.0252	45.7145	-0.158558	-0.219553	0.6984	0.990209	no
gene:SpnNT_01669	truA	Chromosome:1671440-1673392	110.58	ΔORF2+peptide	OK	53.3634	42.5992	-0.325024	-0.436869	0.45495	0.921244	no
gene:SpnNT_01669	truA	Chromosome:1671440-1673392	ΔORF2	ΔORF2+peptide	OK	51.0252	42.5992	-0.260383	-0.356742	0.53645	0.959314	no
gene:SpnNT_01669	truA	Chromosome:1671440-1673392	110.58+peptide	ΔORF2+peptide	OK	45.7145	42.5992	-0.101825	-0.135902	0.81115	0.994748	no
gene:SpnNT_01670	NA	Chromosome:1673704-1674856	110.58	ΔORF2	OK	130.336	117.53	-0.149205	-0.335334	0.5563	0.966729	no
gene:SpnNT_01670	NA	Chromosome:1673704-1674856	110.58	110.58+peptide	OK	130.336	168.952	0.374374	0.84059	0.14385	0.579018	no
gene:SpnNT_01670	NA	Chromosome:1673704-1674856	ΔORF2	110.58+peptide	OK	117.53	168.952	0.523579	1.19396	0.0349	0.247144	no
gene:SpnNT_01670	NA	Chromosome:1673704-1674856	110.58	ΔORF2+peptide	OK	130.336	172.621	0.405371	0.910129	0.11305	0.510701	no
gene:SpnNT_01670	NA	Chromosome:1673704-1674856	ΔORF2	ΔORF2+peptide	OK	117.53	172.621	0.554576	1.26456	0.0257	0.200735	no
gene:SpnNT_01670	NA	Chromosome:1673704-1674856	110.58+peptide	ΔORF2+peptide	OK	168.952	172.621	0.0309972	0.0706114	0.90175	0.994748	no
gene:SpnNT_01671	NA	Chromosome:1675095-1675725	110.58	ΔORF2	OK	130.356	132.265	0.0209696	0.0449594	0.9395	0.994855	no
gene:SpnNT_01671	NA	Chromosome:1675095-1675725	110.58	110.58+peptide	OK	130.356	161.279	0.307103	0.659351	0.24515	0.742067	no
gene:SpnNT_01671	NA	Chromosome:1675095-1675725	ΔORF2	110.58+peptide	OK	132.265	161.279	0.286134	0.617533	0.28565	0.78895	no
gene:SpnNT_01671	NA	Chromosome:1675095-1675725	110.58	ΔORF2+peptide	OK	130.356	196.081	0.588995	1.27497	0.02635	0.204232	no
gene:SpnNT_01671	NA	Chromosome:1675095-1675725	ΔORF2	ΔORF2+peptide	OK	132.265	196.081	0.568026	1.23609	0.03265	0.236051	no
gene:SpnNT_01671	NA	Chromosome:1675095-1675725	110.58+peptide	ΔORF2+peptide	OK	161.279	196.081	0.281892	0.614307	0.2828	0.785349	no
gene:SpnNT_01672	NA	Chromosome:1675727-1676066	110.58	ΔORF2	OK	455.522	421.37	-0.112432	-0.240063	0.6773	0.98524	no
gene:SpnNT_01672	NA	Chromosome:1675727-1676066	110.58	110.58+peptide	OK	455.522	535.737	0.234006	0.507318	0.3748	0.872472	no
gene:SpnNT_01672	NA	Chromosome:1675727-1676066	ΔORF2	110.58+peptide	OK	421.37	535.737	0.346438	0.725629	0.2037	0.688187	no
gene:SpnNT_01672	NA	Chromosome:1675727-1676066	110.58	ΔORF2+peptide	OK	455.522	523.402	0.200399	0.4385	0.43935	0.916225	no
gene:SpnNT_01672	NA	Chromosome:1675727-1676066	ΔORF2	ΔORF2+peptide	OK	421.37	523.402	0.312831	0.660921	0.24125	0.736034	no
gene:SpnNT_01672	NA	Chromosome:1675727-1676066	110.58+peptide	ΔORF2+peptide	OK	535.737	523.402	-0.0336073	-0.0720689	0.8983	0.994748	no
gene:SpnNT_01673	cmk	Chromosome:1676196-1676868	110.58	ΔORF2	OK	155.65	158.024	0.0218394	0.0483316	0.9357	0.994855	no
gene:SpnNT_01673	cmk	Chromosome:1676196-1676868	110.58	110.58+peptide	OK	155.65	186.502	0.260887	0.584212	0.30875	0.814396	no

gene:SpnNT_01673	cmk	Chromosome:1676196-1676868	ΔORF2	110.58+peptide	OK	158.024	186.502	0.239048	0.531607	0.3663	0.864634	no
gene:SpnNT_01673	cmk	Chromosome:1676196-1676868	110.58	ΔORF2+peptide	OK	155.65	196.835	0.33868	0.759431	0.18085	0.648746	no
gene:SpnNT_01673	cmk	Chromosome:1676196-1676868	ΔORF2	ΔORF2+peptide	OK	158.024	196.835	0.31684	0.705537	0.2275	0.71915	no
gene:SpnNT_01673	cmk	Chromosome:1676196-1676868	110.58+peptide	ΔORF2+peptide	OK	186.502	196.835	0.0777928	0.175312	0.7575	0.994748	no
gene:SpnNT_01674	NA	Chromosome:1676876-1677353	110.58	ΔORF2	OK	159.272	157.647	-0.0147968	-0.0309937	0.95725	0.994855	no
gene:SpnNT_01674	NA	Chromosome:1676876-1677353	110.58	110.58+peptide	OK	159.272	172.973	0.119056	0.253409	0.65555	0.982966	no
gene:SpnNT_01674	NA	Chromosome:1676876-1677353	ΔORF2	110.58+peptide	OK	157.647	172.973	0.133853	0.281244	0.6205	0.980887	no
gene:SpnNT_01674	NA	Chromosome:1676876-1677353	110.58	ΔORF2+peptide	OK	159.272	171.518	0.106866	0.227105	0.69155	0.98828	no
gene:SpnNT_01674	NA	Chromosome:1676876-1677353	ΔORF2	ΔORF2+peptide	OK	157.647	171.518	0.121662	0.25524	0.6531	0.98212	no
gene:SpnNT_01674	NA	Chromosome:1676876-1677353	110.58+peptide	ΔORF2+peptide	OK	172.973	171.518	-0.0121904	-0.0259895	0.9673	0.994855	no
gene:SpnNT_01675	NA	Chromosome:1677398-1677611	110.58	ΔORF2	OK	263.196	302.393	0.200288	0.345977	0.5478	0.962667	no
gene:SpnNT_01675	NA	Chromosome:1677398-1677611	110.58	110.58+peptide	OK	263.196	377.227	0.519297	0.906785	0.1103	0.506069	no
gene:SpnNT_01675	NA	Chromosome:1677398-1677611	ΔORF2	110.58+peptide	OK	302.393	377.227	0.31901	0.57204	0.30875	0.814396	no
gene:SpnNT_01675	NA	Chromosome:1677398-1677611	110.58	ΔORF2+peptide	OK	263.196	383.021	0.541288	0.955024	0.10345	0.488069	no
gene:SpnNT_01675	NA	Chromosome:1677398-1677611	ΔORF2	ΔORF2+peptide	OK	302.393	383.021	0.341001	0.618191	0.289	0.793193	no
gene:SpnNT_01675	NA	Chromosome:1677398-1677611	110.58+peptide	ΔORF2+peptide	OK	377.227	383.021	0.0219912	0.0403454	0.9432	0.994855	no
gene:SpnNT_01676	csbB	Chromosome:1677661-1679648	110.58	ΔORF2	OK	65.562	67.8873	0.0502829	0.0349154	0.95045	0.994855	no
gene:SpnNT_01676	csbB	Chromosome:1677661-1679648	110.58	110.58+peptide	OK	65.562	41.2256	-0.66932	-0.478513	0.40505	0.894933	no
gene:SpnNT_01676	csbB	Chromosome:1677661-1679648	ΔORF2	110.58+peptide	OK	67.8873	41.2256	-0.719603	-0.582408	0.31895	0.825137	no
gene:SpnNT_01676	csbB	Chromosome:1677661-1679648	110.58	ΔORF2+peptide	OK	65.562	52.1348	-0.330613	-0.243085	0.66135	0.982966	no
gene:SpnNT_01676	csbB	Chromosome:1677661-1679648	ΔORF2	ΔORF2+peptide	OK	67.8873	52.1348	-0.380896	-0.31965	0.5777	0.969538	no
gene:SpnNT_01676	csbB	Chromosome:1677661-1679648	110.58+peptide	ΔORF2+peptide	OK	41.2256	52.1348	0.338707	0.296789	0.62255	0.980887	no
gene:SpnNT_01677	galE_1	Chromosome:1677661-1679648	110.58	ΔORF2	OK	460.32	466.165	0.0182048	0.0372101	0.94905	0.994855	no
gene:SpnNT_01677	galE_1	Chromosome:1677661-1679648	110.58	110.58+peptide	OK	460.32	214.207	-1.10363	-2.26399	1.00E-04	0.0025332	yes
gene:SpnNT_01677	galE_1	Chromosome:1677661-1679648	ΔORF2	110.58+peptide	OK	466.165	214.207	-1.12184	-2.32462	5.00E-05	0.0013612	yes
gene:SpnNT_01677	galE_1	Chromosome:1677661-1679648	110.58	ΔORF2+peptide	OK	460.32	219.209	-1.07033	-2.19072	0.00015	0.00355289	yes
gene:SpnNT_01677	galE_1	Chromosome:1677661-1679648	ΔORF2	ΔORF2+peptide	OK	466.165	219.209	-1.08854	-2.25042	0.00015	0.00355289	yes
gene:SpnNT_01677	galE_1	Chromosome:1677661-1679648	110.58+peptide	ΔORF2+peptide	OK	214.207	219.209	0.0332993	0.0690984	0.90405	0.994748	no
gene:SpnNT_01678	hcnC	Chromosome:1679903-1681007	110.58	ΔORF2	OK	35.6011	30.3762	-0.228978	-0.462116	0.4105	0.898351	no
gene:SpnNT_01678	hcnC	Chromosome:1679903-1681007	110.58	110.58+peptide	OK	35.6011	37.0436	0.0573056	0.116345	0.84025	0.994748	no
gene:SpnNT_01678	hcnC	Chromosome:1679903-1681007	ΔORF2	110.58+peptide	OK	30.3762	37.0436	0.286283	0.574312	0.3158	0.821686	no
gene:SpnNT_01678	hcnC	Chromosome:1679903-1681007	110.58	ΔORF2+peptide	OK	35.6011	33.7092	-0.0787782	-0.159488	0.7772	0.994748	no
gene:SpnNT_01678	hcnC	Chromosome:1679903-1681007	ΔORF2	ΔORF2+peptide	OK	30.3762	33.7092	0.1502	0.300485	0.59755	0.97629	no
gene:SpnNT_01678	hcnC	Chromosome:1679903-1681007	110.58+peptide	ΔORF2+peptide	OK	37.0436	33.7092	-0.136084	-0.273846	0.6366	0.980887	no
gene:SpnNT_01679	NA	Chromosome:1681023-1682485	110.58	ΔORF2	OK	60.243	54.0361	-0.156871	-0.263319	0.65125	0.981475	no
gene:SpnNT_01679	NA	Chromosome:1681023-1682485	110.58	110.58+peptide	OK	60.243	57.1389	-0.0763204	-0.130159	0.8221	0.994748	no
gene:SpnNT_01679	NA	Chromosome:1681023-1682485	ΔORF2	110.58+peptide	OK	54.0361	57.1389	0.0805508	0.134537	0.81635	0.994748	no
gene:SpnNT_01679	NA	Chromosome:1681023-1682485	110.58	ΔORF2+peptide	OK	60.243	54.0409	-0.156744	-0.265033	0.64985	0.981475	no
gene:SpnNT_01679	NA	Chromosome:1681023-1682485	ΔORF2	ΔORF2+peptide	OK	54.0361	54.0409	0.00012754	0.000211273	0.9985	0.999412	no
gene:SpnNT_01679	NA	Chromosome:1681023-1682485	110.58+peptide	ΔORF2+peptide	OK	57.1389	54.0409	-0.0804233	-0.135298	0.81395	0.994748	no
gene:SpnNT_01680	trmK	Chromosome:1681023-1682485	110.58	ΔORF2	OK	46.2645	45.8035	-0.0144489	-0.0191397	0.9734	0.99536	no
gene:SpnNT_01680	trmK	Chromosome:1681023-1682485	110.58	110.58+peptide	OK	46.2645	42.6165	-0.118496	-0.150629	0.79165	0.994748	no
gene:SpnNT_01680	trmK	Chromosome:1681023-1682485	ΔORF2	110.58+peptide	OK	45.8035	42.6165	-0.104047	-0.134613	0.81385	0.994748	no
gene:SpnNT_01680	trmK	Chromosome:1681023-1682485	110.58	ΔORF2+peptide	OK	46.2645	46.1909	-0.0022984	-0.00306189	0.9947	0.999116	no
gene:SpnNT_01680	trmK	Chromosome:1681023-1682485	ΔORF2	ΔORF2+peptide	OK	45.8035	46.1909	0.0121505	0.0165036	0.97665	0.99536	no

gene:SpnNT_01680	trmK	Chromosome:1681023-1682485	110.58+peptide	ΔORF2+peptide	OK	42.6165	46.1909	0.116198	0.151148	0.79555	0.994748	no
gene:SpnNT_01681	NA	Chromosome:1682586-1684923	110.58	ΔORF2	OK	170.357	129.608	-0.394412	-0.890802	0.11565	0.516049	no
gene:SpnNT_01681	NA	Chromosome:1682586-1684923	110.58	110.58+peptide	OK	170.357	245.854	0.529238	1.17745	0.04355	0.285874	no
gene:SpnNT_01681	NA	Chromosome:1682586-1684923	ΔORF2	110.58+peptide	OK	129.608	245.854	0.923649	2.08645	0.00045	0.00885292	yes
gene:SpnNT_01681	NA	Chromosome:1682586-1684923	110.58	ΔORF2+peptide	OK	170.357	227.528	0.417484	0.930348	0.109	0.502305	no
gene:SpnNT_01681	NA	Chromosome:1682586-1684923	ΔORF2	ΔORF2+peptide	OK	129.608	227.528	0.811896	1.83712	0.0019	0.0281715	yes
gene:SpnNT_01681	NA	Chromosome:1682586-1684923	110.58+peptide	ΔORF2+peptide	OK	245.854	227.528	-0.111754	-0.249078	0.66305	0.982966	no
gene:SpnNT_01682	plsC	Chromosome:1685055-1685805	110.58	ΔORF2	OK	41.3136	47.3822	0.197728	0.388546	0.4969	0.94388	no
gene:SpnNT_01682	plsC	Chromosome:1685055-1685805	110.58	110.58+peptide	OK	41.3136	36.6296	-0.173608	-0.335818	0.55055	0.964281	no
gene:SpnNT_01682	plsC	Chromosome:1685055-1685805	ΔORF2	110.58+peptide	OK	47.3822	36.6296	-0.371336	-0.723096	0.1945	0.676266	no
gene:SpnNT_01682	plsC	Chromosome:1685055-1685805	110.58	ΔORF2+peptide	OK	41.3136	35.5278	-0.217668	-0.419323	0.4577	0.921722	no
gene:SpnNT_01682	plsC	Chromosome:1685055-1685805	ΔORF2	ΔORF2+peptide	OK	47.3822	35.5278	-0.415396	-0.805541	0.14725	0.584811	no
gene:SpnNT_01682	plsC	Chromosome:1685055-1685805	110.58+peptide	ΔORF2+peptide	OK	36.6296	35.5278	-0.0440603	-0.0841413	0.87965	0.994748	no
gene:SpnNT_01683	NA	Chromosome:1686097-1686712	110.58	ΔORF2	OK	804.133	948.256	0.237842	0.521034	0.37135	0.868447	no
gene:SpnNT_01683	NA	Chromosome:1686097-1686712	110.58	110.58+peptide	OK	804.133	1020.73	0.344092	0.761763	0.1861	0.660358	no
gene:SpnNT_01683	NA	Chromosome:1686097-1686712	ΔORF2	110.58+peptide	OK	948.256	1020.73	0.10625	0.226844	0.69265	0.98828	no
gene:SpnNT_01683	NA	Chromosome:1686097-1686712	110.58	ΔORF2+peptide	OK	804.133	982.792	0.289451	0.646087	0.26095	0.761099	no
gene:SpnNT_01683	NA	Chromosome:1686097-1686712	ΔORF2	ΔORF2+peptide	OK	948.256	982.792	0.0516082	0.111029	0.8451	0.994748	no
gene:SpnNT_01683	NA	Chromosome:1686097-1686712	110.58+peptide	ΔORF2+peptide	OK	1020.73	982.792	-0.0546418	-0.118754	0.8343	0.994748	no
gene:SpnNT_01684	rpsO	Chromosome:1687526-1687796	110.58	ΔORF2	OK	5457.84	5116.55	-0.0931595	-0.203515	0.71525	0.992053	no
gene:SpnNT_01684	rpsO	Chromosome:1687526-1687796	110.58	110.58+peptide	OK	5457.84	9902.81	0.859508	1.85008	0.0015	0.0233488	yes
gene:SpnNT_01684	rpsO	Chromosome:1687526-1687796	ΔORF2	110.58+peptide	OK	5116.55	9902.81	0.952668	2.09371	2.00E-04	0.00450928	yes
gene:SpnNT_01684	rpsO	Chromosome:1687526-1687796	110.58	ΔORF2+peptide	OK	5457.84	9670.08	0.825198	1.81964	0.0018	0.0270557	yes
gene:SpnNT_01684	rpsO	Chromosome:1687526-1687796	ΔORF2	ΔORF2+peptide	OK	5116.55	9670.08	0.918358	2.06981	3.00E-04	0.00631878	yes
gene:SpnNT_01684	rpsO	Chromosome:1687526-1687796	110.58+peptide	ΔORF2+peptide	OK	9902.81	9670.08	-0.0343098	-0.0761203	0.89765	0.994748	no
gene:SpnNT_01685	NA	Chromosome:1687936-1688566	110.58	ΔORF2	OK	1.25946	1.14009	-0.143659	-0.131546	0.82385	0.994748	no
gene:SpnNT_01685	NA	Chromosome:1687936-1688566	110.58	110.58+peptide	OK	1.25946	1.03963	-0.27673	-0.256776	0.66055	0.982966	no
gene:SpnNT_01685	NA	Chromosome:1687936-1688566	ΔORF2	110.58+peptide	OK	1.14009	1.03963	-0.133072	-0.119036	0.83855	0.994748	no
gene:SpnNT_01685	NA	Chromosome:1687936-1688566	110.58	ΔORF2+peptide	OK	1.25946	1.66062	0.398916	0.380871	0.54215	0.961494	no
gene:SpnNT_01685	NA	Chromosome:1687936-1688566	ΔORF2	ΔORF2+peptide	OK	1.14009	1.66062	0.542574	0.49837	0.4361	0.914104	no
gene:SpnNT_01685	NA	Chromosome:1687936-1688566	110.58+peptide	ΔORF2+peptide	OK	1.03963	1.66062	0.675646	0.628929	0.32675	0.831577	no
gene:SpnNT_01686	NA	Chromosome:1688804-1689179	110.58	ΔORF2	OK	7.50329	9.03794	0.26847	0.333358	0.56475	0.968621	no
gene:SpnNT_01686	NA	Chromosome:1688804-1689179	110.58	110.58+peptide	OK	7.50329	6.60942	-0.182999	-0.222305	0.70265	0.990367	no
gene:SpnNT_01686	NA	Chromosome:1688804-1689179	ΔORF2	110.58+peptide	OK	9.03794	6.60942	-0.451469	-0.562095	0.32955	0.835301	no
gene:SpnNT_01686	NA	Chromosome:1688804-1689179	110.58	ΔORF2+peptide	OK	7.50329	7.49372	-0.00184069	-0.002177	0.97505	0.99536	no
gene:SpnNT_01686	NA	Chromosome:1688804-1689179	ΔORF2	ΔORF2+peptide	OK	9.03794	7.49372	-0.270311	-0.327229	0.5718	0.969025	no
gene:SpnNT_01686	NA	Chromosome:1688804-1689179	110.58+peptide	ΔORF2+peptide	OK	6.60942	7.49372	0.181158	0.21478	0.7123	0.991034	no
gene:SpnNT_01687	NA	Chromosome:1689435-1689576	110.58	ΔORF2	NOTEST	2.1137	2.90847	0.460489	0	1	1	no
gene:SpnNT_01687	NA	Chromosome:1689435-1689576	110.58	110.58+peptide	OK	2.1137	13.3925	2.66358	4.36692	0.17185	0.632011	no
gene:SpnNT_01687	NA	Chromosome:1689435-1689576	ΔORF2	110.58+peptide	OK	2.90847	13.3925	2.20309	4.65702	0.23065	0.723891	no
gene:SpnNT_01687	NA	Chromosome:1689435-1689576	110.58	ΔORF2+peptide	OK	2.1137	18.3983	3.12173	5.20056	0.1436	0.57877	no
gene:SpnNT_01687	NA	Chromosome:1689435-1689576	ΔORF2	ΔORF2+peptide	OK	2.90847	18.3983	2.66124	5.77872	0.1704	0.62897	no
gene:SpnNT_01687	NA	Chromosome:1689435-1689576	110.58+peptide	ΔORF2+peptide	OK	13.3925	18.3983	0.458147	1.46171	0.67285	0.984845	no
gene:SpnNT_01688	NA	Chromosome:1689582-1689759	110.58	ΔORF2	OK	4.30752	11.859	1.46105	0.729659	0.19365	0.674905	no
gene:SpnNT_01688	NA	Chromosome:1689582-1689759	110.58	110.58+peptide	OK	4.30752	9.03371	1.06846	0.526868	0.2987	0.80426	no

gene:SpnNT_01688	NA	Chromosome:1689582-1689759	ΔORF2	110.58+peptide	OK	11.859	9.03371	-0.392586	-0.262874	0.6616	0.982966	no
gene:SpnNT_01688	NA	Chromosome:1689582-1689759	110.58	ΔORF2+peptide	OK	4.30752	19.3545	2.16774	1.25006	0.07275	0.397264	no
gene:SpnNT_01688	NA	Chromosome:1689582-1689759	ΔORF2	ΔORF2+peptide	OK	11.859	19.3545	0.706694	0.666302	0.3821	0.879865	no
gene:SpnNT_01688	NA	Chromosome:1689582-1689759	110.58+peptide	ΔORF2+peptide	OK	9.03371	19.3545	1.09928	0.991991	0.1851	0.658055	no
gene:SpnNT_01689	thrS	Chromosome:1689816-1691760	110.58	ΔORF2	OK	46.9044	44.9151	-0.0625203	-0.138708	0.80555	0.994748	no
gene:SpnNT_01689	thrS	Chromosome:1689816-1691760	110.58	110.58+peptide	OK	46.9044	117.489	1.32473	2.98323	5.00E-05	0.0013612	yes
gene:SpnNT_01689	thrS	Chromosome:1689816-1691760	ΔORF2	110.58+peptide	OK	44.9151	117.489	1.38725	3.09362	5.00E-05	0.0013612	yes
gene:SpnNT_01689	thrS	Chromosome:1689816-1691760	110.58	ΔORF2+peptide	OK	46.9044	123.541	1.3972	3.1586	5.00E-05	0.0013612	yes
gene:SpnNT_01689	thrS	Chromosome:1689816-1691760	ΔORF2	ΔORF2+peptide	OK	44.9151	123.541	1.45972	3.26758	5.00E-05	0.0013612	yes
gene:SpnNT_01689	thrS	Chromosome:1689816-1691760	110.58+peptide	ΔORF2+peptide	OK	117.489	123.541	0.0724645	0.164694	0.7684	0.994748	no
gene:SpnNT_01690	graS	Chromosome:1691808-1693453	110.58	ΔORF2	OK	17.7467	19.3593	0.125474	0.170213	0.76695	0.994748	no
gene:SpnNT_01690	graS	Chromosome:1691808-1693453	110.58	110.58+peptide	OK	17.7467	14.6937	-0.272359	-0.368932	0.53075	0.957488	no
gene:SpnNT_01690	graS	Chromosome:1691808-1693453	ΔORF2	110.58+peptide	OK	19.3593	14.6937	-0.397833	-0.535436	0.36025	0.861019	no
gene:SpnNT_01690	graS	Chromosome:1691808-1693453	110.58	ΔORF2+peptide	OK	17.7467	18.0461	0.0241351	0.0333456	0.9553	0.994855	no
gene:SpnNT_01690	graS	Chromosome:1691808-1693453	ΔORF2	ΔORF2+peptide	OK	19.3593	18.0461	-0.101339	-0.139077	0.81005	0.994748	no
gene:SpnNT_01690	graS	Chromosome:1691808-1693453	110.58+peptide	ΔORF2+peptide	OK	14.6937	18.0461	0.296494	0.4063	0.49745	0.944017	no
gene:SpnNT_01691	graR	Chromosome:1691808-1693453	110.58	ΔORF2	OK	34.014	32.1762	-0.0801343	-0.113236	0.846	0.994748	no
gene:SpnNT_01691	graR	Chromosome:1691808-1693453	110.58	110.58+peptide	OK	34.014	25.2624	-0.429136	-0.59523	0.29395	0.798429	no
gene:SpnNT_01691	graR	Chromosome:1691808-1693453	ΔORF2	110.58+peptide	OK	32.1762	25.2624	-0.349002	-0.481257	0.4073	0.896593	no
gene:SpnNT_01691	graR	Chromosome:1691808-1693453	110.58	ΔORF2+peptide	OK	34.014	26.3391	-0.368923	-0.512012	0.3713	0.868447	no
gene:SpnNT_01691	graR	Chromosome:1691808-1693453	ΔORF2	ΔORF2+peptide	OK	32.1762	26.3391	-0.288789	-0.398457	0.49595	0.943191	no
gene:SpnNT_01691	graR	Chromosome:1691808-1693453	110.58+peptide	ΔORF2+peptide	OK	25.2624	26.3391	0.0602134	0.0816183	0.88715	0.994748	no
gene:SpnNT_01692	NA	Chromosome:1693608-1694682	110.58	ΔORF2	OK	10.5428	10.3497	-0.0266808	-0.0457626	0.93515	0.994855	no
gene:SpnNT_01692	NA	Chromosome:1693608-1694682	110.58	110.58+peptide	OK	10.5428	8.94469	-0.237161	-0.405607	0.46985	0.927937	no
gene:SpnNT_01692	NA	Chromosome:1693608-1694682	ΔORF2	110.58+peptide	OK	10.3497	8.94469	-0.210481	-0.355765	0.53325	0.958798	no
gene:SpnNT_01692	NA	Chromosome:1693608-1694682	110.58	ΔORF2+peptide	OK	10.5428	9.26433	-0.186505	-0.32013	0.57505	0.969538	no
gene:SpnNT_01692	NA	Chromosome:1693608-1694682	ΔORF2	ΔORF2+peptide	OK	10.3497	9.26433	-0.159825	-0.271101	0.63675	0.980887	no
gene:SpnNT_01692	NA	Chromosome:1693608-1694682	110.58+peptide	ΔORF2+peptide	OK	8.94469	9.26433	0.0506559	0.0856831	0.88365	0.994748	no
gene:SpnNT_01693	NA	Chromosome:1694772-1695075	110.58	ΔORF2	OK	57.8572	121.726	1.07307	1.78387	0.00255	0.0352222	yes
gene:SpnNT_01693	NA	Chromosome:1694772-1695075	110.58	110.58+peptide	OK	57.8572	85.6597	0.56612	0.940748	0.1246	0.533791	no
gene:SpnNT_01693	NA	Chromosome:1694772-1695075	ΔORF2	110.58+peptide	OK	121.726	85.6597	-0.506945	-0.848365	0.16045	0.612755	no
gene:SpnNT_01693	NA	Chromosome:1694772-1695075	110.58	ΔORF2+peptide	OK	57.8572	126.026	1.12316	1.90178	0.00135	0.021343	yes
gene:SpnNT_01693	NA	Chromosome:1694772-1695075	ΔORF2	ΔORF2+peptide	OK	121.726	126.026	0.0500907	0.0854382	0.8793	0.994748	no
gene:SpnNT_01693	NA	Chromosome:1694772-1695075	110.58+peptide	ΔORF2+peptide	OK	85.6597	126.026	0.557036	0.949723	0.11825	0.520872	no
gene:SpnNT_01694	ywnA	Chromosome:1695143-1695581	110.58	ΔORF2	OK	198.151	181.736	-0.124753	-0.264427	0.63905	0.980887	no
gene:SpnNT_01694	ywnA	Chromosome:1695143-1695581	110.58	110.58+peptide	OK	198.151	130.71	-0.60023	-1.24413	0.0337	0.241514	no
gene:SpnNT_01694	ywnA	Chromosome:1695143-1695581	ΔORF2	110.58+peptide	OK	181.736	130.71	-0.475477	-0.981318	0.0896	0.449782	no
gene:SpnNT_01694	ywnA	Chromosome:1695143-1695581	110.58	ΔORF2+peptide	OK	198.151	129.269	-0.616221	-1.2688	0.02775	0.211953	no
gene:SpnNT_01694	ywnA	Chromosome:1695143-1695581	ΔORF2	ΔORF2+peptide	OK	181.736	129.269	-0.491467	-1.00765	0.07655	0.408994	no
gene:SpnNT_01694	ywnA	Chromosome:1695143-1695581	110.58+peptide	ΔORF2+peptide	OK	130.71	129.269	-0.0159906	-0.0321058	0.955	0.994855	no
gene:SpnNT_01695	NA	Chromosome:1695656-1696499	110.58	ΔORF2	OK	61.2343	60.4259	-0.0191739	-0.0397982	0.94535	0.994855	no
gene:SpnNT_01695	NA	Chromosome:1695656-1696499	110.58	110.58+peptide	OK	61.2343	39.0163	-0.650265	-1.31585	0.0253	0.198438	no
gene:SpnNT_01695	NA	Chromosome:1695656-1696499	ΔORF2	110.58+peptide	OK	60.4259	39.0163	-0.631091	-1.27202	0.02965	0.220185	no
gene:SpnNT_01695	NA	Chromosome:1695656-1696499	110.58	ΔORF2+peptide	OK	61.2343	41.5538	-0.559361	-1.13351	0.0534	0.327284	no
gene:SpnNT_01695	NA	Chromosome:1695656-1696499	ΔORF2	ΔORF2+peptide	OK	60.4259	41.5538	-0.540187	-1.09034	0.0606	0.353734	no

gene:SpnNT_01695	NA	Chromosome:1695656-1696499	110.58+peptide	ΔORF2+peptide	OK	39.0163	41.5538	0.0909041	0.179122	0.76205	0.994748	no
gene:SpnNT_01696	dtxR	Chromosome:1696610-1697261	110.58	ΔORF2	OK	48.3649	42.3285	-0.192333	-0.369431	0.518	0.953876	no
gene:SpnNT_01696	dtxR	Chromosome:1696610-1697261	110.58	110.58+peptide	OK	48.3649	44.7254	-0.112868	-0.218155	0.70435	0.990367	no
gene:SpnNT_01696	dtxR	Chromosome:1696610-1697261	ΔORF2	110.58+peptide	OK	42.3285	44.7254	0.0794658	0.151804	0.78625	0.994748	no
gene:SpnNT_01696	dtxR	Chromosome:1696610-1697261	110.58	ΔORF2+peptide	OK	48.3649	41.3119	-0.227404	-0.434978	0.46085	0.924377	no
gene:SpnNT_01696	dtxR	Chromosome:1696610-1697261	ΔORF2	ΔORF2+peptide	OK	42.3285	41.3119	-0.0350704	-0.0663166	0.90725	0.994748	no
gene:SpnNT_01696	dtxR	Chromosome:1696610-1697261	110.58+peptide	ΔORF2+peptide	OK	44.7254	41.3119	-0.114536	-0.2179	0.7059	0.990422	no
gene:SpnNT_01697	NA	Chromosome:1697437-1697956	110.58	ΔORF2	OK	25.0484	27.9138	0.156258	0.264161	0.65495	0.982966	no
gene:SpnNT_01697	NA	Chromosome:1697437-1697956	110.58	110.58+peptide	OK	25.0484	13.6786	-0.872796	-1.3953	0.0139	0.127371	no
gene:SpnNT_01697	NA	Chromosome:1697437-1697956	ΔORF2	110.58+peptide	OK	27.9138	13.6786	-1.02905	-1.64852	0.00395	0.0500308	no
gene:SpnNT_01697	NA	Chromosome:1697437-1697956	110.58	ΔORF2+peptide	OK	25.0484	12.3103	-1.02485	-1.61354	0.00675	0.0753176	no
gene:SpnNT_01697	NA	Chromosome:1697437-1697956	ΔORF2	ΔORF2+peptide	OK	27.9138	12.3103	-1.18111	-1.8633	0.00185	0.0276489	yes
gene:SpnNT_01697	NA	Chromosome:1697437-1697956	110.58+peptide	ΔORF2+peptide	OK	13.6786	12.3103	-0.152056	-0.228409	0.68675	0.98828	no
gene:SpnNT_01698	NA	Chromosome:1698214-1698628	110.58	ΔORF2	OK	10.2268	9.94777	-0.039911	-0.0533986	0.92825	0.994748	no
gene:SpnNT_01698	NA	Chromosome:1698214-1698628	110.58	110.58+peptide	OK	10.2268	6.95773	-0.555666	-0.723139	0.22245	0.712122	no
gene:SpnNT_01698	NA	Chromosome:1698214-1698628	ΔORF2	110.58+peptide	OK	9.94777	6.95773	-0.515755	-0.672889	0.2459	0.743308	no
gene:SpnNT_01698	NA	Chromosome:1698214-1698628	110.58	ΔORF2+peptide	OK	10.2268	6.32423	-0.693395	-0.851705	0.1544	0.599419	no
gene:SpnNT_01698	NA	Chromosome:1698214-1698628	ΔORF2	ΔORF2+peptide	OK	9.94777	6.32423	-0.653484	-0.804481	0.16775	0.623936	no
gene:SpnNT_01698	NA	Chromosome:1698214-1698628	110.58+peptide	ΔORF2+peptide	OK	6.95773	6.32423	-0.137728	-0.165607	0.7762	0.994748	no
gene:SpnNT_01699	NA	Chromosome:1698646-1698832	110.58	ΔORF2	OK	7.75777	5.97606	-0.376448	-0.21661	0.68565	0.988184	no
gene:SpnNT_01699	NA	Chromosome:1698646-1698832	110.58	110.58+peptide	OK	7.75777	7.88775	0.0239719	0.0158013	0.90565	0.994748	no
gene:SpnNT_01699	NA	Chromosome:1698646-1698832	ΔORF2	110.58+peptide	OK	5.97606	7.88775	0.40042	0.239075	0.68695	0.98828	no
gene:SpnNT_01699	NA	Chromosome:1698646-1698832	110.58	ΔORF2+peptide	OK	7.75777	3.5595	-1.12397	-0.887291	0.3039	0.809208	no
gene:SpnNT_01699	NA	Chromosome:1698646-1698832	ΔORF2	ΔORF2+peptide	OK	5.97606	3.5595	-0.74752	-0.514825	0.501	0.944652	no
gene:SpnNT_01699	NA	Chromosome:1698646-1698832	110.58+peptide	ΔORF2+peptide	OK	7.88775	3.5595	-1.14794	-0.97385	0.3016	0.807672	no
gene:SpnNT_01700	dtd	Chromosome:1699098-1699542	110.58	ΔORF2	OK	193.304	176.906	-0.127883	-0.270378	0.6318	0.980887	no
gene:SpnNT_01700	dtd	Chromosome:1699098-1699542	110.58	110.58+peptide	OK	193.304	166.689	-0.213708	-0.451242	0.42535	0.908126	no
gene:SpnNT_01700	dtd	Chromosome:1699098-1699542	ΔORF2	110.58+peptide	OK	176.906	166.689	-0.0858252	-0.180821	0.74685	0.994748	no
gene:SpnNT_01700	dtd	Chromosome:1699098-1699542	110.58	ΔORF2+peptide	OK	193.304	172.059	-0.167969	-0.348867	0.53635	0.959314	no
gene:SpnNT_01700	dtd	Chromosome:1699098-1699542	ΔORF2	ΔORF2+peptide	OK	176.906	172.059	-0.0400859	-0.0830803	0.87945	0.994748	no
gene:SpnNT_01700	dtd	Chromosome:1699098-1699542	110.58+peptide	ΔORF2+peptide	OK	166.689	172.059	0.0457393	0.0946781	0.86365	0.994748	no
gene:SpnNT_01701	relA_1	Chromosome:1699571-1701794	110.58	ΔORF2	OK	133.064	129.154	-0.0430243	-0.0977732	0.8647	0.994748	no
gene:SpnNT_01701	relA_1	Chromosome:1699571-1701794	110.58	110.58+peptide	OK	133.064	104.225	-0.352416	-0.802544	0.17095	0.62976	no
gene:SpnNT_01701	relA_1	Chromosome:1699571-1701794	ΔORF2	110.58+peptide	OK	129.154	104.225	-0.309392	-0.70409	0.22425	0.715065	no
gene:SpnNT_01701	relA_1	Chromosome:1699571-1701794	110.58	ΔORF2+peptide	OK	133.064	101.78	-0.386666	-0.885548	0.12525	0.535526	no
gene:SpnNT_01701	relA_1	Chromosome:1699571-1701794	ΔORF2	ΔORF2+peptide	OK	129.154	101.78	-0.343642	-0.786475	0.1671	0.622922	no
gene:SpnNT_01701	relA_1	Chromosome:1699571-1701794	110.58+peptide	ΔORF2+peptide	OK	104.225	101.78	-0.0342501	-0.0785525	0.89045	0.994748	no
gene:SpnNT_01702	pksB	Chromosome:1702787-1703417	110.58	ΔORF2	OK	52.8786	47.0407	-0.168774	-0.320612	0.571	0.96884	no
gene:SpnNT_01702	pksB	Chromosome:1702787-1703417	110.58	110.58+peptide	OK	52.8786	42.5207	-0.314517	-0.597653	0.29805	0.803352	no
gene:SpnNT_01702	pksB	Chromosome:1702787-1703417	ΔORF2	110.58+peptide	OK	47.0407	42.5207	-0.145743	-0.269883	0.627	0.980887	no
gene:SpnNT_01702	pksB	Chromosome:1702787-1703417	110.58	ΔORF2+peptide	OK	52.8786	34.6934	-0.608023	-1.15056	0.04405	0.287717	no
gene:SpnNT_01702	pksB	Chromosome:1702787-1703417	ΔORF2	ΔORF2+peptide	OK	47.0407	34.6934	-0.439249	-0.810168	0.1516	0.593484	no
gene:SpnNT_01702	pksB	Chromosome:1702787-1703417	110.58+peptide	ΔORF2+peptide	OK	42.5207	34.6934	-0.293506	-0.541507	0.3376	0.842043	no
gene:SpnNT_01703	pepO	Chromosome:1703539-1705432	110.58	ΔORF2	OK	202.19	203.073	0.0062856	0.0142606	0.9793	0.995765	no
gene:SpnNT_01703	pepO	Chromosome:1703539-1705432	110.58	110.58+peptide	OK	202.19	167.047	-0.275454	-0.627124	0.2784	0.781125	no

gene:SpnNT_01703	pepO	Chromosome:1703539-1705432	ΔORF2	110.58+peptide	OK	203.073	167.047	-0.28174	-0.638652	0.2635	0.764218	no
gene:SpnNT_01703	pepO	Chromosome:1703539-1705432	110.58	ΔORF2+peptide	OK	202.19	183.939	-0.136486	-0.311282	0.5941	0.975539	no
gene:SpnNT_01703	pepO	Chromosome:1703539-1705432	ΔORF2	ΔORF2+peptide	OK	203.073	183.939	-0.142772	-0.3242	0.57025	0.968621	no
gene:SpnNT_01703	pepO	Chromosome:1703539-1705432	110.58+peptide	ΔORF2+peptide	OK	167.047	183.939	0.138968	0.316667	0.58335	0.971331	no
gene:SpnNT_01704	adcC_1	Chromosome:1705722-1707290	110.58	ΔORF2	OK	31.148	24.9116	-0.322321	-0.467076	0.41095	0.898655	no
gene:SpnNT_01704	adcC_1	Chromosome:1705722-1707290	110.58	110.58+peptide	OK	31.148	29.9664	-0.0557952	-0.0815382	0.88105	0.994748	no
gene:SpnNT_01704	adcC_1	Chromosome:1705722-1707290	ΔORF2	110.58+peptide	OK	24.9116	29.9664	0.266526	0.384999	0.5017	0.944784	no
gene:SpnNT_01704	adcC_1	Chromosome:1705722-1707290	110.58	ΔORF2+peptide	OK	31.148	28.6648	-0.119856	-0.173935	0.7646	0.994748	no
gene:SpnNT_01704	adcC_1	Chromosome:1705722-1707290	ΔORF2	ΔORF2+peptide	OK	24.9116	28.6648	0.202465	0.29047	0.6197	0.980887	no
gene:SpnNT_01704	adcC_1	Chromosome:1705722-1707290	110.58+peptide	ΔORF2+peptide	OK	29.9664	28.6648	-0.0640609	-0.0926694	0.8769	0.994748	no
gene:SpnNT_01705	mntB	Chromosome:1705722-1707290	110.58	ΔORF2	OK	16.7027	13.8473	-0.27048	-0.327922	0.58025	0.969538	no
gene:SpnNT_01705	mntB	Chromosome:1705722-1707290	110.58	110.58+peptide	OK	16.7027	20.6904	0.30888	0.392835	0.50535	0.945292	no
gene:SpnNT_01705	mntB	Chromosome:1705722-1707290	ΔORF2	110.58+peptide	OK	13.8473	20.6904	0.57936	0.734399	0.2057	0.690314	no
gene:SpnNT_01705	mntB	Chromosome:1705722-1707290	110.58	ΔORF2+peptide	OK	16.7027	17.8149	0.0930033	0.116674	0.84375	0.994748	no
gene:SpnNT_01705	mntB	Chromosome:1705722-1707290	ΔORF2	ΔORF2+peptide	OK	13.8473	17.8149	0.363483	0.454529	0.439	0.916119	no
gene:SpnNT_01705	mntB	Chromosome:1705722-1707290	110.58+peptide	ΔORF2+peptide	OK	20.6904	17.8149	-0.215877	-0.284096	0.6322	0.980887	no
gene:SpnNT_01706	psaA	Chromosome:1707321-1708251	110.58	ΔORF2	OK	25.103	22.1365	-0.181434	-0.338516	0.5525	0.964853	no
gene:SpnNT_01706	psaA	Chromosome:1707321-1708251	110.58	110.58+peptide	OK	25.103	24.5707	-0.0309213	-0.0579653	0.9215	0.994748	no
gene:SpnNT_01706	psaA	Chromosome:1707321-1708251	ΔORF2	110.58+peptide	OK	22.1365	24.5707	0.150512	0.282582	0.623	0.980887	no
gene:SpnNT_01706	psaA	Chromosome:1707321-1708251	110.58	ΔORF2+peptide	OK	25.103	23.9398	-0.0684468	-0.127809	0.82475	0.994748	no
gene:SpnNT_01706	psaA	Chromosome:1707321-1708251	ΔORF2	ΔORF2+peptide	OK	22.1365	23.9398	0.112987	0.211298	0.71565	0.992121	no
gene:SpnNT_01706	psaA	Chromosome:1707321-1708251	110.58+peptide	ΔORF2+peptide	OK	24.5707	23.9398	-0.0375255	-0.0705102	0.9059	0.994748	no
gene:SpnNT_01707	tpx	Chromosome:1708373-1708865	110.58	ΔORF2	OK	549.273	620.865	0.176757	0.393446	0.482	0.935166	no
gene:SpnNT_01707	tpx	Chromosome:1708373-1708865	110.58	110.58+peptide	OK	549.273	823.782	0.58474	1.29313	0.0224	0.182567	no
gene:SpnNT_01707	tpx	Chromosome:1708373-1708865	ΔORF2	110.58+peptide	OK	620.865	823.782	0.407983	0.927185	0.09995	0.479559	no
gene:SpnNT_01707	tpx	Chromosome:1708373-1708865	110.58	ΔORF2+peptide	OK	549.273	920.118	0.744295	1.64278	0.0033	0.0432593	yes
gene:SpnNT_01707	tpx	Chromosome:1708373-1708865	ΔORF2	ΔORF2+peptide	OK	620.865	920.118	0.567538	1.28715	0.02155	0.177402	no
gene:SpnNT_01707	tpx	Chromosome:1708373-1708865	110.58+peptide	ΔORF2+peptide	OK	823.782	920.118	0.159555	0.359424	0.52275	0.954832	no
gene:SpnNT_01708	NA	Chromosome:1708960-1711669	110.58	ΔORF2	OK	24.2811	21.2433	-0.19282	-0.413171	0.47035	0.928389	no
gene:SpnNT_01708	NA	Chromosome:1708960-1711669	110.58	110.58+peptide	OK	24.2811	22.542	-0.107216	-0.231427	0.68655	0.98828	no
gene:SpnNT_01708	NA	Chromosome:1708960-1711669	ΔORF2	110.58+peptide	OK	21.2433	22.542	0.0856039	0.184064	0.7476	0.994748	no
gene:SpnNT_01708	NA	Chromosome:1708960-1711669	110.58	ΔORF2+peptide	OK	24.2811	20.7209	-0.228747	-0.491407	0.3907	0.884993	no
gene:SpnNT_01708	NA	Chromosome:1708960-1711669	ΔORF2	ΔORF2+peptide	OK	21.2433	20.7209	-0.0359263	-0.076884	0.8926	0.994748	no
gene:SpnNT_01708	NA	Chromosome:1708960-1711669	110.58+peptide	ΔORF2+peptide	OK	22.542	20.7209	-0.12153	-0.261985	0.6455	0.980887	no
gene:SpnNT_01709	NA	Chromosome:1711670-1712372	110.58	ΔORF2	OK	23.2151	20.7303	-0.16332	-0.288853	0.61505	0.979616	no
gene:SpnNT_01709	NA	Chromosome:1711670-1712372	110.58	110.58+peptide	OK	23.2151	18.5617	-0.322732	-0.569349	0.32025	0.825861	no
gene:SpnNT_01709	NA	Chromosome:1711670-1712372	ΔORF2	110.58+peptide	OK	20.7303	18.5617	-0.159412	-0.277514	0.62705	0.980887	no
gene:SpnNT_01709	NA	Chromosome:1711670-1712372	110.58	ΔORF2+peptide	OK	23.2151	19.4355	-0.256368	-0.451042	0.4309	0.912485	no
gene:SpnNT_01709	NA	Chromosome:1711670-1712372	ΔORF2	ΔORF2+peptide	OK	20.7303	19.4355	-0.0930484	-0.161554	0.77275	0.994748	no
gene:SpnNT_01709	NA	Chromosome:1711670-1712372	110.58+peptide	ΔORF2+peptide	OK	18.5617	19.4355	0.066364	0.114943	0.83575	0.994748	no
gene:SpnNT_01710	NA	Chromosome:1712621-1715033	110.58	ΔORF2	OK	8.94726	7.99567	-0.162226	-0.306142	0.5942	0.975539	no
gene:SpnNT_01710	NA	Chromosome:1712621-1715033	110.58	110.58+peptide	OK	8.94726	7.65403	-0.225225	-0.422725	0.4629	0.9258	no
gene:SpnNT_01710	NA	Chromosome:1712621-1715033	ΔORF2	110.58+peptide	OK	7.99567	7.65403	-0.0629994	-0.118604	0.82915	0.994748	no
gene:SpnNT_01710	NA	Chromosome:1712621-1715033	110.58	ΔORF2+peptide	OK	8.94726	6.50747	-0.45935	-0.853529	0.1409	0.572766	no
gene:SpnNT_01710	NA	Chromosome:1712621-1715033	ΔORF2	ΔORF2+peptide	OK	7.99567	6.50747	-0.297124	-0.553743	0.3199	0.825861	no

gene:SpnNT_01710	NA	Chromosome:1712621-1715033	110.58+peptide	ΔORF2+peptide	OK	7.65403	6.50747	-0.234125	-0.434023	0.44965	0.920402	no
gene:SpnNT_01711	NA	Chromosome:1715175-1716527	110.58	ΔORF2	OK	1.23192	0.789963	-0.641047	-0.331622	0.5907	0.974909	no
gene:SpnNT_01711	NA	Chromosome:1715175-1716527	110.58	110.58+peptide	OK	1.23192	0.99716	-0.305007	-0.161785	0.7611	0.994748	no
gene:SpnNT_01711	NA	Chromosome:1715175-1716527	ΔORF2	110.58+peptide	OK	0.789963	0.99716	0.33604	0.152874	0.80875	0.994748	no
gene:SpnNT_01711	NA	Chromosome:1715175-1716527	110.58	ΔORF2+peptide	OK	1.23192	2.01372	0.708961	0.405265	0.52185	0.954832	no
gene:SpnNT_01711	NA	Chromosome:1715175-1716527	ΔORF2	ΔORF2+peptide	OK	0.789963	2.01372	1.35001	0.648175	0.4096	0.8977	no
gene:SpnNT_01711	NA	Chromosome:1715175-1716527	110.58+peptide	ΔORF2+peptide	OK	0.99716	2.01372	1.01397	0.49741	0.42715	0.908839	no
gene:SpnNT_01712	NA	Chromosome:1715175-1716527	110.58	ΔORF2	OK	3.6806	7.00944	0.929359	0.631126	0.30725	0.81331	no
gene:SpnNT_01712	NA	Chromosome:1715175-1716527	110.58	110.58+peptide	OK	3.6806	13.3112	1.85463	1.38282	0.05675	0.33895	no
gene:SpnNT_01712	NA	Chromosome:1715175-1716527	ΔORF2	110.58+peptide	OK	7.00944	13.3112	0.925273	0.805413	0.17685	0.641766	no
gene:SpnNT_01712	NA	Chromosome:1715175-1716527	110.58	ΔORF2+peptide	OK	3.6806	10.3983	1.49833	0.981862	0.1313	0.551334	no
gene:SpnNT_01712	NA	Chromosome:1715175-1716527	ΔORF2	ΔORF2+peptide	OK	7.00944	10.3983	0.568973	0.418353	0.4676	0.92732	no
gene:SpnNT_01712	NA	Chromosome:1715175-1716527	110.58+peptide	ΔORF2+peptide	OK	13.3112	10.3983	-0.3563	-0.292865	0.60595	0.976937	no
gene:SpnNT_01713	NA	Chromosome:1715175-1716527	110.58	ΔORF2	NOTEST	0	0	0	0	1	1	no
gene:SpnNT_01713	NA	Chromosome:1715175-1716527	110.58	110.58+peptide	NOTEST	0	0	0	0	1	1	no
gene:SpnNT_01713	NA	Chromosome:1715175-1716527	ΔORF2	110.58+peptide	NOTEST	0	0	0	0	1	1	no
gene:SpnNT_01713	NA	Chromosome:1715175-1716527	110.58	ΔORF2+peptide	NOTEST	0	0	0	0	1	1	no
gene:SpnNT_01713	NA	Chromosome:1715175-1716527	ΔORF2	ΔORF2+peptide	NOTEST	0	0	0	0	1	1	no
gene:SpnNT_01713	NA	Chromosome:1715175-1716527	110.58+peptide	ΔORF2+peptide	NOTEST	0	0	0	0	1	1	no
gene:SpnNT_01714	NA	Chromosome:1715175-1716527	110.58	ΔORF2	OK	1.31236	1.36496	0.0566872	0.0353638	0.95395	0.994855	no
gene:SpnNT_01714	NA	Chromosome:1715175-1716527	110.58	110.58+peptide	OK	1.31236	2.3518	0.8416	0.591258	0.3447	0.848904	no
gene:SpnNT_01714	NA	Chromosome:1715175-1716527	ΔORF2	110.58+peptide	OK	1.36496	2.3518	0.784912	0.524206	0.3964	0.890558	no
gene:SpnNT_01714	NA	Chromosome:1715175-1716527	110.58	ΔORF2+peptide	OK	1.31236	3.53077	1.42782	1.01096	0.1124	0.510174	no
gene:SpnNT_01714	NA	Chromosome:1715175-1716527	ΔORF2	ΔORF2+peptide	OK	1.36496	3.53077	1.37113	0.922187	0.1425	0.576108	no
gene:SpnNT_01714	NA	Chromosome:1715175-1716527	110.58+peptide	ΔORF2+peptide	OK	2.3518	3.53077	0.586218	0.454008	0.45215	0.921244	no
gene:SpnNT_01715	gpmA_2	Chromosome:1716576-1717269	110.58	ΔORF2	OK	1097.03	1196.99	0.125796	0.278731	0.6154	0.979616	no
gene:SpnNT_01715	gpmA_2	Chromosome:1716576-1717269	110.58	110.58+peptide	OK	1097.03	1550.32	0.498955	1.09875	0.0508	0.317427	no
gene:SpnNT_01715	gpmA_2	Chromosome:1716576-1717269	ΔORF2	110.58+peptide	OK	1196.99	1550.32	0.373159	0.833635	0.1377	0.565186	no
gene:SpnNT_01715	gpmA_2	Chromosome:1716576-1717269	110.58	ΔORF2+peptide	OK	1097.03	1594.91	0.539862	1.1913	0.0361	0.252912	no
gene:SpnNT_01715	gpmA_2	Chromosome:1716576-1717269	ΔORF2	ΔORF2+peptide	OK	1196.99	1594.91	0.414066	0.926998	0.10005	0.479559	no
gene:SpnNT_01715	gpmA_2	Chromosome:1716576-1717269	110.58+peptide	ΔORF2+peptide	OK	1550.32	1594.91	0.0409067	0.0910057	0.8794	0.994748	no
gene:SpnNT_01716	iga_6	Chromosome:1717427-1723247	110.58	ΔORF2	OK	47.9103	57.4892	0.262955	0.596578	0.29905	0.80426	no
gene:SpnNT_01716	iga_6	Chromosome:1717427-1723247	110.58	110.58+peptide	OK	47.9103	82.1344	0.777649	1.72266	0.0027	0.0368289	yes
gene:SpnNT_01716	iga_6	Chromosome:1717427-1723247	ΔORF2	110.58+peptide	OK	57.4892	82.1344	0.514694	1.13969	0.0503	0.314903	no
gene:SpnNT_01716	iga_6	Chromosome:1717427-1723247	110.58	ΔORF2+peptide	OK	47.9103	125.371	1.38779	3.11058	5.00E-05	0.0013612	yes
gene:SpnNT_01716	iga_6	Chromosome:1717427-1723247	ΔORF2	ΔORF2+peptide	OK	57.4892	125.371	1.12484	2.52014	5.00E-05	0.0013612	yes
gene:SpnNT_01716	iga_6	Chromosome:1717427-1723247	110.58+peptide	ΔORF2+peptide	OK	82.1344	125.371	0.610146	1.33552	0.0199	0.166429	no
gene:SpnNT_01717	ileS	Chromosome:1723429-1726222	110.58	ΔORF2	OK	62.1887	64.7173	0.0574982	0.131086	0.8182	0.994748	no
gene:SpnNT_01717	ileS	Chromosome:1723429-1726222	110.58	110.58+peptide	OK	62.1887	116.391	0.904253	2.06143	0.00045	0.00885292	yes
gene:SpnNT_01717	ileS	Chromosome:1723429-1726222	ΔORF2	110.58+peptide	OK	64.7173	116.391	0.846755	1.92258	0.0011	0.0181562	yes
gene:SpnNT_01717	ileS	Chromosome:1723429-1726222	110.58	ΔORF2+peptide	OK	62.1887	128.555	1.04766	2.37974	5.00E-05	0.0013612	yes
gene:SpnNT_01717	ileS	Chromosome:1723429-1726222	ΔORF2	ΔORF2+peptide	OK	64.7173	128.555	0.990163	2.24015	1.00E-04	0.0025332	yes
gene:SpnNT_01717	ileS	Chromosome:1723429-1726222	110.58+peptide	ΔORF2+peptide	OK	116.391	128.555	0.143408	0.324429	0.5697	0.968621	no
gene:SpnNT_01718	divIVA	Chromosome:1726474-1727359	110.58	ΔORF2	OK	373.883	386.731	0.0487448	0.112275	0.8407	0.994748	no
gene:SpnNT_01718	divIVA	Chromosome:1726474-1727359	110.58	110.58+peptide	OK	373.883	344.8	-0.116825	-0.269025	0.6308	0.980887	no

gene:SpnNT_01718	divIVA	Chromosome:1726474-1727359	ΔORF2	110.58+peptide	OK	386.731	344.8	-0.16557	-0.381031	0.5006	0.944482	no
gene:SpnNT_01718	divIVA	Chromosome:1726474-1727359	110.58	ΔORF2+peptide	OK	373.883	378.371	0.0172141	0.0396571	0.94345	0.994855	no
gene:SpnNT_01718	divIVA	Chromosome:1726474-1727359	ΔORF2	ΔORF2+peptide	OK	386.731	378.371	-0.0315307	-0.0725925	0.89805	0.994748	no
gene:SpnNT_01718	divIVA	Chromosome:1726474-1727359	110.58+peptide	ΔORF2+peptide	OK	344.8	378.371	0.134039	0.308526	0.58345	0.971331	no
gene:SpnNT_01719	NA	Chromosome:1727367-1728952	110.58	ΔORF2	OK	232.156	289.264	0.317292	0.425809	0.45865	0.922502	no
gene:SpnNT_01719	NA	Chromosome:1727367-1728952	110.58	110.58+peptide	OK	232.156	214.122	-0.116662	-0.149321	0.7974	0.994748	no
gene:SpnNT_01719	NA	Chromosome:1727367-1728952	ΔORF2	110.58+peptide	OK	289.264	214.122	-0.433954	-0.565441	0.3314	0.837459	no
gene:SpnNT_01719	NA	Chromosome:1727367-1728952	110.58	ΔORF2+peptide	OK	232.156	297.801	0.359252	0.499371	0.38745	0.881894	no
gene:SpnNT_01719	NA	Chromosome:1727367-1728952	ΔORF2	ΔORF2+peptide	OK	289.264	297.801	0.0419597	0.0595701	0.9157	0.994748	no
gene:SpnNT_01719	NA	Chromosome:1727367-1728952	110.58+peptide	ΔORF2+peptide	OK	214.122	297.801	0.475914	0.640968	0.276	0.778493	no
gene:SpnNT_01720	NA	Chromosome:1727367-1728952	110.58	ΔORF2	OK	205.283	244.236	0.250661	0.120017	0.8775	0.994748	no
gene:SpnNT_01720	NA	Chromosome:1727367-1728952	110.58	110.58+peptide	OK	205.283	224.21	0.127241	0.0630261	0.9323	0.994855	no
gene:SpnNT_01720	NA	Chromosome:1727367-1728952	ΔORF2	110.58+peptide	OK	244.236	224.21	-0.12342	-0.0614616	0.9285	0.994748	no
gene:SpnNT_01720	NA	Chromosome:1727367-1728952	110.58	ΔORF2+peptide	OK	205.283	300.149	0.548067	0.274557	0.8091	0.994748	no
gene:SpnNT_01720	NA	Chromosome:1727367-1728952	ΔORF2	ΔORF2+peptide	OK	244.236	300.149	0.297406	0.149804	0.85555	0.994748	no
gene:SpnNT_01720	NA	Chromosome:1727367-1728952	110.58+peptide	ΔORF2+peptide	OK	224.21	300.149	0.420827	0.220116	0.8078	0.994748	no
gene:SpnNT_01721	sepF	Chromosome:1727367-1728952	110.58	ΔORF2	OK	677.613	680.299	0.00570745	0.00944657	0.9866	0.997339	no
gene:SpnNT_01721	sepF	Chromosome:1727367-1728952	110.58	110.58+peptide	OK	677.613	615.908	-0.137747	-0.237566	0.6803	0.986361	no
gene:SpnNT_01721	sepF	Chromosome:1727367-1728952	ΔORF2	110.58+peptide	OK	680.299	615.908	-0.143454	-0.233169	0.68405	0.98747	no
gene:SpnNT_01721	sepF	Chromosome:1727367-1728952	110.58	ΔORF2+peptide	OK	677.613	654.214	-0.0506992	-0.0851824	0.88825	0.994748	no
gene:SpnNT_01721	sepF	Chromosome:1727367-1728952	ΔORF2	ΔORF2+peptide	OK	680.299	654.214	-0.0564067	-0.0895723	0.8816	0.994748	no
gene:SpnNT_01721	sepF	Chromosome:1727367-1728952	110.58+peptide	ΔORF2+peptide	OK	615.908	654.214	0.0870477	0.143548	0.8052	0.994748	no
gene:SpnNT_01722	NA	Chromosome:1728961-1729633	110.58	ΔORF2	OK	245.812	274.065	0.156965	0.359542	0.5253	0.955327	no
gene:SpnNT_01722	NA	Chromosome:1728961-1729633	110.58	110.58+peptide	OK	245.812	214.457	-0.196864	-0.449483	0.43055	0.912416	no
gene:SpnNT_01722	NA	Chromosome:1728961-1729633	ΔORF2	110.58+peptide	OK	274.065	214.457	-0.353829	-0.808402	0.1587	0.608196	no
gene:SpnNT_01722	NA	Chromosome:1728961-1729633	110.58	ΔORF2+peptide	OK	245.812	274.793	0.160788	0.368164	0.51475	0.951747	no
gene:SpnNT_01722	NA	Chromosome:1728961-1729633	ΔORF2	ΔORF2+peptide	OK	274.065	274.793	0.00382301	0.00875958	0.9887	0.997703	no
gene:SpnNT_01722	NA	Chromosome:1728961-1729633	110.58+peptide	ΔORF2+peptide	OK	214.457	274.793	0.357652	0.816838	0.15345	0.597144	no
gene:SpnNT_01723	ftsZ	Chromosome:1729637-1730897	110.58	ΔORF2	OK	667.923	634.528	-0.0739981	-0.161638	0.7766	0.994748	no
gene:SpnNT_01723	ftsZ	Chromosome:1729637-1730897	110.58	110.58+peptide	OK	667.923	594.328	-0.168425	-0.373511	0.5167	0.953204	no
gene:SpnNT_01723	ftsZ	Chromosome:1729637-1730897	ΔORF2	110.58+peptide	OK	634.528	594.328	-0.0944266	-0.206638	0.717	0.992452	no
gene:SpnNT_01723	ftsZ	Chromosome:1729637-1730897	110.58	ΔORF2+peptide	OK	667.923	585.67	-0.189594	-0.420678	0.46285	0.9258	no
gene:SpnNT_01723	ftsZ	Chromosome:1729637-1730897	ΔORF2	ΔORF2+peptide	OK	634.528	585.67	-0.115596	-0.253093	0.6576	0.982966	no
gene:SpnNT_01723	ftsZ	Chromosome:1729637-1730897	110.58+peptide	ΔORF2+peptide	OK	594.328	585.67	-0.0211698	-0.0470609	0.934	0.994855	no
gene:SpnNT_01724	ftsA	Chromosome:1730913-1732287	110.58	ΔORF2	OK	490.111	498.173	0.0235393	0.0525741	0.9238	0.994748	no
gene:SpnNT_01724	ftsA	Chromosome:1730913-1732287	110.58	110.58+peptide	OK	490.111	438.386	-0.160908	-0.360718	0.5251	0.955327	no
gene:SpnNT_01724	ftsA	Chromosome:1730913-1732287	ΔORF2	110.58+peptide	OK	498.173	438.386	-0.184448	-0.415546	0.45555	0.921244	no
gene:SpnNT_01724	ftsA	Chromosome:1730913-1732287	110.58	ΔORF2+peptide	OK	490.111	467.024	-0.0696117	-0.155824	0.7797	0.994748	no
gene:SpnNT_01724	ftsA	Chromosome:1730913-1732287	ΔORF2	ΔORF2+peptide	OK	498.173	467.024	-0.093151	-0.209551	0.70385	0.990367	no
gene:SpnNT_01724	ftsA	Chromosome:1730913-1732287	110.58+peptide	ΔORF2+peptide	OK	438.386	467.024	0.0912966	0.206153	0.7133	0.991272	no
gene:SpnNT_01725	NA	Chromosome:1732505-1733210	110.58	ΔORF2	OK	176.834	222.669	0.332504	0.741101	0.19415	0.675407	no
gene:SpnNT_01725	NA	Chromosome:1732505-1733210	110.58	110.58+peptide	OK	176.834	212.21	0.2631	0.593346	0.296	0.800323	no
gene:SpnNT_01725	NA	Chromosome:1732505-1733210	ΔORF2	110.58+peptide	OK	222.669	212.21	-0.0694038	-0.155596	0.7872	0.994748	no
gene:SpnNT_01725	NA	Chromosome:1732505-1733210	110.58	ΔORF2+peptide	OK	176.834	253.57	0.519991	1.17941	0.0379	0.261474	no
gene:SpnNT_01725	NA	Chromosome:1732505-1733210	ΔORF2	ΔORF2+peptide	OK	222.669	253.57	0.187488	0.422707	0.45825	0.922405	no

gene:SpnNT_01725	NA	Chromosome:1732505-1733210	110.58+peptide	ΔORF2+peptide	OK	212.21	253.57	0.256892	0.586196	0.3019	0.807816	no
gene:SpnNT_01726	NA	Chromosome:1733300-1735272	110.58	ΔORF2	OK	37.6567	29.222	-0.365851	-0.348305	0.5503	0.964281	no
gene:SpnNT_01726	NA	Chromosome:1733300-1735272	110.58	110.58+peptide	OK	37.6567	44.7172	0.247924	0.233829	0.69265	0.98828	no
gene:SpnNT_01726	NA	Chromosome:1733300-1735272	ΔORF2	110.58+peptide	OK	29.222	44.7172	0.613776	0.565788	0.3334	0.839028	no
gene:SpnNT_01726	NA	Chromosome:1733300-1735272	110.58	ΔORF2+peptide	OK	37.6567	55.61	0.562439	0.550648	0.3532	0.856141	no
gene:SpnNT_01726	NA	Chromosome:1733300-1735272	ΔORF2	ΔORF2+peptide	OK	29.222	55.61	0.92829	0.886738	0.1316	0.551535	no
gene:SpnNT_01726	NA	Chromosome:1733300-1735272	110.58+peptide	ΔORF2+peptide	OK	44.7172	55.61	0.314515	0.297612	0.61425	0.979616	no
gene:SpnNT_01727	murF	Chromosome:1733300-1735272	110.58	ΔORF2	OK	53.0038	44.0078	-0.268337	-0.526827	0.3574	0.858392	no
gene:SpnNT_01727	murF	Chromosome:1733300-1735272	110.58	110.58+peptide	OK	53.0038	72.8306	0.458449	0.921961	0.1075	0.498451	no
gene:SpnNT_01727	murF	Chromosome:1733300-1735272	ΔORF2	110.58+peptide	OK	44.0078	72.8306	0.726786	1.45029	0.0128	0.120144	no
gene:SpnNT_01727	murF	Chromosome:1733300-1735272	110.58	ΔORF2+peptide	OK	53.0038	71.4379	0.430594	0.861162	0.1351	0.559237	no
gene:SpnNT_01727	murF	Chromosome:1733300-1735272	ΔORF2	ΔORF2+peptide	OK	44.0078	71.4379	0.698931	1.38713	0.0169	0.146377	no
gene:SpnNT_01727	murF	Chromosome:1733300-1735272	110.58+peptide	ΔORF2+peptide	OK	72.8306	71.4379	-0.0278552	-0.0566573	0.92115	0.994748	no
gene:SpnNT_01728	ddl	Chromosome:1735356-1736400	110.58	ΔORF2	OK	165.283	168.659	0.0291651	0.0665692	0.90635	0.994748	no
gene:SpnNT_01728	ddl	Chromosome:1735356-1736400	110.58	110.58+peptide	OK	165.283	151.54	-0.125243	-0.284489	0.61475	0.979616	no
gene:SpnNT_01728	ddl	Chromosome:1735356-1736400	ΔORF2	110.58+peptide	OK	168.659	151.54	-0.154408	-0.348538	0.5382	0.959938	no
gene:SpnNT_01728	ddl	Chromosome:1735356-1736400	110.58	ΔORF2+peptide	OK	165.283	172.715	0.0634537	0.145277	0.80095	0.994748	no
gene:SpnNT_01728	ddl	Chromosome:1735356-1736400	ΔORF2	ΔORF2+peptide	OK	168.659	172.715	0.0342887	0.0780037	0.89385	0.994748	no
gene:SpnNT_01728	ddl	Chromosome:1735356-1736400	110.58+peptide	ΔORF2+peptide	OK	151.54	172.715	0.188697	0.427214	0.45765	0.921722	no
gene:SpnNT_01729	recR	Chromosome:1736575-1737172	110.58	ΔORF2	OK	177.793	173.591	-0.0344994	-0.0756018	0.8965	0.994748	no
gene:SpnNT_01729	recR	Chromosome:1736575-1737172	110.58	110.58+peptide	OK	177.793	163.056	-0.124825	-0.273426	0.63455	0.980887	no
gene:SpnNT_01729	recR	Chromosome:1736575-1737172	ΔORF2	110.58+peptide	OK	173.591	163.056	-0.0903254	-0.19746	0.7329	0.994748	no
gene:SpnNT_01729	recR	Chromosome:1736575-1737172	110.58	ΔORF2+peptide	OK	177.793	150.588	-0.239587	-0.523541	0.3582	0.859746	no
gene:SpnNT_01729	recR	Chromosome:1736575-1737172	ΔORF2	ΔORF2+peptide	OK	173.591	150.588	-0.205087	-0.447261	0.4361	0.914104	no
gene:SpnNT_01729	recR	Chromosome:1736575-1737172	110.58+peptide	ΔORF2+peptide	OK	163.056	150.588	-0.114762	-0.250173	0.6659	0.98367	no
gene:SpnNT_01730	penA	Chromosome:1737182-1739225	110.58	ΔORF2	OK	55.6712	61.4553	0.142607	0.321714	0.5767	0.969538	no
gene:SpnNT_01730	penA	Chromosome:1737182-1739225	110.58	110.58+peptide	OK	55.6712	45.9805	-0.275907	-0.613362	0.2825	0.785208	no
gene:SpnNT_01730	penA	Chromosome:1737182-1739225	ΔORF2	110.58+peptide	OK	61.4553	45.9805	-0.418514	-0.92986	0.10575	0.49453	no
gene:SpnNT_01730	penA	Chromosome:1737182-1739225	110.58	ΔORF2+peptide	OK	55.6712	54.642	-0.0269191	-0.0608105	0.91785	0.994748	no
gene:SpnNT_01730	penA	Chromosome:1737182-1739225	ΔORF2	ΔORF2+peptide	OK	61.4553	54.642	-0.169526	-0.382737	0.50575	0.945292	no
gene:SpnNT_01730	penA	Chromosome:1737182-1739225	110.58+peptide	ΔORF2+peptide	OK	45.9805	54.642	0.248987	0.553934	0.3337	0.839335	no
gene:SpnNT_01731	ybbH	Chromosome:1739439-1740291	110.58	ΔORF2	OK	99.98	102.771	0.0397274	0.0868842	0.8778	0.994748	no
gene:SpnNT_01731	ybbH	Chromosome:1739439-1740291	110.58	110.58+peptide	OK	99.98	92.8336	-0.106993	-0.234453	0.67795	0.98524	no
gene:SpnNT_01731	ybbH	Chromosome:1739439-1740291	ΔORF2	110.58+peptide	OK	102.771	92.8336	-0.14672	-0.322313	0.5722	0.969077	no
gene:SpnNT_01731	ybbH	Chromosome:1739439-1740291	110.58	ΔORF2+peptide	OK	99.98	107.934	0.110435	0.242095	0.67155	0.984845	no
gene:SpnNT_01731	ybbH	Chromosome:1739439-1740291	ΔORF2	ΔORF2+peptide	OK	102.771	107.934	0.0707071	0.155393	0.78515	0.994748	no
gene:SpnNT_01731	ybbH	Chromosome:1739439-1740291	110.58+peptide	ΔORF2+peptide	OK	92.8336	107.934	0.217427	0.478787	0.4092	0.897533	no
gene:SpnNT_01732	bglK_1	Chromosome:1740306-1741191	110.58	ΔORF2	OK	43.7883	41.5087	-0.0771314	-0.155314	0.7843	0.994748	no
gene:SpnNT_01732	bglK_1	Chromosome:1740306-1741191	110.58	110.58+peptide	OK	43.7883	159.813	1.86777	3.69834	5.00E-05	0.0013612	yes
gene:SpnNT_01732	bglK_1	Chromosome:1740306-1741191	ΔORF2	110.58+peptide	OK	41.5087	159.813	1.9449	3.84792	5.00E-05	0.0013612	yes
gene:SpnNT_01732	bglK_1	Chromosome:1740306-1741191	110.58	ΔORF2+peptide	OK	43.7883	85.6458	0.967837	2.01437	0.00105	0.0174849	yes
gene:SpnNT_01732	bglK_1	Chromosome:1740306-1741191	ΔORF2	ΔORF2+peptide	OK	41.5087	85.6458	1.04497	2.17293	0.00015	0.00355289	yes
gene:SpnNT_01732	bglK_1	Chromosome:1740306-1741191	110.58+peptide	ΔORF2+peptide	OK	159.813	85.6458	-0.899931	-1.83816	0.00165	0.0253528	yes
gene:SpnNT_01733	nanA_2	Chromosome:1741208-1742126	110.58	ΔORF2	OK	27.6617	23.503	-0.235047	-0.443588	0.4272	0.908839	no
gene:SpnNT_01733	nanA_2	Chromosome:1741208-1742126	110.58	110.58+peptide	OK	27.6617	70.0052	1.33957	2.62798	5.00E-05	0.0013612	yes

gene:SpnNT_01733	nanA_2	Chromosome:1741208-1742126	ΔORF2	110.58+peptide	OK	23.503	70.0052	1.57462	3.04777	5.00E-05	0.0013612	yes
gene:SpnNT_01733	nanA_2	Chromosome:1741208-1742126	110.58	ΔORF2+peptide	OK	27.6617	45.2554	0.710199	1.39396	0.01435	0.129952	no
gene:SpnNT_01733	nanA_2	Chromosome:1741208-1742126	ΔORF2	ΔORF2+peptide	OK	23.503	45.2554	0.945246	1.83047	0.0023	0.0329123	yes
gene:SpnNT_01733	nanA_2	Chromosome:1741208-1742126	110.58+peptide	ΔORF2+peptide	OK	70.0052	45.2554	-0.629372	-1.26965	0.0274	0.209891	no
gene:SpnNT_01734	NA	Chromosome:1742294-1742939	110.58	ΔORF2	OK	11.3009	11.2533	-0.00608268	-0.00962097	0.9911	0.998249	no
gene:SpnNT_01734	NA	Chromosome:1742294-1742939	110.58	110.58+peptide	OK	11.3009	37.4874	1.72997	2.75727	5.00E-05	0.0013612	yes
gene:SpnNT_01734	NA	Chromosome:1742294-1742939	ΔORF2	110.58+peptide	OK	11.2533	37.4874	1.73605	2.75784	5.00E-05	0.0013612	yes
gene:SpnNT_01734	NA	Chromosome:1742294-1742939	110.58	ΔORF2+peptide	OK	11.3009	19.1942	0.764233	1.25501	0.03355	0.24057	no
gene:SpnNT_01734	NA	Chromosome:1742294-1742939	ΔORF2	ΔORF2+peptide	OK	11.2533	19.1942	0.770316	1.26057	0.03135	0.229306	no
gene:SpnNT_01734	NA	Chromosome:1742294-1742939	110.58+peptide	ΔORF2+peptide	OK	37.4874	19.1942	-0.965736	-1.59334	0.0068	0.0755543	no
gene:SpnNT_01735	NA	Chromosome:1743225-1743570	110.58	ΔORF2	OK	20.4733	16.2322	-0.334885	-0.476702	0.4071	0.896303	no
gene:SpnNT_01735	NA	Chromosome:1743225-1743570	110.58	110.58+peptide	OK	20.4733	70.6763	1.78748	2.54165	5.00E-05	0.0013612	yes
gene:SpnNT_01735	NA	Chromosome:1743225-1743570	ΔORF2	110.58+peptide	OK	16.2322	70.6763	2.12236	2.9225	5.00E-05	0.0013612	yes
gene:SpnNT_01735	NA	Chromosome:1743225-1743570	110.58	ΔORF2+peptide	OK	20.4733	23.9667	0.227286	0.333676	0.55965	0.968621	no
gene:SpnNT_01735	NA	Chromosome:1743225-1743570	ΔORF2	ΔORF2+peptide	OK	16.2322	23.9667	0.562171	0.797611	0.16115	0.614534	no
gene:SpnNT_01735	NA	Chromosome:1743225-1743570	110.58+peptide	ΔORF2+peptide	OK	70.6763	23.9667	-1.56019	-2.2112	2.00E-04	0.00450928	yes
gene:SpnNT_01736	araQ_2	Chromosome:1743629-1744469	110.58	ΔORF2	OK	17.266	14.4405	-0.257812	-0.4484	0.4367	0.914104	no
gene:SpnNT_01736	araQ_2	Chromosome:1743629-1744469	110.58	110.58+peptide	OK	17.266	44.7436	1.37375	2.46417	5.00E-05	0.0013612	yes
gene:SpnNT_01736	araQ_2	Chromosome:1743629-1744469	ΔORF2	110.58+peptide	OK	14.4405	44.7436	1.63156	2.8624	5.00E-05	0.0013612	yes
gene:SpnNT_01736	araQ_2	Chromosome:1743629-1744469	110.58	ΔORF2+peptide	OK	17.266	25.4204	0.558051	1.01434	0.0773	0.411995	no
gene:SpnNT_01736	araQ_2	Chromosome:1743629-1744469	ΔORF2	ΔORF2+peptide	OK	14.4405	25.4204	0.815863	1.44956	0.01265	0.119248	no
gene:SpnNT_01736	araQ_2	Chromosome:1743629-1744469	110.58+peptide	ΔORF2+peptide	OK	44.7436	25.4204	-0.815696	-1.49676	0.00935	0.0961526	no
gene:SpnNT_01737	lacF_2	Chromosome:1744484-1745372	110.58	ΔORF2	OK	16.4568	14.7038	-0.162494	-0.286793	0.61895	0.980887	no
gene:SpnNT_01737	lacF_2	Chromosome:1744484-1745372	110.58	110.58+peptide	OK	16.4568	44.2138	1.42581	2.5639	5.00E-05	0.0013612	yes
gene:SpnNT_01737	lacF_2	Chromosome:1744484-1745372	ΔORF2	110.58+peptide	OK	14.7038	44.2138	1.5883	2.8403	5.00E-05	0.0013612	yes
gene:SpnNT_01737	lacF_2	Chromosome:1744484-1745372	110.58	ΔORF2+peptide	OK	16.4568	25.9579	0.657488	1.19425	0.03745	0.259324	no
gene:SpnNT_01737	lacF_2	Chromosome:1744484-1745372	ΔORF2	ΔORF2+peptide	OK	14.7038	25.9579	0.819982	1.481	0.00995	0.0998195	no
gene:SpnNT_01737	lacF_2	Chromosome:1744484-1745372	110.58+peptide	ΔORF2+peptide	OK	44.2138	25.9579	-0.76832	-1.41512	0.01415	0.128942	no
gene:SpnNT_01738	yesO_1	Chromosome:1745602-1746931	110.58	ΔORF2	OK	52.5236	44.4044	-0.242261	-0.524846	0.366	0.864634	no
gene:SpnNT_01738	yesO_1	Chromosome:1745602-1746931	110.58	110.58+peptide	OK	52.5236	334.428	2.67066	4.69466	5.00E-05	0.0013612	yes
gene:SpnNT_01738	yesO_1	Chromosome:1745602-1746931	ΔORF2	110.58+peptide	OK	44.4044	334.428	2.91292	5.08098	5.00E-05	0.0013612	yes
gene:SpnNT_01738	yesO_1	Chromosome:1745602-1746931	110.58	ΔORF2+peptide	OK	52.5236	99.6813	0.924358	2.05776	0.00055	0.0103545	yes
gene:SpnNT_01738	yesO_1	Chromosome:1745602-1746931	ΔORF2	ΔORF2+peptide	OK	44.4044	99.6813	1.16662	2.56513	5.00E-05	0.0013612	yes
gene:SpnNT_01738	yesO_1	Chromosome:1745602-1746931	110.58+peptide	ΔORF2+peptide	OK	334.428	99.6813	-1.7463	-3.09969	5.00E-05	0.0013612	yes
gene:SpnNT_01739	ptsG_2	Chromosome:1747055-1747977	110.58	ΔORF2	OK	24.3651	21.9032	-0.153672	-0.176974	0.7629	0.994748	no
gene:SpnNT_01739	ptsG_2	Chromosome:1747055-1747977	110.58	110.58+peptide	OK	24.3651	54.2219	1.15406	1.19858	0.0345	0.245504	no
gene:SpnNT_01739	ptsG_2	Chromosome:1747055-1747977	ΔORF2	110.58+peptide	OK	21.9032	54.2219	1.30773	1.35487	0.0169	0.146377	no
gene:SpnNT_01739	ptsG_2	Chromosome:1747055-1747977	110.58	ΔORF2+peptide	OK	24.3651	47.9587	0.976978	1.17718	0.04445	0.289753	no
gene:SpnNT_01739	ptsG_2	Chromosome:1747055-1747977	ΔORF2	ΔORF2+peptide	OK	21.9032	47.9587	1.13065	1.35789	0.02115	0.174547	no
gene:SpnNT_01739	ptsG_2	Chromosome:1747055-1747977	110.58+peptide	ΔORF2+peptide	OK	54.2219	47.9587	-0.177084	-0.190247	0.7319	0.994748	no
gene:SpnNT_01740	malX_1	Chromosome:1747055-1747977	110.58	ΔORF2	OK	37.3784	30.9712	-0.271276	-0.397248	0.49515	0.942739	no
gene:SpnNT_01740	malX_1	Chromosome:1747055-1747977	110.58	110.58+peptide	OK	37.3784	104.784	1.48714	2.1965	1.00E-04	0.0025332	yes
gene:SpnNT_01740	malX_1	Chromosome:1747055-1747977	ΔORF2	110.58+peptide	OK	30.9712	104.784	1.75842	2.55491	5.00E-05	0.0013612	yes
gene:SpnNT_01740	malX_1	Chromosome:1747055-1747977	110.58	ΔORF2+peptide	OK	37.3784	60.9233	0.704789	1.04915	0.0708	0.390464	no
gene:SpnNT_01740	malX_1	Chromosome:1747055-1747977	ΔORF2	ΔORF2+peptide	OK	30.9712	60.9233	0.976065	1.42896	0.01475	0.132477	no

gene:SpnNT_01740	malX_1	Chromosome:1747055-1747977	110.58+peptide	ΔORF2+peptide	OK	104.784	60.9233	-0.78235	-1.15524	0.04295	0.284067	no
gene:SpnNT_01741	nanE	Chromosome:1747995-1748694	110.58	ΔORF2	OK	46.5412	38.7912	-0.26278	-0.507543	0.3728	0.869822	no
gene:SpnNT_01741	nanE	Chromosome:1747995-1748694	110.58	110.58+peptide	OK	46.5412	129.382	1.47506	2.7862	5.00E-05	0.0013612	yes
gene:SpnNT_01741	nanE	Chromosome:1747995-1748694	ΔORF2	110.58+peptide	OK	38.7912	129.382	1.73784	3.24917	5.00E-05	0.0013612	yes
gene:SpnNT_01741	nanE	Chromosome:1747995-1748694	110.58	ΔORF2+peptide	OK	46.5412	80.4118	0.788898	1.58241	0.00665	0.0743916	no
gene:SpnNT_01741	nanE	Chromosome:1747995-1748694	ΔORF2	ΔORF2+peptide	OK	38.7912	80.4118	1.05168	2.08536	0.00015	0.00355289	yes
gene:SpnNT_01741	nanE	Chromosome:1747995-1748694	110.58+peptide	ΔORF2+peptide	OK	129.382	80.4118	-0.68616	-1.32904	0.0211	0.174244	no
gene:SpnNT_01742	afr	Chromosome:1748866-1749970	110.58	ΔORF2	OK	13.1615	11.8236	-0.154652	-0.273878	0.63215	0.980887	no
gene:SpnNT_01742	afr	Chromosome:1748866-1749970	110.58	110.58+peptide	OK	13.1615	267.059	4.34277	6.67757	5.00E-05	0.0013612	yes
gene:SpnNT_01742	afr	Chromosome:1748866-1749970	ΔORF2	110.58+peptide	OK	11.8236	267.059	4.49742	6.90785	5.00E-05	0.0013612	yes
gene:SpnNT_01742	afr	Chromosome:1748866-1749970	110.58	ΔORF2+peptide	OK	13.1615	31.6975	1.26805	2.374	1.00E-04	0.0025332	yes
gene:SpnNT_01742	afr	Chromosome:1748866-1749970	ΔORF2	ΔORF2+peptide	OK	11.8236	31.6975	1.4227	2.65925	5.00E-05	0.0013612	yes
gene:SpnNT_01742	afr	Chromosome:1748866-1749970	110.58+peptide	ΔORF2+peptide	OK	267.059	31.6975	-3.07472	-4.92144	5.00E-05	0.0013612	yes
gene:SpnNT_01743	nanB	Chromosome:1749981-1752075	110.58	ΔORF2	OK	7.6902	7.08523	-0.118206	-0.212273	0.7155	0.992053	no
gene:SpnNT_01743	nanB	Chromosome:1749981-1752075	110.58	110.58+peptide	OK	7.6902	92.0018	3.58057	5.63211	5.00E-05	0.0013612	yes
gene:SpnNT_01743	nanB	Chromosome:1749981-1752075	ΔORF2	110.58+peptide	OK	7.08523	92.0018	3.69878	5.8095	5.00E-05	0.0013612	yes
gene:SpnNT_01743	nanB	Chromosome:1749981-1752075	110.58	ΔORF2+peptide	OK	7.6902	17.5018	1.18641	2.25284	1.00E-04	0.0025332	yes
gene:SpnNT_01743	nanB	Chromosome:1749981-1752075	ΔORF2	ΔORF2+peptide	OK	7.08523	17.5018	1.30462	2.47199	5.00E-05	0.0013612	yes
gene:SpnNT_01743	nanB	Chromosome:1749981-1752075	110.58+peptide	ΔORF2+peptide	OK	92.0018	17.5018	-2.39416	-3.92217	5.00E-05	0.0013612	yes
gene:SpnNT_01744	ycjP	Chromosome:1752092-1753810	110.58	ΔORF2	OK	10.3001	7.60595	-0.437455	-0.486675	0.39345	0.888728	no
gene:SpnNT_01744	ycjP	Chromosome:1752092-1753810	110.58	110.58+peptide	OK	10.3001	77.8059	2.91723	3.02256	5.00E-05	0.0013612	yes
gene:SpnNT_01744	ycjP	Chromosome:1752092-1753810	ΔORF2	110.58+peptide	OK	7.60595	77.8059	3.35468	3.50547	5.00E-05	0.0013612	yes
gene:SpnNT_01744	ycjP	Chromosome:1752092-1753810	110.58	ΔORF2+peptide	OK	10.3001	15.7195	0.609901	0.692475	0.23025	0.723674	no
gene:SpnNT_01744	ycjP	Chromosome:1752092-1753810	ΔORF2	ΔORF2+peptide	OK	7.60595	15.7195	1.04736	1.20137	0.0379	0.261474	no
gene:SpnNT_01744	ycjP	Chromosome:1752092-1753810	110.58+peptide	ΔORF2+peptide	OK	77.8059	15.7195	-2.30733	-2.45461	5.00E-05	0.0013612	yes
gene:SpnNT_01745	ycjO	Chromosome:1752092-1753810	110.58	ΔORF2	OK	11.848	8.73711	-0.43942	-0.573681	0.32625	0.831527	no
gene:SpnNT_01745	ycjO	Chromosome:1752092-1753810	110.58	110.58+peptide	OK	11.848	89.9142	2.9239	3.45167	5.00E-05	0.0013612	yes
gene:SpnNT_01745	ycjO	Chromosome:1752092-1753810	ΔORF2	110.58+peptide	OK	8.73711	89.9142	3.36332	3.99952	5.00E-05	0.0013612	yes
gene:SpnNT_01745	ycjO	Chromosome:1752092-1753810	110.58	ΔORF2+peptide	OK	11.848	17.7058	0.579572	0.759326	0.19665	0.677815	no
gene:SpnNT_01745	ycjO	Chromosome:1752092-1753810	ΔORF2	ΔORF2+peptide	OK	8.73711	17.7058	1.01899	1.34712	0.02335	0.187285	no
gene:SpnNT_01745	ycjO	Chromosome:1752092-1753810	110.58+peptide	ΔORF2+peptide	OK	89.9142	17.7058	-2.34433	-2.79594	5.00E-05	0.0013612	yes
gene:SpnNT_01746	yesO_2	Chromosome:1753888-1755226	110.58	ΔORF2	OK	14.8111	13.3027	-0.154953	-0.282047	0.62265	0.980887	no
gene:SpnNT_01746	yesO_2	Chromosome:1753888-1755226	110.58	110.58+peptide	OK	14.8111	150.936	3.3492	5.26966	5.00E-05	0.0013612	yes
gene:SpnNT_01746	yesO_2	Chromosome:1753888-1755226	ΔORF2	110.58+peptide	OK	13.3027	150.936	3.50415	5.4638	5.00E-05	0.0013612	yes
gene:SpnNT_01746	yesO_2	Chromosome:1753888-1755226	110.58	ΔORF2+peptide	OK	14.8111	20.5624	0.473334	0.896085	0.11255	0.510502	no
gene:SpnNT_01746	yesO_2	Chromosome:1753888-1755226	ΔORF2	ΔORF2+peptide	OK	13.3027	20.5624	0.628287	1.17401	0.04095	0.275139	no
gene:SpnNT_01746	yesO_2	Chromosome:1753888-1755226	110.58+peptide	ΔORF2+peptide	OK	150.936	20.5624	-2.87586	-4.61388	5.00E-05	0.0013612	yes
gene:SpnNT_01747	tabA	Chromosome:1755244-1755697	110.58	ΔORF2	OK	13.3745	12.3127	-0.119343	-0.168323	0.77425	0.994748	no
gene:SpnNT_01747	tabA	Chromosome:1755244-1755697	110.58	110.58+peptide	OK	13.3745	84.2924	2.65592	3.71246	5.00E-05	0.0013612	yes
gene:SpnNT_01747	tabA	Chromosome:1755244-1755697	ΔORF2	110.58+peptide	OK	12.3127	84.2924	2.77526	3.72638	5.00E-05	0.0013612	yes
gene:SpnNT_01747	tabA	Chromosome:1755244-1755697	110.58	ΔORF2+peptide	OK	13.3745	20.0661	0.585277	0.887001	0.1209	0.526535	no
gene:SpnNT_01747	tabA	Chromosome:1755244-1755697	ΔORF2	ΔORF2+peptide	OK	12.3127	20.0661	0.70462	1.01889	0.0733	0.399046	no
gene:SpnNT_01747	tabA	Chromosome:1755244-1755697	110.58+peptide	ΔORF2+peptide	OK	84.2924	20.0661	-2.07064	-2.96606	5.00E-05	0.0013612	yes
gene:SpnNT_01748	NA	Chromosome:1755835-1755931	110.58	ΔORF2	NOTEST	12.2753	10.3577	-0.245047	0	1	1	no
gene:SpnNT_01748	NA	Chromosome:1755835-1755931	110.58	110.58+peptide	NOTEST	12.2753	0	#NAME?	0	1	1	no

gene:SpnNT_01748	NA	Chromosome:1755835-1755931	ΔORF2	110.58+peptide	NOTEST	10.3577	0	#NAME?	0	1	1	no
gene:SpnNT_01748	NA	Chromosome:1755835-1755931	110.58	ΔORF2+peptide	NOTEST	12.2753	0	#NAME?	0	1	1	no
gene:SpnNT_01748	NA	Chromosome:1755835-1755931	ΔORF2	ΔORF2+peptide	NOTEST	10.3577	0	#NAME?	0	1	1	no
gene:SpnNT_01748	NA	Chromosome:1755835-1755931	110.58+peptide	ΔORF2+peptide	NOTEST	0	0	0	0	1	1	no
gene:SpnNT_01749	NA	Chromosome:1756726-1757542	110.58	ΔORF2	OK	189.286	179.558	-0.0761201	-0.17322	0.7624	0.994748	no
gene:SpnNT_01749	NA	Chromosome:1756726-1757542	110.58	110.58+peptide	OK	189.286	207.59	0.133168	0.298025	0.59905	0.97629	no
gene:SpnNT_01749	NA	Chromosome:1756726-1757542	ΔORF2	110.58+peptide	OK	179.558	207.59	0.209288	0.465007	0.41305	0.899511	no
gene:SpnNT_01749	NA	Chromosome:1756726-1757542	110.58	ΔORF2+peptide	OK	189.286	152.889	-0.308082	-0.69723	0.2158	0.702836	no
gene:SpnNT_01749	NA	Chromosome:1756726-1757542	ΔORF2	ΔORF2+peptide	OK	179.558	152.889	-0.231962	-0.521097	0.3584	0.859746	no
gene:SpnNT_01749	NA	Chromosome:1756726-1757542	110.58+peptide	ΔORF2+peptide	OK	207.59	152.889	-0.44125	-0.975263	0.0849	0.438605	no
gene:SpnNT_01750	lytA_8	Chromosome:1757914-1761481	110.58	ΔORF2	OK	51.2615	52.4537	0.0331691	0.0760872	0.89475	0.994748	no
gene:SpnNT_01750	lytA_8	Chromosome:1757914-1761481	110.58	110.58+peptide	OK	51.2615	42.1628	-0.281908	-0.641451	0.2595	0.759841	no
gene:SpnNT_01750	lytA_8	Chromosome:1757914-1761481	ΔORF2	110.58+peptide	OK	52.4537	42.1628	-0.315077	-0.718185	0.2051	0.69003	no
gene:SpnNT_01750	lytA_8	Chromosome:1757914-1761481	110.58	ΔORF2+peptide	OK	51.2615	41.3465	-0.310112	-0.707255	0.22135	0.710681	no
gene:SpnNT_01750	lytA_8	Chromosome:1757914-1761481	ΔORF2	ΔORF2+peptide	OK	52.4537	41.3465	-0.343281	-0.784285	0.1728	0.632681	no
gene:SpnNT_01750	lytA_8	Chromosome:1757914-1761481	110.58+peptide	ΔORF2+peptide	OK	42.1628	41.3465	-0.0282044	-0.0639215	0.91345	0.994748	no
gene:SpnNT_01751	nanA_3	Chromosome:1762152-1763166	110.58	ΔORF2	OK	21.0527	17.238	-0.288415	-0.533571	0.35265	0.855356	no
gene:SpnNT_01751	nanA_3	Chromosome:1762152-1763166	110.58	110.58+peptide	OK	21.0527	113.375	2.42902	4.08935	5.00E-05	0.0013612	yes
gene:SpnNT_01751	nanA_3	Chromosome:1762152-1763166	ΔORF2	110.58+peptide	OK	17.238	113.375	2.71743	4.53037	5.00E-05	0.0013612	yes
gene:SpnNT_01751	nanA_3	Chromosome:1762152-1763166	110.58	ΔORF2+peptide	OK	21.0527	25.7724	0.291821	0.546119	0.33945	0.844054	no
gene:SpnNT_01751	nanA_3	Chromosome:1762152-1763166	ΔORF2	ΔORF2+peptide	OK	17.238	25.7724	0.580235	1.07285	0.0615	0.357873	no
gene:SpnNT_01751	nanA_3	Chromosome:1762152-1763166	110.58+peptide	ΔORF2+peptide	OK	113.375	25.7724	-2.1372	-3.59641	5.00E-05	0.0013612	yes
gene:SpnNT_01752	nanA_4	Chromosome:1763233-1765036	110.58	ΔORF2	OK	18.6605	14.6971	-0.34446	-0.681086	0.2343	0.728033	no
gene:SpnNT_01752	nanA_4	Chromosome:1763233-1765036	110.58	110.58+peptide	OK	18.6605	66.5538	1.83453	3.39752	5.00E-05	0.0013612	yes
gene:SpnNT_01752	nanA_4	Chromosome:1763233-1765036	ΔORF2	110.58+peptide	OK	14.6971	66.5538	2.17899	3.98893	5.00E-05	0.0013612	yes
gene:SpnNT_01752	nanA_4	Chromosome:1763233-1765036	110.58	ΔORF2+peptide	OK	18.6605	27.485	0.558654	1.13678	0.04585	0.295648	no
gene:SpnNT_01752	nanA_4	Chromosome:1763233-1765036	ΔORF2	ΔORF2+peptide	OK	14.6971	27.485	0.903114	1.81222	0.0024	0.0337543	yes
gene:SpnNT_01752	nanA_4	Chromosome:1763233-1765036	110.58+peptide	ΔORF2+peptide	OK	66.5538	27.485	-1.27588	-2.39362	1.00E-04	0.0025332	yes
gene:SpnNT_01753	NA	Chromosome:1765151-1765379	110.58	ΔORF2	OK	3.09982	4.25841	0.458129	0.274453	0.6201	0.980887	no
gene:SpnNT_01753	NA	Chromosome:1765151-1765379	110.58	110.58+peptide	OK	3.09982	14.4083	2.21664	1.51497	0.0274	0.209891	no
gene:SpnNT_01753	NA	Chromosome:1765151-1765379	ΔORF2	110.58+peptide	OK	4.25841	14.4083	1.75851	1.30014	0.0338	0.241834	no
gene:SpnNT_01753	NA	Chromosome:1765151-1765379	110.58	ΔORF2+peptide	OK	3.09982	11.0733	1.83683	1.2597	0.05	0.313773	no
gene:SpnNT_01753	NA	Chromosome:1765151-1765379	ΔORF2	ΔORF2+peptide	OK	4.25841	11.0733	1.3787	1.02343	0.0856	0.439454	no
gene:SpnNT_01753	NA	Chromosome:1765151-1765379	110.58+peptide	ΔORF2+peptide	OK	14.4083	11.0733	-0.379807	-0.351246	0.54455	0.961568	no
gene:SpnNT_01754	axeA	Chromosome:1766231-1767188	110.58	ΔORF2	OK	66.4541	61.2679	-0.117228	-0.249441	0.6561	0.982966	no
gene:SpnNT_01754	axeA	Chromosome:1766231-1767188	110.58	110.58+peptide	OK	66.4541	57.0677	-0.219686	-0.46089	0.41735	0.902665	no
gene:SpnNT_01754	axeA	Chromosome:1766231-1767188	ΔORF2	110.58+peptide	OK	61.2679	57.0677	-0.102458	-0.213685	0.7076	0.990441	no
gene:SpnNT_01754	axeA	Chromosome:1766231-1767188	110.58	ΔORF2+peptide	OK	66.4541	40.0215	-0.731584	-1.51636	0.0097	0.0982125	no
gene:SpnNT_01754	axeA	Chromosome:1766231-1767188	ΔORF2	ΔORF2+peptide	OK	61.2679	40.0215	-0.614356	-1.26605	0.02825	0.214152	no
gene:SpnNT_01754	axeA	Chromosome:1766231-1767188	110.58+peptide	ΔORF2+peptide	OK	57.0677	40.0215	-0.511898	-1.041	0.0731	0.398512	no
gene:SpnNT_01755	recG	Chromosome:1767508-1769524	110.58	ΔORF2	OK	144.29	130.943	-0.140031	-0.318827	0.5649	0.968621	no
gene:SpnNT_01755	recG	Chromosome:1767508-1769524	110.58	110.58+peptide	OK	144.29	110.377	-0.386524	-0.882967	0.1161	0.516779	no
gene:SpnNT_01755	recG	Chromosome:1767508-1769524	ΔORF2	110.58+peptide	OK	130.943	110.377	-0.246493	-0.558763	0.3235	0.829061	no
gene:SpnNT_01755	recG	Chromosome:1767508-1769524	110.58	ΔORF2+peptide	OK	144.29	102.583	-0.492182	-1.13316	0.0423	0.281043	no
gene:SpnNT_01755	recG	Chromosome:1767508-1769524	ΔORF2	ΔORF2+peptide	OK	130.943	102.583	-0.352151	-0.804449	0.1568	0.603558	no

gene:SpnNT_01755	recG	Chromosome:1767508-1769524	110.58+peptide	ΔORF2+peptide	OK	110.377	102.583	-0.105658	-0.242169	0.6694	0.984845	no
gene:SpnNT_01756	alr	Chromosome:1769542-1771004	110.58	ΔORF2	OK	154.747	140.104	-0.143415	-0.304545	0.58855	0.974014	no
gene:SpnNT_01756	alr	Chromosome:1769542-1771004	110.58	110.58+peptide	OK	154.747	121.509	-0.348857	-0.74332	0.19495	0.676386	no
gene:SpnNT_01756	alr	Chromosome:1769542-1771004	ΔORF2	110.58+peptide	OK	140.104	121.509	-0.205441	-0.430012	0.44805	0.919648	no
gene:SpnNT_01756	alr	Chromosome:1769542-1771004	110.58	ΔORF2+peptide	OK	154.747	96.4993	-0.681325	-1.45702	0.011	0.107477	no
gene:SpnNT_01756	alr	Chromosome:1769542-1771004	ΔORF2	ΔORF2+peptide	OK	140.104	96.4993	-0.53791	-1.12987	0.0482	0.306138	no
gene:SpnNT_01756	alr	Chromosome:1769542-1771004	110.58+peptide	ΔORF2+peptide	OK	121.509	96.4993	-0.332469	-0.700667	0.2195	0.707834	no
gene:SpnNT_01757	acpS	Chromosome:1769542-1771004	110.58	ΔORF2	OK	107.143	87.4952	-0.292265	-0.192558	0.73275	0.994748	no
gene:SpnNT_01757	acpS	Chromosome:1769542-1771004	110.58	110.58+peptide	OK	107.143	82.477	-0.377476	-0.275949	0.61715	0.980654	no
gene:SpnNT_01757	acpS	Chromosome:1769542-1771004	ΔORF2	110.58+peptide	OK	87.4952	82.477	-0.0852109	-0.0578647	0.9136	0.994748	no
gene:SpnNT_01757	acpS	Chromosome:1769542-1771004	110.58	ΔORF2+peptide	OK	107.143	76.944	-0.477659	-0.353432	0.53075	0.957488	no
gene:SpnNT_01757	acpS	Chromosome:1769542-1771004	ΔORF2	ΔORF2+peptide	OK	87.4952	76.944	-0.185394	-0.127214	0.81495	0.994748	no
gene:SpnNT_01757	acpS	Chromosome:1769542-1771004	110.58+peptide	ΔORF2+peptide	OK	82.477	76.944	-0.100183	-0.0770341	0.88605	0.994748	no
gene:SpnNT_01758	aroF_1	Chromosome:1771042-1772074	110.58	ΔORF2	OK	287.971	310.488	0.108613	0.250256	0.654	0.982798	no
gene:SpnNT_01758	aroF_1	Chromosome:1771042-1772074	110.58	110.58+peptide	OK	287.971	167.96	-0.777812	-1.7888	0.00195	0.0288477	yes
gene:SpnNT_01758	aroF_1	Chromosome:1771042-1772074	ΔORF2	110.58+peptide	OK	310.488	167.96	-0.886425	-2.03623	3.00E-04	0.00631878	yes
gene:SpnNT_01758	aroF_1	Chromosome:1771042-1772074	110.58	ΔORF2+peptide	OK	287.971	164.405	-0.808674	-1.86065	0.0013	0.020854	yes
gene:SpnNT_01758	aroF_1	Chromosome:1771042-1772074	ΔORF2	ΔORF2+peptide	OK	310.488	164.405	-0.917287	-2.10811	0.00015	0.00355289	yes
gene:SpnNT_01758	aroF_1	Chromosome:1771042-1772074	110.58+peptide	ΔORF2+peptide	OK	167.96	164.405	-0.0308624	-0.0707956	0.8958	0.994748	no
gene:SpnNT_01759	aroF_2	Chromosome:1772075-1773107	110.58	ΔORF2	OK	149.59	150.559	0.00930712	0.0212026	0.97135	0.99536	no
gene:SpnNT_01759	aroF_2	Chromosome:1772075-1773107	110.58	110.58+peptide	OK	149.59	94.9315	-0.656059	-1.48387	0.0091	0.0941721	no
gene:SpnNT_01759	aroF_2	Chromosome:1772075-1773107	ΔORF2	110.58+peptide	OK	150.559	94.9315	-0.665366	-1.49655	0.0087	0.0910378	no
gene:SpnNT_01759	aroF_2	Chromosome:1772075-1773107	110.58	ΔORF2+peptide	OK	149.59	90.4627	-0.725623	-1.62833	0.006	0.0690632	no
gene:SpnNT_01759	aroF_2	Chromosome:1772075-1773107	ΔORF2	ΔORF2+peptide	OK	150.559	90.4627	-0.73493	-1.64019	0.00525	0.0625141	no
gene:SpnNT_01759	aroF_2	Chromosome:1772075-1773107	110.58+peptide	ΔORF2+peptide	OK	94.9315	90.4627	-0.0695636	-0.154182	0.78975	0.994748	no
gene:SpnNT_01760	NA	Chromosome:1773187-1775701	110.58	ΔORF2	OK	439.745	430.036	-0.0322114	-0.0692479	0.9038	0.994748	no
gene:SpnNT_01760	NA	Chromosome:1773187-1775701	110.58	110.58+peptide	OK	439.745	323.65	-0.442235	-0.965484	0.09105	0.453591	no
gene:SpnNT_01760	NA	Chromosome:1773187-1775701	ΔORF2	110.58+peptide	OK	430.036	323.65	-0.410023	-0.894632	0.11715	0.518813	no
gene:SpnNT_01760	NA	Chromosome:1773187-1775701	110.58	ΔORF2+peptide	OK	439.745	287.385	-0.613685	-1.3427	0.01885	0.159272	no
gene:SpnNT_01760	NA	Chromosome:1773187-1775701	ΔORF2	ΔORF2+peptide	OK	430.036	287.385	-0.581474	-1.27147	0.0243	0.193603	no
gene:SpnNT_01760	NA	Chromosome:1773187-1775701	110.58+peptide	ΔORF2+peptide	OK	323.65	287.385	-0.171451	-0.38093	0.5023	0.944788	no
gene:SpnNT_01761	NA	Chromosome:1775846-1776134	110.58	ΔORF2	OK	37.8374	31.068	-0.284384	-0.412435	0.47705	0.932178	no
gene:SpnNT_01761	NA	Chromosome:1775846-1776134	110.58	110.58+peptide	OK	37.8374	123.277	1.70402	2.45386	5.00E-05	0.0013612	yes
gene:SpnNT_01761	NA	Chromosome:1775846-1776134	ΔORF2	110.58+peptide	OK	31.068	123.277	1.9884	2.78126	5.00E-05	0.0013612	yes
gene:SpnNT_01761	NA	Chromosome:1775846-1776134	110.58	ΔORF2+peptide	OK	37.8374	57.5737	0.605597	0.944815	0.11165	0.508705	no
gene:SpnNT_01761	NA	Chromosome:1775846-1776134	ΔORF2	ΔORF2+peptide	OK	31.068	57.5737	0.88998	1.34209	0.0247	0.195721	no
gene:SpnNT_01761	NA	Chromosome:1775846-1776134	110.58+peptide	ΔORF2+peptide	OK	123.277	57.5737	-1.09842	-1.64379	0.00445	0.054829	no
gene:SpnNT_01762	ytrB	Chromosome:1776144-1776795	110.58	ΔORF2	OK	18.7608	19.72	0.0719365	0.121221	0.836	0.994748	no
gene:SpnNT_01762	ytrB	Chromosome:1776144-1776795	110.58	110.58+peptide	OK	18.7608	44.0443	1.23123	2.11285	0.00025	0.00542231	yes
gene:SpnNT_01762	ytrB	Chromosome:1776144-1776795	ΔORF2	110.58+peptide	OK	19.72	44.0443	1.1593	1.96472	0.00085	0.0148124	yes
gene:SpnNT_01762	ytrB	Chromosome:1776144-1776795	110.58	ΔORF2+peptide	OK	18.7608	29.7076	0.66311	1.15987	0.04785	0.304652	no
gene:SpnNT_01762	ytrB	Chromosome:1776144-1776795	ΔORF2	ΔORF2+peptide	OK	19.72	29.7076	0.591173	1.02072	0.0851	0.43912	no
gene:SpnNT_01762	ytrB	Chromosome:1776144-1776795	110.58+peptide	ΔORF2+peptide	OK	44.0443	29.7076	-0.568123	-0.999857	0.08175	0.42738	no
gene:SpnNT_01763	NA	Chromosome:1776798-1778224	110.58	ΔORF2	OK	7.9083	8.84343	0.161238	0.159015	0.78835	0.994748	no
gene:SpnNT_01763	NA	Chromosome:1776798-1778224	110.58	110.58+peptide	OK	7.9083	15.789	0.997478	1.01255	0.0938	0.462897	no

gene:SpnNT_01763	NA	Chromosome:1776798-1778224	ΔORF2	110.58+peptide	OK	8.84343	15.789	0.83624	0.862749	0.14445	0.579833	no
gene:SpnNT_01763	NA	Chromosome:1776798-1778224	110.58	ΔORF2+peptide	OK	7.9083	13.7903	0.802216	0.823161	0.1736	0.634017	no
gene:SpnNT_01763	NA	Chromosome:1776798-1778224	ΔORF2	ΔORF2+peptide	OK	8.84343	13.7903	0.640978	0.668702	0.25505	0.754219	no
gene:SpnNT_01763	NA	Chromosome:1776798-1778224	110.58+peptide	ΔORF2+peptide	OK	15.789	13.7903	-0.195262	-0.210422	0.7196	0.99385	no
gene:SpnNT_01764	NA	Chromosome:1776798-1778224	110.58	ΔORF2	OK	10.4749	12.7925	0.288354	0.381505	0.5087	0.947472	no
gene:SpnNT_01764	NA	Chromosome:1776798-1778224	110.58	110.58+peptide	OK	10.4749	20.9037	0.996816	1.33614	0.0215	0.177102	no
gene:SpnNT_01764	NA	Chromosome:1776798-1778224	ΔORF2	110.58+peptide	OK	12.7925	20.9037	0.708463	0.979556	0.0869	0.443008	no
gene:SpnNT_01764	NA	Chromosome:1776798-1778224	110.58	ΔORF2+peptide	OK	10.4749	17.2582	0.720342	0.980092	0.0855	0.439454	no
gene:SpnNT_01764	NA	Chromosome:1776798-1778224	ΔORF2	ΔORF2+peptide	OK	12.7925	17.2582	0.431989	0.606875	0.28455	0.787874	no
gene:SpnNT_01764	NA	Chromosome:1776798-1778224	110.58+peptide	ΔORF2+peptide	OK	20.9037	17.2582	-0.276474	-0.394163	0.4891	0.939082	no
gene:SpnNT_01765	NA	Chromosome:1778591-1778744	110.58	ΔORF2	OK	2.29829	5.39703	1.2316	2.06385	0.45525	0.921244	no
gene:SpnNT_01765	NA	Chromosome:1778591-1778744	110.58	110.58+peptide	OK	2.29829	12.1215	2.39894	4.33759	0.1847	0.657346	no
gene:SpnNT_01765	NA	Chromosome:1778591-1778744	ΔORF2	110.58+peptide	OK	5.39703	12.1215	1.16733	2.56072	0.35045	0.854722	no
gene:SpnNT_01765	NA	Chromosome:1778591-1778744	110.58	ΔORF2+peptide	NOTEST	2.29829	1.27028	-0.855412	0	1	1	no
gene:SpnNT_01765	NA	Chromosome:1778591-1778744	ΔORF2	ΔORF2+peptide	OK	5.39703	1.27028	-2.08702	-2.2356	0.31695	0.822753	no
gene:SpnNT_01765	NA	Chromosome:1778591-1778744	110.58+peptide	ΔORF2+peptide	OK	12.1215	1.27028	-3.25435	-3.59109	0.1087	0.501534	no
gene:SpnNT_01766	NA	Chromosome:1778883-1779228	110.58	ΔORF2	OK	16.9922	23.1605	0.446791	0.634328	0.27685	0.780052	no
gene:SpnNT_01766	NA	Chromosome:1778883-1779228	110.58	110.58+peptide	OK	16.9922	37.4459	1.13993	1.65868	0.0052	0.0621443	no
gene:SpnNT_01766	NA	Chromosome:1778883-1779228	ΔORF2	110.58+peptide	OK	23.1605	37.4459	0.693144	1.02371	0.0792	0.419057	no
gene:SpnNT_01766	NA	Chromosome:1778883-1779228	110.58	ΔORF2+peptide	OK	16.9922	40.8718	1.26623	1.87808	0.00185	0.0276489	yes
gene:SpnNT_01766	NA	Chromosome:1778883-1779228	ΔORF2	ΔORF2+peptide	OK	23.1605	40.8718	0.819442	1.23437	0.03745	0.259324	no
gene:SpnNT_01766	NA	Chromosome:1778883-1779228	110.58+peptide	ΔORF2+peptide	OK	37.4459	40.8718	0.126299	0.195605	0.7346	0.994748	no
gene:SpnNT_01767	der	Chromosome:1779309-1780620	110.58	ΔORF2	OK	184.805	175.203	-0.0769752	-0.175814	0.76015	0.994748	no
gene:SpnNT_01767	der	Chromosome:1779309-1780620	110.58	110.58+peptide	OK	184.805	176.164	-0.0690913	-0.15873	0.7754	0.994748	no
gene:SpnNT_01767	der	Chromosome:1779309-1780620	ΔORF2	110.58+peptide	OK	175.203	176.164	0.0078839	0.0180155	0.97545	0.99536	no
gene:SpnNT_01767	der	Chromosome:1779309-1780620	110.58	ΔORF2+peptide	OK	184.805	166.199	-0.153093	-0.351802	0.53265	0.958424	no
gene:SpnNT_01767	der	Chromosome:1779309-1780620	ΔORF2	ΔORF2+peptide	OK	175.203	166.199	-0.0761177	-0.173979	0.7593	0.994748	no
gene:SpnNT_01767	der	Chromosome:1779309-1780620	110.58+peptide	ΔORF2+peptide	OK	176.164	166.199	-0.0840016	-0.193123	0.73665	0.994748	no
gene:SpnNT_01768	nfrA2	Chromosome:1780633-1782240	110.58	ΔORF2	OK	132.093	121.559	-0.119895	-0.19417	0.73945	0.994748	no
gene:SpnNT_01768	nfrA2	Chromosome:1780633-1782240	110.58	110.58+peptide	OK	132.093	118.267	-0.159509	-0.26218	0.64935	0.981429	no
gene:SpnNT_01768	nfrA2	Chromosome:1780633-1782240	ΔORF2	110.58+peptide	OK	121.559	118.267	-0.0396145	-0.0638406	0.913	0.994748	no
gene:SpnNT_01768	nfrA2	Chromosome:1780633-1782240	110.58	ΔORF2+peptide	OK	132.093	117.13	-0.173439	-0.287041	0.6219	0.980887	no
gene:SpnNT_01768	nfrA2	Chromosome:1780633-1782240	ΔORF2	ΔORF2+peptide	OK	121.559	117.13	-0.0535439	-0.0868605	0.88235	0.994748	no
gene:SpnNT_01768	nfrA2	Chromosome:1780633-1782240	110.58+peptide	ΔORF2+peptide	OK	118.267	117.13	-0.0139294	-0.022935	0.96715	0.994855	no
gene:SpnNT_01769	dnaI	Chromosome:1780633-1782240	110.58	ΔORF2	OK	84.7301	88.7755	0.0672859	0.103792	0.8579	0.994748	no
gene:SpnNT_01769	dnaI	Chromosome:1780633-1782240	110.58	110.58+peptide	OK	84.7301	76.9506	-0.138943	-0.211378	0.7126	0.991034	no
gene:SpnNT_01769	dnaI	Chromosome:1780633-1782240	ΔORF2	110.58+peptide	OK	88.7755	76.9506	-0.206229	-0.320735	0.581	0.96988	no
gene:SpnNT_01769	dnaI	Chromosome:1780633-1782240	110.58	ΔORF2+peptide	OK	84.7301	74.8875	-0.17815	-0.273004	0.63255	0.980887	no
gene:SpnNT_01769	dnaI	Chromosome:1780633-1782240	ΔORF2	ΔORF2+peptide	OK	88.7755	74.8875	-0.245436	-0.384628	0.4998	0.944017	no
gene:SpnNT_01769	dnaI	Chromosome:1780633-1782240	110.58+peptide	ΔORF2+peptide	OK	76.9506	74.8875	-0.0392067	-0.0605698	0.9168	0.994748	no
gene:SpnNT_01770	NA	Chromosome:1782240-1783410	110.58	ΔORF2	OK	74.1245	77.079	0.0563859	0.125374	0.8246	0.994748	no
gene:SpnNT_01770	NA	Chromosome:1782240-1783410	110.58	110.58+peptide	OK	74.1245	65.6657	-0.174812	-0.387239	0.4934	0.941456	no
gene:SpnNT_01770	NA	Chromosome:1782240-1783410	ΔORF2	110.58+peptide	OK	77.079	65.6657	-0.231198	-0.512262	0.37235	0.869546	no
gene:SpnNT_01770	NA	Chromosome:1782240-1783410	110.58	ΔORF2+peptide	OK	74.1245	72.3051	-0.0358537	-0.0793523	0.8909	0.994748	no
gene:SpnNT_01770	NA	Chromosome:1782240-1783410	ΔORF2	ΔORF2+peptide	OK	77.079	72.3051	-0.0922397	-0.204194	0.71995	0.993917	no

gene:SpnNT_01770	NA	Chromosome:1782240-1783410	110.58+peptide	ΔORF2+peptide	OK	65.6657	72.3051	0.138958	0.306475	0.59145	0.975468	no
gene:SpnNT_01771	nrdR	Chromosome:1783410-1783884	110.58	ΔORF2	OK	101.527	103.817	0.0321797	0.0636806	0.9121	0.994748	no
gene:SpnNT_01771	nrdR	Chromosome:1783410-1783884	110.58	110.58+peptide	OK	101.527	80.276	-0.338818	-0.670823	0.24285	0.738169	no
gene:SpnNT_01771	nrdR	Chromosome:1783410-1783884	ΔORF2	110.58+peptide	OK	103.817	80.276	-0.370998	-0.724752	0.21	0.696723	no
gene:SpnNT_01771	nrdR	Chromosome:1783410-1783884	110.58	ΔORF2+peptide	OK	101.527	84.6479	-0.262314	-0.511989	0.3596	0.860914	no
gene:SpnNT_01771	nrdR	Chromosome:1783410-1783884	ΔORF2	ΔORF2+peptide	OK	103.817	84.6479	-0.294493	-0.567354	0.31515	0.821264	no
gene:SpnNT_01771	nrdR	Chromosome:1783410-1783884	110.58+peptide	ΔORF2+peptide	OK	80.276	84.6479	0.0765046	0.147459	0.79105	0.994748	no
gene:SpnNT_01772	ytrA	Chromosome:1784023-1784389	110.58	ΔORF2	OK	70.2607	67.7259	-0.0530111	-0.0936503	0.8651	0.994748	no
gene:SpnNT_01772	ytrA	Chromosome:1784023-1784389	110.58	110.58+peptide	OK	70.2607	67.0348	-0.0678086	-0.11771	0.8382	0.994748	no
gene:SpnNT_01772	ytrA	Chromosome:1784023-1784389	ΔORF2	110.58+peptide	OK	67.7259	67.0348	-0.0147975	-0.0254475	0.96405	0.994855	no
gene:SpnNT_01772	ytrA	Chromosome:1784023-1784389	110.58	ΔORF2+peptide	OK	70.2607	53.6246	-0.389823	-0.660932	0.25315	0.751355	no
gene:SpnNT_01772	ytrA	Chromosome:1784023-1784389	ΔORF2	ΔORF2+peptide	OK	67.7259	53.6246	-0.336811	-0.565969	0.3196	0.825861	no
gene:SpnNT_01772	ytrA	Chromosome:1784023-1784389	110.58+peptide	ΔORF2+peptide	OK	67.0348	53.6246	-0.322014	-0.532574	0.34805	0.852869	no
gene:SpnNT_01773	skfE	Chromosome:1784393-1785089	110.58	ΔORF2	OK	50.091	48.0117	-0.0611677	-0.119417	0.8378	0.994748	no
gene:SpnNT_01773	skfE	Chromosome:1784393-1785089	110.58	110.58+peptide	OK	50.091	47.3877	-0.0800396	-0.156211	0.78735	0.994748	no
gene:SpnNT_01773	skfE	Chromosome:1784393-1785089	ΔORF2	110.58+peptide	OK	48.0117	47.3877	-0.0188719	-0.0367835	0.9498	0.994855	no
gene:SpnNT_01773	skfE	Chromosome:1784393-1785089	110.58	ΔORF2+peptide	OK	50.091	40.4143	-0.309686	-0.599315	0.29115	0.795269	no
gene:SpnNT_01773	skfE	Chromosome:1784393-1785089	ΔORF2	ΔORF2+peptide	OK	48.0117	40.4143	-0.248518	-0.480323	0.4013	0.894186	no
gene:SpnNT_01773	skfE	Chromosome:1784393-1785089	110.58+peptide	ΔORF2+peptide	OK	47.3877	40.4143	-0.229646	-0.443709	0.43755	0.914985	no
gene:SpnNT_01774	NA	Chromosome:1785105-1785873	110.58	ΔORF2	OK	60.2606	64.2402	0.0922614	0.188427	0.7365	0.994748	no
gene:SpnNT_01774	NA	Chromosome:1785105-1785873	110.58	110.58+peptide	OK	60.2606	54.2292	-0.152147	-0.307485	0.5927	0.975468	no
gene:SpnNT_01774	NA	Chromosome:1785105-1785873	ΔORF2	110.58+peptide	OK	64.2402	54.2292	-0.244408	-0.498513	0.3871	0.881745	no
gene:SpnNT_01774	NA	Chromosome:1785105-1785873	110.58	ΔORF2+peptide	OK	60.2606	51.7021	-0.220993	-0.445527	0.433	0.913334	no
gene:SpnNT_01774	NA	Chromosome:1785105-1785873	ΔORF2	ΔORF2+peptide	OK	64.2402	51.7021	-0.313254	-0.637343	0.25995	0.760041	no
gene:SpnNT_01774	NA	Chromosome:1785105-1785873	110.58+peptide	ΔORF2+peptide	OK	54.2292	51.7021	-0.068846	-0.138621	0.80965	0.994748	no
gene:SpnNT_01775	NA	Chromosome:1786225-1788315	110.58	ΔORF2	OK	79.7883	79.8857	0.00175923	0.0029853	0.9954	0.999258	no
gene:SpnNT_01775	NA	Chromosome:1786225-1788315	110.58	110.58+peptide	OK	79.7883	50.8082	-0.651117	-1.10427	0.05375	0.328508	no
gene:SpnNT_01775	NA	Chromosome:1786225-1788315	ΔORF2	110.58+peptide	OK	79.8857	50.8082	-0.652876	-1.08925	0.0589	0.346586	no
gene:SpnNT_01775	NA	Chromosome:1786225-1788315	110.58	ΔORF2+peptide	OK	79.7883	47.5617	-0.746376	-1.26539	0.02645	0.204524	no
gene:SpnNT_01775	NA	Chromosome:1786225-1788315	ΔORF2	ΔORF2+peptide	OK	79.8857	47.5617	-0.748135	-1.24777	0.03	0.222281	no
gene:SpnNT_01775	NA	Chromosome:1786225-1788315	110.58+peptide	ΔORF2+peptide	OK	50.8082	47.5617	-0.0952587	-0.158788	0.7826	0.994748	no
gene:SpnNT_01776	ecsA_3	Chromosome:1786225-1788315	110.58	ΔORF2	OK	87.9737	93.9269	0.0944662	0.141714	0.80435	0.994748	no
gene:SpnNT_01776	ecsA_3	Chromosome:1786225-1788315	110.58	110.58+peptide	OK	87.9737	59.8727	-0.555175	-0.833445	0.1472	0.584811	no
gene:SpnNT_01776	ecsA_3	Chromosome:1786225-1788315	ΔORF2	110.58+peptide	OK	93.9269	59.8727	-0.649641	-0.970392	0.0938	0.462897	no
gene:SpnNT_01776	ecsA_3	Chromosome:1786225-1788315	110.58	ΔORF2+peptide	OK	87.9737	57.1971	-0.621132	-0.927763	0.1043	0.490371	no
gene:SpnNT_01776	ecsA_3	Chromosome:1786225-1788315	ΔORF2	ΔORF2+peptide	OK	93.9269	57.1971	-0.715598	-1.06358	0.06575	0.373171	no
gene:SpnNT_01776	ecsA_3	Chromosome:1786225-1788315	110.58+peptide	ΔORF2+peptide	OK	59.8727	57.1971	-0.0659571	-0.0981	0.86685	0.994748	no
gene:SpnNT_01777	NA	Chromosome:1788437-1788629	110.58	ΔORF2	NOTEST	0	0	0	0	1	1	no
gene:SpnNT_01777	NA	Chromosome:1788437-1788629	110.58	110.58+peptide	NOTEST	0	0	0	0	1	1	no
gene:SpnNT_01777	NA	Chromosome:1788437-1788629	ΔORF2	110.58+peptide	NOTEST	0	0	0	0	1	1	no
gene:SpnNT_01777	NA	Chromosome:1788437-1788629	110.58	ΔORF2+peptide	NOTEST	0	0	0	0	1	1	no
gene:SpnNT_01777	NA	Chromosome:1788437-1788629	ΔORF2	ΔORF2+peptide	NOTEST	0	0	0	0	1	1	no
gene:SpnNT_01777	NA	Chromosome:1788437-1788629	110.58+peptide	ΔORF2+peptide	NOTEST	0	0	0	0	1	1	no
gene:SpnNT_01778	NA	Chromosome:1789624-1790242	110.58	ΔORF2	OK	62.3996	65.9396	0.0796085	0.158886	0.77965	0.994748	no
gene:SpnNT_01778	NA	Chromosome:1789624-1790242	110.58	110.58+peptide	OK	62.3996	56.1111	-0.15325	-0.302322	0.59675	0.97629	no

gene:SpnNT_01778	NA	Chromosome:1789624-1790242	ΔORF2	110.58+peptide	OK	65.9396	56.1111	-0.232858	-0.460003	0.42645	0.908569	no
gene:SpnNT_01778	NA	Chromosome:1789624-1790242	110.58	ΔORF2+peptide	OK	62.3996	56.307	-0.148221	-0.291463	0.60465	0.976761	no
gene:SpnNT_01778	NA	Chromosome:1789624-1790242	ΔORF2	ΔORF2+peptide	OK	65.9396	56.307	-0.22783	-0.448621	0.4332	0.913334	no
gene:SpnNT_01778	NA	Chromosome:1789624-1790242	110.58+peptide	ΔORF2+peptide	OK	56.1111	56.307	0.00502841	0.00978988	0.98665	0.997339	no
gene:SpnNT_01779	gmuE	Chromosome:1790284-1791172	110.58	ΔORF2	OK	40.4655	40.4268	-0.00137829	-0.00268107	0.9968	0.999412	no
gene:SpnNT_01779	gmuE	Chromosome:1790284-1791172	110.58	110.58+peptide	OK	40.4655	30.3231	-0.416273	-0.809393	0.1582	0.607521	no
gene:SpnNT_01779	gmuE	Chromosome:1790284-1791172	ΔORF2	110.58+peptide	OK	40.4268	30.3231	-0.414895	-0.799323	0.15575	0.601103	no
gene:SpnNT_01779	gmuE	Chromosome:1790284-1791172	110.58	ΔORF2+peptide	OK	40.4655	27.9681	-0.532908	-1.02959	0.07425	0.401777	no
gene:SpnNT_01779	gmuE	Chromosome:1790284-1791172	ΔORF2	ΔORF2+peptide	OK	40.4268	27.9681	-0.53153	-1.01764	0.0726	0.396941	no
gene:SpnNT_01779	gmuE	Chromosome:1790284-1791172	110.58+peptide	ΔORF2+peptide	OK	30.3231	27.9681	-0.116635	-0.22321	0.69395	0.98828	no
gene:SpnNT_01780	bglF_2	Chromosome:1791320-1793258	110.58	ΔORF2	OK	20.4755	18.313	-0.161032	-0.326092	0.56685	0.968621	no
gene:SpnNT_01780	bglF_2	Chromosome:1791320-1793258	110.58	110.58+peptide	OK	20.4755	27.0281	0.40056	0.829023	0.1509	0.593025	no
gene:SpnNT_01780	bglF_2	Chromosome:1791320-1793258	ΔORF2	110.58+peptide	OK	18.313	27.0281	0.561592	1.17047	0.039	0.266402	no
gene:SpnNT_01780	bglF_2	Chromosome:1791320-1793258	110.58	ΔORF2+peptide	OK	20.4755	23.2587	0.183871	0.375743	0.5191	0.954494	no
gene:SpnNT_01780	bglF_2	Chromosome:1791320-1793258	ΔORF2	ΔORF2+peptide	OK	18.313	23.2587	0.344903	0.709641	0.21495	0.701601	no
gene:SpnNT_01780	bglF_2	Chromosome:1791320-1793258	110.58+peptide	ΔORF2+peptide	OK	27.0281	23.2587	-0.216689	-0.455998	0.42945	0.911557	no
gene:SpnNT_01781	scrB	Chromosome:1793633-1796034	110.58	ΔORF2	OK	31.5461	29.1802	-0.11247	-0.204465	0.72315	0.994748	no
gene:SpnNT_01781	scrB	Chromosome:1793633-1796034	110.58	110.58+peptide	OK	31.5461	34.8536	0.143846	0.263363	0.64455	0.980887	no
gene:SpnNT_01781	scrB	Chromosome:1793633-1796034	ΔORF2	110.58+peptide	OK	29.1802	34.8536	0.256316	0.469815	0.4089	0.897533	no
gene:SpnNT_01781	scrB	Chromosome:1793633-1796034	110.58	ΔORF2+peptide	OK	31.5461	27.9508	-0.174572	-0.315393	0.57655	0.969538	no
gene:SpnNT_01781	scrB	Chromosome:1793633-1796034	ΔORF2	ΔORF2+peptide	OK	29.1802	27.9508	-0.0621014	-0.112321	0.8401	0.994748	no
gene:SpnNT_01781	scrB	Chromosome:1793633-1796034	110.58+peptide	ΔORF2+peptide	OK	34.8536	27.9508	-0.318417	-0.579964	0.3105	0.816346	no
gene:SpnNT_01782	degA	Chromosome:1793633-1796034	110.58	ΔORF2	OK	20.1418	18.6022	-0.114721	-0.127386	0.82895	0.994748	no
gene:SpnNT_01782	degA	Chromosome:1793633-1796034	110.58	110.58+peptide	OK	20.1418	21.728	0.109364	0.120747	0.83725	0.994748	no
gene:SpnNT_01782	degA	Chromosome:1793633-1796034	ΔORF2	110.58+peptide	OK	18.6022	21.728	0.224085	0.248061	0.6683	0.984776	no
gene:SpnNT_01782	degA	Chromosome:1793633-1796034	110.58	ΔORF2+peptide	OK	20.1418	18.9859	-0.0852667	-0.0961067	0.8734	0.994748	no
gene:SpnNT_01782	degA	Chromosome:1793633-1796034	ΔORF2	ΔORF2+peptide	OK	18.6022	18.9859	0.0294542	0.03329	0.9522	0.994855	no
gene:SpnNT_01782	degA	Chromosome:1793633-1796034	110.58+peptide	ΔORF2+peptide	OK	21.728	18.9859	-0.194631	-0.218682	0.7067	0.990422	no
gene:SpnNT_01783	mvaA	Chromosome:1796069-1798516	110.58	ΔORF2	OK	82.3225	71.463	-0.20409	-0.321369	0.5782	0.969538	no
gene:SpnNT_01783	mvaA	Chromosome:1796069-1798516	110.58	110.58+peptide	OK	82.3225	111.277	0.434797	0.705958	0.21995	0.70824	no
gene:SpnNT_01783	mvaA	Chromosome:1796069-1798516	ΔORF2	110.58+peptide	OK	71.463	111.277	0.638887	0.983609	0.0898	0.450098	no
gene:SpnNT_01783	mvaA	Chromosome:1796069-1798516	110.58	ΔORF2+peptide	OK	82.3225	105.981	0.364448	0.578962	0.3189	0.825137	no
gene:SpnNT_01783	mvaA	Chromosome:1796069-1798516	ΔORF2	ΔORF2+peptide	OK	71.463	105.981	0.568539	0.858257	0.14035	0.571974	no
gene:SpnNT_01783	mvaA	Chromosome:1796069-1798516	110.58+peptide	ΔORF2+peptide	OK	111.277	105.981	-0.0703485	-0.109223	0.85025	0.994748	no
gene:SpnNT_01784	pksG	Chromosome:1796069-1798516	110.58	ΔORF2	OK	75.871	77.8775	0.0376592	0.0570574	0.91765	0.994748	no
gene:SpnNT_01784	pksG	Chromosome:1796069-1798516	110.58	110.58+peptide	OK	75.871	106.247	0.485803	0.728344	0.21105	0.697158	no
gene:SpnNT_01784	pksG	Chromosome:1796069-1798516	ΔORF2	110.58+peptide	OK	77.8775	106.247	0.448144	0.665013	0.25935	0.759841	no
gene:SpnNT_01784	pksG	Chromosome:1796069-1798516	110.58	ΔORF2+peptide	OK	75.871	125.553	0.726678	1.13349	0.0499	0.313446	no
gene:SpnNT_01784	pksG	Chromosome:1796069-1798516	ΔORF2	ΔORF2+peptide	OK	77.8775	125.553	0.689019	1.06286	0.0658	0.373293	no
gene:SpnNT_01784	pksG	Chromosome:1796069-1798516	110.58+peptide	ΔORF2+peptide	OK	106.247	125.553	0.240874	0.367543	0.5258	0.955748	no
gene:SpnNT_01785	NA	Chromosome:1798708-1798933	110.58	ΔORF2	OK	33.2002	26.3506	-0.333359	-0.410993	0.4696	0.927886	no
gene:SpnNT_01785	NA	Chromosome:1798708-1798933	110.58	110.58+peptide	OK	33.2002	26.1361	-0.345147	-0.416231	0.4595	0.923646	no
gene:SpnNT_01785	NA	Chromosome:1798708-1798933	ΔORF2	110.58+peptide	OK	26.3506	26.1361	-0.0117875	-0.0139706	0.9735	0.99536	no
gene:SpnNT_01785	NA	Chromosome:1798708-1798933	110.58	ΔORF2+peptide	OK	33.2002	28.3521	-0.227738	-0.285493	0.6278	0.980887	no
gene:SpnNT_01785	NA	Chromosome:1798708-1798933	ΔORF2	ΔORF2+peptide	OK	26.3506	28.3521	0.105622	0.129951	0.8285	0.994748	no

gene:SpnNT_01785	NA	Chromosome:1798708-1798933	110.58+peptide	ΔORF2+peptide	OK	26.1361	28.3521	0.117409	0.141311	0.8085	0.994748	no
gene:SpnNT_01786	NA	Chromosome:1799000-1799390	110.58	ΔORF2	OK	23.411	25.1275	0.10208	0.159015	0.77795	0.994748	no
gene:SpnNT_01786	NA	Chromosome:1799000-1799390	110.58	110.58+peptide	OK	23.411	24.1701	0.0460404	0.0725802	0.8999	0.994748	no
gene:SpnNT_01786	NA	Chromosome:1799000-1799390	ΔORF2	110.58+peptide	OK	25.1275	24.1701	-0.0560399	-0.0875075	0.8768	0.994748	no
gene:SpnNT_01786	NA	Chromosome:1799000-1799390	110.58	ΔORF2+peptide	OK	23.411	26.8626	0.198417	0.313929	0.5846	0.971855	no
gene:SpnNT_01786	NA	Chromosome:1799000-1799390	ΔORF2	ΔORF2+peptide	OK	25.1275	26.8626	0.0963366	0.150967	0.79065	0.994748	no
gene:SpnNT_01786	NA	Chromosome:1799000-1799390	110.58+peptide	ΔORF2+peptide	OK	24.1701	26.8626	0.152376	0.241688	0.6711	0.984845	no
gene:SpnNT_01787	NA	Chromosome:1799686-1800136	110.58	ΔORF2	OK	32.9779	35.2887	0.0977041	0.167192	0.7739	0.994748	no
gene:SpnNT_01787	NA	Chromosome:1799686-1800136	110.58	110.58+peptide	OK	32.9779	32.2213	-0.0334856	-0.0574471	0.92195	0.994748	no
gene:SpnNT_01787	NA	Chromosome:1799686-1800136	ΔORF2	110.58+peptide	OK	35.2887	32.2213	-0.13119	-0.225071	0.6965	0.989512	no
gene:SpnNT_01787	NA	Chromosome:1799686-1800136	110.58	ΔORF2+peptide	OK	32.9779	37.7273	0.194107	0.33401	0.5703	0.968621	no
gene:SpnNT_01787	NA	Chromosome:1799686-1800136	ΔORF2	ΔORF2+peptide	OK	35.2887	37.7273	0.0964032	0.165889	0.77825	0.994748	no
gene:SpnNT_01787	NA	Chromosome:1799686-1800136	110.58+peptide	ΔORF2+peptide	OK	32.2213	37.7273	0.227593	0.39265	0.49845	0.944017	no
gene:SpnNT_01788	yjjP	Chromosome:1800137-1800896	110.58	ΔORF2	OK	35.3186	39.1804	0.149704	0.288548	0.6052	0.976847	no
gene:SpnNT_01788	yjjP	Chromosome:1800137-1800896	110.58	110.58+peptide	OK	35.3186	34.3364	-0.0406897	-0.0779683	0.88885	0.994748	no
gene:SpnNT_01788	yjjP	Chromosome:1800137-1800896	ΔORF2	110.58+peptide	OK	39.1804	34.3364	-0.190394	-0.366128	0.5172	0.953322	no
gene:SpnNT_01788	yjjP	Chromosome:1800137-1800896	110.58	ΔORF2+peptide	OK	35.3186	38.1438	0.111017	0.213706	0.70635	0.990422	no
gene:SpnNT_01788	yjjP	Chromosome:1800137-1800896	ΔORF2	ΔORF2+peptide	OK	39.1804	38.1438	-0.0386867	-0.0747393	0.89645	0.994748	no
gene:SpnNT_01788	yjjP	Chromosome:1800137-1800896	110.58+peptide	ΔORF2+peptide	OK	34.3364	38.1438	0.151707	0.29136	0.6072	0.977869	no
gene:SpnNT_01789	stkP	Chromosome:1801010-1803727	110.58	ΔORF2	OK	366.586	363.76	-0.0111663	-0.0213821	0.97155	0.99536	no
gene:SpnNT_01789	stkP	Chromosome:1801010-1803727	110.58	110.58+peptide	OK	366.586	290.697	-0.334635	-0.638378	0.2606	0.760924	no
gene:SpnNT_01789	stkP	Chromosome:1801010-1803727	ΔORF2	110.58+peptide	OK	363.76	290.697	-0.323468	-0.617534	0.28595	0.789224	no
gene:SpnNT_01789	stkP	Chromosome:1801010-1803727	110.58	ΔORF2+peptide	OK	366.586	298.919	-0.294397	-0.570117	0.31545	0.821264	no
gene:SpnNT_01789	stkP	Chromosome:1801010-1803727	ΔORF2	ΔORF2+peptide	OK	363.76	298.919	-0.283231	-0.548912	0.34045	0.844744	no
gene:SpnNT_01789	stkP	Chromosome:1801010-1803727	110.58+peptide	ΔORF2+peptide	OK	290.697	298.919	0.0402378	0.0776821	0.8921	0.994748	no
gene:SpnNT_01790	NA	Chromosome:1801010-1803727	110.58	ΔORF2	OK	388.003	378.783	-0.0346969	-0.0365299	0.953	0.994855	no
gene:SpnNT_01790	NA	Chromosome:1801010-1803727	110.58	110.58+peptide	OK	388.003	350.928	-0.144896	-0.15544	0.78515	0.994748	no
gene:SpnNT_01790	NA	Chromosome:1801010-1803727	ΔORF2	110.58+peptide	OK	378.783	350.928	-0.110199	-0.119417	0.83005	0.994748	no
gene:SpnNT_01790	NA	Chromosome:1801010-1803727	110.58	ΔORF2+peptide	OK	388.003	308.633	-0.330175	-0.344738	0.5521	0.964668	no
gene:SpnNT_01790	NA	Chromosome:1801010-1803727	ΔORF2	ΔORF2+peptide	OK	378.783	308.633	-0.295478	-0.311473	0.58915	0.974146	no
gene:SpnNT_01790	NA	Chromosome:1801010-1803727	110.58+peptide	ΔORF2+peptide	OK	350.928	308.633	-0.185279	-0.199017	0.72835	0.994748	no
gene:SpnNT_01791	rsmB	Chromosome:1803741-1805983	110.58	ΔORF2	OK	137.985	122.547	-0.171176	-0.317338	0.5905	0.974909	no
gene:SpnNT_01791	rsmB	Chromosome:1803741-1805983	110.58	110.58+peptide	OK	137.985	113.921	-0.27647	-0.508623	0.37255	0.869703	no
gene:SpnNT_01791	rsmB	Chromosome:1803741-1805983	ΔORF2	110.58+peptide	OK	122.547	113.921	-0.105293	-0.193954	0.73115	0.994748	no
gene:SpnNT_01791	rsmB	Chromosome:1803741-1805983	110.58	ΔORF2+peptide	OK	137.985	97.2139	-0.505275	-0.950364	0.10065	0.48079	no
gene:SpnNT_01791	rsmB	Chromosome:1803741-1805983	ΔORF2	ΔORF2+peptide	OK	122.547	97.2139	-0.334098	-0.629234	0.27315	0.775481	no
gene:SpnNT_01791	rsmB	Chromosome:1803741-1805983	110.58+peptide	ΔORF2+peptide	OK	113.921	97.2139	-0.228805	-0.427532	0.46055	0.924377	no
gene:SpnNT_01792	fmt	Chromosome:1803741-1805983	110.58	ΔORF2	OK	109.589	89.9052	-0.285624	-0.369032	0.524	0.954832	no
gene:SpnNT_01792	fmt	Chromosome:1803741-1805983	110.58	110.58+peptide	OK	109.589	77.5328	-0.499221	-0.619412	0.28165	0.784843	no
gene:SpnNT_01792	fmt	Chromosome:1803741-1805983	ΔORF2	110.58+peptide	OK	89.9052	77.5328	-0.213597	-0.26194	0.651	0.981475	no
gene:SpnNT_01792	fmt	Chromosome:1803741-1805983	110.58	ΔORF2+peptide	OK	109.589	66.1938	-0.727332	-0.929925	0.10785	0.499897	no
gene:SpnNT_01792	fmt	Chromosome:1803741-1805983	ΔORF2	ΔORF2+peptide	OK	89.9052	66.1938	-0.441708	-0.557777	0.3381	0.842454	no
gene:SpnNT_01792	fmt	Chromosome:1803741-1805983	110.58+peptide	ΔORF2+peptide	OK	77.5328	66.1938	-0.228111	-0.277106	0.63475	0.980887	no
gene:SpnNT_01793	priA	Chromosome:1805995-1808392	110.58	ΔORF2	OK	95.6909	84.6643	-0.176628	-0.398293	0.4803	0.934672	no
gene:SpnNT_01793	priA	Chromosome:1805995-1808392	110.58	110.58+peptide	OK	95.6909	70.9296	-0.431993	-0.968908	0.09295	0.46026	no

gene:SpnNT_01793	priA	Chromosome:1805995-1808392	ΔORF2	110.58+peptide	OK	84.6643	70.9296	-0.255365	-0.569809	0.31305	0.819111	no
gene:SpnNT_01793	priA	Chromosome:1805995-1808392	110.58	ΔORF2+peptide	OK	95.6909	59.3957	-0.688023	-1.56014	0.00765	0.0829614	no
gene:SpnNT_01793	priA	Chromosome:1805995-1808392	ΔORF2	ΔORF2+peptide	OK	84.6643	59.3957	-0.511395	-1.15354	0.0442	0.288553	no
gene:SpnNT_01793	priA	Chromosome:1805995-1808392	110.58+peptide	ΔORF2+peptide	OK	70.9296	59.3957	-0.25603	-0.574417	0.31725	0.823011	no
gene:SpnNT_01794	rpoZ	Chromosome:1808457-1808772	110.58	ΔORF2	OK	729.508	861.724	0.240302	0.532448	0.3515	0.854885	no
gene:SpnNT_01794	rpoZ	Chromosome:1808457-1808772	110.58	110.58+peptide	OK	729.508	639.299	-0.190433	-0.416826	0.4658	0.926452	no
gene:SpnNT_01794	rpoZ	Chromosome:1808457-1808772	ΔORF2	110.58+peptide	OK	861.724	639.299	-0.430735	-0.956419	0.0957	0.468573	no
gene:SpnNT_01794	rpoZ	Chromosome:1808457-1808772	110.58	ΔORF2+peptide	OK	729.508	854.649	0.22841	0.495133	0.38065	0.878145	no
gene:SpnNT_01794	rpoZ	Chromosome:1808457-1808772	ΔORF2	ΔORF2+peptide	OK	861.724	854.649	-0.0118929	-0.0261455	0.96315	0.994855	no
gene:SpnNT_01794	rpoZ	Chromosome:1808457-1808772	110.58+peptide	ΔORF2+peptide	OK	639.299	854.649	0.418842	0.909784	0.11065	0.507143	no
gene:SpnNT_01795	gmk	Chromosome:1808796-1809423	110.58	ΔORF2	OK	285.645	316.258	0.146879	0.330315	0.56115	0.968621	no
gene:SpnNT_01795	gmk	Chromosome:1808796-1809423	110.58	110.58+peptide	OK	285.645	282.875	-0.0140542	-0.0315795	0.9563	0.994855	no
gene:SpnNT_01795	gmk	Chromosome:1808796-1809423	ΔORF2	110.58+peptide	OK	316.258	282.875	-0.160933	-0.363541	0.5237	0.954832	no
gene:SpnNT_01795	gmk	Chromosome:1808796-1809423	110.58	ΔORF2+peptide	OK	285.645	273.627	-0.0620126	-0.139238	0.81005	0.994748	no
gene:SpnNT_01795	gmk	Chromosome:1808796-1809423	ΔORF2	ΔORF2+peptide	OK	316.258	273.627	-0.208892	-0.471528	0.41565	0.901067	no
gene:SpnNT_01795	gmk	Chromosome:1808796-1809423	110.58+peptide	ΔORF2+peptide	OK	282.875	273.627	-0.0479583	-0.108163	0.84975	0.994748	no
gene:SpnNT_01796	rny	Chromosome:1809552-1811166	110.58	ΔORF2	OK	493.869	460.063	-0.102297	-0.225296	0.68885	0.98828	no
gene:SpnNT_01796	rny	Chromosome:1809552-1811166	110.58	110.58+peptide	OK	493.869	579.403	0.230439	0.50739	0.36545	0.864634	no
gene:SpnNT_01796	rny	Chromosome:1809552-1811166	ΔORF2	110.58+peptide	OK	460.063	579.403	0.332735	0.727822	0.1918	0.672222	no
gene:SpnNT_01796	rny	Chromosome:1809552-1811166	110.58	ΔORF2+peptide	OK	493.869	591.564	0.260406	0.57693	0.30785	0.813881	no
gene:SpnNT_01796	rny	Chromosome:1809552-1811166	ΔORF2	ΔORF2+peptide	OK	460.063	591.564	0.362702	0.798229	0.15555	0.601087	no
gene:SpnNT_01796	rny	Chromosome:1809552-1811166	110.58+peptide	ΔORF2+peptide	OK	579.403	591.564	0.029967	0.0659348	0.9056	0.994748	no
gene:SpnNT_01797	hsdR_3	Chromosome:1811289-1814277	110.58	ΔORF2	OK	20.1591	19.6463	-0.0371733	-0.0806843	0.88345	0.994748	no
gene:SpnNT_01797	hsdR_3	Chromosome:1811289-1814277	110.58	110.58+peptide	OK	20.1591	25.7914	0.355457	0.776719	0.17945	0.645843	no
gene:SpnNT_01797	hsdR_3	Chromosome:1811289-1814277	ΔORF2	110.58+peptide	OK	19.6463	25.7914	0.39263	0.855831	0.1362	0.561488	no
gene:SpnNT_01797	hsdR_3	Chromosome:1811289-1814277	110.58	ΔORF2+peptide	OK	20.1591	32.6853	0.697209	1.52795	0.009	0.0935059	no
gene:SpnNT_01797	hsdR_3	Chromosome:1811289-1814277	ΔORF2	ΔORF2+peptide	OK	19.6463	32.6853	0.734382	1.60542	0.0054	0.0637793	no
gene:SpnNT_01797	hsdR_3	Chromosome:1811289-1814277	110.58+peptide	ΔORF2+peptide	OK	25.7914	32.6853	0.341752	0.752212	0.19505	0.676386	no
gene:SpnNT_01798	NA	Chromosome:1814384-1815386	110.58	ΔORF2	OK	7.04768	9.63297	0.450832	0.729017	0.2101	0.696723	no
gene:SpnNT_01798	NA	Chromosome:1814384-1815386	110.58	110.58+peptide	OK	7.04768	11.4589	0.701243	1.12219	0.05335	0.327284	no
gene:SpnNT_01798	NA	Chromosome:1814384-1815386	ΔORF2	110.58+peptide	OK	9.63297	11.4589	0.25041	0.40424	0.48715	0.938799	no
gene:SpnNT_01798	NA	Chromosome:1814384-1815386	110.58	ΔORF2+peptide	OK	7.04768	23.3197	1.72633	2.9752	5.00E-05	0.0013612	yes
gene:SpnNT_01798	NA	Chromosome:1814384-1815386	ΔORF2	ΔORF2+peptide	OK	9.63297	23.3197	1.27549	2.2206	5.00E-04	0.0095781	yes
gene:SpnNT_01798	NA	Chromosome:1814384-1815386	110.58+peptide	ΔORF2+peptide	OK	11.4589	23.3197	1.02508	1.76326	0.0034	0.0443928	yes
gene:SpnNT_01799	NA	Chromosome:1815387-1816071	110.58	ΔORF2	OK	6.49145	9.71989	0.582399	0.864802	0.13265	0.554518	no
gene:SpnNT_01799	NA	Chromosome:1815387-1816071	110.58	110.58+peptide	OK	6.49145	11.2493	0.793226	1.16066	0.0481	0.305947	no
gene:SpnNT_01799	NA	Chromosome:1815387-1816071	ΔORF2	110.58+peptide	OK	9.71989	11.2493	0.210827	0.323323	0.5753	0.969538	no
gene:SpnNT_01799	NA	Chromosome:1815387-1816071	110.58	ΔORF2+peptide	OK	6.49145	19.2523	1.56841	2.41497	5.00E-05	0.0013612	yes
gene:SpnNT_01799	NA	Chromosome:1815387-1816071	ΔORF2	ΔORF2+peptide	OK	9.71989	19.2523	0.986016	1.59973	0.00535	0.0633027	no
gene:SpnNT_01799	NA	Chromosome:1815387-1816071	110.58+peptide	ΔORF2+peptide	OK	11.2493	19.2523	0.775189	1.23584	0.03095	0.226886	no
gene:SpnNT_01800	NA	Chromosome:1816073-1819446	110.58	ΔORF2	OK	10.6206	12.7564	0.264354	0.285259	0.61985	0.980887	no
gene:SpnNT_01800	NA	Chromosome:1816073-1819446	110.58	110.58+peptide	OK	10.6206	15.3542	0.531758	0.573429	0.32635	0.831527	no
gene:SpnNT_01800	NA	Chromosome:1816073-1819446	ΔORF2	110.58+peptide	OK	12.7564	15.3542	0.267405	0.291397	0.62375	0.980887	no
gene:SpnNT_01800	NA	Chromosome:1816073-1819446	110.58	ΔORF2+peptide	OK	10.6206	21.5839	1.02308	1.18675	0.043	0.284112	no
gene:SpnNT_01800	NA	Chromosome:1816073-1819446	ΔORF2	ΔORF2+peptide	OK	12.7564	21.5839	0.758731	0.89086	0.12455	0.533791	no

gene:SpnNT_01800	NA	Chromosome:1816073-1819446	110.58+peptide	ΔORF2+peptide	OK	15.3542	21.5839	0.491326	0.576436	0.32635	0.831527	no
gene:SpnNT_01801	NA	Chromosome:1816073-1819446	110.58	ΔORF2	OK	16.1843	16.4121	0.0201666	0.0354945	0.9506	0.994855	no
gene:SpnNT_01801	NA	Chromosome:1816073-1819446	110.58	110.58+peptide	OK	16.1843	18.9305	0.226117	0.397303	0.4844	0.936747	no
gene:SpnNT_01801	NA	Chromosome:1816073-1819446	ΔORF2	110.58+peptide	OK	16.4121	18.9305	0.205951	0.355285	0.5299	0.957488	no
gene:SpnNT_01801	NA	Chromosome:1816073-1819446	110.58	ΔORF2+peptide	OK	16.1843	22.2284	0.457807	0.801349	0.1637	0.618328	no
gene:SpnNT_01801	NA	Chromosome:1816073-1819446	ΔORF2	ΔORF2+peptide	OK	16.4121	22.2284	0.437641	0.752214	0.19165	0.672051	no
gene:SpnNT_01801	NA	Chromosome:1816073-1819446	110.58+peptide	ΔORF2+peptide	OK	18.9305	22.2284	0.23169	0.39758	0.48415	0.936747	no
gene:SpnNT_01802	relK	Chromosome:1819661-1819916	110.58	ΔORF2	OK	79.7935	87.18	0.127726	0.203444	0.724	0.994748	no
gene:SpnNT_01802	relK	Chromosome:1819661-1819916	110.58	110.58+peptide	OK	79.7935	77.2968	-0.045863	-0.0728006	0.90105	0.994748	no
gene:SpnNT_01802	relK	Chromosome:1819661-1819916	ΔORF2	110.58+peptide	OK	87.18	77.2968	-0.173589	-0.2771	0.6252	0.980887	no
gene:SpnNT_01802	relK	Chromosome:1819661-1819916	110.58	ΔORF2+peptide	OK	79.7935	70.9602	-0.169261	-0.259282	0.65705	0.982966	no
gene:SpnNT_01802	relK	Chromosome:1819661-1819916	ΔORF2	ΔORF2+peptide	OK	87.18	70.9602	-0.296987	-0.457326	0.41895	0.903984	no
gene:SpnNT_01802	relK	Chromosome:1819661-1819916	110.58+peptide	ΔORF2+peptide	OK	77.2968	70.9602	-0.123398	-0.189409	0.7376	0.994748	no
gene:SpnNT_01803	NA	Chromosome:1819919-1820174	110.58	ΔORF2	OK	65.7705	78.6149	0.257363	0.403768	0.4804	0.934687	no
gene:SpnNT_01803	NA	Chromosome:1819919-1820174	110.58	110.58+peptide	OK	65.7705	50.7915	-0.372854	-0.552519	0.33235	0.837993	no
gene:SpnNT_01803	NA	Chromosome:1819919-1820174	ΔORF2	110.58+peptide	OK	78.6149	50.7915	-0.630217	-0.933605	0.10155	0.483329	no
gene:SpnNT_01803	NA	Chromosome:1819919-1820174	110.58	ΔORF2+peptide	OK	65.7705	41.38	-0.668508	-0.957576	0.0897	0.450098	no
gene:SpnNT_01803	NA	Chromosome:1819919-1820174	ΔORF2	ΔORF2+peptide	OK	78.6149	41.38	-0.925871	-1.32584	0.0197	0.165072	no
gene:SpnNT_01803	NA	Chromosome:1819919-1820174	110.58+peptide	ΔORF2+peptide	OK	50.7915	41.38	-0.295654	-0.403543	0.4754	0.930746	no
gene:SpnNT_01804	NA	Chromosome:1820305-1821403	110.58	ΔORF2	OK	72.787	65.7034	-0.147713	-0.324441	0.5704	0.968621	no
gene:SpnNT_01804	NA	Chromosome:1820305-1821403	110.58	110.58+peptide	OK	72.787	77.3649	0.0879988	0.193511	0.73625	0.994748	no
gene:SpnNT_01804	NA	Chromosome:1820305-1821403	ΔORF2	110.58+peptide	OK	65.7034	77.3649	0.235711	0.517286	0.36485	0.864498	no
gene:SpnNT_01804	NA	Chromosome:1820305-1821403	110.58	ΔORF2+peptide	OK	72.787	76.6252	0.0741376	0.163087	0.7745	0.994748	no
gene:SpnNT_01804	NA	Chromosome:1820305-1821403	ΔORF2	ΔORF2+peptide	OK	65.7034	76.6252	0.22185	0.487037	0.399	0.892547	no
gene:SpnNT_01804	NA	Chromosome:1820305-1821403	110.58+peptide	ΔORF2+peptide	OK	77.3649	76.6252	-0.0138612	-0.030466	0.95615	0.994855	no
gene:SpnNT_01805	NA	Chromosome:1821412-1822189	110.58	ΔORF2	OK	49.0325	42.2429	-0.215027	-0.426206	0.4647	0.926394	no
gene:SpnNT_01805	NA	Chromosome:1821412-1822189	110.58	110.58+peptide	OK	49.0325	45.0719	-0.121509	-0.242849	0.6673	0.983929	no
gene:SpnNT_01805	NA	Chromosome:1821412-1822189	ΔORF2	110.58+peptide	OK	42.2429	45.0719	0.0935182	0.184517	0.74715	0.994748	no
gene:SpnNT_01805	NA	Chromosome:1821412-1822189	110.58	ΔORF2+peptide	OK	49.0325	44.8377	-0.129025	-0.256707	0.6568	0.982966	no
gene:SpnNT_01805	NA	Chromosome:1821412-1822189	ΔORF2	ΔORF2+peptide	OK	42.2429	44.8377	0.0860016	0.16894	0.77155	0.994748	no
gene:SpnNT_01805	NA	Chromosome:1821412-1822189	110.58+peptide	ΔORF2+peptide	OK	45.0719	44.8377	-0.00751664	-0.0148863	0.97895	0.995765	no
gene:SpnNT_01806	rsfS	Chromosome:1822371-1822722	110.58	ΔORF2	OK	221.472	212.241	-0.0614211	-0.12437	0.8294	0.994748	no
gene:SpnNT_01806	rsfS	Chromosome:1822371-1822722	110.58	110.58+peptide	OK	221.472	319.024	0.526536	1.07617	0.0578	0.342262	no
gene:SpnNT_01806	rsfS	Chromosome:1822371-1822722	ΔORF2	110.58+peptide	OK	212.241	319.024	0.587958	1.1806	0.03995	0.270497	no
gene:SpnNT_01806	rsfS	Chromosome:1822371-1822722	110.58	ΔORF2+peptide	OK	221.472	203.914	-0.119166	-0.240895	0.6729	0.984845	no
gene:SpnNT_01806	rsfS	Chromosome:1822371-1822722	ΔORF2	ΔORF2+peptide	OK	212.241	203.914	-0.0577446	-0.114725	0.8401	0.994748	no
gene:SpnNT_01806	rsfS	Chromosome:1822371-1822722	110.58+peptide	ΔORF2+peptide	OK	319.024	203.914	-0.645702	-1.29443	0.02175	0.178712	no
gene:SpnNT_01807	NA	Chromosome:1822722-1823945	110.58	ΔORF2	OK	89.6109	79.5603	-0.171625	-0.215404	0.70535	0.990422	no
gene:SpnNT_01807	NA	Chromosome:1822722-1823945	110.58	110.58+peptide	OK	89.6109	91.8666	0.0358658	0.0422667	0.94005	0.994855	no
gene:SpnNT_01807	NA	Chromosome:1822722-1823945	ΔORF2	110.58+peptide	OK	79.5603	91.8666	0.207491	0.248982	0.65395	0.982798	no
gene:SpnNT_01807	NA	Chromosome:1822722-1823945	110.58	ΔORF2+peptide	OK	89.6109	86.1729	-0.0564402	-0.0715402	0.90195	0.994748	no
gene:SpnNT_01807	NA	Chromosome:1822722-1823945	ΔORF2	ΔORF2+peptide	OK	79.5603	86.1729	0.115185	0.149096	0.79195	0.994748	no
gene:SpnNT_01807	NA	Chromosome:1822722-1823945	110.58+peptide	ΔORF2+peptide	OK	91.8666	86.1729	-0.0923059	-0.111768	0.84985	0.994748	no
gene:SpnNT_01808	nadD	Chromosome:1822722-1823945	110.58	ΔORF2	OK	153.543	137.727	-0.156832	-0.282725	0.63275	0.980887	no
gene:SpnNT_01808	nadD	Chromosome:1822722-1823945	110.58	110.58+peptide	OK	153.543	172.106	0.164659	0.297835	0.61135	0.979429	no

gene:SpnNT_01808	nadD	Chromosome:1822722-1823945	ΔORF2	110.58+peptide	OK	137.727	172.106	0.321491	0.578012	0.31305	0.819111	no
gene:SpnNT_01808	nadD	Chromosome:1822722-1823945	110.58	ΔORF2+peptide	OK	153.543	123.564	-0.313385	-0.556204	0.33715	0.84149	no
gene:SpnNT_01808	nadD	Chromosome:1822722-1823945	ΔORF2	ΔORF2+peptide	OK	137.727	123.564	-0.156553	-0.276244	0.6296	0.980887	no
gene:SpnNT_01808	nadD	Chromosome:1822722-1823945	110.58+peptide	ΔORF2+peptide	OK	172.106	123.564	-0.478044	-0.846249	0.1449	0.580489	no
gene:SpnNT_01809	yhbY	Chromosome:1823996-1824308	110.58	ΔORF2	OK	87.5678	79.5774	-0.138042	-0.238533	0.67465	0.985063	no
gene:SpnNT_01809	yhbY	Chromosome:1823996-1824308	110.58	110.58+peptide	OK	87.5678	95.4253	0.12397	0.21741	0.7004	0.990367	no
gene:SpnNT_01809	yhbY	Chromosome:1823996-1824308	ΔORF2	110.58+peptide	OK	79.5774	95.4253	0.262013	0.450281	0.42995	0.91188	no
gene:SpnNT_01809	yhbY	Chromosome:1823996-1824308	110.58	ΔORF2+peptide	OK	87.5678	108.483	0.308992	0.544914	0.3378	0.842186	no
gene:SpnNT_01809	yhbY	Chromosome:1823996-1824308	ΔORF2	ΔORF2+peptide	OK	79.5774	108.483	0.447034	0.772368	0.1821	0.651805	no
gene:SpnNT_01809	yhbY	Chromosome:1823996-1824308	110.58+peptide	ΔORF2+peptide	OK	95.4253	108.483	0.185021	0.324436	0.56575	0.968621	no
gene:SpnNT_01810	NA	Chromosome:1824521-1825628	110.58	ΔORF2	OK	117.768	108.808	-0.114162	-0.257232	0.65285	0.981932	no
gene:SpnNT_01810	NA	Chromosome:1824521-1825628	110.58	110.58+peptide	OK	117.768	139.16	0.240795	0.542996	0.35325	0.856141	no
gene:SpnNT_01810	NA	Chromosome:1824521-1825628	ΔORF2	110.58+peptide	OK	108.808	139.16	0.354957	0.801005	0.168	0.623973	no
gene:SpnNT_01810	NA	Chromosome:1824521-1825628	110.58	ΔORF2+peptide	OK	117.768	104.935	-0.166457	-0.375086	0.51415	0.951174	no
gene:SpnNT_01810	NA	Chromosome:1824521-1825628	ΔORF2	ΔORF2+peptide	OK	108.808	104.935	-0.0522944	-0.117922	0.8366	0.994748	no
gene:SpnNT_01810	NA	Chromosome:1824521-1825628	110.58+peptide	ΔORF2+peptide	OK	139.16	104.935	-0.407252	-0.919072	0.10925	0.502305	no
gene:SpnNT_01811	NA	Chromosome:1825630-1826158	110.58	ΔORF2	OK	103.977	88.8699	-0.226502	-0.451476	0.4258	0.908216	no
gene:SpnNT_01811	NA	Chromosome:1825630-1826158	110.58	110.58+peptide	OK	103.977	111.912	0.1061	0.214124	0.69965	0.990367	no
gene:SpnNT_01811	NA	Chromosome:1825630-1826158	ΔORF2	110.58+peptide	OK	88.8699	111.912	0.332602	0.654774	0.253	0.751101	no
gene:SpnNT_01811	NA	Chromosome:1825630-1826158	110.58	ΔORF2+peptide	OK	103.977	98.505	-0.0779998	-0.156424	0.77505	0.994748	no
gene:SpnNT_01811	NA	Chromosome:1825630-1826158	ΔORF2	ΔORF2+peptide	OK	88.8699	98.505	0.148502	0.290598	0.6092	0.978849	no
gene:SpnNT_01811	NA	Chromosome:1825630-1826158	110.58+peptide	ΔORF2+peptide	OK	111.912	98.505	-0.1841	-0.364588	0.52105	0.954832	no
gene:SpnNT_01812	NA	Chromosome:1826172-1827081	110.58	ΔORF2	OK	128.161	129.801	0.0183379	0.0413982	0.94235	0.994855	no
gene:SpnNT_01812	NA	Chromosome:1826172-1827081	110.58	110.58+peptide	OK	128.161	139.172	0.118909	0.267887	0.6396	0.980887	no
gene:SpnNT_01812	NA	Chromosome:1826172-1827081	ΔORF2	110.58+peptide	OK	129.801	139.172	0.100571	0.226586	0.68895	0.98828	no
gene:SpnNT_01812	NA	Chromosome:1826172-1827081	110.58	ΔORF2+peptide	OK	128.161	121.207	-0.0804946	-0.181275	0.755	0.994748	no
gene:SpnNT_01812	NA	Chromosome:1826172-1827081	ΔORF2	ΔORF2+peptide	OK	129.801	121.207	-0.0988325	-0.222583	0.69905	0.990367	no
gene:SpnNT_01812	NA	Chromosome:1826172-1827081	110.58+peptide	ΔORF2+peptide	OK	139.172	121.207	-0.199403	-0.448163	0.43095	0.912485	no
gene:SpnNT_01813	ykuT	Chromosome:1827137-1827698	110.58	ΔORF2	OK	66.137	69.5865	0.07335	0.144386	0.7985	0.994748	no
gene:SpnNT_01813	ykuT	Chromosome:1827137-1827698	110.58	110.58+peptide	OK	66.137	61.4085	-0.107019	-0.20943	0.71745	0.992553	no
gene:SpnNT_01813	ykuT	Chromosome:1827137-1827698	ΔORF2	110.58+peptide	OK	69.5865	61.4085	-0.180369	-0.35407	0.5276	0.956161	no
gene:SpnNT_01813	ykuT	Chromosome:1827137-1827698	110.58	ΔORF2+peptide	OK	66.137	68.5012	0.0506731	0.099564	0.86325	0.994748	no
gene:SpnNT_01813	ykuT	Chromosome:1827137-1827698	ΔORF2	ΔORF2+peptide	OK	69.5865	68.5012	-0.022677	-0.0446962	0.93375	0.994855	no
gene:SpnNT_01813	ykuT	Chromosome:1827137-1827698	110.58+peptide	ΔORF2+peptide	OK	61.4085	68.5012	0.157692	0.308987	0.59055	0.974909	no
gene:SpnNT_01814	sstT	Chromosome:1827836-1829042	110.58	ΔORF2	OK	54.7437	50.2406	-0.123839	-0.262739	0.6378	0.980887	no
gene:SpnNT_01814	sstT	Chromosome:1827836-1829042	110.58	110.58+peptide	OK	54.7437	42.1565	-0.376938	-0.790144	0.1661	0.621332	no
gene:SpnNT_01814	sstT	Chromosome:1827836-1829042	ΔORF2	110.58+peptide	OK	50.2406	42.1565	-0.253099	-0.53055	0.35175	0.854885	no
gene:SpnNT_01814	sstT	Chromosome:1827836-1829042	110.58	ΔORF2+peptide	OK	54.7437	38.5525	-0.505871	-1.04767	0.06755	0.380099	no
gene:SpnNT_01814	sstT	Chromosome:1827836-1829042	ΔORF2	ΔORF2+peptide	OK	50.2406	38.5525	-0.382032	-0.791197	0.1686	0.62514	no
gene:SpnNT_01814	sstT	Chromosome:1827836-1829042	110.58+peptide	ΔORF2+peptide	OK	42.1565	38.5525	-0.128933	-0.263974	0.64505	0.980887	no
gene:SpnNT_01815	NA	Chromosome:1829109-1829961	110.58	ΔORF2	OK	10.2192	9.87202	-0.0498719	-0.0792891	0.88645	0.994748	no
gene:SpnNT_01815	NA	Chromosome:1829109-1829961	110.58	110.58+peptide	OK	10.2192	10.1292	-0.0127752	-0.0203608	0.96995	0.995283	no
gene:SpnNT_01815	NA	Chromosome:1829109-1829961	ΔORF2	110.58+peptide	OK	9.87202	10.1292	0.0370966	0.0612282	0.9148	0.994748	no
gene:SpnNT_01815	NA	Chromosome:1829109-1829961	110.58	ΔORF2+peptide	OK	10.2192	6.85843	-0.575339	-0.888194	0.1129	0.510701	no
gene:SpnNT_01815	NA	Chromosome:1829109-1829961	ΔORF2	ΔORF2+peptide	OK	9.87202	6.85843	-0.525467	-0.838205	0.13915	0.569233	no

gene:SpnNT_01815	NA	Chromosome:1829109-1829961	110.58+peptide	ΔORF2+peptide	OK	10.1292	6.85843	-0.562563	-0.899606	0.11155	0.508705	no
gene:SpnNT_01816	NA	Chromosome:1830103-1830484	110.58	ΔORF2	OK	138.847	124.071	-0.162333	-0.316234	0.586	0.972738	no
gene:SpnNT_01816	NA	Chromosome:1830103-1830484	110.58	110.58+peptide	OK	138.847	122.153	-0.184814	-0.357266	0.5342	0.958867	no
gene:SpnNT_01816	NA	Chromosome:1830103-1830484	ΔORF2	110.58+peptide	OK	124.071	122.153	-0.0224812	-0.0433295	0.9388	0.994855	no
gene:SpnNT_01816	NA	Chromosome:1830103-1830484	110.58	ΔORF2+peptide	OK	138.847	120.346	-0.206311	-0.388938	0.4921	0.941165	no
gene:SpnNT_01816	NA	Chromosome:1830103-1830484	ΔORF2	ΔORF2+peptide	OK	124.071	120.346	-0.0439783	-0.0826734	0.8806	0.994748	no
gene:SpnNT_01816	NA	Chromosome:1830103-1830484	110.58+peptide	ΔORF2+peptide	OK	122.153	120.346	-0.0214971	-0.0401227	0.94195	0.994855	no
gene:SpnNT_01817	NA	Chromosome:1830581-1830803	110.58	ΔORF2	OK	891.883	849.88	-0.0695943	-0.13635	0.80585	0.994748	no
gene:SpnNT_01817	NA	Chromosome:1830581-1830803	110.58	110.58+peptide	OK	891.883	1385.44	0.635418	1.12801	0.03955	0.269038	no
gene:SpnNT_01817	NA	Chromosome:1830581-1830803	ΔORF2	110.58+peptide	OK	849.88	1385.44	0.705013	1.27914	0.02255	0.183627	no
gene:SpnNT_01817	NA	Chromosome:1830581-1830803	110.58	ΔORF2+peptide	OK	891.883	1064.16	0.254788	0.473648	0.39365	0.888833	no
gene:SpnNT_01817	NA	Chromosome:1830581-1830803	ΔORF2	ΔORF2+peptide	OK	849.88	1064.16	0.324382	0.617649	0.27255	0.774448	no
gene:SpnNT_01817	NA	Chromosome:1830581-1830803	110.58+peptide	ΔORF2+peptide	OK	1385.44	1064.16	-0.38063	-0.659972	0.2232	0.712784	no
gene:SpnNT_01818	trxA	Chromosome:1830818-1831133	110.58	ΔORF2	OK	2548.96	3493.83	0.4549	0.895318	0.11435	0.512816	no
gene:SpnNT_01818	trxA	Chromosome:1830818-1831133	110.58	110.58+peptide	OK	2548.96	5133.57	1.01005	1.73054	0.00345	0.0449116	yes
gene:SpnNT_01818	trxA	Chromosome:1830818-1831133	ΔORF2	110.58+peptide	OK	3493.83	5133.57	0.555154	0.943962	0.09455	0.46485	no
gene:SpnNT_01818	trxA	Chromosome:1830818-1831133	110.58	ΔORF2+peptide	OK	2548.96	3251.39	0.351148	0.721891	0.1976	0.679305	no
gene:SpnNT_01818	trxA	Chromosome:1830818-1831133	ΔORF2	ΔORF2+peptide	OK	3493.83	3251.39	-0.103752	-0.210983	0.70995	0.990919	no
gene:SpnNT_01818	trxA	Chromosome:1830818-1831133	110.58+peptide	ΔORF2+peptide	OK	5133.57	3251.39	-0.658906	-1.15699	0.0427	0.282842	no
gene:SpnNT_01819	queC	Chromosome:1831437-1831812	110.58	ΔORF2	OK	7.51032	5.27635	-0.509334	-0.597566	0.3023	0.80833	no
gene:SpnNT_01819	queC	Chromosome:1831437-1831812	110.58	110.58+peptide	OK	7.51032	6.15237	-0.287732	-0.34332	0.5546	0.966389	no
gene:SpnNT_01819	queC	Chromosome:1831437-1831812	ΔORF2	110.58+peptide	OK	5.27635	6.15237	0.221602	0.251033	0.67605	0.98524	no
gene:SpnNT_01819	queC	Chromosome:1831437-1831812	110.58	ΔORF2+peptide	OK	7.51032	4.92312	-0.609303	-0.699621	0.2306	0.723891	no
gene:SpnNT_01819	queC	Chromosome:1831437-1831812	ΔORF2	ΔORF2+peptide	OK	5.27635	4.92312	-0.0999682	-0.109377	0.85275	0.994748	no
gene:SpnNT_01819	queC	Chromosome:1831437-1831812	110.58+peptide	ΔORF2+peptide	OK	6.15237	4.92312	-0.321571	-0.357027	0.5488	0.964037	no
gene:SpnNT_01820	aqpZ2	Chromosome:1832227-1832896	110.58	ΔORF2	OK	83.1138	75.5899	-0.136896	-0.283635	0.6236	0.980887	no
gene:SpnNT_01820	aqpZ2	Chromosome:1832227-1832896	110.58	110.58+peptide	OK	83.1138	59.9724	-0.470791	-0.960534	0.09605	0.46955	no
gene:SpnNT_01820	aqpZ2	Chromosome:1832227-1832896	ΔORF2	110.58+peptide	OK	75.5899	59.9724	-0.333895	-0.677316	0.2311	0.72461	no
gene:SpnNT_01820	aqpZ2	Chromosome:1832227-1832896	110.58	ΔORF2+peptide	OK	83.1138	56.6589	-0.552786	-1.12051	0.05645	0.338391	no
gene:SpnNT_01820	aqpZ2	Chromosome:1832227-1832896	ΔORF2	ΔORF2+peptide	OK	75.5899	56.6589	-0.41589	-0.838234	0.1467	0.584218	no
gene:SpnNT_01820	aqpZ2	Chromosome:1832227-1832896	110.58+peptide	ΔORF2+peptide	OK	59.9724	56.6589	-0.081995	-0.16287	0.77365	0.994748	no
gene:SpnNT_01821	NA	Chromosome:1833253-1833769	110.58	ΔORF2	OK	46.2333	44.6272	-0.0510108	-0.0937207	0.8693	0.994748	no
gene:SpnNT_01821	NA	Chromosome:1833253-1833769	110.58	110.58+peptide	OK	46.2333	37.8877	-0.287206	-0.523836	0.3692	0.866165	no
gene:SpnNT_01821	NA	Chromosome:1833253-1833769	ΔORF2	110.58+peptide	OK	44.6272	37.8877	-0.236195	-0.428125	0.4569	0.921525	no
gene:SpnNT_01821	NA	Chromosome:1833253-1833769	110.58	ΔORF2+peptide	OK	46.2333	36.4905	-0.341413	-0.618209	0.2853	0.788813	no
gene:SpnNT_01821	NA	Chromosome:1833253-1833769	ΔORF2	ΔORF2+peptide	OK	44.6272	36.4905	-0.290403	-0.522627	0.36015	0.860974	no
gene:SpnNT_01821	NA	Chromosome:1833253-1833769	110.58+peptide	ΔORF2+peptide	OK	37.8877	36.4905	-0.0542076	-0.0968743	0.868	0.994748	no
gene:SpnNT_01822	NA	Chromosome:1833814-1834048	110.58	ΔORF2	OK	34.8703	30.0477	-0.214742	-0.281057	0.62815	0.980887	no
gene:SpnNT_01822	NA	Chromosome:1833814-1834048	110.58	110.58+peptide	OK	34.8703	37.989	0.123585	0.165971	0.7741	0.994748	no
gene:SpnNT_01822	NA	Chromosome:1833814-1834048	ΔORF2	110.58+peptide	OK	30.0477	37.989	0.338327	0.444721	0.4433	0.917631	no
gene:SpnNT_01822	NA	Chromosome:1833814-1834048	110.58	ΔORF2+peptide	OK	34.8703	44.532	0.352844	0.477579	0.4158	0.901203	no
gene:SpnNT_01822	NA	Chromosome:1833814-1834048	ΔORF2	ΔORF2+peptide	OK	30.0477	44.532	0.567586	0.751683	0.2064	0.691266	no
gene:SpnNT_01822	NA	Chromosome:1833814-1834048	110.58+peptide	ΔORF2+peptide	OK	37.989	44.532	0.229259	0.311742	0.6037	0.976761	no
gene:SpnNT_01823	NA	Chromosome:1834088-1834880	110.58	ΔORF2	OK	55.0325	68.8996	0.324211	0.659954	0.2472	0.743921	no
gene:SpnNT_01823	NA	Chromosome:1834088-1834880	110.58	110.58+peptide	OK	55.0325	38.5496	-0.513567	-1.02203	0.07045	0.390391	no

gene:SpnNT_01823	NA	Chromosome:1834088-1834880	ΔORF2	110.58+peptide	OK	68.8996	38.5496	-0.837778	-1.6607	0.0048	0.058266	no
gene:SpnNT_01823	NA	Chromosome:1834088-1834880	110.58	ΔORF2+peptide	OK	55.0325	44.8433	-0.295391	-0.59377	0.2821	0.785039	no
gene:SpnNT_01823	NA	Chromosome:1834088-1834880	ΔORF2	ΔORF2+peptide	OK	68.8996	44.8433	-0.619602	-1.2405	0.0312	0.228336	no
gene:SpnNT_01823	NA	Chromosome:1834088-1834880	110.58+peptide	ΔORF2+peptide	OK	38.5496	44.8433	0.218176	0.42735	0.43935	0.916225	no
gene:SpnNT_01824	yadH	Chromosome:1835304-1837830	110.58	ΔORF2	OK	14.7748	11.4717	-0.36506	-0.351468	0.5368	0.959314	no
gene:SpnNT_01824	yadH	Chromosome:1835304-1837830	110.58	110.58+peptide	OK	14.7748	12.2221	-0.273639	-0.274433	0.6348	0.980887	no
gene:SpnNT_01824	yadH	Chromosome:1835304-1837830	ΔORF2	110.58+peptide	OK	11.4717	12.2221	0.0914206	0.0867199	0.8835	0.994748	no
gene:SpnNT_01824	yadH	Chromosome:1835304-1837830	110.58	ΔORF2+peptide	OK	14.7748	11.9637	-0.304473	-0.299321	0.59865	0.97629	no
gene:SpnNT_01824	yadH	Chromosome:1835304-1837830	ΔORF2	ΔORF2+peptide	OK	11.4717	11.9637	0.0605873	0.0564529	0.92195	0.994748	no
gene:SpnNT_01824	yadH	Chromosome:1835304-1837830	110.58+peptide	ΔORF2+peptide	OK	12.2221	11.9637	-0.0308332	-0.0298463	0.9592	0.994855	no
gene:SpnNT_01825	drrA	Chromosome:1835304-1837830	110.58	ΔORF2	OK	18.6577	17.4003	-0.100661	-0.111349	0.84695	0.994748	no
gene:SpnNT_01825	drrA	Chromosome:1835304-1837830	110.58	110.58+peptide	OK	18.6577	17.3044	-0.108631	-0.119073	0.83905	0.994748	no
gene:SpnNT_01825	drrA	Chromosome:1835304-1837830	ΔORF2	110.58+peptide	OK	17.4003	17.3044	-0.00796948	-0.00886667	0.9872	0.997359	no
gene:SpnNT_01825	drrA	Chromosome:1835304-1837830	110.58	ΔORF2+peptide	OK	18.6577	19.1873	0.0403849	0.0449718	0.9405	0.994855	no
gene:SpnNT_01825	drrA	Chromosome:1835304-1837830	ΔORF2	ΔORF2+peptide	OK	17.4003	19.1873	0.141046	0.1595	0.77965	0.994748	no
gene:SpnNT_01825	drrA	Chromosome:1835304-1837830	110.58+peptide	ΔORF2+peptide	OK	17.3044	19.1873	0.149015	0.166913	0.77135	0.994748	no
gene:SpnNT_01826	moeZ_2	Chromosome:1835304-1837830	110.58	ΔORF2	OK	9.56667	12.1851	0.349024	0.393664	0.4929	0.941402	no
gene:SpnNT_01826	moeZ_2	Chromosome:1835304-1837830	110.58	110.58+peptide	OK	9.56667	8.13421	-0.234016	-0.243536	0.66735	0.983929	no
gene:SpnNT_01826	moeZ_2	Chromosome:1835304-1837830	ΔORF2	110.58+peptide	OK	12.1851	8.13421	-0.58304	-0.619652	0.2863	0.789581	no
gene:SpnNT_01826	moeZ_2	Chromosome:1835304-1837830	110.58	ΔORF2+peptide	OK	9.56667	10.3493	0.113443	0.127489	0.82335	0.994748	no
gene:SpnNT_01826	moeZ_2	Chromosome:1835304-1837830	ΔORF2	ΔORF2+peptide	OK	12.1851	10.3493	-0.235582	-0.271346	0.6415	0.980887	no
gene:SpnNT_01826	moeZ_2	Chromosome:1835304-1837830	110.58+peptide	ΔORF2+peptide	OK	8.13421	10.3493	0.347459	0.36809	0.5196	0.954494	no
gene:SpnNT_01827	sdpR	Chromosome:1838097-1838775	110.58	ΔORF2	OK	35.7424	34.2072	-0.0633335	-0.118554	0.83875	0.994748	no
gene:SpnNT_01827	sdpR	Chromosome:1838097-1838775	110.58	110.58+peptide	OK	35.7424	33.3078	-0.101774	-0.190511	0.7438	0.994748	no
gene:SpnNT_01827	sdpR	Chromosome:1838097-1838775	ΔORF2	110.58+peptide	OK	34.2072	33.3078	-0.0384407	-0.0718182	0.90365	0.994748	no
gene:SpnNT_01827	sdpR	Chromosome:1838097-1838775	110.58	ΔORF2+peptide	OK	35.7424	30.0574	-0.249917	-0.463148	0.41715	0.902489	no
gene:SpnNT_01827	sdpR	Chromosome:1838097-1838775	ΔORF2	ΔORF2+peptide	OK	34.2072	30.0574	-0.186584	-0.345124	0.5436	0.961568	no
gene:SpnNT_01827	sdpR	Chromosome:1838097-1838775	110.58+peptide	ΔORF2+peptide	OK	33.3078	30.0574	-0.148143	-0.274019	0.628	0.980887	no
gene:SpnNT_01828	pepF1_2	Chromosome:1838834-1840631	110.58	ΔORF2	OK	94.3632	77.1605	-0.290362	-0.658828	0.25585	0.754781	no
gene:SpnNT_01828	pepF1_2	Chromosome:1838834-1840631	110.58	110.58+peptide	OK	94.3632	89.761	-0.0721354	-0.163801	0.77565	0.994748	no
gene:SpnNT_01828	pepF1_2	Chromosome:1838834-1840631	ΔORF2	110.58+peptide	OK	77.1605	89.761	0.218226	0.495214	0.3854	0.881745	no
gene:SpnNT_01828	pepF1_2	Chromosome:1838834-1840631	110.58	ΔORF2+peptide	OK	94.3632	69.6469	-0.438165	-0.991688	0.0886	0.448365	no
gene:SpnNT_01828	pepF1_2	Chromosome:1838834-1840631	ΔORF2	ΔORF2+peptide	OK	77.1605	69.6469	-0.147803	-0.334303	0.56275	0.968621	no
gene:SpnNT_01828	pepF1_2	Chromosome:1838834-1840631	110.58+peptide	ΔORF2+peptide	OK	89.761	69.6469	-0.366029	-0.828527	0.15155	0.593484	no
gene:SpnNT_01829	rsmE	Chromosome:1840642-1841386	110.58	ΔORF2	OK	53.8172	41.6038	-0.371354	-0.73005	0.1988	0.681625	no
gene:SpnNT_01829	rsmE	Chromosome:1840642-1841386	110.58	110.58+peptide	OK	53.8172	47.0548	-0.193725	-0.381954	0.4972	0.943998	no
gene:SpnNT_01829	rsmE	Chromosome:1840642-1841386	ΔORF2	110.58+peptide	OK	41.6038	47.0548	0.177629	0.34543	0.54265	0.961518	no
gene:SpnNT_01829	rsmE	Chromosome:1840642-1841386	110.58	ΔORF2+peptide	OK	53.8172	43.7854	-0.297617	-0.587716	0.3089	0.814396	no
gene:SpnNT_01829	rsmE	Chromosome:1840642-1841386	ΔORF2	ΔORF2+peptide	OK	41.6038	43.7854	0.0737371	0.143615	0.80265	0.994748	no
gene:SpnNT_01829	rsmE	Chromosome:1840642-1841386	110.58+peptide	ΔORF2+peptide	OK	47.0548	43.7854	-0.103892	-0.202923	0.7247	0.994748	no
gene:SpnNT_01830	prmA	Chromosome:1841387-1842341	110.58	ΔORF2	OK	113.959	103.082	-0.144725	-0.315041	0.5745	0.969538	no
gene:SpnNT_01830	prmA	Chromosome:1841387-1842341	110.58	110.58+peptide	OK	113.959	116.715	0.0344763	0.0765747	0.89195	0.994748	no
gene:SpnNT_01830	prmA	Chromosome:1841387-1842341	ΔORF2	110.58+peptide	OK	103.082	116.715	0.179201	0.386685	0.4915	0.940662	no
gene:SpnNT_01830	prmA	Chromosome:1841387-1842341	110.58	ΔORF2+peptide	OK	113.959	90.1591	-0.337974	-0.751417	0.1904	0.668744	no
gene:SpnNT_01830	prmA	Chromosome:1841387-1842341	ΔORF2	ΔORF2+peptide	OK	103.082	90.1591	-0.193249	-0.417391	0.45595	0.921244	no

gene:SpnNT_01830	prmA	Chromosome:1841387-1842341	110.58+peptide	ΔORF2+peptide	OK	116.715	90.1591	-0.372451	-0.820534	0.1554	0.600813	no
gene:SpnNT_01831	NA	Chromosome:1842475-1842904	110.58	ΔORF2	OK	105.106	94.6374	-0.151366	-0.29221	0.6022	0.976761	no
gene:SpnNT_01831	NA	Chromosome:1842475-1842904	110.58	110.58+peptide	OK	105.106	108.699	0.0484844	0.0942146	0.8632	0.994748	no
gene:SpnNT_01831	NA	Chromosome:1842475-1842904	ΔORF2	110.58+peptide	OK	94.6374	108.699	0.199851	0.383189	0.49905	0.944017	no
gene:SpnNT_01831	NA	Chromosome:1842475-1842904	110.58	ΔORF2+peptide	OK	105.106	89.9404	-0.224808	-0.434494	0.44185	0.917253	no
gene:SpnNT_01831	NA	Chromosome:1842475-1842904	ΔORF2	ΔORF2+peptide	OK	94.6374	89.9404	-0.0734415	-0.140077	0.80095	0.994748	no
gene:SpnNT_01831	NA	Chromosome:1842475-1842904	110.58+peptide	ΔORF2+peptide	OK	108.699	89.9404	-0.273292	-0.524607	0.3526	0.855356	no
gene:SpnNT_01832	NA	Chromosome:1842905-1843973	110.58	ΔORF2	OK	60.5401	57.3378	-0.0784024	-0.167465	0.76715	0.994748	no
gene:SpnNT_01832	NA	Chromosome:1842905-1843973	110.58	110.58+peptide	OK	60.5401	55.535	-0.124492	-0.264881	0.6373	0.980887	no
gene:SpnNT_01832	NA	Chromosome:1842905-1843973	ΔORF2	110.58+peptide	OK	57.3378	55.535	-0.0460897	-0.0982849	0.8599	0.994748	no
gene:SpnNT_01832	NA	Chromosome:1842905-1843973	110.58	ΔORF2+peptide	OK	60.5401	57.9614	-0.0627979	-0.134178	0.81655	0.994748	no
gene:SpnNT_01832	NA	Chromosome:1842905-1843973	ΔORF2	ΔORF2+peptide	OK	57.3378	57.9614	0.0156045	0.033417	0.95455	0.994855	no
gene:SpnNT_01832	NA	Chromosome:1842905-1843973	110.58+peptide	ΔORF2+peptide	OK	55.535	57.9614	0.0616942	0.131604	0.8228	0.994748	no
gene:SpnNT_01833	NA	Chromosome:1843991-1844462	110.58	ΔORF2	OK	64.1379	68.0667	0.0857719	0.161092	0.77575	0.994748	no
gene:SpnNT_01833	NA	Chromosome:1843991-1844462	110.58	110.58+peptide	OK	64.1379	66.0384	0.0421271	0.0793399	0.89115	0.994748	no
gene:SpnNT_01833	NA	Chromosome:1843991-1844462	ΔORF2	110.58+peptide	OK	68.0667	66.0384	-0.0436448	-0.0818476	0.8892	0.994748	no
gene:SpnNT_01833	NA	Chromosome:1843991-1844462	110.58	ΔORF2+peptide	OK	64.1379	69.3573	0.112869	0.213452	0.71025	0.990984	no
gene:SpnNT_01833	NA	Chromosome:1843991-1844462	ΔORF2	ΔORF2+peptide	OK	68.0667	69.3573	0.0270975	0.0510247	0.9317	0.994855	no
gene:SpnNT_01833	NA	Chromosome:1843991-1844462	110.58+peptide	ΔORF2+peptide	OK	66.0384	69.3573	0.0707424	0.133579	0.8166	0.994748	no
gene:SpnNT_01834	NA	Chromosome:1844752-1845205	110.58	ΔORF2	OK	246.257	239.51	-0.0400796	-0.0861615	0.88195	0.994748	no
gene:SpnNT_01834	NA	Chromosome:1844752-1845205	110.58	110.58+peptide	OK	246.257	232.669	-0.0818868	-0.17798	0.75505	0.994748	no
gene:SpnNT_01834	NA	Chromosome:1844752-1845205	ΔORF2	110.58+peptide	OK	239.51	232.669	-0.0418072	-0.0910166	0.87395	0.994748	no
gene:SpnNT_01834	NA	Chromosome:1844752-1845205	110.58	ΔORF2+peptide	OK	246.257	211.218	-0.221433	-0.472088	0.4129	0.899511	no
gene:SpnNT_01834	NA	Chromosome:1844752-1845205	ΔORF2	ΔORF2+peptide	OK	239.51	211.218	-0.181354	-0.387249	0.50515	0.945292	no
gene:SpnNT_01834	NA	Chromosome:1844752-1845205	110.58+peptide	ΔORF2+peptide	OK	232.669	211.218	-0.139547	-0.301222	0.6067	0.977784	no
gene:SpnNT_01835	NA	Chromosome:1845241-1845421	110.58	ΔORF2	OK	1006.82	1254	0.31674	0.563322	0.30105	0.807023	no
gene:SpnNT_01835	NA	Chromosome:1845241-1845421	110.58	110.58+peptide	OK	1006.82	1383.87	0.458905	0.855101	0.12765	0.541728	no
gene:SpnNT_01835	NA	Chromosome:1845241-1845421	ΔORF2	110.58+peptide	OK	1254	1383.87	0.142165	0.259965	0.63825	0.980887	no
gene:SpnNT_01835	NA	Chromosome:1845241-1845421	110.58	ΔORF2+peptide	OK	1006.82	1528.13	0.601969	1.09179	0.04905	0.310335	no
gene:SpnNT_01835	NA	Chromosome:1845241-1845421	ΔORF2	ΔORF2+peptide	OK	1254	1528.13	0.285229	0.508168	0.3565	0.857875	no
gene:SpnNT_01835	NA	Chromosome:1845241-1845421	110.58+peptide	ΔORF2+peptide	OK	1383.87	1528.13	0.143064	0.267091	0.6344	0.980887	no
gene:SpnNT_01836	NA	Chromosome:1845557-1846017	110.58	ΔORF2	OK	24.3886	28.0968	0.204197	0.178961	0.7578	0.994748	no
gene:SpnNT_01836	NA	Chromosome:1845557-1846017	110.58	110.58+peptide	OK	24.3886	26.0621	0.0957463	0.0896552	0.87665	0.994748	no
gene:SpnNT_01836	NA	Chromosome:1845557-1846017	ΔORF2	110.58+peptide	OK	28.0968	26.0621	-0.10845	-0.0892988	0.87985	0.994748	no
gene:SpnNT_01836	NA	Chromosome:1845557-1846017	110.58	ΔORF2+peptide	OK	24.3886	29.2307	0.261275	0.238201	0.67675	0.98524	no
gene:SpnNT_01836	NA	Chromosome:1845557-1846017	ΔORF2	ΔORF2+peptide	OK	28.0968	29.2307	0.0570778	0.0460312	0.93685	0.994855	no
gene:SpnNT_01836	NA	Chromosome:1845557-1846017	110.58+peptide	ΔORF2+peptide	OK	26.0621	29.2307	0.165528	0.141104	0.8015	0.994748	no
gene:SpnNT_01837	NA	Chromosome:1845557-1846017	110.58	ΔORF2	OK	32.0371	79.6295	1.31356	1.38058	0.02025	0.168819	no
gene:SpnNT_01837	NA	Chromosome:1845557-1846017	110.58	110.58+peptide	OK	32.0371	53.0605	0.727895	0.768025	0.208	0.693596	no
gene:SpnNT_01837	NA	Chromosome:1845557-1846017	ΔORF2	110.58+peptide	OK	79.6295	53.0605	-0.585666	-0.663904	0.2594	0.759841	no
gene:SpnNT_01837	NA	Chromosome:1845557-1846017	110.58	ΔORF2+peptide	OK	32.0371	64.2509	1.00398	1.04487	0.077	0.410729	no
gene:SpnNT_01837	NA	Chromosome:1845557-1846017	ΔORF2	ΔORF2+peptide	OK	79.6295	64.2509	-0.309585	-0.345431	0.54405	0.961568	no
gene:SpnNT_01837	NA	Chromosome:1845557-1846017	110.58+peptide	ΔORF2+peptide	OK	53.0605	64.2509	0.276081	0.309405	0.6107	0.979429	no
gene:SpnNT_01838	rarA	Chromosome:1846308-1847580	110.58	ΔORF2	OK	48.5624	44.6008	-0.122768	-0.26142	0.64915	0.981391	no
gene:SpnNT_01838	rarA	Chromosome:1846308-1847580	110.58	110.58+peptide	OK	48.5624	44.2684	-0.133562	-0.285628	0.6211	0.980887	no

gene:SpnNT_01838	rarA	Chromosome:1846308-1847580	ΔORF2	110.58+peptide	OK	44.6008	44.2684	-0.0107933	-0.0228668	0.96755	0.994855	no
gene:SpnNT_01838	rarA	Chromosome:1846308-1847580	110.58	ΔORF2+peptide	OK	48.5624	34.1844	-0.5065	-1.06252	0.06335	0.365076	no
gene:SpnNT_01838	rarA	Chromosome:1846308-1847580	ΔORF2	ΔORF2+peptide	OK	44.6008	34.1844	-0.383732	-0.797748	0.1549	0.600007	no
gene:SpnNT_01838	rarA	Chromosome:1846308-1847580	110.58+peptide	ΔORF2+peptide	OK	44.2684	34.1844	-0.372939	-0.778493	0.1692	0.626833	no
gene:SpnNT_01839	SpnNT_01839	Chromosome:1847843-1847919	110.58	ΔORF2	OK	4185.36	3587.46	-0.222388	-0.182523	0.75185	0.994748	no
gene:SpnNT_01839	SpnNT_01839	Chromosome:1847843-1847919	110.58	110.58+peptide	OK	4185.36	7311.16	0.80475	0.658	0.20225	0.6861	no
gene:SpnNT_01839	SpnNT_01839	Chromosome:1847843-1847919	ΔORF2	110.58+peptide	OK	3587.46	7311.16	1.02714	0.841105	0.12355	0.531549	no
gene:SpnNT_01839	SpnNT_01839	Chromosome:1847843-1847919	110.58	ΔORF2+peptide	OK	4185.36	6136.63	0.552096	0.639838	0.412	0.899511	no
gene:SpnNT_01839	SpnNT_01839	Chromosome:1847843-1847919	ΔORF2	ΔORF2+peptide	OK	3587.46	6136.63	0.774484	0.900301	0.27215	0.774266	no
gene:SpnNT_01839	SpnNT_01839	Chromosome:1847843-1847919	110.58+peptide	ΔORF2+peptide	OK	7311.16	6136.63	-0.252655	-0.291491	0.68505	0.988044	no
gene:SpnNT_01840	NA	Chromosome:1847981-1849238	110.58	ΔORF2	OK	8.91383	8.01124	-0.154019	-0.264524	0.64255	0.980887	no
gene:SpnNT_01840	NA	Chromosome:1847981-1849238	110.58	110.58+peptide	OK	8.91383	21.1463	1.24629	2.28095	5.00E-05	0.0013612	yes
gene:SpnNT_01840	NA	Chromosome:1847981-1849238	ΔORF2	110.58+peptide	OK	8.01124	21.1463	1.40031	2.53261	5.00E-05	0.0013612	yes
gene:SpnNT_01840	NA	Chromosome:1847981-1849238	110.58	ΔORF2+peptide	OK	8.91383	18.8916	1.08363	1.9658	7.00E-04	0.012687	yes
gene:SpnNT_01840	NA	Chromosome:1847981-1849238	ΔORF2	ΔORF2+peptide	OK	8.01124	18.8916	1.23765	2.21918	1.00E-04	0.0025332	yes
gene:SpnNT_01840	NA	Chromosome:1847981-1849238	110.58+peptide	ΔORF2+peptide	OK	21.1463	18.8916	-0.162661	-0.312714	0.58475	0.971855	no
gene:SpnNT_01841	acpB	Chromosome:1849553-1851026	110.58	ΔORF2	OK	10.0727	17.0036	0.755396	1.35295	0.0197	0.165072	no
gene:SpnNT_01841	acpB	Chromosome:1849553-1851026	110.58	110.58+peptide	OK	10.0727	16.3652	0.700181	1.28289	0.02835	0.214414	no
gene:SpnNT_01841	acpB	Chromosome:1849553-1851026	ΔORF2	110.58+peptide	OK	17.0036	16.3652	-0.055215	-0.101573	0.85465	0.994748	no
gene:SpnNT_01841	acpB	Chromosome:1849553-1851026	110.58	ΔORF2+peptide	OK	10.0727	22.839	1.18105	2.21869	1.00E-04	0.0025332	yes
gene:SpnNT_01841	acpB	Chromosome:1849553-1851026	ΔORF2	ΔORF2+peptide	OK	17.0036	22.839	0.425657	0.803001	0.15615	0.601939	no
gene:SpnNT_01841	acpB	Chromosome:1849553-1851026	110.58+peptide	ΔORF2+peptide	OK	16.3652	22.839	0.480872	0.930393	0.1072	0.498292	no
gene:SpnNT_01842	NA	Chromosome:1851474-1851639	110.58	ΔORF2	OK	889.96	848.772	-0.0683631	-0.123081	0.82835	0.994748	no
gene:SpnNT_01842	NA	Chromosome:1851474-1851639	110.58	110.58+peptide	OK	889.96	1186.64	0.41507	0.716815	0.19825	0.680978	no
gene:SpnNT_01842	NA	Chromosome:1851474-1851639	ΔORF2	110.58+peptide	OK	848.772	1186.64	0.483433	0.829074	0.1453	0.580581	no
gene:SpnNT_01842	NA	Chromosome:1851474-1851639	110.58	ΔORF2+peptide	OK	889.96	921.92	0.0509011	0.0919179	0.87195	0.994748	no
gene:SpnNT_01842	NA	Chromosome:1851474-1851639	ΔORF2	ΔORF2+peptide	OK	848.772	921.92	0.119264	0.213734	0.7103	0.990984	no
gene:SpnNT_01842	NA	Chromosome:1851474-1851639	110.58+peptide	ΔORF2+peptide	OK	1186.64	921.92	-0.364169	-0.626243	0.2763	0.779031	no
gene:SpnNT_01843	NA	Chromosome:1851722-1852289	110.58	ΔORF2	OK	1080.54	1807.31	0.742086	1.57308	0.0062	0.0706832	no
gene:SpnNT_01843	NA	Chromosome:1851722-1852289	110.58	110.58+peptide	OK	1080.54	1532.89	0.5045	1.10816	0.05435	0.331404	no
gene:SpnNT_01843	NA	Chromosome:1851722-1852289	ΔORF2	110.58+peptide	OK	1807.31	1532.89	-0.237586	-0.48653	0.39835	0.892005	no
gene:SpnNT_01843	NA	Chromosome:1851722-1852289	110.58	ΔORF2+peptide	OK	1080.54	1687.95	0.643515	1.44683	0.01175	0.113121	no
gene:SpnNT_01843	NA	Chromosome:1851722-1852289	ΔORF2	ΔORF2+peptide	OK	1807.31	1687.95	-0.0985704	-0.20597	0.7168	0.992452	no
gene:SpnNT_01843	NA	Chromosome:1851722-1852289	110.58+peptide	ΔORF2+peptide	OK	1532.89	1687.95	0.139015	0.300685	0.6029	0.976761	no
gene:SpnNT_01844	NA	Chromosome:1852300-1852471	110.58	ΔORF2	OK	207.168	290.09	0.485703	0.707453	0.2154	0.702231	no
gene:SpnNT_01844	NA	Chromosome:1852300-1852471	110.58	110.58+peptide	OK	207.168	329.351	0.668828	0.984521	0.0853	0.439454	no
gene:SpnNT_01844	NA	Chromosome:1852300-1852471	ΔORF2	110.58+peptide	OK	290.09	329.351	0.183125	0.279141	0.6276	0.980887	no
gene:SpnNT_01844	NA	Chromosome:1852300-1852471	110.58	ΔORF2+peptide	OK	207.168	313.691	0.598546	0.915573	0.1202	0.524705	no
gene:SpnNT_01844	NA	Chromosome:1852300-1852471	ΔORF2	ΔORF2+peptide	OK	290.09	313.691	0.112844	0.179265	0.76055	0.994748	no
gene:SpnNT_01844	NA	Chromosome:1852300-1852471	110.58+peptide	ΔORF2+peptide	OK	329.351	313.691	-0.0702815	-0.113063	0.84945	0.994748	no
gene:SpnNT_01845	NA	Chromosome:1852501-1853110	110.58	ΔORF2	OK	1645.72	2180.19	0.40574	0.888443	0.1188	0.522242	no
gene:SpnNT_01845	NA	Chromosome:1852501-1853110	110.58	110.58+peptide	OK	1645.72	2077.88	0.336393	0.730158	0.20525	0.69003	no
gene:SpnNT_01845	NA	Chromosome:1852501-1853110	ΔORF2	110.58+peptide	OK	2180.19	2077.88	-0.0693466	-0.148335	0.79165	0.994748	no
gene:SpnNT_01845	NA	Chromosome:1852501-1853110	110.58	ΔORF2+peptide	OK	1645.72	2122.88	0.367303	0.815729	0.15	0.591436	no
gene:SpnNT_01845	NA	Chromosome:1852501-1853110	ΔORF2	ΔORF2+peptide	OK	2180.19	2122.88	-0.0384368	-0.0840666	0.8781	0.994748	no

gene:SpnNT_01845	NA	Chromosome:1852501-1853110	110.58+peptide	ΔORF2+peptide	OK	2077.88	2122.88	0.0309098	0.0670147	0.901	0.994748	no
gene:SpnNT_01846	NA	Chromosome:1853140-1853344	110.58	ΔORF2	OK	6808.41	8391.94	0.301687	0.669053	0.2336	0.72774	no
gene:SpnNT_01846	NA	Chromosome:1853140-1853344	110.58	110.58+peptide	OK	6808.41	9640.64	0.501811	1.04991	0.05965	0.35037	no
gene:SpnNT_01846	NA	Chromosome:1853140-1853344	ΔORF2	110.58+peptide	OK	8391.94	9640.64	0.200124	0.414216	0.45405	0.921244	no
gene:SpnNT_01846	NA	Chromosome:1853140-1853344	110.58	ΔORF2+peptide	OK	6808.41	8856.67	0.379446	0.834658	0.13505	0.559237	no
gene:SpnNT_01846	NA	Chromosome:1853140-1853344	ΔORF2	ΔORF2+peptide	OK	8391.94	8856.67	0.0777599	0.169022	0.76365	0.994748	no
gene:SpnNT_01846	NA	Chromosome:1853140-1853344	110.58+peptide	ΔORF2+peptide	OK	9640.64	8856.67	-0.122364	-0.251472	0.64095	0.980887	no
gene:SpnNT_01847	NA	Chromosome:1853765-1854221	110.58	ΔORF2	OK	10.243	10.4136	0.0238224	0.0327027	0.95405	0.994855	no
gene:SpnNT_01847	NA	Chromosome:1853765-1854221	110.58	110.58+peptide	OK	10.243	4.902	-1.0632	-1.39378	0.0173	0.148957	no
gene:SpnNT_01847	NA	Chromosome:1853765-1854221	ΔORF2	110.58+peptide	OK	10.4136	4.902	-1.08702	-1.3819	0.0192	0.161709	no
gene:SpnNT_01847	NA	Chromosome:1853765-1854221	110.58	ΔORF2+peptide	OK	10.243	5.74516	-0.834221	-1.07669	0.0617	0.358718	no
gene:SpnNT_01847	NA	Chromosome:1853765-1854221	ΔORF2	ΔORF2+peptide	OK	10.4136	5.74516	-0.858044	-1.07491	0.0647	0.368967	no
gene:SpnNT_01847	NA	Chromosome:1853765-1854221	110.58+peptide	ΔORF2+peptide	OK	4.902	5.74516	0.228978	0.27597	0.631	0.980887	no
gene:SpnNT_01848	comC	Chromosome:1854290-1854950	110.58	ΔORF2	OK	4.60956	3.16288	-0.543389	-0.699315	0.215	0.701601	no
gene:SpnNT_01848	comC	Chromosome:1854290-1854950	110.58	110.58+peptide	OK	4.60956	4.34875	-0.0840289	-0.111809	0.8482	0.994748	no
gene:SpnNT_01848	comC	Chromosome:1854290-1854950	ΔORF2	110.58+peptide	OK	3.16288	4.34875	0.45936	0.589525	0.306	0.812428	no
gene:SpnNT_01848	comC	Chromosome:1854290-1854950	110.58	ΔORF2+peptide	OK	4.60956	2.91692	-0.660184	-0.842252	0.13715	0.56381	no
gene:SpnNT_01848	comC	Chromosome:1854290-1854950	ΔORF2	ΔORF2+peptide	OK	3.16288	2.91692	-0.116795	-0.14412	0.804	0.994748	no
gene:SpnNT_01848	comC	Chromosome:1854290-1854950	110.58+peptide	ΔORF2+peptide	OK	4.34875	2.91692	-0.576155	-0.733033	0.2031	0.687222	no
gene:SpnNT_01849	NA	Chromosome:1855001-1856153	110.58	ΔORF2	OK	104.114	168.95	0.698433	1.4988	0.0101	0.100938	no
gene:SpnNT_01849	NA	Chromosome:1855001-1856153	110.58	110.58+peptide	OK	104.114	82.8365	-0.329822	-0.72815	0.2052	0.69003	no
gene:SpnNT_01849	NA	Chromosome:1855001-1856153	ΔORF2	110.58+peptide	OK	168.95	82.8365	-1.02826	-2.15867	0.00025	0.00542231	yes
gene:SpnNT_01849	NA	Chromosome:1855001-1856153	110.58	ΔORF2+peptide	OK	104.114	90.7529	-0.198144	-0.441634	0.43995	0.916416	no
gene:SpnNT_01849	NA	Chromosome:1855001-1856153	ΔORF2	ΔORF2+peptide	OK	168.95	90.7529	-0.896577	-1.89852	0.00145	0.0226781	yes
gene:SpnNT_01849	NA	Chromosome:1855001-1856153	110.58+peptide	ΔORF2+peptide	OK	82.8365	90.7529	0.131678	0.286634	0.6223	0.980887	no
gene:SpnNT_01850	NA	Chromosome:1856167-1856791	110.58	ΔORF2	OK	79.571	144.959	0.865335	1.72206	0.0025	0.0347143	yes
gene:SpnNT_01850	NA	Chromosome:1856167-1856791	110.58	110.58+peptide	OK	79.571	58.4435	-0.4452	-0.875709	0.13405	0.556999	no
gene:SpnNT_01850	NA	Chromosome:1856167-1856791	ΔORF2	110.58+peptide	OK	144.959	58.4435	-1.31054	-2.54251	5.00E-05	0.0013612	yes
gene:SpnNT_01850	NA	Chromosome:1856167-1856791	110.58	ΔORF2+peptide	OK	79.571	65.5777	-0.279038	-0.554905	0.33845	0.842721	no
gene:SpnNT_01850	NA	Chromosome:1856167-1856791	ΔORF2	ΔORF2+peptide	OK	144.959	65.5777	-1.14437	-2.2439	0.00025	0.00542231	yes
gene:SpnNT_01850	NA	Chromosome:1856167-1856791	110.58+peptide	ΔORF2+peptide	OK	58.4435	65.5777	0.166163	0.322147	0.58445	0.971855	no
gene:SpnNT_01851	NA	Chromosome:1856813-1856927	110.58	ΔORF2	OK	79.0369	69.6067	-0.1833	-0.139519	0.82465	0.994748	no
gene:SpnNT_01851	NA	Chromosome:1856813-1856927	110.58	110.58+peptide	OK	79.0369	28.3927	-1.47701	-1.62005	0.2173	0.70491	no
gene:SpnNT_01851	NA	Chromosome:1856813-1856927	ΔORF2	110.58+peptide	OK	69.6067	28.3927	-1.29371	-1.35909	0.27105	0.772864	no
gene:SpnNT_01851	NA	Chromosome:1856813-1856927	110.58	ΔORF2+peptide	OK	79.0369	34.1198	-1.21192	-1.32234	0.2479	0.74404	no
gene:SpnNT_01851	NA	Chromosome:1856813-1856927	ΔORF2	ΔORF2+peptide	OK	69.6067	34.1198	-1.02862	-1.07542	0.2818	0.784843	no
gene:SpnNT_01851	NA	Chromosome:1856813-1856927	110.58+peptide	ΔORF2+peptide	OK	28.3927	34.1198	0.265088	1.87536	0.83145	0.994748	no
gene:SpnNT_01852	trpA	Chromosome:1857228-1861553	110.58	ΔORF2	OK	40.8558	41.2672	0.0144533	0.011859	0.98395	0.996246	no
gene:SpnNT_01852	trpA	Chromosome:1857228-1861553	110.58	110.58+peptide	OK	40.8558	21.4801	-0.92754	-0.840785	0.1424	0.576108	no
gene:SpnNT_01852	trpA	Chromosome:1857228-1861553	ΔORF2	110.58+peptide	OK	41.2672	21.4801	-0.941994	-0.788663	0.16755	0.623891	no
gene:SpnNT_01852	trpA	Chromosome:1857228-1861553	110.58	ΔORF2+peptide	OK	40.8558	18.6883	-1.1284	-0.985579	0.09615	0.46955	no
gene:SpnNT_01852	trpA	Chromosome:1857228-1861553	ΔORF2	ΔORF2+peptide	OK	41.2672	18.6883	-1.14286	-0.926842	0.11435	0.512816	no
gene:SpnNT_01852	trpA	Chromosome:1857228-1861553	110.58+peptide	ΔORF2+peptide	OK	21.4801	18.6883	-0.200862	-0.179507	0.75765	0.994748	no
gene:SpnNT_01853	trpB	Chromosome:1857228-1861553	110.58	ΔORF2	OK	49.8131	49.7432	-0.00202613	-0.00258987	0.99665	0.999412	no
gene:SpnNT_01853	trpB	Chromosome:1857228-1861553	110.58	110.58+peptide	OK	49.8131	22.5156	-1.1456	-1.44881	0.01255	0.118903	no

gene:SpnNT_01853	trpB	Chromosome:1857228-1861553	ΔORF2	110.58+peptide	OK	49.7432	22.5156	-1.14357	-1.4266	0.0163	0.143069	no
gene:SpnNT_01853	trpB	Chromosome:1857228-1861553	110.58	ΔORF2+peptide	OK	49.8131	19.4153	-1.35933	-1.70617	0.0035	0.0454723	yes
gene:SpnNT_01853	trpB	Chromosome:1857228-1861553	ΔORF2	ΔORF2+peptide	OK	49.7432	19.4153	-1.3573	-1.68082	0.0053	0.0629381	no
gene:SpnNT_01853	trpB	Chromosome:1857228-1861553	110.58+peptide	ΔORF2+peptide	OK	22.5156	19.4153	-0.213731	-0.262037	0.65885	0.982966	no
gene:SpnNT_01854	trpF	Chromosome:1857228-1861553	110.58	ΔORF2	OK	51.5898	53.3163	0.0474902	0.0376898	0.9463	0.994855	no
gene:SpnNT_01854	trpF	Chromosome:1857228-1861553	110.58	110.58+peptide	OK	51.5898	24.4698	-1.07608	-0.8454	0.13775	0.565214	no
gene:SpnNT_01854	trpF	Chromosome:1857228-1861553	ΔORF2	110.58+peptide	OK	53.3163	24.4698	-1.12357	-0.854397	0.145	0.580489	no
gene:SpnNT_01854	trpF	Chromosome:1857228-1861553	110.58	ΔORF2+peptide	OK	51.5898	21.1421	-1.28697	-1.01392	0.0785	0.416361	no
gene:SpnNT_01854	trpF	Chromosome:1857228-1861553	ΔORF2	ΔORF2+peptide	OK	53.3163	21.1421	-1.33446	-1.01743	0.083	0.431508	no
gene:SpnNT_01854	trpF	Chromosome:1857228-1861553	110.58+peptide	ΔORF2+peptide	OK	24.4698	21.1421	-0.210886	-0.159287	0.78155	0.994748	no
gene:SpnNT_01855	trpC	Chromosome:1857228-1861553	110.58	ΔORF2	OK	38.6076	36.7726	-0.0702543	-0.0543236	0.9244	0.994748	no
gene:SpnNT_01855	trpC	Chromosome:1857228-1861553	110.58	110.58+peptide	OK	38.6076	17.5905	-1.13409	-0.940056	0.10735	0.498339	no
gene:SpnNT_01855	trpC	Chromosome:1857228-1861553	ΔORF2	110.58+peptide	OK	36.7726	17.5905	-1.06383	-0.823825	0.16415	0.618436	no
gene:SpnNT_01855	trpC	Chromosome:1857228-1861553	110.58	ΔORF2+peptide	OK	38.6076	15.584	-1.30881	-1.04105	0.0806	0.423392	no
gene:SpnNT_01855	trpC	Chromosome:1857228-1861553	ΔORF2	ΔORF2+peptide	OK	36.7726	15.584	-1.23856	-0.925048	0.1231	0.531005	no
gene:SpnNT_01855	trpC	Chromosome:1857228-1861553	110.58+peptide	ΔORF2+peptide	OK	17.5905	15.584	-0.174729	-0.139201	0.8055	0.994748	no
gene:SpnNT_01856	trpD	Chromosome:1857228-1861553	110.58	ΔORF2	OK	44.8064	48.3487	0.109775	0.120264	0.82665	0.994748	no
gene:SpnNT_01856	trpD	Chromosome:1857228-1861553	110.58	110.58+peptide	OK	44.8064	19.1235	-1.22836	-1.28617	0.0285	0.215053	no
gene:SpnNT_01856	trpD	Chromosome:1857228-1861553	ΔORF2	110.58+peptide	OK	48.3487	19.1235	-1.33813	-1.41105	0.0144	0.130225	no
gene:SpnNT_01856	trpD	Chromosome:1857228-1861553	110.58	ΔORF2+peptide	OK	44.8064	17.1226	-1.3878	-1.42857	0.01515	0.134962	no
gene:SpnNT_01856	trpD	Chromosome:1857228-1861553	ΔORF2	ΔORF2+peptide	OK	48.3487	17.1226	-1.49757	-1.55213	0.00765	0.0829614	no
gene:SpnNT_01856	trpD	Chromosome:1857228-1861553	110.58+peptide	ΔORF2+peptide	OK	19.1235	17.1226	-0.159443	-0.158661	0.77985	0.994748	no
gene:SpnNT_01857	trpG	Chromosome:1861563-1863488	110.58	ΔORF2	OK	42.8747	52.3628	0.288415	0.306133	0.58715	0.973049	no
gene:SpnNT_01857	trpG	Chromosome:1861563-1863488	110.58	110.58+peptide	OK	42.8747	21.5134	-0.994892	-1.02489	0.07545	0.405594	no
gene:SpnNT_01857	trpG	Chromosome:1861563-1863488	ΔORF2	110.58+peptide	OK	52.3628	21.5134	-1.28331	-1.41044	0.0168	0.14609	no
gene:SpnNT_01857	trpG	Chromosome:1861563-1863488	110.58	ΔORF2+peptide	OK	42.8747	25.7933	-0.733132	-0.727579	0.2206	0.709102	no
gene:SpnNT_01857	trpG	Chromosome:1861563-1863488	ΔORF2	ΔORF2+peptide	OK	52.3628	25.7933	-1.02155	-1.07629	0.07575	0.406374	no
gene:SpnNT_01857	trpG	Chromosome:1861563-1863488	110.58+peptide	ΔORF2+peptide	OK	21.5134	25.7933	0.26176	0.267775	0.6462	0.980887	no
gene:SpnNT_01858	trpE	Chromosome:1861563-1863488	110.58	ΔORF2	OK	40.9494	42.3194	0.0474775	0.0881732	0.8794	0.994748	no
gene:SpnNT_01858	trpE	Chromosome:1861563-1863488	110.58	110.58+peptide	OK	40.9494	16.219	-1.33616	-2.35026	1.00E-04	0.0025332	yes
gene:SpnNT_01858	trpE	Chromosome:1861563-1863488	ΔORF2	110.58+peptide	OK	42.3194	16.219	-1.38363	-2.42569	5.00E-05	0.0013612	yes
gene:SpnNT_01858	trpE	Chromosome:1861563-1863488	110.58	ΔORF2+peptide	OK	40.9494	17.7342	-1.20731	-2.03705	0.00075	0.0133173	yes
gene:SpnNT_01858	trpE	Chromosome:1861563-1863488	ΔORF2	ΔORF2+peptide	OK	42.3194	17.7342	-1.25479	-2.11069	0.00045	0.00885292	yes
gene:SpnNT_01858	trpE	Chromosome:1861563-1863488	110.58+peptide	ΔORF2+peptide	OK	16.219	17.7342	0.128849	0.207204	0.7185	0.993483	no
gene:SpnNT_01859	NA	Chromosome:1863859-1864291	110.58	ΔORF2	OK	494.895	490.325	-0.0133852	-0.0295	0.95555	0.994855	no
gene:SpnNT_01859	NA	Chromosome:1863859-1864291	110.58	110.58+peptide	OK	494.895	262.837	-0.912956	-1.97531	0.00065	0.0118958	yes
gene:SpnNT_01859	NA	Chromosome:1863859-1864291	ΔORF2	110.58+peptide	OK	490.325	262.837	-0.899571	-2.01143	0.00065	0.0118958	yes
gene:SpnNT_01859	NA	Chromosome:1863859-1864291	110.58	ΔORF2+peptide	OK	494.895	201.946	-1.29316	-2.71603	5.00E-05	0.0013612	yes
gene:SpnNT_01859	NA	Chromosome:1863859-1864291	ΔORF2	ΔORF2+peptide	OK	490.325	201.946	-1.27977	-2.77236	5.00E-05	0.0013612	yes
gene:SpnNT_01859	NA	Chromosome:1863859-1864291	110.58+peptide	ΔORF2+peptide	OK	262.837	201.946	-0.380199	-0.809061	0.1617	0.614666	no
gene:SpnNT_01860	ccpA_1	Chromosome:1864898-1865900	110.58	ΔORF2	OK	80.6354	124.035	0.621268	1.32061	0.02435	0.193766	no
gene:SpnNT_01860	ccpA_1	Chromosome:1864898-1865900	110.58	110.58+peptide	OK	80.6354	52.3366	-0.623593	-1.29393	0.02525	0.198283	no
gene:SpnNT_01860	ccpA_1	Chromosome:1864898-1865900	ΔORF2	110.58+peptide	OK	124.035	52.3366	-1.24486	-2.55942	5.00E-05	0.0013612	yes
gene:SpnNT_01860	ccpA_1	Chromosome:1864898-1865900	110.58	ΔORF2+peptide	OK	80.6354	75.7377	-0.0904018	-0.193502	0.7309	0.994748	no
gene:SpnNT_01860	ccpA_1	Chromosome:1864898-1865900	ΔORF2	ΔORF2+peptide	OK	124.035	75.7377	-0.711669	-1.50849	0.00825	0.0878705	no

gene:SpnNT_01860	ccpA_1	Chromosome:1864898-1865900	110.58+peptide	ΔORF2+peptide	OK	52.3366	75.7377	0.533191	1.10337	0.0527	0.325273	no
gene:SpnNT_01861	NA	Chromosome:1866184-1866376	110.58	ΔORF2	NOTEST	0	0	0	0	1	1	no
gene:SpnNT_01861	NA	Chromosome:1866184-1866376	110.58	110.58+peptide	NOTEST	0	0	0	0	1	1	no
gene:SpnNT_01861	NA	Chromosome:1866184-1866376	ΔORF2	110.58+peptide	NOTEST	0	0	0	0	1	1	no
gene:SpnNT_01861	NA	Chromosome:1866184-1866376	110.58	ΔORF2+peptide	NOTEST	0	0	0	0	1	1	no
gene:SpnNT_01861	NA	Chromosome:1866184-1866376	ΔORF2	ΔORF2+peptide	NOTEST	0	0	0	0	1	1	no
gene:SpnNT_01861	NA	Chromosome:1866184-1866376	110.58+peptide	ΔORF2+peptide	NOTEST	0	0	0	0	1	1	no
gene:SpnNT_01862	NA	Chromosome:1866471-1867182	110.58	ΔORF2	OK	13.5395	18.1119	0.419761	0.705599	0.22315	0.712784	no
gene:SpnNT_01862	NA	Chromosome:1866471-1867182	110.58	110.58+peptide	OK	13.5395	29.2577	1.11164	1.92002	0.00105	0.0174849	yes
gene:SpnNT_01862	NA	Chromosome:1866471-1867182	ΔORF2	110.58+peptide	OK	18.1119	29.2577	0.691881	1.1992	0.0348	0.246835	no
gene:SpnNT_01862	NA	Chromosome:1866471-1867182	110.58	ΔORF2+peptide	OK	13.5395	30.7707	1.18438	2.08161	0.00045	0.00885292	yes
gene:SpnNT_01862	NA	Chromosome:1866471-1867182	ΔORF2	ΔORF2+peptide	OK	18.1119	30.7707	0.764619	1.34874	0.01775	0.151736	no
gene:SpnNT_01862	NA	Chromosome:1866471-1867182	110.58+peptide	ΔORF2+peptide	OK	29.2577	30.7707	0.072738	0.132208	0.81505	0.994748	no
gene:SpnNT_01863	phnV_2	Chromosome:1867184-1868876	110.58	ΔORF2	OK	5.03717	5.65945	0.168047	0.283284	0.61795	0.980887	no
gene:SpnNT_01863	phnV_2	Chromosome:1867184-1868876	110.58	110.58+peptide	OK	5.03717	9.21273	0.871015	1.49271	0.0096	0.0977279	no
gene:SpnNT_01863	phnV_2	Chromosome:1867184-1868876	ΔORF2	110.58+peptide	OK	5.65945	9.21273	0.702968	1.20291	0.03595	0.25213	no
gene:SpnNT_01863	phnV_2	Chromosome:1867184-1868876	110.58	ΔORF2+peptide	OK	5.03717	10.3039	1.03251	1.81885	0.0015	0.0233488	yes
gene:SpnNT_01863	phnV_2	Chromosome:1867184-1868876	ΔORF2	ΔORF2+peptide	OK	5.65945	10.3039	0.864463	1.5204	0.00855	0.0898983	no
gene:SpnNT_01863	phnV_2	Chromosome:1867184-1868876	110.58+peptide	ΔORF2+peptide	OK	9.21273	10.3039	0.161495	0.289183	0.61995	0.980887	no
gene:SpnNT_01864	malK	Chromosome:1868887-1869898	110.58	ΔORF2	OK	7.94446	8.40417	0.0811557	0.134477	0.80795	0.994748	no
gene:SpnNT_01864	malK	Chromosome:1868887-1869898	110.58	110.58+peptide	OK	7.94446	13.9645	0.813743	1.36604	0.0163	0.143069	no
gene:SpnNT_01864	malK	Chromosome:1868887-1869898	ΔORF2	110.58+peptide	OK	8.40417	13.9645	0.732587	1.23433	0.0298	0.220924	no
gene:SpnNT_01864	malK	Chromosome:1868887-1869898	110.58	ΔORF2+peptide	OK	7.94446	15.6714	0.980111	1.67815	0.00415	0.0519126	no
gene:SpnNT_01864	malK	Chromosome:1868887-1869898	ΔORF2	ΔORF2+peptide	OK	8.40417	15.6714	0.898956	1.54508	0.00885	0.092313	no
gene:SpnNT_01864	malK	Chromosome:1868887-1869898	110.58+peptide	ΔORF2+peptide	OK	13.9645	15.6714	0.166368	0.289981	0.61165	0.979429	no
gene:SpnNT_01865	NA	Chromosome:1870000-1871068	110.58	ΔORF2	OK	10.0907	12.7379	0.336095	0.586388	0.299	0.80426	no
gene:SpnNT_01865	NA	Chromosome:1870000-1871068	110.58	110.58+peptide	OK	10.0907	23.5938	1.22537	2.18541	3.00E-04	0.00631878	yes
gene:SpnNT_01865	NA	Chromosome:1870000-1871068	ΔORF2	110.58+peptide	OK	12.7379	23.5938	0.889278	1.59987	0.00645	0.0727121	no
gene:SpnNT_01865	NA	Chromosome:1870000-1871068	110.58	ΔORF2+peptide	OK	10.0907	27.7767	1.46084	2.6754	5.00E-05	0.0013612	yes
gene:SpnNT_01865	NA	Chromosome:1870000-1871068	ΔORF2	ΔORF2+peptide	OK	12.7379	27.7767	1.12475	2.07889	8.00E-04	0.0140342	yes
gene:SpnNT_01865	NA	Chromosome:1870000-1871068	110.58+peptide	ΔORF2+peptide	OK	23.5938	27.7767	0.235471	0.446122	0.4445	0.917673	no
gene:SpnNT_01866	NA	Chromosome:1871092-1871752	110.58	ΔORF2	OK	9.45289	11.1011	0.231873	0.355009	0.527	0.956161	no
gene:SpnNT_01866	NA	Chromosome:1871092-1871752	110.58	110.58+peptide	OK	9.45289	31.1104	1.71857	2.72946	5.00E-05	0.0013612	yes
gene:SpnNT_01866	NA	Chromosome:1871092-1871752	ΔORF2	110.58+peptide	OK	11.1011	31.1104	1.48669	2.33466	5.00E-05	0.0013612	yes
gene:SpnNT_01866	NA	Chromosome:1871092-1871752	110.58	ΔORF2+peptide	OK	9.45289	28.9144	1.61296	2.66311	0.00015	0.00355289	yes
gene:SpnNT_01866	NA	Chromosome:1871092-1871752	ΔORF2	ΔORF2+peptide	OK	11.1011	28.9144	1.38108	2.25262	0.00015	0.00355289	yes
gene:SpnNT_01866	NA	Chromosome:1871092-1871752	110.58+peptide	ΔORF2+peptide	OK	31.1104	28.9144	-0.105609	-0.179609	0.74755	0.994748	no
gene:SpnNT_01867	pfbA	Chromosome:1872773-1874900	110.58	ΔORF2	OK	0.592261	1.19559	1.01342	1.28857	0.0308	0.226786	no
gene:SpnNT_01867	pfbA	Chromosome:1872773-1874900	110.58	110.58+peptide	NOTEST	0.592261	0.646243	0.125845	0	1	1	no
gene:SpnNT_01867	pfbA	Chromosome:1872773-1874900	ΔORF2	110.58+peptide	OK	1.19559	0.646243	-0.88757	-1.13181	0.05015	0.314264	no
gene:SpnNT_01867	pfbA	Chromosome:1872773-1874900	110.58	ΔORF2+peptide	NOTEST	0.592261	0.474587	-0.319559	0	1	1	no
gene:SpnNT_01867	pfbA	Chromosome:1872773-1874900	ΔORF2	ΔORF2+peptide	OK	1.19559	0.474587	-1.33297	-1.54331	0.01115	0.108378	no
gene:SpnNT_01867	pfbA	Chromosome:1872773-1874900	110.58+peptide	ΔORF2+peptide	NOTEST	0.646243	0.474587	-0.445404	0	1	1	no
gene:SpnNT_01868	arnB	Chromosome:1876063-1877290	110.58	ΔORF2	OK	155.58	143.127	-0.120362	-0.273268	0.6228	0.980887	no
gene:SpnNT_01868	arnB	Chromosome:1876063-1877290	110.58	110.58+peptide	OK	155.58	197.889	0.347035	0.790564	0.16175	0.614678	no

gene:SpnNT_01868	arnB	Chromosome:1876063-1877290	ΔORF2	110.58+peptide	OK	143.127	197.889	0.467397	1.05526	0.06255	0.3611	no
gene:SpnNT_01868	arnB	Chromosome:1876063-1877290	110.58	ΔORF2+peptide	OK	155.58	183.403	0.237366	0.545552	0.33025	0.836198	no
gene:SpnNT_01868	arnB	Chromosome:1876063-1877290	ΔORF2	ΔORF2+peptide	OK	143.127	183.403	0.357728	0.814729	0.1496	0.59128	no
gene:SpnNT_01868	arnB	Chromosome:1876063-1877290	110.58+peptide	ΔORF2+peptide	OK	197.889	183.403	-0.10967	-0.250622	0.6514	0.981476	no
gene:SpnNT_01869	wcaJ	Chromosome:1877332-1878025	110.58	ΔORF2	OK	172.602	147.503	-0.226709	-0.500704	0.3736	0.87076	no
gene:SpnNT_01869	wcaJ	Chromosome:1877332-1878025	110.58	110.58+peptide	OK	172.602	220.899	0.355935	0.797434	0.1623	0.615876	no
gene:SpnNT_01869	wcaJ	Chromosome:1877332-1878025	ΔORF2	110.58+peptide	OK	147.503	220.899	0.582645	1.28221	0.02475	0.195998	no
gene:SpnNT_01869	wcaJ	Chromosome:1877332-1878025	110.58	ΔORF2+peptide	OK	172.602	192.816	0.159772	0.360114	0.5269	0.956161	no
gene:SpnNT_01869	wcaJ	Chromosome:1877332-1878025	ΔORF2	ΔORF2+peptide	OK	147.503	192.816	0.386481	0.855477	0.13385	0.556521	no
gene:SpnNT_01869	wcaJ	Chromosome:1877332-1878025	110.58+peptide	ΔORF2+peptide	OK	220.899	192.816	-0.196164	-0.440494	0.43255	0.913334	no
gene:SpnNT_01870	yheH	Chromosome:1878197-1881684	110.58	ΔORF2	OK	91.9308	86.1094	-0.0943775	-0.156116	0.77955	0.994748	no
gene:SpnNT_01870	yheH	Chromosome:1878197-1881684	110.58	110.58+peptide	OK	91.9308	100.859	0.133715	0.220296	0.69935	0.990367	no
gene:SpnNT_01870	yheH	Chromosome:1878197-1881684	ΔORF2	110.58+peptide	OK	86.1094	100.859	0.228093	0.379949	0.5022	0.944784	no
gene:SpnNT_01870	yheH	Chromosome:1878197-1881684	110.58	ΔORF2+peptide	OK	91.9308	106.271	0.209125	0.347408	0.54595	0.961863	no
gene:SpnNT_01870	yheH	Chromosome:1878197-1881684	ΔORF2	ΔORF2+peptide	OK	86.1094	106.271	0.303502	0.509877	0.36635	0.864634	no
gene:SpnNT_01870	yheH	Chromosome:1878197-1881684	110.58+peptide	ΔORF2+peptide	OK	100.859	106.271	0.0754095	0.12616	0.8277	0.994748	no
gene:SpnNT_01871	yheI	Chromosome:1878197-1881684	110.58	ΔORF2	OK	79.438	71.1417	-0.159134	-0.243619	0.6636	0.982966	no
gene:SpnNT_01871	yheI	Chromosome:1878197-1881684	110.58	110.58+peptide	OK	79.438	86.5171	0.123155	0.188474	0.73365	0.994748	no
gene:SpnNT_01871	yheI	Chromosome:1878197-1881684	ΔORF2	110.58+peptide	OK	71.1417	86.5171	0.282289	0.433319	0.44815	0.919688	no
gene:SpnNT_01871	yheI	Chromosome:1878197-1881684	110.58	ΔORF2+peptide	OK	79.438	89.6974	0.175236	0.265783	0.63835	0.980887	no
gene:SpnNT_01871	yheI	Chromosome:1878197-1881684	ΔORF2	ΔORF2+peptide	OK	71.1417	89.6974	0.33437	0.508654	0.37835	0.875802	no
gene:SpnNT_01871	yheI	Chromosome:1878197-1881684	110.58+peptide	ΔORF2+peptide	OK	86.5171	89.6974	0.0520812	0.0792005	0.8866	0.994748	no
gene:SpnNT_01872	NA	Chromosome:1881889-1882135	110.58	ΔORF2	OK	306.7	232.645	-0.398696	-0.710844	0.2058	0.690314	no
gene:SpnNT_01872	NA	Chromosome:1881889-1882135	110.58	110.58+peptide	OK	306.7	284.078	-0.11054	-0.200207	0.7173	0.992452	no
gene:SpnNT_01872	NA	Chromosome:1881889-1882135	ΔORF2	110.58+peptide	OK	232.645	284.078	0.288156	0.502855	0.3699	0.866446	no
gene:SpnNT_01872	NA	Chromosome:1881889-1882135	110.58	ΔORF2+peptide	OK	306.7	286.053	-0.100546	-0.18351	0.7467	0.994748	no
gene:SpnNT_01872	NA	Chromosome:1881889-1882135	ΔORF2	ΔORF2+peptide	OK	232.645	286.053	0.29815	0.524012	0.3583	0.859746	no
gene:SpnNT_01872	NA	Chromosome:1881889-1882135	110.58+peptide	ΔORF2+peptide	OK	284.078	286.053	0.00999355	0.0178344	0.9751	0.99536	no
gene:SpnNT_01873	NA	Chromosome:1882338-1882608	110.58	ΔORF2	OK	188.292	180.72	-0.059221	-0.106933	0.8518	0.994748	no
gene:SpnNT_01873	NA	Chromosome:1882338-1882608	110.58	110.58+peptide	OK	188.292	184.039	-0.0329669	-0.0588442	0.91805	0.994748	no
gene:SpnNT_01873	NA	Chromosome:1882338-1882608	ΔORF2	110.58+peptide	OK	180.72	184.039	0.0262541	0.0463338	0.9346	0.994855	no
gene:SpnNT_01873	NA	Chromosome:1882338-1882608	110.58	ΔORF2+peptide	OK	188.292	194.13	0.0440463	0.0767664	0.8941	0.994748	no
gene:SpnNT_01873	NA	Chromosome:1882338-1882608	ΔORF2	ΔORF2+peptide	OK	180.72	194.13	0.103267	0.178044	0.75295	0.994748	no
gene:SpnNT_01873	NA	Chromosome:1882338-1882608	110.58+peptide	ΔORF2+peptide	OK	184.039	194.13	0.0770132	0.131388	0.81225	0.994748	no
gene:SpnNT_01874	NA	Chromosome:1882638-1882794	110.58	ΔORF2	OK	98.5981	149.93	0.604658	0.762796	0.1903	0.668572	no
gene:SpnNT_01874	NA	Chromosome:1882638-1882794	110.58	110.58+peptide	OK	98.5981	163.439	0.729117	0.928763	0.11455	0.513187	no
gene:SpnNT_01874	NA	Chromosome:1882638-1882794	ΔORF2	110.58+peptide	OK	149.93	163.439	0.12446	0.163419	0.774	0.994748	no
gene:SpnNT_01874	NA	Chromosome:1882638-1882794	110.58	ΔORF2+peptide	OK	98.5981	231.465	1.23116	1.67524	0.01025	0.102049	no
gene:SpnNT_01874	NA	Chromosome:1882638-1882794	ΔORF2	ΔORF2+peptide	OK	149.93	231.465	0.626505	0.88262	0.15565	0.601087	no
gene:SpnNT_01874	NA	Chromosome:1882638-1882794	110.58+peptide	ΔORF2+peptide	OK	163.439	231.465	0.502046	0.715902	0.25865	0.759455	no
gene:SpnNT_01875	NA	Chromosome:1883089-1884823	110.58	ΔORF2	OK	22.5665	23.5808	0.0634305	0.13013	0.8139	0.994748	no
gene:SpnNT_01875	NA	Chromosome:1883089-1884823	110.58	110.58+peptide	OK	22.5665	22.553	-0.000863472	-0.00175982	0.9984	0.999412	no
gene:SpnNT_01875	NA	Chromosome:1883089-1884823	ΔORF2	110.58+peptide	OK	23.5808	22.553	-0.064294	-0.130982	0.815	0.994748	no
gene:SpnNT_01875	NA	Chromosome:1883089-1884823	110.58	ΔORF2+peptide	OK	22.5665	29.7963	0.40095	0.828224	0.15185	0.594089	no
gene:SpnNT_01875	NA	Chromosome:1883089-1884823	ΔORF2	ΔORF2+peptide	OK	23.5808	29.7963	0.33752	0.696906	0.2257	0.716568	no

gene:SpnNT_01875	NA	Chromosome:1883089-1884823	110.58+peptide	ΔORF2+peptide	OK	22.553	29.7963	0.401814	0.824143	0.15705	0.604166	no
gene:SpnNT_01876	exoA	Chromosome:1885019-1885847	110.58	ΔORF2	OK	88.3174	83.1176	-0.0875429	-0.18751	0.74135	0.994748	no
gene:SpnNT_01876	exoA	Chromosome:1885019-1885847	110.58	110.58+peptide	OK	88.3174	84.5822	-0.062344	-0.132912	0.8179	0.994748	no
gene:SpnNT_01876	exoA	Chromosome:1885019-1885847	ΔORF2	110.58+peptide	OK	83.1176	84.5822	0.0251989	0.0537493	0.927	0.994748	no
gene:SpnNT_01876	exoA	Chromosome:1885019-1885847	110.58	ΔORF2+peptide	OK	88.3174	74.677	-0.242033	-0.509458	0.37505	0.87259	no
gene:SpnNT_01876	exoA	Chromosome:1885019-1885847	ΔORF2	ΔORF2+peptide	OK	83.1176	74.677	-0.15449	-0.325349	0.56415	0.968621	no
gene:SpnNT_01876	exoA	Chromosome:1885019-1885847	110.58+peptide	ΔORF2+peptide	OK	84.5822	74.677	-0.179689	-0.376708	0.5043	0.945292	no
gene:SpnNT_01877	NA	Chromosome:1885858-1886106	110.58	ΔORF2	OK	1.46612	13.3638	3.18826	2.11161	0.0613	0.356867	no
gene:SpnNT_01877	NA	Chromosome:1885858-1886106	110.58	110.58+peptide	OK	1.46612	9.05529	2.62676	3.03048	0.06575	0.373171	no
gene:SpnNT_01877	NA	Chromosome:1885858-1886106	ΔORF2	110.58+peptide	OK	13.3638	9.05529	-0.561497	-0.42375	0.32985	0.835739	no
gene:SpnNT_01877	NA	Chromosome:1885858-1886106	110.58	ΔORF2+peptide	NOTEST	1.46612	3.18702	1.12021	0	1	1	no
gene:SpnNT_01877	NA	Chromosome:1885858-1886106	ΔORF2	ΔORF2+peptide	OK	13.3638	3.18702	-2.06805	-1.55148	0.1369	0.563312	no
gene:SpnNT_01877	NA	Chromosome:1885858-1886106	110.58+peptide	ΔORF2+peptide	OK	9.05529	3.18702	-1.50655	-3.02306	0.1114	0.508272	no
gene:SpnNT_01878	NA	Chromosome:1885858-1886106	110.58	ΔORF2	NOTEST	0	0	0	0	1	1	no
gene:SpnNT_01878	NA	Chromosome:1885858-1886106	110.58	110.58+peptide	NOTEST	0	0	0	0	1	1	no
gene:SpnNT_01878	NA	Chromosome:1885858-1886106	ΔORF2	110.58+peptide	NOTEST	0	0	0	0	1	1	no
gene:SpnNT_01878	NA	Chromosome:1885858-1886106	110.58	ΔORF2+peptide	NOTEST	0	0	0	0	1	1	no
gene:SpnNT_01878	NA	Chromosome:1885858-1886106	ΔORF2	ΔORF2+peptide	NOTEST	0	0	0	0	1	1	no
gene:SpnNT_01878	NA	Chromosome:1885858-1886106	110.58+peptide	ΔORF2+peptide	NOTEST	0	0	0	0	1	1	no
gene:SpnNT_01879	xpt	Chromosome:1887119-1888963	110.58	ΔORF2	OK	6.90117	6.19504	-0.155728	-0.111908	0.8438	0.994748	no
gene:SpnNT_01879	xpt	Chromosome:1887119-1888963	110.58	110.58+peptide	OK	6.90117	2.71367	-1.3466	-0.928307	0.12845	0.543366	no
gene:SpnNT_01879	xpt	Chromosome:1887119-1888963	ΔORF2	110.58+peptide	OK	6.19504	2.71367	-1.19087	-0.792889	0.1913	0.671187	no
gene:SpnNT_01879	xpt	Chromosome:1887119-1888963	110.58	ΔORF2+peptide	OK	6.90117	1.11346	-2.63179	-1.53901	0.08195	0.428175	no
gene:SpnNT_01879	xpt	Chromosome:1887119-1888963	ΔORF2	ΔORF2+peptide	OK	6.19504	1.11346	-2.47606	-1.41181	0.09175	0.456039	no
gene:SpnNT_01879	xpt	Chromosome:1887119-1888963	110.58+peptide	ΔORF2+peptide	OK	2.71367	1.11346	-1.28519	-0.713593	0.3065	0.81251	no
gene:SpnNT_01880	pucK	Chromosome:1887119-1888963	110.58	ΔORF2	OK	10.8195	10.5242	-0.0399265	-0.0658925	0.90935	0.994748	no
gene:SpnNT_01880	pucK	Chromosome:1887119-1888963	110.58	110.58+peptide	OK	10.8195	4.64346	-1.22036	-1.88958	0.00145	0.0226781	yes
gene:SpnNT_01880	pucK	Chromosome:1887119-1888963	ΔORF2	110.58+peptide	OK	10.5242	4.64346	-1.18043	-1.81914	0.00245	0.0342374	yes
gene:SpnNT_01880	pucK	Chromosome:1887119-1888963	110.58	ΔORF2+peptide	OK	10.8195	2.84398	-1.92765	-2.87043	5.00E-05	0.0013612	yes
gene:SpnNT_01880	pucK	Chromosome:1887119-1888963	ΔORF2	ΔORF2+peptide	OK	10.5242	2.84398	-1.88772	-2.79871	5.00E-05	0.0013612	yes
gene:SpnNT_01880	pucK	Chromosome:1887119-1888963	110.58+peptide	ΔORF2+peptide	OK	4.64346	2.84398	-0.707289	-0.995395	0.07965	0.42059	no
gene:SpnNT_01881	dpnB	Chromosome:1889049-1890673	110.58	ΔORF2	OK	37.3722	64.3406	0.783763	1.30717	0.02415	0.192642	no
gene:SpnNT_01881	dpnB	Chromosome:1889049-1890673	110.58	110.58+peptide	OK	37.3722	49.4372	0.40363	0.686347	0.2339	0.72774	no
gene:SpnNT_01881	dpnB	Chromosome:1889049-1890673	ΔORF2	110.58+peptide	OK	64.3406	49.4372	-0.380133	-0.639689	0.2692	0.770939	no
gene:SpnNT_01881	dpnB	Chromosome:1889049-1890673	110.58	ΔORF2+peptide	OK	37.3722	69.577	0.896643	1.56908	0.0065	0.0732129	no
gene:SpnNT_01881	dpnB	Chromosome:1889049-1890673	ΔORF2	ΔORF2+peptide	OK	64.3406	69.577	0.11288	0.195369	0.73765	0.994748	no
gene:SpnNT_01881	dpnB	Chromosome:1889049-1890673	110.58+peptide	ΔORF2+peptide	OK	49.4372	69.577	0.493013	0.871297	0.13	0.547452	no
gene:SpnNT_01882	dpnA_2	Chromosome:1889049-1890673	110.58	ΔORF2	OK	21.6197	29.9376	0.469608	0.516127	0.36945	0.866165	no
gene:SpnNT_01882	dpnA_2	Chromosome:1889049-1890673	110.58	110.58+peptide	OK	21.6197	25.7427	0.251812	0.284188	0.6164	0.980293	no
gene:SpnNT_01882	dpnA_2	Chromosome:1889049-1890673	ΔORF2	110.58+peptide	OK	29.9376	25.7427	-0.217797	-0.234734	0.67265	0.984845	no
gene:SpnNT_01882	dpnA_2	Chromosome:1889049-1890673	110.58	ΔORF2+peptide	OK	21.6197	34.7646	0.685267	0.800252	0.1676	0.6239	no
gene:SpnNT_01882	dpnA_2	Chromosome:1889049-1890673	ΔORF2	ΔORF2+peptide	OK	29.9376	34.7646	0.215659	0.239763	0.6695	0.984845	no
gene:SpnNT_01882	dpnA_2	Chromosome:1889049-1890673	110.58+peptide	ΔORF2+peptide	OK	25.7427	34.7646	0.433456	0.495157	0.3848	0.881745	no
gene:SpnNT_01883	dpnM	Chromosome:1890698-1891553	110.58	ΔORF2	OK	61.8022	93.611	0.599019	1.21007	0.03645	0.254549	no
gene:SpnNT_01883	dpnM	Chromosome:1890698-1891553	110.58	110.58+peptide	OK	61.8022	62.7109	0.0210575	0.0435136	0.93755	0.994855	no

gene:SpnNT_01883	dpnM	Chromosome:1890698-1891553	ΔORF2	110.58+peptide	OK	93.611	62.7109	-0.577962	-1.16041	0.04055	0.273431	no
gene:SpnNT_01883	dpnM	Chromosome:1890698-1891553	110.58	ΔORF2+peptide	OK	61.8022	85.5813	0.469636	0.984433	0.0856	0.439454	no
gene:SpnNT_01883	dpnM	Chromosome:1890698-1891553	ΔORF2	ΔORF2+peptide	OK	93.611	85.5813	-0.129383	-0.263295	0.6368	0.980887	no
gene:SpnNT_01883	dpnM	Chromosome:1890698-1891553	110.58+peptide	ΔORF2+peptide	OK	62.7109	85.5813	0.448579	0.934121	0.0968	0.470343	no
gene:SpnNT_01884	paaI	Chromosome:1891709-1892114	110.58	ΔORF2	OK	55.8486	45.1237	-0.307637	-0.531997	0.3562	0.857745	no
gene:SpnNT_01884	paaI	Chromosome:1891709-1892114	110.58	110.58+peptide	OK	55.8486	139.188	1.31744	2.41864	5.00E-05	0.0013612	yes
gene:SpnNT_01884	paaI	Chromosome:1891709-1892114	ΔORF2	110.58+peptide	OK	45.1237	139.188	1.62508	2.9022	5.00E-05	0.0013612	yes
gene:SpnNT_01884	paaI	Chromosome:1891709-1892114	110.58	ΔORF2+peptide	OK	55.8486	124.495	1.15649	2.13753	0.00015	0.00355289	yes
gene:SpnNT_01884	paaI	Chromosome:1891709-1892114	ΔORF2	ΔORF2+peptide	OK	45.1237	124.495	1.46413	2.63151	5.00E-05	0.0013612	yes
gene:SpnNT_01884	paaI	Chromosome:1891709-1892114	110.58+peptide	ΔORF2+peptide	OK	139.188	124.495	-0.160947	-0.308674	0.57575	0.969538	no
gene:SpnNT_01885	NA	Chromosome:1892183-1893665	110.58	ΔORF2	OK	4.39927	3.89535	-0.175511	-0.279082	0.62725	0.980887	no
gene:SpnNT_01885	NA	Chromosome:1892183-1893665	110.58	110.58+peptide	OK	4.39927	4.26164	-0.0458568	-0.0736168	0.89695	0.994748	no
gene:SpnNT_01885	NA	Chromosome:1892183-1893665	ΔORF2	110.58+peptide	OK	3.89535	4.26164	0.129654	0.207632	0.71265	0.991034	no
gene:SpnNT_01885	NA	Chromosome:1892183-1893665	110.58	ΔORF2+peptide	OK	4.39927	3.95439	-0.153811	-0.244434	0.6721	0.984845	no
gene:SpnNT_01885	NA	Chromosome:1892183-1893665	ΔORF2	ΔORF2+peptide	OK	3.89535	3.95439	0.0217005	0.0344034	0.9518	0.994855	no
gene:SpnNT_01885	NA	Chromosome:1892183-1893665	110.58+peptide	ΔORF2+peptide	OK	4.26164	3.95439	-0.107954	-0.172778	0.75605	0.994748	no
gene:SpnNT_01886	galK	Chromosome:1893683-1894862	110.58	ΔORF2	OK	4.56849	4.42366	-0.0464779	-0.0717709	0.89755	0.994748	no
gene:SpnNT_01886	galK	Chromosome:1893683-1894862	110.58	110.58+peptide	OK	4.56849	5.25377	0.201634	0.315446	0.57555	0.969538	no
gene:SpnNT_01886	galK	Chromosome:1893683-1894862	ΔORF2	110.58+peptide	OK	4.42366	5.25377	0.248112	0.387909	0.49505	0.942686	no
gene:SpnNT_01886	galK	Chromosome:1893683-1894862	110.58	ΔORF2+peptide	OK	4.56849	4.28474	-0.0925104	-0.14077	0.80195	0.994748	no
gene:SpnNT_01886	galK	Chromosome:1893683-1894862	ΔORF2	ΔORF2+peptide	OK	4.42366	4.28474	-0.0460326	-0.0700039	0.9021	0.994748	no
gene:SpnNT_01886	galK	Chromosome:1893683-1894862	110.58+peptide	ΔORF2+peptide	OK	5.25377	4.28474	-0.294144	-0.453006	0.4395	0.916288	no
gene:SpnNT_01887	lacR_3	Chromosome:1895025-1896033	110.58	ΔORF2	OK	20.6445	19.2764	-0.0989182	-0.184881	0.74665	0.994748	no
gene:SpnNT_01887	lacR_3	Chromosome:1895025-1896033	110.58	110.58+peptide	OK	20.6445	14.2338	-0.536437	-0.980557	0.0964	0.470071	no
gene:SpnNT_01887	lacR_3	Chromosome:1895025-1896033	ΔORF2	110.58+peptide	OK	19.2764	14.2338	-0.437519	-0.795892	0.1737	0.634017	no
gene:SpnNT_01887	lacR_3	Chromosome:1895025-1896033	110.58	ΔORF2+peptide	OK	20.6445	14.7812	-0.481987	-0.882598	0.12985	0.547347	no
gene:SpnNT_01887	lacR_3	Chromosome:1895025-1896033	ΔORF2	ΔORF2+peptide	OK	19.2764	14.7812	-0.383069	-0.698072	0.2295	0.722182	no
gene:SpnNT_01887	lacR_3	Chromosome:1895025-1896033	110.58+peptide	ΔORF2+peptide	OK	14.2338	14.7812	0.0544495	0.0971454	0.86805	0.994748	no
gene:SpnNT_01888	xylB	Chromosome:1896078-1897071	110.58	ΔORF2	OK	39.2779	33.0758	-0.247944	-0.491752	0.3814	0.879103	no
gene:SpnNT_01888	xylB	Chromosome:1896078-1897071	110.58	110.58+peptide	OK	39.2779	34.5472	-0.185148	-0.361011	0.51955	0.954494	no
gene:SpnNT_01888	xylB	Chromosome:1896078-1897071	ΔORF2	110.58+peptide	OK	33.0758	34.5472	0.0627961	0.122153	0.8237	0.994748	no
gene:SpnNT_01888	xylB	Chromosome:1896078-1897071	110.58	ΔORF2+peptide	OK	39.2779	29.5608	-0.410035	-0.771444	0.16885	0.62589	no
gene:SpnNT_01888	xylB	Chromosome:1896078-1897071	ΔORF2	ΔORF2+peptide	OK	33.0758	29.5608	-0.162091	-0.304286	0.57715	0.969538	no
gene:SpnNT_01888	xylB	Chromosome:1896078-1897071	110.58+peptide	ΔORF2+peptide	OK	34.5472	29.5608	-0.224887	-0.415771	0.45245	0.921244	no
gene:SpnNT_01889	adhR	Chromosome:1897105-1897459	110.58	ΔORF2	OK	39.3844	34.8799	-0.17523	-0.278058	0.6244	0.980887	no
gene:SpnNT_01889	adhR	Chromosome:1897105-1897459	110.58	110.58+peptide	OK	39.3844	22.1566	-0.829887	-1.27077	0.0305	0.22497	no
gene:SpnNT_01889	adhR	Chromosome:1897105-1897459	ΔORF2	110.58+peptide	OK	34.8799	22.1566	-0.654657	-0.997491	0.0861	0.440466	no
gene:SpnNT_01889	adhR	Chromosome:1897105-1897459	110.58	ΔORF2+peptide	OK	39.3844	30.1931	-0.383405	-0.568899	0.3164	0.822432	no
gene:SpnNT_01889	adhR	Chromosome:1897105-1897459	ΔORF2	ΔORF2+peptide	OK	34.8799	30.1931	-0.208175	-0.307457	0.58395	0.971794	no
gene:SpnNT_01889	adhR	Chromosome:1897105-1897459	110.58+peptide	ΔORF2+peptide	OK	22.1566	30.1931	0.446482	0.639275	0.2639	0.764605	no
gene:SpnNT_01890	czcD	Chromosome:1897702-1898593	110.58	ΔORF2	OK	7.10909	4.51188	-0.655935	-0.955693	0.0961	0.46955	no
gene:SpnNT_01890	czcD	Chromosome:1897702-1898593	110.58	110.58+peptide	OK	7.10909	5.15593	-0.46343	-0.683481	0.23525	0.729189	no
gene:SpnNT_01890	czcD	Chromosome:1897702-1898593	ΔORF2	110.58+peptide	OK	4.51188	5.15593	0.192505	0.271002	0.6446	0.980887	no
gene:SpnNT_01890	czcD	Chromosome:1897702-1898593	110.58	ΔORF2+peptide	OK	7.10909	5.79716	-0.294317	-0.430994	0.44395	0.917631	no
gene:SpnNT_01890	czcD	Chromosome:1897702-1898593	ΔORF2	ΔORF2+peptide	OK	4.51188	5.79716	0.361619	0.505785	0.369	0.866165	no

gene:SpnNT_01890	czcD	Chromosome:1897702-1898593	110.58+peptide	ΔORF2+peptide	OK	5.15593	5.79716	0.169113	0.239199	0.6716	0.984845	no
gene:SpnNT_01891	kstR2	Chromosome:1898725-1899262	110.58	ΔORF2	OK	7.83654	7.62146	-0.0401503	-0.0561763	0.92245	0.994748	no
gene:SpnNT_01891	kstR2	Chromosome:1898725-1899262	110.58	110.58+peptide	OK	7.83654	6.38851	-0.294737	-0.404689	0.4789	0.933291	no
gene:SpnNT_01891	kstR2	Chromosome:1898725-1899262	ΔORF2	110.58+peptide	OK	7.62146	6.38851	-0.254586	-0.349815	0.53545	0.958867	no
gene:SpnNT_01891	kstR2	Chromosome:1898725-1899262	110.58	ΔORF2+peptide	OK	7.83654	4.32418	-0.85779	-1.07371	0.0676	0.380217	no
gene:SpnNT_01891	kstR2	Chromosome:1898725-1899262	ΔORF2	ΔORF2+peptide	OK	7.62146	4.32418	-0.81764	-1.02407	0.07505	0.404272	no
gene:SpnNT_01891	kstR2	Chromosome:1898725-1899262	110.58+peptide	ΔORF2+peptide	OK	6.38851	4.32418	-0.563054	-0.694611	0.2304	0.723783	no
gene:SpnNT_01892	pnuC	Chromosome:1899428-1900235	110.58	ΔORF2	OK	18.8931	12.2215	-0.62844	-1.06592	0.06425	0.368	no
gene:SpnNT_01892	pnuC	Chromosome:1899428-1900235	110.58	110.58+peptide	OK	18.8931	18.3601	-0.0412863	-0.0706181	0.90285	0.994748	no
gene:SpnNT_01892	pnuC	Chromosome:1899428-1900235	ΔORF2	110.58+peptide	OK	12.2215	18.3601	0.587153	0.999731	0.0885	0.448031	no
gene:SpnNT_01892	pnuC	Chromosome:1899428-1900235	110.58	ΔORF2+peptide	OK	18.8931	14.4831	-0.383492	-0.651128	0.2532	0.751355	no
gene:SpnNT_01892	pnuC	Chromosome:1899428-1900235	ΔORF2	ΔORF2+peptide	OK	12.2215	14.4831	0.244947	0.414031	0.4699	0.927937	no
gene:SpnNT_01892	pnuC	Chromosome:1899428-1900235	110.58+peptide	ΔORF2+peptide	OK	18.3601	14.4831	-0.342206	-0.583274	0.3126	0.818913	no
gene:SpnNT_01893	opuCB	Chromosome:1900579-1902821	110.58	ΔORF2	OK	231.423	243.597	0.0739688	0.134677	0.80755	0.994748	no
gene:SpnNT_01893	opuCB	Chromosome:1900579-1902821	110.58	110.58+peptide	OK	231.423	207.831	-0.155122	-0.275907	0.623	0.980887	no
gene:SpnNT_01893	opuCB	Chromosome:1900579-1902821	ΔORF2	110.58+peptide	OK	243.597	207.831	-0.229091	-0.402035	0.471	0.928415	no
gene:SpnNT_01893	opuCB	Chromosome:1900579-1902821	110.58	ΔORF2+peptide	OK	231.423	242.699	0.0686376	0.121688	0.8266	0.994748	no
gene:SpnNT_01893	opuCB	Chromosome:1900579-1902821	ΔORF2	ΔORF2+peptide	OK	243.597	242.699	-0.00533124	-0.00932647	0.98685	0.997339	no
gene:SpnNT_01893	opuCB	Chromosome:1900579-1902821	110.58+peptide	ΔORF2+peptide	OK	207.831	242.699	0.22376	0.383071	0.5012	0.944758	no
gene:SpnNT_01894	opuCA	Chromosome:1900579-1902821	110.58	ΔORF2	OK	214.204	236.315	0.141725	0.15249	0.7975	0.994748	no
gene:SpnNT_01894	opuCA	Chromosome:1900579-1902821	110.58	110.58+peptide	OK	214.204	218.713	0.0300489	0.033311	0.9514	0.994855	no
gene:SpnNT_01894	opuCA	Chromosome:1900579-1902821	ΔORF2	110.58+peptide	OK	236.315	218.713	-0.111676	-0.122196	0.83285	0.994748	no
gene:SpnNT_01894	opuCA	Chromosome:1900579-1902821	110.58	ΔORF2+peptide	OK	214.204	241.69	0.17417	0.181114	0.75525	0.994748	no
gene:SpnNT_01894	opuCA	Chromosome:1900579-1902821	ΔORF2	ΔORF2+peptide	OK	236.315	241.69	0.0324446	0.0333527	0.95565	0.994855	no
gene:SpnNT_01894	opuCA	Chromosome:1900579-1902821	110.58+peptide	ΔORF2+peptide	OK	218.713	241.69	0.144121	0.152236	0.7828	0.994748	no
gene:SpnNT_01895	NA	Chromosome:1902835-1903600	110.58	ΔORF2	OK	165.684	177.794	0.101767	0.209964	0.70855	0.990475	no
gene:SpnNT_01895	NA	Chromosome:1902835-1903600	110.58	110.58+peptide	OK	165.684	194.286	0.229745	0.449443	0.41835	0.903487	no
gene:SpnNT_01895	NA	Chromosome:1902835-1903600	ΔORF2	110.58+peptide	OK	177.794	194.286	0.127978	0.25181	0.64835	0.981391	no
gene:SpnNT_01895	NA	Chromosome:1902835-1903600	110.58	ΔORF2+peptide	OK	165.684	190.021	0.197718	0.401667	0.4792	0.933501	no
gene:SpnNT_01895	NA	Chromosome:1902835-1903600	ΔORF2	ΔORF2+peptide	OK	177.794	190.021	0.0959504	0.196144	0.7326	0.994748	no
gene:SpnNT_01895	NA	Chromosome:1902835-1903600	110.58+peptide	ΔORF2+peptide	OK	194.286	190.021	-0.0320271	-0.0621356	0.9158	0.994748	no
gene:SpnNT_01896	NA	Chromosome:1903603-1904047	110.58	ΔORF2	OK	204.454	250.621	0.293728	0.580649	0.3024	0.80833	no
gene:SpnNT_01896	NA	Chromosome:1903603-1904047	110.58	110.58+peptide	OK	204.454	201.582	-0.0204119	-0.0386562	0.94365	0.994855	no
gene:SpnNT_01896	NA	Chromosome:1903603-1904047	ΔORF2	110.58+peptide	OK	250.621	201.582	-0.31414	-0.588754	0.2988	0.80426	no
gene:SpnNT_01896	NA	Chromosome:1903603-1904047	110.58	ΔORF2+peptide	OK	204.454	234.711	0.199104	0.370293	0.4861	0.93803	no
gene:SpnNT_01896	NA	Chromosome:1903603-1904047	ΔORF2	ΔORF2+peptide	OK	250.621	234.711	-0.0946238	-0.174221	0.74705	0.994748	no
gene:SpnNT_01896	NA	Chromosome:1903603-1904047	110.58+peptide	ΔORF2+peptide	OK	201.582	234.711	0.219516	0.389325	0.4726	0.928558	no
gene:SpnNT_01897	NA	Chromosome:1904728-1905025	110.58	ΔORF2	OK	1206.65	1282.94	0.0884497	0.197828	0.72665	0.994748	no
gene:SpnNT_01897	NA	Chromosome:1904728-1905025	110.58	110.58+peptide	OK	1206.65	943.701	-0.354602	-0.778676	0.17155	0.631262	no
gene:SpnNT_01897	NA	Chromosome:1904728-1905025	ΔORF2	110.58+peptide	OK	1282.94	943.701	-0.443052	-0.982126	0.08955	0.449782	no
gene:SpnNT_01897	NA	Chromosome:1904728-1905025	110.58	ΔORF2+peptide	OK	1206.65	916.517	-0.396771	-0.882091	0.1213	0.52727	no
gene:SpnNT_01897	NA	Chromosome:1904728-1905025	ΔORF2	ΔORF2+peptide	OK	1282.94	916.517	-0.48522	-1.08921	0.0555	0.335918	no
gene:SpnNT_01897	NA	Chromosome:1904728-1905025	110.58+peptide	ΔORF2+peptide	OK	943.701	916.517	-0.0421683	-0.0929236	0.8731	0.994748	no
gene:SpnNT_01898	pepA_2	Chromosome:1905046-1906111	110.58	ΔORF2	OK	253.963	255.904	0.010982	0.0248367	0.9653	0.994855	no
gene:SpnNT_01898	pepA_2	Chromosome:1905046-1906111	110.58	110.58+peptide	OK	253.963	169.445	-0.5838	-1.32659	0.02195	0.180017	no

gene:SpnNT_01898	pepA_2	Chromosome:1905046-1906111	ΔORF2	110.58+peptide	OK	255.904	169.445	-0.594782	-1.34241	0.01745	0.149953	no
gene:SpnNT_01898	pepA_2	Chromosome:1905046-1906111	110.58	ΔORF2+peptide	OK	253.963	166.629	-0.607977	-1.39073	0.0158	0.139615	no
gene:SpnNT_01898	pepA_2	Chromosome:1905046-1906111	ΔORF2	ΔORF2+peptide	OK	255.904	166.629	-0.618959	-1.40616	0.0126	0.119205	no
gene:SpnNT_01898	pepA_2	Chromosome:1905046-1906111	110.58+peptide	ΔORF2+peptide	OK	169.445	166.629	-0.0241769	-0.0551889	0.91965	0.994748	no
gene:SpnNT_01899	galE_2	Chromosome:1906511-1907213	110.58	ΔORF2	OK	1.63119	1.11629	-0.547211	-0.549355	0.34585	0.849858	no
gene:SpnNT_01899	galE_2	Chromosome:1906511-1907213	110.58	110.58+peptide	OK	1.63119	1.24003	-0.39555	-0.398791	0.48805	0.938811	no
gene:SpnNT_01899	galE_2	Chromosome:1906511-1907213	ΔORF2	110.58+peptide	OK	1.11629	1.24003	0.151661	0.147664	0.8015	0.994748	no
gene:SpnNT_01899	galE_2	Chromosome:1906511-1907213	110.58	ΔORF2+peptide	OK	1.63119	1.98163	0.280765	0.294058	0.60955	0.97901	no
gene:SpnNT_01899	galE_2	Chromosome:1906511-1907213	ΔORF2	ΔORF2+peptide	OK	1.11629	1.98163	0.827976	0.835239	0.1583	0.60755	no
gene:SpnNT_01899	galE_2	Chromosome:1906511-1907213	110.58+peptide	ΔORF2+peptide	OK	1.24003	1.98163	0.676315	0.685181	0.2402	0.735646	no
gene:SpnNT_01900	feuB	Chromosome:1907635-1910290	110.58	ΔORF2	OK	17.7916	12.1188	-0.553955	-0.427368	0.4406	0.916647	no
gene:SpnNT_01900	feuB	Chromosome:1907635-1910290	110.58	110.58+peptide	OK	17.7916	21.6927	0.28601	0.260259	0.6439	0.980887	no
gene:SpnNT_01900	feuB	Chromosome:1907635-1910290	ΔORF2	110.58+peptide	OK	12.1188	21.6927	0.839965	0.676467	0.2178	0.705473	no
gene:SpnNT_01900	feuB	Chromosome:1907635-1910290	110.58	ΔORF2+peptide	OK	17.7916	16.8532	-0.0781731	-0.0679915	0.90335	0.994748	no
gene:SpnNT_01900	feuB	Chromosome:1907635-1910290	ΔORF2	ΔORF2+peptide	OK	12.1188	16.8532	0.475782	0.369719	0.4967	0.943637	no
gene:SpnNT_01900	feuB	Chromosome:1907635-1910290	110.58+peptide	ΔORF2+peptide	OK	21.6927	16.8532	-0.364183	-0.334749	0.5467	0.962122	no
gene:SpnNT_01901	fepD	Chromosome:1907635-1910290	110.58	ΔORF2	OK	19.8184	14.0689	-0.494329	-0.41686	0.45065	0.920951	no
gene:SpnNT_01901	fepD	Chromosome:1907635-1910290	110.58	110.58+peptide	OK	19.8184	20.6852	0.061763	0.0564191	0.9166	0.994748	no
gene:SpnNT_01901	fepD	Chromosome:1907635-1910290	ΔORF2	110.58+peptide	OK	14.0689	20.6852	0.556092	0.488469	0.38335	0.880579	no
gene:SpnNT_01901	fepD	Chromosome:1907635-1910290	110.58	ΔORF2+peptide	OK	19.8184	18.1528	-0.126648	-0.112417	0.83095	0.994748	no
gene:SpnNT_01901	fepD	Chromosome:1907635-1910290	ΔORF2	ΔORF2+peptide	OK	14.0689	18.1528	0.36768	0.314492	0.5677	0.968621	no
gene:SpnNT_01901	fepD	Chromosome:1907635-1910290	110.58+peptide	ΔORF2+peptide	OK	20.6852	18.1528	-0.188411	-0.175008	0.74855	0.994748	no
gene:SpnNT_01902	fhuC	Chromosome:1907635-1910290	110.58	ΔORF2	OK	30.3625	21.3978	-0.504829	-0.465974	0.4442	0.917631	no
gene:SpnNT_01902	fhuC	Chromosome:1907635-1910290	110.58	110.58+peptide	OK	30.3625	31.4149	0.0491575	0.047364	0.9361	0.994855	no
gene:SpnNT_01902	fhuC	Chromosome:1907635-1910290	ΔORF2	110.58+peptide	OK	21.3978	31.4149	0.553987	0.51189	0.40465	0.894933	no
gene:SpnNT_01902	fhuC	Chromosome:1907635-1910290	110.58	ΔORF2+peptide	OK	30.3625	29.183	-0.0571592	-0.0562265	0.92375	0.994748	no
gene:SpnNT_01902	fhuC	Chromosome:1907635-1910290	ΔORF2	ΔORF2+peptide	OK	21.3978	29.183	0.44767	0.421595	0.488	0.938811	no
gene:SpnNT_01902	fhuC	Chromosome:1907635-1910290	110.58+peptide	ΔORF2+peptide	OK	31.4149	29.183	-0.106317	-0.104708	0.86415	0.994748	no
gene:SpnNT_01903	yclQ	Chromosome:1910385-1911351	110.58	ΔORF2	OK	21.0637	15.4171	-0.450228	-0.616568	0.27495	0.777354	no
gene:SpnNT_01903	yclQ	Chromosome:1910385-1911351	110.58	110.58+peptide	OK	21.0637	21.977	0.0612321	0.0909865	0.8695	0.994748	no
gene:SpnNT_01903	yclQ	Chromosome:1910385-1911351	ΔORF2	110.58+peptide	OK	15.4171	21.977	0.51146	0.722603	0.21315	0.698345	no
gene:SpnNT_01903	yclQ	Chromosome:1910385-1911351	110.58	ΔORF2+peptide	OK	21.0637	23.7733	0.174578	0.259069	0.6395	0.980887	no
gene:SpnNT_01903	yclQ	Chromosome:1910385-1911351	ΔORF2	ΔORF2+peptide	OK	15.4171	23.7733	0.624806	0.881691	0.13325	0.556318	no
gene:SpnNT_01903	yclQ	Chromosome:1910385-1911351	110.58+peptide	ΔORF2+peptide	OK	21.977	23.7733	0.113346	0.17451	0.7578	0.994748	no
gene:SpnNT_01904	NA	Chromosome:1911575-1913096	110.58	ΔORF2	OK	291.567	237.669	-0.294874	-0.201518	0.72655	0.994748	no
gene:SpnNT_01904	NA	Chromosome:1911575-1913096	110.58	110.58+peptide	OK	291.567	292.701	0.00560426	0.00423353	0.99345	0.998522	no
gene:SpnNT_01904	NA	Chromosome:1911575-1913096	ΔORF2	110.58+peptide	OK	237.669	292.701	0.300478	0.207885	0.7053	0.990422	no
gene:SpnNT_01904	NA	Chromosome:1911575-1913096	110.58	ΔORF2+peptide	OK	291.567	306.158	0.0704508	0.0498408	0.9322	0.994855	no
gene:SpnNT_01904	NA	Chromosome:1911575-1913096	ΔORF2	ΔORF2+peptide	OK	237.669	306.158	0.365324	0.239084	0.69205	0.98828	no
gene:SpnNT_01904	NA	Chromosome:1911575-1913096	110.58+peptide	ΔORF2+peptide	OK	292.701	306.158	0.0648465	0.0464845	0.93555	0.994855	no
gene:SpnNT_01905	rluB	Chromosome:1911575-1913096	110.58	ΔORF2	OK	174.687	151.434	-0.206086	-0.354291	0.53975	0.961134	no
gene:SpnNT_01905	rluB	Chromosome:1911575-1913096	110.58	110.58+peptide	OK	174.687	167.633	-0.0594675	-0.102511	0.8602	0.994748	no
gene:SpnNT_01905	rluB	Chromosome:1911575-1913096	ΔORF2	110.58+peptide	OK	151.434	167.633	0.146618	0.250659	0.6659	0.98367	no
gene:SpnNT_01905	rluB	Chromosome:1911575-1913096	110.58	ΔORF2+peptide	OK	174.687	145.861	-0.260181	-0.446241	0.43575	0.914093	no
gene:SpnNT_01905	rluB	Chromosome:1911575-1913096	ΔORF2	ΔORF2+peptide	OK	151.434	145.861	-0.0540959	-0.0920229	0.8694	0.994748	no

gene:SpnNT_01905	rluB	Chromosome:1911575-1913096	110.58+peptide	ΔORF2+peptide	OK	167.633	145.861	-0.200714	-0.342345	0.555	0.966389	no
gene:SpnNT_01906	scpB	Chromosome:1911575-1913096	110.58	ΔORF2	OK	130.629	110.885	-0.23641	-0.284027	0.6253	0.980887	no
gene:SpnNT_01906	scpB	Chromosome:1911575-1913096	110.58	110.58+peptide	OK	130.629	118	-0.146679	-0.174168	0.76045	0.994748	no
gene:SpnNT_01906	scpB	Chromosome:1911575-1913096	ΔORF2	110.58+peptide	OK	110.885	118	0.0897302	0.103978	0.8527	0.994748	no
gene:SpnNT_01906	scpB	Chromosome:1911575-1913096	110.58	ΔORF2+peptide	OK	130.629	130.891	0.00289317	0.00369379	0.99525	0.999258	no
gene:SpnNT_01906	scpB	Chromosome:1911575-1913096	ΔORF2	ΔORF2+peptide	OK	110.885	130.891	0.239303	0.297058	0.60175	0.976761	no
gene:SpnNT_01906	scpB	Chromosome:1911575-1913096	110.58+peptide	ΔORF2+peptide	OK	118	130.891	0.149573	0.183362	0.74525	0.994748	no
gene:SpnNT_01907	scpA	Chromosome:1913114-1917291	110.58	ΔORF2	OK	155.928	135.304	-0.204674	-0.195963	0.7346	0.994748	no
gene:SpnNT_01907	scpA	Chromosome:1913114-1917291	110.58	110.58+peptide	OK	155.928	159.861	0.0359354	0.0366908	0.948	0.994855	no
gene:SpnNT_01907	scpA	Chromosome:1913114-1917291	ΔORF2	110.58+peptide	OK	135.304	159.861	0.240609	0.2328	0.6818	0.986574	no
gene:SpnNT_01907	scpA	Chromosome:1913114-1917291	110.58	ΔORF2+peptide	OK	155.928	147.735	-0.0778715	-0.078122	0.8909	0.994748	no
gene:SpnNT_01907	scpA	Chromosome:1913114-1917291	ΔORF2	ΔORF2+peptide	OK	135.304	147.735	0.126802	0.120761	0.83305	0.994748	no
gene:SpnNT_01907	scpA	Chromosome:1913114-1917291	110.58+peptide	ΔORF2+peptide	OK	159.861	147.735	-0.113807	-0.115497	0.8405	0.994748	no
gene:SpnNT_01908	NA	Chromosome:1913114-1917291	110.58	ΔORF2	OK	82.3944	75.1998	-0.131818	-0.0846554	0.8851	0.994748	no
gene:SpnNT_01908	NA	Chromosome:1913114-1917291	110.58	110.58+peptide	OK	82.3944	80.1349	-0.0401165	-0.0258343	0.9652	0.994855	no
gene:SpnNT_01908	NA	Chromosome:1913114-1917291	ΔORF2	110.58+peptide	OK	75.1998	80.1349	0.091702	0.0578067	0.91905	0.994748	no
gene:SpnNT_01908	NA	Chromosome:1913114-1917291	110.58	ΔORF2+peptide	OK	82.3944	83.9135	0.0263568	0.0188814	0.97265	0.99536	no
gene:SpnNT_01908	NA	Chromosome:1913114-1917291	ΔORF2	ΔORF2+peptide	OK	75.1998	83.9135	0.158175	0.110372	0.8427	0.994748	no
gene:SpnNT_01908	NA	Chromosome:1913114-1917291	110.58+peptide	ΔORF2+peptide	OK	80.1349	83.9135	0.0664733	0.0465351	0.9305	0.994855	no
gene:SpnNT_01909	ykuL	Chromosome:1913114-1917291	110.58	ΔORF2	OK	164.476	137.26	-0.260965	-0.16488	0.76125	0.994748	no
gene:SpnNT_01909	ykuL	Chromosome:1913114-1917291	110.58	110.58+peptide	OK	164.476	158.817	-0.0505122	-0.0349875	0.9492	0.994855	no
gene:SpnNT_01909	ykuL	Chromosome:1913114-1917291	ΔORF2	110.58+peptide	OK	137.26	158.817	0.210453	0.12733	0.82555	0.994748	no
gene:SpnNT_01909	ykuL	Chromosome:1913114-1917291	110.58	ΔORF2+peptide	OK	164.476	144.746	-0.184356	-0.129188	0.81405	0.994748	no
gene:SpnNT_01909	ykuL	Chromosome:1913114-1917291	ΔORF2	ΔORF2+peptide	OK	137.26	144.746	0.0766095	0.0467625	0.9342	0.994855	no
gene:SpnNT_01909	ykuL	Chromosome:1913114-1917291	110.58+peptide	ΔORF2+peptide	OK	158.817	144.746	-0.133844	-0.0889699	0.87895	0.994748	no
gene:SpnNT_01910	NA	Chromosome:1913114-1917291	110.58	ΔORF2	OK	179.76	154.77	-0.215942	-0.175221	0.76325	0.994748	no
gene:SpnNT_01910	NA	Chromosome:1913114-1917291	110.58	110.58+peptide	OK	179.76	175.205	-0.0370287	-0.0305414	0.9556	0.994855	no
gene:SpnNT_01910	NA	Chromosome:1913114-1917291	ΔORF2	110.58+peptide	OK	154.77	175.205	0.178914	0.141733	0.809	0.994748	no
gene:SpnNT_01910	NA	Chromosome:1913114-1917291	110.58	ΔORF2+peptide	OK	179.76	143.26	-0.327438	-0.254675	0.6597	0.982966	no
gene:SpnNT_01910	NA	Chromosome:1913114-1917291	ΔORF2	ΔORF2+peptide	OK	154.77	143.26	-0.111496	-0.0836503	0.88925	0.994748	no
gene:SpnNT_01910	NA	Chromosome:1913114-1917291	110.58+peptide	ΔORF2+peptide	OK	175.205	143.26	-0.29041	-0.220941	0.70325	0.990367	no
gene:SpnNT_01911	rdgB	Chromosome:1913114-1917291	110.58	ΔORF2	OK	152.469	139.661	-0.126585	-0.150607	0.7891	0.994748	no
gene:SpnNT_01911	rdgB	Chromosome:1913114-1917291	110.58	110.58+peptide	OK	152.469	134.406	-0.181924	-0.212446	0.7016	0.990367	no
gene:SpnNT_01911	rdgB	Chromosome:1913114-1917291	ΔORF2	110.58+peptide	OK	139.661	134.406	-0.0553392	-0.0637699	0.91205	0.994748	no
gene:SpnNT_01911	rdgB	Chromosome:1913114-1917291	110.58	ΔORF2+peptide	OK	152.469	133.705	-0.189468	-0.22684	0.688	0.98828	no
gene:SpnNT_01911	rdgB	Chromosome:1913114-1917291	ΔORF2	ΔORF2+peptide	OK	139.661	133.705	-0.0628831	-0.0742422	0.8972	0.994748	no
gene:SpnNT_01911	rdgB	Chromosome:1913114-1917291	110.58+peptide	ΔORF2+peptide	OK	134.406	133.705	-0.00754389	-0.00874441	0.9881	0.997703	no
gene:SpnNT_01912	murI	Chromosome:1913114-1917291	110.58	ΔORF2	OK	121.615	113.334	-0.101743	-0.0950459	0.8645	0.994748	no
gene:SpnNT_01912	murI	Chromosome:1913114-1917291	110.58	110.58+peptide	OK	121.615	101.357	-0.262881	-0.240264	0.66385	0.982966	no
gene:SpnNT_01912	murI	Chromosome:1913114-1917291	ΔORF2	110.58+peptide	OK	113.334	101.357	-0.161138	-0.144969	0.8022	0.994748	no
gene:SpnNT_01912	murI	Chromosome:1913114-1917291	110.58	ΔORF2+peptide	OK	121.615	107.117	-0.183134	-0.171831	0.76115	0.994748	no
gene:SpnNT_01912	murI	Chromosome:1913114-1917291	ΔORF2	ΔORF2+peptide	OK	113.334	107.117	-0.0813907	-0.0751092	0.8994	0.994748	no
gene:SpnNT_01912	murI	Chromosome:1913114-1917291	110.58+peptide	ΔORF2+peptide	OK	101.357	107.117	0.0797474	0.0720377	0.89955	0.994748	no
gene:SpnNT_01913	NA	Chromosome:1917497-1917746	110.58	ΔORF2	OK	1105.71	1450.62	0.391699	0.823295	0.15425	0.599191	no
gene:SpnNT_01913	NA	Chromosome:1917497-1917746	110.58	110.58+peptide	OK	1105.71	2173.53	0.975068	2.02762	5.00E-04	0.0095781	yes

gene:SpnNT_01913	NA	Chromosome:1917497-1917746	ΔORF2	110.58+peptide	OK	1450.62	2173.53	0.58337	1.20361	0.031	0.227126	no
gene:SpnNT_01913	NA	Chromosome:1917497-1917746	110.58	ΔORF2+peptide	OK	1105.71	1936.86	0.808745	1.7631	0.0024	0.0337543	yes
gene:SpnNT_01913	NA	Chromosome:1917497-1917746	ΔORF2	ΔORF2+peptide	OK	1450.62	1936.86	0.417047	0.901373	0.1087	0.501534	no
gene:SpnNT_01913	NA	Chromosome:1917497-1917746	110.58+peptide	ΔORF2+peptide	OK	2173.53	1936.86	-0.166323	-0.355432	0.52515	0.955327	no
gene:SpnNT_01914	treA	Chromosome:1918001-1919627	110.58	ΔORF2	OK	7.20296	6.27306	-0.19942	-0.347645	0.5253	0.955327	no
gene:SpnNT_01914	treA	Chromosome:1918001-1919627	110.58	110.58+peptide	OK	7.20296	6.53008	-0.141489	-0.247385	0.65895	0.982966	no
gene:SpnNT_01914	treA	Chromosome:1918001-1919627	ΔORF2	110.58+peptide	OK	6.27306	6.53008	0.0579312	0.10079	0.8543	0.994748	no
gene:SpnNT_01914	treA	Chromosome:1918001-1919627	110.58	ΔORF2+peptide	OK	7.20296	6.34053	-0.183986	-0.317786	0.579	0.969538	no
gene:SpnNT_01914	treA	Chromosome:1918001-1919627	ΔORF2	ΔORF2+peptide	OK	6.27306	6.34053	0.0154341	0.02653	0.9616	0.994855	no
gene:SpnNT_01914	treA	Chromosome:1918001-1919627	110.58+peptide	ΔORF2+peptide	OK	6.53008	6.34053	-0.0424971	-0.0732594	0.89985	0.994748	no
gene:SpnNT_01915	treB	Chromosome:1919804-1921772	110.58	ΔORF2	OK	5.77862	5.23273	-0.14316	-0.24629	0.66025	0.982966	no
gene:SpnNT_01915	treB	Chromosome:1919804-1921772	110.58	110.58+peptide	OK	5.77862	6.5196	0.17406	0.30307	0.59595	0.976247	no
gene:SpnNT_01915	treB	Chromosome:1919804-1921772	ΔORF2	110.58+peptide	OK	5.23273	6.5196	0.317219	0.556675	0.32735	0.83262	no
gene:SpnNT_01915	treB	Chromosome:1919804-1921772	110.58	ΔORF2+peptide	OK	5.77862	5.19863	-0.152593	-0.262917	0.64355	0.980887	no
gene:SpnNT_01915	treB	Chromosome:1919804-1921772	ΔORF2	ΔORF2+peptide	OK	5.23273	5.19863	-0.00943372	-0.0163792	0.97585	0.99536	no
gene:SpnNT_01915	treB	Chromosome:1919804-1921772	110.58+peptide	ΔORF2+peptide	OK	6.5196	5.19863	-0.326653	-0.574131	0.3096	0.814895	no
gene:SpnNT_01916	treR	Chromosome:1921956-1922667	110.58	ΔORF2	OK	94.0188	105.06	0.160196	0.342722	0.55115	0.964385	no
gene:SpnNT_01916	treR	Chromosome:1921956-1922667	110.58	110.58+peptide	OK	94.0188	88.3042	-0.0904667	-0.192776	0.7318	0.994748	no
gene:SpnNT_01916	treR	Chromosome:1921956-1922667	ΔORF2	110.58+peptide	OK	105.06	88.3042	-0.250662	-0.534383	0.35085	0.854885	no
gene:SpnNT_01916	treR	Chromosome:1921956-1922667	110.58	ΔORF2+peptide	OK	94.0188	99.1101	0.076083	0.162781	0.77365	0.994748	no
gene:SpnNT_01916	treR	Chromosome:1921956-1922667	ΔORF2	ΔORF2+peptide	OK	105.06	99.1101	-0.0841128	-0.180044	0.7575	0.994748	no
gene:SpnNT_01916	treR	Chromosome:1921956-1922667	110.58+peptide	ΔORF2+peptide	OK	88.3042	99.1101	0.16655	0.355085	0.53115	0.957488	no
gene:SpnNT_01917	NA	Chromosome:1922785-1924078	110.58	ΔORF2	OK	6.59443	6.82306	0.0491725	0.0829393	0.88565	0.994748	no
gene:SpnNT_01917	NA	Chromosome:1922785-1924078	110.58	110.58+peptide	OK	6.59443	8.88849	0.430692	0.730401	0.2048	0.689595	no
gene:SpnNT_01917	NA	Chromosome:1922785-1924078	ΔORF2	110.58+peptide	OK	6.82306	8.88849	0.381519	0.649428	0.26165	0.761932	no
gene:SpnNT_01917	NA	Chromosome:1922785-1924078	110.58	ΔORF2+peptide	OK	6.59443	9.92346	0.589596	1.01327	0.0762	0.408056	no
gene:SpnNT_01917	NA	Chromosome:1922785-1924078	ΔORF2	ΔORF2+peptide	OK	6.82306	9.92346	0.540424	0.932329	0.1028	0.486105	no
gene:SpnNT_01917	NA	Chromosome:1922785-1924078	110.58+peptide	ΔORF2+peptide	OK	8.88849	9.92346	0.158905	0.275699	0.62655	0.980887	no
gene:SpnNT_01918	oppF	Chromosome:1924152-1925079	110.58	ΔORF2	OK	1538.23	1473.41	-0.0621161	-0.135046	0.8103	0.994748	no
gene:SpnNT_01918	oppF	Chromosome:1924152-1925079	110.58	110.58+peptide	OK	1538.23	1349.32	-0.189036	-0.412904	0.4707	0.928415	no
gene:SpnNT_01918	oppF	Chromosome:1924152-1925079	ΔORF2	110.58+peptide	OK	1473.41	1349.32	-0.12692	-0.276395	0.62885	0.980887	no
gene:SpnNT_01918	oppF	Chromosome:1924152-1925079	110.58	ΔORF2+peptide	OK	1538.23	1460.82	-0.0744904	-0.162648	0.77275	0.994748	no
gene:SpnNT_01918	oppF	Chromosome:1924152-1925079	ΔORF2	ΔORF2+peptide	OK	1473.41	1460.82	-0.0123743	-0.026938	0.9641	0.994855	no
gene:SpnNT_01918	oppF	Chromosome:1924152-1925079	110.58+peptide	ΔORF2+peptide	OK	1349.32	1460.82	0.114546	0.250528	0.6651	0.983559	no
gene:SpnNT_01919	oppD	Chromosome:1925089-1926157	110.58	ΔORF2	OK	925.114	860.182	-0.104988	-0.230975	0.68585	0.988184	no
gene:SpnNT_01919	oppD	Chromosome:1925089-1926157	110.58	110.58+peptide	OK	925.114	810.402	-0.190993	-0.424033	0.4548	0.921244	no
gene:SpnNT_01919	oppD	Chromosome:1925089-1926157	ΔORF2	110.58+peptide	OK	860.182	810.402	-0.0860046	-0.189112	0.73745	0.994748	no
gene:SpnNT_01919	oppD	Chromosome:1925089-1926157	110.58	ΔORF2+peptide	OK	925.114	788.638	-0.230268	-0.512734	0.37135	0.868447	no
gene:SpnNT_01919	oppD	Chromosome:1925089-1926157	ΔORF2	ΔORF2+peptide	OK	860.182	788.638	-0.12528	-0.276267	0.63015	0.980887	no
gene:SpnNT_01919	oppD	Chromosome:1925089-1926157	110.58+peptide	ΔORF2+peptide	OK	810.402	788.638	-0.0392752	-0.0874065	0.88225	0.994748	no
gene:SpnNT_01920	oppC	Chromosome:1926165-1928579	110.58	ΔORF2	OK	779.701	744.902	-0.0658711	-0.0963241	0.86315	0.994748	no
gene:SpnNT_01920	oppC	Chromosome:1926165-1928579	110.58	110.58+peptide	OK	779.701	691.75	-0.17267	-0.25353	0.65195	0.98163	no
gene:SpnNT_01920	oppC	Chromosome:1926165-1928579	ΔORF2	110.58+peptide	OK	744.902	691.75	-0.106799	-0.15533	0.7841	0.994748	no
gene:SpnNT_01920	oppC	Chromosome:1926165-1928579	110.58	ΔORF2+peptide	OK	779.701	687.141	-0.182316	-0.264205	0.6404	0.980887	no
gene:SpnNT_01920	oppC	Chromosome:1926165-1928579	ΔORF2	ΔORF2+peptide	OK	744.902	687.141	-0.116445	-0.167193	0.77115	0.994748	no

gene:SpnNT_01920	oppC	Chromosome:1926165-1928579	110.58+peptide	ΔORF2+peptide	OK	691.75	687.141	-0.00964576	-0.0139041	0.97925	0.995765	no
gene:SpnNT_01921	gsiC	Chromosome:1926165-1928579	110.58	ΔORF2	OK	508.801	496.359	-0.0357186	-0.056163	0.92295	0.994748	no
gene:SpnNT_01921	gsiC	Chromosome:1926165-1928579	110.58	110.58+peptide	OK	508.801	435.361	-0.224889	-0.348375	0.5442	0.961568	no
gene:SpnNT_01921	gsiC	Chromosome:1926165-1928579	ΔORF2	110.58+peptide	OK	496.359	435.361	-0.18917	-0.29388	0.6093	0.978849	no
gene:SpnNT_01921	gsiC	Chromosome:1926165-1928579	110.58	ΔORF2+peptide	OK	508.801	470.604	-0.112587	-0.176558	0.75665	0.994748	no
gene:SpnNT_01921	gsiC	Chromosome:1926165-1928579	ΔORF2	ΔORF2+peptide	OK	496.359	470.604	-0.0768685	-0.120898	0.83005	0.994748	no
gene:SpnNT_01921	gsiC	Chromosome:1926165-1928579	110.58+peptide	ΔORF2+peptide	OK	435.361	470.604	0.112302	0.17401	0.75985	0.994748	no
gene:SpnNT_01922	amiA	Chromosome:1928645-1930625	110.58	ΔORF2	OK	559.618	610.616	0.125823	0.274542	0.619	0.980887	no
gene:SpnNT_01922	amiA	Chromosome:1928645-1930625	110.58	110.58+peptide	OK	559.618	554.074	-0.0143655	-0.0310382	0.95715	0.994855	no
gene:SpnNT_01922	amiA	Chromosome:1928645-1930625	ΔORF2	110.58+peptide	OK	610.616	554.074	-0.140188	-0.300641	0.60555	0.976937	no
gene:SpnNT_01922	amiA	Chromosome:1928645-1930625	110.58	ΔORF2+peptide	OK	559.618	662.005	0.242399	0.528241	0.34395	0.848207	no
gene:SpnNT_01922	amiA	Chromosome:1928645-1930625	ΔORF2	ΔORF2+peptide	OK	610.616	662.005	0.116576	0.252126	0.65545	0.982966	no
gene:SpnNT_01922	amiA	Chromosome:1928645-1930625	110.58+peptide	ΔORF2+peptide	OK	554.074	662.005	0.256765	0.549972	0.33185	0.837894	no
gene:SpnNT_01923	NA	Chromosome:1931311-1932505	110.58	ΔORF2	OK	109.908	111.966	0.0267698	0.0603156	0.91485	0.994748	no
gene:SpnNT_01923	NA	Chromosome:1931311-1932505	110.58	110.58+peptide	OK	109.908	148.984	0.438863	0.983138	0.08615	0.440466	no
gene:SpnNT_01923	NA	Chromosome:1931311-1932505	ΔORF2	110.58+peptide	OK	111.966	148.984	0.412093	0.920325	0.1074	0.498339	no
gene:SpnNT_01923	NA	Chromosome:1931311-1932505	110.58	ΔORF2+peptide	OK	109.908	158.035	0.523948	1.18762	0.03635	0.254335	no
gene:SpnNT_01923	NA	Chromosome:1931311-1932505	ΔORF2	ΔORF2+peptide	OK	111.966	158.035	0.497178	1.12339	0.0484	0.307111	no
gene:SpnNT_01923	NA	Chromosome:1931311-1932505	110.58+peptide	ΔORF2+peptide	OK	148.984	158.035	0.085085	0.191142	0.7386	0.994748	no
gene:SpnNT_01924	gtfA	Chromosome:1932642-1934085	110.58	ΔORF2	OK	3.82116	3.68221	-0.053439	-0.0823015	0.8859	0.994748	no
gene:SpnNT_01924	gtfA	Chromosome:1932642-1934085	110.58	110.58+peptide	OK	3.82116	4.04486	0.0820771	0.127802	0.8232	0.994748	no
gene:SpnNT_01924	gtfA	Chromosome:1932642-1934085	ΔORF2	110.58+peptide	OK	3.68221	4.04486	0.135516	0.213118	0.7088	0.990577	no
gene:SpnNT_01924	gtfA	Chromosome:1932642-1934085	110.58	ΔORF2+peptide	OK	3.82116	3.60927	-0.0823032	-0.124178	0.83015	0.994748	no
gene:SpnNT_01924	gtfA	Chromosome:1932642-1934085	ΔORF2	ΔORF2+peptide	OK	3.68221	3.60927	-0.0288642	-0.0439578	0.93995	0.994855	no
gene:SpnNT_01924	gtfA	Chromosome:1932642-1934085	110.58+peptide	ΔORF2+peptide	OK	4.04486	3.60927	-0.16438	-0.253037	0.661	0.982966	no
gene:SpnNT_01925	araQ_3	Chromosome:1934396-1935230	110.58	ΔORF2	OK	4.82057	3.51713	-0.454806	-0.638373	0.27515	0.777354	no
gene:SpnNT_01925	araQ_3	Chromosome:1934396-1935230	110.58	110.58+peptide	OK	4.82057	3.56444	-0.435529	-0.610337	0.2879	0.791968	no
gene:SpnNT_01925	araQ_3	Chromosome:1934396-1935230	ΔORF2	110.58+peptide	OK	3.51713	3.56444	0.0192775	0.0260376	0.9604	0.994855	no
gene:SpnNT_01925	araQ_3	Chromosome:1934396-1935230	110.58	ΔORF2+peptide	OK	4.82057	2.48023	-0.958729	-1.2901	0.02715	0.208707	no
gene:SpnNT_01925	araQ_3	Chromosome:1934396-1935230	ΔORF2	ΔORF2+peptide	OK	3.51713	2.48023	-0.503922	-0.655379	0.25565	0.7547	no
gene:SpnNT_01925	araQ_3	Chromosome:1934396-1935230	110.58+peptide	ΔORF2+peptide	OK	3.56444	2.48023	-0.5232	-0.679516	0.23125	0.724662	no
gene:SpnNT_01926	ugpA	Chromosome:1935243-1936110	110.58	ΔORF2	OK	3.42691	3.38688	-0.0169512	-0.0229007	0.9684	0.994855	no
gene:SpnNT_01926	ugpA	Chromosome:1935243-1936110	110.58	110.58+peptide	OK	3.42691	3.8332	0.161638	0.21982	0.6961	0.989512	no
gene:SpnNT_01926	ugpA	Chromosome:1935243-1936110	ΔORF2	110.58+peptide	OK	3.38688	3.8332	0.17859	0.243341	0.67155	0.984845	no
gene:SpnNT_01926	ugpA	Chromosome:1935243-1936110	110.58	ΔORF2+peptide	OK	3.42691	1.96166	-0.804836	-0.993615	0.0884	0.447871	no
gene:SpnNT_01926	ugpA	Chromosome:1935243-1936110	ΔORF2	ΔORF2+peptide	OK	3.38688	1.96166	-0.787885	-0.97423	0.09475	0.46531	no
gene:SpnNT_01926	ugpA	Chromosome:1935243-1936110	110.58+peptide	ΔORF2+peptide	OK	3.8332	1.96166	-0.966474	-1.2017	0.0447	0.290805	no
gene:SpnNT_01927	msmE	Chromosome:1936123-1937383	110.58	ΔORF2	OK	6.23832	4.8256	-0.37045	-0.590565	0.3123	0.818454	no
gene:SpnNT_01927	msmE	Chromosome:1936123-1937383	110.58	110.58+peptide	OK	6.23832	5.56075	-0.165879	-0.268436	0.6373	0.980887	no
gene:SpnNT_01927	msmE	Chromosome:1936123-1937383	ΔORF2	110.58+peptide	OK	4.8256	5.56075	0.204571	0.32823	0.56335	0.968621	no
gene:SpnNT_01927	msmE	Chromosome:1936123-1937383	110.58	ΔORF2+peptide	OK	6.23832	3.50366	-0.832294	-1.28555	0.02905	0.217452	no
gene:SpnNT_01927	msmE	Chromosome:1936123-1937383	ΔORF2	ΔORF2+peptide	OK	4.8256	3.50366	-0.461844	-0.707813	0.22215	0.711509	no
gene:SpnNT_01927	msmE	Chromosome:1936123-1937383	110.58+peptide	ΔORF2+peptide	OK	5.56075	3.50366	-0.666416	-1.03557	0.07	0.388882	no
gene:SpnNT_01928	rafA	Chromosome:1937474-1939637	110.58	ΔORF2	OK	3.69535	3.27358	-0.174841	-0.281818	0.6264	0.980887	no
gene:SpnNT_01928	rafA	Chromosome:1937474-1939637	110.58	110.58+peptide	OK	3.69535	3.10983	-0.248873	-0.405442	0.4781	0.933077	no

gene:SpnNT_01928	rafA	Chromosome:1937474-1939637	ΔORF2	110.58+peptide	OK	3.27358	3.10983	-0.0740323	-0.11978	0.8383	0.994748	no
gene:SpnNT_01928	rafA	Chromosome:1937474-1939637	110.58	ΔORF2+peptide	OK	3.69535	2.47224	-0.579893	-0.928461	0.10925	0.502305	no
gene:SpnNT_01928	rafA	Chromosome:1937474-1939637	ΔORF2	ΔORF2+peptide	OK	3.27358	2.47224	-0.405052	-0.644228	0.2676	0.768869	no
gene:SpnNT_01928	rafA	Chromosome:1937474-1939637	110.58+peptide	ΔORF2+peptide	OK	3.10983	2.47224	-0.331019	-0.531967	0.34895	0.853357	no
gene:SpnNT_01929	cdhR	Chromosome:1939744-1941537	110.58	ΔORF2	OK	11.9662	11.5516	-0.0508721	-0.0647317	0.90945	0.994748	no
gene:SpnNT_01929	cdhR	Chromosome:1939744-1941537	110.58	110.58+peptide	OK	11.9662	12.5394	0.0674998	0.0855318	0.88245	0.994748	no
gene:SpnNT_01929	cdhR	Chromosome:1939744-1941537	ΔORF2	110.58+peptide	OK	11.5516	12.5394	0.118372	0.148112	0.79335	0.994748	no
gene:SpnNT_01929	cdhR	Chromosome:1939744-1941537	110.58	ΔORF2+peptide	OK	11.9662	13.0665	0.126914	0.164218	0.7721	0.994748	no
gene:SpnNT_01929	cdhR	Chromosome:1939744-1941537	ΔORF2	ΔORF2+peptide	OK	11.5516	13.0665	0.177786	0.227035	0.68045	0.986397	no
gene:SpnNT_01929	cdhR	Chromosome:1939744-1941537	110.58+peptide	ΔORF2+peptide	OK	12.5394	13.0665	0.0594141	0.0755545	0.894	0.994748	no
gene:SpnNT_01930	birA	Chromosome:1939744-1941537	110.58	ΔORF2	OK	10.5144	12.6713	0.269202	0.353879	0.53915	0.960848	no
gene:SpnNT_01930	birA	Chromosome:1939744-1941537	110.58	110.58+peptide	OK	10.5144	11.1795	0.0884969	0.111699	0.8444	0.994748	no
gene:SpnNT_01930	birA	Chromosome:1939744-1941537	ΔORF2	110.58+peptide	OK	12.6713	11.1795	-0.180705	-0.234955	0.68745	0.98828	no
gene:SpnNT_01930	birA	Chromosome:1939744-1941537	110.58	ΔORF2+peptide	OK	10.5144	13.0266	0.309094	0.406893	0.4815	0.935095	no
gene:SpnNT_01930	birA	Chromosome:1939744-1941537	ΔORF2	ΔORF2+peptide	OK	12.6713	13.0266	0.0398922	0.0542423	0.9242	0.994748	no
gene:SpnNT_01930	birA	Chromosome:1939744-1941537	110.58+peptide	ΔORF2+peptide	OK	11.1795	13.0266	0.220597	0.287219	0.6198	0.980887	no
gene:SpnNT_01931	SpnNT_01931	Chromosome:1941661-1941751	110.58	ΔORF2	OK	4813.41	5614.11	0.221997	0.278025	0.6021	0.976761	no
gene:SpnNT_01931	SpnNT_01931	Chromosome:1941661-1941751	110.58	110.58+peptide	OK	4813.41	10036.6	1.06013	1.33279	0.01565	0.138756	no
gene:SpnNT_01931	SpnNT_01931	Chromosome:1941661-1941751	ΔORF2	110.58+peptide	OK	5614.11	10036.6	0.838134	1.04758	0.0532	0.327128	no
gene:SpnNT_01931	SpnNT_01931	Chromosome:1941661-1941751	110.58	ΔORF2+peptide	OK	4813.41	13864.1	1.52622	2.25727	0.00045	0.00885292	yes
gene:SpnNT_01931	SpnNT_01931	Chromosome:1941661-1941751	ΔORF2	ΔORF2+peptide	OK	5614.11	13864.1	1.30422	1.91349	0.00175	0.0265167	yes
gene:SpnNT_01931	SpnNT_01931	Chromosome:1941661-1941751	110.58+peptide	ΔORF2+peptide	OK	10036.6	13864.1	0.46609	0.687435	0.25275	0.751038	no
gene:SpnNT_01932	SpnNT_01932	Chromosome:1941755-1941831	110.58	ΔORF2	OK	1496.03	2401.78	0.682963	0.442598	0.422	0.905409	no
gene:SpnNT_01932	SpnNT_01932	Chromosome:1941755-1941831	110.58	110.58+peptide	OK	1496.03	2569.71	0.78046	0.484295	0.3538	0.856246	no
gene:SpnNT_01932	SpnNT_01932	Chromosome:1941755-1941831	ΔORF2	110.58+peptide	OK	2401.78	2569.71	0.0974967	0.0678544	0.9167	0.994748	no
gene:SpnNT_01932	SpnNT_01932	Chromosome:1941755-1941831	110.58	ΔORF2+peptide	OK	1496.03	1670.58	0.159208	0.131905	0.8797	0.994748	no
gene:SpnNT_01932	SpnNT_01932	Chromosome:1941755-1941831	ΔORF2	ΔORF2+peptide	OK	2401.78	1670.58	-0.523755	-0.544779	0.57645	0.969538	no
gene:SpnNT_01932	SpnNT_01932	Chromosome:1941755-1941831	110.58+peptide	ΔORF2+peptide	OK	2569.71	1670.58	-0.621252	-0.581788	0.4814	0.935095	no
gene:SpnNT_01933	SpnNT_01933	Chromosome:1941865-1941936	110.58	ΔORF2	OK	3251.67	2643.67	-0.29864	-0.203223	0.73155	0.994748	no
gene:SpnNT_01933	SpnNT_01933	Chromosome:1941865-1941936	110.58	110.58+peptide	OK	3251.67	4366.58	0.425322	0.31007	0.5792	0.969538	no
gene:SpnNT_01933	SpnNT_01933	Chromosome:1941865-1941936	ΔORF2	110.58+peptide	OK	2643.67	4366.58	0.723962	0.48083	0.3922	0.887166	no
gene:SpnNT_01933	SpnNT_01933	Chromosome:1941865-1941936	110.58	ΔORF2+peptide	OK	3251.67	4428.28	0.445563	0.473084	0.59715	0.97629	no
gene:SpnNT_01933	SpnNT_01933	Chromosome:1941865-1941936	ΔORF2	ΔORF2+peptide	OK	2643.67	4428.28	0.744203	0.659733	0.38605	0.881745	no
gene:SpnNT_01933	SpnNT_01933	Chromosome:1941865-1941936	110.58+peptide	ΔORF2+peptide	OK	4366.58	4428.28	0.020241	0.0202966	0.9292	0.994748	no
gene:SpnNT_01934	SpnNT_01934	Chromosome:1941955-1942028	110.58	ΔORF2	OK	860.225	745.404	-0.206691	-24.5046	0.50045	0.944471	no
gene:SpnNT_01934	SpnNT_01934	Chromosome:1941955-1942028	110.58	110.58+peptide	OK	860.225	886.358	0.0431763	5.60861	0.5196	0.954494	no
gene:SpnNT_01934	SpnNT_01934	Chromosome:1941955-1942028	ΔORF2	110.58+peptide	OK	745.404	886.358	0.249867	29.996	0.6107	0.979429	no
gene:SpnNT_01934	SpnNT_01934	Chromosome:1941955-1942028	110.58	ΔORF2+peptide	OK	860.225	454.631	-0.920019	-120.921	0.52185	0.954832	no
gene:SpnNT_01934	SpnNT_01934	Chromosome:1941955-1942028	ΔORF2	ΔORF2+peptide	OK	745.404	454.631	-0.713328	-86.4941	0.6185	0.980887	no
gene:SpnNT_01934	SpnNT_01934	Chromosome:1941955-1942028	110.58+peptide	ΔORF2+peptide	OK	886.358	454.631	-0.963195	-128.561	0.62945	0.980887	no
gene:SpnNT_01935	SpnNT_01935	Chromosome:1942031-1942105	110.58	ΔORF2	OK	1308.52	1597.9	0.288242	35.9637	0.79475	0.994748	no
gene:SpnNT_01935	SpnNT_01935	Chromosome:1942031-1942105	110.58	110.58+peptide	OK	1308.52	2128.04	0.701588	88.6304	0.485	0.937286	no
gene:SpnNT_01935	SpnNT_01935	Chromosome:1942031-1942105	ΔORF2	110.58+peptide	OK	1597.9	2128.04	0.413347	58.4385	0.6898	0.98828	no
gene:SpnNT_01935	SpnNT_01935	Chromosome:1942031-1942105	110.58	ΔORF2+peptide	OK	1308.52	425.086	-1.62211	-195.41	0.25165	0.749765	no
gene:SpnNT_01935	SpnNT_01935	Chromosome:1942031-1942105	ΔORF2	ΔORF2+peptide	OK	1597.9	425.086	-1.91035	-254.654	0.217	0.704472	no

gene:SpnNT_01935	SpnNT_01935	Chromosome:1942031-1942105	110.58+peptide	ΔORF2+peptide	OK	2128.04	425.086	-2.3237	-314.185	0.1343	0.557156	no
gene:SpnNT_01936	SpnNT_01936	Chromosome:1942115-1942205	110.58	ΔORF2	OK	5344.54	7126.95	0.415219	0.568008	0.3036	0.809137	no
gene:SpnNT_01936	SpnNT_01936	Chromosome:1942115-1942205	110.58	110.58+peptide	OK	5344.54	10024.9	0.90745	1.21416	0.0269	0.207514	no
gene:SpnNT_01936	SpnNT_01936	Chromosome:1942115-1942205	ΔORF2	110.58+peptide	OK	7126.95	10024.9	0.492231	0.711257	0.20375	0.688187	no
gene:SpnNT_01936	SpnNT_01936	Chromosome:1942115-1942205	110.58	ΔORF2+peptide	OK	5344.54	13934.3	1.3825	2.0155	0.00075	0.0133173	yes
gene:SpnNT_01936	SpnNT_01936	Chromosome:1942115-1942205	ΔORF2	ΔORF2+peptide	OK	7126.95	13934.3	0.967283	1.54719	0.0098	0.0986919	no
gene:SpnNT_01936	SpnNT_01936	Chromosome:1942115-1942205	110.58+peptide	ΔORF2+peptide	OK	10024.9	13934.3	0.475052	0.737353	0.2045	0.689595	no
gene:SpnNT_01937	SpnNT_01937	Chromosome:1942215-1942289	110.58	ΔORF2	NOTEST	200.052	174.353	-0.198359	0	1	1	no
gene:SpnNT_01937	SpnNT_01937	Chromosome:1942215-1942289	110.58	110.58+peptide	NOTEST	200.052	255.132	0.350874	0	1	1	no
gene:SpnNT_01937	SpnNT_01937	Chromosome:1942215-1942289	ΔORF2	110.58+peptide	NOTEST	174.353	255.132	0.549233	0	1	1	no
gene:SpnNT_01937	SpnNT_01937	Chromosome:1942215-1942289	110.58	ΔORF2+peptide	NOTEST	200.052	0	#NAME?	0	1	1	no
gene:SpnNT_01937	SpnNT_01937	Chromosome:1942215-1942289	ΔORF2	ΔORF2+peptide	NOTEST	174.353	0	#NAME?	0	1	1	no
gene:SpnNT_01937	SpnNT_01937	Chromosome:1942215-1942289	110.58+peptide	ΔORF2+peptide	NOTEST	255.132	0	#NAME?	0	1	1	no
gene:SpnNT_01938	SpnNT_01938	Chromosome:1942301-1942377	110.58	ΔORF2	OK	12294.1	14891.2	0.276485	0.334992	0.56045	0.968621	no
gene:SpnNT_01938	SpnNT_01938	Chromosome:1942301-1942377	110.58	110.58+peptide	OK	12294.1	15899.1	0.370974	0.443684	0.43255	0.913334	no
gene:SpnNT_01938	SpnNT_01938	Chromosome:1942301-1942377	ΔORF2	110.58+peptide	OK	14891.2	15899.1	0.0944891	0.115047	0.8426	0.994748	no
gene:SpnNT_01938	SpnNT_01938	Chromosome:1942301-1942377	110.58	ΔORF2+peptide	OK	12294.1	15668.1	0.349856	0.391487	0.48395	0.936747	no
gene:SpnNT_01938	SpnNT_01938	Chromosome:1942301-1942377	ΔORF2	ΔORF2+peptide	OK	14891.2	15668.1	0.0733706	0.0833934	0.88115	0.994748	no
gene:SpnNT_01938	SpnNT_01938	Chromosome:1942301-1942377	110.58+peptide	ΔORF2+peptide	OK	15899.1	15668.1	-0.0211186	-0.0237307	0.9742	0.99536	no
gene:SpnNT_01939	SpnNT_01939	Chromosome:1942380-1942456	110.58	ΔORF2	NOTEST	0	0	0	0	1	1	no
gene:SpnNT_01939	SpnNT_01939	Chromosome:1942380-1942456	110.58	110.58+peptide	OK	0	953.721	Inf	NA	0.015	0.133716	no
gene:SpnNT_01939	SpnNT_01939	Chromosome:1942380-1942456	ΔORF2	110.58+peptide	OK	0	953.721	Inf	NA	0.015	0.133716	no
gene:SpnNT_01939	SpnNT_01939	Chromosome:1942380-1942456	110.58	ΔORF2+peptide	OK	0	1635.88	Inf	NA	0.0012	0.0193683	yes
gene:SpnNT_01939	SpnNT_01939	Chromosome:1942380-1942456	ΔORF2	ΔORF2+peptide	OK	0	1635.88	Inf	NA	0.0012	0.0193683	yes
gene:SpnNT_01939	SpnNT_01939	Chromosome:1942380-1942456	110.58+peptide	ΔORF2+peptide	OK	953.721	1635.88	0.778425	0.468611	0.47475	0.930496	no
gene:SpnNT_01940	SpnNT_01940	Chromosome:1942470-1942544	110.58	ΔORF2	OK	200.052	687.692	1.78139	131.292	0.4336	0.913361	no
gene:SpnNT_01940	SpnNT_01940	Chromosome:1942470-1942544	110.58	110.58+peptide	OK	200.052	808.887	2.01557	140.869	0.2344	0.728172	no
gene:SpnNT_01940	SpnNT_01940	Chromosome:1942470-1942544	ΔORF2	110.58+peptide	OK	687.692	808.887	0.234175	21.9788	0.7149	0.992053	no
gene:SpnNT_01940	SpnNT_01940	Chromosome:1942470-1942544	110.58	ΔORF2+peptide	NOTEST	200.052	0	#NAME?	0	1	1	no
gene:SpnNT_01940	SpnNT_01940	Chromosome:1942470-1942544	ΔORF2	ΔORF2+peptide	OK	687.692	0	#NAME?	NA	0.12215	0.528994	no
gene:SpnNT_01940	SpnNT_01940	Chromosome:1942470-1942544	110.58+peptide	ΔORF2+peptide	OK	808.887	0	#NAME?	NA	0.02495	0.197107	no
gene:SpnNT_01941	SpnNT_01941	Chromosome:1942553-1942639	110.58	ΔORF2	OK	3218.94	2893.25	-0.153898	-0.147611	0.7825	0.994748	no
gene:SpnNT_01941	SpnNT_01941	Chromosome:1942553-1942639	110.58	110.58+peptide	OK	3218.94	9176.72	1.51139	1.71319	0.0031	0.0411306	yes
gene:SpnNT_01941	SpnNT_01941	Chromosome:1942553-1942639	ΔORF2	110.58+peptide	OK	2893.25	9176.72	1.66529	1.75071	0.0036	0.0465411	yes
gene:SpnNT_01941	SpnNT_01941	Chromosome:1942553-1942639	110.58	ΔORF2+peptide	OK	3218.94	10529.6	1.70979	2.11905	0.00135	0.021343	yes
gene:SpnNT_01941	SpnNT_01941	Chromosome:1942553-1942639	ΔORF2	ΔORF2+peptide	OK	2893.25	10529.6	1.86369	2.11355	0.0018	0.0270557	yes
gene:SpnNT_01941	SpnNT_01941	Chromosome:1942553-1942639	110.58+peptide	ΔORF2+peptide	OK	9176.72	10529.6	0.198399	0.289756	0.65855	0.982966	no
gene:SpnNT_01942	SpnNT_01942	Chromosome:1942646-1942718	110.58	ΔORF2	OK	700.432	523.909	-0.418928	-72.8472	0.75595	0.994748	no
gene:SpnNT_01942	SpnNT_01942	Chromosome:1942646-1942718	110.58	110.58+peptide	OK	700.432	305.974	-1.19484	-138.835	0.45295	0.921244	no
gene:SpnNT_01942	SpnNT_01942	Chromosome:1942646-1942718	ΔORF2	110.58+peptide	OK	523.909	305.974	-0.775911	-84.9569	0.66385	0.982966	no
gene:SpnNT_01942	SpnNT_01942	Chromosome:1942646-1942718	110.58	ΔORF2+peptide	OK	700.432	0	#NAME?	NA	0.0494	0.311514	no
gene:SpnNT_01942	SpnNT_01942	Chromosome:1942646-1942718	ΔORF2	ΔORF2+peptide	OK	523.909	0	#NAME?	NA	0.1092	0.502305	no
gene:SpnNT_01942	SpnNT_01942	Chromosome:1942646-1942718	110.58+peptide	ΔORF2+peptide	NOTEST	305.974	0	#NAME?	0	1	1	no
gene:SpnNT_01943	SpnNT_01943	Chromosome:1942740-1942813	110.58	ΔORF2	OK	2234.8	3083.5	0.464427	79.1289	0.60605	0.976937	no
gene:SpnNT_01943	SpnNT_01943	Chromosome:1942740-1942813	110.58	110.58+peptide	OK	2234.8	7053.02	1.6581	2.40018	0.04945	0.311514	no

gene:SpnNT_01943	SpnNT_01943	Chromosome:1942740-1942813	ΔORF2	110.58+peptide	OK	3083.5	7053.02	1.19367	1.72792	0.1212	0.52727	no
gene:SpnNT_01943	SpnNT_01943	Chromosome:1942740-1942813	110.58	ΔORF2+peptide	OK	2234.8	4847.26	1.11703	206.367	0.1947	0.676386	no
gene:SpnNT_01943	SpnNT_01943	Chromosome:1942740-1942813	ΔORF2	ΔORF2+peptide	OK	3083.5	4847.26	0.6526	151.328	0.4227	0.905982	no
gene:SpnNT_01943	SpnNT_01943	Chromosome:1942740-1942813	110.58+peptide	ΔORF2+peptide	OK	7053.02	4847.26	-0.541071	-0.783241	0.42465	0.907534	no
gene:SpnNT_01944	SpnNT_01944	Chromosome:1942830-1942914	110.58	ΔORF2	OK	3648.18	5592.52	0.61632	0.6314	0.25	0.747607	no
gene:SpnNT_01944	SpnNT_01944	Chromosome:1942830-1942914	110.58	110.58+peptide	OK	3648.18	16616.8	2.18739	2.47626	0.00025	0.00542231	yes
gene:SpnNT_01944	SpnNT_01944	Chromosome:1942830-1942914	ΔORF2	110.58+peptide	OK	5592.52	16616.8	1.57107	1.95553	0.00115	0.0187457	yes
gene:SpnNT_01944	SpnNT_01944	Chromosome:1942830-1942914	110.58	ΔORF2+peptide	OK	3648.18	18361.2	2.33141	2.78453	5.00E-05	0.0013612	yes
gene:SpnNT_01944	SpnNT_01944	Chromosome:1942830-1942914	ΔORF2	ΔORF2+peptide	OK	5592.52	18361.2	1.71509	2.27935	0.00065	0.0118958	yes
gene:SpnNT_01944	SpnNT_01944	Chromosome:1942830-1942914	110.58+peptide	ΔORF2+peptide	OK	16616.8	18361.2	0.144019	0.229536	0.7228	0.994748	no
gene:SpnNT_01945	SpnNT_01945	Chromosome:1942918-1942991	110.58	ΔORF2	OK	826.952	2203.52	1.41393	0.631901	0.17935	0.645837	no
gene:SpnNT_01945	SpnNT_01945	Chromosome:1942918-1942991	110.58	110.58+peptide	OK	826.952	2265.49	1.45395	0.568168	0.1544	0.599419	no
gene:SpnNT_01945	SpnNT_01945	Chromosome:1942918-1942991	ΔORF2	110.58+peptide	OK	2203.52	2265.49	0.0400151	0.0322266	0.9013	0.994748	no
gene:SpnNT_01945	SpnNT_01945	Chromosome:1942918-1942991	110.58	ΔORF2+peptide	OK	826.952	1257.6	0.604795	0.270289	0.38565	0.881745	no
gene:SpnNT_01945	SpnNT_01945	Chromosome:1942918-1942991	ΔORF2	ΔORF2+peptide	OK	2203.52	1257.6	-0.809136	-107.397	0.44105	0.916711	no
gene:SpnNT_01945	SpnNT_01945	Chromosome:1942918-1942991	110.58+peptide	ΔORF2+peptide	OK	2265.49	1257.6	-0.849151	-0.683867	0.40405	0.894692	no
gene:SpnNT_01946	SpnNT_01946	Chromosome:1943015-1943088	110.58	ΔORF2	OK	1869.72	2297.02	0.296945	0.244988	0.75635	0.994748	no
gene:SpnNT_01946	SpnNT_01946	Chromosome:1943015-1943088	110.58	110.58+peptide	OK	1869.72	4145.2	1.14862	0.742958	0.17915	0.645832	no
gene:SpnNT_01946	SpnNT_01946	Chromosome:1943015-1943088	ΔORF2	110.58+peptide	OK	2297.02	4145.2	0.851678	0.887427	0.31305	0.819111	no
gene:SpnNT_01946	SpnNT_01946	Chromosome:1943015-1943088	110.58	ΔORF2+peptide	OK	1869.72	2863.53	0.614977	0.507373	0.49315	0.941402	no
gene:SpnNT_01946	SpnNT_01946	Chromosome:1943015-1943088	ΔORF2	ΔORF2+peptide	OK	2297.02	2863.53	0.318032	52.7238	0.74695	0.994748	no
gene:SpnNT_01946	SpnNT_01946	Chromosome:1943015-1943088	110.58+peptide	ΔORF2+peptide	OK	4145.2	2863.53	-0.533647	-0.556046	0.49985	0.944017	no
gene:SpnNT_01947	SpnNT_01947	Chromosome:1943090-1943163	110.58	ΔORF2	OK	2446.07	2686.07	0.135032	0.0823454	0.90255	0.994748	no
gene:SpnNT_01947	SpnNT_01947	Chromosome:1943090-1943163	110.58	110.58+peptide	OK	2446.07	4185.67	0.774994	0.471949	0.38485	0.881745	no
gene:SpnNT_01947	SpnNT_01947	Chromosome:1943090-1943163	ΔORF2	110.58+peptide	OK	2686.07	4185.67	0.639962	0.402791	0.46365	0.926089	no
gene:SpnNT_01947	SpnNT_01947	Chromosome:1943090-1943163	110.58	ΔORF2+peptide	OK	2446.07	3211.87	0.392948	0.328528	0.6633	0.982966	no
gene:SpnNT_01947	SpnNT_01947	Chromosome:1943090-1943163	ΔORF2	ΔORF2+peptide	OK	2686.07	3211.87	0.257916	0.229912	0.7767	0.994748	no
gene:SpnNT_01947	SpnNT_01947	Chromosome:1943090-1943163	110.58+peptide	ΔORF2+peptide	OK	4185.67	3211.87	-0.382046	-0.339554	0.64925	0.981391	no
gene:SpnNT_01948	SpnNT_01948	Chromosome:1943168-1943280	110.58	ΔORF2	HIDATA	6781220	0	0	0	1	1	no
gene:SpnNT_01948	SpnNT_01948	Chromosome:1943168-1943280	110.58	110.58+peptide	OK	6781220	8093820	0.255275	0.370716	0.51645	0.95301	no
gene:SpnNT_01948	SpnNT_01948	Chromosome:1943168-1943280	ΔORF2	110.58+peptide	HIDATA	0	8093820	0	0	1	1	no
gene:SpnNT_01948	SpnNT_01948	Chromosome:1943168-1943280	110.58	ΔORF2+peptide	OK	6781220	9576870	0.498009	0.771244	0.19305	0.674611	no
gene:SpnNT_01948	SpnNT_01948	Chromosome:1943168-1943280	ΔORF2	ΔORF2+peptide	HIDATA	0	9576870	0	0	1	1	no
gene:SpnNT_01948	SpnNT_01948	Chromosome:1943168-1943280	110.58+peptide	ΔORF2+peptide	OK	8093820	9576870	0.242734	0.391595	0.5136	0.95097	no
gene:SpnNT_01949	SpnNT_01949	Chromosome:1943361-1946260	110.58	ΔORF2	HIDATA	0	0	0	0	1	1	no
gene:SpnNT_01949	SpnNT_01949	Chromosome:1943361-1946260	110.58	110.58+peptide	HIDATA	0	0	0	0	1	1	no
gene:SpnNT_01949	SpnNT_01949	Chromosome:1943361-1946260	ΔORF2	110.58+peptide	HIDATA	0	0	0	0	1	1	no
gene:SpnNT_01949	SpnNT_01949	Chromosome:1943361-1946260	110.58	ΔORF2+peptide	HIDATA	0	0	0	0	1	1	no
gene:SpnNT_01949	SpnNT_01949	Chromosome:1943361-1946260	ΔORF2	ΔORF2+peptide	HIDATA	0	0	0	0	1	1	no
gene:SpnNT_01949	SpnNT_01949	Chromosome:1943361-1946260	110.58+peptide	ΔORF2+peptide	HIDATA	0	0	0	0	1	1	no
gene:SpnNT_01950	SpnNT_01950	Chromosome:1946384-1946459	110.58	ΔORF2	OK	2140.53	1755.33	-0.286228	-0.168089	0.7508	0.994748	no
gene:SpnNT_01950	SpnNT_01950	Chromosome:1946384-1946459	110.58	110.58+peptide	OK	2140.53	2972.4	0.473659	0.307658	0.5559	0.966729	no
gene:SpnNT_01950	SpnNT_01950	Chromosome:1946384-1946459	ΔORF2	110.58+peptide	OK	1755.33	2972.4	0.759887	0.479442	0.3854	0.881745	no
gene:SpnNT_01950	SpnNT_01950	Chromosome:1946384-1946459	110.58	ΔORF2+peptide	OK	2140.53	1656.28	-0.370021	-0.315102	0.67575	0.98524	no
gene:SpnNT_01950	SpnNT_01950	Chromosome:1946384-1946459	ΔORF2	ΔORF2+peptide	OK	1755.33	1656.28	-0.0837929	-0.0679484	0.8926	0.994748	no

gene:SpnNT_01950	SpnNT_01950	Chromosome:1946384-1946459	110.58+peptide	ΔORF2+peptide	OK	2972.4	1656.28	-0.84368	-0.847356	0.34565	0.849649	no
gene:SpnNT_01951	SpnNT_01951	Chromosome:1946513-1948050	110.58	ΔORF2	HIDATA	0	0	0	0	1	1	no
gene:SpnNT_01951	SpnNT_01951	Chromosome:1946513-1948050	110.58	110.58+peptide	HIDATA	0	0	0	0	1	1	no
gene:SpnNT_01951	SpnNT_01951	Chromosome:1946513-1948050	ΔORF2	110.58+peptide	HIDATA	0	0	0	0	1	1	no
gene:SpnNT_01951	SpnNT_01951	Chromosome:1946513-1948050	110.58	ΔORF2+peptide	HIDATA	0	0	0	0	1	1	no
gene:SpnNT_01951	SpnNT_01951	Chromosome:1946513-1948050	ΔORF2	ΔORF2+peptide	HIDATA	0	0	0	0	1	1	no
gene:SpnNT_01951	SpnNT_01951	Chromosome:1946513-1948050	110.58+peptide	ΔORF2+peptide	HIDATA	0	0	0	0	1	1	no
gene:SpnNT_01952	SpnNT_01952	Chromosome:1948306-1948378	110.58	ΔORF2	NOTEST	233.349	193.357	-0.271219	0	1	1	no
gene:SpnNT_01952	SpnNT_01952	Chromosome:1948306-1948378	110.58	110.58+peptide	NOTEST	233.349	349.297	0.581964	0	1	1	no
gene:SpnNT_01952	SpnNT_01952	Chromosome:1948306-1948378	ΔORF2	110.58+peptide	NOTEST	193.357	349.297	0.853184	0	1	1	no
gene:SpnNT_01952	SpnNT_01952	Chromosome:1948306-1948378	110.58	ΔORF2+peptide	OK	233.349	7769.78	5.05731	484.715	0.0899	0.450255	no
gene:SpnNT_01952	SpnNT_01952	Chromosome:1948306-1948378	ΔORF2	ΔORF2+peptide	OK	193.357	7769.78	5.32853	424.392	0.0879	0.446544	no
gene:SpnNT_01952	SpnNT_01952	Chromosome:1948306-1948378	110.58+peptide	ΔORF2+peptide	OK	349.297	7769.78	4.47535	635.244	0.155	0.600007	no
gene:SpnNT_01953	rlmCD_2	Chromosome:1948439-1949543	110.58	ΔORF2	OK	26.5374	28.9373	0.124902	0.245279	0.6633	0.982966	no
gene:SpnNT_01953	rlmCD_2	Chromosome:1948439-1949543	110.58	110.58+peptide	OK	26.5374	21.868	-0.279207	-0.540403	0.342	0.846102	no
gene:SpnNT_01953	rlmCD_2	Chromosome:1948439-1949543	ΔORF2	110.58+peptide	OK	28.9373	21.868	-0.404109	-0.781243	0.17205	0.632215	no
gene:SpnNT_01953	rlmCD_2	Chromosome:1948439-1949543	110.58	ΔORF2+peptide	OK	26.5374	27.1424	0.0325179	0.0638632	0.9152	0.994748	no
gene:SpnNT_01953	rlmCD_2	Chromosome:1948439-1949543	ΔORF2	ΔORF2+peptide	OK	28.9373	27.1424	-0.0923841	-0.18122	0.75765	0.994748	no
gene:SpnNT_01953	rlmCD_2	Chromosome:1948439-1949543	110.58+peptide	ΔORF2+peptide	OK	21.868	27.1424	0.311724	0.602693	0.2872	0.790568	no
gene:SpnNT_01954	butA	Chromosome:1949850-1950627	110.58	ΔORF2	OK	13.2004	17.914	0.440502	0.75378	0.1779	0.644329	no
gene:SpnNT_01954	butA	Chromosome:1949850-1950627	110.58	110.58+peptide	OK	13.2004	12.857	-0.0380321	-0.0641456	0.90855	0.994748	no
gene:SpnNT_01954	butA	Chromosome:1949850-1950627	ΔORF2	110.58+peptide	OK	17.914	12.857	-0.478534	-0.822548	0.1438	0.578995	no
gene:SpnNT_01954	butA	Chromosome:1949850-1950627	110.58	ΔORF2+peptide	OK	13.2004	15.9079	0.269161	0.455328	0.4262	0.908545	no
gene:SpnNT_01954	butA	Chromosome:1949850-1950627	ΔORF2	ΔORF2+peptide	OK	17.914	15.9079	-0.171341	-0.29543	0.6053	0.976847	no
gene:SpnNT_01954	butA	Chromosome:1949850-1950627	110.58+peptide	ΔORF2+peptide	OK	12.857	15.9079	0.307193	0.521952	0.36715	0.864634	no
gene:SpnNT_01955	recX	Chromosome:1950636-1951413	110.58	ΔORF2	OK	17.6839	18.7196	0.082107	0.142962	0.80215	0.994748	no
gene:SpnNT_01955	recX	Chromosome:1950636-1951413	110.58	110.58+peptide	OK	17.6839	17.2139	-0.0388687	-0.0686712	0.90565	0.994748	no
gene:SpnNT_01955	recX	Chromosome:1950636-1951413	ΔORF2	110.58+peptide	OK	18.7196	17.2139	-0.120976	-0.21047	0.71415	0.991753	no
gene:SpnNT_01955	recX	Chromosome:1950636-1951413	110.58	ΔORF2+peptide	OK	17.6839	15.8205	-0.160649	-0.279395	0.63115	0.980887	no
gene:SpnNT_01955	recX	Chromosome:1950636-1951413	ΔORF2	ΔORF2+peptide	OK	18.7196	15.8205	-0.242756	-0.415942	0.47895	0.933291	no
gene:SpnNT_01955	recX	Chromosome:1950636-1951413	110.58+peptide	ΔORF2+peptide	OK	17.2139	15.8205	-0.12178	-0.211627	0.7154	0.992053	no
gene:SpnNT_01956	NA	Chromosome:1951501-1952035	110.58	ΔORF2	OK	625.485	736.634	0.235971	0.539317	0.33815	0.842454	no
gene:SpnNT_01956	NA	Chromosome:1951501-1952035	110.58	110.58+peptide	OK	625.485	563.426	-0.150751	-0.345743	0.5445	0.961568	no
gene:SpnNT_01956	NA	Chromosome:1951501-1952035	ΔORF2	110.58+peptide	OK	736.634	563.426	-0.386722	-0.877878	0.12855	0.543614	no
gene:SpnNT_01956	NA	Chromosome:1951501-1952035	110.58	ΔORF2+peptide	OK	625.485	618.043	-0.0172698	-0.0397291	0.9427	0.994855	no
gene:SpnNT_01956	NA	Chromosome:1951501-1952035	ΔORF2	ΔORF2+peptide	OK	736.634	618.043	-0.253241	-0.576593	0.30345	0.809137	no
gene:SpnNT_01956	NA	Chromosome:1951501-1952035	110.58+peptide	ΔORF2+peptide	OK	563.426	618.043	0.133481	0.304966	0.59295	0.975539	no
gene:SpnNT_01957	NA	Chromosome:1952392-1952746	110.58	ΔORF2	OK	6.98643	12.6702	0.858813	0.952135	0.1181	0.520735	no
gene:SpnNT_01957	NA	Chromosome:1952392-1952746	110.58	110.58+peptide	OK	6.98643	5.495	-0.346437	-0.38464	0.5183	0.953876	no
gene:SpnNT_01957	NA	Chromosome:1952392-1952746	ΔORF2	110.58+peptide	OK	12.6702	5.495	-1.20525	-1.34324	0.0261	0.202893	no
gene:SpnNT_01957	NA	Chromosome:1952392-1952746	110.58	ΔORF2+peptide	OK	6.98643	8.39883	0.265634	0.338333	0.6344	0.980887	no
gene:SpnNT_01957	NA	Chromosome:1952392-1952746	ΔORF2	ΔORF2+peptide	OK	12.6702	8.39883	-0.593179	-0.759308	0.2789	0.781158	no
gene:SpnNT_01957	NA	Chromosome:1952392-1952746	110.58+peptide	ΔORF2+peptide	OK	5.495	8.39883	0.61207	0.785007	0.26555	0.766773	no
gene:SpnNT_01958	NA	Chromosome:1952759-1954086	110.58	ΔORF2	OK	6.24183	5.3866	-0.212595	-0.228726	0.70095	0.990367	no
gene:SpnNT_01958	NA	Chromosome:1952759-1954086	110.58	110.58+peptide	OK	6.24183	11.7056	0.907155	1.01962	0.1	0.479559	no

gene:SpnNT_01958	NA	Chromosome:1952759-1954086	ΔORF2	110.58+peptide	OK	5.3866	11.7056	1.11975	1.34254	0.02215	0.180866	no
gene:SpnNT_01958	NA	Chromosome:1952759-1954086	110.58	ΔORF2+peptide	OK	6.24183	17.9938	1.52746	1.7794	0.0094	0.0963647	no
gene:SpnNT_01958	NA	Chromosome:1952759-1954086	ΔORF2	ΔORF2+peptide	OK	5.3866	17.9938	1.74006	2.17345	0.00025	0.00542231	yes
gene:SpnNT_01958	NA	Chromosome:1952759-1954086	110.58+peptide	ΔORF2+peptide	OK	11.7056	17.9938	0.620308	0.822629	0.15085	0.593025	no
gene:SpnNT_01959	NA	Chromosome:1952759-1954086	110.58	ΔORF2	NOTEST	0.346865	0	#NAME?	0	1	1	no
gene:SpnNT_01959	NA	Chromosome:1952759-1954086	110.58	110.58+peptide	NOTEST	0.346865	0.743401	1.09977	0	1	1	no
gene:SpnNT_01959	NA	Chromosome:1952759-1954086	ΔORF2	110.58+peptide	NOTEST	0	0.743401	Inf	0	1	1	no
gene:SpnNT_01959	NA	Chromosome:1952759-1954086	110.58	ΔORF2+peptide	NOTEST	0.346865	0	#NAME?	0	1	1	no
gene:SpnNT_01959	NA	Chromosome:1952759-1954086	ΔORF2	ΔORF2+peptide	NOTEST	0	0	0	0	1	1	no
gene:SpnNT_01959	NA	Chromosome:1952759-1954086	110.58+peptide	ΔORF2+peptide	NOTEST	0.743401	0	#NAME?	0	1	1	no
gene:SpnNT_01960	NA	Chromosome:1952759-1954086	110.58	ΔORF2	OK	3.44244	2.66417	-0.369747	-0.266318	0.63545	0.980887	no
gene:SpnNT_01960	NA	Chromosome:1952759-1954086	110.58	110.58+peptide	OK	3.44244	6.6016	0.939384	0.722563	0.19895	0.681625	no
gene:SpnNT_01960	NA	Chromosome:1952759-1954086	ΔORF2	110.58+peptide	OK	2.66417	6.6016	1.30913	1.09019	0.07575	0.406374	no
gene:SpnNT_01960	NA	Chromosome:1952759-1954086	110.58	ΔORF2+peptide	OK	3.44244	4.75938	0.467341	0.310297	0.59195	0.975468	no
gene:SpnNT_01960	NA	Chromosome:1952759-1954086	ΔORF2	ΔORF2+peptide	OK	2.66417	4.75938	0.837088	0.588947	0.3408	0.844885	no
gene:SpnNT_01960	NA	Chromosome:1952759-1954086	110.58+peptide	ΔORF2+peptide	OK	6.6016	4.75938	-0.472043	-0.353532	0.54695	0.962122	no
gene:SpnNT_01961	groL	Chromosome:1954185-1955808	110.58	ΔORF2	OK	651.924	639.024	-0.0288319	-0.0627234	0.91005	0.994748	no
gene:SpnNT_01961	groL	Chromosome:1954185-1955808	110.58	110.58+peptide	OK	651.924	1108.89	0.766341	1.62907	0.0046	0.0562022	no
gene:SpnNT_01961	groL	Chromosome:1954185-1955808	ΔORF2	110.58+peptide	OK	639.024	1108.89	0.795173	1.7056	0.0024	0.0337543	yes
gene:SpnNT_01961	groL	Chromosome:1954185-1955808	110.58	ΔORF2+peptide	OK	651.924	1093.62	0.746343	1.59677	0.0063	0.0714508	no
gene:SpnNT_01961	groL	Chromosome:1954185-1955808	ΔORF2	ΔORF2+peptide	OK	639.024	1093.62	0.775174	1.6736	0.00415	0.0519126	no
gene:SpnNT_01961	groL	Chromosome:1954185-1955808	110.58+peptide	ΔORF2+peptide	OK	1108.89	1093.62	-0.0199987	-0.042205	0.94185	0.994855	no
gene:SpnNT_01962	NA	Chromosome:1955823-1956108	110.58	ΔORF2	OK	448.371	402.111	-0.157099	-0.314766	0.57825	0.969538	no
gene:SpnNT_01962	NA	Chromosome:1955823-1956108	110.58	110.58+peptide	OK	448.371	821.581	0.873711	1.78491	0.0015	0.0233488	yes
gene:SpnNT_01962	NA	Chromosome:1955823-1956108	ΔORF2	110.58+peptide	OK	402.111	821.581	1.03081	2.09185	2.00E-04	0.00450928	yes
gene:SpnNT_01962	NA	Chromosome:1955823-1956108	110.58	ΔORF2+peptide	OK	448.371	766.084	0.77281	1.5916	0.0042	0.052488	no
gene:SpnNT_01962	NA	Chromosome:1955823-1956108	ΔORF2	ΔORF2+peptide	OK	402.111	766.084	0.929909	1.90221	0.00115	0.0187457	yes
gene:SpnNT_01962	NA	Chromosome:1955823-1956108	110.58+peptide	ΔORF2+peptide	OK	821.581	766.084	-0.100901	-0.210626	0.70575	0.990422	no
gene:SpnNT_01963	ssb_2	Chromosome:1956377-1956773	110.58	ΔORF2	OK	19.3955	15.7269	-0.302485	-0.441839	0.43385	0.913361	no
gene:SpnNT_01963	ssb_2	Chromosome:1956377-1956773	110.58	110.58+peptide	OK	19.3955	15.3256	-0.33978	-0.490779	0.38115	0.87899	no
gene:SpnNT_01963	ssb_2	Chromosome:1956377-1956773	ΔORF2	110.58+peptide	OK	15.7269	15.3256	-0.0372953	-0.0516841	0.92695	0.994748	no
gene:SpnNT_01963	ssb_2	Chromosome:1956377-1956773	110.58	ΔORF2+peptide	OK	19.3955	15.9742	-0.279973	-0.405815	0.472	0.928525	no
gene:SpnNT_01963	ssb_2	Chromosome:1956377-1956773	ΔORF2	ΔORF2+peptide	OK	15.7269	15.9742	0.0225116	0.0312976	0.95815	0.994855	no
gene:SpnNT_01963	ssb_2	Chromosome:1956377-1956773	110.58+peptide	ΔORF2+peptide	OK	15.3256	15.9742	0.0598068	0.0823071	0.88815	0.994748	no
gene:SpnNT_01964	ydfG	Chromosome:1956850-1957612	110.58	ΔORF2	OK	217.003	206.007	-0.0750237	-0.170753	0.76915	0.994748	no
gene:SpnNT_01964	ydfG	Chromosome:1956850-1957612	110.58	110.58+peptide	OK	217.003	211.901	-0.0343259	-0.0779987	0.89345	0.994748	no
gene:SpnNT_01964	ydfG	Chromosome:1956850-1957612	ΔORF2	110.58+peptide	OK	206.007	211.901	0.0406978	0.0920047	0.87555	0.994748	no
gene:SpnNT_01964	ydfG	Chromosome:1956850-1957612	110.58	ΔORF2+peptide	OK	217.003	222.706	0.0374251	0.0851534	0.88175	0.994748	no
gene:SpnNT_01964	ydfG	Chromosome:1956850-1957612	ΔORF2	ΔORF2+peptide	OK	206.007	222.706	0.112449	0.254543	0.6603	0.982966	no
gene:SpnNT_01964	ydfG	Chromosome:1956850-1957612	110.58+peptide	ΔORF2+peptide	OK	211.901	222.706	0.071751	0.162158	0.78475	0.994748	no
gene:SpnNT_01965	pheT_2	Chromosome:1957653-1958280	110.58	ΔORF2	OK	187.135	181.654	-0.0428843	-0.09555	0.8661	0.994748	no
gene:SpnNT_01965	pheT_2	Chromosome:1957653-1958280	110.58	110.58+peptide	OK	187.135	186.192	-0.007289	-0.01624	0.97695	0.99546	no
gene:SpnNT_01965	pheT_2	Chromosome:1957653-1958280	ΔORF2	110.58+peptide	OK	181.654	186.192	0.0355953	0.0794555	0.8853	0.994748	no
gene:SpnNT_01965	pheT_2	Chromosome:1957653-1958280	110.58	ΔORF2+peptide	OK	187.135	191.23	0.0312348	0.0690716	0.90245	0.994748	no
gene:SpnNT_01965	pheT_2	Chromosome:1957653-1958280	ΔORF2	ΔORF2+peptide	OK	181.654	191.23	0.0741191	0.164207	0.76945	0.994748	no

gene:SpnNT_01965	pheT_2	Chromosome:1957653-1958280	110.58+peptide	ΔORF2+peptide	OK	186.192	191.23	0.0385238	0.0853447	0.87945	0.994748	no
gene:SpnNT_01966	ytpP	Chromosome:1958295-1958909	110.58	ΔORF2	OK	133.901	134.957	0.0113316	0.00811428	0.98745	0.997484	no
gene:SpnNT_01966	ytpP	Chromosome:1958295-1958909	110.58	110.58+peptide	OK	133.901	134.72	0.00879896	0.00674587	0.9901	0.997703	no
gene:SpnNT_01966	ytpP	Chromosome:1958295-1958909	ΔORF2	110.58+peptide	OK	134.957	134.72	-0.0025326	-0.00198786	0.99705	0.999412	no
gene:SpnNT_01966	ytpP	Chromosome:1958295-1958909	110.58	ΔORF2+peptide	OK	133.901	149.967	0.163483	0.125243	0.82495	0.994748	no
gene:SpnNT_01966	ytpP	Chromosome:1958295-1958909	ΔORF2	ΔORF2+peptide	OK	134.957	149.967	0.152151	0.119331	0.833	0.994748	no
gene:SpnNT_01966	ytpP	Chromosome:1958295-1958909	110.58+peptide	ΔORF2+peptide	OK	134.72	149.967	0.154684	0.131826	0.81705	0.994748	no
gene:SpnNT_01967	NA	Chromosome:1958295-1958909	110.58	ΔORF2	OK	921.862	933.491	0.0180856	0.0368008	0.94845	0.994855	no
gene:SpnNT_01967	NA	Chromosome:1958295-1958909	110.58	110.58+peptide	OK	921.862	762.395	-0.274012	-0.542538	0.33535	0.840108	no
gene:SpnNT_01967	NA	Chromosome:1958295-1958909	ΔORF2	110.58+peptide	OK	933.491	762.395	-0.292098	-0.589935	0.29735	0.80235	no
gene:SpnNT_01967	NA	Chromosome:1958295-1958909	110.58	ΔORF2+peptide	OK	921.862	750.823	-0.296077	-0.57956	0.30225	0.80833	no
gene:SpnNT_01967	NA	Chromosome:1958295-1958909	ΔORF2	ΔORF2+peptide	OK	933.491	750.823	-0.314163	-0.626996	0.2677	0.768869	no
gene:SpnNT_01967	NA	Chromosome:1958295-1958909	110.58+peptide	ΔORF2+peptide	OK	762.395	750.823	-0.0220655	-0.0428944	0.94105	0.994855	no
gene:SpnNT_01968	NA	Chromosome:1959238-1960972	110.58	ΔORF2	OK	22.968	24.3476	0.0841536	0.173425	0.7629	0.994748	no
gene:SpnNT_01968	NA	Chromosome:1959238-1960972	110.58	110.58+peptide	OK	22.968	23.0662	0.00615967	0.0125664	0.98245	0.996246	no
gene:SpnNT_01968	NA	Chromosome:1959238-1960972	ΔORF2	110.58+peptide	OK	24.3476	23.0662	-0.0779939	-0.159759	0.7846	0.994748	no
gene:SpnNT_01968	NA	Chromosome:1959238-1960972	110.58	ΔORF2+peptide	OK	22.968	29.9982	0.385252	0.802831	0.156	0.601537	no
gene:SpnNT_01968	NA	Chromosome:1959238-1960972	ΔORF2	ΔORF2+peptide	OK	24.3476	29.9982	0.301099	0.630112	0.2744	0.77651	no
gene:SpnNT_01968	NA	Chromosome:1959238-1960972	110.58+peptide	ΔORF2+peptide	OK	23.0662	29.9982	0.379093	0.785112	0.17275	0.632681	no
gene:SpnNT_01969	NA	Chromosome:1961701-1962376	110.58	ΔORF2	OK	112.795	105.004	-0.103248	-0.220886	0.69865	0.990209	no
gene:SpnNT_01969	NA	Chromosome:1961701-1962376	110.58	110.58+peptide	OK	112.795	107.425	-0.0703754	-0.149995	0.78985	0.994748	no
gene:SpnNT_01969	NA	Chromosome:1961701-1962376	ΔORF2	110.58+peptide	OK	105.004	107.425	0.0328728	0.0699622	0.9019	0.994748	no
gene:SpnNT_01969	NA	Chromosome:1961701-1962376	110.58	ΔORF2+peptide	OK	112.795	111.83	-0.0123862	-0.0264622	0.96165	0.994855	no
gene:SpnNT_01969	NA	Chromosome:1961701-1962376	ΔORF2	ΔORF2+peptide	OK	105.004	111.83	0.090862	0.193837	0.7324	0.994748	no
gene:SpnNT_01969	NA	Chromosome:1961701-1962376	110.58+peptide	ΔORF2+peptide	OK	107.425	111.83	0.0579892	0.123248	0.82515	0.994748	no
gene:SpnNT_01970	NA	Chromosome:1962381-1962828	110.58	ΔORF2	OK	135.891	131.462	-0.0478001	-0.0968086	0.86995	0.994748	no
gene:SpnNT_01970	NA	Chromosome:1962381-1962828	110.58	110.58+peptide	OK	135.891	136.165	0.00291101	0.00591058	0.9928	0.998522	no
gene:SpnNT_01970	NA	Chromosome:1962381-1962828	ΔORF2	110.58+peptide	OK	131.462	136.165	0.0507111	0.102402	0.86125	0.994748	no
gene:SpnNT_01970	NA	Chromosome:1962381-1962828	110.58	ΔORF2+peptide	OK	135.891	140.897	0.0521889	0.106273	0.8582	0.994748	no
gene:SpnNT_01970	NA	Chromosome:1962381-1962828	ΔORF2	ΔORF2+peptide	OK	131.462	140.897	0.099989	0.202488	0.73465	0.994748	no
gene:SpnNT_01970	NA	Chromosome:1962381-1962828	110.58+peptide	ΔORF2+peptide	OK	136.165	140.897	0.0492779	0.100046	0.8625	0.994748	no
gene:SpnNT_01971	bcrC	Chromosome:1962941-1963804	110.58	ΔORF2	OK	59.3664	52.9733	-0.164379	-0.176413	0.7624	0.994748	no
gene:SpnNT_01971	bcrC	Chromosome:1962941-1963804	110.58	110.58+peptide	OK	59.3664	56.2171	-0.0786364	-0.0855012	0.88545	0.994748	no
gene:SpnNT_01971	bcrC	Chromosome:1962941-1963804	ΔORF2	110.58+peptide	OK	52.9733	56.2171	0.0857427	0.0902164	0.8767	0.994748	no
gene:SpnNT_01971	bcrC	Chromosome:1962941-1963804	110.58	ΔORF2+peptide	OK	59.3664	45.8866	-0.371573	-0.429318	0.4526	0.921244	no
gene:SpnNT_01971	bcrC	Chromosome:1962941-1963804	ΔORF2	ΔORF2+peptide	OK	52.9733	45.8866	-0.207194	-0.230715	0.67725	0.98524	no
gene:SpnNT_01971	bcrC	Chromosome:1962941-1963804	110.58+peptide	ΔORF2+peptide	OK	56.2171	45.8866	-0.292937	-0.330807	0.56185	0.968621	no
gene:SpnNT_01972	NA	Chromosome:1962941-1963804	110.58	ΔORF2	OK	238.665	258.118	0.113044	0.198979	0.7341	0.994748	no
gene:SpnNT_01972	NA	Chromosome:1962941-1963804	110.58	110.58+peptide	OK	238.665	192.94	-0.306835	-0.506959	0.3797	0.877496	no
gene:SpnNT_01972	NA	Chromosome:1962941-1963804	ΔORF2	110.58+peptide	OK	258.118	192.94	-0.419879	-0.707346	0.21885	0.707128	no
gene:SpnNT_01972	NA	Chromosome:1962941-1963804	110.58	ΔORF2+peptide	OK	238.665	181.004	-0.398967	-0.688593	0.2395	0.734795	no
gene:SpnNT_01972	NA	Chromosome:1962941-1963804	ΔORF2	ΔORF2+peptide	OK	258.118	181.004	-0.512011	-0.902676	0.1233	0.53117	no
gene:SpnNT_01972	NA	Chromosome:1962941-1963804	110.58+peptide	ΔORF2+peptide	OK	192.94	181.004	-0.0921317	-0.152436	0.79345	0.994748	no
gene:SpnNT_01973	NA	Chromosome:1963869-1964901	110.58	ΔORF2	OK	4.57333	4.07317	-0.167093	-0.243378	0.66975	0.984845	no
gene:SpnNT_01973	NA	Chromosome:1963869-1964901	110.58	110.58+peptide	OK	4.57333	3.05161	-0.583672	-0.832763	0.15055	0.592892	no

gene:SpnNT_01973	NA	Chromosome:1963869-1964901	ΔORF2	110.58+peptide	OK	4.07317	3.05161	-0.416578	-0.581201	0.3198	0.825861	no
gene:SpnNT_01973	NA	Chromosome:1963869-1964901	110.58	ΔORF2+peptide	OK	4.57333	1.72839	-1.40381	-1.89477	0.00255	0.0352222	yes
gene:SpnNT_01973	NA	Chromosome:1963869-1964901	ΔORF2	ΔORF2+peptide	OK	4.07317	1.72839	-1.23672	-1.63605	0.0073	0.0801595	no
gene:SpnNT_01973	NA	Chromosome:1963869-1964901	110.58+peptide	ΔORF2+peptide	OK	3.05161	1.72839	-0.820141	-1.06657	0.07095	0.390523	no
gene:SpnNT_01974	NA	Chromosome:1964907-1965261	110.58	ΔORF2	OK	1.78332	2.01615	0.177042	0.149361	0.81145	0.994748	no
gene:SpnNT_01974	NA	Chromosome:1964907-1965261	110.58	110.58+peptide	OK	1.78332	0.90139	-0.984338	-0.615215	0.33415	0.839627	no
gene:SpnNT_01974	NA	Chromosome:1964907-1965261	ΔORF2	110.58+peptide	OK	2.01615	0.90139	-1.16138	-0.680459	0.2054	0.69003	no
gene:SpnNT_01974	NA	Chromosome:1964907-1965261	110.58	ΔORF2+peptide	OK	1.78332	0.862461	-1.04803	-0.953031	0.30225	0.80833	no
gene:SpnNT_01974	NA	Chromosome:1964907-1965261	ΔORF2	ΔORF2+peptide	OK	2.01615	0.862461	-1.22507	-0.980102	0.2264	0.717936	no
gene:SpnNT_01974	NA	Chromosome:1964907-1965261	110.58+peptide	ΔORF2+peptide	OK	0.90139	0.862461	-0.0636926	-0.0386384	0.88405	0.994748	no
gene:SpnNT_01975	NA	Chromosome:1965300-1965729	110.58	ΔORF2	OK	1.91783	1.29098	-0.571001	-0.475471	0.4349	0.913666	no
gene:SpnNT_01975	NA	Chromosome:1965300-1965729	110.58	110.58+peptide	OK	1.91783	1.78215	-0.105852	-0.0910778	0.8758	0.994748	no
gene:SpnNT_01975	NA	Chromosome:1965300-1965729	ΔORF2	110.58+peptide	OK	1.29098	1.78215	0.465149	0.373624	0.51405	0.951123	no
gene:SpnNT_01975	NA	Chromosome:1965300-1965729	110.58	ΔORF2+peptide	OK	1.91783	0.286753	-2.74159	-1.97791	0.1172	0.518859	no
gene:SpnNT_01975	NA	Chromosome:1965300-1965729	ΔORF2	ΔORF2+peptide	OK	1.29098	0.286753	-2.17059	-1.4906	0.1408	0.572715	no
gene:SpnNT_01975	NA	Chromosome:1965300-1965729	110.58+peptide	ΔORF2+peptide	OK	1.78215	0.286753	-2.63574	-1.85037	0.1177	0.519495	no
gene:SpnNT_01976	NA	Chromosome:1965767-1966217	110.58	ΔORF2	OK	2.66304	3.3654	0.337701	0.343323	0.56025	0.968621	no
gene:SpnNT_01976	NA	Chromosome:1965767-1966217	110.58	110.58+peptide	OK	2.66304	1.53599	-0.793909	-0.719993	0.22165	0.710949	no
gene:SpnNT_01976	NA	Chromosome:1965767-1966217	ΔORF2	110.58+peptide	OK	3.3654	1.53599	-1.13161	-1.02496	0.08935	0.449387	no
gene:SpnNT_01976	NA	Chromosome:1965767-1966217	110.58	ΔORF2+peptide	OK	2.66304	0.993813	-1.42203	-1.29806	0.06375	0.366737	no
gene:SpnNT_01976	NA	Chromosome:1965767-1966217	ΔORF2	ΔORF2+peptide	OK	3.3654	0.993813	-1.75973	-1.60427	0.0291	0.217703	no
gene:SpnNT_01976	NA	Chromosome:1965767-1966217	110.58+peptide	ΔORF2+peptide	OK	1.53599	0.993813	-0.628122	-0.521349	0.4149	0.900591	no
gene:SpnNT_01977	yeeN	Chromosome:1966340-1967057	110.58	ΔORF2	OK	688.215	747.252	0.118736	0.26663	0.6381	0.980887	no
gene:SpnNT_01977	yeeN	Chromosome:1966340-1967057	110.58	110.58+peptide	OK	688.215	551.705	-0.318961	-0.724959	0.19565	0.677365	no
gene:SpnNT_01977	yeeN	Chromosome:1966340-1967057	ΔORF2	110.58+peptide	OK	747.252	551.705	-0.437697	-0.993241	0.07575	0.406374	no
gene:SpnNT_01977	yeeN	Chromosome:1966340-1967057	110.58	ΔORF2+peptide	OK	688.215	519.821	-0.404845	-0.921379	0.10245	0.485149	no
gene:SpnNT_01977	yeeN	Chromosome:1966340-1967057	ΔORF2	ΔORF2+peptide	OK	747.252	519.821	-0.523581	-1.1897	0.0364	0.254335	no
gene:SpnNT_01977	yeeN	Chromosome:1966340-1967057	110.58+peptide	ΔORF2+peptide	OK	551.705	519.821	-0.085884	-0.197579	0.7274	0.994748	no
gene:SpnNT_01978	ply_3	Chromosome:1967937-1969353	110.58	ΔORF2	OK	522.99	579.396	0.147768	0.31808	0.5683	0.968621	no
gene:SpnNT_01978	ply_3	Chromosome:1967937-1969353	110.58	110.58+peptide	OK	522.99	341.601	-0.61447	-1.34416	0.02065	0.171608	no
gene:SpnNT_01978	ply_3	Chromosome:1967937-1969353	ΔORF2	110.58+peptide	OK	579.396	341.601	-0.762238	-1.66616	0.00395	0.0500308	no
gene:SpnNT_01978	ply_3	Chromosome:1967937-1969353	110.58	ΔORF2+peptide	OK	522.99	393.701	-0.409684	-0.891504	0.11275	0.510701	no
gene:SpnNT_01978	ply_3	Chromosome:1967937-1969353	ΔORF2	ΔORF2+peptide	OK	579.396	393.701	-0.557452	-1.21216	0.03095	0.226886	no
gene:SpnNT_01978	ply_3	Chromosome:1967937-1969353	110.58+peptide	ΔORF2+peptide	OK	341.601	393.701	0.204786	0.452681	0.4211	0.904877	no
gene:SpnNT_01979	NA	Chromosome:1969363-1970375	110.58	ΔORF2	OK	214.077	231.203	0.11103	0.143811	0.79685	0.994748	no
gene:SpnNT_01979	NA	Chromosome:1969363-1970375	110.58	110.58+peptide	OK	214.077	172.432	-0.312101	-0.399125	0.4878	0.938811	no
gene:SpnNT_01979	NA	Chromosome:1969363-1970375	ΔORF2	110.58+peptide	OK	231.203	172.432	-0.423131	-0.548728	0.33355	0.839119	no
gene:SpnNT_01979	NA	Chromosome:1969363-1970375	110.58	ΔORF2+peptide	OK	214.077	182.884	-0.227206	-0.237543	0.6616	0.982966	no
gene:SpnNT_01979	NA	Chromosome:1969363-1970375	ΔORF2	ΔORF2+peptide	OK	231.203	182.884	-0.338236	-0.356928	0.50005	0.944123	no
gene:SpnNT_01979	NA	Chromosome:1969363-1970375	110.58+peptide	ΔORF2+peptide	OK	172.432	182.884	0.0848956	0.0888287	0.86075	0.994748	no
gene:SpnNT_01980	NA	Chromosome:1969363-1970375	110.58	ΔORF2	OK	201.383	227.537	0.176159	0.31572	0.5827	0.971068	no
gene:SpnNT_01980	NA	Chromosome:1969363-1970375	110.58	110.58+peptide	OK	201.383	155.206	-0.375753	-0.668847	0.2551	0.754219	no
gene:SpnNT_01980	NA	Chromosome:1969363-1970375	ΔORF2	110.58+peptide	OK	227.537	155.206	-0.551912	-0.983593	0.0922	0.457756	no
gene:SpnNT_01980	NA	Chromosome:1969363-1970375	110.58	ΔORF2+peptide	OK	201.383	231.043	0.19822	0.348804	0.54985	0.964281	no
gene:SpnNT_01980	NA	Chromosome:1969363-1970375	ΔORF2	ΔORF2+peptide	OK	227.537	231.043	0.0220615	0.0388669	0.94755	0.994855	no

gene:SpnNT_01980	NA	Chromosome:1969363-1970375	110.58+peptide	ΔORF2+peptide	OK	155.206	231.043	0.573973	1.00453	0.09015	0.450991	no
gene:SpnNT_01981	NA	Chromosome:1970387-1970846	110.58	ΔORF2	OK	243.861	274.62	0.171375	0.368349	0.52195	0.954832	no
gene:SpnNT_01981	NA	Chromosome:1970387-1970846	110.58	110.58+peptide	OK	243.861	186.466	-0.387145	-0.828368	0.1557	0.601087	no
gene:SpnNT_01981	NA	Chromosome:1970387-1970846	ΔORF2	110.58+peptide	OK	274.62	186.466	-0.55852	-1.19021	0.04335	0.285562	no
gene:SpnNT_01981	NA	Chromosome:1970387-1970846	110.58	ΔORF2+peptide	OK	243.861	224.861	-0.117024	-0.25209	0.6599	0.982966	no
gene:SpnNT_01981	NA	Chromosome:1970387-1970846	ΔORF2	ΔORF2+peptide	OK	274.62	224.861	-0.288399	-0.618705	0.2842	0.787265	no
gene:SpnNT_01981	NA	Chromosome:1970387-1970846	110.58+peptide	ΔORF2+peptide	OK	186.466	224.861	0.270121	0.576889	0.32015	0.825861	no
gene:SpnNT_01982	NA	Chromosome:1971106-1971370	110.58	ΔORF2	NOTEST	0.775747	0.211631	-1.87403	0	1	1	no
gene:SpnNT_01982	NA	Chromosome:1971106-1971370	110.58	110.58+peptide	NOTEST	0.775747	0	#NAME?	0	1	1	no
gene:SpnNT_01982	NA	Chromosome:1971106-1971370	ΔORF2	110.58+peptide	NOTEST	0.211631	0	#NAME?	0	1	1	no
gene:SpnNT_01982	NA	Chromosome:1971106-1971370	110.58	ΔORF2+peptide	NOTEST	0.775747	0.753633	-0.0417227	0	1	1	no
gene:SpnNT_01982	NA	Chromosome:1971106-1971370	ΔORF2	ΔORF2+peptide	NOTEST	0.211631	0.753633	1.83231	0	1	1	no
gene:SpnNT_01982	NA	Chromosome:1971106-1971370	110.58+peptide	ΔORF2+peptide	NOTEST	0	0.753633	Inf	0	1	1	no
gene:SpnNT_01983	NA	Chromosome:1971554-1972010	110.58	ΔORF2	OK	4.85532	3.74399	-0.37499	-0.428332	0.45645	0.921244	no
gene:SpnNT_01983	NA	Chromosome:1971554-1972010	110.58	110.58+peptide	OK	4.85532	5.16387	0.0888872	0.104366	0.8527	0.994748	no
gene:SpnNT_01983	NA	Chromosome:1971554-1972010	ΔORF2	110.58+peptide	OK	3.74399	5.16387	0.463878	0.518742	0.34905	0.853357	no
gene:SpnNT_01983	NA	Chromosome:1971554-1972010	110.58	ΔORF2+peptide	OK	4.85532	1.72438	-1.49349	-1.61886	0.0335	0.240474	no
gene:SpnNT_01983	NA	Chromosome:1971554-1972010	ΔORF2	ΔORF2+peptide	OK	3.74399	1.72438	-1.11849	-1.16271	0.0896	0.449782	no
gene:SpnNT_01983	NA	Chromosome:1971554-1972010	110.58+peptide	ΔORF2+peptide	OK	5.16387	1.72438	-1.58237	-1.68269	0.0271	0.208567	no
gene:SpnNT_01984	NA	Chromosome:1972073-1972493	110.58	ΔORF2	OK	3.25427	2.64002	-0.301786	-0.295079	0.6185	0.980887	no
gene:SpnNT_01984	NA	Chromosome:1972073-1972493	110.58	110.58+peptide	OK	3.25427	2.70539	-0.266495	-0.26819	0.6534	0.982346	no
gene:SpnNT_01984	NA	Chromosome:1972073-1972493	ΔORF2	110.58+peptide	OK	2.64002	2.70539	0.0352917	0.0344788	0.9531	0.994855	no
gene:SpnNT_01984	NA	Chromosome:1972073-1972493	110.58	ΔORF2+peptide	OK	3.25427	3.32899	0.0327503	0.0334742	0.9616	0.994855	no
gene:SpnNT_01984	NA	Chromosome:1972073-1972493	ΔORF2	ΔORF2+peptide	OK	2.64002	3.32899	0.334537	0.331642	0.5651	0.968621	no
gene:SpnNT_01984	NA	Chromosome:1972073-1972493	110.58+peptide	ΔORF2+peptide	OK	2.70539	3.32899	0.299245	0.305582	0.6027	0.976761	no
gene:SpnNT_01985	NA	Chromosome:1973028-1973289	110.58	ΔORF2	OK	47.7176	44.9332	-0.0867394	-0.127923	0.8233	0.994748	no
gene:SpnNT_01985	NA	Chromosome:1973028-1973289	110.58	110.58+peptide	OK	47.7176	51.3624	0.10619	0.157847	0.78635	0.994748	no
gene:SpnNT_01985	NA	Chromosome:1973028-1973289	ΔORF2	110.58+peptide	OK	44.9332	51.3624	0.19293	0.284148	0.6262	0.980887	no
gene:SpnNT_01985	NA	Chromosome:1973028-1973289	110.58	ΔORF2+peptide	OK	47.7176	37.084	-0.363723	-0.514626	0.3715	0.868488	no
gene:SpnNT_01985	NA	Chromosome:1973028-1973289	ΔORF2	ΔORF2+peptide	OK	44.9332	37.084	-0.276983	-0.388635	0.49545	0.942995	no
gene:SpnNT_01985	NA	Chromosome:1973028-1973289	110.58+peptide	ΔORF2+peptide	OK	51.3624	37.084	-0.469913	-0.664048	0.24025	0.735646	no
gene:SpnNT_01986	NA	Chromosome:1973347-1974875	110.58	ΔORF2	OK	21.765	22.7102	0.0613308	0.0604902	0.9129	0.994748	no
gene:SpnNT_01986	NA	Chromosome:1973347-1974875	110.58	110.58+peptide	OK	21.765	20.6529	-0.0756671	-0.0767228	0.89205	0.994748	no
gene:SpnNT_01986	NA	Chromosome:1973347-1974875	ΔORF2	110.58+peptide	OK	22.7102	20.6529	-0.136998	-0.134044	0.81715	0.994748	no
gene:SpnNT_01986	NA	Chromosome:1973347-1974875	110.58	ΔORF2+peptide	OK	21.765	26.7489	0.297471	0.302004	0.5989	0.97629	no
gene:SpnNT_01986	NA	Chromosome:1973347-1974875	ΔORF2	ΔORF2+peptide	OK	22.7102	26.7489	0.236141	0.231322	0.6919	0.98828	no
gene:SpnNT_01986	NA	Chromosome:1973347-1974875	110.58+peptide	ΔORF2+peptide	OK	20.6529	26.7489	0.373138	0.375629	0.5221	0.954832	no
gene:SpnNT_01987	NA	Chromosome:1973347-1974875	110.58	ΔORF2	OK	26.1047	33.2394	0.348585	0.604003	0.2952	0.799613	no
gene:SpnNT_01987	NA	Chromosome:1973347-1974875	110.58	110.58+peptide	OK	26.1047	25.8273	-0.0154146	-0.0263115	0.96385	0.994855	no
gene:SpnNT_01987	NA	Chromosome:1973347-1974875	ΔORF2	110.58+peptide	OK	33.2394	25.8273	-0.364	-0.633766	0.27345	0.775829	no
gene:SpnNT_01987	NA	Chromosome:1973347-1974875	110.58	ΔORF2+peptide	OK	26.1047	33.7731	0.371565	0.645913	0.2577	0.757683	no
gene:SpnNT_01987	NA	Chromosome:1973347-1974875	ΔORF2	ΔORF2+peptide	OK	33.2394	33.7731	0.0229798	0.0407782	0.94595	0.994855	no
gene:SpnNT_01987	NA	Chromosome:1973347-1974875	110.58+peptide	ΔORF2+peptide	OK	25.8273	33.7731	0.38698	0.675987	0.2331	0.727059	no
gene:SpnNT_01988	NA	Chromosome:1975283-1975541	110.58	ΔORF2	NOTEST	1.15257	1.04498	-0.141374	0	1	1	no
gene:SpnNT_01988	NA	Chromosome:1975283-1975541	110.58	110.58+peptide	OK	1.15257	2.58806	1.16702	0.770414	0.30605	0.812428	no

gene:SpnNT_01988	NA	Chromosome:1975283-1975541	ΔORF2	110.58+peptide	OK	1.04498	2.58806	1.3084	0.95724	0.2595	0.759841	no
gene:SpnNT_01988	NA	Chromosome:1975283-1975541	110.58	ΔORF2+peptide	NOTEST	1.15257	0.94684	-0.283656	0	1	1	no
gene:SpnNT_01988	NA	Chromosome:1975283-1975541	ΔORF2	ΔORF2+peptide	NOTEST	1.04498	0.94684	-0.142282	0	1	1	no
gene:SpnNT_01988	NA	Chromosome:1975283-1975541	110.58+peptide	ΔORF2+peptide	OK	2.58806	0.94684	-1.45068	-1.16258	0.28295	0.78546	no
gene:SpnNT_01989	NA	Chromosome:1975664-1976024	110.58	ΔORF2	OK	2.63782	2.21604	-0.251358	-0.209393	0.7144	0.99189	no
gene:SpnNT_01989	NA	Chromosome:1975664-1976024	110.58	110.58+peptide	OK	2.63782	1.97991	-0.413908	-0.345314	0.56525	0.968621	no
gene:SpnNT_01989	NA	Chromosome:1975664-1976024	ΔORF2	110.58+peptide	OK	2.21604	1.97991	-0.162549	-0.129546	0.8138	0.994748	no
gene:SpnNT_01989	NA	Chromosome:1975664-1976024	110.58	ΔORF2+peptide	OK	2.63782	1.3493	-0.96713	-0.922757	0.26885	0.770416	no
gene:SpnNT_01989	NA	Chromosome:1975664-1976024	ΔORF2	ΔORF2+peptide	OK	2.21604	1.3493	-0.715772	-0.643777	0.4062	0.895895	no
gene:SpnNT_01989	NA	Chromosome:1975664-1976024	110.58+peptide	ΔORF2+peptide	OK	1.97991	1.3493	-0.553222	-0.498437	0.52275	0.954832	no
gene:SpnNT_01990	NA	Chromosome:1976179-1976275	110.58	ΔORF2	NOTEST	0	10.3577	Inf	0	1	1	no
gene:SpnNT_01990	NA	Chromosome:1976179-1976275	110.58	110.58+peptide	NOTEST	0	0	0	0	1	1	no
gene:SpnNT_01990	NA	Chromosome:1976179-1976275	ΔORF2	110.58+peptide	NOTEST	10.3577	0	#NAME?	0	1	1	no
gene:SpnNT_01990	NA	Chromosome:1976179-1976275	110.58	ΔORF2+peptide	NOTEST	0	0	0	0	1	1	no
gene:SpnNT_01990	NA	Chromosome:1976179-1976275	ΔORF2	ΔORF2+peptide	NOTEST	10.3577	0	#NAME?	0	1	1	no
gene:SpnNT_01990	NA	Chromosome:1976179-1976275	110.58+peptide	ΔORF2+peptide	NOTEST	0	0	0	0	1	1	no
gene:SpnNT_01991	lytA_9	Chromosome:1976799-1977756	110.58	ΔORF2	OK	284.411	274.215	-0.0526676	-0.119984	0.8315	0.994748	no
gene:SpnNT_01991	lytA_9	Chromosome:1976799-1977756	110.58	110.58+peptide	OK	284.411	297.39	0.064381	0.141698	0.79945	0.994748	no
gene:SpnNT_01991	lytA_9	Chromosome:1976799-1977756	ΔORF2	110.58+peptide	OK	274.215	297.39	0.117049	0.260049	0.6416	0.980887	no
gene:SpnNT_01991	lytA_9	Chromosome:1976799-1977756	110.58	ΔORF2+peptide	OK	284.411	221.285	-0.362072	-0.813605	0.15265	0.595267	no
gene:SpnNT_01991	lytA_9	Chromosome:1976799-1977756	ΔORF2	ΔORF2+peptide	OK	274.215	221.285	-0.309404	-0.702107	0.21115	0.697158	no
gene:SpnNT_01991	lytA_9	Chromosome:1976799-1977756	110.58+peptide	ΔORF2+peptide	OK	297.39	221.285	-0.426453	-0.935164	0.09895	0.476835	no
gene:SpnNT_01992	NA	Chromosome:1978131-1979172	110.58	ΔORF2	OK	17.3527	16.5547	-0.0679196	-0.124338	0.82535	0.994748	no
gene:SpnNT_01992	NA	Chromosome:1978131-1979172	110.58	110.58+peptide	OK	17.3527	13.5277	-0.359243	-0.647554	0.25275	0.751038	no
gene:SpnNT_01992	NA	Chromosome:1978131-1979172	ΔORF2	110.58+peptide	OK	16.5547	13.5277	-0.291323	-0.524863	0.35955	0.860914	no
gene:SpnNT_01992	NA	Chromosome:1978131-1979172	110.58	ΔORF2+peptide	OK	17.3527	16.1371	-0.10478	-0.190496	0.73625	0.994748	no
gene:SpnNT_01992	NA	Chromosome:1978131-1979172	ΔORF2	ΔORF2+peptide	OK	16.5547	16.1371	-0.0368602	-0.0669801	0.9047	0.994748	no
gene:SpnNT_01992	NA	Chromosome:1978131-1979172	110.58+peptide	ΔORF2+peptide	OK	13.5277	16.1371	0.254463	0.455395	0.4317	0.913083	no
gene:SpnNT_01993	NA	Chromosome:1979202-1979502	110.58	ΔORF2	OK	76.7749	83.3771	0.119017	0.202247	0.72475	0.994748	no
gene:SpnNT_01993	NA	Chromosome:1979202-1979502	110.58	110.58+peptide	OK	76.7749	54.9406	-0.482761	-0.794546	0.171	0.629768	no
gene:SpnNT_01993	NA	Chromosome:1979202-1979502	ΔORF2	110.58+peptide	OK	83.3771	54.9406	-0.601778	-0.992079	0.0878	0.446382	no
gene:SpnNT_01993	NA	Chromosome:1979202-1979502	110.58	ΔORF2+peptide	OK	76.7749	64.3219	-0.255324	-0.430451	0.45855	0.922502	no
gene:SpnNT_01993	NA	Chromosome:1979202-1979502	ΔORF2	ΔORF2+peptide	OK	83.3771	64.3219	-0.374341	-0.632205	0.27515	0.777354	no
gene:SpnNT_01993	NA	Chromosome:1979202-1979502	110.58+peptide	ΔORF2+peptide	OK	54.9406	64.3219	0.227436	0.37216	0.523	0.954832	no
gene:SpnNT_01994	recA	Chromosome:1979826-1980993	110.58	ΔORF2	OK	314.366	343.58	0.128204	0.291991	0.61335	0.979616	no
gene:SpnNT_01994	recA	Chromosome:1979826-1980993	110.58	110.58+peptide	OK	314.366	294.756	-0.0929243	-0.212662	0.7094	0.990851	no
gene:SpnNT_01994	recA	Chromosome:1979826-1980993	ΔORF2	110.58+peptide	OK	343.58	294.756	-0.221128	-0.506332	0.37425	0.871501	no
gene:SpnNT_01994	recA	Chromosome:1979826-1980993	110.58	ΔORF2+peptide	OK	314.366	340.68	0.115974	0.26373	0.64885	0.981391	no
gene:SpnNT_01994	recA	Chromosome:1979826-1980993	ΔORF2	ΔORF2+peptide	OK	343.58	340.68	-0.0122295	-0.0278249	0.9641	0.994855	no
gene:SpnNT_01994	recA	Chromosome:1979826-1980993	110.58+peptide	ΔORF2+peptide	OK	294.756	340.68	0.208899	0.477584	0.3947	0.889826	no
gene:SpnNT_01995	cinA	Chromosome:1981047-1982304	110.58	ΔORF2	OK	94.3237	96.5323	0.0333913	0.0752837	0.8934	0.994748	no
gene:SpnNT_01995	cinA	Chromosome:1981047-1982304	110.58	110.58+peptide	OK	94.3237	58.1269	-0.698414	-1.55369	0.00715	0.07871	no
gene:SpnNT_01995	cinA	Chromosome:1981047-1982304	ΔORF2	110.58+peptide	OK	96.5323	58.1269	-0.731806	-1.62707	0.00455	0.0556951	no
gene:SpnNT_01995	cinA	Chromosome:1981047-1982304	110.58	ΔORF2+peptide	OK	94.3237	64.388	-0.550828	-1.22904	0.0346	0.246082	no
gene:SpnNT_01995	cinA	Chromosome:1981047-1982304	ΔORF2	ΔORF2+peptide	OK	96.5323	64.388	-0.584219	-1.30283	0.02485	0.196435	no

gene:SpnNT_01995	cinA	Chromosome:1981047-1982304	110.58+peptide	ΔORF2+peptide	OK	58.1269	64.388	0.147587	0.324836	0.5722	0.969077	no
gene:SpnNT_01996	brpA	Chromosome:1982387-1983404	110.58	ΔORF2	OK	213.75	242.909	0.184491	0.421529	0.45545	0.921244	no
gene:SpnNT_01996	brpA	Chromosome:1982387-1983404	110.58	110.58+peptide	OK	213.75	231.827	0.117129	0.26901	0.62945	0.980887	no
gene:SpnNT_01996	brpA	Chromosome:1982387-1983404	ΔORF2	110.58+peptide	OK	242.909	231.827	-0.0673627	-0.153948	0.78965	0.994748	no
gene:SpnNT_01996	brpA	Chromosome:1982387-1983404	110.58	ΔORF2+peptide	OK	213.75	253.396	0.245469	0.564352	0.3116	0.817436	no
gene:SpnNT_01996	brpA	Chromosome:1982387-1983404	ΔORF2	ΔORF2+peptide	OK	242.909	253.396	0.0609775	0.139498	0.808	0.994748	no
gene:SpnNT_01996	brpA	Chromosome:1982387-1983404	110.58+peptide	ΔORF2+peptide	OK	231.827	253.396	0.12834	0.295134	0.59785	0.97629	no
gene:SpnNT_01997	NA	Chromosome:1983411-1984363	110.58	ΔORF2	OK	152.929	153.621	0.00651594	0.00941981	0.9868	0.997339	no
gene:SpnNT_01997	NA	Chromosome:1983411-1984363	110.58	110.58+peptide	OK	152.929	136.134	-0.167831	-0.241099	0.6699	0.984845	no
gene:SpnNT_01997	NA	Chromosome:1983411-1984363	ΔORF2	110.58+peptide	OK	153.621	136.134	-0.174347	-0.238511	0.67565	0.985207	no
gene:SpnNT_01997	NA	Chromosome:1983411-1984363	110.58	ΔORF2+peptide	OK	152.929	170.676	0.158401	0.239458	0.67975	0.986036	no
gene:SpnNT_01997	NA	Chromosome:1983411-1984363	ΔORF2	ΔORF2+peptide	OK	153.621	170.676	0.151885	0.217569	0.70805	0.990441	no
gene:SpnNT_01997	NA	Chromosome:1983411-1984363	110.58+peptide	ΔORF2+peptide	OK	136.134	170.676	0.326231	0.464426	0.41975	0.904223	no
gene:SpnNT_01998	NA	Chromosome:1983411-1984363	110.58	ΔORF2	OK	231.986	282.329	0.283342	0.463912	0.419	0.903984	no
gene:SpnNT_01998	NA	Chromosome:1983411-1984363	110.58	110.58+peptide	OK	231.986	219.546	-0.0795156	-0.12715	0.8287	0.994748	no
gene:SpnNT_01998	NA	Chromosome:1983411-1984363	ΔORF2	110.58+peptide	OK	282.329	219.546	-0.362858	-0.587995	0.32395	0.829371	no
gene:SpnNT_01998	NA	Chromosome:1983411-1984363	110.58	ΔORF2+peptide	OK	231.986	285.734	0.300637	0.4864	0.403	0.894399	no
gene:SpnNT_01998	NA	Chromosome:1983411-1984363	ΔORF2	ΔORF2+peptide	OK	282.329	285.734	0.0172948	0.0283648	0.96195	0.994855	no
gene:SpnNT_01998	NA	Chromosome:1983411-1984363	110.58+peptide	ΔORF2+peptide	OK	219.546	285.734	0.380153	0.608873	0.31385	0.819701	no
gene:SpnNT_01999	NA	Chromosome:1984448-1985069	110.58	ΔORF2	OK	13.0683	14.3535	0.135328	0.216026	0.7048	0.990367	no
gene:SpnNT_01999	NA	Chromosome:1984448-1985069	110.58	110.58+peptide	OK	13.0683	8.06631	-0.696092	-1.07238	0.06625	0.375036	no
gene:SpnNT_01999	NA	Chromosome:1984448-1985069	ΔORF2	110.58+peptide	OK	14.3535	8.06631	-0.83142	-1.2804	0.0297	0.220307	no
gene:SpnNT_01999	NA	Chromosome:1984448-1985069	110.58	ΔORF2+peptide	OK	13.0683	9.89041	-0.40197	-0.626075	0.28605	0.789224	no
gene:SpnNT_01999	NA	Chromosome:1984448-1985069	ΔORF2	ΔORF2+peptide	OK	14.3535	9.89041	-0.537298	-0.836545	0.15125	0.593484	no
gene:SpnNT_01999	NA	Chromosome:1984448-1985069	110.58+peptide	ΔORF2+peptide	OK	8.06631	9.89041	0.294122	0.44268	0.4542	0.921244	no
gene:SpnNT_02000	NA	Chromosome:1985990-1986164	110.58	ΔORF2	OK	46.3542	47.8921	0.0470856	0.0529452	0.92215	0.994748	no
gene:SpnNT_02000	NA	Chromosome:1985990-1986164	110.58	110.58+peptide	OK	46.3542	25.6852	-0.851767	-0.824144	0.1483	0.587736	no
gene:SpnNT_02000	NA	Chromosome:1985990-1986164	ΔORF2	110.58+peptide	OK	47.8921	25.6852	-0.898852	-0.87854	0.1289	0.544217	no
gene:SpnNT_02000	NA	Chromosome:1985990-1986164	110.58	ΔORF2+peptide	OK	46.3542	52.5523	0.181054	0.208243	0.7279	0.994748	no
gene:SpnNT_02000	NA	Chromosome:1985990-1986164	ΔORF2	ΔORF2+peptide	OK	47.8921	52.5523	0.133968	0.156313	0.7995	0.994748	no
gene:SpnNT_02000	NA	Chromosome:1985990-1986164	110.58+peptide	ΔORF2+peptide	OK	25.6852	52.5523	1.03282	1.02678	0.0987	0.47598	no
gene:SpnNT_02001	NA	Chromosome:1986405-1986847	110.58	ΔORF2	NOTEST	34.1925	0	#NAME?	0	1	1	no
gene:SpnNT_02001	NA	Chromosome:1986405-1986847	110.58	110.58+peptide	NOTEST	34.1925	0	#NAME?	0	1	1	no
gene:SpnNT_02001	NA	Chromosome:1986405-1986847	ΔORF2	110.58+peptide	NOTEST	0	0	0	0	1	1	no
gene:SpnNT_02001	NA	Chromosome:1986405-1986847	110.58	ΔORF2+peptide	NOTEST	34.1925	0	#NAME?	0	1	1	no
gene:SpnNT_02001	NA	Chromosome:1986405-1986847	ΔORF2	ΔORF2+peptide	NOTEST	0	0	0	0	1	1	no
gene:SpnNT_02001	NA	Chromosome:1986405-1986847	110.58+peptide	ΔORF2+peptide	NOTEST	0	0	0	0	1	1	no
gene:SpnNT_02002	ndk	Chromosome:1986405-1986847	110.58	ΔORF2	OK	813.222	843.322	0.0524347	0.119756	0.83065	0.994748	no
gene:SpnNT_02002	ndk	Chromosome:1986405-1986847	110.58	110.58+peptide	OK	813.222	678.083	-0.262187	-0.602103	0.29365	0.797915	no
gene:SpnNT_02002	ndk	Chromosome:1986405-1986847	ΔORF2	110.58+peptide	OK	843.322	678.083	-0.314622	-0.721955	0.20495	0.689755	no
gene:SpnNT_02002	ndk	Chromosome:1986405-1986847	110.58	ΔORF2+peptide	OK	813.222	701.484	-0.213239	-0.491031	0.3958	0.890245	no
gene:SpnNT_02002	ndk	Chromosome:1986405-1986847	ΔORF2	ΔORF2+peptide	OK	843.322	701.484	-0.265674	-0.611295	0.2869	0.790183	no
gene:SpnNT_02002	ndk	Chromosome:1986405-1986847	110.58+peptide	ΔORF2+peptide	OK	678.083	701.484	0.0489478	0.113254	0.8405	0.994748	no
gene:SpnNT_02003	rpoC	Chromosome:1986947-1990625	110.58	ΔORF2	OK	383.74	368.628	-0.057966	-0.121587	0.8317	0.994748	no
gene:SpnNT_02003	rpoC	Chromosome:1986947-1990625	110.58	110.58+peptide	OK	383.74	402.936	0.0704224	0.150732	0.7926	0.994748	no

gene:SpnNT_02003	rpoC	Chromosome:1986947-1990625	ΔORF2	110.58+peptide	OK	368.628	402.936	0.128388	0.267079	0.63975	0.980887	no
gene:SpnNT_02003	rpoC	Chromosome:1986947-1990625	110.58	ΔORF2+peptide	OK	383.74	403.089	0.0709674	0.152773	0.7886	0.994748	no
gene:SpnNT_02003	rpoC	Chromosome:1986947-1990625	ΔORF2	ΔORF2+peptide	OK	368.628	403.089	0.128933	0.269669	0.6308	0.980887	no
gene:SpnNT_02003	rpoC	Chromosome:1986947-1990625	110.58+peptide	ΔORF2+peptide	OK	402.936	403.089	0.000545054	0.00116315	0.9982	0.999412	no
gene:SpnNT_02004	rpoB	Chromosome:1990658-1994270	110.58	ΔORF2	OK	318.565	316.133	-0.011055	-0.0234742	0.96525	0.994855	no
gene:SpnNT_02004	rpoB	Chromosome:1990658-1994270	110.58	110.58+peptide	OK	318.565	357.233	0.165281	0.357728	0.528	0.956161	no
gene:SpnNT_02004	rpoB	Chromosome:1990658-1994270	ΔORF2	110.58+peptide	OK	316.133	357.233	0.176336	0.37139	0.5124	0.950409	no
gene:SpnNT_02004	rpoB	Chromosome:1990658-1994270	110.58	ΔORF2+peptide	OK	318.565	363.867	0.191826	0.416473	0.46085	0.924377	no
gene:SpnNT_02004	rpoB	Chromosome:1990658-1994270	ΔORF2	ΔORF2+peptide	OK	316.133	363.867	0.202881	0.428558	0.4451	0.917756	no
gene:SpnNT_02004	rpoB	Chromosome:1990658-1994270	110.58+peptide	ΔORF2+peptide	OK	357.233	363.867	0.026545	0.0571426	0.9163	0.994748	no
gene:SpnNT_02005	NA	Chromosome:1994769-1996503	110.58	ΔORF2	OK	29.1896	31.4409	0.107186	0.225637	0.69315	0.98828	no
gene:SpnNT_02005	NA	Chromosome:1994769-1996503	110.58	110.58+peptide	OK	29.1896	27.8344	-0.0685884	-0.142891	0.8016	0.994748	no
gene:SpnNT_02005	NA	Chromosome:1994769-1996503	ΔORF2	110.58+peptide	OK	31.4409	27.8344	-0.175774	-0.369145	0.5189	0.954444	no
gene:SpnNT_02005	NA	Chromosome:1994769-1996503	110.58	ΔORF2+peptide	OK	29.1896	35.8107	0.294933	0.623858	0.2837	0.786627	no
gene:SpnNT_02005	NA	Chromosome:1994769-1996503	ΔORF2	ΔORF2+peptide	OK	31.4409	35.8107	0.187747	0.400435	0.4902	0.93986	no
gene:SpnNT_02005	NA	Chromosome:1994769-1996503	110.58+peptide	ΔORF2+peptide	OK	27.8344	35.8107	0.363521	0.767099	0.18435	0.656278	no
gene:SpnNT_02006	SpnNT_02006	Chromosome:1996729-1996803	110.58	ΔORF2	OK	1191.99	3340.99	1.48691	1.49831	0.16295	0.61656	no
gene:SpnNT_02006	SpnNT_02006	Chromosome:1996729-1996803	110.58	110.58+peptide	OK	1191.99	2100.5	0.817365	0.658225	0.4281	0.909574	no
gene:SpnNT_02006	SpnNT_02006	Chromosome:1996729-1996803	ΔORF2	110.58+peptide	OK	3340.99	2100.5	-0.669546	-0.421209	0.4238	0.907047	no
gene:SpnNT_02006	SpnNT_02006	Chromosome:1996729-1996803	110.58	ΔORF2+peptide	OK	1191.99	6826.98	2.51788	5.13922	0.0567	0.338806	no
gene:SpnNT_02006	SpnNT_02006	Chromosome:1996729-1996803	ΔORF2	ΔORF2+peptide	OK	3340.99	6826.98	1.03097	0.931562	0.20265	0.686593	no
gene:SpnNT_02006	SpnNT_02006	Chromosome:1996729-1996803	110.58+peptide	ΔORF2+peptide	OK	2100.5	6826.98	1.70052	1.27389	0.0782	0.415273	no
gene:SpnNT_02007	corC	Chromosome:1996998-1998330	110.58	ΔORF2	OK	240.709	285.915	0.248299	0.551927	0.3408	0.844885	no
gene:SpnNT_02007	corC	Chromosome:1996998-1998330	110.58	110.58+peptide	OK	240.709	228.58	-0.074592	-0.167839	0.7664	0.994748	no
gene:SpnNT_02007	corC	Chromosome:1996998-1998330	ΔORF2	110.58+peptide	OK	285.915	228.58	-0.322891	-0.70967	0.22075	0.709102	no
gene:SpnNT_02007	corC	Chromosome:1996998-1998330	110.58	ΔORF2+peptide	OK	240.709	239.761	-0.00569431	-0.0130024	0.982	0.996246	no
gene:SpnNT_02007	corC	Chromosome:1996998-1998330	ΔORF2	ΔORF2+peptide	OK	285.915	239.761	-0.253993	-0.566118	0.3202	0.825861	no
gene:SpnNT_02007	corC	Chromosome:1996998-1998330	110.58+peptide	ΔORF2+peptide	OK	228.58	239.761	0.0688977	0.155458	0.78525	0.994748	no
gene:SpnNT_02008	endA	Chromosome:1998620-1999445	110.58	ΔORF2	OK	144.602	138.898	-0.0580595	-0.130823	0.81785	0.994748	no
gene:SpnNT_02008	endA	Chromosome:1998620-1999445	110.58	110.58+peptide	OK	144.602	129.918	-0.154485	-0.344364	0.5536	0.965961	no
gene:SpnNT_02008	endA	Chromosome:1998620-1999445	ΔORF2	110.58+peptide	OK	138.898	129.918	-0.0964256	-0.214349	0.7163	0.992428	no
gene:SpnNT_02008	endA	Chromosome:1998620-1999445	110.58	ΔORF2+peptide	OK	144.602	119.336	-0.277051	-0.618465	0.29065	0.795003	no
gene:SpnNT_02008	endA	Chromosome:1998620-1999445	ΔORF2	ΔORF2+peptide	OK	138.898	119.336	-0.218991	-0.487503	0.39875	0.892547	no
gene:SpnNT_02008	endA	Chromosome:1998620-1999445	110.58+peptide	ΔORF2+peptide	OK	129.918	119.336	-0.122566	-0.269991	0.6393	0.980887	no
gene:SpnNT_02009	NA	Chromosome:1999483-2000948	110.58	ΔORF2	OK	591.167	546.729	-0.112739	-0.0574459	0.9259	0.994748	no
gene:SpnNT_02009	NA	Chromosome:1999483-2000948	110.58	110.58+peptide	OK	591.167	689.708	0.222421	0.116817	0.8363	0.994748	no
gene:SpnNT_02009	NA	Chromosome:1999483-2000948	ΔORF2	110.58+peptide	OK	546.729	689.708	0.33516	0.177129	0.78015	0.994748	no
gene:SpnNT_02009	NA	Chromosome:1999483-2000948	110.58	ΔORF2+peptide	OK	591.167	582.734	-0.0207287	-0.00998816	0.98725	0.997359	no
gene:SpnNT_02009	NA	Chromosome:1999483-2000948	ΔORF2	ΔORF2+peptide	OK	546.729	582.734	0.0920107	0.0445684	0.9514	0.994855	no
gene:SpnNT_02009	NA	Chromosome:1999483-2000948	110.58+peptide	ΔORF2+peptide	OK	689.708	582.734	-0.243149	-0.121034	0.85195	0.994748	no
gene:SpnNT_02010	murA1	Chromosome:1999483-2000948	110.58	ΔORF2	OK	256.241	235.462	-0.122011	-0.268103	0.6354	0.980887	no
gene:SpnNT_02010	murA1	Chromosome:1999483-2000948	110.58	110.58+peptide	OK	256.241	214.793	-0.254557	-0.557822	0.32905	0.834967	no
gene:SpnNT_02010	murA1	Chromosome:1999483-2000948	ΔORF2	110.58+peptide	OK	235.462	214.793	-0.132546	-0.286383	0.61995	0.980887	no
gene:SpnNT_02010	murA1	Chromosome:1999483-2000948	110.58	ΔORF2+peptide	OK	256.241	197.165	-0.378097	-0.839584	0.1459	0.581915	no
gene:SpnNT_02010	murA1	Chromosome:1999483-2000948	ΔORF2	ΔORF2+peptide	OK	235.462	197.165	-0.256086	-0.560473	0.328	0.833593	no

gene:SpnNT_02010	murA1	Chromosome:1999483-2000948	110.58+peptide	ΔORF2+peptide	OK	214.793	197.165	-0.12354	-0.269646	0.6386	0.980887	no
gene:SpnNT_02011	NA	Chromosome:2001259-2003298	110.58	ΔORF2	OK	93.5879	89.902	-0.0579693	-0.0974777	0.8648	0.994748	no
gene:SpnNT_02011	NA	Chromosome:2001259-2003298	110.58	110.58+peptide	OK	93.5879	62.3899	-0.585009	-0.973842	0.0947	0.46531	no
gene:SpnNT_02011	NA	Chromosome:2001259-2003298	ΔORF2	110.58+peptide	OK	89.902	62.3899	-0.52704	-0.871798	0.13265	0.554518	no
gene:SpnNT_02011	NA	Chromosome:2001259-2003298	110.58	ΔORF2+peptide	OK	93.5879	57.1818	-0.710767	-1.19044	0.04115	0.275917	no
gene:SpnNT_02011	NA	Chromosome:2001259-2003298	ΔORF2	ΔORF2+peptide	OK	89.902	57.1818	-0.652798	-1.08635	0.0601	0.351911	no
gene:SpnNT_02011	NA	Chromosome:2001259-2003298	110.58+peptide	ΔORF2+peptide	OK	62.3899	57.1818	-0.125758	-0.207222	0.72365	0.994748	no
gene:SpnNT_02012	coaD	Chromosome:2001259-2003298	110.58	ΔORF2	OK	73.2722	76.065	0.0539666	0.042341	0.94235	0.994855	no
gene:SpnNT_02012	coaD	Chromosome:2001259-2003298	110.58	110.58+peptide	OK	73.2722	48.9193	-0.582863	-0.471264	0.42255	0.905849	no
gene:SpnNT_02012	coaD	Chromosome:2001259-2003298	ΔORF2	110.58+peptide	OK	76.065	48.9193	-0.63683	-0.500806	0.3838	0.880921	no
gene:SpnNT_02012	coaD	Chromosome:2001259-2003298	110.58	ΔORF2+peptide	OK	73.2722	52.0095	-0.494492	-0.418872	0.4816	0.935095	no
gene:SpnNT_02012	coaD	Chromosome:2001259-2003298	ΔORF2	ΔORF2+peptide	OK	76.065	52.0095	-0.548459	-0.450685	0.43685	0.914104	no
gene:SpnNT_02012	coaD	Chromosome:2001259-2003298	110.58+peptide	ΔORF2+peptide	OK	48.9193	52.0095	0.0883711	0.0750603	0.89585	0.994748	no
gene:SpnNT_02013	rsmD	Chromosome:2001259-2003298	110.58	ΔORF2	OK	130.593	122.27	-0.095001	-0.111441	0.8433	0.994748	no
gene:SpnNT_02013	rsmD	Chromosome:2001259-2003298	110.58	110.58+peptide	OK	130.593	89.7015	-0.541869	-0.642279	0.26095	0.761099	no
gene:SpnNT_02013	rsmD	Chromosome:2001259-2003298	ΔORF2	110.58+peptide	OK	122.27	89.7015	-0.446868	-0.541844	0.34105	0.844955	no
gene:SpnNT_02013	rsmD	Chromosome:2001259-2003298	110.58	ΔORF2+peptide	OK	130.593	80.6715	-0.694941	-0.823766	0.16045	0.612755	no
gene:SpnNT_02013	rsmD	Chromosome:2001259-2003298	ΔORF2	ΔORF2+peptide	OK	122.27	80.6715	-0.59994	-0.727496	0.2094	0.695632	no
gene:SpnNT_02013	rsmD	Chromosome:2001259-2003298	110.58+peptide	ΔORF2+peptide	OK	89.7015	80.6715	-0.153072	-0.18769	0.75025	0.994748	no
gene:SpnNT_02014	asnA	Chromosome:2003362-2004355	110.58	ΔORF2	OK	875.789	975.827	0.156042	0.346551	0.5418	0.961494	no
gene:SpnNT_02014	asnA	Chromosome:2003362-2004355	110.58	110.58+peptide	OK	875.789	400.932	-1.12722	-2.54235	5.00E-05	0.0013612	yes
gene:SpnNT_02014	asnA	Chromosome:2003362-2004355	ΔORF2	110.58+peptide	OK	975.827	400.932	-1.28327	-2.89707	5.00E-05	0.0013612	yes
gene:SpnNT_02014	asnA	Chromosome:2003362-2004355	110.58	ΔORF2+peptide	OK	875.789	384.745	-1.18668	-2.68034	5.00E-05	0.0013612	yes
gene:SpnNT_02014	asnA	Chromosome:2003362-2004355	ΔORF2	ΔORF2+peptide	OK	975.827	384.745	-1.34272	-3.03572	5.00E-05	0.0013612	yes
gene:SpnNT_02014	asnA	Chromosome:2003362-2004355	110.58+peptide	ΔORF2+peptide	OK	400.932	384.745	-0.0594543	-0.136586	0.80245	0.994748	no
gene:SpnNT_02015	ybhL	Chromosome:2004465-2005149	110.58	ΔORF2	OK	498.984	590.819	0.243723	0.558014	0.3275	0.83284	no
gene:SpnNT_02015	ybhL	Chromosome:2004465-2005149	110.58	110.58+peptide	OK	498.984	432.592	-0.205984	-0.473592	0.40515	0.894933	no
gene:SpnNT_02015	ybhL	Chromosome:2004465-2005149	ΔORF2	110.58+peptide	OK	590.819	432.592	-0.449707	-1.02601	0.0741	0.40113	no
gene:SpnNT_02015	ybhL	Chromosome:2004465-2005149	110.58	ΔORF2+peptide	OK	498.984	468.311	-0.0915245	-0.211466	0.7063	0.990422	no
gene:SpnNT_02015	ybhL	Chromosome:2004465-2005149	ΔORF2	ΔORF2+peptide	OK	590.819	468.311	-0.335248	-0.768574	0.1798	0.64657	no
gene:SpnNT_02015	ybhL	Chromosome:2004465-2005149	110.58+peptide	ΔORF2+peptide	OK	432.592	468.311	0.11446	0.263511	0.6435	0.980887	no
gene:SpnNT_02016	NA	Chromosome:2005204-2005945	110.58	ΔORF2	OK	119.132	146.931	0.302574	0.658715	0.25175	0.749765	no
gene:SpnNT_02016	NA	Chromosome:2005204-2005945	110.58	110.58+peptide	OK	119.132	109.527	-0.121279	-0.264555	0.64575	0.980887	no
gene:SpnNT_02016	NA	Chromosome:2005204-2005945	ΔORF2	110.58+peptide	OK	146.931	109.527	-0.423852	-0.912797	0.11645	0.517109	no
gene:SpnNT_02016	NA	Chromosome:2005204-2005945	110.58	ΔORF2+peptide	OK	119.132	141.245	0.245633	0.541866	0.3477	0.852489	no
gene:SpnNT_02016	NA	Chromosome:2005204-2005945	ΔORF2	ΔORF2+peptide	OK	146.931	141.245	-0.0569406	-0.123974	0.8288	0.994748	no
gene:SpnNT_02016	NA	Chromosome:2005204-2005945	110.58+peptide	ΔORF2+peptide	OK	109.527	141.245	0.366912	0.800452	0.17265	0.632681	no
gene:SpnNT_02017	acyP	Chromosome:2005978-2006257	110.58	ΔORF2	OK	14.383	13.7226	-0.0678134	-0.0812551	0.88805	0.994748	no
gene:SpnNT_02017	acyP	Chromosome:2005978-2006257	110.58	110.58+peptide	OK	14.383	15.0653	0.0668608	0.0802404	0.886	0.994748	no
gene:SpnNT_02017	acyP	Chromosome:2005978-2006257	ΔORF2	110.58+peptide	OK	13.7226	15.0653	0.134674	0.159079	0.78045	0.994748	no
gene:SpnNT_02017	acyP	Chromosome:2005978-2006257	110.58	ΔORF2+peptide	OK	14.383	11.7987	-0.285745	-0.323451	0.57745	0.969538	no
gene:SpnNT_02017	acyP	Chromosome:2005978-2006257	ΔORF2	ΔORF2+peptide	OK	13.7226	11.7987	-0.217931	-0.243225	0.6746	0.985063	no
gene:SpnNT_02017	acyP	Chromosome:2005978-2006257	110.58+peptide	ΔORF2+peptide	OK	15.0653	11.7987	-0.352605	-0.394069	0.4843	0.936747	no
gene:SpnNT_02018	misCB	Chromosome:2006334-2007261	110.58	ΔORF2	OK	101.107	112.903	0.159202	0.355304	0.5285	0.956161	no
gene:SpnNT_02018	misCB	Chromosome:2006334-2007261	110.58	110.58+peptide	OK	101.107	111.34	0.139092	0.310703	0.57755	0.969538	no

gene:SpnNT_02018	misCB	Chromosome:2006334-2007261	ΔORF2	110.58+peptide	OK	112.903	111.34	-0.0201097	-0.0450329	0.93655	0.994855	no
gene:SpnNT_02018	misCB	Chromosome:2006334-2007261	110.58	ΔORF2+peptide	OK	101.107	114.437	0.178673	0.399073	0.4783	0.933133	no
gene:SpnNT_02018	misCB	Chromosome:2006334-2007261	ΔORF2	ΔORF2+peptide	OK	112.903	114.437	0.0194708	0.0435975	0.9395	0.994855	no
gene:SpnNT_02018	misCB	Chromosome:2006334-2007261	110.58+peptide	ΔORF2+peptide	OK	111.34	114.437	0.0395805	0.0887057	0.8762	0.994748	no
gene:SpnNT_02019	pflA_2	Chromosome:2007335-2008130	110.58	ΔORF2	OK	437.254	423.803	-0.0450793	-0.102097	0.85575	0.994748	no
gene:SpnNT_02019	pflA_2	Chromosome:2007335-2008130	110.58	110.58+peptide	OK	437.254	474.007	0.116437	0.26532	0.6332	0.980887	no
gene:SpnNT_02019	pflA_2	Chromosome:2007335-2008130	ΔORF2	110.58+peptide	OK	423.803	474.007	0.161516	0.369007	0.51165	0.949897	no
gene:SpnNT_02019	pflA_2	Chromosome:2007335-2008130	110.58	ΔORF2+peptide	OK	437.254	443.927	0.0218504	0.0495995	0.92995	0.99482	no
gene:SpnNT_02019	pflA_2	Chromosome:2007335-2008130	ΔORF2	ΔORF2+peptide	OK	423.803	443.927	0.0669297	0.152324	0.78905	0.994748	no
gene:SpnNT_02019	pflA_2	Chromosome:2007335-2008130	110.58+peptide	ΔORF2+peptide	OK	474.007	443.927	-0.0945862	-0.216593	0.7026	0.990367	no
gene:SpnNT_02020	lysA	Chromosome:2008243-2009506	110.58	ΔORF2	OK	209.374	225.242	0.105395	0.239908	0.67505	0.985207	no
gene:SpnNT_02020	lysA	Chromosome:2008243-2009506	110.58	110.58+peptide	OK	209.374	135.395	-0.62891	-1.43109	0.0143	0.129589	no
gene:SpnNT_02020	lysA	Chromosome:2008243-2009506	ΔORF2	110.58+peptide	OK	225.242	135.395	-0.734305	-1.67042	0.00435	0.0540026	no
gene:SpnNT_02020	lysA	Chromosome:2008243-2009506	110.58	ΔORF2+peptide	OK	209.374	143.512	-0.54491	-1.24647	0.0317	0.230965	no
gene:SpnNT_02020	lysA	Chromosome:2008243-2009506	ΔORF2	ΔORF2+peptide	OK	225.242	143.512	-0.650305	-1.4871	0.00945	0.0967261	no
gene:SpnNT_02020	lysA	Chromosome:2008243-2009506	110.58+peptide	ΔORF2+peptide	OK	135.395	143.512	0.0840001	0.192025	0.7363	0.994748	no
gene:SpnNT_02021	purR	Chromosome:2009572-2010400	110.58	ΔORF2	OK	471.639	505.515	0.100073	0.227094	0.68855	0.98828	no
gene:SpnNT_02021	purR	Chromosome:2009572-2010400	110.58	110.58+peptide	OK	471.639	509.123	0.110333	0.248703	0.66075	0.982966	no
gene:SpnNT_02021	purR	Chromosome:2009572-2010400	ΔORF2	110.58+peptide	OK	505.515	509.123	0.0102599	0.0234125	0.9658	0.994855	no
gene:SpnNT_02021	purR	Chromosome:2009572-2010400	110.58	ΔORF2+peptide	OK	471.639	486.238	0.0439801	0.099334	0.86275	0.994748	no
gene:SpnNT_02021	purR	Chromosome:2009572-2010400	ΔORF2	ΔORF2+peptide	OK	505.515	486.238	-0.0560926	-0.128261	0.82075	0.994748	no
gene:SpnNT_02021	purR	Chromosome:2009572-2010400	110.58+peptide	ΔORF2+peptide	OK	509.123	486.238	-0.0663525	-0.150692	0.79485	0.994748	no
gene:SpnNT_02022	yhaM	Chromosome:2010666-2012854	110.58	ΔORF2	OK	131.706	129.689	-0.0222671	-0.0350125	0.95075	0.994855	no
gene:SpnNT_02022	yhaM	Chromosome:2010666-2012854	110.58	110.58+peptide	OK	131.706	145.904	0.147696	0.229496	0.68965	0.98828	no
gene:SpnNT_02022	yhaM	Chromosome:2010666-2012854	ΔORF2	110.58+peptide	OK	129.689	145.904	0.169963	0.267971	0.64215	0.980887	no
gene:SpnNT_02022	yhaM	Chromosome:2010666-2012854	110.58	ΔORF2+peptide	OK	131.706	124.439	-0.0818798	-0.12552	0.82925	0.994748	no
gene:SpnNT_02022	yhaM	Chromosome:2010666-2012854	ΔORF2	ΔORF2+peptide	OK	129.689	124.439	-0.0596128	-0.0926899	0.8713	0.994748	no
gene:SpnNT_02022	yhaM	Chromosome:2010666-2012854	110.58+peptide	ΔORF2+peptide	OK	145.904	124.439	-0.229576	-0.35284	0.54585	0.961816	no
gene:SpnNT_02023	NA	Chromosome:2010666-2012854	110.58	ΔORF2	OK	97.3613	89.2036	-0.126246	-0.200203	0.7312	0.994748	no
gene:SpnNT_02023	NA	Chromosome:2010666-2012854	110.58	110.58+peptide	OK	97.3613	94.7263	-0.0395826	-0.0611778	0.917	0.994748	no
gene:SpnNT_02023	NA	Chromosome:2010666-2012854	ΔORF2	110.58+peptide	OK	89.2036	94.7263	0.0866636	0.132973	0.81935	0.994748	no
gene:SpnNT_02023	NA	Chromosome:2010666-2012854	110.58	ΔORF2+peptide	OK	97.3613	96.1816	-0.0175872	-0.0283431	0.96105	0.994855	no
gene:SpnNT_02023	NA	Chromosome:2010666-2012854	ΔORF2	ΔORF2+peptide	OK	89.2036	96.1816	0.108659	0.173732	0.7664	0.994748	no
gene:SpnNT_02023	NA	Chromosome:2010666-2012854	110.58+peptide	ΔORF2+peptide	OK	94.7263	96.1816	0.0219954	0.0342612	0.953	0.994855	no
gene:SpnNT_02024	thiN	Chromosome:2012855-2014137	110.58	ΔORF2	OK	90.7142	91.3908	0.0107206	0.0161164	0.9789	0.995765	no
gene:SpnNT_02024	thiN	Chromosome:2012855-2014137	110.58	110.58+peptide	OK	90.7142	102.815	0.180652	0.285443	0.61715	0.980654	no
gene:SpnNT_02024	thiN	Chromosome:2012855-2014137	ΔORF2	110.58+peptide	OK	91.3908	102.815	0.169931	0.261356	0.64785	0.981391	no
gene:SpnNT_02024	thiN	Chromosome:2012855-2014137	110.58	ΔORF2+peptide	OK	90.7142	101.216	0.15803	0.252124	0.65755	0.982966	no
gene:SpnNT_02024	thiN	Chromosome:2012855-2014137	ΔORF2	ΔORF2+peptide	OK	91.3908	101.216	0.14731	0.228646	0.68685	0.98828	no
gene:SpnNT_02024	thiN	Chromosome:2012855-2014137	110.58+peptide	ΔORF2+peptide	OK	102.815	101.216	-0.0226214	-0.0370333	0.9472	0.994855	no
gene:SpnNT_02025	rpe	Chromosome:2012855-2014137	110.58	ΔORF2	OK	103.807	98.8462	-0.0706535	-0.1119	0.8442	0.994748	no
gene:SpnNT_02025	rpe	Chromosome:2012855-2014137	110.58	110.58+peptide	OK	103.807	94.4891	-0.135691	-0.21095	0.7139	0.991721	no
gene:SpnNT_02025	rpe	Chromosome:2012855-2014137	ΔORF2	110.58+peptide	OK	98.8462	94.4891	-0.0650376	-0.100768	0.86435	0.994748	no
gene:SpnNT_02025	rpe	Chromosome:2012855-2014137	110.58	ΔORF2+peptide	OK	103.807	87.2332	-0.250961	-0.388836	0.5015	0.944784	no
gene:SpnNT_02025	rpe	Chromosome:2012855-2014137	ΔORF2	ΔORF2+peptide	OK	98.8462	87.2332	-0.180307	-0.278427	0.62945	0.980887	no

gene:SpnNT_02025	rpe	Chromosome:2012855-2014137	110.58+peptide	ΔORF2+peptide	OK	94.4891	87.2332	-0.115269	-0.174878	0.7668	0.994748	no
gene:SpnNT_02026	rsgA	Chromosome:2014147-2015026	110.58	ΔORF2	OK	71.4408	71.1247	-0.0063988	-0.0136734	0.98145	0.996246	no
gene:SpnNT_02026	rsgA	Chromosome:2014147-2015026	110.58	110.58+peptide	OK	71.4408	72.1352	0.0139559	0.0299179	0.95895	0.994855	no
gene:SpnNT_02026	rsgA	Chromosome:2014147-2015026	ΔORF2	110.58+peptide	OK	71.1247	72.1352	0.0203547	0.0435133	0.93815	0.994855	no
gene:SpnNT_02026	rsgA	Chromosome:2014147-2015026	110.58	ΔORF2+peptide	OK	71.4408	80.2099	0.167032	0.35759	0.53895	0.960753	no
gene:SpnNT_02026	rsgA	Chromosome:2014147-2015026	ΔORF2	ΔORF2+peptide	OK	71.1247	80.2099	0.173431	0.370253	0.51755	0.953833	no
gene:SpnNT_02026	rsgA	Chromosome:2014147-2015026	110.58+peptide	ΔORF2+peptide	OK	72.1352	80.2099	0.153076	0.327848	0.57205	0.969077	no
gene:SpnNT_02027	rsmA	Chromosome:2015027-2015900	110.58	ΔORF2	OK	81.5232	81.611	0.00155261	0.00333432	0.99545	0.999258	no
gene:SpnNT_02027	rsmA	Chromosome:2015027-2015900	110.58	110.58+peptide	OK	81.5232	75.2984	-0.114591	-0.246329	0.6612	0.982966	no
gene:SpnNT_02027	rsmA	Chromosome:2015027-2015900	ΔORF2	110.58+peptide	OK	81.611	75.2984	-0.116144	-0.248075	0.66335	0.982966	no
gene:SpnNT_02027	rsmA	Chromosome:2015027-2015900	110.58	ΔORF2+peptide	OK	81.5232	67.1556	-0.279704	-0.5932	0.301	0.807023	no
gene:SpnNT_02027	rsmA	Chromosome:2015027-2015900	ΔORF2	ΔORF2+peptide	OK	81.611	67.1556	-0.281257	-0.59279	0.30095	0.807023	no
gene:SpnNT_02027	rsmA	Chromosome:2015027-2015900	110.58+peptide	ΔORF2+peptide	OK	75.2984	67.1556	-0.165113	-0.348323	0.5447	0.961568	no
gene:SpnNT_02028	NA	Chromosome:2016030-2016973	110.58	ΔORF2	OK	39.0647	32.9027	-0.247661	-0.229943	0.6858	0.988184	no
gene:SpnNT_02028	NA	Chromosome:2016030-2016973	110.58	110.58+peptide	OK	39.0647	21.4445	-0.86526	-0.775331	0.1775	0.643591	no
gene:SpnNT_02028	NA	Chromosome:2016030-2016973	ΔORF2	110.58+peptide	OK	32.9027	21.4445	-0.617599	-0.530696	0.3541	0.856303	no
gene:SpnNT_02028	NA	Chromosome:2016030-2016973	110.58	ΔORF2+peptide	OK	39.0647	13.4119	-1.54235	-1.01288	0.15135	0.593484	no
gene:SpnNT_02028	NA	Chromosome:2016030-2016973	ΔORF2	ΔORF2+peptide	OK	32.9027	13.4119	-1.29469	-0.830948	0.20455	0.689595	no
gene:SpnNT_02028	NA	Chromosome:2016030-2016973	110.58+peptide	ΔORF2+peptide	OK	21.4445	13.4119	-0.677092	-0.427119	0.4777	0.933072	no
gene:SpnNT_02029	bceA_2	Chromosome:2016030-2016973	110.58	ΔORF2	OK	23.544	28.4924	0.275219	0.432589	0.4536	0.921244	no
gene:SpnNT_02029	bceA_2	Chromosome:2016030-2016973	110.58	110.58+peptide	OK	23.544	12.5668	-0.905743	-1.28621	0.03275	0.236644	no
gene:SpnNT_02029	bceA_2	Chromosome:2016030-2016973	ΔORF2	110.58+peptide	OK	28.4924	12.5668	-1.18096	-1.72935	0.0047	0.0572109	no
gene:SpnNT_02029	bceA_2	Chromosome:2016030-2016973	110.58	ΔORF2+peptide	OK	23.544	14.5406	-0.695273	-1.02356	0.0808	0.423764	no
gene:SpnNT_02029	bceA_2	Chromosome:2016030-2016973	ΔORF2	ΔORF2+peptide	OK	28.4924	14.5406	-0.970492	-1.4768	0.0121	0.11539	no
gene:SpnNT_02029	bceA_2	Chromosome:2016030-2016973	110.58+peptide	ΔORF2+peptide	OK	12.5668	14.5406	0.21047	0.291036	0.62105	0.980887	no
gene:SpnNT_02030	NA	Chromosome:2016974-2019005	110.58	ΔORF2	OK	36.0865	35.4547	-0.0254816	-0.0553076	0.92305	0.994748	no
gene:SpnNT_02030	NA	Chromosome:2016974-2019005	110.58	110.58+peptide	OK	36.0865	20.4201	-0.82147	-1.75159	0.00335	0.0437836	yes
gene:SpnNT_02030	NA	Chromosome:2016974-2019005	ΔORF2	110.58+peptide	OK	35.4547	20.4201	-0.795988	-1.67578	0.0047	0.0572109	no
gene:SpnNT_02030	NA	Chromosome:2016974-2019005	110.58	ΔORF2+peptide	OK	36.0865	17.4762	-1.04607	-2.19686	2.00E-04	0.00450928	yes
gene:SpnNT_02030	NA	Chromosome:2016974-2019005	ΔORF2	ΔORF2+peptide	OK	35.4547	17.4762	-1.02058	-2.11703	0.00025	0.00542231	yes
gene:SpnNT_02030	NA	Chromosome:2016974-2019005	110.58+peptide	ΔORF2+peptide	OK	20.4201	17.4762	-0.224595	-0.458373	0.4311	0.912549	no
gene:SpnNT_02031	NA	Chromosome:2019273-2020173	110.58	ΔORF2	OK	42.5172	67.3726	0.664114	1.3143	0.0224	0.182567	no
gene:SpnNT_02031	NA	Chromosome:2019273-2020173	110.58	110.58+peptide	OK	42.5172	46.2335	0.12089	0.240666	0.676	0.98524	no
gene:SpnNT_02031	NA	Chromosome:2019273-2020173	ΔORF2	110.58+peptide	OK	67.3726	46.2335	-0.543224	-1.05889	0.0641	0.367622	no
gene:SpnNT_02031	NA	Chromosome:2019273-2020173	110.58	ΔORF2+peptide	OK	42.5172	62.0389	0.545125	1.11765	0.05545	0.335771	no
gene:SpnNT_02031	NA	Chromosome:2019273-2020173	ΔORF2	ΔORF2+peptide	OK	67.3726	62.0389	-0.118989	-0.238573	0.67605	0.98524	no
gene:SpnNT_02031	NA	Chromosome:2019273-2020173	110.58+peptide	ΔORF2+peptide	OK	46.2335	62.0389	0.424235	0.855776	0.13935	0.569287	no
gene:SpnNT_02032	rnmV	Chromosome:2020244-2021578	110.58	ΔORF2	OK	44.6688	42.4047	-0.0750455	-0.0864019	0.8775	0.994748	no
gene:SpnNT_02032	rnmV	Chromosome:2020244-2021578	110.58	110.58+peptide	OK	44.6688	60.8698	0.446458	0.518963	0.36505	0.864498	no
gene:SpnNT_02032	rnmV	Chromosome:2020244-2021578	ΔORF2	110.58+peptide	OK	42.4047	60.8698	0.521503	0.599934	0.3016	0.807673	no
gene:SpnNT_02032	rnmV	Chromosome:2020244-2021578	110.58	ΔORF2+peptide	OK	44.6688	45.9031	0.0393218	0.0452366	0.9365	0.994855	no
gene:SpnNT_02032	rnmV	Chromosome:2020244-2021578	ΔORF2	ΔORF2+peptide	OK	42.4047	45.9031	0.114367	0.130239	0.821	0.994748	no
gene:SpnNT_02032	rnmV	Chromosome:2020244-2021578	110.58+peptide	ΔORF2+peptide	OK	60.8698	45.9031	-0.407136	-0.467999	0.4176	0.902759	no
gene:SpnNT_02033	ycfH	Chromosome:2020244-2021578	110.58	ΔORF2	OK	67.9819	66.4584	-0.0326974	-0.0593635	0.91765	0.994748	no
gene:SpnNT_02033	ycfH	Chromosome:2020244-2021578	110.58	110.58+peptide	OK	67.9819	81.7021	0.265224	0.474813	0.40525	0.894933	no

gene:SpnNT_02033	ycfH	Chromosome:2020244-2021578	ΔORF2	110.58+peptide	OK	66.4584	81.7021	0.297922	0.534571	0.35165	0.854885	no
gene:SpnNT_02033	ycfH	Chromosome:2020244-2021578	110.58	ΔORF2+peptide	OK	67.9819	69.7164	0.0363478	0.0654371	0.90725	0.994748	no
gene:SpnNT_02033	ycfH	Chromosome:2020244-2021578	ΔORF2	ΔORF2+peptide	OK	66.4584	69.7164	0.0690451	0.12459	0.8247	0.994748	no
gene:SpnNT_02033	ycfH	Chromosome:2020244-2021578	110.58+peptide	ΔORF2+peptide	OK	81.7021	69.7164	-0.228877	-0.407313	0.47405	0.929818	no
gene:SpnNT_02034	NA	Chromosome:2021882-2022713	110.58	ΔORF2	OK	20.8269	16.5744	-0.329493	-0.581663	0.3049	0.811048	no
gene:SpnNT_02034	NA	Chromosome:2021882-2022713	110.58	110.58+peptide	OK	20.8269	28.5683	0.455966	0.827529	0.1423	0.576108	no
gene:SpnNT_02034	NA	Chromosome:2021882-2022713	ΔORF2	110.58+peptide	OK	16.5744	28.5683	0.785459	1.41304	0.01205	0.115248	no
gene:SpnNT_02034	NA	Chromosome:2021882-2022713	110.58	ΔORF2+peptide	OK	20.8269	23.1351	0.151637	0.27459	0.6257	0.980887	no
gene:SpnNT_02034	NA	Chromosome:2021882-2022713	ΔORF2	ΔORF2+peptide	OK	16.5744	23.1351	0.481131	0.863652	0.122	0.528994	no
gene:SpnNT_02034	NA	Chromosome:2021882-2022713	110.58+peptide	ΔORF2+peptide	OK	28.5683	23.1351	-0.304329	-0.562167	0.3205	0.826085	no
gene:SpnNT_02035	rpmH	Chromosome:2022996-2023131	110.58	ΔORF2	OK	1121.76	990.964	-0.178862	-0.291771	0.61145	0.979429	no
gene:SpnNT_02035	rpmH	Chromosome:2022996-2023131	110.58	110.58+peptide	OK	1121.76	1792.84	0.676484	1.04648	0.0647	0.368967	no
gene:SpnNT_02035	rpmH	Chromosome:2022996-2023131	ΔORF2	110.58+peptide	OK	990.964	1792.84	0.855346	1.33303	0.02335	0.187285	no
gene:SpnNT_02035	rpmH	Chromosome:2022996-2023131	110.58	ΔORF2+peptide	OK	1121.76	1767.02	0.655551	0.936024	0.0893	0.449387	no
gene:SpnNT_02035	rpmH	Chromosome:2022996-2023131	ΔORF2	ΔORF2+peptide	OK	990.964	1767.02	0.834413	1.19897	0.03695	0.257219	no
gene:SpnNT_02035	rpmH	Chromosome:2022996-2023131	110.58+peptide	ΔORF2+peptide	OK	1792.84	1767.02	-0.0209336	-0.0288521	0.95815	0.994855	no
gene:SpnNT_02036	alaA	Chromosome:2023275-2024490	110.58	ΔORF2	OK	190.611	189.391	-0.00926332	-0.0213983	0.9709	0.99536	no
gene:SpnNT_02036	alaA	Chromosome:2023275-2024490	110.58	110.58+peptide	OK	190.611	167.109	-0.189844	-0.437762	0.4415	0.917252	no
gene:SpnNT_02036	alaA	Chromosome:2023275-2024490	ΔORF2	110.58+peptide	OK	189.391	167.109	-0.180581	-0.416493	0.4647	0.926394	no
gene:SpnNT_02036	alaA	Chromosome:2023275-2024490	110.58	ΔORF2+peptide	OK	190.611	169.078	-0.172941	-0.398732	0.48195	0.935166	no
gene:SpnNT_02036	alaA	Chromosome:2023275-2024490	ΔORF2	ΔORF2+peptide	OK	189.391	169.078	-0.163678	-0.377457	0.50175	0.944784	no
gene:SpnNT_02036	alaA	Chromosome:2023275-2024490	110.58+peptide	ΔORF2+peptide	OK	167.109	169.078	0.0169027	0.0389102	0.94515	0.994855	no
gene:SpnNT_02037	NA	Chromosome:2024645-2024939	110.58	ΔORF2	OK	28.5358	29.7281	0.0590524	0.0844443	0.8849	0.994748	no
gene:SpnNT_02037	NA	Chromosome:2024645-2024939	110.58	110.58+peptide	OK	28.5358	34.3695	0.268353	0.383983	0.5071	0.946135	no
gene:SpnNT_02037	NA	Chromosome:2024645-2024939	ΔORF2	110.58+peptide	OK	29.7281	34.3695	0.209301	0.293277	0.61145	0.979429	no
gene:SpnNT_02037	NA	Chromosome:2024645-2024939	110.58	ΔORF2+peptide	OK	28.5358	23.6585	-0.270412	-0.373068	0.5153	0.951959	no
gene:SpnNT_02037	NA	Chromosome:2024645-2024939	ΔORF2	ΔORF2+peptide	OK	29.7281	23.6585	-0.329464	-0.445758	0.43325	0.913334	no
gene:SpnNT_02037	NA	Chromosome:2024645-2024939	110.58+peptide	ΔORF2+peptide	OK	34.3695	23.6585	-0.538765	-0.729347	0.18885	0.665974	no
gene:SpnNT_02038	NA	Chromosome:2025101-2025554	110.58	ΔORF2	OK	113.641	104.813	-0.116658	-0.231768	0.68335	0.98747	no
gene:SpnNT_02038	NA	Chromosome:2025101-2025554	110.58	110.58+peptide	OK	113.641	181.53	0.675724	1.37711	0.0178	0.152065	no
gene:SpnNT_02038	NA	Chromosome:2025101-2025554	ΔORF2	110.58+peptide	OK	104.813	181.53	0.792382	1.59893	0.00545	0.0642542	no
gene:SpnNT_02038	NA	Chromosome:2025101-2025554	110.58	ΔORF2+peptide	OK	113.641	151.408	0.413961	0.84058	0.1426	0.576108	no
gene:SpnNT_02038	NA	Chromosome:2025101-2025554	ΔORF2	ΔORF2+peptide	OK	104.813	151.408	0.530618	1.06691	0.0598	0.351074	no
gene:SpnNT_02038	NA	Chromosome:2025101-2025554	110.58+peptide	ΔORF2+peptide	OK	181.53	151.408	-0.261763	-0.540242	0.3441	0.848346	no
gene:SpnNT_02039	NA	Chromosome:2025588-2026977	110.58	ΔORF2	OK	148.049	159.183	0.104613	0.241246	0.66665	0.983929	no
gene:SpnNT_02039	NA	Chromosome:2025588-2026977	110.58	110.58+peptide	OK	148.049	96.1383	-0.622891	-1.43073	0.0121	0.11539	no
gene:SpnNT_02039	NA	Chromosome:2025588-2026977	ΔORF2	110.58+peptide	OK	159.183	96.1383	-0.727503	-1.66966	0.00295	0.0393393	yes
gene:SpnNT_02039	NA	Chromosome:2025588-2026977	110.58	ΔORF2+peptide	OK	148.049	102.432	-0.531405	-1.21511	0.03665	0.255537	no
gene:SpnNT_02039	NA	Chromosome:2025588-2026977	ΔORF2	ΔORF2+peptide	OK	159.183	102.432	-0.636018	-1.45314	0.01235	0.117432	no
gene:SpnNT_02039	NA	Chromosome:2025588-2026977	110.58+peptide	ΔORF2+peptide	OK	96.1383	102.432	0.0914851	0.208205	0.71325	0.991272	no
gene:SpnNT_02040	ansB	Chromosome:2027042-2028005	110.58	ΔORF2	OK	28.3755	27.9492	-0.0218371	-0.0423287	0.9406	0.994855	no
gene:SpnNT_02040	ansB	Chromosome:2027042-2028005	110.58	110.58+peptide	OK	28.3755	18.7624	-0.596801	-1.12314	0.04665	0.299189	no
gene:SpnNT_02040	ansB	Chromosome:2027042-2028005	ΔORF2	110.58+peptide	OK	27.9492	18.7624	-0.574964	-1.0799	0.0542	0.330796	no
gene:SpnNT_02040	ansB	Chromosome:2027042-2028005	110.58	ΔORF2+peptide	OK	28.3755	19.1288	-0.568904	-1.07194	0.06155	0.358005	no
gene:SpnNT_02040	ansB	Chromosome:2027042-2028005	ΔORF2	ΔORF2+peptide	OK	27.9492	19.1288	-0.547066	-1.02875	0.0711	0.391184	no

gene:SpnNT_02040	ansB	Chromosome:2027042-2028005	110.58+peptide	ΔORF2+peptide	OK	18.7624	19.1288	0.0278975	0.0510192	0.9298	0.99482	no
gene:SpnNT_02041	ccpA_2	Chromosome:2028345-2029356	110.58	ΔORF2	OK	343.194	340.45	-0.0115819	-0.0263481	0.96275	0.994855	no
gene:SpnNT_02041	ccpA_2	Chromosome:2028345-2029356	110.58	110.58+peptide	OK	343.194	626.226	0.867661	1.93744	0.00085	0.0148124	yes
gene:SpnNT_02041	ccpA_2	Chromosome:2028345-2029356	ΔORF2	110.58+peptide	OK	340.45	626.226	0.879243	1.98354	9.00E-04	0.0154579	yes
gene:SpnNT_02041	ccpA_2	Chromosome:2028345-2029356	110.58	ΔORF2+peptide	OK	343.194	620.192	0.853692	1.90716	0.00075	0.0133173	yes
gene:SpnNT_02041	ccpA_2	Chromosome:2028345-2029356	ΔORF2	ΔORF2+peptide	OK	340.45	620.192	0.865273	1.95297	9.00E-04	0.0154579	yes
gene:SpnNT_02041	ccpA_2	Chromosome:2028345-2029356	110.58+peptide	ΔORF2+peptide	OK	626.226	620.192	-0.0139693	-0.0309564	0.9553	0.994855	no
gene:SpnNT_02042	vraR	Chromosome:2029779-2030379	110.58	ΔORF2	OK	53.9183	49.3122	-0.12883	-0.245228	0.6668	0.983929	no
gene:SpnNT_02042	vraR	Chromosome:2029779-2030379	110.58	110.58+peptide	OK	53.9183	30.9734	-0.799748	-1.47779	0.00965	0.0978573	no
gene:SpnNT_02042	vraR	Chromosome:2029779-2030379	ΔORF2	110.58+peptide	OK	49.3122	30.9734	-0.670919	-1.22772	0.03475	0.246835	no
gene:SpnNT_02042	vraR	Chromosome:2029779-2030379	110.58	ΔORF2+peptide	OK	53.9183	28.8913	-0.900139	-1.65235	0.0047	0.0572109	no
gene:SpnNT_02042	vraR	Chromosome:2029779-2030379	ΔORF2	ΔORF2+peptide	OK	49.3122	28.8913	-0.771309	-1.40232	0.01645	0.144001	no
gene:SpnNT_02042	vraR	Chromosome:2029779-2030379	110.58+peptide	ΔORF2+peptide	OK	30.9734	28.8913	-0.100391	-0.17763	0.75325	0.994748	no
gene:SpnNT_02043	desK	Chromosome:2030379-2032214	110.58	ΔORF2	OK	36.9408	37.9422	0.0385867	0.065401	0.91065	0.994748	no
gene:SpnNT_02043	desK	Chromosome:2030379-2032214	110.58	110.58+peptide	OK	36.9408	21.3774	-0.789133	-1.31698	0.02455	0.194885	no
gene:SpnNT_02043	desK	Chromosome:2030379-2032214	ΔORF2	110.58+peptide	OK	37.9422	21.3774	-0.827719	-1.36639	0.02055	0.170994	no
gene:SpnNT_02043	desK	Chromosome:2030379-2032214	110.58	ΔORF2+peptide	OK	36.9408	20.7901	-0.82932	-1.36596	0.02	0.167053	no
gene:SpnNT_02043	desK	Chromosome:2030379-2032214	ΔORF2	ΔORF2+peptide	OK	37.9422	20.7901	-0.867907	-1.41439	0.01605	0.141158	no
gene:SpnNT_02043	desK	Chromosome:2030379-2032214	110.58+peptide	ΔORF2+peptide	OK	21.3774	20.7901	-0.0401875	-0.0645612	0.9123	0.994748	no
gene:SpnNT_02044	NA	Chromosome:2030379-2032214	110.58	ΔORF2	OK	35.2381	40.9199	0.215667	0.271936	0.6319	0.980887	no
gene:SpnNT_02044	NA	Chromosome:2030379-2032214	110.58	110.58+peptide	OK	35.2381	15.1974	-1.21331	-1.3458	0.02845	0.214799	no
gene:SpnNT_02044	NA	Chromosome:2030379-2032214	ΔORF2	110.58+peptide	OK	40.9199	15.1974	-1.42898	-1.60164	0.0109	0.106978	no
gene:SpnNT_02044	NA	Chromosome:2030379-2032214	110.58	ΔORF2+peptide	OK	35.2381	20.2343	-0.800336	-0.961587	0.0919	0.456439	no
gene:SpnNT_02044	NA	Chromosome:2030379-2032214	ΔORF2	ΔORF2+peptide	OK	40.9199	20.2343	-1.016	-1.23577	0.033	0.237796	no
gene:SpnNT_02044	NA	Chromosome:2030379-2032214	110.58+peptide	ΔORF2+peptide	OK	15.1974	20.2343	0.412974	0.445379	0.44415	0.917631	no
gene:SpnNT_02045	yxlF_4	Chromosome:2032215-2033469	110.58	ΔORF2	OK	7.89238	7.12879	-0.146804	-0.217268	0.709	0.990577	no
gene:SpnNT_02045	yxlF_4	Chromosome:2032215-2033469	110.58	110.58+peptide	OK	7.89238	6.78797	-0.217481	-0.329377	0.5655	0.968621	no
gene:SpnNT_02045	yxlF_4	Chromosome:2032215-2033469	ΔORF2	110.58+peptide	OK	7.12879	6.78797	-0.0706775	-0.104499	0.85515	0.994748	no
gene:SpnNT_02045	yxlF_4	Chromosome:2032215-2033469	110.58	ΔORF2+peptide	OK	7.89238	5.63103	-0.487061	-0.719915	0.21365	0.698761	no
gene:SpnNT_02045	yxlF_4	Chromosome:2032215-2033469	ΔORF2	ΔORF2+peptide	OK	7.12879	5.63103	-0.340257	-0.491529	0.4035	0.89449	no
gene:SpnNT_02045	yxlF_4	Chromosome:2032215-2033469	110.58+peptide	ΔORF2+peptide	OK	6.78797	5.63103	-0.26958	-0.39807	0.4928	0.941402	no
gene:SpnNT_02046	NA	Chromosome:2032215-2033469	110.58	ΔORF2	OK	9.25304	2.32068	-1.99538	-0.812588	0.5506	0.964281	no
gene:SpnNT_02046	NA	Chromosome:2032215-2033469	110.58	110.58+peptide	OK	9.25304	6.61227	-0.484782	-0.149042	0.8048	0.994748	no
gene:SpnNT_02046	NA	Chromosome:2032215-2033469	ΔORF2	110.58+peptide	OK	2.32068	6.61227	1.5106	0.512674	0.55005	0.964281	no
gene:SpnNT_02046	NA	Chromosome:2032215-2033469	110.58	ΔORF2+peptide	OK	9.25304	4.39785	-1.07313	-0.478539	0.6633	0.982966	no
gene:SpnNT_02046	NA	Chromosome:2032215-2033469	ΔORF2	ΔORF2+peptide	OK	2.32068	4.39785	0.922253	0.521208	0.72155	0.994748	no
gene:SpnNT_02046	NA	Chromosome:2032215-2033469	110.58+peptide	ΔORF2+peptide	OK	6.61227	4.39785	-0.588346	-0.212289	0.775	0.994748	no
gene:SpnNT_02047	NA	Chromosome:2032215-2033469	110.58	ΔORF2	OK	17.6513	33.1687	0.910044	0.458938	0.43265	0.913334	no
gene:SpnNT_02047	NA	Chromosome:2032215-2033469	110.58	110.58+peptide	OK	17.6513	6.48583	-1.44441	-0.795627	0.46525	0.926405	no
gene:SpnNT_02047	NA	Chromosome:2032215-2033469	ΔORF2	110.58+peptide	OK	33.1687	6.48583	-2.35446	-1.60144	0.2652	0.766341	no
gene:SpnNT_02047	NA	Chromosome:2032215-2033469	110.58	ΔORF2+peptide	OK	17.6513	1.14215	-3.94995	-1.9301	0.23215	0.724785	no
gene:SpnNT_02047	NA	Chromosome:2032215-2033469	ΔORF2	ΔORF2+peptide	OK	33.1687	1.14215	-4.85999	-2.78108	0.21375	0.698761	no
gene:SpnNT_02047	NA	Chromosome:2032215-2033469	110.58+peptide	ΔORF2+peptide	OK	6.48583	1.14215	-2.50554	-1.61141	0.47535	0.930746	no
gene:SpnNT_02048	SpnNT_02048	Chromosome:2034071-2034147	110.58	ΔORF2	NOTEST	166.61	0	#NAME?	0	1	1	no
gene:SpnNT_02048	SpnNT_02048	Chromosome:2034071-2034147	110.58	110.58+peptide	NOTEST	166.61	0	#NAME?	0	1	1	no

gene:SpnNT_02048	SpnNT_02048	Chromosome:2034071-2034147	ΔORF2	110.58+peptide	NOTEST	0	0	0	0	1	1	no
gene:SpnNT_02048	SpnNT_02048	Chromosome:2034071-2034147	110.58	ΔORF2+peptide	OK	166.61	891.535	2.41982	156.015	0.1658	0.621332	no
gene:SpnNT_02048	SpnNT_02048	Chromosome:2034071-2034147	ΔORF2	ΔORF2+peptide	OK	0	891.535	Inf	NA	0.02305	0.18636	no
gene:SpnNT_02048	SpnNT_02048	Chromosome:2034071-2034147	110.58+peptide	ΔORF2+peptide	OK	0	891.535	Inf	NA	0.02305	0.18636	no
gene:SpnNT_02049	SpnNT_02049	Chromosome:2034161-2034235	110.58	ΔORF2	OK	569.541	338.986	-0.748575	-46.5338	0.60315	0.976761	no
gene:SpnNT_02049	SpnNT_02049	Chromosome:2034161-2034235	110.58	110.58+peptide	OK	569.541	1280.98	1.16937	112.638	0.4134	0.899866	no
gene:SpnNT_02049	SpnNT_02049	Chromosome:2034161-2034235	ΔORF2	110.58+peptide	OK	338.986	1280.98	1.91795	126.107	0.2788	0.781158	no
gene:SpnNT_02049	SpnNT_02049	Chromosome:2034161-2034235	110.58	ΔORF2+peptide	OK	569.541	316.779	-0.846322	-76.4455	0.57125	0.968844	no
gene:SpnNT_02049	SpnNT_02049	Chromosome:2034161-2034235	ΔORF2	ΔORF2+peptide	OK	338.986	316.779	-0.0977472	-6.23091	0.81725	0.994748	no
gene:SpnNT_02049	SpnNT_02049	Chromosome:2034161-2034235	110.58+peptide	ΔORF2+peptide	OK	1280.98	316.779	-2.0157	-206.703	0.1758	0.63937	no
gene:SpnNT_02050	SpnNT_02050	Chromosome:2034244-2034330	110.58	ΔORF2	OK	3011.46	2367.34	-0.347195	-0.485112	0.57405	0.969538	no
gene:SpnNT_02050	SpnNT_02050	Chromosome:2034244-2034330	110.58	110.58+peptide	OK	3011.46	7944.69	1.39953	1.50785	0.0098	0.0986919	no
gene:SpnNT_02050	SpnNT_02050	Chromosome:2034244-2034330	ΔORF2	110.58+peptide	OK	2367.34	7944.69	1.74672	2.95476	0.00835	0.0885762	no
gene:SpnNT_02050	SpnNT_02050	Chromosome:2034244-2034330	110.58	ΔORF2+peptide	OK	3011.46	7707.87	1.35587	1.43958	0.01295	0.120861	no
gene:SpnNT_02050	SpnNT_02050	Chromosome:2034244-2034330	ΔORF2	ΔORF2+peptide	OK	2367.34	7707.87	1.70306	2.78088	0.0096	0.0977279	no
gene:SpnNT_02050	SpnNT_02050	Chromosome:2034244-2034330	110.58+peptide	ΔORF2+peptide	OK	7944.69	7707.87	-0.0436579	-0.0512976	0.9303	0.994855	no
gene:SpnNT_02051	SpnNT_02051	Chromosome:2034337-2034409	110.58	ΔORF2	NOTEST	0	0	0	0	1	1	no
gene:SpnNT_02051	SpnNT_02051	Chromosome:2034337-2034409	110.58	110.58+peptide	OK	0	601.635	Inf	NA	0.11495	0.514628	no
gene:SpnNT_02051	SpnNT_02051	Chromosome:2034337-2034409	ΔORF2	110.58+peptide	OK	0	601.635	Inf	NA	0.11495	0.514628	no
gene:SpnNT_02051	SpnNT_02051	Chromosome:2034337-2034409	110.58	ΔORF2+peptide	OK	0	487.103	Inf	NA	0.0582	0.3437	no
gene:SpnNT_02051	SpnNT_02051	Chromosome:2034337-2034409	ΔORF2	ΔORF2+peptide	OK	0	487.103	Inf	NA	0.0582	0.3437	no
gene:SpnNT_02051	SpnNT_02051	Chromosome:2034337-2034409	110.58+peptide	ΔORF2+peptide	OK	601.635	487.103	-0.304661	-32.3577	0.78305	0.994748	no
gene:SpnNT_02052	SpnNT_02052	Chromosome:2034431-2034504	110.58	ΔORF2	OK	2299.83	2036.24	-0.175621	-0.154346	0.8585	0.994748	no
gene:SpnNT_02052	SpnNT_02052	Chromosome:2034431-2034504	110.58	110.58+peptide	OK	2299.83	4861.14	1.07977	0.770402	0.17405	0.634412	no
gene:SpnNT_02052	SpnNT_02052	Chromosome:2034431-2034504	ΔORF2	110.58+peptide	OK	2036.24	4861.14	1.25539	1.534	0.15785	0.606532	no
gene:SpnNT_02052	SpnNT_02052	Chromosome:2034431-2034504	110.58	ΔORF2+peptide	OK	2299.83	2650.95	0.204981	0.180149	0.821	0.994748	no
gene:SpnNT_02052	SpnNT_02052	Chromosome:2034431-2034504	ΔORF2	ΔORF2+peptide	OK	2036.24	2650.95	0.380601	70.4538	0.69135	0.98828	no
gene:SpnNT_02052	SpnNT_02052	Chromosome:2034431-2034504	110.58+peptide	ΔORF2+peptide	OK	4861.14	2650.95	-0.874788	-1.06894	0.27385	0.775958	no
gene:SpnNT_02053	SpnNT_02053	Chromosome:2034521-2034605	110.58	ΔORF2	OK	3904	5089.88	0.382679	0.416726	0.46275	0.9258	no
gene:SpnNT_02053	SpnNT_02053	Chromosome:2034521-2034605	110.58	110.58+peptide	OK	3904	11667.9	1.57952	1.85765	0.0024	0.0337543	yes
gene:SpnNT_02053	SpnNT_02053	Chromosome:2034521-2034605	ΔORF2	110.58+peptide	OK	5089.88	11667.9	1.19684	1.40855	0.01605	0.141158	no
gene:SpnNT_02053	SpnNT_02053	Chromosome:2034521-2034605	110.58	ΔORF2+peptide	OK	3904	14437.8	1.88682	2.45931	0.00025	0.00542231	yes
gene:SpnNT_02053	SpnNT_02053	Chromosome:2034521-2034605	ΔORF2	ΔORF2+peptide	OK	5089.88	14437.8	1.50414	1.96215	0.0036	0.0465411	yes
gene:SpnNT_02053	SpnNT_02053	Chromosome:2034521-2034605	110.58+peptide	ΔORF2+peptide	OK	11667.9	14437.8	0.3073	0.449514	0.4706	0.928415	no
gene:SpnNT_02054	SpnNT_02054	Chromosome:2034609-2034682	110.58	ΔORF2	OK	858.712	1269.04	0.563487	73.574	0.6366	0.980887	no
gene:SpnNT_02054	SpnNT_02054	Chromosome:2034609-2034682	110.58	110.58+peptide	OK	858.712	3637.05	2.08252	1.90392	0.2488	0.745378	no
gene:SpnNT_02054	SpnNT_02054	Chromosome:2034609-2034682	ΔORF2	110.58+peptide	OK	1269.04	3637.05	1.51904	1.38876	0.17815	0.644701	no
gene:SpnNT_02054	SpnNT_02054	Chromosome:2034609-2034682	110.58	ΔORF2+peptide	OK	858.712	2408.9	1.48813	210.319	0.3308	0.836657	no
gene:SpnNT_02054	SpnNT_02054	Chromosome:2034609-2034682	ΔORF2	ΔORF2+peptide	OK	1269.04	2408.9	0.924643	134.179	0.4389	0.916056	no
gene:SpnNT_02054	SpnNT_02054	Chromosome:2034609-2034682	110.58+peptide	ΔORF2+peptide	OK	3637.05	2408.9	-0.594393	-0.543419	0.4736	0.929491	no
gene:SpnNT_02055	SpnNT_02055	Chromosome:2034706-2034779	110.58	ΔORF2	OK	3404.6	4667.82	0.455262	0.381134	0.53705	0.959321	no
gene:SpnNT_02055	SpnNT_02055	Chromosome:2034706-2034779	110.58	110.58+peptide	OK	3404.6	6507.26	0.934564	0.805602	0.1871	0.661936	no
gene:SpnNT_02055	SpnNT_02055	Chromosome:2034706-2034779	ΔORF2	110.58+peptide	OK	4667.82	6507.26	0.479302	0.421074	0.4686	0.927504	no
gene:SpnNT_02055	SpnNT_02055	Chromosome:2034706-2034779	110.58	ΔORF2+peptide	OK	3404.6	2302.61	-0.564216	-0.656567	0.52395	0.954832	no
gene:SpnNT_02055	SpnNT_02055	Chromosome:2034706-2034779	ΔORF2	ΔORF2+peptide	OK	4667.82	2302.61	-1.01948	-1.22876	0.2069	0.692058	no

gene:SpnNT_02055	SpnNT_02055	Chromosome:2034706-2034779	110.58+peptide	ΔORF2+peptide	OK	6507.26	2302.61	-1.49878	-1.92318	0.0816	0.426936	no
gene:SpnNT_02056	SpnNT_02056	Chromosome:2034781-2034854	110.58	ΔORF2	OK	431.625	1986.87	2.20265	1.72615	0.2622	0.762711	no
gene:SpnNT_02056	SpnNT_02056	Chromosome:2034781-2034854	110.58	110.58+peptide	OK	431.625	5757.93	3.7377	4.24376	0.2243	0.715065	no
gene:SpnNT_02056	SpnNT_02056	Chromosome:2034781-2034854	ΔORF2	110.58+peptide	OK	1986.87	5757.93	1.53505	0.990055	0.0914	0.454989	no
gene:SpnNT_02056	SpnNT_02056	Chromosome:2034781-2034854	110.58	ΔORF2+peptide	OK	431.625	4044.29	3.22804	528.641	0.2261	0.717505	no
gene:SpnNT_02056	SpnNT_02056	Chromosome:2034781-2034854	ΔORF2	ΔORF2+peptide	OK	1986.87	4044.29	1.02539	0.803572	0.2529	0.751101	no
gene:SpnNT_02056	SpnNT_02056	Chromosome:2034781-2034854	110.58+peptide	ΔORF2+peptide	OK	5757.93	4044.29	-0.509664	-0.578678	0.4914	0.940662	no
gene:SpnNT_02057	SpnNT_02057	Chromosome:2034859-2034971	110.58	ΔORF2	HIDATA	5564230	0	0	0	1	1	no
gene:SpnNT_02057	SpnNT_02057	Chromosome:2034859-2034971	110.58	110.58+peptide	OK	5564230	6758500	0.28052	0.438779	0.43685	0.914104	no
gene:SpnNT_02057	SpnNT_02057	Chromosome:2034859-2034971	ΔORF2	110.58+peptide	HIDATA	0	6758500	0	0	1	1	no
gene:SpnNT_02057	SpnNT_02057	Chromosome:2034859-2034971	110.58	ΔORF2+peptide	OK	5564230	7851340	0.496757	0.818185	0.16605	0.621332	no
gene:SpnNT_02057	SpnNT_02057	Chromosome:2034859-2034971	ΔORF2	ΔORF2+peptide	HIDATA	0	7851340	0	0	1	1	no
gene:SpnNT_02057	SpnNT_02057	Chromosome:2034859-2034971	110.58+peptide	ΔORF2+peptide	OK	6758500	7851340	0.216237	0.362723	0.5467	0.962122	no
gene:SpnNT_02058	SpnNT_02058	Chromosome:2035052-2037951	110.58	ΔORF2	HIDATA	0	0	0	0	1	1	no
gene:SpnNT_02058	SpnNT_02058	Chromosome:2035052-2037951	110.58	110.58+peptide	HIDATA	0	0	0	0	1	1	no
gene:SpnNT_02058	SpnNT_02058	Chromosome:2035052-2037951	ΔORF2	110.58+peptide	HIDATA	0	0	0	0	1	1	no
gene:SpnNT_02058	SpnNT_02058	Chromosome:2035052-2037951	110.58	ΔORF2+peptide	HIDATA	0	0	0	0	1	1	no
gene:SpnNT_02058	SpnNT_02058	Chromosome:2035052-2037951	ΔORF2	ΔORF2+peptide	HIDATA	0	0	0	0	1	1	no
gene:SpnNT_02058	SpnNT_02058	Chromosome:2035052-2037951	110.58+peptide	ΔORF2+peptide	HIDATA	0	0	0	0	1	1	no
gene:SpnNT_02059	SpnNT_02059	Chromosome:2038075-2038150	110.58	ΔORF2	OK	1104.16	1468.36	0.411262	0.278976	0.74745	0.994748	no
gene:SpnNT_02059	SpnNT_02059	Chromosome:2038075-2038150	110.58	110.58+peptide	OK	1104.16	2465.07	1.15869	1.18201	0.2707	0.772705	no
gene:SpnNT_02059	SpnNT_02059	Chromosome:2038075-2038150	ΔORF2	110.58+peptide	OK	1468.36	2465.07	0.747424	0.422193	0.41255	0.899511	no
gene:SpnNT_02059	SpnNT_02059	Chromosome:2038075-2038150	110.58	ΔORF2+peptide	OK	1104.16	2451.42	1.15067	148.604	0.2794	0.782225	no
gene:SpnNT_02059	SpnNT_02059	Chromosome:2038075-2038150	ΔORF2	ΔORF2+peptide	OK	1468.36	2451.42	0.73941	0.501572	0.4272	0.908839	no
gene:SpnNT_02059	SpnNT_02059	Chromosome:2038075-2038150	110.58+peptide	ΔORF2+peptide	OK	2465.07	2451.42	-0.00801385	-0.00817519	0.92195	0.994748	no
gene:SpnNT_02060	SpnNT_02060	Chromosome:2038204-2039741	110.58	ΔORF2	HIDATA	0	0	0	0	1	1	no
gene:SpnNT_02060	SpnNT_02060	Chromosome:2038204-2039741	110.58	110.58+peptide	HIDATA	0	0	0	0	1	1	no
gene:SpnNT_02060	SpnNT_02060	Chromosome:2038204-2039741	ΔORF2	110.58+peptide	HIDATA	0	0	0	0	1	1	no
gene:SpnNT_02060	SpnNT_02060	Chromosome:2038204-2039741	110.58	ΔORF2+peptide	HIDATA	0	0	0	0	1	1	no
gene:SpnNT_02060	SpnNT_02060	Chromosome:2038204-2039741	ΔORF2	ΔORF2+peptide	HIDATA	0	0	0	0	1	1	no
gene:SpnNT_02060	SpnNT_02060	Chromosome:2038204-2039741	110.58+peptide	ΔORF2+peptide	HIDATA	0	0	0	0	1	1	no
gene:SpnNT_02061	SpnNT_02061	Chromosome:2039997-2040069	110.58	ΔORF2	OK	660.442	616.806	-0.0986155	-11.9186	0.63045	0.980887	no
gene:SpnNT_02061	SpnNT_02061	Chromosome:2039997-2040069	110.58	110.58+peptide	OK	660.442	1236.28	0.904501	98.9781	0.4987	0.944017	no
gene:SpnNT_02061	SpnNT_02061	Chromosome:2039997-2040069	ΔORF2	110.58+peptide	OK	616.806	1236.28	1.00312	148.465	0.46595	0.926452	no
gene:SpnNT_02061	SpnNT_02061	Chromosome:2039997-2040069	110.58	ΔORF2+peptide	OK	660.442	0	#NAME?	NA	0.11515	0.515092	no
gene:SpnNT_02061	SpnNT_02061	Chromosome:2039997-2040069	ΔORF2	ΔORF2+peptide	OK	616.806	0	#NAME?	NA	0.1201	0.524705	no
gene:SpnNT_02061	SpnNT_02061	Chromosome:2039997-2040069	110.58+peptide	ΔORF2+peptide	OK	1236.28	0	#NAME?	NA	0.00965	0.0978573	no
gene:SpnNT_02062	NA	Chromosome:2040162-2040642	110.58	ΔORF2	OK	10.2345	12.4124	0.278342	0.397501	0.49975	0.944017	no
gene:SpnNT_02062	NA	Chromosome:2040162-2040642	110.58	110.58+peptide	OK	10.2345	7.3642	-0.474844	-0.654544	0.2502	0.747694	no
gene:SpnNT_02062	NA	Chromosome:2040162-2040642	ΔORF2	110.58+peptide	OK	12.4124	7.3642	-0.753186	-1.02761	0.0746	0.403172	no
gene:SpnNT_02062	NA	Chromosome:2040162-2040642	110.58	ΔORF2+peptide	OK	10.2345	11.8625	0.212959	0.307917	0.58965	0.974359	no
gene:SpnNT_02062	NA	Chromosome:2040162-2040642	ΔORF2	ΔORF2+peptide	OK	12.4124	11.8625	-0.0653832	-0.0934755	0.8671	0.994748	no
gene:SpnNT_02062	NA	Chromosome:2040162-2040642	110.58+peptide	ΔORF2+peptide	OK	7.3642	11.8625	0.687803	0.949057	0.0957	0.468573	no
gene:SpnNT_02063	nusG	Chromosome:2040763-2041300	110.58	ΔORF2	OK	783.132	946.213	0.272908	0.586584	0.30795	0.813881	no
gene:SpnNT_02063	nusG	Chromosome:2040763-2041300	110.58	110.58+peptide	OK	783.132	1273.15	0.701077	1.5414	0.0075	0.0820125	no

gene:SpnNT_02063	nusG	Chromosome:2040763-2041300	ΔORF2	110.58+peptide	OK	946.213	1273.15	0.428168	0.899406	0.1174	0.519044	no
gene:SpnNT_02063	nusG	Chromosome:2040763-2041300	110.58	ΔORF2+peptide	OK	783.132	1243.11	0.666631	1.48373	0.00915	0.0945404	no
gene:SpnNT_02063	nusG	Chromosome:2040763-2041300	ΔORF2	ΔORF2+peptide	OK	946.213	1243.11	0.393722	0.836335	0.1417	0.575128	no
gene:SpnNT_02063	nusG	Chromosome:2040763-2041300	110.58+peptide	ΔORF2+peptide	OK	1273.15	1243.11	-0.0344461	-0.0748055	0.8941	0.994748	no
gene:SpnNT_02064	NA	Chromosome:2041354-2041531	110.58	ΔORF2	OK	78.3578	67.2621	-0.220283	-0.269016	0.6323	0.980887	no
gene:SpnNT_02064	NA	Chromosome:2041354-2041531	110.58	110.58+peptide	OK	78.3578	105.873	0.43419	0.568482	0.32405	0.829371	no
gene:SpnNT_02064	NA	Chromosome:2041354-2041531	ΔORF2	110.58+peptide	OK	67.2621	105.873	0.654473	0.809626	0.14985	0.591417	no
gene:SpnNT_02064	NA	Chromosome:2041354-2041531	110.58	ΔORF2+peptide	OK	78.3578	111.314	0.50648	0.660583	0.2571	0.756914	no
gene:SpnNT_02064	NA	Chromosome:2041354-2041531	ΔORF2	ΔORF2+peptide	OK	67.2621	111.314	0.726763	0.895969	0.1157	0.516049	no
gene:SpnNT_02064	NA	Chromosome:2041354-2041531	110.58+peptide	ΔORF2+peptide	OK	105.873	111.314	0.0722899	0.0956835	0.86485	0.994748	no
gene:SpnNT_02065	pbpF_1	Chromosome:2041745-2043941	110.58	ΔORF2	OK	128.529	123.069	-0.0626254	-0.14289	0.80375	0.994748	no
gene:SpnNT_02065	pbpF_1	Chromosome:2041745-2043941	110.58	110.58+peptide	OK	128.529	107.251	-0.2611	-0.592412	0.30365	0.809137	no
gene:SpnNT_02065	pbpF_1	Chromosome:2041745-2043941	ΔORF2	110.58+peptide	OK	123.069	107.251	-0.198475	-0.454651	0.42405	0.907286	no
gene:SpnNT_02065	pbpF_1	Chromosome:2041745-2043941	110.58	ΔORF2+peptide	OK	128.529	104.413	-0.299789	-0.684613	0.23375	0.72774	no
gene:SpnNT_02065	pbpF_1	Chromosome:2041745-2043941	ΔORF2	ΔORF2+peptide	OK	123.069	104.413	-0.237164	-0.546875	0.33565	0.840108	no
gene:SpnNT_02065	pbpF_1	Chromosome:2041745-2043941	110.58+peptide	ΔORF2+peptide	OK	107.251	104.413	-0.0386887	-0.0887028	0.8796	0.994748	no
gene:SpnNT_02066	truC	Chromosome:2044027-2044903	110.58	ΔORF2	OK	99.0939	95.7637	-0.0493183	-0.107222	0.8523	0.994748	no
gene:SpnNT_02066	truC	Chromosome:2044027-2044903	110.58	110.58+peptide	OK	99.0939	79.5325	-0.317252	-0.680267	0.2483	0.744389	no
gene:SpnNT_02066	truC	Chromosome:2044027-2044903	ΔORF2	110.58+peptide	OK	95.7637	79.5325	-0.267934	-0.581944	0.3137	0.819635	no
gene:SpnNT_02066	truC	Chromosome:2044027-2044903	110.58	ΔORF2+peptide	OK	99.0939	75.0603	-0.400746	-0.859519	0.14495	0.580489	no
gene:SpnNT_02066	truC	Chromosome:2044027-2044903	ΔORF2	ΔORF2+peptide	OK	95.7637	75.0603	-0.351428	-0.763492	0.18365	0.654852	no
gene:SpnNT_02066	truC	Chromosome:2044027-2044903	110.58+peptide	ΔORF2+peptide	OK	79.5325	75.0603	-0.0834942	-0.178909	0.7555	0.994748	no
gene:SpnNT_02067	gap	Chromosome:2044970-2045978	110.58	ΔORF2	OK	4314.76	3839.25	-0.168456	-0.334337	0.55135	0.964385	no
gene:SpnNT_02067	gap	Chromosome:2044970-2045978	110.58	110.58+peptide	OK	4314.76	5081.88	0.236083	0.465523	0.411	0.898655	no
gene:SpnNT_02067	gap	Chromosome:2044970-2045978	ΔORF2	110.58+peptide	OK	3839.25	5081.88	0.404539	0.80398	0.1638	0.61835	no
gene:SpnNT_02067	gap	Chromosome:2044970-2045978	110.58	ΔORF2+peptide	OK	4314.76	4384.3	0.0230661	0.0458379	0.9342	0.994855	no
gene:SpnNT_02067	gap	Chromosome:2044970-2045978	ΔORF2	ΔORF2+peptide	OK	3839.25	4384.3	0.191523	0.383647	0.5058	0.945292	no
gene:SpnNT_02067	gap	Chromosome:2044970-2045978	110.58+peptide	ΔORF2+peptide	OK	5081.88	4384.3	-0.213016	-0.42389	0.4551	0.921244	no
gene:SpnNT_02068	macB_2	Chromosome:2046154-2047120	110.58	ΔORF2	OK	18.1401	16.037	-0.177776	-0.318555	0.5812	0.969923	no
gene:SpnNT_02068	macB_2	Chromosome:2046154-2047120	110.58	110.58+peptide	OK	18.1401	19.1601	0.0789251	0.143259	0.8022	0.994748	no
gene:SpnNT_02068	macB_2	Chromosome:2046154-2047120	ΔORF2	110.58+peptide	OK	16.037	19.1601	0.256701	0.464004	0.41485	0.900591	no
gene:SpnNT_02068	macB_2	Chromosome:2046154-2047120	110.58	ΔORF2+peptide	OK	18.1401	18.5595	0.032975	0.0598469	0.91905	0.994748	no
gene:SpnNT_02068	macB_2	Chromosome:2046154-2047120	ΔORF2	ΔORF2+peptide	OK	16.037	18.5595	0.210751	0.380903	0.5021	0.944784	no
gene:SpnNT_02068	macB_2	Chromosome:2046154-2047120	110.58+peptide	ΔORF2+peptide	OK	19.1601	18.5595	-0.0459501	-0.0841446	0.88275	0.994748	no
gene:SpnNT_02069	NA	Chromosome:2047180-2047519	110.58	ΔORF2	OK	189.774	181.636	-0.0632367	-0.125231	0.8309	0.994748	no
gene:SpnNT_02069	NA	Chromosome:2047180-2047519	110.58	110.58+peptide	OK	189.774	185.376	-0.033831	-0.0675326	0.905	0.994748	no
gene:SpnNT_02069	NA	Chromosome:2047180-2047519	ΔORF2	110.58+peptide	OK	181.636	185.376	0.0294058	0.058555	0.91815	0.994748	no
gene:SpnNT_02069	NA	Chromosome:2047180-2047519	110.58	ΔORF2+peptide	OK	189.774	170.079	-0.158077	-0.30414	0.5934	0.975539	no
gene:SpnNT_02069	NA	Chromosome:2047180-2047519	ΔORF2	ΔORF2+peptide	OK	181.636	170.079	-0.0948399	-0.182056	0.7512	0.994748	no
gene:SpnNT_02069	NA	Chromosome:2047180-2047519	110.58+peptide	ΔORF2+peptide	OK	185.376	170.079	-0.124246	-0.240293	0.67355	0.984881	no
gene:SpnNT_02070	NA	Chromosome:2047677-2048028	110.58	ΔORF2	OK	290.598	322.776	0.151507	0.318443	0.5779	0.969538	no
gene:SpnNT_02070	NA	Chromosome:2047677-2048028	110.58	110.58+peptide	OK	290.598	233.45	-0.315909	-0.650044	0.2569	0.756517	no
gene:SpnNT_02070	NA	Chromosome:2047677-2048028	ΔORF2	110.58+peptide	OK	322.776	233.45	-0.467416	-0.959998	0.10035	0.480231	no
gene:SpnNT_02070	NA	Chromosome:2047677-2048028	110.58	ΔORF2+peptide	OK	290.598	259.552	-0.163002	-0.334436	0.55775	0.968033	no
gene:SpnNT_02070	NA	Chromosome:2047677-2048028	ΔORF2	ΔORF2+peptide	OK	322.776	259.552	-0.314509	-0.644086	0.27065	0.772705	no

gene:SpnNT_02070	NA	Chromosome:2047677-2048028	110.58+peptide	ΔORF2+peptide	OK	233.45	259.552	0.152907	0.306888	0.59825	0.97629	no
gene:SpnNT_02071	nadC	Chromosome:2048158-2049031	110.58	ΔORF2	OK	35.1737	27.0687	-0.377875	-0.721431	0.19645	0.677648	no
gene:SpnNT_02071	nadC	Chromosome:2048158-2049031	110.58	110.58+peptide	OK	35.1737	35.7934	0.0251944	0.0487697	0.93	0.99482	no
gene:SpnNT_02071	nadC	Chromosome:2048158-2049031	ΔORF2	110.58+peptide	OK	27.0687	35.7934	0.403069	0.773411	0.16935	0.627212	no
gene:SpnNT_02071	nadC	Chromosome:2048158-2049031	110.58	ΔORF2+peptide	OK	35.1737	30.0256	-0.228305	-0.438994	0.43505	0.913689	no
gene:SpnNT_02071	nadC	Chromosome:2048158-2049031	ΔORF2	ΔORF2+peptide	OK	27.0687	30.0256	0.14957	0.285117	0.609	0.978849	no
gene:SpnNT_02071	nadC	Chromosome:2048158-2049031	110.58+peptide	ΔORF2+peptide	OK	35.7934	30.0256	-0.253499	-0.489932	0.3903	0.884392	no
gene:SpnNT_02072	NA	Chromosome:2049266-2050577	110.58	ΔORF2	OK	6.77849	5.39073	-0.330482	-0.543452	0.3416	0.84575	no
gene:SpnNT_02072	NA	Chromosome:2049266-2050577	110.58	110.58+peptide	OK	6.77849	5.90619	-0.198735	-0.327701	0.56135	0.968621	no
gene:SpnNT_02072	NA	Chromosome:2049266-2050577	ΔORF2	110.58+peptide	OK	5.39073	5.90619	0.131747	0.211853	0.714	0.991753	no
gene:SpnNT_02072	NA	Chromosome:2049266-2050577	110.58	ΔORF2+peptide	OK	6.77849	5.39335	-0.329782	-0.547401	0.33555	0.840108	no
gene:SpnNT_02072	NA	Chromosome:2049266-2050577	ΔORF2	ΔORF2+peptide	OK	5.39073	5.39335	0.000699484	0.0011319	0.99225	0.99849	no
gene:SpnNT_02072	NA	Chromosome:2049266-2050577	110.58+peptide	ΔORF2+peptide	OK	5.90619	5.39335	-0.131047	-0.212622	0.7114	0.991007	no
gene:SpnNT_02073	NA	Chromosome:2050942-2051137	110.58	ΔORF2	OK	106.392	156.497	0.556738	0.807492	0.16535	0.620808	no
gene:SpnNT_02073	NA	Chromosome:2050942-2051137	110.58	110.58+peptide	OK	106.392	188.143	0.822432	1.16464	0.0425	0.282086	no
gene:SpnNT_02073	NA	Chromosome:2050942-2051137	ΔORF2	110.58+peptide	OK	156.497	188.143	0.265694	0.368534	0.5123	0.950409	no
gene:SpnNT_02073	NA	Chromosome:2050942-2051137	110.58	ΔORF2+peptide	OK	106.392	167.941	0.658562	0.991896	0.0967	0.470343	no
gene:SpnNT_02073	NA	Chromosome:2050942-2051137	ΔORF2	ΔORF2+peptide	OK	156.497	167.941	0.101824	0.14982	0.79705	0.994748	no
gene:SpnNT_02073	NA	Chromosome:2050942-2051137	110.58+peptide	ΔORF2+peptide	OK	188.143	167.941	-0.16387	-0.235249	0.6763	0.98524	no
gene:SpnNT_02074	gmuR	Chromosome:2051596-2052325	110.58	ΔORF2	OK	33.222	42.9031	0.368943	0.702968	0.2196	0.707834	no
gene:SpnNT_02074	gmuR	Chromosome:2051596-2052325	110.58	110.58+peptide	OK	33.222	31.7154	-0.0669557	-0.125576	0.8209	0.994748	no
gene:SpnNT_02074	gmuR	Chromosome:2051596-2052325	ΔORF2	110.58+peptide	OK	42.9031	31.7154	-0.435899	-0.825526	0.1451	0.580489	no
gene:SpnNT_02074	gmuR	Chromosome:2051596-2052325	110.58	ΔORF2+peptide	OK	33.222	45.9363	0.467497	0.898208	0.12065	0.526145	no
gene:SpnNT_02074	gmuR	Chromosome:2051596-2052325	ΔORF2	ΔORF2+peptide	OK	42.9031	45.9363	0.0985537	0.191296	0.7328	0.994748	no
gene:SpnNT_02074	gmuR	Chromosome:2051596-2052325	110.58+peptide	ΔORF2+peptide	OK	31.7154	45.9363	0.534453	1.02054	0.0776	0.413426	no
gene:SpnNT_02075	gmuD_2	Chromosome:2052482-2053893	110.58	ΔORF2	OK	103.224	99.6993	-0.0501162	-0.0891924	0.88	0.994748	no
gene:SpnNT_02075	gmuD_2	Chromosome:2052482-2053893	110.58	110.58+peptide	OK	103.224	85.613	-0.269871	-0.467354	0.42835	0.909958	no
gene:SpnNT_02075	gmuD_2	Chromosome:2052482-2053893	ΔORF2	110.58+peptide	OK	99.6993	85.613	-0.219754	-0.387409	0.5095	0.948051	no
gene:SpnNT_02075	gmuD_2	Chromosome:2052482-2053893	110.58	ΔORF2+peptide	OK	103.224	94.5344	-0.126861	-0.227474	0.702	0.990367	no
gene:SpnNT_02075	gmuD_2	Chromosome:2052482-2053893	ΔORF2	ΔORF2+peptide	OK	99.6993	94.5344	-0.0767446	-0.140269	0.81405	0.994748	no
gene:SpnNT_02075	gmuD_2	Chromosome:2052482-2053893	110.58+peptide	ΔORF2+peptide	OK	85.613	94.5344	0.14301	0.253975	0.6657	0.98367	no
gene:SpnNT_02076	gmuD_3	Chromosome:2052482-2053893	110.58	ΔORF2	OK	110.715	99.6069	-0.152539	-0.190271	0.74055	0.994748	no
gene:SpnNT_02076	gmuD_3	Chromosome:2052482-2053893	110.58	110.58+peptide	OK	110.715	89.9907	-0.29901	-0.384168	0.5022	0.944784	no
gene:SpnNT_02076	gmuD_3	Chromosome:2052482-2053893	ΔORF2	110.58+peptide	OK	99.6069	89.9907	-0.14647	-0.185332	0.7473	0.994748	no
gene:SpnNT_02076	gmuD_3	Chromosome:2052482-2053893	110.58	ΔORF2+peptide	OK	110.715	79.1823	-0.483607	-0.583801	0.3052	0.811188	no
gene:SpnNT_02076	gmuD_3	Chromosome:2052482-2053893	ΔORF2	ΔORF2+peptide	OK	99.6069	79.1823	-0.331068	-0.394295	0.49745	0.944017	no
gene:SpnNT_02076	gmuD_3	Chromosome:2052482-2053893	110.58+peptide	ΔORF2+peptide	OK	89.9907	79.1823	-0.184598	-0.225844	0.69535	0.98895	no
gene:SpnNT_02077	licC_2	Chromosome:2053910-2055206	110.58	ΔORF2	OK	99.1275	94.4603	-0.0695762	-0.152695	0.7881	0.994748	no
gene:SpnNT_02077	licC_2	Chromosome:2053910-2055206	110.58	110.58+peptide	OK	99.1275	80.6518	-0.297578	-0.646671	0.25915	0.759841	no
gene:SpnNT_02077	licC_2	Chromosome:2053910-2055206	ΔORF2	110.58+peptide	OK	94.4603	80.6518	-0.228002	-0.507605	0.37525	0.872746	no
gene:SpnNT_02077	licC_2	Chromosome:2053910-2055206	110.58	ΔORF2+peptide	OK	99.1275	76.1492	-0.380456	-0.818729	0.1542	0.599175	no
gene:SpnNT_02077	licC_2	Chromosome:2053910-2055206	ΔORF2	ΔORF2+peptide	OK	94.4603	76.1492	-0.31088	-0.685054	0.2391	0.734427	no
gene:SpnNT_02077	licC_2	Chromosome:2053910-2055206	110.58+peptide	ΔORF2+peptide	OK	80.6518	76.1492	-0.0828785	-0.180825	0.7575	0.994748	no
gene:SpnNT_02078	licB	Chromosome:2055210-2055824	110.58	ΔORF2	OK	204.961	174.623	-0.231108	-0.359865	0.5283	0.956161	no
gene:SpnNT_02078	licB	Chromosome:2055210-2055824	110.58	110.58+peptide	OK	204.961	157.816	-0.377106	-0.581427	0.3107	0.816676	no

gene:SpnNT_02078	licB	Chromosome:2055210-2055824	ΔORF2	110.58+peptide	OK	174.623	157.816	-0.145998	-0.227405	0.6926	0.98828	no
gene:SpnNT_02078	licB	Chromosome:2055210-2055824	110.58	ΔORF2+peptide	OK	204.961	124.783	-0.715929	-1.07678	0.06235	0.360898	no
gene:SpnNT_02078	licB	Chromosome:2055210-2055824	ΔORF2	ΔORF2+peptide	OK	174.623	124.783	-0.484821	-0.736286	0.2048	0.689595	no
gene:SpnNT_02078	licB	Chromosome:2055210-2055824	110.58+peptide	ΔORF2+peptide	OK	157.816	124.783	-0.338823	-0.509746	0.37685	0.875032	no
gene:SpnNT_02079	gmuA	Chromosome:2055210-2055824	110.58	ΔORF2	OK	119.988	103.691	-0.210605	-0.251489	0.6726	0.984845	no
gene:SpnNT_02079	gmuA	Chromosome:2055210-2055824	110.58	110.58+peptide	OK	119.988	88.3766	-0.441153	-0.506099	0.402	0.894197	no
gene:SpnNT_02079	gmuA	Chromosome:2055210-2055824	ΔORF2	110.58+peptide	OK	103.691	88.3766	-0.230549	-0.271298	0.6383	0.980887	no
gene:SpnNT_02079	gmuA	Chromosome:2055210-2055824	110.58	ΔORF2+peptide	OK	119.988	93.03	-0.367122	-0.442485	0.4669	0.92732	no
gene:SpnNT_02079	gmuA	Chromosome:2055210-2055824	ΔORF2	ΔORF2+peptide	OK	103.691	93.03	-0.156517	-0.194029	0.74035	0.994748	no
gene:SpnNT_02079	gmuA	Chromosome:2055210-2055824	110.58+peptide	ΔORF2+peptide	OK	88.3766	93.03	0.0740314	0.0879062	0.8809	0.994748	no
gene:SpnNT_02080	adhE	Chromosome:2056744-2059396	110.58	ΔORF2	OK	140.1	104.93	-0.417034	-0.931711	0.10205	0.484479	no
gene:SpnNT_02080	adhE	Chromosome:2056744-2059396	110.58	110.58+peptide	OK	140.1	576.489	2.04084	3.73814	5.00E-05	0.0013612	yes
gene:SpnNT_02080	adhE	Chromosome:2056744-2059396	ΔORF2	110.58+peptide	OK	104.93	576.489	2.45787	4.52156	5.00E-05	0.0013612	yes
gene:SpnNT_02080	adhE	Chromosome:2056744-2059396	110.58	ΔORF2+peptide	OK	140.1	549.67	1.97211	3.91393	5.00E-05	0.0013612	yes
gene:SpnNT_02080	adhE	Chromosome:2056744-2059396	ΔORF2	ΔORF2+peptide	OK	104.93	549.67	2.38915	4.7658	5.00E-05	0.0013612	yes
gene:SpnNT_02080	adhE	Chromosome:2056744-2059396	110.58+peptide	ΔORF2+peptide	OK	576.489	549.67	-0.0687278	-0.116333	0.8414	0.994748	no
gene:SpnNT_02081	NA	Chromosome:2059695-2060536	110.58	ΔORF2	OK	735.946	980.583	0.414039	0.776288	0.18035	0.647661	no
gene:SpnNT_02081	NA	Chromosome:2059695-2060536	110.58	110.58+peptide	OK	735.946	504.162	-0.545714	-1.02903	0.07465	0.403276	no
gene:SpnNT_02081	NA	Chromosome:2059695-2060536	ΔORF2	110.58+peptide	OK	980.583	504.162	-0.959753	-1.7945	0.00225	0.0324089	yes
gene:SpnNT_02081	NA	Chromosome:2059695-2060536	110.58	ΔORF2+peptide	OK	735.946	603.248	-0.286848	-0.545672	0.34475	0.848904	no
gene:SpnNT_02081	NA	Chromosome:2059695-2060536	ΔORF2	ΔORF2+peptide	OK	980.583	603.248	-0.700887	-1.32186	0.025	0.197145	no
gene:SpnNT_02081	NA	Chromosome:2059695-2060536	110.58+peptide	ΔORF2+peptide	OK	504.162	603.248	0.258866	0.491045	0.39445	0.889648	no
gene:SpnNT_02082	yfkJ	Chromosome:2059695-2060536	110.58	ΔORF2	OK	274.491	331.176	0.270842	0.300374	0.59895	0.97629	no
gene:SpnNT_02082	yfkJ	Chromosome:2059695-2060536	110.58	110.58+peptide	OK	274.491	166.899	-0.717786	-0.808881	0.1624	0.616078	no
gene:SpnNT_02082	yfkJ	Chromosome:2059695-2060536	ΔORF2	110.58+peptide	OK	331.176	166.899	-0.988628	-1.06251	0.06415	0.367748	no
gene:SpnNT_02082	yfkJ	Chromosome:2059695-2060536	110.58	ΔORF2+peptide	OK	274.491	193.497	-0.504442	-0.591136	0.30915	0.814396	no
gene:SpnNT_02082	yfkJ	Chromosome:2059695-2060536	ΔORF2	ΔORF2+peptide	OK	331.176	193.497	-0.775284	-0.863287	0.13205	0.552891	no
gene:SpnNT_02082	yfkJ	Chromosome:2059695-2060536	110.58+peptide	ΔORF2+peptide	OK	166.899	193.497	0.213343	0.241421	0.67255	0.984845	no
gene:SpnNT_02083	NA	Chromosome:2060582-2060882	110.58	ΔORF2	OK	1819.69	2231.33	0.294209	0.63567	0.26125	0.761443	no
gene:SpnNT_02083	NA	Chromosome:2060582-2060882	110.58	110.58+peptide	OK	1819.69	1930.41	0.0852126	0.177879	0.7474	0.994748	no
gene:SpnNT_02083	NA	Chromosome:2060582-2060882	ΔORF2	110.58+peptide	OK	2231.33	1930.41	-0.208997	-0.433414	0.43725	0.914608	no
gene:SpnNT_02083	NA	Chromosome:2060582-2060882	110.58	ΔORF2+peptide	OK	1819.69	1540.94	-0.239883	-0.510791	0.3601	0.860974	no
gene:SpnNT_02083	NA	Chromosome:2060582-2060882	ΔORF2	ΔORF2+peptide	OK	2231.33	1540.94	-0.534093	-1.1295	0.0471	0.301339	no
gene:SpnNT_02083	NA	Chromosome:2060582-2060882	110.58+peptide	ΔORF2+peptide	OK	1930.41	1540.94	-0.325096	-0.665172	0.23735	0.73229	no
gene:SpnNT_02084	tkt_1	Chromosome:2060998-2062975	110.58	ΔORF2	OK	275.478	299.604	0.121121	0.270263	0.62935	0.980887	no
gene:SpnNT_02084	tkt_1	Chromosome:2060998-2062975	110.58	110.58+peptide	OK	275.478	190.538	-0.531854	-1.19851	0.0329	0.237205	no
gene:SpnNT_02084	tkt_1	Chromosome:2060998-2062975	ΔORF2	110.58+peptide	OK	299.604	190.538	-0.652975	-1.46268	0.01075	0.105743	no
gene:SpnNT_02084	tkt_1	Chromosome:2060998-2062975	110.58	ΔORF2+peptide	OK	275.478	214.764	-0.359187	-0.811155	0.15125	0.593484	no
gene:SpnNT_02084	tkt_1	Chromosome:2060998-2062975	ΔORF2	ΔORF2+peptide	OK	299.604	214.764	-0.480307	-1.07819	0.06255	0.3611	no
gene:SpnNT_02084	tkt_1	Chromosome:2060998-2062975	110.58+peptide	ΔORF2+peptide	OK	190.538	214.764	0.172668	0.391489	0.49055	0.940215	no
gene:SpnNT_02085	ulaG	Chromosome:2063088-2064180	110.58	ΔORF2	OK	2.09205	2.06688	-0.0174611	-0.0228354	0.9793	0.995765	no
gene:SpnNT_02085	ulaG	Chromosome:2063088-2064180	110.58	110.58+peptide	OK	2.09205	1.86002	-0.169597	-0.219414	0.6995	0.990367	no
gene:SpnNT_02085	ulaG	Chromosome:2063088-2064180	ΔORF2	110.58+peptide	OK	2.06688	1.86002	-0.152136	-0.197051	0.73545	0.994748	no
gene:SpnNT_02085	ulaG	Chromosome:2063088-2064180	110.58	ΔORF2+peptide	OK	2.09205	1.54436	-0.437905	-0.554948	0.32895	0.834909	no
gene:SpnNT_02085	ulaG	Chromosome:2063088-2064180	ΔORF2	ΔORF2+peptide	OK	2.06688	1.54436	-0.420444	-0.533411	0.3584	0.859746	no

gene:SpnNT_02085	ulaG	Chromosome:2063088-2064180	110.58+peptide	ΔORF2+peptide	OK	1.86002	1.54436	-0.268308	-0.336953	0.56045	0.968621	no
gene:SpnNT_02086	licR	Chromosome:2064312-2065965	110.58	ΔORF2	OK	1.77812	2.54579	0.51776	0.740573	0.1989	0.681625	no
gene:SpnNT_02086	licR	Chromosome:2064312-2065965	110.58	110.58+peptide	OK	1.77812	1.5414	-0.206115	-0.287017	0.61945	0.980887	no
gene:SpnNT_02086	licR	Chromosome:2064312-2065965	ΔORF2	110.58+peptide	OK	2.54579	1.5414	-0.723876	-1.01396	0.08545	0.439454	no
gene:SpnNT_02086	licR	Chromosome:2064312-2065965	110.58	ΔORF2+peptide	OK	1.77812	1.61646	-0.137519	-0.191202	0.73755	0.994748	no
gene:SpnNT_02086	licR	Chromosome:2064312-2065965	ΔORF2	ΔORF2+peptide	OK	2.54579	1.61646	-0.655279	-0.916446	0.11395	0.512073	no
gene:SpnNT_02086	licR	Chromosome:2064312-2065965	110.58+peptide	ΔORF2+peptide	OK	1.5414	1.61646	0.0685968	0.0935065	0.8748	0.994748	no
gene:SpnNT_02087	araD	Chromosome:2066143-2066848	110.58	ΔORF2	OK	2.29293	1.56245	-0.553379	-0.592309	0.31535	0.821264	no
gene:SpnNT_02087	araD	Chromosome:2066143-2066848	110.58	110.58+peptide	OK	2.29293	2.08558	-0.13674	-0.151377	0.80035	0.994748	no
gene:SpnNT_02087	araD	Chromosome:2066143-2066848	ΔORF2	110.58+peptide	OK	1.56245	2.08558	0.416639	0.455476	0.42335	0.906634	no
gene:SpnNT_02087	araD	Chromosome:2066143-2066848	110.58	ΔORF2+peptide	OK	2.29293	2.54433	0.150096	0.170245	0.77575	0.994748	no
gene:SpnNT_02087	araD	Chromosome:2066143-2066848	ΔORF2	ΔORF2+peptide	OK	1.56245	2.54433	0.703475	0.787456	0.1792	0.645832	no
gene:SpnNT_02087	araD	Chromosome:2066143-2066848	110.58+peptide	ΔORF2+peptide	OK	2.08558	2.54433	0.286836	0.333176	0.568	0.968621	no
gene:SpnNT_02088	ulaE	Chromosome:2066849-2067713	110.58	ΔORF2	OK	1.11965	0.866633	-0.369559	-0.352331	0.5279	0.956161	no
gene:SpnNT_02088	ulaE	Chromosome:2066849-2067713	110.58	110.58+peptide	OK	1.11965	0.949807	-0.237346	-0.229699	0.68135	0.986574	no
gene:SpnNT_02088	ulaE	Chromosome:2066849-2067713	ΔORF2	110.58+peptide	OK	0.866633	0.949807	0.132213	0.129004	0.8223	0.994748	no
gene:SpnNT_02088	ulaE	Chromosome:2066849-2067713	110.58	ΔORF2+peptide	OK	1.11965	0.492202	-1.18573	-1.03564	0.1001	0.479559	no
gene:SpnNT_02088	ulaE	Chromosome:2066849-2067713	ΔORF2	ΔORF2+peptide	OK	0.866633	0.492202	-0.816169	-0.717612	0.2436	0.73908	no
gene:SpnNT_02088	ulaE	Chromosome:2066849-2067713	110.58+peptide	ΔORF2+peptide	OK	0.949807	0.492202	-0.948382	-0.844535	0.17035	0.628962	no
gene:SpnNT_02089	ulaD	Chromosome:2067716-2068382	110.58	ΔORF2	OK	1.41762	1.35328	-0.0670157	-0.0634651	0.9137	0.994748	no
gene:SpnNT_02089	ulaD	Chromosome:2067716-2068382	110.58	110.58+peptide	OK	1.41762	1.33556	-0.0860242	-0.0815633	0.88095	0.994748	no
gene:SpnNT_02089	ulaD	Chromosome:2067716-2068382	ΔORF2	110.58+peptide	OK	1.35328	1.33556	-0.0190084	-0.0179628	0.9572	0.994855	no
gene:SpnNT_02089	ulaD	Chromosome:2067716-2068382	110.58	ΔORF2+peptide	OK	1.41762	0.907029	-0.644253	-0.600419	0.3278	0.833442	no
gene:SpnNT_02089	ulaD	Chromosome:2067716-2068382	ΔORF2	ΔORF2+peptide	OK	1.35328	0.907029	-0.577237	-0.536236	0.3965	0.890558	no
gene:SpnNT_02089	ulaD	Chromosome:2067716-2068382	110.58+peptide	ΔORF2+peptide	OK	1.33556	0.907029	-0.558229	-0.519171	0.4043	0.894933	no
gene:SpnNT_02090	ulaC	Chromosome:2068398-2068884	110.58	ΔORF2	OK	0.955669	0.94344	-0.0185801	-0.0143056	0.92235	0.994748	no
gene:SpnNT_02090	ulaC	Chromosome:2068398-2068884	110.58	110.58+peptide	OK	0.955669	1.2176	0.349461	0.286249	0.64765	0.981391	no
gene:SpnNT_02090	ulaC	Chromosome:2068398-2068884	ΔORF2	110.58+peptide	OK	0.94344	1.2176	0.368041	0.277447	0.63215	0.980887	no
gene:SpnNT_02090	ulaC	Chromosome:2068398-2068884	110.58	ΔORF2+peptide	OK	0.955669	1.75605	0.877747	0.757352	0.2313	0.724662	no
gene:SpnNT_02090	ulaC	Chromosome:2068398-2068884	ΔORF2	ΔORF2+peptide	OK	0.94344	1.75605	0.896327	0.705866	0.2215	0.710815	no
gene:SpnNT_02090	ulaC	Chromosome:2068398-2068884	110.58+peptide	ΔORF2+peptide	OK	1.2176	1.75605	0.528287	0.443952	0.4457	0.918272	no
gene:SpnNT_02091	ulaB	Chromosome:2068959-2069241	110.58	ΔORF2	OK	2.26329	2.52624	0.158566	0.104777	0.8677	0.994748	no
gene:SpnNT_02091	ulaB	Chromosome:2068959-2069241	110.58	110.58+peptide	OK	2.26329	3.45847	0.611711	0.418329	0.4428	0.917631	no
gene:SpnNT_02091	ulaB	Chromosome:2068959-2069241	ΔORF2	110.58+peptide	OK	2.52624	3.45847	0.453145	0.318604	0.56295	0.968621	no
gene:SpnNT_02091	ulaB	Chromosome:2068959-2069241	110.58	ΔORF2+peptide	OK	2.26329	3.96244	0.807964	0.667358	0.3186	0.824882	no
gene:SpnNT_02091	ulaB	Chromosome:2068959-2069241	ΔORF2	ΔORF2+peptide	OK	2.52624	3.96244	0.649398	0.558826	0.42145	0.905117	no
gene:SpnNT_02091	ulaB	Chromosome:2068959-2069241	110.58+peptide	ΔORF2+peptide	OK	3.45847	3.96244	0.196253	0.179276	0.7869	0.994748	no
gene:SpnNT_02092	ulaA_1	Chromosome:2069263-2070721	110.58	ΔORF2	OK	1.10347	0.74056	-0.575365	-0.661597	0.2481	0.74413	no
gene:SpnNT_02092	ulaA_1	Chromosome:2069263-2070721	110.58	110.58+peptide	OK	1.10347	0.981118	-0.169555	-0.202385	0.72595	0.994748	no
gene:SpnNT_02092	ulaA_1	Chromosome:2069263-2070721	ΔORF2	110.58+peptide	OK	0.74056	0.981118	0.40581	0.465061	0.41495	0.900591	no
gene:SpnNT_02092	ulaA_1	Chromosome:2069263-2070721	110.58	ΔORF2+peptide	OK	1.10347	0.851961	-0.373194	-0.438094	0.4469	0.919157	no
gene:SpnNT_02092	ulaA_1	Chromosome:2069263-2070721	ΔORF2	ΔORF2+peptide	OK	0.74056	0.851961	0.202171	0.228153	0.68785	0.98828	no
gene:SpnNT_02092	ulaA_1	Chromosome:2069263-2070721	110.58+peptide	ΔORF2+peptide	OK	0.981118	0.851961	-0.20364	-0.238215	0.67955	0.985855	no
gene:SpnNT_02093	NA	Chromosome:2070851-2071475	110.58	ΔORF2	OK	99.6122	98.5505	-0.0154594	-0.0323331	0.9552	0.994855	no
gene:SpnNT_02093	NA	Chromosome:2070851-2071475	110.58	110.58+peptide	OK	99.6122	70.3075	-0.502645	-1.03028	0.07075	0.390464	no

gene:SpnNT_02093	NA	Chromosome:2070851-2071475	ΔORF2	110.58+peptide	OK	98.5505	70.3075	-0.487185	-0.995207	0.08685	0.442925	no
gene:SpnNT_02093	NA	Chromosome:2070851-2071475	110.58	ΔORF2+peptide	OK	99.6122	62.2946	-0.677216	-1.36725	0.01735	0.149192	no
gene:SpnNT_02093	NA	Chromosome:2070851-2071475	ΔORF2	ΔORF2+peptide	OK	98.5505	62.2946	-0.661757	-1.33164	0.02215	0.180866	no
gene:SpnNT_02093	NA	Chromosome:2070851-2071475	110.58+peptide	ΔORF2+peptide	OK	70.3075	62.2946	-0.174572	-0.34478	0.54175	0.961494	no
gene:SpnNT_02094	NA	Chromosome:2071525-2072512	110.58	ΔORF2	OK	380.701	389.038	0.0312539	0.0707771	0.901	0.994748	no
gene:SpnNT_02094	NA	Chromosome:2071525-2072512	110.58	110.58+peptide	OK	380.701	460.743	0.275303	0.615167	0.2811	0.784473	no
gene:SpnNT_02094	NA	Chromosome:2071525-2072512	ΔORF2	110.58+peptide	OK	389.038	460.743	0.24405	0.547208	0.3398	0.844213	no
gene:SpnNT_02094	NA	Chromosome:2071525-2072512	110.58	ΔORF2+peptide	OK	380.701	418.537	0.136698	0.308489	0.5833	0.971331	no
gene:SpnNT_02094	NA	Chromosome:2071525-2072512	ΔORF2	ΔORF2+peptide	OK	389.038	418.537	0.105444	0.238793	0.6766	0.98524	no
gene:SpnNT_02094	NA	Chromosome:2071525-2072512	110.58+peptide	ΔORF2+peptide	OK	460.743	418.537	-0.138606	-0.309724	0.58035	0.969538	no
gene:SpnNT_02095	misCA	Chromosome:2072530-2073701	110.58	ΔORF2	OK	109.359	118.762	0.118994	0.180042	0.7506	0.994748	no
gene:SpnNT_02095	misCA	Chromosome:2072530-2073701	110.58	110.58+peptide	OK	109.359	120.825	0.14384	0.223419	0.6984	0.990209	no
gene:SpnNT_02095	misCA	Chromosome:2072530-2073701	ΔORF2	110.58+peptide	OK	118.762	120.825	0.0248462	0.0362911	0.95135	0.994855	no
gene:SpnNT_02095	misCA	Chromosome:2072530-2073701	110.58	ΔORF2+peptide	OK	109.359	130.123	0.250794	0.395452	0.4891	0.939082	no
gene:SpnNT_02095	misCA	Chromosome:2072530-2073701	ΔORF2	ΔORF2+peptide	OK	118.762	130.123	0.131799	0.195085	0.73105	0.994748	no
gene:SpnNT_02095	misCA	Chromosome:2072530-2073701	110.58+peptide	ΔORF2+peptide	OK	120.825	130.123	0.106953	0.162328	0.77735	0.994748	no
gene:SpnNT_02096	rnpA	Chromosome:2072530-2073701	110.58	ΔORF2	OK	380.054	506.167	0.413411	0.661621	0.2617	0.761932	no
gene:SpnNT_02096	rnpA	Chromosome:2072530-2073701	110.58	110.58+peptide	OK	380.054	414.623	0.125598	0.191726	0.74415	0.994748	no
gene:SpnNT_02096	rnpA	Chromosome:2072530-2073701	ΔORF2	110.58+peptide	OK	506.167	414.623	-0.287813	-0.439818	0.4567	0.921405	no
gene:SpnNT_02096	rnpA	Chromosome:2072530-2073701	110.58	ΔORF2+peptide	OK	380.054	579.343	0.608215	0.983077	0.0892	0.449387	no
gene:SpnNT_02096	rnpA	Chromosome:2072530-2073701	ΔORF2	ΔORF2+peptide	OK	506.167	579.343	0.194804	0.315246	0.59015	0.974817	no
gene:SpnNT_02096	rnpA	Chromosome:2072530-2073701	110.58+peptide	ΔORF2+peptide	OK	414.623	579.343	0.482617	0.744193	0.20215	0.685961	no
gene:SpnNT_02097	ackA	Chromosome:2073849-2075040	110.58	ΔORF2	OK	148.981	164.208	0.140392	0.319768	0.5736	0.969538	no
gene:SpnNT_02097	ackA	Chromosome:2073849-2075040	110.58	110.58+peptide	OK	148.981	208.109	0.482211	1.09708	0.0559	0.337095	no
gene:SpnNT_02097	ackA	Chromosome:2073849-2075040	ΔORF2	110.58+peptide	OK	164.208	208.109	0.341819	0.781091	0.16975	0.627807	no
gene:SpnNT_02097	ackA	Chromosome:2073849-2075040	110.58	ΔORF2+peptide	OK	148.981	234.691	0.655632	1.49611	0.00965	0.0978573	no
gene:SpnNT_02097	ackA	Chromosome:2073849-2075040	ΔORF2	ΔORF2+peptide	OK	164.208	234.691	0.515241	1.18095	0.0378	0.261334	no
gene:SpnNT_02097	ackA	Chromosome:2073849-2075040	110.58+peptide	ΔORF2+peptide	OK	208.109	234.691	0.173421	0.39703	0.48145	0.935095	no
gene:SpnNT_02098	NA	Chromosome:2075090-2076044	110.58	ΔORF2	OK	100.528	106.826	0.0876703	0.195542	0.73185	0.994748	no
gene:SpnNT_02098	NA	Chromosome:2075090-2076044	110.58	110.58+peptide	OK	100.528	94.0313	-0.0963818	-0.213555	0.7078	0.990441	no
gene:SpnNT_02098	NA	Chromosome:2075090-2076044	ΔORF2	110.58+peptide	OK	106.826	94.0313	-0.184052	-0.409383	0.47115	0.928525	no
gene:SpnNT_02098	NA	Chromosome:2075090-2076044	110.58	ΔORF2+peptide	OK	100.528	113.514	0.17527	0.391197	0.4888	0.939082	no
gene:SpnNT_02098	NA	Chromosome:2075090-2076044	ΔORF2	ΔORF2+peptide	OK	106.826	113.514	0.0875998	0.196286	0.7295	0.994748	no
gene:SpnNT_02098	NA	Chromosome:2075090-2076044	110.58+peptide	ΔORF2+peptide	OK	94.0313	113.514	0.271652	0.604644	0.29575	0.800006	no
gene:SpnNT_02099	NA	Chromosome:2076104-2076467	110.58	ΔORF2	OK	7.5047	6.14147	-0.289211	-0.34047	0.5561	0.966729	no
gene:SpnNT_02099	NA	Chromosome:2076104-2076467	110.58	110.58+peptide	OK	7.5047	9.88462	0.397391	0.498241	0.389	0.88343	no
gene:SpnNT_02099	NA	Chromosome:2076104-2076467	ΔORF2	110.58+peptide	OK	6.14147	9.88462	0.686601	0.830406	0.16155	0.614666	no
gene:SpnNT_02099	NA	Chromosome:2076104-2076467	110.58	ΔORF2+peptide	OK	7.5047	8.45863	0.17263	0.206915	0.7162	0.992395	no
gene:SpnNT_02099	NA	Chromosome:2076104-2076467	ΔORF2	ΔORF2+peptide	OK	6.14147	8.45863	0.461841	0.535592	0.36285	0.86287	no
gene:SpnNT_02099	NA	Chromosome:2076104-2076467	110.58+peptide	ΔORF2+peptide	OK	9.88462	8.45863	-0.224761	-0.27705	0.6355	0.980887	no
gene:SpnNT_02100	NA	Chromosome:2076473-2076692	110.58	ΔORF2	OK	6.03182	5.2457	-0.201458	-0.320324	0.81185	0.994748	no
gene:SpnNT_02100	NA	Chromosome:2076473-2076692	110.58	110.58+peptide	OK	6.03182	4.81778	-0.324225	-0.274543	0.68295	0.987294	no
gene:SpnNT_02100	NA	Chromosome:2076473-2076692	ΔORF2	110.58+peptide	OK	5.2457	4.81778	-0.122767	-0.104613	0.9025	0.994748	no
gene:SpnNT_02100	NA	Chromosome:2076473-2076692	110.58	ΔORF2+peptide	OK	6.03182	2.05623	-1.55259	-1.70444	0.2006	0.683352	no
gene:SpnNT_02100	NA	Chromosome:2076473-2076692	ΔORF2	ΔORF2+peptide	OK	5.2457	2.05623	-1.35113	-1.49916	0.23795	0.732745	no

gene:SpnNT_02100	NA	Chromosome:2076473-2076692	110.58+peptide	ΔORF2+peptide	OK	4.81778	2.05623	-1.22837	-0.912683	0.2657	0.766773	no
gene:SpnNT_02101	NA	Chromosome:2076828-2078632	110.58	ΔORF2	OK	5.29274	7.45881	0.494932	0.337466	0.5673	0.968621	no
gene:SpnNT_02101	NA	Chromosome:2076828-2078632	110.58	110.58+peptide	OK	5.29274	5.06306	-0.0640054	-0.0387234	0.94815	0.994855	no
gene:SpnNT_02101	NA	Chromosome:2076828-2078632	ΔORF2	110.58+peptide	OK	7.45881	5.06306	-0.558937	-0.361862	0.52295	0.954832	no
gene:SpnNT_02101	NA	Chromosome:2076828-2078632	110.58	ΔORF2+peptide	OK	5.29274	4.1569	-0.348505	-0.182628	0.75505	0.994748	no
gene:SpnNT_02101	NA	Chromosome:2076828-2078632	ΔORF2	ΔORF2+peptide	OK	7.45881	4.1569	-0.843437	-0.464625	0.39605	0.890349	no
gene:SpnNT_02101	NA	Chromosome:2076828-2078632	110.58+peptide	ΔORF2+peptide	OK	5.06306	4.1569	-0.2845	-0.1445	0.7999	0.994748	no
gene:SpnNT_02102	NA	Chromosome:2076828-2078632	110.58	ΔORF2	OK	5.55422	3.64603	-0.607257	-0.395525	0.49645	0.943435	no
gene:SpnNT_02102	NA	Chromosome:2076828-2078632	110.58	110.58+peptide	OK	5.55422	6.16037	0.149432	0.114769	0.8386	0.994748	no
gene:SpnNT_02102	NA	Chromosome:2076828-2078632	ΔORF2	110.58+peptide	OK	3.64603	6.16037	0.756689	0.513879	0.3627	0.86287	no
gene:SpnNT_02102	NA	Chromosome:2076828-2078632	110.58	ΔORF2+peptide	OK	5.55422	4.14436	-0.422436	-0.315779	0.67545	0.985207	no
gene:SpnNT_02102	NA	Chromosome:2076828-2078632	ΔORF2	ΔORF2+peptide	OK	3.64603	4.14436	0.184821	0.12287	0.86855	0.994748	no
gene:SpnNT_02102	NA	Chromosome:2076828-2078632	110.58+peptide	ΔORF2+peptide	OK	6.16037	4.14436	-0.571868	-0.452007	0.5441	0.961568	no
gene:SpnNT_02103	NA	Chromosome:2076828-2078632	110.58	ΔORF2	OK	5.90873	3.61404	-0.709235	-0.352284	0.7368	0.994748	no
gene:SpnNT_02103	NA	Chromosome:2076828-2078632	110.58	110.58+peptide	OK	5.90873	7.80109	0.400828	0.245263	0.73445	0.994748	no
gene:SpnNT_02103	NA	Chromosome:2076828-2078632	ΔORF2	110.58+peptide	OK	3.61404	7.80109	1.11006	0.541039	0.44185	0.917253	no
gene:SpnNT_02103	NA	Chromosome:2076828-2078632	110.58	ΔORF2+peptide	OK	5.90873	3.69177	-0.678535	-0.441566	0.7473	0.994748	no
gene:SpnNT_02103	NA	Chromosome:2076828-2078632	ΔORF2	ΔORF2+peptide	OK	3.61404	3.69177	0.0307005	0.0155458	0.9239	0.994748	no
gene:SpnNT_02103	NA	Chromosome:2076828-2078632	110.58+peptide	ΔORF2+peptide	OK	7.80109	3.69177	-1.07936	-0.680242	0.46575	0.926452	no
gene:SpnNT_02104	NA	Chromosome:2076828-2078632	110.58	ΔORF2	OK	6.17728	6.66061	0.108681	0.0710487	0.8964	0.994748	no
gene:SpnNT_02104	NA	Chromosome:2076828-2078632	110.58	110.58+peptide	OK	6.17728	5.78951	-0.09353	-0.0602485	0.914	0.994748	no
gene:SpnNT_02104	NA	Chromosome:2076828-2078632	ΔORF2	110.58+peptide	OK	6.66061	5.78951	-0.202211	-0.138383	0.81345	0.994748	no
gene:SpnNT_02104	NA	Chromosome:2076828-2078632	110.58	ΔORF2+peptide	OK	6.17728	8.56802	0.47199	0.246211	0.6122	0.97955	no
gene:SpnNT_02104	NA	Chromosome:2076828-2078632	ΔORF2	ΔORF2+peptide	OK	6.66061	8.56802	0.363308	0.197025	0.693	0.98828	no
gene:SpnNT_02104	NA	Chromosome:2076828-2078632	110.58+peptide	ΔORF2+peptide	OK	5.78951	8.56802	0.56552	0.303574	0.54935	0.964281	no
gene:SpnNT_02105	NA	Chromosome:2076828-2078632	110.58	ΔORF2	OK	10.8335	8.09558	-0.4203	-0.295366	0.59365	0.975539	no
gene:SpnNT_02105	NA	Chromosome:2076828-2078632	110.58	110.58+peptide	OK	10.8335	6.25993	-0.791288	-0.46125	0.3826	0.880283	no
gene:SpnNT_02105	NA	Chromosome:2076828-2078632	ΔORF2	110.58+peptide	OK	8.09558	6.25993	-0.370988	-0.207938	0.70425	0.990367	no
gene:SpnNT_02105	NA	Chromosome:2076828-2078632	110.58	ΔORF2+peptide	OK	10.8335	6.74675	-0.68324	-0.531597	0.4874	0.938799	no
gene:SpnNT_02105	NA	Chromosome:2076828-2078632	ΔORF2	ΔORF2+peptide	OK	8.09558	6.74675	-0.26294	-0.191161	0.8026	0.994748	no
gene:SpnNT_02105	NA	Chromosome:2076828-2078632	110.58+peptide	ΔORF2+peptide	OK	6.25993	6.74675	0.108048	0.0644544	0.92685	0.994748	no
gene:SpnNT_02106	NA	Chromosome:2078633-2080539	110.58	ΔORF2	OK	2.97887	3.10354	0.0591515	0.057726	0.9225	0.994748	no
gene:SpnNT_02106	NA	Chromosome:2078633-2080539	110.58	110.58+peptide	OK	2.97887	3.4158	0.197458	0.192994	0.72935	0.994748	no
gene:SpnNT_02106	NA	Chromosome:2078633-2080539	ΔORF2	110.58+peptide	OK	3.10354	3.4158	0.138307	0.13544	0.8149	0.994748	no
gene:SpnNT_02106	NA	Chromosome:2078633-2080539	110.58	ΔORF2+peptide	OK	2.97887	3.22447	0.114296	0.112095	0.8437	0.994748	no
gene:SpnNT_02106	NA	Chromosome:2078633-2080539	ΔORF2	ΔORF2+peptide	OK	3.10354	3.22447	0.0551441	0.0541869	0.9264	0.994748	no
gene:SpnNT_02106	NA	Chromosome:2078633-2080539	110.58+peptide	ΔORF2+peptide	OK	3.4158	3.22447	-0.0831629	-0.0818456	0.88485	0.994748	no
gene:SpnNT_02107	xpsE	Chromosome:2078633-2080539	110.58	ΔORF2	OK	4.55298	4.17938	-0.12352	-0.137879	0.8043	0.994748	no
gene:SpnNT_02107	xpsE	Chromosome:2078633-2080539	110.58	110.58+peptide	OK	4.55298	5.36549	0.236901	0.273937	0.63505	0.980887	no
gene:SpnNT_02107	xpsE	Chromosome:2078633-2080539	ΔORF2	110.58+peptide	OK	4.17938	5.36549	0.360421	0.421107	0.46335	0.925853	no
gene:SpnNT_02107	xpsE	Chromosome:2078633-2080539	110.58	ΔORF2+peptide	OK	4.55298	3.51645	-0.372691	-0.386603	0.507	0.946083	no
gene:SpnNT_02107	xpsE	Chromosome:2078633-2080539	ΔORF2	ΔORF2+peptide	OK	4.17938	3.51645	-0.249171	-0.260631	0.6471	0.981307	no
gene:SpnNT_02107	xpsE	Chromosome:2078633-2080539	110.58+peptide	ΔORF2+peptide	OK	5.36549	3.51645	-0.609592	-0.657606	0.2594	0.759841	no
gene:SpnNT_02108	NA	Chromosome:2080614-2080980	110.58	ΔORF2	OK	471.203	680.59	0.530439	1.16495	0.0467	0.299217	no
gene:SpnNT_02108	NA	Chromosome:2080614-2080980	110.58	110.58+peptide	OK	471.203	476.055	0.0147793	0.0325672	0.9563	0.994855	no

gene:SpnNT_02108	NA	Chromosome:2080614-2080980	ΔORF2	110.58+peptide	OK	680.59	476.055	-0.51566	-1.12654	0.0536	0.327897	no
gene:SpnNT_02108	NA	Chromosome:2080614-2080980	110.58	ΔORF2+peptide	OK	471.203	591.156	0.327192	0.734831	0.20635	0.691266	no
gene:SpnNT_02108	NA	Chromosome:2080614-2080980	ΔORF2	ΔORF2+peptide	OK	680.59	591.156	-0.203247	-0.452397	0.43285	0.913334	no
gene:SpnNT_02108	NA	Chromosome:2080614-2080980	110.58+peptide	ΔORF2+peptide	OK	476.055	591.156	0.312413	0.697784	0.2314	0.724662	no
gene:SpnNT_02109	adh	Chromosome:2081130-2081835	110.58	ΔORF2	OK	36.9298	32.7652	-0.172621	-0.323101	0.57335	0.969538	no
gene:SpnNT_02109	adh	Chromosome:2081130-2081835	110.58	110.58+peptide	OK	36.9298	47.77	0.371317	0.693938	0.228	0.719706	no
gene:SpnNT_02109	adh	Chromosome:2081130-2081835	ΔORF2	110.58+peptide	OK	32.7652	47.77	0.543939	1.0139	0.078	0.414883	no
gene:SpnNT_02109	adh	Chromosome:2081130-2081835	110.58	ΔORF2+peptide	OK	36.9298	48.0002	0.378254	0.696667	0.22205	0.711509	no
gene:SpnNT_02109	adh	Chromosome:2081130-2081835	ΔORF2	ΔORF2+peptide	OK	32.7652	48.0002	0.550876	1.01203	0.07535	0.405389	no
gene:SpnNT_02109	adh	Chromosome:2081130-2081835	110.58+peptide	ΔORF2+peptide	OK	47.77	48.0002	0.00693707	0.0127255	0.98105	0.99608	no
gene:SpnNT_02110	nagA	Chromosome:2082350-2083502	110.58	ΔORF2	OK	146.647	168.925	0.204032	0.46487	0.4154	0.901067	no
gene:SpnNT_02110	nagA	Chromosome:2082350-2083502	110.58	110.58+peptide	OK	146.647	85.0196	-0.786483	-1.76265	0.00265	0.0362222	yes
gene:SpnNT_02110	nagA	Chromosome:2082350-2083502	ΔORF2	110.58+peptide	OK	168.925	85.0196	-0.990515	-2.23712	5.00E-05	0.0013612	yes
gene:SpnNT_02110	nagA	Chromosome:2082350-2083502	110.58	ΔORF2+peptide	OK	146.647	72.1432	-1.02341	-2.28338	5.00E-05	0.0013612	yes
gene:SpnNT_02110	nagA	Chromosome:2082350-2083502	ΔORF2	ΔORF2+peptide	OK	168.925	72.1432	-1.22745	-2.75964	5.00E-05	0.0013612	yes
gene:SpnNT_02110	nagA	Chromosome:2082350-2083502	110.58+peptide	ΔORF2+peptide	OK	85.0196	72.1432	-0.236931	-0.524203	0.3518	0.854885	no
gene:SpnNT_02111	oatA	Chromosome:2083654-2085472	110.58	ΔORF2	OK	131.897	142.566	0.112219	0.257938	0.6438	0.980887	no
gene:SpnNT_02111	oatA	Chromosome:2083654-2085472	110.58	110.58+peptide	OK	131.897	126.989	-0.0547026	-0.126	0.8282	0.994748	no
gene:SpnNT_02111	oatA	Chromosome:2083654-2085472	ΔORF2	110.58+peptide	OK	142.566	126.989	-0.166922	-0.383569	0.4933	0.941402	no
gene:SpnNT_02111	oatA	Chromosome:2083654-2085472	110.58	ΔORF2+peptide	OK	131.897	126.081	-0.0650566	-0.149638	0.7894	0.994748	no
gene:SpnNT_02111	oatA	Chromosome:2083654-2085472	ΔORF2	ΔORF2+peptide	OK	142.566	126.081	-0.177276	-0.406789	0.46695	0.92732	no
gene:SpnNT_02111	oatA	Chromosome:2083654-2085472	110.58+peptide	ΔORF2+peptide	OK	126.989	126.081	-0.010354	-0.0238091	0.96605	0.994855	no
gene:SpnNT_02112	tgt	Chromosome:2085572-2086715	110.58	ΔORF2	OK	148.505	118.246	-0.328727	-0.740367	0.19685	0.678125	no
gene:SpnNT_02112	tgt	Chromosome:2085572-2086715	110.58	110.58+peptide	OK	148.505	202.965	0.450721	1.01226	0.075	0.404272	no
gene:SpnNT_02112	tgt	Chromosome:2085572-2086715	ΔORF2	110.58+peptide	OK	118.246	202.965	0.779447	1.75512	0.00255	0.0352222	yes
gene:SpnNT_02112	tgt	Chromosome:2085572-2086715	110.58	ΔORF2+peptide	OK	148.505	202.734	0.449078	1.01449	0.0779	0.414519	no
gene:SpnNT_02112	tgt	Chromosome:2085572-2086715	ΔORF2	ΔORF2+peptide	OK	118.246	202.734	0.777805	1.76176	0.00265	0.0362222	yes
gene:SpnNT_02112	tgt	Chromosome:2085572-2086715	110.58+peptide	ΔORF2+peptide	OK	202.965	202.734	-0.00164233	-0.00370933	0.99455	0.999116	no
gene:SpnNT_02113	NA	Chromosome:2086778-2087702	110.58	ΔORF2	OK	69.6908	67.6809	-0.0422204	-0.0896307	0.8745	0.994748	no
gene:SpnNT_02113	NA	Chromosome:2086778-2087702	110.58	110.58+peptide	OK	69.6908	74.3038	0.0924682	0.197744	0.72535	0.994748	no
gene:SpnNT_02113	NA	Chromosome:2086778-2087702	ΔORF2	110.58+peptide	OK	67.6809	74.3038	0.134689	0.289581	0.61335	0.979616	no
gene:SpnNT_02113	NA	Chromosome:2086778-2087702	110.58	ΔORF2+peptide	OK	69.6908	90.6583	0.37947	0.818306	0.1526	0.595249	no
gene:SpnNT_02113	NA	Chromosome:2086778-2087702	ΔORF2	ΔORF2+peptide	OK	67.6809	90.6583	0.42169	0.914323	0.11285	0.510701	no
gene:SpnNT_02113	NA	Chromosome:2086778-2087702	110.58+peptide	ΔORF2+peptide	OK	74.3038	90.6583	0.287001	0.627054	0.2719	0.774059	no
gene:SpnNT_02114	pcp_2	Chromosome:2087729-2088373	110.58	ΔORF2	OK	1035.22	977.313	-0.0830476	-0.121828	0.8327	0.994748	no
gene:SpnNT_02114	pcp_2	Chromosome:2087729-2088373	110.58	110.58+peptide	OK	1035.22	1808.69	0.805003	1.14066	0.04595	0.296002	no
gene:SpnNT_02114	pcp_2	Chromosome:2087729-2088373	ΔORF2	110.58+peptide	OK	977.313	1808.69	0.888051	1.26151	0.0294	0.218948	no
gene:SpnNT_02114	pcp_2	Chromosome:2087729-2088373	110.58	ΔORF2+peptide	OK	1035.22	1530.05	0.563634	0.733285	0.1981	0.680846	no
gene:SpnNT_02114	pcp_2	Chromosome:2087729-2088373	ΔORF2	ΔORF2+peptide	OK	977.313	1530.05	0.646682	0.843117	0.1523	0.594786	no
gene:SpnNT_02114	pcp_2	Chromosome:2087729-2088373	110.58+peptide	ΔORF2+peptide	OK	1808.69	1530.05	-0.241368	-0.306123	0.59985	0.976542	no
gene:SpnNT_02115	pcp_3	Chromosome:2087729-2088373	110.58	ΔORF2	OK	245.52	203.603	-0.270079	-0.434648	0.44495	0.917756	no
gene:SpnNT_02115	pcp_3	Chromosome:2087729-2088373	110.58	110.58+peptide	OK	245.52	319.13	0.378306	0.567939	0.311	0.816778	no
gene:SpnNT_02115	pcp_3	Chromosome:2087729-2088373	ΔORF2	110.58+peptide	OK	203.603	319.13	0.648385	0.941854	0.0974	0.471791	no
gene:SpnNT_02115	pcp_3	Chromosome:2087729-2088373	110.58	ΔORF2+peptide	OK	245.52	348.369	0.504777	0.776474	0.16815	0.624353	no
gene:SpnNT_02115	pcp_3	Chromosome:2087729-2088373	ΔORF2	ΔORF2+peptide	OK	203.603	348.369	0.774856	1.15147	0.04375	0.286757	no

gene:SpnNT_02115	pcp_3	Chromosome:2087729-2088373	110.58+peptide	ΔORF2+peptide	OK	319.13	348.369	0.126471	0.177021	0.74805	0.994748	no
gene:SpnNT_02116	NA	Chromosome:2088534-2088786	110.58	ΔORF2	OK	165.587	200.379	0.275143	0.485987	0.40075	0.893669	no
gene:SpnNT_02116	NA	Chromosome:2088534-2088786	110.58	110.58+peptide	OK	165.587	208.015	0.329099	0.59448	0.30625	0.81251	no
gene:SpnNT_02116	NA	Chromosome:2088534-2088786	ΔORF2	110.58+peptide	OK	200.379	208.015	0.0539563	0.0977012	0.86325	0.994748	no
gene:SpnNT_02116	NA	Chromosome:2088534-2088786	110.58	ΔORF2+peptide	OK	165.587	253.974	0.617091	1.12639	0.05505	0.333965	no
gene:SpnNT_02116	NA	Chromosome:2088534-2088786	ΔORF2	ΔORF2+peptide	OK	200.379	253.974	0.341949	0.625706	0.2741	0.776305	no
gene:SpnNT_02116	NA	Chromosome:2088534-2088786	110.58+peptide	ΔORF2+peptide	OK	208.015	253.974	0.287993	0.53984	0.3519	0.854885	no
gene:SpnNT_02117	NA	Chromosome:2088987-2090130	110.58	ΔORF2	OK	1159.57	1116.42	-0.054708	-0.118037	0.8321	0.994748	no
gene:SpnNT_02117	NA	Chromosome:2088987-2090130	110.58	110.58+peptide	OK	1159.57	1202.43	0.0523575	0.112967	0.84095	0.994748	no
gene:SpnNT_02117	NA	Chromosome:2088987-2090130	ΔORF2	110.58+peptide	OK	1116.42	1202.43	0.107065	0.232255	0.688	0.98828	no
gene:SpnNT_02117	NA	Chromosome:2088987-2090130	110.58	ΔORF2+peptide	OK	1159.57	1334.25	0.202437	0.43519	0.43965	0.91631	no
gene:SpnNT_02117	NA	Chromosome:2088987-2090130	ΔORF2	ΔORF2+peptide	OK	1116.42	1334.25	0.257145	0.555769	0.3266	0.831577	no
gene:SpnNT_02117	NA	Chromosome:2088987-2090130	110.58+peptide	ΔORF2+peptide	OK	1202.43	1334.25	0.150079	0.324369	0.57345	0.969538	no
gene:SpnNT_02118	cbbZC	Chromosome:2090259-2090916	110.58	ΔORF2	OK	75.9261	75.0676	-0.0164052	-0.0333697	0.95055	0.994855	no
gene:SpnNT_02118	cbbZC	Chromosome:2090259-2090916	110.58	110.58+peptide	OK	75.9261	68.3218	-0.152249	-0.310749	0.5885	0.974014	no
gene:SpnNT_02118	cbbZC	Chromosome:2090259-2090916	ΔORF2	110.58+peptide	OK	75.0676	68.3218	-0.135844	-0.275035	0.6392	0.980887	no
gene:SpnNT_02118	cbbZC	Chromosome:2090259-2090916	110.58	ΔORF2+peptide	OK	75.9261	83.0181	0.128832	0.265327	0.64115	0.980887	no
gene:SpnNT_02118	cbbZC	Chromosome:2090259-2090916	ΔORF2	ΔORF2+peptide	OK	75.0676	83.0181	0.145237	0.296665	0.6131	0.979616	no
gene:SpnNT_02118	cbbZC	Chromosome:2090259-2090916	110.58+peptide	ΔORF2+peptide	OK	68.3218	83.0181	0.28108	0.576127	0.3169	0.822753	no
gene:SpnNT_02119	norM_2	Chromosome:2090919-2092200	110.58	ΔORF2	OK	75.6968	69.8779	-0.115397	-0.257452	0.64905	0.981391	no
gene:SpnNT_02119	norM_2	Chromosome:2090919-2092200	110.58	110.58+peptide	OK	75.6968	69.1768	-0.129944	-0.288616	0.6119	0.979429	no
gene:SpnNT_02119	norM_2	Chromosome:2090919-2092200	ΔORF2	110.58+peptide	OK	69.8779	69.1768	-0.0145474	-0.0322229	0.9573	0.994855	no
gene:SpnNT_02119	norM_2	Chromosome:2090919-2092200	110.58	ΔORF2+peptide	OK	75.6968	71.5323	-0.0816366	-0.182426	0.7459	0.994748	no
gene:SpnNT_02119	norM_2	Chromosome:2090919-2092200	ΔORF2	ΔORF2+peptide	OK	69.8779	71.5323	0.0337601	0.0752325	0.8958	0.994748	no
gene:SpnNT_02119	norM_2	Chromosome:2090919-2092200	110.58+peptide	ΔORF2+peptide	OK	69.1768	71.5323	0.0483075	0.107172	0.85245	0.994748	no
gene:SpnNT_02120	thrC	Chromosome:2092260-2093745	110.58	ΔORF2	OK	187.491	200.763	0.0986709	0.224324	0.6909	0.98828	no
gene:SpnNT_02120	thrC	Chromosome:2092260-2093745	110.58	110.58+peptide	OK	187.491	215.297	0.199508	0.455643	0.422	0.905409	no
gene:SpnNT_02120	thrC	Chromosome:2092260-2093745	ΔORF2	110.58+peptide	OK	200.763	215.297	0.100837	0.228207	0.6871	0.98828	no
gene:SpnNT_02120	thrC	Chromosome:2092260-2093745	110.58	ΔORF2+peptide	OK	187.491	208.338	0.152109	0.349418	0.53525	0.958867	no
gene:SpnNT_02120	thrC	Chromosome:2092260-2093745	ΔORF2	ΔORF2+peptide	OK	200.763	208.338	0.0534384	0.12163	0.8296	0.994748	no
gene:SpnNT_02120	thrC	Chromosome:2092260-2093745	110.58+peptide	ΔORF2+peptide	OK	215.297	208.338	-0.047399	-0.108377	0.85075	0.994748	no
gene:SpnNT_02121	NA	Chromosome:2093820-2095098	110.58	ΔORF2	OK	61.938	79.4828	0.359819	0.790172	0.16795	0.623964	no
gene:SpnNT_02121	NA	Chromosome:2093820-2095098	110.58	110.58+peptide	OK	61.938	56.8577	-0.123469	-0.266918	0.6417	0.980887	no
gene:SpnNT_02121	NA	Chromosome:2093820-2095098	ΔORF2	110.58+peptide	OK	79.4828	56.8577	-0.483288	-1.04102	0.0672	0.378942	no
gene:SpnNT_02121	NA	Chromosome:2093820-2095098	110.58	ΔORF2+peptide	OK	61.938	73.111	0.239265	0.527716	0.3626	0.86287	no
gene:SpnNT_02121	NA	Chromosome:2093820-2095098	ΔORF2	ΔORF2+peptide	OK	79.4828	73.111	-0.120554	-0.264893	0.64305	0.980887	no
gene:SpnNT_02121	NA	Chromosome:2093820-2095098	110.58+peptide	ΔORF2+peptide	OK	56.8577	73.111	0.362734	0.784606	0.1671	0.622922	no
gene:SpnNT_02122	NA	Chromosome:2095359-2095836	110.58	ΔORF2	OK	107.638	204.762	0.927756	1.77637	0.00235	0.0334454	yes
gene:SpnNT_02122	NA	Chromosome:2095359-2095836	110.58	110.58+peptide	OK	107.638	155.484	0.530572	1.02901	0.07095	0.390523	no
gene:SpnNT_02122	NA	Chromosome:2095359-2095836	ΔORF2	110.58+peptide	OK	204.762	155.484	-0.397184	-0.733165	0.1896	0.667533	no
gene:SpnNT_02122	NA	Chromosome:2095359-2095836	110.58	ΔORF2+peptide	OK	107.638	199.831	0.892588	1.81707	0.00135	0.021343	yes
gene:SpnNT_02122	NA	Chromosome:2095359-2095836	ΔORF2	ΔORF2+peptide	OK	204.762	199.831	-0.0351683	-0.0678165	0.90185	0.994748	no
gene:SpnNT_02122	NA	Chromosome:2095359-2095836	110.58+peptide	ΔORF2+peptide	OK	155.484	199.831	0.362016	0.707243	0.2054	0.69003	no
gene:SpnNT_02123	NA	Chromosome:2095989-2096271	110.58	ΔORF2	OK	414.019	411.634	-0.00833395	-0.0172516	0.9762	0.99536	no
gene:SpnNT_02123	NA	Chromosome:2095989-2096271	110.58	110.58+peptide	OK	414.019	406.543	-0.0262882	-0.0544008	0.9202	0.994748	no

gene:SpnNT_02123	NA	Chromosome:2095989-2096271	ΔORF2	110.58+peptide	OK	411.634	406.543	-0.0179542	-0.0370512	0.94675	0.994855	no
gene:SpnNT_02123	NA	Chromosome:2095989-2096271	110.58	ΔORF2+peptide	OK	414.019	338.407	-0.290938	-0.600232	0.293	0.797831	no
gene:SpnNT_02123	NA	Chromosome:2095989-2096271	ΔORF2	ΔORF2+peptide	OK	411.634	338.407	-0.282604	-0.581427	0.3112	0.816845	no
gene:SpnNT_02123	NA	Chromosome:2095989-2096271	110.58+peptide	ΔORF2+peptide	OK	406.543	338.407	-0.26465	-0.544321	0.34265	0.846557	no
gene:SpnNT_02124	NA	Chromosome:2096332-2097902	110.58	ΔORF2	OK	27.1526	27.3031	0.00797234	0.00253221	0.9801	0.995963	no
gene:SpnNT_02124	NA	Chromosome:2096332-2097902	110.58	110.58+peptide	OK	27.1526	27.8537	0.0367749	0.0118816	0.97535	0.99536	no
gene:SpnNT_02124	NA	Chromosome:2096332-2097902	ΔORF2	110.58+peptide	OK	27.3031	27.8537	0.0288026	0.00948875	0.9747	0.99536	no
gene:SpnNT_02124	NA	Chromosome:2096332-2097902	110.58	ΔORF2+peptide	OK	27.1526	26.5712	-0.0312262	-0.00979572	0.9762	0.99536	no
gene:SpnNT_02124	NA	Chromosome:2096332-2097902	ΔORF2	ΔORF2+peptide	OK	27.3031	26.5712	-0.0391985	-0.0125242	0.97255	0.99536	no
gene:SpnNT_02124	NA	Chromosome:2096332-2097902	110.58+peptide	ΔORF2+peptide	OK	27.8537	26.5712	-0.0680011	-0.0221051	0.9659	0.994855	no
gene:SpnNT_02125	NA	Chromosome:2096332-2097902	110.58	ΔORF2	OK	63.0102	63.3624	0.00804035	0.0172337	0.9757	0.99536	no
gene:SpnNT_02125	NA	Chromosome:2096332-2097902	110.58	110.58+peptide	OK	63.0102	64.7771	0.039898	0.0860718	0.8771	0.994748	no
gene:SpnNT_02125	NA	Chromosome:2096332-2097902	ΔORF2	110.58+peptide	OK	63.3624	64.7771	0.0318576	0.0699863	0.8989	0.994748	no
gene:SpnNT_02125	NA	Chromosome:2096332-2097902	110.58	ΔORF2+peptide	OK	63.0102	62.0728	-0.0216246	-0.0466423	0.93315	0.994855	no
gene:SpnNT_02125	NA	Chromosome:2096332-2097902	ΔORF2	ΔORF2+peptide	OK	63.3624	62.0728	-0.0296649	-0.0651572	0.90555	0.994748	no
gene:SpnNT_02125	NA	Chromosome:2096332-2097902	110.58+peptide	ΔORF2+peptide	OK	64.7771	62.0728	-0.0615225	-0.136051	0.81405	0.994748	no
gene:SpnNT_02126	NA	Chromosome:2097999-2098314	110.58	ΔORF2	OK	51.0173	44.8054	-0.187313	-0.300325	0.60285	0.976761	no
gene:SpnNT_02126	NA	Chromosome:2097999-2098314	110.58	110.58+peptide	OK	51.0173	58.2992	0.19249	0.312827	0.5796	0.969538	no
gene:SpnNT_02126	NA	Chromosome:2097999-2098314	ΔORF2	110.58+peptide	OK	44.8054	58.2992	0.379804	0.615344	0.2863	0.789581	no
gene:SpnNT_02126	NA	Chromosome:2097999-2098314	110.58	ΔORF2+peptide	OK	51.0173	53.0157	0.055433	0.0891515	0.88045	0.994748	no
gene:SpnNT_02126	NA	Chromosome:2097999-2098314	ΔORF2	ΔORF2+peptide	OK	44.8054	53.0157	0.242746	0.389229	0.5147	0.951747	no
gene:SpnNT_02126	NA	Chromosome:2097999-2098314	110.58+peptide	ΔORF2+peptide	OK	58.2992	53.0157	-0.137057	-0.222755	0.6943	0.988457	no
gene:SpnNT_02127	NA	Chromosome:2098347-2098737	110.58	ΔORF2	OK	58.0863	52.516	-0.145442	-0.257646	0.65635	0.982966	no
gene:SpnNT_02127	NA	Chromosome:2098347-2098737	110.58	110.58+peptide	OK	58.0863	57.5425	-0.013571	-0.0240586	0.9662	0.994855	no
gene:SpnNT_02127	NA	Chromosome:2098347-2098737	ΔORF2	110.58+peptide	OK	52.516	57.5425	0.131871	0.231528	0.6849	0.988009	no
gene:SpnNT_02127	NA	Chromosome:2098347-2098737	110.58	ΔORF2+peptide	OK	58.0863	55.7424	-0.0594229	-0.105598	0.85605	0.994748	no
gene:SpnNT_02127	NA	Chromosome:2098347-2098737	ΔORF2	ΔORF2+peptide	OK	52.516	55.7424	0.0860195	0.151382	0.79425	0.994748	no
gene:SpnNT_02127	NA	Chromosome:2098347-2098737	110.58+peptide	ΔORF2+peptide	OK	57.5425	55.7424	-0.0458519	-0.080753	0.88875	0.994748	no
gene:SpnNT_02128	NA	Chromosome:2098903-2099671	110.58	ΔORF2	OK	53.8763	56.5545	0.0699919	0.137965	0.80725	0.994748	no
gene:SpnNT_02128	NA	Chromosome:2098903-2099671	110.58	110.58+peptide	OK	53.8763	57.9798	0.1059	0.210908	0.7048	0.990367	no
gene:SpnNT_02128	NA	Chromosome:2098903-2099671	ΔORF2	110.58+peptide	OK	56.5545	57.9798	0.0359079	0.0728097	0.8976	0.994748	no
gene:SpnNT_02128	NA	Chromosome:2098903-2099671	110.58	ΔORF2+peptide	OK	53.8763	50.8739	-0.0827232	-0.162446	0.76915	0.994748	no
gene:SpnNT_02128	NA	Chromosome:2098903-2099671	ΔORF2	ΔORF2+peptide	OK	56.5545	50.8739	-0.152715	-0.305172	0.59225	0.975468	no
gene:SpnNT_02128	NA	Chromosome:2098903-2099671	110.58+peptide	ΔORF2+peptide	OK	57.9798	50.8739	-0.188623	-0.380942	0.4978	0.944017	no
gene:SpnNT_02129	NA	Chromosome:2099856-2100636	110.58	ΔORF2	OK	45.3372	42.3996	-0.0966471	-0.188878	0.74495	0.994748	no
gene:SpnNT_02129	NA	Chromosome:2099856-2100636	110.58	110.58+peptide	OK	45.3372	42.0672	-0.108002	-0.210752	0.7161	0.992361	no
gene:SpnNT_02129	NA	Chromosome:2099856-2100636	ΔORF2	110.58+peptide	OK	42.3996	42.0672	-0.0113546	-0.0221941	0.9683	0.994855	no
gene:SpnNT_02129	NA	Chromosome:2099856-2100636	110.58	ΔORF2+peptide	OK	45.3372	42.8906	-0.0800346	-0.156288	0.7877	0.994748	no
gene:SpnNT_02129	NA	Chromosome:2099856-2100636	ΔORF2	ΔORF2+peptide	OK	42.3996	42.8906	0.0166125	0.0324944	0.95595	0.994855	no
gene:SpnNT_02129	NA	Chromosome:2099856-2100636	110.58+peptide	ΔORF2+peptide	OK	42.0672	42.8906	0.0279671	0.0546222	0.9251	0.994748	no
gene:SpnNT_02130	NA	Chromosome:2100652-2100856	110.58	ΔORF2	OK	35.0582	28.999	-0.273752	-0.322513	0.57485	0.969538	no
gene:SpnNT_02130	NA	Chromosome:2100652-2100856	110.58	110.58+peptide	OK	35.0582	26.2479	-0.417552	-0.48151	0.3951	0.890116	no
gene:SpnNT_02130	NA	Chromosome:2100652-2100856	ΔORF2	110.58+peptide	OK	28.999	26.2479	-0.143801	-0.160205	0.77855	0.994748	no
gene:SpnNT_02130	NA	Chromosome:2100652-2100856	110.58	ΔORF2+peptide	OK	35.0582	49.4525	0.496289	0.52901	0.3256	0.830907	no
gene:SpnNT_02130	NA	Chromosome:2100652-2100856	ΔORF2	ΔORF2+peptide	OK	28.999	49.4525	0.77004	0.79686	0.1437	0.57877	no

gene:SpnNT_02130	NA	Chromosome:2100652-2100856	110.58+peptide	ΔORF2+peptide	OK	26.2479	49.4525	0.913841	0.930104	0.08775	0.446301	no
gene:SpnNT_02131	xerC_4	Chromosome:2100859-2101993	110.58	ΔORF2	OK	41.5713	42.9693	0.0477181	0.0972277	0.8677	0.994748	no
gene:SpnNT_02131	xerC_4	Chromosome:2100859-2101993	110.58	110.58+peptide	OK	41.5713	38.1463	-0.124043	-0.251958	0.6654	0.98367	no
gene:SpnNT_02131	xerC_4	Chromosome:2100859-2101993	ΔORF2	110.58+peptide	OK	42.9693	38.1463	-0.171761	-0.352783	0.5432	0.961568	no
gene:SpnNT_02131	xerC_4	Chromosome:2100859-2101993	110.58	ΔORF2+peptide	OK	41.5713	38.4737	-0.111717	-0.226899	0.69655	0.989512	no
gene:SpnNT_02131	xerC_4	Chromosome:2100859-2101993	ΔORF2	ΔORF2+peptide	OK	42.9693	38.4737	-0.159435	-0.327433	0.5691	0.968621	no
gene:SpnNT_02131	xerC_4	Chromosome:2100859-2101993	110.58+peptide	ΔORF2+peptide	OK	38.1463	38.4737	0.0123262	0.0252346	0.96345	0.994855	no
gene:SpnNT_02132	relA_2	Chromosome:2102033-2102471	110.58	ΔORF2	OK	108.974	147.951	0.441139	0.866461	0.1245	0.533791	no
gene:SpnNT_02132	relA_2	Chromosome:2102033-2102471	110.58	110.58+peptide	OK	108.974	121.08	0.151973	0.297847	0.5952	0.975667	no
gene:SpnNT_02132	relA_2	Chromosome:2102033-2102471	ΔORF2	110.58+peptide	OK	147.951	121.08	-0.289165	-0.559862	0.3154	0.821264	no
gene:SpnNT_02132	relA_2	Chromosome:2102033-2102471	110.58	ΔORF2+peptide	OK	108.974	130.455	0.259576	0.51194	0.35675	0.858005	no
gene:SpnNT_02132	relA_2	Chromosome:2102033-2102471	ΔORF2	ΔORF2+peptide	OK	147.951	130.455	-0.181563	-0.35369	0.52375	0.954832	no
gene:SpnNT_02132	relA_2	Chromosome:2102033-2102471	110.58+peptide	ΔORF2+peptide	OK	121.08	130.455	0.107603	0.209164	0.7125	0.991034	no
gene:SpnNT_02133	NA	Chromosome:2102686-2103647	110.58	ΔORF2	OK	36.6832	54.1227	0.561115	0.288004	0.7535	0.994748	no
gene:SpnNT_02133	NA	Chromosome:2102686-2103647	110.58	110.58+peptide	OK	36.6832	21.6393	-0.761465	-0.377693	0.67775	0.98524	no
gene:SpnNT_02133	NA	Chromosome:2102686-2103647	ΔORF2	110.58+peptide	OK	54.1227	21.6393	-1.32258	-0.63439	0.36755	0.864955	no
gene:SpnNT_02133	NA	Chromosome:2102686-2103647	110.58	ΔORF2+peptide	OK	36.6832	29.4489	-0.316906	-0.157492	0.86915	0.994748	no
gene:SpnNT_02133	NA	Chromosome:2102686-2103647	ΔORF2	ΔORF2+peptide	OK	54.1227	29.4489	-0.87802	-0.421915	0.62055	0.980887	no
gene:SpnNT_02133	NA	Chromosome:2102686-2103647	110.58+peptide	ΔORF2+peptide	OK	21.6393	29.4489	0.44456	0.207288	0.7865	0.994748	no
gene:SpnNT_02134	NA	Chromosome:2102686-2103647	110.58	ΔORF2	OK	52.5948	77.7814	0.564507	1.0709	0.06025	0.352475	no
gene:SpnNT_02134	NA	Chromosome:2102686-2103647	110.58	110.58+peptide	OK	52.5948	37.8409	-0.474973	-0.883664	0.12825	0.54287	no
gene:SpnNT_02134	NA	Chromosome:2102686-2103647	ΔORF2	110.58+peptide	OK	77.7814	37.8409	-1.03948	-1.92161	9.00E-04	0.0154579	yes
gene:SpnNT_02134	NA	Chromosome:2102686-2103647	110.58	ΔORF2+peptide	OK	52.5948	48.6446	-0.11264	-0.215477	0.7105	0.990984	no
gene:SpnNT_02134	NA	Chromosome:2102686-2103647	ΔORF2	ΔORF2+peptide	OK	77.7814	48.6446	-0.677147	-1.28666	0.0261	0.202893	no
gene:SpnNT_02134	NA	Chromosome:2102686-2103647	110.58+peptide	ΔORF2+peptide	OK	37.8409	48.6446	0.362332	0.67515	0.2472	0.743921	no
gene:SpnNT_02135	NA	Chromosome:2103705-2104959	110.58	ΔORF2	OK	4.27385	4.2254	-0.016448	-0.0257195	0.96535	0.994855	no
gene:SpnNT_02135	NA	Chromosome:2103705-2104959	110.58	110.58+peptide	OK	4.27385	4.11853	-0.0534076	-0.0831474	0.8834	0.994748	no
gene:SpnNT_02135	NA	Chromosome:2103705-2104959	ΔORF2	110.58+peptide	OK	4.2254	4.11853	-0.0369596	-0.0573529	0.9218	0.994748	no
gene:SpnNT_02135	NA	Chromosome:2103705-2104959	110.58	ΔORF2+peptide	OK	4.27385	4.1106	-0.0561889	-0.0862112	0.87975	0.994748	no
gene:SpnNT_02135	NA	Chromosome:2103705-2104959	ΔORF2	ΔORF2+peptide	OK	4.2254	4.1106	-0.0397408	-0.0607819	0.91625	0.994748	no
gene:SpnNT_02135	NA	Chromosome:2103705-2104959	110.58+peptide	ΔORF2+peptide	OK	4.11853	4.1106	-0.00278129	-0.00423607	0.9896	0.997703	no
gene:SpnNT_02136	SpnNT_02136	Chromosome:2105099-2105185	110.58	ΔORF2	OK	482.92	230.415	-1.06755	-47.2324	0.34325	0.847277	no
gene:SpnNT_02136	SpnNT_02136	Chromosome:2105099-2105185	110.58	110.58+peptide	OK	482.92	515.072	0.0929891	0.0653061	0.889	0.994748	no
gene:SpnNT_02136	SpnNT_02136	Chromosome:2105099-2105185	ΔORF2	110.58+peptide	OK	230.415	515.072	1.16054	0.815024	0.31715	0.823011	no
gene:SpnNT_02136	SpnNT_02136	Chromosome:2105099-2105185	110.58	ΔORF2+peptide	OK	482.92	359.926	-0.424083	-19.849	0.68535	0.988184	no
gene:SpnNT_02136	SpnNT_02136	Chromosome:2105099-2105185	ΔORF2	ΔORF2+peptide	OK	230.415	359.926	0.643464	27.3831	0.47315	0.928984	no
gene:SpnNT_02136	SpnNT_02136	Chromosome:2105099-2105185	110.58+peptide	ΔORF2+peptide	OK	515.072	359.926	-0.517072	-0.363135	0.639	0.980887	no
gene:SpnNT_02137	SpnNT_02137	Chromosome:2105192-2105266	110.58	ΔORF2	OK	6534.65	8042.7	0.299571	0.294767	0.60005	0.976542	no
gene:SpnNT_02137	SpnNT_02137	Chromosome:2105192-2105266	110.58	110.58+peptide	OK	6534.65	6273.95	-0.0587349	-0.0556716	0.9325	0.994855	no
gene:SpnNT_02137	SpnNT_02137	Chromosome:2105192-2105266	ΔORF2	110.58+peptide	OK	8042.7	6273.95	-0.358306	-0.350805	0.55135	0.964385	no
gene:SpnNT_02137	SpnNT_02137	Chromosome:2105192-2105266	110.58	ΔORF2+peptide	OK	6534.65	2867.3	-1.18842	-1.60044	0.12805	0.542797	no
gene:SpnNT_02137	SpnNT_02137	Chromosome:2105192-2105266	ΔORF2	ΔORF2+peptide	OK	8042.7	2867.3	-1.48799	-2.14431	0.064	0.367209	no
gene:SpnNT_02137	SpnNT_02137	Chromosome:2105192-2105266	110.58+peptide	ΔORF2+peptide	OK	6273.95	2867.3	-1.12968	-1.50725	0.15505	0.600007	no
gene:SpnNT_02138	SpnNT_02138	Chromosome:2105277-2105352	110.58	ΔORF2	OK	552.078	881.874	0.675699	0.336693	0.54435	0.961568	no
gene:SpnNT_02138	SpnNT_02138	Chromosome:2105277-2105352	110.58	110.58+peptide	OK	552.078	3771.06	2.77203	3.27004	0.25185	0.749892	no

gene:SpnNT_02138	SpnNT_02138	Chromosome:2105277-2105352	ΔORF2	110.58+peptide	OK	881.874	3771.06	2.09633	0.962266	0.0248	0.196158	no
gene:SpnNT_02138	SpnNT_02138	Chromosome:2105277-2105352	110.58	ΔORF2+peptide	OK	552.078	861.14	0.641375	72.9171	0.66295	0.982966	no
gene:SpnNT_02138	SpnNT_02138	Chromosome:2105277-2105352	ΔORF2	ΔORF2+peptide	OK	881.874	861.14	-0.0343241	-0.0171034	0.819	0.994748	no
gene:SpnNT_02138	SpnNT_02138	Chromosome:2105277-2105352	110.58+peptide	ΔORF2+peptide	OK	3771.06	861.14	-2.13065	-2.51354	0.2418	0.736324	no
gene:SpnNT_02139	SpnNT_02139	Chromosome:2105364-2105437	110.58	ΔORF2	OK	6039.26	7770.51	0.363636	0.345619	0.54025	0.961372	no
gene:SpnNT_02139	SpnNT_02139	Chromosome:2105364-2105437	110.58	110.58+peptide	OK	6039.26	14642.1	1.27768	1.26747	0.02905	0.217452	no
gene:SpnNT_02139	SpnNT_02139	Chromosome:2105364-2105437	ΔORF2	110.58+peptide	OK	7770.51	14642.1	0.914046	0.962	0.1021	0.484541	no
gene:SpnNT_02139	SpnNT_02139	Chromosome:2105364-2105437	110.58	ΔORF2+peptide	OK	6039.26	17766.2	1.55669	1.59729	0.0096	0.0977279	no
gene:SpnNT_02139	SpnNT_02139	Chromosome:2105364-2105437	ΔORF2	ΔORF2+peptide	OK	7770.51	17766.2	1.19306	1.30452	0.03585	0.251833	no
gene:SpnNT_02139	SpnNT_02139	Chromosome:2105364-2105437	110.58+peptide	ΔORF2+peptide	OK	14642.1	17766.2	0.27901	0.323119	0.58665	0.973049	no
gene:SpnNT_02140	SpnNT_02140	Chromosome:2105439-2105522	110.58	ΔORF2	OK	1466.87	1069.89	-0.455283	-0.310032	0.57025	0.968621	no
gene:SpnNT_02140	SpnNT_02140	Chromosome:2105439-2105522	110.58	110.58+peptide	OK	1466.87	2701.7	0.881127	0.690696	0.20085	0.683494	no
gene:SpnNT_02140	SpnNT_02140	Chromosome:2105439-2105522	ΔORF2	110.58+peptide	OK	1069.89	2701.7	1.33641	0.958347	0.0893	0.449387	no
gene:SpnNT_02140	SpnNT_02140	Chromosome:2105439-2105522	110.58	ΔORF2+peptide	OK	1466.87	314.581	-2.22124	-2.31617	0.24325	0.739024	no
gene:SpnNT_02140	SpnNT_02140	Chromosome:2105439-2105522	ΔORF2	ΔORF2+peptide	OK	1069.89	314.581	-1.76595	-1.58787	0.28285	0.785349	no
gene:SpnNT_02140	SpnNT_02140	Chromosome:2105439-2105522	110.58+peptide	ΔORF2+peptide	OK	2701.7	314.581	-3.10237	-3.68751	0.22825	0.720148	no
gene:SpnNT_02141	SpnNT_02141	Chromosome:2105533-2105606	110.58	ΔORF2	OK	0	723.607	Inf	NA	0.1261	0.537759	no
gene:SpnNT_02141	SpnNT_02141	Chromosome:2105533-2105606	110.58	110.58+peptide	OK	0	2039.46	Inf	NA	0.00205	0.0300896	yes
gene:SpnNT_02141	SpnNT_02141	Chromosome:2105533-2105606	ΔORF2	110.58+peptide	OK	723.607	2039.46	1.49491	1.25148	0.3549	0.857011	no
gene:SpnNT_02141	SpnNT_02141	Chromosome:2105533-2105606	110.58	ΔORF2+peptide	OK	0	1605.94	Inf	NA	0.0041	0.0516317	no
gene:SpnNT_02141	SpnNT_02141	Chromosome:2105533-2105606	ΔORF2	ΔORF2+peptide	OK	723.607	1605.94	1.15014	138.085	0.4337	0.913361	no
gene:SpnNT_02141	SpnNT_02141	Chromosome:2105533-2105606	110.58+peptide	ΔORF2+peptide	OK	2039.46	1605.94	-0.344773	-0.288632	0.72185	0.994748	no
gene:SpnNT_02142	SpnNT_02142	Chromosome:2105609-2105683	110.58	ΔORF2	NOTEST	200.052	329.265	0.718878	0	1	1	no
gene:SpnNT_02142	SpnNT_02142	Chromosome:2105609-2105683	110.58	110.58+peptide	OK	200.052	1025.85	2.35837	176.082	0.19355	0.674905	no
gene:SpnNT_02142	SpnNT_02142	Chromosome:2105609-2105683	ΔORF2	110.58+peptide	OK	329.265	1025.85	1.63949	170.401	0.30075	0.806713	no
gene:SpnNT_02142	SpnNT_02142	Chromosome:2105609-2105683	110.58	ΔORF2+peptide	NOTEST	200.052	633.559	1.66311	0	1	1	no
gene:SpnNT_02142	SpnNT_02142	Chromosome:2105609-2105683	ΔORF2	ΔORF2+peptide	NOTEST	329.265	633.559	0.944228	0	1	1	no
gene:SpnNT_02142	SpnNT_02142	Chromosome:2105609-2105683	110.58+peptide	ΔORF2+peptide	OK	1025.85	633.559	-0.695264	-93.3466	0.63835	0.980887	no
gene:SpnNT_02143	SpnNT_02143	Chromosome:2105693-2105783	110.58	ΔORF2	OK	5627.91	7633.41	0.439727	0.628899	0.2671	0.768422	no
gene:SpnNT_02143	SpnNT_02143	Chromosome:2105693-2105783	110.58	110.58+peptide	OK	5627.91	10488.2	0.898098	1.23918	0.0248	0.196158	no
gene:SpnNT_02143	SpnNT_02143	Chromosome:2105693-2105783	ΔORF2	110.58+peptide	OK	7633.41	10488.2	0.458371	0.673353	0.2353	0.729189	no
gene:SpnNT_02143	SpnNT_02143	Chromosome:2105693-2105783	110.58	ΔORF2+peptide	OK	5627.91	14308.7	1.34622	2.0703	7.00E-04	0.012687	yes
gene:SpnNT_02143	SpnNT_02143	Chromosome:2105693-2105783	ΔORF2	ΔORF2+peptide	OK	7633.41	14308.7	0.906491	1.50881	0.01835	0.155749	no
gene:SpnNT_02143	SpnNT_02143	Chromosome:2105693-2105783	110.58+peptide	ΔORF2+peptide	OK	10488.2	14308.7	0.448121	0.710911	0.23405	0.72774	no
gene:SpnNT_02144	SpnNT_02144	Chromosome:2105864-2105940	110.58	ΔORF2	OK	1639.3	1873.61	0.192737	0.151534	0.828	0.994748	no
gene:SpnNT_02144	SpnNT_02144	Chromosome:2105864-2105940	110.58	110.58+peptide	OK	1639.3	4149.01	1.33968	0.824781	0.10465	0.490961	no
gene:SpnNT_02144	SpnNT_02144	Chromosome:2105864-2105940	ΔORF2	110.58+peptide	OK	1873.61	4149.01	1.14695	1.1353	0.1618	0.61469	no
gene:SpnNT_02144	SpnNT_02144	Chromosome:2105864-2105940	110.58	ΔORF2+peptide	OK	1639.3	1263.71	-0.37542	-0.295162	0.7029	0.990367	no
gene:SpnNT_02144	SpnNT_02144	Chromosome:2105864-2105940	ΔORF2	ΔORF2+peptide	OK	1873.61	1263.71	-0.568157	-71.698	0.5923	0.975468	no
gene:SpnNT_02144	SpnNT_02144	Chromosome:2105864-2105940	110.58+peptide	ΔORF2+peptide	OK	4149.01	1263.71	-1.7151	-1.69767	0.07805	0.414981	no
gene:SpnNT_02145	SpnNT_02145	Chromosome:2105972-2106045	110.58	ΔORF2	OK	5461.4	3763.42	-0.537225	-0.395877	0.4682	0.927504	no
gene:SpnNT_02145	SpnNT_02145	Chromosome:2105972-2106045	110.58	110.58+peptide	OK	5461.4	3047.96	-0.841428	-0.646094	0.25405	0.752855	no
gene:SpnNT_02145	SpnNT_02145	Chromosome:2105972-2106045	ΔORF2	110.58+peptide	OK	3763.42	3047.96	-0.304203	-0.201687	0.71705	0.992452	no
gene:SpnNT_02145	SpnNT_02145	Chromosome:2105972-2106045	110.58	ΔORF2+peptide	OK	5461.4	5969.11	0.128246	0.1614	0.8438	0.994748	no
gene:SpnNT_02145	SpnNT_02145	Chromosome:2105972-2106045	ΔORF2	ΔORF2+peptide	OK	3763.42	5969.11	0.665471	0.604913	0.38945	0.883687	no

gene:SpnNT_02145	SpnNT_02145	Chromosome:2105972-2106045	110.58+peptide	ΔORF2+peptide	OK	3047.96	5969.11	0.969674	0.93974	0.2131	0.698345	no
gene:SpnNT_02146	SpnNT_02146	Chromosome:2106049-2106122	110.58	ΔORF2	OK	612.652	2850.19	2.21792	1.93131	0.2397	0.735065	no
gene:SpnNT_02146	SpnNT_02146	Chromosome:2106049-2106122	110.58	110.58+peptide	OK	612.652	5750.37	3.23051	3.52978	0.2264	0.717936	no
gene:SpnNT_02146	SpnNT_02146	Chromosome:2106049-2106122	ΔORF2	110.58+peptide	OK	2850.19	5750.37	1.01259	0.68957	0.17595	0.639561	no
gene:SpnNT_02146	SpnNT_02146	Chromosome:2106049-2106122	110.58	ΔORF2+peptide	OK	612.652	4634.67	2.91933	361.185	0.231	0.72461	no
gene:SpnNT_02146	SpnNT_02146	Chromosome:2106049-2106122	ΔORF2	ΔORF2+peptide	OK	2850.19	4634.67	0.701409	0.610783	0.42625	0.908545	no
gene:SpnNT_02146	SpnNT_02146	Chromosome:2106049-2106122	110.58+peptide	ΔORF2+peptide	OK	5750.37	4634.67	-0.311186	-0.340024	0.684	0.98747	no
gene:SpnNT_02147	SpnNT_02147	Chromosome:2106127-2106239	110.58	ΔORF2	OK	4884850	5152900	0.0770703	0.122821	0.82895	0.994748	no
gene:SpnNT_02147	SpnNT_02147	Chromosome:2106127-2106239	110.58	110.58+peptide	OK	4884850	5865700	0.263989	0.424856	0.45005	0.920586	no
gene:SpnNT_02147	SpnNT_02147	Chromosome:2106127-2106239	ΔORF2	110.58+peptide	OK	5152900	5865700	0.186919	0.302752	0.58825	0.973845	no
gene:SpnNT_02147	SpnNT_02147	Chromosome:2106127-2106239	110.58	ΔORF2+peptide	OK	4884850	6858090	0.489491	0.829196	0.1625	0.616279	no
gene:SpnNT_02147	SpnNT_02147	Chromosome:2106127-2106239	ΔORF2	ΔORF2+peptide	OK	5152900	6858090	0.412421	0.703612	0.23145	0.724662	no
gene:SpnNT_02147	SpnNT_02147	Chromosome:2106127-2106239	110.58+peptide	ΔORF2+peptide	OK	5865700	6858090	0.225502	0.389083	0.50285	0.944788	no
gene:SpnNT_02148	SpnNT_02148	Chromosome:2106320-2109219	110.58	ΔORF2	HIDATA	0	0	0	0	1	1	no
gene:SpnNT_02148	SpnNT_02148	Chromosome:2106320-2109219	110.58	110.58+peptide	HIDATA	0	0	0	0	1	1	no
gene:SpnNT_02148	SpnNT_02148	Chromosome:2106320-2109219	ΔORF2	110.58+peptide	HIDATA	0	0	0	0	1	1	no
gene:SpnNT_02148	SpnNT_02148	Chromosome:2106320-2109219	110.58	ΔORF2+peptide	HIDATA	0	0	0	0	1	1	no
gene:SpnNT_02148	SpnNT_02148	Chromosome:2106320-2109219	ΔORF2	ΔORF2+peptide	HIDATA	0	0	0	0	1	1	no
gene:SpnNT_02148	SpnNT_02148	Chromosome:2106320-2109219	110.58+peptide	ΔORF2+peptide	HIDATA	0	0	0	0	1	1	no
gene:SpnNT_02149	SpnNT_02149	Chromosome:2109343-2109418	110.58	ΔORF2	OK	2494.72	1476.78	-0.756421	-0.750737	0.40285	0.894399	no
gene:SpnNT_02149	SpnNT_02149	Chromosome:2109343-2109418	110.58	110.58+peptide	OK	2494.72	1471.25	-0.761842	-0.495504	0.4034	0.89446	no
gene:SpnNT_02149	SpnNT_02149	Chromosome:2109343-2109418	ΔORF2	110.58+peptide	OK	1476.78	1471.25	-0.00542062	-0.00466742	0.88005	0.994748	no
gene:SpnNT_02149	SpnNT_02149	Chromosome:2109343-2109418	110.58	ΔORF2+peptide	OK	2494.72	6393.7	1.35777	1.18672	0.11125	0.507941	no
gene:SpnNT_02149	SpnNT_02149	Chromosome:2109343-2109418	ΔORF2	ΔORF2+peptide	OK	1476.78	6393.7	2.11419	3.89975	0.04855	0.307914	no
gene:SpnNT_02149	SpnNT_02149	Chromosome:2109343-2109418	110.58+peptide	ΔORF2+peptide	OK	1471.25	6393.7	2.11961	1.65381	0.05005	0.313937	no
gene:SpnNT_02150	SpnNT_02150	Chromosome:2109472-2111009	110.58	ΔORF2	HIDATA	0	0	0	0	1	1	no
gene:SpnNT_02150	SpnNT_02150	Chromosome:2109472-2111009	110.58	110.58+peptide	HIDATA	0	0	0	0	1	1	no
gene:SpnNT_02150	SpnNT_02150	Chromosome:2109472-2111009	ΔORF2	110.58+peptide	HIDATA	0	0	0	0	1	1	no
gene:SpnNT_02150	SpnNT_02150	Chromosome:2109472-2111009	110.58	ΔORF2+peptide	HIDATA	0	0	0	0	1	1	no
gene:SpnNT_02150	SpnNT_02150	Chromosome:2109472-2111009	ΔORF2	ΔORF2+peptide	HIDATA	0	0	0	0	1	1	no
gene:SpnNT_02150	SpnNT_02150	Chromosome:2109472-2111009	110.58+peptide	ΔORF2+peptide	HIDATA	0	0	0	0	1	1	no
gene:SpnNT_02151	SpnNT_02151	Chromosome:2111265-2111337	110.58	ΔORF2	OK	1127.27	1003.52	-0.167761	-0.0945362	0.5664	0.968621	no
gene:SpnNT_02151	SpnNT_02151	Chromosome:2111265-2111337	110.58	110.58+peptide	OK	1127.27	907.608	-0.31269	-0.176207	0.7106	0.991007	no
gene:SpnNT_02151	SpnNT_02151	Chromosome:2111265-2111337	ΔORF2	110.58+peptide	OK	1003.52	907.608	-0.144929	-16.8149	0.44245	0.917631	no
gene:SpnNT_02151	SpnNT_02151	Chromosome:2111265-2111337	110.58	ΔORF2+peptide	OK	1127.27	974.205	-0.210533	-0.11864	0.8605	0.994748	no
gene:SpnNT_02151	SpnNT_02151	Chromosome:2111265-2111337	ΔORF2	ΔORF2+peptide	OK	1003.52	974.205	-0.0427721	-5.32583	0.5825	0.970941	no
gene:SpnNT_02151	SpnNT_02151	Chromosome:2111265-2111337	110.58+peptide	ΔORF2+peptide	OK	907.608	974.205	0.102157	14.9698	0.73985	0.994748	no
gene:SpnNT_02152	NA	Chromosome:2111562-2113296	110.58	ΔORF2	OK	23.6924	25.9272	0.130047	0.268959	0.63495	0.980887	no
gene:SpnNT_02152	NA	Chromosome:2111562-2113296	110.58	110.58+peptide	OK	23.6924	23.123	-0.0350937	-0.0718423	0.9008	0.994748	no
gene:SpnNT_02152	NA	Chromosome:2111562-2113296	ΔORF2	110.58+peptide	OK	25.9272	23.123	-0.16514	-0.338663	0.5608	0.968621	no
gene:SpnNT_02152	NA	Chromosome:2111562-2113296	110.58	ΔORF2+peptide	OK	23.6924	31.314	0.402386	0.839594	0.1358	0.560574	no
gene:SpnNT_02152	NA	Chromosome:2111562-2113296	ΔORF2	ΔORF2+peptide	OK	25.9272	31.314	0.272339	0.569284	0.31315	0.819209	no
gene:SpnNT_02152	NA	Chromosome:2111562-2113296	110.58+peptide	ΔORF2+peptide	OK	23.123	31.314	0.437479	0.904996	0.11425	0.512718	no
gene:SpnNT_02153	NA	Chromosome:2113487-2113922	110.58	ΔORF2	OK	276.193	297.199	0.105755	0.233727	0.684	0.98747	no
gene:SpnNT_02153	NA	Chromosome:2113487-2113922	110.58	110.58+peptide	OK	276.193	232	-0.25155	-0.552968	0.3417	0.845838	no

gene:SpnNT_02153	NA	Chromosome:2113487-2113922	ΔORF2	110.58+peptide	OK	297.199	232	-0.357305	-0.78426	0.1788	0.645815	no
gene:SpnNT_02153	NA	Chromosome:2113487-2113922	110.58	ΔORF2+peptide	OK	276.193	325.068	0.235065	0.520815	0.3682	0.865399	no
gene:SpnNT_02153	NA	Chromosome:2113487-2113922	ΔORF2	ΔORF2+peptide	OK	297.199	325.068	0.12931	0.286064	0.61985	0.980887	no
gene:SpnNT_02153	NA	Chromosome:2113487-2113922	110.58+peptide	ΔORF2+peptide	OK	232	325.068	0.486615	1.07072	0.0684	0.383191	no
gene:SpnNT_02154	gltX	Chromosome:2113935-2115396	110.58	ΔORF2	OK	164.765	163.185	-0.0139002	-0.031985	0.95295	0.994855	no
gene:SpnNT_02154	gltX	Chromosome:2113935-2115396	110.58	110.58+peptide	OK	164.765	142.199	-0.212499	-0.490278	0.38975	0.884062	no
gene:SpnNT_02154	gltX	Chromosome:2113935-2115396	ΔORF2	110.58+peptide	OK	163.185	142.199	-0.198599	-0.458009	0.4234	0.906634	no
gene:SpnNT_02154	gltX	Chromosome:2113935-2115396	110.58	ΔORF2+peptide	OK	164.765	152.436	-0.112206	-0.258731	0.6437	0.980887	no
gene:SpnNT_02154	gltX	Chromosome:2113935-2115396	ΔORF2	ΔORF2+peptide	OK	163.185	152.436	-0.0983055	-0.226581	0.6877	0.98828	no
gene:SpnNT_02154	gltX	Chromosome:2113935-2115396	110.58+peptide	ΔORF2+peptide	OK	142.199	152.436	0.100294	0.231783	0.684	0.98747	no
gene:SpnNT_02155	pgi	Chromosome:2115519-2116869	110.58	ΔORF2	OK	590.738	617.676	0.0643301	0.1434	0.80595	0.994748	no
gene:SpnNT_02155	pgi	Chromosome:2115519-2116869	110.58	110.58+peptide	OK	590.738	642.555	0.121301	0.270284	0.6379	0.980887	no
gene:SpnNT_02155	pgi	Chromosome:2115519-2116869	ΔORF2	110.58+peptide	OK	617.676	642.555	0.0569714	0.126985	0.8256	0.994748	no
gene:SpnNT_02155	pgi	Chromosome:2115519-2116869	110.58	ΔORF2+peptide	OK	590.738	662.449	0.165289	0.368234	0.52025	0.954744	no
gene:SpnNT_02155	pgi	Chromosome:2115519-2116869	ΔORF2	ΔORF2+peptide	OK	617.676	662.449	0.100959	0.224993	0.6985	0.990209	no
gene:SpnNT_02155	pgi	Chromosome:2115519-2116869	110.58+peptide	ΔORF2+peptide	OK	642.555	662.449	0.0439878	0.0979883	0.86365	0.994748	no
gene:SpnNT_02156	NA	Chromosome:2117038-2117728	110.58	ΔORF2	OK	15.8551	14.478	-0.131086	-0.221006	0.69815	0.990209	no
gene:SpnNT_02156	NA	Chromosome:2117038-2117728	110.58	110.58+peptide	OK	15.8551	14.0081	-0.178688	-0.300408	0.5981	0.97629	no
gene:SpnNT_02156	NA	Chromosome:2117038-2117728	ΔORF2	110.58+peptide	OK	14.478	14.0081	-0.0476028	-0.0796437	0.88625	0.994748	no
gene:SpnNT_02156	NA	Chromosome:2117038-2117728	110.58	ΔORF2+peptide	OK	15.8551	16.076	0.019954	0.0338142	0.95365	0.994855	no
gene:SpnNT_02156	NA	Chromosome:2117038-2117728	ΔORF2	ΔORF2+peptide	OK	14.478	16.076	0.15104	0.2547	0.64805	0.981391	no
gene:SpnNT_02156	NA	Chromosome:2117038-2117728	110.58+peptide	ΔORF2+peptide	OK	14.0081	16.076	0.198642	0.334023	0.56025	0.968621	no
gene:SpnNT_02157	NA	Chromosome:2117866-2119633	110.58	ΔORF2	OK	371.005	354.495	-0.0656718	-0.146134	0.79905	0.994748	no
gene:SpnNT_02157	NA	Chromosome:2117866-2119633	110.58	110.58+peptide	OK	371.005	268.303	-0.467577	-1.03283	0.07125	0.391681	no
gene:SpnNT_02157	NA	Chromosome:2117866-2119633	ΔORF2	110.58+peptide	OK	354.495	268.303	-0.401905	-0.887828	0.1177	0.519495	no
gene:SpnNT_02157	NA	Chromosome:2117866-2119633	110.58	ΔORF2+peptide	OK	371.005	212.961	-0.800849	-1.80049	0.0021	0.0307204	yes
gene:SpnNT_02157	NA	Chromosome:2117866-2119633	ΔORF2	ΔORF2+peptide	OK	354.495	212.961	-0.735177	-1.65295	0.00435	0.0540026	no
gene:SpnNT_02157	NA	Chromosome:2117866-2119633	110.58+peptide	ΔORF2+peptide	OK	268.303	212.961	-0.333272	-0.743716	0.19375	0.674905	no
gene:SpnNT_02158	NA	Chromosome:2119883-2120093	110.58	ΔORF2	OK	6.00392	7.96422	0.407629	0.304007	0.60965	0.979051	no
gene:SpnNT_02158	NA	Chromosome:2119883-2120093	110.58	110.58+peptide	OK	6.00392	5.13436	-0.22572	-0.161545	0.77535	0.994748	no
gene:SpnNT_02158	NA	Chromosome:2119883-2120093	ΔORF2	110.58+peptide	OK	7.96422	5.13436	-0.633348	-0.457662	0.4326	0.913334	no
gene:SpnNT_02158	NA	Chromosome:2119883-2120093	110.58	ΔORF2+peptide	OK	6.00392	1.28856	-2.22014	-1.96988	0.24785	0.74404	no
gene:SpnNT_02158	NA	Chromosome:2119883-2120093	ΔORF2	ΔORF2+peptide	OK	7.96422	1.28856	-2.62777	-2.36649	0.2306	0.723891	no
gene:SpnNT_02158	NA	Chromosome:2119883-2120093	110.58+peptide	ΔORF2+peptide	OK	5.13436	1.28856	-1.99442	-1.69319	0.2793	0.782112	no
gene:SpnNT_02159	NA	Chromosome:2120421-2122116	110.58	ΔORF2	OK	266.424	253.505	-0.0717095	-0.160127	0.7734	0.994748	no
gene:SpnNT_02159	NA	Chromosome:2120421-2122116	110.58	110.58+peptide	OK	266.424	167.743	-0.667477	-1.47876	0.01	0.100168	no
gene:SpnNT_02159	NA	Chromosome:2120421-2122116	ΔORF2	110.58+peptide	OK	253.505	167.743	-0.595767	-1.32886	0.01825	0.155101	no
gene:SpnNT_02159	NA	Chromosome:2120421-2122116	110.58	ΔORF2+peptide	OK	266.424	163.99	-0.700116	-1.58006	0.0052	0.0621443	no
gene:SpnNT_02159	NA	Chromosome:2120421-2122116	ΔORF2	ΔORF2+peptide	OK	253.505	163.99	-0.628407	-1.42823	0.01105	0.107885	no
gene:SpnNT_02159	NA	Chromosome:2120421-2122116	110.58+peptide	ΔORF2+peptide	OK	167.743	163.99	-0.0326396	-0.0735789	0.8975	0.994748	no
gene:SpnNT_02160	mutS	Chromosome:2122262-2124797	110.58	ΔORF2	OK	232.098	198.585	-0.22498	-0.50031	0.38595	0.881745	no
gene:SpnNT_02160	mutS	Chromosome:2122262-2124797	110.58	110.58+peptide	OK	232.098	223.183	-0.0565093	-0.125467	0.8299	0.994748	no
gene:SpnNT_02160	mutS	Chromosome:2122262-2124797	ΔORF2	110.58+peptide	OK	198.585	223.183	0.168471	0.37332	0.51575	0.952522	no
gene:SpnNT_02160	mutS	Chromosome:2122262-2124797	110.58	ΔORF2+peptide	OK	232.098	204.246	-0.18443	-0.414064	0.4714	0.928525	no
gene:SpnNT_02160	mutS	Chromosome:2122262-2124797	ΔORF2	ΔORF2+peptide	OK	198.585	204.246	0.0405509	0.0908584	0.8725	0.994748	no

gene:SpnNT_02160	mutS	Chromosome:2122262-2124797	110.58+peptide	ΔORF2+peptide	OK	223.183	204.246	-0.12792	-0.28616	0.61555	0.979616	no
gene:SpnNT_02161	argR_3	Chromosome:2124847-2125294	110.58	ΔORF2	OK	602.096	778.166	0.370083	0.786471	0.1678	0.623936	no
gene:SpnNT_02161	argR_3	Chromosome:2124847-2125294	110.58	110.58+peptide	OK	602.096	847.899	0.493897	1.08622	0.05775	0.342262	no
gene:SpnNT_02161	argR_3	Chromosome:2124847-2125294	ΔORF2	110.58+peptide	OK	778.166	847.899	0.123814	0.263021	0.6452	0.980887	no
gene:SpnNT_02161	argR_3	Chromosome:2124847-2125294	110.58	ΔORF2+peptide	OK	602.096	903.475	0.585491	1.30279	0.0232	0.186653	no
gene:SpnNT_02161	argR_3	Chromosome:2124847-2125294	ΔORF2	ΔORF2+peptide	OK	778.166	903.475	0.215407	0.462605	0.41505	0.900659	no
gene:SpnNT_02161	argR_3	Chromosome:2124847-2125294	110.58+peptide	ΔORF2+peptide	OK	847.899	903.475	0.0915936	0.203723	0.7202	0.994053	no
gene:SpnNT_02162	argS	Chromosome:2125429-2127121	110.58	ΔORF2	OK	88.6047	97.1234	0.132435	0.299227	0.60045	0.976678	no
gene:SpnNT_02162	argS	Chromosome:2125429-2127121	110.58	110.58+peptide	OK	88.6047	71.9667	-0.300054	-0.669616	0.24435	0.740499	no
gene:SpnNT_02162	argS	Chromosome:2125429-2127121	ΔORF2	110.58+peptide	OK	97.1234	71.9667	-0.432489	-0.962229	0.09395	0.463158	no
gene:SpnNT_02162	argS	Chromosome:2125429-2127121	110.58	ΔORF2+peptide	OK	88.6047	85.904	-0.0446578	-0.101026	0.85785	0.994748	no
gene:SpnNT_02162	argS	Chromosome:2125429-2127121	ΔORF2	ΔORF2+peptide	OK	97.1234	85.904	-0.177093	-0.399373	0.4814	0.935095	no
gene:SpnNT_02162	argS	Chromosome:2125429-2127121	110.58+peptide	ΔORF2+peptide	OK	71.9667	85.904	0.255397	0.568907	0.3221	0.827899	no
gene:SpnNT_02163	NA	Chromosome:2127510-2127975	110.58	ΔORF2	OK	51.9297	53.4804	0.042453	0.0766669	0.8957	0.994748	no
gene:SpnNT_02163	NA	Chromosome:2127510-2127975	110.58	110.58+peptide	OK	51.9297	48.8181	-0.0891419	-0.161389	0.7852	0.994748	no
gene:SpnNT_02163	NA	Chromosome:2127510-2127975	ΔORF2	110.58+peptide	OK	53.4804	48.8181	-0.131595	-0.236744	0.68865	0.98828	no
gene:SpnNT_02163	NA	Chromosome:2127510-2127975	110.58	ΔORF2+peptide	OK	51.9297	52.7645	0.0230078	0.0416396	0.94225	0.994855	no
[truncated: 156,928 more chars]
