# Supplementary material for: Foreign peptide triggers boost in pneumococcal metabolism and growth
Source: BMC Microbiol. 2018 Mar 27;18:23. doi: 10.1186/s12866-018-1167-y (PMC5870813; doi:10.1186/s12866-018-1167-y)
Supplement: Supplementary file 3 — Table S2. RNA-Seq data for wild type with and without ORF 2 peptide. Table shows only significant changes in expression. A significant change in expression was observed for 210 genes of which 159 were upregulated by the ORF 2 peptide and 51 were downregulated by the peptide. (PDF 209 kb) [file 12866_2018_1167_MOESM3_ESM.pdf]

gene_id	gene_short_nam	locus	sample_1	sample_2	status	value_1	value_2	log2_fold_chan	test_stat	p_value	q_value	signif
gene:SpnNT_00049	mtcA1	Chromosome:37404-37902	110.58	110.58+peptide	OK	402.16	181.215	-1.15007	-2.43141	5.00E-05	0.0013612	yes
gene:SpnNT_00050	NA	Chromosome:37926-38742	110.58	110.58+peptide	OK	208.649	86.799	-1.26533	-2.72967	5.00E-05	0.0013612	yes
gene:SpnNT_00072	purC	Chromosome:59090-59798	110.58	110.58+peptide	OK	10.348	1.11288	-3.21699	-3.72326	5.00E-05	0.0013612	yes
gene:SpnNT_00073	purL	Chromosome:59999-63725	110.58	110.58+peptide	OK	17.7458	2.77224	-2.67836	-5.03976	5.00E-05	0.0013612	yes
gene:SpnNT_00074	purF	Chromosome:63817-65260	110.58	110.58+peptide	OK	16.8536	3.35618	-2.32816	-3.86726	5.00E-05	0.0013612	yes
gene:SpnNT_00075	purM	Chromosome:65296-66861	110.58	110.58+peptide	OK	20.8546	3.78042	-2.46375	-3.42677	5.00E-05	0.0013612	yes
gene:SpnNT_00078	purH	Chromosome:67478-69026	110.58	110.58+peptide	OK	44.911	10.4894	-2.09814	-4.05106	5.00E-05	0.0013612	yes
gene:SpnNT_00079	purD	Chromosome:69147-70410	110.58	110.58+peptide	OK	45.0527	8.84793	-2.3482	-4.40533	5.00E-05	0.0013612	yes
gene:SpnNT_00081	purK	Chromosome:70812-72379	110.58	110.58+peptide	OK	34.6546	6.52987	-2.40792	-3.77105	5.00E-05	0.0013612	yes
gene:SpnNT_00087	bga	Chromosome:79347-81608	110.58	110.58+peptide	OK	8.88371	28.9271	1.70319	2.38439	5.00E-05	0.0013612	yes
gene:SpnNT_00089	agaC_1	Chromosome:81635-83343	110.58	110.58+peptide	OK	6.69293	28.0236	2.06593	2.31094	5.00E-05	0.0013612	yes
gene:SpnNT_00091	manX_1	Chromosome:83349-83742	110.58	110.58+peptide	OK	9.18859	38.8093	2.07849	2.61476	5.00E-05	0.0013612	yes
gene:SpnNT_00092	agaS	Chromosome:84039-85206	110.58	110.58+peptide	OK	7.26321	30.5708	2.07348	3.3307	5.00E-05	0.0013612	yes
gene:SpnNT_00093	lytA_1	Chromosome:85504-86692	110.58	110.58+peptide	OK	6.42341	22.2158	1.79018	2.82361	5.00E-05	0.0013612	yes
gene:SpnNT_00099	deoD_1	Chromosome:96812-97577	110.58	110.58+peptide	OK	136.808	56.3856	-1.27876	-2.66644	5.00E-05	0.0013612	yes
gene:SpnNT_00108	rpsD	Chromosome:106058-106670	110.58	110.58+peptide	OK	1549.31	3497.97	1.17489	2.54089	5.00E-05	0.0013612	yes
gene:SpnNT_00112	NA	Chromosome:111742-112729	110.58	110.58+peptide	OK	412.141	970.812	1.23605	2.35608	5.00E-05	0.0013612	yes
gene:SpnNT_00175	NA	Chromosome:169980-171243	110.58	110.58+peptide	OK	73.5206	285.471	1.95712	4.02675	5.00E-05	0.0013612	yes
gene:SpnNT_00186	mccF	Chromosome:180722-181754	110.58	110.58+peptide	OK	65.9014	29.602	-1.15462	-2.38305	5.00E-05	0.0013612	yes
gene:SpnNT_00238	NA	Chromosome:217561-218899	110.58	110.58+peptide	OK	193.78	79.3568	-1.288	-2.89832	5.00E-05	0.0013612	yes
gene:SpnNT_00288	manZ_2	Chromosome:277000-277912	110.58	110.58+peptide	OK	388.93	918.111	1.23916	2.79625	5.00E-05	0.0013612	yes
gene:SpnNT_00289	manY	Chromosome:277935-278739	110.58	110.58+peptide	OK	248.599	556.784	1.1633	2.65157	5.00E-05	0.0013612	yes
gene:SpnNT_00290	manX_2	Chromosome:278766-279756	110.58	110.58+peptide	OK	248.993	583.152	1.22777	2.79259	5.00E-05	0.0013612	yes
gene:SpnNT_00293	pbuO	Chromosome:282348-283767	110.58	110.58+peptide	OK	151.235	46.415	-1.70413	-3.78017	5.00E-05	0.0013612	yes
gene:SpnNT_00294	NA	Chromosome:283819-284527	110.58	110.58+peptide	OK	19.2161	5.35942	-1.84217	-2.88877	5.00E-05	0.0013612	yes
gene:SpnNT_00301	rpsI	Chromosome:289496-289889	110.58	110.58+peptide	OK	2354.32	5131.41	1.12405	2.44871	5.00E-05	0.0013612	yes
gene:SpnNT_00332	clpC_1	Chromosome:316451-318557	110.58	110.58+peptide	OK	215.75	557.608	1.36989	2.46591	5.00E-05	0.0013612	yes
gene:SpnNT_00377	alsT	Chromosome:366821-368144	110.58	110.58+peptide	OK	26.1388	69.5722	1.41232	2.85478	5.00E-05	0.0013612	yes
gene:SpnNT_00415	rpmB	Chromosome:423743-423932	110.58	110.58+peptide	OK	4161.6	13394.7	1.68645	3.40749	5.00E-05	0.0013612	yes
gene:SpnNT_00466	hrcA	Chromosome:481476-482511	110.58	110.58+peptide	OK	368.212	944.415	1.35888	3.00978	5.00E-05	0.0013612	yes
gene:SpnNT_00467	grpE	Chromosome:482537-483062	110.58	110.58+peptide	OK	656.282	2919.89	2.15352	3.89856	5.00E-05	0.0013612	yes
gene:SpnNT_00573	rplA	Chromosome:594064-594754	110.58	110.58+peptide	OK	1017.05	2052.78	1.01319	2.22403	5.00E-05	0.0013612	yes
gene:SpnNT_00675	NA	Chromosome:727863-728208	110.58	110.58+peptide	OK	29.4116	101.479	1.78672	2.71493	5.00E-05	0.0013612	yes
gene:SpnNT_00689	pyrE	Chromosome:740845-741478	110.58	110.58+peptide	OK	29.4782	74.5554	1.33866	2.48934	5.00E-05	0.0013612	yes
gene:SpnNT_00690	NA	Chromosome:741710-742061	110.58	110.58+peptide	OK	26.3529	102.51	1.95974	2.98838	5.00E-05	0.0013612	yes
gene:SpnNT_00712	pox5	Chromosome:762226-764002	110.58	110.58+peptide	OK	501.471	1377.08	1.45738	3.07853	5.00E-05	0.0013612	yes
gene:SpnNT_00713	NA	Chromosome:764112-764460	110.58	110.58+peptide	OK	463.174	1456.29	1.65267	3.46962	5.00E-05	0.0013612	yes
gene:SpnNT_00732	braC	Chromosome:776627-777788	110.58	110.58+peptide	OK	152.651	394.455	1.36962	3.03792	5.00E-05	0.0013612	yes
gene:SpnNT_00733	livH_1	Chromosome:778055-778925	110.58	110.58+peptide	OK	47.0072	155.177	1.72296	3.56881	5.00E-05	0.0013612	yes
gene:SpnNT_00736	livF	Chromosome:778928-781359	110.58	110.58+peptide	OK	80.994	263.852	1.70384	2.22266	5.00E-05	0.0013612	yes
gene:SpnNT_00742	mapP	Chromosome:787672-788488	110.58	110.58+peptide	OK	17.2089	47.3627	1.46059	2.74554	5.00E-05	0.0013612	yes
gene:SpnNT_00755	NA	Chromosome:800842-801082	110.58	110.58+peptide	OK	1504.52	3434.57	1.19082	2.6103	5.00E-05	0.0013612	yes
gene:SpnNT_00793	NA	Chromosome:840349-841111	110.58	110.58+peptide	OK	28.1171	81.0592	1.52753	2.93295	5.00E-05	0.0013612	yes
gene:SpnNT_00794	glyQ	Chromosome:841303-842221	110.58	110.58+peptide	OK	56.7058	136.885	1.27139	2.69917	5.00E-05	0.0013612	yes

gene:SpnNT_00795	glyS	Chromosome:842480-844517	110.58	110.58+peptide	OK	71.6761	179.763	1.32653	2.97157	5.00E-05	0.0013612	yes
gene:SpnNT_00796	NA	Chromosome:844558-844816	110.58	110.58+peptide	OK	493.496	1763.59	1.83741	3.61296	5.00E-05	0.0013612	yes
gene:SpnNT_00853	rpsU	Chromosome:891009-891186	110.58	110.58+peptide	OK	5245.19	16486.3	1.6522	3.16128	5.00E-05	0.0013612	yes
gene:SpnNT_00873	glnH_2	Chromosome:907827-908643	110.58	110.58+peptide	OK	431.854	163.028	-1.40542	-3.13879	5.00E-05	0.0013612	yes
gene:SpnNT_00883	alaS	Chromosome:917808-920427	110.58	110.58+peptide	OK	69.1295	150.043	1.118	2.52603	5.00E-05	0.0013612	yes
gene:SpnNT_00910	rplJ	Chromosome:951105-951606	110.58	110.58+peptide	OK	330.379	961.294	1.54086	3.4987	5.00E-05	0.0013612	yes
gene:SpnNT_00911	rplL	Chromosome:951681-952186	110.58	110.58+peptide	OK	1743.95	4822.11	1.46731	3.09817	5.00E-05	0.0013612	yes
gene:SpnNT_00992	gdhA	Chromosome:1022113-1023460	110.58	110.58+peptide	OK	480.103	1679.11	1.80629	3.7587	5.00E-05	0.0013612	yes
gene:SpnNT_01021	pyrP	Chromosome:1044618-1045902	110.58	110.58+peptide	OK	48.1634	205.416	2.09254	4.59883	5.00E-05	0.0013612	yes
gene:SpnNT_01032	pyrR	Chromosome:1056577-1057099	110.58	110.58+peptide	OK	43.4849	169.244	1.96052	3.83957	5.00E-05	0.0013612	yes
gene:SpnNT_01033	pyrB	Chromosome:1057117-1058041	110.58	110.58+peptide	OK	54.8878	205.724	1.90615	4.10718	5.00E-05	0.0013612	yes
gene:SpnNT_01034	carA	Chromosome:1058090-1059170	110.58	110.58+peptide	OK	51.7615	183.712	1.82749	3.9601	5.00E-05	0.0013612	yes
gene:SpnNT_01035	carB	Chromosome:1059482-1062659	110.58	110.58+peptide	OK	51.943	175.052	1.75278	3.9137	5.00E-05	0.0013612	yes
gene:SpnNT_01079	fhs1	Chromosome:1108271-1109942	110.58	110.58+peptide	OK	167.968	45.4634	-1.88541	-4.1678	5.00E-05	0.0013612	yes
gene:SpnNT_01185	NA	Chromosome:1224162-1224507	110.58	110.58+peptide	OK	489.216	1127.88	1.20507	2.70217	5.00E-05	0.0013612	yes
gene:SpnNT_01186	rplU	Chromosome:1224522-1224837	110.58	110.58+peptide	OK	497.099	1133.64	1.18935	2.62461	5.00E-05	0.0013612	yes
gene:SpnNT_01255	queT	Chromosome:1293058-1293568	110.58	110.58+peptide	OK	76.3541	261.646	1.77684	3.71331	5.00E-05	0.0013612	yes
gene:SpnNT_01258	hemH	Chromosome:1294622-1295717	110.58	110.58+peptide	OK	8.01384	27.9902	1.80436	3.03475	5.00E-05	0.0013612	yes
gene:SpnNT_01267	yhdG	Chromosome:1307718-1309110	110.58	110.58+peptide	OK	151.693	76.7204	-0.983471	-2.18349	5.00E-05	0.0013612	yes
gene:SpnNT_01289	NA	Chromosome:1324644-1325358	110.58	110.58+peptide	OK	15.3587	44.1889	1.52463	2.75464	5.00E-05	0.0013612	yes
gene:SpnNT_01290	pepF1_1	Chromosome:1325359-1327162	110.58	110.58+peptide	OK	28.1247	73.9095	1.39392	3.05388	5.00E-05	0.0013612	yes
gene:SpnNT_01295	NA	Chromosome:1331968-1332202	110.58	110.58+peptide	OK	4956.51	10543.4	1.08894	2.23333	5.00E-05	0.0013612	yes
gene:SpnNT_01308	rplT	Chromosome:1345184-1345544	110.58	110.58+peptide	OK	2149.68	4917.87	1.19391	2.5585	5.00E-05	0.0013612	yes
gene:SpnNT_01310	infC	Chromosome:1345828-1346416	110.58	110.58+peptide	OK	185.44	499.628	1.4299	3.22458	5.00E-05	0.0013612	yes
gene:SpnNT_01352	ydaF_5	Chromosome:1391460-1392030	110.58	110.58+peptide	OK	162.249	67.4202	-1.26696	-2.58771	5.00E-05	0.0013612	yes
gene:SpnNT_01391	NA	Chromosome:1428017-1428713	110.58	110.58+peptide	OK	77.9114	21.3319	-1.86882	-3.32339	5.00E-05	0.0013612	yes
gene:SpnNT_01474	rpsT	Chromosome:1510832-1511069	110.58	110.58+peptide	OK	2626.2	6286.19	1.25921	2.79228	5.00E-05	0.0013612	yes
gene:SpnNT_01507	fliY	Chromosome:1542792-1543629	110.58	110.58+peptide	OK	366.202	118.564	-1.62698	-3.66647	5.00E-05	0.0013612	yes
gene:SpnNT_01508	glnQ_3	Chromosome:1543641-1544271	110.58	110.58+peptide	OK	288.811	83.7272	-1.78636	-3.8098	5.00E-05	0.0013612	yes
gene:SpnNT_01509	yecS_2	Chromosome:1544280-1544922	110.58	110.58+peptide	OK	211.6	51.9972	-2.02483	-4.14391	5.00E-05	0.0013612	yes
gene:SpnNT_01532	lmrA	Chromosome:1568158-1569589	110.58	110.58+peptide	OK	200.835	51.7142	-1.95738	-4.24422	5.00E-05	0.0013612	yes
gene:SpnNT_01534	sarA_5	Chromosome:1570011-1571970	110.58	110.58+peptide	OK	1132.49	437.484	-1.37219	-2.85131	5.00E-05	0.0013612	yes
gene:SpnNT_01537	NA	Chromosome:1575387-1575585	110.58	110.58+peptide	OK	2068.24	9578.11	2.21134	3.68634	5.00E-05	0.0013612	yes
gene:SpnNT_01548	rpsR	Chromosome:1582275-1582515	110.58	110.58+peptide	OK	2010.07	4559.14	1.18151	2.61354	5.00E-05	0.0013612	yes
gene:SpnNT_01549	ssb_1	Chromosome:1582546-1583017	110.58	110.58+peptide	OK	736.644	1989.34	1.43325	3.11065	5.00E-05	0.0013612	yes
gene:SpnNT_01550	rpsF	Chromosome:1583028-1583319	110.58	110.58+peptide	OK	1032.11	3008.46	1.54343	3.32732	5.00E-05	0.0013612	yes
gene:SpnNT_01660	yhjX	Chromosome:1663456-1664683	110.58	110.58+peptide	OK	91.0081	26.1712	-1.79801	-3.80155	5.00E-05	0.0013612	yes
gene:SpnNT_01689	thrS	Chromosome:1689816-1691760	110.58	110.58+peptide	OK	46.9044	117.489	1.32473	2.98323	5.00E-05	0.0013612	yes
gene:SpnNT_01732	bglK_1	Chromosome:1740306-1741191	110.58	110.58+peptide	OK	43.7883	159.813	1.86777	3.69834	5.00E-05	0.0013612	yes
gene:SpnNT_01733	nanA_2	Chromosome:1741208-1742126	110.58	110.58+peptide	OK	27.6617	70.0052	1.33957	2.62798	5.00E-05	0.0013612	yes
gene:SpnNT_01734	NA	Chromosome:1742294-1742939	110.58	110.58+peptide	OK	11.3009	37.4874	1.72997	2.75727	5.00E-05	0.0013612	yes
gene:SpnNT_01735	NA	Chromosome:1743225-1743570	110.58	110.58+peptide	OK	20.4733	70.6763	1.78748	2.54165	5.00E-05	0.0013612	yes
gene:SpnNT_01736	araQ_2	Chromosome:1743629-1744469	110.58	110.58+peptide	OK	17.266	44.7436	1.37375	2.46417	5.00E-05	0.0013612	yes
gene:SpnNT_01737	lacF_2	Chromosome:1744484-1745372	110.58	110.58+peptide	OK	16.4568	44.2138	1.42581	2.5639	5.00E-05	0.0013612	yes
gene:SpnNT_01738	yesO_1	Chromosome:1745602-1746931	110.58	110.58+peptide	OK	52.5236	334.428	2.67066	4.69466	5.00E-05	0.0013612	yes

gene:SpnNT_01741	nanE	Chromosome:1747995-1748694	110.58	110.58+peptide	OK	46.5412	129.382	1.47506	2.7862	5.00E-05	0.0013612	yes
gene:SpnNT_01742	afr	Chromosome:1748866-1749970	110.58	110.58+peptide	OK	13.1615	267.059	4.34277	6.67757	5.00E-05	0.0013612	yes
gene:SpnNT_01743	nanB	Chromosome:1749981-1752075	110.58	110.58+peptide	OK	7.6902	92.0018	3.58057	5.63211	5.00E-05	0.0013612	yes
gene:SpnNT_01744	ycjP	Chromosome:1752092-1753810	110.58	110.58+peptide	OK	10.3001	77.8059	2.91723	3.02256	5.00E-05	0.0013612	yes
gene:SpnNT_01745	ycjO	Chromosome:1752092-1753810	110.58	110.58+peptide	OK	11.848	89.9142	2.9239	3.45167	5.00E-05	0.0013612	yes
gene:SpnNT_01746	yesO_2	Chromosome:1753888-1755226	110.58	110.58+peptide	OK	14.8111	150.936	3.3492	5.26966	5.00E-05	0.0013612	yes
gene:SpnNT_01747	tabA	Chromosome:1755244-1755697	110.58	110.58+peptide	OK	13.3745	84.2924	2.65592	3.71246	5.00E-05	0.0013612	yes
gene:SpnNT_01751	nanA_3	Chromosome:1762152-1763166	110.58	110.58+peptide	OK	21.0527	113.375	2.42902	4.08935	5.00E-05	0.0013612	yes
gene:SpnNT_01752	nanA_4	Chromosome:1763233-1765036	110.58	110.58+peptide	OK	18.6605	66.5538	1.83453	3.39752	5.00E-05	0.0013612	yes
gene:SpnNT_01761	NA	Chromosome:1775846-1776134	110.58	110.58+peptide	OK	37.8374	123.277	1.70402	2.45386	5.00E-05	0.0013612	yes
gene:SpnNT_01840	NA	Chromosome:1847981-1849238	110.58	110.58+peptide	OK	8.91383	21.1463	1.24629	2.28095	5.00E-05	0.0013612	yes
gene:SpnNT_01866	NA	Chromosome:1871092-1871752	110.58	110.58+peptide	OK	9.45289	31.1104	1.71857	2.72946	5.00E-05	0.0013612	yes
gene:SpnNT_01884	paaI	Chromosome:1891709-1892114	110.58	110.58+peptide	OK	55.8486	139.188	1.31744	2.41864	5.00E-05	0.0013612	yes
gene:SpnNT_02014	asnA	Chromosome:2003362-2004355	110.58	110.58+peptide	OK	875.789	400.932	-1.12722	-2.54235	5.00E-05	0.0013612	yes
gene:SpnNT_02080	adhE	Chromosome:2056744-2059396	110.58	110.58+peptide	OK	140.1	576.489	2.04084	3.73814	5.00E-05	0.0013612	yes
gene:SpnNT_02191	malX_2	Chromosome:2158441-2159713	110.58	110.58+peptide	OK	46.9078	109.266	1.21994	2.6541	5.00E-05	0.0013612	yes
gene:SpnNT_02277	NA	Chromosome:2250554-2251103	110.58	110.58+peptide	OK	672.631	2404.55	1.83788	3.49749	5.00E-05	0.0013612	yes
gene:SpnNT_02285	tsf	Chromosome:2256639-2257680	110.58	110.58+peptide	OK	1013.59	2137.71	1.07659	2.3306	5.00E-05	0.0013612	yes
gene:SpnNT_00882	NA	Chromosome:917301-917787	110.58	110.58+peptide	OK	24.0687	61.3143	1.34907	2.37662	1.00E-04	0.0025332	yes
gene:SpnNT_00909	mtaD	Chromosome:949389-950856	110.58	110.58+peptide	OK	158.001	77.8727	-1.02075	-2.33226	1.00E-04	0.0025332	yes
gene:SpnNT_01184	rpmA	Chromosome:1223852-1224146	110.58	110.58+peptide	OK	4293.04	9441.48	1.13701	2.16613	1.00E-04	0.0025332	yes
gene:SpnNT_01677	galE_1	Chromosome:1677661-1679648	110.58	110.58+peptide	OK	460.32	214.207	-1.10363	-2.26399	1.00E-04	0.0025332	yes
gene:SpnNT_01740	malX_1	Chromosome:1747055-1747977	110.58	110.58+peptide	OK	37.3784	104.784	1.48714	2.1965	1.00E-04	0.0025332	yes
gene:SpnNT_01858	trpE	Chromosome:1861563-1863488	110.58	110.58+peptide	OK	40.9494	16.219	-1.33616	-2.35026	1.00E-04	0.0025332	yes
gene:SpnNT_02242	NA	Chromosome:2211026-2211926	110.58	110.58+peptide	OK	903.499	423.261	-1.09398	-2.44296	1.00E-04	0.0025332	yes
gene:SpnNT_00180	ribBA	Chromosome:175396-176602	110.58	110.58+peptide	OK	17.7309	39.3466	1.14997	2.23849	0.00015	0.00355289	yes
gene:SpnNT_00734	livH_2	Chromosome:778928-781359	110.58	110.58+peptide	OK	50.1429	171.942	1.77781	2.25877	0.00015	0.00355289	yes
gene:SpnNT_01483	rpiA	Chromosome:1518271-1518955	110.58	110.58+peptide	OK	79.9838	161.506	1.01381	2.18373	0.00015	0.00355289	yes
gene:SpnNT_02256	NA	Chromosome:2227043-2228453	110.58	110.58+peptide	OK	16.1381	36.3938	1.17322	2.30989	0.00015	0.00355289	yes
gene:SpnNT_01107	NA	Chromosome:1134891-1135368	110.58	110.58+peptide	OK	7.74417	22.5453	1.54165	2.0868	2.00E-04	0.00450928	yes
gene:SpnNT_02299	recF	Chromosome:2270375-2271473	110.58	110.58+peptide	OK	27.5876	56.684	1.03892	2.12214	2.00E-04	0.00450928	yes
gene:SpnNT_01109	gatC_2	Chromosome:1135759-1136158	110.58	110.58+peptide	OK	6.21124	20.9831	1.75628	2.24944	0.00025	0.00542231	yes
gene:SpnNT_01307	gloA	Chromosome:1344745-1345126	110.58	110.58+peptide	OK	686.521	1352.23	0.977968	2.05785	0.00025	0.00542231	yes
gene:SpnNT_01762	ytrB	Chromosome:1776144-1776795	110.58	110.58+peptide	OK	18.7608	44.0443	1.23123	2.11285	0.00025	0.00542231	yes
gene:SpnNT_01944	SpnNT_01944	Chromosome:1942830-1942914	110.58	110.58+peptide	OK	3648.18	16616.8	2.18739	2.47626	0.00025	0.00542231	yes
gene:SpnNT_00090	manZ_1	Chromosome:81635-83343	110.58	110.58+peptide	OK	8.43764	31.8994	1.91862	2.22249	3.00E-04	0.00631878	yes
gene:SpnNT_00409	gutB	Chromosome:416594-417638	110.58	110.58+peptide	OK	15.5773	32.0804	1.04225	1.97382	3.00E-04	0.00631878	yes
gene:SpnNT_00737	NA	Chromosome:781666-782323	110.58	110.58+peptide	OK	49.8849	107.722	1.11064	2.26881	3.00E-04	0.00631878	yes
gene:SpnNT_01174	gapN	Chromosome:1206672-1210242	110.58	110.58+peptide	OK	227.797	71.7947	-1.6658	-2.18888	3.00E-04	0.00631878	yes
gene:SpnNT_01865	NA	Chromosome:1870000-1871068	110.58	110.58+peptide	OK	10.0907	23.5938	1.22537	2.18541	3.00E-04	0.00631878	yes
gene:SpnNT_00572	rplK	Chromosome:593430-593856	110.58	110.58+peptide	OK	575.984	1080.78	0.907976	2.08136	0.00035	0.00717609	yes
gene:SpnNT_00697	NA	Chromosome:747790-748927	110.58	110.58+peptide	OK	469.544	916.696	0.965184	2.12487	0.00035	0.00717609	yes
gene:SpnNT_01056	mcrB	Chromosome:1081545-1084780	110.58	110.58+peptide	OK	17.7517	6.37854	-1.47666	-2.15732	0.00035	0.00717609	yes
gene:SpnNT_01340	lytB_5	Chromosome:1381240-1383175	110.58	110.58+peptide	OK	77.5008	40.8185	-0.924989	-2.06277	0.00035	0.00717609	yes
gene:SpnNT_01482	deoB	Chromosome:1517046-1518258	110.58	110.58+peptide	OK	75.4671	143.151	0.923618	2.09084	4.00E-04	0.00811252	yes

gene:SpnNT_02298	NA	Chromosome:2270004-2270373	110.58	110.58+peptide	OK	70.2538	155.59	1.1471	2.1542	4.00E-04	0.00811252	yes
gene:SpnNT_00208	rplC	Chromosome:202823-203450	110.58	110.58+peptide	OK	576.013	1087.21	0.916459	2.06158	0.00045	0.00885292	yes
gene:SpnNT_00236	rplQ	Chromosome:216634-217021	110.58	110.58+peptide	OK	1420.94	2667.74	0.908765	2.03415	0.00045	0.00885292	yes
gene:SpnNT_00688	pyrF	Chromosome:740110-740812	110.58	110.58+peptide	OK	21.8499	48.9777	1.1645	2.14871	0.00045	0.00885292	yes
gene:SpnNT_00747	sodA	Chromosome:794107-794713	110.58	110.58+peptide	OK	782.562	1477.46	0.916841	2.04824	0.00045	0.00885292	yes
gene:SpnNT_01006	rplS	Chromosome:1031622-1031970	110.58	110.58+peptide	OK	2100.75	4044.99	0.945235	2.13139	0.00045	0.00885292	yes
gene:SpnNT_01116	NA	Chromosome:1141691-1142309	110.58	110.58+peptide	OK	191.292	365.159	0.932749	2.06644	0.00045	0.00885292	yes
gene:SpnNT_01476	deoD_2	Chromosome:1511668-1512379	110.58	110.58+peptide	OK	80.9349	157.402	0.959625	2.05173	0.00045	0.00885292	yes
gene:SpnNT_01717	ileS	Chromosome:1723429-1726222	110.58	110.58+peptide	OK	62.1887	116.391	0.904253	2.06143	0.00045	0.00885292	yes
gene:SpnNT_00080	purE	Chromosome:70812-72379	110.58	110.58+peptide	OK	39.2474	7.45245	-2.39681	-2.23309	5.00E-04	0.0095781	yes
gene:SpnNT_00227	rpmD	Chromosome:211505-211688	110.58	110.58+peptide	OK	2079.09	4116.71	0.985541	2.08471	5.00E-04	0.0095781	yes
gene:SpnNT_00468	dnaK	Chromosome:483541-485365	110.58	110.58+peptide	OK	509.029	977.915	0.941962	2.02434	5.00E-04	0.0095781	yes
gene:SpnNT_01648	dps	Chromosome:1651393-1651912	110.58	110.58+peptide	OK	1788.5	893.843	-1.00066	-2.16178	5.00E-04	0.0095781	yes
gene:SpnNT_01913	NA	Chromosome:1917497-1917746	110.58	110.58+peptide	OK	1105.71	2173.53	0.975068	2.02762	5.00E-04	0.0095781	yes
gene:SpnNT_01126	ptsH	Chromosome:1152548-1152812	110.58	110.58+peptide	OK	2470.72	4742.86	0.940824	1.98734	0.00055	0.0103545	yes
gene:SpnNT_01506	NA	Chromosome:1542167-1542515	110.58	110.58+peptide	OK	975.316	505.705	-0.947575	-2.01389	0.00055	0.0103545	yes
gene:SpnNT_00741	ptsG_1	Chromosome:785440-787621	110.58	110.58+peptide	OK	126.397	230.823	0.868826	1.98367	6.00E-04	0.0111203	yes
gene:SpnNT_01424	NA	Chromosome:1463857-1464598	110.58	110.58+peptide	OK	23.5461	49.6505	1.07632	2.02965	6.00E-04	0.0111203	yes
gene:SpnNT_00179	ribH	Chromosome:174928-175396	110.58	110.58+peptide	OK	23.3309	54.833	1.2328	2.11014	0.00065	0.0118958	yes
gene:SpnNT_01098	udk	Chromosome:1125772-1126411	110.58	110.58+peptide	OK	177.633	331.094	0.898342	1.99623	0.00065	0.0118958	yes
gene:SpnNT_01218	NA	Chromosome:1251675-1252005	110.58	110.58+peptide	OK	376.057	721.575	0.940196	1.97871	0.00065	0.0118958	yes
gene:SpnNT_01859	NA	Chromosome:1863859-1864291	110.58	110.58+peptide	OK	494.895	262.837	-0.912956	-1.97531	0.00065	0.0118958	yes
gene:SpnNT_00300	rplM	Chromosome:289030-289477	110.58	110.58+peptide	OK	500.827	897.461	0.841539	1.92007	0.00075	0.0133173	yes
gene:SpnNT_01654	rebM	Chromosome:1657217-1657976	110.58	110.58+peptide	OK	54.1741	109.599	1.01657	1.95549	0.00075	0.0133173	yes
gene:SpnNT_02220	rpmF	Chromosome:2186953-2187136	110.58	110.58+peptide	OK	317.142	706.736	1.15604	1.97685	0.00075	0.0133173	yes
gene:SpnNT_02286	rpsB	Chromosome:2257758-2258538	110.58	110.58+peptide	OK	1486.16	2779.98	0.903485	1.93744	0.00075	0.0133173	yes
gene:SpnNT_01172	glgA	Chromosome:1206672-1210242	110.58	110.58+peptide	OK	230.259	84.6431	-1.4438	-2.02289	8.00E-04	0.0140342	yes
gene:SpnNT_01192	pta	Chromosome:1228170-1229145	110.58	110.58+peptide	OK	54.4825	102.776	0.915638	1.98181	8.00E-04	0.0140342	yes
gene:SpnNT_02041	ccpA_2	Chromosome:2028345-2029356	110.58	110.58+peptide	OK	343.194	626.226	0.867661	1.93744	0.00085	0.0148124	yes
gene:SpnNT_00207	rpsJ	Chromosome:202298-202607	110.58	110.58+peptide	OK	754.256	1362.39	0.853012	1.88489	9.00E-04	0.0154579	yes
gene:SpnNT_00449	pyrG	Chromosome:458614-460222	110.58	110.58+peptide	OK	57.6678	106.3	0.882307	1.96976	0.001	0.0168447	yes
gene:SpnNT_00800	NA	Chromosome:848077-849457	110.58	110.58+peptide	OK	298.017	543.081	0.865771	1.92993	0.00105	0.0174849	yes
gene:SpnNT_01862	NA	Chromosome:1866471-1867182	110.58	110.58+peptide	OK	13.5395	29.2577	1.11164	1.92002	0.00105	0.0174849	yes
gene:SpnNT_01309	rpmI	Chromosome:1345595-1345796	110.58	110.58+peptide	OK	259.59	546.813	1.07481	1.86226	0.0011	0.0181562	yes
gene:SpnNT_00174	NA	Chromosome:169217-169688	110.58	110.58+peptide	OK	189.46	101.032	-0.907079	-1.84816	0.00115	0.0187457	yes
gene:SpnNT_00235	rpoA	Chromosome:215687-216623	110.58	110.58+peptide	OK	733.176	1300.51	0.826841	1.82859	0.00115	0.0187457	yes
gene:SpnNT_02178	ykuR	Chromosome:2139388-2140519	110.58	110.58+peptide	OK	242.179	133.936	-0.85453	-1.91636	0.0012	0.0193683	yes
gene:SpnNT_01425	axe1-6A_2	Chromosome:1464619-1465444	110.58	110.58+peptide	OK	30.3663	61.1156	1.00907	1.96038	0.00125	0.0201258	yes
gene:SpnNT_00684	thiF	Chromosome:735263-737628	110.58	110.58+peptide	OK	26.4662	58.4142	1.14217	1.96431	0.0013	0.020854	yes
gene:SpnNT_00137	NA	Chromosome:133970-134105	110.58	110.58+peptide	OK	24.4409	0	#NAME?	NA	0.00135	0.021343	yes
gene:SpnNT_00694	yxlF_1	Chromosome:743676-745025	110.58	110.58+peptide	OK	15.3044	37.8852	1.30769	1.91586	0.00145	0.0226781	yes
gene:SpnNT_01880	pucK	Chromosome:1887119-1888963	110.58	110.58+peptide	OK	10.8195	4.64346	-1.22036	-1.88958	0.00145	0.0226781	yes
gene:SpnNT_02221	rpmGA	Chromosome:2187151-2187301	110.58	110.58+peptide	OK	208.13	541.849	1.38041	1.87796	0.00145	0.0226781	yes
gene:SpnNT_01684	rpsO	Chromosome:1687526-1687796	110.58	110.58+peptide	OK	5457.84	9902.81	0.859508	1.85008	0.0015	0.0233488	yes
gene:SpnNT_01962	NA	Chromosome:1955823-1956108	110.58	110.58+peptide	OK	448.371	821.581	0.873711	1.78491	0.0015	0.0233488	yes

gene:SpnNT_00076	purN	Chromosome:65296-66861	110.58	110.58+peptide	OK	19.9024	4.39661	-2.17848	-1.97865	0.0017	0.025969	yes
gene:SpnNT_00211	rplB	Chromosome:204411-205245	110.58	110.58+peptide	OK	811.342	1469.84	0.857275	1.84142	0.0017	0.025969	yes
gene:SpnNT_00676	NA	Chromosome:728290-730312	110.58	110.58+peptide	OK	7.17119	14.287	0.994417	1.7811	0.00175	0.0265167	yes
gene:SpnNT_01220	dnaG	Chromosome:1253130-1254891	110.58	110.58+peptide	OK	82.7642	141.719	0.775955	1.78736	0.0018	0.0270557	yes
gene:SpnNT_02300	guaB	Chromosome:2271523-2273002	110.58	110.58+peptide	OK	436.452	251.856	-0.793223	-1.78501	0.0018	0.0270557	yes
gene:SpnNT_01422	NA	Chromosome:1462678-1463500	110.58	110.58+peptide	OK	15.4466	31.4143	1.02413	1.84538	0.0019	0.0281715	yes
gene:SpnNT_01758	aroF_1	Chromosome:1771042-1772074	110.58	110.58+peptide	OK	287.971	167.96	-0.777812	-1.7888	0.00195	0.0288477	yes
gene:SpnNT_00268	glmS	Chromosome:249253-251062	110.58	110.58+peptide	OK	250.502	446.921	0.835196	1.85995	0.00205	0.0300896	yes
gene:SpnNT_02141	SpnNT_02141	Chromosome:2105533-2105606	110.58	110.58+peptide	OK	0	2039.46	Inf	NA	0.00205	0.0300896	yes
gene:SpnNT_00145	mnmA	Chromosome:141928-143050	110.58	110.58+peptide	OK	1157.91	662.917	-0.804626	-1.74253	0.0021	0.0307204	yes
gene:SpnNT_00942	tetM	Chromosome:976980-978900	110.58	110.58+peptide	OK	6.95905	13.7842	0.986055	1.84028	0.00215	0.0312429	yes
gene:SpnNT_01045	NA	Chromosome:1072398-1073442	110.58	110.58+peptide	OK	46.8885	83.9414	0.840149	1.77715	0.0022	0.0317584	yes
gene:SpnNT_02176	gluP	Chromosome:2138176-2139377	110.58	110.58+peptide	OK	256.401	126.177	-1.02295	-1.7674	0.0023	0.0329123	yes
gene:SpnNT_02053	SpnNT_02053	Chromosome:2034521-2034605	110.58	110.58+peptide	OK	3904	11667.9	1.57952	1.85765	0.0024	0.0337543	yes
gene:SpnNT_00735	lptB	Chromosome:778928-781359	110.58	110.58+peptide	OK	63.984	193.34	1.59536	1.85586	0.00245	0.0342374	yes
gene:SpnNT_00797	azr_1	Chromosome:844932-846177	110.58	110.58+peptide	OK	56.5963	101.89	0.848228	1.75868	0.00245	0.0342374	yes
gene:SpnNT_02309	SpnNT_02309	Chromosome:2282383-2282457	110.58	110.58+peptide	OK	0	1801.88	Inf	NA	0.00245	0.0342374	yes
gene:SpnNT_01010	lytA_6	Chromosome:1033596-1034817	110.58	110.58+peptide	OK	98.4461	52.3515	-0.911104	-1.73787	0.0025	0.0347143	yes
gene:SpnNT_00278	rpsG	Chromosome:264620-265091	110.58	110.58+peptide	OK	1680.18	2888.11	0.781509	1.70685	0.00265	0.0362222	yes
gene:SpnNT_00568	brnQ	Chromosome:589460-590786	110.58	110.58+peptide	OK	38.9221	70.3582	0.854131	1.73731	0.00265	0.0362222	yes
gene:SpnNT_02110	nagA	Chromosome:2082350-2083502	110.58	110.58+peptide	OK	146.647	85.0196	-0.786483	-1.76265	0.00265	0.0362222	yes
gene:SpnNT_01716	iga_6	Chromosome:1717427-1723247	110.58	110.58+peptide	OK	47.9103	82.1344	0.777649	1.72266	0.0027	0.0368289	yes
gene:SpnNT_01941	SpnNT_01941	Chromosome:1942553-1942639	110.58	110.58+peptide	OK	3218.94	9176.72	1.51139	1.71319	0.0031	0.0411306	yes
gene:SpnNT_00088	sorB_1	Chromosome:79347-81608	110.58	110.58+peptide	OK	10.7965	72.4926	2.74727	2.02404	0.0032	0.0422439	yes
gene:SpnNT_01533	NA	Chromosome:1569606-1569840	110.58	110.58+peptide	OK	60.0944	23.528	-1.35285	-1.83649	0.0033	0.0432593	yes
gene:SpnNT_02030	NA	Chromosome:2016974-2019005	110.58	110.58+peptide	OK	36.0865	20.4201	-0.82147	-1.75159	0.00335	0.0437836	yes
gene:SpnNT_00798	azr_2	Chromosome:846194-846800	110.58	110.58+peptide	OK	14.1691	30.4331	1.10289	1.74522	0.00345	0.0449116	yes
gene:SpnNT_01818	trxA	Chromosome:1830818-1831133	110.58	110.58+peptide	OK	2548.96	5133.57	1.01005	1.73054	0.00345	0.0449116	yes
gene:SpnNT_01423	NA	Chromosome:1463557-1463845	110.58	110.58+peptide	OK	12.0279	30.6747	1.35066	1.7699	0.00355	0.0460763	yes
gene:SpnNT_01480	punA	Chromosome:1515677-1516487	110.58	110.58+peptide	OK	55.1816	94.708	0.779298	1.63514	0.00365	0.0469562	yes
gene:SpnNT_00214	rpsC	Chromosome:205998-206652	110.58	110.58+peptide	OK	953.89	1584.25	0.731902	1.59461	0.0039	0.0495409	yes
