# Supplementary material for: Foreign peptide triggers boost in pneumococcal metabolism and growth
Source: BMC Microbiol. 2018 Mar 27;18:23. doi: 10.1186/s12866-018-1167-y (PMC5870813; doi:10.1186/s12866-018-1167-y)
Supplement: Supplementary file 4 — Table S3. RNA-Seq data for ΔORF 2 mutant with and without ORF 2 peptide. Table shows only significant changes in expression. A significant change in expression was observed for 249 genes of which 177 were upregulated by the ORF 2 peptide and 72 were downregulated by the peptide. (PDF 231 kb) [file 12866_2018_1167_MOESM4_ESM.pdf]

gene_id	gene_short_nam	locus	sample_1	sample_2	status	value_1	value_2	log2_fold_cha	test_stat	p_value	q_value	signif
gene:SpnNT_00049	mtcA1	Chromosome:37404-37902	ΔORF2	ΔORF2+peptide	OK	439.302	145.759	-1.59162	-3.3603	5.00E-05	0.0013612	yes
gene:SpnNT_00050	NA	Chromosome:37926-38742	ΔORF2	ΔORF2+peptide	OK	276.308	119.6	-1.20807	-2.70647	5.00E-05	0.0013612	yes
gene:SpnNT_00072	purC	Chromosome:59090-59798	ΔORF2	ΔORF2+peptide	OK	12.6205	0.860569	-3.87433	-4.46227	5.00E-05	0.0013612	yes
gene:SpnNT_00073	purL	Chromosome:59999-63725	ΔORF2	ΔORF2+peptide	OK	18.2885	1.84753	-3.30727	-6.07412	5.00E-05	0.0013612	yes
gene:SpnNT_00074	purF	Chromosome:63817-65260	ΔORF2	ΔORF2+peptide	OK	16.4773	2.03969	-3.01406	-4.72476	5.00E-05	0.0013612	yes
gene:SpnNT_00075	purM	Chromosome:65296-66861	ΔORF2	ΔORF2+peptide	OK	19.9093	2.80511	-2.82731	-3.72202	5.00E-05	0.0013612	yes
gene:SpnNT_00078	purH	Chromosome:67478-69026	ΔORF2	ΔORF2+peptide	OK	45.9005	7.86369	-2.54523	-4.86151	5.00E-05	0.0013612	yes
gene:SpnNT_00079	purD	Chromosome:69147-70410	ΔORF2	ΔORF2+peptide	OK	45.9717	6.3938	-2.846	-5.21531	5.00E-05	0.0013612	yes
gene:SpnNT_00081	purK	Chromosome:70812-72379	ΔORF2	ΔORF2+peptide	OK	34.9025	5.63025	-2.63206	-4.06154	5.00E-05	0.0013612	yes
gene:SpnNT_00099	deoD_1	Chromosome:96812-97577	ΔORF2	ΔORF2+peptide	OK	138.184	55.624	-1.31281	-2.73137	5.00E-05	0.0013612	yes
gene:SpnNT_00108	rpsD	Chromosome:106058-106670	ΔORF2	ΔORF2+peptide	OK	1567.8	3427.95	1.1286	2.47772	5.00E-05	0.0013612	yes
gene:SpnNT_00175	NA	Chromosome:169980-171243	ΔORF2	ΔORF2+peptide	OK	64.294	281.989	2.13288	4.63781	5.00E-05	0.0013612	yes
gene:SpnNT_00180	ribBA	Chromosome:175396-176602	ΔORF2	ΔORF2+peptide	OK	20.2701	58.4787	1.52856	3.0871	5.00E-05	0.0013612	yes
gene:SpnNT_00182	ribD_1	Chromosome:176621-178342	ΔORF2	ΔORF2+peptide	OK	18.9401	55.5954	1.55353	2.63954	5.00E-05	0.0013612	yes
gene:SpnNT_00186	mccF	Chromosome:180722-181754	ΔORF2	ΔORF2+peptide	OK	72.7026	29.62	-1.29544	-2.67785	5.00E-05	0.0013612	yes
gene:SpnNT_00207	rpsJ	Chromosome:202298-202607	ΔORF2	ΔORF2+peptide	OK	661.585	1486.78	1.1682	2.59162	5.00E-05	0.0013612	yes
gene:SpnNT_00208	rplC	Chromosome:202823-203450	ΔORF2	ΔORF2+peptide	OK	546.627	1201.88	1.13667	2.57271	5.00E-05	0.0013612	yes
gene:SpnNT_00220	rplE	Chromosome:208370-208913	ΔORF2	ΔORF2+peptide	OK	459.71	908.625	0.982961	2.24404	5.00E-05	0.0013612	yes
gene:SpnNT_00221	rpsN2	Chromosome:208930-209200	ΔORF2	ΔORF2+peptide	OK	357.605	858.283	1.26309	2.6616	5.00E-05	0.0013612	yes
gene:SpnNT_00227	rpmD	Chromosome:211505-211688	ΔORF2	ΔORF2+peptide	OK	1883.51	4250	1.17404	2.48974	5.00E-05	0.0013612	yes
gene:SpnNT_00235	rpoA	Chromosome:215687-216623	ΔORF2	ΔORF2+peptide	OK	778.703	1727.15	1.14925	2.53042	5.00E-05	0.0013612	yes
gene:SpnNT_00238	NA	Chromosome:217561-218899	ΔORF2	ΔORF2+peptide	OK	200.705	90.6627	-1.1465	-2.59236	5.00E-05	0.0013612	yes
gene:SpnNT_00288	manZ_2	Chromosome:277000-277912	ΔORF2	ΔORF2+peptide	OK	427.606	1009.84	1.23977	2.79029	5.00E-05	0.0013612	yes
gene:SpnNT_00289	manY	Chromosome:277935-278739	ΔORF2	ΔORF2+peptide	OK	228.845	568.053	1.31165	2.91833	5.00E-05	0.0013612	yes
gene:SpnNT_00290	manX_2	Chromosome:278766-279756	ΔORF2	ΔORF2+peptide	OK	256.28	678.385	1.40438	3.18925	5.00E-05	0.0013612	yes
gene:SpnNT_00293	pbuO	Chromosome:282348-283767	ΔORF2	ΔORF2+peptide	OK	179.721	59.3433	-1.5986	-3.58142	5.00E-05	0.0013612	yes
gene:SpnNT_00294	NA	Chromosome:283819-284527	ΔORF2	ΔORF2+peptide	OK	28.9423	7.61873	-1.92556	-3.12185	5.00E-05	0.0013612	yes
gene:SpnNT_00301	rpsI	Chromosome:289496-289889	ΔORF2	ΔORF2+peptide	OK	2181.5	4867.35	1.15782	2.53449	5.00E-05	0.0013612	yes
gene:SpnNT_00305	yicI	Chromosome:291297-293511	ΔORF2	ΔORF2+peptide	OK	75.5642	36.7024	-1.04183	-2.29096	5.00E-05	0.0013612	yes
gene:SpnNT_00377	alsT	Chromosome:366821-368144	ΔORF2	ΔORF2+peptide	OK	23.1184	80.6922	1.80339	3.73998	5.00E-05	0.0013612	yes
gene:SpnNT_00409	gutB	Chromosome:416594-417638	ΔORF2	ΔORF2+peptide	OK	14.9401	39.6159	1.40689	2.69077	5.00E-05	0.0013612	yes
gene:SpnNT_00449	pyrG	Chromosome:458614-460222	ΔORF2	ΔORF2+peptide	OK	56.612	116.749	1.04423	2.33071	5.00E-05	0.0013612	yes
gene:SpnNT_00466	hrcA	Chromosome:481476-482511	ΔORF2	ΔORF2+peptide	OK	367.162	997.246	1.44153	3.23795	5.00E-05	0.0013612	yes
gene:SpnNT_00467	grpE	Chromosome:482537-483062	ΔORF2	ΔORF2+peptide	OK	547.778	1178.29	1.10503	2.53065	5.00E-05	0.0013612	yes
gene:SpnNT_00568	brnQ	Chromosome:589460-590786	ΔORF2	ΔORF2+peptide	OK	37.6157	85.4873	1.18438	2.45086	5.00E-05	0.0013612	yes
gene:SpnNT_00572	rplK	Chromosome:593430-593856	ΔORF2	ΔORF2+peptide	OK	643.026	1423.74	1.14673	2.63079	5.00E-05	0.0013612	yes
gene:SpnNT_00573	rplA	Chromosome:594064-594754	ΔORF2	ΔORF2+peptide	OK	1091.84	2390.52	1.13057	2.50444	5.00E-05	0.0013612	yes
gene:SpnNT_00684	thiF	Chromosome:735263-737628	ΔORF2	ΔORF2+peptide	OK	39.1386	96.6196	1.30372	2.47695	5.00E-05	0.0013612	yes
gene:SpnNT_00689	pyrE	Chromosome:740845-741478	ΔORF2	ΔORF2+peptide	OK	28.2391	75.1248	1.4116	2.67184	5.00E-05	0.0013612	yes
gene:SpnNT_00712	pox5	Chromosome:762226-764002	ΔORF2	ΔORF2+peptide	OK	581.371	1643.76	1.49947	3.13698	5.00E-05	0.0013612	yes
gene:SpnNT_00713	NA	Chromosome:764112-764460	ΔORF2	ΔORF2+peptide	OK	702.588	1720.02	1.29167	2.75667	5.00E-05	0.0013612	yes
gene:SpnNT_00732	braC	Chromosome:776627-777788	ΔORF2	ΔORF2+peptide	OK	145.867	400.962	1.45881	3.30474	5.00E-05	0.0013612	yes
gene:SpnNT_00733	livH_1	Chromosome:778055-778925	ΔORF2	ΔORF2+peptide	OK	40.6202	190.524	2.2297	4.69299	5.00E-05	0.0013612	yes
gene:SpnNT_00734	livH_2	Chromosome:778928-781359	ΔORF2	ΔORF2+peptide	OK	44.2395	191.035	2.11043	2.71399	5.00E-05	0.0013612	yes

gene:SpnNT_00735	lptB	Chromosome:778928-781359	ΔORF2	ΔORF2+peptide	OK	54.0367	242.483	2.16587	2.70374	5.00E-05	0.0013612	yes
gene:SpnNT_00736	livF	Chromosome:778928-781359	ΔORF2	ΔORF2+peptide	OK	70.9691	305.866	2.10764	2.74127	5.00E-05	0.0013612	yes
gene:SpnNT_00737	NA	Chromosome:781666-782323	ΔORF2	ΔORF2+peptide	OK	51.514	120.381	1.22457	2.50122	5.00E-05	0.0013612	yes
gene:SpnNT_00742	mapP	Chromosome:787672-788488	ΔORF2	ΔORF2+peptide	OK	15.4265	56.3811	1.8698	3.49007	5.00E-05	0.0013612	yes
gene:SpnNT_00755	NA	Chromosome:800842-801082	ΔORF2	ΔORF2+peptide	OK	1750.59	3761.82	1.10359	2.52906	5.00E-05	0.0013612	yes
gene:SpnNT_00793	NA	Chromosome:840349-841111	ΔORF2	ΔORF2+peptide	OK	33.2494	121.831	1.87348	3.71747	5.00E-05	0.0013612	yes
gene:SpnNT_00794	glyQ	Chromosome:841303-842221	ΔORF2	ΔORF2+peptide	OK	56.911	167.381	1.55635	3.35773	5.00E-05	0.0013612	yes
gene:SpnNT_00795	glyS	Chromosome:842480-844517	ΔORF2	ΔORF2+peptide	OK	76.9567	233.648	1.60222	3.61186	5.00E-05	0.0013612	yes
gene:SpnNT_00796	NA	Chromosome:844558-844816	ΔORF2	ΔORF2+peptide	OK	422.93	1644.1	1.95881	3.95877	5.00E-05	0.0013612	yes
gene:SpnNT_00853	rpsU	Chromosome:891009-891186	ΔORF2	ΔORF2+peptide	OK	5858.35	14743.1	1.33148	2.73288	5.00E-05	0.0013612	yes
gene:SpnNT_00873	glnH_2	Chromosome:907827-908643	ΔORF2	ΔORF2+peptide	OK	504.752	144.532	-1.80419	-4.06414	5.00E-05	0.0013612	yes
gene:SpnNT_00882	NA	Chromosome:917301-917787	ΔORF2	ΔORF2+peptide	OK	26.0851	88.3408	1.75985	3.17247	5.00E-05	0.0013612	yes
gene:SpnNT_00883	alaS	Chromosome:917808-920427	ΔORF2	ΔORF2+peptide	OK	68.9088	172.886	1.32706	3.00846	5.00E-05	0.0013612	yes
gene:SpnNT_00910	rplJ	Chromosome:951105-951606	ΔORF2	ΔORF2+peptide	OK	364.225	1346.82	1.88666	4.24481	5.00E-05	0.0013612	yes
gene:SpnNT_00911	rplL	Chromosome:951681-952186	ΔORF2	ΔORF2+peptide	OK	1755.08	4738.12	1.43278	3.08398	5.00E-05	0.0013612	yes
gene:SpnNT_00992	gdhA	Chromosome:1022113-1023460	ΔORF2	ΔORF2+peptide	OK	433.525	1844.27	2.08886	4.38611	5.00E-05	0.0013612	yes
gene:SpnNT_01021	pyrP	Chromosome:1044618-1045902	ΔORF2	ΔORF2+peptide	OK	48.2595	220.529	2.19208	4.83514	5.00E-05	0.0013612	yes
gene:SpnNT_01032	pyrR	Chromosome:1056577-1057099	ΔORF2	ΔORF2+peptide	OK	43.039	155.552	1.85368	3.58094	5.00E-05	0.0013612	yes
gene:SpnNT_01033	pyrB	Chromosome:1057117-1058041	ΔORF2	ΔORF2+peptide	OK	49.8619	234.304	2.23237	4.85398	5.00E-05	0.0013612	yes
gene:SpnNT_01034	carA	Chromosome:1058090-1059170	ΔORF2	ΔORF2+peptide	OK	47.9782	186.535	1.959	4.26707	5.00E-05	0.0013612	yes
gene:SpnNT_01035	carB	Chromosome:1059482-1062659	ΔORF2	ΔORF2+peptide	OK	48.4182	191.72	1.98538	4.45518	5.00E-05	0.0013612	yes
gene:SpnNT_01045	NA	Chromosome:1072398-1073442	ΔORF2	ΔORF2+peptide	OK	56.2964	129.722	1.20431	2.61162	5.00E-05	0.0013612	yes
gene:SpnNT_01079	fhs1	Chromosome:1108271-1109942	ΔORF2	ΔORF2+peptide	OK	176.686	44.3548	-1.99403	-4.4375	5.00E-05	0.0013612	yes
gene:SpnNT_01185	NA	Chromosome:1224162-1224507	ΔORF2	ΔORF2+peptide	OK	552.513	1275.25	1.2067	2.71005	5.00E-05	0.0013612	yes
gene:SpnNT_01186	rplU	Chromosome:1224522-1224837	ΔORF2	ΔORF2+peptide	OK	564.915	1222.76	1.11404	2.48581	5.00E-05	0.0013612	yes
gene:SpnNT_01255	queT	Chromosome:1293058-1293568	ΔORF2	ΔORF2+peptide	OK	125.322	307.193	1.29351	2.68837	5.00E-05	0.0013612	yes
gene:SpnNT_01258	hemH	Chromosome:1294622-1295717	ΔORF2	ΔORF2+peptide	OK	7.9104	32.18	2.02434	3.37712	5.00E-05	0.0013612	yes
gene:SpnNT_01289	NA	Chromosome:1324644-1325358	ΔORF2	ΔORF2+peptide	OK	16.3367	57.3418	1.81147	3.31124	5.00E-05	0.0013612	yes
gene:SpnNT_01290	pepF1_1	Chromosome:1325359-1327162	ΔORF2	ΔORF2+peptide	OK	28.5891	86.9644	1.60496	3.50845	5.00E-05	0.0013612	yes
gene:SpnNT_01308	rplT	Chromosome:1345184-1345544	ΔORF2	ΔORF2+peptide	OK	1837.9	4965.77	1.43396	3.15665	5.00E-05	0.0013612	yes
gene:SpnNT_01309	rpmI	Chromosome:1345595-1345796	ΔORF2	ΔORF2+peptide	OK	243.651	704.198	1.53117	2.76272	5.00E-05	0.0013612	yes
gene:SpnNT_01310	infC	Chromosome:1345828-1346416	ΔORF2	ΔORF2+peptide	OK	192.905	618.089	1.67992	3.7963	5.00E-05	0.0013612	yes
gene:SpnNT_01340	lytB_5	Chromosome:1381240-1383175	ΔORF2	ΔORF2+peptide	OK	86.4958	38.7124	-1.15983	-2.60735	5.00E-05	0.0013612	yes
gene:SpnNT_01352	ydaF_5	Chromosome:1391460-1392030	ΔORF2	ΔORF2+peptide	OK	182.9	64.8783	-1.49525	-3.08987	5.00E-05	0.0013612	yes
gene:SpnNT_01391	NA	Chromosome:1428017-1428713	ΔORF2	ΔORF2+peptide	OK	72.8028	22.7837	-1.67599	-2.96699	5.00E-05	0.0013612	yes
gene:SpnNT_01425	axe1-6A_2	Chromosome:1464619-1465444	ΔORF2	ΔORF2+peptide	OK	24.6223	70.0003	1.50739	2.95599	5.00E-05	0.0013612	yes
gene:SpnNT_01481	NA	Chromosome:1516502-1517045	ΔORF2	ΔORF2+peptide	OK	39.1681	92.5697	1.24086	2.37192	5.00E-05	0.0013612	yes
gene:SpnNT_01482	deoB	Chromosome:1517046-1518258	ΔORF2	ΔORF2+peptide	OK	70.2211	156.559	1.15673	2.5996	5.00E-05	0.0013612	yes
gene:SpnNT_01483	rpiA	Chromosome:1518271-1518955	ΔORF2	ΔORF2+peptide	OK	71.227	172.706	1.27782	2.73282	5.00E-05	0.0013612	yes
gene:SpnNT_01507	fliY	Chromosome:1542792-1543629	ΔORF2	ΔORF2+peptide	OK	451.573	131.326	-1.78181	-4.03679	5.00E-05	0.0013612	yes
gene:SpnNT_01508	glnQ_3	Chromosome:1543641-1544271	ΔORF2	ΔORF2+peptide	OK	304.164	86.172	-1.81956	-3.87652	5.00E-05	0.0013612	yes
gene:SpnNT_01509	yecS_2	Chromosome:1544280-1544922	ΔORF2	ΔORF2+peptide	OK	251.826	57.3928	-2.13348	-4.40838	5.00E-05	0.0013612	yes
gene:SpnNT_01532	lmrA	Chromosome:1568158-1569589	ΔORF2	ΔORF2+peptide	OK	215.149	59.3975	-1.85686	-4.08393	5.00E-05	0.0013612	yes
gene:SpnNT_01534	sarA_5	Chromosome:1570011-1571970	ΔORF2	ΔORF2+peptide	OK	1228.19	478.031	-1.36136	-2.89053	5.00E-05	0.0013612	yes
gene:SpnNT_01537	NA	Chromosome:1575387-1575585	ΔORF2	ΔORF2+peptide	OK	1912.69	7866.66	2.04015	3.54653	5.00E-05	0.0013612	yes

gene:SpnNT_01548	rpsR	Chromosome:1582275-1582515	ΔORF2	ΔORF2+peptide	OK	1780.01	4748.41	1.41556	3.09013	5.00E-05	0.0013612	yes
gene:SpnNT_01549	ssb_1	Chromosome:1582546-1583017	ΔORF2	ΔORF2+peptide	OK	726.469	1929.31	1.40911	3.15691	5.00E-05	0.0013612	yes
gene:SpnNT_01550	rpsF	Chromosome:1583028-1583319	ΔORF2	ΔORF2+peptide	OK	1118.24	2997.52	1.42254	3.20452	5.00E-05	0.0013612	yes
gene:SpnNT_01648	dps	Chromosome:1651393-1651912	ΔORF2	ΔORF2+peptide	OK	2550.58	769.4	-1.72902	-3.90776	5.00E-05	0.0013612	yes
gene:SpnNT_01660	yhjX	Chromosome:1663456-1664683	ΔORF2	ΔORF2+peptide	OK	118.852	31.3166	-1.92417	-4.09856	5.00E-05	0.0013612	yes
gene:SpnNT_01689	thrS	Chromosome:1689816-1691760	ΔORF2	ΔORF2+peptide	OK	44.9151	123.541	1.45972	3.26758	5.00E-05	0.0013612	yes
gene:SpnNT_01716	iga_6	Chromosome:1717427-1723247	ΔORF2	ΔORF2+peptide	OK	57.4892	125.371	1.12484	2.52014	5.00E-05	0.0013612	yes
gene:SpnNT_01738	yesO_1	Chromosome:1745602-1746931	ΔORF2	ΔORF2+peptide	OK	44.4044	99.6813	1.16662	2.56513	5.00E-05	0.0013612	yes
gene:SpnNT_01742	afr	Chromosome:1748866-1749970	ΔORF2	ΔORF2+peptide	OK	11.8236	31.6975	1.4227	2.65925	5.00E-05	0.0013612	yes
gene:SpnNT_01743	nanB	Chromosome:1749981-1752075	ΔORF2	ΔORF2+peptide	OK	7.08523	17.5018	1.30462	2.47199	5.00E-05	0.0013612	yes
gene:SpnNT_01859	NA	Chromosome:1863859-1864291	ΔORF2	ΔORF2+peptide	OK	490.325	201.946	-1.27977	-2.77236	5.00E-05	0.0013612	yes
gene:SpnNT_01880	pucK	Chromosome:1887119-1888963	ΔORF2	ΔORF2+peptide	OK	10.5242	2.84398	-1.88772	-2.79871	5.00E-05	0.0013612	yes
gene:SpnNT_01884	paaI	Chromosome:1891709-1892114	ΔORF2	ΔORF2+peptide	OK	45.1237	124.495	1.46413	2.63151	5.00E-05	0.0013612	yes
gene:SpnNT_02014	asnA	Chromosome:2003362-2004355	ΔORF2	ΔORF2+peptide	OK	975.827	384.745	-1.34272	-3.03572	5.00E-05	0.0013612	yes
gene:SpnNT_02080	adhE	Chromosome:2056744-2059396	ΔORF2	ΔORF2+peptide	OK	104.93	549.67	2.38915	4.7658	5.00E-05	0.0013612	yes
gene:SpnNT_02110	nagA	Chromosome:2082350-2083502	ΔORF2	ΔORF2+peptide	OK	168.925	72.1432	-1.22745	-2.75964	5.00E-05	0.0013612	yes
gene:SpnNT_02178	ykuR	Chromosome:2139388-2140519	ΔORF2	ΔORF2+peptide	OK	246.442	115.38	-1.09486	-2.4424	5.00E-05	0.0013612	yes
gene:SpnNT_02191	malX_2	Chromosome:2158441-2159713	ΔORF2	ΔORF2+peptide	OK	44.1236	112.476	1.35	2.95901	5.00E-05	0.0013612	yes
gene:SpnNT_02242	NA	Chromosome:2211026-2211926	ΔORF2	ΔORF2+peptide	OK	936.982	360.667	-1.37735	-3.11527	5.00E-05	0.0013612	yes
gene:SpnNT_02285	tsf	Chromosome:2256639-2257680	ΔORF2	ΔORF2+peptide	OK	1020.96	2196.16	1.10505	2.38325	5.00E-05	0.0013612	yes
gene:SpnNT_00168	NA	Chromosome:164295-164865	ΔORF2	ΔORF2+peptide	OK	21.6603	50.4205	1.21896	2.16993	1.00E-04	0.0025332	yes
gene:SpnNT_00236	rplQ	Chromosome:216634-217021	ΔORF2	ΔORF2+peptide	OK	1431.66	2867.4	1.00205	2.27847	1.00E-04	0.0025332	yes
gene:SpnNT_00300	rplM	Chromosome:289030-289477	ΔORF2	ΔORF2+peptide	OK	479.3	969.344	1.01608	2.31766	1.00E-04	0.0025332	yes
gene:SpnNT_00443	NA	Chromosome:454495-454999	ΔORF2	ΔORF2+peptide	OK	26.0881	68.56	1.39398	2.50181	1.00E-04	0.0025332	yes
gene:SpnNT_00800	NA	Chromosome:848077-849457	ΔORF2	ΔORF2+peptide	OK	328.938	650.852	0.984514	2.2042	1.00E-04	0.0025332	yes
gene:SpnNT_01076	panT	Chromosome:1106241-1107340	ΔORF2	ΔORF2+peptide	OK	267.875	118.403	-1.17786	-2.36801	1.00E-04	0.0025332	yes
gene:SpnNT_01174	gapN	Chromosome:1206672-1210242	ΔORF2	ΔORF2+peptide	OK	223.619	65.4001	-1.77368	-2.31145	1.00E-04	0.0025332	yes
gene:SpnNT_01424	NA	Chromosome:1463857-1464598	ΔORF2	ΔORF2+peptide	OK	18.4118	46.6984	1.34275	2.44167	1.00E-04	0.0025332	yes
gene:SpnNT_01717	ileS	Chromosome:1723429-1726222	ΔORF2	ΔORF2+peptide	OK	64.7173	128.555	0.990163	2.24015	1.00E-04	0.0025332	yes
gene:SpnNT_01840	NA	Chromosome:1847981-1849238	ΔORF2	ΔORF2+peptide	OK	8.01124	18.8916	1.23765	2.21918	1.00E-04	0.0025332	yes
gene:SpnNT_02256	NA	Chromosome:2227043-2228453	ΔORF2	ΔORF2+peptide	OK	19.1329	43.0643	1.17043	2.33535	1.00E-04	0.0025332	yes
gene:SpnNT_02286	rpsB	Chromosome:2257758-2258538	ΔORF2	ΔORF2+peptide	OK	1336.94	2697.22	1.01253	2.19112	1.00E-04	0.0025332	yes
gene:SpnNT_00179	ribH	Chromosome:174928-175396	ΔORF2	ΔORF2+peptide	OK	27.242	66.7966	1.29394	2.25169	0.00015	0.00355289	yes
gene:SpnNT_00209	rplD	Chromosome:203474-204394	ΔORF2	ΔORF2+peptide	OK	449.29	934.73	1.0569	2.19834	0.00015	0.00355289	yes
gene:SpnNT_00690	NA	Chromosome:741710-742061	ΔORF2	ΔORF2+peptide	OK	24.3133	66.0763	1.44239	2.29575	0.00015	0.00355289	yes
gene:SpnNT_00697	NA	Chromosome:747790-748927	ΔORF2	ΔORF2+peptide	OK	456.786	897.205	0.97392	2.17855	0.00015	0.00355289	yes
gene:SpnNT_01677	galE_1	Chromosome:1677661-1679648	ΔORF2	ΔORF2+peptide	OK	466.165	219.209	-1.08854	-2.25042	0.00015	0.00355289	yes
gene:SpnNT_01732	bglK_1	Chromosome:1740306-1741191	ΔORF2	ΔORF2+peptide	OK	41.5087	85.6458	1.04497	2.17293	0.00015	0.00355289	yes
gene:SpnNT_01741	nanE	Chromosome:1747995-1748694	ΔORF2	ΔORF2+peptide	OK	38.7912	80.4118	1.05168	2.08536	0.00015	0.00355289	yes
gene:SpnNT_01758	aroF_1	Chromosome:1771042-1772074	ΔORF2	ΔORF2+peptide	OK	310.488	164.405	-0.917287	-2.10811	0.00015	0.00355289	yes
gene:SpnNT_01866	NA	Chromosome:1871092-1871752	ΔORF2	ΔORF2+peptide	OK	11.1011	28.9144	1.38108	2.25262	0.00015	0.00355289	yes
gene:SpnNT_00174	NA	Chromosome:169217-169688	ΔORF2	ΔORF2+peptide	OK	175.788	85.0357	-1.0477	-2.1012	2.00E-04	0.00450928	yes
gene:SpnNT_00211	rplB	Chromosome:204411-205245	ΔORF2	ΔORF2+peptide	OK	768.608	1529.09	0.992355	2.11998	2.00E-04	0.00450928	yes
gene:SpnNT_00229	NA	Chromosome:212285-213596	ΔORF2	ΔORF2+peptide	OK	731.366	1476.72	1.01373	2.15669	2.00E-04	0.00450928	yes
gene:SpnNT_00688	pyrF	Chromosome:740110-740812	ΔORF2	ΔORF2+peptide	OK	18.9494	45.2581	1.25603	2.30127	2.00E-04	0.00450928	yes

gene:SpnNT_01422	NA	Chromosome:1462678-1463500	ΔORF2	ΔORF2+peptide	OK	13.1531	33.2372	1.3374	2.39023	2.00E-04	0.00450928	yes
gene:SpnNT_01480	punA	Chromosome:1515677-1516487	ΔORF2	ΔORF2+peptide	OK	54.2866	109.197	1.00826	2.12918	2.00E-04	0.00450928	yes
gene:SpnNT_02298	NA	Chromosome:2270004-2270373	ΔORF2	ΔORF2+peptide	OK	91.0769	208.139	1.19239	2.23597	2.00E-04	0.00450928	yes
gene:SpnNT_00268	glmS	Chromosome:249253-251062	ΔORF2	ΔORF2+peptide	OK	196.938	384.973	0.967017	2.17649	0.00025	0.00542231	yes
gene:SpnNT_00415	rpmB	Chromosome:423743-423932	ΔORF2	ΔORF2+peptide	OK	6004.64	11754	0.969004	2.08469	0.00025	0.00542231	yes
gene:SpnNT_00675	NA	Chromosome:727863-728208	ΔORF2	ΔORF2+peptide	OK	26.0683	70.8279	1.44202	2.3133	0.00025	0.00542231	yes
gene:SpnNT_01184	rpmA	Chromosome:1223852-1224146	ΔORF2	ΔORF2+peptide	OK	3604.68	7564.47	1.06937	2.1021	0.00025	0.00542231	yes
gene:SpnNT_01557	NA	Chromosome:1588906-1589071	ΔORF2	ΔORF2+peptide	OK	3133.46	1377.05	-1.18617	-2.12281	0.00025	0.00542231	yes
gene:SpnNT_01850	NA	Chromosome:1856167-1856791	ΔORF2	ΔORF2+peptide	OK	144.959	65.5777	-1.14437	-2.2439	0.00025	0.00542231	yes
gene:SpnNT_01958	NA	Chromosome:1952759-1954086	ΔORF2	ΔORF2+peptide	OK	5.3866	17.9938	1.74006	2.17345	0.00025	0.00542231	yes
gene:SpnNT_02030	NA	Chromosome:2016974-2019005	ΔORF2	ΔORF2+peptide	OK	35.4547	17.4762	-1.02058	-2.11703	0.00025	0.00542231	yes
gene:SpnNT_02300	guaB	Chromosome:2271523-2273002	ΔORF2	ΔORF2+peptide	OK	503.579	252.145	-0.997967	-2.24374	0.00025	0.00542231	yes
gene:SpnNT_00226	rpsE	Chromosome:210997-211492	ΔORF2	ΔORF2+peptide	OK	408.264	765.445	0.906796	2.0754	3.00E-04	0.00631878	yes
gene:SpnNT_00468	dnaK	Chromosome:483541-485365	ΔORF2	ΔORF2+peptide	OK	496.75	1032.5	1.05555	2.27337	3.00E-04	0.00631878	yes
gene:SpnNT_01305	pyrDB	Chromosome:1342747-1343686	ΔORF2	ΔORF2+peptide	OK	25.7697	53.396	1.05106	2.08666	3.00E-04	0.00631878	yes
gene:SpnNT_01684	rpsO	Chromosome:1687526-1687796	ΔORF2	ΔORF2+peptide	OK	5116.55	9670.08	0.918358	2.06981	3.00E-04	0.00631878	yes
gene:SpnNT_00233	rpsM	Chromosome:214878-215244	ΔORF2	ΔORF2+peptide	OK	860.354	1614.11	0.907734	2.023	0.00035	0.00717609	yes
gene:SpnNT_00237	NA	Chromosome:217285-217552	ΔORF2	ΔORF2+peptide	OK	101.693	37.5363	-1.43786	-2.18368	0.00035	0.00717609	yes
gene:SpnNT_01172	glgA	Chromosome:1206672-1210242	ΔORF2	ΔORF2+peptide	OK	224.084	71.5362	-1.64729	-2.2509	0.00035	0.00717609	yes
gene:SpnNT_00216	rpmC	Chromosome:207078-207285	ΔORF2	ΔORF2+peptide	OK	359.624	781.305	1.1194	2.08856	4.00E-04	0.00811252	yes
gene:SpnNT_00112	NA	Chromosome:111742-112729	ΔORF2	ΔORF2+peptide	OK	467.321	976.022	1.0625	2.155	0.00045	0.00885292	yes
gene:SpnNT_01858	trpE	Chromosome:1861563-1863488	ΔORF2	ΔORF2+peptide	OK	42.3194	17.7342	-1.25479	-2.11069	0.00045	0.00885292	yes
gene:SpnNT_01104	recN	Chromosome:1130179-1131847	ΔORF2	ΔORF2+peptide	OK	54.4027	99.7871	0.875175	1.98143	5.00E-04	0.0095781	yes
gene:SpnNT_01220	dnaG	Chromosome:1253130-1254891	ΔORF2	ΔORF2+peptide	OK	80.6101	147.745	0.874081	2.00947	5.00E-04	0.0095781	yes
gene:SpnNT_01476	deoD_2	Chromosome:1511668-1512379	ΔORF2	ΔORF2+peptide	OK	80.5487	152.982	0.925426	2.00075	5.00E-04	0.0095781	yes
gene:SpnNT_01798	NA	Chromosome:1814384-1815386	ΔORF2	ΔORF2+peptide	OK	9.63297	23.3197	1.27549	2.2206	5.00E-04	0.0095781	yes
gene:SpnNT_00662	NA	Chromosome:717769-717940	ΔORF2	ΔORF2+peptide	OK	8223.76	3977.4	-1.04797	-2.08069	0.00055	0.0103545	yes
gene:SpnNT_00942	tetM	Chromosome:976980-978900	ΔORF2	ΔORF2+peptide	OK	8.32522	17.3085	1.05592	1.99765	0.00055	0.0103545	yes
gene:SpnNT_01192	pta	Chromosome:1228170-1229145	ΔORF2	ΔORF2+peptide	OK	57.4289	109.157	0.926552	2.01888	0.00055	0.0103545	yes
gene:SpnNT_01267	yhdG	Chromosome:1307718-1309110	ΔORF2	ΔORF2+peptide	OK	162.867	87.2489	-0.900488	-2.00464	0.00055	0.0103545	yes
gene:SpnNT_01306	pyrK	Chromosome:1343696-1344497	ΔORF2	ΔORF2+peptide	OK	26.5699	54.8662	1.04613	2.01059	0.00055	0.0103545	yes
gene:SpnNT_00741	ptsG_1	Chromosome:785440-787621	ΔORF2	ΔORF2+peptide	OK	119.809	218.797	0.868856	1.9572	6.00E-04	0.0111203	yes
gene:SpnNT_01219	sigA	Chromosome:1252018-1253128	ΔORF2	ΔORF2+peptide	OK	114.519	205.836	0.845901	1.93281	6.00E-04	0.0111203	yes
gene:SpnNT_01259	pepT	Chromosome:1295871-1297095	ΔORF2	ΔORF2+peptide	OK	123.321	64.443	-0.936328	-2.10406	6.00E-04	0.0111203	yes
gene:SpnNT_01506	NA	Chromosome:1542167-1542515	ΔORF2	ΔORF2+peptide	OK	1026.67	529.337	-0.95572	-2.07026	6.00E-04	0.0111203	yes
gene:SpnNT_00002	dnaN	Chromosome:1705-2842	ΔORF2	ΔORF2+peptide	OK	79.4161	39.3211	-1.01413	-2.13255	0.00065	0.0118958	yes
gene:SpnNT_01944	SpnNT_01944	Chromosome:1942830-1942914	ΔORF2	ΔORF2+peptide	OK	5592.52	18361.2	1.71509	2.27935	0.00065	0.0118958	yes
gene:SpnNT_00224	rplF	Chromosome:210003-210540	ΔORF2	ΔORF2+peptide	OK	902.549	1641.03	0.862523	1.94335	7.00E-04	0.012687	yes
gene:SpnNT_02220	rpmF	Chromosome:2186953-2187136	ΔORF2	ΔORF2+peptide	OK	290.427	690.505	1.24948	2.05156	7.00E-04	0.012687	yes
gene:SpnNT_02299	recF	Chromosome:2270375-2271473	ΔORF2	ΔORF2+peptide	OK	31.7465	61.1515	0.945792	1.93301	7.00E-04	0.012687	yes
gene:SpnNT_00080	purE	Chromosome:70812-72379	ΔORF2	ΔORF2+peptide	OK	36.4253	5.05896	-2.84803	-2.35216	0.00075	0.0133173	yes
gene:SpnNT_00217	rpsQ	Chromosome:207309-207570	ΔORF2	ΔORF2+peptide	OK	386.838	746.959	0.949299	1.9288	0.00075	0.0133173	yes
gene:SpnNT_00752	NA	Chromosome:798585-799389	ΔORF2	ΔORF2+peptide	OK	184.939	99.1721	-0.899039	-1.99671	0.00075	0.0133173	yes
gene:SpnNT_00909	mtaD	Chromosome:949389-950856	ΔORF2	ΔORF2+peptide	OK	173.123	92.3677	-0.906334	-2.06472	0.00075	0.0133173	yes
gene:SpnNT_00083	purB	Chromosome:72678-73977	ΔORF2	ΔORF2+peptide	OK	129.114	72.3035	-0.836508	-1.86874	8.00E-04	0.0140342	yes

gene:SpnNT_01865	NA	Chromosome:1870000-1871068	ΔORF2	ΔORF2+peptide	OK	12.7379	27.7767	1.12475	2.07889	8.00E-04	0.0140342	yes
gene:SpnNT_00234	rpsK	Chromosome:215261-215645	ΔORF2	ΔORF2+peptide	OK	790.313	1437.84	0.863411	1.9008	0.00085	0.0148124	yes
gene:SpnNT_00798	azr_2	Chromosome:846194-846800	ΔORF2	ΔORF2+peptide	OK	13.362	29.9439	1.16413	1.89026	9.00E-04	0.0154579	yes
gene:SpnNT_02041	ccpA_2	Chromosome:2028345-2029356	ΔORF2	ΔORF2+peptide	OK	340.45	620.192	0.865273	1.95297	9.00E-04	0.0154579	yes
gene:SpnNT_00219	rplX	Chromosome:208041-208347	ΔORF2	ΔORF2+peptide	OK	475.815	878.016	0.883846	1.9057	0.00095	0.0161266	yes
gene:SpnNT_00456	glnR	Chromosome:469600-469957	ΔORF2	ΔORF2+peptide	OK	442.64	225.409	-0.973592	-2.0595	0.00095	0.0161266	yes
gene:SpnNT_01161	NA	Chromosome:1191404-1192583	ΔORF2	ΔORF2+peptide	OK	15.3053	30.7774	1.00784	1.93587	0.00095	0.0161266	yes
gene:SpnNT_01478	NA	Chromosome:1513573-1514566	ΔORF2	ΔORF2+peptide	OK	46.0825	87.0004	0.916804	1.95307	0.001	0.0168447	yes
gene:SpnNT_01628	NA	Chromosome:1640172-1640304	ΔORF2	ΔORF2+peptide	OK	29.3391	0	#NAME?	NA	0.001	0.0168447	yes
gene:SpnNT_01654	rebM	Chromosome:1657217-1657976	ΔORF2	ΔORF2+peptide	OK	56.5152	114.184	1.01465	1.96649	0.001	0.0168447	yes
gene:SpnNT_00215	rplP	Chromosome:206655-207069	ΔORF2	ΔORF2+peptide	OK	804.393	1462.76	0.862717	1.8704	0.00105	0.0174849	yes
gene:SpnNT_01126	ptsH	Chromosome:1152548-1152812	ΔORF2	ΔORF2+peptide	OK	2243.68	4094.72	0.867894	1.87918	0.00105	0.0174849	yes
gene:SpnNT_00287	pepC	Chromosome:275610-276945	ΔORF2	ΔORF2+peptide	OK	332.343	187.824	-0.823291	-1.88632	0.0011	0.0181562	yes
gene:SpnNT_01218	NA	Chromosome:1251675-1252005	ΔORF2	ΔORF2+peptide	OK	369.793	658.834	0.833197	1.78988	0.0011	0.0181562	yes
gene:SpnNT_00145	mnmA	Chromosome:141928-143050	ΔORF2	ΔORF2+peptide	OK	1116.8	575.111	-0.957456	-2.03887	0.00115	0.0187457	yes
gene:SpnNT_01105	pphA	Chromosome:1131848-1132577	ΔORF2	ΔORF2+peptide	OK	52.498	98.716	0.911021	1.88101	0.00115	0.0187457	yes
gene:SpnNT_01962	NA	Chromosome:1955823-1956108	ΔORF2	ΔORF2+peptide	OK	402.111	766.084	0.929909	1.90221	0.00115	0.0187457	yes
gene:SpnNT_00231	infA	Chromosome:214501-214720	ΔORF2	ΔORF2+peptide	OK	459.058	876.709	0.933421	1.84929	0.0012	0.0193683	yes
gene:SpnNT_01939	SpnNT_01939	Chromosome:1942380-1942456	ΔORF2	ΔORF2+peptide	OK	0	1635.88	Inf	NA	0.0012	0.0193683	yes
gene:SpnNT_00524	bglH_1	Chromosome:532252-533668	ΔORF2	ΔORF2+peptide	OK	11.172	5.09793	-1.1319	-1.90892	0.00125	0.0201258	yes
gene:SpnNT_00797	azr_1	Chromosome:844932-846177	ΔORF2	ΔORF2+peptide	OK	45.919	84.6997	0.883267	1.8747	0.0013	0.020854	yes
gene:SpnNT_01098	udk	Chromosome:1125772-1126411	ΔORF2	ΔORF2+peptide	OK	168.173	296.938	0.820216	1.82633	0.00135	0.021343	yes
gene:SpnNT_01383	NA	Chromosome:1419155-1419377	ΔORF2	ΔORF2+peptide	OK	1.80092	0	#NAME?	NA	0.00135	0.021343	yes
gene:SpnNT_01467	tmpC	Chromosome:1504982-1506035	ΔORF2	ΔORF2+peptide	OK	455.187	808.361	0.82854	1.8655	0.0014	0.0220273	yes
gene:SpnNT_02176	gluP	Chromosome:2138176-2139377	ΔORF2	ΔORF2+peptide	OK	268.358	130.437	-1.04081	-1.8414	0.0014	0.0220273	yes
gene:SpnNT_01849	NA	Chromosome:1855001-1856153	ΔORF2	ΔORF2+peptide	OK	168.95	90.7529	-0.896577	-1.89852	0.00145	0.0226781	yes
gene:SpnNT_00747	sodA	Chromosome:794107-794713	ΔORF2	ΔORF2+peptide	OK	980.597	1745.16	0.831629	1.85973	0.0015	0.0233488	yes
gene:SpnNT_01011	lytB_3	Chromosome:1034829-1035948	ΔORF2	ΔORF2+peptide	OK	240.783	124.891	-0.947059	-1.85914	0.00155	0.0240131	yes
gene:SpnNT_01479	NA	Chromosome:1514569-1515214	ΔORF2	ΔORF2+peptide	OK	65.8172	122.091	0.891421	1.84765	0.00155	0.0240131	yes
gene:SpnNT_01268	resA	Chromosome:1309231-1309798	ΔORF2	ΔORF2+peptide	OK	521.245	289.972	-0.846049	-1.77458	0.0016	0.0247585	yes
gene:SpnNT_00763	gor	Chromosome:805656-807003	ΔORF2	ΔORF2+peptide	OK	449.731	257.34	-0.80539	-1.80919	0.00165	0.0253528	yes
gene:SpnNT_00510	valS	Chromosome:514725-518944	ΔORF2	ΔORF2+peptide	OK	102.152	190.079	0.895884	1.84246	0.0017	0.025969	yes
gene:SpnNT_00171	NA	Chromosome:165859-167248	ΔORF2	ΔORF2+peptide	OK	31.908	57.9627	0.861205	1.8407	0.00175	0.0265167	yes
gene:SpnNT_01091	NA	Chromosome:1120660-1121457	ΔORF2	ΔORF2+peptide	OK	16.185	36.0856	1.15677	1.81416	0.00175	0.0265167	yes
gene:SpnNT_01931	SpnNT_01931	Chromosome:1941661-1941751	ΔORF2	ΔORF2+peptide	OK	5614.11	13864.1	1.30422	1.91349	0.00175	0.0265167	yes
gene:SpnNT_01941	SpnNT_01941	Chromosome:1942553-1942639	ΔORF2	ΔORF2+peptide	OK	2893.25	10529.6	1.86369	2.11355	0.0018	0.0270557	yes
gene:SpnNT_01697	NA	Chromosome:1697437-1697956	ΔORF2	ΔORF2+peptide	OK	27.9138	12.3103	-1.18111	-1.8633	0.00185	0.0276489	yes
gene:SpnNT_00172	NA	Chromosome:167259-168546	ΔORF2	ΔORF2+peptide	OK	29.5316	54.5019	0.884046	1.84722	0.0019	0.0281715	yes
gene:SpnNT_01681	NA	Chromosome:1682586-1684923	ΔORF2	ΔORF2+peptide	OK	129.608	227.528	0.811896	1.83712	0.0019	0.0281715	yes
gene:SpnNT_00277	rpsL	Chromosome:264187-264601	ΔORF2	ΔORF2+peptide	OK	593.925	1045.38	0.815672	1.79763	0.00195	0.0288477	yes
gene:SpnNT_01175	amyX	Chromosome:1210785-1213065	ΔORF2	ΔORF2+peptide	OK	44.5446	75.5187	0.761583	1.73101	0.002	0.0294545	yes
gene:SpnNT_00278	rpsG	Chromosome:264620-265091	ΔORF2	ΔORF2+peptide	OK	1529.43	2684.82	0.81183	1.76016	0.00205	0.0300896	yes
gene:SpnNT_01477	ply_2	Chromosome:1513036-1513549	ΔORF2	ΔORF2+peptide	OK	59.5698	111.493	0.9043	1.78265	0.00215	0.0312429	yes
gene:SpnNT_02221	rpmGA	Chromosome:2187151-2187301	ΔORF2	ΔORF2+peptide	OK	192.672	476.583	1.30658	1.74723	0.00215	0.0312429	yes
gene:SpnNT_01127	ptsI	Chromosome:1152826-1154551	ΔORF2	ΔORF2+peptide	OK	882.941	1614.13	0.870366	1.80279	0.0022	0.0317584	yes

gene:SpnNT_01403	NA	Chromosome:1438818-1439130	ΔORF2	ΔORF2+peptide	OK	90.7894	42.4229	-1.09768	-1.72901	0.0022	0.0317584	yes
gene:SpnNT_01733	nanA_2	Chromosome:1741208-1742126	ΔORF2	ΔORF2+peptide	OK	23.503	45.2554	0.945246	1.83047	0.0023	0.0329123	yes
gene:SpnNT_01752	nanA_4	Chromosome:1763233-1765036	ΔORF2	ΔORF2+peptide	OK	14.6971	27.485	0.903114	1.81222	0.0024	0.0337543	yes
gene:SpnNT_00051	prs	Chromosome:38886-39855	ΔORF2	ΔORF2+peptide	OK	175.744	295.962	0.751939	1.73058	0.0025	0.0347143	yes
gene:SpnNT_00242	NA	Chromosome:221710-221977	ΔORF2	ΔORF2+peptide	OK	1.95671	0	#NAME?	NA	0.0025	0.0347143	yes
gene:SpnNT_01385	NA	Chromosome:1420638-1421514	ΔORF2	ΔORF2+peptide	OK	27.9325	51.8411	0.892151	1.7406	0.00255	0.0352222	yes
gene:SpnNT_00799	apbE	Chromosome:846859-847783	ΔORF2	ΔORF2+peptide	OK	14.8627	30.1688	1.02136	1.79944	0.00265	0.0362222	yes
gene:SpnNT_02112	tgt	Chromosome:2085572-2086715	ΔORF2	ΔORF2+peptide	OK	118.246	202.734	0.777805	1.76176	0.00265	0.0362222	yes
gene:SpnNT_00214	rpsC	Chromosome:205998-206652	ΔORF2	ΔORF2+peptide	OK	899.993	1581.19	0.813023	1.75575	0.00275	0.037317	yes
gene:SpnNT_01464	NA	Chromosome:1501293-1502250	ΔORF2	ΔORF2+peptide	OK	63.8211	111.145	0.800342	1.74531	0.0028	0.0378	yes
gene:SpnNT_02179	dapH	Chromosome:2140586-2141285	ΔORF2	ΔORF2+peptide	OK	559.697	328.531	-0.768615	-1.735	0.00285	0.0382781	yes
gene:SpnNT_00230	adk	Chromosome:213746-214385	ΔORF2	ΔORF2+peptide	OK	223.317	382.157	0.775073	1.74968	0.0029	0.0388304	yes
gene:SpnNT_01138	NA	Chromosome:1162650-1163846	ΔORF2	ΔORF2+peptide	OK	37.198	19.6856	-0.918086	-1.70015	0.0031	0.0411306	yes
gene:SpnNT_01158	iga_3	Chromosome:1180786-1182703	ΔORF2	ΔORF2+peptide	OK	22.4138	39.5477	0.819204	1.75325	0.0032	0.0422439	yes
gene:SpnNT_00754	rpsP	Chromosome:800550-800823	ΔORF2	ΔORF2+peptide	OK	1606.29	2723.71	0.761841	1.64035	0.00335	0.0437836	yes
gene:SpnNT_00996	NA	Chromosome:1024591-1025918	ΔORF2	ΔORF2+peptide	OK	5.523	15.9621	1.53113	1.82476	0.00345	0.0449116	yes
gene:SpnNT_00228	rplO	Chromosome:211832-212273	ΔORF2	ΔORF2+peptide	OK	961.026	1592.69	0.72882	1.64391	0.0036	0.0465411	yes
gene:SpnNT_02053	SpnNT_02053	Chromosome:2034521-2034605	ΔORF2	ΔORF2+peptide	OK	5089.88	14437.8	1.50414	1.96215	0.0036	0.0465411	yes
gene:SpnNT_00480	lcnD_2	Chromosome:493871-495233	ΔORF2	ΔORF2+peptide	OK	3.21897	1.28575	-1.32399	-1.76452	0.00365	0.0469562	yes
gene:SpnNT_00934	NA	Chromosome:967320-968568	ΔORF2	ΔORF2+peptide	OK	11.1523	20.9064	0.906602	1.68224	0.00365	0.0469562	yes
gene:SpnNT_01160	iga_5	Chromosome:1185949-1191166	ΔORF2	ΔORF2+peptide	OK	50.05	83.7541	0.74279	1.6905	0.00365	0.0469562	yes
gene:SpnNT_00223	rpsH	Chromosome:209413-209812	ΔORF2	ΔORF2+peptide	OK	1059.91	1746.97	0.720911	1.63724	0.0037	0.0473672	yes
gene:SpnNT_01244	glyA	Chromosome:1283531-1284788	ΔORF2	ΔORF2+peptide	OK	333.679	201.656	-0.726563	-1.66279	0.00375	0.0479605	yes
gene:SpnNT_00686	NA	Chromosome:737837-739377	ΔORF2	ΔORF2+peptide	OK	55.0296	105.49	0.938825	1.63572	0.0038	0.0485054	yes
gene:SpnNT_00335	dexB	Chromosome:321065-322676	ΔORF2	ΔORF2+peptide	OK	37.7615	62.8778	0.735637	1.61649	0.0039	0.0495409	yes
