# Supplementary material for: Foreign peptide triggers boost in pneumococcal metabolism and growth
Source: BMC Microbiol. 2018 Mar 27;18:23. doi: 10.1186/s12866-018-1167-y (PMC5870813; doi:10.1186/s12866-018-1167-y)
Supplement: Supplementary file 5 — Table S4. RNA-Seq data for wild type and ΔORF 2 mutant without ORF 2 peptide. Table shows only significant differences in expression. A significant difference in expression was observed for 20 genes of which 6 were more highly expressed in the wildtype and 14 were more highly expressed in the mutant. (PDF 108 kb) [file 12866_2018_1167_MOESM5_ESM.pdf]

gene_id	gene_short_n	locus	sample_1	sample_2	status	value_1	value_2	log2_fold_chang	test_stat	p_value	q_value	signif
gene:SpnNT_00337	sarA_2	Chromosome:325122-327081	110.58	ΔORF2	OK	91.3827	235.318	1.36462	3.11995	5.00E-05	0.0013612	yes
gene:SpnNT_00338	glf_1	Chromosome:327403-327805	110.58	ΔORF2	OK	78.8182	212.336	1.42975	2.80976	5.00E-05	0.0013612	yes
gene:SpnNT_00339	glf_2	Chromosome:327814-328321	110.58	ΔORF2	OK	108.31	337.58	1.64007	3.51808	5.00E-05	0.0013612	yes
gene:SpnNT_00543	NA	Chromosome:555058-562378	110.58	ΔORF2	OK	19.1381	77.7203	2.02184	4.49705	5.00E-05	0.0013612	yes
gene:SpnNT_00592	NA	Chromosome:608806-611115	110.58	ΔORF2	OK	6.74045	2.06949	-1.70357	-2.48878	5.00E-05	0.0013612	yes
gene:SpnNT_00601	NA	Chromosome:622378-627283	110.58	ΔORF2	OK	4.05112	1.48794	-1.445	-2.46408	5.00E-05	0.0013612	yes
gene:SpnNT_00827	NA	Chromosome:868544-869231	110.58	ΔORF2	OK	7.82022	20.0891	1.36113	1.9602	0.00055	0.0103545	yes
gene:SpnNT_00825	NA	Chromosome:866845-868210	110.58	ΔORF2	OK	3.72251	8.99838	1.27339	1.93535	0.00095	0.0161266	yes
gene:SpnNT_00833	dpnA_1	Chromosome:873787-875163	110.58	ΔORF2	OK	25.0897	51.8324	1.04676	1.92661	0.00095	0.0161266	yes
gene:SpnNT_00635	NA	Chromosome:682068-683400	110.58	ΔORF2	OK	775.627	423.834	-0.871862	-1.88679	0.00105	0.0174849	yes
gene:SpnNT_00522	licT_1	Chromosome:529544-530384	110.58	ΔORF2	OK	1.37887	4.49977	1.70636	2.08442	0.0014	0.0220273	yes
gene:SpnNT_00597	NA	Chromosome:612435-617772	110.58	ΔORF2	OK	5.10598	1.83198	-1.47879	-1.87643	0.00185	0.0276489	yes
gene:SpnNT_00722	NA	Chromosome:769595-770886	110.58	ΔORF2	OK	3477.66	7162.75	1.0424	1.85037	0.00185	0.0276489	yes
gene:SpnNT_01337	NA	Chromosome:1375635-1376055	110.58	ΔORF2	OK	225.268	429.361	0.93055	1.8249	0.00185	0.0276489	yes
gene:SpnNT_02122	NA	Chromosome:2095359-2095836	110.58	ΔORF2	OK	107.638	204.762	0.927756	1.77637	0.00235	0.0334454	yes
gene:SpnNT_00589	NA	Chromosome:607913-608147	110.58	ΔORF2	OK	2.82204	0	#NAME?	NA	0.0024	0.0337543	yes
gene:SpnNT_01850	NA	Chromosome:1856167-1856791	110.58	ΔORF2	OK	79.571	144.959	0.865335	1.72206	0.0025	0.0347143	yes
gene:SpnNT_01693	NA	Chromosome:1694772-1695075	110.58	ΔORF2	OK	57.8572	121.726	1.07307	1.78387	0.00255	0.0352222	yes
gene:SpnNT_00989	immR_2	Chromosome:1019703-1019925	110.58	ΔORF2	OK	92.6937	29.9242	-1.63116	-1.95923	0.00285	0.0382781	yes
gene:SpnNT_01474	rpsT	Chromosome:1510832-1511069	110.58	ΔORF2	OK	2626.2	4561.21	0.796438	1.67398	0.0037	0.0473672	yes
