# Supplementary material for: Foreign peptide triggers boost in pneumococcal metabolism and growth
Source: BMC Microbiol. 2018 Mar 27;18:23. doi: 10.1186/s12866-018-1167-y (PMC5870813; doi:10.1186/s12866-018-1167-y)
Supplement: Supplementary file 6 — Table S5. Complete proteomic data. (PDF 3560 kb) [file 12866_2018_1167_MOESM6_ESM.pdf]

	A
1	Fasta headers
2	pep chromosome:ASM81700v1:Chromosome:186:1547:1 gene:SpnNT_00001 transcript:AJD70948 gene_biotype:protein_coding transcript_biotype:protein_coding gene_symbol:dnaA description:Chromosomal replication initiator pro
3	pep chromosome:ASM81700v1:Chromosome:1706:2842:1 gene:SpnNT_00002 transcript:AJD70949 gene_biotype:protein_coding transcript_biotype:protein_coding gene_symbol:dnaN description:DNA polymerase III subunit beta
4	pep chromosome:ASM81700v1:Chromosome:2907:3101:1 gene:SpnNT_00003 transcript:AJD70950 gene_biotype:protein_coding transcript_biotype:protein_coding description:hypothetical protein
5	pep chromosome:ASM81700v1:Chromosome:9837:10793:1 gene:SpnNT_00021 transcript:AJD70968 gene_biotype:protein_coding transcript_biotype:protein_coding description:Helix-turn-helix domain protein
6	pep chromosome:ASM81700v1:Chromosome:10942:11946:1 gene:SpnNT_00022 transcript:AJD70969 gene_biotype:protein_coding transcript_biotype:protein_coding description:hypothetical protein
7	pep chromosome:ASM81700v1:Chromosome:13780:14895:1 gene:SpnNT_00026 transcript:AJD70973 gene_biotype:protein_coding transcript_biotype:protein_coding gene_symbol:ychF description:Ribosome-binding ATPase YchF
8	pep chromosome:ASM81700v1:Chromosome:15536:19051:1 gene:SpnNT_00028 transcript:AJD70975 gene_biotype:protein_coding transcript_biotype:protein_coding gene_symbol:mfd description:Transcription-repair-coupling facto
9	pep chromosome:ASM81700v1:Chromosome:19368:19736:1 gene:SpnNT_00030 transcript:AJD70977 gene_biotype:protein_coding transcript_biotype:protein_coding description:Septum formation initiator
10	pep chromosome:ASM81700v1:Chromosome:22402:22944:1 gene:SpnNT_00034 transcript:AJD70981 gene_biotype:protein_coding transcript_biotype:protein_coding gene_symbol:hpt description:Hypoxanthine-guanine phosphoribo
11	pep chromosome:ASM81700v1:Chromosome:22960:24918:1 gene:SpnNT_00035 transcript:AJD70982 gene_biotype:protein_coding transcript_biotype:protein_coding gene_symbol:ftsH description:ATP-dependent zinc metalloprote
12	pep chromosome:ASM81700v1:Chromosome:32814:34142:1 gene:SpnNT_00044 transcript:AJD70985 gene_biotype:protein_coding transcript_biotype:protein_coding gene_symbol:purA description:Adenylosuccinate synthetase
13	pep chromosome:ASM81700v1:Chromosome:34343:34810:1 gene:SpnNT_00045 transcript:AJD70986 gene_biotype:protein_coding transcript_biotype:protein_coding gene_symbol:tadA description:tRNA-specific adenosine deamina
14	pep chromosome:ASM81700v1:Chromosome:34997:35440:1 gene:SpnNT_00046 transcript:AJD70987 gene_biotype:protein_coding transcript_biotype:protein_coding gene_symbol:dut description:Deoxyuridine 5-triphosphate nucleo
15	pep chromosome:ASM81700v1:Chromosome:37405:37902:1 gene:SpnNT_00049 transcript:AJD70990 gene_biotype:protein_coding transcript_biotype:protein_coding gene_symbol:mtcA1 description:Beta-carbonic anhydrase 1
16	pep chromosome:ASM81700v1:Chromosome:38887:39855:1 gene:SpnNT_00051 transcript:AJD70992 gene_biotype:protein_coding transcript_biotype:protein_coding gene_symbol:prs description:Ribose-phosphate pyrophosphokin
17	pep chromosome:ASM81700v1:Chromosome:40650:40871:1 gene:SpnNT_00053 transcript:AJD70994 gene_biotype:protein_coding transcript_biotype:protein_coding description:hypothetical protein
18	pep chromosome:ASM81700v1:Chromosome:41338:44007:1 gene:SpnNT_00054 transcript:AJD70995 gene_biotype:protein_coding transcript_biotype:protein_coding gene_symbol:polA description:DNA polymerase I
19	pep chromosome:ASM81700v1:Chromosome:44092:44529:1 gene:SpnNT_00055 transcript:AJD70996 gene_biotype:protein_coding transcript_biotype:protein_coding description:acetyl coenzyme A synthetase (ADP forming), alpha
20	pep chromosome:ASM81700v1:Chromosome:45916:47085:1 gene:SpnNT_00057 transcript:AJD70998 gene_biotype:protein_coding transcript_biotype:protein_coding gene_symbol:patA description:Putative N-acetyl-LL-diaminopime
21	pep chromosome:ASM81700v1:Chromosome:47849:48841:1 gene:SpnNT_00059 transcript:AJD71000 gene_biotype:protein_coding transcript_biotype:protein_coding gene_symbol:plsX description:Phosphate acyltransferase
22	pep chromosome:ASM81700v1:Chromosome:48847:49080:1 gene:SpnNT_00060 transcript:AJD71001 gene_biotype:protein_coding transcript_biotype:protein_coding gene_symbol:acpP description:Acyl carrier protein
23	pep chromosome:ASM81700v1:Chromosome:50603:51496:1 gene:SpnNT_00064 transcript:AJD71005 gene_biotype:protein_coding transcript_biotype:protein_coding description:Helix-turn-helix domain protein
24	pep chromosome:ASM81700v1:Chromosome:59091:59798:1 gene:SpnNT_00072 transcript:AJD71013 gene_biotype:protein_coding transcript_biotype:protein_coding gene_symbol:purC description:Phosphoribosylaminoimidazole-s
25	pep chromosome:ASM81700v1:Chromosome:60000:63725:1 gene:SpnNT_00073 transcript:AJD71014 gene_biotype:protein_coding transcript_biotype:protein_coding gene_symbol:purL description:Phosphoribosylformylglycinamidi
26	pep chromosome:ASM81700v1:Chromosome:63818:65260:1 gene:SpnNT_00074 transcript:AJD71015 gene_biotype:protein_coding transcript_biotype:protein_coding gene_symbol:purF description:Amidophosphoribosyltransferase
27	pep chromosome:ASM81700v1:Chromosome:65297:66319:1 gene:SpnNT_00075 transcript:AJD71016 gene_biotype:protein_coding transcript_biotype:protein_coding gene_symbol:purM description:Phosphoribosylformylglycinamidi
28	pep chromosome:ASM81700v1:Chromosome:67479:69026:1 gene:SpnNT_00078 transcript:AJD71019 gene_biotype:protein_coding transcript_biotype:protein_coding gene_symbol:purH description:Bifunctional purine biosynthesis p
29	pep chromosome:ASM81700v1:Chromosome:69148:70410:1 gene:SpnNT_00079 transcript:AJD71020 gene_biotype:protein_coding transcript_biotype:protein_coding gene_symbol:purD description:Phosphoribosylamine--glycine lig
30	pep chromosome:ASM81700v1:Chromosome:70813:71301:1 gene:SpnNT_00080 transcript:AJD71021 gene_biotype:protein_coding transcript_biotype:protein_coding gene_symbol:purE description:N5-carboxyaminoimidazole ribon
31	pep chromosome:ASM81700v1:Chromosome:71288:72379:1 gene:SpnNT_00081 transcript:AJD71022 gene_biotype:protein_coding transcript_biotype:protein_coding gene_symbol:purK description:N5-carboxyaminoimidazole ribon
32	pep chromosome:ASM81700v1:Chromosome:72389:72616:1 gene:SpnNT_00082 transcript:AJD71023 gene_biotype:protein_coding transcript_biotype:protein_coding description:hypothetical protein
33	pep chromosome:ASM81700v1:Chromosome:72679:73977:1 gene:SpnNT_00083 transcript:AJD71024 gene_biotype:protein_coding transcript_biotype:protein_coding gene_symbol:purB description:Adenylosuccinate lyase
34	pep chromosome:ASM81700v1:Chromosome:78284:79000:-1 gene:SpnNT_00086 transcript:AJD71027 gene_biotype:protein_coding transcript_biotype:protein_coding gene_symbol:frlR description:HTH-type transcriptional regulator
35	pep chromosome:ASM81700v1:Chromosome:89873:94723:1 gene:SpnNT_00096 transcript:AJD71037 gene_biotype:protein_coding transcript_biotype:protein_coding gene_symbol:iga_1 description:Immunoglobulin A1 protease pre
36	pep chromosome:ASM81700v1:Chromosome:96813:97577:1 gene:SpnNT_00099 transcript:AJD71040 gene_biotype:protein_coding transcript_biotype:protein_coding gene_symbol:deoD_1 description:Purine nucleoside phosphoryla
37	pep chromosome:ASM81700v1:Chromosome:99760:100425:1 gene:SpnNT_00102 transcript:AJD71043 gene_biotype:protein_coding transcript_biotype:protein_coding gene_symbol:ktrA description:Ktr system potassium uptake pro
38	pep chromosome:ASM81700v1:Chromosome:104117:104815:1 gene:SpnNT_00106 transcript:AJD71047 gene_biotype:protein_coding transcript_biotype:protein_coding gene_symbol:saeR description:exoprotein expression protein
39	pep chromosome:ASM81700v1:Chromosome:104812:105864:1 gene:SpnNT_00107 transcript:AJD71048 gene_biotype:protein_coding transcript_biotype:protein_coding gene_symbol:saeS description:Histidine protein kinase SaeS
40	pep chromosome:ASM81700v1:Chromosome:106059:106670:1 gene:SpnNT_00108 transcript:AJD71049 gene_biotype:protein_coding transcript_biotype:protein_coding gene_symbol:rpsD description:30S ribosomal protein S4
41	pep chromosome:ASM81700v1:Chromosome:111743:112729:1 gene:SpnNT_00112 transcript:AJD71053 gene_biotype:protein_coding transcript_biotype:protein_coding description:putative rhodanese-related sulfurtransferase
42	pep chromosome:ASM81700v1:Chromosome:114038:115102:-1 gene:SpnNT_00115 transcript:AJD71056 gene_biotype:protein_coding transcript_biotype:protein_coding description:hypothetical protein
43	pep chromosome:ASM81700v1:Chromosome:115164:116075:-1 gene:SpnNT_00116 transcript:AJD71057 gene_biotype:protein_coding transcript_biotype:protein_coding description:hypothetical protein
44	pep chromosome:ASM81700v1:Chromosome:116068:116661:-1 gene:SpnNT_00117 transcript:AJD71058 gene_biotype:protein_coding transcript_biotype:protein_coding description:putative membrane protein
45	pep chromosome:ASM81700v1:Chromosome:119536:121386:1 gene:SpnNT_00121 transcript:AJD71062 gene_biotype:protein_coding transcript_biotype:protein_coding gene_symbol:pglF description:UDP-N-acetyl-alpha-D-glucosa
46	pep chromosome:ASM81700v1:Chromosome:122807:123679:-1 gene:SpnNT_00126 transcript:AJD71067 gene_biotype:protein_coding transcript_biotype:protein_coding gene_symbol:sdhA description:L-serine dehydratase, alpha c
47	pep chromosome:ASM81700v1:Chromosome:123688:124359:-1 gene:SpnNT_00127 transcript:AJD71068 gene_biotype:protein_coding transcript_biotype:protein_coding gene_symbol:sdhB description:L-serine dehydratase, beta ch
48	pep chromosome:ASM81700v1:Chromosome:124601:125188:1 gene:SpnNT_00128 transcript:AJD71069 gene_biotype:protein_coding transcript_biotype:protein_coding gene_symbol:lytN description:putative cell wall hydrolase LytN
49	pep chromosome:ASM81700v1:Chromosome:125240:126103:-1 gene:SpnNT_00129 transcript:AJD71070 gene_biotype:protein_coding transcript_biotype:protein_coding description:Helix-turn-helix domain protein
50	pep chromosome:ASM81700v1:Chromosome:139271:141529:1 gene:SpnNT_00143 transcript:AJD71084 gene_biotype:protein_coding transcript_biotype:protein_coding gene_symbol:lytB_1 description:Putative endo-beta-N-acetylg
51	pep chromosome:ASM81700v1:Chromosome:141929:143050:1 gene:SpnNT_00145 transcript:AJD71086 gene_biotype:protein_coding transcript_biotype:protein_coding gene_symbol:mnmA description:tRNA-specific 2-thiouridylase
52	pep chromosome:ASM81700v1:Chromosome:143656:145569:1 gene:SpnNT_00147 transcript:AJD71088 gene_biotype:protein_coding transcript_biotype:protein_coding gene_symbol:mnmG description:Glucose-inhibited division pr
53	pep chromosome:ASM81700v1:Chromosome:145921:147600:-1 gene:SpnNT_00148 transcript:AJD71089 gene_biotype:protein_coding transcript_biotype:protein_coding description:Ribonuclease J 1
54	pep chromosome:ASM81700v1:Chromosome:147602:147835:-1 gene:SpnNT_00149 transcript:AJD71090 gene_biotype:protein_coding transcript_biotype:protein_coding description:hypothetical protein

	A
55	pep chromosome:ASM81700v1:Chromosome:150514:151197:1 gene:SpnNT_00154 transcript:AJD71095 gene_biotype:protein_coding transcript_biotype:protein_coding description:UGMP family protein
56	pep chromosome:ASM81700v1:Chromosome:151621:152631:1 gene:SpnNT_00156 transcript:AJD71097 gene_biotype:protein_coding transcript_biotype:protein_coding gene_symbol:gcp description:t(6)A37 threonylcarbamoyladen
57	pep chromosome:ASM81700v1:Chromosome:152735:153598:1 gene:SpnNT_00157 transcript:AJD71098 gene_biotype:protein_coding transcript_biotype:protein_coding description:hypothetical protein
58	pep chromosome:ASM81700v1:Chromosome:154096:156291:1 gene:SpnNT_00158 transcript:AJD71099 gene_biotype:protein_coding transcript_biotype:protein_coding description:type 2 lantibiotic biosynthesis protein LanM
59	pep chromosome:ASM81700v1:Chromosome:159207:160037:1 gene:SpnNT_00163 transcript:AJD71104 gene_biotype:protein_coding transcript_biotype:protein_coding gene_symbol:tcyA description:L-cystine-binding protein TcyA
60	pep chromosome:ASM81700v1:Chromosome:160191:161045:1 gene:SpnNT_00164 transcript:AJD71105 gene_biotype:protein_coding transcript_biotype:protein_coding gene_symbol:metQ description:D-methionine-binding lipoprot
61	pep chromosome:ASM81700v1:Chromosome:161145:162518:1 gene:SpnNT_00165 transcript:AJD71106 gene_biotype:protein_coding transcript_biotype:protein_coding gene_symbol:dapE description:Succinyl-diaminopimelate des
62	pep chromosome:ASM81700v1:Chromosome:162511:163572:1 gene:SpnNT_00166 transcript:AJD71107 gene_biotype:protein_coding transcript_biotype:protein_coding gene_symbol:metN description:Methionine import ATP-bindin
63	pep chromosome:ASM81700v1:Chromosome:168607:169140:1 gene:SpnNT_00173 transcript:AJD71114 gene_biotype:protein_coding transcript_biotype:protein_coding description:hypothetical protein
64	pep chromosome:ASM81700v1:Chromosome:169218:169688:1 gene:SpnNT_00174 transcript:AJD71115 gene_biotype:protein_coding transcript_biotype:protein_coding description:Putative NrdI-like protein
65	pep chromosome:ASM81700v1:Chromosome:169981:171243:-1 gene:SpnNT_00175 transcript:AJD71116 gene_biotype:protein_coding transcript_biotype:protein_coding description:Mn2+ and Fe2+ transporters of the NRAMP famil
66	pep chromosome:ASM81700v1:Chromosome:172655:174604:1 gene:SpnNT_00178 transcript:AJD71119 gene_biotype:protein_coding transcript_biotype:protein_coding gene_symbol:mutL description:DNA mismatch repair protein
67	pep chromosome:ASM81700v1:Chromosome:174929:175396:-1 gene:SpnNT_00179 transcript:AJD71120 gene_biotype:protein_coding transcript_biotype:protein_coding gene_symbol:ribH description:6,7-dimethyl-8-ribityllumazine
68	pep chromosome:ASM81700v1:Chromosome:176622:177257:-1 gene:SpnNT_00181 transcript:AJD71122 gene_biotype:protein_coding transcript_biotype:protein_coding gene_symbol:ribE description:Riboflavin synthase
69	pep chromosome:ASM81700v1:Chromosome:177242:178342:-1 gene:SpnNT_00182 transcript:AJD71123 gene_biotype:protein_coding transcript_biotype:protein_coding gene_symbol:ribD_1 description:Riboflavin biosynthesis prot
70	pep chromosome:ASM81700v1:Chromosome:178746:179339:1 gene:SpnNT_00183 transcript:AJD71124 gene_biotype:protein_coding transcript_biotype:protein_coding gene_symbol:ruvA description:Holliday junction ATP-depende
71	pep chromosome:ASM81700v1:Chromosome:180723:181754:-1 gene:SpnNT_00186 transcript:AJD71127 gene_biotype:protein_coding transcript_biotype:protein_coding gene_symbol:mccF description:Microcin C7 self-immunity pro
72	pep chromosome:ASM81700v1:Chromosome:182009:182692:-1 gene:SpnNT_00187 transcript:AJD71128 gene_biotype:protein_coding transcript_biotype:protein_coding description:hypothetical protein
73	pep chromosome:ASM81700v1:Chromosome:182704:183648:-1 gene:SpnNT_00188 transcript:AJD71129 gene_biotype:protein_coding transcript_biotype:protein_coding gene_symbol:corA description:Magnesium transport protein
74	pep chromosome:ASM81700v1:Chromosome:183780:186611:1 gene:SpnNT_00189 transcript:AJD71130 gene_biotype:protein_coding transcript_biotype:protein_coding gene_symbol:uvrA description:Excinuclease ABC subunit A
75	pep chromosome:ASM81700v1:Chromosome:186604:187665:1 gene:SpnNT_00190 transcript:AJD71131 gene_biotype:protein_coding transcript_biotype:protein_coding description:putative peptidase
76	pep chromosome:ASM81700v1:Chromosome:190103:190501:1 gene:SpnNT_00193 transcript:AJD71134 gene_biotype:protein_coding transcript_biotype:protein_coding gene_symbol:spxA_1 description:Regulatory protein spx
77	pep chromosome:ASM81700v1:Chromosome:190567:191136:1 gene:SpnNT_00194 transcript:AJD71135 gene_biotype:protein_coding transcript_biotype:protein_coding description:hypothetical protein
78	pep chromosome:ASM81700v1:Chromosome:191223:191489:1 gene:SpnNT_00195 transcript:AJD71136 gene_biotype:protein_coding transcript_biotype:protein_coding description:hypothetical protein
79	pep chromosome:ASM81700v1:Chromosome:191493:191912:1 gene:SpnNT_00196 transcript:AJD71137 gene_biotype:protein_coding transcript_biotype:protein_coding gene_symbol:yrrK description:Putative Holliday junction resol
80	pep chromosome:ASM81700v1:Chromosome:191928:192233:1 gene:SpnNT_00197 transcript:AJD71138 gene_biotype:protein_coding transcript_biotype:protein_coding description:hypothetical protein
81	pep chromosome:ASM81700v1:Chromosome:192540:193790:1 gene:SpnNT_00198 transcript:AJD71139 gene_biotype:protein_coding transcript_biotype:protein_coding gene_symbol:fgs_1 description:Folylpolyglutamate synthase
82	pep chromosome:ASM81700v1:Chromosome:193874:194332:1 gene:SpnNT_00199 transcript:AJD71140 gene_biotype:protein_coding transcript_biotype:protein_coding description:Bacterial lipoprotein
83	pep chromosome:ASM81700v1:Chromosome:194478:196010:1 gene:SpnNT_00200 transcript:AJD71141 gene_biotype:protein_coding transcript_biotype:protein_coding gene_symbol:cls description:Cardiolipin synthase
84	pep chromosome:ASM81700v1:Chromosome:196268:197806:1 gene:SpnNT_00202 transcript:AJD71143 gene_biotype:protein_coding transcript_biotype:protein_coding description:hypothetical protein
85	pep chromosome:ASM81700v1:Chromosome:197926:200133:1 gene:SpnNT_00203 transcript:AJD71144 gene_biotype:protein_coding transcript_biotype:protein_coding gene_symbol:nrdD_1 description:Anaerobic ribonucleoside-tri
86	pep chromosome:ASM81700v1:Chromosome:201415:202032:1 gene:SpnNT_00206 transcript:AJD71147 gene_biotype:protein_coding transcript_biotype:protein_coding description:uridine/cytidine kinase
87	pep chromosome:ASM81700v1:Chromosome:202299:202607:1 gene:SpnNT_00207 transcript:AJD71148 gene_biotype:protein_coding transcript_biotype:protein_coding gene_symbol:rpsJ description:ribosomal protein s10
88	pep chromosome:ASM81700v1:Chromosome:202824:203450:1 gene:SpnNT_00208 transcript:AJD71149 gene_biotype:protein_coding transcript_biotype:protein_coding gene_symbol:rplC description:50S ribosomal protein L3
89	pep chromosome:ASM81700v1:Chromosome:203475:204098:1 gene:SpnNT_00209 transcript:AJD71150 gene_biotype:protein_coding transcript_biotype:protein_coding gene_symbol:rplD description:50S ribosomal protein L4
90	pep chromosome:ASM81700v1:Chromosome:204098:204394:1 gene:SpnNT_00210 transcript:AJD71151 gene_biotype:protein_coding transcript_biotype:protein_coding gene_symbol:rplW description:50S ribosomal protein L23
91	pep chromosome:ASM81700v1:Chromosome:204412:205245:1 gene:SpnNT_00211 transcript:AJD71152 gene_biotype:protein_coding transcript_biotype:protein_coding gene_symbol:rplB description:ribosomal protein l2
92	pep chromosome:ASM81700v1:Chromosome:205349:205630:1 gene:SpnNT_00212 transcript:AJD71153 gene_biotype:protein_coding transcript_biotype:protein_coding gene_symbol:rpsS description:30S ribosomal protein S19
93	pep chromosome:ASM81700v1:Chromosome:205642:205986:1 gene:SpnNT_00213 transcript:AJD71154 gene_biotype:protein_coding transcript_biotype:protein_coding gene_symbol:rplV description:50S ribosomal protein L22
94	pep chromosome:ASM81700v1:Chromosome:205999:206652:1 gene:SpnNT_00214 transcript:AJD71155 gene_biotype:protein_coding transcript_biotype:protein_coding gene_symbol:rpsC description:ribosomal protein s3
95	pep chromosome:ASM81700v1:Chromosome:206656:207069:1 gene:SpnNT_00215 transcript:AJD71156 gene_biotype:protein_coding transcript_biotype:protein_coding gene_symbol:rplP description:50S ribosomal protein L16
96	pep chromosome:ASM81700v1:Chromosome:207079:207285:1 gene:SpnNT_00216 transcript:AJD71157 gene_biotype:protein_coding transcript_biotype:protein_coding gene_symbol:rpmC description:50S ribosomal protein L29
97	pep chromosome:ASM81700v1:Chromosome:207310:207570:1 gene:SpnNT_00217 transcript:AJD71158 gene_biotype:protein_coding transcript_biotype:protein_coding gene_symbol:rpsQ description:30s ribosomal protein s17
98	pep chromosome:ASM81700v1:Chromosome:207596:207964:1 gene:SpnNT_00218 transcript:AJD71159 gene_biotype:protein_coding transcript_biotype:protein_coding gene_symbol:rplN description:50S ribosomal protein L14
99	pep chromosome:ASM81700v1:Chromosome:208042:208347:1 gene:SpnNT_00219 transcript:AJD71160 gene_biotype:protein_coding transcript_biotype:protein_coding gene_symbol:rplX description:50S ribosomal protein L24
100	pep chromosome:ASM81700v1:Chromosome:208371:208913:1 gene:SpnNT_00220 transcript:AJD71161 gene_biotype:protein_coding transcript_biotype:protein_coding gene_symbol:rplE description:50S ribosomal protein L5
101	pep chromosome:ASM81700v1:Chromosome:208931:209200:1 gene:SpnNT_00221 transcript:AJD71162 gene_biotype:protein_coding transcript_biotype:protein_coding gene_symbol:rpsN2 description:Alternate 30S ribosomal prote
102	pep chromosome:ASM81700v1:Chromosome:209414:209812:1 gene:SpnNT_00223 transcript:AJD71164 gene_biotype:protein_coding transcript_biotype:protein_coding gene_symbol:rpsH description:30S ribosomal protein S8
103	pep chromosome:ASM81700v1:Chromosome:210004:210540:1 gene:SpnNT_00224 transcript:AJD71165 gene_biotype:protein_coding transcript_biotype:protein_coding gene_symbol:rplF description:50s ribosomal protein l6
104	pep chromosome:ASM81700v1:Chromosome:210624:210980:1 gene:SpnNT_00225 transcript:AJD71166 gene_biotype:protein_coding transcript_biotype:protein_coding gene_symbol:rplR description:ribosomal protein l18
105	pep chromosome:ASM81700v1:Chromosome:210998:211492:1 gene:SpnNT_00226 transcript:AJD71167 gene_biotype:protein_coding transcript_biotype:protein_coding gene_symbol:rpsE description:ribosomal protein s5
106	pep chromosome:ASM81700v1:Chromosome:211506:211688:1 gene:SpnNT_00227 transcript:AJD71168 gene_biotype:protein_coding transcript_biotype:protein_coding gene_symbol:rpmD description:50S ribosomal protein L30
107	pep chromosome:ASM81700v1:Chromosome:211833:212273:1 gene:SpnNT_00228 transcript:AJD71169 gene_biotype:protein_coding transcript_biotype:protein_coding gene_symbol:rplO description:50S ribosomal protein L15
108	pep chromosome:ASM81700v1:Chromosome:212286:213596:1 gene:SpnNT_00229 transcript:AJD71170 gene_biotype:protein_coding transcript_biotype:protein_coding description:preprotein translocase subunit SecY

	A
109	pep chromosome:ASM81700v1:Chromosome:213747:214385:1 gene:SpnNT_00230 transcript:AJD71171 gene_biotype:protein_coding transcript_biotype:protein_coding gene_symbol:adk description:Adenylate kinase
110	pep chromosome:ASM81700v1:Chromosome:214502:214720:1 gene:SpnNT_00231 transcript:AJD71172 gene_biotype:protein_coding transcript_biotype:protein_coding gene_symbol:infA description:Translation initiation factor IF-1
111	pep chromosome:ASM81700v1:Chromosome:214745:214861:1 gene:SpnNT_00232 transcript:AJD71173 gene_biotype:protein_coding transcript_biotype:protein_coding gene_symbol:rpmJ description:Ribosomal protein II
112	pep chromosome:ASM81700v1:Chromosome:214879:215244:1 gene:SpnNT_00233 transcript:AJD71174 gene_biotype:protein_coding transcript_biotype:protein_coding gene_symbol:rpsM description:30s ribosomal protein s13
113	pep chromosome:ASM81700v1:Chromosome:215262:215645:1 gene:SpnNT_00234 transcript:AJD71175 gene_biotype:protein_coding transcript_biotype:protein_coding gene_symbol:rpsK description:30S ribosomal protein S11
114	pep chromosome:ASM81700v1:Chromosome:215688:216623:1 gene:SpnNT_00235 transcript:AJD71176 gene_biotype:protein_coding transcript_biotype:protein_coding gene_symbol:rpoA description:DNA-directed RNA polymerase
115	pep chromosome:ASM81700v1:Chromosome:216635:217021:1 gene:SpnNT_00236 transcript:AJD71177 gene_biotype:protein_coding transcript_biotype:protein_coding gene_symbol:rplQ description:ribosomal protein l17
116	pep chromosome:ASM81700v1:Chromosome:217286:217552:1 gene:SpnNT_00237 transcript:AJD71178 gene_biotype:protein_coding transcript_biotype:protein_coding description:ACT domain-containing protein
117	pep chromosome:ASM81700v1:Chromosome:217562:218899:1 gene:SpnNT_00238 transcript:AJD71179 gene_biotype:protein_coding transcript_biotype:protein_coding description:hypothetical protein
118	pep chromosome:ASM81700v1:Chromosome:219143:219835:1 gene:SpnNT_00239 transcript:AJD71180 gene_biotype:protein_coding transcript_biotype:protein_coding gene_symbol:gpmA_1 description:2,3-bisphosphoglycerate-de
119	pep chromosome:ASM81700v1:Chromosome:225172:225918:1 gene:SpnNT_00248 transcript:AJD71189 gene_biotype:protein_coding transcript_biotype:protein_coding gene_symbol:srlR description:Glucitol operon repressor
120	pep chromosome:ASM81700v1:Chromosome:235374:237875:1 gene:SpnNT_00258 transcript:AJD71199 gene_biotype:protein_coding transcript_biotype:protein_coding gene_symbol:leuS description:Leucine--tRNA ligase
121	pep chromosome:ASM81700v1:Chromosome:238069:238764:1 gene:SpnNT_00259 transcript:AJD71200 gene_biotype:protein_coding transcript_biotype:protein_coding gene_symbol:ydaF_1 description:Putative ribosomal N-acetylt
122	pep chromosome:ASM81700v1:Chromosome:238776:239192:1 gene:SpnNT_00260 transcript:AJD71201 gene_biotype:protein_coding transcript_biotype:protein_coding description:ribosomal-protein-alanine acetyltransferase
123	pep chromosome:ASM81700v1:Chromosome:241085:242083:1 gene:SpnNT_00261 transcript:AJD71202 gene_biotype:protein_coding transcript_biotype:protein_coding gene_symbol:ruvB description:Holliday junction ATP-depende
124	pep chromosome:ASM81700v1:Chromosome:244458:245717:1 gene:SpnNT_00265 transcript:AJD71206 gene_biotype:protein_coding transcript_biotype:protein_coding description:Putative zinc metalloprotease
125	pep chromosome:ASM81700v1:Chromosome:245730:247583:1 gene:SpnNT_00266 transcript:AJD71207 gene_biotype:protein_coding transcript_biotype:protein_coding gene_symbol:proS description:Proline--tRNA ligase
126	pep chromosome:ASM81700v1:Chromosome:247682:249061:1 gene:SpnNT_00267 transcript:AJD71208 gene_biotype:protein_coding transcript_biotype:protein_coding gene_symbol:gmuD_1 description:6-phospho-beta-glucosidas
127	pep chromosome:ASM81700v1:Chromosome:249254:251062:1 gene:SpnNT_00268 transcript:AJD71209 gene_biotype:protein_coding transcript_biotype:protein_coding gene_symbol:glmS description:Glutamine--fructose-6-phosph
128	pep chromosome:ASM81700v1:Chromosome:258293:262063:-1 gene:SpnNT_00275 transcript:AJD71216 gene_biotype:protein_coding transcript_biotype:protein_coding gene_symbol:pulA description:Pullulanase precursor
129	pep chromosome:ASM81700v1:Chromosome:264188:264601:1 gene:SpnNT_00277 transcript:AJD71218 gene_biotype:protein_coding transcript_biotype:protein_coding gene_symbol:rpsL description:30S ribosomal protein S12
130	pep chromosome:ASM81700v1:Chromosome:264621:265091:1 gene:SpnNT_00278 transcript:AJD71219 gene_biotype:protein_coding transcript_biotype:protein_coding gene_symbol:rpsG description:30S ribosomal protein S7
131	pep chromosome:ASM81700v1:Chromosome:265516:267597:1 gene:SpnNT_00279 transcript:AJD71220 gene_biotype:protein_coding transcript_biotype:protein_coding gene_symbol:fus description:Elongation factor G
132	pep chromosome:ASM81700v1:Chromosome:267701:272092:1 gene:SpnNT_00280 transcript:AJD71221 gene_biotype:protein_coding transcript_biotype:protein_coding gene_symbol:polC_1 description:DNA polymerase III PolC-typ
133	pep chromosome:ASM81700v1:Chromosome:272194:272457:1 gene:SpnNT_00281 transcript:AJD71222 gene_biotype:protein_coding transcript_biotype:protein_coding description:addiction module antitoxin, RelB/DinJ family
134	pep chromosome:ASM81700v1:Chromosome:272923:274164:1 gene:SpnNT_00284 transcript:AJD71225 gene_biotype:protein_coding transcript_biotype:protein_coding gene_symbol:pepS description:Aminopeptidase PepS
135	pep chromosome:ASM81700v1:Chromosome:274672:275394:1 gene:SpnNT_00286 transcript:AJD71227 gene_biotype:protein_coding transcript_biotype:protein_coding gene_symbol:rsuA_1 description:Ribosomal small subunit pse
136	pep chromosome:ASM81700v1:Chromosome:275611:276945:1 gene:SpnNT_00287 transcript:AJD71228 gene_biotype:protein_coding transcript_biotype:protein_coding gene_symbol:pepC description:Aminopeptidase C
137	pep chromosome:ASM81700v1:Chromosome:277001:277912:-1 gene:SpnNT_00288 transcript:AJD71229 gene_biotype:protein_coding transcript_biotype:protein_coding gene_symbol:manZ_2 description:PTS system mannose-spe
138	pep chromosome:ASM81700v1:Chromosome:277936:278739:-1 gene:SpnNT_00289 transcript:AJD71230 gene_biotype:protein_coding transcript_biotype:protein_coding gene_symbol:manY description:PTS system mannose-specif
139	pep chromosome:ASM81700v1:Chromosome:278767:279756:-1 gene:SpnNT_00290 transcript:AJD71231 gene_biotype:protein_coding transcript_biotype:protein_coding gene_symbol:manX_2 description:EIIAB-Man
140	pep chromosome:ASM81700v1:Chromosome:279989:281008:-1 gene:SpnNT_00291 transcript:AJD71232 gene_biotype:protein_coding transcript_biotype:protein_coding gene_symbol:adhA description:Alcohol dehydrogenase 1
141	pep chromosome:ASM81700v1:Chromosome:281347:282159:-1 gene:SpnNT_00292 transcript:AJD71233 gene_biotype:protein_coding transcript_biotype:protein_coding gene_symbol:yidA_1 description:Sugar phosphatase YidA
142	pep chromosome:ASM81700v1:Chromosome:282349:283767:1 gene:SpnNT_00293 transcript:AJD71234 gene_biotype:protein_coding transcript_biotype:protein_coding gene_symbol:pbuO description:Guanine/hypoxanthine perme
143	pep chromosome:ASM81700v1:Chromosome:284651:285598:1 gene:SpnNT_00295 transcript:AJD71236 gene_biotype:protein_coding transcript_biotype:protein_coding gene_symbol:sulA description:Dihydropteroate synthase
144	pep chromosome:ASM81700v1:Chromosome:285600:286922:1 gene:SpnNT_00296 transcript:AJD71237 gene_biotype:protein_coding transcript_biotype:protein_coding gene_symbol:fgs_2 description:Folylpolyglutamate synthase
145	pep chromosome:ASM81700v1:Chromosome:286903:287457:1 gene:SpnNT_00297 transcript:AJD71238 gene_biotype:protein_coding transcript_biotype:protein_coding gene_symbol:folE description:GTP cyclohydrolase 1
146	pep chromosome:ASM81700v1:Chromosome:287500:288312:1 gene:SpnNT_00298 transcript:AJD71239 gene_biotype:protein_coding transcript_biotype:protein_coding gene_symbol:sulD description:Bifunctional folate synthesis pr
147	pep chromosome:ASM81700v1:Chromosome:289031:289477:1 gene:SpnNT_00300 transcript:AJD71241 gene_biotype:protein_coding transcript_biotype:protein_coding gene_symbol:rplM description:50S ribosomal protein L13
148	pep chromosome:ASM81700v1:Chromosome:289497:289889:1 gene:SpnNT_00301 transcript:AJD71242 gene_biotype:protein_coding transcript_biotype:protein_coding gene_symbol:rpsI description:30S ribosomal protein S9
149	pep chromosome:ASM81700v1:Chromosome:291298:293511:1 gene:SpnNT_00305 transcript:AJD71246 gene_biotype:protein_coding transcript_biotype:protein_coding gene_symbol:yicI description:Alpha-xylosidase
150	pep chromosome:ASM81700v1:Chromosome:308209:309210:1 gene:SpnNT_00321 transcript:AJD71262 gene_biotype:protein_coding transcript_biotype:protein_coding gene_symbol:kdgR description:Kdg operon repressor
151	pep chromosome:ASM81700v1:Chromosome:311235:311429:-1 gene:SpnNT_00326 transcript:AJD71267 gene_biotype:protein_coding transcript_biotype:protein_coding description:transcriptional repressor DicA
152	pep chromosome:ASM81700v1:Chromosome:311595:312545:1 gene:SpnNT_00327 transcript:AJD71268 gene_biotype:protein_coding transcript_biotype:protein_coding gene_symbol:rsmH description:Ribosomal RNA small subunit
153	pep chromosome:ASM81700v1:Chromosome:312557:312874:1 gene:SpnNT_00328 transcript:AJD71269 gene_biotype:protein_coding transcript_biotype:protein_coding description:cell division protein FtsL
154	pep chromosome:ASM81700v1:Chromosome:312878:315130:1 gene:SpnNT_00329 transcript:AJD71270 gene_biotype:protein_coding transcript_biotype:protein_coding gene_symbol:pbpX_1 description:Penicillin-binding protein 2X
155	pep chromosome:ASM81700v1:Chromosome:315132:316112:1 gene:SpnNT_00330 transcript:AJD71271 gene_biotype:protein_coding transcript_biotype:protein_coding gene_symbol:mraY description:Phospho-N-acetylmuramoyl-p
156	pep chromosome:ASM81700v1:Chromosome:316452:318557:1 gene:SpnNT_00332 transcript:AJD71273 gene_biotype:protein_coding transcript_biotype:protein_coding gene_symbol:clpC_1 description:putative ATP-dependent Clp
157	pep chromosome:ASM81700v1:Chromosome:318853:319335:-1 gene:SpnNT_00333 transcript:AJD71274 gene_biotype:protein_coding transcript_biotype:protein_coding gene_symbol:luxS description:S-ribosylhomocysteine lyase
158	pep chromosome:ASM81700v1:Chromosome:319430:320914:-1 gene:SpnNT_00334 transcript:AJD71275 gene_biotype:protein_coding transcript_biotype:protein_coding description:hypothetical protein
159	pep chromosome:ASM81700v1:Chromosome:321066:322676:1 gene:SpnNT_00335 transcript:AJD71276 gene_biotype:protein_coding transcript_biotype:protein_coding gene_symbol:dexB description:Glucan 1,6-alpha-glucosidase
160	pep chromosome:ASM81700v1:Chromosome:322952:324919:1 gene:SpnNT_00336 transcript:AJD71277 gene_biotype:protein_coding transcript_biotype:protein_coding gene_symbol:sarA_1 description:76 kDa cell surface lipoprote
161	pep chromosome:ASM81700v1:Chromosome:325123:327081:1 gene:SpnNT_00337 transcript:AJD71278 gene_biotype:protein_coding transcript_biotype:protein_coding gene_symbol:sarA_2 description:76 kDa cell surface lipoprote
162	pep chromosome:ASM81700v1:Chromosome:328433:328804:-1 gene:SpnNT_00340 transcript:AJD71281 gene_biotype:protein_coding transcript_biotype:protein_coding gene_symbol:doc_1 description:Death on curing protein

	A
163	pep chromosome:ASM81700v1:Chromosome:328801:329058:-1 gene:SpnNT_00341 transcript:AJD71282 gene_biotype:protein_coding transcript_biotype:protein_coding description:Phd_YefM
164	pep chromosome:ASM81700v1:Chromosome:329277:331256:1 gene:SpnNT_00342 transcript:AJD71283 gene_biotype:protein_coding transcript_biotype:protein_coding gene_symbol:sarA_3 description:76 kDa cell surface lipoprote
165	pep chromosome:ASM81700v1:Chromosome:333071:335227:-1 gene:SpnNT_00345 transcript:AJD71286 gene_biotype:protein_coding transcript_biotype:protein_coding gene_symbol:ponA description:Penicillin-binding protein 1A
166	pep chromosome:ASM81700v1:Chromosome:336496:336825:1 gene:SpnNT_00348 transcript:AJD71289 gene_biotype:protein_coding transcript_biotype:protein_coding gene_symbol:gpsB description:Guiding PBP1-shuttling protei
167	pep chromosome:ASM81700v1:Chromosome:337311:338468:1 gene:SpnNT_00349 transcript:AJD71290 gene_biotype:protein_coding transcript_biotype:protein_coding gene_symbol:rlmL description:Ribosomal RNA large subunit
168	pep chromosome:ASM81700v1:Chromosome:338481:339851:1 gene:SpnNT_00350 transcript:AJD71291 gene_biotype:protein_coding transcript_biotype:protein_coding description:putative membrane protein
169	pep chromosome:ASM81700v1:Chromosome:339927:341351:1 gene:SpnNT_00351 transcript:AJD71292 gene_biotype:protein_coding transcript_biotype:protein_coding gene_symbol:gnd description:6-phosphogluconate dehydroge
170	pep chromosome:ASM81700v1:Chromosome:341363:342049:1 gene:SpnNT_00352 transcript:AJD71293 gene_biotype:protein_coding transcript_biotype:protein_coding gene_symbol:arlR_1 description:Response regulator ArlR
171	pep chromosome:ASM81700v1:Chromosome:343572:344534:1 gene:SpnNT_00354 transcript:AJD71295 gene_biotype:protein_coding transcript_biotype:protein_coding gene_symbol:lytA_2 description:Autolysin
172	pep chromosome:ASM81700v1:Chromosome:344553:345551:1 gene:SpnNT_00355 transcript:AJD71296 gene_biotype:protein_coding transcript_biotype:protein_coding gene_symbol:lytA_3 description:Autolysin
173	pep chromosome:ASM81700v1:Chromosome:348882:349760:1 gene:SpnNT_00360 transcript:AJD71301 gene_biotype:protein_coding transcript_biotype:protein_coding description:mevalonate kinase
174	pep chromosome:ASM81700v1:Chromosome:349742:350695:1 gene:SpnNT_00361 transcript:AJD71302 gene_biotype:protein_coding transcript_biotype:protein_coding description:diphosphomevalonate decarboxylase
175	pep chromosome:ASM81700v1:Chromosome:353456:354451:1 gene:SpnNT_00365 transcript:AJD71306 gene_biotype:protein_coding transcript_biotype:protein_coding gene_symbol:liaS description:Sensor histidine kinase LiaS
176	pep chromosome:ASM81700v1:Chromosome:354465:355097:1 gene:SpnNT_00366 transcript:AJD71307 gene_biotype:protein_coding transcript_biotype:protein_coding gene_symbol:liaR description:Transcriptional regulatory prote
177	pep chromosome:ASM81700v1:Chromosome:357782:359065:1 gene:SpnNT_00370 transcript:AJD71311 gene_biotype:protein_coding transcript_biotype:protein_coding gene_symbol:tig description:Trigger factor
178	pep chromosome:ASM81700v1:Chromosome:359113:361479:-1 gene:SpnNT_00371 transcript:AJD71312 gene_biotype:protein_coding transcript_biotype:protein_coding gene_symbol:recD description:Exodeoxyribonuclease V alph
179	pep chromosome:ASM81700v1:Chromosome:361607:362221:-1 gene:SpnNT_00372 transcript:AJD71313 gene_biotype:protein_coding transcript_biotype:protein_coding gene_symbol:spsB description:Signal peptidase IB
180	pep chromosome:ASM81700v1:Chromosome:363192:363494:1 gene:SpnNT_00374 transcript:AJD71315 gene_biotype:protein_coding transcript_biotype:protein_coding description:hypothetical protein
181	pep chromosome:ASM81700v1:Chromosome:363491:364039:1 gene:SpnNT_00375 transcript:AJD71316 gene_biotype:protein_coding transcript_biotype:protein_coding description:Colicin V production protein
182	pep chromosome:ASM81700v1:Chromosome:364143:366479:1 gene:SpnNT_00376 transcript:AJD71317 gene_biotype:protein_coding transcript_biotype:protein_coding gene_symbol:mutS2 description:MutS2 protein
183	pep chromosome:ASM81700v1:Chromosome:366822:368144:1 gene:SpnNT_00377 transcript:AJD71318 gene_biotype:protein_coding transcript_biotype:protein_coding gene_symbol:alsT description:Amino-acid carrier protein AlsT
184	pep chromosome:ASM81700v1:Chromosome:369944:371218:-1 gene:SpnNT_00380 transcript:AJD71321 gene_biotype:protein_coding transcript_biotype:protein_coding gene_symbol:serS description:Serine--tRNA ligase
185	pep chromosome:ASM81700v1:Chromosome:371806:373170:-1 gene:SpnNT_00382 transcript:AJD71323 gene_biotype:protein_coding transcript_biotype:protein_coding gene_symbol:yclM description:Aspartokinase 3
186	pep chromosome:ASM81700v1:Chromosome:375571:393276:1 gene:SpnNT_00384 transcript:AJD71325 gene_biotype:protein_coding transcript_biotype:protein_coding description:hypothetical protein
187	pep chromosome:ASM81700v1:Chromosome:393273:398615:1 gene:SpnNT_00385 transcript:AJD71326 gene_biotype:protein_coding transcript_biotype:protein_coding description:hypothetical protein
188	pep chromosome:ASM81700v1:Chromosome:401142:401927:1 gene:SpnNT_00390 transcript:AJD71331 gene_biotype:protein_coding transcript_biotype:protein_coding gene_symbol:fabM description:Trans-2-decenoyl-[acyl-carrier-
189	pep chromosome:ASM81700v1:Chromosome:404066:404500:1 gene:SpnNT_00392 transcript:AJD71333 gene_biotype:protein_coding transcript_biotype:protein_coding gene_symbol:marR description:Multiple antibiotic resistance p
190	pep chromosome:ASM81700v1:Chromosome:404500:405474:1 gene:SpnNT_00393 transcript:AJD71334 gene_biotype:protein_coding transcript_biotype:protein_coding gene_symbol:fabH description:3-oxoacyl-[acyl-carrier-protein]
191	pep chromosome:ASM81700v1:Chromosome:405534:405758:1 gene:SpnNT_00394 transcript:AJD71335 gene_biotype:protein_coding transcript_biotype:protein_coding gene_symbol:acpA description:Acyl carrier protein
192	pep chromosome:ASM81700v1:Chromosome:405877:406851:1 gene:SpnNT_00395 transcript:AJD71336 gene_biotype:protein_coding transcript_biotype:protein_coding description:Nitronate monooxygenase
193	pep chromosome:ASM81700v1:Chromosome:406844:407764:1 gene:SpnNT_00396 transcript:AJD71337 gene_biotype:protein_coding transcript_biotype:protein_coding gene_symbol:fabD description:Malonyl CoA-acyl carrier protei
194	pep chromosome:ASM81700v1:Chromosome:407798:408529:1 gene:SpnNT_00397 transcript:AJD71338 gene_biotype:protein_coding transcript_biotype:protein_coding gene_symbol:fabG description:3-oxoacyl-[acyl-carrier-protein]
195	pep chromosome:ASM81700v1:Chromosome:408551:409786:1 gene:SpnNT_00398 transcript:AJD71339 gene_biotype:protein_coding transcript_biotype:protein_coding gene_symbol:fabF description:3-oxoacyl-[acyl-carrier-protein]
196	pep chromosome:ASM81700v1:Chromosome:409789:410274:1 gene:SpnNT_00399 transcript:AJD71340 gene_biotype:protein_coding transcript_biotype:protein_coding gene_symbol:accB description:Biotin carboxyl carrier protein o
197	pep chromosome:ASM81700v1:Chromosome:410705:412072:1 gene:SpnNT_00401 transcript:AJD71342 gene_biotype:protein_coding transcript_biotype:protein_coding gene_symbol:accC description:Biotin carboxylase
198	pep chromosome:ASM81700v1:Chromosome:412109:412975:1 gene:SpnNT_00402 transcript:AJD71343 gene_biotype:protein_coding transcript_biotype:protein_coding gene_symbol:accD description:Acetyl-coenzyme A carboxylas
199	pep chromosome:ASM81700v1:Chromosome:412972:413739:1 gene:SpnNT_00403 transcript:AJD71344 gene_biotype:protein_coding transcript_biotype:protein_coding gene_symbol:accA description:Acetyl-coenzyme A carboxylas
200	pep chromosome:ASM81700v1:Chromosome:414878:415300:-1 gene:SpnNT_00406 transcript:AJD71347 gene_biotype:protein_coding transcript_biotype:protein_coding description:transcription antitermination factor
201	pep chromosome:ASM81700v1:Chromosome:415293:415682:-1 gene:SpnNT_00407 transcript:AJD71348 gene_biotype:protein_coding transcript_biotype:protein_coding description:hypothetical protein
202	pep chromosome:ASM81700v1:Chromosome:415704:416264:-1 gene:SpnNT_00408 transcript:AJD71349 gene_biotype:protein_coding transcript_biotype:protein_coding gene_symbol:efp description:Elongation factor P
203	pep chromosome:ASM81700v1:Chromosome:416595:417638:-1 gene:SpnNT_00409 transcript:AJD71350 gene_biotype:protein_coding transcript_biotype:protein_coding gene_symbol:gutB description:Sorbitol dehydrogenase
204	pep chromosome:ASM81700v1:Chromosome:417762:419204:-1 gene:SpnNT_00410 transcript:AJD71351 gene_biotype:protein_coding transcript_biotype:protein_coding gene_symbol:gatB description:Aspartyl/glutamyl-tRNA(Asn/G
205	pep chromosome:ASM81700v1:Chromosome:419204:420670:-1 gene:SpnNT_00411 transcript:AJD71352 gene_biotype:protein_coding transcript_biotype:protein_coding gene_symbol:gatA description:Glutamyl-tRNA(Gln) amidotra
206	pep chromosome:ASM81700v1:Chromosome:420670:420972:-1 gene:SpnNT_00412 transcript:AJD71353 gene_biotype:protein_coding transcript_biotype:protein_coding gene_symbol:gatC_1 description:Glutamyl-tRNA(Gln) amido
207	pep chromosome:ASM81700v1:Chromosome:421185:422729:1 gene:SpnNT_00413 transcript:AJD71354 gene_biotype:protein_coding transcript_biotype:protein_coding gene_symbol:prfC description:Peptide chain release factor 3
208	pep chromosome:ASM81700v1:Chromosome:423744:423932:1 gene:SpnNT_00415 transcript:AJD71356 gene_biotype:protein_coding transcript_biotype:protein_coding gene_symbol:rpmB description:50S ribosomal protein L28
209	pep chromosome:ASM81700v1:Chromosome:424089:424454:1 gene:SpnNT_00416 transcript:AJD71357 gene_biotype:protein_coding transcript_biotype:protein_coding description:hypothetical protein
210	pep chromosome:ASM81700v1:Chromosome:424457:426124:1 gene:SpnNT_00417 transcript:AJD71358 gene_biotype:protein_coding transcript_biotype:protein_coding description:dihydroxyacetone kinase
211	pep chromosome:ASM81700v1:Chromosome:426324:428024:1 gene:SpnNT_00418 transcript:AJD71359 gene_biotype:protein_coding transcript_biotype:protein_coding gene_symbol:ilvB description:Acetolactate synthase large sub
212	pep chromosome:ASM81700v1:Chromosome:428017:428493:1 gene:SpnNT_00419 transcript:AJD71360 gene_biotype:protein_coding transcript_biotype:protein_coding gene_symbol:ilvH description:Putative acetolactate synthase
213	pep chromosome:ASM81700v1:Chromosome:428559:429581:1 gene:SpnNT_00420 transcript:AJD71361 gene_biotype:protein_coding transcript_biotype:protein_coding gene_symbol:ilvC description:Ketol-acid reductoisomerase
214	pep chromosome:ASM81700v1:Chromosome:430299:431549:1 gene:SpnNT_00422 transcript:AJD71363 gene_biotype:protein_coding transcript_biotype:protein_coding gene_symbol:ilvA description:L-threonine dehydratase biosyn
215	pep chromosome:ASM81700v1:Chromosome:432420:432635:1 gene:SpnNT_00423 transcript:AJD71364 gene_biotype:protein_coding transcript_biotype:protein_coding description:hypothetical protein
216	pep chromosome:ASM81700v1:Chromosome:432744:433484:-1 gene:SpnNT_00424 transcript:AJD71365 gene_biotype:protein_coding transcript_biotype:protein_coding gene_symbol:glnQ_1 description:Glutamine transport ATP-bi

	A
217	pep chromosome:ASM81700v1:Chromosome:433484:435049:-1 gene:SpnNT_00425 transcript:AJD71366 gene_biotype:protein_coding transcript_biotype:protein_coding gene_symbol:yecS_1 description:Inner membrane amino-aci
218	pep chromosome:ASM81700v1:Chromosome:435188:437077:1 gene:SpnNT_00426 transcript:AJD71367 gene_biotype:protein_coding transcript_biotype:protein_coding description:hypothetical protein
219	pep chromosome:ASM81700v1:Chromosome:441053:443377:1 gene:SpnNT_00430 transcript:AJD71371 gene_biotype:protein_coding transcript_biotype:protein_coding gene_symbol:pflB description:Formate acetyltransferase
220	pep chromosome:ASM81700v1:Chromosome:448491:449840:-1 gene:SpnNT_00437 transcript:AJD71378 gene_biotype:protein_coding transcript_biotype:protein_coding gene_symbol:trkA description:Trk system potassium uptake
221	pep chromosome:ASM81700v1:Chromosome:451806:453488:1 gene:SpnNT_00441 transcript:AJD71382 gene_biotype:protein_coding transcript_biotype:protein_coding gene_symbol:ykoD_1 description:Putative HMP/thiamine impo
222	pep chromosome:ASM81700v1:Chromosome:454496:454999:1 gene:SpnNT_00443 transcript:AJD71384 gene_biotype:protein_coding transcript_biotype:protein_coding description:Putative tRNA (cytidine(34)-2-O)-methyltransferas
223	pep chromosome:ASM81700v1:Chromosome:457638:458225:1 gene:SpnNT_00448 transcript:AJD71389 gene_biotype:protein_coding transcript_biotype:protein_coding gene_symbol:rpoE description:RNAP delta factor
224	pep chromosome:ASM81700v1:Chromosome:458615:460222:1 gene:SpnNT_00449 transcript:AJD71390 gene_biotype:protein_coding transcript_biotype:protein_coding gene_symbol:pyrG description:CTP synthase
225	pep chromosome:ASM81700v1:Chromosome:467665:468861:1 gene:SpnNT_00454 transcript:AJD71395 gene_biotype:protein_coding transcript_biotype:protein_coding gene_symbol:pgk description:Phosphoglycerate kinase
226	pep chromosome:ASM81700v1:Chromosome:469601:469957:1 gene:SpnNT_00456 transcript:AJD71397 gene_biotype:protein_coding transcript_biotype:protein_coding gene_symbol:glnR description:HTH-type transcriptional regula
227	pep chromosome:ASM81700v1:Chromosome:469994:471340:1 gene:SpnNT_00457 transcript:AJD71398 gene_biotype:protein_coding transcript_biotype:protein_coding gene_symbol:glnA description:Glutamine synthetase
228	pep chromosome:ASM81700v1:Chromosome:474840:476387:-1 gene:SpnNT_00462 transcript:AJD71403 gene_biotype:protein_coding transcript_biotype:protein_coding gene_symbol:hsdS_2 description:Type I restriction enzyme E
229	pep chromosome:ASM81700v1:Chromosome:476387:477850:-1 gene:SpnNT_00463 transcript:AJD71404 gene_biotype:protein_coding transcript_biotype:protein_coding description:putative type I restriction enzymeP M protein
230	pep chromosome:ASM81700v1:Chromosome:477863:480196:-1 gene:SpnNT_00464 transcript:AJD71405 gene_biotype:protein_coding transcript_biotype:protein_coding gene_symbol:hsdR_1 description:Type-1 restriction enzyme
231	pep chromosome:ASM81700v1:Chromosome:481477:482511:1 gene:SpnNT_00466 transcript:AJD71407 gene_biotype:protein_coding transcript_biotype:protein_coding gene_symbol:hrcA description:Heat-inducible transcription rep
232	pep chromosome:ASM81700v1:Chromosome:482538:483062:1 gene:SpnNT_00467 transcript:AJD71408 gene_biotype:protein_coding transcript_biotype:protein_coding gene_symbol:grpE description:HSP-70 cofactor
233	pep chromosome:ASM81700v1:Chromosome:483542:485365:1 gene:SpnNT_00468 transcript:AJD71409 gene_biotype:protein_coding transcript_biotype:protein_coding gene_symbol:dnaK description:Heat shock protein 70
234	pep chromosome:ASM81700v1:Chromosome:486124:487260:1 gene:SpnNT_00469 transcript:AJD71410 gene_biotype:protein_coding transcript_biotype:protein_coding gene_symbol:dnaJ description:Chaperone protein DnaJ
235	pep chromosome:ASM81700v1:Chromosome:487986:488273:-1 gene:SpnNT_00470 transcript:AJD71411 gene_biotype:protein_coding transcript_biotype:protein_coding description:hypothetical protein
236	pep chromosome:ASM81700v1:Chromosome:488283:488693:-1 gene:SpnNT_00471 transcript:AJD71412 gene_biotype:protein_coding transcript_biotype:protein_coding description:HIT-like protein
237	pep chromosome:ASM81700v1:Chromosome:488761:489492:1 gene:SpnNT_00472 transcript:AJD71413 gene_biotype:protein_coding transcript_biotype:protein_coding gene_symbol:ecsA_1 description:ABC-type transporter ATP-b
238	pep chromosome:ASM81700v1:Chromosome:492278:493618:1 gene:SpnNT_00478 transcript:AJD71419 gene_biotype:protein_coding transcript_biotype:protein_coding gene_symbol:citS description:Sensor protein CitS
239	pep chromosome:ASM81700v1:Chromosome:503102:503896:1 gene:SpnNT_00492 transcript:AJD71433 gene_biotype:protein_coding transcript_biotype:protein_coding description:Phosphotransferase enzyme family protein
240	pep chromosome:ASM81700v1:Chromosome:503893:504528:1 gene:SpnNT_00493 transcript:AJD71434 gene_biotype:protein_coding transcript_biotype:protein_coding gene_symbol:trmB description:tRNA (guanine-N(7)-)-methyltra
241	pep chromosome:ASM81700v1:Chromosome:504654:505133:1 gene:SpnNT_00494 transcript:AJD71435 gene_biotype:protein_coding transcript_biotype:protein_coding gene_symbol:rimP description:ribosome maturation factor rim
242	pep chromosome:ASM81700v1:Chromosome:505189:506313:1 gene:SpnNT_00495 transcript:AJD71436 gene_biotype:protein_coding transcript_biotype:protein_coding gene_symbol:nusA description:transcription termination factor
243	pep chromosome:ASM81700v1:Chromosome:506335:506628:1 gene:SpnNT_00496 transcript:AJD71437 gene_biotype:protein_coding transcript_biotype:protein_coding description:hypothetical protein
244	pep chromosome:ASM81700v1:Chromosome:506937:509729:1 gene:SpnNT_00498 transcript:AJD71439 gene_biotype:protein_coding transcript_biotype:protein_coding gene_symbol:infB description:Translation initiation factor IF-2
245	pep chromosome:ASM81700v1:Chromosome:509980:510330:1 gene:SpnNT_00499 transcript:AJD71440 gene_biotype:protein_coding transcript_biotype:protein_coding gene_symbol:rbfA description:Ribosome-binding factor A
246	pep chromosome:ASM81700v1:Chromosome:511991:512224:1 gene:SpnNT_00503 transcript:AJD71444 gene_biotype:protein_coding transcript_biotype:protein_coding description:hypothetical protein
247	pep chromosome:ASM81700v1:Chromosome:512224:513558:1 gene:SpnNT_00504 transcript:AJD71445 gene_biotype:protein_coding transcript_biotype:protein_coding description:Hemerythrin HHE cation binding domain protein
248	pep chromosome:ASM81700v1:Chromosome:513568:513822:1 gene:SpnNT_00505 transcript:AJD71446 gene_biotype:protein_coding transcript_biotype:protein_coding description:hypothetical protein
249	pep chromosome:ASM81700v1:Chromosome:513918:514313:1 gene:SpnNT_00506 transcript:AJD71447 gene_biotype:protein_coding transcript_biotype:protein_coding description:hypothetical protein
250	pep chromosome:ASM81700v1:Chromosome:516293:518944:1 gene:SpnNT_00510 transcript:AJD71451 gene_biotype:protein_coding transcript_biotype:protein_coding gene_symbol:valS description:Valine--tRNA ligase
251	pep chromosome:ASM81700v1:Chromosome:519039:520136:1 gene:SpnNT_00511 transcript:AJD71452 gene_biotype:protein_coding transcript_biotype:protein_coding description:hypothetical protein
252	pep chromosome:ASM81700v1:Chromosome:520129:520872:1 gene:SpnNT_00512 transcript:AJD71453 gene_biotype:protein_coding transcript_biotype:protein_coding description:hypothetical protein
253	pep chromosome:ASM81700v1:Chromosome:524053:524856:1 gene:SpnNT_00515 transcript:AJD71456 gene_biotype:protein_coding transcript_biotype:protein_coding description:cell filamentation protein Fic
254	pep chromosome:ASM81700v1:Chromosome:530402:532240:1 gene:SpnNT_00523 transcript:AJD71464 gene_biotype:protein_coding transcript_biotype:protein_coding gene_symbol:bglF_1 description:EIIBCA-Bgl
255	pep chromosome:ASM81700v1:Chromosome:532253:533668:1 gene:SpnNT_00524 transcript:AJD71465 gene_biotype:protein_coding transcript_biotype:protein_coding gene_symbol:bglH_1 description:Aryl-phospho-beta-D-glucos
256	pep chromosome:ASM81700v1:Chromosome:534260:535306:1 gene:SpnNT_00525 transcript:AJD71466 gene_biotype:protein_coding transcript_biotype:protein_coding gene_symbol:pheS description:Phenylalanine--tRNA ligase al
257	pep chromosome:ASM81700v1:Chromosome:535892:538297:1 gene:SpnNT_00527 transcript:AJD71468 gene_biotype:protein_coding transcript_biotype:protein_coding gene_symbol:pheT_1 description:Phenylalanine--tRNA ligase
258	pep chromosome:ASM81700v1:Chromosome:544592:546805:1 gene:SpnNT_00533 transcript:AJD71474 gene_biotype:protein_coding transcript_biotype:protein_coding gene_symbol:pnp_1 description:Polyribonucleotide nucleotidy
259	pep chromosome:ASM81700v1:Chromosome:547450:548334:1 gene:SpnNT_00535 transcript:AJD71476 gene_biotype:protein_coding transcript_biotype:protein_coding description:putative acetyltransferase
260	pep chromosome:ASM81700v1:Chromosome:548416:549759:1 gene:SpnNT_00536 transcript:AJD71477 gene_biotype:protein_coding transcript_biotype:protein_coding gene_symbol:cysS description:Cysteine--tRNA ligase
261	pep chromosome:ASM81700v1:Chromosome:551685:552659:1 gene:SpnNT_00539 transcript:AJD71480 gene_biotype:protein_coding transcript_biotype:protein_coding description:flagellar assembly protein H
262	pep chromosome:ASM81700v1:Chromosome:555059:562378:1 gene:SpnNT_00543 transcript:AJD71484 gene_biotype:protein_coding transcript_biotype:protein_coding description:hypothetical protein
263	pep chromosome:ASM81700v1:Chromosome:562529:563806:1 gene:SpnNT_00544 transcript:AJD71485 gene_biotype:protein_coding transcript_biotype:protein_coding description:lipoprotein releasing system, transmembrane prot
264	pep chromosome:ASM81700v1:Chromosome:563933:564580:1 gene:SpnNT_00545 transcript:AJD71486 gene_biotype:protein_coding transcript_biotype:protein_coding description:putative ABC transporter ATP-binding protein
265	pep chromosome:ASM81700v1:Chromosome:564632:566011:1 gene:SpnNT_00546 transcript:AJD71487 gene_biotype:protein_coding transcript_biotype:protein_coding description:FtsX-like permease family protein
266	pep chromosome:ASM81700v1:Chromosome:566943:568271:1 gene:SpnNT_00548 transcript:AJD71489 gene_biotype:protein_coding transcript_biotype:protein_coding gene_symbol:hssS description:Heme sensor protein HssS
267	pep chromosome:ASM81700v1:Chromosome:568412:569293:1 gene:SpnNT_00549 transcript:AJD71490 gene_biotype:protein_coding transcript_biotype:protein_coding gene_symbol:fba description:Fructose-bisphosphate aldolase
268	pep chromosome:ASM81700v1:Chromosome:569613:570803:-1 gene:SpnNT_00550 transcript:AJD71491 gene_biotype:protein_coding transcript_biotype:protein_coding gene_symbol:nagAa description:Naphthalene 1,2-dioxygenas
269	pep chromosome:ASM81700v1:Chromosome:574084:576306:1 gene:SpnNT_00554 transcript:AJD71495 gene_biotype:protein_coding transcript_biotype:protein_coding gene_symbol:recJ description:Single-stranded-DNA-specific e
270	pep chromosome:ASM81700v1:Chromosome:576481:578142:1 gene:SpnNT_00555 transcript:AJD71496 gene_biotype:protein_coding transcript_biotype:protein_coding gene_symbol:rnjB description:Ribonuclease J 2

	A
271	pep chromosome:ASM81700v1:Chromosome:578211:578990:1 gene:SpnNT_00556 transcript:AJD71497 gene_biotype:protein_coding transcript_biotype:protein_coding gene_symbol:axe1-6A_1 description:Carbohydrate acetyl este
272	pep chromosome:ASM81700v1:Chromosome:579102:580322:1 gene:SpnNT_00557 transcript:AJD71498 gene_biotype:protein_coding transcript_biotype:protein_coding gene_symbol:femX description:Lipid II:glycine glycyltransferas
273	pep chromosome:ASM81700v1:Chromosome:580327:581559:1 gene:SpnNT_00558 transcript:AJD71499 gene_biotype:protein_coding transcript_biotype:protein_coding gene_symbol:femA description:Aminoacyltransferase FemA
274	pep chromosome:ASM81700v1:Chromosome:584203:585036:1 gene:SpnNT_00562 transcript:AJD71503 gene_biotype:protein_coding transcript_biotype:protein_coding description:phosphodiesterase
275	pep chromosome:ASM81700v1:Chromosome:586193:586798:1 gene:SpnNT_00564 transcript:AJD71505 gene_biotype:protein_coding transcript_biotype:protein_coding description:Putative NAD(P)H nitroreductase
276	pep chromosome:ASM81700v1:Chromosome:586811:588211:1 gene:SpnNT_00565 transcript:AJD71506 gene_biotype:protein_coding transcript_biotype:protein_coding gene_symbol:pepV description:Beta-Ala-Xaa dipeptidase
277	pep chromosome:ASM81700v1:Chromosome:590884:591921:1 gene:SpnNT_00569 transcript:AJD71510 gene_biotype:protein_coding transcript_biotype:protein_coding gene_symbol:pepA_1 description:Glutamyl aminopeptidase
278	pep chromosome:ASM81700v1:Chromosome:592396:593112:1 gene:SpnNT_00571 transcript:AJD71512 gene_biotype:protein_coding transcript_biotype:protein_coding gene_symbol:vanYB description:D-alanyl-D-alanine carboxyp
279	pep chromosome:ASM81700v1:Chromosome:593431:593856:1 gene:SpnNT_00572 transcript:AJD71513 gene_biotype:protein_coding transcript_biotype:protein_coding gene_symbol:rplK description:50S ribosomal protein L11
280	pep chromosome:ASM81700v1:Chromosome:594065:594754:1 gene:SpnNT_00573 transcript:AJD71514 gene_biotype:protein_coding transcript_biotype:protein_coding gene_symbol:rplA description:50S ribosomal protein L1
281	pep chromosome:ASM81700v1:Chromosome:600586:602001:1 gene:SpnNT_00580 transcript:AJD71521 gene_biotype:protein_coding transcript_biotype:protein_coding gene_symbol:ply_1 description:Thiol-activated cytolysin
282	pep chromosome:ASM81700v1:Chromosome:602011:602757:1 gene:SpnNT_00581 transcript:AJD71522 gene_biotype:protein_coding transcript_biotype:protein_coding description:N-acetylmuramoyl-L-alanine amidase
283	pep chromosome:ASM81700v1:Chromosome:620753:621598:-1 gene:SpnNT_00599 transcript:AJD71540 gene_biotype:protein_coding transcript_biotype:protein_coding description:hypothetical protein
284	pep chromosome:ASM81700v1:Chromosome:634935:635234:1 gene:SpnNT_00605 transcript:AJD71546 gene_biotype:protein_coding transcript_biotype:protein_coding description:hypothetical protein
285	pep chromosome:ASM81700v1:Chromosome:637696:638589:1 gene:SpnNT_00608 transcript:AJD71549 gene_biotype:protein_coding transcript_biotype:protein_coding description:Adenosine monophosphate-protein transferase So
286	pep chromosome:ASM81700v1:Chromosome:638893:661572:1 gene:SpnNT_00609 transcript:AJD71550 gene_biotype:protein_coding transcript_biotype:protein_coding description:hypothetical protein
287	pep chromosome:ASM81700v1:Chromosome:666053:666529:1 gene:SpnNT_00616 transcript:AJD71557 gene_biotype:protein_coding transcript_biotype:protein_coding gene_symbol:pezA_1 description:Antitoxin PezA
288	pep chromosome:ASM81700v1:Chromosome:667508:668071:1 gene:SpnNT_00618 transcript:AJD71559 gene_biotype:protein_coding transcript_biotype:protein_coding description:hypothetical protein
289	pep chromosome:ASM81700v1:Chromosome:668281:669999:1 gene:SpnNT_00620 transcript:AJD71561 gene_biotype:protein_coding transcript_biotype:protein_coding description:hypothetical protein
290	pep chromosome:ASM81700v1:Chromosome:676629:676826:1 gene:SpnNT_00630 transcript:AJD71571 gene_biotype:protein_coding transcript_biotype:protein_coding gene_symbol:immR_1 description:HTH-type transcriptional re
291	pep chromosome:ASM81700v1:Chromosome:677097:677237:1 gene:SpnNT_00631 transcript:AJD71572 gene_biotype:protein_coding transcript_biotype:protein_coding description:hypothetical protein
292	pep chromosome:ASM81700v1:Chromosome:678905:680521:1 gene:SpnNT_00633 transcript:AJD71574 gene_biotype:protein_coding transcript_biotype:protein_coding description:hypothetical protein
293	pep chromosome:ASM81700v1:Chromosome:680514:681953:1 gene:SpnNT_00634 transcript:AJD71575 gene_biotype:protein_coding transcript_biotype:protein_coding description:Recombinase
294	pep chromosome:ASM81700v1:Chromosome:683856:684143:-1 gene:SpnNT_00637 transcript:AJD71578 gene_biotype:protein_coding transcript_biotype:protein_coding description:addiction module antitoxin, RelB/DinJ family
295	pep chromosome:ASM81700v1:Chromosome:694784:695341:1 gene:SpnNT_00645 transcript:AJD71586 gene_biotype:protein_coding transcript_biotype:protein_coding description:putative methyltransferase
296	pep chromosome:ASM81700v1:Chromosome:695334:695588:1 gene:SpnNT_00646 transcript:AJD71587 gene_biotype:protein_coding transcript_biotype:protein_coding description:hypothetical protein
297	pep chromosome:ASM81700v1:Chromosome:699622:700188:1 gene:SpnNT_00650 transcript:AJD71591 gene_biotype:protein_coding transcript_biotype:protein_coding gene_symbol:msrAB_1 description:Peptide methionine sulfox
298	pep chromosome:ASM81700v1:Chromosome:700199:701311:1 gene:SpnNT_00651 transcript:AJD71592 gene_biotype:protein_coding transcript_biotype:protein_coding gene_symbol:msrAB_2 description:Peptide methionine sulfox
299	pep chromosome:ASM81700v1:Chromosome:702494:704185:1 gene:SpnNT_00653 transcript:AJD71594 gene_biotype:protein_coding transcript_biotype:protein_coding gene_symbol:yehU description:Sensor histidine kinase YehU
300	pep chromosome:ASM81700v1:Chromosome:704301:704864:1 gene:SpnNT_00654 transcript:AJD71595 gene_biotype:protein_coding transcript_biotype:protein_coding description:hypothetical protein
301	pep chromosome:ASM81700v1:Chromosome:704886:710822:1 gene:SpnNT_00655 transcript:AJD71596 gene_biotype:protein_coding transcript_biotype:protein_coding gene_symbol:iga_2 description:Immunoglobulin A1 protease
302	pep chromosome:ASM81700v1:Chromosome:712313:714034:1 gene:SpnNT_00657 transcript:AJD71598 gene_biotype:protein_coding transcript_biotype:protein_coding gene_symbol:pabB description:Para-aminobenzoate synthase
303	pep chromosome:ASM81700v1:Chromosome:714035:714652:1 gene:SpnNT_00658 transcript:AJD71599 gene_biotype:protein_coding transcript_biotype:protein_coding description:2-succinyl-6-hydroxy-2, 4-cyclohexadiene-1-carbo
304	pep chromosome:ASM81700v1:Chromosome:714702:715700:1 gene:SpnNT_00659 transcript:AJD71600 gene_biotype:protein_coding transcript_biotype:protein_coding gene_symbol:lytB_2 description:Putative endo-beta-N-acetylg
305	pep chromosome:ASM81700v1:Chromosome:715827:716786:1 gene:SpnNT_00660 transcript:AJD71601 gene_biotype:protein_coding transcript_biotype:protein_coding gene_symbol:glcK description:Glucokinase
306	pep chromosome:ASM81700v1:Chromosome:716883:717722:1 gene:SpnNT_00661 transcript:AJD71602 gene_biotype:protein_coding transcript_biotype:protein_coding gene_symbol:thyA description:Thymidylate synthase
307	pep chromosome:ASM81700v1:Chromosome:717770:717940:-1 gene:SpnNT_00662 transcript:AJD71603 gene_biotype:protein_coding transcript_biotype:protein_coding description:hypothetical protein
308	pep chromosome:ASM81700v1:Chromosome:718061:718945:1 gene:SpnNT_00663 transcript:AJD71604 gene_biotype:protein_coding transcript_biotype:protein_coding gene_symbol:miaA description:tRNA dimethylallyltransferase
309	pep chromosome:ASM81700v1:Chromosome:720169:720792:1 gene:SpnNT_00665 transcript:AJD71606 gene_biotype:protein_coding transcript_biotype:protein_coding description:hypothetical protein
310	pep chromosome:ASM81700v1:Chromosome:720807:721736:1 gene:SpnNT_00666 transcript:AJD71607 gene_biotype:protein_coding transcript_biotype:protein_coding gene_symbol:rnz description:Ribonuclease Z
311	pep chromosome:ASM81700v1:Chromosome:722566:723534:-1 gene:SpnNT_00668 transcript:AJD71609 gene_biotype:protein_coding transcript_biotype:protein_coding gene_symbol:hcaR description:Hca operon transcriptional ac
312	pep chromosome:ASM81700v1:Chromosome:723807:724187:1 gene:SpnNT_00670 transcript:AJD71611 gene_biotype:protein_coding transcript_biotype:protein_coding gene_symbol:moeZ_1 description:putative adenylyltransferase
313	pep chromosome:ASM81700v1:Chromosome:725430:727271:1 gene:SpnNT_00673 transcript:AJD71614 gene_biotype:protein_coding transcript_biotype:protein_coding gene_symbol:typA description:Tyrosine phosphorylated protei
314	pep chromosome:ASM81700v1:Chromosome:731059:732411:1 gene:SpnNT_00678 transcript:AJD71619 gene_biotype:protein_coding transcript_biotype:protein_coding gene_symbol:murD description:UDP-N-acetylmuramoylalanin
315	pep chromosome:ASM81700v1:Chromosome:732415:733473:1 gene:SpnNT_00679 transcript:AJD71620 gene_biotype:protein_coding transcript_biotype:protein_coding gene_symbol:murG description:UDP-N-acetylglucosamine--N
316	pep chromosome:ASM81700v1:Chromosome:733483:734682:1 gene:SpnNT_00680 transcript:AJD71621 gene_biotype:protein_coding transcript_biotype:protein_coding gene_symbol:divIB description:Cell division protein DivIB
317	pep chromosome:ASM81700v1:Chromosome:737838:738671:1 gene:SpnNT_00686 transcript:AJD71627 gene_biotype:protein_coding transcript_biotype:protein_coding description:Fluoroquinolones export ATP-binding proteinc
318	pep chromosome:ASM81700v1:Chromosome:740111:740812:1 gene:SpnNT_00688 transcript:AJD71629 gene_biotype:protein_coding transcript_biotype:protein_coding gene_symbol:pyrF description:Orotidine 5-phosphate decarbo
319	pep chromosome:ASM81700v1:Chromosome:740846:741478:1 gene:SpnNT_00689 transcript:AJD71630 gene_biotype:protein_coding transcript_biotype:protein_coding gene_symbol:pyrE description:Orotate phosphoribosyltransfer
320	pep chromosome:ASM81700v1:Chromosome:745677:747167:1 gene:SpnNT_00696 transcript:AJD71637 gene_biotype:protein_coding transcript_biotype:protein_coding gene_symbol:lysS description:Lysine--tRNA ligase
321	pep chromosome:ASM81700v1:Chromosome:747791:748927:1 gene:SpnNT_00697 transcript:AJD71638 gene_biotype:protein_coding transcript_biotype:protein_coding description:Lactate 2-monooxygenase
322	pep chromosome:ASM81700v1:Chromosome:749484:750152:1 gene:SpnNT_00698 transcript:AJD71639 gene_biotype:protein_coding transcript_biotype:protein_coding gene_symbol:tenA_1 description:Thiaminase-2
323	pep chromosome:ASM81700v1:Chromosome:750379:751161:1 gene:SpnNT_00699 transcript:AJD71640 gene_biotype:protein_coding transcript_biotype:protein_coding gene_symbol:thiM_1 description:Hydroxyethylthiazole kinase
324	pep chromosome:ASM81700v1:Chromosome:757833:758624:-1 gene:SpnNT_00708 transcript:AJD71649 gene_biotype:protein_coding transcript_biotype:protein_coding gene_symbol:thiD description:Hydroxymethylpyrimidine/phos

	A
325	pep chromosome:ASM81700v1:Chromosome:758990:759385:1 gene:SpnNT_00709 transcript:AJD71650 gene_biotype:protein_coding transcript_biotype:protein_coding gene_symbol:copY description:CopAB ATPases metal-fist typ
326	pep chromosome:ASM81700v1:Chromosome:759396:759767:1 gene:SpnNT_00710 transcript:AJD71651 gene_biotype:protein_coding transcript_biotype:protein_coding description:hypothetical protein
327	pep chromosome:ASM81700v1:Chromosome:759777:762020:1 gene:SpnNT_00711 transcript:AJD71652 gene_biotype:protein_coding transcript_biotype:protein_coding gene_symbol:pacS description:Cation-transporting ATPase P
328	pep chromosome:ASM81700v1:Chromosome:762227:764002:1 gene:SpnNT_00712 transcript:AJD71653 gene_biotype:protein_coding transcript_biotype:protein_coding gene_symbol:pox5 description:Pyruvate oxidase
329	pep chromosome:ASM81700v1:Chromosome:764113:764460:1 gene:SpnNT_00713 transcript:AJD71654 gene_biotype:protein_coding transcript_biotype:protein_coding description:Glyoxalase-like domain protein
330	pep chromosome:ASM81700v1:Chromosome:766167:767111:1 gene:SpnNT_00718 transcript:AJD71659 gene_biotype:protein_coding transcript_biotype:protein_coding gene_symbol:gmuF description:putative mannose-6-phospha
331	pep chromosome:ASM81700v1:Chromosome:768707:769456:1 gene:SpnNT_00721 transcript:AJD71662 gene_biotype:protein_coding transcript_biotype:protein_coding gene_symbol:mta description:Multidrug transporter activation
332	pep chromosome:ASM81700v1:Chromosome:771470:772645:-1 gene:SpnNT_00725 transcript:AJD71666 gene_biotype:protein_coding transcript_biotype:protein_coding description:dihydrolipoamide dehydrogenase
333	pep chromosome:ASM81700v1:Chromosome:772704:773549:-1 gene:SpnNT_00726 transcript:AJD71667 gene_biotype:protein_coding transcript_biotype:protein_coding description:Fatty acid-binding protein
334	pep chromosome:ASM81700v1:Chromosome:773674:774231:1 gene:SpnNT_00727 transcript:AJD71668 gene_biotype:protein_coding transcript_biotype:protein_coding gene_symbol:mtrR description:HTH-type transcriptional regula
335	pep chromosome:ASM81700v1:Chromosome:774250:774717:1 gene:SpnNT_00728 transcript:AJD71669 gene_biotype:protein_coding transcript_biotype:protein_coding gene_symbol:ribD_2 description:Riboflavin biosynthesis prote
336	pep chromosome:ASM81700v1:Chromosome:774806:775435:1 gene:SpnNT_00729 transcript:AJD71670 gene_biotype:protein_coding transcript_biotype:protein_coding gene_symbol:upp description:Uracil phosphoribosyltransferas
337	pep chromosome:ASM81700v1:Chromosome:775609:776199:1 gene:SpnNT_00730 transcript:AJD71671 gene_biotype:protein_coding transcript_biotype:protein_coding gene_symbol:clpP description:ATP-dependent Clp protease p
338	pep chromosome:ASM81700v1:Chromosome:776278:776526:1 gene:SpnNT_00731 transcript:AJD71672 gene_biotype:protein_coding transcript_biotype:protein_coding description:hypothetical protein
339	pep chromosome:ASM81700v1:Chromosome:776628:777788:1 gene:SpnNT_00732 transcript:AJD71673 gene_biotype:protein_coding transcript_biotype:protein_coding gene_symbol:braC description:Leucine-, isoleucine-, valine-, t
340	pep chromosome:ASM81700v1:Chromosome:779885:780649:1 gene:SpnNT_00735 transcript:AJD71676 gene_biotype:protein_coding transcript_biotype:protein_coding gene_symbol:lptB description:Lipopolysaccharide export syste
341	pep chromosome:ASM81700v1:Chromosome:780649:781359:1 gene:SpnNT_00736 transcript:AJD71677 gene_biotype:protein_coding transcript_biotype:protein_coding gene_symbol:livF description:LIV-I protein F
342	pep chromosome:ASM81700v1:Chromosome:781667:782323:1 gene:SpnNT_00737 transcript:AJD71678 gene_biotype:protein_coding transcript_biotype:protein_coding description:putative manganese-dependent inorganic pyropho
343	pep chromosome:ASM81700v1:Chromosome:782549:783526:1 gene:SpnNT_00738 transcript:AJD71679 gene_biotype:protein_coding transcript_biotype:protein_coding gene_symbol:prfB description:Peptide chain release factor 2
344	pep chromosome:ASM81700v1:Chromosome:783544:784236:1 gene:SpnNT_00739 transcript:AJD71680 gene_biotype:protein_coding transcript_biotype:protein_coding gene_symbol:ftsE description:Cell division ATP-binding protei
345	pep chromosome:ASM81700v1:Chromosome:784229:785155:1 gene:SpnNT_00740 transcript:AJD71681 gene_biotype:protein_coding transcript_biotype:protein_coding gene_symbol:ftsX description:Cell division protein FtsX
346	pep chromosome:ASM81700v1:Chromosome:785441:787621:1 gene:SpnNT_00741 transcript:AJD71682 gene_biotype:protein_coding transcript_biotype:protein_coding gene_symbol:ptsG_1 description:EIICBA-Glc
347	pep chromosome:ASM81700v1:Chromosome:787673:788488:1 gene:SpnNT_00742 transcript:AJD71683 gene_biotype:protein_coding transcript_biotype:protein_coding gene_symbol:mapP description:Maltose 6-phosphate phospha
348	pep chromosome:ASM81700v1:Chromosome:788628:789971:1 gene:SpnNT_00743 transcript:AJD71684 gene_biotype:protein_coding transcript_biotype:protein_coding gene_symbol:cshB description:DEAD-box ATP-dependent RN
349	pep chromosome:ASM81700v1:Chromosome:790186:791376:1 gene:SpnNT_00744 transcript:AJD71685 gene_biotype:protein_coding transcript_biotype:protein_coding gene_symbol:metK description:S-adenosylmethionine synthas
350	pep chromosome:ASM81700v1:Chromosome:791929:792864:1 gene:SpnNT_00745 transcript:AJD71686 gene_biotype:protein_coding transcript_biotype:protein_coding gene_symbol:pyrDA description:putative dihydroorotate dehyd
351	pep chromosome:ASM81700v1:Chromosome:794108:794713:1 gene:SpnNT_00747 transcript:AJD71688 gene_biotype:protein_coding transcript_biotype:protein_coding gene_symbol:sodA description:Superoxide dismutase [Mn]
352	pep chromosome:ASM81700v1:Chromosome:794869:795399:1 gene:SpnNT_00748 transcript:AJD71689 gene_biotype:protein_coding transcript_biotype:protein_coding description:hypothetical protein
353	pep chromosome:ASM81700v1:Chromosome:796989:798530:1 gene:SpnNT_00751 transcript:AJD71692 gene_biotype:protein_coding transcript_biotype:protein_coding description:putative ABC transporter ATP-binding protein
354	pep chromosome:ASM81700v1:Chromosome:798586:799389:-1 gene:SpnNT_00752 transcript:AJD71693 gene_biotype:protein_coding transcript_biotype:protein_coding description:Putative peptidyl-prolyl cis-trans isomerase
355	pep chromosome:ASM81700v1:Chromosome:800551:800823:1 gene:SpnNT_00754 transcript:AJD71695 gene_biotype:protein_coding transcript_biotype:protein_coding gene_symbol:rpsP description:30S ribosomal protein S16
356	pep chromosome:ASM81700v1:Chromosome:800843:801082:1 gene:SpnNT_00755 transcript:AJD71696 gene_biotype:protein_coding transcript_biotype:protein_coding description:putative RNA-binding protein (contains KH domain
357	pep chromosome:ASM81700v1:Chromosome:802478:802996:1 gene:SpnNT_00757 transcript:AJD71698 gene_biotype:protein_coding transcript_biotype:protein_coding gene_symbol:rimM description:Ribosome maturation factor R
358	pep chromosome:ASM81700v1:Chromosome:802986:803705:1 gene:SpnNT_00758 transcript:AJD71699 gene_biotype:protein_coding transcript_biotype:protein_coding gene_symbol:trmD description:tRNA (guanine-N(1)-)-methyltr
359	pep chromosome:ASM81700v1:Chromosome:803717:804055:1 gene:SpnNT_00759 transcript:AJD71700 gene_biotype:protein_coding transcript_biotype:protein_coding gene_symbol:nrdD_2 description:Anaerobic ribonucleoside-tri
360	pep chromosome:ASM81700v1:Chromosome:804085:804405:-1 gene:SpnNT_00760 transcript:AJD71701 gene_biotype:protein_coding transcript_biotype:protein_coding description:hypothetical protein
361	pep chromosome:ASM81700v1:Chromosome:804503:804718:1 gene:SpnNT_00761 transcript:AJD71702 gene_biotype:protein_coding transcript_biotype:protein_coding description:hypothetical protein
362	pep chromosome:ASM81700v1:Chromosome:805657:807003:-1 gene:SpnNT_00763 transcript:AJD71704 gene_biotype:protein_coding transcript_biotype:protein_coding gene_symbol:gor description:Glutathione reductase
363	pep chromosome:ASM81700v1:Chromosome:807289:808488:1 gene:SpnNT_00764 transcript:AJD71705 gene_biotype:protein_coding transcript_biotype:protein_coding gene_symbol:yknX description:Putative efflux system compor
364	pep chromosome:ASM81700v1:Chromosome:808472:809173:1 gene:SpnNT_00765 transcript:AJD71706 gene_biotype:protein_coding transcript_biotype:protein_coding gene_symbol:macB_1 description:Macrolide export ATP-bind
365	pep chromosome:ASM81700v1:Chromosome:809175:810434:1 gene:SpnNT_00766 transcript:AJD71707 gene_biotype:protein_coding transcript_biotype:protein_coding gene_symbol:yknZ description:putative ABC transporter perm
366	pep chromosome:ASM81700v1:Chromosome:810558:812555:1 gene:SpnNT_00767 transcript:AJD71708 gene_biotype:protein_coding transcript_biotype:protein_coding gene_symbol:metG description:Methionine--tRNA ligase
367	pep chromosome:ASM81700v1:Chromosome:813202:813522:1 gene:SpnNT_00768 transcript:AJD71709 gene_biotype:protein_coding transcript_biotype:protein_coding description:lineage-specific thermal regulator protein
368	pep chromosome:ASM81700v1:Chromosome:814416:815342:1 gene:SpnNT_00770 transcript:AJD71711 gene_biotype:protein_coding transcript_biotype:protein_coding gene_symbol:ydhF description:Oxidoreductase YdhF
369	pep chromosome:ASM81700v1:Chromosome:815440:815676:1 gene:SpnNT_00771 transcript:AJD71712 gene_biotype:protein_coding transcript_biotype:protein_coding description:hypothetical protein
370	pep chromosome:ASM81700v1:Chromosome:815669:816367:1 gene:SpnNT_00772 transcript:AJD71713 gene_biotype:protein_coding transcript_biotype:protein_coding gene_symbol:cpnA description:Cyclopentanol dehydrogenase
371	pep chromosome:ASM81700v1:Chromosome:816378:816878:1 gene:SpnNT_00773 transcript:AJD71714 gene_biotype:protein_coding transcript_biotype:protein_coding description:NUDIX domain protein
372	pep chromosome:ASM81700v1:Chromosome:816856:817725:1 gene:SpnNT_00774 transcript:AJD71715 gene_biotype:protein_coding transcript_biotype:protein_coding gene_symbol:ppsA description:Phosphoenolpyruvate synthas
373	pep chromosome:ASM81700v1:Chromosome:817700:818140:1 gene:SpnNT_00775 transcript:AJD71716 gene_biotype:protein_coding transcript_biotype:protein_coding description:ASCH domain protein
374	pep chromosome:ASM81700v1:Chromosome:818263:820809:1 gene:SpnNT_00776 transcript:AJD71717 gene_biotype:protein_coding transcript_biotype:protein_coding gene_symbol:pepN description:Aminopeptidase N
375	pep chromosome:ASM81700v1:Chromosome:820918:821592:1 gene:SpnNT_00777 transcript:AJD71718 gene_biotype:protein_coding transcript_biotype:protein_coding gene_symbol:arlR_2 description:Response regulator ArlR
376	pep chromosome:ASM81700v1:Chromosome:821582:822916:1 gene:SpnNT_00778 transcript:AJD71719 gene_biotype:protein_coding transcript_biotype:protein_coding gene_symbol:arlS description:Signal transduction histidine-pro
377	pep chromosome:ASM81700v1:Chromosome:823377:824306:1 gene:SpnNT_00780 transcript:AJD71721 gene_biotype:protein_coding transcript_biotype:protein_coding gene_symbol:yhbU_1 description:putative protease YhbU pre
378	pep chromosome:ASM81700v1:Chromosome:824506:826956:1 gene:SpnNT_00781 transcript:AJD71722 gene_biotype:protein_coding transcript_biotype:protein_coding gene_symbol:dinG description:family exonuclease family helic

	A
379	pep chromosome:ASM81700v1:Chromosome:828227:828781:1 gene:SpnNT_00783 transcript:AJD71724 gene_biotype:protein_coding transcript_biotype:protein_coding gene_symbol:yajL description:Chaperone protein YajL
380	pep chromosome:ASM81700v1:Chromosome:828857:829429:1 gene:SpnNT_00784 transcript:AJD71725 gene_biotype:protein_coding transcript_biotype:protein_coding gene_symbol:gph description:Phosphoglycolate phosphatase
381	pep chromosome:ASM81700v1:Chromosome:829444:831390:1 gene:SpnNT_00785 transcript:AJD71726 gene_biotype:protein_coding transcript_biotype:protein_coding gene_symbol:gyrB description:DNA gyrase subunit B
382	pep chromosome:ASM81700v1:Chromosome:831472:833199:1 gene:SpnNT_00786 transcript:AJD71727 gene_biotype:protein_coding transcript_biotype:protein_coding gene_symbol:ezrA description:Septation ring formation regula
383	pep chromosome:ASM81700v1:Chromosome:834829:835911:-1 gene:SpnNT_00789 transcript:AJD71730 gene_biotype:protein_coding transcript_biotype:protein_coding gene_symbol:cshA_1 description:DEAD-box ATP-dependent
384	pep chromosome:ASM81700v1:Chromosome:835908:836885:-1 gene:SpnNT_00790 transcript:AJD71731 gene_biotype:protein_coding transcript_biotype:protein_coding gene_symbol:ycjS description:putative oxidoreductase YcjS
385	pep chromosome:ASM81700v1:Chromosome:837679:839121:1 gene:SpnNT_00791 transcript:AJD71732 gene_biotype:protein_coding transcript_biotype:protein_coding gene_symbol:xynD description:Bifunctional xylanase/deacetyl
386	pep chromosome:ASM81700v1:Chromosome:839235:840077:1 gene:SpnNT_00792 transcript:AJD71733 gene_biotype:protein_coding transcript_biotype:protein_coding gene_symbol:yvgN description:Glyoxal reductase
387	pep chromosome:ASM81700v1:Chromosome:841304:842221:1 gene:SpnNT_00794 transcript:AJD71735 gene_biotype:protein_coding transcript_biotype:protein_coding gene_symbol:glyQ description:Glycine--tRNA ligase alpha sub
388	pep chromosome:ASM81700v1:Chromosome:842481:844517:1 gene:SpnNT_00795 transcript:AJD71736 gene_biotype:protein_coding transcript_biotype:protein_coding gene_symbol:glyS description:Glycine--tRNA ligase beta subu
389	pep chromosome:ASM81700v1:Chromosome:844559:844816:1 gene:SpnNT_00796 transcript:AJD71737 gene_biotype:protein_coding transcript_biotype:protein_coding description:hypothetical protein
390	pep chromosome:ASM81700v1:Chromosome:844933:846177:-1 gene:SpnNT_00797 transcript:AJD71738 gene_biotype:protein_coding transcript_biotype:protein_coding gene_symbol:azr_1 description:NADPH azoreductase
391	pep chromosome:ASM81700v1:Chromosome:848078:849457:1 gene:SpnNT_00800 transcript:AJD71741 gene_biotype:protein_coding transcript_biotype:protein_coding description:putative NADH oxidase
392	pep chromosome:ASM81700v1:Chromosome:852312:852806:1 gene:SpnNT_00805 transcript:AJD71746 gene_biotype:protein_coding transcript_biotype:protein_coding description:hypothetical protein
393	pep chromosome:ASM81700v1:Chromosome:853330:853686:1 gene:SpnNT_00807 transcript:AJD71748 gene_biotype:protein_coding transcript_biotype:protein_coding gene_symbol:mgsR description:Modulator of the general stres
394	pep chromosome:ASM81700v1:Chromosome:853844:854644:1 gene:SpnNT_00808 transcript:AJD71749 gene_biotype:protein_coding transcript_biotype:protein_coding gene_symbol:yxeN description:putative amino-acid permease
395	pep chromosome:ASM81700v1:Chromosome:854644:855387:1 gene:SpnNT_00809 transcript:AJD71750 gene_biotype:protein_coding transcript_biotype:protein_coding gene_symbol:tcyC description:L-cystine import ATP-binding p
396	pep chromosome:ASM81700v1:Chromosome:855755:856666:1 gene:SpnNT_00811 transcript:AJD71752 gene_biotype:protein_coding transcript_biotype:protein_coding gene_symbol:trxB description:Thioredoxin reductase
397	pep chromosome:ASM81700v1:Chromosome:856705:857433:-1 gene:SpnNT_00812 transcript:AJD71753 gene_biotype:protein_coding transcript_biotype:protein_coding description:Putative TrmH family tRNA/rRNA methyltransfera
398	pep chromosome:ASM81700v1:Chromosome:857479:858090:-1 gene:SpnNT_00813 transcript:AJD71754 gene_biotype:protein_coding transcript_biotype:protein_coding gene_symbol:def_1 description:Peptide deformylase
399	pep chromosome:ASM81700v1:Chromosome:859692:860519:1 gene:SpnNT_00817 transcript:AJD71758 gene_biotype:protein_coding transcript_biotype:protein_coding gene_symbol:ybiV description:Sugar phosphatase YbiV
400	pep chromosome:ASM81700v1:Chromosome:861240:861965:1 gene:SpnNT_00819 transcript:AJD71760 gene_biotype:protein_coding transcript_biotype:protein_coding description:hypothetical protein
401	pep chromosome:ASM81700v1:Chromosome:861962:862897:1 gene:SpnNT_00820 transcript:AJD71761 gene_biotype:protein_coding transcript_biotype:protein_coding gene_symbol:pbpX_2 description:Putative penicillin-binding p
402	pep chromosome:ASM81700v1:Chromosome:863845:864543:-1 gene:SpnNT_00822 transcript:AJD71763 gene_biotype:protein_coding transcript_biotype:protein_coding gene_symbol:yvoA description:HTH-type transcriptional repre
403	pep chromosome:ASM81700v1:Chromosome:864685:866247:1 gene:SpnNT_00823 transcript:AJD71764 gene_biotype:protein_coding transcript_biotype:protein_coding gene_symbol:guaA description:GMP synthase [glutamine-hyd
404	pep chromosome:ASM81700v1:Chromosome:866846:868210:-1 gene:SpnNT_00825 transcript:AJD71766 gene_biotype:protein_coding transcript_biotype:protein_coding description:hypothetical protein
405	pep chromosome:ASM81700v1:Chromosome:873788:874978:-1 gene:SpnNT_00833 transcript:AJD71774 gene_biotype:protein_coding transcript_biotype:protein_coding gene_symbol:dpnA_1 description:Modification methylase Dp
406	pep chromosome:ASM81700v1:Chromosome:876153:877439:1 gene:SpnNT_00836 transcript:AJD71777 gene_biotype:protein_coding transcript_biotype:protein_coding gene_symbol:yhbU_2 description:putative protease YhbU pre
407	pep chromosome:ASM81700v1:Chromosome:881104:881313:1 gene:SpnNT_00840 transcript:AJD71781 gene_biotype:protein_coding transcript_biotype:protein_coding description:DNA-binding transcriptional repressor PuuR
408	pep chromosome:ASM81700v1:Chromosome:881907:883367:1 gene:SpnNT_00842 transcript:AJD71783 gene_biotype:protein_coding transcript_biotype:protein_coding gene_symbol:pncB2 description:Nicotinate phosphoribosyltra
409	pep chromosome:ASM81700v1:Chromosome:883364:884188:1 gene:SpnNT_00843 transcript:AJD71784 gene_biotype:protein_coding transcript_biotype:protein_coding gene_symbol:nadE description:NH(3)-dependent NAD(+) syn
410	pep chromosome:ASM81700v1:Chromosome:890163:890870:1 gene:SpnNT_00852 transcript:AJD71793 gene_biotype:protein_coding transcript_biotype:protein_coding gene_symbol:nagB description:Glucosamine-6-phosphate de
411	pep chromosome:ASM81700v1:Chromosome:891010:891186:1 gene:SpnNT_00853 transcript:AJD71794 gene_biotype:protein_coding transcript_biotype:protein_coding gene_symbol:rpsU description:30S ribosomal protein S21
412	pep chromosome:ASM81700v1:Chromosome:891316:892251:1 gene:SpnNT_00854 transcript:AJD71795 gene_biotype:protein_coding transcript_biotype:protein_coding gene_symbol:hprK description:HPr kinase/phosphorylase
413	pep chromosome:ASM81700v1:Chromosome:893033:893416:1 gene:SpnNT_00856 transcript:AJD71797 gene_biotype:protein_coding transcript_biotype:protein_coding description:putative protein containing a divergent version of t
414	pep chromosome:ASM81700v1:Chromosome:893432:893821:1 gene:SpnNT_00857 transcript:AJD71798 gene_biotype:protein_coding transcript_biotype:protein_coding description:Gas vesicle protein
415	pep chromosome:ASM81700v1:Chromosome:893905:895035:1 gene:SpnNT_00858 transcript:AJD71799 gene_biotype:protein_coding transcript_biotype:protein_coding gene_symbol:hemN description:Oxygen-independent copropo
416	pep chromosome:ASM81700v1:Chromosome:895040:895777:1 gene:SpnNT_00859 transcript:AJD71800 gene_biotype:protein_coding transcript_biotype:protein_coding description:acyl-CoA thioester hydrolase, YbgC/YbaW family
417	pep chromosome:ASM81700v1:Chromosome:895788:896561:1 gene:SpnNT_00860 transcript:AJD71801 gene_biotype:protein_coding transcript_biotype:protein_coding gene_symbol:yutF description:putative hydrolase YutF
418	pep chromosome:ASM81700v1:Chromosome:899832:900110:1 gene:SpnNT_00864 transcript:AJD71805 gene_biotype:protein_coding transcript_biotype:protein_coding description:hypothetical protein
419	pep chromosome:ASM81700v1:Chromosome:900874:902178:1 gene:SpnNT_00866 transcript:AJD71807 gene_biotype:protein_coding transcript_biotype:protein_coding gene_symbol:rsmF description:Ribosomal RNA small subunit
420	pep chromosome:ASM81700v1:Chromosome:902752:903630:1 gene:SpnNT_00867 transcript:AJD71808 gene_biotype:protein_coding transcript_biotype:protein_coding gene_symbol:pstS1 description:Phosphate-binding protein Ps
421	pep chromosome:ASM81700v1:Chromosome:903630:904547:1 gene:SpnNT_00868 transcript:AJD71809 gene_biotype:protein_coding transcript_biotype:protein_coding gene_symbol:pstC_1 description:Phosphate transport system
422	pep chromosome:ASM81700v1:Chromosome:905432:906235:1 gene:SpnNT_00870 transcript:AJD71811 gene_biotype:protein_coding transcript_biotype:protein_coding gene_symbol:pstB3_1 description:Phosphate import ATP-bin
423	pep chromosome:ASM81700v1:Chromosome:906248:907006:1 gene:SpnNT_00871 transcript:AJD71812 gene_biotype:protein_coding transcript_biotype:protein_coding gene_symbol:pstB3_2 description:Phosphate import ATP-bin
424	pep chromosome:ASM81700v1:Chromosome:907018:907671:1 gene:SpnNT_00872 transcript:AJD71813 gene_biotype:protein_coding transcript_biotype:protein_coding gene_symbol:phoU_2 description:phosphate transport system
425	pep chromosome:ASM81700v1:Chromosome:907828:908643:1 gene:SpnNT_00873 transcript:AJD71814 gene_biotype:protein_coding transcript_biotype:protein_coding gene_symbol:glnH_2 description:Glutamine-binding periplasm
426	pep chromosome:ASM81700v1:Chromosome:908663:909934:-1 gene:SpnNT_00874 transcript:AJD71815 gene_biotype:protein_coding transcript_biotype:protein_coding gene_symbol:yjiR description:putative HTH-type transcription
427	pep chromosome:ASM81700v1:Chromosome:910136:910807:1 gene:SpnNT_00875 transcript:AJD71816 gene_biotype:protein_coding transcript_biotype:protein_coding gene_symbol:aldC description:Alpha-acetolactate decarboxyla
428	pep chromosome:ASM81700v1:Chromosome:911681:912586:1 gene:SpnNT_00877 transcript:AJD71818 gene_biotype:protein_coding transcript_biotype:protein_coding gene_symbol:murB description:UDP-N-acetylenolpyruvoylgluc
429	pep chromosome:ASM81700v1:Chromosome:912746:913903:1 gene:SpnNT_00878 transcript:AJD71819 gene_biotype:protein_coding transcript_biotype:protein_coding gene_symbol:potA description:Spermidine/putrescine import
430	pep chromosome:ASM81700v1:Chromosome:917302:917787:1 gene:SpnNT_00882 transcript:AJD71823 gene_biotype:protein_coding transcript_biotype:protein_coding description:hypothetical protein
431	pep chromosome:ASM81700v1:Chromosome:917809:920427:1 gene:SpnNT_00883 transcript:AJD71824 gene_biotype:protein_coding transcript_biotype:protein_coding gene_symbol:alaS description:Alanine--tRNA ligase
432	pep chromosome:ASM81700v1:Chromosome:922150:922899:1 gene:SpnNT_00885 transcript:AJD71826 gene_biotype:protein_coding transcript_biotype:protein_coding gene_symbol:ecsA_2 description:ABC-type transporter ATP-b

	A
433	pep chromosome:ASM81700v1:Chromosome:925201:926364:1 gene:SpnNT_00887 transcript:AJD71828 gene_biotype:protein_coding transcript_biotype:protein_coding gene_symbol:rlmI description:Ribosomal RNA large subunit m
434	pep chromosome:ASM81700v1:Chromosome:926361:927038:1 gene:SpnNT_00888 transcript:AJD71829 gene_biotype:protein_coding transcript_biotype:protein_coding gene_symbol:aroD description:3-dehydroquinate dehydratase
435	pep chromosome:ASM81700v1:Chromosome:927028:927882:1 gene:SpnNT_00889 transcript:AJD71830 gene_biotype:protein_coding transcript_biotype:protein_coding gene_symbol:aroE description:Shikimate dehydrogenase
436	pep chromosome:ASM81700v1:Chromosome:927901:928968:1 gene:SpnNT_00890 transcript:AJD71831 gene_biotype:protein_coding transcript_biotype:protein_coding gene_symbol:aroB description:3-dehydroquinate synthase
437	pep chromosome:ASM81700v1:Chromosome:928978:930144:1 gene:SpnNT_00891 transcript:AJD71832 gene_biotype:protein_coding transcript_biotype:protein_coding gene_symbol:aroC description:Chorismate synthase
438	pep chromosome:ASM81700v1:Chromosome:930154:931257:1 gene:SpnNT_00892 transcript:AJD71833 gene_biotype:protein_coding transcript_biotype:protein_coding gene_symbol:tyrC description:Arogenate dehydrogenase
439	pep chromosome:ASM81700v1:Chromosome:931268:931606:1 gene:SpnNT_00893 transcript:AJD71834 gene_biotype:protein_coding transcript_biotype:protein_coding description:hypothetical protein
440	pep chromosome:ASM81700v1:Chromosome:931698:932981:1 gene:SpnNT_00894 transcript:AJD71835 gene_biotype:protein_coding transcript_biotype:protein_coding gene_symbol:aroA description:3-phosphoshikimate 1-carboxy
441	pep chromosome:ASM81700v1:Chromosome:933447:934295:1 gene:SpnNT_00896 transcript:AJD71837 gene_biotype:protein_coding transcript_biotype:protein_coding gene_symbol:pheA description:Prephenate dehydratase
442	pep chromosome:ASM81700v1:Chromosome:934292:935566:1 gene:SpnNT_00897 transcript:AJD71838 gene_biotype:protein_coding transcript_biotype:protein_coding gene_symbol:msrR description:Regulatory protein MsrR
443	pep chromosome:ASM81700v1:Chromosome:935854:936690:1 gene:SpnNT_00898 transcript:AJD71839 gene_biotype:protein_coding transcript_biotype:protein_coding description:LPS biosynthesis protein
444	pep chromosome:ASM81700v1:Chromosome:940249:941283:1 gene:SpnNT_00902 transcript:AJD71843 gene_biotype:protein_coding transcript_biotype:protein_coding description:hypothetical protein
445	pep chromosome:ASM81700v1:Chromosome:941483:942220:1 gene:SpnNT_00903 transcript:AJD71844 gene_biotype:protein_coding transcript_biotype:protein_coding gene_symbol:mecA description:Adapter protein MecA
446	pep chromosome:ASM81700v1:Chromosome:942371:943657:1 gene:SpnNT_00904 transcript:AJD71845 gene_biotype:protein_coding transcript_biotype:protein_coding gene_symbol:hom description:Homoserine dehydrogenase
447	pep chromosome:ASM81700v1:Chromosome:943659:944528:1 gene:SpnNT_00905 transcript:AJD71846 gene_biotype:protein_coding transcript_biotype:protein_coding gene_symbol:thrB description:Homoserine kinase
448	pep chromosome:ASM81700v1:Chromosome:944823:945761:1 gene:SpnNT_00906 transcript:AJD71847 gene_biotype:protein_coding transcript_biotype:protein_coding gene_symbol:msrAB1 description:Peptide methionine sulfoxid
449	pep chromosome:ASM81700v1:Chromosome:945821:947545:1 gene:SpnNT_00907 transcript:AJD71848 gene_biotype:protein_coding transcript_biotype:protein_coding gene_symbol:bmrA description:Multidrug resistance ABC tran
450	pep chromosome:ASM81700v1:Chromosome:947547:949295:1 gene:SpnNT_00908 transcript:AJD71849 gene_biotype:protein_coding transcript_biotype:protein_coding description:putative ABC transporter ATP-binding protein
451	pep chromosome:ASM81700v1:Chromosome:949390:950856:1 gene:SpnNT_00909 transcript:AJD71850 gene_biotype:protein_coding transcript_biotype:protein_coding gene_symbol:mtaD description:5-methylthioadenosine/S-ade
452	pep chromosome:ASM81700v1:Chromosome:951106:951606:1 gene:SpnNT_00910 transcript:AJD71851 gene_biotype:protein_coding transcript_biotype:protein_coding gene_symbol:rplJ description:50S ribosomal protein L10
453	pep chromosome:ASM81700v1:Chromosome:951682:952050:1 gene:SpnNT_00911 transcript:AJD71852 gene_biotype:protein_coding transcript_biotype:protein_coding gene_symbol:rplL description:50S ribosomal protein L7/L12
454	pep chromosome:ASM81700v1:Chromosome:962416:963210:1 gene:SpnNT_00926 transcript:AJD71867 gene_biotype:protein_coding transcript_biotype:protein_coding gene_symbol:aphA description:Aminoglycoside 3-phosphotra
455	pep chromosome:ASM81700v1:Chromosome:983576:984412:1 gene:SpnNT_00949 transcript:AJD71890 gene_biotype:protein_coding transcript_biotype:protein_coding description:hypothetical protein
456	pep chromosome:ASM81700v1:Chromosome:1006943:1007638:1 gene:SpnNT_00976 transcript:AJD71917 gene_biotype:protein_coding transcript_biotype:protein_coding description:hypothetical protein
457	pep chromosome:ASM81700v1:Chromosome:1022114:1023460:-1 gene:SpnNT_00992 transcript:AJD71933 gene_biotype:protein_coding transcript_biotype:protein_coding gene_symbol:gdhA description:NADP-specific glutamate d
458	pep chromosome:ASM81700v1:Chromosome:1026025:1027236:-1 gene:SpnNT_00997 transcript:AJD71938 gene_biotype:protein_coding transcript_biotype:protein_coding gene_symbol:lytA_5 description:Autolysin
459	pep chromosome:ASM81700v1:Chromosome:1028608:1028850:-1 gene:SpnNT_01000 transcript:AJD71941 gene_biotype:protein_coding transcript_biotype:protein_coding gene_symbol:rpmE2 description:50S ribosomal protein L3
460	pep chromosome:ASM81700v1:Chromosome:1028946:1029881:-1 gene:SpnNT_01001 transcript:AJD71942 gene_biotype:protein_coding transcript_biotype:protein_coding gene_symbol:nrnA_1 description:putative bifunctional oligo
461	pep chromosome:ASM81700v1:Chromosome:1029985:1030428:1 gene:SpnNT_01002 transcript:AJD71943 gene_biotype:protein_coding transcript_biotype:protein_coding description:Flavodoxin
462	pep chromosome:ASM81700v1:Chromosome:1030544:1030810:1 gene:SpnNT_01003 transcript:AJD71944 gene_biotype:protein_coding transcript_biotype:protein_coding description:chorismate mutase
463	pep chromosome:ASM81700v1:Chromosome:1031623:1031970:1 gene:SpnNT_01006 transcript:AJD71947 gene_biotype:protein_coding transcript_biotype:protein_coding gene_symbol:rplS description:50S ribosomal protein L19
464	pep chromosome:ASM81700v1:Chromosome:1033597:1034817:1 gene:SpnNT_01010 transcript:AJD71950 gene_biotype:protein_coding transcript_biotype:protein_coding gene_symbol:lytA_6 description:Autolysin
465	pep chromosome:ASM81700v1:Chromosome:1034830:1035948:1 gene:SpnNT_01011 transcript:AJD71951 gene_biotype:protein_coding transcript_biotype:protein_coding gene_symbol:lytB_3 description:Putative endo-beta-N-acet
466	pep chromosome:ASM81700v1:Chromosome:1039846:1040652:-1 gene:SpnNT_01016 transcript:AJD71956 gene_biotype:protein_coding transcript_biotype:protein_coding description:Putative hydrolase
467	pep chromosome:ASM81700v1:Chromosome:1040665:1041969:-1 gene:SpnNT_01017 transcript:AJD71957 gene_biotype:protein_coding transcript_biotype:protein_coding description:putative dGTPase
468	pep chromosome:ASM81700v1:Chromosome:1042041:1042418:1 gene:SpnNT_01018 transcript:AJD71958 gene_biotype:protein_coding transcript_biotype:protein_coding description:hypothetical protein
469	pep chromosome:ASM81700v1:Chromosome:1042509:1042841:1 gene:SpnNT_01019 transcript:AJD71959 gene_biotype:protein_coding transcript_biotype:protein_coding description:putative DNA-binding protein
470	pep chromosome:ASM81700v1:Chromosome:1042853:1044424:1 gene:SpnNT_01020 transcript:AJD71960 gene_biotype:protein_coding transcript_biotype:protein_coding gene_symbol:ffh description:signal recognition particle prot
471	pep chromosome:ASM81700v1:Chromosome:1044619:1045902:-1 gene:SpnNT_01021 transcript:AJD71961 gene_biotype:protein_coding transcript_biotype:protein_coding gene_symbol:pyrP description:Uracil transporter
472	pep chromosome:ASM81700v1:Chromosome:1046303:1047016:-1 gene:SpnNT_01022 transcript:AJD71962 gene_biotype:protein_coding transcript_biotype:protein_coding gene_symbol:rsmG description:Ribosomal RNA small sub
473	pep chromosome:ASM81700v1:Chromosome:1047110:1047670:1 gene:SpnNT_01023 transcript:AJD71963 gene_biotype:protein_coding transcript_biotype:protein_coding description:LemA family protein
474	pep chromosome:ASM81700v1:Chromosome:1047672:1048571:1 gene:SpnNT_01024 transcript:AJD71964 gene_biotype:protein_coding transcript_biotype:protein_coding gene_symbol:htpX description:peptidase m48 family protei
475	pep chromosome:ASM81700v1:Chromosome:1053140:1053682:1 gene:SpnNT_01029 transcript:AJD71969 gene_biotype:protein_coding transcript_biotype:protein_coding description:hypothetical protein
476	pep chromosome:ASM81700v1:Chromosome:1053682:1054311:1 gene:SpnNT_01030 transcript:AJD71970 gene_biotype:protein_coding transcript_biotype:protein_coding gene_symbol:pdg description:UV-endonuclease
477	pep chromosome:ASM81700v1:Chromosome:1056578:1057099:1 gene:SpnNT_01032 transcript:AJD71972 gene_biotype:protein_coding transcript_biotype:protein_coding gene_symbol:pyrR description:Bifunctional protein PyrR
478	pep chromosome:ASM81700v1:Chromosome:1057118:1058041:1 gene:SpnNT_01033 transcript:AJD71973 gene_biotype:protein_coding transcript_biotype:protein_coding gene_symbol:pyrB description:Aspartate carbamoyltransfe
479	pep chromosome:ASM81700v1:Chromosome:1058091:1059170:1 gene:SpnNT_01034 transcript:AJD71974 gene_biotype:protein_coding transcript_biotype:protein_coding gene_symbol:carA description:Carbamoyl-phosphate synth
480	pep chromosome:ASM81700v1:Chromosome:1059483:1062659:1 gene:SpnNT_01035 transcript:AJD71975 gene_biotype:protein_coding transcript_biotype:protein_coding gene_symbol:carB description:Carbamoyl-phosphate synth
481	pep chromosome:ASM81700v1:Chromosome:1062934:1063743:-1 gene:SpnNT_01036 transcript:AJD71976 gene_biotype:protein_coding transcript_biotype:protein_coding description:LPS biosynthesis protein
482	pep chromosome:ASM81700v1:Chromosome:1063745:1064548:-1 gene:SpnNT_01037 transcript:AJD71977 gene_biotype:protein_coding transcript_biotype:protein_coding description:LPS biosynthesis protein
483	pep chromosome:ASM81700v1:Chromosome:1064558:1066045:-1 gene:SpnNT_01038 transcript:AJD71978 gene_biotype:protein_coding transcript_biotype:protein_coding description:Polysaccharide biosynthesis protein
484	pep chromosome:ASM81700v1:Chromosome:1066381:1067088:1 gene:SpnNT_01039 transcript:AJD71979 gene_biotype:protein_coding transcript_biotype:protein_coding gene_symbol:ispD2 description:Putative 2-C-methyl-D-eryt
485	pep chromosome:ASM81700v1:Chromosome:1067093:1068115:1 gene:SpnNT_01040 transcript:AJD71980 gene_biotype:protein_coding transcript_biotype:protein_coding gene_symbol:yjjN description:Putative L-galactonate oxido
486	pep chromosome:ASM81700v1:Chromosome:1068132:1069001:1 gene:SpnNT_01041 transcript:AJD71981 gene_biotype:protein_coding transcript_biotype:protein_coding description:thiamine kinase

	A
487	pep chromosome:ASM81700v1:Chromosome:1069881:1070570:1 gene:SpnNT_01043 transcript:AJD71983 gene_biotype:protein_coding transcript_biotype:protein_coding gene_symbol:gtaB_1 description:UTP--glucose-1-phosphat
488	pep chromosome:ASM81700v1:Chromosome:1072399:1073442:1 gene:SpnNT_01045 transcript:AJD71985 gene_biotype:protein_coding transcript_biotype:protein_coding description:putative hydrophobic domain protein
489	pep chromosome:ASM81700v1:Chromosome:1073609:1075696:1 gene:SpnNT_01046 transcript:AJD71986 gene_biotype:protein_coding transcript_biotype:protein_coding gene_symbol:topA description:DNA topoisomerase 1
490	pep chromosome:ASM81700v1:Chromosome:1076369:1077001:1 gene:SpnNT_01048 transcript:AJD71988 gene_biotype:protein_coding transcript_biotype:protein_coding description:CutC-like protein
491	pep chromosome:ASM81700v1:Chromosome:1081546:1083477:1 gene:SpnNT_01056 transcript:AJD71996 gene_biotype:protein_coding transcript_biotype:protein_coding gene_symbol:mcrB description:5-methylcytosine-specific re
492	pep chromosome:ASM81700v1:Chromosome:1084954:1085940:1 gene:SpnNT_01058 transcript:AJD71998 gene_biotype:protein_coding transcript_biotype:protein_coding gene_symbol:guaC description:GMP reductase
493	pep chromosome:ASM81700v1:Chromosome:1088433:1089131:1 gene:SpnNT_01061 transcript:AJD72000 gene_biotype:protein_coding transcript_biotype:protein_coding gene_symbol:rnc description:Ribonuclease 3
494	pep chromosome:ASM81700v1:Chromosome:1089122:1092661:1 gene:SpnNT_01062 transcript:AJD72001 gene_biotype:protein_coding transcript_biotype:protein_coding gene_symbol:smc description:Chromosome partition protei
495	pep chromosome:ASM81700v1:Chromosome:1092658:1093452:1 gene:SpnNT_01063 transcript:AJD72002 gene_biotype:protein_coding transcript_biotype:protein_coding gene_symbol:ybjI description:Flavin mononucleotide phosp
496	pep chromosome:ASM81700v1:Chromosome:1093452:1094270:1 gene:SpnNT_01064 transcript:AJD72003 gene_biotype:protein_coding transcript_biotype:protein_coding gene_symbol:yidA_2 description:Sugar phosphatase YidA
497	pep chromosome:ASM81700v1:Chromosome:1094274:1095563:1 gene:SpnNT_01065 transcript:AJD72004 gene_biotype:protein_coding transcript_biotype:protein_coding gene_symbol:ftsY description:Signal recognition particle re
498	pep chromosome:ASM81700v1:Chromosome:1095609:1097096:-1 gene:SpnNT_01066 transcript:AJD72005 gene_biotype:protein_coding transcript_biotype:protein_coding gene_symbol:zwf description:Glucose-6-phosphate 1-dehy
499	pep chromosome:ASM81700v1:Chromosome:1097245:1097985:-1 gene:SpnNT_01067 transcript:AJD72006 gene_biotype:protein_coding transcript_biotype:protein_coding gene_symbol:artM_1 description:Arginine transport ATP-b
500	pep chromosome:ASM81700v1:Chromosome:1097985:1100150:-1 gene:SpnNT_01068 transcript:AJD72007 gene_biotype:protein_coding transcript_biotype:protein_coding gene_symbol:glnP_2 description:Glutamine transport syste
501	pep chromosome:ASM81700v1:Chromosome:1101353:1103341:1 gene:SpnNT_01070 transcript:AJD72009 gene_biotype:protein_coding transcript_biotype:protein_coding gene_symbol:uvrB description:Excinuclease ABC subunit
502	pep chromosome:ASM81700v1:Chromosome:1104442:1104909:1 gene:SpnNT_01073 transcript:AJD72012 gene_biotype:protein_coding transcript_biotype:protein_coding gene_symbol:mutX_2 description:8-oxo-dGTP diphosphata
503	pep chromosome:ASM81700v1:Chromosome:1104906:1105421:-1 gene:SpnNT_01074 transcript:AJD72013 gene_biotype:protein_coding transcript_biotype:protein_coding gene_symbol:niaR description:putative transcription repres
504	pep chromosome:ASM81700v1:Chromosome:1106789:1107340:-1 gene:SpnNT_01077 transcript:AJD72016 gene_biotype:protein_coding transcript_biotype:protein_coding gene_symbol:coaBC_1 description:DNA/pantothenate me
505	pep chromosome:ASM81700v1:Chromosome:1107352:1108041:-1 gene:SpnNT_01078 transcript:AJD72017 gene_biotype:protein_coding transcript_biotype:protein_coding gene_symbol:coaBC_2 description:DNA/pantothenate me
506	pep chromosome:ASM81700v1:Chromosome:1108272:1109942:1 gene:SpnNT_01079 transcript:AJD72018 gene_biotype:protein_coding transcript_biotype:protein_coding gene_symbol:fhs1 description:Formate--tetrahydrofolate lig
507	pep chromosome:ASM81700v1:Chromosome:1110270:1111445:1 gene:SpnNT_01080 transcript:AJD72019 gene_biotype:protein_coding transcript_biotype:protein_coding gene_symbol:yfhQ description:putative A/G-specific adenin
508	pep chromosome:ASM81700v1:Chromosome:1111501:1112205:1 gene:SpnNT_01081 transcript:AJD72020 gene_biotype:protein_coding transcript_biotype:protein_coding gene_symbol:yycF description:Transcriptional regulatory pr
509	pep chromosome:ASM81700v1:Chromosome:1112198:1113547:1 gene:SpnNT_01082 transcript:AJD72021 gene_biotype:protein_coding transcript_biotype:protein_coding gene_symbol:yycG description:Sensor histidine kinase Yyc
510	pep chromosome:ASM81700v1:Chromosome:1113549:1114358:1 gene:SpnNT_01083 transcript:AJD72022 gene_biotype:protein_coding transcript_biotype:protein_coding gene_symbol:yycJ description:Putative metallo-hydrolase
511	pep chromosome:ASM81700v1:Chromosome:1114449:1114691:1 gene:SpnNT_01084 transcript:AJD72023 gene_biotype:protein_coding transcript_biotype:protein_coding description:hypothetical protein
512	pep chromosome:ASM81700v1:Chromosome:1116026:1117012:-1 gene:SpnNT_01087 transcript:AJD72026 gene_biotype:protein_coding transcript_biotype:protein_coding gene_symbol:ldh description:L-lactate dehydrogenase
513	pep chromosome:ASM81700v1:Chromosome:1117206:1119674:1 gene:SpnNT_01088 transcript:AJD72027 gene_biotype:protein_coding transcript_biotype:protein_coding gene_symbol:gyrA description:DNA gyrase subunit A
514	pep chromosome:ASM81700v1:Chromosome:1119674:1120417:1 gene:SpnNT_01089 transcript:AJD72028 gene_biotype:protein_coding transcript_biotype:protein_coding description:sortase
515	pep chromosome:ASM81700v1:Chromosome:1122960:1124234:1 gene:SpnNT_01094 transcript:AJD72033 gene_biotype:protein_coding transcript_biotype:protein_coding description:hypothetical protein
516	pep chromosome:ASM81700v1:Chromosome:1124244:1125122:1 gene:SpnNT_01095 transcript:AJD72034 gene_biotype:protein_coding transcript_biotype:protein_coding gene_symbol:truB description:tRNA pseudouridine synthas
517	pep chromosome:ASM81700v1:Chromosome:1125773:1126411:1 gene:SpnNT_01098 transcript:AJD72037 gene_biotype:protein_coding transcript_biotype:protein_coding gene_symbol:udk description:Uridine kinase
518	pep chromosome:ASM81700v1:Chromosome:1126539:1127879:1 gene:SpnNT_01099 transcript:AJD72038 gene_biotype:protein_coding transcript_biotype:protein_coding gene_symbol:xseA description:Exodeoxyribonuclease 7 lar
519	pep chromosome:ASM81700v1:Chromosome:1127857:1128069:1 gene:SpnNT_01100 transcript:AJD72039 gene_biotype:protein_coding transcript_biotype:protein_coding gene_symbol:xseB description:Exodeoxyribonuclease 7 sm
520	pep chromosome:ASM81700v1:Chromosome:1128066:1128941:1 gene:SpnNT_01101 transcript:AJD72040 gene_biotype:protein_coding transcript_biotype:protein_coding description:Farnesyl diphosphate synthase
521	pep chromosome:ASM81700v1:Chromosome:1129742:1130173:1 gene:SpnNT_01103 transcript:AJD72042 gene_biotype:protein_coding transcript_biotype:protein_coding gene_symbol:argR_1 description:Arginine hydroxamate res
522	pep chromosome:ASM81700v1:Chromosome:1130180:1131847:1 gene:SpnNT_01104 transcript:AJD72043 gene_biotype:protein_coding transcript_biotype:protein_coding gene_symbol:recN description:Recombination protein N
523	pep chromosome:ASM81700v1:Chromosome:1131849:1132577:1 gene:SpnNT_01105 transcript:AJD72044 gene_biotype:protein_coding transcript_biotype:protein_coding gene_symbol:pphA description:Serine/threonine-protein ph
524	pep chromosome:ASM81700v1:Chromosome:1132656:1134479:1 gene:SpnNT_01106 transcript:AJD72045 gene_biotype:protein_coding transcript_biotype:protein_coding gene_symbol:lepA description:Elongation factor 4
525	pep chromosome:ASM81700v1:Chromosome:1135407:1135712:1 gene:SpnNT_01108 transcript:AJD72047 gene_biotype:protein_coding transcript_biotype:protein_coding description:Phosphotransferase system, galactitol-specific
526	pep chromosome:ASM81700v1:Chromosome:1138540:1138965:1 gene:SpnNT_01112 transcript:AJD72051 gene_biotype:protein_coding transcript_biotype:protein_coding gene_symbol:lacA description:Galactose-6-phosphate isom
527	pep chromosome:ASM81700v1:Chromosome:1138996:1139511:1 gene:SpnNT_01113 transcript:AJD72052 gene_biotype:protein_coding transcript_biotype:protein_coding gene_symbol:lacB description:Galactose-6-phosphate isom
528	pep chromosome:ASM81700v1:Chromosome:1139522:1140451:1 gene:SpnNT_01114 transcript:AJD72053 gene_biotype:protein_coding transcript_biotype:protein_coding gene_symbol:lacC_1 description:Tagatose-6-phosphate ki
529	pep chromosome:ASM81700v1:Chromosome:1140453:1141433:1 gene:SpnNT_01115 transcript:AJD72054 gene_biotype:protein_coding transcript_biotype:protein_coding gene_symbol:lacD description:Tagatose 1,6-diphosphate a
530	pep chromosome:ASM81700v1:Chromosome:1141692:1142309:1 gene:SpnNT_01116 transcript:AJD72055 gene_biotype:protein_coding transcript_biotype:protein_coding description:hypothetical protein
531	pep chromosome:ASM81700v1:Chromosome:1147648:1148409:-1 gene:SpnNT_01122 transcript:AJD72061 gene_biotype:protein_coding transcript_biotype:protein_coding gene_symbol:lacR_1 description:Lactose phosphotransfer
532	pep chromosome:ASM81700v1:Chromosome:1148591:1149553:-1 gene:SpnNT_01123 transcript:AJD72062 gene_biotype:protein_coding transcript_biotype:protein_coding gene_symbol:nrdF description:Ribonucleoside-diphosphat
533	pep chromosome:ASM81700v1:Chromosome:1149741:1151900:-1 gene:SpnNT_01124 transcript:AJD72063 gene_biotype:protein_coding transcript_biotype:protein_coding gene_symbol:nrdE2 description:Ribonucleoside-diphospha
534	pep chromosome:ASM81700v1:Chromosome:1151981:1152199:-1 gene:SpnNT_01125 transcript:AJD72064 gene_biotype:protein_coding transcript_biotype:protein_coding gene_symbol:nrdH description:Glutaredoxin-like protein N
535	pep chromosome:ASM81700v1:Chromosome:1152549:1152812:1 gene:SpnNT_01126 transcript:AJD72065 gene_biotype:protein_coding transcript_biotype:protein_coding gene_symbol:ptsH description:Phosphocarrier protein HPr
536	pep chromosome:ASM81700v1:Chromosome:1152827:1154551:1 gene:SpnNT_01127 transcript:AJD72066 gene_biotype:protein_coding transcript_biotype:protein_coding gene_symbol:ptsI description:Phosphoenolpyruvate-protein
537	pep chromosome:ASM81700v1:Chromosome:1160203:1161192:1 gene:SpnNT_01135 transcript:AJD72074 gene_biotype:protein_coding transcript_biotype:protein_coding gene_symbol:dhaK description:PTS-dependent dihydroxya
538	pep chromosome:ASM81700v1:Chromosome:1161214:1161789:1 gene:SpnNT_01136 transcript:AJD72075 gene_biotype:protein_coding transcript_biotype:protein_coding gene_symbol:dhaL description:PTS-dependent dihydroxya
539	pep chromosome:ASM81700v1:Chromosome:1161789:1162163:1 gene:SpnNT_01137 transcript:AJD72076 gene_biotype:protein_coding transcript_biotype:protein_coding gene_symbol:dhaM description:PTS-dependent dihydroxya
540	pep chromosome:ASM81700v1:Chromosome:1162651:1163652:1 gene:SpnNT_01138 transcript:AJD72077 gene_biotype:protein_coding transcript_biotype:protein_coding description:hypothetical protein

	A
541	pep chromosome:ASM81700v1:Chromosome:1163649:1163846:1 gene:SpnNT_01139 transcript:AJD72078 gene_biotype:protein_coding transcript_biotype:protein_coding description:hypothetical protein
542	pep chromosome:ASM81700v1:Chromosome:1163885:1164598:1 gene:SpnNT_01140 transcript:AJD72079 gene_biotype:protein_coding transcript_biotype:protein_coding gene_symbol:yfnB description:Putative HAD-hydrolase Yfn
543	pep chromosome:ASM81700v1:Chromosome:1165770:1166234:1 gene:SpnNT_01143 transcript:AJD72082 gene_biotype:protein_coding transcript_biotype:protein_coding gene_symbol:mutX_3 description:8-oxo-dGTP diphosphata
544	pep chromosome:ASM81700v1:Chromosome:1166247:1167515:1 gene:SpnNT_01144 transcript:AJD72083 gene_biotype:protein_coding transcript_biotype:protein_coding gene_symbol:pyrC description:Dihydroorotase
545	pep chromosome:ASM81700v1:Chromosome:1167529:1167789:1 gene:SpnNT_01145 transcript:AJD72084 gene_biotype:protein_coding transcript_biotype:protein_coding description:hypothetical protein
546	pep chromosome:ASM81700v1:Chromosome:1169315:1170283:1 gene:SpnNT_01147 transcript:AJD72086 gene_biotype:protein_coding transcript_biotype:protein_coding gene_symbol:acoA description:Acetoin:2,6-dichlorophenoli
547	pep chromosome:ASM81700v1:Chromosome:1175690:1176760:-1 gene:SpnNT_01154 transcript:AJD72093 gene_biotype:protein_coding transcript_biotype:protein_coding gene_symbol:xerS description:Tyrosine recombinase XerS
548	pep chromosome:ASM81700v1:Chromosome:1178910:1179689:-1 gene:SpnNT_01156 transcript:AJD72095 gene_biotype:protein_coding transcript_biotype:protein_coding gene_symbol:rnhB description:Ribonuclease HII
549	pep chromosome:ASM81700v1:Chromosome:1179676:1180527:-1 gene:SpnNT_01157 transcript:AJD72096 gene_biotype:protein_coding transcript_biotype:protein_coding gene_symbol:rbgA description:Ribosome biogenesis GTP
550	pep chromosome:ASM81700v1:Chromosome:1185950:1191166:-1 gene:SpnNT_01160 transcript:AJD72099 gene_biotype:protein_coding transcript_biotype:protein_coding gene_symbol:iga_5 description:Immunoglobulin A1 protea
551	pep chromosome:ASM81700v1:Chromosome:1192601:1196251:-1 gene:SpnNT_01162 transcript:AJD72101 gene_biotype:protein_coding transcript_biotype:protein_coding gene_symbol:addA description:ATP-dependent helicase/n
552	pep chromosome:ASM81700v1:Chromosome:1196248:1199523:-1 gene:SpnNT_01163 transcript:AJD72102 gene_biotype:protein_coding transcript_biotype:protein_coding gene_symbol:rexB description:ATP-dependent helicase/de
553	pep chromosome:ASM81700v1:Chromosome:1201492:1202796:-1 gene:SpnNT_01165 transcript:AJD72104 gene_biotype:protein_coding transcript_biotype:protein_coding gene_symbol:eno description:Enolase
554	pep chromosome:ASM81700v1:Chromosome:1202960:1203406:1 gene:SpnNT_01166 transcript:AJD72105 gene_biotype:protein_coding transcript_biotype:protein_coding description:hypothetical protein
555	pep chromosome:ASM81700v1:Chromosome:1208854:1210242:-1 gene:SpnNT_01174 transcript:AJD72113 gene_biotype:protein_coding transcript_biotype:protein_coding gene_symbol:gapN description:NADP-dependent glyceral
556	pep chromosome:ASM81700v1:Chromosome:1210786:1213065:-1 gene:SpnNT_01175 transcript:AJD72114 gene_biotype:protein_coding transcript_biotype:protein_coding gene_symbol:amyX description:Pullulanase
557	pep chromosome:ASM81700v1:Chromosome:1213177:1215135:-1 gene:SpnNT_01176 transcript:AJD72115 gene_biotype:protein_coding transcript_biotype:protein_coding gene_symbol:ligA description:DNA ligase
558	pep chromosome:ASM81700v1:Chromosome:1216486:1217349:-1 gene:SpnNT_01178 transcript:AJD72117 gene_biotype:protein_coding transcript_biotype:protein_coding description:hypothetical protein
559	pep chromosome:ASM81700v1:Chromosome:1217352:1219253:-1 gene:SpnNT_01179 transcript:AJD72118 gene_biotype:protein_coding transcript_biotype:protein_coding gene_symbol:yheS_2 description:putative ABC transporte
560	pep chromosome:ASM81700v1:Chromosome:1219679:1219954:-1 gene:SpnNT_01180 transcript:AJD72119 gene_biotype:protein_coding transcript_biotype:protein_coding gene_symbol:hup description:DNA-binding protein HU
561	pep chromosome:ASM81700v1:Chromosome:1220061:1220900:-1 gene:SpnNT_01181 transcript:AJD72120 gene_biotype:protein_coding transcript_biotype:protein_coding description:DegV domain-containing protein
562	pep chromosome:ASM81700v1:Chromosome:1221041:1221889:1 gene:SpnNT_01182 transcript:AJD72121 gene_biotype:protein_coding transcript_biotype:protein_coding description:hypothetical protein
563	pep chromosome:ASM81700v1:Chromosome:1221982:1222899:-1 gene:SpnNT_01183 transcript:AJD72122 gene_biotype:protein_coding transcript_biotype:protein_coding gene_symbol:ribF description:Riboflavin biosynthesis prote
564	pep chromosome:ASM81700v1:Chromosome:1223853:1224146:-1 gene:SpnNT_01184 transcript:AJD72123 gene_biotype:protein_coding transcript_biotype:protein_coding gene_symbol:rpmA description:50S ribosomal protein L27
565	pep chromosome:ASM81700v1:Chromosome:1224163:1224507:-1 gene:SpnNT_01185 transcript:AJD72124 gene_biotype:protein_coding transcript_biotype:protein_coding description:putative ribosomal protein
566	pep chromosome:ASM81700v1:Chromosome:1224523:1224837:-1 gene:SpnNT_01186 transcript:AJD72125 gene_biotype:protein_coding transcript_biotype:protein_coding gene_symbol:rplU description:50S ribosomal protein L21
567	pep chromosome:ASM81700v1:Chromosome:1226053:1226352:1 gene:SpnNT_01188 transcript:AJD72127 gene_biotype:protein_coding transcript_biotype:protein_coding description:Nucleoid-associated protein
568	pep chromosome:ASM81700v1:Chromosome:1228171:1229145:-1 gene:SpnNT_01192 transcript:AJD72131 gene_biotype:protein_coding transcript_biotype:protein_coding gene_symbol:pta description:Phosphate acetyltransferase
569	pep chromosome:ASM81700v1:Chromosome:1230884:1231555:-1 gene:SpnNT_01195 transcript:AJD72134 gene_biotype:protein_coding transcript_biotype:protein_coding gene_symbol:yjbM description:GTP pyrophosphokinase Yj
570	pep chromosome:ASM81700v1:Chromosome:1231683:1232252:1 gene:SpnNT_01196 transcript:AJD72135 gene_biotype:protein_coding transcript_biotype:protein_coding description:hypothetical protein
571	pep chromosome:ASM81700v1:Chromosome:1232365:1233324:1 gene:SpnNT_01197 transcript:AJD72136 gene_biotype:protein_coding transcript_biotype:protein_coding gene_symbol:prs2 description:Ribose-phosphate pyrophos
572	pep chromosome:ASM81700v1:Chromosome:1233334:1234449:1 gene:SpnNT_01198 transcript:AJD72137 gene_biotype:protein_coding transcript_biotype:protein_coding gene_symbol:iscS_1 description:Cysteine desulfurase
573	pep chromosome:ASM81700v1:Chromosome:1234454:1234801:1 gene:SpnNT_01199 transcript:AJD72138 gene_biotype:protein_coding transcript_biotype:protein_coding description:hypothetical protein
574	pep chromosome:ASM81700v1:Chromosome:1235098:1235739:1 gene:SpnNT_01201 transcript:AJD72140 gene_biotype:protein_coding transcript_biotype:protein_coding gene_symbol:rex description:Redox-sensing transcriptional
575	pep chromosome:ASM81700v1:Chromosome:1235757:1236446:1 gene:SpnNT_01202 transcript:AJD72141 gene_biotype:protein_coding transcript_biotype:protein_coding description:Putative glutamine amidotransferasec
576	pep chromosome:ASM81700v1:Chromosome:1237166:1239457:-1 gene:SpnNT_01204 transcript:AJD72143 gene_biotype:protein_coding transcript_biotype:protein_coding gene_symbol:pcrA description:ATP-dependent DNA helica
577	pep chromosome:ASM81700v1:Chromosome:1240834:1241694:-1 gene:SpnNT_01207 transcript:AJD72146 gene_biotype:protein_coding transcript_biotype:protein_coding gene_symbol:map description:Methionine aminopeptidase
578	pep chromosome:ASM81700v1:Chromosome:1241710:1242987:-1 gene:SpnNT_01208 transcript:AJD72147 gene_biotype:protein_coding transcript_biotype:protein_coding description:Cobalt-dependent inorganic pyrophosphatase
579	pep chromosome:ASM81700v1:Chromosome:1243547:1244806:-1 gene:SpnNT_01210 transcript:AJD72149 gene_biotype:protein_coding transcript_biotype:protein_coding gene_symbol:murAB description:UDP-N-acetylglucosamin
580	pep chromosome:ASM81700v1:Chromosome:1247237:1247395:-1 gene:SpnNT_01212 transcript:AJD72151 gene_biotype:protein_coding transcript_biotype:protein_coding description:hypothetical protein
581	pep chromosome:ASM81700v1:Chromosome:1247405:1248715:-1 gene:SpnNT_01213 transcript:AJD72152 gene_biotype:protein_coding transcript_biotype:protein_coding gene_symbol:obg description:Spo0B-associated GTP-bind
582	pep chromosome:ASM81700v1:Chromosome:1249177:1250502:-1 gene:SpnNT_01216 transcript:AJD72155 gene_biotype:protein_coding transcript_biotype:protein_coding gene_symbol:mgtA description:GDP-mannose-dependent
583	pep chromosome:ASM81700v1:Chromosome:1250518:1251561:-1 gene:SpnNT_01217 transcript:AJD72156 gene_biotype:protein_coding transcript_biotype:protein_coding gene_symbol:mshA description:D-inositol 3-phosphate gly
584	pep chromosome:ASM81700v1:Chromosome:1251676:1252005:-1 gene:SpnNT_01218 transcript:AJD72157 gene_biotype:protein_coding transcript_biotype:protein_coding description:FeS assembly SUF system protein
585	pep chromosome:ASM81700v1:Chromosome:1252019:1253128:-1 gene:SpnNT_01219 transcript:AJD72158 gene_biotype:protein_coding transcript_biotype:protein_coding gene_symbol:sigA description:Sigma-A
586	pep chromosome:ASM81700v1:Chromosome:1253131:1254891:-1 gene:SpnNT_01220 transcript:AJD72159 gene_biotype:protein_coding transcript_biotype:protein_coding gene_symbol:dnaG description:DNA primase
587	pep chromosome:ASM81700v1:Chromosome:1257050:1257808:-1 gene:SpnNT_01222 transcript:AJD72161 gene_biotype:protein_coding transcript_biotype:protein_coding gene_symbol:proV description:Glycine betaine/L-proline tr
588	pep chromosome:ASM81700v1:Chromosome:1258740:1259744:-1 gene:SpnNT_01224 transcript:AJD72163 gene_biotype:protein_coding transcript_biotype:protein_coding description:ABC-type uncharacterized transport system, pe
589	pep chromosome:ASM81700v1:Chromosome:1261898:1264594:-1 gene:SpnNT_01227 transcript:AJD72166 gene_biotype:protein_coding transcript_biotype:protein_coding gene_symbol:ppc description:Phosphoenolpyruvate carbo
590	pep chromosome:ASM81700v1:Chromosome:1278622:1279188:-1 gene:SpnNT_01239 transcript:AJD72178 gene_biotype:protein_coding transcript_biotype:protein_coding description:hypothetical protein
591	pep chromosome:ASM81700v1:Chromosome:1279456:1280814:-1 gene:SpnNT_01240 transcript:AJD72179 gene_biotype:protein_coding transcript_biotype:protein_coding gene_symbol:rlmCD_1 description:23S rRNA (uracil-C(5))
592	pep chromosome:ASM81700v1:Chromosome:1280888:1281862:-1 gene:SpnNT_01241 transcript:AJD72180 gene_biotype:protein_coding transcript_biotype:protein_coding description:putative secreted protein
593	pep chromosome:ASM81700v1:Chromosome:1281936:1282550:-1 gene:SpnNT_01242 transcript:AJD72181 gene_biotype:protein_coding transcript_biotype:protein_coding description:hypothetical protein
594	pep chromosome:ASM81700v1:Chromosome:1282550:1283524:-1 gene:SpnNT_01243 transcript:AJD72182 gene_biotype:protein_coding transcript_biotype:protein_coding description:37-kD nucleoid-associated bacterial protein

	A
595	pep chromosome:ASM81700v1:Chromosome:1283532:1284788:-1 gene:SpnNT_01244 transcript:AJD72183 gene_biotype:protein_coding transcript_biotype:protein_coding gene_symbol:glyA description:Serine hydroxymethyltransfe
596	pep chromosome:ASM81700v1:Chromosome:1284849:1285280:-1 gene:SpnNT_01245 transcript:AJD72184 gene_biotype:protein_coding transcript_biotype:protein_coding description:putative acetyltransferase
597	pep chromosome:ASM81700v1:Chromosome:1285282:1285884:-1 gene:SpnNT_01246 transcript:AJD72185 gene_biotype:protein_coding transcript_biotype:protein_coding gene_symbol:rimN description:t(6)A37 threonylcarbamoyla
598	pep chromosome:ASM81700v1:Chromosome:1285868:1286707:-1 gene:SpnNT_01247 transcript:AJD72186 gene_biotype:protein_coding transcript_biotype:protein_coding gene_symbol:prmC description:Release factor glutamine r
599	pep chromosome:ASM81700v1:Chromosome:1286707:1287786:-1 gene:SpnNT_01248 transcript:AJD72187 gene_biotype:protein_coding transcript_biotype:protein_coding gene_symbol:prfA description:Peptide chain release factor
600	pep chromosome:ASM81700v1:Chromosome:1288496:1288678:1 gene:SpnNT_01250 transcript:AJD72189 gene_biotype:protein_coding transcript_biotype:protein_coding gene_symbol:xylH description:2-hydroxymuconate tautome
601	pep chromosome:ASM81700v1:Chromosome:1288815:1290188:1 gene:SpnNT_01251 transcript:AJD72190 gene_biotype:protein_coding transcript_biotype:protein_coding gene_symbol:mnmE description:tRNA modification GTPas
602	pep chromosome:ASM81700v1:Chromosome:1290235:1291170:-1 gene:SpnNT_01252 transcript:AJD72191 gene_biotype:protein_coding transcript_biotype:protein_coding gene_symbol:dapA description:4-hydroxy-tetrahydrodipicol
603	pep chromosome:ASM81700v1:Chromosome:1291226:1292302:-1 gene:SpnNT_01253 transcript:AJD72192 gene_biotype:protein_coding transcript_biotype:protein_coding gene_symbol:asd description:Aspartate-semialdehyde deh
604	pep chromosome:ASM81700v1:Chromosome:1294204:1294581:1 gene:SpnNT_01257 transcript:AJD72195 gene_biotype:protein_coding transcript_biotype:protein_coding gene_symbol:mscL description:Large-conductance mechar
605	pep chromosome:ASM81700v1:Chromosome:1295872:1297095:1 gene:SpnNT_01259 transcript:AJD72197 gene_biotype:protein_coding transcript_biotype:protein_coding gene_symbol:pepT description:Peptidase T
606	pep chromosome:ASM81700v1:Chromosome:1307719:1309110:-1 gene:SpnNT_01267 transcript:AJD72205 gene_biotype:protein_coding transcript_biotype:protein_coding gene_symbol:yhdG description:putative amino acid perme
607	pep chromosome:ASM81700v1:Chromosome:1309232:1309798:-1 gene:SpnNT_01268 transcript:AJD72206 gene_biotype:protein_coding transcript_biotype:protein_coding gene_symbol:resA description:Thiol-disulfide oxidoreducta
608	pep chromosome:ASM81700v1:Chromosome:1313438:1315201:-1 gene:SpnNT_01275 transcript:AJD72213 gene_biotype:protein_coding transcript_biotype:protein_coding gene_symbol:ugpQ description:Glycerophosphoryl diester
609	pep chromosome:ASM81700v1:Chromosome:1315210:1315800:-1 gene:SpnNT_01276 transcript:AJD72214 gene_biotype:protein_coding transcript_biotype:protein_coding gene_symbol:polC_2 description:DNA polymerase III PolC
610	pep chromosome:ASM81700v1:Chromosome:1315898:1316389:-1 gene:SpnNT_01277 transcript:AJD72215 gene_biotype:protein_coding transcript_biotype:protein_coding description:hypothetical protein
611	pep chromosome:ASM81700v1:Chromosome:1316466:1317158:-1 gene:SpnNT_01278 transcript:AJD72216 gene_biotype:protein_coding transcript_biotype:protein_coding gene_symbol:mtnN description:5-methylthioadenosine/S-a
612	pep chromosome:ASM81700v1:Chromosome:1317175:1317489:-1 gene:SpnNT_01279 transcript:AJD72217 gene_biotype:protein_coding transcript_biotype:protein_coding description:hypothetical protein
613	pep chromosome:ASM81700v1:Chromosome:1317501:1318046:-1 gene:SpnNT_01280 transcript:AJD72218 gene_biotype:protein_coding transcript_biotype:protein_coding gene_symbol:nudF description:ADP-ribose pyrophosphata
614	pep chromosome:ASM81700v1:Chromosome:1318056:1319435:-1 gene:SpnNT_01281 transcript:AJD72219 gene_biotype:protein_coding transcript_biotype:protein_coding gene_symbol:glmU description:Bifunctional protein GlmU
615	pep chromosome:ASM81700v1:Chromosome:1319579:1320379:1 gene:SpnNT_01282 transcript:AJD72220 gene_biotype:protein_coding transcript_biotype:protein_coding description:Lyzozyme M1 (1,4-beta-N-acetylmuramidase)
616	pep chromosome:ASM81700v1:Chromosome:1321316:1321798:1 gene:SpnNT_01284 transcript:AJD72222 gene_biotype:protein_coding transcript_biotype:protein_coding gene_symbol:ybaK description:Cys-tRNA(Pro)/Cys-tRNA(C
617	pep chromosome:ASM81700v1:Chromosome:1323637:1324578:-1 gene:SpnNT_01288 transcript:AJD72226 gene_biotype:protein_coding transcript_biotype:protein_coding gene_symbol:prsA description:Foldase protein PrsA precu
618	pep chromosome:ASM81700v1:Chromosome:1324645:1325358:-1 gene:SpnNT_01289 transcript:AJD72227 gene_biotype:protein_coding transcript_biotype:protein_coding description:Putative O-methyltransferase
619	pep chromosome:ASM81700v1:Chromosome:1325360:1327162:-1 gene:SpnNT_01290 transcript:AJD72228 gene_biotype:protein_coding transcript_biotype:protein_coding gene_symbol:pepF1_1 description:Oligoendopeptidase F,
620	pep chromosome:ASM81700v1:Chromosome:1328207:1329067:-1 gene:SpnNT_01292 transcript:AJD72230 gene_biotype:protein_coding transcript_biotype:protein_coding gene_symbol:tehB description:putative S-adenosyl-L-meth
621	pep chromosome:ASM81700v1:Chromosome:1329083:1329550:-1 gene:SpnNT_01293 transcript:AJD72231 gene_biotype:protein_coding transcript_biotype:protein_coding gene_symbol:smpB description:SsrA-binding protein
622	pep chromosome:ASM81700v1:Chromosome:1329513:1331867:-1 gene:SpnNT_01294 transcript:AJD72232 gene_biotype:protein_coding transcript_biotype:protein_coding gene_symbol:rnr description:Ribonuclease R
623	pep chromosome:ASM81700v1:Chromosome:1335585:1336190:-1 gene:SpnNT_01298 transcript:AJD72236 gene_biotype:protein_coding transcript_biotype:protein_coding gene_symbol:coaE description:Dephospho-CoA kinase
624	pep chromosome:ASM81700v1:Chromosome:1336190:1337014:-1 gene:SpnNT_01299 transcript:AJD72237 gene_biotype:protein_coding transcript_biotype:protein_coding gene_symbol:mutM description:Formamidopyrimidine-DNA
625	pep chromosome:ASM81700v1:Chromosome:1337063:1337962:-1 gene:SpnNT_01300 transcript:AJD72238 gene_biotype:protein_coding transcript_biotype:protein_coding gene_symbol:era description:GTPase Era
626	pep chromosome:ASM81700v1:Chromosome:1338355:1338852:-1 gene:SpnNT_01302 transcript:AJD72240 gene_biotype:protein_coding transcript_biotype:protein_coding gene_symbol:ybeY description:Endoribonuclease YbeY
627	pep chromosome:ASM81700v1:Chromosome:1338957:1340612:1 gene:SpnNT_01303 transcript:AJD72241 gene_biotype:protein_coding transcript_biotype:protein_coding description:hypothetical protein
628	pep chromosome:ASM81700v1:Chromosome:1340673:1342649:-1 gene:SpnNT_01304 transcript:AJD72242 gene_biotype:protein_coding transcript_biotype:protein_coding gene_symbol:lytB_4 description:Putative endo-beta-N-ace
629	pep chromosome:ASM81700v1:Chromosome:1342748:1343686:-1 gene:SpnNT_01305 transcript:AJD72243 gene_biotype:protein_coding transcript_biotype:protein_coding gene_symbol:pyrDB description:Dihydroorotate dehydroge
630	pep chromosome:ASM81700v1:Chromosome:1344746:1345126:-1 gene:SpnNT_01307 transcript:AJD72245 gene_biotype:protein_coding transcript_biotype:protein_coding gene_symbol:gloA description:Lactoylglutathione lyase
631	pep chromosome:ASM81700v1:Chromosome:1345185:1345544:-1 gene:SpnNT_01308 transcript:AJD72246 gene_biotype:protein_coding transcript_biotype:protein_coding gene_symbol:rplT description:50S ribosomal protein L20
632	pep chromosome:ASM81700v1:Chromosome:1345596:1345796:-1 gene:SpnNT_01309 transcript:AJD72247 gene_biotype:protein_coding transcript_biotype:protein_coding gene_symbol:rpmI description:50S ribosomal protein L35
633	pep chromosome:ASM81700v1:Chromosome:1345829:1346416:-1 gene:SpnNT_01310 transcript:AJD72248 gene_biotype:protein_coding transcript_biotype:protein_coding gene_symbol:infC description:Translation initiation factor I
634	pep chromosome:ASM81700v1:Chromosome:1348584:1349360:-1 gene:SpnNT_01313 transcript:AJD72251 gene_biotype:protein_coding transcript_biotype:protein_coding description:Protein involved in cell division
635	pep chromosome:ASM81700v1:Chromosome:1349425:1349646:-1 gene:SpnNT_01314 transcript:AJD72252 gene_biotype:protein_coding transcript_biotype:protein_coding description:hypothetical protein
636	pep chromosome:ASM81700v1:Chromosome:1351605:1352453:-1 gene:SpnNT_01316 transcript:AJD72254 gene_biotype:protein_coding transcript_biotype:protein_coding gene_symbol:soj description:Sporulation initiation inhibitor
637	pep chromosome:ASM81700v1:Chromosome:1360649:1361056:-1 gene:SpnNT_01324 transcript:AJD72262 gene_biotype:protein_coding transcript_biotype:protein_coding description:hypothetical protein
638	pep chromosome:ASM81700v1:Chromosome:1381241:1383175:-1 gene:SpnNT_01340 transcript:AJD72278 gene_biotype:protein_coding transcript_biotype:protein_coding gene_symbol:lytB_5 description:Putative endo-beta-N-ace
639	pep chromosome:ASM81700v1:Chromosome:1394641:1395609:-1 gene:SpnNT_01357 transcript:AJD72295 gene_biotype:protein_coding transcript_biotype:protein_coding gene_symbol:ybeZ description:PhoH-like protein
640	pep chromosome:ASM81700v1:Chromosome:1395695:1395910:-1 gene:SpnNT_01358 transcript:AJD72296 gene_biotype:protein_coding transcript_biotype:protein_coding description:hypothetical protein
641	pep chromosome:ASM81700v1:Chromosome:1395919:1396773:-1 gene:SpnNT_01359 transcript:AJD72297 gene_biotype:protein_coding transcript_biotype:protein_coding gene_symbol:rnpS1 description:s1 rna-binding protein
642	pep chromosome:ASM81700v1:Chromosome:1396833:1397390:-1 gene:SpnNT_01360 transcript:AJD72298 gene_biotype:protein_coding transcript_biotype:protein_coding gene_symbol:frr description:Ribosome-releasing factor
643	pep chromosome:ASM81700v1:Chromosome:1397399:1398136:-1 gene:SpnNT_01361 transcript:AJD72299 gene_biotype:protein_coding transcript_biotype:protein_coding gene_symbol:pyrH description:Uridylate kinase
644	pep chromosome:ASM81700v1:Chromosome:1398220:1399554:-1 gene:SpnNT_01362 transcript:AJD72300 gene_biotype:protein_coding transcript_biotype:protein_coding gene_symbol:trmFO description:Methylenetetrahydrofolate
645	pep chromosome:ASM81700v1:Chromosome:1399898:1401220:-1 gene:SpnNT_01363 transcript:AJD72301 gene_biotype:protein_coding transcript_biotype:protein_coding description:hypothetical protein
646	pep chromosome:ASM81700v1:Chromosome:1401220:1401783:-1 gene:SpnNT_01364 transcript:AJD72302 gene_biotype:protein_coding transcript_biotype:protein_coding description:hypothetical protein
647	pep chromosome:ASM81700v1:Chromosome:1405060:1406115:-1 gene:SpnNT_01368 transcript:AJD72306 gene_biotype:protein_coding transcript_biotype:protein_coding gene_symbol:bspRIM description:Modification methylase B
648	pep chromosome:ASM81700v1:Chromosome:1406333:1407202:-1 gene:SpnNT_01369 transcript:AJD72307 gene_biotype:protein_coding transcript_biotype:protein_coding gene_symbol:rsmI description:Ribosomal RNA small subu

	A
649	pep chromosome:ASM81700v1:Chromosome:1407205:1407522:-1 gene:SpnNT_01370 transcript:AJD72308 gene_biotype:protein_coding transcript_biotype:protein_coding description:Initiation-control protein YabA
650	pep chromosome:ASM81700v1:Chromosome:1408449:1409087:-1 gene:SpnNT_01372 transcript:AJD72310 gene_biotype:protein_coding transcript_biotype:protein_coding gene_symbol:tmk description:Thymidylate kinase
651	pep chromosome:ASM81700v1:Chromosome:1410108:1411370:-1 gene:SpnNT_01374 transcript:AJD72312 gene_biotype:protein_coding transcript_biotype:protein_coding gene_symbol:proA description:Gamma-glutamyl phosphat
652	pep chromosome:ASM81700v1:Chromosome:1414063:1415943:-1 gene:SpnNT_01378 transcript:AJD72316 gene_biotype:protein_coding transcript_biotype:protein_coding gene_symbol:lytB_6 description:Putative endo-beta-N-ace
653	pep chromosome:ASM81700v1:Chromosome:1415946:1416833:-1 gene:SpnNT_01379 transcript:AJD72317 gene_biotype:protein_coding transcript_biotype:protein_coding gene_symbol:rluD_2 description:Ribosomal large subunit
654	pep chromosome:ASM81700v1:Chromosome:1417281:1418189:-1 gene:SpnNT_01381 transcript:AJD72319 gene_biotype:protein_coding transcript_biotype:protein_coding gene_symbol:cysB description:Cys regulon transcriptional
655	pep chromosome:ASM81700v1:Chromosome:1419649:1420458:1 gene:SpnNT_01384 transcript:AJD72322 gene_biotype:protein_coding transcript_biotype:protein_coding gene_symbol:yidA_3 description:Sugar phosphatase YidA
656	pep chromosome:ASM81700v1:Chromosome:1420639:1421514:-1 gene:SpnNT_01385 transcript:AJD72323 gene_biotype:protein_coding transcript_biotype:protein_coding description:N-carbamoyl-D-amino acid hydrolase
657	pep chromosome:ASM81700v1:Chromosome:1422606:1423733:-1 gene:SpnNT_01387 transcript:AJD72325 gene_biotype:protein_coding transcript_biotype:protein_coding gene_symbol:nspC description:Carboxynorspermidine/carl
658	pep chromosome:ASM81700v1:Chromosome:1423733:1424992:-1 gene:SpnNT_01388 transcript:AJD72326 gene_biotype:protein_coding transcript_biotype:protein_coding description:Homospermidine synthase
659	pep chromosome:ASM81700v1:Chromosome:1424989:1425849:-1 gene:SpnNT_01389 transcript:AJD72327 gene_biotype:protein_coding transcript_biotype:protein_coding gene_symbol:speE description:Spermidine synthase
660	pep chromosome:ASM81700v1:Chromosome:1425850:1427310:-1 gene:SpnNT_01390 transcript:AJD72328 gene_biotype:protein_coding transcript_biotype:protein_coding gene_symbol:speA description:Arginine decarboxylase
661	pep chromosome:ASM81700v1:Chromosome:1431034:1431792:-1 gene:SpnNT_01393 transcript:AJD72331 gene_biotype:protein_coding transcript_biotype:protein_coding gene_symbol:bceA_1 description:Bacitracin export ATP-bi
662	pep chromosome:ASM81700v1:Chromosome:1433532:1435661:-1 gene:SpnNT_01398 transcript:AJD72336 gene_biotype:protein_coding transcript_biotype:protein_coding gene_symbol:rpsA_1 description:30S ribosomal protein S1
663	pep chromosome:ASM81700v1:Chromosome:1436121:1436252:1 gene:SpnNT_01399 transcript:AJD72337 gene_biotype:protein_coding transcript_biotype:protein_coding description:hypothetical protein
664	pep chromosome:ASM81700v1:Chromosome:1437155:1437970:1 gene:SpnNT_01401 transcript:AJD72339 gene_biotype:protein_coding transcript_biotype:protein_coding description:hypothetical protein
665	pep chromosome:ASM81700v1:Chromosome:1439763:1440635:-1 gene:SpnNT_01405 transcript:AJD72343 gene_biotype:protein_coding transcript_biotype:protein_coding description:hypothetical protein
666	pep chromosome:ASM81700v1:Chromosome:1442393:1443898:-1 gene:SpnNT_01406 transcript:AJD72344 gene_biotype:protein_coding transcript_biotype:protein_coding gene_symbol:pyk description:Pyruvate kinase
667	pep chromosome:ASM81700v1:Chromosome:1443957:1444964:-1 gene:SpnNT_01407 transcript:AJD72345 gene_biotype:protein_coding transcript_biotype:protein_coding gene_symbol:pfkA description:6-phosphofructokinase
668	pep chromosome:ASM81700v1:Chromosome:1448494:1450767:1 gene:SpnNT_01409 transcript:AJD72347 gene_biotype:protein_coding transcript_biotype:protein_coding gene_symbol:pepX description:Xaa-Pro dipeptidyl-peptidas
669	pep chromosome:ASM81700v1:Chromosome:1450784:1451254:1 gene:SpnNT_01410 transcript:AJD72348 gene_biotype:protein_coding transcript_biotype:protein_coding gene_symbol:argR_2 description:Arginine regulator
670	pep chromosome:ASM81700v1:Chromosome:1459785:1460021:-1 gene:SpnNT_01418 transcript:AJD72356 gene_biotype:protein_coding transcript_biotype:protein_coding description:hypothetical protein
671	pep chromosome:ASM81700v1:Chromosome:1464620:1465444:-1 gene:SpnNT_01425 transcript:AJD72363 gene_biotype:protein_coding transcript_biotype:protein_coding gene_symbol:axe1-6A_2 description:Carbohydrate acetyl
672	pep chromosome:ASM81700v1:Chromosome:1465580:1466794:-1 gene:SpnNT_01426 transcript:AJD72364 gene_biotype:protein_coding transcript_biotype:protein_coding gene_symbol:thiI description:putative tRNA sulfurtransfera
673	pep chromosome:ASM81700v1:Chromosome:1466803:1468005:-1 gene:SpnNT_01427 transcript:AJD72365 gene_biotype:protein_coding transcript_biotype:protein_coding gene_symbol:iscS_2 description:Cysteine desulfurase
674	pep chromosome:ASM81700v1:Chromosome:1468576:1470879:-1 gene:SpnNT_01429 transcript:AJD72367 gene_biotype:protein_coding transcript_biotype:protein_coding gene_symbol:ftsK_2 description:DNA translocase FtsK
675	pep chromosome:ASM81700v1:Chromosome:1470965:1472917:-1 gene:SpnNT_01430 transcript:AJD72368 gene_biotype:protein_coding transcript_biotype:protein_coding gene_symbol:fruA description:EIIABC-Fru
676	pep chromosome:ASM81700v1:Chromosome:1472914:1473825:-1 gene:SpnNT_01431 transcript:AJD72369 gene_biotype:protein_coding transcript_biotype:protein_coding gene_symbol:lacC_2 description:Tagatose-6-phosphate ki
677	pep chromosome:ASM81700v1:Chromosome:1473822:1474562:-1 gene:SpnNT_01432 transcript:AJD72370 gene_biotype:protein_coding transcript_biotype:protein_coding gene_symbol:lacR_2 description:Lactose phosphotransfer
678	pep chromosome:ASM81700v1:Chromosome:1477186:1478427:1 gene:SpnNT_01436 transcript:AJD72372 gene_biotype:protein_coding transcript_biotype:protein_coding gene_symbol:dacA description:D-alanyl-D-alanine carboxyp
679	pep chromosome:ASM81700v1:Chromosome:1478658:1480070:-1 gene:SpnNT_01437 transcript:AJD72373 gene_biotype:protein_coding transcript_biotype:protein_coding gene_symbol:sufB_1 description:FeS cluster assembly pro
680	pep chromosome:ASM81700v1:Chromosome:1480128:1480568:-1 gene:SpnNT_01438 transcript:AJD72374 gene_biotype:protein_coding transcript_biotype:protein_coding gene_symbol:nifU description:NifU-like protein
681	pep chromosome:ASM81700v1:Chromosome:1480555:1481781:-1 gene:SpnNT_01439 transcript:AJD72375 gene_biotype:protein_coding transcript_biotype:protein_coding gene_symbol:csd description:putative cysteine desulfurase
682	pep chromosome:ASM81700v1:Chromosome:1481792:1483054:-1 gene:SpnNT_01440 transcript:AJD72376 gene_biotype:protein_coding transcript_biotype:protein_coding gene_symbol:sufB_2 description:FeS cluster assembly pro
683	pep chromosome:ASM81700v1:Chromosome:1483082:1483852:-1 gene:SpnNT_01441 transcript:AJD72377 gene_biotype:protein_coding transcript_biotype:protein_coding description:putative ABC transporter ATP-binding protein
684	pep chromosome:ASM81700v1:Chromosome:1484224:1485879:-1 gene:SpnNT_01443 transcript:AJD72379 gene_biotype:protein_coding transcript_biotype:protein_coding gene_symbol:dnaX_2 description:DNA polymerase III subu
685	pep chromosome:ASM81700v1:Chromosome:1485879:1486376:-1 gene:SpnNT_01444 transcript:AJD72380 gene_biotype:protein_coding transcript_biotype:protein_coding gene_symbol:msrC description:Free methionine-R-sulfoxid
686	pep chromosome:ASM81700v1:Chromosome:1487982:1489184:-1 gene:SpnNT_01448 transcript:AJD72384 gene_biotype:protein_coding transcript_biotype:protein_coding gene_symbol:rpsA_2 description:30S Ribosomal protein S
687	pep chromosome:ASM81700v1:Chromosome:1489557:1489787:-1 gene:SpnNT_01451 transcript:AJD72385 gene_biotype:protein_coding transcript_biotype:protein_coding description:hypothetical protein
688	pep chromosome:ASM81700v1:Chromosome:1489955:1490599:-1 gene:SpnNT_01452 transcript:AJD72386 gene_biotype:protein_coding transcript_biotype:protein_coding gene_symbol:pcp_1 description:Pyrrolidone-carboxylate pe
689	pep chromosome:ASM81700v1:Chromosome:1490614:1491537:-1 gene:SpnNT_01453 transcript:AJD72387 gene_biotype:protein_coding transcript_biotype:protein_coding description:hypothetical protein
690	pep chromosome:ASM81700v1:Chromosome:1491534:1492223:-1 gene:SpnNT_01454 transcript:AJD72388 gene_biotype:protein_coding transcript_biotype:protein_coding description:hypothetical protein
691	pep chromosome:ASM81700v1:Chromosome:1493048:1494070:-1 gene:SpnNT_01456 transcript:AJD72390 gene_biotype:protein_coding transcript_biotype:protein_coding gene_symbol:ilvE description:Branched-chain-amino-acid
692	pep chromosome:ASM81700v1:Chromosome:1494205:1496640:-1 gene:SpnNT_01457 transcript:AJD72391 gene_biotype:protein_coding transcript_biotype:protein_coding gene_symbol:parC description:DNA topoisomerase 4 subu
693	pep chromosome:ASM81700v1:Chromosome:1497096:1499039:-1 gene:SpnNT_01460 transcript:AJD72394 gene_biotype:protein_coding transcript_biotype:protein_coding gene_symbol:parE description:DNA topoisomerase 4 subu
694	pep chromosome:ASM81700v1:Chromosome:1500674:1501210:-1 gene:SpnNT_01463 transcript:AJD72397 gene_biotype:protein_coding transcript_biotype:protein_coding description:Transposase;pep chromosome:ASM81700v1:C
695	pep chromosome:ASM81700v1:Chromosome:1503304:1504839:-1 gene:SpnNT_01466 transcript:AJD72400 gene_biotype:protein_coding transcript_biotype:protein_coding gene_symbol:mglA description:Galactose/methyl galactosi
696	pep chromosome:ASM81700v1:Chromosome:1504983:1506035:-1 gene:SpnNT_01467 transcript:AJD72401 gene_biotype:protein_coding transcript_biotype:protein_coding gene_symbol:tmpC description:Purine nucleoside receptor
697	pep chromosome:ASM81700v1:Chromosome:1506125:1506514:-1 gene:SpnNT_01468 transcript:AJD72402 gene_biotype:protein_coding transcript_biotype:protein_coding gene_symbol:cdd description:Cytidine deaminase
698	pep chromosome:ASM81700v1:Chromosome:1506501:1507163:-1 gene:SpnNT_01469 transcript:AJD72403 gene_biotype:protein_coding transcript_biotype:protein_coding gene_symbol:deoC1 description:Deoxyribose-phosphate a
699	pep chromosome:ASM81700v1:Chromosome:1507181:1508458:-1 gene:SpnNT_01470 transcript:AJD72404 gene_biotype:protein_coding transcript_biotype:protein_coding gene_symbol:pdp description:Pyrimidine-nucleoside phosp
700	pep chromosome:ASM81700v1:Chromosome:1508455:1509045:-1 gene:SpnNT_01471 transcript:AJD72405 gene_biotype:protein_coding transcript_biotype:protein_coding gene_symbol:rsmC description:Ribosomal RNA small sub
701	pep chromosome:ASM81700v1:Chromosome:1510833:1511069:1 gene:SpnNT_01474 transcript:AJD72408 gene_biotype:protein_coding transcript_biotype:protein_coding gene_symbol:rpsT description:30S ribosomal protein S20
702	pep chromosome:ASM81700v1:Chromosome:1511669:1512379:-1 gene:SpnNT_01476 transcript:AJD72410 gene_biotype:protein_coding transcript_biotype:protein_coding gene_symbol:deoD_2 description:Purine nucleoside phosp

	A
703	pep chromosome:ASM81700v1:Chromosome:1515678:1516487:-1 gene:SpnNT_01480 transcript:AJD72414 gene_biotype:protein_coding transcript_biotype:protein_coding gene_symbol:punA description:Purine nucleoside phospho
704	pep chromosome:ASM81700v1:Chromosome:1517047:1518258:-1 gene:SpnNT_01482 transcript:AJD72416 gene_biotype:protein_coding transcript_biotype:protein_coding gene_symbol:deoB description:Phosphopentomutase
705	pep chromosome:ASM81700v1:Chromosome:1518272:1518955:-1 gene:SpnNT_01483 transcript:AJD72417 gene_biotype:protein_coding transcript_biotype:protein_coding gene_symbol:rpiA description:Ribose-5-phosphate isomer
706	pep chromosome:ASM81700v1:Chromosome:1520251:1521171:-1 gene:SpnNT_01485 transcript:AJD72419 gene_biotype:protein_coding transcript_biotype:protein_coding gene_symbol:folD description:Bifunctional protein FolD pro
707	pep chromosome:ASM81700v1:Chromosome:1521262:1521996:-1 gene:SpnNT_01486 transcript:AJD72420 gene_biotype:protein_coding transcript_biotype:protein_coding gene_symbol:artM_2 description:Arginine transport ATP-b
708	pep chromosome:ASM81700v1:Chromosome:1522869:1523099:1 gene:SpnNT_01488 transcript:AJD72422 gene_biotype:protein_coding transcript_biotype:protein_coding description:hypothetical protein
709	pep chromosome:ASM81700v1:Chromosome:1523323:1525581:1 gene:SpnNT_01489 transcript:AJD72423 gene_biotype:protein_coding transcript_biotype:protein_coding gene_symbol:clpE description:ATP-dependent Clp proteas
710	pep chromosome:ASM81700v1:Chromosome:1527328:1527627:1 gene:SpnNT_01492 transcript:AJD72426 gene_biotype:protein_coding transcript_biotype:protein_coding description:hypothetical protein
711	pep chromosome:ASM81700v1:Chromosome:1529692:1530888:-1 gene:SpnNT_01496 transcript:AJD72430 gene_biotype:protein_coding transcript_biotype:protein_coding gene_symbol:tuf description:Elongation factor Tu
712	pep chromosome:ASM81700v1:Chromosome:1533764:1534633:-1 gene:SpnNT_01498 transcript:AJD72432 gene_biotype:protein_coding transcript_biotype:protein_coding gene_symbol:gla description:Glyceroaquaporin
713	pep chromosome:ASM81700v1:Chromosome:1540339:1542057:1 gene:SpnNT_01505 transcript:AJD72439 gene_biotype:protein_coding transcript_biotype:protein_coding gene_symbol:pgcA description:Phosphoglucomutase
714	pep chromosome:ASM81700v1:Chromosome:1542168:1542515:1 gene:SpnNT_01506 transcript:AJD72440 gene_biotype:protein_coding transcript_biotype:protein_coding description:putative bacteriocin transport accessory protein
715	pep chromosome:ASM81700v1:Chromosome:1542793:1543629:-1 gene:SpnNT_01507 transcript:AJD72441 gene_biotype:protein_coding transcript_biotype:protein_coding gene_symbol:fliY description:Sulfate starvation-induced pr
716	pep chromosome:ASM81700v1:Chromosome:1543642:1544271:-1 gene:SpnNT_01508 transcript:AJD72442 gene_biotype:protein_coding transcript_biotype:protein_coding gene_symbol:glnQ_3 description:Glutamine transport ATP
717	pep chromosome:ASM81700v1:Chromosome:1544281:1544922:-1 gene:SpnNT_01509 transcript:AJD72443 gene_biotype:protein_coding transcript_biotype:protein_coding gene_symbol:yecS_2 description:Inner membrane amino-
718	pep chromosome:ASM81700v1:Chromosome:1545068:1546297:-1 gene:SpnNT_01510 transcript:AJD72444 gene_biotype:protein_coding transcript_biotype:protein_coding description:putative PEP-CTERM system TPR-repeat lipo
719	pep chromosome:ASM81700v1:Chromosome:1547597:1548610:1 gene:SpnNT_01512 transcript:AJD72446 gene_biotype:protein_coding transcript_biotype:protein_coding gene_symbol:pgl description:6-phosphogluconolactonase
720	pep chromosome:ASM81700v1:Chromosome:1548656:1549075:-1 gene:SpnNT_01513 transcript:AJD72447 gene_biotype:protein_coding transcript_biotype:protein_coding gene_symbol:atpC description:F-ATPase epsilon subunit
721	pep chromosome:ASM81700v1:Chromosome:1549086:1550492:-1 gene:SpnNT_01514 transcript:AJD72448 gene_biotype:protein_coding transcript_biotype:protein_coding gene_symbol:atpD description:ATP synthase subunit beta
722	pep chromosome:ASM81700v1:Chromosome:1550578:1551456:-1 gene:SpnNT_01515 transcript:AJD72449 gene_biotype:protein_coding transcript_biotype:protein_coding gene_symbol:atpG description:F-ATPase gamma subunit
723	pep chromosome:ASM81700v1:Chromosome:1551472:1552977:-1 gene:SpnNT_01516 transcript:AJD72450 gene_biotype:protein_coding transcript_biotype:protein_coding gene_symbol:atpA description:ATP synthase subunit alph
724	pep chromosome:ASM81700v1:Chromosome:1552992:1553528:-1 gene:SpnNT_01517 transcript:AJD72451 gene_biotype:protein_coding transcript_biotype:protein_coding gene_symbol:atpH description:F-type ATPase subunit delt
725	pep chromosome:ASM81700v1:Chromosome:1553528:1554022:-1 gene:SpnNT_01518 transcript:AJD72452 gene_biotype:protein_coding transcript_biotype:protein_coding gene_symbol:atpF description:F-type ATPase subunit b
726	pep chromosome:ASM81700v1:Chromosome:1554036:1554752:-1 gene:SpnNT_01519 transcript:AJD72453 gene_biotype:protein_coding transcript_biotype:protein_coding gene_symbol:atpB description:F-ATPase subunit 6
727	pep chromosome:ASM81700v1:Chromosome:1557277:1557759:-1 gene:SpnNT_01523 transcript:AJD72457 gene_biotype:protein_coding transcript_biotype:protein_coding gene_symbol:greA description:Transcript cleavage factor
728	pep chromosome:ASM81700v1:Chromosome:1557830:1559485:-1 gene:SpnNT_01524 transcript:AJD72458 gene_biotype:protein_coding transcript_biotype:protein_coding description:putative aminodeoxychorismate lyase
729	pep chromosome:ASM81700v1:Chromosome:1560057:1560521:-1 gene:SpnNT_01526 transcript:AJD72460 gene_biotype:protein_coding transcript_biotype:protein_coding description:ribosomal-protein-alanine acetyltransferase
730	pep chromosome:ASM81700v1:Chromosome:1560531:1561865:-1 gene:SpnNT_01527 transcript:AJD72461 gene_biotype:protein_coding transcript_biotype:protein_coding gene_symbol:murC description:UDP-N-acetylmuramate--L
731	pep chromosome:ASM81700v1:Chromosome:1561877:1562494:-1 gene:SpnNT_01528 transcript:AJD72462 gene_biotype:protein_coding transcript_biotype:protein_coding description:hypothetical protein
732	pep chromosome:ASM81700v1:Chromosome:1562542:1565640:-1 gene:SpnNT_01529 transcript:AJD72463 gene_biotype:protein_coding transcript_biotype:protein_coding description:ATP-dependent helicase HepA
733	pep chromosome:ASM81700v1:Chromosome:1566907:1568001:-1 gene:SpnNT_01531 transcript:AJD72465 gene_biotype:protein_coding transcript_biotype:protein_coding gene_symbol:metI description:Cystathionine gamma-synth
734	pep chromosome:ASM81700v1:Chromosome:1570012:1571970:-1 gene:SpnNT_01534 transcript:AJD72468 gene_biotype:protein_coding transcript_biotype:protein_coding gene_symbol:sarA_5 description:76 kDa cell surface lipop
735	pep chromosome:ASM81700v1:Chromosome:1572172:1573794:-1 gene:SpnNT_01535 transcript:AJD72469 gene_biotype:protein_coding transcript_biotype:protein_coding gene_symbol:ytgP description:putative cell division protein
736	pep chromosome:ASM81700v1:Chromosome:1573900:1575345:1 gene:SpnNT_01536 transcript:AJD72470 gene_biotype:protein_coding transcript_biotype:protein_coding gene_symbol:murE description:UDP-N-acetylmuramoyl-L-a
737	pep chromosome:ASM81700v1:Chromosome:1575388:1575585:-1 gene:SpnNT_01537 transcript:AJD72471 gene_biotype:protein_coding transcript_biotype:protein_coding description:CsbD-like protein
738	pep chromosome:ASM81700v1:Chromosome:1576918:1577853:-1 gene:SpnNT_01540 transcript:AJD72474 gene_biotype:protein_coding transcript_biotype:protein_coding gene_symbol:ppaC description:putative manganese-deper
739	pep chromosome:ASM81700v1:Chromosome:1579017:1579376:-1 gene:SpnNT_01543 transcript:AJD72477 gene_biotype:protein_coding transcript_biotype:protein_coding gene_symbol:yugI description:General stress protein 13
740	pep chromosome:ASM81700v1:Chromosome:1579378:1580778:-1 gene:SpnNT_01544 transcript:AJD72478 gene_biotype:protein_coding transcript_biotype:protein_coding description:Putative bifunctional phosphatase/peptidyl-prol
741	pep chromosome:ASM81700v1:Chromosome:1582276:1582515:-1 gene:SpnNT_01548 transcript:AJD72482 gene_biotype:protein_coding transcript_biotype:protein_coding gene_symbol:rpsR description:30S ribosomal protein S18
742	pep chromosome:ASM81700v1:Chromosome:1582547:1583017:-1 gene:SpnNT_01549 transcript:AJD72483 gene_biotype:protein_coding transcript_biotype:protein_coding gene_symbol:ssb_1 description:Helix-destabilizing protein
743	pep chromosome:ASM81700v1:Chromosome:1583029:1583319:-1 gene:SpnNT_01550 transcript:AJD72484 gene_biotype:protein_coding transcript_biotype:protein_coding gene_symbol:rpsF description:30S ribosomal protein S6
744	pep chromosome:ASM81700v1:Chromosome:1583472:1584815:-1 gene:SpnNT_01551 transcript:AJD72485 gene_biotype:protein_coding transcript_biotype:protein_coding gene_symbol:asnS description:Asparagine--tRNA ligase
745	pep chromosome:ASM81700v1:Chromosome:1585192:1586379:-1 gene:SpnNT_01553 transcript:AJD72487 gene_biotype:protein_coding transcript_biotype:protein_coding description:Aspartate aminotransferase
746	pep chromosome:ASM81700v1:Chromosome:1586376:1586807:-1 gene:SpnNT_01554 transcript:AJD72488 gene_biotype:protein_coding transcript_biotype:protein_coding description:hypothetical protein
747	pep chromosome:ASM81700v1:Chromosome:1587185:1587733:1 gene:SpnNT_01555 transcript:AJD72489 gene_biotype:protein_coding transcript_biotype:protein_coding description:arsenical resistance protein ArsH
748	pep chromosome:ASM81700v1:Chromosome:1588100:1588828:-1 gene:SpnNT_01556 transcript:AJD72490 gene_biotype:protein_coding transcript_biotype:protein_coding description:hypothetical protein
749	pep chromosome:ASM81700v1:Chromosome:1589137:1590732:-1 gene:SpnNT_01558 transcript:AJD72492 gene_biotype:protein_coding transcript_biotype:protein_coding description:hypothetical protein
750	pep chromosome:ASM81700v1:Chromosome:1592074:1594770:-1 gene:SpnNT_01561 transcript:AJD72495 gene_biotype:protein_coding transcript_biotype:protein_coding description:Calcium-transporting ATPase
751	pep chromosome:ASM81700v1:Chromosome:1595074:1596258:1 gene:SpnNT_01562 transcript:AJD72496 gene_biotype:protein_coding transcript_biotype:protein_coding gene_symbol:fieF description:Ferrous-iron efflux pump Fie
752	pep chromosome:ASM81700v1:Chromosome:1596401:1598272:-1 gene:SpnNT_01563 transcript:AJD72497 gene_biotype:protein_coding transcript_biotype:protein_coding description:putative ABC transporter ATP-binding protein
753	pep chromosome:ASM81700v1:Chromosome:1598269:1599453:-1 gene:SpnNT_01564 transcript:AJD72498 gene_biotype:protein_coding transcript_biotype:protein_coding gene_symbol:cca description:CCA-adding enzyme
754	pep chromosome:ASM81700v1:Chromosome:1599465:1600232:-1 gene:SpnNT_01565 transcript:AJD72499 gene_biotype:protein_coding transcript_biotype:protein_coding gene_symbol:dapB description:4-hydroxy-tetrahydrodipico
755	pep chromosome:ASM81700v1:Chromosome:1600509:1601357:-1 gene:SpnNT_01566 transcript:AJD72500 gene_biotype:protein_coding transcript_biotype:protein_coding description:DegV domain-containing protein
756	pep chromosome:ASM81700v1:Chromosome:1601359:1601733:-1 gene:SpnNT_01567 transcript:AJD72501 gene_biotype:protein_coding transcript_biotype:protein_coding description:hypothetical protein

	A
757	pep chromosome:ASM81700v1:Chromosome:1601807:1603159:-1 gene:SpnNT_01568 transcript:AJD72502 gene_biotype:protein_coding transcript_biotype:protein_coding gene_symbol:glmM description:Phosphoglucosamine muta
758	pep chromosome:ASM81700v1:Chromosome:1603183:1603962:-1 gene:SpnNT_01569 transcript:AJD72503 gene_biotype:protein_coding transcript_biotype:protein_coding description:YbbR-like protein
759	pep chromosome:ASM81700v1:Chromosome:1603949:1604806:-1 gene:SpnNT_01570 transcript:AJD72504 gene_biotype:protein_coding transcript_biotype:protein_coding description:DNA integrity scanning protein DisA
760	pep chromosome:ASM81700v1:Chromosome:1604942:1605910:1 gene:SpnNT_01571 transcript:AJD72505 gene_biotype:protein_coding transcript_biotype:protein_coding gene_symbol:yumC description:Ferredoxin--NADP reducta
761	pep chromosome:ASM81700v1:Chromosome:1608417:1611614:-1 gene:SpnNT_01578 transcript:AJD72512 gene_biotype:protein_coding transcript_biotype:protein_coding description:Phage-related protein
762	pep chromosome:ASM81700v1:Chromosome:1627781:1628671:-1 gene:SpnNT_01602 transcript:AJD72536 gene_biotype:protein_coding transcript_biotype:protein_coding gene_symbol:hhaIM description:Modification methylase Hh
763	pep chromosome:ASM81700v1:Chromosome:1629832:1631169:-1 gene:SpnNT_01606 transcript:AJD72540 gene_biotype:protein_coding transcript_biotype:protein_coding description:putative P-loop ATPase
764	pep chromosome:ASM81700v1:Chromosome:1632166:1632672:-1 gene:SpnNT_01608 transcript:AJD72542 gene_biotype:protein_coding transcript_biotype:protein_coding description:hypothetical protein
765	pep chromosome:ASM81700v1:Chromosome:1638418:1638657:1 gene:SpnNT_01621 transcript:AJD72555 gene_biotype:protein_coding transcript_biotype:protein_coding description:hypothetical protein
766	pep chromosome:ASM81700v1:Chromosome:1639077:1639244:1 gene:SpnNT_01624 transcript:AJD72558 gene_biotype:protein_coding transcript_biotype:protein_coding description:hypothetical protein
767	pep chromosome:ASM81700v1:Chromosome:1641586:1641954:1 gene:SpnNT_01632 transcript:AJD72566 gene_biotype:protein_coding transcript_biotype:protein_coding description:Helix-turn-helix domain protein
768	pep chromosome:ASM81700v1:Chromosome:1641954:1642193:1 gene:SpnNT_01633 transcript:AJD72567 gene_biotype:protein_coding transcript_biotype:protein_coding description:hypothetical protein
769	pep chromosome:ASM81700v1:Chromosome:1642469:1642945:1 gene:SpnNT_01635 transcript:AJD72569 gene_biotype:protein_coding transcript_biotype:protein_coding description:hypothetical protein
770	pep chromosome:ASM81700v1:Chromosome:1642942:1643109:1 gene:SpnNT_01636 transcript:AJD72570 gene_biotype:protein_coding transcript_biotype:protein_coding description:hypothetical protein
771	pep chromosome:ASM81700v1:Chromosome:1643552:1644148:1 gene:SpnNT_01638 transcript:AJD72572 gene_biotype:protein_coding transcript_biotype:protein_coding description:CD20-like family protein
772	pep chromosome:ASM81700v1:Chromosome:1644298:1645425:1 gene:SpnNT_01639 transcript:AJD72573 gene_biotype:protein_coding transcript_biotype:protein_coding gene_symbol:xerD_2 description:Tyrosine recombinase Xe
773	pep chromosome:ASM81700v1:Chromosome:1646421:1647398:-1 gene:SpnNT_01641 transcript:AJD72575 gene_biotype:protein_coding transcript_biotype:protein_coding description:LPPG:FO 2-phospho-L-lactate transferase
774	pep chromosome:ASM81700v1:Chromosome:1647395:1648285:-1 gene:SpnNT_01642 transcript:AJD72576 gene_biotype:protein_coding transcript_biotype:protein_coding description:glmZ(sRNA)-inactivating NTPase
775	pep chromosome:ASM81700v1:Chromosome:1648337:1648717:-1 gene:SpnNT_01643 transcript:AJD72577 gene_biotype:protein_coding transcript_biotype:protein_coding gene_symbol:yabJ description:Enamine/imine deaminase
776	pep chromosome:ASM81700v1:Chromosome:1648728:1649315:-1 gene:SpnNT_01644 transcript:AJD72578 gene_biotype:protein_coding transcript_biotype:protein_coding gene_symbol:engB description:putative GTP-binding prote
777	pep chromosome:ASM81700v1:Chromosome:1649324:1650556:-1 gene:SpnNT_01645 transcript:AJD72579 gene_biotype:protein_coding transcript_biotype:protein_coding gene_symbol:clpX description:ATP-dependent Clp proteas
778	pep chromosome:ASM81700v1:Chromosome:1650758:1651264:-1 gene:SpnNT_01647 transcript:AJD72581 gene_biotype:protein_coding transcript_biotype:protein_coding gene_symbol:dhfR description:Dihydrofolate reductase
779	pep chromosome:ASM81700v1:Chromosome:1651394:1651912:-1 gene:SpnNT_01648 transcript:AJD72582 gene_biotype:protein_coding transcript_biotype:protein_coding gene_symbol:dps description:DNA protection during starva
780	pep chromosome:ASM81700v1:Chromosome:1652408:1653880:-1 gene:SpnNT_01649 transcript:AJD72583 gene_biotype:protein_coding transcript_biotype:protein_coding gene_symbol:lytB_7 description:Putative endo-beta-N-ace
781	pep chromosome:ASM81700v1:Chromosome:1653950:1654708:-1 gene:SpnNT_01650 transcript:AJD72584 gene_biotype:protein_coding transcript_biotype:protein_coding gene_symbol:tpiA description:Triosephosphate isomerase
782	pep chromosome:ASM81700v1:Chromosome:1654807:1655484:-1 gene:SpnNT_01651 transcript:AJD72585 gene_biotype:protein_coding transcript_biotype:protein_coding gene_symbol:dnaD description:DNA replication protein Dn
783	pep chromosome:ASM81700v1:Chromosome:1656619:1657131:-1 gene:SpnNT_01653 transcript:AJD72587 gene_biotype:protein_coding transcript_biotype:protein_coding gene_symbol:apt description:Adenine phosphoribosyltrans
784	pep chromosome:ASM81700v1:Chromosome:1657218:1657976:-1 gene:SpnNT_01654 transcript:AJD72588 gene_biotype:protein_coding transcript_biotype:protein_coding gene_symbol:rebM description:Demethylrebeccamycin-D-
785	pep chromosome:ASM81700v1:Chromosome:1658477:1659607:-1 gene:SpnNT_01655 transcript:AJD72589 gene_biotype:protein_coding transcript_biotype:protein_coding gene_symbol:sugC description:Trehalose import ATP-bind
786	pep chromosome:ASM81700v1:Chromosome:1660057:1660632:-1 gene:SpnNT_01657 transcript:AJD72590 gene_biotype:protein_coding transcript_biotype:protein_coding gene_symbol:yecD description:Isochorismatase family pro
787	pep chromosome:ASM81700v1:Chromosome:1660632:1661420:-1 gene:SpnNT_01658 transcript:AJD72591 gene_biotype:protein_coding transcript_biotype:protein_coding gene_symbol:codY description:GTP-sensing transcriptiona
788	pep chromosome:ASM81700v1:Chromosome:1661684:1663258:-1 gene:SpnNT_01659 transcript:AJD72592 gene_biotype:protein_coding transcript_biotype:protein_coding gene_symbol:cshA_2 description:DEAD-box ATP-depende
789	pep chromosome:ASM81700v1:Chromosome:1664877:1666193:1 gene:SpnNT_01661 transcript:AJD72594 gene_biotype:protein_coding transcript_biotype:protein_coding gene_symbol:lpd description:Dihydrolipoyl dehydrogenase
790	pep chromosome:ASM81700v1:Chromosome:1666292:1667635:1 gene:SpnNT_01662 transcript:AJD72595 gene_biotype:protein_coding transcript_biotype:protein_coding description:UDP-N-acetylmuramate--L-alanine ligase
791	pep chromosome:ASM81700v1:Chromosome:1667635:1668417:1 gene:SpnNT_01663 transcript:AJD72596 gene_biotype:protein_coding transcript_biotype:protein_coding description:cobyric acid synthase
792	pep chromosome:ASM81700v1:Chromosome:1668543:1669625:1 gene:SpnNT_01664 transcript:AJD72597 gene_biotype:protein_coding transcript_biotype:protein_coding gene_symbol:pepQ description:Xaa-Pro dipeptidase
793	pep chromosome:ASM81700v1:Chromosome:1671880:1672653:-1 gene:SpnNT_01668 transcript:AJD72601 gene_biotype:protein_coding transcript_biotype:protein_coding gene_symbol:pdxK description:Pyridoxine kinase
794	pep chromosome:ASM81700v1:Chromosome:1675728:1676066:-1 gene:SpnNT_01672 transcript:AJD72605 gene_biotype:protein_coding transcript_biotype:protein_coding description:putative alkylphosphonate utilization operon pro
795	pep chromosome:ASM81700v1:Chromosome:1676197:1676868:-1 gene:SpnNT_01673 transcript:AJD72606 gene_biotype:protein_coding transcript_biotype:protein_coding gene_symbol:cmk description:Cytidylate kinase
796	pep chromosome:ASM81700v1:Chromosome:1676877:1677353:-1 gene:SpnNT_01674 transcript:AJD72607 gene_biotype:protein_coding transcript_biotype:protein_coding description:hypothetical protein
797	pep chromosome:ASM81700v1:Chromosome:1677399:1677611:1 gene:SpnNT_01675 transcript:AJD72608 gene_biotype:protein_coding transcript_biotype:protein_coding description:hypothetical protein
798	pep chromosome:ASM81700v1:Chromosome:1678629:1679648:-1 gene:SpnNT_01677 transcript:AJD72610 gene_biotype:protein_coding transcript_biotype:protein_coding gene_symbol:galE_1 description:UDP-glucose 4-epimeras
799	pep chromosome:ASM81700v1:Chromosome:1681024:1681821:-1 gene:SpnNT_01679 transcript:AJD72612 gene_biotype:protein_coding transcript_biotype:protein_coding description:metal-binding protein
800	pep chromosome:ASM81700v1:Chromosome:1681808:1682485:-1 gene:SpnNT_01680 transcript:AJD72613 gene_biotype:protein_coding transcript_biotype:protein_coding gene_symbol:trmK description:tRNA (adenine(22)-N(1))-m
801	pep chromosome:ASM81700v1:Chromosome:1682587:1684923:-1 gene:SpnNT_01681 transcript:AJD72614 gene_biotype:protein_coding transcript_biotype:protein_coding description:Calcium-transporting ATPase
802	pep chromosome:ASM81700v1:Chromosome:1685056:1685805:1 gene:SpnNT_01682 transcript:AJD72615 gene_biotype:protein_coding transcript_biotype:protein_coding gene_symbol:plsC description:1-acyl-sn-glycerol-3-phospha
803	pep chromosome:ASM81700v1:Chromosome:1687527:1687796:-1 gene:SpnNT_01684 transcript:AJD72617 gene_biotype:protein_coding transcript_biotype:protein_coding gene_symbol:rpsO description:30S ribosomal protein S15
804	pep chromosome:ASM81700v1:Chromosome:1689817:1691760:1 gene:SpnNT_01689 transcript:AJD72622 gene_biotype:protein_coding transcript_biotype:protein_coding gene_symbol:thrS description:Threonine--tRNA ligase
805	pep chromosome:ASM81700v1:Chromosome:1692776:1693453:-1 gene:SpnNT_01691 transcript:AJD72624 gene_biotype:protein_coding transcript_biotype:protein_coding gene_symbol:graR description:Glycopeptide resistance-as
806	pep chromosome:ASM81700v1:Chromosome:1695144:1695581:-1 gene:SpnNT_01694 transcript:AJD72627 gene_biotype:protein_coding transcript_biotype:protein_coding gene_symbol:ywnA description:Putative HTH-type transcri
807	pep chromosome:ASM81700v1:Chromosome:1695657:1696499:-1 gene:SpnNT_01695 transcript:AJD72628 gene_biotype:protein_coding transcript_biotype:protein_coding description:phosphodiesterase
808	pep chromosome:ASM81700v1:Chromosome:1696611:1697261:1 gene:SpnNT_01696 transcript:AJD72629 gene_biotype:protein_coding transcript_biotype:protein_coding gene_symbol:dtxR description:Tox regulatory factor
809	pep chromosome:ASM81700v1:Chromosome:1699099:1699542:-1 gene:SpnNT_01700 transcript:AJD72633 gene_biotype:protein_coding transcript_biotype:protein_coding gene_symbol:dtd description:D-tyrosyl-tRNA(Tyr) deacylas
810	pep chromosome:ASM81700v1:Chromosome:1699572:1701794:-1 gene:SpnNT_01701 transcript:AJD72634 gene_biotype:protein_coding transcript_biotype:protein_coding gene_symbol:relA_1 description:Bifunctional (p)ppGpp syn

	A
811	pep chromosome:ASM81700v1:Chromosome:1702788:1703417:1 gene:SpnNT_01702 transcript:AJD72635 gene_biotype:protein_coding transcript_biotype:protein_coding gene_symbol:pksB description:putative polyketide biosynth
812	pep chromosome:ASM81700v1:Chromosome:1703540:1705432:-1 gene:SpnNT_01703 transcript:AJD72636 gene_biotype:protein_coding transcript_biotype:protein_coding gene_symbol:pepO description:Neutral endopeptidase
813	pep chromosome:ASM81700v1:Chromosome:1707322:1708251:1 gene:SpnNT_01706 transcript:AJD72639 gene_biotype:protein_coding transcript_biotype:protein_coding gene_symbol:psaA description:Pneumococcal surface adh
814	pep chromosome:ASM81700v1:Chromosome:1708374:1708865:1 gene:SpnNT_01707 transcript:AJD72640 gene_biotype:protein_coding transcript_biotype:protein_coding gene_symbol:tpx description:putative thiol peroxidase
815	pep chromosome:ASM81700v1:Chromosome:1711671:1712372:-1 gene:SpnNT_01709 transcript:AJD72642 gene_biotype:protein_coding transcript_biotype:protein_coding description:putative ABC transporter ATP-binding protein
816	pep chromosome:ASM81700v1:Chromosome:1716577:1717269:-1 gene:SpnNT_01715 transcript:AJD72648 gene_biotype:protein_coding transcript_biotype:protein_coding gene_symbol:gpmA_2 description:2,3-bisphosphoglycerat
817	pep chromosome:ASM81700v1:Chromosome:1717428:1723247:-1 gene:SpnNT_01716 transcript:AJD72649 gene_biotype:protein_coding transcript_biotype:protein_coding gene_symbol:iga_6 description:Immunoglobulin A1 protea
818	pep chromosome:ASM81700v1:Chromosome:1723430:1726222:-1 gene:SpnNT_01717 transcript:AJD72650 gene_biotype:protein_coding transcript_biotype:protein_coding gene_symbol:ileS description:Isoleucine--tRNA ligase
819	pep chromosome:ASM81700v1:Chromosome:1726475:1727359:-1 gene:SpnNT_01718 transcript:AJD72651 gene_biotype:protein_coding transcript_biotype:protein_coding gene_symbol:divIVA description:Cell division protein DivIV
820	pep chromosome:ASM81700v1:Chromosome:1728413:1728952:-1 gene:SpnNT_01721 transcript:AJD72654 gene_biotype:protein_coding transcript_biotype:protein_coding gene_symbol:sepF description:Cell division protein SepF
821	pep chromosome:ASM81700v1:Chromosome:1728962:1729633:-1 gene:SpnNT_01722 transcript:AJD72655 gene_biotype:protein_coding transcript_biotype:protein_coding description:pyridoxal phosphate enzyme, YggS family
822	pep chromosome:ASM81700v1:Chromosome:1729638:1730897:-1 gene:SpnNT_01723 transcript:AJD72656 gene_biotype:protein_coding transcript_biotype:protein_coding gene_symbol:ftsZ description:Cell division protein FtsZ
823	pep chromosome:ASM81700v1:Chromosome:1730914:1732287:-1 gene:SpnNT_01724 transcript:AJD72657 gene_biotype:protein_coding transcript_biotype:protein_coding gene_symbol:ftsA description:Cell division protein FtsA
824	pep chromosome:ASM81700v1:Chromosome:1733899:1735272:-1 gene:SpnNT_01727 transcript:AJD72660 gene_biotype:protein_coding transcript_biotype:protein_coding gene_symbol:murF description:UDP-N-acetylmuramoyl-tri
825	pep chromosome:ASM81700v1:Chromosome:1735357:1736400:-1 gene:SpnNT_01728 transcript:AJD72661 gene_biotype:protein_coding transcript_biotype:protein_coding gene_symbol:ddl description:D-alanine--D-alanine ligase
826	pep chromosome:ASM81700v1:Chromosome:1736576:1737172:-1 gene:SpnNT_01729 transcript:AJD72662 gene_biotype:protein_coding transcript_biotype:protein_coding gene_symbol:recR description:Recombination protein Rec
827	pep chromosome:ASM81700v1:Chromosome:1737183:1739225:-1 gene:SpnNT_01730 transcript:AJD72663 gene_biotype:protein_coding transcript_biotype:protein_coding gene_symbol:penA description:Penicillin-binding protein 2
828	pep chromosome:ASM81700v1:Chromosome:1739440:1740291:1 gene:SpnNT_01731 transcript:AJD72664 gene_biotype:protein_coding transcript_biotype:protein_coding gene_symbol:ybbH description:putative HTH-type transcrip
829	pep chromosome:ASM81700v1:Chromosome:1740307:1741191:-1 gene:SpnNT_01732 transcript:AJD72665 gene_biotype:protein_coding transcript_biotype:protein_coding gene_symbol:bglK_1 description:Beta-glucoside kinase
830	pep chromosome:ASM81700v1:Chromosome:1741209:1742126:-1 gene:SpnNT_01733 transcript:AJD72666 gene_biotype:protein_coding transcript_biotype:protein_coding gene_symbol:nanA_2 description:N-acetylneuraminate lya
831	pep chromosome:ASM81700v1:Chromosome:1745603:1746931:-1 gene:SpnNT_01738 transcript:AJD72671 gene_biotype:protein_coding transcript_biotype:protein_coding gene_symbol:yesO_1 description:Putative ABC transporte
832	pep chromosome:ASM81700v1:Chromosome:1747996:1748694:-1 gene:SpnNT_01741 transcript:AJD72674 gene_biotype:protein_coding transcript_biotype:protein_coding gene_symbol:nanE description:Putative N-acetylmannosa
833	pep chromosome:ASM81700v1:Chromosome:1748867:1749970:-1 gene:SpnNT_01742 transcript:AJD72675 gene_biotype:protein_coding transcript_biotype:protein_coding gene_symbol:afr description:1,5-anhydro-D-fructose reduc
834	pep chromosome:ASM81700v1:Chromosome:1753889:1755226:-1 gene:SpnNT_01746 transcript:AJD72679 gene_biotype:protein_coding transcript_biotype:protein_coding gene_symbol:yesO_2 description:Putative ABC transporte
835	pep chromosome:ASM81700v1:Chromosome:1756727:1757542:-1 gene:SpnNT_01749 transcript:AJD72682 gene_biotype:protein_coding transcript_biotype:protein_coding description:PD-(D/E)XK nuclease family transposase
836	pep chromosome:ASM81700v1:Chromosome:1757915:1761481:-1 gene:SpnNT_01750 transcript:AJD72683 gene_biotype:protein_coding transcript_biotype:protein_coding gene_symbol:lytA_8 description:Autolysin
837	pep chromosome:ASM81700v1:Chromosome:1767509:1769524:-1 gene:SpnNT_01755 transcript:AJD72688 gene_biotype:protein_coding transcript_biotype:protein_coding gene_symbol:recG description:ATP-dependent DNA helica
838	pep chromosome:ASM81700v1:Chromosome:1769543:1770646:-1 gene:SpnNT_01756 transcript:AJD72689 gene_biotype:protein_coding transcript_biotype:protein_coding gene_symbol:alr description:Alanine racemase
839	pep chromosome:ASM81700v1:Chromosome:1770636:1771004:-1 gene:SpnNT_01757 transcript:AJD72690 gene_biotype:protein_coding transcript_biotype:protein_coding gene_symbol:acpS description:Holo-[acyl-carrier-protein] s
840	pep chromosome:ASM81700v1:Chromosome:1771043:1772074:-1 gene:SpnNT_01758 transcript:AJD72691 gene_biotype:protein_coding transcript_biotype:protein_coding gene_symbol:aroF_1 description:Phospho-2-dehydro-3-de
841	pep chromosome:ASM81700v1:Chromosome:1772076:1773107:-1 gene:SpnNT_01759 transcript:AJD72692 gene_biotype:protein_coding transcript_biotype:protein_coding gene_symbol:aroF_2 description:Phospho-2-dehydro-3-de
842	pep chromosome:ASM81700v1:Chromosome:1773188:1775701:-1 gene:SpnNT_01760 transcript:AJD72693 gene_biotype:protein_coding transcript_biotype:protein_coding description:preprotein translocase subunit SecA
843	pep chromosome:ASM81700v1:Chromosome:1779310:1780620:-1 gene:SpnNT_01767 transcript:AJD72700 gene_biotype:protein_coding transcript_biotype:protein_coding gene_symbol:der description:GTP-binding protein EngA
844	pep chromosome:ASM81700v1:Chromosome:1780634:1781347:-1 gene:SpnNT_01768 transcript:AJD72701 gene_biotype:protein_coding transcript_biotype:protein_coding gene_symbol:nfrA2 description:FMN reductase [NAD(P)H]
845	pep chromosome:ASM81700v1:Chromosome:1781344:1782240:-1 gene:SpnNT_01769 transcript:AJD72702 gene_biotype:protein_coding transcript_biotype:protein_coding gene_symbol:dnaI description:Primosomal protein DnaI
846	pep chromosome:ASM81700v1:Chromosome:1782241:1783410:-1 gene:SpnNT_01770 transcript:AJD72703 gene_biotype:protein_coding transcript_biotype:protein_coding description:Replication initiation/membrane attachment pro
847	pep chromosome:ASM81700v1:Chromosome:1783411:1783884:-1 gene:SpnNT_01771 transcript:AJD72704 gene_biotype:protein_coding transcript_biotype:protein_coding gene_symbol:nrdR description:Transcriptional repressor N
848	pep chromosome:ASM81700v1:Chromosome:1784024:1784389:1 gene:SpnNT_01772 transcript:AJD72705 gene_biotype:protein_coding transcript_biotype:protein_coding gene_symbol:ytrA description:HTH-type transcriptional repr
849	pep chromosome:ASM81700v1:Chromosome:1784394:1785089:1 gene:SpnNT_01773 transcript:AJD72706 gene_biotype:protein_coding transcript_biotype:protein_coding gene_symbol:skfE description:SkfA peptide export ATP-bin
850	pep chromosome:ASM81700v1:Chromosome:1786226:1787425:-1 gene:SpnNT_01775 transcript:AJD72708 gene_biotype:protein_coding transcript_biotype:protein_coding description:ABC-2 family transporter protein
851	pep chromosome:ASM81700v1:Chromosome:1787422:1788315:-1 gene:SpnNT_01776 transcript:AJD72709 gene_biotype:protein_coding transcript_biotype:protein_coding gene_symbol:ecsA_3 description:ABC-type transporter AT
852	pep chromosome:ASM81700v1:Chromosome:1790285:1791172:-1 gene:SpnNT_01779 transcript:AJD72712 gene_biotype:protein_coding transcript_biotype:protein_coding gene_symbol:gmuE description:Putative fructokinase
853	pep chromosome:ASM81700v1:Chromosome:1791321:1793258:-1 gene:SpnNT_01780 transcript:AJD72713 gene_biotype:protein_coding transcript_biotype:protein_coding gene_symbol:bglF_2 description:EIIBCA-Bgl
854	pep chromosome:ASM81700v1:Chromosome:1793634:1795088:1 gene:SpnNT_01781 transcript:AJD72714 gene_biotype:protein_coding transcript_biotype:protein_coding gene_symbol:scrB description:Sucrose-6-phosphate hydro
855	pep chromosome:ASM81700v1:Chromosome:1796070:1797344:-1 gene:SpnNT_01783 transcript:AJD72716 gene_biotype:protein_coding transcript_biotype:protein_coding gene_symbol:mvaA description:3-hydroxy-3-methylglutary
856	pep chromosome:ASM81700v1:Chromosome:1797344:1798516:-1 gene:SpnNT_01784 transcript:AJD72717 gene_biotype:protein_coding transcript_biotype:protein_coding gene_symbol:pksG description:Polyketide biosynthesis 3-h
857	pep chromosome:ASM81700v1:Chromosome:1801011:1802990:-1 gene:SpnNT_01789 transcript:AJD72722 gene_biotype:protein_coding transcript_biotype:protein_coding gene_symbol:stkP description:Serine/threonine-protein kin
858	pep chromosome:ASM81700v1:Chromosome:1802987:1803727:-1 gene:SpnNT_01790 transcript:AJD72723 gene_biotype:protein_coding transcript_biotype:protein_coding description:Putative protein phosphatase 2C-type
859	pep chromosome:ASM81700v1:Chromosome:1803742:1805055:-1 gene:SpnNT_01791 transcript:AJD72724 gene_biotype:protein_coding transcript_biotype:protein_coding gene_symbol:rsmB description:Ribosomal RNA small sub
860	pep chromosome:ASM81700v1:Chromosome:1805048:1805983:-1 gene:SpnNT_01792 transcript:AJD72725 gene_biotype:protein_coding transcript_biotype:protein_coding gene_symbol:fmt description:Methionyl-tRNA formyltransfe
861	pep chromosome:ASM81700v1:Chromosome:1805996:1808392:-1 gene:SpnNT_01793 transcript:AJD72726 gene_biotype:protein_coding transcript_biotype:protein_coding gene_symbol:priA description:Primosomal protein N
862	pep chromosome:ASM81700v1:Chromosome:1808458:1808772:-1 gene:SpnNT_01794 transcript:AJD72727 gene_biotype:protein_coding transcript_biotype:protein_coding gene_symbol:rpoZ description:DNA-directed RNA polyme
863	pep chromosome:ASM81700v1:Chromosome:1808797:1809423:-1 gene:SpnNT_01795 transcript:AJD72728 gene_biotype:protein_coding transcript_biotype:protein_coding gene_symbol:gmk description:Guanylate kinase
864	pep chromosome:ASM81700v1:Chromosome:1809553:1811166:-1 gene:SpnNT_01796 transcript:AJD72729 gene_biotype:protein_coding transcript_biotype:protein_coding gene_symbol:rny description:Ribonuclease Y

	A
865	pep chromosome:ASM81700v1:Chromosome:1811290:1814277:-1 gene:SpnNT_01797 transcript:AJD72730 gene_biotype:protein_coding transcript_biotype:protein_coding gene_symbol:hsdR_3 description:Type-1 restriction enzym
866	pep chromosome:ASM81700v1:Chromosome:1814385:1815386:-1 gene:SpnNT_01798 transcript:AJD72731 gene_biotype:protein_coding transcript_biotype:protein_coding description:hypothetical protein
867	pep chromosome:ASM81700v1:Chromosome:1816074:1817345:-1 gene:SpnNT_01800 transcript:AJD72733 gene_biotype:protein_coding transcript_biotype:protein_coding description:EcoKI restriction-modification system protein H
868	pep chromosome:ASM81700v1:Chromosome:1817338:1819446:-1 gene:SpnNT_01801 transcript:AJD72734 gene_biotype:protein_coding transcript_biotype:protein_coding description:putative type I restriction enzymeP M protein
869	pep chromosome:ASM81700v1:Chromosome:1819920:1820174:-1 gene:SpnNT_01803 transcript:AJD72736 gene_biotype:protein_coding transcript_biotype:protein_coding description:prevent-host-death family protein
870	pep chromosome:ASM81700v1:Chromosome:1821413:1822189:-1 gene:SpnNT_01805 transcript:AJD72738 gene_biotype:protein_coding transcript_biotype:protein_coding description:Glycine/sarcosine N-methyltransferase
871	pep chromosome:ASM81700v1:Chromosome:1822372:1822722:-1 gene:SpnNT_01806 transcript:AJD72739 gene_biotype:protein_coding transcript_biotype:protein_coding gene_symbol:rsfS description:Ribosomal silencing factor R
872	pep chromosome:ASM81700v1:Chromosome:1822723:1823316:-1 gene:SpnNT_01807 transcript:AJD72740 gene_biotype:protein_coding transcript_biotype:protein_coding description:putative nicotinate-nucleotide adenylyltransfera
873	pep chromosome:ASM81700v1:Chromosome:1823316:1823945:-1 gene:SpnNT_01808 transcript:AJD72741 gene_biotype:protein_coding transcript_biotype:protein_coding gene_symbol:nadD description:Nicotinate-nucleotide aden
874	pep chromosome:ASM81700v1:Chromosome:1823997:1824308:-1 gene:SpnNT_01809 transcript:AJD72742 gene_biotype:protein_coding transcript_biotype:protein_coding gene_symbol:yhbY description:RNA-binding protein YhbY
875	pep chromosome:ASM81700v1:Chromosome:1824522:1825628:-1 gene:SpnNT_01810 transcript:AJD72743 gene_biotype:protein_coding transcript_biotype:protein_coding description:GTPase YqeH
876	pep chromosome:ASM81700v1:Chromosome:1827138:1827698:-1 gene:SpnNT_01813 transcript:AJD72746 gene_biotype:protein_coding transcript_biotype:protein_coding gene_symbol:ykuT description:putative MscS family protei
877	pep chromosome:ASM81700v1:Chromosome:1830104:1830484:1 gene:SpnNT_01816 transcript:AJD72749 gene_biotype:protein_coding transcript_biotype:protein_coding description:accessory regulator family protein
878	pep chromosome:ASM81700v1:Chromosome:1830582:1830803:1 gene:SpnNT_01817 transcript:AJD72750 gene_biotype:protein_coding transcript_biotype:protein_coding description:hypothetical protein
879	pep chromosome:ASM81700v1:Chromosome:1830819:1831133:1 gene:SpnNT_01818 transcript:AJD72751 gene_biotype:protein_coding transcript_biotype:protein_coding gene_symbol:trxA description:Thioredoxin
880	pep chromosome:ASM81700v1:Chromosome:1834089:1834880:-1 gene:SpnNT_01823 transcript:AJD72756 gene_biotype:protein_coding transcript_biotype:protein_coding description:hypothetical protein
881	pep chromosome:ASM81700v1:Chromosome:1838835:1840631:-1 gene:SpnNT_01828 transcript:AJD72761 gene_biotype:protein_coding transcript_biotype:protein_coding gene_symbol:pepF1_2 description:Oligoendopeptidase F,
882	pep chromosome:ASM81700v1:Chromosome:1840643:1841386:-1 gene:SpnNT_01829 transcript:AJD72762 gene_biotype:protein_coding transcript_biotype:protein_coding gene_symbol:rsmE description:Ribosomal RNA small subu
883	pep chromosome:ASM81700v1:Chromosome:1841388:1842341:-1 gene:SpnNT_01830 transcript:AJD72763 gene_biotype:protein_coding transcript_biotype:protein_coding gene_symbol:prmA description:Ribosomal protein L11 me
884	pep chromosome:ASM81700v1:Chromosome:1842476:1842904:-1 gene:SpnNT_01831 transcript:AJD72764 gene_biotype:protein_coding transcript_biotype:protein_coding description:nucleoside triphosphate pyrophosphohydrolase
885	pep chromosome:ASM81700v1:Chromosome:1842906:1843973:-1 gene:SpnNT_01832 transcript:AJD72765 gene_biotype:protein_coding transcript_biotype:protein_coding description:Zn-dependent protease
886	pep chromosome:ASM81700v1:Chromosome:1843992:1844462:-1 gene:SpnNT_01833 transcript:AJD72766 gene_biotype:protein_coding transcript_biotype:protein_coding description:hypothetical protein
887	pep chromosome:ASM81700v1:Chromosome:1846309:1847580:1 gene:SpnNT_01838 transcript:AJD72771 gene_biotype:protein_coding transcript_biotype:protein_coding gene_symbol:rarA description:Replication-associated recon
888	pep chromosome:ASM81700v1:Chromosome:1851723:1852289:1 gene:SpnNT_01843 transcript:AJD72775 gene_biotype:protein_coding transcript_biotype:protein_coding description:hypothetical protein
889	pep chromosome:ASM81700v1:Chromosome:1852502:1853110:1 gene:SpnNT_01845 transcript:AJD72777 gene_biotype:protein_coding transcript_biotype:protein_coding description:hypothetical protein
890	pep chromosome:ASM81700v1:Chromosome:1853141:1853344:1 gene:SpnNT_01846 transcript:AJD72778 gene_biotype:protein_coding transcript_biotype:protein_coding description:CsbD-like protein
891	pep chromosome:ASM81700v1:Chromosome:1857229:1858005:-1 gene:SpnNT_01852 transcript:AJD72784 gene_biotype:protein_coding transcript_biotype:protein_coding gene_symbol:trpA description:Tryptophan synthase alpha
892	pep chromosome:ASM81700v1:Chromosome:1857998:1859221:-1 gene:SpnNT_01853 transcript:AJD72785 gene_biotype:protein_coding transcript_biotype:protein_coding gene_symbol:trpB description:Tryptophan synthase beta c
893	pep chromosome:ASM81700v1:Chromosome:1859199:1859798:-1 gene:SpnNT_01854 transcript:AJD72786 gene_biotype:protein_coding transcript_biotype:protein_coding gene_symbol:trpF description:N-(5-phosphoribosyl)anthran
894	pep chromosome:ASM81700v1:Chromosome:1859785:1860552:-1 gene:SpnNT_01855 transcript:AJD72787 gene_biotype:protein_coding transcript_biotype:protein_coding gene_symbol:trpC description:Indole-3-glycerol phosphate
895	pep chromosome:ASM81700v1:Chromosome:1860549:1861553:-1 gene:SpnNT_01856 transcript:AJD72788 gene_biotype:protein_coding transcript_biotype:protein_coding gene_symbol:trpD description:Anthranilate phosphoribosyl
896	pep chromosome:ASM81700v1:Chromosome:1861564:1862130:-1 gene:SpnNT_01857 transcript:AJD72789 gene_biotype:protein_coding transcript_biotype:protein_coding gene_symbol:trpG description:Anthranilate synthase comp
897	pep chromosome:ASM81700v1:Chromosome:1862127:1863488:-1 gene:SpnNT_01858 transcript:AJD72790 gene_biotype:protein_coding transcript_biotype:protein_coding gene_symbol:trpE description:Anthranilate synthase comp
898	pep chromosome:ASM81700v1:Chromosome:1864899:1865900:-1 gene:SpnNT_01860 transcript:AJD72792 gene_biotype:protein_coding transcript_biotype:protein_coding gene_symbol:ccpA_1 description:Glucose-resistance amy
899	pep chromosome:ASM81700v1:Chromosome:1868888:1869898:-1 gene:SpnNT_01864 transcript:AJD72796 gene_biotype:protein_coding transcript_biotype:protein_coding gene_symbol:malK description:Maltose/maltodextrin impor
900	pep chromosome:ASM81700v1:Chromosome:1870001:1871068:-1 gene:SpnNT_01865 transcript:AJD72797 gene_biotype:protein_coding transcript_biotype:protein_coding description:2-aminoethylphosphonate ABC transporter sub
901	pep chromosome:ASM81700v1:Chromosome:1871093:1871752:-1 gene:SpnNT_01866 transcript:AJD72798 gene_biotype:protein_coding transcript_biotype:protein_coding description:hypothetical protein
902	pep chromosome:ASM81700v1:Chromosome:1876064:1877290:-1 gene:SpnNT_01868 transcript:AJD72800 gene_biotype:protein_coding transcript_biotype:protein_coding gene_symbol:arnB description:UDP-4-amino-4-deoxy-L-ar
903	pep chromosome:ASM81700v1:Chromosome:1878198:1879949:-1 gene:SpnNT_01870 transcript:AJD72802 gene_biotype:protein_coding transcript_biotype:protein_coding gene_symbol:yheH description:putative multidrug resistanc
904	pep chromosome:ASM81700v1:Chromosome:1879939:1881684:-1 gene:SpnNT_01871 transcript:AJD72803 gene_biotype:protein_coding transcript_biotype:protein_coding gene_symbol:yheI description:putative multidrug resistance
905	pep chromosome:ASM81700v1:Chromosome:1885020:1885847:1 gene:SpnNT_01876 transcript:AJD72808 gene_biotype:protein_coding transcript_biotype:protein_coding gene_symbol:exoA description:Exodeoxyribonuclease
906	pep chromosome:ASM81700v1:Chromosome:1889050:1889916:-1 gene:SpnNT_01881 transcript:AJD72813 gene_biotype:protein_coding transcript_biotype:protein_coding gene_symbol:dpnB description:Type-2 restriction enzyme
907	pep chromosome:ASM81700v1:Chromosome:1890699:1891553:-1 gene:SpnNT_01883 transcript:AJD72815 gene_biotype:protein_coding transcript_biotype:protein_coding gene_symbol:dpnM description:Modification methylase Dp
908	pep chromosome:ASM81700v1:Chromosome:1900580:1902100:-1 gene:SpnNT_01893 transcript:AJD72825 gene_biotype:protein_coding transcript_biotype:protein_coding gene_symbol:opuCB description:Glycine betaine/carnitine/
909	pep chromosome:ASM81700v1:Chromosome:1902093:1902821:-1 gene:SpnNT_01894 transcript:AJD72826 gene_biotype:protein_coding transcript_biotype:protein_coding gene_symbol:opuCA description:Carnitine transport ATP-b
910	pep chromosome:ASM81700v1:Chromosome:1902836:1903600:-1 gene:SpnNT_01895 transcript:AJD72827 gene_biotype:protein_coding transcript_biotype:protein_coding description:putative ester cyclase
911	pep chromosome:ASM81700v1:Chromosome:1903604:1904047:-1 gene:SpnNT_01896 transcript:AJD72828 gene_biotype:protein_coding transcript_biotype:protein_coding description:transcriptional regulator SlyA
912	pep chromosome:ASM81700v1:Chromosome:1904729:1905025:-1 gene:SpnNT_01897 transcript:AJD72829 gene_biotype:protein_coding transcript_biotype:protein_coding description:hypothetical protein
913	pep chromosome:ASM81700v1:Chromosome:1905047:1906111:-1 gene:SpnNT_01898 transcript:AJD72830 gene_biotype:protein_coding transcript_biotype:protein_coding gene_symbol:pepA_2 description:Glutamyl aminopeptidas
914	pep chromosome:ASM81700v1:Chromosome:1910386:1911351:1 gene:SpnNT_01903 transcript:AJD72835 gene_biotype:protein_coding transcript_biotype:protein_coding gene_symbol:yclQ description:putative ABC transporter sol
915	pep chromosome:ASM81700v1:Chromosome:1911818:1912540:-1 gene:SpnNT_01905 transcript:AJD72837 gene_biotype:protein_coding transcript_biotype:protein_coding gene_symbol:rluB description:Ribosomal large subunit pse
916	pep chromosome:ASM81700v1:Chromosome:1912527:1913096:-1 gene:SpnNT_01906 transcript:AJD72838 gene_biotype:protein_coding transcript_biotype:protein_coding gene_symbol:scpB description:Segregation and condensa
917	pep chromosome:ASM81700v1:Chromosome:1914574:1915035:-1 gene:SpnNT_01909 transcript:AJD72841 gene_biotype:protein_coding transcript_biotype:protein_coding gene_symbol:ykuL description:CBS domain-containing pro
918	pep chromosome:ASM81700v1:Chromosome:1915032:1915553:-1 gene:SpnNT_01910 transcript:AJD72842 gene_biotype:protein_coding transcript_biotype:protein_coding description:phosphodiesterase

	A
919	pep chromosome:ASM81700v1:Chromosome:1915529:1916500:-1 gene:SpnNT_01911 transcript:AJD72843 gene_biotype:protein_coding transcript_biotype:protein_coding gene_symbol:rdgB description:dITP/XTP pyrophosphatase
920	pep chromosome:ASM81700v1:Chromosome:1916497:1917291:-1 gene:SpnNT_01912 transcript:AJD72844 gene_biotype:protein_coding transcript_biotype:protein_coding gene_symbol:murI description:Glutamate racemase
921	pep chromosome:ASM81700v1:Chromosome:1917498:1917746:-1 gene:SpnNT_01913 transcript:AJD72845 gene_biotype:protein_coding transcript_biotype:protein_coding description:hypothetical protein
922	pep chromosome:ASM81700v1:Chromosome:1924153:1925079:-1 gene:SpnNT_01918 transcript:AJD72850 gene_biotype:protein_coding transcript_biotype:protein_coding gene_symbol:oppF description:Stage 0 sporulation protein
923	pep chromosome:ASM81700v1:Chromosome:1925090:1926157:-1 gene:SpnNT_01919 transcript:AJD72851 gene_biotype:protein_coding transcript_biotype:protein_coding gene_symbol:oppD description:Stage 0 sporulation protein
924	pep chromosome:ASM81700v1:Chromosome:1926166:1927092:-1 gene:SpnNT_01920 transcript:AJD72852 gene_biotype:protein_coding transcript_biotype:protein_coding gene_symbol:oppC description:Stage 0 sporulation protein
925	pep chromosome:ASM81700v1:Chromosome:1927092:1928579:-1 gene:SpnNT_01921 transcript:AJD72853 gene_biotype:protein_coding transcript_biotype:protein_coding gene_symbol:gsiC description:Glutathione transport syster
926	pep chromosome:ASM81700v1:Chromosome:1928646:1930625:-1 gene:SpnNT_01922 transcript:AJD72854 gene_biotype:protein_coding transcript_biotype:protein_coding gene_symbol:amiA description:Oligopeptide-binding protei
927	pep chromosome:ASM81700v1:Chromosome:1940602:1941537:1 gene:SpnNT_01930 transcript:AJD72862 gene_biotype:protein_coding transcript_biotype:protein_coding gene_symbol:birA description:Bifunctional protein BirA
928	pep chromosome:ASM81700v1:Chromosome:1951502:1952035:1 gene:SpnNT_01956 transcript:AJD72866 gene_biotype:protein_coding transcript_biotype:protein_coding description:hypothetical protein
929	pep chromosome:ASM81700v1:Chromosome:1954186:1955808:-1 gene:SpnNT_01961 transcript:AJD72871 gene_biotype:protein_coding transcript_biotype:protein_coding gene_symbol:groL description:Stress protein H5
930	pep chromosome:ASM81700v1:Chromosome:1955824:1956108:-1 gene:SpnNT_01962 transcript:AJD72872 gene_biotype:protein_coding transcript_biotype:protein_coding description:co-chaperonin GroES
931	pep chromosome:ASM81700v1:Chromosome:1956851:1957612:-1 gene:SpnNT_01964 transcript:AJD72874 gene_biotype:protein_coding transcript_biotype:protein_coding gene_symbol:ydfG description:NADP-dependent 3-hydroxy
932	pep chromosome:ASM81700v1:Chromosome:1957654:1958280:-1 gene:SpnNT_01965 transcript:AJD72875 gene_biotype:protein_coding transcript_biotype:protein_coding gene_symbol:pheT_2 description:Phenylalanine--tRNA liga
933	pep chromosome:ASM81700v1:Chromosome:1958296:1958613:-1 gene:SpnNT_01966 transcript:AJD72876 gene_biotype:protein_coding transcript_biotype:protein_coding gene_symbol:ytpP description:Thioredoxin-like protein Ytp
934	pep chromosome:ASM81700v1:Chromosome:1961702:1962376:-1 gene:SpnNT_01969 transcript:AJD72879 gene_biotype:protein_coding transcript_biotype:protein_coding description:putative membrane protein
935	pep chromosome:ASM81700v1:Chromosome:1966341:1967057:-1 gene:SpnNT_01977 transcript:AJD72887 gene_biotype:protein_coding transcript_biotype:protein_coding gene_symbol:yeeN description:putative transcriptional regu
936	pep chromosome:ASM81700v1:Chromosome:1967938:1969353:-1 gene:SpnNT_01978 transcript:AJD72888 gene_biotype:protein_coding transcript_biotype:protein_coding gene_symbol:ply_3 description:Thiol-activated cytolysin
937	pep chromosome:ASM81700v1:Chromosome:1969364:1969774:-1 gene:SpnNT_01979 transcript:AJD72889 gene_biotype:protein_coding transcript_biotype:protein_coding description:hypothetical protein;pep chromosome:ASM817
938	pep chromosome:ASM81700v1:Chromosome:1969767:1970375:-1 gene:SpnNT_01980 transcript:AJD72890 gene_biotype:protein_coding transcript_biotype:protein_coding description:hypothetical protein;pep chromosome:ASM817
939	pep chromosome:ASM81700v1:Chromosome:1970388:1970846:-1 gene:SpnNT_01981 transcript:AJD72891 gene_biotype:protein_coding transcript_biotype:protein_coding description:hypothetical protein;pep chromosome:ASM817
940	pep chromosome:ASM81700v1:Chromosome:1976800:1977756:-1 gene:SpnNT_01991 transcript:AJD72901 gene_biotype:protein_coding transcript_biotype:protein_coding gene_symbol:lytA_9 description:Autolysin
941	pep chromosome:ASM81700v1:Chromosome:1979827:1980993:-1 gene:SpnNT_01994 transcript:AJD72904 gene_biotype:protein_coding transcript_biotype:protein_coding gene_symbol:recA description:Recombinase A
942	pep chromosome:ASM81700v1:Chromosome:1981048:1982304:-1 gene:SpnNT_01995 transcript:AJD72905 gene_biotype:protein_coding transcript_biotype:protein_coding gene_symbol:cinA description:Exported protein 10
943	pep chromosome:ASM81700v1:Chromosome:1982388:1983404:-1 gene:SpnNT_01996 transcript:AJD72906 gene_biotype:protein_coding transcript_biotype:protein_coding gene_symbol:brpA description:Biofilm regulatory protein A
944	pep chromosome:ASM81700v1:Chromosome:1983412:1983930:-1 gene:SpnNT_01997 transcript:AJD72907 gene_biotype:protein_coding transcript_biotype:protein_coding description:putative acetyltransferase YhhY
945	pep chromosome:ASM81700v1:Chromosome:1983920:1984363:-1 gene:SpnNT_01998 transcript:AJD72908 gene_biotype:protein_coding transcript_biotype:protein_coding description:ADP-binding protein
946	pep chromosome:ASM81700v1:Chromosome:1986434:1986847:-1 gene:SpnNT_02002 transcript:AJD72912 gene_biotype:protein_coding transcript_biotype:protein_coding gene_symbol:ndk description:Nucleoside diphosphate kina
947	pep chromosome:ASM81700v1:Chromosome:1986948:1990625:-1 gene:SpnNT_02003 transcript:AJD72913 gene_biotype:protein_coding transcript_biotype:protein_coding gene_symbol:rpoC description:DNA-directed RNA polyme
948	pep chromosome:ASM81700v1:Chromosome:1990659:1994270:-1 gene:SpnNT_02004 transcript:AJD72914 gene_biotype:protein_coding transcript_biotype:protein_coding gene_symbol:rpoB description:DNA-directed RNA polyme
949	pep chromosome:ASM81700v1:Chromosome:1996999:1998330:-1 gene:SpnNT_02007 transcript:AJD72916 gene_biotype:protein_coding transcript_biotype:protein_coding gene_symbol:corC description:Magnesium and cobalt efflu
950	pep chromosome:ASM81700v1:Chromosome:1998621:1999445:-1 gene:SpnNT_02008 transcript:AJD72917 gene_biotype:protein_coding transcript_biotype:protein_coding gene_symbol:endA description:Competence-specific nucle
951	pep chromosome:ASM81700v1:Chromosome:1999665:2000948:-1 gene:SpnNT_02010 transcript:AJD72919 gene_biotype:protein_coding transcript_biotype:protein_coding gene_symbol:murA1 description:UDP-N-acetylglucosamin
952	pep chromosome:ASM81700v1:Chromosome:2001260:2002297:-1 gene:SpnNT_02011 transcript:AJD72920 gene_biotype:protein_coding transcript_biotype:protein_coding description:ATP-dependent protease LonB
953	pep chromosome:ASM81700v1:Chromosome:2002281:2002769:-1 gene:SpnNT_02012 transcript:AJD72921 gene_biotype:protein_coding transcript_biotype:protein_coding gene_symbol:coaD description:Phosphopantetheine adeny
954	pep chromosome:ASM81700v1:Chromosome:2002759:2003298:-1 gene:SpnNT_02013 transcript:AJD72922 gene_biotype:protein_coding transcript_biotype:protein_coding gene_symbol:rsmD description:Ribosomal RNA small sub
955	pep chromosome:ASM81700v1:Chromosome:2003363:2004355:-1 gene:SpnNT_02014 transcript:AJD72923 gene_biotype:protein_coding transcript_biotype:protein_coding gene_symbol:asnA description:Aspartate--ammonia ligase
956	pep chromosome:ASM81700v1:Chromosome:2004466:2005149:-1 gene:SpnNT_02015 transcript:AJD72924 gene_biotype:protein_coding transcript_biotype:protein_coding gene_symbol:ybhL description:Inner membrane protein Yb
957	pep chromosome:ASM81700v1:Chromosome:2005205:2005945:-1 gene:SpnNT_02016 transcript:AJD72925 gene_biotype:protein_coding transcript_biotype:protein_coding description:Putative TrmH family tRNA/rRNA methyltransfe
958	pep chromosome:ASM81700v1:Chromosome:2006335:2007261:1 gene:SpnNT_02018 transcript:AJD72927 gene_biotype:protein_coding transcript_biotype:protein_coding gene_symbol:misCB description:Membrane protein YidC 1
959	pep chromosome:ASM81700v1:Chromosome:2007336:2008130:-1 gene:SpnNT_02019 transcript:AJD72928 gene_biotype:protein_coding transcript_biotype:protein_coding gene_symbol:pflA_2 description:Pyruvate formate-lyase-ac
960	pep chromosome:ASM81700v1:Chromosome:2008244:2009506:-1 gene:SpnNT_02020 transcript:AJD72929 gene_biotype:protein_coding transcript_biotype:protein_coding gene_symbol:lysA description:Diaminopimelate decarboxy
961	pep chromosome:ASM81700v1:Chromosome:2009573:2010400:-1 gene:SpnNT_02021 transcript:AJD72930 gene_biotype:protein_coding transcript_biotype:protein_coding gene_symbol:purR description:Pur operon repressor
962	pep chromosome:ASM81700v1:Chromosome:2010667:2011608:-1 gene:SpnNT_02022 transcript:AJD72931 gene_biotype:protein_coding transcript_biotype:protein_coding gene_symbol:yhaM description:3-5 exoribonuclease YhaM
963	pep chromosome:ASM81700v1:Chromosome:2011598:2012854:-1 gene:SpnNT_02023 transcript:AJD72932 gene_biotype:protein_coding transcript_biotype:protein_coding description:RmuC family protein
964	pep chromosome:ASM81700v1:Chromosome:2012856:2013518:-1 gene:SpnNT_02024 transcript:AJD72933 gene_biotype:protein_coding transcript_biotype:protein_coding gene_symbol:thiN description:Thiamine pyrophosphokinas
965	pep chromosome:ASM81700v1:Chromosome:2013481:2014137:-1 gene:SpnNT_02025 transcript:AJD72934 gene_biotype:protein_coding transcript_biotype:protein_coding gene_symbol:rpe description:Ribulose-phosphate 3-epime
966	pep chromosome:ASM81700v1:Chromosome:2014148:2015026:-1 gene:SpnNT_02026 transcript:AJD72935 gene_biotype:protein_coding transcript_biotype:protein_coding gene_symbol:rsgA description:Putative ribosome biogenes
967	pep chromosome:ASM81700v1:Chromosome:2015028:2015900:-1 gene:SpnNT_02027 transcript:AJD72936 gene_biotype:protein_coding transcript_biotype:protein_coding gene_symbol:rsmA description:Ribosomal RNA small subu
968	pep chromosome:ASM81700v1:Chromosome:2016332:2016973:-1 gene:SpnNT_02029 transcript:AJD72938 gene_biotype:protein_coding transcript_biotype:protein_coding gene_symbol:bceA_2 description:Bacitracin export ATP-bi
969	pep chromosome:ASM81700v1:Chromosome:2016975:2019005:-1 gene:SpnNT_02030 transcript:AJD72939 gene_biotype:protein_coding transcript_biotype:protein_coding description:bacteriocin-associated integral membrane prot
970	pep chromosome:ASM81700v1:Chromosome:2019274:2020173:-1 gene:SpnNT_02031 transcript:AJD72940 gene_biotype:protein_coding transcript_biotype:protein_coding description:Helix-turn-helix domain protein
971	pep chromosome:ASM81700v1:Chromosome:2020805:2021578:-1 gene:SpnNT_02033 transcript:AJD72942 gene_biotype:protein_coding transcript_biotype:protein_coding gene_symbol:ycfH description:putative deoxyribonuclease
972	pep chromosome:ASM81700v1:Chromosome:2023276:2024490:-1 gene:SpnNT_02036 transcript:AJD72945 gene_biotype:protein_coding transcript_biotype:protein_coding gene_symbol:alaA description:Glutamate-pyruvate aminot

	A
973	pep chromosome:ASM81700v1:Chromosome:2025102:2025554:1 gene:SpnNT_02038 transcript:AJD72947 gene_biotype:protein_coding transcript_biotype:protein_coding description:Putative universal stress protein
974	pep chromosome:ASM81700v1:Chromosome:2025589:2026977:-1 gene:SpnNT_02039 transcript:AJD72948 gene_biotype:protein_coding transcript_biotype:protein_coding description:Putative bifunctional phosphatase/peptidyl-prol
975	pep chromosome:ASM81700v1:Chromosome:2028346:2029356:-1 gene:SpnNT_02041 transcript:AJD72950 gene_biotype:protein_coding transcript_biotype:protein_coding gene_symbol:ccpA_2 description:Glucose-resistance amy
976	pep chromosome:ASM81700v1:Chromosome:2029780:2030379:-1 gene:SpnNT_02042 transcript:AJD72951 gene_biotype:protein_coding transcript_biotype:protein_coding gene_symbol:vraR description:Response regulator protein
977	pep chromosome:ASM81700v1:Chromosome:2040764:2041300:-1 gene:SpnNT_02063 transcript:AJD72958 gene_biotype:protein_coding transcript_biotype:protein_coding gene_symbol:nusG description:transcription termination ar
978	pep chromosome:ASM81700v1:Chromosome:2041355:2041531:-1 gene:SpnNT_02064 transcript:AJD72959 gene_biotype:protein_coding transcript_biotype:protein_coding description:preprotein translocase subunit SecE
979	pep chromosome:ASM81700v1:Chromosome:2041746:2043941:-1 gene:SpnNT_02065 transcript:AJD72960 gene_biotype:protein_coding transcript_biotype:protein_coding gene_symbol:pbpF_1 description:Penicillin-binding protein
980	pep chromosome:ASM81700v1:Chromosome:2044971:2045978:-1 gene:SpnNT_02067 transcript:AJD72962 gene_biotype:protein_coding transcript_biotype:protein_coding gene_symbol:gap description:Glyceraldehyde-3-phosphate
981	pep chromosome:ASM81700v1:Chromosome:2047678:2048028:-1 gene:SpnNT_02070 transcript:AJD72965 gene_biotype:protein_coding transcript_biotype:protein_coding description:Transposase;pep chromosome:ASM81700v1:C
982	pep chromosome:ASM81700v1:Chromosome:2051597:2052325:-1 gene:SpnNT_02074 transcript:AJD72969 gene_biotype:protein_coding transcript_biotype:protein_coding gene_symbol:gmuR description:Glucomannan utilization p
983	pep chromosome:ASM81700v1:Chromosome:2055211:2055519:1 gene:SpnNT_02078 transcript:AJD72973 gene_biotype:protein_coding transcript_biotype:protein_coding gene_symbol:licB description:Lichenan-specific phosphotra
984	pep chromosome:ASM81700v1:Chromosome:2055516:2055824:1 gene:SpnNT_02079 transcript:AJD72974 gene_biotype:protein_coding transcript_biotype:protein_coding gene_symbol:gmuA description:Oligo-beta-mannoside-spe
985	pep chromosome:ASM81700v1:Chromosome:2056745:2059396:-1 gene:SpnNT_02080 transcript:AJD72975 gene_biotype:protein_coding transcript_biotype:protein_coding gene_symbol:adhE description:Aldehyde-alcohol dehydrog
986	pep chromosome:ASM81700v1:Chromosome:2059696:2060106:-1 gene:SpnNT_02081 transcript:AJD72976 gene_biotype:protein_coding transcript_biotype:protein_coding description:tRNA(1-methyladenosine) methyltransferase
987	pep chromosome:ASM81700v1:Chromosome:2060093:2060536:-1 gene:SpnNT_02082 transcript:AJD72977 gene_biotype:protein_coding transcript_biotype:protein_coding gene_symbol:yfkJ description:Low molecular weight protei
988	pep chromosome:ASM81700v1:Chromosome:2060583:2060882:-1 gene:SpnNT_02083 transcript:AJD72978 gene_biotype:protein_coding transcript_biotype:protein_coding description:preprotein translocase subunit YajC
989	pep chromosome:ASM81700v1:Chromosome:2060999:2062975:-1 gene:SpnNT_02084 transcript:AJD72979 gene_biotype:protein_coding transcript_biotype:protein_coding gene_symbol:tkt_1 description:Transketolase
990	pep chromosome:ASM81700v1:Chromosome:2071526:2072512:-1 gene:SpnNT_02094 transcript:AJD72989 gene_biotype:protein_coding transcript_biotype:protein_coding description:putative RNA-binding protein (contains KH dom
991	pep chromosome:ASM81700v1:Chromosome:2072531:2073361:-1 gene:SpnNT_02095 transcript:AJD72990 gene_biotype:protein_coding transcript_biotype:protein_coding gene_symbol:misCA description:Stage III sporulation prote
992	pep chromosome:ASM81700v1:Chromosome:2073850:2075040:-1 gene:SpnNT_02097 transcript:AJD72992 gene_biotype:protein_coding transcript_biotype:protein_coding gene_symbol:ackA description:Acetate kinase
993	pep chromosome:ASM81700v1:Chromosome:2075091:2076044:-1 gene:SpnNT_02098 transcript:AJD72993 gene_biotype:protein_coding transcript_biotype:protein_coding description:N-6 DNA Methylase
994	pep chromosome:ASM81700v1:Chromosome:2080615:2080980:-1 gene:SpnNT_02108 transcript:AJD73003 gene_biotype:protein_coding transcript_biotype:protein_coding description:hypothetical protein
995	pep chromosome:ASM81700v1:Chromosome:2082351:2083502:-1 gene:SpnNT_02110 transcript:AJD73005 gene_biotype:protein_coding transcript_biotype:protein_coding gene_symbol:nagA description:N-acetylglucosamine-6-pho
996	pep chromosome:ASM81700v1:Chromosome:2083655:2085472:-1 gene:SpnNT_02111 transcript:AJD73006 gene_biotype:protein_coding transcript_biotype:protein_coding gene_symbol:oatA description:O-acetyltransferase OatA
997	pep chromosome:ASM81700v1:Chromosome:2085573:2086715:-1 gene:SpnNT_02112 transcript:AJD73007 gene_biotype:protein_coding transcript_biotype:protein_coding gene_symbol:tgt description:Queuine tRNA-ribosyltransfer
998	pep chromosome:ASM81700v1:Chromosome:2088535:2088786:-1 gene:SpnNT_02116 transcript:AJD73011 gene_biotype:protein_coding transcript_biotype:protein_coding description:MarR family protein
999	pep chromosome:ASM81700v1:Chromosome:2088988:2090130:-1 gene:SpnNT_02117 transcript:AJD73012 gene_biotype:protein_coding transcript_biotype:protein_coding description:LysM domain protein
1000	pep chromosome:ASM81700v1:Chromosome:2090260:2090916:-1 gene:SpnNT_02118 transcript:AJD73013 gene_biotype:protein_coding transcript_biotype:protein_coding gene_symbol:cbbZC description:Phosphoglycolate phosph
1001	pep chromosome:ASM81700v1:Chromosome:2092261:2093745:-1 gene:SpnNT_02120 transcript:AJD73015 gene_biotype:protein_coding transcript_biotype:protein_coding gene_symbol:thrC description:Threonine synthase
1002	pep chromosome:ASM81700v1:Chromosome:2093821:2095098:-1 gene:SpnNT_02121 transcript:AJD73016 gene_biotype:protein_coding transcript_biotype:protein_coding description:glutamate--cysteine ligase
1003	pep chromosome:ASM81700v1:Chromosome:2095360:2095836:-1 gene:SpnNT_02122 transcript:AJD73017 gene_biotype:protein_coding transcript_biotype:protein_coding description:hypothetical protein
1004	pep chromosome:ASM81700v1:Chromosome:2095990:2096271:1 gene:SpnNT_02123 transcript:AJD73018 gene_biotype:protein_coding transcript_biotype:protein_coding description:hypothetical protein
1005	pep chromosome:ASM81700v1:Chromosome:2096538:2097902:1 gene:SpnNT_02125 transcript:AJD73020 gene_biotype:protein_coding transcript_biotype:protein_coding description:DNA segregation ATPase FtsK/SpoIIIE
1006	pep chromosome:ASM81700v1:Chromosome:2098348:2098737:1 gene:SpnNT_02127 transcript:AJD73022 gene_biotype:protein_coding transcript_biotype:protein_coding description:hypothetical protein
1007	pep chromosome:ASM81700v1:Chromosome:2098904:2099671:1 gene:SpnNT_02128 transcript:AJD73023 gene_biotype:protein_coding transcript_biotype:protein_coding description:Calcineurin-like phosphoesterase
1008	pep chromosome:ASM81700v1:Chromosome:2113488:2113922:-1 gene:SpnNT_02153 transcript:AJD73032 gene_biotype:protein_coding transcript_biotype:protein_coding description:cytidine deaminase
1009	pep chromosome:ASM81700v1:Chromosome:2113936:2115396:-1 gene:SpnNT_02154 transcript:AJD73033 gene_biotype:protein_coding transcript_biotype:protein_coding gene_symbol:gltX description:Glutamate--tRNA ligase
1010	pep chromosome:ASM81700v1:Chromosome:2115520:2116869:-1 gene:SpnNT_02155 transcript:AJD73034 gene_biotype:protein_coding transcript_biotype:protein_coding gene_symbol:pgi description:Glucose-6-phosphate isomer
1011	pep chromosome:ASM81700v1:Chromosome:2117867:2119633:-1 gene:SpnNT_02157 transcript:AJD73036 gene_biotype:protein_coding transcript_biotype:protein_coding description:putative ABC transporter ATP-binding protein
1012	pep chromosome:ASM81700v1:Chromosome:2120422:2122116:-1 gene:SpnNT_02159 transcript:AJD73038 gene_biotype:protein_coding transcript_biotype:protein_coding description:putative ABC transporter ATP-binding protein
1013	pep chromosome:ASM81700v1:Chromosome:2122263:2124797:-1 gene:SpnNT_02160 transcript:AJD73039 gene_biotype:protein_coding transcript_biotype:protein_coding gene_symbol:mutS description:DNA mismatch repair prote
1014	pep chromosome:ASM81700v1:Chromosome:2124848:2125294:-1 gene:SpnNT_02161 transcript:AJD73040 gene_biotype:protein_coding transcript_biotype:protein_coding gene_symbol:argR_3 description:Arginine regulator
1015	pep chromosome:ASM81700v1:Chromosome:2125430:2127121:1 gene:SpnNT_02162 transcript:AJD73041 gene_biotype:protein_coding transcript_biotype:protein_coding gene_symbol:argS description:Arginine--tRNA ligase
1016	pep chromosome:ASM81700v1:Chromosome:2129333:2130664:1 gene:SpnNT_02166 transcript:AJD73045 gene_biotype:protein_coding transcript_biotype:protein_coding gene_symbol:phoR description:Alkaline phosphatase synth
1017	pep chromosome:ASM81700v1:Chromosome:2136173:2137189:1 gene:SpnNT_02174 transcript:AJD73053 gene_biotype:protein_coding transcript_biotype:protein_coding gene_symbol:gpsA description:Glycerol-3-phosphate dehyd
1018	pep chromosome:ASM81700v1:Chromosome:2137211:2138110:1 gene:SpnNT_02175 transcript:AJD73054 gene_biotype:protein_coding transcript_biotype:protein_coding gene_symbol:gtaB_2 description:UTP--glucose-1-phosphat
1019	pep chromosome:ASM81700v1:Chromosome:2138838:2139377:-1 gene:SpnNT_02177 transcript:AJD73056 gene_biotype:protein_coding transcript_biotype:protein_coding description:putative 5-formyltetrahydrofolate cyclo-ligase
1020	pep chromosome:ASM81700v1:Chromosome:2139389:2140519:-1 gene:SpnNT_02178 transcript:AJD73057 gene_biotype:protein_coding transcript_biotype:protein_coding gene_symbol:ykuR description:N-acetyldiaminopimelate de
1021	pep chromosome:ASM81700v1:Chromosome:2140587:2141285:-1 gene:SpnNT_02179 transcript:AJD73058 gene_biotype:protein_coding transcript_biotype:protein_coding gene_symbol:dapH description:2,3,4,5-tetrahydropyridine-2
1022	pep chromosome:ASM81700v1:Chromosome:2142491:2144953:-1 gene:SpnNT_02181 transcript:AJD73060 gene_biotype:protein_coding transcript_biotype:protein_coding gene_symbol:pbpF_2 description:Penicillin-binding protein
1023	pep chromosome:ASM81700v1:Chromosome:2145095:2146351:1 gene:SpnNT_02182 transcript:AJD73061 gene_biotype:protein_coding transcript_biotype:protein_coding gene_symbol:tyrS description:Tyrosine--tRNA ligase
1024	pep chromosome:ASM81700v1:Chromosome:2146429:2148492:-1 gene:SpnNT_02183 transcript:AJD73062 gene_biotype:protein_coding transcript_biotype:protein_coding gene_symbol:copA description:putative copper-importing P
1025	pep chromosome:ASM81700v1:Chromosome:2148494:2148772:-1 gene:SpnNT_02184 transcript:AJD73063 gene_biotype:protein_coding transcript_biotype:protein_coding description:hypothetical protein
1026	pep chromosome:ASM81700v1:Chromosome:2148924:2149772:1 gene:SpnNT_02185 transcript:AJD73064 gene_biotype:protein_coding transcript_biotype:protein_coding gene_symbol:rlmA description:23S rRNA (guanine(745)-N(

	A
1027	pep chromosome:ASM81700v1:Chromosome:2154091:2156349:-1 gene:SpnNT_02189 transcript:AJD73068 gene_biotype:protein_coding transcript_biotype:protein_coding gene_symbol:malP description:Maltodextrin phosphorylase
1028	pep chromosome:ASM81700v1:Chromosome:2156375:2157892:-1 gene:SpnNT_02190 transcript:AJD73069 gene_biotype:protein_coding transcript_biotype:protein_coding gene_symbol:malQ description:4-alpha-glucanotransferase
1029	pep chromosome:ASM81700v1:Chromosome:2158442:2159713:1 gene:SpnNT_02191 transcript:AJD73070 gene_biotype:protein_coding transcript_biotype:protein_coding gene_symbol:malX_2 description:Maltose/maltodextrin-bind
1030	pep chromosome:ASM81700v1:Chromosome:2163031:2164017:1 gene:SpnNT_02195 transcript:AJD73074 gene_biotype:protein_coding transcript_biotype:protein_coding gene_symbol:malR description:Maltose operon transcriptio
1031	pep chromosome:ASM81700v1:Chromosome:2165228:2166991:-1 gene:SpnNT_02197 transcript:AJD73076 gene_biotype:protein_coding transcript_biotype:protein_coding gene_symbol:aspS description:Aspartate--tRNA ligase
1032	pep chromosome:ASM81700v1:Chromosome:2169133:2169339:-1 gene:SpnNT_02202 transcript:AJD73081 gene_biotype:protein_coding transcript_biotype:protein_coding description:DNA-binding transcriptional repressor PuuR
1033	pep chromosome:ASM81700v1:Chromosome:2169349:2169663:-1 gene:SpnNT_02203 transcript:AJD73082 gene_biotype:protein_coding transcript_biotype:protein_coding description:hypothetical protein
1034	pep chromosome:ASM81700v1:Chromosome:2170124:2171443:-1 gene:SpnNT_02204 transcript:AJD73083 gene_biotype:protein_coding transcript_biotype:protein_coding gene_symbol:bag_1 description:Beta antigen
1035	pep chromosome:ASM81700v1:Chromosome:2173434:2173625:-1 gene:SpnNT_02206 transcript:AJD73085 gene_biotype:protein_coding transcript_biotype:protein_coding description:hypothetical protein;pep chromosome:ASM817
1036	pep chromosome:ASM81700v1:Chromosome:2173622:2174911:-1 gene:SpnNT_02207 transcript:AJD73086 gene_biotype:protein_coding transcript_biotype:protein_coding gene_symbol:hisS description:Histidine--tRNA ligase
1037	pep chromosome:ASM81700v1:Chromosome:2177402:2177743:1 gene:SpnNT_02211 transcript:AJD73090 gene_biotype:protein_coding transcript_biotype:protein_coding description:FeS assembly SUF system protein
1038	pep chromosome:ASM81700v1:Chromosome:2177997:2179700:-1 gene:SpnNT_02212 transcript:AJD73091 gene_biotype:protein_coding transcript_biotype:protein_coding gene_symbol:ilvD description:Dihydroxy-acid dehydratase
1039	pep chromosome:ASM81700v1:Chromosome:2183082:2183366:-1 gene:SpnNT_02216 transcript:AJD73095 gene_biotype:protein_coding transcript_biotype:protein_coding description:PTS system ascorbate-specific transporter sub
1040	pep chromosome:ASM81700v1:Chromosome:2183370:2185400:-1 gene:SpnNT_02217 transcript:AJD73096 gene_biotype:protein_coding transcript_biotype:protein_coding gene_symbol:cmtB description:Mannitol-specific cryptic ph
1041	pep chromosome:ASM81700v1:Chromosome:2185592:2186599:1 gene:SpnNT_02218 transcript:AJD73097 gene_biotype:protein_coding transcript_biotype:protein_coding description:SPFH domain / Band 7 family protein
1042	pep chromosome:ASM81700v1:Chromosome:2186602:2186802:1 gene:SpnNT_02219 transcript:AJD73098 gene_biotype:protein_coding transcript_biotype:protein_coding description:hypothetical protein
1043	pep chromosome:ASM81700v1:Chromosome:2186954:2187136:1 gene:SpnNT_02220 transcript:AJD73099 gene_biotype:protein_coding transcript_biotype:protein_coding gene_symbol:rpmF description:50S ribosomal protein L32
1044	pep chromosome:ASM81700v1:Chromosome:2187152:2187301:1 gene:SpnNT_02221 transcript:AJD73100 gene_biotype:protein_coding transcript_biotype:protein_coding gene_symbol:rpmGA description:50S ribosomal protein L3
1045	pep chromosome:ASM81700v1:Chromosome:2187431:2187814:-1 gene:SpnNT_02222 transcript:AJD73101 gene_biotype:protein_coding transcript_biotype:protein_coding description:hypothetical protein
1046	pep chromosome:ASM81700v1:Chromosome:2187816:2188403:-1 gene:SpnNT_02223 transcript:AJD73102 gene_biotype:protein_coding transcript_biotype:protein_coding description:putative protein encoded in hypervariable junct
1047	pep chromosome:ASM81700v1:Chromosome:2188390:2188647:-1 gene:SpnNT_02224 transcript:AJD73103 gene_biotype:protein_coding transcript_biotype:protein_coding description:hypothetical protein
1048	pep chromosome:ASM81700v1:Chromosome:2205880:2210709:-1 gene:SpnNT_02241 transcript:AJD73120 gene_biotype:protein_coding transcript_biotype:protein_coding gene_symbol:iga_7 description:Immunoglobulin A1 protea
1049	pep chromosome:ASM81700v1:Chromosome:2211027:2211926:-1 gene:SpnNT_02242 transcript:AJD73121 gene_biotype:protein_coding transcript_biotype:protein_coding description:FtsH protease regulator HflK
1050	pep chromosome:ASM81700v1:Chromosome:2212168:2212353:1 gene:SpnNT_02243 transcript:AJD73122 gene_biotype:protein_coding transcript_biotype:protein_coding description:hypothetical protein
1051	pep chromosome:ASM81700v1:Chromosome:2214343:2215848:-1 gene:SpnNT_02245 transcript:AJD73124 gene_biotype:protein_coding transcript_biotype:protein_coding gene_symbol:adcA_2 description:putative zinc transport sy
1052	pep chromosome:ASM81700v1:Chromosome:2216657:2217361:-1 gene:SpnNT_02247 transcript:AJD73126 gene_biotype:protein_coding transcript_biotype:protein_coding gene_symbol:adcC_2 description:putative zinc transport sy
1053	pep chromosome:ASM81700v1:Chromosome:2217361:2217801:-1 gene:SpnNT_02248 transcript:AJD73127 gene_biotype:protein_coding transcript_biotype:protein_coding gene_symbol:adcR description:Transcriptional repressor A
1054	pep chromosome:ASM81700v1:Chromosome:2217956:2219224:-1 gene:SpnNT_02249 transcript:AJD73128 gene_biotype:protein_coding transcript_biotype:protein_coding description:D-alanyl-lipoteichoic acid biosynthesis protein
1055	pep chromosome:ASM81700v1:Chromosome:2219217:2219456:-1 gene:SpnNT_02250 transcript:AJD73129 gene_biotype:protein_coding transcript_biotype:protein_coding gene_symbol:dltC description:D-alanine--poly(phosphoribit
1056	pep chromosome:ASM81700v1:Chromosome:2220711:2222261:-1 gene:SpnNT_02252 transcript:AJD73131 gene_biotype:protein_coding transcript_biotype:protein_coding gene_symbol:dltA description:D-alanine--poly(phosphoribit
1057	pep chromosome:ASM81700v1:Chromosome:2228636:2229508:1 gene:SpnNT_02257 transcript:AJD73136 gene_biotype:protein_coding transcript_biotype:protein_coding gene_symbol:hslO description:Heat shock protein 33
1058	pep chromosome:ASM81700v1:Chromosome:2229495:2230475:1 gene:SpnNT_02258 transcript:AJD73137 gene_biotype:protein_coding transcript_biotype:protein_coding gene_symbol:dus description:putative tRNA-dihydrouridine
1059	pep chromosome:ASM81700v1:Chromosome:2233160:2233471:-1 gene:SpnNT_02261 transcript:AJD73140 gene_biotype:protein_coding transcript_biotype:protein_coding description:hypothetical protein
1060	pep chromosome:ASM81700v1:Chromosome:2233606:2235882:-1 gene:SpnNT_02262 transcript:AJD73141 gene_biotype:protein_coding transcript_biotype:protein_coding gene_symbol:bag_2 description:Beta antigen
1061	pep chromosome:ASM81700v1:Chromosome:2235965:2236492:-1 gene:SpnNT_02263 transcript:AJD73142 gene_biotype:protein_coding transcript_biotype:protein_coding description:Putative protein-S-isoprenylcysteine methyltran
1062	pep chromosome:ASM81700v1:Chromosome:2237938:2238591:-1 gene:SpnNT_02265 transcript:AJD73144 gene_biotype:protein_coding transcript_biotype:protein_coding gene_symbol:phoB description:Phosphate regulon transcri
1063	pep chromosome:ASM81700v1:Chromosome:2238846:2241278:-1 gene:SpnNT_02266 transcript:AJD73145 gene_biotype:protein_coding transcript_biotype:protein_coding gene_symbol:clpC_2 description:ATP-dependent Clp prote
1064	pep chromosome:ASM81700v1:Chromosome:2241280:2241738:-1 gene:SpnNT_02267 transcript:AJD73146 gene_biotype:protein_coding transcript_biotype:protein_coding gene_symbol:ctsR description:Class three stress gene rep
1065	pep chromosome:ASM81700v1:Chromosome:2242585:2243592:-1 gene:SpnNT_02269 transcript:AJD73148 gene_biotype:protein_coding transcript_biotype:protein_coding description:Putative thiamine biosynthesis protein
1066	pep chromosome:ASM81700v1:Chromosome:2244352:2244642:-1 gene:SpnNT_02271 transcript:AJD73150 gene_biotype:protein_coding transcript_biotype:protein_coding description:hypothetical protein
1067	pep chromosome:ASM81700v1:Chromosome:2246333:2246599:-1 gene:SpnNT_02273 transcript:AJD73152 gene_biotype:protein_coding transcript_biotype:protein_coding description:hypothetical protein
1068	pep chromosome:ASM81700v1:Chromosome:2246601:2247953:-1 gene:SpnNT_02274 transcript:AJD73153 gene_biotype:protein_coding transcript_biotype:protein_coding gene_symbol:dnaC description:Replicative DNA helicase
1069	pep chromosome:ASM81700v1:Chromosome:2247997:2248449:-1 gene:SpnNT_02275 transcript:AJD73154 gene_biotype:protein_coding transcript_biotype:protein_coding gene_symbol:rplI description:ribosomal protein l9
1070	pep chromosome:ASM81700v1:Chromosome:2248446:2250419:-1 gene:SpnNT_02276 transcript:AJD73155 gene_biotype:protein_coding transcript_biotype:protein_coding gene_symbol:nrnA_2 description:Bifunctional oligoribonuc
1071	pep chromosome:ASM81700v1:Chromosome:2250555:2251103:-1 gene:SpnNT_02277 transcript:AJD73156 gene_biotype:protein_coding transcript_biotype:protein_coding description:Ribosome-associated factor Y
1072	pep chromosome:ASM81700v1:Chromosome:2253196:2253831:1 gene:SpnNT_02280 transcript:AJD73159 gene_biotype:protein_coding transcript_biotype:protein_coding gene_symbol:yigZ description:IMPACT family member Yig
1073	pep chromosome:ASM81700v1:Chromosome:2254116:2255036:1 gene:SpnNT_02281 transcript:AJD73160 gene_biotype:protein_coding transcript_biotype:protein_coding gene_symbol:cysK description:Cysteine synthase
1074	pep chromosome:ASM81700v1:Chromosome:2256640:2257680:-1 gene:SpnNT_02285 transcript:AJD73164 gene_biotype:protein_coding transcript_biotype:protein_coding gene_symbol:tsf description:Elongation factor Ts
1075	pep chromosome:ASM81700v1:Chromosome:2257759:2258538:-1 gene:SpnNT_02286 transcript:AJD73165 gene_biotype:protein_coding transcript_biotype:protein_coding gene_symbol:rpsB description:30S ribosomal protein S2
1076	pep chromosome:ASM81700v1:Chromosome:2258762:2259940:-1 gene:SpnNT_02287 transcript:AJD73166 gene_biotype:protein_coding transcript_biotype:protein_coding description:Secreted 45 kDa protein precursor
1077	pep chromosome:ASM81700v1:Chromosome:2260203:2261936:1 gene:SpnNT_02288 transcript:AJD73167 gene_biotype:protein_coding transcript_biotype:protein_coding description:Transposase;pep chromosome:ASM81700v1:C
1078	pep chromosome:ASM81700v1:Chromosome:2262585:2263403:-1 gene:SpnNT_02290 transcript:AJD73169 gene_biotype:protein_coding transcript_biotype:protein_coding gene_symbol:mreC description:Cell shape protein MreC
1079	pep chromosome:ASM81700v1:Chromosome:2264249:2265088:-1 gene:SpnNT_02292 transcript:AJD73171 gene_biotype:protein_coding transcript_biotype:protein_coding gene_symbol:ecfA2 description:Energy-coupling factor tra
1080	pep chromosome:ASM81700v1:Chromosome:2265073:2265900:-1 gene:SpnNT_02293 transcript:AJD73172 gene_biotype:protein_coding transcript_biotype:protein_coding gene_symbol:ecfA1 description:Energy-coupling factor tra

	A
1081	pep chromosome:ASM81700v1:Chromosome:2266453:2267274:-1 gene:SpnNT_02295 transcript:AJD73174 gene_biotype:protein_coding transcript_biotype:protein_coding description:cytoskeletal protein RodZ
1082	pep chromosome:ASM81700v1:Chromosome:2267316:2268599:-1 gene:SpnNT_02296 transcript:AJD73175 gene_biotype:protein_coding transcript_biotype:protein_coding gene_symbol:albF description:Putative zinc protease AlbF
1083	pep chromosome:ASM81700v1:Chromosome:2268596:2269846:-1 gene:SpnNT_02297 transcript:AJD73176 gene_biotype:protein_coding transcript_biotype:protein_coding gene_symbol:albE description:Antilisterial bacteriocin subt
1084	pep chromosome:ASM81700v1:Chromosome:2270005:2270373:1 gene:SpnNT_02298 transcript:AJD73177 gene_biotype:protein_coding transcript_biotype:protein_coding description:S4 domain protein YaaA
1085	pep chromosome:ASM81700v1:Chromosome:2271524:2273002:-1 gene:SpnNT_02300 transcript:AJD73179 gene_biotype:protein_coding transcript_biotype:protein_coding gene_symbol:guaB description:Inosine-5-monophosphate
1086	pep chromosome:ASM81700v1:Chromosome:2273154:2274179:-1 gene:SpnNT_02301 transcript:AJD73180 gene_biotype:protein_coding transcript_biotype:protein_coding gene_symbol:trpS2 description:Tryptophan--tRNA ligase 2
1087	pep chromosome:ASM81700v1:Chromosome:2274385:2276007:1 gene:SpnNT_02302 transcript:AJD73181 gene_biotype:protein_coding transcript_biotype:protein_coding gene_symbol:yheS_3 description:putative ABC transporter
1088	pep chromosome:ASM81700v1:Chromosome:2276069:2278621:1 gene:SpnNT_02303 transcript:AJD73182 gene_biotype:protein_coding transcript_biotype:protein_coding description:putative membrane protein
1089	pep chromosome:ASM81700v1:Chromosome:2282586:2283338:-1 gene:SpnNT_02311 transcript:AJD73188 gene_biotype:protein_coding transcript_biotype:protein_coding gene_symbol:agrA_2 description:Accessory gene regulato
1090	pep chromosome:ASM81700v1:Chromosome:2283335:2284660:-1 gene:SpnNT_02312 transcript:AJD73189 gene_biotype:protein_coding transcript_biotype:protein_coding description:sensory histidine kinase DcuS
1091	pep chromosome:ASM81700v1:Chromosome:2285750:2286931:1 gene:SpnNT_02316 transcript:AJD73192 gene_biotype:protein_coding transcript_biotype:protein_coding gene_symbol:htrA description:Serine protease Do-like HtrA

	B	C	D	E	F
1	Protein IDs	Majority protein IDs	Student's T-test Significant ORF2_ORF2_PEP	Student's T-test Significant WT_WT_PEP	Student's T-test Significant ORF2_WT
2	AJD70948	AJD70948			
3	AJD70949	AJD70949			
4	AJD70950	AJD70950			
5	AJD70968	AJD70968			
6	AJD70969	AJD70969			
7	AJD70973	AJD70973			
8	AJD70975	AJD70975			
9	AJD70977	AJD70977			+
10	AJD70981	AJD70981			
11	AJD70982	AJD70982			
12	AJD70985	AJD70985			
13	AJD70986	AJD70986			
14	AJD70987	AJD70987			
15	AJD70990	AJD70990			
16	AJD70992	AJD70992			
17	AJD70994	AJD70994			
18	AJD70995	AJD70995			
19	AJD70996	AJD70996			
20	AJD70998	AJD70998			
21	AJD71000	AJD71000			
22	AJD71001	AJD71001			
23	AJD71005	AJD71005			
24	AJD71013	AJD71013			
25	AJD71014	AJD71014			
26	AJD71015	AJD71015			
27	AJD71016	AJD71016			
28	AJD71019	AJD71019			
29	AJD71020	AJD71020			
30	AJD71021	AJD71021			
31	AJD71022	AJD71022			
32	AJD71023	AJD71023			
33	AJD71024	AJD71024			
34	AJD71027	AJD71027			
35	AJD71037	AJD71037			
36	AJD71040	AJD71040			
37	AJD71043	AJD71043			
38	AJD71047	AJD71047			
39	AJD71048	AJD71048			
40	AJD71049	AJD71049			
41	AJD71053	AJD71053			
42	AJD71056	AJD71056			
43	AJD71057	AJD71057			
44	AJD71058	AJD71058			
45	AJD71062	AJD71062			
46	AJD71067	AJD71067			
47	AJD71068	AJD71068			
48	AJD71069	AJD71069			
49	AJD71070	AJD71070			
50	AJD71084	AJD71084			
51	AJD71086	AJD71086			
52	AJD71088	AJD71088			
53	AJD71089	AJD71089			
54	AJD71090	AJD71090			

	B	C	D	E	F
55	AJD71095	AJD71095			
56	AJD71097	AJD71097			
57	AJD71098	AJD71098		+	
58	AJD71099	AJD71099			
59	AJD71104	AJD71104			
60	AJD71105	AJD71105			
61	AJD71106	AJD71106			
62	AJD71107	AJD71107			
63	AJD71114	AJD71114			
64	AJD71115	AJD71115			
65	AJD71116	AJD71116			
66	AJD71119	AJD71119			
67	AJD71120	AJD71120	+	+	+
68	AJD71122	AJD71122	+	+	
69	AJD71123	AJD71123			
70	AJD71124	AJD71124			
71	AJD71127	AJD71127			
72	AJD71128	AJD71128			
73	AJD71129	AJD71129			
74	AJD71130	AJD71130			
75	AJD71131;AJD7113	AJD71131			
76	AJD71134	AJD71134			
77	AJD71135	AJD71135			
78	AJD71136	AJD71136			
79	AJD71137	AJD71137			
80	AJD71138	AJD71138			
81	AJD71139	AJD71139			
82	AJD71140	AJD71140			
83	AJD71141	AJD71141			
84	AJD71143	AJD71143			
85	AJD71144	AJD71144			
86	AJD71147	AJD71147			
87	AJD71148	AJD71148			
88	AJD71149	AJD71149			
89	AJD71150	AJD71150			
90	AJD71151	AJD71151			
91	AJD71152	AJD71152			
92	AJD71153	AJD71153			
93	AJD71154	AJD71154			
94	AJD71155	AJD71155			
95	AJD71156	AJD71156			
96	AJD71157	AJD71157			
97	AJD71158	AJD71158			
98	AJD71159	AJD71159			
99	AJD71160	AJD71160			
100	AJD71161	AJD71161			
101	AJD71162	AJD71162			
102	AJD71164	AJD71164			
103	AJD71165	AJD71165			
104	AJD71166	AJD71166			
105	AJD71167	AJD71167			
106	AJD71168	AJD71168			
107	AJD71169	AJD71169			
108	AJD71170	AJD71170			

	B	C	D	E	F
109	AJD71171	AJD71171			
110	AJD71172	AJD71172			
111	AJD71173	AJD71173			
112	AJD71174	AJD71174			
113	AJD71175	AJD71175			
114	AJD71176	AJD71176			
115	AJD71177	AJD71177			
116	AJD71178	AJD71178			
117	AJD71179	AJD71179			
118	AJD71180	AJD71180			
119	AJD71189	AJD71189			
120	AJD71199	AJD71199			
121	AJD71200	AJD71200			
122	AJD71201	AJD71201			
123	AJD71202	AJD71202			
124	AJD71206	AJD71206			
125	AJD71207	AJD71207			
126	AJD71208	AJD71208			
127	AJD71209	AJD71209			
128	AJD71216	AJD71216			
129	AJD71218	AJD71218			
130	AJD71219	AJD71219			
131	AJD71220	AJD71220			
132	AJD71221	AJD71221			
133	AJD71222	AJD71222			
134	AJD71225	AJD71225			
135	AJD71227	AJD71227			
136	AJD71228	AJD71228			
137	AJD71229	AJD71229			
138	AJD71230	AJD71230			
139	AJD71231	AJD71231			
140	AJD71232	AJD71232			
141	AJD71233	AJD71233			
142	AJD71234	AJD71234			
143	AJD71236	AJD71236			
144	AJD71237	AJD71237			
145	AJD71238	AJD71238			
146	AJD71239	AJD71239			
147	AJD71241	AJD71241			
148	AJD71242	AJD71242			
149	AJD71246	AJD71246			
150	AJD71262	AJD71262			
151	AJD71267	AJD71267			
152	AJD71268	AJD71268			
153	AJD71269	AJD71269			
154	AJD71270	AJD71270			
155	AJD71271	AJD71271	+		+
156	AJD71273	AJD71273			
157	AJD71274	AJD71274			
158	AJD71275	AJD71275			
159	AJD71276	AJD71276			
160	AJD71277	AJD71277			
161	AJD71278	AJD71278			
162	AJD71281	AJD71281			

	B	C	D	E	F
163	AJD71282	AJD71282			
164	AJD71283	AJD71283			
165	AJD71286	AJD71286			
166	AJD71289	AJD71289			
167	AJD71290	AJD71290			
168	AJD71291	AJD71291			
169	AJD71292	AJD71292			
170	AJD71293	AJD71293			
171	AJD71295	AJD71295			
172	AJD71296	AJD71296			
173	AJD71301	AJD71301			
174	AJD71302	AJD71302			
175	AJD71306	AJD71306			
176	AJD71307	AJD71307			
177	AJD71311	AJD71311			
178	AJD71312	AJD71312			
179	AJD71313	AJD71313			
180	AJD71315	AJD71315			
181	AJD71316	AJD71316			
182	AJD71317	AJD71317			
183	AJD71318	AJD71318			
184	AJD71321	AJD71321			
185	AJD71323	AJD71323			
186	AJD71325	AJD71325			
187	AJD71326	AJD71326			
188	AJD71331	AJD71331			
189	AJD71333	AJD71333			
190	AJD71334	AJD71334			
191	AJD71335	AJD71335			
192	AJD71336	AJD71336			
193	AJD71337	AJD71337			
194	AJD71338	AJD71338			
195	AJD71339	AJD71339			
196	AJD71340	AJD71340			
197	AJD71342	AJD71342			
198	AJD71343	AJD71343			
199	AJD71344	AJD71344			
200	AJD71347	AJD71347			
201	AJD71348	AJD71348			
202	AJD71349	AJD71349			
203	AJD71350	AJD71350			
204	AJD71351	AJD71351			
205	AJD71352	AJD71352			
206	AJD71353	AJD71353			
207	AJD71354	AJD71354			
208	AJD71356	AJD71356			
209	AJD71357	AJD71357			
210	AJD71358	AJD71358			
211	AJD71359	AJD71359			
212	AJD71360	AJD71360	+		
213	AJD71361	AJD71361			
214	AJD71363	AJD71363			
215	AJD71364	AJD71364			
216	AJD71365	AJD71365			

	B	C	D	E	F
217	AJD71366	AJD71366			
218	AJD71367	AJD71367			
219	AJD71371	AJD71371			
220	AJD71378	AJD71378			
221	AJD71382	AJD71382			
222	AJD71384	AJD71384			
223	AJD71389	AJD71389			
224	AJD71390	AJD71390			
225	AJD71395	AJD71395			
226	AJD71397	AJD71397			
227	AJD71398	AJD71398			
228	AJD71403	AJD71403			
229	AJD71404	AJD71404			
230	AJD71405	AJD71405			
231	AJD71407	AJD71407			
232	AJD71408	AJD71408			
233	AJD71409	AJD71409			
234	AJD71410	AJD71410			
235	AJD71411	AJD71411			
236	AJD71412	AJD71412			
237	AJD71413	AJD71413			
238	AJD71419	AJD71419	+	+	+
239	AJD71433	AJD71433			
240	AJD71434	AJD71434	+	+	+
241	AJD71435	AJD71435			
242	AJD71436	AJD71436			
243	AJD71437	AJD71437			
244	AJD71439	AJD71439			
245	AJD71440	AJD71440			
246	AJD71444	AJD71444			
247	AJD71445	AJD71445			
248	AJD71446	AJD71446			
249	AJD71447	AJD71447			
250	AJD71451	AJD71451			
251	AJD71452	AJD71452			
252	AJD71453	AJD71453			
253	AJD71456	AJD71456			
254	AJD71464	AJD71464			
255	AJD71465	AJD71465			
256	AJD71466	AJD71466			
257	AJD71468	AJD71468			
258	AJD71474	AJD71474			
259	AJD71476	AJD71476			
260	AJD71477	AJD71477			
261	AJD71480	AJD71480			
262	AJD71484	AJD71484			+
263	AJD71485	AJD71485			
264	AJD71486	AJD71486			
265	AJD71487	AJD71487			
266	AJD71489	AJD71489	+		
267	AJD71490	AJD71490			
268	AJD71491	AJD71491			
269	AJD71495	AJD71495			
270	AJD71496	AJD71496			

	B	C	D	E	F
271	AJD71497	AJD71497			
272	AJD71498	AJD71498			
273	AJD71499	AJD71499			
274	AJD71503	AJD71503			
275	AJD71505	AJD71505			
276	AJD71506	AJD71506			
277	AJD71510	AJD71510			
278	AJD71512	AJD71512			
279	AJD71513	AJD71513			
280	AJD71514	AJD71514			
281	AJD71521	AJD71521			
282	AJD71522	AJD71522			
283	AJD71540	AJD71540			
284	AJD71546	AJD71546			
285	AJD71549;AJD7151	AJD71549			
286	AJD71550	AJD71550			
287	AJD71557	AJD71557			
288	AJD71559	AJD71559			
289	AJD71561	AJD71561			
290	AJD71571	AJD71571			
291	AJD71572	AJD71572		+	
292	AJD71574	AJD71574			+
293	AJD71575	AJD71575			
294	AJD71578	AJD71578			
295	AJD71586	AJD71586			
296	AJD71587	AJD71587			
297	AJD71591	AJD71591			
298	AJD71592	AJD71592			
299	AJD71594	AJD71594			
300	AJD71595	AJD71595			
301	AJD71596	AJD71596			
302	AJD71598	AJD71598			
303	AJD71599	AJD71599			
304	AJD71600	AJD71600			
305	AJD71601	AJD71601			
306	AJD71602	AJD71602			
307	AJD71603	AJD71603			
308	AJD71604	AJD71604			
309	AJD71606	AJD71606			
310	AJD71607	AJD71607			
311	AJD71609	AJD71609			
312	AJD71611	AJD71611			
313	AJD71614	AJD71614			
314	AJD71619	AJD71619			
315	AJD71620	AJD71620			
316	AJD71621	AJD71621		+	
317	AJD71627	AJD71627			
318	AJD71629	AJD71629			
319	AJD71630	AJD71630			
320	AJD71637	AJD71637			
321	AJD71638	AJD71638			
322	AJD71639	AJD71639			
323	AJD71640	AJD71640			
324	AJD71649	AJD71649			

	B	C	D	E	F
325	AJD71650	AJD71650			
326	AJD71651	AJD71651			
327	AJD71652	AJD71652			
328	AJD71653	AJD71653			
329	AJD71654	AJD71654			
330	AJD71659	AJD71659			
331	AJD71662	AJD71662			
332	AJD71666	AJD71666			
333	AJD71667	AJD71667			
334	AJD71668	AJD71668	+		
335	AJD71669	AJD71669			
336	AJD71670	AJD71670			
337	AJD71671	AJD71671			
338	AJD71672	AJD71672			
339	AJD71673	AJD71673			
340	AJD71676	AJD71676			
341	AJD71677	AJD71677			
342	AJD71678	AJD71678			
343	AJD71679	AJD71679			
344	AJD71680	AJD71680			
345	AJD71681	AJD71681			
346	AJD71682	AJD71682			
347	AJD71683	AJD71683			
348	AJD71684	AJD71684			
349	AJD71685	AJD71685			
350	AJD71686	AJD71686			
351	AJD71688	AJD71688			
352	AJD71689	AJD71689			
353	AJD71692	AJD71692			
354	AJD71693	AJD71693			
355	AJD71695	AJD71695			
356	AJD71696	AJD71696			
357	AJD71698	AJD71698			
358	AJD71699	AJD71699			
359	AJD71700	AJD71700			
360	AJD71701	AJD71701			
361	AJD71702	AJD71702			
362	AJD71704	AJD71704			
363	AJD71705	AJD71705			
364	AJD71706	AJD71706			
365	AJD71707	AJD71707			
366	AJD71708	AJD71708			
367	AJD71709	AJD71709			
368	AJD71711	AJD71711			
369	AJD71712	AJD71712			
370	AJD71713	AJD71713			
371	AJD71714	AJD71714			
372	AJD71715	AJD71715			
373	AJD71716	AJD71716		+	
374	AJD71717	AJD71717			
375	AJD71718	AJD71718			
376	AJD71719	AJD71719			
377	AJD71721	AJD71721			
378	AJD71722	AJD71722			

	B	C	D	E	F
379	AJD71724	AJD71724			
380	AJD71725	AJD71725			
381	AJD71726	AJD71726			
382	AJD71727	AJD71727			
383	AJD71730	AJD71730			
384	AJD71731	AJD71731			
385	AJD71732	AJD71732			
386	AJD71733	AJD71733			
387	AJD71735	AJD71735			
388	AJD71736	AJD71736			
389	AJD71737	AJD71737			
390	AJD71738	AJD71738			
391	AJD71741	AJD71741			
392	AJD71746	AJD71746			
393	AJD71748	AJD71748			
394	AJD71749	AJD71749			
395	AJD71750	AJD71750			
396	AJD71752	AJD71752			
397	AJD71753	AJD71753			
398	AJD71754	AJD71754			
399	AJD71758	AJD71758			
400	AJD71760	AJD71760			
401	AJD71761	AJD71761			
402	AJD71763	AJD71763	+		+
403	AJD71764	AJD71764			
404	AJD71766	AJD71766			
405	AJD71774	AJD71774			
406	AJD71777	AJD71777			
407	AJD71781	AJD71781			
408	AJD71783	AJD71783			
409	AJD71784	AJD71784			
410	AJD71793	AJD71793			
411	AJD71794	AJD71794			
412	AJD71795	AJD71795			
413	AJD71797	AJD71797			
414	AJD71798	AJD71798			
415	AJD71799	AJD71799			
416	AJD71800	AJD71800			
417	AJD71801	AJD71801			
418	AJD71805	AJD71805			
419	AJD71807	AJD71807			
420	AJD71808	AJD71808			
421	AJD71809	AJD71809			
422	AJD71811	AJD71811			
423	AJD71812	AJD71812			
424	AJD71813	AJD71813			
425	AJD71814	AJD71814			
426	AJD71815	AJD71815			
427	AJD71816	AJD71816			
428	AJD71818	AJD71818			
429	AJD71819	AJD71819			
430	AJD71823	AJD71823			
431	AJD71824	AJD71824			
432	AJD71826	AJD71826			

	B	C	D	E	F
433	AJD71828	AJD71828			
434	AJD71829	AJD71829			
435	AJD71830	AJD71830			
436	AJD71831	AJD71831			
437	AJD71832	AJD71832			
438	AJD71833	AJD71833			
439	AJD71834	AJD71834			
440	AJD71835	AJD71835			
441	AJD71837	AJD71837			
442	AJD71838	AJD71838			
443	AJD71839	AJD71839			
444	AJD71843	AJD71843			
445	AJD71844	AJD71844			
446	AJD71845	AJD71845			
447	AJD71846	AJD71846			
448	AJD71847	AJD71847			
449	AJD71848	AJD71848			
450	AJD71849	AJD71849			
451	AJD71850	AJD71850			
452	AJD71851	AJD71851			
453	AJD71852	AJD71852			
454	AJD71867	AJD71867			
455	AJD71890	AJD71890			
456	AJD71917	AJD71917			
457	AJD71933	AJD71933			
458	AJD71938	AJD71938			
459	AJD71941	AJD71941			
460	AJD71942	AJD71942			
461	AJD71943	AJD71943			
462	AJD71944	AJD71944			
463	AJD71947	AJD71947			
464	AJD71950	AJD71950			+
465	AJD71951	AJD71951			
466	AJD71956	AJD71956			
467	AJD71957	AJD71957			
468	AJD71958	AJD71958			
469	AJD71959	AJD71959			
470	AJD71960	AJD71960			
471	AJD71961	AJD71961		+	
472	AJD71962	AJD71962			
473	AJD71963	AJD71963			
474	AJD71964	AJD71964			
475	AJD71969	AJD71969			
476	AJD71970	AJD71970			
477	AJD71972	AJD71972			
478	AJD71973	AJD71973			
479	AJD71974	AJD71974			
480	AJD71975	AJD71975			
481	AJD71976	AJD71976			
482	AJD71977	AJD71977			
483	AJD71978	AJD71978	+		+
484	AJD71979	AJD71979			
485	AJD71980	AJD71980			
486	AJD71981	AJD71981			

	B	C	D	E	F
487	AJD71983	AJD71983			
488	AJD71985	AJD71985			
489	AJD71986	AJD71986			
490	AJD71988	AJD71988			
491	AJD71996	AJD71996			
492	AJD71998	AJD71998			
493	AJD72000	AJD72000			
494	AJD72001	AJD72001			
495	AJD72002	AJD72002			
496	AJD72003	AJD72003			
497	AJD72004	AJD72004			
498	AJD72005	AJD72005			
499	AJD72006	AJD72006			
500	AJD72007	AJD72007			
501	AJD72009	AJD72009			
502	AJD72012	AJD72012			
503	AJD72013	AJD72013			
504	AJD72016	AJD72016			
505	AJD72017	AJD72017			
506	AJD72018	AJD72018			
507	AJD72019	AJD72019			
508	AJD72020	AJD72020			
509	AJD72021	AJD72021			
510	AJD72022	AJD72022			
511	AJD72023	AJD72023			
512	AJD72026	AJD72026			
513	AJD72027	AJD72027			
514	AJD72028	AJD72028			
515	AJD72033	AJD72033			
516	AJD72034	AJD72034			
517	AJD72037	AJD72037			
518	AJD72038	AJD72038			
519	AJD72039	AJD72039			
520	AJD72040	AJD72040			
521	AJD72042	AJD72042			
522	AJD72043	AJD72043			
523	AJD72044	AJD72044			
524	AJD72045	AJD72045			
525	AJD72047	AJD72047			
526	AJD72051	AJD72051			
527	AJD72052	AJD72052			
528	AJD72053	AJD72053			
529	AJD72054	AJD72054			
530	AJD72055	AJD72055			
531	AJD72061	AJD72061			
532	AJD72062	AJD72062			
533	AJD72063	AJD72063			
534	AJD72064	AJD72064			
535	AJD72065	AJD72065			
536	AJD72066	AJD72066			
537	AJD72074	AJD72074			
538	AJD72075	AJD72075			
539	AJD72076	AJD72076			
540	AJD72077	AJD72077			

	B	C	D	E	F
541	AJD72078	AJD72078			
542	AJD72079	AJD72079			
543	AJD72082	AJD72082			
544	AJD72083	AJD72083			
545	AJD72084	AJD72084			
546	AJD72086	AJD72086			
547	AJD72093	AJD72093			
548	AJD72095	AJD72095			
549	AJD72096	AJD72096			
550	AJD72099	AJD72099			
551	AJD72101	AJD72101			
552	AJD72102	AJD72102			
553	AJD72104	AJD72104			
554	AJD72105	AJD72105			
555	AJD72113	AJD72113			
556	AJD72114	AJD72114			
557	AJD72115	AJD72115			
558	AJD72117	AJD72117			
559	AJD72118	AJD72118			
560	AJD72119	AJD72119			
561	AJD72120	AJD72120			
562	AJD72121	AJD72121			
563	AJD72122	AJD72122			
564	AJD72123	AJD72123			
565	AJD72124	AJD72124			
566	AJD72125	AJD72125			
567	AJD72127	AJD72127			
568	AJD72131	AJD72131			
569	AJD72134	AJD72134			
570	AJD72135	AJD72135			
571	AJD72136	AJD72136			
572	AJD72137	AJD72137			
573	AJD72138	AJD72138			
574	AJD72140	AJD72140			
575	AJD72141	AJD72141			
576	AJD72143	AJD72143			
577	AJD72146	AJD72146			
578	AJD72147	AJD72147			
579	AJD72149	AJD72149			
580	AJD72151	AJD72151			
581	AJD72152	AJD72152			
582	AJD72155	AJD72155			
583	AJD72156	AJD72156			
584	AJD72157	AJD72157			
585	AJD72158	AJD72158			
586	AJD72159	AJD72159			
587	AJD72161	AJD72161			
588	AJD72163	AJD72163			
589	AJD72166	AJD72166			
590	AJD72178	AJD72178			
591	AJD72179	AJD72179			
592	AJD72180	AJD72180			
593	AJD72181	AJD72181			
594	AJD72182	AJD72182			

	B	C	D	E	F
595	AJD72183	AJD72183			
596	AJD72184	AJD72184			
597	AJD72185	AJD72185			
598	AJD72186	AJD72186			
599	AJD72187	AJD72187			
600	AJD72189	AJD72189			
601	AJD72190	AJD72190			
602	AJD72191	AJD72191			
603	AJD72192	AJD72192			
604	AJD72195	AJD72195			
605	AJD72197	AJD72197			
606	AJD72205	AJD72205			
607	AJD72206	AJD72206			
608	AJD72213	AJD72213			
609	AJD72214	AJD72214			
610	AJD72215	AJD72215			
611	AJD72216	AJD72216			
612	AJD72217	AJD72217			
613	AJD72218	AJD72218			
614	AJD72219	AJD72219			
615	AJD72220	AJD72220			
616	AJD72222	AJD72222			
617	AJD72226	AJD72226			
618	AJD72227	AJD72227			
619	AJD72228	AJD72228			
620	AJD72230	AJD72230			
621	AJD72231	AJD72231			
622	AJD72232	AJD72232			
623	AJD72236	AJD72236			
624	AJD72237	AJD72237			
625	AJD72238	AJD72238			
626	AJD72240	AJD72240			
627	AJD72241	AJD72241			
628	AJD72242	AJD72242			
629	AJD72243	AJD72243	+		
630	AJD72245	AJD72245			
631	AJD72246	AJD72246			
632	AJD72247	AJD72247			
633	AJD72248	AJD72248			
634	AJD72251	AJD72251			
635	AJD72252	AJD72252			
636	AJD72254	AJD72254			
637	AJD72262	AJD72262			
638	AJD72278	AJD72278			
639	AJD72295	AJD72295			
640	AJD72296	AJD72296			
641	AJD72297	AJD72297			
642	AJD72298	AJD72298			
643	AJD72299	AJD72299			
644	AJD72300	AJD72300			
645	AJD72301	AJD72301			
646	AJD72302	AJD72302			
647	AJD72306	AJD72306			
648	AJD72307	AJD72307			

	B	C	D	E	F
649	AJD72308	AJD72308			
650	AJD72310	AJD72310			
651	AJD72312	AJD72312			
652	AJD72316	AJD72316			
653	AJD72317	AJD72317			
654	AJD72319	AJD72319			
655	AJD72322	AJD72322			
656	AJD72323	AJD72323			
657	AJD72325	AJD72325			
658	AJD72326	AJD72326			
659	AJD72327	AJD72327			
660	AJD72328	AJD72328			
661	AJD72331	AJD72331			
662	AJD72336	AJD72336			
663	AJD72337	AJD72337			
664	AJD72339	AJD72339			
665	AJD72343	AJD72343			
666	AJD72344	AJD72344			
667	AJD72345	AJD72345			
668	AJD72347	AJD72347			
669	AJD72348	AJD72348			
670	AJD72356	AJD72356			
671	AJD72363	AJD72363			
672	AJD72364	AJD72364			
673	AJD72365	AJD72365			
674	AJD72367	AJD72367			
675	AJD72368	AJD72368			
676	AJD72369	AJD72369			
677	AJD72370	AJD72370			
678	AJD72372	AJD72372			
679	AJD72373	AJD72373			
680	AJD72374	AJD72374			
681	AJD72375	AJD72375			
682	AJD72376	AJD72376			
683	AJD72377	AJD72377			
684	AJD72379	AJD72379			
685	AJD72380	AJD72380			
686	AJD72384	AJD72384			
687	AJD72385	AJD72385			
688	AJD72386	AJD72386			
689	AJD72387	AJD72387			
690	AJD72388	AJD72388			
691	AJD72390	AJD72390			
692	AJD72391	AJD72391			
693	AJD72394	AJD72394			
694	AJD72397;AJD7195	AJD72397;AJD71954;AJD71871;AJD71285;AJD73183;AJD71901;AJD71192			
695	AJD72400	AJD72400			
696	AJD72401	AJD72401			
697	AJD72402	AJD72402			
698	AJD72403	AJD72403			
699	AJD72404	AJD72404			
700	AJD72405	AJD72405			
701	AJD72408	AJD72408			
702	AJD72410	AJD72410			

	B	C	D	E	F
703	AJD72414	AJD72414			
704	AJD72416	AJD72416			
705	AJD72417	AJD72417			
706	AJD72419	AJD72419			
707	AJD72420	AJD72420			
708	AJD72422	AJD72422			
709	AJD72423	AJD72423			
710	AJD72426	AJD72426			
711	AJD72430	AJD72430			
712	AJD72432	AJD72432			
713	AJD72439	AJD72439			
714	AJD72440	AJD72440			
715	AJD72441	AJD72441			
716	AJD72442	AJD72442			
717	AJD72443	AJD72443			
718	AJD72444	AJD72444			
719	AJD72446	AJD72446			
720	AJD72447	AJD72447			
721	AJD72448	AJD72448			
722	AJD72449	AJD72449			
723	AJD72450	AJD72450			
724	AJD72451	AJD72451			
725	AJD72452	AJD72452			
726	AJD72453	AJD72453			
727	AJD72457	AJD72457			
728	AJD72458	AJD72458			
729	AJD72460	AJD72460			
730	AJD72461	AJD72461			
731	AJD72462	AJD72462			
732	AJD72463	AJD72463			
733	AJD72465	AJD72465			
734	AJD72468	AJD72468			
735	AJD72469	AJD72469			
736	AJD72470	AJD72470			
737	AJD72471	AJD72471			
738	AJD72474	AJD72474			
739	AJD72477	AJD72477			
740	AJD72478	AJD72478			
741	AJD72482	AJD72482			
742	AJD72483	AJD72483			
743	AJD72484	AJD72484			
744	AJD72485	AJD72485			
745	AJD72487	AJD72487			
746	AJD72488	AJD72488			
747	AJD72489	AJD72489			
748	AJD72490	AJD72490			
749	AJD72492	AJD72492			
750	AJD72495	AJD72495			
751	AJD72496	AJD72496			
752	AJD72497	AJD72497			
753	AJD72498	AJD72498			
754	AJD72499	AJD72499			
755	AJD72500	AJD72500			
756	AJD72501	AJD72501			

	B	C	D	E	F
757	AJD72502	AJD72502			
758	AJD72503	AJD72503			
759	AJD72504	AJD72504			
760	AJD72505	AJD72505			
761	AJD72512	AJD72512	+		
762	AJD72536	AJD72536	+		
763	AJD72540	AJD72540			
764	AJD72542	AJD72542			
765	AJD72555	AJD72555			
766	AJD72558	AJD72558			
767	AJD72566	AJD72566			
768	AJD72567	AJD72567			
769	AJD72569	AJD72569			
770	AJD72570	AJD72570			
771	AJD72572	AJD72572			
772	AJD72573	AJD72573			
773	AJD72575	AJD72575			
774	AJD72576	AJD72576			
775	AJD72577	AJD72577			
776	AJD72578	AJD72578			
777	AJD72579	AJD72579			
778	AJD72581	AJD72581			
779	AJD72582	AJD72582			
780	AJD72583	AJD72583			
781	AJD72584	AJD72584			
782	AJD72585	AJD72585			
783	AJD72587	AJD72587			
784	AJD72588	AJD72588			
785	AJD72589	AJD72589			
786	AJD72590	AJD72590			
787	AJD72591	AJD72591			
788	AJD72592	AJD72592			
789	AJD72594	AJD72594			
790	AJD72595	AJD72595			
791	AJD72596	AJD72596			
792	AJD72597	AJD72597			
793	AJD72601	AJD72601			
794	AJD72605	AJD72605			
795	AJD72606	AJD72606			
796	AJD72607	AJD72607			
797	AJD72608	AJD72608			+
798	AJD72610	AJD72610			
799	AJD72612	AJD72612			
800	AJD72613	AJD72613			
801	AJD72614	AJD72614			
802	AJD72615	AJD72615			
803	AJD72617	AJD72617			
804	AJD72622	AJD72622			
805	AJD72624	AJD72624			
806	AJD72627	AJD72627			
807	AJD72628	AJD72628			
808	AJD72629	AJD72629			
809	AJD72633	AJD72633			
810	AJD72634	AJD72634			

	B	C	D	E	F
811	AJD72635	AJD72635			
812	AJD72636	AJD72636			
813	AJD72639	AJD72639			
814	AJD72640	AJD72640			
815	AJD72642	AJD72642			
816	AJD72648	AJD72648			
817	AJD72649	AJD72649			
818	AJD72650	AJD72650			
819	AJD72651	AJD72651			
820	AJD72654	AJD72654			
821	AJD72655	AJD72655			
822	AJD72656	AJD72656			
823	AJD72657	AJD72657			
824	AJD72660	AJD72660			
825	AJD72661	AJD72661			
826	AJD72662	AJD72662			
827	AJD72663	AJD72663			
828	AJD72664	AJD72664			
829	AJD72665	AJD72665			
830	AJD72666	AJD72666			
831	AJD72671	AJD72671			
832	AJD72674	AJD72674			
833	AJD72675	AJD72675	+	+	+
834	AJD72679	AJD72679			
835	AJD72682	AJD72682			
836	AJD72683	AJD72683			
837	AJD72688	AJD72688			
838	AJD72689	AJD72689			
839	AJD72690	AJD72690			
840	AJD72691	AJD72691			
841	AJD72692	AJD72692			
842	AJD72693	AJD72693			
843	AJD72700	AJD72700			
844	AJD72701	AJD72701			
845	AJD72702	AJD72702			
846	AJD72703	AJD72703			
847	AJD72704	AJD72704			
848	AJD72705	AJD72705			
849	AJD72706	AJD72706			
850	AJD72708	AJD72708			
851	AJD72709	AJD72709			
852	AJD72712	AJD72712			
853	AJD72713	AJD72713			
854	AJD72714	AJD72714			
855	AJD72716	AJD72716			
856	AJD72717	AJD72717			
857	AJD72722	AJD72722			
858	AJD72723	AJD72723			
859	AJD72724	AJD72724			
860	AJD72725	AJD72725			
861	AJD72726	AJD72726			
862	AJD72727	AJD72727			
863	AJD72728	AJD72728			
864	AJD72729	AJD72729			

	B	C	D	E	F
865	AJD72730	AJD72730			
866	AJD72731	AJD72731			
867	AJD72733	AJD72733			+
868	AJD72734	AJD72734			
869	AJD72736	AJD72736			
870	AJD72738	AJD72738			
871	AJD72739	AJD72739			
872	AJD72740	AJD72740			
873	AJD72741	AJD72741			
874	AJD72742	AJD72742			
875	AJD72743	AJD72743			
876	AJD72746	AJD72746			
877	AJD72749	AJD72749	+		
878	AJD72750	AJD72750			
879	AJD72751	AJD72751			
880	AJD72756	AJD72756			
881	AJD72761	AJD72761			
882	AJD72762	AJD72762			
883	AJD72763	AJD72763			
884	AJD72764	AJD72764			
885	AJD72765	AJD72765	+		+
886	AJD72766	AJD72766			
887	AJD72771	AJD72771			
888	AJD72775	AJD72775			
889	AJD72777	AJD72777			
890	AJD72778	AJD72778			
891	AJD72784	AJD72784			
892	AJD72785	AJD72785			
893	AJD72786	AJD72786			
894	AJD72787	AJD72787			
895	AJD72788	AJD72788			
896	AJD72789	AJD72789			
897	AJD72790	AJD72790			
898	AJD72792	AJD72792			
899	AJD72796	AJD72796			
900	AJD72797	AJD72797			
901	AJD72798	AJD72798			
902	AJD72800	AJD72800			
903	AJD72802	AJD72802			
904	AJD72803	AJD72803			
905	AJD72808	AJD72808			
906	AJD72813	AJD72813			
907	AJD72815	AJD72815			
908	AJD72825	AJD72825			
909	AJD72826	AJD72826			
910	AJD72827	AJD72827			
911	AJD72828	AJD72828			
912	AJD72829	AJD72829			
913	AJD72830	AJD72830			
914	AJD72835	AJD72835			
915	AJD72837	AJD72837			
916	AJD72838	AJD72838			
917	AJD72841	AJD72841			
918	AJD72842	AJD72842			

	B	C	D	E	F
919	AJD72843	AJD72843			
920	AJD72844	AJD72844			
921	AJD72845	AJD72845			
922	AJD72850	AJD72850			
923	AJD72851	AJD72851			
924	AJD72852	AJD72852			
925	AJD72853	AJD72853			
926	AJD72854	AJD72854			
927	AJD72862	AJD72862			
928	AJD72866	AJD72866			
929	AJD72871	AJD72871			
930	AJD72872	AJD72872			
931	AJD72874	AJD72874			
932	AJD72875	AJD72875			
933	AJD72876	AJD72876			
934	AJD72879	AJD72879			
935	AJD72887	AJD72887			
936	AJD72888	AJD72888			
937	AJD72889;AJD7152	AJD72889;AJD71520			
938	AJD72890;AJD7151	AJD72890;AJD71519			
939	AJD72891;AJD7151	AJD72891;AJD71518			
940	AJD72901;AJD725	AJD72901			
941	AJD72904	AJD72904			
942	AJD72905	AJD72905			
943	AJD72906	AJD72906			
944	AJD72907	AJD72907			
945	AJD72908	AJD72908			
946	AJD72912	AJD72912			
947	AJD72913	AJD72913			
948	AJD72914	AJD72914			
949	AJD72916	AJD72916			
950	AJD72917	AJD72917			
951	AJD72919	AJD72919			
952	AJD72920	AJD72920			
953	AJD72921	AJD72921	+		+
954	AJD72922	AJD72922			
955	AJD72923	AJD72923			
956	AJD72924	AJD72924			
957	AJD72925	AJD72925			
958	AJD72927	AJD72927			
959	AJD72928	AJD72928			
960	AJD72929	AJD72929			
961	AJD72930	AJD72930			
962	AJD72931	AJD72931			
963	AJD72932	AJD72932			
964	AJD72933	AJD72933			
965	AJD72934	AJD72934			
966	AJD72935	AJD72935			
967	AJD72936	AJD72936			
968	AJD72938	AJD72938			
969	AJD72939	AJD72939			
970	AJD72940	AJD72940			
971	AJD72942	AJD72942			
972	AJD72945	AJD72945			

	B	C	D	E	F
973	AJD72947	AJD72947			
974	AJD72948	AJD72948			
975	AJD72950	AJD72950			
976	AJD72951	AJD72951			
977	AJD72958	AJD72958			
978	AJD72959	AJD72959			
979	AJD72960	AJD72960			
980	AJD72962	AJD72962			
981	AJD72965;AJD7242[illegible]	AJD72965;AJD72425;AJD72103			
982	AJD72969	AJD72969	+		+
983	AJD72973	AJD72973			
984	AJD72974	AJD72974			
985	AJD72975	AJD72975			
986	AJD72976	AJD72976			
987	AJD72977	AJD72977			
988	AJD72978	AJD72978			
989	AJD72979	AJD72979			
990	AJD72989	AJD72989			
991	AJD72990	AJD72990	+	+	
992	AJD72992	AJD72992			
993	AJD72993	AJD72993			
994	AJD73003	AJD73003			
995	AJD73005	AJD73005			
996	AJD73006	AJD73006			
997	AJD73007	AJD73007			
998	AJD73011	AJD73011			
999	AJD73012	AJD73012			
1000	AJD73013	AJD73013			
1001	AJD73015	AJD73015			
1002	AJD73016	AJD73016			
1003	AJD73017	AJD73017			
1004	AJD73018	AJD73018			
1005	AJD73020	AJD73020			
1006	AJD73022	AJD73022			
1007	AJD73023	AJD73023			
1008	AJD73032	AJD73032			
1009	AJD73033	AJD73033			
1010	AJD73034	AJD73034			
1011	AJD73036;AJD728[illegible]	AJD73036			
1012	AJD73038	AJD73038			
1013	AJD73039	AJD73039			
1014	AJD73040	AJD73040			
1015	AJD73041	AJD73041			
1016	AJD73045	AJD73045			+
1017	AJD73053	AJD73053			
1018	AJD73054	AJD73054			
1019	AJD73056	AJD73056			
1020	AJD73057	AJD73057			
1021	AJD73058	AJD73058			
1022	AJD73060	AJD73060			
1023	AJD73061	AJD73061			
1024	AJD73062	AJD73062			
1025	AJD73063	AJD73063			
1026	AJD73064	AJD73064	+		

	B	C	D	E	F
1027	AJD73068	AJD73068			
1028	AJD73069	AJD73069			
1029	AJD73070	AJD73070			
1030	AJD73074	AJD73074			
1031	AJD73076	AJD73076			
1032	AJD73081	AJD73081			
1033	AJD73082	AJD73082			
1034	AJD73083	AJD73083			
1035	AJD73085;AJD7138	AJD73085;AJD7138	+	+	
1036	AJD73086	AJD73086			
1037	AJD73090	AJD73090			
1038	AJD73091	AJD73091			
1039	AJD73095	AJD73095			
1040	AJD73096	AJD73096			
1041	AJD73097	AJD73097			
1042	AJD73098	AJD73098			
1043	AJD73099	AJD73099			
1044	AJD73100	AJD73100			
1045	AJD73101	AJD73101			
1046	AJD73102	AJD73102			
1047	AJD73103	AJD73103			
1048	AJD73120	AJD73120			
1049	AJD73121	AJD73121			
1050	AJD73122	AJD73122			
1051	AJD73124	AJD73124			
1052	AJD73126	AJD73126			
1053	AJD73127	AJD73127			
1054	AJD73128	AJD73128			
1055	AJD73129	AJD73129			
1056	AJD73131	AJD73131			
1057	AJD73136	AJD73136			
1058	AJD73137	AJD73137			
1059	AJD73140	AJD73140			
1060	AJD73141	AJD73141			
1061	AJD73142	AJD73142			
1062	AJD73144	AJD73144			
1063	AJD73145	AJD73145			
1064	AJD73146	AJD73146			
1065	AJD73148	AJD73148			
1066	AJD73150	AJD73150			
1067	AJD73152	AJD73152			
1068	AJD73153	AJD73153			
1069	AJD73154	AJD73154			
1070	AJD73155	AJD73155			
1071	AJD73156	AJD73156			
1072	AJD73159	AJD73159			
1073	AJD73160	AJD73160			
1074	AJD73164	AJD73164			
1075	AJD73165	AJD73165			
1076	AJD73166	AJD73166			
1077	AJD73167;AJD7306	AJD73167;AJD73067;AJD73031;AJD72915;AJD72878;AJD72807;AJD72438;AJD72431;AJD72351;AJD72253;AJD72234;AJD72200;AJD72160;AJD71999;AJD71971;AJD71967			
1078	AJD73169	AJD73169			
1079	AJD73171	AJD73171			
1080	AJD73172	AJD73172			

	B	C	D	E	F
1081	AJD73174	AJD73174			
1082	AJD73175	AJD73175			
1083	AJD73176	AJD73176			
1084	AJD73177	AJD73177			
1085	AJD73179	AJD73179			
1086	AJD73180	AJD73180			
1087	AJD73181	AJD73181			
1088	AJD73182	AJD73182			
1089	AJD73188	AJD73188			
1090	AJD73189	AJD73189			+
1091	AJD73192	AJD73192			

	G	H	I	J
1	Student's T-test Significant ORF2_PEP_WT_PEP	-Log Student's T-test p-value ORF2_ORF2_PEP	Student's T-test q-value ORF2_ORF2_PEP	Student's T-test Difference ORF2_ORF2_PEP
2		0.281799571	0.852426332	-0.164819929
3		0.343673489	0.811783582	0.21658304
4		0.200697074	0.883424731	-0.124721103
5		0.739313158	0.580355556	-0.503960927
6		0.042721981	0.965476	0.034286075
7		0.602357951	0.828446667	-0.152460734
8		0.62890558	0.549830065	0.640856637
9		2.865733389	0.058782609	-1.135011249
10		0.393783528	0.874472419	-0.116626316
11		0.176330813	0.873706522	-0.141357422
12		0.632509354	0.737971347	-0.293450885
13		0.73984953	0.488188976	0.68206003
14		1.060723498	0.716962025	-0.277581957
15		0.686521212	0.589309091	0.458198759
16		1.085850532	0.553084337	-0.455668343
17		0.014005817	0.982467681	0.018619325
18		0.774325471	0.675426573	-0.335711585
19		0.160577677	0.959561129	0.035469479
20		0.366704413	0.851633491	-0.152427249
21		0.85893902	0.55477707	-0.517262353
22		0.430752293	0.855746522	-0.139588038
23		0.062343407	0.958901879	-0.046464284
24		0.099727031	0.917063232	0.097561942
25		0.248793684	0.814531646	0.246038225
26		0.041409693	0.962935252	-0.049879074
27		0.038467399	0.973519288	0.02795389
28		0.19077088	0.876758621	-0.155464172
29		0.269476897	0.874294985	-0.141511917
30		0.116203958	0.942325167	0.061646779
31		1.707633589	0.595297297	0.338473002
32		1.206335052	0.242466667	0.945810954
33		0.220513651	0.873655367	-0.139719433
34		0.359569287	0.808369231	-0.264699512
35		2.158771106	0.674725424	0.264848073
36		0.141405555	0.937789709	0.060363558
37		0.08020645	0.960215417	0.045216242
38		0.335009408	0.742802083	-0.376136356
39		0.581926203	0.617933884	-0.455049091
40		0.272198754	0.87279564	-0.118999057
41		0.536862031	0.811556503	-0.216381285
42		0.185346638	0.890927939	-0.105736627
43		0.049803283	0.956862661	-0.071735806
44	+	0.901222108	0.424035714	0.685922199
45		0.70723991	0.587411168	0.485100004
46		0.03356277	0.981852797	-0.023134232
47		0.269430428	0.812835073	-0.294851727
48		1.117004553	0.810317627	0.150946935
49		0.496125395	0.673090909	-0.427770191
50		2.049567583	0.582055249	0.362700992
51		0.579029762	0.813149826	-0.166892793
52		0.124409027	0.905541463	0.107527839
53		2.896343057	0.198526316	-0.690420363
54		0.298850391	0.873582538	0.11591

	G	H	I	J
55	+	0.181351954	0.869348252	-0.152619044
56		2.054014822	0.147833333	-0.887737062
57		2.053890174	0.132255319	-0.922000249
58		0.46954883	0.652469697	0.471257104
59		0.148807804	0.955423693	-0.042274687
60		0.171148693	0.905508516	-0.091611014
61		0.097069214	0.905400245	0.131574419
62		0.424575157	0.809992958	-0.184708913
63		0.203727949	0.868730823	-0.142613305
64		0.142333048	0.916538012	-0.080993864
65		0.195016216	0.850462992	-0.205085542
66		0.088558738	0.94829548	-0.062998454
67		7.621646143	0	-3.351281484
68		5.823568498	0.001454545	-1.793219037
69		0.324830086	0.814584838	-0.210611979
70		0.214177818	0.814021277	0.267092599
71		0.03222651	0.984512621	-0.018396589
72		0.008729792	0.989672591	-0.009584851
73		0.378559999	0.748164948	0.337640974
74		0.582266201	0.583832461	-0.56251653
75		0.823172397	0.712993631	-0.298729791
76		0.508149352	0.74884878	-0.274284999
77		0.318833004	0.810694737	-0.205067741
78		0.059794794	0.96600993	0.027256436
79		0.48453539	0.810683012	-0.176211463
80		0.122308761	0.884010624	-0.164464739
81		0.248754087	0.825400335	-0.209891637
82		1.38156961	0.5224	-0.453170141
83		0.411629857	0.554961039	-1.057796054
84		0.084200892	0.933380571	-0.087954415
85		0.391921472	0.811090559	-0.214479234
86		0.056143335	0.957071355	-0.046006944
87		1.271189607	0.429148148	-0.582251867
88		0.626424382	0.579840796	-0.51859559
89		0.773130193	0.72510559	-0.295542187
90		2.258604875	0.36537931	-0.551652696
91		1.042775311	0.417333333	-0.653556612
92		1.337259886	0.487100775	-0.503236135
93		1.013187956	0.400884211	-0.708009508
94		1.051958119	0.406524272	-0.665395313
95		0.842893279	0.529362319	-0.574763404
96		1.456049945	0.745020202	-0.219559987
97		2.325586233	0.349297297	-0.585391151
98		1.891629402	0.424707965	-0.502937741
99		0.492851785	0.783239437	-0.255727132
100		0.212714729	0.915612602	-0.071271049
101		0.485195287	0.702045016	-0.379251268
102		0.846124402	0.584290155	-0.451599121
103		0.27643549	0.854134969	-0.158793132
104		0.413135273	0.808362031	-0.248677572
105		1.058277829	0.602556522	-0.3705616
106		2.145330165	0.107294118	-1.043825785
107		0.630877179	0.813310345	-0.183749729
108		0.198282488	0.87442836	-0.16371727

	G	H	I	J
109		0.697888886	0.808321569	-0.185774909
110		0.546638149	0.814642468	-0.177607854
111		0.177800071	0.884346912	-0.123417748
112		1.409974123	0.261206349	-0.821835624
113		1.996715438	0.19154717	-0.807563146
114		0.709465024	0.753024155	-0.242238363
115		0.649792306	0.74642487	-0.26568879
116		0.084241476	0.954937634	0.05258645
117		1.467687687	0.734298592	-0.238621182
118		0.312690789	0.878666667	-0.125253042
119		0.220914333	0.869487603	0.132852342
120		0.002212321	0.996195572	-0.003178914
121		0.079698898	0.942393771	-0.073431015
122		0.053618759	0.957733051	0.05892902
123		0.867696407	0.464295082	-0.6445357
124		0.061965773	0.958866667	0.04586135
125		1.050041799	0.576502415	-0.403087192
126		0.244425437	0.81326087	-0.250575384
127		0.614694676	0.677865724	-0.37030877
128		1.36047936	0.7215625	-0.261043125
129		0.826626593	0.554871795	-0.529038747
130		0.179764873	0.875572065	-0.176110374
131		0.582158809	0.736634561	-0.300497055
132		0.042525608	0.974299803	0.025303523
133		0.104667383	0.919309551	-0.086167865
134		0.226594284	0.875667145	-0.14254464
135		0.853628969	0.423243243	-0.714272605
136		0.484612617	0.74715736	-0.291125615
137		0.411744073	0.804586139	-0.219913695
138		0.606883979	0.731654596	-0.293313344
139		0.441519758	0.804741935	-0.218800651
140		0.580506362	0.827734694	-0.157946269
141		0.045710689	0.982023483	0.019579146
142		0.077249572	0.935166478	0.087772581
143		0.085445305	0.961611452	-0.033918169
144		0.071796161	0.958673913	-0.059697045
145		0.049652201	0.964581745	-0.033287472
146		0.049828258	0.955676724	0.075642268
147		1.34995221	0.195854545	-0.974647098
148		0.947881292	0.459152542	-0.625685798
149		0.337799784	0.826161074	-0.181368722
150		0.227749791	0.87744868	-0.148731232
151		0.926918457	0.652332075	0.33942922
152		0.771802837	0.523651007	-0.582787196
153		0.007542702	0.993104089	-0.005331039
154		0.619109317	0.593990783	-0.487639109
155		8.81559378	0	-2.598408805
156		0.069066138	0.966918699	-0.03265614
157		2.049166776	0.17736	-0.831705729
158		0.656052869	0.579597765	-0.543507894
159		0.010298494	0.989820225	0.008958181
160		0.363031973	0.921909308	0.068232642
161		2.125760552	0.48768254	0.446804047
162		0.156929601	0.870218534	-0.16190105

	G	H	I	J
163		0.466390198	0.843830065	-0.152652317
164		1.029224322	0.809459916	0.186233309
165		0.202842089	0.9167277	-0.073289871
166		0.012326646	0.987166197	-0.010508431
167		0.153833684	0.88485298	-0.136523777
168		0.384405923	0.873561119	-0.127000597
169		0.217661471	0.866766917	-0.165323893
170		1.93212802	0.110736842	-1.070653068
171		0.954403001	0.718571429	0.272989697
172		0.149474267	0.886676963	0.125899421
173		2.33242992	0.115428571	-0.935549842
174		0.082154442	0.939139353	-0.074591107
175		0.534285276	0.673718519	0.414290958
176		0.723650964	0.791516279	-0.222882377
177		0.728767875	0.748274809	-0.25236045
178		0.05598263	0.956250811	-0.069514381
179		0.00532087	0.992465549	0.008582221
180		1.051458357	0.749866667	-0.226596408
181		0.631365816	0.620497992	-0.425085704
182		0.658847532	0.750553398	-0.249099943
183		0.290146569	0.811795501	-0.271446228
184		0.187386579	0.907047388	-0.087861591
185		0.493568965	0.731069519	-0.309107463
186		0.928980128	0.617383966	0.374213749
187		1.975649063	0.673653137	0.280426025
188		0.212397847	0.868324022	0.140475591
189		0.747397026	0.676544218	-0.333570904
190		0.004479051	0.994857143	0.005185021
191		0.602406623	0.876228404	0.11486117
192		0.799173878	0.733217877	-0.268834432
193		0.014446655	0.990286785	0.007817586
194		0.502699327	0.740417582	-0.310374366
195		0.502439861	0.747845758	-0.289759954
196		0.298784515	0.811146444	0.275620566
197		0.65912758	0.644275862	-0.392989477
198		0.482150251	0.723987952	-0.35090616
199		0.76181805	0.525260274	-0.592713038
200		1.674264115	0.2005	-0.85053889
201		0.973219255	0.722630303	-0.274006314
202		0.457856462	0.873847988	-0.124749502
203		0.585976735	0.789044289	-0.237410227
204		0.514750254	0.80996063	-0.200760947
205		0.193908537	0.891346008	-0.104449378
206		0.202725876	0.89982801	-0.091194577
207		1.671903674	0.343684211	-0.648803499
208		0.776228347	0.517757576	-0.625673082
209		0.715814982	0.604684211	-0.431153827
210		0.784533547	0.745979899	-0.245559057
211		0.352079364	0.874285714	-0.112604353
212		2.580955485	0.012	-1.626541138
213		0.074872192	0.955309168	-0.05201043
214		0.425859385	0.808752643	-0.233386781
215		0.563878499	0.811266667	-0.208055284
216		0.446466523	0.808848723	-0.209992727

	G	H	I	J
217		0.005004953	0.996507407	-0.003193961
218		0.383307168	0.879165714	0.118404388
219		0.355781915	0.813678639	-0.2163709
220		0.370324954	0.552506329	1.30225669
221		0.833234077	0.402391753	-0.800056458
222		0.560640533	0.744519481	-0.28089269
223		0.18833101	0.921542112	0.076216168
224		1.183644794	0.52441791	-0.501611074
225		0.30265213	0.855046154	-0.153940201
226		0.151568915	0.915090909	0.078497993
227		0.813078735	0.590142077	-0.471192254
228		0.438902844	0.6756337	-0.468377007
229		0.234396695	0.85431677	-0.175630993
230		0.60194807	0.793689095	0.233137767
231		0.09620433	0.941882353	0.065602832
232		1.196462655	0.639206349	-0.329702589
233		5.375621269	0.583678392	-0.307150523
234		0.108892831	0.956085745	0.046630859
235		0.077626237	0.958578616	-0.043853124
236		0.310950225	0.934210046	-0.05709415
237		0.34575381	0.796831409	-0.293157154
238		19.27311386	0	-20.69751718
239		0.590140782	0.683934426	-0.354914559
240		25.67712702	0	-22.38170666
241		0.283472235	0.886007742	0.099274105
242		1.205143805	0.557918129	-0.4320524
243		0.716044435	0.783400468	-0.2270298
244		0.070882537	0.960976842	0.046176063
245		0.568030957	0.848174757	-0.143104977
246		0.838468782	0.618103586	0.374581019
247		0.190748658	0.877014245	-0.151919259
248		0.354364824	0.890602287	-0.089814928
249		1.637335817	0.489384615	-0.470364677
250		0.095195734	0.939503386	-0.07329411
251		0.889848145	0.550780488	-0.498496797
252		0.847370029	0.583528302	-0.427316242
253		0.471976593	0.768765957	-0.267273585
254		0.039997136	0.967168357	0.041510052
255		0.239637907	0.816443649	0.246938917
256		0.430750707	0.735040431	-0.332431793
257		0.33518992	0.813380531	-0.202120251
258		0.546407406	0.810290976	-0.182194392
259		2.722628606	0.080592593	-1.061590619
260		0.340740151	0.809395712	-0.232394748
261		0.070063651	0.960239669	0.039907667
262	+	0.063470988	0.918112941	0.162766774
263		0.852501364	0.548533333	-0.508199056
264		0.458961532	0.812038241	-0.20019065
265		0.014809909	0.987496241	0.009818395
266	+	18.80575571	0	20.3950225
267		0.251112589	0.886176166	-0.103588952
268		0.324962513	0.809181651	-0.21798261
269		0.140189215	0.882536842	0.143306732
270		0.696222154	0.691607843	-0.327998903

	G	H	I	J
271		0.077451673	0.958559304	-0.057759179
272		0.052148227	0.966902834	-0.035025703
273		0.495002966	0.813158879	-0.189772924
274		0.212085569	0.88470801	-0.109138489
275		0.466613808	0.802872727	-0.246166017
276		0.536603446	0.813971134	-0.207869212
277		0.645790644	0.747365729	-0.262922075
278		0.157996477	0.874522572	-0.153764513
279		0.630936535	0.864712991	-0.122866948
280		0.883910022	0.554494118	-0.489203559
281		0.21691951	0.852992248	-0.183422301
282		0.133144269	0.957737811	-0.046678543
283		0.282975943	0.816233766	-0.298060311
284	+	0.287067764	0.851324921	0.1658535
285		0.040379197	0.973077228	-0.027849197
286		0.117538167	0.953897268	0.051788966
287		1.293102221	0.591428571	-0.359551112
288		0.139648082	0.876167568	-0.160957972
289		0.208332435	0.808528455	0.365181181
290	+	0.652478625	0.836281967	-0.144976086
291		0.890195006	0.390408602	-0.787840949
292		0.324376363	0.736953488	0.444192674
293		0.346053871	0.811553903	-0.214836968
294		0.369192937	0.851325914	0.153772566
295		0.704466732	0.553988024	-0.557742013
296		0.448748705	0.687747508	-0.416912503
297		0.560106631	0.69374026	-0.357320362
298		0.258673428	0.827400341	0.210472107
299		1.247679947	0.185098039	-1.0839937
300		0.110927654	0.913838359	0.106195238
301		0.67832427	0.675458333	-0.351104948
302		0.120253553	0.885034121	0.158989165
303		1.353176905	0.348307692	-0.69993443
304		1.934084526	0.725098765	0.244986428
305		0.14308621	0.894892768	-0.112618128
306		0.534415787	0.579281553	-0.571498871
307		1.017200352	0.572384181	-0.444160461
308		0.248069343	0.8615	-0.161683189
309		0.085730952	0.956084836	0.046940698
310		0.212224989	0.862191781	-0.17429373
311		0.387393648	0.745333333	0.334957335
312		0.325949601	0.810619329	-0.241043939
313		0.522449238	0.747990099	-0.275207943
314		0.848923501	0.768331754	0.224263721
315		0.00285784	0.997080481	0.003597683
316		0.38993433	0.593900452	-0.732132382
317		0.412074291	0.816792793	0.190351486
318		1.331681821	0.190592593	-0.999701818
319		3.76633231	0.182538462	-0.672544267
320		0.201326729	0.884100529	-0.118979984
321		0.53860539	0.776715294	-0.250136269
322		0.184198095	0.8975822	-0.09675365
323		0.974376592	0.829469115	-0.141580582
324		0.318367504	0.808	-0.250007629

	G	H	I	J
325		0.843825243	0.806331839	0.204820421
326		0.245329268	0.853980769	-0.182614856
327		0.06688333	0.983012488	-0.013152017
328		1.234814957	0.46707438	-0.542817858
329		0.705134028	0.732133333	-0.277941174
330		0.083059474	0.965418327	-0.026046965
331		0.380604264	0.73757265	0.379190021
332		0.664922467	0.611559322	-0.433449215
333		0.17187698	0.95484492	-0.041869058
334	+	3.213852528	0.001333333	2.545367347
335		0.113845235	0.955254818	0.045994653
336		0.65345542	0.810453782	-0.202303145
337		0.19681445	0.885420513	-0.109569126
338		0.340096973	0.719134796	-0.474893146
339		3.15391765	0.553267081	-0.362152735
340		0.983467794	0.365932584	-0.773767259
341		2.681795808	0.552904762	-0.365037282
342		0.169394445	0.949557018	0.049278259
343	+	1.94018002	0.379472527	0.559595956
344		0.137621994	0.898345342	-0.112064785
345		0.546287014	0.734801061	-0.292325126
346		0.31663882	0.877604618	-0.12749015
347		0.697476747	0.563704545	-0.537718667
348		0.84663197	0.590586957	-0.461158541
349		0.152076482	0.956935065	-0.045246548
350		0.412179012	0.828059801	-0.166402817
351		0.402491541	0.812145581	-0.183299171
352		0.053924234	0.960911392	0.054807027
353		0.741934567	0.583520833	-0.482704163
354		0.362722654	0.809649667	-0.26577674
355		1.44025472	0.365190476	-0.649537404
356		0.908396685	0.646356589	-0.349067264
357		1.108556535	0.675901754	-0.303362317
358		0.88471426	0.584449198	-0.451148139
359		1.400889408	0.261935484	-0.82813708
360		0.427325213	0.726504451	-0.370470259
361		0.340159702	0.858570992	-0.144837697
362		0.298518559	0.815429752	-0.268730587
363		0.410020334	0.853803432	-0.144085354
364		0.116237873	0.917405405	0.090280533
365		0.249258726	0.856161994	0.170610428
366		0.353121481	0.826780822	-0.18370035
367		0.003733842	0.996650046	0.002426995
368		1.491112549	0.372536585	-0.64115927
369		0.16214361	0.962053498	-0.031318453
370		0.35720953	0.853535604	-0.147774378
371		0.621110525	0.72502439	-0.314872106
372		1.58966664	0.133333333	-1.077512741
373	+	0.508081395	0.614564103	0.513761944
374		0.413133991	0.81145733	-0.245174832
375		0.652658703	0.642144487	0.39361657
376		0.32784186	0.817488172	-0.267243703
377		0.755293772	0.521351724	-0.601232953
378		0.480121216	0.665776119	-0.450566398

	G	H	I	J
379		0.03765758	0.981295129	0.01352183
380		0.39756119	0.813410909	-0.196262995
381		0.353575955	0.810776557	-0.208692975
382		0.177294113	0.891720812	-0.108532164
383		1.000529668	0.369255814	-0.774589539
384		0.457634003	0.723352025	-0.372424867
385		0.464532035	0.751085995	-0.286332448
386		0.258517828	0.883930667	0.111153497
387		0.97508511	0.525361702	-0.529319763
388		0.888397513	0.644560311	-0.353120592
389		3.943445374	0.302909091	-0.557268567
390		0.274367838	0.847674797	0.180029339
391		0.073115142	0.956228695	-0.057061725
392		0.559903669	0.811497872	-0.213207669
393		0.627653183	0.815621429	-0.168497086
394		0.197881994	0.867838565	-0.171687868
395		0.021260734	0.985267633	-0.020148383
396		0.121142678	0.940442938	0.065859901
397		0.371996873	0.812443609	-0.211082458
398		0.085846357	0.9608	0.033540302
399		0.334660886	0.803029478	0.289402432
400		0.797312704	0.674945205	-0.328189002
401		0.423514791	0.748764268	0.303456412
402		5.020457669	0.005882353	-1.285534965
403		0.36111293	0.809474542	-0.239357842
404		0.193411661	0.854236559	-0.192821927
405		0.375100191	0.697838188	-0.462119208
406		0.069513667	0.95828512	-0.043322245
407		0.232873667	0.87411797	0.127133687
408		0.576716539	0.749927007	-0.260658688
409		0.834806832	0.835456628	-0.13946067
410		0.20730678	0.909454545	-0.083776898
411		0.092401788	0.914559424	0.119041443
412		0.069277741	0.955354144	-0.05821334
413		0.147556643	0.891144304	-0.118035634
414		0.526457383	0.813325758	-0.189444012
415		0.422601072	0.740273684	-0.326189465
416		0.11837569	0.904821516	0.112364875
417		0.10142524	0.957488083	-0.03725391
418		0.593043481	0.824228571	-0.155653636
419		0.196163036	0.853411402	0.192752414
420		0.077362627	0.957797101	-0.039969126
421		0.438137872	0.64807722	-0.509548399
422		1.015039993	0.581117647	-0.409167396
423		0.016468217	0.987751412	-0.009999169
424		0.06671145	0.964664653	-0.030949487
425		0.223477589	0.870311724	-0.132348802
426		0.957337318	0.407176471	-0.705336041
427		0.272225862	0.814958707	-0.229066849
428		0.341513254	0.826251291	-0.187770844
429		0.315231481	0.746536341	-0.375473022
430		1.65072128	0.105375	-1.257012473
431		0.986718246	0.675676056	-0.313160578
432		0.068908003	0.959335505	-0.06041654

	G	H	I	J
433		0.072908419	0.9389819	0.088951111
434		0.17600179	0.937768279	-0.058501985
435		0.059328952	0.93855157	-0.102708605
436		0.208454798	0.876573066	-0.147297329
437		0.159109123	0.887478927	-0.118309233
438		0.171893755	0.883381014	0.122098075
439		0.083737107	0.960755418	-0.037085851
440		0.446274873	0.866981928	-0.129535039
441		0.468051358	0.746922693	-0.290914959
442		0.07375369	0.958765381	-0.042777803
443		1.065659917	0.368626506	-0.757500119
444		0.155033456	0.914411622	-0.090352376
445		0.370135674	0.874724559	-0.108321296
446		0.488813845	0.809573333	-0.233920203
447		0.278200328	0.808816176	-0.238034566
448		0.772749961	0.86998209	0.11612214
449		0.140511485	0.915958333	-0.078248978
450		1.212747228	0.57775	0.384364446
451		0.447236869	0.920687204	0.065259933
452		2.16024531	0.42722807	-0.483525594
453		0.88935001	0.726686391	-0.274390327
454		0.805010616	0.727374631	-0.280889299
455		0.024023007	0.985027027	0.018467797
456		0.514107913	0.682572391	-0.389816496
457		1.961783344	0.392478261	-0.549484253
458		0.613582908	0.803140271	0.223594877
459		1.712599353	0.619934426	-0.315933651
460		1.083013394	0.418716981	-0.637839423
461		0.579714167	0.825759729	0.157437219
462		0.207218429	0.922217443	-0.076068878
463		1.720044608	0.201241379	-0.830606249
464		0.153265895	0.883372032	-0.135831197
465		1.152014752	0.552049383	0.452150981
466		0.504888859	0.87458728	0.101882087
467		0.973715607	0.518045802	-0.553153568
468		0.005604428	0.993207829	-0.008246316
469		0.049135255	0.966860631	0.038020876
470		0.405139631	0.812836502	-0.207177056
471		1.511455725	0.243934426	-0.82067087
472		0.033911255	0.965122244	0.041372723
473		0.958924231	0.721113772	0.273116854
474		0.319205337	0.807512415	-0.294520908
475		0.992262088	0.485	-0.580950207
476		0.665158395	0.645784615	0.391676797
477		1.879753958	0.089857143	-1.254268646
478		1.520163148	0.370588235	-0.629169464
479		1.345827606	0.312588235	-0.769065645
480		3.191636481	0.107030303	-0.888834212
481		0.141428502	0.913918465	-0.090808021
482		1.007418808	0.459586207	-0.610465368
483		4.412128045	0.002	-1.632285436
484		0.682937521	0.815905579	-0.203109317
485		0.689477844	0.527135135	-0.627786212
486		0.431384803	0.850028986	-0.150506549

	G	H	I	J
487		1.027073535	0.688653333	-0.296370612
488		0.615230719	0.64534375	-0.411442439
489		0.011558396	0.988176858	-0.01138009
490		0.378347024	0.814226981	-0.247631921
491		0.109807521	0.924721839	-0.081163194
492		0.055886627	0.961903787	-0.03990131
493		0.013277636	0.987267925	0.012190501
494		0.473033414	0.745928571	-0.296169917
495		0.746627193	0.687892977	-0.326392068
496		0.818116737	0.806534413	-0.186525133
497		0.352672052	0.810564315	-0.247770733
498		0.113187906	0.918990566	-0.092358271
499		0.776953339	0.87119337	0.101185905
500		0.043454247	0.966535323	-0.031484816
501		0.116985765	0.896266667	-0.131802241
502		0.146875568	0.914098676	0.090478473
503		0.764951045	0.682818792	-0.32609325
504		0.545584709	0.737889855	-0.314149857
505		0.645705714	0.642211765	-0.403931724
506		0.130960077	0.914434783	0.096679688
507		1.668182592	0.108410256	1.172611237
508		0.426376278	0.853249608	-0.144291772
509		0.497069789	0.588086486	-0.656175825
510		0.66237936	0.666846442	-0.377002292
511		0.351480394	0.873733728	-0.130972332
512		0.28258543	0.8534272	-0.170447244
513		0.028671093	0.973395257	0.033163706
514		0.047106757	0.964971029	0.032462014
515		0.005558207	0.993634169	0.005641725
516		0.142660439	0.884091644	-0.1522789
517		0.051111153	0.960218717	0.055675507
518		0.673850404	0.599415929	-0.449586656
519		0.837432213	0.584488263	-0.427742852
520		1.232600412	0.579563981	-0.379067103
521		1.048077002	0.58094	-0.409240087
522		0.141763289	0.898465347	-0.108732224
523		1.497771671	0.528027211	-0.444594913
524		0.280125636	0.847043619	-0.176447974
525	+	0.549275962	0.80831793	-0.183314429
526		0.301173003	0.876371553	-0.131336424
527		0.061218633	0.967194444	0.026384989
528		0.07986145	0.954010741	-0.053807576
529		0.128884265	0.949856986	0.053474214
530		1.03569372	0.585102804	0.394414266
531		0.382391107	0.792915332	-0.275391049
532		0.704571783	0.812090278	-0.160090764
533		0.618140187	0.701858065	-0.337086995
534		0.270438367	0.88314094	-0.111773173
535		0.264667001	0.974839092	-0.018919627
536		0.935624465	0.674886598	-0.31317393
537		0.066142415	0.966675917	-0.025698768
538		0.470485075	0.82736772	-0.161004172
539		0.052270469	0.962413934	-0.041508993
540		0.1504388	0.883440108	-0.146388796

	G	H	I	J
541		0.177803921	0.868168067	-0.155555937
542		0.024496428	0.984579767	0.021886614
543		0.60801269	0.806183267	-0.195523156
544		0.010681201	0.99320186	0.004929013
545		0.771228573	0.810624339	-0.16093021
546		0.708973838	0.738148571	-0.281807794
547	+	0.995474127	0.244610169	-1.108195199
548		0.215942486	0.876688478	-0.14230601
549		0.468115661	0.685801325	-0.405162599
550		0.537584279	0.827883362	0.16237704
551		0.13019916	0.89995098	0.109355927
552		0.264016472	0.811917137	-0.254286024
553		0.244830946	0.913918072	-0.077557246
554		0.666685711	0.690840391	-0.332999759
555		0.144660629	0.873275035	-0.18133778
556		0.117115296	0.91830289	-0.083353678
557		0.473217389	0.615915966	0.535665512
558		1.078388389	0.348394366	-0.826450772
559		0.767844062	0.724381538	-0.294545492
560		0.029156453	0.984049103	0.010273404
561		1.355201969	0.550605263	-0.44247161
562		0.040257222	0.96288391	-0.045802858
563		0.280921336	0.81168559	-0.306066301
564		2.173546232	0.579082927	-0.340575536
565		0.764952652	0.577049505	-0.463822683
566		0.47331779	0.810437956	-0.187244839
567		0.281347631	0.893675	-0.089576085
568		0.6490643	0.735935135	-0.278255251
569		0.495387529	0.743949622	-0.28732321
570		0.555407811	0.592092593	0.526836183
571		0.154555128	0.877761151	0.179475784
572		0.119880636	0.854623457	-0.340571298
573		1.012641675	0.557371429	-0.45354716
574		0.111559908	0.918166282	0.085014979
575		0.042128736	0.984351013	0.014968872
576		0.143454624	0.89591468	-0.114484575
577		0.971045276	0.752794258	-0.223745558
578		0.136973373	0.885872892	-0.137078603
579		0.045649349	0.964257028	-0.035221736
580		0.048472584	0.985290445	-0.009509405
581		1.598943383	0.420037383	-0.541630427
582		0.722362942	0.346293333	1.160931269
583	+	1.319025496	0.131043478	1.236293581
584		1.780515041	0.594328889	-0.333872689
585		0.386754541	0.835039735	-0.166641447
586		0.532993377	0.674569343	-0.409825219
587		0.60058998	0.726175227	-0.316491445
588		0.305190817	0.883256545	0.101803674
589		0.07157573	0.958383246	-0.044979095
590		0.15695604	0.883105882	0.130281236
591		0.238438659	0.825159322	-0.22032547
592		2.03550019	0.811583012	0.158230252
593		0.368685439	0.859394495	0.141609404
594		0.227397475	0.875512329	-0.127404743

	G	H	I	J
595		0.124963468	0.943660044	0.056928211
596		0.076156279	0.963494867	-0.03571913
597		0.731906524	0.639543307	-0.383722305
598	+	0.097053154	0.885397154	-0.189428753
599		0.39918317	0.83749835	-0.163422055
600		0.240427001	0.916880649	-0.067416297
601		0.615127428	0.823116358	-0.155507406
602		0.535416907	0.812966038	-0.18782213
603		0.430833059	0.80742515	-0.217231962
604		0.595057342	0.547788079	-0.679215961
605		0.313989475	0.904893773	0.079235925
606		0.030762903	0.965042254	0.04821078
607		0.042869475	0.982726388	-0.018211365
608		0.171479217	0.882790945	0.131234911
609		1.101763945	0.710269231	0.279214011
610		0.379323533	0.850851613	-0.156167348
611		1.948017789	0.40712	-0.529370202
612		0.093342941	0.922188319	-0.108958774
613		1.807856839	0.586804124	-0.359202067
614		0.248950612	0.873080764	-0.145017836
615		0.141210513	0.882841823	-0.151435852
616		0.075053538	0.958300211	-0.047524346
617		0.405047238	0.81181913	-0.183885786
618		0.205485133	0.810345083	0.30731625
619		0.842405152	0.673508651	-0.324843301
620		0.087580735	0.938413408	0.072990841
621		0.207118542	0.861416413	-0.176564111
622		1.727846522	0.465886179	-0.483446969
623		0.028241307	0.983097656	0.022481706
624		0.319748756	0.736088643	-0.425833172
625		0.545950007	0.581915789	-0.59485711
626		0.050622326	0.981335945	0.019556681
627		0.672936314	0.739386301	-0.276951684
628		0.324749605	0.884484375	0.098820792
629		6.29022879	0	-2.245675617
630		0.183863432	0.919133641	-0.069549984
631		0.467215523	0.749568627	-0.28548283
632		0.637873443	0.4708	-0.820315043
633		1.791825795	0.349369863	-0.638036304
634		0.98655251	0.548858896	-0.479149712
635		0.439198248	0.817995662	-0.2344674
636		0.033004087	0.96695846	-0.048355314
637		1.209228346	0.426678899	0.593611611
638		2.330125159	0.742099217	0.213011848
639		0.712609426	0.641862595	-0.37961536
640		0.449995689	0.826546689	-0.167867025
641		0.435518303	0.8075	0.210758421
642		0.33083392	0.919281324	-0.067595164
643		0.384519262	0.809889831	-0.244152069
644		0.057504674	0.957150571	0.047185686
645		0.678427421	0.3656	-1.167698754
646		0.049478529	0.950551043	-0.095595466
647		0.231829065	0.812614853	0.239437951
648	+	1.322227425	0.070333333	1.992374844

	G	H	I	J
649		0.185230793	0.927304348	0.065170924
650		0.055760132	0.957015873	-0.057116614
651		0.017255533	0.984400756	0.012538486
652		0.061261361	0.959555089	0.048925824
653		1.353232332	0.111111111	-1.412531959
654		0.145211901	0.875694051	-0.181507746
655		0.384506159	0.794073394	-0.274902556
656		0.503458117	0.81364271	0.211102592
657		0.726934653	0.708345048	-0.312868118
658		0.088461068	0.95428884	-0.057797114
659		0.941859381	0.551775	-0.491999944
660		0.203622719	0.878042796	-0.146849738
661		0.813390494	0.612391489	-0.396645228
662		0.585990006	0.718314465	-0.332398309
663		0.043674252	0.982977384	-0.021089342
664		0.038555656	0.964072362	0.039632797
665		0.307709885	0.866956522	0.141979429
666		0.460555161	0.806791252	-0.211313883
667		0.580517058	0.736550964	-0.29409239
668		0.324534538	0.851156894	-0.159622192
669		0.113041262	0.921980998	-0.094028049
670		0.191667572	0.831701493	0.245260663
671		1.594129701	0.105714286	-1.253569285
672		0.277485062	0.860260606	0.152679443
673		0.304132374	0.717097792	-0.545426051
674		0.017634086	0.985649289	0.013066186
675		0.045936519	0.964323353	0.032744514
676		0.104684937	0.95641453	0.046106127
677		0.198645292	0.864760181	0.176417669
678		0.046539657	0.965594349	0.03633817
679		0.801875016	0.670840149	-0.346636242
680		0.228638394	0.846311688	-0.198451784
681		1.059530899	0.735050847	-0.253077613
682		0.330611376	0.876636905	-0.133929359
683		0.027180685	0.982802303	-0.016079161
684		0.050269194	0.940265487	-0.112522761
685		1.714535929	0.303223881	-0.700230069
686		1.224028651	0.738023392	-0.25128852
687		1.41027764	0.807732252	-0.172441906
688		0.602402174	0.812844961	-0.188487795
689		0.548063333	0.525464789	-0.792362637
690		0.119908322	0.885183099	-0.145083321
691		0.074807864	0.98031619	0.011618508
692		1.053982831	0.557065089	-0.455365923
693		0.458011748	0.735783198	0.322838253
694		0.260947859	0.944119337	-0.048982196
695		0.897788766	0.685631579	-0.305250168
696		0.418146568	0.825919732	-0.167966419
697		0.249518327	0.936182232	-0.057908588
698		0.786975251	0.883594126	0.09192933
699		0.220958324	0.882870027	-0.116026772
700		0.843809023	0.677351536	0.320864571
701		0.180864514	0.919772781	-0.076993518
702		0.522244669	0.805914989	-0.231450399

	G	H	I	J
703		0.59953323	0.722858859	-0.316338433
704		0.409381297	0.812564885	-0.207574844
705		0.479149785	0.809574692	-0.177893957
706		0.63381043	0.586306977	-0.488034142
707		0.003407586	0.995295853	-0.002362569
708		0.326042097	0.872495238	0.112904655
709		0.242289874	0.885433766	-0.105694877
710		0.598115491	0.874302671	0.118037542
711		0.642760303	0.752086747	-0.248880598
712		0.148730393	0.886596939	-0.122625563
713		0.13099526	0.935687075	-0.066659504
714		0.571125779	0.763780952	-0.252184762
715		0.539543759	0.809115304	-0.212878121
716		0.227825689	0.874223529	0.150822957
717		0.171807921	0.875630682	-0.16133817
718		0.021871862	0.980823642	-0.015834384
719		0.34439986	0.81204947	-0.199754503
720		1.066565982	0.67371223	-0.31137085
721		0.033382425	0.981295019	0.014841715
722		0.35854059	0.813439446	-0.19019254
723		0.162005843	0.935767389	-0.063928392
724		0.346101301	0.812648045	-0.214998669
725		0.351641274	0.811938462	-0.223787732
726		22.70182073	0	-20.94210709
727		1.807253328	0.619824	-0.309230593
728		0.061166612	0.961973223	-0.040547477
729		0.733793056	0.725364706	0.288678063
730		0.715037094	0.676808362	-0.344175975
731		0.09564141	0.933934166	-0.077747345
732		0.494074651	0.6825	0.400171068
733		0.007664026	0.987718574	-0.014593548
734		1.291339829	0.733172043	0.236287435
735		0.431964421	0.850768254	-0.147038142
736		0.091374705	0.981787402	0.018478182
737		1.09096147	0.818336207	-0.186397764
738		0.035659831	0.982104662	-0.012390561
739		1.58192464	0.279875	-0.754182604
740		1.683661366	0.681836299	-0.280277464
741		1.085443751	0.618563265	-0.349752426
742		1.553519006	0.527194245	-0.451167425
743		0.727363394	0.813128205	-0.200069639
744		0.292783131	0.81140824	-0.23649576
745		0.368417433	0.81513879	0.195272658
746		0.394974563	0.746805063	-0.32307752
747		0.294521229	0.808365256	-0.305454466
748		0.163100458	0.882581486	-0.126782523
749		0.160739435	0.894802005	0.108048969
750		0.42817158	0.812900971	-0.208844079
751		0.056835241	0.954434024	-0.074698978
752		0.152809547	0.900742927	-0.101655112
753		0.290734927	0.808579256	-0.255602519
754		0.2001204	0.88651928	-0.109274122
755		1.833213232	0.560528736	-0.383389579
756		1.160679614	0.59296861	-0.370325512

	G	H	I	J
757		0.318975638	0.884707447	-0.104240629
758		0.025355607	0.98494186	0.019544178
759		0.025817996	0.982197507	-0.016300837
760		0.39306586	0.806252252	-0.261454476
761		4.254728403	0.001846154	-1.832877053
762		2.202972954	0.0198	-1.676073922
763		0.346048981	0.836604269	0.170164956
764		0.872517733	0.520676471	-0.573026657
765	+	0.465492158	0.750625	-0.280131446
766		0.168007325	0.888270064	0.113916185
767		0.444592192	0.8112	-0.201520496
768		1.294877596	0.794779043	0.195929633
769		0.469052852	0.737743869	-0.319754071
770		0.153254895	0.925110092	-0.070039961
771		0.091625251	0.917351788	-0.095862707
772		0.111382029	0.907975728	-0.111789915
773		0.062152677	0.952886463	-0.070884705
774		0.11228869	0.920337693	-0.095778359
775		0.000629708	0.999551471	-0.000360913
776		0.598057964	0.718985075	-0.316546334
777		0.04109405	0.985103582	0.015943527
778		0.069605811	0.958240664	-0.04218928
779		0.114281144	0.949410734	0.054277844
780		1.351762049	0.868198198	0.111463547
781		0.408113715	0.869695291	-0.112182405
782		0.900156996	0.342914286	-0.961237801
783		0.863527279	0.717409524	-0.292749405
784		0.085426487	0.934990909	-0.083314472
785		0.660701918	0.67175	-0.370731566
786		0.893716964	0.825560137	-0.14921697
787		0.17558389	0.885817715	-0.115230136
788		1.456543019	0.362814815	-0.657563527
789		0.324833828	0.852434505	-0.161255731
790		0.272409444	0.809439689	0.264121585
791		0.239109199	0.877886463	-0.1431889
792		0.05468957	0.985326886	-0.01464759
793		0.129602692	0.937167228	-0.063251919
794		0.191180104	0.919029446	0.074741152
795		0.237970255	0.874650407	-0.122208701
796		0.200591026	0.810405594	-0.278274748
797	+	1.943386501	0.089548387	-1.193715201
798		0.119664084	0.937863382	0.064122942
799		0.239605177	0.872135211	-0.134620031
800		0.563572882	0.617300813	-0.459022098
801		1.928852591	0.353888889	-0.622252146
802		0.029909544	0.981168627	-0.02442593
803		1.060181072	0.322666667	-0.873388926
804		1.357888227	0.522041958	-0.465328005
805		0.106450476	0.916126168	-0.093043645
806		0.677194398	0.604329004	-0.439265357
807		0.21049449	0.885056701	-0.108516481
808		0.191619844	0.869307153	-0.149044673
809		0.40817497	0.680657143	0.483013365
810		0.39867862	0.745412935	0.316016939

	G	H	I	J
811		0.403197745	0.795898618	-0.269116296
812		0.312009926	0.887669211	-0.093201107
813		0.824457346	0.6029869	-0.40493732
814		0.059364118	0.98520038	-0.010146883
815		0.843049275	0.738603175	-0.253223207
816		0.688908099	0.811617261	-0.17634413
817		0.259848296	0.889296343	0.094640944
818		0.170234825	0.884343461	-0.128074646
819		0.610596004	0.817812636	-0.21169281
820		0.211686116	0.876587896	-0.148408042
821		0.003837095	0.997367933	0.003710853
822		0.130540374	0.98461657	0.012599097
823		0.436839459	0.815040853	-0.183695475
824		1.664844042	0.423781818	-0.527393129
825		0.058867124	0.963616718	0.036842134
826		0.029416136	0.985326867	0.018445545
827		0.594350575	0.583893617	0.56026819
828		0.369821763	0.817234783	-0.254051844
829		0.565403852	0.824493243	-0.158228556
830		2.41243465	0.52075	-0.40411928
831		0.681387949	0.738564644	-0.267965741
832		0.55101788	0.676043321	-0.398335775
833		2.170622605	0.046857143	-1.423506843
834		1.448415531	0.0916	1.507467482
835		1.716672056	0.108756757	-1.166087257
836		1.346304427	0.850474684	0.12379943
837		0.022649385	0.984435374	-0.022614585
838		0.213271614	0.869488889	0.137725406
839		0.642676057	0.582955665	-0.504972034
840		0.187578821	0.876098551	-0.160972171
841		0.316118059	0.828546075	-0.190471861
842		0.149453661	0.896373599	-0.108922746
843		0.126671892	0.906246041	-0.105534236
844		0.548874748	0.748594872	-0.278575685
845		0.244082852	0.854347418	-0.174719916
846		1.606450817	0.090758621	1.393693288
847		1.25012218	0.392851064	-0.642090056
848		0.29107457	0.91409546	-0.066846636
849		0.023911283	0.987442036	-0.009188758
850		0.152380275	0.895034483	0.104417589
851		0.099626764	0.915024038	-0.112204234
852		1.760181727	0.362025316	-0.617175844
853		0.467125245	0.677144928	-0.442159017
854		0.392808625	0.809400881	0.253473282
855		3.078548877	0.113463415	-0.84842915
856		1.350262449	0.622129032	-0.328085793
857		0.959434232	0.585204301	0.437581168
858		0.00141405	1	-0.00052113
859		0.448970509	0.733658263	-0.337321811
860		0.575182539	0.783813084	0.24091212
861		0.008298264	0.993716418	0.00638983
862		0.393288467	0.854948678	-0.144574695
863		0.063729433	0.966234343	-0.031885359
864		0.132521855	0.914538922	-0.092918184

	G	H	I	J
865		0.101214107	0.917566237	-0.096926795
866		0.205492173	0.804985859	0.370180766
867	+	0	0.999082569	0
868		2.874599319	0.1159	-0.871731228
869		0.140763771	0.925712974	0.072232776
870		0.147295805	0.957937238	-0.03665034
871		0.503212909	0.805904762	-0.205199136
872		0.746842437	0.460068376	-0.734710905
873		0.222704292	0.855485452	0.174834357
874		1.110256419	0.404323232	-0.659877989
875		0.480200098	0.726390093	-0.358832677
876		1.781410319	0.0712	-1.440129598
877		3.249284242	0.046181818	-1.1335623
878		0.080328595	0.941266888	-0.071530448
879		0.130573695	0.956485623	-0.042619493
880		0.468109638	0.739757225	-0.336695989
881		0.159367919	0.89580597	-0.105376773
882		0.741466547	0.65724812	-0.364103105
883		1.137961686	0.824848485	-0.141450246
884		0.320556322	0.847583062	-0.169106166
885	+	22.39659345	0	-21.30843862
886		0.63379268	0.737668508	0.285003026
887		0.016523439	0.981690249	-0.020062341
888		0.345288252	0.811841996	-0.250618829
889		0.59167086	0.812592593	0.202213711
890		0.978573515	0.735085106	-0.246562534
891		0.306900962	0.85327673	-0.159984377
892		0.180614967	0.883695187	-0.128818936
893		0.117356096	0.877886131	0.254961862
894		0.51885759	0.803399209	0.203153822
895		0.155147591	0.896819095	-0.109977298
896		1.008365535	0.849073955	-0.130198797
897		0.831492746	0.585010101	-0.448048274
898		0.073943929	0.937208989	-0.085247464
899		1.180503837	0.736653061	-0.252842585
900		0.939836379	0.686323432	-0.301860174
901		0.764467025	0.679531915	-0.339107513
902		0.596600015	0.810334694	-0.20059522
903		0.908438235	0.484352	-0.606470744
904		0.062859652	0.965505506	0.029448615
905		0.04315805	0.982776149	-0.019319534
906		0.719718617	0.563790698	-0.535170661
907		0.232994841	0.842655791	0.199703005
908		0.182690823	0.881740295	-0.129493713
909		0.416704347	0.876187234	-0.115446515
910		0.776031541	0.584021164	-0.475734923
911		0.518846598	0.763724466	-0.260351181
912		0.024611567	0.983807507	0.017553965
913		0.07196234	0.959896907	-0.038982179
914		0.056315436	0.965431705	-0.029359394
915		0.575207562	0.735089888	-0.30031946
916		1.239877295	0.347844156	-0.735186047
917		0.241785154	0.916078886	-0.067804972
918		0.630939319	0.938901786	0.04825041

	G	H	I	J
919		0.027474739	0.985950758	0.010671192
920		0.035914602	0.965366935	-0.042664422
921		0.237399782	0.812	-0.358757867
922		0.291393667	0.851987241	-0.167800056
923		0.546279431	0.732589812	-0.295761108
924		0.130064364	0.927001145	0.07319514
925		0.07323946	0.956241901	0.057462268
926		2.054510222	0.807871743	0.164311727
927		0.702820976	0.513954887	-0.670553207
928		0.457291629	0.677204301	0.444763819
929		0.385206745	0.868982036	-0.132106569
930		0.104850417	0.954174292	0.052638584
931		0.730041199	0.731190616	-0.286841922
932		0.198521363	0.955013815	-0.039409637
933		0.426718048	0.809185841	-0.245568169
934		0.365145684	0.794482759	-0.283337275
935		0.853846963	0.811646316	-0.191349665
936		0.000531856	1	-0.000447803
937		0.114597807	0.913492823	-0.100497564
938		0.022471361	0.981466029	0.016934925
939		0.614322893	0.724571429	0.315685484
940		2.332580019	0.739586207	0.226689445
941		0.441696028	0.74521	-0.3005867
942		0.437578257	0.804875	-0.247044881
943		0.255023224	0.896138101	-0.087534587
944		1.202719754	0.406851485	-0.627188577
945		0.830784195	0.861080425	-0.120102988
946		0.04905758	0.982250246	-0.021153556
947		0.322634434	0.815555556	-0.208573659
948		0.556124094	0.742215223	-0.28506173
949		0.327274168	0.775443396	-0.322632472
950		0.417766208	0.810494465	-0.197763231
951		0.012549156	0.994554622	0.005456077
952		2.996708327	0.554812903	0.369787004
953		23.31980829	0	-22.02440156
954		0.088161999	0.895895131	-0.17028851
955		0.179116299	0.875524966	-0.136614694
956		0.141451576	0.8823083	0.143119812
957		0.487450806	0.735872	-0.308287938
958		0.047741879	0.957654886	0.053589079
959		0.155175456	0.870947961	-0.171185811
960		0.227520462	0.85089172	-0.189414978
961		0.249085147	0.815633094	-0.242964003
962		0.082030407	0.966455005	-0.029842377
963		0.568577879	0.616033195	-0.464086533
964		0.102091251	0.938606539	-0.070697149
965		0.063285185	0.949608791	0.075406392
966		0.97636524	0.560531792	-0.461650636
967		0.804293085	0.603400881	-0.411059697
968		0.231228847	0.809730849	-0.335532506
969		1.191766484	0.465983193	-0.554768244
970		0.249001464	0.811362764	-0.274770525
971		0.14736115	0.949951542	-0.05190489
972		0.18390511	0.87883815	-0.160411411

	G	H	I	J
973		0.005192507	0.997101664	-0.002323574
974		0.358578432	0.81144592	0.217663023
975		0.305497078	0.877023256	-0.130936941
976		0.058953091	0.963113846	-0.039218903
977		0.449347951	0.897007407	-0.079122119
978		0.349380833	0.736863636	-0.404529148
979		0.07326611	0.959940991	-0.046283298
980		0.268417426	0.8544875	-0.166508993
981		0.485214369	0.639873518	-0.483882692
982		4.111564695	0.0016	-2.342860964
983		0.981899813	0.827733333	-0.145971934
984	+	0.723204763	0.80625618	-0.21219635
985		3.360772232	0.078307692	-0.989788691
986		0.19399889	0.888603581	-0.108228683
987		0.969108517	0.837123967	0.138259676
988		0.411160461	0.582179775	-0.866563161
989		0.465513845	0.79725	-0.253205405
990		0.460766442	0.809814815	-0.192591773
991	+	3.124257146	0.016842105	-1.359483719
992		0.535948034	0.812762295	-0.206900279
993		0.879843476	0.675724138	-0.319201999
994		0.941666785	0.283446154	-1.046708849
995		0.230810608	0.879577424	-0.142873552
996		0.27295163	0.87705848	0.137499068
997		0.301235887	0.741581152	0.417112774
998		1.254247097	0.374444444	-0.665336397
999		0.22346624	0.914502326	0.069938236
1000		0.041086128	0.966914513	-0.03178872
1001		0.720040708	0.837842105	-0.142888175
1002		0.670588639	0.581244019	-0.484032101
1003		0.324105317	0.722269939	-0.485159132
1004		0.49542842	0.614426778	-0.51532703
1005		0.61411466	0.591050228	0.488610797
1006		0.155374328	0.938454955	-0.060472912
1007		0.699629812	0.58922449	-0.488636229
1008		0.720336528	0.461808696	0.762484021
1009		0.591329238	0.752029056	-0.25669882
1010		2.351889784	0.48853125	-0.434123145
1011		0.106700017	0.935494881	-0.073705673
1012		0.136190005	0.914442563	-0.095254262
1013		0.204174634	0.874032787	-0.133085463
1014		0.272081499	0.820051836	-0.30409495
1015		0.675760939	0.727082569	-0.305182987
1016		1.276501806	0.129909091	-1.306792577
1017		0.581339712	0.741556196	-0.303191927
1018		0.213942153	0.91879575	0.073273341
1019		0.20994931	0.868233983	0.140304777
1020		0.025076734	0.984421252	-0.012035582
1021		0.587591757	0.826565517	-0.160743078
1022		0.509132263	0.75182266	-0.275581148
1023		0.231202809	0.877002915	-0.146082348
1024		0.883576986	0.591671795	-0.437742445
1025		0.615509853	0.619983806	-0.433490753
1026		4.50209757	0.0032	-1.52093188

	G	H	I	J
1027		2.566986086	0.593761468	-0.319271088
1028		1.593483623	0.402916667	-0.573248121
1029		1.879420814	0.870511236	-0.097770055
1030		0.842473959	0.526142857	-0.571512858
1031		0.173245402	0.921352381	-0.079332987
1032		0.073961775	0.958546638	-0.057936986
1033		0.069551618	0.982364878	0.016942342
1034		0.42741087	0.851826645	0.14966011
1035		4.546424644	0.003	-1.497462379
1036		0.206756557	0.883874181	-0.114870707
1037		0.712215636	0.615216667	-0.413041645
1038		0.129227333	0.942111111	-0.058840646
1039		0.68464383	0.899734969	0.073984994
1040		0.018693815	0.982366472	-0.030138652
1041		0.031412025	0.982471513	0.024027507
1042		0.797046989	0.549610063	-0.536796358
1043		1.007013153	0.838273476	-0.136773851
1044		2.021187722	0.120837209	-0.984074699
1045		0.259484856	0.955476596	-0.038285149
1046		0.400019204	0.815037433	-0.190014521
1047		0.036513946	0.9839	0.014828576
1048		0.382187719	0.882511749	0.096702364
1049		0.22857835	0.865331316	0.16394594
1050		0.073673165	0.967508629	-0.031567256
1051		0.666521939	0.811034735	-0.172428767
1052		1.398078719	0.406693878	-0.595607969
1053		0.020388183	0.990194393	0.006318622
1054		0.3521592	0.748968825	0.326417499
1055		0.056418034	0.961242084	0.039186902
1056		0.101499683	0.897120198	-0.139347076
1057		0.941331316	0.753546539	-0.224545373
1058		2.106968648	0.529576642	-0.421109517
1059		0.748417545	0.524177778	0.628614638
1060		0.215281271	0.870414465	0.137239032
1061		0.226001095	0.868588721	-0.131500668
1062		0.454009319	0.737152174	-0.324617386
1063		0.283743837	0.874945185	-0.139935388
1064		0.56695504	0.605413793	-0.484580782
1065		0.839677072	0.61690535	-0.382132848
1066		0.188226435	0.847533225	-0.22659768
1067		0.707491032	0.893927409	-0.078637229
1068		0.84802146	0.578901099	-0.468422148
1069		0.710638804	0.739497268	-0.271718555
1070		0.834930362	0.603158798	-0.398526933
1071		0.122106932	0.95997479	-0.038576762
1072		0.08315273	0.955273885	-0.048213323
1073		0.62280286	0.897816377	0.077088038
1074		3.197339975	0.791287671	-0.180260976
1075		2.055817109	0.172897959	-0.841843287
1076		0.018623767	0.98051145	0.017970403
1077	AJD71803;AJD71517;AJD71454;AJD71332;AJD71324;AJI	0.098568591	0.915238095	-0.095510483
1078		0.497404489	0.809477912	-0.207647747
1079		2.98E-05	0.9999045	-5.02E-05
1080		0.220954878	0.811675048	-0.309401406

	G	H	I	J
1081		0.078839887	0.941272727	0.072950151
1082		2.107009206	0.419115385	-0.505438063
1083		0.267186869	0.814717668	-0.237482283
1084		0.83776594	0.362727273	-0.882730696
1085		0.19403149	0.868366158	-0.144149356
1086		0.167332024	0.893531486	-0.108213213
1087		0.122791398	0.889914141	-0.132378048
1088		2.902826885	0.581066667	-0.322083791
1089		0.34111218	0.811507431	-0.259402593
1090	+	0.280871121	0.80682495	-0.273415248
1091		1.484138403	0.75002445	-0.21295823

	K	L	M	N
1	Student's T-test Test statistic ORF2_ORF2_PEP	-Log Student's T-test p-value WT_WT_PEP	Student's T-test q-value WT_WT_PEP	Student's T-test Difference WT_WT_PEP
2	-0.21912791	0.295807823	0.911531587	0.133763419
3	0.277055159	0.311111063	0.923772114	-0.121164746
4	-0.165433616	2.110739325	0.420965517	-0.605242835
5	-0.585036742	0.185565665	0.949231623	-0.112236235
6	0.043579609	0.382989477	0.881263581	-0.199039247
7	-0.242897773	0.318746027	0.931929825	-0.111981074
8	0.628707717	0.560995858	0.791522388	0.291534
9	-1.430517386	0.35613402	0.869664596	-0.216736476
10	-0.183371222	1.296583846	0.581531915	-0.451024161
11	-0.172018354	0.274660038	0.912971714	-0.158691406
12	-0.398293883	0.216656659	0.955917695	-0.100793203
13	0.68973278	0.430292918	0.762609836	-0.374797185
14	-0.425596844	1.578658087	0.707251142	-0.313573625
15	0.540680834	1.370609973	0.515139241	0.590239207
16	-0.611146514	0.077235774	0.980895455	-0.064856211
17	0.01938017	0.293162311	0.826433915	-0.333068848
18	-0.458278754	0.064102599	0.984682105	-0.038748423
19	0.060363292	0.541381576	0.885793103	-0.167128033
20	-0.221491942	0.294402153	0.954352021	-0.081122716
21	-0.621986269	0.124677452	0.97722795	-0.056530635
22	-0.214218797	1.793039191	0.700415842	-0.320887884
23	-0.060223235	0.624959094	0.853898901	-0.197351032
24	0.112274698	0.288478474	0.858610169	0.254393472
25	0.268153638	0.049477233	0.981161157	0.037545098
26	-0.053645024	0.006583779	0.998435107	-0.005647447
27	0.036853442	0.27842203	0.908149109	0.151782354
28	-0.187174176	0.035793666	0.992024072	-0.026506848
29	-0.195277012	0.430163783	0.82782808	0.303984324
30	0.087696466	0.037828457	0.990665343	0.022645526
31	0.536817936	0.341130762	0.948688889	0.094359928
32	0.973336887	0.474692535	0.690557692	-0.534744475
33	-0.183240228	0.274468341	0.918112977	0.132458793
34	-0.31815062	0.343891936	0.821871148	0.3429771
35	0.452309956	0.224039383	0.95556701	-0.083153195
36	0.090533444	0.118229101	0.977519451	0.057092879
37	0.063784203	0.246863668	0.944784314	0.105320401
38	-0.376324686	0.444108465	0.774389776	0.353980382
39	-0.510570971	0.281951028	0.854013921	-0.297279358
40	-0.173124613	0.321659871	0.974578199	-0.054905362
41	-0.310030861	0.338437336	0.825889447	-0.303379695
42	-0.144744195	0.145019156	0.949116919	-0.123829524
43	-0.070443914	0.454195109	0.721776062	-0.441230562
44	0.742069535	0.028217912	0.9820818	0.050968806
45	0.5642727	1.34055604	0.560946237	-0.5399189
46	-0.031095978	0.028989907	0.985090909	-0.038189146
47	-0.304538778	0.231283235	0.85915565	-0.303832584
48	0.260398123	0.23047784	0.954846061	-0.067791833
49	-0.46701854	0.207574147	0.882177024	0.253264321
50	0.583223383	1.344550191	0.825076487	0.218783061
51	-0.259132083	0.047431867	0.987849946	-0.05286471
52	0.129091981	0.000359839	1	0.000175476
53	-1.019883991	0.286730995	0.943173975	0.103866577
54	0.173269496	0.149145253	0.986752643	0.031823052

	K	L	M	N
55	-0.181897524	0.199307426	0.885829703	0.288965861
56	-1.112670494	0.092592783	0.973152225	-0.073527442
57	-1.139181117	4.057308319	0.023111111	-1.31421513
58	0.481689382	0.451338307	0.567657895	-0.890007655
59	-0.069117806	0.024540819	0.994831589	0.009771347
60	-0.128306243	0.17512414	0.957642214	-0.088604397
61	0.130270882	0.01460659	0.99299516	-0.023247189
62	-0.262772468	0.069007738	0.980045267	0.031849967
63	-0.181297279	0.208848125	0.911341693	-0.160463121
64	-0.112109489	0.288547009	0.941724138	-0.107930077
65	-0.22101573	0.680793902	0.549365854	-0.68815528
66	-0.082284005	0.010923644	0.996428975	0.008041594
67	-4.028976131	3.713827157	0.047333333	-1.188654582
68	-2.415698082	4.381471266	0.012	-1.521065394
69	-0.267687072	0.590501629	0.751211268	0.341111289
70	0.263306878	1.785138175	0.164666667	-1.212265439
71	-0.026214815	0.498777119	0.84874463	0.231138017
72	-0.010912998	0.034847637	0.985848332	-0.03328938
73	0.372527153	0.101470313	0.980886576	-0.03158972
74	-0.571988336	0.864898476	0.521060241	-0.730738958
75	-0.428171422	0.597319498	0.773944272	-0.299053616
76	-0.360049141	0.038272039	0.981474542	-0.036745495
77	-0.261731462	0.127271944	0.975716312	0.06600825
78	0.040946282	0.435269533	0.947095238	0.080686145
79	-0.261236673	1.925698269	0.614390244	-0.372228199
80	-0.16170084	0.097953433	0.953413497	0.100195567
81	-0.245133562	0.297336698	0.86304	0.245743222
82	-0.643250814	0.274724948	0.887263941	-0.20287429
83	-0.625568652	0.031837864	0.981410042	0.06358401
84	-0.098993089	0.376225736	0.81185567	-0.298843808
85	-0.285562412	0.065420047	0.988051227	-0.042079926
86	-0.057743157	0.140841422	0.953969543	-0.093406677
87	-0.747051856	0.106260301	0.955724841	-0.110701243
88	-0.562764958	0.200599405	0.88619883	-0.277732213
89	-0.419232426	0.244053779	0.916099502	-0.165043301
90	-0.820797791	0.402266535	0.884405253	-0.176663081
91	-0.757364444	0.580388338	0.72848583	-0.391633564
92	-0.686895254	0.279439618	0.853834452	-0.283907784
93	-0.78526116	0.884911425	0.731917355	-0.334266875
94	-0.767357405	0.237791143	0.924403561	-0.12916459
95	-0.657846914	0.523575433	0.722486056	-0.41214625
96	-0.368817561	2.401504689	0.564589147	-0.407800886
97	-0.862727619	0.630311886	0.752622837	-0.326716105
98	-0.740045954	0.033746796	0.983338071	0.037481308
99	-0.340992154	0.62689697	0.730672566	-0.400952233
100	-0.111699606	0.141338222	0.97541217	0.05563524
101	-0.433316121	0.870871689	0.590318471	-0.494142532
102	-0.569633649	0.175479428	0.943463687	0.119362937
103	-0.212621287	0.139670742	0.945913272	-0.109484143
104	-0.319157646	0.169099347	0.925848889	-0.15148947
105	-0.526701094	0.350678731	0.878973737	-0.208044476
106	-1.244469658	0.834222502	0.532883721	-0.728860219
107	-0.283324255	0.452327314	0.854578947	-0.216350979
108	-0.195654232	0.044612181	0.98212356	-0.045888689

	K	L	M	N
109	-0.29066542	3.378248058	0.637529412	-0.329939736
110	-0.26901205	4.498958594	0.274307692	-0.684124417
111	-0.158449676	0.246714828	0.9088	-0.160550435
112	-0.949735087	0.495354939	0.752114695	-0.378056632
113	-1.039557251	0.631205765	0.733581749	-0.357532289
114	-0.356657813	0.665581036	0.774846154	-0.295362049
115	-0.374195045	0.468132543	0.859344111	-0.225286484
116	0.071822871	0.091016807	0.98712381	-0.035334057
117	-0.395740681	0.023392175	0.994634981	0.009679794
118	-0.185304239	0.173889001	0.948504648	0.101358626
119	0.177323758	0.490616391	0.8130625	-0.265277863
120	-0.003206341	0.043989346	0.981265514	-0.033529705
121	-0.087288697	0.313503669	0.840995215	-0.293230693
122	0.065695181	0.287317352	0.859046809	0.256448322
123	-0.708185955	0.242056688	0.883032882	-0.236271116
124	0.059595383	0.39220595	0.854468271	0.228119108
125	-0.557752397	0.033139019	0.990361493	0.020040512
126	-0.268976327	0.352447083	0.86165368	0.233543396
127	-0.45981173	0.334002687	0.850695238	-0.276083204
128	-0.421618451	0.783865229	0.825885714	-0.244314618
129	-0.623127789	1.239870492	0.561535354	-0.542003208
130	-0.196944493	0.712612007	0.814042105	-0.236679077
131	-0.396281953	0.178792044	0.944039161	-0.118608687
132	0.035510905	2.49454924	0.578647887	0.386446423
133	-0.106139351	1.021913889	0.719299611	-0.312403149
134	-0.187140212	0.255864015	0.915357711	-0.148914761
135	-0.743990606	0.161516546	0.914582278	-0.193460041
136	-0.369304134	0.223347483	0.944039381	-0.110182868
137	-0.294183725	0.614511613	0.776248447	-0.295893351
138	-0.394116983	0.108694391	0.975828571	-0.072913064
139	-0.298470185	0.214599159	0.916971168	-0.14477751
140	-0.248320591	0.647533458	0.561230769	-0.683367623
141	0.029960176	0.133327662	0.960598311	0.076203028
142	0.095418918	0.118235884	0.951724696	0.13157781
143	-0.052358903	0.102847124	0.983345133	0.047619078
144	-0.074094705	0.230510822	0.880669173	0.233285692
145	-0.044909049	0.288077033	0.923629851	-0.122772641
146	0.072294608	0.905879646	0.555433962	0.612614102
147	-1.0287523	0.112090943	0.952751295	-0.115737703
148	-0.716826712	0.479972749	0.692830918	-0.530447854
149	-0.245344971	0.028718323	0.988193867	0.025391261
150	-0.192685612	0.223689728	0.912621664	0.154962752
151	0.481016297	0.123553329	0.974211268	0.065032111
152	-0.64418686	0.244974962	0.880328657	-0.250548681
153	-0.007173125	0.00520023	0.998881197	-0.007733451
154	-0.541874729	1.041945235	0.55872	-0.583183712
155	-3.648678713	0.134343572	0.956399002	-0.088513268
156	-0.048495873	0.125441713	0.955203065	-0.100947274
157	-1.066908957	1.587807956	0.725741935	-0.288790385
158	-0.58660408	0.477161673	0.809298153	-0.272830327
159	0.011194454	0.549117117	0.852372506	-0.206472397
160	0.116652428	0.014986369	0.998531455	0.005894767
161	0.691614953	0.065182997	0.985985216	0.039149814
162	-0.178306859	0.950693483	0.552904348	-0.575381809

	K	L	M	N
163	-0.232784224	0.771448766	0.895106457	-0.136767281
164	0.308117218	0.578657173	0.895873905	0.143909242
165	-0.113127908	0.128599699	0.98076082	-0.053393258
166	-0.013219677	0.059100745	0.985361142	-0.057638168
167	-0.160594243	0.139999081	0.949191946	0.116437276
168	-0.19508981	0.265194149	0.947341238	0.087219026
169	-0.203088389	0.016864182	0.993484733	-0.01280933
170	-1.221824435	0.192418383	0.915118644	0.19925499
171	0.41246177	0.855812639	0.883211786	-0.146968206
172	0.151476577	0.259248095	0.880689394	-0.218979306
173	-1.19227134	0.240910314	0.914119601	0.167383618
174	-0.089113205	0.022185471	0.994898833	-0.018262227
175	0.470580978	0.568267105	0.814308094	-0.251504474
176	-0.336459971	0.615982343	0.643818182	-0.527586195
177	-0.369517921	3.856793438	0.474810811	-0.474123213
178	-0.07320433	0.509469758	0.731096296	-0.391203774
179	0.008145585	0.501862793	0.67478453	-0.583032184
180	-0.362515782	2.372996554	0.553948718	-0.426691691
181	-0.503983954	0.214534599	0.887752277	-0.22838317
182	-0.358489646	0.391863181	0.814297872	0.301035987
183	-0.299803932	0.701115192	0.608477987	-0.541059282
184	-0.127384061	0.071109565	0.980016667	-0.035405265
185	-0.385525035	0.089575288	0.982625551	0.048803118
186	0.515196144	1.017224315	0.860440605	0.175308228
187	0.469798837	0.017737002	0.995702448	-0.007212321
188	0.181854216	0.377920026	0.882671429	-0.168378194
189	-0.452436316	0.730466804	0.787840491	0.273458905
190	0.005780748	0.234522584	0.88415748	-0.248890559
191	0.192661341	0.559187294	0.948697051	-0.080540551
192	-0.394257065	0.101287605	0.97465896	0.065006892
193	0.011368772	0.151108878	0.980355456	-0.048043569
194	-0.388582674	0.061458552	0.984807818	0.051672618
195	-0.371961637	0.482668293	0.851488372	-0.226782269
196	0.305621916	0.086750802	0.954417085	0.123694738
197	-0.486783507	0.193262769	0.939462857	-0.123014026
198	-0.413369028	0.427419625	0.853690265	-0.223522822
199	-0.64734032	0.242868343	0.883229862	-0.242297067
200	-1.021251691	0.994189656	0.516926829	-0.675618066
201	-0.415081393	0.233830712	0.902870017	-0.189225303
202	-0.198282807	2.713475518	0.61595	-0.354131063
203	-0.337687729	0.636591201	0.814802139	-0.247481028
204	-0.291078338	0.884632183	0.732556777	0.309182697
205	-0.145268453	0.203882674	0.948633609	-0.106230206
206	-0.133313806	3.17347477	0.567054545	-0.410692003
207	-0.860297009	0.73683848	0.696037383	-0.399454753
208	-0.670883217	0.864876393	0.556571429	-0.593095356
209	-0.527855298	0.914322203	0.820893855	-0.232668771
210	-0.367360769	0.268128519	0.933354839	-0.11794832
211	-0.174947506	0.076752752	0.98740678	0.037395477
212	-1.699248308	0.10130444	0.976189349	-0.072939131
213	-0.068785513	0.085367562	0.980707727	0.056030485
214	-0.308798249	0.068910084	0.983565217	-0.049561607
215	-0.3045032	1.198552634	0.7464	-0.267036862
216	-0.290953411	0.168075935	0.952176623	-0.094393836

	K	L	M	N
217	-0.004442501	0.996537506	0.756066007	-0.273481157
218	0.18469293	0.099093674	0.985861472	0.041154226
219	-0.279656685	0.208080995	0.93173913	-0.126234902
220	0.621565887	0.009758802	0.992725869	0.042074415
221	-0.780792681	0.953828637	0.4376	-0.862353431
222	-0.375266344	1.344861969	0.572085106	-0.532643424
223	0.114804626	2.194297781	0.82465896	-0.208911684
224	-0.665528873	0.361633969	0.881729084	-0.200698641
225	-0.213190522	0.361715309	0.887160584	-0.175169839
226	0.111510207	0.115736863	0.986934759	0.036097633
227	-0.578363533	0.007602577	0.999136961	-0.007106357
228	-0.467782663	0.408972942	0.754966887	-0.39528656
229	-0.215933932	0.132211227	0.952611995	-0.08412785
230	0.335287477	0.094371465	0.975009174	0.064112769
231	0.086732967	0.699840349	0.725818182	-0.377225876
232	-0.495467717	2.339642718	0.564888889	-0.46544838
233	-0.563453966	0.276454325	0.953583435	-0.066874398
234	0.070353995	0.187627813	0.954292683	0.102804184
235	-0.061866949	0.056473182	0.986755839	0.043611738
236	-0.098342944	1.367071983	0.842236145	-0.189381282
237	-0.333328077	0.018049327	0.99198845	-0.01684422
238	-24.10979423	3.707604529	0.0115	-1.634911007
239	-0.442590142	0.061132289	0.985836207	-0.048184077
240	-34.22597405	4.700525129	0.011333333	-1.514814589
241	0.152461568	0.770503682	0.915723229	-0.122090869
242	-0.603727289	0.465041452	0.827264484	-0.25767835
243	-0.340488528	1.183104405	0.570424242	-0.483958986
244	0.062459963	0.12910153	0.955227102	0.092859268
245	-0.228842256	0.104463104	0.992084	-0.019208908
246	0.503128019	0.462168391	0.884214149	0.174013138
247	-0.184574124	0.207375853	0.931692308	-0.126997842
248	-0.146283464	3.486784121	0.755236111	-0.235882653
249	-0.684513614	0.743232779	0.702208955	-0.411971834
250	-0.092870315	0.39878498	0.85975	-0.220567703
251	-0.614434325	0.262710078	0.909042882	-0.172544479
252	-0.55013927	0.041013295	0.99155468	0.019108878
253	-0.3470181	0.269480093	0.913677852	-0.162238651
254	0.047724728	0.004633769	0.996511585	0.004054175
255	0.264827244	0.797162637	0.55640678	0.625546985
256	-0.386066595	0.055030337	0.987913978	0.049227185
257	-0.263076911	0.516209098	0.820123596	-0.276757558
258	-0.274218295	0.074503821	0.987675214	-0.04039277
259	-1.350636758	0.54010777	0.752951049	0.352934943
260	-0.288903153	0.022087095	0.991533333	-0.023543676
261	0.056282373	0.409341994	0.914473333	-0.139572567
262	0.113401114	0.179249563	0.877357873	0.358617147
263	-0.614027893	0.879983882	0.553977528	0.678133859
264	-0.28321113	0.029338809	0.991608821	-0.012467066
265	0.013450374	0.061214489	0.984703899	-0.039929708
266	23.23085084	1.078950147	0.523636364	-0.661196391
267	-0.153594711	0.813131126	0.880958084	-0.163240857
268	-0.273599248	0.377982859	0.820629738	0.336506102
269	0.159419541	0.04748233	0.985036876	-0.061425527
270	-0.439618487	0.614732044	0.832472906	-0.227504942

	K	L	M	N
271	-0.07446786	0.083999251	0.9755	0.071620729
272	-0.047187302	0.045413074	0.993034205	0.024646971
273	-0.277097934	0.350854016	0.874774848	-0.210511314
274	-0.153295687	0.306712446	0.942286525	0.103028827
275	-0.327742729	0.4460721	0.785081081	-0.328767565
276	-0.301164171	0.899749233	0.816664879	-0.226953083
277	-0.37093105	0.088198802	0.9746097	-0.0691647
278	-0.173707901	0.120195487	0.94538764	-0.154461543
279	-0.205009731	1.401465907	0.884976492	-0.135746002
280	-0.606177948	0.342845705	0.912454849	-0.148015128
281	-0.215932908	0.085965011	0.976624553	-0.083208296
282	-0.073389924	0.842292981	0.915465313	-0.125568602
283	-0.312286516	0.337937364	0.827807107	0.3066917
284	0.221169185	0.744378722	0.826191919	-0.223014408
285	-0.037339578	0.363734333	0.906211604	-0.152100457
286	0.077440556	0.374560995	0.862188506	-0.244791879
287	-0.536344493	0.173304062	0.917550152	-0.163508733
288	-0.16950002	0.676893069	0.560928571	-0.722704569
289	0.299313131	0.13802851	0.921666667	-0.181159337
290	-0.236022844	1.895853781	0.640542373	-0.353376812
291	-0.794304567	3.608454496	0.051272727	-1.205735101
292	0.401790672	0.408577893	0.734210909	-0.445680406
293	-0.276173148	0.341081777	0.850234742	-0.268107308
294	0.223241329	1.442971028	0.553770492	0.474995083
295	-0.609701365	0.147293135	0.939805158	-0.143802431
296	-0.444244526	0.780470905	0.577027778	-0.556013955
297	-0.437696684	0.131316369	0.954950495	0.087430742
298	0.24886366	0.039214695	0.980091858	-0.050170263
299	-1.055111174	0.545361823	0.692020408	-0.50344891
300	0.122825824	0.154294988	0.950761777	-0.110920376
301	-0.456771778	1.739884188	0.708818182	-0.306937536
302	0.157799038	0.443961335	0.768335484	0.359776391
303	-0.852813005	0.053737714	0.980836485	0.071953244
304	0.418041998	0.255623437	0.953013624	0.095024957
305	-0.13940154	0.545817249	0.907092784	0.139062458
306	-0.55776467	0.271414765	0.82431	-0.358865102
307	-0.591253594	0.280167551	0.885176044	0.193927341
308	-0.208610679	0.202158063	0.948440367	-0.092247009
309	0.066684719	0.20067386	0.974519573	-0.059505887
310	-0.207915806	1.282963606	0.5545	-0.496705161
311	0.373935511	2.01926969	0.41936	0.643178728
312	-0.291360271	0.363872393	0.838653659	-0.275289536
313	-0.363505659	0.704535869	0.731090909	-0.341639201
314	0.347673296	0.210936854	0.951293478	0.100366592
315	0.003864843	0.934670401	0.569139073	-0.497874366
316	-0.538119653	4.105345534	0.004	-2.665047328
317	0.266551389	0.783093458	0.78952439	-0.266429053
318	-1.037523199	2.863808941	0.183047619	-0.895688587
319	-1.053844653	1.152364761	0.73584058	-0.286649704
320	-0.160443905	0.03676287	0.990384845	-0.024564531
321	-0.343480264	0.246035738	0.915932432	-0.170524385
322	-0.135878558	0.426931525	0.852856512	-0.223297331
323	-0.242987697	0.104423344	0.987880724	0.035631604
324	-0.295501476	0.200081004	0.923392481	0.144210815

	K	L	M	N
325	0.323539225	0.257075029	0.853788546	0.295716816
326	-0.224463422	0.022832558	0.982828774	-0.056951523
327	-0.022994796	0.148528093	0.915552632	-0.232099321
328	-0.708536011	0.093581541	0.974376471	-0.074374093
329	-0.393377584	2.083297544	0.573955556	-0.409702937
330	-0.042249258	1.507817342	0.860180995	-0.174336327
331	0.397308883	0.474622487	0.818874652	0.284908507
332	-0.51803414	0.407138621	0.813149051	-0.301021576
333	-0.07008018	1.325662685	0.885140625	0.148822149
334	2.316275426	0.305843737	0.91190566	-0.13752683
335	0.070301096	0.094191468	0.991377953	0.016014523
336	-0.306872837	0.09922507	0.979220068	0.059175915
337	-0.150749943	0.052694937	0.987817603	0.04439248
338	-0.422881495	0.780207813	0.515857143	0.788648393
339	-0.617421602	4.353916018	0.453151515	-0.485874388
340	-0.815614947	0.152184929	0.930737151	-0.156075795
341	-0.608867498	0.674998346	0.828413793	-0.255012088
342	0.079977524	0.274842701	0.893859155	0.182434294
343	0.803960507	0.701974282	0.862906725	-0.187431124
344	-0.137146257	1.136670215	0.771925234	-0.252365536
345	-0.382703987	0.619327808	0.691737374	-0.460925208
346	-0.188246987	0.181286086	0.946352632	-0.097580804
347	-0.595542756	1.354555165	0.4884	-0.632888794
348	-0.577275867	0.044516463	0.981600819	0.035709593
349	-0.073307714	0.151679994	0.986615721	-0.039359623
350	-0.242164032	0.371985299	0.885711111	-0.177713818
351	-0.258138774	0.477924077	0.825741772	-0.256693734
352	0.063195898	0.067655508	0.981826281	-0.060591804
353	-0.571075503	0.004554596	0.998079851	-0.004225625
354	-0.319822447	0.003047323	0.998441367	-0.002249824
355	-0.828239019	0.521111566	0.724963855	-0.414942635
356	-0.488425229	2.191068557	0.408518519	-0.617150625
357	-0.458924262	0.548486967	0.881343396	-0.164421082
358	-0.576212542	1.055326216	0.696176471	-0.361765756
359	-0.951996305	0.921634606	0.71087037	-0.3604304
360	-0.409389739	0.166175821	0.91172457	0.185204612
361	-0.20991626	1.284301527	0.727356322	-0.291792976
362	-0.301241621	0.107416095	0.945878628	0.132979711
363	-0.217413398	0.218858106	0.953756458	0.072513368
364	0.113254501	0.268768275	0.916160514	0.148525238
365	0.216216123	0.055605981	0.981985462	0.037170622
366	-0.250346556	0.458691141	0.888433645	-0.167985068
367	0.003358252	0.794400068	0.908012987	-0.120533413
368	-0.828951245	0.695266107	0.612814815	-0.534733031
369	-0.054306897	0.923479858	0.884879852	-0.145776537
370	-0.21531165	1.421691784	0.517692308	-0.583634694
371	-0.41562233	0.480514881	0.832967901	-0.247386085
372	-1.14842208	0.063657875	0.984509249	-0.051710341
373	0.518728802	4.320336894	0.012	-1.963269764
374	-0.316257941	0.332045364	0.876668024	-0.216374079
375	0.485875533	0.139155363	0.952249661	-0.120011436
376	-0.310323532	0.615863243	0.72140625	-0.373151355
377	-0.650464306	0.793557806	0.624674699	-0.483511183
378	-0.474551452	0.167302431	0.925885125	-0.149982876

	K	L	M	N
379	0.021544234	0.007320989	0.997324675	0.003366682
380	-0.269906025	0.95125503	0.639291429	-0.427927653
381	-0.272688767	0.19926326	0.944856745	-0.111242082
382	-0.145509716	0.017235013	0.992604785	0.014287101
383	-0.820981076	0.018980877	0.991478942	0.026144028
384	-0.420483132	0.527267272	0.853296037	-0.220258501
385	-0.361034154	0.108954096	0.961963899	-0.082070457
386	0.16281974	0.225842981	0.981521244	-0.027214474
387	-0.654376753	0.242213057	0.915872054	-0.17074373
388	-0.489907468	0.429476253	0.868273859	0.202366723
389	-0.91364841	3.464929091	0.363243243	-0.627078798
390	0.230336719	0.77851871	0.641517241	-0.466688368
391	-0.072479803	0.027205045	0.991854043	-0.023054123
392	-0.309524634	0.470023235	0.89448951	-0.150381936
393	-0.264620397	0.728974435	0.822084507	-0.247029622
394	-0.201092138	0.345341491	0.842973621	-0.276097192
395	-0.024244189	0.040522861	0.980674948	-0.043704563
396	0.092909839	0.102672184	0.986549356	0.038117515
397	-0.278612179	0.364243256	0.880795181	-0.202592002
398	0.051958153	0.84388265	0.904069204	-0.131652832
399	0.327140157	0.230439516	0.91307483	-0.17990303
400	-0.454292047	0.629330549	0.749939394	-0.321152581
401	0.363839093	0.719669021	0.715837104	-0.387678358
402	-1.830547631	0.273908392	0.814107527	-0.401322047
403	-0.299505218	0.124799404	0.953490494	-0.098941803
404	-0.213015384	0.596651097	0.727677966	-0.399610096
405	-0.435852777	0.323208347	0.816441026	-0.325585471
406	-0.059457503	0.51360038	0.748040134	-0.350740645
407	0.174615418	0.047299437	0.984953586	-0.046347936
408	-0.359199431	0.10645304	0.973757785	-0.063203812
409	-0.235842761	0.177509457	0.973899179	-0.05834304
410	-0.125812158	0.129680687	0.983153252	-0.043874317
411	0.121135232	0.386780476	0.729797357	-0.568504333
412	-0.072029498	0.032467629	0.987745711	0.032948812
413	-0.145003173	0.111732553	0.952446078	-0.09209209
414	-0.280270676	0.569276031	0.851518868	-0.218257056
415	-0.379432233	0.554639867	0.671846154	-0.537619273
416	0.130079682	0.007573542	0.998819119	-0.006806903
417	-0.058324545	0.373232551	0.905542169	0.153182348
418	-0.246322852	0.797965653	0.875739837	-0.168117523
419	0.214129312	0.311171945	0.883106227	-0.188122855
420	-0.057847838	0.024578661	0.993646154	-0.013517804
421	-0.487101362	0.169778721	0.882533081	0.315236621
422	-0.559132362	0.228841076	0.924472674	-0.12972535
423	-0.014063402	0.132172554	0.953921182	0.085809708
424	-0.046211431	0.074149177	0.986142402	0.03929117
425	-0.177447655	0.41791219	0.859818594	-0.230234146
426	-0.768975126	0.495143178	0.778320988	-0.322007073
427	-0.266161944	0.649704509	0.788593939	0.280864292
428	-0.251871622	0.355342968	0.884176583	-0.190875159
429	-0.366701382	0.270614322	0.8591693	-0.292074415
430	-1.260637806	1.25407466	0.491631579	-0.633284251
431	-0.459750828	0.64500318	0.8406618	-0.219819387
432	-0.073548245	0.098940461	0.966293269	0.08411683

	K	L	M	N
433	0.093730706	0.018356579	0.99139122	0.024574492
434	-0.092364997	0.082016179	0.98312277	-0.035072962
435	-0.09098326	0.022398553	0.992478392	0.03936153
436	-0.186382656	0.221843076	0.916199678	-0.163001378
437	-0.148900168	0.25650631	0.91155832	0.143356747
438	0.155708728	1.232242551	0.641209302	0.396238327
439	-0.0557981	0.613309528	0.877371663	-0.178632736
440	-0.203406764	0.74784485	0.812319559	0.243205176
441	-0.365458564	0.520759902	0.704986047	0.477017932
442	-0.059944044	0.967135166	0.698187192	0.372023688
443	-0.828801379	0.220690605	0.884839216	-0.258888245
444	-0.123747959	0.125360569	0.980960362	-0.052117242
445	-0.171195039	2.538259829	0.57179085	-0.375567966
446	-0.320392813	0.271399995	0.915119869	-0.154907227
447	-0.274211862	0.14875232	0.942926554	0.135351393
448	0.200017107	0.141645751	0.987641138	-0.040152868
449	-0.109075824	0.037037196	0.990998004	-0.02461603
450	0.556281725	0.245269308	0.938820144	0.114548577
451	0.114729147	0.307034389	0.952859671	0.074076547
452	-0.73697348	2.28718641	0.398285714	-0.60768191
453	-0.408667145	2.130267333	0.574769231	-0.38380856
454	-0.40767584	0.27246345	0.918244648	-0.133197572
455	0.024004252	0.768120144	0.722482213	-0.343225479
456	-0.44872301	1.200486476	0.421066667	-0.844420963
457	-0.795316095	0.571136821	0.814929134	-0.252099567
458	0.326594182	0.028329822	0.989795837	0.025741365
459	-0.508289821	5.109526028	0.448738462	-0.477473789
460	-0.755334251	0.273701364	0.880801587	-0.229073207
461	0.247635747	0.001579954	0.9991875	-0.000601663
462	-0.11690661	0.094289304	0.980781354	0.05092197
463	-1.014717955	0.034680279	0.980242051	-0.044311735
464	-0.159900184	0.008219305	0.99408596	0.028959274
465	0.61659833	0.000724125	1	-0.003017002
466	0.170448065	0.133210517	0.986291262	-0.037414763
467	-0.672007657	0.213199409	0.915265574	-0.173412959
468	-0.008203781	0.19760285	0.885507692	-0.274550968
469	0.048852737	1.093332992	0.479722222	-0.699697495
470	-0.281409159	0.055159761	0.979635246	-0.033337063
471	-0.969458968	3.710281746	0.012	-1.967867745
472	0.044392763	0.323523184	0.812583554	-0.340941747
473	0.412959737	0.714728513	0.849235154	0.207649231
474	-0.324796696	0.146820398	0.946919205	0.109176848
475	-0.696023242	1.792637766	0.329371429	-0.808245765
476	0.4870467	0.024811738	0.989128713	-0.0280749
477	-1.320225268	0.045881886	0.987554098	-0.066869948
478	-0.822943762	4.117129072	0.165882353	-0.84979248
479	-0.900720251	2.111278463	0.366461538	-0.713036431
480	-1.251265678	1.39005732	0.5836	-0.44226265
481	-0.120944975	0.335938251	0.952599483	0.07764456
482	-0.720096627	1.636203073	0.315483871	-0.888760037
483	-2.064375267	0.883808034	0.57114094	-0.51363288
484	-0.310267839	0.188097779	0.923975976	0.148203532
485	-0.644211957	0.274939636	0.856051392	-0.267810822
486	-0.226911021	0.152923242	0.974527316	-0.063976924

	K	L	M	N
487	-0.44470977	1.420650499	0.566827068	-0.455531226
488	-0.490372035	1.192341805	0.554947368	-0.521929423
489	-0.013542769	1.055913052	0.571	-0.477536096
490	-0.310253297	0.220249632	0.895298246	-0.2044025
491	-0.103891743	0.094237861	0.975864959	0.069426219
492	-0.052606514	0.016854234	0.993703985	-0.010522419
493	0.014903807	0.06841911	0.980840223	-0.062022315
494	-0.370726424	0.254727255	0.883264865	-0.202142504
495	-0.445677619	0.517423714	0.775783439	-0.324895435
496	-0.298916907	0.402888655	0.916013201	-0.137452655
497	-0.303772189	0.895564397	0.725092437	-0.338436339
498	-0.113830656	0.012566125	0.9952772	-0.010290782
499	0.17754579	0.091333135	0.954358974	0.10219468
500	-0.041477938	0.043742758	0.980583942	0.045230018
501	-0.141523215	0.315095062	0.824490028	-0.373363707
502	0.121989034	0.383249626	0.868316667	0.212273492
503	-0.447957435	0.869516412	0.726677824	-0.340269301
504	-0.400793084	0.412755817	0.857175966	-0.217150158
505	-0.492095254	1.804840919	0.335777778	0.795864529
506	0.123011802	0.239130291	0.904372414	0.184354782
507	1.221314281	0.130039658	0.938447932	0.156487359
508	-0.219291246	0.898612135	0.695113208	0.372609668
509	-0.577052287	1.654655538	0.08875	-1.637216568
510	-0.474981394	0.297942186	0.882358423	0.185520808
511	-0.196269796	0.498843878	0.917507438	-0.131082111
512	-0.224221587	0.069826553	0.988635268	-0.050260332
513	0.036552295	0.686235859	0.724156522	-0.381892098
514	0.043438119	0.631964565	0.803633136	-0.27351443
515	0.006649225	0.634890378	0.695548718	-0.457738664
516	-0.165991337	0.079874125	0.973275986	-0.090737449
517	0.062380751	0.101991155	0.954041312	0.092668109
518	-0.531583745	0.541576613	0.689454545	-0.482893414
519	-0.548766282	0.303072761	0.876269388	-0.227310181
520	-0.552511106	0.154553499	0.951207547	0.111151165
521	-0.563372354	1.083579148	0.732946939	-0.313125398
522	-0.13596612	2.015412111	0.394243902	-0.697583093
523	-0.645277884	3.002407557	0.426782609	-0.587082545
524	-0.228771158	0.031311971	0.989753968	0.02471945
525	-0.275760775	2.470634488	0.411255814	-0.635742188
526	-0.190278655	0.500904977	0.862055046	-0.217709012
527	0.040168732	0.559430067	0.866702703	-0.187868754
528	-0.071783713	1.952201264	0.425666667	-0.6567546
529	0.080969188	0.230760723	0.956317949	0.081026713
530	0.547696858	0.051288349	0.993012048	0.022993935
531	-0.33228597	0.017340792	0.993499513	0.023126178
532	-0.258610427	2.187977332	0.732315789	-0.263568242
533	-0.433957709	0.209688853	0.954931507	-0.101803886
534	-0.165116855	5.048595281	0.458387097	-0.482038074
535	-0.035664371	1.479444569	0.738661871	-0.271336873
536	-0.454908513	0.652245009	0.831326733	-0.225135803
537	-0.040024554	0.672432108	0.867909091	-0.178516176
538	-0.242779434	0.585472188	0.815946667	-0.253252029
539	-0.05277837	0.440472502	0.847791469	-0.240614997
540	-0.165811424	0.613474736	0.7504	0.330242157

	K	L	M	N
541	-0.182678222	0.587826755	0.850915556	-0.203484005
542	0.026913891	0.608814264	0.886478261	-0.151567459
543	-0.295096776	2.813273094	0.5655625	-0.397427029
544	0.007475618	0.040015962	0.994727619	0.009075165
545	-0.263038666	0.129788132	0.975647332	0.059961955
546	-0.397713917	0.019871228	0.992141762	-0.013872147
547	-0.974769208	1.801829948	0.186363636	1.099163479
548	-0.184297872	2.29362411	0.166947368	-1.028972838
549	-0.44414122	0.109028511	0.966905162	0.079137378
550	0.250434852	0.254762826	0.922433483	-0.128605101
551	0.132534058	0.500568892	0.81281039	0.26262135
552	-0.279236132	0.745321259	0.636781065	-0.488343769
553	-0.122446253	0.167289525	0.980662883	0.049256855
554	-0.439215973	3.824588308	0.414285714	-0.574377272
555	-0.182993257	0.251572333	0.907808219	0.176043404
556	-0.107901729	0.189952146	0.9314	-0.136936188
557	0.514853894	0.174910467	0.915160256	0.187325795
558	-0.872048403	1.228953665	0.3856	-0.90118938
559	-0.417604855	0.115465049	0.980183036	-0.049353282
560	0.016460237	0.289915378	0.913856049	0.132246229
561	-0.629822942	0.478033175	0.858477419	-0.206365373
562	-0.050583268	0.066360073	0.9867473	-0.046678755
563	-0.315704826	0.299598259	0.851466042	-0.28935602
564	-0.558841471	1.3831319	0.695595745	-0.352278815
565	-0.562730914	0.553731373	0.752893617	-0.352853563
566	-0.271811981	1.007603448	0.729102881	-0.321813795
567	-0.140571678	1.742051098	0.655711111	-0.352526347
568	-0.386385974	0.19520192	0.924769231	-0.139666875
569	-0.368498251	0.5721239	0.796605341	-0.286200205
570	0.543420433	0.250654128	0.862363257	0.272461997
571	0.187270451	0.05001024	0.982238532	-0.031927533
572	-0.214162542	0.1633793	0.906633694	-0.184188419
573	-0.598836901	0.092721979	0.953808354	-0.104765574
574	0.107561376	0.025680892	0.99093412	-0.022953457
575	0.023878532	0.525248283	0.91497351	-0.130670759
576	-0.140945824	0.324261838	0.889259786	-0.174700631
577	-0.354604954	0.856135619	0.885601463	-0.145152834
578	-0.154101574	0.623418337	0.725172414	-0.396789975
579	-0.045359511	0.025186896	0.992399232	-0.013157527
580	-0.016668425	1.718146673	0.811388235	-0.219680574
581	-0.752948744	0.089475427	0.98040404	0.055765152
582	0.861733349	0.394124311	0.750337838	0.417260276
583	1.147581267	0.298563118	0.814971722	-0.348266178
584	-0.534384583	1.989193809	0.4032	-0.635533227
585	-0.239031889	0.52603059	0.893460317	-0.148744795
586	-0.467311335	0.322509152	0.87783871	-0.215927548
587	-0.413372101	0.411768603	0.883650624	0.163231108
588	0.157624325	0.093206783	0.987191489	-0.036572986
589	-0.061532435	0.447755008	0.858637555	0.214905421
590	0.157294987	0.937532474	0.855444444	-0.176580005
591	-0.248260259	0.693334168	0.609276074	-0.535491731
592	0.285895585	0.154727588	0.97532093	0.057757484
593	0.210079771	0.398979754	0.861506276	-0.212052027
594	-0.173764124	0.006784966	0.999300935	-0.005577935

	K	L	M	N
595	0.084266329	0.235696451	0.941754584	0.109615962
596	-0.053063392	0.40460497	0.91461563	-0.129957411
597	-0.493675077	1.134221691	0.58581295	-0.473486582
598	-0.15346173	0.401436673	0.720372093	-0.489633984
599	-0.237348644	0.1434021	0.98221041	0.044210434
600	-0.109134272	1.218439767	0.786344411	-0.240842395
601	-0.247560443	0.063053376	0.981759336	0.034945806
602	-0.279427426	0.697492708	0.811036269	-0.235743205
603	-0.295187087	0.367744087	0.885183206	-0.186489317
604	-0.632350016	0.120021615	0.954743169	-0.134789997
605	0.129699431	1.112725402	0.824265985	-0.208580441
606	0.045781931	0.213673924	0.88184493	0.27513059
607	-0.027939834	0.016307345	0.993704545	0.010022057
608	0.162812461	0.171876178	0.944402266	-0.125204934
609	0.430298647	1.542257457	0.552850394	-0.458105511
610	-0.22710442	1.604156736	0.720346457	-0.285605325
611	-0.772921528	0.617647379	0.852324074	-0.209165785
612	-0.116192347	0.795817066	0.56439604	-0.674737506
613	-0.567710526	2.452698722	0.5688	-0.45117357
614	-0.194402884	0.201705844	0.954445964	-0.102930493
615	-0.164809596	1.169429633	0.564869565	-0.570817947
616	-0.064790553	2.501744109	0.1822	-0.95748753
617	-0.259105246	1.393881164	0.698190955	-0.340713925
618	0.276082855	0.088370092	0.955481943	0.115750843
619	-0.456689499	0.667945608	0.73001626	-0.366978963
620	0.09001063	0.019077937	0.992721311	-0.016856935
621	-0.207749283	0.261441581	0.858008439	-0.270647897
622	-0.705980382	0.333737226	0.878482474	0.218905555
623	0.028816062	0.859885074	0.770352201	0.270777596
624	-0.39162975	0.114165438	0.944903226	-0.161499659
625	-0.573549621	0.185594335	0.907676898	-0.186708238
626	0.030598544	1.375594146	0.547936	-0.47910987
627	-0.388319766	1.363870518	0.529929412	-0.568090015
628	0.155724195	0.238518332	0.951466993	0.070240657
629	-2.882713936	2.50741032	0.063076923	-1.362650341
630	-0.106561285	2.102375495	0.69742439	-0.310848448
631	-0.360956719	0.26102433	0.916363057	-0.147872925
632	-0.708612687	0.672351114	0.562702703	-0.729199727
633	-0.865337193	1.156077586	0.579216783	-0.466540655
634	-0.616502546	0.129932157	0.951470588	-0.11896748
635	-0.312289724	0.785957398	0.884605825	-0.158237669
636	-0.047488165	0.425351103	0.720162162	-0.535079108
637	0.746174913	1.106881722	0.555619048	0.557162391
638	0.377100786	0.046433627	0.986870968	-0.02722168
639	-0.486762753	0.028208635	0.99328031	0.015831206
640	-0.248263477	3.406893019	0.50985	-0.463819292
641	0.289925801	0.441716012	0.844721154	0.244944043
642	-0.114435664	0.017196109	0.999108614	-0.004730225
643	-0.308977737	0.128911332	0.956168367	-0.098584069
644	0.059163524	0.078138742	0.979667804	-0.06545639
645	-0.838007884	0.526350896	0.746552901	-0.352759467
646	-0.079992917	0.871057509	0.556884956	-0.606287003
647	0.257132512	0.628571076	0.679247312	0.483171039
648	1.394533741	0.623081479	0.457079365	-1.308909098

	K	L	M	N
649	0.101488633	0.313148446	0.862630252	0.238046646
650	-0.065590788	0.309837484	0.855886957	-0.253967073
651	0.016657184	0.070670034	0.98556427	-0.04901335
652	0.061879392	2.067300954	0.646592179	-0.344714271
653	-1.231227162	0.553641175	0.620452381	-0.606579675
654	-0.183384956	0.069161268	0.975378092	-0.092218187
655	-0.332509399	0.932298987	0.613842424	-0.456334008
656	0.300310451	0.103840923	0.982287006	-0.05418947
657	-0.430213574	2.501637074	0.31369697	-0.733054267
658	-0.077690663	0.282359944	0.918940639	-0.130167855
659	-0.619197304	0.263233595	0.890792185	-0.19059266
660	-0.184653526	0.542627452	0.601493671	0.663348092
661	-0.518602356	0.335920289	0.82094186	-0.364739948
662	-0.423825579	1.058978354	0.561061224	-0.582376056
663	-0.031350349	1.177718332	0.774234921	-0.253288905
664	0.045780433	0.107600759	0.955160248	0.098804262
665	0.202232198	0.078749419	0.982544715	0.026635276
666	-0.294406724	0.063629638	0.984315457	-0.038622326
667	-0.390388235	0.013240643	0.994305213	0.011270735
668	-0.222465626	0.276062808	0.913554502	0.14332623
669	-0.115037852	0.215545532	0.910104235	-0.172013389
670	0.240585821	0.251837275	0.857297297	-0.309707854
671	-1.242633096	0.159047502	0.922918276	0.160445743
672	0.207279103	0.086561956	0.981426667	0.052803463
673	-0.424884157	0.343968485	0.784566265	-0.392997318
674	0.017241646	0.147348046	0.946139276	-0.128910065
675	0.043340258	0.028779745	0.992039139	0.019248539
676	0.069142218	0.142999356	0.979799087	-0.052721447
677	0.204583825	0.14669062	0.939641026	-0.141866684
678	0.046583777	0.36208364	0.911582382	-0.139866723
679	-0.472307047	0.445099654	0.813945652	-0.2881368
680	-0.230333654	0.158359957	0.913098827	0.229797999
681	-0.396117153	1.374876059	0.730477612	-0.282656352
682	-0.197021037	0.223391067	0.924205128	-0.137572183
683	-0.022711561	0.049133587	0.981049112	-0.042682436
684	-0.086165439	0.58434947	0.586347826	-0.696724998
685	-0.910334735	0.430877997	0.801781711	-0.322472254
686	-0.402711527	0.285792181	0.931936324	-0.112633175
687	-0.299044177	1.817847689	0.67547541	-0.342137867
688	-0.286527197	0.401169877	0.918286585	-0.118997786
689	-0.653026167	1.624344039	0.083714286	-1.745630688
690	-0.150446969	0.638475557	0.695729167	-0.461199866
691	0.020849313	0.083160468	0.992941402	0.010632621
692	-0.606436905	1.022378982	0.56744	-0.481815762
693	0.387033206	0.402043365	0.884028881	-0.167465846
694	-0.084470173	2.619298943	0.724816667	-0.273434957
695	-0.44279276	0.358718682	0.907901235	-0.127798716
696	-0.244574675	0.379509759	0.882155598	-0.183757782
697	-0.096831946	1.27903062	0.725524664	-0.319670995
698	0.16331015	0.037142619	0.992475362	0.013602151
699	-0.161685178	0.187978103	0.951205333	-0.09871165
700	0.45290847	0.192975994	0.942903409	-0.120021184
701	-0.114669626	0.279056943	0.929861671	-0.112860786
702	-0.323127473	0.288134233	0.930787879	0.111950133

	K	L	M	N
703	-0.413048817	0.396062587	0.889617021	0.16214286
704	-0.282523	1.258629919	0.728534351	-0.292017619
705	-0.262482404	2.434886901	0.724855895	-0.282489777
706	-0.546416504	0.190433776	0.9143104	-0.176277161
707	-0.003203692	0.579318498	0.842902913	0.225435681
708	0.172828927	1.779466018	0.727070175	-0.295999951
709	-0.154661638	0.043711174	0.992828829	0.023372014
710	0.196931922	0.19327218	0.981177528	-0.045363108
711	-0.356372849	0.589698128	0.820678363	-0.272462209
712	-0.148824834	0.088031731	0.974712264	-0.077898873
713	-0.095623491	0.121366522	0.953346341	-0.085779826
714	-0.350273256	0.521428759	0.722112	-0.414773517
715	-0.306748196	0.422301404	0.811322751	0.288779153
716	0.1944518	0.051693211	0.990997038	0.018624624
717	-0.184254656	0.112100201	0.953841764	0.115790897
718	-0.021026335	0.011558707	0.995045283	-0.00951555
719	-0.263042945	0.710048011	0.788430769	-0.276305305
720	-0.464693654	0.07766711	0.987948608	0.040346993
721	0.022578813	0.245549043	0.949572414	0.100107405
722	-0.257331358	0.411930121	0.883705882	0.170613395
723	-0.097352862	0.25154236	0.949763441	0.092801412
724	-0.276317652	0.151170762	0.947549072	-0.107802921
725	-0.284801613	2.524438159	0.577832258	-0.372443517
726	-29.04076864	1.962171644	0.0856	1.451680713
727	-0.503398983	3.758581008	0.548227273	-0.433496475
728	-0.054702214	0.188735055	0.954846348	0.081288232
729	0.407624995	0.722074558	0.749542857	-0.319203907
730	-0.457011984	0.011708536	0.994111216	-0.009442012
731	-0.096523829	0.410512719	0.861312073	-0.232742098
732	0.449439573	0.353695551	0.811390029	0.358879301
733	-0.012631909	1.482854528	0.242666667	1.120905558
734	0.386041642	0.271552469	0.91370607	0.147287793
735	-0.223003765	1.280368687	0.557941748	0.529032601
736	0.032096805	0.22573019	0.910291925	-0.151450899
737	-0.310602287	3.309373045	0.421824561	-0.542863846
738	-0.019880571	1.306012254	0.940941176	-0.088199827
739	-0.933553408	1.470273089	0.414090909	-0.765336355
740	-0.460056937	1.716026335	0.814951351	-0.206589805
741	-0.50794652	0.626676604	0.74500339	-0.324921078
742	-0.657042181	1.45468593	0.691957672	-0.348210653
743	-0.310066233	5.493236077	0.53645977	-0.415353563
744	-0.278036547	0.041074431	0.981822268	-0.036378225
745	0.263817628	0.261769946	0.925129909	-0.129459805
746	-0.368862653	0.117554057	0.946509908	-0.12412601
747	-0.321321098	0.393215658	0.74552381	-0.423429913
748	-0.156739155	0.668987644	0.723089286	-0.393850327
749	0.14094259	0.070710136	0.980452915	0.06232516
750	-0.2869071	0.020529116	0.994305927	-0.012126711
751	-0.076514089	0.199347153	0.896390244	-0.215594822
752	-0.133365112	0.12795126	0.955490291	-0.081280178
753	-0.290090469	0.213759876	0.933117904	0.125857035
754	-0.151138431	0.438447232	0.884899048	0.175551309
755	-0.598874786	1.111856563	0.556373832	-0.551323149
756	-0.536683024	0.323596548	0.916700965	-0.139443503

	K	L	M	N
757	-0.161849446	0.072399253	0.986802632	-0.050413979
758	0.025366936	0.210654217	0.932568082	0.128655752
759	-0.022588417	0.01519698	0.995565217	0.008913252
760	-0.324644133	0.295840508	0.881528	-0.221576267
761	-2.188325595	1.34063346	0.306625	-1.000398424
762	-1.622514393	0.131777766	0.912662461	-0.237250858
763	0.23629988	0.464078265	0.73595572	-0.410918978
764	-0.663900338	0.231833288	0.878987654	0.274239222
765	-0.356274697	1.528910521	0.420085106	-0.732210371
766	0.147859035	0.00393444	0.998340741	-0.003133562
767	-0.282439891	0.855929931	0.81565651	-0.235651652
768	0.330544878	2.158315669	0.560967033	-0.473295848
769	-0.387660688	1.220565489	0.573970149	0.474173652
770	-0.102967457	1.715368841	0.568870748	-0.414047665
771	-0.107500047	0.00891146	0.996642857	0.008496602
772	-0.126689595	0.219779346	0.859430998	0.316645092
773	-0.077432392	0.120376984	0.952061935	-0.109007094
774	-0.116062552	0.012300532	0.99516525	0.009577645
775	-0.0005188	0.625767822	0.907611584	-0.133892483
776	-0.412955931	0.200191064	0.931028902	0.127695719
777	0.024974473	0.96711444	0.747780822	0.284611384
778	-0.058403888	0.06896122	0.980981289	-0.034783257
779	0.079655789	2.020655045	0.613242236	0.372229894
780	0.202250529	0.515585634	0.851943925	-0.222686768
781	-0.178872408	1.308456831	0.752418118	-0.268672943
782	-0.877698953	0.40634482	0.729735849	-0.466712952
783	-0.426160308	1.888167601	0.562156863	-0.469830407
784	-0.096526682	0.317532326	0.822806122	-0.324481116
785	-0.469643261	0.117870435	0.95651816	-0.083109538
786	-0.251679041	2.820681696	0.57137037	-0.420437707
787	-0.150971948	0.172627667	0.96746283	-0.06628863
788	-0.837268676	0.769427319	0.683336898	-0.43211725
789	-0.2240985	0.124816934	0.955909206	-0.096038606
790	0.288472937	0.308761519	0.854053812	-0.266384761
791	-0.190606837	1.009653664	0.563670103	-0.597760306
792	-0.024541425	0.539275828	0.907622291	-0.118126975
793	-0.091826387	0.127154071	0.979410309	-0.028418647
794	0.113478048	2.855194112	0.727253333	-0.279001872
795	-0.170854442	0.158238302	0.97493772	0.060320748
796	-0.260859341	0.423965251	0.77178481	-0.361838235
797	-1.30079006	0.446389644	0.724759494	-0.481296115
798	0.090876395	0.067938417	0.983365546	-0.037599776
799	-0.182962093	1.224421796	0.769967427	-0.256765789
800	-0.50749333	0.451063484	0.833171216	0.254956139
801	-0.865480758	0.447506499	0.85213363	-0.221809811
802	-0.030999783	0.139324763	0.957933162	-0.096836302
803	-0.892011824	0.204845913	0.888097015	-0.248024411
804	-0.652923048	0.720599127	0.733875519	-0.35955747
805	-0.111885763	0.701561093	0.690495238	-0.41568968
806	-0.525091426	0.934291867	0.494724638	0.774904039
807	-0.152390142	0.192187784	0.954199181	0.104041206
808	-0.18271426	0.241982586	0.92297006	0.131394492
809	0.461130539	1.39841648	0.329529412	0.930628883
810	0.365363067	0.848883637	0.552396694	0.59638426

	K	L	M	N
811	-0.333062232	0.174686763	0.915405676	-0.207034641
812	-0.147669951	0.131193964	0.974904368	-0.065189785
813	-0.527495831	0.264066849	0.893848858	0.185449176
814	-0.018051945	0.120851651	0.953767089	-0.100172043
815	-0.38100459	0.133956707	0.95377129	0.08110004
816	-0.278421896	1.335632069	0.888089385	-0.140421549
817	0.144517382	2.880342396	0.544825397	-0.403249529
818	-0.159977389	0.356836527	0.823672316	-0.336556541
819	-0.313410219	3.318091813	0.461970149	-0.505395465
820	-0.188174474	0.802290107	0.815704918	-0.236516741
821	0.004463505	0.105592863	0.985460946	-0.04510498
822	0.023445862	2.082518247	0.783233533	0.222582499
823	-0.263427998	0.009020167	0.997416357	0.003715727
824	-0.745691493	0.2983986	0.915264659	-0.139795303
825	0.050659682	0.149261167	0.956150376	0.086297989
826	0.025584255	0.25772129	0.912423676	-0.143278334
827	0.575222598	0.016467608	0.99294186	-0.021329244
828	-0.313054745	0.514519988	0.824920455	0.278512319
829	-0.247586571	0.453884947	0.889303371	0.168707742
830	-0.652032536	1.2559605	0.770779221	-0.2548364
831	-0.380443562	0.15768713	0.955753086	-0.080154631
832	-0.464724488	0.43836925	0.816175342	-0.291769028
833	-1.486126144	21.50714759	0	-20.98047108
834	1.303000577	0.019311691	0.991681592	0.038307614
835	-1.23020466	0.01912681	0.9915	-0.024176915
836	0.22228379	0.443271277	0.9121625	0.123484717
837	-0.02662657	0.298413327	0.883698842	0.208055708
838	0.179752149	0.498895877	0.850817967	0.228293737
839	-0.559507056	0.691936565	0.709706422	0.400524563
840	-0.189979555	0.040597934	0.980276574	-0.042273204
841	-0.248927973	0.333737022	0.884177122	0.184538947
842	-0.138485248	0.161740816	0.950272251	-0.09920311
843	-0.12845745	0.091790507	0.979776286	-0.054270638
844	-0.371177667	0.062145705	0.987097744	-0.045904795
845	-0.218048128	0.106026985	0.95622023	-0.113057454
846	1.311778447	1.55764344	0.264965517	0.991181056
847	-0.790816255	0.112141169	0.951599466	0.131440481
848	-0.111490011	1.313080754	0.753639576	-0.270671209
849	-0.014478938	0.1700428	0.955219024	0.08219507
850	0.135602766	0.36309828	0.907760518	0.1388109
851	-0.121338497	0.54259212	0.74854485	-0.339083142
852	-0.842384547	0.984435086	0.70472	0.371114519
853	-0.465123773	0.057682491	0.984805167	0.049770355
854	0.318357696	0.989236244	0.559175573	0.526769638
855	-1.200251531	0.023950578	0.993578947	-0.01885181
856	-0.504296896	0.226657311	0.95434375	-0.085800383
857	0.576954807	0.645346447	0.757065789	0.309908125
858	-0.000832569	0.430877277	0.883210054	-0.163030624
859	-0.394699021	0.100016366	0.954160692	-0.101887597
860	0.339793265	0.273283903	0.911	0.150586446
861	0.00835074	0.13995446	0.955135959	0.093712913
862	-0.216178339	2.338796947	0.483323944	-0.528992759
863	-0.046716577	0.156090819	0.955268542	-0.0913709
864	-0.120372276	0.001803437	1	0.001074049

	K	L	M	N
865	-0.112490531	1.163076328	0.45025	-0.728865729
866	0.298887039	0.810322567	0.407846154	1.110274633
867	0	0.487479287	0.678108696	0.587882572
868	-1.202719527	0.037517526	0.982779592	0.037594689
869	0.103138239	0.355675083	0.862447489	-0.248868942
870	-0.061323399	0.759451659	0.884970646	-0.160581165
871	-0.294256823	2.047207946	0.471253333	-0.542824427
872	-0.718256548	0.741205764	0.572328767	-0.570914163
873	0.211735073	0.308349714	0.913032258	0.157353719
874	-0.776439124	0.196102158	0.883387597	0.282452265
875	-0.418266887	0.256341803	0.876614754	0.253336377
876	-1.387376592	0.067743664	0.979727891	-0.07003763
877	-1.483163798	0.0983353	0.954671756	-0.117414263
878	-0.086181511	0.153167454	0.976305489	-0.06473732
879	-0.068040573	0.309588854	0.890600707	-0.175391727
880	-0.399601503	0.088422369	0.973997661	-0.074575848
881	-0.138292874	0.105535323	0.976444959	0.064047496
882	-0.478737568	0.013339466	0.994192564	-0.013902664
883	-0.246580071	0.603036142	0.934303207	-0.098749161
884	-0.230778774	1.009886072	0.722307692	-0.330782996
885	-28.98643818	0.714232144	0.567449541	-0.69809511
886	0.390629308	0.304665051	0.907664957	0.161569383
887	-0.0217602	0.07435136	0.980394678	0.056263818
888	-0.303863716	0.021363896	0.992639692	0.015475167
889	0.301060981	0.233018902	0.950583679	0.102142334
890	-0.383155485	0.856460414	0.725304721	-0.347810533
891	-0.219705437	0.437763677	0.905112263	-0.147491879
892	-0.163646499	0.057072081	0.985982418	0.059239705
893	0.191417118	1.710544223	0.251130435	-1.024038527
894	0.294085708	0.893667287	0.749812081	0.285989973
895	-0.141007948	0.410556696	0.81408377	0.289027532
896	-0.226780963	0.786771598	0.899340278	-0.134499232
897	-0.564089545	0.056159365	0.987164179	0.045313517
898	-0.092189349	0.071654483	0.980877828	-0.065858629
899	-0.402530668	0.025349855	0.991276637	0.020436817
900	-0.443245838	1.080903091	0.641132948	-0.41127989
901	-0.460015165	0.158213802	0.953336942	-0.110532125
902	-0.299744797	0.733740111	0.855757303	-0.194228066
903	-0.694720012	0.406926022	0.834696078	0.262872272
904	0.043902855	0.000946407	1	0.001058578
905	-0.029271393	0.098394409	0.980792793	0.054673513
906	-0.600218353	0.151028083	0.950237017	0.108295441
907	0.23272407	0.537978043	0.767305466	0.324826558
908	-0.164783554	0.15055563	0.93320292	-0.153381983
909	-0.183621003	0.046435048	0.982572008	-0.030959023
910	-0.57386514	0.139816081	0.913885434	-0.246647093
911	-0.349689676	0.040479499	0.986311887	0.076215956
912	0.023403352	2.506144319	0.452655738	-0.556750404
913	-0.055753889	0.241062447	0.90899845	0.147001478
914	-0.042622392	0.487577085	0.7732875	-0.329982122
915	-0.394884637	0.837772282	0.420813559	-0.988722695
916	-0.855530626	0.073576311	0.974167832	-0.082505756
917	-0.109742235	1.57553458	0.851821176	-0.180954615
918	0.089517676	0.477827961	0.950923695	-0.081292046

	K	L	M	N
919	0.01676306	0.352400535	0.881502683	0.173270331
920	-0.046299546	0.865157537	0.469058824	0.848460939
921	-0.316572533	0.382830386	0.77151634	-0.401231554
922	-0.223813834	0.401732099	0.884972763	-0.185789108
923	-0.385635166	0.062250191	0.987282162	-0.048419952
924	0.101969963	0.131902968	0.94583727	0.112609016
925	0.072844295	0.12558702	0.957062901	-0.102038913
926	0.295994279	0.411898415	0.952253558	0.075650533
927	-0.670852676	0.070912698	0.974895282	-0.077528636
928	0.462863545	0.578405148	0.689402844	0.459638808
929	-0.201138272	0.105948524	0.98180602	-0.049891578
930	0.076266336	0.333966433	0.890484956	-0.170888901
931	-0.405333288	0.16688402	0.9526	-0.107609431
932	-0.067832755	4.123984843	0.621365269	-0.327816857
933	-0.319453461	2.64845919	0.694135922	-0.300680161
934	-0.332945526	0.02605992	0.991424575	0.030706829
935	-0.307032411	1.458465979	0.698021053	-0.345663918
936	-0.000568704	0.089354594	0.978537143	0.063116921
937	-0.120350486	0.544069851	0.821344928	-0.278495789
938	0.022177367	0.185115022	0.944732394	-0.1188861
939	0.415142117	0.07197425	0.98170424	-0.032457987
940	0.398416583	0.014997907	0.997525581	-0.003544066
941	-0.366526949	0.803813853	0.770355987	-0.281504737
942	-0.322924128	0.090947294	0.98431636	-0.04306115
943	-0.135606396	0.86700852	0.838953545	-0.206885232
944	-0.770974572	1.039300118	0.641777778	-0.419320636
945	-0.207454754	1.509513318	0.72132766	-0.301088545
946	-0.032292735	2.115472465	0.719372549	-0.273373286
947	-0.265502557	0.241244115	0.924568835	-0.134041044
948	-0.378177976	0.294810717	0.886232727	-0.189468808
949	-0.344453697	0.113505759	0.95443522	-0.1009655
950	-0.274648146	0.049322458	0.990836653	0.021543715
951	0.008388269	0.215643929	0.95522	0.076420466
952	0.624445858	0.759650804	0.860872727	0.193874783
953	-30.90115502	0.334881696	0.834152334	-0.29353057
954	-0.139470602	0.134957855	0.912430769	-0.212893804
955	-0.1693367	0.111210596	0.960574879	0.083098518
956	0.159864987	0.151826068	0.913653666	0.196744919
957	-0.383481283	0.251549257	0.886521739	-0.23738416
958	0.059338901	0.541164068	0.721380952	-0.403478834
959	-0.182955595	1.523493621	0.476164384	-0.600118425
960	-0.223748828	0.264244368	0.894935652	-0.181560728
961	-0.26643536	0.167696468	0.952587755	-0.109772788
962	-0.046993625	0.196833882	0.949779006	0.108139462
963	-0.512131751	0.237032664	0.886085343	-0.221101125
964	-0.092813244	0.470205806	0.727672131	0.449794981
965	0.080691037	0.02712115	0.993354709	0.030327055
966	-0.600215952	1.018668779	0.696018779	-0.356673559
967	-0.529188701	0.397442285	0.852821429	0.232860141
968	-0.303496102	0.229772229	0.932843023	-0.121949938
969	-0.711991778	0.619799745	0.728252788	-0.356309467
970	-0.284453344	0.068606523	0.975763066	-0.084288491
971	-0.08132388	0.181584969	0.970898204	-0.06427998
972	-0.188261795	0.02819147	0.990803181	0.026793798

	K	L	M	N
973	-0.003555219	0.258761827	0.947079498	-0.102142334
974	0.281361434	0.562952811	0.734558824	-0.367268244
975	-0.190467488	0.025383953	0.993875728	0.01668969
976	-0.052885926	0.511752442	0.790083333	-0.302671432
977	-0.135685877	0.514973644	0.864210526	-0.214687347
978	-0.396885067	0.374254744	0.843304348	-0.266297446
979	-0.063179655	0.264270842	0.922879518	0.128889508
980	-0.217594566	0.227963788	0.940506401	0.113631778
981	-0.494394849	0.438214028	0.733093633	-0.434755749
982	-2.478749976	0.727148509	0.5595	0.712806702
983	-0.249633018	0.607518644	0.885380671	-0.169038773
984	-0.324103689	1.203585429	0.567416667	-0.553001192
985	-1.366522298	1.096061188	0.523308642	-0.641044193
986	-0.148902436	0.188604776	0.949718085	0.098252614
987	0.237947532	2.090810636	0.677059459	-0.331566705
988	-0.586897364	0.884736029	0.276740741	-1.744757122
989	-0.333712574	0.001261562	0.999543698	-0.000840929
990	-0.275762572	0.115738491	0.9824	-0.045943366
991	-1.642923385	2.513296575	0.0448	1.53611713
992	-0.300068191	0.003919081	0.998279371	-0.002775616
993	-0.455337902	0.770082122	0.692766839	-0.42001258
994	-0.928925755	0.268605316	0.788587156	-0.517812941
995	-0.188425923	0.007828583	0.997107176	-0.004978604
996	0.192039648	0.109742928	0.976317241	0.060446845
997	0.377800194	0.994996119	0.549413793	-0.561861674
998	-0.808976112	0.089059006	0.973165899	-0.068458133
999	0.111027052	1.080259504	0.812816537	-0.213793013
1000	-0.040965729	0.239095404	0.953505495	-0.098777983
1001	-0.236363451	0.078637223	0.993373615	-0.02145852
1002	-0.55406117	0.423299107	0.861937107	-0.207794613
1003	-0.417582265	0.319190988	0.815169811	0.349984699
1004	-0.514479577	0.050976757	0.980221994	0.035408656
1005	0.540984665	0.1099665	0.953621212	0.105125427
1006	-0.092504314	2.732753213	0.573931034	-0.378661897
1007	-0.564715497	1.54929094	0.354315789	-0.827587763
1008	0.720950324	0.358010859	0.771041009	0.407869127
1009	-0.357508345	0.263311223	0.917187184	-0.164267646
1010	-0.687719977	0.120746028	0.983052411	-0.03179529
1011	-0.096711419	0.14344523	0.95471161	-0.087022569
1012	-0.123375784	0.053156516	0.981047228	-0.03406673
1013	-0.173519504	0.039099248	0.985125506	-0.03184594
1014	-0.310630554	0.005362134	0.997764925	-0.006371816
1015	-0.415669832	0.161017283	0.954993835	-0.07943217
1016	-1.161435889	0.820137002	0.710341014	0.374439663
1017	-0.398470908	0.100340734	0.973518692	-0.069526884
1018	0.114267307	0.892287332	0.735781022	0.306846619
1019	0.18107719	0.568806621	0.842208232	-0.22632938
1020	-0.018007787	0.387139151	0.923040238	0.113633898
1021	-0.252269702	0.180764988	0.95339777	-0.078386307
1022	-0.361350578	0.297705004	0.882649541	-0.192559348
1023	-0.191289983	0.364724761	0.857185185	-0.23346795
1024	-0.56496822	0.570610414	0.752463158	0.344995499
1025	-0.505772476	0.836203095	0.788838906	-0.261375427
1026	-1.986091181	0.088235916	0.981474634	0.057345708

	K	L	M	N
1027	-0.541017142	0.091924219	0.982057395	-0.049190097
1028	-0.78233386	0.904158604	0.549064516	-0.570614285
1029	-0.182718619	2.888259119	0.856896406	-0.155784819
1030	-0.655609047	0.411594672	0.721923077	0.47503874
1031	-0.116091084	0.029362524	0.994087464	-0.015998628
1032	-0.073470472	0.10774404	0.974143541	-0.075621287
1033	0.028746793	0.012280795	0.99679702	-0.004144245
1034	0.225542469	0.351348013	0.925870206	-0.112984339
1035	-1.972198241	3.248933906	0.0112	-1.963662253
1036	-0.157794799	0.301154661	0.911867717	-0.138680988
1037	-0.51331941	0.483955055	0.771887147	-0.332631853
1038	-0.087028508	0.021625663	0.989880275	-0.0254275
1039	0.133014327	0.634751074	0.882431655	-0.14990192
1040	-0.028388673	0.596796432	0.570656934	-0.700355742
1041	0.031199636	0.315776867	0.885263969	0.201348199
1042	-0.621374459	1.457476273	0.423183673	0.734578874
1043	-0.2366587	0.288509917	0.959927711	-0.062611262
1044	-1.179611217	1.014128419	0.404754717	-0.899833679
1045	-0.068035254	0.005694847	0.998591497	0.002036624
1046	-0.264332862	0.271478181	0.95619403	0.071344376
1047	0.023019366	0.167394817	0.951479224	0.116263072
1048	0.157112449	0.690187501	0.829994236	0.253547245
1049	0.205245474	0.184496493	0.954912418	0.092344708
1050	-0.047988797	0.214345032	0.93853505	0.118541929
1051	-0.272095964	0.035161356	0.990256917	0.021647559
1052	-0.777120868	0.01806677	0.994617284	0.010240555
1053	0.010389686	0.443422449	0.954908616	0.076160431
1054	0.356224042	0.130135683	0.961102781	-0.077556186
1055	0.052138081	0.680703396	0.818054945	-0.24739986
1056	-0.136804419	0.393860152	0.832318408	-0.272477256
1057	-0.353894099	2.407550392	0.270857143	-0.807937622
1058	-0.659457455	0.548659026	0.858359447	-0.213689592
1059	0.664012308	0.200931393	0.879724696	-0.303854624
1060	0.179832944	0.595267751	0.814209945	-0.260243734
1061	-0.177234181	0.443265619	0.820712468	0.267924203
1062	-0.387234106	2.443681962	0.307466667	0.761353599
1063	-0.196296126	0.129339519	0.972504084	0.062766181
1064	-0.523840321	0.173541843	0.914325153	0.168960359
1065	-0.510077421	0.925806644	0.689116751	-0.387271033
1066	-0.2294764	0.169610597	0.9164	-0.165064706
1067	-0.14085319	1.151228132	0.645393258	-0.395648744
1068	-0.583202337	1.524433983	0.415764706	-0.714003669
1069	-0.387755558	0.10963411	0.954129032	0.097584407
1070	-0.523718933	0.197539173	0.914965079	-0.168909497
1071	-0.062073521	0.11110809	0.981273138	0.052920659
1072	-0.06739042	0.680705771	0.698471204	-0.445901023
1073	0.136939735	0.3251038	0.974692033	0.05558798
1074	-0.3322192	0.40682202	0.912215054	-0.121888902
1075	-1.076171851	0.59965135	0.738787004	-0.350156784
1076	0.021492659	0.416832308	0.817544444	-0.30325826
1077	-0.11040781	0.192551978	0.91251634	-0.184574551
1078	-0.296000689	1.972517773	0.573205882	-0.413246367
1079	-4.67E-05	0.352195216	0.814212534	-0.330114577
1080	-0.286405171	0.496096514	0.572545455	-0.771746318

	K	L	M	N
1081	0.086595006	0.200675471	0.915206723	-0.189861086
1082	-0.758555835	0.494778381	0.747188612	-0.378085878
1083	-0.269951008	0.601908389	0.749044674	-0.332476086
1084	-0.819959074	1.147821829	0.23408	-1.397427665
1085	-0.17971773	0.019716133	0.992495164	-0.017947515
1086	-0.142798608	0.000137607	0.999614679	9.01E-05
1087	-0.144716425	0.235035936	0.883688213	-0.233499527
1088	-0.553025925	0.299090925	0.824260652	-0.331188838
1089	-0.309003843	1.123874983	0.695525773	-0.365558836
1090	-0.297368613	0.034364858	0.979728115	-0.047514598
1091	-0.360247705	0.275262189	0.953409623	-0.08183225

	O	P	Q	R
1	Student's T-test Test statistic WT_WT_PEP	-Log Student's T-test p-value ORF2_WT	Student's T-test q-value ORF2_WT	Student's T-test Difference ORF2_WT
2	0.192015528	2.263343457	0.372540541	-0.6929334
3	-0.180601596	0.356842795	0.708289593	0.196615007
4	-0.866007871	1.189008674	0.493674419	-0.491558711
5	-0.150768704	0.688689342	0.552292359	-0.405405045
6	-0.269946855	0.021959157	0.972645038	0.018842273
7	-0.171003341	0.141025483	0.915109731	-0.054511388
8	0.384708585	0.83818924	0.410046512	0.663538191
9	-0.280040823	5.292697483	0.025142857	-1.845865673
10	-0.632311191	0.385862004	0.731451303	0.168909709
11	-0.212154985	0.408747428	0.647581982	-0.252128601
12	-0.145608423	0.795016798	0.638042553	-0.282926136
13	-0.413031114	0.183204433	0.687314642	-0.218905979
14	-0.499162456	0.259722239	0.865407966	-0.076711019
15	0.768615086	1.233996148	0.363959184	-0.748370065
16	-0.080040231	0.34932181	0.670350168	-0.231913673
17	-0.335338661	0.19037691	0.670406612	-0.237301932
18	-0.053772187	1.14023985	0.553863636	0.424588097
19	-0.256372537	0.168540345	0.911933541	0.055897607
20	-0.130903094	0.706698343	0.708652695	-0.191099803
21	-0.083785468	0.772437954	0.54447541	-0.45174408
22	-0.518147539	1.046940782	0.643049002	-0.247737885
23	-0.298703253	1.527291258	0.556012862	-0.386754142
24	0.288508777	0.095127208	0.844387685	-0.1003223
25	0.048571437	0.041614193	0.944143141	-0.037903256
26	-0.00710518	0.542620078	0.574713043	-0.372716904
27	0.206634979	0.990191187	0.57760452	-0.357565138
28	-0.034753233	0.79869729	0.551960526	-0.400846905
29	0.366012199	0.329205583	0.718276151	-0.177410338
30	0.031768898	0.101957581	0.895033613	0.064370685
31	0.151343131	1.393086593	0.644	-0.269299189
32	-0.515077115	1.114712558	0.361172414	0.922164281
33	0.187321016	1.296530465	0.50360181	-0.479609383
34	0.362586645	0.244059171	0.708382398	-0.201629215
35	-0.127119397	0.266517254	0.847772321	0.089920892
36	0.083339735	2.215399052	0.484854369	-0.495590846
37	0.154905954	0.183986402	0.846081818	0.095645905
38	0.404391038	0.414753883	0.572756447	-0.376944224
39	-0.311412831	0.372873879	0.631706422	-0.309836282
40	-0.095427469	0.45296	0.718235294	-0.176420424
41	-0.337249288	0.81061944	0.500387097	0.494328181
42	-0.148383661	2.618954078	0.295591837	-1.002344979
43	-0.459769638	1.097523718	0.350047619	-0.811823951
44	0.044878251	0.779320761	0.349707317	-0.840014352
45	-0.720729735	0.274207689	0.715501449	0.188097212
46	-0.03961478	0.135693822	0.804433735	0.123694526
47	-0.289850748	0.062116163	0.856106667	0.093535529
48	-0.108870192	0.960083204	0.771180662	0.138124042
49	0.253025621	0.008263807	0.984310861	-0.010525174
50	0.364195964	0.800129671	0.737144399	-0.160959032
51	-0.058730252	0.211357877	0.770825921	0.143346151
52	0.000263307	0.483343714	0.631082405	0.299733904
53	0.158176688	3.041612517	0.377108911	-0.704960293
54	0.054504071	0.418866769	0.774484848	-0.137585322

	O	P	Q	R
55	0.264673198	1.517531093	0.264	1.159215503
56	-0.092186875	2.164879796	0.354754098	-0.877241558
57	-1.745918267	1.124958075	0.474041237	0.523865806
58	-0.621592166	0.60661072	0.325405405	0.921847449
59	0.015277594	0.700623788	0.719022346	-0.173784044
60	-0.12605773	0.220737098	0.826936768	-0.107556873
61	-0.022098839	0.172406315	0.677009585	-0.2295244
62	0.047591725	0.518580513	0.688784929	-0.208673689
63	-0.196710052	0.355591285	0.633419355	-0.269458135
64	-0.163053005	0.272711412	0.862529284	-0.079126782
65	-0.671283562	1.385234705	0.287	1.058471468
66	0.01066146	0.842080682	0.559744409	-0.391242557
67	-1.590725991	5.808985888	0.0295	-1.766399595
68	-1.968637844	0.007248612	0.986481481	-0.006501728
69	0.431772074	0.806639671	0.553304348	0.420807097
70	-1.273139689	0.182055968	0.676675325	0.232186
71	0.319327604	0.565595692	0.643188406	-0.251389186
72	-0.039698117	0.44655803	0.631227766	-0.295290417
73	-0.051149698	0.898582	0.549466667	-0.425146103
74	-0.756386625	0.230304991	0.66357268	0.249053107
75	-0.397653172	0.148229064	0.848767677	-0.092763053
76	-0.043667451	0.225296448	0.718303371	0.182136112
77	0.094261039	1.127663318	0.529921397	-0.46404775
78	0.137473871	0.77693798	0.714183942	-0.184007221
79	-0.589764575	0.756166189	0.671577889	-0.224576314
80	0.113193231	0.892452645	0.432885906	-0.612744437
81	0.285844954	0.712002198	0.556812903	-0.396897422
82	-0.24832132	0.23819852	0.771843236	0.141135746
83	0.052741553	0.694461413	0.245375	-1.351328532
84	-0.346910113	0.085225656	0.866158798	-0.079040103
85	-0.057248196	0.378242676	0.718918919	-0.180550469
86	-0.123073285	0.336499852	0.671221298	0.22969034
87	-0.123665738	0.787098177	0.458227027	0.551289241
88	-0.26062567	0.24665411	0.681292994	-0.221161948
89	-0.210344714	0.331255219	0.724022222	-0.174686856
90	-0.251454681	0.021414805	0.98189434	0.012952805
91	-0.467670729	0.769017144	0.552892308	-0.434770372
92	-0.30287062	0.025138966	0.967804598	0.021207598
93	-0.471055011	0.321845393	0.707910314	-0.195720249
94	-0.177502599	0.517252007	0.628414414	-0.301984575
95	-0.466029256	0.424629625	0.633688213	-0.267345005
96	-0.656362668	0.227690168	0.889892518	-0.065007104
97	-0.427466582	0.372721347	0.719799714	-0.181657791
98	0.041963912	0.233525651	0.758099476	0.153423309
99	-0.485586903	0.140005039	0.844054795	-0.098390791
100	0.085100775	0.070215992	0.963676301	0.023231506
101	-0.607373129	0.03700363	0.946632411	-0.035765754
102	0.15444754	0.533494896	0.603742268	0.337641186
103	-0.135873761	0.331244242	0.717123404	0.181347105
104	-0.176641173	0.010514491	0.98379124	-0.009038501
105	-0.271509362	0.521287745	0.641959514	0.275516934
106	-0.745591321	0.155967714	0.718770115	-0.192161772
107	-0.297952351	0.267871705	0.824212014	0.109066857
108	-0.052853231	0.645822936	0.552052117	-0.402649562

	O	P	Q	R
109	-0.574361871	0.884185104	0.712889543	-0.18570794
110	-1.104079907	0.008374888	0.993434381	0.003832287
111	-0.207328588	0.178983999	0.843972665	-0.096855587
112	-0.435517395	0.118140691	0.845133106	-0.098148558
113	-0.45318845	0.151030324	0.835143519	-0.104025735
114	-0.405007042	0.602699766	0.643175758	-0.273205651
115	-0.308944117	0.323628235	0.737302826	0.163934072
116	-0.05475778	0.283468941	0.806455304	0.116689682
117	0.015009989	1.117971062	0.638236559	-0.28262096
118	0.138180376	0.103070085	0.926942249	0.046283722
119	-0.348956182	0.697841633	0.555282051	-0.396551556
120	-0.043393341	0.056480578	0.913344398	0.057849672
121	-0.321876847	0.505721957	0.559444068	0.413995319
122	0.289406453	0.353890385	0.6366	-0.271397273
123	-0.259555116	0.04658436	0.917826804	0.055665758
124	0.297460207	0.895561996	0.580100279	-0.354885525
125	0.02807011	1.022404013	0.579748503	-0.368280411
126	0.292780225	1.410324773	0.347488372	-0.794649124
127	-0.318241528	0.373644858	0.633174242	-0.268394258
128	-0.365907852	0.12509997	0.915958635	-0.054207484
129	-0.708581124	0.024454123	0.97644697	-0.015435113
130	-0.350898186	0.052929428	0.957129594	-0.026252323
131	-0.154690092	0.332902553	0.710383481	-0.191781998
132	0.631879393	0.689683291	0.670425249	-0.223766327
133	-0.462070937	0.009385062	0.987350649	-0.006693098
134	-0.199389168	0.641833728	0.621307506	-0.316231622
135	-0.198346954	0.391530662	0.556113074	-0.432076984
136	-0.156338034	0.590582539	0.646209205	-0.279539956
137	-0.397683296	0.535982709	0.645527721	-0.276972877
138	-0.096575658	0.836515041	0.554570342	-0.429958767
139	-0.186005093	0.240325479	0.782876238	-0.13294771
140	-0.656054685	3.241636768	0.465789474	-0.516256332
141	0.105572801	0.070676447	0.947001972	-0.034057193
142	0.142019549	0.416268634	0.550504918	-0.415401883
143	0.070536142	0.305073587	0.777040201	0.136884265
144	0.252984778	0.028234986	0.949718781	0.034146839
145	-0.17937439	0.521939139	0.661041667	-0.2376425
146	0.698104882	0.355845603	0.557972973	-0.424495909
147	-0.129513816	0.123249433	0.804877698	-0.123138004
148	-0.515363209	0.08215025	0.854322296	-0.090098275
149	0.031295407	0.157591051	0.837259516	-0.102407455
150	0.19674745	0.253487106	0.758491634	-0.148919
151	0.092556763	0.016349372	0.983801312	-0.01054128
152	-0.269195719	0.516070164	0.552613027	-0.44359631
153	-0.007654181	2.242945926	0.260818182	-1.08991517
154	-0.707707567	0.033250242	0.954466019	-0.028676139
155	-0.117096304	6.374190217	0.0096	-2.371354845
156	-0.124584318	0.085792646	0.901931034	0.061519199
157	-0.467630233	2.492766577	0.342060606	-0.858548906
158	-0.352733233	0.814695686	0.444636943	-0.596558041
159	-0.301149587	0.456919678	0.639103704	-0.259222666
160	0.009261638	0.176172215	0.92655102	-0.047630098
161	0.054418399	1.658423331	0.575832402	0.35114691
162	-0.683248439	0.000368802	1	0.000434028

	O	P	Q	R
163	-0.229859575	1.585758639	0.644016985	-0.276333067
164	0.230524257	0.454451714	0.793822168	0.123701943
165	-0.080837574	0.068768314	0.955167473	-0.026871575
166	-0.06747651	0.803996017	0.571630769	-0.37991333
167	0.141218055	0.380043935	0.645831933	-0.286069658
168	0.136209465	2.181970968	0.555659574	-0.403441111
169	-0.016764484	0.2584982	0.738177898	-0.165487501
170	0.21644522	2.032594611	0.248228571	-1.183897442
171	-0.247221039	0.265420968	0.884494715	0.067036735
172	-0.254911101	0.155687506	0.815824852	0.11503813
173	0.211388579	2.346271065	0.327333333	-0.940206104
174	-0.02317987	0.272620743	0.716553672	-0.182113012
175	-0.349205823	0.082780632	0.860337719	0.086565865
176	-0.564659893	0.525306589	0.549363296	0.439218097
177	-0.796312209	1.14359931	0.629680365	-0.296672609
178	-0.44829687	0.043147538	0.926334012	-0.04999521
179	-0.549049866	0.350387908	0.569465839	-0.401138094
180	-0.679326516	1.483425108	0.615512195	-0.312250137
181	-0.243165986	0.394945604	0.633403774	-0.266737408
182	0.353144358	1.203403223	0.548848	-0.437123405
183	-0.598635323	0.228063224	0.646507216	-0.292264938
184	-0.051843302	1.114335325	0.584775956	-0.347208659
185	0.069359828	1.065868542	0.531914163	-0.460716248
186	0.292593414	3.342121204	0.332222222	0.831450992
187	-0.011242787	3.403375219	0.420946565	0.618503147
188	-0.239541563	0.369133595	0.718580645	-0.177757051
189	0.391834723	1.343308689	0.528189655	-0.459337446
190	-0.263693892	0.133170882	0.782944513	-0.136179394
191	-0.140953193	3.449267608	0.634213884	-0.25270462
192	0.087564671	0.004665637	0.99611819	0.002827326
193	-0.076849863	0.520985441	0.732622101	-0.166001002
194	0.064105465	0.425985835	0.636561404	0.271769842
195	-0.312676914	1.293565337	0.469791045	-0.515286128
196	0.119913613	0.471719274	0.540216667	-0.470568339
197	-0.162142879	1.239302972	0.471564767	-0.524176704
198	-0.300323607	0.795998054	0.482442308	-0.513014264
199	-0.263487948	0.211762516	0.689064567	-0.218371497
200	-0.760276025	0.569764349	0.549603239	-0.454299927
201	-0.225725418	0.506570703	0.595756614	0.346794764
202	-0.594518622	0.437898926	0.805330144	-0.116596646
203	-0.354199298	0.840926106	0.58848913	-0.347801844
204	0.4455539	0.123077767	0.917701339	-0.052767648
205	-0.14891428	0.169941739	0.858293275	-0.085346434
206	-0.687172106	1.598414659	0.619932367	-0.308524874
207	-0.507442122	0.157330568	0.797932203	-0.125693851
208	-0.675272702	0.505896289	0.573773585	-0.392073101
209	-0.362184176	0.323882428	0.689232704	-0.212639703
210	-0.171425653	0.455229536	0.717920455	-0.179472605
211	0.054996742	0.519119363	0.803807692	-0.117701424
212	-0.094490225	1.467038695	0.345057471	-0.791649712
213	0.075273099	0.577143933	0.636743083	-0.271179199
214	-0.064897382	0.534942902	0.631791045	-0.261513604
215	-0.421312995	0.564677732	0.697405239	-0.201493157
216	-0.1303761	0.162522199	0.858167401	0.085264842

	O	P	Q	R
217	-0.416228603	0.822826591	0.634	-0.258891424
218	0.0626832	0.038110973	0.977982971	-0.014526791
219	-0.168500438	0.783869516	0.579259701	-0.371255027
220	0.021172572	0.088617387	0.629545852	0.365877787
221	-0.852537333	0.014765222	0.971544933	0.020301819
222	-0.714775254	0.397172423	0.694625387	-0.205656264
223	-0.368686294	0.185587546	0.883805291	-0.068031099
224	-0.267396354	0.619528422	0.615172749	-0.319710626
225	-0.243746895	0.354386443	0.731733696	0.167135874
226	0.058290205	1.511835357	0.492166667	-0.487172021
227	-0.008648856	0.715828007	0.53507563	-0.462579939
228	-0.417263754	0.012239571	0.975631179	0.017668406
229	-0.11266405	0.567288485	0.635971897	-0.306215286
230	0.084917299	0.656649142	0.714489146	0.182725059
231	-0.482429122	0.185415216	0.781424318	0.135549121
232	-0.725797493	1.547194168	0.596654354	-0.333390766
233	-0.110728945	4.242191041	0.53004329	-0.448135588
234	0.1424354	0.031721207	0.97528585	-0.016483943
235	0.05593043	0.6489438	0.632553271	-0.259899987
236	-0.323104831	1.711792592	0.660888099	-0.236117257
237	-0.02042027	0.344170154	0.637706349	-0.278224733
238	-1.945026774	19.90064492	0	-20.15731261
239	-0.061238539	1.189578336	0.445031447	-0.582288954
240	-2.00875345	23.58594901	0	-20.08740955
241	-0.208742762	0.885261793	0.689539432	0.206649568
242	-0.337481529	0.31215105	0.781427509	0.132471297
243	-0.64973379	0.180586253	0.845875142	0.095515993
244	0.119330925	0.359725047	0.704979228	-0.194060855
245	-0.033721162	1.257652624	0.671496732	-0.217596478
246	0.256341819	0.509182538	0.771275032	-0.138661491
247	-0.169014944	0.604780707	0.593045333	-0.346516927
248	-0.427470177	2.91762965	0.584607735	-0.342802684
249	-0.518675417	1.019991013	0.554396887	0.431766298
250	-0.292274382	0.059574385	0.929536906	0.046058019
251	-0.221268578	1.489153051	0.390725664	-0.688447316
252	0.028684186	0.954411098	0.571779817	-0.376394696
253	-0.214154629	0.317020265	0.788044064	-0.127607769
254	0.005067323	0.097347516	0.847780515	-0.094616572
255	0.676997371	0.02401891	0.96108599	0.025923411
256	0.059771516	0.772794639	0.505018182	-0.490443548
257	-0.363696728	0.591652816	0.61554902	-0.321740468
258	-0.057718129	0.810637225	0.662303887	-0.237866084
259	0.429701109	3.349508228	0.250344828	-1.20448833
260	-0.026977728	0.69254522	0.572490798	-0.381295522
261	-0.212169307	0.639751051	0.677797101	-0.215898726
262	0.274856385	2.237670423	0.011	-2.940858841
263	0.73152467	2.160735292	0.152	-1.402129703
264	-0.019187972	0.656696228	0.681748808	-0.211953269
265	-0.05415006	0.974640579	0.578380665	-0.371219847
266	-0.770578995	1.681554586	0.262536585	-1.130796856
267	-0.268038672	0.968493672	0.637974499	-0.250392702
268	0.371632253	0.659186829	0.55003125	-0.442509121
269	-0.063689509	0.151976182	0.779531756	0.138511234
270	-0.330874402	0.2524102	0.758711168	-0.148292965

	O	P	Q	R
271	0.087658125	0.662685755	0.587538462	-0.353497399
272	0.035491527	1.123822455	0.556107744	-0.399401347
273	-0.273640073	1.293365112	0.507082569	-0.481079949
274	0.159236384	0.476473023	0.709762406	-0.193596734
275	-0.387971858	0.644211064	0.563100346	0.411596722
276	-0.354297475	0.884137775	0.639215352	-0.28216913
277	-0.087250348	0.17312474	0.834317497	-0.103875478
278	-0.155503018	0.175339666	0.748779841	0.161911435
279	-0.242138171	0.361971397	0.864153355	0.074132495
280	-0.213612313	0.09909515	0.897194969	-0.06329155
281	-0.096690143	0.044335064	0.932330979	0.045469708
282	-0.215869913	0.447163727	0.799076179	-0.120646583
283	0.339104275	0.539325852	0.530205128	-0.476523929
284	-0.338352801	2.275976665	0.33815	-0.806417677
285	-0.220751841	0.655950612	0.63154386	-0.292695787
286	-0.307094224	0.181856495	0.800057971	0.123552958
287	-0.186266989	0.851636085	0.554715719	-0.402314504
288	-0.685744291	1.740361907	0.358237288	-0.893967311
289	-0.179135112	0.524490621	0.416	0.674926334
290	-0.564516117	8.608668518	0.14592	1.314170202
291	-1.59248269	1.797824053	0.333381818	0.939963871
292	-0.443575313	2.422849786	0.023384615	2.003107283
293	-0.315087735	0.778099346	0.531052632	0.471886105
294	0.671305216	0.197606499	0.866105946	-0.078084098
295	-0.162824389	0.787031612	0.455756098	-0.581314511
296	-0.629771805	0.725982575	0.455076923	-0.577590095
297	0.115350408	0.102010342	0.859851366	-0.08470175
298	-0.052507979	0.075383934	0.857707371	-0.088838153
299	-0.527178768	1.668221727	0.224571429	-1.290275786
300	-0.141532748	0.058842321	0.926707441	-0.049172507
301	-0.497698918	1.120538427	0.647565762	-0.273776372
302	0.408100839	0.204218287	0.671250814	-0.232435226
303	0.073142893	0.315943983	0.627380531	-0.306934569
304	0.144492583	2.483441532	0.549693431	-0.410675473
305	0.222288109	0.63055874	0.628618102	-0.295430925
306	-0.336527492	0.674867944	0.396	-0.709263908
307	0.242979179	0.483197615	0.649637343	0.249057346
308	-0.134347238	0.144362322	0.860109409	-0.083525764
309	-0.095799133	0.376426343	0.731744218	-0.167129517
310	-0.674198596	0.111760917	0.85309699	0.091140959
311	0.895031263	0.012788326	0.983151174	0.011474609
312	-0.326950904	0.272896625	0.671452145	-0.230505413
313	-0.453072415	0.435420014	0.6776704	-0.21723832
314	0.144242871	0.035197562	0.975821801	-0.015700658
315	-0.622505408	0.213190753	0.707486405	-0.202575684
316	-2.646276544	0.416940159	0.409228346	0.712273916
317	-0.390081763	0.327167238	0.738872483	0.162816366
318	-1.224149297	0.562702504	0.565006897	-0.412977642
319	-0.442448558	1.364562887	0.595893899	-0.335587184
320	-0.03331755	0.564983589	0.656199288	-0.243636237
321	-0.215282398	0.264210103	0.769709184	-0.143185722
322	-0.300031192	1.223270673	0.629778846	0.306202147
323	0.056627964	0.269575057	0.86592136	-0.073538674
324	0.181544451	0.455635459	0.630254545	-0.304990133

	O	P	Q	R
325	0.29922409	2.00586602	0.299137255	0.997372097
326	-0.041484578	0.505145094	0.45005	0.610575994
327	-0.208364597	1.39320989	0.331622642	0.969032711
328	-0.093188719	0.514948236	0.634372093	-0.269068188
329	-0.644203179	0.253581321	0.804760529	-0.119770262
330	-0.303857166	1.517099473	0.639808765	-0.265390184
331	0.362122536	0.775362294	0.551221122	-0.402288861
332	-0.357562423	0.149618449	0.822033019	-0.112572352
333	0.261441174	1.646771988	0.718285714	-0.180594338
334	-0.197397004	0.678559518	0.371857143	0.737581677
335	0.02840423	6.240403471	0.36988	-0.700293435
336	0.081641949	0.274395668	0.785793612	0.129686356
337	0.055211906	0.235925221	0.735153324	0.168734868
338	0.755978729	0.268521372	0.636009524	-0.323986477
339	-0.826874293	0.534200226	0.846154195	0.092844645
340	-0.172636763	0.028638559	0.948266405	0.033392376
341	-0.366469389	0.154068912	0.895597062	0.063027276
342	0.23241663	0.627728227	0.6284375	0.29783482
343	-0.292970669	0.442231443	0.714230548	-0.183773465
344	-0.39962353	0.151793056	0.841043678	-0.100608826
345	-0.525030586	0.496575019	0.638950298	0.27325609
346	-0.13612615	0.933498973	0.631454965	-0.299840291
347	-0.801332549	0.120226022	0.854753591	0.088149812
348	0.045366492	1.089537727	0.468098522	-0.517368529
349	-0.065359915	0.654039969	0.722479109	0.172407362
350	-0.247891763	0.366051599	0.716776978	-0.183934318
351	-0.339102458	0.35310537	0.723573997	0.173526764
352	-0.073181748	0.13814087	0.82831345	-0.109065586
353	-0.005164789	0.666665243	0.488242991	-0.509004381
354	-0.00298881	1.748393215	0.376923077	-0.711858326
355	-0.46706688	0.155033881	0.776468514	0.141998715
356	-0.885337082	0.270013877	0.777696361	0.136966493
357	-0.253759111	0.504613726	0.712558824	-0.187837177
358	-0.517450999	0.435277144	0.631189542	-0.296553294
359	-0.50108383	0.299823702	0.7057474	-0.195678499
360	0.196211614	0.180364852	0.708030257	0.205229229
361	-0.456665513	0.947764539	0.607604061	-0.326147079
362	0.13708461	0.08843987	0.841139588	-0.102776421
363	0.113816752	1.402708521	0.608425316	-0.322143979
364	0.201711889	0.377824161	0.696461538	0.204138862
365	0.050098907	1.318857048	0.424110345	-0.612102297
366	-0.249361146	0.548464148	0.656365591	-0.245351368
367	-0.207133772	1.292538695	0.577797619	-0.36542405
368	-0.593074682	0.321271291	0.636662813	-0.273357391
369	-0.247681128	4.372659071	0.54013278	-0.435725742
370	-0.77010734	0.184516046	0.800622437	0.123015722
371	-0.331341582	0.423243937	0.646225	-0.282668008
372	-0.064935973	1.456235281	0.346	-0.887176514
373	-2.292133582	0.146244154	0.716290698	0.196809557
374	-0.274093173	0.42352306	0.661591696	-0.238608254
375	-0.14350605	1.28095971	0.378627451	0.725415124
376	-0.462251342	0.171589204	0.758496749	-0.154373805
377	-0.583358484	0.522409026	0.55396139	-0.44435925
378	-0.174933956	0.865721303	0.435424837	-0.605883069

	O	P	Q	R
379	0.005115014	3.077300236	0.53133913	-0.450949139
380	-0.56728894	0.113269804	0.853158129	-0.090787252
381	-0.152834456	0.577970545	0.632146552	-0.290468004
382	0.018123466	0.381310332	0.686964119	-0.210132811
383	0.026678011	1.156106906	0.337314286	-0.878324297
384	-0.312731169	1.781304549	0.321226667	-0.849772135
385	-0.104363773	0.648808693	0.630359447	-0.30315272
386	-0.049471504	0.264947263	0.847869222	0.092263116
387	-0.214410225	1.177460585	0.451264368	-0.55846405
388	0.280941266	1.293440577	0.533299578	-0.453373167
389	-0.982147611	0.743113832	0.674546053	-0.220194287
390	-0.567765992	0.099451	0.857984632	-0.086509705
391	-0.028892435	0.515271269	0.633199226	-0.26905081
392	-0.230462477	0.520878475	0.717456091	-0.178313573
393	-0.363791137	0.888320504	0.628404494	-0.295895047
394	-0.321886772	0.044698558	0.925505595	0.049815708
395	-0.049412364	0.118813957	0.828	0.109865824
396	0.059523224	0.355879086	0.753936675	0.154009077
397	-0.269593229	0.131777119	0.867522581	-0.0774214
398	-0.224846076	0.736768992	0.737155914	-0.160296334
399	-0.217900048	0.741836852	0.464439791	0.542661667
400	-0.4225052	0.154657343	0.841647871	0.100624508
401	-0.494670954	0.475915101	0.642779661	0.285729514
402	-0.355593292	4.59528366	0.025866667	-1.841522217
403	-0.122831229	0.763424371	0.549654676	-0.421668794
404	-0.47738303	0.045416373	0.919774359	-0.053808636
405	-0.344480359	0.173453891	0.687360759	-0.223617978
406	-0.421554894	1.622838013	0.379456311	-0.71445783
407	-0.054415724	0.044466306	0.954399221	-0.02905443
408	-0.087148226	0.568259707	0.634387947	-0.262034098
409	-0.092309832	0.410393495	0.832590012	0.102420171
410	-0.069541982	2.201640321	0.4643125	-0.519154231
411	-0.484842451	0.019654797	0.955145366	-0.031165653
412	0.038367428	0.238774009	0.719649928	-0.185968187
413	-0.113118623	0.725195731	0.561651877	-0.406812456
414	-0.315926785	0.606421152	0.66060312	-0.23627917
415	-0.548814591	0.163779695	0.7568	-0.158175786
416	-0.008411167	0.167133949	0.784437884	-0.133251826
417	0.22314849	0.082968286	0.935158107	0.042734994
418	-0.273866775	2.941252836	0.474686869	-0.504986869
419	-0.245818093	0.038494759	0.937636181	0.043265873
420	-0.019511374	0.818372576	0.638560886	-0.253745397
421	0.253903029	0.468434414	0.455469613	-0.579578188
422	-0.176212787	1.125728886	0.529628319	-0.466909832
423	0.114152356	0.347444099	0.737924324	-0.164262136
424	0.056514299	0.046445888	0.968597129	-0.020181444
425	-0.304493631	0.837645898	0.572682927	-0.37716696
426	-0.395780816	0.402643543	0.645971014	-0.282101949
427	0.388983628	0.314122345	0.687892063	-0.215392219
428	-0.257351389	0.336469331	0.716383099	-0.179900275
429	-0.303581374	0.571680828	0.47744898	0.539674759
430	-0.784796978	1.214366999	0.281021277	-1.085627026
431	-0.325781194	0.50251836	0.703314985	-0.198701647
432	0.102529352	0.94453493	0.540773663	-0.450325012

	O	P	Q	R
433	0.025434286	0.224980306	0.675132686	-0.228076299
434	-0.053243868	0.464506003	0.754900925	0.152887768
435	0.035312774	0.259142867	0.612655087	-0.34290123
436	-0.202567682	0.51759515	0.635086247	-0.306804445
437	0.194475803	0.015238208	0.982457786	-0.011286418
438	0.57049537	0.809578774	0.604342857	-0.333414502
439	-0.275859315	0.756667082	0.696958525	-0.199530919
440	0.361417677	0.221037471	0.855148559	-0.086771435
441	0.503853784	0.187109077	0.735512195	0.170538584
442	0.517837142	0.836565338	0.634779528	-0.267914878
443	-0.26255169	1.487545876	0.344070175	-0.928023444
444	-0.078931911	0.96410967	0.60402046	-0.328564962
445	-0.618759149	0.465932212	0.791770732	-0.12449752
446	-0.20807091	0.13354889	0.859777293	0.083321465
447	0.157838242	0.183803095	0.758725849	-0.154619005
448	-0.065742944	1.052277696	0.678882068	-0.213948356
449	-0.033441678	0.463197211	0.715941349	-0.186663098
450	0.164409368	0.200263418	0.848737908	0.092217339
451	0.122316515	0.119340376	0.954366472	-0.028532452
452	-0.883593659	0.292002953	0.76467433	0.144461102
453	-0.613859928	0.128530853	0.919260021	-0.051779429
454	-0.187722018	0.704578401	0.642016129	-0.271752463
455	-0.464326237	0.599498798	0.633358852	0.310717689
456	-0.915199281	0.881051438	0.416421053	-0.65008333
457	-0.350192655	0.347242095	0.759029677	0.147654851
458	0.031398377	0.164667047	0.858589011	-0.084582859
459	-0.832086139	1.873847663	0.614456311	-0.310341305
460	-0.266665978	0.386054375	0.636299065	-0.311550988
461	-0.000954795	1.961914946	0.630805195	-0.28315311
462	0.072466274	3.241128558	0.402252101	-0.651994281
463	-0.046508823	0.240514074	0.696314815	-0.209491942
464	0.016902997	1.286154778	0.043333333	-1.873022503
465	-0.001569273	0.616608535	0.257422222	-1.221486621
466	-0.061437489	0.040465164	0.983058601	0.012885412
467	-0.207613098	1.117101477	0.452423529	-0.56458007
468	-0.25745087	0.497801368	0.428190476	-0.646001604
469	-0.799455993	0.320957216	0.666202703	0.235040453
470	-0.046388379	1.973214054	0.458527607	-0.559363047
471	-2.163304945	0.64939767	0.549076305	0.449257745
472	-0.353023665	1.319341423	0.329041096	0.864664714
473	0.318114018	1.973225185	0.639229122	-0.278558307
474	0.137895013	1.009455958	0.400455285	-0.674683889
475	-1.009630089	0.984272586	0.452074534	-0.581098557
476	-0.031305826	0.838179198	0.530144681	-0.464191013
477	-0.065404091	0.441737051	0.551648352	-0.438636992
478	-1.285080948	0.96937799	0.594352941	-0.34072219
479	-0.971089183	0.281819835	0.715732946	-0.188105689
480	-0.633000246	1.7309445	0.549903226	-0.433894475
481	0.128789654	0.385340799	0.731344262	-0.168065389
482	-1.04093696	0.037115126	0.946567755	-0.03606987
483	-0.624561673	3.97685189	0.041894737	-1.688187493
484	0.180956161	0.538476413	0.690041074	-0.209272808
485	-0.291677969	0.892839878	0.37306422	-0.727897008
486	-0.096216282	1.457698729	0.576761905	-0.352371216

	O	P	Q	R
487	-0.649486039	0.152601663	0.854880531	0.087447272
488	-0.684444759	0.74785392	0.563395189	-0.407023748
489	-0.626073804	0.044807217	0.941249751	-0.038834042
490	-0.231118675	0.5463752	0.632930456	-0.312556797
491	0.089409787	0.636588027	0.611291771	-0.325734244
492	-0.014674505	0.389658244	0.707438806	-0.194065094
493	-0.074528	0.591607893	0.573655172	-0.388182958
494	-0.241699349	0.033632442	0.953984436	0.029321882
495	-0.403201311	0.634002418	0.586093151	0.354094187
496	-0.209081551	1.835221871	0.596361702	-0.334019343
497	-0.476435872	0.394585798	0.672806557	0.224512524
498	-0.013136525	0.395517327	0.635526627	-0.275593652
499	0.111220568	1.187064461	0.412729927	0.634702259
500	0.051998664	0.205307797	0.758765685	0.149107827
501	-0.365626602	0.212544447	0.667803056	0.242024104
502	0.281914817	0.505410585	0.654816399	-0.245374044
503	-0.475118039	0.271251068	0.757522078	-0.150870429
504	-0.291886944	1.000163461	0.547313433	-0.425843133
505	1.001805409	1.864792475	0.347058824	-0.788651784
506	0.223886827	0.196854722	0.775566085	-0.138038211
507	0.162037636	1.394098213	0.356602151	0.763214323
508	0.509721169	0.441886967	0.739662198	-0.160729514
509	-1.428211831	0.497765784	0.548220472	-0.45236418
510	0.240591218	0.622686141	0.556142349	-0.420906915
511	-0.209088105	2.259532359	0.452659091	-0.543495602
512	-0.065774591	0.295588774	0.731295736	-0.171169069
513	-0.483606029	0.427022076	0.630748359	-0.297919379
514	-0.379444437	0.372402961	0.709703148	-0.194665273
515	-0.527183634	0.165118139	0.782237037	-0.134944704
516	-0.098540736	0.2012859	0.686674961	-0.217095269
517	0.10988033	0.391284275	0.637401835	-0.259690603
518	-0.514395787	0.034914813	0.94550149	-0.037679036
519	-0.274678375	1.455563036	0.394	-0.680447049
520	0.141796071	0.247323548	0.770690355	0.142087089
521	-0.468029564	0.668890224	0.645909465	-0.275084813
522	-0.945943954	0.108087598	0.864107759	-0.079617606
523	-0.908618677	0.866730333	0.627349451	-0.291983287
524	0.031742105	0.82324598	0.569609907	-0.381444719
525	-0.928148871	2.704540753	0.434282051	-0.576321708
526	-0.306577594	1.238623762	0.614790932	-0.320116043
527	-0.281877713	0.867567916	0.636835165	-0.252448612
528	-0.90087918	0.381817106	0.666881356	-0.233547423
529	0.125334302	0.763443589	0.708761035	-0.194087346
530	0.034756346	1.665552683	0.550023715	-0.430072361
531	0.023992195	0.221063853	0.705137778	-0.198487176
532	-0.451115595	3.197304549	0.555142857	-0.383581585
533	-0.145511373	0.65841234	0.633832061	-0.26365153
534	-0.837705729	0.05044006	0.973012416	0.018379423
535	-0.440194636	0.046217862	0.982568461	0.012745751
536	-0.332322068	0.831497362	0.63547025	-0.262621138
537	-0.280023309	0.129531445	0.950290766	-0.031656053
538	-0.353339626	0.083394782	0.930307692	-0.045418633
539	-0.317941356	0.240314808	0.776922306	0.137525558
540	0.427374507	0.297179551	0.67292916	-0.228596793

	O	P	Q	R
541	-0.302079007	0.000248137	1	0.000227186
542	-0.242159882	0.309165009	0.715871157	-0.189159393
543	-0.657677436	2.047469678	0.574018927	-0.374386893
544	0.015625092	2.730629926	0.527947137	-0.455758413
545	0.088338994	0.664577619	0.687683043	-0.209515254
546	-0.018657741	0.714559753	0.673655172	-0.220356199
547	1.211672622	0.743153551	0.323792208	-0.889493518
548	-1.259139041	0.961741379	0.541371901	0.450690375
549	0.101983452	0.508831861	0.550575163	-0.409092585
550	-0.180130019	1.145417108	0.697472393	0.197600047
551	0.348485671	0.514970688	0.666558376	0.230626424
552	-0.575811641	0.677696463	0.54517551	-0.453550551
553	0.079784511	0.695421819	0.719334266	-0.174003601
554	-0.93178043	0.326187873	0.713554913	-0.186604394
555	0.221165304	0.322442046	0.634808511	-0.318247477
556	-0.172840798	0.005756196	0.991752081	-0.004663891
557	0.20155647	0.747624239	0.376224299	0.739815606
558	-0.955304764	0.785350305	0.3744	-0.743299908
559	-0.074392212	0.792020494	0.643221987	-0.27997801
560	0.189561423	0.583083647	0.678587097	-0.216547224
561	-0.292071131	0.306407548	0.7708	-0.140352885
562	-0.061640859	0.896424193	0.461234043	-0.542447408
563	-0.314203354	0.020168539	0.962420849	-0.025547451
564	-0.534696746	0.191806131	0.89925445	0.061132431
565	-0.432857965	1.54537186	0.373962963	-0.70810318
566	-0.47097131	0.341570579	0.75068254	0.15573989
567	-0.556102158	0.337189338	0.866787234	-0.073266983
568	-0.176528774	0.083034837	0.898979079	-0.062764274
569	-0.381956051	0.519782196	0.63689505	0.272633235
570	0.283964523	0.21263726	0.708993902	-0.204177645
571	-0.04374889	0.132373515	0.759100654	0.158412298
572	-0.194278862	0.008003849	0.973792578	0.019611147
573	-0.113446355	0.332508208	0.646213382	0.255917655
574	-0.028207943	0.255776354	0.70923486	-0.194927004
575	-0.210228018	0.99107523	0.730203857	-0.16671541
576	-0.23664992	0.259727855	0.768963057	-0.143226412
577	-0.244658406	2.129155506	0.628993228	-0.291420195
578	-0.481720905	0.418237371	0.584520776	-0.364852905
579	-0.01925685	0.529429882	0.662839161	-0.238373862
580	-0.375957649	1.687509707	0.655671429	-0.238797506
581	0.076087447	0.574981146	0.637830882	-0.255855136
582	0.423063564	0.519326777	0.325684211	0.929413266
583	-0.345482862	1.159894792	0.3586	-0.90979788
584	-0.884544782	0.116638215	0.94872549	-0.031629562
585	-0.233061028	0.352892261	0.794886999	-0.123543845
586	-0.271353341	0.633450304	0.547466667	-0.445246379
587	0.238737238	0.056024393	0.94100998	0.039248996
588	-0.056515703	0.32061177	0.848439462	-0.090435028
589	0.295844153	0.847758436	0.613062657	-0.322977702
590	-0.291344949	1.757720761	0.404067797	-0.665446175
591	-0.592985592	0.108012957	0.810271429	-0.120899412
592	0.089219086	1.697773689	0.643115619	-0.267476188
593	-0.284735734	0.11538564	0.912108221	0.05694898
594	-0.007127117	0.157917325	0.848411173	0.09148958

	O	P	Q	R
595	0.157797091	0.136689126	0.89284122	-0.064545102
596	-0.200450594	1.92109151	0.552271698	0.418465084
597	-0.633742741	0.871989429	0.555801527	-0.429560767
598	-0.460900025	1.640570204	0.075636364	-1.628967073
599	0.071235444	0.3594051	0.786828431	-0.127807617
600	-0.388933125	2.139698119	0.633416847	-0.28167767
601	0.049776082	0.133991529	0.888460401	0.066818449
602	-0.348404709	0.698601419	0.672091354	-0.2196526
603	-0.255564906	0.088759119	0.920299795	-0.051886453
604	-0.144773878	1.507120795	0.2563	-1.153660668
605	-0.341704643	1.043565676	0.724359116	-0.168796963
606	0.266878478	0.840235459	0.341235294	-0.906835344
607	0.014045582	0.843734285	0.644311475	-0.272906621
608	-0.158206849	0.478753029	0.640820926	0.275556564
609	-0.663381778	1.181394781	0.604473008	0.32777977
610	-0.464110001	1.39843036	0.587302452	-0.343858931
611	-0.311330316	2.075589194	0.398944	-0.651180903
612	-0.70437657	0.073628879	0.847642058	-0.096429613
613	-0.713868869	3.563930632	0.454072289	-0.549491035
614	-0.145249639	0.569388624	0.676919614	-0.216511197
615	-0.721377488	0.122619523	0.805312575	-0.122798284
616	-1.234129908	3.368771024	0.265714286	1.078452428
617	-0.521968512	0.345670528	0.816170213	-0.111441718
618	0.117038078	0.229304095	0.62878733	0.319689433
619	-0.468022195	0.687057583	0.580833333	0.357471042
620	-0.020864136	0.433742704	0.661444248	-0.243215773
621	-0.287723395	0.034004152	0.94184	0.040855196
622	0.276535827	2.313161611	0.337101449	-0.85269165
623	0.402069191	0.14632204	0.845626274	0.095849991
624	-0.155250774	0.280247325	0.605561224	-0.349295086
625	-0.205953224	0.59879453	0.456581818	-0.590227551
626	-0.668308913	0.019938881	0.98398869	0.012086233
627	-0.748657396	0.198114703	0.840440873	-0.099429872
628	0.112656367	1.957233177	0.551509025	0.40957684
629	-1.52786617	3.654727601	0.243666667	-1.147159364
630	-0.515967026	0.369881151	0.816014218	-0.111856249
631	-0.199537421	0.303762144	0.776	-0.137513479
632	-0.686804684	0.755744293	0.349283951	-0.846962187
633	-0.630375271	0.044995044	0.9545625	-0.029836019
634	-0.139250806	0.11198141	0.849319774	-0.095570034
635	-0.260089927	1.215143818	0.536702929	-0.451087316
636	-0.492254495	0.027896562	0.939555556	0.042683283
637	0.699882496	1.578594839	0.33656338	0.857697593
638	-0.038326585	1.65948828	0.632499055	-0.256897397
639	0.022696448	1.351405299	0.451955056	-0.549934387
640	-0.768030525	1.267626286	0.574538012	-0.363114675
641	0.321942637	0.053629239	0.945520792	0.035895454
642	-0.007941014	0.49932946	0.824427732	-0.107011795
643	-0.123895371	0.49662447	0.604640827	0.339095857
644	-0.080829496	0.461862322	0.636054688	-0.27131017
645	-0.426217708	0.042490774	0.940123629	-0.039843453
646	-0.685320229	0.346477262	0.549912351	-0.468198776
647	0.541853686	0.262289001	0.631713043	-0.30585395
648	-0.834646919	0.489950736	0.29752	1.153383891

	O	P	Q	R
649	0.285439998	2.112157024	0.453028571	-0.54546886
650	-0.29551405	0.387879685	0.633457077	0.310910543
651	-0.064943613	0.243152825	0.787779141	-0.129383723
652	-0.560430741	0.116036764	0.914640415	-0.055189768
653	-0.582202935	1.18887087	0.43618543	-0.598868264
654	-0.093245689	0.164049374	0.741586115	-0.166502423
655	-0.588593431	0.340840367	0.634735632	-0.271754159
656	-0.077687994	0.18581426	0.848749436	-0.093261507
657	-1.030741996	0.012790391	0.98528	0.008017646
658	-0.186267748	0.570749888	0.655613596	-0.244874107
659	-0.235721115	0.651167843	0.599486911	-0.339437909
660	0.60204736	0.736557246	0.582168675	-0.373001946
661	-0.370934357	0.223671635	0.663469459	0.248844994
662	-0.710433998	0.404937595	0.642531646	0.287133959
663	-0.402952611	0.28396572	0.818965762	-0.11098014
664	0.116417114	0.212006393	0.731813953	-0.172607846
665	0.042605569	1.372165174	0.607664122	-0.32361412
666	-0.053536614	1.026349015	0.573644315	-0.364961412
667	0.014181202	0.771677486	0.5729683	-0.366345935
668	0.198226675	0.935796488	0.57660355	-0.36810006
669	-0.207374709	0.230883929	0.717275109	-0.190282398
670	-0.303560055	1.248432274	0.356	-0.896618949
671	0.178352941	1.015517261	0.348096386	-0.821029239
672	0.072596141	0.214308371	0.839821306	0.098784129
673	-0.388775639	0.014170926	0.963188462	-0.025021235
674	-0.152849347	0.170130253	0.79519708	0.127104865
675	0.026169529	1.143542114	0.557426573	-0.406488207
676	-0.081839653	2.958258644	0.449604938	-0.559633467
677	-0.16132436	0.080921139	0.865736501	0.081101735
678	-0.207380479	0.172632384	0.873959703	0.071465174
679	-0.357938465	0.342010605	0.709651652	-0.195659002
680	0.213865092	1.376038809	0.347323077	-0.8834326
681	-0.450081639	0.773164293	0.669735099	-0.222688039
682	-0.182062011	0.03772309	0.963251688	0.024060991
683	-0.052538479	1.134727793	0.454355556	0.549873988
684	-0.635032849	0.076496473	0.758675258	0.164660136
685	-0.379301282	0.857975575	0.549333333	-0.440632502
686	-0.168026251	0.069219186	0.954403101	0.02733273
687	-0.54663914	0.445982057	0.790836186	-0.125431273
688	-0.186885746	0.317512265	0.829850816	-0.104587131
689	-1.456277417	0.037804355	0.855564928	0.100001229
690	-0.530436249	0.454555709	0.563910828	-0.401101642
691	0.019420101	0.626306357	0.864980728	-0.0746585
692	-0.62452167	0.413609609	0.639606426	-0.277394825
693	-0.241966541	0.136550698	0.835829099	0.103790495
694	-0.474739267	2.604708974	0.603489583	-0.325691011
695	-0.193460064	0.95536236	0.626774049	-0.294962353
696	-0.254967473	0.05127974	0.95414549	-0.027932273
697	-0.489764535	1.78630428	0.550516556	-0.393162621
698	0.021587291	0.469014433	0.795898058	-0.122206158
699	-0.138525226	0.676682645	0.638880342	-0.284959793
700	-0.159452965	0.750131801	0.638388778	0.27117602
701	-0.167431588	0.022023907	0.986510678	0.007024129
702	0.167549325	0.756655052	0.632407407	-0.301825841

	O	P	Q	R
703	0.235594828	0.064127355	0.928985859	-0.045761744
704	-0.455453958	1.033459651	0.613758025	-0.317962011
705	-0.484438508	0.348921348	0.862139434	0.079928504
706	-0.201364187	0.412200467	0.596598425	-0.34888098
707	0.324601694	0.903881609	0.629724444	0.293131722
708	-0.484710005	0.61440378	0.757966102	0.148260964
709	0.033811873	0.410200081	0.758323077	-0.145765093
710	-0.076191015	0.036101856	0.983755639	0.011121538
711	-0.372267271	0.566888492	0.662954386	-0.23856269
712	-0.093825197	1.465998471	0.367298969	-0.742340724
713	-0.11117444	0.794032847	0.635264151	-0.304246267
714	-0.4670558	0.63389026	0.5524	-0.422781626
715	0.35276969	1.245433436	0.551345455	-0.415953318
716	0.029567588	0.293413828	0.775727617	-0.138327916
717	0.129551375	0.744931746	0.462687831	-0.546056747
718	-0.012128779	0.561345935	0.632174713	0.303848267
719	-0.392332208	0.629451622	0.646821577	-0.277053409
720	0.058301575	0.84732846	0.589473118	-0.344952477
721	0.149022141	0.344924124	0.816275208	-0.112213559
722	0.246559485	1.084611404	0.574848101	-0.380442725
723	0.141456463	0.539681383	0.75485639	-0.1518116
724	-0.138077328	0.06894237	0.918074074	0.053651386
725	-0.614049258	0.319953056	0.720108417	0.181963815
726	1.44551739	19.84984285	0	-21.9256009
727	-0.735702902	2.867196133	0.576449438	-0.348773744
728	0.120523389	0.740301747	0.647848921	-0.246574826
729	-0.435468163	0.308849714	0.723445215	0.175035901
730	-0.012124145	0.540606077	0.616850856	-0.322001139
731	-0.305195005	0.343493623	0.66070669	-0.239316728
732	0.375347198	1.724139492	0.25774359	-1.148465051
733	1.143730564	0.014194818	0.963083734	-0.025378545
734	0.20111439	0.179002412	0.86664232	-0.076587677
735	0.703016096	1.038936055	0.643422764	-0.270246294
736	-0.194429246	0.209847109	0.77649562	0.138173209
737	-0.869087891	0.15818849	0.927060266	-0.047836304
738	-0.162884051	0.489344866	0.840691244	-0.098082225
739	-0.922269654	0.733613792	0.546052045	-0.431045532
740	-0.356575972	1.023191073	0.668536913	-0.224273894
741	-0.425281315	0.691405729	0.630177677	-0.300248464
742	-0.534673808	0.062790697	0.947000987	0.034437815
743	-0.742134536	1.160986455	0.662347518	-0.236776776
744	-0.044585826	1.158856436	0.425267606	-0.620290544
745	-0.182194411	1.273605181	0.593383378	-0.339109209
746	-0.137243262	0.975935649	0.458391304	-0.546474245
747	-0.425822628	0.722371668	0.449832402	0.566589567
748	-0.489528926	0.074554595	0.918728586	-0.054025014
749	0.07566378	0.162248943	0.797813333	-0.12580257
750	-0.01717195	0.766595838	0.645962264	-0.277629852
751	-0.229003193	0.623097736	0.452416185	-0.579276827
752	-0.109037221	0.490681186	0.639495362	-0.258742015
753	0.169439659	0.948959289	0.454681319	-0.551688724
754	0.255149265	0.851641966	0.63522673	0.305916892
755	-0.696106669	0.114795191	0.861405876	0.08257654
756	-0.20195596	1.863617947	0.647090909	-0.269441817

	O	P	Q	R
757	-0.066672268	0.268032502	0.782950739	0.131162855
758	0.171245026	0.344662176	0.722514604	-0.175199085
759	0.01266914	0.529763103	0.676175041	-0.21889072
760	-0.268355857	0.986652164	0.41447482	-0.636189143
761	-1.040367464	2.214818309	0.24	-1.205737856
762	-0.198071435	0.635937082	0.362222222	-0.783008364
763	-0.446196058	0.931342518	0.4098125	0.661460453
764	0.27593669	1.013706871	0.326481013	-0.855740017
765	-0.907827928	1.547134755	0.356956522	-0.762046814
766	-0.004054125	0.250211071	0.758982005	0.148575889
767	-0.361718968	1.03889747	0.61438191	-0.321157667
768	-0.724914808	0.6477505	0.750378808	-0.153632906
769	0.645611152	1.817260507	0.350984127	-0.877450519
770	-0.628181366	0.032246801	0.972662857	-0.017984178
771	0.010254462	0.803617967	0.474331658	-0.526248084
772	0.288758874	0.05700021	0.865811966	-0.080548392
773	-0.128597155	0.040456876	0.940052104	-0.041467455
774	0.012449016	0.479901105	0.629977324	-0.303843392
775	-0.219887189	0.225157682	0.862530435	0.080008825
776	0.16790517	1.584670588	0.48715493	-0.492013084
777	0.426639473	0.041505829	0.974645161	0.01627371
778	-0.050783247	0.017099099	0.983468662	-0.010042826
779	0.594187885	0.281527973	0.839587156	-0.098358578
780	-0.313568357	0.035108415	0.983996269	0.008893543
781	-0.429048012	0.058185386	0.968590604	-0.020574994
782	-0.452737222	0.124607321	0.688100156	-0.228691949
783	-0.703325932	1.199236026	0.573945289	-0.371722539
784	-0.341526744	0.21363368	0.731313187	0.173609204
785	-0.107916184	1.060399884	0.47206	-0.519349204
786	-0.689136771	1.224410502	0.666462585	-0.226899677
787	-0.101406091	0.416491504	0.719726496	-0.180036969
788	-0.53963732	1.450911309	0.425805556	-0.610458374
789	-0.120574502	0.142283982	0.847824719	-0.093427446
790	-0.303380468	0.302653079	0.639064	0.281696955
791	-0.711653206	0.253118896	0.716023495	0.191571554
792	-0.194389016	1.251190431	0.710070229	-0.192749871
793	-0.048471632	2.648239214	0.448325581	-0.548438814
794	-0.487472469	1.047205617	0.638550832	-0.252713521
795	0.09267215	0.5012629	0.678480769	-0.216270659
796	-0.402950685	0.916320792	0.362851064	-0.774877124
797	-0.477339287	3.484090757	0.015636364	-2.172329373
798	-0.053505874	0.929201947	0.62815859	-0.29162195
799	-0.409734388	0.004200919	0.998442804	-0.001937442
800	0.332380364	0.558349476	0.585304582	-0.352941725
801	-0.302279528	0.618567124	0.635146771	-0.269031101
802	-0.125590164	1.367780233	0.468372549	-0.511843152
803	-0.248981405	0.063614026	0.889767932	0.068774753
804	-0.471319173	0.333226145	0.72937931	-0.171559652
805	-0.51313216	0.990264575	0.454595238	-0.569478989
806	0.801561903	0.432739621	0.611336634	-0.330183029
807	0.144535266	1.30293109	0.525262222	-0.46735594
808	0.180430033	0.664680328	0.575610619	-0.372635735
809	1.015729561	1.022376942	0.416144928	-0.635785421
810	0.67333097	1.229460289	0.428615385	-0.61481709

	O	P	Q	R
811	-0.212321124	0.096602901	0.82463306	-0.113523483
812	-0.0941372	0.119994347	0.912829167	0.056942198
813	0.23197503	1.258551634	0.468514851	-0.515353733
814	-0.122406203	0.846681343	0.571954023	0.364856296
815	0.110329412	0.545428937	0.662444062	-0.235045327
816	-0.248547278	2.46893418	0.564361111	-0.395425161
817	-0.6678295	0.668561013	0.696877898	0.201465183
818	-0.363955391	0.792394281	0.478646154	-0.529354095
819	-0.820743913	1.933178513	0.489056604	-0.489454269
820	-0.358604562	0.494430667	0.638976234	-0.255779902
821	-0.068141341	0.310399761	0.758020672	-0.148805406
822	0.38784318	1.06723166	0.783105068	-0.12850931
823	0.005787933	0.759822536	0.663950791	-0.23666403
824	-0.198564989	0.017871047	0.983555974	0.011687173
825	0.11865992	0.142669187	0.865229437	0.079390208
826	-0.194650036	0.34406464	0.71519883	0.188230091
827	-0.02244913	0.165942752	0.706563338	0.203668594
828	0.364889281	0.909778785	0.475878173	-0.524692747
829	0.24962111	1.198203694	0.639928144	-0.266902924
830	-0.408799577	0.254801541	0.861997828	-0.079666562
831	-0.114320747	0.654456855	0.663103448	-0.233905792
832	-0.359074343	0.082117585	0.863163597	-0.082543479
833	-27.49079735	20.77994575	0	20.38956261
834	0.032150761	0.092291218	0.718255977	0.198515362
835	-0.025705005	2.358711352	0.0664	-1.631000731
836	0.195656239	2.181128523	0.63306367	-0.253943549
837	0.258891644	0.586099545	0.5722375	-0.388240178
838	0.316620797	0.672150465	0.633869767	0.303540124
839	0.499458103	1.446595288	0.361555556	-0.767276552
840	-0.048521011	0.374776016	0.64762578	-0.283549203
841	0.247430819	0.514954684	0.633318095	-0.265702354
842	-0.133434041	0.938376141	0.554366197	-0.411681917
843	-0.075228335	0.267057469	0.717001414	-0.182439592
844	-0.059688984	0.30467673	0.718834286	-0.183077494
845	-0.125010756	0.133138931	0.803779112	0.123165766
846	1.090070893	0.88202486	0.339820896	0.909067578
847	0.138817609	1.191281837	0.358065934	-0.774815453
848	-0.431819645	0.15781607	0.938579477	0.040883594
849	0.118591489	0.224583249	0.829606519	0.105385886
850	0.206336862	1.592321283	0.481073171	-0.502543979
851	-0.419840494	0.456422801	0.615312039	-0.326295005
852	0.519008377	2.168163591	0.40614876	-0.65355746
853	0.061223228	0.328431798	0.604666667	-0.346288893
854	0.655068213	0.518198641	0.638515021	-0.288825777
855	-0.024300892	0.117358101	0.866515508	0.076807022
856	-0.130497845	1.328127167	0.636151659	-0.300953759
857	0.415280532	0.37152116	0.716282087	-0.17959955
858	-0.240800487	0.072674271	0.945867195	0.035624822
859	-0.1152184	0.920326998	0.461668449	-0.542718675
860	0.204514123	0.018888336	0.984863551	-0.009317822
861	0.123102344	0.587923325	0.636075294	-0.306774987
862	-0.80074338	1.864643741	0.551172932	-0.418372684
863	-0.124936117	0.767574952	0.660778157	-0.231498718
864	0.001526518	0.439855954	0.69743951	-0.201702542

	O	P	Q	R
865	-0.834479731	0.008692474	0.985862197	0.00828743
866	0.892927293	0.340307679	0.443189873	-0.645505905
867	0.544575127	16.28057888	0	-20.88737975
868	0.043912605	1.928860663	0.364449438	-0.758576499
869	-0.30540303	0.469440503	0.648628159	0.250689401
870	-0.262018057	1.953128383	0.574826979	-0.360151715
871	-0.795250246	0.469591331	0.68778882	-0.207221137
872	-0.628311561	0.748016344	0.736584574	-0.161898295
873	0.217471124	0.04252588	0.954581147	0.028562122
874	0.25995375	0.171693733	0.708556213	0.200677236
875	0.275575297	0.843225537	0.387824561	-0.709997177
876	-0.079712403	2.168212302	0.082956522	-1.551718818
877	-0.123624597	0.617864499	0.504840183	-0.497675154
878	-0.097107772	0.72515642	0.581285285	-0.372893863
879	-0.234364071	0.146727234	0.864875944	0.078634686
880	-0.09151734	0.868828749	0.461290323	-0.545530107
881	0.087729977	0.083197075	0.920741044	-0.05105061
882	-0.016148143	0.343718713	0.673168614	-0.229539024
883	-0.169490357	1.246456088	0.708596386	-0.190396839
884	-0.480724993	2.306803483	0.40304918	-0.652223587
885	-0.688591551	19.12205416	0	-21.9882679
886	0.220777103	0.153166259	0.865483387	0.076786677
887	0.072237287	0.097347446	0.847927928	-0.095987956
888	0.020549316	1.127205532	0.487885714	-0.500612471
889	0.149517285	0.112539554	0.927858012	-0.046171824
890	-0.480753221	1.692995378	0.450736842	-0.555374146
891	-0.224092739	0.046224646	0.951820137	-0.030855391
892	0.067607498	0.571718169	0.618862651	-0.317514631
893	-1.144978395	0.042661558	0.864554371	0.083091524
894	0.421793569	1.25830453	0.57641543	-0.36557134
895	0.349878367	1.462179217	0.432851613	-0.589457194
896	-0.227170328	2.116752873	0.635945525	-0.26067543
897	0.057199598	1.41196642	0.407784615	-0.645132913
898	-0.078603536	0.572983111	0.54495935	-0.457099915
899	0.026104867	0.75650073	0.669606723	-0.225720723
900	-0.569308925	0.14809742	0.859614458	-0.083831787
901	-0.14233623	0.48688566	0.628736142	-0.299503326
902	-0.303440928	1.666147854	0.528661017	-0.452977075
903	0.329138002	0.956224583	0.434103896	-0.601669735
904	0.001200206	0.171224193	0.806983294	-0.119079802
905	0.077079166	0.389421177	0.757693316	0.150992288
906	0.138439117	0.292683052	0.66660371	-0.235723919
907	0.407735814	0.010915197	0.984474323	0.0093814
908	-0.170258196	0.056315146	0.920862986	-0.051797655
909	-0.041886585	0.296518006	0.756746114	0.149997075
910	-0.207575962	0.239978352	0.601859008	0.361388736
911	0.065075633	0.534314218	0.436157895	0.628455268
912	-0.843342186	0.585052203	0.719581662	-0.179667791
913	0.194405935	0.562392821	0.66266899	-0.237211439
914	-0.399861879	1.231991088	0.45360452	0.552473068
915	-0.862925807	0.881251723	0.575894118	-0.367833032
916	-0.090372551	2.272175104	0.242941176	-1.191686418
917	-0.315202899	1.299619672	0.705904762	-0.1887171
918	-0.139822299	0.403216454	0.926680203	-0.045789295

	O	P	Q	R
919	0.240361419	0.096709434	0.909968717	0.058425479
920	0.814998177	1.075181617	0.364375	-0.759640588
921	-0.410016787	0.279272556	0.604352332	-0.353402456
922	-0.26048117	0.312755562	0.758089239	-0.15220197
923	-0.061810514	0.719974758	0.596610526	-0.340095944
924	0.135522253	0.437458133	0.673257525	-0.22747612
925	-0.125462352	0.185951696	0.806260454	-0.119082769
926	0.129288808	0.814699273	0.775830212	0.133073171
927	-0.085989633	0.910859402	0.366568421	-0.769116084
928	0.512393481	0.02848636	0.946138889	-0.037777371
929	-0.073524452	0.06924954	0.950507874	-0.032592773
930	-0.234895404	0.408928756	0.731885559	0.16699579
931	-0.142159062	0.096542302	0.911858628	0.0571359
932	-0.58317948	1.177218812	0.748106525	-0.154148102
933	-0.515917165	1.151310509	0.556738676	-0.406032986
934	0.033604996	1.020318223	0.378037736	-0.725089179
935	-0.531902101	0.983297468	0.672268412	0.218602922
936	0.082620077	0.36198369	0.6870375	-0.210897234
937	-0.370267203	0.042086946	0.952062622	0.031199349
938	-0.156541765	0.565253763	0.672106667	-0.225156572
939	-0.048748776	0.326208993	0.747712766	-0.15808932
940	-0.006089591	2.073518573	0.67682825	-0.211279975
941	-0.408198997	0.968453807	0.570438746	0.362027062
942	-0.063589485	0.980595518	0.576597183	-0.357403649
943	-0.327495129	0.149318073	0.927247967	-0.046503915
944	-0.571722909	0.520261548	0.613870647	-0.327254401
945	-0.480010169	0.000452729	0.999376147	0.000166787
946	-0.463596523	0.506087514	0.756964981	-0.147946252
947	-0.182760316	0.575770693	0.634939096	-0.270577749
948	-0.24313082	0.470154335	0.637546961	-0.258278317
949	-0.120265443	0.066213215	0.873575372	-0.075323105
950	0.032785568	1.035581886	0.61253	-0.320701387
951	0.11823902	0.16389682	0.91842562	0.052898619
952	0.304721345	0.11981562	0.946128079	-0.033585231
953	-0.329838657	19.14516344	0	-20.92815717
954	-0.191278391	0.046433941	0.854051168	0.097928365
955	0.105905784	0.784515207	0.560952381	-0.405069775
956	0.194715971	0.228363804	0.661410959	-0.244818581
957	-0.264011364	0.959170513	0.403233333	0.68115849
958	-0.465471468	0.109690924	0.836679769	0.105161455
959	-0.79812367	0.914298267	0.484135266	-0.509826024
960	-0.228956123	0.448123628	0.635877934	-0.310112
961	-0.144243795	0.315611251	0.670839138	-0.229864544
962	0.149395189	0.612969026	0.709167173	0.194665273
963	-0.248210024	0.337204103	0.637615202	-0.317324109
964	0.470467915	0.54327139	0.552279412	-0.43312433
965	0.033957254	0.454543509	0.627542601	-0.303162469
966	-0.508429265	0.482136277	0.634347992	-0.266887241
967	0.302582997	0.747696151	0.573079545	-0.363859812
968	-0.169076942	0.162046646	0.681148325	-0.229113897
969	-0.449908242	0.63165376	0.574895238	-0.389069027
970	-0.088695792	0.38621976	0.571177143	-0.378752814
971	-0.100025269	0.149786139	0.932967742	0.042935477
972	0.032107913	0.315269597	0.660793162	-0.239617666

	O	P	Q	R
973	-0.152968008	0.034711783	0.97300286	0.018093321
974	-0.445762901	0.634090746	0.571802469	0.384364658
975	0.022837936	0.127287706	0.89144	-0.065338135
976	-0.384435072	0.346800189	0.69379845	0.207363341
977	-0.305471509	0.945776459	0.641258182	0.249118381
978	-0.323397152	0.451898108	0.55594386	-0.426071803
979	0.182052972	0.297011141	0.763667519	-0.144983927
980	0.160597194	0.144561187	0.865135539	-0.076014625
981	-0.450121146	0.197989623	0.663884157	-0.245572196
982	0.70047569	4.908806836	0.010222222	-2.645497852
983	-0.263859633	8.453360884	0.343928571	-0.877855725
984	-0.712380906	4.044037432	0.205333333	-1.257152345
985	-0.760312541	2.179165907	0.263826087	-1.072683122
986	0.138190373	2.543628229	0.391965217	-0.669988208
987	-0.543776553	2.698292126	0.587306233	-0.338668823
988	-1.094392814	0.571535623	0.262418605	-1.246815999
989	-0.001155548	1.258248139	0.511569507	-0.474929386
990	-0.070486374	0.392944075	0.757097025	0.148275799
991	1.632541595	2.101532316	0.154461538	-1.384578281
992	-0.003737207	0.001242464	1	0.000758489
993	-0.530222572	0.359175638	0.6625	0.24383884
994	-0.391001308	0.829405467	0.316692308	-1.036874983
995	-0.006925355	1.655806257	0.430547945	-0.601665285
996	0.085189379	0.108590423	0.919418033	-0.051209768
997	-0.682685815	0.316288227	0.630343249	-0.31317287
998	-0.086969531	0.645946681	0.557838188	0.398968167
999	-0.347252747	0.088659809	0.96490682	-0.02194998
1000	-0.146703872	0.208241098	0.791531136	-0.127879037
1001	-0.035706642	0.712489768	0.778970149	-0.131603877
1002	-0.285107414	0.992766103	0.418757576	-0.646211412
1003	0.355889466	0.874122116	0.345727273	-0.812626521
1004	0.047195509	0.268689129	0.63267658	-0.269919501
1005	0.121703881	0.077100055	0.846923077	-0.099260754
1006	-0.629310609	4.384446302	0.432186667	-0.579910702
1007	-0.981605251	1.27767421	0.490729858	0.495873981
1008	0.402494599	0.619511807	0.405587302	-0.688721763
1009	-0.21448544	0.452849276	0.68784326	-0.209517585
1010	-0.052892688	2.992220802	0.54804428	-0.411902322
1011	-0.118017522	0.537177385	0.638664234	-0.254727893
1012	-0.046577527	0.572419334	0.645071575	-0.275618235
1013	-0.040314494	0.926488359	0.512864865	-0.480859545
1014	-0.007018378	0.867106512	0.412851064	-0.638608509
1015	-0.114174728	1.049931734	0.573317919	-0.363023122
1016	0.500271619	2.838265636	0.036470588	-1.781821781
1017	-0.091306297	0.151974399	0.830235706	-0.10745536
1018	0.443910125	0.246964828	0.85572536	0.086154302
1019	-0.324239825	0.171252186	0.831869767	-0.105528726
1020	0.179139375	3.148813056	0.451353293	-0.550916884
1021	-0.11619289	0.016628241	0.984505576	-0.007892821
1022	-0.246373967	0.35340838	0.67777561	-0.222452799
1023	-0.295719522	0.689329748	0.55392	-0.405490028
1024	0.430637441	1.436324637	0.41357037	-0.632505205
1025	-0.389557479	3.846416557	0.249081081	-1.127463023
1026	0.07722269	4.099110595	0.072380952	-1.542241414

	O	P	Q	R
1027	-0.070229739	0.574716144	0.758833333	-0.147869322
1028	-0.669615687	0.077427402	0.878809322	-0.072343614
1029	-0.288480043	0.01047199	1	-0.000939051
1030	0.459034248	2.352549307	0.2452	-1.222309113
1031	-0.023119465	0.065089294	0.949636899	-0.032108307
1032	-0.098649181	0.642482702	0.586975207	-0.354674657
1033	-0.006719682	3.36532321	0.487215311	-0.487413194
1034	-0.17533993	1.709367976	0.556563758	-0.394079844
1035	-2.050044401	0.199999174	0.714932179	-0.190728929
1036	-0.197866898	0.06184099	0.939521912	0.038960351
1037	-0.400876253	0.131300392	0.824816901	0.111360762
1038	-0.027914277	1.885196575	0.327435897	-0.829239315
1039	-0.241534554	0.198080856	0.937730924	-0.040465249
1040	-0.642136903	0.367213021	0.579454545	0.390284008
1041	0.257954395	0.860868739	0.585751351	-0.347326914
1042	0.897316959	2.497032305	0.252903226	-1.201508204
1043	-0.105415302	0.004908026	1	0.001187642
1044	-0.890847984	0.030764645	0.940553661	-0.042544683
1045	0.003270115	0.019987589	0.986917516	0.006399367
1046	0.116465967	0.526304937	0.66129806	-0.240777334
1047	0.149651905	1.12659651	0.572372093	-0.363843494
1048	0.366667418	0.877886427	0.731216438	-0.165588167
1049	0.131523901	0.513135841	0.663440415	-0.235941145
1050	0.162800742	1.85068668	0.45663388	-0.535746045
1051	0.030138503	0.281947425	0.824141176	-0.108646393
1052	0.014685495	0.180983473	0.812133175	-0.116413328
1053	0.131117877	0.858937533	0.739374332	-0.158111148
1054	-0.106143164	0.171889575	0.757477004	-0.157120599
1055	-0.359169649	1.666719095	0.397645161	-0.658645418
1056	-0.333216022	0.903497662	0.414823529	-0.644483778
1057	-1.092017572	0.707329086	0.609191919	0.327687158
1058	-0.308699336	0.281918438	0.815420428	-0.113310496
1059	-0.271793369	0.508361394	0.524642857	-0.490728166
1060	-0.361492733	0.382222186	0.723412742	-0.173388375
1061	0.341514778	0.53528462	0.643283096	-0.276105457
1062	1.052447357	1.690608145	0.375854545	-0.700428645
1063	0.091268234	0.689450947	0.661683849	-0.233293109
1064	0.189853533	0.329643486	0.612581281	-0.334558487
1065	-0.527010553	0.298220023	0.749115538	-0.157889896
1066	-0.185751107	0.016956154	0.966817658	-0.022731781
1067	-0.561803078	0.879781429	0.63374902	-0.267139223
1068	-0.892973326	0.968684498	0.42927027	0.613394207
1069	0.116364223	0.429010357	0.661662609	0.239530139
1070	-0.199051853	0.130383551	0.840297143	-0.100152969
1071	0.077661674	0.624242525	0.707630303	0.194354587
1072	-0.530643903	0.140625626	0.831888631	0.105892181
1073	0.096567146	0.053166683	0.98393597	0.011517631
1074	-0.190894285	0.140163324	0.941002997	0.038419088
1075	-0.440870255	0.255310931	0.74648	0.160594304
1076	-0.361862722	0.067631155	0.907511482	-0.060460832
1077	-0.207536415	0.611526065	0.56330137	-0.410264333
1078	-0.642707914	1.045694361	0.643570526	-0.277016534
1079	-0.358427828	0.29337529	0.570903427	0.407837338
1080	-0.61716356	0.469703809	0.415685714	-0.671120962

	O	P	Q	R
1081	-0.21415715	0.124540585	0.781719255	-0.139196608
1082	-0.435369552	0.735063741	0.554310078	-0.436863369
1083	-0.427027372	0.473799365	0.578141643	-0.369370778
1084	-1.14264153	0.046898472	0.859655398	-0.090194066
1085	-0.021954994	0.434534194	0.636416988	-0.26949713
1086	0.00012452	0.247756411	0.776485	-0.136936824
1087	-0.255010364	0.294251332	0.631232775	-0.269183265
1088	-0.337107257	0.243787423	0.740198126	0.163778729
1089	-0.528195423	0.763084102	0.547899642	-0.421589533
1090	-0.048005193	3.32809523	0.015333333	-2.103653802
1091	-0.130378207	1.328917235	0.634796117	-0.262522803

	S	T	U	V
1	Student's T-test Test statistic ORF2_WT	-Log Student's T-test p-value ORF2_PEP_WT_PEP	Student's T-test q-value ORF2_PEP_WT_PEP	Student's T-test Difference ORF2_PEP_WT_PEP
2	-0.132852305	0.942734919	0.433568627	-0.394350052
3	0.037463923	0.280368479	0.809745856	-0.141132779
4	-0.093669634	3.625778183	0.05762963	-0.972080443
5	-0.076395174	0.015299523	0.980165725	-0.013680352
6	0.00355516	0.462327104	0.67653833	-0.214483049
7	-0.010582789	0.037711321	0.980455067	-0.014031728
8	0.122126002	0.369791365	0.61032613	0.314215554
9	-0.349894993	2.232437934	0.092846154	-0.9275909
10	0.032481363	0.522274129	0.738229814	-0.165488137
11	-0.047702401	0.437624303	0.624204159	-0.269462585
12	-0.054491061	0.149909168	0.879916182	-0.090268453
13	-0.039931512	2.135528251	0.058	-1.275763194
14	-0.014966295	0.322457824	0.823494845	-0.112702688
15	-0.139437578	1.682050797	0.239401575	-0.616329617
16	-0.043776056	0.259062977	0.797789773	0.15889846
17	-0.043102639	0.685278444	0.351888889	-0.588990106
18	0.081327142	2.166568354	0.154876404	0.721551259
19	0.010891262	0.656734915	0.75289955	-0.146699905
20	-0.037166094	0.304035816	0.828089947	-0.119795269
21	-0.085018219	0.015179176	0.98617603	0.008987639
22	-0.04822458	2.219453187	0.324761905	-0.42903773
23	-0.074922612	1.19076033	0.311040816	-0.537640889
24	-0.018591562	0.057996526	0.932665289	0.05650923
25	-0.007117891	0.280517136	0.703273927	-0.246396383
26	-0.069821365	0.377023045	0.595563636	-0.328485277
27	-0.068679284	0.3781173	0.676630137	-0.233736674
28	-0.076043158	0.383908809	0.63454479	-0.271889581
29	-0.033863647	0.381948105	0.636730561	0.268085904
30	0.012287815	0.053280403	0.965227139	0.025369432
31	-0.052589461	2.857970544	0.243	-0.513412264
32	0.16804806	0.519498509	0.424414716	-0.558391147
33	-0.091759311	0.386235595	0.706423645	-0.207431157
34	-0.037703759	0.439929349	0.523431373	0.406047397
35	0.017480462	1.661535491	0.527971154	-0.258080377
36	-0.096102045	1.694591672	0.299648352	-0.498861525
37	0.018358702	0.401674317	0.775123696	0.155750063
38	-0.069523131	0.34230685	0.595821138	0.353172514
39	-0.057617858	0.125814676	0.838399002	-0.152066549
40	-0.034030475	0.712641138	0.812612022	-0.112326728
41	0.092728871	0.717079355	0.457288754	0.407329771
42	-0.189891595	2.482606962	0.068324324	-1.020437876
43	-0.149393855	1.357927239	0.104561404	-1.181318707
44	-0.15056481	2.03248524	0.044631579	-1.474967745
45	0.035527889	1.700551985	0.147291139	-0.836921692
46	0.023101274	0.106730453	0.880708625	0.108639611
47	0.016857822	0.066077191	0.918323561	0.084554672
48	0.027181563	0.287081001	0.873178698	-0.080614726
49	-0.001935271	0.738530493	0.314980769	0.670509338
50	-0.031506247	1.763898662	0.468596491	-0.304876963
51	0.027153029	0.362394559	0.653314587	0.257374234
52	0.056580889	0.395015699	0.7234176	0.192381541
53	-0.136260827	0.232698317	0.871173077	0.089326647
54	-0.026701187	1.205291629	0.598928	-0.22167227

	S	T	U	V
55	0.211226532	2.399806383	0.030461538	1.600800408
56	-0.166058235	0.074464082	0.926503719	-0.063031938
57	0.099309543	0.183375027	0.829396178	0.131650925
58	0.159825264	0.262779442	0.599345309	-0.439417309
59	-0.033877532	0.472801346	0.809038462	-0.12173801
60	-0.020676478	0.198413774	0.855764706	-0.104550256
61	-0.041484594	0.31980884	0.589336082	-0.384346008
62	-0.04015996	0.015452552	0.987300935	0.007885191
63	-0.050450853	0.555358491	0.579949791	-0.287307951
64	-0.015441214	0.173020847	0.860953771	-0.106062995
65	0.193267491	0.603314552	0.380536232	0.57540173
66	-0.074457667	0.610727172	0.543483568	-0.32020251
67	-0.337142044	0.589004519	0.482843243	0.396227307
68	-0.001224769	0.718804129	0.578219409	0.265651915
69	0.079657266	2.352296333	0.072916667	0.972530365
70	0.042107936	1.851982978	0.071354839	-1.247172038
71	-0.048150408	0.003098039	1	-0.001854579
72	-0.05559183	0.358921702	0.611215686	-0.318994946
73	-0.080771214	1.539576374	0.163752577	-0.794376797
74	0.045689394	0.05899648	0.930610572	0.08083068
75	-0.017683307	0.182765761	0.870944114	-0.093086878
76	0.034133354	0.860151664	0.424577181	0.419675615
77	-0.088504027	0.349515453	0.732509434	-0.192971759
78	-0.03588885	0.449134351	0.798128852	-0.130577511
79	-0.04353642	1.890296555	0.351107143	-0.42059305
80	-0.113854334	0.290354009	0.62325283	-0.348084132
81	-0.074988079	0.051256266	0.933950617	0.058737437
82	0.02689109	0.728380414	0.465026549	0.391431597
83	-0.224628186	0.091557254	0.837058971	-0.229948468
84	-0.014787001	0.316765156	0.643685714	-0.289929496
85	-0.034605189	0.010561251	0.988324627	-0.00815116
86	0.043305709	0.321216413	0.747358548	0.182290607
87	0.102522812	2.412486865	0.069473684	1.022839864
88	-0.041123163	0.011432102	0.984527514	0.019701428
89	-0.0333747	0.061594048	0.938178499	-0.04418797
90	0.002485507	1.544457196	0.381221818	0.38794242
91	-0.081990031	0.176899248	0.810615595	-0.172847324
92	0.004004502	0.256973867	0.721642512	0.240535948
93	-0.037149025	0.199115132	0.799915493	0.178022385
94	-0.057147517	0.317134116	0.698325581	0.234246148
95	-0.050502689	0.087071531	0.893454545	-0.104727851
96	-0.012699524	1.691756604	0.536978622	-0.253248003
97	-0.034791255	0.129779293	0.899216216	0.077017254
98	0.029087228	1.266607087	0.238	0.693842358
99	-0.018655064	0.359556132	0.671875862	-0.243615892
100	0.004536115	0.417390904	0.776898678	0.150137795
101	-0.006693542	0.173250005	0.824262812	-0.150657018
102	0.063581598	2.616261309	0.07872	0.908603244
103	0.034588143	0.331673866	0.696633333	0.230656094
104	-0.001706416	0.096618194	0.899106622	0.088149601
105	0.052401302	1.239657485	0.358879377	0.438034058
106	-0.035022115	0.148305216	0.852369458	0.122803794
107	0.021079703	0.144550736	0.896906002	0.076465607
108	-0.075689165	0.329799489	0.643260323	-0.28482098

	S	T	U	V
109	-0.036295713	2.162446942	0.425786667	-0.329872767
110	0.000743037	3.063510404	0.242797203	-0.502684275
111	-0.018563155	0.163845211	0.836465823	-0.133988274
112	-0.018454684	0.386205815	0.580997904	0.345630434
113	-0.019735003	0.590240164	0.524394089	0.346005122
114	-0.052251259	1.052845442	0.473643836	-0.326329337
115	0.031381557	0.446682	0.696834725	0.204336378
116	0.022537154	0.049662455	0.964622398	0.028769175
117	-0.05488759	0.138134122	0.938678825	-0.034319984
118	0.008952612	0.571285253	0.593783231	0.272895389
119	-0.074863233	2.891749516	0.10662069	-0.794681761
120	0.010770677	0.022793294	0.973660524	0.027498881
121	0.076713823	0.225597618	0.775340206	0.194195641
122	-0.050777951	0.062090995	0.928136898	-0.07387797
123	0.010254113	0.597491613	0.449069182	0.463930342
124	-0.067978252	0.233856932	0.796080692	-0.172627767
125	-0.07072093	0.089634654	0.923524711	0.054847293
126	-0.148444277	0.36187272	0.611230769	-0.310530345
127	-0.050389329	0.204473222	0.799679775	-0.174168693
128	-0.010491053	0.119758809	0.935173116	-0.037478977
129	-0.002956207	0.026377323	0.970566699	-0.028399573
130	-0.005068952	0.082183889	0.912053393	-0.086821026
131	-0.036488287	0.012730767	0.986804124	-0.009893629
132	-0.043289288	0.364549416	0.799248247	0.137376573
133	-0.001275872	0.41540928	0.666532174	-0.232928382
134	-0.060207874	0.645094833	0.530787589	-0.322601742
135	-0.078470907	0.076974	0.915324444	0.08873558
136	-0.053371245	0.14783573	0.871966942	-0.098597209
137	-0.05271977	0.804283778	0.475693989	-0.352952533
138	-0.081414009	0.467045571	0.681972973	-0.209558487
139	-0.025409208	0.078098042	0.923448421	-0.058924569
140	-0.100819479	1.129716022	0.147816092	-1.041677687
141	-0.006578597	0.039475355	0.970330739	0.022566689
142	-0.076031458	0.467406749	0.535130024	-0.371596654
143	0.026343212	1.077577075	0.60907393	0.218421512
144	0.006293595	0.533328665	0.553020501	0.327129576
145	-0.045506476	0.87420035	0.488364583	-0.327127669
146	-0.076666772	0.109472746	0.874131148	0.112475925
147	-0.022868952	0.90095859	0.265797468	0.735771391
148	-0.016662878	0.003497174	0.995455556	0.005139669
149	-0.019479778	0.136862923	0.871634638	0.104352527
150	-0.028372039	0.206249945	0.814755798	0.154774984
151	-0.002017843	0.862719628	0.54371897	-0.284938388
152	-0.081869143	0.100688078	0.880148492	-0.111357795
153	-0.204033691	1.561188972	0.101555556	-1.092317581
154	-0.005405343	0.114480655	0.870700361	-0.124220742
155	-0.448246406	0.392662666	0.797570413	0.138540692
156	0.011679956	0.009962741	0.987402043	-0.006771935
157	-0.163594303	1.049205499	0.486978723	-0.315633562
158	-0.11051796	0.502496408	0.55759292	-0.325880475
159	-0.049199597	1.263545942	0.349	-0.474653244
160	-0.009323695	0.434675776	0.824905263	-0.109967973
161	0.068337912	0.094570816	0.928452026	-0.056507323
162	8.04E-05	0.59413684	0.474225989	-0.413046731

	S	T	U	V
163	-0.054049439	1.053299395	0.556630631	-0.260448032
164	0.024119003	0.37463244	0.871518072	0.081377877
165	-0.005224634	0.014695469	0.986793296	-0.006974962
166	-0.072283588	0.55015049	0.476112045	-0.427043067
167	-0.053538905	0.035382873	0.963056269	-0.033108605
168	-0.078655043	0.591984913	0.695404682	-0.189221488
169	-0.031389937	0.014645351	0.980556391	-0.012972938
170	-0.219224476	0.080134341	0.914401776	0.086010615
171	0.013124433	1.454497043	0.427355263	-0.352921168
172	0.021738422	0.247612563	0.732244898	-0.229840597
173	-0.177908132	0.233070023	0.799679325	0.162727356
174	-0.03444958	0.145748129	0.853034653	-0.125784132
175	0.016065196	1.90529427	0.243518797	-0.579229567
176	0.08120762	0.26100221	0.814802685	0.134514279
177	-0.057562678	2.778391976	0.243391304	-0.518435372
178	-0.009234121	0.420263533	0.5538	-0.371684604
179	-0.072752806	0.932977904	0.196934579	-0.992752499
180	-0.060823754	3.261219087	0.238534351	-0.51234542
181	-0.050229763	0.054675494	0.922840459	-0.070034875
182	-0.083765708	0.12917746	0.870431655	0.113012526
183	-0.052815953	1.820558305	0.248162162	-0.561877992
184	-0.066982172	0.858226746	0.528555024	-0.294752333
185	-0.087726349	0.154274249	0.869561677	-0.102805667
186	0.160223614	2.970431258	0.1625	0.632545471
187	0.120362215	2.325628833	0.42240273	0.3308648
188	-0.034064192	1.096998158	0.350137931	-0.486610836
189	-0.088137074	0.28307919	0.799357542	0.147692362
190	-0.025233381	0.406234684	0.546193548	-0.390254974
191	-0.049980934	3.197702238	0.281954023	-0.448106342
192	0.000542898	0.83452354	0.484918206	0.33666865
193	-0.032199368	0.71323832	0.633211896	-0.221862157
194	0.051298736	1.255301151	0.254842105	0.633816825
195	-0.098260088	0.981341538	0.377605948	-0.452308443
196	-0.085934042	0.697604395	0.349526316	-0.622494168
197	-0.099723762	0.373396197	0.654130511	-0.254201253
198	-0.09592447	0.867630116	0.452361371	-0.385630925
199	-0.040255558	0.123875633	0.857173594	0.132044474
200	-0.084176617	0.398335055	0.627314501	-0.279379103
201	0.065042626	1.61883203	0.351391304	0.431575775
202	-0.022750519	2.45961033	0.396129496	-0.345978207
203	-0.066545458	1.273970462	0.435521036	-0.357872645
204	-0.010216436	1.303421727	0.350334764	0.457175997
205	-0.016410878	0.148307249	0.883246243	-0.087127262
206	-0.060200269	2.773027156	0.17172	-0.6280223
207	-0.023644262	0.238724554	0.824113548	0.123654895
208	-0.072936817	0.375975034	0.574004228	-0.359495375
209	-0.04019476	0.026048602	0.983328244	-0.014154646
210	-0.034602848	0.116772968	0.925115589	-0.051861869
211	-0.023031295	0.058004814	0.957002997	0.032298406
212	-0.1482008	1.096695676	0.237821138	0.761952294
213	-0.051804741	0.262095561	0.794528736	-0.163138284
214	-0.049908307	0.10587231	0.907848214	-0.077688429
215	-0.038918666	1.072901487	0.554428894	-0.260474735
216	0.016370721	0.389101038	0.715102439	0.200863732

	S	T	U	V
217	-0.050062371	1.665124105	0.276941176	-0.529178619
218	-0.002828673	0.238511721	0.866012092	-0.091776954
219	-0.070650331	0.507853303	0.595854839	-0.281119029
220	0.055929892	0.244017751	0.522699752	-0.894304487
221	0.003706784	0.026521343	0.962209073	-0.041995154
222	-0.039260789	1.036113808	0.368046154	-0.457406998
223	-0.013214689	2.667853591	0.378622222	-0.353158951
224	-0.06075771	0.027334366	0.977661538	-0.018798192
225	0.032066949	0.274171012	0.803339806	0.145906236
226	-0.093584921	3.487680124	0.2312	-0.529572381
227	-0.086627246	0.001745576	1	0.001505958
228	0.003212832	0.074413738	0.914075388	0.090758853
229	-0.05812215	0.321377029	0.718061389	-0.214712143
230	0.035526814	0.016562651	0.98340965	0.013700061
231	0.025597503	0.657554523	0.546301624	-0.307279587
232	-0.064882793	1.886111739	0.308020725	-0.469136556
233	-0.088167777	1.789047541	0.603848907	-0.207859463
234	-0.003180037	0.071169421	0.941117409	0.039689382
235	-0.04992647	0.259948971	0.786947368	-0.172435125
236	-0.046380413	3.796840383	0.350961039	-0.368404388
237	-0.051893133	0.001895757	1	-0.001911799
238	-3.789789517	1.978305736	0.069727273	-1.094706429
239	-0.110002318	0.437801755	0.612629981	-0.275558472
240	-3.87284892	2.411881068	0.119178082	0.779482524
241	0.040286113	0.045790184	0.980599424	-0.014715407
242	0.025542379	0.526337755	0.567251613	0.306845347
243	0.018322778	0.351994847	0.774124629	-0.161413193
244	-0.037011119	0.222622417	0.813262873	-0.14737765
245	-0.042622156	0.455561266	0.838894147	-0.093700409
246	-0.027018255	0.63593693	0.522164589	-0.339229372
247	-0.065512881	0.547805437	0.553614512	-0.32159551
248	-0.067383458	4.271933063	0.240248062	-0.488870409
249	0.082339784	1.173713724	0.345863014	0.490159141
250	0.0087216	0.151044528	0.871637664	-0.101215574
251	-0.130045073	0.617097046	0.497030612	-0.362494999
252	-0.072062618	0.114721909	0.913302326	0.070030424
253	-0.024647843	0.024074365	0.976158301	-0.022572835
254	-0.017633288	0.173479373	0.834912325	-0.132072449
255	0.004820127	0.392435972	0.547588785	0.404531479
256	-0.091839302	0.120967105	0.871030588	-0.10878457
257	-0.061014859	0.851090052	0.449782051	-0.396377775
258	-0.046105025	0.191082589	0.871054282	-0.096064462
259	-0.228393407	0.271063849	0.737990698	0.210037231
260	-0.072116275	0.179717185	0.81029904	-0.17244445
261	-0.041738134	1.208251097	0.400968421	-0.395378961
262	-0.496427746	2.39721534	0.002285714	-2.745008469
263	-0.257150237	0.328277805	0.714012987	-0.215796789
264	-0.041026818	0.044747425	0.968266145	-0.024229685
265	-0.071159855	0.954553805	0.407847222	-0.42096795
266	-0.207825788	21.55135142	0	-22.18701575
267	-0.048649651	1.328326134	0.475457064	-0.310044607
268	-0.082773567	0.117743142	0.872595745	0.111979591
269	0.025846323	0.047962351	0.932864529	-0.066221025
270	-0.028253642	0.097681429	0.928024948	-0.047799004

	S	T	U	V
271	-0.067009772	0.309653906	0.713194127	-0.224117491
272	-0.076662284	0.802958482	0.484860892	-0.339728673
273	-0.09202119	1.321732009	0.316	-0.501818339
274	-0.037271189	0.038951757	0.97563285	0.018570582
275	0.077258827	0.599404052	0.537314286	0.328995175
276	-0.054503754	1.138231493	0.494849741	-0.301253001
277	-0.019822653	0.130031105	0.887211073	0.089881897
278	0.030149135	0.122939838	0.835768844	0.161214405
279	0.01455724	0.399565949	0.896198646	0.061253442
280	-0.01207682	0.49393864	0.603920319	0.277896881
281	0.008472021	0.169821667	0.825829016	0.145683712
282	-0.023530514	1.288376506	0.632574627	-0.199536641
283	-0.087687166	0.115068124	0.865801932	0.128228082
284	-0.153600871	5.916063809	0.0176	-1.195285585
285	-0.055967686	1.203658649	0.378934307	-0.416947047
286	0.023427647	0.387425263	0.744146119	-0.173027886
287	-0.076491785	0.273895957	0.741156105	-0.206272125
288	-0.167413932	1.470672775	0.067666667	-1.455713908
289	0.11991006	0.076551481	0.889873708	0.128585815
290	0.257131791	6.652083659	0.020727273	1.105769475
291	0.175734987	0.612314028	0.408899654	0.522069719
292	0.358226003	1.315574463	0.113791045	1.113234202
293	0.088605622	0.717452992	0.452338558	0.418615765
294	-0.015129378	0.514768891	0.634155556	0.243138419
295	-0.107694598	0.166365518	0.814930481	-0.167374929
296	-0.106567959	1.024789858	0.248939597	-0.716691547
297	-0.015940006	0.728475103	0.481584906	0.360049354
298	-0.016346405	0.442701467	0.55751663	-0.349480523
299	-0.234327862	0.803234724	0.294022222	-0.709730996
300	-0.009271842	0.341265676	0.653556338	-0.266288122
301	-0.053220637	0.418829359	0.668124352	-0.22960896
302	-0.042522324	0.025768486	0.968996094	-0.031648
303	-0.05655178	0.59590437	0.450119874	0.464953105
304	-0.080225806	3.139243457	0.212109091	-0.560636944
305	-0.056391488	0.069399972	0.934930894	-0.043750339
306	-0.12791447	0.423235767	0.482554348	-0.496630139
307	0.047464718	2.019948766	0.109269841	0.887145148
308	-0.015981049	0.020213761	0.984601332	-0.014089584
309	-0.032127608	1.369749844	0.522945274	-0.273576101
310	0.017161424	0.389963092	0.676048027	-0.231270472
311	0.002160717	0.462092241	0.572806794	0.319696003
312	-0.042987462	0.400146321	0.634254545	-0.26475101
313	-0.041505552	0.496899432	0.594752515	-0.283669578
314	-0.003043854	0.334991379	0.798242678	-0.139597787
315	-0.037572005	1.214193263	0.238830769	-0.704047733
316	0.122936779	1.213252558	0.109625	-1.22064103
317	0.031188403	0.946362541	0.523842752	-0.293964174
318	-0.076985634	0.382837651	0.609283465	-0.308964411
319	-0.065126768	0.149021015	0.929033226	0.05030738
320	-0.04672481	0.238697597	0.811382069	-0.149220785
321	-0.027372058	0.081360705	0.930148547	-0.063573837
322	0.059443385	0.268646769	0.773191111	0.179658466
323	-0.014372232	0.504277727	0.827916883	0.103673511
324	-0.057361826	0.103761549	0.896031674	0.089228312

	S	T	U	V
325	0.186748754	1.988619132	0.068888889	1.088268492
326	0.109315102	0.493123543	0.368335878	0.736239327
327	0.17830837	0.969639724	0.245959184	0.750085407
328	-0.051210237	0.278154435	0.743407634	0.199375576
329	-0.023032591	0.987664035	0.567760171	-0.251532025
330	-0.05191767	3.989358439	0.303784946	-0.413679547
331	-0.076214336	0.528789593	0.4535125	-0.496570375
332	-0.021256741	0.018300475	0.980283254	0.019855287
333	-0.035609305	0.037470439	0.980885957	0.010096868
334	0.132557232	5.463208538	0.001	-1.9453125
335	-0.137642228	4.008181779	0.1055	-0.730273565
336	0.024926112	1.282837518	0.402448399	0.391165415
337	0.031840331	0.733052295	0.521744361	0.322696474
338	-0.058736831	0.782411055	0.240977778	0.939555062
339	0.018257615	0.135021006	0.94879112	-0.030877007
340	0.00617171	0.79365863	0.312673267	0.65108384
341	0.012211235	0.580649678	0.72352488	0.17305247
342	0.056820303	1.42149517	0.35188755	0.430990855
343	-0.035371568	5.233364629	0.048	-0.930800544
344	-0.019123293	0.528842462	0.6352282	-0.240909576
345	0.051895321	0.105286615	0.883824074	0.104656008
346	-0.057876244	0.647467462	0.583716075	-0.269930946
347	0.016690428	0.005898495	0.990415584	-0.007020315
348	-0.098020723	0.023363005	0.978460058	-0.020500395
349	0.033570916	1.489541918	0.660307155	0.178294288
350	-0.035186529	0.554404861	0.692141653	-0.195245319
351	0.033237295	0.16021533	0.870803828	0.1001322
352	-0.020548939	0.246051454	0.737738318	-0.224464417
353	-0.094353884	0.042888758	0.964091089	-0.030525843
354	-0.135085209	0.752277519	0.42222973	-0.448331409
355	0.02648401	0.596950491	0.491667532	0.376593484
356	0.026253392	0.29271118	0.815194595	-0.131116867
357	-0.036260182	0.156107177	0.929264957	-0.048895942
358	-0.055748757	0.858302362	0.63753176	-0.20717091
359	-0.03702895	0.385789445	0.634043956	0.272028181
360	0.037601061	1.167210982	0.228745763	0.7609041
361	-0.062788532	2.586835231	0.278366864	-0.473102358
362	-0.018914021	0.302203542	0.643130125	0.298933877
363	-0.062628225	0.291828126	0.836216352	-0.105545256
364	0.03891276	0.422950329	0.633520295	0.262383567
365	-0.115800027	2.102359435	0.146930233	-0.745542102
366	-0.046995268	0.61222553	0.633804067	-0.229636086
367	-0.070637749	2.08203187	0.281909091	-0.488384459
368	-0.050851744	0.22503634	0.798984485	-0.166931152
369	-0.085802352	4.983825621	0.171456311	-0.550183826
370	0.023346106	1.078138143	0.486148148	-0.312844594
371	-0.053219977	0.63092837	0.644838366	-0.215181986
372	-0.16474731	0.150344292	0.837269521	0.138625887
373	0.035603993	5.997615243	0.001333333	-2.280222151
374	-0.045339924	0.304848677	0.729774245	-0.209807502
375	0.135689557	0.310647711	0.724415335	0.211787118
376	-0.028802102	0.385324241	0.642876565	-0.260281457
377	-0.082058733	0.454095031	0.568136461	-0.32663748
378	-0.112488433	0.316182633	0.633576642	-0.305299547

	S	T	U	V
379	-0.088248474	1.799995573	0.313365854	-0.461104287
380	-0.017110721	1.316742225	0.466895349	-0.322451909
381	-0.055316123	0.357011233	0.730725552	-0.193017112
382	-0.04001435	0.118013819	0.893377423	-0.087313546
383	-0.16110097	0.059743938	0.929064378	-0.07759073
384	-0.159807189	1.477740165	0.218902655	-0.697605769
385	-0.057854474	0.113635155	0.883344111	-0.098890728
386	0.017920176	0.154897484	0.922234801	-0.046104855
387	-0.105698629	0.266123856	0.746569697	-0.199888017
388	-0.086939877	0.181975162	0.862855758	0.102114148
389	-0.042695598	2.612962188	0.46635503	-0.290004518
390	-0.016236842	1.775934311	0.169373737	-0.733227412
391	-0.051208292	0.316702813	0.698116473	-0.235043208
392	-0.034510308	0.282782509	0.824241335	-0.11548784
393	-0.057067272	1.992815371	0.376842105	-0.374427583
394	0.009226835	0.065066883	0.9303147	-0.054593616
395	0.020518243	0.088814588	0.908321788	0.086309645
396	0.029646929	0.356822562	0.812827211	0.126266691
397	-0.014809802	0.092644932	0.921973742	-0.068930944
398	-0.031336919	1.748296691	0.451210191	-0.325489468
399	0.100713397	0.084942395	0.921679825	0.073356205
400	0.019141621	0.193464847	0.852801968	0.107660929
401	0.054043891	0.622169242	0.476125714	-0.405405256
402	-0.346433987	0.926906535	0.210422018	-0.957309299
403	-0.079632126	0.423952614	0.610669202	-0.281252755
404	-0.009919591	0.380335312	0.642156584	-0.260596805
405	-0.040537489	0.064150628	0.918917391	-0.08708424
406	-0.135156267	2.619052831	0.066823529	-1.021876229
407	-0.005551228	0.245208846	0.753027027	-0.202536053
408	-0.050107482	0.111105694	0.922169045	-0.064579222
409	0.020021198	0.924474761	0.67556701	0.183537801
410	-0.100510508	2.653017037	0.27202439	-0.47925165
411	-0.005612436	0.584219908	0.34831441	-0.718711429
412	-0.034912664	0.105027802	0.891063927	-0.094806035
413	-0.076814979	0.573504498	0.494408269	-0.380868912
414	-0.04546581	0.864219085	0.562914661	-0.265092214
415	-0.029379077	0.362660947	0.574067797	-0.369605594
416	-0.025057782	0.310708602	0.681376271	-0.252423604
417	0.008231968	1.01687716	0.593368635	0.233171251
418	-0.098479781	3.321556659	0.237792	-0.517450756
419	0.008012428	0.653348799	0.522962217	-0.337609397
420	-0.049093143	0.521661769	0.644475177	-0.227294074
421	-0.103692641	0.134617697	0.798997214	0.245206833
422	-0.089019155	0.385430501	0.729814173	-0.187467787
423	-0.031517366	0.103146789	0.920731278	-0.068453259
424	-0.003914289	0.090689159	0.926239334	0.050059213
425	-0.071890999	1.088313068	0.351016393	-0.475052304
426	-0.052994594	0.092560214	0.891981797	0.101227019
427	-0.040629187	0.472512008	0.593763265	0.294538922
428	-0.034344813	0.325102694	0.745596358	-0.183004591
429	0.098654057	0.610440019	0.358139535	0.623073366
430	-0.196003629	1.238887619	0.34892827	-0.461898804
431	-0.03827587	0.276856982	0.83755194	-0.105360455
432	-0.085465929	0.381798998	0.611210938	-0.305791643

	S	T	U	V
433	-0.042070645	0.251753584	0.681826825	-0.292452918
434	0.029649508	0.671345318	0.70797377	0.176316791
435	-0.06164609	0.113384962	0.823225641	-0.200831095
436	-0.058011442	0.511636789	0.557501104	-0.322508494
437	-0.002146912	0.420154094	0.643232975	0.250379562
438	-0.063830954	0.08411055	0.92707234	-0.05927425
439	-0.038814525	1.201684243	0.456954683	-0.341077805
440	-0.016807404	1.138476646	0.522020101	0.285968781
441	0.031765042	1.550267193	0.124540541	0.938471476
442	-0.051769237	0.247550927	0.810319559	0.146886614
443	-0.171967324	0.394175254	0.528626506	-0.42941157
444	-0.063266464	0.764019248	0.546381609	-0.290329827
445	-0.02428208	2.481782507	0.348357447	-0.39174419
446	0.015894469	0.305255069	0.784878477	0.162334442
447	-0.028957497	0.23842888	0.742831804	0.218766954
448	-0.041802974	4.167551707	0.348108108	-0.370223363
449	-0.03595553	0.212876231	0.829013123	-0.13303015
450	0.017774842	0.412874281	0.7397375	-0.177598529
451	-0.005603707	0.08483569	0.969077073	-0.019715839
452	0.027703057	0.063636853	0.969918129	0.020304786
453	-0.010043096	0.552159116	0.74005547	-0.161197662
454	-0.052239279	0.269464597	0.827598415	-0.124060737
455	0.059058895	0.075815965	0.929997927	-0.050975588
456	-0.120185879	1.961414191	0.070325581	-1.104687797
457	0.028447277	1.219015308	0.355795276	0.445039537
458	-0.016252334	0.429275333	0.608191205	-0.282436371
459	-0.060712939	4.189627706	0.244358621	-0.471881443
460	-0.058026391	0.110989361	0.887640553	0.097215229
461	-0.055537997	1.848832069	0.328613953	-0.441191991
462	-0.12654357	1.718789669	0.279047619	-0.525003433
463	-0.039042437	0.7347835	0.352082645	0.576802572
464	-0.317942901	1.129191824	0.074382979	-1.708232032
465	-0.203417374	0.905469447	0.11369697	-1.676654604
466	0.002519875	0.720415201	0.795240288	-0.126411438
467	-0.106564852	0.212891876	0.791272727	-0.184839461
468	-0.114813673	1.309130698	0.151347826	-0.912306256
469	0.044154078	0.80798944	0.372882129	-0.502677917
470	-0.107704876	0.81412089	0.457248485	-0.385523054
471	0.08389026	0.992251301	0.264	-0.697939131
472	0.160013594	0.468468504	0.473779037	0.482350243
473	-0.054658386	0.978650465	0.470758621	-0.34402593
474	-0.125318254	0.385518458	0.632859745	-0.270986133
475	-0.10888595	1.811907149	0.1484	-0.808394114
476	-0.087532433	1.38743934	0.153644444	-0.88394271
477	-0.080232557	0.797737268	0.281684211	0.748761707
478	-0.065527299	1.512761715	0.271209877	-0.561345206
479	-0.035573717	0.169042989	0.83652725	-0.132076475
480	-0.0839965	0.018612815	0.981166038	0.012677087
481	-0.032323883	0.00071984	1	0.000387192
482	-0.006748033	0.408240265	0.594453441	-0.314364539
483	-0.31670072	1.221890643	0.292157303	-0.569534938
484	-0.040314629	0.193285493	0.825823452	0.142040041
485	-0.133474872	0.380569456	0.568112069	-0.367921617
486	-0.068384001	0.831175612	0.56749345	-0.26584159

	S	T	U	V
487	0.016733594	0.227303765	0.890338673	-0.071713342
488	-0.076950936	0.91547547	0.350158996	-0.517510732
489	-0.007312932	0.962397275	0.348116183	-0.504990048
490	-0.059173203	0.295388347	0.675834768	-0.269327376
491	-0.061908572	0.250839528	0.786382482	-0.175144831
492	-0.037118697	0.286314529	0.786151603	-0.164686203
493	-0.07283561	0.773157722	0.405658537	-0.462395774
494	0.005523472	0.147914446	0.852739827	0.123349296
495	0.066997461	0.730885358	0.48448	0.35559082
496	-0.065212286	1.036490667	0.521903382	-0.284946866
497	0.042671573	0.19867612	0.824160207	0.133846919
498	-0.051800584	0.310695471	0.740950617	-0.193526162
499	0.119297415	1.436278332	0.243352941	0.635711034
500	0.028145732	0.28783137	0.720032258	0.225822661
501	0.044252619	0.00032395	0.999573921	0.000462638
502	-0.046870151	0.19398748	0.837187817	-0.123579025
503	-0.028801798	0.364391529	0.763683109	-0.16504648
504	-0.081207593	0.57888189	0.541797647	-0.328843435
505	-0.149233515	0.638099522	0.467883382	0.411144469
506	-0.026115345	0.056367704	0.934046891	-0.050363117
507	0.14286723	0.182716778	0.753894737	-0.252909554
508	-0.031082253	0.895033445	0.468069364	0.356171926
509	-0.083174329	1.045512324	0.110967742	-1.433404922
510	-0.078767327	0.303011452	0.802188889	0.141616186
511	-0.105135146	3.335368911	0.218596491	-0.543605381
512	-0.032592665	0.068664899	0.932260062	-0.050982157
513	-0.055938734	1.106382808	0.244719424	-0.712975184
514	-0.037168469	1.336829441	0.316504854	-0.500641717
515	-0.025340553	0.843130317	0.326767773	-0.598325094
516	-0.039900987	0.15504942	0.830511749	-0.155553818
517	-0.048957611	0.21741859	0.750087481	-0.222698
518	-0.00700029	0.063602133	0.928661686	-0.070985794
519	-0.12849666	0.838881606	0.37998524	-0.480014377
520	0.027103845	1.766829628	0.231862069	0.632305357
521	-0.052771674	0.455770111	0.729360759	-0.178970125
522	-0.015079531	1.683212143	0.213035714	-0.668468475
523	-0.056302815	1.978219585	0.329757009	-0.434470918
524	-0.072622828	0.258853763	0.776112094	-0.180277295
525	-0.111774941	4.185676102	0.045333333	-1.028749466
526	-0.062079159	0.980327511	0.420972603	-0.406488631
527	-0.048917965	1.95084562	0.306708333	-0.466702355
528	-0.044239531	3.313349045	0.079510204	-0.836494446
529	-0.037790493	0.467899084	0.739895865	-0.166534848
530	-0.083202956	2.744874765	0.105311475	-0.801492691
531	-0.036947687	0.086733226	0.896578531	0.100030051
532	-0.075350322	3.225492199	0.248266667	-0.487059063
533	-0.050640418	0.037076067	0.964254652	-0.02836842
534	0.003581888	1.627559472	0.423537415	-0.351885478
535	0.002499714	2.52412827	0.537490566	-0.239671495
536	-0.050772639	0.42125668	0.738464396	-0.174583011
537	-0.006213714	0.517334443	0.713068404	-0.184473462
538	-0.008730321	0.339982644	0.800166898	-0.13766649
539	0.026243975	0.07561927	0.924584127	-0.061580446
540	-0.042850474	0.334235257	0.674976109	0.248034159

	S	T	U	V
541	4.28E-05	0.074209165	0.933779609	-0.047700882
542	-0.035906094	1.017671929	0.45593808	-0.362613466
543	-0.073038052	2.926667218	0.207407407	-0.576290766
544	-0.088971985	2.502777838	0.302251366	-0.451612261
545	-0.040581528	0.029941232	0.983700474	0.011376911
546	-0.042687092	0.062690053	0.934685714	0.047579447
547	-0.157839582	2.270845724	0.047619048	1.31786516
548	0.085589804	0.709105524	0.440296774	-0.435976452
549	-0.075897547	0.091890061	0.916679912	0.075207392
550	0.038726963	0.153893931	0.875066823	-0.093382094
551	0.04419939	0.539031715	0.50205598	0.383891847
552	-0.084818602	0.943010443	0.275418182	-0.687608295
553	-0.033914408	0.154724961	0.924261603	-0.047189501
554	-0.035521817	2.41367074	0.314373206	-0.427981906
555	-0.058544869	0.037354022	0.957466135	0.039133708
556	-0.000883608	0.074878421	0.922940314	-0.058246401
557	0.133875928	0.404621294	0.547334873	0.391475889
558	-0.134913149	1.373562912	0.170470588	-0.818038517
559	-0.053938476	0.070877782	0.949428571	-0.034785801
560	-0.041756872	0.290503328	0.853980344	-0.094574398
561	-0.026988649	0.185504947	0.870554622	0.095753352
562	-0.101640721	0.879706124	0.350973451	-0.543323305
563	-0.004694512	0.006755852	0.988089219	-0.00883717
564	0.01191807	0.134191518	0.923809725	0.049429152
565	-0.133752931	0.994381081	0.305312169	-0.59713406
566	0.029929285	0.045477551	0.970301263	0.021170934
567	-0.014375133	1.227742831	0.458638806	-0.336217244
568	-0.011885329	0.125338069	0.901457399	0.075824102
569	0.051873828	0.478334845	0.61118591	0.273756239
570	-0.037841245	0.403580336	0.508941772	-0.458551831
571	0.028991716	0.090915542	0.922702929	-0.052991019
572	0.003356044	0.079129329	0.871274463	0.175994025
573	0.047907393	0.953754633	0.30808377	0.604699241
574	-0.036618241	0.530155436	0.567514894	-0.30289544
575	-0.032714149	1.304061054	0.47649162	-0.312355042
576	-0.027362138	0.277661019	0.738411043	-0.203442468
577	-0.057195838	0.920231821	0.627947566	-0.212827471
578	-0.067495023	1.250882414	0.2608	-0.624564277
579	-0.045661892	0.402504474	0.691986532	-0.216309653
580	-0.046888313	6.001099213	0.244777778	-0.448968675
581	-0.048995442	0.802438544	0.486514589	0.341540443
582	0.158271387	0.168544027	0.80857538	0.185742272
583	-0.166413835	3.061910372	0.000666667	-2.494357639
584	-0.006197278	0.781854616	0.49585567	-0.3332901
585	-0.023955098	0.243914599	0.841364764	-0.105647193
586	-0.083086339	0.356383511	0.664787456	-0.251348707
587	0.007470778	1.709892727	0.281132948	0.518971549
588	-0.017647718	0.730205052	0.61905303	-0.228811688
589	-0.062002615	0.096119963	0.923406926	-0.063093185
590	-0.1267248	3.087086423	0.069466667	-0.972307417
591	-0.022284731	0.607063192	0.459844311	-0.436065674
592	-0.052409445	2.187178042	0.378249057	-0.367948956
593	0.010975603	0.653098439	0.553592841	-0.296712452
594	0.017495609	0.331337894	0.715236246	0.213316388

	S	T	U	V
595	-0.012451416	0.02217746	0.981926346	-0.011857351
596	0.081291587	1.09501657	0.474497238	0.324226803
597	-0.081474543	1.248279823	0.311842365	-0.519325044
598	-0.288456119	1.99874696	0.029333333	-1.929172304
599	-0.024767194	0.210304359	0.880311266	0.079824871
600	-0.055322011	2.293169902	0.304427807	-0.455103768
601	0.012865459	1.065940074	0.557306667	0.257271661
602	-0.042532207	0.782474973	0.568725702	-0.267573675
603	-0.009943141	0.030254194	0.974967118	-0.021143807
604	-0.210208777	0.578215841	0.378089552	-0.609234704
605	-0.03313848	2.658641148	0.291683616	-0.456613329
606	-0.162195516	0.737859536	0.313627451	-0.679915534
607	-0.052712063	0.652138511	0.608139806	-0.244673199
608	0.05223335	0.020830637	0.979478927	0.01911672
609	0.063449238	1.477776527	0.368818533	-0.409539753
610	-0.066719658	2.072530405	0.300972376	-0.473296907
611	-0.124825146	1.676726605	0.448205788	-0.330976486
612	-0.017589194	0.898152809	0.29298324	-0.662208345
613	-0.107354984	3.37224863	0.148090909	-0.641462538
614	-0.041722688	0.289276164	0.773741456	-0.174423854
615	-0.022802872	0.904486946	0.347408072	-0.542180379
616	0.205657878	0.27047788	0.78556623	0.168489244
617	-0.021663201	0.63578466	0.586115702	-0.268269857
618	0.057281209	0.091031462	0.876308411	0.128124025
619	0.067819644	0.731412519	0.521181598	0.31533538
620	-0.046214728	0.449304058	0.568363636	-0.333063549
621	0.007530298	0.0521396	0.935562819	-0.05322859
622	-0.16207355	0.288186417	0.799422914	-0.150339127
623	0.018233185	0.939838586	0.474625	0.344145881
624	-0.063085607	0.051889275	0.927731629	-0.084961573
625	-0.107382132	0.158323618	0.813394844	-0.182078679
626	0.002319168	2.348332496	0.275856287	-0.486580319
627	-0.019103947	0.793644785	0.458060976	-0.390568203
628	0.079644783	2.377558089	0.352980237	0.380996704
629	-0.21884151	0.272283073	0.693364706	-0.264134089
630	-0.021774017	1.96645582	0.402352113	-0.353154712
631	-0.026456164	0.000102202	0.999651376	9.64E-05
632	-0.151311688	0.612001069	0.327716981	-0.755846871
633	-0.005696646	0.24933727	0.811967391	0.141659631
634	-0.01794632	0.390371284	0.635358559	0.264612198
635	-0.086352746	1.531205741	0.402228571	-0.374857585
636	0.007706901	0.350386764	0.546927739	-0.44404051
637	0.160297343	2.619955834	0.105288136	0.821248372
638	-0.050360211	1.664604394	0.303195652	-0.497130924
639	-0.104704647	0.288567586	0.799340029	-0.154487822
640	-0.070172439	4.88558765	0.114823529	-0.659066942
641	0.006847946	0.107698432	0.916030939	0.070081075
642	-0.020968159	0.224841422	0.923717597	-0.044146856
643	0.063626491	1.145057072	0.352373333	0.484663857
644	-0.05139454	0.590062767	0.485723238	-0.383952247
645	-0.007468372	0.401145774	0.412965517	0.775095834
646	-0.083531592	1.122724982	0.160294737	-0.978890313
647	-0.055643873	0.075252667	0.924652542	-0.062120861
648	0.188076679	1.651626856	0.033714286	-2.147900052

	S	T	U	V
649	-0.105320067	0.593272767	0.495023136	-0.372593138
650	0.057930227	0.120551185	0.871642857	0.114060084
651	-0.02476799	0.296088893	0.745696049	-0.190935559
652	-0.010650419	1.015213589	0.376749064	-0.448829863
653	-0.112953278	0.099012781	0.838143758	0.20708402
654	-0.030813287	0.049402658	0.921933333	-0.077212863
655	-0.05073946	1.134765004	0.358682353	-0.453185611
656	-0.017925015	1.053549715	0.453895385	-0.358553569
657	0.001536842	1.088594729	0.40325448	-0.412168503
658	-0.04696746	0.724420803	0.521781022	-0.317244848
659	-0.064435189	0.040852807	0.956954092	-0.038030624
660	-0.070801855	0.312534778	0.568373102	0.437195884
661	0.045560094	0.438660537	0.611606178	0.280750275
662	0.053896511	0.048504882	0.953497497	0.037156211
663	-0.021471488	1.683284148	0.42789404	-0.343179703
664	-0.032351968	0.114645033	0.871575472	-0.113436381
665	-0.062877077	1.493982246	0.348991453	-0.438958274
666	-0.070116629	0.374848572	0.730292994	-0.192269855
667	-0.069721466	0.076651421	0.923003168	-0.06098281
668	-0.070497657	0.113480081	0.920171429	-0.065151638
669	-0.035607746	0.412133695	0.633869646	-0.268267737
670	-0.164940321	1.584218629	0.068275862	-1.451587465
671	-0.150235602	0.68608893	0.353193548	0.592985789
672	0.019033024	0.001395127	0.999061638	-0.001091851
673	-0.004458479	0.065829873	0.898326209	0.127407498
674	0.023989005	0.016745804	0.9842849	-0.014871385
675	-0.078007553	0.830024288	0.42710231	-0.419984182
676	-0.108857121	2.879786892	0.15243956	-0.658461041
677	0.015098196	0.27499214	0.713303079	-0.237182617
678	0.013834576	0.143655901	0.871201912	-0.104739719
679	-0.037229275	0.194265621	0.830643979	-0.137159559
680	-0.163603321	0.377918277	0.522741463	-0.455182817
681	-0.04320323	1.469426253	0.546175926	-0.252266778
682	0.00460345	0.033601345	0.975295257	0.020418167
683	0.104006843	1.336840394	0.308168421	0.523270713
684	0.028414873	0.298726903	0.584475	-0.419542101
685	-0.083409896	0.067864536	0.923542017	-0.062874688
686	0.005312673	0.531500413	0.741316614	0.165988074
687	-0.02443834	3.511829046	0.451322684	-0.295127233
688	-0.020327597	0.087134395	0.946341759	-0.035097122
689	0.016834151	1.661647768	0.145589744	-0.853266822
690	-0.074036629	0.965596379	0.261350649	-0.717218187
691	-0.01475263	1.009118156	0.865549637	-0.075644387
692	-0.052229026	0.83109918	0.523589242	-0.303844664
693	0.019598824	0.862007362	0.451043478	-0.386513604
694	-0.063975278	6.815482139	0.153376344	-0.550143772
695	-0.057000288	0.288372131	0.831137255	-0.117510902
696	-0.005375408	0.076028343	0.936314928	-0.043723636
697	-0.076392967	4.832445242	0.114197183	-0.654925028
698	-0.023849041	1.812304352	0.607931298	-0.200533337
699	-0.054606199	0.649182885	0.584140787	-0.26764467
700	0.052225904	0.27473432	0.785384164	-0.169709735
701	0.00137418	0.047643148	0.963833828	-0.028843138
702	-0.057901762	0.090038722	0.938071795	0.04157469

	S	T	U	V
703	-0.008699228	1.37781139	0.35289243	0.432719549
704	-0.061409352	1.1463614	0.404028369	-0.402404785
705	0.015663943	0.053278817	0.965511286	-0.024667316
706	-0.064699105	0.034974487	0.962477137	-0.037123998
707	0.056578803	1.285381725	0.30959596	0.520929972
708	0.028944235	1.048371341	0.555559551	-0.260643641
709	-0.028224079	0.028303111	0.980583493	-0.016698201
710	0.002176715	1.055507954	0.728398104	-0.152279112
711	-0.045795746	0.593206637	0.600128257	-0.262144301
712	-0.139521475	1.277702974	0.239516129	-0.697614034
713	-0.058432168	0.622016043	0.536056872	-0.323366589
714	-0.079164363	1.228304573	0.281	-0.585370382
715	-0.079950516	0.103558938	0.8976	0.085703956
716	-0.026577495	0.605287584	0.589226337	-0.27052625
717	-0.101320743	0.310234571	0.665446367	-0.26892768
718	0.057671915	0.572700114	0.555429844	0.310167101
719	-0.053031026	0.785970697	0.479313351	-0.353604211
720	-0.066039722	0.018698719	0.988746965	0.006765366
721	-0.021807926	0.053522109	0.961966502	-0.026947869
722	-0.073088642	0.030405452	0.976	-0.01963679
723	-0.029545046	0.009834421	0.990591288	0.004918204
724	0.010151007	0.24833473	0.799194326	0.160847134
725	0.034654194	0.070507454	0.951313942	0.033308029
726	-4.098723631	0.54127355	0.459975976	0.468186908
727	-0.068519628	3.136623038	0.266343949	-0.473039627
728	-0.047632078	0.214797433	0.824821429	-0.124739117
729	0.033350627	1.438012318	0.351412245	-0.432846069
730	-0.060835556	0.01875514	0.982763705	0.012732824
731	-0.045056875	0.846775548	0.449012658	-0.394311481
732	-0.211127179	1.990547446	0.069125	-1.189756817
733	-0.004515942	1.296187713	0.113314286	1.110120561
734	-0.014807936	0.396110881	0.754379518	-0.165587319
735	-0.052470083	0.869173373	0.432938111	0.405824449
736	0.02621499	0.12057548	0.948630303	-0.031755871
737	-0.009343566	2.817534917	0.329074074	-0.404302385
738	-0.019245391	1.195898315	0.679986395	-0.173891491
739	-0.081161999	0.709909261	0.433155844	-0.442199283
740	-0.043749656	2.212783241	0.712333333	-0.150586234
741	-0.057447763	0.596648363	0.585261411	-0.275417116
742	0.006622778	0.497719345	0.790295652	0.137394587
743	-0.04623215	3.477964259	0.27453012	-0.452060699
744	-0.11662532	0.610915254	0.465657971	-0.420173009
745	-0.065687047	1.55101831	0.23025641	-0.663841671
746	-0.102745897	0.344807474	0.594465863	-0.347522736
747	0.104662872	0.391284249	0.5216	0.44861412
748	-0.010257072	0.441946535	0.57968	-0.321092818
749	-0.023701028	0.255435789	0.786815675	-0.171526379
750	-0.053454445	0.131412443	0.894824064	-0.080912484
751	-0.105847553	0.902032907	0.272819876	-0.72017267
752	-0.049248856	0.440943862	0.650070671	-0.238367081
753	-0.103535805	0.228511725	0.798885714	-0.17022917
754	0.058859827	1.847091325	0.238151515	0.590742323
755	0.015654711	0.203978306	0.873488263	-0.08535703
756	-0.052859634	0.055641643	0.949869084	-0.038559808

	S	T	U	V
757	0.025179206	0.416293321	0.728857143	0.184989505
758	-0.033515713	0.084528708	0.92515027	-0.066087511
759	-0.042075774	0.400658608	0.720758842	-0.193676631
760	-0.118514405	1.504017689	0.247788079	-0.596310933
761	-0.224037402	0.405021712	0.5555625	-0.373259226
762	-0.139098164	0.522605605	0.380205882	0.655814701
763	0.122507479	0.119921533	0.898480271	0.080376519
764	-0.156007749	0.007787195	0.987981395	-0.008474138
765	-0.143342885	3.355373633	0.036266667	-1.214125739
766	0.02829512	0.038548459	0.963869565	0.031526142
767	-0.062013464	1.088379238	0.455111111	-0.355288823
768	-0.029996248	4.961053298	0.068848485	-0.822858387
769	-0.164843021	0.137168792	0.890130286	-0.083522797
770	-0.003460412	1.740276376	0.403123675	-0.361991882
771	-0.098280472	0.570294298	0.473577465	-0.421888775
772	-0.014612829	0.311867719	0.611465116	0.347886615
773	-0.007728714	0.073261399	0.921193443	-0.079589844
774	-0.057294306	0.293173954	0.739458462	-0.198487388
775	0.01554269	0.12226039	0.929374866	-0.053522746
776	-0.09460727	0.055008663	0.937283249	-0.04777103
777	0.003166143	0.978699838	0.529448441	0.284941567
778	-0.001929736	0.004415092	0.998360777	-0.002636804
779	-0.019096522	0.712508698	0.633264706	0.219593472
780	0.001747326	0.928763624	0.484431579	-0.325256772
781	-0.004012041	0.754655029	0.697410596	-0.177065531
782	-0.040071307	0.317436012	0.666486111	0.265832901
783	-0.071675268	2.299687262	0.242089552	-0.548803541
784	0.03254114	0.051674772	0.927912773	-0.067557441
785	-0.098271128	0.385435255	0.675548718	-0.231727176
786	-0.044386289	4.309771622	0.236892562	-0.498120414
787	-0.034617031	0.239938956	0.825798408	-0.131095462
788	-0.115931421	0.612660234	0.483518717	-0.385012097
789	-0.017773087	0.037840286	0.964847059	-0.028210322
790	0.052104521	0.275429361	0.701756579	-0.248809391
791	0.0360067	0.450641705	0.626288931	-0.262999852
792	-0.037841723	1.78424129	0.473640449	-0.296229257
793	-0.106471275	2.625640651	0.246273973	-0.513605542
794	-0.049167019	4.590239027	0.153037037	-0.606456545
795	-0.041521658	0.077989873	0.948506533	-0.033741209
796	-0.141568984	0.963579164	0.229277311	-0.858440611
797	-0.39659432	2.276894672	0.04075	-1.459910287
798	-0.056337227	0.980411931	0.426636066	-0.393344667
799	-0.000375612	0.241396712	0.830153846	-0.124083201
800	-0.066426788	0.464012961	0.547023256	0.361036513
801	-0.051530838	0.237252037	0.82541457	0.131411235
802	-0.097816367	1.063662084	0.304778378	-0.584253523
803	0.012749355	0.623998834	0.347547511	0.694139269
804	-0.032810426	0.09965639	0.919895425	-0.065789117
805	-0.10687313	1.9338113	0.112430769	-0.892125024
806	-0.061632005	1.16966625	0.176461538	0.883986367
807	-0.089527957	0.608646341	0.607777778	-0.254798253
808	-0.070447437	0.137780149	0.879592121	-0.092196571
809	-0.118655297	0.123377844	0.829546875	-0.188169903
810	-0.115951243	0.34226748	0.60920396	-0.334449768

	S	T	U	V
811	-0.020860701	0.05244668	0.934779247	-0.051441828
812	0.010988185	0.246681533	0.870750594	0.08495352
813	-0.098178955	0.088968291	0.919232635	0.075032764
814	0.069676479	0.631373578	0.580579832	0.274831136
815	-0.045093495	0.211839538	0.859171742	0.09927792
816	-0.077305407	2.840993932	0.366651341	-0.35950258
817	0.039074986	1.978165034	0.470236311	-0.29642529
818	-0.098752469	1.174659766	0.235377049	-0.73783599
819	-0.094636405	5.116786962	0.072	-0.783156925
820	-0.04872573	0.883722493	0.475283747	-0.343888601
821	-0.028559998	0.281049035	0.743981707	-0.19762124
822	-0.025346237	1.082961387	0.851832099	0.081474092
823	-0.045809403	0.105257279	0.923803758	-0.049252828
824	0.00223566	1.372822344	0.379292419	0.399284999
825	0.015215866	0.22661628	0.829803408	0.128846063
826	0.03589009	0.044882083	0.965654224	0.026506212
827	0.037106514	0.401437263	0.55432287	-0.37792884
828	-0.098583349	0.01087708	0.987705499	0.007871416
829	-0.05198953	0.129031288	0.920593886	0.060033374
830	-0.015521912	0.272282906	0.890541284	0.069616318
831	-0.045121559	0.077441517	0.933882837	-0.046094682
832	-0.015362286	0.02884673	0.973316505	0.024023268
833	3.858688433	1.097587287	0.213333333	0.832598368
834	0.034192195	1.617461254	0.072	-1.270644506
835	-0.29697772	0.640944113	0.422508475	-0.48909039
836	-0.049975335	1.337152407	0.548394495	-0.254258262
837	-0.07281335	0.199446801	0.813722071	-0.157569885
838	0.057995236	0.848504841	0.450247619	0.394108454
839	-0.14382823	0.143998443	0.838012484	0.138220045
840	-0.05305928	0.176836826	0.813730458	-0.164850235
841	-0.050600282	0.167439929	0.858712195	0.109308455
842	-0.078457965	0.75650099	0.454355828	-0.40196228
843	-0.034475707	0.207693637	0.825164295	-0.131175995
844	-0.034787928	0.070819188	0.932692784	0.049593396
845	0.022983175	0.215426397	0.789722543	0.184828228
846	0.163165447	0.658054164	0.404181818	0.506555345
847	-0.143749751	0.001153237	0.999882136	-0.001284917
848	0.008012434	0.680019116	0.730415214	-0.162940979
849	0.020286238	0.808812739	0.657204904	0.196769714
850	-0.096566088	1.116856399	0.351593496	-0.468150669
851	-0.061119559	0.730134374	0.358875	-0.553173913
852	-0.125433345	0.75260852	0.49826087	0.334732903
853	-0.06333792	0.208138671	0.816299065	0.145640479
854	-0.054789782	0.016537869	0.984136882	-0.015529421
855	0.014628164	2.830011411	0.073652174	0.906384362
856	-0.05855333	0.136611467	0.920623773	-0.058668349
857	-0.034409855	0.535624566	0.568592593	-0.307272593
858	0.006876892	0.50812983	0.798746853	-0.126884672
859	-0.101812665	0.337947416	0.626278195	-0.307284461
860	-0.001801628	0.147909335	0.872478673	-0.099643495
861	-0.058303326	0.377435553	0.695236181	-0.219451904
862	-0.081221159	3.461841683	0.086588235	-0.802790748
863	-0.044851794	0.593228577	0.568892704	-0.29098426
864	-0.038673676	0.162974131	0.862019417	-0.107710308

	S	T	U	V
865	0.001555308	0.850130461	0.313970149	-0.623651505
866	-0.110411039	0.055401134	0.924078007	0.094587962
867	-3.757931172	24.91933831	0	-20.29949718
868	-0.144026093	0.187258402	0.82082024	0.150749418
869	0.047709429	0.097047968	0.919819582	-0.070412318
870	-0.070266068	4.730554147	0.239619048	-0.48408254
871	-0.039771275	2.302258583	0.241605839	-0.544846429
872	-0.03165111	0.000992044	1	0.001898448
873	0.005450861	0.012212326	0.985459662	0.011081484
874	0.036703754	1.449165332	0.101963636	1.14300749
875	-0.130000501	0.125042746	0.880265116	-0.097828123
876	-0.282154887	0.169379648	0.809397524	-0.18162685
877	-0.09201267	0.888662735	0.351950617	0.518472883
878	-0.070739922	0.784662156	0.475441595	-0.366100735
879	0.015092326	0.112908792	0.928766595	-0.054137548
880	-0.102029504	0.426510173	0.609226054	-0.283409966
881	-0.009764848	0.180709897	0.841957764	0.118373659
882	-0.043321069	0.142918073	0.857282742	0.120661418
883	-0.037386249	1.575181599	0.727566188	-0.147695753
884	-0.125423455	2.6737346	0.104133333	-0.813900418
885	-4.080036605	2.403604941	0.042352941	-1.377924389
886	0.014773275	0.065681868	0.937224156	-0.046646966
887	-0.017871208	0.019956739	0.98040652	-0.019661797
888	-0.09512856	0.327500935	0.696362416	-0.234518475
889	-0.008954157	0.33062459	0.797883024	-0.146243201
890	-0.10648456	3.19726504	0.152390244	-0.656622145
891	-0.005889165	0.04682695	0.975895349	-0.018362893
892	-0.060172624	0.130538302	0.856166871	-0.12945599
893	0.014588047	1.188895438	0.11426087	-1.195908864
894	-0.070617981	0.795216827	0.554063927	-0.282735189
895	-0.112169007	0.229528884	0.777664706	-0.190452364
896	-0.051258824	2.295679668	0.496738462	-0.264975866
897	-0.122034437	0.207850494	0.813596752	-0.151771122
898	-0.084684049	0.596598193	0.458967359	-0.43771108
899	-0.043751878	0.065148729	0.938390144	0.047558679
900	-0.016052637	0.52010543	0.69745937	-0.193251504
901	-0.056557806	0.12869924	0.907897436	-0.070927938
902	-0.087482675	2.49650423	0.303553191	-0.446609921
903	-0.112330942	0.384054634	0.637144928	0.267673281
904	-0.02256127	0.191417763	0.823026596	-0.147469838
905	0.02916226	0.577563705	0.640856115	0.224985335
906	-0.044064281	0.622609823	0.473558739	0.407742182
907	0.001771126	0.147505117	0.838099502	0.134504954
908	-0.009712414	0.077139257	0.919146899	-0.075685925
909	0.028734758	0.795903545	0.61042998	0.234484567
910	0.06416557	0.731221106	0.349050847	0.590476566
911	0.112687117	1.445563764	0.128105263	0.965022405
912	-0.034860903	2.043002492	0.148423529	-0.753972159
913	-0.045535561	0.076292626	0.929917012	-0.051227782
914	0.104807691	0.458504512	0.633768807	0.25185034
915	-0.070312901	0.895014146	0.188566038	-1.056236267
916	-0.221913794	0.698592278	0.377984848	-0.539006127
917	-0.037082661	1.911164739	0.467964706	-0.301866743
918	-0.009062872	1.526625567	0.666038128	-0.175331752

	S	T	U	V
919	0.011177123	1.009468067	0.61019883	0.221024619
920	-0.140351263	0.089681769	0.875415205	0.131484773
921	-0.063736722	0.29866948	0.594831643	-0.395876143
922	-0.029192427	0.297062553	0.775255539	-0.170191023
923	-0.064794886	0.118775565	0.888363636	-0.092754788
924	-0.043377662	0.255566542	0.770155224	-0.188062244
925	-0.022644166	0.374085582	0.632994475	-0.27858395
926	0.02615049	0.421384043	0.930012931	0.044411977
927	-0.140553218	0.161133303	0.814623482	-0.176091512
928	-0.006910232	0.020497792	0.978412331	-0.022902383
929	-0.006301024	0.116235292	0.922875131	0.049622218
930	0.032182662	0.092724866	0.92558811	-0.056531694
931	0.010940181	0.459139013	0.646945133	0.236368391
932	-0.030354404	5.336738557	0.257019608	-0.442555322
933	-0.077939412	1.591076543	0.327605634	-0.461144977
934	-0.134047746	0.575748684	0.47478022	-0.411045074
935	0.042639423	0.175593388	0.907104677	0.064288669
936	-0.040070959	0.210303529	0.813967828	-0.147332509
937	0.005926144	0.181926062	0.826812665	-0.146798876
938	-0.043316383	0.576890228	0.515717172	-0.360977597
939	-0.030317106	1.435842562	0.308927835	-0.506232791
940	-0.041669474	4.285003863	0.271558282	-0.441513485
941	0.069455832	0.637945945	0.48396748	0.381109026
942	-0.068629847	0.271804768	0.799322034	-0.153419918
943	-0.009078487	0.529219899	0.738764431	-0.16585456
944	-0.061668054	0.201427879	0.838919192	-0.119386461
945	3.25E-05	1.387996629	0.657006993	-0.18081877
946	-0.028774039	2.666046409	0.343577982	-0.400165982
947	-0.051690758	0.30026916	0.738807988	-0.196045134
948	-0.049084635	0.253605972	0.799071633	-0.162685394
949	-0.013907503	0.159023166	0.824183908	0.146343867
950	-0.061923351	0.205062299	0.858227106	-0.101394441
951	0.010314985	0.331110239	0.815657296	0.123863008
952	-0.006575584	0.951923753	0.63368972	-0.209497452
953	-3.897793952	2.300163884	0.114833333	0.802713818
954	0.01701215	0.034461265	0.949770161	0.055323071
955	-0.076748833	0.250341362	0.773798817	-0.185356564
956	-0.044949553	0.162925499	0.809063712	-0.191193475
957	0.126085142	1.634397791	0.171524752	0.752062268
958	0.019602367	0.400406972	0.568923077	-0.351906459
959	-0.095975484	1.519472752	0.128693333	-0.938758638
960	-0.058220504	0.453678719	0.593240246	-0.30225775
961	-0.043204137	0.099133472	0.892382688	-0.09667333
962	0.037719534	0.899557394	0.483399464	0.332647112
963	-0.058555804	0.072582349	0.924650759	-0.074338701
964	-0.080318398	0.093695121	0.902203807	0.0873678
965	-0.057032091	0.309205087	0.610959538	-0.348241806
966	-0.050687346	0.352235536	0.774809524	-0.161910163
967	-0.069191383	0.464005928	0.611177866	0.280060026
968	-0.041197483	0.017032896	0.98544	-0.015531328
969	-0.073212516	0.333852377	0.739253499	-0.19061025
970	-0.069513874	0.159545557	0.809353425	-0.188270781
971	0.008397769	0.068476117	0.957832	0.030560388
972	-0.044920001	0.053759533	0.937963115	-0.052412457

	S	T	U	V
973	0.003489747	0.262367432	0.870605418	-0.081725438
974	0.072391394	0.302109734	0.740788732	-0.20056661
975	-0.012569339	0.145616667	0.890020619	0.082285775
976	0.039364519	0.078908678	0.922520376	-0.056089189
977	0.048384736	0.309063609	0.82255527	0.113553153
978	-0.078240698	0.299272323	0.653164912	-0.287840101
979	-0.027815131	0.04742591	0.963242063	0.030188878
980	-0.014601554	0.342134457	0.723153846	0.204126146
981	-0.044602569	0.181375786	0.797350427	-0.196445253
982	-0.487600305	0.343426587	0.562622807	0.410169813
983	-0.172944867	4.885681208	0.058956522	-0.900922563
984	-0.239801235	6.147670931	0.0008	-1.597957187
985	-0.200727204	2.295844938	0.151228916	-0.723938624
986	-0.129079481	1.11925014	0.353232	-0.463506911
987	-0.066511361	6.268274387	0.064142857	-0.808495204
988	-0.204835103	1.332875259	0.051090909	-2.125009961
989	-0.090815178	0.398574475	0.68235533	-0.222564909
990	0.028662206	0.917761732	0.521126214	0.294924206
991	-0.253748091	3.837305068	0.012888889	1.511022568
992	0.000145624	0.421135202	0.700046281	0.204883152
993	0.045954439	0.360405668	0.798454936	0.143028259
994	-0.18247407	0.281002639	0.562584615	-0.507979075
995	-0.114878569	1.138761114	0.352145749	-0.463770337
996	-0.009887505	0.219678875	0.826853437	-0.12826199
997	-0.05761305	1.577777421	0.070731707	-1.292147319
998	0.075039035	2.431622057	0.069428571	0.99584643
999	-0.004310297	1.263421755	0.482139785	-0.305681229
1000	-0.024347495	0.444892301	0.710671031	-0.1948683
1001	-0.02581967	0.033276271	0.979597366	-0.010174221
1002	-0.120289691	0.88890193	0.46113253	-0.369973924
1003	-0.147368433	0.012204575	0.984343184	0.02251731
1004	-0.049712746	0.349707937	0.63631769	0.280816184
1005	-0.018127585	1.075840711	0.349183333	-0.482746124
1006	-0.113621482	4.648328553	0.059692308	-0.898099687
1007	0.09468558	0.138509829	0.828478372	0.156922446
1008	-0.123769781	1.253945199	0.134077922	-1.043336656
1009	-0.040146806	0.177305558	0.845710037	-0.117086411
1010	-0.080720699	0.025868793	0.981907981	-0.009574466
1011	-0.048678088	0.483112323	0.610103647	-0.26804479
1012	-0.052599013	0.367072357	0.702200988	-0.214430703
1013	-0.090875007	0.765090904	0.469841642	-0.379620022
1014	-0.118117429	0.311679172	0.611047619	-0.340885374
1015	-0.069804354	0.25256555	0.812946237	-0.137272305
1016	-0.326087563	0.076278564	0.903785235	-0.10058954
1017	-0.020357044	0.21467089	0.824414853	0.126209683
1018	0.016738382	0.973814181	0.484628272	0.31972758
1019	-0.020114561	1.227608599	0.35014978	-0.472162882
1020	-0.107352504	1.623071191	0.350823529	-0.425247404
1021	-0.001528141	0.205362644	0.890836782	0.07446395
1022	-0.04210795	0.22126717	0.820229333	-0.139431
1023	-0.076413361	1.220949441	0.334211982	-0.492875629
1024	-0.119852905	0.20624053	0.813927322	0.150232739
1025	-0.215683387	2.024488216	0.097207547	-0.955347697
1026	-0.291326734	0.063259837	0.948196787	0.036036174

	S	T	U	V
1027	-0.02883365	0.298396658	0.825705179	0.122211668
1028	-0.013534003	0.109200346	0.916911504	-0.069709778
1029	-0.000186655	0.716386335	0.896825397	-0.058953815
1030	-0.227640798	0.126429158	0.824419437	-0.175757514
1031	-0.006198276	0.058108948	0.958169492	0.031226052
1032	-0.067137784	0.718963667	0.477091922	-0.372358958
1033	-0.095377946	4.086742728	0.230033333	-0.508499781
1034	-0.076492962	3.161596816	0.150190476	-0.656724294
1035	-0.035389363	1.086812337	0.265559748	-0.656928804
1036	0.007450224	0.026721007	0.981008596	0.01515007
1037	0.020898701	0.26795248	0.753497006	0.191770554
1038	-0.156552113	1.522372925	0.16444898	-0.79582617
1039	-0.007960413	1.667140993	0.521425743	-0.264352163
1040	0.071202786	0.16999625	0.749383686	-0.279933082
1041	-0.06650831	0.220202596	0.799864023	-0.170006222
1042	-0.224721579	0.076486697	0.924514039	0.069867028
1043	0.000233649	0.384876795	0.872521739	0.075350232
1044	-0.007752273	0.040652953	0.953827655	0.041696337
1045	0.001251908	0.243016504	0.929307527	0.046721141
1046	-0.046093656	0.073823582	0.969536657	0.020581563
1047	-0.070093265	0.517235084	0.61096325	-0.262408998
1048	-0.032439935	0.015347654	0.985799813	-0.008743286
1049	-0.045169148	0.532414484	0.568417391	-0.307542377
1050	-0.103137199	1.161419917	0.421360825	-0.385636859
1051	-0.021030439	0.176062666	0.88065811	0.085429933
1052	-0.022135749	1.509124029	0.31314	0.489435196
1053	-0.030993865	0.472055126	0.852766378	-0.08826934
1054	-0.029282648	1.074901286	0.309766497	-0.561094284
1055	-0.125250718	4.124987499	0.05632	-0.945232179
1056	-0.11939404	0.957611338	0.242524823	-0.777613958
1057	0.062500536	1.189876825	0.553927602	-0.255705092
1058	-0.021902957	0.245282005	0.860068044	0.094109429
1059	-0.089741563	1.508536342	0.0696	-1.423197428
1060	-0.03329862	1.274798238	0.282537143	-0.570871141
1061	-0.052560066	0.18084408	0.837233083	0.123319414
1062	-0.132855269	0.661963263	0.476333333	0.38554234
1063	-0.045069157	0.04948562	0.961561072	-0.030591541
1064	-0.061385251	0.40937627	0.592606557	0.318982654
1065	-0.030186099	0.2767953	0.790610709	-0.163028081
1066	-0.004158653	0.033161233	0.96321592	0.038801193
1067	-0.051688588	3.444452812	0.176571429	-0.584150738
1068	0.114445566	0.895284276	0.458071429	0.367812686
1069	0.045529392	1.256438326	0.268325	0.608833101
1070	-0.018906135	0.161545893	0.838055	0.129464467
1071	0.037677367	1.123191423	0.52222	0.285852008
1072	0.020002435	0.515763141	0.585746362	-0.291795519
1073	0.002268045	0.060831703	0.983094697	-0.009982427
1074	0.007522879	0.472472317	0.83674842	0.096791161
1075	0.030492411	1.296913625	0.244478873	0.652280807
1076	-0.011349107	0.542129635	0.502375635	-0.381689496
1077	-0.076833849	0.614688898	0.422558923	-0.499328401
1078	-0.053752913	1.619454014	0.309226131	-0.482615153
1079	0.072781401	0.075865028	0.922860898	0.077772988
1080	-0.118133566	0.977160955	0.152893617	-1.133465873

	S	T	U	V
1081	-0.025631864	0.750560139	0.455058104	-0.402007845
1082	-0.082199722	0.619364014	0.550022883	-0.309511185
1083	-0.068754768	0.896325547	0.379824176	-0.464364582
1084	-0.015851155	0.468001843	0.427734219	-0.604891035
1085	-0.050937846	0.1752448	0.828556567	-0.143295288
1086	-0.026166955	0.041030397	0.964885827	-0.028633542
1087	-0.049872662	0.393338397	0.56369163	-0.370304743
1088	0.031002782	0.152583121	0.823129987	0.154673682
1089	-0.079616509	1.260039497	0.310276923	-0.527745777
1090	-0.38383009	3.321065618	0.0035	-1.877753152
1091	-0.051254818	0.695285187	0.785348837	-0.131396823

	W	X	Y	Z	AA	AB	AC
1	Student's T-test Test statistic ORF2_PEP_WT_PEP	TOP3 ORF2_i01_1	TOP3 ORF2_i01_2	TOP3 ORF2_i01_3	TOP3 ORF2_i02_1	TOP3 ORF2_i02_2	TOP3 ORF2_i02_3
2	-0.535839367	24.85724	23.7813	24.22833	24.58927	23.97395	24.37029
3	-0.196883497	27.18251	27.67983	26.10091	27.18951	27.52705	26.38193
4	-1.375999353	24.5075	23.43802	23.89127	24.23618	22.75064	23.92647
5	-0.016882201	24.06293	22.79934	22.40927	24.0882	22.65654	22.82056
6	-0.297742643	26.39467	26.35475	25.95539	26.17571	25.08701	25.11632
7	-0.022192882	26.56642	25.98466	26.30341	26.60307	26.08863	26.32368
8	0.354915457	23.88704	26.23227	25.69345	24.48907	23.75209	26.81874
9	-1.171225791	21.45844	20.45173	19.97823	21.25865	20.03595	20.24544
10	-0.252794157	27.57863	27.08646	27.60034	27.52959	27.04825	27.64879
11	-0.341502625	28.77962	27.59933	27.52254	28.9472	27.57542	27.48023
12	-0.12251056	27.27634	26.58753	26.4618	27.06693	26.39571	26.42116
13	-1.393514122	22.9879	22.54041	22.68228	22.81042	20.56729	23.04513
14	-0.172227629	25.98535	25.88014	26.37486	25.84272	26.04488	26.49132
15	-0.832479989	26.61329	24.34009	26.49634	25.74434	24.45758	26.57887
16	0.208887928	26.47932	24.95536	25.23117	26.37984	25.10779	25.63058
17	-0.62179804	24.46735	22.09343	22.30639	24.2538	22.24478	22.09223
18	0.985165159	25.9129	26.09911	26.33953	25.67537	26.22014	25.10282
19	-0.238522399	29.80412	29.86189	30.06374	29.75248	29.84614	30.08027
20	-0.178220412	27.00237	26.48048	26.48155	26.81198	26.28209	26.36752
21	0.012739826	25.86008	24.85096	25.07478	25.65467	25.05807	24.59323
22	-0.674949843	26.30571	26.19739	26.92913	25.98348	26.16885	26.95718
23	-0.697576017	24.37129	24.10523	23.87649	24.00308	24.28028	23.47986
24	0.066209731	24.93486	23.11701	22.37655	24.68791	22.88098	23.52552
25	-0.280508042	25.1956	25.03036	25.03474	25.18347	25.13894	23.07484
26	-0.366358469	22.93829	24.33636	21.85336	22.93371	21.71634	24.01128
27	-0.299061702	22.97163	23.05482	22.85292	23.80838	23.46593	23.45528
28	-0.330134764	24.33471	22.83859	23.04462	24.16984	23.2739	22.96076
29	0.326793533	25.56203	24.03856	24.64429	25.28329	24.51485	24.9891
30	0.037755366	25.44541	24.7895	25.54472	25.55615	24.79861	25.73379
31	-0.811051238	25.25122	24.83302	24.88442	25.24119	24.76209	24.40378
32	-0.545341654	25.31852	25.2872	26.69419	25.28355	25.77051	25.3044
33	-0.278179985	26.42644	25.52626	25.16403	26.37863	25.48318	25.70182
34	0.43484956	27.90768	25.94661	26.81541	27.73497	25.73014	27.56457
35	-0.429011125	26.58012	26.6444	26.42389	26.87268	26.36617	26.47573
36	-0.719371548	26.46268	26.13613	25.99197	26.27031	26.07141	26.03426
37	0.229451027	26.43615	26.51608	27.1233	26.48717	26.39711	27.43749
38	0.367546704	21.87815	22.11204	21.92358	21.91657	21.78231	24.36039
39	-0.157393893	23.86255	21.96884	22.70682	23.84218	22.57167	22.76282
40	-0.192732376	28.94017	28.17456	27.81345	28.95517	28.06955	27.77933
41	0.509863013	30.78597	31.01337	31.53074	30.68176	30.77296	31.36162
42	-1.282354285	28.04583	26.92449	27.19734	27.74771	26.80857	27.21858
43	-1.135964378	25.3463	25.13915	23.41639	24.81364	23.7651	22.97309
44	-1.476668716	22.54522	22.30264	23.11131	21.63253	22.047	23.40082
45	-1.016020311	27.42517	25.85788	27.03267	27.42833	25.8667	27.21388
46	0.122582743	25.13438	24.48295	23.89287	25.20373	25.37059	24.11964
47	0.087359726	22.35108	22.18427	22.72737	23.41265	21.24376	23.20844
48	-0.129718507	32.123	31.74007	31.73896	32.09471	31.6591	31.61949
49	0.683327879	24.68477	24.38803	22.12325	24.36453	24.48421	22.71463
50	-0.495939438	35.08836	35.14796	34.45882	35.05809	35.15193	34.46404
51	0.313572106	28.5508	27.97665	27.81678	28.28082	27.83512	27.76585
52	0.265793059	26.83724	27.1264	25.3535	25.77674	27.24991	25.53525
53	0.135270816	26.87719	26.54588	27.51653	27.1494	26.39132	26.18197
54	-0.36302293	30.55505	30.16322	30.97306	30.44902	30.55915	31.34637

	W	X	Y	Z	AA	AB	AC
55	1.639432131	23.40333	22.67447	23.16181	23.82768	22.68896	22.69567
56	-0.077627289	23.10905	22.39779	22.52678	23.43797	22.15855	22.78139
57	0.166717423	21.24341	20.95141	20.1962	20.01066	21.07656	21.44983
58	-0.362436356	28.24713	28.22701	28.23608	27.87332	28.04537	28.27732
59	-0.195436389	28.9873	28.73973	28.55799	28.82857	28.65389	28.46007
60	-0.146236604	27.71371	26.78552	26.64753	27.26332	26.65853	26.75957
61	-0.373139426	25.37233	23.84696	24.09498	25.31656	23.77312	24.31713
62	0.011644921	25.12732	24.59584	24.43313	25.41145	24.40884	24.63202
63	-0.380013136	27.40082	26.07277	26.26719	27.27489	25.80569	26.44551
64	-0.142284644	25.75105	25.72265	26.34656	25.75325	25.81236	26.32262
65	0.586383037	23.73877	25.50032	25.60775	23.35616	25.48653	25.42553
66	-0.418380363	25.27052	24.54914	24.61539	25.04671	23.56699	24.34141
67	0.473083121	21.26221	20.91191	20.00001	20.83743	21.1759	20.69763
68	0.382461615	20.64547	20.99124	20.28038	20.5313	19.44458	20.30026
69	1.224866616	21.51339	22.69226	21.77832	22.31341	22.87369	22.32363
70	-1.309314449	24.29568	23.06464	21.99745	23.97759	22.43918	21.3147
71	-0.002630803	25.56602	24.63832	24.95619	25.14468	24.64414	25.10888
72	-0.354173602	26.32002	25.1123	25.00208	26.20394	24.58311	24.66127
73	-0.956080071	25.00759	22.85213	23.07669	24.68189	23.50243	23.52504
74	0.081029603	24.52518	23.58148	25.60409	24.7884	22.25693	25.26509
75	-0.131962352	28.15285	27.27211	27.99612	27.66864	27.44956	27.94901
76	0.545896551	24.5034	23.89637	24.85316	24.17455	23.81336	24.90546
77	-0.258113142	26.81665	25.99087	25.43298	26.87405	25.83561	25.36027
78	-0.204911563	30.15445	30.12829	30.64215	29.95927	29.90459	30.56833
79	-0.646790361	27.71333	27.02236	27.46863	27.58424	27.07042	27.48514
80	-0.341403743	25.36737	24.73431	26.81369	25.33087	24.74481	26.19917
81	0.064335606	25.29149	23.56222	24.02509	25.08158	23.80416	24.3264
82	0.499353866	25.4842	25.00981	24.92924	25.8891	24.79754	24.56145
83	-0.159649702	26.95673	24.85912	29.12376	25.96726	22.49147	29.14869
84	-0.321088799	24.85326	23.91137	24.59735	24.3914	24.18399	23.8512
85	-0.010634705	27.05199	25.82915	26.18638	26.77151	26.03357	26.01955
86	0.242895295	22.60959	22.78017	23.1121	23.23023	22.17219	23.40294
87	1.273500222	29.60625	28.32087	29.6239	29.62689	28.26129	29.60354
88	0.017982787	29.28053	28.23204	28.39382	29.11928	27.77774	28.37275
89	-0.058051669	29.9594	29.2234	29.57737	29.77603	29.0132	29.61121
90	0.586721074	30.56576	30.25386	30.95074	30.29535	29.7274	30.93591
91	-0.193701701	30.80997	29.42333	29.40812	30.54076	29.15582	29.51263
92	0.268067479	29.52374	28.57772	28.92236	29.25431	28.46188	28.92521
93	0.205834696	28.15811	26.41235	27.12665	28.05998	26.43124	27.1519
94	0.283846269	30.00436	28.37158	28.95764	29.90115	28.16247	28.78816
95	-0.110413916	28.79637	27.37324	27.77882	28.46122	27.50723	27.72059
96	-0.423252773	32.00002	32.091	32.27497	31.76249	31.82301	32.28261
97	0.10555603	28.25145	28.12691	28.83865	28.2917	28.02642	28.85562
98	0.832109781	28.84264	28.29237	29.16984	28.53107	28.17327	29.19172
99	-0.302418213	30.97619	29.51507	30.17797	30.61371	29.62178	30.06115
100	0.225032012	31.18893	30.60069	30.84286	31.02677	30.42558	30.76013
101	-0.177616087	28.89785	27.58284	27.40048	28.96315	27.51849	27.28014
102	1.207172612	30.3395	28.57948	30.39019	30.19288	28.62472	30.14873
103	0.285182485	30.91005	29.71887	30.2737	30.67041	29.6832	30.34524
104	0.104549043	31.37268	30.2074	30.81737	31.30226	30.01269	31.08921
105	0.613386707	30.81982	29.68974	30.62511	30.59844	29.73659	30.67393
106	0.148807928	29.17912	28.04751	28.02996	28.95182	26.90001	28.0834
107	0.108130264	30.6177	29.91405	30.48907	30.45213	29.8429	30.45782
108	-0.322532795	25.63316	24.50693	24.06358	25.23401	24.54057	24.21547

	W	X	Y	Z	AA	AB	AC
109	-0.543976893	30.04271	29.96004	30.51717	30.00363	29.80883	30.39682
110	-0.806790016	28.21103	27.75147	28.12714	28.06729	27.30446	28.04209
111	-0.162383808	30.3586	29.88194	29.22228	30.23851	29.66273	29.29304
112	0.380074746	28.43634	27.09175	27.30579	28.41043	26.98417	27.372
113	0.435617334	28.2801	27.05827	27.97116	28.19539	26.74073	27.84028
114	-0.479940441	29.21413	28.46152	29.20895	28.9119	28.71064	29.17369
115	0.285511425	30.23051	29.13137	30.06775	29.99798	29.04403	30.02746
116	0.040609381	26.03917	25.55848	26.2455	25.87049	25.48334	26.31334
117	-0.057506851	27.51889	27.10578	27.39378	27.46081	27.17967	27.4337
118	0.369787231	26.06898	25.42887	25.54784	26.14738	25.4186	25.6721
119	-1.128897363	22.68868	23.39217	22.44499	22.16216	22.88672	23.25283
120	0.029820253	26.94083	28.6186	26.21257	26.93133	26.06771	26.4899
121	0.226372469	25.41475	24.36817	24.42577	25.61115	24.37552	24.29842
122	-0.079150412	24.28932	23.60563	23.1834	23.03447	23.87057	23.11389
123	0.520731409	24.63453	23.81925	24.11059	24.1115	22.12212	22.99954
124	-0.213490695	26.07725	25.5391	25.1662	25.96872	25.5971	24.97701
125	0.075267192	27.73017	26.95015	26.83852	27.62974	26.84431	26.75801
126	-0.349900443	23.73384	21.88306	23.90738	23.64223	22.75358	23.44901
127	-0.20515636	27.97546	26.47613	26.98961	27.09652	26.54329	26.59426
128	-0.060441335	25.78674	25.10392	25.29385	25.34793	25.08725	25.24842
129	-0.032338679	28.97426	27.70516	28.19476	28.97589	27.53222	28.22897
130	-0.097314635	28.50949	28.10217	27.69986	28.17621	28.12775	27.70294
131	-0.012866226	31.53368	30.37855	30.28024	31.42727	30.4531	30.38658
132	0.20491506	25.84153	25.48093	24.68945	25.82033	25.69131	24.68813
133	-0.306342137	24.20078	25.17847	25.91637	24.35551	25.19283	25.61607
134	-0.426482301	29.29167	28.72192	28.02019	29.25324	28.44691	28.03155
135	0.095838943	23.26093	22.70212	21.66318	24.13055	22.57631	23.95595
136	-0.129414732	28.14602	27.07213	27.20679	27.91933	26.95797	27.0093
137	-0.478481798	29.03586	27.92374	28.13055	28.83459	27.57653	28.43445
138	-0.293663746	25.05518	24.09073	24.28284	24.92979	23.96776	24.07979
139	-0.075638619	30.10621	28.99005	29.27094	29.87312	28.99451	29.23855
140	-0.996551822	27.43487	27.07941	26.96943	27.295	27.52434	27.13873
141	0.032074224	26.96519	26.23563	26.56403	26.86871	26.41974	26.78747
142	-0.422973323	25.06915	23.93677	24.40425	25.44329	23.01323	24.33237
143	0.353209448	26.98145	26.59092	27.20885	27.06755	26.84131	27.59136
144	0.408518654	24.96935	25.03288	22.66501	24.41794	24.98439	24.31406
145	-0.462697591	28.46708	27.23449	27.60516	28.31563	27.17458	27.50617
146	0.12626321	23.04374	22.92937	21.64538	25.2472	24.10225	21.85198
147	0.770220114	28.80371	26.53026	27.4401	28.53414	26.48247	27.32499
148	0.00512233	29.52521	27.64053	28.64734	28.94212	27.50513	28.6545
149	0.130972803	24.14491	23.558	23.29688	24.44043	23.94588	23.51818
150	0.191597134	25.69415	25.29744	25.14866	25.818	25.01065	24.98235
151	-0.417745964	24.13061	25.08178	24.85961	24.2457	25.48245	25.23037
152	-0.121375561	25.51681	23.59529	24.06562	25.01207	23.01555	23.63638
153	-1.149466522	22.44159	23.07118	21.89241	23.49126	22.64157	22.25736
154	-0.135832833	26.50879	24.44735	24.41161	25.00762	24.15084	24.33488
155	0.20929608	20.04071	19.9568	19.86497	20.40018	20.45691	19.27262
156	-0.009219506	26.35839	26.61884	26.15018	26.24971	25.74389	25.86993
157	-0.467962281	26.11387	25.21287	25.47914	25.10526	24.96435	25.91429
158	-0.400463933	25.80944	24.63806	24.71618	26.08399	23.03789	24.87105
159	-0.651296315	26.82339	25.35949	25.54802	26.95094	25.93112	25.99715
160	-0.177771566	27.29414	27.15333	27.37169	27.21464	27.23597	27.31743
161	-0.078014441	28.31346	28.55568	28.2776	28.24244	28.36497	28.25286
162	-0.486197574	24.31977	22.10496	24.08708	24.09757	23.37959	24.22875

	W	X	Y	Z	AA	AB	AC
163	-0.404695969	26.57886	26.80952	27.01511	26.68223	26.59256	27.06257
164	0.135910767	31.65482	31.31808	31.92811	31.60178	31.22371	31.64243
165	-0.010494616	26.79912	26.21876	26.18232	26.55357	26.24828	26.09001
166	-0.483437465	28.23776	28.73672	29.48232	28.60231	28.93376	29.50397
167	-0.039802809	25.29275	24.98862	23.41017	25.1735	24.88081	24.08133
168	-0.28644953	27.06953	26.4683	26.26172	26.84362	26.33993	26.61811
169	-0.016071173	30.18633	29.79018	28.82182	30.00193	29.84735	28.84458
170	0.09581321	22.01186	20.97737	20.56785	20.72734	19.73606	20.80675
171	-0.540197293	27.43155	27.64483	27.6406	27.27167	27.89435	27.60563
172	-0.257785043	27.29288	28.38015	28.80086	27.27993	28.41898	29.08205
173	0.205534959	23.00123	21.95119	22.51306	23.11868	21.95117	22.78141
174	-0.150046655	25.29641	24.56853	24.29073	25.13609	23.74558	24.01085
175	-0.820754878	24.72588	23.53525	22.88224	24.79234	22.24607	22.42918
176	0.187003191	24.83814	25.34049	25.70283	24.91939	25.07264	25.01069
177	-0.813340399	31.01197	30.95035	31.80338	30.82283	30.85499	31.73543
178	-0.40770758	25.11041	23.66081	22.86692	24.83018	23.01032	25.26188
179	-0.903667429	25.88447	23.50788	23.88978	25.81766	24.12263	23.93887
180	-0.827583948	27.34064	26.83808	27.28105	27.09024	26.75088	27.27118
181	-0.07271124	22.94648	21.88545	22.06453	21.82997	21.67786	22.39947
182	0.134802125	26.31208	25.72062	25.53956	26.29482	25.67241	25.93826
183	-0.79527759	23.08827	24.66532	21.36388	22.71699	21.25643	21.18154
184	-0.427741557	27.98986	27.08476	27.4071	27.92999	27.0493	27.41873
185	-0.134741617	27.39303	26.83677	25.75264	27.14313	26.49259	25.79392
186	0.960108959	35.8327	36.28096	35.14282	35.79969	36.32676	35.2414
187	0.55060877	29.32257	28.89728	28.64586	29.05983	28.87581	28.59328
188	-0.640020873	24.985	24.5235	24.43888	25.25603	24.3117	24.87131
189	0.203681302	25.92158	24.77792	25.29881	25.60691	24.83973	25.09872
190	-0.413407138	25.93574	24.95137	24.39615	25.69131	24.83868	24.5314
191	-0.739596337	32.30757	32.14386	32.23156	32.30227	32.07717	32.28353
192	0.467250772	27.57349	27.47186	27.80106	27.28316	26.88992	28.02059
193	-0.334400253	26.8193	26.10365	26.1295	26.78868	25.75755	25.99376
194	0.785421133	27.95848	26.82194	27.57092	27.71996	26.84831	27.72361
195	-0.593032238	29.67907	28.52754	28.52972	29.65775	28.50539	28.88841
196	-0.644162513	26.99247	27.31739	29.05266	27.04877	28.09228	28.83871
197	-0.314094488	26.9001	25.54931	25.4607	26.35658	25.32056	25.4891
198	-0.517352268	24.42547	24.12677	23.40813	24.46199	23.89362	23.42074
199	0.145032508	25.27278	23.53855	23.89314	24.91387	23.19205	24.00976
200	-0.339525722	25.2667	24.18729	24.94454	25.82157	23.83219	24.9475
201	0.64110302	27.76995	27.64077	28.11865	27.63373	27.66395	27.99867
202	-0.575662098	30.91667	30.64025	31.23062	30.69143	30.65306	31.22213
203	-0.532982909	25.69932	24.86316	24.92457	25.37783	25.22283	24.58763
204	0.639079191	27.75843	27.65685	27.04104	28.03575	27.62265	27.56471
205	-0.119228783	27.83988	26.93462	27.07093	27.8743	26.99419	27.23204
206	-0.941957464	28.36124	27.99003	28.41532	27.97752	27.69072	28.53517
207	0.172401896	24.57119	24.86555	24.29914	24.58245	22.75873	24.17664
208	-0.384473385	28.89097	27.09652	26.91225	28.29506	26.93646	26.89597
209	-0.020490023	27.41356	27.43433	28.76648	27.16713	27.35498	28.88731
210	-0.077406679	27.71555	27.15009	27.63837	27.69926	26.99218	27.84676
211	0.046008308	26.54026	26.60613	26.47634	26.61453	26.44345	26.19513
212	0.839453092	22.93956	21.03938	21.03842	20.42555	21.19679	20.77058
213	-0.213235929	30.18621	29.59633	29.06076	30.08431	29.55029	29.05212
214	-0.099825411	27.70436	26.71639	26.70034	27.51062	26.54794	26.60075
215	-0.405952332	29.22352	28.76954	29.69426	29.04543	28.87841	29.77533
216	0.272729054	26.3602	25.44646	26.22656	26.07991	25.44384	26.34278

	W	X	Y	Z	AA	AB	AC
217	-0.747502004	26.2516	25.20749	25.40358	26.18126	25.23342	25.4312
218	-0.138769423	24.43963	23.97053	24.02136	24.37724	24.19689	24.28417
219	-0.365829376	28.81153	28.17872	27.60905	28.76605	28.1068	27.56532
220	-0.437847476	28.4079	21.47109	27.83947	28.2975	21.68719	21.98861
221	-0.039757198	24.26383	22.78924	24.17443	23.9086	23.40115	22.00018
222	-0.605665574	24.24853	24.41942	25.51261	24.27746	24.62915	25.31207
223	-0.591856721	29.02181	29.05212	29.85291	28.99704	28.95693	29.80687
224	-0.025340852	26.59775	25.90531	25.33879	26.58987	26.08377	25.56153
225	0.200323162	31.82458	30.69374	30.78972	31.717	30.71284	31.33465
226	-0.859286512	25.71832	24.78827	25.03462	25.07352	24.47265	25.38128
227	0.001894504	27.37742	26.12134	26.13431	27.25066	26.16917	25.62113
228	0.095585844	24.35881	26.48275	25.03694	23.05814	26.47266	25.24552
229	-0.270104771	25.64453	24.94253	24.41459	25.69545	25.19356	24.25708
230	0.017397223	24.89398	25.14752	24.47942	24.38872	24.79032	24.61852
231	-0.414834154	24.35588	24.60103	24.86863	24.74387	23.44777	23.8289
232	-0.702403225	29.97754	30.13865	30.54254	29.75197	30.0791	30.5755
233	-0.359988185	30.72461	30.58476	30.55048	30.76111	30.42293	30.54211
234	0.056304796	27.53911	27.29744	26.70858	27.59019	27.24122	26.81062
235	-0.220479452	25.85924	25.06122	25.27977	25.4872	25.22578	25.69912
236	-0.640526957	29.5702	29.37865	29.77667	29.47673	29.4548	29.76501
237	-0.002263061	24.87466	23.69192	24.27136	25.06781	23.65462	23.81482
238	-1.246943512	0	0	0	0	0	0
239	-0.346398981	24.98744	24.94476	24.27434	24.77677	24.42532	23.78167
240	1.066198525	0	0	0	0	0	0
241	-0.023920425	28.24468	28.20698	28.71051	28.10834	28.13352	28.76128
242	0.390880648	26.98504	27.11599	28.09189	27.16599	27.39468	28.30221
243	-0.22864659	26.69108	25.98522	26.00293	26.19033	25.57118	25.84821
244	-0.190249387	28.90306	29.21655	28.93986	28.78064	28.20766	28.53922
245	-0.156850132	28.93349	28.97995	29.45242	28.76593	29.0396	29.44513
246	-0.439083952	25.98786	26.34515	26.35102	25.38855	26.24415	26.43681
247	-0.407268542	23.70683	23.82382	22.80516	22.92735	22.56507	22.62799
248	-0.829009072	26.47525	26.43752	26.8904	26.4041	26.22792	26.79897
249	0.654020527	25.63188	24.82937	25.18391	25.54812	25.17842	25.17356
250	-0.132518897	28.24439	27.56482	26.42138	27.34505	27.33829	26.4121
251	-0.45424368	26.94816	25.86974	25.7403	26.67445	25.52944	25.76355
252	0.095508062	23.18207	22.43554	21.38383	22.86956	23.05183	22.1415
253	-0.027243995	24.97551	25.11849	25.62535	24.86489	25.45494	25.75298
254	-0.164083238	26.16644	27.2042	25.20891	25.39175	24.84122	23.43182
255	0.415729872	25.19695	25.46348	23.12213	24.91905	25.69584	23.17462
256	-0.128673244	25.93931	25.21666	25.19114	25.7938	24.65397	24.17781
257	-0.524399663	27.15878	25.99403	25.90356	26.98259	25.86708	26.00237
258	-0.136457425	27.39586	26.66781	27.03713	27.11077	26.71552	27.04212
259	0.2524953	22.97259	21.67791	21.63138	21.5894	21.89437	22.24787
260	-0.194611471	25.14041	24.47813	24.01842	24.88276	23.85211	23.93576
261	-0.567287994	25.75956	25.09494	25.26679	25.3579	24.42887	25.50374
262	-2.083232781	25.89408	25.92325	25.73137	31.97153	25.7852	25.64373
263	-0.272700403	24.99015	22.86544	22.59954	24.41352	23.27373	23.23192
264	-0.034919766	26.09145	25.36388	25.18912	26.04083	25.31017	25.18102
265	-0.561619268	26.80994	25.99127	25.81907	26.67542	26.03801	25.73567
266	-28.5713522	20.58392	19.51042	19.29515	20.64356	20.39329	23.14594
267	-0.481412667	31.16543	30.75334	31.47	31.01506	30.5084	31.62893
268	0.129557907	23.4942	22.7937	22.25654	24.02435	23.10244	22.36128
269	-0.066490923	23.57218	25.44598	24.17378	23.97897	24.78923	23.39993
270	-0.069859464	27.03716	26.11922	25.7354	26.93725	26.93338	26.02773

	W	X	Y	Z	AA	AB	AC
271	-0.274210495	26.43933	25.41283	25.73877	26.09469	25.89241	25.50878
272	-0.466001677	24.55933	23.60837	23.76782	24.2198	23.2206	23.16749
273	-0.683697967	25.16641	24.64805	24.13357	24.91831	24.60491	24.13077
274	0.027730192	24.72707	25.37694	24.82962	24.6704	23.88761	24.27641
275	0.42366543	27.86318	26.17612	26.94996	27.57316	26.62468	26.8365
276	-0.458650484	29.94759	29.45719	29.76204	29.82512	29.38209	30.31912
277	0.117105759	26.4343	25.79502	25.97052	26.37783	25.75581	25.57475
278	0.160486061	26.23021	25.47604	25.21202	25.23651	25.00514	24.39008
279	0.107347088	31.03331	30.67559	31.22594	30.91466	30.54246	31.17327
280	0.360364902	30.15984	28.87864	28.99838	30.03271	28.6969	28.89953
281	0.172891459	25.54627	24.57202	23.68547	25.55098	24.86691	23.67986
282	-0.335464983	27.5389	27.4246	27.88191	27.32269	27.21826	28.05443
283	0.138488266	24.64679	23.08317	23.30622	24.73768	21.6721	23.30392
284	-1.814289353	25.17721	24.09065	25.11201	24.89466	23.90646	25.05983
285	-0.588665758	24.03531	23.39832	23.13111	24.21938	24.35038	22.74992
286	-0.245630167	29.23844	28.36169	28.43314	28.95514	28.41552	28.68456
287	-0.250603914	23.94212	23.20324	23.53996	23.45598	23.62273	22.80967
288	-1.291386618	22.39952	21.79521	21.64224	22.91562	21.20753	23.19228
289	0.114777701	23.84639	23.78398	22.73258	26.08901	26.229	21.88531
290	1.758112534	26.72726	26.53179	26.8422	26.26229	26.41275	26.79727
291	0.560303553	23.69784	24.82509	25.11869	24.44678	22.35197	23.33355
292	1.090502479	22.73796	22.82796	23.85501	21.9511	23.23788	22.47297
293	0.518691129	24.98197	23.92873	24.04211	25.07756	24.26651	23.54025
294	0.333178702	26.42782	26.74857	26.89074	26.47835	26.7465	26.76165
295	-0.185813485	23.77211	22.36467	22.42032	23.66463	23.96825	21.85533
296	-0.793553139	24.67195	22.00693	22.08503	24.01661	22.58036	22.67667
297	0.473069761	24.14743	22.86222	23.8513	24.24863	22.05049	23.41375
298	-0.401012053	24.79759	24.68651	24.28783	24.67929	22.70305	24.10415
299	-0.725441231	24.30313	20.64375	20.99644	23.52296	21.05824	21.06879
300	-0.313884123	25.76078	24.0479	25.1806	25.42869	24.65733	23.92444
301	-0.304124595	28.42457	27.82069	27.95563	28.2142	27.67904	28.16192
302	-0.033988869	25.68386	23.8743	23.29632	26.16477	25.26114	25.29752
303	0.52090958	23.91069	24.1299	22.59294	23.675	23.97541	21.58211
304	-0.883568171	27.00126	26.52666	26.41711	26.67883	26.40363	26.74687
305	-0.059830521	27.51841	27.15011	26.06311	27.52516	27.56684	26.32222
306	-0.474350998	25.11389	22.88609	22.90875	24.56827	23.462	22.34858
307	1.106917949	29.00877	28.05004	28.26888	28.94664	27.507	28.12518
308	-0.018957426	22.70658	21.68091	21.8271	22.87606	21.6299	22.69582
309	-0.438253837	25.54504	25.11176	26.13738	25.18346	25.04374	25.92054
310	-0.299644519	24.64535	22.95505	23.21025	24.53457	23.14907	23.60609
311	0.385830255	24.2497	23.42589	25.03697	24.42906	24.11575	24.16166
312	-0.328946605	26.46547	26.17061	25.13424	26.33686	25.81642	25.25298
313	-0.365787572	27.98906	26.94595	27.37443	27.92662	26.78728	27.03906
314	-0.203676305	26.85572	26.43565	26.26002	26.70365	26.21038	26.53974
315	-0.828813196	24.66359	23.40537	23.85475	25.16043	23.11645	22.52254
316	-1.103424092	26.11502	23.58556	26.49617	25.88132	22.40986	26.55354
317	-0.435253856	25.72352	25.60525	24.65591	25.79338	25.54889	25.43398
318	-0.355738629	23.30439	22.80149	23.1846	23.56968	22.09098	25.10448
319	0.079515212	25.20143	24.94051	25.42389	25.20431	25.041	25.59074
320	-0.195779186	27.60728	26.74119	26.80303	27.58041	26.74108	26.85262
321	-0.080488845	29.2849	28.90896	28.82923	29.41178	28.8208	28.81027
322	0.228582354	25.92604	26.43869	26.78457	25.806	26.25887	26.81345
323	0.172920915	26.38641	26.34092	26.69953	26.36987	26.23037	26.57546
324	0.108093448	26.12785	25.00601	25.03993	26.77794	25.03093	26.06612

	W	X	Y	Z	AA	AB	AC
325	1.244857396	27.90763	28.24902	28.11551	27.57651	28.29907	27.79505
326	0.603821813	26.11985	24.68285	25.03725	25.3554	24.64812	24.60612
327	0.798354231	26.40225	26.79406	27.01131	26.49491	26.65395	26.54729
328	0.246650435	31.20088	30.45539	31.17522	31.11261	30.47169	31.49972
329	-0.389730594	30.01187	30.00303	30.55217	29.84859	29.50569	30.72782
330	-0.712294129	27.91424	27.30692	27.91778	27.51891	27.20754	27.88618
331	-0.517486549	24.97115	25.16353	25.51811	24.30165	25.12136	25.18973
332	0.022619363	23.56021	22.1482	21.98032	22.99931	22.22154	22.97183
333	0.016947859	26.38141	26.43709	26.20341	26.4056	26.38062	26.29981
334	-2.490574432	25.57217	22.62674	21.68689	25.52998	22.56222	21.6683
335	-1.13752407	26.10759	25.60671	25.58551	26.03768	25.70184	25.47233
336	0.569841075	27.97885	27.23454	27.59974	27.90423	27.17337	27.5859
337	0.440297219	28.91005	28.42496	29.28221	28.46172	28.51587	29.3198
338	0.820002984	23.57586	20.95099	22.11084	23.39194	21.33881	21.60337
339	-0.052434332	28.37701	28.46502	28.68283	28.38972	28.49491	28.65577
340	0.690610003	24.07642	21.9959	22.84333	23.97121	21.60457	23.22385
341	0.266627344	26.39912	26.18136	26.29552	26.30146	26.1534	26.4446
342	0.624226501	27.30404	27.53413	26.76443	27.24602	27.31075	26.99305
343	-1.453220897	30.14655	29.18246	29.36374	29.63937	29.24974	28.65362
344	-0.333232609	26.3578	25.26567	25.00554	26.35027	25.16584	24.43891
345	0.119372105	27.72763	26.95159	26.80595	27.06465	26.82047	26.66465
346	-0.37807477	27.13607	26.65879	26.83422	27.14845	26.54712	26.74741
347	-0.007725053	22.67702	22.63662	21.56845	22.63118	21.90013	21.7802
348	-0.025395985	26.25061	24.91635	25.14516	26.17535	25.58365	24.99252
349	0.30948336	27.52545	27.04927	27.46195	27.38688	26.90365	27.66936
350	-0.289581904	30.31368	29.74029	30.1282	30.07645	29.65146	30.54819
351	0.133904232	30.65166	29.91058	30.823	30.33437	30.32159	30.84645
352	-0.253783761	24.9878	23.72945	23.07845	24.33722	23.59503	23.32673
353	-0.040466514	24.61821	23.83358	25.3178	24.8765	23.07769	23.66047
354	-0.548514422	27.94103	26.48095	26.58368	27.79512	26.99846	26.95562
355	0.460637863	30.85263	29.4466	30.84644	30.64927	29.39807	30.77424
356	-0.188816818	26.56057	26.01637	26.71392	26.20014	25.79572	26.983
357	-0.07838555	27.42242	26.70689	27.48524	27.24325	26.79169	27.42944
358	-0.327338127	25.07915	23.80266	24.82916	24.80038	22.94895	25.4617
359	0.330743103	24.96125	24.69713	25.23524	23.84793	25.02463	25.34564
360	0.856070122	24.92103	22.82224	24.06429	25.16557	22.62334	23.09635
361	-0.7476954	28.00585	27.31915	28.35077	27.85552	27.22094	28.27665
362	0.320822146	30.08571	27.89611	28.55056	29.71676	27.83907	28.70975
363	-0.16067503	26.84493	27.32703	27.46381	26.46299	27.21395	27.52759
364	0.332496257	24.26528	23.18146	24.2197	24.20097	23.2047	23.66218
365	-0.999737737	24.79511	23.62282	23.67343	25.27594	24.03462	24.61019
366	-0.33283745	27.22367	26.40962	26.24792	27.44068	26.53629	26.08548
367	-0.737546698	24.68815	24.7129	24.87991	24.44721	23.37792	24.91889
368	-0.206529976	23.99486	24.05786	23.5829	23.74804	23.81382	22.52935
369	-0.936971035	26.79663	26.39888	26.8818	26.57853	26.42085	26.7128
370	-0.467276833	25.80444	25.24226	25.19378	25.64238	25.17135	25.09085
371	-0.319285096	23.40588	22.3439	23.39558	23.48987	23.38397	21.85559
372	0.160677046	22.51097	22.98055	21.27747	23.27885	21.35935	22.31292
373	-2.855531984	19.17295	20.4156	20.20545	18.65885	21.17051	20.63755
374	-0.261969495	28.96586	27.9435	27.71845	28.88053	27.94587	27.83045
375	0.265039857	25.97486	26.81261	25.84402	25.41495	26.44698	27.17781
376	-0.321792818	24.10776	23.02835	23.74669	23.93997	22.46767	21.95264
377	-0.388691112	24.4607	22.9332	23.24525	24.7251	23.43859	23.05709
378	-0.330068901	23.96412	24.72078	23.13277	23.22806	24.57	23.33398

	W	X	Y	Z	AA	AB	AC
379	-0.687261298	26.66851	27.00767	26.86602	27.09129	26.8976	27.30254
380	-0.4954961	25.61847	25.51132	25.55068	25.43869	24.45442	25.4642
381	-0.259612024	26.52955	25.19489	25.75675	26.19695	25.50948	25.29295
382	-0.111442618	28.03091	27.35081	27.02881	27.62691	26.93423	27.08484
383	-0.079828264	24.56245	23.3264	23.47366	24.63577	24.24504	22.30713
384	-0.872525703	26.51804	25.17704	24.46449	26.19142	24.79466	24.61763
385	-0.118830013	26.19116	25.7643	25.31815	26.23033	25.00041	25.63816
386	-0.074661063	27.69408	27.33737	28.32451	27.67167	27.15981	27.99489
387	-0.243565957	26.31795	25.44033	25.40294	26.29317	25.23257	25.30579
388	0.140619978	27.07164	26.83619	26.72626	27.5396	26.69255	27.06669
389	-0.499381723	27.35248	27.16086	26.96587	27.02874	26.93757	26.95675
390	-0.946634811	24.91511	24.25212	24.44977	24.63154	23.22995	23.32852
391	-0.284305942	30.31295	29.23622	29.3658	30.21974	29.30906	29.11896
392	-0.170794303	27.36645	27.02638	27.84545	26.92978	27.21866	27.85428
393	-0.595705491	27.93167	27.7025	28.15639	27.88748	27.59019	28.20474
394	-0.067692707	26.31236	24.45107	26.1802	25.93682	25.8334	24.28338
395	0.100017477	26.63991	25.09621	25.6216	26.31892	25.19686	25.66795
396	0.191502378	29.68462	30.16459	30.67799	29.65608	30.04173	30.95623
397	-0.088504216	26.01632	25.25474	25.03182	26.05788	25.39089	25.1545
398	-0.52218527	29.40834	29.57409	30.01145	29.4135	29.50405	30.08701
399	0.089306238	24.4463	22.33246	22.16035	24.1385	23.17107	23.83249
400	0.148254804	25.20377	24.32917	25.09739	24.59405	24.41152	24.95925
401	-0.487719661	24.0843	22.92164	22.64139	23.37663	22.36027	22.19315
402	-0.88638849	20.43462	20.36823	19.67786	19.8587	20.24907	19.60997
403	-0.347506192	28.49831	27.62728	27.33303	28.44123	27.67965	27.21181
404	-0.320750968	25.36887	22.8654	24.5996	24.53956	24.98321	26.06472
405	-0.087483006	23.27363	21.76652	20.86307	22.54864	24.73499	21.94497
406	-1.303529923	22.72755	23.08904	21.82314	22.43067	22.52518	22.81368
407	-0.238864745	23.92913	24.00344	24.92374	23.60579	23.32145	24.68319
408	-0.089519477	26.10726	25.27666	25.17188	25.86072	25.16346	24.86348
409	0.300183857	26.72888	26.64849	26.69084	26.43103	26.52827	26.70244
410	-0.758543442	27.71595	27.5214	28.1768	27.56441	27.26683	27.89311
411	-0.643950914	28.46371	27.09639	26.16292	27.93635	27.24505	26.49738
412	-0.112592437	26.97093	25.5236	25.56083	26.37681	25.3525	25.3968
413	-0.458242023	28.41357	27.57425	27.11721	28.30972	27.55103	27.3338
414	-0.396142316	28.22204	27.76315	27.48894	28.07051	27.45885	27.80488
415	-0.384849209	23.7261	21.80929	22.39527	23.37823	22.59575	22.50006
416	-0.294748125	23.75419	22.51484	23.09991	22.38199	22.16097	22.02599
417	0.368992546	26.71832	27.00701	27.13698	26.46175	26.78888	27.08896
418	-0.836710243	25.87984	25.46382	25.76787	25.79569	25.31253	25.51882
419	-0.440870627	23.83428	22.38527	25.05085	23.76055	22.62501	23.73958
420	-0.31897268	26.85786	26.23785	26.15937	26.90214	26.13915	26.23401
421	0.203047235	23.18945	25.34252	25.08405	23.30322	25.06209	22.29746
422	-0.259507318	26.74968	25.89202	26.14319	26.61435	25.78255	25.76484
423	-0.091143822	27.6918	26.82415	27.18097	27.45719	26.80542	27.23839
424	0.07085466	27.66216	27.22063	27.55516	27.58882	27.1655	27.53957
425	-0.628695441	28.54806	27.29555	27.74512	28.12497	27.16326	27.52211
426	0.11125887	24.18266	22.61749	23.60902	22.16662	22.83732	22.31317
427	0.36932734	24.34454	22.4556	24.42599	23.97854	23.11279	24.28089
428	-0.244324686	24.13273	23.32509	23.44003	24.25061	23.72205	23.50302
429	0.612959714	22.28932	22.44826	23.24443	22.8722	22.38544	23.98924
430	-0.636318008	24.11613	21.05553	23.29389	21.78099	20.73082	23.21105
431	-0.15877802	26.79335	26.23717	26.00455	26.78908	26.34459	26.61026
432	-0.353302527	23.65134	23.09473	23.52638	24.0947	24.01855	24.50916

	W	X	Y	Z	AA	AB	AC
433	-0.294988694	24.18462	25.49368	23.60205	24.43911	24.91961	23.89536
434	0.277237762	27.22081	27.19185	27.57447	27.07695	26.98026	27.56826
435	-0.17077545	23.78332	20.54014	21.25108	23.44067	22.89845	20.86203
436	-0.400035815	27.20725	26.17981	26.04993	27.12172	26.13916	26.21785
437	0.32209148	28.41477	26.97264	27.22668	28.16012	27.13953	27.4744
438	-0.077780479	26.27042	25.03413	25.36647	25.70931	24.79805	25.28396
439	-0.508618574	31.03023	31.3042	31.71489	30.89612	31.22447	31.74155
440	0.440735423	26.74312	26.54166	26.25784	27.0535	26.55658	26.65435
441	1.055786255	25.12468	24.27485	23.89118	25.02926	24.26253	23.91128
442	0.195766666	26.52584	26.46692	26.39614	26.57018	25.59953	25.67253
443	-0.429243078	23.43823	21.8527	22.45716	23.22906	22.12669	22.30044
444	-0.412904243	28.20379	27.31057	27.12072	28.04788	27.21832	26.93929
445	-0.638468236	27.44846	27.33512	27.70499	27.31352	27.18231	27.73713
446	0.221601815	28.38049	27.41431	27.70059	28.2206	27.41928	27.57312
447	0.247264096	27.35425	25.71532	26.91829	27.5417	25.78723	26.23649
448	-0.650097414	30.34759	30.0584	30.25365	30.23765	29.92273	30.28777
449	-0.175610926	25.78409	25.37888	25.04238	25.83775	25.19725	25.127
450	-0.25390293	24.87782	25.58305	25.12131	25.44646	25.0208	24.77094
451	-0.033617092	29.16887	28.8525	28.87603	29.02906	28.76752	28.75489
452	0.032941275	29.52562	29.30968	29.97166	29.14336	29.29335	30.01848
453	-0.250191024	32.8592	32.59875	33.42618	32.69833	32.46377	33.53978
454	-0.177999184	28.18389	27.48902	27.66613	28.05158	27.1079	27.56807
455	-0.06813796	22.22647	23.08937	23.33239	21.88658	22.7653	23.31299
456	-1.249869405	23.84304	21.80852	21.97618	21.99949	21.65008	21.79455
457	0.61788924	30.57634	29.81427	30.13169	30.44767	29.62579	29.9576
458	-0.349722101	27.61697	28.26151	28.15746	27.77743	28.25478	28.25735
459	-0.80232267	32.27292	31.9415	32.26737	31.96643	31.6588	32.4812
460	0.11662077	25.88944	25.72267	23.78265	25.96265	25.50243	24.19983
461	-0.668176721	29.00879	29.06538	29.49112	29.31173	29.13307	29.72142
462	-0.748441624	27.94346	28.37802	28.06039	27.76738	27.81385	28.52521
463	0.629992958	28.71127	27.28445	28.48643	28.44356	27.63126	28.65734
464	-1.224972838	25.07561	24.47199	24.14392	25.05083	22.95756	24.14922
465	-1.093260525	25.40218	24.95697	24.89625	25.69775	25.93902	25.21647
466	-0.21337499	27.33892	27.16306	27.18595	27.35002	26.99014	27.25388
467	-0.215480602	24.1681	22.98104	23.36088	23.80137	23.22564	22.62598
468	-0.98267932	24.87834	21.56159	23.19577	23.74018	21.45127	22.06967
469	-0.600352398	23.92143	23.70167	24.64963	24.4104	24.09438	24.46919
470	-0.509118417	27.61045	27.18453	26.6066	27.51502	27.16031	26.4573
471	-0.773709862	22.04471	21.96786	22.44866	22.41744	22.1717	23.13369
472	0.486984005	24.81983	23.0996	22.57517	23.75277	22.63018	23.44806
473	-0.491311554	27.00507	27.087	26.96773	27.01613	27.43578	26.90705
474	-0.329899326	25.67534	23.97023	23.40041	25.21748	23.56112	23.31825
475	-1.012784469	26.82118	25.0508	25.87889	26.65549	25.74354	26.22549
476	-0.984734823	21.77703	22.65454	22.5363	22.50261	20.78894	21.91178
477	0.743375856	24.94941	23.16354	24.14309	24.61592	22.7396	24.26061
478	-0.761792844	27.35717	26.4211	26.35094	27.30045	26.52351	27.18952
479	-0.162680816	24.81753	23.46967	24.53115	24.40947	23.26802	24.91905
480	0.017195128	25.54593	25.12201	25.35236	25.17755	24.50297	25.40814
481	0.000566359	25.68837	25.3294	24.74516	25.91978	24.76764	24.64454
482	-0.367136626	23.17682	21.12311	20.74284	22.49107	21.26704	21.1348
483	-0.728880032	20.63505	20.75261	19.90307	19.32986	19.71463	19.92977
484	0.177849094	26.88922	26.5403	26.71314	26.88439	26.40478	26.81617
485	-0.391021209	24.98298	23.80768	23.3461	25.14096	23.06374	22.59972
486	-0.394038498	27.73081	27.14501	27.22526	27.5311	27.04133	27.37064

	W	X	Y	Z	AA	AB	AC
487	-0.11359417	26.58726	25.95056	25.70153	26.61784	25.69966	25.73858
488	-0.633858129	24.48493	23.83369	23.12017	24.4629	23.07952	23.26178
489	-0.633165897	26.06605	24.49439	24.30626	25.75435	24.72049	24.81563
490	-0.300466165	24.47659	23.09471	23.89584	23.60257	23.1575	23.82597
491	-0.220255326	25.38884	24.36614	23.83948	24.55371	24.17141	24.0182
492	-0.21997059	26.95501	26.83843	25.79762	26.95886	26.6008	26.47739
493	-0.563439925	23.9257	23.44051	22.76962	23.42739	22.95673	22.89853
494	0.149070398	25.2389	23.40471	24.29967	24.96796	24.07519	25.05209
495	0.469601029	24.05954	22.86744	24.03043	23.37415	23.27136	24.33646
496	-0.432428491	26.42069	26.67353	26.97601	26.29233	26.6067	26.88106
497	0.17273764	25.44029	25.77048	26.3598	24.91283	25.59609	26.3569
498	-0.250274756	27.38517	26.2919	26.03636	27.413	26.26863	25.86913
499	0.816313961	29.31308	29.7038	29.46289	29.29213	29.38442	29.3521
500	0.268994583	29.3073	28.38966	28.01647	29.0798	28.23488	27.94619
501	0.000468551	25.71328	23.20653	24.8727	25.0519	24.3882	23.19876
502	-0.162811838	26.05	25.50724	25.02989	25.68268	24.30312	25.89626
503	-0.234174412	23.66667	22.36701	23.44298	23.3352	22.77847	22.64378
504	-0.419507096	23.78718	22.69128	23.21641	23.53363	22.42576	23.20077
505	0.495641318	24.11031	22.69971	23.94784	23.26132	22.04897	24.11958
506	-0.061147911	27.79453	26.76757	26.65285	27.85331	26.90973	27.06941
507	-0.239000275	20.77481	21.42619	22.42219	22.62543	20.84422	21.30009
508	0.493674604	27.33772	26.73883	27.71043	27.43054	26.49559	27.58663
509	-1.107334244	21.48298	21.53461	25.11387	21.50599	21.64706	21.11794
510	0.201130641	25.57123	23.10654	24.96113	25.5625	24.7195	25.49238
511	-0.871180852	25.30724	25.19901	24.67894	24.54725	24.42168	23.95042
512	-0.066025812	31.97081	30.88449	31.02762	31.75063	30.74208	30.89703
513	-0.811115369	26.85332	25.64413	24.94795	26.51376	25.61163	25.0632
514	-0.684423	27.07802	26.13703	25.76203	26.48428	25.55812	25.47175
515	-0.673075555	24.67639	23.76231	23.91507	24.30786	23.52609	23.00376
516	-0.173576643	25.15416	23.80927	23.68869	25.02861	22.50909	24.78196
517	-0.241161774	24.00708	22.2328	23.64383	24.42098	23.03716	23.16233
518	-0.078253902	22.47875	24.25002	24.00046	23.16391	24.42358	23.97396
519	-0.590270973	26.60582	25.61947	25.64944	26.49515	26.13902	25.50355
520	0.857148606	24.38251	23.80266	25.25696	24.44681	23.84991	24.81671
521	-0.260909575	26.34503	25.08202	25.56451	26.28462	24.93257	25.53236
522	-0.878924984	23.05011	24.1384	21.86242	23.15458	22.67773	22.96732
523	-0.668425117	26.04395	25.55069	26.40905	25.81064	25.37096	26.21048
524	-0.226490135	27.50875	26.6617	26.38101	27.3748	26.69832	26.35624
525	-1.48592599	24.44512	24.06844	24.93312	23.92569	23.95986	24.75217
526	-0.552089768	26.28992	26.75742	27.17669	26.17053	26.67005	27.01898
527	-0.704107659	27.25314	26.78834	26.61069	27.13303	26.7511	26.59297
528	-1.209056606	25.34025	25.42362	24.2222	25.31213	24.07373	24.56641
529	-0.248743437	27.87107	27.44907	27.33815	27.99512	27.48266	27.18292
530	-1.121721459	22.76176	23.67575	23.25231	23.58259	23.08274	23.49872
531	0.107568203	25.88142	24.64446	24.40172	25.60106	25.65912	24.62832
532	-0.792925167	27.65894	27.70383	28.1047	27.65253	27.60577	27.9238
533	-0.036756057	27.85413	26.89241	26.95009	27.50564	26.55934	26.91003
534	-0.549275179	27.0814	26.73436	27.56164	27.28985	26.72721	27.71164
535	-0.421538084	32.69161	32.72064	32.5755	32.52749	32.57007	32.55697
536	-0.251926387	28.23542	27.46588	27.67549	28.23044	27.48998	27.83576
537	-0.273842307	26.62391	27.1117	27.1672	26.66823	26.94067	26.91666
538	-0.202257877	24.96941	25.08735	25.61128	25.2031	25.2617	25.43492
539	-0.076930691	24.26207	24.49212	23.95863	23.83466	23.41009	23.90406
540	0.298835791	26.22787	25.4286	25.38445	26.08581	24.68803	25.95525

	W	X	Y	Z	AA	AB	AC
541	-0.064735893	23.16999	22.19548	23.2995	23.05371	21.76624	23.27081
542	-0.514327244	25.56676	26.07741	26.39067	25.61012	24.45021	26.43184
543	-0.891452302	25.67144	25.45862	25.36706	25.69535	25.49666	25.13546
544	-0.716774781	28.23473	28.19228	28.07061	28.42227	28.13464	27.4762
545	0.017945325	23.2963	22.71584	22.96502	22.67436	22.97056	23.15284
546	0.061265845	25.1233	24.4878	24.42536	25.09367	24.7349	24.05515
547	1.449145953	21.79348	21.90037	21.33838	21.35166	26.04576	20.75849
548	-0.529981416	22.78468	21.81834	22.07189	22.30977	22.90284	22.37806
549	0.09324873	24.88023	23.86256	22.94213	24.76389	24.40674	23.25753
550	-0.126294994	27.62533	28.23363	27.88853	27.49386	28.10548	27.93198
551	0.451598038	23.81023	24.54578	23.34848	23.99629	24.09836	24.64986
552	-0.754517351	23.81959	22.64212	23.49372	24.5272	23.3197	22.3929
553	-0.076062445	32.12726	31.50258	31.73124	32.02399	31.47521	31.71321
554	-0.682797755	24.58681	25.28538	24.83347	23.17435	25.22151	24.52378
555	0.04486454	25.11402	23.15178	24.10507	24.09515	25.70208	23.79944
556	-0.074028401	26.65264	26.07252	25.79595	26.48748	25.8623	25.62515
557	0.413474732	26.69263	25.63035	23.8024	26.548	25.8576	26.16969
558	-0.939474792	20.40208	21.00731	19.87872	22.57601	20.21583	19.79037
559	-0.051134835	25.13019	24.33385	24.16452	24.84294	24.14447	24.57903
560	-0.147517427	34.74124	35.29681	35.19801	34.70709	35.3751	34.76086
561	0.135132361	25.43344	24.8373	25.23606	25.41703	24.6597	25.26066
562	-0.645119485	24.9584	24.09656	23.62823	24.7941	24.36314	22.89835
563	-0.009305254	25.96893	24.08582	24.68634	25.32551	22.90219	24.00724
564	0.076866828	30.88302	30.21038	30.70316	30.65676	30.08481	30.64183
565	-0.707964753	26.75397	26.10179	27.14681	25.37651	26.54425	27.35283
566	0.031750179	29.71035	29.01863	30.10268	29.43964	28.97388	30.05288
567	-0.505189473	28.84839	28.86376	29.33181	28.7403	29.0673	29.23787
568	0.10341158	26.55081	26.06976	25.89314	26.13446	25.756	25.42127
569	0.353746113	22.03317	22.89282	21.95269	22.32306	22.33588	23.68499
570	-0.447620289	24.64407	22.76618	23.17159	24.89165	22.80109	23.28674
571	-0.073793352	27.85201	27.43782	26.96357	27.79296	31.00732	26.94706
572	0.132994504	29.6037	22.62942	22.2454	25.3287	22.76156	23.91815
573	0.704199618	27.59897	26.53928	26.81009	27.59192	26.48213	26.92766
574	-0.388439557	24.31725	24.24719	25.19962	24.54506	23.96142	25.0361
575	-0.482706377	26.04066	25.84219	25.66153	25.98184	25.70946	25.5264
576	-0.249596754	24.9439	24.75794	24.97795	25.37023	25.28156	23.99563
577	-0.338014606	28.65863	28.82608	29.17921	28.94908	28.71841	29.19345
578	-0.777514463	25.24003	23.8439	22.75637	25.02956	24.0127	23.55684
579	-0.289219954	26.26354	25.55239	25.16834	25.60051	25.45098	24.6496
580	-0.803534509	24.51775	24.27807	24.45724	24.26167	24.17673	24.59872
581	0.467631931	25.60697	24.68341	24.96151	25.81169	24.91144	24.88718
582	0.1976552	27.52747	22.22069	22.35766	27.28301	24.21695	22.69995
583	-2.24528642	22.81644	23.25019	20.84387	21.8948	21.40856	21.87139
584	-0.457058983	25.99163	25.9947	26.36152	25.78902	26.01798	26.24549
585	-0.15484305	27.95122	27.00416	27.08842	27.27659	26.71277	26.88819
586	-0.307454422	23.94623	23.39516	22.81252	24.57667	23.22005	22.23931
587	0.741471445	24.5131	22.58343	24.10217	24.02241	22.7159	23.2436
588	-0.343752928	26.0754	25.81636	25.29606	25.53327	25.49441	25.29728
589	-0.084488421	27.95508	27.32347	27.17257	27.75546	26.99342	27.01819
590	-1.32009924	25.31928	24.52325	23.51462	25.19451	24.17714	23.58261
591	-0.505284039	25.52495	24.35138	24.01599	25.134	23.97627	23.79976
592	-0.595713708	28.29054	28.23086	28.18282	28.14129	28.3479	28.2937
593	-0.404432646	24.31377	23.97281	23.53544	23.84952	23.69517	23.85318
594	0.271456227	27.45164	26.43609	26.63462	27.21339	26.40523	26.5994

	W	X	Y	Z	AA	AB	AC
595	-0.017260587	30.49789	29.70058	29.79304	30.46721	29.63645	29.7844
596	0.481283338	25.61363	24.81077	25.04777	25.53965	25.07552	24.94967
597	-0.690071159	23.99514	23.8301	23.85437	25.05511	24.44251	23.81878
598	-1.661915051	20.99058	20.40205	20.30252	20.02369	19.8653	23.3429
599	0.121658532	27.19761	27.37764	27.8926	27.30283	27.26987	27.7682
600	-0.710747322	29.44831	29.09166	29.46785	29.2467	28.8092	29.32617
601	0.401628887	26.31027	25.86259	26.38481	26.26042	25.84922	26.20706
602	-0.391245003	26.95688	26.46957	26.23622	26.89477	26.33401	26.19549
603	-0.028323977	28.68126	28.34622	27.42966	28.60353	27.96851	27.68244
604	-0.593565909	25.50719	23.72738	24.34177	25.27376	22.17003	25.32064
605	-0.730151734	28.6583	28.28877	28.56969	28.58342	28.31024	28.86629
606	-0.687947145	23.1273	21.43684	22.08958	22.31295	21.59241	20.98038
607	-0.353121426	27.55491	26.7263	26.67853	27.41349	26.89906	26.74599
608	0.023300421	23.65005	23.5719	23.88013	24.52617	23.40446	23.55863
609	-0.605974032	26.81202	27.01641	27.01594	26.65678	27.18581	27.14857
610	-0.719609673	26.74532	26.94258	27.39729	26.35491	26.73153	27.26992
611	-0.525878325	25.46024	24.93403	26.18345	25.88428	25.12319	25.7244
612	-0.727137975	25.33908	23.61455	23.29321	24.89524	24.41688	23.19423
613	-0.994586703	24.58938	24.98981	25.33391	24.56755	25.05909	25.01323
614	-0.229157582	26.00961	25.30842	25.39045	25.94758	25.11258	25.18673
615	-0.64969603	24.8761	23.88863	23.69555	25.34376	23.7539	24.27694
616	0.219764372	23.68353	22.39599	22.21772	22.33202	22.02923	22.87618
617	-0.374885527	29.09309	28.44174	29.00494	29.03471	28.42167	28.63585
618	0.124790155	24.95724	24.81179	22.41739	24.35649	25.0659	21.45841
619	0.433167032	26.86642	26.00566	26.2687	26.60255	26.0417	26.39005
620	-0.391862731	26.59939	25.47726	25.57302	26.30011	25.44698	25.69956
621	-0.061306651	25.13623	23.69062	24.56892	25.29195	23.40662	24.1316
622	-0.207137658	25.9818	25.37098	24.86598	25.83249	26.04637	25.75248
623	0.487196594	23.65844	24.86818	24.97512	23.69302	25.25451	24.92849
624	-0.077914067	24.41842	23.87023	21.67107	24.54566	21.59111	22.10596
625	-0.190599998	25.85073	23.13464	23.75726	25.54037	22.87776	23.35235
626	-0.751727868	26.84503	26.46869	27.21119	26.89812	26.46554	26.95198
627	-0.510141617	22.96779	22.48105	22.98347	22.81006	23.04325	22.24164
628	0.620294014	28.21435	28.38266	28.6039	28.08912	28.47763	28.7607
629	-0.288429999	20.86282	20.88015	20.5764	20.28341	20.79523	20.17514
630	-0.567342937	28.88111	28.29383	29.25612	28.73485	28.44683	29.11706
631	0.000117321	29.3812	28.62446	29.09969	29.26007	28.75417	29.23604
632	-0.671658293	27.69796	24.68252	25.5015	27.29794	24.47039	25.12283
633	0.191442928	26.51324	26.0453	26.64865	26.69558	25.24972	26.57491
634	0.326381967	24.90087	23.82199	23.27793	24.72462	23.49372	23.31616
635	-0.57076294	26.11629	26.12039	26.73134	25.85473	25.81809	26.60128
636	-0.41551266	25.52758	23.90995	23.69237	25.21634	24.21143	22.74292
637	1.127898097	22.76578	24.16414	24.11594	24.4887	23.7685	23.77813
638	-0.714902968	28.35275	28.14591	28.18118	28.03828	28.06896	28.25267
639	-0.211114818	24.27037	23.35377	23.2622	24.1689	23.10465	22.95611
640	-1.08665989	24.95692	23.74757	25.03108	25.01686	24.50732	25.03169
641	0.093799974	26.23171	25.69507	25.49964	25.95232	25.18678	25.23832
642	-0.075907921	30.71921	30.24525	30.83549	30.42705	30.26216	30.77173
643	0.645191134	27.34599	26.73881	27.49902	27.3269	26.08595	27.55317
644	-0.464475036	26.65055	25.47467	25.99003	26.65267	25.83089	25.34178
645	0.557338905	23.94056	23.83351	23.49889	22.73439	22.51394	22.92035
646	-0.964646601	25.211	20.7271	21.33566	24.34037	21.57868	20.85158
647	-0.077219094	22.57279	25.09039	23.51233	22.26196	24.40614	22.91957
648	-1.592091343	28.77121	24.47492	26.41914	27.71904	24.25995	23.39493

	W	X	Y	Z	AA	AB	AC
649	-0.456798647	28.09328	28.9939	29.14433	28.53572	28.5467	28.22682
650	0.132119985	24.88549	23.47355	24.95143	23.75823	23.62184	24.79784
651	-0.244647897	26.55763	26.30873	25.82995	26.56494	25.94246	25.91363
652	-0.59508655	24.9513	25.05544	24.32686	24.49806	23.97999	24.21295
653	0.160742469	22.65358	21.6613	22.70942	21.01555	22.3948	21.8264
654	-0.072690525	24.42899	22.90614	22.67499	24.72134	22.95729	23.05046
655	-0.615366571	26.06322	24.97078	25.05048	25.56824	24.3853	24.91451
656	-0.513975754	24.30298	23.26825	24.67654	24.7044	24.41404	24.13242
657	-0.571078425	23.37	22.71224	22.45778	23.57183	22.93205	22.6014
658	-0.433976301	26.17532	25.54413	24.99237	26.08066	24.60594	25.47542
659	-0.045710952	21.70359	22.87967	22.26731	23.40988	21.6799	21.85138
660	0.39233588	27.32314	26.34604	26.37911	27.64546	26.17165	26.63794
661	0.35067585	24.57528	23.51127	24.00883	24.28803	23.68556	23.88592
662	0.047924521	25.51043	26.00498	26.20125	25.94767	26.18957	24.62965
663	-0.541499058	27.12886	26.71421	27.11429	27.09698	26.25373	27.13891
664	-0.129193326	24.7312	24.81715	25.59662	25.39022	22.92799	24.95796
665	-0.638994135	26.87701	26.52355	26.11246	26.82902	26.13851	26.13774
666	-0.262223272	31.15516	30.36301	30.24809	31.00996	30.19307	30.28569
667	-0.076821248	29.26586	28.7485	28.53925	29.44909	28.83945	29.02653
668	-0.090597396	27.12885	26.71702	26.21572	27.18436	26.5691	26.21601
669	-0.334606708	26.05771	24.88888	24.69593	26.29208	24.91982	24.94691
670	-1.329402224	23.1407	20.39458	20.7299	21.93816	20.62371	21.71081
671	0.624281417	24.58987	24.10553	22.45239	21.71344	21.34066	22.42723
672	-0.001422762	26.19985	25.50794	25.53157	25.97627	25.03614	25.27815
673	0.105513325	25.94513	23.33657	22.12263	25.64104	22.31059	22.12627
674	-0.018388937	25.10052	24.7891	24.97309	24.84353	24.9034	24.77244
675	-0.541065844	25.91958	25.93266	25.07867	25.86292	25.04281	25.00215
676	-0.983431407	25.63055	24.98158	25.03592	25.5855	25.25722	24.91731
677	-0.272532859	24.62564	24.54246	24.20464	25.67472	23.68947	23.41757
678	-0.133371034	25.36208	25.90154	25.60431	25.26341	25.44843	24.97314
679	-0.174250195	28.07764	27.25296	27.08617	27.80667	27.27675	27.14141
680	-0.43410325	23.61214	22.48136	22.86148	22.58072	22.22312	23.58683
681	-0.414389834	26.57277	26.0136	25.46668	26.21321	25.93865	25.72782
682	0.028553627	28.74013	27.90427	28.28479	28.68477	27.89913	28.57908
683	0.705272295	29.00304	29.21898	29.88766	28.86494	29.17246	29.8636
684	-0.377367766	27.72896	22.35717	22.23346	22.27639	22.5207	22.38177
685	-0.074909735	26.25977	25.69407	26.04866	26.54956	25.66604	26.42349
686	0.254185965	30.69436	30.34456	30.35468	30.60937	30.06743	30.27708
687	-0.522878599	28.18798	27.99119	28.41898	28.06184	28.12502	28.49822
688	-0.05397852	27.9077	27.59876	27.27776	27.78068	27.37122	27.16674
689	-1.020903711	23.84404	23.65752	23.05054	24.07588	20.77551	19.69705
690	-0.77824717	26.19765	23.92311	24.8919	25.44728	23.39286	24.17368
691	-0.139298622	29.74659	29.52766	29.57197	29.69873	29.39688	29.65133
692	-0.434286674	24.11347	24.08465	22.92947	24.43563	24.43913	23.32886
693	-0.517319274	23.8415	24.64164	23.93701	24.31582	24.91943	25.54847
694	-0.978250969	25.04117	24.88448	24.50066	24.84405	24.67346	25.00442
695	-0.173731146	25.65264	24.65672	25.22268	25.29968	24.34424	24.82684
696	-0.061386145	28.69001	27.7681	28.00855	28.5582	27.84283	28.14059
697	-1.079007986	25.94847	25.40816	25.57153	25.78736	25.50442	25.57566
698	-0.349386839	27.26439	27.33346	26.84535	26.93462	27.03308	27.13204
699	-0.376051065	25.72876	24.74849	24.72297	25.45225	24.39039	24.78294
700	-0.221797219	25.75812	25.95854	25.54376	25.71077	25.51634	25.62433
701	-0.040205729	31.66959	31.2999	31.1769	31.69883	31.23813	31.15606
702	0.061808006	26.59762	25.96428	26.18322	26.52856	25.91904	25.45333

	W	X	Y	Z	AA	AB	AC
703	0.622023327	27.67071	28.5173	28.64507	27.63112	28.94563	28.74899
704	-0.568058972	28.78395	28.22061	27.72603	28.54867	28.13735	27.42497
705	-0.036972033	26.42847	26.09556	26.21266	26.22496	26.43732	26.79914
706	-0.042367254	23.76946	23.68857	22.07039	24.4616	22.73108	22.01049
707	0.696490557	25.83454	25.04321	26.57545	25.95816	25.57711	25.84178
708	-0.404618866	29.26762	29.34025	29.44971	29.15223	29.62228	29.77662
709	-0.023596309	27.24295	26.60565	26.61423	27.28738	26.59168	26.74143
710	-0.260836805	29.08842	29.27432	29.55046	29.14675	29.01553	29.6926
711	-0.363026397	33.79882	32.90287	33.04403	33.66419	32.69751	33.0893
712	-0.836971347	28.39495	27.42476	27.1027	28.27189	27.24472	27.21434
713	-0.423117397	28.18936	27.71941	27.59455	28.52838	27.69959	27.86961
714	-0.742767833	32.80271	31.72735	32.33339	32.46097	31.65379	32.23606
715	0.105394328	28.45642	27.68004	27.63428	28.31672	27.79842	27.86693
716	-0.372775433	27.86201	27.03851	28.30049	27.47243	27.29392	28.03581
717	-0.30553669	26.63989	24.87036	25.32109	26.37709	24.64908	25.44872
718	0.402830175	25.09643	24.63294	25.49847	25.2072	25.9816	25.69962
719	-0.476412394	27.38624	26.50741	25.98229	27.20975	26.29903	26.15214
720	0.010806562	27.173	27.76174	28.09991	27.69552	27.68991	28.33835
721	-0.039521169	28.95786	28.61651	28.46715	28.84372	28.27953	28.52112
722	-0.026980027	26.83616	25.96463	25.88291	26.60495	25.98683	25.76749
723	0.007313069	29.2698	28.93351	28.61465	29.37838	29.00419	28.67205
724	0.207979366	24.97808	25.21636	24.52822	25.08142	24.724	24.56484
725	0.049457828	28.18358	27.3283	26.64638	28.10288	26.78035	26.76709
726	0.505831011	0	0	0	0	0	0
727	-0.770955467	30.8263	30.27573	30.7294	30.54727	30.44341	30.83747
728	-0.168648927	29.18421	28.47438	28.18687	29.11716	28.56857	28.25337
729	-0.627647118	23.73784	23.11057	23.16342	23.64595	23.79516	24.12075
730	0.017287812	26.4493	26.44752	25.86583	26.31882	26.73137	26.55719
731	-0.52192081	26.12574	24.63461	25.33162	25.81691	24.6374	25.50009
732	-1.309139174	24.79112	22.93364	25.52361	24.70934	23.70045	23.70347
733	1.082931047	22.90374	21.25439	24.36739	21.69983	23.50723	21.26735
734	-0.239216297	31.20034	31.35889	30.7331	31.07503	31.35009	31.0067
735	0.535457458	21.45372	22.10009	22.22355	22.66437	21.8273	21.70626
736	-0.052826854	26.2812	26.4037	26.45932	26.19253	26.38563	26.44456
737	-0.667205854	29.97607	29.98906	30.17973	29.80499	29.73519	30.05052
738	-0.296093852	29.91944	29.87936	30.44	29.97282	29.93487	30.35647
739	-0.534734031	23.5001	21.94826	21.10173	22.70649	22.43509	22.02014
740	-0.274930508	26.70747	26.09386	26.73272	26.78417	26.18201	26.55009
741	-0.376086115	29.16441	28.44622	29.18154	29.15413	28.27835	29.27485
742	0.216961019	27.43617	26.69504	27.49166	27.32679	26.15312	27.36426
743	-0.754052035	31.82639	32.06227	32.52555	31.80092	32.03934	32.66061
744	-0.495429844	27.94011	26.64679	26.51485	27.64457	26.34103	26.53267
745	-0.856587633	27.67733	27.536	27.06929	27.9454	27.62303	27.01833
746	-0.365483435	23.01919	22.19423	22.08829	23.99045	22.52035	22.16397
747	0.437312156	30.07358	30.4095	31.24538	29.71272	30.26687	31.31616
748	-0.381445573	23.11714	23.19607	22.969	22.89205	22.55745	23.66635
749	-0.218578461	27.24334	27.16673	25.75712	27.07021	26.63263	26.02829
750	-0.109552825	26.93092	26.41001	26.07352	26.79806	26.46091	25.75787
751	-0.761903505	23.81491	21.89374	23.16981	24.50653	22.62311	22.08124
752	-0.316062332	25.14156	25.66108	24.23791	25.07154	24.37518	24.69808
753	-0.210079846	23.9986	22.57587	23.0748	23.64163	21.99812	22.7111
754	0.826468935	27.17092	26.19715	27.09233	27.18411	26.33926	27.0462
755	-0.127138206	25.07257	25.06668	24.88971	24.67266	24.80144	25.26195
756	-0.051355903	28.18315	28.77123	28.86797	28.2219	28.54773	28.477

	W	X	Y	Z	AA	AB	AC
757	0.261868013	28.02488	27.43161	27.40864	27.96204	27.59869	27.4764
758	-0.083564033	26.91115	25.99295	25.47236	26.79748	26.11777	26.34245
759	-0.267960575	25.74117	25.11781	24.86653	25.73421	24.68681	24.88492
760	-0.792216794	26.01364	24.62023	25.49163	26.6954	24.84356	25.30338
761	-0.403231475	23.11099	22.37976	23.82193	22.10902	21.99099	22.53742
762	0.589492274	20.3453	22.68516	20.80452	21.86046	19.41212	21.07672
763	0.10614618	23.76359	23.83442	24.30263	24.02337	24.00119	25.45721
764	-0.009689751	22.36145	23.33236	21.7277	23.05187	22.97779	23.02303
765	-1.565549693	25.93915	25.16123	25.05736	25.83889	24.71427	24.75923
766	0.039857966	27.29937	27.9901	28.36549	27.2742	27.68945	28.30765
767	-0.514043356	27.10549	26.88294	27.76864	27.21077	26.89886	27.52662
768	-1.304061738	24.58944	24.6917	24.34067	24.36589	24.43577	24.14026
769	-0.113321179	22.89941	21.70516	21.4823	22.3474	21.5029	21.56071
770	-0.567719489	26.47405	26.1508	25.88776	26.60974	25.15219	26.46138
771	-0.485823126	29.59381	27.73034	27.90424	29.41603	27.75018	28.3934
772	0.351507511	25.14069	25.15258	25.37485	25.41364	25.02887	25.19649
773	-0.088438002	25.45038	23.79262	23.17096	24.74865	23.78681	23.44135
774	-0.250007987	26.84335	25.43867	25.53595	26.69474	25.59063	25.40517
775	-0.080083073	30.65315	30.99591	31.54544	30.82046	31.04415	31.8643
776	-0.058677073	25.46582	24.6946	24.1612	25.3032	24.16029	24.43943
777	0.427951466	27.00346	27.03103	26.79844	27.39246	26.86391	26.81829
778	-0.003741349	25.50655	25.3358	26.17162	25.31455	25.77543	26.06234
779	0.331754248	30.17749	30.05131	30.601	30.37676	30.01532	30.83718
780	-0.46680436	27.48576	27.86275	27.503	27.48199	27.49032	27.49957
781	-0.283286114	32.24717	31.92915	32.29379	32.20306	32.14836	32.61666
782	0.305966592	26.96953	22.49512	25.73059	26.56665	25.02594	25.33471
783	-0.820535995	28.43474	27.60365	28.15961	28.18159	27.75496	28.2847
784	-0.069550184	25.1365	24.73284	24.30122	25.12723	25.10987	25.13762
785	-0.299040459	27.73763	26.49485	26.5113	27.69152	26.52156	26.52046
786	-0.843325331	26.75263	26.69129	26.47817	26.71557	26.3243	26.43097
787	-0.179745523	26.6306	25.54671	25.48729	26.28733	25.53321	25.59926
788	-0.470516348	27.00957	25.94814	26.53505	26.90725	25.71467	26.1607
789	-0.036874383	31.34856	30.28415	30.40865	31.13801	30.31803	30.51565
790	-0.280212588	25.39383	25.55522	25.00136	25.19301	25.62543	24.86173
791	-0.339053042	26.50211	25.34798	26.17485	25.84305	25.27624	25.91627
792	-0.485193989	29.44026	29.06885	29.68436	29.31601	29.27917	29.64753
793	-0.799483091	25.86801	25.35917	24.92543	25.7129	25.35345	24.45431
794	-1.003967964	28.5456	28.1483	27.80448	28.54897	28.1201	27.74461
795	-0.051124484	27.1497	26.6965	27.50355	26.89725	26.687	27.40296
796	-0.853852228	26.85289	25.19563	24.36034	26.73142	25.09934	24.77533
797	-1.532545931	22.82271	20.87766	21.69927	22.06508	21.70657	21.04438
798	-0.539850194	29.65269	29.14988	28.7635	29.53577	29.09256	28.81712
799	-0.173307948	24.53257	24.81654	25.21458	24.02465	24.78333	25.02525
800	0.415041657	23.27743	22.12309	23.45341	21.98377	21.83509	23.26455
801	0.179503849	25.75061	24.99135	24.5941	25.49401	25.1575	24.61611
802	-0.712735	25.00833	23.74054	23.97677	24.64446	24.00874	23.72299
803	0.651246306	27.74716	25.7466	27.40038	27.58023	26.30827	27.423
804	-0.087840559	27.04451	26.44458	26.1522	26.80081	26.41195	26.27584
805	-1.096885921	23.21799	23.44856	21.38311	23.55302	22.49555	23.00199
806	0.929502583	24.24841	24.20379	23.53873	23.77002	24.46112	24.28544
807	-0.358018261	25.27946	24.28402	24.44147	24.83172	24.15884	24.67354
808	-0.121210789	23.53016	22.23413	22.15074	22.61843	22.00531	23.66026
809	-0.173105685	24.36182	25.12143	25.88116	25.80525	25.92499	25.2784
810	-0.357127784	25.23856	24.43333	25.64945	25.43748	24.68034	24.30667

	W	X	Y	Z	AA	AB	AC
811	-0.060243482	25.24113	25.61664	26.11126	25.91113	25.23365	26.25713
812	0.131670076	30.07528	29.62349	29.54935	29.9285	29.60549	29.71824
813	0.092062102	23.29906	24.51669	23.69429	23.93767	24.31242	23.62523
814	0.380626395	31.49099	31.62258	31.79253	31.44108	31.47322	31.69319
815	0.14326045	23.52241	23.37157	22.97294	23.19879	22.60752	23.2882
816	-0.605637502	30.83221	30.71641	31.23723	30.71765	30.60095	31.29425
817	-0.492125558	27.82353	27.11333	26.85189	27.69732	26.77485	26.89073
818	-0.842969635	27.77258	27.27774	26.52063	27.55318	26.88384	26.57553
819	-1.261282551	29.31626	28.93651	29.59294	29.1811	28.8729	29.42377
820	-0.480383694	26.50507	25.94779	27.24024	26.4168	25.92168	27.27284
821	-0.246099382	26.28585	26.37541	26.41975	25.97024	25.36929	26.34902
822	0.149768754	30.27764	30.166	30.28525	30.29344	30.10117	30.36047
823	-0.07271552	28.44965	27.99037	27.59368	28.24369	27.95935	27.47296
824	0.586295259	25.16865	25.2132	25.14284	25.11344	24.22683	25.3861
825	0.17493641	26.76859	26.37009	25.66669	26.62201	25.93936	25.53197
826	0.037255301	24.38722	25.00855	24.4141	24.09543	23.82949	24.8797
827	-0.404613236	27.14155	25.01313	24.53123	27.09832	24.52788	24.25679
828	0.010496072	26.01295	25.08241	25.37815	26.07915	24.35946	24.86299
829	0.088294588	25.50739	24.75554	25.24404	25.71577	25.20369	25.2437
830	0.114203582	26.73133	26.09121	26.18928	26.54425	26.04656	26.35411
831	-0.064021314	26.64107	25.88645	25.68984	26.49591	25.82865	26.08824
832	0.030282851	26.18403	24.26759	24.66524	26.06482	24.67899	24.43225
833	0.880861887	20.22073	21.46594	18.58007	20.62705	21.04316	19.69403
834	-1.257828886	22.70928	26.22949	23.94643	21.89816	26.25197	21.93633
835	-0.549126463	22.03466	20.22257	22.02339	22.08958	19.78709	19.01625
836	-0.411727896	26.62245	26.71775	26.61971	26.31171	26.59457	26.54459
837	-0.191574397	24.8155	25.02037	23.40944	24.89101	24.2571	23.14021
838	0.52201076	24.79842	25.04117	25.85549	25.50433	25.01209	25.68795
839	0.157832584	22.47772	22.66032	22.46109	22.51147	20.60573	22.08889
840	-0.188551092	29.13261	27.9676	27.73954	29.30487	27.80554	28.14195
841	0.143776622	27.84796	27.2009	26.67262	27.94625	26.46501	27.16826
842	-0.513138321	28.12962	27.33187	27.08514	28.14506	27.03908	27.1994
843	-0.172751901	26.78766	25.44771	25.36559	26.74563	25.36607	25.31933
844	0.065494547	25.83626	24.93901	24.72427	25.70875	25.39663	25.23036
845	0.216377448	26.336	25.18362	25.0914	26.0113	25.14256	25.08632
846	0.564962935	21.64385	22.03226	22.36927	22.57945	25.47938	22.27057
847	-0.001459129	23.66615	24.57546	22.71409	22.9517	22.29124	23.4994
848	-0.260936108	26.35136	26.16952	26.57321	26.40132	25.93072	26.49581
849	0.311376401	27.64737	27.01151	27.53665	27.48055	26.903	27.38018
850	-0.626591944	25.71456	25.00591	24.89366	25.49535	25.08209	24.80253
851	-0.614329631	24.28542	23.11829	23.03736	24.69725	23.53462	23.00447
852	0.454265775	24.39773	24.89268	25.36671	25.04703	25.04002	24.64013
853	0.184963314	21.89605	21.51001	21.10524	23.88805	21.49667	24.00789
854	-0.018792727	22.71244	23.09834	22.45483	21.94125	22.51436	23.75681
855	1.230315313	22.66055	23.26176	22.69513	21.74145	22.93974	22.98594
856	-0.087944118	25.54048	25.25127	25.11674	25.53347	25.08688	25.27631
857	-0.393143716	26.35147	25.4252	25.12547	26.26072	25.6887	25.05511
858	-0.204286254	28.74581	28.21735	28.74724	28.50502	28.20179	28.75931
859	-0.33946996	24.70388	23.34802	23.54747	24.40879	23.14432	22.87989
860	-0.130332548	27.57744	27.15444	27.47804	27.67792	27.01532	27.20086
861	-0.286962467	24.53528	23.35676	24.26883	24.18027	23.7815	23.49821
862	-1.185080952	28.32759	28.01383	28.2761	27.89158	27.59266	28.26672
863	-0.389779023	25.48614	25.32124	25.46147	25.3066	25.06759	25.46492
864	-0.141242971	27.32616	26.77806	26.11236	26.96694	26.80367	26.15115

	W	X	Y	Z	AA	AB	AC
865	-0.69070711	24.14031	22.81537	23.58003	23.99463	22.90373	23.60696
866	0.08461765	25.22457	26.76131	23.2189	24.61749	26.83282	22.74514
867	-30.95762683	0	0	0	0	0	0
868	0.18256355	23.72205	23.16127	22.67946	23.49923	22.95824	23.27411
869	-0.091091379	23.76521	23.46623	23.51369	23.3435	23.44858	24.11143
870	-0.833605799	26.61641	26.25021	26.38673	26.21201	26.13618	26.52963
871	-0.816282239	26.7114	26.94988	26.80369	26.3969	26.79739	27.09019
872	0.001645685	26.01817	25.74664	25.81285	25.23566	25.61904	25.70675
873	0.013615928	25.14522	24.55137	25.66157	24.93456	24.3598	25.60381
874	1.145405965	26.0838	25.42327	25.49895	25.52271	24.69054	25.24326
875	-0.122049488	23.22021	21.99163	23.79885	23.091	21.74443	22.33712
876	-0.195689857	22.52142	19.57153	19.91541	22.72861	19.27952	20.27535
877	0.629133841	21.766	23.31836	23.2594	22.70138	21.90643	23.57657
878	-0.487422095	27.88685	26.72591	28.36681	27.62992	26.75445	28.52211
879	-0.079273605	31.91944	32.07254	32.52425	31.90193	31.89759	32.48902
880	-0.349784668	25.34674	23.66523	23.63872	24.74909	24.06962	23.78529
881	0.154985554	27.19297	26.28632	26.00648	27.11839	26.16734	26.13309
882	0.14534011	23.89525	22.96756	24.69318	23.7306	23.26842	24.29582
883	-0.263515603	26.43814	26.44776	26.28739	26.25918	26.14326	26.39837
884	-1.126568836	22.64486	21.98402	22.25807	22.8696	22.81251	22.13783
885	-1.5148732	0	0	0	0	0	0
886	-0.061411507	26.34563	26.63134	26.91781	26.21108	26.26818	26.85362
887	-0.023304568	23.90167	21.83553	22.83311	24.2646	23.15187	22.38687
888	-0.286971771	28.41399	28.0022	27.53214	28.74703	27.91882	27.43645
889	-0.210030752	34.01677	34.29911	34.36588	33.98697	34.26334	34.75214
890	-1.002222739	33.18134	32.32264	32.88204	32.72173	32.23857	32.92147
891	-0.02866083	26.19345	26.10267	25.86735	26.50554	26.5514	26.85126
892	-0.146446632	26.48248	25.43636	25.5734	26.46209	25.47547	25.28461
893	-1.084338943	26.66492	23.58197	20.94526	26.09072	23.15609	22.45258
894	-0.408582153	25.20387	24.59251	24.67182	25.19382	25.26959	24.86358
895	-0.225155378	24.87928	24.88907	23.85561	25.02172	24.38643	24.3545
896	-0.455576162	27.34885	27.08472	27.16142	27.36428	27.22407	27.28469
897	-0.18974843	25.22745	23.99321	24.09019	24.71227	23.57642	23.69284
898	-0.503550664	24.97322	23.58826	24.16491	24.92713	22.31543	23.15844
899	0.06203175	23.83702	23.56491	23.47601	23.91258	23.17182	23.40392
900	-0.283709046	25.05249	24.04212	24.69426	24.87259	24.32933	24.42401
901	-0.099469453	24.3544	24.01461	23.24967	23.22772	24.25136	24.1505
902	-0.710172968	27.90314	27.16154	27.03091	27.74421	26.96743	26.9652
903	0.32704618	25.78488	24.98287	24.82036	25.51176	27.30486	25.90097
904	-0.181462627	25.10988	25.98162	25.20478	26.08558	26.0362	25.40867
905	0.323923836	27.66927	27.52849	28.0193	27.65198	27.21814	27.96917
906	0.489512336	23.94479	22.34403	22.4993	23.59177	24.28358	24.6987
907	0.156775505	21.87277	23.26062	23.23796	23.41962	22.59959	23.31057
908	-0.087748868	27.85089	26.66788	26.50263	27.70253	26.85397	26.33148
909	0.355751913	28.24124	28.33474	28.56492	28.1036	28.21543	28.72985
910	0.63699106	27.05795	27.244	28.29608	26.88079	26.9172	28.19951
911	1.04762974	26.07833	25.20493	25.57285	26.36992	24.95936	25.54625
912	-0.999961128	22.83791	22.31275	23.58672	23.43883	23.17816	23.16172
913	-0.068495188	27.75768	27.16323	27.23047	27.76627	27.17185	27.48773
914	0.331188489	27.03101	26.5276	26.78477	27.0384	26.32077	26.69636
915	-0.913076708	25.50582	23.93313	24.96343	25.22171	24.71412	24.40779
916	-0.596652587	23.13022	21.55991	21.48255	21.99256	21.61596	20.9926
917	-0.497336448	25.69066	25.37117	26.0782	25.61883	25.91631	25.99475
918	-0.305729261	28.34524	28.26521	28.35784	28.35735	28.28099	28.28991

	W	X	Y	Z	AA	AB	AC
919	0.353233093	26.65604	26.3732	26.27216	26.59077	26.21311	25.95509
920	0.125462804	23.54377	23.20929	22.8188	22.18246	22.88447	22.52397
921	-0.367456463	29.01417	26.45181	25.95666	28.49298	26.36748	26.27283
922	-0.227131244	28.05113	27.26625	27.24818	28.09574	27.74445	27.28055
923	-0.116028725	29.01737	28.13608	27.80717	28.77123	28.06464	27.7168
924	-0.231588832	26.40923	26.42363	25.95698	26.32952	26.17462	26.18924
925	-0.332247495	28.85407	28.02746	27.57356	28.64397	27.72142	27.74956
926	0.080883857	31.77651	32.01133	31.64489	31.73741	31.96079	31.90244
927	-0.18861853	25.76045	26.81824	25.21888	25.65891	26.73509	25.22197
928	-0.025733135	24.9186	24.16224	22.74281	22.49333	23.52809	23.38281
929	0.074807878	30.20595	29.2789	29.63642	30.15109	29.24693	29.66098
930	-0.077599629	30.36973	30.84074	31.16124	30.41605	30.77937	31.0518
931	0.317627239	23.98093	23.01283	23.18853	23.47375	23.22589	23.63568
932	-0.782597906	27.27201	26.81436	26.98651	27.15978	26.89939	27.01624
933	-0.670817608	25.21725	23.88306	25.33248	24.31671	24.25648	25.21163
934	-0.480012727	24.4233	22.82858	22.79697	23.76096	22.09665	22.72532
935	0.099384636	27.10826	27.21009	27.3049	27.10418	26.97604	27.23021
936	-0.186926144	28.86889	28.05791	27.54483	28.75223	28.09653	27.593
937	-0.177890937	27.39214	26.19439	27.00628	27.32413	25.84597	27.15158
938	-0.444310307	25.38067	25.44074	25.88588	25.53704	25.33992	25.889
939	-0.701122494	26.8627	26.49905	26.20215	27.16405	26.80854	25.70412
940	-0.760198117	28.38367	28.21896	28.24946	28.07591	28.0518	28.19985
941	0.473131441	27.46968	27.35804	27.9524	27.34506	27.10612	28.27593
942	-0.206808362	25.65117	24.93082	24.48553	25.47148	25.16278	24.8446
943	-0.25383166	26.14581	25.99584	25.79043	26.17299	25.81572	25.83268
944	-0.160835658	23.98436	22.03183	23.02645	23.94977	22.43213	22.94398
945	-0.311049679	25.71406	25.7409	25.64688	25.6335	25.91395	25.99278
946	-0.656411628	29.80978	29.03196	29.37806	29.6556	28.95165	29.61074
947	-0.249847447	28.85836	28.2256	27.73199	28.75136	27.76835	27.53491
948	-0.210817862	28.93208	27.85266	27.81848	28.70833	27.77179	27.75786
949	0.169276728	25.24281	24.21548	23.92613	24.63765	22.86704	24.07024
950	-0.144302945	24.57174	23.51105	24.11016	24.73853	24.74521	24.26899
951	0.185964706	25.96029	26.07868	25.71109	25.78331	25.55303	25.76262
952	-0.335491325	25.98912	25.84795	25.70597	25.92068	25.82775	25.94006
953	1.07471368	0	0	0	0	0	0
954	0.051765153	21.42794	25.2139	22.83967	23.33246	25.25381	22.87984
955	-0.228012976	28.83693	27.49727	27.91916	28.66867	27.63086	27.88716
956	-0.197874605	23.06997	22.00124	21.52471	22.42093	21.80096	21.30042
957	0.940506096	24.25099	24.48283	23.46109	24.14514	24.34887	22.40235
958	-0.388851429	25.78828	24.36431	23.61997	25.95133	25.05368	24.63256
959	-1.049085345	20.66127	20.98623	22.25724	22.38088	21.36242	22.1698
960	-0.370788492	27.2143	25.78986	25.43326	26.90703	25.8678	25.5218
961	-0.111427053	26.58344	25.1372	25.36411	26.58522	24.99188	25.44737
962	0.471124576	25.59458	25.04809	25.35353	25.81768	25.53353	25.4065
963	-0.084782504	24.21241	22.22256	22.82536	24.07895	22.53177	22.81946
964	0.102810609	23.78923	23.91376	24.52405	24.55369	24.14169	25.22224
965	-0.350468279	26.33743	25.36464	25.22186	26.309	24.53307	25.25387
966	-0.22918166	21.39338	21.59506	22.64674	21.54645	21.1013	21.89399
967	0.355892287	24.29851	22.82813	24.61856	24.13655	23.89892	24.45442
968	-0.019008361	25.36338	23.06151	24.34963	24.34892	21.68608	24.58818
969	-0.25285351	22.55946	21.33321	22.80071	21.44724	21.19188	21.9881
970	-0.194572404	24.64804	22.62909	22.37468	24.28212	23.903	21.87307
971	0.046055269	26.10651	26.62906	26.70824	26.16701	26.41713	26.51729
972	-0.061500446	28.32508	27.07705	26.89358	27.76931	26.74683	26.46824

	W	X	Y	Z	AA	AB	AC
973	-0.129175593	27.31733	27.17622	27.87216	27.14187	27.08506	28.00162
974	-0.253956242	25.26632	24.75735	24.33322	25.20427	24.47058	24.32782
975	0.114050316	28.18922	27.18412	27.62075	27.84847	27.1378	27.25741
976	-0.073504571	23.72287	23.22997	23.39894	23.6982	22.60491	23.69518
977	0.171783044	28.63795	28.47637	28.6016	28.53975	28.34459	28.57111
978	-0.313179903	24.38753	23.34454	23.17947	24.2419	22.41091	23.2291
979	0.041438426	25.37356	24.97115	24.4323	25.45935	24.81064	24.60795
980	0.266311479	33.68353	32.46588	32.78887	33.54377	32.454	32.87539
981	-0.209799502	26.4389	24.52818	24.45852	25.78043	23.87738	23.61544
982	0.396735733	21.90495	20.46297	19.76974	20.73123	19.93566	20.63123
983	-1.395504942	25.53844	25.66251	25.56473	25.49072	25.6376	25.72199
984	-2.270990964	25.01754	24.79572	24.85029	24.90663	24.82024	24.78556
985	-1.001294191	26.49056	25.2824	25.56976	26.81861	25.81166	25.33148
986	-0.622741573	25.87341	24.8747	25.09964	25.56308	25.19484	24.49628
987	-1.345600979	27.8932	27.41123	27.69453	27.6853	27.82056	27.79567
988	-1.431288107	25.94616	22.41751	21.64157	21.9809	21.61251	21.61612
989	-0.293980557	28.96155	28.19173	27.68181	28.65973	27.96682	28.12096
990	0.433708265	26.84992	26.64478	26.17076	26.77217	26.12856	26.06774
991	1.876119331	20.78274	21.9112	20.82544	20.40085	20.30928	19.62101
992	0.281998835	28.27139	27.68758	27.41046	28.08825	27.41359	27.47772
993	0.210628212	24.41634	24.20804	23.65086	24.10899	22.9402	23.71356
994	-0.397170806	24.88921	22.87844	21.61942	24.73192	23.53961	22.14076
995	-0.625580105	27.2481	25.88104	26.03625	26.9527	25.55632	25.86555
996	-0.1729193	26.10332	25.45687	25.71959	25.88125	25.38301	25.43892
997	-1.256350489	25.4788	24.34737	22.59962	25.42699	24.30007	24.10029
998	1.255233993	24.59486	21.9631	22.9369	22.34324	22.83204	22.03069
999	-0.472204912	30.99546	31.02963	31.15275	30.90555	30.93769	31.21512
1000	-0.27588393	24.23145	23.02408	24.24144	23.49609	23.2671	23.77533
1001	-0.016728863	28.32668	28.06639	27.90047	28.28901	28.1283	28.16988
1002	-0.505960729	24.62887	24.11036	23.11685	24.74711	24.67683	23.44354
1003	0.019783973	23.92612	20.57238	21.34323	23.86582	21.83761	21.21808
1004	0.326459031	24.86945	22.00154	23.94909	24.77838	22.13223	22.37407
1005	-0.633546558	23.58845	22.47338	25.767	23.35365	22.43737	23.86658
1006	-1.376882416	26.52785	26.0424	26.28538	26.27442	26.15411	26.29133
1007	0.166667946	22.07928	22.35582	21.44931	21.81859	21.89014	22.20446
1008	-1.037399696	21.27341	23.39782	22.82653	24.2062	23.5235	23.07923
1009	-0.15300609	27.65788	26.74343	26.82263	27.05365	26.87516	26.9777
1010	-0.015199076	29.26424	29.07891	29.1158	29.1578	28.88916	28.94857
1011	-0.349885289	26.6578	25.93593	25.43999	26.77883	26.02753	25.52966
1012	-0.280477701	26.59629	26.33846	25.62001	26.49734	25.91261	25.32323
1013	-0.496016494	25.74658	25.15422	24.30056	26.06086	24.92725	25.24149
1014	-0.347813501	24.94539	22.83198	22.64145	24.6089	22.89618	23.15474
1015	-0.188034977	27.59285	26.98688	26.7838	27.44831	26.74128	26.96963
1016	-0.101928893	21.07349	20.76104	23.12459	19.82521	20.16535	22.94255
1017	0.16996003	27.65483	26.77434	27.5129	27.52901	26.54503	27.49137
1018	0.465558511	29.03262	28.45732	28.79527	28.88238	28.49059	28.78067
1019	-0.644613201	25.36559	24.53563	24.85807	25.56739	24.56336	24.83394
1020	-0.634408024	28.10896	28.01546	27.40565	28.11593	27.94969	27.2662
1021	0.114800174	29.69164	29.3729	29.84091	29.44676	29.54173	29.89957
1022	-0.183173971	25.47882	24.90499	23.99519	25.3355	24.45929	24.17168
1023	-0.662780988	27.72297	26.79404	26.73697	27.47481	26.7729	26.7637
1024	0.188074728	24.44382	22.97721	23.98476	24.08027	22.59977	24.28293
1025	-1.159293625	26.96349	25.45176	25.84015	26.85282	25.50248	25.48104
1026	0.050918241	19.90229	19.05713	20.29378	21.06786	19.50128	20.25006

	W	X	Y	Z	AA	AB	AC
1027	0.180155944	27.1525	27.37413	27.16788	27.00629	27.37769	27.31522
1028	-0.093852563	24.96053	24.53234	24.77386	24.12712	24.59439	24.97743
1029	-0.108512196	27.79245	27.73634	27.76834	27.73902	27.76502	27.81591
1030	-0.169579411	22.55079	23.02481	21.59578	22.82959	22.26145	22.04737
1031	0.044931172	27.06652	26.28334	26.59675	26.89413	26.19116	26.17947
1032	-0.481893511	23.02985	22.99242	23.49885	23.69234	23.36282	24.04576
1033	-0.851539678	23.68631	23.49132	23.54856	23.6164	23.91331	23.22857
1034	-1.000121531	24.53366	25.28032	25.40648	24.91276	24.95052	25.30572
1035	-0.769381625	20.49152	20.11032	20.47352	20.16668	20.51392	19.97644
1036	0.021666826	27.01486	26.26446	26.06505	26.75847	26.1324	26.1488
1037	0.237956964	24.40317	23.64066	24.58189	24.25218	23.18956	24.56261
1038	-0.953946546	28.59077	27.78922	27.71936	28.47119	27.64815	28.09574
1039	-0.437830212	25.84834	25.77016	25.96661	25.77856	25.6847	25.76759
1040	-0.241784177	24.44778	24.49207	24.96957	24.47638	24.58121	25.02302
1041	-0.207381232	25.11417	24.62116	23.75128	24.80872	24.39055	24.67782
1042	0.083442607	21.03106	22.96312	21.98413	22.19335	21.21729	22.64466
1043	0.127822103	30.73549	30.50711	30.88538	30.67564	30.45642	30.99896
1044	0.048165206	29.38774	27.16038	28.57664	28.49795	27.31928	28.54128
1045	0.080433929	26.55517	26.72341	26.70651	26.43719	26.61797	26.83437
1046	0.034149382	25.76559	25.28015	25.68845	25.6452	23.90122	25.76933
1047	-0.351261464	24.14394	24.08489	24.32056	24.32808	23.75909	23.78278
1048	-0.012531013	27.00784	27.60542	26.98406	26.88544	27.38849	26.92339
1049	-0.392706199	30.13695	29.34294	28.83413	30.02419	29.11532	28.86108
1050	-0.552654381	24.89805	25.37851	24.16776	24.90556	24.88127	24.6101
1051	0.122971603	26.37111	25.85207	26.10233	26.30449	25.79831	26.44789
1052	0.692369913	22.59906	21.97164	22.04244	21.94719	22.07735	21.42461
1053	-0.14980597	25.99933	26.01946	26.29481	25.69942	25.76695	26.29745
1054	-0.697281699	25.0628	23.59923	25.82727	24.77114	23.50781	24.86811
1055	-1.391933972	27.83171	27.06879	27.3743	27.79987	25.83967	27.3979
1056	-0.810098709	25.39554	25.24274	24.49696	25.40788	23.82698	23.47014
1057	-0.406636327	27.28504	26.87725	26.98681	27.09008	26.62285	26.98124
1058	0.14224129	27.14947	26.70516	26.58508	27.10891	26.62475	26.65808
1059	-1.291701664	24.61645	26.55365	26.71189	25.80381	26.20623	26.9529
1060	-0.738163893	31.25756	30.47495	30.78534	30.97496	30.41802	30.32621
1061	0.159200647	24.85103	23.8774	24.39295	24.72277	24.66322	24.55791
1062	0.481616942	22.10071	21.27819	20.2034	22.32275	22.13455	21.53356
1063	-0.042347526	26.33496	25.74011	25.63565	26.18094	25.82424	25.17418
1064	0.370609279	23.67993	21.88541	21.36611	23.26319	22.66199	22.09632
1065	-0.21646286	26.20335	24.822	25.30004	25.91722	25.10703	25.46036
1066	0.042453977	22.22865	22.40134	23.85827	21.42784	22.48112	24.13461
1067	-0.927737814	27.01786	26.94134	26.81518	26.97154	26.91517	26.90057
1068	0.504778335	23.63343	23.65273	21.84486	24.01314	23.68984	23.59886
1069	0.766136304	27.41478	26.37733	27.03926	27.17741	26.24096	27.02201
1070	0.158257138	25.02858	24.34352	24.69799	24.80308	24.04623	24.36682
1071	0.439587022	29.98271	29.2557	29.61019	29.85154	29.265	29.69336
1072	-0.376354665	24.65874	24.28389	24.03639	24.29378	24.00636	23.64271
1073	-0.017835358	25.5545	25.73005	25.64587	25.53645	25.38972	25.77268
1074	0.161920143	32.30992	32.20258	32.24226	32.26183	32.0618	32.29789
1075	0.806865431	30.06878	29.07423	29.63363	29.76181	28.99001	29.76753
1076	-0.450886292	30.67492	31.91022	31.95528	30.49839	31.75773	31.87254
1077	-0.547666274	24.19125	23.29293	23.09362	24.15192	23.23301	23.08701
1078	-0.695809918	24.68601	24.03136	24.14247	24.25494	23.91232	23.13184
1079	0.088546248	22.43459	21.35883	24.66472	21.75513	21.0245	20.54468
1080	-0.976781358	25.83518	24.56621	23.9257	25.03381	24.31348	22.46367

	W	X	Y	Z	AA	AB	AC
1081	-0.512077099	26.30945	25.53348	23.92154	26.38985	25.33697	25.3451
1082	-0.410628669	23.42012	23.40228	23.66311	23.64413	23.7833	24.22931
1083	-0.588968409	24.63858	24.4204	21.87968	25.00312	23.54	24.29122
1084	-0.54222515	23.13708	22.12188	20.35679	20.49146	20.05431	20.37709
1085	-0.173078015	29.02435	28.06697	27.83678	28.70846	27.63928	27.66105
1086	-0.038233413	26.60743	26.39287	25.9525	26.53448	25.62571	25.66709
1087	-0.397217619	27.65368	26.01281	25.76084	27.23454	25.63633	25.91493
1088	0.171951258	25.66851	25.20074	25.2963	25.36535	25.08833	25.21979
1089	-0.69909865	24.66998	24.9321	23.07034	24.82357	23.94224	24.491
1090	-2.019445105	21.73116	21.47204	20.09116	21.3506	20.01228	19.80548
1091	-0.219474704	28.37162	27.98302	28.12453	28.2912	27.91489	28.06428

	AD	AE	AF	AG	AH	AI	AJ
1	TOP3 ORF2_i03_1	TOP3 ORF2_i03_2	TOP3 ORF2_i03_3	TOP3 ORF2_PEP_i01_1	TOP3 ORF2_PEP_i01_2	TOP3 ORF2_PEP_i01_3	TOP3 ORF2_PEP_i02_1
2	25.26221	24.74997	23.66921	25.23281	24.10699	23.97227	25.12652
3	27.13615	27.46831	26.1431	27.56573	26.35047	26.53826	27.51538
4	24.04724	24.17153	23.65148	24.41776	23.02954	24.05616	24.59439
5	22.27303	22.01184	22.45305	24.24894	23.01039	23.01916	24.18758
6	26.15094	24.87598	26.1069	26.56374	25.7934	24.93699	26.51833
7	26.37122	26.21651	26.38083	26.70192	26.29787	26.39269	27.02338
8	24.17198	24.82985	26.10758	24.06059	25.58596	22.94511	24.37522
9	20.54779	19.90251	19.55491	22.01793	21.51597	21.09286	22.62287
10	27.5039	27.00936	27.50871	27.7044	27.10675	27.8357	27.73982
11	28.85388	27.49182	27.38286	29.05825	27.83287	27.52345	28.90585
12	27.15219	26.2613	26.20613	27.67616	26.70544	26.44892	27.72171
13	22.42517	20.05062	22.74976	21.02612	21.21051	20.58981	23.13255
14	25.7529	26.16528	26.6188	26.18864	26.30742	26.82978	26.101
15	25.22393	26.47889	26.645	26.14686	25.43237	25.09475	25.91408
16	25.82603	25.53379	25.534	26.71005	25.76436	25.5282	26.6837
17	24.47259	22.81941	22.65138	24.01901	22.7362	24.26312	23.34791
18	25.68525	26.10243	26.40463	26.08916	25.26196	26.53599	26.00363
19	29.65408	29.77805	30.10975	29.58956	29.84901	30.16935	29.60733
20	26.77104	26.26405	26.31547	27.28381	26.27888	26.47713	27.34796
21	24.95132	23.03302	24.64576	25.91495	25.28303	25.64931	26.04772
22	26.11864	26.17037	26.7329	26.1902	26.52141	26.77402	26.21124
23	24.54377	23.65598	23.82689	24.66577	24.59756	22.99672	24.91867
24	24.11981	23.48285	23.25341	24.20941	23.68787	23.16029	24.40118
25	24.91586	25.27853	23.704	25.10448	25.50296	22.70887	25.33871
26	22.54043	22.17263	23.66722	22.76382	22.16266	22.1923	22.45631
27	23.48532	22.57255	22.4289	23.81028	23.02678	22.41992	23.17834
28	23.75434	23.21399	23.37587	24.46148	23.55109	23.00713	25.024
29	25.39041	24.7726	25.16981	25.58933	24.75427	24.15298	25.56659
30	25.8491	24.8846	25.79343	25.43899	24.8817	25.52113	25.61919
31	24.71992	25.01238	24.64359	24.64627	24.54283	24.17583	24.97873
32	25.31066	25.2563	25.75068	25.16647	24.67821	25.21057	23.89174
33	26.30499	25.48858	25.37199	26.78548	25.61875	25.4006	26.62926
34	27.51656	26.71617	27.0905	28.13133	26.97919	26.73883	27.90706
35	26.53225	26.40993	26.28489	26.35195	26.50229	26.05559	26.11936
36	26.25829	25.99145	25.84478	26.71327	26.01153	25.4751	26.60261
37	26.264	26.54951	27.27935	26.76378	26.17371	27.17853	26.74248
38	22.98717	23.73385	24.51304	24.16687	22.10473	24.04762	24.24749
39	23.66512	22.17492	22.26045	24.17049	22.58652	22.31364	24.2816
40	28.92302	27.86646	27.78011	28.57658	28.22245	28.39387	28.59304
41	30.38145	30.51646	31.21996	30.9451	31.07102	31.2508	30.95459
42	27.60375	26.94973	27.04909	28.07536	27.05605	27.23646	28.12597
43	25.08497	24.90812	22.96422	25.24207	23.77876	26.46059	25.1523
44	22.16871	23.64765	23.49737	21.6766	24.16058	20.94177	21.57043
45	27.09334	25.89811	25.42366	27.25131	26.02438	25.56297	26.60597
46	25.33645	24.55514	24.42036	25.45552	24.30446	24.36645	25.20875
47	23.80298	21.86896	21.68604	23.54819	23.20992	23.11656	23.62819
48	32.0084	31.60845	31.68738	31.50481	31.7432	31.81277	31.48394
49	24.18937	24.34003	24.806	23.52155	24.7155	25.43326	23.38931
50	34.92287	35.07791	34.45013	34.46352	34.83065	34.3199	34.45987
51	28.07118	27.67797	27.81679	28.66529	28.00799	27.93386	28.69905
52	25.7851	25.39988	25.07999	26.09194	25.62348	25.36357	26.31302
53	27.04944	26.39855	27.3237	27.17608	27.77566	27.54238	27.2963
54	30.49392	30.24	31.16566	30.3783	30.32165	30.81163	30.42296

	AD	AE	AF	AG	AH	AI	AJ
55	22.74996	22.48588	22.66489	23.25711	22.5981	23.15328	23.47285
56	22.73403	22.30937	21.88771	23.64271	22.77968	24.30869	24.06023
57	21.673	21.39907	21.43428	22.52142	23.07843	21.29595	21.49542
58	27.84565	28.00471	28.12936	27.80147	23.89647	28.37496	27.73527
59	28.79228	28.62018	28.49303	29.03989	28.80499	28.48298	29.06996
60	27.3749	26.53296	26.48633	27.58586	26.935	26.6719	27.64304
61	24.89093	23.4649	23.44314	25.92788	22.66441	24.84354	25.87695
62	25.16255	24.40042	24.74298	25.49235	24.6356	25.16064	25.27392
63	27.36614	26.13022	26.51399	27.41927	26.9442	26.22997	27.33302
64	25.71068	25.55001	26.27218	25.87078	25.36291	26.77676	25.83117
65	26.21064	25.46855	25.36776	23.91255	25.72314	25.49653	23.83183
66	25.34172	24.75655	24.4266	25.4427	24.46619	24.49341	25.52435
67	20.56837	19.73164	21.51605	24.70163	23.08628	23.07781	24.77806
68	20.81431	21.07155	19.76049	22.2848	21.56338	22.12217	22.36866
69	21.76803	22.04903	23.64944	22.88501	23.04747	22.0407	22.77606
70	23.67714	21.17761	20.94808	22.97598	23.08409	20.90925	22.93927
71	25.4407	24.50624	24.52765	25.57606	24.76136	24.95771	25.63051
72	25.94422	24.93633	24.67882	26.55048	24.98725	24.58182	26.24603
73	24.36045	23.16628	23.8162	24.38788	23.79402	21.8747	24.22463
74	24.71039	23.199	24.91193	25.02166	23.55682	25.48537	24.7014
75	27.548	27.25271	27.89949	28.1895	27.21599	28.37503	28.31204
76	24.49651	23.66186	24.25893	24.55228	24.22421	25.11298	25.04296
77	26.66337	26.15994	25.41416	27.14013	26.20232	25.89065	26.95647
78	29.7316	30.0367	30.59381	29.74072	29.99926	30.76893	30.00819
79	27.23483	26.67011	27.53761	27.62418	27.1994	27.43206	28.08446
80	24.94782	24.33375	27.11411	25.7727	24.77747	26.64193	25.60281
81	25.04499	24.35351	24.02611	25.64255	24.21121	22.82742	25.57648
82	25.51436	24.31161	24.92112	25.91822	25.11052	25.57357	25.94333
83	25.168	22.96106	23.50401	26.20267	22.76083	28.81232	26.11909
84	25.00912	22.93333	24.03268	24.9811	24.80263	23.79781	25.37572
85	26.58536	25.35136	26.2332	27.1137	26.06298	25.88962	27.17686
86	23.01105	22.02935	23.81938	22.10497	23.41967	22.70839	22.32021
87	29.57905	28.26778	29.59342	30.03743	29.06947	30.0822	30.08792
88	29.01084	28.21245	28.06581	30.4976	28.10417	28.39944	30.6087
89	29.69637	29.01163	29.6785	30.41193	29.38306	29.62675	30.44092
90	30.33244	30.05019	30.90355	31.00531	31.01584	31.41666	30.99832
91	30.44806	29.0948	29.46061	31.57037	30.11239	29.74769	31.54196
92	29.41737	28.74091	28.65305	30.30548	29.24569	28.95563	30.24026
93	28.02943	26.19867	26.88799	28.88472	26.82767	27.88168	28.91093
94	29.82078	28.19224	28.89725	30.72355	29.00146	29.31188	30.76974
95	28.19625	27.52099	27.45735	29.70666	27.69866	27.82368	29.83772
96	31.82475	31.88338	32.30315	32.00935	32.3538	32.42617	31.99288
97	28.13	27.91477	28.86472	29.61563	28.78931	28.90296	29.27773
98	28.61463	28.13935	29.19587	29.59907	28.84255	29.35616	29.60854
99	30.342	29.41336	30.09641	31.03022	29.87377	30.16594	31.00634
100	30.98588	30.42916	30.7921	31.2403	30.60826	30.78354	31.28713
101	28.9757	27.3595	27.23848	29.35639	28.17879	27.46137	29.2324
102	29.92313	28.70599	29.98063	30.58427	29.6902	30.11683	30.57452
103	30.68749	29.61761	30.38067	31.19333	30.08232	30.21086	31.16323
104	31.02501	30.01254	31.21597	31.90951	30.35218	30.88296	31.64825
105	30.48849	29.66967	30.55782	31.11264	30.30783	30.73045	31.15155
106	28.70217	26.76314	28.01349	29.79283	28.5542	29.2516	29.74668
107	30.40843	29.91731	30.69399	30.85727	30.2172	30.36396	30.8658
108	25.20801	24.34543	24.59154	25.97167	24.86213	23.6824	25.81449

	AD	AE	AF	AG	AH	AI	AJ
109	29.86156	29.89146	30.54052	30.25242	30.15187	30.66574	30.27972
110	27.82179	27.18075	28.33545	28.15673	27.86183	28.32318	28.20699
111	30.25696	29.60312	29.14552	30.78927	29.88972	29.05227	30.71185
112	28.4242	26.8764	27.18935	29.55417	27.92838	27.83243	29.64307
113	28.0179	26.84524	27.81865	29.11051	27.8138	28.44086	29.14031
114	28.62145	28.37858	28.96667	29.7536	28.88385	28.94333	29.64894
115	30.03523	28.84238	30.02525	30.33336	29.59359	29.90673	30.35286
116	25.69752	25.40805	26.2632	26.06942	25.17539	26.52765	25.98763
117	27.25909	26.91846	27.1031	27.83017	27.30789	27.43205	27.81357
118	26.04613	25.4671	25.65613	26.38512	25.375	25.71514	26.38165
119	23.38781	21.70872	23.431	22.36352	21.97308	23.10113	23.07157
120	26.87059	28.20283	26.08142	29.66262	26.49219	26.15043	27.72165
121	25.40748	23.98956	24.2297	25.83682	24.48496	24.43332	25.76395
122	22.89914	23.40137	22.96651	24.62112	22.19508	23.04956	24.36918
123	22.49287	24.65652	23.16306	24.43771	24.52755	24.63123	25.24707
124	26.17095	25.95323	25.00823	26.54887	26.33547	25.00568	25.98462
125	27.49694	26.86124	26.77	28.28116	27.13142	27.23289	28.13039
126	23.82043	21.5766	22.46042	23.97062	23.60457	22.25501	23.89198
127	27.20891	26.49769	26.55293	28.34799	27.13747	27.01421	28.15493
128	25.71889	25.02769	25.47906	25.83319	25.45926	25.66504	25.69869
129	29.14273	27.51921	28.16036	29.90318	28.35734	28.09967	29.95744
130	27.91744	27.8864	27.4846	29.31549	26.69667	28.61817	29.14462
131	31.35234	30.30633	30.24494	31.64963	30.86304	30.3812	31.73683
132	25.81427	25.676	25.61843	26.1325	25.38541	25.15818	25.97053
133	24.04381	25.08026	25.76661	24.42958	24.86174	26.03087	24.74105
134	29.18391	28.65644	27.89149	29.36419	28.8306	28.17894	29.37832
135	23.93986	21.53272	22.0959	24.67967	22.83894	22.70901	24.92766
136	27.77861	27.00045	26.95126	28.44754	26.97946	27.32191	28.51207
137	28.57291	27.6096	28.14156	29.15949	27.9777	28.2945	29.14505
138	25.04334	23.75494	24.32412	25.55941	24.36168	24.61671	25.23198
139	29.67466	28.92181	29.22038	30.33881	29.22851	29.32397	30.29043
140	27.55559	27.36254	27.61978	27.78966	27.03154	27.6751	27.83688
141	27.11063	26.43129	26.8549	27.1941	26.42001	26.6302	27.09236
142	25.0844	23.27494	23.56933	25.37738	22.99908	24.07664	25.10762
143	26.70583	26.60194	27.40963	27.04269	26.88186	27.32624	27.15158
144	25.00816	24.71884	24.72096	24.94132	24.63451	23.54494	25.1544
145	27.97682	27.86278	27.59618	28.60743	27.60325	27.33448	28.53769
146	24.57011	24.11176	21.79456	24.27512	22.48093	22.05421	24.29293
147	28.4905	26.62555	27.42863	29.76199	27.67539	28.18085	29.81496
148	28.52557	27.72835	28.46852	30.06373	28.34425	28.63939	30.17248
149	23.85204	23.68214	23.38206	24.71812	24.1636	23.42783	24.55022
150	25.71595	24.88446	24.58559	26.36076	25.12554	24.82584	26.32574
151	24.58216	24.58982	24.65738	23.99445	24.17851	24.69363	23.83335
152	24.39268	22.92845	23.75062	25.78702	24.94217	24.0956	25.45743
153	23.38401	22.34436	22.60757	23.35734	22.60086	22.21984	22.77775
154	25.21035	23.89679	24.15129	25.46503	24.56481	23.86987	25.20925
155	19.14239	19.1048	19.75973	23.04501	22.29597	21.87847	22.47831
156	25.99734	25.38698	26.24093	26.50081	25.61652	26.26306	26.47603
157	24.15372	24.23318	25.86834	25.9205	26.05209	26.67101	26.15138
158	25.50509	23.78863	24.30118	26.30853	24.83824	24.83328	26.3275
159	26.31687	25.87331	27.27461	26.96502	26.3998	26.02054	26.86636
160	27.2419	26.94057	27.27306	27.35905	27.11216	27.10285	27.51297
161	28.19248	28.46341	28.21792	27.24991	28.14188	28.16999	27.27489
162	23.33231	23.93846	22.60793	24.59166	22.5745	24.55668	23.79905

	AD	AE	AF	AG	AH	AI	AJ
163	26.50014	26.7106	27.39271	26.30499	26.77363	27.20767	26.72096
164	31.37135	31.55169	31.7693	31.5748	31.17049	31.44424	31.59319
165	26.69462	26.27334	26.01278	26.78862	26.72058	26.22233	26.69741
166	28.66439	28.72205	29.79425	28.36589	28.79986	29.83245	28.32145
167	25.54749	23.7835	23.79534	25.43589	24.68772	23.87184	25.58377
168	26.92481	26.39091	26.31156	27.12186	26.70177	26.40078	27.17119
169	29.85697	29.75047	28.73127	30.53903	29.93968	28.82659	30.53508
170	19.74026	20.90445	21.18083	23.2098	21.50525	20.9522	23.13341
171	26.95421	27.41955	27.79917	26.80402	27.53507	27.60801	26.8878
172	27.23186	28.39468	29.07049	27.27704	27.93017	28.91094	27.50731
173	23.11221	21.35789	22.62846	24.03382	22.80222	23.16046	24.09506
174	25.19596	23.7394	23.8241	25.08336	24.61992	23.59407	25.72408
175	23.49738	21.91314	23.48545	22.63663	23.29425	22.88961	22.59355
176	24.82835	24.56394	25.66537	25.19618	25.13515	25.96764	25.18642
177	30.87124	30.60355	31.51719	31.39587	31.20616	31.72124	31.30851
178	25.1555	24.33163	23.82962	25.41457	23.33783	24.08907	25.46477
179	24.85049	23.69862	23.63227	26.38809	23.597	23.5833	26.07526
180	26.83462	26.595	27.22224	27.44859	27.02424	27.40552	27.47215
181	22.63915	21.30791	21.50906	23.97506	22.35745	22.59311	21.65876
182	26.4201	25.92797	25.55452	26.83248	26.0018	25.74637	26.55577
183	22.48029	21.8341	21.53436	22.83369	22.46347	21.78155	22.53012
184	27.83025	27.06352	27.43542	28.16665	27.20128	27.37505	28.14805
185	27.11474	26.7446	26.18457	27.89927	26.7523	26.25832	27.76423
186	36.04858	36.28109	35.10647	35.28372	35.95292	34.90063	35.26426
187	29.14853	28.84045	28.70412	28.78662	28.7292	28.48148	28.7554
188	25.35724	24.47618	24.32768	25.39884	24.47238	24.55109	25.27968
189	25.7971	24.50223	25.26385	26.34717	25.10121	25.51257	26.19188
190	25.62171	24.60111	24.54257	26.49839	24.42478	24.45209	26.21075
191	32.22488	32.07158	32.23727	32.1206	31.85919	32.25253	32.15508
192	27.32094	26.84536	27.74338	28.15305	27.37041	27.98685	27.88552
193	26.43523	25.88223	26.3477	26.6463	25.95475	26.10207	26.78947
194	27.66864	26.81154	27.83148	28.4068	26.86626	28.04606	28.37051
195	29.51456	28.40478	28.93099	30.11345	29.07612	28.86281	30.10601
196	26.88337	27.35973	28.2914	27.17239	27.17867	28.81521	26.29042
197	26.37647	25.41779	25.2261	27.13443	25.65581	25.77519	27.16808
198	24.13341	21.87154	23.2512	24.90944	23.43964	23.94118	25.06512
199	24.9006	23.08652	24.24163	26.04743	23.98813	24.35674	25.786
200	24.39303	24.61019	25.10942	26.26847	24.88464	25.7004	26.39111
201	27.59593	27.52426	27.98092	28.12993	27.57814	28.58444	28.04832
202	30.6477	30.76564	31.25739	31.12273	30.73664	31.32078	31.12214
203	25.29039	24.63443	24.20689	25.69139	24.95164	24.97997	25.78022
204	27.9511	27.32433	27.45018	28.24195	27.67592	27.33834	28.40553
205	27.63852	27.11253	27.07166	28.02283	27.17472	27.1694	28.1615
206	27.86354	27.56618	28.4359	27.85445	28.00154	28.77606	27.82334
207	24.46143	24.5962	23.32815	25.18409	24.84175	24.64113	24.93835
208	28.12461	26.51151	26.806	29.54037	27.86528	27.26445	29.16341
209	27.01056	27.29439	28.71097	28.0199	27.89278	28.8024	28.31408
210	27.5751	27.01018	27.49643	28.19483	27.00769	27.83497	27.77166
211	26.50228	26.60559	26.13488	27.09654	26.36532	26.25181	26.94787
212	21.89049	21.05119	21.47439	24.82036	22.21562	21.97519	24.13205
213	30.2685	29.51222	29.06584	30.31109	29.5768	29.11075	30.40589
214	27.30795	26.59315	26.50136	28.09648	26.85148	26.76544	27.91668
215	28.89458	28.84167	29.61431	29.27317	28.99501	29.64601	29.18043
216	25.96281	25.51811	26.24521	26.80421	25.84573	25.95058	26.41825

	AD	AE	AF	AG	AH	AI	AJ
217	26.0052	25.31986	25.32534	26.10532	25.33292	25.42914	26.00706
218	24.59323	23.39166	24.26991	24.07703	23.60426	23.9242	24.30818
219	28.57108	27.99189	27.472	29.17146	28.24846	27.59792	29.09303
220	28.4054	21.87886	27.93242	28.61571	21.98315	21.43009	28.53965
221	23.46692	22.96822	21.34725	24.40276	22.17922	22.54995	24.54386
222	24.08779	24.69743	25.18427	25.11437	24.19495	25.55766	25.19483
223	28.89845	28.94988	29.80537	28.92605	29.12735	29.52703	29.01646
224	26.47563	25.7412	24.94276	27.0667	26.2394	25.83824	27.09703
225	31.55135	30.57498	31.9019	31.81776	31.00454	31.24181	31.89753
226	25.10962	23.71556	25.13618	24.88707	25.01138	24.96518	25.03391
227	27.13343	25.50932	25.74393	27.66154	26.7319	26.26205	27.4958
228	24.50894	26.61021	25.28038	25.23313	26.86123	25.43905	25.70565
229	25.35326	24.86946	24.18681	25.97276	24.66968	25.0437	26.24571
230	24.77164	24.75625	24.96493	25.32721	24.26372	23.88192	25.04972
231	24.93506	24.03326	24.17058	25.0849	23.8626	23.69389	25.01084
232	29.71677	29.9674	30.1	30.01529	30.42996	30.93286	29.98019
233	30.49626	30.46989	30.52778	30.84523	31.05037	30.81157	30.88937
234	27.59116	27.14965	26.87561	27.44893	27.38525	26.77588	27.40235
235	25.6009	24.95322	25.36279	26.1799	24.83209	25.13096	26.07147
236	29.3926	29.34794	29.58813	29.63623	29.32995	29.76229	29.69664
237	24.86556	22.47539	24.23999	25.33655	23.7838	24.62695	25.41043
238	0	0	0	20.16272	21.21923	19.56831	22.33085
239	24.86728	23.5375	23.56976	25.26095	24.39066	24.16815	25.7362
240	0	0	0	22.369	21.47504	22.37111	23.16522
241	28.0028	28.17959	28.74809	28.10228	28.05775	28.80873	28.05285
242	27.78059	27.20331	28.2172	27.74211	27.77081	28.29168	27.92597
243	26.39536	25.75025	25.56091	26.60405	26.36736	26.10726	26.67672
244	29.1059	28.37829	28.28075	29.49453	28.10516	28.16483	29.44448
245	28.74232	28.86839	29.48018	29.07975	29.0189	29.44953	29.11376
246	26.22629	26.26019	26.46101	25.81137	26.26742	26.19993	24.26847
247	23.82436	22.49196	21.6193	24.06728	22.77311	22.86859	23.85243
248	26.3155	26.25599	26.67591	26.33326	26.66665	26.95958	26.38774
249	25.35859	24.50393	25.06482	26.13162	25.2391	25.85768	26.03668
250	27.2049	27.36634	26.30113	27.92795	26.96602	26.7192	28.04348
251	26.35033	25.42435	25.47244	27.45138	26.10474	26.54923	27.46049
252	22.90306	22.14328	22.19341	23.73257	22.52323	22.45565	23.559
253	24.6547	25.07137	25.94887	25.07834	25.28595	26.35572	25.17991
254	25.07943	24.54928	25.48769	25.54189	24.73025	25.19354	25.83424
255	25.05836	25.4581	24.4504	24.89128	24.82189	22.25122	24.94309
256	26.24343	24.48139	25.06503	26.73613	25.37713	24.81001	26.51564
257	27.00808	26.36541	26.03366	27.39159	26.40039	25.97532	27.33591
258	27.33103	26.64885	27.15664	27.62867	26.89916	27.20888	27.74245
259	21.99245	21.02288	21.68953	24.22487	22.75564	22.68288	23.12091
260	24.67008	24.24811	23.65759	25.73757	24.16252	24.20134	25.43515
261	25.15999	24.60026	25.16702	25.39679	24.95685	25.55897	25.11783
262	25.96411	25.88408	25.93987	25.97995	25.81422	25.43236	25.88744
263	24.62072	24.05241	23.50848	25.09599	23.98532	23.54133	24.51154
264	25.94142	25.16863	25.20874	26.19875	25.41816	25.51088	26.3801
265	26.70489	26.21791	25.76395	26.91915	26.03238	25.8712	26.83086
266	20.20133	20.0914	19.69019	0	0	0	0
267	30.84657	30.92003	31.3114	31.32095	30.65377	31.44726	31.33667
268	23.83099	23.15127	22.04474	24.32416	22.74988	23.02973	23.5536
269	24.97063	25.59315	23.32474	24.41438	25.19542	24.83523	23.79251
270	26.98106	25.81837	25.92885	27.17893	26.89287	26.53834	27.24036

	AD	AE	AF	AG	AH	AI	AJ
271	26.27804	26.07335	25.40362	26.98454	25.85117	25.2424	26.72957
272	24.18376	24.2514	23.51689	24.5043	23.20289	23.97065	24.2497
273	24.99986	24.72637	24.27738	25.57086	24.63858	24.58392	25.16617
274	23.84768	24.46531	24.41339	24.39116	24.93042	25.05983	24.29871
275	27.52038	26.39598	26.97386	27.84554	26.82807	27.23117	27.87542
276	29.44988	29.18143	30.05932	29.77104	29.52245	30.54278	30.08538
277	26.46364	25.58133	25.7218	26.78954	26.35257	25.79109	26.89847
278	25.55304	24.71808	24.83395	26.40489	24.69754	25.03245	26.47691
279	30.89675	30.49175	31.08566	31.10794	31.00272	31.09317	31.06453
280	29.9177	28.79309	29.02321	30.71504	29.07986	29.48677	30.59567
281	25.50365	24.76834	23.97986	25.66636	24.90235	23.95987	25.62083
282	27.31693	27.27817	27.92516	27.55924	27.36407	27.94555	27.5976
283	23.79099	22.11051	23.70402	24.86549	23.05591	22.54454	24.57414
284	24.41319	23.94118	23.54136	24.86562	24.06244	23.9383	24.75806
285	23.48041	23.41933	24.00012	24.32944	23.64773	23.27596	24.03881
286	28.60963	28.58616	28.56597	29.14693	28.2953	28.45667	29.07276
287	23.59413	23.65752	22.73334	23.9416	23.96253	23.67643	23.94473
288	22.35042	21.84785	20.877	22.95822	20.71556	22.58076	23.1103
289	26.3183	23.68737	22.66	24.12068	26.37461	23.86918	24.24719
290	26.40708	26.27441	26.70121	26.84119	26.40605	27.05324	26.67901
291	23.62191	24.09664	25.16774	25.50503	25.32738	25.35818	25.25027
292	23.08368	25.60398	21.5656	22.58071	22.0925	22.42237	21.52865
293	24.91338	24.27829	23.64004	24.9514	25.26571	23.69822	24.79329
294	26.20766	27.11617	27.04521	26.27736	26.83868	26.97043	26.19451
295	22.99136	23.79779	22.6942	24.2621	24.10435	21.42087	24.04721
296	23.68447	22.66182	22.24042	24.76638	22.86392	23.17727	24.51436
297	24.23012	24.10922	22.65245	24.6805	23.39325	23.69789	24.47466
298	25.16348	24.64842	24.68413	24.9879	22.86839	23.89443	25.19727
299	20.87346	22.86045	21.46365	21.91327	23.87821	22.30135	23.48351
300	25.40231	24.50689	23.69132	25.47763	24.01635	24.1789	25.64502
301	28.27493	27.44384	27.81894	28.29052	27.95254	28.37071	28.35592
302	26.5535	24.25343	25.51181	25.81539	24.15642	23.407	26.01741
303	23.48978	23.19297	23.01652	24.20911	23.78142	22.76901	23.81147
304	26.69487	26.49913	26.42989	26.54381	26.17208	26.32501	26.4952
305	27.45663	27.05527	26.06227	27.94715	27.16622	26.42006	27.83294
306	24.61018	23.07749	23.26848	25.67088	23.75706	23.94184	25.76352
307	28.61079	27.82722	28.16677	29.66139	28.23433	28.40686	29.32327
308	21.99072	22.26603	22.68297	23.18945	22.50674	22.15654	23.55422
309	25.73991	24.37876	25.73796	25.64432	25.33873	25.51096	25.63947
310	24.39204	23.00228	22.9919	24.63942	22.87229	23.43373	24.40542
311	24.39751	22.60967	22.94061	23.77302	23.7648	23.64368	24.31257
312	26.58429	25.23171	24.45366	26.5744	26.12519	25.3408	26.75466
313	27.45065	27.06593	26.92978	28.34665	26.93768	27.29175	28.4772
314	26.65702	26.30004	26.21013	26.65856	25.93899	26.55455	26.65716
315	24.38376	23.26841	23.07635	24.93132	23.24913	24.13203	24.69263
316	23.37172	21.86078	26.59784	25.93623	23.24141	26.98949	25.96462
317	25.61828	25.52398	24.45869	25.70492	24.96965	24.93458	25.70441
318	24.59751	23.7985	24.15208	25.18012	23.85783	24.9359	24.91297
319	24.77007	24.84326	25.62077	26.00698	25.45386	25.88066	25.75138
320	27.49223	26.58776	26.76255	27.93815	26.56932	26.90152	27.83871
321	29.36863	28.63318	28.70032	30.06034	29.11106	28.92084	29.95103
322	25.71442	26.13871	26.69456	26.31075	26.00894	27.0726	26.35279
323	26.3052	26.01543	26.68491	26.53989	26.50349	26.61035	26.7323
324	25.87972	24.8613	24.29161	26.64663	26.05776	25.16862	26.15932

	AD	AE	AF	AG	AH	AI	AJ
325	27.46643	28.37327	28.09565	27.45212	27.71443	27.63379	27.49802
326	25.66525	25.24141	24.9715	26.16695	24.89259	24.54691	26.219
327	26.50331	26.77368	26.76391	26.77601	26.71948	26.6349	26.53142
328	30.801	30.3153	31.20859	32.1653	30.69301	31.47841	32.32982
329	29.66963	29.58301	30.54334	30.40833	30.00723	30.81815	30.19673
330	27.39915	27.24869	27.59128	27.6282	27.50046	27.62612	27.54699
331	25.34268	26.041	24.99397	25.67343	25.70181	25.05844	25.67072
332	23.28652	22.80335	22.04217	24.00253	21.93677	22.14647	23.91236
333	26.59685	26.2285	26.14356	26.61788	26.30138	26.38491	26.70017
334	24.81669	22.01042	21.74493	20.55295	21.17634	20.04047	21.33568
335	25.9167	25.61897	25.43467	26.18405	25.52354	25.34555	26.11018
336	27.83028	27.16499	27.58477	28.22609	27.42458	27.69184	28.23685
337	28.36951	28.56332	29.42863	29.25652	28.27491	29.48153	29.06937
338	22.19246	22.73191	23.84214	24.91143	20.50401	22.46251	24.67845
339	28.19377	28.40604	28.59923	29.01499	28.6601	28.95965	28.94798
340	23.74245	22.24782	23.21884	25.13439	22.36853	24.1123	24.46657
341	26.0484	26.40998	26.02347	26.78936	26.62312	26.44608	26.74027
342	27.1924	27.17773	26.76243	27.05769	27.57014	26.89751	26.87765
343	29.87269	28.977	29.28068	28.85874	28.96992	28.64287	28.88897
344	26.27221	25.39546	24.54463	26.14544	24.67576	25.51593	26.08519
345	27.4785	26.78289	26.17332	27.91914	27.36024	26.52392	27.89277
346	27.17272	26.30258	26.77921	27.51739	26.71327	26.75467	27.47394
347	20.6948	21.00797	21.86049	24.72908	22.04684	21.63885	22.44181
348	26.02181	25.30838	24.63115	26.85285	25.29439	25.36204	26.73804
349	27.29809	26.84106	27.55894	27.40414	27.22213	27.66901	27.30601
350	29.84267	29.65746	30.66181	30.47256	29.74951	30.40166	30.54568
351	30.09392	30.05706	30.86164	30.84795	29.91593	31.06407	30.7808
352	23.63984	23.69806	22.61625	25.14391	23.10401	22.78473	24.25447
353	24.96117	26.15829	25.01018	25.36566	24.83763	25.04808	25.48248
354	27.69325	26.55868	26.61215	28.50928	26.81028	26.78559	28.36236
355	30.49529	29.35857	30.78643	31.363	30.31359	31.30435	31.28314
356	27.1624	25.73138	26.9561	26.91578	26.30994	27.40749	26.85013
357	26.39029	26.70597	27.47227	27.21637	27.2843	27.67857	27.2927
358	24.13724	25.15414	24.72546	24.93492	24.71025	25.17843	25.1559
359	24.02308	23.4228	25.52748	25.96213	24.66937	25.99162	25.9558
360	24.56146	23.23867	22.79813	25.79141	23.40635	23.95196	24.42336
361	27.83963	27.14277	28.32977	27.78561	27.91637	28.39596	27.76496
362	29.37235	27.83736	28.75632	30.11216	28.24309	28.62431	30.14358
363	26.61331	26.94364	27.40929	26.94576	27.15991	27.71902	27.04892
364	24.2606	22.92963	23.37092	24.12124	23.94484	23.66293	24.20635
365	24.73417	24.50419	23.78344	24.75658	24.30811	23.75687	25.08214
366	27.29385	26.52344	26.21866	27.48232	26.5589	26.26461	27.40855
367	24.39681	24.4349	24.97619	24.69328	23.74032	24.64373	24.7963
368	23.88789	23.73937	24.51255	25.1179	23.59818	24.72353	25.09161
369	26.52372	26.33747	26.51343	26.61134	26.40703	26.76885	26.72977
370	25.32828	25.40595	24.89688	25.98996	25.04263	25.19378	26.02614
371	24.00962	23.34639	23.48646	22.74726	23.61549	23.98754	23.26627
372	21.09695	20.71114	20.65899	23.67062	22.25401	22.15652	23.96232
373	18.06533	17.83252	18.53722	19.51814	18.75316	18.17061	19.17447
374	28.86417	27.89958	27.82386	29.29752	28.22699	27.85099	29.23277
375	25.32722	26.79866	27.34122	26.04239	26.79721	26.30182	25.93316
376	22.659	22.16699	23.21619	23.97874	23.03206	23.63168	24.20002
377	24.73083	24.92746	23.36463	25.57606	23.93687	24.68864	25.73967
378	22.03892	24.61432	23.00483	23.32394	25.95847	24.1792	22.62095

	AD	AE	AF	AG	AH	AI	AJ
379	27.07774	26.87682	26.98871	26.92857	26.55815	27.22879	27.09795
380	25.4646	23.91048	25.55301	25.70036	24.82045	25.44826	25.67392
381	26.53502	25.58272	25.4835	26.69777	25.7865	26.17042	26.7463
382	27.59621	26.97499	26.96565	28.14691	27.35486	26.50493	28.23041
383	24.31808	23.24942	22.96314	25.19462	22.93163	23.50237	25.11022
384	25.51017	24.50146	24.75913	26.47996	24.81164	25.08636	26.63583
385	26.39099	25.22323	24.82807	26.74403	25.7179	24.91841	26.80347
386	27.88996	27.00551	27.92894	27.85827	27.14454	27.41575	27.91046
387	26.01599	24.63749	25.18696	27.12193	25.62787	25.56506	27.03075
388	27.16668	26.77703	27.55974	27.79411	27.02587	27.18369	28.1551
389	27.14043	26.94137	27.75331	27.71527	27.68314	27.63471	27.83349
390	24.87402	23.84518	23.61505	24.56582	23.52533	23.56256	24.65438
391	30.13592	29.27806	29.62193	30.63938	29.29984	29.10922	30.6193
392	27.18487	27.22644	27.98349	27.48966	27.44162	27.95043	27.38436
393	27.70364	27.56602	28.2843	28.2062	27.7631	28.47313	28.15903
394	25.2644	24.2526	25.84667	26.13964	24.50378	25.96443	26.13365
395	26.24666	24.97222	25.69825	26.6613	24.72394	25.88761	26.72712
396	29.74677	30.00704	30.6696	29.78885	29.98726	30.59054	29.70636
397	25.97089	25.25439	25.27903	26.68425	25.57863	25.29043	26.50783
398	29.23891	29.44955	29.93136	29.30815	29.51522	29.80462	29.23597
399	22.77438	22.33018	23.05534	23.378	21.70635	23.50832	23.04402
400	25.19268	24.04084	24.82324	25.65394	24.74092	25.34696	25.44164
401	23.31654	22.60114	22.51	23.57042	23.19676	22.74339	23.03943
402	20.14434	20.11561	20.69857	21.40713	21.55554	20.58024	22.09807
403	28.3055	27.47447	27.23693	28.93274	27.61244	27.363	29.00999
404	24.38491	22.83817	24.86213	24.4573	24.91999	24.12992	25.03281
405	22.32431	21.27467	21.71112	23.43338	21.79176	23.89385	23.36827
406	22.26878	22.80055	22.33475	23.70427	22.19179	22.57321	22.44183
407	24.70981	23.98158	24.3165	23.84405	24.22303	24.65038	23.38108
408	25.72635	25.23283	25.18411	26.51234	25.1459	25.49288	26.25105
409	26.57901	26.47963	26.73875	26.69371	26.45452	26.93974	26.82052
410	27.37969	27.05321	28.09448	27.87687	27.44535	28.04031	27.78867
411	27.53597	26.20066	26.17595	27.88604	26.13966	25.84186	28.81792
412	26.38599	25.70375	25.40244	26.88089	25.58538	25.35267	26.75466
413	28.12411	27.0191	27.08626	28.63869	27.64338	26.88619	28.6599
414	27.95864	27.99264	27.36094	28.51896	27.87545	27.64938	28.52832
415	23.19415	21.9293	22.33829	24.25938	22.11331	22.86212	23.85266
416	22.14967	21.91675	23.3593	23.17241	21.99017	22.78391	23.76396
417	26.66196	27.32265	27.29587	26.67016	26.9539	27.35749	26.83632
418	25.19542	25.04874	25.32415	25.99139	25.18873	25.81893	25.75825
419	23.78703	22.2582	24.97136	23.92367	23.58258	23.18528	24.23422
420	26.79997	26.05435	26.19118	27.0251	26.03961	26.18563	27.05476
421	24.74439	24.52958	24.94376	25.232	26.02963	23.08169	24.63851
422	26.44401	25.76406	25.62772	27.28837	26.23522	26.34235	27.2593
423	27.37225	26.5629	27.25234	27.95696	26.85685	26.92758	27.72391
424	27.57966	27.06442	27.66804	28.04523	27.01093	27.55078	27.98377
425	27.87565	27.38972	27.56921	28.55987	27.52383	27.67017	28.57591
426	22.99731	23.26806	22.24886	23.89075	22.17909	22.01104	23.64384
427	23.31101	22.7566	24.3547	24.40905	23.24587	24.44857	23.94792
428	24.32679	22.87268	24.10179	24.36782	24.47797	23.41761	24.29536
429	24.86706	22.9068	26.00576	23.29725	25.70058	23.61606	23.27563
430	21.47567	21.81858	24.58594	24.04156	23.20853	24.6147	23.54712
431	26.67528	26.17124	25.98047	27.27644	26.52063	26.38908	27.31304
432	23.55338	23.52882	24.28803	23.72836	24.41226	21.83208	24.17352

	AD	AE	AF	AG	AH	AI	AJ
433	24.92911	25.36371	23.249	24.70859	25.72013	22.82869	24.25741
434	27.1715	26.99619	27.66384	27.38449	27.0037	27.76066	27.37545
435	23.42385	21.70728	22.68977	23.63718	20.76643	22.55664	23.91925
436	26.92773	26.21568	26.02496	27.76094	26.21478	26.10293	27.53171
437	28.20008	26.88072	27.31201	28.26981	27.2942	27.65935	28.41395
438	25.97867	24.59984	25.54281	26.24571	24.63177	25.37094	25.93489
439	30.86842	31.09452	31.76584	31.02912	31.25583	31.77097	30.98998
440	26.78701	26.57252	26.39521	27.06577	26.52739	26.56457	27.30835
441	24.97472	24.60535	23.49711	25.46943	24.31934	24.35042	25.57833
442	26.50519	25.3987	25.88537	26.57722	26.0373	26.06698	26.75064
443	23.12901	22.0154	21.58729	24.65676	22.19391	22.90267	24.53028
444	27.97575	27.26478	27.15961	28.2227	27.31496	26.98755	28.15829
445	27.09018	26.89649	27.34142	27.57616	27.3688	27.82211	27.38907
446	28.01856	27.31201	27.61385	28.65309	27.41723	27.99159	28.64052
447	27.02512	25.96249	26.80007	27.71727	26.02751	25.72846	27.79502
448	30.34451	29.79145	30.24436	30.21191	29.98143	30.11867	30.06916
449	25.83534	25.20215	25.34992	26.25508	25.19659	24.76707	26.10559
450	25.27132	25.39239	25.3113	25.36713	25.30758	24.43324	25.22876
451	29.03762	28.67251	28.84311	28.95106	28.83221	28.69313	28.9642
452	29.2215	29.06924	30.05185	30.16997	29.71532	30.22008	30.07388
453	32.64191	32.53112	33.59469	33.19371	32.99073	33.51013	33.15299
454	27.8973	27.03097	27.49263	28.38261	27.692	27.82224	28.45904
455	22.33945	23.80147	23.27035	22.62201	23.03371	22.98421	23.14537
456	23.2786	22.34819	21.59216	23.59031	22.06298	22.03097	23.99961
457	30.3474	29.53968	30.39549	31.12091	30.17521	30.68116	31.10744
458	27.77329	27.86264	28.18521	27.19999	28.01566	28.38169	27.17432
459	31.91177	31.61794	32.41025	32.29658	32.33379	32.63206	32.27846
460	25.68069	25.24869	24.88487	26.32019	26.10663	24.64363	26.58723
461	29.11804	29.2115	29.43101	28.79494	29.07068	29.54379	28.91878
462	27.71363	28.18696	28.44155	27.87116	28.18129	28.81038	27.95661
463	28.37272	27.0633	28.60961	30.0715	27.79707	29.08751	29.63627
464	24.72682	22.6162	23.94601	24.48717	24.73125	24.8312	24.52131
465	25.11565	25.10099	25.11288	24.86726	24.28019	26.01836	24.64824
466	27.0929	26.94471	27.25574	27.26734	26.59089	27.30733	27.20346
467	23.818	23.28118	22.68355	24.98424	23.56872	23.56559	24.85357
468	23.39247	22.07955	22.51041	24.28001	22.04985	23.00943	22.94685
469	24.37233	24.52288	25.23469	24.83795	24.44162	24.64404	24.71699
470	27.50502	26.75003	26.98923	28.02701	26.94752	27.13631	28.00631
471	21.43619	22.21932	22.05758	24.16636	22.39602	23.45024	24.09644
472	23.41111	23.36535	22.37601	24.77112	23.09835	22.80679	23.74242
473	27.35416	26.90087	26.95996	26.27234	27.33503	27.156	26.20539
474	24.94049	23.83763	24.25089	25.6894	24.78858	23.81983	25.44607
475	26.27594	25.45931	26.19329	27.27194	25.34305	26.96542	27.23675
476	22.57431	22.02901	21.4013	22.70507	21.45865	20.94997	22.6942
477	24.10726	21.72913	23.95891	25.85408	24.50145	25.24695	26.02857
478	27.13062	26.37087	26.54782	28.22589	27.54002	26.58016	28.22774
479	25.10656	23.05886	24.19795	26.00113	24.19023	24.70612	25.89494
480	25.37077	25.23803	24.66652	26.55485	25.984	25.50759	26.54394
481	25.90414	24.52142	24.82062	25.83462	25.0295	24.84455	25.76327
482	21.92859	21.31111	21.23528	23.17554	21.5812	21.74378	22.70968
483	20.69649	20.08769	20.38422	22.74977	21.80082	20.6454	22.36097
484	26.64239	26.36867	26.35841	27.41307	26.49424	26.74295	27.40673
485	24.20068	22.63553	23.29403	25.65096	24.05087	23.44192	25.86687
486	27.4365	27.06816	27.15541	27.99568	27.0564	27.46847	27.98191

	AD	AE	AF	AG	AH	AI	AJ
487	26.31805	25.70944	25.5895	26.68041	26.1461	26.25626	26.50065
488	23.93843	23.95805	22.66788	24.74943	23.92015	22.9172	25.0427
489	26.03185	24.32385	24.88714	26.0851	24.50202	25.18693	25.74772
490	24.02632	23.04212	23.39825	24.9702	23.63599	23.40868	24.31157
491	24.98839	24.7824	23.94775	25.14913	24.53687	24.1033	25.48214
492	27.02763	26.41759	26.12111	27.41342	26.36891	26.74704	27.32402
493	23.69874	23.14475	24.94497	24.70306	23.58674	22.91655	23.92764
494	24.10765	24.15723	23.99871	25.17061	23.22993	24.44604	25.21845
495	23.80758	23.43346	24.45776	24.40743	24.26815	23.61172	24.69482
496	26.28548	26.50539	26.97597	26.53892	26.80947	27.18194	26.63186
497	25.11147	25.04475	26.41259	25.63003	25.03406	26.83807	25.60929
498	27.80819	26.1069	25.97972	27.39229	26.02066	26.21763	27.37511
499	29.23617	29.78822	29.39314	29.47858	29.1897	29.24973	29.35263
500	29.11501	28.20896	28.09175	29.27771	28.22968	28.02773	29.2124
501	24.91062	24.59815	24.91383	25.77273	24.74477	24.02085	26.02666
502	25.73466	25.43029	25.85242	26.03057	25.28946	25.23892	26.08904
503	22.60277	22.71866	23.7674	23.10136	23.27777	24.16545	22.90908
504	23.71177	22.52728	23.56796	24.24564	23.1067	23.28293	24.32869
505	23.57619	23.25248	23.83495	24.02627	23.57916	24.09626	24.1329
506	27.82847	26.87975	26.75071	27.93076	26.57832	26.63664	28.09832
507	22.36244	20.88234	21.55641	21.93592	19.99482	18.87997	21.89072
508	27.08936	27.33127	27.32237	27.56475	27.12021	27.63319	27.67039
509	22.10152	22.18032	20.9969	22.10708	22.16846	25.15148	21.82995
510	25.26089	25.62076	24.67251	25.71384	25.37378	25.1314	25.53416
511	24.76513	24.72418	24.88191	25.3769	24.53121	24.8615	25.0694
512	31.60285	30.79162	30.85402	32.1096	30.95916	30.97294	32.20032
513	26.55264	25.60109	24.78793	27.06983	25.50105	25.02357	26.73429
514	26.65941	25.86716	25.6201	26.62341	26.14029	25.72073	26.5811
515	24.46091	23.50557	24.49397	24.59801	23.78564	22.57125	25.30624
516	24.60065	23.8488	23.56341	25.39753	24.0841	23.69818	25.42566
517	23.24185	22.38834	23.43321	24.50683	21.93475	23.45197	24.05765
518	22.44168	24.15468	24.243	23.1404	24.59544	24.64467	23.12743
519	25.88027	24.89209	25.47719	26.37966	27.39832	25.50364	26.19138
520	23.83797	24.0645	24.27539	24.82351	24.1841	24.713	24.57059
521	26.07862	25.26616	25.15806	26.36819	25.74714	25.77383	26.69077
522	23.03761	22.89362	22.89527	23.62593	22.48468	22.84193	23.37858
523	25.53477	25.54186	26.37591	26.36136	25.8061	26.75507	26.38131
524	27.24123	26.83743	26.0667	27.89257	26.74369	26.59389	27.71924
525	24.19572	23.97842	24.7165	24.51614	24.43821	24.9644	24.33223
526	26.1303	26.69837	27.06565	26.37501	27.20132	27.54931	26.50179
527	27.24207	26.48841	26.78403	27.3231	26.66866	26.49559	27.36761
528	24.83299	25.37696	23.97354	25.17749	24.02533	25.34232	25.32644
529	27.87492	27.45908	27.23184	27.93166	27.44311	27.2562	27.99686
530	23.33833	22.47147	23.5443	23.42226	22.84215	22.81301	22.15571
531	25.26719	25.39011	24.45713	26.49214	24.95927	25.0561	26.34882
532	27.36445	27.43107	27.915	27.80716	27.65933	28.37045	27.7286
533	27.43367	27.11996	26.63091	28.4054	26.99818	26.90497	28.46267
534	27.0887	26.82469	27.59581	27.00695	26.96996	27.76866	27.02174
535	32.57144	32.51916	32.53606	32.65231	32.60454	32.53748	32.6945
536	28.24928	27.42984	27.95866	28.76411	27.63378	28.08831	28.68562
537	26.63386	26.99018	27.19634	26.59089	26.73711	27.42994	26.58354
538	25.26159	25.70502	25.85067	25.72017	25.19866	26.0491	25.46904
539	24.16437	23.33658	23.76449	24.20406	24.62247	24.50922	24.10607
540	25.56833	24.14115	24.14723	26.12551	25.54144	23.44343	25.95183

	AD	AE	AF	AG	AH	AI	AJ
541	23.095	21.23412	23.72522	22.31262	23.38605	22.26952	22.45914
542	25.67241	24.46178	26.38972	25.57084	25.32121	26.18164	25.70889
543	25.30677	25.28869	24.71743	25.78378	25.20474	25.6981	25.85368
544	28.4306	27.88737	27.47763	28.22184	28.24829	28.18843	27.97313
545	22.97095	22.65115	22.7713	23.15955	23.32849	22.89198	23.34072
546	24.89649	24.76393	24.3705	25.0956	25.12292	24.27555	24.88982
547	22.53861	21.29598	21.4373	22.77161	24.38575	22.39747	22.67356
548	22.3575	22.35523	21.56701	23.48532	21.71558	23.13044	21.98145
549	25.57722	23.48782	22.72134	25.20153	24.35128	23.81885	25.33618
550	27.60849	28.10192	27.9942	27.21236	27.9511	27.95042	27.26523
551	23.78055	23.48106	24.53246	24.42322	22.34561	24.78638	24.12183
552	24.50437	23.21356	23.09176	24.545	23.08012	23.57878	24.77334
553	32.03698	31.39741	31.66874	32.19623	31.78386	31.60614	32.21687
554	23.48852	24.70416	24.5026	24.34655	24.79908	24.98424	24.3377
555	25.53948	22.24301	23.05302	23.89329	25.41983	24.88839	24.77546
556	26.77698	26.35662	27.1153	26.83731	26.65833	25.50256	26.90425
557	26.56247	25.90714	23.62621	26.88213	23.88825	24.99484	26.23942
558	18.7758	20.8606	20.52036	21.33563	19.67617	21.87839	20.87979
559	25.01068	24.13505	23.86585	25.25798	24.90561	24.70787	25.1566
560	34.35066	34.88134	34.8348	34.95129	35.0753	35.01997	34.90114
561	25.28438	25.04055	25.05016	26.19068	24.80066	25.23177	26.26902
562	24.46429	24.45955	22.35201	25.18047	24.32057	23.06801	24.52321
563	25.46076	23.30087	24.2251	26.00718	24.18737	23.81413	26.0442
564	30.63657	30.12128	30.55245	31.01179	30.78608	30.87015	30.9929
565	25.63562	26.80277	26.93428	27.08329	26.67393	27.45909	27.12037
566	29.54243	29.12885	29.97546	30.0478	29.31977	29.94092	29.93028
567	28.66089	28.69136	29.24972	28.83446	28.95368	29.5455	28.88099
568	26.40604	25.86958	25.59168	26.7645	26.06847	25.70659	26.9992
569	22.6712	21.68775	21.98203	23.49465	23.03599	22.83798	22.05114
570	24.6224	23.13795	23.46156	24.49661	22.45793	22.32285	24.94929
571	27.62054	27.4054	26.87199	28.37448	27.4093	27.17769	28.21759
572	24.33936	22.58673	23.75204	26.22573	22.89946	23.74593	29.81966
573	27.46225	26.40648	26.88041	28.01275	26.71656	27.63139	28.05548
574	24.19382	24.05204	24.85093	25.45902	23.49028	24.86712	25.26344
575	26.03167	25.32431	25.48888	25.77254	26.03456	25.37097	25.92957
576	25.00952	24.41999	24.69529	25.7776	25.26419	23.90334	25.47795
577	28.75317	28.70671	29.16523	28.86454	29.01902	29.21354	29.86603
578	25.13118	23.94601	23.47458	25.36555	23.7228	23.76526	24.85611
579	25.9167	25.22226	24.39405	25.9201	25.41931	25.13232	26.1317
580	24.14537	24.52998	24.5368	24.49644	24.33806	24.49366	24.49824
581	25.61812	25.20051	24.86278	26.30446	25.5529	25.26487	26.26847
582	27.13386	25.62402	23.45868	24.02962	22.26853	26.37464	23.68335
583	20.90739	23.59111	21.33748	22.54108	20.54601	19.46693	22.60351
584	25.88268	25.96117	26.41135	26.34876	26.23002	26.75923	26.43352
585	27.24368	26.94102	26.80558	28.00087	26.85252	27.05873	27.82829
586	24.15145	22.75099	22.4578	24.90312	23.82644	22.85644	24.62637
587	24.05232	22.93777	23.88293	24.1084	24.02303	23.5688	24.57782
588	25.55448	25.29488	25.59931	25.76107	25.42983	25.23023	25.82407
589	27.9396	27.31692	27.07096	28.19893	27.20449	27.11913	28.11352
590	25.00373	24.39369	23.42971	25.03277	24.25941	23.76763	24.66764
591	24.83897	23.35785	25.20708	25.82473	24.33757	24.78704	25.75447
592	28.23319	28.0533	28.2835	28.16272	28.19453	28.13508	28.10455
593	23.52534	24.21013	24.25217	24.32991	23.34382	23.86983	23.93188
594	27.2024	26.42552	26.43773	27.6422	26.33417	26.53162	27.57772

	AD	AE	AF	AG	AH	AI	AJ
595	30.30387	29.69386	29.82718	30.37435	29.64384	29.6187	30.47869
596	25.44177	24.88111	25.00594	25.51166	25.22177	24.93073	25.57713
597	24.63521	23.08872	24.76464	24.40942	25.33637	25.09707	24.56629
598	20.45024	20.6981	23.79488	23.29884	21.89716	20.33825	23.31167
599	27.083	27.18022	28.26542	27.38537	27.37131	28.44928	27.46101
600	29.21122	28.80128	29.32062	29.25868	29.10211	29.68685	29.3232
601	26.10441	25.6926	26.33237	26.60154	25.93332	26.30029	26.64495
602	26.85035	26.7596	26.13373	27.34726	26.62817	26.22913	27.12726
603	28.38944	27.92963	27.51954	28.95987	28.35529	27.47928	28.83369
604	25.97564	22.61001	24.42616	26.28974	25.19013	24.04875	26.2107
605	28.43578	28.29605	28.86116	28.67562	28.24127	28.63598	28.55747
606	21.88342	24.27903	20.47287	23.11661	21.0266	22.76617	24.0256
607	27.29319	26.60552	26.63414	27.27102	26.95736	26.73623	27.25446
608	25.02218	23.56949	24.19465	23.55704	22.75772	22.66084	24.02233
609	26.96919	26.99161	27.11548	26.43431	26.35487	27.35168	26.56191
610	26.27269	26.64338	27.35721	26.67823	26.94947	27.48686	26.65335
611	25.09826	25.21605	26.23731	26.24825	25.77869	26.08352	26.19403
612	24.48396	24.80863	22.9996	25.28731	23.82269	23.46323	25.33286
613	25.14229	24.96951	25.34494	25.50053	25.18168	25.25706	25.96596
614	25.90342	25.09988	25.33856	26.54544	25.21901	25.22608	26.31631
615	25.62763	23.56941	22.95973	25.1464	25.2334	24.75483	25.13496
616	22.15732	22.93717	22.22133	23.08778	22.11513	22.42944	23.20494
617	28.74074	28.46522	28.61252	29.25301	28.24461	29.42083	29.29723
618	24.76237	24.3474	21.87758	24.71511	23.62003	24.08332	24.41052
619	26.26531	25.92028	26.40724	27.22256	26.02798	26.75849	27.26619
620	26.2414	26.1904	25.68452	27.10908	25.41132	25.32284	26.81706
621	25.57323	23.43651	24.64591	25.555	24.66992	24.09274	25.26437
622	25.97317	25.08967	25.10558	26.30049	26.10032	26.05516	26.56774
623	23.4955	24.4591	24.50072	24.30996	25.217	24.37495	23.94073
624	24.69051	22.40845	23.1428	25.17045	23.70412	22.21881	24.98935
625	25.41043	23.84307	23.00511	26.16282	23.21983	23.87848	25.98706
626	26.91386	26.62911	26.82996	27.0612	26.38812	27.15745	26.65489
627	23.42515	23.52665	22.58654	23.68301	22.63222	22.7302	23.27367
628	27.64471	28.21361	28.34072	27.94244	28.12649	28.71358	28.14778
629	22.0231	19.64586	20.09724	23.16425	22.50567	22.6885	23.25886
630	28.7444	28.63873	29.17555	28.97492	28.53461	29.25356	28.9395
631	29.22729	28.48869	29.06738	30.28246	28.34988	29.42818	30.23324
632	27.06233	24.62346	25.53554	28.89923	26.10335	25.9002	28.29391
633	26.64188	25.64616	26.27369	27.43363	26.3766	26.66606	27.50921
634	23.89012	22.92043	23.22166	24.47189	23.99705	23.73441	25.25558
635	25.87273	24.95168	27.082	25.93799	26.58838	26.82789	25.83063
636	25.31322	25.10369	24.14618	25.8749	24.09736	25.42349	25.77625
637	22.50527	24.66721	23.77668	22.52328	22.78209	23.52682	23.48096
638	27.90974	28.11102	28.22868	28.01349	27.83965	28.09632	27.91101
639	24.24137	23.43647	22.89727	24.82156	23.35145	23.38272	24.53757
640	24.65559	24.06477	24.8049	25.09224	24.78551	25.10689	24.90535
641	25.82709	25.01248	25.63376	25.98522	24.55764	25.15998	26.18869
642	30.37062	30.27592	30.7181	30.54697	30.54376	30.70658	30.51119
643	27.17535	26.22722	27.59466	27.70202	26.86923	27.6173	27.70916
644	26.59564	25.74441	25.40388	26.88467	25.60015	25.36533	26.76282
645	22.59685	23.78736	22.36844	29.20865	22.53153	28.06814	24.28839
646	22.53827	22.50108	20.52855	24.66145	22.06021	20.64614	22.57145
647	21.58774	22.01143	22.15105	22.88903	21.87127	23.09178	23.3385
648	27.0462	30.60777	23.46192	24.94916	23.48335	23.59192	24.72685

	AD	AE	AF	AG	AH	AI	AJ
649	28.55876	28.68918	28.9258	28.11423	28.85911	28.33169	28.60421
650	24.21155	23.01577	23.87963	25.2345	23.13687	25.06121	24.7256
651	26.31666	25.59919	25.93047	26.97167	26.05097	25.37396	26.93616
652	24.64869	25.55883	24.71058	24.63403	25.52689	23.66005	24.20933
653	22.69202	21.7928	22.38421	24.0008	22.70938	26.10512	23.73601
654	24.50293	23.20891	23.17164	25.23943	22.52135	23.04175	25.33222
655	26.30812	24.66138	24.12865	26.06778	25.44777	25.3277	26.16896
656	23.7916	24.23457	24.24605	24.50466	23.85532	23.66066	24.84468
657	22.85612	23.07096	21.96626	23.72901	23.14601	22.5663	23.17925
658	26.12363	25.2366	24.98465	25.95627	25.20629	25.43121	26.02581
659	22.6314	22.06552	22.19649	23.5958	22.25814	22.78578	23.61554
660	27.12231	26.201	26.43729	27.77773	26.38095	26.51903	27.78828
661	24.56758	23.48993	22.25834	24.76117	24.20345	24.26287	24.32616
662	26.06839	26.07466	24.75319	26.21371	26.28992	24.56981	25.98214
663	26.91908	26.26378	27.09791	26.82994	26.92025	27.39388	26.33488
664	24.74864	23.39612	25.17013	25.17548	24.37006	23.65153	25.28503
665	26.74182	26.09027	26.07978	26.76955	26.38838	26.15969	26.82101
666	30.92782	30.1633	30.17485	31.46173	30.43631	30.313	31.31359
667	29.19282	28.7614	28.57764	29.85626	28.80229	28.95079	30.45274
668	27.30513	26.51923	26.26948	27.35501	26.73545	26.34326	27.52052
669	26.20317	24.7049	24.54273	26.17253	25.39285	24.84564	25.98686
670	22.50261	20.64617	21.65924	22.86932	21.77137	21.90657	19.69503
671	24.03041	22.52346	23.19808	24.82381	23.67215	22.5038	23.37187
672	26.0783	25.18704	25.08214	26.12128	24.91047	25.32687	26.04801
673	25.27752	22.97112	23.73436	26.22688	25.17012	22.43771	26.08555
674	23.77561	23.61236	24.87736	24.88151	25.12155	24.77644	24.73272
675	26.14408	24.79407	25.35022	26.03452	25.49374	24.82655	25.84412
676	25.27348	24.98179	25.22089	25.62254	25.19874	24.80356	25.55879
677	25.58639	24.14177	23.5752	24.97835	23.74239	24.3829	24.82796
678	25.67138	25.25844	25.2742	25.38132	24.74585	26.12834	25.52721
679	27.94587	27.34773	27.2424	28.54412	27.71326	27.48014	28.60494
680	22.846	22.48874	23.10963	24.12099	22.23598	22.05099	22.76299
681	26.20365	25.81152	25.77035	26.58337	26.26663	25.83675	26.56461
682	28.49564	27.78981	28.39657	28.98625	28.18772	28.23986	28.97577
683	28.94958	29.08974	29.7543	29.0807	29.01001	30.03156	29.03128
684	22.0257	26.87761	21.14806	24.10844	23.19733	22.66856	24.69484
685	26.07696	26.40475	25.74002	27.0922	25.41548	27.63845	26.47444
686	30.76647	29.98291	30.3162	31.00912	30.49309	30.50294	30.94683
687	28.19721	27.99988	28.46469	28.43319	28.37509	28.36292	28.32162
688	27.80167	27.41384	27.35846	28.21811	27.53744	27.30238	28.33713
689	23.96741	19.55075	20.36356	23.32748	23.35051	23.00524	23.53527
690	24.80956	23.28645	24.81856	26.15353	24.20061	23.76591	25.96291
691	29.7274	29.43619	29.54158	29.6169	29.51905	29.75663	29.57418
692	24.87905	24.31503	24.28272	25.2199	24.54139	24.04767	25.11738
693	23.18554	24.71314	25.51403	24.30806	24.43509	22.70763	24.65338
694	25.15395	24.59919	24.96434	24.93806	24.99818	24.96215	24.81366
695	25.35686	24.97637	25.02883	25.88145	25.03296	25.1848	25.69285
696	28.70707	28.04188	28.05037	28.91717	27.91585	28.30596	28.97445
697	25.4789	25.36225	25.49043	25.9674	25.62628	25.42509	25.85275
698	27.22497	27.10471	27.06293	26.97366	27.04359	27.01255	27.00691
699	25.90383	24.4602	24.63838	25.7138	24.96577	25.33452	25.18767
700	25.52205	26.27683	25.36545	25.68547	24.92332	25.48218	25.62329
701	31.6814	31.22434	31.12091	31.74158	31.96782	30.83553	31.60279
702	26.80082	25.67672	25.55135	26.6738	25.94024	25.95311	26.70838

	AD	AE	AF	AG	AH	AI	AJ
703	27.49435	27.91721	28.90928	28.06243	27.88081	29.07388	28.74076
704	28.55095	27.86938	27.71228	28.8093	28.53455	27.72605	28.87407
705	26.53036	26.42373	26.64671	26.24153	26.19102	26.97975	26.53074
706	23.21823	22.04903	22.44036	24.31241	23.7477	22.27895	23.94426
707	25.68429	25.69842	26.29019	25.78352	25.06792	25.66288	25.84177
708	29.1923	29.5225	29.85344	29.0336	28.96364	29.79396	29.12659
709	27.0282	26.58219	26.4636	27.41942	26.8359	26.32565	27.38211
710	29.14993	29.13484	29.47415	29.07534	29.18436	29.39672	29.01183
711	33.52378	32.62646	32.94189	34.00282	33.35472	33.02316	33.83449
712	28.23748	27.26823	27.07797	28.71944	26.83578	27.22464	28.63835
713	28.45445	27.57313	27.46818	28.58937	27.70877	27.73438	28.55561
714	32.18122	31.51663	32.25937	33.01619	31.98994	32.4006	32.93818
715	28.14332	27.80343	27.79063	28.79677	27.73599	28.01776	28.79632
716	27.23377	27.18893	28.44069	27.76611	26.58727	28.20159	27.70061
717	26.29936	24.88209	25.47307	26.81418	24.59135	25.4496	26.81211
718	25.08439	24.6396	25.51414	25.43728	24.04081	25.89552	25.68962
719	27.05448	26.24799	26.22105	27.50566	26.54319	26.30295	27.51804
720	27.21241	27.40392	28.25424	27.81289	27.55677	28.43207	28.19631
721	28.85513	28.32361	28.44405	29.08643	28.28406	28.38251	29.10703
722	26.69706	25.9161	25.74662	26.99825	25.96923	26.2353	27.08649
723	29.22249	28.95263	28.71529	29.53878	28.85954	28.74771	29.55677
724	24.58477	23.93602	25.29638	25.6888	24.08865	25.17124	25.98738
725	28.08377	27.19635	26.32691	28.079	27.69555	27.04138	28.01087
726	0	0	0	20.84009	19.96251	20.72988	21.15999
727	30.21186	30.40567	30.72552	30.76652	30.78629	31.20853	30.71459
728	29.04981	28.21971	28.37356	29.44224	28.49161	27.9829	29.37844
729	23.01227	23.52704	24.445	23.55722	22.80609	23.45458	23.92279
730	26.20425	25.9399	25.69692	27.21575	25.69077	26.5027	27.45463
731	25.51056	23.75503	25.73128	26.12618	25.30541	24.55359	25.8307
732	23.78971	23.182	25.5266	24.59437	23.53041	23.52053	24.4603
733	24.31162	21.49323	20.71829	22.44874	24.28286	20.79755	23.12594
734	30.87283	31.33431	30.67183	31.0722	30.61655	30.6828	31.09222
735	21.59397	22.25653	21.49431	21.89647	22.28424	21.9096	21.79992
736	26.35072	26.49934	26.79575	26.31435	26.60334	26.53117	26.23806
737	29.77478	29.62686	30.13546	29.87841	30.14104	30.58351	29.9821
738	29.82545	29.90041	30.51851	29.93337	30.02404	30.51018	29.96057
739	22.38856	21.88253	22.20387	23.39908	22.28067	22.7006	23.76283
740	26.82421	26.07029	26.46767	26.95698	26.63627	26.76394	26.83648
741	29.2759	28.27182	29.24081	29.65676	29.11782	29.24161	29.65964
742	27.29852	26.58115	27.21658	27.69245	27.22826	27.55722	27.89366
743	31.77798	32.05323	32.54453	32.061	32.31925	32.62582	32.0322
744	27.49823	26.23431	26.62016	28.2725	26.48161	26.73775	28.21866
745	27.66432	27.67581	27.21698	27.63112	27.38808	26.50204	28.13169
746	22.0008	22.38256	22.59877	23.85463	22.86733	22.2689	23.47191
747	29.58372	30.24495	31.60386	30.21679	29.91003	32.25348	30.22602
748	23.32063	23.05344	23.01263	24.31592	22.20037	24.09157	24.25695
749	26.84976	26.79224	25.86371	27.0361	26.15534	25.79033	27.3984
750	26.90407	26.37617	26.05057	27.1615	26.57053	26.12339	27.40071
751	23.83548	22.77261	21.61342	24.33611	23.30336	22.28799	24.12988
752	25.06931	24.26138	24.33766	25.67361	24.27567	24.28806	25.62374
753	24.14885	23.25901	22.02903	24.10967	23.44641	23.16115	24.75871
754	27.02691	26.37438	27.33142	27.32997	26.37327	27.23527	27.43145
755	24.91861	24.76752	25.26348	25.61435	24.87646	25.57496	25.37698
756	28.15847	28.30965	28.70107	28.52716	28.18671	29.5034	28.67035

	AD	AE	AF	AG	AH	AI	AJ
757	28.02969	27.44763	27.50466	28.21214	27.68751	27.53939	28.11302
758	26.84295	25.94791	25.69435	27.04637	26.1524	25.75256	27.00125
759	25.62003	24.93565	24.963	25.8736	24.87487	24.78767	25.91344
760	26.48423	25.76391	25.20119	26.79327	25.90773	25.48018	26.4961
761	23.43691	22.33503	22.42156	24.61073	25.57736	23.68801	24.3217
762	21.32534	22.45631	19.38086	23.66582	23.40063	21.73561	24.45653
763	24.35026	24.96449	24.57609	24.14925	24.19546	24.46403	24.26274
764	24.30565	24.31427	24.03424	24.37201	22.8647	23.45873	23.94237
765	24.91152	24.72366	24.66328	26.41002	25.13473	25.56971	26.1122
766	27.33704	27.45558	28.49767	27.18896	28.07514	28.28723	26.61532
767	26.89894	26.44634	27.59676	27.4562	26.94443	27.96666	27.5181
768	24.60676	24.23951	24.42996	24.04755	24.25439	24.36066	24.53575
769	23.36996	20.78196	22.42714	23.47604	21.94274	21.79929	22.83326
770	26.06919	25.8272	25.98161	26.36672	26.41803	25.8957	26.03082
771	29.15065	27.5244	28.27472	29.72242	27.78996	28.25435	29.65566
772	22.19525	24.80128	25.24261	25.03645	24.90503	25.66573	23.92213
773	24.58641	23.81592	22.76213	25.04346	23.49834	23.56457	25.07059
774	26.68702	25.83229	25.3328	26.9449	25.64415	25.40495	27.03024
775	30.56239	30.83926	31.35198	30.82322	30.79309	31.61466	30.70681
776	25.2257	24.42417	24.46404	25.85121	25.1398	24.5257	25.62411
777	27.20033	26.9054	26.93758	27.29687	27.04145	27.15905	27.44959
778	24.99259	25.30524	26.0806	25.69256	25.21905	26.24508	25.5689
779	30.13733	29.73761	30.83259	30.51314	29.69449	30.613	30.55585
780	27.61351	27.56419	27.47564	27.36349	27.45085	27.55076	27.44228
781	31.79567	32.13579	32.30021	32.20691	31.97662	32.58722	32.1799
782	25.14111	22.00791	25.55213	25.22781	26.16278	26.30371	26.84616
783	28.30006	27.52507	28.44796	28.7624	28.14323	28.57798	28.84824
784	25.05967	24.76303	24.15886	26.09927	24.2572	24.42067	26.18029
785	27.51637	26.51125	26.43601	28.18886	26.79723	26.79475	28.07482
786	26.79908	26.33729	26.39919	26.8599	26.77153	26.57486	27.02105
787	26.37091	25.3166	25.73383	26.69847	25.65963	25.53192	26.8364
788	26.57157	25.58288	26.12653	27.88997	26.43877	26.80237	27.79285
789	30.92379	30.30174	30.52497	31.49358	30.39838	30.71791	31.45136
790	25.84057	25.62172	23.196	25.76779	24.1713	23.69346	25.78225
791	25.686	24.71706	25.47855	26.01583	25.47242	26.11555	25.87069
792	29.25536	29.13597	29.44841	29.4146	29.28229	29.72908	29.31748
793	25.57694	25.16038	25.09317	25.8198	25.41351	24.80111	25.74226
794	28.66955	27.81578	27.71283	28.4113	28.46688	27.90237	28.15305
795	26.57006	26.04764	27.37158	26.85929	26.67349	27.51942	27.21491
796	26.60147	24.68663	24.67206	27.34647	25.53697	25.95386	27.36989
797	21.50413	23.46636	21.24723	23.06929	21.62691	23.72163	23.28878
798	29.44534	29.10539	28.66412	29.69492	28.93176	28.56848	29.72519
799	23.96936	24.0865	24.53617	24.093	24.9498	25.39514	24.08094
800	21.23339	21.98969	22.00525	23.65816	21.95588	23.56803	23.36058
801	25.55783	24.82849	24.64014	26.35096	25.73121	25.04449	26.27139
802	24.65709	24.3853	23.5174	25.06159	24.62699	23.27632	24.79735
803	27.11382	25.49999	27.18396	29.21862	26.60089	27.09539	29.43812
804	27.05875	26.34619	26.018	27.5952	26.7234	26.91549	27.69929
805	23.36014	22.82075	22.7115	23.58833	21.44496	23.20024	23.20465
806	23.79444	23.83511	24.44824	24.56804	23.73092	26.04873	24.12519
807	24.96851	23.81881	24.65594	25.18412	24.09942	24.7397	25.07678
808	23.76482	23.39046	23.08424	24.00258	22.44582	22.44788	23.67845
809	22.79598	24.83576	25.72606	23.16098	25.63216	25.84475	25.97814
810	25.03981	25.92113	24.39185	25.82826	24.20955	23.54877	25.87922

	AD	AE	AF	AG	AH	AI	AJ
811	24.87355	25.04306	26.69436	26.54703	25.11571	26.50297	26.61015
812	29.72251	29.59954	29.90759	30.28187	29.51333	29.80871	30.29411
813	23.38199	23.99968	24.1116	24.93815	23.83096	23.82886	25.04161
814	31.42266	31.43343	31.72176	31.58852	31.47574	31.77965	31.55476
815	23.41968	22.77447	23.42953	22.78811	23.49121	22.98282	23.43447
816	30.62255	30.46025	31.30206	30.96627	30.96322	31.30895	30.94688
817	27.71923	26.79444	26.72267	26.97338	27.09967	27.07912	26.9568
818	27.78056	28.18439	26.59261	28.19248	27.09535	26.56569	28.13593
819	28.94265	28.27817	29.48334	29.57798	29.28111	29.53734	29.43769
820	26.26735	25.58354	27.18683	26.9086	26.11833	27.15497	26.77368
821	25.14542	25.81856	26.48738	26.95232	25.24945	26.61705	26.60029
822	30.25135	30.12935	30.26713	30.20232	30.29197	30.15474	30.24659
823	28.20084	27.80221	27.53692	28.72016	27.80235	27.74029	28.72289
824	25.28853	23.84592	25.10053	25.96014	24.93857	25.51242	25.77166
825	26.64283	26.07642	25.66978	26.47943	25.83792	25.56899	26.86912
826	24.24181	24.28966	25.3524	24.69848	23.90601	24.90166	24.29334
827	26.63555	25.39899	24.63563	26.2605	25.19193	24.23762	25.17218
828	26.29281	24.24925	24.70955	26.11088	24.70396	25.43595	26.18176
829	25.19646	24.76983	24.97762	25.32149	25.28913	25.20252	25.8931
830	26.2988	25.61364	25.84024	26.66139	26.55027	26.61732	26.64941
831	26.49899	25.85723	25.34933	26.86976	26.00045	26.08974	26.83368
832	24.39664	24.4997	24.26061	26.22276	24.50493	24.47164	26.25514
833	20.41883	21.00297	20.45327	23.03074	21.50203	20.19432	22.38723
834	23.01553	26.02122	22.24119	22.12564	22.05839	21.60109	21.90152
835	19.25388	19.90587	20.67669	21.29589	21.87097	22.00815	21.95911
836	26.50236	26.70334	26.42751	26.48019	26.59544	26.471	26.4949
837	25.10402	23.50554	24.03028	25.06854	24.18677	24.12156	25.24416
838	24.75729	25.42738	25.04177	25.25452	24.73298	25.6983	25.2369
839	22.57112	20.45073	22.35262	23.16196	23.20754	21.99767	23.24076
840	28.90307	27.57649	27.71622	29.40786	28.23957	27.67513	29.48133
841	27.76791	26.80077	26.88924	28.06276	26.78954	27.1693	28.14501
842	28.13081	26.83481	27.09961	28.55233	27.27848	27.26985	28.40696
843	26.55841	25.01789	25.35914	26.72658	25.76863	25.48475	26.6939
844	25.60453	24.44116	24.89421	26.28292	25.37001	25.02763	26.04727
845	26.02048	25.15055	24.89782	26.4138	25.35533	24.87835	26.42952
846	21.20483	21.59605	25.07337	20.88429	20.71446	21.49983	22.78826
847	24.08433	23.9617	23.6158	24.85727	24.19178	22.78561	24.44649
848	26.49348	25.94176	26.49809	26.48791	26.39397	26.51636	26.44498
849	27.54389	26.92986	27.5725	27.64139	27.04453	27.39621	27.63798
850	25.53373	24.20042	24.60162	25.76275	24.5041	24.42134	25.67878
851	24.70472	22.90088	23.25474	25.03483	23.70706	23.01833	25.13598
852	25.16574	24.83607	23.95657	25.6367	25.77841	25.03005	25.74118
853	21.1927	20.85967	22.33174	22.3023	21.89564	22.17228	22.65839
854	23.7843	23.96564	23.02743	23.43094	23.11892	22.17625	23.12302
855	22.61094	22.82873	23.48655	23.58671	23.75855	23.53611	23.04435
856	25.71556	24.95942	25.28552	25.9525	25.46053	25.28666	26.18641
857	26.25274	25.46584	25.04435	25.85237	24.79646	24.78876	25.76239
858	28.28724	28.08063	28.74401	28.57953	28.36616	28.65178	28.37523
859	24.55956	23.26538	23.33483	25.13646	23.62988	23.41016	25.16344
860	27.51328	26.96121	27.18581	27.75558	26.84863	26.75121	27.77368
861	24.57009	23.37998	23.30756	24.54973	22.94979	22.97717	24.56168
862	27.94774	27.83328	28.34312	28.56854	27.90096	28.52324	28.65312
863	25.29959	25.24074	24.86002	25.87945	25.03111	25.57536	25.9359
864	27.08348	26.52083	25.97229	27.47709	26.7063	25.97099	27.53804

	AD	AE	AF	AG	AH	AI	AJ
865	24.97528	23.04663	24.79243	24.34328	22.91973	23.58367	24.67093
866	24.72828	26.69585	22.81307	25.33157	26.50672	23.19	25.25896
867	0	0	0	0	0	0	0
868	22.83962	22.35168	22.9144	23.88447	23.12872	23.82838	24.5446
869	24.19867	22.97446	23.75327	23.21642	23.20031	23.11514	23.40749
870	26.36584	25.92683	26.12719	26.5	26.11728	26.4807	26.4752
871	26.43565	26.87456	25.82317	26.76959	26.47274	27.61286	26.86763
872	25.73759	25.78718	25.76223	25.76972	25.56532	25.8859	29.19323
873	25.12065	24.3079	25.23594	25.33109	24.20321	25.29869	25.42577
874	25.3787	23.18351	25.68557	26.45592	25.47455	25.46234	26.55776
875	22.38776	23.9936	21.34507	23.54663	22.34545	23.14895	23.18295
876	22.68727	21.22251	21.19955	23.75745	21.90008	22.51622	23.50989
877	22.25554	22.12054	23.29214	23.67474	23.6519	24.15561	24.05623
878	27.57252	26.89746	28.44054	28.00167	27.00262	28.2152	27.93624
879	31.90852	31.89976	32.44328	32.13128	32.02318	32.48673	31.99318
880	23.96581	24.50819	24.28334	25.88425	23.64269	24.64181	25.56413
881	27.16804	26.10419	26.07471	27.29839	26.32663	26.22663	27.40168
882	22.72661	23.61216	24.4173	24.067	23.84447	24.25523	24.42841
883	26.54617	25.9916	26.43376	26.4873	26.40466	26.60916	26.42117
884	22.59821	21.53636	22.9985	22.84589	21.94125	22.81631	22.79144
885	0	0	0	20.42794	20.97754	21.22715	20.69062
886	25.85016	26.13242	26.79356	25.6673	25.69408	26.96485	26.09555
887	24.55059	22.52834	22.5429	24.16107	22.36243	22.80003	24.3618
888	28.73872	27.13795	27.41144	29.13205	28.04949	27.42127	29.16325
889	33.89751	34.18198	34.28591	33.59269	34.11817	34.22193	33.47588
890	32.44779	32.14634	32.75338	33.17226	32.71013	32.74803	33.17403
891	26.47413	25.02361	26.69156	26.39942	26.27931	26.63232	26.50785
892	26.26285	25.58163	25.82195	27.38577	25.6043	25.26056	26.69588
893	23.38337	22.98727	21.70514	23.28745	24.20612	21.42531	26.667
894	25.30667	24.61673	24.51234	25.21304	24.27497	24.27296	25.33509
895	24.94597	24.55673	24.71356	25.92144	24.1362	24.40547	25.69291
896	27.09572	27.22225	27.16235	27.63202	27.34081	27.29061	27.56482
897	24.70008	23.98697	23.83121	25.58271	24.50203	24.15423	25.5074
898	23.77351	23.50922	23.25598	24.88385	23.3977	22.98468	25.10716
899	23.76042	23.63297	23.61098	24.44803	23.83203	23.67684	24.03843
900	25.24319	24.3117	25.14631	25.13543	24.9779	25.07431	25.24511
901	24.73573	23.70588	24.53403	24.67571	24.86866	24.46589	24.83964
902	27.77061	27.22032	26.91096	27.8744	27.45378	27.1532	27.8908
903	25.4297	27.0837	25.82788	26.23215	27.32553	25.8832	26.11504
904	25.37496	25.69852	25.51726	25.26656	26.14653	25.49103	25.22971
905	27.55859	27.32536	28.09277	27.65526	27.45148	28.34364	27.71362
906	23.50287	23.71418	22.48345	24.60185	23.32326	24.92936	24.34053
907	22.10982	22.24467	21.51888	23.5621	22.33423	22.61923	23.17473
908	27.4679	27.1215	26.7266	28.01583	26.89165	26.57432	28.04729
909	28.09902	27.81586	28.7094	28.34798	28.25009	28.79388	28.32308
910	26.85953	27.00535	28.75983	27.49752	27.7521	29.20629	27.45362
911	25.86035	24.24738	25.82559	26.06997	25.83262	25.54294	26.10028
912	22.64982	23.08516	23.29871	23.9595	22.60007	22.23107	23.43661
913	27.76071	27.13359	26.9786	28.13329	26.83137	27.14542	28.07472
914	27.2158	26.75029	26.48872	27.18559	26.03028	26.98316	27.31575
915	25.43414	24.42733	24.85386	25.86736	24.53205	24.69627	25.84246
916	21.7974	20.83628	21.29978	23.70349	22.18299	21.58292	23.3739
917	25.51997	25.82766	25.74376	25.83403	25.63179	26.11019	25.95377
918	28.38636	28.1562	28.26213	28.1577	28.27969	28.40926	28.12422

	AD	AE	AF	AG	AH	AI	AJ
919	26.83192	26.09313	26.13171	26.89551	26.2791	26.04989	26.68178
920	23.29834	23.29483	22.52347	24.06704	22.5624	22.47005	25.0003
921	28.22149	25.94451	26.08987	29.31597	26.64991	26.12091	29.11765
922	27.94682	27.45536	27.24374	28.6163	27.16574	27.63594	28.59339
923	28.63381	27.74047	27.98156	29.40374	28.33852	27.83297	29.33379
924	26.53196	25.95864	25.80084	26.86806	26.32432	25.59756	26.706
925	28.58556	27.54765	27.62276	28.91567	27.78436	27.47051	28.91312
926	31.73137	31.91361	31.65963	31.6428	31.83684	31.53899	31.64878
927	25.29477	22.75668	25.02509	25.8299	27.28829	25.11685	25.83083
928	25.22901	23.33088	22.35745	22.88286	23.17973	23.74821	23.39616
929	29.92338	29.24109	29.62898	30.12958	29.51092	29.6634	30.09879
930	30.40446	30.7588	31.08912	30.16785	30.82731	31.23263	30.03951
931	24.08522	23.70499	23.43929	24.15133	23.95086	23.38991	24.26516
932	26.98429	26.56119	26.92625	27.01923	26.7907	27.01295	27.14765
933	25.06534	23.99948	25.18869	25.2708	24.17252	25.48342	25.23801
934	23.70943	22.56233	22.38183	24.52497	22.89205	22.82674	24.14386
935	27.05613	27.05994	27.38983	27.35415	27.55957	27.65632	26.87162
936	28.74206	27.85163	27.47811	28.88364	27.99266	27.47157	28.96666
937	27.03223	25.98907	27.00702	27.83403	26.08651	26.67241	27.75739
938	25.51481	25.46114	25.39327	26.00923	24.7455	26.37789	25.29845
939	27.13114	26.46381	26.23325	27.40672	26.18618	25.57668	26.2927
940	28.01029	28.18705	28.20262	27.69695	28.14913	28.21377	27.83114
941	27.23763	27.18562	28.33689	27.82558	26.86864	28.76346	27.88928
942	25.51125	25.25366	24.53462	26.07518	25.37604	24.83352	26.15346
943	25.87895	25.46771	25.57759	26.27143	25.60306	26.11041	26.43429
944	22.91593	23.03123	24.01426	24.19219	23.34722	24.09112	24.04519
945	25.60897	25.56915	26.13861	25.88895	26.01408	25.92234	25.85808
946	29.42894	28.59742	29.40885	29.50033	29.04366	29.47789	29.60916
947	28.76365	28.04153	27.69807	29.28719	27.96795	28.03708	29.28992
948	28.53435	27.68256	27.78583	29.17625	27.94467	28.1427	29.1383
949	24.95539	22.5545	22.7352	25.00413	24.1973	22.66182	25.11201
950	24.52597	24.27257	23.88134	24.93629	23.98863	24.90466	25.18863
951	25.82877	25.36374	25.89378	25.97017	25.2686	26.00137	26.10664
952	26.02367	25.76254	25.84112	25.2957	25.44104	25.92114	25.2583
953	0	0	0	22.44934	21.89212	22.4289	22.77844
954	23.07334	25.30914	20.34594	22.80626	24.96236	22.88133	23.06557
955	28.46995	27.50943	27.88308	29.18152	27.57702	27.81561	29.19551
956	22.81597	21.79395	21.51128	23.3653	20.92224	21.84429	23.19795
957	23.50375	23.47972	22.24771	24.34064	23.2625	23.28713	23.8742
958	25.3807	24.72523	23.77608	25.92593	24.66383	24.2077	25.749
959	22.95531	22.46399	21.30038	22.14273	21.87507	20.67042	23.90264
960	26.80785	25.88746	25.48667	27.31536	25.74478	25.69173	27.37526
961	26.39979	25.13221	25.12363	27.07864	24.5587	25.75392	27.13929
962	25.51952	25.21559	25.21241	25.85352	25.00627	25.5374	25.84682
963	24.87408	22.61733	23.21271	24.71549	22.75672	23.3464	24.53765
964	24.66575	24.34597	24.36174	24.68856	23.9694	25.66634	24.92371
965	25.49121	25.09742	25.00859	26.79131	25.33308	24.33899	26.71837
966	23.15384	21.68909	22.35737	22.98815	23.02271	22.61144	22.42606
967	23.60818	23.66348	24.43771	25.0992	24.18654	24.25055	24.93769
968	21.24187	24.96955	25.70503	24.55039	24.22665	24.28507	25.04879
969	21.32974	21.1689	23.08468	22.3843	22.16211	23.27952	22.5459
970	22.51634	22.35503	22.59591	24.3582	22.39708	22.93534	24.53023
971	26.0658	26.44653	26.51701	26.14138	26.31485	26.91254	26.08605
972	27.63428	26.6524	26.56729	28.41341	26.88089	26.64787	28.40453

	AD	AE	AF	AG	AH	AI	AJ
973	27.28946	27.25373	27.64356	27.24239	26.98709	27.78639	27.39431
974	24.88822	24.55286	24.22817	25.23883	24.07761	24.6021	25.08174
975	27.86661	27.26206	27.56553	28.23046	27.67973	27.5724	28.12192
976	23.64064	22.48735	23.75671	23.70775	23.57225	23.69898	23.69833
977	28.70166	28.16726	28.49779	28.61745	28.47812	28.73732	28.82676
978	24.06062	20.8421	23.84039	25.21903	23.59833	23.31841	24.62066
979	25.32189	24.59222	24.31069	25.49556	25.37835	24.67928	25.41196
980	33.47022	32.5078	32.98508	34.01174	32.70026	32.74422	33.95533
981	25.72366	23.75868	24.24248	26.54602	25.12745	24.13788	26.32758
982	20.53209	20.54969	20.68117	24.7489	21.90385	22.32419	24.89775
983	25.86285	25.55332	25.64851	26.10367	25.97132	25.45259	25.93482
984	24.73306	25.72999	24.75515	25.70799	25.04884	24.65381	25.03318
985	25.95054	25.84624	25.12078	27.16179	26.80258	26.50241	27.14944
986	25.36492	25.22657	24.97907	26.02761	24.86328	24.80649	26.14498
987	27.86337	27.60716	27.53238	27.90264	27.46916	27.56472	27.72099
988	23.80809	21.74743	23.28274	27.01851	21.50553	22.65291	27.0027
989	28.54482	28.05093	28.106	29.41971	27.94504	28.21808	29.32272
990	26.81802	25.87881	26.13382	27.10974	25.9143	26.55072	27.1096
991	20.09641	21.08857	20.7597	21.90344	22.74934	21.61536	21.38971
992	28.06462	27.42037	27.3691	28.53816	27.82003	27.78552	28.42248
993	24.20896	23.36219	23.48062	24.82561	23.67659	23.97226	24.50265
994	23.58352	22.84781	22.54028	26.44819	24.67875	23.03964	26.06751
995	26.49215	25.8354	26.41067	27.01003	26.48212	25.89874	26.84966
996	25.7624	25.12129	24.88129	26.02237	24.98817	25.27694	25.84137
997	25.29658	23.39437	24.14935	25.30146	22.48566	21.85221	25.67738
998	22.20674	23.29595	22.97968	23.97939	23.58013	23.23316	23.61741
999	30.77707	30.78858	31.13683	30.43191	31.16021	31.2755	30.46403
1000	23.80112	22.45119	23.94114	24.031	23.37017	23.60692	24.09512
1001	28.11716	27.70422	28.0986	28.35319	28.28031	27.76517	28.5361
1002	24.83052	24.86606	22.21706	25.1925	25.02971	24.14322	25.218
1003	23.62561	21.42023	23.32412	24.71021	22.03629	22.45504	24.5913
1004	23.34677	21.81556	23.55577	24.62786	22.82015	24.03441	24.53979
1005	23.0505	22.44966	22.49654	22.9766	22.67172	22.69912	23.61035
1006	26.28875	26.00099	26.26811	26.9489	25.93756	26.09164	26.89888
1007	22.03262	20.95931	22.598	22.74594	21.18025	23.62812	21.96437
1008	20.98436	23.90316	20.89073	20.16322	22.07349	21.66873	21.596
1009	27.46241	26.37855	27.0124	27.95075	26.89904	26.81129	28.08151
1010	29.19668	28.75533	28.84923	29.99754	29.51519	29.10203	29.83116
1011	26.65356	26.02462	26.28857	26.32829	25.88713	25.38698	26.61223
1012	26.63284	25.69877	25.47733	26.78318	26.02283	25.4	26.82773
1013	25.78858	24.82659	25.19847	26.11901	24.8856	25.12059	26.14048
1014	24.10152	23.1103	22.96355	25.15402	23.00345	22.49463	25.22032
1015	27.22348	26.6607	26.79504	28.06399	26.85893	27.33095	28.16432
1016	20.69815	20.74532	19.22882	21.98566	21.64772	22.97623	21.70352
1017	27.36783	26.63329	27.39347	28.287	26.82495	27.62536	28.19471
1018	28.90834	28.31254	28.86969	29.10421	28.57194	28.36688	29.1103
1019	25.78101	24.26091	24.80851	25.30158	24.96832	24.91223	24.99014
1020	28.08812	27.61988	27.36815	28.17414	27.72712	27.32896	28.3167
1021	29.07827	29.42872	29.62776	29.71805	29.68759	29.5586	29.60988
1022	25.31039	24.05389	23.92249	25.47379	25.03289	24.41574	25.47306
1023	27.43458	26.61694	26.79874	28.15045	26.78756	26.74125	27.95764
1024	24.11134	22.94616	23.06682	24.54302	23.24684	23.8724	24.55421
1025	26.58156	25.67377	25.82971	27.41581	26.43155	26.81983	27.11781
1026	19.83429	20.68702	19.97274	21.28007	22.08759	21.86572	21.32209

	AD	AE	AF	AG	AH	AI	AJ
1027	26.98062	27.30783	27.3368	27.83301	27.4306	27.29556	27.76032
1028	24.84283	23.81515	24.64434	25.64333	24.24105	25.56819	25.79577
1029	27.70017	27.66389	27.75399	27.91634	27.87781	27.78825	27.95369
1030	22.57274	22.73534	22.10258	22.31415	22.67405	22.16217	23.92486
1031	26.89405	26.32861	26.02937	27.09183	26.57473	26.44808	27.00928
1032	23.19866	22.58506	22.09229	23.27261	22.01985	23.18526	23.63014
1033	23.69948	23.73144	23.44391	23.70122	23.57419	23.47328	23.88828
1034	25.16017	25.92003	25.30866	24.79075	24.83712	24.88225	25.45266
1035	20.59272	20.53949	19.74419	23.06993	21.74178	20.69247	21.54372
1036	27.19539	26.28724	26.16747	27.22425	26.162	26.36491	27.08771
1037	24.32133	23.61435	22.82839	24.58337	24.21369	23.93596	24.69633
1038	28.27949	27.64502	27.75441	28.38264	27.97349	27.814	28.52012
1039	25.55076	25.74018	25.68462	25.70527	25.67604	25.92259	25.49993
1040	24.10442	24.73962	22.16455	24.785	24.95424	25.22326	25.2707
1041	24.39459	23.90387	24.10083	25.20222	24.45259	23.92993	25.28224
1042	22.5955	22.49016	20.59714	22.11718	21.90044	23.02276	22.52607
1043	30.83205	30.36454	30.86702	30.89267	30.80298	30.9648	30.76712
1044	28.31885	27.08125	28.53029	30.09076	28.87888	28.74472	30.04821
1045	26.41663	26.43755	26.54521	26.76455	26.42079	26.80265	26.64248
1046	25.56948	25.0142	25.9253	25.53123	25.64427	25.96262	25.7335
1047	24.43441	23.6113	24.30088	24.42129	24.15898	23.97375	24.12528
1048	27.01682	27.21452	26.86522	26.64104	27.32515	27.13946	26.84582
1049	29.96817	29.07136	28.81822	30.12014	29.03501	28.60531	30.09895
1050	25.20719	24.34467	24.31997	24.93013	24.85395	24.8317	24.71708
1051	26.12444	25.61201	26.30067	26.61851	25.99094	26.27729	26.60876
1052	23.5103	21.58256	21.62867	23.15494	22.40113	22.2986	23.41694
1053	25.67769	25.60266	26.10071	25.78273	25.83827	26.17016	25.71105
1054	25.20753	22.98278	25.05433	25.28794	24.00277	23.38523	25.01696
1055	27.78814	26.90637	27.38026	27.57036	26.84907	27.30635	26.95114
1056	25.32081	23.26856	24.54439	26.02564	23.22727	23.57131	26.14176
1057	27.59184	26.77727	27.0078	27.62984	27.07177	27.07741	27.66597
1058	26.71975	26.72129	26.43705	27.67087	27.19529	26.8233	27.47606
1059	25.90179	26.23661	26.87753	24.22988	26.40534	26.55035	23.88535
1060	30.95383	31.37883	30.21182	31.48002	31.05212	29.8807	31.30731
1061	25.06597	23.67616	25.20959	25.1318	24.99158	24.68015	25.27589
1062	21.16574	21.21495	21.63773	22.32985	21.83092	22.84131	21.23244
1063	26.56257	25.96769	25.50858	26.70184	25.94868	25.82848	26.45649
1064	22.7491	21.37759	23.82637	23.78638	22.12512	22.94863	23.80252
1065	25.71148	24.9936	25.28028	26.63009	25.2622	25.64988	26.33031
1066	22.01005	21.53617	24.24767	23.21255	21.98194	24.87519	23.05104
1067	26.94361	26.85563	26.63049	27.11225	27.04055	26.90145	26.95702
1068	23.7085	22.04786	23.48936	24.15782	23.62016	23.49386	24.29037
1069	27.16619	26.34685	26.77632	27.74003	26.85073	26.85202	27.67327
1070	23.62653	25.00365	24.30542	25.43531	23.65341	24.80172	25.60286
1071	29.74481	29.17296	29.70571	29.86928	29.47639	29.67312	29.93819
1072	24.1215	23.88217	24.01125	24.86624	23.78215	23.66312	24.80326
1073	25.6543	25.40428	25.65892	25.51673	25.40032	25.57346	25.35632
1074	32.22298	32.02684	32.29124	32.30407	32.46815	32.46083	32.30113
1075	29.75611	28.79643	29.72602	30.59334	29.49743	31.06693	30.51825
1076	30.59798	31.72208	31.86665	30.36117	31.91451	32.08251	30.38224
1077	24.30919	24.11859	23.48174	24.99952	23.12349	23.11791	25.27855
1078	24.10509	23.89609	24.03335	24.68301	24.1485	24.38202	25.1032
1079	22.39168	24.57696	21.35751	22.90476	23.88242	21.51497	22.6139
1080	24.58544	24.77134	22.5469	25.77947	23.17906	25.26937	25.71181

	AD	AE	AF	AG	AH	AI	AJ
1081	26.46627	25.7115	25.5839	26.208	25.72052	24.25737	26.17386
1082	23.57488	22.99464	23.55484	24.2284	23.95951	24.26427	24.55824
1083	24.17486	24.25749	24.15732	24.67631	24.0963	24.60717	24.77071
1084	23.15657	20.53332	19.73454	23.31751	21.89542	21.18297	21.62628
1085	28.57933	27.62047	27.96367	29.31388	27.90625	27.9748	29.25256
1086	26.4182	26.41624	25.43381	26.79556	25.80239	27.21761	26.66875
1087	27.16256	26.54111	26.2141	27.9573	26.01142	26.11816	27.95273
1088	25.3738	25.10474	25.31674	25.77176	25.52832	25.54245	25.92128
1089	25.0283	24.44897	23.10815	25.1438	24.56322	23.56615	25.23682
1090	20.30188	19.52946	19.17612	21.53284	19.50365	21.34002	21.46695
1091	28.24547	27.88746	28.19391	28.59466	28.50609	28.19108	28.41799

	AK	AL	AM	AN	AO	AP	AQ	AR
1	TOP3 ORF2_PEP_i02_2	TOP3 ORF2_PEP_i02_3	TOP3 ORF2_PEP_i03_1	TOP3 ORF2_PEP_i03_2	TOP3 ORF2_PEP_i03_3	TOP3 WT_i01_1	TOP3 WT_i01_2	TOP3 WT_i01_3
2	24.12089	24.05561	25.09779	24.99455	24.25772	25.82214	25.24436	24.79498
3	26.34526	26.39027	27.51561	26.17306	26.46602	27.11242	27.21713	26.79257
4	23.97351	24.24438	24.15639	24.21695	23.05376	24.52866	24.58981	24.82173
5	23.09156	23.46214	24.39839	22.49561	22.19661	23.8498	23.15458	23.02081
6	25.49019	25.73644	26.4434	25.14497	25.28164	25.88914	25.84841	24.77592
7	26.09856	26.52253	26.65651	25.951	26.56609	26.9113	26.61772	25.99545
8	23.56036	23.68553	24.41824	25.27525	26.30813	24.04738	24.39741	24.44017
9	21.28725	21.53153	22.00886	20.84053	20.73095	22.70664	22.14872	21.88705
10	27.16291	27.6692	27.70146	27.0283	27.61512	27.8384	27.43163	26.68353
11	27.6963	27.49788	29.03971	27.76567	27.58513	28.88582	27.79206	28.04928
12	26.55362	26.51384	27.73874	26.56979	26.54191	27.46268	26.64713	26.83892
13	21.47089	20.85112	21.78333	20.60337	23.05274	21.15221	22.61168	22.98475
14	26.31983	26.85525	25.94816	26.29619	26.8082	26.32076	26.39777	25.94861
15	25.05533	25.10054	25.67185	25.13208	24.90667	26.93295	26.99797	26.31778
16	25.92121	26.01338	26.60608	25.2969	26.25501	26.65543	25.32869	24.78843
17	22.12342	22.15376	24.0661	22.14235	22.38191	24.61934	23.04028	21.82131
18	26.50174	26.5193	25.87105	26.49108	27.28969	26.18793	25.52388	25.50531
19	29.85773	30.08442	29.69639	29.76472	30.01279	30.06666	30.16454	29.40988
20	26.12874	26.57595	27.29734	26.16693	26.59165	27.06994	26.33826	26.7876
21	24.46997	24.97189	26.02789	24.66677	25.34572	25.70499	24.6212	26.00382
22	26.69548	26.82558	26.30247	26.45798	26.84156	26.37669	26.9743	26.62157
23	23.87077	23.70884	24.842	23.92038	23.04034	24.88808	24.16076	24.39657
24	22.52613	23.31786	24.40875	23.18876	22.6006	24.74205	24.05796	23.81949
25	24.27155	23.3713	25.23017	24.90426	23.90971	25.52733	25.05979	24.26803
26	24.30477	22.8094	23.24214	24.4693	22.21783	22.92657	23.4684	23.4931
27	23.37757	23.44849	23.9169	22.52881	22.13707	23.72203	22.69889	23.87645
28	23.07642	22.90877	24.47522	23.11662	22.74506	24.43012	23.6334	23.56723
29	24.89245	25.09378	25.52428	24.85138	25.21347	25.72727	25.13392	25.07888
30	24.95362	25.9966	25.38283	24.60672	25.43971	25.80676	25.97318	24.82606
31	24.28375	24.21736	24.86073	24.42979	24.57008	25.53396	24.90931	25.07474
32	25.20156	25.33882	21.28523	25.43995	25.25116	23.76521	25.18188	26.33286
33	25.48822	25.37989	26.74783	25.67001	25.38336	26.84753	25.46858	26.33689
34	27.08866	26.48952	28.13303	26.79777	27.13951	28.22651	27.15408	26.64698
35	26.52153	26.08569	26.08295	26.35424	26.13284	26.75097	26.47898	26.11173
36	26.07432	25.63944	26.48917	25.99398	25.51858	27.0795	26.28676	26.98676
37	26.25293	27.39534	26.66171	26.05443	26.8603	26.98656	26.95398	26.05005
38	21.96565	23.81502	22.33778	23.68437	22.22279	23.8593	22.24785	23.4529
39	23.27579	22.34933	24.84595	23.1877	22.8998	23.87872	22.44772	22.68303
40	28.16425	28.37331	28.60338	28.10389	28.34202	28.40616	28.35805	28.68692
41	30.72097	31.73578	30.89215	30.66548	31.97584	31.0449	30.88158	29.52951
42	26.76242	27.09877	28.07942	26.89699	27.16528	29.13908	27.49212	28.39677
43	24.046	22.85563	25.0695	22.99853	23.45322	26.04067	24.11768	25.00611
44	21.46158	21.60126	21.55618	23.40805	21.80352	26.01351	22.46155	23.27389
45	25.9986	25.32948	27.06728	25.72131	25.31254	26.70972	26.11535	26.44692
46	24.48349	24.5899	25.33172	24.14795	24.8361	25.27192	25.32814	22.98892
47	23.51683	21.52407	24.05941	21.23481	21.30123	20.30896	20.65795	20.88934
48	31.62936	31.79624	31.59247	31.63462	31.72363	31.70348	31.81016	31.54147
49	24.73057	24.96802	23.2619	24.65445	25.27021	23.03262	25.06398	23.3125
50	34.67602	34.4287	34.47807	34.65899	34.24009	34.91604	35.13262	35.02898
51	28.00053	27.93753	28.2598	27.98129	27.80864	28.43231	28.23034	26.92225
52	26.58413	25.2215	26.69169	26.00249	25.28443	25.95039	26.12312	25.53359
53	27.62788	27.61816	27.12757	27.72955	27.75418	27.20321	27.49663	27.76946
54	30.50922	31.07767	30.26769	30.24977	30.86336	30.83835	30.61565	30.61325

	AK	AL	AM	AN	AO	AP	AQ	AR
55	22.96246	21.49083	22.92986	22.86285	24.99888	23.06839	22.7113	21.10487
56	23.81142	22.03727	23.21712	24.2894	23.18576	24.10536	22.91103	22.77321
57	21.68057	21.89582	22.62613	20.80685	22.33183	21.14606	20.51308	19.65751
58	28.34735	28.26419	27.70257	28.3074	28.21497	28.25372	28.54882	28.14905
59	28.68657	28.42466	29.08349	28.55762	28.36334	29.34214	28.87745	28.50659
60	26.721	26.66446	27.64355	26.66593	26.51614	27.63715	26.69511	26.75171
61	22.62375	23.57393	25.22598	23.02968	23.56974	25.78582	23.40872	22.68495
62	24.61934	24.99617	25.51536	24.02279	24.86075	24.9477	24.62436	25.39945
63	26.88684	26.25365	27.45706	25.88823	26.14852	28.02869	26.28824	26.43305
64	25.59614	26.68905	25.90705	25.22507	26.71138	25.67057	26.18735	25.91568
65	25.87726	25.37804	26.41919	25.74896	25.62029	23.98538	23.10953	22.76893
66	24.09053	24.04625	25.41353	24.43933	24.56572	25.81367	24.66442	24.97316
67	23.36848	24.57532	25.18571	23.60486	24.48452	22.32589	23.11196	22.26373
68	21.80415	23.11309	22.7459	22.07979	21.8966	20.94349	20.20562	20.6989
69	22.8361	21.41573	23.08632	22.32262	22.4467	21.14638	21.89043	21.44257
70	21.8291	20.96482	23.14955	22.31085	22.32533	23.87423	21.41259	22.51098
71	24.63991	24.5411	25.43501	24.48213	24.6746	25.83433	24.29913	25.00998
72	24.95912	24.58736	26.4535	25.03867	24.12412	26.42187	25.22141	25.30142
73	23.3996	21.85608	24.22629	23.38045	23.80628	24.40088	24.17964	24.40171
74	23.71424	26.18507	24.85196	24.18606	26.20256	24.84776	23.18152	25.36777
75	27.52745	28.32477	28.27851	27.28033	28.37345	28.46299	28.03914	26.90398
76	23.58781	25.14064	24.41975	23.64152	25.31001	25.1667	24.28472	23.5359
77	26.12353	25.69489	26.93713	25.88695	25.56144	27.34875	26.49912	26.31981
78	30.23672	30.47334	29.82247	29.84254	30.58172	30.67138	30.32915	30.12893
79	27.13668	27.58812	28.04298	26.87039	27.39431	27.78116	27.56655	27.36078
80	23.86748	26.87324	25.14239	24.73048	27.65761	26.69407	25.45579	26.14613
81	24.12811	24.5632	25.23881	24.33294	24.88386	25.39746	24.94781	24.18132
82	25.06785	25.58776	25.83053	25.07898	25.38618	25.83142	24.58065	24.43569
83	22.73272	28.73644	25.95625	29.29055	29.08939	26.0711	26.23716	29.04583
84	24.90502	24.19662	24.9724	22.94879	22.57521	25.06548	23.33914	24.49115
85	25.96943	26.02636	27.16609	26.39826	26.1891	26.88092	25.9612	26.36181
86	23.37624	24.07752	22.8339	22.22933	23.51082	21.91349	22.09277	22.62031
87	29.03256	30.06591	30.10549	29.08442	30.15786	29.2667	29.24566	27.34513
88	28.11237	28.33587	30.53127	28.37145	28.17172	30.35993	28.25973	27.97381
89	29.3126	29.569	30.5517	29.37038	29.54066	30.30712	29.77201	29.01018
90	30.61141	31.29363	30.90907	30.53896	31.19088	30.41007	30.98215	30.03952
91	30.03872	29.69377	31.55472	29.94384	29.53265	31.02767	29.84266	30.03431
92	28.98725	29.15623	30.09523	29.01732	29.00257	29.78273	28.90658	27.94051
93	26.82522	27.90866	29.03664	26.72704	27.82586	27.49243	27.09052	27.48032
94	28.93827	29.25978	30.77536	28.99647	29.30768	29.887	29.01912	29.12589
95	27.73564	27.75298	29.78662	27.76739	27.87559	29.2347	27.7821	27.51447
96	32.39794	32.41342	32.02409	32.33544	32.26832	31.68916	32.52292	32.04202
97	28.37233	28.97683	29.19733	28.55811	28.87853	28.27361	28.84247	28.02439
98	28.76615	29.18003	29.22104	28.83636	29.2673	29.27526	28.66666	27.56095
99	29.76909	30.18439	31.10194	29.892	30.0955	31.0185	30.29624	29.52313
100	30.51862	30.73284	31.26721	30.54347	30.71215	30.99362	30.86889	30.49355
101	28.14146	27.41941	29.29824	28.07626	27.46558	28.77004	27.8688	27.37299
102	29.60097	30.16374	30.54752	29.59838	30.07321	29.57306	29.86478	28.86236
103	29.9778	30.05908	31.11861	29.96529	29.94586	30.91756	29.82373	29.75523
104	30.44743	31.05127	31.79337	30.27809	30.93018	31.72629	30.79048	30.31499
105	30.17233	30.73822	31.11522	30.24646	30.61998	30.71407	30.32019	29.35033
106	28.44177	29.09149	29.84505	28.35398	28.98743	29.10718	29.18722	26.78585
107	30.16853	30.56194	30.82259	30.1049	30.48496	30.64602	30.25011	29.72001
108	24.95471	24.36415	25.81119	24.55167	23.79972	26.03604	25.05207	23.79251

	AK	AL	AM	AN	AO	AP	AQ	AR
109	29.9037	30.64321	30.29867	29.87303	30.62635	30.57481	30.44109	30.21614
110	27.72767	28.29317	27.93465	27.62611	28.30961	28.07425	27.9666	27.64651
111	29.92013	29.08043	30.49528	29.72303	29.11146	30.42754	29.61383	29.59751
112	27.92214	27.69692	29.4365	27.8338	27.63954	28.40553	27.82022	26.98874
113	27.92261	28.41113	29.15138	27.67484	28.37035	28.45604	27.90172	27.03113
114	28.60015	28.86507	29.5187	28.68025	28.93378	30.05518	28.85036	28.54395
115	29.64342	30.00549	30.40405	29.60023	29.95345	29.95638	29.63807	29.06858
116	25.02859	26.35071	25.99209	24.9815	26.29283	26.2189	25.86544	25.51383
117	27.33398	27.4459	27.74377	27.19893	27.4146	28.19245	27.17562	27.28914
118	25.36376	25.79873	26.41767	25.48877	25.65456	26.26623	25.63943	25.33261
119	22.73605	22.93087	22.4956	22.3008	23.18678	23.04281	24.12265	23.33128
120	26.16161	26.51738	27.27185	26.26181	26.20488	27.7159	26.17008	27.03986
121	24.06353	24.76126	25.69685	24.0618	23.67892	25.28677	24.30329	23.93678
122	22.45456	23.72307	24.67612	23.05124	21.69401	24.64745	24.06794	22.56985
123	24.33068	22.61157	24.7642	24.20874	23.15206	24.83555	23.03082	22.57922
124	25.24735	24.83296	26.01944	25.16184	24.90878	26.2888	25.74637	26.25845
125	27.08886	27.18188	28.2069	26.99814	27.25522	28.0388	27.03469	27.24093
126	24.32799	23.34053	24.03406	21.62096	22.43601	24.1697	23.80355	23.10384
127	26.58159	26.61558	28.22012	26.57493	26.62076	28.28128	27.13715	26.40819
128	26.04499	25.4925	25.54316	25.28466	25.42163	25.44114	25.90369	25.13122
129	28.35143	28.11082	29.85313	28.26583	28.29608	28.51551	28.17509	28.29274
130	26.52771	28.52105	29.12106	26.76267	28.4844	28.45343	27.32998	28.08069
131	30.79669	30.61873	31.73647	30.67955	30.60537	31.65342	30.75447	30.40867
132	25.13233	25.49419	25.82565	25.17021	24.82366	25.74018	25.53473	26.03039
133	24.86587	25.90814	24.66996	24.72312	25.8959	24.94207	25.50187	24.93406
134	28.8341	28.05784	29.35948	28.74863	28.02813	29.82785	28.63275	28.70013
135	22.8789	24.46429	24.1944	22.83852	22.75457	24.7912	24.31164	23.59372
136	26.82006	27.66787	28.65811	27.05048	27.20449	28.39039	27.3156	27.39753
137	28.09606	28.23634	29.22045	27.97949	28.12993	29.24667	28.01651	28.34219
138	24.45084	24.39336	25.27201	23.91555	24.36676	25.71312	24.45616	24.11613
139	29.31474	29.14014	30.33618	29.11729	29.16936	30.26259	29.23922	29.09181
140	26.94178	27.31112	27.84042	27.42554	27.54916	28.0261	27.71427	28.07465
141	26.34716	26.32156	27.07554	26.63399	26.34647	27.31859	26.58913	26.42937
142	23.51319	23.70314	25.29843	23.0767	24.18561	25.9068	24.65486	24.21507
143	26.69397	27.47902	26.99134	26.67284	27.06458	27.15124	27.09922	26.09386
144	24.47256	24.5539	25.03945	24.02736	25.00045	25.15284	25.18532	22.72236
145	27.5509	27.47336	28.40442	27.38953	27.13743	28.54354	27.91019	27.68163
146	22.12404	22.34567	24.28576	23.68559	23.07132	24.66041	24.32052	23.21903
147	27.54047	28.21531	29.7096	27.41878	28.11482	28.62266	27.2358	27.15746
148	28.21518	28.6947	30.32096	28.04601	28.77173	29.73447	28.1463	27.50655
149	23.83315	22.91962	24.30771	24.24894	23.28365	24.58305	23.10739	23.84828
150	25.05261	24.96683	26.13453	24.51309	25.1709	26.29624	25.17793	25.10495
151	24.78122	24.67551	23.97486	24.66787	25.00562	25.61328	24.40036	24.90671
152	23.66632	24.36536	25.43783	23.49413	23.9127	25.31814	23.21418	23.92803
153	22.70529	22.13356	23.5364	22.67604	22.17221	25.06436	22.50849	23.87121
154	24.49137	26.53298	25.42411	24.56741	26.38347	25.18301	24.67091	24.74245
155	22.23384	22.57802	22.72948	22.28413	21.86156	23.39336	22.19787	21.9621
156	25.81125	25.83693	26.62613	25.80713	25.97223	26.63172	26.41415	25.36623
157	25.30725	26.49497	25.7186	25.66507	26.54949	26.45947	25.95425	26.09759
158	25.19731	24.56833	26.39802	24.95111	24.22078	26.47364	24.41083	25.337
159	25.72359	25.91261	26.95775	25.21246	25.93613	26.92044	26.27845	25.79471
160	26.87288	27.14614	27.39275	26.88455	27.04528	27.54326	26.96064	27.52059
161	28.16362	28.2144	27.42672	28.10285	28.11531	28.43411	27.9762	27.65257
162	22.48657	24.07429	24.8132	22.5487	24.10887	24.25397	24.17535	22.23592

	AK	AL	AM	AN	AO	AP	AQ	AR
163	27.14381	27.43416	26.72415	27.02593	27.38286	26.87517	27.17293	27.00418
164	31.09277	31.33423	31.63551	31.07547	31.46448	31.52601	31.13354	31.78135
165	26.32457	25.97904	26.8354	26.19056	25.97392	26.87988	26.39727	26.16783
166	28.6684	29.91243	28.15685	28.7853	29.9295	28.55327	29.70263	29.88337
167	24.52217	24.40228	25.63058	24.07618	23.97179	25.73306	24.3623	24.29923
168	26.47774	26.50631	27.13956	26.50859	26.34371	27.30636	26.8497	27.05075
169	29.81284	28.73764	30.43286	29.69915	28.79594	30.52043	29.58706	29.16352
170	21.98486	20.63217	21.2779	21.70697	21.88608	23.48048	21.03721	21.57189
171	27.53254	27.47367	26.53695	27.40749	27.41909	27.21951	27.67838	27.50079
172	27.88813	28.8434	27.69399	27.81894	28.94885	27.73744	28.22336	28.6156
173	23.31786	23.39711	24.32695	22.675	23.02675	23.98631	23.09901	23.10522
174	24.23121	23.44157	25.37836	24.16713	24.23926	24.90792	24.13316	24.38638
175	22.80779	22.8473	23.15009	21.86966	23.68944	24.10395	23.46004	22.49626
176	25.52838	25.33768	25.07114	25.05116	25.47404	25.76578	25.48781	22.90418
177	31.06548	31.90251	31.13013	30.97399	31.73829	31.56824	31.48259	31.14266
178	23.78347	23.21307	25.59576	23.33192	24.45244	25.38902	23.81303	23.8635
179	23.64375	23.33515	26.06351	23.63117	22.9482	26.59825	24.17972	23.83488
180	26.91018	27.37302	27.52449	26.85486	27.25024	27.67468	27.19407	27.57557
181	21.81365	21.66266	23.83124	22.05348	22.14024	23.2788	21.59197	21.69312
182	26.03981	26.21239	26.8828	25.66262	25.68818	27.17656	26.32013	25.71658
183	23.0163	22.06947	23.01594	22.23857	22.61506	22.73435	22.55413	23.4261
184	27.29766	27.32499	28.05228	27.17421	27.25951	28.38626	27.5626	27.91139
185	26.53649	26.26671	27.71444	26.7261	26.32012	27.74515	26.80406	26.87392
186	36.01084	34.97435	35.32513	35.99073	34.98996	35.27406	34.97292	34.62227
187	28.74977	28.38601	28.67553	28.61918	28.38071	28.52352	27.97218	28.42938
188	24.20536	24.55562	25.54156	23.47499	23.80371	25.35987	24.45627	25.37256
189	25.10949	25.41176	26.11394	24.91529	25.40569	26.3172	25.1389	26.02575
190	24.43071	23.83849	26.38459	24.43536	24.38821	26.52607	25.06107	24.58689
191	31.66054	32.33164	32.26619	31.75278	32.44739	32.60361	32.39516	32.55557
192	27.25622	28.04455	27.85359	27.11766	27.70141	28.10457	27.57798	27.17528
193	26.01333	25.87498	26.92003	26.00171	25.88461	26.7747	26.33122	26.42781
194	26.72021	28.22978	28.30589	26.65295	28.14978	28.00748	27.37562	26.26821
195	28.6176	28.59533	30.06051	29.01004	28.80419	30.19275	29.2238	29.59406
196	26.92053	28.24092	27.11537	26.95824	28.70445	27.48891	27.67585	29.82221
197	25.69147	25.63862	27.04805	25.70309	25.81888	26.86955	26.04385	25.86065
198	23.19476	23.81433	24.48573	23.41295	23.88786	24.66847	23.3091	24.23573
199	23.71204	24.40581	26.02276	23.71612	24.34829	25.67912	24.47677	23.71048
200	24.43191	25.68963	26.42054	24.64937	26.33122	26.72663	25.29761	23.79926
201	27.48306	28.49064	28.03147	27.54441	28.50247	28.14384	28.14021	26.0715
202	30.66556	31.21932	31.08563	30.59691	31.27792	31.2629	30.87913	30.72923
203	25.03867	24.93095	25.70928	24.75616	25.10544	26.07829	25.17884	25.35897
204	27.65874	27.92802	28.41202	27.48312	27.06824	28.18799	27.23409	27.28435
205	26.87581	27.11284	28.17515	26.93324	27.08322	28.02829	27.10675	27.05623
206	28.07792	28.74874	27.69604	28.02658	28.65169	28.33451	28.61453	28.21007
207	24.20577	24.96666	25.29775	24.80375	24.59946	25.10636	23.55918	23.86357
208	27.65705	26.99105	29.13768	27.48424	26.99688	28.73457	27.65512	27.25887
209	27.61102	28.83163	28.15146	27.35224	28.94457	28.4617	27.97741	27.78088
210	27.57624	27.86287	28.05028	27.17954	27.85587	28.28018	27.57402	27.30051
211	26.70665	26.11706	27.01041	26.57006	26.06631	26.9977	26.33777	26.47693
212	21.93895	22.06295	24.29502	22.92882	22.09625	21.5873	21.20397	22.23955
213	29.55111	29.03205	30.37666	29.59478	28.88553	30.5219	29.68221	29.55965
214	26.74472	26.69607	27.83385	26.72571	26.6529	27.93184	26.68316	26.92569
215	29.40374	29.93154	29.22051	28.96942	29.98971	29.61156	29.30539	28.85778
216	25.35012	25.72408	26.29006	27.07846	26.05433	26.71074	25.37444	25.87893

	AK	AL	AM	AN	AO	AP	AQ	AR
217	25.47636	24.95593	26.52522	25.29642	25.25932	26.19449	25.34456	25.98377
218	24.26719	23.93004	24.27987	24.2034	23.8848	24.34509	24.33702	24.15671
219	28.19502	27.63926	29.10947	28.2161	27.74905	29.23137	28.17853	28.26382
220	22.01116	21.95977	28.36443	22.02097	21.2632	28.34904	22.39903	27.93231
221	24.14927	25.42389	24.36293	22.53646	25.37198	23.94772	21.90337	24.45679
222	25.00392	25.49672	25.06895	23.99764	25.26772	24.92174	24.49158	25.74793
223	29.02524	29.46709	29.01418	29.01997	29.53207	29.3614	29.36899	29.64707
224	26.15488	26.31448	26.97345	25.99294	26.07398	26.91209	25.87459	26.29094
225	31.0874	31.16851	31.92545	30.96788	31.37535	31.5248	30.79025	30.65149
226	24.5429	24.54342	25.07886	24.97959	24.68125	25.45122	25.87143	25.56434
227	26.55405	26.15906	27.58691	26.31754	26.53259	27.86913	26.64711	26.12713
228	26.63792	24.20792	24.93739	26.94715	25.30031	24.74889	24.04792	24.88197
229	25.0022	24.55951	25.89024	24.71333	24.04081	25.98675	24.68684	24.7583
230	24.2105	23.95695	25.16838	24.57616	24.27851	24.75638	24.22202	24.40821
231	24.19857	23.67678	24.88734	23.78707	24.19257	24.94245	23.66647	24.18885
232	30.39444	30.85137	30.01233	30.27739	30.92297	30.75476	30.70737	30.11507
233	30.82695	30.81136	30.8309	30.91854	30.85999	30.85582	30.97941	31.36359
234	27.30762	26.66973	27.5264	27.1958	26.67194	27.71809	27.27261	26.62738
235	24.93001	25.20584	26.1805	25.05754	25.33559	26.25382	25.5257	25.03398
236	29.39708	29.5995	29.78415	29.34243	29.71631	30.04274	29.88825	29.5191
237	23.54099	23.56035	25.32532	23.71246	24.29768	25.24965	24.04298	23.58797
238	19.73118	20.4189	22.34025	20.82141	19.6848	21.89212	20.33641	19.23272
239	24.90659	23.89107	25.51572	24.5659	23.92385	25.44359	24.93455	25.5018
240	22.15475	22.34913	22.83736	22.29144	22.42231	20.14095	20.6592	20.62835
241	27.99934	28.66755	28.10301	27.8672	28.5436	28.07302	28.46238	27.80876
242	27.87055	28.57096	27.50871	27.84359	28.62098	27.59909	27.11382	27.33668
243	26.16197	25.704	26.3833	26.24216	25.7919	26.19429	26.08629	25.4977
244	28.24976	28.32257	29.29217	28.87157	27.99127	29.82476	28.22317	28.45533
245	29.1397	29.60758	29.00805	29.09759	29.4805	29.38506	29.35851	29.28196
246	26.31511	26.33994	25.60804	25.99258	25.52693	26.16718	26.63258	26.36409
247	22.71688	23.09493	23.51586	22.0482	22.82183	24.08178	23.16305	22.86789
248	26.44209	26.80939	26.2577	26.61791	26.81558	26.98068	26.87503	26.82267
249	25.22683	25.76181	26.0776	24.89957	25.47499	25.4176	25.00491	23.70118
250	26.93271	26.83589	27.93987	26.89816	26.59478	27.98064	26.77369	26.81639
251	25.78422	25.97243	27.35579	25.67708	25.90388	27.69436	26.34197	26.35525
252	22.49588	22.20925	23.59849	23.12695	22.44889	22.90813	22.73541	22.68291
253	24.92263	26.4302	25.00393	25.08924	26.52663	25.25815	25.73519	25.29276
254	24.88267	25.37735	25.54051	24.96138	24.9253	25.53864	25.36676	24.95153
255	24.81157	24.50711	24.88191	24.95708	24.25133	24.92425	24.46679	24.3952
256	25.31706	24.6385	26.51174	24.83727	25.01096	26.51419	24.96793	25.42573
257	26.05289	26.00024	27.31947	26.28248	26.37636	27.41752	26.26097	26.65986
258	26.84983	26.84542	27.76705	26.97604	26.82799	27.516	27.04675	27.09039
259	22.55388	22.269	23.78533	22.43691	22.44327	24.07764	22.41158	22.75483
260	24.43098	23.52455	25.33157	24.21844	23.93282	25.60832	24.54848	23.97112
261	24.09718	25.4881	25.33394	24.68088	25.34935	25.58341	25.63915	25.43306
262	25.53176	25.54981	31.46357	25.88858	25.72462	31.90994	27.67544	28.94934
263	23.59741	23.82276	24.53629	24.68656	24.3525	25.21863	26.72377	24.02051
264	25.34775	25.55581	26.34539	25.1311	25.40903	26.08006	25.43414	25.67299
265	25.59245	25.87812	26.91607	25.83493	25.7926	27.03454	25.85683	26.73869
266	0	0	0	0	0	20.67339	22.89965	21.10062
267	30.91469	31.53106	31.25419	30.53033	31.56256	31.5253	30.98594	31.4449
268	23.03386	22.74861	23.70859	23.22151	22.65141	24.64284	23.12296	22.78217
269	25.10767	24.19132	24.16696	23.71028	22.54507	24.49836	24.07029	23.27279
270	26.19639	26.66644	27.20083	25.84094	26.71534	27.25236	26.3048	26.54854

	AK	AL	AM	AN	AO	AP	AQ	AR
271	25.38278	25.76023	26.86785	25.21105	25.33207	27.16804	25.79177	25.79683
272	24.13886	24.11147	24.27449	23.06119	23.29714	24.72219	23.67567	24.43341
273	24.62697	24.37447	25.23368	24.62482	24.4941	25.9056	24.74092	25.0647
274	24.34341	24.75172	24.35554	24.03922	25.30667	24.74512	24.18652	24.3885
275	26.73184	27.1079	27.87343	26.75355	26.88238	27.41367	27.22202	25.75054
276	29.52286	30.44674	29.8829	29.27941	30.20103	30.26441	29.93135	29.78557
277	25.96122	25.53383	26.76181	26.23427	25.71849	26.9297	26.00793	25.35365
278	24.73809	24.97559	26.93612	23.85264	24.92471	26.38904	24.43739	24.53295
279	30.88512	31.13426	31.0474	30.73361	31.07645	30.81219	30.90937	30.84404
280	29.08831	29.7194	30.58576	28.91743	29.61462	29.90474	29.37379	28.8877
281	24.71054	24.05631	25.85212	24.25022	24.78556	25.56169	24.48886	23.5909
282	27.35531	27.8641	27.50141	27.27661	27.91725	27.81317	27.85051	27.45989
283	22.69752	24.09897	24.22579	22.73791	24.23767	24.24174	23.90441	22.04114
284	23.97582	24.36805	24.68076	23.61308	24.38175	25.72133	24.94256	24.99576
285	23.98227	23.8926	23.93724	22.73808	23.19278	24.35423	23.47018	23.74191
286	28.19317	28.45665	29.148	28.06405	28.55061	29.55956	27.9208	28.39116
287	23.06558	24.06925	23.93162	23.51253	23.6904	24.49047	24.50521	23.542
288	23.36401	20.71323	23.1881	20.92015	22.12596	22.48569	23.20558	22.91875
289	23.81385	21.62992	24.32393	23.40327	22.16265	24.29791	22.44412	23.62276
290	26.22315	26.8015	26.73604	26.58304	26.93783	25.14883	25.52621	25.09362
291	24.81122	25.5472	25.03152	25.05828	21.86171	23.08823	23.33093	23.74808
292	21.85571	22.44673	22.04473	26.17926	22.18774	20.48133	22.32895	19.66428
293	24.89376	23.71808	24.84914	24.6221	23.81069	23.66518	23.56563	22.95223
294	27.03089	26.82569	25.52395	26.62709	26.7501	26.33752	27.02037	26.89801
295	24.25331	23.205	23.91119	24.48875	22.85555	23.95044	24.21682	22.60999
296	22.82804	22.45016	24.41827	22.57982	22.77823	24.49423	22.94222	23.21831
297	23.42981	23.5202	24.23552	23.95836	23.39131	24.36098	22.90428	23.53109
298	24.35359	23.69976	24.98612	23.74492	24.12784	25.46325	23.7676	23.76275
299	21.78414	23.23506	23.78437	23.73602	22.43089	22.81894	22.82393	22.31715
300	23.95778	23.64811	25.75611	24.65699	24.30763	25.51583	24.79057	24.39018
301	27.8149	30.21464	28.16874	27.7859	27.99983	28.72535	28.26091	28.25835
302	25.50459	25.6294	26.12479	24.01286	23.7979	26.34443	23.73639	25.92475
303	24.11554	24.29317	24.61612	24.22383	24.04506	22.73318	24.54739	23.40912
304	26.28973	26.1747	26.39347	26.19145	26.60792	27.1707	26.72236	27.25315
305	26.99654	26.26466	27.7985	26.97274	26.33477	27.76304	27.14603	26.95905
306	22.96374	23.14216	25.87994	23.23361	23.03448	25.54556	23.75461	24.41361
307	28.26397	28.51126	29.40025	28.359	28.34839	28.75728	27.98504	27.12297
308	21.70834	21.71397	22.86318	22.26935	21.84642	22.65722	22.42974	21.87723
309	24.88684	25.61291	25.53826	25.08643	25.11818	26.00429	25.43898	25.55218
310	22.86494	23.62272	24.83873	23.4944	23.88361	24.51964	23.4939	23.57486
311	22.03229	24.67382	24.41987	23.36141	22.37074	23.80299	24.52013	23.14046
312	25.64387	25.37463	26.93199	25.60304	25.26707	27.24298	24.69459	25.61206
313	27.2295	27.19095	28.32553	27.22148	26.9649	28.24202	27.32003	27.23849
314	25.86713	26.07211	26.59903	25.92598	25.88049	26.92165	26.8643	26.15848
315	22.62684	23.1911	24.94619	22.67813	22.97191	24.52668	23.46491	23.84134
316	23.74571	27.27272	26.00865	23.03774	27.26443	25.85452	22.623	23.31948
317	25.04834	24.61663	25.7912	24.96187	24.91711	25.80094	24.90861	24.94205
318	24.51307	25.25529	24.93605	22.22368	25.78612	24.53874	23.90661	23.93702
319	25.85937	26.17814	25.97767	25.42302	26.15778	25.94823	25.85322	25.13714
320	26.68298	26.99852	27.95544	26.62308	26.73125	27.82109	26.72092	27.20363
321	28.92309	28.54854	30.015	28.93903	28.55036	29.91818	29.02654	28.51694
322	25.96211	26.95331	25.89613	25.97409	26.91537	26.21059	25.8559	26.18649
323	26.47885	26.65356	26.50323	26.34414	26.51649	26.71014	26.71543	26.39466
324	25.612	25.88476	26.22441	25.02487	24.5531	26.06467	25.81603	24.58681

	AK	AL	AM	AN	AO	AP	AQ	AR
325	28.13965	27.80124	27.77498	28.01588	28.00464	27.56619	27.59557	25.87604
326	25.05449	24.85505	26.67568	24.77796	24.78266	26.38963	24.65844	22.69625
327	26.83858	26.63845	26.56398	26.64971	26.71052	26.34574	26.81162	24.17258
328	30.86687	31.65697	32.10612	30.43705	31.39221	32.10906	30.44074	31.28061
329	29.83554	30.72476	30.30774	29.75561	30.89253	30.73512	30.01493	29.99662
330	27.55321	27.8533	27.60131	27.15251	27.76301	28.01328	27.91551	27.79129
331	22.83026	24.39722	25.6846	22.53465	25.67935	25.95756	24.01871	25.97713
332	22.46501	23.78579	23.94048	22.8822	22.84289	23.37815	22.13723	22.34777
333	26.1448	26.2688	26.63573	25.91165	26.48838	26.69214	26.52286	26.63393
334	20.21396	21.65202	20.41171	19.98198	19.94492	22.50317	22.52181	22.12377
335	25.86588	25.34659	26.12388	25.22146	25.34692	26.45617	26.31172	26.42222
336	27.45532	27.55116	28.2124	27.39024	27.68892	27.96371	27.46405	26.8008
337	28.25972	29.38847	29.06548	28.18334	29.28286	29.46864	28.85628	27.64924
338	21.94638	23.79498	23.93205	21.03904	22.74351	24.06838	22.01743	22.44046
339	28.54015	28.96027	28.96427	28.49572	28.98055	28.49308	28.20195	28.49542
340	22.3351	23.92516	24.69019	22.87686	23.97917	22.23471	22.12164	23.50844
341	26.78365	26.23127	26.71219	26.96682	26.24989	26.16075	26.82476	25.74376
342	27.32454	26.83879	27.16274	27.04868	27.06373	26.99192	27.60759	25.86184
343	28.25217	29.07221	29.41925	28.27879	28.94655	29.891	29.84418	29.87984
344	25.10648	25.70943	26.18144	24.69287	25.69239	25.6042	25.39035	25.71552
345	27.47611	26.676	27.86909	27.23035	26.15296	27.10177	26.46279	26.83548
346	26.43054	26.84647	27.5129	26.47183	26.75298	27.59353	26.6456	27.20892
347	22.59765	21.32319	22.65695	22.2349	21.92704	22.70874	21.52175	21.93862
348	25.48301	25.46314	26.80384	25.60177	25.57632	26.39609	25.33069	25.9697
349	27.08057	27.48796	27.46666	27.14591	27.31947	27.5375	27.25988	27.00944
350	29.72898	30.73159	30.49438	29.62632	30.36718	30.98845	30.14582	29.72241
351	29.93109	31.07654	30.7961	30.02941	31.10806	31.01882	30.32235	29.73374
352	22.63239	23.2651	24.52937	23.73823	23.06338	24.40565	23.20128	23.3996
353	25.04423	24.52666	25.57053	25.54481	24.43813	25.5631	23.90004	26.10078
354	26.61366	26.81082	28.31003	26.99176	26.81714	28.56158	27.39474	27.70926
355	30.25255	31.27345	31.27298	30.18139	31.20892	31.11128	30.36138	29.15427
356	26.38643	26.54435	26.8925	26.80166	27.15292	25.95797	27.17189	26.43568
357	27.28104	27.76187	27.01687	27.31845	27.52755	27.1609	27.89485	27.0577
358	25.08056	24.55824	25.62511	24.70737	25.0484	25.00258	24.9941	23.565
359	24.3959	26.1149	25.94501	24.20113	26.30257	25.45858	24.86074	24.28374
360	23.70297	23.78426	24.04314	23.65744	23.86443	23.76446	23.21647	22.43723
361	27.93816	28.35118	27.71821	27.52575	28.24839	28.15209	28.37211	27.56484
362	28.38607	28.79918	30.03457	28.18217	28.65746	30.20282	28.4906	27.84574
363	27.00669	27.65364	27.02452	27.08901	27.45584	27.38432	27.59981	27.22621
364	22.71496	23.8199	24.11799	22.14993	23.74478	24.00684	24.02999	22.84781
365	23.78359	23.79641	25.02821	23.42458	23.56191	25.2664	24.16572	25.65984
366	26.60011	26.5843	27.5013	26.54739	26.68544	27.55824	26.78208	26.82539
367	24.27697	25.23516	24.29094	24.16473	24.96962	24.94552	25.03858	24.51507
368	24.34502	24.13216	24.97568	24.21584	23.43714	24.96975	22.90469	23.81867
369	26.51006	26.71476	26.5797	26.42687	26.6976	27.00664	27.00629	27.22498
370	24.89905	25.53334	26.13221	24.96855	25.32048	26.07246	25.21039	25.0046
371	23.52717	23.91699	23.10512	23.73529	23.64997	23.82381	23.70304	22.57133
372	22.58994	22.79814	24.2773	22.19049	21.98545	23.23212	21.90054	22.66155
373	18.00648	17.95799	19.16427	20.51669	18.81032	19.29069	19.10852	18.16639
374	28.11715	28.1299	29.29426	28.1848	27.74447	29.34368	28.15291	28.09657
375	25.20113	26.53371	25.73405	25.10351	25.9488	26.40559	26.24073	24.89105
376	22.26193	22.37965	24.17541	22.7075	23.32345	24.3052	22.57518	23.26729
377	23.78423	23.92661	25.73479	23.28762	23.61945	25.33719	23.27865	24.34636
378	24.80525	23.86408	23.31775	24.78981	23.80341	23.44018	25.19752	23.93472

	AK	AL	AM	AN	AO	AP	AQ	AR
379	26.60442	27.51145	27.27567	26.61436	26.83584	27.52787	27.39597	27.4064
380	25.48943	25.50505	25.60851	24.95179	25.53447	25.89413	25.59142	25.63684
381	25.20671	25.44497	26.75008	25.50977	25.64754	26.78247	25.74129	25.69167
382	27.26804	27.03429	28.04812	27.30413	26.67848	28.28845	26.94296	27.31731
383	25.39511	23.31772	25.19558	25.68713	23.71804	25.28168	23.46722	23.86021
384	25.45987	24.6268	26.87952	24.82807	25.07782	26.67409	25.56564	26.25771
385	25.51667	25.72711	26.66026	25.72096	25.35298	26.58062	25.86814	25.4828
386	27.12766	27.65576	27.92885	27.17339	27.7917	27.68777	27.47959	27.47723
387	26.01375	25.69073	26.87453	25.37191	25.30053	26.9887	25.64685	25.8007
388	26.87204	27.91871	28.07025	26.85877	26.73593	27.92728	26.80438	27.33625
389	27.53331	27.52839	27.63645	27.5553	28.13273	27.76481	26.99609	27.19188
390	23.45361	24.21601	24.57238	23.6079	23.36302	24.47604	23.29845	25.42977
391	29.36726	28.99039	30.62915	29.27941	29.17824	30.64787	29.15684	30.06397
392	27.24544	28.27297	27.21304	27.35735	28.19979	27.71538	27.79395	26.99933
393	27.75284	28.34255	28.07308	27.54754	28.22593	28.94058	28.19888	27.7583
394	25.51278	25.60691	25.97212	24.42237	25.65041	26.53537	26.10925	24.60294
395	24.94781	25.67275	26.6909	24.94339	25.3851	26.79251	25.29002	25.09237
396	30.02714	30.62063	29.63816	30.01205	30.64092	30.15278	30.35357	29.68489
397	25.46025	25.06104	26.44881	25.11247	25.16648	26.23926	24.74859	25.43046
398	29.58922	29.98354	29.31976	29.44611	30.1138	29.79457	29.98132	29.80503
399	23.29573	22.64543	23.1058	23.57577	21.37703	23.29645	22.48624	22.66139
400	24.27608	25.2182	25.56938	24.29114	25.06735	25.38996	24.08333	23.80611
401	21.88275	21.44316	23.49767	21.70605	22.19431	23.28883	21.78347	22.70597
402	21.37897	21.62964	22.01982	21.11516	20.9422	22.25077	20.88685	23.55624
403	27.64896	27.39386	28.91638	27.74384	27.34124	29.06271	27.59162	27.93826
404	24.98255	25.47585	24.67021	23.50298	25.07035	25.52394	24.68398	23.63999
405	21.57534	21.6175	24.94337	21.77751	22.20001	23.29111	22.36781	23.77246
406	22.55645	22.68426	23.11989	22.24074	21.69078	23.81786	22.68741	24.08885
407	24.05183	23.98929	23.45699	24.30814	24.42565	23.74913	24.13289	23.8789
408	25.28668	25.34135	26.32025	25.44113	25.14111	26.16867	25.10435	25.68428
409	26.45514	27.04957	26.74273	26.5127	27.11385	26.78464	26.85632	26.13645
410	27.27584	27.96072	27.72361	27.22073	28.08776	28.55766	28.03056	27.96325
411	26.41345	26.04158	28.58689	26.53223	25.98337	29.21536	26.60108	26.29601
412	25.31514	25.47163	26.95561	25.46119	25.42041	27.01957	25.55012	25.61823
413	27.67481	26.91038	28.72826	27.50909	26.9407	28.84097	27.80869	27.32432
414	27.77386	27.69777	28.59231	27.6343	27.55521	28.5633	28.08271	27.65617
415	22.73587	22.07166	23.99244	22.84271	22.07198	24.06051	22.57778	22.26257
416	22.1661	21.63334	23.61331	21.43144	21.79769	23.0346	23.35758	22.48632
417	26.78334	27.28975	26.64466	26.9059	27.37614	27.08783	27.15904	26.00101
418	25.35639	25.80957	25.8288	25.38009	25.57562	26.16751	25.97151	25.84651
419	22.77441	23.07861	24.32533	22.66396	22.9093	24.03935	22.71854	24.37624
420	26.21123	26.31658	27.01245	25.98205	26.1082	26.97528	26.24432	26.73325
421	25.79589	22.38964	25.18923	25.48285	25.24302	25.20809	26.02468	24.99422
422	26.14475	26.08682	27.11253	26.02347	25.97212	27.46476	26.55048	26.37817
423	26.84055	26.8083	27.87195	26.78429	26.70502	28.07291	27.27723	26.87795
424	27.03952	27.53577	27.93503	26.81986	27.4016	27.98939	27.39075	27.04521
425	27.07846	27.81905	28.54102	27.18561	27.47086	28.58371	27.45546	28.32728
426	24.53404	23.0317	25.17593	24.3096	23.81255	23.8345	22.58845	22.15051
427	22.78108	24.56923	24.34059	22.75845	24.58149	24.35268	23.82152	23.52051
428	23.88399	23.31996	24.67464	23.71558	23.21181	24.37419	23.56344	24.16828
429	24.37089	24.10999	23.1326	22.58077	24.304	22.83063	23.43349	22.62279
430	23.44302	24.52815	23.12125	23.32166	23.55573	24.45127	24.34919	23.0955
431	26.54445	26.40247	27.27125	26.41707	26.29003	27.11197	26.15343	26.58149
432	24.40424	23.52717	24.13025	24.76707	23.83391	24.41277	23.89362	22.69367

	AK	AL	AM	AN	AO	AP	AQ	AR
433	25.26668	23.16005	24.44763	25.66631	23.22019	24.54675	25.75497	23.42871
434	27.01346	27.65094	27.3014	26.91424	27.5663	27.3765	27.4386	26.61458
435	19.91674	22.78291	23.75212	21.1658	23.02388	24.17202	21.18114	22.85394
436	26.12475	26.17579	27.37044	25.99065	26.13777	27.75226	26.24202	26.48787
437	27.34047	27.66602	28.56304	26.51374	27.12515	28.02658	27.63045	26.79477
438	24.41121	25.30494	25.77792	24.4694	25.33801	26.18782	25.79591	25.18181
439	31.22218	31.78434	31.00018	31.20493	31.71647	31.44391	31.8079	31.50355
440	26.66087	26.53541	27.17734	26.47907	26.40884	27.15767	26.43948	26.33477
441	24.93567	24.41716	25.36398	23.64335	24.11151	25.21299	23.03861	23.03266
442	25.99388	26.10283	26.56613	26.02641	25.28402	26.70955	26.42268	26.19542
443	22.50312	22.5759	24.61171	22.4161	22.56303	25.02344	22.93052	22.78395
444	27.40896	27.15178	28.22344	27.51359	27.07261	28.19333	27.60049	27.79793
445	27.2402	27.7822	27.21957	26.94465	27.68176	27.74945	27.55361	27.24563
446	27.30905	27.91455	28.6275	27.22068	27.98387	28.51525	27.45681	27.18241
447	27.42647	26.1841	27.86893	26.10376	26.63173	27.9136	25.97539	25.9524
448	29.83779	30.07465	30.14098	29.82599	30.18241	30.66181	30.5343	30.02872
449	25.53015	25.21638	26.22084	25.28001	24.88729	26.22007	25.37839	25.49026
450	24.65711	24.31876	25.4237	24.28518	24.31466	25.08228	24.58117	24.3819
451	28.8261	28.71892	28.97686	28.85101	28.60129	29.10486	29.14391	28.63011
452	29.65225	30.13531	30.03694	29.67261	30.28012	29.68623	29.69646	28.89395
453	32.94196	33.52803	33.1784	32.93048	33.39682	33.14511	33.04047	32.92096
454	27.43137	27.72874	28.40124	27.5043	27.59397	28.57822	27.86999	27.68357
455	23.33125	23.6415	22.19721	22.07721	22.82568	23.18676	22.22964	22.9656
456	22.2521	22.00695	23.36542	22.18724	22.30359	24.62236	22.75527	22.53818
457	30.14664	30.74822	31.12197	30.06483	30.61491	30.56463	29.64444	29.55061
458	28.00176	28.20967	27.12523	27.86972	28.15625	27.48875	28.30319	28.65806
459	32.21764	32.56526	32.35492	32.09328	32.59959	32.35778	32.56179	32.3228
460	25.9492	24.85986	26.43674	26.1376	25.5734	26.61812	24.89288	24.69022
461	28.99645	29.64749	28.82252	28.83962	29.44087	29.46919	29.53202	29.91413
462	27.83157	28.53257	27.93715	28.05863	28.33569	28.70877	28.53312	29.46107
463	28.3742	29.01434	29.46436	28.97395	28.31619	29.78062	28.26385	27.28297
464	24.59127	24.00768	24.50969	22.96436	23.71671	24.90597	24.2576	29.39907
465	24.01703	25.35786	24.5064	24.55295	25.12051	24.66853	24.28577	30.45998
466	26.80334	27.32619	27.21002	26.91893	27.0309	27.4808	27.40632	26.81075
467	23.75896	23.22025	24.9034	22.94387	23.12553	24.7692	23.19115	23.68844
468	22.05026	23.33531	24.06829	21.79332	21.4201	25.754	22.7513	22.55886
469	24.35	24.24216	24.93966	24.29023	22.57177	24.97759	24.48321	24.69805
470	26.89654	26.7651	28.07005	26.70279	27.09145	27.96268	27.34216	27.77326
471	22.38684	22.09662	24.01824	22.9493	21.72313	22.34814	22.55926	20.40946
472	22.76551	23.19447	24.7624	22.48563	21.47903	23.56513	21.61296	22.70934
473	27.00475	26.91208	26.21333	27.13292	26.94384	27.57532	27.49641	27.21503
474	23.53885	23.37841	25.41877	24.72805	24.01458	25.72816	24.96758	24.52782
475	25.45619	27.09759	27.36983	25.79082	27.0009	27.72131	26.72577	25.6679
476	21.1765	21.72402	21.66974	21.13864	21.13394	22.84593	22.10782	21.93079
477	23.20872	24.8797	25.82339	24.39861	25.01441	25.11558	24.73371	22.89995
478	27.20764	26.67686	28.22003	27.27305	26.90314	27.56223	27.49711	26.8529
479	24.70699	24.49993	25.87053	24.1671	24.66287	24.7669	24.64887	24.47743
480	25.77121	25.27915	26.78489	26.289	25.66917	26.18529	25.39934	25.3347
481	25.54579	24.6853	25.8467	24.81165	24.79697	25.55673	25.15088	25.09871
482	22.36477	22.34653	23.09709	21.36372	21.52257	22.50977	21.23623	21.33405
483	21.25795	21.49175	22.81864	21.47705	21.52161	22.83957	20.95793	22.41494
484	26.97294	26.48072	27.09245	26.48782	26.35453	27.36915	27.01767	26.4231
485	23.67757	23.47641	25.55595	23.81151	23.18946	25.56861	24.47163	23.85985
486	26.95952	27.29896	27.95544	26.97679	27.36561	28.10931	27.62657	27.36398

	AK	AL	AM	AN	AO	AP	AQ	AR
487	26.10677	26.15463	26.73686	25.88696	26.11112	26.35167	26.5501	25.22963
488	24.35513	23.70051	25.01045	23.75906	23.05569	24.79043	24.02768	23.37395
489	24.4028	24.5615	25.98358	24.13471	24.89806	25.82343	25.13473	24.48818
490	23.46972	23.55697	25.10475	23.09427	23.19641	24.05735	24.21694	23.50998
491	24.34008	23.99026	25.42617	24.04798	23.71086	25.45229	24.2188	24.58096
492	25.71491	26.24772	27.36616	25.79624	26.57515	27.32892	25.85411	26.95086
493	23.29568	22.80651	24.11102	22.96605	22.78399	24.09252	23.9342	23.10619
494	24.77637	24.15775	25.27993	24.53005	25.15851	25.02145	24.34193	23.84884
495	23.17844	24.13236	23.89643	24.23712	24.14924	24.24253	23.55212	22.37998
496	26.72345	27.17352	26.54478	26.6811	27.01085	26.79648	27.05383	27.3689
497	25.52292	26.91245	25.3867	25.44787	26.85375	25.87192	25.91751	24.85831
498	26.42519	26.27627	27.43446	26.42493	26.42368	27.65849	26.45366	26.38615
499	29.4009	29.42103	29.35423	29.25609	29.31238	29.84346	29.12445	27.66894
500	28.29778	28.1081	29.31224	28.20457	28.00317	29.23888	28.34026	27.61546
501	23.30098	23.64498	25.73652	24.21115	24.58156	24.95595	22.87277	23.62992
502	25.44178	24.97202	25.86861	24.84833	24.89351	26.3811	25.74343	25.28277
503	22.84827	23.64786	23.28402	23.14113	23.88283	23.68886	23.40984	23.18514
504	22.87997	23.41401	24.2684	22.40137	23.56168	24.30959	23.6556	23.60809
505	22.18662	24.35023	24.18604	24.21045	23.71881	23.97943	24.97628	23.49297
506	26.37824	26.77982	27.90582	26.59221	26.7361	28.12473	26.66564	26.8389
507	20.47921	20.15991	21.56891	18.90523	19.82591	20.06141	20.13976	20.54461
508	27.02991	27.54751	27.07266	27.16133	27.54141	27.74189	27.4556	26.92915
509	21.91489	25.16401	21.93716	21.4679	21.84583	21.59972	22.82745	22.83381
510	25.40012	25.59049	25.71198	24.51466	25.39003	25.75481	25.99926	24.73849
511	24.50374	25.0782	24.95215	24.60509	24.67631	25.46692	25.5392	24.64588
512	30.97234	30.86681	32.1936	30.91188	30.86854	32.13089	31.09122	30.92596
513	25.25524	24.97374	26.98553	25.18349	24.55043	26.54011	26.17804	25.77262
514	25.94961	25.26424	26.56573	25.98119	25.51943	26.76629	26.29272	26.10538
515	23.74782	23.67215	25.06453	23.68296	23.17255	25.06391	23.54095	23.82122
516	23.58961	23.65337	25.51754	23.76786	23.22129	25.46181	24.00784	22.49931
517	22.95831	22.45344	24.45524	22.92931	22.319	23.84924	23.90733	22.92735
518	24.3957	24.45564	23.4407	24.60388	24.77248	22.52626	24.67118	23.73881
519	26.88358	25.61773	26.18186	26.45215	25.50337	26.5773	27.37903	25.56083
520	24.88678	24.6259	24.69938	24.50674	25.135	24.62045	24.69247	23.4759
521	25.65722	25.66508	26.55896	25.69815	25.76776	26.37903	25.80817	25.36356
522	22.66711	22.42366	24.14045	22.26955	23.82375	24.24437	22.86197	23.0395
523	26.11555	26.64929	26.3573	25.63269	26.791	26.39385	26.28226	25.80658
524	26.6618	26.48889	27.81752	26.51551	26.28109	28.02344	26.83705	27.01931
525	24.15991	25.0326	24.30304	24.28482	24.59352	24.62658	24.90495	25.36245
526	27.13401	26.78294	26.47888	26.74573	26.39094	26.7143	27.03465	27.35036
527	26.55302	26.55449	27.24549	26.62819	26.57016	27.63514	26.87976	26.87397
528	24.47004	24.71734	25.19019	24.76948	24.58747	25.62343	24.73407	25.70378
529	27.32225	27.20679	27.98702	27.03172	27.22796	28.15721	27.57269	27.54169
530	22.51046	23.48379	22.1716	22.79493	23.46433	23.62362	23.88516	23.91991
531	24.67194	24.71278	26.16078	25.66269	24.34455	26.57825	25.55574	24.90404
532	27.66888	28.14858	27.73965	27.60756	28.07068	28.26432	28.33171	28.03898
533	26.87264	26.94857	28.25569	27.11556	26.92629	28.01046	26.98685	27.13955
534	26.97446	27.86667	27.21542	27.12676	27.67064	27.21369	27.24201	27.18577
535	32.59954	32.60675	32.64483	32.59026	32.50901	32.73176	32.90194	32.20781
536	27.77733	28.30539	28.57363	27.60917	27.952	28.65217	27.47986	28.13589
537	26.94021	27.47408	26.5954	26.86965	27.25919	26.96849	27.11466	26.87963
538	24.85027	25.91611	25.54647	25.23428	25.84998	25.79021	25.82431	24.92173
539	22.34291	24.52448	24.43567	23.61982	23.13596	24.65279	24.55072	23.10935
540	25.93022	25.5614	26.00755	24.99689	25.38594	25.58291	25.61927	25.51596

	AK	AL	AM	AN	AO	AP	AQ	AR
541	23.71046	22.29556	22.53873	23.45437	23.78362	22.91335	22.72391	21.77317
542	25.0414	26.37574	25.63801	24.80825	26.20796	25.62352	25.92344	26.31294
543	25.6492	25.90364	25.80216	25.23101	24.77087	25.95859	25.43651	25.57721
544	28.26898	27.53995	28.19234	28.19886	27.45014	28.48769	28.46479	28.53653
545	23.23449	22.69888	23.04796	23.21387	22.70075	22.70315	23.58916	22.94201
546	24.93738	24.38812	26.07363	24.94143	24.76292	25.01857	24.78834	24.52397
547	22.52732	21.67943	23.14357	25.14447	23.71061	21.99469	21.47515	24.18573
548	22.13214	22.10027	22.94478	22.87087	21.46523	21.79997	21.7935	20.84881
549	24.35521	23.46361	25.1465	24.61249	23.26027	25.14169	23.62232	23.47695
550	27.90896	28.01904	27.24962	27.95222	28.01306	27.56928	27.88956	27.74054
551	23.25415	23.42456	24.43818	24.99069	23.47425	23.91346	22.80659	23.73224
552	22.58886	23.15014	24.72591	24.59933	22.25204	24.91757	24.58955	23.60964
553	31.66853	31.62718	32.1891	31.63619	31.45054	32.25012	31.78612	31.78837
554	24.86945	25.2404	24.76912	24.94479	25.02624	24.97379	24.79856	24.74861
555	23.30029	23.70252	24.29706	25.2446	22.91367	24.91155	24.23106	24.80062
556	26.60676	25.66174	27.08846	26.75087	25.48484	27.23471	26.34427	25.50182
557	23.90154	24.75432	26.45597	24.71823	24.14081	26.25135	23.98543	24.01007
558	21.8145	20.1651	21.89531	21.83318	21.98709	22.3731	21.61108	21.77395
559	24.20861	24.52864	25.35849	24.30716	24.42652	25.3031	24.91048	24.34372
560	34.73465	34.83368	34.57277	35.13995	34.82471	35.33647	35.6482	35.16294
561	24.85514	25.40607	25.94238	25.95635	25.54946	25.76724	24.94991	25.20681
562	24.07979	22.52498	24.86586	24.14935	23.7146	25.19215	24.32574	24.42055
563	24.06085	24.46854	26.03239	24.06745	24.03524	25.45263	24.32737	23.75789
564	30.6413	30.66386	31.03166	30.64276	30.91496	30.58093	30.69007	30.12605
565	25.83408	27.83679	27.33488	25.94359	27.53719	27.36586	26.80633	27.13904
566	29.21896	30.01238	29.941	29.26912	29.94976	29.73409	29.65429	28.97194
567	28.77467	29.49507	28.76575	28.88034	29.36713	29.0496	29.22752	29.08154
568	25.80429	25.80252	27.02705	26.28554	25.73888	27.36378	25.40082	25.58455
569	22.02471	22.33488	22.99369	23.31328	22.06318	21.88342	21.64794	21.99336
570	22.62577	22.69229	24.29637	21.98005	22.22056	25.04429	23.46904	22.79136
571	27.29248	27.24181	28.24041	27.29655	27.03308	28.42606	27.33574	27.34081
572	23.9994	22.76414	25.17253	22.72968	22.87365	25.88119	23.68177	23.62204
573	26.73044	27.70587	27.96541	26.51536	27.44785	27.76745	26.91602	25.39999
574	23.8183	24.6035	24.36452	23.30878	24.46334	24.39342	23.72551	25.01831
575	25.91112	25.38827	25.83294	25.91446	25.3178	26.01687	25.83329	25.82315
576	23.80501	25.767	25.76353	24.56255	24.16121	25.53601	24.22905	24.83766
577	29.01435	29.34251	28.80077	28.91924	29.12366	29.2184	29.30759	29.09814
578	23.49422	23.99446	25.53927	23.4485	24.03872	25.53188	23.70207	24.64164
579	25.53997	24.59488	26.09036	25.13827	24.56847	26.05747	25.53116	25.36735
580	24.22973	24.45809	24.28587	24.25069	24.53713	24.83617	24.57337	24.59642
581	26.23402	25.13869	26.16587	25.30782	25.18119	25.96651	25.05288	24.61514
582	22.64325	22.65741	23.4519	24.29731	22.66789	24.12575	22.93716	22.9886
583	19.86866	19.93057	22.26347	20.77837	18.796	23.5598	22.46811	21.94662
584	26.02746	26.85017	26.46688	25.94927	26.59509	25.88323	26.37246	26.11991
585	26.83018	27.17023	27.75981	26.94989	26.96088	27.64425	26.92601	27.15855
586	23.36604	22.89159	24.36091	23.62019	22.78752	24.59562	23.18521	23.62394
587	23.6077	23.46088	24.25767	23.58392	23.71384	23.58686	23.40417	23.48423
588	25.41086	25.23747	26.00935	24.90742	25.23493	25.77749	25.57829	25.74731
589	27.13899	26.95029	28.23803	27.19143	26.79567	28.3073	26.91465	27.60752
590	24.30964	23.78748	24.86124	24.39403	22.88617	24.96397	24.70539	24.9291
591	24.48272	23.86312	25.73598	23.47982	23.92371	25.76152	23.79271	24.52703
592	28.13953	28.02469	28.06189	28.06157	27.74848	28.29436	28.41371	28.97141
593	23.41173	23.71458	24.50639	23.35924	23.46566	24.59446	23.375	23.74995
594	26.37836	26.5438	27.64483	26.59643	26.70353	27.45028	26.36819	26.23045

	AK	AL	AM	AN	AO	AP	AQ	AR
595	29.60929	29.69265	30.44032	29.62034	29.71395	30.62867	29.88233	29.65774
596	25.0883	24.31306	25.69904	25.37487	24.97075	24.82928	24.8085	24.54333
597	24.5529	24.11245	24.66891	23.38434	24.81034	24.80713	25.54115	24.41327
598	19.78191	20.75983	23.21077	19.51542	19.46127	23.6874	20.72083	22.68019
599	27.45327	27.98436	27.27797	27.43269	27.99291	27.95599	27.55069	28.01721
600	28.90571	29.45645	29.22778	28.93807	29.43092	29.54569	29.46082	29.62339
601	25.9487	26.18943	26.6137	25.98197	26.18941	26.68184	26.15966	25.61492
602	26.73594	26.43766	27.12027	26.63428	26.26103	27.12836	26.40842	26.76504
603	28.26807	27.73866	28.81841	28.30015	27.75189	28.8984	27.98881	27.85096
604	25.15292	24.92741	26.02563	24.76596	22.85428	26.43038	25.32337	25.20349
605	28.12751	28.74816	28.62716	28.12008	28.42333	28.80247	28.59386	28.88182
606	20.81631	21.7115	20.58446	22.61312	21.08051	24.40757	21.41545	22.9936
607	26.89653	26.60278	27.32033	26.99674	26.67958	27.72271	27.26348	26.85113
608	23.52071	24.73739	24.22152	24.60397	24.11501	23.1756	23.85401	24.07567
609	26.20635	27.40082	26.6445	26.74418	26.70028	26.83599	27.05831	26.43438
610	26.8482	27.51325	26.48453	27.19952	27.30692	27.22715	27.23651	27.49258
611	26.01588	26.36691	26.07622	25.54418	26.31786	26.71822	25.98696	25.70225
612	24.44351	22.4162	25.36519	24.16187	23.73316	25.51487	23.85914	22.43525
613	25.08119	25.63081	25.24075	25.24856	25.13601	25.41959	25.51411	25.79342
614	25.02861	25.22138	26.52834	25.22317	25.29408	26.198	25.50059	25.68512
615	23.28746	23.57672	25.22555	23.81643	23.17882	24.80762	24.46219	23.52271
616	22.31996	22.41747	23.11907	22.634	21.95043	21.87539	21.51434	21.50373
617	28.14936	29.04848	29.31351	28.06966	29.30877	29.30403	28.80964	28.67669
618	21.84814	22.89699	24.95834	22.17013	22.58615	24.47637	24.05653	22.01759
619	26.01396	26.56791	27.25004	25.87228	26.71209	26.87827	25.41528	25.86176
620	25.4669	24.95856	26.85597	25.28546	25.32853	27.22588	25.84461	25.59726
621	23.72255	24.02792	25.33253	24.08407	24.72157	25.5099	24.24819	22.74096
622	26.14942	25.79276	26.14962	25.76947	25.48457	26.94505	25.65652	27.00565
623	25.00388	24.93495	24.16715	23.83163	23.85048	23.92803	24.32139	24.25671
624	22.27019	22.59206	25.05446	22.64194	23.63534	25.03771	22.62436	22.84771
625	24.4545	24.06285	26.04976	24.23022	24.07993	25.87762	24.73603	24.30158
626	26.4333	26.96049	27.12338	26.29877	26.95986	27.2272	26.90885	26.60274
627	22.70404	22.99081	24.08785	23.19486	23.2615	23.47943	23.40491	22.7136
628	28.08669	28.49324	28.15125	28.03022	28.14631	28.14979	27.87593	27.48342
629	22.56369	22.20108	23.68645	22.34458	23.13737	21.96576	21.81648	21.71807
630	28.66134	29.19377	28.95198	28.30064	29.10409	29.23698	28.82261	28.98954
631	28.34181	29.38529	30.16088	28.37626	29.15034	29.87841	28.84612	29.01654
632	25.72748	25.52657	28.5183	24.81485	25.59341	28.35348	26.3869	25.79969
633	26.39143	26.97778	27.54705	26.37792	26.75177	26.97138	26.30127	25.75696
634	24.40061	24.22037	23.75801	24.17519	23.86672	25.02743	24.02965	23.16583
635	26.30626	26.5872	25.89327	26.29107	26.996	26.44395	26.71016	27.03554
636	22.45124	23.11291	25.62748	23.92535	24.0099	25.92197	24.67072	23.71915
637	23.89988	23.64907	22.75336	22.63395	23.43845	22.45263	22.32897	23.02654
638	27.85561	28.0036	28.01139	27.61859	28.02242	28.42014	28.18787	28.87047
639	23.52326	23.5207	24.8154	23.57952	23.57547	24.92941	24.03572	23.61661
640	24.5083	24.93492	24.90605	24.34531	24.74293	25.29001	24.7141	24.78293
641	25.23688	25.17156	26.02226	25.04479	25.01333	26.47299	25.60385	25.22882
642	30.45403	30.72439	30.53541	30.44008	30.77145	30.70107	30.8028	30.43998
643	26.15225	27.91418	27.66938	26.39818	27.71274	27.65524	26.90555	25.78359
644	25.33359	25.12968	26.83286	25.83441	25.51632	27.17111	25.96603	25.64672
645	22.16622	22.2482	23.53618	22.41994	24.23632	24.29908	22.61525	23.16913
646	22.43411	21.58813	23.79674	21.49826	21.21617	22.25619	22.61112	22.69729
647	22.29654	22.71812	23.05038	21.93349	23.16934	23.24505	21.96577	24.06366
648	26.69605	23.00409	24.83657	23.63664	23.29906	28.69623	27.78839	24.17552

	AK	AL	AM	AN	AO	AP	AQ	AR
649	28.65049	28.35312	28.68841	28.71835	28.80834	28.90343	28.78322	28.93703
650	23.4309	23.77972	24.89914	22.93611	23.90532	24.85382	24.10596	23.33461
651	25.74872	25.58346	26.96112	25.80715	25.4176	26.88068	25.75311	25.77497
652	25.25731	23.74496	25.51091	24.87596	24.08292	24.67139	24.86837	24.98137
653	21.9203	22.30514	26.9215	21.96234	22.18227	24.42138	22.57433	22.63358
654	22.83743	23.71964	25.23738	22.46809	22.85897	24.99892	23.55322	23.37608
655	25.21506	24.98359	26.11054	24.18237	25.02102	26.00733	25.61907	24.00181
656	23.79772	23.87445	24.00242	23.69724	23.63378	24.71972	24.72444	24.01713
657	23.67851	22.73859	23.73895	23.03755	22.5403	22.93501	22.14634	22.68554
658	25.0389	25.26515	26.28485	25.54198	24.98842	26.17402	25.50938	25.58717
659	22.61507	22.01653	23.65664	22.1147	22.45495	23.26224	22.11617	22.1814
660	26.52096	26.57692	27.7194	26.18349	26.11884	27.61129	26.51495	27.09784
661	23.69039	24.12325	24.66246	23.82142	23.9894	24.5521	24.83457	22.2604
662	26.18692	26.29608	26.00063	26.10705	26.72513	26.3529	24.80314	25.37674
663	26.92213	27.1339	26.32393	26.82106	27.23756	27.14817	27.50761	26.71702
664	24.12674	24.01202	25.06437	25.38547	24.30861	25.3515	24.67654	24.53357
665	25.92493	25.50856	26.90292	25.81098	25.96631	26.7755	26.44024	26.74571
666	30.45646	30.21028	31.36627	30.57416	30.29097	31.5238	30.46073	30.63809
667	29.13133	28.71561	29.96731	28.67786	28.49318	30.03697	28.9974	28.65585
668	26.61755	26.37839	27.48919	26.77939	26.34274	27.87968	26.64249	26.92595
669	25.15898	24.44855	26.09011	25.31087	24.692	26.41301	24.61183	25.49264
670	21.56598	20.0049	22.87082	20.38102	20.07352	23.51008	22.60845	22.57658
671	24.09017	25.89027	24.53326	23.58669	25.19115	24.9023	22.28497	24.19248
672	24.96388	25.01501	26.16042	24.95513	25.00221	25.62702	25.79279	24.8916
673	21.93147	22.79535	26.19442	24.82022	22.71235	24.073	22.81137	25.38435
674	25.01661	23.6084	24.8593	24.8189	23.71439	24.55158	25.22512	23.75951
675	24.82317	25.15001	26.46449	25.16539	25.03048	26.26216	25.51551	25.82938
676	25.28903	24.76355	25.7912	24.73191	24.70995	26.27732	25.43438	25.79796
677	24.56778	23.82338	25.05287	23.11871	23.37576	24.92392	25.46758	23.74799
678	25.3981	25.95913	25.63388	23.59328	26.06276	25.80424	25.26626	25.0073
679	27.47229	27.31976	28.58427	27.4295	27.14904	28.57339	27.41466	27.23784
680	22.20345	22.81067	24.90006	22.8386	23.65236	25.4099	22.88259	22.80128
681	26.23232	26.13269	26.37284	26.06383	25.94292	26.68193	26.07241	26.02909
682	28.05607	28.31308	28.90865	27.9996	28.31254	29.0715	28.23202	27.65017
683	28.91936	29.86744	29.10397	28.95519	29.9495	29.26116	29.37514	27.85876
684	23.35215	23.67914	23.13906	23.06985	22.65314	23.53552	22.3618	22.60797
685	26.50694	27.35268	26.47636	26.4663	27.74253	27.39739	25.59574	25.90038
686	30.39524	30.45111	30.96003	30.45117	30.46513	30.7586	30.42321	30.00912
687	28.2701	28.6066	28.42576	28.21539	28.48632	28.72586	28.73763	27.92157
688	27.49372	27.49714	28.09288	27.55383	27.3406	28.12134	27.76339	27.28522
689	20.78579	22.48864	23.39859	23.28347	22.93852	23.98231	23.52607	23.4187
690	24.70954	23.5338	25.81699	24.07007	24.03343	25.97689	24.05181	24.12412
691	29.3879	29.60125	29.60576	29.43383	29.69826	29.78224	29.69962	29.55174
692	24.17321	24.00457	24.91113	24.58506	24.30599	24.97131	24.67116	24.38258
693	23.95557	24.48672	24.76367	24.51695	23.88395	24.5231	25.23847	24.7826
694	24.83849	24.83367	24.97366	24.73491	25.01378	25.36959	25.25426	25.2903
695	25.19198	25.28989	25.96531	24.78135	25.09151	25.73475	25.21883	25.23192
696	27.87454	28.15324	28.84746	28.1151	28.21553	28.80133	27.88305	28.09094
697	25.53332	25.4889	25.91825	25.36501	25.47137	26.59375	25.96699	25.6172
698	27.04893	27.24956	26.8639	26.94976	26.95933	27.43939	27.57516	26.76778
699	24.90915	24.87291	25.344	24.5996	24.94504	25.72861	25.42705	25.261
700	25.9896	25.2651	25.87051	25.40217	24.14677	25.46845	25.5231	25.07312
701	31.6571	30.91882	31.73545	31.69346	30.80644	31.53476	31.54391	30.96147
702	26.04228	26.69972	26.9289	25.96196	25.8496	26.67889	26.04774	26.40726

	AK	AL	AM	AN	AO	AP	AQ	AR
703	28.60174	29.33842	28.05542	28.37545	29.19779	28.21473	28.31133	27.54072
704	28.45143	27.61166	28.72722	28.55827	27.54982	28.84661	28.30568	28.52958
705	26.62663	27.11462	26.2954	25.98446	27.4358	26.64857	26.19739	26.14696
706	23.71782	22.50451	24.15799	23.63309	22.53479	24.3031	23.43664	22.17292
707	25.3886	26.77334	25.98905	25.38834	26.62898	25.53558	25.83014	25.35591
708	29.3698	29.98697	28.9721	29.21184	29.70231	29.40526	29.30062	29.03931
709	27.15358	26.4514	27.43909	26.6504	26.451	27.48508	26.84879	26.49843
710	29.08938	29.36565	28.82257	29.23664	29.28219	29.29705	29.59186	29.02014
711	33.25584	33.02521	33.94317	33.09961	32.98975	33.95669	33.48606	32.93505
712	27.11881	27.83257	28.76719	26.97551	27.22838	29.38649	27.87441	28.208
713	27.75726	27.61608	28.4948	27.54952	27.69081	29.10604	27.67985	28.12287
714	31.8072	32.37854	32.96203	31.64289	32.30556	33.72296	32.79212	31.59829
715	27.60949	27.8531	28.67393	27.58618	28.33656	29.07583	27.85711	27.93314
716	26.78611	28.01676	27.61594	26.68604	28.14872	28.10918	27.81221	27.47954
717	24.87496	25.47914	26.74094	25.13205	25.51846	27.09033	25.06084	26.38189
718	24.53901	25.77126	25.2913	25.3774	25.45468	25.31565	24.53477	24.38431
719	26.56101	26.18708	27.51847	26.53979	26.18198	27.35172	26.67737	26.36962
720	28.00565	28.32206	27.90141	27.95685	28.24734	28.68794	27.90484	27.60917
721	28.29506	28.35729	29.13302	28.32456	28.20513	29.23083	28.52679	28.56737
722	25.89417	26.17533	27.16054	25.70549	25.88969	27.26085	26.01249	26.49476
723	28.92224	28.81158	29.5137	28.71062	28.6774	29.40806	28.8532	29.06832
724	24.47142	24.94676	25.8055	24.4348	24.25052	25.5275	25.03235	24.24584
725	27.49543	26.8537	27.9471	27.47516	26.83151	27.2221	26.97515	26.95264
726	20.17243	21.95098	21.75122	20.64544	21.26641	24.02693	21.289	21.01216
727	30.67328	31.21517	30.62431	30.65266	31.14437	31.03113	30.80458	31.08707
728	28.38788	28.21978	29.36949	28.55357	27.96669	29.26221	28.30255	28.74216
729	23.70778	23.02921	23.59399	22.89601	22.99224	24.16791	23.2561	23.86626
730	26.17489	26.73688	27.49257	26.15816	25.88233	27.47648	25.67954	26.46564
731	25.07352	24.70855	25.82324	25.01484	25.30693	26.26471	24.68973	25.63916
732	23.32453	23.69282	24.8814	23.22066	23.03338	26.28954	25.2131	25.66887
733	21.32129	20.7434	23.53656	23.95539	21.44266	21.14833	24.18025	22.35809
734	30.77808	30.92695	31.02843	30.53422	30.74509	31.34174	30.46378	31.6029
735	22.17316	21.81223	22.18669	22.53006	22.05108	22.13863	22.27493	22.21844
736	26.61447	26.29755	26.21891	26.46052	26.36806	26.17174	26.83769	26.43217
737	30.00033	30.21537	29.90352	29.93944	30.30653	30.0912	30.32382	29.61234
738	29.98279	30.37762	29.87882	29.83207	30.35939	30.23997	30.24492	30.20737
739	22.24531	22.334	23.7725	22.83509	23.64433	23.01439	21.70345	22.02097
740	26.56301	26.84771	26.76224	26.69526	26.8731	26.86146	26.98919	26.61424
741	28.88226	29.36711	29.59305	28.65711	29.26043	29.93732	29.1983	28.73244
742	27.03962	27.73476	27.68038	27.11294	27.68451	27.33679	27.1896	26.7642
743	32.29369	32.73234	32.06297	32.34088	32.6233	32.29131	32.46556	32.33071
744	26.33323	26.72482	28.18781	26.38231	26.7625	28.43725	26.82145	27.45038
745	27.21671	27.17636	27.96006	27.54198	26.12099	28.01828	27.35477	27.88369
746	22.88538	21.45193	24.40857	22.77523	21.88243	23.6245	22.57238	22.83734
747	29.81425	32.2774	30.19328	29.92048	32.39408	30.20659	31.09025	28.97348
748	22.97823	22.63743	23.63438	22.12675	22.68421	23.96455	21.85161	22.53858
749	26.34971	26.2837	27.15284	26.12472	26.14046	27.21419	26.21747	27.50212
750	26.52444	26.14231	27.34017	26.43922	25.93944	27.19179	26.40457	26.7787
751	22.21749	21.83729	24.34843	22.31493	22.20766	24.76174	22.99001	23.82838
752	24.76046	24.35507	25.63564	24.64993	24.50641	26.03413	24.25488	24.66065
753	22.47464	22.62316	23.98207	22.46187	22.71974	24.34984	23.06802	23.49932
754	26.4383	27.33745	27.48099	26.16633	26.9531	27.03836	26.46728	25.96314
755	25.22894	25.78767	25.45518	24.68196	25.56864	25.76096	25.16512	23.86704
756	28.55653	29.37442	28.70973	28.48174	29.56106	28.6367	28.8328	28.92248

	AK	AL	AM	AN	AO	AP	AQ	AR
757	27.60679	27.38955	28.24454	27.5946	27.43487	28.08756	27.75925	27.06001
758	26.08959	25.46848	26.92546	25.91606	25.5913	27.04998	26.10421	26.17772
759	24.74478	24.98506	25.84029	24.94052	24.7366	25.98621	25.15432	25.18209
760	25.40779	25.67475	26.47401	25.31595	25.22047	26.93024	26.60328	25.60569
761	25.93257	24.41601	24.58711	23.74138	23.76466	24.65428	24.10648	23.60004
762	21.55275	21.91588	23.23449	22.92748	21.54227	22.45461	23.04956	19.29292
763	24.15048	24.15135	23.43387	24.16326	24.77136	23.52413	24.05024	24.04425
764	22.95742	24.818	23.98043	23.63346	24.25848	24.14734	23.57679	24.84896
765	24.95613	24.92819	26.06468	24.31635	24.79777	27.12004	25.29264	25.75363
766	27.92426	28.15155	26.81518	27.88647	28.24717	27.02523	28.50189	27.12476
767	26.7462	27.90345	27.36061	26.62444	27.62894	27.71557	27.08865	27.80503
768	23.95562	24.53698	24.07547	24.07483	24.23535	24.746	24.33591	25.08432
769	21.86512	22.29232	22.49813	22.14794	22.09989	23.09619	22.65541	22.30193
770	26.51363	25.5297	26.28628	25.95154	26.25185	26.38344	26.10057	25.92923
771	27.79096	28.20225	29.556	27.59998	28.02896	29.99984	28.26384	28.98506
772	24.45313	25.31411	25.36176	24.57516	25.31886	25.05145	25.42427	24.9552
773	23.52299	22.57839	25.26719	23.50915	24.13852	25.12385	23.59361	23.73925
774	25.52662	25.50218	27.0549	25.73643	25.37826	27.04522	25.68855	25.67841
775	30.96829	31.50703	30.79623	30.80516	31.66581	31.03555	31.09014	30.88562
776	24.8723	24.29123	25.70946	25.02785	24.14572	25.61342	25.35564	24.71683
777	27.20872	26.45385	26.35878	26.91998	26.91911	27.28442	26.9427	26.99842
778	24.8958	26.33063	25.6569	25.1264	26.18911	26.0757	25.84613	25.1319
779	29.84333	30.52717	30.47732	29.70751	30.34628	30.4539	30.13474	30.54068
780	27.41419	27.53061	27.35636	27.31136	27.55366	27.28457	27.70114	27.85352
781	32.17131	32.7315	32.24291	31.89683	32.68627	32.52274	31.97559	31.89792
782	24.83784	26.14767	25.67356	25.96495	26.31035	25.4156	26.53223	23.99118
783	27.65068	28.31985	28.8489	27.84104	28.33477	29.08973	28.20204	28.34233
784	24.10346	23.90817	26.17694	23.80619	25.32447	25.90026	24.4206	24.53752
785	26.76098	26.88577	28.0765	26.90227	26.79635	28.33289	26.93575	27.1232
786	26.47427	26.43589	26.9012	26.67969	26.55308	27.074	27.21451	26.25986
787	25.49449	25.48033	26.8278	25.36708	25.64668	26.4187	25.44655	26.18206
788	26.30547	26.34521	27.79088	26.27472	26.8342	27.5064	26.38443	27.14286
789	30.28071	30.67745	31.45973	30.21445	30.52129	31.55058	30.87603	30.02593
790	25.45773	23.82158	25.74742	25.62971	23.84053	25.86597	24.01534	23.91434
791	24.89142	26.37373	26.36569	25.08427	26.04122	26.66427	25.75668	24.80382
792	29.25071	29.60249	29.3365	29.04538	29.42924	29.83236	29.44117	29.50674
793	25.35319	25.02438	25.72015	25.22057	24.97807	25.88218	25.82014	25.96072
794	27.89077	27.83338	28.27519	27.81697	27.68764	28.37395	28.56166	28.21135
795	26.66261	27.71722	26.71088	26.59711	27.4712	27.31504	27.04671	26.74955
796	25.06889	24.47083	27.15301	25.18139	23.39827	27.46417	24.86773	26.62628
797	22.209	24.2632	22.18904	24.16063	22.64834	23.68873	23.8949	25.78994
798	29.00463	28.55922	29.70016	28.92555	28.53935	29.99247	29.07619	29.51826
799	24.82097	25.27729	24.38768	24.04159	25.15411	24.37847	24.78435	24.6446
800	22.07632	21.6524	23.98167	21.75512	23.28872	23.32215	22.56409	22.89891
801	25.30997	25.23498	26.21773	25.60694	25.46274	25.99181	25.10734	24.81155
802	24.11664	23.31873	24.90951	24.25442	23.51993	25.34043	24.40966	24.68787
803	26.94436	27.02377	29.18517	26.72316	27.6344	27.92728	26.78508	26.03488
804	26.47945	26.50103	27.60531	26.68588	26.53573	27.49384	26.26488	26.46735
805	22.28404	23.61663	23.5037	22.56277	23.4247	24.31593	22.83507	23.14008
806	24.08007	26.21021	23.84957	23.85371	24.07227	24.36117	24.25517	25.89244
807	24.48776	24.37869	25.35124	24.10737	24.66388	25.70605	24.8824	24.56258
808	22.8359	23.00506	24.02543	22.64671	22.69215	23.74376	23.78312	22.57141
809	22.01064	24.58102	24.68066	24.51715	24.97824	26.20518	26.06986	25.69038
810	24.49707	24.50697	25.71739	24.56527	23.50198	25.82115	24.69331	26.43161

	AK	AL	AM	AN	AO	AP	AQ	AR
811	25.57446	25.69062	26.18918	24.73299	26.44087	27.25507	25.4547	25.71409
812	29.47552	29.83416	30.226	29.43558	29.69952	30.44983	29.58519	29.37582
813	24.85357	23.1551	24.97173	24.17833	23.72478	24.91274	24.41585	24.05987
814	31.46699	31.71695	31.52621	31.42455	31.64941	31.66274	31.72414	30.32495
815	23.21712	23.49916	23.86195	23.8726	23.71668	22.90328	23.31054	23.50649
816	30.78106	31.31131	30.97974	30.74636	31.36686	31.47951	31.20351	31.22034
817	27.10905	27.09826	27.18255	27.00526	27.03215	27.00849	26.86684	26.97877
818	27.23257	26.59355	28.02741	27.64476	26.80596	28.00882	27.11291	29.08355
819	29.23526	29.36717	29.56298	28.49518	29.43816	29.99457	29.29297	29.5606
820	25.86147	26.94897	27.05235	25.63641	27.22303	27.22969	26.62146	26.45945
821	24.64227	26.67947	25.95516	24.92483	26.56668	26.26395	25.50693	25.9335
822	30.33747	30.11814	30.16573	30.28707	30.21438	30.34743	30.61784	30.25158
823	27.8125	27.85842	28.78137	27.73503	27.72994	28.67958	27.87546	28.12565
824	25.10217	25.54156	25.63618	25.23525	25.53462	25.57066	24.83188	25.02171
825	26.12204	25.73069	26.77769	25.93625	25.63403	26.50436	25.84995	25.87639
826	23.84658	25.09228	24.36453	24.27824	24.95123	24.56036	24.64456	23.57391
827	24.22237	24.37498	26.05152	24.12788	24.55768	25.07872	24.60449	25.04687
828	25.53736	25.16525	26.07621	24.91996	25.18184	26.39497	25.28953	25.40802
829	25.36452	25.06658	25.65824	25.11177	25.13077	25.13766	25.3735	25.20345
830	26.54367	26.61999	26.69287	26.66749	26.34409	26.55492	26.44773	26.05751
831	25.97812	26.15706	26.9319	25.90834	25.97836	26.71871	25.88132	26.25023
832	24.63571	25.09613	26.13496	24.66411	25.0495	25.06331	26.24947	23.78146
833	20.63136	22.02377	23.12755	22.59486	20.82576	0	0	0
834	22.05425	22.6834	22.24793	23.68919	22.321	21.96432	25.48727	24.1529
835	21.92283	22.85454	21.34932	21.22623	21.01773	22.85424	22.48046	21.16166
836	26.38292	26.21131	26.38692	26.51765	26.38947	26.64465	26.6322	26.96132
837	22.90482	23.76443	24.89293	24.02694	24.16685	25.0815	23.51728	24.78387
838	23.87163	25.74137	24.95276	24.3737	26.02419	25.23118	23.60323	25.25608
839	22.68106	20.85143	22.84131	23.11102	21.63168	22.41311	22.83758	22.52485
840	27.99691	27.9558	29.47236	27.75315	27.75454	29.58539	28.30636	28.03303
841	27.37973	27.05618	28.11491	27.09568	26.66004	28.16321	27.0905	27.5502
842	27.06648	27.1485	28.33504	26.85403	27.06403	28.58208	27.48788	27.89412
843	25.44655	25.34562	26.79295	25.38263	25.27562	26.61302	25.80761	25.73467
844	24.72739	25.1401	26.1357	25.66915	24.8822	26.23528	25.28882	25.0089
845	25.10328	24.75588	26.64885	25.81374	25.0938	26.42154	25.20941	23.91559
846	21.5422	21.04875	21.21638	21.72101	20.29061	20.97076	21.79751	21.99133
847	24.10955	23.74556	24.10169	24.24804	24.65269	24.89585	23.91434	24.11393
848	26.04041	26.50546	26.47675	26.14876	26.44229	26.42893	26.3249	26.14092
849	27.01226	27.41192	27.6663	27.04369	27.23393	27.6668	27.57852	26.50813
850	24.72961	24.45104	25.9071	24.56763	24.36776	25.9474	24.89629	25.5226
851	22.81623	23.25037	24.98236	22.93404	22.66837	24.65399	23.52696	23.50858
852	25.1563	25.88109	25.99724	24.24837	25.42792	25.86613	25.64438	25.02328
853	23.67028	22.28697	21.9776	23.48915	21.81483	22.22001	23.80268	21.67659
854	22.67176	21.86662	23.42583	22.79595	22.36484	23.62878	22.54699	23.4931
855	23.97997	23.85398	23.45099	23.31148	24.32451	23.05106	23.57506	21.92721
856	25.44498	25.99493	25.87325	25.47047	25.04869	25.80194	25.03302	25.73921
857	25.10485	24.23853	25.86111	25.33024	24.99664	26.33368	25.43713	25.67085
858	28.14759	28.85792	28.39308	28.15792	28.76389	28.78105	28.85536	27.98743
859	23.55248	23.38531	25.0131	23.646	23.2912	24.95134	23.55381	23.99167
860	26.46845	26.97985	27.85266	26.44887	26.71719	27.68802	27.49188	26.81385
861	23.98072	23.65857	24.38386	23.83618	23.92327	24.8353	24.06484	24.39321
862	27.54169	28.22839	28.58016	27.58109	28.21661	29.0333	28.41427	28.41913
863	24.63372	24.89903	25.8537	24.7974	25.1896	26.09174	25.59765	25.12906
864	26.74898	26.01073	27.49575	26.66751	25.93581	27.43138	26.62582	26.71438

	AK	AL	AM	AN	AO	AP	AQ	AR
865	22.89493	24.31866	23.87993	23.21819	24.89839	24.16831	22.86662	23.8386
866	26.22079	22.69937	24.66728	23.59794	22.83317	25.85674	28.40001	23.80087
867	0	0	0	0	0	21.44014	21.04131	24.64764
868	23.37009	23.72353	24.67933	24.39891	23.68761	24.3798	24.01125	23.31097
869	24.08422	24.29816	24.00245	23.07681	23.52396	23.71806	23.0981	23.86421
870	26.25401	26.34706	26.57041	26.05957	26.07665	26.8438	26.87397	26.31508
871	26.12287	27.26025	26.58837	26.82366	27.21166	27.58755	27.1897	26.31191
872	25.67492	25.73791	29.16377	25.69861	25.34914	26.3187	25.87244	25.70691
873	23.15941	25.21745	25.40638	23.91152	25.39379	25.43268	24.78642	24.35093
874	24.69435	26.46932	26.27494	25.49018	25.76986	25.88818	25.56431	24.61934
875	23.20071	23.1656	23.77124	22.02473	22.7529	24.64983	24.91544	21.98446
876	21.90872	21.9918	23.41663	21.38992	21.97164	23.24909	22.60566	22.36762
877	23.47815	24.16298	24.25756	23.08921	23.87205	23.78139	23.85282	21.9676
878	26.84063	28.49087	27.95325	26.70863	28.29123	28.62573	27.84613	27.83964
879	31.96523	32.48824	31.93915	31.92326	32.48964	32.28956	32.749	31.40885
880	23.83771	24.00044	25.46758	23.99741	24.00628	25.80864	24.62472	24.26004
881	26.32004	26.05529	27.3351	26.2723	25.96386	27.12395	26.01629	26.51271
882	23.45242	24.18662	23.67238	24.81666	24.16065	24.59447	24.3393	23.00309
883	26.48847	26.59266	26.55394	26.15878	26.50256	26.74799	26.4953	26.36238
884	22.20876	21.98524	23.29302	22.28002	23.19998	23.2173	23.31687	22.88408
885	22.2482	21.89672	22.39273	20.79035	21.12469	24.25784	21.06294	20.99166
886	25.91693	26.83413	25.94203	25.44482	26.8791	26.66518	26.43224	25.65865
887	22.75169	22.43509	24.23607	22.70898	22.35887	23.99898	23.0119	22.76892
888	27.97766	27.44506	29.13322	27.94293	27.3294	29.10652	28.14908	28.14128
889	34.02727	34.76507	33.52716	33.95827	34.54322	34.33684	34.82781	34.00985
890	32.64243	32.96085	33.09972	32.47661	32.85029	33.93153	33.35492	32.70374
891	26.98412	26.42784	25.95153	25.68972	26.82871	26.55919	26.62778	26.15241
892	25.66117	25.6686	26.45249	25.39311	25.41833	27.53654	25.45293	26.09914
893	23.84344	22.51168	22.95109	21.07652	22.70405	24.13185	22.28015	24.06411
894	24.40661	24.70395	25.27816	24.14357	24.77418	25.51471	25.11468	24.3194
895	24.16527	24.43464	25.4593	24.49233	23.88511	26.03238	24.62344	25.30617
896	27.46575	27.12308	27.45953	27.16201	27.08152	27.67731	27.55763	27.46099
897	24.35838	23.70377	25.42683	24.51463	24.09309	25.62533	23.91684	24.99664
898	23.3572	22.91209	25.1827	23.53238	23.07557	25.07045	23.18296	24.37769
899	23.58147	23.58227	23.95197	24.06064	23.47454	24.53459	23.67908	23.85708
900	24.97347	24.90826	25.40671	24.19032	24.92122	25.22526	24.56982	24.75507
901	23.55773	24.22814	24.56147	23.67563	24.403	24.69564	23.70615	24.23483
902	27.37521	27.21166	27.92435	27.42405	27.17219	28.03925	28.04626	27.99335
903	27.47607	25.64784	26.05808	27.41183	25.95547	26.0786	27.2975	26.1413
904	26.04764	25.54216	25.28904	25.86438	25.27539	25.04129	26.78287	25.26673
905	27.10905	27.71577	27.61222	27.45517	28.15074	28.06978	27.61591	26.8994
906	23.77336	24.17377	24.8786	22.45746	23.40102	23.31523	24.26223	22.89198
907	21.44271	23.32724	21.56716	21.61826	22.13152	23.06226	22.97691	22.60881
908	26.84096	26.39351	27.98593	26.83138	26.80994	28.18989	26.99925	26.01438
909	28.10744	28.77411	28.41351	28.27843	28.56456	28.44446	28.66219	27.10122
910	27.54123	28.62787	27.27562	27.7046	28.44303	28.53577	28.0161	25.54879
911	25.15848	25.37061	26.21092	25.74576	25.97654	25.92011	26.22698	22.50365
912	22.26913	23.02147	23.60291	22.6648	23.60621	23.39401	23.47037	23.43918
913	27.26548	27.09233	28.04297	27.11221	27.10319	28.35537	27.03585	27.83509
914	26.1301	26.68342	27.3598	26.55208	26.87776	27.09563	26.29354	25.29453
915	24.61934	24.87334	25.96129	24.85936	24.91273	25.85116	25.05672	24.58204
916	21.90541	21.27924	22.7841	21.67673	22.83516	23.98995	22.78903	22.16616
917	25.44018	26.25269	25.80477	25.48713	25.85701	26.17808	25.98966	25.81236
918	28.24596	28.3176	28.15906	28.32859	28.2449	28.53225	28.52482	28.17297

	AK	AL	AM	AN	AO	AP	AQ	AR
919	26.3215	26.21224	26.41055	26.0908	26.07972	26.46848	26.86882	25.2605
920	21.76515	21.90586	24.33437	22.38739	22.17081	23.56452	22.76033	22.74128
921	26.89462	25.85465	29.22523	26.747	26.11467	28.62768	26.88073	26.67565
922	27.21358	27.33126	28.61946	27.20315	27.46359	28.54382	27.5211	27.47129
923	28.31669	27.94639	29.24774	28.09075	28.0204	29.37914	28.13626	28.26085
924	26.33628	25.32805	26.54636	26.20433	25.20495	27.38314	26.26679	25.79819
925	27.66812	27.15526	28.78361	27.70058	27.41761	28.78649	27.7371	27.82354
926	31.69745	31.56253	31.68507	31.70753	31.53919	31.73396	31.43905	31.95527
927	27.1666	25.08929	26.1012	27.07641	25.02568	26.09396	27.28725	25.20893
928	21.76595	22.82528	24.03991	24.50606	21.79819	24.47824	24.72522	23.32023
929	29.5751	29.72307	30.27395	29.55704	29.63085	29.99658	29.80663	29.24973
930	30.81126	31.1414	30.12612	30.82676	31.22474	30.33507	31.33135	30.29878
931	23.85559	23.79866	24.29226	23.95048	22.67444	24.13906	23.44394	22.70474
932	26.85628	26.9554	27.15088	26.89218	27.14944	27.16391	27.18357	26.91526
933	23.90901	25.09434	25.3935	25.07584	25.04379	25.39197	25.21703	25.12525
934	22.87106	22.57686	23.93868	23.46341	22.59777	25.11425	23.36979	23.84944
935	27.28274	27.67303	26.76997	27.27769	27.71662	26.66065	27.50887	26.62526
936	27.90358	27.44622	28.97263	27.94673	27.40553	29.06093	27.78165	28.28572
937	25.82015	27.07356	27.4665	25.78875	27.34798	27.40451	26.44023	26.87167
938	25.27376	26.08049	25.39535	24.13599	26.3734	25.78493	25.40741	26.3127
939	26.3644	25.52228	26.94454	26.16225	25.77189	27.34963	26.33519	26.60945
940	27.84992	27.89686	27.85616	27.92715	28.11834	28.36009	28.70845	28.39976
941	26.91133	29.05122	27.96675	26.99912	28.69726	27.79728	27.15358	26.83368
942	24.63697	25.10383	26.32373	24.90641	24.66018	26.04522	24.90962	25.08185
943	25.54269	25.96954	26.34915	25.49069	25.69428	26.10603	25.75666	25.9803
944	22.43994	24.26666	24.42083	23.4146	23.7569	23.84701	23.337	23.56348
945	25.70388	25.88162	25.69998	25.97217	26.09862	26.00663	25.8976	25.5676
946	28.93344	29.47928	29.50606	29.03428	29.47929	29.64596	29.68169	29.3868
947	27.93213	27.77078	29.21123	27.8118	27.94295	28.97904	28.13457	28.49456
948	27.95685	27.97157	29.13733	27.83108	28.11075	29.164	27.78504	28.1707
949	24.24093	23.69465	25.37785	24.26874	23.5507	24.85212	24.12799	23.15949
950	24.0982	23.93848	25.03883	24.3036	24.00812	25.02595	24.32327	24.93049
951	25.43395	25.92708	26.37729	25.28664	25.51447	26.0021	25.68931	25.90982
952	25.27807	25.76248	25.41715	25.34728	25.80965	26.00639	25.72789	26.14694
953	21.31963	21.71985	22.74498	21.96469	20.92167	21.97495	18.92908	20.17066
954	25.51182	22.57236	21.87641	24.85444	22.67806	22.24071	24.93561	22.56284
955	27.59761	27.73475	29.18544	27.45738	27.7872	29.34157	28.11862	28.01369
956	21.36783	21.11842	22.98291	21.17142	20.981	24.05936	22.21168	21.40479
957	24.35939	23.91679	24.19807	23.87866	23.97966	23.45164	23.18362	21.98313
958	24.23769	24.72061	25.86676	23.80414	23.63416	25.66057	24.62965	24.27299
959	20.40794	22.61305	22.47657	21.63497	22.3548	22.50241	22.4783	21.92322
960	25.82998	25.5631	27.2554	26.11804	25.7271	27.4555	25.94826	26.10836
961	24.90021	25.77835	27.13621	24.78038	25.82582	26.63956	25.43641	25.94812
962	25.06414	25.32075	25.78175	25.18893	25.37043	25.72504	25.16258	24.75712
963	22.84146	23.46471	24.98323	23.44855	23.47721	24.72017	23.11903	23.30063
964	23.90273	25.06523	24.27681	23.84937	23.81226	24.96421	25.65074	22.98791
965	24.5347	24.37954	26.7597	25.2344	23.84834	26.73598	25.56602	24.93066
966	21.92847	22.6524	22.15829	22.02714	21.71742	22.20259	21.84533	22.91925
967	24.21315	24.74876	25.03346	23.2319	23.94274	24.88796	24.32161	24.65176
968	24.71827	24.47789	24.98998	22.08705	23.94988	24.609	23.25967	24.59971
969	22.25427	22.31861	22.04131	22.27286	22.63797	23.15893	22.10357	21.74296
970	22.4178	22.37774	24.75497	23.30134	22.57751	24.69087	23.95224	22.71108
971	26.30013	26.89774	26.19154	26.32234	26.87515	26.47591	26.56352	26.0425
972	26.78329	26.73983	28.38849	26.67885	26.64059	28.44693	26.80552	26.97918

	AK	AL	AM	AN	AO	AP	AQ	AR
973	27.215	27.88678	27.29591	27.21411	27.77994	27.91335	27.48046	26.71993
974	24.5059	24.41166	25.18103	24.04754	22.92344	25.0162	24.47697	23.54324
975	27.28786	27.17096	28.26885	27.47786	27.30038	28.23298	27.38104	27.19067
976	22.81531	23.57952	23.99861	22.31187	23.20512	23.52464	23.0882	22.59971
977	28.37957	28.66795	28.74248	28.24316	28.55735	28.38588	28.70269	27.78045
978	21.58675	23.19111	24.87632	23.42926	23.33744	24.76726	23.22591	23.28712
979	24.36669	24.19186	25.51054	24.97167	24.2904	25.1189	25.58325	24.4932
980	32.6608	32.93158	33.96341	32.66201	32.64376	33.50771	32.62337	33.04678
981	23.75347	24.38066	26.24081	25.41111	24.85364	26.62795	24.20657	23.23386
982	22.0126	21.67244	22.9581	22.4581	23.30855	23.42562	22.22209	22.96729
983	25.57553	25.53298	25.95683	25.68784	25.77883	26.57777	26.31163	26.67592
984	25.30463	25.06202	25.53641	25.23128	24.72579	26.49156	25.50784	26.8237
985	26.73389	26.80884	27.23863	26.65574	26.07683	26.59188	26.26206	27.9627
986	24.8867	25.0805	25.8589	25.1145	24.8636	26.18085	25.62926	26.0735
987	27.36683	27.42053	27.69855	27.36688	27.54877	28.28829	27.83632	28.07034
988	22.01809	21.70773	26.65495	21.71387	21.57779	27.82624	22.07026	23.62897
989	27.89198	28.35363	29.34106	27.77431	28.29667	29.49401	28.46506	28.4228
990	26.31754	26.41215	27.16006	26.08942	26.53437	26.63039	26.3305	26.00012
991	23.50537	21.65541	22.09901	21.07916	22.03374	21.76204	23.63297	21.15541
992	27.60927	27.43863	28.42199	27.45547	27.57364	28.37637	27.34653	27.50298
993	23.82528	23.84583	24.17123	24.13698	24.00613	24.07425	23.24205	24.20087
994	22.82302	22.82341	25.66164	24.02479	22.62438	26.69089	23.1259	23.35917
995	26.34043	25.83763	27.08031	26.45676	25.60835	27.37379	26.80873	26.41922
996	25.16067	25.32593	26.02687	24.41541	25.45275	25.96033	25.77548	25.68605
997	22.65186	24.8461	25.83691	22.74137	23.94648	25.469	23.79657	24.68872
998	23.81068	22.44135	24.09167	23.39199	23.02544	21.69566	22.06687	22.35312
999	31.21082	31.13627	30.46946	31.03982	31.12123	30.68347	31.30593	30.82851
1000	23.00539	24.24427	24.32057	22.81207	23.02953	24.29482	23.91214	23.56704
1001	28.43345	28.1407	28.44103	27.95995	28.17681	28.50644	28.10214	28.13879
1002	23.59087	24.62088	25.06795	24.21535	23.91501	25.21271	24.76032	24.44935
1003	20.9973	22.86534	24.50337	22.48898	20.85181	24.11737	22.67274	22.00408
1004	22.57561	24.13291	24.41869	22.16928	24.14209	24.02604	23.44493	22.91218
1005	22.34897	22.56937	23.69342	22.22054	22.29556	22.81955	25.50813	23.38079
1006	26.21696	26.06272	26.71557	25.89402	25.91135	26.96624	26.80535	26.83672
1007	21.55746	23.51667	22.02384	21.53171	23.6369	20.83742	22.36504	21.61322
1008	22.97083	22.83556	20.72006	21.72521	23.46949	22.59681	24.86845	23.64452
1009	27.26974	26.8492	27.76636	26.83783	26.82837	28.00851	27.06957	26.94297
1010	29.55562	29.1558	29.75253	29.2478	29.00515	29.79907	29.20596	29.51044
1011	25.82621	27.4624	26.66601	25.88479	25.94579	26.8086	25.79998	26.01045
1012	26.18458	25.40499	26.82732	26.24415	25.25938	27.00643	25.86026	26.34844
1013	24.96616	24.95557	26.21352	25.03665	25.00479	26.75917	25.09361	25.32871
1014	23.66911	22.4622	25.30362	23.52108	23.16243	24.986	23.68293	23.01982
1015	26.61732	27.44879	27.97154	26.68398	26.80881	27.96588	26.87676	27.50742
1016	25.15443	23.22017	21.00697	20.80884	21.82211	22.45287	23.472	22.92825
1017	26.6465	27.67332	28.13419	26.68111	27.56367	28.2481	27.04472	26.76142
1018	28.27133	28.57847	29.04429	28.27829	28.54425	29.18315	28.55363	28.13431
1019	24.78368	25.05158	25.65246	23.41321	24.23845	25.46737	24.8786	23.93932
1020	27.76808	27.44542	28.18102	27.64143	27.46349	28.46576	28.09015	28.30154
1021	29.66641	30.16744	29.7548	29.07008	30.14211	29.97782	29.80746	28.78584
1022	24.87591	24.33093	25.40599	24.5908	24.51335	25.82978	24.75732	24.44395
1023	26.71866	26.55505	27.99429	26.47979	27.04571	28.38563	26.46926	27.86534
1024	23.78698	23.72721	24.28178	24.39378	24.02654	24.80948	24.40129	24.05097
1025	25.20567	26.72784	27.0164	24.79466	26.54863	27.58906	27.09392	26.9201
1026	21.10459	21.12588	22.31329	22.17777	20.97785	21.05246	21.25955	21.63683

	AK	AL	AM	AN	AO	AP	AQ	AR
1027	27.42706	27.70655	27.75971	27.2377	27.44189	27.92598	27.55178	26.89939
1028	24.63074	24.8565	25.72725	24.60264	25.36176	24.81394	23.69642	25.42798
1029	27.70265	27.81409	27.98982	27.75946	27.81294	27.83755	27.70176	27.82508
1030	24.81626	22.72568	23.97859	22.56367	21.70464	24.48767	24.90471	22.99813
1031	26.3562	26.13543	27.13293	26.21865	26.21026	27.07612	26.29795	26.55535
1032	23.05686	24.15012	23.42537	23.72957	22.5497	24.05726	22.21468	23.47289
1033	23.45304	23.54935	23.60911	23.24899	23.70936	24.53526	23.71002	24.16448
1034	25.08178	25.5815	25.20572	24.86883	24.73077	25.79689	25.45361	25.43478
1035	21.85854	22.36779	22.07933	20.90182	21.83059	21.42231	20.12486	19.91953
1036	25.79868	26.50849	27.27758	26.03465	26.6097	26.93616	25.99139	26.04224
1037	23.69269	24.71153	24.92994	23.14784	25.20015	24.60761	24.50907	23.09717
1038	27.94086	27.5203	28.56066	28.18265	27.62822	28.77731	28.06051	29.83049
1039	25.70699	25.549	25.69736	25.60962	25.75887	25.59282	26.13836	25.89196
1040	22.67096	26.55221	24.78027	22.35764	22.6756	24.39834	21.87462	24.27794
1041	24.16574	23.71257	25.20223	24.08194	23.51728	25.25233	24.33693	24.71315
1042	21.40754	23.24805	21.98673	23.59072	22.7481	22.76243	23.3397	23.14372
1043	30.78391	30.82774	30.84236	30.71551	30.95648	30.90565	30.52918	30.60114
1044	28.55713	28.83016	29.6979	28.75034	28.67224	29.46818	28.67548	27.24426
1045	26.57337	26.67052	26.6114	26.50784	26.62498	26.78544	26.77909	26.22038
1046	25.70415	25.45908	25.55054	25.35538	25.32829	25.92381	25.87414	25.24799
1047	24.10052	23.77535	24.58381	23.95123	23.54227	24.93216	24.46086	24.17511
1048	27.20884	27.04479	26.66582	27.09945	27.05049	27.1609	27.31733	27.69069
1049	28.92663	28.418	30.09323	28.82579	28.57378	30.06008	29.19427	29.44834
1050	24.90005	24.87015	24.44724	24.37438	25.0725	25.43375	24.91545	25.80782
1051	26.02147	26.21741	26.63677	25.98849	26.10555	26.67408	26.09068	25.9315
1052	22.67656	21.95627	23.23034	22.55352	22.45599	21.88098	21.90022	22.03194
1053	25.93357	26.29669	25.88318	25.83144	25.95454	26.16767	26.38575	26.00344
1054	23.4248	23.00965	24.8703	24.21288	24.7327	24.77665	24.66261	24.91654
1055	26.89044	28.00583	27.59096	26.74883	27.12134	28.16453	27.42678	28.0543
1056	22.73701	24.4524	26.1772	25.00498	24.89056	26.13822	25.36699	24.14385
1057	27.10184	27.12371	27.58058	26.94696	27.04301	27.37238	26.93606	25.81207
1058	27.30329	26.6827	27.46444	27.06407	26.8195	27.22133	27.38752	26.26331
1059	25.94117	26.00581	24.20139	26.43931	26.54474	25.39609	26.04213	28.47019
1060	30.23827	29.86958	31.34057	30.32917	30.04864	31.13176	31.46781	30.80046
1061	24.40276	24.68223	24.97327	23.54118	24.52165	25.45744	24.31557	25.30073
1062	21.99181	20.53335	21.28591	22.90387	21.56369	22.41594	21.72869	22.8196
1063	25.60629	25.45655	26.68064	25.82593	25.68344	26.4267	26.1596	25.89634
1064	22.31758	24.05378	23.9596	21.90386	22.36976	23.98007	22.08759	22.82443
1065	25.06271	25.78226	26.66293	25.3902	25.46397	26.35177	25.3741	25.20883
1066	22.0225	23.69814	22.64536	21.8262	23.05218	21.58031	22.66606	23.49636
1067	27.06179	26.93407	27.04029	26.99186	26.65983	27.62204	27.38693	26.67658
1068	23.79771	23.43323	24.27695	22.86531	23.95899	23.44167	21.87966	21.71091
1069	26.79246	26.90881	27.60391	26.68468	26.90067	27.27276	26.85386	25.81915
1070	24.14525	24.88056	25.41967	25.03811	24.83168	25.54973	24.229	24.41664
1071	29.32673	29.58867	29.84222	29.34738	29.5672	29.80472	29.5362	28.92273
1072	23.77697	23.89764	25.0514	23.93315	23.59678	25.09555	24.2502	23.6399
1073	25.58768	25.75354	25.38383	25.63851	25.4426	25.61851	25.1408	25.69972
1074	32.44456	32.49793	32.31701	32.36535	32.38065	32.45323	32.32288	31.79315
1075	29.351	31.06879	30.55242	29.44562	31.05735	30.03053	29.72725	28.54887
1076	31.91033	31.9187	30.36088	31.8818	31.88193	30.57233	31.81104	32.08179
1077	23.16442	23.03922	24.75856	23.49366	22.84352	25.32844	23.59467	23.63643
1078	24.15451	23.79868	24.16475	23.96672	23.66092	24.13821	24.2717	24.25015
1079	21.85116	21.85346	22.82037	21.60645	21.06156	22.04045	22.86382	21.1664
1080	24.63409	22.43589	25.97245	24.89492	22.94927	26.50612	25.6549	23.31332

	AK	AL	AM	AN	AO	AP	AQ	AR
1081	25.46469	25.22384	26.40285	25.40614	25.08422	26.52398	26.22635	25.90277
1082	23.92525	24.10389	24.2734	23.23109	24.2715	25.14674	24.58758	23.05519
1083	23.2753	25.09468	24.78832	24.088	23.10324	25.33514	24.1196	23.77837
1084	23.25712	21.385	23.55375	21.15447	20.53511	23.63468	19.94544	19.66605
1085	27.68742	27.87483	29.14781	27.69537	27.54478	29.33854	27.83108	28.04805
1086	25.82323	25.54525	26.56524	26.24555	25.35868	26.47218	25.69883	27.05748
1087	25.89202	25.9888	27.86162	25.62695	25.91328	27.95371	26.31525	26.20364
1088	25.4366	25.50295	25.81992	25.5407	25.46906	25.92841	25.47557	25.10082
1089	23.46232	24.81512	25.34416	24.14328	24.5744	25.14136	24.027	24.57044
1090	19.88124	19.76538	21.73785	20.28647	20.41651	22.17867	20.70197	24.5693
1091	28.36857	28.16428	28.58085	27.97062	28.19888	28.74103	28.12582	28.43754

	AS	AT	AU	AV	AW	AX	AY	AZ	BA
1	TOP3 WT_i02_1	TOP3 WT_i02_2	TOP3 WT_i02_3	TOP3 WT_i03_1	TOP3 WT_i03_2	TOP3 WT_i03_3	TOP3 WT_PEP_i01_1	TOP3 WT_PEP_i01_2	TOP3 WT_PEP_i01_3
2	25.20144	24.99385	24.59296	25.32924	25.02222	24.71699	25.04807	24.58146	24.46492
3	27.07418	26.16176	26.782	27.18903	26.06695	26.6437	27.03449	27.28644	26.75965
4	24.96952	24.53756	23.66681	24.59	23.45396	23.88632	24.96959	24.88221	25.47637
5	24.05171	22.89596	22.43781	23.80468	23.18071	22.82733	23.8462	22.65144	23.5975
6	26.74069	26.14182	24.84637	26.50268	25.4279	25.87516	26.09014	26.38812	26.10411
7	26.81884	26.23855	25.96375	26.60452	26.42536	25.75351	26.88387	26.47298	26.2648
8	24.06899	24.20598	25.49014	23.82796	25.56512	23.96709	24.01039	23.55444	24.53117
9	22.85705	21.6538	22.16209	23.04651	21.51105	22.07353	23.36547	21.77412	22.08442
10	27.7841	27.39394	26.47231	27.68221	27.19625	26.51146	27.95562	28.0069	27.40228
11	28.85961	27.76055	27.86208	28.92766	27.74533	28.01966	28.9955	28.17048	27.92566
12	27.39154	26.54904	26.78592	27.52157	26.55237	26.62624	27.61067	26.62841	26.89242
13	23.22988	23.13451	23.04573	20.69858	21.96486	23.00693	22.94547	23.16172	23.67948
14	26.03036	26.49822	25.94217	26.41104	26.28841	26.0093	26.20346	26.97727	26.78346
15	27.26667	26.96674	26.0152	27.0034	25.65467	26.15827	25.80618	26.30188	26.12955
16	26.09889	26.14513	26.428	26.68843	25.73592	24.89617	26.66416	25.40802	26.14861
17	24.39594	22.74858	22.4501	24.77548	23.49788	22.18818	24.62641	24.15891	23.96705
18	26.02975	24.66789	25.47587	26.11294	24.96229	25.25504	26.04927	25.47203	25.429
19	29.9747	30.11223	29.34765	30.02935	30.04649	29.29595	29.99549	30.3392	29.69461
20	27.02509	26.43718	26.61411	27.21314	26.29945	26.71166	26.92101	26.87658	26.63781
21	25.74063	25.48318	25.05721	25.46908	24.67624	25.03123	25.5198	25.40821	25.62538
22	26.70799	26.74158	26.66673	26.43616	26.69024	26.57804	26.70833	27.28291	27.20865
23	24.62382	24.23312	23.87502	24.89074	24.36726	24.18829	25.0453	24.59096	24.68369
24	24.19811	23.33779	22.50203	24.80566	23.19232	22.62639	24.40139	24.36973	22.22502
25	25.34327	24.92605	24.36341	25.28724	24.12344	23.99891	24.96597	25.15037	23.82747
26	23.03117	23.57444	23.69645	22.52449	23.57717	23.2323	23.89095	23.52109	24.06084
27	23.78176	23.06176	23.44704	23.95656	23.46163	23.30771	23.65916	23.24799	23.56216
28	24.53461	23.38535	23.72809	24.76609	22.84595	23.68341	24.48959	23.66949	23.37503
29	25.49884	24.01982	25.2415	25.62441	25.00108	24.63589	25.65931	24.3443	24.52375
30	25.64012	25.30023	24.77617	25.85434	25.31728	24.32184	25.58653	25.58239	25.20668
31	25.26183	24.96838	25.29391	25.33736	24.95816	24.83767	25.38665	24.97718	24.92157
32	24.12531	25.846	22.3891	22.95778	25.25703	25.82136	23.98687	25.08771	26.07294
33	26.88054	25.93951	26.09418	26.68731	25.89416	26.01372	26.44542	26.05028	26.13312
34	27.98675	26.76243	26.81094	28.09301	26.48569	26.6709	28.59973	26.32954	25.97668
35	26.66506	26.203	25.89778	27.02942	26.69011	25.95373	26.51478	26.933	26.49877
36	26.88077	26.08228	26.71618	27.13166	26.07516	26.28253	26.86252	26.31668	26.58213
37	26.85433	26.92545	26.02207	27.02961	26.80665	26.00064	26.49186	26.75222	26.5053
38	23.62467	23.60003	23.38786	23.27053	22.77153	22.38492	23.85315	21.98798	22.02799
39	24.1097	22.4949	22.67421	24.79551	22.56381	22.95631	24.85042	22.68639	22.33144
40	28.37226	28.27468	28.61015	28.31522	28.26982	28.59634	28.58254	28.34131	28.80846
41	31.10941	31.05056	29.15124	31.11671	30.93794	28.9935	31.07674	31.44868	29.49079
42	29.00512	27.35659	28.22341	29.18817	27.52287	28.24204	29.1325	27.6574	28.50466
43	26.12047	24.07878	25.01893	26.16205	24.18863	24.98407	25.98287	24.32671	27.64095
44	24.64564	21.37793	23.44664	25.47822	21.84334	23.37267	24.69434	22.16391	23.26969
45	26.53224	25.8773	26.47165	27.07308	25.98816	26.33245	27.33574	27.68629	26.19384
46	25.52269	24.90632	24.08054	25.29964	24.56039	23.44431	25.22669	24.50298	23.24342
47	23.52936	23.70626	23.02884	23.09946	23.89824	22.52531	22.49979	23.22856	22.84875
48	31.79149	31.64958	31.57751	31.83805	31.67001	31.4547	32.35879	31.65122	31.58989
49	23.11361	24.99475	25.05083	23.07217	24.75517	23.79394	24.73806	23.00881	22.3306
50	34.88597	35.10055	35.0423	35.002	35.10321	35.05707	34.37222	35.14717	35.0254
51	28.26587	28.10427	26.61853	28.78485	28.22423	26.9192	28.37644	28.68798	26.86284
52	25.97937	25.40888	25.70601	25.98399	25.07313	25.68792	26.13503	25.31066	25.62243
53	27.32147	27.58296	27.76763	27.24055	27.61155	27.78514	27.41312	26.85627	27.94819
54	30.99294	30.91436	31.08588	30.89156	30.44154	30.79017	30.93069	30.68329	30.71155

	AS	AT	AU	AV	AW	AX	AY	AZ	BA
55	22.6271	22.59532	21.65367	20.877	22.61534	18.66672	20.30264	20.09051	19.99978
56	24.20267	22.88319	22.64226	24.52281	23.62481	23.57248	23.20932	23.42956	23.41161
57	19.84831	21.12857	20.39904	21.16672	20.85876	20.00156	22.40753	22.40803	21.77299
58	28.08829	28.45485	24.27157	28.12453	28.49276	22.20573	28.50368	28.93956	28.86665
59	29.21942	28.82662	28.52561	29.23325	28.78926	28.37676	29.0784	29.00168	28.58353
60	27.41562	26.58361	26.79613	27.66811	26.79149	26.85145	27.51204	26.7229	27.02083
61	26.07164	23.20427	22.90635	25.70072	25.31133	25.51195	25.17089	25.11743	24.11395
62	24.92612	24.3337	25.23931	25.24436	24.41596	25.66166	25.06782	25.03005	24.9587
63	27.8371	26.32483	26.2941	27.98042	26.22499	26.29095	27.44958	27.02494	26.35014
64	25.78516	26.16308	26.0856	25.76616	26.16666	26.21326	25.64561	26.48487	26.54683
65	24.42059	25.61203	24.01781	24.10699	25.74288	22.87163	23.82934	26.08081	25.44121
66	25.42419	24.30366	25.33257	25.39949	24.26127	25.26378	25.74494	24.64528	24.82135
67	22.72269	22.32242	21.94448	23.22432	22.32329	22.35995	23.88257	23.89217	23.37649
68	21.03859	19.16673	21.6854	20.31882	19.87555	19.96499	22.29879	21.82463	22.2164
69	21.7229	22.39386	22.16369	21.99356	21.54009	22.88046	21.0597	21.89663	21.05927
70	22.61453	21.33809	22.62297	23.12378	21.93475	21.37047	23.7937	21.93212	24.49922
71	25.76774	24.66758	25.25148	25.66172	24.84504	25.45834	25.35104	24.60708	24.92642
72	26.26946	24.97878	25.18629	26.48542	25.20709	25.02797	26.6357	24.82915	25.40746
73	24.51889	23.73547	24.23536	24.27484	23.96805	24.10017	24.61165	23.80804	24.41438
74	24.41653	23.5235	23.16991	24.75459	23.36621	23.97322	25.87996	23.45325	25.64834
75	28.37369	27.98171	26.97349	28.4906	27.81526	26.9825	28.11646	28.65163	27.48333
76	25.09198	24.77133	23.47836	24.5795	23.56834	22.44755	24.40995	24.05867	24.00082
77	26.83128	26.15366	26.4066	26.79996	26.08643	26.27873	27.37362	26.24771	26.3489
78	30.54518	30.45369	30.20082	30.45467	30.44563	30.1458	30.43492	30.14707	30.09993
79	27.90808	27.28402	27.64943	28.02681	27.15857	27.07246	28.08291	28.04821	27.95501
80	26.73823	26.17388	26.2803	26.8239	25.59255	25.19576	27.31049	24.25703	26.90085
81	25.28939	24.89786	24.67898	25.67434	23.76754	24.25292	25.26423	25.29026	22.72757
82	25.18914	24.58074	24.16228	25.61091	24.74862	25.00873	26.10713	24.52174	24.83312
83	26.11153	23.37878	28.34897	26.49348	27.83884	28.81637	26.05181	26.75308	28.101
84	25.14096	23.84389	24.75574	25.05843	22.85423	23.92604	25.3022	24.63504	24.65026
85	26.93705	25.91642	26.42417	26.70647	25.92936	26.56962	27.12521	25.87201	26.5474
86	22.15051	23.17182	23.79888	22.42048	22.2379	23.69363	22.41703	23.19971	23.30543
87	29.1863	29.21072	27.39125	29.24516	29.19706	27.43342	29.435	29.0161	27.86212
88	30.19051	28.0475	28.16272	29.51005	28.0958	27.85566	30.71937	28.2071	28.25856
89	30.31673	29.67389	29.00334	30.43388	29.77498	28.82717	30.5458	29.98991	29.35985
90	30.63655	30.94705	29.85284	30.32354	30.9137	29.79319	30.95641	31.21587	30.18542
91	30.9909	29.69298	29.8064	31.0619	29.61682	29.69339	31.55967	30.40498	29.92921
92	29.95504	28.89688	27.9598	29.72269	29.08788	28.03356	30.64971	29.09916	28.36905
93	27.61072	27.04988	27.64271	27.5315	26.95159	27.36814	28.28554	26.97321	28.13737
94	29.87716	28.93911	29.10163	29.89897	28.9362	29.02842	30.2029	29.16589	29.28867
95	28.95186	27.52778	27.89462	29.05785	27.71379	27.541	29.69307	28.21852	27.93017
96	31.99256	32.37683	32.01172	31.8202	32.40678	31.96827	32.43951	32.7308	32.54948
97	29.56403	28.75017	27.89639	29.03858	28.41372	28.1318	28.97166	29.77881	29.2778
98	29.0884	28.79471	27.6043	29.21696	28.81114	27.75156	29.53221	28.56737	27.43185
99	30.88103	30.05471	29.48865	30.99565	29.89367	29.55158	31.43429	30.76703	29.95244
100	31.02603	30.73587	30.45863	31.07968	30.74993	30.43681	31.26814	30.63439	30.47741
101	28.83352	27.72315	27.24824	28.80184	27.62279	27.29717	29.15768	28.54228	28.06235
102	29.61285	29.66921	28.43143	29.54975	29.59568	28.68736	30.13961	29.02403	28.80042
103	30.63052	29.79705	29.59466	30.82859	29.70378	29.604	31.20352	29.82488	29.74148
104	31.66169	30.5305	30.20787	31.57518	30.47949	29.85	32.08709	30.68039	30.28412
105	30.63164	30.14989	29.28695	30.58157	30.16974	29.17558	30.85859	30.39612	29.75135
106	29.27538	28.9709	26.60967	29.10754	28.72028	26.63604	29.75105	29.47649	28.31397
107	30.70762	30.16954	29.73827	30.69867	30.07977	29.80177	30.98794	30.61634	29.90251
108	26.16792	25.07894	24.49152	25.88971	24.95927	24.49456	26.05275	25.1494	24.7474

	AS	AT	AU	AV	AW	AX	AY	AZ	BA
109	30.37851	30.34644	30.04462	30.45668	30.25748	29.97833	30.76064	30.75036	30.70528
110	28.40215	27.64137	27.66185	27.86891	27.84612	27.6992	28.76201	28.49252	28.84801
111	30.49949	29.56498	29.42861	30.37925	29.55689	29.46629	31.08126	29.49987	29.75662
112	28.63625	27.74253	26.95691	28.24991	27.48227	26.6914	29.09975	28.3382	27.30815
113	28.27349	27.78365	27.17231	28.34121	27.67993	27.06447	28.83516	28.4046	27.46103
114	29.83051	29.14183	28.52618	29.77081	28.77518	28.61237	29.80613	29.38578	29.35236
115	30.08582	29.60457	29.06746	29.92717	29.49517	29.08336	30.52697	29.7375	29.27934
116	26.1573	25.78665	25.21509	26.17071	25.73704	25.16392	25.92123	26.02012	25.9
117	28.01192	27.22614	27.64828	27.88671	27.13538	27.35124	27.63688	27.40244	27.72762
118	26.0295	25.37488	25.28597	26.27217	25.6012	25.23457	26.20309	25.61745	25.03852
119	23.08071	23.75893	22.64308	22.99468	23.88034	22.06956	23.61775	23.803	23.81188
120	27.63552	26.54091	26.9754	27.39583	25.91055	26.51109	27.50542	26.4865	26.7257
121	25.30236	24.31061	22.47651	25.50431	24.16399	23.10995	25.30854	24.94548	24.23259
122	24.46079	23.72482	23.04287	24.3324	24.00003	21.96071	23.71088	24.19832	22.8304
123	24.49423	22.83424	22.44787	24.5529	23.47882	23.35535	24.38775	23.80894	23.85303
124	26.05145	26.52184	25.51339	26.51791	25.39715	25.35638	26.15501	25.60833	26.42827
125	28.04045	27.02208	27.36345	28.13667	26.95253	27.36398	27.97919	27.19253	27.29697
126	23.56571	24.61261	22.98816	23.63435	24.62923	23.87126	24.41458	24.15362	23.52847
127	28.19331	27.11062	26.52525	28.09958	26.41357	26.18139	28.42025	27.13207	27.05931
128	25.96276	25.59274	24.67304	25.24279	25.60871	25.02552	26.00272	25.89512	25.70208
129	28.61497	28.11427	28.3338	28.38799	28.14917	27.98892	29.89214	28.67093	28.27585
130	28.40206	27.51101	28.26395	28.37552	27.47876	27.94773	28.57121	28.20788	28.24111
131	31.48796	30.45283	30.59616	31.63781	30.48111	30.61665	31.82622	30.66813	30.7518
132	25.59033	25.7418	25.9574	25.81279	25.42217	25.5045	25.71001	24.96522	25.12363
133	24.98384	25.27967	24.74698	24.88837	25.30334	24.83075	24.83975	25.75363	25.89194
134	29.46487	28.66169	28.46177	29.49503	28.59027	28.50905	29.788	28.66097	28.98195
135	23.30788	21.84172	22.50741	24.80981	22.40005	22.18277	24.37082	22.5713	23.76775
136	28.19221	26.98959	27.33028	28.34026	27.2275	27.37437	27.94641	27.67266	27.74982
137	29.15819	28.03815	28.31588	29.31763	27.88597	28.43137	29.34958	28.43931	28.84366
138	25.76926	24.46613	24.29599	25.6673	24.31702	24.59701	25.39677	24.75246	25.16272
139	30.18885	29.12453	29.15725	30.25286	29.08986	29.0798	30.43202	29.43168	29.16079
140	27.98106	27.74293	27.99035	27.99815	27.17448	27.924	27.74782	27.7219	28.62045
141	27.22909	26.49966	26.36172	27.36411	26.26528	26.48716	27.30936	26.33148	26.60438
142	26.02698	23.02982	24.45829	25.96695	24.04525	23.56235	25.37731	24.34286	24.02801
143	27.37311	26.87023	26.51939	27.39177	27.00447	26.26359	27.00258	26.81427	26.56825
144	25.64983	25.39785	23.74584	24.81686	23.20415	24.64923	25.05909	22.63508	24.34431
145	28.55717	27.85424	27.38615	28.61786	28.00595	27.32094	28.32292	28.12227	28.27836
146	24.22365	22.71617	22.57986	24.74932	22.5037	24.14415	24.14942	23.37639	22.51824
147	28.51396	27.29463	26.96536	28.62761	27.24917	27.10194	29.00021	27.40462	27.13771
148	29.73816	28.29446	27.40453	29.83169	28.38847	27.40351	30.262	29.85027	27.92976
149	24.67555	23.46272	23.07888	24.8363	23.52498	23.62505	24.56591	24.40083	23.65161
150	25.95358	25.02983	24.83161	26.2547	24.69311	25.1356	26.0183	24.99339	24.65133
151	24.9168	24.70328	24.50938	25.36745	23.94808	24.58941	25.00727	23.93461	24.96113
152	25.26422	24.83573	23.80381	25.32927	23.14352	25.06896	25.33958	25.02454	23.62185
153	24.51565	23.73086	24.00729	24.39506	22.46665	23.38099	25.42172	23.3407	23.51205
154	25.31575	24.47641	24.22544	25.19549	24.09457	24.47359	25.61109	24.37146	24.79457
155	22.58671	22.78903	21.73195	22.15882	21.08914	21.43233	22.27368	22.21227	22.40951
156	26.40019	26.53358	25.30906	26.54097	26.21951	24.64711	26.49432	26.44906	25.38053
157	26.32847	25.73484	26.11667	26.31071	25.90575	25.86421	26.18863	26.98839	26.34469
158	26.08907	25.2091	24.82855	25.95903	25.12079	24.69254	26.07182	25.90543	25.6411
159	26.81322	25.76071	26.50995	26.98684	26.4785	26.86506	26.42207	26.78007	27.01912
160	27.5405	26.91832	27.34586	27.48398	26.78783	27.3704	27.48108	27.02499	27.428
161	28.38892	28.00466	27.43649	28.43388	27.94159	27.45206	28.39259	28.13687	27.30724
162	24.78365	24.26735	22.41017	22.87177	23.9752	23.11915	24.63888	24.77046	24.05375

	AS	AT	AU	AV	AW	AX	AY	AZ	BA
163	26.85159	27.35594	27.31776	27.06486	27.04766	27.14122	26.85374	26.96932	27.38832
164	31.51546	31.12218	31.71234	31.61049	30.88128	31.66529	31.45678	31.11543	31.52267
165	26.78246	26.20774	25.90555	26.63752	26.2252	26.1112	26.88407	26.27716	26.2598
166	28.70063	29.58812	29.86252	28.56913	29.43567	29.80141	28.44081	29.23815	30.63393
167	25.5305	25.27994	24.27885	25.57143	24.25971	24.21311	25.48994	24.57173	24.04938
168	27.14752	26.71502	27.06463	27.28308	26.58992	26.85248	27.32719	26.69959	26.74087
169	30.32422	29.52026	29.24374	30.44692	29.56221	28.95192	30.21901	30.05213	29.25899
170	22.88937	22.12056	20.90282	22.68846	20.63139	21.98567	22.60329	21.04456	20.75804
171	27.36181	27.67024	27.44382	27.33366	27.4586	27.39142	27.40987	27.77798	27.93862
172	27.64526	28.13168	28.68452	27.40088	28.12191	28.35589	27.04639	28.84662	28.91541
173	24.02082	22.96853	22.64122	24.31923	22.96738	23.76944	23.98573	22.99336	22.18521
174	25.23023	24.48275	24.95075	25.44761	23.73404	24.17383	25.53713	24.28848	24.22668
175	23.57268	23.32781	22.3265	23.37632	23.28242	22.78186	23.41522	23.93039	22.95183
176	25.08046	25.48188	23.37482	25.37087	25.37742	23.14565	25.32823	25.83474	24.64844
177	31.48809	31.64445	31.40745	31.44501	31.35683	31.30567	31.54514	31.89297	32.21745
178	25.10444	24.13199	24.02864	25.44436	23.51132	23.22092	25.72087	24.20335	24.66179
179	26.4061	23.73075	24.16739	26.19571	23.88848	23.95163	26.84112	25.71509	24.4718
180	27.57798	27.18382	27.25074	27.62656	27.14985	26.80091	27.621	27.91062	28.24492
181	23.83288	22.2734	22.20871	21.95749	21.95507	21.86908	23.89144	21.62876	21.56658
182	26.96915	25.86848	26.04611	27.07961	25.94382	26.19401	27.17506	26.03026	25.57162
183	22.82747	19.98252	23.47876	22.94929	21.51462	23.28431	23.17279	22.95952	23.61407
184	28.29886	27.36855	27.6644	28.15905	27.23282	27.74986	28.1712	27.42499	27.87893
185	27.59091	26.51615	27.06763	27.62683	26.65698	26.72081	27.49973	26.95233	26.79379
186	35.19661	34.89991	34.63147	35.31792	34.913	34.74925	34.98389	34.71727	34.65857
187	28.66796	27.88209	28.3166	28.68629	27.76415	28.27903	28.38685	28.16147	28.79652
188	25.4451	24.22769	24.49309	25.21968	24.30011	25.27296	25.23044	25.18755	25.06077
189	25.97361	25.14454	25.92049	25.70339	25.32623	25.69078	25.69226	24.77985	25.32717
190	26.39375	24.67841	24.10898	26.49802	24.43626	24.04622	26.45905	25.13695	25.44575
191	32.54322	32.3093	32.42031	32.67122	32.24062	32.41501	32.70586	32.3725	32.52682
192	27.86961	27.54357	26.81768	27.61531	27.59768	26.62264	27.92438	26.94085	27.03761
193	26.7714	26.33718	26.07887	26.63286	26.3962	26.00135	26.79644	26.35755	26.37844
194	27.80607	27.44586	25.99181	27.8582	27.39625	26.35946	27.68395	26.94991	26.46028
195	30.06481	29.01176	28.97375	30.07037	29.05348	29.09102	30.33879	29.38943	29.62355
196	27.62503	27.30634	30.19838	27.47071	27.31408	29.21039	27.49816	27.32151	29.0228
197	26.84535	26.34014	26.00368	27.06935	25.85195	25.92978	27.09496	26.50444	26.29611
198	24.72018	23.3641	24.86304	24.59195	23.29909	24.55834	24.70873	24.29535	24.53559
199	25.41523	24.32313	22.92953	25.36012	24.03785	23.08202	25.41229	24.23812	24.06273
200	26.36467	25.20173	24.13276	26.27286	24.7704	24.63522	26.53654	25.42392	26.03108
201	28.16621	27.95055	26.28942	28.05526	27.95033	26.03833	27.64862	28.00578	27.44957
202	31.33791	30.87948	30.95518	31.4114	30.84192	30.7771	31.41907	31.48546	31.48779
203	25.80501	24.48675	25.1967	25.81242	24.99239	25.02787	25.81064	25.20777	25.32519
204	28.08477	27.69071	27.6867	28.06598	27.44549	27.19987	27.87885	27.14749	27.08627
205	27.93148	27.11852	27.28036	28.0639	27.06263	26.88861	27.94306	27.22998	27.28292
206	28.4257	28.31483	28.53778	28.33244	28.39979	28.44269	28.82891	28.93289	29.03299
207	25.09113	23.93965	23.85557	25.19348	23.80007	24.3617	25.70728	24.56703	24.58795
208	28.83387	27.52723	27.13469	28.66749	27.3613	26.82487	29.57435	28.51264	27.94876
209	28.43887	27.84708	27.66831	28.56621	27.87434	27.33867	28.16413	28.10279	28.17265
210	28.10998	27.57252	27.00432	28.09666	27.70891	27.09207	28.09352	28.02611	27.4482
211	26.8574	26.26936	26.5828	26.91825	26.30762	26.43005	27.20658	25.8563	26.38125
212	23.78144	22.18273	21.72552	22.20915	21.97841	22.0431	22.04891	22.52094	22.31867
213	30.50836	29.52769	29.41201	30.57109	29.61963	29.41465	30.4528	29.46657	29.60498
214	27.86896	26.88799	26.95325	27.85726	26.57742	26.85089	27.75046	26.8609	26.85771
215	29.61542	29.34847	29.9997	29.60198	29.17863	29.03155	29.90886	29.47706	29.66378
216	26.407	25.64184	25.3493	26.30285	25.48126	25.71213	26.41745	25.54895	25.9724

	AS	AT	AU	AV	AW	AX	AY	AZ	BA
217	26.19512	25.71482	25.58596	26.11078	25.81087	25.7486	26.36744	25.69015	26.02452
218	24.36831	23.95366	24.36707	24.28593	24.04798	23.81359	24.46478	24.26864	24.05194
219	29.19636	27.94459	28.25488	29.25448	27.9082	28.1815	29.14873	28.37479	28.28037
220	28.45805	22.43574	22.57411	28.31556	22.16987	21.98183	28.55072	22.80779	27.90154
221	23.99115	22.3112	22.72199	24.33606	22.08818	22.38063	24.11314	24.79166	21.95869
222	24.63462	24.58922	25.35274	24.90288	24.20014	25.37879	24.93253	25.32516	25.36888
223	29.1956	29.24669	29.34888	29.22811	29.28789	29.26904	29.4861	29.47098	29.46393
224	26.82279	25.84001	25.84483	26.90823	25.50334	26.11717	26.72867	25.43838	26.54962
225	31.3574	30.80608	31.04912	31.4739	30.73796	31.20556	31.77924	31.18378	31.58906
226	25.30484	25.2907	25.18619	25.63489	25.25548	25.25549	25.53486	25.80515	25.03603
227	27.52279	26.60594	26.15945	27.7054	26.59264	25.99434	27.42429	26.86874	26.13631
228	24.79062	26.62937	24.52333	25.33673	26.80443	25.13208	24.58223	26.82672	25.79786
229	25.84353	24.81867	25.22849	26.10718	25.00924	24.87419	25.84745	24.75607	25.24468
230	24.86174	24.64635	24.72268	25.2106	24.1282	24.2106	24.91393	23.95947	23.84471
231	25.09776	23.89139	23.70533	25.13542	23.02806	24.10931	25.13815	24.27044	24.47411
232	30.70225	30.5181	30.07369	30.42729	30.51663	30.03483	30.43486	31.27446	31.13657
233	30.69891	30.89544	31.21545	30.89949	30.927	31.27805	30.8371	31.20642	31.371
234	27.61287	27.32024	26.76818	27.60788	27.39095	26.63372	27.62192	27.13832	26.53113
235	26.45699	25.28786	25.38963	26.33018	25.37441	25.21577	26.40329	25.49359	25.2573
236	29.95746	29.74782	29.56513	30.01698	29.66908	29.46923	29.89803	29.95944	30.00806
237	25.11065	24.76356	24.17452	25.27262	23.68852	23.56967	25.24328	24.11089	24.4008
238	21.29224	19.71429	18.93083	20.16023	20.33467	19.52231	22.29432	21.40946	21.71966
239	25.49993	24.06541	24.51136	25.81914	24.30184	24.32784	25.56691	24.54838	25.25023
240	19.60742	20.43457	19.65162	20.05342	20.57603	19.03514	21.78221	21.53392	21.36366
241	28.15459	28.397	27.92728	28.28432	28.29104	27.83755	28.12385	28.40457	28.22883
242	27.40981	27.48564	27.6738	27.66484	27.61287	27.1681	28.28697	27.11319	27.05108
243	26.93077	25.88483	25.37747	26.23446	25.57442	25.35558	26.66088	26.94013	26.4076
244	29.63766	28.26597	28.77356	29.55915	28.97394	28.38493	29.44193	28.51187	28.47931
245	29.25399	29.38987	29.07385	29.34136	29.27178	29.30941	29.16832	29.0562	29.68038
246	26.09055	26.56636	26.42901	26.00161	26.47079	26.22678	26.01325	26.56452	26.36738
247	23.58295	22.70238	23.35621	23.68999	22.78192	23.28432	24.35479	23.28881	23.29292
248	26.94418	26.8295	26.66872	26.93215	26.68167	26.83218	27.06519	27.15351	27.27562
249	25.26871	24.84203	23.77934	25.39962	24.66159	24.51171	25.9747	25.16245	24.5795
250	27.73534	26.54998	26.81045	27.66845	26.58468	26.86426	27.93775	27.00014	26.93746
251	27.41046	26.22392	26.04913	27.58317	26.26774	26.0428	27.35533	26.65893	26.78549
252	23.48303	22.56951	22.75384	23.34623	22.71466	22.49791	23.15529	22.92095	22.65755
253	25.27084	25.68184	25.45952	24.80667	25.36612	25.74449	24.65092	26.03127	26.37524
254	26.00553	25.38909	25.56441	25.37142	24.95993	25.06498	25.77061	24.89158	24.83651
255	24.86678	25.41908	23.36401	24.90624	24.73391	25.22936	24.77357	24.9737	22.51828
256	26.61482	25.34673	25.40785	26.81476	25.18655	24.89798	26.27213	25.34697	25.17565
257	27.51223	26.07906	26.61552	27.45399	25.91578	26.29629	27.46933	26.8288	27.13528
258	27.76745	26.94197	27.01307	27.93773	26.87768	27.05549	27.97364	26.76767	27.38021
259	23.87456	22.79433	22.52686	23.63434	22.76199	22.72266	23.42881	22.884	21.79868
260	25.50562	24.46779	24.51758	25.63568	23.94886	24.11159	25.82068	24.45306	24.14548
261	25.51591	24.85889	25.46272	25.57405	25.40401	24.81096	25.64575	24.9147	25.85186
262	31.68064	27.59545	29.00467	31.89459	27.50548	28.98938	28.28645	27.64224	29.54075
263	24.39902	23.9845	25.78038	24.63694	26.78025	24.63107	25.3362	23.53896	24.68203
264	26.01215	25.38683	25.6736	26.1261	25.36965	25.64733	26.23858	25.47459	25.63456
265	26.97176	26.08856	26.62507	27.18857	25.9602	26.6329	27.21307	26.42863	26.53082
266	21.41914	21.16687	21.67574	22.2633	21.05657	21.4771	21.58941	22.88372	21.49713
267	31.47625	30.994	31.54789	31.52188	30.97092	31.40564	31.3025	31.45919	31.95486
268	24.35278	23.26275	22.96836	24.11879	23.42871	22.36275	24.24169	22.81127	22.02187
269	24.13155	24.33275	23.86858	24.27351	25.63302	23.92115	24.11803	23.8097	27.5302
270	27.10416	25.94458	26.13952	27.10041	26.30812	26.15058	27.02236	26.79931	26.6981

	AS	AT	AU	AV	AW	AX	AY	AZ	BA
271	26.99343	25.73443	26.06748	27.33738	25.36643	25.76748	26.81561	25.80718	25.8004
272	24.59394	23.80547	24.31625	24.46045	23.73435	24.34835	24.77392	24.21251	24.36267
273	25.58123	24.34305	24.94561	25.85964	24.45602	25.03857	26.05314	25.45443	24.68733
274	24.76187	25.05371	24.32937	24.924	24.678	25.16972	24.53844	24.48582	24.2219
275	26.94412	26.66866	25.50732	27.5401	26.65578	25.50723	27.47379	27.22753	26.18808
276	30.52508	29.7702	30.12387	30.46809	29.67488	29.37983	29.96663	30.21772	30.28596
277	26.80052	25.9723	25.39447	26.70904	26.01906	25.4232	26.93873	26.01244	25.43181
278	26.1072	23.88172	24.31806	26.32243	24.81805	24.39102	26.63211	23.90708	25.11819
279	30.74352	30.83738	30.8588	30.88448	30.79189	30.69055	31.13808	30.87912	30.93596
280	29.78611	29.04947	28.99142	29.8833	29.08546	29.00764	29.95953	29.23524	29.36394
281	25.44453	24.13012	24.91244	25.68655	24.05522	23.87382	25.69427	24.74108	23.82554
282	28.02906	27.54947	27.42703	27.82357	27.5701	27.52406	27.83159	27.90627	27.85674
283	24.52594	24.42659	23.27287	23.48418	24.40513	24.34211	24.6616	24.37085	22.98236
284	25.59809	24.89074	25.93209	25.5047	24.73317	25.07588	25.51163	25.18804	25.69624
285	24.2741	23.11219	23.93381	24.11221	24.00246	24.41745	24.32336	24.75622	23.5324
286	29.43377	27.62815	28.29384	29.43706	27.64047	28.43346	29.07477	28.66383	29.24294
287	24.10365	24.13311	23.20859	24.0453	23.7262	22.42501	24.65804	24.20043	23.11135
288	22.9961	22.81074	24.40717	22.7994	22.81255	21.83739	23.24937	23.51188	22.91652
289	22.98113	21.85253	24.05162	24.21211	24.04776	23.64764	24.05834	23.98062	22.09064
290	24.90005	25.61405	25.13995	25.28856	25.43759	24.97989	25.35396	26.0561	25.3567
291	23.11989	22.3563	22.4757	23.14193	23.80504	23.13443	24.02306	25.22481	24.77912
292	20.04997	20.93039	22.11135	20.12218	20.0661	23.55361	22.10532	21.55169	20.85084
293	24.02026	25.17478	22.89062	23.64265	24.95586	23.55467	24.09204	25.07557	23.16468
294	26.519	27.31083	27.37469	26.70508	26.55719	26.40272	25.39066	26.44691	26.78032
295	24.08352	25.09662	22.58979	24.12555	23.68095	22.4068	24.76885	24.06073	23.18105
296	24.7217	22.62311	23.0844	24.67435	23.04859	23.01564	24.76622	24.69315	24.19647
297	23.82052	23.12403	23.16212	24.10948	23.50233	23.8131	23.98548	23.45266	24.14982
298	25.71321	26.17749	23.4073	25.10665	23.70072	23.45504	25.13283	25.26279	23.15611
299	23.97095	22.56665	23.94013	24.17945	23.31531	22.47084	24.35477	22.70802	22.43516
300	25.24694	24.40457	24.68988	25.4503	24.55989	23.99468	25.69045	24.70083	24.75329
301	28.43136	28.02078	27.93366	28.62878	28.08105	27.9175	28.7117	28.54763	28.7758
302	26.29901	24.22773	25.03675	25.71889	25.20953	25.4911	25.1866	25.14057	24.42731
303	23.79908	24.67467	22.48038	24.42404	24.28515	21.97473	24.68126	22.15757	24.91538
304	27.02753	26.58245	27.38705	26.98553	26.65861	27.30695	27.17794	26.51652	27.37436
305	27.62657	27.12705	27.12403	27.62792	27.06442	26.94078	26.99869	27.38387	27.0935
306	26.05927	22.54865	23.45612	26.11325	23.82029	22.91575	25.7651	24.2661	24.38555
307	28.62568	27.97433	27.64139	28.6224	27.96326	27.57741	28.3601	27.98936	26.93717
308	21.93477	22.15244	22.79315	22.63152	23.00211	21.62963	22.72889	22.45576	22.59039
309	25.77391	25.51211	25.06072	25.83543	25.54193	25.58319	25.80011	25.75337	25.49402
310	23.9432	22.75848	22.89087	24.18024	23.16836	23.13678	24.54415	23.86206	23.46909
311	23.07898	24.13091	24.3258	24.33672	23.89697	24.03058	23.41078	23.23864	22.91512
312	26.79703	25.84781	25.91137	26.54501	25.72532	25.14464	27.18115	26.04603	25.9823
313	28.1786	27.19169	27.10935	28.20933	27.06693	26.90744	28.44289	27.51322	27.67412
314	26.95262	26.14523	26.15099	26.90578	26.10418	26.11044	26.77742	26.6566	26.42894
315	24.26251	24.11623	23.52422	25.51774	22.96015	23.06105	24.69107	24.2004	24.4
316	25.78743	23.60816	23.41307	25.8335	23.58051	22.44168	26.22385	27.53092	26.70965
317	25.57603	24.49437	25.14202	25.87928	24.95305	25.20019	25.78651	25.39874	25.45408
318	24.39174	22.79898	23.8227	24.74399	24.47474	23.70597	24.33414	25.08843	25.30494
319	25.74207	25.46347	25.35798	25.63301	25.60191	24.91922	25.96721	25.80467	25.54593
320	27.83419	26.63957	27.19332	27.89887	26.8082	27.24108	28.05424	26.862	27.4263
321	29.85063	28.97577	28.45382	29.92804	28.92762	28.45922	29.93707	29.30992	28.99162
322	25.78125	25.94331	26.07714	25.74638	26.03329	25.98514	25.51665	27.1306	26.48874
323	26.57389	26.44633	26.58231	26.60767	26.41749	25.82201	26.42237	26.7838	26.10099
324	26.36275	25.90094	25.74831	26.27162	25.55052	25.52466	26.46752	25.76727	25.40801

	AS	AT	AU	AV	AW	AX	AY	AZ	BA
325	27.86454	27.93936	25.91161	26.75132	27.82478	25.57238	26.2906	27.77155	25.17843
326	26.13347	25.20932	21.95026	26.23713	24.79041	22.76765	26.22772	25.85581	22.19542
327	26.23004	26.73226	23.85671	26.2262	26.86357	23.98467	26.41303	26.94367	25.0556
328	31.941	30.76634	31.01526	31.83112	30.48303	30.79486	31.84872	30.57446	30.85753
329	30.52026	30.35433	29.91438	30.54974	29.91377	29.52392	30.49825	30.53579	30.7258
330	27.76761	27.88491	27.75853	28.06833	27.59932	27.58043	27.9696	28.05024	28.2954
331	25.61687	25.36262	25.82662	26.52917	25.68667	25.28843	26.32157	24.99938	25.71369
332	23.93635	21.83979	22.93224	23.26472	22.77295	22.4174	23.50997	24.21181	23.46515
333	26.60886	26.5104	26.34502	26.61654	26.17649	26.59599	26.44057	26.22572	26.55999
334	22.86749	22.33932	22.22973	22.21464	22.37724	22.40294	23.31347	22.28865	22.263
335	26.40261	26.26686	26.48515	26.46105	26.37031	26.60854	26.37722	26.44927	26.7622
336	27.88425	27.68827	26.76832	28.04515	27.48285	26.79208	27.82645	27.325	26.98269
337	29.38436	28.86934	27.74059	29.35482	28.91302	27.52117	28.87827	28.72927	28.03677
338	24.03757	20.98486	21.50567	23.40849	23.88859	22.30276	20.82489	23.12286	22.41947
339	28.51859	28.05432	28.55728	28.52602	28.09207	28.48997	28.98487	28.82955	29.077
340	22.21889	22.29938	24.6033	22.71059	23.29786	23.62904	22.42294	23.48722	24.35433
341	26.28099	26.58416	25.73226	26.17537	26.67919	25.50881	26.46233	26.92075	26.24887
342	27.06598	27.60999	26.07195	27.07994	27.2689	26.04635	26.88615	26.75726	26.0532
343	29.16197	29.35564	29.78695	28.90187	29.35594	29.84241	29.73667	29.73869	30.22809
344	25.36682	25.31075	25.91894	25.54431	25.16149	25.68945	26.1602	25.70781	25.97726
345	27.25218	26.4539	26.54694	27.6685	26.29819	25.39058	28.18583	26.41954	27.26711
346	27.52997	26.6565	27.22509	27.65104	26.40498	27.10949	27.50552	26.64206	27.3308
347	22.26055	21.34622	21.70074	21.33468	21.59771	21.55449	23.17988	21.52508	21.91262
348	26.59378	25.38424	26.15908	26.68862	25.03095	26.12813	26.73837	25.40999	25.80395
349	27.37178	26.81441	26.89694	27.43658	26.86681	26.94964	27.10607	27.25535	27.32326
350	30.90302	30.1101	29.74373	31.02601	29.99667	29.63941	30.76314	30.59633	30.27496
351	30.91635	30.21319	29.75525	30.75092	30.14738	29.48052	30.57018	31.22654	29.8914
352	24.10262	23.46863	23.68107	25.03733	23.39401	23.30025	24.4152	23.4097	23.97837
353	25.34742	24.21027	25.68281	25.0698	25.47257	24.74813	25.50539	24.22591	25.45817
354	28.42582	27.38306	27.50809	28.45751	27.02375	27.56183	28.42688	27.4379	27.56542
355	31.01147	30.26658	29.09235	31.03586	30.21504	29.08132	31.48458	30.59014	29.94254
356	26.32596	26.32518	26.09531	26.0597	26.44882	26.06639	26.99195	27.62735	26.98533
357	27.4042	27.55556	26.96122	27.43834	27.03436	26.83085	27.26895	27.22229	27.43815
358	25.46592	24.88896	24.60841	25.22907	25.17008	24.6837	25.05281	24.78374	25.39144
359	25.25602	24.72565	24.65473	25.47293	24.54519	24.58872	24.8631	25.89012	24.99268
360	25.49556	22.93006	23.62696	23.7385	23.93023	22.30454	25.00261	23.09512	22.21579
361	28.08423	28.21559	28.6555	28.23513	28.27885	27.71805	28.12101	28.47134	28.69793
362	29.97434	28.67255	27.88136	30.22995	28.35819	28.03344	29.8099	28.69216	27.60286
363	27.10358	27.55548	27.38711	27.25919	27.63977	27.55036	27.08358	26.95199	27.99244
364	24.01466	23.52451	22.94752	23.79258	23.39099	22.9033	24.09903	22.63168	23.23781
365	25.11101	23.95254	24.84224	25.30245	24.50938	25.73324	25.25017	24.61339	25.13193
366	27.34036	26.47349	26.65655	27.4245	26.5816	26.54557	27.40167	26.96472	26.79409
367	25.00889	24.79282	24.97335	24.94703	24.7726	25.12783	25.0039	24.87385	25.17952
368	25.30999	24.00192	23.37369	25.33054	23.974	22.6436	25.22072	24.60663	24.41859
369	27.02467	27.00188	27.07337	27.03571	26.67941	27.03271	26.98513	27.0168	27.39073
370	25.73713	25.14314	25.00528	26.17592	24.58157	23.73854	25.7795	25.64279	25.92055
371	24.15934	23.57545	22.22348	23.33773	23.87635	23.99075	23.99984	23.90811	23.12395
372	23.44908	22.19115	22.40757	23.59854	22.46447	22.26676	23.5515	23.05882	22.78506
373	18.6924	19.17078	21.15965	18.52163	18.3129	20.50173	22.05556	20.84272	21.38038
374	29.16383	28.00027	28.12705	29.14894	27.91371	28.07279	29.43151	28.30943	28.60244
375	26.38948	25.8271	24.60791	26.15759	24.85317	25.23698	26.21917	25.51687	25.32073
376	24.0471	22.7317	22.70539	24.00715	22.36668	22.66893	24.3447	23.23333	22.78733
377	25.32258	23.35517	24.49478	25.48515	23.037	24.22521	24.81514	25.05091	24.5611
378	23.7106	25.27391	23.67336	23.64035	24.9377	24.2524	23.71116	25.48228	24.22152

	AS	AT	AU	AV	AW	AX	AY	AZ	BA
379	27.46541	27.34707	26.83254	27.67721	27.33896	27.84399	27.46169	26.88195	28.04841
380	25.78994	25.54162	25.08704	25.77444	24.68696	23.78058	25.74739	25.68953	25.92753
381	26.73064	25.58364	25.82106	26.89588	25.5854	25.86399	26.70059	25.96341	26.16422
382	28.2464	26.91574	27.28371	28.18026	26.54696	27.7628	28.12408	27.14197	26.96548
383	25.95251	23.49157	24.38521	26.24975	23.43069	24.86719	26.05582	23.79385	25.00028
384	26.6066	25.41522	26.07222	26.46028	24.96909	26.16115	26.57081	26.41367	26.10936
385	26.47854	25.88006	25.66109	26.25916	25.84545	25.2573	26.75224	26.20493	25.76144
386	27.55767	27.47912	27.66746	27.70738	27.73227	27.38788	27.68019	27.53506	27.76686
387	26.89258	26.06437	25.66229	26.85176	25.58397	25.36813	26.95959	25.86445	26.02584
388	28.1172	26.96883	28.17697	27.92291	26.82333	27.43958	27.64787	26.98184	27.29496
389	27.80173	27.02222	27.6646	27.78622	26.87375	27.11782	27.96583	28.11244	28.15937
390	24.60362	23.39678	23.75882	24.8057	23.52408	24.6266	24.67673	24.2779	25.42431
391	30.46929	29.13968	29.95608	30.51753	29.24065	29.82817	30.83465	29.25834	29.71102
392	27.71982	27.72937	27.35663	27.83617	27.88401	27.20595	27.60996	28.39955	27.74778
393	28.71587	28.02897	27.74212	28.72313	27.88455	27.69758	28.68648	28.08226	28.53058
394	25.49851	25.0833	24.52572	26.47147	24.58726	24.49873	25.53628	25.84105	25.68239
395	26.48973	25.62082	24.94201	26.64958	25.4819	24.11087	26.53542	25.80945	24.98581
396	30.0761	30.40494	29.65789	30.00624	30.35835	29.52378	29.93786	30.28991	29.62455
397	26.27994	25.57478	25.31459	26.18557	25.16609	25.16798	26.55865	25.61201	25.54584
398	29.71999	29.97418	29.68187	29.73791	29.90478	29.46128	29.64159	29.93646	30.11525
399	24.12676	23.16578	21.62645	22.12759	22.08142	21.78505	23.82195	23.05987	22.33352
400	25.34275	24.83521	24.52943	25.37661	24.5809	23.80198	25.21396	25.00778	24.60763
401	23.72342	22.09686	22.11045	22.8082	22.5949	22.32139	23.34495	23.35062	22.59462
402	21.86395	22.62365	20.84016	22.04554	22.38436	21.27916	20.61047	23.94432	21.11028
403	29.10566	27.61589	27.73888	29.19367	27.57425	27.7823	28.98888	28.17426	27.89164
404	25.51524	24.57639	23.91127	25.03814	24.2457	23.85619	26.01578	24.85012	24.13551
405	23.1429	20.87492	21.80812	23.70304	21.66726	21.82689	23.99188	21.53251	22.15642
406	22.5428	22.44868	24.47982	22.45609	23.1132	23.60875	23.87489	23.27461	24.34291
407	24.10619	24.31936	25.30877	24.20894	23.97196	24.05999	24.11247	23.94189	25.23733
408	26.16466	25.09519	25.42293	26.63024	25.07449	25.60026	25.85218	25.79907	25.61422
409	26.45098	26.67525	26.04799	26.68544	26.83561	26.13287	26.43236	26.84937	26.76821
410	28.54799	27.97958	27.69792	28.56867	27.99864	27.99401	28.3914	28.2465	28.11892
411	28.84432	26.54951	25.04696	28.52508	26.33229	26.18426	29.52258	27.30032	27.0598
412	26.99493	25.38974	25.31458	27.22392	25.64391	25.59238	26.98993	26.29971	25.4472
413	28.97633	27.89921	27.22365	28.92881	27.53422	27.65417	28.8951	27.98328	27.72899
414	28.59801	27.98845	27.2341	28.71109	27.78478	27.62846	28.47394	28.41224	28.13681
415	23.82804	22.45347	21.49297	24.10015	22.50788	22.00665	24.13925	22.79427	23.8814
416	23.38801	22.90206	22.09306	23.01487	23.06018	21.2262	22.9421	23.45632	21.77412
417	27.01915	27.45095	26.79073	27.13038	27.26229	26.19638	26.80059	27.00932	26.32678
418	26.29055	25.78372	25.9886	26.39462	25.7278	25.68094	26.38161	26.47281	26.38026
419	24.17225	23.35278	23.58428	24.0162	22.80191	22.96119	24.29364	23.43433	23.88327
420	26.93605	26.06337	26.72271	27.08508	26.26216	26.83738	27.20156	26.05956	26.80933
421	24.87662	26.22687	22.30788	25.39222	25.94299	22.73916	24.65467	23.83121	25.6433
422	27.30677	26.08429	25.96244	27.17255	26.06369	26.00145	27.00169	26.49039	26.54949
423	27.92564	27.17889	26.76793	27.94037	27.03282	26.79003	27.82194	26.8499	27.05006
424	27.92188	27.24043	27.12212	28.0556	27.34459	27.11561	27.97103	27.42822	27.27669
425	28.49758	27.2582	28.42282	28.65037	27.17764	28.25509	28.38884	27.87802	28.95215
426	23.97801	22.70782	23.18795	24.20397	22.8759	23.25233	23.92419	23.22752	23.56797
427	24.34501	23.94311	24.01678	24.41144	23.60394	22.9442	24.22692	23.59412	23.32581
428	24.42869	23.87172	23.70269	24.59104	23.60467	22.98919	24.75067	23.80936	23.43587
429	22.97718	23.59213	21.86682	22.49825	23.76682	22.56334	22.66252	24.91136	23.28431
430	23.2865	24.26827	21.86437	22.97231	23.9475	23.60433	24.02587	24.26411	24.46298
431	27.18803	26.12638	26.5354	27.21149	25.98839	26.49774	27.10381	26.85952	26.84942
432	24.45347	24.11443	24.41161	24.51724	24.94962	24.87159	23.87525	23.73948	24.20817

	AS	AT	AU	AV	AW	AX	AY	AZ	BA
433	24.64553	25.95232	24.12344	24.7629	25.69993	23.21437	24.3516	25.72635	23.15475
434	27.23932	27.46033	26.73441	27.27716	27.43118	26.49605	27.30186	27.39535	26.84949
435	24.00974	21.21098	22.26512	23.90198	22.07723	22.01055	24.36984	21.21284	23.0985
436	27.60403	26.20998	26.30648	27.7678	26.13904	26.33584	27.592	26.58883	26.72997
437	28.19242	27.4289	27.04556	28.21023	27.4006	27.15302	27.9805	27.00593	27.18036
438	25.9604	25.9006	25.3738	26.10584	25.92388	25.15433	25.22735	25.59165	25.46732
439	31.31961	31.77744	31.26324	31.33266	31.69242	31.29527	31.27134	32.16467	31.81677
440	27.31252	26.76074	26.23446	27.26279	26.42392	26.41639	26.7878	26.2379	26.35776
441	25.46662	24.45615	24.38109	25.19331	23.81779	23.43688	24.90207	23.5631	23.01332
442	26.66525	26.14334	26.25329	26.65438	26.12856	26.25917	26.62018	24.77241	25.97564
443	24.49653	22.34232	22.88785	24.46338	23.047	22.51321	24.94066	23.40567	23.76728
444	28.23481	27.38861	27.6776	28.18701	27.3366	27.78141	28.32561	27.74771	27.70654
445	27.6413	27.51369	27.05723	27.85887	27.35452	27.19581	27.63841	27.892	28.01429
446	28.43845	27.43718	27.0774	28.40703	27.39228	26.99609	28.23857	27.98021	27.3376
447	27.41298	26.22379	26.96251	27.60265	26.60236	26.08683	27.54726	26.07255	25.56209
448	30.69336	30.58029	29.98205	30.45189	30.49143	29.98978	30.49987	30.31489	30.29695
449	26.16951	25.23517	25.16676	26.22142	25.00944	25.54373	26.0321	25.38736	25.04291
450	25.20889	25.35616	25.94952	25.35264	24.63893	25.41397	25.20329	24.52405	24.81367
451	28.94613	28.97792	28.6061	29.08013	29.1122	28.65755	29.02691	29.02008	28.61708
452	29.85118	29.63037	28.63152	29.66608	29.6564	28.59241	30.10323	30.42828	30.11244
453	32.9385	32.94624	32.93491	33.06562	32.95239	32.87554	33.2011	33.28391	34.32877
454	28.3863	27.62771	27.4243	28.4271	27.56759	27.36848	28.44075	27.92434	27.76531
455	22.5689	22.43746	23.29373	22.39267	22.2238	21.92934	22.52464	22.73269	23.32295
456	22.8377	22.55744	22.15594	24.05198	23.11512	21.50757	24.47284	23.31169	23.79496
457	30.41532	29.63401	29.75441	30.54848	29.74168	29.65346	30.78836	30.19784	29.95867
458	27.24816	28.35838	28.59003	27.41267	28.2789	28.56975	27.05146	28.31304	28.96548
459	32.47168	32.44474	32.36794	32.30051	32.33995	32.15405	32.80572	33.12893	33.05426
460	26.48737	24.67848	25.50842	26.36382	25.55293	24.88564	26.41433	25.8881	24.9007
461	29.3799	29.52542	29.75988	29.40985	29.4059	29.64417	29.15819	29.45789	30.05006
462	28.47906	28.44007	28.77571	28.53118	28.64731	29.1221	28.24314	28.4137	28.72582
463	29.35987	28.11023	27.72481	29.15245	28.14771	27.32285	29.65052	28.49189	27.72754
464	24.62026	23.02615	29.27384	24.85513	24.45	29.20732	24.77719	23.61344	29.51315
465	24.62623	24.04139	30.53559	25.18224	24.24313	30.38867	24.47088	24.63985	30.6486
466	27.34675	27.02653	26.77627	27.43317	27.4002	26.77855	27.16205	27.15988	27.05443
467	24.69932	23.23515	23.27478	24.24672	23.16117	24.76103	24.84615	24.09712	23.30913
468	24.95737	23.52013	22.24588	25.13028	21.70748	22.06792	24.3195	23.66454	22.82313
469	24.93747	23.96387	23.12804	24.70001	23.88737	22.48565	25.04782	25.21599	24.66585
470	28.06474	27.06844	27.85691	28.10604	27.08402	27.5545	28.34763	27.43969	27.30666
471	22.44471	21.1325	22.2193	22.94295	20.32362	21.47389	23.96406	23.78379	23.90376
472	23.38527	20.99901	21.52859	23.52696	21.87583	22.493	24.69534	22.34926	21.59019
473	27.55418	27.28343	27.08193	27.53856	27.39613	26.99977	26.59947	27.58817	27.35032
474	25.57032	24.62152	23.65928	25.79897	25.41107	23.95926	25.35415	24.64093	24.52409
475	27.55338	26.66886	25.94176	27.57857	25.95698	25.7193	27.87358	27.61965	27.48718
476	23.56935	22.63602	21.67892	23.22758	22.54442	21.81271	23.35396	20.03206	22.76241
477	25.01255	24.39856	23.08771	25.06367	24.48564	22.81785	25.51966	24.72708	23.40411
478	27.6436	26.95845	26.79627	27.53891	26.8604	26.54862	28.33472	28.00483	28.05337
479	24.79554	23.69047	24.33099	24.80316	23.94674	24.01109	25.65116	24.86253	24.60343
480	25.70172	25.9808	25.3116	25.86247	25.20442	25.30898	26.53575	25.39479	26.40494
481	25.50596	25.29185	25.01779	25.52635	25.40674	25.29866	25.75347	25.00633	25.24629
482	22.63233	21.22519	21.62381	22.32701	20.82708	21.01983	23.14439	23.22465	21.65489
483	22.68165	21.18309	20.77089	22.91218	21.62014	21.2467	22.82335	22.63635	22.29168
484	27.27393	26.91418	26.55677	27.18964	27.06543	25.69104	27.56982	26.84774	25.91758
485	25.60583	23.51807	23.63583	25.80121	23.54786	23.61363	25.58399	24.10744	24.52654
486	28.21898	27.51946	27.38762	28.11377	27.40585	27.13003	27.98194	27.53057	27.64038

	AS	AT	AU	AV	AW	AX	AY	AZ	BA
487	26.2719	26.1306	25.25229	26.29313	25.85243	25.19364	26.36118	26.85476	26.3636
488	24.59439	23.88838	23.67916	24.92089	23.82787	23.36782	25.00754	24.14762	24.77057
489	25.7551	25.02592	24.59439	25.86371	24.68563	24.37841	26.11744	25.22154	25.38109
490	25.1582	23.53794	23.72102	24.71552	23.52246	22.89347	25.13053	25.02835	22.91975
491	25.21663	24.48453	24.93624	25.45678	23.70935	24.93234	25.49828	24.02689	24.78087
492	27.24833	26.21639	26.76679	27.37982	26.29068	26.90516	27.23485	26.82441	26.70745
493	23.97311	24.45017	22.89238	25.19796	23.16694	23.88713	24.32286	23.18211	24.90118
494	25.0069	24.62327	22.825	24.99286	24.24256	24.13541	25.05577	25.36306	24.03981
495	23.5173	23.86177	22.85028	23.59266	23.99817	22.45654	23.8038	24.23376	23.48416
496	26.7227	26.54162	27.04663	27.1246	26.93085	27.03772	26.59255	27.00935	27.49409
497	25.54044	25.8371	24.92782	25.75241	25.69423	24.58485	25.24693	26.37606	25.99156
498	27.6038	26.33315	26.59556	27.67418	26.27722	26.65713	27.58834	26.7697	26.36163
499	29.59364	29.06051	27.5752	29.6973	29.09221	27.55793	29.42074	29.11298	27.75835
500	29.08235	28.36584	27.66588	29.25167	28.10521	27.3825	29.3198	28.25118	27.57475
501	25.71181	24.77559	23.95057	25.9609	22.8552	23.96305	25.97918	24.80553	24.77697
502	26.20721	25.6644	25.21667	26.31993	25.53318	25.34623	26.20557	25.39172	25.18647
503	23.33144	23.32616	22.22922	23.75965	23.15885	22.5916	24.23951	23.72634	23.07165
504	24.22087	22.80373	23.23971	24.02947	23.55548	23.07208	24.02975	23.47272	23.71322
505	23.97296	24.81768	24.57939	23.9077	23.87055	24.35226	23.82841	22.53004	22.11152
506	28.16022	26.67003	26.79376	28.27248	26.87183	27.35111	28.08484	26.22512	26.87412
507	21.51553	20.41224	20.84502	21.85345	20.15417	21.79901	21.80746	21.33556	20.04073
508	27.60268	27.64318	27.21446	27.54837	27.55284	26.80114	27.81011	26.61805	26.44208
509	22.47641	22.6906	21.80916	22.52094	22.58039	22.41399	26.70513	22.63419	22.43886
510	25.55782	25.99144	25.04832	25.61449	25.93329	24.1177	25.87901	25.4648	24.63533
511	25.56025	25.23583	25.07828	25.56834	25.08227	25.19026	25.48199	25.56557	25.3445
512	32.02379	31.03107	30.86196	32.20276	30.96193	30.8321	32.08365	31.35098	30.98785
513	26.76137	26.11094	24.86346	26.72705	25.44448	25.85884	26.95263	26.82534	27.14047
514	26.74486	25.90521	25.95939	26.85144	25.5728	26.1918	27.15567	26.22445	26.56995
515	25.09146	24.11341	23.31702	25.15184	23.53642	23.23018	25.69687	23.86011	24.29919
516	25.4404	23.96409	24.01077	25.50957	24.46012	23.58456	25.35144	23.52586	24.54142
517	24.31809	23.88704	22.72735	23.61787	23.89983	22.7707	24.2152	23.17298	22.10024
518	22.86448	24.72623	23.94702	22.75266	24.50731	23.73522	22.68608	24.89795	24.75105
519	26.85463	26.9294	25.65415	26.75447	27.09886	25.57734	26.72983	27.62466	26.20075
520	24.31801	24.46359	23.74943	24.51898	24.38726	23.22852	24.14321	22.31185	24.21366
521	26.11496	25.74124	25.45932	26.29402	25.88591	25.6735	26.55817	26.09753	26.00379
522	23.46826	22.37165	22.76478	23.53451	22.57491	22.53367	23.35982	24.39926	24.12205
523	26.47621	26.92203	25.81813	26.10048	25.93763	25.739	26.46552	26.96228	27.0445
524	27.87103	26.6659	26.88481	27.85801	26.61324	26.78637	28.00556	26.96538	26.67893
525	25.08552	24.79298	24.97356	24.6447	24.62676	25.14443	25.10859	25.38836	26.37999
526	26.63133	27.22371	27.22232	26.69703	26.93721	27.04803	26.62676	27.22254	28.14758
527	27.50447	27.00754	26.87349	27.66099	26.79914	26.68132	27.60518	26.93277	27.51457
528	25.42092	24.74706	24.57935	25.6543	24.67411	24.08674	25.4722	25.82352	26.25588
529	28.01439	27.53642	27.54629	28.13928	27.54342	27.58022	28.13419	27.48022	27.47577
530	23.15689	23.29895	23.67089	23.54053	23.98975	23.99291	22.94975	24.13964	23.8834
531	26.28699	24.42793	24.3702	26.54438	24.79547	24.25394	26.45672	24.96572	24.5495
532	28.00858	28.04272	28.02784	27.97622	28.14857	27.97338	28.00448	28.44779	28.51865
533	27.9887	27.05715	27.09872	27.84236	26.95495	27.15033	28.01644	27.07652	27.33191
534	27.20103	26.99166	27.08246	27.18389	27.04999	27.29937	27.64142	27.5682	27.92185
535	32.74157	32.8642	32.32156	32.61742	32.65445	32.11352	33.00586	32.97053	32.74059
536	28.38177	27.70606	28.15646	28.50886	28.12612	27.78716	28.60768	27.87313	28.34265
537	26.69496	27.09769	26.98401	26.66284	27.09391	27.03744	26.74693	27.28904	27.754
538	25.53646	25.80065	24.59867	25.54084	26.04404	24.73688	25.12507	26.03839	26.07905
539	23.6199	23.76668	23.28461	24.32324	23.58547	22.9966	24.17009	24.32914	23.82214
540	26.10897	24.72419	26.52306	25.81397	25.17946	24.6163	25.8002	25.06989	24.63367

	AS	AT	AU	AV	AW	AX	AY	AZ	BA
541	22.63628	22.72623	23.06145	22.57725	23.09749	23.29889	22.61522	23.47817	22.74773
542	25.97912	25.69373	25.55467	26.0279	25.80224	25.83579	25.84328	25.75596	26.59693
543	25.91239	25.87004	25.69667	26.06134	25.4934	25.5008	26.20113	26.05595	26.33325
544	28.31266	28.48836	28.54031	28.46307	28.52336	28.6114	28.14217	28.53923	28.82458
545	22.4515	23.38562	23.30704	23.45007	23.57304	22.65236	23.23869	22.77425	22.97525
546	25.44084	24.75909	24.65478	25.35995	24.85191	24.53685	25.58014	23.79407	24.96028
547	23.05927	23.02737	24.4199	22.53628	23.93228	21.83479	21.87225	21.88285	22.05708
548	22.95291	21.0597	21.63556	22.69344	21.69465	22.01056	23.00328	23.02811	24.22945
549	25.02729	24.05916	24.36584	25.0233	23.95821	24.90653	25.0595	24.12637	24.63722
550	27.34212	27.76643	27.65774	27.60812	27.77362	27.85758	26.8967	28.42057	28.21101
551	24.07626	24.08067	23.80685	24.17072	24.20002	23.38062	23.99849	23.01364	23.00982
552	23.81883	23.68426	23.22912	24.551	24.1054	22.58152	25.2143	24.88683	24.84983
553	32.28191	31.66837	31.6729	32.34222	31.66256	31.7901	32.13523	31.8639	31.82809
554	24.93768	24.7079	24.25543	24.571	24.26185	24.7452	24.89463	25.23267	25.32013
555	24.90464	25.15921	23.47708	24.0366	24.02011	24.12644	24.67104	23.61509	24.56655
556	26.83468	26.21524	25.4887	26.98831	26.60958	25.5696	26.99246	26.6163	25.95028
557	26.4666	24.08882	24.90287	26.11105	23.85274	24.46921	26.09149	24.27214	24.41117
558	20.68115	19.93586	21.34251	22.11058	21.91568	18.97289	22.65249	22.03463	22.08702
559	24.93283	24.33198	24.41701	25.19671	24.58674	24.70379	24.81774	24.84229	24.54328
560	34.96166	35.29312	34.23911	35.3071	35.53703	34.60922	34.72302	35.01638	34.21558
561	25.85377	24.27551	25.01659	25.82266	25.67646	24.9135	25.55059	25.04663	25.57135
562	24.63495	24.18706	24.57623	25.47091	23.9735	24.11557	24.57952	24.05869	24.66482
563	25.56677	24.21649	23.33316	25.53487	24.3135	23.68999	26.16235	24.11699	24.05883
564	30.80451	30.51228	30.17426	30.51351	30.49095	30.04752	31.37812	30.73328	30.68813
565	27.20094	27.10989	28.48091	27.00757	26.41514	27.49607	27.04414	26.7986	28.67009
566	29.84333	29.46323	28.92382	29.76033	29.49259	28.69952	30.26856	29.808	29.63543
567	28.85882	29.09053	29.04564	29.11451	29.07328	28.80936	29.04748	29.71401	29.90732
568	26.5709	25.78377	25.60555	26.97992	25.80255	25.1658	26.64042	25.84664	25.75962
569	21.69588	22.07819	22.3329	22.23387	22.04148	23.20284	22.94724	22.0743	22.18233
570	24.32785	23.98576	23.00431	25.04093	23.85722	23.10008	24.73452	23.4281	23.03461
571	28.30765	27.18279	27.15475	28.35991	27.17359	27.19166	28.02921	27.61759	27.50997
572	24.96304	23.82257	22.77121	25.41215	24.02024	22.81433	25.07454	23.23669	23.98158
573	27.63884	26.79615	25.88378	27.6616	26.75331	25.57881	27.32361	27.53536	25.77022
574	24.08264	24.3083	25.08248	25.17672	23.878	26.49239	24.3723	24.81659	25.18737
575	26.04572	25.9149	26.03406	25.97281	25.79772	25.66887	26.10671	25.90218	26.56482
576	25.75448	24.95253	24.78337	25.63065	24.27581	24.74148	25.41652	24.67242	25.33124
577	29.32087	29.03452	28.95861	29.49981	29.31726	29.01752	29.16927	29.73966	29.57272
578	25.63792	23.5917	23.37683	25.18611	24.7429	23.8638	25.16775	24.65429	25.1474
579	25.72874	25.12946	25.54688	26.04209	25.62541	25.33518	26.26929	25.28567	25.37604
580	24.73261	24.21629	24.65542	24.74002	24.38656	24.91465	24.99385	24.99212	24.84522
581	25.63312	25.87346	24.63927	25.83887	25.73892	25.48814	25.90192	25.11673	25.11248
582	23.71484	22.70728	23.07199	27.25799	23.08207	24.27189	23.50988	23.19544	23.03568
583	24.0855	23.13686	21.82601	23.33334	24.15447	21.59871	24.03994	24.84109	22.03712
584	25.93836	26.4448	26.0489	25.80118	26.29964	26.03174	25.9616	27.18636	27.3368
585	27.67923	26.98867	27.19373	27.43309	26.92269	27.0773	27.72368	27.13372	27.32981
586	24.6352	22.83685	23.80261	24.55662	23.38545	22.93589	24.40573	23.57471	23.93692
587	23.86779	23.14046	23.01595	23.75161	23.58114	23.86816	22.90995	23.87968	23.62289
588	25.80893	25.5177	25.67579	25.76201	25.0034	25.90445	26.05134	25.27153	25.6708
589	28.27241	27.25245	27.74444	28.2934	27.46881	27.5915	28.11991	26.9887	27.27205
590	25.27243	25.20512	24.97635	25.13057	25.1099	24.83473	24.8859	25.62651	25.49537
591	25.72143	23.25048	24.50796	25.81786	23.1721	24.74325	25.85123	24.82788	25.05995
592	28.2266	28.3449	28.75655	28.20775	28.34716	28.90196	28.28334	28.2097	28.95952
593	24.21383	23.25783	23.52622	24.35662	24.13515	23.48591	24.76695	23.95745	24.21197
594	27.23728	26.68143	26.18969	27.39746	26.33	26.09783	27.41993	26.58814	26.07715

	AS	AT	AU	AV	AW	AX	AY	AZ	BA
595	30.52279	29.8724	29.6344	30.60363	29.81032	29.67312	30.44212	29.91581	29.58885
596	24.57801	24.97271	24.27408	24.91208	25.31848	24.36318	24.86119	25.05634	24.60564
597	24.35097	25.03237	24.54337	24.80883	23.60383	24.24971	24.92122	25.3893	25.12497
598	23.91518	23.65659	21.37119	23.57535	23.79247	21.13176	24.02592	22.33815	23.68774
599	27.80685	27.5032	27.28174	27.47973	27.28729	27.60496	27.84108	27.62399	27.26415
600	29.52903	29.343	29.44168	29.64188	29.34721	29.32541	29.42822	29.61094	30.23792
601	26.35643	26.19063	25.3277	26.74784	25.95717	25.36617	26.35677	26.17356	25.49257
602	27.02288	26.33323	27.05955	27.21094	26.25362	26.62543	27.04851	26.79672	27.55526
603	28.70154	27.89713	27.62248	28.49368	27.82715	27.73706	28.71013	28.3236	27.98708
604	26.21417	25.32445	24.92896	26.3731	25.11002	24.8276	26.95099	24.66902	25.17418
605	28.80293	28.57931	28.66054	28.90127	28.39506	28.77162	28.807	28.78976	29.38635
606	24.55769	21.5508	24.06408	21.70557	21.62272	24.01882	23.63878	21.7583	23.17404
607	27.6224	27.05023	27.00296	27.68527	27.16409	26.645	27.86155	27.0381	26.83121
608	24.36219	22.78592	24.37399	23.64697	23.8766	22.74669	24.47045	23.32941	24.16952
609	26.94567	27.02367	26.01576	27.2407	26.46593	25.94139	26.74575	27.57108	27.20889
610	26.95151	27.07774	27.12931	27.06502	27.08952	27.54021	27.2884	27.68822	27.99879
611	26.6544	25.9876	25.65196	26.79494	26.12107	26.10445	26.61381	26.15077	26.70145
612	25.76961	23.8413	22.89828	25.47383	23.98964	24.13132	25.7614	25.34394	24.53451
613	25.16303	25.84572	25.51314	25.55068	25.77958	25.37586	25.53451	26.09	26.33036
614	26.16352	25.10031	25.63149	26.29107	25.33184	25.34391	26.20118	25.38318	25.74545
615	25.11171	24.52632	23.2478	24.76602	25.18817	23.46429	25.35436	24.97554	24.48357
616	21.53384	20.64599	21.22502	20.79891	21.85711	22.19009	22.71255	22.27291	21.76508
617	29.15425	28.65562	28.54571	29.22482	28.27516	28.80753	29.44254	29.03148	29.27374
618	24.09966	24.27574	22.82733	24.41529	22.6403	22.36856	24.44086	24.72562	23.73581
619	26.90216	25.70357	25.1766	26.97012	25.29636	25.34654	26.55727	26.44073	25.78037
620	26.83997	25.57033	25.87914	26.9196	26.08615	25.43862	26.84458	25.75803	25.39439
621	25.16328	24.34318	24.42695	25.33239	24.8053	22.94372	25.41179	25.07096	23.47088
622	27.01683	25.63616	26.81749	26.68761	25.48878	26.43866	26.56668	25.49538	25.85113
623	23.87102	24.02953	24.93739	24.29644	24.30831	25.02162	24.15164	24.05357	23.42912
624	24.58605	22.87594	22.98039	24.80354	23.28312	22.54905	25.65632	22.47342	23.3058
625	25.66539	23.82504	24.19198	25.8231	23.47049	24.19256	25.81787	24.69094	25.23088
626	27.2424	26.77788	26.9007	27.40574	26.62258	25.41661	27.46779	27.09872	27.16703
627	23.41438	22.03727	22.94549	23.11221	22.92889	22.92429	23.82667	23.82125	24.28369
628	28.03352	28.09362	27.60585	28.23998	28.01102	27.54807	27.73788	28.13459	27.57811
629	22.16558	21.74022	21.88565	21.55855	21.55804	21.25545	23.12919	22.54352	23.13902
630	29.06221	28.64441	28.87171	29.18843	28.4806	28.99868	29.28595	29.16448	29.51499
631	29.77569	28.80988	28.85707	29.77274	28.78929	28.63089	29.94467	28.80044	29.30543
632	28.20334	26.09836	25.46858	28.2761	25.85309	25.17758	28.72736	27.46801	26.8215
633	26.92161	26.1349	25.82657	26.72764	26.25004	25.66728	27.42658	26.64296	26.23044
634	24.60585	23.86459	23.47471	23.96265	22.76756	23.52934	24.10726	23.78397	23.15135
635	26.25367	26.70148	26.6783	26.40013	26.67416	26.31091	26.75589	26.66573	27.10312
636	25.90484	22.58588	24.35363	25.76742	22.49642	24.0595	25.87081	25.2263	24.03685
637	22.78177	22.44731	22.42619	24.11232	22.42899	24.30636	22.8513	22.01718	22.3902
638	28.33461	28.18367	28.57108	28.56831	27.94174	28.52339	27.82904	28.59616	29.1243
639	24.48394	23.4058	23.95675	24.52993	23.51681	24.16553	24.43479	23.7237	24.1146
640	25.32988	24.90978	24.89948	25.32528	24.82918	25.00407	25.57098	25.44028	25.61986
641	26.29907	25.45534	24.69836	25.98357	24.84768	25.36445	25.68767	25.97752	25.60568
642	30.64554	30.73894	30.36212	30.79966	30.77495	30.32357	30.86569	30.62527	30.77515
643	27.52672	26.80416	25.75407	27.54693	26.85003	25.66893	27.13363	26.73121	26.57219
644	26.98209	25.86908	25.94965	27.06216	25.67341	25.80606	27.08431	26.37436	26.05473
645	24.10696	22.30914	22.48062	24.08889	22.62725	22.85655	24.08099	23.14935	22.62466
646	24.35799	21.74137	22.15732	23.17545	22.59345	22.23591	23.52335	23.29648	22.87738
647	23.16288	23.43107	24.288	22.98903	21.79936	24.32125	23.01163	22.08544	23.59157
648	24.62607	22.37927	22.21363	25.04722	26.83139	24.01689	28.93347	24.27258	27.51624

	AS	AT	AU	AV	AW	AX	AY	AZ	BA
649	28.97472	29.32233	30.10229	28.90304	29.42626	29.27139	28.64979	28.36636	30.7032
650	24.43663	23.96895	22.29306	24.23808	24.01439	22.55165	24.73683	24.41247	23.58069
651	26.89967	25.93974	25.82171	27.17889	25.77601	26.10332	26.58784	26.35555	25.55156
652	24.2802	24.73482	25.02303	24.33713	24.73967	24.80345	24.87991	24.9351	25.13682
653	22.59964	22.61539	22.6379	22.44736	21.82384	22.76648	23.29527	22.58995	25.52081
654	24.83848	22.82453	22.97346	24.68912	22.71793	23.14947	25.10629	22.63802	24.10737
655	26.09364	25.43899	24.45374	26.34124	25.54052	25.00013	26.07594	25.85707	25.89487
656	24.5791	24.07483	24.16239	24.78906	23.80603	23.7375	24.64182	24.6603	24.6331
657	22.93764	23.56624	22.47493	23.21265	23.06309	22.44506	23.75027	23.23719	24.28134
658	25.92192	25.35131	25.98069	25.94929	25.34318	25.60563	26.13579	25.67577	25.92299
659	23.08034	21.92616	22.86618	23.4838	22.27245	22.55136	23.90976	22.3514	22.16487
660	27.63816	26.32918	27.39769	27.8751	26.27928	26.87747	27.71684	25.95842	22.21403
661	24.6052	22.93559	22.26933	24.47167	24.17335	21.92894	24.73134	24.1016	23.90145
662	26.0509	24.98546	26.01182	26.25932	24.64163	24.31368	26.49124	25.09469	26.02187
663	26.93841	27.19637	26.61561	26.83498	27.3194	26.44899	27.0907	27.4444	27.2417
664	25.39564	24.7061	25.05356	25.18718	24.21015	24.17526	25.05095	24.88246	24.77632
665	27.03416	26.60074	26.93887	26.91867	26.17371	26.81508	26.87875	26.38919	26.78712
666	31.3584	30.45405	30.63574	31.50295	30.43006	30.80177	31.49629	30.50025	30.621
667	30.20281	28.77368	28.75378	30.35902	29.13166	28.78649	30.1993	29.33959	29.21617
668	27.54798	26.37062	26.86688	27.59052	26.70167	26.91201	27.33391	26.58469	26.98768
669	26.40631	25.37868	24.61424	26.20765	24.76907	25.07124	26.54245	25.01921	25.29098
670	23.23968	20.87237	22.43583	23.11641	21.67465	21.3814	23.22011	22.14486	23.61679
671	24.67154	23.10061	23.73146	23.94855	23.04832	23.89009	23.29959	22.16645	24.11943
672	25.95522	25.52403	25.14671	25.58498	25.46164	25.00435	26.15491	25.53597	25.21451
673	23.52974	23.2135	22.75557	23.82853	22.83175	25.2626	23.56867	22.8259	25.66922
674	24.87901	23.29647	23.81577	24.61072	25.16414	25.20115	25.20636	23.86985	24.14779
675	26.40534	25.42362	25.42092	26.20173	26.05569	25.67122	26.68538	25.24216	25.74477
676	26.12577	25.44059	25.75542	25.90541	25.27659	25.90749	26.13088	25.70732	25.89829
677	24.80305	24.26083	22.99728	24.66768	23.63954	24.22007	25.21926	23.34433	24.59606
678	25.39996	25.68402	24.49951	25.77025	25.45765	25.22454	25.28514	25.70167	25.21661
679	28.41147	27.32544	27.08186	28.57806	27.4362	26.87961	28.65346	27.81641	27.42877
680	25.17607	23.23695	23.52826	24.91955	22.89137	22.89494	25.33691	22.43023	22.83837
681	26.61788	26.0411	25.89303	26.36433	26.32023	25.70244	26.6405	26.34552	26.36388
682	28.84965	28.31216	27.61501	28.95499	28.22897	27.64318	29.0128	28.3859	27.96339
683	29.16606	29.32047	27.79392	29.13084	29.32203	27.62706	29.00844	29.45011	28.23491
684	23.51218	22.83599	23.27815	23.9618	22.60994	23.36452	28.20847	22.85133	23.657
685	27.28766	27.06865	25.21372	26.65945	26.82675	26.87926	27.27301	26.30998	27.42345
686	30.67945	30.3296	29.94487	30.78012	30.32823	29.91388	30.98174	30.47813	30.03472
687	28.56044	28.35361	28.00115	28.57786	28.35537	27.84039	28.76947	28.70865	28.73654
688	27.9115	27.57028	27.29784	28.03564	27.45175	27.18114	27.88703	27.81659	27.65679
689	23.9284	20.22783	19.49386	23.64361	20.23956	19.6219	23.81224	23.50517	23.48949
690	26.09433	24.30115	25.20329	25.73786	24.53571	24.52579	26.25858	25.1748	24.80665
691	29.71261	29.72858	29.51047	29.85536	29.67545	29.45418	29.62534	29.70164	29.74149
692	24.81883	23.71787	23.59403	25.12021	23.12157	24.90701	25.09222	24.58213	24.90834
693	24.20177	24.35284	23.99559	24.55227	23.88768	24.14814	25.09703	24.44313	24.04841
694	25.14381	24.94091	25.34159	25.25636	24.96212	25.038	25.45034	25.54835	25.5129
695	25.54492	25.32065	25.03089	25.94518	25.09383	24.89856	25.5483	25.42105	25.20909
696	28.83021	27.80289	28.09096	28.85634	27.709	27.99429	28.88091	27.97461	28.75028
697	26.15243	25.83522	25.63444	26.55051	25.82903	25.48606	26.38554	26.16887	26.57667
698	27.27436	27.60267	26.89928	27.34655	27.39605	26.73417	27.117	27.52664	27.34475
699	25.59616	24.88368	25.02348	25.61376	24.97693	24.8822	25.87571	25.23666	26.06588
700	25.76781	25.70749	24.88157	25.52661	24.60263	26.28483	25.60456	26.13933	25.52093
701	31.46843	31.48483	31.14852	31.53869	31.44225	31.07997	32.00921	31.57257	31.05042
702	26.77011	25.79384	26.36145	26.86644	25.78096	26.68468	26.64523	26.23055	26.31503

	AS	AT	AU	AV	AW	AX	AY	AZ	BA
703	28.11265	29.16748	28.51915	28.49991	28.82662	27.69892	27.9292	28.33755	28.48931
704	28.6934	28.14525	28.20065	28.71136	28.16558	28.23774	29.06004	28.31928	28.83617
705	26.47011	26.397	26.08087	26.55516	26.48474	26.09876	26.60596	26.67348	26.62077
706	24.26479	22.6173	23.40218	23.90604	22.6034	22.87278	24.21422	23.84453	22.40326
707	25.94876	25.06424	25.1328	25.9559	25.54152	25.50011	25.54051	25.34098	26.02349
708	29.46663	29.10729	29.78127	29.24654	28.93775	29.55792	29.53956	29.30263	29.89491
709	27.32885	26.78329	26.66231	27.48145	26.7729	26.60808	27.53171	26.70584	26.54295
710	29.3358	29.404	28.96418	29.34129	29.48836	28.98423	29.26866	29.58241	29.27972
711	33.83525	33.34875	32.79696	33.95384	33.29472	32.82858	34.29339	33.73931	33.35682
712	29.18062	27.14369	28.02291	29.32073	27.73597	28.04528	28.98276	27.83809	28.62518
713	28.5886	27.80886	28.05201	28.62019	27.72057	28.13588	28.893	27.44184	28.31224
714	33.48131	32.5652	31.43468	33.57038	32.4663	31.34526	33.63441	33.29755	32.55923
715	29.00931	27.81535	28.2613	28.89112	27.82972	28.56088	28.89388	27.3405	27.87189
716	28.09265	27.88618	27.45902	28.3014	27.50661	27.46473	27.91163	28.13329	27.81235
717	27.10361	24.63659	26.05043	27.13457	25.59238	25.82462	26.69481	24.93392	26.07585
718	25.89997	25.4031	24.45377	25.7376	24.89804	23.99254	25.32565	25.03034	24.35897
719	27.4855	26.70552	26.3814	27.33239	26.58952	26.66084	27.5854	27.07859	27.40309
720	28.00953	28.15624	27.53228	28.47566	28.8914	27.46652	27.60937	28.1651	28.12368
721	29.03024	28.43464	28.46118	29.25401	28.38261	28.43091	29.07362	28.45206	28.45062
722	26.89997	26.07836	26.40172	27.0721	26.26644	26.34006	26.92666	26.21913	26.29602
723	29.48923	28.8943	29.20454	29.57769	28.69405	28.93991	29.34224	28.62171	28.93421
724	25.53978	24.48996	23.84657	25.67138	23.8	24.27384	24.98633	24.6766	24.67517
725	27.49087	26.85175	26.92261	27.51531	27.0444	26.80312	27.61864	27.27879	27.72285
726	22.71996	21.83856	21.25891	22.52744	22.02708	20.63037	20.35002	19.2555	22.30805
727	30.95756	30.71851	30.93132	30.93538	30.7073	30.96874	31.12429	31.47723	31.42731
728	29.20009	28.77861	28.87229	29.25739	28.46196	28.76956	29.34532	28.30881	28.6731
729	23.85578	22.76286	23.3071	24.01228	22.51452	23.23987	24.05286	23.82387	23.46407
730	27.23987	25.52857	27.09123	27.17218	25.45588	26.99972	26.60858	26.46732	26.26681
731	26.02026	24.71941	25.21827	26.0097	25.26163	25.37422	26.49498	25.19587	25.32303
732	25.014	26.13418	25.64505	25.22652	23.23265	25.7722	24.84393	23.67608	26.41125
733	21.9251	22.39009	21.12987	22.55549	24.53525	21.529	22.04558	21.26761	21.50152
734	31.06921	30.62817	31.73809	31.25276	30.76996	31.42581	31.03042	30.40861	32.08892
735	22.13417	22.36111	22.49709	21.84958	22.2424	22.03596	21.97135	21.14791	22.57086
736	26.22511	26.8777	24.27104	26.5608	26.7212	26.47175	26.15613	26.86239	26.48275
737	30.08366	30.24467	29.6817	30.08415	30.12403	29.45762	30.58438	30.79358	30.63879
738	30.17254	30.15808	30.20656	30.25511	30.2084	29.93713	30.15275	30.34165	30.25641
739	23.08921	23.39338	22.95997	22.86181	23.2838	21.73919	24.14273	23.89547	23.09347
740	26.8225	26.75476	26.46881	27.00726	26.53492	26.37783	26.90817	26.77493	26.89675
741	29.81071	29.08325	28.71854	29.76477	29.17868	28.56624	30.42171	29.48404	28.99237
742	27.47604	27.08389	26.4065	27.3743	27.06017	26.56187	27.59739	27.43737	27.21901
743	32.17027	32.4105	32.47357	32.27764	32.49828	32.50397	32.56896	32.76369	32.97777
744	28.27983	26.65941	27.42977	28.33112	26.61388	27.53223	28.2867	26.98372	27.43732
745	27.84075	27.45347	28.24726	27.92435	27.37479	28.38112	28.03612	26.98055	28.58255
746	23.74536	22.15725	23.35332	23.98024	22.01161	23.59487	24.00762	22.14229	23.16568
747	30.13627	30.96009	28.48182	30.18041	30.75004	28.57847	29.70888	31.67597	30.01721
748	23.49251	23.36726	23.91683	23.52647	23.32717	22.28601	23.68437	23.95657	23.00657
749	27.12434	25.83743	27.61241	27.12316	25.98585	25.91929	26.80545	26.27571	26.33168
750	27.30926	26.52958	26.33816	27.11657	26.28649	26.30566	27.26369	26.30282	26.43413
751	24.39728	22.51209	23.83485	24.59629	21.86992	22.7338	24.67161	23.40601	22.9754
752	25.51796	24.83021	24.9001	25.62041	24.72978	24.63424	25.26089	25.37742	24.89916
753	24.65818	23.51485	23.12931	23.87057	22.9243	23.38782	24.19983	22.79039	23.39926
754	26.96825	26.50247	26.29832	27.15409	26.36811	26.24937	26.57461	26.23757	25.9596
755	25.55491	25.05029	24.06346	25.59208	25.22864	23.68893	25.59764	26.05449	25.47233
756	28.65015	28.57573	28.85543	28.7339	28.77924	28.67671	28.26597	28.8771	29.64206

	AS	AT	AU	AV	AW	AX	AY	AZ	BA
757	27.97159	27.63993	26.78692	28.12935	27.66018	26.60898	28.01098	27.64347	27.08734
758	26.88124	26.11012	26.12276	27.03843	26.04508	26.16663	27.11337	26.0407	25.90986
759	25.79198	24.9535	25.12634	26.12683	25.05177	25.1471	25.86294	25.12865	25.27202
760	27.02877	26.53127	25.26753	27.15108	26.2966	24.7284	26.80653	25.8136	26.45826
761	24.15218	22.41772	24.07024	24.6753	25.00718	22.31186	24.75343	24.42285	24.05442
762	22.86743	22.6234	19.72591	22.73716	22.7763	20.86659	24.00793	22.68348	20.56568
763	22.8692	21.47738	24.40912	23.38443	24.50805	25.05332	24.76852	24.20749	24.37186
764	24.44434	25.51101	24.84482	24.59942	21.56925	23.28811	22.44585	25.18026	24.40114
765	26.55958	24.68178	25.78924	26.74002	25.02287	25.6672	26.84566	26.10999	27.13913
766	28.01018	27.66195	27.12365	27.13489	28.13673	28.16008	27.26066	27.91341	27.18061
767	27.68671	27.27055	27.29052	27.9616	27.0306	27.37653	27.64489	27.54174	28.08179
768	24.72603	23.91656	24.59876	24.59238	24.6572	24.56549	25.36745	24.8305	25.50624
769	23.69142	23.02758	22.42757	23.6917	22.67868	22.40352	22.82533	21.95272	21.68842
770	26.61255	25.82513	25.6214	26.71537	25.78908	25.79899	26.59286	26.71181	26.81848
771	29.71691	28.22734	28.7406	29.778	28.19041	28.57201	29.84156	28.3708	28.7942
772	25.74697	25.35426	24.93934	25.5649	25.32144	21.91335	25.36367	25.38419	24.95238
773	25.00327	23.45093	23.42434	24.58623	23.51246	23.49449	24.6794	22.92288	23.92715
774	26.98782	26.48111	25.78361	27.09308	25.66824	25.66917	26.78785	26.36955	26.16858
775	31.01308	30.98846	31.08209	30.98923	31.08386	30.78894	30.71392	31.00047	31.2597
776	25.42182	24.94961	24.92655	25.64431	25.21457	24.92382	25.77679	24.86695	24.86202
777	27.63496	26.92128	27.23422	26.55605	26.80825	26.42413	26.99899	26.69752	26.39404
778	25.93022	25.95138	25.12324	25.67993	25.98488	24.91173	25.99773	25.42405	25.32869
779	30.56163	30.18728	30.76364	30.4525	30.01831	30.53914	30.02092	29.56433	30.45227
780	27.14303	27.51183	27.6588	27.40035	27.53026	27.81319	26.91488	27.78719	28.41632
781	32.44453	32.29808	32.39522	32.59838	31.92387	31.7987	32.52118	32.22311	32.85731
782	26.72535	25.16688	24.00783	26.82221	24.95777	23.26288	25.29607	26.06992	25.02319
783	28.83815	28.21339	28.39368	28.98249	28.19439	27.78163	29.23771	28.67919	29.05146
784	25.85873	23.80539	23.58401	25.77134	24.05916	24.02736	26.12904	25.03258	23.03025
785	28.09789	26.89599	27.18896	28.2064	26.68957	27.14446	28.07846	27.3307	27.3327
786	26.76919	26.68567	26.64297	26.90649	26.62532	26.79257	26.94432	27.18754	27.55081
787	26.29107	25.72941	26.00611	26.43491	25.48007	26.13716	26.3788	25.82159	26.21975
788	27.51965	25.91803	27.14779	27.50309	26.14189	26.78635	27.89799	26.57085	27.66737
789	31.3146	30.8989	29.93419	31.42546	30.74779	29.83092	31.41405	31.06741	30.05757
790	25.6924	25.25764	23.35273	25.73218	25.34416	24.57883	25.60232	24.72669	25.33017
791	25.88663	25.41363	24.01641	26.24766	25.51031	24.91854	26.13749	27.22098	25.96925
792	29.66277	29.54189	29.53625	29.82244	29.3438	29.32327	29.4245	29.98318	29.93116
793	25.91504	25.68845	25.78457	26.04039	25.74854	25.59968	25.75693	25.64956	25.93012
794	28.61858	28.56576	28.37738	28.11593	28.28063	28.2794	28.7413	28.76457	28.79924
795	27.8323	27.12075	26.90881	27.6002	27.17365	26.52564	27.38542	26.95501	26.94744
796	27.21209	25.52712	25.31567	27.28459	25.14721	26.50414	27.3805	26.07914	26.50904
797	23.26288	23.97938	24.75697	22.92971	22.249	25.43284	23.50632	24.37318	26.14052
798	29.83038	28.99752	29.37348	29.85162	28.92845	29.28262	29.93722	28.9257	29.77581
799	24.59795	24.49149	24.31834	24.5556	24.70914	24.52643	24.35125	25.23315	24.70749
800	22.96849	22.5537	22.28155	23.23895	21.62143	22.89288	23.18601	22.7918	23.00153
801	25.66916	25.16801	24.89754	25.84219	24.73961	25.82421	26.08857	25.32692	26.08301
802	25.3044	24.28666	24.46079	25.24219	24.44221	24.09401	25.63884	24.42085	25.23373
803	27.83594	26.55874	26.08836	27.88245	26.43889	25.83281	28.1826	27.80276	25.84879
804	27.18158	25.98146	26.59068	27.39086	25.97628	26.74993	27.68574	26.34812	27.02354
805	24.39868	23.10078	22.42922	24.30065	23.58131	23.01622	23.68148	23.97153	24.48524
806	24.83201	24.37787	22.57443	23.98636	23.57964	25.69787	22.97954	24.09716	22.46955
807	25.57722	25.11642	24.58123	25.6364	24.54032	24.71589	25.39476	25.1435	24.87242
808	23.75224	22.63991	22.89398	23.64199	23.75132	23.01455	23.66063	23.3969	22.55144
809	25.29825	25.44505	25.15684	25.77894	26.13652	25.6719	24.74977	25.46381	24.03949
810	25.70141	24.95848	26.3341	25.66615	24.72129	26.30449	25.90771	23.86315	24.20345

	AS	AT	AU	AV	AW	AX	AY	AZ	BA
811	26.94984	25.65745	23.88374	27.05113	25.71654	24.32105	26.67567	25.8538	25.01748
812	30.29651	29.43423	29.18156	30.32816	29.34287	29.22336	29.93075	29.94149	29.72719
813	24.42201	23.19066	24.51443	25.437	24.56758	23.99669	24.69915	24.38852	23.73611
814	31.58426	31.71269	30.25655	31.62216	31.672	30.24826	31.62704	31.86079	30.4804
815	22.74639	23.75579	22.84577	23.28831	23.95144	24.3925	23.22503	23.72887	23.37762
816	31.35393	31.21297	31.19342	31.36815	31.17583	31.13473	31.52093	31.25381	31.55006
817	27.08815	26.92415	26.84504	27.05664	26.96336	26.84339	27.00921	27.35198	27.59052
818	27.81435	26.92382	26.80742	27.95662	27.05706	29.14066	28.08266	27.02849	29.27898
819	29.99726	29.66539	29.47036	29.89148	29.07518	29.48493	29.9634	30.29243	30.32278
820	27.26973	26.53736	26.41853	27.30906	26.46952	26.32936	27.19762	26.62354	27.32684
821	26.41988	26.62094	25.90462	26.63004	26.46791	25.81239	25.89231	26.65977	26.61605
822	30.34328	30.57468	30.10298	30.38943	30.55706	30.1041	30.24461	30.16955	29.97146
823	28.59337	27.78094	27.8986	28.57074	27.81606	28.03926	28.35788	28.02141	27.80609
824	25.26652	24.28299	24.59256	25.45521	24.62982	24.72949	25.45335	25.32501	24.94586
825	26.46247	25.4341	26.02811	26.80779	25.58416	26.02591	26.58255	25.54757	25.90889
826	24.79902	24.93763	23.52156	24.32475	24.69448	23.74803	24.29976	25.09752	24.2885
827	26.9185	24.36481	25.05964	26.5828	24.65249	25.09776	25.32482	25.16784	27.1916
828	26.28394	24.98248	26.08786	26.47517	24.98786	25.83913	26.04111	25.32023	25.6293
829	25.86564	25.56858	25.25854	25.66372	25.64834	25.29673	24.90712	25.45375	25.07947
830	26.22807	26.27498	25.93429	26.47064	26.28535	26.17293	26.18351	27.0636	26.80217
831	26.69485	26.0458	26.26408	26.60887	25.77078	26.20623	26.89418	26.45444	26.06482
832	26.03852	24.85834	24.18233	25.07531	24.79781	24.14622	25.13531	24.87283	24.7334
833	0	0	0	0	0	0	21.84838	20.57772	22.02009
834	22.93097	25.45465	22.55958	22.42382	25.42195	22.06751	22.70306	22.43087	24.22901
835	22.46536	22.04051	21.06166	23.30948	22.9706	21.34503	22.96218	22.349	22.74713
836	26.8215	26.5976	27.12193	26.88783	26.59802	27.06443	26.27094	26.84928	27.16464
837	25.02032	23.86452	25.21243	25.35021	24.10972	24.72779	24.94607	24.50898	24.59972
838	24.61386	25.07057	25.61052	25.16867	24.69857	25.14135	24.64985	24.60415	24.4838
839	23.03527	23.24025	23.07477	23.36692	22.90771	21.6847	22.20741	21.39595	23.66024
840	29.49642	28.06131	28.05223	29.60463	27.70322	27.99724	29.36921	27.85379	28.40195
841	28.0514	26.91544	26.97652	28.12209	27.14719	27.1337	27.88668	26.76919	26.65379
842	28.42579	27.28476	27.56023	28.46155	27.28929	27.71483	28.51763	27.56263	28.06739
843	26.70681	25.52432	25.62384	26.57925	25.45825	25.56162	26.64601	25.94556	25.74029
844	25.95701	25.02939	24.36949	26.18622	25.01944	25.32833	26.00559	25.50656	25.19509
845	26.40057	25.17047	24.50379	26.47648	25.18278	24.53095	26.52821	25.22168	24.83011
846	22.57733	20.91317	21.99254	23.20846	20.98214	21.63419	22.4021	20.73075	20.54025
847	24.68369	24.53817	23.73239	25.58195	22.28775	24.58513	25.33036	23.18911	23.54708
848	26.28749	26.33021	26.16262	26.37245	26.53578	25.90401	26.67704	26.11222	27.12781
849	27.51392	27.43909	26.52044	27.71122	27.42584	26.69309	27.3854	27.31544	26.8182
850	25.82571	25.29544	25.49411	25.97875	25.14416	25.74832	25.78863	25.14305	25.4896
851	25.0345	23.56325	23.54702	24.87181	23.35315	23.41513	24.96917	24.39177	24.03058
852	25.97243	24.84681	25.34826	26.08034	25.51201	24.93106	25.46997	24.52938	25.14453
853	21.49843	22.02006	23.37662	22.51725	21.83199	22.46099	22.79436	22.32342	22.87126
854	23.50975	22.89971	23.50709	23.47254	23.86859	22.92829	23.766	22.43696	22.45049
855	22.88368	22.62609	21.83892	22.62018	23.27263	22.72469	22.42449	22.45198	22.42794
856	25.82195	25.38897	25.31931	25.87328	26.10374	25.39282	26.09845	25.42572	25.76718
857	26.14028	25.65171	25.86472	26.32042	25.28136	25.58584	26.36204	25.19474	25.20392
858	28.87936	28.60736	27.77593	28.81119	28.47382	27.79629	28.62515	29.00421	28.60825
859	25.08069	23.55554	23.95037	25.39246	23.51979	24.08093	25.28539	23.91892	24.39837
860	27.77963	27.21457	26.65086	27.95022	27.25209	27.00707	27.78216	27.46038	27.00929
861	24.53368	24.45089	23.8664	24.64358	22.86007	23.99149	24.67232	24.08078	24.13999
862	28.94049	27.9915	28.37508	28.76801	28.05113	28.26507	29.32687	29.10614	29.37225
863	25.92523	25.306	24.79449	26.02097	25.26539	25.46129	26.18722	25.68813	25.42323
864	27.28634	26.57252	26.30269	27.4761	26.47491	26.64611	27.35371	26.85632	26.62373

	AS	AT	AU	AV	AW	AX	AY	AZ	BA
865	24.70429	23.96204	23.31213	24.26589	23.56497	23.09793	24.82985	23.00467	25.68234
866	25.50852	26.93029	23.23262	25.77437	27.19593	22.74762	24.85077	26.65661	23.49011
867	19.39052	20.27784	19.96342	20.28765	21.84569	19.09222	20.43352	19.57132	19.84834
868	24.58328	23.37153	22.65612	24.56798	24.10398	23.24233	24.47965	22.71166	22.65812
869	23.25956	21.84246	23.76679	23.20125	24.08166	23.48675	23.87219	22.25202	23.78865
870	26.78328	26.95288	26.06179	26.86635	26.69526	26.39999	26.80899	26.86013	27.03779
871	27.54788	26.59598	26.64279	26.99959	26.66092	26.2115	27.59942	27.59304	27.19201
872	26.18358	25.70376	25.75709	26.15275	25.6468	25.54118	26.14493	29.59426	26.17209
873	25.29962	25.4212	24.46021	25.44716	25.28711	24.17845	25.23283	24.85946	24.51335
874	25.67406	25.52486	22.94704	25.81925	25.57545	23.29174	25.96027	24.46916	22.64896
875	24.05878	23.19086	22.73091	23.757	22.68512	22.32726	23.88322	22.9567	22.00732
876	23.71277	22.16387	21.98197	22.97227	22.49257	21.82081	23.41879	22.88571	23.17731
877	24.0801	23.51734	22.00683	24.57165	22.9929	21.90482	24.23055	24.37333	22.11633
878	28.31616	27.70389	27.6256	28.53322	27.9002	27.76204	28.33882	27.87064	28.36019
879	32.27514	32.49947	31.27396	32.14443	32.42385	31.28434	32.32017	32.88288	31.813
880	25.8285	23.30518	25.0794	25.19367	23.72478	25.09684	25.12314	24.76703	24.90656
881	26.92985	26.09649	26.39761	27.09318	26.01075	26.53015	27.13332	26.00004	26.16651
882	24.37414	24.19653	23.18971	24.51414	24.01371	23.44767	24.03061	25.49421	23.61916
883	26.61859	26.74161	26.27655	26.76631	26.4715	26.17898	26.58479	26.84228	26.60946
884	23.43554	22.41573	23.38564	23.08865	23.33105	22.63511	22.86388	23.35042	24.05415
885	22.50003	21.26809	22.399	21.83088	22.98712	20.59685	24.30922	22.21202	22.16285
886	26.67118	26.68927	25.95928	26.81244	26.7199	25.70459	25.81938	27.10301	26.12329
887	23.85369	22.35915	22.56819	23.96356	23.07608	23.25887	23.42937	22.92269	22.71993
888	29.08871	28.00146	28.08916	29.12825	28.04827	28.09153	28.96708	28.34326	28.0723
889	34.38465	34.75477	33.72083	34.37772	34.32802	33.72465	33.92188	34.66462	34.1205
890	33.69008	32.9819	32.66073	33.85859	32.90593	32.52624	33.78869	34.00429	33.25599
891	26.61885	26.4143	25.95944	26.64828	26.32625	25.23216	26.51113	26.29408	26.44171
892	26.50811	25.46503	26.07644	26.56231	25.36683	26.17115	27.11123	25.27916	25.94899
893	24.52045	22.07357	22.21082	23.40908	23.69183	23.83763	24.11416	23.65411	23.77741
894	25.67884	25.30958	25.46981	25.69256	25.13637	25.28512	25.13906	25.20763	25.47102
895	25.74436	24.21887	25.63301	25.8318	24.3811	25.13685	25.7372	24.94426	24.81185
896	27.8615	27.49953	27.29249	27.60752	27.23046	27.10699	27.82684	27.56101	27.70723
897	25.52239	24.6666	24.94271	25.58864	24.28999	24.06772	25.52103	24.91835	24.86032
898	25.10174	23.9473	23.95664	25.12185	22.79006	24.23132	24.88669	24.38764	23.82451
899	24.30064	23.7083	23.09654	23.97216	23.68672	23.56701	24.28532	23.88426	23.00522
900	25.14434	23.68167	24.49672	25.18885	24.54482	25.26393	25.70465	24.53599	25.06747
901	24.94974	24.50407	24.52233	24.74757	24.83932	22.71976	24.95395	24.41655	24.07955
902	27.90668	27.92407	27.22945	27.93075	27.56435	27.11695	27.83759	27.99063	27.94913
903	25.72286	27.37132	26.2299	25.98682	27.22501	26.0087	26.00962	25.44145	25.9433
904	25.45216	26.66056	25.20545	25.29961	26.67876	25.10177	25.34146	26.95726	25.40917
905	27.83179	27.74316	26.95898	27.75177	27.78016	27.02318	27.9199	27.68321	26.95381
906	23.28797	24.40337	24.73922	23.32942	23.63213	23.32265	24.18789	23.00003	24.22197
907	21.74775	22.54273	22.56868	23.49426	21.99351	22.49518	22.50083	22.92293	22.06494
908	27.99935	26.92504	26.33363	28.13828	26.82311	26.26863	28.30856	27.19984	26.31722
909	28.39302	28.61177	27.73081	28.38742	28.6889	27.4443	28.20298	28.57902	27.55245
910	28.37323	27.99646	25.0032	28.41912	28.07524	23.99985	27.76631	27.8982	26.15318
911	25.88082	25.7135	22.80235	25.97451	26.01634	22.9706	25.95714	25.56583	23.59585
912	23.19726	22.93877	23.21925	23.30515	23.3957	22.80709	23.81707	24.08783	23.90157
913	28.14038	26.97576	27.63185	28.08132	26.88146	27.64795	28.24751	27.18382	27.45324
914	26.91228	26.1395	24.97868	27.09403	26.29185	25.78141	27.30941	25.98822	26.16352
915	25.82094	24.84833	25.08103	25.62816	25.10397	24.79947	25.78619	25.48902	29.49868
916	23.93881	21.94597	21.98278	23.95235	22.26984	22.39755	23.54144	22.10689	23.95408
917	25.9003	26.08155	25.9621	25.98263	25.78708	25.766	25.87457	26.38103	26.05402
918	28.32109	28.43057	28.24376	28.41391	28.3224	28.15157	28.4359	28.65055	28.37354

	AS	AT	AU	AV	AW	AX	AY	AZ	BA
919	26.84803	26.48071	25.5364	26.75754	26.66305	25.70775	26.17963	26.19964	25.83569
920	23.96253	23.28252	26.3775	24.38408	22.97867	23.06473	24.08925	23.55799	22.10014
921	28.91678	26.78375	26.51899	28.70885	26.60784	26.27213	28.87854	28.00234	26.69161
922	28.19152	27.25753	27.57272	28.44474	27.04758	27.65172	28.36868	27.6324	27.85812
923	29.20226	28.17812	28.08101	29.32986	28.17924	28.18325	29.42016	28.27829	28.14047
924	27.39204	26.34129	25.52091	27.01898	26.22686	25.87375	27.16486	25.90023	25.64353
925	28.98232	27.70929	27.80227	28.9822	27.65598	27.91857	29.23147	27.75162	28.00948
926	31.60247	31.46197	31.97652	31.71435	31.36604	31.89067	31.61816	31.62235	31.76799
927	25.94375	27.02069	25.4357	25.73816	27.0889	25.59476	25.87885	27.56954	25.70259
928	24.67598	23.53366	21.9288	24.07622	22.79615	22.95072	23.7937	23.1487	22.4115
929	30.13872	29.72551	29.29627	30.09471	29.73714	29.22177	30.14673	29.87819	29.39344
930	30.50901	31.13628	30.15482	30.27859	31.1686	30.15586	30.37448	31.45966	30.79293
931	23.85074	23.90565	22.61307	23.87112	23.69424	23.01031	24.15737	23.79912	23.05276
932	27.11414	27.19606	27.10089	27.31197	27.02149	27.00006	27.23328	27.50338	27.74821
933	25.13391	24.98686	25.15689	25.32629	25.13383	24.65339	25.31901	25.48219	25.64007
934	24.87892	22.8864	23.28026	24.87231	22.67408	22.88571	25.0114	22.81131	23.58001
935	26.84774	27.35056	26.6977	26.86676	27.32686	26.58775	26.89943	27.71331	27.40235
936	28.92017	27.76394	28.19036	29.09737	27.74558	28.03754	29.00045	27.87153	28.08072
937	27.4594	26.16195	26.09244	27.2564	26.40005	26.57536	27.40124	26.31368	27.13307
938	25.6148	25.38352	26.30483	25.97176	24.69342	26.39552	25.44363	25.56665	26.60784
939	27.13796	26.28007	26.64145	27.31044	26.3101	26.51733	27.08087	26.6071	26.90627
940	28.35806	28.53111	28.26023	28.18651	28.51895	28.15798	28.35087	28.4108	28.75385
941	27.80765	27.1664	26.8313	27.63202	27.07758	26.70964	27.50579	27.97223	27.52249
942	25.99269	25.2729	25.35635	26.06795	25.0694	25.26658	25.66273	25.76243	25.05309
943	26.01029	25.69965	25.99106	26.31806	25.3212	25.91299	26.33871	25.84997	26.29997
944	24.13412	22.97334	23.65623	24.01497	22.20326	23.54583	23.44232	24.69561	23.85424
945	25.90984	25.94129	25.20231	26.02706	26.06907	25.33589	26.01974	26.31667	25.97171
946	29.60879	29.13407	29.32377	29.54586	29.43402	29.44356	29.60235	29.93175	30.08218
947	28.92792	27.88664	28.37582	29.05881	27.81142	28.14027	29.26593	28.37824	28.29406
948	29.05733	27.63986	28.26127	29.13603	27.72484	28.22938	29.29383	28.10747	28.30855
949	24.96712	23.70029	22.98729	24.74134	24.10695	23.23975	25.09521	23.53331	23.41207
950	24.81977	24.42153	24.65548	24.93201	24.40193	24.00144	25.06308	24.43418	24.98962
951	26.03461	25.50578	25.20368	26.10878	25.65413	25.35102	26.01969	25.97204	25.25824
952	25.92128	25.50236	26.06778	25.58794	25.72578	26.47478	25.95082	25.39639	25.81498
953	21.63407	19.7812	21.14208	22.18474	20.77843	21.75821	21.46178	21.41587	20.93958
954	22.51729	25.1796	23.21471	21.48713	24.95816	21.69861	23.12075	24.5694	22.29218
955	29.24825	27.84871	28.15551	29.33109	27.75429	28.1364	29.04855	27.88624	28.27786
956	23.73998	21.1327	21.10938	23.55605	21.42863	21.80024	23.25505	22.48947	21.6646
957	23.64069	21.89271	22.01131	23.17924	24.4456	22.40408	23.85147	22.48612	22.17632
958	25.8812	24.26641	24.07111	25.34841	24.09479	24.12057	25.59915	25.23967	24.35706
959	22.37062	21.72501	23.02076	22.96432	21.53758	22.60372	23.39647	22.37314	23.27191
960	27.2546	25.64893	26.13618	27.2539	25.82319	26.07811	27.13791	26.46112	26.42193
961	26.60353	24.99087	25.7217	26.80276	25.04419	25.64648	26.49798	25.83164	25.95634
962	25.45683	25.28862	24.77862	25.92452	25.04973	24.80637	25.51027	24.51567	24.33591
963	24.87905	23.68231	23.17457	24.2603	22.39039	22.72409	24.98192	23.82965	22.96776
964	25.02613	25.74598	26.24534	25.28442	24.03138	23.48013	24.98274	23.73583	23.77755
965	26.46637	25.51309	25.05391	26.7371	25.33273	25.00967	26.60109	26.03493	25.0348
966	21.86922	22.0519	21.8846	22.95644	22.21882	21.83107	23.15698	22.00236	22.51808
967	24.8039	24.21245	23.34394	24.61058	24.71656	23.67043	24.6775	24.02683	24.31178
968	24.34698	24.11851	24.49118	24.43821	23.1052	24.40773	24.42785	24.75955	24.41962
969	22.51453	21.78118	22.92397	22.69766	21.79405	21.68869	23.29704	21.36906	22.72742
970	23.8999	22.8586	22.45793	24.66559	22.37844	22.9714	24.95917	22.30406	23.453
971	26.32007	26.46273	26.09175	26.47427	26.71945	26.03795	25.95693	26.89627	26.4634
972	28.25541	26.60579	27.00845	28.42202	26.71234	27.05497	28.16411	27.08261	27.13345

	AS	AT	AU	AV	AW	AX	AY	AZ	BA
973	27.80196	27.55479	26.78566	27.75861	27.54276	27.06066	27.39817	27.88064	27.27142
974	25.38993	23.97764	23.30342	25.27749	24.44498	23.13966	25.29009	24.83115	24.05284
975	28.17639	27.33496	27.16402	28.2725	27.36769	27.39979	28.19436	27.27638	27.20197
976	23.93806	23.39272	23.37974	23.83064	22.5858	22.02899	23.79529	23.32468	23.39164
977	28.60074	28.62077	27.71194	28.34314	28.47311	27.6773	28.96416	28.89928	28.0814
978	24.59713	22.96574	23.33168	24.72908	23.26207	23.20521	24.75753	23.64874	23.72927
979	25.46052	25.06263	25.48207	25.14839	24.52759	24.30807	25.27636	24.44724	24.81187
980	33.47369	32.61765	33.10106	33.51431	32.59412	32.97997	33.4115	32.41529	32.78623
981	25.96394	24.29044	24.55361	25.92323	25.71189	24.12233	25.91223	26.09047	24.1848
982	24.96245	22.74232	21.86834	25.06381	23.28564	22.47063	25.1134	21.68199	22.20041
983	26.35161	26.65377	26.65852	26.77552	26.33808	26.23853	26.39908	26.48801	27.24753
984	26.40327	25.33165	26.79211	26.34886	25.21043	26.79912	26.56811	26.41915	27.66607
985	26.70134	25.44346	27.91423	26.78815	26.3763	27.83609	27.25195	27.02097	28.19272
986	25.83421	25.27712	25.85667	26.62714	25.40199	25.82166	26.24486	26.16918	25.62744
987	28.30972	27.78962	28.11881	28.26164	27.72924	27.94745	28.38214	28.3253	28.76731
988	27.68969	21.71981	21.6272	27.602	21.84249	21.26772	26.9726	26.1563	26.23337
989	29.41135	28.17099	28.50044	29.45492	28.08304	28.55609	29.01974	28.81453	28.31124
990	26.58156	26.17719	25.84017	26.67323	26.22363	25.67332	26.54949	26.26383	26.25641
991	21.44529	23.71365	20.90149	20.95324	23.4422	21.25013	20.99416	20.15039	20.00274
992	28.34832	27.5633	27.09456	28.33151	27.29745	27.33523	28.22741	27.51139	27.04271
993	23.84283	23.33004	23.7487	24.42567	23.22306	21.80772	24.1676	23.88119	23.72563
994	26.3446	24.5033	22.3615	25.97161	23.57548	22.17038	26.91821	26.31355	23.37211
995	27.43722	26.8213	26.48372	27.36189	26.64131	26.34598	27.38267	26.90542	26.28718
996	25.9123	25.28153	25.36316	26.05212	25.05532	25.12254	26.0657	25.77728	25.07019
997	25.45046	24.82781	24.95864	25.35614	22.75413	24.61053	25.84432	24.81733	25.14602
998	22.35478	22.11477	23.41138	22.44501	22.38185	22.76904	23.57813	22.95445	22.44587
999	30.77431	31.2429	31.16829	30.89891	31.22285	31.01106	30.95931	31.42264	31.57935
1000	24.32843	23.29016	23.03687	24.14347	23.51002	23.29689	24.08378	23.71106	23.70512
1001	28.42929	28.23491	27.85907	28.50359	28.15512	28.0558	28.16752	28.46568	27.99596
1002	25.42753	23.97262	25.17374	25.28659	23.79057	24.37968	25.11408	24.28684	24.70565
1003	24.04988	22.61451	23.63642	23.80816	23.3071	22.23659	23.15785	23.41057	21.44233
1004	23.98126	23.50717	22.89628	24.19598	23.43736	22.85092	23.83009	24.26268	23.3818
1005	22.71187	22.32537	24.01526	23.14231	23.09128	23.38194	24.00464	22.82089	23.58425
1006	27.11916	26.45134	26.64944	27.21412	26.43646	26.87373	27.13691	27.20474	27.31716
1007	21.61619	21.93483	21.53142	20.8837	21.00383	21.13902	21.30133	23.03578	22.24538
1008	21.95398	23.72901	21.7704	23.78775	24.72851	23.20399	23.94944	22.03709	23.09695
1009	27.77597	27.17042	26.78202	27.81923	26.83213	26.46863	28.207	27.16449	27.1302
1010	29.71069	29.20235	29.5664	29.55092	29.02985	29.38716	29.49783	29.18612	29.56297
1011	26.80356	26.03071	26.38171	26.79249	25.84442	27.15713	26.78737	26.08057	26.32288
1012	26.75393	25.75843	26.13461	26.97822	25.82455	25.91257	26.91665	26.21748	26.06766
1013	26.35284	24.78963	25.75368	26.55021	25.37012	25.57436	26.39558	25.22693	25.64275
1014	25.23433	23.33774	23.25142	25.01626	24.75166	23.72133	25.43347	23.77419	23.89307
1015	27.90852	26.77314	27.24076	27.97141	26.75575	27.46953	27.90867	27.23602	27.58646
1016	22.32919	23.37275	22.89643	22.26173	22.73355	22.15413	22.86783	21.56509	22.06025
1017	28.20906	26.99341	26.76736	28.29298	27.00198	26.55014	27.68439	27.34853	27.21713
1018	29.09243	28.6619	28.2572	28.95807	28.43215	28.48119	28.9824	27.92376	28.17592
1019	25.76959	25.14337	24.81978	25.46734	25.23878	24.80002	25.33064	25.56189	25.57406
1020	28.41	28.25141	28.42283	28.44871	27.99853	28.50738	27.75485	28.21899	28.7055
1021	29.8525	29.60549	29.12039	30.01707	29.61836	29.21438	29.6604	30.09315	29.56843
1022	25.54018	24.46063	24.55124	25.23348	24.67796	24.13975	25.75132	25.11741	24.33708
1023	28.20967	26.53448	27.4054	28.2694	26.30184	27.32404	27.91167	27.49785	27.38544
1024	24.16665	23.77942	24.13993	25.06266	24.09001	23.68521	24.43949	24.89499	23.73879
1025	27.55091	26.87376	27.11481	27.45436	26.79065	26.93638	27.21161	27.55389	28.14065
1026	22.86317	21.38261	21.94714	21.1481	20.89613	22.26064	21.48405	21.51276	21.29303

	AS	AT	AU	AV	AW	AX	AY	AZ	BA
1027	27.53705	27.38662	26.9371	27.62141	27.49388	26.99659	26.96099	28.26891	27.27783
1028	25.32247	23.297	25.39997	25.17427	23.25611	25.5309	24.98325	24.84196	25.65853
1029	27.7758	27.62759	27.84881	27.77213	27.67316	27.68171	27.95274	27.82195	27.9997
1030	23.46246	23.11096	22.43211	24.07499	24.95198	22.29822	24.20369	22.23421	22.39904
1031	26.95435	26.14162	26.40824	26.98991	26.05483	26.27401	26.84478	25.91023	26.44974
1032	23.74836	23.10597	23.3092	23.94973	24.27386	23.55816	23.46668	24.03032	23.36386
1033	24.44709	24.03245	23.88816	24.06502	24.01775	23.88578	24.0093	23.958	24.34383
1034	25.68856	25.32738	25.51448	26.04656	25.31446	25.74831	25.9246	26.04772	25.4134
1035	22.62169	19.29837	19.03249	20.50571	21.28913	20.11127	23.28466	22.65095	23.21962
1036	27.1842	26.05306	26.13734	27.21517	25.88835	26.2356	26.68474	26.54862	26.32789
1037	24.42086	23.71982	23.29043	24.51708	23.73699	22.49288	24.5223	24.46542	24.68852
1038	28.633	27.93974	29.81429	28.71062	27.88507	29.80547	28.54242	27.79111	29.98597
1039	25.64797	26.02983	25.87917	25.59019	25.89091	25.49451	26.00285	26.38447	25.80142
1040	24.71617	24.85052	24.55508	24.40532	21.9676	24.44049	25.02338	25.26411	26.59441
1041	25.39144	24.05935	24.59767	25.43357	24.3051	24.79941	25.45	24.04952	24.20741
1042	23.08633	22.25998	24.01408	22.50185	23.26882	24.15307	22.95737	21.72682	23.42324
1043	30.75508	30.68303	30.59225	30.82974	30.74745	30.6684	31.04342	30.49305	30.44292
1044	29.53914	28.4071	26.61716	29.3072	28.30029	26.23776	30.1707	29.6297	28.29005
1045	26.63593	26.72255	26.1321	26.84441	26.87951	26.217	26.67141	26.79055	26.38311
1046	25.686	25.87109	25.46248	25.76226	25.37723	25.52092	25.5564	25.66896	25.62158
1047	24.91274	24.24957	23.76835	25.27112	24.21998	24.05062	25.23532	24.32832	23.77208
1048	27.1463	27.08058	27.36307	27.12514	27.2481	27.24937	26.26215	27.33892	27.4065
1049	30.07259	29.19265	29.5298	30.09356	29.17712	29.5274	30.16088	29.13748	29.58089
1050	25.29656	24.5589	25.8369	25.24703	25.02591	25.41249	25.97935	24.90448	25.36324
1051	26.74661	25.93056	25.77216	26.78274	26.08712	25.87571	26.81014	25.83334	26.02826
1052	22.48578	23.11009	22.56643	21.88227	21.7128	22.26104	21.65393	22.63235	22.11418
1053	26.02972	26.23211	25.93484	25.98265	26.17176	25.97357	25.79085	26.03672	26.42146
1054	24.85392	24.34114	25.22756	25.14888	23.3995	24.96828	25.18804	24.26611	24.35541
1055	28.05386	28.0206	28.74353	28.08139	27.20173	27.56808	28.05454	28.3503	28.11388
1056	25.86418	25.29735	25.15714	25.87777	23.59081	25.33806	25.77584	25.34774	24.9847
1057	27.3065	26.8306	25.75915	27.31295	27.00019	25.94109	27.73372	27.43981	27.45312
1058	26.94754	27.14666	26.21777	26.993	27.23552	26.31668	27.11751	27.25883	26.67932
1059	25.61778	26.64436	28.44074	25.48953	26.65213	27.52447	25.52726	27.20571	28.73138
1060	31.33894	31.22064	30.5453	31.15001	30.02472	30.66238	31.60262	30.73022	31.63135
1061	25.31699	24.02985	24.31037	25.45756	24.82688	24.48654	25.30433	24.11775	24.7583
1062	22.02804	22.16821	21.68131	23.13179	21.87517	22.04671	21.77693	21.70131	21.83738
1063	26.51032	26.29947	25.57626	26.31417	25.98665	25.85905	26.64599	26.19777	25.53562
1064	24.28289	21.89225	23.25358	23.79839	21.84276	21.95507	22.86819	22.07116	22.08977
1065	26.01968	25.18278	25.42439	26.38694	25.07028	25.19757	26.38601	25.8981	25.76661
1066	23.58483	21.90408	22.24303	24.21886	22.23456	22.60223	23.62376	22.54319	22.82381
1067	27.65698	27.38029	26.44736	27.42174	27.36613	26.4376	28.01044	27.87783	27.44159
1068	23.2616	22.28801	23.24733	23.41565	21.8132	23.10001	23.51641	23.735	22.41039
1069	27.34448	26.69639	25.83218	27.21547	26.66452	25.70654	27.46322	26.66714	25.49084
1070	25.53251	24.0494	24.12158	25.58649	23.77915	23.85871	25.32407	24.52729	24.60495
1071	29.65121	29.51196	28.95351	29.80618	29.48256	28.86372	29.80193	29.31715	29.08167
1072	25.07652	23.41894	23.43636	24.79653	23.4686	22.80115	24.87105	24.07149	24.28027
1073	25.55422	25.6852	25.847	25.53267	25.62136	25.54364	25.44583	25.68837	25.40262
1074	32.29912	32.4424	31.78387	32.40754	32.31902	31.75035	32.52148	32.51098	32.04587
1075	29.8507	29.47672	28.42924	30.03758	29.52756	28.50075	29.9391	30.33288	29.01513
1076	30.4775	31.79945	32.20808	30.52382	31.65701	32.26892	30.9357	32.08589	32.65639
1077	25.23105	23.64488	23.6426	25.09668	23.28969	23.18722	25.26134	23.4334	24.29997
1078	24.5168	24.32522	24.088	24.74611	24.23689	24.11357	24.71533	24.98079	25.34958
1079	20.84134	20.79078	21.60027	21.39952	24.02653	21.70895	21.70991	21.47183	22.57686
1080	26.58978	25.26187	22.77115	26.90421	24.69928	22.38119	26.82829	26.80231	26.43438

	AS	AT	AU	AV	AW	AX	AY	AZ	BA
1081	26.3264	26.0582	25.54347	26.48134	23.17367	25.61465	26.58877	25.73088	25.5291
1082	24.8196	24.11073	22.82404	24.71917	23.99448	22.94083	24.75003	25.17709	23.89999
1083	25.17932	23.94552	24.12408	25.01932	24.67955	23.50612	25.17717	24.37811	24.94386
1084	22.87157	19.80293	19.65395	22.87816	20.75599	21.56604	24.03404	21.52286	20.8619
1085	29.27412	27.57982	28.18543	29.24782	27.81295	28.20801	29.22624	27.88239	28.26714
1086	26.82089	26.46631	25.9195	26.51515	25.48443	25.84599	26.79887	26.20932	25.72419
1087	27.91425	25.78264	26.41025	27.98914	25.76964	26.21502	28.08845	26.3837	26.31861
1088	25.56471	25.16122	23.08787	25.68429	25.27823	24.87916	25.97904	25.57575	25.74631
1089	25.24855	24.79961	24.68445	25.33921	24.12773	24.37063	25.13162	24.95914	25.26533
1090	22.42049	21.88206	23.7073	22.22481	22.78978	21.92868	22.16113	22.69444	23.41151
1091	28.65062	27.98858	28.33018	28.8494	27.94325	28.37267	28.59916	28.46892	28.49186

	BB	BC	BD	BE	BF	BG	BH	BI	BJ
1	TOP3 WT_PEP_i02_1	TOP3 WT_PEP_i02_2	TOP3 WT_PEP_i02_3	TOP3 WT_PEP_i03_1	TOP3 WT_PEP_i03_2	TOP3 WT_PEP_i03_3	Number of proteins	Peptides	Razor + unique pep
2	25.03381	25.60956	24.67386	25.64168	25.04223	24.41872	1	21	21
3	27.04943	27.22324	26.58203	27.01656	26.49902	26.67938	1	16	16
4	24.92709	24.67728	25.21334	24.73516	24.62895	24.98156	1	3	3
5	23.39214	24.20808	23.23479	23.46898	22.8019	23.03249	1	9	9
6	25.50541	25.87621	25.994	26.02907	25.85064	26.00174	1	20	20
7	26.61414	26.55307	26.32839	26.66609	26.41246	26.14105	1	21	21
8	24.14061	23.72509	25.02111	24.18045	24.17347	24.04971	1	19	19
9	23.21774	22.18343	21.81071	23.10845	22.0225	22.43023	1	3	3
10	27.96741	27.8728	27.18547	27.81898	27.70505	27.13856	1	15	15
11	29.12996	27.99287	27.98025	29.08642	28.08225	27.96688	1	47	47
12	27.55363	26.74521	26.8971	27.5192	26.7096	26.7263	1	41	41
13	22.37736	21.66775	23.22923	23.23834	21.47399	23.42896	1	3	3
14	26.03776	26.72109	26.71647	26.21441	26.43752	26.57736	1	11	11
15	26.60024	26.36112	25.71387	26.56536	25.77156	24.75176	1	6	6
16	26.56839	25.84346	25.6032	26.61458	25.26997	25.22841	1	19	19
17	24.18994	24.16188	21.90567	24.21992	22.0906	23.21432	1	5	5
18	25.96518	25.23174	25.24861	26.14877	24.96225	25.56278	1	40	40
19	29.97423	30.29575	29.74594	29.95962	30.32548	29.62127	1	21	21
20	26.82451	26.79377	26.88068	27.01639	26.66546	26.61034	1	26	26
21	25.59396	25.15068	25.2183	25.29715	25.07845	25.40443	1	13	13
22	26.55734	27.23864	26.97673	26.4735	27.22755	27.00763	1	2	2
23	24.90364	24.71329	24.06893	24.76097	24.58527	24.04777	1	10	10
24	24.03289	23.15835	22.47092	23.74224	23.13807	23.45363	1	10	10
25	25.22219	24.97681	24.02789	25.17634	25.14702	24.06549	1	24	24
26	23.72972	22.60157	23.94249	22.02586	22.1706	23.63178	1	3	3
27	23.6015	22.40975	24.17441	23.53466	23.30312	22.45504	1	5	5
28	24.43641	23.6722	23.52414	24.5783	23.70077	23.36686	1	15	15
29	25.74566	24.349	23.40783	25.89894	24.2525	25.04447	1	18	18
30	25.49543	25.41997	24.89173	25.51787	24.96304	24.94854	1	13	13
31	25.4022	24.93989	24.98199	25.2962	24.49948	24.92092	1	12	12
32	24.49054	25.28483	26.2071	24.1506	25.1862	26.02243	1	4	4
33	26.68414	25.64266	26.06206	26.56559	25.67026	25.71676	1	36	36
34	28.20181	26.06684	25.7323	28.20643	25.98922	26.64794	1	23	23
35	26.55983	26.69971	26.14136	26.60105	26.32933	26.25134	1	73	73
36	26.84099	26.09707	26.99731	26.83482	26.07648	26.39975	1	15	15
37	26.86753	26.6177	26.04263	26.69673	26.64365	26.06383	1	11	11
38	23.49016	21.39367	23.57198	24.12177	22.76396	22.20311	1	4	4
39	24.64855	23.03587	23.47089	24.89184	22.27983	23.08419	1	8	8
40	28.55491	28.31994	28.5132	28.52804	28.28479	28.45054	1	27	27
41	31.145	31.45294	29.6129	31.07457	31.1562	30.08796	1	46	46
42	29.13208	27.54723	28.5272	29.26394	27.51689	28.39875	1	20	20
43	26.2617	24.48437	25.10959	26.18769	24.75367	24.94091	1	15	15
44	25.26588	22.86181	22.86096	24.4719	22.49104	23.37512	1	3	3
45	27.39249	27.404	25.97156	26.81695	27.3883	26.21698	1	27	27
46	25.42673	26.59348	23.43108	25.04847	24.14592	24.12781	1	10	10
47	22.95667	24.24961	23.05721	22.39646	21.92109	21.22006	1	2	2
48	32.12614	31.55137	31.49561	32.02568	31.491	31.35687	1	9	9
49	23.31085	22.81967	22.97101	23.81713	25.18938	25.72468	1	6	6
50	34.55177	35.03403	34.94835	34.41781	34.99085	34.81209	1	127	127
51	28.27748	28.49971	26.65189	28.71925	28.31549	26.58655	1	30	30
52	26.27788	25.47053	25.65771	26.13855	25.46168	25.37034	1	34	34
53	27.40202	27.52014	26.71708	27.45712	27.80448	27.72539	1	30	30
54	31.03122	30.69897	30.68949	30.86285	30.5717	30.71752	1	9	9

	BB	BC	BD	BE	BF	BG	BH	BI	BJ
55	21.72166	22.82694	21.98743	21.685	22.81422	21.89083	1	3	3
56	23.92365	24.21542	23.5926	22.93611	24.41276	22.76854	1	7	7
57	21.64344	21.40577	21.63771	22.52146	21.45207	21.29856	1	2	2
58	28.48612	23.85631	28.39315	28.54059	28.69213	28.32122	1	5	5
59	29.04695	28.86248	28.6091	29.08594	28.8472	28.49388	1	33	33
60	27.71639	26.87565	26.70722	27.70429	27.11857	26.60993	1	27	27
61	25.55414	24.13758	22.7623	25.06592	24.89688	23.97589	1	14	14
62	25.0109	25.00519	25.05007	24.80792	24.39501	25.18029	1	12	12
63	27.46166	26.93924	26.45991	27.62374	27.25438	26.58294	1	3	3
64	25.585	26.35453	26.51666	25.47599	25.96322	26.35216	1	8	8
65	23.67299	25.91136	23.25159	23.65055	25.89734	25.09399	1	4	4
66	25.91346	24.65178	24.85117	25.57019	24.55626	24.60942	1	26	26
67	24.24195	23.43646	24.38069	23.86089	23.90622	22.3192	1	5	5
68	21.71133	21.8035	21.78829	22.45327	22.09089	21.40057	1	3	3
69	21.24399	22.34824	22.0044	21.62436	22.49824	20.3691	1	3	3
70	24.52921	21.92744	24.26987	23.57879	22.91198	24.27045	1	3	3
71	25.53427	24.76241	24.52802	25.5691	24.82207	24.61468	1	14	14
72	26.64392	25.12318	24.96966	26.62565	24.88497	25.27961	1	11	11
73	24.42098	24.29812	24.31849	24.25533	23.98369	23.98866	1	6	6
74	24.66036	22.83394	25.74877	24.50701	24.37676	26.06927	1	23	23
75	28.20068	28.31739	27.82683	28.24412	28.25367	27.62075	2	22	22
76	24.44605	24.31971	23.70598	24.95099	24.09993	23.263	1	4	4
77	26.87145	25.97826	26.15908	26.72768	26.13041	26.29314	1	20	20
78	30.51592	30.37251	30.01228	30.45342	30.41717	30.19585	1	10	10
79	28.12814	27.77443	27.89823	28.06919	27.59141	27.61037	1	15	15
80	26.61168	25.12027	25.80926	26.83466	25.34575	26.00888	1	3	3
81	25.24592	24.32739	24.48037	25.22025	24.65571	23.66423	1	13	13
82	25.93795	24.64499	24.36833	26.29498	24.79346	24.47238	1	2	2
83	26.02258	25.86495	28.44666	25.99434	26.0876	28.44779	1	11	11
84	25.50921	23.65013	24.38952	25.33013	23.76811	23.93005	1	6	6
85	27.19171	26.1399	26.19085	27.1966	25.81996	25.98211	1	23	23
86	22.51036	22.62664	22.86345	22.43374	22.68932	22.89478	1	5	5
87	29.39802	28.99217	27.76297	29.42431	28.97925	27.64778	1	9	9
88	30.77307	28.24595	27.8752	30.91362	28.12648	27.83595	1	21	21
89	30.47515	29.92614	29.06823	30.45067	29.77357	29.01538	1	19	19
90	30.79383	30.63237	30.23363	30.87429	30.51674	30.08001	1	16	16
91	31.60696	30.23964	29.87709	31.67717	30.10312	29.89391	1	29	29
92	30.5302	28.97722	28.00929	30.26997	28.89427	28.04197	1	13	13
93	28.06957	27.00587	27.95854	28.11139	26.86716	27.81755	1	10	10
94	30.07541	29.10462	29.10111	30.10559	29.0318	28.89999	1	25	25
95	29.67645	28.15795	27.80534	29.7891	28.03919	27.6177	1	8	8
96	32.13939	32.74194	32.52917	32.15081	32.72897	32.49057	1	17	17
97	29.55248	28.25699	28.68689	28.84524	28.36284	28.14288	1	13	13
98	29.65039	28.36677	27.27314	29.69599	28.22735	27.68753	1	14	14
99	31.4755	30.50748	29.75891	31.41151	30.42979	29.57478	1	18	18
100	31.17807	30.52286	30.29097	31.16065	30.45867	30.35114	1	23	23
101	29.089	28.48973	27.70718	29.27308	28.292	27.37251	1	14	14
102	29.95055	28.87912	28.59613	29.9537	28.95839	28.47026	1	18	18
103	31.15968	29.71681	29.67997	31.10646	29.61098	29.5967	1	18	18
104	31.99446	30.62935	30.36146	31.86869	30.68303	29.91131	1	17	17
105	30.86862	30.18526	29.73444	30.81513	30.07566	29.56719	1	19	19
106	29.82313	29.17298	27.9651	29.61234	29.03907	27.80566	1	7	7
107	30.98325	30.49142	29.71075	31.04469	30.30872	29.71334	1	14	14
108	26.075	25.0294	23.99536	26.00596	25.02138	24.29889	1	6	6

	BB	BC	BD	BE	BF	BG	BH	BI	BJ
109	30.55606	30.70084	30.60989	30.55022	30.59373	30.43654	1	27	27
110	28.79318	28.47961	28.49237	28.73716	28.23943	28.1198	1	4	4
111	30.80544	29.43991	29.46819	30.80523	29.65644	29.46639	1	4	4
112	28.90614	28.13721	26.92486	28.84745	28.01819	26.79632	1	18	18
113	28.71357	28.29281	27.30557	28.74529	28.08162	27.08208	1	8	8
114	29.84851	29.26316	29.10711	29.8162	29.18103	29.00435	1	36	36
115	30.45654	29.56334	29.28017	30.44286	29.54601	29.12142	1	15	15
116	25.80653	25.92726	25.67485	25.82124	25.62349	25.45217	1	5	5
117	27.66032	27.32347	27.49332	27.77138	27.3018	27.51251	1	23	23
118	26.17458	25.8329	24.86448	26.15583	25.40775	24.82975	1	10	10
119	23.03744	23.78215	23.13367	22.60881	23.82581	23.69103	1	9	9
120	27.72486	26.44642	26.81225	27.46559	26.43757	26.5926	1	50	50
121	25.22564	23.61176	23.83648	25.39316	24.60836	23.87164	1	9	9
122	23.83789	23.16147	22.71102	23.60037	24.23473	22.21375	1	3	3
123	24.84397	22.20029	24.12528	24.41276	23.1678	22.93561	1	5	5
124	26.22468	25.32355	25.57675	26.54763	25.0866	24.64786	1	24	24
125	27.98677	27.22054	27.10227	28.06151	27.07658	27.09686	1	42	42
126	23.75533	24.1483	23.18995	23.73953	22.1914	23.15533	1	4	4
127	28.32655	27.02708	26.99033	28.30282	26.98997	26.58671	1	43	43
128	25.34768	25.83539	25.26456	25.59405	25.82359	25.31525	1	23	23
129	29.78439	28.40875	28.12684	29.80056	28.42165	28.06939	1	14	14
130	28.49311	28.0464	27.98659	28.56444	27.73177	28.13073	1	15	15
131	31.75826	30.66678	30.44692	31.83158	30.65483	30.55202	1	74	74
132	25.56689	25.03194	25.29711	25.58771	25.19872	25.37503	1	35	35
133	24.76509	25.75173	25.23575	24.78611	25.74246	25.45613	1	4	4
134	29.73478	28.64104	28.84704	29.77324	28.61422	28.64239	1	39	39
135	24.41347	22.42972	22.9673	24.38448	24.00489	22.57763	1	5	5
136	28.19254	27.60376	27.6034	28.06542	27.52417	27.19118	1	36	36
137	29.43024	28.35835	28.88035	29.3318	28.16356	28.61872	1	14	14
138	25.37437	24.59647	24.87878	24.51128	24.8332	24.54828	1	4	4
139	30.46165	29.42961	29.13557	30.47422	29.25854	29.00569	1	27	27
140	27.77089	27.44061	28.74368	32.62356	27.73925	28.36813	1	26	26
141	27.36688	26.14894	26.34411	27.1172	26.05794	26.57797	1	20	20
142	25.28054	23.77665	24.25465	25.35165	23.7555	24.51499	1	4	4
143	26.96271	27.01704	26.60144	27.11379	26.82556	26.43268	1	21	21
144	24.49646	24.64938	23.58434	24.34508	24.59991	24.71105	1	7	7
145	28.3971	27.92457	27.99866	28.38032	27.77982	27.77862	1	18	18
146	23.55963	22.51237	22.91888	23.75753	22.46962	22.34121	1	8	8
147	29.01415	27.2581	27.09402	28.96636	27.02762	26.90743	1	13	13
148	30.25729	28.98696	28.03438	30.16857	28.83232	26.90062	1	7	7
149	24.41232	23.2766	23.47408	24.56011	22.97367	23.19855	1	17	17
150	26.06535	25.00624	24.97382	25.93683	24.94655	24.49107	1	13	13
151	24.89933	24.81311	24.89994	24.80292	24.42898	24.62215	1	3	3
152	25.57474	24.24612	23.53722	26.22853	24.45346	24.13474	1	8	8
153	25.30648	23.34014	22.00216	25.19784	22.41013	23.47894	1	4	4
154	25.77007	24.88971	24.79479	26.03826	24.39013	26.9662	1	23	23
155	22.2496	22.09522	21.8944	22.84649	21.8275	22.32926	1	2	2
156	26.58225	26.50598	25.50497	26.49684	26.46998	25.08711	1	40	38
157	26.22688	26.29396	26.43991	26.11532	26.45934	26.31394	1	11	11
158	26.16719	25.1777	25.10934	25.94947	24.99356	25.56041	1	23	23
159	26.91216	26.93628	26.67778	26.96074	26.1495	26.40841	1	28	28
160	27.56496	26.85051	27.29503	27.63748	27.1288	27.00749	1	49	47
161	28.44219	28.10231	27.22983	28.51123	27.97754	27.26835	1	41	41
162	24.45856	23.84315	23.27658	23.88828	24.1609	24.18041	1	7	7

	BB	BC	BD	BE	BF	BG	BH	BI	BJ
163	27.04447	27.33373	27.5202	27.28135	27.24573	27.42533	1	6	6
164	31.43744	31.09361	31.35278	31.42428	30.93491	31.31488	1	80	80
165	26.7992	26.28618	26.19095	26.9072	26.26521	25.92542	1	33	33
166	28.4208	29.5219	30.33794	28.27713	29.42628	30.31857	1	14	14
167	25.71263	24.42118	24.29702	25.70215	24.0575	24.17868	1	14	14
168	27.34436	26.63313	26.69459	27.3698	26.60786	26.65712	1	22	22
169	30.22958	29.93419	29.13607	30.08833	29.75922	28.75804	1	48	48
170	22.83661	21.23417	20.86944	22.41964	22.21005	21.53876	1	3	3
171	27.34781	27.70063	27.79128	27.18977	27.62088	27.60409	1	18	18
172	27.36671	28.79527	29.44757	26.74074	28.76862	28.96001	1	18	18
173	24.15794	23.00953	23.14928	23.90462	23.2274	22.75761	1	5	5
174	25.3825	24.32042	24.02385	25.53746	24.11007	24.18444	1	11	11
175	23.48977	23.78237	23.0909	23.15556	23.32693	23.84841	1	8	8
176	25.45077	25.7186	24.70807	25.26683	25.69153	24.08996	1	10	10
177	31.68197	32.06795	32.09044	31.57061	31.97841	32.06314	1	59	59
178	25.34564	24.75611	23.98869	25.3601	24.66958	23.32195	1	18	18
179	26.80678	24.95408	24.15843	26.61215	24.20295	24.43779	1	10	10
180	27.55858	27.76245	28.02139	27.69058	27.55145	27.51341	1	9	9
181	23.85363	21.68786	22.01618	24.03096	22.32804	21.71252	1	2	2
182	27.03294	25.82566	24.98793	27.21277	25.91232	24.85656	1	33	33
183	23.4328	22.41452	23.42816	23.17798	22.28431	23.13692	1	2	2
184	28.34555	27.42745	27.74411	28.35357	27.50948	27.79717	1	35	35
185	27.5449	26.96111	26.57451	27.47652	26.81615	26.54419	1	23	23
186	34.97356	34.77104	34.66741	34.89176	34.71902	34.61711	1	323	323
187	28.11765	28.08919	28.52861	28.09102	28.03218	28.38263	1	68	13
188	25.45106	25.12444	25.16072	25.13519	25.027	24.28556	1	10	10
189	25.84914	25.1638	25.54697	26.14601	25.11527	25.15932	1	7	7
190	26.43653	24.93364	24.71735	26.40496	24.6961	24.34533	1	9	9
191	32.68462	32.39804	32.55812	32.68136	32.27355	32.67802	1	2	2
192	28.03252	27.1431	27.17667	28.21255	27.23285	26.63873	1	21	21
193	26.78482	26.37388	26.03957	26.70849	26.49383	26.25097	1	21	21
194	27.66802	27.20795	26.80962	27.78601	27.06817	26.40999	1	15	15
195	30.29179	29.26772	29.48983	30.19431	29.32461	29.39681	1	36	36
196	27.73349	27.18765	29.82506	27.57998	27.1402	29.6898	1	13	13
197	27.06897	26.11791	25.71254	27.21186	26.39075	25.52389	1	19	19
198	24.08097	24.11314	24.39831	24.68192	23.95944	24.84824	1	10	10
199	25.63317	24.00846	24.13868	25.69206	23.98886	24.02054	1	9	9
200	26.59192	25.37322	25.75112	26.67293	25.53936	25.36161	1	7	7
201	27.76454	27.88643	27.12637	27.65117	27.73232	27.2439	1	12	12
202	31.42786	31.30777	31.2587	31.47541	31.2703	31.12908	1	20	20
203	25.90708	25.57622	25.67951	26.06272	25.07272	25.52273	1	13	13
204	27.75768	27.37189	26.48644	27.78866	27.30244	27.27758	1	30	30
205	28.15091	27.41909	27.18138	28.07325	27.11661	27.09565	1	32	32
206	28.23859	29.06394	28.64951	28.8287	28.66859	29.06443	1	13	13
207	25.10922	24.32399	24.46578	25.28994	24.47288	23.84175	1	21	21
208	29.2429	28.23377	27.51341	29.28955	27.78713	27.23336	1	9	9
209	28.26313	28.36568	28.21102	28.44151	28.13467	28.19189	1	8	8
210	28.15526	27.61836	27.3276	28.0939	27.56007	27.47769	1	30	30
211	26.95713	26.35085	26.52664	27.1246	26.28549	26.15248	1	32	32
212	22.36621	21.22788	22.09764	22.43429	22.15127	22.44182	1	3	3
213	30.58274	29.57086	29.44312	30.4795	29.46713	29.24521	1	35	35
214	28.11371	26.85496	26.97812	28.02601	26.80785	26.73279	1	29	29
215	29.93222	29.49483	29.58045	29.87854	29.58398	29.43409	1	12	12
216	26.56802	25.64734	25.98378	26.56609	25.33929	25.66471	1	14	14

	BB	BC	BD	BE	BF	BG	BH	BI	BJ
217	26.30917	26.44229	25.97621	26.70878	26.09013	25.54162	1	25	25
218	24.84837	23.78007	23.74582	24.60371	23.66169	23.87997	1	6	6
219	29.33419	28.28192	28.45464	29.27835	28.13948	28.25737	1	64	64
220	28.24295	21.87803	22.67701	28.22857	21.9222	22.02805	1	4	4
221	24.33175	23.87392	25.54047	23.92682	22.51651	24.84533	1	6	6
222	26.31507	24.87569	25.36278	26.26166	24.92213	25.64953	1	9	9
223	29.63636	29.55695	29.86973	29.45842	29.34729	29.54411	1	9	9
224	27.09267	26.09764	26.02568	27.04393	26.25653	26.68715	1	28	28
225	31.94112	30.69292	30.89909	31.87529	30.6698	30.54276	1	45	45
226	25.71115	25.20726	25.22096	25.60385	25.2024	25.16804	1	9	9
227	27.51501	26.96143	26.14708	27.51563	26.9299	25.78949	1	28	28
228	24.26643	26.44268	25.88465	24.448	26.61696	25.58737	1	8	8
229	25.97193	25.22368	25.47875	25.85525	24.75747	24.93505	1	22	22
230	25.62473	24.6011	23.66733	25.34075	24.41375	24.22399	1	27	27
231	25.06222	24.99732	23.92447	24.75698	24.31338	24.22301	1	10	10
232	30.54953	31.14723	30.94403	30.53289	31.12474	30.89473	1	21	21
233	30.811	31.094	31.29612	30.863	30.95042	31.28595	1	84	84
234	27.65183	27.21626	26.48795	27.72625	27.15572	26.4973	1	25	25
235	26.54483	25.31476	25.22051	26.3285	25.11073	24.8023	1	12	12
236	30.17545	29.98206	30.11685	29.85048	29.80252	29.78734	1	15	15
237	25.02292	24.36497	23.41209	25.18738	24.27721	23.5922	1	9	9
238	21.74339	21.68741	21.68705	22.43924	21.36344	21.78605	1	2	2
239	25.62498	25.10422	24.58868	25.57701	24.42849	24.15022	1	13	13
240	22.46411	22.2383	21.32718	21.13985	21.73652	20.83426	1	3	3
241	28.25914	28.33469	28.21499	28.24485	28.31609	28.20774	1	9	9
242	27.95193	27.22884	27.48253	27.75468	29.3933	27.12123	1	33	33
243	26.76883	26.6428	25.82056	26.89071	25.77159	25.58835	1	9	9
244	29.46803	28.33968	28.37329	29.6228	28.75075	28.27507	1	84	84
245	29.38111	29.21001	29.42097	29.41993	29.19368	29.30807	1	14	14
246	25.02172	26.66782	26.15277	25.9145	26.42795	26.25293	1	3	3
247	23.73046	22.58293	22.66917	24.04805	23.66276	23.02358	1	8	8
248	27.08807	27.02383	27.14487	27.05574	26.87883	27.00407	1	9	9
249	25.89925	24.89559	24.4098	25.8212	24.85143	24.70053	1	9	9
250	28.01239	27.26049	26.84445	28.03687	26.77833	26.96112	1	46	46
251	27.46085	26.53377	26.46618	27.50647	26.56009	26.1946	1	26	26
252	23.11679	22.55752	22.2246	23.12232	22.48894	23.27568	1	6	6
253	24.37915	26.20041	25.84764	24.97952	25.84701	25.76456	1	14	14
254	25.9631	25.95843	25.14395	25.78858	26.24593	23.5771	1	8	8
255	24.76138	22.60499	22.97287	25.32172	25.00434	23.74485	1	6	6
256	26.3196	26.0686	24.97609	26.43234	25.00926	25.13287	1	17	17
257	27.65101	26.48346	26.49063	27.47248	26.68782	26.48323	1	42	42
258	27.82082	26.94109	27.03648	27.70516	27.22035	26.76465	1	50	50
259	23.68785	22.57576	22.15068	23.66218	22.17783	22.01657	1	6	6
260	25.71362	24.67276	24.17048	25.75479	24.52014	23.27591	1	16	16
261	25.74897	25.04866	25.87822	25.51116	25.21716	25.72182	1	9	9
262	31.37813	27.53775	29.4168	31.42969	27.53597	29.2096	1	197	184
263	24.90913	23.16386	24.68822	24.71114	24.20842	24.8339	1	12	12
264	26.20999	25.58542	25.63911	25.97564	25.30664	25.45053	1	12	12
265	27.26434	26.01822	26.30043	27.25014	25.93598	26.51485	1	25	25
266	21.33394	23.41316	22.18741	22.02411	23.34097	21.41328	1	2	2
267	31.47841	31.42473	31.60263	31.4921	31.2755	31.35197	1	23	23
268	24.48805	22.32701	22.69213	24.26176	22.45936	22.71041	1	5	5
269	24.13459	24.16903	23.52222	23.82273	23.89139	23.55693	1	13	13
270	27.11452	26.61204	26.56284	27.08987	26.74021	26.26136	1	38	38

	BB	BC	BD	BE	BF	BG	BH	BI	BJ
271	26.98567	26.04734	25.79229	26.96476	25.81741	25.34803	1	14	14
272	24.21926	23.60635	23.81858	24.93026	23.90978	24.03492	1	19	19
273	26.06486	25.15975	24.94982	25.90804	24.84166	24.71093	1	14	14
274	24.78078	24.86674	24.65004	24.60036	24.14085	25.02461	1	13	13
275	27.49445	27.11016	25.98483	27.5258	27.1954	25.96832	1	11	11
276	30.39843	30.25232	30.48123	29.93855	30.19608	30.22895	1	45	45
277	26.87569	26.23576	25.50361	26.78241	26.05578	25.39613	1	20	20
278	26.40464	24.72745	24.61035	26.65196	23.83477	24.70147	1	15	15
279	31.09643	30.75541	30.92755	31.23246	30.74435	30.88455	1	17	17
280	30.08945	29.22587	29.22526	29.95042	29.07919	29.17288	1	18	18
281	25.56522	24.47885	24.10384	25.63773	24.49181	23.95467	1	45	3
282	27.77308	27.92734	27.55548	27.67774	27.91809	27.73066	1	19	19
283	23.84994	22.8038	24.05234	24.38498	22.29533	22.48269	1	6	6
284	25.64732	25.34825	25.21263	25.80288	25.41907	25.57539	1	7	7
285	24.27687	23.62238	23.98525	24.02185	24.14057	24.12854	3	8	8
286	29.13207	28.1574	28.88869	29.12007	27.98426	28.67737	1	142	141
287	24.76277	24.53553	23.43446	24.54275	24.37015	22.03565	1	5	5
288	23.5928	23.95782	23.19929	23.36312	27.31027	21.67665	1	4	4
289	23.96445	22.97023	23.52714	26.53078	22.70035	22.96547	1	8	8
290	25.19682	25.83717	25.65029	25.51573	25.88476	25.4576	1	6	6
291	24.58885	24.96411	24.15576	24.23649	23.56936	23.5106	1	2	2
292	20.3308	22.00488	20.49976	22.23256	21.92737	21.81606	1	4	4
293	23.81384	25.06767	23.52903	23.95587	24.65936	23.47679	1	11	11
294	25.7559	26.54619	26.5295	25.97704	26.76186	26.66208	1	7	7
295	24.28327	23.87549	23.18892	23.70187	24.42156	22.57297	1	5	5
296	24.71773	24.40342	23.36387	24.63753	23.53022	22.51807	1	3	3
297	23.92363	22.31551	23.06972	24.09549	23.10372	23.44504	1	6	6
298	25.20371	24.9032	24.32781	25.3994	24.0764	23.54328	1	15	15
299	24.32153	25.11659	22.2912	24.30016	24.94306	22.46391	1	7	7
300	25.87023	24.8301	24.66571	25.48726	23.87067	24.17257	1	11	11
301	28.69427	28.35004	28.45742	28.78871	28.32028	28.37433	1	156	156
302	25.84743	25.27109	24.36549	26.09915	24.42146	23.99149	1	19	19
303	23.70873	23.56844	24.25472	23.59729	22.97869	21.81808	1	5	5
304	27.06513	26.48263	27.28152	27.06942	26.38918	26.88239	1	14	14
305	27.18979	27.21169	27.02095	27.07444	27.40858	26.74583	1	24	24
306	25.88415	24.76105	23.08878	26.05278	24.11785	23.53554	1	9	9
307	28.79567	27.91324	27.11575	28.70149	27.6832	27.02844	1	11	11
308	22.65148	22.44573	22.43573	22.53473	21.6704	22.42492	1	4	4
309	26.04524	25.44234	25.84748	25.74463	25.3486	25.36249	1	14	14
310	24.12727	23.95196	23.58349	24.25109	24.53473	23.81286	1	7	7
311	23.14699	22.91825	23.63589	23.90433	22.64884	23.6561	1	6	6
312	26.94973	26.03696	25.57997	26.94095	25.86334	25.41796	1	10	10
313	28.52644	27.58723	27.49416	28.59992	27.48511	27.21557	1	37	37
314	26.62186	26.48602	26.37243	26.71589	25.68218	25.66903	1	26	26
315	24.93949	23.80128	23.93771	25.01276	24.43323	24.33977	1	12	12
316	26.09556	27.52018	26.31054	26.09008	27.45404	26.51194	1	10	10
317	25.94213	25.16508	25.59193	25.70488	25.18268	25.06837	1	14	14
318	24.60788	24.68449	25.43524	24.67663	25.11289	25.13705	1	7	7
319	26.14927	25.84826	25.45571	26.12618	25.98449	25.35439	1	13	13
320	27.79775	26.89351	27.1318	27.87652	26.73177	26.80807	1	48	48
321	30.07127	29.28588	28.45253	29.9773	29.10085	28.46501	1	29	29
322	25.28485	26.92763	26.35071	25.24665	26.79148	26.09186	1	10	10
323	26.53268	26.63089	26.04786	26.5452	26.73428	26.15117	1	20	20
324	26.2098	25.70283	25.54714	26.12127	25.26585	24.03872	1	15	15

	BB	BC	BD	BE	BF	BG	BH	BI	BJ
325	27.28203	27.55425	25.7869	27.23358	27.94687	25.19614	1	12	12
326	26.32521	25.76463	21.33241	26.17258	25.0114	22.45996	1	2	2
327	26.87002	27.03775	24.15125	26.41373	26.83738	23.58986	1	31	31
328	31.94264	30.60718	31.56651	32.18503	30.98847	30.76084	1	66	66
329	30.56114	30.47295	30.72683	30.66092	30.39139	30.63732	1	9	9
330	27.89169	27.94854	28.07987	27.76988	27.96949	27.97351	1	16	16
331	25.66848	25.0057	24.87298	25.36814	24.85503	24.89465	1	10	10
332	23.29813	21.69754	23.51457	22.82646	22.07165	23.14051	1	7	7
333	26.22889	26.39941	26.43703	26.22855	26.32496	26.51771	1	13	13
334	23.11804	22.19475	22.6751	23.11242	22.15777	21.69464	1	5	5
335	26.32065	26.34106	26.51498	26.34933	26.17542	26.35038	1	12	12
336	27.9346	27.37356	26.8483	27.86924	27.33359	26.86349	1	19	19
337	28.96711	28.83319	28.01008	29.13959	28.80198	27.96168	1	12	12
338	20.90924	21.75637	21.55332	23.14666	23.50074	20.32282	1	2	2
339	28.96443	28.65233	28.8775	29.02457	28.58818	28.80314	1	31	31
340	23.69664	22.31446	23.36345	22.87745	21.65888	23.85316	1	6	6
341	26.38278	26.93204	26.17835	26.26214	26.76217	25.83573	1	18	18
342	26.88225	27.23108	25.98177	27.4795	26.48416	26.20717	1	13	13
343	29.60133	29.6106	29.70591	29.67838	29.81748	29.58955	1	22	22
344	25.93583	25.20399	25.78238	26.10631	25.40508	25.69425	1	11	11
345	28.1426	27.06182	26.78858	28.05067	26.89097	25.35153	1	15	15
346	27.79821	26.79292	27.31709	27.82397	26.57806	27.11472	1	20	20
347	23.97771	22.41458	22.27435	22.1836	21.95984	22.23184	1	3	3
348	26.72252	25.33967	25.62156	26.77651	25.54603	25.40131	1	26	26
349	27.32634	27.06543	27.07852	27.26078	26.92604	27.15543	1	21	21
350	30.77204	30.48391	30.07873	30.74588	30.25419	29.90589	1	29	29
351	30.84426	30.96842	29.97891	30.73649	30.77395	29.65861	1	21	21
352	25.09635	22.98281	23.85594	24.63286	22.88604	23.2785	1	7	7
353	25.4702	24.44268	25.34301	25.66415	24.24757	25.77588	1	29	29
354	28.48401	27.33191	27.48005	28.46865	27.5114	27.33968	1	23	23
355	31.50289	30.52386	29.6878	31.51646	30.37831	29.43745	1	7	7
356	26.2877	27.49802	26.81642	26.26526	27.12346	26.84575	1	2	2
357	27.1944	27.95576	27.24905	27.26692	27.86805	27.35421	1	13	13
358	25.19225	25.09499	25.0292	25.39686	25.47315	25.44927	1	14	14
359	25.09113	26.12195	25.17495	25.14449	25.32194	24.48982	1	9	9
360	23.49848	23.82593	22.15738	23.71652	23.8208	22.44454	1	3	3
361	28.24731	28.42206	28.68829	28.01347	28.55152	28.68961	1	8	8
362	30.02651	28.34779	27.9641	30.00756	28.4704	27.5709	1	34	34
363	27.13674	27.3585	27.59677	26.86619	27.42569	27.64133	1	25	25
364	23.86424	23.08565	23.1812	23.90671	22.83327	23.28188	1	8	8
365	25.23263	24.36226	24.68063	25.36769	24.58372	24.98586	1	10	10
366	27.51424	27.01167	26.7663	27.51365	26.94767	26.78562	1	48	48
367	25.07805	25.09796	25.20797	24.98607	24.68021	25.09896	1	7	7
368	25.25059	24.19306	25.31883	24.82937	23.22484	24.07681	1	11	11
369	27.00376	27.08768	27.45146	26.95824	26.99951	27.50435	1	2	2
370	25.65817	25.83742	26.00605	25.87005	25.68241	25.52479	1	8	8
371	23.62213	23.26189	24.14707	23.7568	23.84097	23.82698	1	4	4
372	22.94634	23.4453	22.23561	23.00102	21.6097	22.00379	1	6	6
373	21.09689	21.02361	21.077	21.10542	21.00658	21.00597	1	2	2
374	29.47377	28.29607	28.07532	29.56244	28.14752	28.0686	1	75	75
375	25.67275	27.27567	25.20078	26.13246	25.28885	25.06241	1	16	16
376	24.29154	23.09724	23.15586	23.84315	23.48544	23.79439	1	7	7
377	25.0294	24.70148	25.03283	24.93324	24.94982	24.15978	1	11	11
378	23.24955	25.34375	24.09755	23.78307	25.21328	24.3084	1	9	9

	BB	BC	BD	BE	BF	BG	BH	BI	BJ
379	26.95943	27.26597	27.46229	27.44518	27.35412	27.92611	1	12	12
380	25.8304	25.42556	26.30442	26.01365	25.28496	25.41086	1	9	9
381	26.63744	25.82965	25.88085	26.57737	26.36047	25.58322	1	31	31
382	28.30458	27.0342	27.26295	28.28144	27.03531	27.20597	1	29	29
383	25.54647	24.04628	23.58658	25.24537	23.86586	23.61023	1	11	11
384	26.25404	26.31127	26.20441	26.30114	25.9727	26.02692	1	13	13
385	26.72834	26.18493	24.4692	26.63287	25.91305	25.40481	1	26	26
386	27.6424	27.60096	27.59007	27.55155	27.54648	27.50774	1	23	23
387	27.14146	25.9359	25.85196	27.13388	25.7437	25.73928	1	14	14
388	27.49913	26.86905	27.00429	27.75325	26.93631	27.70874	1	49	49
389	27.84697	27.83124	27.97418	27.82394	28.22547	27.92338	1	16	16
390	24.4397	24.45983	25.37005	25.11502	23.44852	24.90799	1	13	13
391	30.65604	29.25954	29.82728	30.70066	29.14145	29.8386	1	42	42
392	27.40503	28.10246	27.5733	27.45963	27.82936	27.46699	1	10	10
393	28.57177	28.40502	28.41951	28.78467	28.19661	28.23636	1	14	14
394	26.27576	24.66544	25.60834	26.37362	25.9279	24.48666	1	7	7
395	26.40019	25.66574	24.78095	26.52979	25.47344	24.68235	1	14	14
396	30.02804	30.25324	29.76311	29.96007	30.31312	29.7056	1	23	23
397	26.36263	25.73487	25.32168	26.30161	25.41566	25.07762	1	13	13
398	29.70422	30.10158	30.12033	29.65317	29.99292	29.98029	1	21	21
399	22.93589	22.2219	22.22253	23.20716	22.35159	22.82183	1	6	6
400	25.35787	24.72274	24.17014	25.40484	25.43443	24.71727	1	11	11
401	23.67936	21.71778	22.83231	23.47059	23.01144	22.92094	1	6	6
402	21.83036	24.25356	24.59495	21.6166	23.25169	20.13032	1	3	3
403	29.04798	27.99065	27.95807	29.01143	27.71	27.72081	1	44	44
404	25.50739	25.40086	24.75592	25.12348	25.18536	23.61292	1	16	16
405	24.00612	22.58788	22.37088	23.58414	22.72427	22.43067	1	4	4
406	23.49249	22.45179	24.21892	23.7151	22.92831	24.1011	1	10	10
407	23.73451	22.16041	25.32823	24.68422	24.25434	24.69985	1	3	3
408	26.22974	25.44901	25.39546	26.40307	25.42783	25.34333	1	22	22
409	26.44749	26.6461	26.31284	26.57891	26.84157	26.25379	1	17	17
410	28.5174	28.16245	27.96543	28.44067	28.02974	27.86063	1	13	13
411	29.45351	26.84982	26.49699	29.45544	26.20533	26.36763	1	7	7
412	26.71292	25.41973	25.27282	26.95997	25.62774	25.32081	1	25	25
413	28.99122	27.827	27.62095	28.87692	27.70462	27.39113	1	4	4
414	28.54258	28.21989	28.08715	28.47006	28.16093	27.7078	1	16	16
415	24.27304	22.35472	24.58727	24.22429	21.95392	21.92042	1	6	6
416	22.64827	22.98857	22.70089	22.90687	23.43933	21.76768	1	3	3
417	26.8939	26.97843	26.56811	26.86175	26.95007	26.33017	1	20	20
418	26.13967	25.82394	26.00242	26.18663	25.87004	26.10742	1	6	6
419	24.3102	23.26409	23.85978	24.27076	23.36169	23.03809	1	9	9
420	27.09021	26.38719	26.77229	27.16564	26.08992	26.40556	1	16	16
421	24.91469	26.66504	22.28999	24.66654	26.54604	21.66413	1	3	3
422	27.11222	26.2975	26.77661	27.24859	26.20706	26.46859	1	23	23
423	27.95668	26.96282	27.11533	27.99609	26.70604	26.63264	1	29	29
424	27.96787	26.97038	27.22153	27.92372	26.91027	27.20225	1	26	26
425	28.49115	27.65411	28.80286	28.5805	27.60514	28.34748	1	28	28
426	23.84632	22.26652	24.31284	24.16419	23.04056	23.32739	1	9	9
427	24.22574	22.89268	23.20862	23.99779	23.37861	23.58111	1	6	6
428	24.7985	23.49862	24.46352	24.39694	24.00177	23.85652	1	8	8
429	22.96669	21.99915	21.77444	22.89056	25.52259	22.76849	1	4	4
430	24.29044	24.3653	23.923	24.47381	24.45836	23.27494	1	4	4
431	27.0652	26.69202	26.50579	27.0353	26.74884	26.51278	1	59	59
432	23.56613	23.14556	25.12605	24.55296	24.15992	25.18745	1	5	5

	BB	BC	BD	BE	BF	BG	BH	BI	BJ
433	25.1404	25.65268	24.59627	24.47541	25.60977	23.20051	1	6	6
434	27.35945	27.41845	26.83882	27.24541	27.26447	26.7105	1	13	13
435	24.23167	21.57571	22.47263	24.22008	21.07309	21.07409	1	3	3
436	27.69889	26.43011	26.72405	27.64124	26.69586	26.21137	1	24	24
437	27.89093	27.26468	27.0145	28.21203	27.18584	26.85754	1	29	29
438	25.65583	25.69554	24.68921	25.44457	25.69127	24.55551	1	13	13
439	31.17645	32.05407	31.81789	31.12926	31.92837	31.68488	1	18	18
440	26.78656	26.54202	26.10749	26.84526	26.36134	26.12776	1	21	21
441	24.41768	24.0842	22.47745	25.11413	22.64142	23.52958	1	7	7
442	26.41072	25.5598	25.95995	26.66244	25.6856	26.43668	1	22	22
443	25.00675	22.06054	23.24688	24.79715	22.5633	23.02997	1	9	9
444	28.21444	27.67822	27.57547	28.32725	27.61394	27.47769	1	28	28
445	27.64497	28.05212	27.95113	27.59219	27.80085	27.96425	1	14	14
446	28.24218	27.90042	27.39279	28.1871	27.74318	27.27502	1	36	36
447	27.46408	26.23417	26.1441	27.70872	26.33202	26.44936	1	12	12
448	30.56361	30.3788	30.21309	30.71087	30.45683	30.3401	1	31	31
449	26.44785	25.6299	25.48608	26.32094	25.14651	25.16264	1	21	21
450	25.26722	24.9535	24.68032	25.46559	25.27103	24.75584	1	13	13
451	28.9448	29.01399	28.54796	29.03349	28.89041	28.4975	1	28	28
452	29.89501	30.10166	29.72713	29.89898	29.92228	29.58472	1	22	22
453	33.23603	33.30231	33.25402	33.263	33.28174	33.12316	1	27	27
454	28.61421	27.69476	27.68244	28.64396	27.75121	27.61509	1	25	25
455	22.75948	21.85206	23.40783	22.84214	23.25755	23.61761	1	7	7
456	24.60605	24.78304	23.00412	24.3911	22.7907	22.58684	1	7	7
457	30.80201	30.11679	29.46465	30.76521	30.08132	29.60108	1	47	47
458	27.06318	28.27896	28.72227	27.12617	28.3616	28.79407	1	23	23
459	32.58715	33.027	32.80764	32.62467	32.83514	32.748	1	20	20
460	26.76664	24.95654	25.39158	26.49626	25.98147	24.94393	1	16	16
461	29.17559	29.64511	30.00093	29.21725	29.50481	29.83603	1	12	12
462	28.53872	28.41616	29.79454	28.5011	28.36718	29.23973	1	16	16
463	29.64629	28.21961	27.11802	29.57335	27.95361	27.16335	1	14	14
464	24.82793	23.95856	29.45536	24.83353	23.54227	29.21328	1	24	24
465	24.95448	24.5489	30.49221	24.65123	23.62941	30.42313	1	34	34
466	27.23179	27.23242	27.40909	27.15611	27.29512	27.0952	1	21	21
467	24.80318	24.07082	23.93208	24.7228	24.05816	22.74824	1	12	12
468	24.45069	24.03641	22.11109	24.493	23.76602	23.4998	1	4	4
469	25.12262	25.03807	22.96017	25.16035	25.23445	25.11321	1	7	7
470	28.22491	27.11609	27.63101	28.44564	27.23041	27.37074	1	38	38
471	23.89198	23.73143	24.07095	23.89165	21.79272	24.53429	1	3	3
472	23.69457	21.96436	22.57074	23.37132	21.7108	22.81801	1	3	3
473	26.61753	27.58702	27.17881	26.7366	27.50846	27.10554	1	17	17
474	25.19352	24.60686	24.39937	25.40018	24.73708	24.40521	1	9	9
475	27.67744	27.3667	27.09131	27.68142	27.12833	26.88244	1	10	10
476	23.03765	22.7368	23.08817	22.08005	22.67557	22.83955	1	3	3
477	25.76941	24.00741	22.86306	25.52124	24.07121	22.33386	1	9	9
478	28.19072	27.884	27.79915	28.28318	27.70535	27.65131	1	19	19
479	25.85025	24.84377	24.6824	25.9564	24.97795	24.46064	1	11	11
480	26.57623	25.73399	25.96706	26.50207	25.4792	25.67566	1	54	54
481	25.37235	25.14347	25.04571	25.39931	25.10741	25.08055	1	17	17
482	23.07072	21.23828	21.66118	23.46949	22.43634	22.83418	1	4	4
483	22.80797	22.2148	21.97184	22.55553	22.49329	21.45495	1	2	2
484	27.48765	27.01746	25.43038	27.39686	26.73042	25.76918	1	12	12
485	25.57044	24.9416	23.74022	25.6029	24.15289	23.80677	1	12	12
486	28.26665	27.69894	27.42041	27.96577	27.59317	27.35353	1	21	21

	BB	BC	BD	BE	BF	BG	BH	BI	BJ
487	26.20063	26.71062	26.19964	26.27441	26.28038	25.97997	1	18	18
488	25.03842	23.96571	24.6948	25.39482	23.9283	24.22015	1	7	7
489	26.17246	25.26038	25.42008	26.33666	24.86778	25.26989	1	41	41
490	24.1011	24.30952	23.20442	24.57269	25.04391	22.86223	1	5	5
491	25.44667	24.30581	24.53104	25.21489	23.88492	24.67372	1	27	27
492	27.17025	26.44719	26.29423	27.1944	26.37017	26.79279	1	18	18
493	23.80377	23.93583	24.79019	23.87738	23.63198	22.8135	1	9	9
494	25.09783	23.68005	23.58784	25.03144	25.11158	23.89012	1	26	26
495	24.31282	23.9999	23.10983	24.50413	23.32432	22.60267	1	8	8
496	26.64789	26.97307	27.75882	26.88777	26.98014	27.51673	1	12	12
497	25.30112	25.94528	25.98654	25.45454	25.91765	25.81084	1	18	18
498	27.56689	26.62234	26.52865	27.70211	26.37697	26.21533	1	33	33
499	29.58228	28.85165	27.70872	29.51029	28.86614	27.48273	1	21	21
500	29.28314	28.19268	27.33445	29.30451	28.17736	27.20311	1	47	47
501	25.01564	24.68675	23.28381	26.14484	22.83948	24.50383	1	22	22
502	26.51034	25.1848	25.0861	26.20054	25.22542	24.79351	1	6	6
503	23.77308	23.34206	23.15572	24.00446	23.29485	23.13552	1	3	3
504	24.17414	23.32005	23.39249	23.85395	24.97658	23.51607	1	11	11
505	24.0358	24.36295	23.18058	23.72608	23.81685	23.1942	1	6	6
506	27.98315	26.70678	26.85221	27.89001	26.58596	26.8873	1	29	29
507	22.27557	19.09121	19.02223	21.75774	20.29439	20.29192	1	2	2
508	27.55642	27.27899	26.68646	27.58153	27.1629	25.99929	1	18	18
509	22.46038	22.33209	25.32214	26.61825	22.71677	25.25961	1	5	5
510	25.87679	24.84457	25.11077	25.61623	24.92227	24.73616	1	10	10
511	25.57559	25.48932	25.13248	25.66766	25.3148	24.97504	1	7	7
512	32.04721	31.26354	30.95794	32.03245	31.04694	30.74346	1	28	28
513	26.59186	25.80725	26.50143	26.73953	25.58002	25.55542	1	37	37
514	27.14998	26.19958	26.08513	27.19125	26.26001	26.01548	1	15	15
515	25.49782	24.27022	23.97751	25.39392	24.29745	23.69298	1	19	19
516	25.51416	24.34533	23.6519	24.96494	24.33595	23.52413	1	12	12
517	24.21587	23.90491	22.65372	24.47667	23.73194	22.59925	1	5	5
518	22.88256	25.08058	24.78981	23.05726	25.08487	24.58504	1	9	9
519	26.49479	27.53671	26.04521	26.51494	27.494	25.79091	1	5	5
520	24.42537	24.13923	24.37269	24.38469	24.13903	24.32454	1	2	2
521	26.65271	26.16819	26.05419	26.60973	25.74086	25.65268	1	11	11
522	23.70515	23.79407	23.31832	23.89101	23.81064	23.27155	1	7	7
523	26.7108	26.90142	26.72717	26.52692	26.8217	26.59959	1	10	10
524	27.98924	26.93352	26.68921	27.82868	26.68747	26.55871	1	38	38
525	25.09362	25.46252	26.06076	25.08029	25.27053	26.03895	1	7	7
526	26.62475	27.59698	27.78382	26.55892	27.41546	26.84152	1	9	9
527	27.66563	26.88596	27.20784	27.62935	26.99252	27.17281	1	13	13
528	25.61382	25.68102	25.52713	25.7146	24.99067	26.0557	1	9	9
529	28.08678	27.52682	27.4354	28.08232	27.37152	27.30935	1	27	27
530	23.62323	24.12591	23.49141	23.3753	23.76866	23.51438	1	4	4
531	26.71952	24.96004	24.2147	26.57401	24.65525	24.41335	1	16	16
532	28.20153	28.44196	28.61396	28.04287	28.49236	28.42083	1	20	20
533	27.96547	27.40425	27.17603	27.91686	27.0237	27.23411	1	47	47
534	27.72633	27.48741	27.795	27.66193	27.22076	27.76534	1	10	10
535	32.92588	33.06384	32.6889	32.83578	32.93578	32.4291	1	12	12
536	28.75603	28.00535	28.66074	28.63936	27.78815	28.2875	1	48	48
537	26.72222	27.2598	26.91579	26.69373	27.26744	27.49136	1	18	18
538	25.38105	26.13101	25.65401	25.40643	25.91263	25.34543	1	13	13
539	24.3305	24.16319	23.16472	24.39001	24.35363	23.33146	1	4	4
540	25.59512	24.78539	25.06237	25.98691	25.37885	24.39952	1	21	21

	BB	BC	BD	BE	BF	BG	BH	BI	BJ
541	22.80971	22.9653	22.91026	22.79792	22.97758	23.33748	1	2	2
542	25.75719	25.97245	26.2967	25.77488	26.23952	25.88056	1	6	6
543	26.26277	25.81143	26.43511	26.12554	25.87591	25.98272	1	9	9
544	28.15295	28.52524	28.61215	28.35721	28.51952	28.67342	1	23	23
545	22.42352	23.46441	23.34836	23.16964	23.00651	23.11366	1	5	5
546	25.2935	24.44311	24.55609	25.90107	24.46811	25.06278	1	10	10
547	21.39317	22.00539	21.18796	23.1754	21.65043	21.34848	1	3	3
548	22.22535	23.00778	22.76164	23.05315	21.78613	22.65499	1	4	4
549	24.98871	24.2382	23.863	24.52381	23.65496	23.77729	1	13	13
550	27.20911	28.21461	28.16797	26.92205	28.072	28.24842	1	124	124
551	24.55572	22.62315	23.56067	24.10765	23.57823	23.35645	1	19	19
552	24.99282	23.80687	24.0642	24.6616	24.14551	22.86005	1	10	10
553	32.09644	31.79441	31.8248	32.06447	31.67356	31.51845	1	54	54
554	25.01368	25.29461	25.1819	25.72948	25.32519	25.17711	1	6	6
555	25.27154	23.9951	23.60677	23.72935	23.49389	25.13359	1	11	11
556	27.07486	26.23447	25.87009	27.27637	26.28101	25.72348	1	44	44
557	24.96794	25.33809	23.76692	24.94448	24.52554	24.13445	1	23	23
558	22.87582	21.10125	22.16137	23.12758	21.64236	21.14497	1	2	2
559	25.30844	24.83629	24.37498	25.035	25.09201	24.32053	1	29	29
560	34.69605	35.31112	35.4704	35.15062	35.09781	35.22365	1	18	18
561	25.53095	26.08522	25.37295	25.54421	25.10752	25.53033	1	13	13
562	24.83243	24.32721	24.44449	25.99202	24.00344	24.41414	1	5	5
563	25.97949	24.6121	24.2739	25.62116	24.42237	23.5497	1	15	15
564	31.29989	30.60792	30.43419	31.17099	30.53093	30.26713	1	12	12
565	26.49138	27.39197	28.26076	27.56558	27.49775	28.47716	1	6	6
566	29.99247	29.66722	29.29109	30.01852	29.60861	29.14957	1	7	7
567	28.90486	29.68332	29.51403	28.8585	29.59443	29.29961	1	9	9
568	26.94279	26.01555	25.80181	26.57206	25.92915	26.0066	1	13	13
569	22.82052	21.65817	21.95313	22.48459	22.047	23.51839	1	5	5
570	25.17774	22.81118	22.50895	24.99563	22.41958	23.05836	1	6	6
571	28.12053	27.59795	27.22025	28.13983	27.45136	27.07362	1	22	22
572	25.13358	23.63921	23.73913	25.5494	24.03442	24.2577	1	10	10
573	27.36936	27.48712	25.87497	27.20111	27.45411	25.32297	1	14	14
574	24.56892	24.68457	25.26047	24.09743	24.47124	24.90545	1	8	8
575	26.06956	25.76018	26.19985	26.09944	25.34297	26.23772	1	15	15
576	25.71607	24.53436	24.77079	25.68481	24.85844	25.32872	1	35	35
577	29.15153	29.23533	29.3875	29.09342	29.44672	29.28296	1	27	27
578	25.5124	24.54469	24.30536	25.39121	24.5797	24.54316	1	9	9
579	25.96273	25.31839	25.21482	26.35093	25.32972	25.37457	1	21	21
580	24.67396	24.79057	24.9738	24.91745	24.69101	24.75065	1	2	2
581	25.77273	25.67737	24.78965	25.73657	25.57392	24.66306	1	22	22
582	23.8011	23.29934	23.09662	24.09532	22.99967	23.36916	1	8	8
583	23.61956	23.52999	21.55985	23.49036	24.31957	21.80634	1	3	3
584	25.87792	27.02028	27.15033	25.94878	27.00792	27.17002	1	3	3
585	27.78526	27.06693	27.30631	27.72985	27.10254	27.18413	1	20	20
586	24.76112	23.87026	23.20652	24.4931	23.86593	23.38646	1	13	13
587	24.12583	22.88728	23.09354	23.61965	22.93083	23.16166	1	7	7
588	26.05894	25.35944	25.84499	26.09842	25.21727	25.53181	1	15	15
589	28.17993	27.30447	27.19066	28.03822	27.25035	27.17403	1	69	69
590	25.19596	25.08319	25.20133	25.25401	25.17641	24.7981	1	6	6
591	25.98531	24.46329	24.7375	25.8621	24.93096	24.39553	1	23	23
592	28.22655	28.19456	28.86633	28.24148	28.18242	28.78069	1	31	31
593	24.54086	23.71247	23.62758	24.66565	23.04737	24.07314	1	7	7
594	27.48754	26.49091	26.10995	27.58291	26.14282	26.13346	1	23	23

	BB	BC	BD	BE	BF	BG	BH	BI	BJ
595	30.38106	29.79428	29.5214	30.4252	29.80218	29.42794	1	43	43
596	24.69659	25.20547	24.35175	25.30633	24.94807	24.73789	1	7	7
597	24.79246	24.83342	26.13267	24.84095	25.24831	24.32872	1	10	10
598	24.54003	21.52194	23.23287	24.12771	21.80087	23.66245	1	2	2
599	27.96788	27.41187	27.41366	27.74327	27.51684	27.30703	1	38	38
600	29.51085	29.62716	30.19506	29.36434	29.46201	29.98917	1	4	4
601	26.29059	25.8883	25.79829	26.34779	26.00101	25.739	1	26	26
602	27.22204	26.72934	27.26662	27.13216	26.22292	26.95559	1	15	15
603	29.02995	28.14591	27.96279	29.01427	27.94606	27.57581	1	27	27
604	26.84031	25.64452	24.73823	26.79998	26.29685	23.83455	1	4	4
605	28.80925	28.69732	29.25427	28.77524	28.54759	29.1993	1	43	43
606	23.3787	21.8062	22.82976	22.94828	21.24195	23.0841	1	2	2
607	28.02237	27.0644	26.77187	27.74261	26.82013	26.76483	1	15	15
608	23.31071	24.04114	23.78842	24.19508	22.53055	24.18921	1	7	7
609	26.81267	27.41729	27.01406	26.72893	27.51166	27.07442	1	11	11
610	27.22352	27.2909	27.5283	27.10054	27.65243	27.6089	1	19	19
611	26.58173	25.84476	26.6637	26.28602	26.27936	26.48273	1	5	5
612	25.73257	24.91572	24.29418	25.45511	23.85694	24.09152	1	7	7
613	25.66099	25.97196	26.57775	26.0452	26.06237	25.74255	1	11	11
614	26.5583	25.29008	25.72746	26.31647	25.47799	25.4721	1	18	18
615	25.42272	24.19733	24.50035	25.47658	24.71972	25.10402	1	15	15
616	23.20387	21.79901	22.08586	23.69144	22.12201	22.10909	1	5	5
617	29.33214	28.6907	29.37225	29.36147	28.64703	29.36853	1	42	42
618	24.13775	22.18289	22.39861	24.12524	21.96388	22.42495	1	5	5
619	26.65746	26.68982	26.04981	26.61275	26.43396	25.63132	1	29	29
620	26.9471	25.73451	26.41732	27.0438	25.63631	25.77726	1	10	10
621	25.27962	24.83942	25.17688	25.5795	23.82286	23.29781	1	9	9
622	27.05724	25.63948	26.33482	26.99029	25.56031	26.22728	1	43	43
623	24.41286	24.14233	24.00875	24.23307	24.42459	23.67751	1	7	7
624	25.41246	22.80449	22.72381	24.90186	22.78619	22.97704	1	7	7
625	25.77626	24.41296	23.82827	25.84288	24.01492	24.14916	1	12	12
626	27.43871	26.9623	27.72812	27.44967	26.83168	27.27268	1	7	7
627	23.4255	23.64737	22.3546	23.62119	24.28015	22.81285	1	10	10
628	28.17787	27.93531	27.52975	27.97907	27.72828	27.60817	1	41	41
629	25.89026	23.14708	22.24238	22.96016	21.81974	23.05629	1	4	4
630	29.33896	29.20572	29.33289	29.26965	28.84361	29.13655	1	12	12
631	29.86901	28.67697	29.31857	29.93755	28.70164	29.15319	1	9	9
632	28.55741	27.26914	26.10115	28.61787	26.79877	25.8187	1	5	5
633	27.41105	26.40228	26.32857	27.3894	26.7225	26.20271	1	9	9
634	24.87413	23.01973	24.94633	24.84022	23.79538	22.97996	1	11	11
635	26.85773	26.42195	26.97786	26.56952	26.6259	26.65472	1	5	5
636	25.9322	25.40318	22.51832	26.12116	24.99925	24.18718	1	9	9
637	22.62838	21.5055	23.01797	22.4853	22.21502	22.18576	1	2	2
638	27.66536	28.62009	29.02322	27.71535	28.36901	28.90372	1	40	40
639	24.45531	23.79435	23.80672	24.38502	23.92399	23.85955	1	13	13
640	25.67306	25.3481	25.58229	25.65668	25.14887	25.21898	1	4	4
641	25.21184	24.73804	24.54681	25.78287	25.20573	24.99346	1	14	14
642	30.8415	30.41184	30.45442	30.86167	30.39297	30.40268	1	31	31
643	27.33316	26.72444	26.41648	27.40133	26.60603	26.46398	1	19	19
644	27.08845	26.0103	25.48112	27.18051	25.99989	25.44176	1	30	30
645	24.44158	23.17086	23.3095	24.18394	23.19829	23.56854	1	13	13
646	23.96914	23.79234	22.53504	24.79775	22.47786	22.01332	1	4	4
647	23.00031	21.84968	22.43127	22.77368	22.11744	24.05654	1	3	3
648	24.82643	28.33047	23.64552	28.74398	27.69163	23.59447	1	12	12

	BB	BC	BD	BE	BF	BG	BH	BI	BJ
649	28.11314	29.48715	27.71909	28.54004	29.44595	29.45658	1	11	11
650	24.37161	23.87538	23.89239	24.69841	23.87122	22.64384	1	7	7
651	26.9262	26.29783	26.07215	27.00036	26.08223	25.69552	1	24	24
652	25.41841	25.34488	24.91632	24.70259	25.04935	25.15846	1	18	18
653	23.48547	25.91432	23.0745	22.12392	22.24193	21.73293	1	7	7
654	25.03704	23.31926	22.61544	25.38959	22.85969	22.87849	1	12	12
655	26.05102	25.70666	25.2626	26.22246	25.89082	25.64202	1	13	13
656	24.59844	23.46223	24.70313	24.31194	24.0167	24.07025	1	8	8
657	23.87472	23.3753	23.96593	23.65558	22.96101	22.96263	1	6	6
658	26.35375	25.90184	25.9602	26.31461	24.62375	25.7054	1	17	17
659	23.95667	22.07857	22.2794	23.37884	22.51793	22.81798	1	6	6
660	27.6284	26.43861	26.78155	27.65396	26.18162	27.07741	1	3	3
661	24.65073	24.34382	24.07779	24.40008	22.31999	22.78703	1	8	8
662	26.61896	25.15731	25.72921	26.16119	26.16601	26.59648	1	32	32
663	27.06096	27.30258	27.36734	27.06166	27.40505	27.03175	1	8	8
664	25.24547	22.47457	25.33883	25.52149	24.04845	25.06171	1	8	8
665	27.07205	26.22148	26.83673	26.75492	26.67946	26.58327	1	18	18
666	31.59227	30.69144	30.77107	31.49416	30.4925	30.49421	1	54	54
667	29.87002	29.11122	28.77841	29.70535	28.83007	28.54611	1	30	30
668	27.385	26.60529	26.65699	27.61783	26.43448	26.54199	1	52	52
669	26.3491	24.96111	25.41484	26.482	25.17231	25.2808	1	7	7
670	25.22558	21.25631	21.31705	23.30657	21.85608	22.25947	1	2	2
671	24.83444	23.09702	23.97198	24.20947	22.55916	24.06876	1	7	7
672	25.91606	25.48579	24.73066	25.9447	25.11581	24.4147	1	18	18
673	23.74011	23.55792	25.67706	23.72208	22.96303	25.5034	1	6	6
674	25.10141	25.40575	23.63607	25.28686	25.20841	23.80116	1	15	15
675	26.37974	26.27198	25.723	26.25982	24.88029	25.42519	1	14	14
676	26.19752	25.62562	25.83471	26.00674	25.30854	25.6858	1	19	19
677	24.86623	25.87465	24.0681	24.82833	23.5098	23.698	1	9	9
678	25.09743	25.99312	25.35284	25.60941	25.88071	25.2356	1	14	14
679	28.75612	27.76233	27.34628	28.80496	27.71454	27.24888	1	43	43
680	24.66748	22.16948	22.80783	25.53794	22.29699	23.58749	1	8	8
681	26.86471	26.32894	26.32473	26.67317	26.39961	26.32528	1	27	27
682	29.07357	28.40877	27.88162	28.98996	28.31942	27.76035	1	22	22
683	28.99597	29.27552	28.10939	28.88293	29.29377	27.98855	1	23	23
684	23.37786	23.12751	22.84367	24.14496	22.69259	23.43501	1	10	10
685	27.19069	26.80343	27.63255	26.82582	26.98319	25.28912	1	7	7
686	30.86043	30.48837	30.03419	30.85533	30.43396	30.01389	1	46	46
687	28.9603	28.54581	28.58488	28.75747	28.66561	28.42442	1	13	13
688	28.024	27.70553	27.51522	28.024	27.67484	27.38509	1	19	19
689	24.55201	23.548	23.56094	24.75072	23.30889	23.26544	1	2	2
690	26.26546	25.49763	25.58935	26.22969	24.60226	24.27731	1	5	5
691	29.72076	29.6327	29.6311	29.58317	29.61043	29.62792	1	23	23
692	25.38453	24.59335	24.50705	25.19983	25.15439	24.21908	1	15	15
693	24.33476	24.00693	24.70372	25.01909	24.72967	24.80691	1	16	16
694	25.60463	25.39389	25.57087	25.58611	25.21018	25.18059	7	11	11
695	25.91668	25.10478	25.46345	26.02012	25.46737	25.01887	1	17	17
696	28.92781	27.85869	28.45275	28.83949	27.73033	28.29794	1	43	43
697	26.60375	26.05961	26.4292	26.18776	25.95787	26.17342	1	9	9
698	27.21263	26.98298	27.04092	27.30282	27.4031	26.98216	1	21	21
699	25.59381	25.20855	25.36521	25.45892	24.14309	25.33344	1	16	16
700	26.24677	25.75262	25.2217	25.69382	25.41935	24.31672	1	8	8
701	31.95506	31.52136	30.91342	32.03872	31.31881	30.83903	1	9	9
702	26.31893	26.53511	26.1333	26.50978	25.87944	25.81644	1	15	15

	BB	BC	BD	BE	BF	BG	BH	BI	BJ
703	27.97656	28.53695	28.13464	28.02744	28.0969	27.90366	1	12	12
704	29.00479	28.42961	28.867	29.09495	28.2578	28.59436	1	27	27
705	26.65185	26.63502	26.71517	26.48848	26.39034	26.8409	1	10	10
706	24.32668	23.73138	22.40538	24.37697	23.27939	22.58384	1	8	8
707	25.58461	24.97401	25.20264	25.60301	24.29934	25.26744	1	12	12
708	29.55912	29.41636	29.69271	29.60317	29.60453	29.89361	1	10	10
709	27.38804	26.79515	26.53232	27.43974	26.88263	26.44045	1	65	65
710	29.27625	29.49406	29.10242	29.34803	29.43239	29.05123	1	12	12
711	34.26208	33.54694	33.04559	34.23358	33.47612	32.93424	1	63	63
712	29.08097	27.69519	28.41901	29.12469	27.58477	28.26852	1	8	8
713	29.19472	27.97409	28.01772	29.20132	27.96229	27.60968	1	48	48
714	33.66533	33.12221	32.05674	33.68615	32.86181	31.82605	1	19	19
715	29.1529	27.33665	28.12524	29.02762	27.01724	27.86883	1	33	33
716	27.85773	27.82728	27.53719	27.64715	27.79835	27.41893	1	14	14
717	26.82232	25.27503	26.27407	26.66496	24.94755	26.14463	1	3	3
718	25.58204	25.23252	24.32597	25.73593	24.65857	24.45539	1	12	12
719	27.47678	26.8969	26.60519	27.60864	26.98318	26.40285	1	13	13
720	28.1341	28.10716	28.30871	27.6294	27.99416	28.29878	1	9	9
721	29.03412	28.40923	28.25924	29.13769	28.28835	28.31269	1	44	44
722	26.75411	26.1321	26.16195	26.82185	26.07682	25.90257	1	20	20
723	29.57885	28.82875	29.03773	29.45933	28.83956	28.65169	1	35	35
724	25.19086	25.07817	24.8196	25.14039	24.94358	23.88675	1	12	12
725	27.63344	27.39657	27.40466	27.56132	27.25571	27.25796	1	14	14
726	20.58732	19.60146	21.63504	21.39499	19.42796	19.70495	1	2	2
727	31.14758	31.23297	31.85667	31.15874	31.24338	31.37489	1	22	22
728	29.15755	28.37483	28.86488	29.20679	28.34163	28.64232	1	51	51
729	24.10058	23.94649	23.19643	24.34146	23.72252	23.20723	1	5	5
730	26.75072	26.68201	26.32897	26.88739	26.41792	26.78436	1	26	26
731	26.26327	25.45138	25.35849	26.50577	25.39335	25.30562	1	11	11
732	24.52857	25.7664	25.75485	24.4986	23.52795	25.95856	1	20	20
733	21.33606	19.92877	20.29324	21.49295	21.4358	22.3618	1	2	2
734	31.11334	30.38231	31.31491	31.13979	30.48069	31.0078	1	68	68
735	21.62086	20.52014	21.05733	22.78662	21.34996	21.96599	1	3	3
736	26.1992	26.58903	26.531	26.07168	26.6826	26.35747	1	19	19
737	30.33112	30.7392	30.35835	30.28629	30.65951	30.19775	1	16	16
738	30.37023	30.27759	30.17468	30.36479	30.24169	30.24407	1	21	21
739	24.14798	23.60115	22.33109	24.14363	23.24767	22.35101	1	4	4
740	26.88928	26.92434	26.98213	27.0399	26.94053	26.93423	1	25	25
741	30.26559	29.20579	28.9631	30.27021	29.29994	29.01179	1	13	13
742	27.59563	27.53951	27.10316	27.52336	27.50767	26.86415	1	11	11
743	32.71551	32.88265	32.95814	32.67598	32.76051	32.85678	1	19	19
744	28.31162	26.91858	27.40688	28.37078	26.90403	27.26311	1	38	38
745	28.02584	27.59325	28.41673	27.88008	27.63772	28.49078	1	24	24
746	23.78208	21.90067	23.52472	24.99152	22.5876	22.89183	1	7	7
747	29.68434	31.9723	29.36976	29.71938	31.67913	29.34134	1	24	24
748	23.83384	23.19173	23.53735	24.00899	24.09189	22.50435	1	5	5
749	27.37487	25.56892	27.33184	27.2919	26.01076	26.9842	1	37	37
750	27.33045	26.50015	26.44889	27.35329	26.33857	26.39792	1	37	37
751	24.81613	23.91381	22.73614	24.77213	23.25613	22.91734	1	6	6
752	25.68289	25.12737	24.99441	25.6783	24.34801	24.54545	1	23	23
753	23.52562	23.61874	23.16591	23.87592	23.62361	23.07021	1	10	10
754	26.88743	26.57901	26.08173	26.86937	26.46055	25.77958	1	12	12
755	25.36312	25.40955	25.21627	25.23841	25.79685	24.78467	1	7	7
756	28.2669	28.99761	29.49316	28.20949	28.72937	29.43647	1	6	6

	BB	BC	BD	BE	BF	BG	BH	BI	BJ
757	28.25102	27.52356	27.10142	28.17035	27.62105	26.74832	1	31	31
758	27.05894	25.94919	25.7074	27.04825	26.10441	25.60615	1	12	12
759	26.01893	24.98204	25.16212	25.88372	24.97507	25.15444	1	7	7
760	26.9803	26.59357	26.00349	26.98594	26.72393	25.77143	1	18	18
761	27.30833	24.92904	24.56881	24.49319	25.40782	24.06096	1	7	7
762	21.23224	22.62713	20.30027	24.19678	22.49588	20.41973	1	2	2
763	23.90979	22.54285	24.53337	23.78113	24.10538	24.79799	1	5	5
764	24.19021	23.34206	24.3335	22.63001	23.36963	24.46922	1	3	3
765	26.82988	26.70037	25.8584	26.87343	26.18631	26.67372	1	2	2
766	27.06689	27.84689	26.82945	28.07588	28.5905	28.14327	1	8	8
767	27.82726	27.38954	28.14614	27.47357	27.28721	27.95449	1	11	11
768	24.88049	24.76031	25.5258	24.88656	24.66185	25.06312	1	6	6
769	23.081	22.66685	22.30829	22.23825	22.82588	22.11969	1	3	3
770	26.82879	26.31384	26.23637	26.55512	26.39229	26.05264	1	3	3
771	29.82821	28.3384	28.79148	29.7055	28.19152	28.53587	1	6	6
772	25.38948	25.4201	22.46782	25.1528	25.3143	21.97664	1	6	6
773	25.37609	24.34475	23.69526	24.83986	23.58685	23.53726	1	8	8
774	26.75822	26.16334	25.61077	26.70466	25.7927	25.65335	1	20	20
775	30.88621	31.4813	31.64168	30.79749	31.14703	31.23421	1	9	9
776	26.04507	24.78069	24.59644	25.90898	24.90342	23.87695	1	19	19
777	27.10713	26.55083	26.20967	27.17956	26.6428	26.46239	1	23	23
778	25.95762	25.54299	25.71496	26.12829	25.71138	25.14244	1	11	11
779	30.02595	29.76029	30.38862	29.98104	29.84554	30.26279	1	14	14
780	27.05038	28.04786	28.24905	27.14722	27.9403	28.34766	1	33	33
781	32.51868	32.20969	32.40823	32.55665	32.26294	32.71528	1	26	26
782	26.98348	25.87989	24.78067	27.09605	25.49819	24.45489	1	5	5
783	29.13346	28.61381	28.91319	29.20599	28.53698	28.89454	1	20	20
784	26.04587	24.88151	24.40228	25.89427	24.95099	24.51789	1	10	10
785	28.24149	27.15018	27.01504	28.08579	27.24642	26.88231	1	24	24
786	27.1109	27.26881	27.3025	27.03344	27.0805	27.27571	1	9	9
787	26.29317	25.63524	26.25818	26.28548	25.86828	25.96214	1	16	16
788	28.08346	26.44758	27.43635	28.07202	26.62021	27.14372	1	28	28
789	31.4291	30.9402	30.22867	31.45655	30.89396	29.98124	1	44	44
790	26.01675	24.45135	24.83121	26.07392	24.26807	24.85057	1	14	14
791	25.91268	26.73911	25.16858	25.89835	26.18907	25.3623	1	14	14
792	29.70804	29.9724	29.79724	29.55303	29.42125	29.28302	1	27	27
793	25.99551	25.48148	26.18812	25.83163	25.78397	26.07815	1	14	14
794	28.75218	28.5591	28.55639	28.74509	28.52353	28.45427	1	8	8
795	27.23342	26.93692	26.91307	27.40388	26.98183	26.97278	1	14	14
796	27.48327	26.01952	26.48423	27.26682	25.90624	26.07679	1	3	3
797	24.47669	23.61962	25.58126	23.61766	23.63597	25.36478	1	5	5
798	29.88226	28.90878	29.58937	29.91788	28.83984	29.41252	1	37	37
799	24.40729	25.03712	25.28449	24.5325	25.04123	24.72275	1	10	10
800	21.80688	21.49626	22.43968	21.79334	22.84874	22.6833	1	3	3
801	26.01283	25.39209	24.88983	26.0664	25.18346	25.00461	1	23	23
802	25.62469	24.1754	24.70015	25.24841	23.92776	24.16991	1	12	12
803	28.37578	26.90711	26.0055	28.32488	27.16829	25.00092	1	9	9
804	27.7069	26.65121	26.91923	27.71039	26.4528	26.83495	1	44	44
805	24.14069	23.15783	23.32145	24.97208	23.5776	23.55123	1	4	4
806	23.3601	23.71122	23.10445	22.95384	24.21731	25.68965	1	7	7
807	25.45734	24.57169	24.64358	25.30564	24.8825	24.11072	1	8	8
808	23.44581	22.48155	23.18782	23.45971	23.58094	22.84492	1	6	6
809	24.58385	25.26528	26.61288	25.33308	22.24845	24.78064	1	3	3
810	25.7284	24.14491	24.36896	25.87019	24.85801	26.31974	1	30	30

	BB	BC	BD	BE	BF	BG	BH	BI	BJ
811	26.8649	25.26236	25.86234	27.36954	25.29288	25.66796	1	8	8
812	29.98106	29.78779	29.4456	30.02857	29.70917	29.25261	1	66	66
813	25.22969	23.9001	23.73218	24.8227	24.17782	23.16151	1	8	8
814	31.77495	31.79167	30.46683	31.65559	31.69837	30.35366	1	31	31
815	23.19446	23.75316	22.87344	23.48027	23.83907	22.49868	1	4	4
816	31.56955	31.23152	31.45513	31.52106	31.14271	31.36142	1	42	42
817	27.00975	27.77321	27.44857	26.96597	27.63371	27.42115	1	117	116
818	28.25873	26.98398	29.09915	28.13148	27.06309	29.00765	1	40	40
819	30.0369	30.1167	30.23754	30.04633	30.02124	29.94396	1	27	27
820	27.04788	26.5924	27.03794	27.16679	26.83591	26.94387	1	16	16
821	26.24141	26.33613	25.98538	26.17769	25.93492	26.12246	1	16	16
822	30.1947	30.19505	30.2113	30.12233	30.23035	29.9458	1	38	38
823	28.4523	28.11974	28.02245	28.45332	28.08944	28.02358	1	29	29
824	25.47196	24.4943	25.15842	25.60816	24.44944	24.7325	1	15	15
825	26.55215	25.57023	25.84614	26.75645	25.49309	25.53947	1	23	23
826	24.04716	24.82799	24.21227	23.9748	25.13298	24.21282	1	8	8
827	25.00726	23.984	26.53948	25.18735	24.15346	25.04223	1	24	24
828	26.03703	25.2754	24.94202	26.13024	24.68946	25.17755	1	12	12
829	25.00704	25.35578	25.06116	25.09173	26.43257	25.10919	1	8	8
830	26.39647	26.72579	26.29998	26.2942	26.22729	26.72694	1	17	17
831	26.97388	26.39756	25.49493	26.75915	26.30302	25.8203	1	22	22
832	25.2631	25.10044	24.83836	25.89613	25.04926	25.92985	1	14	14
833	21.48358	20.21313	20.7704	21.6864	20.3346	19.88995	1	2	2
834	22.81928	25.47583	22.17819	22.06511	25.46204	24.75481	1	7	7
835	22.99419	20.8141	22.18355	23.74191	21.08198	21.03256	1	4	2
836	26.27992	26.53283	27.07005	26.56821	26.50187	26.98039	1	71	71
837	24.78308	23.86138	24.44994	24.88368	22.94878	24.81349	1	19	19
838	24.82022	24.66015	25.18962	24.18592	25.17632	24.56935	1	11	11
839	21.1705	22.76627	22.50107	22.68058	22.6612	22.43723	1	2	2
840	29.72634	27.71256	28.61208	29.57985	27.83308	28.13143	1	29	29
841	27.91657	27.12891	27.15945	27.9693	26.78317	27.22233	1	25	24
842	28.69213	27.43398	27.89053	28.50654	27.28317	27.63935	1	71	71
843	26.54232	25.72681	25.70497	26.45162	25.74366	25.59658	1	28	28
844	26.06892	25.23628	24.87531	26.05047	25.1512	24.74661	1	9	9
845	26.44089	24.70579	24.76663	26.29941	25.17728	24.85907	1	18	18
846	22.32846	20.79958	20.04277	19.77351	20.09812	20.43127	1	3	3
847	25.18457	23.09843	24.38891	25.07829	22.85558	24.47792	1	9	9
848	26.49977	26.25257	26.7137	26.57711	26.15496	26.80816	1	12	12
849	27.36665	27.31244	26.82309	27.41307	27.20235	26.68064	1	14	14
850	25.87505	25.03629	25.20911	25.87369	25.11042	25.07764	1	10	10
851	24.83405	24.64269	23.44217	24.89731	23.90788	23.41052	1	8	8
852	25.86449	24.64555	24.61168	25.43299	25.18663	24.99944	1	14	14
853	23.19851	21.83494	22.10753	22.11999	22.1885	21.51819	1	4	4
854	23.75613	22.41463	21.61699	23.56369	21.89878	23.21024	1	7	7
855	23.01662	23.77374	22.40205	22.74961	23.53963	21.90312	1	4	4
856	25.73615	25.26046	25.92404	25.98198	25.18128	25.87117	1	18	18
857	26.31806	24.85242	25.11871	26.31786	25.07784	25.05123	1	22	22
858	28.54529	28.88888	28.29881	28.69184	28.61736	28.15527	1	19	19
859	25.71334	23.52834	23.83787	25.36638	23.34059	23.6044	1	11	11
860	27.69125	27.16578	26.44034	27.72075	26.93843	26.28453	1	29	29
861	24.83855	23.85095	24.0723	24.37736	23.16731	23.59648	1	19	19
862	29.06008	28.66579	28.98398	29.22416	28.43092	28.84872	1	15	15
863	26.3286	25.7901	25.07795	25.91776	25.36794	24.63322	1	10	10
864	27.39693	26.67572	26.50397	27.34016	26.46625	26.30381	1	41	41

	BB	BC	BD	BE	BF	BG	BH	BI	BJ
865	24.45988	25.19041	24.33468	24.6236	25.25561	22.95954	1	16	16
866	24.92084	24.14272	23.4641	25.29413	23.61462	23.0206	1	16	16
867	20.67217	20.89157	20.61305	20.07607	19.87257	20.71686	1	2	2
868	24.83341	23.35805	23.46447	24.84495	23.72692	23.81165	1	16	16
869	23.56329	24.11181	23.39725	23.44668	24.75868	23.36809	1	4	4
870	26.6935	26.68239	26.96008	26.7181	26.62526	26.85139	1	8	8
871	27.63458	27.38901	27.19328	27.66896	27.23704	27.1259	1	11	11
872	26.16341	26.03178	25.94504	26.3212	25.98439	25.66434	1	13	13
873	25.47982	24.73242	24.69286	25.08057	24.03997	24.61631	1	13	13
874	26.0119	24.95792	22.65762	26.24672	25.182	24.2276	1	4	4
875	24.01847	22.98848	22.71121	24.06398	22.67515	22.71509	1	7	7
876	23.88021	22.81983	21.03879	22.51737	23.04025	21.21871	1	4	4
877	23.66419	23.12732	22.65985	23.09775	24.31338	22.14946	1	3	3
878	28.30554	27.62384	28.30912	28.37002	27.53768	28.01941	1	7	7
879	32.22396	32.71951	31.52353	32.26299	32.59605	31.58504	1	18	18
880	25.17985	24.80441	24.39533	25.22909	25.01885	24.16873	1	11	11
881	27.11687	26.07556	26.10884	27.22624	26.07215	26.23503	1	32	32
882	24.0421	25.40502	23.23805	23.11273	23.7045	23.15151	1	6	6
883	26.68529	26.666	26.39704	26.64181	26.52549	26.59581	1	14	14
884	23.29231	22.71269	23.89609	23.60202	23.3647	23.55075	1	5	5
885	23.92517	22.01016	21.87087	23.77038	22.15112	21.76547	1	3	3
886	25.89366	26.66594	26.04676	25.44971	26.71253	26.04431	1	3	3
887	23.32522	23.26997	23.12297	24.19056	23.29084	22.08142	1	7	7
888	29.17404	28.19226	27.90862	29.20769	28.12211	27.71763	1	14	14
889	33.87535	34.69855	33.98691	33.96103	34.61543	33.7016	1	39	39
890	33.74579	33.82314	32.93793	33.67973	33.58347	32.92492	1	23	23
891	26.41075	26.58861	26.50463	26.54979	26.26243	26.30297	1	8	8
892	27.26549	25.18674	25.69087	27.19507	25.0479	25.97985	1	18	18
893	24.10719	25.43903	24.00915	24.06133	25.7437	24.52976	1	3	3
894	25.20899	24.95475	24.72705	25.15894	24.67614	24.40358	1	9	9
895	25.79558	23.77164	24.60891	25.61446	24.05216	24.97069	1	9	9
896	27.72878	27.42758	27.71247	27.63367	27.44794	27.4594	1	11	11
897	25.30325	23.80178	24.44661	25.38123	24.83772	24.13874	1	17	17
898	25.02203	24.24017	23.58892	24.82765	24.14412	23.45099	1	6	6
899	24.97542	23.63162	22.69213	24.58365	23.84043	23.32014	1	8	8
900	25.50351	25.08512	25.15239	25.52867	25.46615	24.52805	1	16	16
901	25.02045	24.04312	24.34074	24.91096	24.3083	23.84059	1	13	13
902	27.88286	28.12918	28.31192	27.98641	27.91341	27.49802	1	29	29
903	26.31742	27.26664	25.94069	25.82339	27.12683	25.82681	1	16	16
904	25.31072	26.66348	24.30941	25.30696	26.7021	25.4791	1	12	12
905	27.98624	27.54825	26.98907	27.84993	27.464	26.78765	1	24	24
906	23.14547	23.88573	22.76677	23.50997	24.23082	23.26089	1	7	7
907	23.17774	21.5977	21.53178	23.33102	21.69983	21.73985	1	3	3
908	28.2242	26.99948	26.46195	28.3745	26.89022	26.29622	1	28	28
909	28.27451	28.63411	27.91776	28.3092	28.64627	27.62643	1	24	24
910	27.5967	28.49846	25.80644	28.46897	28.11932	25.87999	1	15	15
911	25.75901	25.40215	23.47369	25.62416	25.25349	22.69161	1	6	6
912	23.56252	24.54959	23.21898	23.68239	24.08457	23.27301	1	4	4
913	28.16035	27.01284	27.04642	28.10158	26.95688	27.0994	1	26	26
914	27.3749	26.22449	26.39104	27.31801	26.1824	25.8993	1	23	23
915	25.9269	24.55648	29.27768	25.62993	24.70131	24.80412	1	18	18
916	23.80045	21.93522	22.05212	23.88493	22.53523	22.36463	1	3	3
917	26.00263	26.22187	26.37534	26.00071	26.04437	26.13382	1	8	8
918	28.44121	28.63562	28.15419	28.5173	28.55956	28.07711	1	16	16

	BB	BC	BD	BE	BF	BG	BH	BI	BJ
919	26.1732	26.18256	25.87084	26.39563	26.44163	25.75304	1	14	14
920	24.05664	21.58146	22.63141	24.1754	21.44193	21.84579	1	5	5
921	28.91929	27.49304	26.69491	28.73577	27.4981	26.68989	1	4	4
922	28.48519	27.68115	27.68045	28.33464	27.52743	27.80606	1	26	26
923	29.37966	28.44645	28.08891	29.45306	28.19219	27.9666	1	26	26
924	26.91058	26.64454	25.18909	26.99907	26.68426	25.67232	1	8	8
925	29.17965	27.74694	27.82878	29.28807	27.67175	27.60835	1	28	28
926	31.5232	31.50793	31.69189	31.53597	31.4479	31.74406	1	70	67
927	25.914	27.46119	25.41101	25.85174	27.19144	25.12951	1	7	7
928	23.7112	23.39087	23.70427	23.76829	21.89668	22.52327	1	4	4
929	30.16511	29.69054	29.30906	30.21866	29.76516	29.14919	1	65	65
930	30.22453	31.34498	30.58304	30.30705	31.33653	30.48316	1	13	13
931	24.28633	22.83802	23.41386	24.11194	23.36695	23.17501	1	6	6
932	27.43829	27.4969	27.44213	27.35026	27.34726	27.398	1	12	12
933	25.36361	25.49174	25.49086	25.19369	25.38072	25.46965	1	6	6
934	24.543	22.87226	23.29828	24.45759	23.76371	23.19724	1	6	6
935	27.07814	27.61079	27.36857	27.03229	27.36491	27.11332	1	16	16
936	28.89651	27.89101	27.98278	29.05316	27.68001	27.85903	1	48	48
937	27.78885	26.48165	27.45194	27.41795	26.37104	26.80905	2	10	10
938	25.54515	25.73836	26.67689	25.10561	25.95014	26.3046	2	14	14
939	27.01963	26.60809	26.68683	26.97503	26.38203	26.51788	2	12	12
940	28.27312	28.19229	28.52887	28.27992	28.28444	28.43887	2	22	22
941	27.42238	27.91455	26.93673	27.37519	27.90085	26.99243	1	35	35
942	25.71868	25.32355	25.35459	25.84332	25.23442	25.4973	1	13	13
943	26.48618	25.75689	26.13133	26.33691	25.75961	25.99866	1	19	19
944	23.60951	23.82142	24.06297	23.6468	23.7425	24.17376	1	7	7
945	26.08114	26.36422	25.80243	26.06297	26.2502	25.79803	1	8	8
946	29.51987	29.75728	29.96275	29.59847	29.54352	29.66672	1	12	12
947	29.27175	28.23007	28.06406	29.16098	28.20446	28.14585	1	111	111
948	29.23322	27.96808	28.3693	29.3126	28.09323	28.1874	1	95	95
949	24.78737	23.23483	23.97244	24.80077	23.73172	24.22331	1	9	9
950	24.74926	24.30579	24.32831	24.71477	24.11898	24.61401	1	9	9
951	25.83828	25.49899	25.25147	25.84106	25.38233	25.70935	1	23	23
952	25.98177	25.43287	25.80577	25.95108	25.26941	25.81318	1	14	14
953	21.86826	21.15908	21.15088	20.82029	21.4978	20.68165	1	2	2
954	23.02472	25.04276	22.83294	22.89138	24.87408	22.06251	1	3	3
955	29.03844	27.88991	28.0628	29.06682	27.89791	28.03174	1	32	32
956	23.1873	21.69977	21.35246	23.14004	21.14744	20.73598	1	2	2
957	23.94467	23.37193	24.04849	23.61718	21.90808	22.92421	1	5	5
958	26.15886	25.73223	24.12578	25.98428	24.83545	23.94452	1	8	8
959	22.23546	23.12395	23.60103	22.2095	22.8666	23.44895	1	5	5
960	27.31207	26.31714	26.23695	27.3458	26.19246	25.91569	1	24	24
961	26.48165	25.4683	25.61833	26.39226	25.83827	25.73681	1	15	15
962	25.80038	25.19152	24.77264	25.71533	25.29462	24.83984	1	17	17
963	24.80544	23.57188	23.44023	24.5058	23.27133	22.86645	1	14	14
964	25.1799	25.27775	23.29713	25.24991	24.39679	23.47048	1	11	11
965	26.49238	26.19964	24.16771	26.63221	25.22925	24.6806	1	7	7
966	22.2012	22.39479	22.82771	23.14652	22.49184	22.24978	1	3	3
967	24.98899	24.30949	23.66947	24.56551	23.37002	23.20386	1	8	8
968	24.60323	23.85309	24.33024	24.22167	23.69208	24.16641	1	2	2
969	23.2163	21.79325	22.96322	22.94704	22.28362	23.01539	1	3	3
970	24.01524	22.53577	22.88085	24.82568	23.8186	22.55227	1	8	8
971	26.22766	26.83182	26.26554	26.11278	26.81968	26.1926	1	14	14
972	28.18863	26.75148	26.99891	28.17004	26.76094	26.79932	1	22	22

	BB	BC	BD	BE	BF	BG	BH	BI	BJ
973	27.53745	27.76345	27.47264	27.52138	27.61027	27.08203	1	13	13
974	25.12684	24.57906	24.31286	25.13487	24.56301	23.98421	1	14	14
975	28.30066	27.37938	27.03938	28.28028	27.2531	27.44434	1	28	28
976	24.3664	23.24203	23.93045	23.78667	22.56471	22.69068	1	8	8
977	28.60954	28.78108	27.90278	28.58199	28.65663	27.75133	1	16	16
978	24.69762	23.54016	23.51167	24.87454	23.68512	23.32324	1	2	2
979	25.50765	24.77744	24.63739	25.44101	24.50137	24.62428	1	32	32
980	33.5831	32.43962	33.05639	33.55567	32.37519	32.813	1	47	47
981	25.80766	25.69981	24.14699	25.97252	26.11069	24.62146	3	6	6
982	22.68924	22.89862	21.82125	22.34738	21.60999	22.23067	1	5	5
983	26.46557	26.37767	27.14318	26.48183	26.38095	27.11889	1	9	9
984	26.44109	26.24926	27.30379	26.53134	26.33203	27.17474	1	7	7
985	27.19179	27.18043	28.22489	27.13266	27.10399	28.3462	1	53	53
986	26.15724	25.43372	25.5023	26.37511	25.16747	25.1408	1	6	6
987	28.3746	27.90677	28.5834	28.2903	28.21112	28.49459	1	14	14
988	26.93186	25.24862	25.62213	27.11922	22.53602	24.15705	1	2	2
989	29.2462	28.58306	28.47925	29.31351	28.45205	28.34671	1	53	53
990	26.54921	26.28792	25.91356	26.54589	26.4259	25.75138	1	26	26
991	20.18765	20.80655	19.67393	20.53492	21.43278	20.64824	1	2	2
992	28.23028	27.92864	27.23719	28.31556	27.69873	27.02933	1	29	29
993	24.52028	23.60377	23.42856	24.50474	23.65509	24.18847	1	8	8
994	26.72034	23.14106	23.34153	26.49294	23.27138	23.19204	1	4	4
995	27.34153	27.28164	26.17422	27.20325	26.89105	26.271	1	22	22
996	25.93925	25.63694	24.59949	25.97307	25.53587	25.06702	1	10	10
997	25.39719	25.00931	24.93669	25.78155	25.2827	24.75365	1	8	8
998	21.45661	22.91505	21.63032	22.74004	22.75123	21.73692	1	3	3
999	30.94512	31.41795	31.44226	30.98236	31.35267	30.95871	1	24	24
1000	23.88671	23.87481	23.58012	23.97264	24.00148	23.45314	1	8	8
1001	28.31231	28.46523	28.14103	28.35079	28.39659	27.88317	1	38	38
1002	25.22329	24.56629	25.11876	25.17822	25.20135	24.92878	1	12	12
1003	24.01684	23.52192	20.84588	23.82202	23.63084	21.44874	1	2	2
1004	22.95845	23.67834	22.95439	23.89805	23.43426	22.53539	1	3	3
1005	23.74375	22.22778	23.69645	23.03359	22.86767	23.45133	1	7	7
1006	27.17988	27.1307	27.4715	27.15898	26.94542	27.21522	1	6	6
1007	20.74998	23.10281	22.63819	21.45298	23.21193	22.63458	1	2	2
1008	23.14067	21.38233	23.19377	24.35438	23.92039	21.5376	1	3	3
1009	28.13287	27.06435	26.79864	28.06981	27.03172	26.74879	1	39	39
1010	29.36839	29.30717	29.73899	29.59136	29.45833	29.53784	1	43	43
1011	26.83143	26.04711	27.54933	26.60146	26.07466	26.11742	2	22	22
1012	27.0036	26.06173	25.90603	26.88937	25.95897	25.86256	1	21	21
1013	26.54065	25.34959	25.64301	26.37309	25.10682	25.58053	1	37	37
1014	25.06778	23.57669	24.03615	25.00674	23.26965	23.00108	1	8	8
1015	27.75267	27.08164	27.50174	27.73044	27.09983	27.2866	1	45	45
1016	23.09692	21.52309	22.56928	22.33566	22.26195	22.95088	1	3	3
1017	27.87378	27.28579	27.14838	27.81474	27.1382	26.98398	1	20	20
1018	28.75819	28.11883	27.96567	28.97431	28.00837	28.08497	1	24	24
1019	25.35594	25.05804	25.26524	25.53101	25.04288	24.84142	1	12	12
1020	27.86261	28.31701	28.42321	27.78717	28.11616	28.68807	1	22	22
1021	29.60163	30.01234	29.11639	29.57601	29.75986	29.31657	1	14	14
1022	25.67135	25.14828	23.9595	25.74147	24.77377	24.86715	1	26	26
1023	27.9336	27.43448	27.8436	28.05254	27.4254	27.38169	1	40	40
1024	24.42421	24.19796	22.82571	24.49909	22.70493	23.3555	1	11	11
1025	27.36947	27.34281	27.75508	27.36428	26.62275	27.31577	1	8	8
1026	21.94421	21.32473	21.82836	21.92343	20.91545	21.70449	1	2	2

	BB	BC	BD	BE	BF	BG	BH	BI	BJ
1027	27.11443	27.89945	27.17501	26.97023	27.96093	27.16472	1	48	48
1028	25.55986	24.95752	25.93919	24.99142	24.64072	25.48215	1	10	10
1029	27.95882	27.81017	28.00298	27.9025	27.75575	27.94103	1	35	35
1030	23.43731	22.71615	21.79423	24.18183	25.4726	22.00683	1	6	6
1031	27.20139	26.27334	26.3463	27.03124	26.68334	26.15601	1	36	36
1032	24.26762	23.26334	23.47924	24.31738	22.74889	23.43339	1	4	4
1033	24.1985	24.01894	23.96795	24.53697	23.90608	23.84375	1	2	2
1034	26.09159	25.63291	25.45174	26.11829	25.55569	25.10595	1	15	15
1035	22.97354	21.58472	21.99029	22.99141	21.03317	22.26997	2	2	2
1036	26.5856	26.60456	26.77016	26.56985	26.87387	25.96634	1	24	24
1037	24.29674	24.06814	24.51292	24.44351	23.7875	22.60053	1	3	3
1038	28.87065	27.90389	29.93797	28.94554	27.77967	29.92813	1	36	36
1039	25.70664	26.16341	25.94706	25.6513	26.27411	25.57358	1	3	3
1040	24.93279	25.14982	23.00307	24.51963	25.23383	22.06822	1	5	5
1041	25.41641	24.01416	24.00296	25.45086	23.96675	24.51873	1	13	13
1042	22.89599	21.63582	22.64348	22.52953	21.2792	22.82734	1	3	3
1043	31.02684	30.52278	30.87988	31.08586	30.62132	30.75937	1	3	3
1044	29.96756	29.13499	28.21069	30.03074	28.75525	27.70538	1	6	6
1045	26.55991	26.84629	26.3662	26.61177	26.74076	26.22809	1	5	5
1046	25.81959	25.59881	25.09152	25.8442	25.55458	25.32818	1	17	17
1047	25.07679	24.27834	23.72696	25.19017	23.70408	23.68206	1	3	3
1048	26.29651	27.10044	27.55109	26.35492	27.33108	27.45794	1	108	108
1049	30.00317	29.07125	29.20939	30.07221	29.09415	29.1353	1	38	38
1050	25.58017	24.94403	25.40044	25.50456	24.23257	24.55909	1	3	3
1051	26.87864	25.84583	25.92118	26.8975	25.7005	25.78091	1	28	28
1052	22.33574	21.67342	21.98248	22.11533	22.54856	22.68339	1	3	3
1053	26.05194	25.94075	26.15682	25.88298	25.8477	26.06684	1	6	6
1054	25.33618	24.42659	25.0017	25.16073	24.40909	24.84923	1	14	14
1055	28.12567	28.32735	28.61949	27.98906	27.48484	28.47629	1	6	6
1056	26.08591	25.13279	24.98792	25.93618	25.9551	25.02047	1	13	13
1057	27.86635	27.32411	27.35854	27.88772	27.20176	27.27729	1	18	18
1058	27.362	27.38882	26.87029	27.34753	27.21044	26.4178	1	24	24
1059	25.47681	27.05605	28.75222	25.43666	26.29738	28.52864	1	6	6
1060	31.51662	30.52273	31.42245	31.46272	31.3271	30.46841	1	105	105
1061	25.29594	23.98235	23.68935	25.2011	23.93213	24.80937	1	4	4
1062	20.95946	20.75477	21.44489	22.11418	21.05298	21.40136	1	3	3
1063	26.38355	26.48456	25.37585	26.33895	26.00462	25.49676	1	49	48
1064	23.21164	23.48365	22.59698	23.71314	22.21448	22.14738	1	5	5
1065	26.49685	25.7157	25.44717	26.77953	25.54649	25.66533	1	24	24
1066	22.32794	21.62199	23.88667	23.20048	22.24074	23.74731	1	3	3
1067	27.71021	27.61475	27.01236	27.67687	27.68295	26.92949	1	5	5
1068	23.76493	23.62011	23.00211	23.74911	23.86867	22.91734	1	8	8
1069	27.30563	26.38611	25.81271	27.43991	26.34355	25.61798	1	18	18
1070	25.73637	24.64412	24.86501	25.41177	23.37108	24.15873	1	15	15
1071	29.85191	29.30305	28.86703	29.79662	29.1652	28.87195	1	21	21
1072	25.02747	24.81122	24.77322	24.82232	24.17195	23.16788	1	9	9
1073	25.4252	25.71984	25.52363	25.4405	25.47444	25.62239	1	20	20
1074	32.57048	32.50765	31.93429	32.35635	32.39219	31.82928	1	46	46
1075	29.93796	30.17194	28.97657	29.90124	30.13482	28.87095	1	29	29
1076	30.93694	31.9872	32.51911	30.93457	31.97282	32.10064	1	41	41
1077	25.14821	23.42052	24.30812	25.14363	23.81117	23.48646	24	12	12
1078	24.90549	24.35951	24.03819	24.74442	24.72811	24.58442	1	10	10
1079	22.34492	21.05821	21.99957	22.65405	23.46565	22.1281	1	2	2
1080	26.4527	25.99281	23.54476	26.49519	25.6067	22.8704	1	7	7

	BB	BC	BD	BE	BF	BG	BH	BI	BJ
1081	26.79407	25.58354	25.60051	26.56495	25.55864	25.60912	1	8	8
1082	25.00448	24.53953	23.2619	25.06099	24.06876	23.83838	1	5	5
1083	25.24253	24.79571	24.72385	25.28163	24.44432	23.69211	1	14	14
1084	24.40876	21.18041	21.35662	24.34545	23.6214	22.02021	1	3	3
1085	29.27217	27.78289	28.24325	29.19937	27.69833	28.11558	1	47	47
1086	26.50218	26.11882	26.34115	26.53919	25.55971	26.48652	1	19	19
1087	27.97924	26.77248	26.31547	28.02715	26.31133	26.4586	1	35	35
1088	27.34819	25.28523	25.23523	26.0749	22.87869	25.01764	1	9	9
1089	25.36509	25.67317	24.93331	24.86316	24.6703	24.73787	1	15	15
1090	22.5359	22.37321	24.35569	22.23739	22.21717	20.84427	1	2	2
1091	28.80497	28.35638	28.26493	28.69547	28.22897	28.26495	1	37	37

	BK	BL	BM	BN	BO	BP	BQ
1	Mol. weight [kDa]	Sequence length	Q-value	Score	Intensity	iBAQ	#PSM
2	51.781	453	0	122.71	2511800000	96606000	219
3	42.09	378	0	194.99	8376500000	418820000	298
4	7.4658	64	0	42.69	794630000	264880000	63
5	36.73	318	0	7.911	427450000	20355000	17
6	38.672	334	0	213.63	4787700000	251980000	202
7	41.255	371	0	323.31	11176000000	532180000	610
8	134.96	1171	0	24.719	1185500000	17694000	66
9	14.805	122	0	23.448	67442000	11240000	12
10	20.212	180	0	323.31	22634000000	2057600000	573
11	71.281	652	0	323.31	45626000000	1425800000	1218
12	49.29	442	0	323.31	20552000000	893580000	868
13	17.629	155	0	7.66	152170000	19021000	58
14	15.843	147	0	115.09	4652000000	581500000	275
15	18.385	165	0	48.046	3178700000	454100000	69
16	35.451	322	0	145.28	5621100000	432390000	222
17	8.1021	73	0	65.097	446610000	74435000	42
18	100.58	889	0	323.31	6971100000	142270000	429
19	16.229	145	0	293.73	82416000000	8241600000	992
20	43.199	389	0	284.52	12479000000	623940000	462
21	34.816	330	0	188.22	3040400000	168910000	157
22	8.8077	77	0.003714	2.5235	3770300000	1885100000	53
23	34.476	297	0	91.809	1154700000	67924000	103
24	26.974	235	0	43.452	626450000	39153000	61
25	135.48	1241	0	59.926	1812000000	27454000	118
26	52.78	480	0	11.405	257450000	12260000	9
27	36.502	340	0	6.2369	319430000	22817000	10
28	56.32	515	0	90.398	778170000	31127000	87
29	45.334	420	0	82.503	2165500000	83287000	118
30	16.946	162	0	27.2	2397800000	239780000	164
31	39.36	363	0	33.265	1394700000	116220000	93
32	8.6466	75	0	14.195	1293900000	646930000	19
33	49.591	432	0	181.74	11264000000	363350000	542
34	27.934	238	0	312.88	15305000000	2186400000	590
35	180.69	1616	0	323.31	20941000000	237970000	1121
36	28.246	254	0	301.69	6158400000	559860000	263
37	23.959	221	0	320.77	8000900000	727350000	360
38	26.683	232	0	6.3826	248540000	22594000	10
39	38.846	350	0	65.776	429120000	30652000	28
40	23.028	203	0	323.31	33924000000	2120300000	744
41	37.905	328	0	323.31	2.87E+11	14361000000	3285
42	40.236	354	0	323.31	19113000000	1365200000	641
43	34.256	303	0	323.31	3063300000	218800000	183
44	21.348	197	0	31.939	358960000	44871000	12
45	69.166	616	0	291.65	9445600000	286230000	427
46	30.377	290	0	55.597	1704900000	154990000	118
47	24.343	223	0	24.78	242770000	30347000	15
48	20.863	195	0	323.31	2.23E+11	31889000000	568
49	34.163	287	0.00093897	2.9727	647870000	38110000	8
50	83.709	752	0	323.31	5.41E+12	1.93E+11	19514
51	41.626	373	0	323.31	31993000000	1523500000	1092
52	71.075	637	0	323.31	9322900000	282510000	623
53	60.999	559	0	270.57	17789000000	539060000	632
54	9.2282	77	0	323.31	96324000000	19265000000	2760

	BK	BL	BM	BN	BO	BP	BQ
55	25.347	227	0.0037037	2.4899	216240000	16634000	11
56	36.234	336	0	10.83	345240000	24660000	26
57	34.089	287	0	63.915	72949000	5210600	17
58	85.704	731	0.0099368	1.7065	10523000000	263080000	17
59	30.612	276	0	323.31	47903000000	2993900000	1091
60	31.177	284	0	323.31	15071000000	941930000	630
61	51.519	457	0	67.372	1506700000	65508000	91
62	38.719	353	0	66.148	1749500000	92077000	118
63	19.865	177	0	292.25	4974000000	828990000	104
64	17.6	156	0	85.889	3415100000	487870000	174
65	45.898	420	0	43.606	1315100000	93937000	54
66	73.434	649	0	307.3	2949000000	95128000	228
67	16.766	155	0	231.97	447980000	49775000	26
68	23.317	211	0.00094073	2.9893	31518000	2626500	6
69	40.607	366	0.0018639	2.6107	86268000	5391700	7
70	21.548	197	0	38.709	254440000	42406000	18
71	38.885	343	0	101.05	2332300000	116620000	191
72	25.799	227	0	323.31	3200700000	640130000	188
73	36.447	314	0	217.85	888860000	88886000	103
74	104.23	943	0	35.203	1461300000	31767000	90
75	39.49	353	0	323.31	22218000000	1169400000	575
76	14.89	132	0	181.61	808960000	115570000	59
77	21.015	189	0	323.31	9202100000	707860000	351
78	10.227	88	0	323.31	91631000000	22908000000	1119
79	15.655	139	0	199	14008000000	1556400000	481
80	11.758	101	0	53.256	2550100000	1275100000	234
81	46.578	416	0	58.978	1330100000	88671000	106
82	16.494	152	0	32.856	1432500000	238750000	70
83	58.749	510	0	16.685	6574900000	252880000	36
84	58.032	512	0	34.176	903730000	75311000	67
85	83.832	735	0	61.285	4346000000	90541000	161
86	23.194	205	0.0090992	1.8802	161860000	16186000	7
87	11.582	102	0	100.38	30044000000	4292000000	325
88	22.178	208	0	323.31	44629000000	5578700000	1187
89	22.109	207	0	323.31	85715000000	8571500000	1491
90	10.785	98	0	323.31	1.1E+11	13772000000	1155
91	29.866	277	0	323.31	1.56E+11	9727200000	1161
92	10.75	93	0	323.31	40627000000	6771200000	743
93	12.2	114	0	323.31	15988000000	1998500000	464
94	24.045	217	0	323.31	71077000000	5923100000	978
95	15.436	137	0	204.36	24616000000	8205400000	398
96	8.0153	68	0	323.31	2.2E+11	73241000000	1403
97	10.011	86	0	155.71	33032000000	8258100000	1363
98	13.006	122	0	147.54	25539000000	2837600000	492
99	10.991	101	0	323.31	1.39E+11	34755000000	1558
100	19.774	180	0	323.31	1.43E+11	12995000000	1642
101	10.076	89	0	92.533	22995000000	11497000000	350
102	14.754	132	0	323.31	62021000000	8860100000	767
103	19.413	178	0	323.31	1.04E+11	11585000000	960
104	12.867	118	0	323.31	1.08E+11	18059000000	670
105	17.057	164	0	323.31	1.17E+11	10652000000	1256
106	6.3985	60	0	192.95	23863000000	5965700000	311
107	15.446	146	0	218.34	79180000000	13197000000	1314
108	47.325	436	0	5.935	1473200000	122760000	72

	BK	BL	BM	BN	BO	BP	BQ
109	23.735	212	0	323.31	1.52E+11	11684000000	2191
110	8.2024	72	0	139.61	15246000000	5082000000	411
111	4.4214	38	0	18.285	45639000000	22819000000	312
112	13.421	121	0	135.49	28642000000	4091600000	474
113	13.385	127	0	323.31	17321000000	3464200000	347
114	34.206	311	0	323.31	81704000000	4806100000	1786
115	14.505	128	0	323.31	62189000000	10365000000	1098
116	9.671	88	0	37.545	2633100000	526610000	84
117	46.397	445	0	323.31	15567000000	915710000	463
118	25.696	230	0	143.01	3480800000	386760000	163
119	27.946	248	0	5.9911	220230000	14682000	10
120	94.389	833	0	323.31	23082000000	678890000	879
121	26.993	231	0	64.015	1410500000	156720000	78
122	15.748	138	0	76.491	423150000	84631000	43
123	37.271	332	0	16.295	671180000	39481000	60
124	45.864	419	0	191.83	5308300000	221180000	251
125	68.594	617	0	323.31	37222000000	1240700000	1227
126	53.193	459	0.0063985	2.1152	387840000	16160000	9
127	65.583	602	0	323.31	29522000000	922570000	941
128	140.27	1256	0	112.55	2331900000	31944000	123
129	15.143	137	0	323.31	36159000000	6026600000	800
130	17.756	156	0	323.31	20918000000	2988300000	401
131	76.834	693	0	323.31	5.36E+11	14500000000	6362
132	164.87	1463	0	125.26	3596100000	50649000	171
133	10.029	87	0	125.43	1393300000	348310000	54
134	45.242	413	0	323.31	81318000000	4065900000	1421
135	27.335	240	0	20.364	452590000	32328000	32
136	50.282	444	0	323.31	23863000000	1037500000	799
137	33.785	303	0	323.31	39055000000	2440900000	935
138	27.244	267	0	43.579	1229100000	175590000	59
139	35.443	329	0	323.31	1.23E+11	7222000000	1517
140	35.752	339	0	323.31	31529000000	1970600000	965
141	30.763	270	0	323.31	9428700000	725290000	451
142	49.225	472	0	74.547	1023600000	127950000	49
143	34.518	315	0	323.31	10856000000	638600000	465
144	48.963	440	0	31.141	1033200000	49201000	37
145	20.713	184	0	86.192	19997000000	1817900000	443
146	31.051	270	0	8.7437	449980000	40907000	29
147	16.143	148	0	272.84	24605000000	3514900000	515
148	14.234	130	0	89.696	34528000000	8632000000	478
149	86.055	737	0	81.367	1247400000	33713000	115
150	38.299	333	0	197.36	3131600000	208770000	189
151	7.5175	64	0	85.474	932980000	233250000	38
152	35.987	316	0	116.17	1464000000	97597000	79
153	12.234	105	0	8.898	393600000	56228000	25
154	82.17	750	0	206.86	2712200000	71372000	227
155	35.974	326	0	17.099	63956000	9136500	9
156	77.52	701	0	323.31	10122000000	253040000	669
157	17.923	160	0	112.93	3270300000	363360000	146
158	54.992	494	0	206.53	3724100000	148960000	228
159	62.212	536	0	323.31	8221400000	373700000	493
160	72.62	655	0	323.31	27941000000	755150000	1232
161	72.795	652	0	323.31	28040000000	904510000	856
162	13.887	123	0	6.9975	612310000	102050000	89

	BK	BL	BM	BN	BO	BP	BQ
163	9.5197	85	0	152.53	6008000000	3004000000	171
164	72.769	659	0	323.31	5.14E+11	13167000000	4530
165	79.54	718	0	323.31	11362000000	315600000	639
166	12.575	109	0	323.31	51094000000	7299200000	1769
167	43.231	385	0	193.6	1910100000	90957000	104
168	50.771	456	0	323.31	10122000000	562340000	351
169	52.565	474	0	323.31	2.04E+11	8517200000	3043
170	26.769	228	0.0036832	2.3422	75233000	5787200	9
171	37.155	320	0	323.31	14175000000	833800000	601
172	38.915	332	0	323.31	26915000000	2691500000	848
173	31.498	292	0	16.792	332640000	20790000	18
174	35.559	317	0	100.22	1501900000	88345000	104
175	37.935	331	0	8.9271	316140000	19759000	37
176	23.539	210	0	32.911	2160300000	180020000	96
177	47.34	427	0	323.31	5.05E+11	25274000000	7889
178	88.54	788	0	85.761	2213200000	55329000	144
179	23.513	204	0	118.02	3315000000	331500000	113
180	11.584	100	0	323.31	11360000000	2840100000	303
181	20.309	182	0	19.153	133890000	22315000	24
182	87.58	778	0	323.31	7597000000	223440000	368
183	46.745	440	0	65.129	240300000	21845000	22
184	47.711	424	0	323.31	29860000000	1357300000	1024
185	50.273	454	0	323.31	16993000000	809210000	599
186	627.22	5901	0	323.31	4.49E+12	16583000000	25236
187	187.71	1780	0	323.31	21230000000	299010000	461
188	28.707	261	0	61.346	1909300000	159110000	121
189	16.735	144	0	234.28	2067600000	295370000	116
190	34.931	324	0	320.8	3106100000	345120000	153
191	8.268	74	0	226.12	1.95E+11	97701000000	353
192	34.163	324	0	323.31	16734000000	1115600000	541
193	33.106	306	0	323.31	10017000000	667830000	434
194	25.798	243	0	323.31	17784000000	1482000000	532
195	43.931	411	0	323.31	87181000000	5128300000	2170
196	17.033	161	0	315.52	13089000000	1869800000	454
197	49.58	455	0	216.68	6215000000	282500000	281
198	31.82	288	0	16.458	907880000	64849000	74
199	28.233	255	0	33.933	1634700000	116770000	95
200	15.934	140	0	89.41	2530600000	316320000	97
201	14.133	129	0	178.68	15412000000	1926400000	345
202	20.6	186	0	323.31	1.95E+11	24354000000	2227
203	37.486	347	0	168.64	3040800000	217200000	158
204	53.69	480	0	323.31	19464000000	778580000	610
205	52.094	488	0	323.31	28527000000	1056600000	845
206	11.044	100	0	323.31	31630000000	6326000000	719
207	58.471	514	0	72.032	2056000000	73430000	154
208	6.8841	62	0	30.969	19206000000	4801500000	330
209	12.869	121	0	323.31	18705000000	6235100000	638
210	60.129	555	0	323.31	32268000000	1152400000	1021
211	61.691	566	0	323.31	16873000000	803480000	620
212	17.694	158	0	11.908	136140000	19449000	10
213	37.378	340	0	323.31	1.31E+11	7258400000	2141
214	45.476	416	0	323.31	24250000000	1054300000	831
215	8.1933	71	0	265.8	46133000000	15378000000	861
216	27.902	246	0	213.43	3921900000	301680000	189

	BK	BL	BM	BN	BO	BP	BQ
217	57.3	521	0	323.31	4877900000	162600000	350
218	70.9	629	0	133.59	755360000	26047000	74
219	87.769	774	0	323.31	97047000000	2256900000	2489
220	49.759	449	0.00094518	3.3399	5817700000	252940000	6
221	62.926	560	0	28.085	515240000	16101000	17
222	19.583	167	0	29.562	1416000000	141600000	89
223	22.125	195	0	314.11	35751000000	7150200000	835
224	59.316	535	0	323.31	8478700000	353280000	397
225	41.938	398	0	323.31	4.53E+11	18103000000	6540
226	13.862	118	0	44.614	2099800000	262470000	135
227	50.324	448	0	323.31	14490000000	689980000	488
228	59.459	515	0.0018657	2.665	2011900000	83829000	40
229	56.45	487	0	260.74	3163400000	105450000	232
230	89.211	777	0	129.79	2209900000	49110000	172
231	39.262	344	0	174.73	1080000000	49091000	93
232	19.969	174	0	323.31	1.13E+11	14068000000	862
233	64.859	607	0	323.31	4.29E+11	14306000000	5613
234	40.502	378	0	323.31	15307000000	850420000	624
235	10.947	95	0	302.45	4015800000	803150000	193
236	15.056	136	0	190.71	50528000000	6316000000	668
237	27.025	243	0	323.31	1298400000	144260000	99
238	51.9	446	0	3.9473	33160000	1950600	9
239	31.104	264	0	63.304	1840200000	102230000	114
240	24.378	211	0	30.974	27736000	2311400	13
241	17.763	159	0	148.54	15542000000	1726900000	462
242	42.321	374	0	323.31	24351000000	1217500000	1012
243	11.24	97	0	54.163	3398100000	849520000	110
244	102.92	930	0	323.31	1.36E+11	3171900000	3086
245	13.31	116	0	323.31	34702000000	5783700000	554
246	8.3968	77	0	17.59	2651300000	530260000	108
247	51.315	444	0	33.949	298730000	17572000	42
248	10.114	84	0	188.87	6423000000	1284600000	306
249	14.294	131	0	323.31	2353200000	392200000	143
250	100.83	883	0	323.31	28726000000	638350000	977
251	43.059	365	0	257.06	7963600000	468440000	339
252	29.137	247	0	7.0449	217850000	13615000	33
253	30.95	267	0	124.86	3559400000	254240000	243
254	65.825	612	0.0027907	2.5996	1631500000	95968000	14
255	54.35	471	0	50.566	959230000	43602000	27
256	39.101	348	0	126.2	5435700000	286090000	286
257	87.005	801	0	323.31	16719000000	464430000	855
258	80.999	737	0	323.31	25586000000	731020000	1153
259	33.76	294	0	15.371	210870000	13179000	7
260	50.955	447	0	125.61	1926600000	74101000	167
261	37.352	324	0	323.31	2220700000	148040000	152
262	263.74	2439	0	323.31	1.24E+11	987430000	3346
263	46.553	425	0	163.91	1335400000	66771000	144
264	23.866	215	0	163.3	4638100000	421640000	218
265	49.994	459	0	323.31	8177700000	355550000	406
266	50.485	442	0.0045788	2.1792	53057000	1965100	3
267	31.401	293	0	323.31	2.75E+11	24989000000	3184
268	45.661	396	0	8.7355	346440000	17322000	22
269	82.613	740	0.00093985	2.9835	947660000	26324000	50
270	61.165	553	0	323.31	14445000000	555580000	663

	BK	BL	BM	BN	BO	BP	BQ
271	29.812	259	0	156.89	5177900000	470720000	278
272	47.469	406	0	125.83	1092100000	54604000	109
273	47.326	410	0	170.08	2216300000	130370000	178
274	31.615	277	0	32.469	1240800000	77548000	99
275	22.67	201	0	323.31	10121000000	1445900000	317
276	50.825	466	0	323.31	1.58E+11	7163100000	2640
277	38.122	345	0	118.64	6859400000	326640000	335
278	26.444	238	0	75.591	3168600000	226330000	159
279	14.814	141	0	284.96	1.67E+11	16696000000	1326
280	24.507	229	0	323.31	58575000000	5325000000	860
281	52.928	471	0	55.507	1231800000	45622000	51
282	27.708	248	0	323.31	20019000000	1668200000	724
283	32.586	281	0	25.347	495330000	27519000	24
284	11.777	99	0	57.761	1661800000	184640000	103
285	34.658	297	0	7.1485	549290000	28910000	62
286	801.13	7559	0	323.31	38991000000	92837000	1757
287	18.172	158	0	19.688	532370000	59152000	67
288	21.795	187	0	9.3611	406570000	40657000	25
289	66.963	572	0	19.27	717830000	21113000	31
290	7.6426	65	0	229.49	3684100000	1228000000	195
291	5.1269	46	0	60.289	763360000	381680000	73
292	62.827	538	0	10.366	168420000	5807600	5
293	55.931	479	0	231.78	1046800000	36095000	78
294	10.861	95	0	323.31	4200100000	840020000	100
295	20.757	185	0	31.975	501110000	50111000	29
296	10.18	84	0	4.7524	615620000	153910000	29
297	20.785	188	0	4.2084	604010000	50334000	39
298	41.96	370	0	233.98	1458400000	69450000	108
299	63.837	563	0	9.556	313090000	10436000	14
300	20.212	187	0	203.13	1680900000	152810000	100
301	223.26	1978	0	323.31	84986000000	745490000	3164
302	65.571	573	0	306.81	3075800000	113920000	174
303	22.924	205	0	26.262	434070000	48230000	36
304	37.67	332	0	323.31	8148500000	509280000	448
305	33.421	319	0	208.25	18199000000	1213300000	528
306	32.566	279	0	34.271	1509100000	107790000	83
307	6.006	56	0	323.31	17933000000	5977600000	389
308	33.318	294	0	7.6252	87603000	5840200	8
309	23.632	207	0	222.36	2950400000	295040000	243
310	34.17	309	0	99.838	793100000	41742000	77
311	37.377	322	0.004583	2.2063	491350000	37796000	42
312	14.576	126	0	61.263	5655600000	628400000	187
313	68.183	613	0	323.31	34828000000	1339500000	1166
314	48.518	450	0	323.31	8846900000	402130000	420
315	39.434	352	0	110.98	926910000	42132000	103
316	45.745	399	0	22.367	2570200000	142790000	32
317	30.619	277	0	64.843	2313700000	177980000	169
318	25.409	233	0	43.104	1103800000	84911000	79
319	22.841	210	0	74.581	3616100000	258290000	229
320	56.681	496	0	323.31	26983000000	1173200000	889
321	41.488	378	0	323.31	78630000000	4625300000	1168
322	25.615	222	0	244.15	4539900000	453990000	220
323	27.607	260	0	323.31	11668000000	972330000	608
324	28.323	263	0	209.67	4850300000	440940000	230

	BK	BL	BM	BN	BO	BP	BQ
325	15.148	131	0	150.14	11261000000	1608700000	295
326	13.826	123	0	5.3744	1652600000	550870000	50
327	80.441	747	0	323.31	11661000000	388700000	540
328	65.265	591	0	323.31	4.31E+11	14858000000	4465
329	12.89	115	0	305.99	66985000000	16746000000	605
330	35.389	314	0	323.31	17950000000	1495800000	575
331	28.53	249	0	58.096	1826600000	121770000	104
332	42.622	391	0	14.374	234870000	13049000	26
333	30.665	281	0	240.65	5684100000	406000000	215
334	21.727	185	0	8.3576	171710000	17171000	13
335	17.395	155	0	99.072	4613300000	512590000	271
336	22.871	209	0	323.31	20750000000	1729200000	521
337	21.358	196	0	323.31	32568000000	4071100000	641
338	9.9404	82	0	8.4651	278940000	69734000	15
339	40.429	386	0	323.31	63813000000	3190700000	1600
340	28.279	254	0	11.633	350250000	26942000	19
341	25.717	236	0	266.84	5341700000	356120000	190
342	24.117	218	0	133.07	8378100000	837810000	243
343	36.946	325	0	323.31	38858000000	2428600000	553
344	25.796	230	0	103.66	3852100000	296310000	267
345	34.314	308	0	323.31	7758000000	775800000	280
346	78.078	726	0	323.31	13503000000	519360000	510
347	31.139	271	0	13.01	99417000	7647400	6
348	50.747	447	0	323.31	6162000000	256750000	339
349	43.115	396	0	200.58	9867200000	580420000	439
350	34.527	311	0	323.31	1.75E+11	10301000000	2382
351	22.397	201	0	323.31	1.56E+11	17318000000	2631
352	21.055	176	0	7.2914	575610000	71951000	22
353	58.353	513	0	210.38	3291100000	126580000	263
354	29.052	267	0	323.31	17914000000	1279600000	581
355	10.222	90	0	294.36	69037000000	23012000000	371
356	8.9944	79	0	49.098	4059900000	1015000000	72
357	19.816	172	0	139.02	9694700000	1077200000	328
358	27.633	239	0	149.58	1917700000	174330000	180
359	12.884	112	0	66.77	2204200000	275520000	155
360	12.348	106	0	36.793	579400000	115880000	54
361	8.122	71	0	207.07	15547000000	3886700000	306
362	48.826	448	0	323.31	67529000000	3376500000	1317
363	41.806	399	0	323.31	11828000000	563230000	488
364	25.742	233	0	31.397	505900000	50590000	74
365	45.29	419	0	110.21	1513900000	89054000	127
366	75.692	665	0	323.31	17379000000	511140000	776
367	12.238	106	0	22.99	1307000000	261400000	93
368	35.135	308	0	100.93	1149000000	71810000	53
369	8.8029	78	0	36.8	4279100000	4279100000	100
370	25.249	232	0	90.639	2501400000	208450000	177
371	19.055	166	0	10.258	368430000	61405000	53
372	33.403	289	0	31.56	177030000	10414000	18
373	16.649	146	0	3.6758	17077000	2846100	10
374	95.379	848	0	323.31	1.04E+11	2318300000	2497
375	25.466	224	0	87.424	3798900000	379890000	136
376	50.673	444	0	20.759	404310000	18378000	21
377	35.532	309	0	123.3	1257400000	78590000	108
378	93.507	816	0	5.9907	699730000	14888000	16

	BK	BL	BM	BN	BO	BP	BQ
379	20.232	184	0	323.31	9902200000	1237800000	287
380	21.431	190	0	69.901	2180500000	272560000	122
381	72.268	648	0	323.31	6838200000	201120000	411
382	66.578	575	0	323.31	25428000000	1017100000	742
383	41.316	360	0	44.563	1530400000	90025000	98
384	36.442	325	0	143.25	4791300000	435570000	220
385	54.576	480	0	323.31	6081500000	276430000	265
386	31.405	280	0	323.31	22027000000	1468400000	805
387	34.98	305	0	255.58	7482400000	498830000	276
388	75.395	678	0	323.31	30024000000	750590000	1092
389	9.9011	85	0	231.33	12692000000	2538500000	249
390	45.807	414	0	269.91	1274000000	70780000	174
391	50.241	459	0	323.31	1.23E+11	6152900000	2345
392	18.296	164	0	323.31	12585000000	1797900000	451
393	13.415	118	0	323.31	24109000000	3013700000	683
394	28.975	266	0	30.344	2029900000	202990000	116
395	27.367	247	0	136.95	3440900000	245780000	175
396	33.147	303	0	323.31	1.4E+11	9994100000	2007
397	26.5	242	0	298.58	4112600000	316350000	263
398	22.676	203	0	323.31	83613000000	7601200000	1245
399	30.728	275	0	11.796	205180000	12824000	15
400	27.565	241	0	72.508	1641700000	149240000	147
401	35.89	311	0	18.929	201480000	11193000	30
402	26.829	232	0.0018692	2.7109	103260000	6883800	5
403	57.53	520	0	323.31	60918000000	2030600000	1493
404	54.819	454	0	71.291	1721400000	95635000	104
405	45.747	396	0	17.175	193900000	9694900	13
406	47.899	428	0	83.705	241540000	12077000	43
407	7.7519	69	0	46.57	632270000	158070000	75
408	55.127	486	0	169.9	5684600000	218640000	357
409	30.135	274	0	323.31	8540600000	610040000	499
410	25.73	235	0	323.31	21775000000	2721800000	559
411	6.9981	58	0	134.29	11408000000	5703900000	219
412	34.755	311	0	297.26	6175200000	343060000	217
413	13.707	127	0	227.93	12329000000	1761300000	215
414	14.291	129	0	323.31	21221000000	2652600000	485
415	42.748	376	0	38.189	300120000	17654000	30
416	28.377	245	0	4.0326	182730000	15228000	8
417	28.231	257	0	168.89	12773000000	912340000	413
418	10.38	92	0	40.982	2402700000	2402700000	95
419	48.049	434	0	51.121	610590000	26547000	61
420	31.233	292	0	323.31	7000500000	636410000	310
421	32.695	305	0	4.0326	1249700000	208290000	52
422	30.362	267	0	323.31	9373600000	520750000	458
423	27.954	252	0	323.31	18990000000	1117000000	649
424	25.028	217	0	323.31	18396000000	1226400000	564
425	29.281	271	0	323.31	32644000000	2511100000	870
426	49.282	423	0	13.871	385820000	15433000	42
427	25.04	223	0	13.955	525500000	105100000	39
428	32.985	301	0	9.1863	741190000	46324000	57
429	44.129	385	0	4.4224	450980000	25054000	19
430	18.801	161	0	12.489	440410000	62915000	24
431	96.437	872	0	323.31	24438000000	488770000	1132
432	27.682	249	0	10.519	662010000	50924000	53

	BK	BL	BM	BN	BO	BP	BQ
433	43.85	387	0.0099638	1.7793	1017500000	59850000	13
434	25.651	225	0	185.93	11407000000	1140700000	378
435	31.272	284	0	23.419	264440000	20342000	5
436	39.016	355	0	323.31	14086000000	741380000	500
437	42.772	388	0	323.31	18852000000	992190000	526
438	41.023	367	0	138.88	3234000000	161700000	180
439	12.573	112	0	323.31	2.34E+11	33387000000	1424
440	45.646	427	0	256.41	7141200000	297550000	311
441	31.623	282	0	23.275	1014800000	72488000	53
442	47.656	424	0	218.77	5803900000	252340000	292
443	32.256	278	0	53.518	517030000	43085000	48
444	38.676	344	0	323.31	15566000000	707530000	576
445	28.381	245	0	307.07	14937000000	1067000000	551
446	46.137	428	0	323.31	34493000000	1499700000	808
447	31.548	289	0	212.18	8082200000	898030000	247
448	35.702	312	0	323.31	1.56E+11	8646500000	2389
449	64.245	574	0	295.2	4320600000	148980000	239
450	64.789	582	0	142.73	2272800000	81170000	130
451	54.439	488	0	323.31	71298000000	2640700000	1596
452	17.479	166	0	323.31	96598000000	8049900000	1645
453	12.472	122	0	323.31	8.52E+11	1.07E+11	7412
454	30.974	264	0	323.31	18196000000	1299700000	478
455	32.462	278	0.0037175	2.5246	160990000	8473200	21
456	27.747	231	0	31.614	281470000	23456000	23
457	48.825	448	0	323.31	2.29E+11	9533200000	2719
458	44.918	403	0	323.31	24281000000	1517500000	694
459	9.3584	80	0	323.31	3.28E+11	65503000000	2053
460	34.91	311	0	155.13	4099100000	256190000	209
461	16.001	147	0	323.31	39617000000	7923400000	632
462	10.48	88	0	318.62	26654000000	4442300000	795
463	13.136	115	0	239.44	35429000000	5904800000	537
464	45.466	406	0	323.31	8674500000	510260000	332
465	42.009	372	0	323.31	24231000000	1101400000	627
466	29.754	268	0	323.31	15983000000	998930000	746
467	50.834	434	0	72.151	1135700000	51621000	109
468	15.033	125	0	134.38	439560000	87912000	43
469	13.347	110	0	28.053	1042800000	148980000	57
470	57.752	523	0	323.31	21696000000	803560000	779
471	44.803	427	0	44.35	227420000	22742000	13
472	27.145	237	0.0037106	2.5225	309740000	22124000	9
473	20.66	186	0	323.31	12598000000	969050000	541
474	32.796	299	0	323.31	1719600000	171960000	117
475	20.268	180	0	323.31	6258600000	1043100000	212
476	23.277	209	0	6.0615	118150000	13127000	5
477	19.578	173	0	19.028	1313700000	119430000	60
478	34.616	307	0	298.85	15448000000	1029800000	513
479	39.863	359	0	109.7	1771600000	161050000	111
480	116.03	1058	0	323.31	8069000000	144090000	403
481	32.1	269	0	185.6	2684400000	191740000	184
482	31.887	267	0	3.4195	88450000	5528100	26
483	56.53	495	0.0009434	3.2788	71024000	4439000	9
484	26.256	235	0	63.788	7191800000	553220000	216
485	38.786	340	0	138.19	1638500000	126030000	136
486	33.399	289	0	309.54	15886000000	1059100000	450

	BK	BL	BM	BN	BO	BP	BQ
487	26.865	229	0	165.2	4965000000	331000000	251
488	38.824	347	0	259.57	874060000	79460000	105
489	78.74	695	0	290.72	4931500000	120280000	360
490	23.04	210	0	38.895	692210000	76912000	59
491	74.655	643	0	323.31	2800300000	70006000	235
492	35.971	328	0	323.31	8939200000	470480000	413
493	26.154	232	0	18.481	520860000	34724000	24
494	134.21	1179	0	205.79	1624700000	23210000	107
495	29.433	264	0	14.876	694750000	43422000	62
496	30.323	272	0	323.31	8428000000	702330000	426
497	47.876	429	0	86.327	4338300000	255190000	262
498	56.811	495	0	323.31	13008000000	520340000	509
499	27.351	246	0	323.31	44866000000	4985100000	805
500	78.371	721	0	323.31	55309000000	1907200000	1371
501	75.733	662	0	79.933	1918700000	53298000	86
502	18.055	155	0	137.44	2440500000	271170000	183
503	19.638	171	0	4.0632	356190000	59365000	31
504	19.866	183	0	44.168	682730000	68273000	54
505	25.746	229	0	18.621	525190000	47745000	57
506	59.615	556	0	323.31	16095000000	643800000	555
507	45.295	391	0	19.562	51604000	2866900	10
508	26.815	234	0	296.04	14133000000	942220000	407
509	51.712	449	0	5.3977	425290000	18491000	17
510	29.917	269	0	32.306	1733200000	108320000	82
511	9.2054	80	0	65.019	1550700000	310150000	114
512	35.355	328	0	323.31	2.12E+11	19317000000	1606
513	92.08	822	0	323.31	10235000000	255880000	474
514	28.133	247	0	323.31	8357800000	642910000	368
515	49.163	424	0	165.26	1429800000	59573000	125
516	32.263	292	0	51.577	1244200000	77761000	82
517	24.466	212	0	14.367	411670000	37425000	46
518	50.51	446	0.0099458	1.7355	558000000	20667000	12
519	7.8498	70	0	137.98	4104600000	2052300000	100
520	31.878	291	0	43.873	724410000	90552000	79
521	16.197	143	0	323.31	4373000000	546620000	245
522	62.826	555	0	43.943	370190000	11568000	44
523	27.343	242	0	110.88	5655200000	514110000	219
524	67.757	607	0	323.31	17428000000	544620000	720
525	10.767	101	0	72.674	1264600000	210770000	102
526	15.272	141	0	41.26	5456600000	909430000	138
527	18.957	171	0	190.7	11616000000	1290700000	376
528	33.414	309	0	54.339	1636900000	116920000	57
529	36.422	326	0	323.31	27530000000	1311000000	949
530	21.927	205	0	9.9824	305160000	30516000	26
531	28.389	253	0	156.97	4387800000	258110000	262
532	36.963	320	0	323.31	29476000000	2456300000	943
533	81.554	719	0	323.31	25157000000	698790000	937
534	8.2163	72	0	323.31	8403100000	1680600000	294
535	8.9391	87	0	323.31	3.73E+11	1.24E+11	9887
536	62.889	574	0	323.31	55655000000	1795300000	1468
537	35.228	329	0	189.31	7765100000	647090000	265
538	20.494	191	0	93.162	3198400000	319840000	219
539	13.006	124	0	42.906	646800000	129360000	64
540	37.435	333	0	209.67	3104700000	147850000	193

	BK	BL	BM	BN	BO	BP	BQ
541	7.3509	65	0	3.8653	223740000	111870000	14
542	27.009	237	0	46.18	2664500000	242230000	118
543	17.812	154	0	38.364	3204400000	291310000	161
544	45.332	422	0	323.31	25808000000	1290400000	722
545	9.9882	86	0	6.9718	266240000	88748000	32
546	35.055	322	0	115.61	1556500000	129710000	129
547	41.17	356	0	46.255	239380000	12599000	12
548	28.486	259	0.0045914	2.2702	105960000	8150800	10
549	32.106	283	0	20.616	1149500000	67615000	48
550	194.55	1738	0	323.31	74881000000	832010000	3364
551	139.78	1216	0	55.242	680530000	9451800	78
552	124.86	1091	0	8.3972	666510000	10926000	15
553	47.102	434	0	323.31	7.07E+11	32134000000	6011
554	17.093	148	0	105	1305400000	217560000	60
555	50.222	462	0	34.942	939210000	34785000	42
556	86.579	759	0	323.31	11799000000	368720000	642
557	72.241	652	0	177.88	2882100000	68621000	205
558	34.161	287	0	16.895	56522000	4347900	12
559	72.042	633	0	248.7	2050600000	66147000	194
560	9.65	91	0	323.31	1.55E+12	3.87E+11	4101
561	30.637	279	0	191.85	2492700000	166180000	155
562	31.281	282	0	12.925	763010000	76301000	58
563	34.522	305	0	29.548	2057200000	114290000	113
564	10.462	97	0	102.74	86383000000	14397000000	466
565	12.744	114	0	165.29	6402800000	1280600000	127
566	11.197	104	0	89.699	40772000000	13591000000	894
567	10.935	99	0	323.31	40390000000	10097000000	576
568	35.123	324	0	282.28	5103700000	318980000	241
569	26.211	223	0	14.986	106380000	8865200	22
570	22.365	189	0	42.385	607460000	60746000	24
571	35.125	319	0	323.31	20760000000	1297500000	675
572	40.762	371	0	70.913	3488700000	268360000	83
573	13.526	115	0	222.46	10315000000	1719100000	414
574	24.165	213	0	27.85	1052800000	131600000	59
575	25.723	229	0	102	5347900000	411370000	320
576	86.01	763	0	323.31	3536200000	78583000	344
577	31.664	286	0	323.31	57271000000	4090800000	1142
578	47.649	425	0	117.3	1375700000	68783000	75
579	45.024	419	0	77.269	5382800000	224280000	305
580	5.9887	52	0	6.4624	866360000	433180000	49
581	48.306	436	0	147.94	3974900000	172820000	235
582	50.307	441	0	8.8242	1183800000	62305000	31
583	39.943	347	0.0099819	1.8379	235500000	15700000	5
584	12.386	109	0	144.97	3312000000	1104000000	84
585	42.016	369	0	323.31	15738000000	925760000	422
586	67.278	586	0	25.188	841580000	23377000	82
587	28.353	252	0	65.073	491740000	30734000	30
588	35.157	334	0	269.81	4698600000	276390000	287
589	103.28	898	0	323.31	43790000000	826220000	1531
590	21.946	188	0	15.775	1404800000	117070000	28
591	51.22	452	0	143.04	2796200000	127100000	187
592	35.402	324	0	323.31	43716000000	2185800000	1040
593	22.927	204	0	65.247	575070000	52279000	92
594	37.221	324	0	323.31	10274000000	642140000	466

	BK	BL	BM	BN	BO	BP	BQ
595	45.154	418	0	323.31	1.86E+11	9296200000	2905
596	15.958	143	0	112.52	1662100000	277020000	101
597	22.281	200	0	25.943	1143900000	114390000	102
598	31.708	279	0	17.713	226380000	16170000	24
599	40.629	359	0	323.31	25706000000	1168500000	714
600	6.7467	60	0	90.438	32880000000	10960000000	363
601	50.489	457	0	323.31	8572700000	357200000	458
602	33.883	311	0	119.65	10293000000	735190000	414
603	38.947	358	0	323.31	46081000000	3291500000	1368
604	13.617	125	0	78.818	2189100000	2189100000	118
605	44.803	407	0	323.31	56051000000	2950000000	1695
606	49.853	463	0	13.727	205420000	51356000	17
607	21.138	188	0	93.697	6997900000	583160000	207
608	68.853	587	0	15.509	502640000	16755000	25
609	22.161	196	0	323.31	7689800000	699070000	321
610	18.97	163	0	323.31	13792000000	1724000000	470
611	24.739	230	0	74.533	2879000000	959680000	124
612	11.809	104	0	6.7669	1013100000	144730000	22
613	20.514	181	0	124.06	2699400000	245400000	123
614	49.373	459	0	323.31	4895200000	287950000	297
615	30.08	266	0	80.102	1732900000	101930000	100
616	17.621	160	0	8.2442	100880000	10088000	13
617	34.469	313	0	323.31	74154000000	3707700000	1419
618	26.868	237	0.00093721	2.8617	512030000	34136000	10
619	69.891	600	0	323.31	11392000000	406840000	618
620	32.685	286	0	214.15	4692000000	469200000	160
621	17.747	155	0	140.96	1418000000	283600000	98
622	89.163	784	0	231.44	8394500000	254380000	525
623	22.99	201	0	34.454	866000000	96222000	41
624	31.141	274	0	57.287	730430000	42966000	35
625	34.042	299	0	47.847	2346100000	130340000	141
626	19.305	165	0	213.35	6777600000	1129600000	245
627	63.432	551	0	4.2609	330370000	10324000	20
628	76.359	658	0	323.31	38574000000	918430000	1102
629	33.142	312	0.00093545	2.7788	153300000	11792000	10
630	14.408	126	0	89.089	28820000000	9606600000	514
631	13.665	119	0	270.1	51076000000	12769000000	642
632	7.8362	66	0	132.94	7338700000	3669300000	160
633	22.267	195	0	107.64	5849300000	731160000	154
634	29.778	258	0	39.288	769400000	51294000	62
635	8.3825	73	0	130.46	4120100000	1373400000	167
636	32.365	282	0	56.203	1394100000	82004000	63
637	16.106	135	0.0082042	2.0088	250730000	41789000	67
638	73.354	644	0	323.31	54662000000	1561800000	1471
639	36.255	322	0	104.55	1077000000	59833000	117
640	8.4352	71	0	12.524	1285100000	321270000	44
641	32.477	284	0	164.53	2605900000	144770000	153
642	20.628	185	0	323.31	1.31E+11	11946000000	1550
643	26.471	245	0	323.31	12342000000	1028500000	399
644	49.171	444	0	323.31	8735100000	415960000	364
645	51.399	440	0	28.172	1580100000	63203000	55
646	21.612	187	0	9.4064	287870000	28787000	6
647	39.906	351	0	9.348	219200000	14613000	16
648	32.538	289	0	67.697	5596900000	373120000	39

	BK	BL	BM	BN	BO	BP	BQ
649	12.411	105	0	323.31	24283000000	4856700000	360
650	23.355	212	0	18.121	848520000	56568000	31
651	45.358	420	0	323.31	5379100000	256150000	261
652	71.866	626	0	167.99	1378600000	44471000	120
653	32.882	295	0	4.5292	474670000	31645000	17
654	34.96	302	0	57.922	886140000	59076000	65
655	29.908	269	0	295.96	2718100000	226510000	124
656	33.154	291	0	80.781	790150000	65846000	81
657	41.989	375	0	12.026	318000000	18706000	22
658	46.933	419	0	113.74	3283900000	218920000	231
659	32.944	286	0	9.7082	167810000	15256000	7
660	53.371	486	0	12.716	4921700000	234370000	24
661	28.159	252	0	8.316	631640000	37155000	16
662	78.647	709	0	323.31	5087700000	127190000	357
663	5.1029	43	0	182.8	8135600000	4067800000	343
664	31.148	271	0	30.23	1294500000	117680000	53
665	32.715	290	0	323.31	7680500000	548610000	312
666	54.781	501	0	323.31	2.34E+11	8669400000	3751
667	35.172	335	0	323.31	73151000000	4064000000	1409
668	86.853	757	0	323.31	21248000000	559170000	855
669	17.828	156	0	107.31	2996000000	599190000	184
670	8.7858	78	0.00093809	2.9249	175170000	87583000	15
671	31.579	274	0	19.049	521690000	43474000	39
672	45.185	404	0	62.627	2619400000	119060000	100
673	43.909	400	0	57.878	909280000	45464000	47
674	84.983	767	0	93.804	1357700000	36695000	106
675	66.968	650	0	287.26	3317900000	184330000	159
676	32.651	303	0	226.49	3345300000	223020000	251
677	27.235	246	0	33.626	908030000	69848000	26
678	45.337	413	0	323.31	2811800000	127810000	317
679	52.765	470	0	323.31	48377000000	1860600000	1432
680	15.973	146	0	83.28	513780000	64223000	59
681	45.222	408	0	315.68	11343000000	493180000	611
682	46.223	420	0	323.31	28431000000	1776900000	622
683	28.437	256	0	323.31	62514000000	4167600000	1073
684	60.854	551	0	25.446	962870000	41864000	55
685	18.391	165	0	107.94	4958900000	708420000	185
686	43.895	400	0	323.31	2.71E+11	14254000000	3702
687	8.4637	76	0	323.31	27181000000	5436200000	643
688	23.426	214	0	192.55	18969000000	1185500000	582
689	32.775	307	0.0090827	1.8763	363370000	45422000	15
690	24.973	229	0	139.47	1802600000	257510000	94
691	37.425	340	0	323.31	81590000000	6276100000	1329
692	91.784	811	0	72.851	1587900000	48118000	110
693	71.663	647	0	169.86	1242700000	41422000	81
694	21.445	178	0	5.7488	1683800000	168380000	89
695	55.205	511	0	230.21	3353800000	128990000	324
696	36.783	350	0	323.31	55594000000	3270300000	1519
697	14.189	129	0	244.25	3604200000	450530000	177
698	23.004	220	0	262.05	14117000000	882290000	357
699	46.155	425	0	53.771	1895900000	94793000	81
700	21.752	196	0	54.751	2216800000	201520000	85
701	8.5257	78	0	323.31	1.12E+11	56059000000	421
702	26.125	236	0	323.31	7128000000	594000000	321

	BK	BL	BM	BN	BO	BP	BQ
703	28.888	269	0	269.06	26039000000	2603900000	739
704	44.076	403	0	323.31	40857000000	2403300000	918
705	24.792	227	0	158.02	6183300000	687030000	378
706	33.303	306	0	29.492	485160000	30323000	54
707	26.962	244	0	47.089	2316700000	210610000	101
708	8.8538	76	0	272.78	44125000000	11031000000	870
709	84.013	752	0	323.31	26212000000	624100000	1089
710	11.229	99	0	323.31	46465000000	11616000000	1092
711	43.97	398	0	323.31	1.99E+12	90378000000	15663
712	30.699	289	0	323.31	14746000000	1638500000	183
713	62.73	572	0	323.31	58060000000	1612800000	1865
714	12.895	115	0	323.31	5.12E+11	63941000000	2986
715	31.048	278	0	323.31	43791000000	2736900000	1690
716	23.144	209	0	323.31	15965000000	2280700000	652
717	23.966	213	0	52.927	2826100000	314010000	110
718	47.525	409	0	133.39	2121300000	111640000	160
719	37.723	337	0	317.19	8451600000	650130000	261
720	15.639	139	0	237.99	15705000000	3140900000	424
721	50.934	468	0	323.31	78230000000	3259600000	1773
722	32.324	292	0	323.31	9749800000	609360000	404
723	54.617	501	0	323.31	65267000000	2719500000	1194
724	20.567	178	0	26.349	2043800000	204380000	190
725	17.972	164	0	323.31	15008000000	1072000000	408
726	27.208	238	0	24.761	51292000	17097000	3
727	17.614	160	0	323.31	1.2E+11	17176000000	1032
728	60.823	551	0	323.31	78113000000	2789800000	1912
729	17.499	154	0	70.784	396010000	56573000	61
730	49.916	444	0	323.31	10168000000	442080000	433
731	23.844	205	0	138.58	3045700000	276880000	172
732	118.26	1032	0	102.71	1579700000	32910000	85
733	40.19	364	0.00094251	3.0637	173570000	9642700	3
734	72.546	652	0	323.31	4.19E+11	12685000000	4289
735	60.21	540	0.0036969	2.4525	50838000	3631300	16
736	53.805	481	0	113.08	4302900000	187080000	137
737	6.7675	65	0	323.31	1.05E+11	26160000000	1082
738	33.506	311	0	323.31	1.35E+11	9611800000	1738
739	13.852	119	0	9.6741	249020000	49803000	16
740	52.134	466	0	323.31	14046000000	585260000	535
741	9.2036	79	0	323.31	43656000000	8731100000	649
742	17.394	156	0	323.31	11739000000	1304300000	407
743	11.181	96	0	323.31	3.55E+11	71016000000	3655
744	51.066	447	0	323.31	23142000000	1006200000	729
745	43.013	395	0	323.31	21342000000	1185700000	628
746	16.048	143	0	55.306	291740000	48623000	40
747	19.794	182	0	323.31	1.53E+11	11763000000	1818
748	27.436	242	0	46.067	325360000	27114000	16
749	59.437	531	0	323.31	11149000000	384440000	478
750	97.15	898	0	323.31	11622000000	341830000	545
751	44.506	394	0	46.243	432400000	27025000	44
752	71.006	623	0	323.31	2745200000	88555000	270
753	45.261	394	0	32.584	475680000	19820000	59
754	27.826	255	0	320.44	5427600000	452300000	185
755	31.005	282	0	43.777	2515800000	228710000	128
756	14.413	124	0	260.18	19479000000	3246400000	312

	BK	BL	BM	BN	BO	BP	BQ
757	48.104	450	0	323.31	31091000000	1554500000	928
758	27.833	259	0	323.31	6584700000	658470000	329
759	31.883	285	0	113.71	2130400000	236710000	104
760	35.331	322	0	151.54	5055200000	315950000	260
761	119.2	1065	0.0099728	1.8217	808730000	13707000	27
762	32.988	296	0.0036799	2.3264	210670000	14044000	3
763	51.664	445	1	-2	609760000	27717000	11
764	18.716	168	0	6.7168	537870000	48898000	24
765	9.3004	79	0	7.3489	2410600000	1205300000	91
766	5.7415	55	0	123.43	9740700000	1948100000	246
767	13.685	122	0	323.31	12384000000	1769100000	433
768	9.1	79	0	164.09	1074100000	214820000	82
769	18.306	158	0	6.0142	152570000	21795000	16
770	6.7335	55	0	121.72	2998500000	999500000	117
771	21.288	198	0	132.73	21184000000	4236900000	286
772	44.052	375	0.0082192	2.0556	1255900000	54605000	21
773	36.071	325	0	35.062	917660000	65547000	43
774	33.833	296	0	323.31	8963700000	560230000	343
775	13.731	126	0	323.31	1.21E+11	24162000000	686
776	22.273	195	0	81.97	2610800000	237340000	119
777	45.798	410	0	323.31	9046100000	361840000	411
778	19.703	168	0	228.47	3884100000	388410000	319
779	19.258	172	0	323.31	89706000000	8970600000	1047
780	57.286	490	0	323.31	20322000000	846760000	894
781	26.552	252	0	323.31	4.59E+11	45877000000	3182
782	25.649	225	0	3.8809	2223300000	171020000	12
783	18.728	170	0	323.31	29094000000	2644900000	765
784	27.945	252	0	90.792	2634700000	188200000	119
785	41.821	376	0	323.31	17110000000	855500000	484
786	21.413	191	0	195.57	6338300000	792290000	257
787	29.739	262	0	323.31	6702200000	515560000	409
788	58.817	524	0	323.31	16406000000	781260000	600
789	47.143	438	0	323.31	2.53E+11	10552000000	3376
790	49.603	447	0	96.709	2379300000	95172000	124
791	29.205	260	0	198.51	5219900000	521990000	268
792	40.255	360	0	323.31	66811000000	3711700000	1041
793	27.753	257	0	47.341	3208800000	356530000	158
794	12.314	112	0	171.76	22497000000	5624200000	396
795	24.598	223	0	323.31	9716700000	883330000	335
796	16.807	158	0	92.958	3517000000	703410000	97
797	7.8929	70	0	42.014	432630000	86526000	18
798	37.492	339	0	323.31	79182000000	4948900000	1586
799	29.955	265	0	27.283	1444300000	120360000	77
800	24.935	225	0	19.665	140500000	11708000	9
801	85.522	778	0	323.31	3682400000	122750000	251
802	28.933	249	0	114.31	1695900000	99759000	127
803	10.535	89	0	144.79	11607000000	2901800000	322
804	74.682	647	0	323.31	17041000000	516400000	771
805	25.964	225	0	4.0118	360500000	32772000	31
806	15.824	145	0	42.679	768720000	96090000	51
807	32.365	280	0	64.547	1316600000	109720000	118
808	24.825	216	0	4.7783	299490000	29949000	33
809	16.214	147	0	6.6124	1425500000	237590000	5
810	84.613	740	0	323.31	3747500000	87151000	281

	BK	BL	BM	BN	BO	BP	BQ
811	23.646	209	0	131.18	3447200000	492460000	130
812	71.898	630	0	323.31	2.01E+11	5732100000	3310
813	34.594	309	0	95.901	918270000	70636000	88
814	18.019	163	0	323.31	2.47E+11	24724000000	2281
815	25.726	233	0	33.161	339070000	28255000	32
816	26.051	230	0	323.31	2.95E+11	21102000000	3466
817	213.23	1939	0	323.31	50257000000	534650000	2498
818	105.16	930	0	323.31	29177000000	788570000	926
819	33.374	294	0	323.31	76703000000	5900200000	1271
820	20.637	179	0	323.31	8333200000	1190500000	430
821	25.611	223	0	51.985	3457500000	246970000	160
822	44.443	419	0	323.31	1.76E+11	9278100000	2756
823	49.474	457	0	323.31	26733000000	1336700000	751
824	50.423	457	0	125.67	2752300000	125110000	177
825	38.663	347	0	323.31	8263400000	393490000	435
826	21.733	198	0	15.573	916360000	101820000	74
827	73.786	680	0	196.51	3596200000	112380000	285
828	32.671	283	0	234.58	3241600000	270130000	205
829	31.616	294	0	17.258	1582300000	105490000	22
830	33.425	305	0	272.8	7558500000	503900000	452
831	48.303	442	0	252.32	7339800000	349510000	444
832	25.458	232	0	71.493	2090900000	149350000	151
833	41.144	367	0.00094429	3.3355	49606000	3100300	4
834	49.638	445	0.0054895	2.1617	510890000	26889000	11
835	31.315	271	0.0036934	2.4119	85455000	6573500	1
836	129.61	1188	0	323.31	14840000000	231880000	935
837	75.264	671	0	67.686	1409800000	40281000	104
838	40	367	0	125.72	1497000000	93561000	112
839	13.675	122	0.0045872	2.2412	183550000	26221000	25
840	38.738	343	0	323.31	48949000000	3263300000	1084
841	38.822	343	0	323.31	19540000000	1028400000	579
842	95.013	837	0	323.31	60878000000	1268300000	1537
843	49.063	436	0	242.26	7117100000	323510000	392
844	26.544	237	0	80.83	2286300000	285780000	172
845	34.136	298	0	124.28	3718800000	195730000	225
846	44.562	389	0	5.8375	122280000	6793200	10
847	18.38	157	0	25.472	750360000	107190000	44
848	13.989	121	0	209.8	4055800000	450650000	164
849	25.58	231	0	225.82	13362000000	1113500000	387
850	43.859	399	0	185.52	2516200000	179730000	117
851	33.778	297	0	27.667	900430000	64317000	56
852	31.893	295	0	80.83	2136100000	125650000	184
853	67.75	645	0	4.1937	92237000	5764800	6
854	55.266	484	0	7.293	217260000	9052600	10
855	46.095	424	0	6.6398	226250000	12570000	22
856	43.618	390	0	323.31	3444000000	181260000	260
857	72.246	659	0	116.75	3730500000	109720000	227
858	27.103	246	0	323.31	44959000000	3746600000	932
859	49.009	437	0	159.77	1437200000	68440000	123
860	33.953	311	0	225.86	16399000000	819940000	650
861	90.231	798	0	91.069	990820000	22018000	105
862	11.927	104	0	323.31	37737000000	9434300000	1079
863	23.675	208	0	169.21	3092000000	257670000	254
864	60.23	537	0	323.31	15416000000	550570000	712

	BK	BL	BM	BN	BO	BP	BQ
865	113.98	995	0	99.778	838560000	17114000	88
866	38.561	333	0	99.607	2254700000	132630000	120
867	48.678	423	0.0099548	1.7556	17225000	906580	1
868	80.393	702	0	17.3	702660000	17567000	55
869	9.7339	84	0	11.598	359980000	71996000	43
870	29.773	258	0	156.04	5988200000	598820000	269
871	12.923	116	0	220.11	7842800000	1120400000	348
872	22.44	197	0	65.746	6501400000	500110000	210
873	24.223	209	0	102.82	2074000000	172830000	167
874	11.717	103	0	47.849	2105100000	526270000	100
875	41.131	368	0	29.982	263930000	13891000	25
876	20.706	186	0	77.62	161340000	17926000	31
877	14.612	126	0	5.7594	374010000	41557000	12
878	8.6655	73	0	165.31	13404000000	2680700000	331
879	11.421	104	0	323.31	3.7E+11	46274000000	3166
880	30.933	263	0	40.923	1775900000	118390000	112
881	68.576	598	0	323.31	10731000000	412750000	484
882	27.182	247	0	51.922	722380000	48159000	67
883	34.82	317	0	203.4	8157900000	627530000	305
884	16.082	142	0	7.6026	212370000	35396000	30
885	40.231	355	0	11.563	89983000	6921800	5
886	18.122	156	0	156.2	3172200000	634450000	115
887	46.969	423	0	38.283	335910000	13437000	28
888	21.832	188	0	91.702	20959000000	2095900000	340
889	21.802	202	0	323.31	2.17E+12	1.55E+11	16739
890	7.0479	67	0	323.31	6.15E+11	2.05E+11	2261
891	27.754	258	0	165.67	5142900000	467540000	183
892	44.216	407	0	195.44	7379900000	434110000	307
893	21.266	199	0	49.519	770940000	96367000	17
894	28.866	255	0	134.37	1788800000	149060000	82
895	35.904	334	0	59.877	1601900000	94227000	123
896	20.873	188	0	323.31	9989900000	1110000000	270
897	51.107	453	0	50.89	2139800000	82300000	129
898	37.659	333	0	8.9105	704430000	64039000	23
899	37.443	336	0	34.049	559770000	34986000	55
900	39.211	355	0	81.622	2072900000	94224000	140
901	26.086	219	0	77.704	1211500000	93193000	118
902	45.618	408	0	323.31	31493000000	1259700000	1065
903	65.519	583	0	266.73	4151200000	122090000	139
904	65.212	581	0	92.074	2811000000	133860000	135
905	31.063	275	0	323.31	23396000000	1376300000	685
906	33.585	288	0	15.072	566500000	47208000	35
907	32.953	284	0	35.536	166450000	11889000	6
908	55.556	506	0	323.31	18338000000	797290000	595
909	27.089	242	0	323.31	30617000000	2355200000	789
910	29.343	254	0	323.31	16102000000	2012800000	491
911	16.621	147	0	135.14	3067200000	511190000	127
912	11.2	98	0	5.0913	386560000	48320000	50
913	38.035	354	0	323.31	23727000000	1078500000	882
914	34.826	321	0	323.31	10577000000	587600000	425
915	27.127	240	0	208.82	4347500000	310540000	215
916	21.038	189	0	6.1493	169020000	16902000	10
917	17.299	153	0	183.58	3812300000	476530000	169
918	19.854	173	0	127.55	24317000000	2431700000	641

	BK	BL	BM	BN	BO	BP	BQ
919	36.341	323	0	208.6	5814600000	323030000	368
920	29.137	264	0	5.5847	323030000	20189000	30
921	9.0857	82	0	211.97	9971500000	1661900000	163
922	34.796	308	0	323.31	27611000000	1840700000	781
923	39.437	355	0	323.31	56650000000	2463100000	852
924	34.634	308	0	106.21	5341100000	593460000	255
925	55.339	495	0	323.31	36156000000	1643500000	962
926	72.49	659	0	323.31	6.5E+11	19702000000	5993
927	35.287	311	0	8.5768	2880500000	144020000	16
928	21.338	177	0	7.3978	394430000	32869000	38
929	57.111	540	0	323.31	2.27E+11	7561200000	2768
930	9.9122	94	0	323.31	87664000000	17533000000	1084
931	27.548	253	0	82.215	477690000	39808000	52
932	22.798	208	0	323.31	8348100000	758920000	232
933	12.114	105	0	22.525	1682900000	280480000	103
934	24.887	224	0	58.809	609520000	67725000	67
935	25.831	238	0	323.31	14207000000	1775900000	569
936	52.888	471	0	323.31	65754000000	2435300000	1577
937	15.82	136	0	230.42	7775700000	1110800000	236
938	23.295	202	0	144.46	2940700000	294070000	215
939	17.392	152	0	323.31	6256500000	625650000	239
940	36.602	318	0	323.31	29249000000	1720500000	914
941	41.937	388	0	323.31	25054000000	1473800000	828
942	45.091	418	0	257.8	2591600000	143980000	124
943	37.609	338	0	323.31	6372600000	398290000	391
944	19.339	172	0	14.644	497780000	41482000	26
945	16.432	147	0	140.17	3605700000	515100000	215
946	15.514	137	0	245.29	57733000000	8247600000	637
947	136.95	1225	0	323.31	1.23E+11	1597600000	3288
948	134.34	1203	0	323.31	1.15E+11	1739400000	2929
949	49.636	443	0	119.15	1023900000	102390000	98
950	29.956	274	0	168.87	1089200000	83786000	112
951	45.967	427	0	308.06	6279300000	232570000	359
952	37.493	345	0	90.87	3587200000	256230000	225
953	18.411	162	0.0036866	2.3591	47213000	5901600	6
954	19.844	179	0.0018622	2.6018	462880000	51431000	16
955	37.583	330	0	323.31	47064000000	2241100000	1140
956	24.83	227	0	16.39	171520000	28586000	16
957	27.013	246	0.0082117	2.017	374300000	34027000	6
958	34.169	308	0	67.109	1547300000	171920000	92
959	30.124	264	0.00093633	2.8244	127120000	8474900	7
960	47.131	420	0	323.31	11353000000	567670000	476
961	30.254	275	0	323.31	6010200000	462330000	313
962	35.814	313	0	116.17	3159900000	175550000	179
963	47.973	418	0	43.789	806920000	33622000	70
964	25.092	220	0	62.337	1407300000	108260000	73
965	23.905	218	0	85.88	3058000000	436850000	89
966	32.959	292	0	23.149	82908000	5181700	8
967	32.341	290	0	17.512	879290000	54955000	46
968	23.964	213	0.0037003	2.4757	745350000	62112000	17
969	75.464	676	0	5.1049	96783000	3337300	25
970	35.377	299	0	11.997	479690000	26649000	31
971	28.937	257	0	88.446	6336400000	452600000	288
972	45.741	404	0	323.31	20427000000	1134900000	821

	BK	BL	BM	BN	BO	BP	BQ
973	16.591	150	0	125.66	13453000000	1494800000	436
974	51.84	462	0	143.31	1573200000	68401000	94
975	37.101	336	0	323.31	27098000000	1594000000	708
976	22.203	199	0	42.573	444490000	34191000	49
977	20.347	178	0	323.31	29615000000	2961500000	490
978	7.1404	58	0.0045956	2.3186	581470000	193820000	17
979	80.823	731	0	323.31	3759700000	101610000	311
980	35.855	335	0	323.31	1.25E+12	78362000000	5471
981	13.489	116	0	98.219	1962200000	280310000	54
982	28.018	242	0	4.6374	242240000	22022000	11
983	11.303	102	0	32.78	4309000000	538620000	263
984	11.435	102	0	67.701	2748400000	458060000	100
985	97.239	883	0	323.31	18190000000	478690000	1065
986	15.161	136	0	192.86	1945200000	324200000	72
987	17.041	147	0	323.31	17738000000	1970900000	612
988	11.031	99	0	23.222	2098000000	524500000	16
989	71.14	658	0	323.31	94190000000	3038400000	2230
990	37.037	328	0	248.19	6181400000	363610000	304
991	31.243	276	0	20.357	69040000	11507000	17
992	43.384	396	0	323.31	27413000000	1370700000	722
993	35.676	317	0	28.778	775170000	86130000	84
994	15.117	121	0	4.3843	1311700000	262350000	19
995	41.669	383	0	323.31	15019000000	883450000	514
996	67.268	605	0	156.42	2430800000	135050000	93
997	43.121	380	0	48.26	1280100000	71117000	83
998	9.8112	83	0.0091075	1.9002	204610000	34101000	12
999	40.48	380	0	323.31	1.63E+11	10187000000	1912
1000	24.887	218	0	70.392	614630000	51219000	62
1001	53.743	494	0	323.31	39950000000	1536600000	1185
1002	49.037	425	0	30.377	1722800000	82040000	114
1003	18.83	158	0	24.875	353480000	44185000	7
1004	10.422	93	0	68.429	468040000	93608000	46
1005	51.353	454	0	56.462	294580000	12274000	35
1006	15.06	129	0	269.69	7111000000	1185200000	220
1007	29.399	255	0	3.9774	125320000	8354900	10
1008	16.559	144	0	3.76	243610000	40601000	14
1009	55.875	486	0	323.31	26931000000	928660000	978
1010	50.01	449	0	323.31	1.11E+11	5303800000	2950
1011	65.581	588	0	323.31	8190200000	315010000	337
1012	62.231	564	0	233.63	8399200000	335970000	522
1013	94.74	844	0	323.31	6870400000	180800000	541
1014	17.035	148	0	63.548	875900000	145980000	37
1015	63.445	563	0	323.31	27559000000	725240000	844
1016	49.755	443	0.0037071	2.515	127450000	6372300	5
1017	36.776	338	0	323.31	18737000000	986150000	762
1018	33.178	299	0	323.31	43473000000	3105200000	758
1019	20.529	179	0	38.835	2479900000	309980000	128
1020	41.664	376	0	323.31	26065000000	1629100000	693
1021	23.919	232	0	323.31	80529000000	8947700000	935
1022	89.433	820	0	213.2	3111500000	86430000	177
1023	47.466	418	0	323.31	32018000000	1600900000	1009
1024	77.508	687	0	20.15	901780000	30059000	83
1025	10.046	92	0	96.285	5942800000	990460000	157
1026	32.025	282	0	13.854	28660000	1685900	16

	BK	BL	BM	BN	BO	BP	BQ
1027	85.153	752	0	323.31	26663000000	740640000	1082
1028	58.05	505	0	99.653	2072700000	148050000	176
1029	45.409	423	0	323.31	37020000000	1542500000	1113
1030	37.043	328	0	8.0002	335950000	20997000	8
1031	66.238	587	0	323.31	11125000000	358860000	591
1032	7.8479	68	0	31.857	346830000	86707000	67
1033	12.469	104	0	19.644	502740000	100550000	58
1034	46.739	439	0	211.66	2794300000	127020000	206
1035	7.3581	63	0	23.557	79270000	19817000	6
1036	48.682	429	0	323.31	12302000000	512590000	523
1037	12.811	113	0	39.451	750810000	125130000	45
1038	59.869	567	0	323.31	48264000000	1930600000	1191
1039	10.105	94	0	80.561	2476500000	1238200000	114
1040	77.845	676	0.0090909	1.88	909620000	27564000	13
1041	36.422	335	0	74.704	1713000000	100760000	141
1042	7.5946	66	0	5.4109	134320000	44774000	15
1043	6.7716	60	0	110.02	65489000000	65489000000	187
1044	5.9109	49	0	81.755	32126000000	16063000000	374
1045	14.673	127	0	78.504	4389800000	627110000	133
1046	22.527	195	0	179.62	4111200000	373750000	188
1047	9.9926	85	0	10.995	714750000	357370000	41
1048	179.58	1609	0	323.31	48671000000	559440000	2497
1049	33.66	299	0	323.31	1.03E+11	5421300000	1386
1050	6.7115	61	0	19.037	1258200000	314550000	64
1051	56.297	501	0	323.31	9666400000	439380000	635
1052	26.614	234	0	6.2189	103550000	8629400	11
1053	16.579	146	0	67.732	2923600000	584720000	125
1054	48.792	422	0	58.406	1631000000	65242000	122
1055	8.98	79	0	94.247	9451800000	3150600000	166
1056	57.395	516	0	99.833	1939200000	96960000	101
1057	31.63	290	0	323.31	16010000000	1143600000	457
1058	35.782	326	0	323.31	11584000000	551620000	427
1059	11.631	103	0	149.59	5447400000	907900000	224
1060	83.764	758	0	323.31	4.07E+11	10714000000	5021
1061	20.324	175	0	8.8	1030400000	171730000	82
1062	25.152	217	0.00094162	3.0563	55477000	5043300	13
1063	90.247	810	0	323.31	9127800000	234050000	506
1064	17.571	152	0	8.2511	221410000	20128000	8
1065	37.79	335	0	183.85	4592900000	306200000	292
1066	10.69	96	0	5.5339	210620000	70208000	18
1067	10.212	88	0	64.489	7414500000	1482900000	174
1068	50.203	450	0	13.227	413710000	18805000	32
1069	16.523	150	0	209.71	13270000000	1327000000	413
1070	73.352	657	0	27.89	1433200000	47773000	46
1071	21.085	182	0	323.31	47249000000	7874800000	598
1072	23.705	211	0	35.198	1243200000	103600000	130
1073	31.873	306	0	249.8	4157600000	218820000	262
1074	37.361	346	0	323.31	4.12E+11	21684000000	4784
1075	28.841	259	0	323.31	1.35E+11	9623300000	2029
1076	41.697	392	0	323.31	5.2E+11	27383000000	3663
1077	68.527	577	0	53.182	956950000	35443000	92
1078	29.752	272	0	74.961	1046800000	69785000	106
1079	30.525	279	0.00369	2.3813	136070000	13607000	3
1080	31.146	275	0	12.596	1760000000	135380000	19

	BK	BL	BM	BN	BO	BP	BQ
1081	30.201	273	0	109.6	2957100000	369630000	188
1082	48.922	427	0	18.383	848700000	56580000	98
1083	47.939	416	0	88.313	1144400000	57220000	72
1084	13.979	122	0	5.586	167410000	41852000	8
1085	52.444	492	0	323.31	59119000000	2364800000	1453
1086	38.43	341	0	199.14	6611600000	330580000	304
1087	60.852	540	0	323.31	15473000000	595110000	528
1088	97.197	850	0	4.1369	1750300000	67321000	19
1089	29.958	250	0	27.615	1653300000	110220000	146
1090	51.422	441	0	8.1921	114750000	5737700	5
1091	41.84	393	0	323.31	42998000000	2529300000	1128
