# Supplementary material for: Foreign peptide triggers boost in pneumococcal metabolism and growth
Source: BMC Microbiol. 2018 Mar 27;18:23. doi: 10.1186/s12866-018-1167-y (PMC5870813; doi:10.1186/s12866-018-1167-y)
Supplement: Supplementary file 7 — Table S6. Proteomic data for wild type with and without ORF 2 peptide. Table shows only significant changes in expression. A significant change in expression was observed for 12 proteins of which 11 were upregulated by the ORF 2 peptide (also shown in Table 2 in the main text) and 1 was downregulated by the peptide. (PDF 34 kb) [file 12866_2018_1167_MOESM7_ESM.pdf]

	A
1	Fasta headers
2	pep chromosome:ASM81700v1:Chromosome:152735:153598:1 gene:SpnNT_00157 transcript:AJD71098 gene_biotype:protein_coding transcript_biotype:protein_coding description:hypothetical protein
3	pep chromosome:ASM81700v1:Chromosome:174929:175396:-1 gene:SpnNT_00179 transcript:AJD71120 gene_biotype:protein_coding transcript_biotype:protein_coding gene_symbol:ribH description:6,7-dimethyl-8-ribityllumazine s
4	pep chromosome:ASM81700v1:Chromosome:176622:177257:-1 gene:SpnNT_00181 transcript:AJD71122 gene_biotype:protein_coding transcript_biotype:protein_coding gene_symbol:ribE description:Riboflavin synthase
5	pep chromosome:ASM81700v1:Chromosome:492278:493618:1 gene:SpnNT_00478 transcript:AJD71419 gene_biotype:protein_coding transcript_biotype:protein_coding gene_symbol:citS description:Sensor protein CitS
6	pep chromosome:ASM81700v1:Chromosome:503893:504528:1 gene:SpnNT_00493 transcript:AJD71434 gene_biotype:protein_coding transcript_biotype:protein_coding gene_symbol:trmB description:tRNA (guanine-N(7)-)-methyltra
7	pep chromosome:ASM81700v1:Chromosome:677097:677237:1 gene:SpnNT_00631 transcript:AJD71572 gene_biotype:protein_coding transcript_biotype:protein_coding description:hypothetical protein
8	pep chromosome:ASM81700v1:Chromosome:733483:734682:1 gene:SpnNT_00680 transcript:AJD71621 gene_biotype:protein_coding transcript_biotype:protein_coding gene_symbol:divIB description:Cell division protein DivIB
9	pep chromosome:ASM81700v1:Chromosome:817700:818140:1 gene:SpnNT_00775 transcript:AJD71716 gene_biotype:protein_coding transcript_biotype:protein_coding description:ASCH domain protein
10	pep chromosome:ASM81700v1:Chromosome:1044619:1045902:-1 gene:SpnNT_01021 transcript:AJD71961 gene_biotype:protein_coding transcript_biotype:protein_coding gene_symbol:pyrP description:Uracil transporter
11	pep chromosome:ASM81700v1:Chromosome:1748867:1749970:-1 gene:SpnNT_01742 transcript:AJD72675 gene_biotype:protein_coding transcript_biotype:protein_coding gene_symbol:afr description:1,5-anhydro-D-fructose reduc
12	pep chromosome:ASM81700v1:Chromosome:2072531:2073361:-1 gene:SpnNT_02095 transcript:AJD72990 gene_biotype:protein_coding transcript_biotype:protein_coding gene_symbol:misCA description:Stage III sporulation prote
13	pep chromosome:ASM81700v1:Chromosome:2173434:2173625:-1 gene:SpnNT_02206 transcript:AJD73085 gene_biotype:protein_coding transcript_biotype:protein_coding description:hypothetical protein;pep chromosome:ASM817

	B	C	D	E	F	G
1	Protein IDs	Majority protein IDs	Student's T-test Significant WT_WT_PEP	-Log Student's T-test p-value WT_WT_PEP	Student's T-test q-value WT_WT_PEP	Student's T-test Difference WT_WT_PEP
2	AJD71098	AJD71098	+	4.057308319	0.023111111	-1.31421513
3	AJD71120	AJD71120	+	3.713827157	0.047333333	-1.188654582
4	AJD71122	AJD71122	+	4.381471266	0.012	-1.521065394
5	AJD71419	AJD71419	+	3.707604529	0.0115	-1.634911007
6	AJD71434	AJD71434	+	4.700525129	0.011333333	-1.514814589
7	AJD71572	AJD71572	+	3.608454496	0.051272727	-1.205735101
8	AJD71621	AJD71621	+	4.105345534	0.004	-2.665047328
9	AJD71716	AJD71716	+	4.320336894	0.012	-1.963269764
10	AJD71961	AJD71961	+	3.710281746	0.012	-1.967867745
11	AJD72675	AJD72675	+	21.50714759	0	-20.98047108
12	AJD72990	AJD72990	+	2.513296575	0.0448	1.53611713
13	AJD73085;AJD713[illegible]	AJD73085;AJD713[illegible]	+	3.248933906	0.0112	-1.963662253

	H	I	J	K	L	M	N	O	P
1	Student's T-test Test statistic WT_WT_PEP	TOP3 WT_i01_1	TOP3 WT_i01_2	TOP3 WT_i01_3	TOP3 WT_i02_1	TOP3 WT_i02_2	TOP3 WT_i02_3	TOP3 WT_i03_1	TOP3 WT_i03_2
2	-1.745918267	21.14606	20.51308	19.65751	19.84831	21.12857	20.39904	21.16672	20.85876
3	-1.590725991	22.32589	23.11196	22.26373	22.72269	22.32242	21.94448	23.22432	22.32329
4	-1.968637844	20.94349	20.20562	20.6989	21.03859	19.16673	21.6854	20.31882	19.87555
5	-1.945026774	21.89212	20.33641	19.23272	21.29224	19.71429	18.93083	20.16023	20.33467
6	-2.00875345	20.14095	20.6592	20.62835	19.60742	20.43457	19.65162	20.05342	20.57603
7	-1.59248269	23.08823	23.33093	23.74808	23.11989	22.3563	22.4757	23.14193	23.80504
8	-2.646276544	25.85452	22.623	23.31948	25.78743	23.60816	23.41307	25.8335	23.58051
9	-2.292133582	19.29069	19.10852	18.16639	18.6924	19.17078	21.15965	18.52163	18.3129
10	-2.163304945	22.34814	22.55926	20.40946	22.44471	21.1325	22.2193	22.94295	20.32362
11	-27.49079735	0	0	0	0	0	0	0	0
12	1.632541595	21.76204	23.63297	21.15541	21.44529	23.71365	20.90149	20.95324	23.4422
13	-2.050044401	21.42231	20.12486	19.91953	22.62169	19.29837	19.03249	20.50571	21.28913

	Q	R	S	T	U	V	W	X	Y
1	TOP3 WT_i03_3	TOP3 WT_PEP_i01_1	TOP3 WT_PEP_i01_2	TOP3 WT_PEP_i01_3	TOP3 WT_PEP_i02_1	TOP3 WT_PEP_i02_2	TOP3 WT_PEP_i02_3	TOP3 WT_PEP_i03_1	TOP3 WT_PEP_i03_2
2	20.00156	22.40753	22.40803	21.77299	21.64344	21.40577	21.63771	22.52146	21.45207
3	22.35995	23.88257	23.89217	23.37649	24.24195	23.43646	24.38069	23.86089	23.90622
4	19.96499	22.29879	21.82463	22.2164	21.71133	21.8035	21.78829	22.45327	22.09089
5	19.52231	22.29432	21.40946	21.71966	21.74339	21.68741	21.68705	22.43924	21.36344
6	19.03514	21.78221	21.53392	21.36366	22.46411	22.2383	21.32718	21.13985	21.73652
7	23.13443	24.02306	25.22481	24.77912	24.58885	24.96411	24.15576	24.23649	23.56936
8	22.44168	26.22385	27.53092	26.70965	26.09556	27.52018	26.31054	26.09008	27.45404
9	20.50173	22.05556	20.84272	21.38038	21.09689	21.02361	21.077	21.10542	21.00658
10	21.47389	23.96406	23.78379	23.90376	23.89198	23.73143	24.07095	23.89165	21.79272
11	0	21.84838	20.57772	22.02009	21.48358	20.21313	20.7704	21.6864	20.3346
12	21.25013	20.99416	20.15039	20.00274	20.18765	20.80655	19.67393	20.53492	21.43278
13	20.11127	23.28466	22.65095	23.21962	22.97354	21.58472	21.99029	22.99141	21.03317

	Z	AA	AB	AC	AD	AE	AF	AG	AH	AI	AJ
1	TOP3 WT_PEP_i03_3	Number of proteins	Peptides	Razor + unique pep	Mol. weight [kDa]	Sequence length	Q-value	Score	Intensity	iBAQ	#PSM
2	21.29856	1	2	2	34.089	287	0	63.915	72949000	5210600	17
3	22.3192	1	5	5	16.766	155	0	231.97	447980000	49775000	26
4	21.40057	1	3	3	23.317	211	0.00094073	2.9893	31518000	2626500	6
5	21.78605	1	2	2	51.9	446	0	3.9473	33160000	1950600	9
6	20.83426	1	3	3	24.378	211	0	30.974	27736000	2311400	13
7	23.5106	1	2	2	5.1269	46	0	60.289	763360000	381680000	73
8	26.51194	1	10	10	45.745	399	0	22.367	2570200000	142790000	32
9	21.00597	1	2	2	16.649	146	0	3.6758	17077000	2846100	10
10	24.53429	1	3	3	44.803	427	0	44.35	227420000	22742000	13
11	19.88995	1	2	2	41.144	367	0.00094429	3.3355	49606000	3100300	4
12	20.64824	1	2	2	31.243	276	0	20.357	69040000	11507000	17
13	22.26997	2	2	2	7.3581	63	0	23.557	79270000	19817000	6
