# Supplementary material for: Foreign peptide triggers boost in pneumococcal metabolism and growth
Source: BMC Microbiol. 2018 Mar 27;18:23. doi: 10.1186/s12866-018-1167-y (PMC5870813; doi:10.1186/s12866-018-1167-y)
Supplement: Supplementary file 8 — Table S7. Proteomic data for ΔORF 2 with and without ORF 2 peptide. Table shows only significant changes in expression. A significant change in expression was observed for 22 proteins of which 20 were upregulated by the ORF 2 peptide and 2 were downregulated by the peptide. (PDF 52 kb) [file 12866_2018_1167_MOESM8_ESM.pdf]

	A
1	Fasta headers
2	pep chromosome:ASM81700v1:Chromosome:174929:175396:-1 gene:SpnNT_00179 transcript:AJD71120 gene_biotype:protein_coding transcript_biotype:protein_coding gene_symbol:ribH description:6,7-dimethyl-8-ribityllumazine s
3	pep chromosome:ASM81700v1:Chromosome:176622:177257:-1 gene:SpnNT_00181 transcript:AJD71122 gene_biotype:protein_coding transcript_biotype:protein_coding gene_symbol:ribE description:Riboflavin synthase
4	pep chromosome:ASM81700v1:Chromosome:315132:316112:1 gene:SpnNT_00330 transcript:AJD71271 gene_biotype:protein_coding transcript_biotype:protein_coding gene_symbol:mraY description:Phospho-N-acetylmuramoyl-p
5	pep chromosome:ASM81700v1:Chromosome:428017:428493:1 gene:SpnNT_00419 transcript:AJD71360 gene_biotype:protein_coding transcript_biotype:protein_coding gene_symbol:ilvH description:Putative acetolactate synthase s
6	pep chromosome:ASM81700v1:Chromosome:492278:493618:1 gene:SpnNT_00478 transcript:AJD71419 gene_biotype:protein_coding transcript_biotype:protein_coding gene_symbol:citS description:Sensor protein CitS
7	pep chromosome:ASM81700v1:Chromosome:503893:504528:1 gene:SpnNT_00493 transcript:AJD71434 gene_biotype:protein_coding transcript_biotype:protein_coding gene_symbol:trmB description:tRNA (guanine-N(7)-)-methyltra
8	pep chromosome:ASM81700v1:Chromosome:566943:568271:1 gene:SpnNT_00548 transcript:AJD71489 gene_biotype:protein_coding transcript_biotype:protein_coding gene_symbol:hssS description:Heme sensor protein HssS
9	pep chromosome:ASM81700v1:Chromosome:773674:774231:1 gene:SpnNT_00727 transcript:AJD71668 gene_biotype:protein_coding transcript_biotype:protein_coding gene_symbol:mtrR description:HTH-type transcriptional regula
10	pep chromosome:ASM81700v1:Chromosome:863845:864543:-1 gene:SpnNT_00822 transcript:AJD71763 gene_biotype:protein_coding transcript_biotype:protein_coding gene_symbol:yvoA description:HTH-type transcriptional repre
11	pep chromosome:ASM81700v1:Chromosome:1064558:1066045:-1 gene:SpnNT_01038 transcript:AJD71978 gene_biotype:protein_coding transcript_biotype:protein_coding description:Polysaccharide biosynthesis protein
12	pep chromosome:ASM81700v1:Chromosome:1342748:1343686:-1 gene:SpnNT_01305 transcript:AJD72243 gene_biotype:protein_coding transcript_biotype:protein_coding gene_symbol:pyrDB description:Dihydroorotate dehydroge
13	pep chromosome:ASM81700v1:Chromosome:1554036:1554752:-1 gene:SpnNT_01519 transcript:AJD72453 gene_biotype:protein_coding transcript_biotype:protein_coding gene_symbol:atpB description:F-ATPase subunit 6
14	pep chromosome:ASM81700v1:Chromosome:1608417:1611614:-1 gene:SpnNT_01578 transcript:AJD72512 gene_biotype:protein_coding transcript_biotype:protein_coding description:Phage-related protein
15	pep chromosome:ASM81700v1:Chromosome:1627781:1628671:-1 gene:SpnNT_01602 transcript:AJD72536 gene_biotype:protein_coding transcript_biotype:protein_coding gene_symbol:hhaIM description:Modification methylase Hh
16	pep chromosome:ASM81700v1:Chromosome:1748867:1749970:-1 gene:SpnNT_01742 transcript:AJD72675 gene_biotype:protein_coding transcript_biotype:protein_coding gene_symbol:afr description:1,5-anhydro-D-fructose reduc
17	pep chromosome:ASM81700v1:Chromosome:1830104:1830484:1 gene:SpnNT_01816 transcript:AJD72749 gene_biotype:protein_coding transcript_biotype:protein_coding description:accessory regulator family protein
18	pep chromosome:ASM81700v1:Chromosome:1842906:1843973:-1 gene:SpnNT_01832 transcript:AJD72765 gene_biotype:protein_coding transcript_biotype:protein_coding description:Zn-dependent protease
19	pep chromosome:ASM81700v1:Chromosome:2002281:2002769:-1 gene:SpnNT_02012 transcript:AJD72921 gene_biotype:protein_coding transcript_biotype:protein_coding gene_symbol:coaD description:Phosphopantetheine adeny
20	pep chromosome:ASM81700v1:Chromosome:2051597:2052325:-1 gene:SpnNT_02074 transcript:AJD72969 gene_biotype:protein_coding transcript_biotype:protein_coding gene_symbol:gmuR description:Glucomannan utilization p
21	pep chromosome:ASM81700v1:Chromosome:2072531:2073361:-1 gene:SpnNT_02095 transcript:AJD72990 gene_biotype:protein_coding transcript_biotype:protein_coding gene_symbol:misCA description:Stage III sporulation prote
22	pep chromosome:ASM81700v1:Chromosome:2148924:2149772:1 gene:SpnNT_02185 transcript:AJD73064 gene_biotype:protein_coding transcript_biotype:protein_coding gene_symbol:rlmA description:23S rRNA (guanine(745)-N(
23	pep chromosome:ASM81700v1:Chromosome:2173434:2173625:-1 gene:SpnNT_02206 transcript:AJD73085 gene_biotype:protein_coding transcript_biotype:protein_coding description:hypothetical protein;pep chromosome:ASM817

	B	C	D	E	F
1	Protein IDs	Majority protein IDs	Student's T-test Significant ORF2_ORF2_PEP	-Log Student's T-test p-value ORF2_ORF2_PEP	Student's T-test q-value ORF2_ORF2_PEP
2	AJD71120	AJD71120	+	7.621646143	0
3	AJD71122	AJD71122	+	5.823568498	0.001454545
4	AJD71271	AJD71271	+	8.81559378	0
5	AJD71360	AJD71360	+	2.580955485	0.012
6	AJD71419	AJD71419	+	19.27311386	0
7	AJD71434	AJD71434	+	25.67712702	0
8	AJD71489	AJD71489	+	18.80575571	0
9	AJD71668	AJD71668	+	3.213852528	0.001333333
10	AJD71763	AJD71763	+	5.020457669	0.005882353
11	AJD71978	AJD71978	+	4.412128045	0.002
12	AJD72243	AJD72243	+	6.29022879	0
13	AJD72453	AJD72453	+	22.70182073	0
14	AJD72512	AJD72512	+	4.254728403	0.001846154
15	AJD72536	AJD72536	+	2.202972954	0.0198
16	AJD72675	AJD72675	+	2.170622605	0.046857143
17	AJD72749	AJD72749	+	3.249284242	0.046181818
18	AJD72765	AJD72765	+	22.39659345	0
19	AJD72921	AJD72921	+	23.31980829	0
20	AJD72969	AJD72969	+	4.111564695	0.0016
21	AJD72990	AJD72990	+	3.124257146	0.016842105
22	AJD73064	AJD73064	+	4.50209757	0.0032
23	AJD73085;AJD713[illegible]	AJD73085;AJD713[illegible]	+	4.546424644	0.003

	G	H	I	J	K	L
1	Student's T-test Difference ORF2_ORF2_PEP	Student's T-test Test statistic ORF2_ORF2_PEP	TOP3 ORF2_i01_1	TOP3 ORF2_i01_2	TOP3 ORF2_i01_3	TOP3 ORF2_i02_1
2	-3.351281484	-4.028976131	21.26221	20.91191	20.00001	20.83743
3	-1.793219037	-2.415698082	20.64547	20.99124	20.28038	20.5313
4	-2.598408805	-3.648678713	20.04071	19.9568	19.86497	20.40018
5	-1.626541138	-1.699248308	22.93956	21.03938	21.03842	20.42555
6	-20.69751718	-24.10979423	0	0	0	0
7	-22.38170666	-34.22597405	0	0	0	0
8	20.3950225	23.23085084	20.58392	19.51042	19.29515	20.64356
9	2.545367347	2.316275426	25.57217	22.62674	21.68689	25.52998
10	-1.285534965	-1.830547631	20.43462	20.36823	19.67786	19.8587
11	-1.632285436	-2.064375267	20.63505	20.75261	19.90307	19.32986
12	-2.245675617	-2.882713936	20.86282	20.88015	20.5764	20.28341
13	-20.94210709	-29.04076864	0	0	0	0
14	-1.832877053	-2.188325595	23.11099	22.37976	23.82193	22.10902
15	-1.676073922	-1.622514393	20.3453	22.68516	20.80452	21.86046
16	-1.423506843	-1.486126144	20.22073	21.46594	18.58007	20.62705
17	-1.1335623	-1.483163798	21.766	23.31836	23.2594	22.70138
18	-21.30843862	-28.98643818	0	0	0	0
19	-22.02440156	-30.90115502	0	0	0	0
20	-2.342860964	-2.478749976	21.90495	20.46297	19.76974	20.73123
21	-1.359483719	-1.642923385	20.78274	21.9112	20.82544	20.40085
22	-1.52093188	-1.986091181	19.90229	19.05713	20.29378	21.06786
23	-1.497462379	-1.972198241	20.49152	20.11032	20.47352	20.16668

	M	N	O	P	Q	R	S	T
1	TOP3 ORF2_i02_2	TOP3 ORF2_i02_3	TOP3 ORF2_i03_1	TOP3 ORF2_i03_2	TOP3 ORF2_i03_3	TOP3 ORF2_PEP_i01_1	TOP3 ORF2_PEP_i01_2	TOP3 ORF2_PEP_i01_3
2	21.1759	20.69763	20.56837	19.73164	21.51605	24.70163	23.08628	23.07781
3	19.44458	20.30026	20.81431	21.07155	19.76049	22.2848	21.56338	22.12217
4	20.45691	19.27262	19.14239	19.1048	19.75973	23.04501	22.29597	21.87847
5	21.19679	20.77058	21.89049	21.05119	21.47439	24.82036	22.21562	21.97519
6	0	0	0	0	0	20.16272	21.21923	19.56831
7	0	0	0	0	0	22.369	21.47504	22.37111
8	20.39329	23.14594	20.20133	20.0914	19.69019	0	0	0
9	22.56222	21.6683	24.81669	22.01042	21.74493	20.55295	21.17634	20.04047
10	20.24907	19.60997	20.14434	20.11561	20.69857	21.40713	21.55554	20.58024
11	19.71463	19.92977	20.69649	20.08769	20.38422	22.74977	21.80082	20.6454
12	20.79523	20.17514	22.0231	19.64586	20.09724	23.16425	22.50567	22.6885
13	0	0	0	0	0	20.84009	19.96251	20.72988
14	21.99099	22.53742	23.43691	22.33503	22.42156	24.61073	25.57736	23.68801
15	19.41212	21.07672	21.32534	22.45631	19.38086	23.66582	23.40063	21.73561
16	21.04316	19.69403	20.41883	21.00297	20.45327	23.03074	21.50203	20.19432
17	21.90643	23.57657	22.25554	22.12054	23.29214	23.67474	23.6519	24.15561
18	0	0	0	0	0	20.42794	20.97754	21.22715
19	0	0	0	0	0	22.44934	21.89212	22.4289
20	19.93566	20.63123	20.53209	20.54969	20.68117	24.7489	21.90385	22.32419
21	20.30928	19.62101	20.09641	21.08857	20.7597	21.90344	22.74934	21.61536
22	19.50128	20.25006	19.83429	20.68702	19.97274	21.28007	22.08759	21.86572
23	20.51392	19.97644	20.59272	20.53949	19.74419	23.06993	21.74178	20.69247

	U	V	W	X	Y	Z	AA
1	TOP3 ORF2_PEP_i02_1	TOP3 ORF2_PEP_i02_2	TOP3 ORF2_PEP_i02_3	TOP3 ORF2_PEP_i03_1	TOP3 ORF2_PEP_i03_2	TOP3 ORF2_PEP_i03_3	Number of proteins
2	24.77806	23.36848	24.57532	25.18571	23.60486	24.48452	1
3	22.36866	21.80415	23.11309	22.7459	22.07979	21.8966	1
4	22.47831	22.23384	22.57802	22.72948	22.28413	21.86156	1
5	24.13205	21.93895	22.06295	24.29502	22.92882	22.09625	1
6	22.33085	19.73118	20.4189	22.34025	20.82141	19.6848	1
7	23.16522	22.15475	22.34913	22.83736	22.29144	22.42231	1
8	0	0	0	0	0	0	1
9	21.33568	20.21396	21.65202	20.41171	19.98198	19.94492	1
10	22.09807	21.37897	21.62964	22.01982	21.11516	20.9422	1
11	22.36097	21.25795	21.49175	22.81864	21.47705	21.52161	1
12	23.25886	22.56369	22.20108	23.68645	22.34458	23.13737	1
13	21.15999	20.17243	21.95098	21.75122	20.64544	21.26641	1
14	24.3217	25.93257	24.41601	24.58711	23.74138	23.76466	1
15	24.45653	21.55275	21.91588	23.23449	22.92748	21.54227	1
16	22.38723	20.63136	22.02377	23.12755	22.59486	20.82576	1
17	24.05623	23.47815	24.16298	24.25756	23.08921	23.87205	1
18	20.69062	22.2482	21.89672	22.39273	20.79035	21.12469	1
19	22.77844	21.31963	21.71985	22.74498	21.96469	20.92167	1
20	24.89775	22.0126	21.67244	22.9581	22.4581	23.30855	1
21	21.38971	23.50537	21.65541	22.09901	21.07916	22.03374	1
22	21.32209	21.10459	21.12588	22.31329	22.17777	20.97785	1
23	21.54372	21.85854	22.36779	22.07933	20.90182	21.83059	2

	AB	AC	AD	AE	AF	AG	AH	AI	AJ
1	Peptides	Razor + unique pep	Mol. weight [kDa]	Sequence length	Q-value	Score	Intensity	iBAQ	#PSM
2	5	5	16.766	155	0	231.97	447980000	49775000	26
3	3	3	23.317	211	0.00094073	2.9893	31518000	2626500	6
4	2	2	35.974	326	0	17.099	63956000	9136500	9
5	3	3	17.694	158	0	11.908	136140000	19449000	10
6	2	2	51.9	446	0	3.9473	33160000	1950600	9
7	3	3	24.378	211	0	30.974	27736000	2311400	13
8	2	2	50.485	442	0.0045788	2.1792	53057000	1965100	3
9	5	5	21.727	185	0	8.3576	171710000	17171000	13
10	3	3	26.829	232	0.0018692	2.7109	103260000	6883800	5
11	2	2	56.53	495	0.0009434	3.2788	71024000	4439000	9
12	4	4	33.142	312	0.00093545	2.7788	153300000	11792000	10
13	2	2	27.208	238	0	24.761	51292000	17097000	3
14	7	7	119.2	1065	0.0099728	1.8217	808730000	13707000	27
15	2	2	32.988	296	0.0036799	2.3264	210670000	14044000	3
16	2	2	41.144	367	0.00094429	3.3355	49606000	3100300	4
17	3	3	14.612	126	0	5.7594	374010000	41557000	12
18	3	3	40.231	355	0	11.563	89983000	6921800	5
19	2	2	18.411	162	0.0036866	2.3591	47213000	5901600	6
20	5	5	28.018	242	0	4.6374	242240000	22022000	11
21	2	2	31.243	276	0	20.357	69040000	11507000	17
22	2	2	32.025	282	0	13.854	28660000	1685900	16
23	2	2	7.3581	63	0	23.557	79270000	19817000	6
