# Supplementary material for: Foreign peptide triggers boost in pneumococcal metabolism and growth
Source: BMC Microbiol. 2018 Mar 27;18:23. doi: 10.1186/s12866-018-1167-y (PMC5870813; doi:10.1186/s12866-018-1167-y)
Supplement: Supplementary file 9 — Table S8. Proteomic data for wild type and ΔORF 2 mutant without ORF 2 peptide. Table shows only significant changes in expression. A significant difference in expression was observed for 19 proteins of which 17 were more highly expressed in the wildtype and 2 were more highly expressed in the mutant. (PDF 46 kb) [file 12866_2018_1167_MOESM9_ESM.pdf]

	A
1	Fasta headers
2	pep chromosome:ASM81700v1:Chromosome:19368:19736:1 gene:SpnNT_00030 transcript:AJD70977 gene_biotype:protein_coding transcript_biotype:protein_coding description:Septum formation initiator
3	pep chromosome:ASM81700v1:Chromosome:174929:175396:-1 gene:SpnNT_00179 transcript:AJD71120 gene_biotype:protein_coding transcript_biotype:protein_coding gene_symbol:ribH description:6,7-dimethyl-8-ribityllumazine sy
4	pep chromosome:ASM81700v1:Chromosome:315132:316112:1 gene:SpnNT_00330 transcript:AJD71271 gene_biotype:protein_coding transcript_biotype:protein_coding gene_symbol:mraY description:Phospho-N-acetylmuramoyl-per
5	pep chromosome:ASM81700v1:Chromosome:492278:493618:1 gene:SpnNT_00478 transcript:AJD71419 gene_biotype:protein_coding transcript_biotype:protein_coding gene_symbol:citS description:Sensor protein CitS
6	pep chromosome:ASM81700v1:Chromosome:503893:504528:1 gene:SpnNT_00493 transcript:AJD71434 gene_biotype:protein_coding transcript_biotype:protein_coding gene_symbol:trmB description:tRNA (guanine-N(7)-)-methyltran
7	pep chromosome:ASM81700v1:Chromosome:555059:562378:1 gene:SpnNT_00543 transcript:AJD71484 gene_biotype:protein_coding transcript_biotype:protein_coding description:hypothetical protein
8	pep chromosome:ASM81700v1:Chromosome:678905:680521:1 gene:SpnNT_00633 transcript:AJD71574 gene_biotype:protein_coding transcript_biotype:protein_coding description:hypothetical protein
9	pep chromosome:ASM81700v1:Chromosome:863845:864543:-1 gene:SpnNT_00822 transcript:AJD71763 gene_biotype:protein_coding transcript_biotype:protein_coding gene_symbol:yvoA description:HTH-type transcriptional repres
10	pep chromosome:ASM81700v1:Chromosome:1033597:1034817:1 gene:SpnNT_01010 transcript:AJD71950 gene_biotype:protein_coding transcript_biotype:protein_coding gene_symbol:lytA_6 description:Autolysin
11	pep chromosome:ASM81700v1:Chromosome:1064558:1066045:-1 gene:SpnNT_01038 transcript:AJD71978 gene_biotype:protein_coding transcript_biotype:protein_coding description:Polysaccharide biosynthesis protein
12	pep chromosome:ASM81700v1:Chromosome:1554036:1554752:-1 gene:SpnNT_01519 transcript:AJD72453 gene_biotype:protein_coding transcript_biotype:protein_coding gene_symbol:atpB description:F-ATPase subunit 6
13	pep chromosome:ASM81700v1:Chromosome:1677399:1677611:1 gene:SpnNT_01675 transcript:AJD72608 gene_biotype:protein_coding transcript_biotype:protein_coding description:hypothetical protein
14	pep chromosome:ASM81700v1:Chromosome:1748867:1749970:-1 gene:SpnNT_01742 transcript:AJD72675 gene_biotype:protein_coding transcript_biotype:protein_coding gene_symbol:afr description:1,5-anhydro-D-fructose reducta
15	pep chromosome:ASM81700v1:Chromosome:1816074:1817345:-1 gene:SpnNT_01800 transcript:AJD72733 gene_biotype:protein_coding transcript_biotype:protein_coding description:EcoKI restriction-modification system protein Hs
16	pep chromosome:ASM81700v1:Chromosome:1842906:1843973:-1 gene:SpnNT_01832 transcript:AJD72765 gene_biotype:protein_coding transcript_biotype:protein_coding description:Zn-dependent protease
17	pep chromosome:ASM81700v1:Chromosome:2002281:2002769:-1 gene:SpnNT_02012 transcript:AJD72921 gene_biotype:protein_coding transcript_biotype:protein_coding gene_symbol:coaD description:Phosphopantetheine adenyly
18	pep chromosome:ASM81700v1:Chromosome:2051597:2052325:-1 gene:SpnNT_02074 transcript:AJD72969 gene_biotype:protein_coding transcript_biotype:protein_coding gene_symbol:gmuR description:Glucomannan utilization pro
19	pep chromosome:ASM81700v1:Chromosome:2129333:2130664:1 gene:SpnNT_02166 transcript:AJD73045 gene_biotype:protein_coding transcript_biotype:protein_coding gene_symbol:phoR description:Alkaline phosphatase synthes
20	pep chromosome:ASM81700v1:Chromosome:2283335:2284660:-1 gene:SpnNT_02312 transcript:AJD73189 gene_biotype:protein_coding transcript_biotype:protein_coding description:sensory histidine kinase DcuS

	B	C	D	E	F
1	Protein IDs	Majority protein IDs	Student's T-test Significant ORF2_WT	-Log Student's T-test p-value ORF2_WT	Student's T-test q-value ORF2_WT
2	AJD70977	AJD70977	+	5.292697483	0.025142857
3	AJD71120	AJD71120	+	5.808985888	0.0295
4	AJD71271	AJD71271	+	6.374190217	0.0096
5	AJD71419	AJD71419	+	19.90064492	0
6	AJD71434	AJD71434	+	23.58594901	0
7	AJD71484	AJD71484	+	2.237670423	0.011
8	AJD71574	AJD71574	+	2.422849786	0.023384615
9	AJD71763	AJD71763	+	4.59528366	0.025866667
10	AJD71950	AJD71950	+	1.286154778	0.043333333
11	AJD71978	AJD71978	+	3.97685189	0.041894737
12	AJD72453	AJD72453	+	19.84984285	0
13	AJD72608	AJD72608	+	3.484090757	0.015636364
14	AJD72675	AJD72675	+	20.77994575	0
15	AJD72733	AJD72733	+	16.28057888	0
16	AJD72765	AJD72765	+	19.12205416	0
17	AJD72921	AJD72921	+	19.14516344	0
18	AJD72969	AJD72969	+	4.908806836	0.010222222
19	AJD73045	AJD73045	+	2.838265636	0.036470588
20	AJD73189	AJD73189	+	3.32809523	0.015333333

	G	H	I	J	K	L	M
1	Student's T-test Difference ORF2_WT	Student's T-test Test statistic ORF2_WT	TOP3 ORF2_i01_1	TOP3 ORF2_i01_2	TOP3 ORF2_i01_3	TOP3 ORF2_i02_1	TOP3 ORF2_i02_2
2	-1.845865673	-0.349894993	21.45844	20.45173	19.97823	21.25865	20.03595
3	-1.766399595	-0.337142044	21.26221	20.91191	20.00001	20.83743	21.1759
4	-2.371354845	-0.448246406	20.04071	19.9568	19.86497	20.40018	20.45691
5	-20.15731261	-3.789789517	0	0	0	0	0
6	-20.08740955	-3.87284892	0	0	0	0	0
7	-2.940858841	-0.496427746	25.89408	25.92325	25.73137	31.97153	25.7852
8	2.003107283	0.358226003	22.73796	22.82796	23.85501	21.9511	23.23788
9	-1.841522217	-0.346433987	20.43462	20.36823	19.67786	19.8587	20.24907
10	-1.873022503	-0.317942901	25.07561	24.47199	24.14392	25.05083	22.95756
11	-1.688187493	-0.31670072	20.63505	20.75261	19.90307	19.32986	19.71463
12	-21.9256009	-4.098723631	0	0	0	0	0
13	-2.172329373	-0.39659432	22.82271	20.87766	21.69927	22.06508	21.70657
14	20.38956261	3.858688433	20.22073	21.46594	18.58007	20.62705	21.04316
15	-20.88737975	-3.757931172	0	0	0	0	0
16	-21.9882679	-4.080036605	0	0	0	0	0
17	-20.92815717	-3.897793952	0	0	0	0	0
18	-2.645497852	-0.487600305	21.90495	20.46297	19.76974	20.73123	19.93566
19	-1.781821781	-0.326087563	21.07349	20.76104	23.12459	19.82521	20.16535
20	-2.103653802	-0.38383009	21.73116	21.47204	20.09116	21.3506	20.01228

	N	O	P	Q	R	S	T	U	V
1	TOP3 ORF2_i02_3	TOP3 ORF2_i03_1	TOP3 ORF2_i03_2	TOP3 ORF2_i03_3	TOP3 WT_i01_1	TOP3 WT_i01_2	TOP3 WT_i01_3	TOP3 WT_i02_1	TOP3 WT_i02_2
2	20.24544	20.54779	19.90251	19.55491	22.70664	22.14872	21.88705	22.85705	21.6538
3	20.69763	20.56837	19.73164	21.51605	22.32589	23.11196	22.26373	22.72269	22.32242
4	19.27262	19.14239	19.1048	19.75973	23.39336	22.19787	21.9621	22.58671	22.78903
5	0	0	0	0	21.89212	20.33641	19.23272	21.29224	19.71429
6	0	0	0	0	20.14095	20.6592	20.62835	19.60742	20.43457
7	25.64373	25.96411	25.88408	25.93987	31.90994	27.67544	28.94934	31.68064	27.59545
8	22.47297	23.08368	25.60398	21.5656	20.48133	22.32895	19.66428	20.04997	20.93039
9	19.60997	20.14434	20.11561	20.69857	22.25077	20.88685	23.55624	21.86395	22.62365
10	24.14922	24.72682	22.6162	23.94601	24.90597	24.2576	29.39907	24.62026	23.02615
11	19.92977	20.69649	20.08769	20.38422	22.83957	20.95793	22.41494	22.68165	21.18309
12	0	0	0	0	24.02693	21.289	21.01216	22.71996	21.83856
13	21.04438	21.50413	23.46636	21.24723	23.68873	23.8949	25.78994	23.26288	23.97938
14	19.69403	20.41883	21.00297	20.45327	0	0	0	0	0
15	0	0	0	0	21.44014	21.04131	24.64764	19.39052	20.27784
16	0	0	0	0	24.25784	21.06294	20.99166	22.50003	21.26809
17	0	0	0	0	21.97495	18.92908	20.17066	21.63407	19.7812
18	20.63123	20.53209	20.54969	20.68117	23.42562	22.22209	22.96729	24.96245	22.74232
19	22.94255	20.69815	20.74532	19.22882	22.45287	23.472	22.92825	22.32919	23.37275
20	19.80548	20.30188	19.52946	19.17612	22.17867	20.70197	24.5693	22.42049	21.88206

	W	X	Y	Z	AA	AB	AC	AD	AE	AF
1	TOP3 WT_i02_3	TOP3 WT_i03_1	TOP3 WT_i03_2	TOP3 WT_i03_3	Number of proteins	Peptides	Razor + unique pep	Mol. weight [kDa]	Sequence length	Q-value
2	22.16209	23.04651	21.51105	22.07353	1	3	3	14.805	122	0
3	21.94448	23.22432	22.32329	22.35995	1	5	5	16.766	155	0
4	21.73195	22.15882	21.08914	21.43233	1	2	2	35.974	326	0
5	18.93083	20.16023	20.33467	19.52231	1	2	2	51.9	446	0
6	19.65162	20.05342	20.57603	19.03514	1	3	3	24.378	211	0
7	29.00467	31.89459	27.50548	28.98938	1	197	184	263.74	2439	0
8	22.11135	20.12218	20.0661	23.55361	1	4	4	62.827	538	0
9	20.84016	22.04554	22.38436	21.27916	1	3	3	26.829	232	0.0018692
10	29.27384	24.85513	24.45	29.20732	1	24	24	45.466	406	0
11	20.77089	22.91218	21.62014	21.2467	1	2	2	56.53	495	0.0009434
12	21.25891	22.52744	22.02708	20.63037	1	2	2	27.208	238	0
13	24.75697	22.92971	22.249	25.43284	1	5	5	7.8929	70	0
14	0	0	0	0	1	2	2	41.144	367	0.00094429
15	19.96342	20.28765	21.84569	19.09222	1	2	2	48.678	423	0.0099548
16	22.399	21.83088	22.98712	20.59685	1	3	3	40.231	355	0
17	21.14208	22.18474	20.77843	21.75821	1	2	2	18.411	162	0.0036866
18	21.86834	25.06381	23.28564	22.47063	1	5	5	28.018	242	0
19	22.89643	22.26173	22.73355	22.15413	1	3	3	49.755	443	0.0037071
20	23.7073	22.22481	22.78978	21.92868	1	2	2	51.422	441	0

	AG	AH	AI	AJ
1	Score	Intensity	iBAQ	#PSM
2	23.448	67442000	11240000	12
3	231.97	447980000	49775000	26
4	17.099	63956000	9136500	9
5	3.9473	33160000	1950600	9
6	30.974	27736000	2311400	13
7	323.31	1.24E+11	987430000	3346
8	10.366	168420000	5807600	5
9	2.7109	103260000	6883800	5
10	323.31	8674500000	510260000	332
11	3.2788	71024000	4439000	9
12	24.761	51292000	17097000	3
13	42.014	432630000	86526000	18
14	3.3355	49606000	3100300	4
15	1.7556	17225000	906580	1
16	11.563	89983000	6921800	5
17	2.3591	47213000	5901600	6
18	4.6374	242240000	22022000	11
19	2.515	127450000	6372300	5
20	8.1921	114750000	5737700	5
